# The Landscape of Using Glycosyltransferase Gene Signatures for Overall Survival Prediction in Hepatocellular Carcinoma

**DOI:** 10.1155/2022/5989419

**Published:** 2022-06-21

**Authors:** Qiang Cai, Shizhe Yu, Jian Zhao, Duo Ma, Long Jiang, Xinyi Zhang, Zhiyong Yu

**Affiliations:** ^1^Department of Hepatobiliary and Pancreatic Surgery, Affiliated Hospital of Yunnan University, Kunming 650021, China; ^2^Department Surgery, First Affiliated Hospital of Zhengzhou University, Zhengzhou 400052, China

## Abstract

Hepatocellular carcinoma (HCC) is a heterogeneous disease that occurs in the setting of chronic liver diseases. The role of glycosyltransferase (GT) genes has recently been the focus of research associated with tumor development. However, the prognostic value of GT genes in HCC remains unclear. Therefore, this study aimed to identify GT genes related to HCC prognosis through bioinformatics analysis. We firstly constructed a prognostic signature based on four GT genes using univariate and least absolute shrinkage and selection operator (LASSO) Cox regression analyses in The Cancer Genome Atlas (TCGA) dataset. Next, the risk score of each patient was calculated, and HCC patients were divided into high- and low-risk groups. Kaplan–Meier analysis showed that the survival rate of high-risk patients was significantly lower than that of low-risk patients. Receiver operating characteristic (ROC) curves assessed that risk scores calculated with a four-gene signature could predict 3- and 5-year overall survival (OS) of HCC patients, revealing the prognostic ability of this gene signature. Moreover, univariate and multivariate Cox regression analyses demonstrated that the risk score was an independent prognostic factor of HCC. Finally, functional analysis revealed that immune-related pathways were enriched and the immune status was different between the two risk groups in HCC. In summary, the novel GT gene signature could be used for prognostic prediction of HCC. Thus, targeting the GT genes may serve as an alternative treatment strategy for HCC.

## 1. Introduction

Liver cancer is the sixth most common cancer, with approximately 800,000 new cases and 780,000 deaths per year [1]. Hepatocellular carcinoma (HCC) is the second leading cause of cancer mortality worldwide and always occurs in the context of chronic liver diseases [2,3]. It is reported that the development of HCC was correlated with several risk factors, mainly including nonalcoholic fatty liver disease, chronic viral infection, and alcohol abuse [1,4,5]. Despite significant developments in the diagnosis and treatment of HCC, the majority of patients with HCC are diagnosed at an advanced stage, leading to a poor prognosis [6]. Thus, it is necessary to explore novel prognostic factors for HCC patients.

Glycosylation is a common process of protein modification, mainly N- and O-glycosylation, that is catalyzed by glycosyltransferases (GTs) and exhibits important roles in various physiological and pathological processes [7–10]. Over the past few decades, a growing number of studies have revealed that GTs are essential in the progression of various diseases, such as joint diseases, inflammatory diseases, cancers, and liver diseases [11,12]. Differentially expressed GTs and their corresponding target proteins have been demonstrated to act as tumor biomarkers and therapeutic targets in specific cancers [13]. Additional evidence also reveals a novel role of GTs, such as GLANT3 and B3GT3, in the self-renewal of pancreatic cancer stem cells [14]. Furthermore, Taniguchi and Kizuka discovered that N-glycan is directly associated with cancers, which provided new biomarkers for evaluating the progression, metastasis, and treatment of cancers [15]. However, whether these GT genes are involved in the prognosis of HCC patients remains largely unclear.

The aim of this study was to examine the clinical application of GTs in the prognosis and to facilitate the development of a personalized treatment approach for HCC patients. Therefore, we comprehensively analyzed the expression profiles of GT genes using the RNA sequencing (RNA-seq) data from The Cancer Genome Atlas (TCGA) database and constructed a four-gene signature for predicting the prognosis of HCC. Then, the prognostic value of the four-gene signature was evaluated and validated in the training and validation sets from TCGA database. Intriguingly, this gene signature could effectively predict the prognosis of HCC patients. In addition, we also performed gene set enrichment analysis (GSEA) to study the potential mechanisms of the four-gene signature.

## 2. Materials and Methods

### 2.1. Data Acquisition

The RNA sequencing (RNA-seq) data and paired clinical information of 371 primary HCC samples and 50 normal samples were downloaded from TCGA database (Supplementary Table 1). Principal component analysis (PCA) was performed to observe the distribution of HCC samples and normal samples based on gene expressions. Subsequently, 421 samples were randomly divided into a training set, including 35 normal samples and 260 HCC samples, and a validation set, including 15 normal samples and 111 HCC samples, in a ratio of 7 : 3. The detailed clinical information statistics of HCC patients in the training and validation sets are shown in Table 1. In addition, 210 human GT genes were obtained from the previous literature (Supplementary Table 2) [16].

### 2.2. Construction and Validation of a Prognostic GT Gene Signature

Differentially expressed genes (DEGs) between HCC samples and normal samples were screened by using the “limma” package of R software [17], and |log2FC| ≥ 1 and *P* value ≤0.05 were set as the screening condition. Differentially expressed GT genes were obtained by overlapping DEGs and GT genes. Then, univariate Cox analysis was applied to identify differentially expressed GT genes associated with overall survival (OS) of HCC patients in the training set, and *P* value <0.05 was considered statistically significant [18]. Moreover, least absolute shrinkage and selection operator (LASSO) Cox regression algorithm was used to filter false positive genes and construct a gene signature in the training set using the “glmnet” package in R [19]. Namely, a GT gene signature was established according to genes and their corresponding coefficients obtained by LASSO analysis. Furthermore, the risk scores of each HCC patient were calculated according to the following formula [20]:

(1) $$\begin{array}{c} \text{score}=e\text{ sum}\left(\text{each gene's expression level}\times \text{corresponding coefficient}\right). \end{array}$$

HCC patients were classified into high-risk and low-risk groups based on the median value of risk scores of patients in the training and validation sets, separately. Finally, Kaplan–Meier (KM) survival analysis was performed using the pressminer package in R and overall survival (OS) of high- and low-risk patients was compared using the logarithmic test. Receiver operating characteristic (ROC) curves of time-dependent factors were drawn using the “survivalROC” R package, and the area under the curve (AUC) for 1-year, 3-year, and 5-year OS was calculated to assess the prediction accuracy for the GT gene signature [21].

### 2.3. Relationship between the GT Gene Signature and Clinical Characteristics

To investigate the relationship between the GT gene signature and clinical characteristics, including BMI, treatment, treatment type, prior malignancy, TNM stage, and pathologic stage, the one-way ANOVA test or Wilcoxon test was performed.

### 2.4. Construction of a Predictive Nomogram

To investigate whether the GT gene signature could act as an independently prognostic prediction factor, the GT gene signature and other clinical features were merged to screen the independently prognostic prediction factor via univariate and multivariate Cox regression analyses in the training and validation sets. Moreover, the forest plot was used to show the results of univariate and multivariate Cox regression analyses through the “rms” package in R.

### 2.5. Functional Enrichment Analysis

GSEA was utilized to perform Gene Ontology (GO) and Kyoto Encyclopedia of Genes and Genomes (KEGG) enrichment analyses based on all genes between high-risk and low-risk groups [22].

### 2.6. Tumor Microenvironment Immune Infiltration Analysis

To further investigate the correlation between the GT gene signature and tumor microenvironment immune infiltration, the enrichment scores of infiltrating immune cells between high-risk and low-risk groups were calculated by single-sample gene set enrichment analysis (ssGSEA), which was performed in the “gsva” package of R [23]. Moreover, the immune score, estimate score, and stromal score between high-risk and low-risk groups were generated by the ESTIMATE algorithm.

### 2.7. Statistical Analysis

All bioinformatics analyses were performed with R software. Student's *t*-test was performed to compare differences between groups. We compared the OS among different groups using Kaplan–Meier analysis with the log-rank test. A *P* value <0.05 was considered statistically significant, and all *P* values were two-tailed.

## 3. Results

The aim of our study was to examine the prognostic value of GTs and contribute to the development of therapeutic strategies for patients with HCC. In the present study, we systematically investigated the expression profiles of GT genes and established a four-gene signature for the prognosis of patients with HCC. We also examined the potential mechanisms of the four-gene signature.

### 3.1. Identification of Differentially Expressed Glycosyltransferase (GT) Genes Associated with OS in HCC

**Figure 1:**
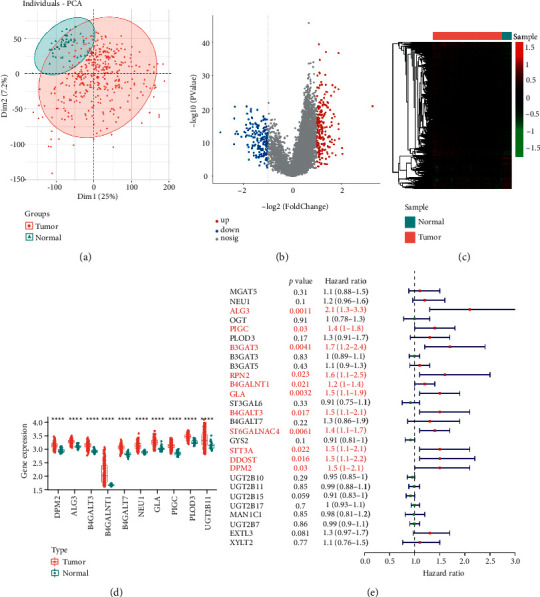
Identification of differentially expressed glycosyltransferase (GT) genes associated with overall survival (OS) in hepatocellular carcinoma (HCC). (a) The principal component analysis (PCA) between the tumor and normal groups in TCGA database. (b) Volcano plots showing the number of DEGs screened from the training set of HCC in TCGA database. (c) DEGs were visualized between tumor and normal groups in the training set from TCGA database using a heatmap. (d) The expressions of top 10 differentially expressed GTs between the tumor and normal samples. (e) Forest plots showing the results of the univariate Cox regression analysis between the gene expression and OS in the training set from the TCGA database.

PCA results of 421 samples from TCGA database revealed that the tumor tissue and the normal tissue samples were distributed in two directions (Figure 1(a)). To further explore genes acting as prognostic factors for the progression of HCC, we first comprehensively analyzed the training set of HCC from TCGA database. As presented in Figure 1(b), a total of 2264 DEGs were identified based on thresholds of |log_2_FC| ≥ 1 and *P* value ≤0.05, of which 1765 were upregulated and 499 were downregulated (Supplementary Table 3). The heatmap showed the expression of DEGs between the tumor and normal groups (Figure 1(c)). Furthermore, these DEGs were combined with 210 GTs downloaded from the literature, and 28 differentially expressed GT genes were obtained (Supplementary Figure 1). Importantly, the expression of the top 10 differentially expressed GT genes was significantly different between the two groups (Figure 1(d), *P* < 0.05). To further identify GTs associated with the survival of patients with HCC, we performed a univariate Cox regression analysis based on the 28 differentially expressed GT genes. As a result, 11 of them were obtained using a cutoff of *P* value <0.05 (Figure 1(e)).

### 3.2. Construction of a Prognostic Model in the Training Set

After the LASSO Cox regression analysis of the training set, we obtained a four-gene signature that included ALG3, B3GAT3, GLA, and ST6GALNAC4 (Supplementary Figure 2). To better demonstrate the prognostic role of the four genes in HCC, we first used Kaplan–Meier analysis in TCGA dataset in which samples were divided into high and low expression based on the gene expression level (Supplementary Figure 3). mRNA expression levels of the four genes in matched HCC and adjacent noncancerous samples in TCGA database were compared, as shown in Supplementary Figure 4. All four of these genes were significantly associated with the OS of HCC patients, which indicated that this gene signature may act as a prognostic factor in HCC. Intriguingly, a protein-protein interaction (PPI) network revealed that there were no direct interactions between the 4 gene signatures (Supplementary Figure 5).

**Figure 2:**
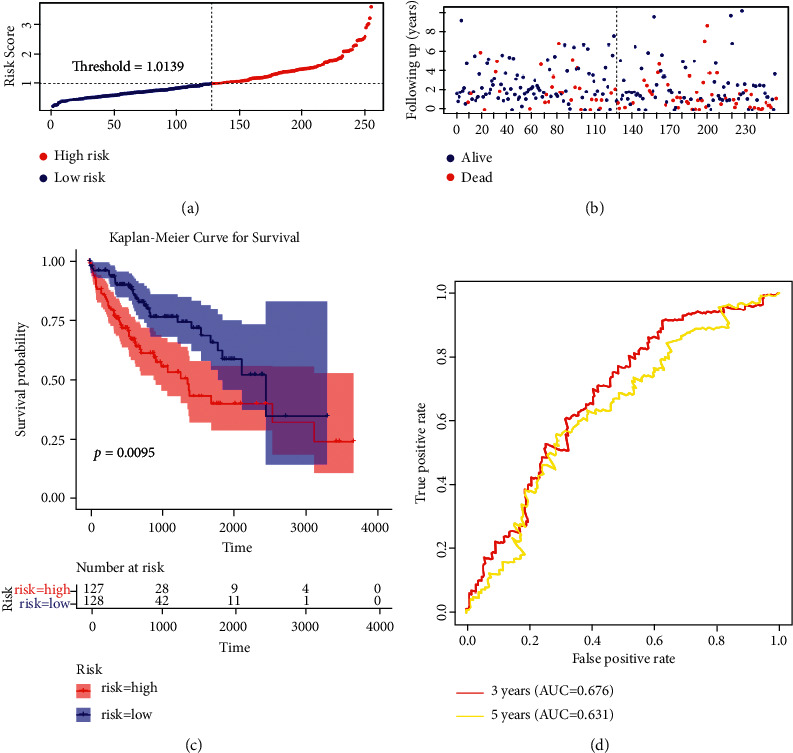
Construction of a prognostic model in the training set of TCGA cohort. (a, b) The distribution of risk score, overall survival (OS), and survival status in the training set of TCGA cohort. (c) Kaplan–Meier analysis to compare OS between HCC patients in high- and low-risk groups in the training set. (d) Receiver operating characteristic (ROC) analysis of the four-gene signature for predicting OS in the training set of TCGA database.

Having shown that this gene signature may act as a prognostic factor regarding the progression of HCC, we investigated the practical prognostic value in HCC. The risk score was calculated according to the gene expression levels and their corresponding coefficients. Based on the median value of risk score, patients with HCC were divided into high- and low-risk groups. The results showed that the number of patients who died of HCC was significantly increased along with an increasing risk score (Figures 2(a) and 2(b)). The OS of patients with HCC was obviously different between the two risk groups; HCC patients with high-risk scores showed a poor OS compared with those with low-risk scores (Figure 2(c), *P*=0.00095). Stratified survival analysis showed that risk factors including nonalcoholic fatty liver disease (NAFLD), hepatitis B virus (HBV) infection, and hepatitis C virus (HCV) infection had no significant effect on the survival of patients in high- and low-risk groups. Interestingly, HCC patients with HBV and HCV infection in the high-risk group showed a significantly worse OS than those in the low-risk group. Moreover, pharmaceutical therapy in the low-risk group contributed to a better OS than in their high-risk counterparts (Supplementary Figure 6). Thereafter, ROC curve analysis was performed to investigate the effectiveness of the prognostic model (Figure 2(d)). The area under the curve (AUC) reached 0.676 at 3 years and 0.631 at 5 years, which indicated that the risk score of the prognostic model had high accuracy. To predict the 3- and 5-year survival probability, we established a nomogram containing the four genes in this signature. As a result, each factor corresponded to a point. In addition, calibration curves revealed the consistence between the actual and the predicted survival probability, indicating the strong predictive performance of the nomogram (Supplementary Figure 7, c-index = 0.66328, calibrated c-index = 0.64057).

### 3.3. Validation of the Four-Gene Signature in the Validation Set

**Figure 3:**
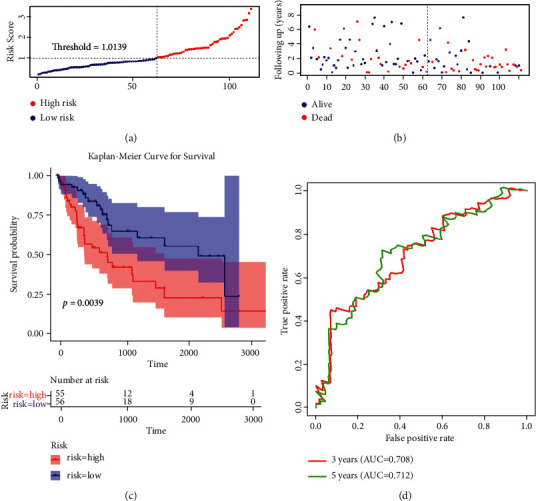
Validation of four-gene signature in the validation set of TCGA database. (a, b) The distribution of the risk score, overall survival (OS), and survival status in the validation set of TCGA cohort. (c) Kaplan–Meier analysis to compare OS between patients with HCC in high- and low-risk groups in the validation set. (d) Receiver operating characteristic analysis of the 4-gene signature for predicting the OS in the validation set of TCGA database.

To obtain additional evidence regarding the prognostic value of the gene signature, we accessed the prognostic model in the validation dataset of TCGA database. Interestingly, survival analyses of 4 genes confirmed that two of these genes correlated with poor OS of HCC (Supplementary Figure 8). To demonstrate the robustness of the validation model from TCGA database, patients with HCC were divided into high- and low-risk groups according to the median value of the risk score (Figures 3(a) and 3(b)). The results were consistent with those obtained from the training set (Figure 3(c), *P*=0.0039). In addition, ROC curve analysis also showed that the AUC of the four-gene signature reached 0.708 at 3 years and 0.712 at 5 years, further revealing the accuracy of the prognostic model (Figure 3(d)).

### 3.4. The Correlation of Risk Scores and Clinicopathological Characteristics in Patients with HCC

**Figure 4:**
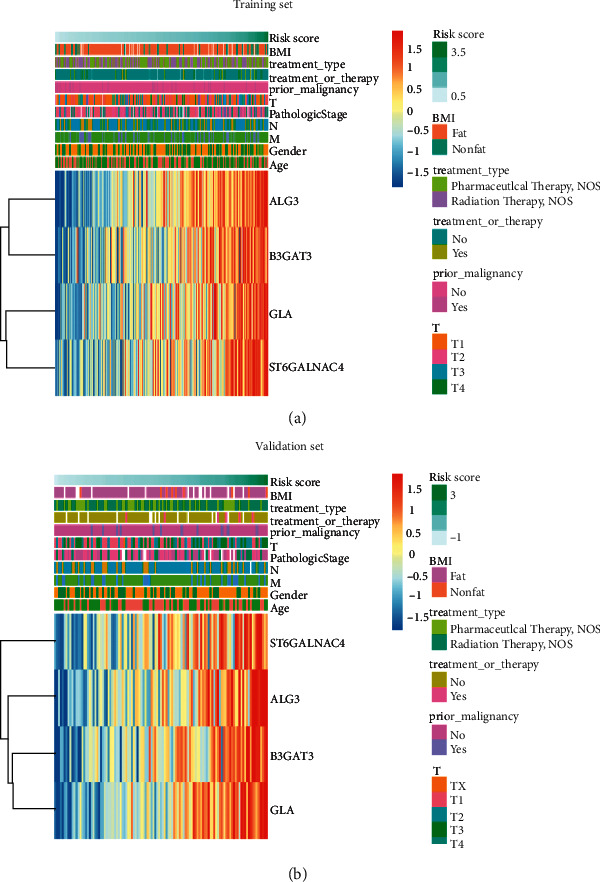
The correlation between the risk scores and clinicopathological characteristics of patients with hepatocellular carcinoma (HCC). (a) The correlation between the four GT gene expression profiles stratified by the risk score and clinicopathological characteristics of patients with HCC in the training set of TCGA database. (b) The correlation between four GT gene expression profiles stratified by the risk score and clinicopathological characteristics of HCC patients in the validation set of TCGA database.

The expressions of these 4 signature genes between the two risk groups and clinical characteristics in TCGA database were visualized with a heatmap (Figures 4(a) and 4(b)). We found that the expression of these signature genes significantly increased as the risk score in TCGA database increased. Interestingly, we found that the four-gene signature was related to the stage (Figure 4(c)), indicating that the four-gene signature might affect the development of HCC.

**Figure 5:**
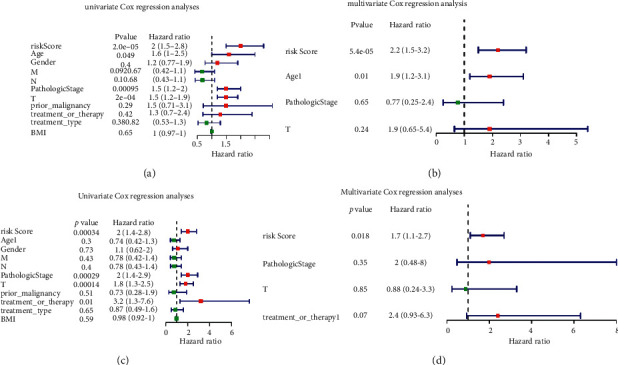
Independent prognostic value of the risk score. Results of the univariate and multivariate Cox regression analyses regarding OS in TCGA database (a, b) in the training set and (c, d) in the validation set.

Furthermore, we performed univariate and multivariate Cox regression analyses after adding clinical characteristics to investigate whether the risk score of the prognostic model was an independent factor for the HCC prognosis (Figures 5(a)–5(d)). In the univariate Cox regression analyses, the risk score was correlated with the OS of patients with HCC both in the training set and the validation set of TCGA database (TCGA training set: HR = 2, 95% CI = 1.5–2.8, *P*=2.2*e* − 05; TCGA validation set: HR = 2, 95% CI = 1.4–2.8, *P*=0.0034). The multivariate Cox regression analyses showed that the risk score of the prognostic model was still an independent factor for the OS of patients with HCC (TCGA training set: HR = 2.2, 95% CI = 1.5–3.2, *P*=5.4*e* − 05; TCGA validation set: HR = 1.7, 95% CI = 1.1–2.7, *P*=0.018).

### 3.5. Functional Analyses in TCGA Database of HCC

**Figure 6:**
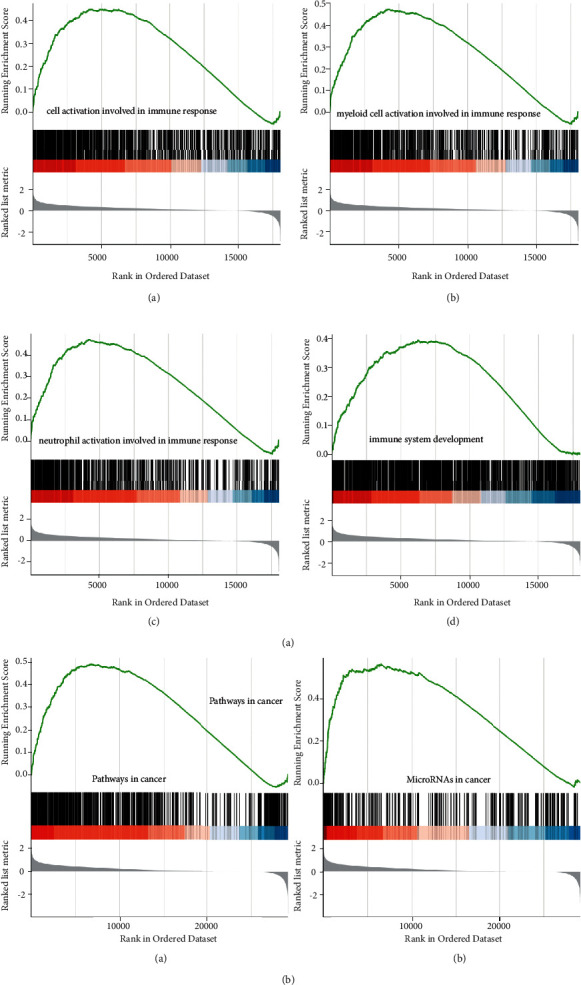
Functional analyses of HCC in TCGA database of HCC. (a) The most significant or shared biological processes of GO enrichment obtained from GSEA. (A) GO: 0002263 cell activation involved in immune response. (B) GO: 0002275 myeloid cell activation involved in immune response. (C) GO: 0002283 neutrophil activation involved in immune response. (D) GO: 0002520 immune system development. (b) The most significant or shared KEGG pathways. (A) hsa05200∼pathways in cancer. (B) hsa05206∼microRNAs in cancer.

Based on these observations, we next investigated the biological progresses related to the risk score. Therefore, we focused on all genes between the two risk groups to perform GSEA. As a result, GO analysis revealed that the genes were involved in several immune-related biological processes, such as GO: 0002263 cell activation involved in immune response, GO: 0002275 myeloid cell activation involved in immune response, GO: 0002283 neutrophil activation involved in immune response, and GO: 0002520 immune system development, which may be highly associated with the development of HCC (Figure 6(a), *P* value <0.05). Notably, the KEGG pathway analysis results further indicated that these genes were involved in cancer-related pathways, such as hsa05200∼pathways in cancer, and hsa05206∼microRNAs in cancer (Figure 6(b), *P* value <0.05). All significantly enriched GO terms and KEGG pathways are shown in Supplementary Tables 4 and 5, respectively.

### 3.6. Correlation between the GT Gene Signature and Tumor Microenvironment Immune Infiltration

**Figure 7:**
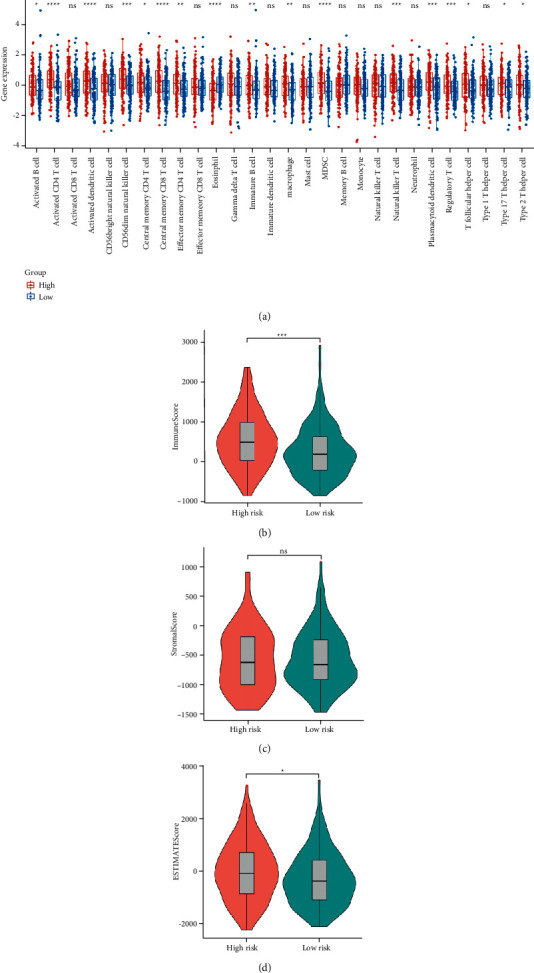
(a) The infiltration proportion of 22 immune cell types in high-risk and low-risk groups in TCGA cohort. ns, not significant; ^*∗*^*P* < 0.05; ^*∗∗*^*P* < 0.01; ^*∗∗∗*^*P* < 0.001; ^*∗∗∗∗*^*P* < 0.0001. (b–d) Immune score, stromal score, and ESTIMATE score calculated by the ESTIMATE algorithm were compared between high-risk and low-risk groups.

To investigate whether the risk score correlated with immune status in HCC, we analyzed the differences in the scores of immune cell enrichment between high-risk and low-risk groups. Interestingly, activated CD4 T cells, activated dendritic cells, CD56dim natural killer cells, central memory CD8 T cells, eosinophils, MDSCs, and natural killer T cells showed obvious and significant differences between the two risk groups (Figure 7(a), all *P* values <0.05). Moreover, the immune score calculated by the ESTIMATE algorithm in the high-risk group was higher than that in the low-risk group, which further revealed that the risk score of the prognostic model was strongly correlated with the immune status of patients with HCC (Figure 7(b), *P* < 0.05). Interestingly, the stromal and ESTIMATE scores showed no significant difference in the high-risk and low-risk groups (Figures 7(c) and 7(d)).

## 4. Discussion

HCC is a common malignancy with a poor prognosis [24,25]. The critical role of glycosylation modification of protein has been demonstrated in several cell biological processes occurring in cancer, such as immune modulation and metastasis, tumor cell invasion, cell signaling and communication, and tumor angiogenesis [8,26,27]. Accordingly, this suggests that GTs catalyzing the glycosylation modification process have a potential application in the diagnosis and prognosis of HCC.

Over the past years, with significant progress in epigenetics and metabolomics, various biomarkers have been identified for many cancers [20,28,29]. In the current study, we focused on whether GTs involved in the progression of HCC are also associated with the prognosis of HCC. By analyzing the expression profiles of HCC patients in TCGA database, we identified a gene signature that could predict the OS of patients with HCC. Four differentially expressed GT genes, including ALG3, B3GAT3, GLA, and ST6GALNAC4, correlated with the prognosis of HCC. Moreover, we constructed a four-gene signature that divided HCC patients into high- and low-risk groups for predicting the prognosis of patients with HCC. Furthermore, the prognostic value of the four-gene signature was assessed by ROC curves and Kaplan–Meier analysis in the training and validation sets. Importantly, the risk score generated from the four-gene signature was demonstrated to be an independent prognostic factor in patients with HCC by univariate and multivariate Cox regression analyses. In addition, GSEA results revealed that differentially expressed genes (DEGs) between high- and low-risk groups were mainly involved in several immune-related biological processes and signaling pathways. Thus, the immune status between the two groups was further evaluated.

Although a previous study has demonstrated that the expressions of GT genes might mediate aberrant glycosylation in many cancers [16], their correlation with the OS of patients with HCC still remains unknown. Surprisingly, 28 GT genes were differentially expressed between tumor tissues and normal tissues, and four of them were demonstrated to be signature genes, which indicated the potential prognostic value of GT genes in HCC. Of the 4 signature GT genes, several genes have been shown to be involved in the progression of tumors. ALG3 is an oncogene, located at the 3q27.1 chromosomal region, which is correlated with multiple malignancies [30]. In non-small-cell lung cancer (NSCLC), the expression of ALG3 in tumor tissues was significantly increased compared with that of normal tissues [31]. Upregulation of ALG3 promoted the metastasis of esophageal squamous cell cancer to lymph nodes and the proliferation of cervical cancer cells [30,32]. ALG3 was also found to promote the proliferation and metastasis of breast cancer cells, and overexpression of ALG3 was associated with poor prognosis [33]. In accordance with previous studies, our results revealed that ALG3 was upregulated in HCC tissues. To our knowledge, this is the first evidence regarding the potential prognostic role of ALG3 in patients with HCC.

An altered expression of the ST6GALNAC family of genes has been reported in several cancers. Aberrant glycosylation caused by the changes in ST6GALNAC4 expression levels promoted the lung cancer metastasis through adhesion to galectins in the metastatic niche [34]. miR-4299 mediated the invasive properties and tumorigenicity of human follicular thyroid carcinoma via targeting ST6GALNAC4 [35]. B3GAT3, as a glycosyltransferase, may mediate the attachment of saccharides to key proteins [36,37]. Moreover, B3GAT3 has been shown to be highly expressed in liver cancer tissues and was associated with poor prognosis, which is consistent with our present study [38]. Furthermore, high expression of B3GAT3 was related to worsened OS in HCC patients without alcohol consumption or hepatitis virus infection. However, high B3GAT3 levels were moderately, but not significantly, correlated with worse OS in patients with positive alcohol intake or positive hepatitis virus status [38]. With respect to GLA, it has been reported that it plays a role in tumor progression, regulation of macrophages, and prevention of fatty liver in mice [39].

In our study, we also explored the potential molecular mechanisms of the four-gene signature, providing new insights into treatment. Although the potential mechanisms of regulating immunity have diverse clinical implications in tumor immunotherapy, the relationship between tumor immunity and GTs remains largely unknown. Based on all the genes between the two risk groups, we performed GSEA and unexpectedly found that several immune-related biological processes were significantly enriched. Therefore, we hypothesized that there were potential associations between GTs and tumor immunity. Interestingly, the fractions of infiltrating immune cells were significantly different between low-risk and high-risk groups. In addition, patients of the high-risk group in TCGA contained higher fractions of central memory CD8 T cells. A previous study has revealed that increased tumor-associated CD8 T cells are related to poor prognosis in HCC patients [40]. Therefore, the attenuated antitumor immune function of high-risk patients may be a reason for their poor prognosis.

Although we identified a novel four-gene prognostic signature in HCC, there are several limitations to this analysis. First, our prognostic signature was only established in TCGA database and was not validated in other independent databases. We tried many external verifications for the gene signature, and unfortunately, the results were poor. Second, this study provided a prognostic model constructed by four genes, which needs to be further verified in clinical trials. In addition, many other important prognostic genes in HCC might have been excluded in the Cox analysis. Third, our study was conducted based on retrospectively collected data and should be verified in prospective studies with real-world data. Notably, the associations between the risk score and immune status have not yet been determined by in vitro and in vivo experiments.

We comprehensively demonstrated a GT gene signature in the training set and validation set of TCGA database for the first time and observed the prognostic value for predicting OS in HCC patients. However, there were obvious difference among the effects of each gene on OS, and this may be due to the deficiency and clinical information of the samples, leading to conflicting or meaningless results. In addition, functional analysis revealed that the DEGs between the high-risk and low-risk groups were primarily involved in several important immune biological processes and pathways, which may provide a new direction for studies on the mechanism of HCC progression. It is unclear how GT genes are involved in HCC prognosis, and this requires further study.

## 5. Conclusions

In conclusion, we revealed the prognostic value of GT genes in the progression of HCC for the first time. A GT gene signature was identified which could effectively divide HCC patients into high- and low-risk groups in order to accurately predict their OS. Our study contributes to the understanding of the molecular mechanisms through which GT genes are involved in the occurrence and development of HCC and provides a unique approach to explore biomarkers for targeted therapy in HCC.

## 6. Disclosure

Qiang Cai and Yu Shizhe are the co-first authors. An earlier version of the manuscript has been presented as a preprint in Research Square (DOI: 10.21203/rs.3.rs-114747/v1).

## Figures and Tables

**Table 1:** The detailed clinical characteristics of patients with hepatocellular carcinoma between the training and validation sets of TCGA database.

	Total (*n* = 371)	Dataset		
		Training set (n = 260)	Validation (*n* = 111)	*P* value
Gender				0.2
Male	250 (67.4%)	181 (69.6%)	69 (62.2%)	
Female	121 (32.6%)	79 (30.4%)	42 (37.8%)	
Age				0.855
<60	169 (45.6%)	117 (45.0%)	52 (46.8%)	
≥60	201 (54.2%)	142 (54.6%)	59 (53.2%)	
Missing	1 (0.3%)	1 (0.4%)	0 (0%)	
Stage				0.12
Stage I	171 (46.1%)	129 (49.6%)	42 (37.8%)	
Stage II	86 (23.2%)	61 (23.5%)	25 (22.5%)	
Stage III	85 (22.9%)	52 (20.0%)	33 (29.7%)	
Stage IV	5 (1.3%)	3 (1.2%)	2 (1.8%)	
Missing	24 (6.5%)	15 (5.8%)	9 (8.1%)	
T				0.077
TX	1 (0.3%)	0 (0%)	1 (0.9%)	
T1	181 (48.8%)	136 (52.3%)	45 (40.5%)	
T2	94 (25.3%)	65 (25.0%)	29 (26.1%)	
T3	80 (21.6%)	48 (18.5%)	32 (28.8%)	
T4	13 (3.5%)	9 (3.5%)	4 (3.6%)	
Missing	2 (0.5%)	2 (0.8%)	0 (0%)	
M				0.826
M0	266 (71.7%)	184 (70.8%)	82 (73.9%)	
M1	4 (1.1%)	3 (1.2%)	1 (0.9%)	
MX	101 (27.2%)	73 (28.1%)	28 (25.2%)	
Treatment				0.149
Pharmaceutical therapy, NOS	180 (48.5%)	133 (51.2%)	47 (42.3%)	
Radiation therapy, NOS	191 (51.5%)	127 (48.8%)	64 (57.7%)	

## Data Availability

The data used to support the findings of this study are included in the article, and the datasets analyzed during the current study are available in The Cancer Genome Atlas database (https://portal.gdc.cancer.gov/projects/TCGA-LIHC).

## Supplementary Materials

Supplementary Materials Figure S1: Venn diagram shows the intersections of co-differentially expressed glycosyltransferase (GT) genes between the 210 GTs obtained from the literature and those obtained from TCGA database. Figure S2: identification of a four-gene signature in the training set of TCGA database constructed using the LASSO method. Figure S3: survival analyses grouped by the optimal cutoff expression value of each gene in the training dataset of TCGA database. (A) ALG3. (B) B3GAT3. (C) GLA. (D) ST6GALNAC4. Figure S4: mRNA expression levels of the four genes in matched HCC and adjacent noncancerous samples in TCGA database were compared. (A) ALG3. (B) B3GAT3. (C) GLA. (D) ST6GALNAC4. Figure S5: the interactions among signature genes displayed in a PPI network. Figure S6: stratified survival analysis between high- and low-risk groups. (A)NAFLD. (B) HBV infection. (C) HCV infection. (D) Both HBV and HCV infections. (E) No treatment and therapy. (F) Pharmaceutical therapy. (G) Radiation therapy, NOS. Figure S7: the nomogram to predict prognostic probabilities in TCGA database. (A) The nomogram for predicting OS in the training set. The calibration plots for predicting (B) 3-year and (C) 5-year survival probabilities. Figure S8: survival analyses grouped by the optimal cutoff expression value of each signature gene in the validation dataset of TCGA database. (A) ALG3. (B) B3GAT3. (C) GLA. (D) ST6GALNAC4. Table S1: the clinical information of 371 primary HCC samples and 50 normal samples. Table S2: combining the clinical information and expression profiles of 210 human glycosyltransferase (GT) genes. Table S3: DEGs of 371 samples in TCGA database. Table S4: all significantly enriched GO terms. Table S5: all significantly enriched KEGG pathways.

## References

[1] Bray F., Ferlay J., Soerjomataram I., Siegel R. L., Torre L. A., Jemal A. (2018). Global cancer statistics 2018: GLOBOCAN estimates of incidence and mortality worldwide for 36 cancers in 185 countries. *CA—a Cancer Journal for Clinicians*.

[2] Degasperi E., Colombo M. (2016). Distinctive features of hepatocellular carcinoma in non-alcoholic fatty liver disease. *Lancet Gastroenterology & Hepatology*.

[3] Sim H. W., Knox J. (2018). Hepatocellular carcinoma in the era of immunotherapy. *Current Problems in Cancer*.

[4] Chen C. J., Yang H. I., Su J. (2006). Risk of hepatocellular carcinoma across a biological gradient of serum hepatitis B virus DNA level. *JAMA*.

[5] Llovet J. M., Zucman-Rossi J., Pikarsky E. (2016). Hepatocellular carcinoma. *Nature Reviews Disease Primers*.

[6] Kinoshita A., Onoda H., Fushiya N., Koike K., Nishino H., Tajiri H. (2015). Staging systems for hepatocellular carcinoma: current status and future perspectives. *World Journal of Hepatology*.

[7] Schäffer C., Messner P. (2017). Emerging facets of prokaryotic glycosylation. *FEMS Microbiology Reviews*.

[8] Pinho S. S., Reis C. A. (2015). Glycosylation in cancer: mechanisms and clinical implications. *Nature Reviews Cancer*.

[9] Yang Y., Zhang X., Yu B. (2015). O-Glycosylation methods in the total synthesis of complex natural glycosides. *Natural Product Reports*.

[10] Wadzinski T. J., Steinauer A., Hie L., Pelletier G., Schepartz A., Miller S. J. (2018). Rapid phenolic O-glycosylation of small molecules and complex unprotected peptides in aqueous solvent. *Nature Chemistry*.

[11] Blomme B., Van Steenkiste C., Callewaert N., Van Vlierberghe H. (2009). Alteration of protein glycosylation in liver diseases. *Journal of Hepatology*.

[12] Dong S., Wang Z., Huang B. (2017). Bioinformatics insight into glycosyltransferase gene expression in gastric cancer: POFUT1 is a potential biomarker. *Biochemical and Biophysical Research Communications*.

[13] Drake RR. (2015). Glycosylation and cancer: moving glycomics to the forefront. *Advances in Cancer Research*.

[14] Barkeer S., Chugh S., Karmakar S. (2018). Novel role of O-glycosyltransferases GALNT3 andB3GNT3 in the self-renewal of pancreatic cancer stem cells. *BMC Cancer*.

[15] Taniguchi N., Kizuka Y. (2015). Glycans and cancer: role of N-glycans in cancer biomarker, progression and metastasis, and therapeutics. *Advances in Cancer Research*.

[16] Ashkani J., Naidoo K. J. (2016). Glycosyltransferase gene expression profiles classify cancer types and propose prognostic subtypes. *Scientific Reports*.

[17] Ritchie M. E., Phipson B., Wu D. (2015). Limma powers differential expression analyses for RNA-sequencing and microarray studies. *Nucleic Acids Research*.

[18] Emura T., Matsui S., Chen H. Y. (2019). Compound.cox: univariate feature selection and compound covariate for predicting survival. *Computer Methods and Programs in Biomedicine*.

[19] Tibshirani R. (1997). The lasso method for variable selection in the Cox model. *Statistics in Medicine*.

[20] Liang J. Y., Wang D. S., Lin H. C. (2020). A novel ferroptosis-related gene signature for overall survival prediction in patients with hepatocellular carcinoma. *International Journal of Biological Sciences*.

[21] Jiang Y. (2020). Receiver operating characteristic (ROC) analysis of image search-and-localize tasks. *Academic Radiology*.

[22] Thomas M. A., Yang L., Carter B. J., Klaper R. D. (2011). Gene set enrichment analysis of microarray data from Pimephales promelas (Rafinesque), a non-mammalian model organism. *BMC Genomics*.

[23] Rooney M. S., Shukla S. A., Wu C. J., Getz G., Hacohen N. (2015). Molecular and genetic properties of tumors associated with local immune cytolytic activity. *Cell*.

[24] Llovet J., Brú C., Bruix J. (1999). Prognosis of hepatocellular carcinoma: the BCLC staging classification. *Seminars in Liver Disease*.

[25] Marquardt J. U., Thorgeirsson S. S. (2014). SnapShot: hepatocellular carcinoma. *Cancer Cell*.

[26] Chen Y., Ding L., Ju H. (2018). In situ cellular glycan analysis. *Accounts of Chemical Research*.

[27] Bhatia R., Gautam S. K., Cannon A. (2019). Cancer-associated mucins: role in immune modulation and metastasis. *Cancer & Metastasis Reviews*.

[28] Mou Y., Zhang Y., Wu J. (2020). The landscape of iron metabolism-related and methylated genes in the prognosis prediction of clear cell renal cell carcinoma. *Frontiers in Oncology*.

[29] Wang P., Wu M., Tu Z. (2020). Identification of RNA: 5-methylcytosine methyltransferases-related signature for predicting prognosis in glioma. *Frontiers in Oncology*.

[30] Shi Z.-Z., Jiang Y.-Y., Hao J.-J. (2013). Identification of putative target genes for amplification within 11q13.2 and 3q27.1 in esophageal squamous cell carcinoma. *Clinical and Translational Oncology*.

[31] Ke S. B., Qiu H., Chen J. M. (2020). ALG3 contributes to the malignancy of non-small cell lung cancer and is negatively regulated by MiR-98-5p. *Pathology, Research & Practice*.

[32] Choi Y. W., Bae S. M., Kim Y. W. (2007). Gene expression profiles in squamous cell cervical carcinoma using array-based comparative genomic hybridization analysis. *International Journal of Gynecological Cancer*.

[33] De Leoz M. L. A., Young L. J. T., An H. J. (2011). High-mannose glycans are elevated during breast cancerprogression. *Molecular & Cellular Proteomics*.

[34] Reticker-Flynn N. E., Bhatia S. N. (2015). Aberrant glycosylation promotes lung cancer metastasis through adhesion to galectins in the metastatic niche. *Cancer Discovery*.

[35] Miao X., Jia L., Zhou H. (2016). miR‐4299 mediates the invasive properties and tumorigenicity of human follicular thyroid carcinoma by targeting ST6GALNAC4. *IUBMB Life*.

[36] Pedersen L. C., Tsuchida K., Kitagawa H., Sugahara K., Darden T. A., Negishi M. (2000). Heparan/chondroitin sulfate biosynthesis. *Journal of Biological Chemistry*.

[37] Job F., Mizumoto S., Smith L. (2016). Functional validation of novel compound heterozygous variants in B3GAT3 resulting in severe osteopenia and fractures: expanding the disease phenotype. *BMC Medical Genetics*.

[38] Zhang Y. L., Ding C., Sun L. (2019). High expression B3GAT3 is related with poor prognosis of liver cancer. *Open Medicine*.

[39] Park J. H., Lee M. K., Yoon J. (2015). Gamma-linolenic acid inhibits hepatic PAI-1 expression by inhibiting p38 MAPK-dependent activator protein and mitochondria-mediated apoptosis pathway. *Apoptosis*.

[40] Ma J., Zheng B., Goswami S. (2019). PD1Hi CD8+ T cells correlate with exhausted signature and poor clinical outcome in hepatocellular carcinoma. *Journal for Immunotherapy of Cancer*.

