# Supplementary material for: The Landscape of Using Glycosyltransferase Gene Signatures for Overall Survival Prediction in Hepatocellular Carcinoma
Source: J Oncol. 2022 Jun 21;2022:5989419. doi: 10.1155/2022/5989419 (PMC9239767; doi:10.1155/2022/5989419)
Supplement: Supplementary Materials — Figure S1: Venn diagram shows the intersections of co-differentially expressed glycosyltransferase (GT) genes between the 210 GTs obtained from the literature and those obtained from TCGA database. Figure S2: identification of a four-gene signature in the training set of TCGA database constructed using the LASSO method. Figure S3: survival analyses grouped by the optimal cutoff expression value of each gene in the training dataset of TCGA database. (A) ALG3. (B) B3GAT3. (C) GLA. (D) ST6GALNAC4. Figure S4: mRNA expression levels of the four genes in matched HCC and adjacent noncancerous samples in TCGA database were compared. (A) ALG3. (B) B3GAT3. (C) GLA. (D) ST6GALNAC4. Figure S5: the interactions among signature genes displayed in a PPI network. Figure S6: stratified survival analysis between high- and low-risk groups. (A)NAFLD. (B) HBV infection. (C) HCV infection. (D) Both HBV and HCV infections. (E) No treatment and therapy. (F) Pharmaceutical therapy. (G) Radiation therapy, NOS. Figure S7: the nomogram to predict prognostic probabilities in TCGA database. (A) The nomogram for predicting OS in the training set. The calibration plots for predicting (B) 3-year and (C) 5-year survival probabilities. Figure S8: survival analyses grouped by the optimal cutoff expression value of each signature gene in the validation dataset of TCGA database. (A) ALG3. (B) B3GAT3. (C) GLA. (D) ST6GALNAC4. Table S1: the clinical information of 371 primary HCC samples and 50 normal samples. Table S2: combining the clinical information and expression profiles of 210 human glycosyltransferase (GT) genes. Table S3: DEGs of 371 samples in TCGA database. Table S4: all significantly enriched GO terms. Table S5: all significantly enriched KEGG pathways. [file 5989419.f1.zip › 5989419.f1/Supplementary Table.1 TCGA-LIHC clinical.pdf]

case_submi	age_at_inc	days_to_de	ethnicity	gender	vital_stat	ajcc_patho	ajcc_patho
1	TCGA-DD-AA	48	'--	not hispar	male	Alive	M0
2	TCGA-DD-A4	32	'--	not hispar	male	Alive	M0
3	TCGA-BC-40	73	'--	not hispar	male	Alive	MX
4	TCGA-MI-A7	61	'--	not hispar	male	Alive	M0
5	TCGA-G3-A7	31	'--	not hispar	male	Alive	MX
6	TCGA-DD-A1	69	'--	not hispar	male	Alive	M0
7	TCGA-CC-A9	68	'--	not hispar	male	Alive	M0
8	TCGA-CC-A3	54	'--	not hispar	male	Alive	M0
9	TCGA-DD-A1	42	1149	not hispar	male	Dead	M0
10	TCGA-WX-AA	61	'--	not hispar	male	Alive	MX
11	TCGA-DD-AA	69	'--	not hispar	male	Alive	M0
12	TCGA-DD-AA	66	'--	not hispar	male	Alive	M0
13	TCGA-5C-A9	58	'--	not hispar	male	Alive	M0
14	TCGA-BC-A3	66	'--	not report	female	Alive	M0
15	TCGA-G3-A2	73	'--	not hispar	male	Alive	M0
16	TCGA-RG-A7	69	'--	not hispar	male	Alive	M0
17	TCGA-G3-AA	83	27	not hispar	female	Dead	M0
18	TCGA-DD-AA	73	'--	not hispar	female	Alive	M0
19	TCGA-DD-AA	32	'--	not hispar	male	Alive	M0
20	TCGA-BC-A1	66	308	not hispar	female	Dead	MX
21	TCGA-DD-A3	78	1694	not hispar	female	Dead	M0
22	TCGA-DD-AA	43	'--	not hispar	male	Alive	M0
23	TCGA-2Y-A9	82	848	not hispar	female	Dead	MX
24	TCGA-DD-A1	57	1560	not hispar	female	Dead	M0
25	TCGA-DD-AA	50	432	not hispar	male	Dead	M0
26	TCGA-DD-A1	68	'--	not hispar	male	Alive	M0
27	TCGA-UB-A7	62	'--	not hispar	female	Alive	M0
28	TCGA-DD-A4	72	'--	not hispar	male	Alive	M0
29	TCGA-DD-AA	54	'--	not hispar	male	Alive	M0
30	TCGA-G3-A7	70	56	not hispar	male	Dead	MX
31	TCGA-DD-AA	35	'--	not hispar	male	Alive	M0
32	TCGA-DD-AA	38	'--	not hispar	male	Alive	M0
33	TCGA-BD-A2	69	'--	not report	male	Alive	MX
34	TCGA-G3-A5	44	768	not hispar	male	Dead	M0
35	TCGA-DD-AA	46	'--	not hispar	male	Alive	M0
36	TCGA-G3-AA	50	'--	not hispar	male	Alive	M0
37	TCGA-FV-A3	75	194	not hispar	male	Dead	MX
38	TCGA-2Y-A9	81	'--	not hispar	female	Alive	MX
39	TCGA-EP-A2	46	596	not hispar	female	Dead	MX
40	TCGA-CC-A8	39	344	not hispar	female	Dead	M0
41	TCGA-DD-A4	25	'--	not hispar	male	Alive	M0
42	TCGA-PD-A5	58	639	not hispar	female	Dead	M0
43	TCGA-CC-52	44	97	not hispar	male	Dead	M0
44	TCGA-DD-A1	73	14	not hispar	male	Dead	M0
45	TCGA-GJ-A6	75	31	not hispar	female	Dead	MX
46	TCGA-CC-52	67	103	not hispar	male	Dead	M0
47	TCGA-DD-AA	48	'--	not hispar	male	Alive	M0
48	TCGA-DD-A1	57	394	not hispar	female	Dead	M0
49	TCGA-XR-A8	58	'--	hispanic c	male	Alive	M0
50	TCGA-DD-AA	70	'--	not hispar	female	Alive	M0

51	TCGA-XR-A8	74	693	hispanic c	male	Dead	MX
52	TCGA-K7-AA	61	'--	not hispar	male	Alive	MX
53	TCGA-DD-AA	51	'--	not hispar	female	Alive	MO
54	TCGA-ED-A7	29	'--	not hispar	male	Alive	MO
55	TCGA-DD-AA	69	195	not hispar	male	Dead	MO
56	TCGA-ZP-A9	72	'--	not hispar	male	Alive	MX
57	TCGA-CC-A7	57	217	not hispar	male	Dead	MO
58	TCGA-MI-A7	63	'--	not hispar	male	Alive	MO
59	TCGA-BC-A1	69	837	not hispar	male	Dead	MX
60	TCGA-G3-A2	52	452	not hispar	female	Dead	MO
61	TCGA-CC-A5	48	272	not hispar	male	Dead	MO
62	TCGA-DD-A7	68	'--	not hispar	female	Alive	MX
63	TCGA-G3-A5	58	'--	not hispar	male	Alive	MO
64	TCGA-DD-AA	73	'--	not hispar	female	Alive	MO
65	TCGA-MR-A5	58	'--	not hispar	male	Alive	MX
66	TCGA-G3-A7	60	'--	not hispar	female	Alive	MX
67	TCGA-RC-A7	53	'--	not hispar	male	Alive	MO
68	TCGA-DD-A1	23	415	not hispar	male	Dead	MO
69	TCGA-5R-AA	57	'--	not hispar	male	Alive	MO
70	TCGA-ED-A8	59	'--	not hispar	female	Alive	MO
71	TCGA-2V-A9	'--	'--	not hispar	male	Alive	MX
72	TCGA-CC-A3	36	315	not hispar	male	Dead	MO
73	TCGA-G3-A5	59	'--	not hispar	male	Alive	MO
74	TCGA-HP-A5	78	91	not report	male	Dead	MO
75	TCGA-RC-A6	74	'--	not hispar	female	Alive	MX
76	TCGA-G3-A2	58	'--	not hispar	male	Alive	MO
77	TCGA-K7-A5	64	'--	not hispar	male	Alive	MX
78	TCGA-2Y-A9	51	1624	not hispar	male	Dead	MX
79	TCGA-DD-AA	55	'--	not hispar	male	Alive	MO
80	TCGA-RC-A7	42	'--	not hispar	male	Alive	MO
81	TCGA-DD-AA	59	'--	not hispar	male	Alive	MX
82	TCGA-ED-A6	51	296	not hispar	female	Dead	MO
83	TCGA-DD-AA	40	'--	not hispar	male	Alive	MO
84	TCGA-DD-AA	72	'--	not hispar	male	Alive	MO
85	TCGA-ZP-A9	66	'--	not hispar	female	Alive	MX
86	TCGA-2Y-A9	85	633	not hispar	female	Dead	MX
87	TCGA-DD-A3	76	2131	not hispar	female	Dead	MO
88	TCGA-CC-A5	45	304	not hispar	male	Dead	MO
89	TCGA-ZP-A9	67	'--	not hispar	female	Alive	MX
90	TCGA-BC-A1	50	91	not hispar	male	Dead	MX
91	TCGA-WX-AA	33	556	not hispar	female	Dead	MX
92	TCGA-G3-A3	61	'--	not hispar	male	Alive	MO
93	TCGA-G3-A3	53	'--	not hispar	male	Alive	MO
94	TCGA-DD-A1	46	'--	not hispar	male	Alive	MO
95	TCGA-DD-AA	52	'--	not hispar	male	Alive	MO
96	TCGA-DD-AA	55	'--	not hispar	male	Alive	MO
97	TCGA-DD-AA	50	'--	not hispar	male	Alive	MO
98	TCGA-G3-A7	65	'--	not hispar	male	Alive	MX
99	TCGA-DD-AA	66	107	not hispar	female	Dead	MO
100	TCGA-BC-A1	52	770	not hispar	female	Dead	MX
101	TCGA-FV-A3	38	366	not hispar	female	Dead	MX

102	TCGA-2Y-A9	64	1271	not hispar	male	Dead	MX
103	TCGA-DD-AA	69	'--	not hispar	female	Alive	M0
104	TCGA-ZS-A9	55	'--	not hispar	male	Alive	MX
105	TCGA-DD-A4	65	'--	not hispar	female	Alive	M0
106	TCGA-DD-AA	68	'--	not hispar	female	Alive	M0
107	TCGA-DD-AA	53	425	not hispar	male	Dead	M0
108	TCGA-DD-AA	66	'--	not hispar	male	Alive	M0
109	TCGA-DD-AA	48	381	not hispar	male	Dead	M0
110	TCGA-NI-A8	74	'--	not hispar	male	Alive	MX
111	TCGA-ZP-A9	64	'--	not hispar	female	Alive	MX
112	TCGA-DD-AA	72	'--	not hispar	male	Alive	M0
113	TCGA-DD-A3	43	601	not report	female	Dead	M0
114	TCGA-DD-AA	66	'--	not hispar	male	Alive	M0
115	TCGA-EP-A2	52	627	not hispar	female	Dead	MX
116	TCGA-ZP-A9	56	'--	not hispar	female	Alive	MX
117	TCGA-DD-A3	64	931	not hispar	female	Dead	M1
118	TCGA-DD-A1	53	2542	not report	male	Dead	M0
119	TCGA-GJ-A9	68	67	not hispar	male	Dead	MX
120	TCGA-DD-AA	63	'--	not hispar	male	Alive	M0
121	TCGA-ED-A7	48	'--	not hispar	female	Alive	M0
122	TCGA-DD-A7	73	'--	not hispar	female	Alive	M0
123	TCGA-5R-AA	65	46	not hispar	female	Dead	M0
124	TCGA-DD-AA	59	'--	not hispar	male	Alive	M0
125	TCGA-DD-A3	29	827	not hispar	female	Dead	M0
126	TCGA-WQ-A9	71	'--	not hispar	female	Alive	M0
127	TCGA-2Y-A9	49	'--	hispanic c	male	Alive	M0
128	TCGA-DD-AA	72	16	not hispar	male	Dead	M0
129	TCGA-KR-A7	57	'--	not report	male	Alive	M0
130	TCGA-BC-A2	62	'--	not report	female	Alive	M0
131	TCGA-DD-AA	50	'--	not hispar	male	Alive	M0
132	TCGA-DD-AA	69	'--	not hispar	male	Alive	M0
133	TCGA-XR-A8	43	'--	hispanic c	female	Alive	MX
134	TCGA-G3-AA	67	'--	not hispar	male	Alive	M0
135	TCGA-K7-A5	66	'--	not hispar	male	Alive	MX
136	TCGA-DD-A3	45	535	not hispar	male	Dead	M0
137	TCGA-2Y-A9	59	555	not hispar	female	Dead	MX
138	TCGA-DD-A4	67	'--	not hispar	male	Alive	M0
139	TCGA-DD-A1	20	'--	not hispar	female	Alive	M0
140	TCGA-DD-A1	71	1005	not hispar	female	Dead	M0
141	TCGA-DD-AA	51	'--	not hispar	male	Alive	M0
142	TCGA-DD-A4	85	9	not hispar	female	Dead	M0
143	TCGA-CC-A7	47	299	not hispar	male	Dead	M0
144	TCGA-WX-AA	64	'--	hispanic c	female	Alive	MX
145	TCGA-DD-A7	72	283	hispanic c	female	Dead	M0
146	TCGA-MI-A7	61	'--	not hispar	male	Alive	MX
147	TCGA-ED-A4	47	'--	not hispar	male	Alive	M0
148	TCGA-DD-AA	51	'--	not hispar	male	Alive	M0
149	TCGA-DD-AA	69	'--	not hispar	male	Alive	M0
150	TCGA-BW-A5	26	'--	hispanic c	female	Alive	M1
151	TCGA-DD-AA	81	410	not hispar	female	Dead	MX
152	TCGA-XR-A8	49	'--	hispanic c	female	Alive	M0

153	TCGA-BC-A1	81		1423	not hispar	male	Dead	MX
154	TCGA-T1-A6	68	'--		not hispar	male	Alive	M0
155	TCGA-KR-A7	65		65	not report	male	Dead	M0
156	TCGA-CC-A8	51		279	not hispar	female	Dead	M0
157	TCGA-DD-A4	80		1210	not hispar	female	Dead	M0
158	TCGA-FV-A4	78		2486	not hispar	male	Dead	M0
159	TCGA-BC-40	74		1490	not hispar	female	Dead	M0
160	TCGA-G3-AA	38	'--		not hispar	male	Alive	M0
161	TCGA-CC-A9	52	'--		not hispar	female	Alive	M0
162	TCGA-DD-AA	45	'--		not hispar	female	Alive	M0
163	TCGA-DD-AA	51	'--		not hispar	male	Alive	M0
164	TCGA-DD-A1	67	'--		not hispar	male	Not Report	M0
165	TCGA-5C-A9	70	'--		not hispar	male	Alive	M0
166	TCGA-CC-A3	61		303	not hispar	male	Dead	M0
167	TCGA-DD-A3	67		419	not report	male	Dead	M0
168	TCGA-CC-52	71		102	not hispar	male	Dead	M0
169	TCGA-DD-AA	58		12	not hispar	male	Dead	M0
170	TCGA-DD-AA	54	'--		not hispar	male	Alive	M0
171	TCGA-DD-AA	68		365	not hispar	male	Dead	M0
172	TCGA-WJ-A8	68	'--		not hispar	female	Alive	MX
173	TCGA-BC-A1	76		711	not hispar	male	Dead	MX
174	TCGA-DD-A1	73		349	not hispar	male	Dead	M0
175	TCGA-FV-A4	52	'--		not hispar	male	Alive	M0
176	TCGA-DD-A1	40		223	not hispar	male	Dead	M1
177	TCGA-CC-A1	50		101	not hispar	male	Dead	M0
178	TCGA-DD-AA	43	'--		not hispar	male	Alive	M0
179	TCGA-DD-A1	72	'--		not report	female	Alive	M0
180	TCGA-DD-A3	75		11	not hispar	male	Dead	M0
181	TCGA-CC-52	48		129	not hispar	male	Dead	M0
182	TCGA-BC-A5	69		547	not hispar	male	Dead	M0
183	TCGA-DD-A1	76		1372	not hispar	male	Dead	M0
184	TCGA-DD-AA	74	'--		not hispar	female	Alive	M0
185	TCGA-BC-A2	75		1397	not report	female	Dead	M0
186	TCGA-G3-AA	58	'--		not hispar	male	Alive	M0
187	TCGA-DD-AA	51	'--		not hispar	male	Alive	M0
188	TCGA-BC-A1	62		34	not hispar	female	Dead	MX
189	TCGA-BC-A6	69	'--		not report	male	Alive	M0
190	TCGA-DD-AA	64		115	not hispar	female	Dead	M0
191	TCGA-RC-A7	66	'--		not hispar	male	Alive	M0
192	TCGA-EP-A2	62		19	not hispar	male	Dead	MX
193	TCGA-ES-A2	80		688	not hispar	male	Dead	MX
194	TCGA-CC-52	60	'--		not hispar	female	Alive	M0
195	TCGA-2Y-A9	64		757	not hispar	female	Dead	MX
196	TCGA-BC-A3	68	'--		not hispar	female	Alive	M0
197	TCGA-DD-A4	65	'--		not hispar	male	Alive	M0
198	TCGA-O8-A7	54	'--		hispanic c	male	Alive	MX
199	TCGA-BC-A6	64	'--		not hispar	male	Alive	M0
200	TCGA-DD-A7	65	'--		not hispar	female	Alive	M0
201	TCGA-5C-AA	61	'--		not hispar	male	Alive	M0
202	TCGA-DD-AA	64	'--		not hispar	male	Alive	M0
203	TCGA-KR-A7	61	'--		not report	female	Alive	M0

204	TCGA-ZS-A9	79	'--	not hispar	female	Alive	MX
205	TCGA-DD-A1	77	'--	not hispar	female	Alive	MO
206	TCGA-ES-A2	54	438	not hispar	male	Dead	MX
207	TCGA-CC-A5	63	347	not hispar	male	Dead	MO
208	TCGA-HP-A5	90	752	not report	female	Dead	MO
209	TCGA-ED-A8	50	56	not hispar	female	Dead	MO
210	TCGA-ZS-A9	64	'--	not hispar	male	Alive	MX
211	TCGA-2Y-A9	68	'--	not hispar	male	Alive	MX
212	TCGA-DD-A1	68	'--	not hispar	male	Alive	MO
213	TCGA-DD-AA	38	'--	not hispar	male	Alive	MO
214	TCGA-DD-A4	54	'--	not hispar	female	Alive	MO
215	TCGA-K7-A6	66	'--	not hispar	male	Alive	MX
216	TCGA-DD-AA	39	'--	not hispar	male	Alive	MO
217	TCGA-DD-AA	44	'--	not hispar	male	Alive	MO
218	TCGA-MR-A8	34	'--	not report	male	Alive	MX
219	TCGA-G3-A7	76	'--	not hispar	male	Alive	MX
220	TCGA-DD-A7	77	'--	not hispar	female	Alive	MO
221	TCGA-ED-A7	61	'--	not hispar	male	Alive	MO
222	TCGA-ED-A9	54	'--	not hispar	male	Alive	MO
223	TCGA-DD-A3	65	233	not report	male	Dead	MO
224	TCGA-G3-A6	65	'--	not hispar	male	Alive	MO
225	TCGA-DD-AA	59	'--	not hispar	male	Alive	MO
226	TCGA-DD-A4	77	802	not hispar	male	Dead	MO
227	TCGA-DD-AA	58	'--	not hispar	male	Alive	MO
228	TCGA-DD-AA	70	'--	not hispar	male	Alive	MO
229	TCGA-G3-A3	80	'--	not hispar	male	Alive	MO
230	TCGA-BC-A1	76	837	not hispar	male	Dead	MX
231	TCGA-FV-A4	84	'--	not hispar	female	Alive	MO
232	TCGA-LG-A9	48	'--	not hispar	male	Alive	MO
233	TCGA-DD-A4	61	'--	not hispar	male	Alive	MO
234	TCGA-DD-AA	53	'--	not hispar	male	Alive	MO
235	TCGA-CC-A3	45	300	not hispar	male	Dead	MO
236	TCGA-UB-A7	56	214	not hispar	male	Dead	MX
237	TCGA-DD-AA	56	'--	not hispar	male	Alive	MO
238	TCGA-ZP-A9	59	1088	not hispar	male	Dead	MX
239	TCGA-ZS-A9	73	1386	not hispar	male	Dead	MX
240	TCGA-4R-AA	66	262	not hispar	male	Dead	MX
241	TCGA-DD-AA	75	'--	not hispar	male	Alive	MO
242	TCGA-BD-A3	62	'--	not hispar	male	Alive	MX
243	TCGA-LG-A9	68	'--	hispanic c	male	Alive	MO
244	TCGA-3K-AA	65	'--	not hispar	male	Alive	MX
245	TCGA-CC-A8	74	140	not hispar	male	Dead	MO
246	TCGA-EP-A1	62	'--	not hispar	male	Alive	MX
247	TCGA-2Y-A9	58	724	hispanic c	male	Dead	MX
248	TCGA-DD-AA	45	'--	not hispar	male	Alive	MO
249	TCGA-DD-AA	43	'--	not hispar	female	Alive	MO
250	TCGA-DD-A1	68	1622	not hispar	male	Dead	MO
251	TCGA-G3-A5	70	'--	not hispar	male	Alive	MO
252	TCGA-NI-A4	71	1791	not hispar	male	Dead	MX
253	TCGA-2Y-A9	45	'--	hispanic c	male	Alive	MX
254	TCGA-2Y-A9	58	1229	not hispar	male	Dead	MX

255	TCGA-DD-AA	55	'--	not hispar	male	Alive	M0
256	TCGA-DD-AA	56	'--	not hispar	male	Alive	M0
257	TCGA-EP-A3	76	'--	not hispar	male	Alive	MX
258	TCGA-2Y-A9	70	36	not hispar	male	Dead	MX
259	TCGA-BC-A1	80	153	not hispar	male	Dead	MX
260	TCGA-ED-A7	20	'--	not hispar	female	Alive	M0
261	TCGA-ED-A6	74	'--	not hispar	male	Alive	M0
262	TCGA-DD-A1	64	558	not hispar	female	Dead	M1
263	TCGA-DD-AA	59	'--	not hispar	female	Alive	M0
264	TCGA-RC-A6	75	'--	not hispar	male	Alive	M0
265	TCGA-G3-AA	53	65	not hispar	female	Dead	M0
266	TCGA-DD-A4	61	2456	not hispar	female	Dead	M0
267	TCGA-CC-52	35	129	not hispar	male	Dead	M0
268	TCGA-MI-A7	64	'--	not hispar	male	Alive	M0
269	TCGA-DD-AA	58	'--	not hispar	male	Alive	M0
270	TCGA-FV-A3	76	'--	not hispar	female	Alive	M0
271	TCGA-ED-A8	60	'--	not hispar	female	Alive	M0
272	TCGA-CC-A7	54	'--	not hispar	male	Alive	M0
273	TCGA-DD-A3	37	612	not hispar	male	Dead	M0
274	TCGA-DD-AA	62	'--	not hispar	male	Alive	M0
275	TCGA-DD-AA	51	'--	not hispar	male	Alive	M0
276	TCGA-RC-A7	59	'--	not hispar	male	Alive	M0
277	TCGA-DD-AA	65	'--	not hispar	male	Alive	M0
278	TCGA-UB-AA	60	'--	not hispar	male	Alive	MX
279	TCGA-DD-A1	55	'--	not hispar	female	Alive	M0
280	TCGA-UB-A7	24	'--	not hispar	male	Alive	MX
281	TCGA-DD-AA	38	'--	not hispar	male	Alive	M0
282	TCGA-QA-A7	48	'--	not hispar	male	Alive	MX
283	TCGA-2Y-A9	66	'--	hispanic c	male	Alive	MX
284	TCGA-2Y-A9	55	'--	not hispar	female	Alive	MX
285	TCGA-DD-AA	51	'--	not hispar	male	Alive	M0
286	TCGA-G3-A3	71	'--	not hispar	male	Alive	M0
287	TCGA-CC-A7	61	278	not hispar	male	Dead	M0
288	TCGA-DD-AA	52	469	not hispar	male	Dead	M0
289	TCGA-DD-A7	71	'--	not hispar	male	Alive	M0
290	TCGA-ED-A6	35	'--	not hispar	male	Alive	M0
291	TCGA-EP-A2	70	'--	not hispar	male	Alive	MX
292	TCGA-G3-A2	64	416	not hispar	male	Dead	M0
293	TCGA-DD-A4	56	899	not hispar	female	Dead	M0
294	TCGA-G3-A2	63	'--	not hispar	female	Alive	M0
295	TCGA-YA-A8	68	412	hispanic c	male	Dead	MX
296	TCGA-2Y-A9	68	'--	not hispar	female	Alive	MX
297	TCGA-DD-A4	75	660	hispanic c	female	Dead	M0
298	TCGA-FV-A4	51	'--	not hispar	female	Alive	M0
299	TCGA-DD-AA	59	'--	not hispar	female	Alive	M0
300	TCGA-UB-A7	51	'--	not hispar	male	Alive	MX
301	TCGA-DD-A3	67	171	not hispar	male	Dead	M0
302	TCGA-DD-A4	67	'--	not hispar	female	Alive	M0
303	TCGA-FV-A3	81	247	not hispar	female	Dead	MX
304	TCGA-DD-A4	60	373	hispanic c	male	Dead	M0
305	TCGA-DD-A4	56	'--	not hispar	female	Alive	M0

306	TCGA-DD-AA	56	'--	not hispar	male	Alive	M0
307	TCGA-UB-A7	67	52	not hispar	male	Dead	MX
308	TCGA-DD-A3	72	3258	not hispar	female	Dead	M0
309	TCGA-CC-A9	57	'--	not hispar	male	Alive	M0
310	TCGA-ED-A4	58	'--	not hispar	male	Alive	M0
311	TCGA-LG-A6	79	'--	not hispar	female	Alive	M0
312	TCGA-RC-A6	24	'--	not report	male	Alive	M0
313	TCGA-DD-AA	49	'--	not hispar	female	Alive	M0
314	TCGA-BW-A5	63	'--	not hispar	male	Alive	MX
315	TCGA-FV-A2	70	1852	not hispar	female	Dead	M0
316	TCGA-CC-52	61	87	not hispar	female	Dead	M0
317	TCGA-DD-A1	23	'--	not hispar	male	Alive	M0
318	TCGA-G3-A3	52	'--	not hispar	male	Alive	M0
319	TCGA-ZP-A9	51	765	not hispar	male	Dead	MX
320	TCGA-ED-A7	53	'--	not hispar	female	Alive	M0
321	TCGA-DD-AA	63	171	not hispar	female	Dead	M0
322	TCGA-ED-A5	60	'--	not hispar	female	Alive	M0
323	TCGA-BC-A1	51	2116	not hispar	female	Dead	MX
324	TCGA-DD-AA	60	'--	not hispar	male	Alive	M0
325	TCGA-2Y-A9	64	'--	not hispar	female	Alive	MX
326	TCGA-CC-A8	18	300	not hispar	male	Dead	M0
327	TCGA-2Y-A9	54	2532	not hispar	female	Dead	MX
328	TCGA-G3-A5	58	'--	not hispar	male	Alive	M0
329	TCGA-FV-A2	75	581	not hispar	male	Dead	M0
330	TCGA-5R-AA	17	'--	not hispar	female	Alive	M0
331	TCGA-RC-A6	20	'--	not report	female	Alive	M0
332	TCGA-WQ-AB	62	'--	not hispar	male	Alive	M0
333	TCGA-DD-AA	65	'--	not hispar	male	Alive	M0
334	TCGA-FV-A2	80	'--	not hispar	male	Alive	MX
335	TCGA-2Y-A9	68	'--	not hispar	male	Alive	MX
336	TCGA-G3-AA	48	'--	hispanic c	male	Alive	M0
337	TCGA-DD-AA	70	'--	not hispar	male	Alive	M0
338	TCGA-DD-A3	66	3125	not hispar	female	Dead	M0
339	TCGA-DD-AA	61	'--	not hispar	male	Alive	M0
340	TCGA-DD-A3	77	643	not hispar	male	Dead	M0
341	TCGA-BD-A3	75	'--	not hispar	female	Alive	M0
342	TCGA-BC-A1	72	1135	not hispar	female	Dead	MX
343	TCGA-DD-A4	46	'--	not hispar	male	Alive	M0
344	TCGA-CC-A7	59	649	not hispar	male	Dead	M0
345	TCGA-DD-A7	66	'--	not hispar	male	Alive	M0
346	TCGA-CC-A1	24	'--	not hispar	female	Alive	M0
347	TCGA-DD-AA	55	'--	not hispar	female	Alive	M0
348	TCGA-CC-A7	58	'--	not hispar	male	Alive	M0
349	TCGA-DD-AA	66	'--	not hispar	female	Alive	M0
350	TCGA-CC-A7	56	'--	not hispar	male	Alive	M0
351	TCGA-DD-AA	69	'--	not hispar	male	Alive	M0
352	TCGA-UB-AA	69	'--	not hispar	female	Alive	MX
353	TCGA-CC-A7	59	262	not hispar	male	Dead	M0
354	TCGA-XR-A8	16	'--	hispanic c	male	Alive	MX
355	TCGA-DD-AA	61	1685	not hispar	male	Dead	M0
356	TCGA-BW-A5	50	'--	not hispar	male	Alive	MX

357	TCGA-G3-AA	51	359	not hispar	male	Dead	M0
358	TCGA-2Y-A9	70	'--	not hispar	male	Alive	MX
359	TCGA-G3-AA	58	'--	not hispar	female	Alive	M0
360	TCGA-DD-AA	60	'--	not hispar	male	Alive	M0
361	TCGA-EP-A3	73	'--	not hispar	male	Alive	MX
362	TCGA-MI-A7	77	'--	not hispar	male	Alive	MX
363	TCGA-G3-A2	68	'--	not hispar	male	Alive	M0
364	TCGA-BC-A8	66	'--	not hispar	female	Alive	M0
365	TCGA-RC-A7	47	'--	not hispar	female	Alive	M0
366	TCGA-CC-A9	55	'--	not hispar	male	Alive	M0
367	TCGA-G3-A2	45	'--	not hispar	female	Alive	M0
368	TCGA-UB-A7	59	'--	not hispar	male	Alive	MX
369	TCGA-GJ-A3	59	'--	not hispar	male	Alive	MX
370	TCGA-DD-AA	73	'--	not hispar	male	Alive	M0
371	TCGA-KR-A7	64	'--	not report	male	Alive	M0

ajcc_pathc	ajcc_pathc	days_to_la	primary_di	prior_mali	prior_trea	tumor_stag	treatment_
N0	Stage I	T1	2752	Hepatocell	no	No	stage i
N0	Stage I	T1	3308	Hepatocell	no	No	stage i
N0	Stage IIIA	T3	849	Hepatocell	no	No	stage iiia
N0	Stage IIIC	T4	507	Hepatocell	no	No	stage iiic
NX	Stage I	T1	430	Hepatocell	no	No	stage i
N0	Stage I	T1	662	Hepatocell	no	No	stage i
N0	Stage IIIA	T3	248	Hepatocell	no	No	stage iiia
N0	Stage IIIA	T3	363	Hepatocell	no	No	stage iiia
'--	Stage II	T2	'--	Hepatocell	no	No	stage ii
NX	Stage II	T2	756	Hepatocell	no	No	stage ii
N0	Stage I	T1	1855	Hepatocell	no	No	stage i
N0	Stage I	T1	658	Hepatocell	no	No	stage i
N0	Stage II	T2	328	Hepatocell	no	No	stage ii
NX	Stage I	T1	8	Hepatocell	no	No	stage i
N0	Stage II	T2	1779	Hepatocell	no	No	stage ii
N0	Stage II	T2	1098	Hepatocell	no	No	stage ii
N0	Stage I	T1	'--	Hepatocell	no	No	stage i
N0	Stage I	T1	137	Hepatocell	no	No	stage i
N0	Stage I	T1	1302	Hepatocell	no	No	stage i
NX	'--	T3	'--	Hepatocell	no	No	not report
NX	Stage I	T1	'--	Hepatocell	no	No	stage i
N0	Stage I	T1	1295	Hepatocell	no	No	stage i
NX	Stage II	T2	'--	Hepatocell	no	No	stage ii
N0	Stage I	T1	'--	Hepatocell	no	No	stage i
N0	Stage II	T2	429	Hepatocell	no	No	stage ii
N0	Stage I	T1	2301	Hepatocell	no	No	stage i
N0	Stage II	T2b	848	Combined h	no	No	stage ii
N0	Stage I	T1	942	Hepatocell	no	No	stage i
N0	Stage I	T1	2015	Hepatocell	no	No	stage i
NX	Stage IIIB	T3b	'--	Hepatocell	yes	No	stage iiib
N0	Stage I	T1	2317	Hepatocell	no	No	stage i
N0	Stage I	T1	1900	Hepatocell	no	No	stage i
NX	'--	T2	1363	Hepatocell	no	No	not report
N0	Stage II	T2	447	Hepatocell	no	No	stage ii
N0	Stage II	T2	2202	Hepatocell	no	No	stage ii
N0	Stage I	T1	372	Hepatocell	no	No	stage i
NX	Stage I	T1	'--	Hepatocell	yes	No	stage i
N0	Stage I	T1	1168	Hepatocell	yes	No	stage i
NX	Stage I	T1	334	Hepatocell	no	No	stage i
N0	Stage IIIA	T3	0	Hepatocell	no	No	stage iiia
N0	Stage I	T1	989	Hepatocell	no	No	stage i
N0	Stage IIIB	T4	'--	Hepatocell	no	No	stage iiib
N0	Stage II	T2	12	Hepatocell	no	No	stage ii
N0	Stage I	T1	'--	Hepatocell	no	No	stage i
NX	Stage II	T2	'--	Hepatocell	no	No	stage ii
N0	Stage IIIC	T4	0	Hepatocell	no	No	stage iiic
N0	Stage I	T1	587	Hepatocell	no	No	stage i
N0	Stage I	T1	'--	Hepatocell	no	No	stage i
NX	Stage I	T1	898	Hepatocell	yes	No	stage i
N0	Stage I	T1	1066	Hepatocell	no	No	stage i

NX	Stage I	T1	'--		Hepatocellno	No	stage i
NX	Stage II	T2a		359	Combined hno	No	stage ii
N0	Stage I	T1		564	Hepatocellno	No	stage i
N0	Stage IIIA	T3a		427	Hepatocellno	No	stage iiia
N0	Stage II	T2	'--		Hepatocellno	No	stage ii
NX	'--	T1		706	Hepatocellno	No	not report
N0	Stage IIIA	T3		0	Hepatocellno	No	stage iiia
N0	Stage II	T2		698	Hepatocellno	No	stage ii
NX	'--	T2	'--		Hepatocellno	No	not report
N0	Stage I	T1	'--		Hepatocellno	No	stage i
N0	Stage IIIB	T4		0	Hepatocellno	No	stage iiib
NX	Stage II	T2		693	Hepatocellno	No	stage ii
NX	Stage I	T1		744	Hepatocellno	No	stage i
N0	Stage I	T1		1219	Hepatocellno	No	stage i
NX	Stage I	T1		229	Hepatocellno	No	stage i
NX	Stage I	T1		632	Hepatocellno	No	stage i
N0	Stage II	T2		588	Hepatocellno	No	stage ii
N0	Stage II	T2	'--		Hepatocellno	No	stage ii
N0	Stage II	T2		520	Hepatocellno	No	stage ii
N0	Stage IIIA	T3a		406	Hepatocellno	No	stage iiia
NX	Stage II	T2	'--		Hepatocellno	No	stage ii
N0	Stage IIIA	T3		3	Hepatocellno	No	stage iiia
NX	Stage I	T1		698	Hepatocellno	No	stage i
NX	Stage I	T1	'--		Hepatocellno	No	stage i
NX	Stage IIIA	T3		22	Hepatocellno	No	stage iiia
N0	Stage I	T1		655	Hepatocellno	No	stage i
NX	Stage I	T1		631	Hepatocellno	No	stage i
NX	Stage I	T1	'--		Hepatocellno	No	stage i
N0	Stage IIIA	T3		1989	Hepatocellno	No	stage iiia
N0	Stage II	T2		468	Hepatocellno	No	stage ii
NX	Stage I	T1		898	Hepatocellno	No	stage i
N0	Stage IIIA	T3a		7	Hepatocellno	No	stage iiia
N0	Stage I	T1		1876	Hepatocellno	No	stage i
N0	Stage I	T1		1531	Hepatocellno	No	stage i
NX	'--	T1		782	Hepatocellno	No	not report
NX	'--	T1	'--		Hepatocellno	No	not report
N0	Stage I	T1	'--		Hepatocellno	No	stage i
N0	Stage IIIA	T3		0	Hepatocellno	No	stage iiia
NX	'--	T1		1091	Hepatocellno	No	not report
NX	'--	T4	'--		Hepatocellno	No	not report
NX	Stage IIIA	T3a	'--		Hepatocellno	No	stage iiia
N0	Stage I	T1		585	Hepatocellno	No	stage i
N0	Stage IIIA	T3a		780	Hepatocellno	No	stage iiia
N0	Stage I	T1		183	Hepatocellno	No	stage i
N0	Stage I	T1		552	Hepatocellno	No	stage i
N0	Stage I	T1		810	Hepatocellno	No	stage i
N0	Stage I	T1		566	Hepatocellno	No	stage i
NX	Stage I	T1		361	Hepatocellno	No	stage i
N0	Stage I	T1		58	Hepatocellno	No	stage i
N0	Stage IIIA	T3a	'--		Hepatocellno	No	stage iiia
NX	Stage I	T1		4	Hepatocellno	No	stage i

N0	Stage I	T1	'--		Hepatocell	no	No	stage i
N0	Stage I	T1		1562	Hepatocell	no	No	stage i
NX	Stage II	T2		341	Hepatocell	no	No	stage ii
N0	Stage IIIE	T3b		917	Hepatocell	no	No	stage iiib
N0	Stage II	T2		1049	Hepatocell	no	No	stage ii
N0	Stage I	T1	'--		Hepatocell	no	No	stage i
N0	Stage IIIA	T3a		672	Hepatocell	no	No	stage iiia
N0	Stage I	T1	'--		Hepatocell	no	No	stage i
NX	Stage I	T1		799	Hepatocell	no	No	stage i
NX	'--	T1		395	Hepatocell	no	No	not report
N0	Stage I	T1		644	Hepatocell	yes	No	stage i
NX	Stage II	T2	'--		Hepatocell	no	No	stage ii
N0	Stage II	T2		170	Hepatocell	no	No	stage ii
NX	Stage IIIA	T3a		357	Hepatocell	no	No	stage iiia
NX	'--	T1		21	Hepatocell	yes	No	not report
N0	Stage IVB	T4	'--		Hepatocell	no	No	stage ivb
N0	Stage IIIA	T3	'--		Hepatocell	no	No	stage iiia
N0	Stage I	T1	'--		Hepatocell	no	No	stage i
N0	Stage I	T1		474	Hepatocell	no	No	stage i
NX	Stage II	T2		6	Hepatocell	no	No	stage ii
N0	Stage I	T1		3478	Hepatocell	no	No	stage i
N0	Stage II	T2	'--		Hepatocell	no	No	stage ii
N0	Stage II	T2		436	Hepatocell	no	No	stage ii
N0	Stage III	T3	'--		Hepatocell	no	No	stage iii
NX	'--	T3a		30	Hepatocell	no	No	not report
N0	Stage IIIA	T3		3675	Hepatocell	no	No	stage iiia
N0	Stage I	T1	'--		Hepatocell	no	No	stage i
N0	Stage I	T1		906	Hepatocell	no	No	stage i
NX	Stage IIIA	T3		1351	Hepatocell	no	No	stage iiia
N0	Stage I	T1		574	Hepatocell	yes	No	stage i
N0	Stage I	T1		722	Hepatocell	no	No	stage i
NX	Stage I	T1		1339	Hepatocell	yes	Yes	stage i
N0	Stage II	T2		354	Hepatocell	no	No	stage ii
NX	Stage I	T1		519	Hepatocell	no	No	stage i
N0	Stage I	T1	'--		Hepatocell	no	No	stage i
N0	Stage I	T1	'--		Hepatocell	no	No	stage i
NX	Stage II	T2		816	Hepatocell	yes	No	stage ii
N0	Stage I	T1		602	Hepatocell	no	No	stage i
N1	Stage IIIC	T1		887	Hepatocell	no	No	stage iiic
N0	Stage I	T1		763	Hepatocell	no	No	stage i
N0	Stage I	T1	'--		Hepatocell	no	No	stage i
N0	Stage II	T2		0	Hepatocell	no	No	stage ii
NX	Stage I	T1		615	Hepatocell	no	No	stage i
N0	Stage I	T1	'--		Hepatocell	no	No	stage i
NX	'--	T2		630	Hepatocell	no	No	not report
N0	Stage II	T2		910	Hepatocell	no	No	stage ii
N0	Stage I	T1		638	Hepatocell	no	No	stage i
N0	Stage I	T1		1633	Hepatocell	no	No	stage i
N0	Stage IV	T2		0	Hepatocell	no	No	stage iv
N0	Stage I	T1	'--		Combined h	no	No	stage i
N0	Stage IIIE	T3		1030	Hepatocell	no	No	stage iiib

NX	'--	T3	'--		Hepatocellno	No	not report
NX	'--	T1		23	Hepatocellyes	No	not report
N0	Stage I	T1	'--		Hepatocellyes	No	stage i
N0	Stage II	T2		0	Hepatocellno	No	stage ii
N0	Stage IIIA	T3	'--		Hepatocellno	No	stage iiia
NX	Stage IIIA	T3		1836	Hepatocellyes	No	stage iiia
N0	Stage IIIA	T3	'--		Hepatocellno	No	stage iiia
N0	Stage II	T2		361	Hepatocellno	No	stage ii
N0	Stage IIIA	T3a		0	Hepatocellno	No	stage iiia
N0	Stage IIIA	T3a		555	Hepatocellno	No	stage iiia
N0	Stage I	T1		1242	Hepatocellno	No	stage i
N0	Stage I	T1		79	Hepatocellno	No	stage i
N0	Stage I	T1		322	Hepatocellno	No	stage i
N0	Stage IIIA	T3		2	Hepatocellno	No	stage iiia
N0	Stage IIIB	T3b	'--		Hepatocellno	No	stage iiib
N0	Stage IIIA	T3		0	Hepatocellno	No	stage iiia
N0	Stage II	T2	'--		Hepatocellno	No	stage ii
N0	Stage I	T1		1345	Hepatocellno	No	stage i
N0	Stage I	T1	'--		Hepatocellno	No	stage i
NX	Stage I	T1		345	Clear cellno	No	stage i
NX	'--	T4	'--		Hepatocellno	No	not report
N0	Stage IIIA	T3		204	Hepatocellno	No	stage iiia
NX	Stage I	T1		12	Hepatocellno	No	stage i
N0	Stage IV	T3a	'--		Hepatocellno	No	stage iv
N0	Stage IIIA	T3		0	Hepatocellno	No	stage iiia
N0	Stage I	T1		1424	Hepatocellno	No	stage i
N0	Stage I	T1		2017	Hepatocellno	No	stage i
N0	Stage II	T2	'--		Hepatocellyes	No	stage ii
N0	Stage II	T2		0	Hepatocellno	No	stage ii
NX	Stage IIIA	T3a		289	Hepatocellyes	No	stage iiia
N0	Stage I	T1	'--		Hepatocellno	No	stage i
N0	Stage I	T1		2324	Hepatocellno	No	stage i
NX	Stage II	T2		421	Hepatocellno	No	stage ii
N0	Stage I	T1		476	Hepatocellno	No	stage i
N0	Stage II	T2		1067	Hepatocellno	No	stage ii
N0	Stage I	T1	'--		Hepatocellno	No	stage i
N0	Stage I	T1		387	Hepatocellno	No	stage i
N0	Stage I	T1	'--		Hepatocellno	No	stage i
N0	Stage I	T1		579	Hepatocellno	No	stage i
NX	Stage I	T1	'--		Hepatocellno	No	stage i
NX	Stage I	T1	'--		Hepatocellyes	No	stage i
N0	Stage IIIC	T4		250	Hepatocellno	No	stage iiic
NX	Stage II	T2	'--		Hepatocellno	No	stage ii
N0	Stage II	T2		680	Hepatocellno	No	stage ii
N0	Stage I	T1		2245	Hepatocellno	No	stage i
NX	Stage I	T1		538	Hepatocellno	No	stage i
NX	Stage II	T2		444	Hepatocellno	No	stage ii
N0	Stage IIIA	T3a		701	Hepatocellno	No	stage iiia
N0	Stage II	T2		20	Combined hno	No	stage ii
N0	Stage I	T1		415	Hepatocellno	No	stage i
N0	Stage II	T2		951	Hepatocellyes	No	stage ii

NX	Stage II	T2		1241	Hepatocellyes	No	stage ii
N0	Stage II	T2		3437	Hepatocellno	No	stage ii
NX	Stage I	T1	'--		Hepatocellno	No	stage i
N0	Stage IIIAT3			0	Hepatocellno	No	stage iiia
NX	'--	TX	'--		Hepatocellno	No	not report
N0	Stage IIIAT3a			6	Hepatocellno	No	stage iiia
NX	Stage II	T2		2412	Hepatocellno	No	stage ii
N0	Stage I	T1		1452	Hepatocellno	No	stage i
N0	Stage II	T2		2415	Hepatocellno	No	stage ii
N0	Stage I	T1		2728	Hepatocellno	No	stage i
N0	Stage II	T2		928	Hepatocellno	No	stage ii
N0	Stage I	T1		512	Hepatocellno	No	stage i
N0	Stage I	T1		1804	Hepatocellno	No	stage i
N0	Stage I	T1		2513	Hepatocellno	No	stage i
N0	Stage I	T1		330	Hepatocellno	No	stage i
NX	Stage I	T1		447	Hepatocellno	No	stage i
N0	Stage I	T1		1085	Hepatocellno	No	stage i
NX	Stage II	T2		6	Hepatocellno	No	stage ii
N0	Stage IIIAT3a			6	Combined hno	No	stage iiia
N0	Stage IIIAT3b		'--		Hepatocellno	No	stage iiia
N0	Stage IIIBT3b			671	Hepatocellyes	No	stage iiib
N0	Stage I	T1		1567	Hepatocellno	No	stage i
NX	Stage IIIAT3a			445	Hepatocellno	No	stage iiia
N0	Stage I	T1		2028	Hepatocellno	No	stage i
N0	Stage IIIAT3a			1145	Hepatocellno	No	stage iiia
N0	Stage I	T1		673	Hepatocellyes	Yes	stage i
NX	'--	T4	'--		Hepatocellno	No	not report
NX	Stage I	T1		10	Hepatocellno	No	stage i
NX	Stage I	T1		425	Hepatocellno	No	stage i
N0	Stage IIIAT3			2398	Hepatocellno	No	stage iiia
N0	Stage I	T1		1531	Hepatocellno	No	stage i
N0	Stage IIIAT3			3	Hepatocellno	No	stage iiia
NX	Stage IIIAT3a		'--		Hepatocellyes	No	stage iiia
N0	Stage II	T2		2455	Hepatocellno	No	stage ii
NX	'--	T1		142	Hepatocellno	No	not report
NX	Stage II	T2	'--		Hepatocellno	No	stage ii
NX	Stage II	T2		216	Hepatocellyes	No	stage ii
N0	Stage II	T2		2102	Hepatocellno	No	stage ii
NX	Stage II	T2		1115	Hepatocellyes	No	stage ii
N0	Stage IIIAT3a			366	Hepatocellno	No	stage iiia
NX	Stage IIIBT3b			396	Hepatocellno	No	stage iiib
N0	Stage IIIAT3			0	Hepatocellno	No	stage iiia
NX	Stage I	T1		570	Hepatocellno	No	stage i
NX	'--	T2	'--		Hepatocellno	No	not report
N0	Stage I	T1		458	Hepatocellno	No	stage i
N0	Stage I	T1		1085	Hepatocellno	No	stage i
N0	Stage IIIAT3		'--		Hepatocellno	No	stage iiia
NX	Stage II	T2		621	Hepatocellno	No	stage ii
NX	Stage IIIAT3		'--		Hepatocellno	No	stage iiia
NX	Stage II	T2		1516	Hepatocellno	No	stage ii
NX	Stage I	T1	'--		Hepatocellno	No	stage i

N0	Stage I	T1		453	Hepatocell	no	No	stage i
N0	Stage I	T1		1823	Hepatocell	no	No	stage i
NX	Stage I	T1		303	Hepatocell	yes	No	stage i
NX	Stage II	T2	'--		Hepatocell	no	No	stage ii
NX	'--	T3	'--		Hepatocell	yes	No	not report
NX	Stage II	T2		390	Hepatocell	no	No	stage ii
NX	Stage I	T1		423	Hepatocell	no	No	stage i
N0	Stage IVB	T4	'--		Hepatocell	no	No	stage ivb
N0	Stage I	T1		141	Hepatocell	no	No	stage i
NX	Stage II	T2		9	Hepatocell	no	No	stage ii
N0	Stage IIIA	T3a	'--		Hepatocell	no	No	stage iiia
N0	Stage I	T1	'--		Hepatocell	no	No	stage i
N0	Stage IIIA	T3	'--		Hepatocell	no	No	stage iiia
N0	Stage I	T1		291	Hepatocell	no	No	stage i
N0	Stage I	T1		636	Hepatocell	no	No	stage i
NX	Stage II	T2		848	Combined h	no	No	stage ii
N0	Stage IIIA	T3a		408	Hepatocell	no	No	stage iiia
N0	Stage IIIA	T3		399	Hepatocell	no	No	stage iiia
N0	Stage IIIA	T3	'--		Hepatocell	no	No	stage iiia
N0	Stage I	T1		2184	Hepatocell	no	No	stage i
N0	Stage I	T1		347	Hepatocell	no	No	stage i
N0	Stage I	T1		472	Hepatocell	no	No	stage i
N0	Stage I	T1		575	Hepatocell	yes	No	stage i
NX	Stage II	T2		327	Hepatocell	no	No	stage ii
N0	Stage II	T2		2425	Hepatocell	no	No	stage ii
NX	Stage II	T2		601	Hepatocell	no	No	stage ii
N0	Stage II	T2		1718	Hepatocell	no	No	stage ii
NX	Stage II	T2		94	Hepatocell	no	No	stage ii
NX	Stage I	T1		260	Hepatocell	no	No	stage i
NX	Stage I	T1		1939	Hepatocell	no	No	stage i
N0	Stage I	T1		1231	Hepatocell	no	No	stage i
N0	Stage I	T1		180	Hepatocell	no	No	stage i
N0	Stage IIIA	T3		0	Hepatocell	no	No	stage iiia
N0	Stage II	T2	'--		Hepatocell	no	No	stage ii
N0	Stage I	T1		728	Hepatocell	no	No	stage i
N0	Stage IIIA	T3a		406	Hepatocell	no	No	stage iiia
N0	Stage I	T1		608	Hepatocell	no	No	stage i
N0	Stage I	T1		37	Hepatocell	no	No	stage i
N0	Stage I	T1	'--		Hepatocell	no	No	stage i
N0	Stage I	T1		1636	Hepatocell	no	No	stage i
N0	Stage IIIA	T3a	'--		Hepatocell	no	No	stage iiia
NX	Stage I	T1		357	Hepatocell	yes	No	stage i
N0	Stage IIIA	T3a	'--		Hepatocell	no	No	stage iiia
NX	Stage II	T2		1	Hepatocell	no	No	stage ii
N0	Stage I	T1		719	Hepatocell	yes	No	stage i
NX	Stage I	T1		486	Hepatocell	no	No	stage i
NX	Stage I	T1	'--		Hepatocell	no	No	stage i
N1	Stage IIIC	T2		1008	Hepatocell	no	No	stage iiic
N0	Stage II	T2		0	Hepatocell	yes	No	stage ii
N0	Stage II	T2	'--		Hepatocell	no	No	stage ii
N0	Stage I	T1		2746	Hepatocell	yes	No	stage i

N0	Stage IIIA	T3		1970	Hepatocellno	No	stage iiia
N0	Stage I	T1	'--		Hepatocellno	No	stage i
N0	Stage II	T2	'--		Hepatocellno	No	stage ii
N0	Stage IIIA	T3		0	Hepatocellno	No	stage iiia
N0	Stage II	T2		819	Hepatocellno	No	stage ii
NX	Stage II	T2		387	Hepatocellyes	No	stage ii
N0	Stage II	T2		0	Hepatocellno	No	stage ii
N0	Stage I	T1		608	Hepatocellno	No	stage i
NX	Stage I	T1		0	Hepatocellyes	No	stage i
N0	Stage II	T2		987	Hepatocellno	No	stage ii
N0	Stage IIIC	T4		0	Hepatocellno	No	stage iiic
N0	Stage III	T3		1495	Hepatocellno	No	stage iii
N0	Stage II	T2		594	Hepatocellno	No	stage ii
NX	'--	T2		743	Hepatocellno	No	not report
N0	Stage II	T2		400	Hepatocellno	No	stage ii
N0	Stage I	T1	'--		Hepatocellno	No	stage i
N0	Stage II	T2		854	Hepatocellno	No	stage ii
NX	'--	T1	'--		Hepatocellyes	No	not report
N0	Stage II	T2		554	Hepatocellno	No	stage ii
N0	Stage I	T1		1731	Hepatocellno	No	stage i
N1	Stage IIIC	T3		0	Hepatocellno	No	stage iiic
NX	Stage I	T1	'--		Hepatocellyes	No	stage i
NX	Stage II	T2		520	Hepatocellno	No	stage ii
N0	Stage I	T1	'--		Hepatocellno	No	stage i
N0	Stage IIIA	T3a		449	Hepatocellno	No	stage iiia
N1	Stage IVA	T1		15	Hepatocellno	No	stage iva
NX	Stage II	T2		395	Hepatocellno	No	stage ii
N0	Stage I	T1		2301	Hepatocellno	No	stage i
N0	Stage I	T1		729	Hepatocellno	No	stage i
NX	Stage I	T1		2442	Hepatocellno	No	stage i
N0	Stage I	T1		480	Hepatocellno	No	stage i
N0	Stage I	T1		9	Hepatocellno	No	stage i
N0	Stage III	T3	'--		Hepatocellno	No	stage iii
N0	Stage I	T1		1450	Hepatocellno	No	stage i
NX	Stage II	T2	'--		Hepatocellno	No	stage ii
N0	Stage I	T1		409	Hepatocellno	No	stage i
NX	'--	T2	'--		Hepatocellno	No	not report
N0	Stage I	T1		1711	Hepatocellno	No	stage i
N0	Stage IIIA	T3		0	Hepatocellno	No	stage iiia
N0	Stage I	T1		44	Hepatocellno	No	stage i
N0	Stage IIIA	T3		219	Hepatocellno	No	stage iiia
N0	Stage I	T1		555	Hepatocellno	No	stage i
N0	Stage IIIA	T3		365	Hepatocellno	No	stage iiia
N0	Stage I	T1		1233	Hepatocellno	No	stage i
N0	Stage II	T2		382	Hepatocellno	No	stage ii
N0	Stage II	T2		1618	Hepatocellno	No	stage ii
NX	Stage I	'--		314	Hepatocellyes	No	stage i
N0	Stage IIIA	T3		0	Hepatocellno	No	stage iiia
N0	Stage IIIA	T3		925	Hepatocellno	No	stage iiia
N0	Stage I	T1	'--		Hepatocellno	No	stage i
NX	Stage IIIA	T3a		20	Hepatocellno	No	stage iiia

N0	Stage IIIC	T4	'--	Hepatocell	no	No	stage iiic
N0	Stage I	T1	697	Hepatocell	no	No	stage i
N0	Stage II	T2	412	Hepatocell	no	No	stage ii
N0	Stage I	T1	478	Hepatocell	no	No	stage i
NX	Stage IIIA	T3a	363	Hepatocell	no	No	stage iiia
NX	'--	'--	747	Hepatocell	yes	No	not report
N0	Stage I	T1	860	Hepatocell	no	No	stage i
N0	Stage IIIC	T4	562	Hepatocell	no	No	stage iiic
N0	Stage I	T1	640	Hepatocell	no	No	stage i
N0	Stage II	T2	211	Hepatocell	no	No	stage ii
N0	Stage IIIA	T3	1553	Hepatocell	no	No	stage iiia
N0	Stage IIIA	T3a	500	Hepatocell	no	No	stage iiia
NX	Stage I	T1	879	Hepatocell	no	No	stage i
N0	Stage I	T1	784	Hepatocell	no	No	stage i
N0	Stage I	T1	829	Combined h	no	No	stage i

treatment_alcohol_hibmi			
no	Radiation	Not Report	22.230987
no	Pharmaceut	Not Report	25.216253
no	Radiation	Not Report	'--
no	Pharmaceut	Not Report	33.632653
no	Radiation	Not Report	'--
no	Radiation	Not Report	35.553243
no	Radiation	Not Report	21.877551
no	Radiation	Not Report	19.571681
no	Radiation	Not Report	24.725183
no	Pharmaceut	Not Report	30.81854
yes	Radiation	Not Report	20.545694
no	Radiation	Not Report	21.367521
no	Pharmaceut	Not Report	25.259516
not report	Radiation	Not Report	19.379197
no	Radiation	Not Report	23.875115
no	Pharmaceut	Not Report	29.407788
no	Radiation	Not Report	23.011177
no	Pharmaceut	Not Report	24
no	Pharmaceut	Not Report	23.93899
no	Radiation	Not Report	'--
no	Pharmaceut	Not Report	20.775623
no	Radiation	Not Report	24.302487
no	Radiation	Not Report	21.786492
no	Radiation	Not Report	27.475629
yes	Radiation	Not Report	22.408179
no	Radiation	Not Report	30.116213
no	Radiation	Not Report	31.221304
yes	Radiation	Not Report	28.734672
no	Radiation	Not Report	25.351541
no	Radiation	Not Report	25.259516
no	Pharmaceut	Not Report	23.624447
no	Pharmaceut	Not Report	21.203105
no	Radiation	Not Report	38.973923
no	Pharmaceut	Not Report	23.620288
no	Radiation	Not Report	34.375
no	Pharmaceut	Not Report	'--
yes	Pharmaceut	Not Report	'--
no	Radiation	Not Report	42.192381
no	Radiation	Not Report	29.752066
no	Pharmaceut	Not Report	15.807896
no	Pharmaceut	Not Report	20.160701
yes	Pharmaceut	Not Report	25.81663
no	Pharmaceut	Not Report	18.938776
no	Radiation	Not Report	40.902292
no	Radiation	Not Report	35.15625
no	Pharmaceut	Not Report	21.453287
no	Pharmaceut	Not Report	18.442546
not report	Radiation	Not Report	30.116002
no	Pharmaceut	Not Report	20.971172
no	Radiation	Not Report	24.87772

no	Pharmaceut	Not Report	20.68515
yes	Pharmaceut	Not Report	'--
no	Pharmaceut	Not Report	16.974654
no	Pharmaceut	Not Report	20.897959
no	Radiation	Not Report	20.703125
no	Pharmaceut	Not Report	26.175195
no	Radiation	Not Report	20.957274
no	Pharmaceut	Not Report	21.777553
no	Pharmaceut	Not Report	'--
no	Radiation	Not Report	22.832879
no	Pharmaceut	Not Report	21.22449
no	Radiation	Not Report	35.294118
no	Radiation	Not Report	23.458562
no	Radiation	Not Report	24.671053
no	Pharmaceut	Not Report	32.458413
no	Radiation	Not Report	30.844416
no	Radiation	Not Report	22.13674
no	Pharmaceut	Not Report	16.302379
no	Pharmaceut	Not Report	29.726516
no	Pharmaceut	Not Report	23.140496
no	Pharmaceut	Not Report	26.061679
no	Pharmaceut	Not Report	18.724174
no	Radiation	Not Report	29.752744
no	Pharmaceut	Not Report	23.959094
not report	Pharmaceut	Not Report	'--
no	Radiation	Not Report	23.054562
no	Pharmaceut	Not Report	23.939481
no	Pharmaceut	Not Report	36.831301
no	Radiation	Not Report	21.513859
yes	Pharmaceut	Not Report	22.460034
no	Pharmaceut	Not Report	24.21875
no	Radiation	Not Report	23.046875
no	Radiation	Not Report	15.987507
no	Pharmaceut	Not Report	28.668892
no	Pharmaceut	Not Report	35.924515
yes	Pharmaceut	Not Report	17.777778
no	Radiation	Not Report	26.827421
no	Radiation	Not Report	21.453287
no	Pharmaceut	Not Report	28.650138
no	Radiation	Not Report	'--
yes	Pharmaceut	Not Report	22.321429
no	Pharmaceut	Not Report	23.183391
yes	Pharmaceut	Not Report	23.355637
not report	Radiation	Not Report	22.530612
no	Pharmaceut	Not Report	21.707923
no	Pharmaceut	Not Report	22.65625
no	Radiation	Not Report	26.81359
no	Pharmaceut	Not Report	23.503556
no	Radiation	Not Report	30.666667
no	Pharmaceut	Not Report	'--
no	Radiation	Not Report	37.421396

no	Radiation	Not Report	41.963531
no	Pharmaceut	Not Report	24.835763
no	Pharmaceut	Not Report	29.337411
yes	Pharmaceut	Not Report	32.861374
no	Pharmaceut	Not Report	22.666667
no	Radiation	Not Report	26.149277
no	Pharmaceut	Not Report	33.346481
no	Pharmaceut	Not Report	19.92513
no	Pharmaceut	Not Report	35.566499
no	Radiation	Not Report	22.89282
no	Radiation	Not Report	25.013521
not report	Pharmaceut	Not Report	22.405877
yes	Pharmaceut	Not Report	22.060354
yes	Radiation	Not Report	'--
no	Radiation	Not Report	44.982699
not report	Pharmaceut	Not Report	21.96712
yes	Pharmaceut	Not Report	27.990363
no	Radiation	Not Report	27.452887
no	Pharmaceut	Not Report	19.705532
not report	Pharmaceut	Not Report	16.588866
no	Radiation	Not Report	18.314256
no	Pharmaceut	Not Report	30.110279
no	Radiation	Not Report	23.507805
not report	Pharmaceut	Not Report	23.620288
no	Radiation	Not Report	27.993022
no	Radiation	Not Report	27.469136
no	Radiation	Not Report	23.422091
no	Radiation	Not Report	37.716263
no	Pharmaceut	Not Report	56.142177
no	Pharmaceut	Not Report	20.661157
no	Pharmaceut	Not Report	23.140496
no	Pharmaceut	Not Report	21.644121
yes	Radiation	Not Report	32.323232
no	Radiation	Not Report	41.097264
not report	Pharmaceut	Not Report	25.458066
no	Radiation	Not Report	27.993022
no	Radiation	Not Report	33.464035
no	Radiation	Not Report	20.284799
no	Radiation	Not Report	26.986001
no	Pharmaceut	Not Report	25.039919
no	Pharmaceut	Not Report	131.83594
no	Radiation	Not Report	20.478267
yes	Radiation	Not Report	26.402355
no	Pharmaceut	Not Report	31.202564
no	Radiation	Not Report	28.293345
yes	Radiation	Not Report	18.28678
no	Radiation	Not Report	27.084159
no	Radiation	Not Report	24.386526
no	Radiation	Not Report	'--
no	Radiation	Not Report	26.314635
no	Pharmaceut	Not Report	28.353057

no	Radiation	Not Report	'--
not report	Pharmaceut	Not Report	18.390675
no	Pharmaceut	Not Report	21.799445
yes	Pharmaceut	Not Report	20.173253
no	Pharmaceut	Not Report	'--
no	Radiation	Not Report	24.447279
no	Radiation	Not Report	'--
no	Pharmaceut	Not Report	21.303949
not report	Radiation	Not Report	20.504934
no	Radiation	Not Report	22.151023
no	Pharmaceut	Not Report	24.977043
not report	Pharmaceut	Not Report	27.531229
no	Pharmaceut	Not Report	25.344353
no	Radiation	Not Report	23.384354
yes	Pharmaceut	Not Report	22.634676
no	Pharmaceut	Not Report	20.3125
no	Pharmaceut	Not Report	21.755469
no	Pharmaceut	Not Report	24.221453
no	Radiation	Not Report	21.258503
no	Pharmaceut	Not Report	22.151023
no	Pharmaceut	Not Report	25.142857
no	Radiation	Not Report	25.142857
not report	Pharmaceut	Not Report	'--
not report	Pharmaceut	Not Report	22.65625
no	Pharmaceut	Not Report	20.324438
no	Radiation	Not Report	23.588329
no	Radiation	Not Report	21.192742
no	Radiation	Not Report	31.600115
no	Pharmaceut	Not Report	'--
no	Radiation	Not Report	31.652184
yes	Pharmaceut	Not Report	29.053288
no	Radiation	Not Report	29.585799
no	Pharmaceut	Not Report	30.486657
no	Radiation	Not Report	23.233456
no	Radiation	Not Report	22.408179
no	Pharmaceut	Not Report	'--
no	Radiation	Not Report	29.320988
no	Pharmaceut	Not Report	26.159334
no	Radiation	Not Report	26.297578
no	Radiation	Not Report	'--
not report	Pharmaceut	Not Report	29.938272
no	Pharmaceut	Not Report	20.93212
no	Radiation	Not Report	35.492458
no	Radiation	Not Report	25.315454
no	Pharmaceut	Not Report	35.188533
no	Radiation	Not Report	28.395062
no	Pharmaceut	Not Report	23.388687
no	Radiation	Not Report	19.382145
no	Pharmaceut	Not Report	23.323418
no	Radiation	Not Report	25.806452
no	Radiation	Not Report	44.288549

no	Radiation	Not Report	'--
no	Radiation	Not Report	26.259585
not report	Pharmaceut	Not Report	26.42357
no	Radiation	Not Report	20.936639
no	Pharmaceut	Not Report	26.709402
no	Pharmaceut	Not Report	16.528926
no	Radiation	Not Report	'--
no	Radiation	Not Report	21.872423
no	Radiation	Not Report	33.304989
no	Pharmaceut	Not Report	24.622961
no	Pharmaceut	Not Report	32.71862
no	Pharmaceut	Not Report	23.62949
no	Pharmaceut	Not Report	27.041644
no	Pharmaceut	Not Report	25.344353
no	Radiation	Not Report	23.427738
no	Radiation	Not Report	25.28257
no	Radiation	Not Report	25.654372
no	Radiation	Not Report	19.777699
not report	Pharmaceut	Not Report	22.145329
no	Pharmaceut	Not Report	22.790358
no	Pharmaceut	Not Report	27.472527
no	Pharmaceut	Not Report	24.314124
no	Pharmaceut	Not Report	34.949435
no	Radiation	Not Report	25.951557
no	Pharmaceut	Not Report	23.828125
no	Radiation	Not Report	32.95068
no	Radiation	Not Report	'--
not report	Pharmaceut	Not Report	20.576132
no	Pharmaceut	Not Report	29.142146
no	Pharmaceut	Not Report	40.260916
no	Radiation	Not Report	24.508946
no	Radiation	Not Report	20.147972
no	Radiation	Not Report	24.983563
no	Pharmaceut	Not Report	28.440955
no	Radiation	Not Report	19.713322
no	Pharmaceut	Not Report	'--
no	Radiation	Not Report	35.224514
no	Radiation	Not Report	28.87544
no	Radiation	Not Report	31.405613
no	Radiation	Not Report	26.218821
yes	Pharmaceut	Not Report	28.393726
no	Radiation	Not Report	18.986591
no	Radiation	Not Report	'--
no	Pharmaceut	Not Report	35.055632
no	Pharmaceut	Not Report	24.023809
no	Pharmaceut	Not Report	23.507805
yes	Radiation	Not Report	23.836735
no	Radiation	Not Report	27.160494
yes	Radiation	Not Report	28.042817
no	Pharmaceut	Not Report	32.770513
no	Pharmaceut	Not Report	30.436716

no	Radiation	Not Report	20.549887
no	Radiation	Not Report	25.209201
no	Pharmaceut	Not Report	25.864512
no	Radiation	Not Report	24.158818
no	Radiation	Not Report	35.629017
no	Radiation	Not Report	16.436555
no	Pharmaceut	Not Report	23.889463
yes	Pharmaceut	Not Report	38.368735
no	Pharmaceut	Not Report	18.195483
not report	Radiation	Not Report	'--
no	Pharmaceut	Not Report	28.650138
yes	Pharmaceut	Not Report	20.79673
no	Pharmaceut	Not Report	22.206331
no	Pharmaceut	Not Report	27.428571
no	Pharmaceut	Not Report	20.964361
no	Radiation	Not Report	'--
no	Radiation	Not Report	19.555556
no	Pharmaceut	Not Report	18.424036
no	Radiation	Not Report	18.196579
no	Radiation	Not Report	23.624447
no	Radiation	Not Report	26.753864
no	Radiation	Not Report	21.007668
no	Pharmaceut	Not Report	24.84098
yes	Radiation	Not Report	29.352355
no	Radiation	Not Report	23.612751
no	Radiation	Not Report	19.135802
yes	Pharmaceut	Not Report	20.515086
no	Pharmaceut	Not Report	33.333333
no	Radiation	Not Report	26.533759
no	Pharmaceut	Not Report	32.889189
no	Radiation	Not Report	24.092971
not report	Radiation	Not Report	35.139302
no	Pharmaceut	Not Report	20.808561
no	Radiation	Not Report	23.030045
no	Radiation	Not Report	33.690052
no	Pharmaceut	Not Report	21.107266
no	Radiation	Not Report	14.526644
yes	Radiation	Not Report	26.491508
no	Radiation	Not Report	22.206331
no	Radiation	Not Report	20.077748
no	Radiation	Not Report	27.141582
no	Radiation	Not Report	40.680473
no	Pharmaceut	Not Report	27.33564
not report	Radiation	Not Report	23.661439
no	Radiation	Not Report	18.017206
no	Pharmaceut	Not Report	27.548209
no	Radiation	Not Report	27.440599
no	Pharmaceut	Not Report	33.749456
no	Radiation	Not Report	'--
yes	Pharmaceut	Not Report	29.666548
no	Radiation	Not Report	33.912513

no	Radiation	Not Report	24.304618
no	Pharmaceut	Not Report	18.612245
no	Pharmaceut	Not Report	16.979592
not report	Pharmaceut	Not Report	21.671258
no	Pharmaceut	Not Report	19.031142
no	Pharmaceut	Not Report	26.037494
not report	Radiation	Not Report	'--
no	Pharmaceut	Not Report	25.15315
not report	Pharmaceut	Not Report	'--
no	Pharmaceut	Not Report	'--
no	Radiation	Not Report	19.650562
no	Radiation	Not Report	21.847009
yes	Radiation	Not Report	23.781213
no	Pharmaceut	Not Report	25.013521
no	Pharmaceut	Not Report	21.907582
no	Radiation	Not Report	23.422368
no	Pharmaceut	Not Report	23.828125
yes	Pharmaceut	Not Report	37.877211
no	Pharmaceut	Not Report	26.491508
no	Radiation	Not Report	17.755682
no	Radiation	Not Report	20.957274
no	Radiation	Not Report	30.477966
no	Pharmaceut	Not Report	31.309074
no	Radiation	Not Report	'--
no	Pharmaceut	Not Report	22.761468
not report	Radiation	Not Report	'--
no	Radiation	Not Report	39.836735
no	Radiation	Not Report	25.711662
no	Radiation	Not Report	'--
no	Radiation	Not Report	33.959184
no	Radiation	Not Report	30.078125
no	Pharmaceut	Not Report	22.985398
no	Pharmaceut	Not Report	20.069204
no	Radiation	Not Report	23.624447
yes	Radiation	Not Report	26.093275
no	Pharmaceut	Not Report	25.155896
no	Radiation	Not Report	'--
no	Pharmaceut	Not Report	37.756023
no	Pharmaceut	Not Report	20.307412
no	Radiation	Not Report	36.50622
no	Pharmaceut	Not Report	'--
no	Pharmaceut	Not Report	21.218317
no	Pharmaceut	Not Report	18.285714
no	Radiation	Not Report	27.303438
no	Radiation	Not Report	19.834711
no	Radiation	Not Report	19.979188
no	Pharmaceut	Not Report	18.612245
no	Pharmaceut	Not Report	20.195578
no	Radiation	Not Report	17.444927
no	Pharmaceut	Not Report	22.405877
no	Radiation	Not Report	'--

no	Radiation	Not Report	30.218858
no	Radiation	Not Report	27.774271
no	Pharmaceut	Not Report	30.116213
no	Radiation	Not Report	28.934069
no	Radiation	Not Report	31.877288
no	Radiation	Not Report	28.066424
no	Pharmaceut	Not Report	25.737081
no	Radiation	Not Report	25.605536
no	Pharmaceut	Not Report	22.37568
no	Pharmaceut	Not Report	23.781213
no	Radiation	Not Report	22.948116
yes	Radiation	Not Report	25.180785
no	Pharmaceut	Not Report	61.673061
no	Pharmaceut	Not Report	23.335466
no	Pharmaceut	Not Report	28.668892
