# Supplementary material for: The Landscape of Using Glycosyltransferase Gene Signatures for Overall Survival Prediction in Hepatocellular Carcinoma
Source: J Oncol. 2022 Jun 21;2022:5989419. doi: 10.1155/2022/5989419 (PMC9239767; doi:10.1155/2022/5989419)
Supplement: Supplementary Materials — Figure S1: Venn diagram shows the intersections of co-differentially expressed glycosyltransferase (GT) genes between the 210 GTs obtained from the literature and those obtained from TCGA database. Figure S2: identification of a four-gene signature in the training set of TCGA database constructed using the LASSO method. Figure S3: survival analyses grouped by the optimal cutoff expression value of each gene in the training dataset of TCGA database. (A) ALG3. (B) B3GAT3. (C) GLA. (D) ST6GALNAC4. Figure S4: mRNA expression levels of the four genes in matched HCC and adjacent noncancerous samples in TCGA database were compared. (A) ALG3. (B) B3GAT3. (C) GLA. (D) ST6GALNAC4. Figure S5: the interactions among signature genes displayed in a PPI network. Figure S6: stratified survival analysis between high- and low-risk groups. (A)NAFLD. (B) HBV infection. (C) HCV infection. (D) Both HBV and HCV infections. (E) No treatment and therapy. (F) Pharmaceutical therapy. (G) Radiation therapy, NOS. Figure S7: the nomogram to predict prognostic probabilities in TCGA database. (A) The nomogram for predicting OS in the training set. The calibration plots for predicting (B) 3-year and (C) 5-year survival probabilities. Figure S8: survival analyses grouped by the optimal cutoff expression value of each signature gene in the validation dataset of TCGA database. (A) ALG3. (B) B3GAT3. (C) GLA. (D) ST6GALNAC4. Table S1: the clinical information of 371 primary HCC samples and 50 normal samples. Table S2: combining the clinical information and expression profiles of 210 human glycosyltransferase (GT) genes. Table S3: DEGs of 371 samples in TCGA database. Table S4: all significantly enriched GO terms. Table S5: all significantly enriched KEGG pathways. [file 5989419.f1.zip › 5989419.f1/Supplementary Table.2 LIHC GTs and clinical information.pdf]

	Time	Status	Age	Gender	M	N	Pathologic
TCGA-2V-A9	NA	0	NA	Male	MX	NX	Stage II
TCGA-2Y-A9	724	1	< 60	Male	MX	NX	NA
TCGA-2Y-A9	1939	0	< 60	Female	MX	NX	Stage I
TCGA-2Y-A9	1271	1	>= 60	Male	MX	N0	Stage I
TCGA-2Y-A9	848	1	>= 60	Female	MX	NX	Stage II
TCGA-2Y-A9	3675	0	< 60	Male	M0	N0	Stage III
TCGA-2Y-A9	1229	1	< 60	Male	MX	NX	Stage I
TCGA-2Y-A9	1731	0	>= 60	Female	MX	N0	Stage I
TCGA-2Y-A9	1516	0	< 60	Male	MX	NX	Stage II
TCGA-2Y-A9	1452	0	>= 60	Male	MX	N0	Stage I
TCGA-2Y-A9	555	1	< 60	Female	MX	N0	Stage I
TCGA-2Y-A9	357	0	>= 60	Female	MX	NX	Stage I
TCGA-2Y-A9	1168	0	>= 60	Female	MX	N0	Stage I
TCGA-2Y-A9	633	1	>= 60	Female	MX	NX	NA
TCGA-2Y-A9	697	0	>= 60	Male	MX	N0	Stage I
TCGA-3K-AA	396	0	>= 60	Male	MX	NX	Stage III
TCGA-4R-AA	262	1	>= 60	Male	MX	NX	Stage II
TCGA-5C-A9	328	0	< 60	Male	M0	N0	Stage II
TCGA-5C-AA	20	0	>= 60	Male	M0	N0	Stage II
TCGA-5R-AA	449	0	< 60	Female	M0	N0	Stage III
TCGA-5R-AA	46	1	>= 60	Female	M0	N0	Stage II
TCGA-BC-A1	1135	1	>= 60	Female	MX	NX	NA
TCGA-BC-A1	308	1	>= 60	Female	MX	NX	NA
TCGA-BC-A1	1423	1	>= 60	Male	MX	NX	NA
TCGA-BC-A1	837	1	>= 60	Male	MX	NX	NA
TCGA-BC-A1	837	1	>= 60	Male	MX	NX	NA
TCGA-BC-A1	91	1	< 60	Male	MX	NX	NA
TCGA-BC-A1	770	1	< 60	Female	MX	N0	Stage III
TCGA-BC-A1	711	1	>= 60	Male	MX	NX	NA
TCGA-BC-A1	34	1	>= 60	Female	MX	N0	Stage I
TCGA-BC-A1	2116	1	< 60	Female	MX	NX	NA
TCGA-BC-A2	1351	0	>= 60	Female	M0	NX	Stage III
TCGA-BC-A2	1397	1	>= 60	Female	M0	NX	Stage II
TCGA-BC-A3	8	0	>= 60	Female	M0	NX	Stage I
TCGA-BC-A5	547	1	>= 60	Male	M0	NX	Stage III
TCGA-BC-A6	444	0	>= 60	Male	M0	NX	Stage II
TCGA-BC-A6	387	0	>= 60	Male	M0	N0	Stage I
TCGA-BD-A2	1363	0	>= 60	Male	MX	NX	NA
TCGA-BD-A3	409	0	>= 60	Female	M0	N0	Stage I
TCGA-BD-A3	1115	0	>= 60	Male	MX	NX	Stage II
TCGA-BW-A5	20	0	< 60	Male	MX	NX	Stage III
TCGA-BW-A5	0	0	< 60	Female	M1	N0	Stage IV
TCGA-CC-52	87	1	>= 60	Female	M0	N0	Stage III
TCGA-CC-52	97	1	< 60	Male	M0	N0	Stage II
TCGA-CC-52	103	1	>= 60	Male	M0	N0	Stage III
TCGA-CC-52	102	1	>= 60	Male	M0	N0	Stage III
TCGA-CC-A1	219	0	< 60	Female	M0	N0	Stage III
TCGA-CC-A1	101	1	< 60	Male	M0	N0	Stage III
TCGA-CC-A3	303	1	>= 60	Male	M0	N0	Stage III
TCGA-CC-A3	363	0	< 60	Male	M0	N0	Stage III

TCGA-CC-A5	347	1	>= 60	Male	M0	N0	Stage III
TCGA-CC-A5	272	1	< 60	Male	M0	N0	Stage III
TCGA-CC-A7	217	1	< 60	Male	M0	N0	Stage III
TCGA-CC-A7	649	1	< 60	Male	M0	N0	Stage III
TCGA-CC-A7	399	0	< 60	Male	M0	N0	Stage III
TCGA-CC-A7	382	0	< 60	Male	M0	N0	Stage II
TCGA-CC-A7	262	1	< 60	Male	M0	N0	Stage III
TCGA-CC-A7	278	1	>= 60	Male	M0	N0	Stage III
TCGA-CC-A8	300	1	< 60	Male	M0	N1	Stage III
TCGA-CC-A8	344	1	< 60	Female	M0	N0	Stage III
TCGA-CC-A9	211	0	< 60	Male	M0	N0	Stage II
TCGA-CC-A9	0	0	< 60	Male	M0	N0	Stage III
TCGA-DD-A1	1149	1	< 60	Male	M0	NA	Stage II
TCGA-DD-A1	2542	1	< 60	Male	M0	N0	Stage III
TCGA-DD-A1	1622	1	>= 60	Male	M0	N0	Stage III
TCGA-DD-A1	3437	0	>= 60	Female	M0	N0	Stage II
TCGA-DD-A1	79	NA	>= 60	Male	M0	N0	Stage I
TCGA-DD-A1	14	1	>= 60	Male	M0	N0	Stage I
TCGA-DD-A1	662	0	>= 60	Male	M0	N0	Stage I
TCGA-DD-A1	1560	1	< 60	Female	M0	N0	Stage I
TCGA-DD-A1	2415	0	>= 60	Male	M0	N0	Stage II
TCGA-DD-A1	2017	0	>= 60	Female	M0	N0	Stage I
TCGA-DD-A1	602	0	< 60	Female	M0	N0	Stage I
TCGA-DD-A1	349	1	>= 60	Male	M0	N0	Stage III
TCGA-DD-A1	1372	1	>= 60	Male	M0	N0	Stage I
TCGA-DD-A1	1495	0	< 60	Male	M0	N0	Stage III
TCGA-DD-A1	183	0	< 60	Male	M0	N0	Stage I
TCGA-DD-A1	1005	1	>= 60	Female	M0	N1	Stage III
TCGA-DD-A1	558	1	>= 60	Female	M1	N0	Stage IV
TCGA-DD-A1	415	1	< 60	Male	M0	N0	Stage II
TCGA-DD-A3	643	1	>= 60	Male	M0	NX	Stage II
TCGA-DD-A3	1694	1	>= 60	Female	M0	NX	Stage I
TCGA-DD-A3	171	1	>= 60	Male	M0	NX	Stage I
TCGA-DD-A3	601	1	< 60	Female	M0	NX	Stage II
TCGA-DD-A3	233	1	>= 60	Male	M0	N0	Stage III
TCGA-DD-A3	2131	1	>= 60	Female	M0	N0	Stage I
TCGA-DD-A3	612	1	< 60	Male	M0	N0	Stage III
TCGA-DD-A3	3125	1	>= 60	Female	M0	N0	Stage III
TCGA-DD-A3	11	1	>= 60	Male	M0	N0	Stage II
TCGA-DD-A3	931	1	>= 60	Female	M1	N0	Stage IV
TCGA-DD-A4	989	0	< 60	Male	M0	N0	Stage I
TCGA-DD-A4	942	0	>= 60	Male	M0	N0	Stage I
TCGA-DD-A4	802	1	>= 60	Male	M0	NX	Stage III
TCGA-DD-A4	917	0	>= 60	Female	M0	N0	Stage III
TCGA-DD-A4	816	0	>= 60	Male	M0	NX	Stage II
TCGA-DD-A4	928	0	< 60	Female	M0	N0	Stage II
TCGA-DD-A4	1711	0	< 60	Male	M0	N0	Stage I
TCGA-DD-A4	899	1	< 60	Female	M0	N0	Stage I
TCGA-DD-A4	2245	0	>= 60	Male	M0	N0	Stage I
TCGA-DD-A4	3308	0	< 60	Male	M0	N0	Stage I
TCGA-DD-A4	373	1	>= 60	Male	M0	N0	Stage II

TCGA-DD-A4	9	1	>= 60	Female	M0	N0	Stage I
TCGA-DD-A4	2456	1	>= 60	Female	M0	N0	Stage I
TCGA-DD-A4	2398	0	>= 60	Male	M0	N0	Stage III
TCGA-DD-A7	728	0	>= 60	Male	M0	N0	Stage I
TCGA-DD-A7	283	1	>= 60	Female	M0	N0	Stage I
TCGA-DD-A7	701	0	>= 60	Female	M0	N0	Stage III
TCGA-DD-A7	44	0	>= 60	Male	M0	N0	Stage I
TCGA-DD-A7	1085	0	>= 60	Female	M0	N0	Stage I
TCGA-DD-A7	3478	0	>= 60	Female	M0	N0	Stage I
TCGA-DD-AA	16	1	>= 60	Male	M0	N0	Stage I
TCGA-DD-AA	347	0	< 60	Male	M0	N0	Stage I
TCGA-DD-AA	2301	0	>= 60	Male	M0	N0	Stage I
TCGA-DD-AA	2324	0	>= 60	Female	M0	N0	Stage I
TCGA-DD-AA	1685	1	>= 60	Male	M0	N0	Stage I
TCGA-DD-AA	381	1	< 60	Male	M0	N0	Stage I
TCGA-DD-AA	365	1	>= 60	Male	M0	N0	Stage I
TCGA-DD-AA	2102	0	>= 60	Male	M0	N0	Stage II
TCGA-DD-AA	9	0	>= 60	Male	M0	N0	Stage I
TCGA-DD-AA	1302	0	< 60	Male	M0	N0	Stage I
TCGA-DD-AA	1876	0	< 60	Male	M0	N0	Stage I
TCGA-DD-AA	1804	0	< 60	Male	M0	N0	Stage I
TCGA-DD-AA	1562	0	>= 60	Female	M0	N0	Stage I
TCGA-DD-AA	1567	0	< 60	Male	M0	N0	Stage I
TCGA-DD-AA	1531	0	< 60	Male	M0	N0	Stage I
TCGA-DD-AA	170	0	>= 60	Male	M0	N0	Stage II
TCGA-DD-AA	1450	0	>= 60	Male	M0	N0	Stage I
TCGA-DD-AA	171	1	>= 60	Female	M0	N0	Stage I
TCGA-DD-AA	137	0	>= 60	Female	M0	N0	Stage I
TCGA-DD-AA	658	0	>= 60	Male	M0	N0	Stage I
TCGA-DD-AA	1295	0	< 60	Male	M0	N0	Stage I
TCGA-DD-AA	1345	0	< 60	Male	M0	N0	Stage I
TCGA-DD-AA	672	0	>= 60	Male	M0	N0	Stage III
TCGA-DD-AA	1219	0	>= 60	Female	M0	N0	Stage I
TCGA-DD-AA	1242	0	< 60	Male	M0	N0	Stage I
TCGA-DD-AA	425	1	< 60	Male	M0	N0	Stage I
TCGA-DD-AA	1231	0	< 60	Male	M0	N0	Stage I
TCGA-DD-AA	115	1	>= 60	Female	M0	N0	Stage I
TCGA-DD-AA	1145	0	>= 60	Male	M0	N0	Stage III
TCGA-DD-AA	1085	0	< 60	Female	M0	N0	Stage I
TCGA-DD-AA	1066	0	>= 60	Female	M0	N0	Stage I
TCGA-DD-AA	1049	0	>= 60	Female	M0	N0	Stage II
TCGA-DD-AA	636	0	< 60	Male	M0	N0	Stage I
TCGA-DD-AA	12	1	< 60	Male	M0	N0	Stage II
TCGA-DD-AA	898	0	< 60	Male	MX	NX	Stage I
TCGA-DD-AA	453	0	< 60	Male	M0	N0	Stage I
TCGA-DD-AA	458	0	< 60	Male	M0	N0	Stage I
TCGA-DD-AA	2028	0	< 60	Male	M0	N0	Stage I
TCGA-DD-AA	474	0	>= 60	Male	M0	N0	Stage I
TCGA-DD-AA	554	0	>= 60	Male	M0	N0	Stage II
TCGA-DD-AA	574	0	< 60	Male	M0	N0	Stage I
TCGA-DD-AA	587	0	< 60	Male	M0	N0	Stage I

TCGA-DD-AA	555	0	< 60	Female	M0	N0	Stage III
TCGA-DD-AA	552	0	< 60	Male	M0	N0	Stage I
TCGA-DD-AA	638	0	< 60	Male	M0	N0	Stage I
TCGA-DD-AA	566	0	< 60	Male	M0	N0	Stage I
TCGA-DD-AA	608	0	< 60	Female	M0	N0	Stage I
TCGA-DD-AA	644	0	>= 60	Male	M0	N0	Stage I
TCGA-DD-AA	722	0	>= 60	Male	M0	N0	Stage I
TCGA-DD-AA	478	0	>= 60	Male	M0	N0	Stage I
TCGA-DD-AA	763	0	< 60	Male	M0	N0	Stage I
TCGA-DD-AA	810	0	< 60	Male	M0	N0	Stage I
TCGA-DD-AA	1531	0	>= 60	Male	M0	N0	Stage I
TCGA-DD-AA	2728	0	< 60	Male	M0	N0	Stage I
TCGA-DD-AA	1823	0	< 60	Male	M0	N0	Stage I
TCGA-DD-AA	2455	0	< 60	Male	M0	N0	Stage II
TCGA-DD-AA	1718	0	< 60	Male	M0	N0	Stage II
TCGA-DD-AA	1970	0	< 60	Male	M0	N0	Stage III
TCGA-DD-AA	1900	0	< 60	Male	M0	N0	Stage I
TCGA-DD-AA	2015	0	< 60	Male	M0	N0	Stage I
TCGA-DD-AA	1989	0	< 60	Male	M0	N0	Stage III
TCGA-DD-AA	1855	0	>= 60	Male	M0	N0	Stage I
TCGA-ED-A4	910	0	< 60	Male	M0	N0	Stage II
TCGA-ED-A4	819	0	< 60	Male	M0	N0	Stage II
TCGA-ED-A5	854	0	>= 60	Female	M0	N0	Stage II
TCGA-ED-A6	406	0	< 60	Male	M0	N0	Stage III
TCGA-ED-A7	6	0	< 60	Female	M0	NX	Stage II
TCGA-ED-A7	390	0	< 60	Female	M0	NX	Stage II
TCGA-ED-A7	6	0	>= 60	Male	M0	NX	Stage II
TCGA-ED-A7	400	0	< 60	Female	M0	N0	Stage II
TCGA-ED-A8	408	0	>= 60	Female	M0	N0	Stage III
TCGA-EP-A1	570	0	>= 60	Male	MX	NX	Stage I
TCGA-EP-A2	608	0	>= 60	Male	MX	N0	Stage I
TCGA-EP-A2	627	1	< 60	Female	MX	NX	Stage III
TCGA-EP-A2	596	1	< 60	Female	MX	NX	Stage I
TCGA-EP-A2	19	1	>= 60	Male	MX	NX	Stage I
TCGA-EP-A3	303	0	>= 60	Male	MX	NX	Stage I
TCGA-EP-A3	363	0	>= 60	Male	MX	NX	Stage III
TCGA-ES-A2	438	1	< 60	Male	MX	NX	Stage I
TCGA-FV-A2	1852	1	>= 60	Female	M0	N0	Stage II
TCGA-FV-A2	729	0	>= 60	Male	MX	N0	Stage I
TCGA-FV-A2	581	1	>= 60	Male	M0	N0	Stage I
TCGA-FV-A3	848	0	>= 60	Female	M0	NX	Stage II
TCGA-FV-A3	247	1	>= 60	Female	MX	N0	Stage II
TCGA-FV-A3	194	1	>= 60	Male	MX	NX	Stage I
TCGA-FV-A3	366	1	< 60	Female	MX	NX	Stage I
TCGA-FV-A4	1	0	< 60	Female	M0	NX	Stage II
TCGA-FV-A4	10	0	>= 60	Female	M0	NX	Stage I
TCGA-FV-A4	12	0	< 60	Male	M0	NX	Stage I
TCGA-G3-A2	1779	0	>= 60	Male	M0	N0	Stage II
TCGA-G3-A2	452	1	< 60	Female	M0	N0	Stage I
TCGA-G3-A2	655	0	< 60	Male	M0	N0	Stage I
TCGA-G3-A3	780	0	< 60	Male	M0	N0	Stage III

TCGA-G3-A3	594	0	< 60	Male	M0	N0	Stage II
TCGA-G3-A3	585	0	>= 60	Male	M0	N0	Stage I
TCGA-G3-A5	768	1	< 60	Male	M0	N0	Stage II
TCGA-G3-A5	698	0	< 60	Male	M0	NX	Stage I
TCGA-G3-A5	520	0	< 60	Male	M0	NX	Stage II
TCGA-G3-A6	671	0	>= 60	Male	M0	N0	Stage III
TCGA-G3-A7	447	0	>= 60	Male	MX	NX	Stage I
TCGA-G3-A7	632	0	>= 60	Female	MX	NX	Stage I
TCGA-G3-A7	361	0	>= 60	Male	MX	NX	Stage I
TCGA-G3-A7	430	0	< 60	Male	MX	NX	Stage I
TCGA-G3-A7	56	1	>= 60	Male	MX	NX	Stage III
TCGA-G3-AA	476	0	< 60	Male	M0	N0	Stage I
TCGA-G3-AA	359	1	< 60	Male	M0	N0	Stage III
TCGA-G3-AA	412	0	< 60	Female	M0	N0	Stage II
TCGA-G3-AA	354	0	>= 60	Male	M0	N0	Stage II
TCGA-GJ-A3	879	0	< 60	Male	MX	NX	Stage I
TCGA-GJ-A6	31	1	>= 60	Female	MX	NX	Stage II
TCGA-GJ-A9	67	1	>= 60	Male	MX	N0	Stage I
TCGA-HP-A5	91	1	>= 60	Male	M0	NX	Stage I
TCGA-K7-A5	519	0	>= 60	Male	MX	NX	Stage I
TCGA-K7-A6	512	0	>= 60	Male	MX	N0	Stage I
TCGA-K7-AA	359	0	>= 60	Male	MX	NX	Stage II
TCGA-KR-A7	65	1	>= 60	Male	M0	N0	Stage I
TCGA-KR-A7	829	0	>= 60	Male	M0	N0	Stage I
TCGA-KR-A7	951	0	>= 60	Female	M0	N0	Stage II
TCGA-KR-A7	906	0	< 60	Male	M0	N0	Stage I
TCGA-LG-A6	387	0	>= 60	Female	M0	NX	Stage II
TCGA-LG-A9	425	0	< 60	Male	M0	NX	Stage I
TCGA-MI-A7	291	0	>= 60	Male	M0	N0	Stage I
TCGA-MI-A7	507	0	>= 60	Male	M0	N0	Stage III
TCGA-MI-A7	698	0	>= 60	Male	M0	N0	Stage II
TCGA-MI-A7	747	0	>= 60	Male	MX	NX	NA
TCGA-MI-A7	630	0	>= 60	Male	MX	NX	NA
TCGA-MR-A5	229	0	< 60	Male	MX	NX	Stage I
TCGA-NI-A4	1791	1	>= 60	Male	MX	NX	Stage III
TCGA-NI-A8	799	0	>= 60	Male	MX	NX	Stage I
TCGA-O8-A7	538	0	< 60	Male	MX	NX	Stage I
TCGA-QA-A7	94	0	< 60	Male	MX	NX	Stage II
TCGA-RC-A6	0	0	< 60	Male	M0	N0	Stage II
TCGA-RC-A7	640	0	< 60	Female	M0	N0	Stage I
TCGA-RC-A7	588	0	< 60	Male	M0	N0	Stage II
TCGA-RC-A7	579	0	>= 60	Male	M0	N0	Stage I
TCGA-RC-A7	468	0	< 60	Male	M0	N0	Stage II
TCGA-RC-A7	472	0	< 60	Male	M0	N0	Stage I
TCGA-T1-A6	23	0	>= 60	Male	M0	NX	NA
TCGA-UB-A7	848	0	>= 60	Female	M0	N0	Stage II
TCGA-UB-A7	601	0	< 60	Male	MX	NX	Stage II
TCGA-UB-A7	500	0	< 60	Male	MX	N0	Stage III
TCGA-UB-A7	52	1	>= 60	Male	MX	N0	Stage I
TCGA-UB-A7	486	0	< 60	Male	MX	NX	Stage I
TCGA-UB-AA	327	0	>= 60	Male	MX	NX	Stage II

TCGA-UB-AA	314	0	>= 60	Female	MX	NX	Stage I
TCGA-WJ-A8	345	0	>= 60	Female	MX	NX	Stage I
TCGA-WQ-A9	30	0	>= 60	Female	M0	NX	NA
TCGA-WQ-AB	395	0	>= 60	Male	M0	NX	Stage II
TCGA-WX-AA	615	0	>= 60	Female	MX	NX	Stage I
TCGA-WX-AA	556	1	< 60	Female	MX	NX	Stage III
TCGA-XR-A8	1339	0	< 60	Female	MX	NX	Stage I
TCGA-XR-A8	1030	0	< 60	Female	M0	N0	Stage III
TCGA-XR-A8	925	0	< 60	Male	MX	N0	Stage III
TCGA-XR-A8	693	1	>= 60	Male	MX	NX	Stage I
TCGA-XR-A8	898	0	< 60	Male	M0	NX	Stage I
TCGA-ZP-A9	1088	1	< 60	Male	MX	NX	NA
TCGA-ZP-A9	782	0	>= 60	Female	MX	NX	NA
TCGA-ZP-A9	21	0	< 60	Female	MX	NX	NA
TCGA-ZP-A9	395	0	>= 60	Female	MX	NX	NA
TCGA-ZS-A9	1386	1	>= 60	Male	MX	NX	Stage II
TCGA-ZS-A9	2412	0	>= 60	Male	MX	NX	Stage II
TCGA-ZS-A9	341	0	< 60	Male	MX	NX	Stage II

T	prior_mali	treatment_	treatment_	BMI	MGAT5	NEU1	ALG3
T2	No	No	Pharmaceut	Fat	3.4152889	7.3568997	7.9721426
T2	No	No	Pharmaceut	Fat	3.9529244	7.5321163	9.262683
T1	No	No	Pharmaceut	Fat	1.5814119	7.2548912	7.860752
T1	No	No	Radiation	Fat	3.0921634	6.9718995	8.5260676
T2	No	No	Radiation	Fat	2.5789976	7.0874138	8.5666414
T3	No	No	Radiation	Fat	1.7469388	10.847243	9.0428312
T1	No	No	Pharmaceut	Fat	2.2070146	6.3800537	8.3105203
T1	No	No	Radiation	Nonfat	5.1225249	7.722981	8.9234338
T2	No	No	Pharmaceut	Fat	2.6221498	7.2433433	8.1323108
T1	No	No	Radiation	Fat	1.5992016	6.4245228	8.9495214
T1	No	No	Radiation	Fat	3.331385	7.6555157	8.3590205
T1	Yes	No	Radiation	Fat	2.5659475	7.3780826	8.623997
T1	Yes	No	Radiation	Fat	2.0180756	6.9128447	8.3087478
T1	No	Yes	Pharmaceut	Nonfat	2.3864987	9.3066608	9.0790935
T1	No	No	Radiation	Fat	3.1078226	6.6015877	8.4111512
T3	No	Yes	Pharmaceut	Fat	2.3664364	8.3307542	9.2708223
T2	Yes	No	Radiation	Fat	2.4940902	7.8254723	9.0392465
T2	No	No	Pharmaceut	Fat	2.4956997	6.4239039	8.8822506
T2	No	No	Pharmaceut	Fat	3.0009654	6.9943336	10.031099
T3	No	No	Pharmaceut	Fat	3.6102858	7.1337621	8.0167214
T2	No	No	Pharmaceut	Fat	3.0275396	7.1398026	8.4564819
T2	No	No	Radiation	NA	1.3611565	6.2898697	7.576955
T3	No	No	Radiation	NA	1.8823335	6.181717	7.3727828
T3	No	No	Radiation	NA	2.9077923	6.9325026	8.1772531
T4	No	No	Radiation	NA	2.5903931	6.7542272	9.4046669
T2	No	No	Pharmaceut	NA	1.6617765	6.1736044	7.438944
T4	No	No	Radiation	NA	1.4220445	6.5689489	7.6019264
T3	No	No	Pharmaceut	NA	1.4976002	6.5277601	7.1709197
T4	No	No	Pharmaceut	Fat	3.4867922	6.9197059	9.2323048
T1	No	No	Pharmaceut	NA	5.0030876	8.3832802	9.4458942
T1	Yes	Yes	Pharmaceut	Fat	2.1643801	6.4549882	7.6726862
T3	No	No	Pharmaceut	Fat	2.9276844	7.1163207	7.9283577
T2	No	No	Pharmaceut	Fat	4.1507519	8.6658936	8.9142863
T1	No	NA	Radiation	Nonfat	2.0181277	8.6134839	8.6787527
T3	Yes	No	Radiation	Fat	3.5626798	6.5677763	9.626766
T2	No	No	Pharmaceut	Fat	2.6421849	7.6562848	9.3590951
T1	No	No	Radiation	Fat	1.6122214	7.0456879	8.2761958
T2	No	No	Radiation	Fat	1.74637	7.7661801	8.1589146
T1	No	No	Pharmaceut	Fat	3.562597	8.2735775	8.8012247
T2	Yes	No	Radiation	Fat	4.0210378	7.0624441	8.4253502
T3	No	No	Radiation	NA	3.1506506	7.4756057	9.2885448
T2	No	No	Radiation	NA	2.6788098	8.2942092	8.5613838
T4	No	No	Radiation	Nonfat	6.3137536	8.3926965	9.2726433
T2	No	No	Pharmaceut	Nonfat	4.8183717	8.8931303	8.6007873
T4	No	No	Pharmaceut	Fat	2.9755097	7.9600961	9.2482847
T3	No	No	Pharmaceut	Fat	2.7690697	9.086411	9.2510778
T3	No	No	Pharmaceut	NA	1.6908969	7.3870139	8.6461045
T3	No	No	Pharmaceut	Fat	3.9532533	7.8194387	9.4992048
T3	No	No	Radiation	Fat	5.3355096	7.282927	8.8367524
T3	No	No	Radiation	Nonfat	3.6891798	7.8132519	9.0563587

T3	No	No	Radiation	Fat	2.6539515	7.9351224	8.6586581
T4	No	No	Pharmaceut	Fat	2.2073794	8.3771827	8.9773797
T3	No	No	Radiation	Fat	2.941327	7.2034415	9.4054895
T3	No	No	Pharmaceut	Fat	2.3181486	7.3972284	8.3130262
T3	No	No	Pharmaceut	Nonfat	3.0755609	6.7971181	9.085063
T2	No	No	Radiation	Nonfat	4.3939218	7.8083171	9.4334055
T3	No	No	Pharmaceut	Fat	3.2661412	7.5257825	8.9484854
T3	No	No	Pharmaceut	Fat	3.0599269	8.9216405	8.7382344
T3	No	No	Radiation	Fat	2.8112272	7.5502244	8.5337092
T3	No	No	Pharmaceut	Nonfat	2.9436395	9.5996642	9.014392
T2	No	No	Pharmaceut	Fat	2.4646462	6.7899535	8.8475185
T3	No	NA	Pharmaceut	Fat	5.4042886	7.8690699	7.5936034
T2	No	No	Radiation	Fat	2.384544	6.6789266	8.0633219
T3	No	Yes	Pharmaceut	Fat	3.1156384	6.9147577	9.1351591
T3	No	Yes	Radiation	Fat	1.5587079	5.9491671	7.3160016
T2	No	No	Radiation	Fat	3.5476179	8.2206327	8.7639954
T1	No	NA	Pharmaceut	Fat	3.2906754	8.3698715	8.2668904
T1	No	No	Radiation	Fat	2.3895044	6.4495332	7.6505329
T1	No	No	Radiation	Fat	2.9558818	6.3857037	9.1555889
T1	No	No	Radiation	Fat	3.2656642	7.235121	8.9460458
T2	No	No	Radiation	Fat	3.0201362	7.2602497	8.7723313
T1	No	No	Radiation	Fat	3.2947145	7.5501541	8.2768088
T1	No	No	Radiation	Fat	4.3901693	8.03686	9.2620727
T3	No	No	Radiation	Fat	3.3783994	8.5201903	8.9467881
T1	No	Yes	Pharmaceut	Fat	2.9438469	7.9683845	9.207937
T3	No	No	Radiation	Fat	3.5441211	8.4911915	9.0489757
T1	No	NA	Radiation	Fat	1.9125839	6.1806522	7.6845407
T1	No	No	Radiation	Fat	2.6263265	9.2580625	9.2662022
T4	No	Yes	Pharmaceut	Fat	2.7619069	8.3039072	8.8837829
T2	No	No	Pharmaceut	Nonfat	2.28372	7.9459325	9.1852007
T2	No	Yes	Radiation	Fat	1.3662281	6.4712674	8.7409562
T1	No	No	Pharmaceut	Fat	3.0150818	7.9335731	9.0016151
T1	No	No	Radiation	Fat	2.1254321	8.8840155	9.2517478
T2	No	NA	Pharmaceut	Fat	1.4359588	6.1810222	7.5075511
T3	No	No	Pharmaceut	Fat	2.8312089	7.4097403	8.7801114
T1	No	No	Radiation	Fat	1.5139403	6.0377675	6.8204208
T3	No	No	Radiation	Nonfat	1.517344	7.3924974	8.7340956
T3	No	No	Pharmaceut	Fat	2.7943114	6.7460061	9.7342808
T2	Yes	No	Radiation	Fat	1.9621796	7.1843929	8.370848
T4	No	NA	Pharmaceut	Fat	3.0041865	8.6446792	8.9227472
T1	No	No	Pharmaceut	Fat	3.9790789	7.5518724	8.7098835
T1	No	Yes	Radiation	Fat	2.7864495	7.1406511	7.9924188
T3	No	No	Pharmaceut	Fat	2.3492212	8.3991552	8.4446209
T3	No	Yes	Pharmaceut	Fat	4.5707411	9.5331984	8.6866679
T2	Yes	No	Radiation	Fat	2.165927	6.6973086	8.0675827
T2	No	No	Pharmaceut	Fat	3.533169	8.2838849	8.7219527
T1	No	No	Pharmaceut	Fat	2.8667623	6.7982647	7.5850999
T1	No	No	Radiation	Fat	3.1427694	8.6089657	9.33713
T1	No	No	Pharmaceut	Fat	4.2926224	7.6182918	8.0812092
T1	No	No	Pharmaceut	Fat	1.5837224	7.0076038	7.5086423
T2	No	Yes	Pharmaceut	Fat	3.4208148	8.4299587	8.6875277

T1	No	No	PharmaceutFat		3.6945031	7.8960791	9.0868639
T1	No	Yes	PharmaceutFat		3.4558418	7.612016	8.1842333
T3	No	No	PharmaceutFat		3.1361182	6.935584	8.2492142
T1	No	No	Radiation	Fat	3.1907799	6.4003609	8.0401941
T1	No	No	PharmaceutFat		1.5597024	8.1162029	7.2155423
T3	No	No	Radiation	Nonfat	3.127348	6.740436	7.5256368
T1	No	No	Radiation	Fat	4.1657856	8.8355229	8.6802943
T1	No	No	Radiation	Fat	2.4843549	6.6621087	8.3229586
T1	No	No	Radiation	Nonfat	2.5258284	7.249516	9.3961071
T1	No	No	Radiation	Fat	2.5441766	9.17724	8.9457972
T1	No	No	Radiation	Fat	2.4710889	6.6414788	7.7235635
T1	No	No	Radiation	Fat	3.8649467	8.1104278	8.7343173
T1	No	No	Radiation	Fat	2.382687	9.1040334	8.5669997
T1	No	No	PharmaceutFat		2.4529576	7.1289237	8.3949593
T1	No	No	PharmaceutNonfat		1.719517	7.4520582	8.5050869
T1	No	No	Radiation	Fat	3.2859836	6.8194388	8.4081734
T2	No	No	Radiation	Fat	2.2978542	8.2591905	8.8783707
T1	No	No	PharmaceutFat		2.7275632	7.578593	9.0409174
T1	No	No	PharmaceutFat		2.845918	7.7119303	8.8380917
T1	No	No	Radiation	Nonfat	2.7277165	6.3611202	8.1686695
T1	No	No	PharmaceutFat		3.4524055	7.9836375	8.6641128
T1	No	No	PharmaceutFat		2.2998022	8.373933	8.6645976
T1	No	No	PharmaceutFat		3.3242014	8.2105127	9.0045052
T1	No	No	Radiation	Fat	3.4215208	7.8690181	8.7994547
T2	No	Yes	PharmaceutFat		3.2865898	9.075734	8.6467728
T1	No	No	Radiation	Fat	2.1635401	7.147905	8.5357109
T1	No	No	Radiation	Fat	2.9987242	8.8355363	9.5036206
T1	No	No	PharmaceutFat		2.1578519	8.1017313	8.7251341
T1	No	No	Radiation	Fat	3.1286024	7.2464353	8.4434063
T1	No	No	Radiation	Fat	3.0301649	8.2856161	8.2497249
T1	No	No	PharmaceutFat		5.2223255	8.2866709	9.6710356
T3	No	No	PharmaceutFat		2.5832732	7.8825698	8.6120879
T1	No	No	Radiation	Fat	2.2322601	7.7820848	8.4054515
T1	No	No	PharmaceutFat		2.5471326	8.3559274	9.26429
T1	No	No	Radiation	Fat	2.0173376	6.9972166	9.1380105
T1	No	No	Radiation	Fat	1.1118341	9.2384247	9.1253753
T1	No	No	PharmaceutFat		1.530955	7.3131811	9.0756796
T3	No	No	PharmaceutFat		1.9903826	7.7577998	8.5454494
T1	No	No	PharmaceutFat		2.6859746	6.9138218	9.1946275
T1	No	No	Radiation	Fat	1.9925474	7.1565873	8.500796
T2	No	No	PharmaceutFat		2.5961461	7.1198029	8.5960668
T1	No	No	PharmaceutFat		2.9669364	8.6091519	9.1560206
T2	No	No	PharmaceutFat		2.8258817	6.8882845	8.7753907
T1	No	No	PharmaceutFat		1.62552	7.6288244	9.7655507
T1	No	No	Radiation	Fat	1.3642492	8.093886	8.8946406
T1	No	No	PharmaceutFat		2.486253	7.0295787	8.1237695
T1	No	No	Radiation	Fat	2.1894021	6.8771795	8.9817326
T1	No	No	PharmaceutNonfat		1.2271376	6.1305359	8.1741104
T2	No	No	PharmaceutFat		1.8585004	7.1307141	8.4039301
T1	Yes	No	PharmaceutFat		3.2734351	7.4739714	8.4839088
T1	No	No	PharmaceutNonfat		3.5908543	8.6212254	8.3065702

T3	No	No	Radiation	Fat	3.8278419	8.8104227	9.4933075
T1	No	No	Pharmaceut	Fat	1.6826113	7.6646237	9.5023164
T1	No	No	Radiation	Fat	2.0405598	7.1275236	8.6469327
T1	No	No	Radiation	Fat	1.8219027	6.7683637	8.0608331
T1	No	No	Pharmaceut	Fat	1.7144509	6.1766678	7.6716622
T1	Yes	No	Radiation	Fat	2.0087961	6.2720201	7.5074978
T1	No	No	Pharmaceut	Fat	1.9981393	8.7639941	9.5951359
T1	No	No	Radiation	Fat	1.0870722	6.5175147	7.7967817
T1	No	No	Pharmaceut	Fat	2.8458993	7.1780903	7.8507078
T1	No	No	Pharmaceut	Fat	2.4272761	7.9485742	8.4191357
T1	No	No	Pharmaceut	Fat	2.5321379	6.6968015	8.9687992
T1	No	No	Pharmaceut	Fat	2.8219205	7.5865556	8.6511386
T1	No	No	Radiation	Fat	3.2622031	7.8718869	9.4015143
T2	No	No	Pharmaceut	Fat	3.1247335	7.773218	8.9102644
T2	No	Yes	Pharmaceut	Fat	2.3079032	6.6608463	8.1029917
T3	No	No	Radiation	Fat	1.875862	8.0417281	8.6748277
T1	No	No	Pharmaceut	Fat	2.1299774	6.8425016	8.6353274
T1	No	No	Radiation	Fat	2.920875	7.3816879	8.8318539
T3	No	No	Radiation	Fat	2.1569984	7.1436422	8.1322228
T1	No	Yes	Radiation	Fat	2.3241212	8.4177725	8.8006034
T2	No	Yes	Radiation	Nonfat	3.4669778	8.2738465	9.3001514
T2	No	No	Pharmaceut	Nonfat	2.7995273	7.1573599	7.9221877
T2	No	No	Pharmaceut	Fat	4.7495581	7.9584692	8.2489446
T3	No	No	Pharmaceut	Fat	3.4826613	8.1379295	9.1551803
T2	No	NA	Pharmaceut	Nonfat	5.1346779	8.7409918	8.9604114
T2	No	No	Radiation	Nonfat	2.2033026	7.0641175	8.4935226
T2	No	No	Radiation	Nonfat	1.6016169	9.1519397	9.6545148
T2	No	No	Pharmaceut	Fat	2.9242191	7.6876069	8.6329856
T3	No	No	Radiation	Nonfat	5.9490572	8.2359644	9.2845518
T1	No	No	Radiation	NA	2.1416027	6.9687082	8.9007415
T1	No	No	Radiation	Nonfat	1.8297151	6.8526455	7.9247758
T3	No	Yes	Radiation	NA	3.0033876	8.8895891	8.3149745
T1	No	No	Radiation	Fat	1.4695347	7.715033	7.9311883
T1	No	No	Radiation	NA	2.9043237	8.0711498	8.5212685
T1	Yes	No	Pharmaceut	Fat	2.6679396	7.0989914	8.6616826
T3	No	No	Radiation	Fat	1.4101584	6.4112192	7.2121345
T1	No	NA	Pharmaceut	Fat	2.0963982	6.8737155	8.6693657
T2	No	No	Pharmaceut	NA	2.6345915	7.1954809	8.6845836
T1	No	No	Radiation	NA	2.6445519	7.1232738	9.2351898
T1	No	No	Radiation	NA	3.303679	7.936831	9.3135964
T2	No	No	Radiation	NA	4.7035447	8.7893517	8.7284301
T2	Yes	No	Radiation	NA	2.9362221	7.8033779	8.8163855
T1	Yes	Yes	Pharmaceut	NA	3.1146282	8.6997234	9.2982469
T1	No	No	Radiation	Fat	3.4056415	7.7706108	8.3239697
T2	No	NA	Radiation	Fat	2.5836967	6.5959892	8.6237111
T1	No	NA	Pharmaceut	Fat	2.942345	7.8571681	7.6688915
T1	No	NA	Pharmaceut	NA	2.3566035	7.9966465	9.0265792
T2	No	No	Radiation	Fat	3.3148954	9.0958242	8.7720403
T1	No	No	Radiation	Fat	3.4094394	7.8445751	9.2256107
T1	No	No	Radiation	Fat	2.5889125	7.3281984	8.2841976
T3	No	Yes	Pharmaceut	Fat	2.4986952	6.5384704	8.1097955

T2	No	Yes	Radiation	Fat	1.3148424	7.1375388	7.5156922
T1	No	No	Pharmaceut	Fat	2.6463841	7.1932763	8.2011883
T2	No	No	Pharmaceut	Fat	2.0446744	7.4292766	8.8446063
T1	No	No	Radiation	Fat	3.5229424	7.3117836	8.6318197
T2	No	No	Pharmaceut	Fat	2.7487246	7.1756972	8.443181
T3	Yes	No	Pharmaceut	Fat	3.0517188	8.252589	8.4936191
T1	No	No	Radiation	Fat	2.2555289	7.2142002	8.013227
T1	No	No	Radiation	Fat	5.5328306	8.1543003	8.4949927
T1	No	No	Pharmaceut	Fat	2.9390115	7.0517371	8.8623548
T1	No	No	Radiation	NA	2.4040887	6.9081286	7.9970082
T3	Yes	No	Radiation	Fat	3.5859879	8.3710299	9.517592
T1	No	No	Radiation	Fat	1.559774	6.5677454	7.9652431
T4	No	No	Radiation	Fat	3.0534035	7.7037918	8.3783943
T2	No	No	Pharmaceut	Fat	2.515732	8.1197927	8.5683725
T2	No	Yes	Radiation	Fat	2.6503837	7.2341127	8.951551
T1	No	No	Pharmaceut	Fat	3.7206425	7.8292976	8.9114132
T2	No	No	Radiation	Fat	2.4472893	8.564825	8.6162701
T1	No	No	Radiation	Fat	3.1593253	7.7737482	8.5894887
T1	No	No	Pharmaceut	Fat	3.0322023	7.0415511	8.4174294
T1	No	No	Radiation	Fat	3.2886727	7.5466726	8.9296028
T1	No	No	Pharmaceut	Fat	2.6680499	7.1933806	8.940963
T2	No	Yes	Pharmaceut	NA	4.8239719	7.8054053	8.8008517
T1	Yes	No	Pharmaceut	Fat	2.6196983	7.7287376	8.5390835
T1	No	No	Pharmaceut	Fat	2.5988968	7.3246897	8.2565048
T2	Yes	No	Radiation	Fat	3.4490809	7.8226643	9.1350883
T1	No	No	Radiation	Fat	2.5578558	8.935936	9.2485964
T2	Yes	No	Pharmaceut	Fat	2.1244779	7.7126295	8.8816142
T1	No	No	Pharmaceut	Fat	2.0634672	7.5479941	8.083448
T1	No	No	Pharmaceut	Fat	2.2702902	7.1023545	8.7849307
T4	No	No	Pharmaceut	Fat	2.7983344	7.4114112	8.4264546
T2	No	No	Pharmaceut	Fat	2.9503509	7.3938911	8.7306629
NA	Yes	No	Radiation	Fat	2.0112412	7.8976577	8.5365488
T2	No	No	Radiation	Fat	2.3301635	8.972222	8.7742331
T1	No	No	Pharmaceut	Fat	1.9901619	6.9388678	7.6497411
T3	No	Yes	Radiation	Fat	1.8794183	7.1941882	7.9267769
T1	No	No	Pharmaceut	Fat	2.5877693	7.5524498	8.1393562
T1	No	No	Radiation	Fat	2.7232862	7.9368026	8.7791463
T2	No	No	Pharmaceut	Fat	3.0275331	7.6135469	9.2517462
T2	No	NA	Radiation	NA	2.6746084	8.2064779	8.9354053
T1	No	No	Pharmaceut	Fat	3.1312919	7.5426729	8.2268153
T2	No	No	Radiation	Fat	1.944984	7.7506822	8.8575827
T1	No	No	Radiation	Fat	2.6675483	6.6149375	8.1939496
T2	No	Yes	Pharmaceut	Fat	3.9838806	7.974018	10.233022
T1	No	No	Radiation	Fat	4.4943414	7.2152838	8.4919018
T1	Yes	NA	Pharmaceut	Nonfat	1.7244905	7.8349653	8.7446491
T2	No	No	Radiation	Fat	3.1207826	8.4604161	8.1388439
T2	No	No	Radiation	Nonfat	3.2652564	7.4958212	8.0177942
T3	No	Yes	Radiation	Fat	3.1368015	7.8245263	8.8209842
T1	No	No	Pharmaceut	Nonfat	3.4508154	8.1992878	9.7587473
T1	No	No	Pharmaceut	Fat	3.1605361	7.3669271	8.7434371
T2	No	Yes	Radiation	Fat	3.6138882	8.3824897	8.367474

NA	Yes	No	Pharmaceut	Nonfat	2.6967626	6.1783923	7.3812689
T1	No	No	Pharmaceut	Fat	3.6407037	7.6457224	9.2095788
T3	No	No	Radiation	Fat	3.7348542	8.9909427	8.9879561
T2	No	No	Radiation	Fat	2.6442044	7.1445119	8.263863
T1	No	Yes	Radiation	Fat	3.7638894	8.66163	8.9152671
T3	No	Yes	Pharmaceut	Fat	3.1069872	7.1503087	9.0591112
T1	Yes	No	Pharmaceut	Fat	2.3723622	7.9059185	7.8390228
T3	No	No	Pharmaceut	Fat	2.6957726	8.7153143	8.891761
T3	No	No	Radiation	Nonfat	2.2013745	8.3917124	8.672818
T1	No	No	Pharmaceut	Fat	1.7353432	6.5729243	8.6707556
T1	Yes	No	Pharmaceut	Fat	3.0401068	7.5991823	8.3148673
T1	No	No	Radiation	Nonfat	3.2094424	7.7804377	8.5352745
T1	No	No	Pharmaceut	Fat	3.1240529	7.0147418	8.3228409
T1	Yes	No	Radiation	Fat	3.4042191	6.7568364	8.6344414
T1	No	No	Radiation	Fat	2.0505005	7.5090541	8.3562382
T2	No	No	Pharmaceut	NA	2.8384277	7.569872	8.7007319
T2	No	No	Radiation	NA	4.4369324	7.7016006	8.858204
T2	No	No	Pharmaceut	Fat	1.9083111	6.7544697	7.9620947

OGT	PIGC	PLOD3	B3GAT3	B3GNT3	B3GNT5	RPN2	B4GALNT1
7.5096169	7.4094009	10.081509	8.4081267	7.17353	3.7295314	10.183334	4.3055143
7.8435518	7.7611868	10.723022	8.1194851	7.4023653	4.9367983	11.049203	5.4134502
7.0997096	7.4178208	9.5384986	6.3825832	5.242286	4.6955604	10.72037	2.8112221
7.6758094	7.4648919	10.721653	7.3973234	7.1403892	4.1218098	10.197543	6.1156206
7.4997461	7.2477359	9.142387	7.4061187	4.4352398	3.931899	10.082802	2.1604164
6.7790553	8.033923	9.669478	7.8176663	5.9871154	7.5093051	10.704826	3.5368467
5.2980128	6.2309536	9.4688685	7.5772612	5.0786261	3.1542859	9.3140374	3.9361497
9.7137403	8.5397513	11.020574	7.1374259	8.2961323	5.3003562	10.44268	2.7371259
6.4085035	6.7469145	9.2475484	7.1216632	7.356737	3.6558188	10.09843	2.5814238
6.5960675	7.0449299	9.8617454	8.0251854	3.4112311	3.8054152	9.3472966	3.5114799
7.1738695	7.8878127	10.512171	7.1694018	7.7448449	4.5888921	10.501227	2.4916426
7.1190045	7.0538869	9.4999107	6.7953974	7.1484954	5.4331872	10.228441	4.5613932
6.7613883	7.9313545	9.2814657	6.320859	3.3827507	3.6835868	10.019253	2.2930343
6.5674969	7.5206719	10.343206	6.9592764	5.5689087	5.4772019	10.929722	2.2083594
6.4136632	6.8652641	10.521386	7.414096	5.1584159	3.4573741	10.18052	5.2422913
6.1742275	7.6068969	10.306695	7.0896543	3.3672103	3.1644453	9.9904703	5.4325168
7.0282514	7.735558	9.0368122	7.2428906	4.1769655	5.6735702	10.033679	5.4010233
8.0168103	7.7597311	10.401114	6.7401877	5.4523907	3.5575203	10.821876	2.7602417
6.3120516	8.1379901	10.170394	7.0505741	8.7553034	5.7558276	10.464138	2.7318353
6.9416014	7.3552224	9.1371824	6.4352266	6.1942967	4.5100178	9.4219368	3.2123689
6.4052831	6.9856843	9.9585271	6.3415208	5.6856655	4.5825715	10.234792	3.7764126
6.1201055	5.8126711	8.2120435	5.830761	4.4062375	3.9906734	9.2762127	2.1805196
5.7255859	5.8656954	8.16943	5.8714269	4.4845206	5.4628061	9.4780661	2.1044554
5.7079976	6.6782015	9.6159186	6.6044291	6.6686771	3.7787282	9.6175457	2.1099713
6.5389266	7.6335904	8.90666	6.288129	3.5929262	4.6414424	9.87711	2.3327982
5.7311449	5.9785644	8.2387065	5.9984968	6.6766156	3.9436599	9.3552429	2.2015248
5.8017361	6.2388847	8.5041898	6.3375154	5.6086988	4.7000155	9.1282523	2.323227
6.0165402	6.3139258	7.9553294	5.6139452	4.9117069	3.8864938	9.0473405	2.1613812
6.7609639	8.008421	9.6299449	7.4804241	4.1920506	5.7375632	10.305588	3.5744483
7.0031679	8.0789887	9.6705511	7.9886282	3.5736497	6.7383871	10.498544	2.3463326
5.6089014	6.5887719	8.8987007	6.7860477	5.1087065	4.9602595	9.2672718	2.5117543
7.961497	7.632165	9.5334158	7.4734115	7.6888162	6.855074	9.9792586	2.4227968
7.0842425	9.5108668	9.7067841	7.5477676	7.5726317	4.8001045	10.96577	5.2414001
6.666209	7.3416823	10.738726	7.3312342	5.5129402	5.7681612	10.451359	2.5433985
6.0191316	8.0039437	10.537145	7.9601129	7.427671	4.9940961	10.031271	2.5458391
4.8718637	6.7505936	8.9735524	7.7700412	8.9798313	3.5005968	10.934241	3.0161531
5.8188781	6.8742838	9.0550616	6.4013662	3.3675843	3.3096535	9.6886075	2.4404628
5.8779186	6.7007739	9.0338275	7.3277692	4.5892896	5.8599798	10.297824	2.1543359
8.3616774	7.3388384	10.495771	7.4853307	6.6974712	6.5058358	10.677035	3.0462196
6.5174923	7.1295589	9.5095779	6.5020028	7.3740618	4.8714953	9.9881885	4.2881125
6.5037389	7.7466278	8.7348673	7.3889899	6.9950098	4.8757842	10.702644	4.1142382
7.826523	9.5054511	9.9245785	6.0568718	8.3064877	3.9322415	10.231649	2.1658859
8.2138963	9.202871	11.138912	7.2751704	11.840873	6.0949276	10.877315	5.2813774
8.1135997	9.3371954	10.019826	6.4578955	9.3514058	6.9882714	11.497129	2.6775642
5.922036	7.9718923	10.40034	7.7482665	4.820134	4.0757824	10.312528	2.9107764
7.9335768	8.3764503	11.662977	6.2064494	9.5436163	4.5140848	10.941794	4.4648819
5.5834242	6.9900134	8.0475045	6.8614345	4.3531641	3.3591668	10.56714	2.1648494
7.8796797	8.9956051	10.522646	7.9056728	6.7360382	5.7868844	10.919786	2.9005958
7.8774864	9.1492543	11.132838	7.2187219	6.5059947	5.436805	10.498269	5.0314845
6.899443	7.6775557	10.313834	7.4914188	8.3273068	5.8463323	10.403345	4.8935353

5.9998634	7.7717489	10.486805	7.2624434	4.3889136	3.7006671	10.855346	2.801202
6.9705027	8.272269	10.703247	8.4982999	8.570193	4.5854335	10.505504	4.4589974
7.7818423	7.6562209	9.8686981	7.2596268	5.9218798	4.3369757	10.734453	2.8287595
6.6922013	7.3650053	9.6296184	8.0560564	6.0818108	4.6299216	9.9168308	2.7502655
6.4222938	7.8322301	9.522357	7.7781523	5.640021	3.2875684	11.677197	2.7446754
7.8152435	7.931573	11.838623	7.7151222	3.5771385	7.2853751	10.754904	6.944344
7.0958375	8.1267567	9.3465986	6.6323377	4.2942791	4.0439108	10.395314	3.8855859
5.9321472	7.6822428	10.446056	7.3056509	3.6244589	3.2331303	10.811747	2.1793856
7.2200597	9.0520278	10.56943	7.5374299	6.2519205	6.9494437	10.921737	2.6124326
7.0725981	8.4532151	11.148588	6.9969675	7.1967091	5.2829117	10.852942	3.9271252
7.4119275	7.1924348	9.9019911	7.0883831	3.6801569	3.5759393	9.5970494	2.4144532
7.669793	7.4659339	10.553647	7.94075	3.3434909	4.1786274	9.9394621	2.6416127
6.3138261	6.6601568	9.0172505	6.5361816	5.4240333	4.8899575	10.039475	2.1821743
5.9701895	6.7533584	9.2613679	8.6266564	5.227834	3.7268284	10.627186	3.88546
5.8959182	6.3156259	8.3673465	5.6014735	4.4636017	3.2840643	9.2803187	2.2282928
6.1005994	7.9724992	10.700141	7.1778169	6.7535418	5.192298	11.548026	2.6962193
6.4776929	6.62342	10.574656	7.3698399	3.6557008	4.4499597	10.559415	2.7359836
5.8526481	6.1298878	9.227084	5.9844632	6.487426	3.5516844	9.4122107	2.2055089
6.8747884	7.9114839	9.8878698	7.2885335	3.3641376	3.9971763	10.983496	2.5437044
6.0939491	7.1445921	11.03406	6.7577964	7.2531479	6.5940747	10.241901	4.7664842
8.2938532	8.5117298	9.9590324	6.5468171	5.4813419	4.2467851	10.170368	4.3173775
6.8379243	7.2863769	10.046924	7.0620456	6.5455398	3.7088204	10.255875	6.8963811
7.4453721	7.8779961	10.752132	7.1416086	11.780438	5.992895	10.546054	2.5589601
8.2041776	8.8012932	10.781186	6.6579655	3.8294378	4.2811669	10.316067	5.1750699
5.9002463	7.993547	10.242383	7.3986936	7.0459427	3.8241437	10.670204	4.4723581
6.8593642	9.2432022	10.944017	6.1016048	6.3765569	6.3707472	10.880423	2.2478108
5.5340338	6.0695539	8.5805422	5.8409711	4.6922316	3.5534783	9.5384665	2.2154876
6.8600268	8.3704156	10.149408	7.1068003	5.3717589	3.4812583	11.071785	2.1629205
5.6573209	7.9418916	10.384894	7.6920812	6.6193471	4.5083166	10.75795	2.7049536
7.8991565	8.4112733	8.9954169	8.1258141	5.8158029	5.2133889	11.194847	4.8800016
5.9327978	7.4741852	9.1838279	7.3366751	3.3911052	3.2652499	10.027778	4.1905971
5.788887	6.3917903	9.4751958	6.9733652	8.4316481	3.2540566	9.852307	4.0481636
6.1243006	6.927397	9.8429156	8.896595	7.6573057	4.9958111	9.8443804	5.0918861
5.6452039	6.1712543	8.573948	6.3745344	3.9880125	3.0767042	8.8115765	2.2335439
5.9608639	7.0307778	10.230941	8.2904944	6.5767833	4.2969204	10.057714	4.7809102
5.4754269	6.1546909	7.6810448	5.6117333	4.3374851	3.0360888	8.6779051	2.1157122
5.9320712	7.1879618	9.5966196	5.7506446	4.4181033	4.5572424	10.731031	3.6173002
5.0027005	7.3514781	10.07927	7.7323611	5.5778623	3.0881682	10.40909	3.4790279
6.0016553	7.6032488	9.8616417	6.5215459	6.1564079	3.626461	9.999937	2.2457228
5.8472523	8.3212812	10.306469	7.6208632	9.1522525	4.5839388	10.73485	2.6033969
7.5590559	7.4728495	10.309497	7.1255505	7.7274189	5.1863137	10.360847	3.6613292
7.8333419	8.1746786	9.0286789	6.3362913	5.0226333	5.1034316	10.396284	2.622307
7.7943268	8.6090642	10.958905	7.2980153	8.5269654	4.3649612	10.334759	4.9120293
8.2465656	9.2895117	11.334474	7.6000762	8.0618774	8.1727149	11.416798	5.2170271
6.9346716	6.8334174	9.4484624	6.4654217	3.4525142	3.3456853	9.9137967	3.7769266
8.0338875	8.1401995	10.98216	7.9317206	6.7263543	5.9284877	10.857349	5.6699087
6.5311622	6.1570574	8.8796526	6.1098819	7.6465182	3.8121642	9.430081	3.2622653
7.1157584	9.5246567	10.822208	6.4591009	5.8620607	4.5363125	10.958349	2.2255313
7.5420028	7.7200987	10.546382	7.3026293	5.5039989	3.6584952	10.818738	5.2202152
6.2453386	6.5932644	8.974462	6.463455	3.981651	3.4368075	9.7253103	2.2121065
6.8712695	7.728596	10.511939	7.9385996	7.6936288	6.322014	10.589853	4.315055

7.9299161	7.3754369	10.0663	7.6043584	8.9719526	4.1507098	10.807384	5.4508626
7.8530641	7.8673251	10.202373	6.9911039	7.7429772	4.67376	9.7679125	2.9491643
7.9111892	6.8612522	10.93054	7.3029617	4.411453	5.5126576	10.357754	2.4483473
7.596109	6.6882802	9.2960065	7.140107	3.7445114	3.6184919	9.5426214	3.0958735
6.3640045	8.0454408	9.8006701	7.1816279	4.1086907	5.0996895	10.16844	2.2306108
6.6306048	6.2721418	9.9277136	6.3246554	5.6871888	3.2810144	10.49974	3.3037477
6.9017364	7.8860245	10.390496	7.1760544	4.2117011	3.8393206	10.566771	4.2148054
6.2914562	7.1178865	9.942491	7.261984	3.4130731	5.3109204	10.444909	2.381633
5.5128986	7.0424607	10.041359	7.4640372	6.9109556	3.3967908	10.159799	3.7742061
7.7155793	8.9377972	9.5652856	7.2592121	4.5241409	3.9609157	10.476559	4.6650457
5.4799401	6.7085961	9.3630004	6.290053	6.043318	3.330177	9.3221547	3.613756
7.2443791	7.7411421	10.931316	7.0327351	5.3617769	4.0049092	10.341465	2.3479932
7.6032202	7.7116208	8.6865045	6.9174184	5.2796747	3.6635695	10.779019	3.3193184
6.8287266	7.4322673	9.1553541	6.1665189	5.3854282	3.1824861	10.313711	2.5274072
6.4137006	6.7958885	10.012198	7.2448756	5.8306652	4.3498587	9.397514	3.3241335
7.2398065	7.5797067	10.603836	7.2148846	5.619824	4.727795	9.87308	2.4611248
5.9100133	7.8514624	9.3483601	7.5953074	6.1921741	3.1376039	9.8091546	4.1971781
6.9129566	7.4682653	9.7472142	6.6347693	4.0004899	3.8708849	10.340194	3.5278195
7.3271533	8.8700518	10.336838	7.2179252	7.5745656	6.4444647	10.032038	2.3181375
5.7569321	7.3457537	8.2691366	6.7446346	5.708947	4.5207625	10.53101	3.3953683
7.2750475	7.9066651	10.391423	7.7466574	5.3197793	5.4530804	10.750576	6.637264
6.4822746	7.666011	10.380223	6.8098497	8.2930761	4.4635697	10.01952	6.4047994
7.0482209	7.7967026	10.569496	8.1534278	8.4840164	4.3103357	10.308994	2.7744587
7.0897906	7.2904113	10.205616	6.5506949	3.4562379	4.6710183	10.778363	2.0932375
7.0824682	8.3028319	10.253491	7.7754522	7.5988931	3.9088441	10.088616	5.0448709
5.9867939	7.5748761	10.66906	7.2130838	6.7818377	4.0277021	10.235258	4.1214433
7.7998577	7.8056394	9.7339024	7.6086558	3.5994388	4.1683071	11.216623	3.0897895
6.6462052	8.442436	10.559893	6.5441067	3.5369596	3.3982585	10.820289	2.5550892
7.0688726	6.8216521	9.9910127	7.2996958	7.3878299	3.556178	10.33879	4.0582016
6.4484728	7.4404364	10.321526	6.6256074	7.3657464	6.5165455	10.168694	2.3905121
7.0681847	9.1423417	10.891276	7.6334053	9.1653766	6.2230611	10.493276	6.1764002
7.2296876	7.3647054	9.4633467	6.8033731	4.0718097	4.2562713	10.101274	3.3562008
8.1075555	7.8381837	9.1376868	6.1341522	6.8905383	6.2750655	9.5760424	2.4375213
7.1213781	8.3654985	10.563963	6.7009889	3.6014607	5.2772259	11.115965	2.4788668
8.1043724	8.316562	9.9134808	7.806248	6.251149	4.7856574	10.50452	6.4010939
6.9817369	8.1440312	10.659626	7.2993517	3.6066336	3.4181123	11.347305	2.7981674
5.9099359	7.6979576	8.9445772	8.0526157	3.3699152	3.2562169	9.7380872	3.6091487
6.9529325	7.5412688	10.234894	6.9773239	3.5486767	3.2909071	9.9777906	2.1424959
6.6140252	9.0137738	10.997173	6.9189538	5.5759226	3.7414555	10.536835	2.240761
5.4338793	7.6389722	9.2960457	6.2254795	5.9772073	4.3882707	9.7047797	2.3413888
6.3905284	7.879658	9.7523553	7.5009852	7.1655244	5.0523752	10.328375	2.7052327
7.8828614	7.7636857	10.337909	7.9427693	4.9866449	6.7061445	10.360521	2.5077283
5.3838297	7.2704707	10.483372	7.0839806	5.079143	3.8851817	9.6078464	2.7219795
4.882697	6.3912169	9.1206485	8.0400764	7.141908	2.9714877	10.112652	3.8445442
5.4231804	7.3799342	8.772703	6.7182157	4.6664807	3.1145024	10.262175	2.5141385
6.9082267	7.6030487	9.5199718	7.0030767	4.5640815	5.1893754	10.291021	2.1551069
6.404727	7.4204094	9.21763	7.373967	3.5210073	4.3332454	9.979114	3.4620248
4.6894038	6.2137063	8.8765992	6.6750948	4.4131505	3.0286915	9.3534032	3.874312
6.514739	7.5732093	9.5088133	6.768807	5.8383308	4.7012249	9.6655179	3.4645748
7.0687901	7.6834978	10.329929	7.5160971	5.4614959	3.5309898	10.301275	3.5521825
7.5916068	9.3349796	10.860559	6.9757286	8.3838843	6.5573857	11.032798	2.633096

5.9781964	9.158359	10.245887	8.021401	10.00487	4.3394231	11.283198	2.8291995
6.8115128	8.4590328	9.5822661	6.3582905	7.2720981	3.6449234	9.665946	2.1403617
6.8158632	7.0161484	9.3123744	7.108142	3.5411592	4.4339992	10.200281	2.1977454
5.3984873	6.6910592	8.7370538	6.7461259	3.4358352	3.4952052	9.7166501	2.6950121
5.5203287	6.9922318	8.4839763	6.4283972	3.3434909	3.3309244	9.4081473	2.1270583
5.8935826	6.3339468	8.2593391	5.8410458	5.4655911	3.04271	9.2825709	2.3135719
6.9129932	8.5261671	10.628408	6.7439482	4.0863922	3.3125976	10.232032	3.2948016
6.4094275	7.189782	8.8688107	6.1471958	3.3773251	3.3387208	8.7264881	2.1520237
8.6254344	8.4064478	9.3437916	8.0651378	9.6456066	4.9680727	9.1582838	2.1457248
7.3413668	7.4330656	9.7521983	7.2851333	4.0379953	4.0811197	9.9574865	3.8120596
5.6558491	6.0909857	10.45039	7.1854824	4.7117168	3.3239149	9.9823262	6.2769102
7.2092305	8.7040509	10.750425	6.9307119	7.5890167	3.315966	10.287054	2.3609555
7.5719804	8.8596187	9.8918798	6.6353248	8.8197941	4.8572286	10.099486	2.4128109
6.8342409	8.3234621	10.902911	7.5759438	6.5914356	6.945737	10.584579	6.0675815
6.50166	6.5619605	10.080494	6.4835973	5.2723198	3.6680229	9.1620182	2.505941
7.8371492	7.8315717	9.8415562	6.7633157	3.4421488	3.4805374	9.7916389	2.8128917
7.4504686	8.4434398	9.3193635	5.9075871	7.100857	3.3161952	10.431131	2.1713889
7.3425008	7.8900712	10.178039	7.0030038	8.2434089	7.2552188	10.246793	3.3179681
7.2419599	7.2767711	8.9876809	5.829946	5.4542573	3.4343971	9.6663823	2.2887532
7.3161759	7.9067116	10.058702	6.7434756	4.0945492	3.726931	9.3508837	2.6475337
7.347475	7.1996436	11.460144	7.6518253	9.09754	7.1575644	10.548452	3.8772946
6.9674	6.8563496	9.3958078	6.1265388	6.3153772	4.2011241	10.00277	2.9583246
8.7454125	9.0652895	9.984647	7.1149166	5.8224651	5.2767963	10.737895	3.4454239
7.4227525	8.4255991	10.5073	7.2688091	9.1885249	4.9634916	10.674731	2.9735577
7.5235132	7.8425903	10.433107	8.3004909	10.015034	6.1097641	11.071985	4.1753783
6.8721994	9.0123173	10.67145	5.3833767	6.4356389	4.1470842	9.2446355	2.1629828
6.21876	8.5383477	9.8089489	7.0499044	6.7730947	3.5569609	10.546176	3.1687166
7.6765305	7.7279886	10.18659	7.2499455	5.7671958	4.9478492	10.280399	4.8303345
8.4017649	8.7300161	10.448717	6.784696	9.7472251	5.1989543	10.665612	4.0614315
6.863743	7.5427987	9.9039056	6.4304582	3.3434909	3.6864185	10.016209	2.8942171
7.0452256	8.162467	10.187847	7.2655468	3.4079408	3.5991164	9.8154002	2.3091038
6.731321	7.5937334	11.003755	7.4443406	7.5009208	5.3078016	9.8811912	4.7915936
5.9479887	7.9082177	8.7870096	6.4439027	3.7567247	4.0072557	9.6988785	2.0958346
7.7791598	7.7616978	10.118169	7.1519058	6.231143	3.3622879	9.9850494	3.7774627
6.0879124	7.718232	10.892844	7.4945259	5.9492228	3.6766641	10.324561	4.4245488
5.2471994	5.8498899	8.2941371	5.7054196	4.8234488	3.2020265	9.3321699	2.1325462
4.5943831	6.8993825	9.3655277	7.7446544	4.1273525	4.3608043	9.9088804	4.6778061
7.6056309	7.5733006	9.8597295	6.7555124	6.438877	5.5432629	10.837176	2.2284324
6.3014419	7.3924089	10.014223	6.8685445	6.260817	3.2152931	10.142336	2.8987853
6.1831012	8.5898783	10.58283	8.2422144	7.608992	6.1156071	10.61281	2.3301
7.6861624	8.111364	11.333276	7.9130275	9.9635449	5.5249839	10.950351	2.5331772
7.013168	7.5418022	10.68695	7.5508962	7.8316387	3.4661873	10.667823	6.650809
7.0217224	7.7490386	11.527424	7.7284001	3.3434909	6.5004617	10.73881	2.3157997
6.3217114	7.6449561	9.4624762	7.0413213	7.5830631	5.8436208	10.829055	3.7285314
5.6006235	7.1195101	9.9110521	7.8894075	6.0784871	3.1693528	9.5236709	2.721475
7.0778591	7.5811236	9.3196863	7.4433809	6.3451773	6.757188	10.871629	2.3071811
7.6586025	7.3891011	9.4884744	7.7967999	3.6912333	5.1971352	11.40066	2.1852122
5.8581818	8.2337925	10.816632	7.025906	9.1054893	5.7574868	10.710654	2.974892
7.5509041	9.1861885	10.533454	6.6586936	8.2434024	6.2025831	10.957572	2.3100381
5.9339222	7.0597709	9.7959276	6.4350447	5.6025584	3.7905905	9.9655833	3.1744602
6.6077851	7.0572666	10.187843	7.3664943	5.0534222	5.9332514	9.7943833	2.6635661

5.132919	6.9783673	9.3885388	6.2391066	3.3434909	3.4827936	8.82851	2.0873843
6.7492431	7.6138915	9.4469223	6.540912	3.4211044	3.5579299	10.161353	3.3000439
5.653942	7.9256395	9.9252981	7.0100705	4.4089667	3.9979733	10.500231	2.1847454
7.4945079	7.7370593	10.927067	6.9530135	9.1184063	4.1885464	10.962036	2.4350432
6.4333076	7.6702268	10.18085	7.0885655	5.8862244	6.1858645	10.225941	4.9777046
6.9158669	7.8468125	10.273293	6.3844586	3.3722231	3.2024095	9.7655631	3.1595436
5.8521625	6.9489109	10.376916	6.9835272	3.4081754	3.072857	9.6501302	2.07352
8.3237977	9.2881408	11.220859	7.4753728	9.5080382	3.4822986	11.196188	4.955809
5.9354995	6.4634037	9.7840557	7.5630469	6.2093487	5.8216552	9.9296463	6.0868918
6.2481044	6.2272049	9.1457718	6.276837	4.5481091	4.0270337	10.209927	2.173315
8.0130455	8.8078592	10.66691	7.8456967	6.6633049	5.5271438	11.10426	7.3048302
5.7534382	6.6786482	9.2297825	7.3453117	4.0015537	4.8393794	8.7363642	2.2163292
8.038541	7.4637492	10.498535	6.6098573	4.0079967	5.4302418	10.574868	4.9605825
7.7680298	7.5099629	9.5647419	6.6357256	3.5688687	4.0821069	10.306731	3.1278461
6.5088192	7.9572964	10.914939	7.7861864	4.6221565	3.2856832	9.9083513	2.7575458
7.1655065	7.5532559	10.303905	7.4565889	7.1621937	6.398255	10.283256	4.3129996
6.2775229	7.911892	9.8899913	7.8280643	6.8869753	6.7000447	10.83573	6.7844306
7.5897414	7.2415724	10.867419	7.4684811	4.9939668	4.7945329	10.421103	3.0738895
7.4485097	7.6178494	9.8062638	7.2808504	5.3652796	5.2051324	10.135846	5.0929891
8.1492952	7.237343	9.8240527	7.5251337	6.6463214	4.4083336	10.415978	5.0699411
5.5081808	7.1533556	9.9148003	7.6359327	6.3434247	3.1723911	9.8415785	3.1204735
8.2898901	7.9957113	10.6361	8.1732272	9.6288995	5.959694	10.794174	4.6157847
7.1685337	7.907703	9.5495159	6.230286	6.6271569	3.6948265	10.147316	3.7578349
6.4562076	7.4088373	10.660629	6.6438968	5.330073	3.3909358	9.568318	2.2400505
5.9471207	7.6451288	10.428494	7.2382493	6.9271608	3.1604589	10.144249	4.4811701
6.6723212	7.0732251	10.151619	9.0223073	7.2041365	4.1840798	10.390894	3.7395694
6.9762963	7.4532634	10.355125	8.2256087	8.5831173	5.4817014	10.991456	2.3133922
7.272252	7.8587554	10.633768	7.363741	4.3280001	5.1347564	10.401326	2.3206761
6.5432787	7.7045246	9.5011708	7.3285585	8.1836451	3.6897333	9.5865759	4.0549256
6.3465917	7.5323846	10.432301	7.3015258	4.8505336	3.5613598	9.9144329	3.9494381
6.1839042	7.2947414	10.485252	6.8579973	3.7420424	3.7032117	10.192703	3.2910671
5.7639398	7.7018155	9.5004192	6.77615	3.3725096	3.4422821	9.7829601	2.9735111
6.6881611	7.3306988	10.312123	7.9312652	3.6870334	6.580674	10.378973	2.4323979
6.1072065	6.5399574	8.5660976	6.0522574	3.496789	3.4644295	9.8127443	2.2277422
6.5871758	6.7073701	9.5538701	6.6745872	6.130331	3.9437715	10.130985	2.1875565
7.4976415	6.9054694	9.5906615	7.177042	5.8890238	4.7297008	9.9397105	3.0371939
5.6692746	7.2538427	9.9161062	7.1203998	7.1202442	3.7757611	9.9595084	4.991712
7.6563104	8.3357578	8.9426232	7.2824437	3.9505237	8.2195344	11.167391	2.5042589
7.1481399	7.3770559	11.317103	7.2987189	9.118197	6.291943	10.348638	3.3079363
6.3081492	7.7361413	9.6530607	7.4463554	8.7321451	4.4605798	9.9660829	3.3291117
6.1969579	7.2976251	9.8867506	8.1090822	3.9739337	4.9313552	9.7694801	3.3862209
6.5177286	6.7875393	10.24435	7.6148858	5.748099	4.309786	9.5614167	2.1538467
7.5197788	8.7979911	10.457136	8.1980966	9.1869961	4.3354957	9.5425993	2.608239
6.1117998	6.796625	10.421196	7.7341953	7.500854	3.032727	9.8968737	3.8693854
6.3613387	7.1907386	10.554264	7.2960814	6.2439125	5.7162106	10.197284	3.0135298
7.1853148	8.4925776	10.535079	6.3485738	7.9735723	5.9194427	10.648356	4.7234142
7.2776141	8.3825273	11.109498	6.7775331	6.2836143	6.4687128	10.657744	2.2157411
8.1256816	7.85712	9.5335558	6.0846585	5.5059115	3.5620561	10.010055	3.9167973
6.3974428	7.7977256	10.998198	7.6086599	6.3411315	4.3397434	10.322727	4.9122675
7.3443541	7.9395312	9.7106633	6.1720481	7.907741	3.2487389	11.5375	2.3099053
7.069263	8.0806541	10.889396	7.3710844	7.4187789	5.0330646	10.802186	6.0903876

6.8223099	6.7878902	8.9579209	6.0349696	5.7569848	4.1090601	9.427799	2.4884796
7.2562325	7.8456885	10.63944	8.3231607	7.6307839	7.1355526	10.134708	4.9796022
8.482847	8.1206675	11.302068	8.1666901	9.249817	5.1742656	9.8390152	3.1557553
6.2913486	6.979661	10.390018	7.2254232	6.6766124	5.4475033	9.8029459	6.4143936
7.0864254	8.8144536	9.6468763	8.1330588	7.8326868	6.6605363	10.2466	5.4259614
7.3198305	7.2610551	9.3215089	7.1742755	6.6676272	4.1800158	9.3346906	2.9001343
7.5662485	8.7000985	10.399738	6.0642907	7.2771529	4.2179101	10.904835	2.3274631
6.7268944	7.8430301	10.655875	7.6306214	5.2885401	7.2673871	10.653105	5.2087117
7.3840103	7.488405	10.556027	6.5758399	7.4847694	6.0897305	9.7898595	2.1425303
7.7915001	7.772304	9.6943651	6.2111135	3.3434909	3.6768817	9.7767249	2.376377
7.1204315	7.6734652	9.8689755	6.8264452	6.2257155	6.5734788	10.486437	2.9812102
6.5469819	7.9795208	10.013527	7.1505497	5.8198611	3.8138897	10.539894	5.6902157
6.6059157	6.880904	9.7570563	6.8264751	5.6989683	4.5779878	10.004443	3.3529634
8.0978123	7.8730537	11.225648	6.9157918	7.0845742	6.6791082	10.516669	5.3295299
7.4120515	8.1860801	9.402763	6.8467056	3.4279888	3.4063461	9.5491798	4.1139825
6.7665144	7.2168824	10.290492	7.7126249	4.5310954	5.2536358	10.283933	5.7239261
7.3281689	8.0786878	9.091579	7.1067902	3.6059912	4.5352296	10.307284	4.3907108
6.919977	7.3334775	9.5945776	6.4723025	4.1812762	4.3058987	9.6895115	2.3684507

GLA	ST3GAL6	B4GALT3	B4GALT7	ST6GALNAC4	GYS2	STT3A	DDOST
8.8714327	3.316717	8.7034021	7.3712482	6.3861924	1.7369525	8.8162659	10.471106
9.2764926	6.5659163	7.8170292	7.93216	5.3949328	5.6920493	8.9837983	11.42548
8.9494012	5.9117266	7.3224872	7.1988966	5.2676725	4.5782173	9.5863059	10.981679
9.3591084	5.8026787	7.8370217	7.208229	6.7750752	5.5544617	8.6019046	10.510858
7.6905742	7.16118	7.5293575	7.3367378	4.5177915	7.985331	9.1640853	10.431496
9.4610646	5.3466087	7.7178753	7.0698499	6.2124518	3.7662996	9.5397308	11.102363
7.6018853	5.4653382	6.8441007	7.4888146	5.2163342	4.5309338	7.4127954	9.2735956
9.3955848	5.3750769	8.804238	7.6113438	6.5217853	5.226146	8.1925342	10.515913
8.6556741	4.3756059	7.5298259	8.0607655	5.7802475	1.6760079	8.453477	9.9724898
8.58221	6.4491866	7.4308052	7.4950686	4.4061769	6.7514115	8.1530837	9.7984669
7.712438	5.0810124	7.8659984	7.0929181	5.2936855	5.0032033	8.9918922	9.7985555
8.8458361	6.744543	8.0507647	8.2202246	5.3964981	3.838449	9.0603939	9.7063242
7.6868542	4.9161161	8.0333228	7.1129622	4.9961253	2.8775607	8.9901029	9.5523616
8.7511287	3.4609799	7.6383976	7.9670681	5.0626871	2.6751657	9.3296224	10.626192
8.25005	6.3298512	8.4152543	7.3521426	5.6311051	6.8121438	8.7757809	10.389837
7.7944667	6.4938477	8.0593524	7.4594447	4.8416119	6.8160005	8.8778371	10.400812
7.7434378	7.0899646	7.3674562	7.6293879	5.6324975	7.0737079	9.7494895	11.160755
8.6597204	6.3270264	8.867952	7.3358258	5.3770007	1.8081954	9.4596396	10.975435
9.4059939	5.3248231	7.987251	7.3883016	7.8600764	2.3384803	8.6042899	11.651322
8.8864074	6.0386524	7.0340775	6.5035826	7.1547509	5.4692716	7.9260778	10.320962
7.8264351	6.8617431	7.1083456	6.8154094	5.7527136	7.0805089	8.6590708	10.387893
6.9743012	7.3701366	6.4019541	5.9053551	3.5500026	7.7626205	7.9183105	9.4134571
6.803033	6.6665716	6.3520233	5.6182433	4.04364	7.3016632	7.5824613	9.0787502
7.2617785	6.665028	7.1900082	7.0539753	5.5261801	5.9363689	7.6660614	9.6600026
7.3168959	6.6984605	8.1099998	7.0062927	4.9119105	7.0498031	8.6977865	9.1740212
8.7300744	7.5615022	6.6203796	5.8489007	3.8128775	7.4785182	7.6544021	9.1851733
7.1786626	6.7848049	6.5998901	5.8998543	5.3778471	7.4261454	7.0163288	8.9020915
6.8802458	7.3269989	6.1740577	5.1826798	3.6958679	8.2052483	7.4472426	8.9298262
8.0708499	5.3112707	8.0427884	7.3053285	4.6180228	4.9788851	8.8243237	11.268709
7.9289204	6.5765246	7.6242875	7.2507559	4.9232809	5.768577	9.0185865	11.085658
7.6561087	6.6754754	7.1846892	6.2626215	5.4246104	6.787907	7.2856052	9.5513481
8.4356973	7.0090089	8.8598907	7.6766666	6.5113335	5.7715541	8.8392247	10.405256
8.7377699	6.2808881	8.6975102	7.3845191	5.0435038	2.0788905	9.3430325	11.345301
8.4240616	6.8058348	7.0720291	7.4347714	4.7350148	6.3571405	8.1341976	10.643144
8.1822271	6.1038993	8.0282299	7.1413707	4.4676493	3.4155007	8.6741003	10.205581
7.629034	5.2898531	8.1453933	7.9138415	5.6744576	2.6144666	8.1675995	10.587302
8.6702971	7.472098	7.766525	6.5498393	4.5209444	5.1624952	8.4236897	10.213639
7.5275096	6.7252709	6.6549594	7.2824167	4.4774456	5.8068842	8.0997234	10.490859
9.0882026	5.9079557	7.7742891	7.2566534	6.340633	5.2412483	9.006603	10.754181
8.5861127	6.612772	7.7120741	6.9025835	6.7099305	5.8074579	8.3687532	9.64168
8.696557	5.7445474	6.9598675	7.5388837	5.7530429	4.2599434	8.8784192	11.00246
8.3540015	5.087858	8.7681598	7.00836	5.7634512	1.8869195	9.8216175	10.418279
9.1101751	4.8132416	8.4468565	8.1472477	7.8261188	1.6140722	9.8961302	11.100538
8.6012567	6.3245944	8.7628597	7.4521135	6.9234751	3.5963616	10.205045	11.035349
9.5295436	6.0634234	8.4662212	7.1984158	6.4459556	3.959192	8.9879131	10.992052
10.026804	5.0929881	8.1448825	6.1228557	7.2418265	2.5915179	9.4672704	11.409363
7.8901935	3.1492094	6.8860164	7.5297807	5.2889913	1.9483916	8.5929383	8.8364802
10.055586	6.3462974	9.2320131	8.0493542	6.9858641	1.6862352	9.3814728	11.266583
8.8286684	4.7374336	9.6960456	7.8872833	6.6653892	3.6953689	9.6614622	10.826504
8.9730888	5.7973435	8.4905612	7.7977304	6.9137241	3.5370705	8.5301887	11.045961

10.056838	6.4239137	8.3739848	6.9369353	4.9021306	3.3136299	9.2570825	10.169521
10.722971	3.4882403	8.4088626	7.9880532	5.7243797	5.322874	8.8902276	11.761104
8.6308564	5.025804	8.0410782	7.0712569	5.9569762	1.7806036	9.3090877	10.486074
7.9608182	6.6933777	6.790288	6.8684355	4.174975	6.2713426	8.3888433	10.117826
8.9395093	5.2784495	7.917941	7.1491336	7.1380504	1.6297279	9.7964359	11.465943
10.655979	5.542179	8.6283867	7.3833073	6.9199823	1.6025975	9.3462105	11.553368
8.5501476	4.4737534	7.9959171	6.6989065	5.5096435	3.4330682	8.6687322	11.506591
7.8806899	6.5404247	8.0443316	7.975658	3.6750417	6.156675	8.9690855	10.191255
7.0482955	6.474734	8.3381589	7.1549243	5.1283139	2.5898865	10.173901	10.840139
9.1915956	4.8012403	8.02743	6.9811681	6.3944184	3.9284502	8.0156925	10.688351
9.7378944	7.67147	7.5246324	6.7089195	3.8355543	6.0654395	8.8700761	9.217496
10.196517	6.1325924	7.910576	7.8979455	7.6315879	1.7066318	8.6690047	10.422202
8.0262578	6.6138885	6.6434415	6.3151699	4.5909086	6.8438875	8.2625786	10.053356
7.9709396	6.7900332	7.2310765	8.0742447	5.0541606	6.4770314	9.7289089	10.611342
7.2283849	7.1110972	6.2168678	5.8891431	4.2260444	7.5821114	7.8244134	9.3629535
9.4204115	4.701857	8.1331319	7.7385456	5.1043097	1.5993479	9.859666	10.845189
8.3089055	5.9258382	8.2203673	5.8202702	5.3720295	7.2716434	7.818749	10.341826
7.0637042	6.9831281	6.3491488	6.2714648	4.6301154	7.1548427	7.7571899	9.1685092
7.9368221	7.53532	8.2580636	6.5495529	4.8678138	6.8322968	9.2817828	9.8052662
7.7158564	7.0341154	7.6658388	7.5008883	5.0945802	6.7305418	9.0495148	10.155743
9.0305355	6.9314046	8.2743201	7.4766909	5.349038	5.24306	9.2649622	10.79363
7.6516252	7.7504378	7.1118194	6.7655077	4.1718223	8.4764458	9.3336045	10.307415
10.40731	6.3002064	8.6462475	7.1879071	7.4456614	2.353942	9.2037651	10.683388
10.707966	6.8568085	9.0595154	7.749275	5.0609	4.8387158	9.2018881	10.523805
8.8009314	5.0411497	8.2317098	7.5624513	5.8509998	4.5511588	9.02314	10.777642
7.7682182	3.6433524	8.7507212	7.3381789	4.9773151	3.222082	10.007855	10.519405
6.941588	6.6696449	6.2925061	5.988049	4.1878281	7.4223407	8.3467957	9.595359
8.5914953	4.887016	8.2846462	7.7767516	6.2783389	2.3239164	9.3648236	10.723406
7.8108954	6.6150117	7.8858777	7.5049653	5.727549	7.2947889	9.1981055	10.686567
11.625374	6.7583718	7.8507746	7.4200393	7.0342139	2.6423833	9.8815531	11.783797
10.196627	5.997597	7.7657594	7.8294668	5.2396241	3.8272455	8.2798838	9.7105145
8.2975789	4.9508116	8.6496273	7.8144779	6.2941146	2.7041034	7.9822091	9.4839437
9.3093332	3.1304756	8.2629439	8.4031178	6.3258045	3.006431	9.1518508	11.071949
6.4579574	7.7096898	6.3074344	6.1286698	3.7427198	7.4655108	7.4650022	8.9035971
9.2158731	5.2176132	7.6958665	7.3580805	6.3793856	3.8124158	8.4721743	9.1290111
6.4995603	7.2415363	6.7221438	5.4836846	3.8330551	7.6443984	7.3491398	8.954766
8.6476254	6.7474796	6.3888503	5.6924011	7.2902047	6.4293849	8.6395718	10.876151
8.0901836	6.3823887	6.9043942	7.6565133	4.5471603	6.3879907	7.8082593	10.426883
7.4902161	7.4656997	7.6596429	6.9639842	4.1588526	5.5417752	8.3658462	10.113733
8.2555092	5.5062423	8.4679641	7.3447667	5.6817776	1.661833	8.5304317	10.158857
9.4092361	7.01811	7.5091166	6.7095742	7.808296	5.8644174	8.4950726	10.576192
9.2561103	8.5206878	8.1920623	6.2687462	4.0965206	3.3152221	8.699819	10.397153
8.975116	7.3427916	8.5417418	7.421243	4.2451207	4.3723772	8.8005049	9.468836
9.6228202	6.4446757	9.2965461	6.4953437	7.2667828	1.9369675	10.138861	10.642442
6.6637736	8.1236109	7.3667544	6.5264196	4.3072748	7.7637701	8.057378	9.80234
8.5105383	7.3196759	8.0395421	7.2777292	5.2689127	5.8570994	9.7780243	10.939665
7.5594447	6.774135	6.0813455	6.2363299	5.745818	6.9080294	7.7434997	10.032265
8.4789956	4.0767189	9.3130478	8.117834	5.3108494	1.7353492	8.6506148	10.374182
9.1100469	5.4053471	8.1286125	7.3417999	5.4245913	6.430294	8.7236863	10.106762
7.3670693	7.8112883	6.713659	6.5547233	3.5778445	5.9137417	7.7547944	10.035573
8.6515589	5.2687092	8.1480217	7.152175	7.8586442	3.1950935	9.5188584	11.246085

9.6956248	5.6860805	7.8463624	7.4248157	7.6555179	3.48775	9.0516794	10.720056
8.0007134	6.9039116	8.0181463	7.3112309	6.2055103	6.6104238	8.5815457	10.453691
8.3659903	7.4620907	7.1424482	7.8895349	5.1561716	7.5602	8.8899604	10.2732
8.2614023	7.269797	7.0329186	7.4402563	5.5348599	6.2032452	8.4092973	10.132278
7.9670588	6.6032323	7.0497804	5.8938806	4.6415152	7.3211652	8.2227107	9.8362702
7.0133017	6.900345	6.733622	6.9587245	5.3295373	8.0793661	9.1697858	10.712637
8.5071975	7.4241057	7.9497008	6.9955084	3.9267685	3.0013054	8.1266188	11.306421
7.6647458	6.2116808	7.0666878	6.6009629	5.0053486	5.5706745	8.9536749	10.011791
9.2956329	3.6262927	7.7094057	7.7161198	5.5068848	1.6165251	8.2420382	9.4411946
9.8559847	6.3615288	8.6989898	7.2452812	5.2018988	1.8252247	9.3051311	10.028699
7.7303898	5.1755617	7.1400909	6.1813842	5.6410011	5.1682568	7.9202237	9.5791627
8.3734498	7.9212491	8.0997103	7.0399124	4.3256225	3.347438	9.0785196	9.9629043
8.8897401	4.8889548	7.5962847	7.3482877	6.2232514	3.7874304	8.9255934	11.454142
8.2781405	6.5111957	7.7796371	7.3251193	6.0528457	4.2151393	8.2887993	9.8139628
9.3109083	6.281572	6.8507107	7.0229768	4.0174269	4.5402261	7.8831805	9.3770762
8.8857288	6.1259082	8.0872431	7.5229681	4.2482529	6.2279266	8.8960318	10.479348
8.4081547	5.7596981	8.0485856	7.3954004	5.0304269	5.4834081	8.2174504	9.5918493
7.5981242	7.4627479	7.293756	7.2813654	5.4417124	5.7932664	9.0001652	10.668785
7.7679182	5.1288485	8.3237995	7.416355	5.1788521	5.2135796	9.1314094	10.222158
7.4003531	5.9812843	6.8393662	7.1132847	4.7244401	5.1160256	7.7014996	8.8068133
10.104365	5.7063658	8.1726788	7.8941318	4.8685155	6.2237842	9.0084173	10.781908
8.5293593	4.7737696	8.3301525	8.5667852	5.6550403	5.7857872	8.5630065	10.308805
8.2236209	6.0129659	7.6068928	8.2432038	5.4090692	6.2468514	8.7383814	10.74753
8.6134323	6.4208839	7.3568207	7.2632077	4.7544007	7.255046	8.1167021	10.857419
9.933529	7.5366694	7.8093824	7.1612902	5.4659173	5.4702764	8.7601273	10.928989
7.2240821	5.1300387	8.3286839	6.271214	5.9147902	4.0204468	7.5890589	10.527151
10.465367	3.6841737	8.353793	7.9441868	6.0068086	2.380849	9.3019159	10.90923
8.2391178	5.9497812	7.7243546	7.2712427	3.6426457	5.2040305	8.9756079	9.7181498
8.6168412	5.6280061	7.7283611	7.286081	6.2507964	5.3224581	8.881114	10.835939
8.0047833	5.3585387	7.5028264	7.2221419	5.0896107	5.7163021	8.6691112	10.34147
9.7009098	4.7883272	8.0552339	7.6484154	8.0357017	2.0417314	7.8252043	11.35267
7.982011	5.7120652	7.3550703	7.4392567	6.0540866	7.4963188	7.4163466	9.6893496
8.8295165	7.266146	7.158616	7.0810903	4.5741774	6.2415365	7.6529039	10.678779
8.420892	4.753957	8.1924483	7.4957629	5.8242898	2.0417992	9.97201	10.949784
9.3585753	6.4425164	8.2542283	8.0214448	5.2575756	4.3690531	9.3010667	10.515269
8.6045386	5.0289949	8.6924841	7.8255818	5.0355204	3.2703286	9.2483614	11.572637
8.5075356	6.1907923	7.9989737	7.8045352	5.4854195	5.8638492	9.0693875	9.8840137
7.7146458	7.442036	7.9211303	7.6099322	3.9650722	7.4522949	8.3615834	10.363275
7.8851619	5.3746361	8.3485646	7.9502211	5.2672209	2.2457436	9.0015999	9.7295927
7.9229743	5.7319844	7.4703781	7.088363	4.1810756	3.0097031	8.030949	9.3443801
7.9106908	5.3893711	7.8112174	8.1254627	5.3118604	4.5115773	9.0845262	9.7482828
8.2879035	6.6315233	7.8758992	8.048804	4.9298704	7.8852805	9.2912122	10.821727
8.0998609	5.3512376	7.6927395	7.8606036	4.9685085	4.2406802	8.4523671	9.3392481
8.4467212	3.8436859	7.2646707	7.4717171	6.9529805	2.0434899	8.2987528	9.5134031
8.687479	2.8348845	7.1654744	7.1397923	6.4030784	2.0662223	8.3797259	10.238819
7.8798787	6.031357	7.9934963	6.8011598	5.0950809	7.212542	8.4324547	9.9925731
9.4730639	6.5657931	6.8654068	6.7942728	4.2209311	6.6289323	8.4473459	9.5143633
8.0005978	5.7539589	5.7864826	6.5254106	3.8099848	4.765162	6.5768479	8.0945761
8.5571873	8.5233523	7.3487094	7.4948579	3.6416488	3.9796953	8.009102	9.6160695
8.7765456	6.8321207	7.4567363	7.5036845	4.6335821	6.8051546	9.3439881	10.62763
8.5418877	5.6446098	9.1042995	7.1706028	5.1985239	2.2992819	10.321363	10.724125

8.5344514	4.794518	8.1710455	8.2068908	5.5833931	2.1468085	10.331235	10.567016
7.8020753	3.7074386	7.7153203	6.7956536	4.5116292	2.8470009	8.7134228	9.2545119
7.5660623	7.0204731	7.0055974	7.3469461	4.6793709	8.3148188	7.9973104	10.696145
7.7673786	6.4281124	6.747782	6.8908289	4.4255849	6.6271363	7.3877561	9.1867539
8.1093364	6.2421613	6.7421638	6.4323655	4.3627308	6.398455	8.1093992	9.3873012
7.1939865	7.0087931	6.7728215	6.7200861	4.4881988	6.3816775	8.3343256	9.7734751
9.5805915	8.0874562	7.9849254	7.6431999	3.3726108	3.8188776	9.0192848	10.13388
6.8183125	7.4302143	6.6983722	6.4264322	3.5752453	5.2172451	7.0200444	9.0913085
7.4593875	7.4264472	7.430714	7.1493021	3.6949253	5.0163486	8.1886367	9.4073484
8.4673987	6.3921522	7.3451399	7.6055007	3.8446883	5.0675929	8.9108059	10.009278
9.0683661	6.2233303	6.6615367	6.4532649	4.3995636	5.2287017	8.3449584	10.537437
8.6048543	5.1758671	8.5433391	7.1957991	5.72987	2.0661778	9.1607267	10.348998
8.354535	5.6795127	8.3933296	7.3090854	4.4334427	4.4525004	9.1065441	10.233227
9.7919985	5.6171653	8.5273971	8.1012342	6.1983347	3.3930906	8.9811443	11.092378
7.8599504	7.0414899	7.0611222	6.6420477	4.6536527	5.7385923	7.8231026	9.9843683
8.958946	6.9481452	8.3988404	7.4839934	3.6740403	7.5900179	9.0688783	10.200116
7.9238481	5.9122301	8.1810358	6.8798576	5.0892919	4.8732157	9.3507121	9.7446597
8.3873833	6.0879921	7.7480563	7.3642452	5.3007003	7.505443	9.0587131	9.4435801
8.6429841	7.2801102	7.2121863	6.0902106	3.9389252	6.0091429	8.3362696	10.169961
8.3041913	6.5430563	8.0353834	6.9845191	3.8830125	7.5055968	8.7388618	9.9302693
9.9254097	6.0834753	7.6560402	7.1196177	6.6123528	4.1350702	9.3896962	10.421263
8.0051239	6.2538497	7.1926364	6.359119	5.2964322	6.1307422	8.1106523	10.190555
8.2165779	4.918108	8.7648706	7.4596099	7.0759656	2.0365311	9.7425753	10.940663
9.0158968	4.9143607	8.2029861	7.6527954	6.2031	1.8463173	9.3747009	10.509127
9.4266092	6.6703892	7.7479826	7.6289469	6.5934442	1.6250727	10.534251	11.230702
8.3185464	5.5562033	7.8173329	6.7745936	3.6867961	3.9425211	8.39707	9.0918199
8.6884844	4.6052496	7.5031999	7.7680899	4.0015531	1.9242164	8.4783797	11.258323
8.8610054	5.913439	7.8615165	7.7049753	5.2892219	5.2398548	9.7397798	11.053708
9.8376636	5.4135392	8.034291	7.4838772	6.4830864	1.6174872	9.9004745	10.924971
8.8917244	7.5995694	7.835373	6.7635995	3.6488828	6.357902	8.7026986	10.370255
7.7335074	7.737287	8.1597686	7.1532247	4.3273747	5.8765946	9.0321517	10.200336
6.8645219	3.4300365	7.960306	7.2704648	6.5888027	2.2064882	8.9617999	10.233679
7.8012279	3.7707505	8.0007942	7.1538515	4.0834053	4.1279132	8.47546	9.5798925
8.4077073	6.5291454	7.8741146	7.4615031	5.0059853	6.0244047	8.6170195	10.441972
8.9663864	5.6052897	7.7539647	7.6648545	6.3934119	4.619696	8.0069216	10.302092
7.021975	7.5380359	6.247983	6.0239801	3.7813935	7.904791	7.7899355	9.4257587
8.6289044	5.5872856	6.9761372	7.2211753	6.3799084	4.2538769	7.7591665	9.8349065
8.4046959	4.6534247	8.1485916	7.0557075	4.835421	5.176581	9.3374843	10.130804
8.512838	5.6761883	7.6395025	7.2717331	5.683937	4.861949	8.3435988	9.7649978
8.6816093	6.538302	8.4766534	7.7155705	5.5695982	7.0031371	9.5851344	10.76114
8.6975427	6.6776047	8.9912906	8.5517477	7.1455818	1.7134536	9.976961	11.829456
9.2189199	4.83193	7.911471	7.7800425	5.784136	5.6716067	9.202626	11.151491
7.7961783	7.6173404	8.0294404	8.2829425	4.8878015	5.6118317	9.1207627	10.686088
8.926546	5.3806884	7.7840466	6.780158	5.6175659	2.3038146	8.96454	11.17686
7.9435205	6.2765297	7.3527333	7.5175612	5.8478726	4.4879499	7.867523	9.9524097
8.0274344	5.4846042	7.4660911	6.7792715	5.3932093	6.0011589	9.5027429	11.21115
8.2834407	6.9557093	7.8555134	8.1093046	6.0137027	5.0748708	9.370825	11.082199
8.6608156	4.0847119	9.1581205	7.6736051	7.3319642	1.6431398	9.2632303	11.292458
9.0289703	6.6411012	8.8710017	7.540199	5.7541967	2.6861855	9.5295111	10.568627
8.3905372	5.8619799	7.3834178	6.6923205	5.37732	5.4878917	8.4069103	10.510839
7.3112681	7.4938657	6.970258	6.8154802	4.7708088	7.7621961	7.6372852	10.188105

6.6577631	6.4077404	7.5518279	6.726853	3.4932272	5.8407704	7.3606867	9.0079278
9.3510389	7.1374129	7.6044042	6.7076596	4.0921705	7.0669074	8.855	10.834108
9.7263318	5.5453348	7.6108852	6.262481	3.6648207	6.0482646	9.5131621	10.179152
8.6204202	5.0694021	7.7758798	7.5467173	6.7400155	4.9128041	8.2591318	11.927529
9.2717756	5.8945031	7.5569993	7.1268917	5.5098831	5.4245264	8.523023	10.034318
7.1136503	7.2852396	8.0359997	6.9693014	3.8206906	8.208328	8.7898556	10.447622
7.8418162	6.4959103	7.2900292	7.4659446	3.5066091	5.9761525	7.8154558	9.4532988
7.8553991	6.7287403	9.1083475	7.3016367	6.789728	4.7642271	9.7927319	11.512393
8.8926785	5.8011052	6.7162844	6.6198936	6.9047662	4.5042041	8.2719385	10.230769
8.1449101	5.8830453	6.6082138	6.8634501	5.5089145	7.1435695	8.6024112	10.383101
8.9197569	6.9280003	9.0774477	7.3700309	6.8372706	2.5622924	9.5311232	11.666582
7.3589484	5.2461399	6.4977133	7.3135758	3.9225403	6.0761784	6.9861414	9.7510795
9.9950119	6.9988575	7.8073668	7.4075919	4.8052246	6.9628279	9.0747654	9.6989055
9.6963805	6.9524988	7.8901261	6.9270648	5.7215365	6.3388245	9.1820435	10.727586
9.0212447	6.7921475	8.460306	7.8236176	5.7645762	5.6975985	8.3141942	10.635548
10.217353	5.9229655	8.0142149	7.2306625	7.2331114	2.2995089	8.995593	10.149394
8.4300531	6.5276684	8.2231615	7.726681	7.0248517	4.8162991	8.6424883	11.661711
8.1088977	7.3426394	7.3862261	7.6878023	5.1987493	7.0991046	9.299892	10.968014
9.7494694	6.44564	7.5506561	6.943377	6.357765	4.8260237	8.310264	10.353532
8.9056349	6.1975589	7.8393408	7.7517962	6.8837355	6.1247118	8.2986105	11.025456
8.9379126	6.1491679	7.4895404	7.2227773	6.2743941	5.0523929	8.0942418	10.251603
9.2967505	5.4813217	7.9439286	7.8406793	7.640827	3.7507505	9.401277	11.239544
9.9206142	7.1320425	7.7280335	6.9136519	5.6792418	4.7497752	8.7657001	10.578999
7.7819367	5.0550892	7.4564777	7.3088593	5.4059706	3.8618364	7.9721712	9.7048734
9.6729762	4.834463	7.9703016	7.7370234	6.6984789	2.5580755	8.2232731	10.120097
8.116161	6.7863735	7.8086279	8.8594555	6.2625633	6.8814091	8.0752972	9.9243802
9.0237211	6.8407214	8.0432148	7.9805862	4.7688184	8.179312	9.6870069	10.805251
8.8076751	7.1066249	8.1707737	7.9265549	4.1822694	6.5586381	8.9863883	9.3190998
8.4031743	6.2575242	7.5456244	8.1809028	6.3547063	5.8318581	8.0822859	10.444994
9.4506718	6.759725	7.5726854	7.0161011	5.3909619	6.9997978	8.2435061	10.327455
7.8852282	6.6814514	7.5132474	7.1819203	5.0292616	5.9729307	8.1838982	9.5443597
8.3666833	7.5392167	7.3279618	6.9472254	4.0990761	5.926098	8.5318901	10.586396
8.9422086	5.9433262	8.0095354	8.2774672	4.8693682	4.6258643	9.4894267	11.107803
8.4192471	7.1563472	6.3762837	6.4967691	4.4603252	7.4473231	8.2895971	9.906043
8.0578418	7.3553573	6.3623056	6.7069951	5.1901413	7.700611	8.0519177	9.5519687
8.1695826	8.0195996	7.4030945	7.8920646	4.9631788	7.2463293	8.5192856	9.6867725
8.0938485	4.4526713	7.7266872	7.484837	6.2707101	3.9124678	8.3697515	10.341363
8.3008	5.8707025	7.7613127	7.7304046	5.4317034	6.3973809	9.6541247	11.840202
9.7637455	4.2488678	8.358016	6.9464253	8.2086253	2.6800215	8.3924999	10.053307
8.625248	6.8974236	7.2987404	6.0046649	6.4858709	5.5138067	9.3498603	10.765891
8.0465926	6.7965027	7.0403888	7.9788738	4.4893483	6.6739242	8.0365662	10.655281
8.2239727	6.2577665	6.6003927	7.2002976	4.0589476	6.3140873	7.3360788	9.9471053
8.4075538	5.3407249	8.9958823	9.0901494	5.3698061	1.6064802	8.4740107	10.039684
7.4187282	5.7617452	6.9330913	7.1622598	4.3320687	5.3077484	8.2968915	9.9851235
8.5201608	5.8365412	7.4002998	7.3047203	4.9145075	5.7254063	8.9427496	10.472606
8.3613287	3.7373689	8.0511319	7.2602665	6.1035724	1.603776	8.9580197	9.7026926
7.8837623	7.1256837	8.5957939	6.9420513	5.4078867	7.1200583	8.9354394	11.239684
9.3163664	7.3257377	7.990699	6.4970864	4.0002444	7.0180905	8.3435173	9.8369404
9.2136013	7.0194184	7.9446058	7.4821348	6.633403	5.8009956	8.6147078	10.886583
8.5055447	4.0839524	7.6634905	6.8171858	4.8854229	3.1104828	9.0321831	11.183601
7.7845572	6.6781144	8.2118094	6.9441507	5.374345	6.2755474	9.282185	10.343295

6.9309545	7.2376344	6.7279514	6.4600288	4.7270785	7.5325308	7.9754805	9.6030579
7.4960343	6.0072747	8.8285647	8.621325	6.7456088	5.9114736	8.8211177	10.985239
9.2810329	5.7512559	8.2298133	7.8008382	6.8788108	3.7750743	9.0556528	10.049834
7.8639763	5.9460009	6.6685308	7.3800097	4.7634323	5.09578	7.2859973	9.3384515
8.8510454	6.4157886	8.6791707	8.0010827	6.9067324	6.1622557	9.0353744	11.049944
10.594173	6.3647444	6.7637172	6.9446048	6.5887912	3.9688137	8.2820785	10.519561
8.3658554	7.0326567	8.2217358	6.6160927	5.1501187	2.8671222	8.9427052	10.32781
8.8408653	7.0349208	7.5860375	7.3373243	6.44357	6.0473713	8.7018454	10.854382
7.1397367	6.7035013	8.4352005	6.8360977	4.1464279	4.212927	8.2340183	9.8676766
8.1614621	8.5256152	8.3489389	7.0300683	3.5648445	7.9067161	8.1944494	9.1541919
8.5369134	6.4944393	8.2033964	7.043448	4.7450138	6.1661167	9.193576	9.8469334
8.9377054	6.6745959	8.3892666	7.3529586	5.9784415	5.7060003	9.0016285	10.996475
7.9185693	7.3191371	6.7988145	6.626041	4.4974869	7.2527029	8.6171767	9.951544
9.5251617	7.0254092	8.3711086	7.5834767	5.2128961	6.6685643	9.3851022	10.038744
8.20801	6.9017409	7.8270305	7.2569368	3.9020258	6.6268974	8.8356773	10.303209
9.2441307	6.3022522	7.2811721	7.3622306	6.4747315	5.4211748	8.179532	10.254721
7.9562826	7.8057895	7.0035051	6.7351768	4.7142859	7.7649821	8.592649	11.005164
8.8100703	8.1742927	7.5998111	6.8366131	4.7312884	6.9262561	8.0207015	10.225647

DPM2	UGT2B10	UGT2B11	UGT2B15	UGT2B17	MAN1C1	UGT2B7	EXTL3
8.2025324	10.5393	11.095323	11.054076	5.7805365	3.8697854	10.19457	4.5746902
7.6557033	12.255591	11.477017	11.950795	13.717624	6.0368103	12.679715	5.7792463
6.2889935	9.9966521	8.889093	12.405769	10.517669	5.0773797	10.660715	4.9977218
7.89944	11.832611	8.5804786	12.252278	7.6648206	4.9473426	13.25816	5.3409993
7.2306512	12.409362	8.3033552	11.089603	8.0978049	6.9668723	13.427154	5.4432542
8.1574649	10.016503	8.3993865	9.877288	10.848402	6.4635741	12.492196	5.1276136
8.2239759	11.253002	7.6576543	11.058827	6.8603617	4.4765422	12.652043	3.899785
8.4076462	9.7041005	7.0045096	9.83243	9.4615174	4.5731694	11.36831	5.9683517
7.8702061	5.5972388	8.2661858	9.0872148	8.1274982	4.0492644	6.1945341	4.9139893
8.2909435	10.070427	7.4289639	11.55616	13.682945	5.5555922	14.512323	4.2546505
7.4912423	9.5768058	10.255303	11.457589	5.6352253	5.2075785	9.8247022	5.2670767
7.3528276	10.585453	9.6993092	12.107661	8.1603755	5.2441771	10.204822	5.0078677
7.6214958	9.9685312	6.9668973	4.5506487	5.7264249	3.0005681	8.5610049	5.3610162
7.3990573	6.9543291	8.1163596	5.945367	5.9267081	6.8000555	6.4365797	4.6471554
8.1505691	11.309308	7.219653	11.426402	12.009332	4.708519	11.878921	4.4829415
7.555944	10.826534	11.2881	12.058409	12.475373	4.6291194	11.62549	4.3020692
8.0879225	13.498786	13.150748	11.220548	8.5608516	6.4426915	12.966427	5.560025
8.1213226	9.2450109	9.3900172	9.5884761	6.331031	3.8941703	8.8102724	5.2055382
9.5797151	10.188231	10.136954	10.490335	5.7129513	5.5197467	12.360856	6.0333614
7.6658818	6.3928176	8.2764869	7.6320407	10.686536	6.4769165	9.086859	5.2050869
7.3903826	11.336876	8.1904867	11.812746	10.781659	6.7116166	13.282258	4.5317631
6.2783086	12.207476	7.1735255	10.761531	6.4259774	6.7870219	13.363423	3.4120527
6.3529497	12.680336	6.834694	11.023557	8.7232853	6.444427	13.486144	4.1537464
7.4120059	10.784399	6.6535669	11.202083	6.6467029	6.1539858	11.632017	4.9931179
6.87156	11.945339	7.8333268	11.595342	12.787444	5.9061797	12.841447	4.6214966
6.4375045	11.488443	7.5968181	10.857683	11.357022	7.0066728	13.191946	3.7061451
7.0742227	11.955205	8.4057574	11.413001	5.6167428	6.3063577	13.366061	3.5221268
6.2807443	12.533854	7.798616	11.191009	6.3610635	6.2653857	13.713215	3.2838181
8.2192254	10.074711	10.061201	10.026827	5.7034406	5.7923473	11.320699	5.007723
8.0517806	10.228072	11.505338	11.03469	12.912533	4.978988	12.576862	6.2830013
7.1180622	10.246065	6.6129187	10.375794	11.772056	6.637041	12.735159	3.9056823
7.7158159	9.9125184	12.908177	10.162822	12.051087	5.0501208	11.611304	5.2475488
8.2069526	8.0008024	12.001763	11.577096	11.67688	5.7093093	9.0426767	3.7971381
8.4739794	10.58061	7.8697203	5.4541861	5.7100356	6.9509497	12.679222	4.7763607
7.9563197	12.465462	8.9665316	11.564201	8.8055442	6.793906	13.379207	6.0848242
8.7302848	8.8162158	11.556978	8.0800966	5.6564383	2.9901812	8.3286118	4.19962
7.0897053	12.068495	6.7580086	10.237573	6.2127581	6.1531593	12.452952	3.9358979
8.1759555	11.113734	11.439567	11.767063	13.184516	5.3907021	12.136302	4.9864784
7.9976964	9.3669999	12.190208	10.575932	7.7145272	6.0267743	12.016355	5.5935617
7.5548044	11.490761	8.8739858	11.653584	8.1013786	4.5156288	12.750335	5.6226572
7.9831733	8.4090568	8.6069717	10.317808	5.5420242	4.7084196	11.961142	4.8468086
7.5541771	6.8660077	8.467263	6.0146103	5.6135889	3.4336144	8.1627647	4.9561465
8.5957245	5.1421825	7.7632398	7.8858283	5.5976507	4.801707	10.211724	7.4529225
7.7217447	8.0602387	9.1845968	9.1943206	6.1969093	6.5196317	9.9162912	5.9276579
7.9570596	9.9197154	9.1275097	10.788681	6.3178024	4.6206702	10.683361	5.2717125
8.7827773	11.011472	11.046815	12.248776	7.1597581	5.7496197	11.176853	6.6214264
8.4477186	6.4293913	8.2540711	4.9393997	5.5420242	2.7486094	7.5173758	4.4933738
9.0414352	6.1818111	9.7350466	8.6537166	11.067695	4.3953373	9.5309911	4.8777131
8.6381348	7.0617148	6.267528	7.3470119	5.6739237	4.2246155	9.5055954	6.1530839
7.9445582	9.8730661	10.88917	11.172803	7.8111432	6.0376711	11.657859	5.1536294

8.3174805	10.79653	9.4334571	8.2292471	5.7781527	4.0205297	10.338267	5.9372532
8.2301683	9.9972065	7.8973966	10.76453	5.6011096	3.7710182	10.59787	6.675185
7.9631608	8.2619128	8.0030416	5.8704776	5.6013971	3.3075491	8.0773777	5.288841
7.7213475	8.8437832	7.493529	11.543792	6.9392381	5.8334884	11.882798	4.86412
9.0226624	4.6706793	7.283179	4.5548225	5.5420242	3.6373743	4.689178	6.6985213
8.505045	4.949217	6.1848036	3.9822369	5.5420242	7.783786	4.4702285	8.1577822
8.3059866	11.807743	11.871189	10.555356	6.0583977	4.38454	11.574117	6.5201287
7.0758739	9.576604	7.5634979	11.473151	6.4794296	2.6844976	9.6456573	4.3722067
8.1988656	5.0839281	7.4807497	6.9177153	5.5420242	3.7121933	8.0193605	6.3411195
8.8736454	6.1930992	6.6847998	4.443887	5.5420242	6.1752485	9.3500286	5.047351
7.6641239	10.281915	7.2152047	9.0772252	5.7103053	5.217706	9.2546968	5.3992576
8.0668173	4.475852	5.9986127	3.7441725	5.5420242	10.583542	3.7864662	7.0431155
6.9762041	13.132374	10.25337	11.972686	7.3046246	5.8973957	13.786028	4.4690279
7.4736626	13.22249	8.4045123	12.177099	10.511185	5.9412201	13.484998	5.0823459
6.410621	13.112705	9.0142036	11.400821	10.382472	6.0186994	13.435008	4.3962859
7.9198842	5.4181339	10.408987	11.112402	9.3159025	5.890707	7.3104357	5.0028391
6.990506	9.8500567	8.3514296	10.726031	5.8612635	5.8796631	11.660994	4.5600113
6.3929688	12.164216	7.4047561	11.202019	12.4322	6.5709044	13.180927	5.3288279
6.8172151	10.935756	7.4193597	11.118034	6.3575209	5.1243117	10.632618	4.6211982
7.2197116	11.45116	7.143214	11.793078	8.0425823	4.8327365	9.8037914	4.9606765
7.2829162	10.921212	8.5882312	8.0018485	8.6004244	5.3866536	11.96948	6.3078527
6.536235	12.901381	7.2775524	12.343044	8.7424162	6.7728744	5.1028932	5.2517519
8.7448575	8.9833655	15.788155	11.806472	9.4986959	4.9422234	10.654956	6.8548051
7.7061513	11.334293	11.439255	9.9243442	7.8226758	4.5063969	12.701599	5.8998597
8.5591656	10.163514	9.699723	11.449187	6.4466834	4.3030733	10.778561	4.9519908
7.761319	8.9291799	8.4279064	7.5572459	5.9851456	4.242113	11.146014	7.0631961
6.5139761	12.621642	8.7136585	11.557756	12.613059	6.1620401	13.288592	3.9833594
9.0371988	8.9411467	12.102674	4.5121012	5.6436616	3.087347	8.448425	5.2727446
6.5979988	10.159878	8.5158278	10.675139	12.429148	5.8221154	12.76135	5.276457
8.6987432	10.118417	11.798087	9.306501	7.2529314	3.1823222	11.747239	5.3976358
8.0240707	10.673299	8.3771823	10.684049	6.7717056	3.126053	10.832895	4.1220349
7.6241306	8.4232374	8.2197409	9.0662613	8.5332601	4.5401668	9.0877289	4.818963
8.5112475	11.701234	12.399798	10.868218	5.5420242	4.1636525	10.767734	5.0115577
6.7624336	11.367355	7.7865693	10.803345	7.783797	5.676341	12.725378	4.1965079
8.4428569	11.000703	7.6260114	10.316071	6.1579342	3.1521441	12.697259	5.7637664
6.3056671	11.782538	7.1449417	10.449071	6.3705467	6.3319885	13.220919	4.2452301
7.2855359	10.742948	14.184638	8.4967724	6.5088799	2.7526147	10.136536	4.8979786
7.6969747	12.201726	8.4374548	12.131508	10.291225	6.1218793	13.389009	4.3524115
7.133981	12.419757	9.6305139	10.816547	11.011898	5.7366091	13.707828	5.058211
8.3530452	4.6854428	6.1924453	3.9427622	5.5694559	4.529549	5.3888793	4.9291816
7.8664003	8.8702015	14.168183	7.5891944	7.1303397	5.9727461	9.9896321	6.2024663
6.9420114	12.075892	13.881525	11.636165	10.856532	4.3028759	13.753312	6.2739079
7.5196133	8.693398	10.949683	10.282542	7.3429145	4.5628665	10.152351	5.0256784
8.7465988	4.9171535	8.9094951	4.7336598	6.1818771	4.057094	7.693721	5.9874916
6.7701422	13.031164	9.8126008	13.035938	7.298859	6.2749676	14.011818	4.2222459
7.4294365	9.7603117	9.8559366	11.044005	9.8562864	5.2346888	10.738963	5.317064
6.6271751	11.431926	7.6616861	11.53326	7.6659885	5.9420125	13.162665	4.2073933
8.4792332	7.1694078	7.7303998	6.7756771	6.705239	4.5418025	6.8431721	6.1730652
6.8864473	10.848345	9.0878071	12.370479	7.4884142	6.4482042	11.913597	5.0598312
6.8326121	11.354008	7.2601812	10.870336	9.1440248	4.9566717	12.319243	4.5451778
8.3154131	10.28721	8.632138	9.1135201	8.8520306	3.9977198	10.65473	6.9578256

8.0067791	9.2765647	9.8240588	6.4462509	5.7329909	5.0701465	10.119787	5.2052279
8.3221663	10.120958	10.552013	11.278208	8.52368	6.7498816	11.364841	5.6876972
7.5935042	11.080331	8.0035434	11.108182	8.7361152	6.2240494	12.850634	5.9356711
7.4072834	12.486294	11.445319	12.306117	12.859457	5.0157029	13.263001	4.63027
7.4979781	11.210936	7.041146	10.174244	8.8414214	5.8130433	12.900757	4.2061511
6.7543524	10.560937	6.3893809	6.7332509	7.1251293	8.2173986	4.057204	5.5771155
7.7045749	12.113608	14.01	12.481157	12.401304	6.0927718	12.216168	7.124692
7.1767469	11.709455	11.922796	11.309326	11.598988	4.0070637	13.257568	4.8245182
8.3766067	8.7938278	8.8985874	8.5805792	6.6941862	3.152968	8.4648016	4.1250693
8.0055515	5.9242716	8.6666706	10.932983	6.0848848	4.2682196	8.5295155	5.1954662
7.0007186	10.133989	6.7055138	11.136586	5.66942	4.2443132	10.74396	4.4458307
7.9039327	8.693562	8.9342405	8.8370442	8.7561637	5.3934454	9.3342926	5.4631131
7.8022926	9.2591906	12.256109	10.203373	5.7141651	4.2494708	10.142937	5.328056
7.7242202	8.3392539	6.6643346	7.6508088	5.5420242	4.8187185	7.9845565	4.3172656
7.7721618	7.0909427	7.6572725	9.6763228	5.7067459	4.6313538	10.72824	3.7650486
7.5578763	9.2311631	6.9002677	10.233455	5.6196991	5.7335403	11.678104	5.0396971
8.1279813	7.9267804	9.3626337	10.633932	6.9973153	2.9890737	9.1346431	4.3770763
7.8620248	12.390989	13.372633	11.54482	7.1129354	3.5989544	13.458242	5.7009578
8.0313129	8.6882636	7.682233	9.8127778	5.9999984	5.481208	10.538033	5.5792129
7.8347773	11.038079	9.8612422	12.172621	5.5804479	4.0315742	12.875645	4.6276185
7.9329822	11.079778	11.152512	12.588395	9.6894219	5.9000556	12.292062	5.0746322
7.5199096	10.099468	9.8157673	10.685492	5.5942434	5.1436682	12.004077	5.046721
7.7541222	10.87232	9.7635976	11.879466	5.6661428	6.4162183	12.319564	5.4668326
8.08597	10.954714	8.9648879	11.538304	7.0353147	6.1654443	12.078217	4.9573398
8.1808972	10.867493	11.880168	6.2969678	5.5420242	3.6739346	12.98688	6.1464156
7.8689757	9.0675364	12.821026	9.513667	7.9660969	4.9128675	10.785383	4.6966903
7.9603759	8.9901568	10.400972	7.5372398	5.6022671	4.4827337	9.1360229	5.0566141
7.8292127	12.689901	14.468831	10.686159	5.5420242	3.4132602	11.633123	5.6562499
7.4629921	11.468866	10.215166	10.650154	5.6578353	5.8037434	12.810568	4.7281629
6.4960959	9.4556514	9.1645279	11.643287	5.7025153	5.4141043	10.478322	4.9380999
9.1327674	7.0700616	12.583243	8.4461201	5.5420242	7.2764768	7.5852581	5.163657
9.1028397	11.743185	10.167025	10.922866	5.781201	3.0819624	11.080978	4.6680414
7.5015504	12.385695	9.6586281	11.403041	5.5842773	5.2858542	12.758309	3.4606875
8.4742333	7.8758631	7.7316691	4.7593908	5.5727199	3.4989892	8.3574249	6.0784777
8.450458	9.4040158	12.129766	9.0540185	5.5420242	3.8869106	10.554672	4.8318665
8.5379069	11.782045	9.1199761	5.5428897	5.7957628	3.8659458	11.397814	7.1338593
8.0934508	9.1572103	6.7601014	5.0730623	5.5710206	5.1362211	10.603083	4.2436903
7.3533863	12.876944	7.8927523	10.207748	7.1636844	6.1712467	13.363781	4.7530395
8.3526352	8.3437347	7.2001889	5.314681	5.5420242	4.7496907	8.0620811	5.4336022
8.3651216	9.7050531	7.4610433	8.7070896	5.5764189	2.9741081	9.9387197	5.5356624
6.8420807	10.093385	8.0992303	12.25332	5.7884488	4.9744294	9.6660183	4.756139
7.7946069	11.758218	8.5168957	10.446724	5.5998791	7.1598151	12.328911	6.8701443
8.1162872	9.6116764	10.469879	11.180324	5.566433	4.1817587	10.947914	4.2125661
9.5205071	10.587543	11.455644	6.7238142	5.682922	2.9497788	11.055648	3.697289
8.9209239	5.8241942	6.4614597	5.2402117	5.5420242	2.6915555	7.5151855	4.1570085
7.9715523	10.610984	7.4324826	9.4156762	5.6027321	4.685179	9.7451817	4.9771609
7.7616722	10.940204	6.9422561	11.128356	6.7550599	3.4957287	10.898006	4.3920459
7.607169	11.106846	7.5964536	10.366572	5.5420242	4.0439098	11.830246	4.027116
7.380842	12.580712	10.236763	12.715572	9.9483717	5.8002852	13.453398	5.9089551
7.2359014	11.807212	8.779703	12.263351	5.7680332	7.0004282	12.672837	5.0050102
8.3340462	8.8543516	9.6340081	8.7670046	5.5696158	4.254646	10.170382	6.76942

9.0156342	5.3716827	6.3256352	6.4941209	5.5420242	3.7298377	6.9905402	5.4499934
7.6184644	8.9669445	7.5981143	5.8942993	5.6517915	3.6638625	8.9053728	6.0498582
7.5394983	12.771114	11.019634	11.876373	12.02827	5.8360752	14.13839	4.5134622
6.8431386	10.869834	7.9036358	12.768222	5.7677936	6.0880823	11.488791	3.930779
7.6896037	12.599393	9.2336154	10.735695	5.6054378	5.8271233	14.063462	3.8832867
6.8906602	11.55584	8.893621	11.653924	5.6209463	5.5629639	11.027541	3.7362581
8.1133965	9.2700329	10.532566	12.080651	5.7014008	5.6183601	10.570246	5.2573651
7.369797	12.424811	8.8616307	10.567907	5.5791426	4.4073489	14.039349	3.6242109
6.5607066	10.77037	12.604652	11.453946	5.5817623	3.4745082	11.553979	4.4378081
7.5603697	12.726594	10.120801	10.100497	7.0722418	6.4882785	10.67185	4.3182428
8.0802029	12.99279	8.6432381	11.615433	6.3843922	5.672196	14.878001	4.3625312
7.820644	8.3736132	8.6039538	10.744216	5.6878823	4.2774766	6.8896712	5.4175604
8.3412117	8.0090909	8.7962007	11.169026	5.6981614	4.7604726	8.776637	5.5991858
8.6984947	8.8000446	9.9864797	10.371407	5.6660451	5.4331974	10.130619	6.0695843
6.5657863	10.610351	7.5049777	7.177763	5.5420242	6.3684173	10.504943	4.3664567
7.5919881	10.974149	8.7153035	12.127513	6.1688373	5.4389752	11.498862	4.6419173
7.1650568	9.3759336	9.1706711	10.231803	5.6367571	4.3257115	9.3471162	5.0409249
6.338092	9.1261943	7.2399507	11.207491	7.10819	5.3172554	10.113378	4.6404304
6.7405771	13.110038	10.255308	12.097423	5.9280672	4.7681113	12.668498	5.0454699
7.9795037	9.9420202	10.334492	12.494105	6.6063384	4.3936444	10.95543	4.8886513
7.201277	6.2409769	9.2705317	9.3784824	5.7554555	5.0824614	9.7918432	5.3498073
6.9108047	11.266742	12.50944	12.692398	6.3457924	6.3146266	12.05372	5.1471802
7.8107294	6.0403283	7.6830643	4.6419022	5.5420242	6.5818976	5.7969212	5.4763396
7.4907584	6.4954928	7.9876459	5.3866714	5.5701424	4.8966844	5.9956191	6.0702591
8.3084089	4.6658681	8.5674178	6.3624043	5.5420242	4.5260214	7.0950863	6.2750362
7.5127521	8.728767	8.9107867	8.4022987	6.6887766	3.6999001	9.5041506	5.2174577
7.6938358	6.138848	6.9007036	4.0536767	5.5420242	2.6656165	6.3260432	4.8469527
6.915353	11.390075	9.5469951	10.484579	5.6945379	5.8193498	10.822812	5.1633914
8.6697762	4.6014068	8.68197	4.8746808	5.5420242	4.0667288	5.014599	6.4780671
7.4073211	11.686687	9.3691111	11.586395	11.957243	6.014082	12.420508	5.1606115
7.4870719	11.92795	8.182593	9.9185606	8.1392589	4.2724617	13.387287	5.6029082
7.9336602	7.9858579	11.63957	11.366441	7.2194066	3.7872036	10.694057	4.5868931
8.0664357	8.0261192	7.2268139	5.2367178	5.6722201	3.0022205	7.7529474	5.3227481
7.7462051	9.5463903	13.156081	13.024044	11.870871	4.5880231	9.995046	5.5935591
8.2612692	10.635041	10.343452	11.437267	7.9152187	5.1919503	12.901849	5.2336901
6.3706458	11.313556	7.774165	11.419573	10.811286	6.7072967	13.306106	4.1346293
7.0450079	10.279654	7.4165378	9.1315651	10.54441	4.3441954	9.9751659	4.0292555
7.738222	10.691055	8.6809137	6.7700214	5.5420242	3.402011	9.8701045	6.2021077
7.2400386	11.15385	8.6894543	11.271719	6.9748434	4.5790939	12.148272	4.8807917
7.7892394	12.572464	11.384153	11.395788	10.380735	5.011573	12.275537	6.1309725
8.1430117	5.3750495	9.3498802	10.096496	6.7085735	3.7621402	8.4072371	5.4691281
6.9511994	12.095117	11.251087	11.604575	7.2521463	5.7925303	12.001023	4.8536782
6.8629881	9.1741289	7.4294939	11.320613	10.933433	8.9521937	11.675021	5.817783
7.0665674	8.485822	8.5810715	12.4323	8.9007683	5.9342766	9.0895269	5.589005
8.1164513	10.672023	8.1389569	10.234088	8.2033502	5.1785647	12.355207	4.7209404
7.0223644	10.951748	8.8811026	10.544152	6.860051	6.366429	10.459186	5.8989934
8.0970208	9.803562	13.673447	8.5090193	6.6495118	3.6843128	9.6342797	5.4970732
8.6245052	6.4245378	12.996584	9.3923208	5.5420242	3.6918587	9.1742498	5.3092644
8.5623344	7.1233387	7.3940625	8.4168045	5.6071502	5.2279519	7.3205496	5.9480446
8.1036896	9.1040327	7.1380451	10.107737	6.3215861	4.5412958	10.542996	5.313294
7.4737761	10.720554	7.7683388	12.042206	5.7214925	5.717196	13.404045	5.8558789

7.2836681	11.364747	7.1449615	10.549728	5.6077374	3.4385938	12.84985	4.0558305
7.2279766	12.673472	8.8474138	11.229421	7.1358328	3.9168911	13.889333	4.9426828
7.1798095	10.725545	9.3889498	12.460794	14.907375	3.7100981	11.571556	4.7375326
8.3849392	12.509255	12.142108	12.424424	7.7915819	4.0309104	12.84309	5.053063
7.6784901	11.37427	8.217339	11.323956	9.2475916	5.6184482	10.789082	5.2029734
7.2737055	10.703946	9.1897192	11.737604	6.3320208	6.0385317	12.665347	5.1602797
6.6773306	9.195247	6.098734	9.8063454	5.6357914	6.9889606	12.299431	4.1586023
8.416852	8.9000521	12.968957	9.5704521	8.3746322	4.6825834	9.7289443	6.4791681
8.0032924	11.01148	9.0177183	10.575524	7.5126924	3.4892033	11.458401	4.4033983
7.6750227	12.473962	8.3630904	11.325392	6.7739185	4.8030543	12.506837	5.5236894
9.9556829	5.8416731	10.661283	4.9598815	6.0389471	4.6539875	6.7685814	7.1655117
6.9608537	11.121437	7.42546	11.471583	5.5934292	3.9753123	12.035229	4.3767422
7.4606649	11.285143	10.699405	9.5107342	5.5420242	5.1865707	12.339048	5.8965714
7.5212795	11.643093	8.8026898	11.883059	7.2512101	5.0415871	12.309179	5.8951905
8.0420799	12.461986	8.2086183	11.27133	7.0679118	4.2209716	12.612282	4.7840765
8.2965219	9.4818235	9.22478	10.754158	6.7446983	5.6253205	10.541932	5.2773986
7.8316434	10.241361	7.4024474	8.0558057	7.9350342	5.9637061	12.397043	4.9319445
7.0655915	11.934144	9.5859298	11.927695	13.835145	7.099693	13.403825	5.0702365
7.4725653	12.005758	9.3034589	11.785412	12.954818	5.6836029	13.764664	4.83127
7.8768817	11.784059	7.5446587	10.94659	6.4852183	6.0233008	10.786932	5.878573
7.6324688	11.704037	8.029008	11.160884	11.351288	4.726849	11.667794	5.927274
8.6912321	10.841485	13.248269	12.040324	8.1199965	4.6254487	12.053657	6.2667195
7.6386482	12.100174	11.883286	12.36023	8.099401	4.696395	14.150447	5.6743723
7.9839122	10.785878	10.881644	10.368512	5.9347954	4.6400726	10.961487	4.8273331
9.008979	8.8597201	8.2052279	7.304424	5.9534849	3.6322009	9.9011805	5.4684808
8.6566988	9.1887182	11.304466	10.832208	9.3185097	4.315293	10.173952	4.9444999
7.5792591	10.860117	8.0850312	7.2068604	5.8779715	4.9991901	11.290391	5.8482824
7.5152369	10.081837	13.576479	8.9004469	7.0372056	3.2196799	12.234808	5.3623585
8.0407669	11.483675	9.3116504	10.114702	6.6127289	3.3625689	13.845276	4.6047768
7.7078781	11.77097	7.964534	10.848706	6.8503484	5.0950489	13.795149	4.8744456
7.2516668	10.348806	9.6287911	9.7123607	6.0838641	4.6417797	11.388391	5.2080408
8.0279999	11.564967	9.8747622	11.770669	9.8024169	3.8035004	12.481085	4.8396078
7.0866708	10.644275	10.612386	11.592458	13.640392	5.6599771	12.134278	5.4472036
6.8774778	12.088518	7.278081	11.404242	8.8079433	5.5716427	12.924982	5.0314027
6.4666348	13.279828	8.2136899	12.34703	8.1554778	7.9581413	4.6653765	4.6121546
7.137903	12.316651	10.628503	11.374433	7.4899729	5.830481	12.326171	4.6758554
8.0272708	6.4411657	10.705761	11.624347	5.7941606	4.2636742	6.7242231	4.6226855
7.9621284	9.3337506	8.9289568	10.164483	12.426428	5.7101762	12.538095	5.4768561
8.7055813	8.8838444	12.914212	9.7754195	8.6426016	4.74718	9.6198632	5.4542015
7.3444338	9.4994238	14.43136	10.746067	7.9678348	4.1593424	9.8381136	4.4397911
6.7332371	8.4985337	6.9068529	10.338896	5.5894792	7.997124	10.542083	5.8662042
7.8764294	11.567329	12.886057	11.806542	5.6720905	5.6296684	12.569134	4.763411
8.8569683	6.9806645	8.8593595	8.0467496	5.5420242	3.4787986	6.4797465	6.3945727
6.8535582	12.369138	12.092863	12.183311	5.5966394	4.5350556	10.724278	4.9669531
6.5245601	11.710117	8.4711666	9.4816037	7.1536264	6.5578351	12.9657	4.7141823
7.905109	5.1826085	9.6494315	9.168716	7.1612361	4.1085898	7.7863201	5.2954027
7.3737117	10.131625	8.2885127	11.277282	13.838746	7.0522086	12.332804	5.4221848
7.6807567	10.740927	10.066624	11.538055	8.1675648	4.4180429	10.710024	5.0544829
8.4769854	12.325018	8.9022873	11.652018	6.4297777	6.2002825	13.328057	5.6185552
7.0031621	10.816737	9.2034903	11.465567	5.7347466	4.911026	9.318105	5.2037239
7.3306322	10.778544	11.34279	11.861773	12.313901	5.9226474	11.598959	5.7274507

6.9249399	11.412153	8.3367101	11.568916	7.5010438	6.2343233	12.989622	5.336638
8.4461745	9.8999396	9.5030011	10.35712	8.9100276	6.1248645	11.326765	6.4596201
9.4435737	7.0027328	6.9689684	7.6676838	7.3677037	3.9887697	9.2499367	5.8202527
7.6326509	10.174056	10.71839	10.415315	10.712309	3.9675927	11.711174	4.2546713
8.81833	8.1484674	9.6681237	12.496215	10.060027	5.8885219	11.423614	5.7568707
7.8393804	4.5983293	10.427159	3.764366	5.5818237	2.8815564	5.6149136	4.7204653
7.6181901	7.8935675	7.9607129	7.1351079	7.121603	3.6432018	8.6968241	4.9312432
7.6164205	8.6311292	9.7046544	9.5886824	10.037844	5.4297733	10.420756	4.6880461
7.0183891	11.262157	9.5599	3.9770047	6.1306116	4.4718563	9.105513	6.0068928
7.4189351	9.683706	6.3606959	6.9113817	6.1997486	7.2366089	9.5608129	4.6334508
7.8545837	13.577263	13.201399	13.683253	12.259208	5.7398359	14.345631	4.8123795
7.8222389	11.522676	13.259435	10.586709	7.8107322	5.2288753	12.332943	5.1925515
6.7045889	12.281673	9.574248	12.420997	12.140844	5.588436	12.66594	5.1480236
7.7884442	9.8482697	9.6878474	11.428311	7.7203409	4.3243929	11.794378	5.7381317
7.3828021	12.031675	12.371245	12.625064	9.3038706	4.793095	13.722131	4.8131353
7.9448811	9.8787618	7.8817359	11.149978	8.5732965	4.6308873	12.32522	4.7039411
7.7363772	11.932598	10.396649	11.256603	13.474774	5.8081535	13.750165	4.0939086
7.1649668	12.65477	7.4131076	11.21594	6.7005011	6.9263273	13.282365	4.9792163

XYLT2
7.707767
8.8927602
7.7549333
7.742587
7.7799835
7.5029044
6.9208506
9.8971872
7.7114206
7.3955923
7.8807729
7.9135893
7.6804252
8.4904585
8.3097237
8.3955536
8.1820606
6.7310921
7.858089
7.2103096
7.8215145
6.8505878
6.6180094
7.5276968
7.4883071
6.2771584
6.732157
6.8060027
8.5549396
8.3840121
6.6519349
8.037511
8.4640742
7.5648845
8.0491943
7.9280725
7.3311222
7.4363797
8.0922813
7.5107642
8.1933744
7.567195
10.404708
8.82275
7.7070255
8.2773839
7.9406645
8.5795007
9.2361349
8.4174849

8.2799936
8.8279196
8.1617045
8.652098
7.1277435
8.389517
8.375367
8.7989995
7.7080819
7.6555979
8.087409
8.9171454
7.6261346
8.3223376
6.7296723
8.7361078
7.7043521
7.3208839
7.9371854
8.0151556
8.6551413
8.1092226
8.1396189
8.2760392
7.2005999
8.6649766
7.0238604
8.8509883
8.0366444
7.876468
6.6806185
6.9841714
8.1208848
7.0268831
7.3780117
6.7149116
6.7575912
7.8171591
8.3470851
8.303155
7.6350236
8.02314
8.2673793
8.4149267
7.2628479
8.6069814
6.779228
8.7846457
8.2356794
8.0995925
8.1752826

7.8628261
8.4049299
7.6575344
7.851991
7.2998119
7.8747333
9.5190272
7.6319349
7.8926134
7.8368329
7.0003759
9.1202801
8.174889
7.6021159
7.2823676
7.3727025
8.8081863
7.3207617
8.4646712
7.2630519
9.1986354
7.5879518
8.9015548
7.7651704
9.0416358
7.2135181
7.7149992
8.2459426
7.5420907
7.8720788
8.2962334
8.8284446
7.5834287
8.1378476
8.5684714
8.6100879
6.9212373
8.9843301
8.297159
7.8013676
8.0779616
8.692981
6.9830911
6.4108372
7.219533
7.6126034
7.4742665
6.8615048
8.1149491
8.2044432
9.14711

8.7140947
7.4317728
7.8830378
6.9371369
6.3532153
7.2568733
8.0537834
7.1854091
8.7144101
8.6080876
6.513965
8.2320827
8.4421212
8.6483202
7.2121627
7.617561
7.3016738
7.9306489
7.9536865
7.781665
7.778938
7.8419359
8.7597782
8.296547
8.9819949
7.6354461
8.1532821
7.7556892
9.2232217
8.5522749
7.9692536
8.1017304
7.5420957
8.6035253
7.5445633
6.9867458
7.0192297
7.8227586
6.8446687
8.2704739
8.8952944
7.9130498
8.8912286
7.8544178
7.9939629
7.272175
7.4357463
8.7057518
8.7020616
7.4427102
8.2028235

6.8799897
7.4226023
6.7082093
8.6664919
7.426146
8.2580095
7.5898742
9.5967036
7.2756242
7.1601038
8.8115728
6.5750931
8.2160989
8.1851503
8.479053
7.9035791
8.3611331
8.3656169
7.590278
7.8654185
7.7765742
8.5482353
7.9367244
7.0841745
7.8121289
8.2874409
7.5691291
8.3093453
7.4045607
7.8158406
7.1979048
7.4882099
9.1202939
7.2935646
7.6197332
7.6374304
8.17407
7.6096321
7.3290996
6.2783207
8.5972223
7.4655922
9.3535292
8.0078297
7.8783996
8.6215693
7.9687761
7.8804752
8.0272444
7.9553134
8.4250028

7.3418244
9.8071797
8.9147762
6.7322542
8.585745
5.8207783
7.8076079
8.02631
8.2112553
7.3539832
7.7721189
8.0609024
7.949667
7.2029493
8.9874442
7.7290011
7.3260189
7.9214867
