# Supplementary material for: The Landscape of Using Glycosyltransferase Gene Signatures for Overall Survival Prediction in Hepatocellular Carcinoma
Source: J Oncol. 2022 Jun 21;2022:5989419. doi: 10.1155/2022/5989419 (PMC9239767; doi:10.1155/2022/5989419)
Supplement: Supplementary Materials — Figure S1: Venn diagram shows the intersections of co-differentially expressed glycosyltransferase (GT) genes between the 210 GTs obtained from the literature and those obtained from TCGA database. Figure S2: identification of a four-gene signature in the training set of TCGA database constructed using the LASSO method. Figure S3: survival analyses grouped by the optimal cutoff expression value of each gene in the training dataset of TCGA database. (A) ALG3. (B) B3GAT3. (C) GLA. (D) ST6GALNAC4. Figure S4: mRNA expression levels of the four genes in matched HCC and adjacent noncancerous samples in TCGA database were compared. (A) ALG3. (B) B3GAT3. (C) GLA. (D) ST6GALNAC4. Figure S5: the interactions among signature genes displayed in a PPI network. Figure S6: stratified survival analysis between high- and low-risk groups. (A)NAFLD. (B) HBV infection. (C) HCV infection. (D) Both HBV and HCV infections. (E) No treatment and therapy. (F) Pharmaceutical therapy. (G) Radiation therapy, NOS. Figure S7: the nomogram to predict prognostic probabilities in TCGA database. (A) The nomogram for predicting OS in the training set. The calibration plots for predicting (B) 3-year and (C) 5-year survival probabilities. Figure S8: survival analyses grouped by the optimal cutoff expression value of each signature gene in the validation dataset of TCGA database. (A) ALG3. (B) B3GAT3. (C) GLA. (D) ST6GALNAC4. Table S1: the clinical information of 371 primary HCC samples and 50 normal samples. Table S2: combining the clinical information and expression profiles of 210 human glycosyltransferase (GT) genes. Table S3: DEGs of 371 samples in TCGA database. Table S4: all significantly enriched GO terms. Table S5: all significantly enriched KEGG pathways. [file 5989419.f1.zip › 5989419.f1/Supplementary Table.3 LIHC DEGs sig.pdf]

	logFC	AveExpr	t	PValue	adj.P.Val	B
HAMP	-6.215808	7.9803831	-12.44219	7.75E-29	8.37E-27	54.771656
MT1H	-5.788072	8.4196697	-12.35767	1.55E-28	1.58E-26	54.086819
MT1G	-5.657436	9.7168437	-9.804198	8.08E-20	1.64E-18	34.25979
CYP1A2	-5.60258	6.3088236	-11.67721	3.87E-26	2.53E-24	48.629267
CLEC4G	-5.498155	5.356383	-29.21474	6.16E-89	2.78E-85	191.36866
FCN3	-4.961	6.7782158	-18.14063	7.11E-50	9.16E-47	102.69591
MARCO	-4.874071	6.3974808	-20.05129	5.78E-57	1.04E-53	118.83587
CYP3A4	-4.804377	9.6838897	-7.346982	2.01E-12	1.31E-11	17.511244
MT1M	-4.791746	8.1573282	-10.60558	1.82E-22	6.08E-21	40.273564
CRHBP	-4.646921	5.0453921	-21.42913	5.15E-62	1.33E-58	130.32261
MT1F	-4.503964	7.7132646	-10.83689	3.02E-23	1.19E-21	42.049144
C9	-4.311951	8.974837	-7.602511	3.93E-13	2.82E-12	19.111325
SLC25A47	-4.264047	8.2930386	-8.811311	1.10E-16	1.33E-15	27.150476
HSD17B13	-4.242039	6.6583481	-7.78241	1.22E-13	9.44E-13	20.25975
CXCL14	-4.214687	5.3417439	-16.28285	6.15E-43	4.83E-40	86.89402
MT1E	-4.109401	8.5390795	-9.029166	2.34E-17	3.12E-16	28.674544
CLEC4M	-3.997868	3.619805	-35.48896	2.70E-108	4.88E-104	234.97084
FCN2	-3.873812	4.9067645	-25.78853	1.82E-77	5.46E-74	165.40891
IGFALS	-3.872062	7.0366122	-10.54879	2.83E-22	8.98E-21	39.840242
CYP2E1	-3.71791	11.604655	-5.48605	8.87E-08	3.13E-07	7.0889155
SLC22A1	-3.691731	8.6356704	-8.053524	2.02E-14	1.77E-13	22.023468
LINC01093	-3.671094	4.6979698	-14.43471	4.50E-36	1.85E-33	71.254356
MT1X	-3.617037	11.294139	-8.829909	9.64E-17	1.18E-15	27.279746
ADH4	-3.589104	10.667143	-6.86597	3.93E-11	2.11E-10	14.602995
CYP2A6	-3.547614	10.239348	-5.628087	4.25E-08	1.56E-07	7.8013475
CYP2C8	-3.520129	9.3770847	-8.228476	6.21E-15	5.81E-14	23.181982
CD5L	-3.416672	5.5281761	-10.89849	1.87E-23	7.72E-22	42.524753
MFSD2A	-3.392646	6.0431828	-8.143452	1.10E-14	9.93E-14	22.617005
THRSP	-3.392096	7.1391778	-6.41281	5.69E-10	2.63E-09	11.994282
HAO2	-3.338697	6.7233038	-8.226465	6.30E-15	5.86E-14	23.168579
APOF	-3.326645	8.2520865	-9.169425	8.53E-18	1.23E-16	29.666848
IGF2	-3.195961	8.0932547	-4.92076	1.44E-06	4.33E-06	4.4028862
CLEC1B	-3.181841	3.8883898	-32.59634	1.24E-99	1.12E-95	215.4961
UROC1	-3.179609	5.6954388	-8.08288	1.66E-14	1.46E-13	22.216763
NAT2	-3.167246	5.625456	-11.0206	7.16E-24	3.19E-22	43.470889
CNDP1	-3.159894	5.0843376	-11.83876	1.05E-26	7.42E-25	49.915392
HGFAC	-3.112478	7.4311156	-6.376246	7.01E-10	3.22E-09	11.78959
SLCO1B3	-3.111214	5.7503006	-7.998139	2.93E-14	2.50E-13	21.660007
MT2A	-3.08796	13.067993	-9.451757	1.10E-18	1.83E-17	31.689594
COLEC10	-3.074711	3.0222671	-20.15592	2.38E-57	4.77E-54	119.71341
DNASE1L3	-3.072888	7.9832144	-11.92929	5.07E-27	3.89E-25	50.638875
CYP2B6	-3.057229	7.9213046	-8.675169	2.86E-16	3.23E-15	26.209007
SFRP5	-3.035619	2.9895428	-8.534941	7.60E-16	8.10E-15	25.248345
NNMT	-3.016543	11.118507	-7.068173	1.14E-11	6.69E-11	15.8086
PCK1	-3.013392	8.8912953	-6.673936	1.24E-10	6.21E-10	13.481501
PZP	-2.914368	5.2332146	-12.78453	4.62E-30	6.27E-28	57.55972
DCN	-2.910262	8.2050329	-8.097004	1.51E-14	1.34E-13	22.309919
FOSB	-2.90134	3.2232898	-9.821334	7.11E-20	1.45E-18	34.385983
BCO2	-2.891362	5.0805045	-12.42829	8.69E-29	9.22E-27	54.658909
C7	-2.86814	6.2602254	-7.144896	7.12E-12	4.28E-11	16.272535

ECM1	-2.847396	5.5914237	-13.56776	6.78E-33	1.75E-30	64.013591
OIT3	-2.834767	5.9132737	-13.78474	1.10E-33	3.29E-31	65.817422
GLYAT	-2.794354	7.5196262	-6.574856	2.22E-10	1.08E-09	12.912009
CFP	-2.769972	6.1539734	-17.23785	1.67E-46	1.88E-43	95.020169
FOS	-2.755256	6.4539919	-8.937139	4.51E-17	5.78E-16	28.028151
CDHR2	-2.738111	5.8494089	-7.446788	1.07E-12	7.18E-12	18.131788
ADH1C	-2.733228	11.628355	-5.574794	5.61E-08	2.02E-07	7.5323179
LY6E	-2.720251	7.08332	-7.989226	3.10E-14	2.64E-13	21.601662
GBA3	-2.683206	7.6812988	-7.736738	1.64E-13	1.25E-12	19.966512
MOGAT2	-2.677641	4.644891	-7.793534	1.13E-13	8.83E-13	20.331346
LCAT	-2.670426	7.899347	-10.47084	5.16E-22	1.56E-20	39.247083
LINC00844	-2.657391	6.5868149	-6.101928	3.30E-09	1.39E-08	10.282394
DBH	-2.646447	4.3835137	-12.63367	1.61E-29	2.00E-27	56.328349
JCHAIN	-2.643082	6.7462476	-6.810095	5.50E-11	2.90E-10	14.274292
BHMT	-2.640443	9.7820589	-5.943794	7.87E-09	3.18E-08	9.4368636
CYP39A1	-2.635113	4.7379074	-9.882526	4.50E-20	9.57E-19	34.8375
CXCL12	-2.633445	5.8526466	-10.6013	1.88E-22	6.25E-21	40.240909
CYP8B1	-2.601008	9.8193868	-5.189342	3.94E-07	1.28E-06	5.6488447
CYP4A11	-2.575567	8.5209298	-7.364374	1.80E-12	1.18E-11	17.618969
CYP26A1	-2.560188	4.0283745	-13.10746	3.18E-31	6.03E-29	60.208906
MT1L	-2.542673	8.9893731	-7.261616	3.44E-12	2.16E-11	16.985057
RDH16	-2.534933	9.1440514	-6.727915	8.98E-11	4.60E-10	13.794395
FXYD1	-2.532594	6.6181809	-7.163074	6.36E-12	3.86E-11	16.382975
AKR1D1	-2.520228	6.2652391	-6.489675	3.65E-10	1.72E-09	12.427461
GYS2	-2.49385	5.188909	-7.843478	8.16E-14	6.48E-13	20.653603
LYVE1	-2.49263	4.1169134	-12.56625	2.80E-29	3.28E-27	55.779477
CLRN3	-2.476514	6.3221738	-6.21431	1.76E-09	7.71E-09	10.893724
AADAT	-2.461244	5.5363108	-11.93939	4.67E-27	3.61E-25	50.719683
SLC10A1	-2.443258	9.0832625	-5.328247	1.98E-07	6.70E-07	6.3148211
HP	-2.426479	15.394764	-5.987432	6.20E-09	2.53E-08	9.6684641
TDO2	-2.426406	9.525062	-6.156418	2.44E-09	1.05E-08	10.577732
ADH1B	-2.424948	9.5700298	-6.031367	4.87E-09	2.01E-08	9.9029738
TAT	-2.397287	7.6886382	-4.790292	2.65E-06	7.69E-06	3.8178624
STAB2	-2.395367	4.5495965	-30.23138	3.23E-92	1.94E-88	198.77876
VIPR1	-2.39167	3.6825504	-18.43975	5.46E-51	7.58E-48	105.23435
SHBG	-2.382047	6.7548308	-7.1171	8.46E-12	5.04E-11	16.104046
CHST4	-2.375392	4.6499269	-9.905218	3.79E-20	8.17E-19	35.005296
AVPR1A	-2.343808	3.7991782	-7.222033	4.41E-12	2.73E-11	16.742522
UGT2B7	-2.341001	11.091728	-6.216245	1.74E-09	7.63E-09	10.904323
AFM	-2.339818	9.3192804	-6.645957	1.46E-10	7.26E-10	13.320044
CYP2C9	-2.335517	10.965959	-5.954092	7.44E-09	3.01E-08	9.4913999
F9	-2.334758	10.353331	-6.04627	4.49E-09	1.87E-08	9.9828218
GHR	-2.333071	6.692006	-9.391397	1.70E-18	2.75E-17	31.254371
INMT	-2.328146	3.5810424	-9.468988	9.66E-19	1.63E-17	31.81411
SERPINA11	-2.322567	9.1811366	-6.875527	3.71E-11	2.00E-10	14.659411
SDS	-2.308576	9.5302666	-4.052861	6.48E-05	0.000157	0.7690384
PTH1R	-2.302608	4.2634339	-16.62387	3.28E-44	3.29E-41	89.7949
ASPG	-2.302032	6.6090089	-7.402649	1.41E-12	9.37E-12	17.856648
KDM8	-2.290803	4.7316441	-10.15859	5.61E-21	1.41E-19	36.891598
SLC22A10	-2.289131	5.5691822	-7.878606	6.47E-14	5.23E-13	20.881068
GCGR	-2.275511	6.6849464	-6.042849	4.57E-09	1.90E-08	9.9644811

FBP1	-2.272513	10.763041	-7.76416	1.37E-13	1.05E-12	20.142436
SPP2	-2.263398	8.9862592	-6.130102	2.82E-09	1.20E-08	10.434844
SAA4	-2.234744	10.331259	-6.068831	3.96E-09	1.66E-08	10.103996
CYP2A7	-2.230861	6.9815484	-5.161117	4.53E-07	1.46E-06	5.5153039
LPA	-2.225712	6.4528656	-9.59818	3.73E-19	6.68E-18	32.751474
GLYATL1	-2.207043	9.342404	-7.413895	1.32E-12	8.75E-12	17.926645
CETP	-2.200592	5.2690435	-12.67253	1.17E-29	1.51E-27	56.645117
SRD5A2	-2.189662	5.990242	-7.669669	2.54E-13	1.88E-12	19.537962
GSTA2	-2.185405	10.291247	-4.209126	3.41E-05	8.58E-05	1.3777238
HSD11B1	-2.184878	10.841297	-4.328243	2.06E-05	5.34E-05	1.855391
GLS2	-2.178683	6.5104049	-7.204285	4.92E-12	3.03E-11	16.63407
DPT	-2.17809	5.1008218	-7.017923	1.56E-11	8.92E-11	15.506658
HPGD	-2.175404	6.2122212	-5.089089	6.43E-07	2.03E-06	5.1772801
ANXA10	-2.172664	7.3658765	-7.12013	8.30E-12	4.96E-11	16.122391
BBOX1	-2.170108	5.9794197	-6.635938	1.55E-10	7.68E-10	13.262352
EGR1	-2.156077	8.1650688	-7.830862	8.86E-14	7.01E-13	20.572076
ANGPTL6	-2.154854	5.4355909	-16.00263	6.83E-42	4.74E-39	84.512188
ACSM5	-2.142652	7.589269	-6.298152	1.10E-09	4.92E-09	11.355367
BCHE	-2.132884	7.7572029	-6.572231	2.25E-10	1.09E-09	12.897004
HBB	-2.132685	8.1095592	-7.770816	1.31E-13	1.01E-12	20.185201
LIFR	-2.123742	4.1630076	-12.90018	1.78E-30	2.71E-28	58.506421
NTF3	-2.122239	3.6624443	-18.66599	7.85E-52	1.18E-48	107.15201
PGLYRP2	-2.116092	8.9417597	-5.668532	3.44E-08	1.28E-07	8.0068957
MFAP4	-2.10157	6.9614286	-5.712078	2.73E-08	1.03E-07	8.229523
KCNN2	-2.084506	3.8259018	-13.54006	8.56E-33	2.17E-30	63.783764
C6	-2.078775	9.5795185	-6.690388	1.12E-10	5.67E-10	13.576669
ALDOB	-2.075889	13.369504	-5.068364	7.11E-07	2.23E-06	5.0807522
ADH1A	-2.047981	11.759646	-5.655724	3.68E-08	1.36E-07	7.9416778
VNN1	-2.033614	7.1008623	-4.999055	9.91E-07	3.05E-06	4.7603512
IL13RA2	-2.022836	3.8492596	-10.4971	4.21E-22	1.28E-20	39.446672
ADGRG7	-2.019121	4.4413332	-13.00533	7.42E-31	1.26E-28	59.3692
ENO3	-2.006235	8.2886259	-7.270024	3.26E-12	2.06E-11	17.036698
IGFBP3	-2.000914	8.4057288	-8.378269	2.24E-15	2.23E-14	24.186198
MBL2	-2.000194	8.2107605	-5.739598	2.36E-08	8.95E-08	8.3709149
NGFR	-1.998039	4.7629491	-7.864267	7.11E-14	5.70E-13	20.78814
SAA1	-1.993736	10.841621	-3.392624	0.0007872	0.0016282	-1.572091
GNMT	-1.988946	8.5927363	-5.130002	5.27E-07	1.68E-06	5.3687964
HBA2	-1.961024	7.3608422	-7.854728	7.57E-14	6.05E-13	20.726378
BMPER	-1.960732	1.5307829	-26.2725	3.99E-79	1.44E-75	169.16682
SAA2	-1.956739	7.2103054	-3.54356	0.000459	0.0009866	-1.07034
DIRAS3	-1.950824	3.1345116	-11.24656	1.20E-24	6.13E-23	45.232882
LINC01554	-1.943003	5.4424562	-3.906369	0.0001163	0.0002725	0.217141
SRPX	-1.941786	4.9331602	-8.680734	2.75E-16	3.12E-15	26.24732
GPD1	-1.939581	7.5144275	-6.255016	1.40E-09	6.21E-09	11.117268
ALB	-1.9241	16.599465	-6.003782	5.67E-09	2.33E-08	9.7555751
ASPDH	-1.923621	5.7811153	-5.882352	1.10E-08	4.34E-08	9.1130263
CPS1	-1.91603	9.9577868	-3.968929	9.08E-05	0.0002159	0.4506019
UGT2B10	-1.900623	10.17506	-5.297334	2.31E-07	7.75E-07	6.1653536
C3P1	-1.880483	9.4295962	-5.350107	1.77E-07	6.05E-07	6.4209534
HRG	-1.875402	13.496712	-3.904006	0.0001174	0.0002749	0.2083911
SMIM24	-1.872396	4.8177087	-4.246013	2.92E-05	7.42E-05	1.5243822

ALDH8A1	-1.866297	8.1837074	-6.851668	4.28E-11	2.29E-10	14.518676
AKR7A3	-1.862631	8.9561438	-5.459247	1.02E-07	3.57E-07	6.9561378
TTR	-1.849654	14.707386	-4.939828	1.31E-06	3.98E-06	4.4895037
HPD	-1.849455	10.886281	-3.574651	0.0004098	0.0008874	-0.964493
DTX1	-1.8336	4.3699536	-6.935306	2.58E-11	1.42E-10	15.013581
MT1A	-1.831368	9.6576226	-4.616288	5.84E-06	1.63E-05	3.0585988
C1QTNF1	-1.830467	4.1605512	-6.961456	2.20E-11	1.23E-10	15.1692
RND3	-1.813958	7.3167638	-9.345787	2.38E-18	3.74E-17	30.926496
GSTZ1	-1.810533	7.7504885	-9.556267	5.08E-19	8.91E-18	32.446644
PRG4	-1.810127	9.0046364	-4.671284	4.56E-06	1.29E-05	3.2959643
HPX	-1.810079	13.102259	-5.849633	1.31E-08	5.12E-08	8.941663
HGF	-1.808458	4.0179289	-9.221853	5.84E-18	8.59E-17	30.03994
SLC27A5	-1.80393	11.717953	-5.599097	4.94E-08	1.80E-07	7.6547467
PRSS8	-1.803249	5.4439329	-4.101924	5.31E-05	0.0001303	0.9579425
FOLH1B	-1.801052	5.287562	-8.482596	1.09E-15	1.13E-14	24.89215
APOA4	-1.799681	5.5782196	-2.991813	0.0030082	0.0056613	-2.80582
ALPL	-1.799512	5.1865948	-6.450756	4.57E-10	2.13E-09	12.207648
ID1	-1.796702	8.8255489	-7.756216	1.45E-13	1.11E-12	20.091428
ACSM3	-1.794145	5.6741171	-7.808744	1.02E-13	8.03E-13	20.429345
AGXT2	-1.78713	7.5926681	-5.965316	7.00E-09	2.84E-08	9.5509226
AOX1	-1.785769	10.517828	-4.647946	5.07E-06	1.42E-05	3.1949409
APOA5	-1.780886	9.4073587	-5.069956	7.05E-07	2.22E-06	5.0881561
CYP1A1	-1.780709	5.4686937	-3.855503	0.0001419	0.0003282	0.0297877
FETUB	-1.778441	9.1190373	-4.451568	1.21E-05	3.24E-05	2.3622601
FABP1	-1.773102	13.862045	-4.016006	7.52E-05	0.0001806	0.6284775
KMO	-1.7663	5.675683	-7.724695	1.78E-13	1.34E-12	19.889378
OXT	-1.766252	4.9773157	-6.291011	1.14E-09	5.12E-09	11.315866
HHIP	-1.762361	2.1817536	-16.1879	1.39E-42	1.05E-39	86.086704
LECT2	-1.760857	9.9706847	-4.196848	3.59E-05	9.01E-05	1.3291581
CYP4F2	-1.758239	8.5856502	-5.101056	6.07E-07	1.92E-06	5.2331628
PHLDA1	-1.75316	5.4403388	-7.544144	5.72E-13	4.01E-12	18.742582
TMEM45A	-1.751528	7.3955139	-5.238809	3.09E-07	1.02E-06	5.8843465
TBX15	-1.750296	5.6505216	-6.235843	1.56E-09	6.89E-09	11.011835
KRT19	-1.750244	5.840539	-3.768173	0.0001987	0.0004507	-0.286698
C8A	-1.750231	10.362426	-6.30437	1.06E-09	4.76E-09	11.389791
ZG16	-1.750128	6.534912	-6.867255	3.90E-11	2.10E-10	14.610577
APOA1	-1.749007	14.747973	-4.557472	7.60E-06	2.09E-05	2.8074401
RCAN1	-1.747094	7.0095382	-10.80306	3.93E-23	1.52E-21	41.788418
LRCOL1	-1.735086	5.9540791	-6.68522	1.16E-10	5.83E-10	13.546758
GSTM1	-1.728758	5.3070507	-2.84012	0.0048243	0.0087735	-3.234773
PLAC8	-1.71089	4.6979141	-11.20103	1.72E-24	8.48E-23	44.876746
ITLN1	-1.706959	4.3694125	-10.79264	4.26E-23	1.64E-21	41.708214
CXCL2	-1.705252	7.8742083	-5.737337	2.39E-08	9.05E-08	8.3592796
ACSL1	-1.699667	10.211324	-6.88318	3.54E-11	1.92E-10	14.704632
SLC7A2	-1.699375	5.372109	-6.666676	1.29E-10	6.48E-10	13.439557
NPY1R	-1.693733	3.6152726	-10.87091	2.31E-23	9.43E-22	42.311681
COLEC11	-1.693458	8.2084256	-5.67832	3.26E-08	1.22E-07	8.056819
HOGA1	-1.692458	5.751196	-6.869565	3.84E-11	2.07E-10	14.624209
CRP	-1.690709	8.9035169	-2.594581	0.0099454	0.0170762	-3.884208
ATF5	-1.686153	9.7217917	-4.500716	9.77E-06	2.64E-05	2.5677271
FCGR2B	-1.677722	4.9784722	-7.899086	5.65E-14	4.61E-13	21.01399

GPM6A	-1.672946	3.8327583	-14.16554	4.40E-35	1.66E-32	68.997209
PLG	-1.672885	11.486635	-5.252448	2.89E-07	9.56E-07	5.9496036
PON1	-1.666513	8.000141	-4.504013	9.63E-06	2.61E-05	2.5815801
S100A8	-1.665574	6.1706458	-7.02016	1.54E-11	8.81E-11	15.520071
TIMD4	-1.659368	4.4420651	-12.24168	4.00E-28	3.78E-26	53.149316
AZGP1	-1.657905	12.311189	-5.724315	2.56E-08	9.66E-08	8.2923282
TACSTD2	-1.656171	4.4263076	-5.826741	1.48E-08	5.76E-08	8.8222146
PBLD	-1.655452	8.0060906	-6.959434	2.23E-11	1.24E-10	15.157148
H19	-1.637615	8.2799253	-3.187708	0.0015886	0.0031307	-2.22089
ADAMTS13	-1.637567	4.5310939	-16.43326	1.69E-43	1.45E-40	88.173261
SERPINE1	-1.633398	5.7132989	-5.166776	4.40E-07	1.42E-06	5.5420304
SOCS2	-1.629943	5.0983628	-8.217346	6.70E-15	6.21E-14	23.107821
CYP2B7P	-1.626067	4.8188997	-3.541371	0.0004627	0.000994	-1.077757
ASS1	-1.609489	11.073942	-7.627048	3.35E-13	2.43E-12	19.26691
OLFML3	-1.607924	4.8987497	-6.824418	5.04E-11	2.67E-10	14.358367
MAT1A	-1.607097	12.024197	-6.243314	1.49E-09	6.62E-09	11.052889
SLC39A5	-1.603291	8.470541	-4.317436	2.16E-05	5.59E-05	1.8115702
GREM2	-1.601161	4.9829987	-4.865509	1.86E-06	5.53E-06	4.1535057
ADAMTSL2	-1.582624	5.9149457	-5.622336	4.38E-08	1.61E-07	7.7722155
SOCS3	-1.580573	5.3971552	-5.934656	8.27E-09	3.33E-08	9.388531
GNAO1	-1.579164	2.9525818	-7.798364	1.10E-13	8.57E-13	20.362447
CFHR3	-1.576051	9.1726776	-4.177756	3.89E-05	9.71E-05	1.2538899
DNAJC12	-1.57588	6.4140451	-5.564769	5.91E-08	2.13E-07	7.4819402
ORM1	-1.563283	14.726579	-5.00205	9.77E-07	3.01E-06	4.7741202
FGB	-1.562707	13.69445	-4.884193	1.71E-06	5.08E-06	4.2375688
SULT2A1	-1.55536	10.238865	-3.902475	0.0001181	0.0002764	0.2027203
IL1RAP	-1.554674	4.9041678	-8.661166	3.16E-16	3.54E-15	26.112654
CSRNP1	-1.552835	5.5076332	-10.51935	3.55E-22	1.09E-20	39.615961
CDA	-1.552764	6.8817609	-5.534646	6.91E-08	2.47E-07	7.3310072
OGDHL	-1.55145	8.896025	-5.088323	6.45E-07	2.04E-06	5.1737035
ALDH6A1	-1.547383	9.0969466	-6.777288	6.69E-11	3.48E-10	14.082208
PROZ	-1.54538	6.7520548	-6.282691	1.20E-09	5.35E-09	11.269884
IGFBP1	-1.544078	11.091413	-4.021476	7.36E-05	0.0001769	0.6492655
FAM151A	-1.541997	4.3062012	-5.479021	9.20E-08	3.24E-07	7.0540449
CYP2C19	-1.535315	5.7845007	-10.62455	1.57E-22	5.38E-21	40.418571
CD4	-1.534099	8.8877428	-7.59331	4.17E-13	2.98E-12	19.05307
HAO1	-1.53342	10.011119	-5.075083	6.88E-07	2.17E-06	5.1120113
GGT5	-1.529604	6.3197885	-5.798056	1.73E-08	6.67E-08	8.6730627
STEAP4	-1.523504	3.2566062	-7.71454	1.90E-13	1.43E-12	19.824397
KLKB1	-1.520002	8.3846317	-6.716805	9.60E-11	4.89E-10	13.729846
LRAT	-1.519885	2.7314915	-14.3592	8.54E-36	3.35E-33	70.620371
PDGFRA	-1.518131	3.5720475	-6.575801	2.21E-10	1.07E-09	12.917408
STEAP3	-1.504528	7.972074	-6.58294	2.12E-10	1.03E-09	12.958236
ADH6	-1.501417	7.5707085	-5.612614	4.61E-08	1.69E-07	7.7230263
ZFP36	-1.500102	9.1406799	-7.693167	2.18E-13	1.63E-12	19.687821
PHGDH	-1.496693	7.1266115	-5.436795	1.14E-07	3.98E-07	6.8453199
SLC28A1	-1.488602	5.5739908	-4.813166	2.38E-06	6.95E-06	3.9194647
CPED1	-1.484928	4.2049949	-7.354495	1.92E-12	1.25E-11	17.55776
TFPI2	-1.48416	4.1836874	-7.829216	8.96E-14	7.08E-13	20.561441
FAM13A	-1.482315	3.1499959	-8.207966	7.14E-15	6.59E-14	23.045361
GADD45B	-1.482096	8.6438723	-6.990599	1.84E-11	1.04E-10	15.343122

HSD17B6	-1.481138	11.532282	-4.312856	2.20E-05	5.68E-05	1.7930278
MAN1C1	-1.473264	5.1915983	-7.0196	1.54E-11	8.84E-11	15.516714
BGN	-1.467138	7.0769844	-4.862338	1.89E-06	5.60E-06	4.1392687
GBP7	-1.46667	6.8185361	-4.478666	1.08E-05	2.89E-05	2.4753033
ESR1	-1.465999	3.0227087	-10.52755	3.33E-22	1.03E-20	39.678402
DMGDH	-1.458084	7.5236243	-6.122212	2.95E-09	1.25E-08	10.392099
PPP1R1A	-1.452283	7.6506288	-3.578559	0.0004039	0.0008757	-0.951126
MFAP3L	-1.444349	2.8728519	-6.762643	7.30E-11	3.79E-10	13.996675
CCL21	-1.444227	6.2592559	-3.16746	0.0016995	0.0033256	-2.282958
SLC13A5	-1.441427	9.3677604	-3.918042	0.0001111	0.0002611	0.2604511
ACADS	-1.440039	7.8088155	-9.748121	1.23E-19	2.42E-18	33.847618
VSIG4	-1.439705	7.1033122	-6.542933	2.67E-10	1.29E-09	12.729866
PHYHD1	-1.439444	6.4141871	-4.766706	2.95E-06	8.52E-06	3.7135373
PLA2G2A	-1.437184	8.2625951	-2.128592	0.0341192	0.0527321	-4.96171
IDO2	-1.433696	4.3648732	-7.455803	1.01E-12	6.80E-12	18.188126
WDR72	-1.43322	3.7239667	-5.643348	3.92E-08	1.45E-07	7.878768
C1R	-1.431898	11.786708	-7.622983	3.44E-13	2.49E-12	19.241115
AQP9	-1.428615	10.550879	-3.389847	0.0007949	0.001643	-1.581134
EPHA2	-1.426848	5.0049304	-6.845101	4.45E-11	2.38E-10	14.480004
SLC22A7	-1.425475	8.7923526	-3.422814	0.0007077	0.0014758	-1.473339
ORM2	-1.424386	13.715739	-5.19332	3.86E-07	1.26E-06	5.6677155
CCBE1	-1.421937	3.0782451	-16.48528	1.08E-43	1.03E-40	88.615831
ACADSB	-1.421639	8.044303	-6.838933	4.62E-11	2.46E-10	14.443702
GPR182	-1.42113	3.1890108	-16.44387	1.54E-43	1.39E-40	88.263478
FTCD	-1.418845	10.127026	-4.727309	3.53E-06	1.01E-05	3.5402541
CD163	-1.418573	7.811607	-6.582405	2.12E-10	1.03E-09	12.955177
SULT1E1	-1.414229	3.7613231	-4.690936	4.17E-06	1.18E-05	3.3813687
OTC	-1.410178	9.4815564	-3.703513	0.0002539	0.0005674	-0.516783
RDH5	-1.40942	6.8769895	-6.577979	2.18E-10	1.06E-09	12.929858
ARG1	-1.409338	11.761205	-3.640921	0.0003209	0.000707	-0.736058
PLIN1	-1.406797	4.2828105	-5.653105	3.73E-08	1.38E-07	7.928355
IGLL5	-1.403713	7.0827875	-3.704043	0.0002534	0.0005665	-0.514913
CPEB3	-1.403664	4.0581377	-11.12327	3.19E-24	1.50E-22	44.269675
IL33	-1.400231	5.5648255	-6.983595	1.92E-11	1.08E-10	15.301275
SLC38A4	-1.397766	8.1957807	-5.057435	7.49E-07	2.35E-06	5.0299816
FITM1	-1.396291	3.4930538	-9.643806	2.66E-19	4.95E-18	33.084093
PAMR1	-1.395417	4.322997	-8.67332	2.90E-16	3.27E-15	26.196276
CCL19	-1.394899	7.1867738	-3.259069	0.0012489	0.0025048	-1.999209
C8B	-1.393841	9.8116079	-5.34328	1.83E-07	6.25E-07	6.38777
SLC46A3	-1.391713	5.761987	-4.923231	1.42E-06	4.28E-06	4.4140934
HAND2	-1.389974	3.6857343	-7.298089	2.74E-12	1.74E-11	17.209356
SLC3A1	-1.388916	4.823176	-4.851893	1.99E-06	5.87E-06	4.0924192
RASGEF1B	-1.386187	3.8515136	-7.341464	2.08E-12	1.35E-11	17.477108
TUBE1	-1.382509	5.2062018	-9.552222	5.24E-19	9.16E-18	32.41726
MT1JP	-1.380935	4.6321376	-7.942172	4.25E-14	3.54E-13	21.294355
FMO3	-1.379251	10.150326	-4.002366	7.94E-05	0.0001902	0.5767445
ACADL	-1.379155	4.7846642	-5.353359	1.74E-07	5.96E-07	6.4367678
PDK4	-1.374258	7.4058432	-4.454212	1.20E-05	3.20E-05	2.3732655
NR1I2	-1.372051	6.5718489	-4.7829	2.74E-06	7.94E-06	3.7851181
ANO1	-1.365982	5.8954383	-4.457713	1.18E-05	3.16E-05	2.3878429
FAM180A	-1.365655	3.5676931	-10.01335	1.68E-20	3.86E-19	35.807455

CP	-1.365517	10.883286	-4.628467	5.53E-06	1.54E-05	3.1109557
ALDH2	-1.364325	11.101955	-8.036075	2.27E-14	1.97E-13	21.908789
CA2	-1.360061	8.3258758	-5.27154	2.62E-07	8.74E-07	6.0411879
IL1RN	-1.359489	6.44448	-5.805732	1.66E-08	6.41E-08	8.7129182
PVALB	-1.358382	2.9414564	-17.4386	2.97E-47	3.57E-44	96.728287
MS4A6A	-1.358165	6.3970713	-6.943328	2.46E-11	1.36E-10	15.061276
PLSCR4	-1.3555	6.5829596	-7.267231	3.32E-12	2.09E-11	17.019538
ABAT	-1.354189	8.9354462	-5.380211	1.52E-07	5.24E-07	6.5676891
SLC1A1	-1.353221	5.7386526	-4.602509	6.22E-06	1.73E-05	2.9995084
ADRA1A	-1.352855	2.3424488	-14.45995	3.63E-36	1.56E-33	71.46632
SLC5A1	-1.352331	2.9618163	-7.625546	3.38E-13	2.45E-12	19.257377
THBS1	-1.342019	5.8934303	-4.637846	5.30E-06	1.48E-05	3.1513539
SLITRK3	-1.339463	1.4437409	-8.217282	6.70E-15	6.21E-14	23.107388
ETFDH	-1.338858	8.720948	-8.253598	5.24E-15	4.95E-14	23.349621
NR4A1	-1.338535	4.5069596	-6.141989	2.64E-09	1.13E-08	10.499332
G0S2	-1.338149	10.044627	-3.898678	0.0001199	0.00028	0.1886708
APOC3	-1.335722	15.076985	-3.603238	0.0003689	0.0008048	-0.866422
DUSP1	-1.335386	9.1094722	-6.004051	5.66E-09	2.33E-08	9.757011
NPC1L1	-1.333992	5.0196966	-3.520822	0.0004985	0.0010643	-1.147209
ATOH8	-1.332004	4.4959711	-7.005559	1.68E-11	9.56E-11	15.432605
CXCR2P1	-1.330606	3.4739876	-6.473607	4.00E-10	1.88E-09	12.336592
EHD3	-1.330125	4.2738196	-9.103514	1.37E-17	1.90E-16	29.199477
PON3	-1.328188	11.397379	-5.661353	3.57E-08	1.32E-07	7.9703271
IYD	-1.327914	5.7808198	-6.257238	1.38E-09	6.13E-09	11.129497
MRC1	-1.326205	6.1968084	-5.419059	1.25E-07	4.34E-07	6.7580421
ACSM2A	-1.322975	7.9021457	-4.218814	3.27E-05	8.26E-05	1.416133
XDH	-1.320274	5.8283337	-4.745596	3.25E-06	9.33E-06	3.6205323
HAL	-1.319332	6.6766525	-3.153746	0.0017786	0.003473	-2.324785
APCS	-1.315611	12.205188	-3.882306	0.0001278	0.0002972	0.1282336
AGXT	-1.315494	10.499173	-3.801328	0.000175	0.0004005	-0.167319
PLPP3	-1.312309	8.8374319	-6.768799	7.04E-11	3.66E-10	14.032613
TPPP2	-1.309314	4.589549	-8.671481	2.94E-16	3.31E-15	26.183621
FGL1	-1.308836	13.111856	-3.775924	0.0001929	0.0004387	-0.258876
MT1B	-1.306917	7.4728342	-4.045995	6.66E-05	0.0001612	0.7427642
TRIB1	-1.306678	7.4460393	-7.938553	4.35E-14	3.62E-13	21.27077
AQP3	-1.302459	8.1126313	-6.017782	5.25E-09	2.16E-08	9.8303195
TSLP	-1.300658	3.9068456	-9.437129	1.22E-18	2.01E-17	31.583985
SLC27A2	-1.297054	9.5345207	-4.829988	2.20E-06	6.46E-06	3.994445
RAB25	-1.293488	2.8657719	-3.694846	0.0002623	0.0005853	-0.54735
SLCO1B1	-1.292606	9.11282	-4.099765	5.36E-05	0.0001314	0.9495876
INHBC	-1.290715	6.1254779	-4.282957	2.50E-05	6.40E-05	1.6724038
KCND3	-1.283816	1.8210684	-7.884657	6.22E-14	5.03E-13	20.92032
ANGPTL1	-1.283796	3.6114766	-5.43392	1.16E-07	4.04E-07	6.8311524
AHSG	-1.281886	14.16213	-3.128779	0.0019315	0.0037508	-2.400499
ACAA2	-1.281781	10.399617	-7.534939	6.07E-13	4.24E-12	18.684604
C11orf96	-1.281524	8.2159483	-4.991567	1.03E-06	3.16E-06	4.7259546
FGG	-1.27938	14.562672	-4.178543	3.87E-05	9.68E-05	1.2569846
CAMK2B	-1.277888	3.2987785	-6.70808	1.01E-10	5.14E-10	13.679204
MASP1	-1.276233	5.95265	-6.787399	6.30E-11	3.29E-10	14.141337
OAT	-1.272961	6.4283812	-3.51943	0.000501	0.001069	-1.1519
EPHX2	-1.271407	9.5422696	-6.699659	1.06E-10	5.39E-10	13.630375

PLIN2	-1.2684	9.4756692	-5.916093	9.15E-09	3.66E-08	9.2905379
MPPED1	-1.267863	5.6727781	-5.146367	4.87E-07	1.56E-06	5.44576
RNF125	-1.267283	4.3444846	-8.560838	6.35E-16	6.85E-15	25.42506
NAAA	-1.265279	6.2212214	-8.184203	8.39E-15	7.66E-14	22.887331
CCL14	-1.264254	5.9574571	-9.130108	1.13E-17	1.58E-16	29.387831
COX6A2	-1.262408	8.8882982	-3.63419	0.000329	0.0007237	-0.759434
HABP2	-1.26171	9.3421371	-3.595437	0.0003797	0.0008262	-0.893255
SKAP1	-1.261709	6.776602	-5.853951	1.28E-08	5.01E-08	8.9642358
SFRP1	-1.260347	3.9972278	-9.202024	6.74E-18	9.85E-17	29.898693
RSPO3	-1.251472	3.210059	-10.04811	1.29E-20	3.05E-19	36.066258
FGA	-1.251343	13.222761	-4.193366	3.64E-05	9.13E-05	1.315407
KBTBD11	-1.249132	5.303088	-10.28448	2.15E-21	5.89E-20	37.837291
GSTA1	-1.249065	11.414512	-3.052905	0.0024736	0.0047137	-2.627133
LRG1	-1.247296	11.115632	-4.914737	1.48E-06	4.45E-06	4.3755843
GCH1	-1.247085	6.0370447	-6.389273	6.51E-10	2.99E-09	11.862416
A1BG	-1.246345	10.589094	-4.35276	1.86E-05	4.84E-05	1.9551602
CTH	-1.245154	8.2868875	-4.079869	5.81E-05	0.0001417	0.8727755
PTGIS	-1.243988	3.4998106	-6.172436	2.23E-09	9.64E-09	10.664936
CLDN2	-1.243538	4.7535963	-2.971061	0.0032127	0.0060204	-2.865744
DHODH	-1.24238	7.8466504	-7.217164	4.54E-12	2.81E-11	16.712753
DPYS	-1.238728	8.7006577	-4.293377	2.39E-05	6.14E-05	1.7143563
HMGCS2	-1.23157	11.4333	-4.014444	7.57E-05	0.0001817	0.6225461
CYP2C18	-1.230836	8.1385858	-4.11033	5.13E-05	0.0001261	0.9905082
EGR2	-1.228402	3.9263405	-6.650055	1.43E-10	7.10E-10	13.34366
HS3ST3B1	-1.22552	3.3978339	-5.996705	5.90E-09	2.41E-08	9.7178451
LINC01370	-1.225017	3.6030936	-3.27154	0.001197	0.0024087	-1.96
EHHADH	-1.223793	7.7537504	-4.720153	3.65E-06	1.04E-05	3.5089122
AGTR1	-1.221814	6.4011833	-5.576753	5.55E-08	2.01E-07	7.5421693
CD1D	-1.218131	4.4849984	-6.641127	1.50E-10	7.46E-10	13.292223
S100A12	-1.217006	3.4179008	-10.64471	1.35E-22	4.65E-21	40.57275
PLEK2	-1.213745	5.9446387	-3.661308	0.0002974	0.0006584	-0.665011
MME	-1.208054	3.8703372	-3.260002	0.001245	0.0024983	-1.996281
PRELP	-1.207504	5.1887805	-4.406956	1.47E-05	3.89E-05	2.1774643
ACOT12	-1.207321	7.2726332	-4.548472	7.91E-06	2.17E-05	2.7692532
SERPINA4	-1.206474	9.79532	-3.95896	9.45E-05	0.000224	0.4131779
SLC6A12	-1.205167	7.0704356	-4.417849	1.40E-05	3.72E-05	2.2224381
SLC39A14	-1.200413	7.5319621	-6.726165	9.08E-11	4.64E-10	13.784222
ACAA1	-1.200142	10.231366	-8.091999	1.56E-14	1.38E-13	22.276894
ABCA8	-1.196174	5.0592964	-5.312755	2.14E-07	7.21E-07	6.2398277
CYP3A43	-1.194207	3.9085847	-5.7843	1.86E-08	7.17E-08	8.6017438
EPB41L4B	-1.191218	5.11963	-6.466716	4.17E-10	1.96E-09	12.297668
AMDHD1	-1.187595	9.0931279	-4.833187	2.17E-06	6.37E-06	4.008729
ITGA9	-1.187484	4.4249165	-7.4679	9.33E-13	6.32E-12	18.263789
NAPSB	-1.185819	5.9330256	-5.242902	3.02E-07	1.00E-06	5.9039116
SAMD5	-1.182603	4.6253991	-6.153305	2.48E-09	1.06E-08	10.560807
NOCT	-1.182411	3.2340649	-5.838513	1.39E-08	5.42E-08	8.883595
MAGI2-AS3	-1.181985	3.7678474	-6.043282	4.56E-09	1.89E-08	9.9668009
LEAP2	-1.176663	10.493956	-4.477889	1.08E-05	2.90E-05	2.4720531
CD14	-1.173304	10.470638	-5.267678	2.67E-07	8.90E-07	6.0226381
GNE	-1.172809	8.4841718	-6.366787	7.41E-10	3.39E-09	11.736777
DUSP6	-1.170581	8.2216112	-6.606088	1.85E-10	9.07E-10	13.090841

SHMT1	-1.168629	7.9936459	-5.338422	1.88E-07	6.39E-07	6.3641796
DUSP5	-1.168505	5.1942674	-5.351538	1.76E-07	6.01E-07	6.4279127
CFHR4	-1.167868	8.1652683	-3.528735	0.0004844	0.0010364	-1.120511
GADD45G	-1.161093	7.9635412	-4.666563	4.66E-06	1.31E-05	3.2754915
ABCC9	-1.16086	4.2984073	-5.149373	4.79E-07	1.54E-06	5.4599196
TCTEX1D1	-1.156648	2.4926351	-8.798747	1.20E-16	1.44E-15	27.06323
CFTR	-1.155152	2.6372961	-4.484906	1.05E-05	2.82E-05	2.5014176
ADGRG6	-1.15313	6.2059427	-5.146461	4.86E-07	1.56E-06	5.4462027
IRF8	-1.151778	4.9981484	-5.981444	6.41E-09	2.61E-08	9.6366037
DAO	-1.151454	8.757005	-3.58892	0.0003889	0.000845	-0.91563
FPR1	-1.151251	4.9003158	-6.025529	5.03E-09	2.08E-08	9.8717352
HMOX1	-1.14864	9.7365472	-4.965738	1.16E-06	3.54E-06	4.6076538
TMEM220	-1.144208	7.0860086	-6.61293	1.77E-10	8.74E-10	13.130106
PRODH2	-1.143662	9.2327542	-3.883047	0.0001274	0.0002965	0.1309652
TM6SF2	-1.141466	5.5900155	-4.866715	1.85E-06	5.50E-06	4.1589253
LUM	-1.134858	6.9069853	-2.708103	0.0071632	0.0126199	-3.590885
MPC1	-1.132066	9.5982468	-7.585633	4.38E-13	3.12E-12	19.0045
PSAT1	-1.13123	8.6614102	-4.563232	7.41E-06	2.04E-05	2.8319156
ERRFI1	-1.126591	7.9997651	-4.969047	1.14E-06	3.49E-06	4.6227811
CCL23	-1.126426	3.8031488	-14.44759	4.03E-36	1.69E-33	71.362531
CLDN10	-1.12605	3.6954882	-4.88495	1.70E-06	5.07E-06	4.2409826
SLC16A4	-1.124649	3.4355559	-5.898122	1.01E-08	4.01E-08	9.1958912
SLC19A3	-1.123714	3.7590338	-6.566215	2.33E-10	1.13E-09	12.862639
ANG	-1.122263	12.777752	-4.533488	8.45E-06	2.30E-05	2.7058228
EFHD1	-1.12055	4.7402621	-3.638661	0.0003236	0.0007126	-0.743911
NDRG2	-1.118082	9.2919751	-6.927939	2.70E-11	1.49E-10	14.969815
TRPM8	-1.116047	5.3549279	-4.762	3.01E-06	8.69E-06	3.6927723
GATM	-1.114867	12.008413	-4.282867	2.50E-05	6.40E-05	1.6720417
WNT11	-1.114183	4.1909307	-4.824704	2.26E-06	6.61E-06	3.9708674
SARDH	-1.112235	6.7128403	-5.907754	9.58E-09	3.82E-08	9.2465904
LDHD	-1.105696	8.8551596	-5.20774	3.60E-07	1.18E-06	5.7362168
RANBP3L	-1.100751	1.8773466	-8.643752	3.56E-16	3.98E-15	25.992971
CD302	-1.099757	7.2771969	-6.743137	8.21E-11	4.22E-10	13.882966
PPP1R3B	-1.099405	6.5368247	-5.331647	1.94E-07	6.60E-07	6.3313081
TNFRSF11B	-1.096262	5.375934	-3.755041	0.0002089	0.0004725	-0.333721
CYP4V2	-1.096212	7.5171251	-6.145558	2.59E-09	1.11E-08	10.518707
RNF152	-1.093843	4.5924308	-6.896247	3.27E-11	1.78E-10	14.78192
FOXO1	-1.091384	6.3561576	-7.208598	4.79E-12	2.95E-11	16.660409
SLC38A2	-1.090582	7.2198013	-6.054442	4.29E-09	1.79E-08	10.026673
C1RL	-1.090136	7.6721052	-7.845685	8.04E-14	6.40E-13	20.667877
ETS2	-1.08894	8.0458405	-7.076316	1.09E-11	6.38E-11	15.857669
DCXR	-1.086132	12.076579	-4.373709	1.70E-05	4.45E-05	2.0408042
RCL1	-1.08556	8.5076818	-6.382123	6.78E-10	3.11E-09	11.822429
ALDH1B1	-1.084147	8.2356324	-5.698283	2.94E-08	1.10E-07	8.1588501
G6PC	-1.081699	9.4685756	-3.203606	0.0015063	0.0029814	-2.171899
SLC2A2	-1.081048	9.8755721	-3.573749	0.0004111	0.00089	-0.967576
SCP2	-1.078946	9.0301275	-5.252292	2.89E-07	9.56E-07	5.9488574
GLOD5	-1.07584	4.8627585	-6.604062	1.87E-10	9.18E-10	13.079222
HEPACAM	-1.075181	3.5682942	-3.484503	0.0005681	0.0012012	-1.269054
PCK2	-1.075015	10.474092	-4.9343	1.35E-06	4.08E-06	4.4643625
CNTFR	-1.074416	3.288875	-3.633426	0.00033	0.0007255	-0.762086

GRAMD1C	-1.074208	5.0724909	-5.783288	1.87E-08	7.20E-08	8.5965068
LYPD2	-1.070215	1.9518703	-8.474176	1.16E-15	1.20E-14	24.834984
GSPT2	-1.064409	5.0529647	-4.394483	1.55E-05	4.09E-05	2.1260892
TKFC	-1.06369	7.4352537	-4.898839	1.59E-06	4.76E-06	4.3036572
UGT2B15	-1.06365	10.067579	-2.739782	0.0065237	0.0115792	-3.506899
HAAO	-1.063139	7.1100267	-5.656441	3.66E-08	1.36E-07	7.9453235
PEMT	-1.058183	8.6291713	-6.742386	8.24E-11	4.24E-10	13.878591
CAT	-1.057609	10.849035	-6.047399	4.46E-09	1.85E-08	9.9888791
SERPINF2	-1.056252	9.7373295	-4.489103	1.03E-05	2.77E-05	2.5190029
A2M	-1.052521	13.169482	-3.789614	0.000183	0.0004177	-0.209605
MRO	-1.052399	2.6920221	-9.510648	7.11E-19	1.23E-17	32.115651
ADRA2B	-1.043524	3.2309493	-9.714765	1.57E-19	3.06E-18	33.603014
NR0B2	-1.042561	8.523304	-3.976196	8.82E-05	0.0002101	0.4779381
ALDH1L1	-1.040672	8.1159892	-2.640213	0.0087278	0.0151369	-3.767742
RIDA	-1.039864	11.372702	-5.535622	6.87E-08	2.45E-07	7.3358875
PANK1	-1.035147	5.2092952	-6.777552	6.68E-11	3.48E-10	14.083749
SLCO4C1	-1.034803	2.4232683	-5.056731	7.52E-07	2.35E-06	5.0267153
MTTP	-1.033388	9.1861548	-3.588703	0.0003892	0.0008456	-0.916374
LHX2	-1.032575	4.1441913	-8.686402	2.64E-16	3.01E-15	26.28636
LEPR	-1.031994	6.2433024	-3.298813	0.0010903	0.0022092	-1.87377
NR4A2	-1.030584	3.3326118	-4.74691	3.23E-06	9.28E-06	3.6263142
HSD17B2	-1.029383	10.064746	-3.685387	0.0002718	0.000605	-0.580633
DEPDC7	-1.027821	7.391935	-5.294692	2.34E-07	7.85E-07	6.1526126
SERPINA7	-1.027333	9.7526428	-2.52084	0.0122365	0.0206351	-4.068316
SULT1B1	-1.024314	3.9568159	-3.912001	0.0001138	0.000267	0.2380231
ASPA	-1.021474	3.9488492	-7.962322	3.71E-14	3.12E-13	21.425811
AR	-1.020541	7.4784617	-3.949012	9.83E-05	0.0002326	0.3759129
IL1RL1	-1.018808	1.3944255	-9.777211	9.88E-20	1.98E-18	34.061281
AOC1	-1.018296	3.0614304	-4.933285	1.36E-06	4.10E-06	4.4597466
SCGB3A1	-1.018236	3.5673962	-6.937371	2.55E-11	1.41E-10	15.025852
ATF3	-1.015607	5.829473	-5.258123	2.80E-07	9.31E-07	5.9767945
UPB1	-1.013505	9.2141163	-3.257779	0.0012544	0.0025155	-2.003258
ST3GAL6	-1.012721	6.2287359	-5.398676	1.39E-07	4.79E-07	6.6580225
EDNRB	-1.010907	5.2216761	-5.399685	1.38E-07	4.76E-07	6.6629708
JUNB	-1.010683	7.9680085	-5.093533	6.29E-07	1.99E-06	5.1980201
MTHFD1	-1.010427	10.18633	-4.969117	1.14E-06	3.49E-06	4.6231036
CFI	-1.009169	10.490882	-5.611461	4.64E-08	1.69E-07	7.7171949
SLC17A2	-1.007935	6.9243655	-3.704465	0.000253	0.0005656	-0.513425
SLC4A4	-1.00349	4.5875747	-4.513066	9.25E-06	2.51E-05	2.6196651
SIGLEC7	-1.001002	3.7347745	-7.428081	1.20E-12	8.03E-12	18.015044
UGT2B17	-1.00095	7.6537939	-2.411167	0.0165147	0.0271542	-4.33272
STK25	1.0000801	7.8071761	10.751117	5.89E-23	2.21E-21	41.388789
KIF14	1.0006313	2.3452257	7.4495939	1.05E-12	7.06E-12	18.149319
EXTL3	1.0006427	5.090318	7.4308867	1.18E-12	7.90E-12	18.032542
THOC6	1.0007184	5.9786944	9.618516	3.21E-19	5.84E-18	32.899626
TBX2	1.0009742	6.1864237	5.8616532	1.23E-08	4.82E-08	9.0045315
TCAP	1.000997	5.2092764	4.9488656	1.26E-06	3.82E-06	4.5306554
CCT2	1.0010359	9.8911234	8.4017821	1.90E-15	1.91E-14	24.344842
TMEM38B	1.0014092	6.0755443	7.8592791	7.35E-14	5.88E-13	20.755841
PAK1	1.0014506	5.8909594	6.3249091	9.41E-10	4.26E-09	11.503686
CXCL10	1.001582	7.3197395	2.8266179	0.0050268	0.0091115	-3.271933

LOX	1.0017442	3.2378321	4.997508	9.99E-07	3.08E-06	4.753241
GMPS	1.0017544	6.8278144	8.1460443	1.09E-14	9.79E-14	22.634176
RAD51C	1.0018945	6.1566822	9.6240829	3.08E-19	5.64E-18	32.940211
CCT4	1.0019226	10.617747	9.3387844	2.50E-18	3.93E-17	30.876234
CDC23	1.0020727	5.5914167	8.7278323	1.98E-16	2.28E-15	26.572172
MLLT6	1.0022308	5.1518351	7.0736152	1.11E-11	6.48E-11	15.84139
SEC61A1	1.0023548	9.4623356	9.0827089	1.59E-17	2.18E-16	29.052343
CSNK1D	1.0023648	8.2090338	9.1051079	1.36E-17	1.88E-16	29.210761
SRGAP2	1.00241	6.4685018	7.8050118	1.05E-13	8.22E-13	20.405284
BOD1	1.0024256	6.5846803	10.21556	3.64E-21	9.42E-20	37.318861
NOP58	1.0024322	9.0641722	8.5593558	6.41E-16	6.91E-15	25.414934
POLRMT	1.0025093	8.0859178	12.147729	8.60E-28	7.72E-26	52.392069
OAZ3	1.0026631	3.9767581	8.5397592	7.35E-16	7.86E-15	25.2812
SGSM3	1.0031935	7.8540666	10.257622	2.64E-21	7.10E-20	37.635056
CPSF6	1.0035218	4.9629751	8.3442743	2.82E-15	2.77E-14	23.957319
PTOV1-AS2	1.0036026	4.0200213	7.133857	7.63E-12	4.57E-11	16.205568
PRKCA	1.0036993	5.530039	6.9712503	2.07E-11	1.16E-10	15.227592
LLGL2	1.0038325	7.1246046	6.9690216	2.10E-11	1.18E-10	15.214299
UBE2Q2	1.0044901	4.178863	5.9330605	8.35E-09	3.35E-08	9.3801011
FAM168B	1.0045629	7.3398971	7.0339311	1.41E-11	8.15E-11	15.602683
GAPDH	1.0045758	13.01566	7.5278761	6.35E-13	4.42E-12	18.640148
B3GNT5	1.0046527	4.4974412	4.9702985	1.14E-06	3.47E-06	4.628505
LUC7L3	1.004898	6.7938045	8.1753131	8.91E-15	8.11E-14	22.828289
SGO2	1.0050154	3.2341346	7.6957937	2.15E-13	1.60E-12	19.704595
SHCBP1	1.0050418	2.7810022	7.1079993	8.95E-12	5.31E-11	16.048984
MVB12A	1.0052873	6.8897219	8.6101905	4.50E-16	4.95E-15	25.7627
HNRNPH1	1.0053086	7.9095241	7.2839802	2.99E-12	1.90E-11	17.122498
NUP107	1.0053129	6.9698839	7.6347577	3.19E-13	2.31E-12	19.315867
RUVBL2	1.0053924	8.3580123	11.481363	1.86E-25	1.08E-23	47.078766
RGP1	1.0053945	5.4179409	8.0571726	1.97E-14	1.73E-13	22.047467
ZNF544	1.0059502	5.1478706	8.8768866	6.91E-17	8.61E-16	27.606985
SEMA5B	1.0060902	4.5105025	8.2710371	4.65E-15	4.43E-14	23.466176
MIF	1.0062227	11.109185	4.5952322	6.42E-06	1.78E-05	2.9683663
MED12	1.006231	5.1976309	6.9466031	2.41E-11	1.34E-10	15.080757
VAMP1	1.0065102	2.8873196	7.1093388	8.88E-12	5.27E-11	16.057086
CPSF3	1.0067013	6.4149189	11.721949	2.70E-26	1.78E-24	48.984847
THEM4	1.006918	4.6861825	9.6420631	2.70E-19	5.00E-18	33.071375
PTGES2	1.0069937	6.8135547	9.5920598	3.90E-19	6.95E-18	32.706918
BAP1	1.0073142	7.3866054	9.6215972	3.14E-19	5.72E-18	32.922088
IFT81	1.0076345	2.6082848	7.4958689	7.80E-13	5.36E-12	18.439043
EEF1D	1.0078695	9.1765947	8.4423245	1.44E-15	1.47E-14	24.619017
UBR5	1.0079957	5.1285414	7.3472676	2.01E-12	1.30E-11	17.513014
DMPK	1.0084265	5.6866231	6.2129267	1.77E-09	7.76E-09	10.886147
LLGL1	1.0084793	3.3618996	7.2313174	4.16E-12	2.59E-11	16.799326
SLC35B2	1.0089496	8.7661365	7.6408742	3.06E-13	2.23E-12	19.354729
GOLT1B	1.0090903	5.7543703	9.0567414	1.92E-17	2.59E-16	28.86896
ZNF579	1.009345	9.3207535	7.1157677	8.53E-12	5.08E-11	16.095984
GUK1	1.009369	11.1935	10.539375	3.04E-22	9.58E-21	39.768491
RIT1	1.0096223	5.369901	8.0065944	2.77E-14	2.37E-13	21.715389
RBM8A	1.009672	7.3018001	10.828237	3.23E-23	1.27E-21	41.982403
FBXO46	1.0096845	6.45469	7.4738002	8.98E-13	6.10E-12	18.300721

ACD	1.0096901	6.0923441	9.7552958	1.16E-19	2.31E-18	33.900287
APOLD1	1.0097266	5.7390004	5.317192	2.09E-07	7.06E-07	6.2612884
IKBKE	1.0097387	3.5239895	5.3746312	1.56E-07	5.38E-07	6.5404385
PIDD1	1.0098176	6.0065629	9.331612	2.63E-18	4.12E-17	30.824772
IK	1.0103107	9.2811483	11.898842	6.48E-27	4.81E-25	50.39533
ERCC2	1.0104865	6.070515	10.028	1.51E-20	3.49E-19	35.916482
HLA-A	1.0117091	10.724184	5.8793497	1.12E-08	4.41E-08	9.0972731
ACACA	1.0119699	3.6968948	7.7667799	1.35E-13	1.04E-12	20.159268
COL8A1	1.011981	4.1531124	5.2404624	3.06E-07	1.01E-06	5.8922487
MDM4	1.0120443	4.4956315	7.6547661	2.80E-13	2.05E-12	19.44307
KXD1	1.0120603	8.2208714	12.588076	2.34E-29	2.83E-27	55.957051
CANX	1.0121439	8.234054	7.965411	3.64E-14	3.07E-13	21.445982
BCAN	1.0121478	2.7114972	6.136115	2.73E-09	1.16E-08	10.467452
SART1	1.0122613	7.9691387	12.590426	2.29E-29	2.80E-27	55.97618
ZNF623	1.0123287	3.8090845	7.9216632	4.86E-14	4.02E-13	21.16078
NKIRAS2	1.0123859	6.6527199	9.6688239	2.21E-19	4.19E-18	33.266828
SMG7	1.0127758	6.8910465	8.6431591	3.58E-16	4.00E-15	25.988896
SEC11C	1.0131339	11.045325	7.7956729	1.12E-13	8.72E-13	20.345117
LINGO1	1.0131432	5.1203897	4.6443351	5.15E-06	1.44E-05	3.1793487
LMAN2	1.0132048	11.167734	12.393153	1.16E-28	1.20E-26	54.374141
CCDC14	1.013253	6.4306139	6.8661956	3.92E-11	2.11E-10	14.604328
DNAAF5	1.0134745	6.3399113	8.2144235	6.84E-15	6.33E-14	23.088354
WHRN	1.0135017	3.2368553	8.1853627	8.32E-15	7.61E-14	22.89504
SEMA7A	1.0140164	2.5505992	5.7809212	1.90E-08	7.29E-08	8.5842494
TRMT112	1.0141038	10.674197	11.669001	4.13E-26	2.69E-24	48.564116
TMEM160	1.0142028	7.3786027	7.8093689	1.02E-13	8.01E-13	20.433372
RPIA	1.0142362	7.063806	9.1917172	7.27E-18	1.06E-16	29.825344
PA2G4	1.0142761	8.2257674	10.582561	2.18E-22	7.10E-21	40.097807
STAG3L4	1.0143496	3.2770232	7.953017	3.95E-14	3.31E-13	21.36508
RPL13P5	1.0144909	4.9081681	6.8485722	4.36E-11	2.33E-10	14.50044
COPS6	1.014498	9.9406763	13.059905	4.72E-31	8.38E-29	59.817686
TCERG1	1.0147368	5.327211	7.9050689	5.43E-14	4.45E-13	21.052863
ATP6V1H	1.0147546	6.8213965	8.16161	9.77E-15	8.85E-14	22.737355
DNAL4	1.0149064	5.6574081	8.1684279	9.33E-15	8.48E-14	22.782587
MYPOP	1.0150864	6.5627859	11.298612	7.96E-25	4.16E-23	45.640839
TMEM165	1.0155967	6.0321299	7.2510577	3.68E-12	2.30E-11	16.920275
AGO2	1.0157822	5.2787616	8.1610369	9.81E-15	8.88E-14	22.733554
UNK	1.0158037	6.464913	8.9862266	3.18E-17	4.19E-16	28.372473
AGPAT1	1.0160008	7.121718	9.3156524	2.96E-18	4.56E-17	30.71034
CALR	1.0160603	12.258943	9.7751212	1.00E-19	2.01E-18	34.045924
STK39	1.0160773	3.9726924	6.1535267	2.47E-09	1.06E-08	10.562011
RPAP1	1.0162669	7.1242356	9.1522307	9.66E-18	1.37E-16	29.544747
ZSCAN16	1.0163155	4.6069085	8.9843009	3.22E-17	4.24E-16	28.358945
ZBTB40	1.0163593	3.8198419	9.1950427	7.09E-18	1.03E-16	29.849006
HSPB11	1.0165582	8.3549581	9.4571757	1.05E-18	1.77E-17	31.728739
CDC42SE1	1.0165625	6.681307	6.6078601	1.83E-10	8.99E-10	13.101008
CENPE	1.0167007	3.8227592	7.7040397	2.03E-13	1.52E-12	19.757269
IQSEC1	1.0167658	6.1628998	8.3452125	2.81E-15	2.76E-14	23.963628
CLIP2	1.0168912	4.8169957	4.5310614	8.54E-06	2.33E-05	2.695569
JRKL	1.0173731	4.4861029	7.3632192	1.81E-12	1.19E-11	17.611811
COX19	1.0174652	5.2668039	9.3621711	2.11E-18	3.34E-17	31.044179

VAMP5	1.0176166	9.5116715	7.8752909	6.61E-14	5.34E-13	20.859577
RPS16	1.0177511	13.149364	8.2000007	7.54E-15	6.94E-14	22.992359
GRPEL2	1.0178668	5.6116651	10.41896	7.68E-22	2.27E-20	38.853466
SNAPC2	1.0179995	7.2413358	10.520125	3.53E-22	1.09E-20	39.62189
P2RX4	1.0180276	6.4349227	6.4173902	5.54E-10	2.56E-09	12.019988
DSTYK	1.0186303	4.4285529	8.1135472	1.35E-14	1.21E-13	22.419166
ANAPC4	1.0186715	7.3507524	9.4320736	1.27E-18	2.09E-17	31.547506
PDCD6	1.0187426	9.4412352	10.920787	1.57E-23	6.60E-22	42.697164
DSCR8	1.0189077	1.9374839	3.4800387	0.0005773	0.001219	-1.28395
EXOSC9	1.0189505	7.1286209	9.9464378	2.78E-20	6.10E-19	35.31058
NUP155	1.0192594	5.1190193	8.516106	8.65E-16	9.14E-15	25.120028
XPOT	1.0193198	6.6030799	6.5325528	2.84E-10	1.36E-09	12.67078
ZBED8	1.0193559	4.1508723	8.6668735	3.03E-16	3.41E-15	26.151916
TEAD3	1.0194638	6.8189223	7.2914307	2.85E-12	1.81E-11	17.16835
IFI27	1.019634	10.892213	2.4171609	0.0162505	0.026754	-4.318562
LRRC59	1.0197168	8.8115949	8.4553883	1.32E-15	1.35E-14	24.707535
UBL7	1.0197285	6.2872893	11.185261	1.95E-24	9.50E-23	44.753487
TAF9	1.0200915	7.7142012	8.7449779	1.75E-16	2.05E-15	26.690687
PPP1CC	1.020201	8.3225524	9.2331427	5.39E-18	7.95E-17	30.120435
OLA1	1.0202946	6.2119589	9.2520666	4.70E-18	7.02E-17	30.255479
ABHD11	1.0204252	6.418525	7.1786521	5.78E-12	3.52E-11	16.477773
MANEAL	1.0205378	6.5189649	5.6783301	3.26E-08	1.22E-07	8.0568695
SCAF1	1.0208074	7.1938186	10.609379	1.77E-22	5.93E-21	40.302604
ZMYM3	1.0209787	5.5996523	7.7537912	1.47E-13	1.12E-12	20.075869
AMDHD2	1.021093	5.4892032	8.8403467	8.95E-17	1.10E-15	27.352368
EIF3E	1.0211552	9.5796576	6.6609968	1.34E-10	6.68E-10	13.406771
DHX37	1.0215108	6.2148819	8.8251234	9.97E-17	1.21E-15	27.246468
CNOT11	1.0216253	7.0039916	8.3089835	3.59E-15	3.48E-14	23.720318
PIK3IP1	1.0216293	5.9763673	5.1291494	5.29E-07	1.69E-06	5.3647925
PLXNA3	1.0218547	3.377998	6.1621182	2.36E-09	1.02E-08	10.608745
ZBED5	1.0227619	5.4382825	7.6095386	3.75E-13	2.70E-12	19.155853
DYNC1H1	1.0228983	8.4691947	9.144794	1.02E-17	1.44E-16	29.491975
HSP90AA1	1.0232381	10.590642	8.3746757	2.29E-15	2.28E-14	24.161979
MMS19	1.0233533	6.7760695	9.5420934	5.64E-19	9.83E-18	32.343715
TAF11	1.0234552	7.5069381	10.090414	9.40E-21	2.28E-19	36.381767
LZTR1	1.0235124	6.5609876	9.4923351	8.13E-19	1.39E-17	31.983012
PUSL1	1.0236422	6.8164504	8.0437264	2.16E-14	1.88E-13	21.959054
RAN	1.0237484	9.45695	8.43115	1.56E-15	1.58E-14	24.543367
SH2B1	1.0237609	5.8946628	9.2493752	4.79E-18	7.13E-17	30.236263
GGH	1.0239191	9.8290089	4.223193	3.22E-05	8.12E-05	1.4335186
PTGFRN	1.0240481	4.912883	5.4455691	1.09E-07	3.82E-07	6.8885806
IFI27L2	1.0242595	7.1396698	4.7485022	3.21E-06	9.21E-06	3.6333162
MYH4	1.0242697	2.6061472	2.4343135	0.0155152	0.0256393	-4.277857
FANCA	1.0245417	4.6005023	7.485561	8.33E-13	5.70E-12	18.374401
RPS6KA4	1.0247885	6.1524793	9.3279557	2.71E-18	4.21E-17	30.798547
PPDPF	1.0249287	11.014765	4.2271059	3.16E-05	8.00E-05	1.4490683
MGAT5	1.0250894	2.7433716	6.8071532	5.59E-11	2.95E-10	14.257043
TMEM164	1.0251023	5.4968864	7.4608041	9.76E-13	6.59E-12	18.219393
TM4SF20	1.0251273	1.8640592	3.2056095	0.0014962	0.0029621	-2.16571
B3GNT3	1.025259	5.8890768	3.1798532	0.0016308	0.0032076	-2.245012
VPS16	1.0253193	7.6992111	11.99626	2.94E-27	2.35E-25	51.175227

IQCC	1.0255824	3.3934906	9.7887437	9.07E-20	1.83E-18	34.146082
TUBG2	1.0256768	5.1932214	8.4272294	1.60E-15	1.62E-14	24.51684
CRTAP	1.0263823	6.4072257	7.2628081	3.41E-12	2.15E-11	16.992377
GON4L	1.0267457	5.0056134	8.2813268	4.34E-15	4.16E-14	23.535019
AP3B1	1.0272072	6.8688144	9.0570797	1.91E-17	2.59E-16	28.871348
PREB	1.0272881	9.151568	8.2781475	4.43E-15	4.23E-14	23.513742
CYB561D1	1.027421	5.6359856	8.6851189	2.67E-16	3.03E-15	26.27752
TTL	1.0282301	3.4765405	9.8645119	5.15E-20	1.08E-18	34.704436
HSDL1	1.0283985	4.3314318	8.3749226	2.29E-15	2.27E-14	24.163643
LENG8	1.0288068	5.654832	7.0868871	1.02E-11	6.01E-11	15.921435
CLCN2	1.0288252	4.5552634	7.8998715	5.62E-14	4.59E-13	21.019093
PRKAB2	1.0288593	7.1218865	6.5513454	2.55E-10	1.23E-09	12.777799
ERMP1	1.028915	5.3733612	6.2758654	1.24E-09	5.55E-09	11.232194
BYSL	1.0296504	7.1587689	8.2985478	3.86E-15	3.72E-14	23.650354
FLYWCH1	1.0297958	5.2903529	8.882285	6.65E-17	8.31E-16	27.644654
NCOR2	1.0300783	8.7625542	7.4541422	1.02E-12	6.86E-12	18.177742
MIF4GD	1.0301357	6.1795034	9.0493996	2.02E-17	2.71E-16	28.817166
PTPN23	1.0303103	5.792847	8.9080584	5.54E-17	7.01E-16	27.824674
PQBP1	1.0305903	8.1246711	11.013425	7.57E-24	3.37E-22	43.415142
PUS1	1.0306363	7.4835634	9.6029293	3.60E-19	6.49E-18	32.786058
BRAT1	1.0306379	8.0240759	11.127709	3.08E-24	1.46E-22	44.304307
CERS5	1.0308294	5.4081245	9.6346896	2.85E-19	5.26E-18	33.01757
VPS11	1.0313898	6.3479251	9.9070915	3.74E-20	8.08E-19	35.019159
STX16	1.0315673	6.3890697	8.4896456	1.04E-15	1.08E-14	24.940047
BTN2A2	1.0318097	5.8956254	7.3187398	2.40E-12	1.55E-11	17.336694
VNN2	1.0318141	6.1984774	3.5105287	0.0005173	0.0011011	-1.18186
FAM110A	1.0319525	6.4251774	7.2353891	4.05E-12	2.53E-11	16.824255
CCDC18-AS1	1.0320155	5.17788	7.8735408	6.69E-14	5.39E-13	20.848232
MXD4	1.0320799	7.2445345	8.5132182	8.83E-16	9.31E-15	25.100369
DNAJB2	1.0322538	8.1863849	10.106423	8.33E-21	2.04E-19	36.501335
CCDC142	1.0325466	5.4601043	9.5551737	5.12E-19	8.98E-18	32.438699
UQCRH	1.0326933	11.077134	8.7267358	1.99E-16	2.30E-15	26.564597
SYNGR2	1.0328729	9.7596975	8.4213454	1.66E-15	1.68E-14	24.477042
HMGN4	1.0329031	6.8124045	5.8050926	1.67E-08	6.43E-08	8.7095988
RCE1	1.0330038	5.3675439	8.4913294	1.03E-15	1.07E-14	24.95149
SFSWAP	1.0331414	6.4356145	9.8257861	6.88E-20	1.41E-18	34.418786
SAAL1	1.0332092	6.1727925	9.9564929	2.58E-20	5.72E-19	35.385146
STIL	1.0332838	2.3211064	7.6879877	2.26E-13	1.68E-12	19.654765
PRIM2	1.0333785	3.966142	9.7731241	1.02E-19	2.04E-18	34.031247
GAL3ST1	1.0334049	5.4319698	3.4815031	0.0005743	0.0012132	-1.279066
HARS2	1.0334197	7.4936869	10.940186	1.35E-23	5.77E-22	42.847303
C15orf41	1.0334365	4.9899396	8.2581392	5.08E-15	4.82E-14	23.379957
IER5	1.0334489	8.5050177	7.1520608	6.81E-12	4.11E-11	16.316043
NAP1L1	1.0335481	6.1255601	7.2200858	4.46E-12	2.76E-11	16.730613
DGCR2	1.0336985	8.8538808	8.0741436	1.76E-14	1.55E-13	22.15919
RCC2	1.0338554	6.8176051	6.4288368	5.18E-10	2.41E-09	12.084285
KIF18A	1.0338835	2.3929788	7.3555642	1.90E-12	1.24E-11	17.564381
CCDC84	1.0339204	6.701195	7.6318323	3.25E-13	2.35E-12	19.297288
RRM1	1.0343738	7.776552	7.4942987	7.88E-13	5.41E-12	18.429192
ITGA2	1.0350156	2.5441755	5.2879757	2.42E-07	8.10E-07	6.1202473
CBX3	1.0352483	9.6298866	8.9519515	4.06E-17	5.24E-16	28.13194

IP6K2	1.0366457	6.7593732	9.7678559	1.06E-19	2.11E-18	33.992536
LAMA5	1.0367626	5.9332785	5.0964671	6.20E-07	1.96E-06	5.2117209
SSNA1	1.0369093	8.6643902	10.22769	3.32E-21	8.67E-20	37.409986
AP4M1	1.0372228	5.2639644	10.280977	2.21E-21	6.04E-20	37.810883
CCNL2	1.0374867	7.2916996	6.697821	1.07E-10	5.43E-10	13.619724
NOP2	1.037741	6.8014353	8.5702884	5.94E-16	6.44E-15	25.489621
RBMX	1.0380803	6.8036392	9.2485798	4.82E-18	7.16E-17	30.230585
ZMAT5	1.0383365	6.5633126	10.313828	1.72E-21	4.78E-20	38.058519
BORCS6	1.0386454	6.990848	10.249811	2.80E-21	7.50E-20	37.576291
SAP130	1.0386708	4.9764307	8.9379592	4.48E-17	5.76E-16	28.033895
METTL5	1.0390412	8.3761938	11.532383	1.24E-25	7.41E-24	47.481759
DXO	1.0390952	5.7695452	10.683581	9.96E-23	3.56E-21	40.870455
EFTUD2	1.0394929	8.6666293	9.7049339	1.69E-19	3.28E-18	33.531005
RCOR3	1.0398088	5.520628	8.0908095	1.57E-14	1.39E-13	22.26905
PLA2G1B	1.0398373	4.1998993	4.4516716	1.21E-05	3.24E-05	2.3626922
DANCR	1.0399918	6.6164224	5.1241909	5.42E-07	1.73E-06	5.3415159
GCN1	1.040271	8.5973614	9.0680546	1.77E-17	2.41E-16	28.948818
EHD2	1.0402883	5.4987897	5.8843161	1.09E-08	4.30E-08	9.1233396
YY1AP1	1.0402888	7.8368696	9.0846008	1.57E-17	2.15E-16	29.065715
FOXRED2	1.0403908	5.2716794	6.0313716	4.87E-09	2.01E-08	9.902998
ZSWIM5	1.0404778	3.8295027	5.6202156	4.43E-08	1.62E-07	7.7614828
YIPF3	1.0405639	9.6263082	10.675597	1.06E-22	3.76E-21	40.809273
RPL27	1.0407163	12.820474	8.9370448	4.51E-17	5.78E-16	28.027491
DNAJB6	1.0407854	7.2615787	9.5321253	6.07E-19	1.05E-17	32.271376
TRAF7	1.0408101	6.8696174	7.0185916	1.55E-11	8.89E-11	15.510668
LASP1	1.0408424	7.8669343	7.7772109	1.26E-13	9.73E-13	20.226311
HES4	1.0411632	6.2820218	5.7437766	2.31E-08	8.76E-08	8.3924342
SLC22A23	1.041188	6.5620251	7.048487	1.29E-11	7.49E-11	15.690128
CABLES2	1.0411944	4.5164656	7.1463103	7.06E-12	4.25E-11	16.281122
TTLL1	1.0415798	4.2692752	8.8990306	5.91E-17	7.44E-16	27.761583
COPA	1.0415912	7.3767675	7.4873751	8.23E-13	5.64E-12	18.385773
G6PC3	1.0416276	5.6184923	8.3167337	3.41E-15	3.31E-14	23.772313
GTPBP3	1.0421534	5.9994816	10.023308	1.56E-20	3.61E-19	35.881559
PSMC3IP	1.0422639	3.8966059	7.4712034	9.13E-13	6.20E-12	18.284463
NPM2	1.042297	4.2960828	4.53465	8.41E-06	2.29E-05	2.7107367
EEF2KMT	1.0426058	5.4651635	10.619969	1.63E-22	5.52E-21	40.383539
ASXL1	1.0426599	6.5572095	8.2289331	6.20E-15	5.79E-14	23.185033
ZFP62	1.0428355	6.4317657	9.5240214	6.44E-19	1.11E-17	32.212594
EMC2	1.0432821	7.5524618	9.2248679	5.72E-18	8.42E-17	30.061432
DEAF1	1.0434445	6.5669697	10.192652	4.33E-21	1.11E-19	37.146918
SLC26A2	1.0435127	2.9190085	7.9276881	4.67E-14	3.88E-13	21.199998
MIEN1	1.0439637	9.05909	11.571184	9.06E-26	5.56E-24	47.78868
ZNF213	1.0442251	5.5623161	7.4572417	9.98E-13	6.74E-12	18.197117
ZWILCH	1.0443393	4.6322252	7.9597028	3.78E-14	3.17E-13	21.408712
BORCS8	1.0450416	6.5398313	11.318763	6.78E-25	3.60E-23	45.798956
BPIFB2	1.0451882	4.070512	2.8215048	0.0051054	0.0092418	-3.285961
SLC39A3	1.0457475	5.3579966	11.871109	8.11E-27	5.88E-25	50.173711
TPGS2	1.0458008	6.1232262	7.5469564	5.62E-13	3.95E-12	18.760307
SCARA3	1.0463395	4.260477	4.9658168	1.16E-06	3.54E-06	4.6080148
TP53BP2	1.0465369	7.028761	6.0799473	3.73E-09	1.56E-08	10.163832
AP3D1	1.0468133	8.8616943	8.4964299	9.92E-16	1.04E-14	24.986161

DRAP1	1.0472522	9.4358828	12.314713	2.20E-28	2.18E-26	53.739289
KLHDC8B	1.047367	6.4567276	7.1102529	8.83E-12	5.24E-11	16.062615
RPS7	1.0474323	12.478736	8.9794373	3.33E-17	4.37E-16	28.324787
LYRM4	1.0476521	5.6117088	9.6936131	1.84E-19	3.55E-18	33.448129
INTS7	1.0477799	5.6001703	8.6986568	2.43E-16	2.77E-15	26.370817
DCTPP1	1.0480332	8.2141283	8.9960397	2.96E-17	3.91E-16	28.441435
IFFO1	1.0480447	7.6440445	10.33644	1.45E-21	4.06E-20	38.229181
P3H1	1.0480547	7.7626453	9.463454	1.01E-18	1.69E-17	31.774107
TPRKB	1.0480955	7.6355848	10.731822	6.85E-23	2.52E-21	41.24056
CREB3L2	1.048113	5.8351577	7.393373	1.50E-12	9.89E-12	17.79897
RIC8A	1.0483989	8.515745	10.560131	2.59E-22	8.29E-21	39.926687
NDOR1	1.0484821	6.9564029	8.9042627	5.69E-17	7.19E-16	27.798143
RHBDF1	1.0486115	6.1291003	6.6461969	1.46E-10	7.25E-10	13.321427
ASPH	1.0486747	6.6535791	4.9093848	1.52E-06	4.55E-06	4.3513468
CYTH2	1.0487862	6.4501097	9.5966737	3.77E-19	6.74E-18	32.740506
ABCC4	1.0488687	2.4578598	5.0029428	9.73E-07	3.00E-06	4.7782242
PRPF6	1.0489325	9.4053891	11.680028	3.78E-26	2.48E-24	48.65168
RNF44	1.0490385	6.2894604	7.0349402	1.40E-11	8.11E-11	15.60874
DPF2	1.0492007	7.2928747	9.7135659	1.59E-19	3.09E-18	33.594231
UGGT1	1.0492569	5.8737871	7.8626628	7.19E-14	5.76E-13	20.777751
TCIRG1	1.049326	8.3102145	6.9775562	1.99E-11	1.12E-10	15.265219
BZW2	1.049376	7.8155334	7.1913368	5.34E-12	3.27E-11	16.555071
TIMM17B	1.0495792	6.2238277	11.155613	2.47E-24	1.19E-22	44.521973
PSMD2	1.0496905	8.8038189	9.509989	7.14E-19	1.23E-17	32.110873
SPRYD3	1.0501081	7.2385885	10.065427	1.14E-20	2.72E-19	36.195323
CCDC159	1.0502806	6.2096646	10.535133	3.14E-22	9.83E-21	39.736175
C12orf73	1.0504459	5.390366	12.442648	7.72E-29	8.37E-27	54.775366
TMEM80	1.0505985	6.3629286	8.8522411	8.23E-17	1.02E-15	27.435184
GPR137	1.0506266	5.6435697	9.1667939	8.70E-18	1.25E-16	29.648157
NOL12	1.0507052	5.0983923	10.946112	1.28E-23	5.53E-22	42.893189
DYNC1I1	1.0509374	3.0958809	4.0302661	7.10E-05	0.0001711	0.6827275
PPP1R13L	1.0514011	6.301294	6.2550381	1.40E-09	6.21E-09	11.117387
HAX1	1.0518137	10.218318	11.895751	6.64E-27	4.91E-25	50.370625
MED25	1.0522744	5.5774345	9.8628373	5.21E-20	1.09E-18	34.692072
PXMP4	1.0526136	6.6735289	9.1360303	1.09E-17	1.52E-16	29.429818
PTGES3	1.0526665	8.563505	11.234215	1.33E-24	6.69E-23	45.136283
SUGP2	1.0527605	5.8935419	7.5076381	7.23E-13	5.00E-12	18.512923
PPIB	1.052878	11.054775	11.585816	8.05E-26	4.99E-24	47.904526
RUVBL1	1.0529836	4.9301592	9.4056856	1.53E-18	2.49E-17	31.357267
HTATIP2	1.0533947	7.939145	6.8944489	3.31E-11	1.80E-10	14.771279
LAMTOR1	1.0535707	9.2629566	12.239019	4.09E-28	3.82E-26	53.12786
SLC22A5	1.0536506	6.2160697	8.2268654	6.28E-15	5.86E-14	23.171248
MORC2	1.0538236	6.9885801	9.4721605	9.43E-19	1.59E-17	31.837049
RPS18	1.0538516	12.634209	7.5055584	7.33E-13	5.06E-12	18.499862
CASP2	1.0541049	4.7096732	7.0928492	9.83E-12	5.81E-11	15.957428
CETN2	1.0542268	8.0420403	9.1676815	8.64E-18	1.24E-16	29.654463
NACC1	1.0545989	6.7183702	8.9861537	3.18E-17	4.19E-16	28.371961
RPL23A	1.054763	11.683469	8.1579174	1.00E-14	9.07E-14	22.712867
SLC29A1	1.0548005	8.8086793	5.4183144	1.25E-07	4.36E-07	6.754381
INTS3	1.0549586	6.0782241	6.4677009	4.14E-10	1.95E-09	12.303231
XYLT2	1.0552208	7.8302645	8.9581447	3.88E-17	5.04E-16	28.175363

UNC45A	1.0553138	7.785079	9.863926	5.17E-20	1.09E-18	34.70011
PSMD14	1.0553576	9.1331505	9.5512866	5.27E-19	9.21E-18	32.410465
RPS20	1.0553584	13.176504	7.9006011	5.59E-14	4.57E-13	21.023832
KIZ	1.0553653	5.5371329	8.5572581	6.51E-16	7.01E-15	25.40061
LSM7	1.0554692	9.70817	9.3781083	1.88E-18	3.01E-17	31.158757
RAD21	1.0554798	9.0114763	6.9638208	2.17E-11	1.21E-10	15.183292
GBA2	1.0555077	6.5987719	9.2562283	4.56E-18	6.82E-17	30.285198
DDOST	1.0556013	10.246379	9.3269312	2.73E-18	4.24E-17	30.7912
KRI1	1.05575	7.4960401	10.604609	1.84E-22	6.12E-21	40.266165
ENSA	1.0557679	7.7211975	10.67815	1.04E-22	3.69E-21	40.828836
ATIC	1.0559366	8.3503833	9.3256447	2.75E-18	4.26E-17	30.781973
GMEB2	1.0559492	5.3476869	10.465577	5.37E-22	1.62E-20	39.207143
FKBP1A	1.056023	7.4361364	10.233093	3.18E-21	8.39E-20	37.450587
TULP3	1.0560562	4.5139246	7.8623529	7.20E-14	5.77E-13	20.775744
SNRPA1	1.0562218	7.8559982	8.5182536	8.53E-16	9.01E-15	25.134651
PORCN	1.0562618	6.1468466	8.7953827	1.23E-16	1.47E-15	27.039883
SREBF2	1.0562932	7.3577311	6.909808	3.01E-11	1.65E-10	14.862244
C1QTNF6	1.0563299	5.6811443	6.9270093	2.71E-11	1.49E-10	14.964293
DBP	1.0565277	6.4044766	6.699203	1.07E-10	5.40E-10	13.627732
SOX9-AS1	1.0567566	5.264791	5.3990502	1.38E-07	4.78E-07	6.6598582
CPQ	1.0568936	9.2599003	7.9890881	3.11E-14	2.64E-13	21.60076
WDR5	1.0573792	6.2410547	9.0559487	1.93E-17	2.60E-16	28.863366
DROSHA	1.0574278	7.4889004	9.1407342	1.05E-17	1.48E-16	29.463177
NOL3	1.0575923	8.0732322	5.5211401	7.41E-08	2.64E-07	7.2635511
F13A1	1.0576286	5.3938759	4.4922593	1.01E-05	2.74E-05	2.532234
RNASE1	1.0576975	7.0018514	5.3634816	1.66E-07	5.67E-07	6.4860597
UNC119	1.0577228	6.4294974	6.0439938	4.55E-09	1.89E-08	9.9706181
BTG3	1.0581062	5.2352634	7.5463505	5.64E-13	3.96E-12	18.756488
TEF	1.0582288	5.2918813	6.5194929	3.07E-10	1.46E-09	12.596543
MEGF8	1.0583848	6.0473398	7.780836	1.23E-13	9.53E-13	20.249625
FBXO44	1.0588616	5.7628346	7.9441678	4.19E-14	3.50E-13	21.307367
FTSJ1	1.0590829	5.8379353	10.047403	1.30E-20	3.07E-19	36.06098
DAXX	1.0592716	6.5480898	10.660364	1.19E-22	4.16E-21	40.692598
SOX18	1.0592848	7.0117223	7.1899222	5.38E-12	3.29E-11	16.546446
FDCSP	1.0600502	3.3524332	2.7165733	0.0069869	0.0123334	-3.568521
NUP133	1.0601612	7.0350905	9.2619842	4.37E-18	6.58E-17	30.326313
TATDN2	1.0602724	6.1952661	10.894043	1.93E-23	7.96E-22	42.490353
PSME3	1.0603347	6.5839102	8.6139351	4.39E-16	4.83E-15	25.788366
OTUD6B	1.0606665	4.1253288	8.2560938	5.15E-15	4.87E-14	23.366292
RBBP7	1.0608365	9.373571	9.3477275	2.34E-18	3.70E-17	30.940429
ITGB3BP	1.0608435	5.2424326	8.5827219	5.45E-16	5.92E-15	25.574631
TMEM258	1.060849	10.920402	11.196217	1.79E-24	8.79E-23	44.839097
ENTPD2	1.060986	5.6033197	5.2095731	3.57E-07	1.17E-06	5.7449365
RPN2	1.0609878	10.146717	11.501293	1.58E-25	9.34E-24	47.236102
EIF3D	1.0611856	10.617985	9.2356304	5.29E-18	7.82E-17	30.138179
SAMD4B	1.0613023	4.4863872	9.5100454	7.14E-19	1.23E-17	32.111282
RHBDD3	1.0615884	7.0521996	9.6480818	2.58E-19	4.82E-18	33.11531
SAMD10	1.0616621	5.5273356	9.8414748	6.12E-20	1.27E-18	34.534442
GAPLINC	1.0618326	3.7020008	4.784323	2.72E-06	7.89E-06	3.7914201
GPR107	1.062013	6.0837578	7.9042069	5.46E-14	4.48E-13	21.047261
RNPC3	1.0623542	5.598285	7.1292732	7.85E-12	4.69E-11	16.177781

FAM122B	1.0624397	4.9599085	7.1177792	8.43E-12	5.02E-11	16.108161
GPS1	1.0624939	8.682368	11.852496	9.43E-27	6.75E-25	50.025065
MAP4K4	1.0625121	6.2986173	7.3953607	1.48E-12	9.79E-12	17.811326
MEF2D	1.0629352	5.8618139	8.921247	5.05E-17	6.42E-16	27.916908
C19orf53	1.0632764	8.7579903	10.254182	2.71E-21	7.28E-20	37.609173
ULK3	1.0634878	7.567294	8.5687683	6.01E-16	6.50E-15	25.479233
KDELR3	1.0635004	6.5711127	4.3387968	1.97E-05	5.12E-05	1.8982776
E2F4	1.0645073	6.7334654	8.8497976	8.37E-17	1.03E-15	27.418165
EIF3H	1.0654127	10.475048	8.2564535	5.14E-15	4.86E-14	23.368695
TSEN15	1.0654354	6.2586489	10.540279	3.02E-22	9.53E-21	39.775374
MYO9B	1.0655149	6.0099883	9.665537	2.27E-19	4.27E-18	33.242806
ATF6B	1.0655605	5.6812288	8.0078143	2.74E-14	2.35E-13	21.723382
POGK	1.0660762	6.2907446	7.3079653	2.57E-12	1.65E-11	17.270224
PRKD2	1.0662076	7.2710709	10.576614	2.28E-22	7.39E-21	40.05242
CTNNA1	1.0663965	8.9859483	9.4375069	1.22E-18	2.01E-17	31.586713
YDJC	1.0669735	6.9549874	8.5650995	6.16E-16	6.66E-15	25.454165
ZNF335	1.0672865	5.6202014	11.448238	2.42E-25	1.39E-23	46.81748
ATXN2L	1.0674165	7.4705114	7.2413433	3.91E-12	2.44E-11	16.860726
TAF10	1.0677096	7.9031932	13.39898	2.79E-32	6.62E-30	62.6149
ARMC1	1.0679331	7.0658308	9.3666673	2.04E-18	3.24E-17	31.076493
LINC00944	1.0682039	2.7540326	4.8629379	1.89E-06	5.59E-06	4.14196
ACTL6A	1.0683829	7.5985665	7.0536713	1.25E-11	7.27E-11	15.721304
COLGALT1	1.0689453	7.1597811	9.142985	1.03E-17	1.46E-16	29.479141
FAAP24	1.0689789	2.5005181	9.6095372	3.43E-19	6.21E-18	32.834193
NF2	1.0690243	5.0651747	9.682933	1.99E-19	3.80E-18	33.369989
DUS1L	1.0692829	9.2797621	11.947242	4.38E-27	3.44E-25	50.782535
HOXA10	1.0694145	2.1336635	4.8000461	2.53E-06	7.36E-06	3.8611387
GOLGA3	1.0694491	6.3466382	10.101695	8.63E-21	2.11E-19	36.466011
RGL3	1.0695333	6.9946794	5.7261489	2.54E-08	9.57E-08	8.3017493
CBX2	1.0697442	1.7530137	6.9802318	1.96E-11	1.10E-10	15.281192
EIF3B	1.069786	9.8246074	10.712084	7.98E-23	2.91E-21	41.089043
LAMTOR2	1.0704757	10.361299	10.741572	6.35E-23	2.35E-21	41.315446
VBP1	1.0705822	6.1927136	9.5066449	7.32E-19	1.26E-17	32.086644
DAPK3	1.0708182	6.9054701	9.328541	2.69E-18	4.19E-17	30.802745
MRPL55	1.0712922	8.8311591	10.006847	1.77E-20	4.05E-19	35.759112
RNF43	1.0714172	6.264975	4.4921196	1.01E-05	2.74E-05	2.5316482
RPLP2	1.0714711	13.826029	8.5479677	6.94E-16	7.45E-15	25.337196
NDUFB9	1.0716933	12.195007	9.1574232	9.31E-18	1.33E-16	29.581607
MED24	1.0717907	7.2963475	8.8781014	6.85E-17	8.54E-16	27.615461
UQCRB	1.0718741	9.3030585	9.2377159	5.21E-18	7.71E-17	30.153056
SLC44A3	1.0725221	5.5471712	4.5118902	9.30E-06	2.52E-05	2.6147165
COPS5	1.0725268	9.6681388	10.921489	1.56E-23	6.58E-22	42.702591
ACVRL1	1.0727452	5.5006587	8.490729	1.03E-15	1.08E-14	24.94741
PDZK1	1.0734083	8.6745989	5.5626217	5.98E-08	2.15E-07	7.4711577
BROX	1.0734219	4.8274151	9.0038034	2.80E-17	3.71E-16	28.496025
RRP1	1.0736042	6.7292315	10.825735	3.29E-23	1.29E-21	41.963114
ZSCAN2	1.0736595	4.4378024	10.659262	1.20E-22	4.19E-21	40.684156
CLDN7	1.0736835	7.5501446	4.0364457	6.93E-05	0.0001672	0.7062905
TRMT6	1.0743624	5.1361905	9.1902484	7.34E-18	1.07E-16	29.814895
ANKRD29	1.0743909	4.4815463	6.3943923	6.32E-10	2.91E-09	11.891067
POGZ	1.0744118	6.8887063	7.8770655	6.53E-14	5.28E-13	20.871082

STRN4	1.0746255	7.1291984	8.7572113	1.61E-16	1.89E-15	26.77533
DEF8	1.0747542	6.5680586	10.265839	2.48E-21	6.71E-20	37.696898
ROMO1	1.0755257	10.707004	9.8375374	6.30E-20	1.30E-18	34.505407
TRMT61A	1.07587	5.9128926	10.744859	6.19E-23	2.29E-21	41.340706
HIGD1B	1.0760392	3.8465292	12.012832	2.57E-27	2.08E-25	51.308113
SEC61G	1.0767614	9.5578399	9.6236162	3.09E-19	5.66E-18	32.936808
UCN	1.0769332	4.789831	7.1065212	9.03E-12	5.36E-11	16.040046
ZMYND19	1.0769549	5.7469511	10.3293	1.53E-21	4.27E-20	38.175273
ANKRD39	1.077274	6.9237679	12.02849	2.27E-27	1.85E-25	51.433726
TSC1	1.077726	6.2120266	8.9245549	4.93E-17	6.28E-16	27.940053
WDR46	1.0779652	5.9168861	11.402787	3.48E-25	1.92E-23	46.459425
CMTM4	1.0779899	4.5807335	6.8130705	5.40E-11	2.85E-10	14.29175
TOPBP1	1.0782885	5.0255539	7.1691001	6.13E-12	3.72E-11	16.419628
EEF1E1	1.078331	6.4173473	7.5429218	5.77E-13	4.04E-12	18.734882
ZNF16	1.0790943	5.0639866	10.179816	4.77E-21	1.21E-19	37.050651
SNX8	1.0796012	6.9126506	8.8232879	1.01E-16	1.23E-15	27.233706
NDUFA1	1.0805943	11.34036	10.3917	9.47E-22	2.75E-20	38.646987
ACSM1	1.0812052	5.8495774	3.1410534	0.0018549	0.0036121	-2.363346
SH3GLB2	1.0815776	6.1433925	9.2672884	4.20E-18	6.35E-17	30.364214
ZP3	1.0816339	3.9714321	6.9780047	1.99E-11	1.12E-10	15.267896
BCL6B	1.0817573	4.4937966	7.6075517	3.80E-13	2.73E-12	19.143262
LYSMD1	1.0823495	5.2792868	9.2400098	5.12E-18	7.60E-17	30.169422
THBS3	1.0825169	6.2258573	8.2478436	5.45E-15	5.14E-14	23.311194
PPIL1	1.0828316	7.979162	8.4688598	1.20E-15	1.24E-14	24.798901
BPGM	1.0832914	6.5876959	9.2596192	4.45E-18	6.67E-17	30.309418
CCDC77	1.0833334	5.9635582	9.232181	5.42E-18	8.00E-17	30.113576
MVP	1.0834666	9.9292291	6.6636267	1.32E-10	6.59E-10	13.42195
INCENP	1.0840808	4.4440668	7.4642226	9.55E-13	6.47E-12	18.240777
PARPBP	1.0840855	2.6426987	8.1476134	1.07E-14	9.70E-14	22.644572
PRKCD	1.0841324	6.5431576	6.6243483	1.66E-10	8.20E-10	13.195692
CYC1	1.0846706	10.034718	9.9481448	2.75E-20	6.04E-19	35.323237
YWHAZ	1.0849434	8.3100544	6.9748932	2.03E-11	1.14E-10	15.249326
STX4	1.0849665	7.8944005	12.80865	3.79E-30	5.34E-28	57.756947
MUS81	1.0849683	7.3846197	11.310895	7.22E-25	3.80E-23	45.737206
FHL3	1.0850071	5.0713881	6.8636023	3.98E-11	2.14E-10	14.589028
B3GAT3	1.0858692	7.0221115	10.040274	1.37E-20	3.20E-19	36.00787
PTH2R	1.0858968	1.9423252	3.7109952	0.0002469	0.0005529	-0.490346
ZNF740	1.0860139	3.3437015	9.9352091	3.03E-20	6.60E-19	35.227356
MGP	1.0865082	7.779036	3.4786201	0.0005802	0.001224	-1.28868
BANF1	1.0865868	8.7342621	13.018249	6.67E-31	1.16E-28	59.475297
MFSD3	1.0865893	7.5987626	7.3100501	2.54E-12	1.63E-11	17.28308
ZNF205	1.0867365	6.8806398	10.654861	1.24E-22	4.32E-21	40.65046
MTX1	1.0867903	7.3366372	11.13117	3.00E-24	1.42E-22	44.331292
TAF1D	1.0868108	7.1532039	7.5681814	4.90E-13	3.46E-12	18.894212
GLS	1.086935	6.0453493	5.5157969	7.61E-08	2.70E-07	7.2369015
RPL7	1.0871267	11.175403	7.1450844	7.11E-12	4.28E-11	16.273681
GNPDA1	1.087195	6.0813253	7.7158971	1.88E-13	1.42E-12	19.833079
INPPL1	1.0874716	7.3682839	7.9128299	5.16E-14	4.24E-13	21.103316
CENPT	1.0875037	5.1851663	10.47796	4.88E-22	1.48E-20	39.301209
OGG1	1.0877652	6.1081737	9.6444437	2.65E-19	4.93E-18	33.088751
CDK4	1.0879587	8.1531299	6.7250007	9.14E-11	4.67E-10	13.777455

ZNF57	1.0881092	4.473313	7.1623618	6.39E-12	3.87E-11	16.378644
RRP36	1.0883478	9.0720078	11.443573	2.51E-25	1.43E-23	46.780701
RPL28	1.088366	9.4465342	8.5195641	8.45E-16	8.94E-15	25.143575
DNAJC9	1.0891593	7.0906744	6.9700276	2.09E-11	1.17E-10	15.220299
NTPCR	1.089285	7.0525014	10.313258	1.73E-21	4.79E-20	38.054216
RASSF1	1.0893277	6.7563048	10.356155	1.24E-21	3.53E-20	38.378126
TNRC18	1.0894167	6.6702538	7.7304016	1.71E-13	1.30E-12	19.925917
PARP2	1.0894206	6.9965334	9.9347841	3.04E-20	6.62E-19	35.224207
ADCK2	1.0895561	6.4979959	9.054821	1.95E-17	2.62E-16	28.85541
IFI27L1	1.089619	6.8947991	9.9881803	2.03E-20	4.62E-19	35.620371
NDRG1	1.0896308	7.7843569	4.9236822	1.42E-06	4.27E-06	4.4161402
PIK3C2B	1.0897832	6.6755749	7.3322446	2.21E-12	1.43E-11	17.420104
DLGAP4	1.0905366	5.9513391	8.9890719	3.11E-17	4.11E-16	28.392465
NDUFS6	1.0906199	10.759466	9.9868548	2.05E-20	4.66E-19	35.610525
ARHGEF2	1.0907257	5.1068435	6.3416866	8.55E-10	3.89E-09	11.59693
PLEKHG2	1.0908848	5.1139078	7.803024	1.06E-13	8.32E-13	20.392473
TRNP1	1.0908872	5.9021423	3.3299404	0.0009794	0.0019973	-1.774537
DESI2	1.0910153	4.6710707	8.3084926	3.61E-15	3.49E-14	23.717026
FBLN1	1.0910418	5.1189219	3.6414848	0.0003202	0.0007059	-0.734097
CFL1	1.0913226	10.112685	11.319355	6.75E-25	3.60E-23	45.803606
PFDN4	1.091593	6.3978993	9.0848198	1.57E-17	2.15E-16	29.067263
RPL32P3	1.0921573	4.3026861	8.0591948	1.95E-14	1.70E-13	22.060771
TMED9	1.0925292	9.7819621	11.435536	2.68E-25	1.52E-23	46.717361
PFDN2	1.0925732	8.4796913	10.440788	6.50E-22	1.94E-20	39.018981
CCDC24	1.0926807	5.3113076	6.8364801	4.69E-11	2.49E-10	14.429271
TUBB4A	1.0927615	3.7635259	3.0832686	0.0022418	0.0043083	-2.537063
HAGHL	1.0928131	5.0618382	5.7800964	1.90E-08	7.32E-08	8.5799794
MZT1	1.0930737	5.2298557	8.5421839	7.22E-16	7.74E-15	25.297737
HMGN1	1.093121	7.9327509	8.2268502	6.28E-15	5.86E-14	23.171147
TPCN1	1.093174	6.4620577	7.5934518	4.16E-13	2.98E-12	19.053969
MSX1	1.0932361	3.9103856	7.0723131	1.12E-11	6.53E-11	15.833542
KATNB1	1.0932909	5.8103361	9.3410914	2.46E-18	3.86E-17	30.89279
CDK5RAP2	1.0934842	7.2565082	9.4841511	8.64E-19	1.47E-17	31.923781
SRC	1.0935752	5.3278177	4.5853132	6.71E-06	1.86E-05	2.925982
CCDC124	1.0936038	8.5105358	11.64621	4.96E-26	3.19E-24	48.383231
DNAJC6	1.0936179	1.7048485	6.8490164	4.35E-11	2.32E-10	14.503056
TSACC	1.093618	4.7714472	8.5890693	5.22E-16	5.68E-15	25.618058
SLC1A4	1.0936294	5.2773294	6.9934131	1.81E-11	1.02E-10	15.359944
ITPA	1.0936439	8.0979385	9.6756108	2.10E-19	3.99E-18	33.316442
GPR35	1.0936583	3.2511476	4.2768941	2.57E-05	6.56E-05	1.6480349
FIBP	1.0938046	7.9260847	13.0981	3.43E-31	6.45E-29	60.131883
SCGN	1.0945093	6.3031664	3.1128119	0.0020355	0.0039346	-2.448625
TRIM47	1.0946941	6.8784002	5.1263255	5.36E-07	1.71E-06	5.351534
CLTA	1.094787	10.400615	10.716778	7.70E-23	2.82E-21	41.125067
SOX4	1.0951215	6.2369538	3.9465012	9.93E-05	0.0002348	0.366523
NUAK1	1.095602	6.5520787	7.483701	8.43E-13	5.76E-12	18.362743
ABCC5	1.0958905	4.3262798	9.7628181	1.10E-19	2.19E-18	33.955528
MTERF3	1.0962756	7.0205259	9.0770889	1.66E-17	2.26E-16	29.01263
AP1S1	1.0963206	6.8624027	11.804712	1.39E-26	9.73E-25	49.643842
ZYX	1.0967036	7.581213	7.0100262	1.64E-11	9.31E-11	15.45935
SNHG17	1.096909	5.8904826	7.2678576	3.31E-12	2.09E-11	17.023387

SNF8	1.097237	6.9431504	12.207	5.31E-28	4.91E-26	52.869589
SLC29A2	1.0973435	4.7410607	6.8050162	5.67E-11	2.98E-10	14.244514
HEIH	1.0974466	8.5298232	9.9168578	3.48E-20	7.54E-19	35.09144
POLR1A	1.097545	4.4368539	9.8278478	6.77E-20	1.39E-18	34.433979
ATOX1	1.0977921	10.683262	8.311214	3.54E-15	3.43E-14	23.735279
CLTCL1	1.0978312	5.2541236	7.0221079	1.52E-11	8.72E-11	15.531748
MAPKAPK2	1.0979361	7.5777629	9.1388742	1.06E-17	1.50E-16	29.449984
SLC2A5	1.0983561	3.5944194	4.3911531	1.57E-05	4.15E-05	2.1123941
CD2BP2	1.0983647	6.5532628	10.506422	3.92E-22	1.20E-20	39.517615
HSPA5	1.0987234	10.163041	8.9654901	3.68E-17	4.80E-16	28.226888
DEDD	1.0997319	6.5042103	11.867002	8.38E-27	6.05E-25	50.140901
KIF22	1.1001811	6.7922381	9.2771164	3.92E-18	5.95E-17	30.434471
CHD1L	1.1003567	7.6046997	8.5745829	5.77E-16	6.25E-15	25.518975
LGALS3	1.1004953	8.6059654	3.9202164	0.0001101	0.000259	0.2685302
ASPHD1	1.100864	4.6442236	4.1756982	3.92E-05	9.79E-05	1.2457946
SLC26A11	1.100995	7.424528	9.0070877	2.74E-17	3.63E-16	28.519126
BMP4	1.1011453	4.6810722	4.2927785	2.40E-05	6.15E-05	1.7119458
FAM20B	1.1018917	5.9278515	9.3620824	2.11E-18	3.34E-17	31.043542
ZNF341	1.1020047	3.9569331	9.4063959	1.53E-18	2.48E-17	31.362384
TMEM65	1.1023173	4.2613584	6.1911315	2.00E-09	8.73E-09	10.766936
TMEM120B	1.1026316	5.0262316	12.171747	7.07E-28	6.41E-26	52.585474
HEATR1	1.1027644	4.328802	7.0274863	1.47E-11	8.44E-11	15.564006
ELFN2	1.1033732	3.807633	5.7001295	2.91E-08	1.09E-07	8.1683002
FBXO32	1.103862	3.8066195	6.0028744	5.70E-09	2.34E-08	9.7507365
CH17-340M2	1.1039496	6.2259679	8.3198287	3.34E-15	3.24E-14	23.793085
COPE	1.1041663	8.5881856	11.277385	9.42E-25	4.87E-23	45.4744
DUXAP8	1.1044515	2.9712482	6.4332832	5.05E-10	2.35E-09	12.109284
ZNF74	1.1046268	5.3708953	9.1555748	9.43E-18	1.35E-16	29.568485
NEDD4L	1.1047196	6.1272382	7.7878329	1.18E-13	9.13E-13	20.294643
SIRT6	1.1048829	6.1571661	10.981434	9.74E-24	4.25E-22	43.166912
TERT	1.105071	4.1519129	4.338068	1.98E-05	5.14E-05	1.8953129
UPF3B	1.1055698	5.6322206	8.7173294	2.13E-16	2.45E-15	26.49964
RHEB	1.1057969	8.8347176	9.9560862	2.59E-20	5.73E-19	35.382129
HDAC5	1.1062424	7.0968851	9.2968814	3.39E-18	5.21E-17	30.575886
ARHGEF37	1.1067594	5.2792121	6.1023719	3.29E-09	1.39E-08	10.284791
UTP14A	1.1068867	5.810695	9.9689755	2.35E-20	5.28E-19	35.477764
RPTOR	1.1077391	6.8982626	9.704357	1.70E-19	3.29E-18	33.526781
UBE2M	1.1082425	8.2044689	11.21754	1.51E-24	7.54E-23	45.005816
FOXD2	1.1087492	4.219243	7.327049	2.28E-12	1.47E-11	17.388002
GMPPB	1.108869	6.6324089	9.9633368	2.45E-20	5.48E-19	35.435919
GNA12	1.1090154	5.6158606	7.5886529	4.30E-13	3.06E-12	19.023603
THEM6	1.1090821	8.2345553	7.7408153	1.60E-13	1.22E-12	19.992643
ARPC1A	1.1092201	9.5713383	12.097356	1.30E-27	1.11E-25	51.986831
TPM1	1.109498	7.8631421	6.3391952	8.67E-10	3.94E-09	11.583072
GDI1	1.1095796	8.1559122	10.473895	5.04E-22	1.52E-20	39.270327
BCAS4	1.1100915	3.3422915	6.892548	3.34E-11	1.82E-10	14.760031
TBCB	1.1103028	8.4322973	9.6203167	3.17E-19	5.77E-18	32.912752
PRR11	1.1116521	4.6107437	7.3033585	2.65E-12	1.69E-11	17.241825
SNRPD1	1.112704	7.7032506	9.6932623	1.85E-19	3.55E-18	33.445561
STT3A	1.1127904	8.6113698	9.6316003	2.91E-19	5.37E-18	32.995035
CDC37	1.11314	9.2770245	13.294421	6.68E-32	1.42E-29	61.750463

RPLP1	1.1133399	14.008624	9.7157548	1.56E-19	3.04E-18	33.610268
HNRNPA1	1.1136198	9.787845	8.6237354	4.10E-16	4.54E-15	25.855569
LRRC20	1.1137596	6.8584615	7.2812833	3.04E-12	1.93E-11	17.105909
INTS1	1.1137618	4.7520619	10.06683	1.12E-20	2.69E-19	36.205786
NPC1	1.1140312	5.9248837	7.9313331	4.56E-14	3.79E-13	21.223733
CCDC86	1.1140538	6.5426245	8.4303327	1.56E-15	1.58E-14	24.537837
RABL6	1.1143029	5.5746838	8.519243	8.47E-16	8.96E-15	25.141388
TPM3	1.1150026	7.3050115	9.2757559	3.95E-18	5.99E-17	30.424742
C9orf16	1.1150638	7.3109857	8.6161459	4.32E-16	4.76E-15	25.803522
SLC35F6	1.1152613	6.8336147	8.8240916	1.00E-16	1.22E-15	27.239294
TPR	1.1154474	7.6200707	7.3884729	1.55E-12	1.02E-11	17.768521
CRTC2	1.1155915	7.9169526	10.602904	1.86E-22	6.19E-21	40.25314
PCSK9	1.1156495	7.7483039	4.8378919	2.12E-06	6.24E-06	4.0297528
AGBL5	1.11594	5.7132449	8.2810057	4.35E-15	4.16E-14	23.532869
RPS6KL1	1.1160746	2.8796259	8.1506195	1.05E-14	9.51E-14	22.66449
CEP250	1.1164177	4.4321276	10.391065	9.52E-22	2.76E-20	38.642181
BRD8	1.1166245	7.5971528	9.3115866	3.05E-18	4.69E-17	30.681205
CDC25A	1.1168296	3.1595732	6.7966545	5.96E-11	3.12E-10	14.195517
NIPAL2	1.1170641	5.2490245	6.1352554	2.74E-09	1.17E-08	10.462789
JOSD2	1.1171092	7.0599541	8.7311427	1.93E-16	2.24E-15	26.595044
PGF	1.1174611	5.2182181	5.9014584	9.91E-09	3.94E-08	9.2134468
MSI1	1.1176126	3.6853061	4.6081953	6.06E-06	1.68E-05	3.0238768
SMPX	1.1181925	3.5701267	4.9451867	1.28E-06	3.88E-06	4.5138956
FADS3	1.1182739	6.4884816	8.2003456	7.52E-15	6.93E-14	22.994653
DGKZ	1.1182801	5.7556701	10.146397	6.15E-21	1.54E-19	36.800289
LOXL4	1.1183425	5.8234493	3.2903228	0.0011225	0.0022698	-1.900684
TMEM161B-A	1.1184042	5.3344128	8.4467545	1.40E-15	1.43E-14	24.649024
SOGA1	1.1186299	3.1067939	7.5668059	4.95E-13	3.49E-12	18.885526
ZBTB45	1.1188607	7.0185935	10.306124	1.82E-21	5.03E-20	38.000407
PPM1G	1.119076	8.4877891	11.1367	2.87E-24	1.37E-22	44.374421
CTTN	1.1190869	6.2009972	9.2853722	3.69E-18	5.62E-17	30.493519
UBE2O	1.1193753	6.0296813	9.9571893	2.57E-20	5.70E-19	35.390311
BCAT2	1.1195151	6.503003	7.2607259	3.46E-12	2.17E-11	16.979595
NPM3	1.1196221	7.2814976	5.5839421	5.35E-08	1.94E-07	7.5783503
C7orf50	1.119773	5.8971842	9.7757047	9.99E-20	2.00E-18	34.050213
KLHL21	1.1198093	6.7443606	6.4326629	5.07E-10	2.35E-09	12.105796
AKIP1	1.1198443	6.8289814	12.097623	1.29E-27	1.11E-25	51.988978
CENPQ	1.1200595	5.3770106	7.4705691	9.17E-13	6.22E-12	18.280493
MRAS	1.1200794	5.4304701	6.350319	8.14E-10	3.71E-09	11.644979
POLR2G	1.1200995	9.5182934	12.365519	1.45E-28	1.49E-26	54.150344
EZH1	1.1201389	6.0125926	9.5563415	5.08E-19	8.91E-18	32.447183
RCN2	1.1203253	6.9596461	8.2761906	4.49E-15	4.29E-14	23.500648
MAPK8IP3	1.1205308	3.2665925	7.0964785	9.61E-12	5.68E-11	15.979348
FGFR4	1.1213817	7.8050828	5.4765314	9.32E-08	3.28E-07	7.0417019
CS	1.1219267	6.3515574	7.9074813	5.34E-14	4.39E-13	21.068542
MCRIP1	1.122121	9.2749418	7.8041068	1.06E-13	8.27E-13	20.399451
TELO2	1.1223158	6.7400679	10.95483	1.20E-23	5.18E-22	42.960719
BOLA2B	1.1227183	6.8785954	9.0393539	2.17E-17	2.91E-16	28.746334
SLC22A31	1.1233344	2.8652903	3.0988887	0.0021304	0.0041044	-2.490403
DVL3	1.1237258	6.1296492	8.3841183	2.15E-15	2.14E-14	24.225639
NUDCD1	1.1239729	5.0566039	8.7110965	2.22E-16	2.55E-15	26.456622

GJA5	1.1240603	3.0078697	6.812882	5.41E-11	2.85E-10	14.290644
LCMT1	1.1243243	7.3272724	10.970956	1.06E-23	4.59E-22	43.085678
VPS33A	1.1248923	5.8872557	12.448427	7.36E-29	8.10E-27	54.822246
LRRC1	1.1250043	4.3783483	6.8310087	4.85E-11	2.57E-10	14.397098
ZKSCAN3	1.1254213	4.1153091	8.6621889	3.13E-16	3.52E-15	26.119692
PCGF1	1.1256362	6.6182217	14.287364	1.57E-35	6.03E-33	70.017849
TMEM201	1.1257256	6.0139033	9.2436745	4.99E-18	7.40E-17	30.195572
PWWP2B	1.1257908	6.40024	5.8885293	1.06E-08	4.21E-08	9.1454673
MRPL13	1.125858	9.4747485	9.6261214	3.03E-19	5.58E-18	32.955075
AIF1L	1.1261904	5.3829737	5.4123744	1.29E-07	4.48E-07	6.7252062
ST6GALNAC4	1.1262003	5.2896101	6.1555608	2.45E-09	1.05E-08	10.573071
GGA3	1.1262839	6.4048058	9.5790745	4.30E-19	7.61E-18	32.612433
RSRP1	1.1268232	6.4964051	7.924506	4.77E-14	3.96E-13	21.179282
SLC6A9	1.1268568	2.2155172	5.639374	4.01E-08	1.48E-07	7.8585924
MAF1	1.1270801	7.6471166	9.0703387	1.74E-17	2.37E-16	28.964948
SLC4A2	1.1271175	6.6307019	6.9679839	2.11E-11	1.18E-10	15.208111
SLC12A9	1.1272755	6.9303126	9.3453138	2.38E-18	3.75E-17	30.9231
METTL18	1.1273953	5.9647856	10.621983	1.61E-22	5.46E-21	40.398941
NME3	1.1274894	9.4584061	9.689571	1.90E-19	3.64E-18	33.41855
ACTA2	1.1275323	9.0099206	5.6193033	4.45E-08	1.63E-07	7.7568652
SOX9	1.1278696	5.3330571	3.575555	0.0004084	0.0008848	-0.961401
WDR4	1.1280338	5.2548511	10.005096	1.79E-20	4.10E-19	35.746094
RPUSD1	1.1285395	7.0615776	10.569466	2.41E-22	7.77E-21	39.997885
ARFRP1	1.1286884	6.3149818	11.774579	1.77E-26	1.20E-24	49.403724
WDR83	1.1290732	6.1346938	11.599519	7.22E-26	4.55E-24	48.013058
LTBP2	1.1292857	4.6290538	4.6280413	5.54E-06	1.55E-05	3.1091238
RAD54L	1.1296598	3.872196	6.7784318	6.65E-11	3.46E-10	14.088891
ZNF83	1.1299247	4.7584067	4.2565407	2.80E-05	7.12E-05	1.5664488
RBM42	1.1299903	8.7651139	12.356624	1.56E-28	1.58E-26	54.078335
CSE1L	1.1309364	8.414406	9.4193747	1.39E-18	2.27E-17	31.455921
BCL2L12	1.1310015	7.6024839	9.6859916	1.95E-19	3.72E-18	33.392362
NCAPG2	1.1310278	4.7187568	7.3469644	2.01E-12	1.31E-11	17.511138
KCTD7	1.1312569	3.2830672	8.9891532	3.11E-17	4.11E-16	28.393036
CCDC93	1.1313981	4.5569243	9.0425424	2.12E-17	2.85E-16	28.768811
ZNF7	1.1314171	6.2510329	11.073824	4.71E-24	2.17E-22	43.884601
RRP12	1.1316624	5.737394	8.497864	9.82E-16	1.03E-14	24.995912
RPL17	1.1318167	9.7967171	9.0842721	1.57E-17	2.16E-16	29.063392
HSPA4	1.1319741	8.5459409	11.764459	1.92E-26	1.30E-24	49.323129
HSPH1	1.1321391	7.9177763	7.480421	8.61E-13	5.87E-12	18.34219
PNMA1	1.1321391	6.7458848	6.1739844	2.21E-09	9.55E-09	10.673375
FTSJ3	1.1321654	7.7392992	11.106888	3.63E-24	1.70E-22	44.142035
GLUL	1.1321827	9.9845209	3.2160781	0.0014444	0.002866	-2.133307
TMCO1	1.1321975	8.0192824	11.777592	1.72E-26	1.17E-24	49.427719
RPS27	1.1324564	13.920303	8.5694155	5.98E-16	6.47E-15	25.483656
SPHK1	1.1336299	5.4612521	3.4103059	0.0007397	0.0015356	-1.514352
PHLDA3	1.1336626	6.2829171	4.1706052	4.00E-05	9.98E-05	1.2257759
TBC1D7	1.1339136	6.858958	11.043335	5.99E-24	2.68E-22	43.647492
CD320	1.1349811	7.8102593	9.3573011	2.19E-18	3.46E-17	31.009188
ZNF696	1.1351291	5.2925772	10.399839	8.90E-22	2.60E-20	38.708611
OGT	1.1356462	6.7189246	7.8665785	7.00E-14	5.62E-13	20.803115
FRZB	1.1356576	5.6072182	5.33024	1.96E-07	6.64E-07	6.3244841

PPP1R37	1.1357485	5.7218346	8.3020273	3.77E-15	3.64E-14	23.673676
TJAP1	1.1360711	6.6759554	9.6244268	3.07E-19	5.64E-18	32.942718
CD151	1.1365242	8.820434	7.6395005	3.09E-13	2.25E-12	19.345999
ATP13A1	1.1372937	7.2466756	11.092408	4.07E-24	1.88E-22	44.029256
GM2A	1.1374262	6.088498	9.677475	2.07E-19	3.94E-18	33.330072
PPP6R1	1.1377531	6.6269237	8.0802227	1.69E-14	1.49E-13	22.199246
PDXK	1.1380681	6.3629463	9.7012436	1.74E-19	3.36E-18	33.503984
ANKRD13D	1.1382439	5.039475	7.9024536	5.52E-14	4.53E-13	21.035868
HAGLR	1.1383077	3.0764574	4.7098032	3.83E-06	1.09E-05	3.4636531
ALKBH2	1.1383771	7.4058438	9.3288969	2.69E-18	4.19E-17	30.805297
ADCK5	1.1384208	6.3300346	10.306658	1.82E-21	5.02E-20	38.00444
MAGEA12	1.1385166	3.3193547	3.283976	0.0011472	0.0023165	-1.920763
TCF19	1.1385774	2.9904954	8.1272892	1.23E-14	1.10E-13	22.510021
GIGYF1	1.1386541	5.8756697	9.6336458	2.87E-19	5.30E-18	33.009956
MCM10	1.138841	2.9861981	7.0491098	1.29E-11	7.46E-11	15.693872
ALKBH6	1.1389525	4.7573442	10.585565	2.13E-22	6.97E-21	40.120733
SMYD5	1.1394521	6.0479678	9.3826494	1.82E-18	2.92E-17	31.191424
CLASRP	1.1400741	7.4130635	10.089355	9.48E-21	2.29E-19	36.373857
DDX39B	1.1408297	6.1292011	6.7523498	7.77E-11	4.01E-10	13.936641
CLGN	1.1411033	4.9116326	3.7221819	0.0002366	0.0005317	-0.450725
DPH7	1.1412441	5.9731509	10.545947	2.89E-22	9.17E-21	39.818562
CDPF1	1.1415691	6.5599007	10.246584	2.87E-21	7.65E-20	37.552017
HAUS1	1.1418902	6.9075535	8.8925051	6.19E-17	7.77E-16	27.716002
PPIA	1.1418919	10.178234	12.191667	6.01E-28	5.51E-26	52.745981
CPNE1	1.1424088	7.523235	8.0087158	2.73E-14	2.34E-13	21.72929
MSH2	1.1426149	5.5322991	9.445493	1.15E-18	1.91E-17	31.644361
ZNF282	1.1428695	5.311804	10.605578	1.82E-22	6.08E-21	40.273563
STX6	1.1430933	5.1656554	9.6559863	2.43E-19	4.58E-18	33.173032
RPS19	1.1432293	11.676046	8.030459	2.36E-14	2.04E-13	21.871909
ERICH5	1.1432421	7.7121834	2.8629451	0.0044989	0.0082269	-3.171577
DHX57	1.1432835	3.781418	11.08797	4.21E-24	1.94E-22	43.994704
ZNF563	1.1433328	5.0389996	9.0994325	1.41E-17	1.95E-16	29.1706
VAT1	1.1433393	7.9444462	6.8796935	3.61E-11	1.95E-10	14.684027
SUB1	1.1437194	8.9515664	10.067998	1.11E-20	2.67E-19	36.2145
PTK2	1.1440339	6.2203838	8.4576462	1.30E-15	1.33E-14	24.722841
EXOSC5	1.1442221	6.9236018	10.569807	2.40E-22	7.76E-21	40.000484
REEP4	1.1443433	6.0667846	7.8112085	1.01E-13	7.92E-13	20.445234
MIIP	1.1444259	6.408396	10.060895	1.18E-20	2.81E-19	36.16153
MFSD5	1.1446378	7.3319453	11.72733	2.59E-26	1.72E-24	49.027641
PXN-AS1	1.1451564	6.4020248	10.29875	1.93E-21	5.30E-20	37.944813
SMARCD1	1.1451897	6.0236971	8.9598505	3.83E-17	4.98E-16	28.187327
PMVK	1.1455016	9.7137374	11.229763	1.37E-24	6.91E-23	45.101442
OSBPL3	1.1459735	3.6242895	7.6849276	2.30E-13	1.71E-12	19.63524
DPM2	1.1460226	7.6419548	10.224788	3.39E-21	8.82E-20	37.388178
CUTA	1.1461558	10.67245	11.223242	1.45E-24	7.25E-23	45.050424
USP22	1.146317	6.1055727	7.5635938	5.05E-13	3.56E-12	18.865248
CHTOP	1.1464531	7.996948	11.276782	9.46E-25	4.88E-23	45.469671
PMF1	1.14698	8.3681388	10.531446	3.23E-22	1.01E-20	39.708095
VSIG10L	1.1470158	6.5072211	4.9124491	1.50E-06	4.49E-06	4.3652201
PLEC	1.1471114	5.481439	8.0325441	2.33E-14	2.02E-13	21.885598
VRK1	1.147222	5.5244875	9.1911273	7.30E-18	1.06E-16	29.821147

MRPS12	1.1481395	8.463722	10.419482	7.65E-22	2.26E-20	38.857423
TATDN1	1.1482132	6.5217763	9.9791553	2.18E-20	4.92E-19	35.553339
TMEM147-AS	1.1482163	3.176959	7.8927544	5.89E-14	4.79E-13	20.972873
E4F1	1.1485571	6.7159037	10.335673	1.45E-21	4.08E-20	38.223393
RNPEPL1	1.1488114	7.9573686	11.420907	3.01E-25	1.68E-23	46.602104
TMEM94	1.1490866	6.0510006	9.7792005	9.74E-20	1.96E-18	34.07591
CENPO	1.149266	4.2415433	7.7965122	1.11E-13	8.68E-13	20.350522
THOC5	1.1493623	4.271393	11.24361	1.23E-24	6.26E-23	45.209819
TAX1BP3	1.1498488	5.6631739	6.1755848	2.19E-09	9.47E-09	10.682099
CCDC130	1.1502619	8.4824079	10.837727	3.00E-23	1.19E-21	42.055581
MALAT1	1.1503562	6.7839758	6.920626	2.82E-11	1.55E-10	14.926402
CASC3	1.1506027	7.3881254	9.1805824	7.87E-18	1.14E-16	29.746151
WNK4	1.1506083	3.3607382	4.9563611	1.21E-06	3.69E-06	4.5648344
TBCC	1.1506838	7.2877649	9.4559502	1.06E-18	1.78E-17	31.719886
NENF	1.1508636	9.6167608	9.7991208	8.39E-20	1.69E-18	34.222425
ODAM	1.1512086	2.5988638	3.1923555	0.0015641	0.0030861	-2.206593
PIEZO2	1.1517521	3.6386865	6.2660201	1.31E-09	5.85E-09	11.177887
ANAPC11	1.1518722	11.268615	10.880161	2.15E-23	8.79E-22	42.383092
RELB	1.1519248	8.4169197	6.9234604	2.77E-11	1.52E-10	14.943223
F2RL3	1.1522703	2.4569942	6.1371506	2.71E-09	1.16E-08	10.473071
EFNA1	1.1525709	11.124251	6.5950766	1.97E-10	9.64E-10	13.027719
ORMDL2	1.1526902	7.5305659	12.742612	6.54E-30	8.74E-28	57.217143
GINS3	1.1528437	4.1946364	7.6437072	3.01E-13	2.20E-12	19.372736
C11orf80	1.1530208	3.1299232	7.2252644	4.32E-12	2.68E-11	16.762286
FLYWCH2	1.1534023	5.6171307	7.0199983	1.54E-11	8.82E-11	15.5191
THAP8	1.1542161	6.1296519	10.844647	2.84E-23	1.13E-21	42.10895
PHYHIPL	1.1543378	3.0982875	4.7652382	2.97E-06	8.57E-06	3.7070583
TAOK2	1.1545154	6.3337369	10.159571	5.57E-21	1.40E-19	36.898933
NAT8B	1.1547032	6.6010203	4.0287513	7.15E-05	0.0001721	0.6769566
GTSE1	1.1547942	3.4141438	7.4405568	1.11E-12	7.45E-12	18.092882
IPO9	1.1549329	5.9180594	9.5026939	7.54E-19	1.29E-17	32.058023
TEPSIN	1.154959	7.596647	11.96185	3.89E-27	3.07E-25	50.899504
HYOU1	1.1554956	6.9174102	6.3445196	8.41E-10	3.83E-09	11.612693
LARP1	1.155525	7.1440941	9.063493	1.83E-17	2.48E-16	28.916612
DEPDC1B	1.156025	3.7583724	6.5727761	2.25E-10	1.09E-09	12.90012
YEATS2	1.156111	3.7575169	8.4403495	1.46E-15	1.49E-14	24.605642
TCHP	1.1561741	5.0431732	9.7236954	1.47E-19	2.87E-18	33.668461
SLC22A12	1.1562675	3.8774887	3.5635103	0.0004268	0.0009219	-1.002517
GRN	1.1565558	11.757507	9.375651	1.91E-18	3.06E-17	31.141084
NAA20	1.1566612	9.3105883	10.305402	1.83E-21	5.05E-20	37.994969
PRKDC	1.1566715	5.4474929	7.2702451	3.26E-12	2.06E-11	17.038054
MYRF	1.1567123	7.5681535	5.3612126	1.67E-07	5.73E-07	6.4750046
DGKQ	1.1568576	4.7243721	9.1626922	8.96E-18	1.28E-16	29.619023
TECPR1	1.1569113	6.7646421	11.095477	3.97E-24	1.85E-22	44.053156
CCDC170	1.1571779	3.1330022	5.1130024	5.72E-07	1.82E-06	5.2890636
MANBAL	1.1576725	7.5148847	12.179743	6.63E-28	6.04E-26	52.649897
C17orf58	1.1577247	6.1413216	9.4533569	1.08E-18	1.81E-17	31.701151
DDX11	1.1579992	5.3529912	7.7251369	1.77E-13	1.34E-12	19.892207
RHOC	1.1583926	9.8418745	9.7384617	1.32E-19	2.60E-18	33.776742
TIGD5	1.159007	5.5584759	10.822194	3.39E-23	1.32E-21	41.935825
ZNF512	1.1590327	5.3036624	8.9693912	3.58E-17	4.67E-16	28.254262

ARFGAP1	1.1592684	6.3895209	11.572667	8.95E-26	5.51E-24	47.800421
CHEK2	1.1593318	5.6572139	7.0874176	1.02E-11	5.99E-11	15.924637
INO80E	1.1593901	7.106317	10.700036	8.77E-23	3.16E-21	40.99662
C1orf131	1.1594149	4.8478323	8.250901	5.34E-15	5.04E-14	23.331608
LYPD8	1.1594812	1.3751417	4.8139661	2.37E-06	6.93E-06	3.9230251
AATF	1.1596649	7.6981052	11.87525	7.84E-27	5.71E-25	50.206791
MESP1	1.1597162	5.2304362	9.088622	1.53E-17	2.10E-16	29.094142
GSDMD	1.1598137	8.1179092	10.661803	1.18E-22	4.13E-21	40.703615
TXN	1.1603634	11.877843	7.9395968	4.32E-14	3.60E-13	21.277571
TMEM45B	1.1605139	8.4782525	3.2907121	0.0011211	0.002267	-1.899451
PTK7	1.1607055	4.2685009	4.5039005	9.63E-06	2.61E-05	2.5811084
CBX4	1.1608531	5.8939626	8.0237607	2.47E-14	2.13E-13	21.827947
MPV17L2	1.1610149	7.5553776	11.210843	1.60E-24	7.89E-23	44.95344
DYNLRB1	1.1610244	6.4038176	11.759239	2.00E-26	1.35E-24	49.281564
ADA	1.1619658	6.9958727	7.6693874	2.55E-13	1.88E-12	19.536165
ZMIZ2	1.1620012	7.2559369	8.9956408	2.97E-17	3.92E-16	28.438631
ZSCAN9	1.1622508	5.3677394	9.9490142	2.73E-20	6.03E-19	35.329683
HCFC1	1.1624859	4.77523	9.2647163	4.28E-18	6.46E-17	30.345833
NDUFAF2	1.162848	7.7558407	10.598466	1.93E-22	6.38E-21	40.219242
FANCE	1.1630669	5.466759	8.0656926	1.86E-14	1.63E-13	22.103536
ARHGAP4	1.164123	7.7674039	5.6035838	4.83E-08	1.76E-07	7.6773961
LEMD2	1.1643416	7.2601695	10.845109	2.83E-23	1.13E-21	42.112512
CAPN12	1.1649376	5.7963235	5.3958866	1.41E-07	4.85E-07	6.6443616
ESPL1	1.1651391	3.7311659	6.0708531	3.92E-09	1.64E-08	10.114876
AARS2	1.1658081	6.5893874	10.223774	3.42E-21	8.86E-20	37.38056
EDC3	1.1658847	5.2022726	11.364669	4.71E-25	2.54E-23	46.159568
PPP1R14B	1.1666043	9.5200833	8.6609789	3.16E-16	3.54E-15	26.11137
EIF2D	1.1666553	7.1865135	11.791013	1.55E-26	1.07E-24	49.534655
IQCE	1.1667185	5.2069013	9.1876544	7.48E-18	1.08E-16	29.796442
AGRN	1.1672143	6.6867341	6.2540578	1.41E-09	6.24E-09	11.111991
HSPA1A	1.1678036	8.4774761	6.1893408	2.02E-09	8.81E-09	10.757156
CUL9	1.1685723	7.4996459	11.064972	5.05E-24	2.31E-22	43.815731
RPS5	1.1692341	11.38232	9.2024128	6.73E-18	9.83E-17	29.901463
E2F5	1.1693525	4.4261284	7.0582865	1.22E-11	7.08E-11	15.749072
VSIG10	1.1695519	5.6626941	8.4959728	9.95E-16	1.04E-14	24.983053
PDZD11	1.1697728	7.9073775	11.85207	9.46E-27	6.75E-25	50.021669
SLC38A6	1.1709108	6.0753253	11.318115	6.82E-25	3.61E-23	45.79387
ABCF2	1.1711493	5.9138078	10.635	1.45E-22	4.99E-21	40.49848
TRMU	1.1715355	6.6680057	10.211045	3.76E-21	9.73E-20	37.284962
LINC00482	1.1715589	5.4169308	7.3639201	1.81E-12	1.18E-11	17.616155
CANT1	1.1716041	5.4437489	9.6540828	2.47E-19	4.63E-18	33.159129
CPD	1.1719657	7.0121785	6.7006146	1.06E-10	5.36E-10	13.635914
SMC4	1.172017	5.6023162	5.5165034	7.59E-08	2.70E-07	7.240424
LDLRAD1	1.1723679	3.0691163	5.1624225	4.50E-07	1.45E-06	5.5214695
BAK1	1.1726153	6.3296802	6.8592862	4.09E-11	2.19E-10	14.563575
SETDB1	1.1726744	7.0675083	8.8146809	1.07E-16	1.30E-15	27.173886
ITGB1BP1	1.1737269	6.9288609	10.973105	1.04E-23	4.52E-22	43.102335
TOMM20	1.1739814	9.0302476	10.169372	5.17E-21	1.30E-19	36.972361
TNNC1	1.1743285	3.6582159	4.0961821	5.44E-05	0.0001331	0.9357295
ALG3	1.1754461	8.5314205	13.093519	3.57E-31	6.63E-29	60.094181
METTL3	1.1757771	6.4457393	9.3377911	2.52E-18	3.95E-17	30.869106

EXOSC4	1.1760154	8.3821404	8.4087544	1.81E-15	1.83E-14	24.391936
SHKBP1	1.1762444	5.9716407	9.0614799	1.85E-17	2.51E-16	28.902401
FAM220A	1.1763302	6.0317043	10.565337	2.49E-22	7.99E-21	39.966388
UBXN11	1.1763421	5.0667129	7.7610648	1.40E-13	1.07E-12	20.122561
FAM193B	1.1763795	4.8178964	9.6242597	3.08E-19	5.64E-18	32.9415
FAT1	1.1768371	7.4482544	5.082984	6.62E-07	2.09E-06	5.1488097
NPM1	1.1771034	10.165335	8.9585584	3.87E-17	5.03E-16	28.178265
RAC3	1.1771661	5.7696212	5.6617745	3.56E-08	1.32E-07	7.9724718
ZNF251	1.1774517	4.0730718	10.365762	1.16E-21	3.30E-20	38.450753
TIMELESS	1.1775852	5.8291523	7.7954913	1.12E-13	8.72E-13	20.343948
CYSTM1	1.1777164	9.9384653	9.3678001	2.02E-18	3.22E-17	31.084636
FAM219A	1.1777331	6.4969653	9.9032616	3.85E-20	8.27E-19	34.990823
ANO10	1.1777893	5.3619429	9.6326634	2.89E-19	5.33E-18	33.002789
PDCD2L	1.1792401	6.4760304	10.266101	2.48E-21	6.71E-20	37.698864
BBC3	1.1794078	7.7197425	8.6582066	3.22E-16	3.61E-15	26.092306
SEMA3G	1.1794624	4.1800767	7.1200524	8.31E-12	4.96E-11	16.121923
POLM	1.1796878	7.0408322	11.23816	1.29E-24	6.50E-23	45.16716
AP1M2	1.1800243	4.7490672	2.8641412	0.0044825	0.0082013	-3.168252
FBXL19	1.1800323	6.0317363	9.0028632	2.82E-17	3.73E-16	28.489412
NOL7	1.1802052	8.6521115	11.85617	9.15E-27	6.58E-25	50.054403
DUSP23	1.180272	11.109092	8.9527029	4.03E-17	5.22E-16	28.137207
HSF1	1.1810647	9.4252807	9.3301402	2.66E-18	4.16E-17	30.814215
GTSF1	1.1817223	2.8659499	3.2361825	0.0013497	0.0026923	-2.070804
MRAP2	1.181821	2.9586781	6.1597068	2.39E-09	1.03E-08	10.595623
SMO	1.182736	8.1574712	6.2274611	1.63E-09	7.19E-09	10.965822
MTFP1	1.1827682	6.3960157	8.5007125	9.63E-16	1.01E-14	25.015282
CAMK2N2	1.1827694	5.3209601	5.3450981	1.82E-07	6.20E-07	6.3966023
CCDC97	1.1828172	7.448738	10.366366	1.15E-21	3.29E-20	38.455315
PIF1	1.1835131	3.7879853	7.4826137	8.49E-13	5.79E-12	18.355929
PSMC4	1.1835627	8.4820195	13.329584	4.98E-32	1.07E-29	62.040992
TRIM28	1.1836807	8.577376	9.6193468	3.19E-19	5.81E-18	32.905682
PKDCC	1.1837161	6.4658023	4.8969284	1.61E-06	4.80E-06	4.2950262
MAPKAPK5-A	1.1839116	5.1218441	11.440016	2.58E-25	1.47E-23	46.752662
CD200	1.1842777	4.1502927	8.1112245	1.37E-14	1.22E-13	22.403819
COX6C	1.184653	11.628721	9.0549473	1.94E-17	2.62E-16	28.856301
SNHG20	1.1848449	4.4656846	9.58045	4.25E-19	7.54E-18	32.622438
PP7080	1.1854689	5.475591	7.0355558	1.40E-11	8.08E-11	15.612437
UBR5-AS1	1.1856373	4.8661187	9.2248419	5.72E-18	8.42E-17	30.061247
FOXK1	1.1860328	4.9099505	8.7618553	1.56E-16	1.84E-15	26.80748
EME1	1.1861737	3.7390128	8.1620261	9.74E-15	8.83E-14	22.740115
ATXN7L3	1.1863047	6.4558747	8.2212492	6.53E-15	6.06E-14	23.133821
CSNK1E	1.1864567	7.2860725	8.39692	1.97E-15	1.97E-14	24.312015
NEIL3	1.1865171	2.1422353	7.4624759	9.65E-13	6.53E-12	18.22985
COL7A1	1.1867977	4.2453573	4.9923863	1.02E-06	3.15E-06	4.7297176
ANXA2R	1.1869208	5.5181052	8.7786595	1.38E-16	1.64E-15	26.923898
RPS10	1.1872126	11.465505	8.5831694	5.43E-16	5.90E-15	25.577692
UBE2A	1.1874919	8.4059629	10.118321	7.61E-21	1.87E-19	36.590254
SPAG4	1.1878152	5.4099179	6.2176225	1.73E-09	7.58E-09	10.911873
CAPN10	1.1879229	6.198992	11.222865	1.45E-24	7.25E-23	45.047471
PECAM1	1.188059	7.5714569	7.9933511	3.02E-14	2.58E-13	21.628659
NAT14	1.1884203	6.0818577	6.8116499	5.45E-11	2.87E-10	14.283415

FUS	1.1885664	8.3364282	9.1085587	1.32E-17	1.83E-16	29.235186
KCTD17	1.188733	4.5813126	5.0320499	8.46E-07	2.63E-06	4.9124163
OCRL	1.1887345	5.4031981	8.2647436	4.86E-15	4.62E-14	23.424095
SLC25A29	1.1888842	6.52278	9.1307123	1.13E-17	1.58E-16	29.392115
NRBP2	1.1890975	7.9040408	5.7604978	2.11E-08	8.07E-08	8.4786609
ZBTB7B	1.1892657	6.4272953	8.0258042	2.43E-14	2.10E-13	21.841356
LSM2	1.1895484	5.8244402	10.246461	2.87E-21	7.65E-20	37.551092
CPSF4	1.19012	7.0305896	12.425453	8.89E-29	9.38E-27	54.635921
CIZ1	1.1901562	6.7698144	9.3749323	1.92E-18	3.07E-17	31.135915
TYMSOS	1.1902875	5.0070231	6.1668469	2.30E-09	9.93E-09	10.634489
SNX27	1.1909568	6.4948637	8.6340898	3.81E-16	4.24E-15	25.926621
CAPN2	1.1912143	9.1114013	7.4042704	1.40E-12	9.28E-12	17.866737
DUT	1.1917807	7.4797691	9.2934564	3.48E-18	5.33E-17	30.551368
COL1A2	1.1919305	6.718794	3.636923	0.0003257	0.0007166	-0.749947
NUP210	1.1922068	5.6739578	7.6097836	3.75E-13	2.70E-12	19.157407
ANKRD65	1.1925011	5.2809826	4.7636895	2.99E-06	8.63E-06	3.700225
SLC16A3	1.1930788	7.3129139	4.781775	2.75E-06	7.98E-06	3.7801404
PSMD10	1.193093	7.6370181	9.8231522	7.01E-20	1.43E-18	34.399378
FGD1	1.1932067	4.7303976	7.716627	1.87E-13	1.41E-12	19.837748
SH3PXD2B	1.1934809	3.663678	8.4546131	1.32E-15	1.35E-14	24.702279
PACS2	1.1935695	5.9542078	11.280797	9.17E-25	4.77E-23	45.501147
SRM	1.1936368	8.2447756	9.561499	4.89E-19	8.60E-18	32.484655
ATP13A2	1.1940114	5.2880808	7.8529092	7.66E-14	6.12E-13	20.714611
RRAGD	1.1941286	5.5402913	6.8464987	4.42E-11	2.36E-10	14.48823
HM13	1.1941708	7.900912	13.08358	3.87E-31	7.06E-29	60.01241
UBL4A	1.1944763	6.7301538	10.399587	8.91E-22	2.60E-20	38.706703
SPATC1L	1.1945418	3.7729812	7.0789785	1.07E-11	6.29E-11	15.873724
NAGPA	1.1947958	6.7395345	10.862308	2.48E-23	1.00E-21	42.24523
CCT5	1.1953321	7.6150027	8.7264525	2.00E-16	2.30E-15	26.562641
PDRG1	1.1953702	7.5620398	10.885264	2.07E-23	8.47E-22	42.422513
LZTS2	1.1955408	7.5912003	7.7960639	1.11E-13	8.70E-13	20.347635
MRPL9	1.1964219	8.9527754	10.540268	3.02E-22	9.53E-21	39.775289
CYB5R1	1.1965992	8.0321875	10.674074	1.07E-22	3.79E-21	40.797602
ANKS3	1.1969948	6.212843	10.375438	1.07E-21	3.09E-20	38.523932
KLHL23	1.1978236	5.1262572	7.0661902	1.16E-11	6.77E-11	15.796655
RALY	1.1987791	7.2789363	10.834295	3.08E-23	1.21E-21	42.029115
INTS8	1.1988435	5.2503235	9.9058599	3.78E-20	8.14E-19	35.010046
PARP1	1.1997128	9.0516625	9.3199252	2.87E-18	4.44E-17	30.740966
BRICD5	1.2000018	4.6902903	7.6732185	2.48E-13	1.84E-12	19.560577
IFT52	1.2002137	4.4205511	8.9245823	4.93E-17	6.28E-16	27.940245
ABHD4	1.2004295	7.310338	8.5906538	5.16E-16	5.62E-15	25.628902
MRPS23	1.2006772	6.5024964	12.067927	1.65E-27	1.39E-25	51.750338
FAM53B	1.2007502	5.3398046	8.8096556	1.11E-16	1.34E-15	27.138975
RTN2	1.2008182	4.938565	5.6500909	3.79E-08	1.40E-07	7.9130295
MICAL1	1.2013856	5.8792168	6.2478579	1.46E-09	6.46E-09	11.077875
TBC1D31	1.2014757	5.0073433	11.045522	5.88E-24	2.65E-22	43.664487
NUP37	1.2015196	6.2794475	10.449853	6.06E-22	1.82E-20	39.087768
RPS21	1.202274	11.608881	8.2960703	3.92E-15	3.78E-14	23.633752
HOMER3	1.2023472	7.3946062	6.048413	4.44E-09	1.85E-08	9.9943189
CEBPA	1.2039073	10.747511	5.570976	5.72E-08	2.06E-07	7.5131213
ZFAS1	1.2039356	7.639322	7.2854797	2.96E-12	1.88E-11	17.131724

GTF2IRD1	1.2051371	4.8422328	7.9115404	5.20E-14	4.28E-13	21.094931
PLXND1	1.2052904	6.6379098	7.5634909	5.05E-13	3.56E-12	18.864598
TINAG	1.2056441	2.3869183	3.8342963	0.0001541	0.000355	-0.04767
DNMT3A	1.2056875	4.9003784	8.4852537	1.07E-15	1.11E-14	24.910206
NVL	1.2059378	6.7676176	11.249719	1.17E-24	6.01E-23	45.257653
BCAM	1.2067685	10.400757	5.5448508	6.55E-08	2.35E-07	7.3820641
WASF2	1.2078656	7.37813	8.2806999	4.36E-15	4.17E-14	23.530823
CORO1B	1.208315	7.0417374	10.585533	2.13E-22	6.97E-21	40.120492
CRELD2	1.208332	8.9597312	9.3176004	2.92E-18	4.50E-17	30.724301
GPRIN1	1.2088052	3.0744488	7.6371947	3.14E-13	2.28E-12	19.331348
LPGAT1	1.2089179	7.8266707	7.5165447	6.83E-13	4.74E-12	18.568885
SLC39A13	1.2095442	8.5038611	11.48727	1.77E-25	1.03E-23	47.125389
DPH2	1.2116753	6.8961337	9.8252259	6.91E-20	1.41E-18	34.414658
DNAJC5	1.2116912	6.6059108	9.8031886	8.14E-20	1.65E-18	34.252363
POLE2	1.2117477	4.0163007	8.2306937	6.12E-15	5.73E-14	23.196771
ARPC1B	1.2118353	9.4426711	7.4969254	7.75E-13	5.33E-12	18.445672
CPLX1	1.2119706	4.0780041	4.9505536	1.25E-06	3.79E-06	4.5383486
ALPK3	1.2128013	3.2057383	5.8073636	1.65E-08	6.36E-08	8.721397
CST1	1.2129369	2.5489894	3.167435	0.0016996	0.0033256	-2.283034
FASTK	1.2134295	9.4663381	12.657206	1.32E-29	1.69E-27	56.520188
NFKB2	1.2148912	6.2038723	9.0569861	1.92E-17	2.59E-16	28.870687
BOLA3	1.2158175	8.2517648	11.010294	7.76E-24	3.44E-22	43.390833
SNAPIN	1.2166017	8.3263267	12.596018	2.19E-29	2.69E-27	56.021698
C20orf96	1.2168327	4.3183655	9.5662127	4.72E-19	8.33E-18	32.518913
MAZ	1.2174623	5.9615477	6.9689086	2.10E-11	1.18E-10	15.213625
MAP2	1.2177027	3.1383531	5.6460296	3.87E-08	1.43E-07	7.8923897
GSDMB	1.2178764	7.7366901	6.4262411	5.26E-10	2.44E-09	12.069697
AACS	1.2188604	6.1322515	7.7211078	1.82E-13	1.37E-12	19.866418
CDC7	1.2195761	4.3862145	7.7271168	1.75E-13	1.32E-12	19.904883
TSSK6	1.2197424	2.9365285	10.022662	1.57E-20	3.62E-19	35.876753
NECTIN1	1.2201019	4.8816431	7.1368535	7.49E-12	4.49E-11	16.223739
PLPPR1	1.2201372	5.0008611	5.5701571	5.75E-08	2.07E-07	7.5090054
TTYH3	1.220252	6.7240375	5.6938752	3.01E-08	1.13E-07	8.1362957
RPL38	1.2203449	12.813105	9.6068755	3.50E-19	6.33E-18	32.814802
LPL	1.2204306	4.1013992	7.4861098	8.30E-13	5.68E-12	18.377841
NUDT2	1.2208968	7.4643434	9.8814484	4.54E-20	9.63E-19	34.82954
THUMPD3-AS	1.221402	6.039042	9.4493915	1.11E-18	1.86E-17	31.672511
IRF3	1.2214167	7.7895405	11.479434	1.89E-25	1.09E-23	47.063541
CPXM1	1.221707	3.6818887	6.1779861	2.16E-09	9.35E-09	10.695192
FKBP11	1.2219914	9.1374074	7.3501855	1.97E-12	1.28E-11	17.531075
CHAF1B	1.22228	4.5499433	7.462276	9.67E-13	6.53E-12	18.2286
CA12	1.2229358	3.215169	3.829225	0.0001571	0.0003617	-0.066136
TRIM50	1.2231613	3.5197953	3.7229745	0.0002359	0.0005302	-0.447914
RNF215	1.2235918	4.9795993	11.600564	7.16E-26	4.53E-24	48.021342
SLC39A7	1.2237659	8.0565855	10.099017	8.81E-21	2.14E-19	36.446005
MAPK3	1.224832	6.3464449	11.046456	5.84E-24	2.63E-22	43.671747
LIN37	1.2262028	6.4941236	13.78578	1.09E-33	3.29E-31	65.826122
MRGBP	1.2266331	6.3044019	12.280462	2.91E-28	2.80E-26	53.462477
TTLL4	1.2266905	4.7190031	6.7986105	5.89E-11	3.09E-10	14.206975
BRMS1	1.2268053	7.9008325	12.63696	1.56E-29	1.96E-27	56.355172
BGLAP	1.2274528	4.7422614	8.3723279	2.33E-15	2.31E-14	24.146158

DDX41	1.2275005	8.1455736	14.822551	1.66E-37	8.57E-35	74.518459
HAUS5	1.2275268	3.602026	10.662441	1.17E-22	4.12E-21	40.708496
SOX13	1.2275296	6.1822068	9.1570635	9.33E-18	1.33E-16	29.579054
SLC12A7	1.2277712	7.2159854	9.2477318	4.85E-18	7.20E-17	30.224531
NUPR1	1.2280947	10.744408	6.409349	5.80E-10	2.68E-09	11.974871
RABIF	1.2283926	5.4380184	13.344018	4.42E-32	9.72E-30	62.160302
RECQL5	1.2284592	4.9907818	12.265872	3.28E-28	3.13E-26	53.344632
ATP6V1F	1.228597	9.9131344	10.861171	2.50E-23	1.01E-21	42.236459
RPL30	1.2286521	13.270493	9.4958654	7.93E-19	1.35E-17	32.008571
MRPL24	1.2287663	10.427564	11.246707	1.20E-24	6.13E-23	45.234068
FBL	1.228799	10.080527	8.7816877	1.35E-16	1.61E-15	26.94489
DAP3	1.228816	10.45252	12.89578	1.84E-30	2.77E-28	58.470346
CPSF1	1.230109	6.8911979	10.324196	1.59E-21	4.43E-20	38.136749
DYNLL1	1.230983	10.921344	12.28591	2.79E-28	2.69E-26	53.506485
MAP2K2	1.2317556	8.653286	12.833263	3.09E-30	4.46E-28	57.958341
XRCC1	1.2317998	8.0870479	10.931732	1.44E-23	6.12E-22	42.78186
DARS2	1.2330955	5.8815817	8.8586627	7.86E-17	9.75E-16	27.479922
ALG1L	1.2337016	5.4177211	6.6979934	1.07E-10	5.43E-10	13.620722
DCTN2	1.2343906	8.8224003	12.433693	8.31E-29	8.87E-27	54.702739
PLAU	1.2348025	6.1357057	6.8462246	4.42E-11	2.36E-10	14.486616
LAMA3	1.2348647	3.2445027	5.2322102	3.19E-07	1.05E-06	5.8528229
C15orf39	1.2353442	6.2164397	7.5855084	4.38E-13	3.12E-12	19.003713
DOCK6	1.2360431	6.3407747	10.750604	5.92E-23	2.21E-21	41.384851
GALNT10	1.2361337	3.9691973	9.8745104	4.78E-20	1.01E-18	34.778278
OLFML2B	1.236216	4.3936769	8.1878923	8.18E-15	7.48E-14	22.91185
CHEK1	1.2369115	4.1154701	8.4162609	1.72E-15	1.74E-14	24.442665
PIR	1.2369509	8.5193453	6.64245	1.49E-10	7.40E-10	13.299842
SPTAN1	1.2370043	8.101609	10.582854	2.17E-22	7.09E-21	40.100041
ANKRD27	1.2372581	5.7125354	8.1660041	9.48E-15	8.61E-14	22.766504
C20orf27	1.2374848	5.9799083	9.9421679	2.88E-20	6.29E-19	35.278927
SCAMP5	1.2379914	3.9408945	5.4100138	1.31E-07	4.53E-07	6.7136192
ERBB3	1.2381662	6.8084329	7.8751597	6.62E-14	5.34E-13	20.858726
CCHCR1	1.238304	5.6669566	10.865079	2.42E-23	9.82E-22	42.266623
BEX2	1.2389445	6.0997016	3.2065534	0.0014914	0.0029547	-2.162792
POC1A	1.2398268	5.3519298	9.612548	3.35E-19	6.09E-18	32.856131
DDIT3	1.2411095	7.0274348	7.6584618	2.73E-13	2.01E-12	19.46659
TNFRSF4	1.2412991	4.0105986	10.263516	2.52E-21	6.82E-20	37.679409
PLCB1	1.2414274	4.9940449	6.2925072	1.13E-09	5.08E-09	11.324138
RAD51	1.2416675	4.4639737	8.0397635	2.22E-14	1.93E-13	21.933014
ALS2CL	1.2417806	5.6207931	6.6148507	1.75E-10	8.65E-10	13.141131
RPSA	1.2418214	11.607825	9.0661375	1.79E-17	2.44E-16	28.935282
ASNS	1.2424461	6.2441904	4.9796708	1.09E-06	3.33E-06	4.6714054
ZNF706	1.2425468	7.6411781	10.233045	3.18E-21	8.39E-20	37.45023
TOR3A	1.2429219	8.3219341	7.5778864	4.60E-13	3.27E-12	18.955524
IRX3	1.24309	5.1747546	4.9886511	1.04E-06	3.20E-06	4.7125751
CCDC167	1.2437256	8.520648	10.01764	1.63E-20	3.75E-19	35.839386
SNHG11	1.2440005	7.2417494	8.5998033	4.84E-16	5.31E-15	25.69154
PHF19	1.2442208	5.0214496	8.2856623	4.21E-15	4.05E-14	23.564041
CBFA2T2	1.2442968	4.4891364	9.8513419	5.68E-20	1.18E-18	34.607228
STXBP6	1.2457411	3.9989791	8.6426628	3.59E-16	4.00E-15	25.985487
C19orf25	1.2461131	6.8150453	11.110683	3.52E-24	1.65E-22	44.171607

CRYGS	1.2463662	5.1679834	7.9230362	4.82E-14	3.99E-13	21.169715
GRK6	1.2464406	6.4946352	11.431797	2.76E-25	1.56E-23	46.6879
ARRDC2	1.2467603	7.6143392	5.703605	2.86E-08	1.07E-07	8.1860974
PIGT	1.2469483	8.4200793	11.578611	8.53E-26	5.27E-24	47.847473
ALYREF	1.2469708	7.7723886	9.0865071	1.55E-17	2.13E-16	29.079191
SLC39A1	1.2473266	8.7341138	9.7481769	1.23E-19	2.42E-18	33.848028
CLIC1	1.2476162	8.5171501	7.8311663	8.84E-14	7.00E-13	20.574039
ABHD12	1.2490454	7.0541879	11.842783	1.02E-26	7.21E-25	49.947531
RAMP1	1.2495407	9.5695847	4.2115634	3.38E-05	8.50E-05	1.3873786
RPL39L	1.2502718	5.3127497	3.8993431	0.0001196	0.0002794	0.1911318
HRAS	1.2514752	6.5765118	11.314133	7.04E-25	3.71E-23	45.762617
APBB3	1.2519332	7.3391115	7.9803983	3.29E-14	2.79E-13	21.543921
AIFM2	1.2519701	7.8134788	9.3120704	3.04E-18	4.68E-17	30.684671
PI4KB	1.2521212	7.1866475	10.170247	5.13E-21	1.30E-19	36.978919
IL3RA	1.2521421	5.1559992	11.732035	2.49E-26	1.66E-24	49.06507
E2F8	1.2521816	2.9123467	7.472497	9.06E-13	6.15E-12	18.292561
SUV39H1	1.252295	5.2971993	10.892268	1.96E-23	8.03E-22	42.476642
RASSF4	1.2527873	7.6927517	7.92238	4.84E-14	4.01E-13	21.165445
LINC01341	1.2533437	5.4290988	7.0093872	1.64E-11	9.35E-11	15.455524
ARPC5	1.2534136	9.0666294	11.479621	1.88E-25	1.09E-23	47.065017
GIPC1	1.2536122	7.7131969	9.6472832	2.59E-19	4.84E-18	33.10948
C1QTNF3	1.2538965	4.0415891	4.2941382	2.38E-05	6.12E-05	1.717426
NAA40	1.254065	4.423506	8.8720181	7.15E-17	8.90E-16	27.573026
PCED1A	1.254409	6.0174797	9.8202654	7.17E-20	1.46E-18	34.37811
SNRPA	1.2547749	7.9961122	12.13905	9.23E-28	8.20E-26	52.322207
HEY1	1.2552696	5.2739721	7.9652462	3.64E-14	3.07E-13	21.444906
MEA1	1.2555414	8.366083	13.120666	2.84E-31	5.46E-29	60.317621
PLXNA1	1.2573734	4.5304043	7.1353395	7.56E-12	4.53E-11	16.214557
TDGF1	1.2573889	4.2727745	3.8598121	0.0001395	0.0003231	0.045572
TMEM81	1.2576985	4.1057691	12.944328	1.23E-30	1.95E-28	58.868416
MYL6B	1.2580695	8.2466959	8.2188391	6.63E-15	6.15E-14	23.117764
RRP9	1.2582403	6.9968649	11.941895	4.57E-27	3.57E-25	50.739728
NCAPH2	1.2588001	6.6623192	10.765945	5.25E-23	1.99E-21	41.502788
ZNF512B	1.2593015	7.2148107	9.0324634	2.28E-17	3.05E-16	28.697775
RAD9A	1.2596558	5.356477	10.584903	2.14E-22	6.99E-21	40.115679
IGF2BP2	1.2597797	3.4689059	4.7954398	2.58E-06	7.52E-06	3.8406934
SP5	1.260688	6.1468964	4.8307449	2.19E-06	6.44E-06	3.9978242
PRUNE1	1.2612295	4.4869636	8.8243252	1.00E-16	1.22E-15	27.240918
TMEM104	1.2619702	6.7103521	11.004024	8.15E-24	3.60E-22	43.342165
ZBED6CL	1.2619872	6.9722974	7.3938748	1.49E-12	9.87E-12	17.80209
EDIL3	1.2624307	2.27831	6.6988945	1.07E-10	5.41E-10	13.625945
RNF187	1.2624338	7.9050796	10.920361	1.57E-23	6.61E-22	42.69387
NAXE	1.2626052	9.0755006	12.498494	4.88E-29	5.61E-27	55.228663
EHMT2	1.2638127	4.6946652	11.156071	2.46E-24	1.19E-22	44.525552
B4GALT3	1.2647981	7.6501848	11.272628	9.78E-25	5.03E-23	45.437116
TPD52L2	1.2654715	6.5991893	10.924891	1.52E-23	6.43E-22	42.728917
PIAS3	1.2657751	5.1295264	7.6961348	2.14E-13	1.60E-12	19.706773
SPDL1	1.2661011	4.6142338	9.6684494	2.22E-19	4.20E-18	33.264091
COMMD4	1.2662126	8.1200222	14.034832	1.33E-34	4.53E-32	67.903872
CCDC107	1.2662139	7.5145433	12.365945	1.45E-28	1.49E-26	54.153792
AKAP8L	1.2662782	7.3217048	12.475927	5.88E-29	6.59E-27	55.04541

MAGED2	1.266299	9.1749509	10.62354	1.59E-22	5.41E-21	40.410842
SFI1	1.2663448	5.8742284	8.3293006	3.13E-15	3.05E-14	23.856685
DONSON	1.2667126	6.2501349	8.8217735	1.02E-16	1.24E-15	27.223178
SIPA1L3	1.2676737	4.0751852	7.7594943	1.42E-13	1.08E-12	20.112476
NCAPH	1.267847	4.0343893	8.1035884	1.44E-14	1.28E-13	22.353385
SQSTM1	1.2679574	9.6332009	6.3890594	6.52E-10	3.00E-09	11.861222
IL32	1.2680127	10.995294	5.335959	1.90E-07	6.47E-07	6.3522232
CKAP2	1.2686798	4.1753232	7.6399433	3.08E-13	2.24E-12	19.348813
TMEM98	1.2703549	7.4329594	4.8894242	1.67E-06	4.97E-06	4.2611552
TMEM147	1.270833	9.5025905	9.4936071	8.06E-19	1.38E-17	31.992221
TMEM101	1.2709025	6.9332295	11.844931	1.00E-26	7.12E-25	49.964675
RAB3B	1.2710912	3.6312337	6.5454202	2.64E-10	1.27E-09	12.744032
CKB	1.2713119	8.4382672	3.4930924	0.0005509	0.0011675	-1.240343
RPL36A	1.2716092	9.3615857	8.0449199	2.14E-14	1.87E-13	21.966897
PDGFB	1.2718249	4.7165143	7.8926889	5.89E-14	4.79E-13	20.972448
REPIN1	1.2720703	9.1421685	10.622211	1.60E-22	5.46E-21	40.40068
CDK16	1.2721088	5.7254622	9.4188354	1.39E-18	2.28E-17	31.452032
IMPDH2	1.2735073	8.2581826	9.8971512	4.03E-20	8.63E-19	34.945626
MAST2	1.2735886	5.9030752	9.6228329	3.11E-19	5.68E-18	32.931097
MAST3	1.2751644	6.0749409	10.04648	1.31E-20	3.08E-19	36.054101
PSMB4	1.2766289	12.050391	14.024022	1.46E-34	4.87E-32	67.813542
MPV17	1.2772907	6.2433509	10.327089	1.55E-21	4.34E-20	38.158583
LIMK2	1.2774573	6.2011341	6.9492153	2.37E-11	1.32E-10	15.096301
ENTPD6	1.2780875	6.3543986	10.127579	7.10E-21	1.75E-19	36.659481
YKT6	1.278697	7.1615207	10.495285	4.27E-22	1.30E-20	39.432905
SNHG12	1.2801868	6.5106538	8.5243664	8.17E-16	8.68E-15	25.176284
PLCG1	1.2814227	5.3251421	8.228061	6.23E-15	5.82E-14	23.179218
RHBDF2	1.2821124	6.9671495	7.4351003	1.15E-12	7.69E-12	18.058827
RBCK1	1.2822531	7.9986955	10.616858	1.67E-22	5.65E-21	40.359764
PGGHG	1.2824012	6.9725282	4.9090401	1.52E-06	4.56E-06	4.3497866
POLG2	1.2826939	6.4912117	10.416132	7.85E-22	2.31E-20	38.832039
B4GALNT1	1.2833791	3.3158695	6.1225403	2.94E-09	1.25E-08	10.393875
B4GALT7	1.2838796	7.140967	13.572728	6.51E-33	1.70E-30	64.054852
TOMM40	1.2844927	6.9846268	11.044361	5.94E-24	2.67E-22	43.655466
TMEM9	1.2857585	8.7530953	11.397936	3.61E-25	1.99E-23	46.421243
CTAG2	1.2860598	2.0887835	3.4395713	0.0006669	0.0013959	-1.41818
GOLM1	1.2873813	5.8799789	4.831093	2.19E-06	6.43E-06	3.9993785
PLXNC1	1.287828	3.4215111	6.7288162	8.94E-11	4.58E-10	13.799635
GOLGA2P5	1.2880406	5.5933027	7.3983959	1.45E-12	9.62E-12	17.830198
MFSD10	1.2892497	6.2759628	6.860805	4.05E-11	2.17E-10	14.572531
BOLA1	1.2895542	8.5078665	11.911411	5.85E-27	4.40E-25	50.495829
CKLF	1.2896808	6.7732649	10.041131	1.36E-20	3.20E-19	36.014253
FDPS	1.2898448	11.121851	7.5072972	7.25E-13	5.01E-12	18.510782
CAPS	1.2901879	6.2223547	7.6011742	3.96E-13	2.84E-12	19.10286
CLN6	1.2907169	7.1128994	11.431598	2.76E-25	1.56E-23	46.686329
NME2	1.2911176	10.821222	7.9742848	3.43E-14	2.90E-13	21.503956
DTNA	1.2916189	3.2623915	7.3249914	2.31E-12	1.49E-11	17.375293
COMMD5	1.2920337	7.9619489	12.104262	1.22E-27	1.07E-25	52.042354
UBE2Q1	1.2920414	7.5316	11.893049	6.79E-27	5.00E-25	50.349021
TRIM65	1.292309	4.0935931	9.9332576	3.07E-20	6.68E-19	35.212897
KNTC1	1.2938865	4.6723931	7.6093351	3.76E-13	2.70E-12	19.154564

TBC1D13	1.2938935	8.4000944	10.229339	3.28E-21	8.59E-20	37.422376
NHP2	1.2941855	9.1735665	12.030124	2.24E-27	1.83E-25	51.446837
TMEM198	1.2942601	5.0032301	7.9487499	4.06E-14	3.40E-13	21.337245
BCAP31	1.2946721	10.350594	11.497953	1.63E-25	9.53E-24	47.209727
FOXD2-AS1	1.2949323	3.2198753	7.5781184	4.60E-13	3.26E-12	18.95699
TLX1	1.2958082	3.0957681	6.5142882	3.16E-10	1.51E-09	12.566989
NDST1	1.2964351	5.9929802	9.0979309	1.43E-17	1.97E-16	29.159978
DVL2	1.2968331	5.952681	10.028729	1.50E-20	3.48E-19	35.921907
FAM3B	1.2969945	5.9232484	3.0808879	0.0022592	0.004339	-2.544156
CD248	1.297004	6.1349607	7.0164173	1.57E-11	8.99E-11	15.497637
TBCE	1.2974518	7.3560914	12.766628	5.36E-30	7.22E-28	57.413359
FANCD2	1.2978686	3.4223743	8.1503622	1.05E-14	9.52E-14	22.662786
MPZL1	1.2979555	6.0114025	8.5214484	8.34E-16	8.83E-15	25.156407
CBX1	1.2990643	6.9554438	8.7784718	1.39E-16	1.64E-15	26.922597
TAF6	1.2995711	7.4924436	7.8762019	6.57E-14	5.31E-13	20.865483
LMNB1	1.2995775	5.7352568	6.292041	1.13E-09	5.09E-09	11.32156
MYO19	1.2996713	6.3014448	9.2775349	3.90E-18	5.93E-17	30.437463
SMG9	1.2997456	5.4798291	11.064069	5.08E-24	2.32E-22	43.808708
CSTB	1.3013405	11.053483	10.30294	1.87E-21	5.14E-20	37.976402
TRIM25	1.3013762	6.1344662	9.4259842	1.32E-18	2.18E-17	31.503581
SNX22	1.3020904	5.214467	6.4951501	3.53E-10	1.67E-09	12.458465
HOXD9	1.3021845	3.3206996	6.3168422	9.85E-10	4.45E-09	11.458919
SH3BP5L	1.3024105	7.5642834	11.146188	2.66E-24	1.27E-22	44.448431
CASC9	1.3035828	2.6356797	4.3130232	2.20E-05	5.68E-05	1.7937026
MLEC	1.3037599	8.1606428	10.649848	1.29E-22	4.48E-21	40.612086
ABCC10	1.3039566	5.1667565	9.8000384	8.34E-20	1.68E-18	34.229178
C8orf76	1.3042635	7.1814983	11.380721	4.14E-25	2.27E-23	46.285798
S100A1	1.3048038	3.9481021	4.4999623	9.80E-06	2.65E-05	2.5645622
KLHL12	1.3055054	6.1823425	11.471656	2.01E-25	1.15E-23	47.002168
CD109	1.3056162	3.7164168	6.2874663	1.16E-09	5.22E-09	11.296268
SLC25A39	1.3057155	9.0424292	13.406181	2.63E-32	6.32E-30	62.674501
MCRS1	1.3067012	7.232687	12.834206	3.07E-30	4.46E-28	57.966058
CUEDC1	1.307092	4.7550218	8.4800827	1.11E-15	1.15E-14	24.875084
TUBA1B	1.3071969	10.788271	5.8638343	1.21E-08	4.77E-08	9.0159497
UXS1	1.307225	6.7096559	10.31133	1.75E-21	4.85E-20	38.039673
UFC1	1.3074656	8.9968355	13.044729	5.35E-31	9.37E-29	59.692917
ANAPC7	1.308286	5.1941925	13.086837	3.77E-31	6.94E-29	60.039202
RASL12	1.3087087	3.8342384	9.5310819	6.12E-19	1.06E-17	32.263806
ENGASE	1.3087951	6.7743744	9.9905515	2.00E-20	4.54E-19	35.637988
CDK5RAP3	1.3100123	9.2354292	12.791793	4.35E-30	5.95E-28	57.619084
DDX49	1.3114413	7.5225856	13.854376	6.09E-34	1.93E-31	66.397648
PABPC1	1.3114863	11.934164	7.8357273	8.58E-14	6.80E-13	20.603506
ARL2	1.3129062	7.4168534	7.8441435	8.12E-14	6.45E-13	20.657909
XPO5	1.3144009	5.6466854	10.356048	1.24E-21	3.53E-20	38.377314
CLCN7	1.3157766	6.7570256	8.8809814	6.71E-17	8.38E-16	27.635557
PRKRIP1	1.3159465	6.3805467	12.931679	1.37E-30	2.15E-28	58.764659
GABRD	1.3159861	3.1956613	11.093487	4.03E-24	1.87E-22	44.037662
LPCAT1	1.3168945	4.2152469	6.4616145	4.29E-10	2.01E-09	12.268877
CDKN2B	1.3172357	3.5198202	7.9020246	5.54E-14	4.53E-13	21.033081
SNRPD2	1.3173882	10.26225	10.058346	1.20E-20	2.85E-19	36.142528
STC2	1.3174402	3.2597348	6.5110249	3.22E-10	1.53E-09	12.548467

TRMT1	1.3174547	7.5876067	12.395562	1.14E-28	1.18E-26	54.393659
USF1	1.317967	5.7385946	14.824559	1.63E-37	8.57E-35	74.535391
NARF	1.3183605	7.537625	11.393589	3.74E-25	2.06E-23	46.387034
FBXL6	1.3187189	6.8559829	9.3886584	1.74E-18	2.80E-17	31.234663
DEPDC1	1.3188236	2.8398223	7.6645525	2.63E-13	1.94E-12	19.505368
TMEM79	1.3192786	5.6240949	12.960148	1.08E-30	1.77E-28	58.998221
APOBEC3B	1.3200106	4.7963956	5.689913	3.07E-08	1.15E-07	8.1160348
ELOVL1	1.320575	7.6224411	11.310052	7.27E-25	3.81E-23	45.73059
CYHR1	1.3206437	7.6463893	12.082628	1.46E-27	1.24E-25	51.868447
CDCA4	1.3234265	3.6303465	9.0066706	2.75E-17	3.64E-16	28.516192
GPATCH4	1.3243952	7.7166698	10.680801	1.02E-22	3.63E-21	40.849151
SKA1	1.3247345	2.2819993	8.4571889	1.30E-15	1.33E-14	24.719741
ZNF687	1.324789	6.230554	8.6452763	3.53E-16	3.94E-15	26.00344
ASPSCR1	1.3251419	7.3559161	7.7357828	1.65E-13	1.25E-12	19.96039
SAE1	1.3256789	7.4590101	11.097741	3.90E-24	1.82E-22	44.070788
TCF3	1.3261889	6.9437453	9.3699979	1.99E-18	3.17E-17	31.100436
LRRC45	1.3265597	6.9279579	8.4900809	1.04E-15	1.08E-14	24.943005
AQP8	1.3274046	3.9179479	4.3037927	2.29E-05	5.89E-05	1.7563837
TMSB10	1.3282717	11.342107	5.6965048	2.97E-08	1.11E-07	8.1497483
UCHL1	1.3282865	5.056766	3.6435209	0.0003178	0.0007009	-0.727017
ABCD1	1.3285231	5.800483	8.5328565	7.71E-16	8.20E-15	25.234138
MAVS	1.3288872	6.642014	10.614141	1.71E-22	5.74E-21	40.338993
NOP56	1.3292106	8.6022846	9.7102056	1.63E-19	3.16E-18	33.569614
VPS28	1.3292914	10.63288	10.965494	1.10E-23	4.77E-22	43.043343
CHML	1.3296747	4.4156581	7.3738687	1.70E-12	1.11E-11	17.677851
NEU1	1.3298773	7.4960567	10.275469	2.30E-21	6.27E-20	37.769402
LYPD1	1.3299082	3.4909189	5.5858488	5.30E-08	1.92E-07	7.5879529
TMCO3	1.3314332	5.6870651	7.9062752	5.39E-14	4.42E-13	21.060703
NAA10	1.3315016	7.6715056	12.324969	2.02E-28	2.03E-26	53.822224
EFNA3	1.3323015	2.4680276	8.0525979	2.03E-14	1.78E-13	22.017376
ALDOA	1.3330987	11.248464	7.5690958	4.87E-13	3.44E-12	18.899987
ANKZF1	1.3339179	6.6656837	13.075396	4.15E-31	7.48E-29	59.945089
GOLPH3L	1.3342682	6.386852	10.0431	1.34E-20	3.16E-19	36.028925
ATP6AP1	1.3345331	9.1530672	12.981589	9.04E-31	1.52E-28	59.174207
GIT1	1.3361191	4.4669476	10.52999	3.27E-22	1.02E-20	39.697
SMYD2	1.33613	8.7885308	7.7686984	1.33E-13	1.03E-12	20.171594
CDC25B	1.3374234	7.1105613	7.5625199	5.08E-13	3.58E-12	18.85847
SFRP4	1.3374363	3.9193906	5.6148967	4.55E-08	1.67E-07	7.7345699
C15orf48	1.3376112	5.6702594	3.9763512	8.82E-05	0.00021	0.4785205
ZNF580	1.3378454	6.4705074	10.510329	3.80E-22	1.17E-20	39.547336
RAP2A	1.3383837	6.3538905	7.7390129	1.62E-13	1.23E-12	19.98109
SEMA6C	1.3391663	5.4543206	7.9013095	5.57E-14	4.55E-13	21.028435
PUF60	1.3399615	9.2732019	12.014529	2.54E-27	2.06E-25	51.321723
TNFRSF25	1.3405438	6.836587	7.8950805	5.80E-14	4.72E-13	20.987976
PDCD5	1.3429394	8.4660204	12.582385	2.45E-29	2.91E-27	55.910731
PGP	1.3429507	5.369227	11.214522	1.55E-24	7.68E-23	44.982215
GNPAT	1.343193	8.5178362	11.551144	1.06E-25	6.39E-24	47.630114
CKAP2L	1.3443057	3.2011201	8.1137367	1.35E-14	1.21E-13	22.420418
NPLOC4	1.3444244	6.6553457	11.791072	1.55E-26	1.07E-24	49.535123
EME2	1.3448327	4.5111906	8.9127103	5.36E-17	6.81E-16	27.857197
PCAT6	1.3454555	4.1053005	8.6139297	4.39E-16	4.83E-15	25.788329

UQCC2	1.3463867	8.6496471	11.915072	5.68E-27	4.29E-25	50.525111
KISS1	1.3467067	4.4187209	6.5976116	1.94E-10	9.51E-10	13.042244
SLC41A3	1.347843	8.112052	11.59216	7.65E-26	4.78E-24	47.954767
KLF13	1.3484344	6.6214592	8.3235121	3.25E-15	3.17E-14	23.817812
ATP6V1C1	1.3484936	6.2135262	9.4371539	1.22E-18	2.01E-17	31.584164
SPARC	1.3493088	10.557855	6.7139703	9.76E-11	4.97E-10	13.713386
SAMD1	1.3522031	5.6354904	11.424184	2.93E-25	1.64E-23	46.627918
TUFT1	1.352581	4.9413372	8.4071413	1.83E-15	1.84E-14	24.381038
SNRNP70	1.3528369	9.4168114	12.316559	2.17E-28	2.16E-26	53.75421
CHKA	1.3529698	6.5059477	9.2516192	4.71E-18	7.03E-17	30.252284
PRPF3	1.3533797	6.0203951	10.829355	3.20E-23	1.26E-21	41.991021
KNSTRN	1.3538941	6.66795	9.7257236	1.45E-19	2.84E-18	33.683329
SOX12	1.3539896	4.3733407	7.4385527	1.12E-12	7.53E-12	18.080372
ILF3	1.3547231	7.5170809	10.537527	3.08E-22	9.66E-21	39.754413
CDKN2AIPNI	1.3549817	6.4148152	12.822595	3.37E-30	4.80E-28	57.871035
LIMK1	1.3553515	4.9959223	7.1424631	7.23E-12	4.35E-11	16.257772
SIRT7	1.3556107	6.9284128	11.008489	7.87E-24	3.48E-22	43.376823
NDRG3	1.3556475	5.695509	9.6972111	1.79E-19	3.46E-18	33.474463
RAB24	1.356746	7.1181724	11.987984	3.15E-27	2.50E-25	51.10889
PDGFRB	1.3571	7.3963806	6.7495663	7.90E-11	4.07E-10	13.920418
JMJD4	1.3579616	6.5468859	13.732527	1.70E-33	5.03E-31	65.382828
LINC01138	1.3579727	4.8150059	9.5969448	3.76E-19	6.73E-18	32.742479
RNF213	1.358897	7.984977	9.0275343	2.36E-17	3.15E-16	28.663051
IP6K1	1.3592911	5.2794842	12.143543	8.90E-28	7.95E-26	52.358367
CDK5	1.3596102	7.5679549	12.105156	1.22E-27	1.07E-25	52.049544
SNRPC	1.3624822	8.1833544	13.227813	1.17E-31	2.39E-29	61.200633
DUSP12	1.362606	7.4885134	12.030524	2.23E-27	1.83E-25	51.450043
CREB3L4	1.3630215	7.3688226	10.787668	4.43E-23	1.69E-21	41.669909
POLA2	1.3637686	5.1067152	10.912722	1.67E-23	7.00E-22	42.634775
TSPAN17	1.3640463	6.3661034	11.917716	5.56E-27	4.25E-25	50.546256
STC1	1.364052	2.8108032	6.4633073	4.25E-10	1.99E-09	12.278429
SHISA4	1.3644596	8.5610386	5.9696349	6.84E-09	2.78E-08	9.5738479
SF3A2	1.3653829	9.7780305	10.133717	6.77E-21	1.68E-19	36.705392
GSTA4	1.3657592	7.5170074	7.1870622	5.48E-12	3.35E-11	16.529012
GTPBP2	1.3661484	5.8971268	8.7906135	1.27E-16	1.52E-15	27.006792
MVD	1.3664966	7.5987245	8.2494879	5.39E-15	5.09E-14	23.322173
TMEM184B	1.3666054	8.3159167	9.5273949	6.29E-19	1.09E-17	32.237061
RPL8	1.3675342	13.238672	8.588202	5.25E-16	5.71E-15	25.612123
NSUN5	1.3679503	8.1863332	14.388508	6.66E-36	2.67E-33	70.866392
SHARPIN	1.368481	8.0323384	11.628395	5.72E-26	3.65E-24	48.241927
HGS	1.3690595	7.8877324	7.6264465	3.37E-13	2.43E-12	19.263094
ARID3A	1.3709057	4.9111244	5.9017968	9.89E-09	3.94E-08	9.2152279
HGH1	1.3713357	7.0441492	9.735204	1.35E-19	2.66E-18	33.752845
TMEM132A	1.3715191	5.3183404	5.8242209	1.50E-08	5.83E-08	8.809089
PYCR2	1.3728764	8.3254885	12.955702	1.12E-30	1.82E-28	58.961738
SPC25	1.3733562	4.2537875	9.2901955	3.56E-18	5.45E-17	30.528031
MAD2L1	1.3738923	4.5177739	8.440506	1.46E-15	1.48E-14	24.606702
COL5A3	1.3757584	5.3829854	5.2158572	3.46E-07	1.13E-06	5.7748472
ACBD6	1.3765953	7.1017279	12.303537	2.41E-28	2.38E-26	53.64894
NPEPL1	1.377247	7.415309	10.824668	3.32E-23	1.30E-21	41.954893
DKK1	1.3772565	2.6101025	3.6009007	0.0003721	0.0008108	-0.874467

SDF2L1	1.3772838	10.973515	10.193489	4.30E-21	1.10E-19	37.1532
UBQLN4	1.3773073	5.1180213	10.236121	3.11E-21	8.25E-20	37.47335
CCDC137	1.3781476	7.0282278	11.998142	2.90E-27	2.33E-25	51.190312
BCL9	1.3784418	3.7202183	8.1456979	1.09E-14	9.80E-14	22.631882
PLXDC1	1.3810814	4.1521425	8.0347108	2.29E-14	1.99E-13	21.899826
TEAD2	1.3812318	4.4670433	6.1304726	2.81E-09	1.20E-08	10.436854
CPE	1.3813037	7.7350417	5.4552623	1.04E-07	3.64E-07	6.9364415
CCT6A	1.382294	9.3004386	11.483268	1.83E-25	1.06E-23	47.093795
CBX8	1.3831935	4.8551716	11.148105	2.62E-24	1.25E-22	44.463384
OXLD1	1.3833457	7.7182607	12.462753	6.55E-29	7.29E-27	54.938488
UHRF1	1.3853664	4.6236924	7.3778294	1.65E-12	1.09E-11	17.702428
MRPL53	1.3858711	7.924013	12.828831	3.21E-30	4.59E-28	57.922067
PSKH1	1.3859711	7.4977456	10.900903	1.83E-23	7.63E-22	42.54338
ORC1	1.3862324	3.0144355	8.377487	2.25E-15	2.24E-14	24.180928
TCOF1	1.3865418	6.3837969	11.497921	1.63E-25	9.53E-24	47.209478
DCAF13	1.3868628	6.2721114	9.8555955	5.50E-20	1.15E-18	34.638617
MAPK11	1.3870861	6.3457626	8.0203446	2.52E-14	2.18E-13	21.805535
CKAP4	1.3893596	7.9906646	8.4638449	1.24E-15	1.28E-14	24.764878
RUSC1-AS1	1.3896268	3.6735744	9.5166663	6.80E-19	1.18E-17	32.159267
STX10	1.3899123	8.0531293	13.645109	3.54E-33	9.84E-31	64.65594
DLL4	1.3911108	3.5147552	9.1339332	1.10E-17	1.55E-16	29.414948
SERPINI1	1.3916027	3.8231618	6.5501371	2.56E-10	1.24E-09	12.770911
SHC1	1.3917377	7.7918145	10.185667	4.57E-21	1.16E-19	37.09452
KMT5C	1.391904	4.6093181	11.067303	4.96E-24	2.28E-22	43.833862
BAX	1.392069	8.37837	11.1903	1.88E-24	9.18E-23	44.792854
PSRC1	1.3921858	4.7040396	8.4137008	1.75E-15	1.77E-14	24.425361
DHX34	1.3941897	3.5756661	12.109426	1.17E-27	1.03E-25	52.083881
LIG1	1.3947315	7.3374267	9.6492973	2.56E-19	4.78E-18	33.124184
WDR62	1.3953255	2.8965959	8.4312109	1.56E-15	1.58E-14	24.543779
RNF157	1.3955276	3.151996	6.4949913	3.54E-10	1.67E-09	12.457566
RHNO1	1.3963716	4.8598089	9.9484619	2.74E-20	6.04E-19	35.325587
NABP2	1.3968356	6.2397139	12.870171	2.28E-30	3.34E-28	58.260526
TTC13	1.3975596	4.994974	11.694981	3.35E-26	2.21E-24	48.770473
P3H4	1.3982715	5.295965	7.4801619	8.62E-13	5.87E-12	18.340567
MRPS21	1.3983536	9.7739483	13.059309	4.74E-31	8.38E-29	59.812784
TDRKH	1.3999884	4.346464	9.2608485	4.41E-18	6.62E-17	30.318199
SMARCA4	1.4004873	7.2400412	10.628006	1.53E-22	5.25E-21	40.444993
KLC2	1.4006711	5.0597067	9.2199882	5.92E-18	8.69E-17	30.026651
TAGLN2	1.400812	9.4899664	9.1360251	1.09E-17	1.52E-16	29.42978
COL1A1	1.4012163	8.2253884	3.7919508	0.0001814	0.0004144	-0.201178
CDH13	1.4025426	4.4410984	11.6026	7.04E-26	4.47E-24	48.03747
COG2	1.4027959	6.4127353	12.002488	2.80E-27	2.26E-25	51.225156
CSAG1	1.4038263	4.6757097	3.5303785	0.0004815	0.0010309	-1.114957
PCNX3	1.4045096	5.0376358	12.733319	7.06E-30	9.37E-28	57.141244
FBLIM1	1.4054108	5.1639901	6.3262846	9.33E-10	4.23E-09	11.511324
SBF1	1.405479	6.2482298	8.8880362	6.39E-17	8.01E-16	27.684798
PPM1F	1.4065813	4.8566989	12.92334	1.47E-30	2.28E-28	58.696278
WDR76	1.4070931	2.9582356	8.2806296	4.36E-15	4.17E-14	23.530352
CRNDE	1.4077363	4.8043063	7.648745	2.91E-13	2.13E-12	19.404768
RBM24	1.4083227	2.9622452	6.5913566	2.01E-10	9.82E-10	13.006411
ZC3H3	1.408561	4.9071203	10.802092	3.96E-23	1.53E-21	41.780954

CLK2	1.409233	6.0068797	10.751892	5.86E-23	2.21E-21	41.394747
PRCC	1.4098603	6.7136564	13.477512	1.45E-32	3.57E-30	63.265219
ATP1B3	1.4101363	8.1194263	6.8345025	4.75E-11	2.52E-10	14.41764
POLR2K	1.4102841	9.0948922	13.176657	1.78E-31	3.54E-29	60.778824
LSM4	1.410819	9.1971991	12.583527	2.43E-29	2.90E-27	55.920027
PKN3	1.4113923	4.4858932	9.1466708	1.01E-17	1.42E-16	29.505291
JAG2	1.4115675	4.5478631	8.701895	2.37E-16	2.71E-15	26.393146
ZNF581	1.4116658	5.8096145	10.984913	9.47E-24	4.16E-22	43.193898
BSG	1.4128884	9.5710114	10.984419	9.51E-24	4.17E-22	43.190063
LEF1	1.4158705	3.7812987	6.2636613	1.33E-09	5.92E-09	11.164886
UNC119B	1.416293	6.2904312	8.8204863	1.03E-16	1.25E-15	27.214231
MFGE8	1.417469	4.553102	7.8392245	8.39E-14	6.66E-13	20.626108
KRT23	1.4184719	6.220045	3.3554596	0.0008964	0.0018382	-1.69254
MARK4	1.4192311	6.2733783	11.664616	4.28E-26	2.77E-24	48.529303
FASN	1.420883	7.7833077	5.8946558	1.03E-08	4.08E-08	9.1776648
MYO15B	1.4210447	5.4574277	7.5842606	4.42E-13	3.14E-12	18.995821
PCBP4	1.4228631	7.7692142	10.631818	1.49E-22	5.10E-21	40.474143
LMNA	1.42297	10.337133	12.798078	4.13E-30	5.70E-28	57.670478
ADRA2C	1.4232812	5.2180037	5.1520806	4.73E-07	1.52E-06	5.472681
PFDN6	1.4236491	7.8680364	12.79792	4.14E-30	5.70E-28	57.669183
TFRC	1.4239464	7.5676446	7.7884563	1.17E-13	9.10E-13	20.298655
MMP14	1.4244945	6.4767719	6.3180731	9.78E-10	4.42E-09	11.465747
SERPINH1	1.425473	7.290358	7.8345263	8.65E-14	6.85E-13	20.595746
CCDC28B	1.4264837	4.6563341	7.8455812	8.04E-14	6.40E-13	20.667206
CTSA	1.4269635	10.062487	10.892548	1.95E-23	8.03E-22	42.478802
MNS1	1.4282058	4.6936317	5.4418818	1.11E-07	3.89E-07	6.8703925
IGSF3	1.4293262	3.4795407	6.5665124	2.33E-10	1.13E-09	12.864337
DNMT1	1.4303013	6.0946378	7.8878051	6.09E-14	4.94E-13	20.940747
PSMG3	1.4306747	7.5419425	11.903493	6.24E-27	4.65E-25	50.432517
IDUA	1.4310317	5.6529647	9.7903233	8.96E-20	1.81E-18	34.1577
KIF11	1.4310713	3.6759946	8.3148124	3.45E-15	3.35E-14	23.75942
PKN1	1.4409045	7.1190794	8.5114667	8.94E-16	9.40E-15	25.088447
PCNA	1.4444647	8.7969327	9.5782295	4.32E-19	7.65E-18	32.606287
IGF2BP1	1.4445492	2.8588478	5.6051547	4.79E-08	1.75E-07	7.6853297
ADCY6	1.4448567	6.0631198	10.335363	1.46E-21	4.09E-20	38.221054
RRS1	1.4451669	7.4923063	8.2625012	4.93E-15	4.69E-14	23.409106
RBM3	1.4462963	8.4584316	10.847	2.79E-23	1.12E-21	42.127102
TESC	1.4468298	7.8874082	3.7862335	0.0001854	0.0004229	-0.221785
PPOX	1.4482048	6.2374913	15.228073	5.22E-39	2.94E-36	77.943857
WFS1	1.4482999	6.9346345	11.124837	3.15E-24	1.49E-22	44.28192
PLA2G6	1.4487878	6.0953002	9.0796039	1.63E-17	2.23E-16	29.0304
MTMR11	1.4491934	5.0290008	6.7825285	6.48E-11	3.38E-10	14.112844
CABYR	1.4505637	3.8393652	5.0776726	6.79E-07	2.14E-06	5.1240658
C12orf75	1.4516844	5.201203	4.6648216	4.70E-06	1.32E-05	3.2679459
RPL22L1	1.4520651	9.1152381	6.8412968	4.56E-11	2.43E-10	14.457609
TAZ	1.4535078	6.6366189	14.698334	4.79E-37	2.33E-34	73.471636
AP5Z1	1.4537027	4.942875	13.237153	1.08E-31	2.26E-29	61.277694
DBN1	1.4538648	5.5975053	6.0772707	3.78E-09	1.59E-08	10.149417
SUCO	1.4540058	7.2829837	10.276341	2.29E-21	6.24E-20	37.775962
ZFP41	1.4543119	4.1034842	11.551567	1.06E-25	6.39E-24	47.633461
UBAP2L	1.4547396	6.0364242	10.360215	1.21E-21	3.43E-20	38.408814

PIGU	1.4548897	5.3151608	13.165031	1.97E-31	3.86E-29	60.683021
ARHGEF11	1.4553582	5.1963673	9.1391868	1.06E-17	1.50E-16	29.452201
MROH1	1.4571385	4.0508424	10.298424	1.93E-21	5.30E-20	37.942356
CNIH4	1.4574376	8.0554082	12.585193	2.39E-29	2.88E-27	55.933586
CIART	1.4604236	5.0965394	7.1333263	7.65E-12	4.58E-11	16.20235
LMNB2	1.460785	5.365176	8.6028951	4.74E-16	5.20E-15	25.712716
LAGE3	1.4613648	9.1429834	10.715638	7.77E-23	2.84E-21	41.116315
CENPW	1.4615867	5.1387144	9.3344243	2.58E-18	4.04E-17	30.844948
KIAA0100	1.4626194	8.0776765	10.314804	1.71E-21	4.75E-20	38.065883
TSEN54	1.4633768	8.0929311	11.796664	1.48E-26	1.03E-24	49.579691
CSPG4	1.4656915	3.8150484	10.061608	1.17E-20	2.79E-19	36.166847
DENND4B	1.4668733	7.0863884	10.766916	5.21E-23	1.98E-21	41.510253
USP21	1.4676436	5.0461694	11.284037	8.94E-25	4.66E-23	45.526542
NME1	1.4682644	9.9102169	10.896882	1.89E-23	7.80E-22	42.512297
CYP7A1	1.4711986	7.2430872	3.2418164	0.0013242	0.0026447	-2.053224
SNHG6	1.4714684	5.5834485	8.3385635	2.94E-15	2.87E-14	23.918925
CD24	1.4721422	8.0821911	3.4537004	0.0006343	0.0013304	-1.371479
CAD	1.4723913	6.5675124	9.8180654	7.29E-20	1.48E-18	34.361904
STIP1	1.4735938	7.4936043	11.936997	4.76E-27	3.67E-25	50.700531
ARHGEF39	1.4740117	3.3926359	10.639476	1.40E-22	4.83E-21	40.53272
CLN3	1.4740545	6.9053214	13.707514	2.10E-33	6.11E-31	65.174745
CEP55	1.4751569	4.0815621	7.7397244	1.61E-13	1.23E-12	19.98565
SPIRE2	1.4796195	5.1966695	10.766938	5.21E-23	1.98E-21	41.510423
HSP90AB1	1.4799923	10.062045	10.429421	7.09E-22	2.10E-20	38.932771
BLVRA	1.4818283	8.4913506	6.9982959	1.76E-11	9.96E-11	15.389143
SOWAHA	1.4822825	5.2195375	7.0792086	1.07E-11	6.28E-11	15.875111
SPAG5	1.4825148	7.1388139	7.6464196	2.96E-13	2.16E-12	19.38998
PITX1	1.4833183	3.3657585	4.9972998	1.00E-06	3.08E-06	4.7522841
COCH	1.4847276	3.3111722	5.7811849	1.89E-08	7.28E-08	8.5856149
ZNF517	1.4853356	3.0628092	11.667063	4.20E-26	2.72E-24	48.548735
TUBG1	1.4857496	6.722151	10.500323	4.11E-22	1.25E-20	39.471221
DNAJB11	1.4865009	9.6779491	15.300924	2.80E-39	1.68E-36	78.560369
OIP5	1.4878729	5.0657183	8.3233967	3.26E-15	3.17E-14	23.817037
FLAD1	1.4884319	6.975079	15.455011	7.48E-40	4.82E-37	79.865333
PRIM1	1.4906761	5.9491139	8.7929622	1.25E-16	1.50E-15	27.023087
MAFG	1.4907474	7.0046554	9.2492083	4.79E-18	7.14E-17	30.235071
RAD51AP1	1.4915589	4.0670181	7.9816441	3.27E-14	2.77E-13	21.552067
PYCR1	1.4920498	5.7614351	4.1465966	4.42E-05	0.0001096	1.1317008
SPON2	1.4923974	11.433191	7.6868942	2.27E-13	1.69E-12	19.647788
POLD1	1.4925753	6.73023	10.560384	2.59E-22	8.29E-21	39.928618
ARL16	1.4931786	7.655582	12.062611	1.72E-27	1.44E-25	51.707641
SLC52A2	1.4937923	7.0465698	9.4311873	1.27E-18	2.10E-17	31.541113
TRAF2	1.493903	7.0631211	11.939953	4.65E-27	3.61E-25	50.724184
TXNRD1	1.4950687	6.7145012	6.5424572	2.68E-10	1.29E-09	12.727155
DCAF4L2	1.4973897	3.1548607	4.0616306	6.26E-05	0.0001519	0.8026553
MAPK8IP2	1.498395	4.2043981	6.7939438	6.06E-11	3.17E-10	14.179643
CACYBP	1.5011328	7.0801609	12.052501	1.87E-27	1.56E-25	51.62645
XRCC3	1.5041107	4.8026914	10.935219	1.40E-23	5.98E-22	42.808846
CUL7	1.5048949	5.8580307	10.973693	1.03E-23	4.51E-22	43.106896
GLA	1.5049106	8.3797459	10.538903	3.05E-22	9.59E-21	39.764895
LAPTM4B	1.506193	8.2104169	5.7886921	1.82E-08	7.00E-08	8.6245026

PVT1	1.5076048	5.3028266	7.1587664	6.54E-12	3.95E-11	16.356787
HOXA13	1.5077923	2.742407	6.6380913	1.53E-10	7.59E-10	13.274745
TSPAN8	1.5081449	8.7967644	3.7227192	0.0002362	0.0005307	-0.448819
PPP1R35	1.5097098	8.9858871	13.603688	5.02E-33	1.33E-30	64.311875
ACLY	1.5138922	7.1319637	9.8657851	5.10E-20	1.07E-18	34.713837
PPP1R16A	1.51512	9.2755875	11.150465	2.57E-24	1.23E-22	44.481801
ILF2	1.5167617	9.8359861	12.801199	4.03E-30	5.63E-28	57.696007
C8orf33	1.5176781	6.3440839	11.741089	2.31E-26	1.55E-24	49.137101
MPZ	1.5194705	2.8212941	4.9158253	1.47E-06	4.42E-06	4.380514
GLMP	1.5207839	9.1791023	10.312843	1.73E-21	4.80E-20	38.051088
RFXANK	1.522565	8.0905209	13.359149	3.89E-32	8.69E-30	62.285407
TRIM16L	1.5238362	5.2898439	5.4893437	8.72E-08	3.08E-07	7.1052708
DSCC1	1.5238464	3.8933554	10.842699	2.89E-23	1.15E-21	42.093927
FAM50A	1.5244677	9.0741844	11.364321	4.72E-25	2.54E-23	46.15683
CENPL	1.5280164	2.6164065	10.419547	7.65E-22	2.26E-20	38.857918
PDLIM7	1.5287815	4.9317365	9.3256887	2.75E-18	4.26E-17	30.782289
C19orf48	1.5299395	7.7295805	9.7322477	1.38E-19	2.71E-18	33.731164
LAMA4	1.5305808	5.6306473	10.104767	8.43E-21	2.06E-19	36.488961
KIF18B	1.5306665	3.0997475	8.136095	1.16E-14	1.04E-13	22.568292
THEM5	1.5316023	7.562657	9.3267825	2.73E-18	4.24E-17	30.790133
RUSC1	1.5316148	6.0374969	10.573547	2.34E-22	7.55E-21	40.029021
FAM222A	1.5320237	5.5198462	8.4311173	1.56E-15	1.58E-14	24.543146
PAQR4	1.5320314	4.8380073	7.6429973	3.02E-13	2.20E-12	19.368224
GINS2	1.5361734	5.6569736	8.7314894	1.93E-16	2.23E-15	26.597439
SEMA3F	1.5362031	4.3888437	13.188891	1.61E-31	3.27E-29	60.879662
TRIM11	1.5390576	7.171698	12.509019	4.48E-29	5.18E-27	55.314156
GPSM1	1.5403536	4.9284932	6.9601865	2.22E-11	1.24E-10	15.161633
PLA2G4C	1.543716	5.9168841	8.4729686	1.17E-15	1.20E-14	24.826785
TTC39A	1.5451079	3.2942764	6.1653874	2.32E-09	1.00E-08	10.626542
MMP9	1.5464688	6.7687292	4.8666514	1.85E-06	5.50E-06	4.1586386
TSPEAR-AS2	1.5475389	4.2781704	5.8171571	1.56E-08	6.04E-08	8.7723187
MINCR	1.5478851	6.8446693	9.3591121	2.16E-18	3.41E-17	31.022199
CCDC34	1.5479069	4.1581935	9.416998	1.41E-18	2.31E-17	31.438787
PSMD4	1.5498885	11.021121	14.16017	4.61E-35	1.70E-32	68.952233
CHAF1A	1.5504031	6.1253187	10.620632	1.62E-22	5.50E-21	40.388608
TLCD1	1.5521265	6.1027766	10.191646	4.36E-21	1.11E-19	37.139373
FANCG	1.5535417	6.5288809	10.430452	7.03E-22	2.09E-20	38.940587
CHTF18	1.5546909	6.2535718	9.8289176	6.72E-20	1.38E-18	34.441864
PIGC	1.5574603	7.5056814	12.659088	1.30E-29	1.68E-27	56.535531
EXO1	1.5575176	3.7225035	9.3633143	2.09E-18	3.32E-17	31.052395
NCAPD2	1.5577858	6.1763129	8.8275946	9.80E-17	1.20E-15	27.263651
TTC9	1.5586027	3.7735005	6.1860826	2.06E-09	8.96E-09	10.739367
PHPT1	1.5602663	10.027674	11.561583	9.78E-26	5.96E-24	47.712703
NAT9	1.5609145	7.7914251	13.519509	1.02E-32	2.55E-30	63.613347
PKM	1.5622239	7.1915365	6.0201946	5.18E-09	2.14E-08	9.8432116
NSMCE2	1.5632181	6.9975344	12.954536	1.13E-30	1.82E-28	58.952169
DSN1	1.5642505	6.7373382	10.70677	8.32E-23	3.02E-21	41.048267
MAGEA6	1.5646385	4.1444029	3.8594084	0.0001398	0.0003235	0.0440925
HES6	1.5651883	5.0373838	10.002275	1.83E-20	4.17E-19	35.725119
PLP2	1.5652316	7.1029438	6.2598367	1.36E-09	6.05E-09	11.143813
MAGEC2	1.5667486	2.7007481	4.2152418	3.32E-05	8.38E-05	1.4019603

VPS45	1.5673129	6.4462885	14.508825	2.40E-36	1.11E-33	71.877073
C1orf198	1.570331	8.475531	8.9005656	5.84E-17	7.37E-16	27.772308
VPS72	1.5721798	7.9452297	13.229596	1.15E-31	2.38E-29	61.215348
ANKRD52	1.5745319	2.8832851	10.413992	7.98E-22	2.35E-20	38.815824
SF3B4	1.576401	5.4533033	12.099964	1.27E-27	1.10E-25	52.007797
ORC6	1.5787876	6.0586478	8.3977282	1.96E-15	1.96E-14	24.317471
ANXA2	1.5791869	9.5341315	8.4231616	1.64E-15	1.66E-14	24.489324
SNRPB	1.5802491	9.03164	12.290183	2.69E-28	2.62E-26	53.541013
SLC50A1	1.5804861	7.7582086	10.368502	1.13E-21	3.25E-20	38.471468
PLOD3	1.5810951	9.8407314	12.960361	1.08E-30	1.77E-28	58.999965
PSPH	1.5816206	7.1858852	8.7672418	1.50E-16	1.77E-15	26.844783
NES	1.582397	6.2129474	8.8044088	1.15E-16	1.39E-15	27.102537
S100A10	1.5909683	10.743999	8.5124731	8.88E-16	9.35E-15	25.095297
OLFML2A	1.59098	3.8319702	9.9485773	2.74E-20	6.04E-19	35.326443
TTK	1.5916736	2.9300956	8.6123459	4.44E-16	4.88E-15	25.777472
MANF	1.5959229	11.052991	11.722348	2.69E-26	1.78E-24	48.988022
NMB	1.596772	6.4766561	9.3308104	2.65E-18	4.14E-17	30.819022
SPATS2	1.5969362	4.5547174	11.365622	4.67E-25	2.53E-23	46.16706
CKS1B	1.5970537	8.6497785	12.288444	2.73E-28	2.65E-26	53.526964
CCNF	1.5971562	3.144742	9.827728	6.78E-20	1.39E-18	34.433097
FOXS1	1.5973551	5.3811045	8.331009	3.09E-15	3.02E-14	23.868161
UGT2B11	1.5993412	9.1705754	4.7293749	3.50E-06	1.00E-05	3.5493098
NXPH4	1.6015378	3.5085368	5.8261205	1.49E-08	5.77E-08	8.8189832
EZH2	1.6035166	5.9307933	10.581123	2.20E-22	7.16E-21	40.08683
COA6	1.605562	8.187168	13.620353	4.36E-33	1.18E-30	64.450273
LINC01089	1.6076554	6.9315268	11.412684	3.21E-25	1.78E-23	46.53735
RFX5	1.613301	6.5503496	10.516439	3.63E-22	1.12E-20	39.593832
SKA3	1.6138106	4.2730906	9.1552252	9.45E-18	1.35E-16	29.566003
GRAMD1A	1.6171493	5.3877324	8.0308713	2.35E-14	2.04E-13	21.874616
SNRPE	1.6176449	8.7768709	14.157078	4.73E-35	1.71E-32	68.926356
CENPH	1.6176662	4.7087504	10.125345	7.22E-21	1.78E-19	36.642771
KIF23	1.6178386	4.7015677	8.3684752	2.39E-15	2.37E-14	24.120201
PYGO2	1.6237135	7.9073455	12.555076	3.07E-29	3.57E-27	55.688549
TONSL	1.6237214	4.8469296	11.592032	7.66E-26	4.78E-24	47.95375
NELFE	1.6255682	8.134795	12.485296	5.44E-29	6.14E-27	55.121475
HMGB2	1.6291205	8.4133943	8.9493111	4.13E-17	5.34E-16	28.113432
RMI2	1.629463	5.7460459	7.4747205	8.93E-13	6.07E-12	18.306484
SAC3D1	1.6313988	7.323338	13.671886	2.83E-33	7.98E-31	64.878489
CDCA3	1.6332779	4.3831384	9.8119027	7.63E-20	1.55E-18	34.316516
ENAH	1.634244	5.3218871	8.8544708	8.10E-17	1.00E-15	27.450716
KRTCAP2	1.6382664	10.151996	15.455616	7.44E-40	4.82E-37	79.870461
PLPPR2	1.6403061	5.6734459	13.160895	2.04E-31	3.95E-29	60.648941
P4HA2	1.6429656	5.8330967	10.23562	3.12E-21	8.27E-20	37.469586
MIR4435-2H	1.6455693	6.4520309	10.746215	6.12E-23	2.27E-21	41.351124
FAM83H	1.647224	5.8284645	10.017716	1.63E-20	3.75E-19	35.839952
SCRIB	1.6486198	8.2133695	11.187553	1.92E-24	9.36E-23	44.77139
HKDC1	1.6486943	6.5406345	5.2305824	3.22E-07	1.06E-06	5.8450522
TRAIP	1.6494804	5.0114701	10.442827	6.40E-22	1.91E-20	39.034454
CKS2	1.6495781	8.2104705	8.8897796	6.31E-17	7.92E-16	27.69697
CCL25	1.6505279	3.9304251	4.4181963	1.40E-05	3.71E-05	2.2238718
DTYMK	1.6508706	8.0554316	12.882039	2.06E-30	3.05E-28	58.357747

TARBP1	1.6509203	6.047753	10.593352	2.00E-22	6.62E-21	40.180181
GABRE	1.651726	6.9165	7.2308793	4.17E-12	2.60E-11	16.796645
CGREF1	1.6543412	6.6441281	6.5618989	2.39E-10	1.16E-09	12.837998
PYGB	1.6587081	6.9339763	10.17502	4.95E-21	1.25E-19	37.014696
TRIP13	1.6612494	2.5709558	8.9579204	3.89E-17	5.04E-16	28.17379
BAIAP2L2	1.6653996	4.4570228	6.6450693	1.47E-10	7.29E-10	13.31493
SLC22A11	1.6657626	3.2878877	4.4488373	1.23E-05	3.27E-05	2.350903
MCM5	1.668328	7.8680106	9.7257105	1.45E-19	2.84E-18	33.683233
LRRC14	1.6694432	6.3094101	14.478844	3.09E-36	1.36E-33	71.625102
SSR2	1.6694467	11.304458	13.359452	3.88E-32	8.69E-30	62.287914
PEA15	1.6697005	8.2293119	12.224333	4.61E-28	4.28E-26	53.009373
TKT	1.671396	10.145939	7.9880578	3.13E-14	2.66E-13	21.594019
ZNF219	1.6726435	7.7404911	12.043234	2.01E-27	1.67E-25	51.55205
C1orf35	1.673877	8.3094792	14.128975	6.00E-35	2.12E-32	68.691149
LINC00665	1.6744447	4.9049357	5.5572009	6.15E-08	2.21E-07	7.4439561
MAPK12	1.6755289	4.5697351	6.9090464	3.03E-11	1.65E-10	14.85773
SNHG7	1.677927	5.9014835	9.9127741	3.59E-20	7.77E-19	35.061213
CCT3	1.6819988	11.131487	15.23194	5.05E-39	2.94E-36	77.976575
RAB11FIP4	1.6849624	5.6392374	8.9695649	3.58E-17	4.67E-16	28.255481
FABP4	1.6894075	3.450643	5.1472061	4.85E-07	1.56E-06	5.4497134
ADAM15	1.6906662	6.8557853	11.003078	8.21E-24	3.62E-22	43.33482
BUB1B	1.6927229	4.2460255	8.6192789	4.23E-16	4.67E-15	25.825004
MND1	1.6929673	4.470055	10.899022	1.86E-23	7.71E-22	42.528844
LAMC1	1.6944712	7.3608919	8.7145058	2.17E-16	2.50E-15	26.48015
MCM4	1.696037	5.8749284	8.780001	1.37E-16	1.63E-15	26.933197
FANCI	1.696051	4.4413194	9.4532366	1.08E-18	1.81E-17	31.700283
ECT2	1.6968441	3.9812558	8.4638365	1.24E-15	1.28E-14	24.764821
KIFC2	1.7006981	5.6718206	9.5289662	6.21E-19	1.08E-17	32.248458
ITGA6	1.7014582	7.2867486	9.6381092	2.78E-19	5.14E-18	33.042521
RGS5	1.7039952	6.2343184	7.5909697	4.23E-13	3.02E-12	19.038262
MCM7	1.704181	9.2009408	10.809196	3.75E-23	1.45E-21	41.835671
C1QL1	1.7042694	3.5643697	4.9956546	1.01E-06	3.10E-06	4.7447262
GINS1	1.7047136	2.6683986	8.6061991	4.63E-16	5.08E-15	25.73535
FAM111B	1.7071154	2.8426188	8.6288209	3.95E-16	4.39E-15	25.890459
CAPG	1.7148466	8.1145031	6.3168212	9.85E-10	4.45E-09	11.458803
SLC6A8	1.7172724	5.0467137	5.5764309	5.56E-08	2.01E-07	7.5405484
HSF4	1.7201886	5.6234946	10.224654	3.39E-21	8.82E-20	37.387174
MSTO1	1.724905	5.1430329	16.134837	2.19E-42	1.58E-39	85.635657
MCAM	1.7263257	6.4389804	9.3647613	2.07E-18	3.29E-17	31.062794
NR2C2AP	1.729321	7.4835495	13.991623	1.92E-34	6.28E-32	67.542872
UPK3A	1.7294512	3.9353736	4.2417991	2.97E-05	7.54E-05	1.507572
PABPC1L	1.7329528	5.8974769	8.8272461	9.82E-17	1.20E-15	27.261228
SCNM1	1.7375793	7.415629	14.478956	3.09E-36	1.36E-33	71.626041
ACTG2	1.7382152	5.643472	7.9779108	3.35E-14	2.83E-13	21.527658
LOXL2	1.7439527	6.0324439	10.405044	8.55E-22	2.50E-20	38.74803
SCAMP3	1.7463042	8.1603097	15.357816	1.72E-39	1.07E-36	79.042037
FLVCR1	1.7488303	5.1764579	11.526027	1.30E-25	7.77E-24	47.431519
LYZ	1.7555134	8.5667116	4.2210206	3.24E-05	8.19E-05	1.4248912
ZNF692	1.7559712	6.9646788	13.439512	1.99E-32	4.84E-30	62.950436
SNCG	1.7587503	4.8896111	6.1437363	2.61E-09	1.12E-08	10.508816
SPSB2	1.7688377	6.4365	13.185023	1.66E-31	3.34E-29	60.847779

NECAB3	1.7719988	7.0503739	12.962395	1.06E-30	1.77E-28	59.016657
KIAA1522	1.7721695	8.2202921	10.04289	1.35E-20	3.16E-19	36.027361
ATAD2	1.7722835	5.2995879	8.9702552	3.56E-17	4.65E-16	28.260325
PTP4A3	1.7810489	4.8731363	8.3609119	2.52E-15	2.49E-14	24.069266
DLGAP5	1.781142	4.1304152	9.3149513	2.97E-18	4.58E-17	30.705315
PODXL	1.7821852	7.6973326	9.2031395	6.69E-18	9.79E-17	29.906637
TBC1D16	1.7850772	5.6271535	12.135987	9.46E-28	8.37E-26	52.297559
TYMS	1.7867089	7.2842152	8.2162564	6.75E-15	6.25E-14	23.100561
CENPU	1.7867157	5.6135748	9.1957192	7.06E-18	1.03E-16	29.85382
IRAK1	1.78688	8.3550317	11.518639	1.38E-25	8.21E-24	47.373131
UBE2S	1.7886825	6.5121039	11.366893	4.63E-25	2.52E-23	46.177052
GBA	1.7890543	7.7045607	12.707423	8.74E-30	1.14E-27	56.929825
HDAC11	1.7903546	5.4210896	12.086561	1.41E-27	1.21E-25	51.90006
TREM2	1.7924814	5.2364157	7.5405874	5.85E-13	4.10E-12	18.720175
CEP131	1.7946871	6.1118565	13.331466	4.90E-32	1.07E-29	62.05655
MCM6	1.7952841	7.1944309	10.037821	1.40E-20	3.25E-19	35.989603
FAM189B	1.7975856	6.5773812	13.365919	3.68E-32	8.51E-30	62.341394
BOP1	1.7979473	8.0089511	10.712052	7.98E-23	2.91E-21	41.088797
MCM3	1.7998523	8.5743931	9.7697571	1.04E-19	2.09E-18	34.006505
CCNE1	1.8026153	5.7733039	6.8869368	3.46E-11	1.88E-10	14.726841
ASPM	1.8089609	4.7181599	9.4139817	1.44E-18	2.36E-17	31.417045
SMG5	1.8112725	8.4939692	12.948154	1.19E-30	1.90E-28	58.899807
DDX39A	1.8156645	8.7381991	12.440915	7.83E-29	8.41E-27	54.761311
ZNF775	1.8171857	6.967304	11.050739	5.65E-24	2.56E-22	43.705048
GPAA1	1.8207287	8.9928373	14.100151	7.66E-35	2.66E-32	68.45
SORT1	1.8249497	5.6619985	9.192283	7.24E-18	1.05E-16	29.82937
ITPKA	1.8272337	6.1347141	7.4584489	9.91E-13	6.69E-12	18.204665
KPNA2	1.8328982	8.6802172	10.585947	2.12E-22	6.97E-21	40.12365
TMEM106C	1.8329854	9.4488584	11.503541	1.56E-25	9.20E-24	47.253859
EFNA4	1.8337809	6.4046151	12.072514	1.59E-27	1.34E-25	51.787187
CYP17A1	1.8365027	5.8053494	3.9093993	0.0001149	0.0002695	0.2283738
KIFC1	1.8382031	4.0868026	9.5780935	4.33E-19	7.65E-18	32.605298
NUDT1	1.8399009	7.0063169	11.515998	1.41E-25	8.36E-24	47.35226
FABP5	1.8431222	6.4947791	8.9369744	4.51E-17	5.78E-16	28.026998
SNHG1	1.8453989	7.8110414	10.750893	5.90E-23	2.21E-21	41.387068
ETV4	1.8472979	4.3010209	5.3433749	1.83E-07	6.25E-07	6.3882298
UCK2	1.8505643	6.9374677	13.011269	7.06E-31	1.21E-28	59.41795
GLI4	1.8547258	5.2252789	13.372144	3.49E-32	8.18E-30	62.392884
GBP2	1.8618239	7.815642	9.8961811	4.06E-20	8.68E-19	34.938452
FEN1	1.8704635	6.4412865	11.781598	1.67E-26	1.14E-24	49.459633
ZIC2	1.8705616	2.1542846	7.7913868	1.15E-13	8.93E-13	20.317519
PGC	1.8720598	3.8235972	3.713292	0.0002447	0.0005485	-0.48222
SLC51B	1.8735359	5.7747074	6.403449	6.00E-10	2.77E-09	11.941795
PAFAH1B3	1.8741635	6.4499069	8.1642462	9.60E-15	8.71E-14	22.754841
ASF1B	1.8779564	5.0287052	9.0453275	2.08E-17	2.79E-16	28.788448
HMGA1	1.8844653	6.5744032	8.6197641	4.21E-16	4.66E-15	25.828331
BUB1	1.8900219	3.7016038	9.623134	3.10E-19	5.67E-18	32.933292
CLDN15	1.8934266	6.6547049	7.386819	1.56E-12	1.03E-11	17.758246
KAT2A	1.8968858	8.6815023	12.9168	1.55E-30	2.39E-28	58.642651
REG1A	1.9016115	3.4572953	3.5871984	0.0003913	0.0008498	-0.921535
PEG10	1.9031593	4.1861564	3.7152255	0.0002429	0.0005449	-0.475376

VWF	1.903682	5.9511003	8.7711726	1.46E-16	1.73E-15	26.872013
MKI67	1.9048906	3.3671198	9.2600909	4.43E-18	6.65E-17	30.312787
CDC25C	1.9110468	3.6509017	10.340812	1.40E-21	3.94E-20	38.262201
ALDH3A1	1.9167748	3.6613399	3.6708743	0.000287	0.000637	-0.63155
PKMYT1	1.9174029	4.9966097	9.968194	2.36E-20	5.30E-19	35.471964
CDC45	1.9218639	5.4978726	10.012901	1.69E-20	3.87E-19	35.804131
TPX2	1.9221449	5.7947426	8.1911026	8.00E-15	7.34E-14	22.933188
RFC4	1.9276547	7.1783661	11.598245	7.29E-26	4.58E-24	48.002964
NCAPG	1.9361326	5.1881855	9.9484713	2.74E-20	6.04E-19	35.325657
AKR1C3	1.9388142	11.717974	11.430841	2.78E-25	1.56E-23	46.68036
ANLN	1.9442425	3.5492541	9.1168384	1.25E-17	1.73E-16	29.293812
DTL	1.9449229	4.5818189	9.6071867	3.49E-19	6.32E-18	32.817069
AFP	1.9452326	4.8291503	3.4795951	0.0005782	0.0012207	-1.285429
PDGFA	1.950454	6.192248	8.2319468	6.07E-15	5.69E-14	23.205126
TP53I3	1.951671	7.5263461	10.369946	1.12E-21	3.22E-20	38.482391
CENPA	1.9529856	3.3267757	9.3745279	1.93E-18	3.08E-17	31.133008
PAGE4	1.9556538	3.4379343	3.8747793	0.0001316	0.0003057	0.1005264
COX4I2	1.9568895	4.0704337	13.6308	4.00E-33	1.09E-30	64.537057
RACGAP1	1.9632421	5.42108	10.849363	2.74E-23	1.10E-21	42.145335
COX7B2	1.9710753	3.0400958	4.7559775	3.10E-06	8.92E-06	3.6662261
NOTCH3	1.9746886	4.3532066	9.1883359	7.45E-18	1.08E-16	29.80129
TOMM40L	1.9809011	7.3765856	16.807191	6.78E-45	7.19E-42	91.354969
COL4A2	1.9925947	9.4682754	9.0482849	2.04E-17	2.73E-16	28.809304
CENPF	2.0109567	4.2730209	9.6551642	2.45E-19	4.60E-18	33.167027
CDC6	2.0176171	4.7150885	9.7676793	1.06E-19	2.11E-18	33.991239
DBNDD1	2.0216097	5.4070118	8.6301572	3.92E-16	4.35E-15	25.899629
MAGEA1	2.0233165	3.1825455	5.1629283	4.49E-07	1.45E-06	5.523858
CRIP3	2.0268571	5.47655	9.1576453	9.29E-18	1.33E-16	29.583184
SPARCL1	2.0305612	8.33367	6.3588572	7.75E-10	3.54E-09	11.692552
CPLX2	2.0354507	4.641607	4.4838983	1.05E-05	2.83E-05	2.4971988
COL4A1	2.036569	6.3602265	9.4694301	9.62E-19	1.62E-17	31.817306
RNASEH2A	2.0483352	7.4903975	12.70768	8.72E-30	1.14E-27	56.931922
SEZ6L2	2.0506519	5.5138671	5.1654538	4.43E-07	1.43E-06	5.5357854
HSPB1	2.0522903	12.108505	11.963997	3.82E-27	3.03E-25	50.916701
TACC3	2.053045	5.6914353	10.553062	2.74E-22	8.72E-21	39.872792
MELK	2.0538013	4.0769513	10.055252	1.23E-20	2.91E-19	36.119467
TPM2	2.0636092	7.7526614	10.445406	6.27E-22	1.88E-20	39.054018
HJURP	2.0666066	3.954033	10.053431	1.24E-20	2.95E-19	36.105894
GAS5	2.0675767	9.6196845	10.784076	4.56E-23	1.74E-21	41.642265
PDZK1IP1	2.0677027	7.3869109	3.8400026	0.0001507	0.0003475	-0.026866
RBP7	2.0750093	6.9962901	10.817473	3.51E-23	1.37E-21	41.899444
KIF4A	2.0769669	4.0990975	9.9920375	1.98E-20	4.50E-19	35.649029
SSX1	2.0821183	2.7337467	4.6138626	5.91E-06	1.64E-05	3.0481883
IQGAP3	2.0900837	5.4027508	10.364209	1.17E-21	3.33E-20	38.439004
HMMR	2.0993441	5.4742599	10.271026	2.38E-21	6.48E-20	37.735942
NUSAP1	2.1000481	5.1642687	9.8018387	8.22E-20	1.66E-18	34.242427
THBS4	2.1014545	4.6599929	7.9221866	4.85E-14	4.01E-13	21.164186
APLN	2.1017078	5.1047617	9.666252	2.25E-19	4.26E-18	33.248032
RECQL4	2.1035901	5.8873192	11.365432	4.68E-25	2.53E-23	46.165567
PBK	2.1049748	4.6781763	9.7480843	1.23E-19	2.42E-18	33.847349
MMP11	2.1079306	5.1245972	8.1970291	7.69E-15	7.07E-14	22.972594

KIF2C	2.1109299	5.2810893	9.8711825	4.90E-20	1.03E-18	34.753696
NEK2	2.1130666	3.3860539	10.057772	1.20E-20	2.86E-19	36.13825
FAM83D	2.1136423	5.1577655	10.196637	4.20E-21	1.08E-19	37.176813
NT5DC2	2.1188302	5.5767289	8.4261224	1.61E-15	1.63E-14	24.509351
MXD3	2.1207453	4.730049	13.885201	4.70E-34	1.51E-31	66.65466
CENPM	2.1337586	4.0967902	10.051294	1.26E-20	2.99E-19	36.089971
HRCT1	2.1432876	5.0330784	7.3481136	2.00E-12	1.30E-11	17.51825
G6PD	2.1622931	5.1876891	8.7031446	2.35E-16	2.69E-15	26.401764
CDCA8	2.1627776	5.7111779	10.23945	3.03E-21	8.06E-20	37.498376
CD34	2.1641471	5.532348	14.749553	3.10E-37	1.55E-34	73.903123
CDKN2C	2.1695186	7.3252316	11.217247	1.52E-24	7.54E-23	45.003525
GMNN	2.1796881	7.9121288	11.561671	9.77E-26	5.96E-24	47.713401
SULT1C2	2.180501	4.0304539	6.2669663	1.31E-09	5.82E-09	11.183104
CTHRC1	2.1942363	5.1078261	7.1929603	5.28E-12	3.24E-11	16.564971
KIF20A	2.2094518	4.3303754	10.118732	7.59E-21	1.87E-19	36.593329
CDCA5	2.219784	5.3311079	11.279956	9.23E-25	4.79E-23	45.494548
TROAP	2.221663	5.5570995	10.104033	8.48E-21	2.07E-19	36.483474
SQLE	2.2274123	7.9728865	9.2546658	4.61E-18	6.89E-17	30.274039
MCM2	2.2286642	6.7784061	9.8768381	4.69E-20	9.95E-19	34.795475
NUF2	2.2302214	4.5342946	10.199781	4.10E-21	1.06E-19	37.200408
ZWINT	2.2395486	6.1279737	10.538028	3.07E-22	9.64E-21	39.758225
NDC80	2.262136	4.3866709	11.183095	1.99E-24	9.64E-23	44.73656
CCNA2	2.2924921	4.168438	9.5981602	3.73E-19	6.68E-18	32.751329
DUSP9	2.2954273	5.0127642	7.2660639	3.35E-12	2.11E-11	17.01237
STMN1	2.3041863	6.8452462	11.591021	7.73E-26	4.81E-24	47.945749
CDKN2A	2.3086496	6.3772245	10.077175	1.04E-20	2.50E-19	36.282954
TK1	2.3280583	8.7361547	10.93221	1.43E-23	6.11E-22	42.785562
AURKB	2.3321705	4.0285478	10.528909	3.30E-22	1.03E-20	39.688772
CCL20	2.3399605	6.2814668	5.2167566	3.44E-07	1.13E-06	5.7791308
ROBO1	2.3436189	5.2037907	8.9561828	3.94E-17	5.10E-16	28.161605
CAP2	2.357957	6.3557691	10.648503	1.31E-22	4.52E-21	40.601794
CDT1	2.363113	5.5138525	10.735779	6.64E-23	2.45E-21	41.270951
SPC24	2.3748572	4.5082653	11.916038	5.64E-27	4.28E-25	50.532841
AURKA	2.3773563	5.909989	12.602233	2.08E-29	2.57E-27	56.072304
CDK1	2.3902052	5.2448277	10.804328	3.89E-23	1.51E-21	41.798173
FOXM1	2.4040654	4.9061321	10.140459	6.44E-21	1.60E-19	36.755844
PLK1	2.4563155	5.0290401	10.247875	2.84E-21	7.60E-20	37.561727
RRM2	2.4909467	4.9379689	11.365836	4.66E-25	2.53E-23	46.16874
REG3A	2.5032985	3.9343029	3.7390074	0.000222	0.0005006	-0.390929
PRC1	2.5057067	6.1941961	11.24293	1.24E-24	6.27E-23	45.204501
ACSL4	2.5205556	7.4970133	6.1487955	2.54E-09	1.09E-08	10.536297
SLC26A6	2.5282796	5.9220964	16.398937	2.27E-43	1.86E-40	87.881311
TGM3	2.56439	3.870227	7.3052212	2.62E-12	1.67E-11	17.253306
ST8SIA6-AS	2.5728875	4.3352924	6.4249501	5.30E-10	2.46E-09	12.062443
S100P	2.6241512	6.9517402	4.7305527	3.48E-06	9.96E-06	3.5544742
EEF1A2	2.6266334	5.2411831	4.770341	2.90E-06	8.39E-06	3.7295862
UBD	2.6307928	9.626763	8.7977376	1.21E-16	1.45E-15	27.056225
COL15A1	2.6323123	4.3504462	12.23957	4.07E-28	3.82E-26	53.13231
NDUFA4L2	2.6387098	4.9288603	13.358904	3.90E-32	8.69E-30	62.283386
THY1	2.6609423	8.2099627	13.847899	6.44E-34	2.00E-31	66.343657
ESM1	2.6835723	3.465503	10.36445	1.17E-21	3.33E-20	38.440833

CDKN3	2.6920551	5.6243115	12.573359	2.64E-29	3.11E-27	55.837277
EPS8L3	2.6921036	3.6929918	7.9135814	5.13E-14	4.23E-13	21.108204
CCNB2	2.7455329	5.2692948	11.555893	1.02E-25	6.22E-24	47.667686
SPP1	2.7465752	9.330539	4.7843079	2.72E-06	7.89E-06	3.7913534
CCNB1	2.7672645	6.2619981	12.496993	4.94E-29	5.64E-27	55.216471
E2F1	2.787895	5.292265	11.781327	1.67E-26	1.14E-24	49.457472
TOP2A	2.8160324	5.0298896	10.658452	1.21E-22	4.21E-21	40.677954
MYBL2	2.8309101	5.3904666	9.6045181	3.56E-19	6.42E-18	32.79763
UBE2T	2.9284109	6.6913501	14.597766	1.13E-36	5.35E-34	72.625048
BIRC5	2.9517795	5.0883125	11.883501	7.34E-27	5.36E-25	50.272711
NQO1	2.9565499	4.9964937	5.8508607	1.30E-08	5.09E-08	8.9480797
SFN	2.9604495	5.2800424	7.1135648	8.65E-12	5.14E-11	16.082653
PTTG1	2.9817642	7.3203345	12.493413	5.09E-29	5.78E-27	55.187394
MUC13	3.0054767	5.0401121	5.8996575	1.00E-08	3.98E-08	9.2039709
SPINK1	3.0109568	9.2422941	4.5189673	9.01E-06	2.45E-05	2.6445283
LCN2	3.0181964	7.5404118	5.5024547	8.15E-08	2.89E-07	7.1704464
CDC20	3.1358123	5.7511957	11.795691	1.49E-26	1.04E-24	49.571935
PLVAP	3.315957	7.1036295	21.325353	1.23E-61	2.77E-58	129.46304
AKR1B10	3.6590449	10.115392	6.0217045	5.14E-09	2.12E-08	9.8512831
MDK	3.7632853	9.053744	12.16454	7.50E-28	6.77E-26	52.527432
GPC3	5.0715746	8.9333733	9.8585566	5.38E-20	1.13E-18	34.660472
