# Supplementary material for: The Landscape of Using Glycosyltransferase Gene Signatures for Overall Survival Prediction in Hepatocellular Carcinoma
Source: J Oncol. 2022 Jun 21;2022:5989419. doi: 10.1155/2022/5989419 (PMC9239767; doi:10.1155/2022/5989419)
Supplement: Supplementary Materials — Figure S1: Venn diagram shows the intersections of co-differentially expressed glycosyltransferase (GT) genes between the 210 GTs obtained from the literature and those obtained from TCGA database. Figure S2: identification of a four-gene signature in the training set of TCGA database constructed using the LASSO method. Figure S3: survival analyses grouped by the optimal cutoff expression value of each gene in the training dataset of TCGA database. (A) ALG3. (B) B3GAT3. (C) GLA. (D) ST6GALNAC4. Figure S4: mRNA expression levels of the four genes in matched HCC and adjacent noncancerous samples in TCGA database were compared. (A) ALG3. (B) B3GAT3. (C) GLA. (D) ST6GALNAC4. Figure S5: the interactions among signature genes displayed in a PPI network. Figure S6: stratified survival analysis between high- and low-risk groups. (A)NAFLD. (B) HBV infection. (C) HCV infection. (D) Both HBV and HCV infections. (E) No treatment and therapy. (F) Pharmaceutical therapy. (G) Radiation therapy, NOS. Figure S7: the nomogram to predict prognostic probabilities in TCGA database. (A) The nomogram for predicting OS in the training set. The calibration plots for predicting (B) 3-year and (C) 5-year survival probabilities. Figure S8: survival analyses grouped by the optimal cutoff expression value of each signature gene in the validation dataset of TCGA database. (A) ALG3. (B) B3GAT3. (C) GLA. (D) ST6GALNAC4. Table S1: the clinical information of 371 primary HCC samples and 50 normal samples. Table S2: combining the clinical information and expression profiles of 210 human glycosyltransferase (GT) genes. Table S3: DEGs of 371 samples in TCGA database. Table S4: all significantly enriched GO terms. Table S5: all significantly enriched KEGG pathways. [file 5989419.f1.zip › 5989419.f1/Supplementary Table.4 risk H_L_AllenrichGO.sig.pdf]

ID	Descriptio	setSize	enrichment	NES	pvalue	p.adjust	qvalues
GO:0000226	microtubul	520	0.4850821	1.6357242	0.000999	0.0199726	0.0159612
GO:0000278	mitotic ce	942	0.4969871	1.6953177	0.000999	0.0199726	0.0159612
GO:0000902	cell morph	994	0.3592589	1.2271772	0.000999	0.0199726	0.0159612
GO:0001816	cytokine p	713	0.4105058	1.3934396	0.000999	0.0199726	0.0159612
GO:0001817	regulation	644	0.4171677	1.4134479	0.000999	0.0199726	0.0159612
GO:0001819	positive r	426	0.4190528	1.4070083	0.000999	0.0199726	0.0159612
GO:0002009	morphogene	520	0.4153179	1.4004754	0.000999	0.0199726	0.0159612
GO:0002263	cell activ	677	0.4479576	1.5195216	0.000999	0.0199726	0.0159612
GO:0002274	myeloid le	622	0.4665462	1.5801816	0.000999	0.0199726	0.0159612
GO:0002275	myeloid ce	522	0.4737107	1.597401	0.000999	0.0199726	0.0159612
GO:0002283	neutrophil	468	0.4721995	1.5868251	0.000999	0.0199726	0.0159612
GO:0002366	leukocyte	674	0.448617	1.5218075	0.000999	0.0199726	0.0159612
GO:0002443	leukocyte	738	0.4264809	1.4481181	0.000999	0.0199726	0.0159612
GO:0002446	neutrophil	479	0.4710272	1.5856378	0.000999	0.0199726	0.0159612
GO:0002520	immune sys	907	0.3939634	1.3431136	0.000999	0.0199726	0.0159612
GO:0002521	leukocyte	490	0.408596	1.3754477	0.000999	0.0199726	0.0159612
GO:0002684	positive r	1000	0.3736783	1.276477	0.000999	0.0199726	0.0159612
GO:0002757	immune res	496	0.4472992	1.50616	0.000999	0.0199726	0.0159612
GO:0002768	immune res	371	0.4711499	1.5753861	0.000999	0.0199726	0.0159612
GO:0006259	DNA metabo	912	0.4479104	1.5268805	0.000999	0.0199726	0.0159612
GO:0006281	DNA repair	490	0.4519938	1.5215369	0.000999	0.0199726	0.0159612
GO:0006325	chromatin	660	0.4046012	1.3724884	0.000999	0.0199726	0.0159612
GO:0006396	RNA proces	863	0.4402512	1.499119	0.000999	0.0199726	0.0159612
GO:0006412	translatio	576	0.4198636	1.4191349	0.000999	0.0199726	0.0159612
GO:0006887	exocytosis	865	0.3948595	1.3447369	0.000999	0.0199726	0.0159612
GO:0006935	chemotaxis	604	0.3902042	1.3214412	0.000999	0.0199726	0.0159612
GO:0006974	cellular r	782	0.4441513	1.5095736	0.000999	0.0199726	0.0159612
GO:0007017	microtubul	708	0.4626713	1.5710151	0.000999	0.0199726	0.0159612
GO:0007346	regulation	594	0.4823632	1.6329771	0.000999	0.0199726	0.0159612
GO:0008284	positive r	845	0.4166532	1.4177571	0.000999	0.0199726	0.0159612
GO:0008380	RNA splici	412	0.4458088	1.4950373	0.000999	0.0199726	0.0159612
GO:0010498	proteasoma	449	0.3957709	1.3296441	0.000999	0.0199726	0.0159612
GO:0010564	regulation	707	0.4834443	1.6414593	0.000999	0.0199726	0.0159612
GO:0010638	positive r	585	0.4303194	1.4553329	0.000999	0.0199726	0.0159612
GO:0010942	positive r	663	0.3911456	1.326772	0.000999	0.0199726	0.0159612
GO:0016032	viral proc	791	0.4407604	1.4978627	0.000999	0.0199726	0.0159612
GO:0016071	mRNA metab	794	0.4538033	1.5423541	0.000999	0.0199726	0.0159612
GO:0016569	covalent c	439	0.3976619	1.336455	0.000999	0.0199726	0.0159612
GO:0019221	cytokine-m	738	0.4348773	1.4766282	0.000999	0.0199726	0.0159612
GO:0022411	cellular c	524	0.4079211	1.3750366	0.000999	0.0199726	0.0159612
GO:0022613	ribonucleo	436	0.4740739	1.5935462	0.000999	0.0199726	0.0159612
GO:0030029	actin fila	714	0.4089635	1.3880958	0.000999	0.0199726	0.0159612
GO:0030031	cell proje	519	0.4017481	1.3546937	0.000999	0.0199726	0.0159612
GO:0030036	actin cyto	625	0.4214767	1.4277111	0.000999	0.0199726	0.0159612
GO:0030097	hemopoiesi	821	0.392668	1.3341164	0.000999	0.0199726	0.0159612
GO:0030099	myeloid ce	369	0.4189205	1.4000407	0.000999	0.0199726	0.0159612
GO:0030155	regulation	646	0.4120994	1.3963667	0.000999	0.0199726	0.0159612
GO:0030163	protein ca	868	0.3697814	1.2593224	0.000999	0.0199726	0.0159612
GO:0030334	regulation	793	0.3859257	1.3117128	0.000999	0.0199726	0.0159612
GO:0031175	neuron pro	928	0.3925822	1.3388482	0.000999	0.0199726	0.0159612

GO:0031344	regulatior	635	0.4201868	1.4236759	0.000999	0.0199726	0.0159612
GO:0032446	protein mc	829	0.3797054	1.290386	0.000999	0.0199726	0.0159612
GO:0032880	regulatior	955	0.3762672	1.2839003	0.000999	0.0199726	0.0159612
GO:0033365	protein lc	864	0.3797372	1.2932779	0.000999	0.0199726	0.0159612
GO:0034622	cellular p	981	0.4224763	1.4424385	0.000999	0.0199726	0.0159612
GO:0034655	nucleobase	609	0.3931907	1.332019	0.000999	0.0199726	0.0159612
GO:0034660	ncRNA meta	498	0.4470508	1.5058503	0.000999	0.0199726	0.0159612
GO:0036230	granulocyt	483	0.4786092	1.6108861	0.000999	0.0199726	0.0159612
GO:0040012	regulatior	918	0.3780954	1.2891784	0.000999	0.0199726	0.0159612
GO:0040017	positive r	520	0.3978778	1.3416664	0.000999	0.0199726	0.0159612
GO:0042110	T cell act	436	0.43632	1.466641	0.000999	0.0199726	0.0159612
GO:0042119	neutrophil	478	0.4786605	1.6111435	0.000999	0.0199726	0.0159612
GO:0042330	taxis	606	0.3889532	1.3178682	0.000999	0.0199726	0.0159612
GO:0043043	peptide bi	600	0.408323	1.3826946	0.000999	0.0199726	0.0159612
GO:0043065	positive r	611	0.4146293	1.4044273	0.000999	0.0199726	0.0159612
GO:0043066	negative r	817	0.3810219	1.2946977	0.000999	0.0199726	0.0159612
GO:0043068	positive r	616	0.4123916	1.3967846	0.000999	0.0199726	0.0159612
GO:0043069	negative r	833	0.3786006	1.2872918	0.000999	0.0199726	0.0159612
GO:0043161	proteasome	397	0.4049859	1.3574745	0.000999	0.0199726	0.0159612
GO:0044087	regulatior	884	0.3876868	1.3204767	0.000999	0.0199726	0.0159612
GO:0044089	positive r	505	0.4023153	1.3558829	0.000999	0.0199726	0.0159612
GO:0044403	symbiont p	837	0.4339019	1.4757125	0.000999	0.0199726	0.0159612
GO:0044419	interspeci	880	0.4295461	1.4632479	0.000999	0.0199726	0.0159612
GO:0044703	multi-orga	916	0.374586	1.2769734	0.000999	0.0199726	0.0159612
GO:0044770	cell cycle	565	0.5061221	1.7096357	0.000999	0.0199726	0.0159612
GO:0045055	regulated	764	0.4085089	1.3874157	0.000999	0.0199726	0.0159612
GO:0045087	innate imm	839	0.3688435	1.2545085	0.000999	0.0199726	0.0159612
GO:0045088	regulatior	423	0.4100719	1.3760692	0.000999	0.0199726	0.0159612
GO:0045597	positive r	910	0.373439	1.2728885	0.000999	0.0199726	0.0159612
GO:0045786	negative r	576	0.4622866	1.5625241	0.000999	0.0199726	0.0159612
GO:0045859	regulatior	735	0.392082	1.331305	0.000999	0.0199726	0.0159612
GO:0046649	lymphocyte	628	0.4302479	1.4576545	0.000999	0.0199726	0.0159612
GO:0048285	organelle	418	0.4910112	1.646548	0.000999	0.0199726	0.0159612
GO:0048534	hematopoie	863	0.391686	1.3337472	0.000999	0.0199726	0.0159612
GO:0048729	tissue mor	639	0.3922143	1.3288335	0.000999	0.0199726	0.0159612
GO:0050865	regulatior	510	0.4120022	1.3887356	0.000999	0.0199726	0.0159612
GO:0050900	leukocyte	412	0.4885808	1.6384749	0.000999	0.0199726	0.0159612
GO:0051129	negative r	664	0.4014995	1.3618941	0.000999	0.0199726	0.0159612
GO:0051270	regulatior	926	0.378176	1.2895104	0.000999	0.0199726	0.0159612
GO:0051272	positive r	505	0.4060149	1.3683513	0.000999	0.0199726	0.0159612
GO:0051338	regulatior	904	0.3870904	1.3190513	0.000999	0.0199726	0.0159612
GO:0051345	positive r	708	0.3840824	1.3041638	0.000999	0.0199726	0.0159612
GO:0051493	regulatior	502	0.4526382	1.5249331	0.000999	0.0199726	0.0159612
GO:0051640	organelle	682	0.4295112	1.4571759	0.000999	0.0199726	0.0159612
GO:0051656	establishm	492	0.4300883	1.4480477	0.000999	0.0199726	0.0159612
GO:0051960	regulatior	869	0.3783157	1.2883931	0.000999	0.0199726	0.0159612
GO:0051962	positive r	508	0.4029339	1.3580328	0.000999	0.0199726	0.0159612
GO:0060284	regulatior	883	0.3646121	1.2419388	0.000999	0.0199726	0.0159612
GO:0060341	regulatior	862	0.4038343	1.3750648	0.000999	0.0199726	0.0159612
GO:0060548	negative r	910	0.3848888	1.3119157	0.000999	0.0199726	0.0159612
GO:0061024	membrane c	756	0.4158083	1.4118423	0.000999	0.0199726	0.0159612

GO:0070647	protein mc	997	0.3768206	1.287348	0.000999	0.0199726	0.0159612
GO:0070925	organelle	778	0.40561	1.3781512	0.000999	0.0199726	0.0159612
GO:0072657	protein lc	584	0.4284209	1.4491192	0.000999	0.0199726	0.0159612
GO:0080135	regulatior	671	0.3913747	1.3279457	0.000999	0.0199726	0.0159612
GO:0097435	supramolec	630	0.4316813	1.4622586	0.000999	0.0199726	0.0159612
GO:0098609	cell-cell	786	0.3865449	1.3135888	0.000999	0.0199726	0.0159612
GO:0120031	plasma mem	508	0.3973005	1.3390462	0.000999	0.0199726	0.0159612
GO:0120035	regulatior	626	0.4167516	1.4118074	0.000999	0.0199726	0.0159612
GO:1901987	regulatior	418	0.5032005	1.6874235	0.000999	0.0199726	0.0159612
GO:1903047	mitotic ce	803	0.510581	1.7349888	0.000999	0.0199726	0.0159612
GO:1903706	regulatior	425	0.4053373	1.3608398	0.000999	0.0199726	0.0159612
GO:1903827	regulatior	500	0.4259996	1.4349391	0.000999	0.0199726	0.0159612
GO:2000145	regulatior	848	0.3873687	1.3182511	0.000999	0.0199726	0.0159612
GO:2000147	positive r	490	0.4058655	1.3662561	0.000999	0.0199726	0.0159612
GO:0000375	RNA splici	330	0.4539719	1.5133973	0.001	0.0199726	0.0159612
GO:0000377	RNA splici	327	0.4533001	1.510233	0.001	0.0199726	0.0159612
GO:0000398	mRNA splic	327	0.4533001	1.510233	0.001	0.0199726	0.0159612
GO:0001666	response t	332	0.4258332	1.4192451	0.001	0.0199726	0.0159612
GO:0002444	myeloid le	530	0.4674437	1.5757348	0.001	0.0199726	0.0159612
GO:0002694	regulatior	473	0.4369675	1.4685885	0.001	0.0199726	0.0159612
GO:0002764	immune res	528	0.4327264	1.4586207	0.001	0.0199726	0.0159612
GO:0006397	mRNA proce	476	0.4340746	1.4597689	0.001	0.0199726	0.0159612
GO:0006913	nucleocytc	331	0.4260477	1.4203715	0.001	0.0199726	0.0159612
GO:0007159	leukocyte	312	0.4695597	1.5637432	0.001	0.0199726	0.0159612
GO:0007264	small GTPa	540	0.3992196	1.3462663	0.001	0.0199726	0.0159612
GO:0010608	posttransc	552	0.4128014	1.3927057	0.001	0.0199726	0.0159612
GO:0010948	negative r	313	0.5055795	1.6836332	0.001	0.0199726	0.0159612
GO:0010975	regulatior	467	0.3914321	1.3148063	0.001	0.0199726	0.0159612
GO:0030198	extracellu	356	0.4328369	1.4454442	0.001	0.0199726	0.0159612
GO:0030335	positive r	472	0.4012219	1.3484089	0.001	0.0199726	0.0159612
GO:0032984	protein-cc	318	0.4185983	1.3942993	0.001	0.0199726	0.0159612
GO:0033044	regulatior	311	0.4757813	1.5838327	0.001	0.0199726	0.0159612
GO:0043254	regulatior	431	0.4116637	1.3823124	0.001	0.0199726	0.0159612
GO:0043299	leukocyte	513	0.4821781	1.6248382	0.001	0.0199726	0.0159612
GO:0043312	neutrophil	465	0.4790194	1.6091483	0.001	0.0199726	0.0159612
GO:0044772	mitotic ce	533	0.5078025	1.7122751	0.001	0.0199726	0.0159612
GO:0045787	positive r	357	0.4692329	1.5667349	0.001	0.0199726	0.0159612
GO:0051169	nuclear tr	334	0.4285299	1.4286781	0.001	0.0199726	0.0159612
GO:0051301	cell divis	558	0.5151651	1.7387381	0.001	0.0199726	0.0159612
GO:0071900	regulatior	475	0.4095166	1.3768037	0.001	0.0199726	0.0159612
GO:0097190	apoptotic	569	0.4267209	1.4412851	0.001	0.0199726	0.0159612
GO:1902903	regulatior	322	0.4241504	1.4125217	0.001	0.0199726	0.0159612
GO:1903311	regulatior	312	0.4246278	1.4141094	0.001	0.0199726	0.0159612
GO:1903829	positive r	314	0.4555183	1.517168	0.001	0.0199726	0.0159612
GO:1990778	protein lc	300	0.4220061	1.402719	0.001	0.0199726	0.0159612
GO:0000280	nuclear di	380	0.5077361	1.6975932	0.001001	0.0199726	0.0159612
GO:0002429	immune res	340	0.491825	1.6399764	0.001001	0.0199726	0.0159612
GO:0006401	RNA catabc	373	0.4840832	1.6183644	0.001001	0.0199726	0.0159612
GO:0006402	mRNA catab	342	0.4797497	1.5996733	0.001001	0.0199726	0.0159612
GO:0007015	actin fila	372	0.412806	1.379619	0.001001	0.0199726	0.0159612
GO:0010639	negative r	352	0.4385073	1.4633162	0.001001	0.0199726	0.0159612

GO:0022407regulation	374	0.4114194	1.375521	0.001001	0.0199726	0.0159612
GO:0031346positive r	358	0.4228624	1.4117852	0.001001	0.0199726	0.0159612
GO:0032535regulation	351	0.4403125	1.4694924	0.001001	0.0199726	0.0159612
GO:0034470ncRNA proc	346	0.4595641	1.5321058	0.001001	0.0199726	0.0159612
GO:0036293response t	341	0.4256929	1.4192079	0.001001	0.0199726	0.0159612
GO:0043900regulation	372	0.4259966	1.4237026	0.001001	0.0199726	0.0159612
GO:0050867positive r	302	0.4718203	1.5691126	0.001001	0.0199726	0.0159612
GO:0051052regulation	381	0.4395869	1.4696386	0.001001	0.0199726	0.0159612
GO:0051249regulation	394	0.4364173	1.4616095	0.001001	0.0199726	0.0159612
GO:0060249anatomical	394	0.4260894	1.4270202	0.001001	0.0199726	0.0159612
GO:0070482response t	364	0.4184834	1.3971553	0.001001	0.0199726	0.0159612
GO:0090150establishm	306	0.4768247	1.5874243	0.001001	0.0199726	0.0159612
GO:1901990regulation	393	0.5119623	1.7137488	0.001001	0.0199726	0.0159612
GO:2001233regulation	381	0.4411492	1.4748617	0.001001	0.0199726	0.0159612
GO:0002696positive r	291	0.4770354	1.5839855	0.001002	0.0199726	0.0159612
GO:0006260DNA replic	261	0.509603	1.686061	0.001002	0.0199726	0.0159612
GO:0007059chromosome	297	0.5358976	1.7780138	0.001002	0.0199726	0.0159612
GO:0045930negative r	297	0.5133994	1.7033687	0.001002	0.0199726	0.0159612
GO:0050863regulation	296	0.4467811	1.4827662	0.001002	0.0199726	0.0159612
GO:0051258protein po	262	0.4324541	1.4306105	0.001002	0.0199726	0.0159612
GO:0032943mononuclea	259	0.4687438	1.5499523	0.001003	0.0199726	0.0159612
GO:0042254ribosome b	276	0.4844862	1.6040262	0.001003	0.0199726	0.0159612
GO:0060326cell chemo	277	0.4308624	1.4272413	0.001003	0.0199726	0.0159612
GO:0070661leukocyte	280	0.4703862	1.5584341	0.001003	0.0199726	0.0159612
GO:1903037regulation	279	0.4533559	1.5021776	0.001003	0.0199726	0.0159612
GO:0034599cellular r	275	0.4299656	1.4226155	0.001004	0.0199726	0.0159612
GO:0006310DNA recomb	252	0.4975075	1.6435606	0.001005	0.0199726	0.0159612
GO:0007018microtubul	267	0.4301357	1.4210671	0.001005	0.0199726	0.0159612
GO:0015931nucleobase	231	0.4294203	1.4149364	0.001005	0.0199726	0.0159612
GO:0016072rRNA metab	233	0.4799686	1.5815269	0.001005	0.0199726	0.0159612
GO:0016458gene silen	233	0.4287165	1.4126481	0.001005	0.0199726	0.0159612
GO:0031503protein-co	270	0.4509537	1.4899719	0.001005	0.0199726	0.0159612
GO:0042113B cell act	232	0.4537552	1.4959773	0.001005	0.0199726	0.0159612
GO:0044839cell cycle	252	0.5407731	1.7864922	0.001005	0.0199726	0.0159612
GO:0051251positive r	252	0.4702522	1.5535201	0.001005	0.0199726	0.0159612
GO:0097193intrinsic	271	0.468251	1.547077	0.001005	0.0199726	0.0159612
GO:0006909phagocytos	250	0.4329329	1.429532	0.001006	0.0199726	0.0159612
GO:0007050cell cycle	228	0.4678385	1.540896	0.001006	0.0199726	0.0159612
GO:0019882antigen pr	218	0.5476268	1.8025386	0.001006	0.0199726	0.0159612
GO:0022618ribonucleo	228	0.4820867	1.5878245	0.001006	0.0199726	0.0159612
GO:0031334positive r	249	0.4426441	1.46104	0.001006	0.0199726	0.0159612
GO:0044843cell cycle	250	0.4923899	1.6258573	0.001006	0.0199726	0.0159612
GO:0046651lymphocyte	257	0.4673724	1.5439926	0.001006	0.0199726	0.0159612
GO:0048872homeostasi	235	0.4354593	1.4344808	0.001006	0.0199726	0.0159612
GO:0050851antigen re	230	0.4830028	1.5915415	0.001006	0.0199726	0.0159612
GO:0051321meiotic ce	228	0.4668076	1.5375005	0.001006	0.0199726	0.0159612
GO:0072331signal tra	256	0.4783348	1.5795691	0.001006	0.0199726	0.0159612
GO:0090068positive r	269	0.50443	1.6663975	0.001006	0.0199726	0.0159612
GO:0140014mitotic nu	254	0.5427286	1.7917296	0.001006	0.0199726	0.0159612
GO:2000027regulation	244	0.4250748	1.4020715	0.001006	0.0199726	0.0159612
GO:0000082G1/S trans	237	0.4933756	1.6261529	0.001007	0.0199726	0.0159612

GO:0000086	G2/M trans	237	0.5515277	1.8178205	0.001007	0.0199726	0.0159612
GO:0002573	myeloid le	195	0.450964	1.4761534	0.001007	0.0199726	0.0159612
GO:0008154	actin poly	194	0.4604253	1.5063615	0.001007	0.0199726	0.0159612
GO:0019080	viral gene	185	0.5270772	1.7160317	0.001007	0.0199726	0.0159612
GO:0022409	positive r	242	0.4357675	1.4377004	0.001007	0.0199726	0.0159612
GO:0050657	nucleic ac	185	0.4693836	1.5281959	0.001007	0.0199726	0.0159612
GO:0050658	RNA transp	185	0.4693836	1.5281959	0.001007	0.0199726	0.0159612
GO:0050670	regulation	198	0.4768961	1.5636924	0.001007	0.0199726	0.0159612
GO:0071103	DNA confor	238	0.5194549	1.7117055	0.001007	0.0199726	0.0159612
GO:0071826	ribonucleo	242	0.4785617	1.5788887	0.001007	0.0199726	0.0159612
GO:0098813	nuclear ch	242	0.5483386	1.8090992	0.001007	0.0199726	0.0159612
GO:0110053	regulation	240	0.4251894	1.4020453	0.001007	0.0199726	0.0159612
GO:1901988	negative r	226	0.5291435	1.7414711	0.001007	0.0199726	0.0159612
GO:2000116	regulation	223	0.4996182	1.6441804	0.001007	0.0199726	0.0159612
GO:2001020	regulation	194	0.4522648	1.4796631	0.001007	0.0199726	0.0159612
GO:0006403	RNA locali	222	0.4873613	1.6031316	0.0010081	0.0199726	0.0159612
GO:0007163	establishm	206	0.4577124	1.5043478	0.0010081	0.0199726	0.0159612
GO:0032944	regulation	199	0.4810876	1.5775643	0.0010081	0.0199726	0.0159612
GO:0050870	positive r	193	0.4788666	1.5652691	0.0010081	0.0199726	0.0159612
GO:1901991	negative r	214	0.5358362	1.7614011	0.0010081	0.0199726	0.0159612
GO:1903039	positive r	207	0.4874132	1.6020198	0.0010081	0.0199726	0.0159612
GO:0000075	cell cycle	203	0.5234447	1.7171849	0.0010091	0.0199726	0.0159612
GO:0000956	nuclear-tr	192	0.4846288	1.5823766	0.0010091	0.0199726	0.0159612
GO:0006364	rRNA proce	200	0.4865545	1.5954504	0.0010091	0.0199726	0.0159612
GO:0006457	protein fo	203	0.4905501	1.6092727	0.0010091	0.0199726	0.0159612
GO:0006612	protein ta	183	0.4799233	1.5598044	0.0010091	0.0199726	0.0159612
GO:0032271	regulation	204	0.4757317	1.561681	0.0010091	0.0199726	0.0159612
GO:0043281	regulation	203	0.5004047	1.6416012	0.0010091	0.0199726	0.0159612
GO:0043903	regulation	208	0.4452576	1.4632842	0.0010091	0.0199726	0.0159612
GO:0071456	cellular r	190	0.4477775	1.4606365	0.0010091	0.0199726	0.0159612
GO:0071824	protein-DN	201	0.4882923	1.6010089	0.0010091	0.0199726	0.0159612
GO:0097529	myeloid le	192	0.4854826	1.5851643	0.0010091	0.0199726	0.0159612
GO:1902749	regulation	200	0.5614131	1.8409178	0.0010091	0.0199726	0.0159612
GO:0000819	sister chr	177	0.5960778	1.9339141	0.0010101	0.0199726	0.0159612
GO:0006302	double-str	210	0.4756288	1.5627674	0.0010101	0.0199726	0.0159612
GO:0006611	protein ex	176	0.4612897	1.4958427	0.0010101	0.0199726	0.0159612
GO:0010389	regulation	187	0.5749022	1.8715702	0.0010101	0.0199726	0.0159612
GO:0010952	positive r	176	0.4621981	1.4987886	0.0010101	0.0199726	0.0159612
GO:0030595	leukocyte	205	0.4764336	1.5633269	0.0010101	0.0199726	0.0159612
GO:0032886	regulation	210	0.4809109	1.5801229	0.0010101	0.0199726	0.0159612
GO:0050852	T cell rec	187	0.4780895	1.5564004	0.0010101	0.0199726	0.0159612
GO:0051168	nuclear ex	191	0.4574818	1.4923574	0.0010101	0.0199726	0.0159612
GO:0051236	establishm	188	0.4690776	1.5276104	0.0010101	0.0199726	0.0159612
GO:0051495	positive r	213	0.4559616	1.4988073	0.0010101	0.0199726	0.0159612
GO:0061013	regulation	191	0.4483578	1.4625937	0.0010101	0.0199726	0.0159612
GO:1905330	regulation	176	0.4765243	1.5452448	0.0010101	0.0199726	0.0159612
GO:0006413	translatio	181	0.5087439	1.651707	0.0010111	0.0199726	0.0159612
GO:0007051	spindle or	160	0.5978072	1.9301382	0.0010111	0.0199726	0.0159612
GO:0031424	keratiniza	160	0.4694449	1.5156952	0.0010111	0.0199726	0.0159612
GO:0048002	antigen pr	181	0.5631701	1.828409	0.0010111	0.0199726	0.0159612
GO:0051054	positive r	209	0.4816575	1.5814322	0.0010111	0.0199726	0.0159612

GO:0051302	regulation	160	0.4742302	1.5311455	0.0010111	0.0199726	0.0159612
GO:0070507	regulation	181	0.5118452	1.6617757	0.0010111	0.0199726	0.0159612
GO:0070663	regulation	209	0.4740239	1.5563688	0.0010111	0.0199726	0.0159612
GO:0002478	antigen pr	168	0.5625774	1.8192582	0.0010121	0.0199726	0.0159612
GO:0008361	regulation	172	0.4452819	1.4427893	0.0010121	0.0199726	0.0159612
GO:0016331	morphogene	148	0.4635461	1.4899696	0.0010121	0.0199726	0.0159612
GO:0019884	antigen pr	175	0.5589302	1.8105896	0.0010121	0.0199726	0.0159612
GO:0022406	membrane c	174	0.4946856	1.6035588	0.0010121	0.0199726	0.0159612
GO:0030041	actin fila	168	0.4459586	1.4421372	0.0010121	0.0199726	0.0159612
GO:0030705	cytoskelet	174	0.4499268	1.4584699	0.0010121	0.0199726	0.0159612
GO:0030832	regulation	168	0.4770487	1.5426762	0.0010121	0.0199726	0.0159612
GO:0038093	Fc recepto	173	0.4787599	1.5518843	0.0010121	0.0199726	0.0159612
GO:0042098	T cell pro	174	0.4580249	1.4847206	0.0010121	0.0199726	0.0159612
GO:0043487	regulation	175	0.4615496	1.4951364	0.0010121	0.0199726	0.0159612
GO:0060759	regulation	166	0.450303	1.45467	0.0010121	0.0199726	0.0159612
GO:1901796	regulation	172	0.4891877	1.5850517	0.0010121	0.0199726	0.0159612
GO:2001252	positive r	163	0.4639489	1.4976836	0.0010121	0.0199726	0.0159612
GO:0002685	regulation	180	0.5085478	1.6488005	0.0010132	0.0199726	0.0159612
GO:0006986	response t	167	0.4604229	1.4876002	0.0010132	0.0199726	0.0159612
GO:0008064	regulation	167	0.4751156	1.5350715	0.0010132	0.0199726	0.0159612
GO:0019083	viral tran	171	0.5281828	1.7102568	0.0010132	0.0199726	0.0159612
GO:0030177	positive r	169	0.4463924	1.4434931	0.0010132	0.0199726	0.0159612
GO:0043902	positive r	180	0.4532859	1.4696317	0.0010132	0.0199726	0.0159612
GO:0065004	protein-DN	164	0.5165892	1.6671453	0.0010132	0.0199726	0.0159612
GO:0071897	DNA biosyn	180	0.4579852	1.4848678	0.0010132	0.0199726	0.0159612
GO:0140056	organelle	165	0.4916741	1.5868867	0.0010132	0.0199726	0.0159612
GO:1903046	meiotic ce	169	0.512651	1.6577527	0.0010132	0.0199726	0.0159612
GO:2001235	positive r	167	0.4970751	1.6060216	0.0010132	0.0199726	0.0159612
GO:0006338	chromatin	159	0.4788793	1.5438081	0.0010142	0.0199726	0.0159612
GO:0032200	telomere c	139	0.5390877	1.727597	0.0010142	0.0199726	0.0159612
GO:0038061	NIK/NF-kap	159	0.472297	1.5225882	0.0010142	0.0199726	0.0159612
GO:0043488	regulation	170	0.4584845	1.4836483	0.0010142	0.0199726	0.0159612
GO:1905475	regulation	179	0.4517577	1.4647563	0.0010142	0.0199726	0.0159612
GO:0000910	cytokinesi	150	0.4807043	1.5454025	0.0010152	0.0199726	0.0159612
GO:0006261	DNA-depend	145	0.5520268	1.7713412	0.0010152	0.0199726	0.0159612
GO:0007088	regulation	158	0.5095105	1.6423007	0.0010152	0.0199726	0.0159612
GO:0010950	positive r	158	0.4624417	1.4905842	0.0010152	0.0199726	0.0159612
GO:0033209	tumor necr	157	0.4996804	1.6106258	0.0010152	0.0199726	0.0159612
GO:0043123	positive r	178	0.4401318	1.4259411	0.0010152	0.0199726	0.0159612
GO:0045931	positive r	145	0.4939646	1.5850315	0.0010152	0.0199726	0.0159612
GO:0051028	mRNA trans	145	0.4853512	1.5573926	0.0010152	0.0199726	0.0159612
GO:0051783	regulation	178	0.498296	1.6143818	0.0010152	0.0199726	0.0159612
GO:2000045	regulation	144	0.4808511	1.5415321	0.0010152	0.0199726	0.0159612
GO:0000070	mitotic si	146	0.6237928	2.0014821	0.0010163	0.0199726	0.0159612
GO:0000723	telomere m	138	0.5379116	1.7228536	0.0010163	0.0199726	0.0159612
GO:0001738	morphogene	138	0.5167398	1.6550436	0.0010163	0.0199726	0.0159612
GO:0001959	regulation	156	0.4521255	1.4562076	0.0010163	0.0199726	0.0159612
GO:0030833	regulation	151	0.4667711	1.5003391	0.0010163	0.0199726	0.0159612
GO:0031570	DNA integr	146	0.5298302	1.6999967	0.0010163	0.0199726	0.0159612
GO:0035567	non-canoni	146	0.4806126	1.5420786	0.0010163	0.0199726	0.0159612
GO:0042129	regulation	146	0.4759037	1.5269696	0.0010163	0.0199726	0.0159612

GO:1902806	regulatior	156	0.4565327	1.4704024	0.0010163	0.0199726	0.0159612
GO:2001242	regulatior	151	0.50869	1.6350786	0.0010163	0.0199726	0.0159612
GO:0007093	mitotic ce	155	0.5528638	1.7785026	0.0010173	0.0199726	0.0159612
GO:0140013	meiotic nu	154	0.5211433	1.6752824	0.0010173	0.0199726	0.0159612
GO:0034605	cellular r	126	0.4987427	1.5894053	0.0010204	0.0199726	0.0159612
GO:0050671	positive r	126	0.5163201	1.6454214	0.0010204	0.0199726	0.0159612
GO:0070585	protein lc	135	0.4985446	1.5945822	0.0010204	0.0199726	0.0159612
GO:0000077	DNA damage	134	0.5054161	1.6142097	0.0010215	0.0199726	0.0159612
GO:0032946	positive r	127	0.5222286	1.6642098	0.0010215	0.0199726	0.0159612
GO:0042770	signal tra	125	0.5366712	1.7095225	0.0010215	0.0199726	0.0159612
GO:0050921	positive r	131	0.4687065	1.4952707	0.0010215	0.0199726	0.0159612
GO:0071166	ribonuclec	125	0.4951984	1.5774142	0.0010215	0.0199726	0.0159612
GO:0072175	epithelial	130	0.4738598	1.5101303	0.0010215	0.0199726	0.0159612
GO:0097530	granulocyt	134	0.4918849	1.5709934	0.0010215	0.0199726	0.0159612
GO:1902850	microtubul	127	0.6131994	1.9541107	0.0010215	0.0199726	0.0159612
GO:0001838	embryonic	122	0.488885	1.553722	0.0010225	0.0199726	0.0159612
GO:0006405	RNA export	132	0.491741	1.5684579	0.0010225	0.0199726	0.0159612
GO:0070665	positive r	132	0.5166476	1.6479	0.0010225	0.0199726	0.0159612
GO:0070972	protein lc	132	0.4879349	1.5563181	0.0010225	0.0199726	0.0159612
GO:0071426	ribonuclec	124	0.4936206	1.5721128	0.0010225	0.0199726	0.0159612
GO:0072655	establishm	132	0.503323	1.6053999	0.0010225	0.0199726	0.0159612
GO:2001251	negative r	122	0.5230096	1.6621731	0.0010225	0.0199726	0.0159612
GO:0001736	establishm	121	0.5240656	1.6636225	0.0010235	0.0199726	0.0159612
GO:0002687	positive r	121	0.5318455	1.6883197	0.0010235	0.0199726	0.0159612
GO:0007164	establishm	121	0.5240656	1.6636225	0.0010235	0.0199726	0.0159612
GO:0000725	recombinat	120	0.5314547	1.6857734	0.0010246	0.0199726	0.0159612
GO:0030010	establishm	133	0.4730204	1.5090321	0.0010246	0.0199726	0.0159612
GO:0006323	DNA packag	128	0.5586251	1.7779703	0.0010256	0.0199726	0.0159612
GO:0000724	double-str	119	0.5272993	1.6700926	0.0010267	0.0199726	0.0159612
GO:0043620	regulatior	118	0.4993221	1.5792045	0.0010277	0.0199726	0.0159612
GO:0000184	nuclear-tr	117	0.5592568	1.7664899	0.0010309	0.0199726	0.0159612
GO:0043618	regulatior	113	0.502799	1.5824223	0.0010309	0.0199726	0.0159612
GO:0071621	granulocyt	117	0.5039788	1.5918868	0.0010309	0.0199726	0.0159612
GO:0032392	DNA geomet	112	0.502599	1.5804422	0.001032	0.0199726	0.0159612
GO:0050864	regulatior	112	0.5063799	1.5923313	0.001032	0.0199726	0.0159612
GO:1990266	neutrophil	112	0.5092309	1.6012964	0.001032	0.0199726	0.0159612
GO:1904375	regulatior	111	0.4800812	1.507802	0.0010331	0.0199726	0.0159612
GO:0006406	mRNA expor	108	0.489609	1.53005	0.0010373	0.0199726	0.0159612
GO:0031109	microtubul	108	0.5382836	1.6821603	0.0010373	0.0199726	0.0159612
GO:0071427	mRNA-conta	108	0.489609	1.53005	0.0010373	0.0199726	0.0159612
GO:0072599	establishm	108	0.5482724	1.7133757	0.0010373	0.0199726	0.0159612
GO:0090175	regulatior	108	0.5258614	1.6433402	0.0010373	0.0199726	0.0159612
GO:0002220	innate imm	109	0.5057364	1.5818136	0.0010384	0.0199726	0.0159612
GO:0006333	chromatin	109	0.5373396	1.6806605	0.0010384	0.0199726	0.0159612
GO:0034728	nucleosome	101	0.5375791	1.673449	0.0010406	0.0199726	0.0159612
GO:1902807	negative r	101	0.5211072	1.6221729	0.0010406	0.0199726	0.0159612
GO:0006275	regulatior	102	0.511545	1.5925455	0.0010417	0.0199726	0.0159612
GO:0007052	mitotic sp	102	0.6231471	1.9399857	0.0010417	0.0199726	0.0159612
GO:0030330	DNA damage	102	0.5322215	1.6569155	0.0010417	0.0199726	0.0159612
GO:0002223	stimulator	106	0.5141454	1.6023312	0.0010428	0.0199726	0.0159612
GO:0045047	protein ta	104	0.5565305	1.7355084	0.0010428	0.0199726	0.0159612

GO:0060071	Wnt signal	106	0.5245027	1.6346096	0.0010428	0.0199726	0.0159612
GO:0071156	regulation	104	0.5174819	1.6137375	0.0010428	0.0199726	0.0159612
GO:0002504	antigen pr	98	0.6075532	1.8864375	0.0010449	0.0199726	0.0159612
GO:0007229	integrin-m	98	0.522312	1.6217655	0.0010449	0.0199726	0.0159612
GO:0030593	neutrophil	99	0.5292264	1.6428593	0.0010471	0.0199726	0.0159612
GO:0044774	mitotic DN	99	0.5283592	1.6401673	0.0010471	0.0199726	0.0159612
GO:0051225	spindle as	99	0.5901843	1.8320888	0.0010471	0.0199726	0.0159612
GO:0051983	regulation	99	0.615445	1.9105047	0.0010471	0.0199726	0.0159612
GO:2000134	negative r	99	0.5230358	1.623642	0.0010471	0.0199726	0.0159612
GO:0002495	antigen pr	97	0.6063155	1.8778792	0.0010482	0.0199726	0.0159612
GO:0006613	cotranslat	96	0.5646389	1.7459474	0.0010482	0.0199726	0.0159612
GO:0014020	primary ne	95	0.5001301	1.546091	0.0010482	0.0199726	0.0159612
GO:0019886	antigen pr	95	0.5978112	1.8480601	0.0010482	0.0199726	0.0159612
GO:0006626	protein ta	94	0.4909554	1.5142957	0.0010549	0.0199726	0.0159612
GO:1902750	negative r	94	0.5318096	1.6403058	0.0010549	0.0199726	0.0159612
GO:1904029	regulation	94	0.5067794	1.5631029	0.0010549	0.0199726	0.0159612
GO:0002181	cytoplasmi	93	0.4948521	1.5235877	0.0010582	0.0199726	0.0159612
GO:0002474	antigen pr	92	0.5112113	1.5728224	0.0010582	0.0199726	0.0159612
GO:0006614	SRP-depend	92	0.5673683	1.7455985	0.0010582	0.0199726	0.0159612
GO:0030838	positive r	92	0.4966427	1.5279998	0.0010582	0.0199726	0.0159612
GO:0097711	ciliary ba	93	0.5252598	1.6172093	0.0010582	0.0199726	0.0159612
GO:0000079	regulation	91	0.5098993	1.5657506	0.0010593	0.0199726	0.0159612
GO:0042102	positive r	91	0.5428771	1.6670158	0.0010593	0.0199726	0.0159612
GO:0048010	vascular e	88	0.494676	1.5138877	0.0010604	0.0199726	0.0159612
GO:1901184	regulation	88	0.4906267	1.5014953	0.0010604	0.0199726	0.0159612
GO:1901989	positive r	88	0.5387175	1.6486705	0.0010604	0.0199726	0.0159612
GO:0042100	B cell pro	90	0.5245556	1.6088034	0.0010627	0.0199726	0.0159612
GO:0044773	mitotic DN	90	0.5073675	1.5560877	0.0010627	0.0199726	0.0159612
GO:2000117	negative r	89	0.5559409	1.7012031	0.0010627	0.0199726	0.0159612
GO:2001243	negative r	90	0.5175729	1.5873874	0.0010627	0.0199726	0.0159612
GO:0001776	leukocyte	86	0.504555	1.5415134	0.0010661	0.0199726	0.0159612
GO:0006890	retrograde	82	0.5063874	1.5420168	0.0010661	0.0199726	0.0159612
GO:0048013	ephrin rec	86	0.5528241	1.6889848	0.0010661	0.0199726	0.0159612
GO:0051304	chromosome	86	0.5790038	1.7689689	0.0010661	0.0199726	0.0159612
GO:0010972	negative r	85	0.531718	1.6220119	0.0010684	0.0199726	0.0159612
GO:0022617	extracellu	78	0.5292919	1.6087294	0.0010695	0.0199726	0.0159612
GO:0031145	anaphase-p	78	0.5593742	1.7001613	0.0010695	0.0199726	0.0159612
GO:0033045	regulation	78	0.6126769	1.8621695	0.0010695	0.0199726	0.0159612
GO:0071158	positive r	78	0.5391988	1.6388402	0.0010695	0.0199726	0.0159612
GO:0043154	negative r	81	0.5577741	1.6952984	0.0010707	0.0199726	0.0159612
GO:0038094	Fc-gamma r	77	0.5506485	1.6691789	0.0010718	0.0199726	0.0159612
GO:0002431	Fc recepto	80	0.5466362	1.6626425	0.001073	0.0199726	0.0159612
GO:0045132	meiotic ch	80	0.5295432	1.6106526	0.001073	0.0199726	0.0159612
GO:1900034	regulation	76	0.5404634	1.6389244	0.001073	0.0199726	0.0159612
GO:0010833	telomere m	75	0.5358896	1.6190484	0.0010764	0.0199726	0.0159612
GO:0014015	positive r	68	0.5357549	1.6065716	0.0010764	0.0199726	0.0159612
GO:0031497	chromatin	84	0.5652075	1.7169886	0.0010764	0.0199726	0.0159612
GO:0032465	regulation	84	0.5300566	1.6102071	0.0010764	0.0199726	0.0159612
GO:0034502	protein lo	75	0.5571664	1.6833306	0.0010764	0.0199726	0.0159612
GO:0042590	antigen pr	75	0.5433599	1.641618	0.0010764	0.0199726	0.0159612
GO:0044786	cell cycle	68	0.5936119	1.7800676	0.0010764	0.0199726	0.0159612

GO:0050000chromosome	75	0.5344525	1.6147066	0.0010764	0.0199726	0.0159612
GO:0061640cytoskelet	84	0.5058944	1.5368072	0.0010764	0.0199726	0.0159612
GO:0072401signal tra	68	0.5655823	1.6960151	0.0010764	0.0199726	0.0159612
GO:0072422signal tra	68	0.5655823	1.6960151	0.0010764	0.0199726	0.0159612
GO:1901992positive r	75	0.5510407	1.6648235	0.0010764	0.0199726	0.0159612
GO:0002433immune res	74	0.5447207	1.6441152	0.0010799	0.0199726	0.0159612
GO:0006278RNA-depenc	67	0.5375569	1.6097019	0.0010799	0.0199726	0.0159612
GO:0032204regulation	74	0.5261134	1.5879534	0.0010799	0.0199726	0.0159612
GO:0038096Fc-gamma r	74	0.5447207	1.6441152	0.0010799	0.0199726	0.0159612
GO:0042273ribosomal	67	0.553798	1.6583356	0.0010799	0.0199726	0.0159612
GO:0045454cell redox	73	0.5260592	1.5866583	0.0010799	0.0199726	0.0159612
GO:0051303establishm	74	0.5392594	1.6276315	0.0010799	0.0199726	0.0159612
GO:0070227lymphocyte	69	0.5302138	1.5911374	0.0010799	0.0199726	0.0159612
GO:0072395signal tra	69	0.5631672	1.6900284	0.0010799	0.0199726	0.0159612
GO:2000573positive r	67	0.5273207	1.57905	0.0010799	0.0199726	0.0159612
GO:0000281mitotic cy	71	0.5480536	1.6483149	0.0010834	0.0199726	0.0159612
GO:0002479antigen pr	71	0.5444492	1.6374744	0.0010834	0.0199726	0.0159612
GO:0009988cell-cell	71	0.5242252	1.576649	0.0010834	0.0199726	0.0159612
GO:0043044ATP-depenc	70	0.5569953	1.6731243	0.0010846	0.0199726	0.0159612
GO:2001021negative r	70	0.5186539	1.5579528	0.0010846	0.0199726	0.0159612
GO:0006801superoxide	66	0.5456864	1.6256839	0.0010858	0.0199726	0.0159612
GO:0033047regulation	66	0.6116437	1.8221808	0.0010858	0.0199726	0.0159612
GO:0030888regulation	61	0.5400491	1.5941875	0.0010893	0.0199726	0.0159612
GO:0031571mitotic G1	61	0.5523824	1.6305946	0.0010893	0.0199726	0.0159612
GO:0032623interleuki	61	0.5436394	1.6047859	0.0010893	0.0199726	0.0159612
GO:0042255ribosome a	59	0.539613	1.5886378	0.0010893	0.0199726	0.0159612
GO:0044819mitotic G1	61	0.5523824	1.6305946	0.0010893	0.0199726	0.0159612
GO:0045576mast cell	59	0.5607951	1.6509987	0.0010893	0.0199726	0.0159612
GO:0051306mitotic si	59	0.6412719	1.887925	0.0010893	0.0199726	0.0159612
GO:1905818regulation	61	0.6169056	1.8210624	0.0010893	0.0199726	0.0159612
GO:0002260lymphocyte	62	0.5274215	1.5569489	0.0010917	0.0199726	0.0159612
GO:0006334nucleosome	65	0.6008183	1.7825571	0.0010917	0.0199726	0.0159612
GO:0007004telomere m	65	0.5414654	1.6064639	0.0010917	0.0199726	0.0159612
GO:0033260nuclear DN	58	0.6183009	1.8137059	0.0010917	0.0199726	0.0159612
GO:0044783G1 DNA dam	62	0.5462586	1.6125562	0.0010917	0.0199726	0.0159612
GO:0046605regulation	57	0.5511584	1.6095903	0.0010965	0.0199726	0.0159612
GO:0051310metaphase	57	0.5990846	1.7495525	0.0010965	0.0199726	0.0159612
GO:0051784negative r	57	0.6347978	1.8538488	0.0010965	0.0199726	0.0159612
GO:1904356regulation	57	0.5344265	1.5607266	0.0010965	0.0199726	0.0159612
GO:0010965regulation	56	0.6419723	1.8691973	0.0011001	0.0199726	0.0159612
GO:0044784metaphase/	56	0.6241369	1.8172667	0.0011001	0.0199726	0.0159612
GO:0072413signal tra	56	0.5778159	1.6823965	0.0011001	0.0199726	0.0159612
GO:1902402signal tra	56	0.5778159	1.6823965	0.0011001	0.0199726	0.0159612
GO:1902403signal tra	56	0.5778159	1.6823965	0.0011001	0.0199726	0.0159612
GO:0044380protein lc	55	0.5345851	1.5516416	0.0011074	0.0199726	0.0159612
GO:0072431signal tra	55	0.5825386	1.6908275	0.0011074	0.0199726	0.0159612
GO:1902400intracellu	55	0.5825386	1.6908275	0.0011074	0.0199726	0.0159612
GO:0006977DNA damage	54	0.574833	1.6637536	0.0011099	0.0199726	0.0159612
GO:0007091metaphase/	54	0.6315914	1.828031	0.0011099	0.0199726	0.0159612
GO:0006949syncytium	52	0.5600458	1.6080206	0.0011123	0.0199726	0.0159612
GO:0032613interleuki	52	0.5572121	1.5998844	0.0011123	0.0199726	0.0159612

GO:0090307	mitotic sp	53	0.6155453	1.7736469	0.0011123	0.0199726	0.0159612
GO:1902099	regulation	53	0.6316308	1.819996	0.0011123	0.0199726	0.0159612
GO:0000768	syncytium	51	0.5774897	1.6511826	0.0011136	0.0199726	0.0159612
GO:0030071	regulation	51	0.6398054	1.8293582	0.0011136	0.0199726	0.0159612
GO:0045839	negative r	51	0.6446184	1.84312	0.0011136	0.0199726	0.0159612
GO:0140253	cell-cell	51	0.5774897	1.6511826	0.0011136	0.0199726	0.0159612
GO:1903307	positive r	51	0.5818903	1.663765	0.0011136	0.0199726	0.0159612
GO:0002448	mast cell	48	0.5847848	1.6647556	0.0011148	0.0199726	0.0159612
GO:0002279	mast cell	47	0.574076	1.6299357	0.0011173	0.0199726	0.0159612
GO:0006636	unsaturate	49	0.5719338	1.6311078	0.0011173	0.0199726	0.0159612
GO:0030865	cortical c	47	0.6137934	1.7427026	0.0011173	0.0199726	0.0159612
GO:0036297	interstran	49	0.5807344	1.6562062	0.0011173	0.0199726	0.0159612
GO:0048146	positive r	49	0.5797392	1.6533681	0.0011173	0.0199726	0.0159612
GO:0051653	spindle lo	47	0.5990245	1.7007703	0.0011173	0.0199726	0.0159612
GO:0007566	embryo imp	43	0.6274275	1.7566954	0.0011312	0.0199726	0.0159612
GO:0030225	macrophage	43	0.5674922	1.5888862	0.0011312	0.0199726	0.0159612
GO:0033046	negative r	43	0.6817233	1.9087147	0.0011312	0.0199726	0.0159612
GO:0043303	mast cell	46	0.576478	1.6274595	0.0011312	0.0199726	0.0159612
GO:0051293	establishm	43	0.5999783	1.6798419	0.0011312	0.0199726	0.0159612
GO:0071675	regulation	43	0.5875169	1.6449522	0.0011312	0.0199726	0.0159612
GO:0007080	mitotic me	44	0.6146782	1.7242654	0.0011338	0.0199726	0.0159612
GO:0051985	negative r	44	0.6816726	1.9121947	0.0011338	0.0199726	0.0159612
GO:0043300	regulation	45	0.6168681	1.7331213	0.0011377	0.0199726	0.0159612
GO:0030866	cortical a	42	0.6392418	1.7800762	0.0011416	0.0199726	0.0159612
GO:0031055	chromatin	30	0.6795626	1.8179887	0.0011416	0.0199726	0.0159612
GO:0043304	regulation	30	0.6223368	1.6648963	0.0011416	0.0199726	0.0159612
GO:0043486	histone ex	42	0.627434	1.7471956	0.0011416	0.0199726	0.0159612
GO:0045066	regulatory	30	0.6424361	1.7186666	0.0011416	0.0199726	0.0159612
GO:1902751	positive r	30	0.6514433	1.7427628	0.0011416	0.0199726	0.0159612
GO:0008608	attachment	31	0.686896	1.8486465	0.0011429	0.0199726	0.0159612
GO:0033003	regulation	41	0.5959928	1.6579074	0.0011429	0.0199726	0.0159612
GO:0033048	negative r	41	0.680479	1.8929274	0.0011429	0.0199726	0.0159612
GO:0034508	centromere	38	0.660609	1.8259085	0.0011429	0.0199726	0.0159612
GO:0045730	respirator	31	0.7030213	1.8920446	0.0011429	0.0199726	0.0159612
GO:2000816	negative r	38	0.6828699	1.887437	0.0011429	0.0199726	0.0159612
GO:0034080	CENP-A con	28	0.6850429	1.8084915	0.0011481	0.0199726	0.0159612
GO:0045589	regulation	28	0.6527158	1.723149	0.0011481	0.0199726	0.0159612
GO:0050690	regulation	28	0.6335611	1.672581	0.0011481	0.0199726	0.0159612
GO:0061641	CENP-A con	28	0.6850429	1.8084915	0.0011481	0.0199726	0.0159612
GO:1905819	negative r	39	0.6828158	1.8906253	0.0011494	0.0199726	0.0159612
GO:0006336	DNA replic	35	0.6555835	1.786447	0.0011507	0.0199726	0.0159612
GO:0042554	superoxide	35	0.6330509	1.7250463	0.0011507	0.0199726	0.0159612
GO:0006270	DNA replic	36	0.6398931	1.7492607	0.0011521	0.0199726	0.0159612
GO:0034724	DNA replic	36	0.6422692	1.7557563	0.0011521	0.0199726	0.0159612
GO:0040001	establishm	33	0.6304137	1.7079157	0.0011521	0.0199726	0.0159612
GO:0045841	negative r	36	0.6738804	1.842171	0.0011521	0.0199726	0.0159612
GO:0007339	binding of	37	0.5811429	1.5928843	0.0011534	0.0199726	0.0159612
GO:0032467	positive r	37	0.5906916	1.619057	0.0011534	0.0199726	0.0159612
GO:1902100	negative r	37	0.6738162	1.8468975	0.0011534	0.0199726	0.0159612
GO:1904358	positive r	37	0.5878898	1.6113774	0.0011534	0.0199726	0.0159612
GO:0007094	mitotic sp	34	0.7004935	1.9015949	0.0011547	0.0199726	0.0159612

GO:0031577	spindle ch	34	0.7004935	1.9015949	0.0011547	0.0199726	0.0159612
GO:0032212	positive r	34	0.607018	1.6478415	0.0011547	0.0199726	0.0159612
GO:0071173	spindle as	34	0.7004935	1.9015949	0.0011547	0.0199726	0.0159612
GO:0071174	mitotic sp	34	0.7004935	1.9015949	0.0011547	0.0199726	0.0159612
GO:0007143	female mei	27	0.6501417	1.7027194	0.0011574	0.0199726	0.0159612
GO:0010971	positive r	27	0.6862868	1.7973835	0.0011574	0.0199726	0.0159612
GO:0001783	B cell apo	24	0.6637282	1.6920109	0.0011751	0.0201602	0.0161112
GO:0022616	DNA strand	26	0.6726491	1.7408723	0.0011751	0.0201602	0.0161112
GO:0048240	sperm capa	25	0.681615	1.7533856	0.0011751	0.0201602	0.0161112
GO:0043302	positive r	23	0.6749081	1.7043268	0.0011806	0.0201775	0.016125
GO:0080154	regulation	23	0.710758	1.7948577	0.0011806	0.0201775	0.016125
GO:0006271	DNA strand	20	0.7037914	1.7225184	0.0012121	0.0206659	0.0165153
GO:0006268	DNA unwind	13	0.7821697	1.7494763	0.0012255	0.0206659	0.0165153
GO:0007100	mitotic ce	14	0.7226124	1.6442073	0.0012255	0.0206659	0.0165153
GO:0048681	negative r	13	0.7423911	1.6605037	0.0012255	0.0206659	0.0165153
GO:0071801	regulation	14	0.7480644	1.7021199	0.0012255	0.0206659	0.0165153
GO:1902969	mitotic DN	14	0.7468689	1.6993998	0.0012255	0.0206659	0.0165153
GO:1904996	positive r	14	0.7526492	1.712552	0.0012255	0.0206659	0.0165153
GO:1904874	positive r	15	0.7615943	1.7500545	0.00123	0.0207029	0.0165448
GO:0000076	DNA replic	16	0.7290102	1.693201	0.0012376	0.0207781	0.016605
GO:1900424	regulation	17	0.7161513	1.6810438	0.0012392	0.0207781	0.016605
GO:0051231	spindle el	9	0.8790786	1.8012716	0.00125	0.0208684	0.0166771
GO:1900426	positive r	9	0.8882071	1.8199764	0.00125	0.0208684	0.0166771
GO:0000727	double-str	11	0.8928008	1.9165653	0.0012547	0.0208684	0.0166771
GO:0090594	inflammato	11	0.8307105	1.7832768	0.0012547	0.0208684	0.0166771
GO:0000022	mitotic sp	8	0.9210718	1.8378553	0.0012563	0.0208684	0.0166771
GO:0006267	pre-replic	7	0.912663	1.7618138	0.001287	0.0212594	0.0169896
GO:0036388	pre-replic	7	0.912663	1.7618138	0.001287	0.0212594	0.0169896
GO:1902299	pre-replic	7	0.912663	1.7618138	0.001287	0.0212594	0.0169896
GO:0051256	mitotic sp	6	0.9331228	1.7389674	0.001328	0.0218963	0.0174986
GO:0007265	Ras protei	423	0.3923055	1.3164508	0.001998	0.0320275	0.025595
GO:0016570	histone mo	425	0.3945123	1.3244969	0.001998	0.0320275	0.025595
GO:0043087	regulation	439	0.3909462	1.313885	0.001998	0.0320275	0.025595
GO:0043549	regulation	808	0.3732496	1.268539	0.001998	0.0320275	0.025595
GO:0050767	regulation	769	0.3691493	1.2539741	0.001998	0.0320275	0.025595
GO:0050769	positive r	445	0.3922241	1.3180151	0.001998	0.0320275	0.025595
GO:0050776	regulation	938	0.3602269	1.2290538	0.001998	0.0320275	0.025595
GO:0051347	positive r	614	0.3829126	1.297137	0.001998	0.0320275	0.025595
GO:0009101	glycoprote	323	0.4039578	1.3453741	0.002	0.0320275	0.025595
GO:0018209	peptidyl-s	308	0.4143544	1.3796511	0.002	0.0320275	0.025595
GO:0045089	positive r	355	0.4063041	1.3569159	0.002	0.0320275	0.025595
GO:0051098	regulation	354	0.4073175	1.360194	0.002	0.0320275	0.025595
GO:0043588	skin devel	345	0.4126722	1.3758156	0.002002	0.0320275	0.025595
GO:0002218	activation	297	0.4210784	1.3970638	0.002004	0.0320275	0.025595
GO:0009615	response t	296	0.4066659	1.3496329	0.002004	0.0320275	0.025595
GO:0072659	protein lo	250	0.4195524	1.3853501	0.0020121	0.0320275	0.025595
GO:0050792	regulation	194	0.4477021	1.4647351	0.0020141	0.0320275	0.025595
GO:0032388	positive r	207	0.4320113	1.419926	0.0020161	0.0320275	0.025595
GO:0036294	cellular r	199	0.4466344	1.4645867	0.0020161	0.0320275	0.025595
GO:0071453	cellular r	216	0.4306853	1.4162437	0.0020161	0.0320275	0.025595
GO:0050920	regulation	201	0.4427464	1.4516733	0.0020182	0.0320275	0.025595

GO:0060491	regulatior	164	0.4383994	1.41481	0.0020263	0.0321	0.0256529
GO:0051101	regulatior	120	0.4756307	1.5086999	0.0020492	0.032404	0.0258959
GO:0001841	neural tub	103	0.492944	1.535628	0.0020855	0.0327559	0.0261771
GO:0032508	DNA duplex	103	0.5085143	1.5841332	0.0020855	0.0327559	0.0261771
GO:0061982	meiosis I	106	0.4772122	1.487229	0.0020855	0.0327559	0.0261771
GO:0002688	regulatior	105	0.4711962	1.4683514	0.0020899	0.0327559	0.0261771
GO:0051261	protein de	105	0.4692561	1.4623056	0.0020899	0.0327559	0.0261771
GO:0042058	regulatior	82	0.499251	1.5202855	0.0021322	0.0333606	0.0266604
GO:0002690	positive r	83	0.5001495	1.5214888	0.002139	0.0333859	0.0266806
GO:0006919	activatior	81	0.4978164	1.513063	0.0021413	0.0333859	0.0266806
GO:0061418	regulatior	73	0.5152172	1.5539575	0.0021598	0.0336154	0.026864
GO:0035036	sperm-egg	52	0.5475533	1.5721517	0.0022247	0.0344181	0.0275054
GO:0043392	negative r	53	0.5372948	1.5481741	0.0022247	0.0344181	0.0275054
GO:0032210	regulatior	51	0.5419376	1.5495305	0.0022272	0.0344181	0.0275054
GO:0090329	regulatior	51	0.5312942	1.5190985	0.0022272	0.0344181	0.0275054
GO:0051438	regulatior	50	0.5422779	1.5476674	0.0022321	0.0344181	0.0275054
GO:0032653	regulatior	49	0.5353912	1.5268913	0.0022346	0.0344181	0.0275054
GO:0008347	glial cell	46	0.5528238	1.5606812	0.0022624	0.034726	0.0277515
GO:0090382	phagosome	43	0.5579312	1.562117	0.0022624	0.034726	0.0277515
GO:0000387	spliceosom	38	0.5719064	1.5807364	0.0022857	0.0348649	0.0278625
GO:0015804	neutral am	38	0.5701456	1.5758697	0.0022857	0.0348649	0.0278625
GO:0033006	regulatior	31	0.6186457	1.6649639	0.0022857	0.0348649	0.0278625
GO:0000132	establishm	29	0.6104498	1.6208779	0.0022989	0.0348649	0.0278625
GO:0033574	response t	39	0.5660261	1.5672503	0.0022989	0.0348649	0.0278625
GO:0045740	positive r	35	0.5801612	1.5809233	0.0023015	0.0348649	0.0278625
GO:0051294	establishm	35	0.5997643	1.6343414	0.0023015	0.0348649	0.0278625
GO:0016572	histone ph	36	0.5714884	1.5622643	0.0023041	0.0348649	0.0278625
GO:2000249	regulatior	37	0.5667036	1.5533069	0.0023068	0.0348649	0.0278625
GO:1990776	response t	26	0.6352015	1.6439549	0.0023502	0.0354601	0.0283382
GO:0071459	protein lc	20	0.6821142	1.6694639	0.0024242	0.0365157	0.0291817
GO:0032930	positive r	19	0.7043096	1.695735	0.002451	0.0366082	0.0292557
GO:0051383	kinetochor	19	0.7126132	1.7157273	0.002451	0.0366082	0.0292557
GO:0071352	cellular r	13	0.7234254	1.6180832	0.002451	0.0366082	0.0292557
GO:1904814	regulatior	13	0.7309359	1.6348818	0.002451	0.0366082	0.0292557
GO:1905523	positive r	19	0.7046176	1.6964765	0.002451	0.0366082	0.0292557
GO:0002467	germinal c	15	0.7214913	1.6579025	0.00246	0.0366816	0.0293144
GO:0070200	establishm	16	0.7213091	1.6753144	0.0024752	0.0368307	0.0294335
GO:0072697	protein lc	12	0.8056555	1.7684195	0.0024783	0.0368307	0.0294335
GO:1990173	protein lc	11	0.7945367	1.705623	0.0025094	0.0370916	0.029642
GO:1900003	regulatior	8	0.8220308	1.6402344	0.0025126	0.0370916	0.029642
GO:1900122	positive r	8	0.8564171	1.7088469	0.0025126	0.0370916	0.029642
GO:1902571	regulatior	8	0.8220308	1.6402344	0.0025126	0.0370916	0.029642
GO:0002246	wound heal	7	0.8340042	1.6099701	0.002574	0.0378102	0.0302163
GO:0044771	meiotic ce	7	0.8812166	1.7011094	0.002574	0.0378102	0.0302163
GO:1901993	regulatior	7	0.8812166	1.7011094	0.002574	0.0378102	0.0302163
GO:0000912	assembly c	6	0.8581879	1.5993188	0.002656	0.0386955	0.0309238
GO:0000915	actomyosir	6	0.8581879	1.5993188	0.002656	0.0386955	0.0309238
GO:0002775	antimicrob	6	0.8844694	1.6482971	0.002656	0.0386955	0.0309238
GO:0002778	antibacter	6	0.8844694	1.6482971	0.002656	0.0386955	0.0309238
GO:2001188	regulatior	6	0.8733954	1.6276595	0.002656	0.0386955	0.0309238
GO:0007343	egg activa	5	0.8886399	1.6037883	0.0027064	0.0392356	0.0313554

GO:0034238	macrophage	5	0.9307701	1.6798236	0.0027064	0.0392356	0.0313554
GO:2000359	regulation	5	0.9222403	1.6644292	0.0027064	0.0392356	0.0313554
GO:0016055	Wnt signal	492	0.3872642	1.3038651	0.002997	0.0429923	0.0343576
GO:0043632	modificati	587	0.380908	1.2885829	0.002997	0.0429923	0.0343576
GO:0044257	cellular p	716	0.3812777	1.2942405	0.002997	0.0429923	0.0343576
GO:0007005	mitochondr	475	0.3846959	1.2933558	0.003	0.0429923	0.0343576
GO:0032956	regulation	318	0.4038532	1.345185	0.003	0.0429923	0.0343576
GO:0045785	positive r	388	0.3964284	1.3258701	0.003003	0.0429923	0.0343576
GO:0018105	peptidyl-s	287	0.4166409	1.3832391	0.003006	0.0429923	0.0343576
GO:0002758	innate imm	278	0.4110321	1.3618308	0.003009	0.0429923	0.0343576
GO:1902105	regulation	259	0.4129189	1.3653613	0.003009	0.0429923	0.0343576
GO:0030217	T cell dif	228	0.4235851	1.3951409	0.0030181	0.0430529	0.034406
GO:0043624	cellular p	211	0.4267405	1.4027768	0.0030272	0.0430885	0.0344344
GO:1903708	positive r	176	0.4355335	1.4123223	0.0030303	0.0430885	0.0344344
GO:0090263	positive r	140	0.4564942	1.4632803	0.0030426	0.0431003	0.0344439
GO:0035148	tube forma	145	0.4597311	1.4751831	0.0030457	0.0431003	0.0344439
GO:2001056	positive r	137	0.4645543	1.4883143	0.0030457	0.0431003	0.0344439
GO:2000241	regulation	131	0.4630147	1.4771124	0.0030644	0.0431066	0.0344489
GO:0007098	centrosome	122	0.4711392	1.4973242	0.0030675	0.0431066	0.0344489
GO:0032273	positive r	124	0.4801612	1.5292463	0.0030675	0.0431066	0.0344489
GO:0043280	positive r	124	0.4636139	1.4765454	0.0030675	0.0431066	0.0344489
GO:0031023	microtubul	129	0.4579811	1.4577005	0.0030706	0.0431066	0.0344489
GO:0032606	type I int	119	0.4736863	1.5002864	0.0030801	0.0431066	0.0344489
GO:1905477	positive r	119	0.4726514	1.4970087	0.0030801	0.0431066	0.0344489
GO:0032479	regulation	117	0.4807359	1.5184706	0.0030928	0.0432163	0.0345366
GO:0071887	leukocyte	103	0.4885915	1.5220693	0.0031283	0.0436434	0.0348779
GO:0007006	mitochondr	105	0.4656764	1.4511504	0.0031348	0.043666	0.034896
GO:0001843	neural tub	88	0.4866405	1.4892962	0.0031813	0.044245	0.0353587
GO:0048145	regulation	79	0.5019394	1.5252623	0.0032154	0.0446493	0.0356818
GO:0031110	regulation	75	0.4995408	1.5092301	0.0032293	0.0447716	0.0357795
GO:0046785	microtubul	73	0.511189	1.5418079	0.0032397	0.0448467	0.0358395
GO:0018149	peptide cr	53	0.5291688	1.5247598	0.003337	0.0461217	0.0368585
GO:0032206	positive r	48	0.546157	1.5547907	0.0033445	0.0461528	0.0368833
GO:1905517	macrophage	47	0.5512559	1.565144	0.003352	0.0461842	0.0369084
GO:0090199	regulation	46	0.5414448	1.5285569	0.0033937	0.0466865	0.0373099
GO:0051445	regulation	44	0.5507189	1.5448497	0.0034014	0.0467201	0.0373367
GO:1901659	glycosyl c	45	0.5433138	1.5264667	0.003413	0.0468072	0.0374063
GO:0030261	chromosome	33	0.605405	1.6401622	0.0034562	0.0472545	0.0377638
GO:0070317	negative r	36	0.5654476	1.5457509	0.0034562	0.0472545	0.0377638
GO:0044068	modulation	25	0.6260809	1.6105297	0.0035253	0.0480507	0.0384
GO:0060143	positive r	25	0.6294007	1.6190695	0.0035253	0.0480507	0.0384
GO:0032928	regulation	22	0.6360387	1.5978704	0.0035419	0.0480565	0.0384046
GO:0033622	integrin a	22	0.6349974	1.5952546	0.0035419	0.0480565	0.0384046
GO:0070199	establishm	22	0.632607	1.5892493	0.0035419	0.0480565	0.0384046
GO:0060713	labyrinthi	20	0.6631693	1.6230964	0.0036364	0.0492627	0.0393687
GO:0090670	RNA locali	19	0.6833741	1.6453296	0.0036765	0.0495042	0.0395616
GO:0090671	telomerase	19	0.6833741	1.6453296	0.0036765	0.0495042	0.0395616
GO:0090672	telomerase	19	0.6833741	1.6453296	0.0036765	0.0495042	0.0395616
GO:0090685	RNA locali	19	0.6833741	1.6453296	0.0036765	0.0495042	0.0395616
GO:0051299	centrosome	15	0.6865512	1.5776143	0.00369	0.0495368	0.0395877
GO:0060263	regulation	15	0.6895944	1.5846071	0.00369	0.0495368	0.0395877

GO:1903405	protein lo	10	0.8061994	1.6949717	0.0037083	0.0495467	0.0395956
GO:1904851	positive r	10	0.8061994	1.6949717	0.0037083	0.0495467	0.0395956
GO:1904867	protein lo	10	0.8061994	1.6949717	0.0037083	0.0495467	0.0395956
GO:0007144	female mei	8	0.8136416	1.6234951	0.0037688	0.0495467	0.0395956
GO:0017185	peptidyl-l	8	0.8149493	1.6261043	0.0037688	0.0495467	0.0395956
GO:2000698	positive r	8	0.8096572	1.6155449	0.0037688	0.0495467	0.0395956
GO:2001179	regulation	8	0.8135596	1.6233313	0.0037688	0.0495467	0.0395956
GO:0006001	fructose c	5	-0.969122	-1.972291	0.0038023	0.0495467	0.0395956
GO:0006522	alanine me	5	-0.981651	-1.997788	0.0038023	0.0495467	0.0395956
GO:0006524	alanine ca	5	-0.981651	-1.997788	0.0038023	0.0495467	0.0395956
GO:0006526	arginine b	5	-0.954931	-1.94341	0.0038023	0.0495467	0.0395956
GO:0006572	tyrosine c	5	-0.950552	-1.934497	0.0038023	0.0495467	0.0395956
GO:0009078	pyruvate f	5	-0.981651	-1.997788	0.0038023	0.0495467	0.0395956
GO:0009080	pyruvate f	5	-0.981651	-1.997788	0.0038023	0.0495467	0.0395956
GO:0010897	negative r	5	-0.933022	-1.898822	0.0038023	0.0495467	0.0395956
GO:0010901	regulation	5	-0.96757	-1.969132	0.0038023	0.0495467	0.0395956
GO:0016098	monoterpen	5	-0.98115	-1.99677	0.0038023	0.0495467	0.0395956
GO:0045329	carnitine	5	-0.931576	-1.89588	0.0038023	0.0495467	0.0395956
GO:0061624	fructose c	5	-0.969122	-1.972291	0.0038023	0.0495467	0.0395956
GO:1901523	icosanoid	5	-0.975549	-1.985371	0.0038023	0.0495467	0.0395956
GO:0006518	peptide me	748	0.3693512	1.2542337	0.003996	0.0504004	0.0402778
GO:0016567	protein ub	751	0.3709271	1.2595719	0.003996	0.0504004	0.0402778
GO:0019941	modificati	579	0.3790095	1.2813111	0.003996	0.0504004	0.0402778
GO:0051603	proteolysi	662	0.3718793	1.2614818	0.003996	0.0504004	0.0402778
GO:0198738	cell-cell	494	0.3881954	1.3069408	0.003996	0.0504004	0.0402778
GO:1901137	carbohydra	708	0.3727992	1.2658515	0.003996	0.0504004	0.0402778
GO:1902533	positive r	999	0.3531234	1.2062149	0.003996	0.0504004	0.0402778
GO:0009100	glycoprote	386	0.3950767	1.3223324	0.004	0.0504004	0.0402778
GO:0010256	endomembra	398	0.3960754	1.3267459	0.004	0.0504004	0.0402778
GO:0019058	viral life	311	0.4025324	1.3399937	0.004	0.0504004	0.0402778
GO:0030098	lymphocyte	336	0.4020134	1.3410221	0.004	0.0504004	0.0402778
GO:0031349	positive r	473	0.3819671	1.2837395	0.004	0.0504004	0.0402778
GO:0060271	cilium ass	330	0.4032548	1.3443228	0.004	0.0504004	0.0402778
GO:0070997	neuron dea	332	0.4000895	1.3334447	0.004	0.0504004	0.0402778
GO:0032970	regulation	360	0.395839	1.3218774	0.004004	0.0504004	0.0402778
GO:0044782	cilium org	347	0.3969124	1.3232626	0.004004	0.0504004	0.0402778
GO:0002933	lipid hydr	6	-0.975053	-2.106555	0.0040161	0.0504004	0.0402778
GO:0006531	aspartate	6	-0.950868	-2.054305	0.0040161	0.0504004	0.0402778
GO:0009698	phenylprop	6	-0.939711	-2.030201	0.0040161	0.0504004	0.0402778
GO:0009804	coumarin m	6	-0.939711	-2.030201	0.0040161	0.0504004	0.0402778
GO:0009820	alkaloid m	6	-0.940933	-2.032841	0.0040161	0.0504004	0.0402778
GO:0042063	gliogenesi	275	0.409129	1.3536739	0.0040161	0.0504004	0.0402778
GO:0042426	choline ca	6	-0.905325	-1.955912	0.0040161	0.0504004	0.0402778
GO:0046113	nucleobase	6	-0.929999	-2.009218	0.0040161	0.0504004	0.0402778
GO:0071356	cellular r	270	0.4088098	1.3507265	0.0040201	0.0504004	0.0402778
GO:0007249	I-kappaB k	254	0.4116448	1.358978	0.0040241	0.0504004	0.0402778
GO:0051402	neuron apo	228	0.4195024	1.3816937	0.0040241	0.0504004	0.0402778
GO:0071219	cellular r	197	0.4236115	1.3879479	0.0040282	0.0504004	0.0402778
GO:2001234	negative r	216	0.4198311	1.3805514	0.0040323	0.0504004	0.0402778
GO:0046104	thymidine	5	0.873854	1.5771033	0.0040595	0.0504041	0.0402808
GO:0046125	pyrimidine	5	0.873854	1.5771033	0.0040595	0.0504041	0.0402808

GO:0061743	motor lear	5	0.8711758	1.5722698	0.0040595	0.0504041	0.0402808
GO:0008037	cell recog	145	0.4498644	1.4435228	0.0040609	0.0504041	0.0402808
GO:1903900	regulation	137	0.4542645	1.4553484	0.0040609	0.0504041	0.0402808
GO:0031123	RNA 3'-enc	134	0.4621514	1.47603	0.0040858	0.0506235	0.0404561
GO:0045580	regulation	132	0.4601963	1.4678429	0.00409	0.0506235	0.0404561
GO:0008637	apoptotic	112	0.4680503	1.4718026	0.004128	0.0510226	0.040775
GO:0048524	positive r	104	0.4666931	1.4553556	0.004171	0.051483	0.041143
GO:0031532	actin cyto	99	0.4868035	1.5111674	0.0041885	0.0516095	0.0412441
GO:0120034	positive r	95	0.4716493	1.4580461	0.0041929	0.0516095	0.0412441
GO:0060606	tube closu	89	0.4865577	1.4888875	0.0042508	0.0522501	0.041756
GO:0032602	chemokine	82	0.4917302	1.4973837	0.0042644	0.0523448	0.0418317
GO:0048144	fibroblast	80	0.4944455	1.5039	0.0042918	0.0526091	0.0420429
GO:0033627	cell adhes	64	0.5080939	1.5052506	0.0043621	0.0533961	0.0426718
GO:0061077	chaperone-	54	0.5277651	1.527524	0.0044395	0.0542552	0.0433584
GO:0006578	amino-acic	7	-0.897423	-2.029131	0.0044444	0.0542552	0.0433584
GO:0031113	regulation	48	0.5295303	1.507458	0.0044593	0.0543481	0.0434326
GO:0030199	collagen f	50	0.5298827	1.5122913	0.0044643	0.0543481	0.0434326
GO:0007019	microtubul	42	0.5452762	1.5184132	0.0045662	0.0555129	0.0443635
GO:0000027	ribosomal	28	0.6111719	1.6134743	0.0045924	0.0557553	0.0445572
GO:0000083	regulation	27	0.6235632	1.6331105	0.0046296	0.0559776	0.0447349
GO:0032673	regulation	27	0.6154821	1.6119461	0.0046296	0.0559776	0.0447349
GO:0051984	positive r	27	0.6190202	1.6212124	0.0046296	0.0559776	0.0447349
GO:0001516	prostaglar	24	0.6276758	1.6001042	0.0047004	0.0566017	0.0452336
GO:0018126	protein hy	24	0.6317131	1.6103963	0.0047004	0.0566017	0.0452336
GO:0046457	prostanoic	24	0.6276758	1.6001042	0.0047004	0.0566017	0.0452336
GO:0001561	fatty acic	8	-0.870609	-1.998637	0.0048544	0.0568968	0.0454695
GO:0006548	histidine	8	-0.915336	-2.101315	0.0048544	0.0568968	0.0454695
GO:0019626	short-chai	8	-0.934021	-2.14421	0.0048544	0.0568968	0.0454695
GO:0052805	imidazole-	8	-0.915336	-2.101315	0.0048544	0.0568968	0.0454695
GO:0070327	thyroid hc	8	-0.859584	-1.973327	0.0048544	0.0568968	0.0454695
GO:0000050	urea cycle	11	-0.866825	-2.211816	0.004878	0.0568968	0.0454695
GO:0033539	fatty acic	11	-0.920081	-2.347706	0.004878	0.0568968	0.0454695
GO:0045919	positive r	11	-0.76758	-1.95858	0.004878	0.0568968	0.0454695
GO:0046185	aldehyde c	11	-0.761947	-1.944207	0.004878	0.0568968	0.0454695
GO:0046950	cellular k	11	-0.759573	-1.93815	0.004878	0.0568968	0.0454695
GO:0070189	kynurenine	11	-0.739603	-1.887194	0.004878	0.0568968	0.0454695
GO:1901569	fatty acic	11	-0.749546	-1.912564	0.004878	0.0568968	0.0454695
GO:0060252	positive r	15	0.6824839	1.568268	0.00492	0.0568968	0.0454695
GO:0002291	T cell act	10	0.7968807	1.6753799	0.0049444	0.0568968	0.0454695
GO:0019695	choline me	9	-0.827043	-1.966479	0.0049505	0.0568968	0.0454695
GO:0034382	chylomicrc	9	-0.944616	-2.246036	0.0049505	0.0568968	0.0454695
GO:0046487	glyoxylate	9	-0.910017	-2.163768	0.0049505	0.0568968	0.0454695
GO:0071830	triglyceri	9	-0.944616	-2.246036	0.0049505	0.0568968	0.0454695
GO:0090209	negative r	9	-0.880881	-2.094491	0.0049505	0.0568968	0.0454695
GO:0097267	omega-hydr	9	-0.936945	-2.227795	0.0049505	0.0568968	0.0454695
GO:1904816	positive r	12	0.7727396	1.6961689	0.0049566	0.0568968	0.0454695
GO:0006511	ubiquitin-	573	0.3782779	1.2784754	0.004995	0.0568968	0.0454695
GO:0019953	sexual rep	758	0.3677215	1.2486036	0.004995	0.0568968	0.0454695
GO:0032504	multicellu	768	0.3652336	1.2406547	0.004995	0.0568968	0.0454695
GO:0048609	multicellu	753	0.3646388	1.2381648	0.004995	0.0568968	0.0454695
GO:0032386	regulation	405	0.3911791	1.3114433	0.005	0.0568968	0.0454695

GO:0043405	regulation	320	0.3906517	1.300665	0.005	0.0568968	0.0454695
GO:0051255	spindle mi	9	0.8178117	1.675733	0.005	0.0568968	0.0454695
GO:0060070	canonical	318	0.3933586	1.310229	0.005	0.0568968	0.0454695
GO:1904869	regulation	9	0.8224563	1.6852501	0.005	0.0568968	0.0454695
GO:1904871	positive r	9	0.8224563	1.6852501	0.005	0.0568968	0.0454695
GO:1904951	positive r	437	0.3819645	1.2834413	0.005	0.0568968	0.0454695
GO:1901214	regulation	298	0.3979231	1.32099	0.005005	0.0568968	0.0454695
GO:0060828	regulation	271	0.4050588	1.3382932	0.0050251	0.0568968	0.0454695
GO:1904776	regulation	8	0.804465	1.6051846	0.0050251	0.0568968	0.0454695
GO:0043122	regulation	224	0.4163015	1.3699953	0.0050352	0.0568968	0.0454695
GO:0060560	developmen	226	0.4162984	1.3700849	0.0050352	0.0568968	0.0454695
GO:0043523	regulation	200	0.4150731	1.3610575	0.0050454	0.0568968	0.0454695
GO:0044706	multi-mult	208	0.4141863	1.361172	0.0050454	0.0568968	0.0454695
GO:0051701	interactio	200	0.416033	1.3642049	0.0050454	0.0568968	0.0454695
GO:1902905	positive r	191	0.4376006	1.4275027	0.0050505	0.0568968	0.0454695
GO:1903320	regulation	213	0.4180743	1.3742665	0.0050505	0.0568968	0.0454695
GO:0120032	regulation	162	0.4348501	1.403483	0.0050607	0.0568968	0.0454695
GO:0051099	positive r	171	0.4296854	1.391322	0.0050659	0.0568968	0.0454695
GO:0061025	membrane f	143	0.4477782	1.4360177	0.005071	0.0568968	0.0454695
GO:0002262	myeloid ce	138	0.4491868	1.4386808	0.0050813	0.0568968	0.0454695
GO:0071706	tumor necr	147	0.4440683	1.4244939	0.0050813	0.0568968	0.0454695
GO:0034620	cellular r	132	0.447341	1.4268397	0.0051125	0.0568968	0.0454695
GO:0006063	uronic aci	17	-0.71688	-2.133498	0.0051282	0.0568968	0.0454695
GO:0006069	ethanol ox	12	-0.849154	-2.24626	0.0051282	0.0568968	0.0454695
GO:0006544	glycine me	17	-0.808171	-2.405188	0.0051282	0.0568968	0.0454695
GO:0006554	lysine cat	12	-0.864207	-2.286081	0.0051282	0.0568968	0.0454695
GO:0006577	amino-acid	17	-0.864376	-2.572458	0.0051282	0.0568968	0.0454695
GO:0010896	regulation	12	-0.760421	-2.011535	0.0051282	0.0568968	0.0454695
GO:0015697	quaternary	12	-0.748791	-1.98077	0.0051282	0.0568968	0.0454695
GO:0019585	glucuronat	17	-0.71688	-2.133498	0.0051282	0.0568968	0.0454695
GO:0032372	negative r	12	-0.82606	-2.18517	0.0051282	0.0568968	0.0454695
GO:0032375	negative r	12	-0.82606	-2.18517	0.0051282	0.0568968	0.0454695
GO:0033700	phospholip	12	-0.725927	-1.920289	0.0051282	0.0568968	0.0454695
GO:0034372	very-low-d	12	-0.793658	-2.099458	0.0051282	0.0568968	0.0454695
GO:0052803	imidazole-	12	-0.888542	-2.350454	0.0051282	0.0568968	0.0454695
GO:0070989	oxidative	17	-0.835127	-2.485413	0.0051282	0.0568968	0.0454695
GO:1902224	ketone bod	12	-0.744446	-1.969277	0.0051282	0.0568968	0.0454695
GO:0006103	2-oxogluta	16	-0.841645	-2.453823	0.0051546	0.0569059	0.0454768
GO:0043649	dicarboxyl	16	-0.719881	-2.098819	0.0051546	0.0569059	0.0454768
GO:0043691	reverse ch	16	-0.745282	-2.172877	0.0051546	0.0569059	0.0454768
GO:0046459	short-chai	16	-0.788035	-2.297524	0.0051546	0.0569059	0.0454768
GO:0006547	histidine	10	-0.90144	-2.196988	0.0051813	0.057059	0.0455991
GO:0006570	tyrosine m	10	-0.943995	-2.300704	0.0051813	0.057059	0.0455991
GO:0007127	meiosis I	101	0.471421	1.4675029	0.0052029	0.0572256	0.0457322
GO:0006493	protein O-	96	0.4629904	1.431635	0.0052411	0.0572897	0.0457834
GO:1990868	response t	94	0.4749897	1.4650511	0.0052743	0.0572897	0.0457834
GO:1990869	cellular r	94	0.4749897	1.4650511	0.0052743	0.0572897	0.0457834
GO:0061687	detoxifica	15	-0.748304	-2.113455	0.005291	0.0572897	0.0457834
GO:0071674	mononuclea	82	0.4801407	1.4620922	0.0053305	0.0572897	0.0457834
GO:2000106	regulation	82	0.4828205	1.4702526	0.0053305	0.0572897	0.0457834
GO:1903557	positive r	83	0.4859757	1.4783711	0.0053476	0.0572897	0.0457834

GO:0000097	sulfur ami	14	-0.777734	-2.118087	0.0053763	0.0572897	0.0457834
GO:0000098	sulfur ami	13	-0.772721	-2.063151	0.0053763	0.0572897	0.0457834
GO:0001867	complement	13	-0.817263	-2.182078	0.0053763	0.0572897	0.0457834
GO:0006525	arginine m	19	-0.674349	-2.064867	0.0053763	0.0572897	0.0457834
GO:0006553	lysine met	13	-0.850306	-2.2703	0.0053763	0.0572897	0.0457834
GO:0009071	serine fan	14	-0.84598	-2.303949	0.0053763	0.0572897	0.0457834
GO:0009437	carnitine	13	-0.825221	-2.203324	0.0053763	0.0572897	0.0457834
GO:0010273	detoxifica	13	-0.725092	-1.935981	0.0053763	0.0572897	0.0457834
GO:0015812	gamma-amir	13	-0.78697	-2.101195	0.0053763	0.0572897	0.0457834
GO:0019373	epoxygenas	19	-0.909565	-2.785102	0.0053763	0.0572897	0.0457834
GO:0019627	urea metab	13	-0.865548	-2.310997	0.0053763	0.0572897	0.0457834
GO:0033540	fatty acic	14	-0.749899	-2.04228	0.0053763	0.0572897	0.0457834
GO:0034370	triglyceri	14	-0.812019	-2.211459	0.0053763	0.0572897	0.0457834
GO:0034374	low-densit	13	-0.720531	-1.923804	0.0053763	0.0572897	0.0457834
GO:0034384	high-densi	13	-0.890737	-2.378251	0.0053763	0.0572897	0.0457834
GO:0035112	genitalia	13	-0.707326	-1.888547	0.0053763	0.0572897	0.0457834
GO:0051917	regulatior	14	-0.845791	-2.303434	0.0053763	0.0572897	0.0457834
GO:0071941	nitrogen c	14	-0.865463	-2.357011	0.0053763	0.0572897	0.0457834
GO:1990169	stress res	13	-0.725092	-1.935981	0.0053763	0.0572897	0.0457834
GO:0002312	B cell act	69	0.5003569	1.5015386	0.0053996	0.0574682	0.0459261
GO:0032418	lysosome l	72	0.5042337	1.5177028	0.0054171	0.057586	0.0460203
GO:0010324	membrane i	63	0.5061478	1.4970846	0.0054466	0.0576756	0.0460918
GO:0032729	positive r	63	0.5083282	1.503534	0.0054466	0.0576756	0.0460918
GO:0006555	methionine	18	-0.687463	-2.041955	0.0054645	0.0576756	0.0460918
GO:0007597	blood coag	18	-0.893118	-2.652807	0.0054645	0.0576756	0.0460918
GO:0034375	high-densi	18	-0.691183	-2.053005	0.0054645	0.0576756	0.0460918
GO:0051957	positive r	18	-0.785195	-2.332245	0.0054645	0.0576756	0.0460918
GO:0001836	release of	56	0.5225809	1.5215716	0.0055006	0.0579874	0.046341
GO:0010803	regulatior	54	0.5231334	1.5141182	0.0055494	0.0584205	0.0466871
GO:0007062	sister chr	53	0.5203289	1.4992881	0.0055617	0.0584205	0.0466871
GO:0032663	regulatior	53	0.5267376	1.5177542	0.0055617	0.0584205	0.0466871
GO:0018208	peptidyl-p	51	0.5235586	1.4969807	0.0055679	0.0584205	0.0466871
GO:0006067	ethanol me	20	-0.793269	-2.461971	0.0056497	0.0592059	0.0473148
GO:0043489	RNA stabil	43	0.5381111	1.5066239	0.0056561	0.0592059	0.0473148
GO:0009163	nucleoside	41	0.546445	1.5200774	0.0057143	0.0596743	0.0476891
GO:0045023	G0 to G1 t	41	0.5481073	1.5247015	0.0057143	0.0596743	0.0476891
GO:0032801	receptor c	32	0.5884288	1.5916049	0.0057274	0.0597408	0.0477423
GO:0070316	regulatior	39	0.5557279	1.5387359	0.0057471	0.0598766	0.0478508
GO:1905521	regulatior	34	0.5761229	1.5639722	0.0057737	0.0600827	0.0480155
GO:0018108	peptidyl-t	348	0.3895369	1.2990438	0.006006	0.0623441	0.0498227
GO:0018212	peptidyl-t	351	0.3888866	1.2978644	0.006006	0.0623441	0.0498227
GO:0034612	response t	287	0.4000814	1.3282618	0.006012	0.0623441	0.0498227
GO:0071216	cellular r	220	0.4075114	1.3407801	0.0060484	0.0626382	0.0500578
GO:0071222	cellular r	190	0.4218905	1.376194	0.0060545	0.0626382	0.0500578
GO:0006399	tRNA metab	164	0.430175	1.3882681	0.006079	0.062819	0.0502022
GO:0034250	positive r	141	0.4440683	1.4217483	0.0060976	0.0628643	0.0502384
GO:1903555	regulatior	142	0.4497529	1.4406731	0.0060976	0.0628643	0.0502384
GO:1904872	regulatior	18	0.6786073	1.6190755	0.006105	0.0628681	0.0502415
GO:2000696	regulatior	19	0.6632186	1.596802	0.0061275	0.0630262	0.0503678
GO:0034501	protein lc	16	0.6897491	1.6020132	0.0061881	0.0635767	0.0508077
GO:0007173	epidermal	115	0.4641315	1.4633092	0.0061983	0.0636082	0.0508329

GO:0060968	regulation	107	0.4596584	1.4340652	0.006237	0.0639311	0.051091
GO:0070203	regulation	11	0.7583187	1.6278742	0.0062735	0.0640241	0.0511653
GO:0009081	branched-c	21	-0.753603	-2.319973	0.0062893	0.0640241	0.0511653
GO:0009083	branched-c	21	-0.753603	-2.319973	0.0062893	0.0640241	0.0511653
GO:0031124	mRNA 3'-en	95	0.4646358	1.4363648	0.0062893	0.0640241	0.0511653
GO:0042135	neurotrans	21	-0.827798	-2.548384	0.0062893	0.0640241	0.0511653
GO:0042738	exogenous	21	-0.87421	-2.691265	0.0062893	0.0640241	0.0511653
GO:0009068	aspartate	23	-0.792428	-2.547059	0.0064516	0.0653768	0.0522463
GO:0009310	amine cata	22	-0.679019	-2.145629	0.0064516	0.0653768	0.0522463
GO:0009404	toxin meta	22	-0.711316	-2.247685	0.0064516	0.0653768	0.0522463
GO:0042537	benzene-co	23	-0.777141	-2.497925	0.0064516	0.0653768	0.0522463
GO:1903747	regulation	70	0.4977789	1.4952477	0.0065076	0.065869	0.0526396
GO:0061844	antimicrob	61	0.5141178	1.5176402	0.0065359	0.0660542	0.0527877
GO:0042274	ribosomal	62	0.5057605	1.4930055	0.0065502	0.0660542	0.0527877
GO:0036498	IRE1-media	60	0.5106746	1.5077194	0.0065574	0.0660542	0.0527877
GO:0072678	T cell mig	56	0.5202505	1.5147863	0.0066007	0.0660542	0.0527877
GO:0015721	bile acid	26	-0.814735	-2.693993	0.0066225	0.0660542	0.0527877
GO:0030194	positive r	26	-0.648244	-2.143476	0.0066225	0.0660542	0.0527877
GO:0032369	negative r	25	-0.701236	-2.297077	0.0066225	0.0660542	0.0527877
GO:0042219	cellular m	24	-0.724386	-2.361788	0.0066225	0.0660542	0.0527877
GO:0042430	indole-con	24	-0.648379	-2.113976	0.0066225	0.0660542	0.0527877
GO:0042573	retinoic a	25	-0.769517	-2.520749	0.0066225	0.0660542	0.0527877
GO:0045922	negative r	25	-0.678903	-2.22392	0.0066225	0.0660542	0.0527877
GO:0071377	cellular r	26	-0.656621	-2.171176	0.0066225	0.0660542	0.0527877
GO:1900048	positive r	26	-0.648244	-2.143476	0.0066225	0.0660542	0.0527877
GO:0045742	positive r	30	0.5850044	1.5650234	0.0068493	0.0682398	0.0545343
GO:1901186	positive r	32	0.5748792	1.5549553	0.0068729	0.0683976	0.0546604
GO:0001773	myeloid de	28	0.596418	1.5745246	0.0068886	0.068478	0.0547246
GO:0045684	positive r	35	0.5570301	1.5178917	0.0069045	0.0685589	0.0547893
GO:0045022	early endo	37	0.5419198	1.4853759	0.0069204	0.0685665	0.0547954
GO:0048246	macrophage	34	0.56787	1.5415685	0.0069284	0.0685665	0.0547954
GO:1901030	positive r	34	0.5616104	1.5245759	0.0069284	0.0685665	0.0547954
GO:0042327	positive r	987	0.3508746	1.1982645	0.006993	0.0691211	0.0552386
GO:0043547	positive r	368	0.3892726	1.3004302	0.007	0.0691211	0.0552386
GO:0040029	regulation	285	0.3968641	1.3170985	0.007014	0.0691828	0.0552879
GO:0006486	protein gl	238	0.4014547	1.322872	0.0070493	0.0693771	0.0554432
GO:0043413	macromolec	238	0.4014547	1.322872	0.0070493	0.0693771	0.0554432
GO:0031396	regulation	187	0.4215509	1.3723414	0.0070707	0.0694849	0.0555293
GO:0007565	female pre	181	0.4237239	1.3756778	0.0070779	0.0694849	0.0555293
GO:1900120	regulation	22	0.6231364	1.5654572	0.0070838	0.0694849	0.0555293
GO:0002285	lymphocyte	170	0.4255901	1.3772023	0.0070994	0.0694849	0.0555293
GO:0032680	regulation	139	0.4448155	1.4254859	0.0070994	0.0694849	0.0555293
GO:0032640	tumor necr	142	0.4404397	1.4108406	0.0071138	0.0695495	0.055581
GO:0051250	negative r	133	0.4419035	1.4097631	0.0071721	0.0700426	0.055975
GO:0008209	androgen m	27	-0.642779	-2.111581	0.0072464	0.0702274	0.0561227
GO:0034377	plasma lip	27	-0.669966	-2.200893	0.0072464	0.0702274	0.0561227
GO:0042730	fibrinolys	27	-0.721577	-2.370441	0.0072464	0.0702274	0.0561227
GO:0042759	long-chain	27	-0.756328	-2.484599	0.0072464	0.0702274	0.0561227
GO:0050820	positive r	27	-0.64385	-2.115101	0.0072464	0.0702274	0.0561227
GO:0071280	cellular r	27	-0.579626	-1.904121	0.0072464	0.0702274	0.0561227
GO:0072378	blood coag	27	-0.787264	-2.586229	0.0072464	0.0702274	0.0561227

GO:0006282	regulation	106	0.4553887	1.4192163	0.0072993	0.070663	0.0564708
GO:0006699	bile acid	34	-0.740857	-2.533384	0.0073529	0.0708737	0.0566392
GO:0019433	triglyceri	34	-0.704868	-2.41032	0.0073529	0.0708737	0.0566392
GO:0097202	activation	13	0.7103771	1.588898	0.0073529	0.0708737	0.0566392
GO:1900025	negative r	13	0.7090795	1.5859958	0.0073529	0.0708737	0.0566392
GO:0010771	negative r	94	0.4665156	1.438914	0.007384	0.0710903	0.0568123
GO:0030316	osteoclast	92	0.4740269	1.4584188	0.0074074	0.0710903	0.0568123
GO:0046460	neutral li	37	-0.691563	-2.415668	0.0074074	0.0710903	0.0568123
GO:0046463	acylglycer	37	-0.691563	-2.415668	0.0074074	0.0710903	0.0568123
GO:0006958	complement	33	-0.603337	-2.046691	0.0074627	0.0713128	0.0569901
GO:0010907	positive r	36	-0.551111	-1.906662	0.0074627	0.0713128	0.0569901
GO:0032892	positive r	33	-0.573467	-1.945364	0.0074627	0.0713128	0.0569901
GO:0042572	retinol me	36	-0.744785	-2.576711	0.0074627	0.0713128	0.0569901
GO:0001580	detection	35	-0.567376	-1.95433	0.0075188	0.0713884	0.0570505
GO:0019432	triglyceri	35	-0.687661	-2.368652	0.0075188	0.0713884	0.0570505
GO:0033762	response t	35	-0.623289	-2.146921	0.0075188	0.0713884	0.0570505
GO:0055090	acylglycer	35	-0.725764	-2.499898	0.0075188	0.0713884	0.0570505
GO:0070328	triglyceri	35	-0.725764	-2.499898	0.0075188	0.0713884	0.0570505
GO:0090207	regulation	35	-0.626118	-2.156667	0.0075188	0.0713884	0.0570505
GO:0032481	positive r	73	0.4949906	1.4929516	0.0075594	0.0715609	0.0571883
GO:0008210	estrogen m	29	-0.749976	-2.464397	0.0075758	0.0715609	0.0571883
GO:0009072	aromatic a	39	-0.757137	-2.661315	0.0075758	0.0715609	0.0571883
GO:0034367	protein-co	29	-0.700725	-2.302562	0.0075758	0.0715609	0.0571883
GO:0006064	glucuronat	5	-0.911679	-1.855386	0.0076046	0.0715609	0.0571883
GO:0019640	glucuronat	5	-0.911679	-1.855386	0.0076046	0.0715609	0.0571883
GO:0051167	xylulose 5	5	-0.911679	-1.855386	0.0076046	0.0715609	0.0571883
GO:1901159	xylulose 5	5	-0.911679	-1.855386	0.0076046	0.0715609	0.0571883
GO:0050854	regulation	61	0.5074353	1.4979138	0.0076253	0.0715609	0.0571883
GO:0009074	aromatic a	28	-0.776827	-2.526048	0.0076336	0.0715609	0.0571883
GO:0034368	protein-li	28	-0.711117	-2.312374	0.0076336	0.0715609	0.0571883
GO:0034369	plasma lip	28	-0.711117	-2.312374	0.0076336	0.0715609	0.0571883
GO:0044837	actomyosin	7	0.7917063	1.5283178	0.007722	0.0723135	0.0577898
GO:0006998	nuclear en	52	0.5203027	1.4939089	0.0077864	0.0727614	0.0581477
GO:0000096	sulfur ami	38	-0.662238	-2.295859	0.007874	0.0727614	0.0581477
GO:0006084	acetyl-CoA	38	-0.567419	-1.96714	0.007874	0.0727614	0.0581477
GO:0006536	glutamate	31	-0.700707	-2.304429	0.007874	0.0727614	0.0581477
GO:0019835	cytolysis	40	-0.562392	-1.9592	0.007874	0.0727614	0.0581477
GO:0033344	cholestero	41	-0.551599	-1.936798	0.007874	0.0727614	0.0581477
GO:0050912	detection	40	-0.538694	-1.876643	0.007874	0.0727614	0.0581477
GO:0065005	protein-li	31	-0.641008	-2.108098	0.007874	0.0727614	0.0581477
GO:0000038	very long-	30	-0.564062	-1.825888	0.0079365	0.0727614	0.0581477
GO:0005978	glycogen b	42	-0.497627	-1.762271	0.0079365	0.0727614	0.0581477
GO:0006775	fat-solubl	42	-0.505432	-1.789912	0.0079365	0.0727614	0.0581477
GO:0009250	glucan bio	42	-0.497627	-1.762271	0.0079365	0.0727614	0.0581477
GO:0046320	regulation	30	-0.650862	-2.106862	0.0079365	0.0727614	0.0581477
GO:0046461	neutral li	42	-0.655826	-2.322507	0.0079365	0.0727614	0.0581477
GO:0046464	acylglycer	42	-0.655826	-2.322507	0.0079365	0.0727614	0.0581477
GO:0002248	connective	6	0.8116601	1.5126096	0.0079681	0.0727614	0.0581477
GO:0016255	attachment	6	0.8175222	1.5235342	0.0079681	0.0727614	0.0581477
GO:1900004	negative r	6	0.8257486	1.538865	0.0079681	0.0727614	0.0581477
GO:1902572	negative r	6	0.8257486	1.538865	0.0079681	0.0727614	0.0581477

GO:2001181	positive r	6	0.8203431	1.5287912	0.0079681	0.0727614	0.0581477
GO:0007169	transmembr	676	0.3574702	1.2125864	0.007992	0.0727614	0.0581477
GO:0007276	gamete ger	615	0.3680867	1.2470608	0.007992	0.0727614	0.0581477
GO:0031400	negative r	552	0.3706946	1.2506461	0.008	0.0727614	0.0581477
GO:0030111	regulation	341	0.3897565	1.2994004	0.008008	0.0727614	0.0581477
GO:0016236	macroautop	281	0.3925418	1.3011877	0.0080241	0.0727614	0.0581477
GO:0006552	leucine ca	6	-0.880801	-1.902928	0.0080321	0.0727614	0.0581477
GO:0048255	mRNA stabi	39	0.5519958	1.5284023	0.008046	0.0727614	0.0581477
GO:0050856	regulation	39	0.5475869	1.5161945	0.008046	0.0727614	0.0581477
GO:0050730	regulation	244	0.4023926	1.3272562	0.0080483	0.0727614	0.0581477
GO:0032633	interleuki	33	0.5634009	1.5263647	0.0080645	0.0727614	0.0581477
GO:0043393	regulation	206	0.4063168	1.3354277	0.0080645	0.0727614	0.0581477
GO:0097064	ncRNA expo	36	0.5511976	1.5067959	0.0080645	0.0727614	0.0581477
GO:0016197	endosomal	200	0.4099218	1.3441657	0.0080727	0.0727614	0.0581477
GO:0034341	response t	187	0.4174147	1.3588761	0.0080808	0.0727614	0.0581477
GO:0035966	response t	189	0.4176955	1.361098	0.0080808	0.0727614	0.0581477
GO:0045619	regulation	162	0.4272147	1.3788396	0.0080972	0.0727614	0.0581477
GO:1902107	positive r	139	0.4412647	1.4141068	0.0081136	0.0727614	0.0581477
GO:0031914	negative r	5	0.8559559	1.5448013	0.0081191	0.0727614	0.0581477
GO:0045590	negative r	5	0.8546503	1.542445	0.0081191	0.0727614	0.0581477
GO:0038127	ERBB signa	137	0.4442211	1.4231721	0.0081218	0.0727614	0.0581477
GO:0008206	bile acid	45	-0.700384	-2.555468	0.0081301	0.0727614	0.0581477
GO:0032371	regulation	45	-0.529548	-1.932144	0.0081301	0.0727614	0.0581477
GO:0032374	regulation	45	-0.529548	-1.932144	0.0081301	0.0727614	0.0581477
GO:0042304	regulation	45	-0.493802	-1.801721	0.0081301	0.0727614	0.0581477
GO:0006997	nucleus or	124	0.443901	1.4137628	0.00818	0.0731341	0.0584456
GO:0018107	peptidyl-t	118	0.4507922	1.4257191	0.008222	0.0733946	0.0586538
GO:0033561	regulation	24	0.6138245	1.5647937	0.0082256	0.0733946	0.0586538
GO:0007063	regulation	21	0.6366235	1.5782352	0.0083037	0.0737948	0.0589736
GO:0042451	purine nuc	21	0.628785	1.5588029	0.0083037	0.0737948	0.0589736
GO:0046129	purine rib	21	0.628785	1.5588029	0.0083037	0.0737948	0.0589736
GO:0061436	establishm	21	0.6324041	1.5677748	0.0083037	0.0737948	0.0589736
GO:0009069	serine fam	44	-0.694678	-2.505071	0.0083333	0.0739105	0.0590661
GO:0071827	plasma lip	44	-0.616946	-2.224761	0.0083333	0.0739105	0.0590661
GO:0070498	interleuki	98	0.4614304	1.4327297	0.0083595	0.0740683	0.0591922
GO:0002455	humoral im	46	-0.488639	-1.782527	0.0084746	0.0747158	0.0597097
GO:0006953	acute-phas	46	-0.552649	-2.016031	0.0084746	0.0747158	0.0597097
GO:0045540	regulation	43	-0.509198	-1.80619	0.0084746	0.0747158	0.0597097
GO:0106118	regulation	43	-0.509198	-1.80619	0.0084746	0.0747158	0.0597097
GO:1903076	regulation	91	0.4706698	1.4452884	0.0084746	0.0747158	0.0597097
GO:0070098	chemokine-	86	0.4734686	1.4465385	0.0085288	0.0751192	0.060032
GO:0071800	podosome a	18	0.6570587	1.5676632	0.008547	0.0751839	0.0600838
GO:0032760	positive r	81	0.4743327	1.4416869	0.0085653	0.0751839	0.0600838
GO:0060218	hematopoie	81	0.4774379	1.4511248	0.0085653	0.0751839	0.0600838
GO:0002281	macrophage	13	0.7043666	1.5754545	0.0085784	0.0751839	0.0600838
GO:1901070	guanosine-	13	0.7044692	1.5756839	0.0085784	0.0751839	0.0600838
GO:0033141	positive r	15	0.6780836	1.5581568	0.0086101	0.075387	0.060246
GO:1900182	positive r	74	0.4924352	1.4863034	0.0086393	0.0755684	0.060391
GO:0070664	negative r	72	0.4921408	1.4813041	0.0086674	0.0757237	0.0605151
GO:0070486	leukocyte	12	0.74061	1.6256443	0.0086741	0.0757237	0.0605151
GO:0007040	lysosome o	60	0.5053073	1.4918729	0.0087432	0.076177	0.0608774

GO:0080171	lytic vacu	60	0.5053073	1.4918729	0.0087432	0.076177	0.0608774
GO:0098883	synapse pr	11	0.7535473	1.6176315	0.0087829	0.0764485	0.0610944
GO:0001934	positive r	937	0.3506148	1.1962151	0.008991	0.0781066	0.0624194
GO:0045860	positive r	502	0.3712415	1.2507084	0.008991	0.0781066	0.0624194
GO:0032808	lacrimal g	7	0.7889397	1.5229771	0.009009	0.0781866	0.0624833
GO:0070646	protein mo	262	0.4012033	1.3272293	0.009018	0.0781886	0.0624849
GO:0010821	regulation	177	0.4206858	1.3648725	0.0090909	0.0786669	0.0628672
GO:1902115	regulation	182	0.4182437	1.3581328	0.0090909	0.0786669	0.0628672
GO:0040020	regulation	27	0.5931946	1.5535751	0.0092593	0.079968	0.0639069
GO:0060142	regulation	27	0.5934647	1.5542826	0.0092593	0.079968	0.0639069
GO:2000736	regulation	112	0.4567425	1.4362446	0.0092879	0.0801377	0.0640425
GO:0006584	catecholam	49	-0.418781	-1.558198	0.0093458	0.0802474	0.0641303
GO:0009712	catechol-c	49	-0.418781	-1.558198	0.0093458	0.0802474	0.0641303
GO:0019369	arachidoni	47	-0.612632	-2.233832	0.0093458	0.0802474	0.0641303
GO:0071320	cellular r	49	-0.503318	-1.872742	0.0093458	0.0802474	0.0641303
GO:1903793	positive r	49	-0.509931	-1.897348	0.0093458	0.0802474	0.0641303
GO:0046697	decidualiz	24	0.6045575	1.5411698	0.0094007	0.0805632	0.0643826
GO:0060561	apoptotic	24	0.6108759	1.557277	0.0094007	0.0805632	0.0643826
GO:0030449	regulation	50	-0.665254	-2.485023	0.009434	0.0807703	0.0645482
GO:0042321	negative r	5	0.8522156	1.538051	0.0094723	0.0810202	0.0647478
GO:0031579	membrane r	21	0.6264157	1.5529294	0.0094899	0.0810932	0.0648061
GO:0071825	protein-li	48	-0.597864	-2.195285	0.0095238	0.0813046	0.0649751
GO:0009066	aspartate	51	-0.608521	-2.272525	0.0096154	0.0818505	0.0654114
GO:0010043	response t	51	-0.523068	-1.9534	0.0096154	0.0818505	0.0654114
GO:2000257	regulation	51	-0.681292	-2.544289	0.0096154	0.0818505	0.0654114
GO:0006576	cellular b	53	-0.48523	-1.849471	0.0097087	0.0820947	0.0656065
GO:0030195	negative r	52	-0.596912	-2.24217	0.0097087	0.0820947	0.0656065
GO:0034381	plasma lip	52	-0.458354	-1.721708	0.0097087	0.0820947	0.0656065
GO:0042364	water-solu	8	-0.781072	-1.793089	0.0097087	0.0820947	0.0656065
GO:0046164	alcohol ca	52	-0.504803	-1.89618	0.0097087	0.0820947	0.0656065
GO:0090303	positive r	53	-0.465466	-1.77414	0.0097087	0.0820947	0.0656065
GO:1900047	negative r	53	-0.582831	-2.221479	0.0097087	0.0820947	0.0656065
GO:0006558	L-phenylal	11	-0.723567	-1.846274	0.0097561	0.0821182	0.0656253
GO:0006559	L-phenylal	11	-0.723567	-1.846274	0.0097561	0.0821182	0.0656253
GO:1902221	erythrose	11	-0.723567	-1.846274	0.0097561	0.0821182	0.0656253
GO:1902222	erythrose	11	-0.723567	-1.846274	0.0097561	0.0821182	0.0656253
GO:0002902	regulation	18	0.6522732	1.5562454	0.009768	0.0821182	0.0656253
GO:0031115	negative r	13	0.6915897	1.5468764	0.0098039	0.0821182	0.0656253
GO:0051382	kinetochor	14	0.6902474	1.570565	0.0098039	0.0821182	0.0656253
GO:0070669	response t	14	0.6884043	1.5663714	0.0098039	0.0821182	0.0656253
GO:0070672	response t	14	0.6910936	1.5724906	0.0098039	0.0821182	0.0656253
GO:2000052	positive r	13	0.7030824	1.572582	0.0098039	0.0821182	0.0656253
GO:1903749	positive r	58	0.5027737	1.4748217	0.0098253	0.08222	0.0657067
GO:0072608	interleuki	10	0.775256	1.6299155	0.0098888	0.0823356	0.0657991
GO:0034371	chylomicro	9	-0.766366	-1.822206	0.009901	0.0823356	0.0657991
GO:0046951	ketone boc	9	-0.823079	-1.957055	0.009901	0.0823356	0.0657991
GO:0050819	negative r	54	-0.588869	-2.255683	0.009901	0.0823356	0.0657991
GO:0050994	regulation	54	-0.576181	-2.207083	0.009901	0.0823356	0.0657991
GO:0000212	meiotic sp	12	0.728849	1.5998288	0.0099133	0.0823356	0.0657991
GO:0038110	interleuki	12	0.7255753	1.5926429	0.0099133	0.0823356	0.0657991
GO:0070202	regulation	12	0.7339057	1.6109282	0.0099133	0.0823356	0.0657991

GO:0010720	positive r	515	0.3698017	1.2469265	0.00999	0.0823786	0.0658334
GO:0032990	cell part	661	0.3641564	1.2353081	0.00999	0.0823786	0.0658334
GO:0045664	regulation	617	0.3661773	1.2403156	0.00999	0.0823786	0.0658334
GO:0048858	cell proje	643	0.3651263	1.2372075	0.00999	0.0823786	0.0658334
GO:0050778	positive r	736	0.3593582	1.2203295	0.00999	0.0823786	0.0658334
GO:0006417	regulation	326	0.3826224	1.274873	0.01	0.0823786	0.0658334
GO:0052547	regulation	405	0.3798973	1.2736208	0.01	0.0823786	0.0658334
GO:0098542	defense re	432	0.3751541	1.2601123	0.01	0.0823786	0.0658334
GO:0001558	regulation	390	0.3759806	1.2581055	0.01001	0.0823786	0.0658334
GO:0070228	regulation	53	0.5121713	1.4757827	0.0100111	0.0823786	0.0658334
GO:0016579	protein de	247	0.3986852	1.3161593	0.0100503	0.0824783	0.0659131
GO:0070085	glycosylat	247	0.3952486	1.3048141	0.0100503	0.0824783	0.0659131
GO:2000242	negative r	49	0.5175678	1.4760603	0.0100559	0.0824783	0.0659131
GO:0046777	protein au	229	0.3973936	1.3092364	0.0100604	0.0824783	0.0659131
GO:0042742	defense re	214	0.4039088	1.3277295	0.0100806	0.0825684	0.0659851
GO:0032890	regulation	55	-0.436111	-1.675319	0.010101	0.082659	0.0660575
GO:0002695	negative r	161	0.4264943	1.3764706	0.0101215	0.0827501	0.0661303
GO:0071901	negative r	126	0.4386721	1.397971	0.0102041	0.083349	0.0666089
GO:0006568	tryptophan	12	-0.716568	-1.895532	0.0102564	0.0833932	0.0666442
GO:0006586	indolalkyl	17	-0.692788	-2.061799	0.0102564	0.0833932	0.0666442
GO:0006707	cholestero	12	-0.712933	-1.885917	0.0102564	0.0833932	0.0666442
GO:0010866	regulation	17	-0.664595	-1.977892	0.0102564	0.0833932	0.0666442
GO:0016127	sterol cat	12	-0.712933	-1.885917	0.0102564	0.0833932	0.0666442
GO:0042026	protein re	31	0.5741964	1.5453375	0.0102857	0.0834934	0.0667243
GO:1902001	fatty acid	16	-0.686021	-2.000102	0.0103093	0.0834934	0.0667243
GO:0048284	organelle	115	0.4492685	1.4164494	0.0103306	0.0834934	0.0667243
GO:1902745	positive r	28	0.587012	1.549693	0.010333	0.0834934	0.0667243
GO:0072583	clathrin-c	39	0.542236	1.5013787	0.0103448	0.0834934	0.0667243
GO:0035376	sterol imp	10	-0.79983	-1.949345	0.0103627	0.0834934	0.0667243
GO:0042758	long-chain	10	-0.822648	-2.004955	0.0103627	0.0834934	0.0667243
GO:0046689	response t	10	-0.788176	-1.92094	0.0103627	0.0834934	0.0667243
GO:0051918	negative r	10	-0.8252	-2.011176	0.0103627	0.0834934	0.0667243
GO:0070508	cholestero	10	-0.79983	-1.949345	0.0103627	0.0834934	0.0667243
GO:0008630	intrinsic	101	0.4557546	1.4187344	0.0104058	0.0836892	0.0668808
GO:0032963	collagen m	101	0.4548709	1.4159837	0.0104058	0.0836892	0.0668808
GO:0009070	serine fam	15	-0.716984	-2.024998	0.010582	0.0847992	0.0677679
GO:0045717	negative r	15	-0.707733	-1.998869	0.010582	0.0847992	0.0677679
GO:0045725	positive r	15	-0.716432	-2.023438	0.010582	0.0847992	0.0677679
GO:0097501	stress res	15	-0.696351	-1.966724	0.010582	0.0847992	0.0677679
GO:0070301	cellular r	88	0.4721577	1.4449734	0.0106045	0.0849025	0.0678504
GO:0019262	N-acetylne	6	0.799368	1.4897021	0.0106242	0.0849299	0.0678723
GO:0001824	blastocyst	90	0.4677261	1.4345082	0.010627	0.0849299	0.0678723
GO:0071260	cellular r	77	0.4870984	1.4765397	0.0107181	0.0852445	0.0681237
GO:0006957	complement	14	-0.741875	-2.02043	0.0107527	0.0852445	0.0681237
GO:0010676	positive r	56	-0.448509	-1.731407	0.0107527	0.0852445	0.0681237
GO:0017001	antibiotic	56	-0.414761	-1.601129	0.0107527	0.0852445	0.0681237
GO:0034380	high-densi	14	-0.704706	-1.919204	0.0107527	0.0852445	0.0681237
GO:0044241	lipid dige	19	-0.644208	-1.972573	0.0107527	0.0852445	0.0681237
GO:0071385	cellular r	56	-0.410975	-1.586514	0.0107527	0.0852445	0.0681237
GO:0090181	regulation	56	-0.53915	-2.081318	0.0107527	0.0852445	0.0681237
GO:0090208	positive r	19	-0.609981	-1.867771	0.0107527	0.0852445	0.0681237

GO:0050871	positive r	70	0.4899442	1.4717135	0.010846	0.0858311	0.0685925
GO:1902030	regulation	70	0.4853326	1.4578611	0.010846	0.0858311	0.0685925
GO:2000779	regulation	66	0.4922382	1.4664535	0.0108578	0.0858478	0.0686059
GO:0031998	regulation	18	-0.652399	-1.937804	0.010929	0.0863339	0.0689943
GO:0098657	import int	801	0.3546558	1.2050905	0.010989	0.086436	0.0690759
GO:0022604	regulation	463	0.3737692	1.2553952	0.011	0.086436	0.0690759
GO:0033674	positive r	542	0.3669741	1.237844	0.011	0.086436	0.0690759
GO:0046513	ceramide b	56	0.5064619	1.4746388	0.0110011	0.086436	0.0690759
GO:0008544	epidermis	389	0.3774394	1.2625268	0.011011	0.086436	0.0690759
GO:0002523	leukocyte	14	0.6705683	1.525788	0.0110294	0.086436	0.0690759
GO:0006596	polyamine	13	0.6902622	1.5439071	0.0110294	0.086436	0.0690759
GO:0010759	positive r	14	0.6737285	1.5329786	0.0110294	0.086436	0.0690759
GO:0030852	regulation	14	0.669158	1.522579	0.0110294	0.086436	0.0690759
GO:0099024	plasma mem	55	0.5100786	1.4805111	0.0110742	0.0867105	0.0692953
GO:0070673	response t	10	0.7641875	1.6066449	0.0111248	0.0870304	0.0695509
GO:0051988	regulation	12	0.7218238	1.5844084	0.0111524	0.0871222	0.0696243
GO:0008360	regulation	143	0.4328627	1.3881836	0.0111562	0.0871222	0.0696243
GO:0010970	transport	156	0.4266837	1.3742647	0.0111789	0.0871461	0.0696434
GO:0099111	microtubul	156	0.4266837	1.3742647	0.0111789	0.0871461	0.0696434
GO:0034614	cellular r	153	0.4282592	1.3759782	0.0111902	0.0871583	0.0696531
GO:0018210	peptidyl-t	125	0.4386852	1.3973962	0.011236	0.0873613	0.0698153
GO:0032675	regulation	130	0.4385655	1.3976519	0.011236	0.0873613	0.0698153
GO:0071635	negative r	9	0.7722544	1.5823841	0.01125	0.087394	0.0698415
GO:0070601	centromeri	11	0.7469924	1.6035601	0.0112923	0.0874722	0.069904
GO:0006071	glycerol m	20	-0.638499	-1.981631	0.0112994	0.0874722	0.069904
GO:1901685	glutathion	20	-0.603014	-1.871502	0.0112994	0.0874722	0.069904
GO:1901687	glutathion	20	-0.603014	-1.871502	0.0112994	0.0874722	0.069904
GO:0051972	regulation	46	0.529036	1.4935256	0.0113122	0.0874949	0.0699222
GO:0014013	regulation	113	0.4546522	1.4308935	0.0113402	0.0876351	0.0700342
GO:0006567	threonine	5	-0.888411	-1.808033	0.0114068	0.0879207	0.0702625
GO:0015747	urate tran	5	-0.90353	-1.838803	0.0114068	0.0879207	0.0702625
GO:0019518	L-threonir	5	-0.888411	-1.808033	0.0114068	0.0879207	0.0702625
GO:0090224	regulation	40	0.5339271	1.4829469	0.0114286	0.0880119	0.0703353
GO:0006409	tRNA expor	32	0.5587784	1.5114052	0.0114548	0.0880123	0.0703356
GO:0071431	tRNA-conta	32	0.5587784	1.5114052	0.0114548	0.0880123	0.0703356
GO:0006415	translatic	102	0.4558332	1.4191029	0.0114583	0.0880123	0.0703356
GO:0032506	cytokineti	39	0.5399067	1.494929	0.0114943	0.0880598	0.0703736
GO:0036003	positive r	29	0.5825126	1.5466987	0.0114943	0.0880598	0.0703736
GO:0090200	positive r	29	0.582427	1.5464712	0.0114943	0.0880598	0.0703736
GO:0005977	glycogen m	65	-0.540189	-2.12561	0.0116279	0.0887777	0.0709473
GO:0015909	long-chair	65	-0.438972	-1.727325	0.0116279	0.0887777	0.0709473
GO:1901607	alpha-amir	62	-0.569689	-2.200425	0.0116279	0.0887777	0.0709473
GO:1903036	positive r	62	-0.410301	-1.584789	0.0116279	0.0887777	0.0709473
GO:0045621	positive r	90	0.4597832	1.4101474	0.0116897	0.0891728	0.071263
GO:0060706	cell diffe	26	0.5827125	1.5081089	0.0117509	0.0895627	0.0715746
GO:0000018	regulation	79	0.4725066	1.4358237	0.0117899	0.0897547	0.0717281
GO:0032753	positive r	23	0.6115645	1.544367	0.0118064	0.0897547	0.0717281
GO:1904376	negative r	23	0.6052495	1.5284199	0.0118064	0.0897547	0.0717281
GO:0019748	secondary	59	-0.497205	-1.903231	0.0119048	0.090194	0.0720792
GO:0046503	glycerolip	61	-0.563221	-2.191271	0.0119048	0.090194	0.0720792
GO:0070988	demethylat	63	-0.476561	-1.831773	0.0119048	0.090194	0.0720792

GO:0071384	cellular r	59	-0.406781	-1.5571	0.0119048	0.090194	0.0720792
GO:0150076	neuroinfla	61	0.4942111	1.4588768	0.0119826	0.0905931	0.0723981
GO:0006897	endocytosi	681	0.3558872	1.2074562	0.011988	0.0905931	0.0723981
GO:0048232	male gamet	490	0.3684708	1.2403754	0.011988	0.0905931	0.0723981
GO:0034248	regulation	382	0.3748122	1.2532998	0.012012	0.0906973	0.0724814
GO:0034976	response t	267	0.395601	1.3069727	0.0120603	0.0909846	0.072711
GO:0001818	negative r	256	0.3944655	1.3026139	0.0120724	0.0909989	0.0727224
GO:0031294	lymphocyte	56	0.5043231	1.4684114	0.0121012	0.0911385	0.072834
GO:0015698	inorganic	162	0.423341	1.3663373	0.0121457	0.0913965	0.0730401
GO:0032635	interleuki	139	0.4327907	1.3869505	0.0121704	0.0915044	0.0731264
GO:0043954	cellular c	53	0.5066944	1.4600014	0.0122358	0.0918131	0.0733731
GO:0030183	B cell dif	126	0.4368199	1.3920684	0.0122449	0.0918131	0.0733731
GO:0038065	collagen-a	13	0.6794852	1.5198024	0.0122549	0.0918131	0.0733731
GO:0051315	attachment	13	0.6821473	1.5257566	0.0122549	0.0918131	0.0733731
GO:0032608	interferon	48	0.5142929	1.4640804	0.0122631	0.0918131	0.0733731
GO:0006301	postreplic	50	0.5135502	1.4656783	0.0122768	0.0918382	0.0733931
GO:0051238	sequesteri	15	0.6645503	1.5270587	0.0123001	0.0918581	0.073409
GO:1902166	negative r	15	0.663565	1.5247946	0.0123001	0.0918581	0.073409
GO:0045727	positive r	118	0.4436971	1.4032794	0.012333	0.0919665	0.0734957
GO:0006073	cellular g	66	-0.535682	-2.098997	0.0123457	0.0919665	0.0734957
GO:0044042	glucan met	66	-0.535682	-2.098997	0.0123457	0.0919665	0.0734957
GO:0071803	positive r	10	0.7563697	1.5902086	0.0123609	0.0919999	0.0735224
GO:0097340	inhibition	12	0.7170012	1.5738227	0.0123916	0.0919999	0.0735224
GO:0097341	zymogen in	12	0.7170012	1.5738227	0.0123916	0.0919999	0.0735224
GO:2000641	regulation	17	0.6560613	1.5399928	0.0123916	0.0919999	0.0735224
GO:0061387	regulation	107	0.4449807	1.3882731	0.012474	0.0925347	0.0739497
GO:0015914	phospholip	70	-0.413925	-1.633765	0.0125	0.0926011	0.0740028
GO:0035088	establishm	45	0.5206822	1.4628823	0.0125142	0.0926011	0.0740028
GO:0061245	establishm	45	0.5206822	1.4628823	0.0125142	0.0926011	0.0740028
GO:0019371	cyclooxyge	11	0.7435201	1.5961062	0.0125471	0.0927668	0.0741352
GO:0050868	negative r	99	0.4541958	1.4099444	0.0125654	0.0927686	0.0741366
GO:0042402	cellular b	21	-0.70272	-2.163331	0.0125786	0.0927686	0.0741366
GO:0051004	regulation	21	-0.710259	-2.186539	0.0125786	0.0927686	0.0741366
GO:0051204	protein in	32	0.5524584	1.4943106	0.0126002	0.0928509	0.0742024
GO:0001893	maternal p	35	0.5469254	1.4903567	0.0126582	0.09297	0.0742976
GO:0006081	cellular a	71	-0.485284	-1.92896	0.0126582	0.09297	0.0742976
GO:0006635	fatty acid	71	-0.721668	-2.868566	0.0126582	0.09297	0.0742976
GO:1901616	organic hy	71	-0.577158	-2.294153	0.0126582	0.09297	0.0742976
GO:0060236	regulation	36	0.538507	1.4721039	0.0126728	0.0930002	0.0743217
GO:0032755	positive r	88	0.4621384	1.4143109	0.0127253	0.0933087	0.0745683
GO:0030854	positive r	7	0.7853479	1.5160434	0.01287	0.0941469	0.0752382
GO:0015740	C4-dicarbo	22	-0.600955	-1.898955	0.0129032	0.0941469	0.0752382
GO:0019400	alditol me	23	-0.613184	-1.970925	0.0129032	0.0941469	0.0752382
GO:0030539	male genit	23	-0.615068	-1.976982	0.0129032	0.0941469	0.0752382
GO:0031639	plasminoge	23	-0.575166	-1.848727	0.0129032	0.0941469	0.0752382
GO:0050995	negative r	23	-0.604144	-1.941869	0.0129032	0.0941469	0.0752382
GO:0006283	transcript	68	0.4840892	1.4516412	0.0129171	0.094171	0.0752574
GO:0007167	enzyme lir	974	0.3446632	1.1766685	0.012987	0.0943709	0.0754171
GO:0071677	positive r	23	0.6036668	1.524423	0.012987	0.0943709	0.0754171
GO:0120039	plasma mem	639	0.3611	1.2234174	0.012987	0.0943709	0.0754171
GO:1904385	cellular r	23	0.59937	1.5135724	0.012987	0.0943709	0.0754171

GO:0007283	spermatoge	473	0.3709189	1.2466082	0.013	0.0943881	0.0754309
GO:0052548	regulation	380	0.3739398	1.2502512	0.013013	0.0944054	0.0754447
GO:0070192	chromosome	59	0.4970582	1.4633551	0.0130719	0.0947552	0.0757243
GO:0006625	protein ta	67	-0.678297	-2.623807	0.0131579	0.0948563	0.0758051
GO:0009064	glutamine	67	-0.530004	-2.050175	0.0131579	0.0948563	0.0758051
GO:0016126	sterol bio	73	-0.494399	-1.973874	0.0131579	0.0948563	0.0758051
GO:0061045	negative r	73	-0.523079	-2.088381	0.0131579	0.0948563	0.0758051
GO:0072662	protein lo	67	-0.678297	-2.623807	0.0131579	0.0948563	0.0758051
GO:0072663	establishm	67	-0.678297	-2.623807	0.0131579	0.0948563	0.0758051
GO:0031345	negative r	171	0.4165531	1.3487995	0.0131712	0.0948563	0.0758051
GO:0071346	cellular r	169	0.4170726	1.3486822	0.0131712	0.0948563	0.0758051
GO:1903305	regulation	156	0.4253219	1.3698785	0.0132114	0.0950029	0.0759222
GO:0006706	steroid ca	26	-0.59273	-1.959915	0.013245	0.0950029	0.0759222
GO:0006730	one-carbon	26	-0.577868	-1.910772	0.013245	0.0950029	0.0759222
GO:0009065	glutamine	26	-0.565153	-1.868729	0.013245	0.0950029	0.0759222
GO:0050996	positive r	24	-0.597377	-1.947688	0.013245	0.0950029	0.0759222
GO:2001300	lipoxin me	6	0.7979642	1.4870859	0.0132802	0.0951017	0.0760012
GO:2001301	lipoxin bi	6	0.7979642	1.4870859	0.0132802	0.0951017	0.0760012
GO:0034101	erythrocyt	113	0.4464852	1.4051901	0.0134021	0.095897	0.0766368
GO:0006904	vesicle do	43	0.5273497	1.4764937	0.0135747	0.0969265	0.0774595
GO:0032070	regulation	10	0.7539854	1.5851959	0.013597	0.0969265	0.0774595
GO:0051291	protein he	99	0.4479648	1.3906016	0.0136126	0.0969265	0.0774595
GO:0007077	mitotic nu	12	0.7097758	1.5579628	0.0136307	0.0969265	0.0774595
GO:1904355	positive r	17	0.6390935	1.5001637	0.0136307	0.0969265	0.0774595
GO:0001782	B cell hom	30	0.5744275	1.5367277	0.0136986	0.0969265	0.0774595
GO:0001844	protein in	30	0.5745295	1.5370005	0.0136986	0.0969265	0.0774595
GO:0002920	regulation	68	-0.570933	-2.220563	0.0136986	0.0969265	0.0774595
GO:0006094	gluconeoge	84	-0.485537	-2.016202	0.0136986	0.0969265	0.0774595
GO:0006695	cholestero	68	-0.490902	-1.909295	0.0136986	0.0969265	0.0774595
GO:0006956	complement	68	-0.633871	-2.465353	0.0136986	0.0969265	0.0774595
GO:0042446	hormone bi	84	-0.375857	-1.560753	0.0136986	0.0969265	0.0774595
GO:0043574	peroxisoma	68	-0.657858	-2.558646	0.0136986	0.0969265	0.0774595
GO:1902653	secondary	68	-0.490902	-1.909295	0.0136986	0.0969265	0.0774595
GO:0032733	positive r	34	0.5438592	1.4763876	0.0138568	0.0979678	0.0782916
GO:0051781	positive r	83	0.4628878	1.4081362	0.0139037	0.0981434	0.078432
GO:1901532	regulation	83	0.463446	1.4098341	0.0139037	0.0981434	0.078432
GO:0000904	cell morph	719	0.3508502	1.1910593	0.013986	0.0986273	0.0788186
GO:0018205	peptidyl-l	366	0.3792587	1.2660162	0.014014	0.0986273	0.0788186
GO:0007492	endoderm d	74	0.4722284	1.4253136	0.0140389	0.0986273	0.0788186
GO:0032945	negative r	67	0.4821104	1.4436688	0.0140389	0.0986273	0.0788186
GO:0050672	negative r	67	0.4821104	1.4436688	0.0140389	0.0986273	0.0788186
GO:1903312	negative r	73	0.4723463	1.4246537	0.0140389	0.0986273	0.0788186
GO:0050663	cytokine s	217	0.3969843	1.3064564	0.0140987	0.0988911	0.0790295
GO:0051607	defense re	217	0.3964709	1.3047668	0.0140987	0.0988911	0.0790295
GO:0006839	mitochondr	222	0.3982135	1.3098878	0.0141129	0.0989128	0.0790468
GO:0002244	hematopoie	161	0.4198582	1.3550534	0.01417	0.0992032	0.0792789
GO:0015807	L-amino ac	64	0.4864674	1.441181	0.0141767	0.0992032	0.0792789
GO:0010977	negative r	143	0.4214813	1.3516837	0.0141988	0.0992637	0.0793272
GO:0045453	bone resor	60	0.4905575	1.4483254	0.0142077	0.0992637	0.0793272
GO:0043433	negative r	153	0.4229945	1.359063	0.0142421	0.0994263	0.0794572
GO:0045913	positive r	76	-0.397938	-1.583035	0.0142857	0.0996524	0.0796379

GO:0016266	O-glycan p	56	0.5021125	1.4619746	0.0143014	0.0996838	0.079663
GO:2001244	positive r	55	0.505002	1.4657763	0.0143965	0.1002675	0.0801294
GO:0006023	aminoglyca	112	0.444799	1.3986879	0.0144479	0.1005442	0.0803506
GO:0008652	cellular a	77	-0.617174	-2.460431	0.0144928	0.1005442	0.0803506
GO:0045833	negative r	79	-0.400144	-1.609002	0.0144928	0.1005442	0.0803506
GO:0055092	sterol hom	79	-0.555696	-2.234487	0.0144928	0.1005442	0.0803506
GO:1900046	regulation	79	-0.506557	-2.036898	0.0144928	0.1005442	0.0803506
GO:0006538	glutamate	8	-0.763874	-1.753608	0.0145631	0.100895	0.0806309
GO:0002002	regulation	11	-0.687911	-1.755295	0.0146341	0.100895	0.0806309
GO:0002003	angiotensi	11	-0.687911	-1.755295	0.0146341	0.100895	0.0806309
GO:0006068	ethanol ca	11	-0.691454	-1.764335	0.0146341	0.100895	0.0806309
GO:0006702	androgen b	11	-0.713456	-1.820477	0.0146341	0.100895	0.0806309
GO:0009086	methionine	11	-0.701999	-1.791242	0.0146341	0.100895	0.0806309
GO:0034379	very-low-d	11	-0.659499	-1.682797	0.0146341	0.100895	0.0806309
GO:0050667	homocystei	11	-0.699941	-1.78599	0.0146341	0.100895	0.0806309
GO:0001961	positive r	46	0.5193368	1.4661437	0.0147059	0.1009978	0.0807131
GO:0006767	water-solu	81	-0.37453	-1.515061	0.0147059	0.1009978	0.0807131
GO:0030301	cholestero	81	-0.491306	-1.987448	0.0147059	0.1009978	0.0807131
GO:0045923	positive r	34	-0.526321	-1.799773	0.0147059	0.1009978	0.0807131
GO:0050818	regulation	81	-0.509192	-2.059801	0.0147059	0.1009978	0.0807131
GO:0042116	macrophage	87	0.4626298	1.415909	0.0148462	0.1014498	0.0810743
GO:0010873	positive r	9	-0.739027	-1.757201	0.0148515	0.1014498	0.0810743
GO:0030033	microvillu	16	0.6410243	1.4888448	0.0148515	0.1014498	0.0810743
GO:0034447	very-low-d	9	-0.74284	-1.766269	0.0148515	0.1014498	0.0810743
GO:1904478	regulation	9	-0.762631	-1.813325	0.0148515	0.1014498	0.0810743
GO:0042769	DNA damage	38	0.5320417	1.4705513	0.0148571	0.1014498	0.0810743
GO:0006112	energy res	78	-0.490493	-1.967501	0.0149254	0.1014498	0.0810743
GO:0007031	peroxisome	78	-0.634221	-2.544031	0.0149254	0.1014498	0.0810743
GO:0030193	regulation	78	-0.515795	-2.068992	0.0149254	0.1014498	0.0810743
GO:0034308	primary al	78	-0.633699	-2.541938	0.0149254	0.1014498	0.0810743
GO:0042632	cholestero	78	-0.567069	-2.274668	0.0149254	0.1014498	0.0810743
GO:0046889	positive r	78	-0.39964	-1.603063	0.0149254	0.1014498	0.0810743
GO:0043207	response t	848	0.3472816	1.1818311	0.014985	0.1014498	0.0810743
GO:0048812	neuron pro	625	0.3565109	1.2076459	0.014985	0.1014498	0.0810743
GO:0051707	response t	846	0.3484207	1.185504	0.014985	0.1014498	0.0810743
GO:0072594	establishm	515	0.3601504	1.2143832	0.014985	0.1014498	0.0810743
GO:0007431	salivary g	37	0.5313107	1.4562968	0.0149942	0.1014498	0.0810743
GO:0010569	regulation	37	0.5321798	1.458679	0.0149942	0.1014498	0.0810743
GO:0031589	cell-subst	338	0.3839847	1.280629	0.015	0.1014498	0.0810743
GO:0045835	negative r	9	0.7616722	1.5607006	0.015	0.1014498	0.0810743
GO:0032642	regulation	76	0.4656857	1.4121652	0.0150215	0.1015176	0.0811285
GO:0051954	positive r	35	-0.524967	-1.808252	0.0150376	0.1015495	0.0811539
GO:0007162	negative r	254	0.390797	1.2901522	0.0150905	0.1018297	0.0813778
GO:0019217	regulation	85	-0.489137	-2.015006	0.0151515	0.1021635	0.0816447
GO:0006565	L-serine c	5	-0.849536	-1.728917	0.0152091	0.1021644	0.0816454
GO:0009403	toxin bios	5	-0.873544	-1.777776	0.0152091	0.1021644	0.0816454
GO:0034196	acylglycer	5	-0.884585	-1.800246	0.0152091	0.1021644	0.0816454
GO:0034197	triglyceri	5	-0.884585	-1.800246	0.0152091	0.1021644	0.0816454
GO:1905065	positive r	5	-0.887411	-1.805998	0.0152091	0.1021644	0.0816454
GO:0048278	vesicle do	63	0.4875873	1.4421863	0.0152505	0.1023652	0.0818059
GO:0045123	cellular e	58	0.4932697	1.4469428	0.0152838	0.102434	0.0818608

GO:1904377	positive r	62	0.487673	1.4396115	0.0152838	0.102434	0.0818608
GO:0051494	negative r	134	0.4294863	1.3717036	0.0153218	0.1025566	0.0819588
GO:0032727	positive r	22	0.6063819	1.523366	0.0153483	0.1025566	0.0819588
GO:0048103	somatic st	22	0.6062676	1.5230789	0.0153483	0.1025566	0.0819588
GO:0090189	regulation	22	0.6063823	1.5233671	0.0153483	0.1025566	0.0819588
GO:0042178	xenobiotic	12	-0.690551	-1.82671	0.0153846	0.1026347	0.0820212
GO:0098856	intestinal	17	-0.626305	-1.863938	0.0153846	0.1026347	0.0820212
GO:0045649	regulation	21	0.613132	1.519998	0.0154211	0.1026347	0.0820212
GO:0051683	establishm	7	0.7822015	1.5099697	0.015444	0.1026347	0.0820212
GO:0085020	protein K6	7	0.7810173	1.5076837	0.015444	0.1026347	0.0820212
GO:0006534	cysteine m	16	-0.681343	-1.986463	0.0154639	0.1026347	0.0820212
GO:0060192	negative r	16	-0.665506	-1.94029	0.0154639	0.1026347	0.0820212
GO:0070875	positive r	16	-0.659352	-1.922348	0.0154639	0.1026347	0.0820212
GO:0072488	ammonium t	16	-0.664521	-1.937417	0.0154639	0.1026347	0.0820212
GO:0006569	tryptophan	10	-0.741611	-1.807453	0.015544	0.1027062	0.0820784
GO:0034378	chylomicro	10	-0.727798	-1.773788	0.015544	0.1027062	0.0820784
GO:0042436	indole-con	10	-0.741611	-1.807453	0.015544	0.1027062	0.0820784
GO:0046218	indolalkyl	10	-0.741611	-1.807453	0.015544	0.1027062	0.0820784
GO:0046322	negative r	10	-0.746723	-1.819912	0.015544	0.1027062	0.0820784
GO:0046439	L-cysteine	10	-0.754519	-1.838911	0.015544	0.1027062	0.0820784
GO:0097006	regulation	82	-0.438784	-1.768118	0.015625	0.1031645	0.0824446
GO:1905953	negative r	40	-0.522737	-1.821056	0.015748	0.1038854	0.0830207
GO:0002577	regulation	20	0.6189935	1.5149769	0.0157576	0.1038854	0.0830207
GO:0014009	glial cell	44	0.528397	1.4822335	0.015873	0.1042463	0.0833092
GO:0030299	intestinal	15	-0.655301	-1.850783	0.015873	0.1042463	0.0833092
GO:0035089	establishm	18	0.6348537	1.5146848	0.015873	0.1042463	0.0833092
GO:0046321	positive r	15	-0.664453	-1.876633	0.015873	0.1042463	0.0833092
GO:0090174	organelle	93	0.4516546	1.390588	0.015873	0.1042463	0.0833092
GO:0035987	endodermal	45	0.5173364	1.4534819	0.0159272	0.1042463	0.0833092
GO:0033623	regulation	13	0.6706471	1.5000342	0.0159314	0.1042463	0.0833092
GO:0035723	interleuki	13	0.6702518	1.49915	0.0159314	0.1042463	0.0833092
GO:0071350	cellular r	13	0.6702518	1.49915	0.0159314	0.1042463	0.0833092
GO:0006914	autophagy	470	0.3671625	1.2339761	0.015984	0.1042463	0.0833092
GO:0009607	response t	879	0.3438654	1.1713288	0.015984	0.1042463	0.0833092
GO:0043604	amide bios	730	0.3492774	1.1860447	0.015984	0.1042463	0.0833092
GO:0061919	process ut	470	0.3671625	1.2339761	0.015984	0.1042463	0.0833092
GO:0070571	negative r	15	0.6507088	1.4952526	0.0159902	0.1042463	0.0833092
GO:0061097	regulation	82	0.4556465	1.387504	0.0159915	0.1042463	0.0833092
GO:0002250	adaptive i	385	0.3732128	1.2490994	0.016	0.1042463	0.0833092
GO:0090559	regulation	77	0.4709028	1.427446	0.0160772	0.1044625	0.0834819
GO:0140029	exocytic p	77	0.4678247	1.4181154	0.0160772	0.1044625	0.0834819
GO:0007076	mitotic ch	16	0.6401454	1.4868035	0.0160891	0.1044625	0.0834819
GO:0019985	translesio	39	0.5320142	1.4730759	0.016092	0.1044625	0.0834819
GO:0032743	positive r	29	0.5719781	1.5187273	0.016092	0.1044625	0.0834819
GO:0051031	tRNA trans	34	0.5424339	1.4725184	0.0161663	0.1048684	0.0838063
GO:0060789	hair folli	5	0.8359047	1.5086137	0.0162382	0.1052579	0.0841175
GO:0003073	regulation	89	-0.377724	-1.570139	0.0163934	0.1059553	0.0846749
GO:0043255	regulation	90	-0.372578	-1.567511	0.0163934	0.1059553	0.0846749
GO:0051289	protein ho	90	-0.358982	-1.510307	0.0163934	0.1059553	0.0846749
GO:1901570	fatty acid	89	-0.334596	-1.390863	0.0163934	0.1059553	0.0846749
GO:0032528	microvillu	24	0.5893149	1.5023126	0.0164512	0.1061744	0.08485

GO:0061217	regulation	25	0.5802072	1.4925243	0.0164512	0.1061744	0.08485
GO:0031295	T cell cos	55	0.4955202	1.4382551	0.0166113	0.1069966	0.085507
GO:0090183	regulation	55	0.4942441	1.4345511	0.0166113	0.1069966	0.085507
GO:0038095	Fc-epsilon	109	0.4475835	1.3999265	0.0166147	0.1069966	0.085507
GO:0061517	macrophage	7	0.7768056	1.4995534	0.016731	0.1075894	0.0859808
GO:0061518	microglial	7	0.7768056	1.4995534	0.016731	0.1075894	0.0859808
GO:0032648	regulation	47	0.5125887	1.4553588	0.0167598	0.1076964	0.0860663
GO:0019319	hexose bio	87	-0.483134	-1.978904	0.0169492	0.108756	0.0869131
GO:1903035	negative r	87	-0.436582	-1.788226	0.0169492	0.108756	0.0869131
GO:0061564	axon devel	495	0.3592298	1.2094829	0.016983	0.1088947	0.0870239
GO:0051090	regulation	406	0.3704704	1.242016	0.017	0.108925	0.0870482
GO:0045814	negative r	85	0.4556437	1.3899464	0.017094	0.109346	0.0873846
GO:0034504	protein lo	249	0.3882293	1.2814324	0.0171026	0.109346	0.0873846
GO:0043406	positive r	244	0.3872425	1.2772847	0.0171026	0.109346	0.0873846
GO:0048588	developmen	215	0.3968526	1.3049637	0.0171371	0.1094877	0.0874978
GO:0002673	regulation	91	-0.517457	-2.198754	0.0172414	0.1098381	0.0877778
GO:0043648	dicarboxyl	91	-0.574975	-2.443156	0.0172414	0.1098381	0.0877778
GO:0044070	regulation	91	-0.372758	-1.583906	0.0172414	0.1098381	0.0877778
GO:0072376	protein ac	91	-0.676842	-2.876004	0.0172414	0.1098381	0.0877778
GO:1901224	positive r	74	0.4679283	1.4123348	0.0172786	0.1099876	0.0878973
GO:0045005	DNA-depend	37	0.5288701	1.4496073	0.017301	0.1099876	0.0878973
GO:0030397	membrane d	16	0.6367541	1.4789268	0.0173267	0.1099876	0.0878973
GO:0051081	nuclear en	16	0.6367541	1.4789268	0.0173267	0.1099876	0.0878973
GO:0051444	negative r	16	0.6371106	1.4797549	0.0173267	0.1099876	0.0878973
GO:0031338	regulation	27	0.5763934	1.5095729	0.0173611	0.1101272	0.0880089
GO:0046782	regulation	63	0.4831744	1.4291338	0.0174292	0.1103413	0.08818
GO:0002634	regulation	9	0.7492553	1.5352578	0.0175	0.1103413	0.08818
GO:0046602	regulation	9	0.7497023	1.5361738	0.0175	0.1103413	0.08818
GO:0010822	positive r	113	0.4390077	1.3816568	0.0175258	0.1103413	0.08818
GO:0001523	retinoid m	92	-0.610495	-2.602602	0.0175439	0.1103413	0.08818
GO:0006835	dicarboxyl	93	-0.332233	-1.410657	0.0175439	0.1103413	0.08818
GO:0015908	fatty acid	92	-0.456099	-1.944393	0.0175439	0.1103413	0.08818
GO:0015918	sterol tra	93	-0.435228	-1.847971	0.0175439	0.1103413	0.08818
GO:0044264	cellular p	93	-0.402407	-1.708614	0.0175439	0.1103413	0.08818
GO:0046364	monosaccha	93	-0.443187	-1.881764	0.0175439	0.1103413	0.08818
GO:0051591	response t	92	-0.452132	-1.927483	0.0175439	0.1103413	0.08818
GO:0090277	positive r	93	-0.358783	-1.523387	0.0175439	0.1103413	0.08818
GO:0060054	positive r	8	0.7668325	1.5300947	0.0175879	0.1105403	0.088339
GO:1901222	regulation	101	0.4402713	1.370536	0.0176899	0.110871	0.0886033
GO:0042226	interleuki	23	0.5922058	1.4954809	0.0177096	0.110871	0.0886033
GO:1903078	positive r	55	0.4935511	1.4325396	0.0177187	0.110871	0.0886033
GO:0006566	threonine	7	-0.770733	-1.742677	0.0177778	0.110871	0.0886033
GO:0019530	taurine me	7	-0.817849	-1.849209	0.0177778	0.110871	0.0886033
GO:0019694	alkanesulf	7	-0.817849	-1.849209	0.0177778	0.110871	0.0886033
GO:0030300	regulation	7	-0.78873	-1.783369	0.0177778	0.110871	0.0886033
GO:0032489	regulation	7	-0.79324	-1.793568	0.0177778	0.110871	0.0886033
GO:0035356	cellular t	7	-0.799172	-1.806979	0.0177778	0.110871	0.0886033
GO:0097052	L-kynureni	7	-0.813405	-1.839161	0.0177778	0.110871	0.0886033
GO:1904729	regulation	7	-0.78873	-1.783369	0.0177778	0.110871	0.0886033
GO:0034446	substrate	99	0.4449023	1.381095	0.017801	0.1109382	0.088657
GO:0051153	regulation	95	0.446075	1.3789863	0.0178197	0.1109767	0.0886878

GO:0070201	regulation	703	0.3475521	1.180338	0.017982	0.1118981	0.0894241
GO:0010506	regulation	309	0.3772561	1.2560193	0.018	0.1118981	0.0894241
GO:1904124	microglial	7	0.7726661	1.4915623	0.018018	0.1118981	0.0894241
GO:1904139	regulation	7	0.7726661	1.4915623	0.018018	0.1118981	0.0894241
GO:0016073	snRNA meta	89	0.4505444	1.3786854	0.0180659	0.112117	0.089599
GO:0006672	ceramide m	85	0.4539252	1.3847042	0.0181624	0.1125587	0.089952
GO:0045639	positive r	85	0.4543639	1.3860425	0.0181624	0.1125587	0.089952
GO:0046931	pore compl	20	0.6148661	1.5048751	0.0181818	0.1125811	0.0899699
GO:0045582	positive r	79	0.463814	1.4094094	0.0182208	0.1125811	0.0899699
GO:0016241	regulation	164	0.4128604	1.3323902	0.0182371	0.1125811	0.0899699
GO:0050770	regulation	178	0.408594	1.3237648	0.0182741	0.1125811	0.0899699
GO:0031641	regulation	41	0.5209119	1.4490504	0.0182857	0.1125811	0.0899699
GO:0071622	regulation	41	0.522295	1.452898	0.0182857	0.1125811	0.0899699
GO:0097028	dendritic	41	0.5232204	1.4554721	0.0182857	0.1125811	0.0899699
GO:0098927	vesicle-me	40	0.5172154	1.4365311	0.0182857	0.1125811	0.0899699
GO:0035967	cellular r	152	0.4169823	1.339639	0.0182927	0.1125811	0.0899699
GO:0051100	negative r	156	0.4183919	1.3475583	0.0182927	0.1125811	0.0899699
GO:0090231	regulation	14	0.6454186	1.4685633	0.0183824	0.1126515	0.0900262
GO:0090266	regulation	14	0.6454186	1.4685633	0.0183824	0.1126515	0.0900262
GO:1903504	regulation	14	0.6454186	1.4685633	0.0183824	0.1126515	0.0900262
GO:1904994	regulation	19	0.6247267	1.5041268	0.0183824	0.1126515	0.0900262
GO:1905516	positive r	13	0.6648945	1.4871672	0.0183824	0.1126515	0.0900262
GO:0050715	positive r	127	0.426722	1.3598544	0.0183861	0.1126515	0.0900262
GO:0046467	membrane l	132	0.4223029	1.3469781	0.0184049	0.1126515	0.0900262
GO:0044766	multi-orga	71	0.4697905	1.4129325	0.0184182	0.1126515	0.0900262
GO:1902579	multi-orga	71	0.4697905	1.4129325	0.0184182	0.1126515	0.0900262
GO:0007292	female gam	120	0.4303388	1.365034	0.0184426	0.1127232	0.0900835
GO:0042136	neurotrans	94	-0.318246	-1.350128	0.0185185	0.1129862	0.0902937
GO:0060191	regulation	94	-0.38742	-1.643592	0.0185185	0.1129862	0.0902937
GO:0002562	somatic di	58	0.4864423	1.4269156	0.018559	0.1129862	0.0902937
GO:0016444	somatic ce	58	0.4864423	1.4269156	0.018559	0.1129862	0.0902937
GO:0060135	maternal p	62	0.4819288	1.4226544	0.018559	0.1129862	0.0902937
GO:0030889	negative r	16	0.6315762	1.4669006	0.0185644	0.1129862	0.0902937
GO:1903902	positive r	60	0.4872615	1.4385943	0.0185792	0.1129862	0.0902937
GO:0015816	glycine tr	12	0.70157	1.5399512	0.0185874	0.1129862	0.0902937
GO:0050853	B cell rec	57	0.4937425	1.441914	0.0186404	0.1132309	0.0904892
GO:0003044	regulation	47	-0.430284	-1.568937	0.0186916	0.1133721	0.0906021
GO:0044273	sulfur com	49	-0.407943	-1.517873	0.0186916	0.1133721	0.0906021
GO:2000401	regulation	56	0.4872716	1.4187631	0.0187019	0.1133721	0.0906021
GO:0030311	poly-N-ace	9	0.7438932	1.5242707	0.01875	0.113509	0.0907115
GO:2000278	regulation	102	0.4387791	1.3660101	0.01875	0.113509	0.0907115
GO:0006821	chloride t	104	0.4355951	1.3583782	0.0187696	0.11355	0.0907443
GO:0046348	amino suga	11	0.7203912	1.5464556	0.0188206	0.1137813	0.0909291
GO:0033625	positive r	8	0.7633383	1.5231225	0.0188442	0.1138221	0.0909617
GO:0036315	cellular r	21	-0.557316	-1.715701	0.0188679	0.1138221	0.0909617
GO:0016321	female mei	5	0.8284343	1.4951313	0.0189445	0.1138221	0.0909617
GO:0032077	positive r	5	0.8262422	1.4911751	0.0189445	0.1138221	0.0909617
GO:0072708	response t	5	0.8271386	1.4927929	0.0189445	0.1138221	0.0909617
GO:1902219	negative r	5	0.8306789	1.4991822	0.0189445	0.1138221	0.0909617
GO:0007035	vacuolar a	21	0.6067241	1.5041125	0.0189798	0.1138221	0.0909617
GO:0019054	modulation	21	0.6044662	1.498515	0.0189798	0.1138221	0.0909617

GO:0000165	MAPK casca	881	0.3417572	1.1639703	0.018981	0.1138221	0.0909617
GO:0002253	activation	572	0.355558	1.2014776	0.018981	0.1138221	0.0909617
GO:0090066	regulation	478	0.3637687	1.2244242	0.018981	0.1138221	0.0909617
GO:1905114	cell surfa	589	0.357174	1.2084645	0.018981	0.1138221	0.0909617
GO:0010976	positive r	262	0.3843298	1.2714096	0.0190381	0.1140675	0.0911578
GO:0030148	sphingolip	93	0.4445412	1.3686868	0.0190476	0.1140675	0.0911578
GO:0006906	vesicle fu	87	0.4485716	1.3728828	0.019088	0.1141556	0.0912282
GO:0070126	mitochondr	88	0.4504599	1.3785703	0.019088	0.1141556	0.0912282
GO:0003333	amino acic	82	0.4527862	1.3787941	0.0191898	0.1146869	0.0916528
GO:0031047	gene siler	156	0.4165362	1.3415815	0.0193089	0.1153216	0.09216
GO:0009067	aspartate	22	-0.528442	-1.669821	0.0193548	0.1153631	0.0921932
GO:0010226	response t	22	-0.552659	-1.746345	0.0193548	0.1153631	0.0921932
GO:0042359	vitamin D	22	-0.542129	-1.713071	0.0193548	0.1153631	0.0921932
GO:1902904	negative r	127	0.423408	1.3492937	0.0194076	0.1155998	0.0923824
GO:1903322	positive r	124	0.424748	1.3527632	0.0194274	0.1156405	0.0924149
GO:0010872	regulation	11	-0.649492	-1.657263	0.0195122	0.1160675	0.0927561
GO:0008033	tRNA proce	118	0.4272049	1.3511197	0.0195272	0.1160793	0.0927655
GO:0007213	G protein-	19	0.6231545	1.5003416	0.0196078	0.1164027	0.093024
GO:0030968	endoplasmi	116	0.4299762	1.3574495	0.0196078	0.1164027	0.093024
GO:0070198	protein lc	27	0.5734284	1.5018074	0.0196759	0.1167289	0.0932847
GO:0006024	glycosamir	108	0.4374265	1.3669772	0.0197095	0.1168504	0.0933818
GO:0030309	poly-N-ace	10	0.7399377	1.5556616	0.0197775	0.1171751	0.0936413
GO:0048535	lymph node	17	0.6326142	1.4849547	0.0198265	0.1173873	0.0938108
GO:0003071	renal syst	25	-0.503728	-1.650088	0.0198675	0.1173956	0.0938174
GO:0005979	regulation	26	-0.513799	-1.698922	0.0198675	0.1173956	0.0938174
GO:0010962	regulation	26	-0.513799	-1.698922	0.0198675	0.1173956	0.0938174
GO:0043525	positive r	54	0.4882366	1.4131156	0.0199778	0.1179033	0.0942232
GO:0016049	cell growt	453	0.3656777	1.2283935	0.01998	0.1179033	0.0942232
GO:0097709	connective	9	0.7427869	1.5220039	0.02	0.1179429	0.0942549
GO:0045408	regulation	22	0.5993584	1.5057214	0.0200708	0.1181063	0.0943854
GO:0015744	succinate	6	-0.851562	-1.839759	0.0200803	0.1181063	0.0943854
GO:0071422	succinate	6	-0.851562	-1.839759	0.0200803	0.1181063	0.0943854
GO:0009120	deoxyribor	8	0.7515588	1.4996184	0.0201005	0.1181063	0.0943854
GO:0033129	positive r	8	0.7526464	1.5017885	0.0201005	0.1181063	0.0943854
GO:2001238	positive r	49	0.5068671	1.4455429	0.0201117	0.1181063	0.0943854
GO:0045927	positive r	245	0.3863183	1.2741483	0.0201207	0.1181063	0.0943854
GO:0045069	regulation	87	0.444498	1.3604153	0.0201485	0.1181131	0.0943908
GO:0070125	mitochondr	87	0.4456715	1.364007	0.0201485	0.1181131	0.0943908
GO:0007338	single fer	137	0.4157189	1.331858	0.0203046	0.1189138	0.0950308
GO:0002224	toll-like	141	0.4158342	1.3313527	0.0203252	0.1189138	0.0950308
GO:0010212	response t	138	0.4170093	1.3356211	0.0203252	0.1189138	0.0950308
GO:0042268	regulation	17	-0.603457	-1.795941	0.0205128	0.1199325	0.0958449
GO:1905446	regulation	7	0.7694727	1.4853979	0.020592	0.1203164	0.0961517
GO:0043252	sodium-inc	16	-0.625037	-1.822302	0.0206186	0.1203923	0.0962123
GO:0044319	wound heal	28	0.5595747	1.4772594	0.0206659	0.1204314	0.0962435
GO:0090505	epiboly ir	28	0.5595747	1.4772594	0.0206659	0.1204314	0.0962435
GO:0150077	regulation	28	0.5686551	1.5012314	0.0206659	0.1204314	0.0962435
GO:0032000	positive r	10	-0.704119	-1.716076	0.0207254	0.1206199	0.0963942
GO:2000121	regulation	10	-0.699362	-1.704482	0.0207254	0.1206199	0.0963942
GO:0045780	positive r	18	0.6229495	1.4862828	0.020757	0.1206459	0.096415
GO:0046852	positive r	18	0.6229495	1.4862828	0.020757	0.1206459	0.096415

GO:0006641triglyceri	97	-0.681659	-2.89398	0.0208333	0.1206948	0.0964541
GO:0015837amine tran	95	-0.331355	-1.396554	0.0208333	0.1206948	0.0964541
GO:0018958phenol-con	96	-0.388678	-1.640881	0.0208333	0.1206948	0.0964541
GO:0022600digestive	95	-0.336788	-1.419455	0.0208333	0.1206948	0.0964541
GO:0062014negative r	95	-0.420086	-1.770529	0.0208333	0.1206948	0.0964541
GO:0030004cellular m	104	0.4352185	1.3572038	0.0208551	0.120742	0.0964917
GO:0070268cornificat	100	0.4349837	1.3530347	0.0208768	0.1207893	0.0965296
GO:0002903negative r	12	0.6923471	1.5197067	0.0210657	0.1215017	0.0970989
GO:2000010positive r	17	0.62987	1.4785132	0.0210657	0.1215017	0.0970989
GO:2000726negative r	17	0.6296194	1.4779249	0.0210657	0.1215017	0.0970989
GO:0060760positive r	53	0.493895	1.4231208	0.0211346	0.1215017	0.0970989
GO:0070555response t	196	0.3978604	1.3036053	0.021148	0.1215017	0.0970989
GO:1900739regulation	26	0.5669802	1.4673922	0.0211516	0.1215017	0.0970989
GO:1900740positive r	26	0.5669802	1.4673922	0.0211516	0.1215017	0.0970989
GO:0034433steroid es	15	-0.641329	-1.811324	0.021164	0.1215017	0.0970989
GO:0034434sterol est	15	-0.641329	-1.811324	0.021164	0.1215017	0.0970989
GO:0034435cholestero	15	-0.641329	-1.811324	0.021164	0.1215017	0.0970989
GO:0043650dicarboxyl	15	-0.604886	-1.708396	0.021164	0.1215017	0.0970989
GO:0070365hepatocyte	15	-0.625529	-1.766697	0.021164	0.1215017	0.0970989
GO:0072698protein lo	50	0.5001188	1.4273449	0.0212054	0.1216393	0.0972089
GO:0000731DNA synthe	49	0.5024195	1.4328588	0.0212291	0.1216393	0.0972089
GO:0061900glial cell	49	0.5051226	1.4405677	0.0212291	0.1216393	0.0972089
GO:0006665sphingolip	148	0.4139941	1.3306954	0.0212551	0.1216764	0.0972385
GO:0006637acyl-CoA m	99	-0.538202	-2.312102	0.0212766	0.1216764	0.0972385
GO:0035383thioester	99	-0.538202	-2.312102	0.0212766	0.1216764	0.0972385
GO:2000678negative r	21	0.6000053	1.487456	0.0213523	0.1220309	0.0975218
GO:1903533regulation	77	0.4587768	1.3906883	0.0214362	0.1224317	0.0978421
GO:0007494midgut dev	13	-0.655353	-1.749779	0.0215054	0.1225903	0.0979689
GO:0055081anion home	56	-0.36251	-1.399422	0.0215054	0.1225903	0.0979689
GO:0060080inhibitory	14	-0.632549	-1.722688	0.0215054	0.1225903	0.0979689
GO:0016925protein su	75	0.4588306	1.386235	0.0215285	0.1226436	0.0980114
GO:1904141positive r	5	0.8214877	1.4825943	0.0216509	0.1232616	0.0985053
GO:0030890positive r	41	0.5157669	1.4347384	0.0217143	0.1235434	0.0987306
GO:0045940positive r	27	-0.571088	-1.876071	0.0217391	0.1236057	0.0987803
GO:0032647regulation	28	0.5587375	1.4750491	0.021814	0.1239521	0.0990571
GO:0051973positive r	36	0.5288526	1.445712	0.0218894	0.124301	0.099336
GO:0060338regulation	34	0.5359263	1.4548525	0.02194	0.1245085	0.0995018
GO:0032201telomere m	27	0.5655611	1.4812032	0.0219907	0.1247053	0.0996591
GO:0006470protein de	306	0.3748756	1.2480197	0.022022	0.1247053	0.0996591
GO:0009112nucleobase	34	-0.508746	-1.739673	0.0220588	0.1247053	0.0996591
GO:0035563positive r	13	0.6586116	1.4731144	0.0220588	0.1247053	0.0996591
GO:0035791platelet-d	14	0.6418297	1.460397	0.0220588	0.1247053	0.0996591
GO:0061577calcium io	13	0.659218	1.4744707	0.0220588	0.1247053	0.0996591
GO:0009062fatty acid	105	-0.759553	-3.281203	0.0222222	0.1253061	0.1001392
GO:0016101diterpenoi	98	-0.611305	-2.583987	0.0222222	0.1253061	0.1001392
GO:0019395fatty acid	98	-0.718745	-3.038135	0.0222222	0.1253061	0.1001392
GO:0060059embryonic	7	-0.761459	-1.721709	0.0222222	0.1253061	0.1001392
GO:0038063collagen-a	10	0.7307447	1.5363341	0.0222497	0.1253061	0.1001392
GO:0070099regulation	10	0.7358031	1.546969	0.0222497	0.1253061	0.1001392
GO:0030308negative r	174	0.4037152	1.3086717	0.0222672	0.1253253	0.1001546
GO:0031581hemidesmos	12	0.6833117	1.4998739	0.0223048	0.1253783	0.1001969

GO:0150078 positive r	12	0.6860931	1.5059791	0.0223048	0.1253783	0.1001969
GO:1902475 L-alpha-am	50	0.4962289	1.4162429	0.0223214	0.1253922	0.1002081
GO:0060147 regulation	82	0.4479467	1.3640572	0.0223881	0.1256078	0.1003803
GO:0060966 regulation	82	0.4479467	1.3640572	0.0223881	0.1256078	0.1003803
GO:0097577 sequesteri	6	0.79011	1.4724489	0.0225764	0.1265052	0.1010974
GO:0009713 catechol-c	20	-0.561498	-1.742652	0.0225989	0.1265052	0.1010974
GO:0042423 catecholam	20	-0.561498	-1.742652	0.0225989	0.1265052	0.1010974
GO:0032272 negative r	68	0.4686537	1.4053546	0.022605	0.1265052	0.1010974
GO:0006690 icosanoid	100	-0.503348	-2.159929	0.0227273	0.1267905	0.1013255
GO:0015748 organophos	100	-0.374923	-1.60884	0.0227273	0.1267905	0.1013255
GO:0032368 regulation	100	-0.37777	-1.621059	0.0227273	0.1267905	0.1013255
GO:0033559 unsaturate	100	-0.496624	-2.131075	0.0227273	0.1267905	0.1013255
GO:0034440 lipid oxid	100	-0.707169	-3.034549	0.0227273	0.1267905	0.1013255
GO:0006353 DNA-templa	71	0.4666104	1.4033679	0.0227519	0.1268482	0.1013716
GO:0044107 cellular a	5	-0.829518	-1.688179	0.0228137	0.1269538	0.101456
GO:0044108 cellular a	5	-0.829518	-1.688179	0.0228137	0.1269538	0.101456
GO:0070141 response t	5	-0.83029	-1.689748	0.0228137	0.1269538	0.101456
GO:0032609 interferon	106	0.42961	1.3388771	0.0229406	0.12758	0.1019564
GO:1905448 positive r	5	0.818843	1.4778212	0.0230041	0.1278337	0.1021592
GO:0051154 negative r	35	0.53385	1.4547268	0.023015	0.1278337	0.1021592
GO:0051348 negative r	255	0.3853383	1.272205	0.0231388	0.1283519	0.1025733
GO:0035655 interleuki	7	0.7632239	1.473335	0.023166	0.1283519	0.1025733
GO:0071351 cellular r	7	0.7632239	1.473335	0.023166	0.1283519	0.1025733
GO:1903026 negative r	7	0.7632722	1.4734283	0.023166	0.1283519	0.1025733
GO:0043011 myeloid de	18	0.6216906	1.483279	0.023199	0.1283692	0.1025871
GO:0001676 long-chain	103	-0.598507	-2.568453	0.0232558	0.1283692	0.1025871
GO:0005976 polysaccha	104	-0.35959	-1.54147	0.0232558	0.1283692	0.1025871
GO:0010906 regulation	106	-0.451992	-1.961832	0.0232558	0.1283692	0.1025871
GO:0051187 cofactor c	58	-0.395596	-1.516568	0.0232558	0.1283692	0.1025871
GO:1901606 alpha-amin	103	-0.766667	-3.290101	0.0232558	0.1283692	0.1025871
GO:0001771 immunologi	14	0.640343	1.4570144	0.0232843	0.1284467	0.1026491
GO:0001706 endoderm f	50	0.4948774	1.4123859	0.0234375	0.1292116	0.1032603
GO:1903862 positive r	10	0.727746	1.5300295	0.0234858	0.1293975	0.1034089
GO:0032509 endosome t	25	0.5730729	1.4741719	0.0235018	0.1294053	0.1034151
GO:0010561 negative r	12	0.6779657	1.4881394	0.023544	0.1295575	0.1035368
GO:0050892 intestinal	38	-0.49729	-1.724015	0.023622	0.1299066	0.1038158
GO:0019079 viral geno	113	0.4294976	1.3517263	0.0237113	0.1303171	0.1041437
GO:0006911 phagocytos	46	0.509994	1.4397678	0.0237557	0.1304533	0.1042526
GO:0007588 excretion	61	-0.376609	-1.465238	0.0238095	0.1304533	0.1042526
GO:0046686 response t	59	-0.389129	-1.489532	0.0238095	0.1304533	0.1042526
GO:0071549 cellular r	30	-0.500205	-1.619181	0.0238095	0.1304533	0.1042526
GO:1990748 cellular d	102	-0.343113	-1.480043	0.0238095	0.1304533	0.1042526
GO:0072089 stem cell	109	0.4308547	1.3476031	0.0238837	0.1304853	0.1042782
GO:0001957 intramembr	6	0.7862581	1.4652705	0.0239044	0.1304853	0.1042782
GO:0036072 direct oss	6	0.7862581	1.4652705	0.0239044	0.1304853	0.1042782
GO:0042088 T-helper 1	42	0.5092695	1.4181467	0.0239726	0.1304853	0.1042782
GO:0023014 signal tra	892	0.3390071	1.1546223	0.023976	0.1304853	0.1042782
GO:0001774 microglial	41	0.5148229	1.4321123	0.024	0.1304853	0.1042782
GO:0002269 leukocyte	41	0.5148229	1.4321123	0.024	0.1304853	0.1042782
GO:0071902 positive r	314	0.3743425	1.2468008	0.024	0.1304853	0.1042782
GO:0014910 regulation	58	0.4790746	1.4053034	0.0240175	0.1304853	0.1042782

GO:0070059	intrinsic	58	0.4796169	1.4068942	0.0240175	0.1304853	0.1042782
GO:0045660	positive r	348	0.3731307	1.2443317	0.024024	0.1304853	0.1042782
GO:1905710	positive r	60	0.4798837	1.416812	0.0240437	0.1304853	0.1042782
GO:0006842	tricarboxy	6	-0.822871	-1.777774	0.0240964	0.1304853	0.1042782
GO:0009744	response t	6	-0.811497	-1.753201	0.0240964	0.1304853	0.1042782
GO:0015746	citrate tr	6	-0.822871	-1.777774	0.0240964	0.1304853	0.1042782
GO:0015942	formate me	6	-0.826457	-1.78552	0.0240964	0.1304853	0.1042782
GO:0034285	response t	6	-0.811497	-1.753201	0.0240964	0.1304853	0.1042782
GO:0051005	negative r	6	-0.838775	-1.812132	0.0240964	0.1304853	0.1042782
GO:0007009	plasma mem	95	0.4365667	1.3495924	0.024109	0.1304853	0.1042782
GO:0032649	regulation	95	0.4360412	1.3479681	0.024109	0.1304853	0.1042782
GO:1902742	apoptotic	33	0.5355928	1.4510269	0.0241935	0.1308631	0.1045801
GO:0006469	negative r	211	0.3907492	1.2844666	0.024218	0.1309155	0.104622
GO:0009750	response t	8	-0.739578	-1.697831	0.0242718	0.1309677	0.1046637
GO:0051715	cytolysis	8	-0.743311	-1.706401	0.0242718	0.1309677	0.1046637
GO:0072530	purine-con	8	-0.732876	-1.682447	0.0242718	0.1309677	0.1046637
GO:0014050	negative r	5	0.8160918	1.472856	0.0243572	0.1312083	0.104856
GO:1901216	positive r	91	0.4404121	1.3523759	0.0243644	0.1312083	0.104856
GO:0046849	bone remod	88	0.4405133	1.34813	0.0243902	0.1312083	0.104856
GO:0060337	type I int	88	0.4403256	1.3475557	0.0243902	0.1312083	0.104856
GO:0071357	cellular r	88	0.4403256	1.3475557	0.0243902	0.1312083	0.104856
GO:0051170	import int	154	0.408975	1.3147028	0.0244151	0.1312623	0.1048992
GO:0035524	proline tr	7	0.7629212	1.4727508	0.024453	0.1313076	0.1049353
GO:1990001	inhibition	7	0.7618233	1.4706314	0.024453	0.1313076	0.1049353
GO:0002381	immunoglob	52	0.494519	1.4198779	0.0244716	0.1313282	0.1049518
GO:0045143	homologous	50	0.4938025	1.4093182	0.0245536	0.1316089	0.1051761
GO:0061098	positive r	50	0.4937214	1.4090865	0.0245536	0.1316089	0.1051761
GO:0035195	gene silen	120	0.4216869	1.3375902	0.0245902	0.1317256	0.1052694
GO:0002335	mature B c	25	0.5707074	1.4680871	0.0246769	0.1321103	0.1055769
GO:1900180	regulation	114	0.4261155	1.3437239	0.0247423	0.1323557	0.105773
GO:0014051	gamma-amin	9	-0.710304	-1.688907	0.0247525	0.1323557	0.105773
GO:0035561	regulation	22	0.5785446	1.4534325	0.0247934	0.1324152	0.1058205
GO:0050857	positive r	22	0.5814656	1.4607706	0.0247934	0.1324152	0.1058205
GO:0006289	nucleotide	102	0.4305176	1.3402902	0.025	0.1334384	0.1066382
GO:0030218	erythrocyt	105	0.4304264	1.3413037	0.0250784	0.1337144	0.1068587
GO:0006026	aminoglyca	64	0.4692721	1.3902392	0.0250818	0.1337144	0.1068587
GO:0050869	negative r	30	0.5509436	1.4739027	0.0251142	0.1338067	0.1069325
GO:0031663	lipopolysa	57	0.4764693	1.3914697	0.0252193	0.1342863	0.1073158
GO:0007219	Notch sign	181	0.401016	1.3019537	0.0252781	0.1344937	0.1074815
GO:0032615	interleuki	56	0.4807256	1.3997036	0.0253025	0.1344937	0.1074815
GO:1990138	neuron pro	162	0.4073567	1.3147478	0.0253036	0.1344937	0.1074815
GO:1902275	regulation	165	0.4048263	1.3065841	0.0253293	0.1345495	0.1075261
GO:0007520	myoblast f	37	0.5226928	1.4326756	0.0253749	0.1346617	0.1076158
GO:0021915	neural tub	157	0.4097009	1.3205939	0.0253807	0.1346617	0.1076158
GO:0006111	regulation	43	-0.467746	-1.659152	0.0254237	0.1348095	0.1077339
GO:0046415	urate meta	12	-0.625464	-1.654535	0.025641	0.1357188	0.1084605
GO:0060333	interferon	85	0.4458974	1.3602154	0.025641	0.1357188	0.1084605
GO:1990090	cellular r	48	0.5004296	1.4246145	0.025641	0.1357188	0.1084605
GO:1903795	regulation	7	0.7563411	1.4600484	0.02574	0.1361617	0.1088145
GO:0046500	S-adenosyl	16	-0.609357	-1.776586	0.0257732	0.136256	0.1088899
GO:0046902	regulation	68	0.4662515	1.3981511	0.0258342	0.1364975	0.1090829

GO:0032482	Rab protei	67	0.4645057	1.3909519	0.0259179	0.1368584	0.1093713
GO:1902165	regulation	17	0.6199451	1.455216	0.0260223	0.1373279	0.1097465
GO:0050771	negative r	66	0.4645072	1.3838384	0.0260586	0.137438	0.1098345
GO:0007064	mitotic si	21	0.5936191	1.4716242	0.0260973	0.1374786	0.109867
GO:0043032	positive r	21	0.5951314	1.4753733	0.0260973	0.1374786	0.109867
GO:0006721	terpenoid	108	-0.628394	-2.748539	0.0263158	0.1385476	0.1107213
GO:0031915	positive r	11	0.7066151	1.5168827	0.0263488	0.1386393	0.1107946
GO:0035666	TRIF-deper	28	0.5502529	1.4526503	0.0264064	0.1387782	0.1109055
GO:2000406	positive r	28	0.5485195	1.448074	0.0264064	0.1387782	0.1109055
GO:0006000	fructose m	15	-0.593359	-1.675841	0.026455	0.1388073	0.1109288
GO:0030730	sequesteri	15	-0.561114	-1.584771	0.026455	0.1388073	0.1109288
GO:0003081	regulation	25	-0.458415	-1.501655	0.0264901	0.1388073	0.1109288
GO:0006760	folic acic	25	-0.4578	-1.49964	0.0264901	0.1388073	0.1109288
GO:0098810	neurotrans	26	-0.451742	-1.493726	0.0264901	0.1388073	0.1109288
GO:0045541	negative r	5	-0.824869	-1.678717	0.026616	0.1392208	0.1112592
GO:0090206	negative r	5	-0.824869	-1.678717	0.026616	0.1392208	0.1112592
GO:0106119	negative r	5	-0.824869	-1.678717	0.026616	0.1392208	0.1112592
GO:0010332	response t	53	0.4845633	1.3962322	0.0266963	0.1394769	0.1114639
GO:0043113	receptor c	53	0.4843093	1.3955004	0.0266963	0.1394769	0.1114639
GO:0010818	T cell che	26	0.5612797	1.4526389	0.027027	0.1411217	0.1127784
GO:0019730	antimicrob	103	0.4280939	1.3336061	0.0271116	0.1414801	0.1130647
GO:0090305	nucleic ac	267	0.3743096	1.2366309	0.0271357	0.1415228	0.1130989
GO:0032612	interleuki	98	0.4319658	1.3412429	0.0271682	0.1416041	0.1131639
GO:0060046	regulation	16	0.6216439	1.4438317	0.0272277	0.1416041	0.1131639
GO:2000369	regulation	16	0.6202853	1.4406763	0.0272277	0.1416041	0.1131639
GO:0009612	response t	204	0.3912143	1.2842361	0.0272452	0.1416041	0.1131639
GO:0060219	camera-typ	17	0.6186797	1.4522458	0.0272615	0.1416041	0.1131639
GO:0090026	positive r	17	0.6192077	1.4534851	0.0272615	0.1416041	0.1131639
GO:0014909	smooth mus	64	0.4652779	1.3784062	0.0272628	0.1416041	0.1131639
GO:0010812	negative r	60	0.4734033	1.3976793	0.0273224	0.1418307	0.113345
GO:0045577	regulation	28	0.5482843	1.4474531	0.0275545	0.1428727	0.1141777
GO:0006284	base-excis	39	0.5141262	1.4235464	0.0275862	0.1428727	0.1141777
GO:0017145	stem cell	39	0.5160137	1.4287727	0.0275862	0.1428727	0.1141777
GO:0070254	mucus secr	11	0.7027629	1.5086131	0.0276035	0.1428727	0.1141777
GO:1905941	positive r	11	0.7047795	1.5129421	0.0276035	0.1428727	0.1141777
GO:0042542	response t	133	0.4118135	1.3137697	0.0276639	0.1431021	0.114361
GO:2000403	positive r	34	0.5287318	1.435322	0.0277136	0.1432757	0.1144998
GO:0090383	phagosome	27	0.5543182	1.4517581	0.0277778	0.1435239	0.1146981
GO:0031498	chromatin	19	0.6024769	1.450557	0.0281863	0.1455499	0.1163172
GO:0035425	autocrine	7	0.7517671	1.4512187	0.028314	0.1460399	0.1167088
GO:0035771	interleuki	7	0.7524769	1.4525889	0.028314	0.1460399	0.1167088
GO:0035794	positive r	58	0.4739676	1.3903227	0.0283843	0.14613	0.1167808
GO:0046794	transport	58	0.4751117	1.3936789	0.0283843	0.14613	0.1167808
GO:0030321	transepith	5	0.8088086	1.4597114	0.0284168	0.14613	0.1167808
GO:0090666	scaRNA loc	5	0.8059759	1.4545991	0.0284168	0.14613	0.1167808
GO:1901164	negative r	5	0.8090792	1.4601999	0.0284168	0.14613	0.1167808
GO:0006183	GTP biosyr	10	0.7213509	1.5165843	0.0284302	0.14613	0.1167808
GO:0097320	plasma mem	21	0.5884305	1.4587613	0.0284698	0.1461645	0.1168083
GO:1903077	negative r	21	0.5900818	1.4628549	0.0284698	0.1461645	0.1168083
GO:0060670	branching	12	0.6731073	1.4774753	0.0285006	0.1462385	0.1168674
GO:0071236	cellular r	135	0.4093757	1.3093779	0.0285714	0.1465033	0.1170791

GO:0031333negative r	127	0.4136595	1.3182275	0.0286006	0.1465033	0.1170791
GO:0034340response t	91	0.4349222	1.3355179	0.0286017	0.1465033	0.1170791
GO:0075733intracellu	55	0.4785245	1.3889248	0.0287929	0.1473978	0.1177939
GO:0072611interleuki	11	0.6986375	1.4997572	0.0288582	0.1476471	0.1179932
GO:0042107cytokine m	112	0.4236344	1.3321348	0.0288958	0.1477541	0.1180787
GO:0006855drug trans	79	-0.327236	-1.315837	0.0289855	0.1480316	0.1183004
GO:1905954positive r	79	-0.331325	-1.332279	0.0289855	0.1480316	0.1183004
GO:0032147activatior	321	0.3717668	1.2378439	0.029	0.1480316	0.1183004
GO:0031529ruffle org	49	0.4919481	1.4029951	0.0290503	0.1482031	0.1184375
GO:0048050post-embry	6	0.7792484	1.4522072	0.0292165	0.1489654	0.1190467
GO:0072311glomerular	18	0.6038917	1.4408131	0.029304	0.1493262	0.119335
GO:0060669embryonic	25	0.5627743	1.4476798	0.0293772	0.1494467	0.1194314
GO:0006805xenobiotic	111	-0.72516	-3.164441	0.0294118	0.1494467	0.1194314
GO:0043217myelin mai	14	0.6294982	1.4323384	0.0294118	0.1494467	0.1194314
GO:0070613regulatior	111	-0.438574	-1.913843	0.0294118	0.1494467	0.1194314
GO:1903830magnesium	14	0.6293325	1.4319615	0.0294118	0.1494467	0.1194314
GO:0061351neural pre	141	0.4101201	1.3130583	0.0294715	0.1496145	0.1195655
GO:0042771intrinsic	44	0.5047028	1.4157677	0.0294785	0.1496145	0.1195655
GO:0060416response t	37	-0.437063	-1.526684	0.0296296	0.1502581	0.1200798
GO:0072010glomerular	21	0.5848489	1.4498821	0.029656	0.1502581	0.1200798
GO:1904666regulatior	21	0.5876769	1.456893	0.029656	0.1502581	0.1200798
GO:0002501peptide ar	5	0.8048196	1.4525122	0.02977	0.1506638	0.120404
GO:0035519protein K2	5	0.7990451	1.4420905	0.02977	0.1506638	0.120404
GO:0032652regulatior	86	0.4405617	1.3460016	0.0298507	0.1509867	0.120662
GO:1902110positive r	54	0.4772645	1.3813586	0.0299667	0.1514177	0.1210064
GO:0007409axonogenes	454	0.3584512	1.2040848	0.02997	0.1514177	0.1210064
GO:0045862positive r	333	0.3696217	1.2320287	0.03	0.1514625	0.1210422
GO:0007435salivary g	34	0.5260144	1.4279451	0.0300231	0.1514625	0.1210422
GO:0007204positive r	303	0.3711181	1.2348739	0.03003	0.1514625	0.1210422
GO:0016447somatic re	48	0.4956773	1.4110858	0.0301003	0.1514696	0.121048
GO:1902563regulatior	11	0.6970066	1.4962562	0.0301129	0.1514696	0.121048
GO:0008306associativ	75	0.4472885	1.3513637	0.0301399	0.1514696	0.121048
GO:0032637interleuki	75	0.4470996	1.350793	0.0301399	0.1514696	0.121048
GO:0000055ribosomal	8	0.7344182	1.4654171	0.0301508	0.1514696	0.121048
GO:0048671negative r	8	0.7361486	1.4688698	0.0301508	0.1514696	0.121048
GO:2000643positive r	8	0.7336086	1.4638017	0.0301508	0.1514696	0.121048
GO:0019218regulatior	112	-0.433707	-1.89059	0.030303	0.1518055	0.1213164
GO:0033630positive r	20	0.5924666	1.4500526	0.030303	0.1518055	0.1213164
GO:0045606positive r	20	0.5890272	1.4416348	0.030303	0.1518055	0.1213164
GO:0061162establishm	20	0.5911812	1.4469067	0.030303	0.1518055	0.1213164
GO:1901136carbohydra	182	0.3984788	1.2939516	0.030303	0.1518055	0.1213164
GO:0042311vasodilati	28	-0.484039	-1.573973	0.0305344	0.1527958	0.1221078
GO:0051220cytoplasmi	24	0.5732553	1.4613725	0.0305523	0.1527958	0.1221078
GO:0071624positive r	25	0.5613986	1.444141	0.0305523	0.1527958	0.1221078
GO:0006027glycosamir	60	0.4671281	1.3791522	0.0306011	0.1529538	0.1222341
GO:0009084glutamine	17	-0.566481	-1.685898	0.0307692	0.153449	0.1226298
GO:0010867positive r	12	-0.617269	-1.632858	0.0307692	0.153449	0.1226298
GO:0010889regulatior	12	-0.614903	-1.626598	0.0307692	0.153449	0.1226298
GO:0052695cellular g	12	-0.599334	-1.585414	0.0307692	0.153449	0.1226298
GO:0045687positive r	41	0.5046116	1.403707	0.0308571	0.1538011	0.1229112
GO:2001022positive r	86	0.4401578	1.3447675	0.0309168	0.1539808	0.1230548

GO:0002756	MyD88-inde	32	0.5318757	1.4386377	0.0309278	0.1539808	0.1230548
GO:0016050	vesicle or	307	0.3704841	1.233329	0.031031	0.154408	0.1233962
GO:0002118	aggressive	7	-0.749771	-1.695281	0.0311111	0.1546931	0.1236241
GO:0009211	pyrimidine	5	0.7982246	1.4406097	0.0311231	0.1546931	0.1236241
GO:0006639	acylglycer	117	-0.619946	-2.699194	0.03125	0.1548905	0.1237818
GO:0034754	cellular h	117	-0.586344	-2.55289	0.03125	0.1548905	0.1237818
GO:0050911	detection	113	-0.543528	-2.379656	0.03125	0.1548905	0.1237818
GO:0098754	detoxifica	117	-0.437061	-1.902927	0.03125	0.1548905	0.1237818
GO:1903317	regulation	113	-0.43833	-1.919081	0.03125	0.1548905	0.1237818
GO:0045637	regulation	209	0.3862564	1.2682006	0.0313448	0.1552738	0.1240881
GO:0032071	regulation	8	0.7293045	1.4552135	0.031407	0.1554954	0.1242652
GO:0098781	ncRNA tran	97	0.4290413	1.3288257	0.0314465	0.1555426	0.1243029
GO:0050873	brown fat	41	-0.450916	-1.583277	0.0314961	0.1555426	0.1243029
GO:0070873	regulation	31	-0.511561	-1.682382	0.0314961	0.1555426	0.1243029
GO:0009451	RNA modifi	151	0.4072366	1.3089779	0.0315041	0.1555426	0.1243029
GO:0016482	cytosolic	142	0.4066705	1.3026692	0.0315041	0.1555426	0.1243029
GO:0002931	response t	46	0.4994716	1.4100621	0.0316742	0.1562958	0.1249048
GO:0016114	terpenoid	15	-0.555334	-1.568445	0.031746	0.1564764	0.1250492
GO:0035850	epithelial	44	0.5031813	1.4114998	0.031746	0.1564764	0.1250492
GO:0046633	alpha-beta	31	0.5348772	1.4395174	0.032	0.1574286	0.1258101
GO:0042089	cytokine b	111	0.4211659	1.3227654	0.0320248	0.1574286	0.1258101
GO:0009913	epidermal	286	0.3706461	1.2297591	0.0320963	0.1574286	0.1258101
GO:0000270	peptidogly	6	-0.797126	-1.722153	0.0321285	0.1574286	0.1258101
GO:0009253	peptidogly	6	-0.797126	-1.722153	0.0321285	0.1574286	0.1258101
GO:0015793	glycerol t	6	-0.773509	-1.67113	0.0321285	0.1574286	0.1258101
GO:0045682	regulation	79	0.4432461	1.3469088	0.0321543	0.1574286	0.1258101
GO:0060964	regulation	79	0.4439444	1.3490306	0.0321543	0.1574286	0.1258101
GO:2000542	negative r	7	0.7501149	1.4480292	0.032175	0.1574286	0.1258101
GO:0055075	potassium	29	0.5397008	1.433024	0.0321839	0.1574286	0.1258101
GO:1903321	negative r	80	0.439458	1.3366507	0.0321888	0.1574286	0.1258101
GO:0060391	positive r	12	0.66748	1.4651233	0.0322181	0.1574286	0.1258101
GO:0009109	coenzyme c	14	-0.621849	-1.69355	0.0322581	0.1574286	0.1258101
GO:0009110	vitamin bi	23	-0.492895	-1.584285	0.0322581	0.1574286	0.1258101
GO:0030858	positive r	53	0.4779129	1.3770697	0.0322581	0.1574286	0.1258101
GO:0032373	positive r	22	-0.51935	-1.641092	0.0322581	0.1574286	0.1258101
GO:0032376	positive r	22	-0.51935	-1.641092	0.0322581	0.1574286	0.1258101
GO:0071467	cellular r	23	-0.468982	-1.507426	0.0322581	0.1574286	0.1258101
GO:0032543	mitochondr	132	0.4098642	1.3073035	0.0327198	0.1595945	0.127541
GO:0098876	vesicle-me	87	0.4374003	1.3386923	0.0328738	0.1602576	0.1280709
GO:1901741	positive r	18	0.6011864	1.4343585	0.032967	0.1606239	0.1283637
GO:0030641	regulation	86	0.4373741	1.3362628	0.033049	0.1609352	0.1286125
GO:0035988	chondrocyt	13	0.6453471	1.4434457	0.0330882	0.1610314	0.1286893
GO:0032607	interferon	30	0.5403966	1.4456871	0.033105	0.1610314	0.1286893
GO:2000008	regulation	38	0.5108705	1.4120347	0.0331429	0.1611254	0.1287645
GO:0002761	regulation	110	0.4195537	1.3154261	0.0331606	0.1611254	0.1287645
GO:1903525	regulation	6	0.7759585	1.4460762	0.0332005	0.1612312	0.128849
GO:0032655	regulation	54	0.4718596	1.3657152	0.0332963	0.1614586	0.1290307
GO:0032722	positive r	54	0.4721461	1.3665443	0.0332963	0.1614586	0.1290307
GO:0090504	epiboly	29	0.5379926	1.4284883	0.0333333	0.1614586	0.1290307
GO:0090630	activation	84	0.4397792	1.3359623	0.0333692	0.1614586	0.1290307
GO:1903901	negative r	75	0.4446293	1.3433295	0.0333692	0.1614586	0.1290307

GO:0032941	secretion	35	0.5190677	1.4144452	0.0333717	0.1614586	0.1290307
GO:2000665	regulation	10	0.7078566	1.4882136	0.0333745	0.1614586	0.1290307
GO:0007033	vacuole or	155	0.4023779	1.2944059	0.0335707	0.1623193	0.1297185
GO:2000096	positive r	9	0.7224027	1.4802358	0.03375	0.1630975	0.1303404
GO:0035194	posttransc	129	0.4121622	1.3118644	0.0337769	0.1631386	0.1303733
GO:0006521	regulation	60	0.4651161	1.373212	0.0338798	0.1635468	0.1306995
GO:0042267	natural ki	56	0.4709178	1.3711469	0.0341034	0.164415	0.1313933
GO:1902686	mitochondr	56	0.4723489	1.3753136	0.0341034	0.164415	0.1313933
GO:0072384	organelle	82	0.4333756	1.3196863	0.0341151	0.164415	0.1313933
GO:0006853	carnitine	11	-0.622297	-1.587871	0.0341463	0.1644762	0.1314422
GO:0015911	plasma mem	5	-0.811956	-1.652437	0.0342205	0.1647414	0.1316542
GO:0002407	dendritic	23	0.5727674	1.4463937	0.0342385	0.1647414	0.1316542
GO:0043516	regulation	31	0.5308977	1.4288075	0.0342857	0.1648794	0.1317645
GO:0097062	dendritic	19	0.5945151	1.4313877	0.0343137	0.1649249	0.1318008
GO:0060401	cytosolic	161	0.3981091	1.2848599	0.034413	0.165048	0.1318992
GO:2000095	regulation	15	0.6184771	1.4211879	0.0344403	0.165048	0.1318992
GO:0006638	neutral li	118	-0.618175	-2.676563	0.0344828	0.165048	0.1318992
GO:0046717	acid secre	118	-0.341152	-1.477115	0.0344828	0.165048	0.1318992
GO:0090316	positive r	150	0.4062905	1.3061715	0.0345178	0.165048	0.1318992
GO:0002720	positive r	51	0.4810166	1.3753429	0.0345212	0.165048	0.1318992
GO:1990089	response t	51	0.4801504	1.3728662	0.0345212	0.165048	0.1318992
GO:0008063	Toll signa	6	0.7646432	1.4249889	0.0345286	0.165048	0.1318992
GO:0038145	macrophage	6	0.7697814	1.4345645	0.0345286	0.165048	0.1318992
GO:0061408	positive r	6	0.7733581	1.44123	0.0345286	0.165048	0.1318992
GO:0032651	regulation	74	0.4477844	1.3515352	0.0345572	0.165048	0.1318992
GO:0016233	telomere c	36	0.5171173	1.4136313	0.0345622	0.165048	0.1318992
GO:0010718	positive r	47	0.4954342	1.4066533	0.0346369	0.165227	0.1320422
GO:0070671	response t	47	0.4948873	1.4051004	0.0346369	0.165227	0.1320422
GO:0006972	hyperosmot	27	0.5442284	1.4253328	0.0347222	0.1655453	0.1322966
GO:0045003	double-str	7	0.7470341	1.4420821	0.034749	0.1655843	0.1323278
GO:0071214	cellular r	315	0.3677361	1.2247654	0.035	0.1666015	0.1331407
GO:0104004	cellular r	315	0.3677361	1.2247654	0.035	0.1666015	0.1331407
GO:0032471	negative r	8	0.7252089	1.4470414	0.0351759	0.1672595	0.1336666
GO:0048102	autophagic	8	0.7268556	1.450327	0.0351759	0.1672595	0.1336666
GO:0002691	regulation	26	0.5510671	1.4262079	0.0352526	0.1675349	0.1338866
GO:0031398	positive r	107	0.4215799	1.315266	0.035343	0.1678079	0.1341048
GO:0008334	histone mR	22	0.5719693	1.4369139	0.0354191	0.1678079	0.1341048
GO:0009226	nucleotide	22	0.5725972	1.4384913	0.0354191	0.1678079	0.1341048
GO:0051446	positive r	22	0.5723761	1.4379359	0.0354191	0.1678079	0.1341048
GO:0044003	modificati	40	0.4995069	1.3873469	0.0354286	0.1678079	0.1341048
GO:0090090	negative r	169	0.3996737	1.2924196	0.035461	0.1678079	0.1341048
GO:0002886	regulation	54	0.4711093	1.3635436	0.0355161	0.1678079	0.1341048
GO:0045651	positive r	14	0.6236248	1.4189744	0.0355392	0.1678079	0.1341048
GO:0070234	positive r	14	0.6268994	1.4264253	0.0355392	0.1678079	0.1341048
GO:0043102	amino acid	7	-0.748852	-1.693204	0.0355556	0.1678079	0.1341048
GO:0071265	L-methioni	7	-0.748852	-1.693204	0.0355556	0.1678079	0.1341048
GO:0071267	L-methioni	7	-0.748852	-1.693204	0.0355556	0.1678079	0.1341048
GO:2000192	negative r	7	-0.740581	-1.674501	0.0355556	0.1678079	0.1341048
GO:0045071	negative r	51	0.4786018	1.3684383	0.0356347	0.1680923	0.1343321
GO:1903978	regulation	15	0.6159237	1.4153206	0.0356704	0.1681711	0.134395
GO:0043030	regulation	49	0.4832325	1.3781389	0.0357542	0.1683937	0.134573

GO:0043029	T cell hom	37	0.5103942	1.3989657	0.0357555	0.1683937	0.134573
GO:0010984	regulation	17	-0.534212	-1.589861	0.0358974	0.1689727	0.1350357
GO:0002455	spliceosom	58	0.4628705	1.3577709	0.0360262	0.1693994	0.1353767
GO:1902108	regulation	58	0.4626669	1.3571736	0.0360262	0.1693994	0.1353767
GO:0000209	protein po	294	0.3680023	1.2215993	0.0360721	0.1694361	0.135406
GO:0031098	stress-act	291	0.3700609	1.2287791	0.0360721	0.1694361	0.135406
GO:0034329	cell junct	237	0.3769752	1.2425001	0.0362538	0.1701993	0.1360159
GO:0009204	deoxyribon	8	0.724115	1.4448587	0.0364322	0.1709465	0.136613
GO:0034109	homotypic	80	0.4359257	1.3259066	0.0364807	0.1710237	0.1366748
GO:0010831	positive r	30	0.5263567	1.408127	0.0365297	0.1710237	0.1366748
GO:0045197	establishm	42	0.4959509	1.3810586	0.0365297	0.1710237	0.1366748
GO:0042138	meiotic DN	5	0.7941501	1.4332563	0.0365359	0.1710237	0.1366748
GO:0006360	transcript	55	0.4673038	1.3563565	0.0365449	0.1710237	0.1366748
GO:1990823	response t	95	0.4241987	1.3113583	0.0366876	0.1713309	0.1369202
GO:1990830	cellular r	95	0.4241987	1.3113583	0.0366876	0.1713309	0.1369202
GO:0002763	positive r	52	0.4771824	1.3701006	0.0367075	0.1713309	0.1369202
GO:0097345	mitochondr	52	0.4779883	1.3724145	0.0367075	0.1713309	0.1369202
GO:0032677	regulation	67	0.4543132	1.3604307	0.0367171	0.1713309	0.1369202
GO:0030149	sphingolip	28	0.5377086	1.4195335	0.0367394	0.1713309	0.1369202
GO:0033008	positive r	14	0.6211327	1.4133039	0.0367647	0.1713309	0.1369202
GO:0043306	positive r	14	0.6211327	1.4133039	0.0367647	0.1713309	0.1369202
GO:0006369	terminatic	35	0.5158968	1.4058047	0.0368239	0.1714271	0.1369971
GO:0032735	positive r	35	0.5138646	1.4002669	0.0368239	0.1714271	0.1369971
GO:0048678	response t	70	0.4507622	1.3540172	0.0368764	0.1715812	0.1371203
GO:0009314	response t	419	0.3601076	1.2079406	0.036963	0.1717892	0.1372865
GO:0009894	regulation	927	0.3328907	1.1351056	0.036963	0.1717892	0.1372865
GO:0002576	platelet c	123	-0.366232	-1.614583	0.037037	0.1717892	0.1372865
GO:0007586	digestion	128	-0.322348	-1.4633	0.037037	0.1717892	0.1372865
GO:0072329	monocarbox	128	-0.767642	-3.484717	0.037037	0.1717892	0.1372865
GO:1905476	negative r	27	0.537498	1.407706	0.037037	0.1717892	0.1372865
GO:0043967	histone H4	65	0.4559645	1.3527929	0.0371179	0.1720745	0.1375145
GO:0043414	macromolec	272	0.371132	1.2268745	0.0371486	0.172127	0.1375564
GO:0051955	regulation	33	-0.48922	-1.659575	0.0373134	0.1728007	0.1380948
GO:0050707	regulation	188	0.3923419	1.2777109	0.0373737	0.1729896	0.1382458
GO:0032611	interleuki	85	0.4317815	1.3171545	0.0373932	0.1729896	0.1382458
GO:0006144	purine nuc	19	-0.534261	-1.635915	0.0376344	0.1740151	0.1390653
GO:0042476	odontogene	126	0.4075857	1.2989039	0.0377551	0.1744791	0.1394362
GO:2000050	regulation	23	0.567597	1.433337	0.0377804	0.1744791	0.1394362
GO:0016441	posttransc	130	0.4081511	1.3007251	0.0377937	0.1744791	0.1394362
GO:0010721	negative r	315	0.3652234	1.2163967	0.038	0.1753406	0.1401246
GO:0046653	tetrahydrc	18	-0.55585	-1.651028	0.0382514	0.1763441	0.1409266
GO:0031146	SCF-depenc	90	0.4269894	1.3095694	0.0382572	0.1763441	0.1409266
GO:0034350	regulation	10	0.7011156	1.4740411	0.0383189	0.1765372	0.1410808
GO:0045116	protein ne	16	0.6095808	1.4158139	0.0383663	0.1766641	0.1411823
GO:0042737	drug catab	133	-0.630567	-2.874831	0.0384615	0.1769191	0.1413861
GO:1902373	negative r	46	0.4930583	1.3919568	0.0384615	0.1769191	0.1413861
GO:0072174	metanephri	6	0.7630093	1.4219441	0.0385126	0.1770624	0.1415006
GO:0051195	negative r	22	-0.475088	-1.501227	0.0387097	0.1778764	0.1421511
GO:0006228	UTP biosyr	9	0.7108286	1.4565198	0.03875	0.1779696	0.1422256
GO:0033013	tetrapyrrc	53	-0.36997	-1.410152	0.038835	0.1779911	0.1422427
GO:0030260	entry intc	132	0.4050321	1.2918911	0.0388548	0.1779911	0.1422427

GO:0044409	entry into	132	0.4050321	1.2918911	0.0388548	0.1779911	0.1422427
GO:0051806	entry into	132	0.4050321	1.2918911	0.0388548	0.1779911	0.1422427
GO:0051828	entry into	132	0.4050321	1.2918911	0.0388548	0.1779911	0.1422427
GO:0019042	viral late	11	0.6877207	1.4763223	0.0388959	0.1780425	0.1422838
GO:1902369	negative r	52	0.47483	1.3633463	0.0389321	0.1780425	0.1422838
GO:0021873	forebrain	8	0.7224405	1.4415175	0.0389447	0.1780425	0.1422838
GO:0006298	mismatch r	32	0.5222068	1.4124849	0.0389462	0.1780425	0.1422838
GO:0006968	cellular d	51	0.47741	1.3650306	0.0389755	0.178085	0.1423178
GO:0038083	peptidyl-t	37	0.5045521	1.3829527	0.0392157	0.1788376	0.1429193
GO:0002023	reduction	5	0.7912501	1.4280225	0.0392422	0.1788376	0.1429193
GO:0006561	proline bi	5	0.7929527	1.4310952	0.0392422	0.1788376	0.1429193
GO:0055129	L-proline	5	0.7929527	1.4310952	0.0392422	0.1788376	0.1429193
GO:1903626	positive r	5	0.7918426	1.4290918	0.0392422	0.1788376	0.1429193
GO:0033280	response t	34	0.5164621	1.4020139	0.039261	0.1788376	0.1429193
GO:0042558	pteridine-	31	-0.453017	-1.489848	0.0393701	0.179151	0.1431697
GO:0071548	response t	40	-0.437219	-1.523138	0.0393701	0.179151	0.1431697
GO:1901028	regulation	43	0.4943904	1.3842128	0.0395928	0.1798469	0.1437259
GO:0009142	nucleoside	158	0.3987526	1.285296	0.0395939	0.1798469	0.1437259
GO:0015851	nucleobase	9	-0.686177	-1.631539	0.039604	0.1798469	0.1437259
GO:0046498	S-adenosyl	9	-0.682751	-1.623393	0.039604	0.1798469	0.1437259
GO:0008543	fibroblast	104	0.4201733	1.3102863	0.0396246	0.1798488	0.1437273
GO:0036314	response t	30	-0.451719	-1.462229	0.0396825	0.1800198	0.143864
GO:0006691	leukotrien	26	-0.439902	-1.454576	0.0397351	0.1800744	0.1439076
GO:0008211	glucocorti	25	-0.435795	-1.427557	0.0397351	0.1800744	0.1439076
GO:0060711	labyrinthi	45	0.4922027	1.3828676	0.039818	0.1803551	0.144132
GO:1902230	negative r	30	0.5227839	1.398569	0.0399543	0.1803551	0.144132
GO:0031329	regulation	820	0.3345356	1.1366755	0.03996	0.1803551	0.144132
GO:0009063	cellular a	121	-0.758353	-3.304728	0.04	0.1803551	0.144132
GO:0032324	molybdopte	7	-0.734481	-1.660709	0.04	0.1803551	0.144132
GO:0043545	molybdopte	7	-0.734481	-1.660709	0.04	0.1803551	0.144132
GO:0051189	prosthetic	7	-0.734481	-1.660709	0.04	0.1803551	0.144132
GO:0055088	lipid home	129	-0.534755	-2.433004	0.04	0.1803551	0.144132
GO:1903825	organic ac	129	-0.317966	-1.446664	0.04	0.1803551	0.144132
GO:1905039	carboxylic	129	-0.317966	-1.446664	0.04	0.1803551	0.144132
GO:0030516	regulation	94	0.4234573	1.3061054	0.0400844	0.180644	0.1443629
GO:0002740	negative r	6	-0.766989	-1.657042	0.0401606	0.180725	0.1444276
GO:0014052	regulation	6	-0.757471	-1.636481	0.0401606	0.180725	0.1444276
GO:0051298	centrosome	66	0.4544107	1.3537595	0.0401737	0.180725	0.1444276
GO:0031297	replicatio	28	0.5341749	1.4102047	0.0401837	0.180725	0.1444276
GO:0031111	negative r	35	0.5095919	1.388624	0.0402762	0.1809838	0.1446344
GO:0006643	membrane l	195	0.3855569	1.2620545	0.040282	0.1809838	0.1446344
GO:0000470	maturation	21	0.5765751	1.4293709	0.0403321	0.1810262	0.1446683
GO:0032469	endoplasmi	21	0.575918	1.427742	0.0403321	0.1810262	0.1446683
GO:0050731	positive r	183	0.394702	1.2828257	0.0403633	0.1810744	0.1447068
GO:0097152	mesenchyma	13	0.6398173	1.4310773	0.0404412	0.1813324	0.144913
GO:0006735	NADH regen	27	0.535164	1.4015933	0.0405093	0.1813631	0.1449375
GO:0061621	canonical	27	0.535164	1.4015933	0.0405093	0.1813631	0.1449375
GO:0061718	glucose ca	27	0.535164	1.4015933	0.0405093	0.1813631	0.1449375
GO:0060766	negative r	15	0.6125413	1.4075482	0.0405904	0.1816349	0.1451548
GO:0042035	regulation	102	0.4197837	1.3068735	0.040625	0.1816982	0.1452053
GO:0098661	inorganic	106	0.4170069	1.2995995	0.0406674	0.1817962	0.1452836

GO:0035307	positive r	43	0.4932303	1.3809649	0.040724	0.1819578	0.1454128
GO:0043506	regulation	80	0.4325544	1.3156526	0.0407725	0.1820832	0.145513
GO:0002286	T cell act	99	0.4226554	1.3120348	0.0408377	0.1822826	0.1456723
GO:0042368	vitamin D	12	-0.591245	-1.564016	0.0410256	0.1829377	0.1461959
GO:1902600	proton tra	128	0.4076903	1.2975809	0.0410256	0.1829377	0.1461959
GO:0034330	cell junct	283	0.3673474	1.2178634	0.0411234	0.1831847	0.1463932
GO:1903959	regulation	26	0.5455959	1.4120479	0.0411281	0.1831847	0.1463932
GO:0071526	semaphorin	40	0.4936665	1.3711257	0.0411429	0.1831847	0.1463932
GO:0045746	negative r	39	0.4992438	1.382339	0.0413793	0.1841452	0.1471609
GO:0002679	respirator	11	0.6801426	1.4600544	0.0414053	0.1841685	0.1471795
GO:0072531	pyrimidine	10	-0.661014	-1.611022	0.0414508	0.1842155	0.147217
GO:0048563	post-embry	8	0.7203135	1.4372734	0.0414573	0.1842155	0.147217
GO:0001889	liver deve	132	-0.304154	-1.382603	0.0416667	0.1849609	0.1478127
GO:0002544	chronic in	19	0.5866367	1.4124191	0.0416667	0.1849609	0.1478127
GO:0046822	regulation	104	0.4164477	1.2986682	0.0417101	0.1850613	0.147893
GO:0032911	negative r	5	0.7855634	1.4177592	0.0419486	0.1857903	0.1484756
GO:0090306	spindle as	5	0.7886286	1.4232912	0.0419486	0.1857903	0.1484756
GO:1905671	regulation	5	0.7848945	1.416552	0.0419486	0.1857903	0.1484756
GO:0043009	chordate e	601	0.3434264	1.1629447	0.041958	0.1857903	0.1484756
GO:0051056	regulation	321	0.3655974	1.2173021	0.042	0.1858835	0.14855
GO:0061615	glycolytic	28	0.5326947	1.4062972	0.0424799	0.1875807	0.1499063
GO:0061620	glycolytic	28	0.5326947	1.4062972	0.0424799	0.1875807	0.1499063
GO:0010359	regulation	9	0.707617	1.4499391	0.0425	0.1875807	0.1499063
GO:1904667	negative r	9	0.705883	1.4463862	0.0425	0.1875807	0.1499063
GO:0032092	positive r	89	0.4278296	1.3091772	0.042508	0.1875807	0.1499063
GO:0048771	tissue rem	161	0.3951823	1.275414	0.0425101	0.1875807	0.1499063
GO:0110110	positive r	78	0.432898	1.3157498	0.0427807	0.1886811	0.1507858
GO:0034637	cellular c	80	-0.343598	-1.38601	0.0428571	0.1888363	0.1509098
GO:0002313	mature B c	19	0.5849831	1.408438	0.0428922	0.1888363	0.1509098
GO:0034138	toll-like	19	0.5847365	1.4078443	0.0428922	0.1888363	0.1509098
GO:0044344	cellular r	131	0.403384	1.2868781	0.0429009	0.1888363	0.1509098
GO:0001837	epithelial	132	0.403245	1.2861909	0.0429448	0.1889358	0.1509893
GO:0009411	response t	133	0.4030243	1.2857303	0.0430328	0.1892293	0.1512238
GO:0045187	regulation	17	0.6006501	1.4099245	0.0433705	0.1900597	0.1518874
GO:0010675	regulation	131	-0.39397	-1.767925	0.0434783	0.1900597	0.1518874
GO:0033865	nucleoside	130	-0.512093	-2.32322	0.0434783	0.1900597	0.1518874
GO:0033875	ribonucleo	130	-0.512093	-2.32322	0.0434783	0.1900597	0.1518874
GO:0034032	purine nuc	130	-0.512093	-2.32322	0.0434783	0.1900597	0.1518874
GO:0035296	regulation	134	-0.303357	-1.385858	0.0434783	0.1900597	0.1518874
GO:0042157	lipoprotei	125	-0.317634	-1.392572	0.0434783	0.1900597	0.1518874
GO:0046683	response t	127	-0.356842	-1.569422	0.0434783	0.1900597	0.1518874
GO:0050880	regulation	134	-0.303357	-1.385858	0.0434783	0.1900597	0.1518874
GO:0061041	regulation	134	-0.383355	-1.751319	0.0434783	0.1900597	0.1518874
GO:0097746	regulation	134	-0.303357	-1.385858	0.0434783	0.1900597	0.1518874
GO:1905952	regulation	130	-0.361436	-1.639733	0.0434783	0.1900597	0.1518874
GO:1905268	negative r	47	0.4837051	1.3733517	0.0435754	0.1901629	0.1519699
GO:0006664	glycolipid	107	0.4124964	1.286927	0.043659	0.1901629	0.1519699
GO:0010762	regulation	29	0.5299378	1.407101	0.0436782	0.1901629	0.1519699
GO:0001993	regulation	8	-0.702289	-1.612228	0.0436893	0.1901629	0.1519699
GO:0006107	oxaloaceta	8	-0.67461	-1.548686	0.0436893	0.1901629	0.1519699
GO:0015739	sialic aci	8	-0.702946	-1.613736	0.0436893	0.1901629	0.1519699

GO:1901033	positive r	8	-0.695647	-1.596981	0.0436893	0.1901629	0.1519699
GO:0009206	purine rib	142	0.4017657	1.2869578	0.0436992	0.1901629	0.1519699
GO:0001991	regulation	18	-0.540409	-1.605163	0.0437158	0.1901629	0.1519699
GO:1902884	positive r	18	-0.518151	-1.53905	0.0437158	0.1901629	0.1519699
GO:0061635	regulation	9	0.7014745	1.4373529	0.04375	0.190171	0.1519764
GO:0001147	actin cort	6	0.7585603	1.4136529	0.0438247	0.190171	0.1519764
GO:0002326	B cell lin	6	0.7603878	1.4170586	0.0438247	0.190171	0.1519764
GO:0044396	actin cort	6	0.7585603	1.4136529	0.0438247	0.190171	0.1519764
GO:1903980	positive r	6	0.7596618	1.4157057	0.0438247	0.190171	0.1519764
GO:0040008	regulation	632	0.3410204	1.1552602	0.043956	0.1905548	0.1522831
GO:1902532	negative r	480	0.3492253	1.1754836	0.043956	0.1905548	0.1522831
GO:0042535	positive r	19	0.5843204	1.4068425	0.0441176	0.1911621	0.1527685
GO:0071918	urea trans	6	-0.745982	-1.611659	0.0441767	0.1912316	0.152824
GO:1901374	acetate es	6	-0.751489	-1.623555	0.0441767	0.1912316	0.152824
GO:0090502	RNA phosph	67	0.4507997	1.3499094	0.0442765	0.19157	0.1530945
GO:0009812	flavonoid	7	-0.723575	-1.636049	0.0444444	0.1921098	0.1535258
GO:0010890	positive r	7	-0.724755	-1.638717	0.0444444	0.1921098	0.1535258
GO:0031397	negative r	70	0.4442917	1.3345809	0.0444685	0.1921181	0.1535325
GO:2000574	regulation	10	0.6917946	1.4544445	0.0444994	0.1921181	0.1535325
GO:0010824	regulation	42	0.4913637	1.3682849	0.0445205	0.1921181	0.1535325
GO:0009642	response t	9	-0.646299	-1.53672	0.0445545	0.1921181	0.1535325
GO:0051006	positive r	9	-0.639909	-1.521526	0.0445545	0.1921181	0.1535325
GO:0001704	formation	109	0.412304	1.2895811	0.0446521	0.1922715	0.1536551
GO:1902410	mitotic cy	24	0.5537712	1.4117028	0.0446533	0.1922715	0.1536551
GO:1904956	regulation	5	0.7832869	1.4136507	0.0446549	0.1922715	0.1536551
GO:0050860	negative r	20	0.5769629	1.4121077	0.0448485	0.1930114	0.1542463
GO:0042090	interleuki	9	0.6992663	1.4328282	0.045	0.1934761	0.1546177
GO:0045075	regulation	9	0.6992663	1.4328282	0.045	0.1934761	0.1546177
GO:1902455	negative r	7	0.7362114	1.4211899	0.045045	0.1935761	0.1546976
GO:0023035	CD40 signa	8	0.7135315	1.4237409	0.0452261	0.1942603	0.1552444
GO:0006098	pentose-ph	14	0.6135261	1.3959962	0.0453431	0.1943023	0.155278
GO:0010248	establishm	14	0.6126712	1.3940509	0.0453431	0.1943023	0.155278
GO:0090177	establishm	14	0.6124789	1.3936133	0.0453431	0.1943023	0.155278
GO:0006720	isoprenoid	126	-0.589919	-2.586911	0.0454545	0.1943023	0.155278
GO:0006766	vitamin me	126	-0.402776	-1.766253	0.0454545	0.1943023	0.155278
GO:0032355	response t	126	-0.264717	-1.160836	0.0454545	0.1943023	0.155278
GO:0035150	regulation	135	-0.30329	-1.390951	0.0454545	0.1943023	0.155278
GO:0044058	regulation	39	-0.43026	-1.51235	0.0454545	0.1943023	0.155278
GO:0046688	response t	39	-0.435959	-1.532384	0.0454545	0.1943023	0.155278
GO:0061008	hepaticobi	135	-0.300961	-1.380273	0.0454545	0.1943023	0.155278
GO:0007032	endosome o	72	0.4402288	1.3250532	0.0455038	0.194331	0.1553009
GO:0061005	cell diffe	54	0.464305	1.3438496	0.045505	0.194331	0.1553009
GO:0009201	ribonucleo	148	0.3995999	1.2844283	0.0455466	0.1944151	0.1553681
GO:1903038	negative r	110	0.4109333	1.2883986	0.0455959	0.1945321	0.1554616
GO:0016573	histone ac	150	0.3981116	1.2798776	0.0456853	0.1948201	0.1556918
GO:0045840	positive r	50	0.4780848	1.3644595	0.0457589	0.1950406	0.155868
GO:0070231	T cell apo	47	0.4830228	1.3714144	0.0458101	0.195143	0.1559498
GO:0051156	glucose 6-	24	0.5505586	1.4035131	0.0458284	0.195143	0.1559498
GO:0016075	rRNA catab	17	0.596106	1.3992579	0.0458488	0.195143	0.1559498
GO:0051222	positive r	399	0.3563728	1.194254	0.045954	0.1954972	0.1562329
GO:0043062	extracellu	409	0.3541708	1.1873649	0.046	0.19554	0.1562671

GO:0043932	ossificati	5	0.7807234	1.4090242	0.0460081	0.19554	0.1562671
GO:0010499	proteasoma	22	0.5646958	1.4186414	0.0460449	0.1956026	0.1563171
GO:0034142	toll-like	33	0.5110291	1.3844791	0.0460829	0.1956709	0.1563717
GO:0002583	regulation	6	0.7548607	1.4067583	0.0464807	0.1972657	0.1576462
GO:0019336	phenol-con	10	-0.657251	-1.601851	0.0466321	0.1978137	0.1580841
GO:0050850	positive r	40	0.4906036	1.3626188	0.0468571	0.1985596	0.1586803
GO:0051443	positive r	31	0.5126389	1.3796674	0.0468571	0.1985596	0.1586803
GO:0050810	regulation	86	-0.336685	-1.381492	0.046875	0.1985596	0.1586803
GO:0002228	natural ki	60	0.4557784	1.3456435	0.0469945	0.1989711	0.1590091
GO:1901797	negative r	28	0.5259626	1.3885245	0.0470723	0.1992056	0.1591965
GO:0034644	cellular r	79	0.4296075	1.3054647	0.0471597	0.1994804	0.1594161
GO:0001953	negative r	36	0.4983876	1.3624305	0.047235	0.1995141	0.159443
GO:0006296	nucleotide	36	0.4982766	1.362127	0.047235	0.1995141	0.159443
GO:0007257	activation	36	0.4988264	1.3636301	0.047235	0.1995141	0.159443
GO:0072332	intrinsic	75	0.4345096	1.3127556	0.0473628	0.1999586	0.1597982
GO:0010001	glial cell	210	0.3801989	1.2492146	0.0474747	0.2002526	0.1600332
GO:0007135	meiosis II	9	0.6967772	1.4277279	0.0475	0.2002526	0.1600332
GO:0061983	meiosis II	9	0.6967772	1.4277279	0.0475	0.2002526	0.1600332
GO:0048469	cell matur	165	0.3920914	1.2654817	0.047619	0.2006593	0.1603582
GO:0097120	receptor l	52	0.4659985	1.3379892	0.0478309	0.2014566	0.1609954
GO:1902017	regulation	51	0.4721001	1.3498484	0.0478842	0.2015854	0.1610983
GO:0032728	positive r	30	0.5163	1.381223	0.0479452	0.2017467	0.1612272
GO:0031112	positive r	31	0.5104396	1.3737484	0.048	0.2018205	0.1612862
GO:0046631	alpha-beta	130	0.4030928	1.2846049	0.0480082	0.2018205	0.1612862
GO:0000726	non-recomb	65	0.4505817	1.3368228	0.0480349	0.2018376	0.1612998
GO:0031935	regulation	24	0.5493457	1.400421	0.0481786	0.2021392	0.1615409
GO:0097151	positive r	6	-0.743432	-1.606149	0.0481928	0.2021392	0.1615409
GO:0098828	modulation	6	-0.743432	-1.606149	0.0481928	0.2021392	0.1615409
GO:0051290	protein he	28	0.524483	1.3846185	0.0482204	0.2021392	0.1615409
GO:2000108	positive r	28	0.5231234	1.3810292	0.0482204	0.2021392	0.1615409
GO:0090494	dopamine u	14	-0.567878	-1.546565	0.0483871	0.2027422	0.1620228
GO:0071774	response t	138	0.398432	1.2761207	0.0487805	0.2041979	0.1631861
GO:2000058	regulation	141	0.3998057	1.2800353	0.0487805	0.2041979	0.1631861
GO:0001573	gangliosic	18	0.5838184	1.3929205	0.04884	0.204351	0.1633085
GO:0009792	embryo dev	617	0.3406363	1.1538032	0.048951	0.204719	0.1636026
GO:0006687	glycosphir	64	0.4517028	1.3381893	0.0490731	0.2051328	0.1639332
GO:0060753	regulation	6	0.752689	1.4027111	0.0491368	0.2052315	0.1640121
GO:0071539	protein lc	31	0.5081106	1.3674806	0.0491429	0.2052315	0.1640121
GO:0045410	positive r	15	0.6036169	1.3870409	0.0492005	0.2053757	0.1641273
GO:0046640	regulation	28	0.5219678	1.3779785	0.0493685	0.2059804	0.1646106
GO:0048859	formation	5	-0.791892	-1.611605	0.0494297	0.2060093	0.1646337
GO:0002934	desmosome	10	0.6791955	1.4279558	0.0494438	0.2060093	0.1646337
GO:0030178	negative r	200	0.382604	1.2545885	0.049445	0.2060093	0.1646337
GO:0050866	negative r	182	0.3890112	1.2632081	0.0494949	0.2060204	0.1646426
GO:0042249	establishm	16	0.6019444	1.3980777	0.049505	0.2060204	0.1646426
GO:0003207	cardiac ch	12	0.6493837	1.4254019	0.0495663	0.2060204	0.1646426
GO:0048642	negative r	12	0.648835	1.4241973	0.0495663	0.2060204	0.1646426
GO:0070242	thymocyte	17	0.5906254	1.386393	0.0495663	0.2060204	0.1646426
GO:1903649	regulation	22	0.558004	1.4018301	0.0495868	0.2060204	0.1646426
GO:0009145	purine nuc	143	0.3985208	1.2780498	0.0496957	0.2063767	0.1649273
GO:0006621	protein re	9	0.6909253	1.4157372	0.05	0.2071349	0.1655333

GO:0044275	cellular c	44	-0.423925	-1.528711	0.05	0.2071349	0.1655333
GO:0045741	positive r	9	0.6905729	1.415015	0.05	0.2071349	0.1655333
GO:1990314	cellular r	9	0.6906087	1.4150884	0.05	0.2071349	0.1655333
GO:2000109	regulation	9	0.6914606	1.4168338	0.05	0.2071349	0.1655333
GO:0000139	Golgi memb	694	0.3943994	1.334615	0.000999	0.0136872	0.010176
GO:0000228	nuclear ch	522	0.4637951	1.5583334	0.000999	0.0136872	0.010176
GO:0000323	lytic vacu	647	0.432286	1.4609996	0.000999	0.0136872	0.010176
GO:0000785	chromatin	457	0.4186568	1.4023008	0.000999	0.0136872	0.010176
GO:0005694	chromosome	935	0.4636541	1.5797279	0.000999	0.0136872	0.010176
GO:0005730	nucleolus	874	0.4402603	1.4981758	0.000999	0.0136872	0.010176
GO:0005764	lysosome	646	0.4324098	1.4613732	0.000999	0.0136872	0.010176
GO:0005765	lysosomal	335	0.4532293	1.5040041	0.000999	0.0136872	0.010176
GO:0005773	vacuole	731	0.4272239	1.4480994	0.000999	0.0136872	0.010176
GO:0005813	centrosome	481	0.4575456	1.5339976	0.000999	0.0136872	0.010176
GO:0005815	microtubul	688	0.4285388	1.4503127	0.000999	0.0136872	0.010176
GO:0005819	spindle	329	0.5362948	1.7783763	0.000999	0.0136872	0.010176
GO:0005912	adherens j	533	0.4453756	1.4972931	0.000999	0.0136872	0.010176
GO:0010008	endosome m	448	0.4105504	1.3742784	0.000999	0.0136872	0.010176
GO:0012506	vesicle me	754	0.4176441	1.4165264	0.000999	0.0136872	0.010176
GO:0015629	actin cyto	481	0.4069294	1.3642986	0.000999	0.0136872	0.010176
GO:0016604	nuclear bo	716	0.3812928	1.2915297	0.000999	0.0136872	0.010176
GO:0030133	transport	373	0.4169472	1.3893752	0.000999	0.0136872	0.010176
GO:0030141	secretory	799	0.3978522	1.3520382	0.000999	0.0136872	0.010176
GO:0030659	cytoplasmi	734	0.4144882	1.4048191	0.000999	0.0136872	0.010176
GO:0044427	chromosoma	818	0.4666317	1.5852302	0.000999	0.0136872	0.010176
GO:0044431	Golgi appa	895	0.3858676	1.3133049	0.000999	0.0136872	0.010176
GO:0044437	vacuolar p	532	0.4553026	1.5305065	0.000999	0.0136872	0.010176
GO:0044440	endosomal	492	0.4091708	1.37304	0.000999	0.0136872	0.010176
GO:0044454	nuclear ch	488	0.4557867	1.5294657	0.000999	0.0136872	0.010176
GO:0048471	perinuclea	674	0.4089539	1.384235	0.000999	0.0136872	0.010176
GO:0070161	anchoring	549	0.4414937	1.4847063	0.000999	0.0136872	0.010176
GO:0098791	Golgi subc	791	0.3970866	1.3493224	0.000999	0.0136872	0.010176
GO:0098852	lytic vacu	336	0.4529792	1.5031886	0.000999	0.0136872	0.010176
GO:0099080	supramolec	865	0.4334044	1.4747889	0.000999	0.0136872	0.010176
GO:0099081	supramolec	864	0.4338437	1.4761222	0.000999	0.0136872	0.010176
GO:0099503	secretory	944	0.3994918	1.361072	0.000999	0.0136872	0.010176
GO:0099512	supramolec	857	0.4341186	1.4768915	0.000999	0.0136872	0.010176
GO:0099513	polymeric	644	0.4645429	1.5697974	0.000999	0.0136872	0.010176
GO:1990904	ribonucleo	830	0.4610462	1.5669051	0.000999	0.0136872	0.010176
GO:0000790	nuclear ch	305	0.4114941	1.3606688	0.001	0.0136872	0.010176
GO:0005874	microtubul	394	0.4716302	1.5723818	0.001	0.0136872	0.010176
GO:0005924	cell-subst	399	0.4650916	1.5517959	0.001	0.0136872	0.010176
GO:0005925	focal adhe	396	0.4651493	1.5516563	0.001	0.0136872	0.010176
GO:0030055	cell-subst	403	0.4633481	1.5467257	0.001	0.0136872	0.010176
GO:0030667	secretory	285	0.4420857	1.458815	0.001	0.0136872	0.010176
GO:0031252	cell leadi	390	0.4417259	1.4730244	0.001	0.0136872	0.010176
GO:0098687	chromosoma	313	0.5377898	1.7796256	0.001	0.0136872	0.010176
GO:0005774	vacuolar m	385	0.4505074	1.5007598	0.001001	0.0136872	0.010176
GO:0005840	ribosome	223	0.4654183	1.5189851	0.001006	0.0136872	0.010176
GO:0044445	cytosolic	231	0.4652137	1.5213413	0.0010081	0.0136872	0.010176
GO:0000793	condensed	205	0.5742884	1.861809	0.0010111	0.0136872	0.010176

GO:0030027	lamellipoc	188	0.456149	1.4740362	0.0010121	0.0136872	0.010176
GO:000077	chromosome	185	0.5714266	1.8460828	0.0010132	0.0136872	0.010176
GO:000568	spliceoson	177	0.497132	1.6054783	0.0010132	0.0136872	0.010176
GO:004439	ribosomal	183	0.4884668	1.577014	0.0010142	0.0136872	0.010176
GO:010100	ficolin-1-	181	0.5020473	1.6210213	0.0010142	0.0136872	0.010176
GO:000577	vacuolar l	164	0.5024128	1.6170882	0.0010152	0.0136872	0.010176
GO:000092	spindle pc	157	0.5589185	1.7933941	0.0010163	0.0136872	0.010176
GO:000172	ruffle	165	0.4902523	1.5778312	0.0010163	0.0136872	0.010176
GO:003049	midbody	162	0.5311219	1.7072652	0.0010163	0.0136872	0.010176
GO:007082	tertiary g	158	0.5217094	1.6748853	0.0010163	0.0136872	0.010176
GO:000576	primary ly	147	0.4986937	1.5948331	0.0010173	0.0136872	0.010176
GO:000587	microtubul	147	0.5075094	1.6230261	0.0010173	0.0136872	0.010176
GO:004258	azurophil	147	0.4986937	1.5948331	0.0010173	0.0136872	0.010176
GO:000078	chromosome	132	0.5004845	1.5843823	0.0010267	0.0136872	0.010176
GO:004533	phagocytic	127	0.495761	1.5660938	0.0010267	0.0136872	0.010176
GO:000077	kinetochor	128	0.5876057	1.8558967	0.0010277	0.0136872	0.010176
GO:190481	ficolin-1-	123	0.5035595	1.5837329	0.0010277	0.0136872	0.010176
GO:001593	large ribc	118	0.5125471	1.6063668	0.0010309	0.0136872	0.010176
GO:000077	condensed	111	0.6393145	1.9916608	0.0010331	0.0136872	0.010176
GO:000588	actin fila	108	0.4948569	1.5408255	0.0010331	0.0136872	0.010176
GO:000077	condensed	98	0.6313346	1.9434287	0.0010363	0.0136872	0.010176
GO:000078	nuclear ch	99	0.5177865	1.5937411	0.0010363	0.0136872	0.010176
GO:003066	Golgi-assc	104	0.489934	1.5167475	0.0010363	0.0136872	0.010176
GO:004247	melanosome	104	0.5286526	1.6366135	0.0010363	0.0136872	0.010176
GO:004877	pigment gr	104	0.5286526	1.6366135	0.0010363	0.0136872	0.010176
GO:002262	cytosolic	106	0.5142657	1.5943598	0.0010373	0.0136872	0.010176
GO:007268	mitotic sp	105	0.5501928	1.7044023	0.0010373	0.0136872	0.010176
GO:004320	lysosomal	92	0.5158979	1.5786258	0.0010406	0.0136872	0.010176
GO:000079	condensed	91	0.5720015	1.7447805	0.0010449	0.0136872	0.010176
GO:000568	U2-type sp	87	0.5287814	1.6078262	0.0010449	0.0136872	0.010176
GO:003557	azurophil	86	0.5324573	1.6158721	0.001046	0.0136872	0.010176
GO:007101	catalytic	83	0.5019626	1.5183896	0.0010526	0.0136872	0.010176
GO:000558	collagen t	84	0.4965627	1.5021722	0.0010537	0.0136872	0.010176
GO:012011	Sm-like pr	75	0.5415644	1.6199664	0.0010571	0.0136872	0.010176
GO:007082	tertiary g	70	0.5724937	1.6972324	0.0010638	0.0136872	0.010176
GO:003053	small nucl	64	0.5642184	1.6601394	0.0010672	0.0136872	0.010176
GO:003068	preriboson	72	0.5492289	1.6312458	0.0010672	0.0136872	0.010176
GO:000565	replicatic	66	0.5705023	1.6759536	0.0010787	0.0136872	0.010176
GO:002262	cytosolic	61	0.5730717	1.6693676	0.0010811	0.0136872	0.010176
GO:009752	spliceoson	58	0.5749518	1.6639075	0.001087	0.0136872	0.010176
GO:000587	spindle mi	57	0.5842177	1.6847563	0.0010917	0.0136872	0.010176
GO:004517	intercellu	57	0.5571049	1.6065689	0.0010917	0.0136872	0.010176
GO:000587	kinesin cc	53	0.56628	1.6177392	0.0011086	0.0136872	0.010176
GO:007100	U2-type pr	50	0.5851707	1.6559583	0.0011173	0.0136872	0.010176
GO:007101	precatalyt	51	0.5868498	1.6608444	0.0011173	0.0136872	0.010176
GO:000030	cyclin-der	40	0.6049936	1.6553746	0.0011299	0.013693	0.0101804
GO:005123	spindle mi	33	0.6288714	1.6680476	0.0011561	0.0138605	0.0103049
GO:000078	condensed	25	0.7417225	1.8633748	0.0011834	0.0138959	0.0103312
GO:000077	condensed	15	0.7816153	1.7689201	0.0012361	0.0138959	0.0103312
GO:000094	condensed	13	0.827699	1.838815	0.0012376	0.0138959	0.0103312
GO:004302	NADPH oxic	13	0.7624207	1.693793	0.0012376	0.0138959	0.0103312

GO:004255	MCM comple	12	0.7868741	1.7238159	0.0012392	0.0138959	0.0103312
GO:004512(	pronucleus	12	0.8006881	1.7540783	0.0012392	0.0138959	0.0103312
GO:004261	MHC class	14	0.8050132	1.8016754	0.0012453	0.0138959	0.0103312
GO:003213	chromosome	5	0.964774	1.7342472	0.0013245	0.0146345	0.0108803
GO:000576	endosome	846	0.3774221	1.2838069	0.001998	0.0216514	0.0160973
GO:199023	transferas	732	0.3732735	1.2651677	0.001998	0.0216514	0.0160973
GO:004258	specific g	155	0.4472304	1.4343522	0.0020284	0.0217715	0.0161865
GO:003557	specific g	88	0.4873803	1.4841942	0.0020899	0.0222196	0.0165197
GO:000079	heterochro	71	0.5281128	1.5643863	0.002139	0.0225299	0.0167504
GO:010100	ficolin-1-	58	0.5296946	1.5329333	0.0021739	0.0226852	0.0168658
GO:004278	polysomal	31	0.6065689	1.5882178	0.0023256	0.0236618	0.0175919
GO:000572(	nuclear he	30	0.6062281	1.5724266	0.0023392	0.0236618	0.0175919
GO:000210	podosome	29	0.6295587	1.6175698	0.0023725	0.0236618	0.0175919
GO:004654(	U4/U6 x U5	29	0.6044589	1.553079	0.0023725	0.0236618	0.0175919
GO:009752	spliceosom	29	0.6044589	1.553079	0.0023725	0.0236618	0.0175919
GO:003439	nuclear pe	129	0.4675041	1.4786899	0.0030801	0.0304496	0.0226385
GO:003436	intermedia	5	-0.961971	-1.939595	0.0040486	0.0396398	0.0294712
GO:000579	Golgi stac	128	0.4542696	1.4347674	0.004111	0.0396398	0.0294712
GO:003190	late endos	122	0.4653098	1.4623657	0.0041152	0.0396398	0.0294712
GO:000584	polysome	71	0.5029583	1.489873	0.0042781	0.0405159	0.0301225
GO:003067(	phagocytic	71	0.5072246	1.5025108	0.0042781	0.0405159	0.0301225
GO:000557	membrane a	7	-0.90973	-2.018571	0.0044053	0.041373	0.0307597
GO:004442(	extracellu	50	0.5450147	1.5423218	0.0044693	0.0416009	0.0309292
GO:000015	nuclear ub	41	0.5482725	1.5049145	0.0045403	0.0416009	0.0309292
GO:004359	nuclear re	41	0.5528961	1.5176056	0.0045403	0.0416009	0.0309292
GO:003436	spherical	8	-0.938237	-2.157234	0.0046512	0.0419014	0.0311526
GO:007155	integral c	26	0.6468158	1.6365104	0.0047281	0.0419014	0.0311526
GO:009855	lumenal si	26	0.6468158	1.6365104	0.0047281	0.0419014	0.0311526
GO:000573	small nucl	20	0.6871797	1.6577513	0.0047904	0.0419014	0.0311526
GO:004261	MHC protei	21	0.6758808	1.6475831	0.0047962	0.0419014	0.0311526
GO:010103	chaperone	21	0.6735995	1.6420221	0.0047962	0.0419014	0.0311526
GO:000566	transcript	341	0.3884387	1.2908105	0.004995	0.0429039	0.0318979
GO:003125	cell proje	324	0.3957987	1.3111177	0.004995	0.0429039	0.0318979
GO:004523	tricarboxy	14	-0.709598	-1.938648	0.0050251	0.0429039	0.0318979
GO:003108	platelet c	13	-0.684678	-1.820364	0.0051546	0.0430317	0.0319929
GO:003436	low-densit	13	-0.683776	-1.817967	0.0051546	0.0430317	0.0319929
GO:004262	chylomicro	13	-0.765928	-2.036387	0.0051546	0.0430317	0.0319929
GO:003436	very-low-c	19	-0.724537	-2.132656	0.0057803	0.0475507	0.0353527
GO:003438	triglyceri	19	-0.724537	-2.132656	0.0057803	0.0475507	0.0353527
GO:007168	endocytic	20	-0.601564	-1.818162	0.005988	0.0485989	0.036132
GO:000563	nuclear en	439	0.378717	1.2673561	0.005994	0.0485989	0.036132
GO:003258	ruffle mem	89	0.4748239	1.4457851	0.0062893	0.0501049	0.0372517
GO:000558	fibrillar	11	0.7513515	1.6095537	0.0063131	0.0501049	0.0372517
GO:009864	banded col	11	0.7513515	1.6095537	0.0063131	0.0501049	0.0372517
GO:003436	high-densi	26	-0.769313	-2.496115	0.0064103	0.05052	0.0375603
GO:003243	actin fila	66	0.5070583	1.4895756	0.0064725	0.0506562	0.0376616
GO:000568	U12-type s	26	0.6077604	1.5376961	0.0070922	0.0542259	0.0403155
GO:003013	clathrin-c	178	0.4240939	1.3694639	0.0070922	0.0542259	0.0403155
GO:004430	calyx of H	24	0.6375586	1.5963063	0.007109	0.0542259	0.0403155
GO:003066	coated ves	172	0.4283815	1.3801036	0.0071211	0.0542259	0.0403155
GO:003066	clathrin-c	109	0.4635967	1.4436178	0.0072389	0.0547532	0.0407075

GO:0034358	plasma lip	36	-0.702268	-2.455396	0.0073529	0.0548792	0.0408013
GO:1990777	lipoprotei	36	-0.702268	-2.455396	0.0073529	0.0548792	0.0408013
GO:0002199	zona pellu	7	0.8106315	1.5539841	0.0077419	0.0574024	0.0426772
GO:0032040	small-subu	34	0.5531901	1.4776197	0.008046	0.0590771	0.0439223
GO:0030658	transport	198	0.4173303	1.352341	0.0080727	0.0590771	0.0439223
GO:0032994	protein-li	38	-0.684237	-2.398133	0.0082645	0.0593174	0.0441009
GO:1990752	microtubul	28	0.5876665	1.50378	0.0082938	0.0593174	0.0441009
GO:0030117	membrane c	93	0.4679827	1.4330892	0.008316	0.0593174	0.0441009
GO:0048475	coated mem	93	0.4679827	1.4330892	0.008316	0.0593174	0.0441009
GO:0005686	U2 snRNP	19	0.6692558	1.5915802	0.0084439	0.0598508	0.0444975
GO:0046581	intercellu	6	-0.838879	-1.78566	0.008658	0.0609848	0.0453406
GO:0005782	peroxisoma	48	-0.775642	-2.872245	0.009009	0.0626738	0.0465963
GO:0031907	microbody	48	-0.775642	-2.872245	0.009009	0.0626738	0.0465963
GO:0044452	nucleolar	167	0.4298751	1.3843497	0.0091463	0.0629458	0.0467986
GO:0005882	intermedia	145	0.4302618	1.3748673	0.009165	0.0629458	0.0467986
GO:0031261	DNA replic	5	0.8839036	1.5888771	0.0092715	0.0629458	0.0467986
GO:0032010	phagolysos	5	0.8859506	1.5925568	0.0092715	0.0629458	0.0467986
GO:0030686	90S preril	29	0.5767465	1.4818755	0.0094899	0.0638889	0.0474997
GO:0045240	dihydrolip	11	-0.682631	-1.709893	0.0095238	0.0638889	0.0474997
GO:0000502	proteasome	61	0.5020641	1.4625212	0.0097297	0.0648841	0.0482396
GO:0032154	cleavage f	49	0.5138336	1.4491236	0.010101	0.0669638	0.0497858
GO:0042641	actomyosin	73	0.4819436	1.4358808	0.0106383	0.0701132	0.0521273
GO:0005938	cell corte	283	0.3911374	1.2905973	0.011	0.0720756	0.0535863
GO:0005798	Golgi-asso	164	0.4226161	1.3602511	0.0111675	0.0727502	0.0540879
GO:0030863	cortical c	113	0.4453089	1.3889741	0.0113636	0.0734093	0.0545779
GO:0030880	RNA polyme	99	0.4531213	1.3947022	0.011399	0.0734093	0.0545779
GO:0032588	trans-Golg	83	0.4717229	1.4269173	0.0115789	0.0741447	0.0551247
GO:0044815	DNA packag	26	0.5887248	1.4895342	0.0118203	0.0752628	0.0559559
GO:0005778	peroxisoma	58	-0.482966	-1.858895	0.0121951	0.0764327	0.0568257
GO:0031903	microbody	58	-0.482966	-1.858895	0.0121951	0.0764327	0.0568257
GO:0009898	cytoplasmi	148	0.4256355	1.3622157	0.0122075	0.0764327	0.0568257
GO:0055029	nuclear DN	96	0.4585465	1.4083857	0.0124481	0.077316	0.0574824
GO:0030118	clathrin c	44	0.521348	1.4455282	0.0124858	0.077316	0.0574824
GO:0008305	integrin c	30	0.55911	1.4502123	0.0128655	0.0789474	0.0586953
GO:0090734	site of DN	65	0.4861369	1.427839	0.0128894	0.0789474	0.0586953
GO:0030139	endocytic	291	0.3886202	1.2825747	0.013	0.0791946	0.0588791
GO:0031256	leading ec	161	0.4219777	1.3561763	0.0132114	0.0800496	0.0595148
GO:0016363	nuclear ma	107	0.4476343	1.3897769	0.0134715	0.0808411	0.0601032
GO:0000428	DNA-direct	97	0.4539196	1.3957371	0.0134855	0.0808411	0.0601032
GO:0032153	cell divis	62	0.4903654	1.4349785	0.0139485	0.0815318	0.0606167
GO:1905369	endopeptic	62	0.4924771	1.441158	0.0139485	0.0815318	0.0606167
GO:0005577	fibrinogen	8	-0.771521	-1.773912	0.0139535	0.0815318	0.0606167
GO:0031093	platelet a	65	-0.433938	-1.699562	0.0140845	0.0815318	0.0606167
GO:0005802	trans-Golg	216	0.4045266	1.3165979	0.0141414	0.0815318	0.0606167
GO:0032155	cell divis	57	0.5006838	1.4438627	0.0141921	0.0815318	0.0606167
GO:0001891	phagocytic	24	0.5946861	1.4889628	0.014218	0.0815318	0.0606167
GO:0030666	endocytic	156	0.422639	1.3551511	0.0142276	0.0815318	0.0606167
GO:0016469	proton-tra	27	0.5731922	1.4571859	0.0142518	0.0815318	0.0606167
GO:1904724	tertiary g	53	0.5004508	1.4296796	0.0144124	0.0817481	0.0607776
GO:0098644	complex of	19	0.6443755	1.5324116	0.0144753	0.0817481	0.0607776
GO:0055038	recycling	75	0.4708791	1.4085276	0.0147992	0.0817481	0.0607776

GO:0016581	NuRD compl	15	0.6625668	1.4994944	0.0148331	0.0817481	0.0607776
GO:0090545	CHD-type c	15	0.6625668	1.4994944	0.0148331	0.0817481	0.0607776
GO:0001931	uropod	12	0.7068336	1.54847	0.0148699	0.0817481	0.0607776
GO:0031254	cell trail	12	0.7068336	1.54847	0.0148699	0.0817481	0.0607776
GO:1990023	mitotic sp	12	0.7113285	1.5583171	0.0148699	0.0817481	0.0607776
GO:0030135	coated ves	271	0.3911826	1.2893579	0.0150301	0.0822276	0.061134
GO:0044456	synapse pa	902	0.3382414	1.1510815	0.015984	0.0866508	0.0644226
GO:0034774	secretory	310	0.3855493	1.2759818	0.016	0.0866508	0.0644226
GO:0005682	U5 snRNP	15	0.6515886	1.4746489	0.0160692	0.0866508	0.0644226
GO:0045334	clathrin-c	59	0.4964786	1.4433538	0.0161987	0.086933	0.0646324
GO:0005885	Arp2/3 pro	11	0.7291996	1.5620995	0.0164141	0.0876717	0.0651816
GO:0031298	replicatio	8	0.7797284	1.5545801	0.0165184	0.0878126	0.0652863
GO:0035371	microtubul	20	0.6207485	1.4974926	0.0167665	0.0887127	0.0659556
GO:0030062	mitochondr	6	-0.798155	-1.698974	0.017316	0.0911923	0.0677991
GO:0034708	methyltran	107	0.4367823	1.3560843	0.0176166	0.0923437	0.0686551
GO:0042827	platelet c	20	-0.548046	-1.656409	0.0179641	0.093055	0.069184
GO:0016607	nuclear sp	377	0.3688624	1.2291396	0.018	0.093055	0.069184
GO:0098857	membrane m	303	0.3832664	1.2676431	0.018	0.093055	0.069184
GO:0001725	stress fib	61	0.4856515	1.414711	0.0183784	0.0941474	0.0699961
GO:0097517	contractil	61	0.4856515	1.414711	0.0183784	0.0941474	0.0699961
GO:0045121	membrane r	302	0.3833821	1.2678639	0.019	0.0961385	0.0714765
GO:0031528	microvillu	23	0.6047233	1.5037928	0.0190024	0.0961385	0.0714765
GO:0031838	haptoglobi	11	-0.666261	-1.668888	0.0190476	0.0961385	0.0714765
GO:0005832	chaperonin	10	0.7250128	1.5190526	0.0191083	0.0961385	0.0714765
GO:0070382	exocytic v	197	0.4056421	1.3126331	0.0192113	0.0962274	0.0715425
GO:0001650	fibrillar	125	0.4223082	1.3334308	0.0194076	0.0967802	0.0719535
GO:0031091	platelet a	89	-0.381106	-1.574173	0.0208333	0.1034325	0.0768993
GO:0005658	alpha DNA	5	0.838572	1.5073904	0.0211921	0.1047519	0.0778803
GO:0005838	proteasome	21	0.6098709	1.4866719	0.0215827	0.1060606	0.0788533
GO:0038037	G protein-	6	-0.765432	-1.629319	0.021645	0.1060606	0.0788533
GO:0098636	protein co	33	0.5432055	1.4408233	0.0219653	0.1071641	0.0796737
GO:0008021	synaptic v	181	0.4085335	1.3190817	0.0223124	0.1083881	0.0805837
GO:0098589	membrane r	314	0.3789974	1.2548799	0.022977	0.1111378	0.082628
GO:0044448	cell corte	164	0.4093388	1.3175161	0.0233503	0.1119819	0.0832556
GO:0098562	cytoplasmi	171	0.4109934	1.3250292	0.0233503	0.1119819	0.0832556
GO:0031616	spindle po	14	0.6534059	1.4623676	0.0236613	0.112837	0.0838913
GO:0022627	cytosolic	40	0.5181805	1.4178379	0.0237288	0.112837	0.0838913
GO:0031985	Golgi cist	99	0.4335936	1.3345962	0.0238342	0.1128619	0.0839099
GO:0033267	axon part	374	0.3642435	1.2137854	0.023976	0.1130585	0.084056
GO:1903439	calcitonin	5	-0.793733	-1.600381	0.0242915	0.1132407	0.0841915
GO:0032580	Golgi cist	75	0.4625351	1.3835684	0.0243129	0.1132407	0.0841915
GO:0044798	nuclear tr	191	0.4019774	1.2990936	0.0243161	0.1132407	0.0841915
GO:0000315	organellar	86	0.4514242	1.3699575	0.0251046	0.1157797	0.0860792
GO:0005761	mitochondr	86	0.4514242	1.3699575	0.0251046	0.1157797	0.0860792
GO:0001772	immunologi	35	0.5315835	1.4241837	0.0254042	0.1157797	0.0860792
GO:0061673	mitotic sp	8	0.7487289	1.492775	0.025413	0.1157797	0.0860792
GO:0034663	endoplasmi	10	0.714151	1.4962951	0.0254777	0.1157797	0.0860792
GO:0043256	laminin co	10	0.7163261	1.5008522	0.0254777	0.1157797	0.0860792
GO:0060205	cytoplasmi	327	0.3716784	1.2318574	0.025974	0.1158512	0.0861323
GO:0099568	cytoplasmi	469	0.3572079	1.1970976	0.025974	0.1158512	0.0861323
GO:0150034	distal axo	280	0.3784111	1.2489355	0.026026	0.1158512	0.0861323

GO:0000151	ubiquitin	266	0.3846904	1.2673723	0.0260521	0.1158512	0.0861323
GO:0030687	preribosom	23	0.5863554	1.4581165	0.0261283	0.1158512	0.0861323
GO:0030119	AP-type me	43	0.504081	1.3917577	0.0261364	0.1158512	0.0861323
GO:0044438	microbody	96	-0.639074	-2.646218	0.0263158	0.1158512	0.0861323
GO:0044439	peroxisoma	96	-0.639074	-2.646218	0.0263158	0.1158512	0.0861323
GO:0032993	protein-DN	111	0.4231625	1.3182811	0.0268595	0.1176058	0.0874368
GO:0005687	U4 snRNP	9	0.726768	1.4843732	0.0269231	0.1176058	0.0874368
GO:0030137	COPI-coate	26	0.5613352	1.4202358	0.0271868	0.1177152	0.0875181
GO:0022624	proteasome	24	0.5791051	1.4499516	0.0272512	0.1177152	0.0875181
GO:0034709	methylosom	12	0.6645166	1.4557656	0.0272615	0.1177152	0.0875181
GO:0005697	telomerase	20	0.604499	1.4582924	0.0275449	0.1184852	0.0880906
GO:0030132	clathrin c	19	0.6085594	1.447236	0.0277443	0.1188889	0.0883908
GO:0031983	vesicle lu	328	0.3709604	1.2298988	0.027972	0.1194109	0.0887788
GO:0030864	cortical a	87	0.4515012	1.3728459	0.0282132	0.1199858	0.0892063
GO:0097441	basal denc	7	0.7729145	1.4816803	0.0283871	0.1202716	0.0894188
GO:0072562	blood micr	109	-0.622755	-2.699004	0.0285714	0.1205993	0.0896624
GO:0097470	ribbon syr	10	0.6975945	1.4616057	0.0292994	0.1232104	0.0916037
GO:0031519	PcG protei	43	0.4986521	1.3767687	0.0295455	0.1236763	0.09195
GO:0005643	nuclear pc	80	0.4535019	1.3644127	0.0296296	0.1236763	0.09195
GO:0010369	chromocent	14	0.6373394	1.4264097	0.0298879	0.1241991	0.0923388
GO:0015935	small ribc	68	0.4638718	1.3656572	0.0300107	0.1241991	0.0923388
GO:0061695	transferas	240	0.3881103	1.2728712	0.0301811	0.1241991	0.0923388
GO:0034707	chloride c	48	0.4909602	1.382938	0.030303	0.1241991	0.0923388
GO:0061200	clathrin-s	8	0.7421065	1.4795716	0.0304956	0.1241991	0.0923388
GO:0061202	clathrin-s	8	0.7421065	1.4795716	0.0304956	0.1241991	0.0923388
GO:1902554	serine/thr	83	0.4503254	1.3621918	0.0305263	0.1241991	0.0923388
GO:0015030	Cajal body	55	0.4875312	1.4010034	0.0307355	0.1246002	0.092637
GO:0060198	clathrin-s	12	0.6629585	1.4523524	0.0309789	0.1251371	0.0930362
GO:0005839	proteasome	19	0.5993846	1.4254172	0.0313631	0.1262364	0.0938535
GO:0042765	GPI-anchor	5	0.8209392	1.4756943	0.0331126	0.1328038	0.0987362
GO:0030427	site of pc	174	0.4004941	1.293722	0.0334686	0.1336008	0.0993287
GO:0033061	DNA recomb	7	0.7624569	1.4616331	0.0335484	0.1336008	0.0993287
GO:0000796	condensin	8	0.7385784	1.4725375	0.0343075	0.1361428	0.1012186
GO:0031228	intrinsic	61	0.4675037	1.3618463	0.0345946	0.1368004	0.1017075
GO:0033176	proton-tra	25	0.562361	1.4127783	0.035503	0.1399015	0.1040131
GO:0030131	clathrin a	27	0.5419985	1.3778845	0.0356295	0.1399108	0.10402
GO:0046658	anchored c	58	0.4724573	1.3672888	0.0369565	0.1446181	0.1075197
GO:0098798	mitochondr	222	0.388167	1.2668387	0.0372233	0.1451581	0.1079212
GO:0000235	astral mic	11	0.6754905	1.4470434	0.0378788	0.1466989	0.1090668
GO:0005818	aster	11	0.6754905	1.4470434	0.0378788	0.1466989	0.1090668
GO:0005770	late endos	238	0.381978	1.2516298	0.0382294	0.1475497	0.1096993
GO:0036477	somatodenc	804	0.3324677	1.1298132	0.038961	0.1498604	0.1114172
GO:0035577	azurophil	55	0.4783809	1.3747086	0.039517	0.1514819	0.1126228
GO:0031234	extrinsic	85	0.4383366	1.3263847	0.04	0.1527488	0.1135647
GO:0018995	host	70	0.4538077	1.3453723	0.0404255	0.1527488	0.1135647
GO:0043657	host cell	70	0.4538077	1.3453723	0.0404255	0.1527488	0.1135647
GO:0005947	mitochondr	5	-0.750878	-1.513973	0.0404858	0.1527488	0.1135647
GO:0030426	growth cor	170	0.3955338	1.2746546	0.040568	0.1527488	0.1135647
GO:0031390	Ctf18 RFC-	8	0.7280361	1.4515188	0.0406607	0.1527488	0.1135647
GO:0099572	postsynapt	334	0.3609361	1.1971122	0.040959	0.1532257	0.1139193
GO:0005638	lamin fila	5	0.8101369	1.4562764	0.0410596	0.1532257	0.1139193

GO:0055037	recycling	161	0.3988004	1.2816877	0.0416667	0.1546778	0.1149989
GO:0031258	lamellipod	22	0.5681302	1.3995287	0.0417661	0.1546778	0.1149989
GO:0097648	G protein-	8	-0.723926	-1.664481	0.0418605	0.1546778	0.1149989
GO:0098892	extrinsic	6	0.7700086	1.4362104	0.0428016	0.1570513	0.1167635
GO:0000118	histone de	55	0.4717211	1.3555704	0.0428101	0.1570513	0.1167635
GO:0019774	proteasome	10	0.680936	1.4267027	0.0433121	0.1570513	0.1167635
GO:0033178	proton-tra	10	0.6816956	1.4282942	0.0433121	0.1570513	0.1167635
GO:0005777	peroxisome	125	-0.580295	-2.607773	0.0434783	0.1570513	0.1167635
GO:0035770	ribonucleo	213	0.3854012	1.2523965	0.0434783	0.1570513	0.1167635
GO:0042579	microbody	125	-0.580295	-2.607773	0.0434783	0.1570513	0.1167635
GO:0098978	glutamater	342	0.3570328	1.1864348	0.043956	0.1582698	0.1176695
GO:0005652	nuclear la	11	0.6643863	1.4232559	0.0441919	0.1583421	0.1177232
GO:1905368	peptidase	84	0.4365066	1.320494	0.0442571	0.1583421	0.1177232
GO:0016600	flotillin	8	0.7215579	1.4386031	0.0444727	0.1586098	0.1179222
GO:0014069	postsynapt	311	0.3645077	1.2060296	0.045	0.1599842	0.118944
GO:0044352	pinosome	8	0.7179639	1.4314374	0.047014	0.1649723	0.1226525
GO:0044354	macropinos	8	0.7179639	1.4314374	0.047014	0.1649723	0.1226525
GO:0098843	postsynapt	8	0.7178353	1.431181	0.047014	0.1649723	0.1226525
GO:0005905	clathrin-c	68	0.4539474	1.3364393	0.0471597	0.1649723	0.1226525
GO:0030894	replisome	26	0.5450194	1.378955	0.0472813	0.1649723	0.1226525
GO:0031430	M band	26	0.5426721	1.3730161	0.0472813	0.1649723	0.1226525
GO:0034719	SMN-Sm pro	17	0.5985306	1.3977543	0.047561	0.1654359	0.1229972
GO:0005604	basement m	89	0.4298161	1.3087413	0.048218	0.1672053	0.1243127
GO:0030479	actin cort	16	0.6023799	1.3867132	0.0489596	0.1683478	0.1251621
GO:0061645	endocytic	16	0.6023799	1.3867132	0.0489596	0.1683478	0.1251621
GO:0000315	organellar	56	0.462549	1.3326765	0.0491803	0.1683478	0.1251621
GO:0005762	mitochondr	56	0.462549	1.3326765	0.0491803	0.1683478	0.1251621
GO:0031300	intrinsic	222	0.3797527	1.2393772	0.0492958	0.1683478	0.1251621
GO:0090533	cation-tra	15	0.6121755	1.3854508	0.0494438	0.1683478	0.1251621
GO:0003682	chromatin	491	0.3875248	1.3092281	0.000999	0.0428202	0.0353684
GO:0003712	transcript	539	0.3854809	1.3066818	0.000999	0.0428202	0.0353684
GO:0004672	protein ki	566	0.3892229	1.3201443	0.000999	0.0428202	0.0353684
GO:0005198	structural	684	0.4173836	1.4248682	0.000999	0.0428202	0.0353684
GO:0008047	enzyme act	476	0.3831493	1.29462	0.000999	0.0428202	0.0353684
GO:0008092	cytoskelet	937	0.4162268	1.4281874	0.000999	0.0428202	0.0353684
GO:0016301	kinase act	722	0.3677827	1.2574008	0.000999	0.0428202	0.0353684
GO:0016462	pyrophosph	823	0.4045579	1.3862604	0.000999	0.0428202	0.0353684
GO:0016817	hydrolase	825	0.4042257	1.385331	0.000999	0.0428202	0.0353684
GO:0016818	hydrolase	825	0.4042257	1.385331	0.000999	0.0428202	0.0353684
GO:0017111	nucleoside	778	0.4179197	1.4299864	0.000999	0.0428202	0.0353684
GO:0019900	kinase bin	698	0.4207701	1.4370696	0.000999	0.0428202	0.0353684
GO:0019901	protein ki	620	0.4317626	1.4693546	0.000999	0.0428202	0.0353684
GO:0019904	protein do	675	0.4274799	1.4589065	0.000999	0.0428202	0.0353684
GO:0030234	enzyme reg	950	0.3521207	1.2083923	0.000999	0.0428202	0.0353684
GO:0050839	cell adhes	466	0.4791019	1.6183259	0.000999	0.0428202	0.0353684
GO:0051020	GTPase bin	509	0.4084611	1.3812136	0.000999	0.0428202	0.0353684
GO:0003779	actin binc	411	0.4195797	1.4118036	0.001	0.0428202	0.0353684
GO:0004674	protein se	418	0.3900294	1.3137681	0.001	0.0428202	0.0353684
GO:0016887	ATPase act	343	0.4054036	1.3567529	0.001	0.0428202	0.0353684
GO:0017016	Ras GTPase	404	0.430043	1.445434	0.001	0.0428202	0.0353684
GO:0031267	small GTPa	417	0.4217262	1.4206905	0.001	0.0428202	0.0353684

GO:0060589	nucleoside	312	0.4267149	1.421961	0.001	0.0428202	0.0353684
GO:0015631	tubulin bi	321	0.4647836	1.5502077	0.001001	0.0428202	0.0353684
GO:0045296	cadherin b	305	0.5118519	1.704152	0.001001	0.0428202	0.0353684
GO:0003924	GTPase act	285	0.449839	1.4940631	0.001003	0.0428202	0.0353684
GO:0005126	cytokine r	266	0.4258568	1.41007	0.001004	0.0428202	0.0353684
GO:0030695	GTPase reg	274	0.4196461	1.3896685	0.001004	0.0428202	0.0353684
GO:0005096	GTPase act	243	0.4257473	1.406672	0.001007	0.0428202	0.0353684
GO:0005125	cytokine a	206	0.4361042	1.424281	0.0010081	0.0428202	0.0353684
GO:0008017	microtubul	237	0.4792171	1.5796122	0.0010081	0.0428202	0.0353684
GO:0140097	catalytic	197	0.4710792	1.536197	0.0010091	0.0428202	0.0353684
GO:0051015	actin fila	189	0.4644482	1.5131811	0.0010101	0.0428202	0.0353684
GO:0017048	Rho GTPase	168	0.4632998	1.497902	0.0010142	0.0428202	0.0353684
GO:0019887	protein ki	163	0.4726582	1.5239588	0.0010142	0.0428202	0.0353684
GO:0008022	protein C-	182	0.447837	1.4518827	0.0010152	0.0428202	0.0353684
GO:0042393	histone bi	182	0.4420676	1.4331783	0.0010152	0.0428202	0.0353684
GO:0003735	structural	154	0.5221051	1.678139	0.0010173	0.0428202	0.0353684
GO:0003697	single-str	105	0.5061651	1.585196	0.0010288	0.0428202	0.0353684
GO:0019903	protein ph	135	0.4653193	1.4779535	0.0010288	0.0428202	0.0353684
GO:0017124	SH3 domain	127	0.5165385	1.6343675	0.0010309	0.0428202	0.0353684
GO:0051082	unfolded p	115	0.4923562	1.5416864	0.0010341	0.0428202	0.0353684
GO:0005178	integrin b	125	0.4771789	1.5044288	0.0010352	0.0428202	0.0353684
GO:0003777	microtubul	81	0.5447498	1.6617431	0.0010537	0.0428202	0.0353684
GO:0008094	DNA-depend	65	0.5477382	1.6250703	0.0010811	0.0429498	0.0354754
GO:0098632	cell-cell	50	0.5851533	1.6770829	0.001105	0.0429498	0.0354754
GO:0098641	cadherin b	19	0.7045445	1.724233	0.0012121	0.0440991	0.0364248
GO:0070182	DNA polyme	17	0.7904225	1.8996367	0.0012151	0.0440991	0.0364248
GO:0005391	sodium:pot	11	0.7943414	1.7194692	0.0012579	0.0440991	0.0364248
GO:0008556	potassium-	11	0.7943414	1.7194692	0.0012579	0.0440991	0.0364248
GO:0050786	RAGE recep	11	0.8132119	1.7603171	0.0012579	0.0440991	0.0364248
GO:0043533	inositol 1	5	0.9360264	1.6783195	0.0013387	0.0460303	0.0380198
GO:0016772	transferas	852	0.355531	1.2182951	0.001998	0.0647439	0.0534768
GO:0003729	mRNA bindi	244	0.4117834	1.3600945	0.0020141	0.0647439	0.0534768
GO:0019902	phosphatas	179	0.4365207	1.4153057	0.0020305	0.0647439	0.0534768
GO:0004386	helicase a	157	0.442875	1.4240103	0.0020346	0.0647439	0.0534768
GO:0043021	ribonucleo	130	0.4597553	1.4578858	0.002064	0.0647439	0.0534768
GO:0003678	DNA helica	76	0.5240131	1.5840859	0.0021164	0.0652436	0.0538895
GO:0003810	protein-gl	8	0.819802	1.6575459	0.002574	0.0736166	0.0608054
GO:0015187	glycine tr	8	0.7819333	1.5809797	0.002574	0.0736166	0.0608054
GO:0016176	superoxide	8	0.8169983	1.6518772	0.002574	0.0736166	0.0608054
GO:0043008	ATP-depend	8	0.7831236	1.5833863	0.002574	0.0736166	0.0608054
GO:0016936	galactosid	6	0.8544344	1.6026293	0.002635	0.0736166	0.0608054
GO:0050543	icosatetra	6	0.8620482	1.6169101	0.002635	0.0736166	0.0608054
GO:0019863	IgE bindin	5	0.9174503	1.6450121	0.0026774	0.0736484	0.0608317
GO:0019207	kinase reg	189	0.4192465	1.365913	0.0030303	0.0820937	0.0678073
GO:1902936	phosphatid	98	0.4653111	1.4444584	0.003112	0.0830495	0.0685967
GO:0048365	Rac GTPase	66	0.5298886	1.5768242	0.0032328	0.0850025	0.0702099
GO:0003688	DNA replic	24	0.6735663	1.7247964	0.0035757	0.0869481	0.0718169
GO:0004003	ATP-depend	21	0.6758179	1.6926966	0.0036101	0.0869481	0.0718169
GO:0008026	ATP-depend	21	0.6758179	1.6926966	0.0036101	0.0869481	0.0718169
GO:0044183	protein bi	21	0.6805387	1.7045206	0.0036101	0.0869481	0.0718169
GO:0070035	purine NTP	21	0.6758179	1.6926966	0.0036101	0.0869481	0.0718169

GO:0044548	S100 prote	14	0.737484	1.6926715	0.0037129	0.0869481	0.0718169
GO:0005522	profilin b	11	0.7583145	1.6414836	0.0037736	0.0869481	0.0718169
GO:0003997	acyl-CoA c	5	-0.936695	-1.838933	0.0039216	0.0869481	0.0718169
GO:0004321	fatty-acyl	5	-0.942901	-1.851118	0.0039216	0.0869481	0.0718169
GO:0015185	gamma-amir	5	-0.965479	-1.895443	0.0039216	0.0869481	0.0718169
GO:0016019	peptidogly	5	-0.922693	-1.811444	0.0039216	0.0869481	0.0718169
GO:0034987	immunoglob	7	0.8257071	1.6102177	0.003937	0.0869481	0.0718169
GO:0050544	arachidoni	5	0.8878695	1.591973	0.0040161	0.0869481	0.0718169
GO:0004024	alcohol de	7	-0.962305	-2.192926	0.0041667	0.0869481	0.0718169
GO:0008172	S-methyltr	7	-0.909166	-2.071832	0.0041667	0.0869481	0.0718169
GO:0015245	fatty acic	7	-0.920692	-2.098097	0.0041667	0.0869481	0.0718169
GO:0016713	oxidoreduc	7	-0.943197	-2.149383	0.0041667	0.0869481	0.0718169
GO:0098631	cell adhes	59	0.5466936	1.6056689	0.0043526	0.0869481	0.0718169
GO:0003857	3-hydroxya	8	-0.909808	-2.148321	0.0044444	0.0869481	0.0718169
GO:0004022	alcohol de	8	-0.959007	-2.264494	0.0044444	0.0869481	0.0718169
GO:0101020	estrogen 1	8	-0.952235	-2.248505	0.0044444	0.0869481	0.0718169
GO:0140161	monocarbox	8	-0.95102	-2.245634	0.0044444	0.0869481	0.0718169
GO:0015355	secondary	10	-0.922596	-2.311639	0.0045662	0.0869481	0.0718169
GO:0003995	acyl-CoA c	11	-0.862208	-2.213506	0.0048309	0.0869481	0.0718169
GO:0043138	3'-5' DNA	19	0.6800194	1.6642126	0.0048485	0.0869481	0.0718169
GO:0017116	single-str	20	0.6712471	1.6584753	0.004878	0.0869481	0.0718169
GO:0043142	single-str	20	0.6712471	1.6584753	0.004878	0.0869481	0.0718169
GO:0016725	oxidoreduc	13	-0.86231	-2.326143	0.0049505	0.0869481	0.0718169
GO:0016918	retinal bi	13	-0.767974	-2.071665	0.0049505	0.0869481	0.0718169
GO:0070330	aromatase	13	-0.82457	-2.224337	0.0049505	0.0869481	0.0718169
GO:0016773	phosphotra	661	0.3623098	1.2352831	0.004995	0.0869481	0.0718169
GO:0044389	ubiquitin-	295	0.4022841	1.3386148	0.005	0.0869481	0.0718169
GO:0060090	molecular	222	0.4075126	1.3403415	0.0050302	0.0869481	0.0718169
GO:0032395	MHC class	9	0.7656463	1.5898843	0.0051216	0.0869481	0.0718169
GO:0004029	aldehyde c	15	-0.69665	-1.970427	0.0051282	0.0869481	0.0718169
GO:0008392	arachidoni	15	-0.912042	-2.579646	0.0051282	0.0869481	0.0718169
GO:0003996	acyl-CoA l	14	-0.804925	-2.22411	0.0051546	0.0869481	0.0718169
GO:0010181	FMN bindir	14	-0.710385	-1.962885	0.0051546	0.0869481	0.0718169
GO:0000062	fatty-acyl	20	-0.663101	-2.012343	0.0054945	0.0870836	0.0719288
GO:0004806	triglyceri	20	-0.789932	-2.397242	0.0054945	0.0870836	0.0719288
GO:0016408	C-acyltrar	18	-0.815882	-2.406607	0.0055249	0.0870836	0.0719288
GO:0017127	cholestero	18	-0.604617	-1.783438	0.0055249	0.0870836	0.0719288
GO:0120020	intermembr	18	-0.604617	-1.783438	0.0055249	0.0870836	0.0719288
GO:0008391	arachidoni	16	-0.914622	-2.570566	0.0055556	0.0870836	0.0719288
GO:0015125	bile acid	16	-0.823591	-2.314722	0.0055556	0.0870836	0.0719288
GO:0015347	sodium-inc	16	-0.730228	-2.052325	0.0055556	0.0870836	0.0719288
GO:0004745	retinol de	19	-0.860071	-2.556	0.0056497	0.0870836	0.0719288
GO:0015645	fatty acic	19	-0.777212	-2.309755	0.0056497	0.0870836	0.0719288
GO:0008483	transamina	21	-0.7831	-2.397357	0.005848	0.0893687	0.0738163
GO:0016769	transferas	23	-0.745031	-2.389428	0.0059524	0.0901937	0.0744977
GO:0031625	ubiquitin	280	0.3998176	1.3253483	0.0060241	0.0905133	0.0747617
GO:0023026	MHC class	16	0.6996081	1.6596078	0.0060827	0.0906326	0.0748602
GO:0016405	CoA-ligase	24	-0.672466	-2.166888	0.006135	0.0906556	0.0748792
GO:0005343	organic ac	27	-0.588875	-1.969619	0.0064516	0.092222	0.076173
GO:0016702	oxidoreduc	27	-0.608077	-2.033842	0.0064516	0.092222	0.076173
GO:0031491	nucleosome	53	0.5434789	1.5694099	0.0066152	0.092222	0.076173

GO:0016701	oxidoreduc	28	-0.593978	-1.995069	0.0066225	0.092222	0.076173
GO:0016878	acid-thiol	28	-0.672719	-2.259549	0.0066225	0.092222	0.076173
GO:0019840	isoprenoic	28	-0.521748	-1.752463	0.0066225	0.092222	0.076173
GO:0033764	steroid de	26	-0.680959	-2.207163	0.0066225	0.092222	0.076173
GO:0016538	cyclin-dep	43	0.5707396	1.5989743	0.0067265	0.092222	0.076173
GO:0016712	oxidoreduc	29	-0.852089	-2.883975	0.0067568	0.092222	0.076173
GO:0071949	FAD bindir	29	-0.60313	-2.041349	0.0067568	0.092222	0.076173
GO:0015175	neutral am	31	0.6180729	1.6431912	0.006993	0.0947235	0.0782391
GO:0016229	steroid de	30	-0.736535	-2.479272	0.0071429	0.0948757	0.0783649
GO:0000217	DNA seconc	24	0.6426535	1.6456381	0.0071514	0.0948757	0.0783649
GO:0017134	fibroblast	23	0.6529437	1.6547803	0.0071942	0.0948757	0.0783649
GO:0003774	motor acti	131	0.4405699	1.3978173	0.0072165	0.0948757	0.0783649
GO:0035325	Toll-like	12	0.7195748	1.5899266	0.0075662	0.0970157	0.0801325
GO:0042379	chemokine	59	0.5280638	1.5509521	0.007617	0.0970157	0.0801325
GO:0004499	N,N-dimeth	5	-0.874968	-1.717749	0.0078431	0.0970157	0.0801325
GO:0016841	ammonia-ly	5	-0.880377	-1.728368	0.0078431	0.0970157	0.0801325
GO:0030976	thiamine p	5	-0.874043	-1.715934	0.0078431	0.0970157	0.0801325
GO:0004715	non-membra	45	0.5507629	1.5508099	0.0079007	0.0970157	0.0801325
GO:0042169	SH2 domair	36	0.5863192	1.5987185	0.0079545	0.0970157	0.0801325
GO:0001882	nucleoside	339	0.3894688	1.3015878	0.008008	0.0970157	0.0801325
GO:0001883	purine nuc	331	0.3883041	1.2973337	0.008008	0.0970157	0.0801325
GO:0008395	steroid hy	35	-0.868307	-3.070755	0.0081301	0.0970157	0.0801325
GO:1901981	phosphatic	145	0.4314033	1.3802886	0.00818	0.0970157	0.0801325
GO:0016709	oxidoreduc	36	-0.749878	-2.660996	0.0081967	0.0970157	0.0801325
GO:0016877	ligase act	38	-0.561991	-2.021668	0.0081967	0.0970157	0.0801325
GO:0019825	oxygen bir	36	-0.776657	-2.756022	0.0081967	0.0970157	0.0801325
GO:0003988	acetyl-CoA	6	-0.86653	-1.826675	0.0082305	0.0970157	0.0801325
GO:0042826	histone de	106	0.4471114	1.3998176	0.0082474	0.0970157	0.0801325
GO:0030170	pyridoxal	45	-0.782213	-2.920953	0.0086207	0.0987432	0.0815594
GO:0070279	vitamin B6	45	-0.782213	-2.920953	0.0086207	0.0987432	0.0815594
GO:0016755	transferas	14	0.6706514	1.5392776	0.0086634	0.0987432	0.0815594
GO:0016175	superoxide	10	0.7444165	1.5741757	0.00894	0.0987432	0.0815594
GO:0010997	anaphase-p	8	0.7493031	1.5150052	0.009009	0.0987432	0.0815594
GO:0016830	carbon-car	44	-0.462114	-1.701578	0.009009	0.0987432	0.0815594
GO:0016861	intramolec	8	0.7471821	1.5107169	0.009009	0.0987432	0.0815594
GO:0004879	nuclear re	47	-0.468696	-1.77917	0.0090909	0.0987432	0.0815594
GO:0016903	oxidoreduc	43	-0.55228	-2.007028	0.0090909	0.0987432	0.0815594
GO:0017046	peptide hc	47	-0.51424	-1.952057	0.0090909	0.0987432	0.0815594
GO:0098531	transcript	47	-0.468696	-1.77917	0.0090909	0.0987432	0.0815594
GO:1901618	organic hy	43	-0.626491	-2.276718	0.0090909	0.0987432	0.0815594
GO:0043515	kinetochor	6	0.8265088	1.5502502	0.0092227	0.0987432	0.0815594
GO:0050542	icosanoid	6	0.8177508	1.5338232	0.0092227	0.0987432	0.0815594
GO:1990226	histone me	6	0.8158601	1.530277	0.0092227	0.0987432	0.0815594
GO:0004185	serine-typ	5	0.8614993	1.5446905	0.0093708	0.0997323	0.0823763
GO:0005132	type I int	12	-0.711568	-1.891662	0.0095694	0.0999883	0.0825877
GO:0005372	water trar	12	-0.753842	-2.004044	0.0095694	0.0999883	0.0825877
GO:0015250	water char	11	-0.768922	-1.974019	0.0096618	0.0999883	0.0825877
GO:0050840	extracellu	55	0.5270371	1.5334507	0.0098253	0.0999883	0.0825877
GO:0004467	long-chair	13	-0.673142	-1.815848	0.009901	0.0999883	0.0825877
GO:0015373	anion:sodi	13	-0.65819	-1.775515	0.009901	0.0999883	0.0825877
GO:0016863	intramolec	13	-0.741947	-2.001456	0.009901	0.0999883	0.0825877

GO:0019841	retinol bi	13	-0.727476	-1.962418	0.009901	0.0999883	0.0825877
GO:0008028	monocarbox	48	-0.718098	-2.71335	0.01	0.0999883	0.0825877
GO:0032549	ribonuclec	332	0.3830753	1.2797942	0.01001	0.0999883	0.0825877
GO:0032550	purine rib	328	0.3843217	1.2836675	0.01001	0.0999883	0.0825877
GO:0042165	neurotrans	52	-0.535608	-2.033242	0.010101	0.1003367	0.0828755
GO:0046875	ephrin rec	27	0.6236421	1.6311869	0.0106257	0.1045125	0.0863246
GO:0016836	hydro-lyas	51	-0.430259	-1.612911	0.0106383	0.1045125	0.0863246
GO:0003684	damaged DN	62	0.5099813	1.503027	0.0108814	0.1063166	0.0878147
GO:0035091	phosphatic	224	0.3990863	1.3122315	0.0110775	0.1076448	0.0889119
GO:0050700	CARD domai	15	0.6641461	1.5463643	0.0111524	0.1077866	0.089029
GO:0003707	steroid hc	56	-0.470239	-1.770609	0.0116279	0.1094247	0.090382
GO:0016597	amino acic	55	-0.677835	-2.545262	0.0116279	0.1094247	0.090382
GO:0016627	oxidoreduc	56	-0.573241	-2.158448	0.0116279	0.1094247	0.090382
GO:0033293	monocarbox	56	-0.428336	-1.612833	0.0116279	0.1094247	0.090382
GO:0051287	NAD bindir	56	-0.476322	-1.793515	0.0116279	0.1094247	0.090382
GO:0009374	biotin bir	5	-0.867189	-1.702479	0.0117647	0.1096888	0.0906001
GO:0015129	lactate tr	6	0.8087349	1.5169124	0.0118577	0.1096888	0.0906001
GO:0120013	intermembr	23	-0.563036	-1.805741	0.0119048	0.1096888	0.0906001
GO:0023023	MHC protei	24	0.6213292	1.5910333	0.011919	0.1096888	0.0906001
GO:0042605	peptide ar	22	0.6369814	1.6046924	0.0119904	0.1096888	0.0906001
GO:0005525	GTP bindir	324	0.3828579	1.2772744	0.012024	0.1096888	0.0906001
GO:0030674	protein bi	165	0.4155862	1.342126	0.0121581	0.1098577	0.0907396
GO:0035173	histone ki	16	0.6570178	1.5585751	0.0121655	0.1098577	0.0907396
GO:0016645	oxidoreduc	25	-0.541031	-1.777626	0.0124224	0.111171	0.0918244
GO:0005200	structural	97	0.4508544	1.3981803	0.0124352	0.111171	0.0918244
GO:0052650	NADP-retir	10	0.7225069	1.5278447	0.0127714	0.1136082	0.0938375
GO:0005501	retinoid k	27	-0.523556	-1.751144	0.0129032	0.1138504	0.0940375
GO:0042287	MHC protei	26	0.6137294	1.6016251	0.012926	0.1138504	0.0940375
GO:0034235	GPI anchor	7	0.7734479	1.5083065	0.0131234	0.1150224	0.0950055
GO:0004300	enoyl-CoA	8	-0.83842	-1.979753	0.0133333	0.1151691	0.0951267
GO:0008401	retinoic a	8	-0.820666	-1.937831	0.0133333	0.1151691	0.0951267
GO:0016634	oxidoreduc	8	-0.831074	-1.962407	0.0133333	0.1151691	0.0951267
GO:0032182	ubiquitin-	92	0.4613388	1.4245947	0.0135417	0.1164063	0.0961486
GO:0015651	quaternary	10	-0.775836	-1.943919	0.0136986	0.1171921	0.0967977
GO:0005324	long-chair	11	-0.678856	-1.742794	0.0144928	0.1233954	0.1019215
GO:0043028	cysteine-t	40	0.5375298	1.492453	0.0145903	0.1236376	0.1021215
GO:0008536	Ran GTPase	36	0.5490434	1.4970785	0.0147727	0.1245926	0.1029103
GO:0050664	oxidoreduc	15	0.6529254	1.5202384	0.0148699	0.1248233	0.1031008
GO:0015370	solute:soc	71	-0.52482	-2.089886	0.0153846	0.1276309	0.1054199
GO:0016594	glycine bi	15	-0.573098	-1.620968	0.0153846	0.1276309	0.1054199
GO:0003823	antigen bi	51	0.5098906	1.4651682	0.0154185	0.1276309	0.1054199
GO:0016835	carbon-oxy	70	-0.370469	-1.453544	0.015625	0.1287442	0.1063394
GO:0032813	tumor necr	45	0.5222268	1.4704593	0.0158014	0.1296001	0.1070464
GO:0016620	oxidoreduc	35	-0.50112	-1.772203	0.0162602	0.1327542	0.1096516
GO:0004860	protein ki	57	0.49842	1.4572481	0.0163577	0.1329434	0.1098078
GO:0016714	oxidoreduc	7	-0.850125	-1.937287	0.0166667	0.1348416	0.1113757
GO:0120015	intermembr	19	-0.599486	-1.78158	0.0169492	0.1363926	0.1126568
GO:0055106	ubiquitin-	17	0.6294977	1.5128832	0.0170109	0.1363926	0.1126568
GO:0005201	extracellu	160	0.4160899	1.339747	0.0172414	0.1370115	0.113168
GO:0050660	flavin ade	75	-0.524892	-2.123059	0.0172414	0.1370115	0.113168
GO:0033038	bitter tas	21	-0.569439	-1.743262	0.0175439	0.1383487	0.1142725

GO:0043236	laminin bi	29	0.5671921	1.5005104	0.0175644	0.1383487	0.1142725
GO:0008198	ferrous ir	23	-0.513647	-1.647343	0.0178571	0.1394641	0.1151938
GO:0000774	adenyl-nuc	9	0.7186463	1.4922876	0.0179257	0.1394641	0.1151938
GO:0030545	receptor r	472	0.3582624	1.2103573	0.017982	0.1394641	0.1151938
GO:0070990	snRNP binc	8	0.7283866	1.4727144	0.018018	0.1394641	0.1151938
GO:0017153	sodium:dic	9	-0.777238	-1.929005	0.0180995	0.1394913	0.1152163
GO:0015378	sodium:chl	10	-0.717178	-1.796946	0.0182648	0.1401611	0.1157695
GO:0005549	odorant bi	24	-0.525583	-1.693587	0.0184049	0.1406324	0.1161587
GO:0016628	oxidoreduc	25	-0.46442	-1.52591	0.0186335	0.1417735	0.1171013
GO:0031492	nucleosoma	25	0.5950985	1.5368711	0.019025	0.1435302	0.1185522
GO:0043027	cysteine-t	25	0.5896694	1.5228502	0.019025	0.1435302	0.1185522
GO:0015036	disulfide	39	0.5346883	1.4752997	0.0192308	0.1441605	0.1190729
GO:0017137	Rab GTPase	162	0.4116796	1.3268474	0.0192698	0.1441605	0.1190729
GO:0043178	alcohol bi	82	-0.380267	-1.542574	0.0196078	0.1454415	0.120131
GO:0005154	epidermal	32	0.562886	1.5014024	0.0196305	0.1454415	0.120131
GO:0015193	L-proline	7	0.7629212	1.4877783	0.019685	0.1454415	0.120131
GO:0004867	serine-typ	84	-0.411537	-1.703869	0.02	0.1459592	0.1205585
GO:0019001	guanyl nuc	342	0.3701134	1.2388429	0.02	0.1459592	0.1205585
GO:0032561	guanyl rib	342	0.3701134	1.2388429	0.02	0.1459592	0.1205585
GO:0043130	ubiquitin	72	0.4679	1.4070624	0.0202343	0.1470688	0.121475
GO:0015141	succinate	6	-0.851562	-1.795122	0.0205761	0.1474639	0.1218014
GO:0043878	glyceralde	6	-0.857491	-1.807621	0.0205761	0.1474639	0.1218014
GO:0004713	protein ty	133	0.4211646	1.3376967	0.0205973	0.1474639	0.1218014
GO:0050661	NADP bindi	50	-0.421349	-1.587624	0.0206186	0.1474639	0.1218014
GO:0030492	hemoglobir	7	-0.819018	-1.8664	0.0208333	0.1484064	0.1225799
GO:0001851	complement	5	0.8350448	1.4972568	0.021419	0.151973	0.1255258
GO:0004497	monooxyger	90	-0.763204	-3.177169	0.0222222	0.1570487	0.1297182
GO:0016624	oxidoreduc	9	-0.745604	-1.850495	0.0226244	0.1591938	0.13149
GO:0004222	metalloenc	100	0.4411917	1.3746848	0.0227038	0.1591938	0.13149
GO:0004303	estradiol	10	-0.669384	-1.677194	0.0228311	0.1592361	0.1315249
GO:0003727	single-str	89	0.4499786	1.3844731	0.0229645	0.1592361	0.1315249
GO:0008134	transcript	642	0.3442927	1.1724646	0.022977	0.1592361	0.1315249
GO:0044769	ATPase act	20	0.6071671	1.5001506	0.0231707	0.1593433	0.1316135
GO:0046961	proton-tra	20	0.6071671	1.5001506	0.0231707	0.1593433	0.1316135
GO:0003713	transcript	309	0.3719289	1.2391744	0.024	0.1638188	0.1353101
GO:0140098	catalytic	335	0.3706685	1.2387231	0.024024	0.1638188	0.1353101
GO:0003923	GPI-anchor	5	0.8209392	1.4719652	0.0240964	0.1638188	0.1353101
GO:0015137	citrate tr	6	-0.822871	-1.73464	0.0246914	0.1655556	0.1367446
GO:0015142	tricarboxy	6	-0.822871	-1.73464	0.0246914	0.1655556	0.1367446
GO:0035473	lipase bir	6	-0.820056	-1.728705	0.0246914	0.1655556	0.1367446
GO:0015101	organic ca	25	-0.448307	-1.472971	0.0248447	0.1655556	0.1367446
GO:0070325	lipoprotei	25	-0.457438	-1.502972	0.0248447	0.1655556	0.1367446
GO:0005496	steroid bi	93	-0.455655	-1.883873	0.025	0.1655556	0.1367446
GO:0015205	nucleobase	7	-0.793995	-1.809376	0.025	0.1655556	0.1367446
GO:0031996	thioestera	11	0.6790206	1.4698403	0.0251572	0.165982	0.1370969
GO:0005164	tumor necr	30	0.5464795	1.454941	0.025522	0.1673217	0.1382035
GO:0000400	four-way j	16	0.6215493	1.474437	0.0255474	0.1673217	0.1382035
GO:0008009	chemokine	46	0.5031694	1.4234086	0.0257559	0.1675726	0.1384107
GO:0030553	cGMP bindi	14	-0.605481	-1.67302	0.0257732	0.1675726	0.1384107
GO:0042562	hormone bi	98	-0.396748	-1.66446	0.0263158	0.1689342	0.1395353
GO:0008242	omega pept	11	0.6706828	1.4517919	0.0264151	0.1689342	0.1395353

GO:0005328	neurotrans	26	-0.442086	-1.432914	0.0264901	0.1689342	0.1395353
GO:0008527	taste rece	26	-0.429681	-1.392709	0.0264901	0.1689342	0.1395353
GO:1901567	fatty acic	26	-0.440701	-1.428426	0.0264901	0.1689342	0.1395353
GO:0005007	fibroblast	5	0.8106171	1.4534574	0.0267738	0.1689342	0.1395353
GO:0015643	toxic subs	10	-0.657663	-1.647828	0.0273973	0.1689342	0.1395353
GO:0016453	C-acetyltr	5	-0.843301	-1.65558	0.027451	0.1689342	0.1395353
GO:0031849	olfactory	5	-0.853695	-1.675987	0.027451	0.1689342	0.1395353
GO:0022853	active ior	51	0.4949987	1.4223765	0.027533	0.1689342	0.1395353
GO:0042625	ATPase cou	51	0.4949987	1.4223765	0.027533	0.1689342	0.1395353
GO:1990446	U1 snRNP b	6	0.7752637	1.4541318	0.027668	0.1689342	0.1395353
GO:0005319	lipid trar	125	-0.549973	-2.392467	0.0277778	0.1689342	0.1395353
GO:0015248	sterol tra	31	-0.449696	-1.547435	0.0277778	0.1689342	0.1395353
GO:0016874	ligase act	117	-0.336109	-1.461911	0.0277778	0.1689342	0.1395353
GO:0019842	vitamin bi	123	-0.590281	-2.577664	0.0277778	0.1689342	0.1395353
GO:0020037	heme bindi	123	-0.620368	-2.70905	0.0277778	0.1689342	0.1395353
GO:0052689	carboxylic	116	-0.398008	-1.719468	0.0277778	0.1689342	0.1395353
GO:0055102	lipase inh	16	-0.593152	-1.667068	0.0277778	0.1689342	0.1395353
GO:0005518	collagen b	67	0.4619064	1.37321	0.0280475	0.1694877	0.1399925
GO:0030020	extracellu	40	0.516179	1.4331725	0.0280584	0.1694877	0.1399925
GO:0008519	ammonium t	30	-0.44522	-1.498669	0.0285714	0.1720058	0.1420724
GO:0070300	phosphatic	19	0.6046393	1.4797349	0.0290909	0.1735585	0.1433549
GO:0001664	G protein-	256	0.3797004	1.2568648	0.0291165	0.1735585	0.1433549
GO:0003714	transcript	227	0.3855198	1.2682358	0.0291751	0.1735585	0.1433549
GO:0097472	cyclin-der	29	0.5515807	1.4592106	0.029274	0.1735585	0.1433549
GO:0004984	olfactory	113	-0.543528	-2.315565	0.0294118	0.1735585	0.1433549
GO:0016616	oxidoreduc	111	-0.522186	-2.214819	0.0294118	0.1735585	0.1433549
GO:0008532	N-acetylla	8	0.7169395	1.4495698	0.029601	0.1736961	0.1434685
GO:0015556	C4-dicarbc	13	-0.578679	-1.561028	0.029703	0.1736961	0.1434685
GO:0017056	structural	25	0.5635893	1.455497	0.0297265	0.1736961	0.1434685
GO:0015294	solute:cat	100	-0.414793	-1.715168	0.030303	0.176488	0.1457746
GO:0030548	acetylchol	10	0.680391	1.4387844	0.0306513	0.1773612	0.1464958
GO:0099602	neurotrans	10	0.680391	1.4387844	0.0306513	0.1773612	0.1464958
GO:0048018	receptor l	440	0.3526357	1.1884985	0.031	0.1785144	0.1474483
GO:0008378	galactosyl	32	0.539745	1.4396778	0.0311778	0.1785144	0.1474483
GO:0016614	oxidoreduc	120	-0.533709	-2.300506	0.03125	0.1785144	0.1474483
GO:0046906	tetrapyrrc	131	-0.597916	-2.598281	0.03125	0.1785144	0.1474483
GO:0038132	neuregulir	5	0.8014584	1.4370357	0.0321285	0.1826316	0.150849
GO:0099511	voltage-ga	8	0.7084341	1.4323728	0.032175	0.1826316	0.150849
GO:0047760	butyrate-C	6	-0.80444	-1.695786	0.0329218	0.18625	0.1538377
GO:0042623	ATPase act	258	0.3777013	1.2503108	0.0330993	0.18625	0.1538377
GO:0019911	structural	9	0.6861044	1.4247135	0.0332907	0.18625	0.1538377
GO:0051880	G-quadrupl	9	0.688169	1.4290008	0.0332907	0.18625	0.1538377
GO:0015293	symporter	139	-0.384702	-1.709547	0.0333333	0.18625	0.1538377
GO:0005092	GDP-dissoc	15	0.6221257	1.4485261	0.0334572	0.18636	0.1539286
GO:0005310	dicarboxyl	37	-0.421906	-1.51119	0.0347826	0.1925837	0.1590692
GO:0032934	sterol bir	55	-0.369172	-1.386238	0.0348837	0.1925837	0.1590692
GO:0070034	telomerase	21	0.5820996	1.4579638	0.0348977	0.1925837	0.1590692
GO:0030276	clathrin b	54	0.4764296	1.3773015	0.0351648	0.1929219	0.1593485
GO:0042288	MHC class	16	0.6103046	1.4477622	0.0352798	0.1929219	0.1593485
GO:0015136	sialic aci	8	-0.702946	-1.65986	0.0355556	0.1929219	0.1593485
GO:0004704	NF-kappaB-	6	0.7542695	1.4147539	0.0355731	0.1929219	0.1593485

GO:0042289	MHC class	6	0.7554619	1.4169905	0.0355731	0.1929219	0.1593485
GO:0004033	aldo-keto	23	-0.455726	-1.461582	0.0357143	0.1929219	0.1593485
GO:0004252	serine-typ	141	-0.434789	-1.927742	0.0357143	0.1929219	0.1593485
GO:0015095	magnesium	14	0.6293325	1.4444426	0.0358911	0.193293	0.159655
GO:0051721	protein ph	31	0.5351205	1.4226563	0.0361305	0.1939982	0.1602375
GO:0043531	ADP bindir	35	0.5286882	1.4318469	0.036405	0.1948867	0.1609714
GO:0004364	glutathior	24	-0.45313	-1.460122	0.0368098	0.1964655	0.1622755
GO:0008430	selenium b	6	-0.797286	-1.680706	0.037037	0.196961	0.1626848
GO:0035255	ionotropic	30	0.5371636	1.4301384	0.037123	0.196961	0.1626848
GO:0005504	fatty acic	33	-0.422333	-1.473054	0.0384615	0.201912	0.1667741
GO:0005506	iron ion b	143	-0.590041	-2.608288	0.0384615	0.201912	0.1667741
GO:0016289	CoA hydrol	20	-0.50288	-1.526113	0.0384615	0.201912	0.1667741
GO:0051861	glycolipic	21	0.575241	1.4407851	0.0385078	0.201912	0.1667741
GO:0004298	threonine-	19	0.5880227	1.4390689	0.0387879	0.2021945	0.1670075
GO:0070003	threonine-	19	0.5880227	1.4390689	0.0387879	0.2021945	0.1670075
GO:0035326	enhancer b	126	0.4091577	1.2921621	0.0392157	0.2038304	0.1683587
GO:0031543	peptidyl-p	12	0.6398248	1.4137161	0.0403531	0.2085834	0.1722845
GO:0070405	ammonium i	66	-0.341686	-1.338068	0.0405405	0.2085834	0.1722845
GO:0016796	exonucleas	54	0.4730913	1.3676508	0.0406593	0.2085834	0.1722845
GO:0019787	ubiquitin-	384	0.351879	1.181703	0.041	0.2085834	0.1722845
GO:0048039	ubiquinone	6	-0.791534	-1.668581	0.0411523	0.2085834	0.1722845
GO:0017049	GTP-Rho bi	20	0.5791172	1.4308465	0.0414634	0.2085834	0.1722845
GO:0005534	galactose	5	0.7899932	1.4164781	0.0414993	0.2085834	0.1722845
GO:0031694	alpha-2A a	5	0.7903282	1.417079	0.0414993	0.2085834	0.1722845
GO:0005342	organic ac	145	-0.475692	-2.086355	0.0416667	0.2085834	0.1722845
GO:0015081	sodium ior	145	-0.357864	-1.569567	0.0416667	0.2085834	0.1722845
GO:0016705	oxidoreduc	145	-0.598862	-2.626568	0.0416667	0.2085834	0.1722845
GO:0016885	ligase act	7	-0.752774	-1.715442	0.0416667	0.2085834	0.1722845
GO:0046943	carboxylic	145	-0.475692	-2.086355	0.0416667	0.2085834	0.1722845
GO:0015301	anion:anic	30	0.530574	1.4125943	0.0417633	0.2085834	0.1722845
GO:0032356	oxidized I	9	0.6798399	1.411705	0.0422535	0.2098592	0.1733383
GO:0032357	oxidized p	9	0.6798399	1.411705	0.0422535	0.2098592	0.1733383
GO:0017069	snRNA binc	37	0.515956	1.4119514	0.042841	0.2121877	0.1752616
GO:0031005	filamin bi	10	0.669232	1.4151871	0.0434227	0.2144747	0.1771506
GO:0003730	mRNA 3'-UT	86	0.4345576	1.3322423	0.0439331	0.2163975	0.1787388
GO:0019829	cation-tra	50	0.483842	1.3867189	0.0441989	0.2171089	0.1793263
GO:0004983	neuropepti	8	-0.673653	-1.59069	0.0444444	0.217122	0.1793372
GO:0016742	hydroxymet	8	-0.677685	-1.600211	0.0444444	0.217122	0.1793372
GO:0008376	acetylgala	43	0.4936785	1.383081	0.044843	0.2184347	0.1804214
GO:0051010	microtubul	17	0.5901582	1.4183377	0.0449575	0.2184347	0.1804214
GO:0016595	glutamate	10	-0.614895	-1.540669	0.0456621	0.221257	0.1827526
GO:0043522	leucine zi	10	0.6661328	1.4086335	0.045977	0.222046	0.1834043
GO:0005253	anion char	85	0.4320949	1.3219292	0.0460733	0.222046	0.1834043
GO:0016891	endoribonu	25	0.5406741	1.3963175	0.0463734	0.2228913	0.1841025
GO:0015179	L-amino ac	51	0.4791877	1.3769437	0.0473568	0.2270081	0.1875028
GO:0008417	fucosyltra	13	0.6241537	1.4078649	0.0475	0.2270856	0.1875668
GO:0003743	translatic	47	0.4837683	1.371939	0.0482063	0.2285234	0.1887545
GO:0003756	protein di	16	0.5958493	1.4134715	0.0486618	0.2285234	0.1887545
GO:0016864	intramolec	16	0.5958493	1.4134715	0.0486618	0.2285234	0.1887545
GO:0022889	serine tra	6	0.7435948	1.3947317	0.0487484	0.2285234	0.1887545
GO:0016790	thiolester	35	-0.373013	-1.319156	0.0487805	0.2285234	0.1887545

GO:003195[illegible]	medium-cha	8	-0.669312	-1.580439	0.0488889	0.2285234	0.1887545
GO:010239[illegible]	decanoate-	8	-0.669312	-1.580439	0.0488889	0.2285234	0.1887545
GO:001650[illegible]	peptidase	35	0.5113444	1.3848747	0.0489192	0.2285234	0.1887545
GO:000369[illegible]	double-str	913	0.3296966	1.1311593	0.048951	0.2285234	0.1887545
GO:001528[illegible]	porin acti	5	0.7844116	1.4064702	0.0495315	0.2306309	0.1904952
GO:003202[illegible]	myosin lig	7	0.7249282	1.4136879	0.0498688	0.2315983	0.1912943

rank	leading_e	core_enri	Category	geneSymbol
4298	tags=39%,	4605/991/	GO_BP	MYBL2/CDC20/BIRC5/PLK1/CCNB1/KIF2C/KIF4
4980	tags=47%,	4605/8877/	GO_BP	MYBL2/SPHK1/SFN/CDC20/BIRC5/PLK1/CCNB1/
5318	tags=34%,	6696/7345/	GO_BP	SPP1/UCHL1/ALDOA/S100A6/RAP1GAP/LIMK1/M
6656	tags=49%,	8877/1001	GO_BP	SPHK1/CD24/S100A9/CRP/LAPTM4B/PAEP/TREM
6695	tags=50%,	8877/1001	GO_BP	SPHK1/CD24/CRP/LAPTM4B/PAEP/TREM2/PYCAR
6206	tags=46%,	8877/5047/	GO_BP	SPHK1/PAEP/TREM2/PYCARD/CYBA/LY96/MDK/M
4582	tags=36%,	768/6692/1	GO_BP	CA9/SPINT1/CTHRC1/SOX4/MDK/SOX9/MMP12/T
5064	tags=46%,	6286/5315/	GO_BP	S100P/PKM/MMP9/S100A9/S100A11/CXCL1/ALD
4258	tags=43%,	6286/5315/	GO_BP	S100P/PKM/SPHK1/MMP9/S100A9/S100A11/CXC
4258	tags=45%,	6286/5315/	GO_BP	S100P/PKM/MMP9/S100A9/S100A11/CXCL1/ALD
4258	tags=46%,	6286/5315/	GO_BP	S100P/PKM/MMP9/S100A9/S100A11/CXCL1/ALD
5064	tags=46%,	6286/5315/	GO_BP	S100P/PKM/MMP9/S100A9/S100A11/CXCL1/ALD
4072	tags=39%,	6286/5315/	GO_BP	S100P/PKM/MMP9/S100A9/S100A11/CRP/CXCL1
4258	tags=46%,	6286/5315/	GO_BP	S100P/PKM/MMP9/S100A9/S100A11/CXCL1/ALD
6206	tags=44%,	2539/4318/	GO_BP	G6PD/MMP9/TESC/TREM2/CCNB2/UBD/SOX4/MDK
6267	tags=45%,	4318/54997	GO_BP	MMP9/TESC/TREM2/UBD/SOX4/MDK/ANXA2/FCER
5207	tags=41%,	100133941/	GO_BP	CD24/TESC/S100A9/CCL20/MUC13/CRP/TREM2/
6128	tags=53%,	6280/56667	GO_BP	S100A9/MUC13/CYBA/LIMK1/LY96/TNFRSF21/I
6475	tags=55%,	100133941/	GO_BP	CD24/MUC13/LIMK1/TNFRSF21/FCER1G/ARPC1B
6454	tags=51%,	8877/6282/	GO_BP	SPHK1/S100A11/PTTG1/HMGA1/CDT1/PKIB/AUR
6444	tags=52%,	9232/3159/	GO_BP	PTTG1/HMGA1/UBE2T/FOXM1/TRIP13/CDCA5/RE
6885	tags=50%,	891/79019/	GO_BP	CCNB1/CENPM/HMGA1/AURKB/CENPA/SOX9/ASF1
7311	tags=61%,	891/3958/	GO_BP	CCNB1/LGALS3/RPP25/TGFB1/BOP1/RBM38/DDX
6706	tags=56%,	5997/6659/	GO_BP	RGS2/SOX4/RPL39L/GAPDH/IGF2BP2/TYMS/KRT
4233	tags=38%,	6286/5315/	GO_BP	S100P/PKM/MMP9/S100A9/S100A11/CXCL1/ALD
3729	tags=30%,	6280/6364/	GO_BP	S100A9/CCL20/CXCL1/RAP1GAP/TREM2/CXCL6/
6444	tags=51%,	2810/1123	GO_BP	SFN/EGLN3/PLK1/CCNB1/PTTG1/HMGA1/PYCARD
4298	tags=37%,	4605/991/7	GO_BP	MYBL2/CDC20/UCHL1/BIRC5/PLK1/CCNB1/KIF2
4980	tags=46%,	8877/2810/	GO_BP	SPHK1/SFN/CDC20/PLK1/CCNB1/PTTG1/ASNS/C
4023	tags=31%,	8877/4318/	GO_BP	SPHK1/MMP9/CDC20/CD24/BIRC5/CCNB1/S100A
7528	tags=66%,	3958/5554	GO_BP	LGALS3/RBM38/DDX39A/SNRPB/SF3B4/ALYREF/
6823	tags=49%,	991/7345/	GO_BP	CDC20/UCHL1/PLK1/CCNB1/PTTG1/TREM2/KCTD
4993	tags=45%,	8877/2810/	GO_BP	SPHK1/SFN/CDC20/PLK1/CCNB1/PTTG1/CDT1/U
5809	tags=45%,	8877/4318/	GO_BP	SPHK1/MMP9/SFN/CCNB1/TREM2/KCTD17/CDT1/
5592	tags=44%,	4605/4318/	GO_BP	MYBL2/MMP9/SFN/S100A9/EGLN3/NQO1/PYCARD
6315	tags=58%,	10053/651	GO_BP	AP1M2/SLC1A5/HMGA1/KRT19/CENPA/CXCL8/SL
7272	tags=63%,	891/1869/	GO_BP	CCNB1/E2F1/LGALS3/IGF2BP2/YWHAZ/FLNA/RB
7115	tags=52%,	891/9212/	GO_BP	CCNB1/AURKB/CDK1/CCNA2/LIF/TGFB1/AURKA/
5327	tags=45%,	8877/4318/	GO_BP	SPHK1/MMP9/CD24/CCL20/BIRC5/CXCL1/TREM2
5981	tags=48%,	4318/4316/	GO_BP	MMP9/MMP7/PLK1/CCNB1/KIF2C/HMGA1/TREM2/
7442	tags=71%,	54913/232	GO_BP	RPP25/BOP1/SNRPB/DCAF13/EXOSC4/NOP56/SN
5429	tags=41%,	2919/226/	GO_BP	CXCL1/ALDOA/KRT19/TMSB10/CAPG/PYCARD/LI
6243	tags=46%,	5347/7957	GO_BP	PLK1/EPS8L3/RAP1GAP/KCTD17/TUBA4A/CDK1/
5429	tags=42%,	2919/226/	GO_BP	CXCL1/ALDOA/KRT19/TMSB10/CAPG/PYCARD/LI
6206	tags=43%,	2539/4318/	GO_BP	G6PD/MMP9/TESC/TREM2/UBD/SOX4/MDK/TOP2A
5724	tags=42%,	2539/4318/	GO_BP	G6PD/MMP9/TESC/TREM2/UBD/EPO/ANXA2/FCER
3727	tags=34%,	100133941/	GO_BP	CD24/TESC/AGR2/PYCARD/MDK/CXCL8/TNFRSF2
6823	tags=47%,	991/7345/	GO_BP	CDC20/UCHL1/PLK1/CCNB1/PTTG1/LPCAT1/LAP
3729	tags=30%,	8877/4318/	GO_BP	SPHK1/MMP9/CCL20/PHLDA2/TMSB10/TREM2/PY
5313	tags=35%,	6696/991/	GO_BP	SPP1/CDC20/S100A9/UCHL1/S100A6/RAP1GAP/

6247	tags=45%,	6696/991/	GO_BP	SPP1/CDC20/S100A9/EPS8L3/RAP1GAP/TREM2/
8345	tags=58%,	8877/991/	GO_BP	SPHK1/CDC20/PLK1/UBD/LIMK1/UBE2T/SOX4/U
5036	tags=37%,	2810/10551	GO_BP	SFN/AGR2/PLK1/CRP/PAEP/TREM2/CDT1/PYCAR
6199	tags=49%,	2810/5347/	GO_BP	SFN/PLK1/CDT1/AURKB/CENPA/E2F1/CDCA5/SO
6277	tags=50%,	79019/916	GO_BP	CENPM/TMSB10/CDT1/CAPG/PYCARD/CYBA/CENP
6225	tags=54%,	5315/80201	GO_BP	PKM/HKDC1/ALDOA/ENO2/PFKP/E2F1/ENO1/SLC
7468	tags=65%,	54913/232	GO_BP	RPP25/BOP1/LAGE3/HRAS/DCAF13/PUSL1/EEF1
4258	tags=46%,	6286/5315/	GO_BP	S100P/PKM/MMP9/S100A9/S100A11/CXCL1/ALD
3774	tags=29%,	8877/4318/	GO_BP	SPHK1/MMP9/CCL20/PHLDA2/TMSB10/TREM2/PY
4982	tags=38%,	8877/4318/	GO_BP	SPHK1/MMP9/CCL20/TREM2/PYCARD/MDK/CXCL8
5381	tags=43%,	100133941/	GO_BP	CD24/PYCARD/SOX4/MDK/TNFRSF21/EPO/FCER1
4258	tags=46%,	6286/5315/	GO_BP	S100P/PKM/MMP9/S100A9/S100A11/CXCL1/ALD
3729	tags=30%,	6280/6364/	GO_BP	S100A9/CCL20/CXCL1/RAP1GAP/TREM2/CXCL6/
6706	tags=55%,	5997/6659/	GO_BP	RGS2/SOX4/RPL39L/GAPDH/IGF2BP2/TYMS/KRT
5483	tags=44%,	4605/4318/	GO_BP	MYBL2/MMP9/SFN/S100A9/EGLN3/NQO1/PYCARD
4096	tags=31%,	8877/4318/	GO_BP	SPHK1/MMP9/SFN/BIRC5/NQO1/PLK1/TREM2/AS
5483	tags=44%,	4605/4318/	GO_BP	MYBL2/MMP9/SFN/S100A9/EGLN3/NQO1/PYCARD
4829	tags=35%,	8877/4318/	GO_BP	SPHK1/MMP9/SFN/BIRC5/NQO1/PLK1/TREM2/AS
6823	tags=49%,	991/7345/	GO_BP	CDC20/UCHL1/PLK1/CCNB1/PTTG1/KCTD17/AUR
6249	tags=45%,	8877/5347/	GO_BP	SPHK1/PLK1/EPS8L3/TMSB10/RAP1GAP/KCTD17
5933	tags=45%,	79574/797	GO_BP	EPS8L3/KCTD17/CDT1/PYCARD/LIMK1/SOX9/IC
6315	tags=57%,	10053/651	GO_BP	AP1M2/SLC1A5/HMGA1/KRT19/CXCL6/CENPA/CX
6315	tags=56%,	10053/651	GO_BP	AP1M2/SLC1A5/S100A9/HMGA1/KRT19/CXCL6/C
4335	tags=26%,	6696/6690/	GO_BP	SPP1/SPINK1/CLDN4/MMP9/MMP7/PLK1/CCNB1/
5809	tags=56%,	2810/991/	GO_BP	SFN/CDC20/PLK1/CCNB1/CCNB2/CDT1/UBD/AUR
4233	tags=40%,	6286/5315/	GO_BP	S100P/PKM/MMP9/S100A9/S100A11/CXCL1/ALD
6062	tags=45%,	6280/6364/	GO_BP	S100A9/CCL20/MUC13/CRP/APOA4/TREM2/PYCA
6156	tags=48%,	6280/5666	GO_BP	S100A9/MUC13/TREM2/PYCARD/CYBA/LY96/MMP
5182	tags=35%,	2810/1001	GO_BP	SFN/CD24/TESC/SPINT1/S100A9/TREM2/CTHRC
4835	tags=43%,	2810/991/	GO_BP	SFN/CDC20/PLK1/CCNB1/PTTG1/CDT1/AURKB/F
6306	tags=46%,	2810/1001	GO_BP	SFN/CD24/TESC/UCHL1/PLK1/CCNB1/CCNB2/PY
6206	tags=48%,	100133941/	GO_BP	CD24/IMPDH1/PYCARD/SOX4/MDK/TNFRSF21/EP
3955	tags=37%,	4605/8877/	GO_BP	MYBL2/SPHK1/CDC20/BIRC5/PLK1/CCNB1/PTTG
6206	tags=43%,	2539/4318/	GO_BP	G6PD/MMP9/TESC/TREM2/CCNB2/UBD/SOX4/MDK
4588	tags=33%,	768/6692/	GO_BP	CA9/SPINT1/GCNT3/CTHRC1/SOX4/MDK/SOX9/M
5383	tags=42%,	8877/1001	GO_BP	SPHK1/CD24/TREM2/PYCARD/MDK/TNFRSF21/EP
3442	tags=37%,	9123/6280/	GO_BP	SLC16A3/S100A9/CCL20/CXCL1/TREM2/PYCARD
6427	tags=49%,	6696/991/	GO_BP	SPP1/CDC20/PLK1/CCNB1/PTTG1/APOA4/HMGA1
3743	tags=29%,	8877/4318/	GO_BP	SPHK1/MMP9/CCL20/PHLDA2/TMSB10/TREM2/PY
4418	tags=36%,	8877/4318/	GO_BP	SPHK1/MMP9/CCL20/TREM2/PYCARD/MDK/CXCL8
6225	tags=46%,	2810/991/1	GO_BP	SFN/CDC20/CD24/TESC/UCHL1/PLK1/CCNB1/CC
5207	tags=39%,	6280/6364/	GO_BP	S100A9/CCL20/EGLN3/APOA4/RAP1GAP/PYCARD
4258	tags=40%,	6280/5347/	GO_BP	S100A9/PLK1/TMSB10/CAPG/PYCARD/LIMK1/TP
6259	tags=48%,	10053/734	GO_BP	AP1M2/UCHL1/PLK1/CCNB1/KIF2C/CDT1/CENPA
6249	tags=46%,	10053/734	GO_BP	AP1M2/UCHL1/PLK1/CCNB1/KIF2C/CDT1/CENPA
4326	tags=28%,	6696/991/	GO_BP	SPP1/CDC20/SPINT1/S100A9/TREM2/LIMK1/RG
4291	tags=29%,	991/6692/	GO_BP	CDC20/SPINT1/S100A9/TREM2/LIMK1/RGS2/MD
4383	tags=29%,	6696/2539/	GO_BP	SPP1/G6PD/CDC20/SPINT1/S100A9/PAEP/TREM
6214	tags=46%,	2810/10551	GO_BP	SFN/AGR2/PLK1/TREM2/CDT1/CYBA/E2F1/CDCA
4096	tags=31%,	8877/4318/	GO_BP	SPHK1/MMP9/SFN/BIRC5/AGR2/NQO1/PLK1/PYC
6379	tags=50%,	8877/2810/	GO_BP	SPHK1/SFN/CD24/PLK1/CCNB1/LAPTM4B/TREM2

7323	tags=50%,	8877/991/7	GO_BP	SPHK1/CDC20/UCHL1/PLK1/UBD/LIMK1/UBE2T/
7508	tags=53%,	4605/991/3	GO_BP	MYBL2/CDC20/BIRC5/PLK1/KRT19/KCTD17/KIF
6345	tags=51%,	2810/1001	GO_BP	SFN/CD24/TESC/AGR2/TREM2/ATP1B3/E2F1/AN
4835	tags=37%,	6696/8877/	GO_BP	SPP1/SPHK1/AGR2/HMGA1/PYCR1/HSPB8/PYCAR
4258	tags=36%,	11004/226/	GO_BP	KIF2C/ALDOA/KRT19/TMSB10/CAPG/PYCARD/LI
3723	tags=28%,	1364/1001	GO_BP	CLDN4/CD24/S100A9/S100A11/APOA4/PYCARD/
6243	tags=45%,	5347/7957	GO_BP	PLK1/EPS8L3/RAP1GAP/KCTD17/TUBA4A/CDK1/
6247	tags=45%,	6696/991/6	GO_BP	SPP1/CDC20/S100A9/EPS8L3/RAP1GAP/TREM2/
4102	tags=45%,	2810/991/5	GO_BP	SFN/CDC20/PLK1/CCNB1/CDT1/UBD/AURKB/TPX
4980	tags=49%,	4605/8877/	GO_BP	MYBL2/SPHK1/SFN/CDC20/BIRC5/PLK1/CCNB1/
7272	tags=53%,	54997/542	GO_BP	TESC/TREM2/MDK/LGALS9/FOXJ1/LGALS3/MMP1
4298	tags=37%,	2810/1055	GO_BP	SFN/AGR2/PLK1/TREM2/CDT1/E2F1/CDCA5/CDK
3743	tags=30%,	8877/4318/	GO_BP	SPHK1/MMP9/CCL20/PHLDA2/TMSB10/TREM2/PY
4418	tags=36%,	8877/4318/	GO_BP	SPHK1/MMP9/CCL20/TREM2/PYCARD/MDK/CXCL8
7311	tags=64%,	10212/662	GO_BP	DDX39A/SNRPB/SF3B4/ALYREF/RALY/SNRPD2/S
7311	tags=64%,	10212/662	GO_BP	DDX39A/SNRPB/SF3B4/ALYREF/RALY/SNRPD2/S
7311	tags=64%,	10212/662	GO_BP	DDX39A/SNRPB/SF3B4/ALYREF/RALY/SNRPD2/S
4796	tags=42%,	768/5315/1	GO_BP	CA9/PKM/CD24/EGLN3/CCNB1/CYBA/E2F1/SLC2
4258	tags=45%,	6286/5315/	GO_BP	S100P/PKM/MMP9/S100A9/S100A11/CXCL1/ALD
5383	tags=44%,	8877/1001	GO_BP	SPHK1/CD24/TREM2/PYCARD/MDK/TNFRSF21/EP
6100	tags=50%,	100133941/	GO_BP	CD24/S100A9/MUC13/CYBA/LIMK1/LY96/TNFRS
7311	tags=61%,	891/3958/5	GO_BP	CCNB1/LGALS3/RBM38/DDX39A/SNRPB/SF3B4/A
6199	tags=51%,	2810/4321/	GO_BP	SFN/MMP12/KPNA2/ECT2/TGFB1/FLNA/DDX39A/
3723	tags=37%,	100133941/	GO_BP	CD24/S100A9/APOA4/PYCARD/MDK/TNFRSF21/E
6278	tags=47%,	79574/590	GO_BP	EPS8L3/RAP1GAP/LIMK1/FOXM1/EPO/STMN1/SH
6338	tags=50%,	5997/6659/	GO_BP	RGS2/SOX4/E2F1/TGFB1/GAPDH/IGF2BP2/TYMS
3862	tags=42%,	2810/991/5	GO_BP	SFN/CDC20/PLK1/CCNB1/PTTG1/CDT1/AURKB/S
5252	tags=35%,	6696/991/6	GO_BP	SPP1/CDC20/S100A9/LIMK1/RGS2/MDK/EPO/DB
3838	tags=34%,	6696/4318/	GO_BP	SPP1/MMP9/SPINT1/MMP7/SOX9/MMP12/ANXA2/
3729	tags=32%,	8877/4318/	GO_BP	SPHK1/MMP9/CCL20/TREM2/PYCARD/MDK/CXCL8
7666	tags=61%,	11004/315	GO_BP	KIF2C/HMGA1/CAPG/STMN1/SH3BP1/MICAL1/KI
6693	tags=56%,	991/5347/8	GO_BP	CDC20/PLK1/CCNB1/PTTG1/HMGA1/CDT1/PKIB/
5216	tags=42%,	9168/8162	GO_BP	TMSB10/CDT1/CAPG/PYCARD/CRYAB/DBN1/HJUR
4258	tags=46%,	6286/5315/	GO_BP	S100P/PKM/MMP9/S100A9/S100A11/CXCL1/ALD
4258	tags=46%,	6286/5315/	GO_BP	S100P/PKM/MMP9/S100A9/S100A11/CXCL1/ALD
4980	tags=50%,	2810/991/5	GO_BP	SFN/CDC20/PLK1/CCNB1/CCNB2/CDT1/UBD/AUR
5055	tags=44%,	8877/2810/	GO_BP	SPHK1/SFN/CCNB1/ASNS/CDT1/AURKB/SOX4/E2
6199	tags=51%,	2810/3159/	GO_BP	SFN/HMGA1/MMP12/KPNA2/ECT2/TGFB1/FLNA/D
4980	tags=48%,	2810/991/3	GO_BP	SFN/CDC20/BIRC5/PLK1/CCNB1/PTTG1/KIF2C/
5011	tags=40%,	2810/1001	GO_BP	SFN/CD24/UCHL1/PLK1/CCNB1/CCNB2/PYCARD/
5704	tags=46%,	4318/2810/	GO_BP	MMP9/SFN/CD24/S100A9/PYCARD/LY96/LCN2/E
5290	tags=44%,	9168/822/2	GO_BP	TMSB10/CAPG/PYCARD/LIMK1/CRYAB/DBN1/ICA
7311	tags=58%,	891/1869/1	GO_BP	CCNB1/E2F1/IGF2BP2/YWHAZ/RBM38/PRKCD/HS
4388	tags=41%,	2810/1055	GO_BP	SFN/AGR2/PLK1/TREM2/CDT1/E2F1/CDK1/NDC8
4322	tags=34%,	54997/105	GO_BP	TESC/AGR2/PLK1/TREM2/ATP1B3/ANXA2/FCER1
4263	tags=40%,	4605/8877/	GO_BP	MYBL2/SPHK1/CDC20/BIRC5/PLK1/CCNB1/PTTG
6128	tags=56%,	56667/398	GO_BP	MUC13/LIMK1/TNFRSF21/FCER1G/ARPC1B/SRC/
6225	tags=61%,	1869/1064	GO_BP	E2F1/IGF2BP2/YWHAZ/RNASEH2A/RBM38/FEN1/
6225	tags=61%,	1869/1064	GO_BP	E2F1/IGF2BP2/YWHAZ/RBM38/PRKCD/HSPB1/SM
4258	tags=36%,	226/9168/8	GO_BP	ALDOA/TMSB10/CAPG/PYCARD/LIMK1/DBN1/ICA
6533	tags=53%,	991/5347/8	GO_BP	CDC20/PLK1/CCNB1/PTTG1/HMGA1/TMSB10/CDT

3723	tags=35%,	100133941/	GO_BP	CD24/PYCARD/MDK/TNFRSF21/EPO/ICAM1/LGAL
5313	tags=40%,	6280/7957	GO_BP	S100A9/EPS8L3/KCTD17/LIMK1/RGS2/LCN2/MD
5216	tags=41%,	6696/9168/	GO_BP	SPP1/TMSB10/CAPG/PYCARD/LIMK1/DBN1/ICAM
7468	tags=67%,	54913/232	GO_BP	RPP25/BOP1/LAGE3/DCAF13/PUSL1/EXOSC4/TS
4796	tags=43%,	768/5315/1	GO_BP	CA9/PKM/CD24/EGLN3/CCNB1/CYBA/E2F1/SLC2
6315	tags=50%,	6690/5047/	GO_BP	SPINK1/PAEP/PYCARD/CYBA/CXCL6/LY96/PGC/
5023	tags=45%,	100133941/	GO_BP	CD24/TREM2/PYCARD/MDK/EPO/CTSC/FCER1G/L
6100	tags=49%,	6282/8162	GO_BP	S100A11/CDT1/PKIB/AURKB/FOXM1/CDC6/KPNA
6656	tags=53%,	100133941/	GO_BP	CD24/PYCARD/MDK/TNFRSF21/EPO/LGALS9/FOX
4863	tags=40%,	6696/5666	GO_BP	SPP1/MUC13/ALDOA/LPCAT1/PKIB/AURKB/SOX9
5182	tags=44%,	768/5315/1	GO_BP	CA9/PKM/CD24/EGLN3/CCNB1/CYBA/E2F1/SLC2
6345	tags=61%,	2810/1001	GO_BP	SFN/CD24/E2F1/CLSTN1/ITGAM/STX3/YWHAZ/I
4102	tags=46%,	2810/991/	GO_BP	SFN/CDC20/PLK1/CCNB1/CDT1/UBD/AURKB/TPX
5734	tags=48%,	4318/2810/	GO_BP	MMP9/SFN/S100A9/PYCARD/E2F1/EPO/ENO1/CT
5023	tags=46%,	100133941/	GO_BP	CD24/TREM2/PYCARD/MDK/EPO/CTSC/FCER1G/L
4835	tags=47%,	6282/3159/	GO_BP	S100A11/HMGA1/CDT1/RRM2/CDC6/RECQL4/CDK
4661	tags=46%,	991/332/5	GO_BP	CDC20/BIRC5/PLK1/CCNB1/PTTG1/KIF2C/CDT1
3862	tags=43%,	2810/991/	GO_BP	SFN/CDC20/PLK1/CCNB1/PTTG1/CDT1/AURKB/S
3723	tags=36%,	100133941/	GO_BP	CD24/PYCARD/MDK/TNFRSF21/EPO/LGALS9/FOX
5227	tags=44%,	9168/822/	GO_BP	TMSB10/CAPG/PYCARD/UBE2S/DBN1/ICAM1/ARP
3692	tags=35%,	100133941/	GO_BP	CD24/IMPDH1/PYCARD/TNFRSF21/EPO/LGALS9/
6353	tags=65%,	54913/232	GO_BP	RPP25/BOP1/DCAF13/EXOSC4/NOP56/RAN/MRTO
3774	tags=37%,	6280/6364/	GO_BP	S100A9/CCL20/CXCL1/CXCL6/MDK/CXCL8/CXCL
3692	tags=35%,	100133941/	GO_BP	CD24/IMPDH1/TREM2/PYCARD/TNFRSF21/EPO/L
3723	tags=36%,	100133941/	GO_BP	CD24/PYCARD/MDK/TNFRSF21/EPO/ICAM1/LGAL
3703	tags=32%,	2539/8877/	GO_BP	G6PD/SPHK1/MMP9/NQO1/APOA4/PYCR1/CYBA/G
4950	tags=43%,	7153/9319/	GO_BP	TOP2A/TRIP13/RECQL4/KPNA2/MCM2/CDC45/MC
6229	tags=43%,	7345/1100	GO_BP	UCHL1/KIF2C/KIF4A/KIF20A/CABYR/DLGAP5/K
6595	tags=53%,	222962/10	GO_BP	SLC29A4/IGF2BP2/DDX39A/SMG5/ABCD1/IGF2B
6353	tags=65%,	54913/232	GO_BP	RPP25/BOP1/DCAF13/EXOSC4/TCOF1/NOP56/GT
6181	tags=48%,	3159/8318/	GO_BP	HMGA1/CDC45/TGFB1/NUP62/EZH2/DNMT1/RAN/
6677	tags=53%,	332/1627/1	GO_BP	BIRC5/DBN1/DNM1/CLSTN1/TYROBP/RCC2/GPC4
6060	tags=47%,	27242/578/	GO_BP	TNFRSF21/BAK1/FOXJ1/MMP14/TYROBP/TGFB1/
4151	tags=51%,	5347/891/	GO_BP	PLK1/CCNB1/CCNB2/AURKB/TPX2/FOXM1/CDC6/
4863	tags=45%,	100133941/	GO_BP	CD24/PYCARD/MDK/EPO/LGALS9/SRC/MMP14/TY
5545	tags=48%,	4318/2810/	GO_BP	MMP9/SFN/CD24/S100A9/PYCARD/E2F1/EPO/EN
3727	tags=39%,	8877/1401/	GO_BP	SPHK1/CRP/TREM2/PYCARD/CYBA/LIMK1/NCF2/
4826	tags=44%,	2810/891/	GO_BP	SFN/CCNB1/FOXM1/SOX4/CXCL8/E2F1/CDKN3/C
6240	tags=69%,	10053/110	GO_BP	AP1M2/KIF2C/TREM2/PYCARD/CYBA/KIF4A/NCF
7442	tags=72%,	23246/662	GO_BP	BOP1/SNRPB/SNRPD2/SNRPD1/MRTO4/SF3A2/RU
5206	tags=43%,	81620/291	GO_BP	CDT1/PYCARD/ICAM1/ARPC1B/PFN2/SRC/ARL2/
4909	tags=47%,	2810/891/	GO_BP	SFN/CCNB1/CDT1/SOX4/RRM2/E2F1/CDC6/CDKN
3692	tags=35%,	100133941/	GO_BP	CD24/IMPDH1/PYCARD/TNFRSF21/EPO/LGALS9/
6117	tags=45%,	2539/9133/	GO_BP	G6PD/CCNB2/CXCL6/SOX9/EPO/FCER1G/LGALS9
6128	tags=57%,	27242/915/	GO_BP	TNFRSF21/CD3D/LGALS3/RIPK2/ADA/CMTM3/LA
3955	tags=31%,	991/5347/	GO_BP	CDC20/PLK1/PTTG1/TOP2A/TRIP13/NUF2/PKMY
4663	tags=46%,	2810/891/	GO_BP	SFN/CCNB1/PYCARD/AURKB/TPX2/FOXM1/SOX4/
5055	tags=48%,	8877/2810/	GO_BP	SPHK1/SFN/CCNB1/CDT1/AURKB/SOX4/E2F1/CD
3132	tags=39%,	4605/8877/	GO_BP	MYBL2/SPHK1/CDC20/BIRC5/PLK1/CCNB1/PTTG
4496	tags=39%,	115908/41	GO_BP	CTHRC1/MDK/SOX9/SULF1/ETV5/DAB2/LIF/SAP
4759	tags=46%,	2810/891/	GO_BP	SFN/CCNB1/CDT1/SOX4/RRM2/E2F1/CDC6/CDKN

4151	tags=52%,	5347/891/	GO_BP	PLK1/CCNB1/CCNB2/AURKB/TPX2/FOXM1/CDC6/
4636	tags=36%,	4318/54997	GO_BP	MMP9/TESC/TREM2/UBD/ANXA2/FCER1G/SRC/LI
5216	tags=45%,	9168/822/	GO_BP	TMSB10/CAPG/PYCARD/DBN1/ICAM1/ARPC1B/PF
6235	tags=71%,	23636/613	GO_BP	NUP62/RPL8/CCL5/NUP210/TRIM11/EIF3B/RPL
3678	tags=36%,	100133941/	GO_BP	CD24/PYCARD/MDK/EPO/ICAM1/LGALS9/SRC/TG
6595	tags=58%,	10644/102	GO_BP	IGF2BP2/DDX39A/SMG5/IGF2BP1/MVP/NUP62/M
6595	tags=58%,	10644/102	GO_BP	IGF2BP2/DDX39A/SMG5/IGF2BP1/MVP/NUP62/M
3491	tags=36%,	100133941/	GO_BP	CD24/PYCARD/TNFRSF21/EPO/LGALS9/FOXJ1/L
4832	tags=47%,	891/79019/	GO_BP	CCNB1/CENPM/HMGA1/CENPA/TOP2A/CDCA5/SOX
7442	tags=71%,	23246/662	GO_BP	BOP1/SNRPB/SNRPD2/SNRPD1/MRTO4/SF3A2/RU
2588	tags=34%,	991/5347/	GO_BP	CDC20/PLK1/CCNB1/PTTG1/KIF2C/CDT1/KIF4A
5290	tags=44%,	9168/822/	GO_BP	TMSB10/CAPG/PYCARD/LIMK1/DBN1/ICAM1/ARP
3862	tags=46%,	2810/991/	GO_BP	SFN/CDC20/PLK1/CCNB1/CDT1/AURKB/SOX4/TR
4398	tags=43%,	4318/2810/	GO_BP	MMP9/SFN/S100A9/EGLN3/BIRC5/PYCARD/CRYA
4835	tags=43%,	2305/8870/	GO_BP	FOXM1/IER3/RMI2/RAD51/CD44/CD74/PRKCD/R
6595	tags=62%,	10644/102	GO_BP	IGF2BP2/DDX39A/SMG5/IGF2BP1/MVP/NUP62/C
4972	tags=42%,	5347/1100	GO_BP	PLK1/KIF2C/CENPA/NDC80/SH3BP1/FOXJ1/SPI
3392	tags=36%,	100133941/	GO_BP	CD24/PYCARD/TNFRSF21/EPO/LGALS9/FOXJ1/L
3678	tags=40%,	100133941/	GO_BP	CD24/PYCARD/MDK/EPO/LGALS9/SRC/TGFB1/LG
3862	tags=47%,	2810/991/	GO_BP	SFN/CDC20/PLK1/CCNB1/CDT1/AURKB/SOX4/TR
3678	tags=40%,	100133941/	GO_BP	CD24/PYCARD/MDK/EPO/ICAM1/LGALS9/SRC/TG
4891	tags=51%,	2810/991/	GO_BP	SFN/CDC20/PLK1/CCNB1/CDT1/AURKB/SOX4/TO
6767	tags=68%,	23381/560	GO_BP	SMG5/SMG9/EXOSC4/RPL8/DHX34/MRTO4/CNOT1
6353	tags=65%,	54913/232	GO_BP	RPP25/BOP1/DCAF13/EXOSC4/NOP56/MRTO4/DH
5165	tags=52%,	8877/1410/	GO_BP	SPHK1/CRYAB/CLGN/CD74/HSPB1/PDIA2/CCT5/
6396	tags=66%,	3684/3689/	GO_BP	ITGAM/ITGB2/ADORA1/PRNP/HRAS/RPL8/NCF1/
5216	tags=47%,	9168/822/	GO_BP	TMSB10/CAPG/PYCARD/DBN1/ICAM1/ARPC1B/PF
4374	tags=43%,	4318/2810/	GO_BP	MMP9/SFN/S100A9/EGLN3/BIRC5/PYCARD/CRYA
5653	tags=52%,	6372/3576/	GO_BP	CXCL6/CXCL8/TOP2A/ANXA2/KPNA2/LGALS9/LG
4694	tags=42%,	768/11239	GO_BP	CA9/EGLN3/CCNB1/E2F1/EPO/ENO1/ICAM1/HIL
6260	tags=56%,	79019/315	GO_BP	CENPM/HMGA1/CDT1/CENPA/SOX9/ASF1B/HJURP
3946	tags=40%,	6280/6364/	GO_BP	S100A9/CCL20/CXCL1/TREM2/CXCL6/MDK/CXCL
4151	tags=55%,	5347/891/	GO_BP	PLK1/CCNB1/AURKB/TPX2/CDC6/TUBA4A/CDK1/
2567	tags=41%,	991/5347/	GO_BP	CDC20/PLK1/CCNB1/PTTG1/KIF2C/CDT1/KIF4A
4936	tags=45%,	2305/9319/	GO_BP	FOXM1/TRIP13/CDCA5/RECQL4/MCM2/CDC45/MC
6187	tags=56%,	2810/7040/	GO_BP	SFN/TGFB1/DDX39A/SMG5/NUP62/GTSE1/ALYRE
4151	tags=56%,	5347/891/	GO_BP	PLK1/CCNB1/AURKB/TPX2/CDC6/TUBA4A/CDK1/
3776	tags=36%,	6280/1123	GO_BP	S100A9/EGLN3/PYCARD/LGALS9/BAK1/MAPK12/
3729	tags=40%,	6280/6364/	GO_BP	S100A9/CCL20/CXCL1/CXCL6/MDK/CXCL8/CXCL
4298	tags=41%,	5347/2297	GO_BP	PLK1/TPX2/TACC3/NEK2/STMN1/ARL2/CCNF/SK
6128	tags=58%,	27242/915/	GO_BP	TNFRSF21/CD3D/LGALS3/RIPK2/ADA/HLA-DRB1
6620	tags=58%,	2810/7040/	GO_BP	SFN/TGFB1/DDX39A/SMG5/NUP62/GTSE1/ALYRE
6595	tags=58%,	10644/102	GO_BP	IGF2BP2/DDX39A/SMG5/IGF2BP1/MVP/NUP62/M
4258	tags=41%,	29108/398	GO_BP	PYCARD/LIMK1/ICAM1/ARPC1B/PFN2/ARL2/PSR
6079	tags=53%,	1869/1064	GO_BP	E2F1/IGF2BP2/YWHAZ/RBM38/PRKCD/HSPB1/IG
4582	tags=44%,	115908/41	GO_BP	CTHRC1/MDK/SOX9/SULF1/ETV5/DAB2/LIF/SAP
6217	tags=67%,	1978/3315/	GO_BP	EIF4EBP1/HSPB1/NCK2/RPL8/CCL5/EIF3B/RPL
4298	tags=52%,	4605/991/	GO_BP	MYBL2/CDC20/BIRC5/PLK1/CCNB1/KIF4A/AURK
2521	tags=12%,	2810/3880/	GO_BP	SFN/KRT19/TGM3/KRT23/DSG2/KRT17/PI3/KRT
4718	tags=60%,	10053/110	GO_BP	AP1M2/KIF2C/TREM2/PYCARD/CYBA/KIF4A/NCF
4831	tags=45%,	81620/557	GO_BP	CDT1/PKIB/AURKB/FOXM1/CDK1/NEK2/SRC/RFC

5225	tags=43%,	2810/5347/GO_BP	SFN/PLK1/AURKB/MDK/KIF20A/CDC6/VEGFB/CD
4298	tags=46%,	5347/2297 GO_BP	PLK1/TPX2/TACC3/NEK2/STMN1/ARL2/CCNF/SK
3692	tags=37%,	100133941/GO_BP	CD24/PYCARD/TNFRSF21/EPO/LGALS9/FOXJ1/L
4718	tags=61%,	10053/110 GO_BP	AP1M2/KIF2C/CYBA/KIF4A/NCF2/FCER1G/KIF2
6123	tags=44%,	6696/3984/GO_BP	SPP1/LIMK1/DBN1/SEMA6A/IQGAP3/ARHGAP4/T
4326	tags=39%,	6692/1159 GO_BP	SPINT1/CTHRC1/SOX4/SOX9/TEAD2/SULF1/SPI
5931	tags=69%,	10053/110 GO_BP	AP1M2/KIF2C/CYBA/KIF4A/NCF2/FCER1G/KIF2
6520	tags=64%,	5347/7277/GO_BP	PLK1/TUBA4A/CDK1/ICAM1/NEK2/STX3/CEP131
5216	tags=43%,	9168/822/ GO_BP	TMSB10/CAPG/PYCARD/DBN1/ICAM1/ARPC1B/PF
6229	tags=51%,	7345/2413 GO_BP	UCHL1/KIF4A/TUBA1C/KIF23/TUBA1B/CEP131/
5216	tags=47%,	9168/822/ GO_BP	TMSB10/CAPG/PYCARD/DBN1/ICAM1/ARPC1B/PF
6823	tags=62%,	3984/2207/GO_BP	LIMK1/FCER1G/ARPC1B/SRC/FCGR3A/PRKCD/LA
3692	tags=37%,	100133941/GO_BP	CD24/PYCARD/TNFRSF21/EPO/LGALS9/FOXJ1/L
6337	tags=56%,	1869/1064 GO_BP	E2F1/IGF2BP2/YWHAZ/RBM38/PRKCD/HSPB1/IG
5435	tags=48%,	8877/1001 GO_BP	SPHK1/CD24/TREM2/PYCARD/MMP12/IKBKE/CSF
5644	tags=54%,	9212/2297 GO_BP	AURKB/TPX2/RMI2/RFC4/AURKA/EXO1/BOP1/CD
6581	tags=55%,	891/81620/GO_BP	CCNB1/CDT1/PKIB/AURKB/NEK2/DLGAP5/LIF/T
3729	tags=42%,	6364/5420 GO_BP	CCL20/TREM2/PYCARD/MDK/CXCL8/ICAM1/LGAL
5147	tags=50%,	10551/440/GO_BP	AGR2/ASNS/HSPB8/CXCL8/KDELR3/BAK1/STC2/
5216	tags=46%,	9168/822/ GO_BP	TMSB10/CAPG/PYCARD/DBN1/ICAM1/ARPC1B/PF
6235	tags=71%,	23636/613 GO_BP	NUP62/RPL8/CCL5/NUP210/TRIM11/RPL36A/PO
7545	tags=65%,	6659/1277/GO_BP	SOX4/COL1A1/SULF1/SRC/DAB2/DEPDC1B/BAMB
6507	tags=54%,	1535/2364 GO_BP	CYBA/LY96/PGC/TOP2A/ANXA2/KPNA2/LGALS9/
4946	tags=51%,	79019/816 GO_BP	CENPM/CDT1/CENPA/SOX9/ASF1B/HJURP/MCM2/
4830	tags=43%,	8877/5570/GO_BP	SPHK1/PKIB/AURKB/TK1/NEK2/SRC/CENPF/RFC
5759	tags=58%,	5347/7277/GO_BP	PLK1/TUBA4A/CDK1/NEK2/STX3/CEP131/SNAP2
3955	tags=36%,	991/5347/ GO_BP	CDC20/PLK1/PTTG1/TOP2A/TRIP13/NUF2/BUB1
5701	tags=53%,	4318/2810/GO_BP	MMP9/SFN/S100A9/PYCARD/E2F1/CTSC/LGALS9
4928	tags=47%,	79019/315 GO_BP	CENPM/HMGA1/CENPA/SOX9/HJURP/CENPH/CDKN
5805	tags=60%,	5570/9212/GO_BP	PKIB/AURKB/RECQL4/NEK2/SRC/CCNE1/RFC4/R
6117	tags=52%,	8877/5420 GO_BP	SPHK1/TREM2/IKBKE/LGALS9/PTP4A3/HAVCR2/
6337	tags=56%,	1869/1064 GO_BP	E2F1/IGF2BP2/YWHAZ/RBM38/PRKCD/HSPB1/IG
4663	tags=41%,	2810/1055 GO_BP	SFN/AGR2/TREM2/E2F1/ITGA3/ARHGEF16/LYPD
4258	tags=36%,	5347/8162 GO_BP	PLK1/CDT1/KIF4A/AURKB/CENPA/KIF20A/CEP5
4936	tags=54%,	3159/8162 GO_BP	HMGA1/CDT1/CDC6/RECQL4/MCM2/CDC45/GINS1
3132	tags=35%,	8877/991/ GO_BP	SPHK1/CDC20/PLK1/CCNB1/PTTG1/CDT1/AURKB
4398	tags=40%,	6280/1123 GO_BP	S100A9/EGLN3/PYCARD/LGALS9/BAK1/RIPK2/E
4486	tags=50%,	8877/2910 GO_BP	SPHK1/PYCARD/TRAIP/GSTP1/TNFRSF12A/BIRC
6454	tags=51%,	10537/627 GO_BP	UBD/S100A4/IKBKE/LGALS9/ECT2/LGALS1/RIP
4826	tags=44%,	8877/891/ GO_BP	SPHK1/CCNB1/ASNS/CDT1/CDCA5/CDC6/CDK1/N
6595	tags=59%,	10644/102 GO_BP	IGF2BP2/DDX39A/SMG5/IGF2BP1/MVP/NUP62/M
3315	tags=35%,	8877/991/ GO_BP	SPHK1/CDC20/PLK1/CCNB1/PTTG1/CDT1/AURKB
3828	tags=39%,	2810/891/ GO_BP	SFN/CCNB1/SOX4/E2F1/CDC6/CDK1/CDC45/CCN
2567	tags=45%,	991/5347/ GO_BP	CDC20/PLK1/CCNB1/PTTG1/KIF2C/CDT1/KIF4A
5805	tags=59%,	5570/9212/GO_BP	PKIB/AURKB/RECQL4/NEK2/SRC/CCNE1/RFC4/R
4582	tags=49%,	115908/23 GO_BP	CTHRC1/SH3BP1/DAB2/SAPCD2/DKK1/GPC4/GPC
5435	tags=49%,	8877/1001 GO_BP	SPHK1/CD24/TREM2/PYCARD/MMP12/IKBKE/CSF
5216	tags=45%,	9168/822/ GO_BP	TMSB10/CAPG/PYCARD/DBN1/ICAM1/ARPC1B/PF
4891	tags=51%,	2810/5347/GO_BP	SFN/PLK1/CCNB1/CDT1/SOX4/TOP2A/E2F1/CDC
6592	tags=61%,	115908/16 GO_BP	CTHRC1/DAB2/DKK1/GPC4/GPC3/PLCB1/FZD6/F
3078	tags=35%,	100133941/GO_BP	CD24/PYCARD/TNFRSF21/EPO/LGALS9/FOXJ1/L

4909	tags=44%,	2810/891/	GO_BP	SFN/CCNB1/SOX4/E2F1/CDC6/CDK1/CDC45/CCN
5701	tags=56%,	4318/6280/	GO_BP	MMP9/S100A9/PYCARD/EPO/ENO1/SRC/PLAUR/C
3862	tags=48%,	2810/991/	GO_BP	SFN/CDC20/PLK1/CCNB1/CDT1/AURKB/SOX4/TO
3955	tags=35%,	991/5347/	GO_BP	CDC20/PLK1/PTTG1/TOP2A/TRIP13/NUF2/BUB1
5171	tags=52%,	26353/141	GO_BP	HSPB8/CRYAB/IRAK1/NUP62/HSF1/HMOX1/MAPK
3228	tags=38%,	100133941/	GO_BP	CD24/PYCARD/EPO/LGALS9/RIPK2/ADA/HAVCR2
6055	tags=55%,	2810/1869/	GO_BP	SFN/E2F1/TSPO/RAC2/YWHAZ/HK2/TOMM40/SLC
4891	tags=49%,	2810/5347/	GO_BP	SFN/PLK1/CCNB1/SOX4/E2F1/CDK1/AURKA/CDC
3228	tags=39%,	100133941/	GO_BP	CD24/PYCARD/EPO/LGALS9/CSF1/RIPK2/ADA/H
3978	tags=46%,	2810/5347/	GO_BP	SFN/PLK1/CCNB1/FOXM1/SOX4/E2F1/CDK1/NDR
3729	tags=38%,	54209/419	GO_BP	TREM2/MDK/CXCL8/LGALS9/VEGFB/RAC2/CSF1/
6595	tags=62%,	10212/233	GO_BP	DDX39A/SMG5/NUP62/ALYREF/RAN/NUP210/NPM
4326	tags=39%,	6692/1159	GO_BP	SPINT1/CTHRC1/SOX4/SOX9/TEAD2/SPINT2/TG
3946	tags=40%,	6280/6364/	GO_BP	S100A9/CCL20/CXCL1/CXCL6/MDK/CXCL8/CXCL
3851	tags=51%,	4605/991/	GO_BP	MYBL2/CDC20/BIRC5/PLK1/CCNB1/KIF4A/AURK
4326	tags=41%,	6692/1159	GO_BP	SPINT1/CTHRC1/SOX4/SOX9/TEAD2/SPINT2/TG
6595	tags=62%,	10212/233	GO_BP	DDX39A/SMG5/NUP62/ALYREF/RAN/NUP210/NPM
3228	tags=38%,	100133941/	GO_BP	CD24/PYCARD/EPO/LGALS9/CSF1/RIPK2/ADA/H
6396	tags=70%,	11015/647	GO_BP	KDELR3/PDIA2/RPL8/CHMP4B/GPAA1/SEC61A1/
6595	tags=62%,	10212/233	GO_BP	DDX39A/SMG5/NUP62/ALYREF/RAN/NUP210/NPM
5725	tags=53%,	2810/1869/	GO_BP	SFN/E2F1/TSPO/RAC2/YWHAZ/HK2/TOMM40/SLC
5888	tags=54%,	991/5347/	GO_BP	CDC20/PLK1/CCNB1/PTTG1/HMGA1/CDT1/AURKB
4582	tags=51%,	115908/16	GO_BP	CTHRC1/DAB2/SAPCD2/DKK1/GPC4/GPC3/FZD6/
4797	tags=51%,	6364/5420	GO_BP	CCL20/TREM2/PYCARD/MDK/CXCL8/ICAM1/LGAL
4582	tags=51%,	115908/16	GO_BP	CTHRC1/DAB2/SAPCD2/DKK1/GPC4/GPC3/FZD6/
3842	tags=43%,	9401/4171/	GO_BP	RECQL4/MCM2/CDC45/MCM4/RMI2/RAD51/TONSL
4972	tags=44%,	5347/1058/	GO_BP	PLK1/CENPA/NDC80/SH3BP1/FOXJ1/SAPCD2/MI
4832	tags=52%,	891/79019/	GO_BP	CCNB1/CENPM/HMGA1/CENPA/TOP2A/CDCA5/SOX
3842	tags=43%,	9401/4171/	GO_BP	RECQL4/MCM2/CDC45/MCM4/RMI2/RAD51/TONSL
4151	tags=46%,	768/11239	GO_BP	CA9/EGLN3/EPO/NCK2/MUC1/CREB3L1/CHEK1/R
6754	tags=82%,	23381/560	GO_BP	SMG5/SMG9/RPL8/DHX34/RPL36A/PPP2R1A/UPF
4151	tags=46%,	768/11239	GO_BP	CA9/EGLN3/EPO/NCK2/MUC1/CREB3L1/CHEK1/H
3442	tags=39%,	6280/6364/	GO_BP	S100A9/CCL20/CXCL1/CXCL6/MDK/CXCL8/CXCL
6264	tags=55%,	3159/7153/	GO_BP	HMGA1/TOP2A/RECQL4/MCM2/CDC45/GINS1/MCM
6060	tags=53%,	27242/230	GO_BP	TNFRSF21/FOXJ1/MMP14/TYROBP/TGFB1/ADA/M
3359	tags=38%,	6280/6364/	GO_BP	S100A9/CCL20/CXCL1/CXCL6/MDK/CXCL8/CXCL
4048	tags=40%,	10551/534	GO_BP	AGR2/PLK1/TREM2/ITGA3/ARHGEF16/LYPD1/LG
6595	tags=61%,	10212/233	GO_BP	DDX39A/SMG5/NUP62/ALYREF/NUP210/UPF3B/N
4127	tags=47%,	11004/141	GO_BP	KIF2C/CRYAB/STMN1/KIF18B/ARL2/SKA1/PSRC
6595	tags=61%,	10212/233	GO_BP	DDX39A/SMG5/NUP62/ALYREF/NUP210/UPF3B/N
6396	tags=78%,	6132/1288	GO_BP	RPL8/CHMP4B/SEC61A1/RPL36A/SEC61G/RPS2/
4582	tags=53%,	115908/16	GO_BP	CTHRC1/DAB2/SAPCD2/DKK1/GPC4/GPC3/FZD6/
5931	tags=54%,	56667/220	GO_BP	MUC13/FCER1G/SRC/PRKCD/RELB/HRAS/SYK/MU
5070	tags=54%,	79019/315	GO_BP	CENPM/HMGA1/CENPA/SOX9/ASF1B/HJURP/MCM2
4832	tags=50%,	79019/315	GO_BP	CENPM/HMGA1/CENPA/SOX9/ASF1B/HJURP/MCM2
3828	tags=43%,	2810/891/	GO_BP	SFN/CCNB1/SOX4/E2F1/CDK1/AURKA/GPNMB/CD
6228	tags=57%,	6282/8162	GO_BP	S100A11/CDT1/CDC6/CDK1/CCNA2/RFC4/DSCC1
4298	tags=57%,	4605/991/	GO_BP	MYBL2/CDC20/BIRC5/PLK1/CCNB1/KIF4A/AURK
3978	tags=45%,	2810/891/	GO_BP	SFN/CCNB1/FOXM1/SOX4/E2F1/CDK1/NDRG1/AU
4151	tags=44%,	56667/220	GO_BP	MUC13/FCER1G/SRC/PRKCD/RELB/HRAS/SYK/MU
6396	tags=80%,	6132/1288	GO_BP	RPL8/CHMP4B/SEC61A1/RPL36A/SEC61G/RPS2/

4582	tags=53%,	115908/16(	GO_BP	CTHRC1/DAB2/DKK1/GPC4/GPC3/FZD6/FZD7/PSI
4825	tags=48%,	2810/891/2	GO_BP	SFN/CCNB1/FOXM1/SOX4/E2F1/CDK1/TGFB1/AUI
4662	tags=63%,	10053/110(	GO_BP	AP1M2/KIF2C/TREM2/PYCARD/KIF4A/FCER1G/K
2362	tags=37%,	2207/3691/	GO_BP	FCER1G/ITGB4/SRC/TIMP1/ITGA3/TYROBP/ITG.
2906	tags=37%,	6280/6364/	GO_BP	S100A9/CCL20/CXCL1/CXCL6/MDK/CXCL8/CXCL
3828	tags=45%,	2810/891/6	GO_BP	SFN/CCNB1/SOX4/TOP2A/E2F1/CDC6/CDK1/ORC
4263	tags=51%,	4605/991/3	GO_BP	MYBL2/CDC20/BIRC5/PLK1/KIF4A/AURKB/TPX2,
2848	tags=43%,	991/5347/8	GO_BP	CDC20/PLK1/CCNB1/PTTG1/KIF2C/CDT1/AURKB,
3828	tags=42%,	2810/891/6	GO_BP	SFN/CCNB1/SOX4/E2F1/CDK1/AURKA/GPNMB/CD(
4662	tags=63%,	10053/110(	GO_BP	AP1M2/KIF2C/TREM2/PYCARD/KIF4A/FCER1G/K
6396	tags=81%,	6132/6746/	GO_BP	RPL8/SSR2/SEC61A1/RPL36A/RPS2/RPL17/RPLI
4274	tags=44%,	6692/1159(	GO_BP	SPINT1/CTHRC1/TEAD2/SPINT2/TGFB1/FOLR1/
4662	tags=62%,	10053/110(	GO_BP	AP1M2/KIF2C/KIF4A/FCER1G/KIF23/CTSV/HLA-
6055	tags=55%,	706/5880/1	GO_BP	TSPO/RAC2/TOMM40/SLC25A6/MICALL2/SAE1/T(
4151	tags=53%,	5347/9212/	GO_BP	PLK1/AURKB/CDC6/CDK1/ORC1/AURKA/DTL/CHEI
2720	tags=33%,	2810/5347/	GO_BP	SFN/PLK1/CCNB1/CCNB2/CDC6/CDKN3/PKMYT1/
6396	tags=68%,	1978/2594(	GO_BP	EIF4EBP1/ZNF385A/RPL8/YBX3/DPH2/EIF3B/RI
6240	tags=66%,	1535/4688/	GO_BP	CYBA/NCF2/FCER1G/CYBB/ITGAV/TAP1/NCF4/II
6396	tags=83%,	6132/29927	GO_BP	RPL8/SEC61A1/RPL36A/RPS2/RPL17/RPLP2/TR.
3725	tags=39%,	29108/3383	GO_BP	PYCARD/ICAM1/ARPC1B/PFN2/CORO1A/NCK2/BA
5888	tags=63%,	5347/7277/	GO_BP	PLK1/TUBA4A/CDK1/NEK2/CEP131/TUBG1/MAPRI
2720	tags=33%,	2810/5347/	GO_BP	SFN/PLK1/CCNB1/CCNB2/CDC6/CDKN3/PKMYT1/
3226	tags=43%,	100133941/	GO_BP	CD24/PYCARD/EPO/LGALS9/RIPK2/HAVCR2/COR(
3954	tags=44%,	1535/4688/	GO_BP	CYBA/NCF2/VEGFB/SULF1/SRC/CYBB/HSPB1/NCI
6676	tags=61%,	4318/10551	GO_BP	MMP9/AGR2/PLAUR/RHBDF2/NCK2/ADORA1/SHKBI
5055	tags=55%,	891/81620/	GO_BP	CCNB1/CDT1/CDCA5/CDC6/CDK1/CDC45/DLGAP5,
3491	tags=33%,	27242/730	GO_BP	TNFRSF21/TYROBP/ADA/MZB1/CLCF1/CD74/PKN
3828	tags=43%,	2810/891/6	GO_BP	SFN/CCNB1/SOX4/E2F1/CDK1/AURKA/CDC25C/G
4075	tags=45%,	4318/2810/	GO_BP	MMP9/SFN/BIRC5/CRYAB/MICAL1/SRC/PLAUR/B
4193	tags=44%,	4318/2056/	GO_BP	MMP9/EPO/ENO1/SRC/PLAUR/CD44/CD74/ARHGEI
3729	tags=37%,	9133/6372/	GO_BP	CCNB2/CXCL6/FCER1G/LGALS9/BAK1/TGFB1/AD.
5199	tags=55%,	23423/110(	GO_BP	TMED3/KIF2C/KIF4A/KDELR3/KIF23/RACGAP1/I
4938	tags=49%,	4318/1009	GO_BP	MMP9/ARPC1B/SRC/DNM1/EFNA3/NCK2/EFNA4/HI
1603	tags=33%,	991/5347/8	GO_BP	CDC20/PLK1/CCNB1/PTTG1/CDT1/AURKB/TACC3,
5808	tags=65%,	9212/990/9	GO_BP	AURKB/CDC6/CDK1/ORC1/AURKA/PSMB9/PSMD14,
2251	tags=36%,	4318/4316/	GO_BP	MMP9/MMP7/MMP12/TIMP1/MMP11/MMP14/MELTF,
5764	tags=69%,	991/5347/8	GO_BP	CDC20/PLK1/CCNB1/PTTG1/AURKB/UBE2S/CDK1,
2518	tags=41%,	991/5347/8	GO_BP	CDC20/PLK1/CCNB1/PTTG1/CDT1/AURKB/TACC3,
4825	tags=53%,	2810/891/6	GO_BP	SFN/CCNB1/SOX4/E2F1/CDK1/TGFB1/AURKA/CD(
4075	tags=46%,	4318/2810/	GO_BP	MMP9/SFN/BIRC5/CRYAB/MICAL1/SRC/PLAUR/B
5207	tags=64%,	3984/2207/	GO_BP	LIMK1/FCER1G/ARPC1B/SRC/FCGR3A/PRKCD/SYI
5207	tags=62%,	3984/2207/	GO_BP	LIMK1/FCER1G/ARPC1B/SRC/FCGR3A/PRKCD/SYI
2256	tags=25%,	5347/9232/	GO_BP	PLK1/PTTG1/TOP2A/TRIP13/NUF2/BUB1/BUB1B,
4826	tags=55%,	26353/141(	GO_BP	HSPB8/CRYAB/NUP62/HSF1/MAPKAPK2/NUP210/I
6493	tags=61%,	5570/9212/	GO_BP	PKIB/AURKB/NEK2/SRC/RAD51/SMG5/PIF1/CCT
3385	tags=35%,	6692/5420	GO_BP	SPINT1/TREM2/MDK/E2F1/TSPO/ETV5/LIF/SERI
4832	tags=56%,	79019/315	GO_BP	CENPM/HMGA1/CENPA/SOX9/ASF1B/HJURP/MCM2,
4258	tags=38%,	5347/9212/	GO_BP	PLK1/AURKB/KIF20A/CDC6/CDC25B/PRC1/KIF2
3659	tags=45%,	5347/8162(	GO_BP	PLK1/CDT1/AURKB/CENPA/CDCA5/CDK1/NDC80/
5931	tags=72%,	1535/4688/	GO_BP	CYBA/NCF2/FCER1G/CYBB/ITGAV/TAP1/NCF4/II
4829	tags=62%,	81620/4171	GO_BP	CDT1/MCM2/CDC45/GINS1/MCM4/RFC4/RAD51/M(

4115	tags=43%,	891/11004/	GO_BP	CCNB1/KIF2C/CDT1/CDCA8/CDCA5/NUF2/NDC80/
2588	tags=32%,	5347/8162(	GO_BP	PLK1/CDT1/KIF4A/AURKB/CENPA/KIF20A/CEP5
2029	tags=38%,	2810/5347/	GO_BP	SFN/PLK1/CCNB1/SOX4/E2F1/CDK1/AURKA/CDC
2029	tags=38%,	2810/5347/	GO_BP	SFN/PLK1/CCNB1/SOX4/E2F1/CDK1/AURKA/CDC
4096	tags=47%,	891/81620/	GO_BP	CCNB1/CDT1/CDCA5/CDC6/CDK1/CDC45/DLGAP5/
5207	tags=64%,	3984/1009	GO_BP	LIMK1/ARPC1B/SRC/FCGR3A/PRKCD/SYK/HCK/W
6493	tags=64%,	5570/9212/	GO_BP	PKIB/AURKB/NEK2/SRC/SMG5/PIF1/CCT5/CCT6/
5805	tags=54%,	5570/9212/	GO_BP	PKIB/AURKB/NEK2/SRC/SMG5/PIF1/CCT5/NABP
5207	tags=64%,	3984/1009	GO_BP	LIMK1/ARPC1B/SRC/FCGR3A/PRKCD/SYK/HCK/W
5821	tags=73%,	23246/511	GO_BP	BOP1/MRTO4/PES1/NPM1/RRS1/BRIX1/NOP2/NO
5377	tags=48%,	1535/4688/	GO_BP	CYBA/NCF2/SH3BGRL3/RAC2/TXNRD1/CYBB/QSO
4115	tags=43%,	891/11004/	GO_BP	CCNB1/KIF2C/CDT1/CDCA8/CDCA5/NUF2/NDC80/
3076	tags=38%,	9212/2724	GO_BP	AURKB/TNFRSF21/LGALS9/BAK1/LGALS3/ADA/C
2029	tags=38%,	2810/5347/	GO_BP	SFN/PLK1/CCNB1/SOX4/E2F1/CDK1/AURKA/CDC
4411	tags=48%,	5570/9212/	GO_BP	PKIB/AURKB/NEK2/SRC/RFC4/DSCC1/CCT5/CCT
2588	tags=37%,	5347/8162(	GO_BP	PLK1/CDT1/KIF4A/CENPA/KIF20A/CEP55/ANLN/
5830	tags=72%,	1535/4688/	GO_BP	CYBA/NCF2/CYBB/ITGAV/TAP1/NCF4/NCF1/PSM
3894	tags=34%,	226/5047/	GO_BP	ALDOA/PAEP/LGALS3/HAVCR2/CLGN/NCK2/CCT5/
4513	tags=54%,	79019/105	GO_BP	CENPM/CENPA/HJURP/CENPH/CENPW/OIP5/CENP
6130	tags=63%,	116028/96(	GO_BP	RMI2/CD44/CD74/MUC1/CDKN2D/ZNF385A/HSF1/
1930	tags=35%,	1728/1401/	GO_BP	NQO1/CRP/APOA4/CYBA/NCF2/TYROBP/TGFB1/I
1603	tags=36%,	991/5347/	GO_BP	CDC20/PLK1/CCNB1/PTTG1/CDT1/AURKB/TACC3/
5339	tags=51%,	27242/730	GO_BP	TNFRSF21/TYROBP/ADA/MZB1/CLCF1/CD74/PKN
3828	tags=48%,	2810/891/	GO_BP	SFN/CCNB1/SOX4/E2F1/CDK1/AURKA/CDC25C/G
5765	tags=64%,	9454/2207/	GO_BP	HOMER3/FCER1G/RIPK2/HAVCR2/PRNP/IL18/HD
6353	tags=75%,	23246/511	GO_BP	BOP1/MRTO4/NPM1/RRS1/BRIX1/RRP7A/NOP2/R
3828	tags=48%,	2810/891/	GO_BP	SFN/CCNB1/SOX4/E2F1/CDK1/AURKA/CDC25C/G
5574	tags=56%,	2207/3965/	GO_BP	FCER1G/LGALS9/NDRG1/RAC2/CPLX2/LAT2/SYK/
2518	tags=47%,	991/5347/	GO_BP	CDC20/PLK1/CCNB1/PTTG1/CDT1/AURKB/TACC3/
1603	tags=38%,	991/5347/	GO_BP	CDC20/PLK1/CCNB1/PTTG1/CDT1/AURKB/TACC3/
3655	tags=39%,	9133/3965/	GO_BP	CCNB2/LGALS9/BAK1/TGFB1/ADA/CORO1A/CD74/
4832	tags=62%,	79019/105	GO_BP	CENPM/CENPA/SOX9/ASF1B/HJURP/MCM2/CHAF1
5771	tags=58%,	5570/9212/	GO_BP	PKIB/AURKB/NEK2/SRC/SMG5/PIF1/CCT5/CCT6/
3595	tags=57%,	81620/417	GO_BP	CDT1/MCM2/CDC45/GINS1/MCM4/RFC4/RAD51/M
3828	tags=47%,	2810/891/	GO_BP	SFN/CCNB1/SOX4/E2F1/CDK1/AURKA/CDC25C/G
4207	tags=49%,	4751/899/	GO_BP	NEK2/CCNF/AURKA/CEP131/KIF11/NUP62/CHEK
2048	tags=39%,	891/11004/	GO_BP	CCNB1/KIF2C/CDT1/CDCA8/CDCA5/NUF2/NDC80/
3132	tags=46%,	991/5347/	GO_BP	CDC20/PLK1/CCNB1/PTTG1/CDT1/AURKB/TRIP1
6427	tags=60%,	5570/9212/	GO_BP	PKIB/AURKB/NEK2/SRC/SMG5/PIF1/CCT5/CCT6/
1603	tags=41%,	991/5347/	GO_BP	CDC20/PLK1/CCNB1/PTTG1/CDT1/AURKB/TACC3/
1603	tags=39%,	991/5347/	GO_BP	CDC20/PLK1/CCNB1/CDT1/AURKB/TACC3/TRIP1
2029	tags=39%,	2810/891/	GO_BP	SFN/CCNB1/SOX4/E2F1/CDK1/AURKA/CDC25C/G
2029	tags=39%,	2810/891/	GO_BP	SFN/CCNB1/SOX4/E2F1/CDK1/AURKA/CDC25C/G
2029	tags=39%,	2810/891/	GO_BP	SFN/CCNB1/SOX4/E2F1/CDK1/AURKA/CDC25C/G
4296	tags=45%,	54443/727	GO_BP	ANLN/TTK/AURKA/CEP131/ABHD17C/NUP62/SPA
2029	tags=40%,	2810/891/	GO_BP	SFN/CCNB1/SOX4/E2F1/CDK1/AURKA/CDC25C/G
2029	tags=40%,	2810/891/	GO_BP	SFN/CCNB1/SOX4/E2F1/CDK1/AURKA/CDC25C/G
2029	tags=39%,	2810/891/	GO_BP	SFN/CCNB1/SOX4/E2F1/CDK1/AURKA/CDC25C/G
3033	tags=50%,	991/5347/	GO_BP	CDC20/PLK1/CCNB1/CDT1/AURKB/TACC3/TRIP1
2852	tags=33%,	54209/573	GO_BP	TREM2/PTGFRN/TYROBP/GDF15/PLEKHO1/ADAM9/
4621	tags=44%,	54209/291(	GO_BP	TREM2/PYCARD/TNFRSF21/FCER1G/LGALS9/TYR

4258	tags=55%,	4605/991/	GO_BP	MYBL2/CDC20/BIRC5/PLK1/KIF4A/AURKB/TPX2/
3033	tags=49%,	991/5347/	GO_BP	CDC20/PLK1/CCNB1/CDT1/AURKB/TRIP13/CDC6/
2852	tags=33%,	54209/573	GO_BP	TREM2/PTGFRN/TYROBP/GDF15/PLEKHO1/ADAM9/
3033	tags=51%,	991/5347/	GO_BP	CDC20/PLK1/CCNB1/CDT1/AURKB/TRIP13/CDC6/
3132	tags=49%,	991/5347/	GO_BP	CDC20/PLK1/CCNB1/PTTG1/CDT1/AURKB/TRIP1
2852	tags=33%,	54209/573	GO_BP	TREM2/PTGFRN/TYROBP/GDF15/PLEKHO1/ADAM9/
4662	tags=51%,	2207/3684/	GO_BP	FCER1G/ITGAM/ITGB2/PTAFR/SCAMP5/SYK/UNC
4815	tags=54%,	2207/3965/	GO_BP	FCER1G/LGALS9/RAC2/CPLX2/LAT2/SYK/UNC13
4815	tags=51%,	2207/3965/	GO_BP	FCER1G/LGALS9/RAC2/CPLX2/LAT2/SYK/UNC13
3428	tags=43%,	9536/5730/	GO_BP	PTGES/PTGDS/ELOVL7/ALOX5AP/FABP5/CD74/A
3223	tags=55%,	54443/949	GO_BP	ANLN/KIF23/ECT2/RACGAP1/PLEK/FMNL2/EZR/
5178	tags=59%,	29089/588	GO_BP	UBE2T/RAD51/FANCI/FANCD2/RAD51AP1/EME1/
1236	tags=27%,	8877/891/	GO_BP	SPHK1/CCNB1/S100A6/E2F1/CDC6/ANXA2/CCNA
3750	tags=49%,	5347/1058/	GO_BP	PLK1/CENPA/NDC80/SAPCD2/MISP/NUSAP1/ARH
2865	tags=44%,	6696/4318/	GO_BP	SPP1/MMP9/EPO/TIMP1/LIF/FBLN1/STC2/DDR1/
4283	tags=44%,	4318/3976/	GO_BP	MMP9/LIF/CSF1/TGFB1/SPI1/C1QC/CSF1R/IL3
1927	tags=47%,	991/5347/	GO_BP	CDC20/PLK1/CCNB1/PTTG1/CDT1/AURKB/TRIP1
4815	tags=52%,	2207/3965/	GO_BP	FCER1G/LGALS9/RAC2/CPLX2/LAT2/SYK/UNC13
3750	tags=49%,	5347/1058/	GO_BP	PLK1/CENPA/NDC80/SAPCD2/MISP/NUSAP1/ARH
3727	tags=51%,	4192/3958/	GO_BP	MDK/LGALS3/CSF1/S100A14/TGFB1/SLAMF8/NB
2048	tags=41%,	891/11004/	GO_BP	CCNB1/KIF2C/CDT1/CDCA8/CDCA5/NUF2/NDC80/
1927	tags=45%,	991/5347/	GO_BP	CDC20/PLK1/CCNB1/PTTG1/CDT1/AURKB/TRIP1
5785	tags=67%,	2207/3965/	GO_BP	FCER1G/LGALS9/RAC2/ITGAM/ITGB2/PTAFR/SY
5412	tags=76%,	54443/949	GO_BP	ANLN/KIF23/ECT2/RACGAP1/PLEK/FMNL2/EZR/
4513	tags=77%,	79019/105	GO_BP	CENPM/CENPA/HJURP/CENPH/CENPW/OIP5/CENP
4815	tags=57%,	2207/3965/	GO_BP	FCER1G/LGALS9/RAC2/SYK/UNC13D/HMOX1/CD3
4811	tags=64%,	79019/105	GO_BP	CENPM/CENPA/HJURP/CENPH/CENPW/OIP5/CENP
3356	tags=57%,	4192/3965/	GO_BP	MDK/LGALS9/TGFB1/FANCD2/TNFRSF18/SOCS1/
4096	tags=63%,	891/983/9	GO_BP	CCNB1/CDK1/CDC25B/RCC2/CDC25C/DTL/SMARC
2848	tags=52%,	891/11004/	GO_BP	CCNB1/KIF2C/CDT1/AURKB/NUF2/NDC80/NEK2/
5104	tags=56%,	2207/3965/	GO_BP	FCER1G/LGALS9/RAC2/SYK/UNC13D/HMOX1/CD3
1927	tags=46%,	991/5347/	GO_BP	CDC20/PLK1/CCNB1/PTTG1/CDT1/AURKB/TRIP1
4513	tags=68%,	79019/105	GO_BP	CENPM/CENPA/HJURP/CENPF/CENPH/CENPW/OIP
3266	tags=58%,	100133941/	GO_BP	CD24/CYBA/NCF2/RAC2/CYBB/SLAMF8/HCK/NCF
1927	tags=47%,	991/5347/	GO_BP	CDC20/PLK1/CCNB1/PTTG1/CDT1/AURKB/TRIP1
4513	tags=79%,	79019/105	GO_BP	CENPM/CENPA/HJURP/CENPH/CENPW/OIP5/CENP
3356	tags=57%,	4192/3965/	GO_BP	MDK/LGALS9/TGFB1/FANCD2/TNFRSF18/SOCS1/
4662	tags=75%,	10053/305	GO_BP	AP1M2/HCK/PACS1/SCRIB/ATP6V1H/RAC1/LCK/
4513	tags=79%,	79019/105	GO_BP	CENPM/CENPA/HJURP/CENPH/CENPW/OIP5/CENP
1927	tags=46%,	991/5347/	GO_BP	CDC20/PLK1/CCNB1/PTTG1/CDT1/AURKB/TRIP1
4667	tags=74%,	79019/105	GO_BP	CENPM/CENPA/ASF1B/HJURP/CENPH/CENPW/OIP
1930	tags=46%,	1401/1535/	GO_BP	CRP/CYBA/NCF2/TYROBP/TGFB1/ITGAM/CYBB/G
2917	tags=56%,	81620/990/	GO_BP	CDT1/CDC6/MCM2/CDC45/MCM4/ORC6/CCNE1/OR
4667	tags=72%,	79019/105	GO_BP	CENPM/CENPA/ASF1B/HJURP/CENPH/CENPW/OIP
3750	tags=52%,	5347/1058/	GO_BP	PLK1/CENPA/NDC80/SAPCD2/MISP/NUSAP1/ARH
3033	tags=56%,	991/5347/	GO_BP	CDC20/PLK1/CCNB1/CDT1/AURKB/TRIP13/NDC8
3894	tags=38%,	226/5047/1	GO_BP	ALDOA/PAEP/CLGN/CCT5/CCT3/ZP3/CCT2/UBAP
4258	tags=38%,	9212/990/	GO_BP	AURKB/CDC6/CDC25B/KIF23/ECT2/RACGAP1/NU
3033	tags=54%,	991/5347/	GO_BP	CDC20/PLK1/CCNB1/CDT1/AURKB/TRIP13/NDC8
2298	tags=38%,	5570/9212/	GO_BP	PKIB/AURKB/NEK2/CCT5/CCT6A/CCT3/MAPK3/D
1603	tags=47%,	991/5347/	GO_BP	CDC20/PLK1/CCNB1/CDT1/AURKB/TRIP13/NDC8

1603	tags=47%,	991/5347/8	GO_BP	CDC20/PLK1/CCNB1/CDT1/AURKB/TRIP13/NDC8
2298	tags=41%,	5570/9212/	GO_BP	PKIB/AURKB/NEK2/CCT5/CCT6A/CCT3/MAPK3/D
1603	tags=47%,	991/5347/8	GO_BP	CDC20/PLK1/CCNB1/CDT1/AURKB/TRIP13/NDC8
1603	tags=47%,	991/5347/8	GO_BP	CDC20/PLK1/CCNB1/CDT1/AURKB/TRIP13/NDC8
3955	tags=52%,	5347/7153/	GO_BP	PLK1/TOP2A/TRIP13/CDC25B/TTK/AURKA/NCAP
4096	tags=67%,	891/983/9	GO_BP	CCNB1/CDK1/CDC25B/RCC2/CDC25C/DTL/SMARC
2121	tags=46%,	9212/2724	GO_BP	AURKB/TNFRSF21/BAK1/ADA/CD74/PKN1/BAX/M
3796	tags=65%,	9837/4173/	GO_BP	GINS1/MCM4/RFC4/RNASEH2A/FEN1/GINS2/MCM
2083	tags=20%,	6690/5047/	GO_BP	SPINK1/PAEP/CABYR/SLC26A6/DEFB1
4520	tags=61%,	2207/3684/	GO_BP	FCER1G/ITGAM/ITGB2/PTAFR/SYK/F2RL1/FGR/
1188	tags=22%,	6690/5047/	GO_BP	SPINK1/PAEP/LHFPL2/PLCB1/ZP3
3446	tags=75%,	9837/4173/	GO_BP	GINS1/MCM4/RFC4/RNASEH2A/FEN1/GINS2/MCM
609	tags=54%,	3159/9401/	GO_BP	HMGA1/RECQL4/MCM2/MCM4/RAD51/MCM6/MCM7
3520	tags=64%,	4751/6790/	GO_BP	NEK2/AURKA/KIF11/NUP62/CHEK1/CEP85/RANB
1	tags=8%, l	6696	GO_BP	SPP1
1317	tags=50%,	6714/9181/	GO_BP	SRC/ARHGEF2/HCK/ASAP1/FSCN1/MSN/LCP1
2732	tags=71%,	4171/8318/	GO_BP	MCM2/CDC45/GINS1/MCM4/RAD51/MCM6/MCM3/F
548	tags=36%,	4192/3383/	GO_BP	MDK/ICAM1/ITGB2/IRAK1/PTAFR
3511	tags=87%,	22948/908/	GO_BP	CCT5/CCT6A/CCT3/RUVBL1/DKC1/CCT2/CCT4/N
3598	tags=75%,	81620/990/	GO_BP	CDT1/CDC6/CDC45/ORC1/TICRR/TIPIN/TOPBP1
1586	tags=35%,	1535/6372/	GO_BP	CYBA/CXCL6/PGC/HAVCR2/GRN/F2RL1
1377	tags=67%,	24137/921	GO_BP	KIF4A/AURKB/PRC1/KIF23/RACGAP1/PPP2R1A
1586	tags=56%,	1535/5225/	GO_BP	CYBA/PGC/HAVCR2/GRN/F2RL1
1227	tags=91%,	4171/8318/	GO_BP	MCM2/CDC45/MCM4/MCM6/GINS2/MCM7/MCM3/CD
1283	tags=64%,	4192/7076/	GO_BP	MDK/TIMP1/TGFB1/GRN/HMOX1/SIGLEC10/HIF1
530	tags=62%,	24137/921	GO_BP	KIF4A/AURKB/PRC1/KIF23/RACGAP1
971	tags=86%,	4171/4173/	GO_BP	MCM2/MCM4/MCM6/MCM7/MCM3/MCM5
971	tags=86%,	4171/4173/	GO_BP	MCM2/MCM4/MCM6/MCM7/MCM3/MCM5
971	tags=86%,	4171/4173/	GO_BP	MCM2/MCM4/MCM6/MCM7/MCM3/MCM5
530	tags=67%,	24137/921	GO_BP	KIF4A/AURKB/KIF23/RACGAP1
5597	tags=41%,	79574/398	GO_BP	EPS8L3/LIMK1/FOXM1/EPO/STMN1/ITGA3/ARHG
7249	tags=53%,	891/9212/9	GO_BP	CCNB1/AURKB/CDK1/CCNA2/LIF/TGFB1/AURKA/
5182	tags=37%,	6364/5909/	GO_BP	CCL20/RAP1GAP/PYCARD/RGS2/RGS1/ICAM1/SH
6306	tags=46%,	2810/1001	GO_BP	SFN/CD24/TESC/UCHL1/PLK1/CCNB1/CCNB2/PY
4383	tags=28%,	6696/991/6	GO_BP	SPP1/CDC20/SPINT1/S100A9/TREM2/LIMK1/RG
4662	tags=31%,	6692/6280/	GO_BP	SPINT1/S100A9/TREM2/LIMK1/RGS2/MDK/E2F1
6206	tags=47%,	100133941/	GO_BP	CD24/S100A9/MUC13/CRP/TREM2/PYCARD/CYBA
6222	tags=45%,	991/10013	GO_BP	CDC20/CD24/PLK1/CCNB1/PKIB/AURKB/TPX2/C
5045	tags=37%,	56667/103	GO_BP	MUC13/B3GNT3/GCNT3/ST6GALNAC4/BACE2/CHS
6169	tags=42%,	5347/891/9	GO_BP	PLK1/CCNB1/AURKB/EPO/IKBKE/CDK1/PFN2/BA
6062	tags=48%,	6280/5666	GO_BP	S100A9/MUC13/PYCARD/CYBA/LY96/MMP12/IKB
6112	tags=46%,	4318/7262/	GO_BP	MMP9/PHLDA2/PLK1/CDT1/CTHRC1/AURKB/E2F1
3847	tags=22%,	1364/2810/	GO_BP	CLDN4/SFN/KRT19/SOX9/TGM3/COL1A1/STMN1/
6062	tags=50%,	6280/5666	GO_BP	S100A9/MUC13/PYCARD/CYBA/LY96/IKBKE/FCE
6695	tags=51%,	3159/2910	GO_BP	HMGA1/PYCARD/LCN2/MMP12/ENO1/IKBKE/LGAL
4322	tags=35%,	54997/105	GO_BP	TESC/AGR2/TREM2/ATP1B3/ANXA2/FCER1G/ITG
5653	tags=53%,	3576/7153/	GO_BP	CXCL8/TOP2A/ANXA2/KPNA2/LGALS9/LGALS1/C
6214	tags=51%,	2810/302/2	GO_BP	SFN/ANXA2/FCER1G/RAC2/DAB2/ECT2/TGFB1/I
4694	tags=42%,	768/11239	GO_BP	CA9/EGLN3/CCNB1/E2F1/EPO/ENO1/ICAM1/HIL
4796	tags=42%,	768/11239	GO_BP	CA9/EGLN3/CCNB1/E2F1/EPO/ENO1/ICAM1/HIL
3774	tags=38%,	54209/419	GO_BP	TREM2/MDK/CXCL8/LGALS9/VEGFB/SEMA6A/RAC

6220	tags=52%,	79574/5909	GO_BP	EPS8L3/RAP1GAP/KCTD17/ICAM1/ANLN/PFN2/SI
3454	tags=31%,	4318/81620	GO_BP	MMP9/CDT1/E2F1/CDCA5/HJURP/NEK2/PLAUR/L
4274	tags=44%,	6692/11590	GO_BP	SPINT1/CTHRC1/SOX4/TEAD2/SPINT2/TGFB1/F(
4527	tags=44%,	3159/7153/	GO_BP	HMGA1/TOP2A/RECQL4/MCM2/CDC45/GINS1/MCM4
3935	tags=29%,	5347/9232/	GO_BP	PLK1/PTTG1/TOP2A/TRIP13/CDC25B/CKS2/CCNI
3729	tags=42%,	4192/3576/	GO_BP	MDK/CXCL8/LGALS9/VEGFB/RAC2/CSF1/S100A14
5097	tags=45%,	11004/822/	GO_BP	KIF2C/CAPG/STMN1/SH3BP1/MICAL1/KIF18B/AI
6676	tags=63%,	4318/10551	GO_BP	MMP9/AGR2/PLAUR/RHBDF2/NCK2/ADORA1/SHKBI
3903	tags=45%,	4192/3576/	GO_BP	MDK/CXCL8/LGALS9/VEGFB/RAC2/CSF1/S100A14
3776	tags=43%,	6280/11239	GO_BP	S100A9/EGLN3/PYCARD/BAK1/ROBO1/S100A8/C/
4496	tags=52%,	768/112399	GO_BP	CA9/EGLN3/EPO/PSMB9/PSMD14/PSMD2/HIF1A/I
3894	tags=33%,	226/5047/1	GO_BP	ALDOA/PAEP/CLGN/CCT5/CCT3/ZP3/CCT2/UBAP2
3158	tags=38%,	1869/4751/	GO_BP	E2F1/NEK2/TMSB4X/MDFI/DDIT3/HMOX1/MAD2L1
5771	tags=57%,	5570/9212/	GO_BP	PKIB/AURKB/NEK2/SRC/SMG5/PIF1/CCT5/CCT6/
6228	tags=63%,	81620/5984	GO_BP	CDT1/RFC4/DSCC1/CDC7/GMNN/CHTF18/TICRR/I
5482	tags=52%,	991/5347/3	GO_BP	CDC20/PLK1/LIMK1/UBE2S/TRIB3/MAGEC2/MAD2
4621	tags=41%,	54209/2910	GO_BP	TREM2/PYCARD/TNFRSF21/FCER1G/LGALS9/TYR(
4127	tags=43%,	54209/706/	GO_BP	TREM2/TSPO/MMP14/CSF1/P2RX4/LAMB1/SCRIB/
2658	tags=37%,	11151/5683	GO_BP	CORO1A/SLAMF8/RAB31/ATP6V1F/RAB34/ATP6V(
7160	tags=92%,	6628/6633/	GO_BP	SNRPB/SNRPD2/SNRPD1/CD2BP2/LSM4/WDR77/SI
2230	tags=34%,	6510/81539	GO_BP	SLC1A5/SLC38A1/RGS2/NFKBIE/SLC6A9/SLC7A8
4815	tags=55%,	2207/3965/	GO_BP	FCER1G/LGALS9/RAC2/SYK/UNC13D/HMOX1/CD3(
3750	tags=48%,	5347/1058/	GO_BP	PLK1/CENPA/NDC80/SAPCD2/MISP/ARHGEF2/MAI
3985	tags=49%,	6696/768/2	GO_BP	SPP1/CA9/EPO/TSPO/AACS/KCNJ11/NME1/CDK4/
6228	tags=66%,	81620/983/	GO_BP	CDT1/CDK1/HRAS/CDC7/CACYBP/PCNA/E2F8/FGI
4964	tags=54%,	5347/1058/	GO_BP	PLK1/CENPA/NDC80/SAPCD2/MISP/ARHGEF2/MAI
2246	tags=39%,	891/9212/9	GO_BP	CCNB1/AURKB/CDK1/CCNA2/AURKA/PKN1/PRKCD/
3465	tags=49%,	4192/7040/	GO_BP	MDK/TGFB1/BAIAP2L2/HRAS/HCK/CSF1R/TRPM2/
1195	tags=31%,	1535/990/6	GO_BP	CYBA/CDC6/SRC/CYBB/AGTRAP/PRKCD/HSF1/IQ(
380	tags=35%,	81620/9212	GO_BP	CDT1/AURKB/CENPA/CDK1/NDC80/TTK/RCC2
3911	tags=74%,	1401/1535/	GO_BP	CRP/CYBA/TYROBP/TGFB1/ITGAM/GSTP1/ITGB2/
3149	tags=63%,	81620/1058	GO_BP	CDT1/CENPA/NUF2/NDC80/CENPF/CENPH/CENPW/
3852	tags=62%,	3561/6850/	GO_BP	IL2RG/SYK/JAK3/SHC1/IL2RA/IL2RB/PTPN2/S'
3505	tags=85%,	22948/908/	GO_BP	CCT5/CCT6A/CCT3/DKC1/CCT2/CCT4/CCT7/TCP
3727	tags=74%,	54209/4192	GO_BP	TREM2/MDK/MMP14/CSF1/P2RX4/CCL5/MAPK3/C:
3729	tags=67%,	2302/100/5	GO_BP	FOXJ1/ADA/PKN1/UNC13D/NFKB2/BCL3/CXCL13/
3505	tags=75%,	22948/7903	GO_BP	CCT5/NABP2/CCT6A/CCT3/DKC1/ACD/CCT2/CCT4
1999	tags=67%,	5347/54443	GO_BP	PLK1/ANLN/SAPCD2/MISP/EZR/EPB41L2/GPSM2/
3505	tags=91%,	22948/908/	GO_BP	CCT5/CCT6A/CCT3/DKC1/CCT2/CCT4/CCT7/TCP
2	tags=12%,	6690	GO_BP	SPINK1
162	tags=25%,	4318/302	GO_BP	MMP9/ANXA2
2	tags=12%,	6690	GO_BP	SPINK1
1283	tags=57%,	7076/7040/	GO_BP	TIMP1/TGFB1/HMOX1/HIF1A
732	tags=57%,	994/7272/9	GO_BP	CDC25B/TTK/CDC25C/CDC25A
732	tags=57%,	994/7272/9	GO_BP	CDC25B/TTK/CDC25C/CDC25A
1999	tags=83%,	54443/9493	GO_BP	ANLN/KIF23/ECT2/RACGAP1/RTKN
1999	tags=83%,	54443/9493	GO_BP	ANLN/KIF23/ECT2/RACGAP1/RTKN
110	tags=17%,	5225	GO_BP	PGC
110	tags=17%,	5225	GO_BP	PGC
996	tags=67%,	3965/3958/	GO_BP	LGALS9/LGALS3/HAVCR2/HLA-DMB
670	tags=20%,	23236	GO_BP	PLCB1

648	tags=60%,	54209/730[illegible]	GO_BP	TREM2/TYROBP/ADAM9
1188	tags=40%,	5047/7784	GO_BP	PAEP/ZP3
5863	tags=43%,	100133941/	GO_BP	CD24/CTHRC1/SOX4/MDK/SOX9/COL1A1/SULF1/[illegible]
7377	tags=50%,	991/7345/[illegible]	GO_BP	CDC20/UCHL1/PLK1/CCNB1/PTTG1/KCTD17/UBD/
6823	tags=48%,	991/7345/[illegible]	GO_BP	CDC20/UCHL1/PLK1/CCNB1/PTTG1/LAPTM4B/TR[illegible]
6963	tags=51%,	4318/2810/	GO_BP	MMP9/SFN/TREM2/PYCARD/E2F1/BAK1/TSPO/PL[illegible]
5313	tags=42%,	9168/822/[illegible]	GO_BP	TMSB10/CAPG/PYCARD/LIMK1/MDK/DBN1/ICAM1/
3703	tags=34%,	100133941/	GO_BP	CD24/AGR2/PYCARD/MDK/EPO/ICAM1/LGALS9/S[illegible]
6144	tags=42%,	5347/891/[illegible]	GO_BP	PLK1/CCNB1/AURKB/EPO/IKBKE/CDK1/PFN2/BA[illegible]
6044	tags=49%,	6280/5666[illegible]	GO_BP	S100A9/MUC13/CYBA/LY96/IKBKE/FCER1G/TYR[illegible]
6206	tags=46%,	54997/542[illegible]	GO_BP	TESC/TREM2/MDK/LGALS9/FOXJ1/MMP14/LIF/C[illegible]
6206	tags=49%,	6659/4192/	GO_BP	SOX4/MDK/FCER1G/LGALS9/FOXJ1/CD3D/TGFB1/
7666	tags=62%,	11004/822/	GO_BP	KIF2C/CAPG/STMN1/SH3BP1/MICAL1/KIF18B/A[illegible]
7379	tags=56%,	54997/542[illegible]	GO_BP	TESC/TREM2/MDK/LGALS9/MMP14/LIF/CSF1/TY[illegible]
7545	tags=65%,	6659/1277/	GO_BP	SOX4/COL1A1/SRC/BAMBI/GPC3/WNK2/ASPM/PS[illegible]
4326	tags=39%,	6692/1159[illegible]	GO_BP	SPINT1/CTHRC1/SOX4/SOX9/TEAD2/SPINT2/TG[illegible]
4398	tags=41%,	6280/1123[illegible]	GO_BP	S100A9/EGLN3/PYCARD/LGALS9/BAK1/RIPK2/R[illegible]
3774	tags=30%,	6690/991/[illegible]	GO_BP	SPINK1/CDC20/PHLDA2/PAEP/TRIP13/SOX9/SU[illegible]
4207	tags=43%,	5347/983/[illegible]	GO_BP	PLK1/CDK1/NEK2/WDR62/ARL2/CCNF/AURKA/CE[illegible]
4712	tags=44%,	29108/338[illegible]	GO_BP	PYCARD/ICAM1/ARPC1B/PFN2/ARL2/PSRC1/COR[illegible]
4374	tags=41%,	6280/1123[illegible]	GO_BP	S100A9/EGLN3/PYCARD/LGALS9/BAK1/ROBO1/S[illegible]
4207	tags=41%,	5347/983/[illegible]	GO_BP	PLK1/CDK1/NEK2/WDR62/ARL2/CCNF/AURKA/CE[illegible]
6418	tags=55%,	29108/432[illegible]	GO_BP	PYCARD/MMP12/IKBKE/TRAIP/RIPK2/HAVCR2/I[illegible]
4900	tags=46%,	2810/1055[illegible]	GO_BP	SFN/AGR2/TREM2/E2F1/ITGA3/ARHGEF16/LGAL[illegible]
6418	tags=56%,	29108/432[illegible]	GO_BP	PYCARD/MMP12/IKBKE/TRAIP/RIPK2/HAVCR2/I[illegible]
3195	tags=36%,	9212/2724[illegible]	GO_BP	AURKB/TNFRSF21/FCER1G/LGALS9/BAK1/LGALS[illegible]
5566	tags=52%,	2810/1869/	GO_BP	SFN/E2F1/BAK1/YWHAZ/HK2/BAX/SLC9A1/SLC3[illegible]
4274	tags=43%,	6692/1159[illegible]	GO_BP	SPINT1/CTHRC1/TEAD2/SPINT2/TGFB1/FOLR1/[illegible]
1295	tags=23%,	8877/891/[illegible]	GO_BP	SPHK1/CCNB1/S100A6/E2F1/CDC6/ANXA2/CCNA[illegible]
4127	tags=44%,	3925/402/[illegible]	GO_BP	STMN1/ARL2/SKA1/PSRC1/SKA3/ARHGEF2/TRIM[illegible]
4127	tags=47%,	3925/402/[illegible]	GO_BP	STMN1/ARL2/PSRC1/TUBG1/MAPRE1/MAP2/PAK1/
3727	tags=21%,	7053/5266/	GO_BP	TGM3/PI3/F13A1/COL3A1/TGM2/SPOCK2/EVPL/[illegible]
4411	tags=48%,	5570/9212/	GO_BP	PKIB/AURKB/NEK2/CCT5/NABP2/CCT6A/CCT3/S
3727	tags=47%,	54209/419[illegible]	GO_BP	TREM2/MDK/LGALS3/MMP14/CSF1/P2RX4/SLAMF[illegible]
4013	tags=43%,	4318/2910[illegible]	GO_BP	MMP9/PYCARD/BAK1/PLAUR/BAX/BMF/NOL3/LMN[illegible]
3631	tags=36%,	991/9319/[illegible]	GO_BP	CDC20/TRIP13/CDC25B/LIF/TTK/CDC25C/PLCB
4479	tags=53%,	3614/7083/	GO_BP	IMPDH1/TK1/DTYMK/ADA/UCK2/UPP1/NME1/NME[illegible]
2861	tags=42%,	891/7153/[illegible]	GO_BP	CCNB1/TOP2A/CDCA5/CDK1/NCAPG/NUSAP1/NCA[illegible]
4824	tags=56%,	6241/1869/	GO_BP	RRM2/E2F1/RAD51/EZH2/CDC7/CHEK1/RBBP8/B[illegible]
5652	tags=72%,	3838/2597/	GO_BP	KPNA2/GAPDH/ITGAV/SCRIB/ZC3H12A/KPNB1/C[illegible]
2852	tags=40%,	54209/730[illegible]	GO_BP	TREM2/TYROBP/GDF15/ADAM9/CD53/FLOT1/EHD
3911	tags=73%,	1401/1535/	GO_BP	CRP/CYBA/TYROBP/TGFB1/ITGAM/GSTP1/ITGB2/
2627	tags=50%,	54751/671[illegible]	GO_BP	FBLIM1/SRC/MZB1/FERMT3/PLEK/COL16A1/CXC[illegible]
4042	tags=64%,	22948/790[illegible]	GO_BP	CCT5/NABP2/CCT6A/CCT3/DKC1/ACD/CCT2/CCT[illegible]
2924	tags=45%,	6692/1065[illegible]	GO_BP	SPINT1/SPINT2/ST14/LEF1/WNT7B/SOCS3/FGF[illegible]
3920	tags=79%,	22948/908/	GO_BP	CCT5/CCT6A/CCT3/RUVBL1/DKC1/CCT2/CCT4/N[illegible]
3920	tags=79%,	22948/908/	GO_BP	CCT5/CCT6A/CCT3/RUVBL1/DKC1/CCT2/CCT4/N[illegible]
3920	tags=79%,	22948/908/	GO_BP	CCT5/CCT6A/CCT3/RUVBL1/DKC1/CCT2/CCT4/N[illegible]
3920	tags=79%,	22948/908/	GO_BP	CCT5/CCT6A/CCT3/RUVBL1/DKC1/CCT2/CCT4/N[illegible]
3520	tags=60%,	4751/6790/	GO_BP	NEK2/AURKA/KIF11/NUP62/CHEK1/CEP85/RANB[illegible]
3266	tags=53%,	5880/5683[illegible]	GO_BP	RAC2/SLAMF8/GRN/RAC1/JCHAIN/RPS19/CLEC7[illegible]

3505	tags=100%,	22948/908/	GO_BP	CCT5/CCT6A/CCT3/DKC1/CCT2/CCT4/CCT7/TCP
3505	tags=100%,	22948/908/	GO_BP	CCT5/CCT6A/CCT3/DKC1/CCT2/CCT4/CCT7/TCP
3505	tags=100%,	22948/908/	GO_BP	CCT5/CCT6A/CCT3/DKC1/CCT2/CCT4/CCT7/TCP
1927	tags=50%,	9319/994/	GO_BP	TRIP13/CDC25B/AURKA/FBXO5
1759	tags=75%,	10609/5351	GO_BP	P3H4/PLOD1/JMJD6/P3H3/PLOD2/PLOD3
2776	tags=50%,	100133941/	GO_BP	CD24/LIF/PROM1/PAX8
202	tags=38%,	29108/272	GO_BP	PYCARD/TNFRSF21/LGALS9
563	tags=100%,	26007/1321	GO_BP	TKFC/GLYCTK/KHK/ALDOB
337	tags=100%,	2875/6490	GO_BP	GPT/AGXT2/DAO/AGXT
337	tags=100%,	2875/6490	GO_BP	GPT/AGXT2/DAO/AGXT
819	tags=100%,	445/16241	GO_BP	ASS1/NAGS/OTC/CPS1
898	tags=100%,	2954/3081/	GO_BP	GSTZ1/HGD/HPD/TAT
337	tags=100%,	2875/6490	GO_BP	GPT/AGXT2/DAO/AGXT
337	tags=100%,	2875/6490	GO_BP	GPT/AGXT2/DAO/AGXT
507	tags=80%,	6653/4405	GO_BP	SORL1/PLIN5/GPLD1/APOC3
591	tags=100%,	336/335/11	GO_BP	APOA2/APOA1/APOA5/APOC3
75	tags=100%,	1544/1571/	GO_BP	CYP1A2/CYP2E1/CYP2C9/CYP3A4
771	tags=80%,	223/8424/	GO_BP	ALDH9A1/BBOX1/ACADM/SHMT1
563	tags=100%,	26007/1321	GO_BP	TKFC/GLYCTK/KHK/ALDOB
339	tags=80%,	66002/4051	GO_BP	CYP4F12/CYP4F3/CYP4F2/CYP4A11
6686	tags=52%,	2539/9536/	GO_BP	G6PD/PTGES/RGS2/BACE2/SOX4/RPL39L/ATP13
7323	tags=49%,	8877/991/	GO_BP	SPHK1/CDC20/PLK1/UBD/LIMK1/UBE2T/SOX4/U
6823	tags=46%,	991/7345/	GO_BP	CDC20/UCHL1/PLK1/CCNB1/PTTG1/KCTD17/UBD
6823	tags=46%,	991/7345/	GO_BP	CDC20/UCHL1/PLK1/CCNB1/PTTG1/TREM2/KCTD
5863	tags=43%,	100133941/	GO_BP	CD24/CTHRC1/SOX4/MDK/SOX9/COL1A1/SULF1/
4995	tags=38%,	2539/5315/	GO_BP	G6PD/PKM/MUC13/HKDC1/B3GNT3/ALDOA/GCNT3
4917	tags=34%,	8877/1001	GO_BP	SPHK1/CD24/S100A9/CCL20/TREM2/PYCARD/UB
5045	tags=37%,	56667/103	GO_BP	MUC13/B3GNT3/GCNT3/ST6GALNAC4/BACE2/MMP
6249	tags=44%,	5347/891/	GO_BP	PLK1/CCNB1/TMED3/LAPTM4B/CCNB2/CDK1/NDR
5706	tags=46%,	6510/3576/	GO_BP	SLC1A5/CXCL8/SLC52A2/TOP2A/ANXA2/KPNA2/
6206	tags=46%,	6659/4192/	GO_BP	SOX4/MDK/FCER1G/LGALS9/BAK1/FOXJ1/TYRO3
6062	tags=45%,	6280/56667	GO_BP	S100A9/MUC13/TREM2/PYCARD/CYBA/LY96/PGC
8957	tags=66%,	5347/7973	GO_BP	PLK1/KCTD17/TUBA4A/CDK1/NEK2/FOXJ1/FLNA
4398	tags=32%,	2539/4605/	GO_BP	G6PD/MYBL2/EGLN3/NQO1/MDK/TNFRSF21/EPO/
5555	tags=43%,	9168/822/	GO_BP	TMSB10/CAPG/PYCARD/LIMK1/MDK/DBN1/ICAM1
8957	tags=65%,	5347/7973	GO_BP	PLK1/KCTD17/TUBA4A/CDK1/NEK2/FOXJ1/FLNA
457	tags=100%,	1543/1577/	GO_BP	CYP1A1/CYP3A5/CYP2E1/CYP2C8/CYP3A4
767	tags=83%,	137362/28	GO_BP	GOT1L1/GOT1/ASPA/ASS1/GOT2
349	tags=83%,	1553/1543/	GO_BP	CYP2A13/CYP1A1/CYP2A7/PON3/CYP2A6
349	tags=83%,	1553/1543/	GO_BP	CYP2A13/CYP1A1/CYP2A7/PON3/CYP2A6
1024	tags=83%,	1644/590/	GO_BP	DDC/BCHE/CYP3A5/CYP1A2/CYP3A4
4163	tags=31%,	6692/5420	GO_BP	SPINT1/TREM2/BACE2/SOX4/MDK/TNFRSF21/E2
417	tags=83%,	55349/501/	GO_BP	CHDH/ALDH7A1/SARDH/DMGDH/BHMT
219	tags=100%,	9615/7498/	GO_BP	GDA/XDH/AOX1/DPYS/ALDH6A1
3758	tags=38%,	8877/6364/	GO_BP	SPHK1/CCL20/PYCARD/CYBA/LCN2/CXCL8/TNFR
6454	tags=49%,	29108/105	GO_BP	PYCARD/UBD/LY96/S100A4/IKBKE/LGALS9/ECT
4398	tags=31%,	2539/4605/	GO_BP	G6PD/MYBL2/EGLN3/NQO1/MDK/TNFRSF21/TYRO
3510	tags=30%,	2919/5420	GO_BP	CXCL1/TREM2/PYCARD/CXCL6/LY96/LCN2/CXCL
4093	tags=37%,	4318/2056/	GO_BP	MMP9/EPO/ENO1/ICAM1/SRC/LGALS3/PLAUR/GS
338	tags=40%,	7083/1841	GO_BP	TK1/DTYMK
338	tags=40%,	7083/1841	GO_BP	TK1/DTYMK

666	tags=40%,	22943/108	GO_BP	DKK1/C1QL1
5732	tags=39%,	226/5047/	GO_BP	ALDOA/PAEP/TNFRSF21/LGALS3/HAVCR2/CLGN/
5876	tags=53%,	3576/7153/	GO_BP	CXCL8/TOP2A/ANXA2/KPNA2/LGALS9/LGALS1/C
7518	tags=67%,	891/10212/	GO_BP	CCNB1/DDX39A/EXOSC4/ALYREF/HSF1/INTS8/L
7153	tags=61%,	4192/3965/	GO_BP	MDK/LGALS9/FOXJ1/TGFB1/RIPK2/ADA/CD74/F
5887	tags=51%,	4318/2810/	GO_BP	MMP9/SFN/PYCARD/E2F1/BAK1/PLAUR/YWHAZ/H
5652	tags=55%,	7153/302/	GO_BP	TOP2A/ANXA2/KPNA2/LGALS9/LGALS1/CD74/CS
4770	tags=46%,	4192/7040/	GO_BP	MDK/TGFB1/FLNA/S1PR2/BAIAP2L2/PLEK/HRAS
5981	tags=52%,	79574/797	GO_BP	EPS8L3/KCTD17/ANLN/RAC2/AGRN/HRAS/CDC42
4274	tags=43%,	6692/1159	GO_BP	SPINT1/CTHRC1/TEAD2/SPINT2/TGFB1/FOLR1/
4643	tags=41%,	6280/5420	GO_BP	S100A9/TREM2/PYCARD/CXCL6/POSTN/LGALS9/
2476	tags=28%,	8877/891/	GO_BP	SPHK1/CCNB1/S100A6/E2F1/CDC6/ANXA2/CCNA
3729	tags=48%,	54997/338	GO_BP	TESC/ICAM1/ITGB4/ADA/ITGB2/FERMT3/ITGAV
4322	tags=52%,	972/3315/	GO_BP	CD74/HSPB1/P3H1/DNAJC5/HSPA6/FKBP1A/HSP
771	tags=86%,	55349/223/	GO_BP	CHDH/ALDH9A1/BBOX1/ACADM/ALDH7A1/SHMT1
4469	tags=50%,	3925/402/	GO_BP	STMN1/ARL2/PSRC1/MAPRE1/MAP2/PAK1/TUBB4
3509	tags=46%,	302/1277/	GO_BP	ANXA2/COL1A1/MMP11/LOXL4/P3H4/COL1A2/CO
3870	tags=43%,	11004/392	GO_BP	KIF2C/STMN1/KIF18B/ARHGEF2/TRIM54/MID1I
5821	tags=79%,	23246/511	GO_BP	BOP1/MRTO4/RRS1/BRIX1/NOP2/NLE1/RPL10L/
1927	tags=41%,	81620/624	GO_BP	CDT1/RRM2/E2F1/CDC6/CDC45/CCNE1/ORC1/TY
1784	tags=37%,	2207/3965/	GO_BP	FCER1G/LGALS9/HAVCR2/SYK/CD86/ZP3/CD3E/
4970	tags=63%,	891/81620/	GO_BP	CCNB1/CDT1/CDC6/DLGAP5/RCC2/FEN1/ESPL1/
3428	tags=50%,	9536/5730/	GO_BP	PTGES/PTGDS/FABP5/CD74/AKR1C3/MIF/PTGES
5913	tags=75%,	112399/10	GO_BP	EGLN3/P3H4/ASPH/PLOD1/P3H1/P4HA2/JMJD6/
3428	tags=50%,	9536/5730/	GO_BP	PTGES/PTGDS/FABP5/CD74/AKR1C3/MIF/PTGES
886	tags=75%,	5194/224/	GO_BP	PEX13/ALDH3A2/PECR/PHYH/SLC27A2/HAO1
1099	tags=75%,	3067/3034/	GO_BP	HDC/HAL/HNMT/AMDHD1/FTCD/UROC1
1199	tags=100%,	5096/1667	GO_BP	PCCB/MMAA/MCEE/ACADS/PHYH/PCK2/PCK1
1099	tags=75%,	3067/3034/	GO_BP	HDC/HAL/HNMT/AMDHD1/FTCD/UROC1
854	tags=62%,	117247/12	GO_BP	SLC16A10/ABCC2/SLC16A2/SERPINA7/TTR
819	tags=64%,	435/445/3	GO_BP	ASL/ASS1/ARG1/NAGS/SLC25A15/OTC/CPS1
1334	tags=91%,	2108/37/2	GO_BP	ETFA/ACADVL/ETFBKMT/ACAD11/ACADS/ETFDH/
328	tags=45%,	8993/3078/	GO_BP	PGLYRP1/CFHR1/CFHR2/GPLD1/CFHR5
1409	tags=73%,	2098/5702	GO_BP	ESD/PDXP/ADH5/HOGA1/HAGH/AGXT2/AGXT/ADH
944	tags=64%,	3155/5689	GO_BP	HMGCL/BDH2/ACSS3/ACAT1/BDH1/HMGCS2/SLC2
917	tags=64%,	125061/16	GO_BP	AFMID/IDO2/AADAT/GOT2/TDO2/KMO/ALDH8A1
459	tags=55%,	38/66002/	GO_BP	ACAT1/CYP4F12/CYP4F3/BDH1/CYP4F2/CYP4A1
2867	tags=47%,	1869/706/	GO_BP	E2F1/TSPO/ETV5/LYN/IL1B/PPP1CC/PRKCI
1090	tags=60%,	3383/3965/	GO_BP	ICAM1/LGALS9/LGALS3/HAVCR2/HLA-DMB/APBB
417	tags=67%,	55349/501/	GO_BP	CHDH/ALDH7A1/BCHE/SARDH/DMGDH/BHMT
905	tags=89%,	3949/348/	GO_BP	LDLR/APOE/APOC2/GPIHBP1/LIPC/APOB/APOC1
1121	tags=78%,	8659/1128	GO_BP	ALDH4A1/HOGA1/GOT2/GRHPR/PRODH2/AGXT2/A
905	tags=89%,	3949/348/	GO_BP	LDLR/APOE/APOC2/GPIHBP1/LIPC/APOB/APOC1
1277	tags=67%,	150094/34	GO_BP	SIK1/APOE/SORL1/PLIN5/GPLD1/APOC3
213	tags=89%,	1557/1136	GO_BP	CYP2C19/CYP2U1/CYP1A1/CYP1A2/CYP4F2/CYP
3505	tags=92%,	22948/908/	GO_BP	CCT5/CCT6A/CCT3/DKC1/CCT2/CCT4/CCT7/TCP
6823	tags=46%,	991/7345/	GO_BP	CDC20/UCHL1/PLK1/CCNB1/PTTG1/KCTD17/UBD
5671	tags=31%,	6690/5347/	GO_BP	SPINK1/PLK1/CCNB1/PTTG1/ALDOA/PAFAH1B3/
5671	tags=32%,	6696/6690/	GO_BP	SPP1/SPINK1/MMP7/PLK1/CCNB1/PTTG1/PAFAH
5671	tags=32%,	6696/6690/	GO_BP	SPP1/SPINK1/MMP7/PLK1/CCNB1/PTTG1/PAFAH
6214	tags=45%,	2810/1410/	GO_BP	SFN/CRYAB/ANXA2/FCER1G/LGALS9/PFN2/SRC/

4982	tags=37%,	100133941/	GO_BP	CD24/UCHL1/RGS2/DUSP9/CDK1/SRC/TRIB3/TG
1684	tags=56%,	24137/921	GO_BP	KIF4A/AURKB/KIF23/RACGAP1/LZTS2
5309	tags=40%,	115908/66	GO_BP	CTHRC1/SOX4/MDK/SOX9/COL1A1/SRC/DAB2/BA
3211	tags=100%,	22948/908/	GO_BP	CCT5/CCT6A/CCT3/DKC1/CCT2/CCT4/CCT7/TCP
3211	tags=100%,	22948/908/	GO_BP	CCT5/CCT6A/CCT3/DKC1/CCT2/CCT4/CCT7/TCP
4405	tags=37%,	2810/5047/	GO_BP	SFN/PAEP/TREM2/PYCARD/SOX4/MDK/E2F1/MMP
4728	tags=34%,	2539/4605/	GO_BP	G6PD/MYBL2/EGLN3/NQO1/MDK/EPO/TYRO3/ATP
5309	tags=42%,	115908/66	GO_BP	CTHRC1/SOX4/MDK/SOX9/COL1A1/SRC/DAB2/BA
1892	tags=62%,	5347/8995	GO_BP	PLK1/SAPCD2/MISP/EPB41L2/GPSM2
6454	tags=49%,	29108/105	GO_BP	PYCARD/UBD/S100A4/IKBKE/LGALS9/ECT2/LGA
4127	tags=32%,	6696/3984/	GO_BP	SPP1/LIMK1/SOX9/DBN1/POSTN/REG1A/PLXNA1
4398	tags=31%,	2539/4605/	GO_BP	G6PD/MYBL2/EGLN3/NQO1/MDK/TYRO3/TYROBP/
2875	tags=26%,	6696/1364/	GO_BP	SPP1/CLDN4/MMP9/MMP7/RGS2/SLC2A1/EPO/LG
5706	tags=48%,	6510/3576/	GO_BP	SLC1A5/CXCL8/SLC52A2/KPNA2/CDK1/ICAM1/L
4258	tags=39%,	29108/398	GO_BP	PYCARD/LIMK1/ICAM1/ARPC1B/PFN2/ARL2/PSR
5182	tags=40%,	8877/991/	GO_BP	SPHK1/CDC20/PLK1/LIMK1/SOX4/UBE2S/TSPO/
6220	tags=52%,	79574/590	GO_BP	EPS8L3/RAP1GAP/KCTD17/ICAM1/ANLN/PFN2/R
6465	tags=50%,	4318/8162	GO_BP	MMP9/CDT1/CTHRC1/CDCA5/ANXA2/STMN1/LGAL
5960	tags=48%,	8877/302/	GO_BP	SPHK1/ANXA2/CORO1A/STX3/SNAP25/CPLX2/TA
6055	tags=49%,	2539/2056/	GO_BP	G6PD/EPO/FCER1G/BAK1/SPI1/SLC7A11/PRMT1
3964	tags=33%,	54209/291	GO_BP	TREM2/PYCARD/CYBA/LY96/FCER1G/LGALS9/TS
5147	tags=48%,	10551/440/	GO_BP	AGR2/ASNS/HSPB8/CXCL8/KDELR3/BAK1/STC2/
936	tags=47%,	9942/7367/	GO_BP	XYLB/UGT2B17/UGT2B4/CRYL1/UGT2B7/DCXR/S
1326	tags=83%,	55902/128/	GO_BP	ACSS2/ADH5/ALDH1B1/ALDH1A1/ALDH2/ADH6/A
1115	tags=53%,	23464/275/	GO_BP	GCAT/AMT/SHMT1/RIDA/SDS/BAAT/AGXT2/AGXT
509	tags=75%,	1428/1236	GO_BP	CRYM/HYKK/DLST/PHYKPL/AADAT/GCDH/ALDH7A
771	tags=71%,	55349/138	GO_BP	CHDH/CRAT/ALDH9A1/CPT1A/POR/BBOX1/ACADM
591	tags=67%,	26291/344/	GO_BP	FGF21/APOC2/SORL1/PLIN5/GPLD1/AADAC/APO
1411	tags=50%,	5174/5440	GO_BP	PDZK1/SLC38A2/SLC25A20/SLC22A3/SLC22A1/
936	tags=47%,	9942/7367/	GO_BP	XYLB/UGT2B17/UGT2B4/CRYL1/UGT2B7/DCXR/S
838	tags=58%,	6469/344/	GO_BP	SHH/APOC2/APOC1/ABCG8/ABCG5/APOA2/APOC3
838	tags=58%,	6469/344/	GO_BP	SHH/APOC2/APOC1/ABCG8/ABCG5/APOA2/APOC3
692	tags=58%,	348/344/3	GO_BP	APOE/APOC2/APOC1/APOA2/APOA1/APOA5/APOC
1063	tags=83%,	1071/348/	GO_BP	CETP/APOE/APOC2/LIPG/LIPC/APOA2/LCAT/AP
1099	tags=58%,	3067/3034/	GO_BP	HDC/HAL/HNMT/MTHFD1/AMDHD1/FTCD/UROC1
457	tags=41%,	1551/6481	GO_BP	CYP3A7/CYP3A43/CYP3A5/CYP1A2/CYP2C8/CYP
944	tags=58%,	3155/5689	GO_BP	HMGCL/BDH2/ACSS3/ACAT1/BDH1/HMGCS2/SLC2
767	tags=50%,	79944/280	GO_BP	L2HGDH/GOT1/AADAT/GOT2/GPT2/PHYH/ADHFE1
767	tags=56%,	122970/28	GO_BP	ACOT4/GOT1/ASPA/MTHFS/GLUL/GLUD1/GOT2/A
1184	tags=75%,	1191/2346	GO_BP	CLU/ABCA5/CETP/APOE/APOC2/LIPG/APOM/LIP
1487	tags=81%,	122970/55	GO_BP	ACOT4/ACSS2/PCCA/ACSM1/PCCB/MMAA/THNSL2
1099	tags=70%,	3067/3034/	GO_BP	HDC/HAL/HNMT/MTHFD1/AMDHD1/FTCD/UROC1
898	tags=70%,	2184/2954/	GO_BP	FAH/GSTZ1/HGD/IYD/PAH/HPD/TAT
3935	tags=28%,	5347/9232/	GO_BP	PLK1/PTTG1/TOP2A/TRIP13/CDC25B/CKS2/CCN
6597	tags=51%,	56667/103	GO_BP	MUC13/B3GNT3/GCNT3/ST6GALNAC4/VEGFB/DPM
2996	tags=36%,	6364/2919/	GO_BP	CCL20/CXCL1/TREM2/CXCL6/CXCL8/CXCL5/REG
2996	tags=36%,	6364/2919/	GO_BP	CCL20/CXCL1/TREM2/CXCL6/CXCL8/CXCL5/REG
1429	tags=73%,	4496/4494/	GO_BP	MT1H/MT1F/MT1B/ABCC2/SLC30A1/MT2A/MT1A/
3442	tags=41%,	6364/4192/	GO_BP	CCL20/MDK/LGALS3/CSF1/S100A14/TGFB1/SLA
3195	tags=37%,	9212/2207/	GO_BP	AURKB/FCER1G/LGALS9/LGALS3/ADA/CDKN2A/C
3964	tags=39%,	29108/153	GO_BP	PYCARD/CYBA/LY96/FCER1G/LGALS9/TYROBP/C

761	tags=50%,	875/23743/	GO_BP	CBS/BHMT2/ADI1/MTHFD1/CTH/CDO1/BHMT
804	tags=62%,	51449/875/	GO_BP	PCYOX1/CBS/TST/CSAD/MAT1A/CTH/CDO1/AGXT
656	tags=62%,	2/8547/222	GO_BP	A2M/FCN3/FCN2/SERPING1/COLEC10/MBL2/MASI
922	tags=63%,	4942/23576	GO_BP	OAT/DDAH1/NOS2/AGMAT/FAH/ASL/ART4/ASS1/
509	tags=69%,	1428/12368	GO_BP	CRYM/HYKK/DLST/PHYKPL/AADAT/GCDH/ALDH7A
972	tags=64%,	275/55258/	GO_BP	AMT/THNSL2/CBS/CSAD/SHMT1/SDS/DAO/CDO1/
1343	tags=62%,	1384/223/1	GO_BP	CRAT/ALDH9A1/CPT1A/POR/BBOX1/ACADM/SHMT
1429	tags=69%,	4496/4494/	GO_BP	MT1H/MT1F/MT1B/MT2A/MT1A/MT1M/MT1G/MT1E/
620	tags=38%,	320/6540/6	GO_BP	APBA1/SLC6A13/SLC6A12/ABAT/SLC6A1
717	tags=79%,	1562/1573/	GO_BP	CYP2C18/CYP2J2/CYP2A13/CYP4F12/EPHX2/CYI
819	tags=69%,	9971/435/4	GO_BP	NR1H4/ASL/ASS1/ARG1/NAGS/SLC25A15/OTC/CI
1354	tags=71%,	26063/1384	GO_BP	DECR2/CRAT/ECI2/ACOX1/ACAA1/HSD17B4/AMA
1063	tags=86%,	1071/348/3	GO_BP	CETP/APOE/APOC2/LIPG/GPIHBP1/LIPC/APOB/
1063	tags=54%,	1071/185/3	GO_BP	CETP/AGTR1/APOE/LIPC/APOB/LPA/APOA2
905	tags=77%,	3949/81693	GO_BP	LDLR/AMN/APOE/APOC2/LIPG/APOM/APOA2/GPLI
1101	tags=23%,	4040/367/5	GO_BP	LRP6/AR/RBP4
207	tags=64%,	2147/2160/	GO_BP	F2/F11/SERPINF2/APOH/CPB2/KLKB1/F12/PLG/
876	tags=71%,	9971/6821/	GO_BP	NR1H4/SUOX/ASL/ASS1/ARG1/NAGS/SLC25A15/
1429	tags=69%,	4496/4494/	GO_BP	MT1H/MT1F/MT1B/MT2A/MT1A/MT1M/MT1G/MT1E/
6060	tags=52%,	7040/3956/	GO_BP	TGFB1/LGALS1/ADA/EXO1/CLCF1/BATF/TFRC/M
6259	tags=58%,	2207/3965/	GO_BP	FCER1G/LGALS9/RAC2/CPLX2/LAT2/SYK/UNC13I
6303	tags=60%,	54209/9212	GO_BP	TREM2/AURKB/FCER1G/SH3BP1/ITGAM/ITGB2/I
3465	tags=37%,	29108/3965	GO_BP	PYCARD/LGALS9/RIPK2/HAVCR2/HRAS/IL18/HL
425	tags=39%,	4482/23743	GO_BP	MSRA/BHMT2/ADI1/MTHFD1/MAT1A/GNMT/BHMT
1049	tags=61%,	2157/7450/	GO_BP	F8/VWF/SERPING1/F2/F11/APOH/KLKB1/F12/F
1170	tags=72%,	23461/1071	GO_BP	ABCA5/CETP/APOE/APOC2/LIPG/APOM/LIPC/AP
555	tags=39%,	135/59272/	GO_BP	ADORA2A/ACE2/AVPR1A/KMO/SLC38A3/ABAT/SL
3626	tags=39%,	4318/2810/	GO_BP	MMP9/SFN/PYCARD/BAK1/PLAUR/BAX/BMF/NOL3/
3865	tags=48%,	8877/29108	GO_BP	SPHK1/PYCARD/TRAIP/GSTP1/BIRC3/SYK/CASP
3659	tags=36%,	991/5347/1	GO_BP	CDC20/PLK1/CDCA5/BUB1/BUB1B/FEN1/DSCC1/I
5765	tags=62%,	9454/8767/	GO_BP	HOMER3/RIPK2/HAVCR2/PRNP/HDAC7/EZR/VTCN
5913	tags=55%,	112399/606	GO_BP	EGLN3/FKBP10/P3H1/FKBP1A/P4HA2/P3H3/PPII
1326	tags=75%,	55902/6817	GO_BP	ACSS2/SULT1A1/ADH5/SULT1A2/ALDH1B1/ALDH
6337	tags=51%,	1869/55544	GO_BP	E2F1/RBM38/IGF2BP1/YBX3/MAPKAPK2/DKC1/YI
3999	tags=51%,	3614/7083/	GO_BP	IMPDH1/TK1/DTYMK/ADA/UCK2/UPP1/NME1/NME
4824	tags=51%,	6241/1869/	GO_BP	RRM2/E2F1/RAD51/EZH2/CDC7/CHEK1/RHNO1/RI
4582	tags=59%,	302/7040/8	GO_BP	ANXA2/TGFB1/MTMR2/CLTA/VLDLR/CAPN1/MYLII
4824	tags=54%,	6241/1869/	GO_BP	RRM2/E2F1/RAD51/EZH2/CDC7/CHEK1/RHNO1/RI
3727	tags=53%,	54209/4192	GO_BP	TREM2/MDK/MMP14/CSF1/P2RX4/SLAMF8/CCL5/I
4950	tags=35%,	6690/10013	GO_BP	SPINK1/CD24/TREM2/EPO/ICAM1/MELK/TYRO3/
4950	tags=35%,	6690/10013	GO_BP	SPINK1/CD24/TREM2/EPO/ICAM1/MELK/TYRO3/
3758	tags=37%,	8877/6364/	GO_BP	SPHK1/CCL20/PYCARD/CYBA/UBD/LCN2/CXCL8/
3510	tags=30%,	2919/54209	GO_BP	CXCL1/TREM2/PYCARD/CXCL6/LY96/LCN2/CXCL
3510	tags=30%,	2919/29108	GO_BP	CXCL1/PYCARD/CXCL6/LY96/LCN2/CXCL8/CXCL
6914	tags=57%,	54913/8270	GO_BP	RPP25/LAGE3/PUSL1/EEF1E1/TSEN54/GTF3C6/
5704	tags=48%,	6659/3872/	GO_BP	SOX4/KRT17/PTAFR/PRKCD/NCK2/LARP6/CDK4/
3964	tags=34%,	54209/2910	GO_BP	TREM2/PYCARD/CYBA/LY96/FCER1G/LGALS9/TSI
3920	tags=78%,	22948/908/	GO_BP	CCT5/CCT6A/CCT3/RUVBL1/DKC1/CCT2/CCT4/NI
1596	tags=32%,	4318/10013	GO_BP	MMP9/CD24/LIF/PROM1/PRKX/STAT1
380	tags=38%,	81620/9212	GO_BP	CDT1/AURKB/CDK1/NDC80/TTK/RCC2
6680	tags=57%,	4318/10551	GO_BP	MMP9/AGR2/SOX9/SRC/PLAUR/TGFB1/RHBDF2/N

6181	tags=52%,	3159/8318/	GO_BP	HMGA1/CDC45/TGFB1/NUP62/DNMT1/TRIM28/SI
3505	tags=91%,	22948/908/	GO_BP	CCT5/CCT6A/CCT3/DKC1/CCT2/CCT4/CCT7/TCP
1488	tags=57%,	549/593/6	GO_BP	AUH/BCKDHA/MCCC2/DBT/HMGCL/HIBCH/HIBADH/
1488	tags=57%,	549/593/6	GO_BP	AUH/BCKDHA/MCCC2/DBT/HMGCL/HIBCH/HIBADH/
7272	tags=66%,	891/10212/	GO_BP	CCNB1/DDX39A/ALYREF/HSF1/CSTF2/SNRPA/UP
972	tags=52%,	275/4128/	GO_BP	AMT/MAOA/HNMT/POR/MAOB/ALDH7A1/SARDH/AL
717	tags=57%,	1562/1573/	GO_BP	CYP2C18/CYP2J2/CYP2A13/CYP2A7/NR1I2/CYP
1115	tags=52%,	23464/280	GO_BP	GCAT/GOT1/ASPA/AADAT/GOT2/GCDH/ALDH7A1/
1817	tags=73%,	125061/19	GO_BP	AFMID/PAOX/CHDH/SAT2/SAT1/IDO2/HNMT/AAD
1056	tags=45%,	2327/1644/	GO_BP	FMO2/DDC/AS3MT/CBS/ACAA1/CYP1A1/AKR7A3/
1036	tags=52%,	116285/16	GO_BP	ACSM1/IDO2/AADAT/ACAA1/GOT2/TDO2/KMO/HA
6181	tags=57%,	2810/1869/	GO_BP	SFN/E2F1/RAC2/YWHAZ/MICALL2/SAE1/BID/UB
4608	tags=43%,	6280/2919/	GO_BP	S100A9/CXCL1/CXCL6/PGC/CXCL8/CXCL5/REG1
6353	tags=74%,	25879/576	GO_BP	DCAF13/DHX37/NPM1/RRS1/RRP7A/RPSA/EMG1/
5147	tags=55%,	10551/110	GO_BP	AGR2/KDELR3/BAK1/BAX/LMNA/DDX11/HYOU1/A
5169	tags=55%,	6364/2910	GO_BP	CCL20/PYCARD/ICAM1/STK39/CCL5/SLC12A2/C
1474	tags=58%,	338821/62	GO_BP	SLCO1B7/RXRA/NR1H4/SLCO1A2/ABCC2/STARD5/
207	tags=35%,	948/2147/	GO_BP	CD36/F2/F7/SERPINF2/APOH/CPB2/F12/PLG/H
838	tags=36%,	6469/344/	GO_BP	SHH/APOC2/IRS2/APOC1/ABCG8/ABCG5/APOA2/
1121	tags=46%,	8659/8776	GO_BP	ALDH4A1/GGACT/PCYOX1/MTHFS/HOGA1/GOT2/S
1024	tags=38%,	1644/1693	GO_BP	DDC/IDO2/SRD5A1/AADAT/ACMSD/GCDH/TDO2/K
717	tags=52%,	1562/1592/	GO_BP	CYP2C18/CYP26A1/BCO2/CYP3A7/CYP1A1/ALDH
1275	tags=48%,	23411/37/	GO_BP	SIRT1/ACADVL/ERLIN1/ETFBKMT/ACACB/DGAT2/
745	tags=38%,	196883/11	GO_BP	ADCY4/ADCY8/FGF21/ADCY1/GJB2/ASS1/ARG1/
207	tags=35%,	948/2147/	GO_BP	CD36/F2/F7/SERPINF2/APOH/CPB2/F12/PLG/H
3729	tags=50%,	4318/10551	GO_BP	MMP9/AGR2/PLAUR/ADORA1/SHKBP1/NUP62/NCF
5500	tags=62%,	4318/10551	GO_BP	MMP9/AGR2/PLAUR/ADORA1/SHKBP1/NUP62/NCF
2688	tags=39%,	29108/105	GO_BP	PYCARD/UBD/TGFB1/SPI1/HAVCR2/BATF/RELB/
4573	tags=49%,	2810/3872/	GO_BP	SFN/KRT17/NME2/PPARD/SULT2B1/TGFB2/VDR/
5125	tags=51%,	6714/1601/	GO_BP	SRC/DAB2/MTMR2/EZR/MSN/MAPK3/MAP2K2/ANK
3727	tags=53%,	4192/3958/	GO_BP	MDK/LGALS3/CSF1/SLAMF8/CKLF/CCL5/MIF/MA
3756	tags=50%,	2810/1869/	GO_BP	SFN/E2F1/BAK1/YWHAZ/BAX/HIP1R/BID/YWHAH/
5456	tags=36%,	8877/4318/	GO_BP	SPHK1/MMP9/CD24/CCL20/PLK1/CCNB1/TREM2/
5182	tags=37%,	6364/5909/	GO_BP	CCL20/RAP1GAP/RGS2/RGS1/ICAM1/SH3BP1/FO
6856	tags=53%,	3159/8318/	GO_BP	HMGA1/CDC45/TGFB1/SPI1/PHF19/NUP62/EZH2/
5045	tags=37%,	56667/103	GO_BP	MUC13/B3GNT3/GCNT3/ST6GALNAC4/VEGFB/GAL
5045	tags=37%,	56667/103	GO_BP	MUC13/B3GNT3/GCNT3/ST6GALNAC4/VEGFB/GAL
5182	tags=41%,	8877/991/	GO_BP	SPHK1/CDC20/PLK1/LIMK1/SOX4/UBE2S/TSPO/
2875	tags=27%,	6696/1364/	GO_BP	SPP1/CLDN4/MMP9/MMP7/RGS2/SLC2A1/EPO/LG
3244	tags=41%,	4318/7262/	GO_BP	MMP9/PHLDA2/ANXA2/ADAM15/LEF1/LDOC1/LOX/
6686	tags=49%,	4192/2207/	GO_BP	MDK/FCER1G/ICAM1/LGALS9/LGALS3/TGFB1/LG
3964	tags=33%,	54209/291	GO_BP	TREM2/PYCARD/CYBA/LY96/FCER1G/LGALS9/TS
6603	tags=50%,	54209/291	GO_BP	TREM2/PYCARD/CYBA/LY96/FCER1G/LGALS9/TS
4917	tags=42%,	4192/2724	GO_BP	MDK/TNFRSF21/LGALS9/FOXJ1/TYRO3/LGALS3/
1040	tags=52%,	51170/646	GO_BP	HSD17B11/SHH/CYP17A1/SRD5A1/HSD17B4/HSD
692	tags=44%,	348/344/5	GO_BP	APOE/APOC2/APOM/APOC4/MTTP/APOB/APOC1/A
782	tags=56%,	5627/710/	GO_BP	PROS1/SERPING1/FGB/FGG/FGA/F2/F11/TMPRS
221	tags=41%,	3248/7907	GO_BP	HPGD/ELOVL6/ASAH2/EPHX2/CYP1A1/ACOT12/C
207	tags=33%,	948/2147/	GO_BP	CD36/F2/F7/SERPINF2/APOH/CPB2/F12/PLG/H
1429	tags=44%,	4496/4494/	GO_BP	MT1H/MT1F/MAP1LC3A/MT1B/MT2A/MT1A/MT1M/
697	tags=59%,	2157/7450/	GO_BP	F8/VWF/SERPING1/F10/FGB/FGG/FGA/F2/F11/

7629	tags=65%,	2305/11602	GO_BP	FOXM1/RMI2/RAD51/RAD51AP1/CHEK1/NPAS2/H
607	tags=44%,	5629/3295/	GO_BP	PROX1/HSD17B4/AMACR/AKR1C4/SCP2/CYP27A1/
763	tags=47%,	80339/348/	GO_BP	PNPLA3/APOE/APOC2/LIPG/SORL1/PLIN5/LIPC/
4398	tags=77%,	29108/578/	GO_BP	PYCARD/BAK1/RIPK2/ASPH/PERP/IFI16/FADD/
2702	tags=62%,	10631/4241	GO_BP	POSTN/MELTF/FBLN1/RCC2/EFNA5/CORO1C/ITG
6013	tags=46%,	6696/10631	GO_BP	SPP1/POSTN/SEMA6A/MELTF/FBLN1/RCC2/ARHG/
3359	tags=30%,	54209/302/	GO_BP	TREM2/ANXA2/FCER1G/SRC/CSF1/TYROBP/TGFB
1277	tags=49%,	150094/116	GO_BP	SIK1/MOGAT1/LDLR/PNPLA3/LPIN2/DGAT2/C3/
1277	tags=49%,	150094/116	GO_BP	SIK1/MOGAT1/LDLR/PNPLA3/LPIN2/DGAT2/C3/
1270	tags=61%,	721/733/11	GO_BP	C4B/C8G/CLU/C1RL/APCS/C1R/C2/C5/SERPING
1379	tags=42%,	10580/9852	GO_BP	SORBS1/EPM2AIP1/SIRT1/IGF1/PPARA/KAT2B/
555	tags=33%,	135/8600/5	GO_BP	ADORA2A/TNFSF11/OXT/ACE2/AVPR1A/KMO/SLC
563	tags=42%,	54884/216/	GO_BP	RETSAT/ALDH1A1/DGAT2/RDH5/CYP1A1/RBP4/A
79	tags=9%,	12779/563/8	GO_BP	GNAT1/AZGP1/RTP3
1277	tags=49%,	150094/116	GO_BP	SIK1/MOGAT1/LDLR/PNPLA3/LPIN2/DGAT2/C3/
854	tags=31%,	1244/107/2	GO_BP	ABCC2/ADCY1/GJB2/ASS1/QDPR/ARG1/PFKFB1/
1397	tags=69%,	9481/23411	GO_BP	SLC25A27/SIRT1/NR1H4/C1QTNF3/ANGPTL8/CE
1397	tags=69%,	9481/23411	GO_BP	SLC25A27/SIRT1/NR1H4/C1QTNF3/ANGPTL8/CE
1320	tags=43%,	26291/150(	GO_BP	FGF21/SIK1/LDLR/APOE/APOC2/SORL1/DGAT2/
6418	tags=56%,	4321/8767/	GO_BP	MMP12/RIPK2/IRAK1/SYK/HMGB2/PRKDC/NFKB2/
648	tags=45%,	6817/51170	GO_BP	SULT1A1/HSD17B11/HSD17B2/SULT1E1/HSD17B
1099	tags=59%,	3067/5092/	GO_BP	HDC/PCBD1/IDO2/FAH/HAL/HNMT/GSTZ1/AADAT/
1170	tags=62%,	23461/1071	GO_BP	ABCA5/CETP/AGTR1/APOE/APOC2/LIPG/GPIHBP
936	tags=80%,	9942/51084	GO_BP	XYLB/CRYL1/DCXR/SORD
936	tags=80%,	9942/51084	GO_BP	XYLB/CRYL1/DCXR/SORD
936	tags=80%,	9942/51084	GO_BP	XYLB/CRYL1/DCXR/SORD
936	tags=80%,	9942/51084	GO_BP	XYLB/CRYL1/DCXR/SORD
5879	tags=49%,	3958/100/1	GO_BP	LGALS3/ADA/CMTM3/PRNP/EZR/PHPT1/CD300A/
1099	tags=71%,	3067/5092/	GO_BP	HDC/PCBD1/IDO2/FAH/HAL/HNMT/GSTZ1/AADAT/
1170	tags=64%,	23461/1071	GO_BP	ABCA5/CETP/AGTR1/APOE/APOC2/LIPG/GPIHBP
1170	tags=64%,	23461/1071	GO_BP	ABCA5/CETP/AGTR1/APOE/APOC2/LIPG/GPIHBP
1999	tags=71%,	54443/9493	GO_BP	ANLN/KIF23/ECT2/RACGAP1/RTKN
3750	tags=42%,	5347/891/9	GO_BP	PLK1/CCNB1/CCNB2/CDK1/REEP4/SPAG4/CHMP4
984	tags=42%,	2729/4482/	GO_BP	GCLC/MSRA/PCYOX1/CBS/TST/BHMT2/ADI1/CSA
1428	tags=42%,	51660/5590	GO_BP	MPC1/ACSS2/PDK2/MLYCD/HMGCS1/ACACB/NUDT
1264	tags=61%,	9971/8659/	GO_BP	NR1H4/ALDH4A1/GCLC/HAL/GOT1/MTHFS/GLUL/
651	tags=28%,	727/3078/3	GO_BP	C5/CFHR1/CFHR2/C7/C8B/F2/GPLD1/C8A/CFHR
1493	tags=44%,	4792/4035/	GO_BP	NFKBIA/LRP1/SIRT1/ABCA5/CETP/SHH/APOE/A
79	tags=8%,	12779/563/8	GO_BP	GNAT1/AZGP1/RTP3
692	tags=39%,	348/344/55	GO_BP	APOE/APOC2/APOM/APOC4/MTTP/APOB/APOC1/A
783	tags=40%,	122970/790	GO_BP	ACOT4/ELOVL6/ACOT1/ACOT2/ELOVL2/ACOX1/A
1464	tags=33%,	8864/10580	GO_BP	PER2/SORBS1/EPM2AIP1/IGF1/PGM1/AGL/ENPP
941	tags=24%,	57834/1583	GO_BP	CYP4F11/CYP11A1/CYP26A1/GC/CYP4F12/CYP2
1464	tags=33%,	8864/10580	GO_BP	PER2/SORBS1/EPM2AIP1/IGF1/PGM1/AGL/ENPP
1197	tags=53%,	37/83752/2	GO_BP	ACADVL/LONP2/MLYCD/ETFBKMT/PRKAG2/PPARA/
883	tags=45%,	2166/80339	GO_BP	FAAH/PNPLA3/ABHD6/APOE/APOC2/LIPG/SORL1/
883	tags=45%,	2166/80339	GO_BP	FAAH/PNPLA3/ABHD6/APOE/APOC2/LIPG/SORL1/
1283	tags=50%,	7076/7040/	GO_BP	TIMP1/TGFB1/HIF1A
3069	tags=67%,	128869/940	GO_BP	PIGU/PIGS/GPAA1/PIGT
2	tags=17%,	6690	GO_BP	SPINK1
2	tags=17%,	6690	GO_BP	SPINK1

202	tags=33%,	29108/396	GO_BP	PYCARD/LGALS9
5207	tags=37%,	4318/1055	GO_BP	MMP9/AGR2/CYBA/SOX9/NCF2/COL1A1/ARPC1B/
5671	tags=31%,	6690/5347/	GO_BP	SPINK1/PLK1/CCNB1/PTTG1/PAFAH1B3/PAEP/G
6339	tags=47%,	6690/2539/	GO_BP	SPINK1/G6PD/SFN/TESC/UCHL1/PLK1/CCNB1/P
4885	tags=39%,	115908/66	GO_BP	CTHRC1/SOX4/MDK/SOX9/COL1A1/SULF1/SRC/I
6405	tags=50%,	7345/2635	GO_BP	UCHL1/HSPB8/SRC/ATP13A2/GAPDH/RIPK2/SQS
1488	tags=83%,	54511/549/	GO_BP	HMGCLL1/AUH/MCCC2/HMGCL/IVD
6199	tags=51%,	1869/5554	GO_BP	E2F1/RBM38/IGF2BP1/YBX3/MAPKAPK2/YBX1/P
4983	tags=49%,	3958/100/	GO_BP	LGALS3/ADA/PRNP/EZR/PHPT1/CD300A/THY1/L
4797	tags=36%,	6690/1001	GO_BP	SPINK1/CD24/TREM2/EPO/ICAM1/SRC/LIF/TGF
1784	tags=33%,	2207/3965/	GO_BP	FCER1G/LGALS9/HAVCR2/SYK/VTCN1/CD86/ZP3
5482	tags=44%,	4318/7262/	GO_BP	MMP9/PHLDA2/PLK1/CTHRC1/AURKB/ANXA2/STM
3798	tags=53%,	23636/590	GO_BP	NUP62/RAN/NUP210/NPM1/NUP93/NUP205/NUP3
7614	tags=58%,	55353/101	GO_BP	LAPTM4B/ARL4C/SQSTM1/YKT6/SCRIB/MICALL2
5726	tags=51%,	6364/1053	GO_BP	CCL20/UBD/ICAM1/LGALS9/GAPDH/CD44/PTAFR
5147	tags=47%,	10551/440/	GO_BP	AGR2/ASNS/HSPB8/CXCL8/KDELR3/BAK1/STC2/
7153	tags=57%,	4192/3965/	GO_BP	MDK/LGALS9/FOXJ1/MMP14/TGFB1/RIPK2/ADA/
7150	tags=56%,	54997/542	GO_BP	TESC/TREM2/MDK/LGALS9/MMP14/LIF/CSF1/TY
2246	tags=60%,	10636/961	GO_BP	RGS14/RIN1/CDK5
1885	tags=80%,	4192/3659/	GO_BP	MDK/IRF1/CTLA4/LAG3
6273	tags=50%,	4318/1055	GO_BP	MMP9/AGR2/SOX9/SRC/PLAUR/TGFB1/RHBDF2/N
607	tags=38%,	5629/30/3	GO_BP	PROX1/ACAA1/HSD17B4/AMACR/AKR1C4/SCP2/C
838	tags=40%,	4792/4035/	GO_BP	NFKBIA/LRP1/SIRT1/CETP/SHH/APOE/APOC2/L
838	tags=40%,	4792/4035/	GO_BP	NFKBIA/LRP1/SIRT1/CETP/SHH/APOE/APOC2/L
1275	tags=33%,	23411/37/	GO_BP	SIRT1/ACADVL/ERLIN1/PRKAG2/APOC2/AVPR1A
6249	tags=48%,	5347/891/	GO_BP	PLK1/CCNB1/CCNB2/GOLM1/CDK1/REEP4/SPAG4
6144	tags=48%,	8877/5347/	GO_BP	SPHK1/PLK1/CDK1/TTK/TGFB1/RIPK2/S1PR2/P
4228	tags=42%,	1364/2810/	GO_BP	CLDN4/SFN/STMN1/ELOVL1/GBA/FA2H/CYP26B1
3033	tags=43%,	113130/69	GO_BP	CDCA5/BUB1/FEN1/ESPL1/NAA10/NSMCE2/DDX1
4451	tags=67%,	3614/100/	GO_BP	IMPDH1/ADA/NME1/NME2/AMD1/GMPS/IMPDH2/P
4451	tags=67%,	3614/100/	GO_BP	IMPDH1/ADA/NME1/NME2/AMD1/GMPS/IMPDH2/P
3787	tags=38%,	1364/2810/	GO_BP	CLDN4/SFN/STMN1/ELOVL1/FA2H/CYP26B1/KDF
1115	tags=43%,	23464/272	GO_BP	GCAT/GCLC/AMT/THNSL2/CBS/TST/SEPSECS/CS
1170	tags=45%,	23461/107	GO_BP	ABCA5/CETP/AGTR1/APOE/APOC2/LIPG/GPIHBP
6128	tags=56%,	8767/8878/	GO_BP	RIPK2/SQSTM1/IRAK1/PLCB1/PSMB9/PSMD14/P
1270	tags=46%,	721/733/1	GO_BP	C4B/C8G/CLU/C1RL/APCS/C1R/C2/C5/SERPING
1363	tags=39%,	8600/3700/	GO_BP	TNFSF11/ITIH4/F8/SERPINA1/APCS/EDNRB/OR
1214	tags=35%,	79071/480	GO_BP	ELOVL6/NFYB/ERLIN1/CYP51A1/SOD1/HMGCS1/
1214	tags=35%,	79071/480	GO_BP	ELOVL6/NFYB/ERLIN1/CYP51A1/SOD1/HMGCS1/
4980	tags=47%,	10551/542	GO_BP	AGR2/TREM2/ITGA3/ARHGEF16/LYPD1/LGALS3/
2996	tags=35%,	6364/2919/	GO_BP	CCL20/CXCL1/TREM2/CXCL6/CXCL8/CXCL5/TFF
1317	tags=44%,	6714/9181/	GO_BP	SRC/ARHGEF2/HCK/ASAP1/FSCN1/MSN/SH3PXD2
3964	tags=37%,	29108/153	GO_BP	PYCARD/CYBA/LY96/FCER1G/LGALS9/TYROBP/C
7545	tags=70%,	10538/569	GO_BP	BATF/PSMB9/PSMD14/TCF3/PSMD2/PSMB2/PSME
3266	tags=54%,	54209/730	GO_BP	TREM2/TYROBP/HAVCR2/SYK/GRN/SBNO2/LBP
4451	tags=77%,	3614/4830/	GO_BP	IMPDH1/NME1/NME2/GMPS/IMPDH2/NME6/NME7/
307	tags=7%,	l 3976	GO_BP	LIF
2790	tags=36%,	5347/983/	GO_BP	PLK1/CDK1/SRC/LIF/ECT2/TGFB1/FLNA/PRKCD
4863	tags=49%,	27242/396	GO_BP	TNFRSF21/LGALS9/FOXJ1/TYROBP/TGFB1/GPNM
2904	tags=67%,	6280/5880/	GO_BP	S100A9/RAC2/CD44/S100A8/MSN/IL1B/STK10/
4662	tags=43%,	55353/553	GO_BP	LAPTM4B/PPT1/CORO1A/GRN/RAB34/TMEM165/R

4662	tags=43%,	55353/553[illegible]	GO_BP	LAPTM4B/PPT1/CORO1A/GRN/RAB34/TMEM165/R[illegible]
575	tags=55%,	54209/368[illegible]	GO_BP	TREM2/ITGAM/DKK1/C1QB/C1QC/C1QA
5456	tags=36%,	8877/4318/	GO_BP	SPHK1/MMP9/CD24/CCL20/PLK1/CCNB1/TREM2/[illegible]
6306	tags=44%,	100133941/	GO_BP	CD24/CCNB1/TPX2/CDC6/EPO/DUSP9/CDK1/CDC[illegible]
2511	tags=43%,	3321/6662/	GO_BP	IGSF3/SOX9/FGFR2
7250	tags=56%,	991/7345/[illegible]	GO_BP	CDC20/UCHL1/CDK1/CCNA2/BIRC3/CDC25A/PSM[illegible]
6055	tags=49%,	4318/2810/	GO_BP	MMP9/SFN/TREM2/PYCARD/E2F1/BAK1/TSPO/PL[illegible]
6296	tags=48%,	5347/7973[illegible]	GO_BP	PLK1/KCTD17/SRC/ARHGEF2/NUP62/HCK/SPAG5[illegible]
3631	tags=37%,	991/9319/[illegible]	GO_BP	CDC20/TRIP13/LIF/TTK/PLCB1/FBXO5/CALR/M[illegible]
2852	tags=37%,	54209/730[illegible]	GO_BP	TREM2/TYROBP/GDF15/ADAM9/CD53/FLOT1/EHD
7568	tags=64%,	6662/8463/	GO_BP	SOX9/TEAD2/PSMB9/PSMD14/TRIM6/TCF3/PSMD[illegible]
1763	tags=35%,	2861/5409/	GO_BP	GPR37/PNMT/NR4A2/EPAS1/NPR1/SULT1A1/HDC[illegible]
1763	tags=35%,	2861/5409/	GO_BP	GPR37/PNMT/NR4A2/EPAS1/NPR1/SULT1A1/HDC[illegible]
381	tags=34%,	2166/1562/	GO_BP	FAAH/CYP2C18/CYP2J2/CYP2A13/CYP4F12/EPH[illegible]
967	tags=24%,	9693/343/[illegible]	GO_BP	RAPGEF2/AQP8/FDX1/SRD5A1/ASS1/PDE2A/ITP[illegible]
692	tags=31%,	5243/135/[illegible]	GO_BP	ABCB1/ADORA2A/TNFSF11/OXT/APOE/ACE2/AVP[illegible]
1781	tags=29%,	6696/3976/	GO_BP	SPP1/LIF/STC2/CTSV/BSG/MAPK3/PPARD
2776	tags=46%,	1410/578/[illegible]	GO_BP	CRYAB/BAK1/SPI1/SCRIB/HNF1B/BAX/LEF1/JA[illegible]
1270	tags=58%,	721/733/1[illegible]	GO_BP	C4B/C8G/CLU/C1R/C2/PROS1/C5/SERPING1/C1[illegible]
644	tags=40%,	100/134	GO_BP	ADA/ADORA1
5255	tags=71%,	302/5538/[illegible]	GO_BP	ANXA2/PPT1/S100A10/FLOT2/CD2/NPC1/FLOT1[illegible]
1170	tags=42%,	23461/107[illegible]	GO_BP	ABCA5/CETP/AGTR1/APOE/APOC2/LIPG/GPIHBP
1115	tags=39%,	23464/448[illegible]	GO_BP	GCAT/MSRA/THNSL2/GOT1/ASPA/AADAT/ASS1/G[illegible]
508	tags=33%,	4494/4490/	GO_BP	MT1F/MT1B/CA2/ASS1/MT2A/MT1A/MT1M/HAMP/[illegible]
1270	tags=59%,	721/733/1[illegible]	GO_BP	C4B/C8G/CLU/C1R/C2/PROS1/C5/SERPING1/C1[illegible]
1160	tags=38%,	6303/3067/	GO_BP	SAT1/HDC/DDC/AGMAT/IDO2/MAOA/NAAA/HNMT/[illegible]
782	tags=38%,	5627/348/[illegible]	GO_BP	PROS1/APOE/SERPING1/VTN/FGB/FGG/PROC/FG[illegible]
905	tags=35%,	3949/948/[illegible]	GO_BP	LDLR/CD36/AMN/APOE/APOC2/LIPG/GPIHBP1/A[illegible]
1741	tags=62%,	326625/25[illegible]	GO_BP	MMAB/MMACHC/PNPO/MMAA/RGN
867	tags=29%,	6799/219/[illegible]	GO_BP	SULT1A2/ALDH1B1/APOE/SULT1E1/TKFC/SULT1[illegible]
1524	tags=32%,	7048/825/[illegible]	GO_BP	TGFBR2/CAPN3/SELP/RREB1/KANK1/CD36/MYLK[illegible]
782	tags=38%,	5627/348/[illegible]	GO_BP	PROS1/APOE/SERPING1/VTN/FGB/FGG/PROC/FG[illegible]
1026	tags=73%,	5092/2184/	GO_BP	PCBD1/FAH/GSTZ1/QDPR/HGD/PAH/HPD/TAT
1026	tags=73%,	5092/2184/	GO_BP	PCBD1/FAH/GSTZ1/QDPR/HGD/PAH/HPD/TAT
1026	tags=73%,	5092/2184/	GO_BP	PCBD1/FAH/GSTZ1/QDPR/HGD/PAH/HPD/TAT
1026	tags=73%,	5092/2184/	GO_BP	PCBD1/FAH/GSTZ1/QDPR/HGD/PAH/HPD/TAT
3655	tags=56%,	9212/100/[illegible]	GO_BP	AURKB/ADA/CD74/BAX/MIF/SLC39A10/NOC2L/L[illegible]
4080	tags=77%,	3925/2291[illegible]	GO_BP	STMN1/MAPRE1/MAP2/TUBB4A/FKBP4/INPP5J/C[illegible]
3149	tags=57%,	1058/1063/	GO_BP	CENPA/CENPF/CENPH/CENPW/CENPE/CENPK/RNF[illegible]
3852	tags=57%,	3561/6850/	GO_BP	IL2RG/SYK/JAK3/SHC1/IL2RA/IL2RB/PTPN2/S[illegible]
2803	tags=64%,	3561/2182/	GO_BP	IL2RG/ACSL4/IL15RA/PLCB1/JAK3/SHC1/IL15[illegible]
2020	tags=54%,	1601/2294[illegible]	GO_BP	DAB2/DKK1/GPC3/MLLT3/CSNK1E/PLEKHA4/CSN[illegible]
6181	tags=59%,	2810/1869/	GO_BP	SFN/E2F1/RAC2/YWHAZ/MICALL2/SAE1/BID/UB[illegible]
1586	tags=50%,	29108/272[illegible]	GO_BP	PYCARD/TNFRSF21/LGALS9/ISG15/F2RL1
692	tags=78%,	348/344/3[illegible]	GO_BP	APOE/APOC2/GPIHBP1/APOB/APOA2/APOA1/APO[illegible]
944	tags=78%,	3155/5689[illegible]	GO_BP	HMGCL/BDH2/ACSS3/ACAT1/BDH1/HMGCS2/SLC2[illegible]
782	tags=37%,	5627/348/[illegible]	GO_BP	PROS1/APOE/SERPING1/VTN/FGB/FGG/PROC/FG[illegible]
951	tags=44%,	26291/154[illegible]	GO_BP	FGF21/FMC1/LONP2/MLYCD/ETFBKMT/LDLR/PPA[illegible]
2567	tags=58%,	6790/7283/	GO_BP	AURKA/TUBG1/ASPM/ESPL1/PPP2R1A/FBXO5/TU[illegible]
3852	tags=67%,	3561/6850/	GO_BP	IL2RG/SYK/JAK3/SHC1/IL2RA/IL2RB/PTPN2/S[illegible]
3505	tags=83%,	22948/908/	GO_BP	CCT5/CCT6A/CCT3/DKC1/CCT2/CCT4/CCT7/TCP

4291	tags=29%,	6692/6280/	GO_BP	SPINT1/S100A9/TREM2/LIMK1/RGS2/MDK/E2F1/
6479	tags=40%,	6696/7345/	GO_BP	SPP1/UCHL1/S100A6/RAP1GAP/LIMK1/MDK/DBN
5313	tags=33%,	6696/991/	GO_BP	SPP1/CDC20/S100A9/LIMK1/RGS2/MDK/SOX9/E
6479	tags=39%,	6696/7345/	GO_BP	SPP1/UCHL1/S100A6/RAP1GAP/LIMK1/MDK/DBN
6062	tags=48%,	6280/56667	GO_BP	S100A9/MUC13/CRP/PYCARD/CYBA/LIMK1/LY96/
6706	tags=51%,	5997/6659/	GO_BP	RGS2/SOX4/GAPDH/IGF2BP2/TYMS/KRT17/EIF4
3776	tags=34%,	6690/4318/	GO_BP	SPINK1/MMP9/SFN/SPINT1/S100A9/EGLN3/BIR
6056	tags=41%,	6280/6364/	GO_BP	S100A9/CCL20/CRP/TREM2/PYCARD/CYBA/CXCL
6227	tags=44%,	6696/2539/	GO_BP	SPP1/G6PD/SPHK1/SFN/S100A9/CYBA/LIMK1/R
3076	tags=38%,	9212/3965/	GO_BP	AURKB/LGALS9/LGALS3/ADA/CD74/JAK3/CCL5/
7250	tags=56%,	991/7345/	GO_BP	CDC20/UCHL1/CDK1/CCNA2/BIRC3/CDC25A/PSM
5045	tags=37%,	56667/103	GO_BP	MUC13/B3GNT3/GCNT3/ST6GALNAC4/VEGFB/B4G
3631	tags=33%,	5047/9319/	GO_BP	PAEP/TRIP13/SULF1/TIMP1/LIF/TTK/ADA/ZP3/
4121	tags=31%,	9212/9833/	GO_BP	AURKB/MELK/NEK2/TYRO3/SRC/ATP13A2/TTK/A
4012	tags=28%,	6280/6364/	GO_BP	S100A9/CCL20/CRP/TREM2/PYCARD/CYBA/CXCL
555	tags=25%,	8864/135/	GO_BP	PER2/ADORA2A/TNFSF11/OXT/ACE2/AVPR1A/IR
5356	tags=43%,	4192/2724	GO_BP	MDK/TNFRSF21/LGALS9/FOXJ1/TYRO3/LGALS3/
5011	tags=40%,	2810/7345/	GO_BP	SFN/UCHL1/PLK1/PYCARD/PKIB/RGS2/DUSP9/G
917	tags=67%,	125061/16	GO_BP	AFMID/IDO2/AADAT/ACMSD/GCDH/TDO2/KMO/HA
1024	tags=53%,	125061/16	GO_BP	AFMID/DDC/IDO2/AADAT/ACMSD/GCDH/TDO2/KM
692	tags=42%,	348/1593/	GO_BP	APOE/CYP27A1/CYP39A1/AKR1D1/CYP7A1
1277	tags=47%,	150094/39	GO_BP	SIK1/LDLR/DGAT2/C3/PLIN5/GPLD1/RGN/THRS
692	tags=42%,	348/1593/	GO_BP	APOE/CYP27A1/CYP39A1/AKR1D1/CYP7A1
5073	tags=58%,	3310/2280/	GO_BP	HSPA6/FKBP1A/HSPA8/PPIH/PPIA/DNAJB2/DNA
884	tags=50%,	1376/5142	GO_BP	CPT2/PRKAG2/ACACB/SLC25A20/CPT1A/IRS2/T
6034	tags=49%,	8877/302/	GO_BP	SPHK1/ANXA2/CDK1/BAK1/CORO1A/STX3/SNAP2
5206	tags=64%,	6714/5880/	GO_BP	SRC/RAC2/TWF2/FSCN1/CARMIL1/WASF2/CORO1
5598	tags=64%,	1601/9094/	GO_BP	DAB2/UNC119/FCHO1/DNAJC6/CLTA/GPR107/PI
905	tags=70%,	4035/3949/	GO_BP	LRP1/LDLR/CD36/STARD5/APOA2/APOA1/APOC3
731	tags=60%,	51390/660	GO_BP	AIG1/CYP4F12/ACADL/CYP4F3/CYP4F2/CYP4A1
854	tags=40%,	1244/210/	GO_BP	ABCC2/ALAD/AQP9/TAT
207	tags=70%,	7056/2147/	GO_BP	THBD/F2/SERPINF2/APOH/CPB2/PLG/HRG
905	tags=70%,	4035/3949/	GO_BP	LRP1/LDLR/CD36/STARD5/APOA2/APOA1/APOC3
4013	tags=40%,	2810/2910	GO_BP	SFN/PYCARD/E2F1/IKBKE/BAK1/CD44/CD74/BC
3702	tags=40%,	4318/4316/	GO_BP	MMP9/MMP7/MMP12/COL1A1/MMP11/MMP14/P3H4/
761	tags=40%,	875/6470/	GO_BP	CBS/SHMT1/MTHFD1/AGXT2/CTH/AGXT
1275	tags=53%,	23411/37/	GO_BP	SIRT1/ACADVL/ERLIN1/ACADL/APOC1/INSIG1/
1796	tags=53%,	3643/1058	GO_BP	INSR/SORBS1/EPM2AIP1/IGF1/IRS1/IRS2/GCK/
1429	tags=67%,	4496/4494/	GO_BP	MT1H/MT1F/MT1B/SLC30A1/MT2A/MT1A/MT1M/M
3648	tags=34%,	8877/1728/	GO_BP	SPHK1/NQO1/PYCR1/LCN2/CDK1/SRC/MAPK13/E
3625	tags=100%,	5973/10007	GO_BP	RENBP/GNPDA1/NPL/NAGK/GNPDA2/AMDHD2
4228	tags=39%,	55723/475	GO_BP	ASF1B/NEK2/GINS1/TM4SF1/ADA/CMTM3/ELF3/
3756	tags=38%,	4316/1535/	GO_BP	MMP7/CYBA/SOX9/SLC2A1/COL1A1/BAK1/ATP1A
651	tags=79%,	735/5199/	GO_BP	C9/CFP/C8G/C5/CFB/C3/CFH/C7/C8B/C8A/CFH
1379	tags=30%,	10580/985	GO_BP	SORBS1/EPM2AIP1/SIRT1/IGF1/PPARA/GPER1/
1295	tags=32%,	2098/6817/	GO_BP	ESD/SULT1A1/ADH5/SULT1A2/PRDX3/HBA2/ALD
692	tags=43%,	348/55937/	GO_BP	APOE/APOM/APOA2/LCAT/APOA1/APOA5
1196	tags=47%,	29881/394	GO_BP	NPC1L1/LDLR/ASAH2/CD36/ABCG8/ABCG5/APOA
854	tags=21%,	1244/2706/	GO_BP	ABCC2/GJB2/FAM107A/FOXO1/ERRFI1/SRD5A1/
1264	tags=38%,	9971/7907	GO_BP	NR1H4/ELOVL6/ACADVL/NFYB/ERLIN1/CYP51A1/
905	tags=47%,	26291/394	GO_BP	FGF21/LDLR/APOC2/DGAT2/PLIN5/GPLD1/AADA

4950	tags=43%,	4323/7040/	GO_BP	MMP14/TGFB1/ADA/CLCF1/CD74/IGLL5/SYK/CD
7545	tags=73%,	5698/1021	GO_BP	PSMB9/PSMD14/TCF3/PSMD2/PSMB2/PSME3/CBF
5188	tags=52%,	2305/1160	GO_BP	FOXM1/RMI2/RAD51/RAD51AP1/CHEK1/HSF1/SI
951	tags=56%,	83752/234	GO_BP	LONP2/MLYCD/ETFBKMT/PPARA/ACACB/CPT1A/I
5212	tags=40%,	8877/6510/	GO_BP	SPHK1/SLC1A5/FXYD2/CRP/TREM2/SLC38A1/PY
4367	tags=32%,	6696/226/	GO_BP	SPP1/ALDOA/LIMK1/MDK/DBN1/ICAM1/POSTN/R
6306	tags=44%,	100133941/	GO_BP	CD24/CCNB1/TPX2/CDC6/EPO/DUSP9/CDK1/CDC
3313	tags=34%,	8877/2583/	GO_BP	SPHK1/B4GALNT1/GAL3ST1/PRKCD/ELOVL1/B4G
5992	tags=31%,	2810/3880/	GO_BP	SFN/KRT19/SOX9/TGM3/KRT23/FABP5/DSG2/CT
1942	tags=43%,	6280/4192/	GO_BP	S100A9/MDK/ITGB2/SLAMF8/S100A8/ADAM8
2087	tags=54%,	54498/672	GO_BP	SMOX/SRM/SMS/OAZ1/ODC1/AMD1/OAZ3
3727	tags=71%,	4192/1435/	GO_BP	MDK/CSF1/CCL5/MAPK3/C3AR1/C5AR1/CMKLR1/
2068	tags=36%,	54997/714/	GO_BP	TESC/C1QC/LEF1/EVI2B/HCLS1
6303	tags=60%,	54209/921	GO_BP	TREM2/AURKB/FCER1G/SH3BP1/ITGAM/ITGB2/I
1918	tags=40%,	8767/3606/	GO_BP	RIPK2/IL18/ALOX5/IL18BP
4531	tags=83%,	891/4751/	GO_BP	CCNB1/NEK2/ECT2/RCC2/RACGAP1/SPAG5/KNST
3928	tags=39%,	226/3383/	GO_BP	ALDOA/ICAM1/FBLIM1/PLXNA1/BAMBI/RHOV/CO
6229	tags=49%,	7345/2413	GO_BP	UCHL1/KIF4A/KIF23/CEP131/HSPB1/WDR34/IF
6229	tags=49%,	7345/2413	GO_BP	UCHL1/KIF4A/KIF23/CEP131/HSPB1/WDR34/IF
3703	tags=31%,	8877/4318/	GO_BP	SPHK1/MMP9/NQO1/APOA4/PYCR1/LCN2/CDK1/S
6169	tags=46%,	8877/5347/	GO_BP	SPHK1/PLK1/CDK1/TTK/TGFB1/RIPK2/S1PR2/P
6686	tags=47%,	5047/5420	GO_BP	PAEP/TREM2/PYCARD/CYBA/FCER1G/LGALS9/FO
1585	tags=44%,	100133941/	GO_BP	CD24/LAPTM4B/TYROBP/HSP90AB1
1511	tags=55%,	699/701/8	GO_BP	BUB1/BUB1B/NAA10/SGO2/PPP2R1A/SGO1
767	tags=40%,	116255/28	GO_BP	MOGAT1/GOT1/TKFC/DGAT2/PCK2/ANGPTL3/MOG
1409	tags=45%,	2098/9446/	GO_BP	ESD/GSTO1/MGST1/MGST2/GSTM5/GSTZ1/GSTA2
1409	tags=45%,	2098/9446/	GO_BP	ESD/GSTO1/MGST1/MGST2/GSTM5/GSTZ1/GSTA2
6436	tags=63%,	5570/9212/	GO_BP	PKIB/AURKB/NEK2/SRC/PIF1/MAPK3/DKC1/ACD
3385	tags=27%,	6692/5420	GO_BP	SPINT1/TREM2/MDK/TNFRSF21/E2F1/CDK1/TSP
1115	tags=60%,	23464/102	GO_BP	GCAT/RIDA/SDS
1251	tags=80%,	116085/10	GO_BP	SLC22A12/SLC17A3/SLC2A9/SLC17A1
1115	tags=60%,	23464/102	GO_BP	GCAT/RIDA/SDS
4927	tags=50%,	5347/2297	GO_BP	PLK1/TPX2/TACC3/PSRC1/NUP62/SPAG5/CHMP4
3798	tags=53%,	23636/590	GO_BP	NUP62/RAN/NUP210/NUP93/NUP205/NUP37/NDC
3798	tags=53%,	23636/590	GO_BP	NUP62/RAN/NUP210/NUP93/NUP205/NUP37/NDC
8602	tags=76%,	56648/638	GO_BP	EIF5A2/MRPL17/MRPS12/MRPL47/MRPL9/MRPS2
6303	tags=69%,	9212/1011	GO_BP	AURKB/KIF20A/CEP55/ANLN/KIF23/ECT2/RACG
4496	tags=52%,	4582/9099	GO_BP	MUC1/CREB3L1/HSF1/DDIT3/HIF1A/MT3/ATF4/
3626	tags=45%,	4318/2910	GO_BP	MMP9/PYCARD/BAK1/PLAUR/BAX/BMF/BID/BBC3
909	tags=35%,	8864/1058	GO_BP	PER2/SORBS1/EPM2AIP1/IGF1/PYGL/PGM1/PRK
1020	tags=25%,	286256/13	GO_BP	LCN12/CPT2/PRKAG2/CD36/ACACB/SLC25A20/A
831	tags=39%,	55258/435/	GO_BP	THNSL2/ASL/GOT1/CBS/GLUL/GLUD1/GAMT/ASS
1524	tags=29%,	7048/4035/	GO_BP	TGFBR2/LRP1/CAPN3/SELP/RREB1/KANK1/CD36
7379	tags=64%,	4192/3965/	GO_BP	MDK/LGALS9/MMP14/TGFB1/RIPK2/ADA/CD74/S
4262	tags=50%,	3880/1065	GO_BP	KRT19/SPINT2/LIF/MDFI/ST14/E2F8/KRT8/AS
6100	tags=49%,	3838/1160	GO_BP	KPNA2/RMI2/TGFB1/RAD51/CLCF1/RAD51AP1/A
1409	tags=35%,	2207/3965/	GO_BP	FCER1G/LGALS9/HAVCR2/SYK/CD86/ZP3/CD3E/
4626	tags=52%,	116372/16	GO_BP	LYPD1/DAB2/SAPCD2/TGFB1/RHOQ/PKDCC/AP2M
1056	tags=32%,	2327/1644/	GO_BP	FMO2/DDC/RAPGEF2/BDH2/AS3MT/ABCC2/CBS/C
905	tags=34%,	3949/2166/	GO_BP	LDLR/FAAH/PNPLA3/ABHD6/APOE/APOC2/LIPG/
634	tags=25%,	221037/25	GO_BP	JMJD1C/MMACHC/PHF8/UTY/CYP51A1/KDM5D/PO

854	tags=22%,	1244/2706/	GO_BP	ABCC2/GJB2/GPER1/FAM107A/FOXO1/ERRFI1/SI
3359	tags=30%,	8877/4318/	GO_BP	SPHK1/MMP9/TREM2/BACE2/CTSC/TYROBP/ITGAI
5212	tags=41%,	8877/1401/	GO_BP	SPHK1/CRP/TREM2/PYCARD/LGALS3BP/CYBA/LII
5109	tags=28%,	6690/891/9	GO_BP	SPINK1/CCNB1/PTTG1/PAFAH1B3/PAEP/GTSF1/I
6242	tags=48%,	8877/5997/	GO_BP	SPHK1/RGS2/SOX4/ATP13A2/GAPDH/IGF2BP2/T
6358	tags=49%,	10551/440/	GO_BP	AGR2/ASNS/CXCL8/KDELR3/BAK1/TRIB3/STC2/
6695	tags=49%,	100133941/	GO_BP	CD24/LAPTM4B/TREM2/PYCARD/TNFRSF21/HOMEI
4786	tags=45%,	100133941/	GO_BP	CD24/SRC/LGALS1/CD320/CD86/VAV1/EFNB1/CI
3939	tags=30%,	1364/706/1	GO_BP	CLDN4/TSPO/CLIC1/FXYD3/TTYH3/SLC34A2/AN
6686	tags=46%,	5047/54209	GO_BP	PAEP/TREM2/PYCARD/CYBA/FCER1G/LGALS9/FO
4210	tags=42%,	3675/28626	GO_BP	ITGA3/TPRN/PRNP/ITPKA/C1QL1/MTMR2/DLG3/
5037	tags=37%,	578/4323/1	GO_BP	BAK1/MMP14/CMTM7/LGALS1/ADA/CLCF1/SLAMF
2818	tags=54%,	1277/1287/	GO_BP	COL1A1/COL4A5/SYK/DDR1/UBASH3B/ITGA2/COI
4616	tags=69%,	11004/8162	GO_BP	KIF2C/CDT1/NUF2/NDC80/CENPE/BOD1/HNRNPU
7076	tags=65%,	29108/1029	GO_BP	PYCARD/TRAIP/RIPK2/RELB/HMGB2/SIRPA/IRF
4830	tags=50%,	5984/51514	GO_BP	RFC4/DTL/POLD1/MAD2L2/ISG15/BRCA1/PCNA/
3362	tags=53%,	6280/3934/	GO_BP	S100A9/LCN2/S100A8/FTH1/SRI/FTL/AP3D1/S
2352	tags=53%,	960/972/45	GO_BP	CD44/CD74/MUC1/ZNF385A/MIF/BCL2L12/KDM1
5488	tags=47%,	6659/3872/	GO_BP	SOX4/KRT17/PTAFR/NCK2/LARP6/CDK4/YBX3/E
909	tags=35%,	8864/10580	GO_BP	PER2/SORBS1/EPM2AIP1/IGF1/PYGL/PGM1/PRK
909	tags=35%,	8864/10580	GO_BP	PER2/SORBS1/EPM2AIP1/IGF1/PYGL/PGM1/PRK
1317	tags=50%,	6714/50807	GO_BP	SRC/ASAP1/FSCN1/MSN/LCP1
3318	tags=58%,	330/83596/	GO_BP	BIRC3/BCL2L12/NOL3/BIRC7/CST7/CARD16/CA
3318	tags=58%,	330/83596/	GO_BP	BIRC3/BCL2L12/NOL3/BIRC7/CST7/CARD16/CA
2938	tags=59%,	6714/1601/	GO_BP	SRC/DAB2/MTMR2/EZR/MSN/MAPK3/MAP2K2/CHM
6566	tags=47%,	6696/3984/	GO_BP	SPP1/LIMK1/DBN1/SEMA6A/ARHGAP4/TNFRSF12
1252	tags=26%,	286410/584	GO_BP	ATP11C/PCTP/CETP/LDLR/APOE/APOC2/GLTPD2
4258	tags=40%,	23616/2302	GO_BP	SH3BP1/FOXJ1/DLG3/SCRIB/FSCN1/MTCL1/MSN
4258	tags=40%,	23616/2302	GO_BP	SH3BP1/FOXJ1/DLG3/SCRIB/FSCN1/MTCL1/MSN
3428	tags=73%,	9536/5730/	GO_BP	PTGES/PTGDS/AKR1C3/PTGES2/PTGS1/TBXAS1/
6656	tags=54%,	4192/27242	GO_BP	MDK/TNFRSF21/LGALS9/FOXJ1/LGALS3/TGFB1/
1817	tags=76%,	125061/196	GO_BP	AFMID/PAOX/CHDH/SAT2/SAT1/IDO2/HNMT/AAD
591	tags=52%,	55908/344/	GO_BP	ANGPTL8/APOC2/GPIHBP1/ANGPTL4/PCSK6/LIP
4663	tags=50%,	2810/1869/	GO_BP	SFN/E2F1/YWHAZ/BAX/BMF/BID/YWHAH/BBC3/P
2626	tags=29%,	6696/3976/	GO_BP	SPP1/LIF/STC2/CTSV/BSG/MAPK3/PPARD/LDOC
1221	tags=38%,	51071/2372	GO_BP	DERA/SHPK/RBKS/ESD/PDXP/ADH5/ALDH4A1/PN
1354	tags=58%,	26063/1384	GO_BP	DECR2/CRAT/ETFA/DECR1/ACADVL/HADHB/ECH1
898	tags=37%,	6817/57026	GO_BP	SULT1A1/PDXP/SULT1A2/FAH/MAOA/ALDH1B1/A
4927	tags=50%,	5347/22974	GO_BP	PLK1/TPX2/TACC3/PSRC1/NUP62/CHMP4B/CHMP
4732	tags=33%,	5047/29108	GO_BP	PAEP/PYCARD/CYBA/FCER1G/LGALS9/MAPK13/T
2068	tags=57%,	54997/5117	GO_BP	TESC/LEF1/EVI2B/HCLS1
1228	tags=36%,	83733/1016	GO_BP	SLC25A18/SLC25A13/SLC1A1/SLC25A30/SLC13
767	tags=39%,	116255/280	GO_BP	MOGAT1/GOT1/TKFC/DGAT2/SORD/PCK2/ANGPTL
838	tags=26%,	55366/6469	GO_BP	LGR4/SHH/SRD5A1/TBX3/SRD5A2/AR
440	tags=48%,	3249/2244/	GO_BP	HPN/FGB/FGG/CLEC3B/FGA/F11/SERPINF2/APO
910	tags=43%,	154791/254	GO_BP	FMC1/ETFBKMT/ACACB/PDE3B/SORL1/PLIN5/AP
6431	tags=60%,	5984/5424/	GO_BP	RFC4/POLD1/POLR2G/PCNA/RFC2/POLR2H/LIG1
5207	tags=35%,	4318/10551	GO_BP	MMP9/AGR2/CYBA/AFP/SOX9/NCF2/COL1A1/ARP
5765	tags=61%,	3958/57402	GO_BP	LGALS3/S100A14/TGFB1/CCL5/CREB3/SERPINE
6479	tags=39%,	6696/7345/	GO_BP	SPP1/UCHL1/S100A6/RAP1GAP/LIMK1/DBN1/PO
923	tags=26%,	1535/990/6	GO_BP	CYBA/CDC6/SRC/AGTRAP/PRKCD/HSF1

5109	tags=28%,	6690/891/9	GO_BP	SPINK1/CCNB1/PTTG1/PAFAH1B3/PAEP/GTSF1/
3776	tags=34%,	6690/4318/	GO_BP	SPINK1/MMP9/SFN/SPINT1/S100A9/EGLN3/BIR
2256	tags=24%,	9319/699/7	GO_BP	TRIP13/BUB1/BUB1B/CCNE1/P3H4/RAD51/NCAP
1642	tags=51%,	5194/7314/	GO_BP	PEX13/UBB/PEX12/ACOT4/PEX1/NOS2/PEX19/D
984	tags=40%,	8659/2729/	GO_BP	ALDH4A1/GCLC/AGMAT/FAH/HAL/ASL/GOT1/MTH
1214	tags=36%,	403313/79	GO_BP	PLPP6/ELOVL6/NPC1L1/NFYB/ERLIN1/CYP51A1
1057	tags=30%,	90102/325/	GO_BP	PHLDB2/APCS/PROS1/APOE/SERPING1/VTN/FGB
1642	tags=51%,	5194/7314/	GO_BP	PEX13/UBB/PEX12/ACOT4/PEX1/NOS2/PEX19/D
1642	tags=51%,	5194/7314/	GO_BP	PEX13/UBB/PEX12/ACOT4/PEX1/NOS2/PEX19/D
6041	tags=46%,	6696/5909/	GO_BP	SPP1/RAP1GAP/PFN2/ITM2C/ITGA3/SEMA6A/AR
4827	tags=46%,	6364/3383/	GO_BP	CCL20/ICAM1/LGALS9/GAPDH/CD44/PTAFR/PRK
6027	tags=43%,	2207/3965/	GO_BP	FCER1G/LGALS9/PFN2/MICAL1/NCS1/RAC2/SYT
692	tags=42%,	51170/348/	GO_BP	HSD17B11/APOE/SULT1E1/SRD5A1/CYP27A1/CY
839	tags=31%,	94081/105	GO_BP	SFXN1/MTHFS/SHMT1/ALDH1L1/MTHFD1/MAT1A/
1121	tags=50%,	23576/484	GO_BP	DDAH1/NOS2/ALDH4A1/FAH/GOT1/GLUL/GLUD1/
764	tags=46%,	5465/1374/	GO_BP	PPARA/CPT1A/APOC2/IRS1/IRS2/PLIN5/FABP1
1117	tags=33%,	241/240	GO_BP	ALOX5AP/ALOX5
1117	tags=33%,	241/240	GO_BP	ALOX5AP/ALOX5
5644	tags=45%,	2539/2056/	GO_BP	G6PD/EPO/SPI1/PRMT1/HMGB2/JAK3/HMOX1/PR
6349	tags=67%,	6809/6616/	GO_BP	STX3/SNAP25/CPLX2/PLEK/YKT6/RAB15/STXBP
3106	tags=70%,	5580/4869/	GO_BP	PRKCD/NPM1/PCNA/DDX11/RPS3/DFFA/GZMA
3893	tags=35%,	6241/302/1	GO_BP	RRM2/ANXA2/COL1A1/ITPR3/COL1A2/SQSTM1/B
182	tags=33%,	5347/891/9	GO_BP	PLK1/CCNB1/CCNB2/CDK1
1871	tags=35%,	5570/9212/	GO_BP	PKIB/AURKB/NEK2/NABP2/MAPK3/PNKP
3655	tags=47%,	578/100/97	GO_BP	BAK1/ADA/CD74/PKN1/BAX/MIF/HIF1A/SH2B2/
4663	tags=53%,	2810/1869/	GO_BP	SFN/E2F1/YWHAZ/BAX/BMF/BID/YWHAH/BBC3/P
933	tags=44%,	721/733/11	GO_BP	C4B/C8G/CLU/C1R/C2/PROS1/C5/SERPING1/C1
878	tags=35%,	5164/1500	GO_BP	PDK2/SIK1/SIRT1/C1QTNF3/PGM1/SLC37A4/SL
1331	tags=35%,	403313/79	GO_BP	PLPP6/ELOVL6/NPC1L1/NFYB/ERLIN1/CYP51A1
1270	tags=56%,	721/733/11	GO_BP	C4B/C8G/CLU/C1RL/APCS/RGCC/C1R/C2/PROS1
1040	tags=18%,	51170/223	GO_BP	HSD17B11/FDX1/CYP11A1/CYP17A1/HSD17B2/P
1642	tags=50%,	5194/7314/	GO_BP	PEX13/UBB/PEX12/ACOT4/PEX1/NOS2/PEX19/D
1331	tags=35%,	403313/79	GO_BP	PLPP6/ELOVL6/NPC1L1/NFYB/ERLIN1/CYP51A1
3964	tags=38%,	54209/291	GO_BP	TREM2/PYCARD/FCER1G/LGALS9/SYK/SASH3/CD
2875	tags=29%,	9212/4192/	GO_BP	AURKB/MDK/CDC6/VEGFB/CDC25B/KIF23/ECT2/
7545	tags=67%,	5698/1021	GO_BP	PSMB9/PSMD14/TCF3/PSMD2/PSMB2/PSME3/CBF
5318	tags=33%,	6696/7345/	GO_BP	SPP1/UCHL1/S100A6/RAP1GAP/LIMK1/MDK/DBN
8327	tags=61%,	6659/3976/	GO_BP	SOX4/LIF/LOXL4/P3H4/TGFB1/SPI1/PHF19/NU
5034	tags=38%,	4318/4323/	GO_BP	MMP9/MMP14/TGFB1/DKK1/ITGB2/ITGAV/LAMB1
4621	tags=46%,	27242/396	GO_BP	TNFRSF21/LGALS9/FOXJ1/TYROBP/TGFB1/GPNM
4621	tags=46%,	27242/396	GO_BP	TNFRSF21/LGALS9/FOXJ1/TYROBP/TGFB1/GPNM
6528	tags=52%,	1869/5554	GO_BP	E2F1/RBM38/IGF2BP1/PKP3/YBX3/MAPKAPK2/D
6487	tags=47%,	1401/5047/	GO_BP	CRP/PAEP/PYCARD/MDK/TNFRSF21/MMP12/POST
7198	tags=53%,	29108/432	GO_BP	PYCARD/MMP12/FLNA/BIRC3/UNC13D/EXOSC4/M
6055	tags=49%,	2810/1869/	GO_BP	SFN/E2F1/BAK1/TSPO/RAC2/YWHAZ/HK2/SFXN3
7545	tags=56%,	6659/7153/	GO_BP	SOX4/TOP2A/ANLN/TGFB1/SPI1/BATF/PLEK/CS
2230	tags=33%,	6510/8153	GO_BP	SLC1A5/SLC38A1/RGS2/SLC7A7/SLC7A1/SLC7A
6041	tags=43%,	6696/8161	GO_BP	SPP1/ITM2C/SEMA6A/ARHGAP4/LGALS1/DKK1/P
4415	tags=47%,	6696/6714/	GO_BP	SPP1/SRC/RAC2/NCDN/ACP5/SYK/CSF1R/TNFRS
4909	tags=41%,	29108/230	GO_BP	PYCARD/FOXJ1/NFKBIE/TRAIP/TONSL/HAVCR2/
764	tags=26%,	10580/985	GO_BP	SORBS1/EPM2AIP1/SIRT1/IGF1/PPARA/GPER1/

6169	tags=50%,	56667/103	GO_BP	MUC13/B3GNT3/GCNT3/ST6GALNAC4/MUC1/CHST
5482	tags=60%,	6280/8440/	GO_BP	S100A9/NCK2/S100A8/DDIT3/BAX/NUPR1/BMF/
5045	tags=43%,	10331/505	GO_BP	B3GNT3/CHST11/TGFB1/GPC4/GPC3/AGRN/SDC3/
831	tags=42%,	55258/435/	GO_BP	THNSL2/ASL/GOT1/CBS/ASPA/GLUL/GLUD1/ASP
1301	tags=32%,	154791/15	GO_BP	FMC1/SIK1/SIRT1/NR1H4/ACADVL/ERLIN1/ORM
1063	tags=42%,	4041/2341	GO_BP	LRP5/SIRT1/NR1H4/CETP/ACSM1/LDLR/HNF4A/
782	tags=29%,	5627/948/	GO_BP	PROS1/CD36/APOE/SERPING1/VTN/FGB/FGG/PR
767	tags=62%,	2805/2752/	GO_BP	GOT1/GLUL/GLUD1/GOT2/ADHFE1
1266	tags=64%,	1215/1511/	GO_BP	CMA1/CTSG/ACE2/CES1/ENPEP/MME/REN
1266	tags=64%,	1215/1511/	GO_BP	CMA1/CTSG/ACE2/CES1/ENPEP/MME/REN
1295	tags=64%,	6817/6799/	GO_BP	SULT1A1/SULT1A2/ALDH1B1/SULT1E1/SULT1B1/
673	tags=55%,	3283/3284/	GO_BP	HSD3B1/HSD3B2/CYP17A1/SRD5A1/SRD5A2/HSD
425	tags=45%,	4548/2374	GO_BP	MTR/BHMT2/ADI1/MTHFD1/BHMT
420	tags=45%,	346/4547/	GO_BP	APOC4/MTTP/APOB/APOC1/APOC3
761	tags=45%,	875/7263/1	GO_BP	CBS/TST/CTH/CPS1/BHMT
3063	tags=43%,	54209/4321	GO_BP	TREM2/MMP12/IKBKE/CSF1/RIPK2/CD74/CASP4/
1226	tags=30%,	9446/5702	GO_BP	GSTO1/PDXP/PCCA/MCCC2/PCCB/GCLC/PNPO/BT
1275	tags=36%,	23411/298	GO_BP	SIRT1/NPC1L1/CLU/ABCA5/LRP6/CETP/LDLR/S
940	tags=38%,	23417/546	GO_BP	MLYCD/PPARA/CPT1A/APOC2/IRS1/AVPR1A/IRS
782	tags=28%,	5627/948/	GO_BP	PROS1/CD36/APOE/SERPING1/VTN/FGB/FGG/PR
3964	tags=33%,	8877/5420	GO_BP	SPHK1/TREM2/CTSC/TYROBP/ITGAM/HAVCR2/IT
895	tags=67%,	185/348/3	GO_BP	AGTR1/APOE/APOC1/APOA2/APOA1/APOA5
2496	tags=50%,	5909/5176	GO_BP	RAP1GAP/STK26/FXYD5/EZR/FSCN1/SLC9A3R1/
692	tags=67%,	348/344/3	GO_BP	APOE/APOC2/APOC4/APOB/APOC1/APOC3
446	tags=67%,	57817/642	GO_BP	HAMP/ABCG8/ABCG5/APOA2/APOA1/APOA5
7642	tags=82%,	6659/5984/	GO_BP	SOX4/RFC4/DTL/POLD1/PCNA/RFC2/PNKP/PARP
1464	tags=32%,	8864/1058	GO_BP	PER2/SORBS1/EPM2AIP1/ADGRF5/IGF1/PID1/P
1560	tags=50%,	196743/58	GO_BP	PAOX/ABCD3/PEX13/UBB/PEX12/ACOT4/PEX1/N
782	tags=29%,	5627/948/	GO_BP	PROS1/CD36/APOE/SERPING1/VTN/FGB/FGG/PR
1326	tags=42%,	55902/681	GO_BP	ACSS2/SULT1A1/IGF1/ADH5/SULT1A2/RETSAT/
1063	tags=42%,	4041/2341	GO_BP	LRP5/SIRT1/NR1H4/CETP/ACSM1/LDLR/HNF4A/
1379	tags=26%,	10580/221	GO_BP	SORBS1/ADGRF5/NR1H4/HTR2B/LDLR/ABHD6/AP
5356	tags=37%,	100133941/	GO_BP	CD24/S100A9/CCL20/CRP/CXCL1/HMGA1/PTGES/
6479	tags=38%,	6696/7345/	GO_BP	SPP1/UCHL1/S100A6/RAP1GAP/LIMK1/DBN1/PO
5356	tags=37%,	100133941/	GO_BP	CD24/S100A9/CCL20/CRP/CXCL1/HMGA1/PTGES/
6217	tags=51%,	2810/1869/	GO_BP	SFN/E2F1/MMP12/KPNA2/TSPO/RAC2/ECT2/TGF
3692	tags=38%,	5361/7040/	GO_BP	PLXNA1/TGFB1/CLCN2/LAMA5/EDA/FGFR1/TGM2/
3842	tags=49%,	116028/58	GO_BP	RMI2/RAD51/RAD51AP1/CHEK1/SIRT6/MAD2L2/
3930	tags=33%,	10551/419	GO_BP	AGR2/MDK/MMP12/COL1A1/POSTN/TYRO3/ITGB4/
3631	tags=56%,	9319/3976/	GO_BP	TRIP13/LIF/TTK/FBXO5/RAD1
4643	tags=39%,	54209/291	GO_BP	TREM2/PYCARD/CXCL6/POSTN/LGALS9/RIPK2/H
1060	tags=29%,	65018/135/	GO_BP	PINK1/ADORA2A/OXT/KCNB1/ACE2/AVPR1A/KMO/
3774	tags=34%,	4192/2724	GO_BP	MDK/TNFRSF21/MMP12/COL1A1/POSTN/LGALS9/
1289	tags=34%,	5164/2341	GO_BP	PDK2/SIRT1/ACADVL/ERLIN1/LONP2/MLYCD/ET
761	tags=60%,	875/6470/1	GO_BP	CBS/SHMT1/SDS
761	tags=80%,	4357/875/1	GO_BP	MPST/CBS/CTH/CYP1A2
1063	tags=80%,	1071/948/	GO_BP	CETP/CD36/MTTP/APOH
1063	tags=80%,	1071/948/	GO_BP	CETP/CD36/MTTP/APOH
1281	tags=60%,	2022/2852/	GO_BP	ENG/GPER1/CTH
7500	tags=71%,	6809/6616/	GO_BP	STX3/SNAP25/CPLX2/PLEK/YKT6/RAB15/STXBP
3835	tags=36%,	4192/3383/	GO_BP	MDK/ICAM1/PODXL2/ITGB2/PTAFR/PLCB1/SELP

3268	tags=37%,	10551/542	GO_BP	AGR2/TREM2/ITGA3/ARHGEF16/LGALS3/STX3/S
5216	tags=44%,	9168/822/	GO_BP	TMSB10/CAPG/PFN2/NEK2/STMN1/CCNF/CORO1A
5226	tags=50%,	4321/8767/	GO_BP	MMP12/RIPK2/IRF3/STAT1/IRF7/HSPD1/MAVS/
4243	tags=59%,	9181/2592	GO_BP	ARHGEF2/ASPM/FZD7/NUMBL/LEF1/FGFR1/LBH/
4326	tags=45%,	6662/7040/	GO_BP	SOX9/TGFB1/MAGED1/HNF1B/SIX2/TACSTD2/PA
1226	tags=58%,	9446/2949/	GO_BP	GSTO1/GSTM5/ACAA1/ACSL1/CYP1A1/PON3/GST
1196	tags=47%,	29881/394	GO_BP	NPC1L1/LDLR/CD36/ABCG8/ABCG5/APOA2/APOA
4283	tags=48%,	3976/1435/	GO_BP	LIF/CSF1/TGFB1/C1QC/IL34/HCLS1/FADD/PTP
3546	tags=86%,	7534/9368/	GO_BP	YWHAZ/SLC9A3R1/CDC42/STK25/COPG1/PDCD10
3118	tags=71%,	29089/273	GO_BP	UBE2T/UBE2S/BRCA1/BARD1/RNF4
984	tags=44%,	2729/875/	GO_BP	GCLC/CBS/TST/CSAD/CTH/CDO1/AGXT
634	tags=50%,	5447/4405	GO_BP	POR/PLIN5/ANGPTL4/APOC1/APOA2/ANGPTL3/A
1796	tags=50%,	3643/1058	GO_BP	INSR/SORBS1/EPM2AIP1/IGF1/IRS1/IRS2/GCK
713	tags=38%,	6006/5145	GO_BP	RHCE/RHCG/SLC25A20/RHBG/SLC22A1/SLC25A4
917	tags=70%,	125061/16	GO_BP	AFMID/IDO2/AADAT/ACMSD/TDO2/KMO/HAAO
692	tags=70%,	348/344/4	GO_BP	APOE/APOC2/MTTP/APOB/APOA2/APOA1/APOC3
917	tags=70%,	125061/16	GO_BP	AFMID/IDO2/AADAT/ACMSD/TDO2/KMO/HAAO
917	tags=70%,	125061/16	GO_BP	AFMID/IDO2/AADAT/ACMSD/TDO2/KMO/HAAO
1197	tags=60%,	37/254013/	GO_BP	ACADVL/ETFBKMT/ACACB/DGAT2/PLIN5/ACADL
761	tags=40%,	875/51380/	GO_BP	CBS/CSAD/CDO1/AGXT
905	tags=34%,	26291/9971	GO_BP	FGF21/NR1H4/ABCA5/CETP/LDLR/AGTR1/CD36/
838	tags=25%,	6469/5465/	GO_BP	SHH/PPARA/APOC2/IRS2/APOC1/ABCG8/ABCG5/
6206	tags=80%,	54209/291	GO_BP	TREM2/PYCARD/CD74/CD68/SLC11A1/WAS/HLA-
3988	tags=39%,	54209/665	GO_BP	TREM2/SOX4/E2F1/TSPO/ETV5/CSF1/CSF1R/AR
1196	tags=53%,	29881/394	GO_BP	NPC1L1/LDLR/CD36/ABCG8/ABCG5/APOA2/APOA
4258	tags=61%,	23616/230	GO_BP	SH3BP1/FOXJ1/SCRIB/FSCN1/MSN/PTK7/SLC9A
940	tags=60%,	8013/23417	GO_BP	NR4A3/MLYCD/PPARA/CPT1A/IRS1/IRS2/PLIN5
5960	tags=49%,	8877/302/1	GO_BP	SPHK1/ANXA2/CORO1A/STX3/SNAP25/CPLX2/TA
2910	tags=33%,	4318/4323/	GO_BP	MMP9/MMP14/DKK1/ITGB2/ITGAV/LAMB1/HNF1B
3668	tags=62%,	54751/671	GO_BP	FBLIM1/SRC/PLEK/CXCL13/KIF14/CDH17/PIEZ
3852	tags=69%,	3561/3601/	GO_BP	IL2RG/IL15RA/PLCB1/JAK3/SHC1/IL15/IL2RB
3852	tags=69%,	3561/3601/	GO_BP	IL2RG/IL15RA/PLCB1/JAK3/SHC1/IL15/IL2RB
6418	tags=47%,	6280/7345/	GO_BP	S100A9/UCHL1/TREM2/HSPB8/PYCARD/ITGB4/S
5356	tags=36%,	100133941/	GO_BP	CD24/S100A9/CCL20/CRP/CXCL1/HMGA1/PTGES
6686	tags=51%,	8877/440/	GO_BP	SPHK1/ASNS/RGS2/SOX4/ELOVL7/B4GALNT1/RP
6418	tags=47%,	6280/7345/	GO_BP	S100A9/UCHL1/TREM2/HSPB8/PYCARD/ITGB4/S
1471	tags=13%,	6696/7070	GO_BP	SPP1/THY1
6652	tags=55%,	100133941/	GO_BP	CD24/SRC/UNC119/NCK2/ADORA1/PRNP/MVP/DL
6686	tags=53%,	1401/9245/	GO_BP	CRP/GCNT3/PYCARD/TNFRSF21/CTSC/FCER1G/I
5566	tags=49%,	2810/5535	GO_BP	SFN/LAPTM4B/E2F1/BAK1/YWHAZ/HK2/BAX/SLC
6122	tags=53%,	2207/6809/	GO_BP	FCER1G/STX3/SNAP25/CPLX2/PLEK/SYK/YKT6/
2861	tags=56%,	113130/64	GO_BP	CDCA5/NCAPG/NUSAP1/NCAPD2/NCAPH/SMC4/AK
4830	tags=51%,	5984/5151	GO_BP	RFC4/DTL/POLD1/MAD2L2/ISG15/PCNA/NPLOC4
6180	tags=76%,	8767/7967	GO_BP	RIPK2/VTCN1/TRAF2/CD86/CD3E/SASH3/CD83/
3798	tags=50%,	23636/590	GO_BP	NUP62/RAN/NUP210/NUP93/NUP205/NUP37/NDC
2013	tags=60%,	1896/3065/	GO_BP	EDA/HDAC1/HDAC2
1800	tags=28%,	5547/1215/	GO_BP	PRCP/CMA1/AVPR2/GAS6/DDAH1/P2RX2/SUCNR1
1379	tags=26%,	10580/985	GO_BP	SORBS1/EPM2AIP1/PDK2/SIK1/SIRT1/C1QTNF3
1130	tags=21%,	55856/509	GO_BP	ACOT13/PCBD1/ACTN2/TRPM8/ALDH9A1/ACACB/
1279	tags=24%,	3248/2341	GO_BP	HPGD/SIRT1/ELOVL6/MGST2/HMGCL/BDH2/CBR4
2496	tags=50%,	5909/5176	GO_BP	RAP1GAP/STK26/FXYD5/USH1C/TWF2/EZR/FSCN

4326	tags=44%,	6662/7040/	GO_BP	SOX9/TGFB1/MAGED1/HNF1B/BASP1/SIX2/TACS'
4786	tags=44%,	100133941/	GO_BP	CD24/SRC/LGALS1/CD86/VAV1/EFNB1/CD3E/PA
3315	tags=31%,	4318/1001	GO_BP	MMP9/CD24/SOX9/LIF/TGFB1/MAGED1/HNF1B/P
7545	tags=63%,	2207/7462/	GO_BP	FCER1G/LAT2/SYK/PSMB9/PSMD14/VAV1/PSMD2
1257	tags=57%,	54209/143	GO_BP	TREM2/CSF1/CSF1R/IL34
1257	tags=57%,	54209/143	GO_BP	TREM2/CSF1/CSF1R/IL34
7076	tags=64%,	29108/102	GO_BP	PYCARD/TRAIP/RIPK2/RELB/HMGB2/SIRPA/IRF
878	tags=30%,	5236/2542/	GO_BP	PGM1/SLC37A4/SLC25A13/GOT1/PPARA/PC/KAT
782	tags=25%,	90102/325/	GO_BP	PHLDB2/APCS/PROS1/APOE/SERPING1/VTN/FGB
6229	tags=37%,	6696/7345/	GO_BP	SPP1/UCHL1/S100A6/RAP1GAP/LIMK1/TNFRSF2
4909	tags=34%,	8877/6280/	GO_BP	SPHK1/S100A9/PYCARD/ICAM1/LGALS9/FOXJ1/
6856	tags=56%,	3159/8318/	GO_BP	HMGA1/CDC45/SPI1/PHF19/EZH2/DNMT1/TRIM2
6074	tags=44%,	5347/6662/	GO_BP	PLK1/SOX9/MMP12/KPNA2/CDK1/COL1A1/SRC/L
4824	tags=36%,	100133941/	GO_BP	CD24/DUSP9/CDK1/SRC/TGFB1/IQGAP3/DKK1/R
6566	tags=43%,	6696/2539/	GO_BP	SPP1/G6PD/LIMK1/RGS2/SOX9/DBN1/POSTN/RE
980	tags=38%,	721/733/1	GO_BP	C4B/C8G/CLU/APCS/CREB3L3/C1R/C2/PROS1/E
1344	tags=38%,	79944/227	GO_BP	L2HGDH/FH/NR1H4/ALDH4A1/GCLC/HAL/GOT1/A
1478	tags=23%,	5243/8864/	GO_BP	ABCB1/PER2/ADORA2A/PDZK1/TNFSF11/OXT/CA
1270	tags=57%,	721/733/1	GO_BP	C4B/C8G/CLU/F8/C1RL/APCS/RGCC/C1R/C2/PR
6222	tags=47%,	8877/5420	GO_BP	SPHK1/TREM2/LGALS9/PTP4A3/HAVCR2/IRAK1/
4936	tags=46%,	5888/4796/	GO_BP	RAD51/TONSL/POLD1/ALYREF/EME1/PCNA/DDX1
4263	tags=56%,	5347/891/	GO_BP	PLK1/CCNB1/CCNB2/CDK1/AKAP8L/CTDNEP1/VR
4263	tags=56%,	5347/891/	GO_BP	PLK1/CCNB1/CCNB2/CDK1/AKAP8L/CTDNEP1/VR
5482	tags=69%,	3984/4085/	GO_BP	LIMK1/MAD2L1/MAD2L2/FBXO5/RPL23/ABL1/RP
5774	tags=56%,	8877/302/1	GO_BP	SPHK1/ANXA2/CORO1A/CPLX2/ANKRD27/VPS18/
5632	tags=57%,	6352/8155	GO_BP	CCL5/TRIM11/POLR2G/SMARCA4/HDAC1/LEF1/N
3510	tags=56%,	2302/100/	GO_BP	FOXJ1/ADA/PKN1/TNFSF13B/TNFAIP3
3520	tags=67%,	4751/3832/	GO_BP	NEK2/KIF11/CHEK1/CEP85/RANBP1/NSFL1C
6055	tags=50%,	4318/2810/	GO_BP	MMP9/SFN/TREM2/PYCARD/E2F1/BAK1/PLAUR/R
717	tags=35%,	1562/348/	GO_BP	CYP2C18/APOE/APOC2/CYP26A1/ALDH1A1/RARR
1464	tags=20%,	8864/135/	GO_BP	PER2/ADORA2A/SLC38A2/SLC25A18/SLC26A1/A
1060	tags=27%,	5020/2862	GO_BP	OXT/LCN12/CPT2/ABCC2/PRKAG2/CD36/PPARA/
1275	tags=32%,	23411/298	GO_BP	SIRT1/NPC1L1/CLU/ABCA5/LRP6/CETP/LDLR/S
909	tags=20%,	5836/5236/	GO_BP	PYGL/PGM1/PRKAG2/AGL/ENPP1/STBD1/UGP2/I
878	tags=29%,	5236/2542/	GO_BP	PGM1/SLC37A4/SLC25A13/GOT1/PPARA/PC/KAT
1060	tags=24%,	5020/9693/	GO_BP	OXT/RAPGEF2/AQP8/FDX1/TEK/PER1/SRD5A1/A
638	tags=25%,	23410/403	GO_BP	SIRT3/LRP1/EGFR/TNFSF11/NR1H4/RASL10B/N
1330	tags=38%,	4321/8324/	GO_BP	MMP12/FZD7/JAML
6222	tags=46%,	8877/5420	GO_BP	SPHK1/TREM2/LGALS9/PTP4A3/HAVCR2/IRAK1/
4863	tags=52%,	2302/7305/	GO_BP	FOXJ1/TYROBP/CD74/PTAFR/SYK/CARD9/IL1B/
3562	tags=40%,	10551/542	GO_BP	AGR2/TREM2/ITGA3/ARHGEF16/LGALS3/STX3/S
1115	tags=57%,	23464/552	GO_BP	GCAT/THNSL2/RIDA/SDS
340	tags=71%,	6777/5138	GO_BP	STAT5B/CSAD/BAAT/GHR/CDO1
340	tags=71%,	6777/5138	GO_BP	STAT5B/CSAD/BAAT/GHR/CDO1
234	tags=71%,	64241/642	GO_BP	ABCG8/ABCG5/APOA2/APOA1/APOA5
692	tags=57%,	19/348/33	GO_BP	ABCA1/APOE/APOA1/APOC3
1397	tags=71%,	9481/2341	GO_BP	SLC25A27/SIRT1/NR1H4/C1QTNF3/DGAT2
509	tags=57%,	51166/280	GO_BP	AADAT/GOT2/KMO/ALDH8A1
234	tags=71%,	64241/642	GO_BP	ABCG8/ABCG5/APOA2/APOA1/APOA5
3887	tags=41%,	4192/1063	GO_BP	MDK/POSTN/TYRO3/SRC/MELTF/FBLN1/RCC2/FL
5291	tags=41%,	2539/5997/	GO_BP	G6PD/RGS2/MMP14/TGFB1/DKK1/GDF15/RBM38/

6494	tags=46%,	2810/1401/GO_BP	SFN/CRP/PAEP/TREM2/PYCARD/SOX4/MDK/TNFR
6418	tags=49%,	7345/54209GO_BP	UCHL1/TREM2/HSPB8/PYCARD/TSPO/ATP13A2/T
566	tags=43%,	54209/1435GO_BP	TREM2/CSF1/P2RX4
566	tags=43%,	54209/1435GO_BP	TREM2/CSF1/P2RX4
9155	tags=83%,	54512/7903GO_BP	EXOSC4/NABP2/INTS8/DKC1/TAF6/POLR2G/POL
3928	tags=38%,	8877/2717/GO_BP	SPHK1/GLA/B4GALNT1/GAL3ST1/PRKCD/ELOVL1
5704	tags=41%,	54997/5420GO_BP	TESC/TREM2/LIF/CSF1/TYROBP/TGFB1/CD74/P
3626	tags=45%,	7203/51248GO_BP	CCT3/PDZD11/NUP93/NUP205/NDC1/GSDMD/NUP
7150	tags=62%,	4192/3965/GO_BP	MDK/LGALS9/TGFB1/RIPK2/ADA/CD74/SYK/IL1
6405	tags=52%,	7345/26353GO_BP	UCHL1/HSPB8/ATP13A2/GAPDH/RIPK2/QSOX1/A
7226	tags=48%,	6696/3984/GO_BP	SPP1/LIMK1/DBN1/PLXNA1/SEMA6A/ARHGAP4/T
4265	tags=39%,	27242/1075GO_BP	TNFRSF21/CTSC/MTMR2/TYMP/ITGAX/S100B/MY
3727	tags=44%,	4192/3576/GO_BP	MDK/CXCL8/RAC2/CSF1/S100A14/CD74/CCL5/M
3491	tags=37%,	54209/1053GO_BP	TREM2/UBD/LGALS9/TGFB1/SPI1/BATF/RELB/T
5928	tags=57%,	6714/1601/GO_BP	SRC/DAB2/CORO1A/MTMR2/EZR/MSN/MAPK3/MAP
6133	tags=51%,	10551/440/GO_BP	AGR2/ASNS/HSPB8/CXCL8/KDELR3/BAK1/STC2/
3590	tags=35%,	9212/1869/GO_BP	AURKB/E2F1/NEK2/STMN1/DAB2/AURKA/DKK1/P
2482	tags=50%,	891/81620/GO_BP	CCNB1/CDT1/NDC80/MAD2L1/LCMT1/XRCC3/DYN
2482	tags=50%,	891/81620/GO_BP	CCNB1/CDT1/NDC80/MAD2L1/LCMT1/XRCC3/DYN
2482	tags=50%,	891/81620/GO_BP	CCNB1/CDT1/NDC80/MAD2L1/LCMT1/XRCC3/DYN
548	tags=26%,	4192/3383/GO_BP	MDK/ICAM1/ITGB2/IRAK1/PTAFR
1188	tags=23%,	10184/2323GO_BP	LHFPL2/PLCB1/ZP3
5023	tags=38%,	5047/29108GO_BP	PAEP/PYCARD/MDK/MMP12/POSTN/LGALS9/SRC/
3335	tags=28%,	8612/8877/GO_BP	PLPP2/SPHK1/ELOVL7/B4GALNT1/GAL3ST1/ALD
6175	tags=61%,	3838/23636GO_BP	KPNA2/NUP62/ALYREF/RAN/NUP210/VPS37C/NU
6175	tags=61%,	3838/23636GO_BP	KPNA2/NUP62/ALYREF/RAN/NUP210/VPS37C/NU
3955	tags=28%,	5347/891/1GO_BP	PLK1/CCNB1/AFP/RGS2/MDK/TOP2A/TRIP13/CD
778	tags=19%,	1191/3067/GO_BP	CLU/HDC/DDC/CD36/ALDH9A1/GCH1/RORA/ASS1
895	tags=28%,	2247/4908/GO_BP	FGF2/NTF3/LRP1/EGFR/ANGPTL8/HTR2B/AGTR1
4950	tags=43%,	7040/9156/GO_BP	TGFB1/EXO1/CLCF1/BATF/TFRC/HMGB2/TCF3/P
4950	tags=43%,	7040/9156/GO_BP	TGFB1/EXO1/CLCF1/BATF/TFRC/HMGB2/TCF3/P
3315	tags=35%,	6696/4316/GO_BP	SPP1/MMP7/RGS2/LGALS9/ITGA3/LIF/AKR1B1/
3491	tags=56%,	27242/7305GO_BP	TNFRSF21/TYROBP/PKN1/CD300A/MNDA/CTLA4/
5327	tags=52%,	7153/302/3GO_BP	TOP2A/ANXA2/KPNA2/LGALS9/LGALS1/CD74/CH
2230	tags=33%,	5997/6536/GO_BP	RGS2/SLC6A9/SLC36A1/SLC38A5
5960	tags=51%,	123920/746GO_BP	CMTM3/LAT2/IGLL5/SYK/BAX/CD300A/SLC39A1
1484	tags=36%,	1215/554/5GO_BP	CMA1/AVPR2/SUCNR1/ADRB1/CTSG/RASL10B/AG
991	tags=27%,	4969/23417GO_BP	OGN/MLYCD/PCYOX1/CBS/HYAL1/NUDT7/TST/AC
5169	tags=46%,	6364/29108GO_BP	CCL20/PYCARD/STK39/CCL5/MSN/IL27RA/ADAM
1589	tags=56%,	10331/9334GO_BP	B3GNT3/B4GALT5/B3GNT5/B3GNT9/B3GNT7
6436	tags=54%,	5570/9212/GO_BP	PKIB/AURKB/NEK2/SRC/RFC4/DSCC1/SMG5/PIF
3939	tags=28%,	1364/706/1GO_BP	CLDN4/TSPO/CLIC1/FXYD3/TTYH3/ANO9/PTAFR
3625	tags=64%,	5973/10007GO_BP	RENBP/GNPDA1/NPL/CHIT1/NAGK/GNPDA2/AMDH
2627	tags=62%,	6714/5341/GO_BP	SRC/PLEK/CXCL13/CDH17/PIEZO1
1124	tags=43%,	6608/4040/GO_BP	SMO/LRP6/HMGCS1/GRAMD1C/RORA/INSIG1/ROR
2256	tags=80%,	5347/7272/GO_BP	PLK1/TTK/NCAPH/NCAPH2
2719	tags=80%,	5580/5111/GO_BP	PRKCD/PCNA/DDX11/RPS3
291	tags=40%,	81620/5603GO_BP	CDT1/MAPK13
1048	tags=60%,	2056/9181/GO_BP	EPO/ARHGEF2/YBX3
3246	tags=52%,	5538/2896/GO_BP	PPT1/GRN/ATP6V0D2/TCIRG1/ATP6V1H/CLN3/S
5652	tags=67%,	3838/23513GO_BP	KPNA2/SCRIB/ZC3H12A/KPNB1/CPSF4/BAD/CAS

4824	tags=33%,	8877/1001	GO_BP	SPHK1/CD24/CCL20/UCHL1/TREM2/PYCARD/RGS
6100	tags=49%,	6280/5666	GO_BP	S100A9/MUC13/CRP/PYCARD/CYBA/LIMK1/LY96
5239	tags=36%,	6696/1401	GO_BP	SPP1/CRP/TMSB10/CAPG/PYCARD/LIMK1/RGS2/
5886	tags=40%,	100133941	GO_BP	CD24/CTHRC1/SOX4/MDK/SOX9/COL1A1/SULF1/
4734	tags=32%,	6280/3984	GO_BP	S100A9/LIMK1/RGS2/MDK/EPO/DBN1/REG1A/PL
3335	tags=29%,	8612/8877	GO_BP	PLPP2/SPHK1/ELOVL7/B4GALNT1/GAL3ST1/ALD
5960	tags=48%,	8877/302/1	GO_BP	SPHK1/ANXA2/CORO1A/STX3/SNAP25/CPLX2/TA
8126	tags=70%,	63875/618	GO_BP	MRPL17/MRPS12/MRPL47/MRPL9/MRPS23/MRPL1
2333	tags=29%,	6510/6535	GO_BP	SLC1A5/SLC6A8/SLC38A1/RGS2/SLC7A7/SLC6A
6181	tags=46%,	7040/2363	GO_BP	TGFB1/NUP62/RAN/LIN28B/NUP210/CNOT11/MA
831	tags=36%,	55258/280	GO_BP	THNSL2/GOT1/GOT2/BHMT2/ADI1/AASS/MTHFD1
857	tags=45%,	2185/1027	GO_BP	PTK2B/CDKN1B/LIG4/ASCL1/SHH/ID2/ADCY1/I
785	tags=23%,	1583/2638	GO_BP	CYP11A1/GC/CYP27A1/CYP1A1/CYP3A4
4900	tags=41%,	9168/822/1	GO_BP	TMSB10/CAPG/CRYAB/PFN2/STMN1/CORO1A/ARH
5182	tags=40%,	8877/991/	GO_BP	SPHK1/CDC20/PLK1/UBE2S/TRIB3/MAGEC2/FAN
895	tags=55%,	185/348/3	GO_BP	AGTR1/APOE/APOC1/APOA2/APOA1/APOA5
7685	tags=62%,	54913/827	GO_BP	RPP25/LAGE3/PUSL1/TSEN54/WDR4/CSTF2/TRM
2868	tags=32%,	6001/3757	GO_BP	RGS10/AGRN/PLCB1/GNA15/GNB1/GRK2
5147	tags=47%,	10551/440	GO_BP	AGR2/ASNS/CXCL8/KDELR3/BAK1/STC2/CLGN/N
3505	tags=48%,	22948/790	GO_BP	CCT5/NABP2/CCT6A/CCT3/DKC1/ACD/CCT2/CCT
4973	tags=42%,	10331/5051	GO_BP	B3GNT3/CHST11/TGFB1/GPC4/GPC3/AGRN/SDC3
1589	tags=50%,	10331/933	GO_BP	B3GNT3/B4GALT5/B3GNT5/B3GNT9/B3GNT7
4367	tags=47%,	7040/4050	GO_BP	TGFB1/LTB/CD248/TNFRSF11A/IL15/FADD/TOX
895	tags=32%,	2621/5667	GO_BP	GAS6/SUCNR1/AGTR1/REN/CYP4F12/SERPINF2/
1379	tags=35%,	10580/985	GO_BP	SORBS1/EPM2AIP1/IGF1/ENPP1/IRS1/IRS2/GC
1379	tags=35%,	10580/985	GO_BP	SORBS1/EPM2AIP1/IGF1/ENPP1/IRS1/IRS2/GC
2351	tags=26%,	4605/1728	GO_BP	MYBL2/NQO1/TYROBP/ITGAM/PRNP/GRN/DDIT3/
6227	tags=42%,	6696/2539	GO_BP	SPP1/G6PD/SPHK1/SFN/S100A9/CYBA/LIMK1/R
1283	tags=33%,	7076/7040	GO_BP	TIMP1/TGFB1/HIF1A
3131	tags=41%,	2302/7305	GO_BP	FOXJ1/TYROBP/CD74/PTAFR/SYK/CARD9/IL1B/
557	tags=67%,	1468/25351	GO_BP	SLC25A10/SLC25A30/SLC13A3/SLC13A5
557	tags=67%,	1468/25351	GO_BP	SLC25A10/SLC25A30/SLC13A3/SLC13A5
410	tags=38%,	7083/1841	GO_BP	TK1/DTYMK/ADA
2217	tags=50%,	891/5595/	GO_BP	CCNB1/MAPK3/RPS6KA4/IL1B
5701	tags=55%,	29108/8161	GO_BP	PYCARD/ITM2C/TNFRSF12A/TRAF2/RBCK1/PPP2
4630	tags=32%,	8877/2810	GO_BP	SPHK1/SFN/S100A9/AGR2/CCNB1/CYBA/LIMK1/
5806	tags=51%,	3576/7153	GO_BP	CXCL8/TOP2A/APOBEC3C/TRIM6/TRIM28/CCL5/
8551	tags=74%,	63875/618	GO_BP	MRPL17/MRPS12/MRPL47/MRPL9/MRPS23/MRPL1
4097	tags=24%,	6690/226/	GO_BP	SPINK1/ALDOA/PAEP/CDK1/LHFPL2/GNPDA1/CL
6026	tags=50%,	6280/1535	GO_BP	S100A9/CYBA/LY96/IKBKE/TYRO3/S100A14/IT
5780	tags=51%,	1535/1410	GO_BP	CYBA/CRYAB/ICAM1/BAK1/ECT2/TGFB1/RAD51/
328	tags=24%,	3078/3080	GO_BP	CFHR1/CFHR2/GPLD1/CFHR5
182	tags=29%,	891/983	GO_BP	CCNB1/CDK1
934	tags=50%,	338821/65	GO_BP	SLCO1B7/SLCO1A2/SLCO2A1/SLC22A9/SLC16A2
4418	tags=46%,	4321/2316	GO_BP	MMP12/FLNA/CD44/DDR1/CD151/RHOC/COL5A1/
4418	tags=46%,	4321/2316	GO_BP	MMP12/FLNA/CD44/DDR1/CD151/RHOC/COL5A1/
1295	tags=25%,	8877/4318	GO_BP	SPHK1/MMP9/TREM2/CTSC/IL18/GRN/NUPR1
764	tags=60%,	5465/1374	GO_BP	PPARA/CPT1A/IRS1/IRS2/PLIN5/FABP1
778	tags=50%,	4780/948/	GO_BP	NFE2L2/CD36/GCH1/FBLN5/RGN
1942	tags=33%,	6696/6850	GO_BP	SPP1/SYK/TFRC/ATP6AP1/TNFRSF11A/ADAM8
1942	tags=33%,	6696/6850	GO_BP	SPP1/SYK/TFRC/ATP6AP1/TNFRSF11A/ADAM8

763	tags=43%,	26291/150	GO_BP	FGF21/SIK1/SIRT1/MOGAT1/CETP/LDLR/PNPLA
1109	tags=19%,	8864/5145	GO_BP	PER2/RHCG/ADORA2A/ADRA2B/OXT/DDC/SYT7/K
1404	tags=26%,	5409/4929	GO_BP	PNMT/NR4A2/PMEL/EPAS1/NPR1/SULT1A1/HDC/
1196	tags=19%,	29881/644	GO_BP	NPC1L1/SGK1/OXT/LDLR/ACO1/WNK3/CD36/HAM
1277	tags=29%,	150094/23	GO_BP	SIK1/SIRT1/NR1H4/C1QTNF3/ACADVL/ERLIN1/
3246	tags=31%,	483/64699	GO_BP	ATP1B3/TMPRSS3/ATP1A1/PPT1/MAFG/SLAMF8/
2521	tags=16%,	3880/2598	GO_BP	KRT19/KRT23/DSG2/KRT17/PI3/KRT80/PKP3/K
1933	tags=50%,	9212/100/	GO_BP	AURKB/ADA/CD74/MIF/SLC39A10/NOC2L
5255	tags=65%,	2207/7305	GO_BP	FCER1G/TYROBP/STX3/GPD1L/HSP90AB1/RANGR
2447	tags=35%,	2539/5997	GO_BP	G6PD/RGS2/DKK1/FZD7/PAK1/GSK3A
3964	tags=45%,	54209/432	GO_BP	TREM2/MMP12/IKBKE/CSF1/RIPK2/CD74/CASP4
4824	tags=42%,	6364/2910	GO_BP	CCL20/PYCARD/CYBA/LCN2/CXCL8/SOX9/EPO/I
4663	tags=54%,	2810/1869	GO_BP	SFN/E2F1/YWHAZ/BID/YWHAH/BBC3/PMAIP1/YW
4663	tags=54%,	2810/1869	GO_BP	SFN/E2F1/YWHAZ/BID/YWHAH/BBC3/PMAIP1/YW
895	tags=53%,	185/348/3	GO_BP	AGTR1/APOE/ACAT1/APOC1/APOA2/LCAT/APOA1
895	tags=53%,	185/348/3	GO_BP	AGTR1/APOE/ACAT1/APOC1/APOA2/LCAT/APOA1
895	tags=53%,	185/348/3	GO_BP	AGTR1/APOE/ACAT1/APOC1/APOA2/LCAT/APOA1
767	tags=53%,	2805/2746	GO_BP	GOT1/GLUD1/GOT2/ACMSD/KMO/GLS2/HAAO/MTH
762	tags=40%,	3172/5629	GO_BP	HNF4A/PROX1/CYP1A1/PCK2/CPS1/PCK1
4296	tags=42%,	7272/6790	GO_BP	TTK/AURKA/CEP131/ABHD17C/NUP62/SPAG5/MA
4830	tags=45%,	5984/5151	GO_BP	RFC4/DTL/POLD1/CDKN2D/MAD2L2/ISG15/PCNA
3359	tags=31%,	8877/5420	GO_BP	SPHK1/TREM2/BACE2/CTSC/TYROBP/ITGAM/ITG
3335	tags=29%,	8612/8877	GO_BP	PLPP2/SPHK1/GLA/ELOVL7/B4GALNT1/GAL3ST1
1326	tags=39%,	55902/516	GO_BP	ACSS2/PDK2/ELOVL6/ACOT13/ACSM1/ACSF2/HM
1326	tags=39%,	55902/516	GO_BP	ACSS2/PDK2/ELOVL6/ACOT13/ACSM1/ACSF2/HM
3158	tags=43%,	7114/1649	GO_BP	TMSB4X/DDIT3/MAD2L2/SRI/ZNF593/ANKRD33/
6055	tags=53%,	5880/3684	GO_BP	RAC2/ITGAM/ITGB2/PRNP/HRAS/MICALL2/SAE1
1407	tags=54%,	1956/1600	GO_BP	EGFR/DAB1/SMO/EDNRB/ASS1/OTC/CPS1
1275	tags=29%,	23411/997	GO_BP	SIRT1/NR1H4/CETP/ABCC2/SLC12A1/GOT1/HNF
210	tags=21%,	135/1137/	GO_BP	ADORA2A/CHRNA4/ABAT
7974	tags=64%,	23636/590	GO_BP	NUP62/RANGAP1/SAE1/TRIM28/NUP210/UBE2I/
566	tags=60%,	54209/143	GO_BP	TREM2/CSF1/P2RX4
3228	tags=34%,	100/23529	GO_BP	ADA/CLCF1/CD74/CD320/TFRC/MIF/SASH3/TNF
895	tags=33%,	185/348/5	GO_BP	AGTR1/APOE/POR/PPARGC1A/APOC1/APOA2/APO
5226	tags=46%,	4321/8767	GO_BP	MMP12/RIPK2/HAVCR2/IRF3/STAT1/IRF7/HSPD
6337	tags=64%,	5570/9212	GO_BP	PKIB/AURKB/NEK2/MAPK3/DKC1/ACD/CCT2/HSP
6810	tags=71%,	4321/9641	GO_BP	MMP12/IKBKE/SCRIB/TRIM6/IRF3/HSP90AB1/I
4759	tags=63%,	5984/2237	GO_BP	RFC4/FEN1/POLD1/POLA2/PCNA/RFC2/PRIM1/P
6512	tags=49%,	8612/1852	GO_BP	PLPP2/DUSP9/CDKN3/DLGAP5/CDC25B/PTP4A3/
302	tags=26%,	1723/6470	GO_BP	DHODH/SHMT1/XDH/MTHFD1/AOX1/DPYS/ALDH6A
3876	tags=54%,	81620/510	GO_BP	CDT1/GMNN/PYGO2/DDX11/KDM1A/MED25/CTBP2
3762	tags=64%,	6714/5157	GO_BP	SRC/PDGFRL/HIP1R/PDGFA/LOX/ABL1/PTPN12/
4179	tags=38%,	2539/274/	GO_BP	G6PD/BIN1/CACNB3/SESTD1/CACNB1
1354	tags=60%,	26063/138	GO_BP	DECR2/CRAT/ETFA/DECR1/PCCA/ACADVL/ABHD2
908	tags=37%,	54884/224	GO_BP	RETSAT/ALDH3A2/CYP2C18/APOE/APOC2/CYP26
1354	tags=61%,	114757/80	GO_BP	CYGB/NR4A3/ADIPOR2/ABCD3/PEX13/ECI1/AUH
1398	tags=57%,	28996/404	GO_BP	HIPK2/LRP6/PROX1/RBP4
2818	tags=60%,	1277/1287	GO_BP	COL1A1/COL4A5/SYK/DDR1/UBASH3B/COL4A2
2674	tags=60%,	54209/609	GO_BP	TREM2/ROBO1/CCL5/HIF1A/RNF113A/PADI2
6198	tags=47%,	6696/2539	GO_BP	SPP1/G6PD/RGS2/CRYAB/ENO1/SEMA6A/SERPIN
2857	tags=50%,	3691/977/	GO_BP	ITGB4/CD151/LAMC1/PLEC/LAMC2/LAMB3

3359	tags=50%,	54209/107	GO_BP	TREM2/CTSC/IL18/NUPR1/IL1B/CCL3
2015	tags=32%,	6510/8153	GO_BP	SLC1A5/SLC38A1/RGS2/SLC7A7/SLC7A1/SLC7A
6686	tags=57%,	7040/2363	GO_BP	TGFB1/NUP62/NUP210/MAP2K2/POLR2G/NUP93/
6686	tags=57%,	7040/2363	GO_BP	TGFB1/NUP62/NUP210/MAP2K2/POLR2G/NUP93/
2284	tags=67%,	3934/2495/	GO_BP	LCN2/FTH1/SRI/FTL
1763	tags=45%,	2861/5409/	GO_BP	GPR37/PNMT/NR4A2/HDC/DDC/GCH1/DBH/PAH/D
1763	tags=45%,	2861/5409/	GO_BP	GPR37/PNMT/NR4A2/HDC/DDC/GCH1/DBH/PAH/D
4477	tags=42%,	9168/822/	GO_BP	TMSB10/CAPG/PFN2/STMN1/PRKCD/MAPRE1/TMS
1010	tags=26%,	4258/1264	GO_BP	MGST2/CYP4F22/FAAH/CYP2C18/AVPR1A/ACOX1
1252	tags=23%,	286410/10	GO_BP	ATP11C/SLC17A3/PCTP/CETP/LDLR/SLC37A4/A
1063	tags=22%,	1071/5020/	GO_BP	CETP/OXT/AGTR1/SHH/PPARA/APOE/APOC2/LIP
553	tags=26%,	1562/5489	GO_BP	CYP2C18/ELOVL2/AVPR1A/ACOX1/ACAA1/HSD17
1354	tags=60%,	114757/80	GO_BP	CYGB/NR4A3/ADIPOR2/ABCD3/PEX13/ECI1/AUH
8642	tags=80%,	6628/1478/	GO_BP	SNRPB/CSTF2/CD3EAP/POLR3K/SNRPE/POLR2H/
297	tags=40%,	1593/1576	GO_BP	CYP27A1/CYP3A4
297	tags=40%,	1593/1576	GO_BP	CYP27A1/CYP3A4
2111	tags=80%,	595/5481/	GO_BP	CCND1/PPID/EGFR/MME
3465	tags=30%,	29108/396	GO_BP	PYCARD/LGALS9/RIPK2/HAVCR2/PRNP/HRAS/IL
182	tags=40%,	891/983	GO_BP	CCNB1/CDK1
5886	tags=51%,	2539/5997/	GO_BP	G6PD/RGS2/DKK1/EZH2/FZD7/BHLHE41/PAK1/G
6334	tags=49%,	2810/5499	GO_BP	SFN/TESC/UCHL1/PLK1/PYCARD/PKIB/LIMK1/R
1918	tags=43%,	3606/240/	GO_BP	IL18/ALOX5/IL18BP
1918	tags=43%,	3606/240/	GO_BP	IL18/ALOX5/IL18BP
2206	tags=43%,	7114/1649/	GO_BP	TMSB4X/DDIT3/ZNF593
2688	tags=44%,	10537/704	GO_BP	UBD/TGFB1/SPI1/BATF/RELB/BATF2/LTBR/BAT
883	tags=36%,	2166/5662	GO_BP	FAAH/ASAH2/ACOT1/PNPLA3/AIG1/ACOT2/CYP2
909	tags=18%,	5836/5236/	GO_BP	PYGL/PGM1/PRKAG2/AGL/ENPP1/STBD1/UGP2/I
1379	tags=32%,	10580/985	GO_BP	SORBS1/EPM2AIP1/PDK2/SIK1/SIRT1/C1QTNF3
1039	tags=26%,	57026/109	GO_BP	PDXP/PRDX3/HBA2/CYP4F11/MTHFS/NNT/NUDT7
1179	tags=58%,	64087/865	GO_BP	MCCC2/ALDH4A1/GCAT/HDC/PCBD1/AMT/HMGCL/
5467	tags=64%,	3958/8486	GO_BP	LGALS3/HAVCR2/NCK2/MSN/CD6/DOCK2/CCL19/
2910	tags=30%,	4318/4323/	GO_BP	MMP9/MMP14/DKK1/ITGB2/ITGAV/LAMB1/HNF1B
1295	tags=40%,	891/983/5	GO_BP	CCNB1/CDK1/PGK1/NUPR1
4153	tags=52%,	55353/550	GO_BP	LAPTM4B/VPS37C/SORT1/LEPROTL1/SNF8/CHMP
5255	tags=67%,	25825/816	GO_BP	BACE2/ITM2C/JAK3/AATF/AGO2/ACOT8/HBEGF/
905	tags=32%,	3949/48/9	GO_BP	LDLR/ACO1/CD36/HAMP/FABP1/ABCG8/ABCG5/A
5327	tags=47%,	3576/7153/	GO_BP	CXCL8/TOP2A/APOBEC3C/TRIM6/TRIM28/CCL5/
6241	tags=59%,	54209/220	GO_BP	TREM2/FCER1G/SH3BP1/ITGAM/ITGB2/IGLL5/R
1434	tags=23%,	10686/135/	GO_BP	CLDN16/ADORA2A/NPR1/OXT/AQP6/AGTR1/ADRA
1429	tags=37%,	4496/1956/	GO_BP	MT1H/EGFR/FOS/MT1F/MT1B/GCLC/SOD1/GOT1/
854	tags=40%,	1956/2908/	GO_BP	EGFR/NR3C1/ABCC2/GJB2/FOXO1/ERRFI1/SRD5
1226	tags=25%,	9446/128/	GO_BP	GSTO1/ADH5/MGST1/PRDX3/MGST2/HBA2/SOD1/
4993	tags=41%,	4072/2192/	GO_BP	EPCAM/FBLN1/TGFB1/RACGAP1/ARHGEF2/SMARC
1482	tags=50%,	1277/1513/	GO_BP	COL1A1/CTSK/MMP2
1482	tags=50%,	1277/1513/	GO_BP	COL1A1/CTSK/MMP2
2852	tags=38%,	8767/8486	GO_BP	RIPK2/HAVCR2/RELB/HRAS/IL18/JAK3/SEMA4A
4824	tags=32%,	8877/1001	GO_BP	SPHK1/CD24/CCL20/UCHL1/TREM2/PYCARD/RGS
3359	tags=32%,	8877/5420	GO_BP	SPHK1/TREM2/CTSC/TYROBP/ITGAM/ITGB2/C1Q
3359	tags=32%,	8877/5420	GO_BP	SPHK1/TREM2/CTSC/TYROBP/ITGAM/ITGB2/C1Q
4982	tags=37%,	100133941/	GO_BP	CD24/CCNB1/CDC6/DUSP9/CDK1/SRC/CKS2/TGF
2472	tags=29%,	4192/1063	GO_BP	MDK/POSTN/PLXNA1/SRC/GSTP1/PLAU/IGFBP3/

4013	tags=41%,	578/57761/	GO_BP	BAK1/TRIB3/NCK2/CASP4/CREB3L1/DDIT3/TRA
4662	tags=30%,	6280/3984/	GO_BP	S100A9/LIMK1/RGS2/MDK/EPO/DBN1/REG1A/PL
3919	tags=42%,	2810/1869/	GO_BP	SFN/E2F1/BAK1/YWHAZ/BAX/SLC9A1/SLC35F6/
1191	tags=50%,	94097/648	GO_BP	SFXN5/SLC13A3/SLC13A5
1104	tags=67%,	10203/502	GO_BP	CALCRL/OXT/KHK/SLC6A1
1191	tags=50%,	94097/648	GO_BP	SFXN5/SLC13A3/SLC13A5
888	tags=83%,	3034/1058	GO_BP	HAL/MTHFS/AMDHD1/FTCD/UROC1
1104	tags=67%,	10203/502	GO_BP	CALCRL/OXT/KHK/SLC6A1
395	tags=67%,	51129/341/	GO_BP	ANGPTL4/APOC1/ANGPTL3/APOC3
5466	tags=43%,	10397/338	GO_BP	NDRG1/ANO9/FCHO1/DMKN/PRKCD/BAIAP2L2/AB
3465	tags=31%,	29108/396	GO_BP	PYCARD/LGALS9/RIPK2/HAVCR2/PRNP/HRAS/IL
4318	tags=42%,	1410/578/	GO_BP	CRYAB/BAK1/SPI1/SCRIB/HNF1B/BAX/LEF1/JA
5011	tags=40%,	2810/54997	GO_BP	SFN/TESC/UCHL1/PLK1/PYCARD/PKIB/RGS2/DU
523	tags=50%,	2646/10891	GO_BP	GCKR/PPARGC1A/KHK/PCK1
207	tags=38%,	8993/2147/	GO_BP	PGLYRP1/F2/HRG
466	tags=62%,	51000/915	GO_BP	SLC35B3/SLC28A2/SLC25A42/SLC28A1/AQP9
644	tags=20%,	134	GO_BP	ADORA1
4258	tags=35%,	4605/1728/	GO_BP	MYBL2/NQO1/TYROBP/ITGAM/DKK1/ITGB2/C1QA
3578	tags=36%,	6696/1159	GO_BP	SPP1/CTHRC1/MDK/SRC/RAC2/P3H4/TGFB1/NCD
7144	tags=56%,	4321/9641/	GO_BP	MMP12/IKBKE/IRAK1/MX2/SCRIB/TRIM6/GBP2/
7144	tags=56%,	4321/9641/	GO_BP	MMP12/IKBKE/IRAK1/MX2/SCRIB/TRIM6/GBP2/
7063	tags=55%,	4321/3838/	GO_BP	MMP12/KPNA2/ECT2/TGFB1/FLNA/SNRPB/PRKCD
3177	tags=43%,	6509/2063	GO_BP	SLC1A4/SLC36A1/SLC36A4
2144	tags=57%,	330/83596/	GO_BP	BIRC3/BCL2L12/NOL3/BIRC7
6454	tags=56%,	9245/7040/	GO_BP	GCNT3/TGFB1/EXO1/CLCF1/BATF/HLA-DQB1/TF
1806	tags=16%,	5347/9232/	GO_BP	PLK1/PTTG1/TRIP13/CCNE1/P3H4/FANCD2/ESP
3954	tags=38%,	100133941/	GO_BP	CD24/SRC/UNC119/ADORA1/PRNP/DLG3/CSF1R/
6181	tags=49%,	7040/2363	GO_BP	TGFB1/NUP62/RAN/LIN28B/NUP210/MAP2K2/PO
5871	tags=60%,	112616/39	GO_BP	CMTM7/LGALS1/ADA/SLAMF8/BCL3/CDH17/GPR1
4239	tags=38%,	5347/983/	GO_BP	PLK1/CDK1/SRC/LIF/ECT2/TGFB1/FLNA/PRKCD
620	tags=33%,	320/18/65	GO_BP	APBA1/ABAT/SLC6A1
3876	tags=41%,	81620/510	GO_BP	CDT1/GMNN/PYGO2/DDX11/KDM1A/MED25/NFATC
5879	tags=59%,	100/12392	GO_BP	ADA/CMTM3/SLC39A10/NFAM1/LCK/RPS3/PRKD2
6431	tags=52%,	5984/55247	GO_BP	RFC4/NEIL3/POLD1/RBBP8/POLR2G/PCNA/RFC2
5644	tags=43%,	2539/2056/	GO_BP	G6PD/EPO/SPI1/PRMT1/HMGB2/JAK3/FLVCR1/H
4535	tags=41%,	7040/3161/	GO_BP	TGFB1/HMMR/GPC4/GPC3/CD44/AGRN/SDC3/VCA
5310	tags=57%,	27242/230	GO_BP	TNFRSF21/FOXJ1/TYROBP/PKN1/HMGB3/CD300A
3964	tags=39%,	23643/704	GO_BP	LY96/TGFB1/RIPK2/IRAK1/PTAFR/HCK/IL18/C
6276	tags=46%,	3880/4192/	GO_BP	KRT19/MDK/SOX9/POSTN/DLGAP5/PTP4A3/MMP1
4603	tags=39%,	4192/3965/	GO_BP	MDK/LGALS9/RIPK2/PRKCD/ACP5/SYK/PLCB1/L
6566	tags=44%,	3984/1627/	GO_BP	LIMK1/DBN1/POSTN/REG1A/PLXNA1/SEMA6A/AR
6792	tags=51%,	891/3159/	GO_BP	CCNB1/HMGA1/CDC45/MKI67/LIF/TGFB1/SPI1/
2852	tags=30%,	5738/9518/	GO_BP	PTGFRN/GDF15/PLEKHO1/CD53/FLOT1/EHD1/CX
4274	tags=34%,	6692/1159	GO_BP	SPINT1/CTHRC1/SOX4/TEAD2/SPINT2/TGFB1/F
1289	tags=33%,	5164/1500	GO_BP	PDK2/SIK1/SIRT1/C1QTNF3/PPARA/KAT2B/FOX
1251	tags=50%,	10786/566	GO_BP	SLC17A3/SLC2A9/SLC17A1/GCKR/ABCG2/G6PC
4413	tags=47%,	3383/960/	GO_BP	ICAM1/CD44/PTAFR/PRKCD/HLA-DRB1/HLA-DQA
4291	tags=35%,	1869/11151	GO_BP	E2F1/CORO1A/MICALL1/ARPC3/SORT1/RAB35/E
645	tags=43%,	338440/57	GO_BP	ANO9/PTAFR/PRNP
676	tags=31%,	10400/259	GO_BP	PEMT/GAMT/BHMT2/MAT1A/GNMT
5566	tags=51%,	2810/1869/	GO_BP	SFN/E2F1/BAK1/YWHAZ/HK2/BAX/SLC9A1/SLC3

6258	tags=57%,	11031/8387	GO_BP	RAB31/RAB34/RAB6B/RAB32/RAB15/RAB25/RAB
2352	tags=47%,	960/972/4	GO_BP	CD44/CD74/MUC1/ZNF385A/MIF/BCL2L12/KDM1
6566	tags=52%,	6696/57556	GO_BP	SPP1/SEMA6A/ARHGAP4/PLXNA3/LINGO1/SEMA4
1511	tags=29%,	991/113130	GO_BP	CDC20/CDCA5/DSCC1/NAA10/NSMCE2/SGO1
3727	tags=43%,	54209/1075	GO_BP	TREM2/CTSC/HAVCR2/TNIP2/IL4R/LBP/CCL3/H
908	tags=37%,	54884/3157	GO_BP	RETSAT/HMGCS1/ALDH3A2/CYP2C18/APOE/APOC
525	tags=27%,	991/1627/1	GO_BP	CDC20/DBN1/CPLX2
4189	tags=50%,	23643/9641	GO_BP	LY96/IKBKE/BIRC3/IRF3/IRF7/CD300LF/UBE2
6037	tags=68%,	6364/29108	GO_BP	CCL20/PYCARD/STK39/CCL5/ADAM8/CXCL13/XC
563	tags=53%,	216/26007/	GO_BP	ALDH1A1/TKFC/GLYCTK/KHK/PFKFB1/SORD/FBP
764	tags=47%,	1149/11488	GO_BP	CIDEA/OSBPL11/PPARA/ENPP1/FITM1/APOC4/P
1484	tags=40%,	1215/56670	GO_BP	CMA1/SUCNR1/CTSG/AGTR1/ACE2/CES1/ENPEP/
759	tags=28%,	2643/10588	GO_BP	GCH1/MTHFS/SHMT1/ALDH1L1/MTHFD1/PIPOX/F
1464	tags=19%,	8864/6530/	GO_BP	PER2/SLC6A2/DRD1/SLC1A2/SLC6A1
1155	tags=80%,	11160/1061	GO_BP	ERLIN2/ERLIN1/SOD1/APOE
1155	tags=80%,	11160/1061	GO_BP	ERLIN2/ERLIN1/SOD1/APOE
1155	tags=80%,	11160/1061	GO_BP	ERLIN2/ERLIN1/SOD1/APOE
6436	tags=58%,	1535/1410/	GO_BP	CYBA/CRYAB/BAK1/FANCD2/HRAS/HSF1/BAX/PR
2246	tags=25%,	2119/7040/	GO_BP	ETV5/TGFB1/FLNA/ITGB2/AGRN/SLC7A11/DLG3
6450	tags=77%,	27347/6352	GO_BP	STK39/CCL5/SLC12A2/CCL26/PIK3CD/CXCL13/
3362	tags=32%,	6280/2919/	GO_BP	S100A9/CXCL1/CXCL6/LCN2/PGC/CXCL8/CXCL5
6486	tags=49%,	5984/54913	GO_BP	RFC4/RPP25/EXO1/BOP1/RNASEH2A/FEN1/POLD
3813	tags=36%,	8877/54209	GO_BP	SPHK1/TREM2/PYCARD/LGALS9/TYROBP/RIPK2/
1188	tags=19%,	6690/23236	GO_BP	SPINK1/PLCB1/ZP3
5598	tags=69%,	1601/9094/	GO_BP	DAB2/UNC119/DNAJC6/HIP1R/DNM2/TNK2/AAK1
3756	tags=26%,	4316/891/4	GO_BP	MMP7/CCNB1/ASNS/CYBA/MDK/SOX9/SLC2A1/CO
1471	tags=24%,	6662/10083	GO_BP	SOX9/USH1C/PROM1/THY1
5765	tags=71%,	57402/6352	GO_BP	S100A14/CCL5/CREB3/SERPINE1/PLA2G7/CCR1
2472	tags=28%,	4192/10631	GO_BP	MDK/POSTN/PLXNA1/SRC/GSTP1/DDR1/PLAU/IG
3774	tags=42%,	4321/1277/	GO_BP	MMP12/COL1A1/POSTN/SRC/MMP14/MELTF/FBLN
5037	tags=50%,	4323/56833	GO_BP	MMP14/SLAMF8/SYK/HMGB3/NCKAP1L/CARD11/N
6340	tags=64%,	3159/55247	GO_BP	HMGA1/NEIL3/FEN1/POLD1/SIRT6/APEX2/PCNA
4243	tags=49%,	2119/9181/	GO_BP	ETV5/ARHGEF2/ASPM/FZD7/NUMBL/LEF1/FGFR1
644	tags=36%,	10551/1535	GO_BP	AGR2/CYBA/ADA/ADORA1
1188	tags=27%,	6662/6714/	GO_BP	SOX9/SRC/ZP3
3648	tags=32%,	8877/1728/	GO_BP	SPHK1/NQO1/PYCR1/LCN2/CRYAB/CDK1/COL1A1
5169	tags=56%,	6364/29108	GO_BP	CCL20/PYCARD/STK39/CCL5/ADAM8/CXCL13/CC
3246	tags=44%,	56833/9296	GO_BP	SLAMF8/ATP6V1F/ATP6V0B/ATP6V0D2/ATP6V1C
2791	tags=47%,	3159/6604/	GO_BP	HMGA1/SMARCD3/SMARCD1/MAP1S/SMARCA4/SET
1917	tags=57%,	29923/6279	GO_BP	HILPDA/S100A8/FZD1/CD68
3723	tags=71%,	3561/3718/	GO_BP	IL2RG/JAK3/CD300LF/IL4R/PTPN2
4797	tags=47%,	2810/1869/	GO_BP	SFN/E2F1/BAK1/YWHAZ/BAX/SLC9A1/SLC35F6/
7686	tags=71%,	3838/23636	GO_BP	KPNA2/NUP62/RAN/NUP210/VPS37C/NUP93/NUP
2768	tags=80%,	65010/6558	GO_BP	SLC26A6/SLC12A2/P2RY6/BEST1
3505	tags=100%,	1736/10576	GO_BP	DKC1/CCT2/CCT4/TCP1/WRAP53
2865	tags=60%,	7076/397/8	GO_BP	TIMP1/ARHGDIB/CALR
4451	tags=80%,	3614/4830/	GO_BP	IMPDH1/NME1/NME2/IMPDH2/NME6/NME7/NME3/
4390	tags=52%,	23149/5080	GO_BP	FCHO1/ASAP1/MICALL1/PLEKHM2/EHD2/BIN2/D
4626	tags=52%,	116372/160	GO_BP	LYPD1/DAB2/TGFB1/RHOQ/PKDCC/AP2M1/CSK/T
2803	tags=50%,	6692/10653	GO_BP	SPINT1/SPINT2/ST14/SOCS3/FGFR2/GRB2
5009	tags=39%,	8877/1728/	GO_BP	SPHK1/NQO1/PYCR1/LCN2/CDK1/SRC/MAPK13/E

4477	tags=38%,	9168/822/1	GO_BP	TMSB10/CAPG/CRYAB/PFN2/STMN1/SRC/DKK1/PI
7144	tags=55%,	4321/9641/	GO_BP	MMP12/IKBKE/IRAK1/MX2/SCRIB/TRIM6/GBP2/I
7686	tags=71%,	3838/23636	GO_BP	KPNA2/NUP62/RAN/NUP210/VPS37C/NUP93/NUP
202	tags=27%,	5047/27242	GO_BP	PAEP/TNFRSF21/LGALS9
6450	tags=49%,	2302/7305/	GO_BP	FOXJ1/TYROBP/CYBB/IGF2BP2/CD74/PTAFR/HSI
1251	tags=25%,	8864/5174/	GO_BP	PER2/PDZK1/SLC17A3/SLC25A18/SLC16A12/SL(
778	tags=19%,	948/32/348	GO_BP	CD36/ACACB/APOE/FITM1/LIPG/APOC4/C3/PLII
5500	tags=39%,	22974/2056	GO_BP	TPX2/EPO/DUSP9/CDK1/SRC/ECT2/IQGAP3/STK
4015	tags=41%,	79574/3383	GO_BP	EPS8L3/ICAM1/PFN2/RCC2/PLEK/HRAS/CSF1R/I
2970	tags=67%,	578/4237/5	GO_BP	BAK1/MFAP2/BAX/HMGN1
2103	tags=33%,	100133941/	GO_BP	CD24/FOXJ1/IQGAP1/PROM1/BASP1/JAG1
2924	tags=44%,	6692/10653	GO_BP	SPINT1/SPINT2/TRIM28/ST14/LEF1/WNT7B/CDI
1113	tags=51%,	9/4257/679	GO_BP	NAT1/MGST1/SULT1A2/FMO2/ACSM1/MGST2/GSTI
3190	tags=43%,	10397/215/	GO_BP	NDRG1/ABCD1/CXCR4/MYRF/FA2H/EPB41L3
797	tags=27%,	717/5627/7	GO_BP	C2/PROS1/C5/SERPING1/C1S/CFI/CPN1/HPN/CI
5194	tags=64%,	54503/2544	GO_BP	ZDHHC13/SLC41A1/TUSC3/CNNM4/MMGT1/NIPAL
4297	tags=33%,	388610/669	GO_BP	TRNP1/SPINT1/MDK/DBN1/MELK/SPINT2/TGFB1/
2665	tags=36%,	29108/960/	GO_BP	PYCARD/CD44/CD74/MUC1/ZNF385A/MIF/NUPR1/
545	tags=19%,	5295/6715/	GO_BP	PIK3R1/SRD5A1/ASS1/SOCS2/F7/GHR/CPS1
2103	tags=29%,	100133941/	GO_BP	CD24/FOXJ1/IQGAP1/PROM1/BASP1/JAG1
3955	tags=57%,	991/5347/2	GO_BP	CDC20/PLK1/UBE2S/MAD2L1/MAD2L2/FBXO5/DNI
2865	tags=80%,	3108/3122/	GO_BP	HLA-DMA/HLA-DRA/HLA-DMB/CALR
146	tags=40%,	29089/2733	GO_BP	UBE2T/UBE2S
3813	tags=36%,	8877/54209	GO_BP	SPHK1/TREM2/PYCARD/LGALS9/TYROBP/RIPK2/I
4797	tags=48%,	2810/1869/	GO_BP	SFN/E2F1/BAK1/YWHAZ/BAX/SLC35F6/BMF/HIP
6479	tags=38%,	6696/7345/	GO_BP	SPP1/UCHL1/S100A6/RAP1GAP/LIMK1/DBN1/STI
3842	tags=32%,	6280/11239	GO_BP	S100A9/EGLN3/PLK1/TREM2/PYCARD/ENO1/CTS(
3692	tags=38%,	5361/7040/	GO_BP	PLXNA1/TGFB1/LAMA5/EDA/FGFR1/TGM2/PDGFA/
5315	tags=36%,	100133941/	GO_BP	CD24/CYBA/EPO/BAK1/ITPR3/NMB/LPAR2/TGFB
4950	tags=46%,	7040/9156/	GO_BP	TGFB1/EXO1/CLCF1/BATF/TFRC/TCF3/PRKDC/M
3835	tags=82%,	3684/3689/	GO_BP	ITGAM/ITGB2/PTAFR/SYK/GRN/CD300A/ABR/FC(
4398	tags=36%,	5538/6616/	GO_BP	PPT1/SNAP25/SLC7A11/NPTX2/NRGN/BRSK1/RG
6757	tags=59%,	1401/29108	GO_BP	CRP/PYCARD/LGALS9/CD74/SYK/TMSB4X/DDIT3/
2206	tags=50%,	5901/4869/	GO_BP	RAN/NPM1/RRS1/ZNF593
1	tags=12%,	6696	GO_BP	SPP1
4277	tags=75%,	1601/8898/	GO_BP	DAB2/MTMR2/EZR/MSN/PTPN23/VPS11
1275	tags=29%,	23411/9971	GO_BP	SIRT1/NR1H4/EGR1/ELOVL6/ACADVL/NFYB/ERL
3509	tags=55%,	8754/6850/	GO_BP	ADAM9/SYK/CCL5/CD3E/FERMT1/NCKAP1L/CXCL
4573	tags=50%,	2810/4831/	GO_BP	SFN/NME2/SULT2B1/VDR/OVOL2/KDF1/NOTCH1/
4258	tags=55%,	23616/2302	GO_BP	SH3BP1/FOXJ1/SCRIB/FSCN1/MSN/PTK7/SLC9A
4070	tags=36%,	5973/4321/	GO_BP	RENBP/MMP12/GLA/NUDT1/TGFB1/ADA/HMMR/GNI
1442	tags=39%,	153/135/36	GO_BP	ADRB1/ADORA2A/ITGA1/SOD1/GCH1/EDNRB/APOI
5140	tags=62%,	1627/4794/	GO_BP	DBN1/NFKBIE/TONSL/FLNA/TMSB4X/MDFI/SRI/(
3362	tags=44%,	4192/3576/	GO_BP	MDK/CXCL8/RAC2/S100A14/CD74/C3AR1/NCKAP
4535	tags=42%,	7040/3161/	GO_BP	TGFB1/HMMR/GPC4/GPC3/CD44/AGRN/SDC3/VCAI
819	tags=47%,	435/2752/2	GO_BP	ASL/GLUL/GLUD1/ASS1/NAGS/GLS2/OTC/CPS1
905	tags=42%,	3949/84649	GO_BP	LDLR/DGAT2/PLIN5/GPLD1/RGN
764	tags=50%,	1149/11488	GO_BP	CIDEA/OSBPL11/PPARA/FITM1/APOC4/PLIN5
677	tags=33%,	7367/7363/	GO_BP	UGT2B17/UGT2B4/UGT2B7/UGT2B15
2013	tags=27%,	6692/4192/	GO_BP	SPINT1/MDK/LIF/SERPINE2/TGFB1/CLCF1/CXCI
4825	tags=44%,	2305/5580/	GO_BP	FOXM1/PRKCD/EEF1E1/ANKRD1/ZNF385A/NPAS2/

4189	tags=44%,	23643/9641GO_BP	LY96/IKBKE/BIRC3/IRF3/IRF7/CD300LF/UBE2I
7806	tags=54%,	8877/55353GO_BP	SPHK1/LAPTM4B/ANXA2/CTSC/DNM1/SERPINE2/(
1060	tags=43%,	5020/552/1GO_BP	OXT/AVPR1A/CRHBP
1254	tags=60%,	1841/7298/GO_BP	DTYMK/TYMS/DCTPP1
1320	tags=39%,	26291/150(GO_BP	FGF21/SIK1/SIRT1/MOGAT1/ABHD2/CETP/LDLR/
908	tags=34%,	54884/223(GO_BP	RETSAT/FDX1/SHH/CYP11A1/CYP2C18/CYP17A1/
8299	tags=100%,	79339/2621GO_BP	OR51B4/OR2B6/OR1F1/OR2C3/OR51E2/OR7A5/OI
1239	tags=36%,	27244/9973GO_BP	SESN1/CCS/MT1H/ESD/MT1F/GSTO1/ADH5/MT1B/
797	tags=27%,	717/5627/7GO_BP	C2/PROS1/C5/SERPING1/C1S/CFI/CPN1/HPN/CI
7115	tags=45%,	54997/542(GO_BP	TESC/TREM2/LGALS3/LIF/CSF1/TYROBP/TGFB1/
3106	tags=62%,	5580/4869/GO_BP	PRKCD/NPM1/DDX11/RPS3/GZMA
7622	tags=68%,	6949/11249GO_BP	TCOF1/GTF3C6/NABP2/INTS8/TAF6/POLR2G/CD3
1500	tags=29%,	664/11221/GO_BP	BNIP3/DUSP10/ADRB1/SLC2A4/SIRT1/NUDT7/PI
1379	tags=35%,	10580/9852GO_BP	SORBS1/EPM2AIP1/IGF1/ENPP1/IRS1/IRS2/GCI
6914	tags=53%,	126789/958GO_BP	PUSL1/APOBEC3B/WDR4/APOBEC3C/RPUSD1/JMJI
6995	tags=53%,	6714/1601/GO_BP	SRC/DAB2/CORO1A/MTMR2/YKT6/EZR/SNX8/RAB(
1736	tags=24%,	7345/54209GO_BP	UCHL1/TREM2/CSF1/SQSTM1/EIF4EBP1/HK2/KCI
890	tags=27%,	3157/1543/GO_BP	HMGCS1/CYP1A1/ALDH8A1/HMGCS2
2103	tags=25%,	4318/10013GO_BP	MMP9/CD24/FOXJ1/LIF/IQGAP1/PROM1/PRKX/B/
6999	tags=71%,	3965/8767/GO_BP	LGALS9/RIPK2/ELF4/SYK/IL18/EBI3/CD3E/IRI
6450	tags=49%,	2302/7305/GO_BP	FOXJ1/TYROBP/CYBB/IGF2BP2/CD74/PTAFR/HSI
4594	tags=22%,	2810/3880/GO_BP	SFN/KRT19/TGM3/KRT23/DSG2/CTSV/KRT17/PI3
103	tags=33%,	8993/11477GO_BP	PGLYRP1/PGLYRP2
103	tags=33%,	8993/11477GO_BP	PGLYRP1/PGLYRP2
842	tags=33%,	364/366	GO_BP AQP7/AQP9
4114	tags=32%,	2810/1515/GO_BP	SFN/CTSV/KRT17/MAFG/CD109/EZH2/NME2/ESRI
6686	tags=57%,	7040/23636GO_BP	TGFB1/NUP62/NUP210/MAP2K2/POLR2G/NUP93/I
4062	tags=86%,	22943/8324GO_BP	DKK1/FZD7/COL5A1/COL5A2/SFRP2/MESP1
2863	tags=28%,	483/476/73GO_BP	ATP1B3/ATP1A1/UPK3A/SLC12A2/SLC12A9/SLC
7621	tags=61%,	3984/6659/GO_BP	LIMK1/SOX4/TSPO/MAD2L1/MAD2L2/ISG15/COPS
2909	tags=50%,	1601/7040/GO_BP	DAB2/TGFB1/NUP93/PARP1/TGFB3/RBPMS
1221	tags=64%,	83594/5702GO_BP	NUDT12/PDXP/CYP4F11/MTHFS/NUDT7/ACAT1/AI
954	tags=30%,	326625/259GO_BP	MMAB/MMACHC/PNPO/MMAA/CYP27A1/RGN/CYP3A4
4119	tags=38%,	2810/10013GO_BP	SFN/CD24/SOX9/FOXJ1/LIF/S1PR2/NME2/PROM
1493	tags=41%,	4792/4035/GO_BP	NFKBIA/LRP1/SIRT1/APOE/LIPG/SCP2/ABCB4/I
1493	tags=41%,	4792/4035/GO_BP	NFKBIA/LRP1/SIRT1/APOE/LIPG/SCP2/ABCB4/I
1100	tags=22%,	11261/3373GO_BP	CHP1/HYAL1/SLC38A3/GPLD1/PCK1
8557	tags=67%,	63875/6183GO_BP	MRPL17/MRPS12/MRPL47/MRPL9/MRPS23/MRPL1:
6625	tags=54%,	22883/6809GO_BP	CLSTN1/STX3/SNAP25/RAB31/SCRIB/RAB34/ANI
3800	tags=44%,	9518/963/1GO_BP	GDF15/CD53/FLOT1/EHD1/CXCL9/EHD2/IL4R/C/
5036	tags=42%,	5538/4097/GO_BP	PPT1/MAFG/SLAMF8/ATP6V1F/SLC26A6/GRN/ATI
1990	tags=38%,	4323/1311/GO_BP	MMP14/COMP/SIRT6/FGFR3/SIX2
5226	tags=43%,	4321/8767/GO_BP	MMP12/RIPK2/HAVCR2/IRF3/STAT1/IRF7/HSPD
5255	tags=42%,	2207/7305/GO_BP	FCER1G/TYROBP/STX3/TAX1BP3/RAB11FIP5/GPI
6174	tags=40%,	54997/542(GO_BP	TESC/TREM2/LIF/CSF1/TYROBP/TGFB1/C1QC/CI
3833	tags=67%,	50807/232(GO_BP	ASAP1/PLEKHM2/DNM2/SH3GLB1
6603	tags=52%,	4192/3965/GO_BP	MDK/LGALS9/RIPK2/ACP5/SYK/PLCB1/LTB/JAK3
4643	tags=43%,	29108/1063GO_BP	PYCARD/POSTN/LGALS9/RIPK2/HAVCR2/CD74/S'
1140	tags=28%,	4321/2316/GO_BP	MMP12/FLNA/CD44/DDR1/CD151/RHOC/COL5A1/
6234	tags=42%,	2302/27237GO_BP	FOXJ1/ARHGEF16/ECT2/RCC2/TAX1BP3/SCRIB/I
5876	tags=56%,	27350/1178GO_BP	APOBEC3C/TRIM6/TRIM28/CCL5/TRIM11/SLPI/

688	tags=14%,	10551/153	GO_BP	AGR2/CYBA/ADA/ADORA1/STK39
202	tags=30%,	5047/2724	GO_BP	PAEP/TNFRSF21/LGALS9
7804	tags=57%,	55353/302/	GO_BP	LAPTM4B/ANXA2/ATP13A2/PPT1/CORO1A/GRN/R
3465	tags=67%,	1601/2294	GO_BP	DAB2/DKK1/GPC3/MLLT3/PLEKHA4/ABL1
6181	tags=47%,	7040/2363	GO_BP	TGFB1/NUP62/RAN/LIN28B/NUP210/MAP2K2/PO
3698	tags=50%,	1728/9056/	GO_BP	NQO1/SLC7A7/SLC7A11/PSMB9/PSMD14/PSMD2/
2974	tags=27%,	3965/8486	GO_BP	LGALS9/HAVCR2/CORO1A/UNC13D/IL18/TUBB/S
4797	tags=46%,	2810/1869/	GO_BP	SFN/E2F1/BAK1/YWHAZ/BAX/SLC35F6/BMF/HIP
6229	tags=50%,	7345/9493/	GO_BP	UCHL1/KIF23/TMEM201/KIF3C/DTNBP1/MAP2/H
884	tags=64%,	5565/1376/	GO_BP	PRKAB2/CPT2/PRKAG2/ACACB/SLC25A20/CPT1A
483	tags=40%,	8660/1099	GO_BP	IRS2/SLC27A5
3228	tags=43%,	3965/5683	GO_BP	LGALS9/SLAMF8/TRPM2/CCL5/CXCR4/CCR1/CAL
3259	tags=42%,	960/972/9	GO_BP	CD44/CD74/EEF1E1/ANKRD1/ZNF385A/MIF/SPR
3753	tags=53%,	3675/5621/	GO_BP	ITGA3/PRNP/ITPKA/MTMR2/CTTN/HOMER1/ZMYN
5315	tags=38%,	1535/2056/	GO_BP	CYBA/EPO/BAK1/ITPR3/TGFB1/CORO1A/P2RX4/
3509	tags=47%,	1601/2294	GO_BP	DAB2/DKK1/GPC3/MLLT3/PLEKHA4/ABL1/SFRP2
1320	tags=39%,	26291/150	GO_BP	FGF21/SIK1/SIRT1/MOGAT1/ABHD2/CETP/LDLR
663	tags=20%,	4843/135/	GO_BP	NOS2/ADORA2A/PLA2G12A/TNFSF11/SLC38A2/S
6199	tags=49%,	2810/5880/	GO_BP	SFN/RAC2/ECT2/TGFB1/ITGAM/FLNA/ITGB2/CE
7150	tags=59%,	2207/972/	GO_BP	FCER1G/CD74/IL18/TRAF2/TRIM6/MIF/MAPK3/
4291	tags=33%,	1869/11151	GO_BP	E2F1/CORO1A/MICALL1/ARPC3/SORT1/RAB35/E
3398	tags=50%,	7187/5716	GO_BP	TRAF3/PELI1/PELI3
3723	tags=83%,	1435/1436/	GO_BP	CSF1/CSF1R/DOK1/MST1R/PTPN2
3953	tags=50%,	3297/3298/	GO_BP	HSF1/HSF2/HSF4
3813	tags=38%,	8877/5420	GO_BP	SPHK1/TREM2/PYCARD/LGALS9/TYROBP/RIPK2/
5771	tags=47%,	5570/9212/	GO_BP	PKIB/AURKB/NEK2/NABP2/PRKDC/MAPK3/ACD/P
4367	tags=43%,	4192/1277/	GO_BP	MDK/COL1A1/DAB2/TGFB1/BAMBI/EZH2/GLIPR2
3072	tags=43%,	302/4001/	GO_BP	ANXA2/LMNB1/RIPK2/PLCB1/CFL1/SNRPA1/MIF
1080	tags=26%,	6513/2056/	GO_BP	SLC2A1/EPO/AKR1B1/PKN1/ARHGEF2/YBX3/SLC
2356	tags=57%,	5888/8438/	GO_BP	RAD51/RAD54L/XRCC3/SWI5
4013	tags=30%,	4316/1535/	GO_BP	MMP7/CYBA/AURKB/SOX9/SLC2A1/EPO/CRYAB/C
4013	tags=30%,	4316/1535/	GO_BP	MMP7/CYBA/AURKB/SOX9/SLC2A1/EPO/CRYAB/C
1861	tags=50%,	578/581/7	GO_BP	BAK1/BAX/TGM2/BCAP31
830	tags=38%,	54209/151	GO_BP	TREM2/CTSV/CDKN2D
3668	tags=42%,	4192/3383/	GO_BP	MDK/ICAM1/PTAFR/PLCB1/IL27RA/THY1/ADAM8
5182	tags=38%,	8877/991/	GO_BP	SPHK1/CDC20/PLK1/UBE2S/TRIB3/MAGEC2/FAN
5485	tags=68%,	6628/5451	GO_BP	SNRPB/EXOSC4/SNRPE/NCBP2/SNRPF/LSM10/SS
4453	tags=68%,	5973/9137	GO_BP	RENBP/UAP1L1/GNPDA1/GFPT2/TSTA3/UXS1/NA
3435	tags=32%,	994/995/2	GO_BP	CDC25B/CDC25C/PLCB1/CDC25A/MSX1/WNT4/LF
6306	tags=62%,	3838/7040/	GO_BP	KPNA2/TGFB1/GAPDH/TYMS/ITGAV/SCRIB/ZC3H
5475	tags=44%,	115908/41	GO_BP	CTHRC1/MDK/SOX9/DAB2/DKK1/GPC3/BICC1/FZ
5830	tags=63%,	6372/2207/	GO_BP	CXCL6/FCER1G/LGALS9/RAC2/ITGAM/ITGB2/PT
3756	tags=50%,	3976/1435/	GO_BP	LIF/CSF1/TGFB1/IL34/HCLS1/FADD/CASP8
6656	tags=79%,	3965/6352/	GO_BP	LGALS9/CCL5/PRELID1/PDCD1/ADAM8/IDO1/LG
425	tags=43%,	23743/552	GO_BP	BHMT2/ADI1/BHMT
425	tags=43%,	23743/552	GO_BP	BHMT2/ADI1/BHMT
425	tags=43%,	23743/552	GO_BP	BHMT2/ADI1/BHMT
483	tags=29%,	8660/8529	GO_BP	IRS2/CYP4F2
5806	tags=61%,	27350/117	GO_BP	APOBEC3C/TRIM6/TRIM28/CCL5/SLPI/ISG15/Z
908	tags=27%,	8877/5420	GO_BP	SPHK1/TREM2/CTSC/GRN
3727	tags=35%,	8877/5420	GO_BP	SPHK1/TREM2/CTSC/HAVCR2/CD74/GRN/VSIG4/

3063	tags=32%,	9133/3965/	GO_BP	CCNB2/LGALS9/TGFB1/CORO1A/JAK3/BAX/TCIR
1320	tags=47%,	26291/9971	GO_BP	FGF21/NR1H4/APOC2/LIPG/GPIHBP1/APOC1/GP
7177	tags=64%,	6633/6632/	GO_BP	SNRPD2/SNRPD1/SF3A2/NOL3/SNRPE/TXNL4A/S
6181	tags=57%,	2810/1869/	GO_BP	SFN/E2F1/BAK1/YWHAZ/BAX/SLC35F6/BMF/NOL
7684	tags=53%,	29089/273	GO_BP	UBE2T/UBE2S/CCNF/DTL/BLMH/RUSC1/PSMB9/T
6730	tags=47%,	8877/2910	GO_BP	SPHK1/PYCARD/FOXM1/CRYAB/DUSP9/LGALS9/M
4792	tags=35%,	23616/547	GO_BP	SH3BP1/FBLIM1/ITGB4/SRC/MMP14/POF1B/ECT
1959	tags=50%,	4521/100/7	GO_BP	NUDT1/ADA/DCTPP1/ITPA
2401	tags=30%,	7301/1192/	GO_BP	TYRO3/CLIC1/SERPINE2/DSG2/LGALS1/FLNA/F
2852	tags=33%,	4323/9518/	GO_BP	MMP14/GDF15/CD53/FLOT1/EHD1/CXCL9/EHD2/
4258	tags=38%,	23616/1741	GO_BP	SH3BP1/DLG3/SCRIB/MTCL1/MSN/PTK7/PDZD11
752	tags=20%,	79703	GO_BP	C11orf80
6166	tags=56%,	2316/6949/	GO_BP	FLNA/TCOF1/MAPK3/CD3EAP/SMARCA4/DDX11/P
4693	tags=39%,	4605/9242/	GO_BP	MYBL2/MSC/PFKP/ICAM1/PTP4A3/LAPTM5/SRM/
4693	tags=39%,	4605/9242/	GO_BP	MYBL2/MSC/PFKP/ICAM1/PTP4A3/LAPTM5/SRM/
2486	tags=25%,	54997/542	GO_BP	TESC/TREM2/LIF/CSF1/TYROBP/TGFB1/CD74/I
4797	tags=48%,	2810/1869/	GO_BP	SFN/E2F1/BAK1/YWHAZ/BAX/SLC35F6/BMF/HIP
6757	tags=61%,	1401/2910	GO_BP	CRP/PYCARD/LGALS9/CD74/SYK/TMSB4X/DDIT3
2510	tags=39%,	8877/2717/	GO_BP	SPHK1/GLA/PPT1/PRKCD/NEU1/GM2A/SMPD2/SM
4520	tags=50%,	2207/6850/	GO_BP	FCER1G/SYK/FGR/IL4R/VAMP8/STXBP1/ADORA2
4520	tags=50%,	2207/6850/	GO_BP	FCER1G/SYK/FGR/IL4R/VAMP8/STXBP1/ADORA2
5170	tags=57%,	6628/1478/	GO_BP	SNRPB/CSTF2/SNRPE/NCBP2/SNRPF/LSM10/CPS
4603	tags=40%,	4192/3965/	GO_BP	MDK/LGALS9/RIPK2/SYK/PLCB1/LTB/IRF1/MAP
2279	tags=23%,	6696/2056/	GO_BP	SPP1/EPO/CDK1/TSPO/TYROBP/LGALS1/FOLR1/
5799	tags=39%,	440/1535/	GO_BP	ASNS/CYBA/AURKB/CRYAB/PLEKHB1/ICAM1/BAK
6544	tags=47%,	7345/5347/	GO_BP	UCHL1/PLK1/APOA4/LPCAT1/LAPTM4B/TREM2/H
1372	tags=30%,	94121/640	GO_BP	SYTL4/SELP/ITIH4/TIMP3/IGF1/CLU/CDC37L1
1084	tags=17%,	6446/5020/	GO_BP	SGK1/OXT/LDLR/ASAH2/ACO1/WNK3/CD36/LRCO
1354	tags=62%,	26063/138	GO_BP	DECR2/CRAT/ETFA/DECR1/PCCA/ACADVL/ABHD2
4626	tags=48%,	116372/16	GO_BP	LYPD1/DAB2/TGFB1/RHOQ/PKDCC/GDI1/ITGB1B
7249	tags=66%,	6688/4582/	GO_BP	SPI1/MUC1/NAA40/RUVBL1/BRCA1/MCRS1/ACTL
7168	tags=52%,	6688/2614	GO_BP	SPI1/PHF19/SNRPB/EZH2/DNMT1/PRMT1/WDR4/
555	tags=27%,	8864/135/	GO_BP	PER2/ADORA2A/ACE2/AVPR1A/KMO/ARG1/SLC38
6453	tags=47%,	1401/5047/	GO_BP	CRP/PAEP/PYCARD/MDK/TNFRSF21/MMP12/POST
3813	tags=36%,	8877/5420	GO_BP	SPHK1/TREM2/PYCARD/LGALS9/TYROBP/RIPK2/
302	tags=37%,	1806/9615/	GO_BP	DPYD/GDA/SHMT1/XDH/MTHFD1/AOX1/TTR
4597	tags=33%,	1277/3691/	GO_BP	COL1A1/ITGB4/SRC/CSF1/TGFB1/COL1A2/LAMB
4091	tags=52%,	1601/2294	GO_BP	DAB2/DKK1/GPC3/MLLT3/CSNK1E/PLEKHA4/CSN
6181	tags=47%,	7040/2363	GO_BP	TGFB1/NUP62/RAN/LIN28B/NUP210/MAP2K2/PO
4337	tags=27%,	6696/2539/	GO_BP	SPP1/G6PD/PAEP/RGS2/SOX9/POSTN/ITM2C/TS
759	tags=39%,	2643/1058	GO_BP	GCH1/MTHFS/SHMT1/ALDH1L1/MTHFD1/PIPOX/F
7680	tags=61%,	899/5698/1	GO_BP	CCNF/PSMB9/PSMD14/PSMD2/PSMB2/PSME3/KIF
2867	tags=50%,	5580/7186/	GO_BP	PRKCD/TRAF2/CCL2/CDK5/PRKCI
5363	tags=62%,	9040/1506	GO_BP	UBE2M/COPS9/DCUN1D5/RBX1/RNF7/NAE1/RPL5
1115	tags=41%,	23464/679	GO_BP	GCAT/SULT1A2/PRDX3/PCBD1/AMT/HBA2/FAH/M
6199	tags=48%,	1869/5554	GO_BP	E2F1/RBM38/IGF2BP1/PKP3/YBX3/MAPKAPK2/D
3315	tags=50%,	6662/7849/	GO_BP	SOX9/PAX8/WNT4
986	tags=41%,	255027/86	GO_BP	MPV17L/CBFA2T3/FYN/PPARA/HDAC6/PPARGC1A
4451	tags=78%,	4830/4831/	GO_BP	NME1/NME2/CAD/NME6/NME7/NME3/NME4
927	tags=19%,	56898/166	GO_BP	BDH2/MMAA/AMN/CLYBL/ALAS1/ALAD/AMBP/CYP
3240	tags=30%,	6510/3576/	GO_BP	SLC1A5/CXCL8/SLC52A2/CDK1/ICAM1/LGALS9/

3240	tags=30%,	6510/3576/	GO_BP	SLC1A5/CXCL8/SLC52A2/CDK1/ICAM1/LGALS9/
3240	tags=30%,	6510/3576/	GO_BP	SLC1A5/CXCL8/SLC52A2/CDK1/ICAM1/LGALS9/
3240	tags=30%,	6510/3576/	GO_BP	SLC1A5/CXCL8/SLC52A2/CDK1/ICAM1/LGALS9/
3176	tags=64%,	3159/3665/	GO_BP	HMGA1/IRF7/PPIA/HCFC1/BANF1/XRCC6/XRCC4
6337	tags=48%,	1869/5554	GO_BP	E2F1/RBM38/IGF2BP1/PKP3/YBX3/MAPKAPK2/D
3559	tags=75%,	259266/92	GO_BP	ASPM/NUMBL/LEF1/FGFR1/FGFR2/AKNA
4701	tags=47%,	9156/1053	GO_BP	EXO1/RNASEH2A/POLD1/PCNA/LIG1/MSH2/MUTY
5765	tags=49%,	3959/2364	GO_BP	LGALS3BP/LY96/NCF2/TYROBP/DCDC2/LSP1/NC
3903	tags=41%,	6714/3055/	GO_BP	SRC/HCK/DDR1/IQGAP1/MAPK3/LYN/FGR/LCK/S
487	tags=20%,	9518	GO_BP	GDF15
2689	tags=60%,	5831/5832/	GO_BP	PYCR1/ALDH18A1/PYCR2
2689	tags=60%,	5831/5832/	GO_BP	PYCR1/ALDH18A1/PYCR2
1079	tags=40%,	3297/581	GO_BP	HSF1/BAX
5652	tags=44%,	6696/7040/	GO_BP	SPP1/TGFB1/STC2/CDKN2D/GDAP1/TNC/IL15/V
1026	tags=29%,	5092/2643/	GO_BP	PCBD1/GCH1/MTHFS/QDPR/SHMT1/ALDH1L1/MTH
854	tags=28%,	1244/2706/	GO_BP	ABCC2/GJB2/FOXO1/ERRFI1/SRD5A1/ASS1/SER
4797	tags=51%,	2810/1869/	GO_BP	SFN/E2F1/BAK1/YWHAZ/BAX/SLC35F6/HIP1R/B
2956	tags=33%,	5315/8020	GO_BP	PKM/HKDC1/ALDOA/IMPDH1/TREM2/ENO2/PFKP/
499	tags=44%,	291/9962/	GO_BP	SLC25A4/SLC23A2/SLC28A1/AQP9
676	tags=33%,	10400/259	GO_BP	PEMT/GAMT/GNMT
4321	tags=32%,	23213/798	GO_BP	SULF1/SHCBP1/GPC1/ESRP1/CREB3L1/IQGAP1/
1124	tags=33%,	6608/4040/	GO_BP	SMO/LRP6/HMGCS1/GRAMD1C/RORA/INSIG1/F7/
339	tags=19%,	4258/6600	GO_BP	MGST2/CYP4F12/CYP4F3/CYP4F2/CYP4A11
785	tags=20%,	1583/1586/	GO_BP	CYP11A1/CYP17A1/SERPINA6/APOA1/HSD11B1
4517	tags=38%,	6692/1065	GO_BP	SPINT1/SPINT2/PLCD3/ST14/LEF1/WNT7B/VAS
6130	tags=63%,	960/972/4	GO_BP	CD44/CD74/MUC1/CDKN2D/ZNF385A/MIF/BCL2L
6422	tags=47%,	7345/5347/	GO_BP	UCHL1/PLK1/APOA4/LAPTM4B/TREM2/HSPB8/PY
1179	tags=56%,	64087/865	GO_BP	MCCC2/ALDH4A1/GCAT/HDC/PCBD1/AMT/DBT/HM
1496	tags=71%,	55034/515	GO_BP	MOCOS/MBIP/MOCS2/MOCS1/GPHN
1496	tags=71%,	55034/515	GO_BP	MOCOS/MBIP/MOCS2/MOCS1/GPHN
1496	tags=71%,	55034/515	GO_BP	MOCOS/MBIP/MOCS2/MOCS1/GPHN
1275	tags=37%,	23411/997	GO_BP	SIRT1/NR1H4/C1QTNF3/ANGPTL8/ORMDL3/CETP
884	tags=18%,	1376/5142	GO_BP	CPT2/PRKAG2/SLC25A13/ACACB/SLC25A20/SLC
884	tags=18%,	1376/5142	GO_BP	CPT2/PRKAG2/SLC25A13/ACACB/SLC25A20/SLC
6566	tags=48%,	3984/1627/	GO_BP	LIMK1/DBN1/SEMA6A/ARHGAP4/TNFRSF12A/PLX
183	tags=33%,	336/335	GO_BP	APOA2/APOA1
120	tags=50%,	4923/18/6	GO_BP	NTSR1/ABAT/SLC6A1
4207	tags=41%,	284403/89	GO_BP	WDR62/CCNF/CEP131/NUP62/SAC3D1/CHMP4B/B
4936	tags=46%,	5888/4796/	GO_BP	RAD51/TONSL/ALYREF/EME1/PCNA/DDX11/BLM/
4900	tags=51%,	3925/9181/	GO_BP	STMN1/ARHGEF2/TRIM54/MAPRE1/MID1IP1/MAP
3335	tags=28%,	8612/8877/	GO_BP	PLPP2/SPHK1/ST6GALNAC4/GLA/ELOVL7/B4GAL
6239	tags=81%,	23246/234	GO_BP	BOP1/PES1/GTPBP4/NHP2/RPL7/SNU13/FTSJ3/
3420	tags=43%,	79734/578/	GO_BP	KCTD17/BAK1/BAX/TGM2/BCAP31/WFS1/PML/AP
4771	tags=34%,	100133941/	GO_BP	CD24/TREM2/EPO/ICAM1/SRC/LIF/TGFB1/RIPK
3158	tags=46%,	6662/6928/	GO_BP	SOX9/HNF1B/HIF1A/ETV6/PAX8/MSX1
2956	tags=59%,	5315/226/	GO_BP	PKM/ALDOA/ENO2/PFKP/ENO1/GAPDH/HK2/PGK1
2956	tags=59%,	5315/226/	GO_BP	PKM/ALDOA/ENO2/PFKP/ENO1/GAPDH/HK2/PGK1
2956	tags=59%,	5315/226/	GO_BP	PKM/ALDOA/ENO2/PFKP/ENO1/GAPDH/HK2/PGK1
2946	tags=40%,	1601/6597/	GO_BP	DAB2/SMARCA4/HDAC1/HEYL/PHB/NCOR2
6450	tags=49%,	2302/7305/	GO_BP	FOXJ1/TYROBP/CYBB/IGF2BP2/CD74/PTAFR/HS
3939	tags=29%,	1364/1192/	GO_BP	CLDN4/CLIC1/FXYD3/TTYH3/ANO9/PTAFR/PRNP

6300	tags=61%,	7040/5486(	GO_BP	TGFB1/PPP1R14D/PRKCD/ADORA1/CD300A/SLC3(
8571	tags=65%,	22943/8767	GO_BP	DKK1/RIPK2/GSTP1/PKN1/SYK/DTNBP1/TRAF2/I
6686	tags=47%,	4192/2207/	GO_BP	MDK/FCER1G/ICAM1/LGALS9/LGALS3/RIPK2/HA'
297	tags=17%,	1593/1576	GO_BP	CYP27A1/CYP3A4
5755	tags=40%,	54997/8441	GO_BP	TESC/C15orf48/NDUFA4L2/COX7B2/SLC2A6/ATI
4792	tags=35%,	23616/547(	GO_BP	SH3BP1/FBLIM1/ITGB4/SRC/MMP14/DSG2/POF1I
688	tags=19%,	5997/3384	GO_BP	RGS2/ANO9/PTAFR/PRNP/STK39
3653	tags=40%,	23616/5361	GO_BP	SH3BP1/PLXNA1/SEMA6A/FLNA/PLXNA3/SEMA4A,
6050	tags=56%,	4323/4100/	GO_BP	MMP14/MAGEA1/TSPAN15/JAG2/JAG1/GDPD5/HE'
3266	tags=55%,	56833/305(	GO_BP	SLAMF8/HCK/GRN/PIK3CD/RPS19/LBP
766	tags=30%,	10560/915	GO_BP	SLC19A2/SLC28A1/AQP9
2970	tags=50%,	578/4237/(	GO_BP	BAK1/MFAP2/BAX/HMGN1
944	tags=17%,	3155/3157/	GO_BP	HMGCL/HMGCS1/HNF4A/LIMS2/TGFBR3/PROX1/SI
4520	tags=58%,	9536/2012(	GO_BP	PTGES/UNC13D/S100A8/CCL5/CXCL13/TNFAIP3,
6199	tags=49%,	2810/1894/	GO_BP	SFN/ECT2/TGFB1/FLNA/PRKCD/NUP62/GTSE1/M
344	tags=40%,	55353/730(	GO_BP	LAPTM4B/TYROBP
1927	tags=60%,	6790/2592(	GO_BP	AURKA/ASPM/FBXO5
908	tags=40%,	55353/289(	GO_BP	LAPTM4B/GRN
4326	tags=30%,	6692/7262/	GO_BP	SPINT1/PHLDA2/CCNB1/KRT19/CCNB2/CTHRC1/(
5960	tags=42%,	79574/590(	GO_BP	EPS8L3/RAP1GAP/FOXM1/EPO/STMN1/SH3BP1/SI
2956	tags=57%,	5315/226/(	GO_BP	PKM/ALDOA/ENO2/PFKP/ENO1/GAPDH/HK2/PGK1,
2956	tags=57%,	5315/226/(	GO_BP	PKM/ALDOA/ENO2/PFKP/ENO1/GAPDH/HK2/PGK1,
645	tags=33%,	338440/57(	GO_BP	ANO9/PTAFR/PRNP
3770	tags=78%,	4085/1045(	GO_BP	MAD2L1/MAD2L2/FBXO5/RPL23/RPS7/BAG2/RPL(
6566	tags=51%,	4318/1159(	GO_BP	MMP9/CTHRC1/ANXA2/LGALS3/TRIB3/BAMBI/RI
3578	tags=33%,	6696/1159(	GO_BP	SPP1/CTHRC1/MDK/BAK1/SRC/TIMP1/RAC2/MMP
4496	tags=36%,	4192/6662/	GO_BP	MDK/SOX9/LIF/CSF1/TGFB1/DKK1/ROBO1/MAGE
878	tags=18%,	5236/178/(	GO_BP	PGM1/AGL/GOT1/ENPP1/UGP2/IRS1/IRS2/GCK/I
4695	tags=53%,	3956/100/(	GO_BP	LGALS1/ADA/BCL3/CDH17/GPR183/MFNG/LFNG/I
3756	tags=53%,	84868/810(	GO_BP	HAVCR2/COLEC12/UNC93B1/F2RL1/TNIP2/FLOT
4321	tags=32%,	3576/1277/	GO_BP	CXCL8/COL1A1/POSTN/SULF1/CD44/SHCBP1/GP(
5172	tags=40%,	4192/6275/	GO_BP	MDK/S100A4/SOX9/COL1A1/DAB2/TGFB1/BAMBI,
5969	tags=44%,	9212/578/(	GO_BP	AURKB/BAK1/MAPK13/PBK/DTL/FEN1/POLD1/CD(
644	tags=18%,	5730/100/1	GO_BP	PTGDS/ADA/ADORA1
1379	tags=27%,	10580/985(	GO_BP	SORBS1/EPM2AIP1/PDK2/SIK1/SIRT1/C1QTNF3,
1487	tags=42%,	122970/79(	GO_BP	ACOT4/PANK3/MPC1/ACSS2/SULT1A1/PDK2/ELO'
1487	tags=42%,	122970/79(	GO_BP	ACOT4/PANK3/MPC1/ACSS2/SULT1A1/PDK2/ELO'
1487	tags=42%,	122970/79(	GO_BP	ACOT4/PANK3/MPC1/ACSS2/SULT1A1/PDK2/ELO'
1464	tags=22%,	8864/153/1	GO_BP	PER2/ADRB1/ADORA2A/EGFR/NPR1/ADRA2B/HTR(
1426	tags=21%,	4035/5565(	GO_BP	LRP1/PIGV/HERC3/PIGP/ANGPTL8/NPC1L1/PPM
1221	tags=20%,	57026/546(	GO_BP	PDXP/CRLS1/OXT/RAPGEF2/SOD1/AQP8/FDX1/T
1464	tags=22%,	8864/153/1	GO_BP	PER2/ADRB1/ADORA2A/EGFR/NPR1/ADRA2B/HTR(
782	tags=27%,	7048/2247/	GO_BP	TGFBR2/FGF2/DUSP10/TFPI/CAPN3/SYTL4/SEL
1464	tags=22%,	8864/153/1	GO_BP	PER2/ADRB1/ADORA2A/EGFR/NPR1/ADRA2B/HTR(
1063	tags=22%,	1071/5020/	GO_BP	CETP/OXT/AGTR1/SHH/CD36/PPARA/ACACB/APO
5859	tags=51%,	3159/6688/	GO_BP	HMGA1/SPI1/DNMT1/BRCA1/OTUB1/NOC2L/KDM1,
3928	tags=34%,	27090/271(	GO_BP	ST6GALNAC4/GLA/B4GALNT1/GAL3ST1/PRKCD/P
2225	tags=28%,	7040/5592(	GO_BP	TGFB1/RCC2/ARHGAP4/PAK1/GNA12/CORO1C/IT(
1442	tags=50%,	153/147/1	GO_BP	ADRB1/ADRA1B/ADRA1A/ADRB2
767	tags=50%,	56954/280(	GO_BP	NIT2/GOT1/GOT2/PCK1
1251	tags=50%,	10786/656(	GO_BP	SLC17A3/SLC17A1/SLC17A4/SLC17A2

750	tags=38%,	2021/1001	GO_BP	ENDOG/HDAC6/RGN
4629	tags=45%,	5315/80201	GO_BP	PKM/HKDC1/ALDOA/IMPDH1/TREM2/ENO2/PFKP/
1794	tags=44%,	1215/5667	GO_BP	CMA1/SUCNR1/CTSG/ACE2/CES1/ENPEP/MME/RE
943	tags=22%,	6647/1001	GO_BP	SOD1/HDAC6/ACOX2/RGN
4306	tags=56%,	8878/3312	GO_BP	SQSTM1/HSPA8/SNF8/NCLN/TAPBP
4205	tags=83%,	644150/84	GO_BP	WIPF3/TIGD5/WIPF1/WAS/WIPF2
1236	tags=50%,	56833/692	GO_BP	SLAMF8/TCF3/PRKDC
4205	tags=83%,	644150/84	GO_BP	WIPF3/TIGD5/WIPF1/WAS/WIPF2
178	tags=33%,	54209/107	GO_BP	TREM2/CTSC
4914	tags=33%,	6696/2539	GO_BP	SPP1/G6PD/SPHK1/SFN/S100A9/AGR2/PHLDA2/
6474	tags=45%,	6690/4318	GO_BP	SPINK1/MMP9/UCHL1/PYCARD/RGS2/FOXM1/HOM
3727	tags=47%,	7305/1536	GO_BP	TYROBP/CYBB/HSPB1/SYK/MAPKAPK2/APP/CLEC
1336	tags=50%,	8170/364/	GO_BP	SLC14A2/AQP7/AQP9
30	tags=33%,	135/6580	GO_BP	ADORA2A/SLC22A1
4952	tags=49%,	54913/915	GO_BP	RPP25/EXO1/BOP1/RNASEH2A/FEN1/RNASE1/TS
1295	tags=71%,	6817/5447	GO_BP	SULT1A1/POR/PPARGC1A/SULT1B1/CYP1A1
603	tags=71%,	1149/1148	GO_BP	CIDEA/OSBPL11/FITM1/APOC4/PLIN5
5482	tags=47%,	3984/6659	GO_BP	LIMK1/SOX4/TSPO/MAD2L1/MAD2L2/ISG15/SVB
3256	tags=50%,	4133/2325	GO_BP	MAP2/MTCL1/DYNLL1/DNAL4/NDE1
4207	tags=45%,	899/22994	GO_BP	CCNF/CEP131/NUP62/CHMP4B/STIL/NPM1/CHMP
2149	tags=44%,	9187/2779	GO_BP	SLC24A1/GNAT1/HMGCS1/CAT
591	tags=44%,	344/33832	GO_BP	APOC2/GPIHBP1/APOH/APOA5
3785	tags=28%,	4318/3691	GO_BP	MMP9/ITGB4/ITGA3/MMP14/TXNRD1/DKK1/ITGB
7508	tags=88%,	10112/551	GO_BP	KIF20A/CEP55/NUP62/CHMP4B/CHMP4C/CHMP3/
3509	tags=80%,	22943/145	GO_BP	DKK1/CSNK1E/CSNK1D/SFRP2
3723	tags=45%,	3958/5621	GO_BP	LGALS3/PRNP/EZR/PHPT1/THY1/PTPN6/LILRB4
3131	tags=56%,	6850/4050	GO_BP	SYK/LTB/MAST2/IRF1/CLEC7A
3131	tags=56%,	6850/4050	GO_BP	SYK/LTB/MAST2/IRF1/CLEC7A
2776	tags=57%,	6928/4017	GO_BP	HNF1B/LOXL2/ZNF706/PAX8
3510	tags=75%,	54209/217	GO_BP	TREM2/FANCD2/SHARPIN/TNIP2/FANCA/TNFAIP
2927	tags=43%,	2539/7086	GO_BP	G6PD/TKT/PGD/TALDO1/PGLS/RPIA
1079	tags=36%,	486/483/5	GO_BP	FXYD2/ATP1B3/BAK1/ATP1A1/BAX
3509	tags=50%,	115908/57	GO_BP	CTHRC1/PTK7/FZD1/DVL3/FZD2/DVL2/SFRP2
908	tags=33%,	54884/315	GO_BP	RETSAT/HMGCS1/ALDH3A2/CYP2C18/APOE/APOC
1226	tags=27%,	9446/5702	GO_BP	GSTO1/PDXP/PCCA/MCCC2/PCCB/GCLC/PNPO/CY
730	tags=14%,	2706/2852	GO_BP	GJB2/GPER1/TXNIP/SRD5A1/ASS1/ESR1/RAMP3
1464	tags=22%,	8864/153/1	GO_BP	PER2/ADRB1/ADORA2A/EGFR/NPR1/ADRA2B/HTR
1084	tags=23%,	6446/5020	GO_BP	SGK1/OXT/WNK3/HAMP/ABCG8/ABCG5/APOA2/AP
1239	tags=38%,	4496/4494	GO_BP	MT1H/MT1F/MAP1LC3A/MT1B/SOD1/ATP7B/MT2A
944	tags=16%,	3155/3157	GO_BP	HMGCL/HMGCS1/HNF4A/LIMS2/TGFBR3/PROX1/S
7508	tags=65%,	55353/175	GO_BP	LAPTM4B/DNM1/SQSTM1/CHMP4B/VPS37C/CHMP4
2103	tags=22%,	4318/1001	GO_BP	MMP9/CD24/FOXJ1/LIF/GLIS2/IQGAP1/PROM1/
4629	tags=45%,	5315/80201	GO_BP	PKM/HKDC1/ALDOA/IMPDH1/TREM2/ENO2/PFKP/
6656	tags=50%,	4192/2724	GO_BP	MDK/TNFRSF21/LGALS9/FOXJ1/LGALS3/TGFB1/
7249	tags=55%,	3976/7040	GO_BP	LIF/TGFB1/SPI1/MUC1/CHEK1/YEATS2/NAA40/
4970	tags=44%,	8877/8162	GO_BP	SPHK1/CDT1/CDCA5/DLGAP5/NUSAP1/TGFB1/AU
6676	tags=57%,	3965/578/	GO_BP	LGALS9/BAK1/LGALS3/ADA/JAK3/CCL5/BAX/HI
2956	tags=50%,	2539/80201	GO_BP	G6PD/HKDC1/TKT/PGD/HK2/G6PC3/TALDO1/HK3
4914	tags=59%,	54512/146	GO_BP	EXOSC4/SLFN13/PELO/EXOSC5/DROSHA/EXOSC3
4405	tags=34%,	2810/5047	GO_BP	SFN/PAEP/TREM2/PYCARD/SOX4/MDK/MMP12/PO
3838	tags=31%,	6696/4318	GO_BP	SPP1/MMP9/SPINT1/MMP7/APOA4/PLTP/SOX9/M

2875	tags=60%,	115908/704	GO_BP	CTHRC1/TGFB1/TGFB3
5764	tags=73%,	5698/5690/	GO_BP	PSMB9/PSMB2/PSMB4/PSMB8/PSMB10/PSMA5/PSI
3510	tags=36%,	23643/5740	GO_BP	LY96/S100A14/ITGAM/RIPK2/ITGB2/IRAK1/F2I
81	tags=33%,	54209/2910	GO_BP	TREM2/PYCARD
898	tags=50%,	1734/2184/	GO_BP	DIO2/FAH/MAOA/MAOB/DBH
4900	tags=41%,	54209/100/	GO_BP	TREM2/ADA/P2RX4/SYK/SLC9A1/P2RY6/CD3E/P2
5182	tags=45%,	991/5347/2	GO_BP	CDC20/PLK1/UBE2S/TRIB3/MAGEC2/RPS2/AXIN
1275	tags=24%,	23411/9971	GO_BP	SIRT1/NR1H4/EGR1/ELOVL6/NFYB/ERLIN1/CYP
2974	tags=25%,	3965/84868	GO_BP	LGALS9/HAVCR2/CORO1A/UNC13D/IL18/TUBB/SI
5644	tags=57%,	960/972/45	GO_BP	CD44/CD74/MUC1/ZNF385A/MIF/RNF34/BCL2L1
3913	tags=33%,	9212/578/5	GO_BP	AURKB/BAK1/MAPK13/PBK/POLD1/CDC25A/CHEK
2128	tags=31%,	4321/10631	GO_BP	MMP12/POSTN/SRC/MMP14/RCC2/CDKN2A/ADAM1
5142	tags=50%,	5984/5424/	GO_BP	RFC4/POLD1/PCNA/RFC2/PARP1/RBX1/RPA2/CUI
6834	tags=61%,	8767/5585/	GO_BP	RIPK2/PKN1/SYK/WNT7B/BIRC7/MLKL/MAP3K6/I
3595	tags=35%,	29108/1869	GO_BP	PYCARD/E2F1/CD44/CD74/MUC1/ZNF385A/BAX/I
4258	tags=29%,	6692/54209	GO_BP	SPINT1/TREM2/BACE2/SOX4/MDK/TNFRSF21/SOI
3955	tags=67%,	699/701/15	GO_BP	BUB1/BUB1B/SGO2/PPP2R1A/RAD51C/MASTL
3955	tags=67%,	699/701/15	GO_BP	BUB1/BUB1B/SGO2/PPP2R1A/RAD51C/MASTL
4096	tags=26%,	6690/2539/	GO_BP	SPINK1/G6PD/CCNB1/PAEP/TRIP13/EPO/CABYR/
873	tags=17%,	1627/22883	GO_BP	DBN1/CLSTN1/TYROBP/GPC4/STX3/SNAP25/HRAS
6220	tags=55%,	79734/5147	GO_BP	KCTD17/DCDC2/RABEP2/DYNLL1/TBC1D30/MAP4/
6418	tags=60%,	8767/3148/	GO_BP	RIPK2/HMGB2/IRF1/IRF3/IRF7/FLOT1/POLR3D/
4469	tags=45%,	402/84722/	GO_BP	ARL2/PSRC1/MAPRE1/PAK1/RAC1/RPS3/KATNB1/
6686	tags=54%,	3965/8767/	GO_BP	LGALS9/RIPK2/ADA/BATF/RELB/ELF4/SYK/IL1
8526	tags=71%,	3297/10213	GO_BP	HSF1/PSMD14/PRKDC/MAD2L2/RBBP8/BRCA1/REI
1226	tags=25%,	3159/8318/	GO_BP	HMGA1/CDC45/DNMT1/TRIM28/SIRT6/NAA40
120	tags=33%,	22999/18	GO_BP	RIMS1/ABAT
120	tags=33%,	22999/18	GO_BP	RIMS1/ABAT
3615	tags=43%,	6241/302/9	GO_BP	RRM2/ANXA2/CD74/RCC1/S100A10/FARSB/CPSF
6656	tags=64%,	3965/1029/	GO_BP	LGALS9/CDKN2A/CCL5/BAX/PRELID1/PDCD1/ADA
1072	tags=29%,	6530/6581/	GO_BP	SLC6A2/SLC22A3/DRD1/SLC22A1
4321	tags=31%,	3576/1277/	GO_BP	CXCL8/COL1A1/POSTN/SULF1/CD44/SHCBP1/GP
6651	tags=48%,	5347/1601/	GO_BP	PLK1/DAB2/PBK/TRIB3/AURKA/GIPC1/RNF19B/
3928	tags=44%,	2583/9334/	GO_BP	B4GALNT1/B4GALT5/NEU1/GM2A/CLN6/ST3GAL5/
4326	tags=30%,	6692/7262/	GO_BP	SPINT1/PHLDA2/CCNB1/KRT19/CCNB2/CTHRC1/
3928	tags=39%,	2717/2583/	GO_BP	GLA/B4GALNT1/GAL3ST1/PRKCD/CTSA/BAX/B4G
3903	tags=83%,	7423/5880/	GO_BP	VEGFB/RAC2/PGF/SWAP70/VEGFA
4077	tags=42%,	6790/22994	GO_BP	AURKA/CEP131/NUP62/SPAG5/STIL/SNX10/CSNI
3131	tags=47%,	7305/972/5	GO_BP	TYROBP/CD74/PTAFR/SYK/IL1B/APP/CLEC7A
6999	tags=68%,	3965/8767/	GO_BP	LGALS9/RIPK2/SYK/IL18/EBI3/CD3E/IRF1/RAS
838	tags=100%,	4089/9573/	GO_BP	SMAD4/GDF3/NODAL/SHH
1396	tags=40%,	1829/5818/	GO_BP	DSG2/NECTIN1/PKP3/PERP
5475	tags=42%,	115908/419	GO_BP	CTHRC1/MDK/SOX9/DAB2/DKK1/GPC3/TAX1BP3/I
5356	tags=42%,	4192/27242	GO_BP	MDK/TNFRSF21/LGALS9/FOXJ1/TYRO3/LGALS3/
3568	tags=50%,	115908/575	GO_BP	CTHRC1/PTK7/FZD1/DVL3/FZD2/DVL2/SFRP2/F
4062	tags=42%,	6659/6604/	GO_BP	SOX4/SMARCD3/TBX2/NOTCH1/MESP1
4198	tags=50%,	7040/22943	GO_BP	TGFB1/DKK1/YBX3/S100B/AKIRIN1/TWIST1
2750	tags=41%,	578/100/37	GO_BP	BAK1/ADA/JAK3/BAX/HIF1A/ADAM8/DFFA
4734	tags=64%,	6714/1601/	GO_BP	SRC/DAB2/MTMR2/EZR/MAP2/MSN/MAPK3/MAP2K
4629	tags=45%,	5315/80201	GO_BP	PKM/HKDC1/ALDOA/IMPDH1/TREM2/ENO2/PFKP/I
2063	tags=44%,	11015/6471	GO_BP	KDELR3/PDIA2/GPAA1/KDELR1

909	tags=25%,	5836/5236/	GO_BP	PYGL/PGM1/AGL/STBD1/TREH/IDNK/TKFC/PPP1
5456	tags=89%,	134/653361	GO_BP	ADORA1/NCF1/TGFA/ADRA2A/ADAM17/EREG/EGF/
364	tags=22%,	890/7040	GO_BP	CCNA2/TGFB1
2901	tags=56%,	1029/6352/	GO_BP	CDKN2A/CCL5/IRF7/TCP1/CCR5
5323	tags=40%,	10053/2342	GO_CC	AP1M2/TMED3/B3GNT3/GCNT3/LPCAT1/RAP1GAP/
6546	tags=54%,	388610/332	GO_CC	TRNP1/BIRC5/PLK1/CCNB1/AURKB/CENPA/TOP2A
4828	tags=44%,	10053/5701	GO_CC	AP1M2/AKR1B10/LPCAT1/LAPTM4B/IMPDH1/IL4
6546	tags=50%,	388610/534	GO_CC	TRNP1/PLK1/HMGA1/CENPA/E2F1/CDCA5/ASF1B/
6569	tags=53%,	4605/38861	GO_CC	MYBL2/TRNP1/BIRC5/PLK1/CCNB1/KIF2C/CENPI
6466	tags=55%,	768/2118/5	GO_CC	CA9/ETV4/PITX1/CAPG/PYCARD/UBD/RGS2/UBE
4828	tags=44%,	10053/5701	GO_CC	AP1M2/AKR1B10/LPCAT1/LAPTM4B/IMPDH1/IL4
5068	tags=47%,	10053/7988	GO_CC	AP1M2/LPCAT1/LAPTM4B/ANXA2/ITM2C/SLC2A6/
4828	tags=43%,	10053/5701	GO_CC	AP1M2/AKR1B10/LPCAT1/LAPTM4B/IMPDH1/IL4
6716	tags=56%,	991/5347/8	GO_CC	CDC20/PLK1/CCNB1/KIF2C/CCNB2/CTAG2/AURKI
6716	tags=51%,	991/5347/8	GO_CC	CDC20/PLK1/CCNB1/KIF2C/CCNB2/CAPG/CTAG2/
4157	tags=45%,	991/332/53	GO_CC	CDC20/BIRC5/PLK1/CCNB1/CAPG/KIF4A/AURKB/
5293	tags=45%,	6282/3159/	GO_CC	S100A11/HMGA1/CYBA/LIMK1/ADGRE5/ANXA2/I
6840	tags=54%,	8877/55353	GO_CC	SPHK1/LAPTM4B/LY96/ANXA2/IKBKE/MMD/NDRG
6039	tags=46%,	10053/2342	GO_CC	AP1M2/TMED3/LPCAT1/CYBA/ADGRE5/MFSD10/F
4256	tags=35%,	226/3880/8	GO_CC	ALDOA/KRT19/CAPG/CYBA/SLC2A1/CRYAB/DBN1/
7140	tags=52%,	6286/81620	GO_CC	S100P/CDT1/LIMK1/TKT/IKBKE/SH3BGRL3/MKI
5920	tags=43%,	23423/4922	GO_CC	TMED3/NTS/NRSN2/KDELR3/ATP13A2/DNM1/SYT
4553	tags=39%,	6286/5315/	GO_CC	S100P/PKM/MMP9/S100A9/S100A11/CXCL1/ALD
6039	tags=46%,	10053/2342	GO_CC	AP1M2/TMED3/LPCAT1/CYBA/ADGRE5/MFSD10/F
6546	tags=53%,	4605/38861	GO_CC	MYBL2/TRNP1/BIRC5/PLK1/CCNB1/KIF2C/CENPI
5323	tags=40%,	10053/5666	GO_CC	AP1M2/MUC13/TMED3/B3GNT3/GCNT3/LPCAT1/R
5068	tags=47%,	10053/7988	GO_CC	AP1M2/LPCAT1/LAPTM4B/IMPDH1/PYCARD/ANXA
6840	tags=53%,	8877/55353	GO_CC	SPHK1/LAPTM4B/LY96/ANXA2/IKBKE/MMD/NDRG
6546	tags=52%,	388610/534	GO_CC	TRNP1/PLK1/CCNB1/AURKB/CENPA/E2F1/CDCA5/
6090	tags=47%,	6696/991/9	GO_CC	SPP1/CDC20/PTGES/S100A6/CYBA/S100A4/PTG
5293	tags=45%,	6282/3159/	GO_CC	S100A11/HMGA1/CYBA/LIMK1/ADGRE5/ANXA2/I
5323	tags=41%,	10053/2342	GO_CC	AP1M2/TMED3/B3GNT3/GCNT3/LPCAT1/RAP1GAP/
5068	tags=47%,	10053/7988	GO_CC	AP1M2/LPCAT1/LAPTM4B/ANXA2/ITM2C/SLC2A6/
4337	tags=32%,	332/5347/1	GO_CC	BIRC5/PLK1/KIF2C/ALDOA/KRT19/KIF4A/AURKI
4337	tags=32%,	332/5347/1	GO_CC	BIRC5/PLK1/KIF2C/ALDOA/KRT19/KIF4A/AURKI
4553	tags=38%,	6286/5315/	GO_CC	S100P/PKM/MMP9/S100A9/S100A11/CXCL1/ALD
4337	tags=32%,	332/5347/1	GO_CC	BIRC5/PLK1/KIF2C/ALDOA/KRT19/KIF4A/AURKI
4115	tags=34%,	332/5347/1	GO_CC	BIRC5/PLK1/KIF2C/KRT19/KIF4A/AURKB/TPX2/
7314	tags=64%,	7153/11683	GO_CC	TOP2A/RPL39L/LGALS3/RPP25/CKAP4/GAPDH/S
6546	tags=49%,	388610/105	GO_CC	TRNP1/CENPA/E2F1/CDCA5/ASF1B/CHAF1B/RAD
4329	tags=39%,	332/5347/1	GO_CC	BIRC5/PLK1/KIF2C/KIF4A/AURKB/TPX2/KIF20A
5293	tags=50%,	3159/1535/	GO_CC	HMGA1/CYBA/LIMK1/ADGRE5/ICAM1/ARPC1B/FBI
5293	tags=50%,	3159/1535/	GO_CC	HMGA1/CYBA/LIMK1/ADGRE5/ICAM1/ARPC1B/FBI
5293	tags=50%,	3159/1535/	GO_CC	HMGA1/CYBA/LIMK1/ADGRE5/ICAM1/ARPC1B/FBI
5998	tags=51%,	79888/1535	GO_CC	LPCAT1/CYBA/ADGRE5/FCER1G/TMC6/ITPR3/LHI
5592	tags=48%,	54997/6282	GO_CC	TESC/S100A11/EPS8L3/S100A6/CAPG/LIMK1/A
4115	tags=45%,	332/5347/8	GO_CC	BIRC5/PLK1/CCNB1/KIF2C/CENPM/CDT1/AURKB/
5068	tags=46%,	10053/7988	GO_CC	AP1M2/LPCAT1/LAPTM4B/ANXA2/ITM2C/SLC2A6/
6706	tags=66%,	116832/613	GO_CC	RPL39L/RPL8/NR0B1/RPL36A/MRPL17/RBM3/MRI
6418	tags=64%,	29108/2026	GO_CC	PYCARD/ENO2/PFKP/RRM2/ENO1/DBN1/RPL39L/
3448	tags=42%,	332/5347/8	GO_CC	BIRC5/PLK1/CCNB1/KIF2C/CENPM/CDT1/AURKB/

5430	tags=51%,	54997/822/	GO_CC	TESC/CAPG/LIMK1/DBN1/SH3BP1/RAC2/ENAH/C
3418	tags=43%,	332/5347/8	GO_CC	BIRC5/PLK1/CCNB1/KIF2C/CENPM/CDT1/AURKB/
7236	tags=71%,	3958/6628/	GO_CC	LGALS3/SNRPB/SF3B4/ALYREF/RALY/SNRPD2/S
6706	tags=69%,	116832/613	GO_CC	RPL39L/RPL8/RPL36A/MRPL17/RBM3/MRPS12/R
4258	tags=50%,	5315/4318/	GO_CC	PKM/MMP9/ALDOA/IMPDH1/FCER1G/LGALS3/GST
4605	tags=50%,	3614/29108	GO_CC	IMPDH1/PYCARD/ANXA2/GLA/CTSC/ATP13A2/FA
4263	tags=47%,	991/5347/8	GO_CC	CDC20/PLK1/CCNB1/AURKB/TPX2/TACC3/CDC6/
5255	tags=50%,	54997/6282	GO_CC	TESC/S100A11/EPS8L3/S100A6/CAPG/ANXA2/S
4313	tags=44%,	332/5347/8	GO_CC	BIRC5/PLK1/CAPG/KIF4A/AURKB/CDCA8/KIF20
4072	tags=46%,	4318/2919/	GO_CC	MMP9/CXCL1/ALDOA/CYBA/FCER1G/TMC6/LGALS
3928	tags=44%,	79888/3614	GO_CC	LPCAT1/IMPDH1/PYCARD/ANXA2/GLA/CTSC/FAB
4115	tags=37%,	332/11004/	GO_CC	BIRC5/KIF2C/KIF4A/AURKB/CDCA8/KIF20A/KI
3928	tags=44%,	79888/3614	GO_CC	LPCAT1/IMPDH1/PYCARD/ANXA2/GLA/CTSC/FAB
6436	tags=60%,	9401/983/4	GO_CC	RECQL4/CDK1/MCM2/MCM4/ORC1/RAD51/MCM6/F
6028	tags=58%,	1535/4688/	GO_CC	CYBA/NCF2/RAC2/CYBB/CORO1A/ITGAV/RAB31/
3851	tags=49%,	332/5347/8	GO_CC	BIRC5/PLK1/CCNB1/KIF2C/CENPM/CDT1/AURKB/
3671	tags=50%,	5315/4318/	GO_CC	PKM/MMP9/ALDOA/IMPDH1/GSTP1/COTL1/MVP/C
6644	tags=70%,	116832/613	GO_CC	RPL39L/RPL8/RPL36A/MRPL17/RBM3/MRPL47/M
3343	tags=51%,	332/5347/8	GO_CC	BIRC5/PLK1/CCNB1/KIF2C/CENPM/CDT1/AURKB/
3170	tags=41%,	1627/6714/	GO_CC	DBN1/SRC/RAC2/MISP/POF1B/PDLIM7/CORO1A/
3343	tags=51%,	332/5347/8	GO_CC	BIRC5/PLK1/CCNB1/KIF2C/CENPM/CDT1/CENPA/
6062	tags=62%,	983/4171/4	GO_CC	CDK1/MCM2/MCM4/ORC1/RAD51/MCM6/FEN1/POL
4909	tags=47%,	23423/1101	GO_CC	TMED3/KDELR3/FOLR1/ZDHHC13/CD74/HLA-DRB
5928	tags=62%,	6510/822/4	GO_CC	SLC1A5/CAPG/ATP1B3/SLC2A1/ANXA2/MMP14/A
5928	tags=62%,	6510/822/4	GO_CC	SLC1A5/CAPG/ATP1B3/SLC2A1/ANXA2/MMP14/A
6217	tags=73%,	116832/613	GO_CC	RPL39L/RPL8/RPL36A/RPS2/RPL17/RPLP2/RPS
4115	tags=49%,	822/9212/2	GO_CC	CAPG/AURKB/TPX2/TACC3/CDK1/KIF23/KIFC1/
4535	tags=51%,	2717/23400	GO_CC	GLA/ATP13A2/PPT1/CTSV/GPC4/GPC3/AGRN/SD
4245	tags=45%,	5347/891/9	GO_CC	PLK1/CCNB1/AURKB/CENPA/NUF2/NDC80/NEK2/
7177	tags=77%,	6628/10262	GO_CC	SNRPB/SF3B4/SNRPD2/SNRPD1/SNRPA1/SF3A2/
3928	tags=49%,	3614/29108	GO_CC	IMPDH1/PYCARD/ANXA2/GLA/CTSC/FABP5/PYGB/
7222	tags=73%,	6628/10189	GO_CC	SNRPB/ALYREF/RALY/SNRPD2/SNRPD1/SNRPA1/
3735	tags=35%,	115908/127	GO_CC	CTHRC1/COL1A1/COL1A2/COL9A2/C1QB/C1QC/C
6194	tags=72%,	6628/6633/	GO_CC	SNRPB/SNRPD2/SNRPD1/SNRPA1/SNRPA/SF3A2/
4072	tags=51%,	1535/2207/	GO_CC	CYBA/FCER1G/TMC6/ITGAM/CYBB/ITGB2/SNAP2
5134	tags=66%,	6628/6633/	GO_CC	SNRPB/SNRPD2/SNRPD1/SNRPA1/SNRPA/SF3A2/
5908	tags=72%,	23246/2587	GO_CC	BOP1/DCAF13/NOP56/MRTO4/PES1/RRS1/RRP9/
4936	tags=59%,	8318/5984/	GO_CC	CDC45/RFC4/TONSL/UHRF1/GINS2/MCM10/POLD
6127	tags=79%,	116832/613	GO_CC	RPL39L/RPL8/RPL36A/RPL17/RPLP2/RPL10L/R
5134	tags=67%,	6628/6633/	GO_CC	SNRPB/SNRPD2/SNRPD1/SNRPA1/SNRPA/SF3A2/
4298	tags=52%,	332/5347/2	GO_CC	BIRC5/PLK1/KIF4A/AURKB/CDK1/PRC1/KIF18B/
2966	tags=40%,	24137/2297	GO_CC	KIF4A/TPX2/CDCA8/KIF20A/CEP55/MICAL1/KI
2647	tags=36%,	11004/2413	GO_CC	KIF2C/KIF4A/KIF20A/KIF23/KIFC1/KIF18B/K
5650	tags=74%,	6628/10262	GO_CC	SNRPB/SF3B4/SNRPD2/SNRPD1/SNRPA1/SF3A2/
5650	tags=75%,	6628/10262	GO_CC	SNRPB/SF3B4/SNRPD2/SNRPD1/SNRPA1/SF3A2/
3935	tags=45%,	891/9133/9	GO_CC	CCNB1/CCNB2/CDK1/CKS2/CCNE1/CCNA2/CCNF/
2918	tags=48%,	5347/9212/	GO_CC	PLK1/AURKB/CDCA8/CDC6/KIF18B/RCC2/AURKA/
2698	tags=60%,	5347/891/9	GO_CC	PLK1/CCNB1/AURKB/CENPA/NUF2/NDC80/BUB1/
729	tags=53%,	5347/891/1	GO_CC	PLK1/CCNB1/CENPA/NUF2/NDC80/BUB1/BUB1B/
463	tags=62%,	5347/891/1	GO_CC	PLK1/CCNB1/NDC80/BUB1/BUB1B/CENPF/SKA1/
1560	tags=46%,	1535/4688/	GO_CC	CYBA/NCF2/CYBB/NCF4/NCF1/DUOX2

971	tags=58%,	4171/4173/	GO_CC	MCM2/MCM4/TONSL/MCM6/MCM7/MCM3/MCM5
1353	tags=58%,	6513/890/1	GO_CC	SLC2A1/CCNA2/CENPF/AURKA/EZH2/HSF1/CBX1
2124	tags=86%,	3108/972/3	GO_CC	HLA-DMA/CD74/HLA-DRB1/HLA-DQA1/HLA-DRA/
386	tags=80%,	332/9212/5	GO_CC	BIRC5/AURKB/CDCA8/AURKA
6013	tags=45%,	8877/337/5	GO_CC	SPHK1/APOA4/LAPTM4B/CYBA/BACE2/LY96/ANX
7576	tags=54%,	5315/991/8	GO_CC	PKM/CDC20/CCNB1/KCTD17/CCNB2/PYCARD/PFK
5198	tags=48%,	2919/1535/	GO_CC	CXCL1/CYBA/LCN2/TMC6/PLAUR/CKAP4/ITGAM/
5998	tags=59%,	1535/11322	GO_CC	CYBA/TMC6/PLAUR/CKAP4/ITGAM/CYBB/ITGB2/
4831	tags=55%,	3159/1058/	GO_CC	HMGA1/CENPA/CBX2/UHRF1/CDKN2A/CBX6/DNMT
4473	tags=48%,	2207/3958/	GO_CC	FCER1G/LGALS3/ITGB2/TRPM2/ATAD3B/CD300A
6516	tags=90%,	6132/190/6	GO_CC	RPL8/NR0B1/RPL36A/RPS21/RPL39/EIF3H/RPL
4203	tags=60%,	1058/29128	GO_CC	CENPA/UHRF1/EME1/TRIM28/SIRT6/CBX1/CBX3
3678	tags=62%,	6714/7171/	GO_CC	SRC/TPM4/FERMT3/ARHGEF2/ASAP1/FSCN1/SH3
5451	tags=76%,	6628/6633/	GO_CC	SNRPB/SNRPD2/SNRPD1/LSM4/EFTUD2/PPIH/SN
5451	tags=76%,	6628/6633/	GO_CC	SNRPB/SNRPD2/SNRPD1/LSM4/EFTUD2/PPIH/SN
6525	tags=57%,	24137/8482	GO_CC	KIF4A/LMNB2/CENPF/LMNB1/UHRF1/CENPW/PRK
692	tags=100%,	344/338/33	GO_CC	APOC2/APOB/APOA1/APOC3
4856	tags=42%,	23423/2321	GO_CC	TMED3/SULF1/HID1/GAL3ST4/RAB34/TRAPPC4/
6357	tags=57%,	55353/302/	GO_CC	LAPTM4B/ANXA2/MMD/ATP13A2/HLA-DMA/HLA-D
6658	tags=72%,	55323/6132	GO_CC	LARP6/RPL8/NR0B1/RPL36A/MCRS1/RPL10L/RP
6357	tags=65%,	1535/5880/	GO_CC	CYBA/RAC2/CYBB/CORO1A/RAB31/TAP1/RAB34/
1204	tags=100%,	733/727/73	GO_CC	C8G/C5/C7/C8B/C8A/C6
3406	tags=38%,	1277/4237/	GO_CC	COL1A1/MFAP2/FBLN1/COL1A2/COL4A5/LAMB1/
6885	tags=73%,	991/27338/	GO_CC	CDC20/UBE2S/BUB1B/CBX2/MAD2L2/PHC2/BRCA
4936	tags=56%,	8318/4796/	GO_CC	CDC45/TONSL/GINS2/MCM10/POLD1/MCM3/POLA
591	tags=100%,	344/55937/	GO_CC	APOC2/APOM/APOA2/PON1/HPR/APOA1/APOC3
4306	tags=73%,	972/3123/3	GO_CC	CD74/HLA-DRB1/HLA-DQA1/HLA-DRA/HLA-DQB1
4306	tags=73%,	972/3123/3	GO_CC	CD74/HLA-DRB1/HLA-DQA1/HLA-DRA/HLA-DQB1
3841	tags=75%,	54913/1052	GO_CC	RPP25/NOP56/DKC1/RRP9/FBL/SNRPF/NHP2/PO
2652	tags=67%,	3108/972/3	GO_CC	HLA-DMA/CD74/HLA-DRB1/HLA-DQA1/HLA-DRA/
4125	tags=81%,	26353/1096	GO_CC	HSPB8/STIP1/CCT5/CCT6A/CCT3/HSPA8/CCT2/
6546	tags=45%,	3159/5307/	GO_CC	HMGA1/PITX1/SOX4/E2F1/SOX9/TEAD2/SPI1/T
5574	tags=39%,	6286/768/5	GO_CC	S100P/CA9/TESC/EPS8L3/MFSD10/REG1A/SRC/
1349	tags=64%,	8803/1743/	GO_CC	SUCLA2/DLST/BCKDHA/FH/DBT/SUCLG2/BCKDHB
1341	tags=69%,	3700/7078/	GO_CC	ITIH4/TIMP3/CDC37L1/RARRES2/CLEC3B/ITIH
905	tags=62%,	3949/348/3	GO_CC	LDLR/APOE/APOC2/SORL1/APOM/APOB/APOF/AP
692	tags=69%,	348/344/33	GO_CC	APOE/APOC2/APOB/APOC1/APOA2/APOH/APOA1/
804	tags=63%,	51449/348/	GO_CC	PCYOX1/APOE/APOC2/APOM/APOC4/APOB/APOC1
804	tags=63%,	51449/348/	GO_CC	PCYOX1/APOE/APOC2/APOM/APOC4/APOB/APOC1
946	tags=35%,	3040/348/3	GO_CC	HBA2/APOE/HBB/APOB/HP/APOA1/HPX
5595	tags=40%,	9123/9536/	GO_CC	SLC16A3/PTGES/S100A6/SMOX/PTGDS/TYRO3/T
5574	tags=51%,	54997/7957	GO_CC	TESC/EPS8L3/SRC/PLEKHO1/ARHGEF2/ITGAV/N
3406	tags=64%,	1277/1278/	GO_CC	COL1A1/COL1A2/COL3A1/COL5A1/COL5A2/LUM/
3406	tags=64%,	1277/1278/	GO_CC	COL1A1/COL1A2/COL3A1/COL5A1/COL5A2/LUM/
692	tags=73%,	1191/1071/	GO_CC	CLU/CETP/APOE/APOC2/APOM/APOC4/LIPC/SAA
4003	tags=44%,	1535/1410/	GO_CC	CYBA/CRYAB/FBLIM1/PDLIM7/MARCKS/TPM4/FH
5134	tags=73%,	6628/10262	GO_CC	SNRPB/SF3B4/SNRPD2/SNRPD1/YBX1/SNRPE/SN
4796	tags=42%,	10053/915/	GO_CC	AP1M2/CD3D/DAB2/FOLR1/FCHO1/CD74/RGS19/
2223	tags=25%,	23413/1081	GO_CC	NCS1/CPLX2/ADORA1/GIT1/ACTG1/ACTB
4771	tags=42%,	10053/2342	GO_CC	AP1M2/TMED3/KDELR3/CD3D/DAB2/FOLR1/FCHO
4594	tags=46%,	10053/915/	GO_CC	AP1M2/CD3D/DAB2/FCHO1/CD74/HLA-DRB1/HLA-

1184	tags=64%,	1191/1071/	GO_CC	CLU/CETP/LDLR/PCYOX1/APOE/APOC2/SORL1/A
1184	tags=64%,	1191/1071/	GO_CC	CLU/CETP/LDLR/PCYOX1/APOE/APOC2/SORL1/A
3211	tags=71%,	7203/1057	GO_CC	CCT3/CCT2/CCT4/TCP1/CCT8
4752	tags=62%,	25879/105	GO_CC	DCAF13/NOP56/RRP9/FBL/EMG1/NOP58/UTP18/
6027	tags=43%,	2348/972/1	GO_CC	FOLR1/CD74/SCGN/SCAMP5/HLA-DRB1/SLC6A9/
1184	tags=61%,	1191/1071/	GO_CC	CLU/CETP/LDLR/PCYOX1/APOE/APOC2/SORL1/A
3750	tags=46%,	11004/146	GO_CC	KIF2C/KIF18B/MISP/CLIP2/SPAG5/MAPRE1/AS
6468	tags=58%,	10053/234	GO_CC	AP1M2/TMED3/DNM1/FCHO1/BAIAP2L2/AP5Z1/C
6468	tags=58%,	10053/234	GO_CC	AP1M2/TMED3/DNM1/FCHO1/BAIAP2L2/AP5Z1/C
5134	tags=84%,	6628/6633/	GO_CC	SNRPB/SNRPD2/SNRPD1/SNRPA1/SF3A2/SNRPE/
1552	tags=83%,	10809/124	GO_CC	STARD10/ABCC2/DPP4/ABCB11/ABCB4
1546	tags=60%,	83594/122	GO_CC	NUDT12/ACOT4/NOS2/CRAT/ECH1/LONP2/HMGCL
1546	tags=60%,	83594/122	GO_CC	NUDT12/ACOT4/NOS2/CRAT/ECH1/LONP2/HMGCL
7789	tags=68%,	10537/547	GO_CC	UBD/FBLIM1/ORC6/DAB2/NFKBIE/TXNRD1/BOP1
3320	tags=16%,	3880/6478	GO_CC	KRT19/MICAL1/KRT23/LMNB2/LMNB1/KRT17/KR
1227	tags=80%,	8318/9837/	GO_CC	CDC45/GINS1/GINS2/GINS4
1942	tags=80%,	4688/4689/	GO_CC	NCF2/NCF4/NCF1/ADAM8
4752	tags=66%,	23246/234	GO_CC	BOP1/PES1/RRP7A/NOC2L/UTP18/FBXW9/RRP36
1349	tags=55%,	1743/593/1	GO_CC	DLST/BCKDHA/DBT/BCKDHB/DHTKD1/OGDHL
3698	tags=49%,	3315/5698/	GO_CC	HSPB1/PSMB9/PSMD14/PSMD2/PSMB2/PSME3/AD
4258	tags=51%,	10112/551	GO_CC	KIF20A/CEP55/ECT2/RACGAP1/PKN1/PLCD3/RH
4003	tags=41%,	1535/1627/	GO_CC	CYBA/DBN1/FBLIM1/PDLIM7/TPM4/FHL3/ZYX/M
6494	tags=49%,	3880/6513/	GO_CC	KRT19/SLC2A1/ENO1/ANXA2/DBN1/MELK/ANLN/
6017	tags=48%,	23423/107	GO_CC	TMED3/CTSC/KDELR3/STK26/FOLR1/ZDHHC13/C
4722	tags=42%,	3880/6513/	GO_CC	KRT19/SLC2A1/DBN1/ANLN/MISP/CORO1A/FLNA
7539	tags=67%,	4172/2364	GO_CC	MCM3/POLA2/TAF6/POLR2G/CD3EAP/POLR3K/PO
6488	tags=63%,	10053/972/	GO_CC	AP1M2/CD74/SCAMP5/HLA-DRB1/RAB31/HLA-DQ
2256	tags=27%,	1058/6415	GO_CC	CENPA/NCAPG/NCAPD2/NCAPH/NCAPG2/SMC4/NC
1864	tags=41%,	5826/2550	GO_CC	ABCD4/MPV17L/ABCD3/PEX13/PEX12/PEX1/PEX
1864	tags=41%,	5826/2550	GO_CC	ABCD4/MPV17L/ABCD3/PEX13/PEX12/PEX1/PEX
3566	tags=29%,	2539/6277/	GO_CC	G6PD/S100A6/RGS2/RGS1/TGM3/GRAMD1A/SRC/
7539	tags=68%,	4172/2364	GO_CC	MCM3/POLA2/TAF6/POLR2G/CD3EAP/POLR3K/PO
4607	tags=50%,	10053/231	GO_CC	AP1M2/FCHO1/BAIAP2L2/CLTA/AP4M1/HIP1R/A
3380	tags=40%,	3691/3675/	GO_CC	ITGB4/ITGA3/ITGAM/ITGB2/ITGAV/ITGA5/ITG
6387	tags=58%,	5888/1009	GO_CC	RAD51/ARPC4/WDR76/MAD2L2/CBX1/RBBP8/ARP
6047	tags=48%,	8877/1535/	GO_CC	SPHK1/CYBA/NCF2/LPAR2/RAC2/CYBB/CORO1A/
5574	tags=45%,	54997/795	GO_CC	TESC/EPS8L3/REG1A/SRC/CD44/PLEKHO1/KCNJ
6230	tags=50%,	24137/106	GO_CC	KIF4A/CENPF/LMNB1/UHRF1/CENPW/PRKCD/CFL
7539	tags=67%,	4172/2364	GO_CC	MCM3/POLA2/TAF6/POLR2G/CD3EAP/POLR3K/PO
4258	tags=45%,	10112/551	GO_CC	KIF20A/CEP55/ANLN/ECT2/RACGAP1/PKN1/PLC
3698	tags=48%,	3315/5698/	GO_CC	HSPB1/PSMB9/PSMD14/PSMD2/PSMB2/PSME3/AD
1135	tags=62%,	2267/2244/	GO_CC	FGL1/FGB/FGG/FGA/SERPINF2
1227	tags=34%,	3479/1191/	GO_CC	IGF1/CLU/F8/SERPINA1/ACTN2/PROS1/VWF/SE
6542	tags=52%,	10053/258	GO_CC	AP1M2/BACE2/POSTN/PCSK1N/SNAP25/CD74/SC
4258	tags=47%,	10112/551	GO_CC	KIF20A/CEP55/ANLN/ECT2/RACGAP1/PKN1/PLC
5718	tags=67%,	23616/111	GO_CC	SH3BP1/CORO1A/TRIP10/RAB31/LCP1/PIP5K1C
6592	tags=53%,	1535/5880/	GO_CC	CYBA/RAC2/CYBB/CORO1A/FCHO1/CD74/HLA-DR
3246	tags=44%,	9296/533/	GO_CC	ATP6V1F/ATP6V0B/ATP6V0D2/ATP6V1C1/TCIRG
3001	tags=40%,	4318/2919/	GO_CC	MMP9/CXCL1/ALDOA/QSOX1/CSTB/LYZ/QPCT/TN
3406	tags=47%,	1277/1278/	GO_CC	COL1A1/COL1A2/COL4A5/COL3A1/COL5A1/COL5
7144	tags=65%,	10397/556	GO_CC	NDRG1/SLC39A4/SCAMP5/PLEKHB2/LAMP5/ABHD

4327	tags=73%,	1107/3065/	GO_CC	CHD3/HDAC1/HDAC2/MTA2/CHD4/RBBP4/RBBP7/
4327	tags=73%,	1107/3065/	GO_CC	CHD3/HDAC1/HDAC2/MTA2/CHD4/RBBP4/RBBP7/
2077	tags=58%,	7430/6404/	GO_CC	EZR/SELPLG/FLOT2/MSN/PIP5K1C/FLOT1/PSTP
2077	tags=58%,	7430/6404/	GO_CC	EZR/SELPLG/FLOT2/MSN/PIP5K1C/FLOT1/PSTP
4090	tags=67%,	9212/1469(	GO_CC	AURKB/KIF18B/RCC2/KIF18A/CENPE/CTTN/HNR
6519	tags=51%,	10053/234	GO_CC	AP1M2/TMED3/CTSC/KDELR3/CD3D/DAB2/FOLR1
6229	tags=36%,	8877/9123/	GO_CC	SPHK1/SLC16A3/HOMER3/CRYAB/NTS/DBN1/KPN
3930	tags=42%,	6286/5315/	GO_CC	S100P/PKM/S100A9/S100A11/CXCL1/ALDOA/IM
3609	tags=67%,	6628/6633/	GO_CC	SNRPB/SNRPD2/SNRPD1/CD2BP2/SNRPE/TXNL4A
4582	tags=47%,	23149/972/	GO_CC	FCHO1/CD74/HLA-DRB1/HLA-DQA1/HLA-DRA/HL
3581	tags=73%,	10095/100	GO_CC	ARPC1B/ARPC4/ARPC2/ARPC3/ARPC5L/ARPC1A/
3245	tags=88%,	8318/5165	GO_CC	CDC45/GINS2/MCM10/GINS4/WDHD1/TIPIN/TIM
3750	tags=50%,	11004/146	GO_CC	KIF2C/KIF18B/CLIP2/SPAG5/MAPRE1/NCKAP5L
1349	tags=67%,	593/1629/	GO_CC	BCKDHA/DBT/SUCLG2/BCKDHB
7889	tags=69%,	26147/662	GO_CC	PHF19/SNRPB/EZH2/SNRPD2/PRMT1/WDR4/SNRP
1361	tags=50%,	6403/3700/	GO_CC	SELP/ITIH4/TIMP3/CDC37L1/RARRES2/CLEC3B
7658	tags=55%,	3984/7086/	GO_CC	LIMK1/TKT/RMI2/CKAP4/CDC25C/DDX39A/SNRP
3980	tags=32%,	100133941/	GO_CC	CD24/ATP1B3/SLC2A1/ANXA2/ICAM1/SULF1/SR
4003	tags=43%,	1535/54751	GO_CC	CYBA/FBLIM1/PDLIM7/TPM4/FHL3/ZYX/MICALL
4003	tags=43%,	1535/54751	GO_CC	CYBA/FBLIM1/PDLIM7/TPM4/FHL3/ZYX/MICALL
3980	tags=32%,	100133941/	GO_CC	CD24/ATP1B3/SLC2A1/ANXA2/ICAM1/SULF1/SR
3439	tags=52%,	6286/768/	GO_CC	S100P/CA9/ITGAV/EZR/MSN/PROM1/SLC9A3R1/
1941	tags=55%,	3042/3045/	GO_CC	HBM/HBD/HBA1/HBA2/HBB/HP
3211	tags=80%,	22948/908/	GO_CC	CCT5/CCT6A/CCT3/CCT2/CCT4/CCT7/TCP1/CCT
4547	tags=33%,	1759/5758	GO_CC	DNM1/SYT13/PPT1/STX3/SNAP25/CDK16/SCAMP
7789	tags=65%,	10537/547	GO_CC	UBD/FBLIM1/ORC6/DAB2/NFKBIE/TXNRD1/TAX1
1372	tags=29%,	94121/640	GO_CC	SYTL4/SELP/IGF1/CLU/F8/SERPINA1/ACTN2/P
2917	tags=100%,	4172/2364	GO_CC	MCM3/POLA2/PRIM1/POLA1/PRIM2
4486	tags=71%,	10213/570	GO_CC	PSMD14/PSMD2/ADRM1/PSMD13/PSMC4/PSMD10/
1104	tags=50%,	10203/956	GO_CC	CALCRL/GABBR2/RAMP3
3380	tags=39%,	3691/3675/	GO_CC	ITGB4/ITGA3/ITGAM/ITGB2/ITGAV/ITGA5/ITG
4547	tags=33%,	1759/5538/	GO_CC	DNM1/PPT1/STX3/SNAP25/CDK16/SCAMP5/SLC6
3980	tags=31%,	100133941/	GO_CC	CD24/ATP1B3/SLC2A1/ANXA2/ICAM1/SULF1/SR
6423	tags=51%,	3880/6513/	GO_CC	KRT19/SLC2A1/ENO1/DBN1/ANLN/SH3BP1/SAPC
6511	tags=46%,	2539/6277/	GO_CC	G6PD/S100A6/RGS2/RGS1/TGM3/GRAMD1A/SRC/
3520	tags=57%,	9212/9787/	GO_CC	AURKB/DLGAP5/MISP/AURKA/NPM1/NDE1/EMD/N
6217	tags=75%,	6187/3921/	GO_CC	RPS2/RPSA/RPS5/RPS21/RPS3/RPS20/RPS19/R
5035	tags=41%,	23423/283	GO_CC	TMED3/HID1/GAL3ST4/RAB34/CANT1/B4GALT5/
6229	tags=38%,	7345/2413	GO_CC	UCHL1/KIF4A/TPX2/NTS/DBN1/REG1A/MICAL1/
1104	tags=60%,	10203/102	GO_CC	CALCRL/RAMP1/RAMP3
4288	tags=41%,	23423/796	GO_CC	TMED3/GAL3ST4/CANT1/B4GALT5/FUT4/CHPF2/
6596	tags=51%,	3159/6659/	GO_CC	HMGA1/SOX4/E2F1/SOX9/TEAD2/E2F5/TLE3/SO
8126	tags=72%,	63875/618	GO_CC	MRPL17/MRPS12/MRPL47/MRPL9/MRPL12/MRPS3
8126	tags=72%,	63875/618	GO_CC	MRPL17/MRPS12/MRPL47/MRPL9/MRPL12/MRPS3
3655	tags=46%,	3383/3958/	GO_CC	ICAM1/LGALS3/HAVCR2/CORO1A/EZR/CD53/CD3
955	tags=50%,	146909/12	GO_CC	KIF18B/MISP/MAPRE1/KIF18A
5165	tags=100%,	51237/105	GO_CC	MZB1/HYOU1/DNAJC10/PDIA6/HSPA5/PPIB/SDF
2857	tags=50%,	3912/3915/	GO_CC	LAMB1/LAMC1/LAMC2/LAMA5/LAMB3
3930	tags=41%,	6286/5315/	GO_CC	S100P/PKM/S100A9/S100A11/CXCL1/ALDOA/IM
5230	tags=34%,	7345/3880/	GO_CC	UCHL1/KRT19/KIF4A/SLC2A1/ENO1/ANXA2/DBN
6229	tags=40%,	4922/1627/	GO_CC	NTS/DBN1/REG1A/MICAL1/ITGA3/RGS10/NCS1/

6927	tags=47%,	991/79734/	GO_CC	CDC20/KCTD17/UBE2S/BUB1B/CKS2/CCNF/CBX2/
6466	tags=87%,	23246/511	GO_CC	BOP1/MRTO4/PES1/RRS1/RRP1/NOC2L/ZNF593/
6437	tags=60%,	10053/231	GO_CC	AP1M2/FCHO1/AP5Z1/AP4M1/HIP1R/AP3M2/AP2
1546	tags=51%,	196743/25	GO_CC	PAOX/MPV17L/ABCD3/PEX13/NUDT12/PEX12/AC
1546	tags=51%,	196743/25	GO_CC	PAOX/MPV17L/ABCD3/PEX13/NUDT12/PEX12/AC
8095	tags=57%,	1058/8318/	GO_CC	CENPA/CDC45/GINS1/TONSL/GINS2/POLD1/MCM
3310	tags=78%,	6628/6633/	GO_CC	SNRPB/SNRPD2/SNRPD1/SNRPE/SNRPF/SNRPG/P
6719	tags=77%,	23423/110	GO_CC	TMED3/KDELR3/PACS1/COPE/KDELR1/COPZ1/CO
4486	tags=67%,	10213/570	GO_CC	PSMD14/PSMD2/PSME3/ADRM1/PSMD13/PSMC4/P
5604	tags=92%,	6628/6633/	GO_CC	SNRPB/SNRPD2/PRMT1/SNRPD1/WDR77/SNRPE/S
5511	tags=75%,	6628/2338	GO_CC	SNRPB/SMG5/DKC1/SNRPE/NHP2/PTGES3/NAT10/
4582	tags=58%,	23149/121	GO_CC	FCHO1/CLTA/HIP1R/CLTB/AP2M1/AP2S1/EPS15
3930	tags=41%,	6286/5315/	GO_CC	S100P/PKM/S100A9/S100A11/CXCL1/ALDOA/IM
5230	tags=48%,	3880/6513/	GO_CC	KRT19/SLC2A1/DBN1/ANLN/MISP/CORO1A/CIB2/
1138	tags=29%,	10652/413	GO_CC	YKT6/MAP2
1036	tags=50%,	2022/721/7	GO_CC	ENG/C4B/C8G/CLU/ACSM1/APCS/CP/HBA2/C1R/
5171	tags=60%,	1759/1488/	GO_CC	DNM1/CTBP2/PACSIN1/CACNA1D/NPHP4/ATP2A2
6885	tags=67%,	84733/261	GO_CC	CBX2/PHF19/CBX6/EZH2/PHC2/UBAP2L/HDAC2/
7063	tags=56%,	9961/2363	GO_CC	MVP/NUP62/MX2/MAD2L1/RAN/RANGAP1/EIF5A2/
3408	tags=57%,	9212/5514	GO_CC	AURKB/CDCA8/OIP5/CBX1/INCENP/SCMH1/CBX5/
7639	tags=76%,	6183/6187/	GO_CC	MRPS12/RPS2/RPSA/RPS5/RPS21/MRPS34/RPS3/
6296	tags=48%,	5315/891/	GO_CC	PKM/CCNB1/CCNB2/PYCARD/PFKP/CDK1/CKS2/C
4178	tags=31%,	1364/1192/	GO_CC	CLDN4/CLIC1/TTYH3/CLIC3/SLC26A6/CLCN2/G
3924	tags=62%,	80331/331	GO_CC	DNAJC5/HSPA8/GAD1/SYT1/RAB3A
3924	tags=62%,	80331/331	GO_CC	DNAJC5/HSPA8/GAD1/SYT1/RAB3A
5915	tags=45%,	891/9133/	GO_CC	CCNB1/CCNB2/PYCARD/CDK1/CKS2/CCNE1/CCNA
5987	tags=58%,	11339/173	GO_CC	OIP5/DKC1/EFTUD2/FBL/NHP2/CDK2/LSM10/SN
3924	tags=42%,	80331/331	GO_CC	DNAJC5/HSPA8/GAD1/SYT1/RAB3A
5764	tags=79%,	5698/5690/	GO_CC	PSMB9/PSMB2/PSMB4/PSMB8/PSMB10/PSMA5/PS
3069	tags=80%,	128869/94	GO_CC	PIGU/PIGS/GPAA1/PIGT
6986	tags=53%,	1627/5967/	GO_CC	DBN1/REG1A/ITGA3/GPRIN1/ARHGAP4/STX3/SN
3635	tags=86%,	7516/7517/	GO_CC	XRCC2/XRCC3/SWI5/RAD51C/RAD51D/SFR1
4723	tags=100%,	64151/991	GO_CC	NCAPG/NCAPD2/NCAPH/NCAPG2/SMC4/NCAPH2/N
6558	tags=54%,	2583/5768/	GO_CC	B4GALNT1/QSOX1/CHST4/SLC39A13/SLC35B2/S
3246	tags=44%,	9296/533/	GO_CC	ATP6V1F/ATP6V0B/ATP6V0D2/ATP6V1C1/TCIRG
6437	tags=67%,	10053/231	GO_CC	AP1M2/FCHO1/AP4M1/HIP1R/AP3M2/AP2M1/AP2
2779	tags=28%,	100133941/	GO_CC	CD24/MELTF/FOLR1/GPC4/GPC3/PRNP/GPC1/FL
8126	tags=61%,	84419/569	GO_CC	C15orf48/NDUFA4L2/TOMM40/SLC25A6/MCUB/B
955	tags=36%,	146909/12	GO_CC	KIF18B/MISP/MAPRE1/KIF18A
955	tags=36%,	146909/12	GO_CC	KIF18B/MISP/MAPRE1/KIF18A
6365	tags=49%,	55353/302/	GO_CC	LAPTM4B/ANXA2/SRC/MMD/ATP13A2/HLA-DMA/S
5339	tags=31%,	7345/1728/	GO_CC	UCHL1/NQO1/PYCARD/CYBA/ENO2/TPX2/SNCG/H
6913	tags=58%,	79888/109	GO_CC	LPCAT1/CKAP4/SYNGR1/DNAJC5/DDOST/CD63/F
6423	tags=48%,	6277/5996/	GO_CC	S100A6/RGS1/TGM3/GRAMD1A/SRC/SNAP25/CDK
4779	tags=46%,	7153/3838/	GO_CC	TOP2A/KPNA2/NUP62/RAN/NUP210/VPS37C/GBP
4779	tags=46%,	7153/3838/	GO_CC	TOP2A/KPNA2/NUP62/RAN/NUP210/VPS37C/GBP
1349	tags=60%,	593/1629/	GO_CC	BCKDHA/DBT/BCKDHB
6986	tags=52%,	1627/5967/	GO_CC	DBN1/REG1A/ITGA3/GPRIN1/ARHGAP4/STX3/SN
1726	tags=62%,	5984/7907	GO_CC	RFC4/DSCC1/CHTF18/RFC2/DDX11
7171	tags=42%,	9123/9454/	GO_CC	SLC16A3/HOMER3/CRYAB/DBN1/KPNA2/SRC/NCS
1582	tags=80%,	84823/400	GO_CC	LMNB2/LMNB1/NARF/LMNA

6851	tags=55%,	10397/556	GO_CC	NDRG1/SLC39A4/TUBG1/SCAMP5/PLEKHB2/LAMP
5206	tags=68%,	960/3685/	GO_CC	CD44/ITGAV/VASP/PLEK2/CFL1/SLC39A6/PLXN
1104	tags=50%,	10203/956	GO_CC	CALCRL/GABBR2/RAMP1/RAMP3
873	tags=33%,	3838/2351	GO_CC	KPNA2/SCRIB
5629	tags=55%,	79885/515	GO_CC	HDAC11/HDAC7/BRMS1/CHD3/HDAC1/HDAC2/PHF
5764	tags=100%,	5698/5690/	GO_CC	PSMB9/PSMB2/PSMB4/PSMB8/PSMB10/PSMB3/PS
2200	tags=50%,	9296/528/	GO_CC	ATP6V1F/ATP6V1C1/ATP6AP1/ATP6V1H/ATP6V1
1546	tags=42%,	83594/519	GO_CC	NUDT12/PEX12/ACOT4/PEX1/NOS2/PEX19/ZADH
7418	tags=54%,	10970/887	GO_CC	CKAP4/SQSTM1/IGF2BP1/TUBB/MEX3A/HSF1/AP
1546	tags=42%,	83594/519	GO_CC	NUDT12/PEX12/ACOT4/PEX1/NOS2/PEX19/ZADH
6700	tags=39%,	1627/3838/	GO_CC	DBN1/KPNA2/PFN2/SRC/NCS1/NDRG1/DNM1/CLS
5220	tags=82%,	84823/400	GO_CC	LMNB2/LMNB1/NARF/PCNA/LMNA/EIF6/SUV39H1/
7274	tags=68%,	3315/5698/	GO_CC	HSPB1/PSMB9/PSMD14/PSMD2/TAF6/PSMB2/PSM
3694	tags=62%,	2319/2360	GO_CC	FLOT2/CORO1C/FLOT1/CBL/CTNNA1
7171	tags=43%,	9123/9454/	GO_CC	SLC16A3/HOMER3/CRYAB/DBN1/SRC/NCS1/DNM1/
1043	tags=38%,	302/4323/	GO_CC	ANXA2/MMP14/CARMIL1
1043	tags=38%,	302/4323/	GO_CC	ANXA2/MMP14/CARMIL1
3703	tags=62%,	1759/1211/	GO_CC	DNM1/CLTA/CLTB/RNF216/DNM2
4772	tags=51%,	8877/1601/	GO_CC	SPHK1/DAB2/LRP12/FCHO1/LRP10/TFRC/CLTA/
7642	tags=81%,	4796/5424/	GO_CC	TONSL/POLD1/MCM3/POLA2/PCNA/PRIM1/DONSO
160	tags=12%,	226/1410/	GO_CC	ALDOA/CRYAB/ENO1
6127	tags=88%,	6628/6633/	GO_CC	SNRPB/SNRPD2/SNRPD1/SNRPE/SNRPF/GEMIN7/
3679	tags=35%,	27283/302/	GO_CC	TINAG/ANXA2/TIMP1/FBLN1/COL9A2/AGRN/COL
6273	tags=81%,	644150/84	GO_CC	WIPF3/TIGD5/WIPF1/HIP1R/CAPZA1/WAS/GMFG/
6273	tags=81%,	644150/84	GO_CC	WIPF3/TIGD5/WIPF1/HIP1R/CAPZA1/WAS/GMFG/
8040	tags=71%,	63875/571	GO_CC	MRPL17/MRPL47/MRPL9/MRPL12/MRPL13/MRPL1
8040	tags=71%,	63875/571	GO_CC	MRPL17/MRPL47/MRPL9/MRPL12/MRPL13/MRPL1
6558	tags=47%,	578/2583/	GO_CC	BAK1/B4GALNT1/ATP13A2/CLSTN1/P2RX4/QSOX
1237	tags=27%,	486/483/4	GO_CC	FXYD2/ATP1B3/ATP1A1/SLC9A1
6567	tags=46%,	3159/8162	GO_MF	HMGA1/CDT1/CENPA/UBE2T/TOP2A/CDCA5/SOX9/
7150	tags=50%,	9242/3159/	GO_MF	MSC/HMGA1/SSX1/SOX4/TRIP13/NFE2L3/CD3D/
5356	tags=35%,	5347/891/	GO_MF	PLK1/CCNB1/CCNB2/LIMK1/AURKB/IKBKE/PKMY
6396	tags=45%,	1364/3159/	GO_MF	CLDN4/HMGA1/KRT19/CTHRC1/TUBA1C/TUBA4A/
5120	tags=38%,	100133941/	GO_MF	CD24/CCNB1/APOA4/CXCL1/RAP1GAP/PYCARD/R
4263	tags=34%,	6280/332/	GO_MF	S100A9/BIRC5/PLK1/KIF2C/ALDOA/EPS8L3/TM
5950	tags=38%,	5315/8877/	GO_MF	PKM/SPHK1/HKDC1/PLK1/CCNB1/CCNB2/LIMK1/
5942	tags=44%,	486/11004/	GO_MF	FXYD2/KIF2C/RAP1GAP/KIF4A/RGS2/ATP1B3/K
5942	tags=44%,	486/11004/	GO_MF	FXYD2/KIF2C/RAP1GAP/KIF4A/RGS2/ATP1B3/K
5942	tags=44%,	486/11004/	GO_MF	FXYD2/KIF2C/RAP1GAP/KIF4A/RGS2/ATP1B3/K
5691	tags=44%,	486/11004/	GO_MF	FXYD2/KIF2C/RAP1GAP/KIF4A/RGS2/ATP1B3/K
6339	tags=50%,	2810/1001	GO_MF	SFN/CD24/PLK1/CCNB1/LAPTM4B/TREM2/CCNB2/
6653	tags=53%,	2810/1001	GO_MF	SFN/CD24/PLK1/CCNB1/TREM2/CCNB2/TPX2/FO
4980	tags=41%,	2810/9232/	GO_MF	SFN/PTTG1/CAPG/PYCARD/CYBA/TEAD2/CABYR/
5120	tags=38%,	6690/2810/	GO_MF	SPINK1/SFN/CDC20/CD24/TESC/SPINT1/BIRC5/
4630	tags=45%,	6696/6286/	GO_MF	SPP1/S100P/PKM/SFN/S100A11/ALDOA/CCNB2/
6523	tags=49%,	332/79574/	GO_MF	BIRC5/EPS8L3/RAP1GAP/ENO1/ANXA2/NCF2/RA
4256	tags=34%,	226/79574/	GO_MF	ALDOA/EPS8L3/TMSB10/CAPG/S100A4/MARCKSL
5974	tags=38%,	5347/3984/	GO_MF	PLK1/LIMK1/AURKB/IKBKE/PKMYT1/CDK1/MELK/
5755	tags=45%,	486/11004/	GO_MF	FXYD2/KIF2C/KIF4A/ATP1B3/KIF20A/TOP2A/M
6625	tags=50%,	332/79574/	GO_MF	BIRC5/EPS8L3/RAP1GAP/ANXA2/NCF2/RAB3IL1/
6625	tags=50%,	332/79574/	GO_MF	BIRC5/EPS8L3/RAP1GAP/ANXA2/NCF2/RAB3IL1/

5120	tags=39%,	5909/5997/	GO_MF	RAP1GAP/RGS2/ATP1B3/RGS1/SH3BP1/RGS10/D
4140	tags=37%,	6280/332/	GO_MF	S100A9/BIRC5/PLK1/KIF2C/ALDOA/KIF4A/RGS
4630	tags=48%,	6286/5315/	GO_MF	S100P/PKM/SFN/S100A11/ALDOA/CCNB2/CAPG/
6290	tags=52%,	5909/5997/	GO_MF	RAP1GAP/RGS2/TUBA1C/RGS1/TUBA4A/TUBA1B/
4824	tags=35%,	6364/2919/	GO_MF	CCL20/CXCL1/PYCARD/CXCL6/CXCL8/EPO/CXCL
6005	tags=45%,	5909/5997/	GO_MF	RAP1GAP/RGS2/RGS1/SH3BP1/RGS10/DEPDC1B/
5113	tags=39%,	5909/5997/	GO_MF	RAP1GAP/RGS2/RGS1/SH3BP1/RGS10/DEPDC1B/
2906	tags=23%,	6696/6364/	GO_MF	SPP1/CCL20/CXCL1/CXCL6/CXCL8/EPO/CXCL5/
4115	tags=36%,	6280/332/	GO_MF	S100A9/BIRC5/PLK1/KIF2C/KIF4A/KIF20A/CR
6433	tags=54%,	3159/7153/	GO_MF	HMGA1/TOP2A/RECQL4/MCM2/CDC45/GINS1/MCM
4337	tags=39%,	822/1627/1	GO_MF	CAPG/DBN1/ARPC1B/MICAL1/MISP/POF1B/CORO
6047	tags=50%,	79574/468	GO_MF	EPS8L3/NCF2/ANLN/SH3BP1/ARHGEF16/ECT2/I
4900	tags=44%,	2810/1001	GO_MF	SFN/CD24/TESC/CCNB1/CCNB2/PKIB/EPO/CKS2
5301	tags=46%,	991/7153/	GO_MF	CDC20/TOP2A/NCF2/MKI67/SRC/DAB2/DNM1/CE
6579	tags=52%,	55723/553	GO_MF	ASF1B/HJURP/MCM2/CKS2/CHAF1B/TONSL/CBX2
6644	tags=73%,	116832/61	GO_MF	RPL39L/RPL8/RPL36A/MRPL17/MRPS12/RPS2/M
4527	tags=46%,	4171/8318/	GO_MF	MCM2/CDC45/MCM4/RAD51/NEIL3/MCM6/MCM10/
5383	tags=49%,	8877/9641/	GO_MF	SPHK1/IKBKE/REG1A/NEK2/LGALS3/EIF4EBP1/
4915	tags=50%,	9232/1535/	GO_MF	PTTG1/CYBA/CABYR/SH3BP1/MICAL1/DNM1/ENA
5117	tags=50%,	1410/8208/	GO_MF	CRYAB/CHAF1B/CLGN/CCT5/SERPINH1/TUBB4B/
4394	tags=44%,	6696/3383/	GO_MF	SPP1/ICAM1/ITGB4/SRC/ITGA3/MMP14/GPNMB/
2864	tags=31%,	11004/241	GO_MF	KIF2C/KIF4A/KIF20A/KIF23/KIFC1/KIF18B/K
4899	tags=54%,	7153/4171/	GO_MF	TOP2A/MCM2/RFC4/RAD51/RAD54L/MCM6/DSCC1
2743	tags=38%,	6282/302/	GO_MF	S100A11/ANXA2/EPCAM/DSG2/CDC42EP1/PKP3/
2702	tags=58%,	6282/302/	GO_MF	S100A11/ANXA2/EPCAM/CDC42EP1/PKP3/BAIAP
3686	tags=82%,	81620/588	GO_MF	CDT1/RAD51/FANCI/FANCD2/NABP2/ACD/PCNA/
332	tags=27%,	486/483/4	GO_MF	FXYD2/ATP1B3/ATP1A1
332	tags=27%,	486/483/4	GO_MF	FXYD2/ATP1B3/ATP1A1
2272	tags=73%,	6286/6280/	GO_MF	S100P/S100A9/S100A4/S100A8/HMGB2/FPR1/S
805	tags=60%,	3710/1103	GO_MF	ITPR3/ADAP1/ADAP2
5982	tags=39%,	5315/8877/	GO_MF	PKM/SPHK1/HKDC1/PLK1/CCNB1/CCNB2/LIMK1/
6646	tags=51%,	10644/729	GO_MF	IGF2BP2/TYMS/RBM38/IGF2BP1/CCT5/LARP6/E
5383	tags=46%,	8877/9641/	GO_MF	SPHK1/IKBKE/CTSC/REG1A/NEK2/LGALS3/TPRN
5603	tags=44%,	9401/4171/	GO_MF	RECQL4/MCM2/CDC45/GINS1/MCM4/RFC4/RAD51
6342	tags=54%,	23246/662	GO_MF	BOP1/SNRPB/NME1/EZH2/YBX3/EIF5A2/SNRPD1
4405	tags=46%,	9401/4171/	GO_MF	RECQL4/MCM2/CDC45/GINS1/MCM4/RFC4/RAD51
1827	tags=38%,	7053/2162/	GO_MF	TGM3/F13A1/TGM2
2230	tags=38%,	6536/2063	GO_MF	SLC6A9/SLC36A1/SLC38A5
1235	tags=50%,	4688/4689/	GO_MF	NCF2/NCF4/NCF1/SH3PXD2B
1585	tags=62%,	5621/1005	GO_MF	PRNP/SAE1/DNAJC5/STX1A/HSP90AB1
1116	tags=67%,	2717/3965/	GO_MF	GLA/LGALS9/LGALS2/LGALS4
1369	tags=83%,	6280/241/	GO_MF	S100A9/ALOX5AP/STX3/S100A8/FABP3
288	tags=40%,	2207/3958	GO_MF	FCER1G/LGALS3
4900	tags=41%,	2810/1001	GO_MF	SFN/CD24/TESC/CCNB1/CCNB2/PKIB/EPO/CKS2
3642	tags=34%,	55353/302/	GO_MF	LAPTM4B/ANXA2/PFN2/ATP13A2/PLEK/ADAP1/P
5960	tags=64%,	79574/468	GO_MF	EPS8L3/NCF2/SH3BP1/RCC2/ARHGAP4/FLNA/PK
2808	tags=58%,	990/4171/	GO_MF	CDC6/MCM2/CDC45/MCM4/ORC6/ORC1/MCM6/MCM
4405	tags=76%,	4171/5984/	GO_MF	MCM2/RFC4/RAD51/MCM6/DSCC1/MCM7/PIF1/MC
4405	tags=76%,	4171/5984/	GO_MF	MCM2/RFC4/RAD51/MCM6/DSCC1/MCM7/PIF1/MC
4039	tags=67%,	1047/972/	GO_MF	CLGN/CD74/HSPB1/HSPA6/HSPA8/PDCL3/CCT2/
4405	tags=76%,	4171/5984/	GO_MF	MCM2/RFC4/RAD51/MCM6/DSCC1/MCM7/PIF1/MC

2197	tags=57%,	6282/6277/	GO_MF	S100A11/S100A6/ANXA2/EZR/CACYBP/IQGAP1/S
2283	tags=73%,	1627/55740	GO_MF	DBN1/ENAH/VASP/FMNL1/RHOQ/WIPF1/ACTG1/C
512	tags=80%,	51/30/33/8	GO_MF	ACOX1/ACAA1/ACADL/ACOX2
1036	tags=100%,	6296/54988	GO_MF	ACSM3/ACSM5/ACSM2B/ACSM2A
333	tags=80%,	140679/654	GO_MF	SLC32A1/SLC6A13/SLC6A12/SLC6A1
392	tags=60%,	8993/929/1	GO_MF	PGLYRP1/CD14/PGLYRP2
2322	tags=57%,	2316/10042	GO_MF	FLNA/IGLL5/JCHAIN/FGR
751	tags=80%,	6280/241/6	GO_MF	S100A9/ALOX5AP/STX3/S100A8
110	tags=86%,	128/130/12	GO_MF	ADH5/ADH6/ADH1A/ADH1C/ADH1B/ADH4
1575	tags=86%,	4548/11185	GO_MF	MTR/INMT/TPMT/MGMT/BHMT2/BHMT
439	tags=71%,	6578/11457	GO_MF	SLCO2A1/SLC22A9/MFSD2A/SLC27A2/SLC27A5
785	tags=57%,	1583/66002	GO_MF	CYP11A1/CYP4F12/CYP4F2/CYP4A11
2743	tags=36%,	6282/302/4	GO_MF	S100A11/ANXA2/EPCAM/DSG2/CDC42EP1/PKP3/I
1148	tags=75%,	3032/3033/	GO_MF	HADHB/HADH/HSD17B4/HSD17B8/CRYL1/EHHADH
220	tags=88%,	128/137872	GO_MF	ADH5/ADHFE1/ADH6/ADH1A/ADH1C/ADH1B/ADH4
457	tags=88%,	1551/64816	GO_MF	CYP3A7/CYP3A43/CYP1A1/CYP3A5/CYP1A2/CYP2
333	tags=50%,	6540/6539/	GO_MF	SLC6A13/SLC6A12/SLC6A1/SLC10A1
333	tags=40%,	6540/6539/	GO_MF	SLC6A13/SLC6A12/SLC6A1/SLC10A1
1197	tags=82%,	27034/37/8	GO_MF	ACAD8/ACADVL/ACAD11/ACADS/IVD/GCDH/ACADI
1911	tags=53%,	9401/4171/	GO_MF	RECQL4/MCM2/CDC45/GINS1/MCM6/GINS2/MCM7/
4405	tags=75%,	4171/5984/	GO_MF	MCM2/RFC4/RAD51/MCM6/DSCC1/MCM7/PIF1/MCI
4405	tags=75%,	4171/5984/	GO_MF	MCM2/RFC4/RAD51/MCM6/DSCC1/MCM7/PIF1/MCI
457	tags=77%,	1551/64816	GO_MF	CYP3A7/CYP3A43/XDH/ACOX2/CYP3A5/AOX1/CYI
445	tags=31%,	116362/837	GO_MF	RBP7/RBP5/RBP4/ADH4
1491	tags=69%,	199974/113	GO_MF	CYP4Z1/CYP2U1/CYP4X1/CYP2C18/CYP2A13/CYI
4967	tags=33%,	5315/8877/	GO_MF	PKM/SPHK1/HKDC1/PLK1/CCNB1/CCNB2/LIMK1/I
6074	tags=46%,	7345/891/5	GO_MF	UCHL1/CCNB1/LAPTM4B/UBE2T/IKBKE/SRC/TUBA
4955	tags=40%,	6714/1601/	GO_MF	SRC/DAB2/ARHGAP4/COL1A2/NCK2/PAG1/RUSC1/
1963	tags=78%,	3872/3117/	GO_MF	KRT17/HLA-DQA1/HLA-DRA/HLA-DQB1/HLA-DPA
1212	tags=60%,	128/8659/2	GO_MF	ADH5/ALDH4A1/ALDH3A2/ALDH1B1/ALDH9A1/ALI
717	tags=80%,	1562/1573/	GO_MF	CYP2C18/CYP2J2/CYP2A13/CYP4F12/CYP2A7/C
1036	tags=71%,	116285/802	GO_MF	ACSM1/ACSF2/ACSL6/ACSM3/ACSL5/ACSL1/SLC2
1432	tags=50%,	4843/55163	GO_MF	NOS2/PNPO/DHODH/POR/IYD/HAO2/HAO1
1197	tags=55%,	37/91452/3	GO_MF	ACADVL/ACBD5/HMGCL/PNPLA3/ACBD4/ECI2/GCI
907	tags=45%,	8228/22122	GO_MF	PNPLA4/CES5A/PNPLA3/LIPG/CES1/CES4A/LIP
1148	tags=61%,	3032/23464	GO_MF	HADHB/GCAT/ACSM1/ACAA1/ACAA2/ACAT1/ACSM
1063	tags=56%,	1071/54762	GO_MF	CETP/GRAMD1C/APOE/STARD5/APOB/ABCG8/ABC
1063	tags=56%,	1071/54762	GO_MF	CETP/GRAMD1C/APOE/STARD5/APOB/ABCG8/ABC
717	tags=81%,	1562/1573/	GO_MF	CYP2C18/CYP2J2/CYP2A13/CYP4F12/CYP1A1/C
934	tags=56%,	338821/657	GO_MF	SLCO1B7/SLCO1A2/AKR1C4/SLCO2B1/ABCB11/SI
934	tags=50%,	338821/657	GO_MF	SLCO1B7/SLCO1A2/SLCO2A1/SLC22A9/SLCO2B1/
290	tags=42%,	5959/130/8	GO_MF	RDH5/ADH6/RDH16/HSD17B6/ADH1A/ADH1C/ADH
476	tags=53%,	116285/233	GO_MF	ACSM1/ACSL6/ACSM3/ACSL5/ACSL1/SLC27A2/A
972	tags=52%,	275/2805/5	GO_MF	AMT/GOT1/AADAT/GOT2/GPT2/GPT/AGXT2/ABAT/
972	tags=52%,	275/2805/5	GO_MF	AMT/GOT1/AADAT/GOT2/GPT2/GPT/GATM/AGXT2/
6821	tags=52%,	7345/55353	GO_MF	UCHL1/LAPTM4B/UBE2T/IKBKE/SRC/TUBA1B/TR
3671	tags=69%,	5315/3108/	GO_MF	PKM/HLA-DMA/CD74/HLA-DRB1/HLA-DRA/HLA-DI
1036	tags=54%,	55902/1162	GO_MF	ACSS2/ACSM1/ACSF2/ACSS3/ACSL6/ACSM3/ACSI
663	tags=33%,	6505/9962/	GO_MF	SLC1A1/SLC23A2/SLC13A3/SLC6A13/SLC1A2/SI
917	tags=33%,	169355/842	GO_MF	IDO2/BBOX1/BCO2/TDO2/ADI1/HAAO/HGD/CDO1/
6545	tags=72%,	1058/29128	GO_MF	CENPA/UHRF1/PHF19/RCC1/SMARCA4/ACTL6A/HI

917	tags=32%,	169355/842	GO_MF	IDO2/BBOX1/BCO2/TDO2/ADI1/HAAO/HGD/CDO1/
1036	tags=50%,	55902/1162	GO_MF	ACSS2/ACSM1/ACSF2/SUCLG2/ACSS3/ACSL6/AC
1362	tags=32%,	6256/733/2	GO_MF	RXRA/C8G/LCN12/CYP26A1/RBP7/SERPINA5/RB
1040	tags=38%,	51170/3294	GO_MF	HSD17B11/HSD17B2/HSD17B4/HSD17B8/AKR1C4/
4127	tags=44%,	891/9133/1	GO_MF	CCNB1/CCNB2/CKS2/CCNE1/CCNA2/CCNF/CDKN2
717	tags=55%,	1562/1551/	GO_MF	CYP2C18/CYP3A7/CYP2J2/CYP2A13/CYP4F12/C
547	tags=34%,	1408/51/79	GO_MF	CRY2/ACOX1/STEAP4/KMO/PRODH2/LDHD/XDH/A
2230	tags=42%,	6510/81539	GO_MF	SLC1A5/SLC38A1/SLC7A11/SFXN3/SLC6A9/SLC
648	tags=43%,	51170/3294	GO_MF	HSD17B11/HSD17B2/SRD5A1/HSD17B4/HSD17B8/
3873	tags=58%,	9401/55247	GO_MF	RECQL4/NEIL3/HMGB3/HMGB2/NR0B1/RBBP8/XR
3727	tags=57%,	3685/2817/	GO_MF	ITGAV/GPC1/FGFR4/RPS2/FGFR1/FGFR3/CXCL1
3142	tags=26%,	11004/2411	GO_MF	KIF2C/KIF4A/KIF20A/KIF23/KIFC1/KIF18B/K
1213	tags=42%,	6280/23643	GO_MF	S100A9/LY96/SYK/S100A8/UNC93B1
2996	tags=42%,	6364/2919/	GO_MF	CCL20/CXCL1/CXCL6/CXCL8/CXCL5/TFF2/S100
1056	tags=80%,	2327/2329/	GO_MF	FMO2/FMO4/FMO5/FMO3
888	tags=80%,	63826/3034	GO_MF	SRR/HAL/SDS/FTCD
355	tags=60%,	26061/5552	GO_MF	HACL1/DHTKD1/OGDHL
3752	tags=40%,	9833/6714/	GO_MF	MELK/SRC/RIPK2/PRKCD/SYK/HCK/JAK3/PKDCC/
3797	tags=56%,	6714/8878/	GO_MF	SRC/SQSTM1/SHCBP1/LAT2/SYK/NUP62/PAG1/S
6174	tags=49%,	84790/7277	GO_MF	TUBA1C/TUBA4A/TUBA1B/RAC2/TUBA3C/DNM1/A
6174	tags=49%,	84790/7277	GO_MF	TUBA1C/TUBA4A/TUBA1B/RAC2/TUBA3C/DNM1/A
785	tags=60%,	1583/1562/	GO_MF	CYP11A1/CYP2C18/CYP17A1/CYP3A7/CYP2J2/C
4163	tags=35%,	55353/302/	GO_MF	LAPTM4B/ANXA2/PFN2/ATP13A2/RACGAP1/PLEK
1149	tags=56%,	1595/2327/	GO_MF	CYP51A1/FMO2/CYP4F11/CYP26A1/MSMO1/KMO/
1036	tags=34%,	116285/802	GO_MF	ACSM1/ACSF2/SUCLG2/ACSS3/ACSL6/ACSM3/AC
946	tags=42%,	3040/875/1	GO_MF	HBA2/CBS/CYP2C18/CYP17A1/CYP26A1/HBB/TD
1148	tags=83%,	39/3032/30	GO_MF	ACAT2/HADHB/ACAA1/ACAA2/ACAT1
3757	tags=35%,	991/7153/3	GO_MF	CDC20/TOP2A/KPNA2/MAGEA1/PKN1/SMG5/ANKR
1115	tags=51%,	23464/3067	GO_MF	GCAT/HDC/DDC/PNPO/PYGL/THNSL2/GOT1/CBS/
1115	tags=51%,	23464/3067	GO_MF	GCAT/HDC/DDC/PNPO/PYGL/THNSL2/GOT1/CBS/
2708	tags=36%,	7053/2162/	GO_MF	TGM3/F13A1/QPCT/TGM2/QPCTL
1560	tags=50%,	1535/4688/	GO_MF	CYBA/NCF2/CYBB/NCF1/DUOX2
146	tags=38%,	991/5347/2	GO_MF	CDC20/PLK1/UBE2S
1099	tags=34%,	3067/1644/	GO_MF	HDC/DDC/HMGCL/MLYCD/MOCS1/GOT1/CYP17A1/
2927	tags=62%,	10007/7167	GO_MF	GNPDA1/TPI1/GPI/GNPDA2/RPIA
1867	tags=36%,	9975/405/8	GO_MF	NR1D2/ARNT/NR4A3/NR5A2/NR4A2/RXRA/NR3C1/
1349	tags=44%,	593/128/86	GO_MF	BCKDHA/ADH5/ALDH4A1/ALDH3A2/ALDH1B1/ALD
1371	tags=28%,	4881/8484/	GO_MF	NPR1/GALR3/CALCRL/NPR2/EDNRB/RAMP1/VIPR
1867	tags=36%,	9975/405/8	GO_MF	NR1D2/ARNT/NR4A3/NR5A2/NR4A2/RXRA/NR3C1/
1072	tags=33%,	6530/6579/	GO_MF	SLC6A2/SLCO1A2/AQP7/SLC22A3/AKR1C4/SLCO
1599	tags=67%,	64946/7272	GO_MF	CENPH/TTK/CENPE/SPDL1
751	tags=67%,	6280/241/6	GO_MF	S100A9/ALOX5AP/STX3/S100A8
3121	tags=83%,	10528/1095	GO_MF	NOP56/CBX1/HSP90AB1/CBX3/PRDM4
1803	tags=80%,	59342/5450	GO_MF	SCPEP1/CPVL/CTSA/CPD
5214	tags=100%,	338376/344	GO_MF	IFNE/IFNA5/IFNA21/IFNW1/IFNA4/IFNK/IFNA
987	tags=42%,	4284/363/3	GO_MF	MIP/AQP6/AQP8/AQP7/AQP9
987	tags=45%,	4284/363/3	GO_MF	MIP/AQP6/AQP8/AQP7/AQP9
3903	tags=45%,	6696/302/3	GO_MF	SPP1/ANXA2/ITGA3/LGALS3/LGALS1/AGRN/ITG
476	tags=38%,	23305/5170	GO_MF	ACSL6/ACSL5/ACSL1/SLC27A2/SLC27A5
1072	tags=31%,	6530/6557/	GO_MF	SLC6A2/SLC12A1/SLC22A3/SLC22A1
2031	tags=77%,	3283/3284/	GO_MF	HSD3B1/HSD3B2/EBP/IDI1/ECI1/ECH1/EBPL/E

445	tags=38%,	733/116362	GO_MF	C8G/RBP7/RBP5/RBP4/ADH4
1092	tags=52%,	338821/516	GO_MF	SLCO1B7/MPC1/SLC16A1/SLC16A12/SLC16A13/S
6174	tags=48%,	84790/7277	GO_MF	TUBA1C/TUBA4A/TUBA1B/RAC2/TUBA3C/DNM1/A
6174	tags=48%,	84790/7277	GO_MF	TUBA1C/TUBA4A/TUBA1B/RAC2/TUBA3C/DNM1/A
1297	tags=19%,	1145/3357/	GO_MF	CHRNE/HTR2B/CBS/BCHE/SLC6A13/CRHBP/CHRN
4127	tags=52%,	6714/1944/	GO_MF	SRC/EFNA3/EFNA4/EFNA5/EFNB1/SHC1/LYN/CD
1318	tags=39%,	1632/549/2	GO_MF	ECI1/AUH/FH/HADHB/TGDS/PCBD1/CA2/NAXD/A
6322	tags=60%,	9401/55247	GO_MF	RECQL4/NEIL3/FEN1/POLD1/HMGB2/FANCG/RBB
7012	tags=50%,	55353/302/	GO_MF	LAPTM4B/ANXA2/PFN2/ITPR3/ATP13A2/RACGAP
3875	tags=73%,	8767/837/6	GO_MF	RIPK2/CASP4/CARD9/CARD10/CARD19/CARD11/
1919	tags=39%,	10424/9975	GO_MF	PGRMC2/NR1D2/NR4A3/NR5A2/NR4A2/RXRA/NR3
1689	tags=40%,	2644/23576	GO_MF	GCHFR/DDAH1/SESN1/NOS2/SESN3/DDC/GCLC/T
1949	tags=50%,	27034/6389	GO_MF	ACAD8/SDHA/PTGR1/DPYD/PTGR2/SDHD/ZADH2/
1362	tags=43%,	6256/9971/	GO_MF	RXRA/NR1H4/ADH5/PCCA/LCN12/DBT/CYP4F11/
927	tags=36%,	3248/23411	GO_MF	HPGD/SIRT1/BDH2/ALDH1B1/SIRT5/NNT/HIBAD
1199	tags=80%,	3141/5095/	GO_MF	HLCS/PCCA/ACACB/PC
21	tags=17%,	9123	GO_MF	SLC16A3
737	tags=52%,	1071/54762	GO_MF	CETP/GRAMD1C/APOE/STARD5/TTPA/MTTP/APOB
3671	tags=50%,	5315/3108/	GO_MF	PKM/HLA-DMA/CD74/HLA-DRB1/HLA-DRA/HLA-D
2652	tags=59%,	3123/3117/	GO_MF	HLA-DRB1/HLA-DQA1/TAP1/SLC7A5/HLA-DRA/H
6174	tags=48%,	84790/7277	GO_MF	TUBA1C/TUBA4A/TUBA1B/RAC2/TUBA3C/DNM1/A
6481	tags=50%,	6714/1601/	GO_MF	SRC/DAB2/ARHGAP4/COL1A2/NCK2/PAG1/RUSC1
3674	tags=56%,	891/9212/9	GO_MF	CCNB1/AURKB/CDK1/AURKA/PKN1/CHEK1/CDK2/
515	tags=40%,	8659/2110/	GO_MF	ALDH4A1/ETFDH/QDPR/SARDH/PRODH2/ALDH1L1
4191	tags=37%,	3880/84790	GO_MF	KRT19/TUBA1C/TUBA4A/ARPC1B/TUBA1B/TUBA3
972	tags=30%,	57016/231/	GO_MF	AKR1B10/AKR1B1/AKR1C3
1362	tags=33%,	6256/733/2	GO_MF	RXRA/C8G/LCN12/CYP26A1/RBP7/SERPINA5/RB
6206	tags=69%,	3872/972/6	GO_MF	KRT17/CD74/TAP1/TUBB/TUBB4B/PILRA/BCAP3
2935	tags=57%,	128869/873	GO_MF	PIGU/GPAA1/THY1/DPEP1
1614	tags=75%,	1632/549/3	GO_MF	ECI1/AUH/HADHB/ECHS1/ECHDC2/EHHADH
717	tags=75%,	1562/1592/	GO_MF	CYP2C18/CYP26A1/CYP3A7/CYP3A5/CYP2C8/CY
512	tags=62%,	55316/51/3	GO_MF	RSAD1/ACOX1/ACAA1/ACADL/ACOX2
4684	tags=43%,	7345/7153/	GO_MF	UCHL1/TOP2A/CKS2/SQSTM1/HSPB1/UBXN11/NU
713	tags=40%,	788/6581/6	GO_MF	SLC25A20/SLC22A3/SLC22A1/SLC25A47
280	tags=45%,	5825/2168/	GO_MF	ABCD3/FABP1/SLC27A2/FABP4/SLC27A5
4075	tags=42%,	332/29108/	GO_MF	BIRC5/PYCARD/BIRC3/RPS6KA1/BEX3/TNFAIP8
4677	tags=53%,	332/1104/5	GO_MF	BIRC5/RCC1/RANGAP1/NUTF2/NXT1/XPO6/CSE1
1560	tags=40%,	1535/4688/	GO_MF	CYBA/NCF2/MICAL1/CYBB/NCF1/DUOX2
1251	tags=27%,	10786/6575	GO_MF	SLC17A3/SLC20A2/SLC6A2/SLC4A4/SLC12A1/S
26	tags=27%,	2904/2731/	GO_MF	GRIN2B/GLDC/SRR/GNMT
3039	tags=45%,	7040/10042	GO_MF	TGFB1/IGLL5/HLA-DRB1/HLA-DQA1/TAP1/SLC7
1148	tags=29%,	3032/23483	GO_MF	HADHB/TGDS/PCBD1/CA2/NEIL1/NAXD/ACO1/TH
6676	tags=62%,	4050/7186/	GO_MF	LTB/TRAF2/TRAF5/TNFSF9/BEX3/EDA/NOL3/ST
1212	tags=40%,	128/8659/2	GO_MF	ADH5/ALDH4A1/ALDH3A2/ALDH1B1/ALDH9A1/AL
4900	tags=47%,	2810/54997	GO_MF	SFN/TESC/PKIB/TRIB3/CDKN2A/HSPB1/CDKN2D
1026	tags=57%,	84105/5092	GO_MF	PCBD2/PCBD1/AGMO/PAH
1063	tags=53%,	1071/54762	GO_MF	CETP/GRAMD1C/APOE/STARD5/APOB/ABCG8/ABC
5363	tags=65%,	991/57761/	GO_MF	CDC20/TRIB3/FBXO5/UBE2L3/CBX8/RPL23/RPS
3727	tags=29%,	115908/127	GO_MF	CTHRC1/COL1A1/POSTN/MFAP2/FBLN1/COL1A2/
747	tags=48%,	9645/27034	GO_MF	MICAL2/ACAD8/SDHA/COQ6/TXNRD3/MICAL3/DP
7782	tags=100%,	50840/5726	GO_MF	TAS2R14/TAS2R38/TAS2R4/TAS2R20/TAS2R1/T

3727	tags=48%,	3675/3958/	GO_MF	ITGA3/LGALS3/LGALS1/AGRN/ADAM9/GPC1/RPS
330	tags=22%,	2395/5264/	GO_MF	FXN/PHYH/HAAO/TF/CDO1
3590	tags=67%,	23640/108(	GO_MF	HSPBP1/HSPH1/PFN1/BAG3/GRPEL2/BAG2
4843	tags=28%,	6696/6364/	GO_MF	SPP1/CCL20/CXCL1/CXCL6/MDK/CXCL8/EPO/CX
3384	tags=75%,	6628/6632/	GO_MF	SNRPB/SNRPD1/SNRPA/SNRPC/PRPF31/SNRPB2
663	tags=44%,	6505/6484(	GO_MF	SLC1A1/SLC13A3/SLC1A2/SLC13A5
1072	tags=40%,	6530/6557/	GO_MF	SLC6A2/SLC12A1/SLC22A3/SLC22A1
8574	tags=100%,	29991/317	GO_MF	OBP2A/VN1R5/OR8D1/OR8B8/OBP2B/VN1R2/VN1
1806	tags=48%,	22949/180(	GO_MF	PTGR1/DPYD/PTGR2/ZADH2/DECR2/DECR1/BDH2
5629	tags=76%,	1058/1104/	GO_MF	CENPA/RCC1/SMARCA4/ACTL6A/HDAC1/HDAC2/H
2935	tags=44%,	332/330/61	GO_MF	BIRC5/BIRC3/RPS6KA1/TNFAIP8/NOL3/LEF1/M
5377	tags=56%,	83442/729(	GO_MF	SH3BGRL3/TXNRD1/PDIA2/GSR/PGK1/PTGES2/S
5814	tags=43%,	302/5866/(	GO_MF	ANXA2/RAB3IL1/MICAL1/NDRG1/CHML/UNC13D/
737	tags=21%,	733/10613/	GO_MF	C8G/ERLIN1/CETP/GRAMD1C/APOE/STARD5/RBP
6718	tags=69%,	10551/848(	GO_MF	AGR2/LINGO1/AREG/ITGA5/TGFA/CNOT9/SHC1/
3177	tags=43%,	6509/2063	GO_MF	SLC1A4/SLC36A1/SLC36A4
626	tags=20%,	710/5037/	GO_MF	SERPING1/PEBP1/SERPINF1/SERPINA10/SERPI
6174	tags=47%,	84790/727	GO_MF	TUBA1C/TUBA4A/TUBA1B/RAC2/TUBA3C/DNM1/A
6174	tags=47%,	84790/727	GO_MF	TUBA1C/TUBA4A/TUBA1B/RAC2/TUBA3C/DNM1/A
4684	tags=46%,	7345/7153/	GO_MF	UCHL1/TOP2A/CKS2/SQSTM1/HSPB1/UBXN11/NU
557	tags=67%,	1468/25351	GO_MF	SLC25A10/SLC25A30/SLC13A3/SLC13A5
886	tags=83%,	224/219/2	GO_MF	ALDH3A2/ALDH1B1/ALDH9A1/ALDH7A1/ALDH2
3752	tags=29%,	9833/7301/	GO_MF	MELK/TYRO3/SRC/TTK/RIPK2/EFNA3/PRKCD/EF
714	tags=32%,	4843/1666/	GO_MF	NOS2/DECR1/FMO2/NNT/CBR4/HIBADH/POR/SRD
496	tags=86%,	51327/6521	GO_MF	AHSP/SLC4A1/HBD/HBB/HP/HPR
2318	tags=80%,	3684/3689/	GO_MF	ITGAM/ITGB2/VSIG4/PHB
1056	tags=48%,	2327/2602(	GO_MF	FMO2/CYP4X1/PCBD1/CYP4F22/CYP4F11/CYP11
1349	tags=44%,	593/594/5	GO_MF	BCKDHA/BCKDHB/DHTKD1/OGDHL
5241	tags=34%,	4318/4316/	GO_MF	MMP9/MMP7/MMP12/MMP11/MMP14/ADAM9/ADAM1
1040	tags=40%,	51170/329	GO_MF	HSD17B11/HSD17B2/HSD17B8/HSD17B6
6199	tags=53%,	23466/2321	GO_MF	CBX6/JMJD6/POLR2G/MCRS1/RBM3/PABPC1/DDX
6686	tags=47%,	3159/5307/	GO_MF	HMGA1/PITX1/E2F1/SOX9/TEAD2/SRC/CCNE1/C
3246	tags=55%,	9296/533/	GO_MF	ATP6V1F/ATP6V0B/ATP6V0D2/ATP6V1C1/TCIRG
3246	tags=55%,	9296/533/	GO_MF	ATP6V1F/ATP6V0B/ATP6V0D2/ATP6V1C1/TCIRG
6596	tags=47%,	3159/6659/	GO_MF	HMGA1/SOX4/NFE2L3/CD3D/CCNE1/PKN1/SMARC
7646	tags=56%,	54913/915(	GO_MF	RPP25/EXO1/RNASEH2A/DDX39A/FEN1/PIF1/RN
3069	tags=80%,	128869/94(	GO_MF	PIGU/PIGS/GPAA1/PIGT
1191	tags=50%,	94097/648	GO_MF	SFXN5/SLC13A3/SLC13A5
1191	tags=50%,	94097/648	GO_MF	SFXN5/SLC13A3/SLC13A5
471	tags=67%,	338328/44(	GO_MF	GPIHBP1/PLIN5/APOB/APOA5
713	tags=20%,	788/6581/	GO_MF	SLC25A20/SLC22A3/SLC25A42/SLC22A1/SLC25
692	tags=32%,	4035/1191/	GO_MF	LRP1/CLU/APOE/APOB/APOA2/APOA1/APOA5/AP
737	tags=24%,	54762/348/	GO_MF	GRAMD1C/APOE/GPER1/RORA/STARD5/SULT1E1/
499	tags=57%,	291/9962/	GO_MF	SLC25A4/SLC23A2/SLC28A1/AQP9
3984	tags=73%,	7186/7188/	GO_MF	TRAF2/TRAF5/RAC1/TRAF3/CDC42/ADRA2A/HAU
4088	tags=50%,	4050/7186/	GO_MF	LTB/TRAF2/TRAF5/TNFSF9/EDA/STAT1/TNFSF1
3478	tags=56%,	3149/3148/	GO_MF	HMGB3/HMGB2/XRCC2/XRCC3/BLM/MSH2/RAD51C
2906	tags=41%,	6364/2919/	GO_MF	CCL20/CXCL1/CXCL6/CXCL8/CXCL5/CKLF/CCL5
734	tags=29%,	5146/5094(	GO_MF	PDE6C/PDE11A/PDE2A/CNGA1
1407	tags=24%,	1956/4881/	GO_MF	EGFR/NPR1/NR3C1/CHRNE/GALR3/ABHD2/CALCR
442	tags=18%,	7345/8015(	GO_MF	UCHL1/ASRGL1

1072	tags=23%,	6530/6505/	GO_MF	SLC6A2/SLC1A1/SLC6A13/SLC1A2/SLC6A12/SL
10303	tags=100%,	54429/903	GO_MF	TAS2R5/PKD2L1/TAS2R14/TAS2R38/TAS2R4/TA
1197	tags=46%,	37/91452/	GO_MF	ACADVL/ACBD5/HMGCL/OXER1/PNPLA3/ACBD4/E
2511	tags=80%,	2264/2260/	GO_MF	FGFR4/FGFR1/FGFR3/FGFR2
508	tags=40%,	445/1828/	GO_MF	ASS1/DSG1/EPHX2/ALB
1115	tags=80%,	39/23464/1	GO_MF	ACAT2/GCAT/ACAA2/ACAT1
29	tags=40%,	132112/83	GO_MF	RTP1/RTP3
3332	tags=37%,	486/483/3	GO_MF	FXYD2/ATP1B3/ANXA5/ATP13A2/ATP1A1/ATP6V
3332	tags=37%,	486/483/3	GO_MF	FXYD2/ATP1B3/ANXA5/ATP13A2/ATP1A1/ATP6V
2108	tags=67%,	6628/6632/	GO_MF	SNRPB/SNRPD1/SNRPA/SNRPC
737	tags=29%,	54762/348/	GO_MF	GRAMD1C/APOE/GLTPD2/STARD5/TMEM30B/ABCA
737	tags=32%,	1071/5476	GO_MF	CETP/GRAMD1C/APOE/STARD5/APOB/ABCG8/ABC
1199	tags=21%,	5095/6408	GO_MF	PCCA/MCCC2/ACSM1/PCCB/GCLC/ACSF2/ACACB/
1229	tags=35%,	348751/73	GO_MF	FTCDNL1/C8G/PCCA/GCAT/HDC/DDC/PNPO/PYGL
1041	tags=35%,	260293/12	GO_MF	CYP4X1/CYP4F22/HBA2/CYP4F11/FLVCR2/IDO2
1177	tags=24%,	54414/110	GO_MF	SIAE/ABHD2/CA2/CES5A/FAAH/ACOT1/PNPLA7/
591	tags=38%,	344/341/3	GO_MF	APOC2/APOC1/APOA2/ANGPTL3/APOA1/APOC3
3008	tags=33%,	4318/4321/	GO_MF	MMP9/MMP12/ITGA3/CD44/ADAM9/DDR1/SERPIN
3664	tags=35%,	1277/1278/	GO_MF	COL1A1/COL1A2/COL9A2/COL4A5/COL16A1/COL
1072	tags=27%,	6006/5145	GO_MF	RHCE/RHCG/SLC6A2/SLC25A20/SLC22A3/RHBG/
4158	tags=58%,	5360/2340	GO_MF	PLTP/ATP13A2/NRGN/MICALL1/PITPNM1/UQCC3
3911	tags=29%,	6364/7345/	GO_MF	CCL20/UCHL1/CXCL1/CTHRC1/CXCL6/CXCL8/HO
7420	tags=51%,	9242/6756/	GO_MF	MSC/SSX1/TRIB3/TONSL/RELB/EZH2/HDAC7/TL
4638	tags=45%,	983/890/51	GO_MF	CDK1/CCNA2/CDK16/CDK4/CDK2/CDK5/CDK18/C
8299	tags=100%,	79339/2621	GO_MF	OR51B4/OR2B6/OR1F1/OR2C3/OR51E2/OR7A5/O
691	tags=26%,	84869/1111	GO_MF	CBR4/HIBADH/HADH/HSD17B2/HSD17B4/HSD17B
1589	tags=50%,	10331/840	GO_MF	B3GNT3/B3GNT5/B3GNT9/B3GNT7
1228	tags=46%,	1468/8373	GO_MF	SLC25A10/SLC25A18/SLC25A13/SLC25A30/SLC
2925	tags=40%,	23636/102	GO_MF	NUP62/NUTF2/UPF3B/NUP93/NUP205/NDC1/NUP
1251	tags=21%,	10786/657	GO_MF	SLC17A3/SLC20A2/SLC6A2/SLC2A9/SLC4A4/SL
5694	tags=70%,	116372/40	GO_MF	LYPD1/LY6E/LYPD6B/CDK5/FNTA/LY6H/LYPD6
5694	tags=70%,	116372/40	GO_MF	LYPD1/LY6E/LYPD6B/CDK5/FNTA/LY6H/LYPD6
3359	tags=21%,	6696/6364/	GO_MF	SPP1/CCL20/CXCL1/CXCL6/MDK/CXCL8/EPO/CX
2577	tags=38%,	10331/797	GO_MF	B3GNT3/COLGALT1/B4GALT5/B3GNT5/B3GNT9/B
691	tags=26%,	84869/1111	GO_MF	CBR4/HIBADH/HADH/HSD17B2/HSD17B4/HSD17B
1041	tags=33%,	260293/12	GO_MF	CYP4X1/CYP4F22/HBA2/CYP4F11/FLVCR2/IDO2
593	tags=40%,	3691/3685	GO_MF	ITGB4/ITGAV
5104	tags=62%,	302/23413/	GO_MF	ANXA2/NCS1/CDK5/CACNA1D/CNR1
1036	tags=83%,	116285/62	GO_MF	ACSM1/ACSM3/ACSM5/ACSM2B/ACSM2A
5255	tags=40%,	486/483/71	GO_MF	FXYD2/ATP1B3/TOP2A/MCM2/ANXA5/ATP13A2/R
1903	tags=33%,	114569/83	GO_MF	MAL2/MARVELD1/PLLP
3323	tags=56%,	80119/4831	GO_MF	PIF1/NME2/DDX11/BLM/LONP1
1421	tags=24%,	6566/1540	GO_MF	SLC16A1/SLC2A12/SLC38A2/SLC17A3/SLC25A1
3148	tags=60%,	1122/1063	GO_MF	CHML/RGS14/GPSM1/GDI1/GPSM2/ARHGDIB/ITG
1228	tags=27%,	83733/108	GO_MF	SLC25A18/SLC26A1/ABCC2/SLC25A13/SLC19A2
737	tags=24%,	10613/1071	GO_MF	ERLIN1/CETP/GRAMD1C/APOE/RORA/STARD5/SC
6337	tags=71%,	6628/2338	GO_MF	SNRPB/SMG5/DKC1/NHP2/WRAP53/NOP10/GAR1/
4582	tags=46%,	1601/5758	GO_MF	DAB2/SYT13/DNAJC6/CLTA/SYT8/BIN1/GPR107
6206	tags=81%,	6890/2030	GO_MF	TAP1/TUBB/TUBB4B/PILRA/BCAP31/CD8A/VCP/
1251	tags=50%,	10786/656	GO_MF	SLC17A3/SLC17A1/SLC17A4/SLC17A2
3233	tags=67%,	9641/3654/	GO_MF	IKBKE/IRAK1/MAP3K14/PPP4C

1885	tags=50%,	3872/972/3	GO_MF	KRT17/CD74/LAG3
424	tags=17%,	1109/22977	GO_MF	AKR1C4/AKR7A3/AKR1D1/ADH4
1045	tags=21%,	51279/8375	GO_MF	C1RL/LONP2/C1R/C2/LPAL2/C1S/DPP4/CFI/F1
5194	tags=64%,	54503/2544	GO_MF	ZDHHC13/SLC41A1/TUSC3/CNNM4/MMGT1/NIPAL
5383	tags=58%,	8877/1978/	GO_MF	SPHK1/EIF4EBP1/SMG5/STAT1/PPME1/STRN4/C
4701	tags=54%,	5315/4199/	GO_MF	PKM/ME1/ATP1A1/ABCD1/TAP1/PGK1/RUVBL1/C
1226	tags=33%,	9446/4257/	GO_MF	GSTO1/MGST1/MGST2/GSTM5/GSTZ1/GSTA2/GST
389	tags=67%,	2878/1734/	GO_MF	GPX3/DIO2/DIO1/SELENBP1
4127	tags=33%,	8878/1741/	GO_MF	SQSTM1/DLG3/SCRIB/FLOT2/FLOT1/CACNG4/FU
957	tags=42%,	128/1629/5	GO_MF	ADH5/DBT/CYP4F11/OXER1/PPARA/HNF4A/ACOX
1041	tags=31%,	260293/126	GO_MF	CYP4X1/CYP4F22/HBA2/CYP4F11/FDX1/CYP11A
1130	tags=50%,	122970/558	GO_MF	ACOT4/ACOT13/HIBCH/ACOT1/ACOT2/NUDT7/AC
3648	tags=48%,	128869/873	GO_MF	PIGU/GPAA1/MANF/GLTP/THY1/LYN/DPEP1/CLI
5764	tags=79%,	5698/5690/	GO_MF	PSMB9/PSMB2/PSMB4/PSMB8/PSMB10/PSMA5/PS
5764	tags=79%,	5698/5690/	GO_MF	PSMB9/PSMB2/PSMB4/PSMB8/PSMB10/PSMA5/PS
7292	tags=46%,	9242/3159/	GO_MF	MSC/HMGA1/SOX9/SPI1/RELB/NPAS2/BHLHE41/
5308	tags=67%,	112399/641	GO_MF	EGLN3/P3H1/P4HA2/P3H3/EGLN2/P3H2/P4HB/O
1352	tags=20%,	112483/114	GO_MF	SAT2/CHRNE/PCTP/SAT1/HTR2B/CETP/DRD1/BC
6337	tags=56%,	9156/2237/	GO_MF	EXO1/FEN1/POLD1/EXOSC4/APEX2/ERI3/USB1/
7345	tags=46%,	29089/2733	GO_MF	UBE2T/UBE2S/TRIM47/TRAIP/DTL/UHRF1/BIRC
1549	tags=83%,	6390/93058	GO_MF	SDHB/COQ10A/SDHD/DHODH/ETFDH
2939	tags=35%,	54443/5585	GO_MF	ANLN/PKN1/CDC42EP1/RTKN/PKN3/KCTD10/CDC
1099	tags=40%,	3965/81035	GO_MF	LGALS9/COLEC12
2868	tags=80%,	7345/333/1	GO_MF	UCHL1/APLP1/ADRA2C/GRK2
985	tags=30%,	10786/8373	GO_MF	SLC17A3/SLC25A18/SFXN5/SLC16A12/SLC16A1
1251	tags=18%,	10786/6329	GO_MF	SLC17A3/SCN4A/SLC20A2/SCN4B/KCNK1/SLC6A
1056	tags=33%,	2327/26029	GO_MF	FMO2/CYP4X1/PCBD1/CYP4F22/CYP4F11/CYP11
1199	tags=71%,	5095/64087	GO_MF	PCCA/MCCC2/PCCB/ACACB/PC
985	tags=30%,	10786/8373	GO_MF	SLC17A3/SLC25A18/SFXN5/SLC16A12/SLC16A1
3457	tags=37%,	23657/293/	GO_MF	SLC7A11/SLC25A6/SLC26A6/SLC4A2/SLC37A1/
4701	tags=78%,	9401/641/4	GO_MF	RECQL4/BLM/MSH2/RPS3/MUTYH/OGG1/MSH6
4701	tags=78%,	9401/641/4	GO_MF	RECQL4/BLM/MSH2/RPS3/MUTYH/OGG1/MSH6
5452	tags=59%,	6627/6626/	GO_MF	SNRPA1/SNRPA/LSM4/EFTUD2/NCBP2/LSM7/LSM
5184	tags=70%,	54751/1400	GO_MF	FBLIM1/CRMP1/MICALL2/NEBL/DPYSL3/TMEM67
7311	tags=56%,	10644/5554	GO_MF	IGF2BP2/RBM38/IGF2BP1/CCT5/EXOSC4/IGF2B
3332	tags=36%,	486/483/30	GO_MF	FXYD2/ATP1B3/ANXA5/ATP13A2/ATP1A1/ATP6V
5895	tags=100%,	4889/2834/	GO_MF	NPY5R/PRLHR/PROKR2/NPY2R/QRFPR/NPY6R/NP
302	tags=38%,	6470/10840	GO_MF	SHMT1/ALDH1L1/FTCD
3831	tags=37%,	10331/2583	GO_MF	B3GNT3/B4GALNT1/GALNT10/B3GNT5/GBGT1/B3
955	tags=24%,	11004/7461	GO_MF	KIF2C/CLIP2/MAPRE1/KIF18A
984	tags=40%,	2729/6505/	GO_MF	GCLC/SLC1A1/GLUL/CPS1
2022	tags=40%,	1649/26574	GO_MF	DDIT3/AATF/DAPK3/ATF4
4178	tags=29%,	1364/1192/	GO_MF	CLDN4/CLIC1/FXYD3/TTYH3/ANO9/CLIC3/SLC2
4952	tags=52%,	54913/9156	GO_MF	RPP25/EXO1/RNASEH2A/FEN1/POP7/POP1/EDC3
2230	tags=33%,	6510/81539	GO_MF	SLC1A5/SLC38A1/SLC7A7/SLC7A1/SLC7A11/SL
4288	tags=62%,	2526/2525/	GO_MF	FUT4/FUT3/FUT2/FUT6/FUT1/POFUT2/FUT8/FU
6201	tags=64%,	8662/8894/	GO_MF	EIF3B/EIF2S2/EIF3D/EIF3I/EIF2S3/DENR/EI
5304	tags=56%,	5768/64714	GO_MF	QSOX1/PDIA2/ERO1A/PDIA6/GLRX2/PDIA3/P4H
5304	tags=56%,	5768/64714	GO_MF	QSOX1/PDIA2/ERO1A/PDIA6/GLRX2/PDIA3/P4H
1981	tags=67%,	6510/81855	GO_MF	SLC1A5/SFXN3/SLC1A4/SLC7A10
1487	tags=34%,	122970/209	GO_MF	ACOT4/ESD/ACOT13/HIBCH/ACOT1/ACOT2/NUDT

476	tags=50%,	23305/517(	GO_MF	ACSL6/ACSL5/ACSL1/SLC27A2
476	tags=50%,	23305/517(	GO_MF	ACSL6/ACSL5/ACSL1/SLC27A2
3626	tags=37%,	29108/107[illegible]	GO_MF	PYCARD/CTSC/FBLN1/PSMD14/BEX3/PSME3/ADR[illegible]
6998	tags=40%,	4605/9242/	GO_MF	MYBL2/MSC/ETV4/KIF2C/HMGA1/PITX1/FOXM1/[illegible]
3894	tags=100%,	10452/741(	GO_MF	TOMM40/VDAC1/TOMM40L/VDAC3/VDAC2
1604	tags=43%,	128239/78[illegible]	GO_MF	IQGAP3/CXCR4/MYO19

A/AURKB/TPX2/CENPA/TACC3/KIF20A/TUBA1C/TUBA4A/CRYAB/NUF2/CDK1/NDC80/NEK2/STMN1/
PTTG1/KIF2C/ASNS/CCNB2/CDT1/UBD/KIF4A/AURKB/TPX2/CENPA/FOXM1/SOX4/TACC3/CDCA8/K
DK/DBN1/ICAM1/POSTN/REG1A/STMN1/FBLIM1/TYRO3/PLXNA1/SRC/SEMA6A/SPINT2/RAC2/MELT
2/PYCARD/CYBA/CXCL6/LY96/MDK/TNFRSF21/HOMER3/MMP12/IKBKE/FCER1G/POSTN/HILPDA/LG
D/CYBA/CXCL6/LY96/MDK/TNFRSF21/HOMER3/MMP12/IKBKE/FCER1G/POSTN/HILPDA/LGALS9/SU
MP12/FCER1G/POSTN/HILPDA/LGALS9/SULF1/SRC/MAPK13/TYROBP/TGFB1/CYBB/GAPDH/RIPK2/
EAD2/TGM3/SH3BP1/SULF1/PLXNA1/SRC/ETV5/SPINT2/DAB2/MMP14/LIF/SAPCD2/CSF1/TGFB1/
OA/LPCAT1/IMPDH1/TREM2/PYCARD/CYBA/LCN2/MDK/ADGRE5/ANXA2/GLA/CTSC/FCER1G/ICAM1/
L1/ALDOA/LPCAT1/IMPDH1/TREM2/PYCARD/CYBA/UBD/CXCL6/LCN2/CXCL8/ADGRE5/ANXA2/GLA/
OA/LPCAT1/IMPDH1/TREM2/PYCARD/CYBA/LCN2/ADGRE5/ANXA2/GLA/CTSC/FCER1G/LGALS9/TMC
OA/LPCAT1/IMPDH1/PYCARD/CYBA/LCN2/ADGRE5/ANXA2/GLA/CTSC/FCER1G/TMC6/LGALS3/PLAU
OA/LPCAT1/IMPDH1/TREM2/PYCARD/CYBA/LCN2/MDK/ADGRE5/ANXA2/GLA/CTSC/FCER1G/ICAM1/
/ALDOA/GCNT3/LPCAT1/IMPDH1/PYCARD/CYBA/CXCL6/LCN2/ADGRE5/ANXA2/GLA/CTSC/FCER1G/
OA/LPCAT1/IMPDH1/PYCARD/CYBA/CXCL6/LCN2/ADGRE5/ANXA2/GLA/CTSC/FCER1G/TMC6/LGALS
/TOP2A/EPO/ANXA2/FCER1G/MELK/ANLN/LGALS9/BAK1/FOXJ1/TYRO3/SRC/CD3D/LGALS3/MMP14
1G/LGALS9/BAK1/FOXJ1/TYRO3/SRC/CD3D/MMP14/LIF/CSF1/TYROBP/TGFB1/CMTM7/SPI1/LGAL
PYCARD/CYBA/LIMK1/LY96/PGC/MDK/CXCL8/TNFRSF21/EPO/MMP12/IKBKE/CTSC/FCER1G/ICAM1
KBKE/FCER1G/ARPC1B/TYRO3/SRC/CD3D/LGALS3/S100A14/ITGAM/FCGR3A/RIPK2/ADA/HAVCR2/
/SRC/CD3D/LGALS3/FCGR3A/RIPK2/ADA/CMTM3/PRKCD/LAT2/RELB/IGLL5/HLA-DRB1/PRNP/HRA
KB/UBE2T/FOXM1/TOP2A/TRIP13/RRM2/CDCA5/CDC6/RECQL4/TK1/KPNA2/CDK1/MCM2/CDC45/NE
CQL4/CDK1/MCM2/CDC45/MCM4/NUDT1/RMI2/RFC4/CHAF1B/RAD51/TONSL/FANCI/EXO1/NEIL3/D
B/CDK1/HJURP/MCM2/CDC45/MKI67/CCNA2/LIF/CHAF1B/CENPH/TGFB1/AURKA/SPI1/CBX2/HDAC
39A/SNRPB/LAGE3/SF3B4/DCAF13/PUSL1/LARP6/EXOSC4/ALYREF/TSEN54/NOP56/ESRP1/RALY/
17/EIF4EBP1/PTAFR/HSPB1/NCK2/IGF2BP1/LARP6/EEF1E1/CDK4/TCOF1/IGF2BP3/ZNF385A/RP
OA/LPCAT1/IMPDH1/PYCARD/LGALS3BP/CYBA/LCN2/ADGRE5/TUBA4A/ANXA2/GLA/CTSC/FCER1G/
MDK/CXCL8/CXCL5/FCER1G/LGALS9/VEGFB/PLXNA1/SRC/PLP2/SEMA6A/ARHGEF16/LGALS3/PLAU
/UBE2T/FOXM1/SOX4/TOP2A/TRIP13/IER3/E2F1/CDCA5/RECQL4/IKBKE/CDK1/MCM2/CDC45/BAK
C/KIF4A/AURKB/TPX2/CENPA/TACC3/KIF20A/TUBA1C/TUBA4A/CRYAB/NUF2/CDK1/NDC80/NEK2/
DT1/UBD/AURKB/TPX2/SOX4/TACC3/TOP2A/TRIP13/E2F1/CDCA5/CDC6/TUBA4A/PKMYT1/CDK1/N
6/CTHRC1/PYCARD/CYBA/FOXM1/SOX4/MDK/E2F1/SOX9/CDC6/EPO/MMP12/MARCKSL1/ANXA2/CXC
TSEN54/ESRP1/RALY/SNRPD2/SNRPD1/SNRPA1/CSTF2/SNRPA/SF3A2/CD2BP2/JMJD6/LSM4/HSPA
17/AURKB/UBE2S/CDK1/BUB1B/DAB2/PBK/TRIB3/CCNF/AURKA/UBXN11/MAD2L1/PSMB9/GIPC1/D
BD/AURKB/TPX2/FOXM1/SOX4/TACC3/KIF20A/TRIP13/RRM2/E2F1/CDCA5/SOX9/CDC6/TUBA4A/P
PYCARD/PKIB/LIMK1/AURKB/E2F1/CDCA5/ANXA2/ICAM1/ARPC1B/PFN2/NEK2/BAK1/DLGAP5/SRC
/UBD/SOX4/TOP2A/E2F1/CTSC/MELK/LGALS9/ITM2C/BAK1/ANXA5/SRC/TSPO/ARHGEF16/PLAUR/
C52A2/TOP2A/ANXA2/IKBKE/IL2RG/KPNA2/CDK1/ICAM1/LGALS9/BUB1/TYRO3/SRC/CCNA2/FBLN
M38/DDX39A/SNRPB/PRKCD/HSPB1/SMG5/IGF2BP1/SF3B4/SMG9/EXOSC4/ALYREF/TSEN54/PKP3/
SPI1/HDAC11/UHRF1/PHF19/PKN1/PRKCD/EZH2/DNMT1/HDAC7/MUC1/CHEK1/PRMT1/YEATS2/TRI
/PYCARD/CXCL6/LCN2/CXCL8/EPO/MMP12/ANXA2/IKBKE/CXCL5/IL2RG/FCER1G/ICAM1/PLP2/TI
CCNB2/CAPG/TOP2A/MMP12/CDK1/STMN1/SH3BP1/MICAL1/TIMP1/TSPO/MMP11/ATP13A2/MMP14/
RPD2/RAN/SNRPD1/MRTO4/SF3A2/DHX37/RUVBL1/CD2BP2/LSM4/EIF3B/PES1/DKC1/NPM1/NOL3/
MK1/MDK/DBN1/ICAM1/ANLN/ARPC1B/PFN2/STMN1/SH3BP1/FOXJ1/MICAL1/SRC/KIF23/RAC2/AT
ICAM1/ANLN/PFN2/NEK2/FOXJ1/SRC/RAC2/RHOV/RCC2/STK26/FLNA/EMP3/AGRN/CEP131/FXYD5
MK1/MDK/DBN1/ICAM1/ANLN/ARPC1B/PFN2/STMN1/SH3BP1/FOXJ1/MICAL1/SRC/KIF23/RAC2/TY
/EPO/ANXA2/FCER1G/MELK/ANLN/LGALS9/BAK1/FOXJ1/TYRO3/SRC/CD3D/LGALS3/MMP14/LIF/C
1G/SRC/LGALS3/LIF/CSF1/TYROBP/TGFB1/SPI1/GPC3/C1QC/CD74/BATF/RELB/EFNA4/NME1/CD
1/SOX9/EPO/MMP12/COL1A1/ICAM1/POSTN/LGALS9/FOXJ1/EPCAM/PLXNA1/SRC/ITGA3/SEMA6A/
TM4B/TREM2/KCTD17/UBD/BACE2/AURKB/PGC/SOX9/UBE2S/MMP12/ANXA2/CTSC/CDK1/BUB1B/TI
CARD/MDK/CXCL8/SOX9/COL1A1/ICAM1/POSTN/LGALS9/PFN2/VEGFB/SH3BP1/SULF1/PLXNA1/SR
CTHRC1/LIMK1/RGS2/MDK/TNFRSF21/EPO/DBN1/POSTN/REG1A/ITM2C/STMN1/PLXNA1/SRC/ITGA

KCTD17/LIMK1/RGS2/LCN2/MDK/EPO/DBN1/ICAM1/ANLN/REG1A/PFN2/ITM2C/PLXNA1/SRC/ITGA
BE2S/CDCA3/TSPO/TRIM47/TRIB3/MAGEC2/CCNF/TRAIP/FANCI/RIPK2/DTL/UHRF1/BIRC3/BLMH
D/SOX4/MDK/TNFRSF21/E2F1/CDCA5/SLC2A1/MMP12/CDK1/NDC80/FCER1G/POSTN/LGALS9/ANXA
X9/MMP12/KPNA2/CDK1/NDC80/COL1A1/ANLN/KDELR3/SRC/TSPO/RAC2/LIF/ECT2/ARL2/TTK/TG
A/SOX9/UBE2S/CRYAB/DBN1/ASF1B/HJURP/MCM2/ICAM1/CDC45/ANLN/ARPC1B/PFN2/STMN1/SRC
2A6/NUDT1/GAPDH/ADA/IGF2BP2/YWHAZ/RNASEH2A/RBM38/FEN1/HK2/PFKFB4/PRKCD/HSPB1/SM
E1/EXOSC4/TCOF1/TSEN54/NOP56/GTF3C6/RAN/NABP2/WDR4/INTS8/LIN28B/SLFN13/CSTF2/MR
OA/LPCAT1/IMPDH1/PYCARD/CYBA/CXCL6/LCN2/CXCL8/ADGRE5/ANXA2/GLA/CTSC/FCER1G/TMC6
CARD/MDK/CXCL8/SOX9/COL1A1/ICAM1/POSTN/LGALS9/PFN2/VEGFB/SH3BP1/SULF1/ANXA5/EPC
/SOX9/COL1A1/ICAM1/POSTN/LGALS9/VEGFB/EPCAM/SRC/ITGA3/SEMA6A/LGALS3/RAC2/DAB2/M
G/ICAM1/LGALS9/FOXJ1/SRC/CD3D/LGALS3/RAC2/TGFB1/GPNMB/LGALS1/RIPK2/ADA/HAVCR2/C
OA/LPCAT1/IMPDH1/PYCARD/CYBA/CXCL6/LCN2/CXCL8/ADGRE5/ANXA2/GLA/CTSC/FCER1G/TMC6
MDK/CXCL8/CXCL5/FCER1G/LGALS9/VEGFB/PLXNA1/SRC/PLP2/SEMA6A/ARHGEF16/LGALS3/PLAU
17/EIF4EBP1/PTAFR/HSPB1/NCK2/SLC7A11/IGF2BP1/LARP6/EEF1E1/CDK4/TCOF1/IGF2BP3/ZN
/UBD/SOX4/TOP2A/E2F1/CTSC/MELK/LGALS9/ITM2C/BAK1/ANXA5/SRC/TSPO/ARHGEF16/PLAUR/
NS/AURKB/MDK/IER3/SOX9/EPO/CRYAB/ENO1/CDK1/FCER1G/ICAM1/CHST11/ANXA5/TYRO3/EPCA
/UBD/SOX4/TOP2A/E2F1/CTSC/MELK/LGALS9/ITM2C/BAK1/ANXA5/SRC/TSPO/ARHGEF16/PLAUR/
NS/AURKB/MDK/IER3/SOX9/EPO/CRYAB/ENO1/CDK1/FCER1G/ICAM1/CHST11/ANXA5/TYRO3/EPCA
KB/UBE2S/CDK1/BUB1B/DAB2/PBK/TRIB3/CCNF/AURKA/UBXN11/MAD2L1/PSMB9/GIPC1/DDIT3/R
/CDT1/CAPG/PYCARD/LIMK1/SOX9/CRYAB/DBN1/HJURP/ICAM1/ANLN/ARPC1B/PFN2/STMN1/FBLI
AM1/ANLN/ARPC1B/PFN2/SRC/RAC2/ATP13A2/CLSTN1/ARL2/TGFB1/PSRC1/CORO1A/FLNA/AGRN/
CL8/SLC52A2/TOP2A/ANXA2/IKBKE/IL2RG/KPNA2/CDK1/ICAM1/LGALS9/BUB1/TYRO3/SRC/CCNA
ENPA/CXCL8/SLC52A2/TOP2A/ANXA2/IKBKE/IL2RG/KPNA2/CDK1/ICAM1/REG1A/LGALS9/BUB1/T
PTTG1/ALDOA/PAFAH1B3/PAEP/GTSF1/AFP/RGS2/MDK/TOP2A/TRIP13/E2F1/SOX9/SLC2A1/EPO/
KB/TPX2/FOXM1/SOX4/TACC3/TRIP13/RRM2/E2F1/CDCA5/CDC6/UBE2S/TUBA4A/CDKN3/PKMYT1/
OA/LPCAT1/IMPDH1/PYCARD/LGALS3BP/CYBA/LCN2/ADGRE5/TUBA4A/ANXA2/GLA/CTSC/FCER1G/
RD/CYBA/UBD/LY96/LCN2/MMP12/IKBKE/NCF2/FCER1G/ICAM1/LGALS9/TYRO3/SRC/LGALS3/CSF
12/IKBKE/FCER1G/LGALS9/TYRO3/SRC/S100A14/ITGAM/RIPK2/HAVCR2/BIRC3/ITGB2/IRAK1/P
1/LIMK1/RGS2/MDK/E2F1/SOX9/EPO/ANXA2/DBN1/COL1A1/REG1A/LGALS9/MAPK12/FOXJ1/PLXN
OXM1/SOX4/CXCL8/TOP2A/TRIP13/RRM2/E2F1/CDC6/CDKN3/CDK1/NDC80/CDC45/NEK2/MAPK12/
CARD/PKIB/RGS2/TPX2/CDC6/EPO/DUSP9/CDKN3/PKMYT1/CDK1/CDC25B/SRC/MMD/CKS2/RAC2/C
O/FCER1G/ICAM1/LGALS9/BAK1/FOXJ1/TYRO3/SRC/CD3D/LGALS3/RAC2/MMP14/TYROBP/TGFB1/
1/KIF2C/CDT1/KIF4A/AURKB/TPX2/TACC3/CDCA8/TOP2A/TRIP13/CDCA5/CDC6/UBE2S/NUF2/PK
/TOP2A/EPO/ANXA2/FCER1G/MELK/ANLN/LGALS9/BAK1/FOXJ1/TYRO3/SRC/CD3D/LGALS3/MMP14
MP12/TEAD2/TGM3/SH3BP1/SULF1/PLXNA1/ITGB4/SRC/ITGA3/ETV5/SPINT2/DAB2/MMP14/LIF/
O/CTSC/FCER1G/LGALS9/FOXJ1/TYRO3/SRC/LGALS3/RAC2/MMP14/SERPINE2/TYROBP/TGFB1/IT
/CXCL6/ATP1B3/MDK/CXCL8/CXCL5/COL1A1/FCER1G/ICAM1/LGALS9/VEGFB/SLC7A7/FOXJ1/EPC
/TMSB10/RAP1GAP/CDT1/CAPG/AURKB/TOP2A/TRIP13/CRYAB/NDC80/MCM2/POSTN/PFN2/ITM2C/
CARD/MDK/CXCL8/SOX9/COL1A1/ICAM1/POSTN/LGALS9/PFN2/VEGFB/STMN1/SH3BP1/SULF1/ANX
/SOX9/COL1A1/ICAM1/POSTN/LGALS9/VEGFB/STMN1/EPCAM/SRC/ITGA3/SEMA6A/LGALS3/RAC2/
NB2/PYCARD/PKIB/LIMK1/RGS2/AURKB/TPX2/CDC6/UBE2S/EPO/DUSP9/CDKN3/PKMYT1/CDK1/NE
/RGS2/ATP1B3/RGS1/ICAM1/LGALS9/PFN2/BAK1/MAPK12/SH3BP1/FOXJ1/RGS10/LPAR2/ARHGEF
X2/TACC3/MDK/DBN1/ICAM1/ARPC1B/PFN2/NEK2/STMN1/SH3BP1/ECT2/ARL2/CCNF/TGFB1/SKA1
/CDCA8/CDCA5/TUBA4A/NUF2/CTSC/CDK1/NDC80/FCER1G/LGALS9/PFN2/NEK2/DLGAP5/KIF23/N
/CDCA8/CDCA5/NUF2/CTSC/NDC80/FCER1G/LGALS9/PFN2/DLGAP5/KIF23/NCS1/RAC2/DNM1/CEN
S2/MDK/TNFRSF21/E2F1/SOX9/EPO/DBN1/CTSC/CDK1/REG1A/ITM2C/PLXNA1/ITGA3/TSPO/SEMA
K/E2F1/EPO/DBN1/REG1A/PLXNA1/ITGA3/TSPO/SEMA6A/MMD/ETV5/LIF/CLSTN1/SERPINE2/CSF
2/LIMK1/RGS2/MDK/TNFRSF21/E2F1/SOX9/EPO/DBN1/CDK1/POSTN/REG1A/ITM2C/PLXNA1/ITGA
5/SNCG/EPO/CRYAB/ANXA2/DBN1/CDK1/NDC80/FCER1G/LGALS9/PFN2/BAK1/SRC/ITGA3/ARHGEF
R1/TREM2/ASNS/AURKB/SOX4/MDK/IER3/SOX9/EPO/CRYAB/ENO1/TEAD2/CDK1/FCER1G/ICAM1/C
/CCNB2/AURKB/KIF20A/E2F1/CEP55/ANXA2/CTSC/CDK1/FCER1G/BAK1/SH3BP1/ETV5/CD3D/NDR

SOX4/UBE2S/CDK1/CDCA3/TSPO/TRIM47/CCNA2/TRIB3/MAGEC2/CCNF/TRAIP/FANCI/RIPK2/DTL
4A/AURKB/TPX2/CENPA/TUBA4A/CDK1/ANLN/NEK2/FOXJ1/SRC/KIF23/WDR62/CENPF/KIFC1/MIS
XA2/FCER1G/ITGA3/ETV5/ARHGEF16/LYPD1/LGALS3/DAB2/MMP14/CLSTN1/TGFB1/ITGAM/GPC4/
D/FOXM1/IER3/EPO/CRYAB/ENO1/BAK1/PBK/RMI2/RAD51/STK26/DKK1/RIPK2/GSTP1/CD44/CDK
MK1/CRYAB/ANXA2/DBN1/COL1A1/ICAM1/ARPC1B/PFN2/STMN1/SH3BP1/MICAL1/SRC/MMP11/RAC
MDK/TNFRSF21/SOX9/EPO/ANXA2/ICAM1/LGALS9/FOXJ1/FBLIM1/TYRO3/EPCAM/SRC/CLIC1/LGA
ICAM1/ANLN/PFN2/NEK2/FOXJ1/RAC2/RCC2/STK26/FLNA/EMP3/AGRN/CEP131/FXYD5/TUBG1/S1
KCTD17/LIMK1/RGS2/MDK/EPO/DBN1/ICAM1/ANLN/REG1A/PFN2/ITM2C/PLXNA1/SRC/ITGA3/SEM
2/SOX4/TRIP13/E2F1/CDCA5/CDC6/TUBA4A/CDK1/NDC80/CDC45/ANLN/NEK2/BUB1/DLGAP5/CDC
PTTG1/KIF2C/CCNB2/CDT1/UBD/KIF4A/AURKB/TPX2/CENPA/FOXM1/SOX4/TACC3/CDCA8/KIF20A
4/LIF/CSF1/TYROBP/TGFB1/SPI1/RIPK2/ADA/C1QC/CD74/FANCD2/SLAMF8/NME1/SYK/IL18/NM
1/NDC80/FCER1G/SRC/ITGA3/ARHGEF16/LYPD1/LGALS3/RAC2/ATP13A2/DAB2/CCNE1/MMP14/LI
CARD/MDK/CXCL8/SOX9/COL1A1/ICAM1/POSTN/LGALS9/PFN2/VEGFB/SH3BP1/SULF1/ANXA5/EPC
/SOX9/COL1A1/ICAM1/POSTN/LGALS9/VEGFB/EPCAM/SRC/ITGA3/SEMA6A/LGALS3/RAC2/DAB2/M
NRPD1/SNRPA1/CSTF2/SNRPA/SF3A2/CD2BP2/JMJD6/LSM4/HSPA8/UPF3B/POLR2G/EFTUD2/PPIH
NRPD1/SNRPA1/CSTF2/SNRPA/SF3A2/CD2BP2/JMJD6/LSM4/HSPA8/UPF3B/POLR2G/EFTUD2/PPIH
NRPD1/SNRPA1/CSTF2/SNRPA/SF3A2/CD2BP2/JMJD6/LSM4/HSPA8/UPF3B/POLR2G/EFTUD2/PPIH
A1/EPO/CRYAB/ENO1/ICAM1/POSTN/HILPDA/REG1A/VEGFB/SRC/NDRG1/MMP14/CCNA2/TGFB1/ST
OA/LPCAT1/IMPDH1/PYCARD/CYBA/CXCL6/LCN2/ADGRE5/ANXA2/GLA/CTSC/FCER1G/LGALS9/TMC
O/CTSC/FCER1G/LGALS9/FOXJ1/TYRO3/SRC/LGALS3/RAC2/MMP14/TYROBP/TGFB1/ITGAM/GPNMB
F21/IKBKE/FCER1G/ARPC1B/TYRO3/SRC/CD3D/LGALS3/S100A14/ITGAM/FCGR3A/RIPK2/ADA/HA
LYREF/TSEN54/ESRP1/RALY/SNRPD2/HSF1/SNRPD1/SNRPA1/CSTF2/SNRPA/SF3A2/CD2BP2/JMJD
SNRPB/PRKCD/SMG5/SYK/NUP62/GTSE1/MX2/ALYREF/SNRPD2/RAN/RANGAP1/TRIM28/SNRPD1/NU
PO/ICAM1/LGALS9/FOXJ1/SRC/LGALS3/RAC2/PODXL2/TGFB1/GPNMB/LGALS1/RIPK2/ADA/HAVCR
3BP1/SRC/ITGA3/ARHGEF16/RAC2/CCNA2/CSF1/DEPDC1B/ECT2/IQGAP3/RHOV/ARHGAP4/COL1A2
/YWHAZ/KRT17/RBM38/EIF4EBP1/PTAFR/PRKCD/HSPB1/NCK2/IGF2BP1/NUP62/LARP6/EXOSC4/C
OX4/TRIP13/RRM2/E2F1/CDC6/CDK1/NDC80/NEK2/BUB1/BUB1B/LIF/CENPF/ORC1/CCNF/TTK/RA
N1/REG1A/ITM2C/PLXNA1/ITGA3/SEMA6A/NCS1/ARHGAP4/LGALS1/DKK1/TNFRSF12A/SNAP25/RO
COL1A1/ICAM1/POSTN/SULF1/MFAP2/ITGB4/TIMP1/ITGA3/MMP11/SPINT2/MMP14/MELTF/LOXL4
/SOX9/COL1A1/ICAM1/POSTN/LGALS9/VEGFB/SRC/ITGA3/SEMA6A/LGALS3/RAC2/DAB2/MMP14/C
F18B/ARHGEF2/SMARCD3/PLEK/TRIM54/ASPH/TWF2/MID1IP1/DNAJC6/KIF18A/CFL1/SMARCD1/C
AURKB/TACC3/TOP2A/TRIP13/CDCA5/CDC6/NDC80/MCM2/CDC45/NEK2/BUB1/DLGAP5/BUB1B/MKI
P/ICAM1/ARPC1B/PFN2/STMN1/FBLIM1/SRC/ARL2/TGFB1/DKK1/PSRC1/CORO1A/PMEPA1/EIF4EB
OA/LPCAT1/IMPDH1/PYCARD/CYBA/LCN2/ADGRE5/ANXA2/GLA/CTSC/FCER1G/LGALS9/TMC6/LGAL
OA/LPCAT1/IMPDH1/PYCARD/CYBA/LCN2/ADGRE5/ANXA2/GLA/CTSC/FCER1G/TMC6/LGALS3/PLAU
KB/TPX2/FOXM1/SOX4/TACC3/TRIP13/RRM2/E2F1/CDCA5/CDC6/UBE2S/TUBA4A/CDKN3/PKMYT1/
F1/CDCA5/CDC6/CDK1/NDC80/CDC45/DLGAP5/CDC25B/SRC/KIF23/CKS2/CCNE1/ECT2/NUSAP1/T
DX39A/SNRPB/PRKCD/SMG5/SYK/NUP62/GTSE1/MX2/ALYREF/SNRPD2/RAN/RANGAP1/TRIM28/SNR
CCNB2/CDT1/KIF4A/AURKB/TPX2/CENPA/TACC3/CDCA8/MDK/KIF20A/TOP2A/TUBA1C/CDCA5/CEP
PKIB/RGS2/CDC6/DUSP9/CDKN3/PKMYT1/CDK1/SRC/CKS2/CCNE1/CCNA2/TRIB3/CCNF/TGFB1/IQ
2F1/EPO/ENO1/IKBKE/CTSC/ICAM1/MELK/LGALS9/ITM2C/BAK1/SRC/LGALS3/PLAUR/TRIB3/TYR
M1/ARPC1B/PFN2/STMN1/SH3BP1/ARL2/PSRC1/CORO1A/FLNA/COTL1/ARHGEF2/PRKCD/NCK2/BAI
PB1/IGF2BP1/SF3B4/EXOSC4/PKP3/IGF2BP3/HSF1/PSMB9/PSMD14/YBX3/MAPKAPK2/DHX34/PCB
O/FCER1G/SRC/ITGA3/ARHGEF16/LGALS3/RAC2/LIF/TYROBP/ECT2/TGFB1/ITGAM/STX3/SQSTM1
G/ANLN/ITGA3/ARHGEF16/LYPD1/LGALS3/DAB2/MMP14/SAPCD2/CLSTN1/MISP/TGFB1/GPC4/STX
1/KIF2C/CDT1/KIF4A/AURKB/TPX2/TACC3/CDCA8/TOP2A/TRIP13/CDCA5/CDC6/UBE2S/NUF2/PK
CD3D/LGALS3/FCGR3A/RIPK2/ADA/CMTM3/PRKCD/LAT2/RELB/IGLL5/HLA-DRB1/PRNP/HRAS/SYK
PRKCD/HSPB1/SMG5/IGF2BP1/SMG9/EXOSC4/PKP3/RNASE6/IGF2BP3/RPL8/PSMB9/PSMD14/YBX3
G5/IGF2BP1/SMG9/EXOSC4/PKP3/IGF2BP3/RPL8/PSMB9/PSMD14/YBX3/MAPKAPK2/DHX34/PCBP4
M1/ARPC1B/PFN2/STMN1/SH3BP1/MICAL1/SRC/RAC2/POF1B/ENAH/RHOV/CORO1A/MARCKS/FLNA/
1/CAPG/AURKB/TOP2A/TRIP13/NDC80/MCM2/PFN2/NEK2/STMN1/BUB1/BUB1B/SRC/TSPO/LIF/CE

S9/FOXJ1/EPCAM/SRC/LGALS3/SPINT2/SERPINE2/TGFB1/GPNMB/LGALS1/RIPK2/ADA/HAVCR2/C
K/EPO/DBN1/ANLN/REG1A/PLXNA1/SRC/ITGA3/RAC2/TNFRSF12A/AGRN/ROBO1/SERPINI1/PLXNA
1/ARPC1B/PFN2/SH3BP1/SEMA6A/ATP13A2/IQGAP3/ARHGAP4/CORO1A/TNFRSF12A/COTL1/PRKCD
EN54/NOP56/WDR4/INTS8/LIN28B/CSTF2/MRTO4/DHX37/PES1/DKC1/TRMU/FTSJ1/METTL1/RPS2
A1/EPO/CRYAB/ENO1/ICAM1/POSTN/HILPDA/REG1A/VEGFB/SRC/NDRG1/MMP14/CCNA2/TGFB1/ST
CXCL8/TOP2A/MMP12/ANXA2/KPNA2/LGALS9/TIMP1/LHFPL2/AURKA/LGALS1/GAPDH/RIPK2/ADA/
GALS9/SRC/MMP14/TYROBP/TGFB1/ITGAM/LGALS1/RIPK2/ADA/HAVCR2/CORO1A/CLCF1/ITGB2/C
2/CDK1/NEK2/SRC/CCNA2/RMI2/RFC4/TGFB1/RAD51/SPI1/CLCF1/DSCC1/PRKCD/SMG5/HRAS/PI
J1/TYRO3/SRC/LGALS3/RAC2/MMP14/TYROBP/TGFB1/GPNMB/LGALS1/RIPK2/ADA/HAVCR2/CORO1
/RECQL4/NEK2/TYRO3/SRC/TFF2/NCS1/RAC2/CCNE1/RFC4/CSF1/AKR1B1/TGFB1/RAD51/EXO1/C
A1/EPO/CRYAB/ENO1/COL1A1/ICAM1/POSTN/HILPDA/REG1A/VEGFB/SRC/NDRG1/MMP14/CCNA2/T
TGB2/SNAP25/ADORA1/PRNP/HRAS/RAB31/SCRIB/RPL8/UBL4A/RAB34/ANXA13/NCF1/CHMP4B/BA
2/SOX4/TRIP13/E2F1/CDCA5/CDC6/TUBA4A/CDK1/NDC80/CDC45/ANLN/NEK2/BUB1/DLGAP5/CDC
SC/ICAM1/LGALS9/ITM2C/BAK1/SRC/LGALS3/PLAUR/GSTP1/TNFRSF12A/YWHAZ/CD44/CD74/ARH
GALS9/SRC/MMP14/TYROBP/TGFB1/ITGAM/LGALS1/RIPK2/ADA/HAVCR2/CORO1A/CLCF1/ITGB2/C
1/MCM2/CDC45/GINS1/MCM4/ORC6/CCNE1/CCNA2/RMI2/RFC4/ORC1/CHAF1B/RAD51/TONSL/EXO1
/KIF4A/AURKB/TACC3/CDCA8/TOP2A/TRIP13/CDCA5/CDC6/NUF2/NDC80/HJURP/NCAPG/NEK2/BU
OX4/TOP2A/TRIP13/E2F1/CDC6/CDK1/NDC80/BUB1/BUB1B/ZWINT/CENPF/ORC1/TTK/TGFB1/AUR
J1/SRC/LGALS3/RAC2/TGFB1/GPNMB/LGALS1/RIPK2/ADA/HAVCR2/CORO1A/CD74/CCDC88B/NCK2
C1B/PFN2/STMN1/ARL2/PSRC1/CORO1A/TUBG1/COTL1/PRKCD/NCK2/BAIAP2L2/VASP/CDC42EP1/
FOXJ1/LGALS3/RAC2/CSF1/TYROBP/TGFB1/GPNMB/RIPK2/ADA/HAVCR2/CORO1A/MZB1/CLCF1/CD
4/DHX37/PES1/DKC1/NPM1/RPS2/RRS1/DDX49/RRP9/ERI3/REXO4/RRP1/BRIX1/RRP7A/PA2G4/F
5/FCER1G/LGALS9/VEGFB/ARHGEF16/LGALS3/RAC2/CSF1/S100A14/CORO1A/GSTP1/ITGB2/CD74
GALS9/FOXJ1/LGALS3/RAC2/CSF1/TYROBP/TGFB1/GPNMB/RIPK2/ADA/HAVCR2/CORO1A/GSTP1/M
S9/FOXJ1/SRC/LGALS3/TGFB1/GPNMB/LGALS1/RIPK2/ADA/HAVCR2/CORO1A/CD44/ITGB2/IRAK1
PX2/LCN2/NCF2/CDK1/MELK/SRC/ETV5/MAPK13/ATP13A2/CCNA2/ECT2/TXNRD1/STK26/CYBB/GS
M4/RMI2/TGFB1/RAD51/TONSL/EXO1/CLCF1/RAD54L/MCM6/FEN1/BATF/GINS2/MCM7/PIF1/MCM3
IF23/KIFC1/KIF18B/CEP131/RACGAP1/KLC2/HSPB1/KIF11/WDR34/IFT57/KIF12/TMEM201/KIF
P1/ADORA1/MVP/NUP62/MX2/ALYREF/IGF2BP3/SLC25A6/RAN/HSF1/EIF5A2/NUTF2/NUP210/SLC
F3C6/SLFN13/MRTO4/DHX37/PES1/DKC1/CD3EAP/SMARCA4/RPS2/PELO/RRS1/DDX49/RRP9/DDX1
TRIM28/SIRT6/LIN28B/NUP210/NAA40/CNOT11/MAP2K2/POLR2G/PABPC1/HDAC1/NUP93/NUP205
/STX3/CEP131/SNAP25/DDX39A/SMG5/HRAS/NUP62/WDR34/DLG3/ALYREF/EZR/SCRIB/IFT57/RA
CMTM7/LGALS1/ADA/EXO1/MZB1/CLCF1/CD74/BATF/PKN1/PRKCD/LAT2/SLAMF8/IGLL5/SYK/HMG
TUBA4A/PKMYT1/CDK1/MELK/NEK2/CDC25B/CCNA2/CENPF/ORC1/RCC2/AURKA/HMMR/CDC25C/DTL
ROBP/TGFB1/LGALS1/RIPK2/ADA/HAVCR2/CORO1A/CLCF1/CD74/CCDC88B/NCK2/IGLL5/SYK/IL1
O1/IKBKE/MELK/BAK1/SRC/PLAUR/TRIB3/CD44/CD74/ARHGEF2/BCL2A1/PRKCD/HSPB1/NCK2/HR
IL2RG/FCER1G/ARPC1B/SH3BP1/TYRO3/SRC/TYROBP/TGFB1/ITGAM/FCGR3A/CORO1A/ITGB2/PRK
DK1/MAPK12/TGFB1/AURKA/CDC25C/PRR11/CDKN2A/PRNP/HRAS/GTSE1/CDK4/MUC1/CDKN2D/ZNF
2/FCER1G/ICAM1/KIF23/CTSV/CYBB/HLA-DMA/RACGAP1/CD74/KLC2/RELB/ITGAV/HLA-DRB1/HL
VBL1/CD2BP2/LSM4/EIF3B/NPM1/NOL3/EIF2S2/HSP90AB1/DYNC1H1/TICRR/RRS1/WDR77/BRIX1
TGFB1/PSRC1/CORO1A/NCK2/BAIAP2L2/VASP/PLEK/SYK/CDC42EP1/HCK/MAPRE1/ASAP1/MMP1/G
3/CDK1/MCM2/CDC45/MCM4/ORC6/CCNE1/CCNA2/ORC1/IQGAP3/AURKA/GPNMB/TYMS/CDC25C/CDK
FOXJ1/LGALS3/RAC2/TYROBP/TGFB1/GPNMB/RIPK2/ADA/HAVCR2/CORO1A/MZB1/CLCF1/CD74/CC
/BAK1/CSF1/TGFB1/SPI1/ADA/CORO1A/CD74/PKN1/SLC7A11/EZH2/PRMT1/HMGB2/JAK3/HMOX1/
T2/IGLL5/HLA-DRB1/PRNP/HRAS/SYK/HLA-DQA1/PAG1/HLA-DRA/EZR/HLA-DQB1/VTCN1/PSMB9/
T1/NEK2/BUB1/CDC25B/BUB1B/CKS2/CCNE1/LIF/P3H4/TTK/RAD51/AURKA/EXO1/NCAPD2/CDC25
E2F1/CDK1/NDRG1/RMI2/RFC4/AURKA/EXO1/CDC25C/BOP1/CD44/CD74/BATF/GTSE1/EEF1E1/MU
CA5/CDC6/CDK1/NDC80/CDC45/DLGAP5/CDC25B/KIF23/CCNE1/ECT2/NUSAP1/TGFB1/RCC2/AURK
1/KIF2C/CDT1/KIF4A/AURKB/TPX2/TACC3/CDCA8/TRIP13/CDCA5/CDC6/UBE2S/NUF2/PKMYT1/N
CD2/CSF1/TGFB1/DKK1/GPC4/GPC3/ROBO1/FZD6/TNFRSF11B/MAGED1/FZD7/PSMB9/PSMD14/HNF
3/CDK1/MCM2/CDC45/MCM4/ORC6/CCNE1/ORC1/IQGAP3/AURKA/GPNMB/TYMS/CDC25C/CDKN2A/MC

TUBA4A/PKMYT1/CDK1/MELK/NEK2/CDC25B/CCNA2/CENPF/ORC1/RCC2/AURKA/HMMR/CDC25C/DTL
F/CSF1/TYROBP/TGFB1/SPI1/GPC3/C1QC/CD74/BATF/RELB/EFNA4/NME1/CD109/NME2/CSF1R/T
N2/SH3BP1/MICAL1/ENAH/CORO1A/COTL1/PRKCD/NCK2/BAIAP2L2/VASP/PLEK/CDC42EP1/HCK/T
36A/POLR2G/SMARCA4/RPS2/HDAC1/NUP93/NUP205/NUP37/LEF1/NDC1/NELFE/POLR2H/NUP188/
FB1/LGALS1/RIPK2/ADA/HAVCR2/CORO1A/CD44/ITGB2/IRAK1/CD74/CCDC88B/PTAFR/NCK2/SYK
X2/ALYREF/IGF2BP3/RAN/HSF1/EIF5A2/NUTF2/NUP210/NPM1/UPF3B/ZC3H3/RRS1/NUP93/NXT1
X2/ALYREF/IGF2BP3/RAN/HSF1/EIF5A2/NUTF2/NUP210/NPM1/UPF3B/ZC3H3/RRS1/NUP93/NXT1
GALS3/RAC2/TYROBP/TGFB1/GPNMB/RIPK2/ADA/HAVCR2/CORO1A/MZB1/CLCF1/CD74/CCDC88B/P
9/RECQL4/ASF1B/CDK1/HJURP/NCAPG/MCM2/CDC45/GINS1/MCM4/RFC4/CHAF1B/NUSAP1/CENPH/
VBL1/CD2BP2/LSM4/EIF3B/NPM1/NOL3/EIF2S2/PELO/HSP90AB1/DYNC1H1/TICRR/RRS1/WDR77/
/AURKB/TACC3/CDCA8/TOP2A/TRIP13/CDCA5/CDC6/NUF2/NDC80/NCAPG/NEK2/BUB1/DLGAP5/BU
C1B/PFN2/STMN1/SH3BP1/CORO1A/FLNA/COTL1/PRKCD/NCK2/BAIAP2L2/VASP/PLEK/CDC42EP1/
IP13/E2F1/CDC6/CDK1/NDC80/BUB1/BUB1B/CENPF/ORC1/TTK/AURKA/GPNMB/CDC25C/DTL/CDKN
B/LGALS9/BAK1/MICAL1/SRC/PLAUR/RIPK2/BIRC3/CD44/ROBO1/SYK/S100A8/ASPH/CASP4/CDK
AD51AP1/EEF1E1/MUC1/CDKN2D/ANKRD1/ZNF385A/CHEK1/NPAS2/HSF1/TRIM28/SIRT6/MIF/WDR
CT5/MX2/ALYREF/IGF2BP3/ZNF385A/RAN/HSF1/CCT6A/CCT3/EIF5A2/NUTF2/NUP210/RUVBL1/D
NT2/SAPCD2/MISP/RHOV/ARHGEF2/MAD2L1/DLG3/SCRIB/RHOG/CFL1/RHOQ/FSCN1/RHOC/MAP2/F
GALS3/RAC2/CSF1/TYROBP/TGFB1/GPNMB/RIPK2/ADA/HAVCR2/CORO1A/MZB1/CLCF1/CD74/CCDC
ALS1/RIPK2/ADA/HAVCR2/CORO1A/CD74/CCDC88B/NCK2/SYK/IL18/VTCN1/TFRC/HLA-DPA1/HLA
IP13/E2F1/CDC6/CDK1/NDC80/BUB1/BUB1B/CENPF/ORC1/TTK/AURKA/GPNMB/CDC25C/CDKN2A/G
FB1/LGALS1/RIPK2/ADA/HAVCR2/CORO1A/CD44/ITGB2/IRAK1/CD74/CCDC88B/PTAFR/NCK2/SYK
P2A/TRIP13/E2F1/CDC6/CDK1/NDC80/CDC45/BUB1/BUB1B/ZWINT/CENPF/ORC1/TTK/TGFB1/AUR
1/LSM4/RPL36A/PPP2R1A/UPF3B/POLR2G/RPS2/PELO/PABPC1/RNPS1/ZC3H12A/NCBP2/RPL17/R
X37/PES1/DKC1/RPS2/RRS1/DDX49/RRP9/ERI3/REXO4/RRP1/RRP7A/PA2G4/FBL/DDX27/NOP2/G
GRN/PFDN4/CCT6A/CCT3/P3H1/DNAJC5/HSPA6/FKBP1A/PDRG1/GNAZ/HSPA8/SUCO/NUDC/PPIH/P
CHMP4B/SSR2/MTCL1/VPS37C/SEC61A1/ZDHHC7/RPL36A/MICALL1/PAK1/SLC51B/RPS2/GDI1/RP
N2/STMN1/ARL2/PSRC1/CORO1A/COTL1/PRKCD/NCK2/BAIAP2L2/VASP/CDC42EP1/HCK/MAPRE1/T
B/LGALS9/BAK1/MICAL1/SRC/PLAUR/BIRC3/CD44/ROBO1/SYK/S100A8/CASP4/CDKN2D/IFT57/R
ALS1/GAPDH/CD74/ITGAV/CSF1R/CFL1/APOBEC3C/TRIM6/CHMP4B/TRIM28/CCL5/CXCR4/TRIM11
PDA/SRC/NDRG1/CCNA2/STC2/CYBB/IRAK1/EIF4EBP1/ANKRD1/PGK1/PSMB9/PSMD14/HMOX1/SLC
/MCM2/CDC45/MCM4/CENPF/CHAF1B/CENPH/RAD51/MCM6/CENPW/SMARCD3/OIP5/MCM7/MCM3/NAP
8/CXCL5/FCER1G/VEGFB/LGALS3/RAC2/MMP14/CSF1/S100A14/ITGB2/CD74/P2RX4/SLAMF8/NBL
NEK2/CDC25B/CENPF/ORC1/RCC2/AURKA/HMMR/CDC25C/DTL/CEP131/TUBG1/SMARCD3/GTSE1/MA
/AURKB/TACC3/CDCA8/TOP2A/TRIP13/CDCA5/CDC6/NUF2/NDC80/NCAPG/NEK2/BUB1/DLGAP5/BU
M4/RMI2/RAD51/TONSL/RAD54L/MCM6/FEN1/GINS2/MCM7/MCM3/RAD51AP1/EME1/CDC7/CHEK1/H
F/RAN/RANGAP1/NUTF2/NUP210/NPM1/UPF3B/RRS1/NUP93/NXT1/NUP205/NUP37/RNPS1/NDC1/X
NEK2/CDC25B/CENPF/ORC1/RCC2/AURKA/HMMR/CDC25C/DTL/CEP131/TUBG1/SMARCD3/MAPRE1/T
FBLN1/RIPK2/EFNA3/ROBO1/SYK/S100A8/ASPH/CASP4/IFT57/GRN/HSF1/CARD9/TRAF2/PSMD14
5/FCER1G/LGALS9/VEGFB/LGALS3/RAC2/CSF1/S100A14/CORO1A/ITGB2/CD74/SLAMF8/NBL1/SY
A1/AURKA/PSRC1/SKA3/CEP131/ARHGEF2/TRIM54/KIF11/NUP62/SPAG5/MAPRE1/MID1IP1/CHEK
/PRNP/HRAS/HLA-DQA1/PAG1/HLA-DRA/EZR/HLA-DQB1/VTCN1/PSMB9/HLA-DPA1/RBCK1/PSMD14
F/RAN/RANGAP1/NUTF2/NUP210/NPM1/UPF3B/RRS1/LZTS2/NUP93/NXT1/NUP205/NUP37/RNPS1/
X2/ALYREF/IGF2BP3/RAN/HSF1/EIF5A2/NUTF2/NUP210/NPM1/UPF3B/ZC3H3/RRS1/NUP93/NXT1
C1/CORO1A/FLNA/NCK2/PLXNA3/BAIAP2L2/VASP/PLEK/HRAS/CDC42EP1/NUP62/HCK/SPAG5/MAP
F2BP1/EXOSC4/PKP3/IGF2BP3/PSMB9/PSMD14/YBX3/MAPKAPK2/DHX34/PCBP4/PSMD2/HSPA8/NP
CD2/TGFB1/DKK1/GPC4/GPC3/FZD6/MAGED1/FZD7/PSMB9/PSMD14/HNF1B/SIRT6/PTK7/PSMD2/I
36A/NPM1/POLR2G/EIF2S2/RPS2/PABPC1/TICRR/PPP1CA/NCBP2/RPL17/RPLP2/EIF3D/RPSA/AT
B/TPX2/TACC3/NUF2/NDC80/NEK2/STMN1/PRC1/KIF23/WDR62/KIFC1/MISP/TTK/AURKA/PSRC1/
80/PKP3/KRT7/ST14/SHARPIN/PERP/CNFN/KRT18/CAPN1/KRT8/CAPNS1/CYP26B1/EVPL
2/FCER1G/KIF23/CTSV/CYBB/HLA-DMA/RACGAP1/CD74/KLC2/ITGAV/HLA-DRB1/HLA-DQA1/KIF1
4/TGFB1/RAD51/CLCF1/DSCC1/PRKCD/HRAS/CCT5/DNMT1/CDC7/NPAS2/HSF1/NABP2/CCT6A/TFR

C25B/PRC1/KIF23/ORC6/KIF18B/ECT2/TGFB1/AURKA/RACGAP1/NUP62/PGF/GIT1/ASPM/GIPC1/
A1/AURKA/PSRC1/SKA3/CEP131/ARHGEF2/TRIM54/KIF11/NUP62/SPAG5/MAPRE1/MID1IP1/CHEK
GALS3/RAC2/CSF1/TYROBP/TGFB1/GPNMB/RIPK2/ADA/HAVCR2/CORO1A/GSTP1/MZB1/CLCF1/CD7
3/CTSV/CYBB/HLA-DMA/RACGAP1/CD74/KLC2/ITGAV/HLA-DRB1/HLA-DQA1/KIF11/TAP1/HLA-DR
NFRSF12A/PLXNA3/PLEK/CDK4/TWF2/TRPV2/SLC12A2/VAV1/SEMA4A/MAP2/SLC12A9/MSN/EFNA5
NT2/TGFB1/FOLR1/VASP/FZD6/SCRIB/IFT57/CFL1/HNF1B/MTHFD1L/PTK7/STIL/HIF1A/FZD1/S
3/CTSV/CYBB/HLA-DMA/RACGAP1/CD74/KLC2/ITGAV/HLA-DRB1/HLA-DQA1/KIF11/TAP1/HLA-DR
/SNAP25/TUBG1/CPLX2/PLEK/MAPRE1/TUBB/YKT6/EZR/TUBB4B/TCTN2/MSN/RAB15/STXBP2/SSN
N2/CORO1A/COTL1/PRKCD/NCK2/BAIAP2L2/VASP/CDC42EP1/HCK/TMSB4X/TWF2/MICALL2/CARMI
CCDC88B/HSPB1/WDR34/TUBB/IFT57/TMEM201/KIF3C/DTNBP1/MAP2/FLOT2/SSNA1/HIF1A/HSPA
N2/SH3BP1/CORO1A/COTL1/PRKCD/NCK2/BAIAP2L2/VASP/PLEK/CDC42EP1/HCK/TMSB4X/TWF2/C
T2/SYK/HCK/PSMB9/WIPF3/PSMD14/WASF2/VAV1/ARPC4/PSMD2/MAPK3/PAK1/HSP90AB1/ARPC2/
GALS3/RAC2/TGFB1/GPNMB/RIPK2/HAVCR2/CORO1A/CCDC88B/NCK2/ELF4/PRNP/SYK/IL18/CD15
F2BP1/EXOSC4/IGF2BP3/PSMB9/PSMD14/YBX3/MAPKAPK2/PCBP4/PSMD2/HSPA8/DKC1/NPM1/YBX
1/TRAIP/RIPK2/GSTP1/BIRC3/IRAK1/CD74/ROBO1/SYK/CASP4/IFNGR2/SCRIB/TRAF2/RBCK1/T
44/CD74/EEF1E1/MUC1/ANKRD1/ZNF385A/CHEK1/RHNO1/MIF/RNF34/CHD3/PPP1R13L/TAF6/RBB
GFB1/PHF19/FEN1/CCT5/DNMT1/MUC1/NABP2/CCT6A/CCT3/TRIM28/ESPL1/SIRT6/MAPK3/DKC1/
S9/VEGFB/LGALS3/RAC2/MMP14/CSF1/S100A14/TGFB1/ADA/CD74/PTAFR/P2RX4/SLAMF8/ADORA
CLGN/HSPB1/NCK2/SERPINH1/CREB3L1/COMP/HSF1/DDIT3/BAX/HSPA6/CREB3/MANF/HSPA8/LMN
N2/SH3BP1/CORO1A/COTL1/PRKCD/NCK2/BAIAP2L2/VASP/PLEK/CDC42EP1/HCK/TMSB4X/TWF2/C
LR2G/SMARCA4/RPS2/HDAC1/NUP93/NUP205/NUP37/LEF1/NDC1/NELFE/POLR2H/NUP188/RPL17/
I/DKK1/GPC3/WNK2/ASPM/PSMB9/PSMD14/PTK7/RUVBL1/PSMD2/EDA/MLLT3/SMARCA4/CSNK1E/F
LHFPL2/AURKA/LGALS1/GAPDH/RIPK2/HAVCR2/CD74/PTAFR/SYK/PLCB1/CSF1R/GRN/CFL1/CHMP
CDC45/MCM4/CENPF/CHAF1B/CENPH/RAD51/MCM6/CENPW/OIP5/MCM7/MCM3/NAP1L1/CENPO/CENP
4/TYMS/DTL/DSCC1/POLD1/SMG5/PIF1/CCT5/CDKN2D/ANKRD1/CCT6A/CCT3/POLA2/MAD2L2/CHT
5/TUBG1/CPLX2/PLEK/MAPRE1/TUBB/YKT6/TUBB4B/TCTN2/RAB15/STXBP2/SSNA1/PPP2R1A/STX
/CDC25B/BUB1B/CKS2/CCNE1/LIF/P3H4/TTK/RAD51/AURKA/NCAPD2/CDC25C/RAD54L/TUBG1/NC
/ITM2C/BAK1/PLAUR/TNFRSF12A/YWHAZ/PRKCD/NCK2/S100A8/MAGED1/DDIT3/TRAF2/RBCK1/BA
2A/CENPW/SMARCD3/OIP5/CENPO/CENPU/HMGB3/CHEK1/SMARCD1/CENPL/HMGB2/SIRT6/RUVBL1/
AD51/EXO1/FEN1/POLD1/SMG5/PIF1/CCT5/NABP2/CCT6A/CCT3/POLA2/SIRT6/PRKDC/MAPK3/DK
BIRC3/IRAK1/RELB/IL18/TMSB4X/HDAC7/PSMB9/TRAF2/RBCK1/PSMD14/PRDX1/CARD10/NFKB2/
F2BP1/EXOSC4/IGF2BP3/PSMB9/PSMD14/YBX3/MAPKAPK2/PCBP4/PSMD2/HSPA8/NPM1/YBX1/PAB
1/LGALS3/DAB2/MMP14/TGFB1/ITGAM/GPC4/STX3/SQSTM1/YWHAZ/GPC3/ITGB2/KCNJ11/SLC7A1
5/CDC6/ANLN/STMN1/CDC25B/PRC1/KIF23/ECT2/NUSAP1/AURKA/UNC119/RACGAP1/NUP62/GIT1
/MCM4/ORC6/CCNE1/RFC4/ORC1/RAD51/TONSL/RNASEH2A/MCM6/FEN1/DSCC1/GINS2/MCM10/POL
/TACC3/TRIP13/CDCA5/CDC6/PKMYT1/NDC80/ANLN/NEK2/BUB1/DLGAP5/BUB1B/MKI67/CENPF/N
FNA3/ROBO1/SYK/S100A8/ASPH/CASP4/IFT57/HSF1/CARD9/TRAF2/PSMD14/BAX/BEX3/PERP/PR
3/SYK/TMSB4X/CASP4/TNFRSF11B/PSMB9/LTB/TNFRSF18/TRAF2/RBCK1/PSMD14/TNFSF9/PSMD2
K2/FLNA/BIRC3/IRAK1/ZDHHC13/CD74/NUP62/CARD9/TRAF2/RBCK1/HMOX1/RHOC/TRAF5/FKBP1
DC80/CDC45/DLGAP5/CDC25B/CCNE1/NUSAP1/TGFB1/RCC2/AURKA/CDC25C/DTL/EIF4EBP1/SMAR
X2/ALYREF/IGF2BP3/HSF1/EIF5A2/NUTF2/NUP210/UPF3B/ZC3H3/NUP93/NXT1/NUP205/NUP37/
/TACC3/TRIP13/CDCA5/CDC6/PKMYT1/NDC80/ANLN/NEK2/BUB1/DLGAP5/BUB1B/MKI67/LIF/CEN
E1/AURKA/GPNMB/CDC25C/CDKN2A/PLCB1/GTSE1/EZH2/CDK4/MUC1/CDKN2D/ZNF385A/ARID3A/P
/AURKB/TACC3/CDCA8/TRIP13/CDCA5/CDC6/NUF2/NDC80/NCAPG/NEK2/BUB1/DLGAP5/BUB1B/PR
AD51/EXO1/FEN1/POLD1/SMG5/PIF1/CCT5/NABP2/CCT6A/CCT3/POLA2/SIRT6/PRKDC/MAPK3/DK
3/FZD6/DLG3/FZD7/PSMB9/PSMD14/MSN/BRSK1/PTK7/PSMD2/FZD1/LAMA5/MLLT3/ARHGEF19/PS
1/TRAIP/RIPK2/GSTP1/BIRC3/IRAK1/CD74/ROBO1/SYK/CASP4/IFNGR2/SCRIB/TRAF2/RBCK1/T
N2/CORO1A/COTL1/PRKCD/NCK2/BAIAP2L2/VASP/CDC42EP1/HCK/TMSB4X/TWF2/CARMIL1/PLEKH
6/CDK1/CDC45/ORC1/AURKA/CDC25C/DTL/GTSE1/MUC1/EME1/ZNF385A/ARID3A/CHEK1/RHNO1/P
ZD7/PSMB9/PSMD14/PTK7/PSMD2/FZD1/MLLT3/ARHGEF19/PLCB3/CSNK1E/PSMB2/LEF1/SFRP5/P
GALS3/RAC2/TGFB1/GPNMB/RIPK2/HAVCR2/CORO1A/CCDC88B/NCK2/PRNP/SYK/IL18/VTCN1/TFR

E1/AURKA/GPNMB/CDC25C/CDKN2A/PLCB1/GTSE1/EZH2/CDK4/MUC1/CDKN2D/ZNF385A/ARID3A/P
D44/CD74/ARHGEF2/BCL2A1/HSPB1/NCK2/S100A8/MUC1/CDKN2D/ZNF385A/CREB3L1/DDIT3/YBX
P2A/TRIP13/E2F1/CDC6/CDK1/NDC80/BUB1/BUB1B/ZWINT/CENPF/ORC1/TTK/TGFB1/AURKA/CDC
/CDC25B/BUB1B/CKS2/CCNE1/LIF/P3H4/TTK/RAD51/AURKA/RAD54L/NCAPH/FANCD2/PLCB1/C11
APK2/HSPA6/NUP210/MAPK3/HSPA8/SLC52A3/HSP90AB1/NUP93/NUP205/NUP37/NDC1/NUP188/M
/CORO1A/CLCF1/CD74/CCDC88B/NCK2/SYK/IL18/VTCN1/CD320/TFRC/HLA-DPA1/HLA-DMB/JAK3
25A6/MICALL2/DDIT3/SAE1/BAX/TOMM34/GDAP1/TIMM17B/BMF/BID/TOMM5/UBE2J2/NPEPPS/HS
25C/DTL/GTSE1/MUC1/EME1/ZNF385A/ARID3A/CHEK1/RHNO1/PRMT1/BAX/PCBP4/CASP2/BRSK1/
AVCR2/CORO1A/CLCF1/CD74/CCDC88B/NCK2/SYK/IL18/VTCN1/CD320/TFRC/HLA-DPA1/HLA-DMB
G1/AURKA/CDC25C/DTL/CD44/CD74/BATF/GTSE1/EEF1E1/MUC1/ANKRD1/ZNF385A/ARID3A/CHEK
S100A14/TGFB1/STX3/CD74/P2RX4/HSPB1/STK39/PGF/TMSB4X/CCL5/CXCR4/CCL26/CREB3/MAP
1/UPF3B/RRS1/NUP93/NXT1/NUP205/NUP37/RNPS1/NDC1/NUP188/NCBP2/U2AF2/SRSF9/XPOT/S
FB1/FOLR1/VASP/FZD6/SCRIB/IFT57/CFL1/HNF1B/MTHFD1L/PTK7/STIL/HIF1A/FZD1/ST14/IF
5/FCER1G/LGALS3/RAC2/CSF1/S100A14/ITGB2/CD74/SLAMF8/SYK/CKLF/S100A8/CSF3R/CCL5/
B/TPX2/CENPA/TACC3/NUF2/NDC80/NEK2/STMN1/PRC1/KIF23/WDR62/KIFC1/SAPCD2/MISP/NUS
FB1/FOLR1/VASP/FZD6/SCRIB/IFT57/CFL1/HNF1B/MTHFD1L/PTK7/STIL/HIF1A/FZD1/ST14/IF
1/UPF3B/RRS1/NUP93/NXT1/NUP205/NUP37/RNPS1/NDC1/NUP188/NCBP2/U2AF2/SRSF9/XPOT/S
AVCR2/CORO1A/CLCF1/CD74/CCDC88B/NCK2/SYK/IL18/VTCN1/CD320/TFRC/HLA-DPA1/HLA-DMB
RPL36A/SEC61G/RPS2/BCAP31/RPL17/RPLP2/TRAM1L1/RPSA/KDELR1/RPS5/RPS21/RPL7/RPL28
1/UPF3B/RRS1/NUP93/NXT1/NUP205/NUP37/RNPS1/NDC1/NUP188/NCBP2/U2AF2/SRSF9/XPOT/S
25A6/MICALL2/DDIT3/SAE1/BAX/TOMM34/GDAP1/TIMM17B/BMF/BID/TOMM5/UBE2J2/NPEPPS/HS
/TOP2A/TRIP13/NDC80/MCM2/BUB1/BUB1B/SRC/CENPF/TTK/SPI1/PIF1/MAD2L1/DNMT1/ESPL1/
DLG3/FZD7/PSMB9/PSMD14/BRSK1/PTK7/PSMD2/FZD1/MLLT3/ARHGEF19/PSMB2/PSME3/DVL3/PL
S9/VEGFB/LGALS3/RAC2/MMP14/CSF1/S100A14/TGFB1/CD74/PTAFR/P2RX4/STK39/PGF/TNFRSF
DLG3/FZD7/PSMB9/PSMD14/BRSK1/PTK7/PSMD2/FZD1/MLLT3/ARHGEF19/PSMB2/PSME3/DVL3/PL
/RAD54L/MCM6/FEN1/GINS2/MCM7/MCM3/RAD51AP1/CDC7/CHEK1/RHNO1/NABP2/MCM5/AP5Z1/PS
SP/ARHGEF2/MAD2L1/SCRIB/CFL1/FSCN1/MAP2/FLOT2/MSN/BRSK1/PTK7/PAK1/HSP90AB1/SPDL
9/ASF1B/CDK1/HJURP/NCAPG/MCM2/CHAF1B/NUSAP1/CENPH/NCAPD2/CDKN2A/CENPW/NCAPH/OIP
/RAD54L/MCM6/FEN1/GINS2/MCM7/MCM3/RAD51AP1/CDC7/CHEK1/NABP2/MCM5/AP5Z1/PSMD14/S
PS6KA1/HSF1/PSMB9/DDIT3/PSMD14/HMOX1/PSMD2/RGS14/HIF1A/E2F4/PSMB2/PSME3/MT3/ATF
3B/RPS2/PABPC1/RNPS1/NCBP2/RPL17/RPLP2/RPSA/RPS5/RPS21/RPL7/RPL28/RPL39/RPL35A/
SF1/PSMB9/DDIT3/PSMD14/HMOX1/PSMD2/HIF1A/E2F4/PSMB2/PSME3/MT3/ATF4/PSMB4/PSMB8/
5/FCER1G/LGALS3/RAC2/CSF1/S100A14/ITGB2/CD74/SYK/CKLF/S100A8/CSF3R/CCL5/VAV1/CC
4/RFC4/RAD51/RAD54L/MCM6/DSCC1/GINS2/MCM7/PIF1/MCM3/HMGB3/MCM5/HMGB2/GINS4/RUVB
ZB1/CLCF1/CD74/PKN1/SLAMF8/IGLL5/SYK/HMGB3/CD320/TFRC/MIF/CD300A/MAD2L2/IL27RA/
5/FCER1G/LGALS3/RAC2/ITGB2/CD74/SLAMF8/SYK/CKLF/S100A8/CSF3R/CCL5/VAV1/CCL26/JA
ALS3/DAB2/MMP14/SAPCD2/MISP/TGFB1/GPC4/STX3/SQSTM1/KCNJ11/PRNP/HRAS/EZR/RHOG/AN
UP93/NXT1/NUP205/NUP37/RNPS1/NDC1/NUP188/NCBP2/U2AF2/SRSF9/AKAP8L/CPSF1/THOC5/N
1/SKA3/TUBG1/ARHGEF2/TRIM54/MAPRE1/MID1IP1/KIF18A/NCKAP5L/MAP2/MAP1S/PAK1/TPPP3
UP93/NXT1/NUP205/NUP37/RNPS1/NDC1/NUP188/NCBP2/U2AF2/SRSF9/AKAP8L/CPSF1/THOC5/N
RPL17/RPLP2/TRAM1L1/RPSA/RPS5/RPS21/RPL7/RPL28/RAB10/RPL39/RPL35A/RPS3/RPL27/SS
FZD7/PSMB9/PSMD14/PTK7/PSMD2/FZD1/MLLT3/ARHGEF19/PSMB2/PSME3/DVL3/PLEKHA4/FZD2/
C1/CARD9/PSMB9/PSMD14/PSMD2/PAK1/PSMB2/PSME3/CD209/LYN/CARD11/MUC3A/PSMB4/PSMB8
/CHAF1B/CENPH/CDKN2A/CENPW/SMARCD3/OIP5/NAP1L1/CENPO/CENPU/SMARCD1/CENPL/HMGB2/
/CHAF1B/CENPH/CENPW/SMARCD3/OIP5/NAP1L1/CENPO/CENPU/SMARCD1/CENPL/HMGB2/RUVBL1/
C25C/CDKN2A/GTSE1/EZH2/CDK4/MUC1/CDKN2D/ZNF385A/ARID3A/PRMT1/BAX/PCBP4/CASP2/PR
/HRAS/CDC7/CACYBP/GMNN/CHTF18/PPP2R1A/PCNA/TICRR/E2F8/RFC2/FGFR1/BLM/FBXO5/DBF4
B/TPX2/TACC3/NUF2/NDC80/NEK2/STMN1/PRC1/KIF23/WDR62/KIFC1/MISP/TTK/AURKA/PSRC1/
RKA/CDC25C/CD44/CD74/BATF/GTSE1/EEF1E1/MUC1/ANKRD1/ZNF385A/ARID3A/PRMT1/BAX/MIF
C1/CARD9/PSMB9/PSMD14/PSMD2/PAK1/PSMB2/PSME3/CD209/LYN/CARD11/MUC3A/PSMB4/PSMB8
RPL17/RPLP2/TRAM1L1/RPSA/RPS5/RPS21/RPL7/RPL28/RPL39/RPL35A/RPS3/RPL27/SSR3/RPL

MB9/PSMD14/PTK7/PSMD2/FZD1/MLLT3/ARHGEF19/PSMB2/PSME3/DVL3/PLEKHA4/FZD2/RAC1/PS
RKA/CDC25C/GTSE1/CDK4/MUC1/ZNF385A/ARID3A/PRMT1/BAX/MIF/PCBP4/CASP2/CNOT11/BRCA
IF23/CTSV/HLA-DMA/RACGAP1/CD74/KLC2/HLA-DRB1/HLA-DQA1/KIF11/HLA-DRA/HLA-DQB1/IF
AM/RCC2/FLNA/ITGB2/FERMT3/ITGAV/PLEK/ADAM9/SYK/HCK/ZYX/NME2/ADAM15/COL16A1/COL3
5/FCER1G/LGALS3/RAC2/ITGB2/CD74/SYK/CKLF/S100A8/CSF3R/CCL5/VAV1/CCL26/JAML/C3AR
1/AURKA/CDC25C/GTSE1/MUC1/EME1/ZNF385A/ARID3A/PRMT1/BAX/PCBP4/CASP2/BRSK1/PRKDC
/NEK2/KIF23/KIFC1/MISP/AURKA/FLNA/RACGAP1/CENPW/KIF11/RCC1/SPAG5/MAPRE1/TUBB/AS
/TACC3/TRIP13/CDCA5/CDC6/NDC80/NEK2/BUB1/DLGAP5/BUB1B/MKI67/CENPF/RMI2/ECT2/TTK
C25C/CDKN2A/GTSE1/EZH2/CDK4/MUC1/CDKN2D/ZNF385A/ARID3A/PRMT1/BAX/PCBP4/CASP2/PR
IF23/CTSV/HLA-DMA/RACGAP1/CD74/KLC2/HLA-DRB1/HLA-DQA1/KIF11/HLA-DRA/HLA-DQB1/IF
P2/TRAM1L1/RPSA/RPS5/RPS21/RPL7/RPL28/RPL39/RPL35A/RPS3/RPL27/SSR3/RPL35/RPS20/
VASP/FZD6/SCRIB/IFT57/CFL1/MTHFD1L/PTK7/STIL/HIF1A/FZD1/ST14/DVL3/FZD2/TULP3/DE
-DMA/RACGAP1/CD74/KLC2/HLA-DRB1/HLA-DQA1/KIF11/HLA-DRA/HLA-DQB1/IFI30/KIF3C/KIF
OMM34/GDAP1/TIMM17B/BID/TOMM5/UBE2J2/NPEPPS/HSPA4/UBE2L3/GSK3A/BAG3/GRPEL2/TIMM
K1/PSMB9/PSMD14/BRSK1/MIIP/PSMD2/CHMP4C/BRCA1/TICRR/PSMB2/PSME3/BLM/PSMB4/PSMB8
SRC/CKS2/CCNE1/CCNA2/CCNF/PSRC1/CDC25C/CDKN2A/CDC25A/CDK4/CDKN2D/CKS1B/HSP90AB1
PL36A/FTSJ1/EIF2S2/TICRR/RPL17/EIF3D/RPSA/RPS21/EIF3I/EIF2S3/DENR/RPL39/RPL35A/
FI30/NCF1/PSMB9/PSMD14/PSMD2/HLA-A/ITGB5/PSMB2/PSME3/BCAP31/ERAP2/PSMB4/PSMB8/P
AM1L1/RPSA/RPS5/RPS21/RPL7/RPL28/RPL39/RPL35A/RPS3/RPL27/SSR3/RPL35/RPS20/RPL18
IAP2L2/VASP/CDC42EP1/HCK/CARMIL1/CCL26/ARPC4/BAIAP2L1/FCHSD1/BIN1/ARPC2/ARPC3/C
E1/TUBB/TUBB4B/TCTN2/SSNA1/PPP2R1A/TUBA1A/SFI1/DYNLL1/DYNC1H1/CSNK1E/CEP89/HAUS
SRC/CKS2/CCNE1/CCNA2/CCNF/PSRC1/CDC25C/CDKN2A/CDC25A/CDK4/CDKN2D/CKS1B/CCNO/BLM
O1A/CCDC88B/NCK2/SYK/IL18/VTCN1/TFRC/HLA-DPA1/HLA-DMB/JAK3/CCL5/CD86/TNFSF9/ZP3
K2/ITGAV/PGF/NCF4/SH2D2A/NCF1/WASF2/MAPKAPK2/VAV1/ITGA5/HIF1A/HGS/MT3/NCKAP1L/R
P1/NUP62/NCF1/AREG/DOK1/RHBDF1/HGS/TGFA/HIP1R/SH3KBP1/CNOT9/SHC1/EPS15L1/MVB12A
/CDC25B/CCNE1/TGFB1/RCC2/CDC25C/DTL/SMARCD3/PLCB1/EZH2/CDC25A/CDK4/CDC7/ESPL1/F
1/PRKCD/CD320/TFRC/BAX/MIF/CD300A/SASH3/TNFRSF4/SLC39A10/MNDA/CTLA4/LEF1/NCKAP1
TSE1/MUC1/EME1/ZNF385A/ARID3A/PRMT1/BAX/PCBP4/CASP2/BRSK1/PRKDC/CNOT11/PCNA/E2F
IRC3/CD44/CDKN2D/RPS6KA1/BEX3/TNFAIP8/RNF34/BCL2L12/NOL3/LEF1/MT3/LAMP3/NLE1/BI
F2/BCL2A1/HSPB1/MUC1/CDKN2D/ZNF385A/CREB3L1/YBX3/MIF/CREB3/HIF1A/BCL2L12/NOL3/H
A/CORO1A/CD74/PKN1/SLC7A11/JAK3/BAX/MIF/HIF1A/TCIRG1/SH2B2/NCKAP1L/PIK3CD/PMAIP
KLC2/KIF11/KIF3C/KIF18A/RAB6B/CENPE/COPE/TMED9/ARF3/ARFGAP1/KDELR1/KIF15/COPZ1/
RAS/GIT1/ARPC4/EFNA5/EFNB1/PAK1/MMP2/EPHB6/ARPC2/PSENEN/ARPC3/EPHB3/ACTG1/LYN/R
/TOP2A/TRIP13/CDC6/NDC80/BUB1/DLGAP5/BUB1B/CENPF/TTK/NCAPD2/NCAPH/MAD2L1/EME1/E
/BRSK1/MIIP/PSMD2/CHMP4C/BRCA1/TICRR/PSMB2/PSME3/BLM/PSMB4/PSMB8/PSMB10/PSMA5/P
/TGFB1/CTSV/CD44/DDR1/ADAM15/BSG/LAMC1/MMP1/FSCN1/CTSK/TIMP2/SH3PXD2B/LCP1/MMP1
/BUB1B/AURKA/MAD2L1/PSMB9/PSMD14/PSMD2/PSMB2/PSME3/FBXO5/ANAPC7/CDK2/PSMB4/PSMB
/TRIP13/CDCA5/CDC6/NDC80/BUB1/DLGAP5/BUB1B/CENPF/RMI2/TTK/FEN1/MAD2L1/ESPL1/LCM
C25C/GTSE1/MUC1/ZNF385A/ARID3A/PRMT1/BAX/PCBP4/CASP2/CNOT11/BRCA1/PCNA/E2F8/E2F
IRC3/CD44/CDKN2D/RPS6KA1/BEX3/TNFAIP8/RNF34/BCL2L12/NOL3/LEF1/MT3/LAMP3/NLE1/BI
K/HCK/WIPF3/WASF2/VAV1/ARPC4/MAPK3/PAK1/HSP90AB1/ARPC2/ARPC3/WIPF1/ACTG1/NCKAP1
K/HCK/WIPF3/WASF2/VAV1/ARPC4/MAPK3/PAK1/HSP90AB1/ARPC2/ARPC3/WIPF1/ACTG1/NCKAP1
/CCNE1/P3H4/TTK/NCAPH/FANCD2/EME1/ESPL1/SGO2/PPP2R1A/SGO1/NDC1/SMC4/NCAPH2
MAPK3/HSPA8/HSP90AB1/NUP93/NUP205/NUP37/NDC1/NUP188/MLST8/HSPH1/FKBP4/CHORDC1/N
5/CCT6A/CCT3/MAPK3/DKC1/ACD/CCT2/HSP90AB1/CCT4/PNKP/NHP2/CCT7/TCP1/PARP1/TELO2/
PINE2/CSF1/TGFB1/CLCF1/P2RX4/CXCR4/IL34/BIN1/HDAC1/HDAC2/LYN/IL1B/PPP1CC/PRKCI/
/CHAF1B/CENPH/CDKN2A/CENPW/OIP5/NAP1L1/CENPO/CENPU/CENPL/HMGB2/SIRT6/RUVBL1/NPM
3/ECT2/AURKA/RACGAP1/NUP62/GIT1/GIPC1/CHMP4C/MYO19/E2F8/INCENP/KIF14/RAB11FIP4/
TTK/RCC2/CCT5/EZH2/NABP2/CCT6A/CCT3/DKC1/ACD/CCT2/SPDL1/CCT4/CCT7/TCP1/MSH2/RUV
FI30/NCF1/PSMB9/PSMD14/PSMD2/HLA-A/ITGB5/PSMB2/PSME3/PSMB4/PSMB8/PSMB10/PSMA5/P
CM6/FEN1/POLD1/MCM7/MCM3/CDC7/MCM5/POLA2/GMNN/PCNA/E2F8/RFC2/FGFR1/FBXO5/DBF4/L

/DLGAP5/CENPF/KIFC1/PSRC1/NUP62/SPAG5/KIF18A/CHMP4B/FAM83D/CENPE/CHMP4C/DYNC1H1
5/ANLN/STMN1/KIF23/ECT2/NUSAP1/UNC119/RACGAP1/NUP62/CFL1/CHMP4B/RHOC/ESPL1/CHMP
25C/DTL/GTSE1/MUC1/ZNF385A/ARID3A/CHEK1/PRMT1/BAX/PCBP4/CASP2/PRKDC/CNOT11/BRCA
25C/DTL/GTSE1/MUC1/ZNF385A/ARID3A/CHEK1/PRMT1/BAX/PCBP4/CASP2/PRKDC/CNOT11/BRCA
/CDC25B/CCNE1/TGFB1/RCC2/CDC25C/DTL/SMARCD3/PLCB1/CDC25A/CDK4/CDC7/ESPL1/NSMCE2
IPF3/WASF2/VAV1/ARPC4/MAPK3/PAK1/HSP90AB1/ARPC2/ARPC3/WIPF1/ACTG1/NCKAP1L/MYO1G
A/CCT3/MAPK3/DKC1/ACD/CCT2/HSP90AB1/PPIA/CCT4/PNKP/NHP2/CCT7/TCP1/TELO2/CCT8/PT
2/CCT6A/CCT3/SIRT6/MAPK3/DKC1/ACD/CCT2/CCT4/PNKP/CCT7/TCP1/PML/PARP1/CCT8/GNL3/
IPF3/WASF2/VAV1/ARPC4/MAPK3/PAK1/HSP90AB1/ARPC2/ARPC3/WIPF1/ACTG1/NCKAP1L/MYO1G
C2L/GTPBP4/NHP2/NLE1/RPL10L/RPL7/RPL35A/SNU13/FTSJ3/RPL35/PPAN/RPLP0/LAS1L/RPL3
X1/PDIA2/NCF4/GSR/NCF1/DDIT3/PRDX1/PTGES2/SLC11A1/GLRX3/TXN/ERO1A/GPX1/DNAJC10/
/DLGAP5/CENPF/KIFC1/PSRC1/NUP62/SPAG5/KIF18A/CHMP4B/FAM83D/CENPE/CHMP4C/DYNC1H1
D74/PKN1/JAK3/CCL5/BAX/MIF/HIF1A/SLC39A10/PRELID1/PDCD1/NOC2L/ADAM8/KDELR1/LYN/
25C/DTL/GTSE1/MUC1/ZNF385A/ARID3A/CHEK1/PRMT1/BAX/PCBP4/CASP2/PRKDC/CNOT11/BRCA
6A/CCT3/CHTF18/MAPK3/DKC1/ACD/FGFR4/CCT2/HSP90AB1/RFC2/CCT4/PNKP/CCT7/TCP1/PRKD
/STMN1/KIF23/ECT2/NUSAP1/UNC119/RACGAP1/NUP62/CFL1/CHMP4B/RHOC/ESPL1/CHMP4C/LZT
B9/PSMD14/PSMD2/HLA-A/ITGB5/PSMB2/PSME3/PSMB4/PSMB8/PSMB10/PSMA5/PSMD13/PSMC4/P
/CCT3/MSN/ZP3/CCT2/UBAP2L/CCT4/CCT7/CD209/TCP1/FUT3/FOLR2/SPESP1/CD6/CCT8/HVCN1
O/CENPU/CENPL/RUVBL1/VPS72/CHD3/NPM1/SMARCA4/ACTL6A/HDAC1/ITGB3BP/HDAC2/NASP/AC
/MIF/MAD2L2/BCL2L12/PARPBP/DDIAS/BID/OTUB1/FBXO5/AUNIP/KDM1A/TAF9/THOC5/PSMD10/
TGAM/CYBB/GSTP1/ITGB2/SOD3/PRKCD/ACP5/SYK/NCF4/NCF1/PRDX1/SH3PXD2B/DUOX2/F2RL1/
/TRIP13/CDC6/NDC80/BUB1/DLGAP5/BUB1B/CENPF/TTK/MAD2L1/ESPL1/LCMT1/NAA10/MAD2L2/
1/CD320/TFRC/MIF/CD300A/SASH3/TNFRSF4/SLC39A10/MNDA/CTLA4/NCKAP1L/LYN/CARD11/TN
TSE1/MUC1/ZNF385A/ARID3A/PRMT1/BAX/PCBP4/CASP2/PRKDC/CNOT11/PCNA/E2F8/E2F4/CNOT
AC7/EZR/VTCN1/CARD9/VSIG4/TRAF2/CD86/CD3E/SASH3/CD83/LAG3/SLC11A1/CARD11/IL1B/R
PSA/NLE1/RPL10L/RPS5/VCX/PPAN/RPS19/RPLP0/RRP7BP/RPL38/RPL23A/MRPS7/TRAF7/MPV17
TSE1/MUC1/ZNF385A/ARID3A/PRMT1/BAX/PCBP4/CASP2/PRKDC/CNOT11/PCNA/E2F8/E2F4/CNOT
/UNC13D/HMOX1/CD300A/STXBP2/PIK3CD/PLSCR1/LYN/S100A13/FGR/LCP2/CD300LF/IL4R/BTK
/TRIP13/CDC6/NDC80/BUB1/DLGAP5/BUB1B/CENPF/TTK/MAD2L1/ESPL1/LCMT1/MAD2L2/CENPE/
/TRIP13/CDC6/NDC80/BUB1/DLGAP5/BUB1B/CENPF/TTK/MAD2L1/ESPL1/LCMT1/MAD2L2/CENPE/
/PKN1/JAK3/BAX/MIF/HIF1A/TCIRG1/SH2B2/NCKAP1L/PMAIP1/LYN/IL2RA/TNFSF13B/SIT1/FA
B/CENPH/CENPW/OIP5/NAP1L1/CENPO/CENPU/CENPL/HMGB2/RUVBL1/NPM1/CHAF1A/ITGB3BP/NA
A/CCT3/MAPK3/DKC1/ACD/CCT2/HSP90AB1/CCT4/PNKP/NHP2/CCT7/TCP1/TELO2/CCT8/PTGES3/
CM6/FEN1/POLD1/MCM7/MCM3/CDC7/MCM5/POLA2/GMNN/PCNA/RFC2/FGFR1/DBF4/LIG1/PRIM1/D
TSE1/MUC1/ZNF385A/ARID3A/PRMT1/BAX/PCBP4/CASP2/PRKDC/CNOT11/PCNA/E2F8/E2F4/CNOT
1/CHMP4B/STIL/NPM1/CHMP4C/BRCA1/XRCC3/CEP85/CHORDC1/POC1A/CHMP3/PLK4/KAT2A/CHMP
/CENPF/KIFC1/PSRC1/NUP62/SPAG5/KIF18A/CHMP4B/FAM83D/CENPE/CHMP4C/DYNC1H1/SPDL1/
3/NDC80/BUB1/BUB1B/LIF/CENPF/TTK/MAD2L1/CHEK1/LCMT1/NAA10/MAD2L2/SPDL1/XRCC3/FB
A/CCT3/MAPK3/DKC1/ACD/CCT2/CCT4/PNKP/CCT7/TCP1/PARP1/CCT8/NAT10/HNRNPU/EXOSC10/
/TRIP13/CDC6/NDC80/BUB1/DLGAP5/BUB1B/CENPF/TTK/MAD2L1/ESPL1/LCMT1/MAD2L2/CENPE/
3/CDC6/NDC80/BUB1/DLGAP5/BUB1B/CENPF/TTK/MAD2L1/ESPL1/LCMT1/MAD2L2/CENPE/NSMCE2
TSE1/MUC1/ZNF385A/ARID3A/PRMT1/BAX/PCBP4/CASP2/PRKDC/CNOT11/PCNA/E2F8/E2F4/CNOT
TSE1/MUC1/ZNF385A/ARID3A/PRMT1/BAX/PCBP4/CASP2/PRKDC/CNOT11/PCNA/E2F8/E2F4/CNOT
TSE1/MUC1/ZNF385A/ARID3A/PRMT1/BAX/PCBP4/CASP2/PRKDC/CNOT11/PCNA/E2F8/E2F4/CNOT
G5/MAPRE1/FAM83D/STIL/SNX10/RTKN/CSNK1D/DCTN2/FAM83H/CEP250/NSFL1C/BICD1/HNRNPU
TSE1/MUC1/ZNF385A/ARID3A/PRMT1/BAX/PCBP4/CASP2/PRKDC/CNOT11/PCNA/E2F8/E2F4/CNOT
TSE1/MUC1/ZNF385A/ARID3A/PRMT1/BAX/PCBP4/CASP2/PRKDC/CNOT11/PCNA/E2F8/E2F4/CNOT
TSE1/MUC1/ZNF385A/ARID3A/PRMT1/BAX/PCBP4/CASP2/CNOT11/PCNA/E2F8/E2F4/CNOT9/CDK2
3/CDC6/NDC80/BUB1/DLGAP5/BUB1B/CENPF/TTK/MAD2L1/ESPL1/LCMT1/MAD2L2/CENPE/NSMCE2
/CD109/CD53/FLOT1/EHD1/CXCL9/EHD2/ADAM12/VASH2/MYOF/SBNO2/IL4R
OBP/PRKCD/SYK/JAK3/SASH3/ISG15/F2RL1/CD83/BCL3/CD47/CLEC7A/HSPD1/FCGR2B/TUSC2/I

/NEK2/KIF23/KIFC1/MISP/FLNA/RACGAP1/KIF11/CHMP4B/CHMP4C/MSTO1/KPNB1/KIF2A/ZNF20
/NDC80/BUB1/DLGAP5/BUB1B/CENPF/TTK/MAD2L1/ESPL1/LCMT1/MAD2L2/CENPE/NSMCE2/SPDL1
/CD109/CD53/FLOT1/EHD1/CXCL9/EHD2/ADAM12/VASH2/MYOF/SBNO2/IL4R
/NDC80/BUB1/DLGAP5/BUB1B/CENPF/TTK/MAD2L1/ESPL1/LCMT1/MAD2L2/CENPE/NSMCE2/SPDL1
3/NDC80/BUB1/BUB1B/CENPF/TTK/MAD2L1/CHEK1/LCMT1/NAA10/MAD2L2/SPDL1/XRCC3/FBXO5/
/CD109/CD53/FLOT1/EHD1/CXCL9/EHD2/ADAM12/VASH2/MYOF/SBNO2/IL4R
13D/ZP3/RAB15/STX1A/F2RL1/CDK5/FGR/IL4R/SYT1/DNM1L/STX4/RAB3D/VAMP8/CD177/RAB3A
D/HMOX1/CD300A/STXBP2/SPON2/PIK3CD/LYN/S100A13/FGR/IL4R/BTK/CBL/VAMP8/MILR1/C12
D/HMOX1/CD300A/STXBP2/PIK3CD/LYN/S100A13/FGR/IL4R/BTK/CBL/VAMP8/MILR1/C12orf4/S
BCD1/ELOVL1/AKR1C3/ALOX5/FADS3/MIF/PTGES2/DEGS1/PTGS1/SCD5/IL1B/TBXAS1/FADS1/HP
FMNL1/RHOQ/EPB41L2/WIPF3/TIGD5/WIPF1/NCKAP1L/RTKN/EHD2/CDK5/WAS/VIL1/CIT/LLGL1/
FAAP24/FANCG/FANCE/XRCC3/FANCA/USP1/MCM8/RFWD3/RPA2/RAD51D/DCLRE1B/FAAP20/RPS27
2/TGFB1/CD74/CDK4/SIRT6/MIF/PRKDC
GEF2/MAD2L1/ASPM/ESPL1/DYNC1H1/SPDL1/DYNLT1/GPSM2/KPNB1/MAP4/LLGL1/ACTL8/NDE1/N
/BSG/TRIM28/MMP2/PPARD/ARHGDIB/FKBP4/IL1B/CALCA/SYDE1/AGO2/CALR
4/HCLS1/PARP1/IL15/CDC42/APP/FADD/PTPN2/CASP8/VEGFA/TLR2/INHA
3/NDC80/BUB1/BUB1B/CENPF/TTK/MAD2L1/ESPL1/LCMT1/NAA10/MAD2L2/SPDL1/XRCC3/FBXO5
D/HMOX1/CD300A/STXBP2/PIK3CD/LYN/S100A13/FGR/IL4R/BTK/CBL/VAMP8/MILR1/C12orf4/S
GEF2/MAD2L1/ESPL1/DYNC1H1/SPDL1/DYNLT1/GPSM2/KPNB1/MAP4/LLGL1/ACTL8/NDE1/NSFL1C
L1/PLCB1/CCL5/CREB3/MAPK3/SERPINE1/C3AR1/LYN/PLA2G7/CCR1/C5AR1/CMKLR1/S100A7/CX
/KIFC1/PSRC1/NUP62/KIF18A/CHMP4B/CENPE/CHMP4C/SPDL1/RRS1/KIF14/KPNB1
3/NDC80/BUB1/BUB1B/CENPF/TTK/MAD2L1/ESPL1/LCMT1/NAA10/MAD2L2/SPDL1/XRCC3/FBXO5
K/UNC13D/HMOX1/CD300A/STXBP2/F2RL1/LYN/FGR/ABR/IL4R/STX4/FCGR2B/VAMP8/C12orf4/C
FMNL1/RHOQ/EPB41L2/WIPF3/TIGD5/WIPF1/NCKAP1L/RTKN/EHD2/CDK5/WAS/VIL1/CIT/LLGL1/
O/CENPU/CENPL/RUVBL1/NPM1/ITGB3BP/NASP/CENPI/CENPK/RBBP4/HELLS/CENPN/RBBP7/CENP
OOA/STXBP2/LYN/FGR/IL4R/VAMP8/C12orf4/STXBP1/ADORA2B/IL13RA2/CD84
O/CENPU/CENPL/RUVBL1/VPS72/NPM1/ITGB3BP/NASP/CENPI/CENPK/RBBP4/CENPN/RBBP7/CENP
IRF1/CTLA4/PLA2G2D/LAG3/FANCA/CBFB/IL2RA/DROSHA/KAT2A/LILRB4/BTN2A2
D3/CDC25A/CDK4/CDC7/NPM1/FBXO5/APP/DBF4B/RAD51C/HSPA2/MTA3/RAB11A/MECP2
ECT2/RCC2/RACGAP1/SPAG5/CENPE/SGO1/KNSTRN/CDC42/ZNF207
OOA/STXBP2/PLSCR1/LYN/FGR/CD300LF/IL4R/VAMP8/MILR1/C12orf4/STXBP1/ADORA2B/IL13R
3/NDC80/BUB1/BUB1B/CENPF/TTK/MAD2L1/LCMT1/NAA10/MAD2L2/SPDL1/XRCC3/FBXO5
5/CENPO/CENPU/CENPL/RUVBL1/CENPE/NPM1/ITGB3BP/NASP/CENPI/CENPK/RBBP4/HELLS/RNF4
4/CD52/GRN/NCF1/PIK3CD/SLC11A1/RAC1/JCHAIN/RPS19/CLEC7A/LBP
3/NDC80/BUB1/BUB1B/CENPF/TTK/MAD2L1/LCMT1/MAD2L2/SPDL1/XRCC3/FBXO5
O/CENPU/CENPL/RUVBL1/NPM1/ITGB3BP/NASP/CENPI/CENPK/RBBP4/CENPN/RBBP7/CENPQ/MIS1
IRF1/CTLA4/LAG3/FANCA/CBFB/IL2RA/DROSHA/KAT2A/LILRB4/BTN2A2
AP2M1/AP2S1/AP1B1/PAK2/AP2A2/AP1S1/AP1S3/AP1S2/AP2B1/DOCK2/AP2A1/KPNA6/CD247/AR
O/CENPU/CENPL/RUVBL1/NPM1/ITGB3BP/NASP/CENPI/CENPK/RBBP4/CENPN/RBBP7/CENPQ/MIS1
3/NDC80/BUB1/BUB1B/CENPF/TTK/MAD2L1/LCMT1/MAD2L2/SPDL1/XRCC3/FBXO5
5/CENPO/CENPU/CENPL/RUVBL1/NPM1/ITGB3BP/NASP/CENPI/CENPK/RBBP4/CABIN1/CENPN/RBB
STP1/ITGB2/PRKCD/ACP5/SYK/NCF1/DUOX2/F2RL1/AATF
C1/MCM6/MCM10/MCM7/MCM3/MCM5/POLA2/TICRR/CDK2/PRIM1/POLE4/POLA1/PRIM2
5/CENPO/CENPU/CENPL/RUVBL1/NPM1/ITGB3BP/NASP/CENPI/CENPK/RBBP4/CABIN1/CENPN/RBB
GEF2/MAD2L1/ESPL1/SPDL1/DYNLT1/GPSM2/KPNB1/ACTL8/NDE1/NSFL1C/DCTN1
O/BUB1/BUB1B/CENPF/TTK/MAD2L1/LCMT1/MAD2L2/SPDL1/XRCC3/FBXO5/DYNC1LI1/ZNF207/RA
2L/CCT4/CCT7/TCP1/CCT8/SPA17/VDAC2
P62/GIPC1/KIF14/CIT/CDC42/CSPP1/KIF20B/RHOA
O/BUB1/BUB1B/CENPF/TTK/MAD2L1/LCMT1/MAD2L2/SPDL1/XRCC3/FBXO5/DYNC1LI1/ZNF207/RA
KC1/ACD/CCT2/CCT4/PNKP/CCT7/TCP1
O/BUB1/BUB1B/CENPF/TTK/MAD2L1/LCMT1/MAD2L2/SPDL1/XRCC3

0/BUB1/BUB1B/CENPF/TTK/MAD2L1/LCMT1/MAD2L2/SPDL1/XRCC3
KC1/ACD/CCT2/CCT4/PNKP/CCT7/TCP1
0/BUB1/BUB1B/CENPF/TTK/MAD2L1/LCMT1/MAD2L2/SPDL1/XRCC3
0/BUB1/BUB1B/CENPF/TTK/MAD2L1/LCMT1/MAD2L2/SPDL1/XRCC3
H/HSF1/PPP2R1A/FBXO5/NCAPH2/RAD51C/SPIRE1/MASTL
D3/CDC25A/CDK4/CDC7/FBXO5/APP/DBF4B/RAD51C/HSPA2/MTA3/RAB11A/MECP2
IF/SLC39A10/NOC2L/LYN
7/MCM3/GINS3/GINS4/PCNA/LIG1/POLA1/POLD3/DNA2/DCLRE1B/NBN

IL4R/STX4/VAMP8/CD177/HLA-F/STXBP1/ADORA2B

7/MCM3/GINS3/GINS4/PCNA/LIG1/POLA1/POLD3/DNA2

P1/NDE1/NSFL1C

GFR1/LIG1/POLA1

HP2/CCT7/TCP1/RUVBL2/CCT8/NOP10
/RAD9A/NAE1/TIMELESS/DNA2/CLSPN

C7/MCM5/GINS4
A

EF16/RAC2/CCNA2/CSF1/ECT2/IQGAP3/RHOV/ARHGAP4/COL1A2/SQSTM1/CDKN2A/TAX1BP3/ARHG
SPI1/HDAC11/UHRF1/PHF19/PKN1/PRKCD/EZH2/DNMT1/HDAC7/MUC1/CHEK1/PRMT1/YEATS2/BRM
3BP1/FOXJ1/PLXNA1/RGS10/ARHGEF16/DEPDC1B/ECT2/ARL2/IQGAP3/RCC2/ARHGAP4/AGRN/RAC
CARD/PKIB/RGS2/TPX2/CDC6/EPO/DUSP9/CDKN3/PKMYT1/CDK1/CDC25B/SRC/MMD/CKS2/RAC2/C
S2/MDK/TNFRSF21/E2F1/SOX9/EPO/DBN1/CDK1/REG1A/ITM2C/PLXNA1/ITGA3/TSPO/SEMA6A/MM
/EPO/DBN1/REG1A/PLXNA1/ITGA3/TSPO/SEMA6A/MMD/ETV5/LIF/SERPINE2/CSF1/ECT2/TGFB1/
/LIMK1/LY96/PGC/TNFRSF21/MMP12/IKBKE/COL1A1/FCER1G/ICAM1/ARPC1B/LGALS9/FOXJ1/TY
DC6/UBE2S/EPO/DUSP9/CDK1/NEK2/CDC25B/SRC/MMD/CKS2/RFC4/TRIB3/MAGEC2/CSF1/ECT2/T
T11/ITM2C/VEGFB/GAL3ST1/VCAN/B3GAT3/GAL3ST4/PLCB1/NUDT14/TUSC3/DPM2/MUC1/CHST4/
K1/MAPK12/SRC/MAPK13/LIF/TTK/TGFB1/AURKA/DKK1/RIPK2/CD44/PKN1/PRKCD/NCK2/SYK/ST
KE/FCER1G/TYRO3/SRC/S100A14/ITGAM/RIPK2/HAVCR2/BIRC3/ITGB2/IRAK1/PRKCD/RELB/HRA
/CDCA5/ANXA2/HJURP/NEK2/STMN1/BAK1/SRC/LGALS3/PLAUR/DAB2/LIF/TRIB3/TGFB1/BAMBI/
ITGB4/ITGA3/KRT23/DSG2/CTSV/DKK1/COL1A2/KRT17/PI3/KRT80/ADAM9/CD109/ELOVL1/FZD6
R1G/TYRO3/SRC/S100A14/ITGAM/RIPK2/HAVCR2/BIRC3/ITGB2/IRAK1/PRKCD/RELB/HRAS/SYK/
S9/STMN1/SRC/FLNA/BIRC3/HSPB1/CCT5/UNC13D/EXOSC4/MX2/APOBEC3B/IFNGR2/RNASE6/CFL
A3/ARHGEF16/LYPD1/LGALS3/DAB2/MMP14/TGFB1/STX3/SQSTM1/FLNA/KCNJ11/TSPAN15/PRNP/
D74/CSF1R/CFL1/APOBEC3C/TRIM6/CHMP4B/TRIM28/CCL5/CXCR4/TRIM11/SLPI/ISG15/CHMP4C
TGAM/FLNA/ITGB2/CEP131/PRKCD/B3GAT3/PRNP/HRAS/SYK/MTMR2/GTSE1/SPAG5/EZR/RAN/MIC
PDA/SRC/NDRG1/CCNA2/STC2/CYBB/IRAK1/EIF4EBP1/ANKRD1/PGK1/PSMB9/PSMD14/HMOX1/SLC
PDA/SRC/NDRG1/CCNA2/STC2/CYBB/IRAK1/EIF4EBP1/ANKRD1/PGK1/PSMB9/PSMD14/HMOX1/SLC
2/CSF1/S100A14/TGFB1/GSTP1/STX3/CD74/P2RX4/ROBO1/HSPB1/SLAMF8/PLXNA3/NBL1/STK39

RC/RAC2/RCC2/AGRN/PRKCD/HRAS/CDC42EP1/TWF2/DCDC2/TRPM2/RHOG/PLEK2/RHOQ/FSCN1/WA
IF/TGFB1/NME1/TMSB4X/MDFI/DDIT3/HMGB2/TRIM6/HMOX1/TRIM28/MAD2L2/ACD/LEF1/BCL3/S
OLR1/VASP/FZD6/SCRIB/IFT57/CFL1/MTHFD1L/PTK7/STIL/HIF1A/FZD1/ST14/IFT52/DVL3/FZ
4/RFC4/RAD51/MCM6/DSCC1/GINS2/MCM7/PIF1/MCM3/MCM5/GINS4/RUVBL1/CHTF18/CHD3/ERCC
E1/P3H4/RAD51/AURKA/CDC25C/RAD54L/FANCD2/CDC25A/C11orf80/EME1/PSMC3IP/ESPL1/MND
4/CD74/SLAMF8/NBL1/STK39/PGF/CCL5/MIF/CREB3/MAPK3/SERPINE1/ZNF580/C3AR1/F2RL1/N
RHGEF2/PLEK/TRIM54/ASPH/TWF2/MID1IP1/DNAJC6/KIF18A/CFL1/CARMIL1/NCKAP5L/HSPA8/M
P1/NUP62/NCF1/AREG/DOK1/RHBDF1/HGS/TGFA/HIP1R/SH3KBP1/CNOT9/SHC1/EPS15L1/MVB12A
4/CD74/STK39/PGF/CCL5/CREB3/MAPK3/SERPINE1/ZNF580/C3AR1/F2RL1/NCKAP1L/CXCL13/RA
ASP4/IFT57/TRAF2/BAX/BEX3/HIP1R/BID/BBC3/PMAIP1/F3/LCK/CASP1/PML/HSPE1/FADD/RAC
PSMB2/PSME3/PSMB4/PSMB8/PSMB10/PSMA5/PSMD13/PSMC4/PSMD10/PSMD11/PSMD1/PSMA7/PSM
2L/CCT4/CCT7/TCP1/FOLR2/SPESP1/CCT8/HVCN1/SPA17/VDAC2
2/LEF1/SRI/CPNE1/HDAC2/KDM1A/ZNF593/IFI16/TFAP4/ANKRD33/HEY1/GZMA/ZNF675/MSX1
A/CCT3/MAPK3/DKC1/ACD/CCT2/CCT4/PNKP/CCT7/TCP1/CCT8/NAT10/HNRNPU/EXOSC10/HNRNPA
E2F8/RFC2/FGFR1/BLM/FBXO5/DBF4/CDK2/DONSON/TIPIN/TIMELESS/DBF4B/CHEK2/NBN/STOML
2L1/MAD2L2/RPS2/FBXO5/AXIN1/UBE2L3/DCUN1D5/DNM1L/RPL23/ABL1/RPS7/BAG2/RPL5/MAST
OBP/SYK/JAK3/SASH3/CD83/BCL3/CD47/CLEC7A/HSPD1/FCGR2B/TUSC2/IL20RB/TLR2/IDO1/XC
/NDN/ADGRG1/RELN/MBOAT7/CCL2/CDK5/TGFB2/GPR183/CSPG4/CCL3/SRGAP2/HEXB/CDK5R1
OB/ATP6V0D2/RAB32/ATP6V1C1/TCIRG1/RAB7B/ATP6V1H/RAB20/ATP6V1E1/ATP6V0C/ATP6V0E1
NRPE/SNRPF/SNRPC/GEMIN7/SRSF12/PRPF3/PRPF6/SNRPG/PRPF31/PRPF19/SART1/SART3/TGS1
5/SLC1A4/SLC43A2/SLC7A8/SLC3A2/SLC7A10/SLC36A1/SLC38A5
OOA/STXBP2/LYN/FGR/IL4R/VAMP8/C12orf4/STXBP1/ADORA2B/IL13RA2/CD84
D2L1/SPDL1/DYNLT1/GPSM2/ACTL8/NDE1/NSFL1C/DCTN1
/PSPH/HSF1/MSN/CAD/NASP/GBA/CALR/CBL/BAD/THBS1/CSNK2B
FR1/DBF4/CDK2/RAC1/DBF4B/DNA2/GLI1/STOML2/DHX9/FAF1/EREG/ATAD5/UCN/E2F7/KCTD13/
D2L1/SPDL1/DYNLT1/GPSM2/MAP4/LLGL1/ACTL8/NDE1/NSFL1C/DCTN1/HTT/ACTR1A/LLGL2
/CHEK1/MAPK3/RPS6KA4/CDK2/AKAP8L/IL1B/CDK5
/PHPT1/BAIAP2L1/DAPK3/ARHGDIB/HCLS1/CDK5/CDC42/GMFG/ARHGDIA/SEMA3E/ABL1
GAP1

/PRKCD/SYK/F2RL1/CLEC7A/MAPT/CD177/GNAI3
/CENPE/SMC4/CENPK/RNF4/CENPN
TAT5A
1/CCT8/GNL3/WRAP53
3AR1/C5AR1/CMKLR1/CCL3/CXCL17/AKIRIN1/THBS1
/TNFSF13B/TNFAIP3/ADAM17
4/CCT7/TCP1/CCT8/WRAP53
/RTKN
1/CCT8/WRAP53

TYRO3/SRC/ITGA3/DAB2/CCNE1/DEPDC1B/BAMBI/DKK1/FOLR1/GPC4/GPC3/TAX1BP3/PLCB1/BIC
/AURKB/UBE2S/CDK1/BUB1B/DAB2/PBK/TRIB3/CCNF/AURKA/SQSTM1/DTL/UHRF1/UBXN11/MAD2L
EM2/KCTD17/UBD/AURKB/UBE2S/ANXA2/CTSC/CDK1/BUB1B/ATP13A2/DAB2/PBK/TRIB3/MAGEC2/
AUR/RAC2/ATP13A2/SQSTM1/YWHAZ/HK2/VAT1/PIF1/FUNDC2/MX2/MPV17/MTFP1/TOMM40/TYMP/
/ARPC1B/PFN2/STMN1/SH3BP1/ECT2/TGFB1/RHOV/CORO1A/FLNA/COTL1/PRKCD/NCK2/BAIAP2L2
RC/ITGA3/CSF1/ARL2/TGFB1/LGALS1/RIPK2/ADA/HAVCR2/CORO1A/STX3/FLNA/CD44/ITGB2/IR
K1/MAPK12/SRC/MAPK13/LIF/TTK/TGFB1/AURKA/DKK1/RIPK2/CD44/PKN1/PRKCD/NCK2/SYK/ST
O3/SRC/S100A14/ITGAM/RIPK2/HAVCR2/BIRC3/ITGB2/IRAK1/PRKCD/RELB/HRAS/SYK/HCK/S10
SF1/TYROBP/TGFB1/RIPK2/ADA/C1QC/CD74/FANCD2/SLAMF8/NME1/SYK/IL18/NME2/HMGB3/TNF
/RIPK2/ADA/CD74/BATF/RELB/FANCD2/SYK/IL18/FZD7/TNFRSF18/JAK3/CD86/VAV1/SEMA4A/T
RHGEF2/PLEK/TRIM54/ASPH/TWF2/MID1IP1/DNAJC6/KIF18A/CFL1/CARMIL1/EIF5A2/NCKAP5L/
ROBP/TGFB1/RIPK2/ADA/CD74/SYK/IL18/PRMT1/HMGB2/CD86/TNFSF9/IL34/HIF1A/ATP6AP1/S
MB9/PSMD14/PTK7/RUVBL1/PSMD2/EDA/CSNK1E/FAM53B/PSMB2/PSME3/PLEKHA4/DAPK3/CSNK1D
FB1/FOLR1/VASP/FZD6/SCRIB/IFT57/CFL1/HNF1B/MTHFD1L/PTK7/STIL/HIF1A/FZD1/ST14/ED
OBO1/SYK/S100A8/ASPH/CASP4/IFT57/HSF1/CARD9/TRAF2/BAX/BEX3/PERP/HIP1R/BID/BCAP3
LF1/CDC25B/SRC/TIMP1/LHFPL2/LIF/TTK/AURKA/ADA/CDC25C/PTAFR/PLCB1/CDC25A/ASPM/ZP
P131/KIF11/NUP62/CHEK1/SAC3D1/CHMP4B/BRSK1/STIL/CHD3/NPM1/CHMP4C/BRCA1/XRCC2/SG
O1A/NCK2/BAIAP2L2/VASP/CDC42EP1/HCK/MAPRE1/CARMIL1/CCL26/ARPC4/PAK1/BAIAP2L1/FC
YK/S100A8/CASP4/IFT57/HSF1/CARD9/TRAF2/BAX/BEX3/HIP1R/BID/BCAP31/PRR7/BBC3/PMAI
P131/KIF11/NUP62/CHEK1/SAC3D1/CHMP4B/BRSK1/STIL/CHD3/NPM1/CHMP4C/BRCA1/XRCC2/SG
RAK1/RELB/SYK/HMGB2/PRKDC/NFKB2/ISG15/SIRPA/IRF1/IRF3/STAT1/NPLOC4/POLR3K/POLR2
S3/TGFB1/ITGAM/STX3/SQSTM1/YWHAZ/ITGB2/KCNJ11/PRNP/HRAS/EZR/RHOG/ANXA13/MTCL1/P
RAK1/RELB/SYK/HMGB2/PRKDC/NFKB2/ISG15/SIRPA/IRF1/IRF3/STAT1/NPLOC4/POLR3K/POLR2
3/ADA/CDKN2A/CD74/PKN1/SLC7A11/JAK3/CCL5/BAX/MIF/HIF1A/SLC39A10/IRF3/PRELID1/PD
5F6/BMF/NOL3/HIP1R/BID/DYNLT1/UQCC3/HSPA4/YWHAH/BBC3/PMAIP1/ZNF205/GSK3A/YWHAQ/
VASP/FZD6/SCRIB/IFT57/MTHFD1L/PTK7/STIL/FZD1/ST14/DVL3/FZD2/TULP3/DEAF1/TGFB2/D
2/TGFB1/GSTP1/CD74/CDK4/BAX/SIRT6/MIF/CD300A/PRKDC/FTH1/NUPR1
54/MAPRE1/MID1IP1/MAP2/MAP1S/PAK1/TUBB4A/RANGRF/CAMSAP2/FKBP4/RAC1/STMN3/INPP5J
/TPPP3/FBXO5/TUBB4A/RANGRF/CSNK1D/CAMSAP2/FKBP4/RAC1/INPP5J/TUBG2/RPS3/ZNF207/N
BGN/RNF113A/KRT10/THBS1
IRT6/MAPK3/DKC1/ACD/CCT2/CCT4/PNKP/CCT7/TCP1/PML/CCT8/GNL3/NBN/ERCC1/HNRNPA1/MA
8/CKLF/CCL5/MIF/MAPK3/C3AR1/CCL2/NUP85/C5AR1/CMKLR1/CCL3/EMILIN1/CXCL17/AKIRIN1
A/PRELID1/BID/BBC3/PMAIP1/PSMD10/GPX1/ARRB2/DNM1L/PDCD5/MFF/BAD/BCL2L1
1/CDC25A/ASPM/PPP2R1A/FBXO5/CALR/MSX1/WNT4/LFNG/RAD1
2/TYMP/PUDP/CAD/AMD1/GMPS/IMPDH2/PRTFDC1/NME6/APRT/NME7/GUK1/NME3/DCK/TRMT12/NM
PD2/NCAPH/NCAPG2/SMC4/AKAP8L/NCAPH2/BANF1/CHMP1A
RCA1/CBX3/EHMT2/RBBP4/CBX5/PCGF2/RBBP7/E2F6/RNF2/PPP2R5B/UXT/TFDP1/DAB2IP
PSF4/TNIP1/BAD/CASP8/BCL2L1/ATG7/SERPINB9/CCL8/PABPN1/VAPA/KPNA4/CD4
1/CXCL9/EHD2/IL4R
/PRKCD/ACP5/SYK/F2RL1/AATF/CLEC7A/MAPT/CD177/GNAI3
L13/KIF14/ITGB1BP1/CDH17/PIEZO1
4/CCT7/TCP1/RUVBL2/CCT8/WRAP53/PIH1D1
R2/GRB2/DNAJB6
HP2/CCT7/TCP1/RUVBL2/CCT8/WRAP53/NOP10/EXOSC10
HP2/CCT7/TCP1/RUVBL2/CCT8/WRAP53/NOP10/EXOSC10
HP2/CCT7/TCP1/RUVBL2/CCT8/WRAP53/NOP10/EXOSC10
HP2/CCT7/TCP1/RUVBL2/CCT8/WRAP53/NOP10/EXOSC10
P1/NDE1/NSFL1C
A/LBP

1/CCT8/WRAP53
1/CCT8/WRAP53
1/CCT8/WRAP53

A2/PCSK1N/GAPDH/IGF2BP2/GSTP1/TYMS/KRT17/EIF4EBP1/PTAFR/HSPB1/NCK2/SLC7A11/IGF2
BE2S/CDCA3/TSPO/TRIM47/TRIB3/MAGEC2/CCNF/TRAIP/FANCI/RIPK2/DTL/UHRF1/BIRC3/BLMH
/AURKB/UBE2S/CDK1/BUB1B/DAB2/PBK/TRIB3/CCNF/AURKA/SQSTM1/DTL/UHRF1/UBXN11/MAD2L
17/UBD/AURKB/UBE2S/CTSC/CDK1/BUB1B/DAB2/PBK/TRIB3/CCNF/AURKA/CTSV/SCPEP1/SQSTM1
TYRO3/SRC/ITGA3/DAB2/CCNE1/DEPDC1B/BAMBI/DKK1/FOLR1/GPC4/GPC3/TAX1BP3/PLCB1/BIC
/IMPDH1/TREM2/ENO2/PFKP/ST6GALNAC4/BACE2/RENBP/RRM2/TKT/TK1/ENO1/CHST11/ITM2C/V
D/S100A4/SOX9/EPO/IKBKE/DUSP9/CDK1/ICAM1/LGALS9/SRC/PTP4A3/RAC2/LIF/CSF1/AKR1B1
12/CHST11/ITM2C/VEGFB/SULF1/GAL3ST1/VCAN/B3GAT3/GAL3ST4/PLCB1/GPC1/NUDT14/TUSC3
G1/DNM1/SERPINE2/SQSTM1/YWHAZ/ANO9/FCHO1/DMKN/PRKCD/REEP4/BAIAP2L2/ABCD1/ASAP1/
CDK1/ICAM1/LGALS9/TYRO3/LGALS1/CD74/ITGAV/NUP62/NECTIN1/RAN/TFRC/APOBEC3C/TRIM6
/CD3D/MMP14/TGFB1/CMTM7/SPI1/LGALS1/RIPK2/ADA/CLCF1/CD74/BATF/RELB/FANCD2/SLAMF
/MDK/MMP12/IKBKE/CTSC/FCER1G/TYRO3/SRC/MAPK13/S100A14/ITGAM/RIPK2/HAVCR2/OSMR/B
/CEP131/TUBG1/CCDC28B/WDR34/MAPRE1/TUBB/ASAP1/DCDC2/IFT57/TUBB4B/KIF3C/ABCC4/RA
TYRO3/ATP13A2/CSF1/TYROBP/ITGAM/PPT1/GPNMB/DKK1/GAPDH/CORO1A/CLCF1/ITGB2/C1QA/S
/ARPC1B/PFN2/STMN1/SH3BP1/ATP1A1/DSG2/ECT2/TGFB1/RHOV/CORO1A/FLNA/COTL1/PRKCD/N
/CEP131/TUBG1/CCDC28B/WDR34/MAPRE1/TUBB/ASAP1/DCDC2/IFT57/TUBB4B/KIF3C/ABCC4/RA

F1/SOX9/CDK1/TSPO/ETV5/NDRG1/MMP14/LIF/SERPINE2/CSF1/TGFB1/HDAC11/GSTP1/CLCF1/P

SF21/COL1A1/ICAM1/POSTN/TRAIP/GSTP1/TNFRSF12A/BIRC3/KCNJ11/ARHGEF2/SYK/TMSB4X/C
2/LGALS1/RIPK2/GSTP1/SQSTM1/FLNA/BIRC3/IRAK1/ZDHHC13/CD74/HSPB1/RELB/NUP62/CARD
3/TYROBP/ITGAM/PPT1/GAPDH/CORO1A/CLCF1/PRNP/HRAS/FOXQ1/UNC5B/GRN/DDIT3/HMOX1/BA
8/CXCL5/ICAM1/SRC/TSPO/TGFB1/RIPK2/HAVCR2/GSTP1/IRAK1/PTAFR/ADAM9/HCK/IL18/CDK4
TP1/CD44/CD74/ARHGEF2/BCL2A1/HSPB1/ITGAV/MAPK8IP2/MUC1/UNC5B/CDKN2D/ZNF385A/CRE

YWHAZ/ROBO1/VCAN/NCK2/IGLL5/CCT5/BSG/CCT3/CXCR4/COLEC12/MFGE8/NDN/MSN/ZP3/JMJD6
D74/APOBEC3C/TRIM6/CHMP4B/TRIM28/CCL5/TRIM11/SLPI/ISG15/CHMP4C/PPIH/TRIM59/PABP
IN28B/CSTF2/SNRPA/DKC1/UPF3B/ZC3H3/PABPC1/POLR3K/RNPS1/ERI3/NELFE/FBL/NCBP2/USB
ANCD2/SYK/IL18/TNFRSF18/JAK3/CD86/TNFSF9/CD2/SASH3/SOCS1/IRF1/CTLA4/CD83/PRELID
K2/BAX/SLC35F6/BMF/NOL3/LMNA/GGCT/PRELID1/PIM2/HIP1R/AIFM2/BID/YWHAH/BBC3/PMAIP
F1R/CFL1/CHMP4B/CCL5/TRIM11/CHMP4C/POLR2G/SMARCA4/PPIH/PABPC1/LEF1/NELFE/POLR2H
/HCK/CSF1R/EZR/TRPM2/PLEK2/MICALL2/DTNBP1/PHPT1/PTK7/PAK1/CAPN10/BAIAP2L1/SHC1/
EP1/TWF2/PLEK2/RHOQ/FSCN1/WASF2/F2RL1/ARPC2/DYNLL1/RAC1/MIEN1/PFN1/CROCC/RALA/C
VASP/FZD6/SCRIB/IFT57/MTHFD1L/PTK7/STIL/FZD1/ST14/DVL3/FZD2/TULP3/DEAF1/TGFB2/D
RIPK2/HAVCR2/GSTP1/CD74/SYK/IL18/S100A8/CSF1R/HMOX1/MIF/HIF1A/SIRPA/F2RL1/IL1B/
2/TGFB1/GSTP1/CD74/CDK4/BAX/SIRT6/MIF/CD300A/PRKDC/FTH1/NUPR1/WNT7B/B4GALT7/PDG
/ADAM9/SYK/MUC1/COL16A1/PLAU/CCL5/ITGA5/SERPINE1/CD3E/ITGB5/FERMT1/NCKAP1L/CXCL
A8/CCT2/UNC45A/HSPH1/FKBP4/CHORDC1/DNAJB2/ERO1A/DFFA/HSPE1/PDCD5/SGTB/DNAJC7/PT

A/RANGRF/CAMSAP2/FKBP4/RAC1/INPP5J/RPS3/CLIP3/DRG1/MAPT/ABL1/TBCD/STMN2/DCTN1/E
LGALT1/SERPINH1/COL3A1/COMP/PLOD1/COL5A1/FMOD/PLOD2/PLOD3/COL5A2/LOXL2/TGFB2/LO
P1/KIF18A/NCKAP5L/MAP1S/CAMSAP2/STMN3/KIF2A/KATNB1/CKAP2/CKAP5/STMN2/CCSAP/MAP1
PPAN/RPLP0/RPL38/RPL23A/TRAF7/RPL5/RPF2/RPL6/MRPL11/DHX30/RPL24/RPL11/RPL10/MRP
MS/PCNA/E2F4/FBXO5
SASH3/CD83/LEF1
NSMCE2/DDX11/SFPQ/ANAPC11/RAD21/HNRNPU/RAD18/BECN1/ANAPC5/ANAPC4
2/PTGS1/IL1B/TBXAS1/HPGDS/PTGES3
P3H3/PLOD2/PLOD3/JMJD4/EGLN2/P3H2/P4HB/OGFOD1/VPS33B/HIF1AN/CRTAP
2/PTGS1/IL1B/TBXAS1/HPGDS/PTGES3

IVD/GCDH/ACADM/ACADL

4
7A5

1

1IP

/APOC3
GXT
/APOC3

4A11/CYP2C8/CYP2C9
1/CCT8/GNL3/WRAP53
/AURKB/UBE2S/CDK1/BUB1B/DAB2/PBK/TRIB3/CCNF/AURKA/SQSTM1/DTL/UHRF1/UBXN11/MAD2L
PAEP/GTSF1/AFP/RGS2/MDK/TOP2A/TRIP13/E2F1/SOX9/ASF1B/CDK1/CABYR/FOXJ1/TYRO3/CDC
1B3/PAEP/GTSF1/AFP/RGS2/MDK/TOP2A/TRIP13/E2F1/SOX9/ASF1B/LGALS9/CABYR/FOXJ1/TYR
1B3/PAEP/GTSF1/AFP/RGS2/MDK/TOP2A/TRIP13/E2F1/SOX9/ASF1B/LGALS9/CABYR/FOXJ1/TYR
NCS1/RAC2/ATP13A2/DAB2/ECT2/TGFB1/ITGAM/FLNA/ITGB2/CEP131/CPLX2/PRKCD/B3GAT3/PR

FB1/IQGAP3/DKK1/RIPK2/GSTP1/GDF15/IRAK1/S1PR2/CD74/PKN1/DUSP5/PRKCD/ROBO1/ADORA

MBI/DKK1/FOLR1/GPC3/BICC1/WNK2/FZD6/ASPM/TLE3/FZD7/CTNND2/PSMB9/DDIT3/PSMD14/PT
1/CCT8
1/CCT8
12/POSTN/LGALS9/SRC/PTP4A3/RAC2/ATP13A2/ECT2/TGFB1/ITGAM/AACS/GAPDH/RIPK2/HAVCR
13A2/CSF1/TYROBP/ITGAM/PPT1/GPNMB/DKK1/CORO1A/CLCF1/ITGB2/C1QA/SLC7A11/ADORA1/P
MBI/DKK1/FOLR1/GPC3/BICC1/WNK2/FZD6/ASPM/TLE3/FZD7/CTNND2/PSMB9/DDIT3/PSMD14/PT

LS1/RIPK2/GSTP1/SQSTM1/FLNA/BIRC3/IRAK1/ZDHHC13/CD74/HSPB1/NUP62/CARD9/TRAF2/RB
/SEMA6A/CSF1/TGFB1/ARHGAP4/AURKA/TNFRSF12A/PLXNA3/DDR1/TWF2/TRPV2/HNF1B/AREG/TR
ITGAM/PPT1/CORO1A/CLCF1/PRNP/HRAS/FOXQ1/UNC5B/GRN/DDIT3/HMOX1/BAX/DNAJC5/CASP2/
ALS9/TIMP1/ITGA3/ACSL4/LIF/SERPINE2/AKR1B1/DSG2/FBLN1/TGFB1/STC2/CTSV/HAVCR2/PT
GALS9/TYRO3/SRC/TGFB1/LGALS1/GAPDH/TYMS/CD74/ITGAV/NECTIN1/ALYREF/SCRIB/TFRC/CH
C1/CORO1A/FLNA/NCK2/BAIAP2L2/VASP/PLEK/CDC42EP1/COLGALT1/HCK/MAPRE1/CFL1/S100A1
TRIB3/MAGEC2/FANCI/RIPK2/BIRC3/MAD2L1/FKBP1A/MAD2L2/UBE2I/ISG15/BRCA1/COPS9/RPS
AC2/RCC2/AGRN/PRKCD/HRAS/CDC42EP1/TWF2/DCDC2/TRPM2/RHOG/PLEK2/RHOQ/FSCN1/WASF2/
S3/PLAUR/TRIB3/TGFB1/BAMBI/RIPK2/NME1/MAPRE1/LARP6/RAN/HSF1/S100A10/TRAF2/HMGB2
P1/MX2/YKT6/RAB34/CXCR4/ANKRD27/STX1A/STX6/RAB7B/STX10/PPIA/RABIF/RAB20/CHMP3/F
/HMGB2/JAK3/HMOX1/BAX/PRDX1/FLVCR1/HIF1A/JMJD6/ISG15/STAT1/AMPD3/NCAPG2/NCKAP1L
PO/TYROBP/GPNMB/CYBB/RIPK2/HAVCR2/GSTP1/PTAFR/ARHGEF2/HSPB1/ACP5/SYK/HSF1/CARD9
CLGN/NCK2/CREB3L1/HSF1/DDIT3/BAX/HSPA6/CREB3/HSPA8/LMNA/DDX11/HYOU1/ARFGAP1/EXT
ORD/UGT2B15
DH1A/ADH1C/ADH1B/ADH4
/GLYAT
1/AASS/PIPOX
/ALDH7A1/SHMT1/ACADL/DMGDH/BHMT
A5/APOC3
SLC25A47
ORD/UGT2B15

3
OA1/APOA5/APOC3

2C9/CYP3A4
7A5
/TAT
LDH1L1/ADHFE1
C/APOA2/LCAT/APOA1/APOC3
/MCEE/ACADS/PHYH/ALDH5A1/PCK2/PCK1

E1/P3H4/RAD51/AURKA/RAD54L/FANCD2/C11orf80/EME1/PSMC3IP/ESPL1/MND1/NDC1/FBXO5/P
2/MUC1/CHST4/B4GALT5/GALNT10/B3GNT5/GALNT18/B3GNT7/PLOD3/ST3GAL4/MUC3A/POMT2/MU
1A/TFF2/ROBO1/STK39/CCL5/SLC12A2/CXCR4/CCL26/GPR35/HIF1A/CCL13/ZC3H12A/CCL2/CXC
1A/TFF2/ROBO1/STK39/CCL5/SLC12A2/CXCR4/CCL26/GPR35/HIF1A/CCL13/ZC3H12A/CCL2/CXC
MT1M/MT1G/MT1E/MT1X
MF8/NBL1/PLCB1/CCL5/CCL26/CREB3/MAPK3/SERPINE1/JAML/SIRPA/C3AR1/CCL13/TNFRSF11A
D74/SLC7A11/JAK3/CCL5/BAX/MIF/HIF1A/SLC39A10/PRELID1/PDCD1/IRF7/NOC2L/ADAM8/PIK
YBB/RIPK2/HAVCR2/PTAFR/ARHGEF2/HSPB1/SYK/CARD9/CD86/MAPKAPK2/MIF/CD2/SASH3/SPON

P1/MASP2
ARG1/NAGS/OTC/CPS1
1/AASS/PIPOX
AGXT
1/ACADL
/MT1X

P1A1/CYP2A7/CYP1A2/CYP4F2/CYP2B6/CYP4A11/CYP2E1/CYP2C8/CYP2C9/CYP2A6
PS1/CYP2C9
CR/SCP2/ACOX2/EHHADH
APOA2/LCAT/APOA1/APOA5/APOC3

D1/APOA1/APOC3

/HRG
OTC/CPS1/CYP2C9
/MT1X
AD2L2/IL27RA/BCL3/CDH17/MSH2/SWAP70/GPR183/EXOSC3/MFNG/HSPD1/LFNG/ABL1/FCGR2B/N
D/RAB34/HMOX1/CD300A/STXBP2/PLEKHM2/PIK3CD/LYN/LAMTOR1/S100A13/FGR/KXD1/IL4R/BT
GLL5/RAB31/MFGE8/CD300A/GULP1/HGS/F2RL1/NCKAP1L/MSR1/ITGA2/CDC42/FCGR2B/BIN2/SP
A-DPA1/ZP3/HLA-DPB1/CD2/EBI3/CD276/IL27RA/CD3E/SASH3/BCL3/SLC11A1/IL1B/IL12RB1/

9/KNG1/SERPINC1
OC1/APOA2/LCAT/ALB/APOA1/APOC3
C6A1
/LMNA/GGCT/PRELID1/BID/BBC3/PMAIP1/PSMD10/GPX1/ARRB2/DNM1L/PDCD5/TIMM50/MFF/BAD
4/TRAF2/RBCK1/SHARPIN/NOL3/F2RL1/CPNE1/CASP1/CARD16/IKBKG/RACK1/ZNF675/CLIP3/PE
ESPL1/NAA10/SGO2/PPP2R1A/NSMCE2/SGO1/DDX11/SFPQ/RAD21/ESCO2/RAD51C/BOD1
1/CARD9/VSIG4/TRAF2/CD86/CD3E/SASH3/CD83/LAG3/CARD11/IL1B/RPS3/PRKD2/CLEC7A/ABL
H/FKBP11/FKBP9/PPIA/FKBP4/PPIL1/PTPA/CWC27/NTMT1/FKBP8/EGLN2/AIP/PPIE/PPIB/FKBP
1A1/SULT1E1/SULT1B1/ALDH2/ADH6/SULT2A1/ADH1A/ADH1C/ADH1B/ADH4
BX1/PABPC1/SLC11A1/HNRNPU/DHX9/RBM10/RBM24/LARP1/HNRNPA0/HNRNPD/HNRNPC/SYNCRIP/
2/TYMP/PUDP/CAD/AMD1/GMPS/IMPDH2/PRTFDC1/NME6/APRT/NME7/GUK1/NME3/DCK
BBP8/BRCA1/CBX3/EHMT2/RBBP4/CBX5/PCGF2/RBBP7/E2F6/RNF2/PPP2R5B/UXT/TFDP1/DAB2IP
P/CDK5/AP2M1/AP2S1/MVB12A/PTPN1/SH3GLB1/AP2A2/CLTC/AP2B1/LGMN/BECN1/AP2A1
BBP8/BRCA1/CBX3/EHMT2/RBBP4/CBX5/PCGF2/RBBP7/E2F6/RNF2/PPP2R5B/UXT/TFDP1/DAB2IP
MIF/MAPK3/C3AR1/C5AR1/CMKLR1/CCL3/EMILIN1/CXCL17/AKIRIN1/CYP19A1/THBS1
SRC/LIF/TTK/TGFB1/RIPK2/UNC119/CLCF1/CD44/ITGB2/CD74/ARHGEF2/PRKCD/NCK2/ADORA1/
SRC/LIF/TTK/TGFB1/RIPK2/UNC119/CLCF1/CD44/ITGB2/CD74/ARHGEF2/PRKCD/NCK2/ADORA1/
TNFRSF21/COL1A1/ICAM1/POSTN/TRAIP/GSTP1/TNFRSF12A/BIRC3/KCNJ11/ARHGEF2/ADAM9/SY
8/CXCL5/ICAM1/SRC/TSPO/TGFB1/RIPK2/HAVCR2/GSTP1/IRAK1/PTAFR/ADAM9/SYK/HCK/IL18/
5/ICAM1/SRC/TSPO/TGFB1/RIPK2/HAVCR2/GSTP1/IRAK1/PTAFR/ADAM9/HCK/IL18/CDK4/ANKRD
WDR4/SLFN13/CSTF2/TRMU/FTSJ1/METTL1/POLR3K/PUS1/POP7/URM1/DTD1/TRMT1/SSB/TRMT61
YBX3/EIF5A2/CCL5/MAPK3/SMPD2/NPM1/UPF3B/POLR2G/NSMAF/RBM3/PABPC1/BCL3/EIF3D/ITG
PO/TYROBP/GPNMB/CYBB/RIPK2/HAVCR2/GSTP1/PTAFR/ARHGEF2/HSPB1/ACP5/SYK/HSF1/CARD9
HP2/CCT7/TCP1/RUVBL2/CCT8/NOP10/EXOSC10

CK2/ADORA1/SHKBP1/NUP62/NCF1/AREG/DOK1/IQGAP1/RHBDF1/HGS/TGFA/HIP1R/SH3KBP1/CNO

RT6/NUP210/NAA40/MAP2K2/POLR2G/NUP93/NUP205/NUP37/NDC1/POLR2H/NUP188/ZC3H12A/CD
1/CCT8/WRAP53
/BCKDHB/IVD/ACAT1/ACADSB/ALDH6A1
/BCKDHB/IVD/ACAT1/ACADSB/ALDH6A1
F3B/ZC3H3/PABPC1/RNPS1/NELFE/NCBP2/U2AF2/SRSF9/CPSF1/THOC5/THOC6/CPSF6/EIF4A3/B
DH5A1/DMGDH/ABAT/BHMT
1A2/CYP2B6/CYP2E1/CYP2C8/CYP2C9/CYP2A6/CYP3A4
RIDA/SDS/AASS/MAT1A/PIPOX
AT/ACMSD/TDO2/KMO/ALDH7A1/SARDH/HAAO/DMGDH/BHMT
CTH/PON3/CYP1A2
AO/ALDH8A1/PON3/GSTM1/CYP2E1
E2J2/NPEPPS/YWHAH/BBC3/PMAIP1/UBE2L3/GSK3A/YWHAQ/YWHAB/BAG3/GZMB/PDCD5/MAPT/BAD
A/GAPDH/RNASE6/CCL13/CXCL9/CXCL3/CXCL13/DEFB1/RPL39/PF4V1/REG1B/CXCL11/RPS19/RO
RPS5/RPS21/BYSL/RRP36/RPS19/RRP7BP/TSR3/UTP4/RPL38/MRPS7/NAT10/UTP6/HEATR1/KRI1
RFGAP1/EXTL3/SHC1/WFS1/GSK3A/ZBTB17/DNAJB11/PDIA6/CXXC1/ATP6V0D1/SRPRB/WIPI1/TA
CL26/MSN/IL27RA/ADAM8/MYO1G/CCL2/PIK3CD/CXCL13/CXCR3/CXCL16/CXCL11/XCL2/APP/FAD
/AKR1C4/SLCO2B1/ABCB11/SLC51A/SLCO1B3/SLCO1B1/SLC27A5/AQP9/SLC10A1
RG
CYP4F2/APOC3
ARDH/ACADL/ALDH1L1/DMGDH/BHMT
MO/HAAO
8A1/CYP3A5/ADH6/ADH1A/ADH1C/CYP2C8/ADH1B/CYP3A4
/PLIN5/ACADL/APOC1/INSIG1/CYP7A1/APOC3
CPS1/GCGR/PCK1
RG
1/DOK1/TGFA/HIP1R/CNOT9/ADRA2A/CBL/ARAP1/ADAM17
1/DOK1/TGFA/HIP1R/CNOT9/ADRA2A/CBL/ARAP1/ADAM17/RBPJ/EREG/EGF/NEURL1/PDE6G
CD2/BATF2/LTBR/BATF3
GAL/HPSE/OVOL2/KDF1/TRADD/KRT10/NOTCH1/SFRP4/ALOX15B/CYP27B1
RD27/SNF8/CHMP3/PTPN23/SNX12/WDR91/HOOK2/LMTK2/VPS11/BECN1/SNX3/SNX16
PK3/C3AR1/CCL2/NUP85/C5AR1/CMKLR1/CCL3/CXCL17/AKIRIN1/CYP19A1/THBS1
/BBC3/PMAIP1/ZNF205/GSK3A/YWHAQ/YWHAB/GZMB/BAD/CASP8
PYCARD/TPX2/SOX9/CDC6/EPO/ANXA2/DUSP9/CDK1/ICAM1/LGALS9/PFN2/VEGFB/CDC25B/SRC/M
XJ1/RGS10/ARHGEF16/DEPDC1B/ECT2/RCC2/ARHGAP4/AGRN/RACGAP1/TAX1BP3/CHML/HRAS/ADA
/DNMT1/CHEK1/RAN/SMARCD1/TRIM28/SIRT6/LIN28B/NUP210/NAA40/MAP2K2/BRCA1/POLR2G/C
3ST1/B3GAT3/NUDT14/TUSC3/DPM2/MUC1/CHST4/ALG3/MGAT5/DDOST/B4GALT5/TMEM165/GALNT
3ST1/B3GAT3/NUDT14/TUSC3/DPM2/MUC1/CHST4/ALG3/MGAT5/DDOST/B4GALT5/TMEM165/GALNT
TRIB3/MAGEC2/FANCI/RIPK2/BIRC3/MAD2L1/FKBP1A/MAD2L2/ISG15/BRCA1/RPS2/HSP90AB1/S
ALS9/TIMP1/ITGA3/ACSL4/LIF/AKR1B1/DSG2/FBLN1/TGFB1/STC2/CTSV/HAVCR2/PGF/DDR1/PT
/RUVBL2/PTPRF
ALS1/RIPK2/ADA/EXO1/HAVCR2/CORO1A/CLCF1/BATF/RELB/UNC13D/IL18/TFRC/HLA-DMB/JAK3
PO/TYROBP/GPNMB/CYBB/RIPK2/HAVCR2/GSTP1/PTAFR/ARHGEF2/HSPB1/ACP5/SYK/HSF1/CARD9
PO/TYROBP/GPNMB/CYBB/RIPK2/HAVCR2/GSTP1/PTAFR/ARHGEF2/HSPB1/ACP5/SYK/HSF1/CARD9
TYROBP/TGFB1/GPNMB/HAVCR2/CD74/PKN1/PRNP/PAG1/HMGB3/TNFAIP8L2/VTCN1/VSIG4/JAK3/
17B8/ESR1/AKR1C4/PPARGC1A/UGT2B7/SRD5A2/AKR1D1/HSD17B6/CYP3A4
POA2/LCAT/APOA1/APOA5/APOC3
S6/SERPINF2/APOH/CPB2/KLKB1/F12/PLG/HRG
YP1A2/CYP2E1/CYP2C8/CYP2C9/CYP3A4
RG
MT1G/MT1E/CYP1A1/MT1X/CYP1A2
F7/APOH/KLKB1/F12/F9/KNG1/SERPINC1

SF1/TRIM28/SIRT6/PRKDC/MAD2L2/BRCA1/PCNA/PARPBP/DDX11/OTUB1/AUNIP/KDM1A/WAS/PML
/CYP39A1/ABCB11/ACOX2/BAAT/SLC27A2/AKR1D1/CYP7A1/SLC27A5/CYP8B1
/FABP1/APOB/GPLD1/AADAC/FABP4/APOA1/APOA5/APOC3/CPS1
BAD/CASP8/LGMN
B1BP1/TACSTD2
AP4/PLXNA3/LINGO1/SEMA4A/MAP2/EFNA5/THY1/CORO1C/GDI1/MT3/YWHAH/ITGB1BP1/CDK5/TA
1/GPC3/EFNA4/CD109/CSF1R/TFRC/TCIRG1/ATP6AP1/UBASH3B/TNFRSF11A/FSTL3/TFE3/SNX10
PLIN5/AGMO/GPAM/ACSL1/ANG/PCK2/GPLD1/RGN/THRSP/MOGAT2/PCK1
PLIN5/AGMO/GPAM/ACSL1/ANG/PCK2/GPLD1/RGN/THRSP/MOGAT2/PCK1
1/C1S/CFI/C3/C4BPB/C7/MBL2/C8B/MASP2/C8A/C4BPA/C6
FOXO1/IRS1/IRS2/GCK/DGAT2/PPARGC1A/GPLD1/RGN/IGF2
38A3/ABAT/SLC6A1/CYP4F2/CYP4A11
DH6/RDH16/CYP1A2/TTR/ADH1A/ADH1C/ADH1B/ADH4/CYP3A4

PLIN5/AGMO/GPAM/ACSL1/PCK2/GPLD1/RGN/THRSP/MOGAT2/PCK1
CDO1/CPS1/GCGR/PCK1
TP/HNF4A/APOE/RORA/APOC2/LIPG/GCKR/GPIHBP1/DGAT2/APOC4/ANGPTL4/LIPC/MLXIPL/PLA2
TP/HNF4A/APOE/RORA/APOC2/LIPG/GCKR/GPIHBP1/DGAT2/APOC4/ANGPTL4/LIPC/MLXIPL/PLA2
C3/PLIN5/GPLD1/AADAC/RGN/THRSP/APOA5/APOC3
/IRF1/IRF3/STAT1/POLR3K/POLR2H/IRF7/FLOT1/PQBP1/IFI16/POLR2L/DDX41/POLR2K/XRCC6
4/CYP3A7/HSD17B8/CYP1A1/CYP3A5/CYP1A2/CYP2C8/CYP2C9/CYP3A4
/QDPR/ACMSD/GCDH/TDO2/KMO/HAAO/HGD/IYD/MTHFD1/AMDHD1/PAH/FTCD/UROC1/HPD/TAT
1/APOM/LIPC/APOB/APOC1/LPA/APOA2/LCAT/ALB/APOA1/APOA5/APOC3

SLC39A10/THY1/LYN/CBFB/NFAM1/LCK/PTPN6/RPS3/PRKD2/LILRB4/BTN2A2/FCGR2B/KCNN4/BC
/QDPR/ACMSD/TDO2/KMO/HAAO/HGD/AMDHD1/PAH/FTCD/UROC1/HPD/TAT
1/APOM/LIPC/APOB/APOC1/LPA/APOA2/LCAT/ALB/APOA1/APOA5/APOC3
1/APOM/LIPC/APOB/APOC1/LPA/APOA2/LCAT/ALB/APOA1/APOA5/APOC3

B/PPP2R1A/LMNA/NUP93/AKAP8L/BANF1/TMEM43/NUP155/CTDNEP1/DMPK/VRK1/ANKLE2/EMD/NS
D/MTHFD1/MAT1A/CTH/CDO1/AGXT/CPS1/GNMT/BHMT
7/GSTZ1/AADAT/ACAT1/TDO2/AASS/ACOT12/PIPOX/PDK4/HMGCS2
GLUD1/AADAT/GOT2/NAGS/GLS2/PRODH2/ALDH5A1/ADHFE1/AMDHD1/FTCD/UROC1/TAT
5/C6/HRG
POC2/APOM/APOB/APOC1/ABCG8/ABCG5/APOA2/PON1/APOA1/APOA5/APOC3

POA2/LCAT/APOA1/APOA5/APOC3
CAA1/HSD17B4/CYP4F12/SLC27A2/CYP4F2/SLC27A5
1/UGP2/IRS1/IRS2/GCK/PPP1R3B/IGF2/GYS2
7A1/TTPA/CYP1A1/CYP4F2/CYP3A4
1/UGP2/IRS1/IRS2/GCK/PPP1R3B/IGF2/GYS2
/ACACB/CPT1A/IRS1/IRS2/DGAT2/PLIN5/PPARGC1A/FABP1/ACADL/PDK4
/PLIN5/LIPC/FABP1/APOB/APOA2/GPLD1/AADAC/FABP4/APOA1/APOA5/APOC3/CPS1
/PLIN5/LIPC/FABP1/APOB/APOA2/GPLD1/AADAC/FABP4/APOA1/APOA5/APOC3/CPS1

VEGFB/STMN1/SULF1/TYRO3/SRC/SEMA6A/PTP4A3/PLAUR/DNM1/MPZL1/CSF1/TGFB1/CYBB/RHBD
TSF1/AFP/RGS2/MDK/TOP2A/TRIP13/E2F1/SOX9/ASF1B/CABYR/FOXJ1/TYRO3/CDC25B/B4GALNT
YCARD/PKIB/LIMK1/RGS2/FOXM1/SOX4/DUSP9/BAK1/MICAL1/TSPO/SEMA6A/LGALS3/LIF/PBK/T
TGA3/DAB2/DEPDC1B/BAMBI/DKK1/FOLR1/GPC3/TAX1BP3/BICC1/WNK2/FZD6/ASPM/TLE3/DCDC2
TM1/QSOX1/TOMM40/ATP6V0B/ATP6V0D2/CHMP4B/HMOX1/VPS37C/ATP6V1C1/RRAGD/FUNDC1/HIF

ABPC1/SLC11A1/HNRNPU/DHX9/RBM10/RBM24/LARP1/HNRNPA0/HNRNPD/HNRNPC/SYNCRIP/ELAVL
CK/PTPN6/RPS3/PRKD2/LILRB4/BTN2A2/KCNN4/BCL10/PTPN2/RAB29/DUSP22/NECTIN2
B1/RIPK2/UNC119/CLCF1/CD44/ITGB2/CD74/ARHGEF2/PRKCD/NCK2/ADORA1/PRNP/MVP/SYK/IL
/CD3E/SASH3/CD83/LEF1
N1/BAK1/SRC/LGALS3/DAB2/TRIB3/BAMBI/AURKA/DKK1/RIPK2/PRKCD/SMARCD3/MAPRE1/ADAM1
7/NDC1/NUP188/NCBP2/XPOT/SSB/NUP85/NUP43/RAE1/NUP155/NUP107/SEC13/NOL6
/AP5Z1/CHMP4B/SNX8/RAB6B/VPS37C/MICALL1/CHMP4C/ANKRD27/CORO1C/STX6/HGS/RAB7B/SO
/PRKCD/HLA-DRB1/HLA-DQA1/HCK/ZYX/HLA-DRA/IFNGR2/HLA-DQB1/IFI30/SLC26A6/GBP5/HLA
CLGN/HSPB1/NCK2/SERPINH1/CREB3L1/COMP/HSF1/DDIT3/BAX/HSPA6/CREB3/MANF/HSPA8/LMN
CD74/FANCD2/SLAMF8/SYK/IL18/HMGB3/TNFRSF18/JAK3/CD86/TNFSF9/CD2/SASH3/SOCS1/IRF
ROBP/TGFB1/RIPK2/ADA/CD74/SYK/IL18/CD86/TNFSF9/IL34/ATP6AP1/SASH3/SOCS1/CD83/LE

CK2/ADORA1/MVP/SHKBP1/NUP62/NCF1/AREG/DOK1/IQGAP1/RHBDF1/HGS/TGFA/HIP1R/SH3KBP1
YP27A1/CYP39A1/ABCB11/ACOX2/BAAT/SLC27A2/AKR1D1/CYP7A1/SULT2A1/SLC27A5/CYP8B1
IPG/SCP2/APOC1/ABCG8/ABCG5/APOA2/ABCB4/PON1/APOA1/APOA5/APOC3
IPG/SCP2/APOC1/ABCG8/ABCG5/APOA2/ABCB4/PON1/APOA1/APOA5/APOC3
/MLXIPL/ACADL/APOC1/INSIG1/RGN/PDK4/APOA5/CYP7A1/APOC3
/HMGB2/CHMP4B/PYGO2/PES1/PPP2R1A/CHMP4C/BIN1/LMNA/NUP93/NUP205/NDC1/EMG1/AKAP8L
KN1/PRKCD/STK39/CDC7/CHEK1/TRIM6/SPRED1/CAD/DYRK2/CSNK1E/MARK2/DDIT4/CSNK1D/AXI
/KDF1/UGCG/SRF
1/SFPQ/RAD21
RTFDC1/NME6/APRT/NME7/GUK1/NME3/NME4
RTFDC1/NME6/APRT/NME7/GUK1/NME3/NME4
1/UGCG
AD/SHMT1/RIDA/SDS/BAAT/MTHFD1/AGXT2/CTH/DAO/CDO1/AGXT/GLYAT
1/APOM/APOC4/LIPC/MTTP/APOB/APOC1/LPA/APOA2/LCAT/ALB/APOA1/APOA5/APOC3
SMD2/MAPK3/PSMB2/RPS6KA4/PSME3/TNIP2/PSMB4/IL1B/PSMB8/PSMB10/PSMA5/PSMD13/PSMC4
1/C1S/CFI/C3/C4BPB/C7/MBL2/C8B/MASP2/C8A/C4BPA/HPX/C6
M1/IL6R/ASS1/ORM2/HAMP/SAA4/MBL2/F2/SERPINF2/TFR2/HP/AHSG
ACACB/APOE/POR/SC5D/TM7SF2/GPAM/APOB/SEC14L2/CYP7A1
ACACB/APOE/POR/SC5D/TM7SF2/GPAM/APOB/SEC14L2/CYP7A1
DAB2/MMP14/TGFB1/STX3/SQSTM1/KCNJ11/PRNP/EZR/RHOG/ANXA13/RHOQ/PKDCC/CNPY4/RANGR
2/ROBO1/STK39/CCL5/CXCR4/CCL26/GPR35/HIF1A/CCL13/CCL2/CXCL9/CXCL3/CXCL13/CXCR3/
B/LCP1
YBB/RIPK2/HAVCR2/PTAFR/ARHGEF2/HSPB1/SYK/CARD9/MAPKAPK2/MIF/CD2/SASH3/SPON2/HDA
3/CBFB/PSMB4/PSMB8/PSMB10/PSMA5/PSMD13/PSMC4/PSMD10/PSMD11/PSMD1/PSMA7/PSMB3/PS

GUK1/NME3/NME4

/GTSE1/CCT5/RAN/GLIS2/CCT6A/CCT3/TRIM28/NUTF2/CARD10/DKC1/MCRS1/CCT2/ZC3H12A/CC
B/HAVCR2/GSTP1/PKN1/PRNP/VTCN1/VSIG4/CD86/CD300A/MNDA/CTLA4/PLA2G2D/LYN/IL2RA/P
SEMA4D
AB7B/MT3/CLN3/RAB20/GBA/LAMTOR1/CLN6/VPS18/NAGPA/TMEM199/HPS1/HOOK2/VPS35/HEXB/

AB7B/MT3/CLN3/RAB20/GBA/LAMTOR1/CLN6/VPS18/NAGPA/TMEM199/HPS1/HOOK2/VPS35/HEXB/

PYCARD/TPX2/SOX9/CDC6/EPO/ANXA2/DUSP9/CDK1/ICAM1/LGALS9/PFN2/VEGFB/CDC25B/SRC/M
25B/SRC/MMD/CKS2/CSF1/ECT2/TGFB1/IQGAP3/STK26/DKK1/RIPK2/UNC119/PSRC1/GDF15/IRA

B9/TRAF2/PSMD14/FOXK1/RUVBL1/PSMD2/HIF1A/JOSD2/BRCA1/MCRS1/HGS/ACTL6A/FOXK2/PSM
AUR/RAC2/ATP13A2/SQSTM1/YWHAZ/HK2/VAT1/MICALL2/SAE1/BAX/HIF1A/SLC35F6/BMF/NOL3/
/ASAP1/EZR/DCDC2/FSCN1/CHMP4B/RABEP2/MSN/STIL/NUPR1/LCP1/NPM1/CHMP4C/BRCA1/DYNL
SX1/WNT4/RAD1
1/CXCL9/EHD2/IL4R
2/PSMB2/PSME3/LBH/JAG1/CBFB/NELFB/PSMB4/PSMB8/PSMB10/PSMA5/TGFB2/PSMD13/PSMC4/P
/SULT1A2/DDC/MAOA/GCH1/DRD1/MAOB/DBH/ABAT/PAH/DAO
/SULT1A2/DDC/MAOA/GCH1/DRD1/MAOB/DBH/ABAT/PAH/DAO
X2/CYP1A1/CYP2A7/CYP1A2/CYP4F2/CYP2B6/CYP4A11/CYP2E1/CYP2C8/CYP2C9/CYP2A6
R2/GPD1/CRHBP/CPS1/PCK1/AQP9
R1A/KMO/SLC38A3/ABAT/ABCB4/SLC6A1/APOA1/CYP4F2/CYP4A11

G2/TGFB2/VDR/PAX8
S/CFI/CPN1/CFB/C3/CFH/VTN/CD5L/CFHR1/C4BPB/CFHR2/C7/C8B/F2/CPN2/CPB2/C8A/C4BPA/

/FA2H/MYADM/PACSIN2/DOCK2/CAV2/CAV1/PTPRC/ABCA7
1/APOM/APOC4/LIPC/MTTP/APOB/APOC1/LPA/APOA2/LCAT/ALB/APOA1/APOA5/APOC3
OT2/GCDH/BHMT2/ALDH7A1/ADI1/RIDA/SDS/AASS/MTHFD1/MAT1A/PIPOX/GNMT/BHMT
MT1G/MT1E/ALAD/ARG1/KHK/HAAO/MT1X/OTC/CPS1
S/CFI/CPN1/CFB/C3/CFH/VTN/CD5L/CFHR1/C4BPB/CFHR2/C7/C8B/F2/CPN2/CPB2/C8A/C4BPA/
AADAT/ACMSD/GCDH/TDO2/DBH/KMO/ALDH7A1/SULT1B1/SARDH/HAAO/DMGDH/BHMT
A/F2/F11/TMPRSS6/SERPINF2/APOH/CPB2/KLKB1/ABAT/F12/KNG1/PLG/HRG
POM/DGAT2/APOC4/LIPC/APOB/APOC1/APOA2/CES3/GPLD1/APOA1/APOC3

B1/CYP27A1/CYP39A1/ALDH2/SORD/AKR1D1/HAO1/CYP7A1/SULT2A1/ADH4
/PLPP3/VTN/F2/F7/SERPINF2/APOH/CPB2/F12/PLG/HRG
A/F2/F11/TMPRSS6/SERPINF2/APOH/CPB2/KLKB1/ABAT/F12/KNG1/PLG/HRG

YN/BTK/BCL10
LIP3/TBCD/STMN2/EML2
4/CENPN
TAT5A
/IL2RB/GRB2
K1D
E2J2/NPEPPS/YWHAH/BBC3/PMAIP1/UBE2L3/GSK3A/YWHAQ/YWHAB/GZMB/PDCD5/BAD/CASP8/SH3

C3
7A5
A/F2/F11/TMPRSS6/SERPINF2/APOH/CPB2/KLKB1/ABAT/F12/KNG1/PLG/HRG
RA/ACACB/PDE3B/CPT1A/APOC2/IRS1/RARRES2/SORL1/IRS2/PLIN5/FABP1/APOC1/APOA2/GPLD
BG2
TAT5A
1/CCT8/WRAP53

/EPO/DBN1/REG1A/PLXNA1/ITGA3/TSPO/SEMA6A/MMD/ETV5/LIF/SERPINE2/CSF1/TYROBP/ECT2
1/POSTN/REG1A/STMN1/PLXNA1/SRC/SEMA6A/RAC2/PDLIM7/ENAH/ARHGAP4/AURKA/STK26/EFNA
PO/DBN1/REG1A/ITM2C/PLXNA1/ITGA3/SEMA6A/MMD/ETV5/NCS1/ECT2/ARHGAP4/LGALS1/DKK1/
1/POSTN/REG1A/STMN1/PLXNA1/SRC/SEMA6A/RAC2/PDLIM7/ENAH/ARHGAP4/AURKA/STK26/EFNA
/PGC/TNFRSF21/MMP12/IKBKE/FCER1G/ARPC1B/LGALS9/TYRO3/SRC/CD3D/LGALS3/S100A14/TG
EBP1/PTAFR/HSPB1/NCK2/IGF2BP1/LARP6/CDK4/TCOF1/IGF2BP3/ZNF385A/RPS6KA1/YBX3/EIF
C5/PTTG1/PYCARD/RENBP/CRYAB/LGALS9/BAK1/MAPK12/MICAL1/SRC/TIMP1/PLAUR/SPINT2/AT
6/LCN2/PGC/MMP12/FCER1G/S100A14/TGFB1/GAPDH/RIPK2/HAVCR2/FLNA/BIRC3/BATF/CCDC88
GS2/CRYAB/ENO1/DBN1/REG1A/SEMA6A/MMP14/CLSTN1/SERPINE2/TGFB1/ARHGAP4/PPT1/PSRC1
BAX/MIF/HIF1A/SLC39A10/PRELID1/PDCD1/NOC2L/ADAM8/LYN/BIRC7/CD27/FADD/BTK
B9/TRAF2/PSMD14/FOXK1/RUVBL1/PSMD2/HIF1A/JOSD2/BRCA1/MCRS1/HGS/ACTL6A/FOXK2/PSM
ALNT1/GAL3ST1/B3GAT3/NUDT14/TUSC3/DPM2/MUC1/CHST4/ALG3/MGAT5/DDOST/B4GALT5/TMEM
/WDR77/FBXO5/ARHGDIB/CALR/PRDX4/HPGDS/WNT4/RAD1
URKA/GPNMB/STK26/IRAK1/MVP/SYK/STK39/HCK/DDR1/WNK2/NME2/CSF1R/TRIM28/MAPKAPK2/I
6/LCN2/PGC/FCER1G/S100A14/RIPK2/HAVCR2/PRKCD/SLAMF8/IGLL5/ACP5/SYK/S100A8/LYZ/R
S2/KMO/ARG1/SLC38A3/ABAT/SLC6A1/CYP4F2/CYP4A11
TYROBP/TGFB1/GPNMB/HAVCR2/CD74/PKN1/PRNP/PAG1/HMGB3/TNFAIP8L2/VTCN1/GRN/VSIG4/J
STP1/CDKN2A/DUSP5/PRKCD/HSPB1/NUP62/CDKN2D/DTNBP1/CD300A/RGS14/PPP2R1A/SPRED1/D
AO
O/HAAO

P

JA4/HSPD1/HSPA2/HSP90AA1/HSPA5/HSPA14/PPIE/PPIB/PDCL/HSPA13/PPIC/SNRNP70
HRSP/SLC27A5
5/CPLX2/VAT1/TAP1/RAB34/BAX/GDAP1/ANKRD27/STX1A/STX6/RAB7B/STX10/RAB20/CHMP3/VP
C/ARPC2/CORO1B/RAC1/VIL1/CDC42/BRK1/AKIRIN1/DNM2/ENPP2/ATP7A/NCKAP1
P5K1C/HIP1R/CLTB/AP2M1/AP2S1/DNM2/TNK2/AAK1/SMAP1/CANX/AP2A2/CLTC/GAK/AP2B1/FNB

1

L2A1/MUC1/CDKN2D/ZNF385A/HMOX1/BAX/MIF/CASP2/PRKDC/NUPR1/BRCA1/BCL2L12/DDIAS/DY
/TGFB1/COL1A2/SERPINH1/LARP6/ADAM15/MMP1/CREB3L1/PLOD1/MRC2/CTSK/P3H1/COL5A1/HI

CYP7A1/APOC3
/IGF2
T1G/MT1E/MT1X
CT2/STK26/PRKCD/EZH2/TRPM2/HSF1/PCNA/ZNF580/SIRPA/HDAC2/TXN/RPS3/CBX8/PCGF2/MAP

HNF1B/CCDC24/ZP3/GINS4/RBBP8/ACTL6A/PELO/HOPX/NCAPG2/RRP7A/NLE1/NASP/EMG1/HCFC1
1/TGFB1/ANKRD1/CHEK1/CASP2/SLC9A1/MAPK3/IRF1/RAC1/ITGA2/IL1B/CASP1/MAP3K14/LTBR
R5
KAT2B/FOXO1/IRS1/IRS2/GCK/DGAT2/PPARGC1A/PFKFB1/GPLD1/RGN/IGF2
H1B1/NNT/SULT1E1/HBB/SULT1B1/CAT/ALDH2/PCK2/HP/SULT2A1/PCK1/ADH4

2/APOA1/APOA5
ASS1/SERPINF1/ARG1/KLF9/PCK2/PCK1
/SOD1/LDLR/HMGCS1/ACACB/APOE/POR/SC5D/DGAT2/TM7SF2/GPAM/APOB/ACADL/EPHX2/SEC14L
C/RGN/APOA5

320/TFRC/MIF/MAD2L2/SASH3/TNFRSF4/SLC39A10/TNIP2/NCKAP1L/CARD11/TNFSF13B/CD27/M
B/PSMB4/PSMB8/PSMB10/PSMA5/PSMD13/PSMC4/PSMD10/PSMD11/PSMD1/PSMA7/PSMB3/PSMD4/P
RT6/PRKDC/MAD2L2/PARPBP/DDX11/OTUB1/AUNIP/KDM1A/WAS/PML/PARP1/POLQ/FUS/OGG1/RPA
RS1/IRS2/PLIN5/FABP1
CARD/LGALS3BP/CYBA/LIMK1/RGS2/ATP1B3/CXCL8/TINAG/ANXA2/NCF2/IL2RG/FCER1G/ARPC1B
EG1A/FBLIM1/PLXNA1/SRC/SEMA6A/MELTF/FBLN1/BAMBI/RHOV/RCC2/ARHGAP4/CORO1A/TNFRSF
25B/SRC/MMD/CKS2/CSF1/ECT2/TGFB1/IQGAP3/STK26/DKK1/RIPK2/UNC119/PSRC1/GDF15/IRA
ALT5/CERS5/SMPD2/NSMAF/DEGS1/ORMDL2/B4GALT3/ASAH1/GBA/FA2H/SMPD4/ST3GAL5/RACK1/
SV/DKK1/KRT17/PI3/MAFG/KRT80/ADAM9/USH1C/CD109/ELF3/EZH2/FZD6/NME2/PTHLH/FOXQ1/

CXCL17/AKIRIN1/THBS1

GLL5/RAB31/MFGE8/CD300A/GULP1/F2RL1/NCKAP1L/MSR1/ITGA2/CDC42/FCGR2B/BIN2/SPIRE1

RN/CDC42/HNRNPU/BECN1
RO1A/ITGB2/PLEKHO1/PLXNA3/CDC42EP1/HCK/FGD3/FMNL2/CSF1R/EZR/RHOG/FMNL1/RHOQ/RHO
T57/TMEM201/KIF3C/DTNBP1/MAP2/FLOT2/SSNA1/HIF1A/HSPA8/MAP1S/KLC3/DYNLRB1/IFT52/
T57/TMEM201/KIF3C/DTNBP1/MAP2/FLOT2/SSNA1/HIF1A/HSPA8/MAP1S/KLC3/DYNLRB1/IFT52/
RC/MAPK13/CCNA2/ECT2/STK26/SOD3/PRKCD/EZH2/MPV17/TRPM2/HSF1/NCF1/AKR1C3/TRAF2/P
KN1/PRKCD/STK39/CDC7/CHEK1/TRIM6/SPRED1/CAD/DYRK2/CSNK1E/MARK2/DDIT4/CSNK1D/AXI
XJ1/MAPK13/TYROBP/RIPK2/HAVCR2/CD74/PTAFR/ARHGEF2/SYK/CARD9/MAPKAPK2/IL27RA/SIR

AT2/PCK1
/GSTM1/GSTA1
/GSTM1/GSTA1
/CCT2/HSP90AB1/CCT4/PNKP/TCP1/PARM1/PTGES3/WRAP53/HSP90AA1/NVL/MAPKAPK5/PPARG/H
O/ETV5/LIF/SERPINE2/CSF1/TGFB1/CLCF1/P2RX4/EZH2/CXCR4/IL34/BIN1/HDAC1/HDAC2/LYN

B/CHMP4C/DYNC1H1/GPSM2/RAE1/CHMP1A/RNF4/DRG1/CCSAP/DCTN1/HNRNPU/CLTC/PKD1/BORA
1/NUP188/XPOT/SSB/NUP85/NUP43/RAE1/NUP155/NUP107/SEC13/NOL6
1/NUP188/XPOT/SSB/NUP85/NUP43/RAE1/NUP155/NUP107/SEC13/NOL6
3/MRPL12/MRPS34/TRMT112/MRPL13/GADD45GIP1/MRPL10/MRPL52/MRPL48/DAP3/MRPS7/MRPL5
AP1/NUP62/CHMP4B/CHMP4C/RTKN/ANXA11/CHMP3/KLHDC8B/CHMP1A/SPIRE1/KIF20B/MITD1/RH
GCN1/HSF2/MAPK7/NOTCH1/VEGFA/HSF4/HSPA5/RBPJ
/PMAIP1/DNM1L/PDCD5/MFF/BAD
AG2/AGL/ENPP1/STBD1/UGP2/IRS1/IRS2/GCK/PPP1R3B/PPP1R1A/KHK/LEPR/IGF2/GYS2/GCGR/
POE/CPT1A/IRS2/PLA2G12B/FABP1/ACSL1/SLC27A2/THRSP/FABP4/SLC27A5
1/GOT2/BHMT2/ADI1/SHMT1/NAGS/GLS2/GATM/AASS/MTHFD1/AGXT2/CTH/PAH/OTC/AGXT/CPS1/
/MYLK/PLPP3/VTN/F2/F7/SERPINF2/APOH/CPB2/F12/PLG/HRG
YK/IL18/CD86/TNFSF9/SASH3/SOCS1/CD83/NCKAP1L/ADAM8/CBFB/IL2RA/CD27/IL12RB1/IL15
CL2/SOCS3/PLK4/DNAJB6/CASP8/STK3
LYREF/CHEK1/TFRC/SIRT6/MAD2L2/IL27RA/PARPBP/BLM/KDM1A/WAS/PARP1/MSH2/POLQ/FUS/R
SASH3
1/CSK/TMBIM1/BCL2L1/CLTC/MRAP2
BR4/ACAA1/ACMSD/AKR1C4/CYP2A13/CYP1A1/AKR7A3/CYP2A7/CTH/PON3/CYP1A2/CYP2A6
SORL1/PLIN5/LIPC/FABP1/APOB/APOC1/APOA2/GPLD1/AADAC/FABP4/APOA1/APOA5/APOC3/CPS
R/KDM8/CYP3A7/CYP3A43/CYP1A1/CYP3A5/CYP1A2/CYP2C8/CYP2C9/CYP3A4

RD5A1/ASS1/SERPINF1/ARG1/KLF9/PCK2/PCK1
M/ITGB2/C1QA/IL18/GRN/NUPR1/IL1B/C5AR1/CST7/APP/MAPT/CCL3
MK1/CXCL8/TINAG/ANXA2/NCF2/IL2RG/FCER1G/ARPC1B/TMPRSS3/SH3BP1/TYRO3/SRC/LGALS3/
RGS2/TRIP13/E2F1/SOX9/ASF1B/CABYR/FOXJ1/TYRO3/B4GALNT1/ETV5/RPL39L/KIFC1/GAL3ST
YMS/KRT17/EIF4EBP1/PTAFR/PRKCD/HSPB1/NCK2/SLC7A11/IGF2BP1/PRNP/LARP6/CDK4/TCOF1
CLGN/NCK2/SCAMP5/PDIA2/CASP4/CREB3L1/DDIT3/TRAF2/BAX/CREB3/NUPR1/MANF/HM13/LMNA
R3/IKBKE/LGALS9/FOXJ1/TSPO/TYROBP/TGFB1/TRAIP/GPNMB/HAVCR2/GSTP1/RELB/ABCD1/ACP
D3E/PAK1/CTLA4/PDCD1/LYN/CARD11/RAC1/TNFSF13B/LCK/PTPN6/GRB2/CDC42/CSK/PAK2/TNF
O9/RACGAP1/PTAFR/PRNP/CLIC3/SLC26A6/ANO10/MFSD5/SLC12A2/CLCN2/SLC12A9/SLC1A4/P2
XJ1/MAPK13/TYROBP/RIPK2/HAVCR2/CD74/PTAFR/ARHGEF2/SYK/IL18/CARD9/MAPKAPK2/IL27R
CSF1R/F2RL1/CTTN/KIFC3/HOMER1/ZMYND8/IGF1R/FCGR2B/DCTN1/VPS35/MTHFR/CD177/CTBP2
8/SYK/HMGB3/JAK3/TCF3/BAX/PRKDC/TCIRG1/TPD52/BCL3/NCKAP1L/CARD11/NFAM1/CDH17/PT
L4A2
/BECN1/SEH1L
1/IRF3/IRF7/FLOT1/TRAF3/POLR3D/POLR3C/RNF216/MAVS/IRF5/TLR2/NMI/DHX9/TLR7/TLR8/
NPLOC4/RFC2/MSH2/UBE2A/UBE2L6/RPA2/POLD3/TRIM25/VCP/FAAP20/RPS27A/RAD18/UBA52/R
100A7
A/TAF9
IF5A2/CCL5/MAPK3/NPM1/UPF3B/POLR2G/RBM3/PABPC1/BCL3/EIF3D/ITGA2/EIF2S3/CDC123/E
AG2/AGL/ENPP1/STBD1/UGP2/IRS1/IRS2/GCK/PPP1R3B/PPP1R1A/KHK/LEPR/IGF2/GYS2/GCGR/
AG2/AGL/ENPP1/STBD1/UGP2/IRS1/IRS2/GCK/PPP1R3B/PPP1R1A/KHK/LEPR/IGF2/GYS2/GCGR/

RD18
RD18
P3/PTPN23/SNX12
A/PLXNA3/TWF2/TRPV2/SEMA4A/MAP2/EFNA5/PAK1/CRABP2/NRCAM/GDI1/MT3/CDK5/CTTN/SEMA
/TMEM30B/SCP2/MTTP/APOC1/ABCG8/MFSD2A/APOA2/ABCB4/APOA1/APOA5/APOC3
/PTK7/PDZD11/MARK2/SLC9A3R1/ARF4/CDC42/PRKCI/WDR1/SYNE4/PARD3/RHOA
/PTK7/PDZD11/MARK2/SLC9A3R1/ARF4/CDC42/PRKCI/WDR1/SYNE4/PARD3/RHOA
HPGDS/PTGES3
GPNMB/HAVCR2/CD74/PRNP/PAG1/TNFAIP8L2/VTCN1/VSIG4/JAK3/CD86/CD300A/SOCS1/IRF1/C
AT/ACMSD/TDO2/KMO/ALDH7A1/SARDH/HAAO/DMGDH/BHMT
C/APOC1/APOH/ANGPTL3/APOA5/APOC3
MAIP1/YWHAQ/YWHAB/GZMB/BAD/CASP8/YWHAG/TFDP1
1/DAZAP1/VDR
PO/ALDH3A2/ALDH9A1/ALDH1A1/HOGA1/BCO2/GOT2/TKFC/GLYCTK/HAGH/ALDH7A1/GRHPR/PRODH
/LONP2/MLYCD/BDH2/ETFBKMT/CPT2/HIBCH/PPARA/ACAD11/ACACB/CPT1A/ECHS1/HADH/ACADS/
POE/MAOB/SULT1E1/TKFC/DBH/CYP4F12/SULT1B1/CYP27A1/CYP39A1/LDHD/ALDH2/CYP4F3/SOR
4C/DYNC1H1/GPSM2/RAE1/CHMP1A/DRG1/CCSAP/DCTN1/HNRNPU/CLTC/PKD1/BORA
YROBP/RIPK2/CD74/PTAFR/ARHGEF2/SYK/CARD9/SPON2/F2RL1/RAB7B/IL1B/APP/CLEC7A/LBP/

A3/SLC1A2/ABAT/SLC13A5
3/MOGAT2/PCK1

H/CPB2/KLKB1/F12
OC1/APOA2/GPLD1/APOC3
/COPS7B/ISY1/CDK7/POLR2L/POLR2K/HMGN1/RBX1/RPA2/CUL4B/POLD3/PRPF19/COPS6/RPS27A
C1B/CHST11/VEGFB/STMN1/SULF1/TYRO3/SRC/ITGA3/HTRA3/SEMA6A/PTP4A3/PLAUR/DAB2/DNM
1/PLA2G7/CCR1/S100A7/CXCL17/LGMN/CXCL10/MOSPD2/CCR2
STN/REG1A/STMN1/PLXNA1/SRC/SEMA6A/RAC2/PDLIM7/ENAH/ARHGAP4/AURKA/STK26/EFNA3/TN

RGS2/TRIP13/E2F1/SOX9/ASF1B/CABYR/FOXJ1/TYRO3/B4GALNT1/ETV5/RPL39L/KIFC1/GAL3ST
C5/PTTG1/PYCARD/RENBP/CRYAB/LGALS9/BAK1/MICAL1/SRC/TIMP1/PLAUR/SPINT2/ATP13A2/P
D2/NCAPH/FANCD2/SGO2/PPP2R1A/NDC1/SMC4/NCAPH2
ECR2/CRAT/ECH1/LONP2/HMGCL/MLYCD/ACOT2/NUDT7/ECI2/ACOX1/ACAA1/HSD17B4/AMACR/PEC
FS/ART4/GLUL/GLUD1/AADAT/ASS1/GOT2/ARG1/NAGS/GLS2/PRODH2/ALDH5A1/ADHFE1/AMDHD1/
/SOD1/HMGCS1/PRKAG2/ACACB/APOE/POR/SC5D/CES1/MSMO1/ACAA2/TM7SF2/GPAM/FAXDC2/APO
/FGG/PROC/FGA/F2/F11/TMPRSS6/SERPINF2/APOH/CPB2/KLKB1/ABAT/F12/KNG1/PLG/HRG
ECR2/CRAT/ECH1/LONP2/HMGCL/MLYCD/ACOT2/NUDT7/ECI2/ACOX1/ACAA1/HSD17B4/AMACR/PEC
ECR2/CRAT/ECH1/LONP2/HMGCL/MLYCD/ACOT2/NUDT7/ECI2/ACOX1/ACAA1/HSD17B4/AMACR/PEC
HGAP4/LGALS1/DKK1/PRKCD/PLXNA3/PRNP/ASAP1/CRMP1/LINGO1/SEMA4A/MAP2/THY1/RTN4RL2/
CD/HLA-DRB1/HLA-DQA1/HCK/ZYX/HLA-DRA/IFNGR2/HLA-DQB1/IFI30/SLC26A6/GBP5/HLA-DPA
13/ITGAM/ITGB2/CPLX2/PTAFR/SCAMP5/SYK/UNC13D/GIT1/GIPC1/DTNBP1/HMOX1/CD300A/ZP3/
P39A1/CYP1A2/AKR1D1/CYP7A1/HSD17B6/CYP3A4
FTCD/GNMT
GOT2/ARG1/GLS2/PRODH2/ADHFE1/DAO
/APOA2/AADAC/ANGPTL3/APOA5

DX1/FLVCR1/HIF1A/JMJD6/ISG15/STAT1/AMPD3/NCAPG2/NCKAP1L/HCLS1/LYN/LYAR/PTBP3/MB
2/STX1A/RAB3B/RAB10/VPS18/PPFIA3/RALB/RAB3D/VPS45/CFTR/CTBP2/RAB3A/VPS11/STXBP1/

IRC3/CD74/HRAS/NUP62/RCC1/S100A10/TRAF2/C1QTNF1/GNB1/CHMP3/COL6A2/FARSB/CPSF6/P

NCKAP1L/LYN/TNFSF13B/ABL1/TNFAIP3/BCL10
MAIP1/YWHAQ/YWHAB/GZMB/BAD/CASP8/YWHAG/TFDP1
S/CFI/CPN1/CFB/C3/CFH/VTN/CD5L/CFHR1/C4BPB/CFHR2/C7/C8B/F2/CPN2/CPB2/C8A/C4BPA/
C25A13/GOT1/PPARA/PC/KAT2B/FOXO1/GOT2/GCK/DGAT2/PPARGC1A/GPD1/MST1/LEPR/ENO3/SD
/SOD1/HMGCS1/ACACB/APOE/POR/SC5D/CES1/MSMO1/ACAA2/TM7SF2/GPAM/APOB/INSIG1/SEC14
/FCN3/C5/FCN2/SERPING1/C1S/COLEC10/CFI/CPN1/CFB/C3/CFH/VTN/CD5L/CFHR1/C4BPB/CFH
OR/SRD5A1/HSD17B8/DIO1/PPARGC1A/CYP27A1/SRD5A2/HSD11B1/HSD17B6/CYP3A4
ECR2/CRAT/ECH1/LONP2/HMGCL/MLYCD/ACOT2/NUDT7/ECI2/ACOX1/ACAA1/HSD17B4/AMACR/PEC
/SOD1/HMGCS1/ACACB/APOE/POR/SC5D/CES1/MSMO1/ACAA2/TM7SF2/GPAM/APOB/INSIG1/SEC14
83/BCL3/CLEC7A/HSPD1/TUSC2/IL20RB/TLR2
TGFB1/RACGAP1/NUP62/PGF/GIPC1/YBX1/TGFA/LBH/KIF14/ITGB1BP1/IL1B/PDGFA/TGFB2/FGF
B/PSMB4/PSMB8/PSMB10/PSMA5/PSMD13/PSMC4/PSMD10/PSMD11/PSMD1/PSMA7/PSMB3/PSMD4/P
1/POSTN/STMN1/TYRO3/PLXNA1/SRC/SEMA6A/SPINT2/RAC2/MELTF/FBLN1/POF1B/PDLIM7/ENAH
P62/EZH2/DNMT1/MUC1/CHEK1/PLOD1/RANGAP1/YEATS2/SAE1/TRIM28/NUP210/NAA40/RUVBL1/
/LAMC1/HNF1B/COL5A1/ITGA5/MMP2/ITGB5/PELO/HDAC1/COL5A2/COL4A2/LAMB3/HSBP1/MED12
B/HAVCR2/PKN1/PRNP/VTCN1/VSIG4/CD86/CD300A/MNDA/CTLA4/PLA2G2D/LYN/IL2RA/PTPN6/L
B/HAVCR2/PKN1/PRNP/VTCN1/VSIG4/CD86/CD300A/MNDA/CTLA4/PLA2G2D/LYN/IL2RA/PTPN6/L
HX34/YBX1/PABPC1/RNPS1/NELFE/U2AF2/SLC11A1/SRSF9/SRSF12/BARD1/SUPT5H/RBM42/PTBP
N/LGALS9/SRC/GAPDH/RIPK2/HAVCR2/ITGAV/SCAMP5/SYK/TMSB4X/CSF1R/EZR/VTCN1/GBP5/TR
X2/APOBEC3B/RNASE6/CARD9/APOBEC3C/TRIM6/SLFN13/UNC93B1/NT5C3A/TRIM11/ISG15/SPON
/UCP2/TOMM40/SLC25A6/MID1IP1/MCUB/MICALL2/SAE1/BAX/SLC25A24/SLC9A1/FLVCR1/TOMM3
F1R/PSMB9/PSMD14/TCF3/PRKDC/PSMD2/RRS1/FSTL3/PSMB2/PSME3/CBFB/PSMB4/PSMB8/PSMB1
11/SLC6A9/PRAF2/SLC7A5/SLC1A7/SLC1A4/SLC43A2/SLC1A3/SLC25A22/SLC7A8/SLC3A2/SLC7
LXNA3/ASAP1/CRMP1/LINGO1/SEMA4A/MAP2/THY1/RTN4RL2/GDI1/MT3/HDAC2/RAP1GAP2/YWHAH
F11B/TFRC/CTSK/TCIRG1/ATP6AP1/UBASH3B/TNFRSF11A/SNX10/ADAM8/RAC1/CALCA/TMEM119/
FLNA/IRAK1/CDKN2A/PRNP/EZH2/TMSB4X/FZD6/GLIS2/FOXS1/MDFI/DDIT3/RBCK1/HMOX1/BRMS
KAT2B/FOXO1/IRS1/ENTPD5/IRS2/GCK/DGAT2/MLXIPL/PPARGC1A/GPD1/PFKFB1/GPLD1/RGN/IG

4/B4GALT5/GALNT10/B3GNT5/B3GNT7/ST3GAL4/MUC3A/MUC5B/GALNT6/MUC20/POMGNT1/GALNT7
SLC9A3R1/BID/BCAP31/BBC3/PMAIP1/SFPQ/LCK/RPS3/DNM1L/RACK1/MSX1/RAD9A/IL20RA/RPS
/VCAN/B3GAT3/GPC1/ABCC5/B4GALT5/B3GNT5/CHST1/B3GNT9/B3GNT7/FMOD/CHPF2/B4GALT3/S
G/GAMT/ASS1/GOT2/FOLH1/BHMT2/ADI1/GPT2/SHMT1/NAGS/GLS2/GPT/GATM/AASS/MTHFD1/AGX
DL3/SOD1/ETFBKMT/ACACB/PDE3B/APOE/GPER1/PROX1/APOC2/SORL1/DGAT2/PLIN5/ACADL/APO
APOE/RORA/APOC2/LIPG/GPIHBP1/APOM/DGAT2/ACSM3/LIPC/PLA2G12B/CYP39A1/MTTP/APOB/A
OC/FGA/F2/F11/TMPRSS6/F7/SERPINF2/APOH/CPB2/KLKB1/ABAT/F12/KNG1/PLG/HRG/SERPINC

/ALDH2/SULT2A1
17B6

/TRAF2/TRIM6/CXCR4/HIF1A/IRF3/IRF7/CPNE1/NLRC5/CD300LF/AGPAT2/CASP1/AXL/FADD
D/MMAA/SLC19A2/ENPP1/MTHFS/AMN/CLYBL/PC/SLC23A2/KMO/SHMT1/CYB5A/HAAO/ALDH1L1/MT
YT7/SHH/CD36/APOE/APOC2/STARD5/LIPG/CES1/APOM/LIPC/SCP2/APOB/APOC1/ABCG8/ABCG5/
2/PLIN5/MLXIPL/PPARGC1A/FABP1/RGN/APOA5
OC/FGA/F2/F11/TMPRSS6/F7/SERPINF2/APOH/CPB2/KLKB1/ABAT/F12/KNG1/PLG/HRG/SERPINC
GB2/CD74/C1QA/SYK/GRN/VSIG4/MIF/JMJD6/ZC3H12A/TNIP2/SLC11A1/C5AR1/SBNO2/CST7/IL

RAP2A/TNIK

1/RPS3/USP1/RBX1/RPA2/CUL4B/POLD3/RPS27A/RAD18/UBA52/RFC5/RPA1/HMGA2/DDB1/MRPS2
YGL/PGM1/PRKAG2/AGL/ENPP1/STBD1/UGP2/IRS1/IRS2/GCK/PPP1R3B/PPP1R1A/KHK/LEPR/IGF
OS2/PEX19/DECR2/CRAT/ECH1/SEC16B/LONP2/HMGCL/MLYCD/ACOT2/NUDT7/PEX11A/PEX11G/EC
OC/FGA/F2/F11/TMPRSS6/F7/SERPINF2/APOH/CPB2/KLKB1/ABAT/F12/KNG1/PLG/HRG/SERPINC
ALDH3A2/ALDH1B1/NAAA/CYP11A1/CBR4/ALDH1A1/SULT1E1/DGAT2/AKR1C4/PECR/GPD1/SULT1B
APOE/RORA/APOC2/LIPG/GPIHBP1/APOM/DGAT2/ACSM3/LIPC/PLA2G12B/CYP39A1/MTTP/APOB/A
OE/POR/APOC2/AVPR1A/DGAT2/PLIN5/MLXIPL/PPARGC1A/GPLD1/RGN/APOA1/APOA5/CYP7A1/HS
/TREM2/PYCARD/CYBA/CXCL6/LY96/LCN2/PGC/CXCL8/RGS1/C15orf48/EPO/MMP12/ENO1/IKBKE
STN/REG1A/STMN1/PLXNA1/SRC/SEMA6A/RAC2/PDLIM7/ENAH/ARHGAP4/AURKA/STK26/EFNA3/TN
/TREM2/PYCARD/CYBA/CXCL6/LY96/LCN2/PGC/CXCL8/RGS1/C15orf48/EPO/MMP12/ENO1/IKBKE
B1/YWHAZ/FLNA/HK2/PRKCD/LAMP5/SYK/NUP62/CCT5/MPV17/TOMM40/SCRIB/SLC25A6/RPL8/RA
/PDGFA/TGFB2/PLXND1/FGFR2/TGFB3/SEMA3C/TWSG1
PARPBP/KDM1A/WAS/PARP1/POLQ/FUS/RPA2/PPP4C/TIMELESS/WRAP53/UBQLN4/MAGEF1
/SRC/ITGA3/RAC2/MMP14/MELTF/CSF1/FBLN1/ARL2/RCC2/LGALS1/CORO1A/TNFRSF12A/FLNA/C

AVCR2/GSTP1/CD74/SYK/CSF1R/HMOX1/MIF/HIF1A/SIRPA/F2RL1/IL1B/IL4R/APP/CLEC7A/LBP
/SLC38A3/ABAT/SLC6A1
FOXJ1/EPCAM/PLXNA1/SRC/SEMA6A/LGALS3/SPINT2/MMP14/MELTF/SERPINE2/FBLN1/TGFB1/RC
FBKMT/PRKAG2/PPARA/ACACB/CPT1A/APOC2/IRS1/AVPR1A/GSTZ1/IRS2/DGAT2/PLIN5/MLXIPL/

2/STX1A/STX6/RAB3B/STX10/NDRG4/RAB10/VPS18/SYT1/PPFIA3/STX4/RALB/NSF/RAB3D/STX7
LG/JAML/IL27RA/THY1/SIRPA/ADAM8/CCL2/PIK3CD/CCL28/CD47/ABR/FADD/PTGER4/VCAM1/CD

QSTM1/KCNJ11/PRNP/EZR/RHOG/ANXA13/EPB41L2/CNPY4/GPSM2/RANGRF/VIL1/STAC3/ACSL3/P
/ARHGEF2/PRKCD/TRIM54/MAPRE1/TMSB4X/TWF2/MID1IP1/CARMIL1/WASF2/MAP2/NPM1/BRCA1/
IRF5/DHX9/TLR7/TLR8
TGFB2/RAB10/FGFR2/AKNA/NOTCH1/TEAD3
X8/HOXB7/VEGFA/SIX1
M1
1/APOA5
N2/CASP8/INHA

POA1/APOC3
/IGF2
7

AMN/APOE/APOC2/LIPG/GPIHBP1/APOM/DGAT2/APOC4/LIPC/MTTP/APOB/APOC1/LPA/APOA2/CES
APOA2/CYP4F2/APOC3
DOA/FCGR2B/THBS1/CCL19/YTHDF1/HLA-DOB/NOD1/NOD2/FGL2/LILRB2
EG/IL34/LYN/IL1B/ASCL2/PPP1CC/PRKCI/NF2/NOTCH1/LGI4
1/APOA5
3R1/CDC42/PRKCI/SYNE4/RHOA
/PPARGC1A/FABP1
P1/RAB34/ANKRD27/STX1A/STX6/RAB7B/STX10/RAB20/CHMP3/VPS18/CALR/SYT1/STX4/BNIP1/
/COL5A1/ITGA5/MMP2/ITGB5/COL5A2/COL4A2/LAMB3/HSBP1
O1/PTGER4
/GRB2/STAT5A
/GRB2/STAT5A
RC/TSPO/ATP13A2/TRIB3/GAPDH/RIPK2/SQSTM1/HK2/DRAM1/HSPB1/QSOX1/RGS19/FUNDC2/S10
/TREM2/PYCARD/CYBA/CXCL6/LY96/LCN2/PGC/CXCL8/RGS1/C15orf48/EPO/MMP12/ENO1/IKBKE
L39L/ACSL4/ACSS1/GAL3ST1/PPT1/GAPDH/IGF2BP2/TYMS/KRT17/EIF4EBP1/PTAFR/PRKCD/HSP
RC/TSPO/ATP13A2/TRIB3/GAPDH/RIPK2/SQSTM1/HK2/DRAM1/HSPB1/QSOX1/RGS19/FUNDC2/S10

G3/CSF1R/NCF1/CCL5/RAP2B/THY1/TGFA/RELN/NCAPG2/SHC1/GPRC5B/DVL2/LRP8/VPS25/APP/
CAM1/FOXJ1/CD3D/TGFB1/RIPK2/ADA/EXO1/HLA-DMA/HAVCR2/CLCF1/CD7/C1QB/SUSD4/C1QC/C
9A1/SLC35F6/BMF/NOL3/HIP1R/BID/MT3/DYNLT1/YWHAH/BBC3/PMAIP1/ZNF205/GSK3A/YWHAQ/
SCRIB/RAB15/STXBP2/STX1A/RAB3B/SV2A/LYN/RAB10/VPS18/SYT1/PPFIA3/BTK/STX4/RALB/R
AP8L/NCAPH2/CHMP1A
/RFC2/UBE2L6/RPA2/POLD3/TRIM25/VCP/FAAP20/RPS27A/UBA52/RFC5/RPA1/POLE2/PARP10
CARD11/IL1B/RPS3/PRKD2/CLEC7A/ABL1/GLMN/PTPRC/MAP3K7/IRF4/PDE4B/CD4/ANXA1/CCR2/
1/NUP188/XPOT/SSB/NUP85/NUP43/RAE1/NUP155/NUP107/SEC13/NOL6

/ADRB1/CTSG/RASL10B/AGTR1/ADRA1B/ADRA1A/ACE2/AVPR1A/CES1/ENPEP/MME/ADRB2/REN/CY
/IGF1/PPARA/ENPP1/GPER1/KAT2B/FOXO1/IRS1/IRS2/GCK/DGAT2/PPP1R3B/PPARGC1A/MST1/L
CBR4/TDO2/SHMT1/CAT/ALDH5A1/ACADL/DCXR/MAT1A/CTH/FBP1/DPYS/UPB1/GNMT
/ELOVL2/ACSS3/AVPR1A/ACSL6/GCDH/ACAT1/HSD17B8/ACSL5/ACSL1/BDH1/HMGCS2/CYP2C8/SL
1/SLC9A3R1/RAP2A/GLDN/VIL1/TNIK

TD2/PAX8/HOXB7/VEGFA/SIX1
K1/CTLA4/PDCD1/LYN/CARD11/RAC1/TNFSF13B/LCK/PTPN6/GRB2/CDC42/CSK/PAK2/TNFRSF14/
ROM1/PRKX/BASP1/STAT1/SIX2/PDGFA/TACSTD2/PAX8/HOXB7/WNT4
/MAPK3/PAK1/PSMB2/PSME3/SHC1/LYN/CARD11/RAC1/PSMB4/PSMB8/PSMB10/LCP2/PSMA5/PSMD

1/IRF3/IRF7/FLOT1/TRAF3/POLR3D/POLR3C/RNF216/MAVS/IRF5/TLR2/NMI/DHX9/TLR7/TLR8/
2B/FOXO1/GOT2/GCK/DGAT2/PPARGC1A/GPD1/MST1/LEPR/ENO3/SDS/PFKFB1/SORD/PCK2/FBP1/
/FGG/PROC/FGA/F2/F11/TMPRSS6/SERPINF2/APOH/CPB2/KLKB1/ABAT/F12/KNG1/PLG/HRG
1/DBN1/STMN1/PLXNA1/SRC/TSPO/SEMA6A/RAC2/PDLIM7/ENAH/ARHGAP4/EFNA3/FOLR1/TNFRSF
NFKBIE/TGFB1/TRAIP/TONSL/RIPK2/HAVCR2/FLNA/ITGB2/IRAK1/CDKN2A/ZIC2/ARHGEF2/FANC
8/SIRT6/NAA40/HDAC1/HDAC2/CHRAC1/DNMT3A/ATAD2/RBBP4/BEND3/MTF2/HELLS/PCGF2/RBBP
IF/ECT2/ARL2/TGFB1/FLNA/PRKCD/SYK/NUP62/GTSE1/CCT5/RAN/GLIS2/CCT6A/RANGAP1/CCT3
IPK2/GDF15/IRAK1/S1PR2/CD74/PKN1/DUSP5/ROBO1/ADORA1/ADAM9/HRAS/SYK/STK39/EZH2/M
G1A/PLXNA1/SEMA6A/ARHGAP4/AURKA/TNFRSF12A/PLXNA3/DDR1/TWF2/TRPV2/CXCR4/SEMA4A/S
DNRB/C5/SERPING1/C1S/CFI/CPN1/CFB/C3/CFH/VTN/CD5L/CFHR1/C4BPB/CFHR2/C7/C8B/F2/C
SPA/MTHFS/SUCLG2/GLUL/GLUD1/HOGA1/AADAT/ASS1/GOT2/ACMSD/KMO/GRHPR/GPT2/PHYH/SHM
2/APOE/ACE2/AVPR1A/CRY2/IRS2/KMO/ARG1/SLC38A3/ABAT/ABCB4/SLC6A1/APOA1/CYP4F2/CY
OS1/VWF/FCN3/C5/FCN2/SERPING1/C1S/COLEC10/CFI/F10/CPN1/CFB/C3/CFH/VTN/FGB/CD5L/
IL18/TRAF2/RBCK1/CARD10/EDA/IL1B/CD27/RPS3/CALR/APP/LIMS1/TRIP6/TRADD/TNFSF15/T
1/BLM/DONSON/TIPIN/RFWD3/TIMELESS/DNA2/EME2/SMARCAL1/THOC1/MMS22L
K1/DCTN1/NEK6
K1/DCTN1/NEK6
S7/BAG2/RPL5/RPL11/PARK7
SYT1/RAB3A/VPS11/STXBP1/VPS16/C2CD5/KIF5B/ANXA1/VAMP1
ELFE/POLR2H/TRIM31/NELFB/CCL4/SMARCB1/TFAP4/POLR2L/POLR2K/SUPT5H/CCL3/TRIM21/TA

AC2/YWHAZ/HK2/MICALL2/SAE1/BAX/HIF1A/BMF/HIP1R/BID/UBE2J2/NPEPPS/YWHAH/BBC3/PMA
ES2/BCO2/GPIHBP1/CYP3A7/APOM/DGAT2/AKR1C4/CYP4V2/RDH5/APOB/CYP1A1/APOA2/ALDH8A1
BCC2/SLC25A13/SLC19A2/SLC1A1/APBA1/SLC25A30/AVPR1A/SLC13A3/KMO/GLS2/SLC1A2/ABAT
ACACB/SLC25A20/APOE/CPT1A/IRS2/GOT2/SLCO2A1/SLC22A9/PLA2G12B/FABP1/ACSL1/MFSD2A
YT7/SHH/CD36/GRAMD1C/APOE/APOC2/STARD5/LIPG/CES1/APOM/LIPC/SCP2/APOB/APOC1/ABCG
RS1/IRS2/GCK/PPP1R3B/PPP1R1A/KHK/LEPR/IGF2/GYS2/GCGR/G6PC/GNMT
2B/FOXO1/GOT2/GCK/DGAT2/PPARGC1A/GPD1/MST1/LEPR/ENO3/SDS/PFKFB1/SORD/PCK2/RGN/F
SS1/PDE2A/DUSP1/FOSB/REN/ITPR2/GPD1/CRHBP/PKLR/PFKFB1/CDO1/AGXT/CPS1/PCK1/AQP9
ROB2/GPER1/VSNL1/GLUL/GLUD1/IRS2/SYBU/GCK/FGB/FGG/FGA/MYRIP/TFR2/GPLD1/ANO1/ABA

IL18/TMSB4X/HDAC7/TRAF2/RBCK1/PRDX1/CARD10/EDA/NOL3/ZC3H12A/BCL3/CPNE1/IL1B/CD2
APP/CLEC7A/BCL10/EREG/INPP5D
QSTM1/KCNJ11/PRNP/EZR/RHOG/ANXA13/CNPY4/RANGRF/VIL1/STAC3/ACSL3/PRKCI/STX4/RACK

NA/FERMT3/ITGAV/LAMB1/UNC13D/LAMC1/FZD7/MICALL2/S100A10/CARMIL1/EFNA5/LAMC2/LAM
EZH2/FZD7/CD53/BHLHE41/PAK1/FLOT1/EHD1/CXCL9/EHD2/GSK3A/CYP26B1/MAMSTR/ARRB2/KA

SF21/E2F1/SLC2A1/MMP12/POSTN/LGALS9/ANXA5/ITPR3/SRC/PTP4A3/RAC2/ATP13A2/ECT2/AR
RIB3/GAPDH/RIPK2/HK2/DRAM1/HSPB1/QSOX1/CTSA/ATP6V0B/DDIT3/TPCN1/ATP6V0D2/CHMP4B

R2H/NCBP2/USB1/NHP2/SNAPC2/TAF9/INTS1/CDK7/EXOSC5/SRRT/POLR2L/POLR2K/NABP1/EXOS
/B4GALT5/NEU1/GM2A/CERS5/PPP2R1A/SMPD2/NSMAF/DEGS1/ORMDL2/B4GALT3/ASAH1/CLN3/GB
RMT1/HMGB2/IL34/HIF1A/ATP6AP1/ISG15/STAT1/RAB7B/LEF1/NCKAP1L/EVI2B/HCLS1/JAG1/C
107/CCT8/BAD
8/CD86/TNFSF9/SASH3/SOCS1/CD83/NCKAP1L/ADAM8/CBFB/IL2RA/CD27/IL12RB1/IL4R/PNP/L
TP6V0B/ATP6V0D2/CHMP4B/HMOX1/ATP6V1C1/RRAGD/HIF1A/MAPK3/NUPR1/RRAGC/NPC1/GPSM1/
NFRSF12A/ROBO1/PLXNA3/TWF2/TRPV2/LINGO1/SEMA4A/MAP2/BRSK1/EFNA5/PAK1/CRABP2/NRC
RF/KIF14/CST7/RARG/TMEM98/FIG4/LGI4/ZPR1/PARD3/SLC25A12
APK3/C3AR1/NCKAP1L/RAC1/C5AR1/CMKLR1/LBP/S100A7/CXCL17/AKIRIN1/THBS1
RPM2/F2RL1/BATF2/LYN/LTBR/BATF3/AXL/FCGR2B
2K2/ANKRD27/SNF8/CHMP3/PTPN23/SNX12/WDR91/HOOK2/LMTK2/VPS11/BECN1/SNX3/SNX16/MY
CLGN/NCK2/CREB3L1/HSF1/DDIT3/BAX/HSPA6/CREB3/HSPA8/LMNA/DDX11/HYOU1/ARFGAP1/EXT
RKCD/TMSB4X/ADAM15/CRMP1/MDFI/DDIT3/DTNBP1/HMOX1/BAX/MAP2/MAD2L2/MAPK3/SLPI/GPS
C1LI1
C1LI1
C1LI1

GAPDH/RIPK2/HAVCR2/SCAMP5/SYK/CSF1R/VTCN1/TRIM6/MIF/CD2/MAPK3/CD58/IRF3/F2RL1/F
H3B1/PRKCD/PIGU/SGPP2/GAL3ST4/ELOVL1/DPM2/PIGS/GPAA1/B4GALT5/CERS5/B3GNT5/GBGT1
P93/NUP205/NUP37/NDC1/NUP188/DYNLT1/KPNB1/CD209/TCP1/THOC5/NUP85/NUP43/VPS28/TH
P93/NUP205/NUP37/NDC1/NUP188/DYNLT1/KPNB1/CD209/TCP1/THOC5/NUP85/NUP43/VPS28/TH
C25B/SRC/TTK/AURKA/PAQR5/NCAPH/ASPM/TNFAIP6/HSF1/ZP3/PPP2R1A/FBXO5/NCAPH2/ETV6/
/HBB/DBH/SLC7A2/SHMT1/AGXT2/RGN/ABAT/PAH/AGXT/UPB1
/SELE/ADRA1A/POR/APOC2/AVPR1A/GPIHBP1/ESR1/PLIN5/ANGPTL4/PCSK6/LIPC/APOC1/ANG/A
RKDC/MAD2L2/IL27RA/LEF1/MSH2/SWAP70/XRCC4/EXOSC3/HSPD1/NBN/ERCC1/RNF8/MSH6/TP53
RKDC/MAD2L2/IL27RA/LEF1/MSH2/SWAP70/XRCC4/EXOSC3/HSPD1/NBN/ERCC1/RNF8/MSH6/TP53
DSG2/STC2/CTSV/HAVCR2/BSG/MAPK3/PPARD/LDOC1/PAM/DAZAP1/VDR/UBE2A/ANGPT2/APOL2/W
LYN/BTK/FCGR2B
MP4B/CCL5/TRIM11/CHMP4C/PPIH/PABPC1/PPIA/CHMP3/SRPK1/NOTCH1/TRIM21/TARBP2/VPS37

O/MNDA/CTLA4/NCKAP1L/PIK3CD/LYN/CBFB/BCAR1/NFAM1/LCK/PTPN6/CD79A/BTK/ABL1/FCGR2
TR1/ADRA1B/ADRA1A/ACE2/AVPR1A/CES1/ENPEP/MME/ADRB2/REN/SERPINF2
AT1/CSAD/MAT1A/CTH/CDO1/AGXT
8/CCL2/CXCL13/CCL4/PADI2/STK10/XCL2/APP/FADD/ECM1/CCL3/S100A7/SLC8B1/ADAM17/RHO

1/CCT5/ANKRD1/CCT6A/CCT3/CHTF18/MAPK3/DKC1/ACD/FGFR4/CCT2/HSP90AB1/RFC2/CCT4/PN
/PRNP/CLIC3/SLC26A6/ANO10/SLC12A2/CLCN2/SLC12A9/SLC1A4/P2RY6/SLC12A8/SLC1A3/GAB
D2

C/GPLD1/CYP7A1

LC11A1/CLN6/ATP6V0E1/TMEM199/ATP6V0D1
P8/BCL2L1/ATG7/CCL8/PABPN1/VAPA/KPNA4/CD4

2/FOXM1/SOX9/EPO/CRYAB/DUSP9/IL2RG/CDK1/ICAM1/LGALS9/MAPK12/SRC/SEMA6A/MAPK13/L
/TNFRSF21/IKBKE/FCER1G/ARPC1B/TYRO3/SRC/CD3D/LGALS3/S100A14/ITGAM/FCGR3A/RIPK2/
NTS/DBN1/ICAM1/ARPC1B/PFN2/SH3BP1/SEMA6A/ATP13A2/IQGAP3/ARHGAP4/CORO1A/SCPEP1/T
TYRO3/SRC/ITGA3/DAB2/CCNE1/DEPDC1B/ITGAM/BAMBI/DKK1/FOLR1/GPC4/GPC3/S1PR2/TAX1B
XNA1/ITGA3/TNFRSF12A/ROBO1/SERPINI1/PLXNA3/NME1/ITPKA/EZH2/NME2/TWF2/ANKRD1/TRP
H3B1/PRKCD/SGPP2/ELOVL1/B4GALT5/CERS5/SMPD2/NSMAF/DEGS1/ORMDL2/B4GALT3/ASAH1/GB
P1/RAB34/ANKRD27/STX1A/STX6/RAB7B/STX10/RAB20/VPS18/CALR/SYT1/STX4/STX7/GNAI3/R
2/MRPS34/MRPL13/GADD45GIP1/MRPL10/MRPL52/MRPL48/DAP3/MRPS7/MRPL53/MRPS17/MRPS21
6/SLC7A1/SLC7A11/SLC6A9/STK39/PRAF2/SLC7A5/SLC1A7/SLC1A4/SLC43A2/SLC1A3/SLC25A2
P2K2/POLR2G/PABPC1/NUP93/NUP205/NUP37/NDC1/POLR2H/NUP188/ZC3H12A/NCBP2/CNOT9/ME
/BHMT
GFBP2/PKLR/FABP4

GEF2/PRKCD/TRIM54/MAPRE1/TMSB4X/TWF2/MID1IP1/CARMIL1/WASF2/MAP2/HSPA8/CORO1B/HI
CI/RIPK2/BIRC3/FKBP1A/UBE2I/BRCA1/RPS2/ZC3H12A/HSPBP1/AXIN1/KDM1A/BIRC7/PIAS3/W

U/FTSJ1/METTL1/POLR3K/PUS1/POP7/URM1/TRMT1/SSB/TRMT61A/POP1/CPSF1/TRMT112/TRMT6

CK2/CREB3L1/DDIT3/BAX/CREB3/LMNA/DDX11/HYOU1/ARFGAP1/EXTL3/CCL2/SHC1/ATF4/WFS1/
4/CCT7/TCP1/CCT8/GNL3/WRAP53
/VCAN/B3GAT3/GPC1/ABCC5/B4GALT5/CHST1/B3GNT7/FMOD/CHPF2/B4GALT3/ST3GAL4/EXTL3/B

/PDPN
CYP4F2/CYP4A11
K/PPP1R3B/IGF2
K/PPP1R3B/IGF2
BAX/CASP2/UBE2M/NUPR1/ATF4/CDK5/TGFB2
GS2/SOX9/CRYAB/ENO1/DBN1/POSTN/REG1A/PLXNA1/SEMA6A/MMP14/CLSTN1/SERPINE2/TGFB1/

APP/CLEC7A

R1A/PEA15/PPP1CA/BID/DAPK3/PMAIP1/PML/LTBR/TNFSF12/FADD/BCL10/CTNNA1/THBS1/PAK2
DBN1/CDK1/REG1A/MMP14/CSF1/TNFRSF12A/KRT17/AGRN/PLCB1/EXOSC4/S100A8/TWF2/EZR/TR
SLPI/ISG15/PPIH/PABPC1/PPIA/ZC3H12A/PLSCR1/APOBEC3G/IFI16/BANF1/ILF3/ISG20/APOB
2/MRPS34/MRPL13/GADD45GIP1/MRPL10/MRPL52/MRPL48/DAP3/MRPS7/MRPL53/MRPS17/MRPS21
GN/PLCB1/CCT5/CCT3/MFGE8/ZP3/SYT8/CCT2/UBAP2L/CCT4/SPAG1/CCT7/KDM5B/TCP1/FOLR2/
GAM/RIPK2/HAVCR2/BIRC3/ITGB2/IRAK1/S100A8/CTSK/MAPKAPK2/COLEC12/CD300A/UNC93B1/
RAD54L/FANCD2/HRAS/RAD51AP1/HSF1/RHNO1/NABP2/BAX/PRKDC/BRCA1/XRCC2/TICRR/EEF1D/

/SLCO2B1/SLCO1B3/SLCO1B1
ITGA5/PDCD10/ADAM17/RHOA/PDPN/HBEGF
ITGA5/PDCD10/ADAM17/RHOA/PDPN/HBEGF

3/LPIN2/APOE/CPT1A/APOC2/LIPG/SORL1/DGAT2/C3/PLIN5/LIPC/AGMO/GPAM/FABP1/CAT/APO
CNB1/DRD1/ACE2/AVPR1A/KMO/ARG1/CHRNA4/SLC38A3/ABAT/SLC6A1/AQP9
SULT1A2/DDC/RAPGEF2/FAH/MAOA/GCH1/DRD1/MAOB/SRD5A1/DBH/HPN/DIO1/SULT1B1/IYD/ABA
P/FABP1/ABCG8/ABCG5/APOA2/PBLD/RBP4/MOGAT2/APOA1/APOA5/SLC2A2
SOD1/PID1/ETFBKMT/PPARA/ACACB/APOE/PROX1/ACMSD/GCK/DGAT2/PLIN5/MLXIPL/PPARGC1A/
ATP6V1F/SLC26A6/GRN/ATP6V0B/ATP6V0D2/ATP6V1C1/SLC9A1/TMEM165/MAPK3/TCIRG1/SLC4A
RT7/ST14/PERP/KRT18/CAPN1/KRT8/CAPNS1/CYP26B1/EVPL

F/STX4/MAP1A/CD247/TM9SF4/ABCA7

/TRAF2/TRIM6/CXCR4/HIF1A/IRF3/IRF7/CPNE1/NLRC5/CD300LF/TAF9/AGPAT2/CASP1/AXL/FA
CAM1/LGALS9/SRC/MAPK13/RIPK2/SQSTM1/IRAK1/PLCB1/ANKRD1/PSMB9/PSMD14/CCL5/CCL26/
HAQ/YWHAB/GZMB/BAD/CASP8/YWHAG/TFDP1
HAQ/YWHAB/GZMB/BAD/CASP8/YWHAG/TFDP1
/APOA5
/APOA5
/APOA5
FD1

PRE1/FAM83D/STIL/SNX10/CSNK1D/DCTN2/CEP250/NSFL1C/BICD1/HNRNPU/MAP1A/MARK4/NEDD
/NPLOC4/RFC2/POLA1/UBE2L6/RPA2/POLD3/TRIM25/VCP/FAAP20/RPS27A/UBA52/RFC5/RPA1/P
B2/C1QA/GRN/IL1B/C5AR1/CST7/APP/MAPT/CCL3
/PPT1/ALDH3B1/PRKCD/SGPP2/ELOVL1/CTSA/BAX/B4GALT5/NEU1/GM2A/GLTP/CERS5/PPP2R1A/
GCL/MLYCD/HMGCS1/ACOT1/ACOT2/ACACB/CBR4/SUCLG2/MCEE/NUDT7/ELOVL2/GSTZ1/AADAT/AC
GCL/MLYCD/HMGCS1/ACOT1/ACOT2/ACACB/CBR4/SUCLG2/MCEE/NUDT7/ELOVL2/GSTZ1/AADAT/AC
HEY1/ZNF675/MSX1
/MTCL1/PAK1/SLC51B/GDI1/UBE2J2/NPEPPS/ITGB1BP1/CDK5/UBE2L3/GSK3A/BAG3/PDCD5/CEM

4A/ENPP1/APOE/LIPG/DGAT2/MLXIPL/ANGPTL3/APOA1/OTC/CPS1

NSMCE2/STX1A/NUP93/NUP205/NUP37/NDC1/NUP188/PIAS3/CBX4/NUP85/NUP43/RAE1/ZMIZ2/N

RSF4/SLC39A10/NCKAP1L/CARD11/TNFSF13B/PELI1/GPR183
A1/APOA5/CYP7A1
1/MAVS/IRF5/NMI/DHX9/TLR7/TLR8
90AB1/CCT4/PNKP/TCP1/PARM1/PTGES3/WRAP53/HSP90AA1/NVL/MAPKAPK5/HNRNPD/MYC/MAPK1
RF7/NLRC5/PTPN6/FADD/PTPN2/PTPN1/MAVS/IFNAR2/CDC37/YTHDF2/METTL3/ADAR/MUL1/ZBP1
OLE4/POLA1/PRIM2/RPA2/POLD3/DNA2/RFC5/POLE3/RPA1/POLE2
LGALS3/TGFB1/CDC25C/PPP1R14D/PPP1R14B/DUSP5/PRKCD/B3GAT3/ADORA1/MTMR2/CDC25A/DN
1/TTR/CPS1

PTPN2/PTPN1

/HADHB/ECH1/ACBD5/PCCB/LONP2/MLYCD/BDH2/ETFBKMT/MMAA/ALDH3A2/CPT2/FAAH/HIBCH/PP
A1/ALDH1A1/RARRES2/SRD5A1/BCO2/GPIHBP1/CYP3A7/APOM/DGAT2/AKR1C4/PECR/CYP4V2/RDH
/DECR2/CRAT/ETFA/DECR1/ACADVL/HADHB/ECH1/ACSM1/LONP2/MLYCD/BDH2/ETFBKMT/ALDH3A2

E2/TGFB1/ARHGAP4/PPT1/PSRC1/CDKN2A/PLXNA3/NDRG3/ADAM15/CDKN2D/BDKRB1/SEMA4A/MAP

11/PRAF2/SLC7A5/SLC1A7/SLC43A2/SLC1A3/SLC25A22/SLC7A8/SLC3A2/SLC7A10/SLC7A6
NUP205/NUP37/NDC1/POLR2H/NUP188/ZC3H12A/NUP85/NUP43/RAE1/XPO5/POLR2L/NUP155/AGO
NUP205/NUP37/NDC1/POLR2H/NUP188/ZC3H12A/NUP85/NUP43/RAE1/XPO5/POLR2L/NUP155/AGO

AO
AO
B4X/TWF2/CARMIL1/MAP2/HIP1R/TUBB4A/CAPZA1/MKKS/FHOD3/FKBP4/MYADM/VIL1/PFN1/INPP
/AKR1C4/CYP2J2/CYP2A13/CYP4F12/EPHX2/CYP1A1/CES2/CYP4F3/CYP2A7/PON3/PON1/CYP1A2
POE/APOC2/GLTPD2/TMEM30B/SLC25A42/SLC25A25/SCP2/MTTP/APOC1/ABCG8/MFSD2A/APOA2/A
G/IRS2/REN/SCP2/APOC1/ABCG8/ABCG5/APOA2/ABCB4/PON1/APOA1/CYP4F2/APOA5/APOC3/CYP
B4/AKR1C4/CYP2J2/CYP2A13/SCP2/CYP4F12/ACSL1/EPHX2/CYP1A1/CES2/CYP4F3/CYP2A7/CYP
/DECR2/CRAT/ETFA/DECR1/ACADVL/HADHB/ECH1/ACSM1/LONP2/MLYCD/BDH2/ETFBKMT/ALDH3A2
NCBP2/SNRPF/LSM10/CPSF1/MAZ/CDK7/TTF2/POLR2L/CPSF4/POLR2K/SNRPG/SLBP/SYMPK/CPSF

18/VTCN1/HLA-DPA1/ZP3/HLA-DPB1/CD2/EBI3/CD276/IL27RA/CD3E/TCIRG1/SASH3/ISG15/SI

SK3A/TMEM119/MSX1/NOTCH1/BHLHA15/HDAC5/BMP2/CTDP1/CXCL10/PLPP7/RGS4
GS2/DUSP9/SRC/TRIB3/GSTP1/CDKN2A/PKN1/DUSP5/PRKCD/HSPB1/MVP/PIF1/NUP62/MAD2L1/C

F3
C18/CPT1A/ELOVL2/ACOX1/ACAA1/ACSL6/HSD17B4/CYP2J2/ACSL5/CYP2A13/SCP2/CYP4F12/AC
RS1/IRS2/GCK/PPP1R3B/PPP1R1A/KHK/LEPR/IGF2/GYS2/GCGR/G6PC/GNMT
/IGF1/MLYCD/PPARA/ENPP1/ACACB/IGFBP4/RORA/KAT2B/FOXO1/IRS1/GCKR/IRS2/GCK/DGAT2/
/HBB/ACAT1/AMBP/CAT/ALDH1L1/HP/CYP4F2/ASPDH
IDO2/FAH/HAL/HIBCH/THNSL2/GOT1/CBS/ASPA/HNMT/HIBADH/GLUL/GLUD1/GSTZ1/HOGA1/AADA
DOCK8/PRF1
/COL5A1/ITGA5/MMP2/ITGB5/COL5A2/COL4A2/LAMB3/HSBP1

3/VPS28/MVB12A/CHMP1A/TMEM50A/VPS25/VCP/VPS37B
ABCA7
POA2/MOGAT2/APOA1/APOA5/SLC2A2
SLPI/ISG15/PPIH/PABPC1/PPIA/ZC3H12A/CCL2/PLSCR1/CD209/LAMTOR5/APOBEC3G/IFI16/BA
AB31/MFGE8/CD300A/GULP1/F2RL1/NCKAP1L/MSR1/ITGA2/CDC42/FCGR2B/BIN2/THBS1/XKR8/B
1A/EDNRB/AMN/AVPR1A/GRHPR/ABCG8/ABCG5/AQP9
SLC30A1/MT2A/MT1A/MT1M/MT1G/MT1E/ALAD/ARG1/CYB5A/CAT/HAAO/SORD/MT1X/CYP1A2
A1/ASS1/SERPINF1/ARG1/PCK2/PCK1
CD36/GCH1/NNT/APOE/GSTZ1/HBB/FBLN5/APOM/ABCG2/FABP1/CAT/IYD/HP/RGN/ALB/GSTM1/GS
D3/DAGLA/ASPM/HMGB2/SIRT6/HIF1A/NUMBL/LEF1/FGFR1/FERMT1/SIX2/KDM1A/WNT7B/ETV6/R

/EBI3/IL27RA/LEF1/BCL3/IL18BP/SLC11A1/IL1B/IL12RB1/IL4R
2/FOXM1/SOX9/EPO/CRYAB/DUSP9/IL2RG/CDK1/ICAM1/LGALS9/MAPK12/SRC/SEMA6A/MAPK13/L
A/GRN/C5AR1/CST7/APP/MAPT/CCL3
A/GRN/C5AR1/CST7/APP/MAPT/CCL3
B1/IQGAP3/DKK1/RIPK2/PSRC1/GDF15/IRAK1/S1PR2/CD74/PKN1/DUSP5/ROBO1/ADORA1/ADAM9
CCL5/IQGAP1/P2RY6/SERPINE1/PAK1/PPARD/CORO1B/ITGA2/PDGFA/NDRG4

F2/BAX/CREB3/HYOU1/ATF4/BBC3/PMAIP1/WFS1/PML/ERO1A/DNAJC10/PPP1R15A/GRINA/ERP29
XNA1/ITGA3/MMD/ETV5/ECT2/DKK1/TNFRSF12A/ARHGEF2/ROBO1/SERPINI1/PLXNA3/NME1/NBL1
BMF/HIP1R/BID/MT3/YWHAH/BBC3/PMAIP1/ZNF205/GSK3A/YWHAQ/YWHAB/GZMB/STPG1/RHOT2/S

CD1/ASAP1/TLCD1/COL5A1/CXCR4/MICALL1/BAIAP2L1/BIN1/PLEKHM2/MYRF/FLOT1/PLSCR1/EH
18/HLA-DPA1/ZP3/HLA-DPB1/CD2/EBI3/CD276/IL27RA/CD3E/SASH3/ISG15/SIRPA/ZC3H12A/B
G2/TGFB2/VDR/PAX8/NOTCH1/ZPR1/XKR8
SP9/TRIB3/GSTP1/CDKN2A/PKN1/DUSP5/PRKCD/HSPB1/MVP/NUP62/CDKN2D/DTNBP1/CD300A/RG

/ADORA1/PRNP/GRN/DDIT3/BAX/CASP2/UBE2M/NUPR1/DDIT4/ATF4/CDK5/TGFB2/PARP1/TFAP2A
N/EFNA4/ACP5/SYK/CSF1R/TNFRSF11B/TFRC/CTSK/TCIRG1/SUCO/ATP6AP1/UBASH3B/TNFRSF11
HLA-A/ISG15/IRF1/IRF3/HSP90AB1/STAT1/IFI35/IRF7/NLRC5/PSMB8/PTPN6/ISG20/HLA-B/F
HLA-A/ISG15/IRF1/IRF3/HSP90AB1/STAT1/IFI35/IRF7/NLRC5/PSMB8/PTPN6/ISG20/HLA-B/F
/SYK/NUP62/SNRPD2/RAN/TRIM28/SNRPD1/NUTF2/LMNA/NUP93/NXT1/FAM53B/SNRPE/NUP188/Z

RC/MAD2L2/IL27RA/MSH2/SWAP70/BTK/EXOSC3/HSPD1/NBN/ERCC1/RNF8/XCL1/MSH6/TP53BP1/
L1/NDC1
NCF1/CCL5/TGFA/RELN/NCAPG2/GPRC5B/DVL2/LRP8/ADRA2A/ADAM17/PTPN1/PAK2
LR2G/NUP93/NUP205/NUP37/NDC1/POLR2H/NUP188/ZC3H12A/MEG3/NUP85/NUP43/RAE1/XPO5/S
83/MFNG/LFNG/FCGR2B/NOTCH2/DOCK10/DOCK11/CD19/POU2F2
/NUP62/GTSE1/CCT5/RAN/GLIS2/CCT6A/CCT3/TRIM28/NUTF2/CARD10/DKC1/MCRS1/CCT2/LMNA

4/TDG/CTBP2
/KCNN4/RAB29/PTPRC/NECTIN2/RELA/PRKCB
/POLR2H/PNKP/LIG1/COPS7B/ISY1/PARP1/CDK7/POLR2L/POLR2K/CETN2/HMGN1/RBX1/OGG1/RP
IF1A/JMJD6/ISG15/STAT1/NCAPG2/NCKAP1L/HCLS1/LYN/LYAR/PTBP3/MB/DNASE2/RPS19/ACVR
N/GPC1/CHIT1/SLC9A1/FMOD/GNS/GALNS/BGN/LUM/CSPG5/HPSE/CSPG4/SGSH/CEMIP/HEXB/BCA
/MNDA/CTLA4/LYN/SAMSN1/BTK/FCGR2B/TNFAIP3/INHA/THOC1/INPP5D/CASP3
CL5/MIF/MAPK3/IRF3/LY86/CCL2/LYN/IL1B/CD6/CARD16/LBP/CCL3/TNFAIP3/BCL10/TLR2
4/TGFB1/MAGEA1/ROBO1/TSPAN15/ELF3/HNF1B/DTX3/CFD/PERP/CDH6/YBX1/STAT1/PSENEN/DT
TB/JAK3/MAST2/IRF1/CD47/MAPK11/ARRB2/CMKLR1/CLEC7A/HSPD1/THBS1/IRF5/TLR2/IDO1/C
HGAP4/AURKA/TNFRSF12A/PLXNA3/DDR1/TWF2/TRPV2/CXCR4/SEMA4A/NDN/MAP2/IQGAP1/PAK1/
PHF19/DNMT1/MUC1/CHEK1/TRIM28/SIRT6/NAA40/MAPK3/PYGO2/MAP1S/BRCA1/LMNA/NELFE/RP
CL9/EHD2/ADAM12/MYOF/IL4R
OLR1/VASP/FZD6/SCRIB/IFT57/CFL1/MTHFD1L/PTK7/STIL/HIF1A/FZD1/ST14/IFT52/PLOD3/S
O1/GCK/DGAT2/PPARGC1A/MST1/LEPR/FBP1/GNMT

1/HCK/HLA-DRA/IFNGR2/HLA-DQB1/IFI30/HLA-DPA1/HLA-DPB1/GBP2/HLA-DQB2/HLA-A/SOCS1
HD1/WASF1/EIF4A3/RPS3/KAT2A/APP/CBL/MAPT/STMN2/HSPA5/RAPGEF1

5F6/BMF/NOL3/HIP1R/BID/DYNLT1/YWHAH/BBC3/PMAIP1/ZNF205/GSK3A/YWHAQ/YWHAB/GZMB/S

3B/RAB7B/GDI1/RAB35/RAB24/RAB20/RAB10/RAB4B/RAB3D/RAB5C/SGSM3/RAB3A/RAB11A/DENN
A/TAF9
A/MAP2/THY1/GDI1/MT3/CDK5/SEMA4B/SEMA4D/SEMA4F/SEMA5B/ARHGDIA/SEMA3C/SEMA3E/PTK

SPD1/THBS1
2/CYP26A1/ALDH1A1/RARRES2/SRD5A1/BCO2/GPIHBP1/CYP3A7/APOM/DGAT2/AKR1C4/PECR/CYP

D1/TRAF3/IKBKG/FADD/CASP8/RPS27A/UBA52/UBE2D2
L2/APP/FADD/S100A7/ADAM17/RHOA/TNFRSF14/XCL1/TMEM102/CXCL10/DOCK8/CCR2/WNK1
1/ALDOB
LIN5
MME/REN/SERPINF2
TCD

KDC/XRCC2/KDM1A/PML/PARP1/GPX1/XRCC6/CBL/CHEK2/TMEM109/HSPA5/BCL2L1/TIGAR/POLE3
/SCRIB/SSNA1/THY1/RELN/ITGB1BP1/CDK5
CXCR3/CXCL16/CXCL11/XCL2/GPR183/CCL3/S100A7/ADAM17/XCL1/TMEM102/CXCL10/CCR2/WNK
/REG1A/GAPDH/PI3/S100A8/LYZ/RNASE6/BPIFB2/SLPI/PDZD11/SPON2/PRSS3/CCL13/BCL3/CX
1/RNASE1/EXOSC4/C11orf80/TSEN54/EME1/RNASE6/HSF1/HMGB2/BAX/SLFN13/CSTF2/APEX2/R
HAVCR2/GSTP1/HSPB1/ACP5/GBP5/MNDA/SIRPA/F2RL1/SERPINB1/ZC3H12A/HDAC2/IL1B/S100A

/SMAP1/PIK3CB/SYT11/UBQLN2
L1A1/POSTN/BAK1/SRC/MMP14/SERPINE2/ATP1A1/TGFB1/COL3A1/ANKRD1/PSPH/CHEK1/BDKRB1

/S100A7/CXCL17/LGMN/CXCL10/MOSPD2/CCR2
FBP3/CCL5/IQGAP1/P2RY6/SERPINE1/PAK1/PPARD/CORO1B/ITGA2/PDGFA/NDRG4
1/RCC2/LGALS1/CDKN2A/ADAM15/FZD7/EFNA5/SERPINE1/CORO1C/JAG1/ITGB1BP1/SPOCK1/TAC
FAM1/PTPN6/CD27/BTK/BAD/INHA/INPP5D/IKZF3
/LIG1/RPS3/POLQ/MUTYH/OGG1/RPA2/DNA2/TDG/RPA1/SMUG1/HMGA2/HUWE1/UNG/PRMT6/LIG3/
/LBH/EVI2B/TGFB2/RAB10/NAP1L2/FGFR2/GNL3/SFRP2/AKNA/CDK2AP2/NOTCH1/TEAD3

/BAK1/SRC/MAPK13/ECT2/STK26/ADA/PRKCD/ADAM9/EZH2/TRPM2/HSF1/AREG/HMOX1/PCNA/ZNF
L4/XCL2/APP/FADD/CCL3/S100A7/ADAM17/RHOA/TNFRSF14/XCL1/TMEM102/CXCL10/DOCK8
1/TCIRG1/ATP6V1H/RAB20/ATP6V1E1/ATP6V0C/ATP6V0E1/ATP6V0D1
/SMARCB1/PADI2/SMARCC1

BMF/HIP1R/BID/YWHAH/BBC3/PMAIP1/ZNF205/GSK3A/YWHAQ/YWHAB/GZMB/STPG1/RHOT2/SLC25
205/NUP37/NDC1/NUP188/DYNLT1/KPNB1/CD209/NUP85/NUP43/VPS28/RAE1/MVB12A/NUP155/N

NME4
NM2/SH3GLB1/PACSIN2/PACSIN1/FNBP1L
MBIM1/BCL2L1/CLTC/MRAP2

CT2/STK26/SPI1/CYBB/PRKCD/EZH2/TRPM2/HSF1/SLC9A1/EFNA5/PCNA/ZNF580/SIRPA/DDX11/

MEPA1/EIF4EBP1/ARHGEF2/PRKCD/MAPRE1/TMSB4X/TWF2/CARMIL1/MAP2/LCMT1/ANKRD27/HIP1
HLA-A/ISG15/IRF1/IRF3/HSP90AB1/STAT1/IFI35/IRF7/NLRC5/PSMB8/PTPN6/ISG20/HLA-B/F
205/NUP37/NDC1/NUP188/DYNLT1/KPNB1/CD209/NUP85/NUP43/VPS28/RAE1/MVB12A/NUP155/N

PB1/IGF2BP1/SYK/IL18/IGF2BP3/CARD9/LTB/HMOX1/CD86/MAPKAPK2/EBI3/CD276/CD3E/MAST
C31A1/ABCC2/SLC25A13/SLC19A2/SLC22A3/SLC1A1/SLC25A30/SLC13A3/SLC7A2/SLC47A1/SLC
N5/SCP2/APOB/ABCB4/PON1/APOA1/CYP4F2/CYP4A11
26/RIPK2/GDF15/IRAK1/S1PR2/CD74/PKN1/DUSP5/PRKCD/ADORA1/PRNP/ADAM9/SYK/STK39/IL
RHOG/CARMIL1/CORO1C/CORO1B/SNX10/RAC1/PFN1/TACSTD2/ARFIP2/DBNL/INPPL1/LIMA1/DEF

KN1C/SOCS3/FGFR2/GRB2/DNAJB6
M5/AS3MT/HNF4A/CYP2C18/ACY1/AKR7L/POR/RORA/CYP26A1/ACY3/CES1/ACAA1/BPHL/CYP3A7/

FB/C3/CFH/VTN/CD5L/CFHR1/C4BPB/CFHR2/CLEC3B/C7/C8B/F2/SERPINF2/CPN2/CPB2/C8A/KL
3/NIPAL2/NIPA1/NIPA2
/FLNA/RACGAP1/SHCBP1/ARHGEF2/SMARCD3/IGF2BP1/NAP1L1/DAGLA/FZD6/ASPM/HIF1A/ADGRG
/BCL2L12/DYRK2/DDIT4/BCL3/KDM1A/TAF9/IFI16/PML/MSH2

M1L/RPL23/RPS7/BAG2/RPL5/MASTL

HAVCR2/GSTP1/HSPB1/ACP5/MNDA/SIRPA/SERPINB1/ZC3H12A/HDAC2/IFI16/CASP1/PML/IL1R2
1R/BID/YWHAH/BBC3/PMAIP1/ZNF205/GSK3A/YWHAQ/YWHAB/GZMB/STPG1/RHOT2/SLC25A5/BAD/
MN1/PLXNA1/SRC/SEMA6A/RAC2/PDLIM7/ENAH/ARHGAP4/EFNA3/TNFRSF12A/AGRN/ROBO1/PLXNA
C/LGALS9/BAK1/MAPK12/SRC/DAB2/MMP14/MELTF/TRIB3/FBLN1/AURKA/RIPK2/EFNA3/ROBO1/A
/TGFB2/PLXND1/FGFR2/TGFB3/SEMA3C/TWSG1
1/CORO1A/CIB2/P2RX4/ITGAV/PRNP/PLCB1/CD52/TRPM2/GNA15/DDIT3/TPCN1/BDKRB1/BAX/CX
AD2L2/IL27RA/MSH2/SWAP70/XRCC4/EXOSC3/HSPD1/NBN/ERCC1/RNF8/MSH6/TP53BP1/ATAD5/T
GR2B/CD177
S14/HIF1A/ASIC1/RELN/RIN1/CLN3/CDK5/DEAF1/B4GALT2/NDRG4/PPP1R1B/EIF4A3/APP/RIC8
/CD2/SERPINE1/ZNF580/CD58/F2RL1/BCL3/IL1B/ANXA4/PRKD2/TLR5/FADD/CLEC7A/LBP/CD33

IN1/CYP51A1/SOD1/LDLR/AGTR1/HMGCS1/ACACB/APOE/POR/RORA/PROX1/SC5D/DGAT2/PPARGC1
13/TGFB2/PTPN6/PIEZO1/SFRP2
SFRP4/ALOX15B/CYP27B1
3R1/CDC42/PRKCI/SYNE4/RHOA
PDA1/GPC4/GPC3/CD44/AGRN/SDC3/PRKCD/VCAN/ABCD1/UPP1/GPC1/APOBEC3B/TYMP/NPL/GPD1
E/DRD1/ADRB2/KNG1/CPS1
CCDC22/PSMD10/YWHAB/FAF1/DZIP1/GET4/PKD1/NFKBIL1
1L/RAC1/C5AR1/LBP/S100A7
N/GPC1/SLC9A1/FMOD/GNS/GALNS/BGN/LUM/CSPG5/HPSE/CSPG4/SGSH/CEMIP/HEXB/BCAN/ACAN

R4/IL34/BIN1/HDAC1/HDAC2
/TRIM28/PRKDC/MAD2L2/SPRED1/BRCA1/PCNA/DDX11/PMAIP1/WAS/PARP1/RPS3/CBX8/FUS/BAB

D1/TRAF3/IKBKG/FADD/CASP8/RPS27A/UBA52/UBE2D2
CORO1A/FOLR1/STX3/SQSTM1/SNAP25/CPLX2/TAP1/ZNF385A/RAB34/S100A10/DTNBP1/AREG/CH

/FAAH/PNPLA3/ABHD6/LPIN2/APOE/CPT1A/APOC2/LIPG/SORL1/DGAT2/C3/PLIN5/LIPC/AGMO/G
/HSD17B2/CYP26A1/ALDH1A1/SULT1E1/SRD5A1/BCO2/HSD17B4/CYP3A7/HSD17B8/DHRS2/DGAT2
R8D1/OR10J3/OR1F2P/OR51B5/OR2H2/OR51Q1/OR3A3/OR2B2/OR4K1/OR2W3/OR3A2/OR12D2/OR5
/MGST1/PRDX3/MGST2/HBA2/SOD1/ABCC2/CD36/GCH1/NNT/APOE/SLC30A1/GSTZ1/ACAA1/HBB/M
FB/C3/CFH/VTN/CD5L/CFHR1/C4BPB/CFHR2/CLEC3B/C7/C8B/F2/SERPINF2/TFR2/CPN2/CPB2/C
/SPI1/C1QC/CD74/NME1/NME2/CSF3R/HMGB3/PRMT1/HMGB2/IL34/HIF1A/ATP6AP1/ISG15/UBAS

3EAP/SMARCA4/DDX11/POLR2H/NCBP2/SNAPC2/NPM3/TAF9/SIRT7/INTS1/SMARCB1/CDK7/SRRT/
EX11A/ADRB2/PPARGC1A/ALDH6A1/FABP4/FNDC5
K/PPP1R3B/KHK/IGF2/GCGR
D6/DKC1/TRMU/FTSJ1/METTL1/FBL/NOP2/NHP2/PUS1/URM1/EMG1/TRMT1/SSB/TRMT61A/APOBEC
6B/MAP2/MSN/MAPK3/MAP2K2/AP4M1/ANKRD27/STX6/CORO7/RAB7B/SORT1/STX10/TBC1D10B/SN
NJ11/P2RX4/CSF1R/NOL3/HYOU1

ASP1/STAT1/SIX2/JAG1
F1/IL15/RASAL3/TWSG1/DOCK2/TNFRSF14/XCL1/PTPRC/SH3RF1/CCR2/HLA-E/ARG2/ZBTB7B/CD
PB1/IGF2BP1/SYK/IL18/IGF2BP3/CARD9/LTB/HMOX1/CD86/MAPKAPK2/EBI3/CD276/CD3E/MAST
3/MAFG/KRT80/ADAM9/USH1C/CD109/EZH2/NME2/PKP3/KRT7/ESRP1/SCRIB/AKR1C3/CTSK/ST14

P1/CTSK/PPARD/FERMT1/SULT2B1/CBFB/TGFB2/VDR/MAFF/GAL/HPSE/OVOL2/KDF1/TRADD/KRT1
NUP205/NUP37/NDC1/POLR2H/NUP188/ZC3H12A/NUP85/NUP43/RAE1/POLR2L/NUP155/AGO2/NUP

12A8/KCTD7/SLC12A7
S9/SVBP/OTUB1/FBXO5/GTPBP4/PRR7/U2AF2/PIAS3/CDK5/VPS28/RPS3/ARRB2/RPL23/UBXN1/A

LDH1L1/CYP4F2/ASPDH
4
1/SERPINE1/SULT2B1/VDR/PAX8/OVOL2/KDF1/BAD/NOTCH1/SFRP4/MESP1/ALOX15B/VEZF1
PON1/APOA1
PON1/APOA1

2/MRPS34/MRPL13/UQCC2/GADD45GIP1/MRPL10/MRPL52/MRPL48/DAP3/MRPS7/MRPL53/MPV17L2
XA13/PKDCC/MICALL1/ANKRD27/KRT18/ARF3/CCDC22/RAB10/SNX27/ARF4/ACSL3/LLGL1/CCDC9
APN2
P6V0B/ATP6V0D2/ATP6V1C1/SLC9A1/TMEM165/MAPK3/TCIRG1/SLC4A2/ATP6AP1/ATP6V1H/CLN3

1/MAVS/IRF5/NMI/DHX9/TLR7/TLR8
D1L/HSP90AB1/LEPROTL1/RANGRF/STX4/KCNAB2/MAP1A/CD247/EGF/TM9SF4/ABCA7
D74/NME1/NME2/IL34/ATP6AP1/UBASH3B/FSTL3/TFE3/LEF1/IRF7/EVI2B/HCLS1/MITF/LYN/PI

3/MAST2/IRF1/CD47/MAPK11/ARRB2/CMKLR1/CLEC7A/HSPD1/THBS1/IRF5/TLR2/IDO1/CCL19/T
YK/CSF1R/HMOX1/MIF/HIF1A/IL1B/IL4R/APP/CLEC7A/LBP/ADAM17/MAVS/TLR2/ALOX15B/TWIS
ITGA5
RHOG/TBC1D16/RANGAP1/CORO1C/TBC1D1/TBC1D10B/CXCL13/TBC1D30/TBC1D13/ABR/SYDE1/RU
ISG15/TRIM59/ZC3H12A/TRIM31/PLSCR1/APOBEC3G/CHMP3/IFI16/BANF1/PML/ILF3/ISG20/AP

AB34/CHMP4B/TMEM165/NUPR1/RAB7B/MT3/ATG101/PIP4K2A/CLN3/RAB20/GBA/LAMTOR1/CLN6/

LR2G/NUP93/NUP205/NUP37/NDC1/POLR2H/NUP188/ZC3H12A/MEG3/NUP85/NUP43/RAE1/XPO5/S
OAZ1/ODC1/PSMB2/PSME3/OAZ3/PSMB4/PSMB8/PSMB10/PSMA5/PSMD13/PSMC4/PSMD10/PSMD11/
LAMF7/TUBB4B/VAV1/PRDX1/LAG3/ULBP2/PTPN6/ARRB2/GZMB
1R/BID/YWHAH/BBC3/PMAIP1/ZNF205/GSK3A/YWHAQ/YWHAB/GZMB/STPG1/RHOT2/SLC25A5/BAD/
IF1A/MAP1S/DYNC1H1/BLOC1S4/CLN3/AP3M2/WASF1/CDC42/ACTL8/AP3D1/COPG1/NDE1/MAPT/B
/THRSP

R/CCR5/TRPM4/GPR183
ED1/KDM1A/PMAIP1/KMT5A/PSMD10/MSX1/PTTG1IP
D8/IGF1R/FCGR2B/VPS35
PRNP/PLCB1/TRPM2/MCUB/DDIT3/TPCN1/BDKRB1/BAX/FKBP1A/P2RY6/THY1/UBASH3B/NOL3/PLC

/FAAH/PNPLA3/ABHD6/LPIN2/APOE/CPT1A/APOC2/LIPG/SORL1/DGAT2/C3/PLIN5/LIPC/AGMO/G
GK1/OXT/ABCC2/SLC1A1/APBA1/AVPR1A/KMO/PLA2G12B/GLS2/ABCB11/SLC51A/SLC1A2/ABAT/A
P131/PRKCD/B3GAT3/PRNP/HRAS/GTSE1/RAN/MICALL2/SAE1/TRIM28/NUTF2/MTCL1/OAZ1/PAK1
SASH3/SPON2/F2RL1/GPRC5B/IL1B/HK1/BCL10/MAVS/HLA-F/TNFRSF14/XCL1/MAP3K7/FFAR2/H
HD1/WASF1/EIF4A3/RPS3/KAT2A/APP/CBL/MAPT/STMN2/HSPA5/RAPGEF1

GSTP1/HSPB1/ACP5/MNDA/SIRPA/SERPINB1/ZC3H12A/IFI16/CASP1/PML/GSDMD/ARRB2/APP/HK
NKP/DCLRE1B/NBN/ERCC1/MAPKAPK5/DCLRE1C/TFIP11/HNRNPD/MAPK15/TERF1
/ZNF703/LEF1/HDAC2/JAG1/IL1B/LOXL2/TGFB2/TGFB3/ACVR1/NOTCH1/BCL9L/TWIST1/PDPN
/MSN/TALDO1/LCP1/SIRPA/PPIA/CAPZA1/CD47/TCP1/IL12RB1/RALA/CNN2/CDC42/RPLP0
12A2

OL1A1/BAK1/TSPO/MAPK13/PBK/ATP1A1/AKR1B1/ECT2/TGFB1/RAD51/PTAFR/ARHGEF2/RELB/PO
OL1A1/BAK1/TSPO/MAPK13/PBK/ATP1A1/AKR1B1/ECT2/TGFB1/RAD51/PTAFR/ARHGEF2/RELB/PO

/CCL28/ABR/FADD/PTGER4
CI/RIPK2/BIRC3/FKBP1A/BRCA1/RPS2/HSPBP1/AXIN1/KDM1A/BIRC7/WFS1/UBE2L3/GSK3A/UBE
B/SNRPG/SLBP/CPSF3/EXOSC10/LSM1/SNRPD3/ZNF473/DCP2
GK/GNPDA2/UGDH/NANS/GMDS/AMDHD2/GFPT1/GMPPB/PMM2
NG
12A/KPNB1/CD209/CPSF4/TNIP1/BAD/CASP8/BCL2L1/KPNA6/ATG7/SERPINB9/CCL8/PABPN1/VA
D6/TLE3/PSMB9/DDIT3/PSMD14/MAD2L2/PSMD2/FZD1/IGFBP6/MLLT3/HDAC1/LZTS2/PSMB2/SFR
AFR/SYK/UNC13D/HMOX1/CD300A/STXBP2/F2RL1/LYN/FGR/ABR/IL4R/BTK/STX4/FCGR2B/VAMP8

ALS14/NFKBID/TP53/WNT5A/CD274

C3H12A/PLSCR1/APOBEC3G/IFI16/BANF1/ILF3/ISG20/APOBEC3D/TNIP1/SRPK1/MAVS/OAS3/IF

MIF/ZC3H12A/TNIP2/CST7/IL4R/LBP/CCL3/HSPD1/FCGR2B/THBS1

G1/NCKAP1L/PMAIP1/IL2RA/SIT1/FADD
LD1/APOC3
RSF9/SNRPC/SRSF12/ISY1/PRPF6/SF3A3/SNRPG/PRPF19/SRPK1/DDX23/RBMX/ZRSR2/USP39/LU
3/HIP1R/BID/YWHAH/BBC3/PMAIP1/ZNF205/GSK3A/YWHAQ/YWHAB/GZMB/STPG1/RHOT2/SLC25A5
RAF2/RBCK1/PSMD14/FBXL19/TRIM6/PSMD2/RNF34/UBE2Q2/RNF19B/SHARPIN/BRCA1/PSMB2/PS
APK13/PBK/AKR1B1/STK26/DKK1/RIPK2/GSTP1/IRAK1/PKN1/HRAS/SYK/PLCB1/STK39/MAPK8IP
2/ARL2/FLNC/RCC2/FLNA/VASP/HDAC7/CD151/PKP3/COL16A1/LAMC1/CTNND2/MICALL2/S100A1

ERMT3/PRKCD/HSPB1/SLC7A11/PLEK/SYK/COMP/CCL5/RAP2B/UBASH3B/C1QTNF1/ZNF703/BLOC1
CYP26B1/MAMSTR/IL4R
/MARK2/SLC9A3R1/ARF4/CDC42/PRKCI/WDR1/SYNE4/PARD3/RHOA

OLR2H/ATF4/SMARCB1/CDK7/NCL/POLR2L/POLR2K/HEATR1/TAF1D/UBTF/PIH1D1/BAZ2A/WDR75/
HK2/TFRC/SYNGR1/CACYBP/BSPRY/GFPT2/SNX10/KDM5B/SPOCK2/PML/SOCS3/ADAM23/PADI2/NC
HK2/TFRC/SYNGR1/CACYBP/BSPRY/GFPT2/SNX10/KDM5B/SPOCK2/PML/SOCS3/ADAM23/PADI2/NC
L34/ATP6AP1/LEF1/EVI2B/HCLS1/CCR1
1R/BID/YWHAH/BBC3/PMAIP1/ZNF205/GSK3A/YWHAQ/YWHAB/GZMB/RHOT2/SLC25A5/BAD/CASP8/
/CD2/SERPINE1/ZNF580/CD58/F2RL1/BCL3/IL1B/ANXA4/PRKD2/TLR5/FADD/CLEC7A/LBP/CD33
PDL3B/ASAH1/GBA/SMPD4
B
B
F1/MAZ/TTF2/CPSF4/SNRPG/SLBP/SYMPK/CPSF3/SSU72/PABPN1/FIP1L1/CSTF3/SNRPD3/ZNF47
K11/CLEC7A/HSPD1/IRF5/TLR2/IDO1/CCL19
P2RX4/GRN/RANGAP1/BAX/RTN4RL2/GIPR/LYN/TNC/NREP
1/MAPK13/PBK/ECT2/TGFB1/RAD51/UNC119/DTL/RAD54L/FEN1/FANCD2/SLC7A11/POLD1/HRAS/
SPB8/PYCARD/E2F1/SOX9/ANXA2/CTSC/SLC2A6/TIMP1/TSPO/ATP13A2/DAB2/PBK/SERPINE2/TR
/F8/SERPINA1/ACTN2/SOD1/PROS1/CD36/VWF/SERPING1/ORM1/RARRES2/ORM2/FGB/F5/FGG/CL
L1/HAMP/PPARGC1A/CYP39A1/FABP1/ABCG8/ABCG5/APOA2/PBLD/RBP4/MOGAT2/AKR1D1/APOA1/
/HADHB/ECH1/ACBD5/PCCB/LONP2/MLYCD/XYLB/BDH2/ETFBKMT/FAH/MMAA/ALDH3A2/CPT2/FAAH
P1/AP2M1/CSK/TMBIM1/BCL2L1/CLTC/MRAP2
6A/LEF1/HCFC1/SMARCB1/RUVBL2/USP22/JADE2/KAT2A/BEND3/HAT1/WDR5/PIH1D1/MORF4L2/A
TRIM28/LCMT1/PYGO2/BRCA1/FTSJ1/RAB3B/METTL1/LMNA/PRMT2/NELFE/FBL/NOP2/KDM1A/EMG
A3/ABAT/SLC6A1
N/LGALS9/SRC/GAPDH/RIPK2/HAVCR2/SCAMP5/SYK/TMSB4X/CSF1R/EZR/VTCN1/TRIM6/MIF/CD2
GSTP1/HSPB1/ACP5/GBP5/MNDA/SIRPA/F2RL1/SERPINB1/ZC3H12A/IL1B/IFI16/CASP1/PML/GS

1/NECTIN1/CSF3R/BSG/TNFRSF11B/BAX/TUFT1/SERPINE1/TCIRG1/PERP/LAMA5/EDA/HDAC1/LE
K1D/IFT80/ABL1/SFRP2/SFRP4/RNF213
LR2G/NUP93/NUP205/NUP37/NDC1/POLR2H/NUP188/ZC3H12A/MEG3/NUP85/NUP43/RAE1/XPO5/S
PO/SEMA6A/MELTF/FBLN1/TGFB1/RCC2/ARHGAP4/LGALS1/DKK1/ARHGEF2/PLXNA3/HES4/ASAP1/
TCD
14/PSMB4/PSMB8/PSMB10/PSMA5/PSMD13/PSMC4/PSMD10/PSMD11/PSMD1/PSMA7/FBXL6/PSMB3/

/UBE2F/NEDD8/RPL11
AOA/ALDH1B1/CYP2C18/MTHFS/NNT/POR/MAOB/SULT1E1/GSTZ1/AADAT/HBB/CPN1/QDPR/ACAT1/
HX34/YBX1/PABPC1/SLC11A1/HNRNPU/DHX9/RBM10/RBM24/LARP1/HNRNPA0/HNRNPD/HNRNPC/SY

/PFKFB1/HP/FBP1

1A1/CYP1A2/HPX
TYRO3/SRC/LGALS1/CD74/ITGAV/NECTIN1/TFRC/CD86/CXCR4/ITGA5/SELPLG/HAVCR1/TRIM11/

TYRO3/SRC/LGALS1/CD74/ITGAV/NECTIN1/TFRC/CD86/CXCR4/ITGA5/SELPLG/HAVCR1/TRIM11/
TYRO3/SRC/LGALS1/CD74/ITGAV/NECTIN1/TFRC/CD86/CXCR4/ITGA5/SELPLG/HAVCR1/TRIM11/
TYRO3/SRC/LGALS1/CD74/ITGAV/NECTIN1/TFRC/CD86/CXCR4/ITGA5/SELPLG/HAVCR1/TRIM11/

HX34/DKC1/YBX1/PABPC1/SLC11A1/HNRNPU/DHX9/RBM10/RBM24/LARP1/HNRNPA0/LRPPRC/HNRN

H/RPA2/POLD3/ABL1/TDG/HDAC10/ERCC1/RPA1/MSH6
F1/TCIRG1/MNDA/CXCL9/C5AR1/CCR5/CLEC5A/LBP/CD300C/GNLY/BCL10/VEZF1/ADORA2B/BECN
LA/BTK/IGF1R/ABL1/PTK2/TNK2/VEGFA

DR/TRIM25/PHEX/CYP27B1/CXCL10/ATP2B1/BGLAP/CD4
FD1/PIPOX/FTCD
PINF1/ARG1/PCK2/CPS1/PCK1
ID/YWHAH/BBC3/PMAIP1/ZNF205/GSK3A/YWHAQ/YWHAB/GZMB/SLC25A5/BAD/CASP8/YWHAG/TFDP
ENO1/SLC2A6/DTYMK/TGFB1/GAPDH/TYMS/UCK2/HK2/PFKFB4/NME1/NUP62/PFKFB3/TMSB4X/NME

MAPK3/FGFR4/SPRED1/POLR2G/POLR2H/FGFR1/NCBP2/FGFR3/FIBP/FGFR2/PRKD2/GRB2/POLR2L
RORC/GPLD1/CYP7A1

H2/SOCS3/FGFR2/GRB2/DNAJB6/HEY1/OVOL2/VASH1/CASP8/RBPJ/ADM
12/DDIAS/BID/KDM1A/TAF9/SFRP2/BCL2L1/CCAR2/ATAD5/TRIM32/ING2/TMEM161A/ACKR3
CARD/E2F1/ANXA2/CTSC/SLC2A6/TIMP1/TSPO/ATP13A2/DAB2/PBK/TRIB3/AURKA/GAPDH/RIPK2
GCL/IDO2/FAH/HAL/HIBCH/THNSL2/PCYOX1/GOT1/CBS/ASPA/HNMT/HIBADH/BCKDHB/GLUL/GLUD

/ACSM1/LDLR/GOT1/PNPLA3/HNF4A/APOE/RORA/APOC2/LIPG/GCKR/ACOX1/IRS2/GPIHBP1/APOM
35D1/CPT1A/SLC1A1/SLC25A30/SLC23A2/SLC13A3/IRS2/SLC7A2/SLC25A15/SLC38A3/SLC16A1
35D1/CPT1A/SLC1A1/SLC25A30/SLC23A2/SLC13A3/IRS2/SLC7A2/SLC25A15/SLC38A3/SLC16A1
NA3/TWF2/TRPV2/SEMA4A/MAP2/PAK1/NRCAM/GDI1/MT3/CDK5/CTTN/SEMA4B/SEMA4D/SEMA4F/S

RSK1/STIL/NPM1/CHMP4C/BRCA1/XRCC3/CDK2/CHORDC1/POC1A/CHMP3/PLK4/KAT2A/CHMP1A/CE
RFWD3/DNA2/EME2/SMARCAL1/THOC1/MMS22L
2/TUBB4A/CAMSAP2/FKBP4/INPP5J/KATNB1/CLIP3/CKAP2/TBCD/STMN2/EML2/CAMSAP1/CIB1
NT1/GAL3ST1/PPT1/ALDH3B1/PRKCD/PIGU/SGPP2/GAL3ST4/ELOVL1/DPM2/CTSA/PIGS/BAX/GPA
RPL35/LAS1L/RPL7A/RPF2/RSL1D1/NOL9/WDR12/RPL10A/ZNHIT3/ZNHIT6
P/GRINA
2/UNC119/CLCF1/CD44/CD74/ARHGEF2/ADORA1/PRNP/SYK/IL18/DLG3/CSF1R/HSF1/NCF1/TNFR

/FOXK1/HK3/TPI1/FOXK2/GPI/PFKM/ALDOC/HK1
/FOXK1/HK3/TPI1/FOXK2/GPI/PFKM/ALDOC/HK1
/FOXK1/HK3/TPI1/FOXK2/GPI/PFKM/ALDOC/HK1

PB1/IGF2BP1/SYK/IGF2BP3/CARD9/LTB/HMOX1/CD86/MAPKAPK2/EBI3/CD276/CD3E/MAST2/IRF
/CLIC3/SLC26A6/SLC12A2/CLCN2/SLC12A9/SLC1A4/SLC37A1/SLC12A8/SLC1A3/GABRA3/CLCN7

9A10/HSP90AB1/GBA/CDCA2/NSMF/PTPA/PPP1R15A/ANKLE2/SYMPK/CALM2/PTBP1/CD33/SPPL3/
PAK1/TNFRSF11A/DVL3/WNT7B/BIRC7/MLKL/TNIK/DVL2/RPS3/MAP3K6/ZNF675/SFRP2/DBNL/PT
VCR2/BATF/RELB/IL18/HLA-DMB/JAK3/CD86/APBB1IP/SEMA4A/LCP1/F2RL1/LEF1/ZC3H12A/BC

P1A1/UCP2/ATP6V1F/TMSB4X/ATP6V0B/ATP6V0D2/ATP6V1C1/SLC9A1/TCIRG1/ATP6AP1/CYC1/T
B/ECT2/ARL2/TGFB1/FLNC/RCC2/FLNA/VASP/CSF1R/HDAC7/NECTIN1/CD151/PKP3/COL16A1/LA

/RAC1/PLXND1/SEMA4B/SEMA4D/SEMA4F/SEMA5B/ARHGDIA/SEMA3C/SEMA3E/PLXNC1
Y1/SLC35C1/LFNG/CBFA2T2/RITA1/NOTCH3/ARRDC1/EGF/YTHDF2/CHAC1/METTL3/EGFL7/NEURL

RD5A1/ASS1/QDPR/ACAT1/HAMP/HPN/VTN/ARG1/CYP1A1/BAAT/PCK2/CPB2/RGN/OTC/CPS1/PCK1
/CYP19A1/VCAM1/THBS1/IDO1/ADORA2B
X2/RAN/RANGAP1/TRIM28/NUTF2/ZC3H12A/NCBP2/FERMT1/HCLS1/AKAP8L/IL1B/CDK5/TXN/PPP

SOX4/SOX9/TEAD2/DBN1/ASF1B/COL1A1/CHST11/NEK2/GINS1/SULF1/TM4SF1/SPINT2/MMP14/L
RC/ITGA3/ARHGEF16/RAC2/CSF1/DEPDC1B/ECT2/RHOV/ARHGAP4/SQSTM1/RACGAP1/ARHGEF2/CY
/FOXK1/HK3/TPI1/FOXK2/GPI/PFKM/ALDOC/HK1
/FOXK1/HK3/TPI1/FOXK2/GPI/PFKM/ALDOC/HK1

5
PK2/MAPRE1/RAN/HSF1/TRAF2/FKBP1A/HSP90AB1/HIP1R/FLOT1/CDK5/PLXND1/APP/RALB/STPG
14/LIF/P3H4/TGFB1/GPNMB/NCDN/EFNA4/ACP5/SYK/ELF3/IL18/CSF1R/TNFRSF11B/TFRC/CTSK
D1/BAX/BASP1/FGFR1/JAG1/TGFB2/FGFR2/VDR/PAX8/HOXB7/SEMA3C/TFAP2A/WNT4/ACVR1/TNF
PPP1R3B/PCK2/FBP1/IGF2/GYS2/PCK1
FCGR2B/NOTCH2/DOCK10
1/FADD/PELI1/TNFAIP3/CASP8
C1/ESRP1/CREB3L1/CCL5/IQGAP1/MAPK3/FGFR4/SPRED1/POLR2G/POLR2H/FGFR1/NCBP2/FGFR3
/EZH2/TCOF1/ADAM15/GLIPR2/TRIM28/FAM83D/MAD2L2/HIF1A/MCRIP1/ZNF703/LEF1/HEYL/HD
C25A/CDKN2D/CHEK1/RHNO1/BAX/BRSK1/NPM1/PCNA/BMF/COPS9/E2F4/BCL3/NOC2L/ATF4/KDM1

/IGF1/MLYCD/PPARA/ENPP1/ACACB/IGFBP4/GPER1/RORA/KAT2B/FOXO1/IRS1/GCKR/IRS2/GCK/
VL6/MCCC2/ACOT13/SULT1A2/ACSM1/SLC26A1/ACSF2/HMGCL/MLYCD/HMGCS1/ABHD14B/PAPSS2/
VL6/MCCC2/ACOT13/SULT1A2/ACSM1/SLC26A1/ACSF2/HMGCL/MLYCD/HMGCS1/ABHD14B/PAPSS2/
VL6/MCCC2/ACOT13/SULT1A2/ACSM1/SLC26A1/ACSF2/HMGCL/MLYCD/HMGCS1/ABHD14B/PAPSS2/
2B/ITGA1/GCLC/SOD1/AGTR1/ADRA1B/ADRA1A/GCH1/EDNRB/APOE/DRD1/GPER1/ACE2/AVPR1A/H
1A/LDLR/PPARA/APOE/PEMT/APOLD1/GLUL/LIPG/APOM/DGAT2/MTTP/APOB/APOC1/APOA2/NOTUM
EK/PER1/SRD5A1/ASS1/PDE2A/DUSP1/FOSB/REN/ITPR2/GPD1/CRHBP/PKLR/PFKFB1/CDO1/AGXT
2B/ITGA1/GCLC/SOD1/AGTR1/ADRA1B/ADRA1A/GCH1/EDNRB/APOE/DRD1/GPER1/ACE2/AVPR1A/H
P/RREB1/KANK1/PHLDB2/APCS/PROS1/CD36/APOE/SERPING1/MYLK/PLPP3/VTN/FGB/FGG/PROC/
2B/ITGA1/GCLC/SOD1/AGTR1/ADRA1B/ADRA1A/GCH1/EDNRB/APOE/DRD1/GPER1/ACE2/AVPR1A/H
E/FITM1/APOC2/LIPG/IRS2/APOC4/C3/PLIN5/REN/SCP2/APOB/APOC1/ABCG8/ABCG5/APOA2/AB
A/ATAD2/SET/SMARCB1/MTF2/PIH1D1/UBR5/TWIST1/MLLT6/DNMT3B/CTBP1/UCN/PHF1/BCOR/PR
IGU/GAL3ST4/DPM2/CTSA/PIGS/BAX/GPAA1/B4GALT5/NEU1/GM2A/GLTP/B3GNT5/GBGT1/SMPD2/
GB1BP1/MTA2

ENO1/SLC2A6/TGFB1/GAPDH/HK2/PFKFB4/NME1/NUP62/PFKFB3/TMSB4X/NME2/PGK1/SIRT6/NUP
N

CCNB1/CCNB2/CYBA/LIMK1/RGS2/TKT/CRYAB/ENO1/DBN1/CDK1/REG1A/SEMA6A/MMP14/CLSTN1/
ER3/EPO/ENO1/DUSP9/STMN1/SH3BP1/SRC/ITGA3/SEMA6A/PLAUR/LIF/PBK/SERPINE2/TRIB3/F
7A/LBP/THBS1

EN54/SLFN13/RNASET2/RRS1/ZC3H12A/POP7/RPS21/POP1/EDC3/DROSHA/AGO2/PIWIL4/KRI1/C

P/OTUB1/FBXO5/GTPBP4/PRR7/U2AF2/CDK5/VPS28/RPS3/ARRB2/RPL23/UBXN1/ABL1/TNFAIP3/

4C/BRCA1/XRCC3/CHORDC1/POC1A/CHMP3/PLK4/KAT2A/CHMP1A/FBXW5/TRIM37/CENPJ/RBM14

2/ITGAV/LAMB1/FZD7/HNF1B/COL5A1/ITGA5/MMP2/ITGB5/LEF1/FGFR1/COL5A2/SIX2/ITGA2/F
KLHDC8B/CHMP1A/KIF20B/MITD1/RHOA/ARF1/VPS4A/CHMP6/CNTROB/CHMP4A/BIN3/CHMP2B/CHM

/BTN2A2/PTPN2

3

2/CYP26A1/ALDH1A1/RARRES2/SRD5A1/BCO2/GPIHBP1/CYP3A7/APOM/DGAT2/AKR1C4/PECR/PHY
P4F11/BTD/MMAA/CYP11A1/SLC19A2/ENPP1/MTHFS/AMN/CLYBL/PC/CYP26A1/SLC23A2/KMO/GC/
/IGFBP2/DUSP1/SSTR1/SOCS2/PPARGC1A/CRHBP/CAT/APOB/F7/SLC6A1/CYP1A2
2B/ITGA1/GCLC/SOD1/AGTR1/ADRA1B/ADRA1A/GCH1/EDNRB/APOE/DRD1/GPER1/ACE2/AVPR1A/H
OA1/APOA5
/MT1A/MT1M/MT1G/MT1E/CYP1A1/SORD/MT1X/CYP1A2
RD5A1/ASS1/QDPR/ACAT1/HAMP/HPN/VTN/ARG1/CYP1A1/BAAT/PCK2/CPB2/RGN/OTC/CPS1/PCK1
C/CORO1C/STX6/HGS/SNX10/SNF8/LAMTOR1/RNF26/CHMP3/VPS28/MVB12A/VPS18/CHMP1A/PLEK
PRKX/BASP1/STAT1/SIX2/JAG1
ENO1/SLC2A6/TGFB1/GAPDH/UCK2/HK2/PFKFB4/NME1/NUP62/PFKFB3/TMSB4X/NME2/PGK1/SIRT
GPNMB/HAVCR2/CD74/PRNP/PAG1/TNFAIP8L2/VTCN1/VSIG4/JAK3/CD86/CD300A/SOCS1/IRF1/C
RUVBL1/MAPK3/PYGO2/MRGBP/BRCA1/MCRS1/ACTL6A/LEF1/RPS6KA4/NOC2L/HCFC1/IL1B/TAF9/
RKA/NUP62/ESPL1/NSMCE2/TGFA/IL1B/ANAPC11/RANBP1/NSFL1C/HNRNPU/BTC/BECN1/ANAPC5/
F1A/PRELID1/PDCD1/ADAM8/KDELR1/IL2RA/CD27/DFFA/FADD/BCL10/IDO1/LGALS14/DOCK8/RP
/GPI/PGLS/RPIA/HK1
/EXOSC9/EXOSC10/EXOSC7/EXOSC2
STN/LGALS9/SRC/RAC2/ATP13A2/ECT2/TGFB1/ITGAM/AACS/GAPDH/RIPK2/HAVCR2/FLNA/ITGB2
MP12/ANXA2/COL1A1/ICAM1/POSTN/SULF1/MFAP2/ITGB4/TIMP1/ITGA3/MMP11/SPINT2/MMP14/

MA7/PSMB3/PSMA1/PSMB5/PSMA4/PSMB1/PSMB6/ENC1/PSMA3/PSMB7
RL1/RAB7B/LYN/PELI1/LBP/TNFAIP3

2RX5/CCL4/CCL3/LMCD1/CALM2/PTBP1/SPPL3/HTT/CALM3/CIB1
1/UBE2L3/DCUN1D5/MASTL/UBE2N/STUB1/RAB1A/PIN1
51A1/SOD1/HMGCS1/ACACB/APOE/POR/PROX1/SC5D/PPARGC1A/TM7SF2/GPAM/APOB/INSIG1/SEC
LAMF7/TUBB4B/VAV1/PRDX1/LAG3/ULBP2/PTPN6/ARRB2/GZMB
2/KDM1A/TAF9/PSMD10/PTTG1IP/TWIST1/ATAD5/BDKRB2/ING2/DYRK3
1/RHNO1/BAX/NPM1/PCNA/BMF/COPS9/E2F4/NOC2L/ATF4/KDM1A/PARP1/CRIP1/RUVBL2/CARD16
5/SERPINE1/CORO1C/JAG1/ITGB1BP1
L4B/POLD3/CHD1L/RPS27A/ERCC1/UBA52/RFC5/GTF2H1/RPA1/ERCC3/DDB1
DBNL/PTPN1/MAP3K10/DAXX/CCL19/MAP4K1/MAP4K2/DAB2IP/MAP3K9/MAP3K11/MUL1/MAP3K12/
MIF/NUPR1/PERP/BCL2L12/DYRK2/DDIT4/BCL3/KDM1A/PMAIP1/TAF9/IFI16/PML/E2F2/MSH2/
X9/CDK1/NDRG1/LIF/SERPINE2/TGFB1/HDAC11/GSTP1/CLCF1/C1QA/GPC1/S100A8/GRN/CXCR4/

/CDC25B/AURKA/TYMS/AGRN/C1QA/C1QL1/SLC26A6/B4GALT5/FLVCR1/HIF1A/PPP2R1A/NRCAM/
S/DLG3/SCRIB
/CROCC/WRAP73/TBC1D7/DNM2/TCTEX1D2/ODF2L/VDAC3/MARK4/CENPJ/IFT20/HTT/DZIP1/EVI5
/POLR3C/MAVS/IRF5/TLR2/DHX9/TLR7/TLR8/POLR3F/POLR3A/RNF135/TICAM1
/DRG1/MAPT/STMN2/DCTN1/MECP2/CDK5R1/NIN
8/JAK3/CD86/SEMA4A/CD300A/EBI3/CD3E/TCIRG1/SASH3/SOCS1/IRF1/CD83/PLA2G2D/LEF1/
XO4/AUNIP/POLA1/BARD1/XRCC6/POLQ/BABAM1/XRCC4/OGG1/PRPF19/WRAP53/DCLRE1B/NBN/P

6/PDSS1/XRCC6/RRM1/FARSA/KRT10
AM8/PIK3CD/LYN/HCAR2/IDO1/LGALS14/PIK3CB/NFKBID/ANXA1/TP53/WNT5A/CD274

C1/ESRP1/CREB3L1/CCL5/IQGAP1/MAPK3/FGFR4/SPRED1/POLR2G/POLR2H/FGFR1/NCBP2/FGFR3
GNA12/PDCL3/DDA1/HSP90AB1/CSNK1E/HSPBP1/BCAP31/CSNK1D/AXIN1/CCDC22/GBA/DNAJB2/
/ITGB8/HEXB
SOX4/SOX9/TEAD2/DBN1/ASF1B/COL1A1/CHST11/NEK2/GINS1/SULF1/TM4SF1/ACSL4/SPINT2/
ALT5/NEU1/GM2A/GLTP/SMPD2/B4GALT3/B3GALNT1/ASAH1/CLN3/GBA/CLN6/FA2H/SMPD4/A4GAL

K1D/DCTN2/CEP250/NSFL1C/BICD1/MARK4/NEDD1

SAL3/TWSG1/TNFRSF14/XCL1/PTPRC/SH3RF1/CCR2/HLA-E/ARG2/ZBTB7B/CD274/ZAP70

BICC1/FZD6/TLE3/MDFI/PSMB9/DDIT3/PSMD14/MAD2L2/PSMD2/FZD1/IGFBP6/MLLT3/HDAC1/LZ
SERPINE2/TYROBP/TGFB1/GPNMB/HAVCR2/CD74/PKN1/PRKCD/PRNP/PAG1/HMGB3/TNFAIP8L2/VT
OXF2

2/CHMP3/PTPN23/SNX12/RAB29/VPS11/SNX3
ENO1/SLC2A6/TGFB1/GAPDH/HK2/PFKFB4/NME1/NUP62/PFKFB3/TMSB4X/NME2/PGK1/SIRT6/NUP

R3B/SORD/GBA3/G6PC
/NEURL1

/PYCARD/ST6GALNAC4/TMEM132A/SLC2A1/PKMYT1/CTSC/CHST11/KDELR3/B4GALNT1/CLSTN1/HI
A/E2F1/CDCA5/NUF2/ASF1B/CDK1/NDC80/MCM2/CDC45/NEK2/GINS1/BUB1/BUB1B/MCM4/ORC6/O
I1/PYCARD/PLBD1/ANXA2/NCF2/GLA/CTSC/ITM2C/SLC2A6/SRC/MMD/ATP13A2/DAB2/LAPTM5/FA
/MCM2/CENPF/CHAF1B/RAD51/SPI1/CBX2/UHRF1/CDKN2A/DSCC1/SMARCD3/OIP5/CBX6/MCM7/PL
M/HMGA1/CDT1/KIF4A/AURKB/CENPA/CDCA8/TOP2A/TRIP13/E2F1/CDCA5/RECQL4/NUF2/ASF1B/
2T/CDCA8/TOP2A/NCF2/PKMYT1/HJURP/NEK2/FBLIM1/ITPR3/MKI67/ORC6/DAB2/NFKBIE/SAPCD
I1/PYCARD/PLBD1/ANXA2/NCF2/GLA/CTSC/ITM2C/SLC2A6/SRC/MMD/ATP13A2/DAB2/LAPTM5/FA
/MMD/ATP13A2/DAB2/LAPTM5/CKAP4/HLA-DMA/CD74/P2RX4/DRAM1/HLA-DRB1/ABCD1/LAMP5/HL
I1/PYCARD/PLBD1/ANXA2/NCF2/GLA/CTSC/ITM2C/SLC2A6/SRC/MMD/ATP13A2/DAB2/LAPTM5/FA
B/E2F1/CEP55/CTSC/CDK1/NDC80/CDC45/NEK2/DLGAP5/CDC25B/KIF23/NDRG1/WDR62/CCNE1/C
/AURKB/TPX2/TACC3/TOP2A/E2F1/CEP55/SNCG/CTSC/CDK1/NDC80/CDC45/NEK2/DLGAP5/CDC25
/TPX2/TACC3/CDCA8/KIF20A/CDC6/SNCG/CDK1/NEK2/DLGAP5/CDC25B/BUB1B/PRC1/KIF23/WDR
CAM1/ARPC1B/SH3BP1/FBLIM1/ANXA5/CDCA3/ITGB4/ITGA3/KIF23/NDRG1/PLAUR/RAC2/DAB2/M
1/ATP13A2/RHOV/RCC2/GPNMB/DKK1/HLA-DMA/SLC39A4/PMEPA1/IRAK1/PLIN3/STEAP1/SCAMP5
CER1G/KDELR3/TMC6/ITPR3/LHFPL2/CD3D/LGALS3/PLAUR/RAC2/ATP13A2/DAB2/FABP5/TYROBP
/ANLN/ARPC1B/FBLIM1/MICAL1/SRC/RAC2/MISP/POF1B/PDLIM7/CORO1A/MARCKS/FLNA/TNNT1/
67/ZWINT/RMI2/KIF18B/S100A14/CKAP4/RAD51/TONSL/EXO1/CDC25C/SQSTM1/DDX39A/ZIC2/S
13/PPT1/FOLR1/STX3/SNAP25/CDK16/PLIN3/CD74/SCGN/SCAMP5/HLA-DRB1/SLC6A9/LAMP5/HL
OA/LPCAT1/IMPDH1/PYCARD/LGALS3BP/CYBA/BACE2/LCN2/ADGRE5/ANXA2/NCF2/GLA/CTSC/COL
CER1G/KDELR3/TMC6/ITPR3/LHFPL2/CD3D/LGALS3/PLAUR/RAC2/DAB2/FABP5/TYROBP/CKAP4/I
M/HMGA1/CDT1/AURKB/CENPA/CDCA8/E2F1/CDCA5/RECQL4/NUF2/ASF1B/CDK1/NDC80/HJURP/NC
AP1GAP/PYCARD/ST6GALNAC4/BACE2/TMEM132A/SLC2A1/PKMYT1/CTSC/POSTN/CHST11/KDELR3/
2/GLA/CTSC/ITM2C/SLC2A6/MMD/ATP13A2/DAB2/LAPTM5/FABP5/CKAP4/PYGB/PPT1/CTSV/HLA-
1/ATP13A2/RHOV/RCC2/GPNMB/DKK1/HLA-DMA/SLC39A4/PMEPA1/IRAK1/PLIN3/STEAP1/SCAMP5
/NUF2/ASF1B/CDK1/NDC80/MCM2/CDC45/GINS1/BUB1/BUB1B/MCM4/ORC6/ORC1/CHAF1B/P3H4/R
DS/SNCG/ANXA2/CDKN3/REG1A/ITM2C/BUB1B/SRC/ITGA3/CLIC1/NCS1/NDRG1/NFKBIE/CENPF/C
CAM1/ARPC1B/SH3BP1/FBLIM1/ANXA5/CDCA3/ITGB4/ITGA3/KIF23/NDRG1/PLAUR/RAC2/DAB2/M
/PYCARD/ST6GALNAC4/BACE2/TMEM132A/SLC2A1/PKMYT1/CTSC/POSTN/CHST11/KDELR3/SULF1/
/MMD/ATP13A2/DAB2/LAPTM5/CKAP4/HLA-DMA/CD74/P2RX4/DRAM1/HLA-DRB1/ABCD1/LAMP5/HL
B/TPX2/KIF20A/TUBA1C/SLC2A1/TUBA4A/CRYAB/ENO1/DBN1/CDK1/COL1A1/NEK2/STMN1/MFAP2
B/TPX2/KIF20A/TUBA1C/SLC2A1/TUBA4A/CRYAB/ENO1/DBN1/CDK1/COL1A1/NEK2/STMN1/MFAP2
OA/LPCAT1/IMPDH1/PYCARD/LGALS3BP/CYBA/BACE2/LCN2/ADGRE5/ANXA2/NCF2/GLA/CTSC/COL
B/TPX2/KIF20A/TUBA1C/SLC2A1/TUBA4A/CRYAB/ENO1/DBN1/CDK1/COL1A1/NEK2/STMN1/MFAP2
/KIF20A/TUBA1C/TUBA4A/DBN1/CDK1/NEK2/STMN1/MICAL1/SRC/PRC1/KIF23/KRT23/TUBA1B/N
QSTM1/BOP1/RBM38/SNRPB/SMG5/IGF2BP1/SF3B4/DCAF13/NUP62/TUBB/LARP6/MEX3A/ALYREF/
51/SPI1/UHRF1/SMARCD3/PLCB1/RAD51AP1/RCC1/EZH2/MUC1/EME1/ZNF385A/HMGB3/CREB3L1/
A/TUBA1C/TUBA4A/CDK1/NEK2/STMN1/PRC1/KIF23/TUBA1B/NDRG1/TUBA3C/DNM1/KIFC1/KIF18
LIM1/ANXA5/ITGB4/ITGA3/KIF23/PLAUR/RAC2/DAB2/MMP14/MPZL1/MISP/PDLIM7/ARL2/FLNC/
LIM1/ANXA5/ITGB4/ITGA3/KIF23/PLAUR/RAC2/DAB2/MMP14/MPZL1/MISP/PDLIM7/ARL2/FLNC/
LIM1/ANXA5/ITGB4/ITGA3/KIF23/PLAUR/RAC2/DAB2/MMP14/MPZL1/MISP/PDLIM7/ARL2/FLNC/
FPL2/LGALS3/PLAUR/FABP5/TYROBP/CKAP4/ITGAM/CYBB/STX3/CD44/ITGB2/ALDH3B1/SNAP25/
NXA2/DBN1/REG1A/SH3BP1/ITGB4/SRC/RAC2/PDLIM7/ENAH/CORO1A/STX3/TNFRSF12A/CD44/PL
/CENPA/CDCA8/CDCA5/RECQL4/NUF2/CDK1/NDC80/HJURP/NCAPG/MCM2/NEK2/BUB1/BUB1B/MCM4
/MMD/ATP13A2/DAB2/LAPTM5/CKAP4/HLA-DMA/CD74/P2RX4/DRAM1/HLA-DRB1/ABCD1/LAMP5/HL
PS12/RPS2/MRPL47/MRPL9/MT3/RPL17/RPLP2/MRPS23/RPSA/RPL10L/RPS5/RPS21/MRPL12/RPL
NCS1/BCAS4/SNAP25/EIF4EBP1/CCT5/CASP4/RPL8/UBL4A/CCT6A/DTNBP1/PSMD14/CCT3/PSMD2
/CENPA/TOP2A/CDCA5/NUF2/NDC80/HJURP/NCAPG/NEK2/BUB1/BUB1B/MKI67/ZWINT/CENPF/P3H

ORO1A/STX3/CD44/ITGAV/IGF2BP1/VASP/FGD3/NME2/TWF2/SCRIB/PLEK2/CFL1/FSCN1/CARMIL
/CENPA/CDCA8/CDCA5/NUF2/NDC80/HJURP/NCAPG/NEK2/BUB1/BUB1B/ZWINT/CENPF/CENPH/TTK
NRPD1/SNRPA1/SNRPA/SF3A2/LSM4/HSPA8/EFTUD2/PPIH/RBM3/YBX1/PABPC1/SNRPE/TXNL4A/S
PS2/MRPL47/MRPL9/RPL17/RPLP2/RPSA/RPL10L/RPS5/RPS21/MRPL12/RPL7/RPL28/MRPS34/RP
P1/ITGB2/COTL1/MVP/CSTB/NME2/TRPM2/QPCT/TNFAIP6/ATAD3B/PSMD14/TIMP2/HSPA6/ALOX5
BP5/PYGB/PPT1/CTSV/GPC4/GPC3/AGRN/SDC3/CD74/PRKCD/VCAN/VAT1/GPC1/UNC13D/TUBB/LY
NEK2/DLGAP5/CDC25B/PRC1/WDR62/CENPF/MISP/AURKA/UNC119/PSRC1/TUBG1/FAM110A/KIF11
RC/PDLIM7/TNFRSF12A/PLEKHO1/ARHGEF2/ITGAV/NME1/PLEK/FGD3/NME2/EZR/KIF18A/CFL1/F
A/CEP55/SLC2A1/ANXA2/CDK1/ANLN/NEK2/MICAL1/PRC1/KIF23/CENPF/ECT2/RCC2/AURKA/PSR
3/ITGAM/CYBB/ITGB2/SNAP25/PTAFR/QSOX1/CSTB/LYZ/TRPM2/QPCT/TNFAIP6/CD53/PLAU/ATA
P5/CKAP4/PYGB/STX3/PRKCD/VAT1/UNC13D/TUBB/LYZ/CTSA/TUBB4B/GRN/SYNGR1/DNAJC5/DDO
F23/KIFC1/KIF18B/AURKA/CLIP2/KLC2/TRIM54/KIF11/WDR34/KIF12/KIF3C/KIF18A/KIF21B/
P5/CKAP4/PYGB/STX3/PRKCD/VAT1/UNC13D/TUBB/LYZ/CTSA/TUBB4B/GRN/SYNGR1/DNAJC5/DDO
EN1/POLD1/MCM7/PIF1/MCM3/EZH2/ALYREF/CHEK1/NABP2/MCM5/SIRT6/PRKDC/CBX1/ACD/NSMC
SYK/TAP1/ZYX/NCF4/FMNL1/RAB34/NCF1/ATP6V0B/ATP6V0D2/RAB32/UNC93B1/STXBP2/TCIRG1
/CENPA/NUF2/NDC80/HJURP/NEK2/BUB1/BUB1B/ZWINT/CENPF/CENPH/TTK/SKA1/SPC24/SKA3/S
STB/NME2/QPCT/TNFAIP6/PSMD14/TIMP2/HSPA6/ALOX5/PLEKHO2/MIF/CANT1/CFD/FTH1/PSMD2
RPL9/RPL17/RPLP2/RPL10L/MRPL12/RPL7/RPL28/RPL39/RPL35A/MRPL13/RPL27/RPL35/RPL18
/CENPA/NUF2/NDC80/HJURP/NCAPG/NEK2/BUB1/BUB1B/ZWINT/CENPF/CENPH/SKA1/AURKA/SPC2
FLNA/TPM4/COTL1/HCK/TWF2/EZR/RHOQ/WIPF3/CARMIL1/TPM2/IQGAP1/LCP1/TPM3/PAK1/PALL
/NUF2/NDC80/HJURP/NEK2/BUB1/BUB1B/ZWINT/CENPF/CENPH/SKA1/SPC24/SKA3/SPC25/CENPW
D1/MCM7/PIF1/MCM3/ALYREF/NABP2/MCM5/SIRT6/PRKDC/CBX1/ACD/PCNA/XRCC3/PPP1CA/CBX3
1/HLA-DQA1/NRGN/HLA-DRA/HLA-DQB1/RHOQ/HLA-DPA1/AREG/HLA-DPB1/CLTA/HLA-DQB2/HLA-
TP1A1/GPNMB/STX3/YWHAZ/NAP1L1/BSG/TRPV2/CALU/RAN/TFRC/DTNBP1/SYNGR1/DNAJC5/RAB3
TP1A1/GPNMB/STX3/YWHAZ/NAP1L1/BSG/TRPV2/CALU/RAN/TFRC/DTNBP1/SYNGR1/DNAJC5/RAB3
A/RPL10L/RPS5/RPS21/RPL7/RPL28/RPL39/RPL35A/RPS3/RPL27/RPL35/RPS20/RPL18A/RPS19
KIF18B/MISP/ECT2/NUSAP1/SKA1/RCC2/AURKA/RACGAP1/KIF11/NUP62/SPAG5/MAPRE1/MAD2L1
C3/CD74/VCAN/GPC1/CTSA/IFI30/CTSK/TCN2/EPDR1/HSPA8/NEU1/GM2A/RNASET2/GYG1/FMOD/
BUB1/BUB1B/P3H4/RAD51/AURKA/NCAPD2/TUBG1/NCAPH/RCC1/CENPO/CHEK1/UBE2I/BRCA1/SGO
LSM4/EFTUD2/SNRPE/TXNL4A/SNRPF/LSM7/U2AF2/SF3B5/SNRPC/BUD31/SNRNP40/PPIL1/RBMX2
/PRKCD/VAT1/UNC13D/TUBB/LYZ/CTSA/TUBB4B/GRN/GM2A/FUCA2/STK11IP/RNASET2/MNDA/PTG
SF3A2/EFTUD2/PABPC1/SNRPE/SNRPF/LSM7/BUD31/SNRNP40/PPIL1/LSM2/ISY1/EIF4A3/PRPF6
1QA/COL4A5/EMILIN2/C1QL1/COL16A1/COL3A1/COL5A1/COLEC12/EDA/C1QTNF1/COL5A2/COL6A
CD2BP2/LSM4/EFTUD2/PPIH/SNRPE/TXNL4A/SNRPF/LSM7/LSM10/SF3B5/SNRPC/GEMIN7/SNRNP4
5/PTAFR/TRPM2/CD53/PLAU/CD300A/ITGAX/RAP2B/SIRPA/DYNLL1/NBEAL2/OLR1/ADAM8/SLC11
CD2BP2/LSM4/EFTUD2/PPIH/SNRPE/TXNL4A/SNRPF/LSM7/LSM10/SF3B5/SNRPC/SNRNP40/RBMX2
RRP1/RRP7A/FBL/NOC2L/EMG1/NOP58/ZNF593/UTP18/UTP14A/FBXW9/BYSL/RRP36/SNU13/FTSJ
1/PIF1/MCM3/DNMT1/POLA2/GINS4/PCNA/XRCC2/XRCC3/RFC2/BLM/PRIM1/WDHD1/ZMIZ2/DONSO
PL7/RPL28/RPL39/RPL35A/RPL27/RPL35/RPL18A/RPLP0/RPL18/RPL23/RPL38/RPL23A/RPL7A/
CD2BP2/LSM4/EFTUD2/PPIH/SNRPE/TXNL4A/SNRPF/LSM7/SF3B5/SNRPC/SNRNP40/RBMX2/LSM2/
/MISP/NUSAP1/SKA1/AURKA/PSRC1/SKA3/TUBG1/KIF11/MAPRE1/KIF18A/CENPE/CAPN6/KNTC1/
F23/UNC119/CEP131/QSOX1/CDC7/GSTM3/PPP1R13L/RFXANK/TRIM45/RAB35/AGBL5/USB1/EHD2
LC2/KIF11/KIF12/KIF3C/KIF18A/KIF21B/CENPE/KLC3/KIFC2/KIF14/KIFC3/KIF15/KIF2A
LSM4/EFTUD2/SNRPE/TXNL4A/SNRPF/LSM7/SF3B5/RBMX2/LSM2/RNF113A/PRPF3/SNU13/PRPF6/
LSM4/EFTUD2/SNRPE/TXNL4A/SNRPF/LSM7/SF3B5/RBMX2/LSM2/RNF113A/PRPF3/SNU13/PRPF6/
CDK4/CDKN2D/PCNA/CKS1B/CCNO/CDK2/CDK7/CCND2/CCND3/CCNJL/CCNE2
/UNC119/RACGAP1/MAPRE1/KIF18A/CENPE/KIF14/CTTN/CDC42/APP
BUB1B/AURKA/CENPO/SGO1/CENPK/DSN1/KMT5C/CENPB
CENPO
SKA3

HLA-DQB1/HLA-DPA1/HLA-DMB/HLA-DPB1/HLA-DQB2/HLA-DQA2/HLA-DRB5

A2/IKBKE/IL2RG/SRC/MMD/PTP4A3/NDRG1/ATP13A2/MMP14/KIFC1/CLSTN1/ATP1A1/RHOV/RCC2/
P/UBE2S/CDK1/BUB1B/CKS2/CCNE1/CCNA2/CCNF/CBX2/DTL/PHF19/SNRPB/CHML/POLD1/DCAF13/
CYBB/STX3/ITGB2/ALDH3B1/SNAP25/QSOX1/ITGAV/LYZ/TRPM2/QPCT/CD53/PLAU/CHIT1/TIMP2/
ALDH3B1/SNAP25/ITGAV/TRPM2/CD53/PLAU/DNAJC5/RAP2B/DEGS1/C3AR1/OLR1/SLC2A5/ADAM8/
1/EME1/HSF1/TRIM28/SIRT6/WDR76/CBX1/PHC2/HDAC1/INCENP/CBX3/TCP1/DNMT3A/SIRT7/PM
/ITGAX/TCIRG1/DOK3/FPR1/SIRPA/CD58/DYNLL1/NBEAL2/NCKAP1L/ADAM8/SLC11A1/RAC1/LAM
18A/RPL18/RPL38/LARP4B/RPL7A/RPS23/RPL6/RPL36/RPL19/DHX9/RPL32/RPL30/LARP1/RPL4
/TCP1/DNMT3A/SIRT7/KMT5C/CENPB/BEND3/CBX5/PCGF2/ESCO2/RNF2/NCAPD3
PXD2B/LCP1/PALLD/ADAM8/CTTN/SH3GL1/WDR1/VCL/BIN2/DBNL/PTPN12/VCAM1
RPE/TXNL4A/SNRPF/LSM7/LSM2/PRPF3/SNU13/PRPF6/SNRPG/PRPF31/SART1/LSM8/USP39/PRPF
RPE/TXNL4A/SNRPF/LSM7/LSM2/PRPF3/SNU13/PRPF6/SNRPG/PRPF31/SART1/LSM8/USP39/PRPF
CD/CFL1/ALOX5/MAP2/RUVBL1/NARF/PCNA/LMNA/CAD/NUP93/NUP205/ZNF703/BLM/NONO/ATF4/

CANT1/B4GALT5/FUT4/CHPF2/PSENEN/SORT1/B4GALT3/PITPNM1/ST3GAL4/BCAP31/LYPLA2/CLN
RB1/TSPAN15/LAMP5/HLA-DRA/STARD3NL/HLA-DMB/CHMP4B/VPS37C/TMEM165/MICALL1/CD63/C
S21/VBP1/RPL39/GCN1/MSI2/RPS3/MSI1/EIF3H/AGO2/CALR/RPL18A/FUS/RPL18/VIM/RPL38/D
ATP6V0B/ATP6V0D2/RAB32/TCIRG1/HLA-A/RAB7B/SLC11A1/RAB20/RAB10/ATP6V0C/HLA-B/ATP

COL3A1/LAMC1/COL5A1/LAMC2/LAMA5/ADAMTSL5/COL5A2/TNC/LUM/COL4A2/LAMB3/EMILIN1/CO
1/ANAPC7/CBX4/ANAPC11/BARD1/CBX8/PCGF2/RBX1/RNF7/RNF2/ANAPC5/CDC27/PCGF3/ANAPC4/
2/GINS4/PCNA/PRIM1/WDHD1/ZMIZ2/TIPIN/POLA1/PRIM2/RPA2/POLD3/TIMELESS/PRPF19/SMA

/HLA-DPA1/HLA-DPB1/HLA-DQB2/HLA-A/HM13/BCAP31/HLA-DQA2/HLA-DRB5/HLA-B/CALR/SPPL
/HLA-DPA1/HLA-DPB1/HLA-DQB2/HLA-A/HM13/BCAP31/HLA-DQA2/HLA-DRB5/HLA-B/CALR/SPPL
P7/NOP58/SNRNP40/POP1/SNU13/NOP10/GAR1/POP5
HLA-DQB1/HLA-DPA1/HLA-DMB/HLA-DPB1/HLA-DQB2/HLA-A/HLA-DQA2/HLA-DRB5/HLA-B
HSP90AB1/CCT4/CCT7/TCP1/BAG3/PPP5C/CCT8/PTGES3/BAG2/CDC37
EAD4/E2F5/HES6/ARNTL2/ARNT2/CDK4/TLE3/SOX12/MXD3/GTF3C6/NPAS2/DDIT3/HNF1B/TCF3/
ITGA3/PLAUR/SLC34A2/FOLR1/CD44/PLEKHO1/KCNJ11/ARHGEF2/ITGAV/SLC7A11/NME1/VASP/A
/DHTKD1/OGDHL
3/SERPINA4/APOH/SPP2
OA1
APOA5/APOC3
/APOA2/APOH/APOA1/APOA5/APOC3
/APOA2/APOH/APOA1/APOA5/APOC3

MC6/ITPR3/CLIC1/NUDT1/RAC2/LMNB2/ALOX5AP/CENPF/LMNB1/CYBB/GAPDH/CLGN/DTL/CMTM3/
ME1/PLEK/EZR/CFL1/ITGA5/PDE4A/LCP1/PAK1/CORO1C/PPP1R9B/PIP5K1C/HIP1R/FERMT1/RAC
COL11A1
COL11A1
4/APOB/APOC1/APOA2/APOF/APOH/LCAT/PON1/HPR/APOA1/APOA5/APOC3
L3/ZYX/MICALL2/FSCN1/LCP1/TPM3/LIMCH1/PDLIM3/SH2B2/CORO1B/VIL1/MST1R/SIPA1L3/BA
RPF/LSM7/SF3B5/SNRPG/ZMAT5/PHF5A/ZRSR2/SF3B3/DHX15/SNRNP25/SF3B2/SF3B6/SNRPD3
HLA-DRB1/HLA-DQA1/NRGN/UNC13D/HLA-DRA/HLA-DQB1/TFRC/HLA-DPA1/AREG/DNAJC5/HLA-DP

1/CD74/HLA-DRB1/HLA-DQA1/NRGN/HLA-DRA/HLA-DQB1/TFRC/HLA-DPA1/AREG/DNAJC5/HLA-DP
-DQA1/NRGN/HLA-DRA/HLA-DQB1/TFRC/HLA-DPA1/AREG/DNAJC5/HLA-DPB1/CLTA/HSPA8/HLA-D

POM/APOC4/LIPC/SAA4/APOB/APOC1/LPA/APOA2/APOF/APOH/LCAT/PON1/HPR/APOA1/APOA5/AP
POM/APOC4/LIPC/SAA4/APOB/APOC1/LPA/APOA2/APOF/APOH/LCAT/PON1/HPR/APOA1/APOA5/AP

UTP14A/SNU13/NOC4L/UTP11/UTP4/UTP6/HEATR1/RPS7/NOL6/UTP20/NOL10/UTP23/WDR46/IMP
LAMP5/HLA-DQA1/NRGN/HLA-DRA/SYNGR2/HLA-DQB1/RAB11FIP5/HLA-DPA1/DTNBP1/AREG/SYNG
POM/APOC4/LIPC/SAA4/APOB/APOC1/LPA/APOA2/APOF/APOH/LCAT/PON1/HPR/APOA1/APOA5/AP
PM/NCKAP5L/CAMSAP2/KNSTRN/MYO5A/CKAP5/DCTN1
HMP4B/CLTA/AP4M1/COPE/HIP1R/COPZ1/AP3M2/NECAP2/CLTB/AP2M1/NCALD/AP2S1/EPS15L1/V
HMP4B/CLTA/AP4M1/COPE/HIP1R/COPZ1/AP3M2/NECAP2/CLTB/AP2M1/NCALD/AP2S1/EPS15L1/V
SF3B5/RBMX2/SF3A3/SNRPG/SNRPB2/PHF5A/HTATSF1/SF3B2/SF3B6/SNRPD3

/MLYCD/ACOT2/NUDT7/ECI2/ACOX1/ACAA1/HSD17B4/AMACR/GRHPR/SCP2/PHYH/FABP1/CAT/EPH
/MLYCD/ACOT2/NUDT7/ECI2/ACOX1/ACAA1/HSD17B4/AMACR/GRHPR/SCP2/PHYH/FABP1/CAT/EPH
/TAX1BP3/E2F5/CCDC137/TCOF1/EZR/ANKRD1/NOP56/CD2BP2/APEX2/PES1/DKC1/CDCA7L/CD3E
T80/NME1/NME2/KRT7/IFFO2/SLC1A4/PLEC/NARF/LMNA/KRT18/KRT8/FAM83H/EVPL/NES/MYO5A

/NOC4L/RRP7BP/UTP4/UTP6/HEATR1/KRI1/NOL6/UTP20/FAM207A/BMS1/RSL1D1/IMP4

RM1/PSMB4/PSMB8/PSMB10/PSMA5/DNAJB2/PSMD13/PSMC4/USP14/PSMD10/PSMD11/PSMD1/PSMA
OC/PITPNM1/PSTPIP1/RAB11FIP4/PPP1CC/CIT/PLK4/KLHDC8B/RALA/HTR3A/NDE1/NF2/SPIRE1
ICALL2/FSCN1/LCP1/TPM3/STX1A/LIMCH1/PDLIM3/SH2B2/CORO1B/MST1R/SIPA1L3/BAG3/FHOD
SH3BP1/SAPCD2/MISP/RHOV/CORO1A/MARCKS/AGTRAP/FLNA/CIB2/TPM4/COTL1/TRIP10/MAPRE1
D74/HLA-DRB1/HLA-DQA1/NRGN/PACS1/HLA-DRA/HLA-DQB1/RHOQ/HLA-DPA1/AREG/HLA-DPB1/C
/CIB2/TPM4/COTL1/MAPRE1/EZR/DCDC2/CFL1/EPB41L2/WIPF3/FLOT2/FCHSD1/TIGD5/WIPF1/H
LR2H/PRIM1/TAF9/CDK7/POLA1/POLR2L/KAT2A/PRIM2/POLR2K/POLR3D/POLR3H/POLR3C/TAF12
A1/HLA-DRA/SCAMP3/HLA-DQB1/HLA-DPA1/HLA-DPB1/CLTA/TMEM165/HLA-DQB2/AP4M1/STX6/S
APH2
19/DECR2/ACBD5/MGST1/ALDH3A2/SYT7/PEX11A/PEX11G/ACOX1/PXMP2/ACSL6/HSD17B4/PECR/
19/DECR2/ACBD5/MGST1/ALDH3A2/SYT7/PEX11A/PEX11G/ACOX1/PXMP2/ACSL6/HSD17B4/PECR/
SNAP25/CDK16/RACGAP1/CBX6/NPTX2/HCK/EZR/GNA15/TRAF2/CHMP4B/TRAF5/IQGAP1/GNAZ/CD
LR2H/PRIM1/TAF9/CDK7/POLA1/POLR2L/KAT2A/PRIM2/POLR2K/POLR3D/POLR3H/POLR3C/TAF12
P3M2/NECAP2/CLTB/AP2M1/NCALD/AP2S1/EPS15L1/AP1G2/AP1B1/AP2A2/AP3B1/CLTC/AP1S1/A
AX/ITGB5/LYN/ITGA2/ITGB8/EMILIN1
C2/ARPC3/CBX3/AUNIP/WAS/PARP1/CBX5/RFWD3/RPA2/TIMELESS/ARPC1A/PRPF19/ESCO2/ARPC
FCHO1/CD74/ITGAV/HLA-DRB1/RAB31/SYK/HLA-DQA1/TAP1/ZYX/HLA-DRA/NCF4/HLA-DQB1/FMN
11/ARHGEF2/ITGAV/NME1/VASP/ADORA1/PLEK/LAMP5/EZR/PLEK2/CFL1/ITGA5/PDE4A/LCP1/PA
1/ALOX5/RUVBL1/CAD/ZNF703/BLM/NONO/KRT8/ATN1/XPOT/AKAP8L/SFPQ/DNMT3A/PML/NSMF/R
LR2H/PRIM1/TAF9/CDK7/POLA1/POLR2L/KAT2A/PRIM2/POLR2K/POLR3D/POLR3H/POLR3C/TAF12
D3/RHOC/RHOF/PITPNM1/RTKN/PSTPIP1/RAB11FIP4/PPP1CC/CIT/PLK4/KLHDC8B/RALA/HTR3A/
RM1/PSMB4/PSMB8/PSMB10/PSMA5/DNAJB2/PSMD13/PSMC4/USP14/PSMD10/PSMD11/PSMD1/PSMA

RPING1/ORM1/ORM2/FGB/F5/FGG/FGA/SERPINF2/ALB/IGF2/KNG1/A1BG/PLG/AHSG/HRG
AMP5/HLA-DRB1/RAB31/HLA-DQA1/HLA-DRA/SCAMP3/HLA-DQB1/CHST4/GRN/HLA-DPA1/RAB32/H
D3/RHOC/PITPNM1/RTKN/PSTPIP1/RAB11FIP4/PPP1CC/CIT/PLK4/KLHDC8B/RALA/HTR3A/NDE1/
/MYO1G/CDC42SE2/RACK1/BIN2/DNM2/SNX5/ABCA7/SYT11/ARHGAP25/ANXA1
B1/RAB31/HLA-DQA1/TAP1/HLA-DRA/HLA-DQB1/RAB34/ATP6V0B/HLA-DPA1/ATP6V0D2/RAB32/C
1/ATP6AP1/ATP6V1H/ATP6V1E1/ATP6V0C/ATP6V0E1/TMEM199/ATP6V0D1
FAIP6/CHIT1/TIMP2/CANT1/FTH1/PRSS3/ILF2/ASAH1/CTSS/PTPN6/GSDMD/CNN2/ALDOC/CTSD
A2/LUM/COL4A2/COL11A1
17C/SCAMP3/FZD7/RAB11FIP5/MICALL1/HLA-A/RAP2B/SCAMP4/RAB35/EHD1/RAB11FIP4/RAC1/

MTA3/CSNK2A1/MBD3/GATAD2A
MTA3/CSNK2A1/MBD3/GATAD2A
IP1
IP1
NPU/KIF20B
/FCHO1/CD74/RGS19/HLA-DRB1/HLA-DQA1/NRGN/UNC13D/PACS1/HLA-DRA/HLA-DQB1/TFRC/HLA
A2/PFN2/MICAL1/SRC/ITGA3/RGS10/NCS1/DNM1/FABP5/CLSTN1/ATP1A1/PPT1/GPC4/STX3/C1Q
PDH1/PYCARD/LGALS3BP/LCN2/ANXA2/GLA/CTSC/VEGFB/TIMP1/FABP5/PYGB/TGFB1/GSTP1/COT
/SNRNP40/PRPF6/SNRPG/DDX23
A-DQB1/HLA-DPA1/HLA-DPB1/CLTA/HLA-DQB2/CTLA4/RAB35/HIP1R/HLA-DQA2/FZD2/HLA-DRB5
ARPC5/ACTR3
ELESS
/KNSTRN/MYO5A/CKAP5/DCTN1

D1/RUVBL1/TAF6/MCRS1/WDR77/SNRPE/SNRPF/HDAC2/HCFC1/TRMT61A/KDM5B/TAF9/TRMT6/KDM
/ITIH3/SERPINA4/APOH/SPP2
N/OIP5/PLCB1/ALYREF/GLIS2/AP5Z1/CARMIL1/TCF3/NR0B1/SNRPA1/STK17A/SF3A2/SDCBP2/C
C/TUBA1B/ATP1A1/ITGAM/PPT1/BIRC3/ITGB2/MAL2/LAT2/RGS19/PRNP/HCK/GPC1/TUBB/SERPI
2/FSCN1/LCP1/TPM3/LIMCH1/PDLIM3/SH2B2/CORO1B/MST1R/SIPA1L3/BAG3/FHOD1/CNN2/TRIP
2/FSCN1/LCP1/TPM3/LIMCH1/PDLIM3/SH2B2/CORO1B/MST1R/SIPA1L3/BAG3/FHOD1/CNN2/TRIP
C/TUBA1B/ATP1A1/ITGAM/PPT1/BIRC3/ITGB2/MAL2/LAT2/RGS19/PRNP/HCK/GPC1/TUBB/SERPI
SYTL1/MUC20/DPEP1/SLC26A2/CDHR2

8
5/SLC6A9/LAMP5/MTMR2/UNC13D/SYNGR2/GIPC1/DTNBP1/SYNGR1/DNAJC5/TRAPPC4/BRSK1/RAB
BP3/E2F5/CCDC137/TCOF1/EZR/ANKRD1/NOP56/CD2BP2/APEX2/DKC1/CDCA7L/CD3EAP/B3GNT9/
ROS1/CD36/VWF/SERPING1/ORM1/ORM2/FGB/F5/FGG/SERPINA5/FGA/SERPINF2/ALB/IGF2/KNG1

PSMD11/PSMD1/PSMD4/PSMD3/PSMD7/PSMD8/PSMD9/PSMC3/PSMC5

AX/ITGB5/CTLA4/LYN/ITGA2/ITGB8/EMILIN1
A9/LAMP5/MTMR2/SYNGR2/GIPC1/DTNBP1/SYNGR1/DNAJC5/TRAPPC4/BRSK1/RAB15/CLTA/SYT8/
C/TUBA1B/ATP1A1/ITGAM/PPT1/BIRC3/ITGB2/MAL2/LAT2/RGS19/PRNP/NUP62/HCK/GPC1/TUBB
D2/MISP/CORO1A/FLNA/CIB2/TPM4/COTL1/MAPRE1/ASPH/EZR/DCDC2/CFL1/EPB41L2/WIPF3/FL
SNAP25/CDK16/RACGAP1/CBX6/NPTX2/HCK/EZR/GNA15/TRAF2/CHMP4B/TRAF5/FKBP1A/IQGAP1/
SFL1C
ACK1/MCTS1/RPS7/RPS23/RPS10/RPS15/RPS16/RPS27A/RPS12/RPS4X/UBA52/RPS11/RPS9/RPS
FUT4/CHPF2/PSENEN/SORT1/B4GALT3/PITPNM1/ST3GAL4/BCAP31/UXS1/B4GALT7/B4GALT2/FUT
ITGA3/RGS10/NCS1/GPRIN1/DNM1/ARHGAP4/AURKA/STX3/SNAP25/CPLX2/KCNJ11/P2RX4/HSPB1

PSENEN/SORT1/B4GALT3/PITPNM1/ST3GAL4/BCAP31/UXS1/B4GALT7/B4GALT2/FUT3/GOLGA3/FU
X12/MXD3/GTF3C6/DDIT3/TCF3/TRIM28/HIF1A/PYGO2/BCL9/TAF6/CD3EAP/E2F8/E2F3/E2F4/P
4/MRPL13/MRPL10/MRPL52/MRPL48/DAP3/MRPS7/MRPL53/MPV17L2/MRPS17/MRPS21/MRPS15/MR
4/MRPL13/MRPL10/MRPL52/MRPL48/DAP3/MRPS7/MRPL53/MPV17L2/MRPS17/MRPS21/MRPS15/MR
7/CD3E/CARD11/LCK/SNX27/CD6/GZMB/GZMA/STX7/BCL10

2L1/HSP90B1/HSPA13/P4HB

PDH1/PYCARD/LGALS3BP/LCN2/ANXA2/GLA/CTSC/VEGFB/TIMP1/FABP5/PYGB/TGFB1/ADA/GSTP1
1/MELK/ANLN/SH3BP1/CENPF/SAPCD2/AKR1B1/MISP/RHOV/ADA/CORO1A/MARCKS/AGTRAP/FLNA/
GPRIN1/ARHGAP4/STX3/SNAP25/CPLX2/P2RX4/CBX6/IGF2BP1/ADORA1/LAMP5/NPTX2/GIT1/TWF

/DTL/DCAF13/TRAF2/RBCK1/FBXL19/CACYBP/MAD2L2/RNF19B/SHARPIN/PHC2/BRCA1/MED10/CK
FBXW9/FTSJ3/PPAN/LAS1L/EBNA1BP2/EIF6/NIP7/RPF1/WDR12/ZNF622/RRP15/MAK16/WDR74
M1/AP2S1/VPS18/AP1G2/AP3D1/AP1B1/AP2A2/AP3B1/AP1S1/AP1S2/AP2B1/AP2A1/VPS33A/AP5
OT4/PEX1/NOS2/PEX19/DECR2/CRAT/ECH1/ACBD5/MGST1/LONP2/HMGCL/MLYCD/ALDH3A2/SYT7/
OT4/PEX1/NOS2/PEX19/DECR2/CRAT/ECH1/ACBD5/MGST1/LONP2/HMGCL/MLYCD/ALDH3A2/SYT7/
3/SOX12/DDIT3/POLA2/GINS4/PRKDC/NUPR1/ACD/NPM1/PCNA/LEF1/POLR2H/PRIM1/PARP1/DON
RPF31
PG1/TMEM199/COPG2/PACSIN1/COPB2/COPB1/ARF1/COPA/ACTR1A/CCDC88A/ARCN1/ARFGAP3/SC
SMD10/PSMD11/PSMD1/PSMD4/PSMD3/PSMD7/PSMD8/PSMD9/PSMC3/PSMC5
NRPF/SNRPG/SNRPD3/PRMT5/ERH
/WRAP53/NOP10/GAR1/HNRNPU/NVL/SNRPD3/SMG7/HNRNPC
L1/AP2A2/CLTC/AP2B1/AP2A1
PDH1/PYCARD/LGALS3BP/LCN2/ANXA2/GLA/CTSC/VEGFB/TIMP1/FABP5/PYGB/TGFB1/ADA/GSTP1
/COTL1/DCDC2/CFL1/EPB41L2/WIPF3/FLOT2/FCHSD1/TIGD5/WIPF1/HIP1R/LASP1/FLOT1/CAP1

PZP/PROS1/APOE/FCN3/FCN2/SERPING1/ORM1/C1S/HBB/ORM2/CFB/C3/CFH/ANGPTL4/VTN/FGB/

CBX4/RBBP4/MTF2/CBX8/PCGF2/RBBP7/CSNK2A1/RNF2/CSNK2B/TRIM37/PCGF3/CSNK2A2/ASXL1
/NUTF2/NUP210/NUP93/NXT1/NUP205/NUP37/NDC1/NUP188/KPNB1/XPOT/NUP85/NUP43/AGFG1/
/ESCO2
/RPS20/RPS19/RACK1/MCTS1/DAP3/MRPS7/RPS7/RPS23/RPS10/MRPS17/MRPS21/RPS15/RPS16/
CNE1/CCNA2/CCNF/POLD1/MCM3/CDK4/CDKN2D/POLA2/MAD2L2/TAF6/POLR2G/PCNA/CD3EAP/SOC
ABRA3/GABRR3/BEST1/TTYH2/GABRD/TTYH1/CFTR/GABRB3/CLCNKA

2/CCNF/CDK4/CDKN2D/PCNA/CKS1B/CCNO/CDK2/CDK5/CDK7/CCND2/IKBKG/ACVR1/CCND3/ACVR1
RPC/NOP58/ISG20/PRPF3/PRPF31/FAM118B/SART1/WRAP53/NOP10/GAR1/ZPR1/HSPB7/SART3/T

MA7/PSMB3/PSMA1/PSMB5/PSMA4/PSMB1/PSMB6/PSMA3/PSMB7

AP25/CBX6/IGF2BP1/LAMP5/NPTX2/TWF2/DLG3/NECTIN1/TRPV2/ORAI2/KIF21B/DTNBP1/FSCN1

CAPD3/SMC2
LC35A2/A4GALT/CHST2/TPST1/POMGNT1/CHST12/MFNG/LFNG/B4GALNT2/SYS1/SLC35B1/SLC35A
1/ATP6AP1/ATP6V1H/ATP6V0C/ATP6V0E1/TMEM199/ATP6V0D1
S1/AP1G2/AP1B1/AP2A2/AP3B1/AP1S1/AP2B1/AP2A1/AP4B1/HIP1/AFTPH/AP3M1
OT2/EFNA5/CD2/THY1/RTN4RL2/ULBP2/PRSS22/FOLR2/LYPD3
AX/UQCRH/MRPL17/CYC1/TIMM17B/MRPS12/MRPL47/MRPL9/TOMM5/UQCC3/MCU/DLAT/VDAC1/MRP

QSTM1/ANKRD13D/CD74/HLA-DRB1/TSPAN15/LAMP5/UNC13D/HLA-DRA/STARD3NL/GRN/DDIT3/HL
OMER3/CRYAB/DBN1/REG1A/NRSN2/TMPRSS3/ITPR3/RGS10/ZWINT/ACSL4/NCS1/ATP13A2/DNM1/
PR1/C3AR1/CD68/CPNE1/LAMTOR1/NFAM1/ATP6V0C/RAB3D/VAMP8/RAB5C/PSAP/GLIPR1/MANBA/
16/RACGAP1/HCK/GNA15/IQGAP1/GNAZ/GNA12/GNGT1/PLEKHA4/LYN/GNG5/GNB1/FGR/LCK/STAC
2/NUP93/NUP205/NUP37/NDC1/NUP188/DYNLT1/KPNB1/CD209/NUP85/NUP43/VPS28/RAE1/MVB1
2/NUP93/NUP205/NUP37/NDC1/NUP188/DYNLT1/KPNB1/CD209/NUP85/NUP43/VPS28/RAE1/MVB1

AP25/CBX6/IGF2BP1/LAMP5/NPTX2/TWF2/DLG3/NECTIN1/TRPV2/ORAI2/KIF21B/DTNBP1/FSCN1

1/DNM1/FABP5/CLSTN1/ATP1A1/ARHGEF2/P2RX4/NCK2/SLC6A9/ADORA1/PRNP/ABHD17C/MTMR2/

5/ABHD17C/UNC13D/SCAMP3/FZD7/RAN/RAB11FIP5/MICALL2/TFRC/RAB25/MICALL1/HLA-A/RAP
D1/PIEZO1/CSPG4/PDPN/FAP/EPHA2/ANTXR1/DOCK8/NCKAP1

21A/MTA2/CHD4/RBBP4/SAP30/CBX5/NCOR2/TBL1XR1/RBBP7/RERE/MTA3/DNTTIP1/HDAC10/CSN
MB5/PSMB1/PSMB6/PSMB7
E1
2/DECR2/CRAT/ECH1/ACBD5/MGST1/ISOC1/LONP2/HMGCL/SOD1/MLYCD/ALDH3A2/SYT7/ACAD11/
OBEC3C/IQGAP1/LSM4/MCRIP1/POLR2G/TUBA1A/SOCS1/YBX1/PABPC1/PITPNM1/ZC3H12A/CNOT9
2/DECR2/CRAT/ECH1/ACBD5/MGST1/ISOC1/LONP2/HMGCL/SOD1/MLYCD/ALDH3A2/SYT7/ACAD11/
TN1/CORO1A/GPC4/STX3/YWHAZ/FLNA/SNAP25/CPLX2/EIF4EBP1/ARHGEF2/HRAS/PLCB1/ABHD17
/PRR14/CASK
E3/ADRM1/PSMB4/PSMB8/PSMB10/PSMA5/DNAJB2/TAF9/F3/PSMD13/PSMC4/USP14/PSMD10/PSMD

/FABP5/CLSTN1/ATP1A1/ARHGEF2/P2RX4/NCK2/SLC6A9/ADORA1/PRNP/ABHD17C/MTMR2/NRGN/R

SORT1/RAB35/HIP1R/VLDLR/CTTN/NECAP2/CLTB/AP2M1/AP2S1/EPS15L1/ARRB2/APP/OCRL/DNM
N/POLA1/PRIM2/RPA2/POLD3/PRPF19/RPA1/SMARCAL1/HELB/POLD2/PURB/BCAS2/POLD4/PLRG1

SNRPG/STRAP/GEMIN2/SNRPD3/DDX20/GEMIN8/GEMIN6/SMN2/GEMIN5
4A5/LAMB1/APLP1/LAD1/ENTPD2/CD151/LAMC1/COL5A1/FRAS1/EFNA5/LAMC2/LAMA5/TNC/LOXL
/ACTR3/WIPF2/GMFB/CAPZA2/ACTR2/HIP1
/ACTR3/WIPF2/GMFB/CAPZA2/ACTR2/HIP1
0/MRPL52/MRPL48/MRPL53/MPV17L2/MRPL18/MRPL37/NSUN4/MRPL27/MRPL28/MRPL51/MRPL11/
0/MRPL52/MRPL48/MRPL53/MPV17L2/MRPL18/MRPL37/NSUN4/MRPL27/MRPL28/MRPL51/MRPL11/
1/SLC6A9/ABCD1/FUNDC2/TOMM40/CHST4/MCUB/TMEM201/SYNGR1/VOPP1/DNAJC5/FUNDC1/ABCB

/CDK1/CDC45/CKS2/CENPF/ORC1/CHAF1B/RAD51/EXO1/CBX2/NCAPD2/UHRF1/PHF19/ZIC2/PKN1
CCNE1/TRIB3/TONSL/PKN1/RELB/SMARCD3/HES6/ELF3/EZH2/NME2/HDAC7/MUC1/TLE3/ANKRD1/
T1/CDK1/MELK/NEK2/MAPK12/BUB1/TYRO3/BUB1B/SRC/MMD/MAPK13/CCNE1/CCNA2/PBK/PNCK/C
CRYAB/COL1A1/ARPC1B/POSTN/MFAP2/KRT23/TUBA1B/RPL39L/TUBA3C/MPZL1/FBLN1/LMNB1/MP
GS2/ATP1B3/RGS1/EPO/NCF2/CTSC/SH3BP1/RGS10/CKS2/ALOX5AP/DEPDC1B/FBLN1/ECT2/TGFB
SB10/S100A6/CAPG/PYCARD/KIF4A/RGS2/KIF20A/S100A4/CRYAB/MARCKSL1/ANXA2/DBN1/ANLN
PFKP/AURKB/TK1/IKBKE/PKMYT1/CDK1/MELK/NEK2/MAPK12/BUB1/TYRO3/BUB1B/SRC/MMD/MAPK
IF20A/TOP2A/TUBA1C/RGS1/RECQL4/TUBA4A/MCM2/CDC45/PFN2/GINS1/ANXA5/KIF23/TUBA1B/
IF20A/TOP2A/TUBA1C/RGS1/RECQL4/TUBA4A/MCM2/CDC45/PFN2/GINS1/ANXA5/KIF23/TUBA1B/
IF20A/TOP2A/TUBA1C/RGS1/RECQL4/TUBA4A/MCM2/CDC45/PFN2/GINS1/ANXA5/KIF23/TUBA1B/
IF20A/TOP2A/TUBA1C/RGS1/RECQL4/TUBA4A/MCM2/CDC45/PFN2/GINS1/ANXA5/KIF23/TUBA1B/
/AURKB/TPX2/FOXM1/KIF20A/TOP2A/E2F1/SOX9/CDC6/SLC2A1/TUBA4A/ANXA5/CDC25B/MICAL1
XM1/KIF20A/TOP2A/E2F1/SOX9/TUBA4A/ANXA5/CDC25B/MICAL1/SRC/PRC1/ARHGEF16/NCS1/CK
BAK1/SH3BP1/MICAL1/SRC/ITGA3/ARHGEF16/TCEAL9/PLAUR/DNM1/CCNA2/ATP1A1/ENAH/RCC2/
/CCNB1/PTTG1/APOA4/CXCL1/RAP1GAP/CCNB2/PYCARD/PKIB/RGS2/RENBP/ATP1B3/RGS1/EPO/A
CAPG/PFKP/ENO1/ANXA2/DBN1/ICAM1/ANLN/POSTN/EPCAM/ITGB4/SRC/ITGA3/CLIC1/ARHGEF16
B3IL1/ANLN/SH3BP1/MICAL1/ARHGEF16/NDRG1/ECT2/IQGAP3/RCC2/ARHGAP4/FLNA/PKN1/CHML
1/DBN1/ANLN/ARPC1B/PFN2/MICAL1/MISP/POF1B/PDLIM7/FLNC/ENAH/CORO1A/MARCKS/FLNA/F
/NEK2/MAPK12/BUB1/BUB1B/MAPK13/PBK/PNCK/TTK/AURKA/STK26/RIPK2/SQSTM1/IRAK1/CDK1
CM2/PFN2/ANXA5/KIF23/ATP13A2/KIFC1/RFC4/KIF18B/ATP1A1/RAD51/RAD54L/TNNT1/MCM6/D
/ANLN/SH3BP1/MICAL1/ARHGEF16/NDRG1/ECT2/IQGAP3/RCC2/ARHGAP4/FLNA/PKN1/CHML/ARHG
/ANLN/SH3BP1/MICAL1/ARHGEF16/NDRG1/ECT2/IQGAP3/RCC2/ARHGAP4/FLNA/PKN1/CHML/ARHG

EPDC1B/ECT2/ARL2/ARHGAP4/RACGAP1/CHML/NCAPH/ADAP1/PLCB1/DEPDC1/GMIP/GIT1/ASAP1/

2/KIF20A/CRYAB/STMN1/PRC1/KIF23/NDRG1/DNM1/KIFC1/KIF18B/NUSAP1/SKA1/RCC2/GAPDH/

PFKP/ENO1/ANXA2/DBN1/ANLN/EPCAM/SRC/CLIC1/ARHGEF16/NDRG1/YWHAZ/FLNA/FXYD5/PLIN3

RGS10/NUDT1/RAC2/TUBA3C/DNM1/ARL2/RHOV/ARL4C/TUBG1/RGS19/RAB42/HRAS/RAB31/TUBB/

5/VEGFB/TFF2/LIF/CSF1/S100A14/TGFB1/BAMBI/CLCF1/GDF15/SYK/CKLF/PGF/IL18/LTB/TRA

ECT2/ARL2/ARHGAP4/RACGAP1/CHML/ADAP1/PLCB1/DEPDC1/GMIP/GIT1/ASAP1/ADAP2/TBC1D16

ECT2/ARHGAP4/RACGAP1/CHML/ADAP1/PLCB1/DEPDC1/GMIP/GIT1/ASAP1/ADAP2/TBC1D16/RANG

TIMP1/LIF/CSF1/TGFB1/CMTM7/CLCF1/GDF15/CMTM3/CKLF/IL18/TNFRSF11B/GRN/LTB/AREG/C

YAB/PRC1/KIF23/NDRG1/DNM1/KIFC1/KIF18B/NUSAP1/SKA1/RCC2/GAPDH/PSRC1/CLIP2/FCHO1

4/RFC4/RAD51/EXO1/NEIL3/RAD54L/MCM6/FEN1/DSCC1/GINS2/POLD1/MCM7/NME1/PIF1/MCM3/

1A/MARCKS/FLNA/TPM4/COTL1/USH1C/TWF2/EZR/FMNL1/TMEM201/CFL1/MICALL2/FSCN1/WIPF3

QGAP3/RCC2/ARHGAP4/FLNA/PKN1/ARHGEF2/ITPKA/CDC42EP1/FGD3/FMNL2/FMNL1/ARHGEF39/Y

/RAC2/CCNE1/CCNA2/TRIB3/CCNF/TGFB1/CDKN2A/HSPB1/CDK4/CDKN2D/CCL5/IQGAP1/EFNA5/M

NPF/FBLN1/RAD51/STIP1/CORO1A/KCNJ11/TAX1BP3/HRAS/MAPRE1/MAD2L1/DLG3/EZR/MID1IP1

/NCAPD2/UHRF1/PHF19/PKN1/RCC1/YEATS2/PYGO2/VPS72/NPM1/MLLT3/SMARCA4/CKS1B/STAT1

RPL47/MRPL9/RPL17/RPLP2/MRPS23/RPSA/RPL10L/RPS5/RPS21/MRPL12/RPL7/RPL28/MRPS34/

MCM7/NME1/MCM3/RAD51AP1/HSF1/NABP2/MCM5/HMGB2/RBBP8/POLR2G/YBX1/DDX11/SUB1/REXO

SMG5/MVP/DLG3/CSF1R/TRAF2/JAK3/IQGAP1/SLC9A1/SIRPA/PPP6R1/STAT1/PPP1R9B/PPP1CA/

H/VASP/ADAM9/ADAM15/SH2D2A/DNAJC6/NCF1/WIPF3/WASF2/CD3E/SIRPA/WIPF1/HIP1R/SH3KB

PFDN4/CCT6A/CCT3/HSPA6/PDRG1/HSPA8/NUDC/NPM1/CHAF1A/PPIH/CCT2/HSP90AB1/PPIA/CCT

ITGB2/CIB2/S1PR2/FERMT3/ITGAV/LAMB1/ADAM9/SYK/ADAM15/CD151/COL16A1/COL3A1/COMP/

LC2/KIF11/WDR34/KIF12/KIF3C/KIF18A/KIF21B/CENPE/KLC3/DYNLRB1/DYNLL1/DYNC1H1/KIF

/MCM7/PIF1/MCM5/CHTF18/CHD3/XRCC2/SMARCA4/XRCC3/ERCC6L/RFC2/DDX11/BLM/CDK7/MSH2

BSG/BAIAP2L1/NRCAM/SIRPA/PALLD/KRT18/PPP1CA/PAK4/CD47/GLDN/RAB10/TACSTD2/EMB

2L1/KRT18/PPP1CA/PAK4/RAB10/TACSTD2

SMARCA4/HSP90AB1/LONP1/PTGES3/NAT10/HSP90AA1/CDK2AP1

100B/S100A13

PFKP/AURKB/TK1/IKBKE/PKMYT1/CDK1/MELK/NEK2/MAPK12/UAP1L1/BUB1/TYRO3/BUB1B/SRC/M

XOSC4/IGF2BP3/ZNF385A/ESRP1/YBX3/CSTF2/PCBP4/SLPI/UPF3B/RBM3/EIF2S2/RPS2/PABPC1

/EIF4EBP1/SMG5/MVP/SYK/DLG3/CSF1R/TRAF2/JAK3/IQGAP1/PPP1R18/SLC9A1/SIGLEC10/MAP

/RAD54L/MCM6/DDX39A/DSCC1/GINS2/MCM7/PIF1/MCM3/MCM5/DHX34/GINS4/DHX37/RUVBL1/CH

/NAA10/SNRPA/SEC61A1/OLA1/CD2BP2/PES1/NPM1/PPIH/RBM3/PELO/PPP1CA/ZC3H12A/RPSA/S

/MCM6/DSCC1/GINS2/MCM7/PIF1/MCM3/MCM5/GINS4/RUVBL1/CHTF18/CHD3/ERCC6L/RFC2/DDX1

/RAC2/CCNE1/CCNA2/TRIB3/CCNF/TGFB1/GSTP1/CDKN2A/HSPB1/CDK4/CDKN2D/CCL5/IQGAP1/E

LCB1/TWF2/ASAP1/ADAP2/FZD7/PLEK2/NCF1/TPCN1/SH3PXD2B/SLC9A1/SDCBP2/RAB35/HIP1R/

N1/ARHGEF2/ITPKA/FMNL1/VAV1/IQGAP1/PAK1/CORO1C/DVL3/NCKAP1L/PAK4/ARHGDIB/WAS/DV

10/MCM7/MCM3/MCM5/DDX11/POLA1/MCM8

M5/CHTF18/CHD3/RFC2/POLQ/DNA2/RAD18/RFC5/DHX9

M5/CHTF18/CHD3/RFC2/POLQ/DNA2/RAD18/RFC5/DHX9

PFDN2/PFDN1/DFFA/CALR/HSPA2/HSPA5/HSPA14

M5/CHTF18/CHD3/RFC2/POLQ/DNA2/RAD18/RFC5/DHX9

S100B/ANXA11
TTN

BSG/BAIAP2L1/NRCAM/SIRPA/PALLD/KRT18/PPP1CA/PAK4/RPSA/ITGA2/CD47/GLDN/RAB10/TAC

2C8/CYP3A4

M/ACADL/ACADSB
/MCM5/GINS4/BLM
M5/CHTF18/RFC2/POLQ/DNA2/RAD18/RFC5/DHX9
M5/CHTF18/RFC2/POLQ/DNA2/RAD18/RFC5/DHX9
P1A2/CYP2C8/CYP2C9/CYP3A4

P4F12/CYP3A43/CYP3A5/CYP2A7
PFKP/AURKB/IKBKE/PKMYT1/CDK1/MELK/NEK2/MAPK12/BUB1/TYRO3/BUB1B/SRC/MMD/MAPK13/C
A1B/TRIB3/MAGEC2/AURKA/SQSTM1/YWHAZ/SMG5/TUBB/FZD6/DLG3/SCAMP3/RANGAP1/TRAF2/TR
/MAPK8IP2/ADAP2/SH2D2A/DOK2/SMARCD1/FSCN1/TRIM6/IQGAP1/ARPC4/SLC9A1/SH2D3A/HSPA
1/HLA-DQB2/HLA-DQA2
DH1A1/ALDH7A1/ALDH1L1/ALDH2
YP4F2/CYP2B6/CYP4A11/CYP2E1/CYP2C8/CYP2C9/CYP2A6
27A2/ACSM5/ACSM2B/ACSM2A

DH/SCP2/ACADL/ACOT12/ALDH6A1
C/CES3/AADAC
3/SCP2/ACSM5/ACSM2B/ACSM2A
G5/APOA2/APOA1/APOA5
G5/APOA2/APOA1/APOA5
YP2A7/CYP4F2/CYP2B6/CYP4A11/CYP2E1/CYP2C8/CYP2C9/CYP2A6
LC51A/SLCO1B3/SLCO1B1/SLC10A1
/SLCO1B3/SLCO1B1/SLC22A7
1B/ADH4
CSM5/ACSM2B/ACSM2A/SLC27A5
/ETNPPL/AGXT/TAT
/ABAT/ETNPPL/AGXT/TAT
IB3/MAGEC2/AURKA/SQSTM1/YWHAZ/SMG5/TUBB/FZD6/DLG3/SCAMP3/RANGAP1/TRAF2/TRIM28/C
MB/HSPA8/HSP90AB1/ANXA11/HLA-DOA/HSP90AA1
L5/ACSL1/SLC27A2/ACSM5/ACSM2B/ACSM2A/SLC27A5
LC6A12/SLC6A1/SLC13A5/SLC10A1
/HPD
DAC1/NOC2L/HDAC2/HMGN4/SSRP1/ACTB/MTA2/NAP1L4/SMARCB1/CHD4/RBBP4/SMARCC1/CABIN1

/HPD
SM3/ACSL5/ACSL1/SLC27A2/ACSM5/ACSM2B/ACSM2A/SLC27A5
P5/RBP4/ADH4
/RDH5/SRD5A2/RDH16/HSD11B1/HSD17B6
A/CDK4/CDKN2D/CKS1B/CCNO/CDKN2B/CDKN1C/CDKN2C/CCND2/CCND3/CCNJL/CCNE2/CDK5R1
YP3A43/CYP1A1/CYP3A5/CYP2A7/CYP1A2/CYP2B6/CYP2E1/CYP2C8/CYP2C9/CYP2A6/CYP3A4
COX2/AOX1/DAO
7A5/SLC1A4/SLC43A2/SLC7A8/SLC3A2/SLC7A10/SLC36A1/SLC38A5
/AKR1C4/RDH5/SRD5A2/RDH16/AKR1D1/HSD11B1/HSD17B6/HSD17B13
CC2/XRCC3/BLM/MSH2/RAD51C/ABL1/RAD51D/RAD18
3/S100A13/FIBP/FGFR2/RPS19/GLG1/THBS1
LC2/KIF11/WDR34/KIF12/KIF3C/KIF18A/KIF21B/MYL6B/CENPE/KLC3/DYNLRB1/DYNLL1/DYNC1

A14/CKLF/CCL5/CCL26/STAT1/CCL13/CNIH4/CCL2/CXCL9/CXCL3/CXCL13/DEFB1/CCL28/CCL4/

/LYN/FGR/LCK/SLA/MATK/BTK/ABL1/CSK/PTK2/TNK2
H3PXD2B/SIGLEC10/FGFR1/SYNGR3/LCK/PTPN6/SIT1/AFAP1/RACK1/ABL1/RUFY1/PTK2/INPPL1
RL2/RHOV/ADA/ARL4C/TUBG1/ARL14/RAB42/NME1/ADORA1/HRAS/RAB31/TUBB/MX2/NME2/GUCY2
RL2/RHOV/ADA/ARL4C/TUBG1/ARL14/RAB42/NME1/ADORA1/HRAS/RAB31/TUBB/MX2/NME2/GUCY2
YP2A13/CYP27A1/CYP39A1/CYP3A43/CYP1A1/CYP3A5/CYP2A7/CYP1A2/CYP7A1/CYP2B6/CYP2E1
HB2/PLEK/ADAP1/PLCB1/NRGN/TWF2/ASAP1/ADAP2/NCF4/FZD7/PLEK2/NCF1/TPCN1/IQGAP1/SH
AKR1C4/CYP4F12/CYP27A1/FMO4/FAXDC2/FMO5/CYP1A1/CYP4F3/FMO3/AKR1D1/CYP4F2/CYP7A1
SL5/ACSL1/SLC27A2/ACSM5/ACSM2B/ACSM2A/SLC27A5
O2/CYP3A7/CYP1A1/CYP3A5/CYP2A7/ALB/CYP2E1/CYP8B1/CYP3A4

D1/BHLHE41/BRMS1/GMNN/HIF1A/RFXANK/HSP90AB1/HDAC1/BEX4/LEF1/HDAC2/RAC1/AKAP8L/M
AADAT/GOT2/ALAS1/CSAD/GPT2/SHMT1/GPT/SDS/AGXT2/CTH/ABAT/ALB/ETNPPL/AGXT/TAT
AADAT/GOT2/ALAS1/CSAD/GPT2/SHMT1/GPT/SDS/AGXT2/CTH/ABAT/ALB/ETNPPL/AGXT/TAT

CLYBL/HOGA1/ACMSD/CSAD/SHMT1/PCK2/ALDOB/PCK1

/NR1H4/NR4A1/THRB/PPARA/RORA/ESR1/AR/RORC/NR1I2/NR1I3
H9A1/BCKDHB/ALDH1A1/AKR1C4/ALDH7A1/DHTKD1/ALDH5A1/ALDH1L1/OGDHL/ALDH2/ALDH8A1/A
1/AVPR1A/PIK3R1/CRHBP/LEPR/GHR/GCGR
/NR1H4/NR4A1/THRB/PPARA/RORA/ESR1/AR/RORC/NR1I2/NR1I3
2B1/ABCB11/SLC51A/SLCO1B3/RBP4/SLCO1B1/SLC22A1/AQP9/SLC10A1

2/IFNA8/IFNA6/IFNA16/IFNA10

AV/ADAM9/GPC1/ADGRG1/CD248/RPSA/ITGA2/CTSS/SPOCK2/SPOCK1/PLEKHA2/BGN/LYPD3/ECM1

CI2/EHHADH/GSTA1

SLC16A4/SLCO1A2/SLCO2A1/AKR1C4/SLC22A9/SLC6A13/SLC16A2/SLCO2B1/ABCB11/MFSD2A/SL
RL2/RHOV/ARL4C/TUBG1/ARL14/RAB42/NME1/HRAS/RAB31/TUBB/MX2/NME2/GUCY2C/RABL6/RHO
RL2/RHOV/ARL4C/TUBG1/ARL14/RAB42/NME1/HRAS/RAB31/TUBB/MX2/NME2/GUCY2C/RABL6/RHO
A4/SLC6A12/SLC6A1/GNMT
K5/GRB2/CBL/CHN1/ABL1/PTPN1/CDK5R1
CO1/CA4/CBS/ECHS1/ECHDC2/HSD17B4/CA14/ALAD/CA5A/ENO3/EHHADH/UROC1
P8/BRCA1/PCNA/PNKP/BLM/AUNIP/MSH2/RPS3/XRCC6/POLQ/MUTYH/OGG1/RPA2/CUL4B/DCLRE1B
1/PIGU/PLEKHB2/PLEK/ADAP1/PLCB1/NRGN/TWF2/ASAP1/ADAP2/NCF4/FZD7/PLEK2/NCF1/SNX7
CASP1/CARD16/CARD18/BCL10/MAVS
C1/NR1H4/NR4A1/ABHD2/NR0B2/NR3C2/THRB/PPARA/HNF4A/GPER1/RORA/ESR1/AR/RORC/NR1I2
HNSL2/SLC1A1/GLUL/GLUD1/ASS1/GOT2/TDO2/SHMT1/NAGS/DPYS/OTC/AGXT/CPS1/GNMT/TAT
DECR2/DECR1/ACADVL/BDH2/RETSAT/DHODH/ACAD11/ACADS/SRD5A1/ACOX1/ACAA1/IVD/GCDH/A
PYGL/OXER1/PPARA/HNF4A/ACACB/PC/CYP26A1/STARD5/ACOX1/SCP2/RIDA/FABP1/SERPINA5/A
H/HADH/NUDT6/GLUD1/ALDH1A1/QDPR/HSD17B8/GRHPR/GPD1/CRYL1/ALDH2/SORD/AOX1/ADH4

/ABCG8/ABCG5/APOA2/APOA1/APOA5
MB/HSPA8/HSP90AB1/ANXA11/CD8A/HLA-DOA/HSP90AA1
LA-DQB1/HLA-DPA1/HLA-DPB1/HLA-A/SLC7A8/CD209/HLA-DRB5/HLA-B
RL2/RHOV/ARL4C/TUBG1/ARL14/RAB42/NME1/HRAS/RAB31/TUBB/MX2/GUCY2C/RABL6/RHOG/TUB
/ADAP2/SH2D2A/DOK2/FSCN1/TRIM6/ARPC4/SLC9A1/SH2D3A/HSPA8/STX1A/SH2B2/HIP1R/LASP
VRK1/PRKAA2
/AASS/MTHFD1/PIPOX/DMGDH
C/KRT17/AGRN/TUBG1/TUBB/TUBB4B/MSN/ARPC4/PLEC/TUBB6/TUBA1A/TUBB2A/ARPC2/ARPC3/A

P5/RBP4/ADH4
1/LAG3/CD8A/CLEC7A/VCP/TAPBP/TAP2/CD4/HLA-E/DERL1/LILRB1/LILRB2

P3A4

P62/RBCK1/CXCR4/RNF19B/SHARPIN/CKS1B/NPLOC4/OTUB1/ADRM1/CBX4/DNAJB2/PML/VPS28/R

/NOL3/LEF1/MT3/BIRC7/VIL1/CD27/CASP1/DPEP1/RACK1/BAD/PRDX5
L/RANGRF/KPNB1/XPOT/XPO5/RANBP1/SERGEF/IPO13/XPO7/TNPO1/IPO9/IPO4/RANBP3

LC22A3/SLC1A1/SLC23A2/SLC13A3/SLC28A1/SLC6A13/SLC17A4/SLC1A2/SLC6A12/SLC17A2/SL

A5/HLA-DRA/HLA-DQB1/HLA-DPA1/HLA-DPB1/HLA-A/PPP2R1A/SIRPA/SPON2/SLC7A8/LAG3/CD2
NSL2/CA4/CBS/ECHS1/ECHDC2/HSD17B4/CA14/ALAD/CA5A/ENO3/ETNPPL/EHHADH/UROC1
AT1/BID/TNFSF13B/TNFSF12/TRAF3/FADD/PIDD1/TRADD/TNFSF15/CASP8/TRAF4/TMBIM1/TRIM
DH1A1/AKR1C4/ALDH7A1/ALDH5A1/ALDH1L1/ALDH2/ALDH8A1/ALDH6A1/ADH4
/NPM1/SPRED1/SOCS1/CDKN2B/CDKN1C/SOCS3/PPP1R1B/CDKN2C/GMFG/RACK1/PKIA/PRKRIP1/D

G5/APOA2/APOA1/APOA5
7/RPL5/GLMN/AUP1/RPL11
COL9A2/AGRN/VCAN/COL4A5/EMILIN2/LAMB1/COL16A1/COL3A1/LAMC1/COMP/COL5A1/MFGE8/FR
YD/CHDH/MMACHC/NOS2/ETFA/ACADVL/FMO2/ACAD11/ACADS/POR/MAOB/CRY2/ETFDH/ACOX1/IVD
AS2R43/TAS2R19/TAS2R46/TAS2R31/TAS2R10/TAS2R8/TAS2R9/TAS2R50/TAS2R39/TAS2R16/TA

A/ITGA2/CTSS/PLEKHA2/LYPD3/ECM1/ACHE/THBS1

CL5/NTS/REG1A/VEGFB/TIMP1/SEMA6A/NMB/LYPD1/LGALS3/LIF/CSF1/TGFB1/CMTM7/GPNMB/ST

R4/OR5F1/OR5P2/OR5I1/OR5P3/OR8H1/OR5L2/OR5H1/OR8U1/OR8K3/OR8J1/OR5AP2/OR5A1/OR8
/SRD5A1/PECR/TM7SF2/SRD5A2/AKR1D1
MGN4/ACTB/MTA2/SMARCB1/CHD4/RBBP4/SMARCC1/HMGN1/MBD3/HMGN2/HMGA2/HNRNPC/MBD2
T3/BIRC7/VIL1/CD27/DPEP1
TAB1/GLRX3/TXN/ERO1A/DNAJC10/PDIA6/TXNDC17/TXNDC12/ENOX2/GFER/GLRX2/PDIA3/SCO2/
RAB11FIP5/TBC1D16/MICALL2/TRAPPC4/MICALL1/ATP6AP1/ANKRD27/DENND2D/TBC1D1/GDI1/T
7/ITPR2/SCP2/RBP5/APOA2/APOF/RBP4/APOA1/APOA5/APOC3/ADH4
PLSCR1/ARF4/GRB2/CBL/TNK2/HBEGF/BTC/EREG/EGF/RNF126/VAV3/ATXN2/HIP1/SOCS5/SNX2

NA6/SERPINA5/AMBP/ITIH2/SERPINA7/SERPINA11/SERPINF2/ITIH3/SERPINA4/ITIH1/SERPIN
RL2/RHOV/ARL4C/TUBG1/ARL14/RAB42/NME1/HRAS/RAB31/TUBB/MX2/NME2/GUCY2C/RABL6/RHO
RL2/RHOV/ARL4C/TUBG1/ARL14/RAB42/NME1/HRAS/RAB31/TUBB/MX2/NME2/GUCY2C/RABL6/RHO
P62/RBCK1/CXCR4/RNF19B/SHARPIN/CKS1B/NPLOC4/OTUB1/ADRM1/DNAJB2/VPS28/RAE1/MVB12

NA4/SYK/HCK/DDR1/CSF1R/JAK3/PKDCC/PDGFRL/MAP2K2/FGFR4/EPHB6/DYRK2/EPHB3/FGFR1/F
5A1/QDPR/GRHPR/TM7SF2/FMO4/CAT/FMO5/FMO3/ASPDH

A1/CYP2C18/CYP17A1/CYP26A1/MSMO1/CYP3A7/DBH/KMO/AKR1C4/CYP2J2/CYP2A13/CYP4F12/A

5/MMP1/ADAMDEC1/MMP10/MEP1A/MMP2/ECE2/ADAM8/RCE1/ADAM12/ADAM19/ADAMTS9/ADAM23/M

11/PUS1/U2AF2/SNRPC/SSB/DAZAP1/CBX4/MSI2/EIF4A3/ILF3/MSI1/AGO2/RBPMS/CBX8/PABPC
ENPF/SPI1/HDAC11/YWHAZ/FLNA/TEAD4/CDKN2A/PKN1/E2F5/ARHGEF2/SMARCD3/HES6/ARNT2/D
1/ATP6AP1/ATP6V1H/ATP6V1E1/ATP6V0C/ATP6V0E1/ATP6V0D1
1/ATP6AP1/ATP6V1H/ATP6V1E1/ATP6V0C/ATP6V0E1/ATP6V0D1
D3/ELF3/NME2/ANKRD1/MAGED1/SOX12/PSMC3IP/SMARCD1/HMGB2/TCF3/TRIM28/NFKB2/RUVBL1
ASE1/EXOSC4/TSEN54/RNASE6/WDR4/SLFN13/DHX34/DHX37/NT5C3A/JMJD6/RNASET2/POLR2G/F

A47
OC3
PGRMC1/SHBG/ESR1/GC/SCP2/SERPINA6/APOA2/AR/RORC/APOF/AKR1D1/APOA1/APOA5/HSD11B1

S7/TRAF4
3B/TNFSF12/TRAF3/FADD/TRADD/TNFSF15/CASP8/TRAF4/TRIM37
/ABL1/RAD51D
/CCL26/CCL13/CCL2/CXCL9/CXCL3/CXCL13/CCL28/CCL4/CXCL16/PF4V1/CXCL11/XCL2

L/NPR2/THRB/EDNRB/GPER1/RAMP1/VIPR1/ALDH1A1/AVPR1A/PIK3R1/SHBG/CRHBP/LEPR/CHRNA

C6A1
S2R20/TAS2R1/TAS1R1/TAS2R43/TAS2R19/TAS2R46/TAS2R31/TAS2R10/TAS1R2/TAS2R8/TAS2R
CI2/GCDH/SCP2/ACADL/ACOT12/ALDH6A1

1F/ATP6V0B/ABCC4/ATP6V0D2/ATP6V1C1/TCIRG1/ATP6AP1/ATP13A1/ATP6V1H/ATP6V1E1/ATP6
1F/ATP6V0B/ABCC4/ATP6V0D2/ATP6V1C1/TCIRG1/ATP6AP1/ATP13A1/ATP6V1H/ATP6V1E1/ATP6

9/APOM/SLCO2A1/AKR1C4/APOC4/SLC22A9/FABP1/SLCO2B1/TTPA/MTTP/APOB/ABCA8/ABCG8/AB
G5/APOA2/APOA1/APOA5
MTHFS/SUCLG2/PC/GLUL/ACSS3/ASS1/ACSL6/ACSM3/ACSL5/ACSL1/MTHFD1/SLC27A2/ACSM5/AC
/THNSL2/GOT1/CBS/ACACB/MTHFS/PC/AADAT/GOT2/DBH/RBP7/ALAS1/DHTKD1/GC/CSAD/GPT2/P
/SUOX/CYP11A1/CBS/CYP2C18/CYP17A1/CYP26A1/CYB5D2/HBB/STEAP4/TDO2/CYP3A7/CYP2J2/
PNPLA3/ABHD6/ACOT2/ASPG/LIPG/CES1/ABHD1/BPHL/CES4A/LIPC/PLA2G12B/BCHE/BAAT/CES2

H1/COMP/MRC2/CTSK/P3H1/PAK1/ADGRG1/C1QTNF1/ITGA2/CTSS/PDGFA/SPOCK2/SPOCK1/COCH/
3A1/COL5A1/COL5A2/COL6A3/COL6A2/COL4A2/COL8A2/COL11A1/COL9A3
SLC22A1/SLC25A47
/GRAMD1B/GSDMD/PLEKHN1/PACSIN2/SESTD1
MER3/CXCL5/ITGB4/TFF2/NMB/DNM1/S100A14/BAMBI/FLNA/S1PR2/ADORA1/PRNP/APLP1/CKLF/
E3/ANKRD1/DDIT3/BHLHE41/YBX3/TRIM28/NR0B1/SIRT6/GMNN/PPP1R13L/RBBP8/BASP1/SMARC
DK7/CDK19/CCND3/CDK11B/CDK20/CDK11A
R8D1/OR10J3/OR1F2P/OR51B5/OR2H2/OR51Q1/OR3A3/OR2B2/OR4K1/OR2W3/OR3A2/OR12D2/OR5
8/DHRS2/AKR1C4/GRHPR/GPD1/CRYL1/RDH5/LDHD/ADHFE1/SRD5A2/DCXR/AKR7A3/BDH1/SORD/A

13A3/SLC13A5
188/NUP85/NUP155/NUP107
C2A10/SLC12A1/SLC22A3/SLC1A1/SLC23A2/SLC13A3/SLC28A1/SLC6A13/SLC17A4/SLC1A2/SLC

CL5/NTS/REG1A/VEGFB/TIMP1/SEMA6A/NMB/LGALS3/LIF/CSF1/TGFB1/CMTM7/GPNMB/STC2/DKK
3GNT7/PLOD3/B4GALT3/B3GALNT1/B4GALT7/B4GALT2/A4GALT
8/DHRS2/AKR1C4/GRHPR/GPD1/CRYL1/RDH5/LDHD/ADHFE1/SRD5A2/DCXR/AKR7A3/BDH1/SORD/A
/SUOX/CYP11A1/CBS/CYP2C18/CYP17A1/CYP26A1/CYB5D2/HBB/STEAP4/TDO2/CYP3A7/CYP2J2/

FC4/KIF18B/ATP1A1/RAD51/RAD54L/TNNT1/MCM6/DSCC1/MCM7/ABCD1/KIF11/PIF1/ATP6V1F/W

8/SLC20A2/SLC16A12/SLC6A2/SLC16A13/SLC16A4/SLC2A9/SLC4A4/SLC2A10/SLC12A1/SLC22A
B1BP1/RANBP1/ARHGDIA
/SLC1A1/SLC25A30/SLC13A3/SLC1A2/SLC13A5
P2/APOA2/RORC/APOF/APOA1/APOA5/APOC3
HNRNPU/EXOSC10/SNRPD3/SMG7/DCP2/HNRNPC/TEP1/PARN
/HIP1R/DNER/CLTB/AP2M1/NCALD/AP2S1/SYT12/SYT1/DNM1L/SYT3/AP1B1/CEMIP/SMAP1/AP2A
TAPBP/TAP2/HLA-E/DERL1/LILRB1/LILRB2

0/HPN/CFB/PCSK6/PROC/MST1/MASP1/F2/F11/TMPRSS6/F7/LPA/PROZ/MASP2/HP/KLKB1/F12/H
3/NIPAL2/NIPA1/NIPA2
TTNBP2NL/PTPA/IGBP1/MAPT/ANKLE2/BOD1/PTPN1/MASTL/DAB2IP/ENSA/SMG7/MFHAS1
HORDC1/MSH2/RUVBL2/MYO9B/PPP5C/ABCG1/LONP1/VCP/CYB5R3/MYO6/PRKAG1/MSH6
M1/GSTA1

S/NSF/CANX/CDK5R1
1/SCP2/RIDA/FABP1/APOC1/ACOX2/ALB/FABP4
1/CYP2C18/CYP17A1/CYP26A1/SC5D/BBOX1/MSMO1/CYP3A7/ADI1/CYP2J2/CYP2A13/CYP4F12/A
AA2/GCDH/BAAT/ACOT12
P3/HSPA2/PSAP
MA7/PSMB3/PSMA1/PSMB5/PSMA4/PSMB1/PSMB6/PSMA3/PSMB7
MA7/PSMB3/PSMA1/PSMB5/PSMA4/PSMB1/PSMB6/PSMA3/PSMB7
HMGB2/TCF3/SMARCA4/ACTL6A/HDAC1/LEF1/IRF7/NONO/HDAC2/ATF4/SPIB/ACTB/SFPQ/RAI1/S
GFOD1
HE/SERPINA5/APOC1/CHRNA4/APOA2/APOA5
ISG20/EXOSC5/TATDN1/RAD9A/EXOSC3/REXO2/DCLRE1B/RAD1/EXOSC9/EXOSC10/EXOSC7/TOE1/
3/PJA1/RNF24/RNF145/UBL4A/TRAF2/RBCK1/TRIM6/TRIM28/UBE2M/UBE2Z/TRIM11/RNF34/UBE

42EP2

3/SLC26A1/SLC16A4/SLCO1A2/ABCC2/SFXN1/SLC25A13/SLC19A2/SLC1A1/SLC17A1/SLC25A30/
2/HCN3/SLC4A4/SCNN1D/SLC12A1/SLC22A3/SLC1A1/SLC9B2/SLC23A2/SLC13A3/SLC28A1/SLC6
A1/CYP2C18/CYP17A1/POR/CYP26A1/SC5D/KDM8/BBOX1/MSMO1/CYP3A7/DBH/KMO/AKR1C4/CYP2

3/SLC26A1/SLC16A4/SLCO1A2/ABCC2/SFXN1/SLC25A13/SLC19A2/SLC1A1/SLC17A1/SLC25A30/
SLC26A11/SLC37A2/SLC4A3/SLC4A11/SLC26A2/SLC25A5

10/LSM2/ISG20/SNU13/PRPF31/SNRPB2/HNRNPU/LSM8/EIF5A/SART3/TOE1/PRPF4/SNRNP70/CO
/DPYSL4
P3/ZNF385A/YBX3/PCBP4/RBM3/PABPC1/RNPS1/ZC3H12A/DAZAP1/CPSF1/ILF3/PABPC4/RPS7/R
1F/ATP6V0B/ATP6V0D2/ATP6V1C1/TCIRG1/ATP6AP1/ATP13A1/ATP6V1H/ATP6V1E1/ATP6V0C/AT
Y1R

GNT9/GALNT18/B3GNT7/CHPF2/B3GALNT1/GALNT6/CHPF/B4GALNT3/B4GALNT4/B4GALNT2/GALNT

6A6/ANO10/CLCN2/SLC1A4/GABRA3/CLCN7/GABRR3/VDAC1/LRRC8B/BEST1/TTYH2/GABRD/PIDD1
/DROSHA/AGO2/POP5/RPP30/RNASEH1/DBR1
C6A9/SLC7A5/SLC1A7/SLC1A4/SLC43A2/SLC1A3/SLC25A22/SLC7A8/SLC7A10/SLC7A6/SLC36A1
T11
F3H/AGO2/EIF2B5/MCTS1/EIF3K/EIF3M/COPS5/EIF1AD/EIF3E/EIF6/EIF4G2/EIF4G3/EIF3G/E
B/QSOX2/PDIA4
B/QSOX2/PDIA4

7/ACAA2/GCDH/HAGH/BAAT/ACOT12

M1/CASP1/APP/RACK1/VCP/SFRP2/BAD
SOX4/E2F1/SOX9/CDC6/ENO1/MCM2/CDC45/FOXJ1/NFE2L3/MCM4/ETV5/ORC6/ORC1/RAD51/LMNB

PRC1/KIF23/TUBA1B/WDR62/KIFC1/SAPCD2/KIF18B/MISP/NUSAP1/ARL2/CCNF/TTK/SKA1/AURK
IF20A/TOP2A/TRIP13/TUBA1C/RRM2/E2F1/CDCA5/CEP55/CDC6/UBE2S/TUBA4A/NUF2/CDKN3/PK
F/FBLN1/POF1B/PDLIM7/ECT2/BAMBI/ENAH/RHOV/RCC2/ARHGAP4/AURKA/STK26/DKK1/CORO1A/
ALS9/SULF1/FOXJ1/SRC/TSPO/MAPK13/TYROBP/TGFB1/TRAIP/GPNMB/CYBB/GAPDH/RIPK2/HAVC
LF1/FOXJ1/SRC/TSPO/MAPK13/TYROBP/TGFB1/TRAIP/GPNMB/CYBB/GAPDH/RIPK2/HAVCR2/IGF2
HAVCR2/BIRC3/IRAK1/CD74/CCDC88B/PTAFR/ARHGEF2/HSPB1/SCAMP5/HRAS/SYK/PLCB1/IL18/
DKK1/FOLR1/GPC4/KRT17/GPC3/FLNA/CD44/VASP/PGF/DDR1/FZD6/DLG3/CSF1R/FOXQ1/CD151/
LGALS9/TMC6/LGALS3/PLAUR/RAC2/FABP5/TYROBP/CKAP4/PYGB/TGFB1/ITGAM/CYBB/LGALS1/R
CTSC/FCER1G/LGALS9/TMC6/NDRG1/LGALS3/PLAUR/RAC2/FABP5/CSF1/TYROBP/CKAP4/PYGB/TG
6/LGALS3/PLAUR/RAC2/FABP5/TYROBP/CKAP4/PYGB/ITGAM/CYBB/HAVCR2/GSTP1/CD44/ITGB2/
R/FABP5/TYROBP/CKAP4/PYGB/ITGAM/CYBB/GSTP1/CD44/ITGB2/ALDH3B1/SNAP25/PTAFR/COTL
LGALS9/TMC6/LGALS3/PLAUR/RAC2/FABP5/TYROBP/CKAP4/PYGB/TGFB1/ITGAM/CYBB/LGALS1/R
ICAM1/LGALS9/FOXJ1/TMC6/LGALS3/PLAUR/RAC2/FABP5/TYROBP/CKAP4/PYGB/TGFB1/ITGAM/C
3/PLAUR/FABP5/TYROBP/CKAP4/PYGB/ITGAM/CYBB/GSTP1/CD44/ITGB2/ALDH3B1/SNAP25/PTAF
/LIF/CSF1/TYROBP/TGFB1/CMTM7/SPI1/LGALS1/RIPK2/ADA/EXO1/HAVCR2/CLCF1/GPC3/C1QC/
S1/RIPK2/ADA/CLCF1/GPC3/C1QC/CD74/BATF/RELB/FANCD2/SLAMF8/EFNA4/NME1/SYK/CD109/
/ARPC1B/LGALS9/VEGFB/FOXJ1/TYRO3/SRC/CD3D/LGALS3/RAC2/MMP14/LIF/CSF1/TYROBP/S10
BIRC3/ITGB2/IRAK1/CMTM3/PRKCD/LAT2/RELB/IGLL5/HLA-DRB1/PRNP/HRAS/SYK/HLA-DQA1/H
S/SYK/HLA-DQA1/HCK/PAG1/HLA-DRA/MUC1/EZR/HLA-DQB1/VTCN1/CARD9/PSMB9/HLA-DPA1/WI
K2/GINS1/SRC/MCM4/NUDT1/ORC6/CCNE1/CCNA2/CENPF/RMI2/RFC4/ORC1/CHAF1B/TGFB1/RAD5
TL/UHRF1/RAD54L/RNASEH2A/MCM6/FEN1/GINS2/FANCD2/POLD1/MCM7/PIF1/MCM3/RAD51AP1/E
11/UHRF1/PHF19/CDKN2A/CENPW/PKN1/PRKCD/SMARCD3/OIP5/CBX6/NAP1L1/CENPO/EZH2/DNMT
SNRPD2/HSF1/SNHG7/WDR4/SNRPD1/SNRPA1/INTS8/LIN28B/CSTF2/SNRPA/SNHG12/MRTO4/SF3A
L8/RPS6KA1/YBX3/EIF5A2/CCL5/DPH2/CNOT11/MAPK3/EIF3B/RPL36A/MRPL17/NPM1/UPF3B/PO
LGALS9/PFN2/VEGFB/ANXA5/TMC6/MICAL1/TIMP1/LHFPL2/NCS1/LGALS3/PLAUR/RAC2/ATP13A2
R/RAC2/CSF1/PDLIM7/S100A14/TGFB1/CMTM7/ENAH/GPNMB/CORO1A/EFNA3/GSTP1/STX3/ITGB2
1/MAPK12/MCM4/NUDT1/NDRG1/RMI2/RFC4/CHAF1B/RAD51/TONSL/FANCI/AURKA/EXO1/NEIL3/C
CABYR/STMN1/DLGAP5/PRC1/KIF23/TUBA1B/WDR62/TUBA3C/KIFC1/SAPCD2/KIF18B/MISP/NUSA
DC80/CDC45/ANLN/NEK2/BUB1/DLGAP5/CDC25B/BUB1B/MKI67/ZWINT/CKS2/CCNE1/CENPF/ORC1
L5/CDK1/HILPDA/REG1A/LGALS9/VEGFB/EPCAM/CDC25B/TIMP1/TSPO/PRC1/NMB/ETV5/RAC2/CC
8/RRAGC/PPP2R1A/UPF3B/POLR2G/EFTUD2/PPIH/NOL3/RBM3/YBX1/PABPC1/PPP1R9B/PUF60/WD
DIT3/RBCK1/PSMD14/FBXL19/PSMD2/NUPR1/RHBDF1/RNF34/RNF19B/SHARPIN/HM13/GNA12/DDA
KMYT1/CDK1/NDC80/CDC45/ANLN/NEK2/BUB1/DLGAP5/CDC25B/BUB1B/MKI67/PRC1/KIF23/CCNE
/PLAUR/RAC2/LIF/NUSAP1/ARL2/TGFB1/AURKA/PSRC1/CORO1A/YWHAZ/FLNA/PHF19/FEN1/HK2/
TYROBP/ECT2/TGFB1/ITGAM/PNMA1/DKK1/GAPDH/RIPK2/SQSTM1/TNFRSF12A/YWHAZ/ITGB2/CDK
1/TGFB1/LGALS1/TYMS/CDC25C/IRAK1/CD74/C1QA/ITGAV/SYK/NUP62/RCC1/HCK/TAP1/ZYX/PA
IGF2BP3/ESRP1/RALY/RPL8/SNRPD2/HSF1/PSMB9/DDIT3/PSMD14/YBX3/SNRPD1/MAPKAPK2/SNR
M28/BRMS1/SIRT6/NAA40/RUVBL1/JMJD6/MAPK3/PYGO2/MRGBP/BRCA1/MCRS1/ACTL6A/LMNA/HD
MP1/TFF2/LIF/CSF1/TGFB1/TRAIP/ITGAM/LMNB1/SPI1/RIPK2/GSTP1/STX3/COL1A2/CLCF1/SQ
MELTF/KIF18B/TGFB1/ITGAM/CTSV/DKK1/SQSTM1/C1QB/CD44/C1QC/HK2/ARHGEF2/C1QA/SMARC
EIF2S2/RPS2/HSP90AB1/DYNC1H1/TICRR/RRS1/DDX49/RRP9/WDR77/ERI3/REXO4/RRP1/BRIX1/
P1A1/DSG2/TYROBP/POF1B/PDLIM7/ECT2/TGFB1/ENAH/RHOV/CORO1A/MARCKS/FLNA/TNNT1/TPM
/TUBG1/S1PR2/PRKCD/NCK2/CCDC28B/HRAS/CDC42EP1/WDR34/MAPRE1/TUBB/FGD3/TWF2/ASAP1
ROBP/POF1B/PDLIM7/ECT2/TGFB1/ENAH/RHOV/CORO1A/MARCKS/FLNA/TNNT1/TPM4/S1PR2/RACG
SF1/TYROBP/TGFB1/CMTM7/SPI1/LGALS1/RIPK2/ADA/CLCF1/GPC3/C1QC/CD74/BATF/RELB/FAN
109/NME2/CSF1R/CSF3R/ZNF385A/HMGB3/PRMT1/TFRC/HMGB2/JAK3/WASF2/IL34/FLVCR1/HIF1
LGALS3/SPINT2/RAC2/MMP14/MELTF/SERPINE2/CSF1/FBLN1/ARL2/TGFB1/RCC2/GPNMB/LGALS1
MP1/ATP13A2/DAB2/PBK/SERPINE2/TRIB3/MAGEC2/CCNF/AURKA/PPT1/CTSV/SCPEP1/SQSTM1/D
C/TIMP1/ITGA3/SEMA6A/LGALS3/RAC2/DAB2/MMP14/SERPINE2/CSF1/S100A14/TGFB1/RCC2/AR
3/TSPO/SEMA6A/ACSL4/NCS1/RAC2/GPRIN1/LRP12/PDLIM7/ENAH/ARHGAP4/AURKA/STK26/LGAL

3/SEMA6A/NCS1/RAC2/RCC2/ARHGAP4/LGALS1/DKK1/TNFRSF12A/CD44/AGRN/SNAP25/PRKCD/RO
/DCAF13/NUP62/RUSC1/PJA1/MAD2L1/RNF24/RNF145/RANGAP1/PSMB9/TRAF2/RBCK1/PSMD14/S
5/ITPR3/SRC/ITGA3/MMD/PTP4A3/ARHGEF16/LYPD1/LGALS3/RAC2/ATP13A2/DAB2/CCNE1/MMP1
FB1/RCC2/AURKA/GPC4/YWHAZ/FLNA/CEP131/HK2/PRKCD/LAMP5/HRAS/PDIA2/SYK/ABHD17C/NU
/MCM4/CENPF/CHAF1B/ARL2/CENPH/TGFB1/RAD51/PSRC1/HLA-DMA/CORO1A/PMEPA1/BOP1/FCHO
G5/ABCD1/UPP1/IGF2BP1/NUP62/PFKFB3/SMG9/EXOSC4/APOBEC3B/ENTPD2/PKP3/RNASE6/TYMP
TO4/DHX37/PES1/DKC1/TAF6/POLR2G/TRMU/FTSJ1/CD3EAP/SMARCA4/METTL1/RPS2/PELO/RRS1
/LGALS3/PLAUR/FABP5/TYROBP/CKAP4/PYGB/ITGAM/CYBB/GSTP1/CD44/ITGB2/ALDH3B1/SNAP2
AM/PLXNA1/SRC/TIMP1/ITGA3/SEMA6A/LGALS3/SPINT2/RAC2/DAB2/MMP14/SERPINE2/CSF1/FB
MP14/CSF1/S100A14/TGFB1/GPNMB/CORO1A/STX3/FLNA/CD74/PTAFR/FERMT3/ARHGEF2/P2RX4/
ORO1A/CD7/CD44/CD74/BATF/CCDC88B/RELB/NCK2/FANCD2/ELF4/PRNP/SYK/IL18/PAG1/CD151
/LGALS3/PLAUR/FABP5/TYROBP/CKAP4/PYGB/ITGAM/CYBB/GSTP1/CD44/ITGB2/ALDH3B1/SNAP2
R/RAC2/CSF1/PDLIM7/S100A14/TGFB1/CMTM7/ENAH/GPNMB/CORO1A/EFNA3/GSTP1/STX3/ITGB2
F385A/RPL8/RPS6KA1/YBX3/EIF5A2/CCL5/DPH2/CNOT11/MAPK3/EIF3B/RPL36A/MRPL17/NPM1/
TYROBP/ECT2/TGFB1/ITGAM/PNMA1/GAPDH/RIPK2/SQSTM1/TNFRSF12A/YWHAZ/CDKN2A/ARHGEF2
M/MICAL1/SRC/TIMP1/LGALS3/PLAUR/DAB2/AKR1B1/AURKA/PPT1/DKK1/RIPK2/ADA/CORO1A/GS
TYROBP/ECT2/TGFB1/ITGAM/PNMA1/GAPDH/RIPK2/SQSTM1/TNFRSF12A/YWHAZ/CDKN2A/ARHGEF2
M/MICAL1/SRC/TIMP1/LGALS3/PLAUR/DAB2/AKR1B1/AURKA/PPT1/DKK1/RIPK2/ADA/CORO1A/GS
BCK1/PSMD14/FBXL19/PSMD2/RNF34/RNF19B/SHARPIN/GNA12/DDA1/HSP90AB1/NPLOC4/CSNK1E
M1/SRC/RAC2/ATP13A2/MMP14/CLSTN1/ECT2/ARL2/TGFB1/RCC2/DKK1/PSRC1/CORO1A/GPC4/PM
NCK2/BAIAP2L2/VASP/PLEK/HRAS/SYK/CDC42EP1/NUP62/HCK/SPAG5/MAPRE1/TWF2/ASAP1/COL
2/FBLN1/TGFB1/LGALS1/GAPDH/TYMS/CDC25C/IRAK1/CD74/C1QA/ITGAV/SYK/NUP62/RCC1/HCK
YRO3/SRC/CCNA2/FBLN1/TGFB1/LGALS1/GAPDH/TYMS/CDC25C/IRAK1/CD74/C1QA/ITGAV/SYK/N
ASF1B/CDK1/LGALS9/CABYR/FOXJ1/TYRO3/CDC25B/B4GALNT1/SRC/TIMP1/ITGA3/LHFPL2/ETV5
CDK1/NDC80/MCM2/CDC45/MELK/ANLN/NEK2/BUB1/DLGAP5/CDC25B/BUB1B/MCM4/CKS2/ORC6/CC
LGALS9/PFN2/VEGFB/ANXA5/TMC6/MICAL1/TIMP1/LHFPL2/NCS1/LGALS3/PLAUR/RAC2/SYT13/F
1/TYROBP/S100A14/TGFB1/ITGAM/CYBB/GAPDH/RIPK2/HAVCR2/CORO1A/C1QB/SUSD4/BIRC3/CD
RKCD/RELB/SLAMF8/HRAS/SYK/HCK/S100A8/MUC1/IFNGR2/SCRIB/HMGB3/GRN/GBP5/CARD9/PSM
A1/ITGA3/TSPO/CLIC1/SEMA6A/MMD/ETV5/DAB2/MMP14/LIF/SERPINE2/CSF1/TYROBP/PDLIM7/
BUB1/BUB1B/ZWINT/LIF/CENPF/ORC1/CCNF/TTK/TGFB1/RAD51/AURKA/GPNMB/CDC25C/DTL/PRR
CNE1/CCNA2/TRIB3/CSF1/ECT2/CCNF/TGFB1/IQGAP3/STK26/DKK1/RIPK2/UNC119/PSRC1/GSTP
CMTM7/GPNMB/SPI1/LGALS1/RIPK2/ADA/EXO1/HAVCR2/CORO1A/MZB1/CLCF1/CD7/CD44/ITGB2/
MYT1/NDC80/NCAPG/ANLN/NEK2/BUB1/DLGAP5/CDC25B/BUB1B/MKI67/PRC1/KIF23/ZWINT/CKS2
/LIF/CSF1/TYROBP/TGFB1/CMTM7/SPI1/LGALS1/RIPK2/ADA/CLCF1/GPC3/C1QC/CD74/BATF/PK
SAPCD2/CSF1/TGFB1/TXNRD1/DKK1/FOLR1/GPC4/KRT17/GPC3/FLNA/CD44/ROBO1/VASP/PGF/DD
GAM/GPNMB/LGALS1/RIPK2/ADA/HAVCR2/CORO1A/MZB1/CLCF1/ITGB2/CD74/CCDC88B/PKN1/PTA
AM/SRC/ITGA3/LGALS3/RAC2/MMP14/CSF1/PODXL2/S100A14/TGFB1/ITGAM/ADA/CORO1A/COL1A
NEK2/STMN1/BUB1/BUB1B/SRC/ITGA3/TSPO/SEMA6A/LGALS3/MMP14/MELTF/LIF/CENPF/FBLN1/
A5/EPCAM/PLXNA1/SRC/TIMP1/ITGA3/SEMA6A/LGALS3/SPINT2/RAC2/DAB2/MMP14/SERPINE2/A
DAB2/MMP14/CSF1/S100A14/TGFB1/GPNMB/CORO1A/FLNA/CD74/PTAFR/FERMT3/ARHGEF2/P2RX4
K2/CDC25B/SRC/MMD/CKS2/RAC2/CCNE1/CCNA2/RFC4/TRIB3/MAGEC2/CSF1/ECT2/CCNF/TGFB1/
16/DEPDC1B/FBLN1/ECT2/RCC2/ARHGAP4/RIPK2/EFNA3/UHRF1/PPP1R14D/AGRN/RACGAP1/TAX1
/RHOV/AURKA/PSRC1/CORO1A/SKA3/FLNA/CEP131/COTL1/ARHGEF2/PRKCD/NCK2/PLXNA3/BAIAP
CS1/RAC2/DNM1/CENPF/KIFC1/SAPCD2/MISP/NUSAP1/AURKA/PSRC1/FOLR1/STX3/YWHAZ/CEP13
PF/KIFC1/SAPCD2/MISP/NUSAP1/PSRC1/FOLR1/STX3/YWHAZ/SNAP25/CPLX2/ARHGEF2/LAT2/SY
6A/MMD/ETV5/NCS1/LIF/CLSTN1/SERPINE2/CSF1/ECT2/TGFB1/ARHGAP4/LGALS1/DKK1/GPC4/C
1/ECT2/TGFB1/DKK1/CLCF1/TNFRSF12A/FLNA/AGRN/ARHGEF2/P2RX4/ROBO1/SERPINI1/PLXNA3
3/TSPO/SEMA6A/MMD/ETV5/NCS1/MELTF/LIF/SERPINE2/CSF1/FBLN1/TYROBP/ECT2/TGFB1/RCC
16/NCS1/LYPD1/LGALS3/RAC2/ATP13A2/DAB2/CCNE1/DNM1/MMP14/LIF/SAPCD2/MISP/TYROBP/
HST11/ANXA5/TYRO3/EPCAM/MICAL1/SRC/TIMP1/LGALS3/PLAUR/ATP13A2/DAB2/CSF1/AKR1B1/
G1/DAB2/DNM1/CLSTN1/ITGAM/PPT1/CORO1A/FOLR1/STX3/YWHAZ/ANO9/FCHO1/ITGB2/AGRN/SN

/UHRF1/BIRC3/BLMH/DCAF13/NUP62/CDC25A/RUSC1/PJA1/MAD2L1/RNF24/RNF145/RANGAP1/PS
P/CENPH/AURKA/CORO1A/BOP1/FLNA/TNNT1/CEP131/TUBG1/RACGAP1/CENPW/ARHGEF2/CCDC28B
STX3/SQSTM1/YWHAZ/GPC3/FLNA/ITGB2/AGRN/SNAP25/KCNJ11/SLC7A11/TSPAN15/ADORA1/PRN
N2A/CD74/PKN1/ARHGEF2/PRKCD/HSPB1/NCK2/SLC7A11/ABCD1/HRAS/SYK/PLCB1/NUP62/RAD51
2/LOXL4/KIF18B/POF1B/ARL2/P3H4/ENAH/RHOV/PSRC1/CORO1A/MARCKS/COL1A2/KRT17/FLNA/
LS3/SPINT2/RAC2/CLSTN1/SERPINE2/DSG2/PODXL2/TGFB1/ITGAM/GPNMB/LGALS1/RIPK2/ADA/
PR2/PRKCD/NCK2/CCDC28B/HRAS/CDC42EP1/WDR34/MAPRE1/TUBB/FGD3/TWF2/ASAP1/EZR/DCDC
A6A/NCS1/RAC2/RCC2/ARHGAP4/LGALS1/DKK1/TNFRSF12A/CD44/AGRN/SNAP25/PRKCD/ROBO1/S
25B/BUB1B/CCNE1/CENPF/ORC1/TTK/TGFB1/RCC2/AURKA/GPNMB/HMMR/CDC25C/DTL/CEP131/CD
/TOP2A/TRIP13/RRM2/E2F1/CDCA5/CEP55/CDC6/UBE2S/TUBA4A/NUF2/CDKN3/PKMYT1/CDK1/ND
E2/CSF3R/HMGB3/PRMT1/PSMB9/TNFRSF18/PSMD14/HMGB2/JAK3/TCF3/CD86/TNFSF9/CD2/IL34
F/SAPCD2/MISP/TYROBP/ECT2/TGFB1/ITGAM/GPC4/STX3/SQSTM1/YWHAZ/GPC3/FLNA/ITGB2/CE
AM/PLXNA1/SRC/TIMP1/ITGA3/SEMA6A/LGALS3/SPINT2/RAC2/DAB2/MMP14/SERPINE2/CSF1/FB
MP14/CSF1/S100A14/TGFB1/GPNMB/CORO1A/FLNA/CD74/PTAFR/FERMT3/ARHGEF2/P2RX4/HSPB1
/NOL3/RBM3/YBX1/PABPC1/PUF60/WDR77/RNPS1/SNRPE/POLR2H/TXNL4A/NCBP2/SNRPF/LSM7/U
/NOL3/RBM3/YBX1/PABPC1/PUF60/WDR77/RNPS1/SNRPE/POLR2H/TXNL4A/NCBP2/SNRPF/LSM7/U
/NOL3/RBM3/YBX1/PABPC1/PUF60/WDR77/RNPS1/SNRPE/POLR2H/TXNL4A/NCBP2/SNRPF/LSM7/U
C2/CYBB/ADA/AGTRAP/IRAK1/EIF4EBP1/HK2/SOD3/ADORA1/ARNT2/UCP2/PGF/ADAM15/ANKRD1/
6/LGALS3/PLAUR/RAC2/FABP5/TYROBP/CKAP4/PYGB/ITGAM/CYBB/GSTP1/CD44/ITGB2/ALDH3B1
/LGALS1/RIPK2/ADA/HAVCR2/CORO1A/MZB1/CLCF1/ITGB2/CD74/CCDC88B/PKN1/PTAFR/NCK2/F
VCR2/BIRC3/ITGB2/IRAK1/CMTM3/PRKCD/LAT2/RELB/IGLL5/HLA-DRB1/PRNP/HRAS/SYK/HLA-D
6/LSM4/HSPA8/UPF3B/POLR2G/ZC3H3/EFTUD2/PPIH/NOL3/RBM3/YBX1/PABPC1/PUF60/WDR77/R
TF2/NUP210/RGS14/NPM1/UPF3B/LMNA/RRS1/LZTS2/NUP93/NXT1/FAM53B/NUP205/NUP37/RNPS
2/CORO1A/CD44/ITGB2/IRAK1/CERCAM/CD74/CCDC88B/PTAFR/FERMT3/NCK2/PRNP/SYK/IL18/P
/SQSTM1/CDKN2A/RACGAP1/TAX1BP3/CHML/ARHGEF2/CYTH4/ROBO1/TRIP10/RGS19/RAB42/HRAS
DK4/TCOF1/IGF2BP3/ZNF385A/RAN/RPS6KA1/PSMB9/PSMD14/YBX3/EIF5A2/CCL5/MAPKAPK2/DP
D51/AURKA/GPNMB/CDC25C/DTL/CDKN2A/GTSE1/EZH2/CDK4/MAD2L1/MUC1/CDKN2D/ZNF385A/AR
BO1/SERPINI1/PLXNA3/NME1/ITPKA/EZH2/NME2/TWF2/ASAP1/ANKRD1/TRPV2/GRN/CRMP1/CFL1
/FBLN1/P3H4/TGFB1/ITGAM/CTSV/COL1A2/COL9A2/CD44/ITGB2/AGRN/QSOX1/VCAN/COL4A5/IT
SF1/S100A14/TGFB1/GPNMB/CORO1A/FLNA/CD74/PTAFR/FERMT3/ARHGEF2/P2RX4/HSPB1/ITGAV
ARMIL1/EIF5A2/NCKAP5L/HSPA8/FZD1/MAP1S/MRPL17/SMARCA4/MRPS12/PELO/F2RL1/MRPL47/
67/SRC/LIF/CENPF/RMI2/TTK/TGFB1/SPI1/PHF19/FEN1/SMG5/PIF1/CCT5/MAD2L1/DNMT1/MUC
P1/COTL1/ARHGEF2/PRKCD/NCK2/BAIAP2L2/VASP/PLEK/SYK/CDC42EP1/HCK/MAPRE1/TMSB4X/T
S3/PLAUR/RAC2/FABP5/TYROBP/CKAP4/PYGB/ITGAM/CYBB/CORO1A/GSTP1/CD44/ITGB2/ALDH3B
R/FABP5/TYROBP/CKAP4/PYGB/ITGAM/CYBB/GSTP1/CD44/ITGB2/ALDH3B1/SNAP25/PTAFR/COTL
CDK1/NDC80/MCM2/CDC45/MELK/ANLN/NEK2/BUB1/DLGAP5/CDC25B/BUB1B/MCM4/CKS2/ORC6/CC
GFB1/RCC2/AURKA/PSRC1/CDC25C/DTL/FEN1/RACGAP1/EIF4EBP1/SMARCD3/PLCB1/NUP62/GTSE
PD1/NUTF2/NUP210/RGS14/NPM1/UPF3B/LMNA/RRS1/LZTS2/NUP93/NXT1/FAM53B/NUP205/NUP3
55/CDC6/UBE2S/NUF2/CDK1/NDC80/NCAPG/ANLN/VEGFB/NEK2/STMN1/BUB1/CDC25B/CDCA3/BUB
GAP3/DKK1/RIPK2/PSRC1/GSTP1/CDC25C/GDF15/IRAK1/CDKN2A/S1PR2/CD74/PKN1/DUSP5/PRK
OBP/TGFB1/ITGAM/GSTP1/TNFRSF12A/YWHAZ/CD44/CD74/ARHGEF2/BCL2A1/P2RX4/PRKCD/HSPB
AP2L2/VASP/PLEK/TRIM54/CDC42EP1/COLGALT1/HCK/MAPRE1/TMSB4X/TWF2/MID1IP1/CFL1/S1
P4/SNRPA/PSMD2/JMJD6/HSPA8/NPM1/POLR2G/ZC3H3/RBM3/YBX1/PABPC1/RNPS1/PSMB2/NELFE
/YWHAZ/FLNA/ITGB2/CEP131/KCNJ11/PRKCD/B3GAT3/PRNP/HRAS/ABHD17C/NUP62/GTSE1/CCT5
3/SQSTM1/FLNA/SNAP25/KCNJ11/TSPAN15/PRNP/HRAS/RAB31/PACS1/PKP3/EZR/BSG/SCRIB/RH
MYT1/NDC80/NCAPG/ANLN/NEK2/BUB1/DLGAP5/CDC25B/BUB1B/MKI67/PRC1/KIF23/ZWINT/CKS2
/HLA-DQA1/HCK/PAG1/HLA-DRA/MUC1/EZR/HLA-DQB1/VTCN1/CARD9/PSMB9/HLA-DPA1/WIPF3/R
/MAPKAPK2/LIN28B/SLFN13/DHX34/PCBP4/MRTO4/CNOT11/PSMD2/LSM4/HSPA8/RPL36A/DKC1/P
/MRTO4/CNOT11/PSMD2/LSM4/HSPA8/RPL36A/PPP2R1A/NPM1/UPF3B/POLR2G/YBX1/RPS2/PELO/
TPM4/COTL1/ARHGEF2/PRKCD/NCK2/BAIAP2L2/VASP/PLEK/CDC42EP1/USH1C/HCK/ZYX/TMSB4X/
NPF/CCNF/TTK/SPI1/CORO1A/ARHGEF2/PRKCD/VAT1/TRIM54/PIF1/MAPRE1/TMSB4X/TWF2/MAD2

ORO1A/CD44/ITGB2/IRAK1/FXYD5/CD74/CCDC88B/PTAFR/FERMT3/PRKCD/NCK2/PRNP/SYK/IL18
3/NME1/ITPKA/HRAS/CDC42EP1/EZH2/NME2/TWF2/ANKRD1/TRPV2/PLEK2/GRN/RHOQ/FSCN1/CAR
/NCK2/PLXNA3/BAIAP2L2/VASP/PLEK/CDC42EP1/USH1C/HCK/TMSB4X/CDK4/TWF2/EZR/TRPV2/C
/RRS1/DDX49/RRP9/POLR3K/ERI3/REXO4/RRP1/RRP7A/PA2G4/FBL/USB1/DDX27/NOP2/GTPBP4/
C2/CYBB/ADA/AGTRAP/IRAK1/EIF4EBP1/HK2/SOD3/ADORA1/ARNT2/UCP2/PGF/ADAM15/ANKRD1/
HAVCR2/BIRC3/CD74/PTAFR/ITGAV/SYK/PLCB1/CSF1R/GRN/CFL1/APOBEC3C/TRIM6/CHMP4B/TR
D74/CCDC88B/PTAFR/NCK2/IGLL5/PLEK/SYK/IL18/VTCN1/CD320/RPS6KA1/TFRC/HLA-DPA1/HL
F1/RAD51AP1/CCT5/DNMT1/ALYREF/ANKRD1/CDC7/CHEK1/NPAS2/HSF1/NABP2/CCT6A/TFRC/CCT
A/MZB1/CLCF1/CD74/CCDC88B/PKN1/NCK2/FANCD2/SLAMF8/IGLL5/PRNP/SYK/IL18/PAG1/HMGB
ORO1A/ZG16B/NCDN/CIB2/FEN1/SCGN/P2RX4/HSPB1/POLD1/SMG5/ACP5/ADORA1/SYK/USH1C/PI
GFB1/STC2/CYBB/ADA/AGTRAP/IRAK1/EIF4EBP1/HK2/SOD3/ADORA1/ARNT2/UCP2/PGF/CDK4/AD
X/SSR2/MTCL1/VPS37C/PKDCC/SEC61A1/ZDHHC7/RPL36A/MICALL1/PAK1/BMF/SLC51B/NPC1/RP
25B/BUB1B/CCNE1/CENPF/ORC1/TTK/TGFB1/RCC2/AURKA/GPNMB/HMMR/CDC25C/DTL/CEP131/CD
GEF2/BCL2A1/PRKCD/HSPB1/NCK2/ITGAV/S100A8/MAPK8IP2/MUC1/UNC5B/CDKN2D/ZNF385A/MA
D74/CCDC88B/PTAFR/NCK2/IGLL5/SYK/IL18/VTCN1/CD320/TFRC/HLA-DPA1/HLA-DMB/JAK3/CC
/DTL/RNASEH2A/MCM6/FEN1/DSCC1/GINS2/MCM10/POLD1/MCM7/HRAS/PIF1/MCM3/NAP1L1/ALYR
B1/DLGAP5/BUB1B/MKI67/PRC1/KIF23/ZWINT/CCNE1/CENPF/KIFC1/RMI2/KIF18B/ECT2/NUSAP
KA/GPNMB/CDC25C/CDKN2A/HRAS/GTSE1/EZH2/CDK4/MAD2L1/MUC1/EME1/CDKN2D/ZNF385A/ARI
/FANCD2/PRNP/SYK/IL18/PAG1/TNFAIP8L2/VTCN1/TFRC/HLA-DPA1/HLA-DMB/TNFRSF18/VSIG4
HCK/MAPRE1/TMSB4X/MX2/TWF2/MID1IP1/MICALL2/TRIM6/CARMIL1/PLEKHG2/CCL26/MAP2/ARP
74/CCDC88B/PKN1/PRKCD/NCK2/ELF4/PRNP/SYK/IL18/CD151/VTCN1/CD320/TFRC/HLA-DPA1/H
BL/DDX27/NOP2/AATF/NOC2L/GTPBP4/NHP2/POP7/RPSA/NLE1/RPL10L/EMG1/NOP58/RPS5/ZNF5
/P2RX4/PRKCD/HSPB1/SLAMF8/NBL1/SYK/STK39/CKLF/PGF/TMSB4X/S100A8/CSF3R/TRPM2/RHO
ZB1/CLCF1/CD74/CCDC88B/PKN1/PRKCD/NCK2/ELF4/PRNP/SYK/IL18/CSF1R/CD151/VTCN1/CD3
/CD74/CCDC88B/PTAFR/NCK2/PRNP/SYK/IL18/PAG1/TNFAIP8L2/VTCN1/TFRC/HLA-DPA1/HLA-D
TP1/ALDH3B1/SOD3/PRKCD/HSPB1/FANCD2/SLC7A11/ABCD1/EZH2/NME2/MPV17/NCF4/GSR/TRPM
/RAD51AP1/C11orf80/ALYREF/EME1/CDC7/HMGB3/CHEK1/RHNO1/NABP2/MCM5/TFRC/PSMC3IP/A
3C/KIF18A/KIF21B/DTNBP1/MAP2/FLOT2/SSNA1/HIF1A/HSPA8/MAP1S/CENPE/KLC3/TTLL1/DYN
25A24/NPM1/UPF3B/ZC3H3/RRS1/NUP93/NXT1/NUP205/NUP37/RNPS1/NDC1/NUP188/NCBP2/FLO
1/ERI3/REXO4/RRP1/RRP7A/PA2G4/FBL/DDX27/NOP2/GTPBP4/NHP2/POP7/EMG1/NOP58/UTP18/
/NUP37/NDC1/POLR2H/NUP188/ZC3H12A/NCBP2/CNOT9/SCMH1/KCNQ1/HDAC2/CDK2/CHRAC1/MEG
N/KIF3C/NUP210/SSNA1/NPM1/UPF3B/TNFAIP2/DYNLRB1/IFT52/DYNLL1/RRS1/RELN/NUP93/NX
B3/CD320/TFRC/JAK3/TCF3/BAX/MIF/CD300A/PRKDC/MAD2L2/IL27RA/TCIRG1/SASH3/TPD52/T
/CEP131/TUBG1/SMARCD3/PLCB1/USH1C/GTSE1/MAPRE1/TUBB/CDC25A/CDK4/CDC7/TUBB4B/CHE
8/VTCN1/CD320/TFRC/HLA-DPA1/HLA-DMB/JAK3/CCL5/CD86/VAV1/TNFSF9/MIF/FLOT2/ZP3/HL
AS/S100A8/MUC1/CASP4/CDKN2D/ZNF385A/CREB3L1/DDIT3/TRAF2/YBX3/HMOX1/BAX/MIF/CASP
CD/ITGAV/IL15RA/IGLL5/ADORA1/RAB31/SYK/HCK/UNC13D/NCF4/RAB34/WIPF3/WASF2/VAV1/C
385A/ARID3A/PRMT1/DDIT3/BAX/MIF/PCBP4/CASP2/RRAGD/CNOT11/RRAGC/BRCA1/PCNA/BIN1/
A-DQA1/KIF11/TAP1/HLA-DRA/NCF4/HLA-DQB1/IFI30/RAB34/KIF3C/NCF1/KIF18A/PSMB9/HLA
/RRP7A/SNRPE/TXNL4A/SNRPF/NOP2/EIF3D/RPSA/NLE1/SRSF9/SNRPC/GEMIN7/RPL10L/ATXN2L
BP5/FSCN1/HNF1B/CARMIL1/RHOC/BAX/CCL26/MSN/ARPC4/LCP1/PAK1/BAIAP2L1/FCHSD1/BMF/
N2A/MCM6/EIF4EBP1/MCM10/MCM7/PLCB1/MCM3/RCC1/GTSE1/EZH2/CDC25A/CDK4/MUC1/CDKN2D
DC88B/PKN1/PRKCD/NCK2/ELF4/PRNP/SYK/IL18/CD151/VTCN1/CD320/TFRC/HLA-DPA1/HLA-DM
BAX/MIF/PRDX1/FLVCR1/HIF1A/JMJD6/TCIRG1/SASH3/ISG15/STAT1/AMPD3/UBAP2L/NCAPG2/S
HLA-DPA1/RBCK1/PSMD14/BAX/PHPT1/CD300A/HLA-DPB1/PSMD2/CD276/HLA-DQB2/CD3E/PAK1/
C/RAD54L/TUBG1/NCAPH/FANCD2/PLCB1/CDC25A/C11orf80/ASPM/EME1/HSF1/KIF18A/PSMC3IP
C1/ANKRD1/ZNF385A/ARID3A/CHEK1/RHNO1/PRMT1/BAX/MIF/PCBP4/CASP2/CNOT11/NUPR1/RNF
A/CDC25C/DTL/FEN1/RACGAP1/SMARCD3/PLCB1/NUP62/GTSE1/SPAG5/EZH2/CDC25A/CDK4/MAD2
DC80/NCAPG/ANLN/NEK2/BUB1/DLGAP5/BUB1B/MKI67/PRC1/KIF23/ZWINT/CENPF/KIFC1/KIF18
1B/BAX/PTK7/PSMD2/FZD1/BASP1/MLLT3/STAT1/ARHGEF19/PSMB2/FGFR1/PSME3/DVL3/PLEKHA
M6/EIF4EBP1/MCM10/MCM7/PLCB1/MCM3/RCC1/GTSE1/EZH2/CDC25A/CDK4/MUC1/CDKN2D/ZNF38

/CEP131/TUBG1/SMARCD3/PLCB1/USH1C/MAPRE1/TUBB/CDC25A/CDK4/CDC7/TUBB4B/PSMB9/PSM
FRC/IL34/TCIRG1/ATP6AP1/UBASH3B/F2RL1/TNFRSF11A/PIR/FSTL3/BATF2/TFE3/LEF1/IRF7/
MSB4X/TWF2/CFL1/MICALL2/CARMIL1/PLEKHG2/CCL26/ARPC4/BAIAP2L1/FCHSD1/BIN1/CORO7/
RPL17/RPLP2/EIF3D/RPSA/TRIM31/SSB/RPS5/NELFB/RPS21/RPL7/RPL28/CCL4/NUP85/DENR/N
/IL18/VTCN1/TFRC/HLA-DPA1/HLA-DMB/JAK3/CCL5/CD86/VAV1/TNFSF9/FLOT2/ZP3/HLA-DPB1
/NUP205/NUP37/RNPS1/NDC1/NUP188/NCBP2/FLOT1/U2AF2/SRSF9/XPOT/SSB/ZNF593/AKAP8L/
/NUP205/NUP37/RNPS1/NDC1/NUP188/NCBP2/FLOT1/U2AF2/SRSF9/XPOT/SSB/ZNF593/AKAP8L/
KN1/NCK2/PRNP/SYK/IL18/VTCN1/CD320/TFRC/HLA-DPA1/HLA-DMB/VSIG4/JAK3/CCL5/CD86/T
RAD51/NCAPD2/RAD54L/CDKN2A/MCM6/CENPW/DSCC1/NCAPH/GINS2/OIP5/MCM7/PIF1/MCM3/NAP
BRIX1/RRP7A/SNRPE/TXNL4A/SNRPF/NOP2/EIF3D/RPSA/NLE1/SRSF9/SNRPC/GEMIN7/RPL10L/A
B1B/PRC1/KIF23/ZWINT/CCNE1/CENPF/KIFC1/RMI2/KIF18B/ECT2/NUSAP1/P3H4/TTK/RCC2/PS
HCK/TMSB4X/TWF2/CFL1/S100A10/CARMIL1/WASF2/RHOC/PLEKHG2/CCL26/ARPC4/SH3PXD2B/SL
2A/GTSE1/EZH2/CDK4/MAD2L1/MUC1/CDKN2D/ZNF385A/ARID3A/CHEK1/PRMT1/PSMB9/PSMD14/B
N2D/IFT57/RPS6KA1/HSF1/CARD9/PSMB9/TRAF2/BAX/BEX3/TNFAIP8/RNF34/PERP/BCL2L12/NO
76/PRKDC/MAD2L2/SPRED1/BRCA1/PCNA/BCL2L12/PARPBP/DDIAS/DDX11/BID/OTUB1/FBXO5/AU
KC1/NPM1/UPF3B/ZC3H3/CCT2/RRS1/NUP93/NXT1/NUP205/NUP37/RNPS1/NDC1/NUP188/FBL/NC
LOT2/MTCL1/MSN/BRSK1/PTK7/SLC9A1/PAK1/PDZD11/RHOF/LMNA/HSP90AB1/SPDL1/MARK2/SFR
88B/PKN1/NCK2/PRNP/SYK/IL18/VTCN1/CD320/TFRC/HLA-DPA1/HLA-DMB/VSIG4/JAK3/CCL5/C
-DMB/JAK3/CCL5/CD86/VAV1/TNFSF9/FLOT2/ZP3/HLA-DPB1/EBI3/CD276/EFNB1/CD3E/PAK1/S
TSE1/EZH2/CDK4/MAD2L1/MUC1/CDKN2D/ZNF385A/ARID3A/PRMT1/PSMB9/PSMD14/BAX/LCMT1/P
/IL18/VTCN1/TFRC/HLA-DPA1/HLA-DMB/JAK3/CCL5/CD86/VAV1/TNFSF9/FLOT2/ZP3/HLA-DPB1
KA/CDC25C/DTL/HRAS/GTSE1/MAD2L1/MUC1/EME1/ZNF385A/ARID3A/CHEK1/RHNO1/NABP2/PRMT
PLP2/CNOT9/LSM7/RPSA/SSB/RPS5/RPS21/RPL7/RPL28/LSM2/RPL39/EDC3/RPL35A/EIF4A3/EX
TPBP4/NHP2/POP7/EMG1/NOP58/UTP18/RPS21/UTP14A/RPL7/NPM3/TRMT112/NSUN5/MPHOSPH6/
DCL3/CCT2/HSP90AB1/FKBP9/UNC45A/PPIA/CCT4/HSPBP1/MKKS/HSPH1/CCT7/FKBP4/GNB2/PPI
L17/RPLP2/TRAM1L1/RPSA/ITGB1BP1/RPS5/CDK5/ZDHHC18/RPS21/RPL7/RPL28/ZDHHC1/RPL39
MSB4X/TWF2/CARMIL1/PLEKHG2/CCL26/MAP2/ARPC4/PAK1/BAIAP2L1/FCHSD1/BIN1/ARPC2/ARP
PS6KA1/HSF1/CARD9/TRAF2/BAX/BEX3/TNFAIP8/RNF34/BCL2L12/NOL3/LEF1/HIP1R/BID/MT3/
/SLPI/FUCA2/ISG15/CHMP4C/POLR2G/SMARCA4/PPIH/TRIM59/F2RL1/STAT1/PABPC1/HDAC1/LE
9A1/PSMD2/HIF1A/GNGT1/NOL3/LMNA/S100B/HYOU1/PSMB2/PPARD/PSME3/MT3/NPEPPS/ADAM8/
1L1/CENPO/CENPU/SOX12/MCM5/SMARCD1/HNF1B/CENPL/HMGB2/GMNN/RUVBL1/VPS72/CENPE/TA
1/SYK/PLCB1/CKLF/PGF/S100A8/CSF3R/CCL5/VAV1/CCL26/MIF/CD300A/CREB3/MAPK3/SERPIN
PRE1/TUBB/CDC25A/CDK4/CDC7/TUBB4B/CHEK1/PSMB9/PSMD14/BRSK1/MIIP/SSNA1/PSMD2/PPP
B1B/PRC1/KIF23/ZWINT/CENPF/KIFC1/RMI2/KIF18B/NUSAP1/TTK/PSRC1/NCAPD2/TUBG1/FEN1
SF1/NABP2/MCM5/AP5Z1/PSMD14/SIRT6/GINS4/PRKDC/MAD2L2/RBBP8/BRCA1/NSMCE2/XRCC2/X
PO6/NUP188/CSE1L/NCBP2/U2AF2/SRSF9/XPOT/SSB/ZNF593/AKAP8L/IL1B/CDK5/TXN/CPSF1/T
UBB/CDC25A/CDK4/CDC7/TUBB4B/PSMB9/PSMD14/BRSK1/MIIP/SSNA1/PSMD2/PPP2R1A/CHMP4C/
/BAX/BEX3/MAPK3/PERP/PRELID1/HIP1R/PSME3/BID/BCAP31/PRR7/BBC3/PMAIP1/LYN/ADRM1/
K/STK39/CKLF/PGF/S100A8/CSF3R/TRPM2/CCL5/SLC12A2/VAV1/CXCR4/CCL26/MIF/CREB3/MAP
1/RASSF7/KIF18A/CHMP4B/MAP2/EFNA5/STIL/MAP1S/PAK1/NPM1/CHMP4C/BRCA1/DYNC1H1/XRC
/PHPT1/CD300A/HLA-DPB1/PSMD2/CD276/HLA-DQB2/CD3E/PAK1/THY1/THEMIS2/CTLA4/PSMB2/
NDC1/XPO6/NUP188/CSE1L/NCBP2/U2AF2/SRSF9/XPOT/SSB/ZNF593/AKAP8L/IL1B/CDK5/TXN/C
/NUP205/NUP37/RNPS1/NDC1/NUP188/NCBP2/FLOT1/U2AF2/SRSF9/XPOT/SSB/ZNF593/AKAP8L/
RE1/CFL1/S100A10/CARMIL1/RHOC/CCL26/ARPC4/SH3PXD2B/PAK1/BAIAP2L1/FCHSD1/BIN1/F2
M1/POLR2G/YBX1/PABPC1/PSMB2/ZC3H12A/PSME3/SLC11A1/PSMB4/PSMB8/PSMB10/PSMA5/PSMD
TGAX/FZD1/MLLT3/STAT1/ARHGEF19/PSMB2/FGFR1/PSME3/DVL3/PLEKHA4/SIX2/FZD2/RAC1/PS
F4/RPS5/RPS21/RPL7/EIF3I/RPL28/EIF2S3/DENR/RPL39/RPL35A/CDC123/RPS3/RPL27/EIF3H
FLNA/SPC25/TUBG1/RACGAP1/CENPW/KIF11/NUP62/RCC1/SPAG5/MAPRE1/TUBB/ASPM/EZR/RAN/

1/TAP1/HLA-DRA/NCF4/HLA-DQB1/IFI30/KIF3C/NCF1/KIF18A/PSMB9/HLA-DPA1/HLA-DMB/PSM
C/CCT3/TRIM28/CACYBP/BAX/SIRT6/PRKDC/MAD2L2/CHTF18/MAPK3/DKC1/ACD/FGFR4/BRCA1/P

CHMP4C/RBL1/YBX1/MYO19/TGFA/E2F8/INCENP/LBH/BLM/EVI2B/KIF14/ITGB1BP1/RAB11FIP4/
1/RASSF7/KIF18A/CHMP4B/MAP2/EFNA5/STIL/MAP1S/PAK1/NPM1/CHMP4C/BRCA1/DYNC1H1/XRC
4/CCDC88B/PKN1/NCK2/PRNP/SYK/IL18/VTCN1/CD320/TFRC/HLA-DPA1/HLA-DMB/VSIG4/JAK3/
A/NCF4/HLA-DQB1/IFI30/KIF3C/NCF1/KIF18A/PSMB9/HLA-DPA1/HLA-DMB/PSMD14/HLA-DPB1/
/PAK1/CRABP2/NRCAM/SLC12A8/HSP90AB1/GDI1/E2F4/SLC9A3R1/MT3/RAP1GAP2/KDM1A/RAC1/
T14/LAMA5/IFT52/DVL3/JAG2/FZD2/WNT7B/TULP3/DEAF1/TGFB2/FGFR2/DVL2/PFN1/RALA/PAX
A/NCF4/HLA-DQB1/IFI30/KIF3C/NCF1/KIF18A/PSMB9/HLA-DPA1/HLA-DMB/PSMD14/HLA-DPB1/
A1/PPP2R1A/STX1A/TUBA1A/STX6/RAB3B/SFI1/DYNLL1/DYNC1H1/CSNK1E/STX10/CEP89/HAUS1
L1/PLEKHG2/CCL26/ARPC4/BAIAP2L1/FCHSD1/BIN1/CORO7/ARPC2/GAS7/ARPC3/CORO1B/HIP1R
8/MAP1S/KLC3/TUBA1A/DYNLRB1/IFT52/DYNLL1/DYNC1H1/MYO19/BLOC1S4/CLN3/SFPQ/AP3M2/
FL1/CARMIL1/PLEKHG2/CCL26/ARPC4/BAIAP2L1/FCHSD1/BIN1/F2RL1/ARPC2/ARPC3/CORO1B/H
ARPC3/WIPF1/PSMB2/PSME3/ACTG1/NCKAP1L/MYO1G/SHC1/LYN/CARD11/RAC1/PSMB4/ACTB/CD4
1/VTCN1/TFRC/HLA-DPA1/HLA-DMB/VSIG4/JAK3/CCL5/BAX/CD86/TNFSF9/MSN/ZP3/HLA-DPB1/
1/PABPC1/PSMB2/ZC3H12A/PSME3/SLC11A1/PSMB4/PSMB8/PSMB10/PSMA5/PSMD13/PSMC4/SET/
RIM6/CCL5/CXCR4/HIF1A/SHARPIN/NOL3/SOCS1/IRF3/HSP90AB1/F2RL1/STAT1/IRF7/CPNE1/N
P8/SPRED1/BRCA1/BCL2L12/RRS1/HDAC1/DYRK2/RFC2/BLM/NOP2/NOC2L/HDAC2/CDK2/KDM1A/P
ACD/BRCA1/NSMCE2/CCT2/LMNA/DDX11/NELFE/RPS6KA4/CCT4/PNKP/KDM1A/CCT7/AKAP8L/IL1B
1/NBL1/PLCB1/STK39/PGF/TNFRSF18/HMOX1/BDKRB1/CCL5/MIF/CD300A/MSN/ZP3/CREB3/MAPK
A/HSP90AB1/DDX11/HYOU1/ARFGAP1/UBE2J2/EXTL3/HSPA4/CCL2/SHC1/ATF4/HSPH1/WFS1/DNA
FL1/CARMIL1/PLEKHG2/CCL26/ARPC4/BAIAP2L1/FCHSD1/BIN1/F2RL1/ARPC2/ARPC3/CORO1B/H
RPLP2/RPSA/TRIM31/RPS5/NELFB/RPS21/RPL7/RPL28/CCL4/NUP85/NUP43/RPL39/RPL35A/SMA
AM53B/PSMB2/PSME3/PLEKHA4/DAPK3/CSNK1D/AXIN1/NLE1/GPRC5B/PSMB4/PSMB8/PSMB10/PSM
4B/CCL5/MIF/ZP3/MAPK3/TRIM11/CHMP4C/POLR2G/SMARCA4/PPIH/F2RL1/PABPC1/LY86/LEF1/
U/SOX12/MCM5/HNF1B/CENPL/HMGB2/GMNN/RUVBL1/CENPE/TAF6/NPM1/CHAF1A/SMARCA4/ITGB3
F18/MAPK3/DKC1/ACD/ISG15/FGFR4/PCNA/CCT2/HSP90AB1/NPLOC4/RFC2/PPIA/CCT4/PNKP/NH
1A/TUBA1A/STX6/RAB3B/SFI1/DYNLL1/DYNC1H1/CSNK1E/STX10/CEP89/HAUS1/TUBB4A/CSNK1D
APH/FANCD2/PLCB1/CDC25A/C11orf80/ASPM/EME1/HSF1/KIF18A/PSMC3IP/ESPL1/SGO2/MND1/
X/CASP2/NUPR1/PPP2R1A/PEA15/BMF/PPP1CA/HIP1R/SLC9A3R1/BID/BCAP31/PRR7/DAPK3/YWH
VPS72/CHD3/PAK1/NPM1/SMARCA4/ACTL6A/HDAC1/CBX3/SCMH1/ITGB3BP/HDAC2/NASP/CHRAC1/
C1/ACD/NSMCE2/PCNA/CCT2/HSP90AB1/XRCC3/RFC2/CCT4/PNKP/BLM/NHP2/CCT7/PRIM1/TCP1/
PSMD2/EDA/NOL3/PSMB2/ZC3H12A/PSME3/BCL3/CPNE1/PSMB4/IL1B/PSMB8/PSMB10/PSMA5/PSM
PC1/PSMB2/ZC3H12A/PSME3/SLC11A1/PSMB4/PSMB8/PSMB10/PSMA5/PSMD13/PSMC4/SET/PSMD1
1/PRNP/HRAS/GPC1/EZR/RHOG/ANXA13/RHOQ/MTCL1/PKDCC/PAK1/SLC51B/CNPY4/GDI1/BID/RA
/CFL1/GIPC1/CHMP4B/RHOC/ESPL1/CHMP4C/MYO19/E2F8/LZTS2/INCENP/RAB35/RTKN/KIF14/R
D1/MCM7/MCM3/ALYREF/EME1/CDC7/GINS3/MCM5/POLA2/GMNN/GINS4/CHTF18/PCNA/TICRR/E2F
USAP1/TTK/TGFB1/AURKA/CDC25C/KIF11/NUP62/RCC1/MAD2L1/CHEK1/CHMP4B/ESPL1/LCMT1/N
ELID1/HIP1R/PSME3/BID/BCAP31/PRR7/BBC3/PMAIP1/LYN/ADRM1/F3/LCK/IFI16/CASP1/PML/
/SHARPIN/TNFRSF4/EDA/NOL3/F2RL1/STAT1/TNFRSF11A/PSMB2/KRT18/PSME3/CPNE1/KRT8/PS
A/CANT1/UBE2I/SHARPIN/EDA/SECTM1/IRF3/F2RL1/S100B/PIM2/TGM2/EEF1D/TNIP2/SLC35B2
CD3/PLCB1/NUP62/CDC25A/CDK4/MAD2L1/CDC7/HSF1/ESPL1/NSMCE2/XRCC3/TGFA/FGFR1/FBXO
RNPS1/NDC1/NUP188/NCBP2/U2AF2/SRSF9/AKAP8L/CPSF1/THOC5/NUP85/NUP43/AGFG1/THOC6/
PF/NUSAP1/TTK/TGFB1/AURKA/CDC25C/PLCB1/KIF11/NUP62/RCC1/MAD2L1/CHEK1/CHMP4B/ESP
RMT1/BAX/PCBP4/CASP2/PRKDC/CNOT11/RBL1/PCNA/CTDSPL/E2F8/PRMT2/E2F4/PSME3/BID/CN
C1/KIF23/ZWINT/CENPF/KIFC1/KIF18B/NUSAP1/TTK/PSRC1/NCAPD2/TUBG1/RACGAP1/DSCC1/N
C1/ACD/NSMCE2/PCNA/CCT2/HSP90AB1/XRCC3/RFC2/CCT4/PNKP/BLM/NHP2/CCT7/PRIM1/TCP1/
MB2/PSME3/SLC9A3R1/DVL3/PLEKHA4/FZD2/RAC1/PSMB4/VANGL1/PSMB8/PSMB10/PSMA5/PSMD1
RIM6/CCL5/CXCR4/HIF1A/SHARPIN/NOL3/SOCS1/IRF3/HSP90AB1/F2RL1/STAT1/IRF7/CPNE1/N
G2/CCL26/ARPC4/BAIAP2L1/FCHSD1/BIN1/ARPC2/ARPC3/CORO1B/HIP1R/NCKAP1L/CAPZA1/MKK
RMT1/BAX/PCBP4/CASP2/BRSK1/WDR76/PRKDC/CNOT11/PEA15/BRCA1/PCNA/TICRR/E2F8/E2F4/
SME3/DVL3/PLEKHA4/CSNK1D/FZD2/RAC1/PSMB4/VANGL1/PSMB8/PSMB10/GNB1/PSMA5/PSMD13/
C/HLA-DPA1/HLA-DMB/VSIG4/JAK3/CCL5/CD86/TNFSF9/ZP3/HLA-DPB1/EBI3/CD276/EFNB1/CD

RMT1/BAX/FAM83D/PCBP4/CASP2/PRKDC/CNOT11/RBL1/PCNA/CTDSPL/E2F8/PRMT2/E2F4/PSME3
3/BAX/MIF/CREB3/HIF1A/NUPR1/BCL2L12/BMF/NOL3/HDAC1/DDIAS/HYOU1/SLC9A3R1/BID/BCA
25C/HRAS/GTSE1/MAD2L1/MUC1/EME1/ZNF385A/ARID3A/NABP2/PRMT1/BAX/LCMT1/PCBP4/CASP
orf80/ASPM/EME1/HSF1/KIF18A/PSMC3IP/ESPL1/SGO2/MND1/PPP2R1A/SGO1/NDC1/FBXO5/SMC
LST8/HDAC2/HSPH1/LYN/FKBP4/CHORDC1/NUP85/NUP43/MYOF/RAE1/BAG3/NUP155/HSF2/HSBP1
/CCL5/CD86/TNFSF9/MIF/ZP3/HLA-DPB1/EBI3/CD276/EFNB1/CD3E/SASH3/TNFRSF4/SLC39A10
PA4/YWHAH/BBC3/PMAIP1/UBE2L3/GSK3A/YWHAQ/YWHAB/BAG3/GRPEL2/HK1/GZMB/DNM1L/TIMM4
WDR76/PRKDC/CNOT11/PEA15/BRCA1/PCNA/E2F8/E2F4/BLM/CNOT9/CDK2/THOC5/PML/DONSON/T
/JAK3/CCL5/CD86/TNFSF9/MIF/ZP3/HLA-DPB1/EBI3/CD276/EFNB1/CD3E/SASH3/TNFRSF4/SLC
1/PRMT1/BAX/MIF/PCBP4/CASP2/PRKDC/MAD2L2/CNOT11/SPRED1/BRCA1/PCNA/E2F8/E2F4/BID
K3/SERPINE1/ZNF580/C3AR1/F2RL1/FGFR1/NCKAP1L/CXCL13/RAC1/ITGA2/PLA2G7/CCL4/GPSM
SB/ZNF593/AKAP8L/CPSF1/THOC5/NUP85/NUP43/AGFG1/THOC6/RAE1/EIF4A3/NUP155/CPSF4/N
T52/DVL3/FZD2/TULP3/DEAF1/TGFB2/FGFR2/DVL2/PFN1/RALA/PAX8/KAT2A/TRIM71/WNT4/MED
VAV1/CCL26/CD300A/MAPK3/JAML/C3AR1/CCL13/PIP5K1C/NCKAP1L/ADAM8/CCL2/PIK3CD/CXCL
AP1/TTK/AURKA/PSRC1/FLNA/SPC25/TUBG1/RACGAP1/ARHGEF2/KIF11/NUP62/RCC1/MAD2L1/RA
T52/DVL3/FZD2/TULP3/DEAF1/TGFB2/DVL2/PFN1/RALA/PAX8/KAT2A/TRIM71/WNT4/MED12/OVO
SB/ZNF593/AKAP8L/CPSF1/THOC5/NUP85/NUP43/AGFG1/THOC6/RAE1/CPSF6/EIF4A3/XPO5/NUP
/JAK3/CCL5/CD86/TNFSF9/MIF/ZP3/HLA-DPB1/EBI3/CD276/EFNB1/CD3E/SASH3/TNFRSF4/SLC
/RAB10/RPL39/RPL35A/RPS3/RPL27/SSR3/RPL35/RPS20/RPL18A/PPP1R15A/RPS19/RPLP0/RPL
SB/ZNF593/AKAP8L/CPSF1/THOC5/NUP85/NUP43/AGFG1/THOC6/RAE1/EIF4A3/NUP155/CPSF4/N
PA4/YWHAH/BBC3/PMAIP1/UBE2L3/GSK3A/YWHAQ/YWHAB/BAG3/GRPEL2/HK1/GZMB/TIMM44/PDCD
LCMT1/NAA10/MAD2L2/ACD/BRCA1/SPDL1/XRCC3/OTUB1/FBXO5/NOC2L/KDM1A/ATAD2/DYNC1LI1
EKHA4/FZD2/RAC1/PSMB4/VANGL1/PSMB8/PSMB10/PSMA5/PSMD13/PSMC4/AP2M1/DVL2/PFN1/AP
18/BDKRB1/CCL5/ZP3/CREB3/MAPK3/SERPINE1/ZNF580/THY1/C3AR1/F2RL1/NCKAP1L/ADAM8/C
EKHA4/FZD2/RAC1/PSMB4/VANGL1/PSMB8/PSMB10/PSMA5/PSMD13/PSMC4/AP2M1/DVL2/PFN1/AP
MD14/SIRT6/GINS4/MAD2L2/RBBP8/BRCA1/NSMCE2/XRCC2/XRCC3/PARPBP/BLM/AUNIP/KDM1A/W
1/MARK2/SLC9A3R1/DYNLT1/GPSM2/FERMT1/FAM89B/RAB10/CYP26B1/MAP4/SNX27/SIPA1L3/LL
5/NAP1L1/CENPO/CENPU/CENPL/HMGB2/SIRT6/NAA10/RUVBL1/NPM1/CHAF1A/NCAPG2/NOC2L/IT
IRT6/GINS4/MAD2L2/RBBP8/BRCA1/NSMCE2/XRCC2/XRCC3/PARPBP/BLM/AUNIP/KDM1A/WAS/SWI
4/PSMB4/PSMB8/PSMB10/PSMA5/PSMD13/PSMC4/PSMD10/PSMD11/GCN1/PSMD1/PSMA7/PSMB3/BA
EIF4A3/RPS3/RPL27/RPL35/RPS20/RPL18A/RPS19/RPLP0/RPL18/RPL23/RPL38/RPL23A/GSPT2
PSMB10/PSMA5/PSMD13/PSMC4/PSMD10/PSMD11/GCN1/PSMD1/PSMA7/PSMB3/BAG3/HSF2/PSMD4/
L26/MAPK3/JAML/C3AR1/CCL13/PIP5K1C/NCKAP1L/CCL2/PIK3CD/CXCL9/CXCL3/CXCL13/RAC1/
L1/CHTF18/CHD3/ERCC6L/RFC2/DDX11/BLM/PARP1/CHD4/RUVBL2/MCM8/CETN2/XRCC6/POLQ/RB
SASH3/TNFRSF4/SLC39A10/MNDA/CTLA4/TNIP2/NCKAP1L/LYN/CARD11/NFAM1/TNFSF13B/PTPN6
ML/C3AR1/CCL13/PIP5K1C/NCKAP1L/ADAM8/CCL2/PIK3CD/CXCL9/CXCL3/CXCL13/RAC1/IL1B/C
XA13/RHOQ/EPB41L2/PKDCC/CNPY4/GPSM2/RANGRF/ACTB/CDK5/VIL1/AP2M1/STAC3/ACSL3/PRK
UP85/NUP43/AGFG1/THOC6/RAE1/EIF4A3/NUP155/CPSF4/NUP107/SLBP/CHTOP/SEC13/SYMPK/C
/FBXO5/TUBB4A/RANGRF/CSNK1D/CAMSAP2/FKBP4/RAC1/STMN3/INPP5J/TUBG2/KIF2A/RPS3/ZN
UP85/NUP43/AGFG1/THOC6/RAE1/EIF4A3/NUP155/CPSF4/NUP107/SLBP/CHTOP/SEC13/SYMPK/C
R3/RPL35/RPS20/RPL18A/RPS19/RPLP0/RPL18/SGTB/RPL23/RPL38/RPL23A/RPS7/RPL7A/RPS2
RAC1/PSMB4/VANGL1/PSMB8/PSMB10/PSMA5/PSMD13/PSMC4/AP2M1/DVL2/PFN1/AP2S1/PSMD10/
/PSMB10/PSMA5/NRAS/PSMD13/PSMC4/MUC5B/PSMD10/PSMD11/PSMD1/PSMA7/PSMB3/MUC20/PSM
SIRT6/RUVBL1/MAP1S/CHD3/NPM1/CHAF1A/SMARCA4/NOC2L/ITGB3BP/NASP/MTA2/CENPI/CENPK
VPS72/NPM1/CHAF1A/SMARCA4/ITGB3BP/NASP/CENPI/CENPK/NAP1L4/NAP1L2/SET/SMARCB1/SM
KDC/CNOT11/RBL1/PCNA/CTDSPL/E2F8/PRMT2/E2F4/CNOT9/CDKN2B/CCL2/CDK2/PML/CDKN2C/R
/GTPBP4/CDK2/EHMT2/RAC1/DONSON/TIPIN/TIMELESS/DBF4B/ESCO2/DNA2/CHEK2/NBN/GLI1/S
FLNA/SPC25/TUBG1/RACGAP1/KIF11/NUP62/RCC1/RAN/CHMP4B/STIL/CENPE/CHMP4C/MSTO1/DY
/PCBP4/CASP2/CNOT11/SPRED1/BRCA1/PCNA/E2F8/E2F4/BCL3/CNOT9/CDK2/KDM1A/PMAIP1/KM
/PSMB10/PSMA5/NRAS/PSMD13/PSMC4/MUC5B/PSMD10/PSMD11/PSMD1/PSMA7/PSMB3/MUC20/PSM
35/RPS20/RPL18A/RPS19/RPLP0/RPL18/SGTB/RPL23/RPL38/RPL23A/RPS7/RPL7A/RPS23/RPS1

MB4/VANGL1/PSMB8/PSMB10/PSMA5/PSMD13/PSMC4/AP2M1/DVL2/PFN1/AP2S1/PSMD10/PSMD11/
1/PCNA/BIN1/HSP90AB1/E2F8/E2F4/CNOT9/EVI2B/CDK2/CDK5/PML/ZBTB17/TFAP4/CALR/MED2
I30/KIF3C/KIF18A/HLA-DPA1/HLA-DMB/HLA-DPB1/CLTA/HLA-DQB2/CENPE/DYNLL1/DYNC1H1/L
A1/VAV1/ITGA5/ITGAX/CD63/ISG15/LAMA5/THY1/ITGB5/FERMT1/ITGB1BP1/ITGA2/BCAR1/CD4
1/CCL13/PIP5K1C/NCKAP1L/CCL2/PIK3CD/CXCL9/CXCL3/CXCL13/RAC1/IL1B/CCL4/TGFB2/C5A
/CNOT11/PCNA/TICRR/E2F8/E2F4/BLM/CNOT9/CDK2/PML/DONSON/TIPIN/MSH2/TOPBP1/RFWD3/
PM/SAC3D1/CHMP4B/CHMP4C/MSTO1/DYNC1H1/LZTS2/GPSM2/HAUS1/FBXO5/CSNK1D/KPNB1/HAUS
/RCC2/FEN1/RACGAP1/SPAG5/MAD2L1/ESPL1/LCMT1/NAA10/MAD2L2/CENPE/NSMCE2/DYNC1H1/S
KDC/CNOT11/RBL1/PCNA/CTDSPL/E2F8/PRMT2/E2F4/CNOT9/CDKN2B/CCL2/CDK2/PML/CDKN2C/R
I30/KIF3C/KIF18A/HLA-DPA1/HLA-DMB/HLA-DPB1/CLTA/HLA-DQB2/CENPE/DYNLL1/DYNC1H1/L
RPL18A/RPS19/RPLP0/RPL18/RPL23/RPL38/RPL23A/RPS7/RPL7A/RPS23/RPS10/RPS15/RPS16/
AF1/TGFB2/DVL2/PFN1/RALA/KAT2A/TRIM71/MED12/OVOL2/LUZP1/RARG/ABL1/SFRP2/RPS7/IF
18A/HLA-DPA1/HLA-DMB/HLA-DPB1/CLTA/HLA-DQB2/CENPE/DYNLL1/DYNC1H1/LAG3/HLA-DQA2/
44/PDCD5/TOMM40L/TIMM50/ROMO1/HSPD1/TIMM13/MFF/HSP90AA1/SH3GLB1/TOMM22/BNIP3L/S
/PSMB10/PSMA5/PSMD13/PSMC4/PSMD10/PSMD11/PSMD1/DONSON/PSMA7/PSMB3/TOPBP1/PSMD4/
/CCNO/BLM/GTPBP4/CDKN2B/CDKN1C/ACTB/PSMD10/CDKN2C/CDK7/CCND2/TFAP4
EIF3H/RPL18A/RPLP0/RPL18/DPH7/RPL38/DRG1/MCTS1/GSPT2/RPS23/EIF3K/EIF3M/RPL6/RPL
SMB10/PSMA5/PSMD13/PSMC4/PSMD10/PSMD11/PSMD1/HLA-B/PSMA7/PSMB3/CALR/PSMD4/PSMD3
A/RPS19/RPLP0/RPL18/RPL23/RPL38/RPL23A/RPS7/RPL7A/RPS23/RPS10/RPS15/RPS16/RPL5/
ORO1B/HIP1R/NCKAP1L/ARPC5L/CTTN/WAS/VIL1/PFN1/WASF1/GRB2/CDC42EP2/GMFG/ABI2/ARP
1/TUBB4A/CSNK1D/DCTN2/B9D2/HAUS8/PLK4/ODF2/CETN2/CEP250/HAUS6/NDE1/CKAP5/IQCB1/
/GTPBP4/CDKN2B/CDKN1C/ACTB/PSMD10/CDKN2C/CDK7/CCND2/TFAP4
/HLA-DPB1/EBI3/CD276/EFNB1/CD3E/SASH3/NCKAP1L/CD209/CARD11/IL1B/IL2RA/TNFSF13B/
AC1/IL1B/BCAR1/MAPK11/MYOF/CADM4/PRKD2/CDC42/AXL/PDCD6/EMILIN1/BRK1/MAPKAPK3/HS
/GRB2/CDC42/VPS25/APP/ADRA2A/CBL/ARAP1/PTPN12/PTPN2/ADAM17/IFI6/LGMN/HBEGF/RBPJ
AM83D/NPM1/NSMCE2/FBXO5/LSM10/ANAPC11/CCND2/APP/MTBP/CUL4B/DBF4B/RAD51C/HSPA2/M
L/LYN/CARD11/TNFSF13B/CD79A/BTK/PELI1/GPR183/HSPD1/ABL1/FCGR2B
8/E2F4/BLM/CNOT9/CDK2/PML/DONSON/TIPIN/MSH2/RFWD3/RAD9A/RPA2/BTG2/PIDD1/EME2/CH
RC7/LAMTOR5/VIL1/CD27/IFI16/GPX1/CST7/ARRB2/DNAJB6/DPEP1/CARD16/IGBP1/CARD18/SF
DAC1/DDIAS/HYOU1/BID/NOC2L/NONO/KDM1A/WFS1/TAF9/GPX1/MAPK7/PTTG1IP/GRINA/SFRP2/
1/LYN/IL2RA/TNFSF13B/SIT1/AXL/FADD/HCAR2/GPR183/ABL1/TNFAIP3/BCL10/ADAM17
KIF2A/ARF4/STX18/COPG1/BNIP1/NSF/COPG2/GOLPH3L/KIF22/TMEM115/ERGIC3/COPB2/ARF5/
AC1/ACTB/AP2M1/AP2S1/APH1B/CDC42/ARPC1A/CHN1/ARPC5/ACTR3/PTK2/AP2A2/CDK5R1/RHOA
SPL1/LCMT1/MAD2L2/CENPE/PPP2R1A/NSMCE2/SPDL1/XRCC3
SMD13/PSMC4/PSMD10/PSMD11/PSMD1/DONSON/PSMA7/PSMB3/TOPBP1/PSMD4/PSMD3/RBX1/PSMD
0/FGFR4/MMP2/FLOT1/ADAM8/CAPN1/CAPNS1/CTSS
8/PSMB10/PSMA5/PSMD13/PSMC4/ANAPC11/UBE2D1/PSMD10/PSMD11/PSMD1/PSMA7/UBE2E1/PSM
T1/NAA10/MAD2L2/CENPE/NSMCE2/SPDL1/XRCC3/DDX11/FBXO5/SFPQ/DYNC1LI1/ANAPC11
4/CNOT9/CDK2/PML/ZBTB17/MED25/RPL23/MYBBP1A/BTG2/PIDD1/CHEK2/PLK3/CRLF3/PPP2R5B
RC7/LAMTOR5/VIL1/CD27/GPX1/ARRB2/DNAJB6/DPEP1/CARD16/IGBP1/SFRP2/THBS1/CSNK2A1/
L/MYO1G/LYN/RAC1/ACTB/CD47/FGR/WAS/GRB2/CDC42/ARPC1A/BRK1/ARPC5/ABL1/FCGR2B/ACT
L/MYO1G/PLSCR1/LYN/RAC1/ACTB/CD47/FGR/WAS/GRB2/CDC42/ARPC1A/BRK1/ARPC5/ABL1/FCG

UP85/NUP43/RAE1/BAG3/NUP155/HSBP1/DNAJB6/NUP107/IER5/AKT1S1/DNAJC7/RPA2/MAPT/SE
CCT8/PTGES3/NAT10/WRAP53/NOP10/DCLRE1B/GAR1/HSP90AA1/HNRNPU/EXOSC10/HNRNPA1/MAP
RHEB/CCL3/PRPF19
1/CHAF1A/NOC2L/ITGB3BP/NASP/CENPI/CENPK/NAP1L4/NAP1L2/SET/SMYD3/RBBP4/CABIN1/HE
CIT/CDC42/CETN2/CALM2/CSPP1/PLK3/SH3GLB1/ZFYVE19/BCL2L1/RAB11A/KIF20B/KLHL21/RH
BL2/MCM8/RAD21/MTBP/RPA2/CCT8/GNL3/ESCO2/WRAP53/CENPQ/LEMD2/BOD1
SMD13/PSMC4/PSMD10/PSMD11/PSMD1/HLA-B/PSMA7/PSMB3/CALR/PSMD4/PSMD3/PSMD7/IKBKG/
IG1/PRIM1/DONSON/TIPIN/POLE4/POLA1/PRIM2/RPA2/POLD3/SLBP/DBF4B/DNA2/CHEK2/ZPR1/

/SPDL1/RRS1/KIF14/KPNB1/CHMP1A/KATNB1/NDE1/ACTR3/CENPQ/BOD1/HNRNPU/RAB11A/KIF22
4C/LZTS2/INCENP/RAB35/RTKN/CHMP3/CIT/KLHDC8B
1/PCNA/E2F8/E2F4/CNOT9/CDK2
1/PCNA/E2F8/E2F4/CNOT9/CDK2
/FBXO5/LSM10/ANAPC11/CCND2/APP/MTBP/CUL4B/DBF4B/RAD51C/HSPA2/MTA3/CCND3/ADAM17/
/LYN/RAC1/ACTB/CD47/FGR/WAS/GRB2/CDC42/ARPC1A/BRK1/ARPC5/ABL1/FCGR2B/ACTR3/HSP9
GES3/NAT10/WRAP53/NOP10/GAR1/HSP90AA1/HNRNPU/EXOSC10/HNRNPA1/MAPKAPK5/RPA1/ATR/
NAT10/NBN/HNRNPU/ERCC1/EXOSC10/HNRNPA1/MAPKAPK5/ATR/HDAC8/SMG7/HNRNPD/DCP2/HNRN
/LYN/RAC1/ACTB/CD47/FGR/WAS/GRB2/CDC42/ARPC1A/BRK1/ARPC5/ABL1/FCGR2B/ACTR3/HSP9
8/RPL23A/TRAF7/RPL7A/RPL5/RPF2/NVL/RPL6/PAK1IP1/MRPL11/RSL1D1/EBNA1BP2/NOL9/EIF
PRDX4/PDIA6/HVCN1/TXNDC9/TXNDC12/EGLN2/PRDX5/TMX2/GLRX2/PDIA3/SCO2/P4HB/QSOX2/P
/SPDL1/RRS1/KIF14/KPNB1/CHMP1A/KATNB1/NDE1/ACTR3/CENPQ/BOD1/HNRNPU/RAB11A/KIF22
BIRC7/IL2RA/CD27/DFFA/FADD/BTK
1/PCNA/E2F8/E2F4/CNOT9/CDK2
2/ARRB2/PARM1/CCT8/PTGES3/WRAP53/HSP90AA1/NVL/HNRNPA1/RFC5/MAPKAPK5
S2/INCENP/RAB35/RTKN/CHMP3/CIT/KLHDC8B
SMD10/PSMD11/PSMD1/HLA-B/PSMA7/PSMB3/CALR/PSMD4/PSMD3/PSMD7/IKBKG/PSMD8/PSMA1/V
/SPA17/VDAC2
TB/MTA2/CENPI/CENPK/SMARCB1/CHD4/RBBP4/SMARCC1/CENPN/RBBP7/CENPQ/MBD3/ANP32E/MI
RPS3/POLQ/OGG1/PTTG1IP/SFRP2/UBQLN4/CHEK2/MAGEF1/ERCC1/BCL2L1/UBR5/TWIST1/CCAR2
MT3/AATF
CENPE/NSMCE2/SPDL1/XRCC3
FSF13B/BTK/PELI1/GPR183/FCGR2B/CD38/ATAD5/INPP5D/PTPRC/IKZF3/CASP3/CD22
9/CDK2/PML/RFWD3/RPA2/BTG2/PIDD1/CHEK2/PLK3
PS3/PRKD2/PNP/CLEC7A/ABL1/TNFAIP3/IL20RB/STOML2/GLMN/XCL1/CD247/PTPRC/MAP3K7/TR
L2/RPS10/RPS15/RPL5/RPF2/RPL6/NSUN4/MTERF3/MRPL11/EIF6/RPS14/RPS27/DHX30/NIP7/R
9/CDK2/PML/RFWD3/RPA2/BTG2/PIDD1/CHEK2/PLK3
/CBL/VAMP8/MILR1/C12orf4/STXBP1/ADORA2B/IL13RA2/CD84/NECTIN2/CRLF2/CNR1/RHOH/AD
PPP2R1A/NSMCE2/SPDL1/XRCC3/FBXO5/NCAPH2/DYNC1LI1/ANAPC11
NSMCE2/SPDL1/XRCC3
DD/ABL1/TNFAIP3/BCL10
SP/CENPI/CENPK/NAP1L4/NAP1L2/SET/SMYD3/RBBP4/CABIN1/CENPN/RBBP7/CENPQ/HAT1/MIS1
NAT10/WRAP53/NOP10/GAR1/HSP90AA1/HNRNPU/EXOSC10/HNRNPA1/MAPKAPK5/RPA1/ATR/SMG7/
ONSON/TIPIN/POLE4/POLA1/PRIM2/RPA2/POLD3/SLBP/DBF4B/DNA2/CHEK2
9/CDK2/PML/RFWD3/RPA2/BTG2/PIDD1/CHEK2/PLK3
1A/RANBP1/CEP250/NSFL1C/FBXW5/MARK4/TRIM37/CENPJ/RBM14
RRS1/KIF14/KPNB1
XO5/DYNC1LI1/ZNF207/RAD21/MTBP/NME6
HNRNPA1/MAPKAPK5/ATR/SMG7/DCP2/HNRNPC/MAPK15/TERF1/HNRNPA2B1/XRCC5/PARN/SLX4
NSMCE2/SPDL1/XRCC3
/SPDL1/XRCC3
9/CDK2
9/CDK2
9/CDK2
/MAP1A/MARK4/NEDD1/KIF20B/KLHL21/ABHD17A
9/CDK2
9/CDK2

/SPDL1/XRCC3/FBXO5/DYNC1LI1/ANAPC11/ZNF207/RAD21

L20RB/TLR2/IDO1/XCL1

7/CHMP1A/WRAP73/DRG1/MZT1/CHEK2/CCSAP/HNRNPU/RAB11A/RHOA
/XRCC3/FBXO5/DYNC1LI1/ANAPC11/ZNF207/RAD21

/XRCC3/FBXO5/DYNC1LI1/ANAPC11/ZNF207/RAD21
DYNC1LI1/ZNF207/RAD21/MTBP/NME6

/HLA-F/CACNA1D/STXBP1/ADORA2B/ARF1
orf4/STXBP1/ADORA2B/SERPINB9/IL13RA2/CD84
TXBP1/ADORA2B/IL13RA2/CD84
GDS/PTGES3
CALR/EPB41L1/WDR1/EPB41L3/FMNL3
A/ERCC1/UBA52/FANCF/RNF8/RPA1/MUS81/MSH6/ATR/DCLRE1C/EXO5

SFL1C/ACTR3/SPIRE1/DCTN1

TXBP1/ADORA2B/IL13RA2/CD84
/SPIRE1/DCTN1
CL17/AKIRIN1/THBS1

D177/HLA-F/STXBP1/ADORA2B/IL13RA2/CD84/BCR/ADGRE2/CCR2/PRAM1
CALR/EPB41L1/EPB41L3/FMNL3/TLN1/WIPF2/RAB13/LLGL2/VPS4A/NCKAP1/EPB41
Q/MIS18A/SMARCA5/POLE3

Q/ANP32E/MIS18A/RNF8/SMARCA5/DMAP1/POLE3/SRCAP

A2/CD84/NECTIN2/CRLF2/CNR1

/CENPN/RBBP7/CENPQ/MIS18A/SMARCA5/POLE3

8A/SMARCA5/POLE3

F1
8A/SMARCA5/POLE3

P7/CENPQ/HAT1/MIS18A/SMARCA5/POLE3/IPO4

P7/CENPQ/HAT1/MIS18A/SMARCA5/POLE3/IPO4

D21

D21

EF2/CYTH4/ROBO1/HRAS/RAB31/CDC42EP1/NUP62/FGD3/DNMT1/COL3A1/RHOG/RAB34/CFL1/RHO
S1/SIRT6/NAA40/RUVBL1/JMJD6/MAPK3/PYGO2/MRGBP/BRCA1/MCRS1/ACTL6A/LMNA/HDAC1/PRM
GAP1/TAX1BP3/CHML/PLXNA3/HRAS/ADAP1/PLCB1/DEPDC1/GMIP/EZH2/FGD3/GIT1/ASAP1/ADAP
CNE1/CCNA2/TRIB3/CSF1/ECT2/CCNF/TGFB1/IQGAP3/STK26/DKK1/RIPK2/UNC119/PSRC1/GSTP
D/ETV5/NCS1/LIF/SERPINE2/CSF1/ECT2/TGFB1/ARHGAP4/LGALS1/DKK1/CLCF1/TNFRSF12A/FL
DKK1/CLCF1/TNFRSF12A/FLNA/ARHGEF2/P2RX4/ROBO1/SERPINI1/PLXNA3/SMARCD3/NME1/NBL1
RO3/SRC/CD3D/LGALS3/RAC2/TYROBP/S100A14/TGFB1/ITGAM/FCGR3A/RIPK2/ADA/HAVCR2/COL
GFB1/IQGAP3/STK26/DKK1/RIPK2/UNC119/PSRC1/GDF15/IRAK1/S1PR2/CD74/PKN1/DUSP5/DSC
ALG3/MGAT5/JAK3/DDOST/CANT1/B4GALT5/TMEM165/GALNT10/B3GNT5/GBGT1/FUT4/B3GNT9/SL
K39/CDC7/GPD1L/TRIM6/BAX/MAPKAPK2/MIF/NAA10/PRKDC/SH2D3A/MAD2L2/MAPK3/MAST2/MAP
S/SYK/HCK/S100A8/MUC1/HMGB3/GBP5/CARD9/PSMB9/CTSK/PSMD14/HMGB2/TRIM6/CCL5/MAPKA
AURKA/DKK1/RIPK2/PRKCD/SMARCD3/NME1/MAPRE1/LARP6/TMSB4X/ADAM15/RAN/HSF1/CRMP1/M
/NME2/FOXQ1/PKP3/KRT7/COL3A1/COMP/AKR1C3/LTB/CTSK/COL5A1/FRAS1/ST14/SHARPIN/PER
HCK/S100A8/MUC1/CARD9/PSMB9/CTSK/PSMD14/MAPKAPK2/COLEC12/CD300A/UNC93B1/PRKDC/P
1/CARD9/APOBEC3C/TRIM6/CCL5/CXCR4/SLFN13/UNC93B1/NT5C3A/TRIM11/ISG15/SPON2/IRF1
RAB31/PACS1/PKP3/EZR/BSG/SCRIB/RHOG/RAB34/ANXA13/RHOQ/S100A10/FLOT2/PKDCC/RAB15
/POLR2G/SMARCA4/PPIH/TRIM59/STAT1/PABPC1/HDAC1/LEF1/NELFE/POLR2H/PPIA/ZC3H12A/D
ALL2/SAE1/TRIM28/NUTF2/MAP2/MTCL1/MSN/OAZ1/PAK1/CAPN10/SLC51B/DYNC1H1/ZC3H12A/N
9A1/PSMD2/HIF1A/GNGT1/NOL3/LMNA/S100B/HYOU1/PSMB2/PPARD/PSME3/MT3/NPEPPS/ADAM8/
9A1/PSMD2/HIF1A/ATP6AP1/GNGT1/NOL3/LMNA/S100B/HYOU1/PSMB2/PPARD/PSME3/MT3/NPEPP
/PGF/TMSB4X/CCL5/CXCR4/SEMA4A/CCL26/MIF/CREB3/MAPK3/SERPINE1/ZNF580/C3AR1/F2RL1

SF2/RABEP2/CORO1C/F2RL1/ARPC2/DYNLL1/TBC1D30/RAC1/MIEN1/WAS/PFN1/MAP4/TACSTD2/C
RI/CPNE1/HDAC2/KDM1A/ITGA2/ZNF593/TXN/IFI16/PARP1/TFAP4/ANKRD33/HEY1/GZMA/ZNF67
D2/TULP3/DEAF1/TGFB2/DVL2/PFN1/RALA/KAT2A/TRIM71/MED12/OVOL2/LUZP1/RARG/ABL1/SF
6L/RFC2/DDX11/BLM/PARP1/CHD4/RUVBL2/MCM8/CETN2/XRCC6/POLQ/RBX1/CUL4B/DNA2/CHD1L
1/NDC1/FBXO5/PSMD13/MYBL1/RAD21/RAD51C/HSPA2/RAD51D/EME2/SYCE1L/ERCC1/CCNE2
CKAP1L/CCL2/CXCL13/LYN/RAC1/PLA2G7/CCL4/GPSM3/CCR1/C5AR1/PADI2/CALR/XCL2/CMKLR1
AP1S/F2RL1/CAPZA1/CAMSAP2/STMN3/VIL1/NES/KIF2A/CAPZB/SWAP70/SH3GL1/KATNB1/WDR1/
/GRB2/CDC42/VPS25/APP/ADRA2A/CBL/ARAP1/PTPN12/PTPN2/ADAM17/IFI6/HBEGF/BTC/EREG/
C1/PLA2G7/CCL4/GPSM3/CCR1/C5AR1/CALR/XCL2/CMKLR1/SWAP70/LBP/CCL3/S100A7/CXCL17/
K1/DAP/MAPT/PDCD6/CYCS/MTCH1/HSPD1/PIDD1/VCP/TRADD/BAD/TNFSF15/CASP8/PDCD2
B3/PSMD4/PSMD3/RBX1/PSMD7/PSMD8/VHL/PSMA1/PSMB5/PSMA4/PSMD9/NOTCH1/RPS27A/VEGFA

1/MAPKAPK5/ATR/SMG7/DCP2/HNRNPC/MAPK15/TERF1
2/RFC5/SMARCAL1/MSH6/ATG7/SLFN11/TERF1/E2F7/ZMPSTE24/LIG3/SMC1A/WIZ
L/UBE2N/STUB1/RAB1A/PIN1/RPL11/PARK7
L1

/STRAP/GEMIN2/COIL/SNRPD3/PRMT5/DDX20/GEMIN8/GEMIN6/SMN2/GEMIN5/SRSF10/PRMT7/AA

WIZ

C1/WNK2/FZD6/ASPM/TLE3/DCDC2/FZD7/CTNND2/MDFI/PSMB9/DDIT3/PSMD14/HNF1B/GRK6/PTK
1/PSMB9/GIPC1/DDIT3/RBCK1/PSMD14/FBXL19/VPS37C/UBE2Z/PSMD2/RNF34/UBE2I/RNF19B/S
CCNF/AURKA/PPT1/CTSV/SCPEP1/SQSTM1/DTL/UHRF1/CPVL/UBXN11/MAD2L1/CTSA/EZR/PSMB9/
SLC25A6/MICALL2/ATAD3B/CTSK/SAE1/ATAD3A/BAX/RAB32/FANCG/SLC9A1/FUNDC1/HIF1A/SMI
/VASP/PLEK/HRAS/CDC42EP1/HCK/TMSB4X/TWF2/CSF1R/SMIM22/TRPM2/RHOG/CFL1/RHOQ/S100
AK1/CD74/CCDC88B/PTAFR/NCK2/ITGAV/EMILIN2/ADAM9/SYK/UNC13D/IL18/COL16A1/VTCN1/S
K39/CDC7/GPD1L/TRIM6/BAX/MAPKAPK2/MIF/PRKDC/SH2D3A/MAD2L2/MAPK3/MAST2/MAP2K2/PA
OA8/MUC1/CARD9/PSMB9/CTSK/PSMD14/MAPKAPK2/COLEC12/CD300A/UNC93B1/PSMD2/PAK1/IRF
RSF18/JAK3/CD86/TNFSF9/CD2/IL34/ATP6AP1/SASH3/UBASH3B/SOCS1/IRF1/CTLA4/FSTL3/CD
NFSF9/PRKDC/CD2/JMJD6/CD3E/TCIRG1/SASH3/SOCS1/IRF1/CTLA4/CD83/PLA2G2D/PRELID1/L
HSPA8/MAP1S/MRPL17/MRPS12/F2RL1/MRPL47/MRPL9/MRPS23/CAPZA1/CAMSAP2/MRPL12/STMN3
ASH3/ISG15/SOCS1/STAT1/RAB7B/CD83/LEF1/NCKAP1L/ADAM8/EVI2B/HCLS1/JAG1/CBFB/IL2R
/AXIN1/NLE1/GPRC5B/PSMB4/PSMB8/PSMB10/PSMA5/PSMD13/PSMC4/FGFR2/RNF220/PSMD10/PS
A/IFT52/TGM2/DVL3/FZD2/ITGB1BP1/TULP3/DEAF1/TGFB2/FGFR2/DVL2/PFN1/RALA/PAX8/KAT
1/PRR7/BBC3/PMAIP1/F3/LCK/IFI16/CASP1/PML/RPS3/TFAP4/CTSD/HSPE1/PDCD5/FADD/CLEC
3/NUPR1/PPP2R1A/WDR77/FBXO5/HDAC2/ARHGDIB/OXTR/SYDE1/AGO2/CALR/PRDX4/MSX1/HPGDS
O1/XRCC3/CEP85/HAUS1/CDK2/CHORDC1/POC1A/CHMP3/HAUS8/PLK4/ODF2/CROCC/PARD6B/KAT2
HSD1/BIN1/ARPC2/ARPC3/CORO1B/HIP1R/TPPP3/NCKAP1L/ARPC5L/RAC1/CTTN/WAS/VIL1/PFN1
P1/F3/LCK/CASP1/PML/RPS3/TFAP4/CTSD/HSPE1/PDCD5/FADD/CLEC7A/RACK1/DAP/MAPT/PDCD
O1/XRCC3/CEP85/E2F4/HAUS1/CDK2/CHORDC1/POC1A/CHMP3/HAUS8/PLK4/ODF2/CROCC/PARD6B
H/IRF7/FLOT1/NLRC5/RNF26/PQBP1/IFI16/TRAF3/POLR2L/DDX41/POLR2K/XRCC6/GPATCH3/UB
AK1/SLC51B/CNPY4/BID/RANGRF/YWHAH/BBC3/PMAIP1/ITGB1BP1/CDK5/VIL1/STAC3/YWHAQ/YW
H/IRF7/FLOT1/NLRC5/RNF26/PQBP1/IFI16/TRAF3/POLR2L/DDX41/POLR2K/XRCC6/GPATCH3/UB
CD1/IRF7/NOC2L/ADAM8/PIK3CD/KDELR1/HCLS1/LYN/BIRC7/IL2RA/TCP1/CD27/DFFA/CCR5/AX
YWHAB/CHCHD3/GZMB/PDCD5/STPG1/TIMM50/ROMO1/LETM1/TAZ/RHOT2/SLC25A5/TIMM13/APOO/
VL2/PFN1/RALA/KAT2A/TRIM71/MED12/RARG/ABL1/SFRP2/RPS7/IFT172/BCL10/LMO4/MTHFR/K

/RPS3/KATNB1/CLIP3/DRG1/MAPT/CKAP2/ABL1/TBCD/STMN2/DCTN1/MAP1A/EML2/MECP2/CDK5R
DE1/CLIP3/DRG1/MAPT/CKAP5/MZT1/ABL1/TBCD/STMN2/DCTN1/MAP7D3/EML2/MECP2/CENPJ/CD

PKAPK5
/CYP19A1/THBS1

E4/TYW3

BP1/PRNP/LARP6/EEF1E1/CDK4/TCOF1/CTSA/GSR/IGF2BP3/ZNF385A/RPL8/RPS6KA1/GSTM3/CP
/DCAF13/RUSC1/PJA1/MAD2L1/RNF24/RNF145/PSMB9/TRAF2/RBCK1/PSMD14/SAE1/FBXL19/TRI
1/PSMB9/GIPC1/DDIT3/RBCK1/PSMD14/FBXL19/VPS37C/UBE2Z/PSMD2/RNF34/UBE2I/RNF19B/S
/DTL/UHRF1/CPVL/UBXN11/MAD2L1/CTSA/PSMB9/GIPC1/DDIT3/CTSK/RBCK1/PSMD14/FBXL19/C
C1/WNK2/FZD6/ASPM/TLE3/DCDC2/FZD7/CTNND2/MDFI/PSMB9/DDIT3/PSMD14/HNF1B/GRK6/PTK
EGFB/ELOVL7/UAP1L1/SLC2A6/B4GALNT1/ACSL4/ACSS1/DTYMK/GAL3ST1/TGFB1/PPT1/GAPDH/A
/ECT2/TGFB1/IQGAP3/GPNMB/LGALS1/DKK1/RIPK2/ADA/HAVCR2/CLCF1/FLNA/BIRC3/CD44/GDF
/DPM2/MUC1/CHST4/ALG3/MGAT5/JAK3/DDOST/CANT1/B4GALT5/TMEM165/HIF1A/GALNT10/B3GN
ZNF385A/SYNGR2/TLCD1/SPAG4/UBL4A/DTNBP1/CHMP4B/SYNGR1/COL5A1/CXCR4/VPS37C/SEC61
/CHMP4B/TRIM28/CCL5/CD86/CXCR4/NUP210/ITGA5/SELPLG/VPS37C/HAVCR1/TRIM11/SLPI/IS
8/SYK/IL18/FZD7/HMGB3/TNFRSF18/JAK3/TCF3/BAX/CD86/VAV1/SEMA4A/TNFSF9/PRKDC/CD2/
IRC3/ITGB2/IRAK1/PRKCD/RELB/HRAS/SYK/HCK/IL18/S100A8/MUC1/C2CD4A/HMGB3/GRN/NPAS
BEP2/TCTN2/SSNA1/PPP2R1A/LAMA5/TUBA1A/TTLL1/SFI1/DYNLRB1/IFT52/DYNLL1/DYNC1H1/C
LC7A11/ADORA1/PRNP/HRAS/FOXQ1/UNC5B/GRN/HSF1/DDIT3/TRAF2/CHMP4B/HMOX1/CCL5/BAX/
CK2/BAIAP2L2/VASP/ADORA1/PLEK/HRAS/CDC42EP1/HCK/TMSB4X/TWF2/CSF1R/SMIM22/TRPM2/
BEP2/TCTN2/SSNA1/PPP2R1A/LAMA5/TUBA1A/TTLL1/SFI1/DYNLRB1/IFT52/DYNLL1/DYNC1H1/C

2RX4/C1QA/LAMB1/GPC1/EZH2/S100A8/CSF1R/SCRIB/GRN/AREG/CXCR4/NDN/B4GALT5/IL34/MA

ASP4/ANKRD1/TNFRSF11B/PSMB9/LTB/TNFRSF18/TRAF2/RBCK1/PSMD14/YBX3/CCL5/CCL26/TNF
9/TRAF2/RBCK1/HMOX1/RHOC/TRAF5/FKBP1A/CANT1/UBE2I/SHARPIN/EDA/SIRPA/TRIM59/SECT
X/DNAJC5/CASP2/UBE2M/HIF1A/NUPR1/XRCC2/HSP90AB1/HYOU1/BID/MT3/ADAM8/NONO/CCL2/A
/ANKRD1/HSF1/HMGB2/CCL5/CD86/MIF/MAPK3/SERPINE1/SIRPA/SPON2/IRF3/LY86/PPARD/LDO
B3L1/TRAF2/HMGB2/YBX3/HMOX1/BAX/MIF/CREB3/HIF1A/SERPINE1/RNF34/PEA15/SLC35F6/BR

/LAMA5/NRCAM/PALLD/CCT2/UBAP2L/EPHB3/CCT4/CCT7/CD209/TCP1/FUT3/FOLR2/SPESP1/EMB
C1/PPIA/ZC3H12A/LAMP3/TRIM31/SNF8/PLSCR1/APOBEC3G/CHMP3/IFI16/BANF1/PML/ILF3/IS
1/U2AF2/SRSF9/SSB/RPS21/CPSF1/THOC5/INTS1/THOC6/CPSF6/EIF4A3/EXOSC5/BARD1/CPSF4
1/ZC3H12A/LAG3/NCKAP1L/ADAM8/FANCA/CARD11/CBFB/IL2RA/CD27/IL12RB1/CYP26B1/IL15/
1/ZNF205/GSK3A/PSMD10/GPX1/YWHAQ/YWHAB/ARRB2/GZMB/DNM1L/PDCD5/STPG1/TIMM50/HSPD
/PPIA/NELFB/CHMP3/SMARCB1/TFAP4/POLR2L/POLR2K/SUPT5H/SRPK1/NOTCH1/TRIM21/TARBP2
DAPK3/ARHGDIB/HCLS1/CDC42BPG/BCAR1/RAP2A/CDK5/CTTN/TNIK/PARVG/RALA/CDC42/GMFG/A
DC42/TGFB3/ZMYND8/CDC42EP2/WRAP73/BRK1/AKIRIN1/PALM/ARAP1/DNM2/MARK4/DEF8/CENPJ
VL2/PFN1/RALA/KAT2A/TRIM71/MED12/RARG/ABL1/SFRP2/RPS7/IFT172/BCL10/LMO4/MTHFR/K
IL4R/APP/CLEC7A/LBP/TUSC2/ADAM17/MAVS/TLR2/ALOX15B/TWIST1/SIGIRR/ADORA2B/EPHA2/
FA/PML
13/LYN/ITGB1BP1/ITGA2/TGFB2/PTPN6/PIEZO1/SWAP70/ITGB8/SFRP2/PTK2/ADAM17
GES3/HSPA2/SGTA/TOR2A/CSNK2A1/HSPA5/HSPA14/PPIB/GAK

ML2/MECP2/CDK5R1/NIN
X/LUM/EMILIN1/COL11A1/SFRP2
A
L20/RPL12

1/PSMB9/GIPC1/DDIT3/RBCK1/PSMD14/FBXL19/VPS37C/UBE2Z/PSMD2/RNF34/UBE2I/RNF19B/S
25B/B4GALNT1/SRC/LHFPL2/ETV5/RPL39L/KIFC1/SERPINE2/TTK/GAL3ST1/TGFB1/FAM50A/AUR
O3/CDC25B/B4GALNT1/SRC/ITGA3/ETV5/RPL39L/ACSL4/LIF/KIFC1/SERPINE2/AKR1B1/DSG2/T
O3/CDC25B/B4GALNT1/SRC/ITGA3/ETV5/RPL39L/ACSL4/LIF/KIFC1/SERPINE2/AKR1B1/DSG2/T
NP/HRAS/SYK/MTMR2/NUP62/GTSE1/UNC13D/SPAG5/MX2/GIT1/EZR/RAN/MICALL2/RANGAP1/GIP

1/ADAM9/HRAS/SYK/STK39/NUP62/EZH2/MAGED1/DTNBP1/TRAF2/MAPKAPK2/CXCR4/MIF/CD300A

K7/RUVBL1/MAD2L2/PSMD2/FZD1/PYGO2/BCL9/IGFBP6/EDA/MLLT3/HDAC1/CSNK1E/LZTS2/FAM5

2/YWHAZ/FLNA/ITGB2/CEP131/PRKCD/SCAMP5/B3GAT3/PRNP/ADAM9/HRAS/SYK/GTSE1/CCT5/CS
RNP/HRAS/FOXQ1/UNC5B/GRN/HSF1/DDIT3/TRAF2/CHMP4B/HMOX1/CCL5/BAX/DNAJC5/CASP2/UB
K7/RUVBL1/MAD2L2/PSMD2/FZD1/IGFBP6/EDA/MLLT3/HDAC1/CSNK1E/LZTS2/FAM53B/PSMB2/ZN

CK1/HMOX1/RHOC/TRAF5/FKBP1A/CANT1/UBE2I/SHARPIN/EDA/SIRPA/TRIM59/SECTM1/IRF3/F2
IM28/CXCR4/SEMA4A/SIRT6/NDN/MAP2/IQGAP1/PTK7/EFNA5/PAK1/CRABP2/NRCAM/HSP90AB1/L
UBE2M/HIF1A/NUPR1/XRCC2/HSP90AB1/HYOU1/MT3/ADAM8/NONO/CCL2/ATF4/KIF14/CLN3/CDK5
AFR/PGF/DDR1/PTHLH/BSG/COL16A1/HSF1/TRIM28/ITGA5/SH3PXD2B/MAPK3/MMP2/CAD/PPARD/
MP4B/TRIM28/CD86/CXCR4/ITGA5/SELPLG/HAVCR1/TRIM11/FUCA2/CHMP4C/TNFRSF4/ITGB5/TR
O/CARMIL1/RHOC/CCL26/ARPC4/SH3PXD2B/PAK1/BAIAP2L1/FCHSD1/BIN1/F2RL1/ARPC2/ARPC3
2/HSP90AB1/ZC3H12A/SVBP/HSPBP1/OTUB1/FBXO5/GTPBP4/PRR7/AXIN1/U2AF2/KDM1A/BIRC7/
RABEP2/CORO1C/F2RL1/ARPC2/DYNLL1/TBC1D30/RAC1/MIEN1/WAS/PFN1/MAP4/TACSTD2/CROCC
/TRIM6/TRIM28/FKBP1A/GMNN/PYGO2/ACD/GPSM1/HSP90AB1/DDX11/HIP1R/FLOT1/EIF3D/KDM1
OLR2/SPESP1/STX18/VPS18/CALR/CCR5/SYT1/DNM1L/STX4/BNIP1/NSF/GCA/NSFL1C/STX7/GNA
/PIK3CD/HCLS1/LYN/LYAR/PTBP3/MB/DNASE2/RPS19/AXL/HCAR2/ACVR1B/PTPN2/ADAM17/HOXA
/CD86/MAPKAPK2/MIF/CD2/IL27RA/SASH3/SIRPA/SPON2/ZC3H12A/BCL3/ADAM8/HDAC2/FRMD8/
L3/CCL2/SHC1/ATF4/WFS1/GSK3A/ERO1A/ZBTB17/BAG3/CALR/DNAJB11/PPP1R15A/RACK1/PDIA

SMD13/MYBL1/RAD21/RAD51C/HSPA2/RAD51D/EME2/SYCE1L/CCNE2
C5B/IL15/GALNT6/MUC20/SLC35C1/POMGNT1/MFNG/LFNG/POFUT2/GALNT7/LARGE2/GXYLT2/MUC
L9/CXCL3/CXCL13/CXCR3/CCL4/CCR1/RNF113A/LOX/PADI2/PF4V1/CXCL11/CCR5/XCL2/CMKLR1
L9/CXCL3/CXCL13/CXCR3/CCL4/CCR1/RNF113A/LOX/PADI2/PF4V1/CXCL11/CCR5/XCL2/CMKLR1

/CCL2/LYN/PLA2G7/CD47/CCL4/CALCA/CCR1/C5AR1/XCL2/CMKLR1/RPS19/CCL3/S100A7/CXCL1
3CD/HCLS1/LYN/BIRC7/TCP1/CD27/CCR5/AXL/FADD/BTK/HCAR2
2/ADAM8/HDAC2/FRMD8/APP/FADD/CLEC7A/LBP/CCL3/NFATC4/THBS1/MAVS/TLR2

BN/ERCC1/RNF8/NOTCH2/DOCK10/MSH6/TP53BP1/ATAD5/THOC1/PTPRC/CD180/UNG/SUPT6H/DOC
K/CBL/VAMP8/MILR1/C12orf4/RAB3A/DEF8/BORCS6/ARL8B/STXBP1/ADORA2B/SNAPIN/VPS33A/
IRE1/THBS1/RHOA/XKR8/FNBP1L/BECN1/ARF1/RHOBTB2/SNX3/PPARG/SPIRE2/ABCA7/SYT11/AR
FADD/CLEC7A/HSPD1/ABL1

LI3/TNFAIP3/TRADD/PTPN2/ADAM17/CASP8/PIAS4

1/TNFAIP3/IL20RB/GLMN/XCL1/PTPRC/MAP3K7/TRIM27/IRF4/PDE4B/CD4/ANXA1/CCR2
14/PPIC/P3H2/P4HB/PIN1/OGFOD1/FKBP15/CRTAP

ELAVL1/PAIP1/TARDBP/PARN

PRNP/MVP/SYK/HCK/DDR1/IL18/DLG3/CSF1R/HSF1/NCF1/TNFRSF18/AREG/JAK3/CCL5/ITGA5/M
PRNP/MVP/SYK/HCK/DDR1/IL18/DLG3/CSF1R/HSF1/NCF1/TNFRSF18/AREG/JAK3/CCL5/ITGA5/M
K/TMSB4X/CASP4/ANKRD1/TNFRSF11B/PSMB9/LTB/TNFRSF18/TRAF2/RBCK1/PSMD14/YBX3/CCL5
CDK4/EME1/ANKRD1/HSF1/DDIT3/HMGB2/CCL5/CD86/MIF/MAPK3/SERPINE1/SIRPA/SPON2/IRF3
1/HSF1/HMGB2/CCL5/CD86/MIF/MAPK3/SERPINE1/SIRPA/SPON2/IRF3/LY86/PPARD/LDOC1/ZC3
A/POP1/CPSF1/TRMT112/TRMT6/USP14/FARSB/POLR2L/CPSF4/GTF3C2/KTI12/ZBTB8OS/GTF3C1
A2/EIF2S3/GSK3A/CDC123/EIF4A3/APP/PPP1R15A/UQCC2/RPS6KB2/RACK1/VIM/EIF2B5/KHDRB
/CD86/MAPKAPK2/MIF/CD2/IL27RA/SASH3/SIRPA/SPON2/ZC3H12A/BCL3/ADAM8/HDAC2/FRMD8/

T9/SHC1/BCAR1/VIL1/EPS15L1/MVB12A/ARF4/GRB7/GRB2/CDC42/VPS25/APP/ADRA2A/CBL/ABL

K2/NUP85/ATAD2/NUP43/RAE1/XPO5/POLR2L/NUP155/AGO2/NUP107/NCOR2/POLR2K/TRIM71/SE

ARD1/CPSF4/APP/SLBP/SUPT5H/CHTOP/SYMPK/CPSF3/SARNP/MAGOH/THOC2/POLR2D/SSU72/THO

/CASP8/SH3GLB1/BNIP3L/SREBF1/VPS11/YWHAG/ATG13/TFDP1/HAX1/CSNK2A2/PARL/HUWE1/US
MO1/S100A7/GNLY/PLA2G1B/HMGN2/RPL30/FAM3A
/RPS7/RPS10/RPS15/RPS16/LTV1/UTP20/NOB1/FAM207A/BMS1/NOL10/UTP23/SURF6/WDR46/RP
TDN2/DCTN1/PTPN1/COPS5/HSPA5/PPP2R5B/GFPT1/TLN1/GOSR2/TPP1/DAB2IP/FKBP14/ADD1/S
D/ECM1/GPR183/CCL3/S100A7/ADAM17/RHOA/TNFRSF14/XCL1/TMEM102/CXCL10/DOCK8

MD/CKS2/PLAUR/DAB2/LIF/CSF1/ECT2/TTK/TGFB1/IQGAP3/GPNMB/STK26/DKK1/RIPK2/UNC119
P1/PLCB1/DEPDC1/GMIP/EZH2/GIT1/ASAP1/ADAP2/SCRIB/RHOG/TBC1D16/RANGAP1/S100A10/R
D3EAP/HDAC1/NUP93/NUP205/NUP37/NDC1/POLR2H/NUP188/ZC3H12A/KCNQ1/HDAC2/CHRAC1/AC
10/B3GNT5/GBGT1/FUT4/B3GNT9/SLC51B/NPC1/GALNT18/B3GNT7/GFPT2/MAN1B1/PLOD3/B4GAL
10/B3GNT5/GBGT1/FUT4/B3GNT9/SLC51B/NPC1/GALNT18/B3GNT7/GFPT2/MAN1B1/PLOD3/B4GAL
VBP/HSPBP1/OTUB1/FBXO5/GTPBP4/PRR7/AXIN1/U2AF2/KDM1A/BIRC7/CDK5/WFS1/UBE2L3/DNA
HLH/BSG/COL16A1/HSF1/TRIM28/ITGA5/MAPK3/MMP2/CAD/PPARD/LDOC1/PAM/ARHGDIB/FKBP4/

/CD86/APBB1IP/SEMA4A/MAD2L2/LCP1/IL27RA/F2RL1/LEF1/ZC3H12A/BCL3/SLC11A1/CDH17/I
/MAPKAPK2/MIF/CD2/IL27RA/SASH3/SIRPA/SPON2/ZC3H12A/BCL3/HDAC2/FRMD8/CD47/PTPN6/
/MAPKAPK2/MIF/CD2/IL27RA/SASH3/SIRPA/SPON2/ZC3H12A/BCL3/HDAC2/FRMD8/CD47/PTPN6/
CD86/CD300A/MNDA/SOCS1/IRF1/CTLA4/PLA2G2D/ZC3H12A/LAG3/LYN/CBFB/IL2RA/PTPN6/SAM

/PARP1/RPS3/USP1/CBX8/POLQ/FUS/BABAM1/OGG1/RPA2/PPP4C/TIMELESS/WRAP53/UBQLN4/HD

CSTD2/SEMA4B/SEMA4D/SEMA4F/SEMA5B/ARHGDIA/SEMA3C/SEMA3E/NFATC4/PTK2/IFRD1/CDK5R
/MITF/PIAS3/CCR1/OSTM1/SBNO2/LILRB4/ZNF675/GPR183/CCL3

G12B/ANGPTL3/APOA1/APOA5/APOC3/ACSM2A
G12B/ANGPTL3/APOA1/APOA5/APOC3/ACSM2A

/POLR3D/POLR3H/POLR3C/HSPD1/MAVS/IRF5/TLR2/DHX9/TLR7/POLR2E/ZCCHC3/TLR8/POLR3F/

L10/PTPN2/RAB29/DUSP22/PTPRC/NECTIN2/CD22/RELA/CD19/PRKCB

FL1C/LEMD2/DCTN1

F2/EFNA3/MZB1/CD7/GDF15/EIF4EBP1/SHCBP1/PRKCD/ROBO1/HSPB1/COL4A5/NCK2/ITGAV/EFN
1/SRC/ETV5/RPL39L/KIFC1/TTK/GAL3ST1/TGFB1/FAM50A/AURKA/CTSV/WFDC2/CDC25C/PAQR5/
RIB3/FBLN1/TGFB1/SPI1/DKK1/GSTP1/PMEPA1/PPP1R14D/CDKN2A/PPP1R14B/PKN1/DUSP5/PRK
/FZD7/CTNND2/MDFI/PSMB9/DDIT3/PSMD14/HNF1B/PTK7/RUVBL1/MAD2L2/PSMD2/FZD1/PPP2R1
1A/MAPK3/NUPR1/RRAGC/TCIRG1/CHMP4C/HGS/NPC1/GPSM1/DYNLL1/PIM2/TOMM5/ATG101/MLST

1/PAIP1/TARDBP

18/DLG3/CSF1R/HSF1/NCF1/TNFRSF18/AREG/CCL5/ITGA5/MIF/CD300A/IQGAP1/EFNA5/CD3E/P

5/RAN/HSF1/CRMP1/DTNBP1/TRAF2/BAX/MAP2/FKBP1A/MAPK3/SLPI/UBASH3B/GPSM1/HSP90AB1

RT1/STX10/RAB35/TBC1D10B/EHD1/ARHGAP1/LEPROTL1/SNF8/RAB11FIP4/CCDC22/EHD2/LAMTO
-DPA1/CCL5/CCL26/HLA-DPB1/SEC61A1/STXBP2/GBP2/HLA-DQB2/HLA-A/SIRPA/SOCS1/CD58/I
A/HSP90AB1/DDX11/HYOU1/ARFGAP1/UBE2J2/EXTL3/HSPA4/CCL2/SHC1/ATF4/HSPH1/WFS1/DNA
1/CTLA4/CD83/PRELID1/ZC3H12A/LAG3/NCKAP1L/ADAM8/FANCA/CARD11/CBFB/IL2RA/NFAM1/P
F1/NCKAP1L/ADAM8/EVI2B/HCLS1/CBFB/IL2RA/CD27/CCR1/IL12RB1/IL15/IL4R/PNP/LILRB4/

/CNOT9/SHC1/BCAR1/VIL1/EPS15L1/MVB12A/ARF4/MATK/GRB7/GRB2/CDC42/VPS25/APP/ADRA2

/BANF1/PML/AGFG1/TMEM43/NUP155/CHMP1A/CTDNEP1/NUP107/DMPK/RPS19/VRK1/FAM118B/AN
N1/CDC42BPG/CDK5/GSK3A/CIT/CADM4/PRKD2/GRK2/APP/VRK1/CSNK1G2/CEMIP/ACVR1/CALM2/

/PSMD10/PSMD11/PSMD1/IL1R2/PSMA7/PSMB3/PSMD4/PSMD3/RBX1/PSMD7/IKBKG/PELI1/ZNF67

F/ACTB/CDK5/VIL1/AP2M1/STAC3/ACSL3/PRKCI/STX4/RACK1/CLIP3/STAC/STX7/CSK/VAMP8/T
CCL4/CCR1/RNF113A/PADI2/PF4V1/CXCL11/CCR5/XCL2/CMKLR1/CCRL2

C2/FRMD8/APP/FADD/CLEC7A/LBP/CCL3/NFATC4/THBS1/MAVS/TLR2
MD4/PSMD3/PSMD7/PSMD8/ABL1/PSMA1/PSMB5/PSMA4/PSMD9/PUS7/PSMC3/SRF/PSMC5/OSM/LDB

T4/HCLS1/CCT7/TCP1/PARP1/BAG3
TPN6/LST1/GAL/BTK/PELI1/BTN2A2/FCGR2B/TNFAIP3/TWSG1/IL20RB/GLMN/TNFRSF14/IDO1/X

GNPTAB/TPP1/BECN1/SNAPIN/VPS33A/ARF1

GNPTAB/TPP1/BECN1/SNAPIN/VPS33A/ARF1

MD/CKS2/PLAUR/DAB2/LIF/CSF1/ECT2/TTK/TGFB1/IQGAP3/GPNMB/STK26/DKK1/RIPK2/UNC119
K1/S1PR2/CD74/PKN1/DUSP5/PRKCD/ROBO1/ADORA1/PRNP/ADAM9/HRAS/SYK/STK39/IL18/EZH2

B2/ZC3H12A/PSME3/TNIP2/OTUB1/RCE1/HCFC1/ADRM1/PSMB4/ACTB/PSMB8/PSMB10/PSMA5/PSM
LMNA/MYO19/PRELID1/HIP1R/BID/UBE2J2/NPEPPS/YWHAH/BBC3/PMAIP1/VDAC1/GBA/UBE2L3/C
L1/DYNC1H1/GPSM2/PIP4K2A/TBC1D30/LRFN1/ATXN2L/LRFN4/PLK4/MAP4/CROCC/STX18/KAT2A

SMD10/PSMD11/PSMD1/PSMA7/TACSTD2/PSMB3/PSMD4/PSMD3/PSMD7/SEMA3C/PSMD8/ABL1/PSMA

CFHR5/C6/CFHR4

CFHR5/C6/CFHR4/SERPINC1

GLB1/VPS11/YWHAG/ATG13/TFDP1/CSNK2A2/HUWE1/USP36/NMT1/HTRA2/ARIH2/UBL5/BAP1/TP5

1/AADAC/ANGPTL3/APOA5/APOC3

/TGFB1/AURKA/DKK1/CLCF1/TNFRSF12A/FLNA/S1PR2/ARHGEF2/P2RX4/ROBO1/SERPINI1/PLXNA
3/TNFRSF12A/CD44/AGRN/PLEKHO1/ROBO1/PLXNA3/EFNA4/VASP/NBL1/ITPKA/GPC1/DDR1/TWF2
TNFRSF12A/SNAP25/ARHGEF2/ROBO1/SERPINI1/PLXNA3/NME1/NBL1/ITPKA/HES4/EZH2/NME2/T
3/TNFRSF12A/CD44/AGRN/PLEKHO1/ROBO1/PLXNA3/EFNA4/VASP/NBL1/ITPKA/GPC1/DDR1/TWF2
FB1/ITGAM/FCGR3A/RIPK2/ADA/HAVCR2/CLCF1/C1QB/SUSD4/BIRC3/ITGB2/IRAK1/C1QC/CD74/
5A2/CCL5/DPH2/CNOT11/MAPK3/EIF3B/NPM1/UPF3B/POLR2G/RBM3/PABPC1/TRNAU1AP/PPP1CA/
P13A2/PCSK1N/SERPINE2/FBLN1/PAPLN/WFDC2/GAPDH/RIPK2/EFNA3/GPC3/BIRC3/CD44/PI3/C
B/COTL1/PRKCD/SLAMF8/IGLL5/ACP5/HRAS/SYK/UNC13D/EXOSC4/MX2/S100A8/LYZ/APOBEC3B/
/TNFRSF12A/KRT17/CDKN2A/PLXNA3/NDRG3/DDR1/EXOSC4/S100A8/TWF2/ADAM15/CDKN2D/TRPV

B2/ZC3H12A/PSME3/TNIP2/OTUB1/RCE1/HCFC1/ADRM1/PSMB4/ACTB/PSMB8/PSMB10/PSMA5/PSM
165/GALNT10/B3GNT5/GBGT1/FUT4/B3GNT9/SLC51B/NPC1/GALNT18/B3GNT7/GFPT2/MAN1B1/PL

QGAP1/MAPK3/MAP2K2/PAK1/PRKX/FGFR4/RAP2B/THY1/CAD/MARK2/EPHB3/FGFR1/PIM2/FGFR3/
NASE6/GRN/CARD9/HMGB2/GBP2/SERPINE1/IL27RA/SLPI/ISG15/LYPD8/SPON2/F2RL1/STAB1/B

AK3/HMOX1/CD86/CD300A/MNDA/SOCS1/IRF1/CTLA4/PLA2G2D/ZC3H12A/LAG3/LYN/CBFB/IL2RA
USP2/CDKN2B/TESK1/LYN/CDKN1C/DUSP4/IL1B/GBA/CHORDC1/PTPN6/CDKN2C/TFAP4/SPRY4/ZN

S18/CHCHD3/CALR/SYT1/DNM1L/STX4/BNIP1/MFF/STX7/GNAI3/RAB3A/RCC1L/STOML2/VPS11/S

P1L/AP2A1/PIK3CB/SYT11/UBQLN2

RK2/DDIT4/BID/BCL3/KDM1A/TAF9/IFI16/PML/CRIP1/MSH2/RPS3/RAD9A/ABL1/SFRP2/NFATC4
F1A/MMP10/SUCO/P3H3/MMP2/PLOD2/PLOD3/PPARD/HDAC2/ITGA2/CTSS/ARRB2/TGFB3/CBX8/CT

K7/PPP5C/AXL/STK25/RACK1/ABL1/TNFAIP3/PDCD10/PSAP

/STMN3/PSMC4/BYSL/UBE2A/TBL1XR1/PRPF19/MFNG/DAD1/NBN/CAPN2/ZPR1/PHF6/PSMC3/SRF
/PIEZO1/BAG3/CNN2/TLR5/FADD/BAD/BCL10/PTGER4/CASP8

2/CYP7A1

SH2/BTK/PELI1/GPR183/EXOSC3/BAD/CD38/TP53BP1/ATAD5/INPP5D/PTPRC
SMD3/PSMD7/PSMD8/ABL1/PSMA1/PSMB5/PSMA4/PSMD9/PUS7/PSMC3/PSMC5/OSM/LDB1/YTHDF2/
2/PPP4C/TIMELESS/WRAP53/UBQLN4/SPIRE1/MAGEF1/UBR5/TWIST1/MMS19/TP53BP1/HMGA2/TF

/TMPRSS3/SH3BP1/TYRO3/SRC/LGALS3/DAB2/DNM1/LOXL4/ATP1A1/LRP12/TYROBP/PDLIM7/TGF
12A/FLNA/CD44/ITGB2/PLEKHO1/ROBO1/PLXNA3/ITPKA/CDC42EP1/HCK/UNC13D/FGD3/TWF2/FM
K1/S1PR2/CD74/PKN1/DUSP5/PRKCD/ROBO1/ADORA1/PRNP/ADAM9/HRAS/SYK/STK39/IL18/EZH2
CERS6
PKP3/KRT7/ESRP1/SCRIB/PLOD1/AKR1C3/CTSK/FLOT2/LAMC2/ST14/SHARPIN/CRABP2/PERP/LA

/THBS1/RHOA/XKR8/BECN1/ARF1/RHOBTB2/PPARG/SPIRE2/ABCA7/ARHGAP25/APPL2/RAC3/MYH9

C/SEMA4A/MSN/GNA12/RHOF/CCL13/S100B/MARK2/SLC9A3R1/SH3KBP1/CCL2/DAPK3/S100A13/F
DYNLL1/DYNC1H1/BLOC1S4/CLN3/SFPQ/AP3M2/WASF1/IFT80/BAG3/CDC42/APP/ACTL8/AP3D1/C
DYNLL1/DYNC1H1/BLOC1S4/CLN3/SFPQ/AP3M2/WASF1/IFT80/BAG3/CDC42/APP/ACTL8/AP3D1/C
RDX1/MAPK3/PCNA/ZNF580/MMP2/SIRPA/MT3/HDAC2/CDK2/TXN/RPS3/DPEP1/CBX8/PCGF2/MAPK
N1/CDC42BPG/CDK5/GSK3A/CIT/CADM4/PRKD2/GRK2/APP/VRK1/CSNK1G2/CEMIP/ACVR1/CALM2/
PA/SPON2/F2RL1/RAB7B/ZC3H12A/NCKAP1L/IL1B/GBA/CD47/PTPN6/ARRB2/APP/CLEC7A/LBP/H

NRNPD/MYC/MAPK15/TERF1/HNRNPA2B1/XRCC5/TP53/PARN/MEN1
/IL1B/ASCL2/PPP1CC/PRKCI/WDR1/GPR183/RHEB/NF2/CCL3/PRPF19

3/GSPT2/MRPS17/MRPS21/MRPS15/MRPL18/JMJD4/ETF1/MRPL37/EIF5A/MRPL27/MRPL28/MRPL5
OA/ARF1/VPS4A/SPIRE2/CHMP6/CNTROB/MYH9/CHMP4A/ALKBH4

G6PC/GNMT

BHMT

/IL4R/PNP/LILRB4/AP3D1/AXL/BTK/BTN2A2/SART1/TOX/BAD/NKAP/RHOA/AP3B1/RUNX3/ZMIZ1

PA2/PPP4C/TIMELESS/EXOSC3/WRAP53/UBQLN4/MAGEF1/MMS19/MSH6/TP53BP1/ATAD5/THOC1/P

DAB2/DNM1/LOXL4/LRP12/TYROBP/PDLIM7/TGFB1/ITGAM/RHOV/PPT1/FCGR3A/DKK1/UNC119/CO
1/FAM50A/CTSV/WFDC2/CDC25C/BIRC3/CEP131/CDK16/RACGAP1/NDRG3/NUP62/ASPM/ZNF296/S
/CTSA/IGF2BP3/ZNF385A/RPS6KA1/YBX3/EIF5A2/CCL5/DPH2/PDP1/CNOT11/MAPK3/EIF3B/SMP
/NPLOC4/MAN1B1/DDX11/HYOU1/ARFGAP1/BCAP31/UBE2J2/FLOT1/EXTL3/CCL2/SHC1/ATF4/BBC
5/PRNP/TMSB4X/HDAC7/EZR/HSF1/VSIG4/JAK3/HMOX1/NUTF2/IL27RA/ISG15/TNFRSF4/SIRPA/
RSF14/CCL19/CD5/CAV1
RY6/SLC4A2/SLC37A1/SLC12A8/SLC1A3/GABRA3/CLCN7/FGFR1/GABRR3/ATF4/SLC11A1/VDAC1/
A/SIRPA/SPON2/F2RL1/RAB7B/ZC3H12A/NCKAP1L/IL1B/GBA/CD47/PTPN6/ARRB2/APP/CLEC7A/
/RAB3A/RASSF8
PN6/CD27/CD79A/MSH2/BTK/GPR183/MFNG/LFNG/ABL1/FCGR2B/BAD/VCAM1/PTPN2/ADAM17/ADG

POLR3F/POLR3A/PTPRS/TRIM56/LILRB1/RNF135/TICAM1/CACTIN/TRAF3IP1/POLR3G
FC5/RPA1/UBE2N/POLE2/PARP10

IF4A3/PPP1R15A/UQCC2/RPS6KB2/VIM/EIF2B5/KHDRBS1/LARP4B/NAT10/MPV17L2/THBS1/RPL5
G6PC/GNMT
G6PC/GNMT

4B/SEMA4D/SEMA4F/SEMA5B/SEMA3C/SEMA3E/MAPT/ABL1/DBNL/DNM2/VEGFA/RAB11A/IFRD1/CD

TLA4/PLA2G2D/ZC3H12A/LAG3/CBFB/IL2RA/PTPN6/IL4R/PELI1/BTN2A2/FCGR2B/TWSG1/PTPN2

2/KHK/ALDH8A1/AKR7A3/AGXT2/AGXT/ALDOB/ADH4
IRS1/ECHDC2/ECI2/ETFDH/ACOX1/ACAA1/IVD/ACAA2/IRS2/HSD17B4/GCDH/ACAT1/AMACR/ACAD
D/AKR1D1/HAO1/CYP4F2/CYP7A1/SULT2A1/CYP4A11/ADH4

HSPD1/MAVS/TLR2/TWIST1/DHX9/ADORA2B/EREG/TLR7/IL17D

/ERCC1/COPS5/UBA52/PPIE/POLR2I/POLR2J/RFC5/POLR2D/GPS1/GTF2H1/RPA1/POLR2E/ERCC3
1/LIF/MPZL1/CSF1/TTK/TGFB1/BAMBI/CYBB/DKK1/RHBDF2/EFNA3/FOLR1/PMEPA1/MZB1/COL1A

FRSF12A/CD44/AGRN/PLEKHO1/ROBO1/PLXNA3/EFNA4/VASP/NBL1/ITPKA/GPC1/DDR1/TWF2/MAP

1/FAM50A/CTSV/WFDC2/CDC25C/BIRC3/CEP131/CDK16/RACGAP1/NDRG3/NUP62/ASPM/ZNF296/S
CSK1N/SERPINE2/PAPLN/WFDC2/GAPDH/RIPK2/EFNA3/BIRC3/CD44/PI3/CST1/ROBO1/SERPINI1

R/SCP2/PHYH/CAT/EPHX2/ACOX2/BAAT/SLC27A2/PIPOX/EHHADH/DAO/HAO2/HAO1/AGXT
DAO/FTCD/UROC1/OTC/CPS1/TAT
B/INSIG1/SEC14L2/APOA1/HMGCS2/APOA5/CYP7A1

R/SCP2/PHYH/CAT/EPHX2/ACOX2/BAAT/SLC27A2/PIPOX/EHHADH/DAO/HAO2/HAO1/AGXT
R/SCP2/PHYH/CAT/EPHX2/ACOX2/BAAT/SLC27A2/PIPOX/EHHADH/DAO/HAO2/HAO1/AGXT
/GDI1/MT3/HDAC2/RAP1GAP2/YWHAH/TBC1D30/FKBP4/MYLIP/CDK5/SPOCK1/GSK3A/INPP5J/MAP
1/CCL5/CCL26/HLA-DPB1/STXBP2/GBP2/HLA-DQB2/HLA-A/SIRPA/SOCS1/CD58/IRF1/IRF3/HSP
/RAB15/STXBP2/SYT8/STX1A/F2RL1/LYN/CDK5/FGR/ABR/SYT12/RIMS3/IL4R/VPS18/SYT1/ADR

/DNASE2/RPS19/AXL/ACVR1B/PTPN2/HOXA5/THRA/VEGFA/SMAP1/SRF/INHA/BPGM/MAEA/LDB1/P
/SNPH/VPS33A/RAB8B/VAMP3/RAB3C/VPS33B/EXOC6/UNC13A/EXOC6B

DSS1/YWHAB/GRB2/TMEM120B/SYT1/XRCC6/PPP5C/RRM1/LIMS1/FARSA/TRADD/KRT10/BCL10/CT

CFHR5/HPX/C6/CFHR4
S/PFKFB1/PCK2/FBP1/RBP4/G6PC/GNMT/ALDOB/PCK1
L2/APOA1/HMGCS2/APOA5/CYP7A1
R2/C7/MBL2/C8B/MASP1/F2/MASP2/CPN2/CPB2/C8A/C4BPA/CFHR5/C6/CFHR4

R/SCP2/PHYH/CAT/EPHX2/ACOX2/BAAT/SLC27A2/PIPOX/EHHADH/DAO/HAO2/HAO1/AGXT
L2/APOA1/HMGCS2/APOA5/CYP7A1

R2/CIT/CDC42/TGFB3
SMD3/PSMD7/DPF2/PSMD8/ABL1/PSMA1/PSMB5/PSMA4/PSMD9/PUS7/NOTCH1/PDCD2/PSMC3/PSMC
/RCC2/ARHGAP4/DKK1/EFNA3/TNFRSF12A/FLNA/AGRN/FERMT3/ROBO1/PLXNA3/ITGAV/LAMB1/EF
JMJD6/MAPK3/PYGO2/MRGBP/UBE2I/P3H3/BRCA1/NSMCE2/STX1A/MCRS1/ACTL6A/LMNA/NUP93/P
/COL11A1/EXT1/NOTCH1/MESP1/LDB1/HMGA2/MMP15/PAF1
ST1/GAL/BTK/PELI1/BTN2A2/FCGR2B/TWSG1/IL20RB/GLMN/TNFRSF14/IDO1/XCL1
ST1/GAL/BTK/PELI1/BTN2A2/FCGR2B/TWSG1/IL20RB/GLMN/TNFRSF14/IDO1/XCL1
1/HNRNPU/RBMX/DHX9/RBM10/RBM24/SFSWAP/LARP1/HNRNPA0/HNRNPD/HNRNPC/SYNCRIP/HNRNP
IM6/MIF/CD2/MAPK3/IL27RA/TCIRG1/ISG15/TNFRSF4/SOCS1/CD58/IRF3/F2RL1/SERPINB1/ZC
2/IRF1/IRF3/F2RL1/STAT1/NPLOC4/POLR3K/DDIT4/AGBL5/IRF7/ZC3H12A/CXCL9/NLRC5/PLSC
4/GDAP1/SLC35F6/CYC1/TIMM17B/BMF/NOL3/SLC25A22/HIP1R/BID/TOMM5/UBE2J2/DYNLT1/NP
0/PSMA5/PTPN6/PSMD13/PSMC4/MLF1/PSMD10/PSMD11/PSMD1/SIPA1L3/PSMA7/PSMB3/PSMD4/P
A10/SLC7A6/SLC36A1/SLC11A1/SLC38A5
/FKBP4/MYLIP/CDK5/SPOCK1/GSK3A/INPP5J/DGUOK/SEMA4B/SEMA4D/SEMA4F/SEMA5B/ARHGDIA
ZNF675/IL20RA/RAB3D/TNFAIP3/CSK/TMEM64/DEF8/CD38/TPP1
1/NR0B1/MAD2L2/NUPR1/TMIGD3/TNFRSF4/LRRC14/PRMT2/SFRP5/ZC3H12A/SRI/HEYL/HDAC2/N
F2

/GXYLT2/MUC6/MUC15/XXYLT1/GCNT1/B3GNT8/ST3GAL2/ST6GALNAC2/C1GALT1C1/B3GNT4/GALN
7/BAD/PTPN2/STYXL1/PIAS4/BCL2L1/PLAGL2/BECN1/PRKRA/CAV1/RPL11/NOX1/PARK7
T3GAL4/EXTL3/B4GALT7/IL1B/B4GALT2/XYLT2/UGDH/BGN/CHST2/LUM/CHPF/CSPG5/CHST12/CS
T2/CTH/ABAT/PAH/OTC/CDO1/AGXT/UPB1/CPS1/BHMT
C1/INSIG1/APOA2/GPLD1/CYP7A1/APOC3
BCG8/EPHX2/INSIG1/ABCG5/APOA2/LCAT/ANGPTL3/FABP4/APOA1/APOA5/CYP7A1/APOC3/G6PC
1

HFD1/RGN/AOX1
APOA2/LCAT/ABCB4/PON1/APOA1/APOA5/APOC3

1
4R/APP/LBP/MAPT/CCL3/HSPD1/FCGR2B/THBS1/TLR2

6/MRPS11/POLD2/POLD4/MRPS9/RFC3/MRPS35/UBC/DNAJA1/RPA3
2/GYS2/GCGR/G6PC/GNMT
I2/ACOX1/ACAA1/HSD17B4/AMACR/PECR/SCP2/PHYH/CAT/EPHX2/ACOX2/BAAT/SLC27A2/PIPOX/
1
1/RDH5/CYP1A1/ALDH2/RBP4/ADH6/RDH16/CYP1A2/HAO1/TTR/SULT2A1/ADH1A/ADH1C/ADH1B/A
BCG8/EPHX2/INSIG1/ABCG5/APOA2/LCAT/ANGPTL3/FABP4/APOA1/APOA5/CYP7A1/APOC3/G6PC
D17B13
/CXCL5/FCER1G/ICAM1/REG1A/LGALS9/STMN1/BAK1/SRC/TSPO/S100A14/TGFB1/GAPDH/RIPK2/
FRSF12A/AGRN/ROBO1/PLXNA3/EFNA4/VASP/NBL1/ITPKA/GPC1/DDR1/TWF2/MAPK8IP2/CSF1R/N
/CXCL5/FCER1G/ICAM1/REG1A/LGALS9/STMN1/BAK1/SRC/TSPO/S100A14/TGFB1/GAPDH/RIPK2/
N/NABP2/CCT6A/MICALL2/DDIT3/CCT3/SAE1/CHMP4B/TRIM28/BAX/NUTF2/VPS37C/SEC61A1/HS

D44/ITGB2/CDKN2A/FERMT3/ITGAV/EMILIN2/LAMB1/ADAM9/UNC13D/DDR1/ZYX/ADAM15/COL16A

/ADAM17/MAVS/TLR2/ALOX15B/TWIST1/SIGIRR/ADORA2B/EPHA2/TLR7

C2/GPNMB/LGALS1/HAVCR2/FXYD5/CDKN2A/CD74/PRKCD/PLXNA3/PRNP/PAG1/ADAM15/MUC1/FZD
PPARGC1A/FABP1/ACADL/APOC1/INSIG1/RGN/PDK4/APOA5/CYP7A1/APOC3

/VPS45/CFTR/CTBP2/RAB3A/VPS11/STXBP1/SNPH/VPS33A/CAV2/RAB8B/TSNARE1/VAMP3/RAB3C
177

RKCI/STX4/RACK1/CLIP3
CORO1B/HIP1R/TUBB4A/CAPZA1/MKKS/FHOD3/CAMSAP2/FKBP4/MYADM/WAS/VIL1/PFN1/INPP5J/

3/GPLD1/LCAT/ALB/APOA1/APOA5/APOC3

STX7/GNAI3/RAB3A/RCC1L/VPS11/STXBP1/TAPBP/TAP2/GOSR2/SNAPIN/MFN1/SAMD9L/CAV2/VP

OA8/CTSA/TOMM40/ATP6V0B/DDIT3/TPCN1/CTSK/ATP6V0D2/CHMP4B/HMOX1/SOGA1/MTCL1/VPS3
/CXCL5/FCER1G/ICAM1/REG1A/LGALS9/STMN1/BAK1/SRC/TSPO/S100A14/TGFB1/GAPDH/RIPK2/
B1/NCK2/SLC7A11/IGF2BP1/LARP6/ELOVL1/EEF1E1/CDK4/TCOF1/IGF2BP3/ZNF385A/RPL8/RPS
OA8/CTSA/TOMM40/ATP6V0B/DDIT3/TPCN1/CTSK/ATP6V0D2/CHMP4B/HMOX1/SOGA1/MTCL1/VPS3

ADRA2A/CBL/RACK1/PTPN2/ADAM17/PTPN1/PAK2/SH3BP5L/EREG/EGF/CAV1/DUSP22/PTPRC/NEU
D74/BATF/PKN1/PRKCD/LAT2/C1QA/RELB/IGLL5/HLA-DRB1/HRAS/SYK/HLA-DQA1/UNC13D/TAP1
YWHAB/GZMB/STPG1/RHOT2/SLC25A5/BAD/CASP8/BNIP3L/BCL2L1/YWHAG/HEG1/TFDP1/TMEM102
AB3D/VAMP8/VPS45/CFTR/CTBP2/RAB3A/VPS11/STXBP1/SNPH/SNAPIN/VPS33A/RAB8B/VAMP3/R

CD80

P4F12/AR/SERPINF2/CYP4F2/CYP4A11
EPR/FBP1/IGF2/GNMT

C27A5/CYP2C9

CCL19/CD5/CAV1

13/PSMC4/PSMD10/PSMD11/PSMD1/PSMA7/PSMB3/GRB2/PSMD4/PSMD3/PSMD7/IKBKG/BTK/PSMD8

POLR3F/POLR3A/PTPRS/LILRB1/RNF135/TICAM1/CACTIN/TRAF3IP1/POLR3G
RBP4/G6PC/GNMT/ALDOB/PCK1

12A/AGRN/ROBO1/PLXNA3/EFNA4/VASP/GPC1/DDR1/TWF2/CSF1R/NECTIN1/UNC5B/EZR/BSG/TRP
D2/PRNP/SYK/HCK/IL18/EZH2/TMSB4X/S100A8/FZD6/GLIS2/FOXS1/MDFI/DDIT3/TRAF2/RBCK1
7/DOT1L/HAT1/MBD3/MORF4L2/MECP2/BAZ2A/PPHLN1/DNMT3B/SMARCA5/POLE3/CTBP1/SUV39H1
/TRIM28/NUTF2/CARD10/GBP2/DKC1/MCRS1/CCT2/LMNA/RRS1/LZTS2/NUP93/NXT1/FAM53B/NUP
AGED1/TRAF2/MAPKAPK2/CXCR4/MIF/IQGAP1/MAPK3/MAP2K2/PAK1/PEA15/FPR1/TGFA/TNFRSF1
IRT6/NDN/MAP2/IQGAP1/EFNA5/PAK1/CRABP2/NRCAM/HSP90AB1/PRMT2/GDI1/MT3/CPNE1/CDK5
PN2/CPB2/C8A/KLKB1/C4BPA/F12/CFHR5/DNASE1L3/C6/CFHR4
T1/NAGS/GLS2/PRODH2/ALDH5A1/HAAO/ALDH1L1/ADHFE1/MTHFD1/AMDHD1/FTCD/UROC1/PCK1/T
P4A11
FGG/CFHR1/C4BPB/CFHR2/C7/FGA/MBL2/C8B/MASP1/F2/F11/F7/MASP2/APOH/CPN2/CPB2/C8A/
LR2/TRAF4/RHOA/ACTN4/CCL19/TLR7/MAP3K7/COPS8/RTKN2/AGO1/RELA/NOD1/NOD2/CARD14/T

RBP2/PSMC3/POLR2I/POLR2J/POLR2D/DHX9/IFITM3/NELFA/POLR2E/SUPT4H1/HMGA2/NELFCD/G

IP1/GBA/UBE2L3/ZNF205/GSK3A/YWHAQ/YWHAB/GZMB/DNM1L/PDCD5/MFF/BAD/VPS35/CASP8/SH
/CYP3A5/RBP4/ADH6/RDH16/CYP1A2/TTR/APOA1/APOC3/ADH1A/ADH1C/CYP2C8/ADH1B/ADH4/CY
/SLC13A5/AGXT
/SLC27A2/THRSP/FABP4/CYP4F2/CYP4A11/SLC27A5
8/ABCG5/APOA2/LCAT/ABCB4/PON1/APOA1/APOA5/APOC3

BP1/RBP4/G6PC/GNMT/ALDOB/PCK1

T/RBP4

7/RPS3/AKIP1/CALR/APP/LIMS1/TRIP6/TRADD/TNFSF15/TLR2/TRAF4/ADGRG3/RHOA/ACTN4/CC

1/CLIP3/STAC

A5/CORO1C/ARPC2/EPHB3/KIF14/ITGB1BP1/RAC1/MYADM/PARVG/TACSTD2/AKIP1/CDC42/LAMB3
T2A/IL4R/DMPK/TMEM119/MSX1/NOTCH1/CAPN2/NLN/MESP1/MORF4L2/RBPJ/RBM24/BHLHA15/HD

L2/TGFB1/ITGAM/AACS/GAPDH/RIPK2/HAVCR2/RHBDF2/YWHAZ/FLNA/ITGB2/CEP131/SNAP25/CD
/HMOX1/SOGA1/MTCL1/ATP6V1C1/RRAGD/FOXK1/HIF1A/MAPK3/NUPR1/RRAGC/BMF/NPC1/GPSM1/

C3/NOP10/EXOSC9/EXOSC10/EXOSC7/INTS9/GTF2E2/INTS7/POLR2I/POLR2J/INTS4/POLR2D/TO
A/CLN6/FA2H/FUT3/PLA2G15/SMPD4/FUT6/ST3GAL5/RACK1/ITGB8/CERS6/PSAP/UGCG/HEXB
CR1/FADD/ACVR1B/CASP8/HOXA5/SMAP1/TMEM64/KITLG/HAX1/INPP5D/ZNF16/ACIN1/ANKRD54/

ILRB4/AP3D1/BTN2A2/SART1/BAD/NKAP/RHOA/AP3B1/RUNX3/ZMIZ1/CCL19/PIK3R6/PTPRC/NFK
PIM2/ATG101/MLST8/CAPN1/PIP4K2A/ATP6V1H/ATP6V1E1/GBA/LAMTOR5/CAPNS1/CDK5/LAMTOR
AM/THY1/ZSWIM5/MARK2/GDI1/EPHB3/MT3/CDK5/CTTN/PLXND1/TUBB2B/SEMA4B/SEMA4D/SEMA4

O1D/DNAJC13/RAB7A
L3/CCL2/SHC1/ATF4/WFS1/POMT2/GSK3A/ERO1A/ZBTB17/BAG3/CALR/DNAJB11/PPP1R15A/RACK
M1/CSNK1E/DDX11/PPP1CA/LEF1/PIM2/SRI/GTPBP4/CPNE1/HDAC2/KDM1A/ITGB1BP1/ZNF593/A

ERMT1/FRMD8/IL1B/FGR/MAPK11/CASP1/GSDMD/IL4R/HK1/CLEC5A/CCL3/ABL1/PTGER4/MAVS/T
/SMPD2/NSMAF/DEGS1/ORMDL2/B4GALT3/ST3GAL4/ASAH1/GBA/FA2H/SMPD4/A4GALT/PIGC/PRKD
OC6/RAE1/MVB12A/NUP155/NUP107/SEC13/RPS27A/UBA52/THOC2/RAB29/VPS37B/KPNA6/SEH1L
OC6/RAE1/MVB12A/NUP155/NUP107/SEC13/RPS27A/UBA52/THOC2/RAB29/VPS37B/KPNA6/SEH1L
PAQR8/MCM8/IL4R/MMP19/WNT4/SOHLH2/RAD51C/SPIRE1/PAQR7/ERCC1/HEXB/MASTL

POA2/APOH/ANGPTL3/APOA1/APOA5/APOC3
BP1/ATAD5/THOC1/DCLRE1C/PTPRC
BP1/ATAD5/THOC1/DCLRE1C/PTPRC
NT4

B/PPIE/PPIB/KPNA6/CAV2/LARP1/VPS4A/SRPK2/DDB1/P4HB/VAPA/TBC1D20/ADAR

B/CD79B/CD38/PTPRC/CD22/KLHL6/CD19/PRKCB/VAV3

A/TNFRSF14/XCL1/TMEM102/CXCL10/DOCK8

KP/CCT7/TCP1/PRKD2/ARRB2/PARM1/CCT8/PTGES3/NAT10/WRAP53/HSP90AA1/HNRNPU/NVL/EXO
RA3/CLCN7/GABRR3/BEST1/TTYH2/SLC12A7/SLC26A2/GABRD/TTYH1/CFTR/GABRB3

IF/PBK/TRIB3/FBLN1/TGFB1/IQGAP3/GPNMB/STK26/DKK1/RIPK2/HAVCR2/GSTP1/CD44/GDF15/
ADA/HAVCR2/C1QB/SUSD4/BIRC3/ITGB2/IRAK1/C1QC/CMTM3/PRKCD/LAT2/C1QA/RELB/IGLL5/H
NFRSF12A/PTAFR/COTL1/PRKCD/NCK2/PLXNA3/BAIAP2L2/VASP/ADORA1/PLEK/CDC42EP1/USH1C
P3/P2RX4/ADORA1/PLCB1/MTMR2/BICC1/WNK2/FZD6/MAPK8IP2/ASPM/TLE3/DCDC2/FZD7/CTNND
V2/GRN/P3H1/CXCR4/IQGAP1/PTK7/EFNA5/SF3A2/FZD1/PAK1/CRABP2/ANKRD27/RELN/MARK2/G
A/FA2H/SMPD4/A4GALT/PRKD2/ST3GAL5/RACK1/CSNK1G2/CERS6/HACD1
AB3A/VPS11/STXBP1/TAPBP/TAP2/GOSR2/SNAPIN/SAMD9L/CAV2/VPS4A/VPS16/C2CD5/RAB8B/V
/MRPS15/MRPL18/MRPL37/MRPL27/MRPL28/MRPL51/MRPL11/MRPL15/MRPL4/MRPS24/MRPL33/MR
2/SLC7A8/SLC3A2/SLC7A10/SLC7A6/SLC36A1/SLC38A5/SLC38A6
G3/NUP85/NUP43/RAE1/XPO5/SRRT/HENMT1/POLR2L/NUP155/DROSHA/AGO2/TDRKH/NUP107/NCO

P1R/TUBB4A/CAPZA1/MKKS/FHOD3/CAMSAP2/FKBP4/MYADM/WAS/VIL1/PFN1/INPP5J/TACSTD2/C
FS1/UBE2L3/GSK3A/UBE2D1/PSMD10/ARRB2/DCUN1D5/PELI1/GNL3/TNIP1/PTTG1IP/PDCD6/VCP

/USP14/CPSF4/KTI12/ZBTB80S/NAT10/FAM98B/GTPBP3/ADAT3/PUS7/DUS2/CTU2/POP5/SEPHS1

GSK3A/ERO1A/ZBTB17/CALR/DNAJB11/PPP1R15A/RACK1/PDIA6/CXXC1/TBL2/ATP6V0D1/VCP/SR

4GALT7/IL1B/B4GALT2/XYLT2/UGDH/BGN/CHST2/LUM/CHPF/CSPG5/CHST12/CSPG4/CEMIP/EXT1

ARHGAP4/AURKA/PPT1/PSRC1/TNFRSF12A/KRT17/CDKN2A/PLXNA3/NDRG3/DDR1/EXOSC4/S100A8

/STK3/FAF1/PDIA3/CAV1/PTPRC/STK4/HYAL2/HTRA2
PV2/RPS6KA1/HSF1/FOXS1/YBX3/CXCR4/ZP3/SH3PXD2B/PRKDC/SLC9A1/EFNA5/PAK1/CRABP2/S
EC3D/TNIP1/SRPK1/NOTCH1/MAVS/OAS3/TARBP2/PPIE/PPIB/IFITM3/CCL8/HMGA2/PARP10/MOR
/MRPS15/MRPL18/MRPL37/MRPL27/MRPL28/MRPL51/MRPL11/MRPL15/MRPL4/MRPS24/MRPL33/MR
MST1R/SPESP1/SNU13/HOXD9/CCT8/PLCD4/HVCN1/SPA17/VDAC2/RAB3A/HEXB/TARBP2
IRF1/IRF3/F2RL1/RAB7B/LRRC14/IRF7/TNIP2/FLOT1/SMPDL3B/LYN/CTSS/CD300LF/UBE2D1/T
IKBIP/BLM/KDM1A/SWI5/DNMT3A/IFI16/PML/PARP1/GPX1/MSH2/GRB2/TOPBP1/XRCC6/RRM1/BA

B/ACSL1/APOC1/INSIG1/APOA2/APOH/PCK2/GPLD1/AADAC/RGN/THRSP/FABP4/MOGAT2/APOA1/A

T/PAH/DAO/CYP2E1

ACADL/MST1/APOC1/LEPR/INSIG1/PFKFB1/FBP1/CYP7A1/APOC3
2/ATP6AP1/ATP6V1H/CLN3/SLC11A1/RAB20/ATP6V1E1/KCTD7/CLN6/SLC4A3/SLC4A11/ATP6V0C

DD/ADAM17/MAVS/TLR2
GBP2/PSMD2/HIF1A/MAPK3/SIRPA/CCL13/TNFRSF11A/PSMB2/RPS6KA4/ZC3H12A/PSME3/TNIP2/

1/KIF20B/ABHD17A
OLE2/PARP10

SMPD2/NSMAF/DEGS1/ORMDL2/B4GALT3/B3GALNT1/SMPDL3B/ASAH1/CLN3/GBA/CLN6/FA2H/FUT3
SL6/HSD17B4/GCDH/ACAT1/TDO2/HSD17B8/DGAT2/ACSM3/ACSL5/ACSL1/BAAT/AASS/ACOT12/PI
SL6/HSD17B4/GCDH/ACAT1/TDO2/HSD17B8/DGAT2/ACSM3/ACSL5/ACSL1/BAAT/AASS/ACOT12/PI

IP/MFF/SH3GLB1/BNIP3L/SREBF1/CACNB3/CDK5R1/VPS11/ATG13/HAX1/CIB1/CSNK2A2/PARL/H

UP155/NUP107/TRPM4/GNL3/UBA2/PIAS4/ZMIZ1/SEH1L/SUMO2/AAAS/PARK7/EGR2/RELA/SUMO3

5/HNRNPA2B1/XRCC5/PARN
/WNT5A/LSM14A/CACTIN/UBE2K

AJC6/MGAT5/IGFBP3/PGP/PPP1R37/PPM1M/PHPT1/FKBP1A/CD300A/PDP1/PPP2R1A/PPM1H/SLC3

ARA/LPIN2/ACAD11/AIG1/ACACB/CPT1A/ECHS1/HADH/MCEE/ACADS/NUDT7/IRS1/ECHDC2/ECI2/
5/APOB/CYP1A1/APOA2/ALDH8A1/CYP3A5/RBP4/ADH6/RDH16/CYP1A2/TTR/APOA1/APOC3/ADH1A
/CPT2/HIBCH/PRKAG2/PPARA/ACAD11/ACACB/CPT1A/ECHS1/HADH/ACADS/POR/IRS1/ECHDC2/EC

2/PPP2R1A/PAK1/SMARCA4/PPP1R9B/OSGIN2/PPARD/MT3/CDK5/MEG3/PHB/DNAJB2/TGFB2/GSK3

2/NUP107/NCOR2/POLR2K/TRIM71/SEC13/TARBP2/POLR2I/POLR2J/POLR2D/DHX9/SEH1L/POLR2
2/NUP107/NCOR2/POLR2K/TRIM71/SEC13/TARBP2/POLR2I/POLR2J/POLR2D/DHX9/SEH1L/POLR2

5J/CAPZB/CLIP3/TBCD/STMN2/VDAC2/ADD3/EML2/MTPN/SPTAN1
/GSTA1/CYP4F2/CYP2B6/CYP4A11/CYP2E1/CYP2C8/CYP2C9/CYP2A6
BCB4/APOA1/APOA5/APOC3/G6PC
4A11
1A2/GSTA1/CYP4F2/CYP2B6/CYP4A11/CYP2E1/CYP2C8/CYP2C9/CYP2A6
/CPT2/HIBCH/PRKAG2/PPARA/ACAD11/ACACB/CPT1A/ECHS1/HADH/ACADS/POR/IRS1/ECHDC2/EC
3/TAF1D/UBTF/DHX9/GTF2H1/TBP/SSU72/POLR2E/ERCC3/TAF1B/PABPN1/FIP1L1/CSTF3/SNRPD

RPA/F2RL1/ZC3H12A/BCL3/SLC11A1/IL1B/CD47/IL12RB1/AXL/FADD/CLEC7A/HSPD1/ABL1

DKN2D/DTNBP1/PHPT1/CD300A/MAD2L2/RGS14/PPP2R1A/NPM1/SPRED1/THY1/CORO1C/UBASH3B/

SL1/ACADL/EPHX2/CYP1A1/CYP4F3/ACOT12/SLC27A2/CYP2A7/CYP1A2/GSTA1/CYP4F2/CYP2B6/

PPP1R3B/PPARGC1A/KHK/MST1/LEPR/PFKFB1/RORC/GPLD1/RGN/FBP1/IGF2/PDK4/GCGR/GNMT

T/IVD/QDPR/GOT2/ACMSD/GCDH/ACAT1/TDO2/KMO/ALDH7A1/CSAD/GPT2/ARG1/SHMT1/RIDA/GLS

NF1/SMARCB1/ILF3/ISG20/CDC42/GRK2/APOBEC3D/TNIP1/VCP/SRPK1/NOTCH1/MAVS/CTBP2/HT
ECN1/RHOBTB2/PPARG/ABCA7/ARHGAP25/APPL2/RAC3/MYH9

TA1/ADH4
AB10/FGFR2/NES/BABAM1/TRIM71/NDE1/NF2/ZNF335/OVOL2/SFRP2/KDF1/AKNA/CTNNA1/NOTCH

IF/PBK/TRIB3/FBLN1/TGFB1/IQGAP3/GPNMB/STK26/DKK1/RIPK2/HAVCR2/GSTP1/CD44/GDF15/

/HRAS/SYK/STK39/EZH2/CSF1R/MAGED1/TRAF2/MAPKAPK2/CXCR4/MIF/IQGAP1/MAPK3/MAP2K2/

/PTPN2/PTPN1/TXNDC12/BCL2L1
/ITPKA/EZH2/NME2/TWF2/ANKRD1/TRPV2/GRN/TIMP2/TCF3/P3H1/CXCR4/IQGAP1/PTK7/EFNA5/
LC25A5/BAD/CASP8/BNIP3L

D2/FA2H/TGFB2/MYOF/A4GALT/EPB41L3/BIN2/PSAP/DNM2/UGCG/SH3GLB1/RAB3A/PACSIN2/PAC
CL3/SLC11A1/IL1B/CD47/IL12RB1/AXL/FADD/CLEC7A/HSPD1/ABL1

S14/PPP2R1A/NPM1/SPRED1/THY1/CORO1C/UBASH3B/SOCS1/CEP85/DUSP2/CDKN2B/TESK1/LYN/

/MAPT/CCL3/ABL1/ZNF746/FCGR2B/BAD/CASP8/CAPN2/CDK5R1/DAXX/RHOA
A/SNX10/ADAM8/MITF/RAC1/CALCA/TGFB3/TMEM119/ZNF675/IL20RA/RAB3D/TNFAIP3/CSK
ADD/PTPN2/PTPN1/MAVS/IRF5/OAS3/HLA-F/IFNAR2/CDC37/IFI6/IFITM3/YTHDF2/IP6K2/METT
ADD/PTPN2/PTPN1/MAVS/IRF5/OAS3/HLA-F/IFNAR2/CDC37/IFI6/IFITM3/YTHDF2/IP6K2/METT
C3H12A/CSE1L/SNRPF/FERMT1/SIX2/HTATIP2/KPNB1/HCLS1/GEMIN7/NUP85/PML/BAG3/NUP155

ATAD5/THOC1/PTPRC/UNG/SUPT6H/POU2F2/TNFSF13/FGL2/CD40

RRT/POLR2L/NUP155/DROSHA/AGO2/NUP107/NCOR2/POLR2K/TRIM71/SEC13/TARBP2/POLR2I/PO

/LZTS2/ZC3H12A/CCT4/FERMT1/HCLS1/CCT7/TCP1/BYSL/PARP1/BAG3/LILRB4/CCT8/NF2/PKIA

A2/CUL4B/POLD3/PRPF19/COPS6/CHD1L/RPS27A/ERCC1/COPS5/UBA52/PPIE/POLR2I/POLR2J/R
1B/PTPN2/HOXA5/THRA/VEGFA/SMAP1/SRF/INHA/BPGM/MAEA/LDB1/PKNOX1/RPS14/INPP5D/ZNF
N/ACAN/GPC2/HEXA

X2/JAG2/HEYL/NLE1/JAG1/ITGB1BP1/IL2RA/DNER/TGFB2/PLXND1/APH1B/ANXA4/LLGL1/PRKCI
CL19
NRCAM/HSP90AB1/GDI1/MT3/CPNE1/CDK5/CTTN/RNF157/WASF1/SEMA4B/SEMA4D/SYT1/SYT3/SE
S6KA4/OTUB1/NOC2L/KDM1A/AKAP8L/IL1B/ATAD2/NAP1L2/SET/SMARCB1/PADI2/RUVBL2/PRKD2

ALL2/DVL3/FZD2/TULP3/DEAF1/TGFB2/DVL2/PFN1/GORAB/RALA/KAT2A/TMEM107/SEMA3C/TRIM

/IRF1/IRF3/HSP90AB1/STAT1/IRF7/HLA-DQA2/TRIM31/NLRC5/HLA-DRB5/PML/SOCS3/HLA-B/T

TPG1/RHOT2/SLC25A5/BAD/CASP8/BNIP3L/BCL2L1/YWHAG/TFDP1/TMEM102/NAIF1/CNP/THEM4/

D4B/RAB29/RAB27B/IFT22/RAB9A/RAB40C/RAB13/RAB1A/RAB36/RAB8B/RAB1B/MADD/RAB22A/R

2/IFRD1/CDK5R1/RHOA/EPHB2/RYK/FSTL4/SEMA3F/TRIM46/SEMA6B/SEMA4C/PTPRS/TRAK2/LRP

4V2/RDH5/APOB/CYP1A1/APOA2/ALDH8A1/CYP3A5/RBP4/ADH6/RDH16/CYP1A2/TTR/APOA1/HMGC

/ATR/PTPRC/CXCL10/MYC/TLK2/ZMPSTE24/XRCC5/TP53/ELK1/MEN1

1/WNT5A
CL9/CXCL3/CXCL13/DEFB1/SLC11A1/JCHAIN/RPL39/PF4V1/REG1B/CXCL11/RPS19/ROMO1/S100
BBP8/RNASET2/PCNA/PELO/RRS1/RFC2/POLR3K/ERI3/REXO4/ZC3H12A/NCBP2/USB1/PNKP/NHP2
13/IFI16/CASP1/PML/IL1R2/GSDMD/ARRB2/APP/HK1/CARD16/CLEC7A/CARD18/CCL3/TNFAIP3/

/CXCR4/CASP2/ABHD12/SLC9A1/MAPK3/SMPD2/IRF1/STAT1/SLC1A3/MKKS/TNC/RAC1/ITGA2/IL

STD2/ANGPT2/SEMA3E/NF2/TBCD/THBS1/NOTCH1

TP53/APEX1/FAM168A

580/SIRPA/STAT1/HDAC2/LDHA/TXN/MB/GPX1/RPS3/CBX8/PCGF2/MAPK7/PPP5C/AXL/STK25/RA

A5/BAD/CASP8/BNIP3L/YWHAG/TFDP1/TMEM102
UP107/SEC13/RPS27A/UBA52/VPS37B/KPNA6/SEH1L/CAV2/CAV1/MVB12B/AAAS/TRIM15/NUP214

ZC3H12A/HDAC2/TXN/DNMT3A/CRIP1/RPS3/CBX8/PCGF2/MAPK7/PPP5C/AXL/RPL23/STK25/RACK

R/TUBB4A/CAPZA1/MKKS/KIF14/FHOD3/FKBP4/GBA/MYADM/VIL1/PFN1/INPP5J/CAPZB/CDC42/C
ADD/PTPN2/PTPN1/MAVS/IRF5/OAS3/HLA-F/IFNAR2/CDC37/IFI6/IFITM3/YTHDF2/IP6K2/METT
UP107/SEC13/RPS27A/UBA52/VPS37B/KPNA6/SEH1L/MVB12B/AAAS/TRIM15/NUP214/TSG101/TP

2/IRF1/IRF3/IRF7/BCL3/LAG3/CARD11/IL1B/ASB1/APP/CLEC7A/LBP/BCL10/TNFSF15/THBS1/
25A15/SLC38A3/SLC1A2/SLC13A5/SLC22A1

18/TRAF2/CCL5/MAPKAPK2/CXCR4/CD300A/CARD10/EFNA5/MAPK3/MAP2K2/PAK1/PEA15/FPR1/S
8

CMBL/CYP2J2/EPHX1/CYP2A13/BCHE/CSAD/SULT1B1/ACSL1/EPHX2/CYP1A1/GSTA2/BAAT/NAT2/

KB1/C4BPA/F12/CFHR5/C6/CFHR4

1/NUMBL/LEF1/FGFR1/KIF14/KDM1A/LYN/RAB10/FGFR2/NES/C5AR1/RRM1/TRIM71/NDE1/RERE/

/GSDMD/ARRB2/APP/HK1/CARD16/CLEC7A/CARD18/CCL3/TNFAIP3/CD33/CASP8/S1PR3
CASP8/BNIP3L/YWHAG/TFDP1/TMEM102
3/EFNA4/VASP/GPC1/TWF2/CSF1R/NECTIN1/UNC5B/EZR/BSG/TRPV2/CRMP1/DOK2/LINGO1/CXCR
DAM9/SYK/S100A8/ASPH/CASP4/IFT57/GRN/HSF1/CARD9/TRAF2/PSMD14/BAX/BEX3/MAPK3/NUP

CR4/FKBP1A/GPR35/P2RY6/FPR1/THY1/UBASH3B/NOL3/C3AR1/F2RL1/PLCB3/P2RX5/C1QTNF1/T
HOC1/PTPRC

A/MEIS2/BTG2/MAP1A/MECP2/SRF/IFT20/LGMN
/BCL10/MAVS/PLA2G1B/TLR2/TLR7/IL17D/PTPRC/RAB1A/TLR8/FFAR2/PARK7/HYAL2/ANXA1/NO

A/TM7SF2/GPAM/APOB/ACADL/APOC1/EPHX2/INSIG1/APOA2/RORC/SEC14L2/APOA1/APOA5/CYP7

L/APOBEC3C/CHIT1/PDE4A/SLC9A1/DCTPP1/NT5C3A/NEU1/GM2A/NT5C/ACOT7/FUCA2/PDE7A/AM

/GPC2/HEXA

AM1/MSX1/RAD9A/TIMELESS/EID3/WRAP53/HDAC10/SPIRE1/PIAS4/UIMC1/APBB1/RNF8/DHX9/T

MP4B/TRAPPC4/RAB32/VPS37C/CHMP4C/ANKRD27/STX1A/CORO1C/STX6/HGS/PPP6R1/RAB7B/TGF

PAM/FABP1/CAT/APOB/ACSL1/APOC1/INSIG1/ANG/APOA2/APOH/PCK2/GPLD1/AADAC/RGN/THRSP
/ESR1/AKR1C4/PPARGC1A/RDH5/UGT2B7/CYP1A1/SRD5A2/ALDH8A1/CYP3A5/RBP4/ADH6/RDH16/
1B2/OR11G2/OR10H1/OR5V1/OR1Q1/OR11A1/OR8B8/OR1C1/OR2H1/OR10P1/OR1L3/OR2L13/OR1G
T2A/FBLN5/MT1A/APOM/MT1M/MT1G/ABCG2/MT1E/FABP1/CAT/IYD/AKR7A3/MT1X/HP/RGN/PON3/
8A/KLKB1/C4BPA/F12/CFHR5/C6/CFHR4
H3B/STAT1/RAB7B/HDAC1/FSTL3/TFE3/NCAPG2/LEF1/IRF7/NCKAP1L/EVI2B/HCLS1/JAG1/MITF

NCL/POLR2L/GTF3C2/POLR2K/GTF3C1/NIFK/NABP1/GTF3C5/INTS9/PIH1D1/GTF2E2/INTS7/POL

3G/TRMT112/NSUN5/TRMT6/FTSJ3/HENMT1/APOBEC3D/KTI12/TSR3/NAT10/NOP10/GAR1/GTPBP3
F8/CHMP3/PTPN23/PLEKHJ1/SNX12/WDR91/TBC1D10C/UBE2O/TMEM87B/GGA1/DENND5A/WIPI1/H

274/ZAP70
2/IRF1/IRF3/IRF7/BCL3/LAG3/CARD11/IL1B/ASB1/APP/CLEC7A/LBP/BCL10/THBS1/NMI/GLMN
/SHARPIN/PERP/CNFN/PALLD/HDAC1/KRT18/FGFR1/SLC9A3R1/JAG2/CAPN1/HDAC2/SULT2B1/KR

0/NOTCH1/SFRP4/ALOX15B
107/NCOR2/POLR2K/TRIM71/SEC13/TARBP2/POLR2I/POLR2J/POLR2D/DHX9/SEH1L/POLR2E/PPA

BL1/TNFAIP3/RPS7/BAG2/RPL5/TRIM21/IVNS1ABP/UBR5/GLMN/PRMT3/CAV1/PARP10/HDAC8/RP

/MRPS17/MRPS21/MRPS15/MRPL18/RCC1L/WARS2/MRPL37/RPUSD3/NSUN4/MRPL27/MRPL28/MTER
3/RACK1/NSF/GGA1/CSK/DNM2/SYS1/VPS35/ARFRP1/OPTN/GGA3/RAB11A/GOLPH3L/ARF5/SNX17

/SLC11A1/RAB20/ATP6V1E1/CLN6/SLC4A3/SLC4A11/ATP6V0C/ATP6V0E1/SLC9A7/TMEM199/ATP

AS3/CCR1/LILRB4/FADD/ZNF675/CCL3/PTPN2/CASP8/TMEM64/INHA/KITLG/NOTCH2/HAX1/INPP

LR8/IFNG/RELA/LILRB1/C1QBP/CD40/SLAMF1
T1/ADORA2B/TLR7

NDC1/TBC1D10C/TBC1D22A/TBC1D7/SGSM3/TBC1D22B/ADPRH/RAPGEF1/EPHA2/SGSM1/EVI5L/RA
OBEC3D/TNIP1/TRIM25/SRPK1/MAVS/OAS3/TRIM21/IFITM3/SNX3/CCL8/HMGA2/PARP10/MORC2/

WDR45B/MAP1LC3B/BAG3/VPS18/PIP4K2C/NAGPA/RALB/TMEM199/PACS2/VMP1/HPS1/NPRL3/ATG

RRT/HENMT1/POLR2L/NUP155/DROSHA/AGO2/NUP107/NCOR2/POLR2K/TRIM71/SEC13/TSN/TARBP
PSMD1/PSMA7/PSMB3/PSMD4/PSMD3/PSMD7/PSMD8/PSMA1/PSMB5/PSMA4/PSMD9

CASP8/BNIP3L/YWHAG/TFDP1/TMEM102
ICDL2/RHOT2/COPG2/BICD1/AP3B1/SNAPIN/UXT/HTT/ACTR1A/RAB1A/KIF3A/LRPPRC/KIF5B/TR

B3/P2RX5/SRI/CXCL9/MCU/LYN/LCK/PTPN6/RASA3/PML/ERO1A/CXCL11/FAM155B/CCR5/MYO5A/

PAM/FABP1/CAT/APOB/ACSL1/APOC1/INSIG1/ANG/APOA2/APOH/PCK2/GPLD1/AADAC/RGN/THRSP
BCB4/SLC6A1/CYP4F2/AGXT/CYP4A11/AQP9
/SLC51B/ZC3H12A/BCAP31/UBE2J2/NPEPPS/HCLS1/ITGB1BP1/IL1B/CDK5/UBE2L3/GSK3A/BAG3
LA-E/NOD2/LILRB1/DDX1/NLRP3/TICAM1/WNT5A/MALT1/HLA-G/GATA3

1/CARD16/CLEC7A/CARD18/CCL3/TNFAIP3/CD33/CASP8/S1PR3

LD1/HRAS/STK39/RAD51AP1/CDC25A/ANKRD1/CHEK1/HSF1/RHNO1/RAB11FIP5/YBX3/BAX/CASP2
LD1/HRAS/STK39/RAD51AP1/CDC25A/ANKRD1/CHEK1/HSF1/RHNO1/RAB11FIP5/YBX3/BAX/CASP2

2D1/PSMD10/ARRB2/DCUN1D5/PELI1/PTTG1IP/PDCD6/TBC1D7/BCL10/MASTL/HSPA5/CHFR/NMI/

PA/KPNA4/CD4/HYAL2/SMAD3/DAG1
P5/PSME3/SLC9A3R1/DVL3/FERMT1/AXIN1/PSMB4/PSMB8/PSMB10/PSMA5/PSMD13/PSMC4/GSK3A
/C12orf4/CD177/MAVS/STXBP1/ADORA2B/IL13RA2/CD84/BCR/ADGRE2/CCR2/DNASE1/PRAM1/HL

ITM3/CCL8/HMGA2/PARP10/MORC2/ZC3HAV1/SRPK2/IFITM2/MX1/VAPA/SETDB1/APOBEC3F/FAM1

C7L/SFSWAP/GEMIN2/SRSF1/SRPK2/CELF2/SNRNP200/GEMIN6/SMN2/LUC7L3/DDX1/SRSF10/PSI
/BAD/CASP8/BNIP3L/YWHAG/TFDP1/TMEM102/NAIF1/THEM4/NMT1/FZD9/MUL1/TP53
ME3/UBE2J2/NPEPPS/OTUB1/ANAPC7/SPSB1/CBFB/MYLIP/PSMB4/FBXO32/RNF26/UBE2L3/PSMB8
2/EZR/FZD7/NCF1/PRMT1/CARD9/DTNBP1/TRAF2/MAPKAPK2/PRDX1/SH2D3A/MAPK3/MAP2K2/PAK
0/FSCN1/RHOC/ITGA5/IQGAP1/SLC9A1/EFNA5/PLEC/LAMC2/LAMA5/THY1/CORO1C/CDH6/CDH11/

S4/ACTG1/LYN/ACTB/FIBP/PTPN6

GTF2H1/TBP/POLR2E/ERCC3/TAF1B/PWP1/TTF1/POLR1C/ERCC2/ZMPSTE24/NOL11/ZNRD1
L/SBNO2/MTF2/TRIM25/ILDR1/RARG/SLC25A5/PDCD10/HNRNPU/CTBP2/MRAS/SMARCA5/RNMT/MR
L/SBNO2/MTF2/TRIM25/ILDR1/RARG/SLC25A5/PDCD10/HNRNPU/CTBP2/MRAS/SMARCA5/RNMT/MR

BNIP3L/YWHAG/TFDP1/TMEM102
/BCL10/MAVS/TLR2/TLR7/IL17D/PTPRC/TLR8/FFAR2/PARK7/HYAL2/ANXA1/TLR1/NOD2/AFAP1L

3

RAD51AP1/CDC25A/COL3A1/CDKN2D/CHEK1/HSF1/RHNO1/NABP2/BAX/FANCG/OPN1SW/BRSK1/PRK
IB3/AURKA/GAPDH/RIPK2/IGF2BP2/DTL/YWHAZ/GPC3/FLNA/RBM38/HK2/PFKFB4/PRKCD/DRAM1/
EC3B/FGA/TF/SERPINF2/ITIH3/SERPINA4/APOH/ALB/IGF2/KNG1/A1BG/APOA1/PLG/AHSG/SPP2
APOA5/SLC2A2
/HIBCH/PPARA/LPIN2/ACAD11/AIG1/ACACB/CPT1A/ECHS1/HADH/MCEE/ACADS/NUDT7/IDNK/CYP

PBB1/DMAP1/CTBP1/MORF4L1/EP400/TADA3/TRRAP/NAA60/IRF4/NAA50/ZMPSTE24/IWS1/PHF20
1/TRMT1/KMT5A/EHMT2/TRMT61A/MTA2/DNMT3A/TRMT112/NSUN5/TRMT6/PARP1/SMARCB1/SMYD3

/MAPK3/IL27RA/TNFRSF4/SOCS1/CD58/IRF3/F2RL1/SERPINB1/ZC3H12A/FERMT1/FRMD8/LYN/I
DMD/ARRB2/APP/HK1/CARD16/CLEC7A/CARD18/CCL3/TNFAIP3/CD33/CASP8/S1PR3

F1/PAM/SNX10/JAG2/HDAC2/SSUH2/TNC/TGFB2/FGFR2/TGFB3/CNNM4/MYO5A/MSX1/TFAP2A/NF2

RRT/HENMT1/POLR2L/NUP155/DROSHA/AGO2/NUP107/NCOR2/POLR2K/TRIM71/SEC13/TSN/TARBP
PTHLH/ASPM/COL3A1/CRMP1/LINGO1/SEMA4A/MAP2/EFNA5/TRIM11/PAK1/ADGRG1/THY1/CORO1C

PSMD4/PSMD3/RBX1/PSMD7/RNF7/CUL4B/PSMD8/PSMA1/PSMB5/PSMA4/PSMD9/FBXW5/PSMC3/PSM

DBH/CYP2J2/CYP2A13/SULT1B1/FMO4/RIDA/CAT/HGD/SDS/AASS/ALDH2/CYP3A5/BDH1/PCK2/PI
NCRIP/ELAVL1/PAIP1/TARDBP

FUCA2/TNFRSF4/ITGB5/TRIM59/NPC1/PPIA/DYNLT1/RPSA/TRIM31/CD209/ITGA2/XPR1/GRB2/V

FUCA2/TNFRSF4/ITGB5/TRIM59/NPC1/PPIA/DYNLT1/RPSA/TRIM31/CD209/ITGA2/XPR1/GRB2/V
FUCA2/TNFRSF4/ITGB5/TRIM59/NPC1/PPIA/DYNLT1/RPSA/TRIM31/CD209/ITGA2/XPR1/GRB2/V
FUCA2/TNFRSF4/ITGB5/TRIM59/NPC1/PPIA/DYNLT1/RPSA/TRIM31/CD209/ITGA2/XPR1/GRB2/V

PD/HNRNPC/SYNCRIP/ELAVL1/PAIP1/TARDBP/PARN

1/CCR3/FCMR/PRF1/RELA/CCR2

1/TMEM102
2/PGK1/SIRT6/NUP210/FOXK1/HIF1A/NUPR1/CYC1/HK3/TPI1/FOXK2/CAD/NUP93/NUP205/NUP3

/POLR2K/SPRY4/DSTYK/CBL/TRIM71/HNRNPM/WNT4/PTBP1/THBS1/SULF2/HNRNPA1/POLR2I/POL

/IGF2BP2/YWHAZ/RBM38/HK2/PFKFB4/PRKCD/DRAM1/HSPB1/QSOX1/ABCD1/IGF2BP1/ADAM9/MTM
1/GSTZ1/HOGA1/TST/AADAT/IVD/QDPR/GOT2/ACMSD/GCDH/ACAT1/TDO2/KMO/ALDH7A1/CSAD/GP

/DGAT2/APOC4/ANGPTL4/ACSM3/LIPC/ASGR2/MLXIPL/PLA2G12B/CYP39A1/MTTP/APOB/ABCG8/E
1/SLC1A2/SLC6A12/THRSP/SLC38A4/SLC13A5/SLC27A5/AQP9
1/SLC1A2/SLC6A12/THRSP/SLC38A4/SLC13A5/SLC27A5/AQP9
EMA5B/SEMA3C/SEMA3E/MAPT/ABL1/DBNL/DNM2/VEGFA/RAB11A/IFRD1/CDK5R1/SRF/ADNP/RYK/

TN2/NDE1/CKAP5/FBXW5/TRIM37/CENPJ/SASS6/RBM14

A1/B4GALT5/NEU1/GM2A/GLTP/CERS5/PPP2R1A/B3GNT5/GBGT1/SMPD2/NSMAF/DEGS1/ORMDL2/B

SF18/AREG/CCL5/ITGA5/MIF/IQGAP1/EFNA5/CD3E/TGFA/RELN/NCAPG2/FGFR3/HDAC2/HCLS1/L

1/BCL3/LAG3/CARD11/IL1B/ASB1/APP/CLEC7A/LBP/BCL10/THBS1/NMI/GLMN/INHA/SIGIRR/ER
/GABRR3/SLC26A11/SLC37A2/XPR1/SLC4A11/BEST1/TTYH2/SLC12A7/SLC26A2/GABRD/TTYH1/C

PPP1R7/CALM3/PTPRC/PIN1/PPP1R12A/PPP2R5D/CNEP1R1/LILRB2/DUSP26
PN1/MAP3K10/DAXX/CCL19/MAP4K1/MAP4K2/DAB2IP/MAP3K7/MAP3K9/MAP3K11/MUL1/MAP3K12/
L3/SLC11A1/IL12RB1/IL4R/GPR183/FCGR2B/PTGER4/TMEM98/CCL19/ITGAL/ATP7A/CLEC4E/LO

MCO3/SLC9A3R1/ATP6V1H/SLC36A1/ATP6V1E1/SLC4A11/SLC15A3/ATP6V0C/ATP6V0E1/SLC9A7/
MC1/CTNND2/MICALL2/S100A10/FSCN1/RHOC/ITGA5/IQGAP1/SLC9A1/EFNA5/PLEC/LAMC2/PERP

1/WWP2/HIF1AN/DLK2

1CC/CPSF6/XPO5/BARD1/BAG3/KHDRBS1/PKIA/EMD/MAVS/ZPR1/UBR5/ANP32B/DHX9/DMAP1/EFC

IF/SLC34A2/TGFB1/DKK1/ADA/FOLR1/CMTM3/MAFG/VASP/ARNT2/ELF3/SMG9/PLCD3/FZD6/TCOF
TH4/ROBO1/TRIP10/HRAS/NUP62/GMIP/FGD3/COL3A1/RHOG/RHOQ/ARHGEF39/TIMP2/RHOC/PLEK

1/MFNG/LFNG/ABL1/SPPL3/NVL/TRAF4/STK3/CAV1/PKD1/BMP2/ADD1/PIN1/STK4/RPL11/EPB41
/BAX/HIF1A/TCIRG1/SUCO/ATP6AP1/MMP2/UBASH3B/NOL3/TNFRSF11A/TGM2/SNX10/ADAM8/CAP
AIP3/NOTCH1/VEGFA/MESP1/TWIST1/SIX1/RBPJ

/CCL2/CXCL13/ITGB1BP1/KDM5B/FIBP/FGFR2/PRKD2/GRB2/POLR2L/POLR2K/SPRY4/DSTYK/CBL
AC2/PHLDB1/JAG1/IL1B/LOXL2/TGFB2/FGFR2/HNRNPAB/SDHAF2/TGFB3/HEY1/MSX1/PDCD6/WNT
A/PML/PARP1/CRIP1/TIPIN/GPX1/MSH2/RUVBL2/USP1/UBE2A/HMGN1/CARD16/CUL4B/POLD3/NF

DGAT2/PPP1R3B/PPARGC1A/KHK/MST1/LEPR/PFKFB1/RORC/GPLD1/RGN/FBP1/IGF2/PDK4/GCGR/
SULT4A1/ACOT1/ACOT2/ENPP1/ACACB/CBR4/SUCLG2/MCEE/NUDT7/ELOVL2/SULT1E1/GSTZ1/AAD
SULT4A1/ACOT1/ACOT2/ENPP1/ACACB/CBR4/SUCLG2/MCEE/NUDT7/ELOVL2/SULT1E1/GSTZ1/AAD
SULT4A1/ACOT1/ACOT2/ENPP1/ACACB/CBR4/SUCLG2/MCEE/NUDT7/ELOVL2/SULT1E1/GSTZ1/AAD
BB/DBH/FGB/ADRB2/FGG/FGA/EPHX2/SERPINF2/KNG1/CPS1
/GPLD1/LCAT/APOA1/APOA5/APOC3
/CPS1/PCK1/AQP9
BB/DBH/FGB/ADRB2/FGG/FGA/EPHX2/SERPINF2/KNG1/CPS1
FGA/F2/F11/TMPRSS6/F7/SERPINF2/APOH/CPB2/KLKB1/ABAT/F12/KNG1/PLG/HRG/SERPINC1
BB/DBH/FGB/ADRB2/FGG/FGA/EPHX2/SERPINF2/KNG1/CPS1
CB4/PON1/APOA1/CYP4F2/APOA5/APOC3/CYP4A11
MT6/ASF1A/SUPT6H/KDM3A
B4GALT3/B3GALNT1/ST3GAL4/ASAH1/CLN3/GBA/CLN6/FA2H/SMPD4/A4GALT/PIGC/ST3GAL5/PIG

210/FOXK1/HIF1A/NUPR1/CYC1/HK3/TPI1/FOXK2/NUP93/NUP205/NUP37/DDIT4/GPI/NDC1/NUP

SERPINE2/CSF1/LRP12/TGFB1/ARHGAP4/PPT1/STC2/PSRC1/TNFRSF12A/KRT17/GPC3/GDF15/AG
BLN1/GSTP1/CD44/CD74/DUSP5/ARHGEF2/BCL2A1/PRKCD/HSPB1/PRNP/PLEK/APLP1/NUP62/TMS

PSF3/POP5/TSN/UTP20/NOB1/TSEN34/TSEN15/RNASEK/NOL9/UTP23/RPP30/RNASEH1/SND1/DBR

RPS7/BAG2/RPL5/IVNS1ABP/UBR5/GLMN/PRMT3/CAV1/PARP10/HDAC8/RPL11/NXN/PARK7

GFR2/TBX19/COL4A2/LAMB3/HSBP1/NF2/COL11A1/ACVR1/SFRP2/TWSG1/POFUT2
P2A/CHMP7/CHMP5

H/CYP4V2/RDH5/APOB/CYP1A1/APOA2/ALDH8A1/CYP3A5/RBP4/ADH6/RDH16/CYP1A2/TTR/APOA1
CYP4F12/SHMT1/CYP27A1/CYB5A/TTPA/HAAO/ALDH1L1/CYP1A1/MTHFD1/RGN/AOX1/CYP4F2/CYP

BB/DBH/FGB/ADRB2/FGG/FGA/EPHX2/SERPINF2/KNG1/CPS1

HJ1/VPS25/HOOK2/RAB11A/VPS37B/VPS11/IST1/SNX3/PI4K2A/VPS4A/STAM/TMCC1/RAB22A/AN

6/NUP210/FOXK1/HIF1A/NUPR1/CYC1/HK3/TPI1/FOXK2/CAD/NUP93/NUP205/NUP37/DDIT4/GPI
TLA4/PLA2G2D/ZC3H12A/LAG3/CCL28/CBFB/IL2RA/PTPN6/IL4R/PELI1/BTN2A2/FCGR2B/TWSG1
NAP1L2/SET/SMARCB1/RUVBL2/POLE4/USP22/JADE2/KAT2A/BEND3/PCGF2/DR1/DPF2/TAF12/TA
EREG/EGF/ANAPC4
S6/NFKBID/ARG2/TP53/WNT5A/CD274/FASLG

/CEP131/PRKCD/SCAMP5/B3GAT3/PRNP/ADAM9/HRAS/SYK/GTSE1/CSF1R/EZR/ANKRD1/VTCN1/RA
MELTF/LOXL4/FBLN1/P3H4/TGFB1/ITGAM/CTSV/COL1A2/COL9A2/CD44/ITGB2/AGRN/QSOX1/VCA

14L2/CYP7A1

/CUL4B/POLD3/NFATC4/ERCC1/C9orf116

TAOK1/WNT5A/MAPK8IP3
SX1/MYBBP1A/PTTG1IP/RPS7/CHEK2
B4GALT5/IL34/MAPK3/BIN1/HDAC1/RELN/S100B/LEF1/MYRF/MT3/HDAC2/LYN/CNTNAP1/MXRA8/

GFR1/FBXO5/KIF14/DEFB1/KDM1A/CBFB/CDKN1C/GLDN/IL15/PTBP3/APP/GAL/AXL/BTK/CLEC7A

L/CCDC88A/TCHP/DYNC2LI1/CEP97/MAPK15/ATG3/IFT140/CNTROB/CEP135

C3H12A/BCL3/NCKAP1L/HSPH1/CBFB/IL12RB1/IL15/IL4R/PNP/AP3D1/RASAL3/GPR183/ABL1/P
AS4/ERCC1/UIMC1/RNF8/UBE2N/TP53BP1/HMGA2/DCLRE1C/TFIP11/LIG3/XRCC5/KAT5/SETMAR/

/CCL2/CXCL13/ITGB1BP1/TNC/KDM5B/FIBP/FGFR2/PRKD2/GRB2/POLR2L/POLR2K/SPRY4/DSTYK
AF9/GSK3A/PML/USP14/PSMD10/SMARCC1/RBX1/RPL23/RACK1/UBXN1/VCP/USP5/UBQLN4/RPS7/

MP14/LIF/SLC34A2/TGFB1/DKK1/ADA/FOLR1/CMTM3/MAFG/VASP/ARNT2/ELF3/SMG9/PLCD3/FZD
T/ST3GAL5/ITGB8/PSAP/UGCG/HEXB

TS2/PSMB2/SFRP5/PSME3/SLC9A3R1/DVL3/FERMT1/AXIN1/PSMB4/PSMB8/PSMB10/PSMA5/PSMD1
CN1/GRN/VSIG4/JAK3/HMOX1/CD86/CD300A/MNDA/UBASH3B/SOCS1/IRF1/CTLA4/PLA2G2D/C1QT

210/FOXK1/HIF1A/NUPR1/CYC1/HK3/TPI1/FOXK2/NUP93/NUP205/NUP37/DDIT4/GPI/NDC1/NUP

D1/GAL3ST1/STK26/FOLR1/PMEPA1/AGTRAP/ZDHHC13/CD74/CYTH4/QSOX1/SCAMP5/IL15RA/HLA
RC1/CHAF1B/P3H4/RAD51/TONSL/AURKA/SPI1/NCAPD2/UHRF1/MCM6/TUBG1/FEN1/NCAPH/GINS2
BP5/AKR1B1/CKAP4/PYGB/PPT1/CTSV/ADA/HLA-DMA/GPC4/MPZ/STX3/SQSTM1/GPC3/AGRN/SDC3
CB1/RAD51AP1/RCC1/EZH2/EXOSC4/CDK4/DNMT1/MUC1/EME1/ZNF385A/MAGED1/HMGB3/CREB3L1
CDK1/NDC80/HJURP/NCAPG/MCM2/CDC45/NEK2/GINS1/BUB1/BUB1B/MKI67/MCM4/ZWINT/ORC6/C
2/ORC1/MAGEC2/RPP25/NUSAP1/ARL2/P3H4/CENPH/TRAIP/RAD51/PNMA1/RCC2/TXNRD1/SPC24/
BP5/AKR1B1/CKAP4/PYGB/PPT1/CTSV/ADA/HLA-DMA/GPC4/MPZ/STX3/SQSTM1/GPC3/AGRN/SDC3
A-DQA1/HLA-DRA/STARD3NL/CTSA/HLA-DQB1/TRPM2/GRN/CYB561/HLA-DPA1/HLA-DMB/TPCN1/A
BP5/AKR1B1/CKAP4/PYGB/PPT1/CTSV/ADA/HLA-DMA/GPC4/MPZ/STX3/SQSTM1/GPC3/AGRN/SDC3
ENPF/MISP/ARL2/CCNF/AURKA/UNC119/HMMR/MARCKS/DTL/CEP131/TUBG1/CCDC88B/RELB/SPAT
B/BUB1B/KIF23/NDRG1/WDR62/CCNE1/CENPF/KIFC1/RPP25/MISP/ARL2/CCNF/RAD51/AURKA/UN
62/CENPF/KIFC1/KIF18B/MISP/ECT2/NUSAP1/TTK/SKA1/RCC2/AURKA/UNC119/PSRC1/SKA3/MA
MP14/MPZL1/MISP/POF1B/PDLIM7/ARL2/FLNC/ENAH/MARCKS/YWHAZ/FLNA/CD44/ITGB2/CIB2/T
/HLA-DRB1/PLEKHB2/TSPAN15/LAMP5/RAB31/HLA-DQA1/ABHD17C/MTMR2/PRAF2/HLA-DRA/STAR
/CKAP4/ITGAM/GPNMB/STK26/CYBB/CORO1A/FOLR1/STX3/AGTRAP/CD44/FCHO1/ITGB2/ALDH3B1
CIB2/TPM4/TAX1BP3/COTL1/FERMT3/ARHGEF2/BAIAP2L2/VASP/HCK/FHL3/ZYX/LAD1/TWF2/ASA
NRPN/LAGE3/FANCD2/ELF4/OIP5/PLCB1/ALYREF/CLIC3/ESRP1/GTF3C6/HSF1/GLIS2/AP5Z1/CA
A-DQA1/MTMR2/NRGN/HCK/UNC13D/HLA-DRA/YKT6/SYNGR2/HLA-DQB1/RAB11FIP5/GIPC1/HLA-D
1A1/FCER1G/HILPDA/REG1A/VEGFB/TMC6/ITPR3/TIMP1/LHFPL2/NUDT1/NCS1/LGALS3/PLAUR/P
TGAM/GPNMB/CYBB/CORO1A/FOLR1/STX3/AGTRAP/CD44/FCHO1/ITGB2/ALDH3B1/SNAP25/ZDHHC1
APG/MCM2/CDC45/NEK2/GINS1/BUB1/BUB1B/MCM4/ZWINT/ORC6/CENPF/RFC4/ORC1/CHAF1B/P3H
SULF1/B4GALNT1/MMP11/MMP14/PCSK1N/CLSTN1/HID1/PODXL2/GAL3ST1/TGFB1/STK26/FOLR1/
DMA/GPC4/GPC3/AGRN/SDC3/CD74/P2RX4/PRKCD/DRAM1/VCAN/HLA-DRB1/VAT1/ABCD1/LAMP5/H
/HLA-DRB1/PLEKHB2/TSPAN15/LAMP5/RAB31/HLA-DQA1/ABHD17C/MTMR2/PRAF2/HLA-DRA/STAR
AD51/TONSL/AURKA/SPI1/NCAPD2/UHRF1/MCM6/FEN1/NCAPH/GINS2/MCM10/SMARCD3/POLD1/MC
SF1/AKR1B1/S100A14/CKAP4/TRAIP/RAD51/AURKA/STC2/STK26/CYBB/GAPDH/CDC25C/ANKRD13
MP14/MPZL1/DSG2/MISP/POF1B/PDLIM7/ARL2/FLNC/ENAH/MARCKS/YWHAZ/FLNA/CD44/ITGB2/C
B4GALNT1/PCSK1N/CLSTN1/HID1/GAL3ST1/STK26/FOLR1/PMEPA1/AGTRAP/SNAP25/ZDHHC13/CD
A-DQA1/HLA-DRA/STARD3NL/CTSA/HLA-DQB1/TRPM2/GRN/CYB561/HLA-DPA1/HLA-DMB/TPCN1/A
/MICAL1/SRC/PRC1/KIF23/KRT23/TUBA1B/NDRG1/RAC2/LMNB2/TUBA3C/DNM1/KIFC1/KIF18B/M
/MICAL1/SRC/PRC1/KIF23/KRT23/TUBA1B/NDRG1/RAC2/LMNB2/TUBA3C/DNM1/KIFC1/KIF18B/M
1A1/FCER1G/HILPDA/REG1A/VEGFB/TMC6/ITPR3/TIMP1/LHFPL2/NUDT1/NCS1/LGALS3/PLAUR/D
/MICAL1/SRC/PRC1/KIF23/KRT23/TUBA1B/NDRG1/RAC2/LMNB2/TUBA3C/DNM1/KIFC1/KIF18B/M
DRG1/RAC2/LMNB2/TUBA3C/DNM1/KIFC1/KIF18B/MISP/HID1/POF1B/PDLIM7/NUSAP1/SKA1/LMN
PKP3/NOP56/RALY/RPL8/SNRPD2/HSF1/APOBEC3C/SNRPD1/NR0B1/SNRPA1/SNRPA/IQGAP1/MRTO
SMARCD1/HMGB2/TCF3/TRIM28/BRMS1/SIRT6/RUVBL1/HIF1A/CBX1/CHD3/MCRS1/CHAF1A/SMARC
B/MISP/HID1/NUSAP1/SKA1/RCC2/ARHGAP4/AURKA/PSRC1/CLIP2/SKA3/TTLL4/RASSF3/TUBG1/
ENAH/MARCKS/YWHAZ/FLNA/CD44/ITGB2/CIB2/TPM4/ARHGEF2/HSPB1/ITGAV/VASP/ADAM9/CDC4
ENAH/MARCKS/YWHAZ/FLNA/CD44/ITGB2/TPM4/ARHGEF2/HSPB1/ITGAV/VASP/ADAM9/CDC42EP1/
ENAH/MARCKS/YWHAZ/FLNA/CD44/ITGB2/CIB2/TPM4/ARHGEF2/HSPB1/ITGAV/VASP/ADAM9/CDC4
PTAFR/ITGAV/RAB31/CD109/BSG/TRPM2/RHOG/CD53/PLAU/ABCC4/ATAD3B/SYNGR1/DNAJC5/DDO
EKHO1/TUBG1/KCNJ11/ARHGEF2/ITGAV/IGF2BP1/NME1/VASP/ADORA1/PLEK/LAMP5/FGD3/NME2/
/ZWINT/CENPF/ORC1/CENPH/TTK/SKA1/RAD51/RCC2/AURKA/SPC24/NCAPD2/SKA3/SPC25/MCM6/
A-DQA1/MTMR2/ATP6V1F/HLA-DRA/STARD3NL/CTSA/HLA-DQB1/TRPM2/GRN/CYB561/ATP6V0B/HL
7/RPL28/MRPS34/RPL39/RPL35A/MRPL13/GCN1/RPS3/RPL27/EIF3H/RPL35/RPS20/RPL18A/RPS
/HSPA8/RPL36A/CCT2/RPS2/PLCB3/CCT4/BLOC1S4/RPL17/RPLP2/RPSA/CCT7/RPL10L/RPS5/PF
4/CENPH/SKA1/RAD51/AURKA/SPC24/NCAPD2/SKA3/SPC25/TUBG1/CENPW/NCAPH/FANCD2/RCC1/

1/WASF2/APBB1IP/FLOT2/SLC9A1/DGKZ/PAK1/CORO1C/PALLD/SLC39A6/ARPC2/PPP1R9B/ARPC3
/SKA1/RCC2/AURKA/SPC24/NCAPD2/SKA3/SPC25/CENPW/DSCC1/OIP5/SPAG5/CENPO/MAD2L1/DN
NRPF/LSM7/U2AF2/SF3B5/SNRPC/BUD31/SNRNP40/PPIL1/RBMX2/LSM2/ISY1/RNF113A/EIF4A3/
L39/RPL35A/MRPL13/RPS3/RPL27/RPL35/RPS20/RPL18A/RPS19/RPLP0/RPL18/RPL23/RACK1/M
/PLEKHO2/MIF/CD300A/CANT1/CFD/FTH1/PSMD2/ITGAX/HSPA8/TCIRG1/DOK3/FPR1/MNDA/GYG1
Z/CTSA/IFI30/TUBB4B/GRN/CTSK/TCN2/EPDR1/HSPA8/NEU1/GM2A/FUCA2/STK11IP/RNASET2/M
/NUP62/SPAG5/MAD2L1/ASPM/CKAP2L/HSF1/TMEM201/FAM83D/MTCL1/RGS14/NPM1/SGO1/SPDL1
SCN1/WASF2/ITGA5/PDE4A/IQGAP1/LCP1/PAK1/CORO1C/PALLD/PPP1R9B/WIPF1/S100B/SH2B2/
C1/RACGAP1/SHCBP1/PKN1/NUP62/SPAG5/ASPM/RAN/CRMP1/DTNBP1/CHMP4B/CCDC124/MTCL1/I
D3B/CHIT1/TIMP2/CD300A/CANT1/FTH1/STXBP2/ITGAX/TCIRG1/DOK3/FPR1/RAP2B/SIRPA/CD5
ST/STXBP2/GM2A/FUCA2/CD63/STK11IP/RNASET2/FPR1/MNDA/PTGES2/CCT2/C3AR1/DYNC1H1/N
MAP2/MAP1S/CENPE/PEA15/KLC3/DYNLRB1/DYNLL1/DYNC1H1/KIFC2/DYNLT1/HAUS1/KIF14/DCT
ST/STXBP2/GM2A/FUCA2/CD63/STK11IP/RNASET2/FPR1/MNDA/PTGES2/CCT2/C3AR1/DYNC1H1/N
E2/PCNA/XRCC3/PPP1CA/CBX3/BLM/NHP2/CDK2/KDM1A/SSB/THOC5/PPP1CC/SIRT7/PML/PARP1/
/HLA-A/ITGB5/STX6/RAB7B/ACTG1/ADAM8/SLC11A1/RAB20/ANXA11/WAS/RAB10/ATP6V0C/HLA-
PC25/CENPW/SPAG5/CENPO/MAD2L1/CENPU/HSF1/KIF18A/RANGAP1/SGO2/CENPE/SGO1/DYNLL1/
/HSPA8/MNDA/GYG1/HK3/HSP90AB1/ILF2/AMPD3/GPI/PPIA/ACLY/DYNLT1/CAPN1/C1orf35/ASA
A/RPLP0/RPL18/RPL23/MRPL10/RPL38/MRPL52/RPL23A/MRPL48/MRPL53/MPV17L2/RPL7A/RPL5
4/NCAPD2/SKA3/SPC25/CENPW/SPAG5/CENPO/MAD2L1/CENPU/HSF1/RANGAP1/SGO2/CENPE/SGO1
D/GAS7/TIGD5/ARPC3/WIPF1/MARK2/PDLIM3/SH2B2/CORO1B/ACTG1/DAPK3/HCLS1/PSTPIP1/RA
/SPAG5/CENPO/MAD2L1/CENPU/HSF1/RANGAP1/SGO2/CENPE/SGO1/SPDL1/KNTC1/ERCC6L/NUP37
/NHP2/KDM1A/SSB/THOC5/PPP1CC/SIRT7/PML/PARP1/THOC6/MSH2/CBX5/XRCC6/TELO2/NABP1/
A/TGFA/COPE/VMA21/HLA-DQA2/KDELR1/HLA-DRB5/COPZ1/CLTB/NCALD/HLA-B/AP1G2/CSPG5/C
2/PRDX1/SLC1A4/HSPA8/CD63/ANKRD27/HSP90AB1/RAB35/CCT4/SLC3A2/FLOT1/SYTL1/RAC1/A
2/PRDX1/SLC1A4/HSPA8/CD63/ANKRD27/HSP90AB1/RAB35/CCT4/SLC3A2/FLOT1/SYTL1/RAC1/A
/RPLP0/RPL18/RPL23/RACK1/RPL38/RPL23A/MCTS1/RPS7/RPL7A/RPS23/RPS10/RPS15/RPS16/
/ASPM/DCDC2/CDC7/CKAP2L/HSF1/KIF18A/RANGAP1/YEATS2/ESPL1/FAM83D/CENPE/DYNLL1/AG
NPC2/PLBD2/ASAH1/GBA/CTSS/GNS/GALNS/BGN/LUM/CTSD/CSPG5/HPSE/CSPG4/SGSH/PSAP/HSP
1/RRS1/INCENP/BLM/RSPH1/CENPK/DSN1/KMT5C/CENPB/CHMP1A/TOPBP1/RAD21/NIFK/RAD9A/H
/LSM2/ISY1/RNF113A/EIF4A3/PRPF3/SNU13/PRPF6/SF3A3/CWC27/SNRPG/PRPF31/SNRPB2/PRP
ES2/CCT2/DYNC1H1/NPC2/PA2G4/ACLY/CAP1/RNASE2/GNS/FTL/GALNS/DSN1/PSMD1/PADI2/TRA
/DDX41/SF3A3/CWC27/SNRPG/HNRNPM/HNRNPA3/SNRPB2/PRPF19/SART1/DDX23/HNRNPU/MAGOH/
3/MSR1/GLDN/COL6A2/LOX/LUM/COL4A2/COL8A2/EMILIN1/COL11A1/EMID1/COL9A3/C1QTNF6
O/RBMX2/LSM2/PRPF3/SNU13/PRPF6/SF3A3/SNRPG/PRPF31/SNRPB2/SART1/PHF5A/DDX23/HTAT
A1/CD47/NRAS/SERPINB6/ATP6V0C/TMEM63A/GPR84/LAIR1/CYSTM1/CLEC5A/KCNAB2/VAMP8/CD
/LSM2/PRPF3/SNU13/PRPF6/SF3A3/SNRPG/PRPF31/SNRPB2/SART1/PHF5A/DDX23/HTATSF1/LSM
3/PPAN/NOC4L/RRP7BP/UTP11/LAS1L/UTP4/UTP6/HEATR1/KRI1/RPS7/NOL6/LTV1/UTP20/NOB1
N/TIPIN/POLA1/PRIM2/RPA2/POLD3/TIMELESS/PRPF19/RAD51C/RAD51D/NBN/RAD18/RFC5/SMA
RPL5/RPL6/RPL37/RPL27A/RPL36/UBA52/RPL19/RPL22L1/RPL13A/RPL37A/RPLP1/RPL32/RPL3
PRPF3/SNU13/PRPF6/SF3A3/SNRPG/PRPF31/SNRPB2/SART1/PHF5A/DDX23/HTATSF1/LSM8/SART
CSNK1D/TUBG2/KIF2A/CALM2/BOD1/CCSAP/HNRNPU/RAB11A/KLHL21/CLTC
/NCAPH2/KLHDC8B/PRKCI/DNAJC8

SF3A3/CWC27/SNRPG/PRPF31/SNRPB2/SART1/PHF5A/PRPF38A/LSM8/SF3B3/SF3B2/MFAP1/PRPF
SF3A3/CWC27/SNRPG/PRPF31/SNRPB2/SART1/PHF5A/PRPF38A/LSM8/SF3B3/SF3B2/MFAP1/PRPF

/GPNMB/DKK1/HLA-DMA/HAVCR2/CORO1A/SLC39A4/FOLR1/PMEPA1/SQSTM1/ANKRD13D/IRAK1/TU
/MCM3/EZH2/CDK4/TUSC3/DPM2/CDKN2D/SNRPD2/PRMT1/WDR4/TRAF2/RBCK1/YEATS2/FBXL19/S
/DNAJC5/CANT1/STXBP2/NEU1/SLPI/RAP2B/DEGS1/C3AR1/ILF2/OLR1/SLC2A5/ADAM8/KPNB1/A
/LAMTOR1/CD47/AGPAT2/SLC44A2/TMEM63A/STK10/GPR84/LAIR1/CLEC5A/KCNAB2/HVCN1/VAMP
L/UBA1/KMT5C/CENPB/UBE2A/BEND3/CBX5/CBX8/HELLS/PCGF2/ESCO2/MBD3/RNF2/MECP2/BAZ2
TOR1/SERPINB6/DYNC1LI1/ATP6V0C/TBC1D10C/AP2A2/ATP6AP2/SLC2A3/RHOA/COPB1
1/RPL24/RPL11/RPL10A/RPS28/PNPT1/RPL31/NUFIP2/BTF3

4/SNRPD3/SNRNP200
4/SNRPD3/SNRNP200
KRT8/ATN1/XPOT/AKAP8L/SFPQ/DNMT3A/PML/NSMF/RUVBL2/POLA1/CHMP1A/NUP107/NCOR2/TEL

3/UXS1/B4GALT7/B4GALT2/FUT3/CIT/GOLGA3/FUT2/FUT6/LLGL1/CHPF/OCRL/NAGPA/VRK1/B4G
HMP4C/HGS/NPC1/RAB7B/CD68/LAMP3/SNF8/SLC11A1/HLA-DRB5/LAMTOR5/LAMTOR1/TMEM9/CHM
RG1/LARP4B/PSMA1/RPL7A/RPS23/RPS4X/RPL6/RPL36/HSPA14/RPL19/DHX9/RPL32/RPL30/LAR
6V0E1/CALR/OCRL/STX4/ATP6V0D1/HVCN1/VAMP8/DNM2/DMBT1/TLR2/ANXA3/HLA-F/RAPGEF1/T

L11A1
/CDC23/BCOR/ANAPC15/CUL7/CDC16/CDC26/ANAPC1/PCGF1/BRCC3/PHC1
RCA5/RPA1/SMARCAL1/MMS22L

3/CANX/HLA-F/TAPBP
3/CANX/HLA-F/TAPBP

TRIM28/PRKDC/HIF1A/PYGO2/BCL9/TAF6/RBL1/CD3EAP/RCOR2/HSP90AB1/E2F8/HDAC1/E2F3/S
DORA1/PLEK/LAMP5/MAPRE1/ASAP1/EZR/SLC26A6/PLEK2/CFL1/FSCN1/ITGA5/TCTN2/OPN1SW/M

KCNJ11/P2RX4/TOR4A/IL15RA/SCRN1/PRNP/MVP/PLCB1/NUP62/RCC1/TUBB/MX2/CDK4/MAD2L1/
1/PDE9A/FGR/ARF4/RPS3/SPRY4/NF2/WWC1/DNM2/ADAM17/MYO6/PACSIN2/PACSIN1/TLN1/RHOA

G3/FHOD1/CNN2/TRIP6/PTK2/PDLIM4/MYL9/NEBL/ACTN1/LIMA1

B1/CLTA/HSPA8/HLA-DQB2/STX6/GPR107/TGFA/TMED9/CTLA4/SORT1/RAB35/HIP1R/BCAP31/HL

B1/CLTA/HSPA8/HLA-DQB2/HLA-A/TGFA/COPE/RAB35/VMA21/HIP1R/HLA-DQA2/FZD2/KDELR1/H
QB2/TGFA/RAB35/HIP1R/HLA-DQA2/FZD2/HLA-DRB5/NECAP2/CLTB/AP2M1/NCALD/GAD1/AP2S1/

OC3
OC3

4
R1/DNAJC5/ARFGEF3/HLA-DPB1/CLTA/SYT8/HLA-DQB2/HLA-A/STX1A/STX6/RAB3B/ECE2/TGFA/
OC3

PS18/AP1G2/AP3D1/AP1B1/COPG1/PDCD6/SEC13/COPG2/AP2A2/COPB2/AP3B1/CLTC/AP1S1/AP1
PS18/AP1G2/AP3D1/AP1B1/COPG1/PDCD6/SEC13/COPG2/AP2A2/COPB2/AP3B1/CLTC/AP1S1/AP1

X2/ACOX2/BAAT/PIPOX/EHHADH/DAO/HAO2/HAO1/AGXT
X2/ACOX2/BAAT/PIPOX/EHHADH/DAO/HAO2/HAO1/AGXT
AP/B3GNT9/RRS1/RRP9/DDX11/RRP7A/POLR2H/FBL/EEF1D/NHP2/NOP58/UTP18/DEAF1/POP1/MA
/VIM/KRT20/EPPK1

7/PSMB3/PSMD4/PSMD3/PSMD7/PSMD8/VCP/PSMA1/UBQLN4/PSMB5/PSMA4/PSMD9
/ZFYVE19/MASTL/LIMA1/RAB11A/PLEKHG6/RHOA
1/CNN2/MYO5A/WDR1/TRIP6/PTK2/PDLIM4/MYL9/NEBL/ACTN1/LIMA1
/ASPH/EZR/DCDC2/RHOG/FMNL1/CFL1/RHOQ/GIPC1/EPB41L2/WIPF3/TRAF2/RHOC/FLOT2/CLTA/
LTA/HLA-DQB2/HLA-A/TGFA/COPE/TMED9/SORT1/VMA21/HLA-DQA2/KDELR1/HLA-DRB5/COPZ1/C
IP1R/LASP1/FLOT1/CAP1/CAPZA1/RTKN/GYPC/HCLS1/ACTB/CTTN/MYADM/WAS/NSMF/RIMS3/LLG
/TAF10/ERCC1/MYO6/GTF2E2/POLR2I/POLR2J/POLR2D/GTF2H1/TAF13/TBP/MMS19/POLR2E/GTF
CAMP4/STX10/HLA-DQA2/HLA-DRB5/LLGL1/APP/AP1B1/ARFIP2/M6PR/CLIP3/SYS1/COG1/SCAMP

CAT/ACSL1/SLC27A2/DAO/FNDC5
CAT/ACSL1/SLC27A2/DAO/FNDC5
2/GM2A/MAP2K2/GNA12/GNGT1/PTPN7/PLEKHA4/LYN/GNG5/MIEN1/GNB1/FGR/LCK/RASA3/STAC3
/TAF10/ERCC1/MYO6/GTF2E2/POLR2I/POLR2J/POLR2D/GTF2H1/TAF13/TBP/MMS19/POLR2E/GTF
P2B1/AP2A1/VPS33A

5/VCP/WRAP53/UBQLN4/ACTR3/NBN/RAD18/RNF8/RPA1/SMARCAL1/TP53BP1/PHF1/SLFN11/HUS1
L1/RAB34/NCF1/GIPC1/ATP6V0B/HLA-DPA1/ATP6V0D2/RAB32/COLEC12/FLOT2/HLA-DPB1/CLTA
K1/THY1/CORO1C/SLC39A6/GABRA3/PPP1R9B/PIP5K1C/HIP1R/FERMT1/MYO1G/ATF4/RAC1/PDE9
UVBL2/POLA1/CHMP1A/NCOR2/RAD21/VIM/CXXC1/OGG1/HNRNPM/PHF5A/GHRHR/SRPK1/HAT1/HNR
/TAF10/ERCC1/MYO6/GTF2E2/POLR2I/POLR2J/POLR2D/GTF2H1/TAF13/TBP/MMS19/POLR2E/GTF
NDE1/NF2/SPIRE1/ZFYVE19/MASTL/LIMA1/RAB11A/PLEKHG6/RHOA
7/PSMB3/PSMD4/PSMD3/PSMD7/PSMD8/VCP/PSMA1/UBQLN4/PSMB5/PSMA4/PSMD9

LA-DPB1/CLTA/TMEM165/HLA-DQB2/AP4M1/CHST1/STX6/SCAMP4/CORO7/RAB7B/STX10/PLOD3/P
NF2/SPIRE1/ZFYVE19/MASTL/LIMA1/RAB11A/PLEKHG6/RHOA

OLEC12/HLA-DPB1/HLA-DQB2/TCIRG1/HLA-A/RAB7B/HIP1R/STAB1/HLA-DQA2/FZD2/MSR1/SLC1

EHD2/RAP2A/RAB10/HLA-B/SLC9A7/CLIP3/VAMP8/SCAMP2/CFTR/PDLIM4/OPTN/PACSIN2/GGA3/

-DPA1/AREG/DNAJC5/HLA-DPB1/CLTA/HSPA8/HLA-DQB2/HLA-A/STX6/GPR107/TGFA/COPE/TMED
B/FLNA/C1QC/SNAP25/CDK16/CPLX2/EIF4EBP1/SCGN/ARHGEF2/P2RX4/C1QA/NCK2/SCAMP5/SLC
L1/FERMT3/PRKCD/TOR4A/SERPINI1/QSOX1/VAT1/MVP/UNC13D/TUBB/CSTB/TMSB4X/S100A8/LY

/CLTB/AP2M1/DVL2/AP2S1/CEMIP/DNM2/MYO6/AP2A2/CLTC/AP2B1/HBEGF/AP2A1

5C/RUVBL2/RBBP4/CBX5/MTF2/PRDM4/RBBP7/CXXC1/LAS1L/SNRPG/E2F6/INO80C/PRPF31/ZNF3

D2BP2/DGKZ/HIF1A/VPS72/RNF34/BASP1/EFTUD2/PPIH/LMNA/DPP3/NXT1/NCAPG2/RNPS1/REXO
NH1/PAG1/UNC5B/EZR/BSG/ARID3A/PGK1/KIF18A/RHOQ/S100A10/TRAF2/HMOX1/SELPLG/FLOT2
6/PTK2/PDLIM4/MYL9/NEBL/ACTN1/LIMA1
6/PTK2/PDLIM4/MYL9/NEBL/ACTN1/LIMA1
NH1/PAG1/UNC5B/EZR/BSG/ARID3A/PGK1/KIF18A/RHOQ/S100A10/TRAF2/HMOX1/SELPLG/FLOT2

15/CLTA/SYT8/STX1A/STX6/RAB3B/BIN1/TMED9/STX10/RAB35/MT3/SYTL1/SV2A/SYNGR3/CLN3
RRS1/DDX11/FBL/EEF1D/NOP58/DEAF1/MAP3K14/DSN1/SMARCB1/RAE1/IP6K1/UTP4/INO80C/BT
/A1BG/PLG/AHSG/HRG

STX1A/STX6/RAB3B/BIN1/TMED9/STX10/RAB35/MT3/SV2A/SYNGR3/CLN3/MCTP1/TMEM163/VDAC
/SERPINH1/PAG1/UNC5B/EZR/BSG/ARID3A/PGK1/KIF18A/RHOQ/S100A10/TRAF2/HMOX1/SELPLG
OT2/CLTA/FCHSD1/TNFAIP2/TIGD5/WIPF1/HIP1R/LASP1/GPSM2/FLOT1/CAP1/CAPZA1/RTKN/GY
GNAZ/CD2/GM2A/MAP2K2/HM13/GNA12/GNGT1/PTPN7/PLEKHA4/LYN/GNG5/MIEN1/GNB1/FGR/LCK

14/RPS27/EIF2D/RPS6/RPS24/RPS3A/RPS18/RPS28/RPS17/RPS15A
3/CIT/GOLGA3/FUT2/FUT6/LLGL1/CHPF/NAGPA/B4GALNT3/B4GALNT4/FUT1/ARAP1/NECAB3/FUT
/CBX6/IGF2BP1/ADORA1/LAMP5/NPTX2/DAGLA/GIT1/TWF2/DLG3/NECTIN1/TRPV2/ORAI2/RANGA

T2/FUT6/CHPF/NAGPA/B4GALNT3/B4GALNT4/FUT1/ARAP1/FUT8/B4GALT4/GOLPH3L/TMEM115/TM
PARD/LEF1/NONO/ATF4/MED8/CBFB/TAF9/SFPQ/ASCL2/E2F2/VDR/CDK7/HNRNPAB/KAT2A/GTF3C
PL18/MRPL37/NSUN4/MRPL27/MRPL28/MRPL51/MRPL11/MRPL15/C12orf65/MRPL4/MRPS24/MRPL
PL18/MRPL37/NSUN4/MRPL27/MRPL28/MRPL51/MRPL11/MRPL15/C12orf65/MRPL4/MRPS24/MRPL

/COTL1/FERMT3/PRKCD/TOR4A/SERPINI1/QSOX1/VAT1/MVP/UNC13D/TUBB/CSTB/TMSB4X/S100A
ITGB2/CIB2/TPM4/COTL1/HSPB1/TRIP10/WDR34/MAPRE1/ASPH/EZR/DCDC2/RHOG/IFT57/FMNL1
2/DLG3/NECTIN1/TRPV2/ORAI2/KIF21B/DTNBP1/FSCN1/TIMP2/MAP2/BRSK1/IQGAP1/HSPA8/TH

S1B/DDA1/TSPAN17/DYRK2/ZSWIM5/UBE2J2/ANAPC7/SPSB1/FBXO32/KCTD10/UBE2L3/CBX4/ANA

B1/AP4B1/AP5S1/HIP1/TEPSIN/AFTPH/AP3M1
ACOT2/NUDT7/PEX11A/PEX11G/ECI2/ACOX1/ACAA1/PXMP2/ACSL6/HSD17B4/AMACR/PECR/GRHPR
ACOT2/NUDT7/PEX11A/PEX11G/ECI2/ACOX1/ACAA1/PXMP2/ACSL6/HSD17B4/AMACR/PECR/GRHPR
SON/ZBTB17/POLA1/PRIM2/XRCC6/RPA2/POLD3/NFYC/PRPF19/RPA1/TBP/SMARCAL1/HMGA2/ERC

YL1/CCDC115

/COTL1/FERMT3/PRKCD/TOR4A/SERPINI1/QSOX1/VAT1/MVP/UNC13D/TUBB/CSTB/TMSB4X/S100A
/CAPZA1/RTKN/HCLS1/CTTN/MYADM/WAS/LLGL1/GMFG/WDR1/NF2/ACTR3/DBNL/CAPN2/WIPF2/DS

CD5L/FGG/GC/BCHE/CFHR1/FGA/AMBP/ITIH2/TF/F2/APOA2/SERPINF2/CPN2/HP/ITIH1/C8A/AL

/PHF1/SKP1/BAP1/SUZ12/SCML2/EZH1/JARID2/PCGF1/PHC1
RAE1/NUP155/RANBP1/NUP107/CETN2/SEC13/XPO7/EIF5A/NXT2/KPNA6/SEH1L/RANBP3/ENY2/M

MRPS15/RPS27A/RPS12/RPS4X/UBA52/RPS11/RPS9/RPS14/RPS27/MRPS24/EIF2D/MRPS5/RPS6/
S1/CKS1B/POLR3K/POLR2H/CCNO/PIK3CD/CDK2/CHRAC1/PRIM1/PFKM/CDK5/TAF9/SOCS3/CDK7/

B/CCNJL/CCNE2/CDK5R1/ATG13/WDR41/PHKA2/GTF2H1/PHKG2/MMS19/ERCC3/MAP3K7/CCNG2/UL
OE1/TGS1/HINFP/LSG1/PRPF4/COIL/AK6/ZNF473/RDM1/SMN2/HNRNPA2B1/NOLC1

/TIMP2/MAP2/IQGAP1/THY1/BASP1/PALLD/HSP90AB1/ARPC3/DYNLT1/CYTH2/HCLS1/DCTN2/FKB

4/ZDHHC9/ERGIC3/ACER3/STEAP2/BET1L/ERGIC2/QSOX2/IER3IP1/TBC1D20/ZDHHC20/YIF1A/E

L12/MRPS34/MRPL13/CHCHD3/GRPEL2/NDUFS6/TOMM40L/TIMM50/MRPL10/ROMO1/MRPL52/NDUFS

A-DMB/CHMP4B/CXCR4/VPS37C/TMEM165/MAPK3/TCIRG1/MICALL1/MAP2K2/CD63/CHMP4C/ANKRD
CLSTN1/TGFB1/AURKA/PPT1/CTSV/CYBB/ADA/STX3/FLNA/NCDN/SNAP25/CPLX2/KCNJ11/SCGN/A
BRI3/SLCO4C1/VAPA/NCSTN/SURF4/ARL8A/DNAJC13/GAA/TMEM179B/PIGR/CEACAM6/RAP1B/BST
3/MATK/KCNAB2/GNG4/GNGT2/STAC/GNAI3/RHOA/CAV2/CYTH3/SNX5/GNA13/ATP2A2/GNG2/CARM
2A/NUP155/NUP107/AXL/SEC13/RPS27A/BICD1/UBA52/RAB29/VPS37B/KPNA6/SEH1L/PI4K2A
2A/NUP155/NUP107/AXL/SEC13/RPS27A/BICD1/UBA52/RAB29/VPS37B/KPNA6/SEH1L/PI4K2A

/TIMP2/MAP2/IQGAP1/THY1/BASP1/PALLD/HSP90AB1/ARPC3/DYNLT1/CYTH2/DCTN2/FKBP4/CDK

NRGN/RUSC1/MAPK8IP2/DLG3/DLGAP4/SCRIB/CTNND2/RPL8/DNAJC6/DTNBP1/MAP2/DGKZ/RGS14

2B/FCHSD1/TUBA1A/SCAMP4/RAB35/EHD1/SNF8/RAB11FIP4/MCTP1/RAC1/EHD2/RAP2A/RAB10/T

K2A1/MBD3/GATAD2A/MECOM/SAP130/MORF4L1/HDAC5/SIN3B/HDAC8/ING2/MBD2

ACOT2/HSDL2/VWA8/NUDT7/PEX11A/PEX11G/ECI2/ACOX1/ACAA1/PXMP2/ACSL6/HSD17B4/AMACR
/KPNB1/ATXN2L/RAC1/ACTB/APOBEC3G/RPL28/LSM2/EDC3/GRB7/SAMD4B/BARD1/NCL/HENMT1/C
ACOT2/HSDL2/VWA8/NUDT7/PEX11A/PEX11G/ECI2/ACOX1/ACAA1/PXMP2/ACSL6/HSD17B4/AMACR
C/NPTX2/NRGN/DLG3/DLGAP4/SCRIB/GIPC1/DTNBP1/FLOT2/DGKZ/RGS14/PPP2R1A/PPM1H/NRCA

11/PSMD1/PSMA7/PSMB3/USP22/ATXN7L3/PSMD4/PSMD3/PSMD7/TAF12/PSMD8/VCP/PSMA1/UBQL

USC1/MAPK8IP2/DLG3/SCRIB/CTNND2/RPL8/DNAJC6/DTNBP1/MAP2/DGKZ/RGS14/SHARPIN/NRCA

1L/CEMIP/HSPD1/DNM2/TNK2/AAK1/MYO6/AP2A2/CLTC/AP1S1/AP1S3/AP1S2/AP2B1/AP2A1/EPN
/RPA3

2/CCDC80/VWA1/COL4A2/LAMB3/COL8A2/RELL2/ACHE/THBS2/LAMA4/LOXL1/EFEMP2

MRPL15/C12orf65/MRPL4/MRPL33/MRPL36/MRPL14/MRPL3/MRPL20/MRPL24/MRPL55/MRPL21/MR
MRPL15/C12orf65/MRPL4/MRPL33/MRPL36/MRPL14/MRPL3/MRPL20/MRPL24/MRPL55/MRPL21/MR
6/ARMCX3/GDAP1/STX1A/TIMM17B/STX6/RAB3B/NPC1/P2RX5/STX10/RAB35/BID/SLC25A19/UQC

/NCAPH/RELB/SMARCD3/POLD1/NUP62/RCC1/EZH2/DNMT1/HDAC7/ARID3A/CREB3L1/RAN/HSF1/S
MAGED1/SOX12/RALY/DDIT3/BHLHE41/PSMC3IP/SMARCD1/HMGB2/YBX3/TCF3/TRIM28/NR0B1/SI
CNF/TTK/AURKA/STK26/RIPK2/EFNA3/SQSTM1/IRAK1/CDK16/PKN1/PRKCD/EFNA4/ITPKA/SYK/S
Z/COL1A2/COL9A2/KRT17/AGRN/PI3/TUBG1/TPM4/MAL2/VCAN/COL4A5/NCK2/EMILIN2/LAMB1/N
1/ARHGAP4/RACGAP1/CHML/NCAPH/PRKCD/B3GAT3/ADAP1/PLCB1/DEPDC1/GMIP/GIT1/ASAP1/GU
/ARPC1B/PFN2/STMN1/FBLIM1/MICAL1/PRC1/KIF23/NDRG1/DNM1/KIFC1/CLSTN1/KIF18B/ATP1
13/CCNE1/CCNA2/PBK/PNCK/DTYMK/CCNF/TTK/AURKA/STK26/RIPK2/EFNA3/SQSTM1/UCK2/IRAK
RGS10/MCM4/NUDT1/RAC2/ATP13A2/TUBA3C/DNM1/KIFC1/RFC4/KIF18B/ATP1A1/ARL2/RAD51/R
RGS10/MCM4/NUDT1/RAC2/ATP13A2/TUBA3C/DNM1/KIFC1/RFC4/KIF18B/ATP1A1/ARL2/RAD51/R
RGS10/MCM4/NUDT1/RAC2/ATP13A2/TUBA3C/DNM1/KIFC1/RFC4/KIF18B/ATP1A1/ARL2/RAD51/R
RGS10/MCM4/NUDT1/RAC2/ATP13A2/TUBA3C/DNM1/KIFC1/RFC4/KIF18B/ATP1A1/ARL2/RAD51/R
/SRC/PRC1/ARHGEF16/NCS1/CKS2/RAC2/CCNE1/DNM1/CCNA2/TRIB3/ATP1A1/CCNF/RHOV/RCC2/
S2/RAC2/CCNE1/DNM1/CCNA2/TRIB3/ATP1A1/CCNF/RHOV/RCC2/AURKA/GSTP1/MARCKS/CDC25C/
GAPDH/RIPK2/PMEPA1/CDC25C/SQSTM1/YWHAZ/SHCBP1/BCL2A1/ROBO1/LAT2/VASP/ADAM9/SYK/
NXA2/IKBKE/NCF2/CTSC/SH3BP1/ANXA5/TIMP1/RGS10/CKS2/LGALS3/SPINT2/RAC2/ALOX5AP/C
/NDRG1/MMP14/DSG2/GPNMB/YWHAZ/FLNA/ITGB2/FXYD5/CIB2/PLIN3/S1PR2/FERMT3/P2RX4/KL
/ARHGEF2/CYTH4/ITPKA/CDC42EP1/RCC1/UNC13D/FGD3/FMNL2/FMNL1/RAB11FIP5/RAB34/TBC1
XYD5/TPM4/COTL1/VASP/USH1C/FHL3/TMSB4X/RUSC1/TWF2/FMNL2/EZR/LSP1/FMNL1/TMEM201/
6/PKN1/PRKCD/ITPKA/SYK/STK39/WNK2/CDK4/NME2/CDC7/CHEK1/RPS6KA1/LIMK2/GRK6/MAPKA
DX39A/DSCC1/TOR4A/MCM7/ABCD1/KIF11/PIF1/ATP6V1F/WDR34/TAP1/KIF12/CD320/KIF3C/KI
EF2/ITPKA/CDC42EP1/RCC1/UNC13D/FGD3/FMNL2/FMNL1/RAB11FIP5/RAB34/TBC1D16/MICALL2
EF2/ITPKA/CDC42EP1/RCC1/UNC13D/FGD3/FMNL2/FMNL1/RAB11FIP5/RAB34/TBC1D16/MICALL2

ADAP2/TBC1D16/RANGAP1/RABEP2/IQGAP1/RGS14/ANKRD27/THY1/ARHGAP9/SIRPA/GPSM1/ARHG
PSRC1/CLIP2/ARL4C/TTLL4/FCHO1/RACGAP1/CCDC88B/ARHGEF2/REEP4/NME1/PRNP/TRIM54/KI
/P2RX4/KLC2/VASP/CDC42EP1/TES/MAPRE1/TAGLN2/LAD1/TWF2/FMNL2/ASAP1/YKT6/PKP3/EZR
MX2/RHOG/TUBB4B/GBP5/GNA15/RAN/RAB34/RHOQ/RRAD/RHOC/RAB6B/RAB32/RAB15/GNAZ/RRAG
F2/CCL5/CCL26/TRAF5/ITGA5/TNFSF9/MIF/FKBP1A/BEX3/EFNA5/IL34/EBI3/LEFTY1/SPRED1/
/RANGAP1/RABEP2/IQGAP1/RGS14/ANKRD27/THY1/ARHGAP9/SIRPA/GPSM1/ARHGEF19/TBC1D1/R
AP1/RABEP2/IQGAP1/RGS14/ANKRD27/THY1/ARHGAP9/ARHGEF19/TBC1D1/RIN1/ARHGAP39/GDI1
CL5/CCL26/TNFSF9/MIF/IL34/EBI3/LEFTY1/SECTM1/CCL13/GPI/CCL2/TXLNA/CXCL9/CXCL3/C
/RACGAP1/CCDC88B/ARHGEF2/REEP4/PRNP/TRIM54/KIF11/SPAG5/MAPRE1/MX2/S100A8/EZR/KI
DNMT1/EME1/MCM5/POLA2/GINS4/RUVBL1/CHTF18/APEX2/CHD3/DKC1/RBBP8/PCNA/TOP1MT/XRC
/TPM2/ARPC4/LCP1/MAP1S/TPM3/CORO1C/BIN1/CORO7/ARPC2/GAS7/PPP1R9B/TIGD5/ARPC3/WI
BX3/PLEKHG2/VAV1/IQGAP1/PAK1/CORO1C/PPP6R1/ARHGEF19/DVL3/NCKAP1L/PAK4/RTKN/DAPK
AP2K2/NPM1/SPRED1/SOCS1/CKS1B/HSP90AB1/MARK2/MT3/CCNO/NCKAP1L/MLST8/DBF4/CDKN2B
/SAE1/SDCBP2/IFT52/DYNLL1/PABPC1/DYNLT1/GPSM2/ITGB3BP/DAPK3/ATF4/SNF8/TESK1/PIA
/NCAPG2/LEF1/NOC2L/NASP/SSRP1/KDM5B/CBX4/NPM3/ATAD2/NAP1L2/SET/RBBP4/SMARCC1/BR
RPL39/RPL35A/MRPL13/RPS3/RPL27/RPL35/RPS20/RPL18A/RPS19/RPLP0/RPL18/RPL23/MRPL1
4/FUBP1/POLR2H/BLM/SMC4/PRIM1/SSBP4/JCHAIN/MSH2/POLA1/MCM8/NABP1/RPA2/MAPT/POLR
PPME1/KCNQ1/ANAPC7/STRN4/LCK/PPP1CC/CTTNBP2NL/CADM4/TRAF3/GRB2/PTPA/KAT2A/PPP1R
P1/ARHGAP1/PLSCR1/HCLS1/LYN/CNTNAP1/BCAR1/ADAM12/WAS/ADAM19/PTPN6/CIT/INPP5J/MV
4/MKKS/CLN3/CCT7/PFDN2/TCP1/DNAJB2/SRSF12/DNAJA4/NAP1L4/PFDN1/RUVBL2/CALR/DNAJB
TIMP2/COL5A1/MFGE8/ITGA5/LCP1/JAML/ISG15/LAMA5/TSPAN8/THY1/ITGB5/LYN/TSPAN4/ITG
C2/KIF14/KIFC3/KIF15/DYNC1LI1/KIF2A/DNAL4
/RBBP4/TTF2/POLQ/RAD51C/DNA2/RAD51D/RAD18/RFC5/DHX9/SMARCA5/GTF2H1/SMARCAL1/MSH

MD/MAPK13/CCNE1/CCNA2/PBK/PNCK/DTYMK/CCNF/TTK/AURKA/STK26/RIPK2/EFNA3/SQSTM1/UC
/RNPS1/ZC3H12A/NCBP2/EIF3D/SNRPC/SSB/SRRM3/RPS5/DAZAP1/RPL7/CPSF1/THOC5/DENR/ED
K3/MAST2/SPRED1/SIRPA/SFI1/PPP6R1/STAT1/RCAN3/PPP1R9B/PPP1R35/PPP1CA/SLC9A3R1/P
TF18/CHD3/SMARCA4/DDX49/ERCC6L/RFC2/DDX11/DDX27/BLM/EIF4A3/CHD4/RUVBL2/TTF2/MCM
NRPC/RPN2/ZNF593/PQBP1/GCN1/CPSF6/EIF4A3/XPO5/PRPF6/CBX5/RACK1/TIMM50/LETM1/ZNF
1/BLM/CHD4/RUVBL2/MCM8/XRCC6/POLQ/DNA2/CHD1L/DQX1/NBN/RAD18/ACTR5/RFC5/DHX9

FNA5/MAP2K2/NPM1/SPRED1/SOCS1/CKS1B/HSP90AB1/MARK2/MT3/CCNO/NCKAP1L/MLST8/DBF4/
PLEKHA4/MYO1G/KCNQ1/TULP3/PLEKHA2/VIL1/WDR45B/PFN1/GSDMD/SYT1/MAPT/OBSCN/SNX20/
L2/WASF1/OCRL/ABI2/ARHGDIA/ARFIP2/BRK1/SRGAP2/PAK2/DOCK2/EPS8L1/CAV1/ATP7A/PREX

STD2/EMB

CNE1/CCNA2/PBK/PNCK/CCNF/TTK/AURKA/STK26/RIPK2/EFNA3/SQSTM1/IRAK1/CDK16/HK2/PFK
IM28/CACYBP/CXCR4/TRAF5/HIF1A/HSPA8/RNF34/HM13/BRCA1/UBASH3B/HGS/CCT2/HSP90AB1/
8/EIF3B/STX1A/SH2B2/HIP1R/SLC9A3R1/LASP1/GAS2L1/BCL3/ARHGAP1/SHC1/SPSB1/AXIN1/H

ACYBP/CXCR4/TRAF5/HIF1A/HSPA8/RNF34/HM13/BRCA1/UBASH3B/CCT2/HSP90AB1/TPI1/NPLOC

/MTF2/HMGN1/RNF4/VRK1/DNTTIP1/MBD3/HMGN2/MLLT6/SMARCA5/HMGA2/PHF1/HNRNPC/MBD2/S

H1/MYO19/KIFC2/DYNLT1/MYO1G/MYO1F/KIF14/DCTN2/KIFC3/KIF15/DYNC1LI1/KIF2A/DNAL4/

CXCL16/PF4V1/CXCL11/XCL2/CCRL2

C/RABL6/RHOG/TUBB4B/GBP5/GNA15/RAN/RAB34/RHOQ/RRAD/RHOC/RAB6B/RAB32/RAB15/GNAZ/
C/RABL6/RHOG/TUBB4B/GBP5/GNA15/RAN/RAB34/RHOQ/RRAD/RHOC/RAB6B/RAB32/RAB15/GNAZ/
/CYP2C8/CYP2C9/CYP8B1/CYP2A6/CYP3A4
3PXD2B/SLC9A1/SDCBP2/RNF34/ARHGAP9/RAB35/HIP1R/PLEKHA4/MYO1G/KCNQ1/TULP3/PLEKHA
/CYP4A11/CYP2E1

TA2/PHB/MIER2/SFPQ/PARP1/CHD4/YWHAB/TFAP4/RBBP4/KAT2A/CBX5/NCOR2/RAD9A/MTA3/UHR

OX1/ALDH6A1/ADH4

/ACHE/COL11A1/THBS1/ADAMTS5/VEGFA

C38A3/SLC51A/SLC16A11/SLCO1B3/SLC27A2/SLC6A12/SLC6A1/SLCO1B1/SLC27A5/SLC10A1
G/TUBB4B/GBP5/GNA15/RAN/RAB34/RHOQ/RRAD/RHOC/RAB6B/RAB32/RAB15/GNAZ/OLA1/RRAGD/
G/TUBB4B/GBP5/GNA15/RAN/RAB34/RHOQ/RRAD/RHOC/RAB6B/RAB32/RAB15/GNAZ/OLA1/RRAGD/

/TDG/RAD1/NBN/RAD18/ERCC1/RPA1/MSH6/TP53BP1/DCLRE1C/ERCC3/DDB1/UNG/ERCC2/XRCC5/
/TNFAIP8L3/TPCN1/SNX8/GPAA1/IQGAP1/SH3PXD2B/SLC9A1/SDCBP2/RNF34/THY1/ARHGAP9/RA

/NR1I3

CADM/PECR/TM7SF2/ACADL/ACADSB/SRD5A2/ACOX2/AKR1D1
POC1/ACOX2/ALB/FABP4

B4B/GBP5/GNA15/RAN/RAB34/RHOQ/RRAD/RHOC/RAB6B/RAB32/RAB15/GNAZ/OLA1/RRAGD/GBP2/
1/GAS2L1/BCL3/ARHGAP1/SHC1/SPSB1/HCFC1/FKBP4/CNTNAP1/JCHAIN/AP2M1/SLA/EVPL/DVL2

CTG1/TUBB4A/ACTB/TUBB2B/TUBG2/CROCC/VIM/KRT20/EPB41L3/ARPC5/CCDC6/ACTR3/BICD1/A

AE1/MVB12A/DCUN1D5/IKBKG/RNF4/UBE2L6/UBXN1/TNFAIP3/USP5/NSFL1C/FAAP20/TDG/UBA2/

C6A1/SLC13A5/SLC22A1/SLC10A1

09/HLA-DRB5/TSPAN4/JCHAIN/HLA-B/CD48/LILRB4

37/DAB2IP/CASP3/MADD/TRAF1/TNFSF13/TNFSF18/CD70/FASLG

US2/PRKAR1B/GSKIP/YWHAG/SH3BP5L/GMFB/RPTOR/CIB1

AS1/ZP3/SRPX2/TUFT1/LAMC2/LAMA5/FMOD/COL5A2/COL6A3/TNC/MUC3A/MGP/BGN/COL6A2/VWA
/STEAP4/GCDH/KMO/ACADM/FMO4/PRODH2/LDHD/ACADL/FMO5/XDH/ACADSB/ACOX2/AOX1/DAO/FM
S2R40/TAS2R3/TAS2R7/TAS2R13/TAS2R60

C2/DKK1/CLCF1/GDF15/CMTM3/NBL1/LY6E/CKLF/PGF/IL18/TFF1/PTHLH/GUCA2A/METRNL/TYMP

G1/OR8D2/OR5AK2/OR2C1

P4HB/PDIA4/NXN
BC1D10B/EHD1/SYTL1/ARHGAP1/TBC1D30/RAB11FIP4/RAC1/TBC1D13/SBF1/TRAPPC1/DENND6B/

D1/HRG/SERPINC1
G/TUBB4B/GBP5/GNA15/RAN/RAB34/RHOQ/RRAD/RHOC/RAB6B/RAB32/RAB15/GNAZ/OLA1/RRAGD/
G/TUBB4B/GBP5/GNA15/RAN/RAB34/RHOQ/RRAD/RHOC/RAB6B/RAB32/RAB15/GNAZ/OLA1/RRAGD/
A/IKBKG/UBE2L6/UBXN1/TNFAIP3/USP5/NSFL1C/FAAP20/MARK4/GGA3/UBR5/RNF8/FAF1/UBE2N

GFR3/TESK1/LYN/FGR/LCK/SLA/FGFR2/MST1R/MATK/AXL/BTK/DSTYK/IGF1R/ABL1/CSK/PTK2/T

GMO/CYP4V2/CYP27A1/CYP39A1/FMO4/FAXDC2/CYP3A43/FMO5/CYP1A1/CYP4F3/CYP3A5/CYP2A7

MP19/ADAM28/KEL/ADAM17/ADAMTS5/NLN/COPS5/NRDC/PHEX/ADAMTS14/FAP/ADAMTS10/MMP15/

4/KHDRBS1/LONP1/RPS7/PTBP1/HNRNPU/EXOSC10/RBMX/HNRNPA1/PPIE/POLR2D/DHX9/TLR7/SU
CAF13/NUP62/HES4/HDAC7/TLE3/ANKRD1/ZNF296/HMGB3/PRAME/RAN/MDFI/DDIT3/BHLHE41/YE

/JMJD6/NUPR1/CITED4/BCL9/NPM1/BRCA1/SMARCA4/ACTL6A/PRMT2/WDR77/SUB1/REXO4/PPARD
TSJ1/CD3EAP/METTL1/DDX49/POLR3K/ERI3/POLR2H/FBL/ZC3H12A/USB1/DDX27/NOP2/PUS1/PO

/APOC3/CYP3A4

4/AR/GHR/TTR/GCGR

9/TAS2R50/TAS2R39/TAS2R16/TAS2R40/TAS2R3/TAS2R7/TAS2R13/PKD1L3/TAS2R60

V0C/ATP6V0E1/ATP6V0D1/ATP2A3
V0C/ATP6V0E1/ATP6V0D1/ATP2A3

CB11/ABCG5/MFSD2A/APOA2/SLC51A/APOF/SLCO1B3/SLC27A2/ABCA6/RBP4/ABCB4/SLCO1B1/FA

SM2B/CPS1/ACSM2A/SLC27A5
HYH/SHMT1/TTPA/GPT/SDS/OGDHL/RBP5/AGXT2/CTH/ABAT/ALB/RBP4/ETNPPL/FTCD/SEC14L2/A
CYP2A13/CYP4F12/CYP4V2/CYP27A1/CYB5A/CYP39A1/AMBP/CAT/CYP3A43/CYP1A1/CYP4F3/CYP
/ACOT12/CES3/NOTUM/AADAC/RGN/PON3/PON1

LUM/RELL2

FZD7/GNA15/CCL5/CCL26/GNAZ/HSPA8/FZD1/GNA12/FPR1/NPFFR2/STAT1/CCL13/SLC9A3R1/DV
A4/RCOR2/E2F8/HDAC1/NOC2L/HEYL/ATN1/KMT5A/MTA2/KDM5B/CBX4/MIER2/DNMT3A/KCTD1/DR

1B2/OR11G2/OR10H1/OR5V1/OR1Q1/OR11A1/OR8B8/OR1C1/OR2H1/OR10P1/OR1L3/OR2L13/OR1G
DH6/EHHADH/RDH16/AKR1D1/HSD11B1/HSD17B6/ADH1A/ADH1C/ADH1B/ADH4

6A12/SLC17A2/SLC6A1/SLC13A5/SLC22A1/SLC10A1

1/CLCF1/GDF15/CMTM3/NBL1/CKLF/PGF/IL18/TFF1/PTHLH/GUCA2A/METRNL/TYMP/TNFRSF11B/

DH6/EHHADH/HAO2/RDH16/AKR1D1/HAO1/HSD11B1/HSD17B6/ADH1A/ADH1C/ADH1B/ADH4
CYP2A13/CYP4F12/CYP4V2/CYP27A1/CYB5A/CYP39A1/AMBP/CAT/CYP3A43/CYP1A1/CYP4F3/CYP

DR34/TAP1/CD320/KIF18A/MCM5/ATP6V0B/ABCC4/ATP6V0D2/HSPA6/ATP6V1C1/ABCC5/CHTF18/

3/SLC1A1/SLC17A1/SLC23A2/SLC13A3/SLC28A1/SLC6A13/SLC17A4/SLC16A2/MFSD2A/SLC38A3

2/CLTC/GAK/AP2B1/AP2A1

PR/F9/PLG/HGFAC

GMO/PHYH/CYP4V2/CYP27A1/CYP39A1/FAXDC2/HAAO/CYP3A43/TF/XDH/CYP1A1/CYP4F3/CYP3A5

MARCB1/CHD4/RUVBL2/RBBP4/TBX19/SMARCC1/HOXB7/MSX1/NFYC/MED12/LMO4/MBD3/HOXA5/ME

DCLRE1C/EXOSC2/EXO5/MGME1/DCP2/CNOT2/PNPT1/ERI1/TREX2/APEX1/PARN
2I/UBE2Q2/RNF19B/SHARPIN/BRCA1/NSMCE2/MED10/TRIM59/TSPAN17/UBE2J2/BIRC7/MYLIP/P

SLC23A2/SLC13A3/SLCO2A1/AKR1C4/SLC22A9/SLC7A2/SLC6A13/SLC17A4/SLC16A2/SLCO2B1/S
A13/SLC17A4/ABCB11/SLC1A2/SLC6A12/SLC17A2/SLC6A1/SLC13A5/SLC22A1/SLC10A1
J2/CYP2A13/CYP4F12/AGMO/PHYH/CYP4V2/CYP27A1/CYP39A1/FMO4/FAXDC2/CYP3A43/FMO5/CY

SLC23A2/SLC13A3/SLCO2A1/AKR1C4/SLC22A9/SLC7A2/SLC6A13/SLC17A4/SLC16A2/SLCO2B1/S

IL/SNRPD3/RBM22

PL5/HNRNPU/EXOSC9/HNRNPR/RSL1D1/MEX3D/RBM24/UTP23/KHSRP/PABPC1L/LARP1/HNRNPA0/R
P6V0E1/ATP6V0D1/ATP2A3

7

/TTYH1/CFTR/VDAC3/VDAC2/GABRB3/CLCNKA

/SLC38A5

IF2D/EIF2B4/EIF4E2/EIF3F/EIF2B1/EIF5B/EIF4G1/EIF2B2/EIF2B3/EIF3C/EIF1

1/SPI1/NEIL3/UHRF1/MCM6/ZIC2/FEN1/BATF/MAFG/RELB/MCM10/ELF4/MCM7/HES6/NME1/ARNT

A/GAPDH/PSRC1/SKA3/FLNA/MAP7D1/SPC25/CEP131/TUBG1/RACGAP1/CCDC88B/CENPW/ARHGEF2
MYT1/CDK1/NDC80/NCAPG/MCM2/CDC45/MELK/ANLN/NEK2/STMN1/GINS1/BUB1/DLGAP5/CDC25B/
EFNA3/TNFRSF12A/FLNA/CD44/ITGB2/AGRN/PLEKHO1/FERMT3/ARHGEF2/ROBO1/PLXNA3/ITGAV/
R2/IGF2BP2/GSTP1/BIRC3/IRAK1/CD74/BATF/CCDC88B/PTAFR/ARHGEF2/PRKCD/HSPB1/RELB/I
BP2/GSTP1/BIRC3/IRAK1/CD74/CCDC88B/PTAFR/ARHGEF2/HSPB1/RELB/ITGAV/SCAMP5/ABCD1/
CSF1R/VTCN1/GBP5/CARD9/DDIT3/HLA-DPA1/LTB/TRAF2/HMGB2/TRIM6/HMOX1/CD86/MAPKAPK2
MAGED1/FZD7/SCRIB/IFT57/CFL1/PSMB9/PSMD14/HNF1B/AREG/TRIM28/RHOC/COL5A1/CXCR4/S
IPK2/ADA/EXO1/HAVCR2/CORO1A/GSTP1/CLCF1/CD44/ITGB2/ALDH3B1/SNAP25/CPLX2/BATF/PT
FB1/ITGAM/SPI1/CYBB/HAVCR2/GSTP1/CD44/ITGB2/ALDH3B1/SNAP25/CPLX2/CD74/BATF/PTAF
ALDH3B1/SNAP25/CPLX2/PTAFR/COTL1/PRKCD/LAT2/QSOX1/ITGAV/VAT1/MVP/RAB31/SYK/UNC1
1/PRKCD/QSOX1/ITGAV/VAT1/MVP/RAB31/SYK/UNC13D/TUBB/CSTB/S100A8/LYZ/NME2/CTSA/TR
IPK2/ADA/EXO1/HAVCR2/CORO1A/GSTP1/CLCF1/CD44/ITGB2/ALDH3B1/SNAP25/CPLX2/BATF/PT
YBB/EXO1/HAVCR2/CORO1A/GSTP1/CLCF1/C1QB/SUSD4/CD44/ITGB2/ALDH3B1/C1QC/SNAP25/CP
R/COTL1/PRKCD/QSOX1/ITGAV/VAT1/MVP/RAB31/SYK/UNC13D/TUBB/CSTB/S100A8/LYZ/NME2/C
CD74/BATF/PKN1/RELB/FANCD2/SLAMF8/EFNA4/NME1/PLEK/SYK/CD109/IL18/NME2/CSF1R/CSF
IL18/NME2/CSF1R/TRPM2/FZD7/HMGB3/TFRC/TNFRSF18/JAK3/TCF3/BAX/CD86/VAV1/SEMA4A/T
OA14/TGFB1/ITGAM/FCGR3A/LGALS1/RIPK2/ADA/HAVCR2/CORO1A/MZB1/CLCF1/C1QB/SUSD4/BI
CK/PAG1/S100A8/HLA-DRA/MUC1/EZR/HLA-DQB1/VTCN1/CARD9/PSMB9/HLA-DPA1/CTSK/WIPF3/
PF3/RBCK1/PSMD14/WASF2/BAX/VAV1/PHPT1/CD300A/HLA-DPB1/ARPC4/PSMD2/MAPK3/CD276/H
1/TONSL/FANCI/SPI1/EXO1/NEIL3/TYMS/CLCF1/DTL/UHRF1/RAD54L/RNASEH2A/MCM6/FEN1/BA
ME1/CDKN2D/CDC7/CHEK1/NPAS2/HSF1/RHNO1/NABP2/MCM5/AP5Z1/PSMD14/HMGB2/TRIM28/SIR
1/HDAC7/MUC1/CENPU/HMGB3/CHEK1/PRMT1/SMARCD1/CENPL/YEATS2/HMGB2/TRIM28/BRMS1/SI
2/DHX37/CD2BP2/JMJD6/LSM4/HSPA8/RRAGC/MAP2K2/PES1/DKC1/PPP2R1A/UPF3B/POLR2G/TRM
LR2G/FTSJ1/RBM3/EIF2S2/MRPS12/RPS2/PABPC1/TICRR/MRPL47/TRNAU1AP/PPP1CA/MRPL9/PA
/SYT13/FABP5/TYROBP/CKAP4/PYGB/TGFB1/ITGAM/CYBB/CORO1A/GSTP1/STX3/FLNA/CD44/ITG
/AGRN/CD74/CMTM3/PTAFR/P2RX4/PRKCD/ROBO1/HSPB1/SLAMF8/PLXNA3/ITGAV/EFNA4/VASP/N
DC25C/DTL/UHRF1/RAD54L/RNASEH2A/CD44/MCM6/FEN1/CD74/BATF/BCL2A1/PRKCD/GINS2/MCM
P1/ARL2/CCNF/TTK/SKA1/AURKA/GAPDH/PSRC1/SKA3/FLNA/MAP7D1/SPC25/CEP131/TUBG1/RAC
/NUSAP1/TTK/TGFB1/RCC2/AURKA/GPNMB/PSRC1/HMMR/CDC25C/DTL/CEP131/CDKN2A/TUBG1/EI
NA2/LIF/SAPCD2/CSF1/AKR1B1/TTK/TGFB1/BAMBI/IQGAP3/RIPK2/ADA/HAVCR2/CORO1A/STX3/
R77/RNPS1/SNRPE/POLR2H/TXNL4A/NCBP2/USB1/SNRPF/LSM7/NONO/LSM10/U2AF2/SF3B5/KDM1
1/HSP90AB1/NPLOC4/CSNK1E/MAN1B1/PSMB2/PSME3/HSPBP1/BCAP31/UBE2J2/FBXO5/LAMP3/CS
1/LIF/CENPF/RMI2/ORC1/ECT2/NUSAP1/CCNF/TTK/TGFB1/RAD51/RCC2/AURKA/GPNMB/PSRC1/H
NCK2/PLXNA3/BAIAP2L2/VASP/PLEK/HRAS/PLCB1/CDC42EP1/NUP62/HCK/CCT5/SPAG5/MAPRE1/
N2A/ARHGEF2/PRKCD/ROBO1/C1QA/NCK2/ADORA1/PRNP/SYK/FGD3/EEF1E1/CDK4/S100A8/CASP4
CS1/HLA-DRA/CSF1R/NECTIN1/ALYREF/CENPU/KRT7/MMP1/SCRIB/SLC25A6/RPL8/RAN/CFL1/PS
PA1/DHX34/CSTF2/PCBP4/SNRPA/MRTO4/SF3A2/CD2BP2/CNOT11/PSMD2/HIF1A/JMJD6/LSM4/HS
AC1/PRMT2/LEF1/NELFE/RPS6KA4/FBL/MT3/OTUB1/NOC2L/HDAC2/CDK2/KDM1A/KMT5A/HCFC1/E
STM1/TNFRSF12A/YWHAZ/OSMR/BIRC3/CD44/ITGB2/IRAK1/CD74/BATF/PTAFR/PRKCD/ROBO1/IL
D3/PLEK/TRIM54/FUNDC2/DDR1/ASPH/TWF2/ADAM15/BSG/LAMC1/MMP1/MID1IP1/HSF1/DNAJC6/
RRP7A/PA2G4/SNRPE/FBL/TXNL4A/DDX27/SNRPF/NOP2/AATF/NOC2L/GTPBP4/EIF3D/NHP2/POP7
4/S1PR2/RACGAP1/COTL1/ARHGEF2/PRKCD/TRIP10/NCK2/BAIAP2L2/VASP/ADORA1/PLEK/ITPKA
/EZR/DCDC2/TRPM2/RHOG/IFT57/TUBB4B/PLEK2/KIF3C/RHOQ/FSCN1/ABCC4/CARMIL1/WASF2/R
AP1/COTL1/ARHGEF2/PRKCD/TRIP10/NCK2/BAIAP2L2/VASP/PLEK/ITPKA/HRAS/CDC42EP1/USH1
CD2/SLAMF8/EFNA4/NME1/PLEK/SYK/CD109/IL18/NME2/CSF1R/CSF3R/ZNF385A/TRPM2/FZD7/H
A/JMJD6/TCIRG1/ATP6AP1/ISG15/PRKX/UBASH3B/F2RL1/STAT1/RAB7B/HDAC1/TNFRSF11A/NBE
/RIPK2/ADA/HAVCR2/CORO1A/STX3/FLNA/CD44/ITGB2/IRAK1/FXYD5/CDKN2A/CD74/CCDC88B/P
TL/UHRF1/GPC3/FLNA/CPVL/ADAM9/UBXN11/ABHD17C/GPC1/PJA1/MAD2L1/CTSA/EZR/PSMB9/GI
HGAP4/GPNMB/STK26/ADA/CORO1A/GSTP1/FLNA/CD74/PTAFR/FERMT3/ARHGEF2/P2RX4/ROBO1/H
S1/DKK1/EFNA3/FOLR1/STX3/TNFRSF12A/NCDN/AGRN/SNAP25/TPRN/CDK16/ROBO1/SERPINI1/N

BO1/SERPINI1/PLXNA3/NME1/PRNP/ITPKA/HRAS/CDC42EP1/USH1C/EZH2/NME2/TWF2/ASAP1/EZ
AE1/FBXL19/TRIM6/DTX3/TRIM28/NUP210/FKBP1A/UBE2M/UBE2Z/PRKDC/MAD2L2/PSMD2/HIF1A
4/LIF/SAPCD2/MISP/TYROBP/ECT2/ARL2/TGFB1/ITGAM/AACS/GAPDH/RIPK2/HAVCR2/RHBDF2/G
P62/GTSE1/CCT5/SPAG5/MAPRE1/EZH2/PACS1/MPV17/EZR/TOMM40/SCRIB/SLC25A6/RPL8/GBP5
1/MCM6/SNAP25/TUBG1/SNRPB/CENPW/COTL1/PRKCD/NCK2/OIP5/MCM7/BAIAP2L2/VASP/CDC42E
/IGF2BP3/PGK1/RPL8/HSF1/PSMB9/APOBEC3C/PSMD14/HMGB2/YBX3/BAX/MAPKAPK2/SIRT6/LIN
/DDX49/RRP9/DDX11/POLR3K/ERI3/REXO4/RRP1/RRP7A/PA2G4/POLR2H/FBL/ZC3H12A/NCBP2/U
5/PTAFR/COTL1/PRKCD/QSOX1/ITGAV/VAT1/MVP/RAB31/SYK/UNC13D/IL18/TUBB/CSTB/S100A8
LN1/S100A14/TGFB1/RCC2/ARHGAP4/GPNMB/STK26/ADA/CORO1A/GSTP1/STX3/FLNA/CD74/PKN1
HSPB1/ITGAV/LAMB1/ADAM9/HRAS/STK39/GTSE1/PGF/MAPRE1/TMSB4X/CSF1R/HDAC7/SMIM22/C
/FZD7/TNFAIP8L2/VTCN1/TFRC/HLA-DPA1/HLA-DMB/TNFRSF18/VSIG4/JAK3/CCL5/BAX/CD86/A
5/PTAFR/COTL1/PRKCD/QSOX1/ITGAV/VAT1/MVP/RAB31/SYK/UNC13D/IL18/TUBB/CSTB/S100A8
/AGRN/CD74/CMTM3/PTAFR/P2RX4/PRKCD/ROBO1/HSPB1/SLAMF8/PLXNA3/ITGAV/EFNA4/VASP/N
UPF3B/POLR2G/FTSJ1/RBM3/EIF2S2/MRPS12/RPS2/PABPC1/TICRR/GGCT/MRPL47/TRNAU1AP/PP
/PRKCD/ROBO1/NCK2/PRNP/SYK/FGD3/EEF1E1/CDK4/S100A8/CASP4/ANKRD1/MAGED1/SCRIB/IF
TP1/MPZ/CLCF1/SQSTM1/YWHAZ/FLNA/BIRC3/CD44/IRAK1/CD74/ARHGEF2/BCL2A1/PRKCD/HSPB
/PRKCD/ROBO1/NCK2/PRNP/SYK/FGD3/EEF1E1/CDK4/S100A8/CASP4/ANKRD1/MAGED1/SCRIB/IF
TP1/MPZ/CLCF1/SQSTM1/YWHAZ/FLNA/BIRC3/CD44/IRAK1/CD74/ARHGEF2/BCL2A1/PRKCD/HSPB
/MAN1B1/PSMB2/PSME3/HSPBP1/BCAP31/UBE2J2/FBXO5/CSNK1D/ANAPC7/CDK2/KIF14/SPSB1/P
EPA1/FLNA/AGRN/EIF4EBP1/COTL1/ARHGEF2/PRKCD/NCK2/BAIAP2L2/VASP/PLEK/HRAS/SYK/CD
16A1/MMP1/PLEK2/GBP5/HSF1/RHOQ/S100A10/FSCN1/HNF1B/CARMIL1/WASF2/RHOC/BAX/SEMA4
/TAP1/ZYX/PACS1/HLA-DRA/CSF1R/NECTIN1/ALYREF/CENPU/KRT7/MMP1/SCRIB/SLC25A6/RPL8
UP62/RCC1/HCK/TAP1/ZYX/LYZ/PACS1/HLA-DRA/CSF1R/NECTIN1/ALYREF/CENPU/KRT7/MMP1/S
/RPL39L/ACSL4/LIF/KIFC1/SERPINE2/AKR1B1/DSG2/FBLN1/TTK/GAL3ST1/TGFB1/FAM50A/AUR
NE1/CCNA2/CENPF/ORC1/CCNF/TTK/TGFB1/IQGAP3/RCC2/AURKA/GPNMB/HMMR/TYMS/CDC25C/DT
ABP5/TYROBP/CKAP4/PYGB/TGFB1/ITGAM/CYBB/CORO1A/GSTP1/STX3/FLNA/CD44/ITGB2/ALDH3
44/ITGB2/IRAK1/C1QC/PPP1R14B/PTAFR/ARHGEF2/PRKCD/C1QA/RELB/SLAMF8/ELF4/IGLL5/HL
B9/VSIG4/CTSK/PSMD14/HMGB2/TRIM6/CCL5/MAPKAPK2/VAV1/COLEC12/CD300A/UNC93B1/PRKD
ECT2/TGFB1/BAMBI/AURKA/DKK1/RIPK2/ADA/CLCF1/TNFRSF12A/FLNA/GDF15/S1PR2/CD74/ARH
11/CDKN2A/PRNP/HRAS/GTSE1/EZH2/CDK4/MAD2L1/MUC1/EME1/CDKN2D/ZNF385A/ARID3A/CDC7
1/CDC25C/GDF15/IRAK1/CDKN2A/S1PR2/CD74/PKN1/DUSP5/PRKCD/ROBO1/HSPB1/NCK2/ADORA1
CD74/BATF/CCDC88B/PKN1/PRKCD/LAT2/RELB/NCK2/FANCD2/SLAMF8/ELF4/IGLL5/PRNP/SYK/U
/CCNE1/LIF/CENPF/KIFC1/KIF18B/MISP/NUSAP1/P3H4/TTK/TGFB1/RAD51/AURKA/PSRC1/NCAP
N1/RELB/FANCD2/SLAMF8/EFNA4/NME1/PLEK/SYK/CD109/IL18/NME2/CSF1R/CSF3R/ZNF385A/T
R1/FZD6/DLG3/CSF1R/FOXQ1/CD151/COL3A1/ANKRD1/MAGED1/FZD7/SCRIB/IFT57/CFL1/PSMB9
FR/PRKCD/NCK2/FANCD2/SLAMF8/IGLL5/PRNP/PLEK/SYK/UNC13D/IL18/PAG1/HMGB3/TNFAIP8L
2/CD44/ITGB2/SDC3/CD74/PTAFR/P2RX4/SLAMF8/ITGAV/SLC7A11/ADORA1/NBL1/SYK/PLCB1/S
CCNF/TTK/TGFB1/RCC2/ARHGAP4/SPI1/LGALS1/DKK1/UNC119/CORO1A/PMEPA1/ANKRD13D/EIF4
TP1A1/CSF1/DSG2/FBLN1/S100A14/TGFB1/RCC2/ARHGAP4/GPNMB/STK26/ADA/CORO1A/GSTP1/F
/HSPB1/ITGAV/LAMB1/ADAM9/HRAS/STK39/GTSE1/PGF/MAPRE1/TMSB4X/CSF1R/HDAC7/SMIM22/
IQGAP3/STK26/DKK1/RIPK2/UNC119/PSRC1/GSTP1/CDC25C/GDF15/IRAK1/CDKN2A/S1PR2/CD74
BP3/PTAFR/CHML/NCAPH/PRKCD/ROBO1/PLEK/HRAS/ADAP1/SYK/PLCB1/DEPDC1/GMIP/EZH2/TMS
2L2/VASP/PLEK/TRIM54/HRAS/CDC42EP1/KIF11/NUP62/HCK/SPAG5/MAPRE1/TMSB4X/S100A8/T
1/SNAP25/TUBG1/CPLX2/ARHGEF2/LAT2/PLEK/SYK/NUP62/UNC13D/SPAG5/MAPRE1/TUBB/GIT1/
K/NUP62/UNC13D/SPAG5/GIT1/MAD2L1/STARD3NL/YKT6/EZR/SCRIB/RAN/TMEM201/KIF3C/KIF1
LCF1/TNFRSF12A/YWHAZ/FLNA/AGRN/SNAP25/ARHGEF2/P2RX4/ROBO1/SERPINI1/PLXNA3/SMARC
/SMARCD3/NME1/NBL1/ITPKA/NAP1L1/EZH2/NME2/TWF2/ASPM/ANKRD1/TRPV2/GRN/TIMP2/TCF3
2/ARHGAP4/AURKA/LGALS1/DKK1/CLCF1/TNFRSF12A/FLNA/SNAP25/S1PR2/ARHGEF2/P2RX4/ROB
ECT2/TGFB1/ITGAM/CORO1A/GPC4/STX3/SQSTM1/YWHAZ/GPC3/FLNA/ITGB2/CEP131/CDK16/CPL
AURKA/PPT1/GPNMB/DKK1/RIPK2/ADA/CORO1A/GSTP1/MPZ/CLCF1/SQSTM1/YWHAZ/FLNA/BIRC3/
AP25/CPLX2/DMKN/HK2/PRKCD/TRIP10/REEP4/SLC7A11/IGLL5/BAIAP2L2/ABCD1/RAB31/TAP1/

MB9/TRAF2/RBCK1/PSMD14/SAE1/FBXL19/TRIM6/DTX3/TRIM28/NUP210/FKBP1A/UBE2M/UBE2Z/
/KIF11/NUP62/RCC1/WDR34/HCK/SPAG5/MAPRE1/TUBB/ASAP1/SMIM22/ASPM/EZR/ANKRD1/DCDC
P/HRAS/RAB31/GPC1/PACS1/DLG3/PKP3/EZR/BSG/SCRIB/RHOG/RPL8/UBL4A/RAB34/ANXA13/NC
AP1/EEF1E1/MAPK8IP2/MUC1/EZR/CDKN2D/ANKRD1/ZNF385A/FZD7/CREB3L1/CHEK1/GRN/NPAS2
TNNT1/TUBG1/TPM4/CCDC88B/COTL1/ARHGEF2/PRKCD/NCK2/BAIAP2L2/VASP/PLEK/TRIM54/CDC
HAVCR2/CORO1A/GPC4/MPZ/FLNA/CD44/ITGB2/IRAK1/FXYD5/CERCAM/CD74/CCDC88B/PTAFR/FE
2/TRPM2/RHOG/IFT57/TUBB4B/PLEK2/KIF3C/RHOQ/FSCN1/ABCC4/CARMIL1/WASF2/RABEP2/TCT
ERPINI1/PLXNA3/NME1/PRNP/ITPKA/HRAS/CDC42EP1/USH1C/EZH2/NME2/TWF2/ASAP1/EZR/ANK
KN2A/TUBG1/SMARCD3/PLCB1/GTSE1/MAPRE1/TUBB/EZH2/CDC25A/CDK4/MAD2L1/MUC1/CDKN2D/
C80/NCAPG/MCM2/CDC45/MELK/ANLN/NEK2/STMN1/GINS1/BUB1/DLGAP5/CDC25B/BUB1B/MKI67/
/PSMD2/HIF1A/ATP6AP1/SASH3/ISG15/UBASH3B/SOCS1/IRF1/STAT1/RAB7B/HDAC1/CTLA4/FST
P131/KCNJ11/TAX1BP3/PRKCD/SLC7A11/B3GAT3/PRNP/HRAS/ABHD17C/NUP62/GTSE1/GPC1/CCT
LN1/S100A14/TGFB1/RCC2/ARHGAP4/GPNMB/STK26/ADA/CORO1A/GSTP1/FLNA/CD74/PKN1/PTAF
/ITGAV/LAMB1/ADAM9/HRAS/STK39/GTSE1/PGF/MAPRE1/TMSB4X/CSF1R/HDAC7/SMIM22/CD151/
2AF2/SF3B5/KDM1A/SRSF9/SNRPC/GEMIN7/BUD31/DAZAP1/SNRNP40/RBM19/PPIL1/RBMX2/SFPQ
2AF2/SF3B5/KDM1A/SRSF9/SNRPC/GEMIN7/BUD31/DAZAP1/SNRNP40/RBM19/PPIL1/RBMX2/SFPQ
2AF2/SF3B5/KDM1A/SRSF9/SNRPC/GEMIN7/BUD31/DAZAP1/SNRNP40/RBM19/PPIL1/RBMX2/SFPQ
PGK1/HSF1/PLOD1/PSMB9/PLAU/PSMD14/HMOX1/CXCR4/SLC9A1/PSMD2/FUNDC1/HIF1A/PAK1/GN
/SNAP25/CPLX2/PTAFR/COTL1/PRKCD/LAT2/QSOX1/ITGAV/VAT1/MVP/RAB31/SYK/UNC13D/TUBB
ANCD2/SLAMF8/IGLL5/PRNP/SYK/UNC13D/IL18/PAG1/HMGB3/TNFAIP8L2/VTCN1/CD320/GRN/TF
QA1/HCK/PAG1/S100A8/HLA-DRA/MUC1/EZR/HLA-DQB1/VTCN1/CARD9/PSMB9/HLA-DPA1/CTSK/W
NPS1/NELFE/SNRPE/POLR2H/TXNL4A/NCBP2/SNRPF/LSM7/NONO/LSM10/U2AF2/SF3B5/KDM1A/SR
1/NDC1/SNRPE/XPO6/NUP188/ZC3H12A/CSE1L/NCBP2/SNRPF/FERMT1/SIX2/HTATIP2/KPNB1/HC
AG1/S100A8/EZR/TNFAIP8L2/VTCN1/TFRC/HLA-DPA1/HLA-DMB/VSIG4/JAK3/CCL5/CD86/VAV1/
/RAB31/CDC42EP1/NUP62/GMIP/FGD3/DNMT1/COL3A1/RHOG/RAB34/CFL1/RHOQ/DOK2/RRAD/ARH
H2/LIN28B/NUP210/PCBP4/CNOT11/PSMD2/MAPK3/HSPA8/EIF3B/MAP2K2/DKC1/NPM1/UPF3B/PO
ID3A/CDC7/CHEK1/PRMT1/PSMB9/PSMD14/ESPL1/BAX/MIF/LCMT1/NAA10/PCBP4/CASP2/BRSK1/
/LINGO1/P3H1/CXCR4/SEMA4A/MAP2/BRSK1/IQGAP1/PTK7/EFNA5/SF3A2/FZD1/PAK1/CRABP2/A
GAV/LAMB1/COLGALT1/ELF3/DDR1/SERPINH1/MPV17/ADAM15/BSG/COL16A1/COL3A1/TNFRSF11B
/LAMB1/ADAM9/HRAS/STK39/GTSE1/PGF/MAPRE1/TMSB4X/CSF1R/HDAC7/SMIM22/CD151/GRN/TN
DDIT4/PPP1CA/MRPL9/DVL3/MRPS23/CAPZA1/FRAT2/FZD2/CAMSAP2/CLN3/AXIN1/GBA/MRPL12/
1/CHEK1/NABP2/CCT6A/CCT3/TRIM28/ESPL1/SIRT6/LCMT1/NAA10/NAA40/MAD2L2/MAPK3/PYGO
WF2/ASAP1/MMP1/GBP5/FSCN1/HNF1B/CARMIL1/RHOC/PLEKHG2/BAX/CCL26/MAP2/LCMT1/MSN/A
1/SNAP25/CPLX2/PTAFR/COTL1/PRKCD/LAT2/QSOX1/ITGAV/VAT1/MVP/RAB31/SYK/HCK/UNC13D
1/PRKCD/QSOX1/ITGAV/VAT1/MVP/RAB31/SYK/UNC13D/TUBB/CSTB/S100A8/LYZ/NME2/CTSA/TR
NE1/CCNA2/CENPF/ORC1/CCNF/TTK/TGFB1/IQGAP3/RCC2/AURKA/GPNMB/HMMR/TYMS/CDC25C/DT
1/SPAG5/EZH2/CDC25A/CDK4/MAD2L1/MUC1/ZNF385A/ARID3A/CDC7/CHEK1/HSF1/RHNO1/PRMT1
7/RNPS1/NDC1/SNRPE/XPO6/NUP188/ZC3H12A/CSE1L/NCBP2/SNRPF/FERMT1/SIX2/HTATIP2/KP
1B/PRC1/KIF23/TUBA1B/ETV5/ZWINT/CKS2/ORC6/CCNE1/CCNA2/CENPF/KIFC1/SAPCD2/KIF18B
CD/ROBO1/HSPB1/ADORA1/ADAM9/HRAS/SYK/STK39/NUP62/EZH2/CDC25A/CDK4/CSF1R/CDKN2D/
1/NCK2/ITGAV/ADORA1/HRAS/S100A8/MAPK8IP2/MUC1/UNC5B/CASP4/CDKN2D/ZNF385A/MAGED1
00A10/CARMIL1/WASF2/RHOC/PLEKHG2/CCL26/MAP2/ARPC4/SH3PXD2B/SLC9A1/HSPA8/MAP1S/P
/ZC3H12A/PSME3/NCBP2/U2AF2/SLC11A1/SRSF9/DAZAP1/PSMB4/RBM19/PSMB8/PSMB10/PSMA5/
/EZR/RHOG/RAN/GLIS2/ANXA13/CCT6A/MICALL2/EPB41L2/GPD1L/CCT3/SAE1/TRIM28/NUTF2/M
OG/RAB34/ANXA13/RHOQ/S100A10/EPB41L2/FLOT2/PKDCC/RAB15/AP4M1/PDZD11/LAMA5/RAB3B
/CCNE1/LIF/CENPF/KIFC1/KIF18B/MISP/NUSAP1/P3H4/TTK/TGFB1/RAD51/AURKA/PSRC1/NCAP
BCK1/PSMD14/WASF2/BAX/VAV1/PHPT1/CD300A/HLA-DPB1/ARPC4/PSMD2/MAPK3/CD276/HLA-DQ
PP2R1A/NPM1/UPF3B/RNASET2/POLR2G/YBX1/RPS2/PELO/PABPC1/RNPS1/PSMB2/ZC3H12A/PSME
PABPC1/RNPS1/PSMB2/ZC3H12A/PSME3/NCBP2/RPL17/RPLP2/CNOT9/LSM7/RPSA/SLC11A1/SSB/
TWF2/EZR/RHOG/CFL1/MICALL2/RHOQ/S100A10/FSCN1/CARMIL1/WASF2/RHOC/PLEKHG2/CCL26/
L1/DNMT1/MID1IP1/CHEK1/CARMIL1/CHMP4B/WASF2/ESPL1/MAP2/LCMT1/NAA10/MAD2L2/NUPR1

/PAG1/TNFAIP8L2/VTCN1/TFRC/HLA-DPA1/HLA-DMB/VSIG4/JAK3/CCL5/CD86/VAV1/TNFSF9/CD
MIL1/WASF2/P3H1/CXCR4/IQGAP1/PTK7/EFNA5/SF3A2/FZD1/PAK1/CRABP2/ANKRD27/CORO1C/F
FL1/CARMIL1/PLEKHG2/SLC12A2/VAV1/SEMA4A/CCL26/MAP2/SLC12A9/MSN/ARPC4/EFNA5/PAK1
NHP2/PUS1/POP7/URM1/EMG1/NOP58/TRMT1/SSB/TRMT61A/UTP18/RPS21/UTP14A/RPL7/NPM3/P
PGK1/HSF1/PLOD1/PSMB9/PLAU/PSMD14/HMOX1/CXCR4/SLC9A1/PSMD2/FUNDC1/HIF1A/PAK1/GN
IM28/CCL5/CXCR4/MIF/ZP3/MAPK3/TRIM11/SLPI/FUCA2/PPP2R1A/ISG15/CHMP4C/POLR2G/SMA
A-DMB/JAK3/CCL5/CD86/VAV1/TNFSF9/MIF/FLOT2/ZP3/HLA-DPB1/CD2/EBI3/MAD2L2/CD276/E
3/TRIM28/CACYBP/BAX/SIRT6/GMNN/PRKDC/MAD2L2/CHTF18/MAPK3/IL27RA/DKC1/PPP2R1A/AC
3/TNFAIP8L2/VTCN1/CD320/TFRC/HLA-DPA1/HLA-DMB/TNFRSF18/VSIG4/JAK3/CCL5/CD86/VAV
F1/STK39/CCT5/TFF1/LYZ/CSF1R/TNFRSF11B/NABP2/CCT6A/TFRC/CTSK/CCT3/CHMP4B/POLA2/
AM15/ANKRD1/PGK1/HSF1/PLOD1/PSMB9/PLAU/PSMD14/HMOX1/CXCR4/SLC9A1/PSMD2/FUNDC1/H
S2/GDI1/KRT18/BID/RPL17/RPLP2/HSPA4/TRAM1L1/RPSA/YWHAH/BBC3/PMAIP1/ITGB1BP1/REE
KN2A/TUBG1/SMARCD3/PLCB1/GTSE1/MAPRE1/TUBB/EZH2/CDC25A/CDK4/MAD2L1/MUC1/CDKN2D/
GED1/CREB3L1/DDIT3/TRAF2/RBCK1/HMGB2/YBX3/HMOX1/BAX/MIF/CASP2/CREB3/HIF1A/NUPR1
L5/CD86/VAV1/TNFSF9/MIF/FLOT2/ZP3/HLA-DPB1/CD2/EBI3/MAD2L2/CD276/EFNB1/CD3E/PAK
EF/EME1/CDC7/CHEK1/RHNO1/GINS3/MCM5/POLA2/CACYBP/GMNN/GINS4/CHTF18/PPP2R1A/RBBP
1/P3H4/TTK/SKA1/RCC2/PSRC1/NCAPD2/SKA3/SPC25/TUBG1/FEN1/RACGAP1/CENPW/DSCC1/NCA
D3A/SCRIB/CHEK1/NABP2/PRMT1/PSMB9/PSMD14/TIMP2/BAX/LCMT1/NAA10/PCBP4/CASP2/BRSK
/JAK3/CCL5/CD86/VAV1/TNFSF9/CD300A/FLOT2/ZP3/HLA-DPB1/CD2/EBI3/CD276/EFNB1/CD3E
C4/PAK1/BAIAP2L1/FCHSD1/BIN1/CORO7/ARPC2/GAS7/ARPC3/CORO1B/HIP1R/TPPP3/NCKAP1L/
LA-DMB/VSIG4/JAK3/CCL5/BAX/CD86/TNFSF9/MIF/CD300A/MSN/ZP3/HLA-DPB1/EBI3/CD276/E
93/UTP18/RPS21/UTP14A/RPL7/NPM3/TRMT112/NSUN5/MPHOSPH6/BYSL/RPL35A/LYAR/RRP36/E
G/HMGB2/CCL5/SLC12A2/VAV1/CXCR4/CCL26/MIF/CREB3/MAPK3/SERPINE1/JAML/ZNF580/C3AR
20/TFRC/HLA-DPA1/HLA-DMB/VSIG4/JAK3/CCL5/BAX/CD86/TNFSF9/MIF/CD300A/MSN/ZP3/HLA
MB/VSIG4/JAK3/CCL5/CD86/VAV1/TNFSF9/CD300A/FLOT2/ZP3/HLA-DPB1/EBI3/CD276/EFNB1/
2/HSF1/SRXN1/NCF1/AKR1C3/TRAF2/HMOX1/SLC25A24/PRDX1/HIF1A/MAPK3/FZD1/PCNA/ZNF58
P5Z1/PSMD14/HMGB2/TCF3/SIRT6/GINS4/PRKDC/RUVBL1/MAD2L2/APEX2/IL27RA/MND1/RBBP8/
LRB1/IFT52/DYNLL1/DYNC1H1/DPCD/KIFC2/BLOC1S4/DNAAF5/MKKS/KIF14/KPNB1/CLN3/KIFC3
T1/SLC25A19/SLC35B2/SLC35A2/U2AF2/SLC35E4/SRSF9/XPOT/SSB/ZNF593/AKAP8L/SFPQ/CPS
RPS21/UTP14A/RPL7/NPM3/TRMT112/NSUN5/SIRT7/MPHOSPH6/BYSL/RPL35A/LYAR/SMARCB1/RR
3/DNMT3A/NUP85/ATAD2/NUP43/RAE1/XPO5/SRRT/HENMT1/POLR2L/NUP155/DROSHA/AGO2/BEND
T1/NUP205/NUP37/RNPS1/NDC1/NUP188/NCBP2/U2AF2/SRSF9/XPOT/FKBP4/SSB/ZNF593/AKAP8
NFRSF4/SLC39A10/MNDA/CTLA4/LEF1/TNIP2/BCL3/NCKAP1L/TXLNA/PIK3CD/LYN/CARD11/NFAM
K1/PSMB9/PSMD14/LCMT1/BRSK1/MIIP/SSNA1/PSMD2/PPP2R1A/NPM1/CHMP4C/BRCA1/TUBA1A/S
A-DPB1/EBI3/MAD2L2/CD276/EFNB1/CD3E/PAK1/SASH3/TNFRSF4/SLC39A10/THY1/SIRPA/SOCS
2/PRKDC/CREB3/HIF1A/NUPR1/PERP/BRCA1/BCL2L12/BMF/NOL3/HDAC1/DDIAS/DYRK2/HYOU1/D
OLEC12/MFGE8/CD300A/ARPC4/JMJD6/MAPK3/P2RY6/PAK1/GULP1/CORO1C/SIRPA/SPON2/HSP90
IRF1/PPM1G/HSP90AB1/GAS7/PPP1R9B/E2F8/DDIAS/CGREF1/E2F4/GAS2L1/MLST8/CNOT9/EVI2
-DPA1/HLA-DMB/PSMD14/RAB32/HLA-DPB1/CLTA/PSMD2/HLA-DQB2/HLA-A/CENPE/ITGB5/RAB3B
/RPS5/EIF3I/TAF9/EIF2S3/SRSF12/DENR/EDC3/ISY1/CPSF6/GRB7/RUVBL2/PRPF3/SNU13/PRP
BIN1/ARPC2/ARPC3/SUB1/CORO1B/HIP1R/BID/TPPP3/NCKAP1L/CXCL13/PMAIP1/MITF/ARPC5L/
/ZNF385A/ARID3A/CDC7/PRMT1/MCM5/POLA2/BAX/FAM83D/BCAT1/PCBP4/CASP2/GMNN/PRKDC/C
B/VSIG4/JAK3/CCL5/BAX/CD86/TNFSF9/MIF/CD300A/MSN/ZP3/HLA-DPB1/EBI3/CD276/EFNB1/
H2B2/NCKAP1L/PIK3CD/HCLS1/NLE1/PMAIP1/LYN/CARD11/IL2RA/TNFSF13B/LYAR/PTBP3/MB/S
SLC39A10/MNDA/THY1/THEMIS2/CTLA4/PSMB2/SH2B2/ZC3H12A/PSME3/NCKAP1L/HLA-DQA2/PIK
/ESPL1/SGO2/MND1/PPP2R1A/RBBP8/XRCC2/SGO1/OSGIN2/NDC1/FBXO5/RSPH1/CDK2/SMC4/FAN
34/CHD3/PPP1R13L/PERP/TAF6/RBBP8/SPRED1/BRCA1/PCNA/BCL2L12/RRS1/E2F8/HDAC1/DYRK
L1/MUC1/ZNF385A/ARID3A/CDC7/RHNO1/PRMT1/GIPC1/ESPL1/BAX/FAM83D/PCBP4/CASP2/CNOT
B/MISP/NUSAP1/TTK/TGFB1/AURKA/PSRC1/NCAPD2/CDC25C/FLNA/TUBG1/RACGAP1/DSCC1/NCAP
4/SIX2/FZD2/JAG1/RAC1/PSMB4/PDGFA/VANGL1/PSMB8/PSMB10/PSMA5/TGFB2/PSMD13/PSMC4/
5A/ARID3A/CDC7/PRMT1/MCM5/POLA2/BAX/BCAT1/PCBP4/CASP2/GMNN/PRKDC/CNOT11/RBBP8/R

D14/LCMT1/BRSK1/MIIP/SSNA1/PSMD2/PPP2R1A/CHMP4C/BRCA1/TUBA1A/SFI1/DYNLL1/DYNC1H
SNX10/EVI2B/PIK3CD/HCLS1/MITF/LYN/PIAS3/THOC5/IFI16/CCR1/OSTM1/PARP1/IL15/LTBR/
F2RL1/ARPC2/GAS7/ARPC3/WIPF1/CORO1B/HIP1R/NCKAP1L/CAP1/CAPZA1/MKKS/FHOD3/HCLS1/
UP43/RPL39/RPL35A/SMARCB1/RAE1/RPS3/TFAP4/RPL27/POLR2L/NUP155/RPL35/RPS20/NUP10
/EBI3/CD276/EFNB1/CD3E/PAK1/SASH3/THY1/SIRPA/SOCS1/CTLA4/FSTL3/CD83/PDCD1/SIRPG
SFPQ/CPSF1/THOC5/NUP85/NUP43/AGFG1/THOC6/RAE1/CPSF6/EIF4A3/XPO5/NUP155/CPSF4/NU
SFPQ/CPSF1/THOC5/NUP85/NUP43/AGFG1/THOC6/RAE1/CPSF6/EIF4A3/XPO5/NUP155/CPSF4/NU
NFSF9/MIF/CD300A/ZP3/HLA-DPB1/EBI3/CD276/EFNB1/CD3E/SASH3/TNFRSF4/SLC39A10/MNDA
1L1/CENPO/CENPU/HMGB3/MCM5/CENPL/HMGB2/SIRT6/NAA10/GINS4/RUVBL1/CHTF18/CHD3/NPM
TXN2L/RPS5/EIF3I/TAF9/EIF2S3/SRSF12/DENR/EDC3/ISY1/CPSF6/GRB7/RUVBL2/PRPF3/SNU1
RC1/NCAPD2/TUBG1/FEN1/RACGAP1/DSCC1/NCAPH/FANCD2/NUP62/SPAG5/MAD2L1/EME1/RAN/KI
C9A1/PAK1/BAIAP2L1/FCHSD1/BIN1/F2RL1/ARPC2/ARPC3/LIMCH1/CORO1B/HIP1R/NCKAP1L/CA
AX/LCMT1/PCBP4/CASP2/BRSK1/MIIP/PRKDC/MAD2L2/CNOT11/PSMD2/CHMP4C/BRCA1/RBL1/PCN
L3/LEF1/HIP1R/BID/MT3/BCAP31/PRR7/LAMP3/BBC3/NLE1/PMAIP1/BIRC7/LAMTOR5/F3/LCK/V
NIP/KDM1A/PMAIP1/KMT5A/WAS/TAF9/THOC5/PML/PARP1/PSMD10/RPS3/USP1/CBX8/POLQ/FUS/
BP2/CCT4/FLOT1/NHP2/U2AF2/CCT7/SRSF9/NOP58/XPOT/SSB/ZNF593/AKAP8L/TCP1/SFPQ/CPS
P5/SLC9A3R1/DYNLT1/GPSM2/FERMT1/NCKAP1L/CAP1/WNT7B/CCL4/FAM89B/RAB10/CYP26B1/MA
D86/TNFSF9/MIF/CD300A/ZP3/HLA-DPB1/EBI3/CD276/EFNB1/CD3E/SASH3/TNFRSF4/SLC39A10
ASH3/THY1/SIRPA/SOCS1/CTLA4/CD83/PDCD1/SIRPG/NCKAP1L/ADAM8/CCL2/HSPH1/CD209/LYN
CBP4/CASP2/BRSK1/MIIP/PRKDC/MAD2L2/CNOT11/PSMD2/CHMP4C/BRCA1/RBL1/PCNA/CTDSPL/S
/EBI3/CD276/EFNB1/CD3E/PAK1/SASH3/THY1/SIRPA/SOCS1/CTLA4/CD83/PDCD1/SIRPG/NCKAP
1/BAX/LCMT1/PCBP4/CASP2/BRSK1/WDR76/PRKDC/MAD2L2/CNOT11/PEA15/CHMP4C/BRCA1/PCNA
OSC5/RPS3/SAMD4B/RPL27/AGO2/RPL35/RPS20/RPL18A/RPS19/NT5C3B/RPLP0/RPL18/RPL23/R
BYSL/RPL35A/LYAR/RRP36/EIF4A3/ISG20/EXOSC5/SNU13/RPL27/FTSJ3/RPL35/DDX54/RPS19/
L1/CHORDC1/WFS1/PFDN2/TCP1/VBP1/GNB1/DNAJB2/DNAJA4/ERO1A/PFDN1/RUVBL2/DNAJC10/D
/RPL35A/RPS3/RPL27/SSR3/PRKCI/RPL35/RPS20/RPL18A/RPS19/RPLP0/RPL18/ICMT/SGTB/RP
C3/CORO1B/HIP1R/TPPP3/NCKAP1L/TUBB4A/CAPZA1/RANGRF/MKKS/FHOD3/CAMSAP2/HCLS1/ARP
BCAP31/PRR7/LAMP3/BBC3/NLE1/PMAIP1/BIRC7/LAMTOR5/F3/LCK/VIL1/CD27/CASP1/PML/GPX
F1/NELFE/POLR2H/PPIA/ZC3H12A/DYNLT1/LAMP3/TRIM31/SNF8/PLSCR1/NELFB/APOBEC3G/CCL
PMAIP1/PSMB4/PSMB8/PSMB10/GNB1/PSMA5/DNMT3A/PSMD13/PSMC4/PSMD10/PSMD11/ERO1A/PS
F6/NPM1/CHAF1A/SMARCA4/ITGB3BP/NASP/CENPI/SWI5/PSMC4/CENPK/NAP1L4/NAP1L2/PARP1/
E1/JAML/SIRPA/C3AR1/CCL13/TNFRSF11A/PIP5K1C/NCKAP1L/ADAM8/CCL2/PIK3CD/CXCL9/CXC
2R1A/NPM1/CHMP4C/BRCA1/TUBA1A/SFI1/DYNLL1/DYNC1H1/TICRR/CSNK1E/PSMB2/PSME3/HAUS
/RACGAP1/DSCC1/NCAPH/NUP62/SPAG5/MAD2L1/RAN/KIF18A/CHMP4B/ESPL1/LCMT1/NAA10/SGO
RCC3/PARPBP/DDX11/OTUB1/BLM/AUNIP/KDM1A/WAS/SWI5/SFPQ/PML/PARP1/MSH2/POLA1/BARD
HOC5/NUP85/NUP43/AGFG1/THOC6/RAE1/EIF4A3/XPO5/BARD1/BAG3/NUP155/CALR/CPSF4/NUP1
BRCA1/TUBA1A/SFI1/DYNLL1/DYNC1H1/TICRR/CSNK1E/PSMB2/PSME3/HAUS1/BLM/FBXO5/TUBB4
F3/LCK/IFI16/CASP1/PML/RPS3/TFAP4/APP/CTSD/HSPE1/PDCD5/FADD/CLEC7A/RACK1/DAP/MA
K3/SERPINE1/JAML/ZNF580/C3AR1/F2RL1/CCL13/TNFRSF11A/PIP5K1C/NCKAP1L/ADAM8/CCL2/
C3/MARK2/CEP85/GPSM2/TUBB4A/RANGRF/MKKS/PHLDB1/CAMSAP2/FKBP4/RAC1/CHORDC1/STMN3
ZC3H12A/PSME3/HLA-DQA2/PIK3CD/HLA-DRB5/CARD11/PSMB4/BCAR1/PSMB8/PSMB10/LCP2/PSM
PSF1/THOC5/NUP85/NUP43/AGFG1/THOC6/RAE1/CPSF6/EIF4A3/XPO5/BARD1/BAG3/NUP155/CAL
SFPQ/CPSF1/THOC5/NUP85/NUP43/AGFG1/THOC6/RAE1/CPSF6/EIF4A3/XPO5/NUP155/CPSF4/NU
RL1/ARPC2/DYNC1H1/ARPC3/LIMCH1/CORO1B/HIP1R/GPSM2/NCKAP1L/HCLS1/TESK1/ITGB1BP1/
13/PSMC4/SET/PSMD10/PSMD11/PSMD1/PSMA7/EXOSC5/YWHAB/SAMD4B/PSMB3/AGO2/PSMD4/PSM
MB4/PDGFA/VANGL1/PSMB8/PSMB10/PSMA5/PSMD13/PSMC4/AP2M1/DVL2/PFN1/AP2S1/PSMD10/P
/AGO2/RPL35/RPS20/RPL18A/PPP1R15A/RPS19/RPS6KB2/RPLP0/RPL18/RPL23/EIF2B5/RPL38/
SAC3D1/CHMP4B/ESPL1/STIL/RGS14/CENPE/CHD3/PPP2R1A/CHMP4C/MSTO1/DYNC1H1/LZTS2/GP

D14/HLA-DPB1/CLTA/PSMD2/HLA-DQB2/HLA-A/CENPE/ITGB5/DYNLL1/DYNC1H1/PSMB2/PSME3/B
CNA/CCT2/HSP90AB1/E2F8/RFC2/DDX11/FGFR1/CCT4/PNKP/DBF4/CDK2/CCT7/RAC1/TCP1/WAS/

IL1B/PDGFA/TGFB2/NAP1L2/FGFR2/CIT/CDC42/TGFB3/CETN2/RACK1/SUSD2/SFRP2/KDF1/CALM
C3/MARK2/CEP85/GPSM2/TUBB4A/RANGRF/PHLDB1/CAMSAP2/FKBP4/RAC1/CHORDC1/STMN3/RASS
CCL5/CD86/TNFSF9/MIF/CD300A/ZP3/HLA-DPB1/EBI3/CD276/EFNB1/CD3E/SASH3/TNFRSF4/SL
CLTA/PSMD2/HLA-DQB2/HLA-A/CENPE/ITGB5/DYNLL1/DYNC1H1/PSMB2/PSME3/LAG3/HLA-DQA2/
LAMTOR5/CDK5/CTTN/SEMA4B/SLC12A7/SEMA4D/SEMA4F/SCTR/SEMA5B/AKT1S1/SEMA3C/SEMA3E
8/KAT2A/TRIM71/TFAP2A/WNT4/MED12/OVOL2/LUZP1/RARG/ABL1/SFRP2/KDF1/RPS7/IFT172/B
CLTA/PSMD2/HLA-DQB2/HLA-A/CENPE/ITGB5/DYNLL1/DYNC1H1/PSMB2/PSME3/LAG3/HLA-DQA2/
/TUBB4A/CSNK1D/DCTN2/B9D2/HAUS8/NDRG4/RAB10/PLK4/ODF2/VPS18/SYT1/PPFIA3/CETN2/S
/NCKAP1L/CAPZA1/MKKS/FHOD3/HCLS1/PSTPIP1/ARPC5L/RAC1/CTTN/MYADM/WAS/VIL1/PFN1/W
WASF1/IFT80/BAG3/CDC42/APP/ACTL8/AP3D1/MYO5A/COPG1/NDE1/MAPT/BICDL2/TTC26/HSPB1
IP1R/NCKAP1L/CAPZA1/MKKS/FHOD3/HCLS1/ARPC5L/CTTN/MYADM/WAS/VIL1/CIT/PFN1/WASF1/
7/PSMB8/PSMB10/FGR/LCP2/PSMA5/WAS/PSMD13/PSMC4/PSMD10/PSMD11/PSMD1/PSMA7/PSMB3/
EBI3/CD276/EFNB1/CD3E/SASH3/TNFRSF4/IRF1/CTLA4/PLA2G2D/NCKAP1L/SLC11A1/CD209/CA
PSMD10/PSMD11/PSMD1/PSMA7/EXOSC5/YWHAB/SAMD4B/PSMB3/PSMD4/PSMD3/PABPC4/PSMD7/VI
LRC5/CBFB/CD300LF/TAF9/PTPN6/AGPAT2/CASP1/SOCS3/RNF113A/IL1R2/PADI2/MAPK7/CARD1
MAIP1/KMT5A/EHMT2/SSRP1/MTA2/CDK5/MAPK11/TAF9/PML/PSMD10/CHD4/RBBP4/BARD1/TOPBP
/TCP1/SFPQ/PML/NAP1L2/ANAPC11/SMARCB1/RUVBL2/PRKD2/KAT2A/MTF2/RAD21/CCT8/GNL3/C
3/SERPINE1/IL27RA/ZNF580/THY1/C3AR1/F2RL1/NCKAP1L/ADAM8/CCL2/CXCL13/LYN/CCL28/R
JB2/GSK3A/ERO1A/ZBTB17/BAG3/CALR/DNAJB11/HSPE1/PPP1R15A/RACK1/PDIA6/CXXC1/TBL2/
IP1R/NCKAP1L/CAPZA1/MKKS/FHOD3/HCLS1/ARPC5L/CTTN/MYADM/WAS/VIL1/CIT/PFN1/WASF1/
RCB1/RAE1/RPS3/TFAP4/RPL27/POLR2L/NUP155/RPL35/RPS20/NUP107/POLR2K/RPL18A/RPS19
A5/PSMD13/PSMC4/FGFR2/RNF220/ATP6V0C/PSMD10/PSMD11/PSMD1/PSMA7/PSMB3/CTDNEP1/PS
NELFE/POLR2H/PPIA/HDAC2/NELFB/CHMP3/OXTR/SMARCB1/TFAP4/SYDE1/POLR2L/AGO2/POLR2K
BP/NASP/CENPI/SWI5/PSMC4/CENPK/NAP1L4/NAP1L2/PARP1/SET/SMARCB1/SMYD3/CDK7/RBBP4
P2/CCT7/CHRAC1/LIG1/TCP1/PHB/LOX/POLE4/POLA1/PRKD2/ARRB2/PARM1/POLQ/TELO2/UBE2L
/DCTN2/B9D2/HAUS8/NDRG4/RAB10/PLK4/ODF2/VPS18/SYT1/PPFIA3/CETN2/STX4/RALB/CEP25
PPP2R1A/SGO1/NDC1/FBXO5/SMC4/FANCA/NCAPH2/PSMD13/CYP26B1/TUBG2/CALR/TDRKH/MYBL1
AH/BBC3/PMAIP1/ZNF205/SFPQ/LCK/GSK3A/PML/LTBR/TNFSF12/YWHAQ/YWHAB/RPS3/TFAP4/GZ
ACTB/MTA2/KDM5B/NPM3/CENPI/CENPK/KDM5C/SMARCB1/ZMIZ2/PADI2/CHD4/RUVBL2/RBBP4/SM
PML/PARP1/POLE4/POLA1/PRIM2/XRCC6/TELO2/RPA2/CCT8/GNL3/POLD3/RAD51C/PTGES3/NAT1
D13/CD27/PSMC4/MAP3K14/PSMD10/PSMD11/PSMD1/PSMA7/RPS3/PSMB3/AKIP1/CALR/APP/PSMD
0/PSMD11/PSMD1/PSMA7/EXOSC5/YWHAB/SAMD4B/PSMB3/PSMD4/PSMD3/PABPC4/PSMD7/VIM/TRI
NGRF/YWHAH/BBC3/PMAIP1/ITGB1BP1/ACTB/CDK5/VIL1/AP2M1/STAC3/YWHAQ/YWHAB/ACSL3/PR
AB11FIP4/ANXA11/CHMP3/CIT/KLHDC8B/CDC42/CHMP1A/CETN2/CKAP2/CALM2/ACTR3/CSPP1/SP
8/RFC2/DDX11/FGFR1/PNKP/BLM/FBXO5/DBF4/CDK2/LIG1/PRIM1/WDHD1/DONSON/TIPIN/POLE4
AA10/MAD2L2/CENPE/CHMP4C/NSMCE2/SPDL1/XRCC3/TGFA/KNTC1/PPP1R9B/CEP85/FBXO5/DAPK
RPS3/TFAP4/CTSD/HSPE1/PDCD5/FADD/CLEC7A/RACK1/DAP/MAPT/PDCD6/CYCS/MTCH1/HSPD1/P
MB4/PIAS3/PSMB8/TNFSF13B/PSMB10/PSMA5/COMMD7/PSMD13/CD27/PSMC4/CASP1/MAP3K14/PS
/CARD11/GPRC5B/CCDC22/UNC5CL/IL1B/S100A13/TMEM101/CASP1/MAP3K14/SLC44A2/LTBR/PP
5/LSM10/IL1B/DYNC1LI1/ANAPC11/CCND2/USP22/RANBP1/APP/MTBP/CUL4B/DBF4B/MEIS2/RAD
RAE1/EIF4A3/NUP155/CPSF4/NUP107/CETN2/SLBP/HNRNPA3/CHTOP/SEC13/SYMPK/CPSF3/SARN
L1/LCMT1/NAA10/MAD2L2/CENPE/CHMP4C/NSMCE2/SPDL1/XRCC3/TGFA/KNTC1/PPP1R9B/CEP85/
OT9/CDKN2B/CCL2/CDK2/LSM10/KIF14/PTPN6/PML/CDKN2C/CCND2/MTBP/RFWD3/RPA2/CUL4B/B
CAPH/NUP62/SPAG5/MAD2L1/RAN/KIF18A/CHMP4B/ESPL1/LCMT1/NAA10/MAD2L2/CENPE/PPP2R1
PML/PARP1/POLE4/POLA1/PRIM2/XRCC6/TELO2/RPA2/CCT8/GNL3/POLD3/RAD51C/PTGES3/NAT1
3/PSMC4/AP2M1/RAB10/DVL2/PFN1/AP2S1/PSMD10/PSMD11/PSMD1/PSMA7/PSMB3/ARRB2/CDC42
LRC5/CBFB/CD300LF/PTPN6/AGPAT2/CASP1/SOCS3/RNF113A/IL1R2/PADI2/CARD16/IKBKG/AXL
S/FHOD3/HCLS1/ARPC5L/CTTN/MYADM/WAS/VIL1/PFN1/WASF1/CAPZB/GRB2/ARHGAP18/CDC42EP
BLM/CNOT9/CDK2/THOC5/PML/DONSON/TIPIN/MSH2/TOPBP1/BABAM1/RFWD3/RAD9A/RPA2/NAE1/
PSMC4/AP2M1/DVL2/PFN1/IFT80/AP2S1/PSMD10/PSMD11/PSMD1/PSMA7/PSMB3/ARRB2/CDC42/A
3E/SASH3/IRF1/CTLA4/PLA2G2D/NCKAP1L/CD209/CARD11/IL1B/IL2RA/TNFSF13B/PTPN6/IL12

/BID/CNOT9/CDKN2B/CCL2/CDK2/LSM10/KIF14/PTPN6/PML/CDKN2C/CCND2/MTBP/RFWD3/RPA2/
P31/NOC2L/NONO/BBC3/KDM1A/PMAIP1/WFS1/TAF9/SFPQ/LCK/PARP1/GPX1/RPS3/DNM1L/MAPK7
2/BRSK1/PRKDC/MAD2L2/CNOT11/CHMP4C/BRCA1/PCNA/SPDL1/XRCC3/TICRR/KNTC1/E2F8/E2F4
4/FANCA/NCAPH2/PSMD13/CYP26B1/CALR/TDRKH/MYBL1/RAD21/MSX1/WNT4/RAD51C/HSPA2/RAD
/DNAJB6/NUP107/IER5/AKT1S1/DNAJC7/RBBP7/RPA2/MAPT/PDCD6/SEC13/PTGES3/HSPA2/VCP/
/NCKAP1L/CD209/CARD11/IL1B/IL2RA/TNFSF13B/IL12RB1/IL15/RPS3/CD6/PNP/FADD/PELI1/
4/PDCD5/TOMM40L/TIMM50/ROMO1/MAPT/HSPD1/TIMM13/MFF/BAD/HSP90AA1/CASP8/SH3GLB1/T
IPIN/MSH2/BABAM1/RFWD3/RAD9A/RPA2/PRPF19/BTG2/PIDD1/DOT1L/EME2/CHEK2/CLSPN/RAD1
39A10/NCKAP1L/CD209/CARD11/IL1B/IL2RA/TNFSF13B/IL12RB1/IL15/RPS3/CD6/PNP/FADD/P
/BCL3/CNOT9/CDK2/KDM1A/PMAIP1/KMT5A/PML/PSMD10/TFAP4/GRB2/BABAM1/MSX1/PTTG1IP/P
3/F3/CCR1/TUBB2B/C5AR1/PRKD2/CALR/XCL2/CMKLR1/SWAP70/STX4/LBP/CCL3/S100A7/CXCL1
UP107/SLBP/PRPF31/CHTOP/SEC13/SYMPK/CPSF3/RPS15/NOL6/LTV1/SARNP/MAGOH/THOC2/EIF
12/OVOL2/LUZP1/RARG/ABL1/SFRP2/RPS7/IFT172/BCL10/LMO4/MTHFR/KIF20B/TWIST1/CC2D2
9/CXCL3/CXCL13/RAC1/IL1B/CCL4/TGFB2/C5AR1/PF4V1/CXCL11/XCL2/CMKLR1/WDR1/LBP/CCL
N/CHMP4B/ESPL1/STIL/CENPE/CHMP4C/MSTO1/DYNC1H1/SPDL1/DYNLT1/GPSM2/KPNB1/DCTN2/P
L2/LUZP1/RARG/ABL1/SFRP2/RPS7/IFT172/BCL10/LMO4/MTHFR/KIF20B/TWIST1/CC2D2A/STK3
155/CPSF4/NUP107/SLBP/KHDRBS1/CHTOP/SEC13/SYMPK/CPSF3/RPS15/NOL6/LTV1/SARNP/MAG
39A10/NCKAP1L/CD209/LYN/CARD11/IL1B/IL2RA/TNFSF13B/IL12RB1/IL15/RPS3/CD6/PNP/FA
18/SGTB/RPL23/RPL38/RPL23A/RPS7/RPL7A/RPS23/RPS10/RPS15/SGTA/RPS16/RPL5/RPS27A/
UP107/SLBP/CHTOP/SEC13/SYMPK/CPSF3/RPS15/NOL6/LTV1/SARNP/MAGOH/THOC2/EIF5A/POLR
5/TOMM40L/TIMM50/ROMO1/MAPT/HSPD1/TIMM13/MFF/BAD/HSP90AA1/CASP8/SH3GLB1/TOMM22/
/PARP1/SET/SMARCB1/ZNF207/MTF2/RAD21/NAT10/NBN/HNRNPU/ERCC1/EXOSC10/PIH1D1/HNRN
2S1/PSMD10/PSMD11/PSMD1/PSMA7/PSMB3/ARRB2/CDC42/PSMD4/PSMD3/PSMD7/WDR1/MED12/PS
XCL13/RAC1/ITGA2/PLA2G7/CCL4/GPSM3/CCR1/C5AR1/CALR/XCL2/CMKLR1/APP/SWAP70/FADD/
2S1/PSMD10/PSMD11/PSMD1/PSMA7/PSMB3/ARRB2/CDC42/PSMD4/PSMD3/PSMD7/WDR1/MED12/PS
AS/SWI5/SFPQ/PARP1/MCM8/POLQ/FUS/RFWD3/NABP1/RPA2/PPP4C/TIMELESS/RAD51C/RAD51D/
GL1/CDC42/PRKCI/ACTL8/RACK1/NDE1/NSFL1C/WWC1/HSP90AA1/MPP7/PTK2/DCTN1/ARHGEF11/
GB3BP/SMC4/NASP/AKAP8L/NCAPH2/CENPI/BANF1/CENPK/NAP1L4/NAP1L2/SET/SMYD3/RBBP4/C
5/SFPQ/PARP1/MCM8/POLQ/FUS/RFWD3/NABP1/RPA2/PPP4C/TIMELESS/RAD51C/RAD51D/WRAP53
G3/HSF2/PSMD4/PSMD3/RBX1/MAPK7/PSMD7/PSMD8/VHL/PSMA1/PSMB5/PSMA4/PSMD9/NOTCH1/R
/RPS7/RPL7A/RPS23/RPS10/RPS15/RPS16/RPL5/RPS27A/RPS12/RPS4X/RPL6/EXOSC10/MAGOH/
PSMD3/RBX1/MAPK7/PSMD7/PSMD8/VHL/PSMA1/PSMB5/PSMA4/PSMD9/NOTCH1/RPS27A/VEGFA/HS
IL1B/CCL4/TGFB2/C5AR1/PF4V1/CXCL11/XCL2/CMKLR1/LBP/CCL3/S100A7/CXCL17/AKIRIN1
X1/CUL4B/DNA2/CHD1L/DQX1/RPS27A/NBN/RAD18/UBA52/ACTR5/RFC5/DHX9/GTF2H1/RPA1/SMA
/CD27/SAMSN1/MSH2/BTK/PELI1/GPR183/EXOSC3/FCGR2B/TNFAIP3/BAD/CD38/INHA/TP53BP1/
CL4/TGFB2/C5AR1/PF4V1/CXCL11/XCL2/WDR1/LBP/CCL3
CI/STX4/RACK1/CLIP3/STAC/STX7/CSK/VAMP8/TMBIM1/BCL2L1/RAB11A
PSF3/SARNP/MAGOH/THOC2/EIF5A/POLR2D/RBM15B/SEH1L/THOC1/DHX38/ENY2/PABPN1/SRSF1/
F207/KATNB1/NDE1/CLIP3/DRG1/MAPT/CKAP2/CKAP5/MZT1/ABL1/TBCD/STMN2/CCSAP/DCTN1/M
PSF3/SARNP/MAGOH/THOC2/EIF5A/POLR2D/RBM15B/SEH1L/THOC1/DHX38/ENY2/PABPN1/SRSF1/
3/RPS10/RPS15/SGTA/RPS16/RPL5/RPS27A/RPS12/RPS4X/RPL6/RPL37/RPL27A/HSPA5/RPL36/
PSMD11/PSMD1/PSMA7/PSMB3/ARRB2/CDC42/PSMD4/PSMD3/PSMD7/MED12/PSMD8/ABL1/PSMA1/S
D4/PSMD3/PSMD7/IKBKG/PSMD8/PSMA1/PSMB5/BCL10/PSMA4/PSMD9/PAK2/MUC6/MUC15/PSMC3/
/NAP1L4/NAP1L2/SET/SMARCB1/SMYD3/PADI2/RBBP4/SMARCC1/CABIN1/HELLS/CENPN/RBBP7/S
YD3/RBBP4/SMARCC1/CABIN1/CENPN/RBBP7/CENPQ/HAT1/ANP32E/MIS18A/DAXX/RNF8/SART3/A
FWD3/RPA2/BTN2A2/BTG2/PIDD1/SUSD2/CHEK2/CDK2AP2/PLK3
TOML2/RFC5/DHX9/FAF1/EREG/SMARCAL1/MSH6/ATG7/ATR/ATAD5/TTF1/UCN/CCDC88A/SLFN11/
NC1H1/GPSM2/KPNB1/DCTN2/POC1A/MAP4/TUBG2/RAE1/KIF2A/PTPA/ZNF207/CHMP1A/WRAP73/D
T5A/PML/PSMD10/TFAP4/MSX1/PTTG1IP/BTG2/PIDD1/CHEK2/NBN/PLK3/MYO6
D4/PSMD3/PSMD7/IKBKG/PSMD8/PSMA1/PSMB5/BCL10/PSMA4/PSMD9/PAK2/MUC6/MUC15/PSMC3
O/RPS15/SGTA/RPS16/RPL5/RPS27A/RPS12/RPS4X/RPL6/RPL37/RPL27A/HSPA5/RPL36/UBA52/

PSMD1/PSMA7/PSMB3/ARRB2/CDC42/PSMD4/PSMD3/PSMD7/MED12/PSMD8/ABL1/PSMA1/SFRP2/PS
5/RPL23/MYBBP1A/BTG2/PIDD1/CHEK2/PLK3/CRLF3/PPP2R5B/CDK5R1/FAP/CNOT10/CNOT3/TFD
AG3/HLA-DQA2/CTSE/CAPZA1/DCTN2/HLA-DRB5/DCTN5/CTSS/KIF15/AP2M1/DYNC1LI1/AP2S1/K
7/FGR/CDH17
R1/PF4V1/CXCL11/XCL2
RAD9A/RPA2/NAE1/BTG2/PIDD1/EME2/CHEK2/CLSPN/NBN/PLK3
8/KIF2A/RPS3/ZNF207/CHMP1A/WRAP73/RNF4/HAUS6/DRG1/MZT1/CHEK2/CCSAP/HAUS7/HNRNPU
PDL1/XRCC3/DDX11/FBXO5/KNSTRN/SFPQ/DYNC1LI1/ANAPC11/CDC42/ZNF207
FWD3/RPA2/BTN2A2/BTG2/PIDD1/CHEK2/CDK2AP2/PLK3
AG3/HLA-DQA2/CTSE/CAPZA1/DCTN2/HLA-DRB5/DCTN5/CTSS/KIF15/AP2M1/DYNC1LI1/AP2S1/K
RPL5/RPS27A/RPS12/RPS4X/RPL6/RPL37/RPL27A/RPL36/UBA52/RPL19/RPS11/RPL13A/RPL37A
T172/BCL10/LMO4/MTHFR/KIF20B/TWIST1/CC2D2A/GLMN
CTSE/CAPZA1/DCTN2/HLA-DRB5/DCTN5/CTSS/KIF15/AP2M1/DYNC1LI1/AP2S1/KIF2A/CAPZB/CT
REBF1/TIMM10/AIP/NDUFA13/VPS11/ATG13/HAX1/CSNK2A2/PARL/TIMM23/TIMM8B/HUWE1/PITR
PSMD3/RBX1/PSMD7/RAD21/BABAM1/NAE1/PSMD8/PSMA1/CLSPN/PSMB5/PSMA4/PSMD9/NBN/ZFYV

36/HSPA14/ETF1/RPL19/EIF3E/RPL13A/RPLP1/DHX9/RPL32/RPL30/RBM24/DPH3/EIF3G/RPL29
/PSMD7/IKBKG/SEC13/PSMD8/PSMA1/VAMP8/PSMB5/PSMA4/PSMD9/CANX/HLA-F/PSMC3/TAPBP/T
RPS27A/RPS12/RPS4X/RPL6/RPL37/RPL27A/RPL36/UBA52/RPL19/RPS11/RPL13A/RPL37A/RPLP
C1A/BRK1/ARPC5/ACTR3/SPIRE1
TMEM216/CEP41/HSP90AA1/DCTN1/CEP164/HAUS7/MARK4/HAUS5/DYNC1I2/RAB11A/NEDD1/CENP

IL12RB1/IL15/RPS3/CD6/PNP/FADD/RASAL3
P90AA1/PTK2/PTPN1/VEGFA/PAK2
/BTC/EREG/EGF/DAB2IP/SNX5/STAM/MVB12B/NEURL1/PDE6G/RNF126/RNF115/RAB7A/CHMP6/EP
TA3/CCND3/ADAM17/GLI1/RAB11A/MECP2/STXBP4/ANAPC5/UBE2E2/RPTOR/ATAD5/PAGR1/ANAPC

EK2/CLSPN/NBN/PLK3
RP2/THBS1/CSNK2A1/VEGFA/PAK2/PIH1D1/PRDX5
PTPN1/VDAC2/TXNDC12/IVNS1ABP/BCL2L1/NDUFA13

TAPBP/COG4/COPB1/BET1L/COPA/HTT/LMAN2/KLC1/RAB1A/ERGIC2/ATP9A/KIF3A/RAB1B
/AP2B1/RBPJ/PSEN2/EPHA2/AP2A1/EPHB2/EPHA10

7/RAD21/NAE1/PSMD8/PSMA1/CLSPN/PSMB5/PSMA4/PSMD9/NBN/ZFYVE19/GPR132/PSMC3/PSMC5

B3/PSMD4/PSMD3/PSMD7/PSMD8/PSMA1/PSMB5/PSMA4/PSMD9/RPS27A/UBA52/PSMC3/PSMC5/ANA

/CDK5R1/FAP/CNOT10/CNOT3/TFDP1/DAB2IP/HMGA2
VEGFA/PAK2/PIH1D1/PRDX5
R3/HSP90AA1/PTK2/PLD2/WIPF2/RAPGEF1/ELMO2/CD247/CD3G/PTPRC/CLEC4E/PIK3CB/MYO1C/
R2B/CSK/ACTR3/HSP90AA1/PTK2/PLD2/WIPF2/RAPGEF1/ELMO2/CD247/CD3G/PTPRC/PIK3CB/MY

C13/PTGES3/BAG2/HSP90AA1/HIKESHI/DNAJC2/CCAR2/RPA1/SEH1L/ATR/RPTOR
KAPK5/RPA1/ATR/SMG7/DCP2/HNRNPC/MAPK15/TERF1/HNRNPA2B1/XRCC5/PARN/SLX4/TNKS1BP1

LLS/CENPN/RBBP7/CENPQ/HAT1/MIS18A/DAXX/SART3/ANP32B/SMARCA5/POLE3/IPO4/HMGA2/PA
OA

PSMD8/PSMA1/VAMP8/PSMB5/PSMA4/PSMD9/HLA-F/PSMC3/TAPBP/TAP2/PSMC5/PDIA3/PSMB1/PS
RFC5/POLE3/RPA1/MSH6/POLE2/ATAD5

RAB11A/MECP2
OAA1/PTK2/PLD2/WIPF2/RAPGEF1/ELMO2/CD247/CD3G/PTPRC/PIK3CB/MYO1C/NCKAP1/PLCG1
SMG7/DCP2/HNRNPC/MAPK15/TERF1/XRCC5/TEP1/PARN/TNKS1BP1
PC/MYC/MAPK15/TERF1/HNRNPA2B1
OAA1/PTK2/PLD2/WIPF2/RAPGEF1/ELMO2/CD247/CD3G/PTPRC/PIK3CB/MYO1C/NCKAP1/PLCG1
6/SURF6/DHX30/AAMP/NIP7/RPL24/RPL11/RPL10/WDR12/HEATR3/MRPL20/RPL14/RPL10A/ZNHI
DIA4/NXN

AMP8/PSMB5/PSMA4/PSMD9/HLA-F/PSMC3/TAPBP/TAP2/PSMC5/PDIA3/PSMB1/PSMB6/PSMA3/HLA

S18A/ACTR5/RNF8/SMARCA5/DMAP1/POLE3
/TP53BP1/HMGA2/ATAD5/THOC1/TFIP11/TRIM32/ING2/DYRK3/TMEM161A/HELB/ACKR3

IM27/IRF4/PDE4B/CD4/ANXA1/CCR2
PL24/RPL11/RPL10/ERAL1/MRPL20/RPL12/MRPS2/RPS28/C1QBP/MRPS11

GRE2

8A/DAXX/SART3/ANP32B/SMARCA5/POLE3/IPO4/GRWD1
DCP2/HNRNPC/MAPK15/TERF1

Q/DOK2/ARHGEF39/TIMP2/WASF2/TRIM28/RHOC/PLEKHG2/RAB6B/VAV1/RAB32/ARFGEF3/DOK1/G
T2/LEF1/NELFE/RPS6KA4/FBL/MT3/OTUB1/NOC2L/HDAC2/CDK2/KDM1A/KMT5A/HCFC1/EHMT2/AK
2/SCRIB/RHOG/TBC1D16/RANGAP1/S100A10/RABEP2/CCL5/VAV1/CCL26/IQGAP1/EFNA5/RGS14/
1/CDC25C/GDF15/IRAK1/CDKN2A/S1PR2/CD74/PKN1/DUSP5/PRKCD/ROBO1/HSPB1/NCK2/SLAMF8
NA/SNAP25/ARHGEF2/P2RX4/ROBO1/SERPINI1/PLXNA3/SMARCD3/HES6/NME1/NBL1/ITPKA/NAP1
/ITPKA/NAP1L1/EZH2/NME2/TWF2/ASPM/ANKRD1/TRPV2/GRN/TIMP2/TCF3/P3H1/CXCR4/IQGAP1
1A2/CLCF1/C1QB/SUSD4/BIRC3/ITGB2/IRAK1/C1QC/CD74/CMTM3/PKN1/PTAFR/PRKCD/LAT2/C1
C1/PRKCD/ROBO1/ADORA1/PRNP/ADAM9/HRAS/SYK/STK39/IL18/EZH2/DLG3/CSF1R/MAGED1/NCF
C51B/NPC1/GALNT18/B3GNT7/GFPT2/CHPF2/MAN1B1/PLOD3/B4GALT3/B3GALNT1/ST3GAL4/SOAT
2K2/PAK1/PRKX/HSP90AB1/DYRK2/CSNK1E/MARK2/DDIT4/RPS6KA4/CNOT9/MBOAT4/CSNK1D/CDK
PK2/VAV1/COLEC12/CD300A/UNC93B1/PRKDC/PSMD2/PAK1/MNDA/IRF1/IRF3/F2RL1/RAB7B/LRR
DFI/S100A10/DDIT3/DTNBP1/TRAF2/HMGB2/TRIM6/HMOX1/TRIM28/BAX/MAP2/FKBP1A/GMNN/MA
P/ABCB6/LAMA5/CNFN/EDA/PALLD/HDAC1/KRT18/FERMT1/COL5A2/CAPN1/HDAC2/KRT8/ASAH1/J
SMD2/PAK1/MNDA/IRF1/IRF3/F2RL1/RAB7B/LRRC14/NPLOC4/PSMB2/IRF7/PSME3/TNIP2/FLOT1
/DUOX2/IRF3/F2RL1/STAT1/NPC2/NPLOC4/POLR3K/ODC1/DDIT4/AGBL5/IRF7/PIM2/ZC3H12A/B
/LAMA5/RAB3B/CNPY4/KRT18/SLC9A3R1/FLOT1/RANGRF/ITGB1BP1/EHD2/ACTB/RAP2A/CDK5/MY
YNLT1/LAMP3/TRIM31/SNF8/PLSCR1/NELFB/APOBEC3G/CCL4/CHMP3/IFI16/BANF1/PML/SMARCB
CBP2/BCAP31/UBE2J2/NPEPPS/EHD1/HCLS1/ITGB1BP1/IL1B/EHD2/CDK5/UBE2L3/FGR/GSK3A/C
ATF4/PMAIP1/PSMB4/PSMB8/PSMB10/GNB1/PSMA5/DNMT3A/PSMD13/PSMC4/PSMD10/PSMD11/ERO
S/ADAM8/ATF4/PMAIP1/PSMB4/PSMB8/PSMB10/GNB1/PSMA5/DNMT3A/PSMD13/PSMC4/PSMD10/PS
/ZSWIM5/CORO1B/FGFR1/NCKAP1L/CCL2/CXCL13/LYN/RAC1/ITGA2/PLA2G7/CCL4/GPSM3/F3/CC

ROCC/CAPZB/RALA/CDC42/TGFB3/ZMYND8/CDC42EP2/WRAP73/BRK1/AKIRIN1/PALM/ARAP1/TBC1
5/MSX1/TAF10/DOT1L
RP2/RPS7/IFT172/BCL10/LMO4/MTHFR/KIF20B/TWIST1/CC2D2A/STK3/GLMN
/DQX1/RPS27A/NBN/RAD18/UBA52/ACTR5/RFC5/DHX9/GTF2H1/RPA1

/SWAP70/LBP/CCL3/S100A7/CXCL17/AKIRIN1/CYP19A1/SLC8B1/THBS1/ADAM17
CKAP2/CKAP5/STMN2/CCSAP/MAP1A/ADD3/LIMA1/DSTN/PLEKHH2/GAK/MTPN/SPTAN1/CAMSAP1/C
EGF/DAB2IP/SNX5/STAM/MVB12B/NEURL1/PDE6G/RNF126/RNF115/RAB7A/CHMP6/EPN1/AFAP1L2
AKIRIN1/THBS1/ADAM17/VEGFA

/EGLN2/UBA52/PSMC3/PSMC5/RBPJ

R2/SNUPN/SMN1/USP4

7/RUVBL1/MAD2L2/PSMD2/FZD1/PYGO2/BCL9/PPP2R1A/IGFBP6/EDA/MLLT3/SMARCA4/ARHGEF19
HARPIN/GNA12/ISG15/PDCL3/DDA1/HSP90AB1/NPLOC4/CSNK1E/MAN1B1/PSMB2/PSME3/HSPBP1/
GIPC1/DDIT3/CTSK/RBCK1/PSMD14/FBXL19/CHMP4B/VPS37C/MSN/CLTA/UBE2Z/PSMD2/NUPR1/R
M20/TOMM34/SHARPIN/GDAP1/SLC35F6/TIMM17B/BMF/NOL3/MSTO1/LMNA/MYO19/GGCT/NDUFAF2
A10/FSCN1/CARMIL1/WASF2/RHOC/PLEKHG2/CCL26/PHPT1/ARPC4/SH3PXD2B/SLC9A1/EFNA5/PA
100A10/TFRC/HLA-DPA1/HLA-DMB/TNFRSF18/CARMIL1/JAK3/CCL5/CD86/APBB1IP/VAV1/ITGA5
K1/PRKX/HSP90AB1/DYRK2/CSNK1E/MARK2/DDIT4/RPS6KA4/CNOT9/CSNK1D/CDK2/HCLS1/AXIN1
1/IRF3/F2RL1/RAB7B/LRRC14/NPLOC4/PSMB2/IRF7/PSME3/TNIP2/FLOT1/SMPDL3B/CD209/LYN
83/TFE3/PRELID1/LEF1/IRF7/ZC3H12A/LAG3/NCKAP1L/ADAM8/EVI2B/HCLS1/FANCA/MITF/LYN
EF1/ZC3H12A/BCL3/LAG3/NCKAP1L/JAG2/ADAM8/PRR7/PIK3CD/KDELR1/FANCA/CARD11/CBFB/I
/MRPS34/TRMT112/VIL1/MRPL13/NES/KIF2A/CAPZB/SWAP70/SH3GL1/KATNB1/GADD45GIP1/WDR
A/CD27/CCR1/IL12RB1/IL15/IL4R/PNP/LILRB4/AP3D1/AXL/FADD/BTK/BTN2A2/SART1/TOX/BA
MD11/PSMD1/PSMA7/PSMB3/CTDNEP1/PSMD4/TRPM4/PSMD3/PSMD7/TBL1XR1/CSNK1G2/JRK/PSMD
2A/TRIM71/WNT4/MED12/OVOL2/LUZP1/RARG/ABL1/SFRP2/RPS7/IFT172/BCL10/LMO4/NOTCH1/
7A/RACK1/DAP/MAPT/PDCD6/CYCS/MTCH1/HSPD1/PIDD1/VCP/TRADD/BAD/BCL10/TNFSF15/CASP
/WNT4/LFNG/RAD1/ACVR1B/NOTCH1
A/CHMP1A/RANBP1/CETN2/CEP250/HAUS6/PARD6G/NDE1/CKAP5/NSFL1C/DCTN1/HAUS7/FBXW5/M
/WASF1/RPS3/GRB2/CDC42EP2/GMFG/ABI2/DRG1/MAPT/ARPC1A/BRK1/ARPC5/ACTR3/HSP90AA1/
6/CYCS/MTCH1/HSPD1/PIDD1/VCP/TRADD/BAD/BCL10/TNFSF15/CASP8/PDCD2/PPM1F/NDUFA13/
/KAT2A/CHMP1A/RANBP1/CETN2/CEP250/HAUS6/PARD6G/NDE1/CKAP5/NSFL1C/DCTN1/HAUS7/FB
E2L6/TRIM25/POLR3D/POLR3H/POLR3C/HSPD1/TNFAIP3/RNF216/MAVS/IRF5/TLR2/TRIM21/NMI
HAB/ACSL3/PRKCI/GZMB/PDCD5/STX4/RACK1/CLIP3/CEMIP/MFF/STAC/BAD/CASP8/RAB11A/CAC
E2L6/TRIM25/POLR3D/POLR3H/POLR3C/HSPD1/TNFAIP3/RNF216/MAVS/IRF5/TLR2/TRIM21/NMI
L/FADD/BTK/HCAR2
BAD/HSP90AA1/CASP8/TMEM11/TOMM22/BNIP3L/RCC1L/BCL2L1/TIMM10/NDUFA13/YWHAG/TFDP1
IF20B/TWIST1/CC2D2A/GLMN

1
K5R1

XM1/YBX3/EIF5A2/CCL5/DPH2/CNOT11/MAPK3/HSPA8/EIF3B/RPL36A/MRPL17/HM13/NPM1/UPF3
M6/DTX3/TRIM28/FKBP1A/UBE2M/UBE2Z/PRKDC/MAD2L2/PSMD2/HIF1A/TRIM11/RNF34/UBE2Q2/
HARPIN/GNA12/ISG15/PDCL3/DDA1/HSP90AB1/NPLOC4/CSNK1E/MAN1B1/PSMB2/PSME3/HSPBP1/
HMP4B/VPS37C/UBE2Z/PSMD2/NUPR1/RHBDF1/RNF34/UBE2I/RNF19B/SHARPIN/HM13/GNA12/ISG
7/RUVBL1/MAD2L2/PSMD2/FZD1/PYGO2/BCL9/PPP2R1A/IGFBP6/EDA/MLLT3/SMARCA4/ARHGEF19
DA/GNPDA1/TYMS/GPC4/GPC3/UCK2/AGRN/SDC3/HK2/PFKFB4/VCAN/PIGU/B3GAT3/UPP1/NME1/G
15/IRAK1/ZDHHC13/S1PR2/CD74/PKN1/DUSP5/P2RX4/ROBO1/NCK2/ADORA1/ADAM9/HRAS/SYK/P
T5/GBGT1/FUT4/B3GNT9/SLC51B/NPC1/GALNT18/B3GNT7/GFPT2/CHPF2/MAN1B1/PLOD3/B4GALT
A1/MAPK3/MICALL1/MAP2K2/PPP2R1A/ATP8B2/BAIAP2L1/CHMP4C/CORO1C/STX6/HGS/BIN1/LMN
G15/CHMP4C/TNFRSF4/ITGB5/PPIH/TRIM59/NPC1/HSP90AB1/PABPC1/NUP93/NUP205/NUP37/ND
JMJD6/CD3E/TCIRG1/SASH3/TPD52/SOCS1/IRF1/CTLA4/CD83/PLA2G2D/PRELID1/LEF1/ZC3H12
2/GBP5/CARD9/PSMB9/CTSK/PSMD14/HMGB2/TRIM6/CCL5/MAPKAPK2/VAV1/COLEC12/CD300A/ZP
SNK1E/E2F4/TMEM237/SNX10/CEP89/CCNO/UNC119B/EHD1/HAUS1/SPAG1/RSPH1/TUBB4A/CSNK1
DNAJC5/CASP2/UBE2M/SLC9A1/HIF1A/NUPR1/FZD1/XRCC2/HSP90AB1/HYOU1/DDIT4/BID/MT3/A
RHOG/CFL1/RHOQ/S100A10/FSCN1/CARMIL1/WASF2/RHOC/PLEKHG2/CCL26/PHPT1/ARPC4/SH3PX
SNK1E/E2F4/TMEM237/SNX10/CEP89/CCNO/UNC119B/EHD1/HAUS1/SPAG1/RSPH1/TUBB4A/CSNK1

PK3/ADGRG1/BIN1/HDAC1/RELN/S100B/MBOAT7/LEF1/MYRF/MT3/CCL2/HDAC2/LYN/CNTNAP1/MX

SF9/GBP2/PSMD2/MAPK3/SHARPIN/TNFRSF4/BRCA1/EDA/NOL3/CD58/F2RL1/STAT1/CCL13/TNFR
M1/IRF3/F2RL1/STAT1/HDAC1/S100B/CARD19/PIM2/TGM2/ZC3H12A/EEF1D/TNIP2/BCL3/CPNE1
TF4/KIF14/CLN3/ATN1/CDK5/WFS1/TGFB2/CIT/PARP1/SET/NSMF/NES/C5AR1/MSH2/ARRB2/PRK
C1/ZC3H12A/TNIP2/CD68/CCL2/CXCL9/CXCL3/CXCL13/LYN/IL1B/CASP1/PF4V1/SBNO2/CD6/CX
CA1/BCL2L12/NOL3/LMNA/HDAC1/DDIAS/HYOU1/PRELID1/PSME3/BID/NOC2L/NONO/NLE1/KDM1A

/CD6/APP/CLEC7A/CCT8/HVCN1/SPA17/VDAC2/CDK5R1/ROBO3/DOCK2/CCL19/CD5/EPHB2/CNR1/
G20/APOBEC3D/TNIP1/TRIM25/SRPK1/NOTCH1/MAVS/OAS3/TRIM21/TARBP2/VPS37B/PPIE/PPIB
/APP/SLBP/EXOSC3/SUPT5H/CHTOP/SYMPK/CPSF3/SARNP/EXOSC9/EXOSC10/MAGOH/EXOSC7/THO
DROSHA/KAT2A/IL4R/PNP/LILRB4/AP3D1/BTN2A2/ABL1/SART1/HLA-DOA/BAD/PTPN2/NKAP/RHO
1/RHOT2/SLC25A5/MFF/BAD/VPS35/CASP8/BNIP3L/BCL2L1/YWHAG/IFI6/CCAR2/GCLM/TFDP1/T
/VPS37B/PPIE/POLR2I/PPIB/POLR2J/POLR2D/DHX9/KPNA6/NELFA/POLR2E/CAV2/SUPT4H1/NEL
RHGDIA/SEMA3E/ABL1/ARAP1/PTPN1/PDLIM4/CD2AP/RHOA/MINK1/PARVB/NOTCH2/RHOBTB2/HAX
/SRF/FNBP1L/DPYSL3/EPS8L1/CCL19/HTT/ATP7A/DZIP1/NCKAP1/PLEKHM1/CCDC88A/NEURL1/F
IF20B/TWIST1/CC2D2A/GLMN
TLR7

HARPIN/GNA12/PDCL3/DDA1/HSP90AB1/NPLOC4/CSNK1E/MAN1B1/PSMB2/PSME3/HSPBP1/BCAP31
KA/CTSV/WFDC2/ADA/GNPDA1/CLGN/CDC25C/PAQR5/BIRC3/CEP131/PI3/CDK16/RACGAP1/NCAPH
TK/GAL3ST1/TGFB1/FAM50A/AURKA/STC2/CTSV/WFDC2/ADA/HAVCR2/CDC25C/PAQR5/BIRC3/CEP
TK/GAL3ST1/TGFB1/FAM50A/AURKA/STC2/CTSV/WFDC2/ADA/HAVCR2/CDC25C/PAQR5/BIRC3/CEP
C1/DTNBP1/SAE1/HMOX1/TRIM28/NUTF2/MAP2/CD300A/MTCL1/MSN/STXBP2/MAPK3/OAZ1/LCP1/

/IQGAP1/RGS14/MAPK3/MAP2K2/PPP2R1A/PAK1/PEA15/SPRED1/FPR1/HGS/TGFA/TNFRSF11A/FG

3B/PSMB2/ZNF703/PPP1CA/LEF1/SFRP5/PSME3/SLC9A3R1/DVL3/FERMT1/PLEKHA4/FRAT2/DAPK

F1R/EZR/ANKRD1/VTCN1/RAN/ANXA13/CCT6A/MICALL2/CCT3/SAE1/TRIM6/TRIM28/NUTF2/PHPT
E2M/HIF1A/NUPR1/FZD1/XRCC2/HSP90AB1/HYOU1/DDIT4/MT3/ADAM8/NONO/CCL2/ATF4/KIF14/
F703/PPP1CA/SFRP5/PSME3/SLC9A3R1/DVL3/FERMT1/PLEKHA4/DAPK3/CSNK1D/AXIN1/NLE1/GP

RL1/STAT1/HDAC1/S100B/CARD19/PIM2/TGM2/ZC3H12A/EEF1D/TNIP2/CPNE1/SLC35B2/CARD11
ZTS2/GDI1/MT3/CPNE1/WNT7B/TNC/CDK5/KDM5B/CTTN/RNF157/FGFR2/WASF1/SEMA4B/SEMA4D/
/WFS1/TGFB2/PARP1/SET/NSMF/NES/C5AR1/MSH2/ARRB2/PRKCI/TGFB3/AXL/AKT1S1/NAE1/TFA
LDOC1/PAM/ARHGDIB/FKBP4/ITGA2/DAZAP1/IL1B/MAGED2/ADRA2C/CALCA/PTGFR/BYSL/OXTR/V
IM59/NPC1/PPIA/ZC3H12A/DYNLT1/RPSA/TRIM31/KPNB1/CD209/ITGA2/TCP1/THOC5/XPR1/CHM
/LIMCH1/CORO1B/HIP1R/NCKAP1L/TESK1/ITGB1BP1/ARPC5L/RAC1/CD47/CTTN/WAS/VIL1/PFN1
PIAS3/CDK5/WFS1/UBE2L3/DNAJB2/GSK3A/VPS28/UBE2D1/PSMD10/RPS3/ARRB2/DCUN1D5/DNM1
/CAPZB/RALA/CDC42/TGFB3/ZMYND8/CDC42EP2/WRAP73/BRK1/AKIRIN1/PALM/ARAP1/TBC1D7/D
A/ITGA2/CDK5/TXN/PLXND1/PARP1/APP/MED25/RALB/STPG1/MFNG/LFNG/ABL1/SPPL3/NVL/CTB
I3/RAB3A/RCC1L/VPS11/STXBP1/TAPBP/TAP2/GOSR2/SNAPIN/MFN1/BET1L/NAPG/ATG7/SAMD9L
5/THRA/VEGFA/SMAP1/SRF/INHA/BPGM/KITLG/MAEA/LDB1/PKNOX1/RPS14/INPP5D/ZNF16/ADD1
CD47/PTPN6/ARRB2/APP/AXL/FADD/CLEC7A/LBP/CCL3/TNFAIP3/NFATC4/CD33/BCL10/THBS1/M
6/CXXC1/TBL2/ATP6V0D1/HSPA2/VCP/SRPRB/WIPI1/TATDN2/PTPN2/DCTN1/PTPN1/OPTN/COPS5

6/MUC15/POMGNT2/SLC35C2/XXYLT1/SDF2L1/EOGT/GCNT1/B3GNT8/ST3GAL2/SDF2/TMTC2/ST6G
/CCRL2
/CCRL2

7/AKIRIN1

K11/CD19/TNFSF13
IL13RA2/CD84/PLEKHM1/ADGRE2/VPS33B/FAM98A/CHGA/RASGRP1/MYH9/TFEB
HGAP25/APPL2/RAC3/MYH9/CHMP4A/ALKBH4

IF/CD300A/PKDCC/IQGAP1/EFNA5/MAPK3/CD3E/MAP2K2/PPP2R1A/FGFR4/RAP2B/THY1/PDCL3/S
IF/CD300A/PKDCC/IQGAP1/EFNA5/MAPK3/CD3E/MAP2K2/PPP2R1A/FGFR4/RAP2B/THY1/PDCL3/S
/CCL26/TNFSF9/GBP2/PSMD2/MAPK3/SHARPIN/TNFRSF4/BRCA1/EDA/NOL3/CD58/F2RL1/STAT1/
/LY86/PPARD/LDOC1/ZC3H12A/TNIP2/CD68/CCL2/CXCL9/CXCL3/CXCL13/LYN/IL1B/WFS1/CASP
H12A/TNIP2/CD68/CCL2/CXCL9/CXCL3/CXCL13/LYN/IL1B/CASP1/PF4V1/SBNO2/CD6/CXCL11/C
/EXOSC3/NAT10/FAM98B/FARSA/GTPBP3/ADAT3/PUS7/DUS2/GTF3C5/CTU2/POP5/EXOSC9/EXOSC
S1/ABCG1/LARP4B/NAT10/MPV17L2/THBS1/RPL5/PLK3/JMJD4/RPS4X/RCC1L/TARBP2/EIF5A/EI
CD47/PTPN6/ARRB2/APP/AXL/FADD/CLEC7A/LBP/CCL3/TNFAIP3/NFATC4/CD33/THBS1/MAVS/TL

1/ARAP1/PTPN12/PTK2/PTPN2/ADAM17/IFI6/HBEGF/BTC/EREG/EGF/DAB2IP/SNX5/PLCG1/STAM

C13/TARBP2/POLR2I/POLR2J/PPHLN1/POLR2D/DHX9/SEH1L/POLR2E/MORC2/PPARG/ADAR/AAAS/

C1/DHX38/PABPC1L/PABPN1/SRSF1/SRSF2/PAF1/CASC3/FIP1L1/RBM8A/CSTF3/MAGOHB/ZNF473

P36/NMT1/HTRA2/ARIH2/UBL5/BAP1/TP53

S14/RPS27/DDX52/RPS6/RIOK1/RPS24/ERAL1/RPS28/RPS17/NOL11/TSR1/MRPS11
SR1

/PSRC1/HAVCR2/CLCF1/SQSTM1/CD44/GDF15/IRAK1/S1PR2/CD74/PFKFB4/PKN1/DUSP5/ARHGEF
ABEP2/CCL5/VAV1/CCL26/IQGAP1/RGS14/ANKRD27/THY1/ARHGAP9/CORO1C/F2RL1/ARHGEF19/C
TB/MEG3/DNMT3A/NUP85/IFI16/ATAD2/NUP43/RAE1/ZMIZ2/GPX1/PADI2/XPO5/RBBP4/SRRT/HE
T3/B3GALNT1/ST3GAL4/MT3/EXTL3/B4GALT7/MUC3A/KRTCAP2/RPN2/B4GALT2/POMT2/FUT3/MUC
T3/B3GALNT1/ST3GAL4/MT3/EXTL3/B4GALT7/MUC3A/KRTCAP2/RPN2/B4GALT2/POMT2/FUT3/MUC
JB2/GSK3A/VPS28/UBE2D1/PSMD10/RPS3/ARRB2/DCUN1D5/DNM1L/PELI1/MTBP/RPL23/PTTG1IP
ITGA2/DAZAP1/IL1B/MAGED2/ADRA2C/CALCA/BYSL/OXTR/VDR/UBE2A/SYDE1/AGO2/CALR/TGFB3

L12RB1/MSH2/IL4R/SWAP70/GPR183/EXOSC3/MFNG/HSPD1/LFNG/ABL1/FCGR2B/PTGER4/NBN/TM
ARRB2/APP/AXL/FADD/CLEC7A/LBP/CCL3/TNFAIP3/NFATC4/CD33/THBS1/MAVS/TLR2
ARRB2/APP/AXL/FADD/CLEC7A/LBP/CCL3/TNFAIP3/NFATC4/CD33/THBS1/MAVS/TLR2/TWIST1/D
SN1/LST1/IL4R/GAL/AXL/BTK/PELI1/BTN2A2/FCGR2B/TNFAIP3/TWSG1/PTPN2/IL20RB/HLA-F/

AC10/SPIRE1/MAGEF1/UIMC1/UBR5/TWIST1/APBB1/RNF8/DHX9/TIGAR/UBE2N/MMS19/TP53BP1/

1/RHOA/ACTN4/EPHB2/RYK/FSTL4/SEMA3F/DMTN/TRIM46/SEMA6B/SEMA4C/PTPRS/TRAK2

RELA/POLR1C/POLR3A/TRIM15/XRCC5/RNF135/ZBP1/TICAM1

A4/ADORA1/MVP/HRAS/SHKBP1/SYK/PLCB1/ATP6V1F/NUP62/HCK/GPC1/PGF/DDR1/CDK4/GIT1/C
BIRC3/CEP131/CDK16/RACGAP1/NCAPH/FANCD2/NDRG3/NUP62/ASPM/ZNF296/SPAG4/SLC26A6/T
CD/HSPB1/NCK2/PRNP/MVP/NUP62/CD109/WNK2/MAD2L1/DNMT1/EZR/CDKN2D/MGAT5/IGFBP3/DT
A/IGFBP6/EDA/MLLT3/SMARCA4/HDAC1/CSNK1E/LZTS2/FAM53B/PSMB2/ZNF703/TMEM237/PPP1C
8/CAPN1/PIP4K2A/ATP6V1H/SNF8/CLN3/VDAC1/ATP6V1E1/GBA/LAMTOR5/CAPNS1/CDK5/LAMTOR

PP2R1A/RAP2B/THY1/PDCL3/SOCS1/TGFA/RELN/NCAPG2/FGFR3/SHC1/HDAC2/HCLS1/LYN/GPRC5

/CSNK1E/DDX11/MARK2/PPP1CA/LEF1/LDOC1/PIM2/HIP1R/FLOT1/KDM1A/ITGB1BP1/ACTB/CDK5

R1/WAS/CHMP3/NDRG4/RAB10/VPS28/SNX27/MVB12A/PTPN23/CHMP1A/PLEKHJ1/TMEM50A/VPS25
RF1/IRF3/HSP90AB1/STAT1/RAB7B/CCL13/IRF7/ACTG1/HLA-DQA2/CCL2/TRIM31/DAPK3/NLRC5
JB2/POMT2/GSK3A/ERO1A/ZBTB17/BAG3/CALR/DNAJB11/HSPE1/PPP1R15A/RACK1/PDIA6/CXXC1
TPN6/CD27/IL12RB1/CYP26B1/IL15/DROSHA/KAT2A/IL4R/PNP/LILRB4/AP3D1/AXL/BTK/BTN2A
AP3D1/AXL/FADD/BTK/BTN2A2/SART1/TOX/BAD/CASP8/TMEM64/NKAP/RHOA/AP3B1/RUNX3/ZMIZ

A/CBL/ABL1/ARAP1/PTPN12/HSP90AA1/PTK2/PTPN2/ADAM17/CDC37/IFI6/LGMN/HBEGF/RBPJ/B

KLE2/EMD/WRAP53/NSFL1C/SRPK1/LEMD2/DCTN1/ZPR1/NEK6/RNF8/SEH1L/VPS4A/NECTIN2/SRP
ACVR1B/CSNK2A1/CSNK2B/LMTK2/MAP3K10/DGKQ/CDK5R1/PARD3/SBK1/EGF/RPTOR/CALM3/CSNK

5/PELI3/PSMD8/PSMA1/PSMB5/PSMA4/PSMD9/RPS27A/UBA52/PSMC3/PSMC5/UBE2N/IRAK2/VRK2

MBIM1/BCL2L1/RAB11A/CLTC/EPHA2/MRAP2/EPHB2/CIB1/VPS4A

1/YTHDF2/METTL3/PSMB1/PSMB6/PSMA3/PSMB7/ERCC2/ACE/XRCC5/SETD1A/TP53/PSMD12/KMT2

CL1/CCL8/INPP5D

/PSRC1/HAVCR2/CLCF1/SQSTM1/CD44/GDF15/IRAK1/S1PR2/CD74/PKN1/DUSP5/ARHGEF2/PRKCD
/DLG3/CSF1R/MAGED1/NCF1/TRAF2/CCL5/MAPKAPK2/CXCR4/MIF/CD300A/IQGAP1/CARD10/EFNA

D13/PSMC4/COPS7B/UBE2D1/USP14/PSMD10/PSMD11/PSMD1/PSMA7/USP1/TRAF3/PSMB3/BARD1/
TTN/ZNF205/GSK3A/PSMD10/GPX1/YWHAQ/YWHAB/BAG3/ARRB2/INF2/GZMB/DNM1L/PDCD5/MAPT/
/CHMP1A/PIP4K2C/WRAP73/RNF4/RALB/DRG1/NPRL3/TBC1D7/PRKAA2/DNM2/TCTEX1D2/CCSAP/S

1/PSMB5/PSMA4/PSMD9/PUS7/NOTCH1/HNRNPU/PSMC3/PSMC5/RBM24/OSM/LDB1/YTHDF2/METTL3

3/SMARCD3/NME1/NBL1/ITPKA/NAP1L1/UNC13D/EZH2/NME2/TWF2/ASPM/ANKRD1/TRPV2/GRN/S1
/MAPK8IP2/CSF1R/NECTIN1/UNC5B/EZR/BSG/DCDC2/TRPV2/CTNND2/CRMP1/CFL1/DOK2/DTNBP1
WF2/ASAP1/ASPM/ANKRD1/ESRP1/TRPV2/GRN/CRMP1/CFL1/LINGO1/TIMP2/TCF3/P3H1/CXCR4/S
/MAPK8IP2/CSF1R/NECTIN1/UNC5B/EZR/BSG/DCDC2/TRPV2/CTNND2/CRMP1/CFL1/DOK2/DTNBP1
CMTM3/PTAFR/PRKCD/LAT2/C1QA/RELB/IGLL5/HLA-DRB1/PRNP/HRAS/SYK/HLA-DQA1/HCK/IL18
PA2G4/ZC3H12A/NCBP2/BCL3/EIF3D/CNOT9/DAPK3/ATF4/ITGA2/EIF2S3/MRPL13/GCN1/CDC123
ST1/ROBO1/SERPINI1/ITIH5/SYK/CD109/SERPINH1/CSTB/S100A8/ASPH/CASP4/CDKN2D/IFT57
RNASE6/GRN/CARD9/APOBEC3C/HMGB2/TRIM6/SLFN13/UNC93B1/NT5C3A/GBP2/MAPK3/TRIM11/S
2/RPS6KA1/IGFBP3/BDKRB1/ATAD3A/CXCR4/SEMA4A/MAP2/SLC9A1/EFNA5/MAD2L2/PPP2R1A/PA

D13/PSMC4/UBE2D1/USP14/PSMD10/PSMD11/PSMD1/PSMA7/USP1/TRAF3/PSMB3/BARD1/ARRB2/U
OD3/B4GALT3/B3GALNT1/ST3GAL4/MT3/EXTL3/B4GALT7/MUC3A/KRTCAP2/RPN2/GBA/B4GALT2/P

DAPK3/TESK1/STK17B/LYN/RAP2A/CDK5/PDGFA/FGR/LCK/TNIK/SLA/FGFR2/PRKD2/STK10/BTK/
CL3/SHC1/CXCL13/DEFB1/SLC11A1/JCHAIN/FGR/RPL39/COCH/C5AR1/GSDMD/DROSHA/TLR5/RPS

/CD300LF/PTPN6/SAMSN1/LST1/ABR/CST7/IL4R/GAL/AXL/BTK/PELI1/BTN2A2/FCGR2B/TNFAIP
F675/RGS3/STK38/PKIA/ABL1/SFRP2/TNFAIP3/PTPN1/DUSP7/PRKAR1B/YWHAG/GPS1/HEG1/DUS

TXBP1/TAPBP/TAP2/GOSR2/SNAPIN/MFN1/SAMD9L/CAV2/VPS4A/VPS16/C2CD5/RAB8B/VPS8/TSN

/CHEK2/BAD/PIAS4/TMEM109/BCL2L1
SD/MMP19/VIM/WNT4/EMILIN1/CIITA/RCN3

METTL3/PSMB1/PSMB6/PSMA3/PSMB7/SETD1A/PSMD12/KMT2A/PSME2/MYB/PSMD6/GATA3/PSMC2/
IP11/SPIRE2

B1/ITGAM/RHOV/PPT1/FCGR3A/DKK1/UNC119/CORO1A/SLC39A4/FOLR1/ANKRD13D/GPC3/FCHO1/
NL2/CSF1R/EZR/TRPV2/RHOG/FMNL1/CFL1/RHOQ/S100A10/LINGO1/CARMIL1/RHOC/CXCR4/SEMA
/DLG3/CSF1R/MAGED1/NCF1/TNFAIP8L3/TRAF2/CCL5/MAPKAPK2/CXCR4/MIF/CD300A/IQGAP1/C

MA5/CNFN/EDA/PALLD/HDAC1/PLOD3/KRT18/PPARD/FGFR1/SLC9A3R1/FERMT1/JAG2/CAPN1/HDA

/ALKBH4

GR/PLXND1/VIL1/COCH/LST1/CDC42SE1/FGD6/CDC42/ARHGAP18/CDC42SE2/SEMA4D/CDC42EP2/
OPG1/NDE1/MAPT/BICDL2/TTC26/HSPB11/RHOT2/IFT172/COPG2/TCTEX1D2/BICD1/HNRNPU/MAP
OPG1/NDE1/MAPT/BICDL2/TTC26/HSPB11/RHOT2/IFT172/COPG2/TCTEX1D2/BICD1/HNRNPU/MAP
7/PPP5C/AXL/BTK/STK25/RACK1/ROMO1/MAPT/ABL1/TNFAIP3/PDCD10/PSAP/DNM2
ACVR1B/CSNK2A1/CSNK2B/LMTK2/MAP3K10/DGKQ/CDK5R1/PARD3/SBK1/EGF/RPTOR/CALM3/CSNK
SPD1/TNFAIP3/CSK/CAPN2/MAVS/TLR2/TWIST1/DHX9/ADORA2B/EREG/TLR7/IL17D/INPP5D/TLR

1/MRPL11/MRPL15/C12orf65/MRPL4/MRPS24/MRPL33/MRPS5/MRPL36/MRPL14/OGFOD1/MRPL3/E

/CCL19/PIK3R6/INPP5D/PTPRC/NFKBID/IFNG/ANXA1/PPP2R3C/CD80/RASGRP1/LILRB2/NLRP3/

TPRC/FIGNL1/SUPT6H/ERCC2/HELB/TNFSF13/ACTR2

RO1A/FOLR1/ANKRD13D/GPC3/FCHO1/ITGB2/SNAP25/PRKCD/TRIP10/ITGAV/SCAMP5/IL15RA/IG
PAG4/SLC26A6/HSF1/KIF18A/WIPF3/LIMK2/HMGB2/YBX3/EIF5A2/BAX/NR0B1/SNRPA1/FANCG/R
D2/NPM1/UPF3B/POLR2G/NSMAF/BIN1/RBM3/PABPC1/TRNAU1AP/ORMDL2/PPP1CA/PA2G4/ZC3H12
3/PMAIP1/WFS1/DNAJB2/POMT2/GSK3A/PML/USP14/ERO1A/ZBTB17/DNAJC10/CALR/DNAJB11/PP
IRF3/HSP90AB1/F2RL1/NPLOC4/CD83/LEF1/RPS6KA4/SERPINB1/FGFR1/ZC3H12A/BCL3/LAG3/N

SLC26A11/SLC37A2/SLC4A3/XPR1/SLC4A11/LRRC8B/BEST1/TTYH2/SLC12A7/SLC26A2/GABRD/P
LBP/HSPD1/TNFAIP3/CSK/BCL10/CAPN2/MAVS/TLR2/TWIST1/DHX9/ADORA2B/EREG/TLR7/IL17D

RG3/CD79B/INHA/LYL1/RBPJ/NOTCH2/DOCK10/DCLRE1C/INPP5D/HDAC5/PTPRC/IKZF3

/JMJD4/RPS4X/RCC1L/TARBP2/EIF5A/EIF3E/RPUSD3/NSUN4/RHOA/POLR2D/DHX9/COA3/EIF6/R

K5R1/SRF/IST1/ADNP/RYK/FSTL4/SEMA3F/MEGF8/TRIM46/SEMA6B/SEMA4C/PTPRS/SHTN1/TTL/

/IL20RB/GLMN/TNFRSF14/IDO1/RUNX3/XCL1/DUSP22/LOXL3/NFKBID/CASP3/ANXA1/ARG2/LILR

M/PLIN5/SCP2/FABP1/ACADL/ACOX2/SLC27A2/EHHADH

/DDB1/COPS8/TCEA1/POLR2B/ERCC2/LIG3/COPS7A/POLD2
2/CD7/GPC3/GDF15/IRAK1/EIF4EBP1/SHCBP1/PRKCD/ROBO1/HSPB1/COL4A5/NCK2/ITGAV/EFNA

K8IP2/CSF1R/NECTIN1/UNC5B/EZR/BSG/DCDC2/TRPV2/CTNND2/CRMP1/CFL1/DOK2/DTNBP1/LIN

PAG4/SLC26A6/HSF1/WIPF3/LIMK2/HMGB2/YBX3/EIF5A2/BAX/NR0B1/SNRPA1/FANCG/RUVBL1/P
/ITIH5/SYK/CD109/SERPINH1/CSTB/S100A8/ASPH/CASP4/CDKN2D/IFT57/RPS6KA1/HSF1/CARD

4/TACSTD2/CAPZB/DGUOK/SEMA4B/SEMA4D/SEMA4F/SEMA5B/ARHGDIA/VIM/SEMA3C/PMP22/SEMA
90AB1/STAT1/RAB7B/CCL13/IRF7/ACTG1/HLA-DQA2/CCL2/TRIM31/DAPK3/NLRC5/RAB20/HLA-D
A2A/DNM1L/SYT3/STX4/CSPG5/RAB3D/FCGR2B/VAMP8/C12orf4/NOTCH1/CD177/RAB3A/HLA-F/T

KNOX1/RPS14/INPP5D/ZNF16/ADD1/ACIN1/RPS6/CASP3/ADAR/SMAD5/MFHAS1/RPS24/HOXB6/AN

NNA1/C1QTNF6/HBE1

5/KITLG/OSM/LDB1/YTHDF2/METTL3/PSMB1/PSMB6/PSMA3/PSMB7/SETD1A/PSMD12/KMT2A/PSME
NA4/VASP/ITPKA/GPC1/UNC13D/TWF2/MAPK8IP2/CSF1R/NECTIN1/UNC5B/EZR/BSG/ZNF385A/DC
LOD2/NUP205/NUP37/PLOD3/LEF1/NDC1/NELFE/RPS6KA4/NUP188/TGM2/NOC2L/HDAC2/KDM1A/K

A2B1/ELAVL1/PAIP1/TARDBP/C1QBP/SRSF10/RNF40/SRSF7
3H12A/FERMT1/FRMD8/LYN/CARD11/IL1B/S100A13/FGR/LCP2/MAPK11/CASP1/PML/IL1R2/ANXA
R1/PMAIP1/RNASE2/IL1B/IL2RA/APOBEC3G/RNF26/PQBP1/IFI16/MAP3K14/PML/IL12RB1/IL15
EPPS/HSPA4/YWHAH/MCU/BBC3/PMAIP1/UBE2L3/ZNF205/GSK3A/YWHAQ/YWHAB/BAG3/STARD3/GR
SMD3/PSMD7/DPF2/KCNAB2/ESCO2/PSMD8/ABL1/PSMA1/PSMB5/PSMA4/PSMD9/PUS7/NOTCH1/PDC

/VIM/SEMA3C/PMP22/SEMA3E/CBFA2T2/STMN2/NFATC4/DENND5A/PTK2/RAB29/IFRD1/CDK5R1/R

LRC5/KDM1A/CCDC22/WFS1/COMMD7/PSMD10/ANXA4/TRAF3/ARRB2/CMKLR1/PELI1/ZNF675/DAP/

T3

PG4/CEMIP/EXT1/BCAN/B4GALT4/ACAN/CHST3/CLTC/GPC2/HEXA/AP2A1/EGF/B3GALT6/HS6ST2/

EHHADH/DAO/HAO2/HAO1/AGXT

DH4/CYP3A4

HAVCR2/GSTP1/GPC3/FLNA/BIRC3/IRAK1/PI3/BATF/CCDC88B/PTAFR/COTL1/PRKCD/HSPB1/SLA
ECTIN1/UNC5B/EZR/BSG/DCDC2/TRPV2/CTNND2/CRMP1/CFL1/DOK2/DTNBP1/LINGO1/CXCR4/SEM
HAVCR2/GSTP1/GPC3/FLNA/BIRC3/IRAK1/PI3/BATF/CCDC88B/PTAFR/COTL1/PRKCD/HSPB1/SLA
PA8/RPL36A/TOMM34/AP4M1/DKC1/SEC61G/ACD/GDAP1/TIMM17B/BMF/HGS/CCT2/RPS2/LMNA/TS

1/COL3A1/LAMC1/FZD7/MICALL2/PLAU/S100A10/CARMIL1/EPDR1/ITGA5/IQGAP1/SLC9A1/EFNA

7/TNFAIP8L2/VTCN1/VSIG4/JAK3/CD86/CD300A/EFNA5/MAD2L2/ADAMDEC1/SERPINE1/CORO1C/

/VPS33B/EXOC6/UNC13A/EXOC6B/STX16/STX8/STX12/STX5/STXBP3/STX2/STX11

TACSTD2/CAPZB/KAT2A/SWAP70/KATNB1/GMFG/CLIP3/CKAP2/TBCD/STMN2/ARAP1/ADD3/LIMA1/

S4A/VPS16/C2CD5/RAB8B/VPS8/TSNARE1/VAMP3/KIF5B/ANXA1/VAMP1/NKD2/RAB7A/VAV3

7C/ATP6V1C1/RRAGD/FOXK1/FUNDC1/HIF1A/MAPK3/NUPR1/HSPA8/RRAGC/TCIRG1/MAP1S/CHMP4
HAVCR2/GSTP1/GPC3/FLNA/BIRC3/IRAK1/PI3/BATF/CCDC88B/PTAFR/COTL1/PRKCD/HSPB1/SLA
6KA1/ATIC/YBX3/EIF5A2/CCL5/MTHFD1L/DPH2/B4GALT5/PDP1/CNOT11/MAPK3/EIF3B/RPL36A/
7C/ATP6V1C1/RRAGD/FOXK1/FUNDC1/HIF1A/MAPK3/NUPR1/HSPA8/RRAGC/TCIRG1/MAP1S/CHMP4

RL1/HYAL2/CHMP6/ACE/AFAP1L2/TSG101/WNT3A/ABI1/SOCS5/SNX6
/IL18/PAG1/HLA-DRA/HLA-DQB1/SLAMF7/VTCN1/TFRC/HLA-DPA1/HLA-DMB/TRAF2/JAK3/CD86/
/NAIF1/CNP/THEM4/NMT1
AB3C/DMTN/VPS33B/VAMP1/EXOC6/CADPS2/UNC13A

/PSMA1/PSMB5/BCL10/PSMA4/PSMD9/PAK2/PSMC3/MAP3K1/PSMC5/PIK3CB/PSMB1/PLCG1/MAP3K

V2/GRN/CRMP1/DOK2/LINGO1/CXCR4/SEMA4A/NDN/MAP2/DOK1/BRSK1/B4GALT5/EFNA5/MAPK3/E
/HMOX1/TCF3/BRMS1/NR0B1/TRAF5/FLOT2/NFKB2/MAD2L2/MAPK3/NUPR1/FZD1/TMIGD3/NPM1/T
/MORF4L1/MORC2/HDAC5/TRIM27/PHF1/SETDB1/MBD2/ASF1A/LHX2/MBD1/SUZ12/MSL3/EZH1/JA
188/ZC3H12A/CSE1L/CCT4/BCL3/FERMT1/SIX2/KPNB1/HCLS1/CCT7/CDK5/TXN/TCP1/NUP85/PM
1A/FGFR1/DVL3/ADAM8/SHC1/WNT7B/BIRC7/IL1B/PDGFA/MLKL/MAPK11/MAP3K14/TNIK/DVL2/M
/CTTN/RNF157/GSK3A/WASF1/SEMA4B/SEMA4D/SYT1/APP/GAL/SYT3/SEMA4F/SEMA5B/SEMA3C/S

AT

KLKB1/C4BPA/F12/CFHR5/F9/KNG1/C6/CFHR4/SERPINC1
NFRSF10A

TF2F1/CTDP1/TRIM32/TRIM27/TRIM62/POLR2B

3GLB1/OPTN/CDC37/STOML2/VPS11/YWHAG/ATG13/TFDP1/HTT/CSNK2A2/HUWE1/BIK/DDHD1/PLA
P3A4

L19/TLR7/LITAF/MAP3K7/COPS8/RTKN2/AGO1/RELA/NOD1/NOD2/CARD14/RASSF2/TNFRSF10A

/CALR/AXL/CSPG5/LIMS1/ABL1/SRGAP2/DNM2/PTK2/OLFM4
AC5/BMP2/CTDP1/CXCL10/PIN1/TRIM32

K16/KCNJ11/PRKCD/SLC7A11/SCAMP5/B3GAT3/PRNP/ADAM9/HRAS/SYK/NUP62/GTSE1/CCT5/TMS
FOXK2/PIM2/ZC3H12A/MT3/ATG101/MLST8/TRIM65/CAPN1/PIP4K2A/LAMP3/ATP6V1H/DAPK3/VD

E1/TAF13/TBP/POLR2E/GTF2F1/EXOSC2/RPRD1A/GTF2A2/ZNF143/POU2F1/POLR2B/POU2F2/SNA

CD4/IFNG

BID/IFNG/ANXA1/CD80/RASGRP1/LILRB2/NLRP3/IL12A/ZBTB7B/SOCS5/MYB/ZAP70/MALT1/HLA
1/ATP6V0C/ATP6V0E1/BAG3/IKBKG/PIP4K2C/RALB/ATP6V0D1/RUBCN/RHEB/SUPT5H/PAFAH1B2/
F/SEMA5B/STK25/ARHGDIA/SEMA3C/SEMA3E/MAPT/CHN1/ABL1/KEL/DBNL/PLXNC1/DNM2/PTK2/V

1/PDIA6/CXXC1/TBL2/ATP6V0D1/HSPA2/VCP/SRPRB/WIPI1/TATDN2/PTPN2/DCTN1/PTPN1/OPTN
CTB/DNAJB2/IFI16/NES/EIF4A3/RUVBL2/TFAP4/ARRB2/ANKRD33/CARD16/HEY1/GZMA/ZNF675/

LR2/ALOX15B/TWIST1/GLMN/IL17RA/TNFRSF14/DHX9/CCL19/OSM/IL17RB/PANX2/CLEC4E/CRLF
2/ST3GAL5/PIGT/RACK1/CSNK1G2/CERS6/HACD1
/CAV2/CAV1/THOC1/THOC7/MVB12B/AAAS/THOC3/TRIM15/RAB7A/NUP214/TSG101
/CAV2/CAV1/THOC1/THOC7/MVB12B/AAAS/THOC3/TRIM15/RAB7A/NUP214/TSG101

SC10/HNRNPA1/RFC5/MAPKAPK5/DNAJC2/TFDP1/ATR/PPARG/SMG7/HNRNPD/DCP2/HNRNPC/MYC/M

IRAK1/S1PR2/CD74/PKN1/DUSP5/PRKCD/ROBO1/ITGAV/ADORA1/ADAM9/HRAS/SYK/PLCB1/STK39
LA-DRB1/PRNP/HRAS/SYK/HLA-DQA1/HCK/PAG1/S100A8/HLA-DRA/MUC1/EZR/HLA-DQB1/VTCN1/
/HCK/TMSB4X/CDK4/TWF2/EZR/TRPV2/COMP/CFL1/HNF1B/CARMIL1/HMOX1/PLEKHG2/SLC12A2/V
2/MDFI/PSMB9/DDIT3/PSMD14/HNF1B/GRK6/PTK7/RUVBL1/MAD2L2/PSMD2/FZD1/PYGO2/BCL9/P
DI1/FGFR1/DYNLT1/DVL3/VLDLR/KDM1A/LYN/RNF157/PLXND1/NDRG4/TUBB2B/DVL2/LRP8/PRKC

PS8/TSNARE1/VAMP3/KIF5B/ANXA1/VAMP1/NKD2/RAB7A/VAV3
PS5/MRPL36/MRPL14/MRPL3/ERAL1/AURKAIP1/MRPL20/MRPL24/MRPS16/MRPL55/CHCHD1/MRPS2

R2/POLR2K/TRIM71/PIWIL4/SEC13/TSN/TARBP2/POLR2I/POLR2J/POLR2D/DHX9/CNOT10/SEH1L

APZB/SWAP70/KATNB1/GMFG/CLIP3/CKAP2/EMILIN1/TBCD/STMN2/ARAP1/ADD3/LIMA1/EML2/AR
/TBC1D7/BCL10/PIAS4/MASTL/HSPA5/CHFR/NMI/RASSF5/SART3/UBE2N/PEF1/CAV1/STUB1/RAB

/TRMT12/TP53RK/CTU1/TSEN34/TPRKB/TYW3/TSEN15/DPH3/RPP30/TRMT2B/CDK5RAP1/RTCB/ME

PRB/WIPI1/TATDN2/PTPN2/DCTN1/PTPN1/COPS5/HSPA5/CANX/PPP2R5B/GFPT1/TLN1/GOSR2/TP

/BCAN/B4GALT4/ACAN/CHST3/CLTC/GPC2/HEXA/AP2A1/EGF/B3GALT6/HS6ST2/HS2ST1

/TWF2/ADAM15/CDKN2D/TRPV2/RPS6KA1/IGFBP3/BDKRB1/ATAD3A/CXCR4/SEMA4A/SIRT6/NDN/M

ASH3/BASP1/GDI1/PPARD/LEF1/FGFR1/EXTL3/MKKS/WFS1/RNF157/TGFB2/TAF9/VIL1/FGFR2/C
C2/ZC3HAV1/LARP1/SRPK2/IFITM2/DDB1/MX1/VAPA/TBC1D20/ADAR/SETDB1/APOBEC3F/FAM111
PS5/MRPL36/MRPL14/MRPL3/ERAL1/AURKAIP1/MRPL20/MRPL24/MRPS16/MRPL55/CHCHD1/MRPS2

RAF3/ARRB2/TLR5/IKBKG/FADD/BTK/PELI1/TNIP1/LBP/HSPD1/TNFAIP3/MAPKAPK3/BCL10/CAS
BAM1/CBL/RFWD3/NABP1/RAD9A/XRCC4/SFRP2/BRAT1/CHEK2/RAD1/VCAM1/DNM2/ERCC1/TMEM10

POA5/APOC3/CPS1/G6PC/CYP2E1/SLC27A5/PCK1

/ATP6V0E1/SLC9A7/TMEM199/ATP6V0D1

OTUB1/CCL2/PSMB4/IL1B/CD47/PSMB8/PSMB10/PSMA5/CCL4/MAPK11/TAF9/PSMD13/PSMC4/PSM

/PLA2G15/SMPD4/A4GALT/PRKD2/FUT6/ST3GAL5/RACK1/ITGB8/CSNK1G2/CERS6/HACD1
POX/ACSM5/PDK4/HMGCS2/ACSM2B/ACSM2A/GLYAT
POX/ACSM5/PDK4/HMGCS2/ACSM2B/ACSM2A/GLYAT

UWE1/C2CD5/MYO1C/USP36/DMTN/HTRA2/ARIH2/UBL5/BAP1

/SENP5/NUP214/MUL1/BCL11A/TPR/NUP50/HMG20B/NUP133/RASD2/GNL3L/PIAS2/NUP35/RWDD3

9A10/PPP2R2C/UBASH3B/DUSP23/PPM1G/CTDSPL/PPP6R1/HSP90AB1/PTPN7/RCAN3/PPP1R9B/PP

ETFDH/ACOX1/ACAA1/IVD/ACAA2/ABHD1/IRS2/HSD17B4/GCDH/ACAT1/AMACR/ACADM/PECR/PLIN
/ADH1C/CYP2C8/ADH1B/ADH4/CYP3A4
I2/ETFDH/ACOX1/ACAA1/IVD/ACAA2/IRS2/HSD17B4/GCDH/ACAT1/AMACR/ACADM/DGAT2/PECR/P

A/PML/CDKN2C/SEMA4B/SERTAD3/SEMA4D/GAL/SEMA4F/SEMA5B/NME6/RACK1/MSX1/RBBP7/SEMA

E/PPARG/ADAR/AAAS/AGO1/POLR2B/FXR1/EIF4G1/ELAVL1/ZMPSTE24/NUP214/MYCN/MAEL/TP53
E/PPARG/ADAR/AAAS/AGO1/POLR2B/FXR1/EIF4G1/ELAVL1/ZMPSTE24/NUP214/MYCN/MAEL/TP53

I2/ETFDH/ACOX1/ACAA1/IVD/ACAA2/IRS2/HSD17B4/GCDH/ACAT1/AMACR/ACADM/DGAT2/PECR/P
3/ZNF473/TTF1/PRMT5/SMN2/POLR1C/ERCC2/ZMPSTE24/ZNRD1/TWISTNB/XRN2/PAPOLA/SMN1/C

SOCS1/CEP85/DUSP2/FBXO5/CDKN2B/TESK1/LYN/ITGB1BP1/CDKN1C/DUSP4/DBNDD2/IL1B/GBA/

CYP4A11/CYP2E1/CYP2C8/SLC27A5/CYP2C9/CYP2A6/CYP3A4

2/SARDH/PRODH2/GPT/HAAO/HGD/SDS/ADHFE1/AASS/ALDH8A1/AMDHD1/AGXT2/MAT1A/PIPOX/PA

ATSF1/OAS3/RAB29/TARBP2/CXCR6/PPIE/PPIB/IFITM3/CTBP1/CCL8/HMGA2/PARP10/MORC2/ZC

1/PDCD2/HNRNPU/VEGFA/NKAP/TEAD3/KITLG/ARTN/MECOM/DAGLB/HMGA2/PTPRC/YTHDF2

IRAK1/S1PR2/CD74/PKN1/DUSP5/PRKCD/ROBO1/ITGAV/ADORA1/ADAM9/HRAS/SYK/PLCB1/STK39

PAK1/PEA15/FPR1/CKS1B/HSP90AB1/TGFA/TNFRSF11A/FGFR1/DVL3/MLST8/DBF4/ADAM8/SHC1/

SF3A2/FZD1/PAK1/CRABP2/ANKRD27/NRCAM/RELN/CSNK1E/MARK2/GDI1/FGFR1/DYNLT1/DVL3/V

SIN1/XKR8/FNBP1L/CAV2/CAV1/PTPRC/ATP2A2/ABCA7/NOX1/SYT11

ITGB1BP1/CDKN1C/DUSP4/DBNDD2/IL1B/GBA/CHORDC1/PTPN6/SOCS3/PPP1R1B/SMYD3/CDKN2C/

L3/IFITM2/MX1/ADAR/IRF4/HLA-C/HLA-E/MUL1/ZBP1/WNT5A/LSM14A/CACTIN/UBE2K/BST2/IR
L3/IFITM2/MX1/ADAR/IRF4/HLA-C/HLA-E/MUL1/ZBP1/WNT5A/LSM14A/CACTIN/UBE2K/BST2/IR
/NUP107/RPL23/SNRPG/PTTG1IP/IPO13/PKIA/MAVS/HIKESHI/ZPR1/HNRNPA1/UBR5/PRKAG1/TN

LR2J/POLR2D/DHX9/SEH1L/EIF6/POLR2E/PRKRA/SPOUT1/MIR155HG/METTL3/PPARG/MOV10/PPP

/TMEM98/NVL/MAVS/CD2AP/ZPR1/UBR5/RASSF5

FC5/POLR2D/GPS1/GTF2H1/RPA1/MMS19/POLR2E/ERCC3/DDB1/COPS8/TCEA1/HUS1/POLR2B/ERC
16/ADD1/ACIN1/RPS6/CASP3/ADAR/SMAD5/MFHAS1/ANKRD54/DMTN/DYRK3

/APP/GDPD5/HEY1/SLC35C1/RPS19/GMDS/MFNG/TRAF7/LFNG/CBFA2T2/PDCD10/WWC1/IFT172/T

MA4F/SEMA5B/SEMA3C/SEMA3E/MAPT/ABL1/VCL/RASAL1/DBNL/HSP90AA1/DNM2/LLPH/VEGFA/RA
/KAT2A/MTF2/ZNF335/CHTOP/MTHFR/HNRNPU/VEGFA/SREBF1/PIH1D1/MECP2/UBR5/TWIST1/PPH

71/MED12/OVOL2/LUZP1/RARG/ABL1/SFRP2/RPS7/IFT172/BCL10/LMO4/NOTCH1/FKBP8/MTHFR/

RIM25/CIITA/VCAM1/PTPN2/IRF5/OAS3/TRIM21/HLA-F/CDC37/NMI/NR1H2

NMT1

AB3C/RAB33A/RAB2A/RAB7A/RAB6A/RAB28

4/WNT5A/WNT3A

S2/APOC3/ADH1A/ADH1C/CYP2E1/CYP2C8/CYP2C9/ADH1B/ADH4/CYP3A4

A7
/POP7/KPNB1/RNASE2/RPS21/POP1/CPSF1/EDC3/PARP1/RRP36/CPSF6/ISG20/EXOSC5/DFFA/DN
CD33/CASP8/S1PR3

1B/CASP1/MAP3K14/LTBR/PIEZO1/BAG3/CNN2/TLR5/FADD/ANGPT2/LRP11/PKD1L2/MEIS2/COL1

CK1/ABL1/STK24/TNFAIP3/PDCD10/BAD/PSAP

/TSG101/TPR/NUP50/BST2/NUP133/VPS37A/UBC/NUP35/XPO1

1/TIMELESS/ABL1/TNFAIP3/PDCD10/PSAP/CFTR/HSPA5/HNRNPA1/DAXX/ANKZF1/ADCY6/BECN1/

LIP3/TBCD/STMN2/TMC8/PTGER4/LMO4/STYXL1/VPS35/THRA/VDAC2/ADD3/EML2/RPL13A/STXBP
L3/IFITM2/MX1/ADAR/IRF4/HLA-C/HLA-E/MUL1/TRIM56/ZBP1/WNT5A/LSM14A/CACTIN/UBE2K/
R/NUP50/BST2/NUP133/VPS37A/UBC/NUP35/XPO1

NMI/GLMN/INHA/SIGIRR/EREG/TLR7/INPP5D/PTPRC/TLR8/IRF4/CD4/IFNG/RELA/CCR2/NOD1/T

OCS1/TGFA/MARK2/MT3/NCKAP1L/MLST8/PAK4/SHC1/KIF14/AXIN1/SLC11A1/WNT7B/ITGB1BP1/

CES2/GHR/RORC/AKR7A3/CYP3A5/CES3/CYP2A7/AADAC/PON3/UGT2B15/NR1I2/GSTM1/FMO3/CYP

NF2/ZNF335/BTG2/AKNA/CTNNA1/NOTCH1/VEGFA/PSMG1/TARBP2/TEAD3/RHOA/RAPGEF1/IFT20

4/SEMA4A/NDN/MAP2/DOK1/BRSK1/B4GALT5/EFNA5/MAPK3/EFNB1/MAP1S/PAK1/CRABP2/NRCAM/
R1/RNF19B/PERP/FGFR4/DDA1/CSNK1E/PRELID1/HIP1R/ZC3H12A/PSME3/BID/HSPBP1/BCAP31/

GM2/BCAP31/KISS1/SRI/CXCL9/CXCL13/GIPR/LYN/CCL28/FPR3/CDK5/GNB1/CXCR3/LCK/PTPN6

D1/TLR1/NOD2/AFAP1L2/MAPKBP1/WNT5A/C5AR2/ARRB1/CACTIN/SSC5D

A1

PD3/FMOD/GPD2/MAN1B1/ITPA/PDE9A/NAGK/GBA/APOBEC3G/GNS/GNPDA2/ADAMTS9/GSK3A/GALN

IGAR/CCAR2/UBE2N/MMS19/ATR/HMGA2

A/TMED9/SORT1/STX10/SNX10/BLOC1S4/FBXO5/CSNK1D/SNF8/RAB20/LAMTOR1/RNF26/CHMP3/A

/FABP4/MOGAT2/APOA1/APOA5/APOC3/CPS1/G6PC/CYP2E1/SLC27A5/PCK1
CYP1A2/AKR1D1/TTR/HSD17B6/ADH1A/ADH1C/CYP2C8/CYP2C9/ADH1B/ADH4/CYP3A4
1/OR2A14/OR3A1/OR10H3/OR51D1/OR52B2/OR7C2/OR10H4/OR4D5/OR13D1/OR52K2/OR52A1/OR6
ALB/GSTM1/GSTA1/ADH4

/LYN/CBFB/PIAS3/CCR1/LOX/LILRB4/FADD/ZNF675/CCL3/MEIS2/RARG/ACVR1B/PTPN2/THBS1/

R2I/POLR2J/INTS4/POLR2D/TAF13/TBP/POLR2E/GTF2F1/TAF1B/PWP1/RPRD1A/GTF2A2/ZNF143

/ADAT3/PUS7/WTAP/DUS2/CTU2/BMT2/SEPHS1/TRMT12/RPUSD3/NSUN4/TGS1/CTU1/TPRKB/RNMT
OOK2/SYS1/DCTN1/VPS35/ARFRP1/RGP1/LMTK2/RAB29/TMEM87A/VPS11/CLTC/AP1S1/GAK/GOSR

/INHA/SIGIRR/EREG/TLR7/INPP5D/PTPRC/TLR8/IRF4/CD4/IFNG/RELA/CCR2/NOD1/TLR1/LILR
T8/ASAH1/JAG1/CBFB/CAPNS1/FA2H/CYP26B1/EVPL/VDR/MAFF/KRT20/S100A7/OVOL2/KDF1/KR

RG/AAAS/AGO1/POLR2B/FXR1/EIF4G1/ELAVL1/ZMPSTE24/NUP214/MYCN/MAEL/TP53/TPR/NUP50

L11/NXN/PARK7/RELA/ARRB1/OGT/TRIP12/TRIM44/HMG20B/HSPA1A/GPS2/USP4/RASD2/GNL3L/

F3/DARS2/MRPL51/MRPL11/COA3/YARS2/MRPL15/MRPL4/MRPS24/MRPL33/CDK5RAP1/MRPS5/MRP
/ARF1/STEAP2/VPS26A/VAMP3/ARL3/VPS29/BLZF1/EXOC6/GRIPAP1/GGA2/EXOC6B/ARHGAP44/O

6V0D1/CFTR/SLC4A7/SNAPIN/VPS33A/ATP6V1B2/SLC9A6/SLC9A9/TM9SF4

5D/ACIN1/MYC/BGLAP/CD4/IFNG/ESRRA/RASSF2/LILRB1/GNAS

LGAPB/BCR/TBC1D25/DOCK7/TSC1/RASGRP1/PIP5K1A
ZC3HAV1/SRPK2/IFITM2/MX1/TRIM32/VAPA/TRIM27/SETDB1/APOBEC3F/FAM111A/TRIM15

4B/NSFL1C/WIPI1/HOOK2/VPS35/SH3GLB1/FIG4/HEXB/GNPTAB/VPS11/ATG13/IFT20/TPP1/ATG

2/POLR2I/POLR2J/POLR2D/DHX9/SEH1L/EIF6/POLR2E/PRKRA/SPOUT1/MIR155HG/METTL3/PPAR

IM46/CNIH2/TRAK2/BICD2/RAB6A/NEFH/RASGRP1/FEZ1/NEFL

LETM1/CCL3/CEMIP/ABL1/CALM2/PLCB2/SLC8B1/GRIN2D/MCOLN3/PLA2G1B/JPH1/CACNB3/TRPV

/FABP4/MOGAT2/APOA1/APOA5/APOC3/CPS1/G6PC/CYP2E1/SLC27A5/PCK1

/PDCD5/CEMIP/EMD/MFF/PDCD10/PLK3/SH3GLB1/MAVS/EDEM2/ZPR1/RAB29/KIF20B/CACNB3/CD

/OPN1SW/SLC9A1/MAPK3/ASIC1/NPM1/GNGT1/PCNA/BMF/IRF1/COPS9/E2F4/EEF1D/BLM/NOC2L/
/OPN1SW/SLC9A1/MAPK3/ASIC1/NPM1/GNGT1/PCNA/BMF/IRF1/COPS9/E2F4/EEF1D/BLM/NOC2L/

RASSF5/UBE2N/PEF1/CAV1/STUB1/RAB1A/HUWE1/SKP2/PIN1

/DVL2/PSMD10/PSMD11/PSMD1/PSMA7/RUVBL2/PSMB3/SDHAF2/PSMD4/PSMD3/RBX1/PSMD7/PSMD
A-E

11A

P1/PTBP2/SMN1/CRNKL1/SF1/SF3A1

/RNF187/PSMB10/PSMA5/PSMD13/PSMC4/FBXW9/ANAPC11/UBE2D1/PSMD10/PSMD11/PSMD1/PSMA
1/SIRPA/F2RL1/TNFRSF11A/ZC3H12A/TNIP2/DVL3/PAK4/AXIN1/LYN/WNT7B/BIRC7/UNC5CL/IL
LIMCH1/ZNF703/PIP5K1C/FLOT1/ACTG1/CAPZA1/DAPK3/ITGB1BP1/RAC1/CBFB/CNTNAP1/ITGA2

PL15/GCLM/ARID5B/KLF5
PL15/GCLM/ARID5B/KLF5

2/MAPKBP1/WNT5A/C5AR2/ARRB1/CACTIN/SSC5D

DC/RGS14/HIF1A/NPM1/BRCA1/GNGT1/PCNA/XRCC2/BMF/DYNLRB1/COPS9/TICRR/SLC1A3/TNFRS
HSPB1/QSOX1/ABCD1/IGF2BP1/ADORA1/ADAM9/MTMR2/NUP62/PFKFB3/EXOSC4/CDK4/MAD2L1/CT
/HRG

26A1/IRS1/HOGA1/ECHDC2/ECI2/ETFDH/ACOX1/ACAA1/IVD/ACAA2/ABHD1/IRS2/HSD17B4/GCDH

/KAT5/MSL3/ARRB1/OGT/KANSL2/BRD8/MSL1/KMT2A/BRCA2/KAT7/KANSL1
/KMT5C/FTSJ3/HENMT1/BEND3/MTF2/TDRKH/ICMT/PRDM4/CXXC1/NTMT1/SUV39H2/PIWIL4/ZNF3

L1B/FGR/MAPK11/CASP1/PML/IL1R2/ANXA4/GSDMD/IL4R/TLR5/HK1/CARD16/CLEC5A/CARD18/B

/CTNNA1/OSR2/ADAMTS5/TWIST1/RHOA/PHEX/ADM/ANKRD11

2/POLR2I/POLR2J/POLR2D/DHX9/SEH1L/EIF6/POLR2E/PRKRA/SPOUT1/MIR155HG/METTL3/PPAR
/RTN4RL2/ISL2/GDI1/MT3/DYNLT1/HDAC2/RAP1GAP2/YWHAH/JAG1/ITGB1BP1/FKBP4/MYLIP/IL

C5/FBXL7/FBXO6/FBXO44/SKP2/PSMB1/WWTR1/PSMB6/PSMA3/PSMB7/SKP1/FBXL18/FBXL2/PSMD

POX/CYP2A7/HP/PAH/NR1I2/DPYS/DAO/CYP1A2/HAO1/SULT2A1/CYP2B6/HPD/CYP2E1/CYP2C8/P

PS18/GRK2/CCR5/AXL/CBL/CLEC5A/TRIM25

PS18/GRK2/CCR5/AXL/CBL/CLEC5A/TRIM25
PS18/GRK2/CCR5/AXL/CBL/CLEC5A/TRIM25
PS18/GRK2/CCR5/AXL/CBL/CLEC5A/TRIM25

7/DDIT4/GPI/NDC1/NUP188/UQCC3/LDHA/PFKM/NUP85/NUP43/IMPDH2/PARP1/RAE1/NUP155/AL

R2J/POLR2D

R2/NUP62/PFKFB3/EXOSC4/CTSA/PKP3/EZR/IGF2BP3/HSF1/PSMB9/GIPC1/ATP6V0B/DDIT3/TPC
T2/ARG1/SHMT1/RIDA/GLS2/SARDH/PRODH2/GPT/ALDH5A1/HAAO/HGD/SDS/ADHFE1/ACADSB/AAS

PHX2/INSIG1/ACOX2/ABCG5/APOA2/TM6SF2/LCAT/ANGPTL3/ABCB4/FABP4/APOA1/APOA5/CYP7A

SEMA3F/MEGF8/TRIM46/SEMA6B/SEMA4C/PTPRS/SHTN1/TTL/DPYSL2/WNT5A/OLFM1/WNT3A

4GALT3/B3GALNT1/ST3GAL4/SMPDL3B/ASAH1/CLN3/GBA/CLN6/FA2H/FUT3/PLA2G15/SMPD4/A4G

YN/GPRC5B/SOCS3/DVL2/IL15/LRP8/ARRB2/SEMA4D/ADRA2A/CSPG4/ACVR1/ABL1/ADAM17/TNK2

EG/TLR7/INPP5D/PTPRC/TLR8/IRF4/CD4/IFNG/RELA/CCR2/TLR1/LILRB1/CD80/TICAM1/UBE2J
FTR/GABRB3

TAOK1/WNT5A/FGD2/ROR2/MAPK8IP3/ZEB2/DNAJA1/TLR6/FZD10/PTPN22/SAMD5/MAP4K5/EDN1/
XL3/RPS6/NFKBID/IRF4/IFNG/ANXA1/TSC1/LILRB1/CD80/TP53/FGL2/NLRP3/TNFSF18/ZBTB7B

ATP6V0D1/ATP2A3/COX6B1/COX8A/HVCN1/SLC17A5/SLC15A4/TWIST1/STOML2/ACTN4/COX7B/AT
/LAMA5/THY1/CORO1C/CDH6/F2RL1/CDH11/LIMCH1/ZNF703/NUMBL/PIP5K1C/FLOT1/ACTG1/CAP

AB7/RBM22/AAAS/PARK7/HYAL2/IFNG/PPP1R12A/SUPT6H/PIK3R2/NOLC1/PTPN14/NUP214/HDAC

1/CENPU/SCRIB/IFT57/PSPH/HSF1/SLC39A1/PRMT1/CFL1/MDFI/HNF1B/CCDC24/YBX3/TRIM28/
HG2/VAV1/ARFGEF3/GPR35/ADGRG1/RASGEF1A/ARHGAP9/RHOF/F2RL1/ARHGEF19/RELN/ARHGAP3

/PARK7/DERL1/ACE/PPP2CA/USP33/TICAM1/MEN1/WNT5A/ARRB1/WNT3A
N1/JAG1/MITF/RAC1/CALCA/BGN/IL15/ABR/VDR/TGFB3/TMEM119/AXL/ZNF675/IL20RA/SEMA3C

/TRIM71/HNRNPM/WNT4/PTBP1/THBS1/SULF2/HNRNPA1/POLR2I/POLR2J/POLR2D
4/OVOL2/ACVR1/SFRP2/AKNA/FOXF2/NOTCH1/POFUT2/BCL9L/TWIST1/PDPN/RBPJ/VASN/STRAP/
ATC4/USF1/RAD18/ERCC1/C9orf116/ACTR5/CCAR2/UBE4B/MSH6/ATR/ERCC3/SDF4/METTL3/DDB

GNMT
AT/ACSL6/HSD17B4/GCDH/ACAT1/TDO2/HSD17B8/DGAT2/ACSM3/ACSL5/PANK1/SULT1B1/ACSL1/
AT/ACSL6/HSD17B4/GCDH/ACAT1/TDO2/HSD17B8/DGAT2/ACSM3/ACSL5/PANK1/SULT1B1/ACSL1/
AT/ACSL6/HSD17B4/GCDH/ACAT1/TDO2/HSD17B8/DGAT2/ACSM3/ACSL5/PANK1/SULT1B1/ACSL1/

T/ITGB8/PSAP/PIGX/UGCG/HEXB

188/UQCC3/LDHA/PFKM/NUP85/NUP43/IMPDH2/PARP1/RAE1/NUP155/ALDOC/APP/NUP107/HK1/N

RN/CDKN2A/PLXNA3/NDRG3/PLCB1/DDR1/EXOSC4/CDK4/S100A8/TWF2/ADAM15/EZR/CDKN2D/TRP
B4X/WNK2/HDAC7/MUC1/EZR/CDKN2D/ZNF385A/CREB3L1/DDIT3/GPD1L/TIMP2/YBX3/MIF/CD300

1

/HMGCS2/APOC3/ADH1A/ADH1C/CYP2E1/CYP2C8/CYP2C9/ADH1B/ADH4/CYP3A4
3A4

XA8L1/VPS33B/TMEM127/DNAJC13/RAB7A/CHMP6/TSG101/CHMP4A/VTA1/ALS2CL/FASLG/CHMP2B

/NDC1/NUP188/UQCC3/LDHA/PFKM/NUP85/NUP43/IMPDH2/PARP1/RAE1/NUP155/ALDOC/APP/NUP
/PTPN2/IL20RB/GLMN/TNFRSF14/IDO1/RUNX3/XCL1/DUSP22/LOXL3/NFKBID/CASP3/ANXA1/WNK
F10/HAT1/MBD3/WDR5/PIH1D1/MORF4L2/MECP2/TWIST1/APBB1/DMAP1/CRTC2/POLE3/BRPF1/CT

N/ANXA13/MICALL2/SAE1/TRIM6/TRIM28/NUTF2/PHPT1/MIF/MTCL1/CD2/HIF1A/MAPK3/OAZ1/P
N/COL4A5/ITGAV/LAMB1/COLGALT1/ELF3/DDR1/SERPINH1/MPV17/ADAM15/BSG/COL16A1/COL3A

IL1B/CDK5/DNER/FA2H/CDKN2C/C5AR1/APP/GPR157/VIM/CSPG5/WDR1/EIF2B5/MAPT/RHEB/MED

/MTCH1/GHRHR/GDF11/TUSC2/CFTR/HOXA5/VEGFA/LGI4/MECP2

TGER4/TWSG1/TMEM98/NKAP/STOML2/RHOA/AP3B1/DOCK2/TNFRSF14/RUNX3/CCL19/XCL1/ATP7A
SLX4/MDC1/SMCHD1/BRCC3/UBE2V2/NUDT16L1/RNF168/RIF1/KDM2A/PAXIP1/MLH1/ZBTB7A/POL

/CBL/TRIM71/HNRNPM/WNT4/PTBP1/THBS1/SULF2/HNRNPA1/POLR2I/POLR2J/POLR2D
FOXF2/SGTA/PTK2/CSNK2A1/RPL5/PLK3/MAGEF1/MAP1A/CHFR/GLMN/CCAR2/EGF/CAV1/CSNK2A2

6/TCOF1/CENPU/SCRIB/IFT57/PSPH/HSF1/SLC39A1/PRMT1/CFL1/MDFI/HNF1B/CCDC24/YBX3/T

3/PSMC4/GSK3A/DVL2/IFT80/PSMD10/PSMD11/PSMD1/PSMA7/RUVBL2/PSMB3/SDHAF2/APCDD1/P
NF1/ZC3H12A/LAG3/LYN/CBFB/IL2RA/PDGFA/CD300LF/PTPN6/SAMSN1/LST1/ABR/CST7/IL4R/G

188/UQCC3/LDHA/PFKM/NUP85/NUP43/IMPDH2/PARP1/RAE1/NUP155/ALDOC/APP/NUP107/HK1/N

-DRB1/B3GAT3/HRAS/RAB31/GAL3ST4/HLA-DQA1/HLA-DRA/SCAMP3/YKT6/IFNGR2/RNF24/BSG/H
/MCM10/SMARCD3/POLD1/MCM7/PLCB1/PIF1/MCM3/RAD51AP1/RCC1/CENPO/EZH2/ALYREF/MUC1/
/CPLX2/CD74/P2RX4/PRKCD/DRAM1/LAT2/VCAN/TRIP10/HLA-DRB1/VAT1/ABCD1/ACP5/LAMP5/S
/CHEK1/RAN/HSF1/SMARCD1/HMGB2/TCF3/TRIM28/BRMS1/SIRT6/WDR76/RUVBL1/HIF1A/CBX1/C
ENPF/RFC4/ORC1/CHAF1B/NUSAP1/P3H4/CENPH/TTK/SKA1/RAD51/RCC2/TONSL/AURKA/SPI1/CB
TYMS/BOP1/DTL/FLNA/FEN1/CENPW/TAX1BP3/E2F5/MCM10/FANCD2/CCDC137/ARNTL2/SF3B4/DC
/CPLX2/CD74/P2RX4/PRKCD/DRAM1/LAT2/VCAN/TRIP10/HLA-DRB1/VAT1/ABCD1/ACP5/LAMP5/S
TP6V0D2/SYNGR1/DNAJC5/DDOST/ATP6V1C1/HLA-DPB1/CLTA/TMEM165/HSPA8/NEU1/HLA-DQB2/
/CPLX2/CD74/P2RX4/PRKCD/DRAM1/LAT2/VCAN/TRIP10/HLA-DRB1/VAT1/ABCD1/ACP5/LAMP5/S
C1L/NME1/CCDC28B/DCAF13/MCM3/NUP62/CCT5/SPAG5/MAPRE1/ASPM/RABL6/CKAP2L/IFT57/CH
C119/HMMR/MARCKS/DTL/CEP131/TUBG1/CCDC88B/FAM110A/RELB/SPATC1L/NME1/CCDC28B/DCA
P7D1/TUBG1/RACGAP1/SHCBP1/ARHGEF2/FAM110A/HSPB1/KIF11/NUP62/SPAG5/MAPRE1/MAD2L1
PM4/ARHGEF2/HSPB1/ITGAV/VASP/ADAM9/CDC42EP1/TES/HCK/FHL3/MAPRE1/ZYX/GIT1/NECTIN
D3NL/SCAMP3/NCF4/HLA-DQB1/FZD7/CD320/RAB11FIP5/TFRC/ATP6V0B/HLA-DPA1/HLA-DMB/DT
/SNAP25/ZDHHC13/CD74/PTAFR/SCGN/NCK2/ITGAV/SCAMP5/IL15RA/HLA-DRB1/SLC6A9/BAIAP2
P1/EZR/DCDC2/LSP1/CFL1/MICALL2/RHOQ/EPB41L2/FSCN1/WIPF3/CARMIL1/WASF2/GAS2L3/TP
RMIL1/TCF3/NR0B1/SNRPA1/STK17A/CSTF2/SGO2/SF3A2/SDCBP2/CD2BP2/DGKZ/RGS14/HIF1A/
PA1/DTNBP1/AREG/SYNGR1/DNAJC5/TRAPPC4/ARFGEF3/BRSK1/HLA-DPB1/RAB15/CLTA/SYT8/HL
CSK1N/FABP5/SERPINE2/TYROBP/CKAP4/PYGB/TGFB1/ITGAM/CTSV/CYBB/GSTP1/STX3/CD44/IT
3/CD74/PTAFR/SCGN/ITGAV/SCAMP5/IL15RA/HLA-DRB1/SLC6A9/BAIAP2L2/LAMP5/RAB31/HLA-
4/CENPH/TTK/SKA1/RAD51/RCC2/TONSL/AURKA/SPI1/CBX2/SPC24/NCAPD2/UHRF1/SKA3/FLNA/
GPC4/PMEPA1/GPC3/AGTRAP/AGRN/SNAP25/SDC3/ZDHHC13/CD74/SOD3/CYTH4/QSOX1/VCAN/SCA
LA-DQA1/MTMR2/ATP6V1F/GPC1/UNC13D/TUBB/LYZ/HLA-DRA/STARD3NL/CTSA/HLA-DQB1/TRPM2
D3NL/SCAMP3/NCF4/HLA-DQB1/FZD7/CD320/RAB11FIP5/TFRC/ATP6V0B/HLA-DPA1/HLA-DMB/DT
M7/PLCB1/PIF1/MCM3/RAD51AP1/RCC1/CENPO/EZH2/ALYREF/MUC1/EME1/ZNF385A/HMGB3/CREB
D/FLNA/SNAP25/MAL2/P2RX4/PRKCD/TRIP10/LAMB1/ABCD1/IGF2BP1/NME1/MVP/HRAS/APLP1/M
IB2/TPM4/ARHGEF2/HSPB1/ITGAV/VASP/ADAM9/CDC42EP1/TES/HCK/FHL3/MAPRE1/ZYX/GIT1/N
74/CYTH4/QSOX1/SCAMP5/IL15RA/HLA-DRB1/B3GAT3/HRAS/RAB31/GAL3ST4/HLA-DQA1/HLA-DR
TP6V0D2/SYNGR1/DNAJC5/DDOST/ATP6V1C1/HLA-DPB1/CLTA/TMEM165/HSPA8/NEU1/HLA-DQB2/
ISP/FBLN1/HID1/POF1B/PDLIM7/NUSAP1/SKA1/FLNC/LMNB1/RCC2/ARHGAP4/AURKA/PSRC1/COR
ISP/FBLN1/HID1/POF1B/PDLIM7/NUSAP1/SKA1/FLNC/LMNB1/RCC2/ARHGAP4/AURKA/PSRC1/COR
NM1/PCSK1N/SYT13/FABP5/SERPINE2/TYROBP/CKAP4/PYGB/TGFB1/ITGAM/PPT1/CTSV/CYBB/GS
ISP/FBLN1/HID1/POF1B/PDLIM7/NUSAP1/SKA1/FLNC/LMNB1/RCC2/ARHGAP4/AURKA/PSRC1/COR
B1/RCC2/ARHGAP4/AURKA/PSRC1/CORO1A/CLIP2/KRT17/SKA3/FLNA/TTLL4/RASSF3/TUBG1/TPM
4/SF3A2/RUVBL1/CD2BP2/JMJD6/LSM4/HSPA8/EIF3B/RPL36A/MRPL17/MCRIP1/PES1/DKC1/NPM
A4/ACTL6A/IRF1/COPS9/STAT1/HDAC1/DDX11/E2F4/PPARD/CBX3/DVL3/HDAC2/KDM1A/NASP/CH
RACGAP1/ARHGEF2/KLC2/REEP4/TRIM54/TPGS2/KIF11/GTSE1/CCT5/SPAG5/MAPRE1/TUBB/RUSC
2EP1/TES/HCK/FHL3/MAPRE1/ZYX/GIT1/CD151/EZR/BSG/RHOG/RPL8/CFL1/PLAU/EPB41L2/MRC
TES/HCK/FHL3/MAPRE1/ZYX/GIT1/CD151/EZR/BSG/RHOG/RPL8/CFL1/PLAU/EPB41L2/MRC2/APB
2EP1/TES/HCK/FHL3/MAPRE1/ZYX/GIT1/CD151/EZR/BSG/RHOG/RPL8/CFL1/PLAU/EPB41L2/MRC
ST/CD300A/FLOT2/IQGAP1/STXBP2/ITGAX/TCIRG1/CD63/DOK3/FPR1/RAP2B/RHOF/SIRPA/DEGS
TWF2/EZR/SCRIB/PLEK2/KIF18A/CFL1/FSCN1/CARMIL1/WASF2/APBB1IP/CXCR4/ITGA5/FLOT2/
FEN1/CENPW/DSCC1/OIP5/POLD1/MCM7/PIF1/MCM3/SPAG5/CENPO/EZH2/MAD2L1/DNMT1/ALYREF
A-DPA1/HLA-DMB/TPCN1/ATP6V0D2/SYNGR1/DNAJC5/DDOST/ATP6V1C1/HLA-DPB1/CLTA/RRAGD/
19/RPLP0/RPL18/RPL23/RACK1/GADD45GIP1/MRPL10/RPL38/MRPL52/RPL23A/MCTS1/MRPL48/D
KM/RPS21/TCP1/RPL7/RPL28/HOMER1/KXD1/PSMC4/CASP1/RPL39/RPL35A/EIF4A3/PSMD1/RPS3
SPAG5/CENPO/MAD2L1/CENPU/CHEK1/HSF1/RANGAP1/HMGB2/SGO2/CENPE/UBE2I/PES1/BRCA1/N

/CORO1B/DYNLT1/FLOT1/MYO1G/PSTPIP1/ITGB1BP1/RAC1/BCAR1/CDK5/CTTN/PLXND1/FAM89B/
MT1/CENPU/HSF1/KIF18A/RANGAP1/CENPL/SGO2/CBX1/CENPE/PPP2R1A/SGO1/DYNLL1/SPDL1/K
PRPF3/SNU13/TTF2/PRPF6/DDX41/SF3A3/CWC27/SNRPG/RBM17/HNRNPM/HNRNPA3/PRPF31/ZMAT
RPL10/RPL38/MRPL52/RPL23A/MCTS1/MRPL48/DAP3/MRPS7/MRPL53/MPV17L2/RPS7/RPL7A/RPS
/SIRPA/HK3/CD58/HSP90AB1/DYNLL1/ILF2/AMPD3/NBEAL2/GPI/PPIA/ACLY/DYNLT1/NCKAP1L/
NDA/PTGES2/GYG1/CCT2/DYNC1H1/FMOD/NPC2/PLBD2/PA2G4/ACLY/CAP1/ASAH1/RNASE2/GBA/C
/KNTC1/BEX4/DDX11/CEP85/CEP89/GPSM2/HAUS1/AUNIP/KNSTRN/RASSF1/POC1A/HAUS8/DYNC1
PIP5K1C/HIP1R/SLC9A3R1/FERMT1/ITGB1BP1/RAC1/PDE9A/BCAR1/CTTN/FGR/MYADM/VIL1/SAM
QGAP1/CENPE/CHMP4C/LZTS2/DDX11/GDI1/INCENP/PITPNM1/AGBL5/BCL3/KIF14/RAB11FIP4/A
8/PRSS3/DYNLL1/ILF2/NBEAL2/OLR1/NCKAP1L/ADAM8/ASAH1/SLC11A1/RAC1/CTSS/LAMTOR1/C
PC2/PA2G4/ACLY/CD68/CPNE1/CAP1/RNASE2/ANXA11/LAMTOR1/GNS/FTL/NFAM1/GALNS/DSN1/A
N2/KIFC3/KIF15/HAUS8/DYNC1LI1/MAP4/KIF2A/LRP8/DNAL4/ACTL8/KATNB1/HAUS6/NDE1/TCT
PC2/PA2G4/ACLY/CD68/CPNE1/CAP1/RNASE2/ANXA11/LAMTOR1/GNS/FTL/NFAM1/GALNS/DSN1/A
THOC6/MSH2/CBX5/XRCC6/TELO2/NABP1/RPA2/EID3/PTGES3/NAT10/DNA2/DOT1L/RAD51D/WRAP
B/ATP6V0E1/CALR/CDC42EP2/OCRL/STX4/VIM/ATP6V0D1/HVCN1/VAMP8/GNLY/SRGAP2/DNM2/DM
SPDL1/KNTC1/ERCC6L/NUP37/INCENP/ITGB3BP/DCTN2/KNSTRN/DCTN5/CENPI/NUP85/PPP1CC/C
H1/KPNB1/CTSS/GNS/PSMA5/PSMD13/COMMD9/IMPDH2/PSMD11/GSDMD/ALDOC/PNP/XRCC6/PSMD3
/MRPL18/RPL6/RPL37/RPL27A/RPL36/UBA52/RPL19/MRPL37/RPL22L1/NSUN4/RPL13A/MRPL27/
/SPDL1/KNTC1/ERCC6L/NUP37/INCENP/CBX3/ITGB3BP/KNSTRN/DCTN5/NUP85/PPP1CC/CENPK/N
C1/CTTN/WAS/SPECC1/KPTN/SMTN/VPS18/MYO9B/MYO5A/WDR1
/INCENP/ITGB3BP/KNSTRN/DCTN5/NUP85/PPP1CC/CENPK/NUP43/DYNC1LI1/DSN1/ZNF207/NUP1
RPA2/NAT10/DNA2/RAD51D/DCLRE1B/GAR1/HAT1/NBN/ERCC1/THOC2/RPA1/ATR/TP53BP1/THOC1
OPG1/TMEM199/PDCD6/RASSF9/SEC13/COPG2/SPPL3/CFTR/SREBF1/HLA-F/COPB2/CLTC/AP1S1/
NXA11/YWHAB/CTSD/MYO5A/PDIA6/RAB5C/ERP29/HSP90AA1/HSPA5/CANX/RAB29/PPIB/CLTC/RA
NXA11/YWHAB/CTSD/MYO5A/PDIA6/RAB5C/ERP29/HSP90AA1/HSPA5/CANX/RAB29/PPIB/CLTC/RA
RPL5/RPS27A/RPS12/RPS4X/RPL6/RPL37/RPL27A/RPL36/UBA52/RPL19/RPS11/RPL22L1/RPL13
BL5/GPSM2/KNSTRN/CTTN/MAP4/RAE1/RPS3/CDC42/KAT2A/IKBKG/TBL1XR1/IQCB1/KDF1/CCSAP
90AA1/MAN2B1/HEXB/BCAN/ACAN/LGMN/TPP1/MANBA/GPC2/HEXA
SPA2/RAD1/NOL6/SYCE1L/ADD3/NCAPD3/TEX11
F19/SART1/PHF5A/PRPF38A/HTATSF1/LSM8/PPIE/SF3B3/DHX15/LUC7L/SF3B2/MFAP1/PRPF4/C
PPC1/CCT8/S100A7/GCA/VCP/MAN2B1/CYB5R3/HEXB
RBMX/HNRNPA1/PPIE/SF3B3/HNRNPR/SF3B2/DHX38/SRSF1/DHX8/RBM8A/SF3B6/MAGOHB/SNRPD3

SF1/LSM8/SART3/TGS1/USP39/LUC7L/SF3B2/STRAP/LSM1/PRPF4/ARFGEF1/GEMIN2/SNRNP70/S
33/CD177/ATP6AP2/SLC2A3/TSPAN14
8/SART3/TGS1/USP39/LUC7L/SF3B2/PRPF4/ARFGEF1/SNRNP70/SF3B6/SNRPD3
/FAM207A/BMS1/NOL10/RSL1D1/EBNA1BP2/EIF6/UTP23/WDR46/IMP4/NIP7/RPF1/RIOK1/WDR12
RCA5/DMAP1/RPA1/SMARCAL1/TP53BP1/MMS22L
0/SURF6/RPL29/RPL13/RPL4/RPL41/RPL9/RPL24/RPL11/RPL10/RPL14/RPL10A/ZNF622/RPL12
3/USP39/LUC7L/SF3B2/PRPF4/SNRNP70/SF3B6/SNRPD3

4/MAGOHB/SNRPD3/IK/SNRNP200/BUD13
4/MAGOHB/SNRPD3/PRPF38B/IK/SNRNP200/BUD13

BG1/PLIN3/CD74/KCNJ11/PKN1/STEAP1/SCAMP5/IL15RA/HLA-DRB1/PLEKHB2/TSPAN15/LAMP5/
NRPD1/POLA2/CACYBP/DDOST/NAA10/RUVBL1/MAD2L2/MRGBP/UBE2I/RNF19B/SHARPIN/TAF6/PH
NXA11/LAMTOR1/CD47/PTPN6/AGPAT2/SLC44A2/TMEM63A/GSDMD/STK10/GPR84/CNN2/LAIR1/CT
8/CD33/CD177/TMBIM1/SLC15A4/SLC2A3/TSPAN14/MLEC/ADGRG3/ITGAL/ATP11A/PLD1/SLCO4C
A/NCAPD3/SUV39H1/HMGA2/MORC2

O2/RAD21/VIM/CXXC1/OGG1/HNRNPM/MAPT/PHF5A/GHRHR/SRPK1/HAT1/HNRNPU/PIAS4/CSNK2B/

ALNT3/CLIP3/NSF/B4GALNT4/FUT1/NSFL1C/LPCAT2/ARAP1/NECAB3/FUT8/PLK3/B4GALT4/YIPF
P3/SLC29A3/VPS28/MVB12A/STARD3/VPS18/WDR91/VAMP8/LTV1/FIG4/MCOLN3/VAC14/YIPF2/M
P1/RPL41/RPS6/RPL24/UNK/RPL11/AGO1/RPL10A/FXR1/EIF4G1/RPS28/PNPT1/ATXN2/RPL31/N
APBP/TAP2/RAB9A/ATP7A/RAB8B/HLA-C/VAMP3/RAB22A/ATP6V0A1/HLA-E/RAB7A/APPL2/TLR1/

UB1/E2F4/PPARD/LEF1/NONO/SIX2/ATF4/CDK2/SNF8/HCLS1/KDM1A/MED8/CBFB/MTA2/DEAF1/T
SN/PDE4A/PROM1/RAB25/LCP1/PAK1/GNA12/GNGT1/THY1/CORO1C/SLC39A6/HSP90AB1/ARPC2/G

SPAG4/RAN/OSBPL3/TMEM201/RANGAP1/RRP12/CHMP4B/EIF5A2/CACYBP/BAX/NUTF2/NRM/ALOX5
/PDPN/FAP/EPS8L1/EPHA2/KSR1/CIB1/C2CD5/MYO1C/ABCA7/THEM4/ADGRE2

A-DQA2/FZD2/HLA-DRB5/NECAP2/CLTB/AP2M1/NCALD/DVL2/GAD1/AP2S1/VPS18/SYT1/OCRL/AP

LA-DRB5/COPZ1/NECAP2/CLTB/AP2M1/NCALD/GAD1/AP2S1/HLA-B/SYT1/AP1G2/AP1B1/M6PR/CO
SYT1/AP1G2/AP1B1/M6PR/RASSF9/CEMIP/VAMP8/DBNL/CFTR/RAB3A/MYO6/AP2A2/AP3B1/CLTC/

STX10/RAB35/VMA21/PAM/HLA-DQA2/SV2A/SYNGR3/HLA-DRB5/MCTP1/TMEM163/WFS1/CLTB/NCA

S3/AP1S2/AP2B1/COPB1/AP2A1/VPS33A/COPA/KLHL12/PEF1/AP5B1/AP4B1/IGF2R/AP5S1/ARCN
S3/AP1S2/AP2B1/COPB1/AP2A1/VPS33A/COPA/KLHL12/PEF1/AP5B1/AP4B1/IGF2R/AP5S1/ARCN

P3K14/SIRT7/FBXW9/DSN1/SMARCB1/RAE1/SNU13/POLR2L/POLR2K/IP6K1/RRP7BP/UTP4/INO80

LAMC2/FCHSD1/RHOF/TNFAIP2/CORO1C/GPSM1/DYNC1H1/TIGD5/WIPF1/HIP1R/LASP1/GPSM2/FL
LTB/NCALD/HLA-B/APP/OCRL/AP1G2/CSPG5/COPG1/TMEM199/PDCD6/RASSF9/SEC13/COPG2/SPP
L1/GMFG/WDR1/NF2/ACTR3/DBNL/CAPN2/WIPF2/DSTN/PLEKHH2/ACTN4/SPTAN1/GMFB/MAEA
2F1/TAF3/ERCC3/ZNF768/TADA3/PAF1/TRRAP/RPRD1A/TCEA1/GTF2A2/POLR3F/POLR2B/POLR1C
2/ARFRP1/RGP1/GOLPH3L/CLTC/AP1S1/AP1S3/AP1S2/COG4/COG2/RAB9A/ARL1/AP4B1/IGF2R/V

/MATK/TRAF3/GNB1L/KCNAB2/GNG4/GNGT2/STAC/PALM
2F1/TAF3/ERCC3/ZNF768/TADA3/PAF1/TRRAP/RPRD1A/TCEA1/GTF2A2/POLR3F/POLR2B/POLR1C

/HELB/XRCC5/ACTR2/TP53/SETMAR
/UNC93B1/STXBP2/HLA-DQB2/TCIRG1/HLA-A/ITGB5/STX6/RAB7B/CTLA4/HYOU1/RAB35/HIP1R/
A/FGR/PLXND1/PIEZO1/ARF4/RPS3/SPRY4/EPB41L3/MAPT/CSPG4/NF2/SGCE/PALM/WWC1/DNM2/
NPU/PIAS4/CSNK2B/DGKQ/DCAF7/MAEA/MORC2/THOC1/ARFGEF1/CASK/ENC1/GMCL1/SPARC/HNRN
2F1/TAF3/ERCC3/ZNF768/TADA3/PAF1/TRRAP/RPRD1A/TCEA1/GTF2A2/POLR3F/POLR2B/POLR1C

AM/HLA-DQA2/CLN3/HLA-DRB5/RAC1/GBA/CLTB/RAB10/SMPD4/CHST2/LLGL1/PLEKHJ1/APP/SLC

1A1/RAB20/HLA-DRB5/WNT7B/CACNG4/AP2M1/RAB10/AP2S1/ATP6V0C/HLA-B/ATP6V0E1/CALR/O

HLA-F/RAB11A/ABHD17A/PDIA3/RAB13/SLC9A6/RAB8B/HLA-C/VAMP3/HLA-E/SORCS2/GRIPAP1/

9/CTLA4/SORT1/RAB35/VMA21/HIP1R/BCAP31/HLA-DQA2/FZD2/KDELR1/HLA-DRB5/COPZ1/NECA
6A9/IGF2BP1/ADORA1/PRNP/LAMP5/ITPKA/C1QL1/PLCB1/ABHD17C/MTMR2/NRGN/DAGLA/RUSC1/
Z/NME2/F13A1/CTSA/QPCT/ISLR/TUBB4B/GRN/CHIT1/PSMD14/TIMP2/HSPA6/ALOX5/MIF/CANT1

35/SENP3/WTAP/RNF2/WDR5/TRIM37/RNMT/RBM15B/PELP1/PAGR1/METTL3/TEX10/SNRPD3/RIOK

4/UNC45A/NONO/SMC4/U2AF2/SRSF9/ATXN2L/EHMT2/SNRNP40/AKAP8L/RBM19/PIAS3/CBX4/FIB
/IQGAP1/EFHD2/SLC9A1/EFNA5/CD2/MAPK3/EFNB1/SMPD2/ADGRG1/SPRED1/RAP2B/THY1/CORO1

/IQGAP1/EFHD2/SLC9A1/EFNA5/CD2/MAPK3/EFNB1/SMPD2/ADGRG1/SPRED1/RAP2B/THY1/CORO1

/MCTP1/TMEM163/VDAC1/WFS1/CLTB/RAB10/KCNK9/SYT12/ICA1/SYT1/APP/DNM1L/SYT3/STX4/
BD10/SNRPB2/PAFAH1B2/HEATR1/TIMM13/AKNA/GAR1/SP140/UBTF/NUAK1/SMARCA5/RNMT/ZNF4

1/WFS1/CLTB/RAB10/KCNK9/SYT12/ICA1/SYT1/APP/DNM1L/STX4/RABAC1/ATP6V0D1/RAB3D/MF
/FLOT2/IQGAP1/EFHD2/SLC9A1/EFNA5/CD2/MAPK3/EFNB1/SMPD2/ADGRG1/SPRED1/RAP2B/THY1
PC/PHLDB1/HCLS1/ACTB/CTTN/MYADM/WAS/CLTB/RAB10/NSMF/RIMS3/LLGL1/PPFIA3/ACTL8/GM
/RASA3/STAC3/MATK/TRAF3/RPL27/GNB1L/RASAL3/KCNAB2/EEF1A2/GNG4/GNGT2/STAC/PALM/S

8/B4GALT4/YIPF2/GOLPH3L/TMEM115/TMEM87A/FUT11/BET1L/YIPF1/B3GALT6/GCNT1
P1/KIF21B/DTNBP1/FSCN1/TIMP2/MAP2/BRSK1/IQGAP1/HIF1A/HSPA8/NRCAM/THY1/BASP1/BIN

EM87A/FUT11
2/MED25/GTF3C1/E2F6/NFYC/POU2AF1/TAF12/TAF10/RARG/NFATC4/GTF3C5/HNRNPU/THRA/ERC
33/MRPS5/MRPL36/MRPL14/MRPL3/MRPL20/MRPL24/MRPS16/MRPL55/CHCHD1/MRPS2/MRPL21/ND
33/MRPS5/MRPL36/MRPL14/MRPL3/MRPL20/MRPL24/MRPS16/MRPL55/CHCHD1/MRPS2/MRPL21/ND

8/LYZ/NME2/F13A1/CTSA/QPCT/ISLR/TUBB4B/GRN/CHIT1/PSMD14/TIMP2/HSPA6/ALOX5/MIF/C
/CFL1/RANGAP1/RHOQ/GIPC1/EPB41L2/DTNBP1/WIPF3/TRAF2/RHOC/MAP2/FLOT2/ABHD12/CLTA
Y1/BASP1/PALLD/HSP90AB1/CAD/ARPC3/DYNLT1/CYTH2/ACTG1/SRI/DCTN2/FKBP4/ITGA2/ACTB

PC11/UBE2D1/DCAF16/UBE2E1/FBXL6/BARD1/UBE2A/ASB1/KCTD2/CBX8/DCUN1D5/PCGF2/RBX1/

/SCP2/PHYH/FABP1/CAT/ACSL1/EPHX2/ACOX2/BAAT/SLC27A2/PIPOX/EHHADH/DAO/HAO2/HAO1/
/SCP2/PHYH/FABP1/CAT/ACSL1/EPHX2/ACOX2/BAAT/SLC27A2/PIPOX/EHHADH/DAO/HAO2/HAO1/
C3/NFYA/ATF6B/IRF4/MAX/TERF1/KCNIP3/HELB/XRCC5/GTF2E1/SUZ12/POLD2/SP1/WDR18/PUR

8/LYZ/NME2/F13A1/CTSA/QPCT/ISLR/TUBB4B/GRN/CHIT1/PSMD14/TIMP2/HSPA6/ALOX5/MIF/C
TN/PLEKHH2/SPTAN1/GMFB/MAEA/LLGL2/PPP1R9A/UTRN

B/C4BPA/CFHR3/PON1/HPR/KNG1/A1BG/APOA1/HPX/PLG/AHSG/AFM/HRG/SERPINC1

YO1C/AAAS/IPO7/NUP214/BICD2/DDX19A/TPR/MCM3AP/NUP50/SNUPN/IPO5/NUP133

RPS24/RPS3A/MRPS16/MRPS2/RPS18/RPS28/RPS17/RPS15A/MRPS26/MRPS11/MRPL42/MRPS27/M
CCND2/POLE4/POLA1/POLR2L/KAT2A/PRIM2/POLR2K/IKBKG/LAS1L/POLD3/POLR3D/IGF1R/POLR

K1/CCNJ/SESN2/ERCC2

P4/CDK5/CTTN/STMN3/INPP5J/APP/GDPD5/KATNB1/CBL/MYO5A/CCDC120/MAPT/ARPC5/STMN2/D

NTPD4/TEX261/RER1/UBIAD1/CSGALNACT1

5/NDUFB9/MRPL48/DAP3/MRPS7/DNA2/MRPL53/MPV17L2/TIMM13/APOO/MRPS17/MRPS21/MRPS15

27/HGS/NPC1/RAB7B/CD68/LAMP3/KCNQ1/SNF8/CLN3/SLC11A1/HLA-DRB5/LAMTOR5/CTSS/LAMT
RHGEF2/P2RX4/SERPINI1/CBX6/IGF2BP1/ADORA1/PRNP/LAMP5/ITPKA/ABHD17C/NPTX2/MTMR2/
2
IL2/ESYT1/GNAS/HIP1/GNAI2/DTNA/MYZAP

5/CTTN/STMN3/INPP5J/APP/GDPD5/KATNB1/CBL/MYO5A/CCDC120/MAPT/ARPC5/STMN2/HSP90AA

/SHARPIN/NRCAM/STX1A/GABRA3/PPP1R9B/HIP1R/ARFGAP1/MT3/PRR7/LRFN1/LYN/CDK5/SPOCK

NIK/HLA-B/DENND6B/PLEKHJ1/APP/SLC9A7/MYO5A/CLIP3/RASSF9/ABCG1/RAB4B/STX7/VAMP8/

/PECR/GRHPR/SCP2/PHYH/RIDA/FABP1/CAT/ACSL1/EPHX2/XDH/ACOX2/BAAT/SLC27A2/PIPOX/E
DC42/AGO2/APOBEC3D/TDRKH/RBPMS/PABPC4/RPLP0/SNRPG/TRIM71/MAPT/HNRNPA3/PIWIL4/CH
/PECR/GRHPR/SCP2/PHYH/RIDA/FABP1/CAT/ACSL1/EPHX2/XDH/ACOX2/BAAT/SLC27A2/PIPOX/E
M/STX1A/ARPC2/CDH11/ARHGAP39/PPP1CA/FLOT1/PRR7/SV2A/LYN/ARPC5L/RAC1/ACTB/CDK5/A

N4/PSMB5/PSMA4/PSMD9/PSMC3/PSMC5/ENY2/TADA3/TRRAP/PSMB1/PSMB6/PSMA3/HTRA2/PSMB7

M/STX1A/PPP1R9B/HIP1R/ARFGAP1/MT3/PRR7/LRFN1/LYN/CDK5/SPOCK1/RPL7/ADRA2C/HOMER1

3

PL42/MRPL22/MRPL58/MRPL38/MRPS30/MRPS18A/MRPL43/MRPL50/MRPL45/NDUFAB1/MRPL41/MR
PL42/MRPL22/MRPL58/MRPL38/MRPS30/MRPS18A/MRPL43/MRPL50/MRPL45/NDUFAB1/MRPL41/MR
C3/SLC39A13/SLC35B2/SV2A/MCU/SLC35A2/TMEM163/WFS1/A4GALT/CHST2/TMEM43/SLC22A17/

MARCD1/TRIM28/SIRT6/FAAP24/GMNN/NUPR1/PYGO2/CBX1/NPM1/RBL1/PCNA/MLLT3/CHAF1A/SM
RT6/GMNN/NFKB2/RUVBL1/MED22/JMJD6/NUPR1/CITED4/BCL9/PPP1R13L/RFXANK/NPM1/RBBP8/
TK39/HCK/DDR1/WNK2/CDK4/NME2/CSF1R/CDC7/GUCY2C/CHEK1/RPS6KA1/LIMK2/JAK3/GRK6/TR
UP62/TUBB/ASPH/LAD1/MAPK8IP2/DLG3/MARVELD1/COL16A1/COL3A1/LAMC1/SPAG4/TUBB4B/RP
CA2A/CTSA/ADAP2/NCF4/TBC1D16/NCF1/RANGAP1/IGFBP3/PSMD14/SAE1/RABEP2/CCL5/BEX3/I
A1/MISP/POF1B/PDLIM7/NUSAP1/SKA1/FLNC/ENAH/IQGAP3/RCC2/GAPDH/PSRC1/CORO1A/CLIP2
1/CDK16/HK2/PFKFB4/PKN1/PRKCD/EFNA4/NME1/ITPKA/SYK/STK39/PFKFB3/HCK/DDR1/WNK2/C
HOV/ARL4C/RAD54L/TNNT1/MCM6/DDX39A/TUBG1/DSCC1/TOR4A/GINS2/KLC2/RGS19/MCM7/RAB4
HOV/ARL4C/RAD54L/TNNT1/MCM6/DDX39A/TUBG1/DSCC1/TOR4A/GINS2/KLC2/RGS19/MCM7/RAB4
HOV/ARL4C/RAD54L/TNNT1/MCM6/DDX39A/TUBG1/DSCC1/TOR4A/GINS2/KLC2/RGS19/MCM7/RAB4
HOV/ARL4C/RAD54L/TNNT1/MCM6/DDX39A/TUBG1/DSCC1/TOR4A/GINS2/KLC2/RGS19/MCM7/RAB4
AURKA/GSTP1/MARCKS/CDC25C/SQSTM1/YWHAZ/FLNA/ITGB2/CDKN2A/RACGAP1/PKN1/PTAFR/PRK
SQSTM1/YWHAZ/ITGB2/CDKN2A/RACGAP1/PKN1/PTAFR/PRKCD/HSPB1/RELB/ITGAV/IL15RA/PLEK
NUP62/TUBB/PAG1/DLG3/ADAM15/EZR/CASP4/SH2D2A/FZD7/SLC26A6/CHEK1/DNAJC6/NCF1/CCT
CNE1/CCNA2/PCSK1N/SERPINE2/TRIB3/DEPDC1B/FBLN1/ECT2/ARL2/CCNF/TGFB1/ARHGAP4/PAP
C2/ITGAV/LAMB1/VASP/ADAM9/SYK/CDC42EP1/TES/MAPRE1/TAGLN2/LAD1/TWF2/FMNL2/ASAP1/
D16/MICALL2/RANGAP1/ARHGEF39/YBX3/PLEKHG2/TRAPPC4/VAV1/NUTF2/ARFGEF3/IQGAP1/CLT
KIF18A/CFL1/MICALL2/GIPC1/EPB41L2/FSCN1/WIPF3/WASF2/CXCR4/GAS2L3/TPM2/MSN/PPP1R
PK2/STK17A/BRSK1/PRKDC/MAPK3/MAST2/MAP2K2/PAK1/PRKX/DYRK2/CSNK1E/MARK2/RPS6KA4/
F18A/MCM5/KIF21B/ATP6V0B/ABCC4/ATP6V0D2/HSPA6/ATP6V1C1/ABCC5/OLA1/RUVBL1/CHTF18
/RANGAP1/ARHGEF39/YBX3/PLEKHG2/TRAPPC4/VAV1/NUTF2/IQGAP1/MICALL1/ATP6AP1/PAK1/A
/RANGAP1/ARHGEF39/YBX3/PLEKHG2/TRAPPC4/VAV1/NUTF2/IQGAP1/MICALL1/ATP6AP1/PAK1/A

EF19/TBC1D1/RIN1/ARHGAP39/GDI1/ARFGAP1/HSPBP1/TBC1D10B/GPSM2/NCKAP1L/ARHGAP1/RT
F11/CCT5/SPAG5/MAPRE1/MX2/S100A8/EZR/KIF12/RAB11FIP5/KIF3C/KIF18A/KIF21B/CACYBP
/BSG/NOP56/SCRIB/CTNND2/RAN/RANGAP1/GIPC1/FSCN1/ATIC/CHMP4B/WASF2/BZW2/PRDX1/IQ
D/GBP2/RGS14/RAB25/RIT1/RRAGC/GNA12/RAP2B/GNGT1/TUBB6/RHOF/EFTUD2/TUBA1A/TUBB2A
EDA/NOL3/PDCL3/STAT1/CCL13/BID/CNIH4/CCL2/SHC1/CXCL9/CXCL3/CXCL13/DEFB1/CCL28/I
IN1/ARHGAP39/GDI1/ARFGAP1/TBC1D10B/GPSM2/NCKAP1L/ARHGAP1/RTKN/RAP1GAP2/SIPA1L2/
/ARFGAP1/TBC1D10B/NCKAP1L/ARHGAP1/RAP1GAP2/SIPA1L2/ARHGDIB/TBC1D30/TBC1D13/RASA
XCL13/CCL28/IL1B/TNFSF13B/CCL4/TGFB2/CXCL16/IL15/TNFSF12/PF4V1/TGFB3/CXCL11/XCL
F12/KIF3C/KIF18A/KIF21B/FAM83D/GAS2L3/MAP2/MTCL1/RGS14/MAP1S/MAST2/CENPE/KLC3/C
C3/ERCC6L/RFC2/DDX11/BLM/CHRAC1/LIG1/DNMT3A/ISG20/CHD4/RUVBL2/POLE4/RPS3/POLA1/
PF1/CORO1B/ABLIM2/HIP1R/LASP1/FERMT1/CAPZA1/HCLS1/PSTPIP1/ARPC5L/CTTN/CORO2A/WA
3/ARHGDIB/PKN3/KCTD10/WAS/DVL2/PFN1/WASF1/ABR/CDC42SE1/FGD6/PARD6B/INF2/CDC42SE
/GPRC5B/CDKN1C/SOCS3/PPP1R1B/GCN1/CDKN2C/CCND2/MTCP1/GMFG/RACK1/DBF4B/PKIA/CALM
S3/PFKM/PHB/LCK/BANF1/PPP1CC/CDK7/MSH2/YWHAQ/YWHAB/NCL/AGO2/TOPBP1/SYT1/XRCC6/S
D9/CBX5/MTF2/ZMYND8/CBX8/TBL1XR1/VRK1/DPF2/WRAP53/HAT1/ANP32E/UIMC1/WDR5/PIH1D1
0/RPL38/MRPL52/RPL23A/DAP3/MRPS7/RPS7/RPL7A/RPS23/RPS10/MRPS17/MRPS21/RPS15/RPS
3C/LONP1/SSBP1/HSPD1/RAD51D/MYEF2/HNRNPU/RAD18/ERCC1/TSN/HNRNPA1/POLR2D/DHX9/RP
15A/RACK1/IGBP1/RPA2/MAPT/ANKLE2/VCP/KCNN4/CSK/BAD/CD33/BOD1/PTK2/STYXL1/PTPN1/
B12A/GPX1/ABI3/GRB2/AFAP1/SH3GL1/CBL/ABI2/RAD9A/MAPT/KHDRBS1/CCDC6/ABL1/RUFY1/P
6/GRPEL2/DNAJB11/HSPE1/CCT8/HSPD1/PTGES3/HSPA2/HSP90AA1/PFDN6/HSPA5/TTC1/CANX/H
B1BP1/ITGA2/IL1B/CDH17/ADAM23/IBSP/CALR/ITGB8/EMILIN1/SFRP2/VCAM1/PTPN2/THBS1/A

6/ERCC3

K2/IRAK1/CDK16/HK2/PFKFB4/PKN1/PRKCD/POLD1/EFNA4/NME1/ITPKA/SYK/STK39/PFKFB3/HC
C3/MRPL13/CPSF6/EIF4A3/ILF3/XPO5/RPS3/SAMD4B/HNRNPAB/NCL/AGO2/CALR/RPL35/RBPMS/
PME1/KCNQ1/ANAPC7/STRN4/LCK/PPP1CC/CTTNBP2NL/CADM4/TRAF3/GRB2/PTPA/KAT2A/CHCHD3
8/DDX41/HELLS/XRCC6/POLQ/DDX54/DNA2/DDX23/CHD1L/DQX1/NBN/RAD18/DDX56/DHX57/ACTR
598/PRPF31/SNRPB2/EIF3K/HNRNPU/NVL/CSNK2B/HSPA5/PHF6/ETF1/EIF5A/PRMT3/RPLP1/DHX

CDKN2B/GPRC5B/CDKN1C/SOCS3/PPP1R1B/GCN1/CDKN2C/CCND2/MTCP1/GMFG/WDR91/RACK1/DBF
WIPI1
1/ARHGAP17/RALBP1/NCKAP1/NOX1/SRGAP1/EPS8L2/DOCK7/MAP3K11/VAV3

FB4/PKN1/PRKCD/EFNA4/ITPKA/SYK/STK39/PFKFB3/HCK/DDR1/WNK2/CDK4/NME2/CSF1R/CDC7/
TPI1/STAT1/NPLOC4/GPI/PA2G4/SLC22A18/BID/HSPBP1/UBE2J2/OTUB1/ACTG1/PRR7/AXIN1/L
CFC1/FKBP4/CNTNAP1/LAMTOR5/JCHAIN/LAMTOR1/WWC3/HOMER1/AP2M1/SLA/EVPL/DVL2/AP2S1

4/GPI/PA2G4/SLC22A18/BID/HSPBP1/UBE2J2/OTUB1/ACTG1/AXIN1/LYN/WFS1/UBE2L3/TCP1/P

UPT6H/SUPT16H/HMGN3/L3MBTL1/ACTR6/EZH1

MYO9B/CCDC102A/MYO5A

OLA1/RRAGD/GBP2/RAB25/RIT1/RRAGC/GNA12/RAP2B/TUBB6/RHOF/EFTUD2/TUBA1A/TUBB2A/RA
OLA1/RRAGD/GBP2/RAB25/RIT1/RRAGC/GNA12/RAP2B/TUBB6/RHOF/EFTUD2/TUBA1A/TUBB2A/RA

2/VIL1/WDR45B/PFN1/SNX27/GSDMD/SYT1/BTK/ARFIP2/PLEKHN1/MAPT/OBSCN/SNX11/ARAP1/S

F1BP1/HSP90AA1/HDAC10/USF1

GBP2/RAB25/RIT1/RRAGC/GNA12/RAP2B/TUBB6/RHOF/EFTUD2/TUBA1A/TUBB2A/RAB3B/RASEF/H
GBP2/RAB25/RIT1/RRAGC/GNA12/RAP2B/TUBB6/RHOF/EFTUD2/TUBA1A/TUBB2A/RAB3B/RASEF/H

RAD23A/APEX1
B35/PITPNM1/HIP1R/SNX10/PLEKHA4/MYO1G/KCNQ1/TULP3/PLEKHA2/VIL1/WDR45B/PFN1/SNX2

RAB25/RIT1/RRAGC/GNA12/RAP2B/TUBB6/RHOF/EFTUD2/TUBA1A/TUBB2A/RAB3B/RASEF/HSP90A
/AP2S1/GRB2/VPS18/CBX5/COL8A2/SKAP2/ACTL8/ABI2/MAPT/PDCD6/KHDRBS1/CHN1/COL11A1/

DD3/CD2AP/TUBB3/TLN1

MARK4/GGA3/UBR5/DAXX/RNF8/FAF1/UBE2N/AUP1

1/LUM/COL4A2/LAMB3/MATN3/COL8A2/ECM1/EMILIN1/COL11A1/THBS2/LAMA4/COL9A3/EFEMP2/
03

/TNFRSF11B/CD320/GRN/LTB/HMGB2/AREG/RABEP2/CCL5/SEMA4A/CCL26/TNFSF9/MIF/ZP3/EFN

RIMS3/RUNDC1/LLGL1/DNM1L/TRAPPC3/MYO5A/TBC1D10C/PIFO/BICDL2/NSF/TBC1D22A/DENND3

GBP2/RAB25/RIT1/RRAGC/GNA12/RAP2B/TUBB6/RHOF/EFTUD2/TUBA1A/TUBB2A/RAB3B/RASEF/H
GBP2/RAB25/RIT1/RRAGC/GNA12/RAP2B/TUBB6/RHOF/EFTUD2/TUBA1A/TUBB2A/RAB3B/RASEF/H
/AUP1

NK2

/PAH/FMO3/CYP1A2/AKR1D1/CYP4F2/CYP7A1/CYP2B6/CYP4A11/CYP2E1/CYP2C8/CYP2C9/CYP8B

PITRM1/ADAMTS2

PT4H1/PABPC1L/TLR8/SRA1/STRBP/HNRNPC/AGO1/FXR1/ANXA1/SYNCRIP/RBMS2/PNPT1/DDX1/T
ATS2/HMGB2/TRIM6/TCF3/BRMS1/NR0B1/GMNN/PRKDC/RUVBL1/MAD2L2/HIF1A/TRIM11/UBE2I/P

/HTATIP2/KDM1A/HCFC1/CBFB/BUD31/MTA2/UBE2L3/TAF9/PQBP1/PML/SMARCB1/ZMIZ2/VDR/CD
P7/DTD1/RNASE2/EMG1/TRMT1/PRIM1/TRMT61A/RBMX2/POP1/TRMT112/PTRH2/TRMT6/EDC3/ISY

BP4/APOA1/APOA5/CFHR4/SLC27A5/SLC10A1

GXT/GNMT/AFM/TAT/ADH4
3A5/CYP2A7/CYP1A2/CYP4F2/CYP7A1/CYP2B6/CYP4A11/HRG/CYP2E1/CYP2C8/CYP2C9/CYP8B1/

L3/CNIH4/KISS1/CCL2/CXCL9/CXCL3/CXCL13/DEFB1/WNT7B/CCL28/REEP2/GPRC5B/TULP3/ADR
AP1/BATF3/RUVBL2/SAP30/ZMYND8/HSBP1/NCOR2/DDX54/TBL1XR1/DR1/E2F6/DPF2/MYBBP1A/W

1/OR2A14/OR3A1/OR10H3/OR51D1/OR52B2/OR7C2/OR10H4/OR4D5/OR13D1/OR52K2/OR52A1/OR6

CD320/GRN/LTB/HMGB2/AREG/RABEP2/CCL5/SEMA4A/CCL26/TNFSF9/MIF/ZP3/EFNA5/IL34/EBI

3A5/CYP2A7/CYP1A2/CYP4F2/CYP7A1/CYP2B6/CYP4A11/HRG/CYP2E1/CYP2C8/CYP2C9/CYP8B1/

HSPA8/TCIRG1/CHD3/ATP6AP1/ABCB6/XRCC2/SMARCA4/HSP90AB1/DYNLL1/DYNC1H1/XRCC3/ATP

/SLC16A11/SLC1A2/SLC6A12/SLC17A2/SLC6A1/SLC38A4/SLC13A5/SLC22A1/SLC10A1

/CYP2A7/PAH/AOX1/CYP1A2/CYP4F2/CYP7A1/CDO1/CYP2B6/CYP4A11/CYP2E1/CYP2C8/CYP2C9/

SP1/ARID3B/CCAR1/LDB1/NFYA/ATF6B/HOXB6/HNRNPC/MBD2/RELA/SMAD2/PBX3/SMAD3/TP53/Z

IAS3/KCTD10/RNF26/UBE2L3/CBX4/RNF187/RNF157/ANAPC11/UBE2D1/RNF220/RNF113A/ZMIZ2

LC25A15/ABCB11/MFSD2A/SLC38A3/SLC51A/SLC16A11/SLCO1B3/SLC27A2/SLC1A2/SLC6A12/SL

P1A1/CYP4F3/CYP3A5/CYP2A7/PAH/FMO3/CYP1A2/AKR1D1/CYP4F2/CYP7A1/CYP2B6/CYP4A11/C

LC25A15/ABCB11/MFSD2A/SLC38A3/SLC51A/SLC16A11/SLCO1B3/SLC27A2/SLC1A2/SLC6A12/SL

PL41/HNRNPD/HNRNPC/FXR1/HNRNPA2B1/RBMS2/ELAVL1/SERBP1/GEMIN5/TP53/TARDBP/PARN/R

L2/ARNT2/MCM3/RAD51AP1/HES4/ELF3/EZH2/MAF1/FOXQ1/MUC1/SOX12/ARID3A/MXD3/HMGB3/C

/TRIM54/KIF11/NUP62/RCC1/SPAG5/MAPRE1/TUBB/MAD2L1/ASPM/EZR/MID1IP1/TUBB4B/CHEK1
BUB1B/MKI67/PRC1/KIF23/TUBA1B/MCM4/ZWINT/CKS2/ORC6/WDR62/CCNE1/CCNA2/CENPF/KIFC
UPK3A/LAMB1/EFNA4/VASP/NBL1/ITPKA/CDC42EP1/HCK/GPC1/UNC13D/DDR1/FGD3/TWF2/MAPK8
TGAV/SCAMP5/ABCD1/ACP5/IGF2BP1/PRNP/HRAS/SYK/PLCB1/IL18/TMSB4X/S100A8/CSF1R/HDA
ACP5/IGF2BP1/PRNP/HRAS/SYK/PLCB1/IL18/TMSB4X/CSF1R/HDAC7/EZR/IGF2BP3/VTCN1/GBP5
/MIF/ZP3/HLA-DPB1/PRKDC/NFKB2/CD2/EBI3/HIF1A/MAPK3/CD276/SERPINE1/IL27RA/CD3E/S
IRT6/MTHFD1L/FRAS1/ITGA5/MSN/BRSK1/PTK7/STIL/PSMD2/HIF1A/ITGAX/FZD1/ST14/PAK1/P
AFR/COTL1/PRKCD/LAT2/QSOX1/RELB/ITGAV/VAT1/MVP/RAB31/SYK/UNC13D/IL18/TUBB/CSTB/
R/COTL1/PRKCD/LAT2/C1QA/QSOX1/RELB/ITGAV/VAT1/ADAM9/MVP/RAB31/SYK/UNC13D/IL18/T
3D/TUBB/CSTB/S100A8/LYZ/NME2/CTSA/TRPM2/QPCT/RHOG/TUBB4B/GRN/TNFAIP6/CD53/PLAU/
PM2/QPCT/RHOG/TUBB4B/GRN/TNFAIP6/CD53/PLAU/ATAD3B/CHIT1/PSMD14/TIMP2/SYNGR1/DNA
AFR/COTL1/PRKCD/LAT2/QSOX1/RELB/ITGAV/VAT1/MVP/RAB31/SYK/UNC13D/IL18/TUBB/CSTB/
LX2/CD74/BATF/PTAFR/COTL1/PRKCD/LAT2/C1QA/QSOX1/ITGAV/IGLL5/VAT1/MVP/RAB31/SYK/
TSA/TRPM2/QPCT/RHOG/TUBB4B/GRN/TNFAIP6/CD53/PLAU/ATAD3B/CHIT1/PSMD14/TIMP2/SYNG
3R/ZNF385A/TRPM2/FZD7/HMGB3/PRMT1/PSMB9/TFRC/LTB/TNFRSF18/PSMD14/HMGB2/JAK3/WAS
NFSF9/PRKDC/CD2/IL34/JMJD6/CD3E/TCIRG1/ATP6AP1/SASH3/TPD52/UBASH3B/SOCS1/IRF1/F
RC3/ITGB2/IRAK1/C1QC/CD74/CMTM3/CCDC88B/PTAFR/P2RX4/PRKCD/LAT2/C1QA/RELB/NCK2/I
RBCK1/PSMD14/WASF2/BAX/MAPKAPK2/VAV1/COLEC12/PHPT1/CD300A/HLA-DPB1/ARPC4/UNC93B
LA-DQB2/CD3E/PAK1/SLC39A10/FPR1/MNDA/THY1/THEMIS2/C3AR1/HSP90AB1/ARPC2/CTLA4/AR
TF/DSCC1/PRKCD/GINS2/MCM10/FANCD2/POLD1/MCM7/SMG5/NME1/HRAS/PIF1/MCM3/RAD51AP1/
T6/FAAP24/FANCG/GINS4/PRKDC/RUVBL1/MAD2L2/APEX2/FANCE/ISG15/NPM1/RBBP8/BRCA1/NS
RT6/NAA40/RUVBL1/JMJD6/MAPK3/PYGO2/VPS72/MAP1S/MRGBP/CHD3/PAK1/TAF6/NPM1/BRCA1/
U/ZC3H3/EFTUD2/FTSJ1/PPIH/NOL3/METTL1/RBM3/YBX1/RPS2/PABPC1/RRS1/PPP1R9B/PUF60/
2G4/ZC3H12A/NCBP2/EEF1D/BCL3/GTPBP2/RPL17/RPLP2/EIF3D/MRPS23/CNOT9/RPSA/DAPK3/A
B2/ALDH3B1/SNAP25/CDK16/CPLX2/PTAFR/COTL1/FERMT3/PRKCD/TOR4A/LAT2/QSOX1/ITGAV/S
BL1/HRAS/SYK/STK39/CKLF/GPC1/PGF/TMSB4X/S100A8/CSF1R/NECTIN1/CSF3R/UNC5B/EZR/BS
I10/FANCD2/POLD1/MCM7/PIF1/MCM3/RAD51AP1/GTSE1/EEF1E1/MUC1/EME1/CDKN2D/ANKRD1/ZN
GAP1/CCDC88B/CENPW/ARHGEF2/KLC2/HSPB1/TRIM54/KIF11/NUP62/RCC1/GTSE1/WDR34/SPAG5
F4EBP1/SMARCD3/HRAS/PLCB1/KIF11/NUP62/RCC1/GTSE1/MAPRE1/TUBB/EZH2/CDC25A/CDK4/M
MZB1/CLCF1/OSMR/FLNA/IRAK1/CEP131/S1PR2/CD74/CCDC88B/PTAFR/NCK2/ITGAV/SMARCD3/L
A/SRSF9/SNRPC/GEMIN7/BUD31/DAZAP1/SNRNP40/AKAP8L/RBM19/PPIL1/RBMX2/SFPQ/CPSF1/L
NK1D/ANAPC7/CDK2/KIF14/SPSB1/PMAIP1/PSMB4/GBA/KCTD10/WFS1/PSMB8/RNF187/PSMB10/P
MMR/CDC25C/DTL/CEP131/CDKN2A/TUBG1/FEN1/RACGAP1/SMARCD3/PLCB1/KIF11/NUP62/RCC1/
DNMT1/ASAP1/MUC1/GRN/NABP2/CCT6A/CFL1/MICALL2/S100A10/FSCN1/CCT3/SAE1/CARMIL1/T
/ANKRD1/MAGED1/SCRIB/IFT57/GRN/HSF1/CARD9/AKR1C3/DDIT3/IGFBP3/TRAF2/RBCK1/HMOX1
MB9/TFRC/APOBEC3C/RBCK1/TRIM6/CHMP4B/WASF2/TRIM28/CCL5/BAX/CD86/CXCR4/NUP210/IT
PA8/RPL36A/DKC1/PPP2R1A/NPM1/UPF3B/POLR2G/ZC3H3/EFTUD2/PPIH/NOL3/RBM3/YBX1/RPS2
HMT2/AKAP8L/IL1B/MTA2/CDK5/KDM5B/PHB/MIER2/TAF9/SFPQ/DNMT3A/SIRT7/NAP1L2/KDM5C/
15RA/HLA-DRB1/SYK/PLCB1/HLA-DQA1/STK39/HCK/IL18/TMSB4X/MX2/HLA-DRA/CSF1R/F13A1/
KIF18A/CFL1/SMARCD1/FSCN1/CTSK/HMGB2/CARMIL1/TIMP2/EIF5A2/BAX/NCKAP5L/SH3PXD2B/
/RPSA/NLE1/SRSF9/SNRPC/GEMIN7/RPL10L/EMG1/NOP58/ATXN2L/RPS5/ZNF593/UTP18/RPS21/
/HRAS/CDC42EP1/USH1C/HCK/FHL3/ZYX/TMSB4X/FGD3/TWF2/FMNL2/CSF1R/SMIM22/EZR/ANKRD
ABEP2/TCTN2/SSNA1/PPP2R1A/LAMA5/CORO1C/TUBA1A/TTLL1/SFI1/DYNLRB1/IFT52/F2RL1/AR
C/HCK/FHL3/ZYX/TMSB4X/FGD3/TWF2/FMNL2/CSF1R/SMIM22/EZR/ANKRD1/TRPM2/RHOG/FMNL1/
MGB3/PRMT1/PSMB9/TFRC/TNFRSF18/PSMD14/HMGB2/JAK3/WASF2/TCF3/BAX/CD86/VAV1/SEMA4
AL2/PIR/FSTL3/BATF2/TFE3/NCAPG2/LEF1/IRF7/SNX10/NCKAP1L/PIP4K2A/EVI2B/CDKN2B/PI
TAFR/FERMT3/PRKCD/SERPINI1/NCK2/PLXNA3/ITGAV/EMILIN2/PRNP/ADAM9/SYK/UNC13D/DDR1
PC1/DDIT3/CTSK/TRAF2/RBCK1/PSMD14/FBXL19/TIMP2/CHMP4B/FAM83D/VPS37C/MSN/ABHD12/
SPB1/SLAMF8/PLXNA3/ITGAV/LAMB1/ADORA1/ADAM9/NBL1/HRAS/PLCB1/STK39/GTSE1/PGF/MAP
CK2/PLXNA3/CBX6/LAMB1/EFNA4/NME1/VASP/NBL1/ITPKA/USH1C/NPTX2/GPC1/DDR1/EZH2/NME

R/ANKRD1/DCDC2/TRPM2/TRPV2/RHOG/PLEK2/GRN/CRMP1/CFL1/RHOQ/FSCN1/LINGO1/CARMIL1/
/TRIM11/RNF34/UBE2I/UBE2Q2/RNF19B/SHARPIN/ISG15/BRCA1/NSMCE2/STX1A/PCNA/MED10/T
PC4/STX3/SQSTM1/YWHAZ/GPC3/FLNA/ITGB2/CEP131/SNAP25/CDK16/KCNJ11/TAX1BP3/PRKCD/
/RAN/UBL4A/GLIS2/NABP2/CCT6A/MICALL2/RANGAP1/DDIT3/CCT3/SAE1/CHMP4B/TRIM28/BAX/
P1/USH1C/MCM3/NAP1L1/HCK/MAPRE1/CENPO/TMSB4X/MX2/TWF2/MAPK8IP2/HLA-DRA/CENPU/SC
28B/NUP210/SLFN13/ENTPD6/DHX34/PCBP4/PDE4A/MRTO4/DCTPP1/FOXK1/NT5C3A/CNOT11/PSM
SB1/DDX27/NOP2/GTPBP4/NHP2/PUS1/POP7/URM1/DTD1/SNAPC2/EMG1/NOP58/TRMT1/SSB/TRMT
/LYZ/NME2/CTSA/TRPM2/QPCT/RHOG/TUBB4B/GRN/TNFAIP6/CD53/PLAU/ATAD3B/CHIT1/PSMD14
/PTAFR/FERMT3/ARHGEF2/P2RX4/ROBO1/HSPB1/SLAMF8/PLXNA3/ITGAV/LAMB1/ADORA1/ADAM9/
D151/GRN/TNFAIP6/GLIPR2/MGAT5/PLAU/TNFRSF18/ARHGEF39/CARMIL1/CHMP4B/HMOX1/RHOC/
PBB1IP/VAV1/DDOST/SEMA4A/TNFSF9/FKBP1A/CD300A/FLOT2/MSN/ZP3/HLA-DPB1/PRKDC/CD2/
/LYZ/NME2/CTSA/TRPM2/QPCT/RHOG/TUBB4B/GRN/TNFAIP6/CD53/PLAU/ATAD3B/CHIT1/PSMD14
BL1/HRAS/SYK/STK39/CKLF/GPC1/PGF/TMSB4X/S100A8/CSF1R/NECTIN1/CSF3R/UNC5B/EZR/BS
P1CA/MRPL9/PA2G4/ZC3H12A/NCBP2/EEF1D/BCL3/GTPBP2/RPL17/RPLP2/EIF3D/MRPS23/CNOT9
T57/GRN/HSF1/CARD9/AKR1C3/DDIT3/IGFBP3/TRAF2/RBCK1/HMOX1/PLEKHG2/CCL5/BAX/BRMS1
1/ITGAV/ADORA1/PRNP/ARNT2/HRAS/NUP62/HCK/NME2/MAPK8IP2/MAD2L1/DNMT1/CSF1R/FOXQ1
T57/GRN/HSF1/CARD9/AKR1C3/DDIT3/IGFBP3/TRAF2/RBCK1/HMOX1/PLEKHG2/CCL5/BAX/BRMS1
1/ITGAV/ADORA1/PRNP/ARNT2/HRAS/NUP62/HCK/NME2/MAPK8IP2/MAD2L1/DNMT1/CSF1R/FOXQ1
SMB4/GBA/KCTD10/WFS1/PSMB8/RNF187/PSMB10/PSMA5/DNAJB2/TAF9/PSMD13/PSMC4/GSK3A/P
C42EP1/NUP62/HCK/SPAG5/MAPRE1/TMSB4X/TWF2/ASAP1/NECTIN1/EZR/COL16A1/DCDC2/TRPM2
A/SIRT6/CCL26/MSN/IQGAP1/SRPX2/ARPC4/AMIGO2/SH3PXD2B/EFNA5/LCP1/PAK1/BAIAP2L1/T
/RAN/CFL1/PSMB9/TFRC/APOBEC3C/RBCK1/TRIM6/CHMP4B/WASF2/TRIM28/CCL5/BAX/CD86/CXC
CRIB/SLC25A6/RPL8/RAN/CFL1/PSMB9/TFRC/APOBEC3C/RBCK1/TRIM6/CHMP4B/WASF2/TRIM28/
KA/STC2/CTSV/WFDC2/ADA/GNPDA1/HAVCR2/CLGN/CDC25C/PAQR5/BIRC3/CEP131/PI3/CDK16/R
L/CEP131/CDKN2A/MCM6/TUBG1/EIF4EBP1/MCM10/SMARCD3/MCM7/PLCB1/USH1C/MCM3/RCC1/GT
B1/SNAP25/CPLX2/PTAFR/COTL1/FERMT3/PRKCD/TOR4A/LAT2/QSOX1/ITGAV/SCAMP5/VAT1/PLE
A-DRB1/HRAS/SYK/HLA-DQA1/HCK/UNC13D/ZYX/IL18/TUBB/MX2/S100A8/HLA-DRA/CSF1R/APOB
C/PSMD2/PAK1/MNDA/SOCS1/IRF1/IRF3/HSP90AB1/F2RL1/STAT1/RAB7B/LRRC14/NPLOC4/TRAF
GEF2/P2RX4/ROBO1/SERPINI1/PLXNA3/SMARCD3/NME1/NBL1/ITPKA/SYK/PLCB1/NAP1L1/GPC1/
/SCRIB/CHEK1/RHNO1/NABP2/PRMT1/PSMB9/DDIT3/PSMD14/TIMP2/ESPL1/BAX/MIF/LCMT1/NAA
/PRNP/ADAM9/MVP/HRAS/SYK/STK39/NUP62/IL18/EZH2/CDC25A/CDK4/DLG3/CSF1R/CDKN2D/MA
NC13D/IL18/PAG1/CD151/SLAMF7/FZD7/HMGB3/TNFAIP8L2/VTCN1/CD320/TFRC/HLA-DPA1/HLA
D2/CDC25C/RAD54L/FLNA/TUBG1/RACGAP1/DSCC1/NCAPH/FANCD2/PLCB1/KIF11/NUP62/RCC1/S
RPM2/FZD7/HMGB3/PRMT1/PSMB9/TFRC/LTB/TNFRSF18/PSMD14/HMGB2/JAK3/WASF2/TCF3/BAX/
/PSMD14/HNF1B/AREG/TRIM28/RHOC/COL5A1/CXCR4/SIRT6/MTHFD1L/FRAS1/ITGA5/FKBP1A/MS
2/VTCN1/CD320/GRN/RPS6KA1/TFRC/HLA-DPA1/HLA-DMB/TNFRSF18/VSIG4/JAK3/HMOX1/CCL5/
TK39/HCK/CKLF/GPC1/PGF/SLC7A5/S100A8/CSF3R/BSG/TRPM2/MMP1/DOK2/TNFRSF18/HMOX1/B
EBP1/ARHGEF2/PRKCD/PLXNA3/SCAMP5/VAT1/PRNP/TRIM54/MTMR2/PIF1/MAPRE1/TMSB4X/TWF2
LNA/CD74/PKN1/PTAFR/FERMT3/ARHGEF2/P2RX4/ROBO1/HSPB1/SLAMF8/PLXNA3/ITGAV/LAMB1/
CD151/GRN/TNFAIP6/GLIPR2/MGAT5/PLAU/TNFRSF18/ARHGEF39/CARMIL1/HMOX1/RHOC/BDKRB1
/PKN1/DUSP5/DSCC1/PRKCD/ROBO1/HSPB1/NCK2/SLAMF8/ADORA1/PRNP/ADAM9/MVP/HRAS/SYK/
B4X/S100A8/ASPH/GIT1/ASAP1/ADAP2/CASP4/SCRIB/RHOG/IFT57/GRN/GNA15/HSF1/TBC1D16/
WF2/CSF1R/SMIM22/TRPM2/RHOG/MID1IP1/CHEK1/RASSF7/KIF18A/CFL1/RHOQ/S100A10/FSCN1
MAD2L1/STARD3NL/YKT6/ASPM/EZR/SCRIB/TUBB4B/RAN/TMEM201/RAB34/KIF3C/KIF18A/GIPC1
8A/GIPC1/DTNBP1/AREG/CHMP4B/HMOX1/ESPL1/DNAJC5/TRAPPC4/FAM83D/MAP2/CD300A/STXBP
D3/HES6/NME1/NBL1/ITPKA/MTMR2/NAP1L1/HES4/EZH2/NME2/TWF2/ASAP1/NECTIN1/ASPM/COL
/P3H1/CXCR4/SEMA4A/IQGAP1/SRPX2/PTK7/AMIGO2/EFNA5/SF3A2/IL34/RGS14/HIF1A/ITGAX/
O1/SERPINI1/SLAMF8/PLXNA3/SMARCD3/HES6/NME1/NBL1/ITPKA/NAP1L1/UNC13D/HES4/EZH2/
X2/KCNJ11/TAX1BP3/P2RX4/PRKCD/SLC7A11/B3GAT3/PRNP/HRAS/SYK/ABHD17C/MTMR2/NUP62/
CD44/IRAK1/CD74/ARHGEF2/BCL2A1/PRKCD/HSPB1/ITGAV/SLC7A11/ADORA1/PRNP/ARNT2/HRAS
MX2/ASAP1/YKT6/SYNGR2/SCRIB/TLCD1/SPAG4/UBL4A/DNAJC6/RAB34/S100A10/TFRC/AREG/CH

PRKDC/FOXK1/RUVBL1/MAD2L2/PSMD2/HIF1A/JOSD2/TRIM11/RNF34/UBE2I/UBE2Q2/RNF19B/SH
2/IFT57/TUBB4B/RAB34/KIF3C/SAC3D1/FSCN1/ABCC4/CHMP4B/RABEP2/RAB32/TCTN2/VPS37C/
F1/RHOQ/S100A10/CHMP4B/BAX/RAB32/SSR2/FLOT2/MTCL1/VPS37C/PKDCC/RAB15/SEC61A1/ZD
/HSF1/NCF1/PRMT1/CARD9/DDIT3/DTNBP1/TRAF2/YBX3/TRIM28/BAX/MAPKAPK2/SIRT6/NUP210
42EP1/USH1C/COLGALT1/HCK/MAPRE1/ZYX/SERPINH1/TMSB4X/TWF2/EZR/COL3A1/ANKRD1/RHOG
RMT3/PRKCD/ROBO1/HSPB1/NCK2/ITGAV/SLC7A11/LAMB1/PRNP/PLEK/ADAM9/SYK/CDC42EP1/IL
N2/SSNA1/PPP2R1A/LAMA5/CORO1C/TUBA1A/TTLL1/SFI1/DYNLRB1/IFT52/F2RL1/ARPC2/DYNLL
RD1/DCDC2/TRPM2/TRPV2/RHOG/PLEK2/GRN/CRMP1/CFL1/RHOQ/FSCN1/LINGO1/CARMIL1/WASF2
ZNF385A/ARID3A/CDC7/TUBB4B/CHEK1/PRMT1/PSMB9/PSMD14/ESPL1/BAX/FAM83D/LCMT1/PCBP
PRC1/KIF23/MCM4/ZWINT/CKS2/ORC6/WDR62/CCNE1/CCNA2/CENPF/KIFC1/SAPCD2/KIF18B/ORC
L3/CD83/TFE3/NCAPG2/PRELID1/PSMB2/LEF1/IRF7/ZC3H12A/PSME3/LAG3/NCKAP1L/ADAM8/EV
5/EZR/RHOG/RAN/GLIS2/RAB11FIP5/ANXA13/CCT6A/MICALL2/RANGAP1/RHOQ/EPB41L2/GPD1L/
R/FERMT3/ARHGEF2/P2RX4/ROBO1/HSPB1/SLAMF8/PLXNA3/ITGAV/LAMB1/ADORA1/ADAM9/NBL1/
GRN/TNFAIP6/GLIPR2/MGAT5/PLAU/TNFRSF18/ARHGEF39/CARMIL1/HMOX1/RHOC/BDKRB1/CCL5/
/CPSF1/LSM2/PQBP1/SRSF12/PRCC/ISY1/RNF113A/EIF4A3/PRPF3/SNU13/SRRT/PRPF6/POLR2L
/CPSF1/LSM2/PQBP1/SRSF12/PRCC/ISY1/RNF113A/EIF4A3/PRPF3/SNU13/SRRT/PRPF6/POLR2L
/CPSF1/LSM2/PQBP1/SRSF12/PRCC/ISY1/RNF113A/EIF4A3/PRPF3/SNU13/SRRT/PRPF6/POLR2L
GT1/MMP2/NOL3/LMNA/S100B/PLOD2/HYOU1/PSMB2/DDIT4/PPARD/PSME3/PAM/MT3/NPEPPS/ADA
/CSTB/S100A8/LYZ/NME2/CTSA/TRPM2/QPCT/RHOG/TUBB4B/GRN/TNFAIP6/CD53/PLAU/ATAD3B/
RC/HLA-DPA1/HLA-DMB/TNFRSF18/VSIG4/JAK3/HMOX1/CCL5/CD86/VAV1/TNFSF9/MIF/CD300A/
IPF3/RBCK1/PSMD14/WASF2/BAX/MAPKAPK2/VAV1/COLEC12/PHPT1/CD300A/HLA-DPB1/ARPC4/U
SF9/SNRPC/GEMIN7/BUD31/DAZAP1/SNRNP40/AKAP8L/RBM19/PPIL1/RBMX2/SFPQ/CPSF1/LSM2/
LS1/U2AF2/SRSF9/GEMIN7/XPOT/SSB/ZNF593/AKAP8L/IL1B/CDK5/TXN/CPSF1/THOC5/NUP85/P
ITGA5/TNFSF9/CD300A/SELPLG/FLOT2/MSN/ZP3/HLA-DPB1/EBI3/CD276/EFNB1/CD3E/PAK1/SA
GEF39/TIMP2/HMOX1/WASF2/TRIM28/RHOC/PLEKHG2/RAB6B/VAV1/RAB32/ARFGEF3/DOK1/GPR35
LR2G/RBM3/YBX1/PABPC1/NUP93/TRNAU1AP/NUP205/NUP37/PSMB2/PPP1CA/PA2G4/NDC1/POLR2
MIIP/PRKDC/MAD2L2/CNOT11/PSMD2/NPM1/RBBP8/CHMP4C/BRCA1/RBL1/PCNA/CTDSPL/HSP90AB
NKRD27/NRCAM/THY1/RTN4RL2/RELN/ZSWIM5/MARK2/GDI1/EPHB3/FGFR1/MT3/DYNLT1/DVL3/VL
/LAMC1/MMP1/CREB3L1/COMP/PLOD1/FSCN1/CTSK/TIMP2/COL5A1/ITGA5/SH3PXD2B/NFKB2/ITG
FAIP6/GLIPR2/MGAT5/PLAU/TNFRSF18/ARHGEF39/CARMIL1/HMOX1/RHOC/BDKRB1/CCL5/CXCR4/
STMN3/MRPS34/TRMT112/VIL1/CHMP3/DENR/MAP1LC3B/DVL2/SET/MRPL13/SMARCB1/NES/KIF2A
2/MAP1S/CENPE/DKC1/ACD/BRCA1/NSMCE2/CCT2/LMNA/SPDL1/XRCC3/DDX11/NELFE/RPS6KA4/C
RPC4/HSPA8/LCP1/PAK1/BAIAP2L1/ANKRD27/STX1A/FCHSD1/BMF/BIN1/ARPC2/ARPC3/SUB1/CC
/TUBB/CSTB/S100A8/LYZ/NME2/CTSA/TRPM2/QPCT/RHOG/TUBB4B/GRN/TNFAIP6/CD53/PLAU/AT
PM2/QPCT/RHOG/TUBB4B/GRN/TNFAIP6/CD53/PLAU/ATAD3B/CHIT1/PSMD14/TIMP2/SYNGR1/DNA
L/CEP131/CDKN2A/MCM6/TUBG1/EIF4EBP1/MCM10/SMARCD3/MCM7/PLCB1/USH1C/MCM3/RCC1/GT
/GIPC1/ESPL1/BAX/FAM83D/MIF/PCBP4/CASP2/CNOT11/NPM1/BRCA1/NSMCE2/PCNA/CKS1B/HSP
NB1/HCLS1/U2AF2/SRSF9/GEMIN7/XPOT/SSB/ZNF593/AKAP8L/IL1B/CDK5/TXN/CPSF1/THOC5/N
/MISP/ECT2/NUSAP1/CCNF/TGFB1/SKA1/RCC2/AURKA/UNC119/PSRC1/SPC24/NCAPD2/CDC25C/S
MAGED1/DTNBP1/TRAF2/MAPKAPK2/CXCR4/MIF/CD300A/IQGAP1/RGS14/MAPK3/MAP2K2/PPP2R1A
/CREB3L1/DDIT3/TRAF2/RBCK1/HMGB2/YBX3/HMOX1/BAX/MIF/BEX3/CASP2/PRKDC/CREB3/HIF1
AK1/BAIAP2L1/FCHSD1/BIN1/F2RL1/ARPC2/ARPC3/LIMCH1/CORO1B/HIP1R/NCKAP1L/TUBB4A/C
PSMD13/PSMC4/SRSF12/SET/PSMD10/PSMD11/CPSF6/PSMD1/PSMA7/EXOSC5/YWHAB/SAMD4B/PSM
TCL1/MSN/IQGAP1/CARD10/OAZ1/DKC1/PAK1/MCRS1/SLC51B/CCT2/HSP90AB1/CNPY4/ZC3H12A/
/CNPY4/KRT18/SLC9A3R1/GPSM2/FLOT1/RTKN/RANGRF/ITGB1BP1/EHD2/ACTB/RAP2A/CDK5/MYA
D2/CDC25C/RAD54L/FLNA/TUBG1/RACGAP1/DSCC1/NCAPH/FANCD2/PLCB1/KIF11/NUP62/RCC1/S
B2/CD3E/PAK1/SLC39A10/FPR1/MNDA/THY1/THEMIS2/C3AR1/HSP90AB1/ARPC2/CTLA4/ARPC3/W
3/NCBP2/RPL17/RPLP2/CNOT9/LSM7/RPSA/SLC11A1/RNASE2/SSB/RPS5/PSMB4/RPS21/PSMB8/R
RPS5/PSMB4/RPS21/PSMB8/RPL7/RPL28/PSMB10/PSMA5/LSM2/PSMD13/PSMC4/RPL39/EDC3/RPL
TPM2/ARPC4/SH3PXD2B/SLC9A1/MAD2L2/LCP1/TPM3/PAK1/BAIAP2L1/FCHSD1/RHOF/ITGB5/COR
/ACD/NPM1/SLC35F6/ANKRD27/BRCA1/NOL3/LMNA/SPDL1/XRCC3/PRELID1/CORO1B/HIP1R/OTUB

300A/FLOT2/ZP3/HLA-DPB1/EFNA5/EBI3/MAD2L2/CD276/EFNB1/CD3E/PAK1/SASH3/THY1/UBAS
2RL1/ARPC2/DYNLL1/RELN/MARK2/GDI1/CORO1B/FGFR1/DYNLT1/DVL3/VLDLR/KDM1A/LYN/RAC1
/CRABP2/BAIAP2L1/NRCAM/SLC12A8/FCHSD1/RAB3B/BIN1/HSP90AB1/F2RL1/ARPC2/ARPC3/GDI
OP1/CPSF1/TRMT112/NSUN5/TRMT6/MPHOSPH6/BYSL/RPL35A/LYAR/INTS1/USP14/RNF113A/RRP
GT1/MMP2/NOL3/LMNA/S100B/PLOD2/HYOU1/PSMB2/DDIT4/PPARD/PSME3/PAM/MT3/NPEPPS/ADA
RCA4/PPIH/TRIM59/F2RL1/STAT1/PABPC1/HDAC1/NPLOC4/LY86/LEF1/NELFE/POLR2H/PPIA/ZC
FNB1/CD3E/PAK1/SASH3/TNFRSF4/SLC39A10/THY1/SIRPA/SOCS1/F2RL1/CTLA4/CD83/PDCD1/T
D/NPM1/FGFR4/BRCA1/PCNA/ZNF93/CCT2/HSP90AB1/TICRR/E2F8/PARPBP/RFC2/DDX11/FGFR1/
1/TNFSF9/MIF/CD300A/FLOT2/ZP3/HLA-DPB1/CD2/EBI3/MAD2L2/CD276/EFNB1/IL27RA/CD3E/
BAX/SIRT6/PRDX1/PRKDC/PROM1/HIF1A/TFF3/MAPK3/TCIRG1/DKC1/ATP6AP1/ACD/SASH3/NSMC
IF1A/ATP6AP1/PAK1/GNGT1/MMP2/NOL3/LMNA/S100B/PLOD2/HYOU1/PSMB2/DDIT4/PPARD/PSME
P2/RPS5/CDK5/ZDHHC18/RPS21/RPL7/RPL28/ZDHHC1/RAB10/RPL39/RPL35A/YWHAQ/YWHAB/RPS
ZNF385A/ARID3A/CDC7/TUBB4B/PRMT1/PSMB9/PSMD14/ESPL1/BAX/LCMT1/PCBP4/CASP2/BRSK1
/SERPINE1/RNF34/PPP2R1A/PEA15/SLC35F6/BRCA1/BCL2L12/BMF/NOL3/LMNA/HDAC1/DDIAS/H
1/SASH3/TNFRSF4/SLC39A10/THY1/SIRPA/SOCS1/F2RL1/CTLA4/CD83/PDCD1/TNIP2/SIRPG/NC
8/BRCA1/PCNA/CHAF1A/TOP1MT/TICRR/E2F8/RFC2/DDX11/FGFR1/PNKP/BLM/FBXO5/DBF4/GTPB
PH/FANCD2/OIP5/NUP62/RCC1/SPAG5/MAD2L1/EME1/RAN/KIF18A/CHMP4B/ESPL1/FAM83D/LCMT
1/MIIP/PRKDC/MAD2L2/CNOT11/PSMD2/CHMP4C/BRCA1/RBL1/PCNA/CTDSPL/SPDL1/XRCC3/TICR
/PAK1/SASH3/THY1/SIRPA/SOCS1/IRF1/CTLA4/CD83/PLA2G2D/PRELID1/PDCD1/ZC3H12A/LAG3
FBXO5/TUBB4A/CAPZA1/RANGRF/CSNK1D/MKKS/FHOD3/CAMSAP2/HCLS1/PSTPIP1/ARPC5L/FKBP4
FNB1/CD3E/SASH3/TNFRSF4/SLC39A10/MNDA/IRF1/CTLA4/PLA2G2D/LEF1/NCKAP1L/SLC11A1/C
IF4A3/ISG20/EXOSC5/SNU13/GNL2/RPL27/FTSJ3/DROSHA/VCX/RPL35/PPAN/DDX54/RPS19/RPL
1/F2RL1/CCL13/TNFRSF11A/CORO1B/LEF1/PIP5K1C/FGFR1/NCKAP1L/ADAM8/CCL2/PIK3CD/CXC
-DPB1/IL34/EBI3/CD276/EFNB1/CD3E/TCIRG1/SASH3/TNFRSF4/SLC39A10/MNDA/IRF1/F2RL1/
CD3E/PAK1/SASH3/THY1/SIRPA/SOCS1/IRF1/CTLA4/CD83/PLA2G2D/PDCD1/ZC3H12A/LAG3/SIR
0/MMP2/SIRPA/NOL3/GPX8/GPX7/ZC3H12A/MT3/NONO/HDAC2/ATF4/CDK2/TXN/SFPQ/PML/PARP1
BRCA1/NSMCE2/MCRS1/XRCC2/ACTL6A/XRCC3/PARPBP/LEF1/BLM/NONO/AUNIP/KDM1A/WAS/SWI5
/KIF15/SFPQ/AP3M2/DYNC1LI1/WASF1/MAP4/IFT80/KIF2A/BAG3/CDC42/DNAL4/APP/ACTL8/AP
F1/THOC5/NUP85/NUP43/SLC29A3/AGFG1/THOC6/RAE1/CPSF6/EIF4A3/XPO5/NUP155/CPSF4/NU
P36/EIF4A3/ISG20/EXOSC5/SNU13/RPL27/NCL/FTSJ3/DROSHA/RPL35/GTF3C2/DDX54/RPS19/N
3/CHMP1A/TDRKH/NUP107/NCOR2/POLR2K/HELLS/PCGF2/TRIM71/PIWIL4/SEC13/DOT1L/HAT1/M
L/SFPQ/CPSF1/CACNG4/THOC5/NUP85/NUP43/IFT80/AGFG1/THOC6/RAE1/EIF4A3/NUP155/CPSF
I1/TNFSF13B/CDH17/PTPN6/CD27/SAMSN1/CD79A/MSH2/SWAP70/SKAP2/BTK/PELI1/IGBP1/GPR1
FI1/DYNLL1/DYNC1H1/TICRR/CSNK1E/PSMB2/PSME3/PPME1/HAUS1/BLM/FBXO5/TUBB4A/CDKN2B
1/CTLA4/CD83/PDCD1/TNIP2/SIRPG/NCKAP1L/ADAM8/CCL2/HSPH1/CD209/LYN/CARD11/RAC1/C
DIT4/SLC9A3R1/BID/BCAP31/BCL3/NOC2L/NONO/ATF4/BBC3/KDM1A/PMAIP1/WFS1/TAF9/SFPQ/
AB1/F2RL1/ARPC2/RAB7B/ARPC3/WIPF1/PIP5K1C/TGM2/SIRPG/ACTG1/NCKAP1L/MYO1G/CCL2/M
B/CDKN2B/CDK2/CDKN1C/LAMTOR5/CDK5/LAMTOR1/TGFB2/RASSF1/PML/MLF1/CDC123/CDKN2C/C
/DYNLL1/DYNC1H1/PSMB2/RAB35/PSME3/BCAP31/ERAP2/LAG3/CD68/HLA-DQA2/CTSE/CAPZA1/S
F6/EIF3H/VCX/AGO2/SF3A3/PPAN/RPS19/RPLP0/RRP7BP/SNRPG/RPL38/MAPT/PRPF31/RPL23A/
RAC1/JCHAIN/CTTN/WAS/CDH17/VIL1/PSMC4/PFN1/WASF1/PARP1/PIEZO1/RPS3/GRB2/CDC42EP
NOT11/RBBP8/RBL1/PCNA/CTDSPL/E2F8/PRMT2/E2F4/PIM2/PSME3/BID/FBXO5/DBF4/CNOT9/CD
CD3E/SASH3/TNFRSF4/SLC39A10/MNDA/IRF1/CTLA4/PLA2G2D/LEF1/NCKAP1L/SLC11A1/CD209/
IT1/DNASE2/RPS19/AXL/FADD/HCAR2/GPR183/ABL1/TNFAIP3/BCL10/ACVR1B/PTPN2/ADAM17/N
3CD/LYN/HLA-DRB5/CARD11/CBFB/PSMB4/BCAR1/PSMB8/NFAM1/PSMB10/LCP2/PSMA5/WAS/LCK/
CA/NCAPH2/PSMD13/CYP26B1/TUBG2/CALR/TDRKH/MYBL1/RAD21/MSX1/WNT4/PIWIL4/RAD51C/L
2/RFC2/E2F4/DDIT4/BCL3/BLM/NOP2/NOC2L/CNOT9/HDAC2/CDK2/KDM1A/PMAIP1/KMT5A/EHMT2
11/NPM1/BRCA1/NSMCE2/PCNA/DYNC1H1/XRCC3/TGFA/E2F8/DDX11/E2F4/FGFR1/GPSM2/FBXO5/
H/KIF11/NUP62/RCC1/SPAG5/MAD2L1/CHEK1/RAN/KIF18A/CHMP4B/ESPL1/LCMT1/NAA10/MAD2L
AP2M1/FGFR2/DVL2/PFN1/AP2S1/PSMD10/PSMD11/VDR/PSMD1/PSMA7/TACSTD2/PSMB3/PAX8/AR
BL1/PCNA/CTDSPL/E2F8/PRMT2/E2F4/PIM2/PSME3/BID/FBXO5/DBF4/CNOT9/CDKN2B/CCL2/CDK

I1/TICRR/CSNK1E/PSMB2/PSME3/PPME1/HAUS1/BLM/FBXO5/TUBB4A/CDKN2B/CSNK1D/CDK2/KIF1
BATF3/SBNO2/CDC42/APP/LILRB4/FADD/ZNF675/GPR183/CCL3/PTPN2/CASP8/VEGFA/LBR/TLR2
PSTPIP1/ARPC5L/RAC1/CTTN/MYADM/WAS/VIL1/CIT/PFN1/WASF1/CAPZB/GRB2/ARHGAP18/SWAP
7/POLR2K/RPL18A/RPS19/RPLP0/RPL18/RPL23/RPL38/RPL23A/SUPT5H/MCTS1/CCL3/SEC13/RP
/FLOT1/NCKAP1L/ADAM8/CCL2/CXCL13/HSPH1/CD209/LYN/CARD11/RAC1/CBFB/IL1B/IL2RA/CD
P107/CETN2/SLBP/KHDRBS1/HNRNPA3/CKAP5/CHTOP/SEC13/SYMPK/CPSF3/RPS15/NOL6/MRPL18
P107/CETN2/SLBP/KHDRBS1/HNRNPA3/CKAP5/CHTOP/SEC13/SYMPK/CPSF3/RPS15/NOL6/MRPL18
/IRF1/CTLA4/PLA2G2D/NCKAP1L/CD209/LYN/CARD11/IL1B/IL2RA/TNFSF13B/PTPN6/IL12RB1/
I1/CHAF1A/TOP1MT/ERCC6L/RFC2/DDX11/NCAPG2/BLM/NOC2L/ITGB3BP/SMC4/NASP/AKAP8L/NCA
3/PRPF6/EIF3H/VCX/AGO2/SF3A3/PPAN/RPS19/RPLP0/RRP7BP/SNRPG/RPL38/MAPT/PRPF31/RP
F18A/CHMP4B/ESPL1/FAM83D/LCMT1/NAA10/SGO2/MAD2L2/CENPE/PPP2R1A/CHMP4C/NSMCE2/SG
PZA1/MKKS/FHOD3/HCLS1/TESK1/ITGB1BP1/ARPC5L/RAC1/CD47/CTTN/MYADM/WAS/VIL1/CIT/P
A/CTDSPL/SPDL1/XRCC3/TICRR/E2F8/PRMT2/E2F4/PSMB2/PSME3/BLM/FBXO5/CNOT9/CDKN2B/C
IL1/CD27/IFI16/CASP1/PML/GPX1/RPS3/TFAP4/CST7/ARRB2/DNAJB6/DPEP1/CTSD/HSPE1/CAR
BABAM1/RFWD3/MSX1/RAD9A/OGG1/RPA2/PPP4C/TIMELESS/PTTG1IP/MAPT/EID3/ABL1/WRAP53/
F1/THOC5/NUP85/NUP43/AGFG1/THOC6/RAE1/CPSF6/EIF4A3/RUVBL2/XPO5/PRPF6/NUP155/CPS
P4/SNX27/SIPA1L3/ARF4/PARVG/PARD6B/LLGL1/CDC42/PRKCI/ACTL8/STK25/RACK1/WDR1/PAR
/MNDA/IRF1/CTLA4/PLA2G2D/NCKAP1L/CD209/LYN/CARD11/IL1B/IL2RA/TNFSF13B/PTPN6/IL1
/CARD11/RAC1/CBFB/IL1B/IL2RA/CD47/TNFSF13B/LCK/PTPN6/CD27/IL12RB1/IL15/RPS3/GRB
PDL1/XRCC3/TICRR/E2F8/PRMT2/E2F4/PSMB2/PSME3/BLM/FBXO5/CNOT9/CDKN2B/CCL2/CDK2/P
1L/ADAM8/CCL2/HSPH1/CD209/LYN/CARD11/RAC1/CBFB/IL1B/IL2RA/CD47/TNFSF13B/LCK/PTP
/SPDL1/XRCC3/TICRR/KNTC1/E2F8/E2F4/BLM/CNOT9/CDKN2B/CDK2/THOC5/PML/DYNC1LI1/PRO
PL38/RPL23A/EXOSC3/BTG2/GSPT2/RPS7/RPL7A/RPS23/RPS10/RPS15/RPS16/RPL5/RPS27A/RP
NOC4L/RRP7BP/TSR3/UTP11/LAS1L/UTP4/EXOSC3/NAT10/UTP6/HEATR1/SART1/NOP10/KRI1/RP
FFA/BAG3/CALR/DNAJB6/GRPEL2/DNAJB11/HSPE1/CWC27/PDCD5/SGTB/PRDX4/DNAJC7/PDIA6/E
L23/RPL38/RPL23A/CEMIP/ZDHHC12/MFF/RPS7/RPL7A/RPS23/RPS10/RPS15/SGTA/RPS16/RPL5
C5L/FKBP4/RAC1/CTTN/MYADM/WAS/VIL1/PFN1/WASF1/INPP5J/RPS3/CAPZB/GRB2/ARHGAP18/C
1/RPS3/TFAP4/ARRB2/DNAJB6/DPEP1/CTSD/HSPE1/CARD16/PDCD5/FADD/CLEC7A/RACK1/IGBP1
4/CHMP3/IFI16/BANF1/PML/SMARCB1/ILF3/ISG20/TFAP4/POLR2L/APOBEC3D/POLR2K/TNIP1/T
MD1/PSMA7/PSMB3/PSMD4/PSMD3/RBX1/CARD16/PSMD7/TBL2/SUV39H2/PSMD8/VHL/PSMA1/PSMB
SET/SMARCB1/SMYD3/CDK7/RBBP4/SMARCC1/CABIN1/HELLS/CETN2/RBX1/DR1/RPL23/RNF4/CEN
L3/CXCL13/LYN/RAC1/PLA2G7/IL1B/CD47/CCL4/TGFB2/NUP85/CALCA/CCR1/C5AR1/PF4V1/CXC
1/BLM/FBXO5/TUBB4A/CSNK1D/CDK2/KIF14/DCTN2/PSMB4/PSMB8/PSMB10/PSMA5/PSMD13/PSMC
2/MAD2L2/CENPE/PPP2R1A/CHMP4C/NSMCE2/SGO1/MSTO1/SPDL1/XRCC3/RRS1/DDX11/INCENP/F
1/MCM8/XRCC6/POLQ/FUS/RAD21/BABAM1/RFWD3/NABP1/XRCC4/OGG1/RPA2/PPP4C/TIMELESS/P
07/SLBP/CHTOP/SEC13/EMD/SYMPK/CPSF3/RPS15/NOL6/LTV1/SARNP/MAGOH/THOC2/XPO7/EIF5
A/CSNK1D/CDK2/KIF14/DCTN2/PSMB4/PSMB8/PSMB10/PSMA5/PSMD13/PSMC4/HAUS8/PLK4/PSMD
PT/PDCD6/CYCS/MTCH1/HSPD1/PIDD1/VCP/SFRP2/TRADD/BAD/BCL10/RCN3/TNFSF15/CASP8/PD
PIK3CD/CXCL9/CXCL3/CXCL13/LYN/RAC1/PLA2G7/IL1B/CXCR3/CCL4/TGFB2/GPSM3/NUP85/CAL
/RASSF1/POC1A/CHMP3/GSK3A/PLK4/INPP5J/RAE1/RPS3/KAT2A/CHMP1A/RANBP1/KATNB1/RNF4
A5/WAS/LCK/PTPN6/PSMD13/PSMC4/PSMD10/PSMD11/PSMD1/PSMA7/RPS3/PRKD2/PSMB3/PSMD4/
R/CPSF4/NUP107/SLBP/KHDRBS1/CHTOP/SEC13/EMD/SYMPK/CPSF3/RPS15/NOL6/LTV1/SARNP/M
P107/CETN2/SLBP/KHDRBS1/HNRNPA3/CKAP5/CHTOP/SEC13/SYMPK/CPSF3/RPS15/NOL6/MRPL18
ARPC5L/RAC1/CDK5/CD47/PFDN2/CTTN/WAS/POC1A/VIL1/PLK4/PFN1/WASF1/NES/RPS3/GRB2/F
D3/PABPC4/PSMD7/VIM/TRIM71/PLEKHN1/HNRNPM/EXOSC3/BTG2/PSMD8/PSMA1/MYEF2/PSMB5/P
SMD11/PSMD1/PSMA7/TACSTD2/PSMB3/PAX8/ARRB2/CDC42/PSMD4/HOXB7/PSMD3/PSMD7/WNT4/M
KHDRBS1/RPL23A/MCTS1/RPS7/RPL7A/RPS23/RPS10/EIF3K/RPS15/RPS16/EIF3M/RPL5/RPS27A
SM2/HAUS1/FBXO5/AUNIP/CSNK1D/KPNB1/DCTN2/KNSTRN/POC1A/HAUS8/MAP4/TUBG2/RAE1/KIF

CAP31/ERAP2/LAG3/HLA-DQA2/CTSE/CAPZA1/SLC11A1/CD209/DCTN2/HLA-DRB5/DCTN5/PSMB4/
PML/PARP1/MSH2/RPS3/PRKD2/ARRB2/PARM1/CBX8/FUS/BABAM1/CCT8/GNL3/TIMELESS/EXOSC3

[illegible]2/CSPP1/CDK2AP2/PLK3/SH3GLB1/ZFYVE19/VEGFA/BCL2L1/RAB11A/KIF20B/KLHL21/RHOA/BTC
[illegible]F1/POC1A/CHMP3/GSK3A/PLK4/INPP5J/RAE1/RPS3/KAT2A/CHMP1A/RANBP1/KATNB1/RNF4/CEP2
[illegible]C39A10/MNDA/IRF1/CTLA4/PLA2G2D/NCKAP1L/CD209/LYN/CARD11/IL1B/IL2RA/TNFSF13B/PTP
[illegible]CTSE/CAPZA1/DCTN2/HLA-DRB5/DCTN5/PSMB4/CTSS/PSMB8/KIF15/PSMB10/PSMA5/PSMD13/PSM
[illegible]/MAPT/RARG/ABL1/KCNN4/KEL/DBNL/DNM2/VEGFA/RAB11A/IFRD1/CDK5R1/SRF/RHOA/MTPN/IST
[illegible]CL10/LMO4/MTHFR/KIF20B/TWIST1/CC2D2A/STK3/GLMN/SIX1
[illegible]CTSE/CAPZA1/DCTN2/HLA-DRB5/DCTN5/PSMB4/CTSS/PSMB8/KIF15/PSMB10/PSMA5/PSMD13/PSM
[illegible]TX4/RALB/CEP250/HAUS6/NDE1/NSF/CKAP5/IQCB1/RAB3D/TMEM216/STX7/VAMP8/CEP41/HSP90
[illegible]ASF1/CAPZB/GRB2/ARHGAP18/CDC42EP2/GMFG/ABI2/ARPC1A/BRK1/ARPC5/ACTR3/DBNL/SPIRE1
[illegible]1/RHOT2/IFT172/HOOK2/COPG2/TCTEX1D2/BICD1/HNRNPU/MAP1A/CCDC88C/ARL8B/AP3B1/IFT2
[illegible]NEB/CAPZB/GRB2/ARHGAP18/SWAP70/CDC42EP2/GMFG/ABI2/WDR1/ARPC1A/BRK1/ARPC5/ACTR3/
[illegible]GRB2/CDC42/PSMD4/PSMD3/PSMD7/IKBKG/BTK/ARPC1A/BRK1/PSMD8/ARPC5/ABL1/FCGR2B/PSMA
[illegible]RD11/IL1B/IL2RA/TNFSF13B/PSMB10/PTPN6/IL12RB1/IL15/RPS3/CD6/PNP/FADD/PELI1/RASA
[illegible]M/TRIM71/PLEKHN1/HNRNPM/EXOSC3/PSMD8/PSMA1/MYEF2/PSMB5/PSMA4/PSMD9/RPS27A/HNRNP
[illegible]6/IKBKG/AXL/FADD/ECM1/RACK1/ZNF675/CLIP3/PELI3/TNFAIP3/TRADD/PTPN2/ADAM17/CASP8
[illegible]1/RPL23/MSX1/RBBP7/RAD9A/RPA2/PTTG1IP/TAF12/TAF10/DNA2/RPS7/CHEK2/RAD1/PRKAA2/C
[illegible]HTOP/NBN/HNRNPU/ERCC1/VEGFA/SREBF1/PIH1D1/MECP2/HNRNPA1/PPHLN1/SART3/DNMT3B/MAP
[illegible]AC1/ITGA2/PLA2G7/CXCR3/CCL4/GPSM3/CCR1/ABR/C5AR1/PADI2/STK10/CALR/XCL2/CMKLR1/A
[illegible]ATP6V0D1/HSPD1/HSPA2/VCP/SRPRB/WIPI1/HSP90AA1/TATDN2/PTPN2/THBS1/DCTN1/PTPN1/OP
[illegible]CAPZB/GRB2/ARHGAP18/SWAP70/CDC42EP2/GMFG/ABI2/WDR1/ARPC1A/BRK1/ARPC5/ACTR3/DBNL
[illegible]/RPLP0/RPL18/RPL23/RPL38/RPL23A/SUPT5H/CCL3/SEC13/RPS7/RPL7A/RPS23/RPS10/RPS15/
[illegible]MD4/TRPM4/PSMD3/PSMD7/TBL1XR1/CSNK1G2/JRK/PSMD8/ABL1/VCP/PSMA1/SFRP2/TNFAIP3/PS
[illegible]/CLEC7A/SUPT5H/SRPK1/BAD/ACVR1B/NOTCH1/OPTN/TRIM21/TARBP2/VPS37B/PPIE/POLR2I/PP
[illegible]/CABIN1/HELLS/CETN2/RBX1/DR1/RNF4/CENPN/RBBP7/RPA2/CUL4B/TAF12/CENPQ/CHD1L/HAT1
[illegible]6/RPA2/CCT8/POLD3/TRIM25/PTGES3/NAT10/VCP/WRAP53/NOP10/FAAP20/GAR1/HSP90AA1/RPS
[illegible]0/HAUS6/NDE1/NSF/CKAP5/IQCB1/RAB3D/TMEM216/STX7/VAMP8/CEP41/HSP90AA1/DCTN1/VPS4
[illegible]/RAD21/MSX1/WNT4/RAD51C/HSPA2/RAD51D/EME2/ACTR3/RAD1/SPIRE1/SYCE1L/ERCC1/CCNE2/
[illegible]MB/DNM1L/PDCD5/FADD/RACK1/MSX1/RAD9A/IL20RA/TRAF7/MFF/RPS7/NFATC4/BAD/BCL10/CTN
[illegible]ARCC1/KAT2A/HELLS/DR1/CENPN/RBBP7/INO80C/RERE/SUV39H2/CENPQ/CHD1L/MBD3/ANP32E/M
[illegible]0/DNA2/DOT1L/RAD51D/WRAP53/NOP10/DCLRE1B/GAR1/HSP90AA1/NBN/HNRNPU/ERCC1/EXOSC10
[illegible]4/PSMD3/PSMD7/PPP4C/PSMD8/LIMS1/PSMA1/TRIP6/TRADD/PSMB5/PSMA4/PSMD9/TNFSF15/TLR
[illegible]M71/PLEKHN1/HNRNPM/EXOSC3/PSMD8/PSMA1/MYEF2/PSMB5/PSMA4/PSMD9/RPS27A/HNRNPU/EXO
[illegible]KCI/ZMYND8/GZMB/PDCD5/STX4/RACK1/CLIP3/CEMIP/MFF/STAC/STX7/CSK/VAMP8/BAD/CASP8/
[illegible]IRE1/PLK3/SH3GLB1/ZFYVE19/BCL2L1/RAB11A/KIF20B/MITD1/KLHL21/RHOA
[illegible]/POLA1/PRIM2/POLQ/RFWD3/RPA2/POLD3/TIMELESS/SLBP/DBF4B/SSBP1/DNA2/EME2/CHEK2/CD
[illegible]3/CDKN1C/IL1B/DYNC1LI1/ANAPC11/ZNF207/CHMP1A/RANBP1/RAD21/MTBP/NME6
[illegible]IDD1/VCP/SFRP2/TRADD/BAD/BCL10/TNFSF15/CASP8/PDCD2/PPM1F/NDUFA13/RHOA/ANP32B/LG
[illegible]MD10/PSMD11/LTBR/PSMD1/TNFSF12/PSMA7/TRAF3/PSMB3/PSMD4/PSMD3/CARD16/PSMD7/IKBKG
[illegible]P5C/CARD16/IKBKG/FADD/ECM1/PELI1/TRIM25/ABL1/TRADD/BCL10/CASP8/MAVS/GOLT1B/RHOA
[illegible]51C/HSPA2/ABL1/MTA3/NSFL1C/CCND3/ADAM17/HNRNPU/RAB11A/KIF20B/MECP2/LGMN/BTC/BEC
[illegible]P/MAGOH/THOC2/XPO7/HNRNPA1/EIF5A/POLR2D/DHX9/NXT2/RBM15B/SEH1L/KHSRP/THOC1/DHX3
[illegible]FBXO5/DAPK3/CDKN1C/IL1B/DYNC1LI1/ANAPC11/ZNF207/CHMP1A/CALR/RANBP1/RAD21/MTBP/N
[illegible]TN2A2/BTG2/PIDD1/CHEK2/CCND3/ADAM17/CDK2AP2/PLK3
[illegible]A/CHMP4C/NSMCE2/SGO1/MSTO1/SPDL1/XRCC3/RRS1/INCENP/FBXO5/KIF14/KPNB1/SMC4/KNSTR
[illegible]0/DNA2/RAD51D/WRAP53/NOP10/DCLRE1B/GAR1/HSP90AA1/NBN/HNRNPU/ERCC1/EXOSC10/CCNE2
[illegible]/PSMD4/PSMD3/PSMD7/WDR1/MED12/PSMD8/ABL1/PSMA1/SFRP2/FOXF2/PSMB5/PSMA4/PSMD9/AP
[illegible]/FADD/ECM1/RACK1/ZNF675/CLIP3/PELI3/TNFAIP3/TRADD/PTPN2/ADAM17/CASP8/PTPN1/PIAS
[illegible]2/GMFG/ABI2/ARPC1A/BRK1/ARPC5/ACTR3/DBNL/SPIRE1/ADD3/ARHGAP28/RHOA/MTPN/SPTAN1/
[illegible]TIMELESS/PRPF19/BTG2/PIDD1/DNA2/DOT1L/EME2/CHEK2/CLSPN/RAD1/NBN/PLK3/UIMC1/INTS
[illegible]GO2/PSMD4/PSMD3/PSMD7/WNT4/MED12/PSMD8/ABL1/PSMA1/SFRP2/PLCB2/PSMB5/PSMA4/PSMD9
[illegible]RB1/IL15/RPS3/CD6/PNP/FADD/PELI1

CUL4B/BTN2A2/BTG2/PIDD1/SUSD2/CHEK2/CCND3/ADAM17/CDK2AP2/PLK3/GLI1/ATP2B4/CNOT1
/RACK1/MSX1/RAD9A/IL20RA/PTTG1IP/GRINA/SFRP2/RPS7/BAD/ERP29/PTPN2/STYXL1/PTPN1/
/BLM/CNOT9/CDKN2B/CDK2/PML/DYNC1LI1/PRCC/DONSON/TIPIN/MSH2/ZNF207/TOPBP1/RFWD3/
51D/EME2/ACTR3/RAD1/SPIRE1/SYCE1L/CCNE2/MASTL
STAC/BAG2/HSP90AA1/THBS1/HSF4/HSPA5/HSPA14/HIKESHI/DAXX/POLR2D/DNAJC2/CCAR2/RPA
RASAL3/GPR183
OMM22/BNIP3L/SREBF1/TIMM10/AIP/NDUFA13/VPS11/YWHAG/ATG13/TFDP1/HAX1/CALM3/CSNK2
/NBN/PLK3/UIMC1/INTS7/HINFP/TIPRL/CNOT10/CCAR2/CNOT3/MUS81/TFDP1/ATR/TP53BP1/HM
ELI1/RASAL3/GPR183
RPF19/BTG2/PIDD1/ABL1/CHEK2/NBN/PLK3/UIMC1/MYO6
7/AKIRIN1/THBS1/ADAM17
5A/POLR2D/RBM15B/SEH1L/EIF6/THOC1/DHX38/ENY2/PABPN1/SRSF1/SRSF2/CASC3/FIP1L1/RB
A/STK3/GLMN/SIX1
3/S100A7/CXCL17/AKIRIN1/PTGER4/THBS1/CD177/PLA2G1B
OC1A/MAP4/TUBG2/RAE1/KIF2A/PTPA/ZNF207/CHMP1A/ACTL8/WRAP73/NDE1/DRG1/MZT1/VCP/N
/GLMN/SIX1
OH/THOC2/HNRNPA1/EIF5A/POLR2D/DHX9/RBM15B/SEH1L/EIF6/THOC1/DHX38/ENY2/PABPN1/SR
DD/PELI1/RASAL3/GPR183
RPS12/RPS4X/RPL6/RPL37/RPL27A/HSPA5/RPL36/UBA52/RPL19/RPS11/RPL13A/RPL37A/RPLP1
2D/RBM15B/SEH1L/EIF6/THOC1/DHX38/ENY2/PABPN1/SRSF1/SRSF2/CASC3/FIP1L1/RBM8A/MAG
BNIP3L/SREBF1/TIMM10/AIP/NDUFA13/VPS11/YWHAG/ATG13/TFDP1/HAX1/CALM3/CSNK2A2/PAR
PA1/UBR5/TWIST1/MLLT6/DNMT3B/CTBP1/IK/UCN/PHF1/DCP2/BUB3/HNRNPC/BCOR/PRMT6/ASF1
MD8/ABL1/PSMA1/SFRP2/FOXF2/PSMB5/PSMA4/PSMD9/AP2A2/PSMC3/RHOA/IFT20/CLTC/AP2B1/
LBP/CCL3/S100A7/CXCL17/AKIRIN1/THBS1/ADAM17/VEGFA/RHOA/LGMN/TNFRSF14/KITLG/CCL1
MD8/ABL1/PSMA1/SFRP2/FOXF2/PSMB5/PSMA4/PSMD9/AP2A2/PSMC3/RHOA/IFT20/CLTC/AP2B1/
WRAP53/UBQLN4/SFR1/NBN/MAGEF1
KIF20B/SYNE4/PARD3/RHOA/DOCK2/CCL19/HTT/FRMD4A/KIF26B/ARFGEF1/PKD1/ACTR1A/LLGL2
HMP1A/CABIN1/HELLS/CENPN/RBBP7/SRPK1/CENPQ/HAT1/MIS18A/NCAPD3/DAXX/RNF8/SART3/A
/UBQLN4/SFR1/NBN/MAGEF1
PS27A/VEGFA/HSF4/HSPA5/EGLN2/UBA52/PSMC3
RPL37/RPL27A/RPL36/UBA52/ETF1/RPL19/RPS11/EIF3E/RPL13A/RPL37A/RPLP1/RPL32/RPL30
F4/HSPA5/EGLN2/UBA52/PSMC3

RCAL1/ERCC3/DHX30/DDB1/CHD7/ANXA1/HNRNPA2B1/ERCC2/RAD54B/HELB/XRCC5/TP53/DDX1/A
ATAD5/THOC1/INPP5D/PTPRC/IKZF3/CASP3/CD22/SUPT6H/CD19/VAV3/PPP2R3C/SLA2/NOD2/TN

SRSF2/CASC3/FIP1L1/RBM8A/MAGOHB/SMG7/POLDIP3/THOC7/AAAS/THOC3/HNRNPA2B1/SUPT6H/
AP1A/MAP7D3/EML2/MECP2/CENPJ/CDK5R1
SRSF2/CASC3/FIP1L1/RBM8A/MAGOHB/SMG7/POLDIP3/THOC7/AAAS/THOC3/HNRNPA2B1/SUPT6H/
UBA52/RPL19/RPS11/RPL13A/RPL37A/RPLP1/PMM2/RPL32/RPL30/RPS9/RPS14/RPS27/RPL29/S
FRP2/PSMB5/PSMA4/PSMD9/AP2A2/PSMC3/RHOA/CLTC/AP2B1/PSMC5/AP2A1
PSMC5/CLEC4E/PSMB1/MAP3K7/PSMB6/FFAR2/ICAM2/PSMA3/RELA/PSMB7/SKP1/CLEC4A
UV39H2/CENPQ/HAT1/MIS18A/DAXX/SART3/ANP32B/SMARCA5/POLE3/IPO4/HMGA2/PARP10/GRWD
NP32B/SMARCA5/DMAP1/POLE3/IPO4/SRCAP/GRWD1

HUS1/TERF1/E2F7/KCTD13/ZMPSTE24/LIG3/PPP2CA/SMC1A/TP53/WIZ
RG1/MZT1/VCP/CHEK2/CCSAP/DCTN1/HNRNPU/RAB11A/MECP2/RHOA/CLTC

RPL19/RPS11/RPL13A/RPL37A/RPLP1/PMM2/RPL32/RPL30/RPS9/RPS14/RPS27/RPL29/SRP68/T

MB5/PSMA4/PSMD9/AP2A2/PSMC3/RHOA/CLTC/AP2B1/PSMC5/AP2A1
P1/DAB2IP/HMGA2
IF2A/CAPZB/CTSD/AP1B1/SEC13/HLA-DOA/FCGR2B/DNM2/THBS1/DCTN1/CANX/AP2A2/DYNC1I2/

/HAUS5/RAB11A/RHOA/NEK6

IF2A/CAPZB/CTSD/AP1B1/SEC13/HLA-DOA/FCGR2B/DNM2/DCTN1/CANX/AP2A2/DYNC1I2/KIF22/
/RPLP1/RPL32/RPL30/RPS9/RPS14/RPS27/RPL29/SRP68/TRAM1/RPL13/SSR1/RPL4/RPL41/RPL

SD/AP1B1/SEC13/HLA-DOA/FCGR2B/DNM2/DCTN1/CANX/AP2A2/DYNC1I2/KIF22/CLTC/AP1S1/AP
M1/IMMP1L/TIMM9/TIMM22/USP36/PAM16/HTRA2/ARIH2/UBL5/BAP1
E19/UIMC1/GPR132/PSMC3

/EIF2D/YTHDF2/METTL3/RPL41/RPL9/RPL24/UNK/RPL11/EIF3F/TMA7/RPL10A/EIF4G1/RPS28/
AP2/PSMC5/PDIA3/PSMB1/PSMB6/PSMA3/HLA-C/VAMP3/CD207/PSMB7/HLA-E/CLEC4A/ACE/IKBK
1/RPL32/RPL30/RPS9/RPS14/RPS27/RPL29/SRP68/TRAM1/RPL13/RPL4/RPL41/RPL9/RPS6/RPL

J/CC2D2A/YWHAG/B9D1/NPHP4/ACTR1A/OFD1/TMEM67/CEP72/C2CD3/CNTRL/CEP97/RAB3IP/NPH

N1/AFAP1L2/TSG101/HIP1/SOCS5/SNX6/FASLG
4/PAF1/ZNF16

/FBXL7/HMGA2/VPS4A/PSMB1/PSMB6/PSMA3/HUS1/PSMB7/SKP1

PC5/CDC27/ANAPC4/PSMB1/PSMB6/CDC23/PSMA3/BUB3/ANAPC15/PSMB7

NCKAP1/PLCG1
01C/NCKAP1/PLCG1

RP10/GRWD1

MB6/PSMA3/HLA-C/VAMP3/CD207/PSMB7/HLA-E/CLEC4A

T3/ZNF622/RPL12

.-C/VAMP3/CD207/PSMB7/HLA-E

PR35/RAB15/RAB25/RIT1/RFXANK/DOK3/GNA12/ADGRG1/RASGEF1A/RAP2B/RHOF/RAB3B/F2RL1/
AP8L/IL1B/MTA2/CDK5/KDM5B/PHB/MIER2/TAF9/SFPQ/SIRT7/NAP1L2/KDM5C/SET/SMARCB1/SM
ANKRD27/THY1/ARHGAP9/CORO1C/GPSM1/F2RL1/ARHGEF19/CCL13/TBC1D1/RIN1/ARHGAP39/GDI
/ADORA1/PRNP/ADAM9/MVP/HRAS/SYK/STK39/NUP62/IL18/EZH2/CDC25A/CDK4/DLG3/CSF1R/CD
L1/HES4/EZH2/NME2/TWF2/ASAP1/ASPM/COL3A1/ANKRD1/ESRP1/TRPV2/GRN/CRMP1/CFL1/BHLH
/PTK7/EFNA5/SF3A2/IL34/RGS14/HIF1A/FZD1/PAK1/CRABP2/ANKRD27/NRCAM/XRCC2/BIN1/HD
QA/RELB/SLAMF8/IGLL5/HLA-DRB1/PRNP/HRAS/SYK/HLA-DQA1/HCK/UNC13D/IL18/PAG1/S100A
1/TNFAIP8L3/TRAF2/CCL5/MAPKAPK2/CXCR4/MIF/CD300A/IQGAP1/CARD10/EFNA5/DGKZ/CHTF1
1/MT3/CHST10/AATF/EXTL3/B4GALT7/MUC3A/KRTCAP2/RPN2/B4GALT2/XYLT2/UGDH/POMT2/FUT
2/HCLS1/AXIN1/PKN3/CDK5/TXN/SPOCK2/GSK3A/INPP5J/BGN/PARP1/STK32C/SMYD3/PRKD2/AR
C14/NPLOC4/PSMB2/IRF7/PSME3/TNIP2/LAG3/FLOT1/SMPDL3B/ADAM8/NONO/ULBP2/NLRC5/PLS
D2L2/MAPK3/PYGO2/SLPI/ACD/UBASH3B/GPSM1/HSP90AB1/CSNK1E/DDX11/MARK2/PPP1CA/LEF1
AG1/CBFB/ITGA2/GBA/CAPNS1/PDGFA/FA2H/TGFB2/FGFR2/CYP26B1/EVPL/VDR/GORAB/APCDD1/
/SMPDL3B/NONO/CD209/LYN/CARD11/MUC3A/PSMB4/CTSS/PSMB8/PSMB10/PSMA5/CD300LF/NRAS
CL3/CXCL9/NLRC5/PLSCR1/PMAIP1/RNASE2/IL1B/LAMTOR5/IL2RA/APOBEC3G/RNF26/FGR/CCL4
ADM/VIL1/AP2M1/RAB10/TNIK/STAC3/ACSL3/PRKCI/STX4/RACK1/MYO5A/EPB41L3/PACS2/CLIP
1/ILF3/ISG20/TFAP4/POLR2L/APOBEC3D/POLR2K/TNIP1/TRIM25/SUPT5H/CCL3/SRPK1/NOTCH1
PSF6/PTPN23/STX18/BAG3/IL4R/SYT1/DNM1L/PDCD5/KHDRBS1/CEMIP/EMD/MFF/PDCD10/VAMP8
1A/PSMD1/PSMA7/PSMB3/PSMD4/DNM1L/PSMD3/RBX1/CARD16/PSMD7/CBL/TBL2/SUV39H2/PSMD8
MD11/ERO1A/PSMD1/PSMA7/PSMB3/PSMD4/DNM1L/PSMD3/RBX1/CARD16/PSMD7/CBL/TMEM199/TB
R1/TUBB2B/USP14/C5AR1/PADI2/PRKD2/SEMA4B/CALR/SEMA4D/XCL2/CMKLR1/SWAP70/SEMA4F/

D7/DNM2/TCTEX1D2/ODF2L/VDAC3/MARK4/DEF8/CENPJ/SRF/IFT20/FNBP1L/DPYSL3/EPS8L1/EP

IB1/VPS4A/ADD1
/TSG101/HIP1/SOCS5/SNX6/FASLG

/HDAC1/PLCB3/CSNK1E/LZTS2/FAM53B/MARK2/PSMB2/ZNF703/TMEM237/PPP1CA/LEF1/SFRP5/P
BCAP31/UBE2J2/FBXO5/CSNK1D/ANAPC7/CDK2/KIF14/SPSB1/SNF8/AXIN1/ADRM1/CCDC22/MYLI
HBDF1/TCIRG1/RNF34/UBE2I/RNF19B/SHARPIN/HM13/GNA12/ISG15/CHMP4C/PDCL3/DDA1/HSP9
/MARK2/PRELID1/PIM2/HIP1R/AIFM2/BID/CEP89/TOMM5/UBE2J2/DYNLT1/NPEPPS/UQCC3/HSPA
K1/BAIAP2L1/FCHSD1/RHOF/BIN1/F2RL1/ARPC2/ARHGEF19/ARPC3/LIMCH1/CORO1B/HIP1R/PAM
/TNFSF9/FLOT2/ZP3/IQGAP1/HLA-DPB1/EBI3/CD276/EFNB1/CD3E/PAK1/SASH3/ADGRG1/THY1/
/PKN3/CDK5/TXN/GSK3A/INPP5J/STK32C/SMYD3/PRKD2/ARRB2/MTCP1/PRKCI/GRK2/APP/DMPK/
/CARD11/MUC3A/PSMB4/CTSS/PSMB8/PSMB10/PSMA5/CD300LF/NRAS/PSMD13/PSMC4/MUC5B/UBE
/CARD11/CBFB/PIAS3/IL2RA/NFAM1/PTPN6/CD27/CCR1/IL12RB1/CYP26B1/IL15/DROSHA/KAT2
L2RA/NCAPH2/LCK/CD27/IL12RB1/CYP26B1/IL15/DROSHA/KAT2A/IL4R/PNP/CD8A/LILRB4/AP3
1/MRPL10/MRPL52/CKAP2/NSF/CKAP5/MRPL48/DAP3/MRPS7/MRPL53/GSPT2/STMN2/MRPS17/MRP
D/ACVR1B/CASP8/PDCD2/HOXA5/SMAP1/TMEM64/NKAP/RHOA/AP3B1/RUNX3/ZMIZ1/KITLG/CCL19
8/VCP/PSMA1/SFRP2/PSMB5/PSMA4/PSMD9/VPS35/SFRP4/SULF2/UBR5/PSMC3/GSKIP/PSMC5/RB
MTHFR/VEGFA/KIF20B/TWIST1/CC2D2A/STK3/GLMN/SIX1
8/PDCD2/PPM1F/NDUFA13/RHOA/ANP32B/LGMN

ARK4/HAUS5/TRIM37/CENPJ/SASS6/RBM14
SPIRE1/DCTN1/MECP2/CDK5R1/RHOA/NIN/ARF1/RHOBTB2/GMFB
RHOA/ANP32B
XW5/MARK4/HAUS5/TRIM37/CENPJ/SASS6/RBM14
/DHX9/TLR7/POLR2E/ZCCHC3/PIN1/TLR8/TRIM32/POLR3F/RELA/POLR1C/POLR3A/TRIM15/PTPR
NB3/CDK5R1/YWHAG/AP2B1/EPHA2/TFDP1/EPHB2/CIB1
/DHX9/TLR7/POLR2E/ZCCHC3/PIN1/TLR8/TRIM32/POLR3F/RELA/POLR1C/POLR3A/TRIM15/PTPR

/MFN1/TMEM102/CALM3/NAIF1/CNP/THEM4/TIMM9/TIMM22/NMT1

B/POLR2G/FTSJ1/BIN1/ECE2/RBM3/EIF2S2/MRPS12/RPS2/PABPC1/TICRR/GGCT/PSENEN/MRPL4
RNF19B/SHARPIN/ISG15/BRCA1/PCNA/MED10/TRIM59/SOCS1/RPS2/HSP90AB1/TSPAN17/PSMB2/
BCAP31/UBE2J2/FBXO5/CSNK1D/ANAPC7/CDK2/KIF14/SPSB1/SNF8/AXIN1/ADRM1/CCDC22/MYLI
15/CHMP4C/PDCL3/DDA1/HSP90AB1/NPLOC4/CSNK1E/MAN1B1/PSMB2/PSME3/HSPBP1/BCAP31/UB
/HDAC1/PLCB3/CSNK1E/LZTS2/FAM53B/MARK2/PSMB2/ZNF703/TMEM237/PPP1CA/LEF1/SFRP5/P
AL3ST4/PLCB1/NUP62/PFKFB3/GPC1/TMSB4X/ELOVL1/NUDT14/TUSC3/NME2/DPM2/MUC1/TYMP/G
LCB1/STK39/NUP62/IL18/EZH2/EEF1E1/S100A8/MAPK8IP2/CSF1R/UNC5B/HCST/COL3A1/ANKRD
3/PPARD/B3GALNT1/ST3GAL4/SOAT1/MT3/CHST10/AATF/EXTL3/B4GALT7/MUC3A/KRTCAP2/RPN2
A/PLEKHM2/TMED9/NPLOC4/NUP93/MYRF/SNX10/FLOT1/CSNK1D/SNF8/PLSCR1/CAMSAP2/REEP2/
C1/PPIA/NUP188/ZC3H12A/DYNLT1/LAMP3/CCL2/RPSA/TRIM31/KPNB1/SNF8/PLSCR1/CD209/IT
A/BCL3/LAG3/NCKAP1L/JAG2/ADAM8/PRR7/PIK3CD/KDELR1/FANCA/CARD11/CBFB/IL2RA/NCAPH
3/UNC93B1/PRKDC/PSMD2/MAPK3/NUPR1/SERPINE1/PAK1/MNDA/IRF1/IRF3/F2RL1/RAB7B/LRRC
D/DNAAF5/MKKS/TBC1D30/DCTN2/B9D2/POC1A/HAUS8/PLK4/MAP4/IFT80/GORAB/ODF2/PTPN23/
DAM8/NONO/CCL2/ATF4/KIF14/CLN3/ATN1/GBA/CDK5/WFS1/TGFB2/CIT/PARP1/SET/NSMF/NES/
D2B/SLC9A1/EFNA5/PAK1/BAIAP2L1/FCHSD1/RHOF/BIN1/F2RL1/ARPC2/ARHGEF19/ARPC3/LIMC
D/DNAAF5/MKKS/TBC1D30/DCTN2/B9D2/POC1A/HAUS8/PLK4/MAP4/IFT80/GORAB/ODF2/PTPN23/

RA8/IL1B/CDK5/DNER/FA2H/TGFB2/ASCL2/PPP1CC/CDKN2C/C5AR1/PRKCI/APP/GPR157/VIM/CS

SF11A/PSMB2/KRT18/ZC3H12A/PSME3/CPNE1/CCL2/KRT8/ASAH1/PSMB4/GBA/PIAS3/PSMB8/TNF
/SLC35B2/CARD11/GPRC5B/CCDC22/UNC5CL/IL1B/S100A13/TMEM101/CASP1/MAP3K14/SLC44A2
CI/TGFB3/APP/AXL/FADD/PIGT/AKT1S1/NAE1/TFAP2A/CCL3/BTG2/NFATC4/GABRB3/HSPA5/BCL
CL11/CCR5/CARD16/AXL/VIM/LBP/CCL3/CDK19/ABL1/FCGR2B/TNFAIP3
/IL1B/WFS1/CTTN/TAF9/MAZ/PSMD10/GPX1/FAIM/ARRB2/PCGF2/MAPK7/FADD/PTTG1IP/WNT4/P

DOCK8/NTM/CASP3/ATP8B3/PRF1/MEGF8/CELSR3/TUB
/IFITM3/KPNA6/SNX3/CCL8/CAV2/HMGA2/PARP10/MORC2/ZC3HAV1/LARP1/VPS4A/NECTIN2/SRP
C2/INTS7/POLR2D/TOE1/SSU72/THOC1/DHX38/EXOSC2/PABPC1L/PABPN1/SRSF1/SRSF2/PAF1/C
A/AP3B1/RUNX3/ZMIZ1/CCL19/PIK3R6/ZNF683/PTPRC/METTL3/LOXL3/NFKBID/CLPTM1/SH3RF1
MEM102/BIK/NAIF1/PLA2G6/THEM4/NMT1/PAM16/FZD9/ATG3
FCD/GTF2F1/LARP1/VPS4A/CTDP1/SRPK2/DDB1/P4HB/VAPA/TBC1D20/ADAR/IGF2R/POLR2B/CD4
1
AM98A/DOCK11/EPS8L2/CARMIL2

/UBE2J2/FBXO5/CSNK1D/ANAPC7/CDK2/KIF14/SPSB1/SNF8/AXIN1/ADRM1/CCDC22/MYLIP/PSMB
/FANCD2/NDRG3/PLCB1/NUP62/CCT5/MAPK8IP2/ASPM/ZNF296/SPAG4/SLC26A6/TNFAIP6/HSF1/
131/CDK16/RACGAP1/PTAFR/NCAPH/FANCD2/NDRG3/NUP62/MAPK8IP2/ASPM/BSG/ZNF296/SPAG4
131/CDK16/RACGAP1/PTAFR/NCAPH/FANCD2/NDRG3/NUP62/MAPK8IP2/ASPM/BSG/ZNF296/SPAG4
MAP2K2/PAK1/CAPN10/STX1A/SLC51B/DYNC1H1/GDI1/ZC3H12A/NCBP2/BCAP31/UBE2J2/FERMT1

FR1/DVL3/DUSP2/ADAM8/SHC1/LYN/WNT7B/BIRC7/DUSP4/IL1B/GBA/PDGFA/MLKL/MAPK11/PTPN

3/CSNK1D/FZD2/AXIN1/NLE1/MITF/WNT7B/GPRC5B/PSMB4/PSMB8/PSMB10/PSMA5/PSMD13/PSMC

1/MIF/MTCL1/CD2/HIF1A/MAPK3/OAZ1/DKC1/PAK1/CAPN10/TNFRSF4/SLC51B/CCT2/CD58/IRF3
CLN3/GBA/CDK5/WFS1/TGFB2/PARP1/SET/NSMF/NES/C5AR1/MSH2/ARRB2/PRKCI/TGFB3/PPP5C/
RC5B/PSMB4/PSMB8/PSMB10/PSMA5/PSMD13/PSMC4/GSK3A/FGFR2/DVL2/RNF220/PSMD10/PSMD1

/GPRC5B/CCDC22/UNC5CL/IL1B/S100A13/TMEM101/CASP1/MAP3K14/SLC44A2/LTBR/PPP5C/CAR
SYT1/APP/GAL/SYT3/SEMA4F/SEMA5B/SEMA3C/SEMA3E/MAPT/MED12/RARG/ABL1/VCL/RASAL1/S
P2A/CCL3/BTG2/NFATC4/GABRB3/BCL2L1/EGLN2/ZPR1/MECP2/CDK5R1/RHOA/STXBP1/SIX1/LGM
DR/UBE2A/SYDE1/MAFF/AGO2/CALR/TGFB3
P3/PML/THOC6/GRB2/VPS18/GRK2/CPSF4/CCR5/AXL/CBL/CLEC5A/TNIP1/TRIM25/VAMP8/BAD/T
/WASF1/GPX1/RPS3/GRB2/FHOD1/CDC42/TGFB3/APP/SWAP70/CDC42EP2/KATNB1/GMFG/ABI2/WD
L/PELI1/MTBP/RPL23/GNL3/TNIP1/PTTG1IP/PDCD6/UBXN1/ABL1/VCP/TNFAIP3/RPS7/FOXF2/B
NM2/TCTEX1D2/ODF2L/VDAC3/MARK4/DEF8/CENPJ/SRF/IFT20/FNBP1L/DPYSL3/EPS8L1/EPHA2/
P2/TRAF4/TWIST1/EIF3E/STK3/DHX9/DPH3/CLIC2/EGF/CAV1/HMGA2/CALM3/PKD1/EDF1/BMP2/
/CAV2/VPS4A/NECTIN2/VPS16/C2CD5/SPACA6/RAB8B/VPS8/MX1/VAPA/TSNARE1/VAMP3/KIF5B/
/PIK3CB/ACIN1/RPS6/CASP3/ADAR/SMAD5/NCSTN/MFHAS1/RPS24/HOXB6/ANKRD54/PDE4B/DMTN
AVS/TLR2
/HSPA5/CANX/HSPA14/PPP2R5B/GFPT1/TLN1/DAXX/GOSR2/TPP1/BHLHA15/HSP90B1/HSPA13/DA

ALNAC2/TRAK2/C1GALT1C1/B3GNT4/GALNT3/POMT1/OGT/DPM1

OCS1/EPHB6/TGFA/RELN/DYRK2/NCAPG2/EPHB3/FGFR1/FGFR3/SHC1/HDAC2/HCLS1/TESK1/LYN/
OCS1/EPHB6/TGFA/RELN/DYRK2/NCAPG2/EPHB3/FGFR1/FGFR3/SHC1/HDAC2/HCLS1/TESK1/LYN/
CCL13/TNFRSF11A/PSMB2/KRT18/ZC3H12A/PSME3/CPNE1/CCL2/KRT8/ASAH1/PSMB4/GBA/PIAS3
1/PF4V1/SBNO2/CD6/CXCL11/CCR5/CARD16/AXL/BTK/CLEC7A/VIM/LBP/CCL3/CDK19/ABL1/FCG
CR5/CARD16/AXL/VIM/LBP/CCL3/CDK19/ABL1/TNFAIP3
10/EXOSC7/WARS2/SEPHS1/TRMT12/TP53RK/DARS2/CTU1/TSEN34/TPRKB/TYW3/YARS2/PARS2/H
F3E/RPUSD3/NSUN4/RHOA/POLR2D/DHX9/COA3/EIF6/RPS9/LARP1/YTHDF2/CDK5RAP1/YTHDF1/M
R2

/MVB12B/NEURL1/PDE6G/DGKD/RNF126/RNF115/RAB7A/CHMP6/EPN1/RASSF2/AFAP1L2/TSG101/

SETDB1/AGO1/POLR2B/FXR1/ASF1A/EIF4G1/ELAVL1/ZMPSTE24/NUP214/MYCN/MAEL/TP53

/RPRD1A/POLDIP3/THOC7/THOC3/U2AF1L4/SRSF3/PNPT1/GRSF1/RNF40/SRSF7/PAPOLA/LEO1/T

2/PRKCD/ROBO1/ADORA1/PRNP/ADAM9/HRAS/SYK/PLCB1/STK39/PFKFB3/PGF/IL18/EZH2/MAPK8

CL13/TBC1D1/RIN1/ARHGAP39/GDI1/ARFGAP1/TBC1D10B/DVL3/NCKAP1L/ARHGAP1/CCL2/RAP1G

NMT1/POLR2L/NUP155/DROSHA/KAT2A/AGO2/BEND3/MTF2/NUP107/NCOR2/POLR2K/HELLS/PCGF2

5B/DPAGT1/IL15/A4GALT/STT3A/GALNT6/FUT2/FUT6/MUC20/TMEM258/MVD/SLC35C1/ST3GAL5/

5B/DPAGT1/IL15/A4GALT/STT3A/GALNT6/FUT2/FUT6/MUC20/TMEM258/MVD/SLC35C1/ST3GAL5/

/PDCD6/UBXN1/ABL1/TNFAIP3/RPS7/FOXF2/BAG2/TBC1D7/BCL10/HSP90AA1/RPL5/MASTL/HSPA

EM98/ERCC1/HLA-F/RNF8/CCL19/NOTCH2/DOCK10/MSH6/ITGAL/ATP7A/TP53BP1/ATAD5/THOC1/

HX9/CCL19/TMEM106A/PTPRC/NFKBIL1/TRIM27/SYT11/IFNG/CCR2/AKAP8/HLA-E/NOD1/CLEC4A

RHBDD3/GLMN/INHA/TNFRSF14/IDO1/RUNX3/XCL1/THOC1/INPP5D/DUSP22

HMGA2/TFIP11/SPIRE2/FIGNL1/EYA3/TMEM161A/HELB/ACTR2/FAM168A/SETMAR/WDR70/TRIP12

SF1R/NCF4/SH2D2A/ESRP1/CREB3L1/NCF1/RHOQ/DOK2/ATP6V0B/IGFBP3/AREG/ATP6V0D2/JAK3
NFAIP6/HSF1/KIF18A/WIPF3/LIMK2/HMGB2/YBX3/EIF5A2/BAX/NR0B1/SNRPA1/FANCG/ZP3/RUV
NBP1/GPD1L/PPP1R37/P3H1/BDKRB1/BAX/FKBP1A/CD300A/PRKDC/MAD2L2/RGS14/PPP2R1A/ISG
A/LEF1/SFRP5/PSME3/SLC9A3R1/DVL3/FERMT1/PLEKHA4/DAPK3/CSNK1D/AXIN1/NLE1/GPRC5B/
1/CHMP3/WDR45B/MAP1LC3B/VPS28/ATP6V0C/MVB12A/ATP6V0E1/BAG3/VPS25/PGAM5/IKBKG/PI

B/PDGFA/PTPN6/SAMSN1/SOCS3/DVL2/IL15/CADM4/LRP8/ARRB2/SEMA4D/VPS25/APP/ADRA2A/C

/DNAJB2/PLXND1/NES/LOX/RUVBL2/ARRB2/APP/CARD16/RALB/STPG1/PTPRF/CARD18/MFNG/LFN

/CCDC93/AP3D1/TBC1D10C/UBE2O/RASSF9/TMEM87B/GGA1/VCP/DENND5A/DCTN1/VPS35/RPS27A
/SLC11A1/RAB20/HLA-DRB5/CD47/CCL4/WAS/CASP1/PML/SOCS3/IL12RB1/CXCL16/HLA-B/XCL2
/TBL2/ATP6V0D1/HSPD1/HSPA2/VCP/SRPRB/WIPI1/HSP90AA1/TATDN2/PTPN2/THBS1/DCTN1/PT
2/ABL1/SART1/HLA-DOA/TOX/BAD/PTPN2/NKAP/RHOA/INHA/AP3B1/RUNX3/ZMIZ1/CCL19/PIK3R
1/KITLG/CCL19/PIK3R6/HAX1/INPP5D/PTPRC/ACIN1/NFKBID/CD4/IFNG/ANXA1/PPP2R3C/GNAS

TC/EREG/EGF/DAB2IP/STUB1/SNX5/PLCG1/STAM/MVB12B/NEURL1/PDE6G/DGKD/RNF126/RNF115

K2/USP36/DYRK3/KDM3A/PRKCB/CNEP1R1/ZMPSTE24/CHMP6/NOLC1/SERBP1/PPP2CA/TARDBP/TB
2A2/VRK2/CSNK1G1/ULK1/DMTN/DYRK3/EIF4G1/PRKCB/PPP2R5D/WNK1/MAP3K12

/PSMB1/MAP3K7/PSMB6/PSMA3/RELA/PSMB7/SKP1/NOD1/NOD2/IKBKB

A/PSME2/MYB/PSMD6/GATA3/PSMC2/PSMA6/YAP1/TP73/HSPA9/PSMD5

/ROBO1/ADORA1/PRNP/ADAM9/HRAS/SYK/PLCB1/STK39/PGF/IL18/EZH2/MAPK8IP2/DLG3/CSF1R

5/MAPK3/MAP2K2/CENPE/PAK1/PEA15/FPR1/SOCS1/CKS1B/HSP90AB1/TGFA/TNFRSF11A/RELN/M

ARRB2/USP22/KAT2A/ATXN7L3/PSMD4/PSMD3/PSMD7/IKBKG/BABAM1/TNIP1/INO80C/UBXN1/PSM

RHOT2/SLC25A5/MFF/BAD/VPS35/CASP8/SH3GLB1/OPTN/BNIP3L/SREBF1/BCL2L1/CDC37/STOML

TYXL1/ODF2L/VDAC3/SH3GLB1/HNRNPU/MARK4/TRIM37/CENPJ/RBM14/RHOA/VPS11/IFT20/BECN

/PWP1/PSMB1/PSMB6/PSMA3/PSMB7/KDM3A/LTBP3/SETD1A/PSMD12/GATA4/WNT3A/STAT3/KMT2A

00A10/CARMIL1/TIMP2/TCF3/P3H1/CXCR4/IQGAP1/PTK7/EFNA5/SF3A2/IL34/RGS14/HIF1A/FZ
/LINGO1/WASF2/BAX/CXCR4/SEMA4A/NDN/MAP2/DOK1/BRSK1/IQGAP1/B4GALT5/EFNA5/MAPK3/E
EMA4A/MAP2/BRSK1/IQGAP1/PTK7/EFNA5/SF3A2/FZD1/PAK1/CRABP2/ANKRD27/NRCAM/THY1/RT
/LINGO1/WASF2/CXCR4/SEMA4A/NDN/MAP2/DOK1/BRSK1/IQGAP1/B4GALT5/EFNA5/MAPK3/EFNB1
/PAG1/S100A8/HLA-DRA/MUC1/EZR/HLA-DQB1/HMGB3/VTCN1/GBP5/CARD9/PSMB9/TFRC/HLA-DP
/EIF4A3/ILF3/GRB7/ZNF706/RPS3/SAMD4B/NCL/EIF3H/AGO2/CALR/APP/PPP1R15A/UQCC2/RPS
/GRN/RPS6KA1/HSF1/CARD9/PSMB9/TRAF2/PSMD14/TIMP2/BAX/BEX3/UBE2Z/TNFAIP8/MAPK3/S
ERPINE1/IL27RA/SLPI/ISG15/LYPD8/SPON2/IRF1/IRF3/F2RL1/STAT1/NPLOC4/BATF2/POLR3K
K1/CRABP2/NRCAM/SMARCA4/PPP1R9B/OSGIN2/GDI1/PPARD/LEF1/MT3/PAK4/EXTL3/KIF14/BCA

SP22/KAT2A/ATXN7L3/PSMD4/PSMD3/PSMD7/IKBKG/BABAM1/TNIP1/INO80C/UBXN1/PSMD8/TAF1
OMT2/FUT3/MUC5B/DPAGT1/IL15/A4GALT/STT3A/GALNT6/FUT2/FUT6/MUC20/TMEM258/MVD/SLC

STK25/VRK1/CSNK1G2/IGF1R/ABL1/STK24/PIM3/CALM2/CSK/CHEK2/MAPKAPK3/ACVR1B/PTK2/T
19/ROMO1/LBP/S100A7/GNLY/TUSC2/ADAM17/ADAMTS5/MAVS/DMBT1/OPTN/PLA2G1B/TLR2/ANXA

3/MILR1/TWSG1/PTPN2/IL20RB/HLA-F/RHBDD3/GLMN/INHA/TNFRSF14/IDO1/RUNX3/XCL1/IL13
P18/CAV1/DAB2IP/RPTOR/CIB1/CDK5RAP1

ARE1/VAMP3/KIF5B/ANXA1/VAMP1/NKD2/RAB7A/VAV3/MUL1

PSMA6/YAP1/TP73/HSPA9/PSMD5

ITGB2/SNAP25/KCNJ11/PRKCD/TRIP10/SLC7A1/ITGAV/SLC7A11/SCAMP5/IL15RA/IGLL5/SLC6A
4A/MAP2/MSN/BRSK1/EFNA5/PAK1/CRABP2/GNA12/ANKRD27/NRCAM/THY1/RHOF/CORO1C/ARPC2/
ARD10/EFNA5/DGKZ/MAPK3/MAP2K2/CENPE/PAK1/PEA15/FPR1/SOCS1/CKS1B/HSP90AB1/TGFA/T

C2/SULT2B1/KRT8/ASAH1/JAG1/CBFB/CAPNS1/PDGFA/FA2H/WAS/TGFB2/AGPAT2/FGFR2/CYP26B

WDR1/EPB41L3/FMNL3/SEMA3E/FGD1/CCL3/PALM/ARAP1/PLXNC1/PTK2/VEGFA/HEXB
1A/ARL8B/AP3B1/IFT20/RAB27B/TNPO1/IFT22/SNAPIN/SSX2IP/UXT/HTT/ACTR1A/RAB1A/KIF3
1A/ARL8B/AP3B1/IFT20/RAB27B/TNPO1/IFT22/SNAPIN/SSX2IP/UXT/HTT/ACTR1A/RAB1A/KIF3

2A2/VRK2/CSNK1G1/ULK1/DMTN/DYRK3/EIF4G1/PRKCB/PPP2R5D/WNK1/MAP3K12/GALNT3
8/UCN/SYT11/HYAL2/NOD1/TLR1/NOD2/AFAP1L2/LILRB2/TICAM1/WNT5A/C5AR2/ARRB1/SLAMF1

RAL1/AURKAIP1/MRPL20/MRPL24/MRPS16/MRPL55/CHCHD1/MRPS2/MRPL21/MRPS26/MRPS11/MRP

IL12A/ZBTB7B/SOCS5/MYB/ZAP70/MALT1/HLA-G/GATA3/FLT3LG/IL1RL2

LL5/NME1/ADORA1/HRAS/RAB31/APLP1/SYK/MTMR2/HCK/UNC13D/LRP10/CD151/NCF4/EZR/SCRI
UVBL1/PYGO2/MAST2/SLC4A2/PHC2/TSPAN8/TTLL1/DYNLL1/DPCD/FSTL3/NDC1/BCAP31/JAG2/R
A/NCBP2/BCL3/EIF3D/CNOT9/DAPK3/ATF4/ITGA2/EIF2S3/GSK3A/MRPL13/GCN1/CDC123/EIF4A
P1R15A/TMUB2/UFC1/SGTB/RACK1/PDIA6/CXXC1/ERP27/TBL2/TRIM25/ATP6V0D1/EIF2B5/UBXN
CKAP1L/NLRC5/SLC11A1/RAC1/GBA/TGFB2/PTPN6/PML/IL1R2/ANXA4/ASB1/ARRB2/TGFB3/CMKL

IDD1/TTYH1/CFTR/SFRP4/SLC4A7/VDAC3/VDAC2/GABRB3
/INPP5D/TMEM106A/TLR8/UCN/SYT11/HYAL2/NOD1/TLR1/NOD2/AFAP1L2/LILRB2/TICAM1/WNT5

PS9/LARP1/YTHDF2/CDK5RAP1/YTHDF1/METTL3/UCN/POLDIP3/HNRNPD/PYM1

DPYSL2/WNT5A/OLFM1/WNT3A

B1/PDE5A/CD80/FGL2/LILRB2/ZBTB7B/PDCD1LG2/SOCS5/CD274

4/ADORA1/ADAM9/NBL1/MVP/HRAS/SHKBP1/SYK/PLCB1/ATP6V1F/NUP62/HCK/GPC1/CD109/PGF/

GO1/WASF2/CXCR4/SEMA4A/NDN/MAP2/DOK1/BRSK1/IQGAP1/B4GALT5/EFNA5/MAPK3/EFNB1/MAP

YGO2/MAST2/SLC4A2/PHC2/TSPAN8/TTLL1/DYNLL1/DPCD/FSTL3/NDC1/BCAP31/JAG2/RSPH1/SC
9/PSMB9/TRAF2/PSMD14/TIMP2/BAX/BEX3/UBE2Z/TNFAIP8/SERPINE1/RNF34/SLPI/PERP/BCL2

.3E/CBFA2T2/STMN2/NFATC4/TBC1D7/DENND5A/DNM2/PTK2/ODF2L/RAB29/IFRD1/CDK5R1/RHOA/
RB5/CD47/CCL4/WAS/CASP1/PML/SOCS3/IL12RB1/HLA-B/XCL2/CDC42EP2/STX4/VIM/TRIM25/C
RPV6/CACNA1D/STXBP1/ADORA2B/SNAPIN/ARF1/IL13RA2/CD84/CALM3/CNR1/ATP2A2/CASK/BCR

KRD54/DMTN/DYRK3

2/MYB/PSMD6/GATA3/PSMC2/PSMA6/NUDT21/YAP1/TP73/HSPA9/PSMD5
DC2/LAMC1/TRPV2/FZD7/SCRIB/CTNND2/CRMP1/CFL1/MICALL2/S100A10/DOK2/DTNBP1/LINGO1
MT5A/HCFC1/EHMT2/IL1B/PIAS3/CBX4/LOXL2/TAF9/NUP85/NUP43/NAP1L2/SET/RNF113A/SMAR

.4/GSDMD/IL4R/PNP/TLR5/HK1/CARD16/CLEC5A/CARD18/BTN2A2/CCL3/ABL1/FCGR2B/TNFAIP3/
/ILF3/ISG20/EXOSC5/TRAF3/APOBEC3D/GPATCH3/FADD/TRIM25/POLR3D/POLR3H/POLR3C/TNFA
PEL2/GZMB/TIMM44/PDCD5/TOMM40L/STPG1/TIMM50/ROMO1/LETM1/HSPD1/RHOT2/SLC25A5/TIM
D2/PSMC3/SRF/PSMC5/KITLG/OSM/LDB1/PTPRC/YTHDF2/METTL3/PSMB1/PRRC2C/PSMB6/ADAR/P

HOA/CD38/GAK/ADCY6/DPYSL3/SNAPIN/EPHB2/CIB1/RYK/FSTL4/LPAR1/SEMA3F/TRIM46/SEMA6

TNFAIP3/SFRP4/RNF2/PIAS4/TRIM21/MAP3K10/TRIM37/TWIST1/ADGRG3/SIGIRR/FAF1/XCL1/I

HS2ST1/B3GNT8

MF8/IGLL5/ACP5/ADAM9/HRAS/SYK/HCK/CCT5/UNC13D/IL18/EXOSC4/MX2/CDK4/S100A8/LYZ/A
A4A/NDN/MAP2/DOK1/BRSK1/IQGAP1/B4GALT5/EFNA5/MAPK3/EFNB1/MAP1S/PAK1/CRABP2/ANKR
MF8/IGLL5/ACP5/ADAM9/HRAS/SYK/HCK/CCT5/UNC13D/IL18/EXOSC4/MX2/CDK4/S100A8/LYZ/A
PAN17/NUP93/NXT1/FAM53B/NUP188/ZC3H12A/CSE1L/BID/CCT4/TOMM5/UBE2J2/RPL17/FERMT1

5/LAMC2/SERPINE1/CD3E/CD63/PRKX/LAMA5/EDA/THY1/ITGB5/CORO1C/ARPC2/LIMCH1/EPHB3/

UBASH3B/SOCS1/IRF1/CTLA4/PLA2G2D/C1QTNF1/ZNF703/ZC3H12A/LAG3/GTPBP4/ARHGDIB/JAG

EML2/TRIM37/ARHGAP28/RBM14/PLEKHH2/MTPN/SPTAN1/CORO2B/GMFB/CAMSAP1/ARFGEF1/CIB1

C/BMF/HGS/NPC1/GPSM1/DYNLL1/FOXK2/MARK2/PIM2/ZC3H12A/MT3/RAB24/TOMM5/ATG101/MLS
MF8/IGLL5/ACP5/ADAM9/HRAS/SYK/HCK/CCT5/UNC13D/IL18/EXOSC4/MX2/CDK4/S100A8/LYZ/A
MRPL17/CERS5/SMPD2/NPM1/UPF3B/POLR2G/NSMAF/FTSJ1/DEGS1/RBM3/EIF2S2/MRPS12/RPS2/
C/BMF/HGS/NPC1/GPSM1/DYNLL1/FOXK2/MARK2/PIM2/ZC3H12A/MT3/RAB24/TOMM5/ATG101/MLS

SEMA4A/ZP3/HLA-DPB1/UNC93B1/SIGLEC10/NFKB2/EBI3/MAD2L2/IL27RA/HLA-DQB2/CD3E/TCI

7/PSMB6/PSMA3/RELA/PSMB7/SKP1/PIK3R2/VAV3/IKBKB/PPP3CB/PSMD12/NFATC1/MAPK9/TAB1

FNB1/MAP1S/PAK1/CRABP2/NRCAM/THY1/RTN4RL2/PALLD/HSP90AB1/EPHB6/ISL2/RELN/S100B/
NFRSF4/EDA/SMARCA4/RAB7B/LRRC14/TNFRSF11A/RELN/PRMT2/RPS6KA4/SFRP5/ZC3H12A/FLOT
RID2/PCGF1/SMCHD1
L/BYSL/PARP1/BARD1/BAG3/NUP155/CALR/CTDNEP1/DNAJB6/NUP107/LILRB4/RPL23/MSX1/CCT
ST1R/C5AR1/RPS3/TGFB3/MUC20/ADRA2A/MAP3K6/IKBKG/CSPG4/TRAF7/CXCL17/PDCD10/CSK/M
EMA3E/MAPT/RARG/ABL1/VCL/RASAL1/DBNL/HSP90AA1/DNM2/LLPH/VEGFA/RAB11A/IFRD1/CDK5

2G6/PARK7/USP36/NMT1/HTRA2/ARIH2/UBL5/MUL1/BAP1

B4X/CSF1R/EZR/ANKRD1/SLC25A6/VTCN1/RAN/RAB11FIP5/ANXA13/CCT6A/MICALL2/RANGAP1/T
AC1/ATP6V1E1/GBA/LAMTOR5/CAPNS1/CDK5/LAMTOR1/CTTN/IFI16/GSK3A/OSBPL7/DRAM2/ATP6

PC5/TAF11/GTF2E1/SNAPC1/SP1/METTL16/ICE1/GTF2B/TUT1/INTS3/RPRD2/INTS5/SAGE1/NCB

-G/GATA3
NPRL3/UBQLN4/PRKAA2/VPS35/SH3GLB1/OPTN/GNAI3/BNIP3L/LZTS1/CDC37/CDK5R1/ATG13/IF
EGFA/RAB11A/IFRD1/CDK5R1/METRN/SRF/RHOA/NIN/IST1/EPHB2/YTHDF1/ADNP/RYK/PLXNB2/F

/COPS5/HSPA5/CANX/HSPA14/PPP2R5B/GFPT1/TLN1/DAXX/ANKZF1/GOSR2/TPP1/SDF2L1/BHLHA
RALB/MSX1/PTPRF/CARD18/SYMPK/NFATC4/TDG/TMC8/BAG2

2

APK15/TERF1/HNRNPA2B1/XRCC5/TP53/SH2B1/PARN/MEN1

/NUP62/IL18/EZH2/WNK2/MAPK8IP2/DLG3/CSF1R/EZR/MAGED1/FZD7/HSF1/GLIPR2/NCF1/PRMT
CARD9/PSMB9/HLA-DPA1/VSIG4/CTSK/WIPF3/RBCK1/PSMD14/WASF2/BAX/MAPKAPK2/VAV1/COLE
AV1/SEMA4A/CCL26/MAP2/SLC12A9/MSN/ARPC4/EFNA5/PAK1/CRABP2/BAIAP2L1/NRCAM/SLC12A
PP2R1A/IGFBP6/STX1A/EDA/MLLT3/SMARCA4/ARHGEF19/HDAC1/RELN/PLCB3/CSNK1E/LZTS2/P2
I/ZMYND8/SEMA4D/SYT1/DNM1L/SYT3/KATNB1/STK25/ARHGDIA/SCN1B/MAPT/CBFA2T2/STMN2/R

/MRPL21/MRPS26/MRPS11/MRPL42/MRPL22/MRPL58/MRPL38/MRPS30/MRPS27/MRPS18A/MRPL43/

/EIF6/CNOT3/POLR2E/PRKRA/SND1/SPOUT1/MIR155HG/METTL3/PPARG/MOV10/PPP1R8/ADAR/AA

HGAP28/PLEKHH2/MTPN/SPTAN1/CORO2B/GMFB/CAMSAP1/ARFGEF1/CIB1
1A/HUWE1/SKP2/PIN1

TTL2A/THADA/DUS1L/THUMPD2/PTCD1/DDX1/METTL2B/AARS2/METTL6/GRSF1/RPP38/THUMPD3/Q

P1/BHLHA15/HSP90B1/HSPA13/DAB2IP/FKBP14/STUB1/ADD1/SSR1

AP2/IQGAP1/SLC9A1/EFNA5/MAD2L2/PPP2R1A/PAK1/CRABP2/NRCAM/SMARCA4/HSP90AB1/PPP1R

XCL16/PSMD10/CDC42/SEMA4D/SYT1/SYT3/MAPT/RASAL1/SFRP2/GHRHR/BRAT1/DBNL/TBX2/ADA
A
/MRPL21/MRPS26/MRPS11/MRPL42/MRPL22/MRPL58/MRPL38/MRPS30/MRPS27/MRPS18A/MRPL43/

P8/RPS27A/TLR2/UBA52/UBE2D2/MAP3K1/LGMN/TLR7/HSP90B1/IRAK2/CAV1/DAB2IP/NFKBIL1/
9/UIMC1/HSPA5/BCL2L1/INTS7/LZIC/RNF8/DNMT3B/TIGAR/POLE3/ATR/TP53BP1/HMGA2/DCLRE

D10/PSMD11/PSMD1/IL1R2/PSMA7/PSMB3/PRKCI/XCL2/APP/PSMD4/PSMD3/RBX1/PSMD7/IKBKG/

/NUP98/HDAC4

P1R35/PPP1CA/PPME1/DUSP2/NCKAP1L/DUSP4/GBA/CDCA2/LCK/PTPN6/PPP1CC/PPP1R1B/SET/N

5/SCP2/CYP4F12/PHYH/FABP1/ACADL/ACOX2/CYP4F3/PCK2/SLC27A2/EHHADH/HAO2/HAO1/CYP4

LIN5/SCP2/PPARGC1A/PHYH/CYP4V2/FABP1/ACADL/ACOX2/SLC27A2/EHHADH/HAO2/PDK4/HAO1

3C/SEMA3E/RERG/CDHR2/GNG4/SFRP2/SIPA1/ACVR1B/CCDC85B/NPPB/CRLF3/IFRD1/CDK5R1/ND

/TPR/NUP50/STAT3
/TPR/NUP50/STAT3

LIN5/SCP2/PPARGC1A/PHYH/CYP4V2/FABP1/ACADL/ACOX2/SLC27A2/EHHADH/HAO2/PDK4/HAO1
LP1/NUDT21/NCBP1/MED18/TAF1A/GTF2H3/WDR33/TAF1C/CPSF2/MNAT1/LSM11/MTERF1

CHORDC1/PTPN6/GSK3A/MIDN/SOCS3/PPP1R1B/SMYD3/CDKN2C/TFAP4/VPS25/SPRY4/GMFG/CBL/

H/ALDH6A1/DAO/FTCD/UROC1/OTC/CDO1/AGXT/HPD/TAT

3HAV1/LARP1/SRPK2/IFITM2/DDB1/MX1/VAPA/TBC1D20/ADAR

/NUP62/IL18/EZH2/WNK2/MAPK8IP2/DLG3/CSF1R/EZR/MAGED1/FZD7/HSF1/GLIPR2/NCF1/PRMT

WNT7B/BIRC7/IL1B/PDGFA/MLKL/MAPK11/MAP3K14/TNIK/DVL2/MST1R/PSMD10/C5AR1/CCND2/R

LDLR/CPNE1/HEYL/CSNK1D/KDM1A/LYN/GPRC5B/ADRA2C/RNF157/PLXND1/NDRG4/NAP1L2/TUBB2

TFAP4/VPS25/SPRY4/GMFG/CBL/AKT1S1/RACK1/ZNF675/RGS3/IGF1R/STK38/NF2/PKIA/ABL1/S

F8/HLA-G
F8/HLA-G
PO1/DMAP1/IPO9/NXT2/KPNA6/IPO4/GEMIN2/SNRPD3/TNPO3/EFCAB7/RBM22/DDX20/KPNA4/GEM

1R8/ADAR/AAAS/AGO1/POLR2B/FXR1/EIF4G1/HNRNPA2B1/SMAD2/ELAVL1/ZMPSTE24/NUP214/SM

C2/LIG3/TP53/COPS7A/RAD23A/SLX4/POLD2

BX2/ADAM17/NOTCH1/S1PR3/AAK1/RITA1/MESP1/TSPAN14/RIPPLY2/NOTCH3/NKAP/SLC35C2/ZM

B11A/IFRD1/CDK5R1/SRF/SLC9A6/ADNP/RYK/SEMA3F/MEGF8/ULK1/TRIM46/SEMA6B/LHX2/SEMA
LN1/MLLT6/SART3/DNMT3B/UBE2N/CTBP1/PARP10/MORC2/TADA3/PAF1/UCN/PHF1/ING2/BCOR/S

PHGDH/ATP6AP2/KIF20B/TWIST1/CC2D2A/STK3/GLMN

ASE2/DROSHA/AGO2/CPSF4/RBX1/TATDN1/RAD9A/LAS1L/OGG1/RPA2/CUL4B/POLD3/ENDOD1/EXC

1A1/BTG2/BAD/BCL10/PTGER4/THBS1/CASP8

ADCY5/ATP7A/LARP1/ADCY3/PLA2G4A

1/MTPN/SPTAN1
BST2/IRF8/HLA-G

LR1/LILRB1/CD80/TICAM1/UBE2J1/WNT5A

BIRC7/GPRC5B/IL1B/TXN/MLKL/ADRA2C/MAPK11/TGFB2/CALCA/MAP3K14/TNIK/C5AR1/STK10/

1A2/GSTA1/ACSM2B/CDO1/CYP2B6/CYP2E1/CYP2C8/GLYAT/CYP2C9/CYP2A6/CYP3A4

THY1/PALLD/HSP90AB1/EPHB6/ISL2/RELN/S100B/CDH11/ZSWIM5/MARK2/GDI1/EPHB3/NUMBL/
ADAM8/PRR7/HDAC2/CSNK1D/AXIN1/BBC3/PMAIP1/LYN/ADRM1/CCDC22/IL1B/GBA/CLN6/PHB/D

/RASA3/CALCA/PTGFR/PML/CCR1/OXTR/ERO1A/C5AR1/CXCL11/FAM155B/CCR5/CMKLR1/SWAP70/

S/BGN/LUM/APOBEC3D/PNP/CSPG5/OGG1/HPSE/CSPG4/SGSH/CEMIP/TDG/AMDHD2/PSAP/HEXB/B

P2M1/VPS28/AGFG1/MVB12A/TRAPPC1/VPS18/CHMP1A/CALR/PRKCI/PLEKHJ1/SYT1/VPS25/AP3D

A2/OR6K2/OR13C4/OR2S2/OR2V2/OR7A17/OR6M1/OR5T2/OR1S2/OR7G3/OR4K17/OR4A15/OR12D3

CASP8/MYL9/HOXA5/SMAP1/TMEM64/WDR5/INHA/KITLG/EIF6/NOTCH2/HAX1/LDB1/INPP5D/CIB1

/POU2F1/POLR2B/SPIN1/POU2F2/SNAPC5/GTF3C3/NOL11/TP53/TAF11/GTF2E1/SNAPC1/SP1/GT

/TYW3/RBM15B/DIMT1/DPH3/TRMT2B/CDK5RAP1/METTL3/MRM2/ADAR/METTL2A/TRMT2A/THADA/D
2/BECN1/AP2A1/BET1L/RAB9A/SNX3/ARL1/SNX5/SNX16/VPS26A/TRIM27/MYO1D/VAMP3/KIF5B/

B1/CD80/TICAM1/UBE2J1/WNT5A
T10/PSAP/NOTCH1/UGCG/SFRP4/SLC4A7/MCOLN3/GLI1/ALOX15B/PPHLN1/RBPJ/EPHA2/CYP27B1

/STAT3

PLAA/DNAJA1

L36/QRSL1/MRPL14/LRPPRC/MRPL3/ERAL1/AURKAIP1/MRPL20/MRPL24/MRPS16/MRPL55/CHCHD1
SBPL5/EXOC2

9A/BECN1/SNAPIN/VPS33A/ARF1/ATG7/VPS4A/RAB1A/KIAA1324/VPS16/FEZ2/RAB1B/PIP4K2B/

G/MOV10/PPP1R8/ADAR/AAAS/AGO1/POLR2B/FXR1/EIF4G1/HNRNPA2B1/SMAD2/ELAVL1/ZMPSTE2

6/ATP2B4/PSEN2/CCL19/XCL1/CLIC2/HTT/CAV1/CALM3/PTPRC/CXCL10/ATP2A2/PLCG1/TMEM38

K5R1/UBR5/VPS11/ATG13/ANP32B/DMAP1/CIB1/CSNK2A2/HUWE1/TM9SF4/C2CD5/MYO1C/EFCAB7

ATF4/KDM1A/RAC1/ITGA2/IL1B/GNB1/SWI5/SERPINB6/IFI16/CASP1/MAP3K14/PARP1/NSMF/CR

ATF4/KDM1A/RAC1/ITGA2/IL1B/GNB1/SWI5/SERPINB6/IFI16/CASP1/MAP3K14/PARP1/NSMF/CR

8/EMD/PSMA1/SFRP2/PSMB5/PSMA4/PSMD9/NOTCH1/SFRP4/TMEM64/GLI1/MESP1/PSMC3/STK3/R

.7/UBE2E1/PSMB3/BARD1/UBE2A/PSMD4/PSMD3/RBX1/PSMD7/PELI1/CBL/RNF4/TRIM71/UBE2O/P
.1B/RAP2A/MLKL/MAPK11/TGFB2/CD27/MAP3K14/TNIK/DVL2/LTBR/RPS3/STK10/APP/RELL2/MAP
/ACTB/CTTN/CDH17/PARD6B/CDC42/LAMB3/PRKCI/WDR1/EPB41L3/PARD6G/WNT4/VMP1/LIMS1/A

F11A/E2F4/CDS1/PPP1CA/PITPNM1/EEF1D/PNKP/BCL3/IKBIP/BLM/NOC2L/ATF4/KDM1A/CDK5/T
SA/PKP3/EZR/IGF2BP3/HSF1/PSMB9/GIPC1/ATP6V0B/DDIT3/TPCN1/PSMD14/ATP6V0D2/TIMP2/

/ACAT1/AMACR/ACADM/PECR/PLIN5/SCP2/CYP4F12/PHYH/CRYL1/CYP39A1/FABP1/LDHD/ALDH5A

35/CHTOP/BTG2/RAB3D/DOT1L/EEF2KMT/FAM98B/GTPBP3/WTAP/MBD3/MTHFR/BMT2/MIS18A/WDR

TN2A2/CCL3/ABL1/FCGR2B/TNFAIP3/PTGER4/SRGN/MAVS/TLR2/ALOX15B/TWIST1/GLMN/IL17RA

G/MOV10/PPP1R8/ADAR/AAAS/AGO1/POLR2B/FXR1/EIF4G1/HNRNPA2B1/SMAD2/ELAVL1/ZMPSTE2
.1B/CDK5/SPOCK1/ASCL2/GSK3A/INPP5J/TACSTD2/DGUOK/SEMA4B/CALR/SEMA4D/APP/GAL/SEMA

12/PSME2/PSMD6/PSMC2/PSMA6/CUL2/FBXW11/FBXO27/PSMD5/FBXL15

CK1/CYP2C9/TAT/CYP2A6/CYP3A4

.DOC/APP/NUP107/HK1

N1/PSMD14/ATP6V0D2/TIMP2/YBX3/CHMP4B/HMOX1/BAX/MAPKAPK2/SIRT6/SOGA1/NUP210/DHX3
S/ALDH8A1/AMDHD1/AGXT2/MAT1A/PIPOX/CTH/ABAT/PAH/ALDH6A1/DAO/FTCD/UROC1/OTC/CDO1

.1/APOC3/ACSM2A/G6PC

ALT/PIGC/PRKD2/FUT6/ST3GAL5/PIGT/RACK1/ITGB8/CSNK1G2/CERS6/HACD1

/PTPN1/VEGFA/PAK2/DGKQ/ENPP2/TNFRSF14/HBEGF/KITLG/EREG/CRLF1/OSM/HAX1/EGF

1/WNT5A

PDCD4/FZD8/SERPINB3/MAP3K4/AIDA/MAPK8IP1/MAP2K7
[illegible]/SOCS5/STAT3

[illegible]P7A/ATP6V1B2/SLC9A6/SLC9A9/COX6C/ATP2A2/COX7A2/COX5A/ATP2C1/SLC25A3/SLC30A5/PAR
[illegible]ZA1/DAPK3/ITGB1BP1/RAC1/CBFB/CNTNAP1/ITGA2/ACTB/CTTN/KIFC3/TGFB2/CDH17/PTPN23/P

[illegible]3/SMAD3/TP53/IWS1/TARDBP

MTHFD1L/SSR2/ZP3/PTK7/GINS4/PRKDC/STIL/FLVCR1/HIF1A/FZD1/PYGO2/ST14/HM13/GNA12/
[illegible]9/GDI1/SH2B2/ARFGAP1/DYNLT1/CYTH2/FLOT1/ARHGAP1/SHC1/RAP1GAP2/SIPA1L2/ARHGDIB/K

[illegible]/CSPG4/RAB3D/TNFAIP3/CSK

KLHL12/PEF1/DAB2IP/HMGA2/BMP2/LOXL3/WWTR1
[illegible]1/SCARA3/TRIM32/CASP3/MYC/HUS1/HYAL2/RELA/ERCC2/TMEM161A

BAAT/AASS/ACOT12/PIPOX/ACSM5/PDK4/HMGCS2/ACSM2B/SULT2A1/ACSM2A/GLYAT
BAAT/AASS/ACOT12/PIPOX/ACSM5/PDK4/HMGCS2/ACSM2B/SULT2A1/ACSM2A/GLYAT
BAAT/AASS/ACOT12/PIPOX/ACSM5/PDK4/HMGCS2/ACSM2B/SULT2A1/ACSM2A/GLYAT

ME6/TAZ/SEC13/VCP/NME7/PRKAA2/NME3/PFKFB2/STOML2/SLC25A12/PRKAG1/NME4/TIGAR/BPG

V2/PRAME/RPS6KA1/HSF1/FOXS1/IGFBP3/HNF1B/YBX3/BDKRB1/ATAD3A/CXCR4/SEMA4A/NDN/MA
A/CREB3/RGS14/HIF1A/RNF34/PPP2R1A/SPRED1/BCL2L12/SIRPA/NOL3/TRIM59/HGS/SOCS1/F2

/CHMP2A/CHMP7/VPS37A/CHMP5

107/HK1/NME6/TAZ/SEC13/VCP/NME7/PRKAA2/NME3/PFKFB2/STOML2/SLC25A12/PRKAG1/NME4/
1/ARG2/LILRB1/PDE5A/CD80/FGL2/LILRB2/ZBTB7B/PDCD1LG2/SOCS5/CD274
BP1/MORF4L1/EP400/LDB1/SRCAP/TADA3/TRRAP/SUPT7L/MAP3K7/NAA60/IRF4/BRD7/NAA50/WB

AK1/CAPN10/TNFRSF4/SLC51B/CD58/IRF3/F2RL1/DYNLL1/PPARD/GPI/PPIA/ZC3H12A/BCAP31/
1/TNFRSF11B/LAMC1/MMP1/CREB3L1/COMP/PLOD1/FSCN1/CTSK/TIMP2/COL5A1/ITGA5/SH3PXD2

)12/PRPF19/ABL1/CSK/HDAC10/NOTCH1/TMEM98/TLR2/LGI4/PHGDH/METRN/PARD3/RHOA

./ZNF683/PTPRC/LOXL3/NFKBID/SH3RF1/IRF4/IFNG/ANXA1/CCR2/HLA-E/CLEC4A/ARG2/RPL22/
.M/POLL

:/STUB1/BAG6/BBS7/SUMO2/RPL11/PARK7/RNF144A/NUB1/UBQLN2/ARIH2/TLK2/NKD2/ANKIB1/R

'RIM28/MTHFD1L/SSR2/ZP3/PTK7/GINS4/PRKDC/STIL/FLVCR1/HIF1A/FZD1/PYGO2/ST14/HM13/

'SMD4/PSMD3/RBX1/PSMD7/RACK1/PSMD8/EMD/PSMA1/SFRP2/NFATC4/PSMB5/PSMA4/PSMD9/NOTC
AL/AXL/BTK/PELI1/BTN2A2/EMILIN1/FCGR2B/TNFAIP3/MILR1/TWSG1/PTPN2/IL20RB/HLA-F/R

ME6/TAZ/SEC13/VCP/NME7/PRKAA2/NME3/PFKFB2/STOML2/SLC25A12/PRKAG1/NME4/TIGAR/BPG

LA-DQB1/SLC50A1/CHST4/GBP5/RAB11FIP5/GLIPR2/MGAT5/HLA-DPA1/AREG/TRAPPC4/RAB6B/E
EME1/ZNF385A/HMGB3/CREB3L1/CHEK1/NABP2/MCM5/SMARCD1/HMGB2/TCF3/TRIM28/POLA2/BRM
HKBP1/HLA-DQA1/HCK/GPC1/UNC13D/TUBB/LYZ/HLA-DRA/STARD3NL/CTSA/NCF4/RNASE6/HLA-D
HD3/PHC2/RBL1/PCNA/MCRS1/CHAF1A/SMARCA4/ACTL6A/IRF1/COPS9/STAT1/HDAC1/PARPBP/DD
X2/SPC24/NCAPD2/DTL/UHRF1/SKA3/FLNA/SPC25/CDKN2A/MCM6/TUBG1/FEN1/CENPW/DSCC1/NC
AF13/CCT5/CSTB/EEF1E1/EXOSC4/CDK4/TCOF1/MAF1/TSEN54/EZR/EME1/ANKRD1/ZNF385A/NOP
HKBP1/HLA-DQA1/HCK/GPC1/UNC13D/TUBB/LYZ/HLA-DRA/STARD3NL/CTSA/NCF4/RNASE6/HLA-D
TCIRG1/CD63/STK11IP/SLC35F6/FPR1/NPC1/C3AR1/HSP90AB1/CLCN7/MFSD12/SORT1/GPR137B
HKBP1/HLA-DQA1/MTMR2/ATP6V1F/HCK/GPC1/UNC13D/TUBB/LYZ/HLA-DRA/STARD3NL/CTSA/NCF
EK1/HSF1/SAC3D1/CRMP1/ESPL1/RABEP2/MAPKAPK2/NCKAP5L/NDN/TRAF5/BRSK1/STIL/OLA1/R
F13/MCM3/NUP62/WDR34/CCT5/SPAG5/TAP1/MAPRE1/CENPU/ASPM/EZR/DCDC2/RABL6/CKAP2L/I
/ASPM/DCDC2/CDC7/CKAP2L/HSF1/TMEM201/SAC3D1/KIF18A/CRMP1/RANGAP1/YEATS2/ESPL1/F
1/ADAM15/CD151/PKP3/EZR/BSG/SCRIB/CTNND2/RHOG/RPL8/CFL1/PLAU/EPB41L2/MRC2/APBB1
NBP1/TPCN1/ATP6V0D2/CHMP4B/SNX8/VPS37C/HLA-DPB1/RAB15/CLTA/TMEM165/HLA-DQB2/TCI
L2/LAMP5/RAB31/HLA-DQA1/NRGN/CD109/TAP1/FZD6/HLA-DRA/YKT6/IFNGR2/BSG/SYNGR2/HLA
M2/FLOT2/IQGAP1/ARPC4/MYL6B/SH3PXD2B/GBP2/LCP1/TPM3/PAK1/BAIAP2L1/STX1A/FCHSD1/
VPS72/RNF34/UBE2I/DKC1/ACD/NSMCE2/PCNA/BASP1/EFTUD2/PPIH/LMNA/DPP3/NACC1/NXT1/N
A-DQB2/HLA-A/FGFR4/ANKRD27/STX1A/STX6/RAB3B/BIN1/ECE2/TGFA/COPE/TMED9/SORT1/STX
GB2/ALDH3B1/CEP131/SNAP25/RACGAP1/KCNJ11/PTAFR/COTL1/FERMT3/PRKCD/TOR4A/SERPINI
DQA1/NRGN/CD109/TAP1/FZD6/HLA-DRA/YKT6/IFNGR2/BSG/SYNGR2/HLA-DQB1/TRPM2/RHOG/SL
SPC25/CDKN2A/MCM6/FEN1/CENPW/DSCC1/NCAPH/GINS2/MCM10/SMARCD3/OIP5/CBX6/POLD1/MC
MP5/IL15RA/HLA-DRB1/B3GAT3/HRAS/RAB31/GAL3ST4/HLA-DQA1/GPC1/HLA-DRA/SCAMP3/YKT6
/IFI30/TUBB4B/GRN/CYB561/ATP6V0B/HLA-DPA1/HLA-DMB/TPCN1/CTSK/ATP6V0D2/SYNGR1/DN
NBP1/TPCN1/CTSK/ATP6V0D2/CHMP4B/SNX8/VPS37C/HLA-DPB1/RAB15/CLTA/RRAGD/TMEM165/R
3L1/NABP2/MCM5/SMARCD1/HMGB2/TCF3/TRIM28/POLA2/BRMS1/SIRT6/GINS4/PRKDC/RUVBL1/H
TMR2/MCM3/CDK4/NME2/TWF2/MAD2L1/MAF1/DEF6/EZR/RELT/GBP5/OSBPL3/HSF1/RAB34/RANGA
ECTIN1/ADAM15/CD151/PKP3/EZR/BSG/SCRIB/CTNND2/RHOG/RPL8/CFL1/PLAU/EPB41L2/MRC2/
A/SCAMP3/YKT6/IFNGR2/RNF24/BSG/HLA-DQB1/SLC50A1/CHST4/GRN/GBP5/RAB11FIP5/GLIPR2
TCIRG1/CD63/STK11IP/SLC35F6/FPR1/NPC1/C3AR1/HSP90AB1/CLCN7/MFSD12/SORT1/GPR137B
O1A/CLIP2/COL1A2/SQSTM1/KRT17/SKA3/FLNA/TTLL4/TNNT1/RASSF3/TUBG1/TPM4/RACGAP1/C
O1A/CLIP2/COL1A2/SQSTM1/KRT17/SKA3/FLNA/TTLL4/TNNT1/RASSF3/TUBG1/TPM4/RACGAP1/C
TP1/STX3/CD44/ITGB2/ALDH3B1/CEP131/SNAP25/CDK16/RACGAP1/KCNJ11/PTAFR/COTL1/FERM
O1A/CLIP2/COL1A2/SQSTM1/KRT17/SKA3/FLNA/TTLL4/TNNT1/RASSF3/TUBG1/TPM4/RACGAP1/C
4/RACGAP1/COTL1/ARHGEF2/KLC2/KRT80/REEP4/NME1/TRIM54/TPGS2/KIF11/GTSE1/HCK/CCT5
1/BRCA1/POLR2G/EFTUD2/MCRS1/TUBA1A/PPIH/SOCS1/RBM3/MRPS12/YBX1/RPS2/PABPC1/ILF2
RAC1/EHMT2/BUD31/SSRP1/ACTB/MTA2/TCP1/SWI5/DNMT3A/SIRT7/NSMF/SMARCB1/CPSF6/KMT5
1/ASPM/DCDC2/MID1IP1/TUBB4B/KIF12/TBCB/KIF3C/KIF18A/CCT6A/KIF21B/CCT3/GAS2L3/NC
2/APBB1IP/ITGA5/FLOT2/MSN/IQGAP1/PTK7/SLC9A1/MAPK3/HSPA8/FZD1/PLEC/LCP1/MAP2K2/
B1IP/ITGA5/FLOT2/MSN/IQGAP1/PTK7/SLC9A1/MAPK3/HSPA8/FZD1/PLEC/LCP1/MAP2K2/SLC4A
2/APBB1IP/ITGA5/FLOT2/MSN/IQGAP1/PTK7/SLC9A1/MAPK3/HSPA8/FZD1/PLEC/LCP1/MAP2K2/
1/CD58/C3AR1/DYNLL1/NBEAL2/OLR1/SLC2A5/PAM/RAB24/SRI/CD68/NCKAP1L/ADAM8/LAMP3/C
PDE4A/IQGAP1/SLC9A1/DGKZ/DUOXA2/LCP1/PAK1/BSPRY/THY1/CORO1C/PALLD/DUOX2/SLC39A6
/CENPU/CHEK1/HSF1/NABP2/KIF18A/RANGAP1/MCM5/CENPL/SIRT6/SGO2/PRKDC/CBX1/CENPE/P
TMEM165/HSPA8/RRAGC/NEU1/HLA-DQB2/TCIRG1/CD63/ABCB6/STK11IP/SLC35F6/FPR1/NPC1/C
AP3/MRPS7/LARP4B/MRPL53/MPV17L2/RPS7/RPL7A/RPS23/RPS10/MRPS17/MRPS21/RPS15/RPS1
/BORCS8/GSDMD/RPL27/RPL35/RPS20/RPL18A/RPS19/IKBKG/FADD/RRM1/RPLP0/RPL18/SGTB/R
SMCE2/SGO1/SPDL1/RRS1/KNTC1/ERCC6L/NCAPG2/NUP37/INCENP/CBX3/BLM/RSPH1/ITGB3BP/C

VIL1/WASF1/PIEZO1/KPTN/ABI3/FGD6/NRBP1/APP/SWAP70/STX4/ABI2/FGD1/CSPG4/NF2/BRK1
NTC1/ERCC6L/NUP37/INCENP/CBX3/ITGB3BP/DCTN2/KNSTRN/DCTN5/CENPI/DNMT3A/NUP85/PPP
5/RHEB/SNRPB2/PRPF19/SART1/PHF5A/MYEF2/DDX23/PRKRIP1/PRPF38A/HNRNPU/HTATSF1/LSM
23/RPS10/MRPS17/MRPS21/RPS15/RPS16/MRPS15/RPL5/RPS27A/MRPL18/RPS12/RPS4X/RPL6/R
ADAM8/CAPN1/C1orf35/ASAH1/KPNB1/SLC11A1/RAC1/CTSS/LAMTOR1/GNS/PSMA5/SERPINB6/PS
TSS/GNS/FTL/GALNS/DSN1/BGN/PSMD1/PADI2/TRAPPC1/LUM/CTSD/CSPG5/CCT8/HPSE/CSPG4/S
LI1/DSN1/RAE1/KIF2A/ODF2/TOPBP1/RAD21/KATNB1/IKBKG/NDE1/CKAP2/CKAP5/EMD/NSFL1C/
SN1/INPP5J/ARF4/RPS3/FGD6/SPRY4/MYO5A/ARFIP2/FGD1/NF2/WWC1/DBNL/DNM2/ADAM17/ACT
NXA11/RAP2A/PPP1CC/CIT/KLHDC8B/RALA/CDC42/KATNB1/RACK1/RALB/NAT10/TXNDC9/DNM2/K
D47/NRAS/SERPINB6/PTPN6/DYNC1LI1/ATP6V0C/TMEM63A/GSDMD/GPR84/CNN2/ALDOC/LAIR1/C
TP6V0C/PSMD1/PADI2/TRAPPC1/CCT8/S100A7/GCA/RAB3D/VCP/STX7/VAMP8/RAB5C/PSAP/MAN2
EX1D2/DCTN1/HAUS7/MAP1A/FNTB/HAUS5/FNTA/DYNC1I2/EML2/KIF20B/KIF22
TP6V0C/PSMD1/PADI2/TRAPPC1/CCT8/S100A7/GCA/RAB3D/VCP/STX7/VAMP8/RAB5C/PSAP/MAN2
53/DCLRE1B/GAR1/CHEK2/HAT1/NBN/ERCC1/THOC2/RNF8/RPA1/ATR/TP53BP1/THOC1/DCLRE1C/
BT1/TLR2/ANXA3/HLA-F/RAB11A/RAPGEF1/STXBP1/TAPBP/TAP2/STXBP4/BECN1/TLR7/RAB9A/F
ENPK/NUP43/DYNC1LI1/DSN1/ZNF207/CBX5/NUP107/MTBP/CENPN/NDE1/ZWILCH/CKAP5/SEC13/
/CTSD/PSMD7/GMFG/PRDX4/CCT8/PAFAH1B2/ARPC5/VCP/VCL/PDAP1/BIN2/DBNL/HSP90AA1
MRPL28/RPL37A/MRPL51/MRPL11/RPLP1/RPL32/RPL30/MRPL15/C12orf65/MRPL4/SURF6/RPL29
UP43/DYNC1LI1/DSN1/KMT5C/CENPB/ZNF207/NUP107/CENPN/NDE1/ZWILCH/CKAP5

07/CENPN/NDE1/ZWILCH/CKAP5
/DCLRE1C/DDB1/TFIP11/THOC7/THOC3/POLR2B/TERF1/LRIF1/PPP1CB/ORC4/XRCC5
GOSR2/COPB1/AP2A1/COPA/KLHL12/PEF1/PKD1
B27B/TPP1/HSP90B1/RAB9A/PDIA3/SND1/ATP6V1B2/RAB1A/GNA13/P4HB/CNP/PDIA4/NCSTN/CT
B27B/TPP1/HSP90B1/RAB9A/PDIA3/SND1/ATP6V1B2/RAB1A/GNA13/P4HB/CNP/PDIA4/NCSTN/CT
A/RPL37A/RPLP1/RPL32/RPL30/SURF6/RPS9/RPS14/RPS27/RPL29/EIF2D/RPL13/RPL4/RPL41/
/KATNA1/HNRNPU/EML2/KIF20B/KIF22

ASC3/DHX8/RBM8A/MAGOHB/SNRPD3/TFIP11/IK/SNRNP200/RBM22/BUD13/CCDC12/LUC7L3/LSM3

/TFIP11/SNRNP200/RBM22/HNRNPC/SYNCRIP/HNRNPA2B1/LSM3/CACTIN/BCAS2/XAB2/SYF2/HNR

F3B6/SNRPD3/SNRNP200/DDX20/GEMIN8/GEMIN6/SMN2/LUC7L3/GEMIN5/LSM3

/PDCD11/ZNF622/RRP15/WDR3

/RPL26L1/RPL22/GEMIN5

RAB31/HLA-DQA1/ABHD17C/MTMR2/GPC1/UNC13D/PRAF2/RUSC1/HLA-DRA/STARD3NL/SCAMP3/YK
C2/BRCA1/NSMCE2/POLR2G/PCNA/MED10/MCRS1/CD3EAP/ACTL6A/SOCS1/CKS1B/DDA1/TSPAN17/
SD/STX4/CLEC5A/HPSE/KCNAB2/VCL/HVCN1/VAMP8/CD33/CD177/OLFM4/TMBIM1/SLC15A4/ANXA
1/TNFRSF1B/CD59/MOSPD2/DGAT1/VAMP1/MMP25/UBR4/MCEMP1

DGKQ/DCAF7/EBNA1BP2/EIF6/SUV39H1/MAEA/MORC2/THOC1/ARFGEF1/ANAPC4/PRR14/CASK/ENC

2/GOLPH3L/SULF2/TMEM115/TMEM87A/FUT11/RAB27B/COG2/BET1L/YIPF1/B3GALT6
ITD1/ARL8B/VPS37B/VPS11/GOSR2/RAB27B/IFITM3/ATG9A/VPS33A/YIPF1/LITAF/VPS4A/SLC9
UFIP2/BTF3/NAA38
RAB11FIP1/MCOLN1

AF9/SFPQ/ASCL2/E2F2/PARP1/VDR/CDK7/HNRNPAB/KAT2A/GTF3C2/XRCC6/MED25/GTF3C1/E2F6
ABRA3/PPP1R9B/IQCE/RAB35/PIP5K1C/HIP1R/SLC9A3R1/FERMT1/EHD1/SYTL1/ATF4/RAC1/PDE

/NUP210/GNAZ/MAPK3/LRRC59/SLC52A3/UBE2I/CERS5/PAK1/STX1A/BIN1/NPC1/YBX1/LMNA/NU

1G2/AP1B1/M6PR/RASSF9/CEMIP/VAMP8/WIPI1/DBNL/DNM2/TNK2/CFTR/AAK1/RAB3A/MYO6/AP2

PG1/TMEM199/PDCD6/RASSF9/CEMIP/SEC13/VAMP8/DBNL/COPG2/CFTR/RAB3A/SREBF1/MYO6/HL
AP1S1/AP2B1/HBEGF/BTC/AP2A1/EREG

LD/HLA-B/SYT12/ICA1/SYT1/DNM1L/AP1G2/SYT3/CPE/PDCD6/RASSF9/SEC13/SGTA/RAB3A/SRE

1/SEC23B/CHMP4A/HIP1/TEPSIN/ARFGAP3/AFTPH/AP3M1/SCYL1
1/SEC23B/CHMP4A/HIP1/TEPSIN/ARFGAP3/AFTPH/AP3M1/SCYL1

C/BTBD10/SNRPB2/PAFAH1B2/UTP6/HEATR1/TIMM13/NOP10/AKNA/GAR1/NOL6/POP5/SP140/TAF

OT1/CAP1/CAPZA1/RTKN/GYPC/PHLDB1/HCLS1/GLRX3/ACTB/CTTN/MYADM/WAS/CLTB/RAB10/FGF
L3/CFTR/SREBF1/HLA-F/PACSIN1/ERGIC3/COPB2/CLTC/AP1S1/GOSR2/RAB27B/COPB1/AP2A1/A

/POLR3A/ERCC2/SUPT3H/ZNRD1/TP53/TBPL1/TAF11/GTF2E1/TAF4/TAF2/TWISTNB/ZNFX1/GTF2
AMP3/TMEM79/VPS54/RAB6A/PLD4/TEPSIN/MMP24/AFTPH/COG5/STX16

/POLR3A/ERCC2/SUPT3H/ZNRD1/TP53/TBPL1/TAF11/GTF2E1/TAF4/TAF2/TWISTNB/GTF2B/POLR

RAB24/STAB1/ACTG1/SH3KBP1/ADAM8/HLA-DQA2/FZD2/MSR1/SLC11A1/HSPH1/RAB20/HLA-DRB5
ADAM17/MYO6/PACSIN2/PACSIN1/TLN1/RHOA/PDPN/FAP/EPS8L1/EPHA2/KSR1/MPP2/CIB1/C2CD
PA2B1/AKAP8/LRIF1/MBD1/SMC1A/TP53/TEP1
/POLR3A/ERCC2/SUPT3H/ZNRD1/TP53/TBPL1/TAF11/GTF2E1/TAF4/TAF2/TWISTNB/GTF2B/POLR

9A7/OCRL/AP1B1/STX4/ARFIP2/M6PR/PIFO/CLIP3/ARAP1/DENND5A/WIPI1/DNM2/SYS1/COG1/S

CRL/STX4/ATP6V0D1/WNT4/HVCN1/VAMP8/DNM2/RPS27A/DMBT1/TLR2/ANXA3/UBA52/HLA-F/AP2

ACAP1/LZTR1/CD274/EHD3/ZFYVE27/STX12/HLA-G

P2/CLTB/AP2M1/NCALD/DVL2/GAD1/AP2S1/HLA-B/VPS18/SYT1/APP/OCRL/AP1G2/AP1B1/M6PR/
GIT1/MAPK8IP2/DLG3/ASAP1/NECTIN1/SYNGR2/DLGAP4/SCRIB/CTNND2/RPL8/DNAJC6/GIPC1/D
/CFD/OLA1/PSMD2/HSPA8/NEU1/GM2A/SERPINE1/SLPI/FUCA2/STK11IP/RNASET2/MNDA/PTGES2

1/PRMT5/NCOA6/PHF1/DPY30/ERH/MAX/DYDC2/KMT2B/KDM4A/SETD1A/PHF20/SUZ12/TAF4/MEN1

P/SFPQ/PQBP1/IFI16/SRSF12/PPP1CC/C12orf57/PRCC/SURF2/IL15/THOC6/RNF113A/CPSF6/E
C/TUBA1A/RTN4RL2/NPC1/OLR1/SLC9A3R1/FLOT1/PLLP/RANGRF/KCNQ1/DAPK3/PLSCR1/CLN3/L

C/TUBA1A/RTN4RL2/NPC1/OLR1/SLC9A3R1/FLOT1/PLLP/RANGRF/KCNQ1/DAPK3/PLSCR1/CLN3/L

RABAC1/ATP6V0D1/RAB3D/MFF/STX7/SGTA/VPS45/VDAC2/RAB3A/COPS5/SLC17A5/BCL2L1/STXB
15/UBE2N/SPATA2/IMP4/ACACA/IP6K2/COIL/TRIM27/CC2D1A/YY1AP1/MLLT1/CHCHD1/TERF1/F

F/STX7/SGTA/VPS45/VDAC2/RAB3A/COPS5/SLC17A5/BCL2L1/STXBP5/RAB27B/ATG9A/PSEN2/SN
/CORO1C/TUBA1A/RTN4RL2/NPC1/OLR1/SLC9A3R1/FLOT1/PLLP/RANGRF/KCNQ1/DAPK3/PLSCR1/
FG/WDR1/NF2/EMD/ACTR3/DBNL/DCTN1/CAPN2/CTBP2/WIPF2/EXOC3/DSTN/PLEKHH2/ACTN4/SPT
PPL3/PTPN1/GNAI3/RHOA/CAV2/CISD1/PTPRC/LITAF/CYTH3/SNX5/GNA13/ATP2A2/EPB41/ATP2

1/PALLD/HSP90AB1/DYNLL1/DYNC1H1/CAD/ARPC3/BLOC1S4/DYNLT1/CYTH2/ACTG1/SRI/TUBB4A

C1/BCL9L/TAF1D/PTOV1/GTF2E2/NR1H2/RUNX3/GTF2H1/TAF13/TBP/MMS19/TFDP1/LDB1/GTF2F
UFA7/MRPS26/MRPS11/MRPL42/MRPL22/MRPL58/MRPL38/MRPS30/MRPS27/MRPS18A/MTG2/MRPL4
UFA7/MRPS26/MRPS11/MRPL42/MRPL22/MRPL58/MRPL38/MRPS30/MRPS27/MRPS18A/MTG2/MRPL4

ANT1/CFD/OLA1/PSMD2/HSPA8/NEU1/GM2A/SERPINE1/SLPI/FUCA2/STK11IP/RNASET2/MNDA/PT
/HIF1A/LAMC2/FCHSD1/RHOF/TNFAIP2/CORO1C/GPSM1/DYNLL1/DYNC1H1/TIGD5/WIPF1/HIP1R/
/CDK5/CTTN/ADRA2C/STMN3/INPP5J/GAD1/APP/GDPD5/AP3D1/KATNB1/CBL/MYO5A/CCDC120/AT

IKBKG/UBE2L6/RNF7/CUL4B/DCAF15/PDCD6/MED12/TRAF7/KLHL42/VHL/KCTD5/FBXW5/RNF2/RA

FNDC5/AGXT
FNDC5/AGXT
B/BCAS2/JUP/GTF2B/MED1/POLD4/HP1BP3/HHEX/PLRG1/RPA3/TOP1/SP3/GTF2H3/GTF2F2/HOXA

ANT1/CFD/OLA1/PSMD2/HSPA8/NEU1/GM2A/SERPINE1/SLPI/FUCA2/STK11IP/RNASET2/MNDA/PT

RPS18A/RPS25/MRPS9/EIF2A/MRPS35/MRPS33/MRPS10
3H/POLR3C/TAF12/ACVR1/TAF10/DNA2/CCND3/PRKAA2/ACVR1B/CCNJL/ERCC1/CCNE2/MYO6/GTF

BNL/HSP90AA1/DNM2/LMTK2/ZPR1/TUBB3/KIF20B/CDK5R1/PARD3/EXOC3/APBB1/HNRNPR/ACAP3

/MRPL18/TOMM22/MTX1/NDUFB3/TIMM10/MRPL37/NDUFA13/NSUN4/MRPL27/MRPL28/ANKZF1/MRP

OR1/TMEM9/ANKRD13B/CHMP3/SLC29A3/VPS28/CD79A/MVB12A/CST7/PARM1/STARD3/VPS18/CHM
NRGN/MAPK8IP2/DLG3/ASAP1/NECTIN1/MAF1/YKT6/ZNF385A/TRPM2/CTNND2/IFT57/NCF1/RANG

1/DNM2/LMTK2/ZPR1/TUBB3/KIF20B/CDK5R1/PARD3/EXOC3/APBB1/HNRNPR/ACAP3/NIN/DPYSL3

1/RPL7/ADRA2C/HOMER1/CACNG4/LRFN4/TNIK/MAP4/ABR/NSMF/LRP8/RPS3/ARRB2/DROSHA/KLH

SCAMP2/CFTR/PDLIM4/FIG4/OPTN/PACSIN2/GGA3/HLA-F/LMTK2/RAB11A/RAB29/ABHD17A/ATG9

HHADH/DAO/HAO2/HAO1/FNDC5/AGXT
TOP/LARP4B/SYMPK/VCP/PSMA4/PRKAA2/BTBD2/HNRNPU/SARNP/RPS4X/RPL6/TRIM21/PSMC3/CA
HHADH/DAO/HAO2/HAO1/FNDC5/AGXT
DRA2C/HOMER1/CACNG4/PPP1CC/LRFN4/TNIK/ADAM23/PFN1/PPP1R1B/ABR/EIF4A3/ARF4/VPS18

/RAD23A/PSMD12/ZFAND2B/PSME2/TXNL1/PSMD6/SUPT20H/SEC11A/PSMC2/PSMA6/SPCS2

/CACNG4/LRFN4/TNIK/MAP4/ABR/NSMF/LRP8/RPS3/ARRB2/DROSHA/KLHL17/SEMA4F/SH3GL1/RP

PL49
PL49
CHCHD3/TPST1/SYT1/DMPK/POMGNT1/CHST12/MFNG/LFNG/RHOT2/MFF/APOO/LEMD2/B4GALNT2/S

ARCA4/ACTL6A/STAT1/TICRR/HDAC1/WDR13/DDX11/E2F4/LEF1/NELFE/ZC3H12A/NOC2L/NONO/H
BRCA1/BASP1/MED10/SMARCA4/RCOR2/ACTL6A/IRF3/E2F8/HDAC1/PRMT2/PIR/WDR77/SUB1/REX
IM28/CCL5/MAPKAPK2/STK17A/BRSK1/PKDCC/PDGFRL/PTK7/PRKDC/MAPK3/MAST2/MAP2K2/PAK1
L8/COMP/EPB41L2/COL5A1/NUTF2/MFGE8/FRAS1/MAP2/TPM2/MSN/ZP3/SRPX2/CLTA/ARPC4/TUF
QGAP1/SH3PXD2B/EFNA5/RGS14/NUPR1/GM2A/MAP2K2/ANKRD27/THY1/NSMAF/ARHGAP9/CKS1B/A
/ARL4C/MARCKS/FLNA/TTLL4/TNNT1/FCHO1/FXYD5/TPM4/RACGAP1/CCDC88B/KCNJ11/COTL1/AR
DK4/NME2/CSF1R/CDC7/GUCY2C/PGK1/CHEK1/RPS6KA1/LIMK2/JAK3/GRK6/TRIM28/CCL5/MAPKA
2/ABCD1/HRAS/RAB31/KIF11/PIF1/MCM3/ATP6V1F/WDR34/TAP1/TUBB/MX2/NUDT14/ENTPD2/RH
2/ABCD1/HRAS/RAB31/KIF11/PIF1/MCM3/ATP6V1F/WDR34/TAP1/TUBB/MX2/NUDT14/ENTPD2/RH
2/ABCD1/HRAS/RAB31/KIF11/PIF1/MCM3/ATP6V1F/WDR34/TAP1/TUBB/MX2/NUDT14/ENTPD2/RH
2/ABCD1/HRAS/RAB31/KIF11/PIF1/MCM3/ATP6V1F/WDR34/TAP1/TUBB/MX2/ENTPD2/RHOG/TUBB
CD/HSPB1/RELB/ITGAV/IL15RA/PLEK/ADAM9/MVP/SYK/KIF11/STK39/MAPRE1/CDC25A/TWF2/MA
/ADAM9/MVP/SYK/KIF11/STK39/MAPRE1/CDC25A/TWF2/MAPK8IP2/HDAC7/EZR/CDKN2D/RHOG/HS
6A/CARD9/RHOQ/GIPC1/EPB41L2/DDIT3/WIPF3/HMGB2/WASF2/TRIM28/CACYBP/BAX/NR0B1/CAS
LN/WFDC2/GAPDH/GSTP1/GPC3/PPP1R14D/BIRC3/IRAK1/PI3/CDKN2A/CST1/PPP1R14B/RACGAP1
NECTIN1/ADAM15/YKT6/CD151/PKP3/EZR/BSG/COL16A1/COL3A1/NOP56/SCRIB/CTNND2/COMP/R
A/SH2D3A/RRAGD/RRAGC/LCP1/MICALL1/ATP6AP1/PAK1/ANKRD27/RASGEF1A/CORO1C/PPP6R1/Y
18/ARPC4/PLEC/LCP1/MAP1S/TPM3/BAIAP2L1/CORO1C/BIN1/CORO7/PALLD/ARPC2/MYO19/GAS7
PIM2/PAK4/DAPK3/CSNK1D/CDK2/TESK1/STK17B/CDC42BPG/PKN3/ALPK3/CDK5/MLKL/MAPK11/H
/HSPA8/TCIRG1/CENPE/CHD3/ATP6AP1/ABCB6/ABCF2/XRCC2/SMARCA4/HSP90AB1/DYNLL1/DYNC
NKRD27/RASGEF1A/CORO1C/PPP6R1/DENND2D/ARHGEF19/NXT1/TBC1D1/RIN1/GDI1/XPO6/CSE1L
NKRD27/RASGEF1A/CORO1C/PPP6R1/DENND2D/ARHGEF19/NXT1/TBC1D1/RIN1/GDI1/XPO6/CSE1L

KN/RAP1GAP2/SIPA1L2/ARHGDIB/TBC1D30/HSPH1/ITGB1BP1/DNAJB2/WAS/GPSM3/TBC1D13/RAS
/FAM83D/GAS2L3/NDN/MAP2/MTCL1/BRSK1/GPAA1/RGS14/MAP1S/MAST2/CENPE/BRCA1/KLC3/CA
GAP1/EFHD2/PROM1/OLA1/RUVBL1/LRRC59/HSPA8/PLEC/MICALL1/PPP1R13L/NUDC/BAIAP2L1/C
/RAB3B/RASEF/RAB7B/RRAS/RAB35/ARF3/RAB24/GTPBP2/GTPBP4/TUBB4A/ARHGDIB/RAB20/RAC
L1B/TNFSF13B/CD300LF/CCL4/TGFB2/IL12RB1/CXCL16/IL15/CADM4/TNFSF12/PF4V1/TRAF3/G
ARHGDIB/TBC1D30/ITGB1BP1/WAS/GPSM3/TBC1D13/RASA3/ARHGAP30/AGFG1/ABR/SIPA1L3/CDC
3/ARHGAP30/AGFG1/ABR/SIPA1L3/SYDE1/RUNDC1/LLGL1/ARHGAP18/RANBP1/AGAP1/CDC42EP2/
2
APN6/KIFC2/GAS2L1/KIF14/CAMSAP2/KIFC3/KIF15/MAP1LC3B/MAP4/RAE1/KIF2A/RPS3/ZNF20
DNASE2/MCM8/XRCC6/POLQ/MUTYH/TATDN1/RAD9A/OGG1/POLD3/RAD51C/PTGES3/DNA2/DCLRE1B
S/VIL1/NEB/KPTN/CAPZB/KLHL17/MYO5A/WDR1/ARPC1A/ARPC5/TNNI3/ABL1/ZNF185/ACTR3/DB
2/CDC42EP2/MYO9B/OCRL/ABI2/ARHGDIA/ARFIP2/FMNL3/PARD6G/FGD1/OBSCN/BRK1/ARHGEF3/
2/CCND3/PRKRIP1/CCNJL/DUS2/CCNE2/PAK2/CSNK2B/DGKQ/CDC37/CDK5R1/PRKAR1B/DAXX/STK
H3GL1/ECM1/RABAC1/XRCC4/CEP250/MED12/ABL1/SIPA1/CSK/CIITA/DBNL/BCL10/MCHR1/PIAS
/PHF6/BAZ2A/NCAPD3/APBB1/DAXX/MLLT6/RNF8/SART3/ANP32A/ANP32B/HINFP/SMARCA5/DNAJ
16/MRPS15/RPL5/RPS27A/MRPL18/RPS12/RPS4X/RPL6/RPL37/RPL27A/RPL36/UBA52/RPL19/MR
A1
PDLIM4/MASTL/PARD3/AP3B1/ATP2B4/ANAPC5/CDC27/HSP90B1/SNX3/DAB2IP/GTF2F1/ENSA/AN
TPN12/DNM2/ADAM17/FUT8/INPPL1/SH3BGRL/CD2AP/RAPGEF1/SOCS7/KHDRBS3/ELMO2/DPYSL3/
SPA14/AIP/CDC37/PPIE/PPIB/TAPBP/DNAJC4/HSP90B1/HSPA13/PPIC/HSPB6/TMEM67/AAMP
DAM17/S1PR3/CD177/ADAMTS5/ACTN1/TLN1/ACTN4/EGFL6/FAP

K/DDR1/WNK2/CDK4/NME2/CSF1R/CDC7/GUCY2C/PTDSS2/PGK1/CHEK1/RPS6KA1/LIMK2/JAK3/GR
PABPC4/GNL3/HNRNPM/SLBP/HNRNPA3/SUPT5H/JRK/MRPS7/RPS7/MYEF2/RBM45/RPL5/HNRNPU/T
/CSRNP2/PPP1R15A/SH3GL1/RACK1/IGBP1/RPA2/MAPT/ANKLE2/VCP/KCNN4/CSK/BAD/CD33/BOD
5/RFC5/DDX10/DHX15/DHX9/SMARCA5/DDX55/SMARCAL1/EP400/SRCAP/DHX38/ERCC3/DDX52/DH
9/NAA15/EIF6/SND1/GTPBP6/LARP1/BAG6/SRP68/YTHDF1/SNRPD3/UNK/PRMT5/ERAL1/PYM1/WD

4B/PKIA/CALM2/CCND3/BCL10/PRKRIP1/CCNJL/DUS2/CCNE2/PAK2/CSNK2B/DGKQ/CDC37/CDK5R

GUCY2C/CHEK1/RPS6KA1/LIMK2/JAK3/GRK6/TRIM28/CCL5/MAPKAPK2/STK17A/BRSK1/PKDCC/PD
YN/WFS1/UBE2L3/TCP1/PML/MAP1LC3B/UBE2D1/ATP6V0C/LTBR/PSMD1/TRAF3/RALA/UBE2A/ARR
/GRB2/ARRB2/VPS18/CBX5/CHCHD3/CDC42SE2/KLHL17/COL8A2/SKAP2/TELO2/ACTL8/ABI2/RAC

ML/MAP1LC3B/UBE2D1/ATP6V0C/LTBR/PSMD1/TRAF3/RALA/UBE2A/ARRB2/CALR/DNM1L/RBX1/IK

B3B/RASEF/HSP90AB1/RAB7B/RRAS/RAB35/TGM2/ARF3/RAB24/GTPBP2/EHD1/GTPBP4/SPAG1/TU
B3B/RASEF/HSP90AB1/RAB7B/RRAS/RAB35/TGM2/ARF3/RAB24/GTPBP2/EHD1/GTPBP4/SPAG1/TU

NX20/WIPI1/ZFYVE19/GOLPH3L/TECPR1/SESTD1/PARD3

SP90AB1/RAB7B/RRAS/RAB35/TGM2/ARF3/RAB24/GTPBP2/EHD1/GTPBP4/SPAG1/TUBB4A/RTKN/
SP90AB1/RAB7B/RRAS/RAB35/TGM2/ARF3/RAB24/GTPBP2/EHD1/GTPBP4/SPAG1/TUBB4A/RTKN/

7/GRB7/GSDMD/SYT1/DPEP1/SNX12/BTK/ARFIP2/PLEKHN1/MAPT/OBSCN/SNX11/ARAP1/SNX20/

B1/RAB7B/RRAS/RAB35/TGM2/ARF3/RAB24/GTPBP2/EHD1/GTPBP4/SPAG1/TUBB4A/RTKN/RAB20/
TRADD/BICD1/MAP1A/OPTN/SH3BGRL/AP2A2/RBM14/VPS11/GATAD2A/AP2B1/ARRDC1/AP2A1/MMS

THBS1

A5/IGSF1/IL34/EBI3/LEFTY1/MANF/LYPD6B/SECTM1/TGFA/CCL13/OSGIN2/GPI/CLEC11A/IL18

/TBC1D7/DENND5A/SGSM3/BICD1/RGP1/TBC1D22B/OPTN/RABGGTB/RUSC2/DENND4B/RAB29/YIPF

SP90AB1/RAB7B/RRAS/RAB35/TGM2/ARF3/RAB24/GTPBP2/EHD1/GTPBP4/SPAG1/TUBB4A/RTKN/R
SP90AB1/RAB7B/RRAS/RAB35/TGM2/ARF3/RAB24/GTPBP2/EHD1/GTPBP4/SPAG1/TUBB4A/RTKN/R

1/CYP2A6/CYP3A4

ARDBP
PP1R13L/TAF6/NPM1/RBBP8/BRCA1/RBL1/PCNA/SMARCA4/RCOR2/STAT1/E2F8/HDAC1/E2F3/PRM

K7/MYCBP/PRPF6/SMARCC1/USP22/KAT2A/MED24/PRRX1/HSF2/RBPMS/ATXN7L3/GMEB1/FUS/DR1
1/RTCA/FARSB/EIF4A3/ISG20/EXOSC5/FTSJ3/HENMT1/POLR2L/DDX41/DROSHA/AGO2/PRIM2/PC

CYP2A6/CYP3A4

A2C/PHB/CCL4/HOMER1/CALCA/DVL2/CXCL16/PPP1R1B/PF4V1/RALA/ARRB2/GRK2/CXCL11/XCL2
NT4/RERE/MEIS2/ARID5A/CBFA2T2/LMCD1/MTA3/HNRNPU/PIAS4/CTBP2/HSF4/MAP3K10/MECP2/

A2/OR6K2/OR13C4/OR2S2/OR2V2/OR7A17/OR6M1/OR5T2/OR1S2/OR7G3/OR4K17/OR4A15/OR12D3

3/LEFTY1/MANF/SECTM1/TGFA/CCL13/OSGIN2/GPI/CLEC11A/JAG2/CCL2/TXLNA/CXCL9/CXCL3/

CYP2A6/CYP3A4

13A1/ERCC6L/RFC2/DDX11/BLM/ATP6V1H/KIF14/ATP6V1E1/KIFC3/PSMC4/ABCA2/ATP6V0C/ATP

'CYP8B1/CYP2A6/CYP3A4

.NF704/GATA4/NFATC1/ZBTB7B/KMT2A/HOXC6/HOXB5/GATA3/SMARCE1/SMARCC2

./UBE2E1/FBXL6/TRAF3/BARD1/UBE2A/ASB1/CDC42/RBX1/UFC1/PELI1/CBL/RNF4/UBE2L6/RFWD

.C17A2/SLC6A1/SLCO1B1/SLC38A4/SLC13A5/SLC27A5/AQP9/SLC10A1

:YP2E1/CYP2C8/CYP2C9/CYP8B1/CYP2A6/CYP3A4

.C17A2/SLC6A1/SLCO1B1/SLC38A4/SLC13A5/SLC27A5/AQP9/SLC10A1

.NF40/RBMS1/CARHSP1/TUT1/NUDT21/RBM4

:REB3L1/NPAS2/HSF1/GLIS2/FOXS1/MCM5/DDIT3/BHLHE41/HNF1B/HMGB2/YBX3/TCF3/NDN/PRKD

./RASSF7/RAN/SAC3D1/KIF18A/CRMP1/CHMP4B/ESPL1/GAS2L3/NCKAP5L/MAP2/MTCL1/BRSK1/EI
C1/SAPCD2/PBK/KIF18B/ORC1/MISP/ECT2/NUSAP1/CCNF/TTK/TGFB1/SKA1/RAD51/IQGAP3/RCC2
3IP2/FMNL2/CSF1R/NECTIN1/UNC5B/EZR/BSG/ZNF385A/DCDC2/LAMC1/TRPV2/FZD7/SCRIB/CTNN
AC7/EZR/IGF2BP3/VTCN1/GBP5/HSF1/CARD9/DDIT3/HLA-DPA1/LTB/VSIG4/TRAF2/HMGB2/TRIM6
5/HSF1/CARD9/DDIT3/HLA-DPA1/LTB/VSIG4/TRAF2/HMGB2/TRIM6/JAK3/HMOX1/CD86/MAPKAPK2
SASH3/BRCA1/MNDA/ZNF580/SPON2/CD58/IRF1/IRF3/C3AR1/F2RL1/STAT1/RAB7B/NPLOC4/CD83
PRKX/LAMA5/MLLT3/IFT52/STAT1/ARHGEF19/LZTS2/PLOD3/PSMB2/PPP1CA/LEF1/FGFR1/TGM2/F
/S100A8/LYZ/NME2/CTSA/TRPM2/QPCT/RHOG/TUBB4B/GRN/TNFAIP6/CD53/PLAU/TFRC/HLA-DMB/
TUBB/CSTB/S100A8/LYZ/NME2/CTSA/TRPM2/QPCT/RHOG/TUBB4B/GRN/TNFAIP6/CD53/PLAU/VSIG
/ATAD3B/CHIT1/PSMD14/TIMP2/SYNGR1/HMOX1/DNAJC5/HSPA6/DDOST/ALOX5/PLEKHO2/MIF/CD3
AJC5/HSPA6/DDOST/ALOX5/PLEKHO2/MIF/CD300A/CANT1/IQGAP1/CFD/FTH1/STXBP2/PSMD2/ITG
/S100A8/LYZ/NME2/CTSA/TRPM2/QPCT/RHOG/TUBB4B/GRN/TNFAIP6/CD53/PLAU/TFRC/HLA-DMB/
/UNC13D/IL18/TUBB/CSTB/S100A8/LYZ/NME2/CTSA/HLA-DQB1/SLAMF7/TRPM2/QPCT/RHOG/TUBB
GR1/DNAJC5/HSPA6/DDOST/ALOX5/PLEKHO2/MIF/CD300A/CANT1/IQGAP1/CFD/FTH1/STXBP2/PSM
GF2/TCF3/BAX/CD86/VAV1/SEMA4A/TNFSF9/PRKDC/NFKB2/CD2/IL34/FLVCR1/MAD2L2/PSMD2/HI
F2RL1/RRAS/CTLA4/TNFRSF11A/PIR/FSTL3/CD83/BATF2/PLA2G2D/TFE3/PRELID1/LEF1/IRF7/Z
IGLL5/HLA-DRB1/PRNP/HRAS/SYK/HLA-DQA1/STK39/HCK/UNC13D/PGF/IL18/PAG1/S100A8/HLA-
31/PSMD2/MAPK3/CD276/HLA-DQB2/CD3E/PAK1/SLC39A10/FPR1/MNDA/THY1/THEMIS2/IRF1/IRF
RPC3/WIPF1/PSMB2/SH2B2/ZC3H12A/PSME3/ACTG1/NCKAP1L/HLA-DQA2/MYO1G/SHC1/PIK3CD/PL
/NAP1L1/CCT5/EZH2/EXOSC4/C11orf80/DNMT1/APOBEC3B/ALYREF/EME1/CDKN2D/ANKRD1/CDC7/
SMCE2/POLR2G/PCNA/MCRS1/XRCC2/CHAF1A/ACTL6A/XRCC3/TICRR/PARPBP/NPLOC4/CSNK1E/RFC
/RBL1/MCRS1/CHAF1A/SMARCA4/ACTL6A/LMNA/HDAC1/PRMT2/DDX11/LEF1/NELFE/RPS6KA4/FBL/
/DDX49/RRP9/WDR77/POLR3K/RNPS1/ERI3/REXO4/RRP1/RRP7A/PA2G4/NELFE/SNRPE/POLR2H/FE
ATF4/RPL10L/ITGA2/RPS5/RPS21/MRPL12/RPL7/EIF3I/RPL28/MRPS34/TRMT112/EIF2S3/DENR/
SCAMP5/SCRN1/VAT1/PLEK/MVP/RAB31/SYK/HCK/UNC13D/CD109/TUBB/CSTB/TMSB4X/TAGLN2/S1
SG/TYMP/TRPM2/SCRIB/RHOG/CRMP1/PLAU/DOK2/HMGB2/CCL5/SLC12A2/VAV1/CXCR4/SEMA4A/CC
NF385A/ARID3A/CDC7/CHEK1/NPAS2/HSF1/RHNO1/NABP2/PRMT1/MCM5/DDIT3/AP5Z1/PSMD14/HM
5/MAPRE1/TUBB/MAD2L1/ASPM/EZR/MID1IP1/IFT57/TUBB4B/KIF12/CHEK1/RASSF7/RAN/TMEM2C
MAD2L1/MUC1/EME1/CDKN2D/ZNF385A/ARID3A/CDC7/SCRIB/TUBB4B/CHEK1/HSF1/NABP2/PRMT1/
LAMB1/NME1/ARNT2/HRAS/SYK/NAP1L1/HCK/PGF/DDR1/IL18/EZH2/CDK4/NME2/DNMT1/CSF1R/PT
LSM2/PQBP1/THOC5/SRSF12/PRCC/ISY1/THOC6/RNF113A/PTBP3/EIF4A3/PRPF3/SNU13/SRRT/TT
PSMA5/DNAJB2/TAF9/POMT2/PSMD13/PSMC4/GSK3A/OSBPL7/PML/ANAPC11/UBE2D1/USP14/PSMD1
/GTSE1/SPAG5/MAPRE1/TUBB/EZH2/CDC25A/CDK4/GIT1/MAD2L1/MUC1/CDKN2D/ZNF385A/ARID3A
TRIM28/RHOC/ESPL1/BAX/SIRT6/CCL26/MSN/ARPC4/SH3PXD2B/HIF1A/MAPK3/LCP1/DKC1/PAK1/
./PLEKHG2/CCL5/BAX/BRMS1/VAV1/STK17A/BEX3/CASP2/UBE2M/UBE2Z/PRKDC/SLC9A1/TNFAIP8
TGA5/SELPLG/VPS37C/MSN/IQGAP1/HAVCR1/CREB3/MAPK3/HSPA8/TRIM11/EIF3B/RPL36A/UBE2I
2/PELO/PABPC1/PUF60/WDR77/RNPS1/PSMB2/PPARD/NELFE/SNRPE/POLR2H/ZC3H12A/TXNL4A/PS
/SET/SMARCB1/SMYD3/KMT5C/PADI2/UBE2E1/RUVBL2/POLE4/SAP30/PRKD2/UBE2A/USP22/JADE2
/CSF3R/MUC1/CASP4/IFNGR2/TNFRSF11B/HLA-DQB1/MMP1/SCRIB/IFI30/CFL1/PSMB9/HLA-DPA1
/FUNDC1/HIF1A/HSPA8/FZD1/LCP1/MMP10/MAP1S/MRPL17/SHARPIN/CAPN10/FGFR4/MMP2/SMARC
/UTP14A/RPL7/EIF3I/NPM3/TAF9/TRMT112/EIF2S3/SRSF12/DENR/NSUN5/MPHOSPH6/EDC3/ISY1
D1/TRPM2/RHOG/FMNL1/PLEK2/CFL1/MICALL2/RHOQ/S100A10/EPB41L2/DTNBP1/GPD1L/FSCN1/W
RPC2/DYNLL1/DYNC1H1/PPP1R9B/CSNK1E/E2F4/TMEM237/ABLIM2/SLC9A3R1/SNX10/CEP89/CCNO
/PLEK2/CFL1/MICALL2/RHOQ/S100A10/EPB41L2/DTNBP1/FSCN1/WIPF3/LIMK2/CARMIL1/WASF2/
A/TNFSF9/PRKDC/CD2/IL34/FLVCR1/PSMD2/HIF1A/JMJD6/CD3E/TCIRG1/ATP6AP1/SASH3/ISG1
IK3CD/HCLS1/JAG1/MITF/LYN/CBFB/CDKN1C/PIAS3/PTPN6/THOC5/IFI16/PML/CCR1/LYAR/OSTM
./IL18/PAG1/ADAM15/MUC1/COL16A1/C2CD4A/FZD7/TNFAIP8L2/VTCN1/PLAU/S100A10/TFRC/HL
/CLTA/UBE2Z/MAD2L2/PSMD2/NUPR1/HSPA8/OAZ1/RHBDF1/TCIRG1/RNF34/UBE2I/RNF19B/SHARP
PRE1/TMSB4X/CSF1R/HDAC7/ADAM15/SMIM22/CD151/COL3A1/GRN/TNFAIP6/GLIPR2/MGAT5/PLAU
C2/TWF2/MAPK8IP2/CSF1R/ASAP1/NECTIN1/UNC5B/EZR/BSG/ANKRD1/DCDC2/TRPV2/SCRIB/CTNN

/WASF2/P3H1/RABEP2/CXCR4/SEMA4A/MAP2/BRSK1/IQGAP1/PTK7/EFNA5/SF3A2/FZD1/PAK1/CRA
RIM59/SOCS1/COPS9/RPS2/HSP90AB1/TSPAN17/NUP93/NUP205/NUP37/PSMB2/NDC1/NUP188/SV
/SLC7A11/SCAMP5/B3GAT3/PRNP/ADAM9/HRAS/SYK/ABHD17C/NUP62/GTSE1/GPC1/CCT5/TMSB4X/
/NUTF2/FAM83D/TCTN2/VPS37C/MSN/GPAA1/SEC61A1/CARD10/STIL/GBP2/HSPA8/RPL36A/MICAL
X12/MID1IP1/SNRPD2/GBP5/HSF1/MICALL2/MCM5/HLA-DMB/TRAF2/HNF1B/CENPL/HMGB2/TRIM6
D2/HIF1A/NUPR1/LSM4/HSPA8/NT5C/RPL36A/ACOT7/DKC1/PPP2R1A/NPM1/UPF3B/RNASET2/POL
61A/UTP18/RPS21/UTP14A/RPL7/NPM3/POP1/TAF9/CPSF1/TRMT112/NSUN5/SIRT7/TRMT6/MPHO
/TIMP2/SYNGR1/CCL5/DNAJC5/HSPA6/DDOST/ALOX5/PLEKHO2/MIF/CD300A/CANT1/IQGAP1/CFD
/NBL1/HRAS/PLCB1/STK39/GTSE1/PGF/MAPRE1/TMSB4X/CSF1R/HDAC7/ADAM15/SMIM22/CD151/C
/BDKRB1/CCL5/CXCR4/SEMA4A/CCL26/PHPT1/ITGA5/ZP3/IQGAP1/SRPX2/CREB3/HIF1A/RAB25/M
/EBI3/JMJD6/CD276/LCP1/EFNB1/JAML/CD3E/TCIRG1/PAK1/SASH3/TNFRSF4/THY1/SIRPA/SOCS
/TIMP2/SYNGR1/CCL5/DNAJC5/HSPA6/DDOST/ALOX5/PLEKHO2/MIF/CD300A/CANT1/IQGAP1/CFD
G/TYMP/TRPM2/SCRIB/RHOG/CRMP1/PLAU/DOK2/HMGB2/CCL5/SLC12A2/VAV1/CXCR4/SEMA4A/CC
/RPSA/DAPK3/ATF4/RPL10L/ITGA2/RPS5/RPS21/MRPL12/RPL7/EIF3I/RPL28/MRPS34/TRMT112
/VAV1/STK17A/BEX3/CASP2/UBE2M/UBE2Z/PRKDC/SLC9A1/TNFAIP8/NUPR1/PPP2R1A/PEA15/CA
/MUC1/UNC5B/CDKN2D/ZNF385A/PRAME/VTCN1/CREB3L1/COMP/GRN/RPS6KA1/HSF1/CFL1/TNFRS
/VAV1/STK17A/BEX3/CASP2/UBE2M/UBE2Z/PRKDC/SLC9A1/TNFAIP8/NUPR1/PPP2R1A/PEA15/CA
/MUC1/UNC5B/CDKN2D/ZNF385A/PRAME/VTCN1/CREB3L1/COMP/GRN/RPS6KA1/HSF1/CFL1/TNFRS
ML/ANAPC11/UBE2D1/USP14/PSMD10/PSMD11/PSMD1/PSMA7/UBE2E1/FBXL6/DNAJC10/PSMB3/UE
/MMP1/RHOG/PLEK2/GBP5/HSF1/RHOQ/S100A10/FSCN1/HNF1B/CARMIL1/CHMP4B/WASF2/RHOC/P
HY1/FCHSD1/BMF/HGS/BIN1/F2RL1/ARPC2/DYNLL1/DYNC1H1/ARPC3/LIMCH1/SUB1/EPHB3/CORO
R4/NUP210/ITGA5/SELPLG/VPS37C/MSN/IQGAP1/HAVCR1/CREB3/MAPK3/HSPA8/TRIM11/EIF3B/
/CCL5/BAX/CD86/CXCR4/NUP210/ITGA5/SELPLG/VPS37C/MSN/IQGAP1/HAVCR1/CREB3/MAPK3/HS
ACGAP1/PTAFR/NCAPH/FANCD2/NDRG3/PLCB1/NUP62/CCT5/PGF/DDR1/MAPK8IP2/PTHLH/ASPM/E
SE1/MAPRE1/TUBB/EZH2/CDC25A/CDK4/MAD2L1/MUC1/CDKN2D/ZNF385A/ARID3A/CDC7/TUBB4B/
K/MVP/RAB31/SYK/HCK/UNC13D/CD109/TUBB/CSTB/TMSB4X/TAGLN2/S100A8/LYZ/GIT1/NME2/F
EC3B/ADAM15/MUC1/CASP4/IFNGR2/RNASE6/HLA-DQB1/SLAMF7/SCRIB/IFI30/HMGB3/SLC26A6/
D1/PSMB2/IRF7/PSME3/TNIP2/LAG3/FLOT1/SMPDL3B/ADAM8/NONO/ULBP2/NLRC5/PLSCR1/CD2C
/UNC13D/IL18/EZH2/NME2/TWF2/ASPM/METRNL/ANKRD1/ZNF385A/TRPV2/GRN/RPS6KA1/CD53/GL
10/PCBP4/CASP2/BRSK1/GMNN/MIIP/WDR76/PRKDC/RRAGD/MAD2L2/CNOT11/PSMD2/NUPR1/RRAG
GED1/NCF1/DTNBP1/TRAF2/CCL5/MAPKAPK2/CXCR4/MIF/CD300A/IQGAP1/CARD10/EFNA5/RGS14
-DMB/TNFRSF18/VSIG4/JAK3/TCF3/CCL5/BAX/CD86/APBB1IP/VAV1/DDOST/SEMA4A/TNFSF9/MI
PAG5/MX2/C11orf80/MAD2L1/ASPM/MTFP1/EME1/CHEK1/RAN/HSF1/KIF18A/PSMC3IP/CHMP4B/E
/CD86/VAV1/SEMA4A/TNFSF9/PRKDC/NFKB2/CD2/IL34/FLVCR1/PSMD2/HIF1A/JMJD6/MAPK3/CD3
N/BRSK1/PTK7/STIL/PSMD2/HIF1A/ITGAX/FZD1/ST14/PAK1/PRKX/LAMA5/MLLT3/IFT52/STAT1
/CD86/VAV1/TNFSF9/MIF/CD300A/FLOT2/ZP3/HLA-DPB1/CD2/STXBP2/EBI3/MAD2L2/CD276/EFN
DKRB1/CCL5/SLC12A2/VAV1/CXCR4/CCL26/ITGA5/MIF/CD300A/SELPLG/MSN/ZP3/CD2/CREB3/M
/MAD2L1/DNMT1/ASAP1/MID1IP1/CHEK1/HSF1/CRMP1/LINGO1/CARMIL1/CHMP4B/WASF2/ESPL1/
/ADORA1/ADAM9/NBL1/HRAS/PLCB1/STK39/GTSE1/PGF/MAPRE1/TMSB4X/CSF1R/HDAC7/ADAM15/S
/CCL5/CXCR4/SEMA4A/CCL26/PHPT1/MAP2/ITGA5/ZP3/IQGAP1/SRPX2/CREB3/HIF1A/RAB25/MA
/PIF1/STK39/NUP62/IL18/EZH2/CDC25A/CDK4/MAD2L1/DLG3/CSF1R/CDKN2D/MAGED1/NCF1/DTN
/CARD9/RANGAP1/S100A10/TRAF2/PSMD14/HMGB2/RHOC/RABEP2/CCL5/BAX/VAV1/CCL26/CD300A
/CARMIL1/CHMP4B/WASF2/RHOC/PLEKHG2/CCL26/PHPT1/MAP2/ARPC4/SH3PXD2B/SLC9A1/EFNA5
/DTNBP1/AREG/CHMP4B/HMOX1/ESPL1/DNAJC5/TRAPPC4/FAM83D/MAP2/CD300A/TCTN2/BRSK1/R
2/HIF1A/SYT8/MAP1S/CENPE/ATP6AP1/NPM1/CHMP4C/STX1A/PPP6R1/DYNC1H1/SPDL1/MYO19/T
3A1/TYMP/ANKRD1/ESRP1/TRPV2/GRN/CRMP1/CFL1/BHLHE41/LINGO1/HMGB2/TIMP2/TCF3/P3H1
/FZD1/PAK1/CRABP2/ANKRD27/NRCAM/XRCC2/BIN1/HDAC1/RELN/CSNK1E/S100B/MARK2/GDI1/EF
/NME2/TWF2/ASAP1/PTHLH/ASPM/COL3A1/ANKRD1/ESRP1/TRPV2/GRN/CRMP1/CFL1/S100A10/BHL
/GTSE1/GPC1/CCT5/UNC13D/SPAG5/MX2/GIT1/EZR/SCRIB/RHOG/RAN/GLIS2/RAB11FIP5/ANXA13
/NUP62/HCK/NME2/MAPK8IP2/MAD2L1/DNMT1/CSF1R/FOXQ1/MUC1/UNC5B/CDKN2D/ZNF385A/PRA
MP4B/SYNGR1/BAX/COL5A1/TRAPPC4/CXCR4/MFGE8/CD300A/FLOT2/CLTA/ARPC4/SLC9A1/CD2/H

IARPIN/ISG15/BRCA1/NSMCE2/STX1A/PCNA/MED10/MCRS1/TRIM59/HGS/ACTL6A/SOCS1/COPS9/F
'MSN/MRTO4/SH3PXD2B/STIL/SSNA1/NUPR1/LSM4/LCP1/CENPE/PPP2R1A/NPM1/CHMP4C/LAMA5/E
)HHC7/SH3PXD2B/SSNA1/RPL36A/MICALL1/AP4M1/PAK1/PDZD11/LAMA5/STX1A/THY1/BMF/RAB3E
)/MIF/PRDX1/WDR76/PRKDC/CREB3/MAD2L2/HIF1A/MAPK3/NUPR1/MANF/HSPA8/FZD1/MAP2K2/PA
;/MID1IP1/COMP/PLOD1/KIF18A/CFL1/MICALL2/RHOQ/S100A10/FSCN1/CARMIL1/WASF2/RHOC/F
.18/PAG1/S100A8/DLG3/NECTIN1/PKP3/EZR/BSG/SCRIB/CTNND2/TNFAIP8L2/VTCN1/COMP/TFRC
.1/DYNC1H1/PPP1R9B/CSNK1E/E2F4/TMEM237/ABLIM2/SLC9A3R1/SNX10/CEP89/CCNO/UNC119B/
!/P3H1/RABEP2/CXCR4/SEMA4A/MAP2/BRSK1/IQGAP1/PTK7/EFNA5/SF3A2/FZD1/PAK1/CRABP2/A
'4/CASP2/BRSK1/MIIP/PRKDC/SSNA1/MAD2L2/CNOT11/PSMD2/CENPE/PPP2R1A/NPM1/CHMP4C/BF
!1/MISP/ECT2/NUSAP1/CCNF/TTK/TGFB1/RAD51/IQGAP3/RCC2/AURKA/GPNMB/UNC119/PSRC1/HM
'I2B/HCLS1/FANCA/JAG1/MITF/LYN/CARD11/CBFB/PSMB4/PIAS3/IL2RA/PSMB8/NFAM1/PSMB10/
'CCT3/SAE1/TRIM28/NUTF2/MAP2/MTCL1/MSN/PKDCC/IQGAP1/CARD10/OAZ1/LCP1/DKC1/PAK1/M
'HRAS/PLCB1/STK39/GTSE1/PGF/MAPRE1/TMSB4X/CSF1R/HDAC7/ADAM15/SMIM22/CD151/COL3A1
'CXCR4/SEMA4A/CCL26/PHPT1/ITGA5/ZP3/IQGAP1/SRPX2/CREB3/HIF1A/RAB25/MAPK3/ITGAX/D
./DDX41/SF3A3/CPSF4/POLR2K/DNAJC8/FUS/CWC27/SNRPG/RBM17/HNRNPM/KHDRBS1/HNRNPA3/F
./DDX41/SF3A3/CPSF4/POLR2K/DNAJC8/FUS/CWC27/SNRPG/RBM17/HNRNPM/KHDRBS1/HNRNPA3/F
./DDX41/SF3A3/CPSF4/POLR2K/DNAJC8/FUS/CWC27/SNRPG/RBM17/HNRNPM/KHDRBS1/HNRNPA3/F
IM8/ADSL/PMAIP1/LDHA/ITGA2/PSMB4/PSMB8/PSMB10/GNB1/PSMA5/LOXL2/TGFB2/DNMT3A/PSMD
'CHIT1/PSMD14/TIMP2/SYNGR1/HMOX1/DNAJC5/HSPA6/DDOST/ALOX5/PLEKHO2/MIF/CD300A/CAN
'FLOT2/ZP3/HLA-DPB1/CD2/STXBP2/EBI3/MAD2L2/CD276/EFNB1/IL27RA/CD3E/PAK1/SASH3/TN
INC93B1/PSMD2/MAPK3/CD276/HLA-DQB2/CD3E/PAK1/SLC39A10/FPR1/MNDA/THY1/THEMIS2/IRF
'PQBP1/THOC5/SRSF12/PRCC/ISY1/THOC6/RNF113A/PTBP3/CPSF6/EIF4A3/CDK7/PRPF3/SNU13/
'PP1CC/NUP43/PML/AGFG1/THOC6/RAE1/CPSF6/EIF4A3/XPO5/BARD1/BAG3/NUP155/CALR/CPSF4
ISH3/THY1/SIRPA/SOCS1/IRF1/CTLA4/CD83/PLA2G2D/PDCD1/ZC3H12A/LAG3/SIRPG/TSTA3/NCK
;/RAB15/SH2D3A/RAB25/RIT1/RRAGC/RFXANK/DOK3/GNA12/ADGRG1/RASGEF1A/RAP2B/ARHGAP9/
!H/NUP188/ZC3H12A/PSME3/NCBP2/BCL3/EIF3D/CNOT9/DAPK3/ATF4/SLC11A1/ITGA2/PSMB4/ME
;1/SPDL1/XRCC3/TICRR/E2F8/PRMT2/E2F4/PSMB2/CBX3/PSME3/BLM/FBXO5/CNOT9/EVI2B/CDKN
.DLR/HDAC2/RAP1GAP2/YWHAH/CAMSAP2/KDM1A/LYN/FKBP4/MYLIP/RAP2A/CDK5/CTTN/SPOCK1/F
;AX/LAMC2/LCP1/MMP10/SERPINE1/FGFR4/LAMA5/MMP2/ITGB5/FMOD/PLOD2/PLOD3/BCL3/FERMT
'SEMA4A/CCL26/ITGA5/ZP3/IQGAP1/SRPX2/CREB3/HIF1A/RAB25/MAPK3/ITGAX/LAMC2/P2RY6/S
./CAPZB/SMARCC1/CHMP1A/SWAP70/SH3GL1/KATNB1/RPL23/GADD45GIP1/WDR1/MRPL10/IGF1R/M
!CT4/PNKP/OTUB1/FBXO5/NOC2L/KDM1A/CCT7/AKAP8L/IL1B/TCP1/SFPQ/ATAD2/PML/DYNC1LI1/
)RO1B/HIP1R/BID/TPPP3/NCKAP1L/TUBB4A/CAPZA1/RANGRF/CXCL13/MKKS/KIF14/FHOD3/CAMSA
'AD3B/CHIT1/PSMD14/TIMP2/SYNGR1/HMOX1/DNAJC5/HSPA6/DDOST/ALOX5/PLEKHO2/MIF/CD30C
.JC5/HSPA6/DDOST/ALOX5/PLEKHO2/MIF/CD300A/CANT1/IQGAP1/CFD/FTH1/STXBP2/PSMD2/ITC
'SE1/MAPRE1/TUBB/EZH2/CDC25A/CDK4/MAD2L1/MUC1/CDKN2D/ZNF385A/ARID3A/CDC7/TUBB4B/
'90AB1/DYNC1H1/XRCC3/TGFA/E2F8/DDX11/E2F4/FGFR1/GPSM2/FBXO5/DBF4/CNOT9/EVI2B/CDK
IUP85/BANF1/PPP1CC/NUP43/PML/AGFG1/THOC6/RAE1/CPSF6/EIF4A3/XPO5/BARD1/BAG3/NUP15
;KA3/SPC25/RACGAP1/CENPW/ARHGEF2/NCAPH/REEP4/OIP5/KIF11/NUP62/RCC1/SPAG5/PGF/MAF
./PAK1/PEA15/SPRED1/FPR1/THY1/HGS/CKS1B/HSP90AB1/NPFFR2/TGFA/TNFRSF11A/FGFR1/CCN
.A/NUPR1/SERPINE1/CD3E/RNF34/PPP2R1A/PERP/PEA15/SLC35F6/BRCA1/BCL2L12/BMF/NOL3/L
!APZA1/RANGRF/MKKS/FHOD3/CAMSAP2/HCLS1/TESK1/ITGB1BP1/ARPC5L/FKBP4/RAC1/CD47/CTT
IB3/BARD1/AGO2/CPSF4/PSMD4/PSMD3/PABPC4/PSMD7/VIM/TRIM71/RBM17/PLEKHN1/HNRNPM/KH
'BID/CCT4/BCAP31/UBE2J2/GPSM2/NPEPPS/RANGRF/YWHAH/HCLS1/BBC3/PMAIP1/CCT7/ITGB1BF
.DM/VIL1/AP2M1/RAB10/TNIK/STAC3/ACSL3/PRKCI/STX4/RACK1/MYO5A/EPB41L3/PACS2/CLIP3
;PAG5/C11orf80/MAD2L1/ASPM/EME1/CHEK1/RAN/HSF1/KIF18A/PSMC3IP/CHMP4B/ESPL1/LCMT1
'IPF1/PSMB2/SH2B2/ZC3H12A/PSME3/ACTG1/NCKAP1L/HLA-DQA2/MYO1G/PIK3CD/PLSCR1/CD209
!PL7/RPL28/PSMB10/PSMA5/POP1/LSM2/PSMD13/PSMC4/RPL39/EDC3/RPL35A/SET/PSMD10/PSMD
.35A/SET/PSMD10/PSMD11/EIF4A3/PSMD1/PSMA7/EXOSC5/YWHAB/RPS3/SAMD4B/PSMB3/RPL27/F
!O1C/BIN1/CORO7/F2RL1/ARPC2/GAS7/PPP1R9B/ARPC3/WIPF1/LIMCH1/PDLIM3/CORO1B/HIP1R/
;1/FBXO5/NOC2L/TUBB4A/CAPZA1/MKKS/FHOD3/CAMSAP2/TBC1D30/KDM1A/FKBP4/MYADM/WAS/VI

H3B/SIRPA/SOCS1/IRF1/CTLA4/FSTL3/CD83/PLA2G2D/C1QTNF1/EPHB3/ZNF703/LEF1/PDCD1/
/ITGA2/MIEN1/RNF157/PLXND1/VIL1/NDRG4/TUBB2B/DVL2/PFN1/LRP8/CROCC/RALA/CDC42/K
1/E2F4/PPP1CA/CORO1B/HIP1R/SLC9A3R1/MT3/NCKAP1L/CAPZA1/RAP1GAP2/MKKS/FHOD3/SNF
36/EIF4A3/ISG20/EXOSC5/SNU13/SRRT/RPL27/FTSJ3/DROSHA/AGO2/CPSF4/RPL35/KTI12/DD
M8/ADSL/ATF4/PMAIP1/LDHA/ITGA2/PSMB4/PSMB8/PSMB10/GNB1/PSMA5/LOXL2/TGFB2/DNMT3
3H12A/DYNLT1/LAMP3/TRIM31/HDAC2/ARHGDIB/SNF8/PLSCR1/NELFB/IL1B/IL2RA/APOBEC3G/
NIP2/SIRPG/NCKAP1L/ADAM8/CCL2/HSPH1/CD209/LYN/CARD11/RAC1/CBFB/IL1B/IL2RA/CD47/
CCT4/PNKP/OTUB1/BLM/FBXO5/DBF4/GTPBP4/AUNIP/CDK2/KDM1A/CCT7/EHMT2/RAC1/TCP1/WAS
PAK1/SASH3/TNFRSF4/SLC39A10/MNDA/THY1/SIRPA/SOCS1/IRF1/CTLA4/CD83/PLA2G2D/PRELI
E2/PCNA/UBASH3B/CCT2/HSP90AB1/XRCC3/TNFRSF11A/RFC2/CCT4/SNX10/PNKP/BLM/ACTG1/AD
3/PAM/MT3/NPEPPS/ADAM8/ADSL/HDAC2/ATF4/PMAIP1/LDHA/ITGA2/PSMB4/PSMB8/PSMB10/GN
3/ACSL3/RPL27/SSR3/PRKCI/RPL35/RPS20/RPL18A/GZMB/PDCD5/RPS19/RPLP0/RPL18/ICMT/
/MIIP/PRKDC/SSNA1/MAD2L2/CNOT11/PSMD2/CENPE/PPP2R1A/CHMP4C/BRCA1/RBL1/NSMCE2/P
YOU1/PRELID1/PPP1CA/FGFR1/HIP1R/PSME3/SLC9A3R1/BID/BCAP31/NOC2L/PRR7/NONO/DAPK
KAP1L/ADAM8/CCL2/HSPH1/CD209/LYN/CARD11/RAC1/CBFB/IL1B/IL2RA/CD47/TNFSF13B/FGR/
P4/CDK2/DTD1/NASP/LIG1/PRIM1/EHMT2/RAC1/SSRP1/WDHD1/SET/DONSON/TIPIN/POLE4/RBB
1/NAA10/SGO2/MAD2L2/RGS14/CENPE/UBE2I/PPP2R1A/CHMP4C/BRCA1/NSMCE2/SGO1/MSTO1/D
R/KNTC1/E2F8/DDIAS/PRMT2/E2F4/PSMB2/PSME3/SLC9A3R1/BLM/FBXO5/CNOT9/CDKN2B/CCL2/
/SIRPG/NCKAP1L/ADAM8/CCL2/FANCA/HSPH1/CD209/LYN/CARD11/RAC1/CBFB/IL1B/IL2RA/CD
/RAC1/CTTN/MYADM/WAS/VIL1/CHMP3/PFN1/WASF1/INPP5J/TUBG2/RPS3/CAPZB/GRB2/ZNF207/
D209/LYN/CARD11/IL1B/IL2RA/TNFSF13B/PSMB10/PTPN6/IL12RB1/IMPDH2/CD79A/LST1/IL1
P0/NOC4L/RRP7BP/TSR3/UTP11/LAS1L/MRPL10/CUL4B/UTP4/MYBBP1A/RPL38/RPL23A/EXOSC3/
L9/CXCL3/CXCL13/LYN/CCL28/RAC1/PLA2G7/IL1B/BCAR1/CXCR3/CCL4/TGFB2/GPSM3/NUP85/
CTLA4/PLA2G2D/LEF1/NCKAP1L/SLC11A1/CD209/LYN/CARD11/IL1B/IL2RA/TNFSF13B/PSMB10/
PG/NCKAP1L/ADAM8/CCL2/HSPH1/CD209/LYN/CCL28/CARD11/RAC1/CBFB/IL1B/IL2RA/CD47/T
/ERO1A/GPX1/PYCR2/RPS3/DPEP1/CBX8/PCGF2/MAPK7/PPP5C/AXL/BTK/STK25/RACK1/ROMO1/
/SFPQ/PARP1/MSH2/RUVBL2/MCM8/SWAP70/XRCC6/POLQ/FUS/RAD21/RFWD3/NABP1/XRCC4/RPA
3D1/KATNB1/IGBP1/COPG1/NDE1/MAPT/BICDL2/TTC26/HSPB11/RHOT2/ACTR3/IFT172/COPG2/
P107/CETN2/SLC35C1/SLBP/KHDRBS1/HNRNPA3/CKAP5/CHTOP/SEC13/SLC25A5/SYMPK/CPSF3/
OC4L/RRP7BP/GTF3C1/TSR3/UTP11/NIFK/LAS1L/UTP4/MAPT/EXOSC3/NAT10/UTP6/HEATR1/SA
BD3/TSN/MORF4L2/MECP2/TARBP2/BAZ2A/POLR2I/POLR2J/PPHLN1/POLR2D/DHX9/SMARCA5/CN
4/NUP107/AP3D1/RALB/SLBP/TTC26/HSPB11/MZT1/CHTOP/SEC13/SYMPK/STX7/CPSF3/IFT172/
83/EXOSC3/MFNG/HSPD1/LFNG/ABL1/FCGR2B/TNFAIP3/BAD/VCAM1/PTPN2/ADAM17/CASP8/NBN/
/CSNK1D/CDK2/KIF14/DCTN2/AKAP8L/PSMB4/PSMB8/PSMB10/PSMA5/PSMD13/PSMC4/HAUS8/CIT
BFB/IL1B/IL2RA/CD47/TNFSF13B/LCK/PTPN6/CD27/IL12RB1/IL15/MSH2/RPS3/GRB2/CD6/CD
LCK/IFI16/PML/E2F2/PARP1/ERO1A/CRIP1/GPX1/MSH2/RPS3/DNAJC10/DNM1L/MAPK7/PPP1R1
SR1/SLC11A1/RAB20/LYN/RAC1/ITGA2/ANXA11/IL1B/ACTB/CD47/FGR/CD300LF/WAS/LYAR/IL1
DK7/MSH2/ZBTB17/TFAP4/BARD1/CALR/MED25/PPP1R15A/MTBP/RPL23/GADD45GIP1/MYBBP1A/
LC11A1/CD209/DCTN2/HLA-DRB5/DCTN5/PSMB4/CTSS/PSMB8/KIF15/PSMB10/PSMA5/WAS/PSMD1
MCTS1/PRPF19/MRPS7/PTGES3/TRAF7/MPV17L2/SART1/SRPK1/RPS23/RPS10/DDX23/EIF3K/HS
2/MED25/RBX1/GMFG/ABI2/CLEC7A/RACK1/RPA2/CUL4B/DRG1/MAPT/ARPC1A/BRK1/ARPC5/VCP/
KN2B/CCL2/CDK2/LSM10/KIF14/NASP/PRIM1/PTPN6/PML/CDKN2C/CDK7/CCND2/POLE4/POLA1/
LYN/CARD11/IL1B/IL2RA/TNFSF13B/PSMB10/PTPN6/IL12RB1/IMPDH2/CD79A/LST1/IL15/RPS
OTCH1/HOXA5/THRA/VEGFA/SMAP1/IL20RB/SLC39A3/SRF/INHA/BPGM/KITLG/DOCK10/MAEA/LD
PTPN6/PSMD13/PSMC4/CD79A/PSMD10/PSMD11/PSMD1/PSMA7/RPS3/PRKD2/PSMB3/PSMD4/PSMD
FNG/HSPA2/RAD51D/EME2/ACTR3/RAD1/SPIRE1/NBN/SYCE1L/ERCC1/CCNE2/MASTL
/SSRP1/MTA2/CDK5/MAPK11/TAF9/IFI16/PML/E2F2/PSMD10/MSH2/CHD4/TFAP4/RBBP4/BARD1/
DBF4/CNOT9/CDK2/LSM10/KIF14/RAB11FIP4/IL1B/SFPQ/POC1A/PML/DYNC1LI1/CIT/PLK4/AN
2/CENPE/PPP2R1A/CHMP4C/NSMCE2/SGO1/MSTO1/SPDL1/XRCC3/TGFA/RRS1/KNTC1/PPP1R9B/C
RB2/CDC42/PSMD4/HOXB7/PSMD3/PSMD7/MSX1/SEMA3C/TFAP2A/WNT4/MED12/PSMD8/ACVR1/AB
2/LSM10/KIF14/NASP/PRIM1/PTPN6/PML/CDKN2C/CDK7/CCND2/POLE4/POLA1/MCM8/PRIM2/MT

.4/DCTN2/PSMB4/PSMB8/PSMB10/PSMA5/PSMD13/PSMC4/HAUS8/CIT/PLK4/PSMD10/PSMD11/NES/
2/TMEM64/NKAP/INHA/RBPJ/EPHA2/KITLG/CCL19/NOTCH2
'70/CDC42EP2/GMFG/ABI2/WDR1/ARPC1A/BRK1/ARPC5/ACTR3/DBNL/SPIRE1/ADD3/LIMA1/ARHGA
'S7/RPL7A/RPS23/RPS10/PTBP1/RPS15/RPS16/USF1/RPL5/RPS27A/RPS12/RPS4X/RPL6/RPL37/
)47/TNFSF13B/ADAM19/LCK/PTPN6/CD27/IL12RB1/IL15/PIEZO1/PTPN23/RPS3/GRB2/CD6/CDC4
3/HNRNPU/LTV1/SARNP/MAGOH/THOC2/XPO7/HNRNPA1/EIF5A/POLR2D/DHX9/NXT2/RBM15B/SEH1L
3/HNRNPU/LTV1/SARNP/MAGOH/THOC2/XPO7/HNRNPA1/EIF5A/POLR2D/DHX9/NXT2/RBM15B/SEH1L
'LST1/IL15/RPS3/CD6/PNP/GAL/FADD/BTK/PELI1/RASAL3/GPR183/BTN2A2/ZNF335/FCGR2B
.PH2/CENPI/BANF1/CENPK/NAP1L4/NAP1L2/PARP1/SET/SMYD3/CHD4/RUVBL2/RBBP4/MCM8/CHMP
'L23A/MCTS1/PRPF19/MRPS7/PTGES3/TRAF7/MPV17L2/SART1/VCP/SRPK1/RPS23/RPS10/DDX23/
O1/MSTO1/DYNC1H1/SPDL1/XRCC3/RRS1/DDX11/INCENP/NDC1/FBXO5/KIF14/KPNB1/SMC4/KNST
'FN1/WASF1/TACSTD2/CAPZB/GRB2/FHOD1/CDC42/ARHGAP18/TGFB3/SWAP70/CDC42EP2/GMFG/AB
CL2/CDK2/PSMB4/PSMB8/PSMB10/PSMA5/PSMD13/PSMC4/PML/DYNC1LI1/PSMD10/PSMD11/CDKN2
D16/PDCD5/FADD/CLEC7A/RACK1/IGBP1/DAP/MAPT/PDCD6/CYCS/CARD18/MTCH1/HSPD1/PIDD1/
'SFRP2/UBQLN4/CHEK2/HDAC10/SPIRE1/MAGEF1/PIAS4/ERCC1/UIMC1/BCL2L1/UBR5/TWIST1/AF
F4/NUP107/CETN2/CCT8/SLBP/KHDRBS1/HNRNPA3/EXOSC3/CKAP5/CHTOP/SEC13/SYMPK/WRAP53
D6G/NDE1/CKAP5/ARPC5/NSFL1C/ACTR3/WWC1/HSP90AA1/MPP7/CTNNA1/PTK2/DCTN1/ARHGEF11
.2RB1/LST1/IL15/RPS3/CD6/PNP/GAL/FADD/BTK/PELI1/RASAL3/GPR183/BTN2A2/ZNF335
32/CD6/CDC42/IL4R/PNP/LILRB4/AP3D1/FADD/RASAL3/BTN2A2/HSPD1/SART1/CSK/BAD/BCL10/
'SMB4/PSMB8/PSMB10/PSMA5/PSMD13/PSMC4/PML/DYNC1LI1/PSMD10/PSMD11/CDKN2C/PSMD1/DC
'N6/CD27/IL12RB1/IL15/RPS3/GRB2/CD6/CDC42/IL4R/PNP/LILRB4/AP3D1/FADD/RASAL3/BTN2
C/DONSON/TIPIN/MSH2/ZNF207/TOPBP1/BABAM1/RFWD3/NABP1/RAD9A/RPA2/NAE1/TIMELESS/Z
'S12/RPS4X/RPL6/EXOSC9/EXOSC10/MAGOH/RPL37/RPL27A/DXO/EXOSC7/RPL36/UBA52/NANOS1/
'S7/RPL7A/WDR55/GAR1/SENP3/RPS15/RPS16/RPL5/NOL6/RPF2/BMT2/POP5/NVL/EXOSC9/EXOSC
RP27/CCT8/GNB4/EMC1/NFYC/UBXN1/HSPD1/PTGES3/HSPA2/TBCD/VCP/BAG2/ERP29/HSP90AA1/
/RPS27A/RPS12/RPS4X/RPL6/RPL37/RPL27A/HSPA5/RPL36/UBA52/ZDHHC23/CACNB3/CDK5R1/F
DC42EP2/GMFG/ABI2/CLIP3/DRG1/MAPT/ARPC1A/BRK1/ARPC5/ABL1/TBCD/STMN2/ACTR3/DBNL/
./DAP/MAPT/PDCD6/CYCS/MTCH1/HSPD1/PIDD1/VCP/SFRP2/TRADD/BAD/BCL10/TNFSF15/THBS1/
'RIM25/SUPT5H/CCL3/SRPK1/BAD/NOTCH1/MAVS/OAS3/TRIM21/TARBP2/PSMC3/VPS37B/PPIE/PC
35/BAD/PSMA4/PSMD9/CHCHD2/NOTCH1/RPS27A/PLK3/VEGFA/BNIP3L/EGLN2/UBA52/PSMC3/TWIS
IPN/RBBP7/RPA2/CUL4B/TAF12/CENPQ/CHD1L/HAT1/RPS27A/GTF3C5/ANP32E/THRA/UBTF/MIS18
L11/XCL2/CMKLR1/SWAP70/RPS19/WDR1/LBP/CCL3/S100A7/EMILIN1/CXCL17/AKIRIN1/CYP19A
4/HAUS8/PLK4/PSMD10/PSMD11/PSMD1/DONSON/ODF2/PSMA7/PSMB3/TOPBP1/APP/PSMD4/CETN2
'BXO5/KIF14/KPNB1/SMC4/KNSTRN/AKAP8L/NCAPH2/SFPQ/CENPK/DYNC1LI1/DSN1/ANAPC11/TUE
'RPF19/ESCO2/RAD51C/DNA2/RAD51D/VCP/WRAP53/UBQLN4/DCLRE1B/EME2/CHEK2/SFR1/SPIRE1
A/POLR2D/ANP32B/CCHCR1/RBM15B/SEH1L/EIF6/RANBP3/THOC1/DHX38/PKD1/ENY2/PABPN1/SF
)10/PSMD11/PSMD1/DONSON/ODF2/PSMA7/PSMB3/TOPBP1/APP/PSMD4/CETN2/PSMD3/RBX1/PSMD7
)CD2
.CA/CCR1/CXCL16/C5AR1/PADI2/PF4V1/CALR/CXCL11/CCR5/XCL2/CMKLR1/SWAP70/TRPM4/RPS1
:/IGBP1/CEP250/CLIP3/DRG1/MAPT/CKAP2/ABL1/TBCD/KLHL42/STMN2/NSFL1C/PRKAA2/TCTEX1
'PSMD3/PSMD7/LILRB4/IKBKG/BTK/EIF2B5/BTN2A2/PSMD8/ABL1/PSMA1/KCNN4/CSK/PSMB5/BCL
IAGOH/RITA1/THOC2/XPO7/HNRNPA1/EIF5A/POLR2D/ANP32B/DHX9/CCHCR1/RBM15B/SEH1L/EIF6
3/HNRNPU/LTV1/SARNP/MAGOH/THOC2/XPO7/HNRNPA1/EIF5A/POLR2D/DHX9/NXT2/RBM15B/SEH1L
'HOD1/CDC42/TGFB3/RANBP1/SWAP70/CDC42EP2/KATNB1/GMFG/ABI2/WDR1/DRG1/MAPT/ARPC1A/
'SMA4/PSMD9/RPS27A/HNRNPU/EXOSC9/EXOSC7/TBRG4/UBA52/NANOS1/PSMC3/EXOSC1/HNRNPR/A
IED12/PSMD8/ABL1/PSMA1/SFRP2/PSMB5/PSMA4/TBX2/PSMD9/VEGFA/AP2A2/PSMC3/RHOA/CLTC/
./RPS12/RPS4X/RPL6/C8orf88/RPL37/RPL27A/COPS5/EIF1AD/RPL36/UBA52/RPL19/RPS11/EIF
'2A/RPS3/PTPA/ZNF207/CHMP1A/WRAP73/RNF4/HAUS6/DRG1/NTMT1/CKAP5/MZT1/VCP/CHEK2/CC

'CTSS/PSMB8/KIF15/PSMB10/PSMA5/PSMD13/PSMC4/AP2M1/DYNC1LI1/AP2S1/PSMD10/PSMD11/F
3/DBF4B/PTGES3/DNA2/WRAP53/HSP90AA1/HDAC10/SPIRE1/NBN/NVL/ERCC1/UIMC1/ANXA3/GLI1

:/BECN1/EREG/OSM/CALM3/CIB1/VPS4A/ZNF16/PIN1/EFHC1
250/CLIP3/DRG1/MAPT/CKAP2/ABL1/TBCD/STMN2/NSFL1C/PRKAA2/CDK2AP2/CCSAP/DCTN1/FBXW
PN6/IL12RB1/LST1/IL15/RPS3/CD6/PNP/GAL/FADD/BTK/PELI1/RASAL3/GPR183/BTN2A2/ZNF33
IC4/AP2M1/DYNC1LI1/AP2S1/PSMD10/PSMD11/PSMD1/KIF2A/HLA-B/PSMA7/PSMB3/CAPZB/CALR/
'1/ATP7A/RPTOR/ADNP/ADD1/RYK/FSTL4/UCN/SEMA3F/MEGF8/TRIM46/XK/SEMA6B/SEMA4C/PTPF

IC4/AP2M1/DYNC1LI1/AP2S1/PSMD10/PSMD11/PSMD1/KIF2A/HLA-B/PSMA7/PSMB3/CAPZB/CALR/
)AA1/VCAM1/DCTN1/VPS45/CEP164/HAUS7/CFTR/CTBP2/RAB3A/MARK4/HAUS5/DYNC1I2/RAB11A/
./ADD3/ARHGAP28/RHOA/MTPN/SPTAN1/ARF1/RHOBTB2/GMFB/HAX1/PREX1/ARFGEF1/ADD1/MYO1C
20/ACTN4/RAB27B/FNBP1L/TNPO1/IFT22/SNAPIN/SSX2IP/UXT/HTT/ACTR1A/RAB1A/MYO1C/KIF3
'DBNL/SPIRE1/ADD3/LIMA1/ARHGAP28/DSTN/PLEKHH2/RHOA/MTPN/SPTAN1/ARF1/RHOBTB2/GMFE
.1/ACTR3/PSMB5/BCL10/PSMA4/HSP90AA1/PSMD9/PTK2/PAK2/PLD2/PSMC3/WIPF2/RAPGEF1/MAF
L3/WNT4/BTN2A2/ABL1/CCND3/VCAM1/TWSG1
PU/EXOSC9/DXO/EXOSC7/TBRG4/UBA52/PSMC3/EXOSC1/HNRNPR/ANP32A/DHX9/TNPO1/PSMC5/RBM
3/PTPN1/PIAS4/MAVS/TLR2/IFNAR2/CDC37/SIGIRR/DHX9/NR1H2/SPATA2/IRAK2/CAV1/PTPRC/Y
CSNK2A1/RPL5/MBD3/NBN/RPF2/PLK3/PAK1IP1/CSNK2B/TP53RK/CDK5R1/NUAK1/TWIST1/RFC5/F
PKAPK5/BECN1/UBE2N/ANAPC5/CTBP1/ATR/MORC2/ANAPC4/PAF1/HNRNPD/PHF1/ING2/SETDB1/BF
.PP/SWAP70/FADD/ECM1/LBP/CCL3/S100A7/EMILIN1/CXCL17/AKIRIN1/CYP19A1/PTGER4/SLC8E
PTN/HSPA4L/COPS5/HSPA5/CANX/EDEM2/HSPA14/PPP2R5B/GFPT1/TLN1/DAXX/HSPB7/GOSR2/TPF
./SPIRE1/ADD3/LIMA1/ARHGAP28/DSTN/PLEKHH2/RHOA/MTPN/SPTAN1/ARF1/RHOBTB2/GMFB/HAX
'RPS16/USF1/RPL5/RPS27A/RPS12/RPS4X/RPL6/RPL37/RPL27A/TRIM21/RPL36/UBA52/TARBP2/
SMB5/PSMA4/PSMD9/CSNK2A1/VPS35/SFRP4/TLR2/ATP6AP2/SULF2/UBR5/PSMC3/GSKIP/PSMC5/F
PIB/POLR2J/POLR2D/DHX9/KPNA6/NELFA/POLR2E/CAV2/SUPT4H1/NELFCD/GTF2F1/CIB1/LARP1/
./RPS27A/GTF3C5/THRA/UBTF/MIS18A/UBA52/PIH1D1/PSMC3/BAZ2A/DAXX/SART3/ANP32B/SMAF
S27A/HNRNPU/NVL/EXOSC10/UBA52/HNRNPA1/RFC5/MAPKAPK5/DNAJC2/POLE3/RPA1/TFDP1/POLE
.5/CEP164/HAUS7/CFTR/CTBP2/RAB3A/MARK4/HAUS5/DYNC1I2/RAB11A/NEDD1/CENPJ/CC2D2A/V
'MASTL
NA1/PTPN2/THBS1/STYXL1/CASP8/PIAS4/PAK2/BCL2L1/TPD52L1/PLAGL2/STK3/YWHAG/FAF1/E
IIS18A/PIH1D1/BAZ2A/DAXX/ACTR5/RNF8/SMARCA5/ZMIZ1/DMAP1/POLE3/ELOF1/SUPT4H1/SRCA
)/CCNE2/HNRNPA1/RFC5/MAPKAPK5/POLE3/RPA1/SMARCAL1/POLE2/ATR/DCLRE1C/HDAC8/TFIP11
2/TRAF4/PSMC3/ADGRG3/RHOA/ACTN4/PSMC5/CCL19/TLR7/LITAF/PSMB1/MAP3K7/PSMB6/COPS8
)SC9/EXOSC7/TBRG4/UBA52/PSMC3/EXOSC1/HNRNPR/ANP32A/DHX9/TNPO1/PSMC5/RBM10/MEX3D/
'TMBIM1/BCL2L1/RAB11A/CACNB3/CDK5R1/YWHAG/CLTC/AP2B1/GPC2/EPHA2/MRAP2/TFDP1

)K2AP1/NBN/CCNE2/ZPR1/STOML2/RFC5/POLE3/RPA1/CDC34/SMARCAL1/MSH6/ATG7/RNASEH1/PC

SMN
;/RACK1/ZNF675/CLIP3/PELI3/PSMD8/LIMS1/PSMA1/TNFAIP3/TRADD/PSMB5/PSMA4/PSMD9/TNF
./NEK6/CXXC5/UBE2N/CCL19/TMEM106A/LITAF/LPAR1/MAP3K7/TRIM32/VAPA/ATP2C1/CC2D1A/S
N1/ANAPC5/EREG/UBE2E2/EGF/RPTOR
38/ENY2/PABPN1/SRSF1/SRSF2/CASC3/FIP1L1/RBM8A/MAGOHB/MYO1C/SMG7/LRPPRC/POLDIP3/T
ME6/MSX1/DRG1/WNT4

2N/AKAP8L/NCAPH2/CENPK/DYNC1LI1/DSN1/ANAPC11/TUBG2
2/HNRNPA1/RFC5/MAPKAPK5/POLE3/RPA1/SMARCAL1/POLE2/ATR/DCLRE1C/HDAC8/TFIP11/SMG7/
P2A2/SYNE4/PSMC3/RHOA/IFT20/CLTC/AP2B1/PSMC5/AP2A1
34/MAVS/IFNAR2/CDC37/SIGIRR/NR1H2/SPATA2/IRAK2/CAV1/PTPRC/YTHDF2/METTL3/VRK2/PPA
'ARF1/RHOBTB2/GMFB/HAX1/PREX1/ARFGEF1/ADD1/MYO1C/SPIRE2/NCKAP1/PPP1R9A
57/HINFP/TIPRL/CNOT10/CCAR2/CNOT3/MUS81/TFDP1/ATR/TP53BP1/HMGA2/THOC1/FBXO6
)/SFRP4/AP2A2/RNF213/PSMC3/RHOA/CLTC/AP2B1/PSMC5/AP2A1/RYK/PSMB1/MOV10/MAP3K7/PS

.0/STXBP4/CNOT3/TFDP1/UBE2E2/RPTOR/PAGR1/PKD1
'PIAS4/VDAC2/PTPMT1/TXNDC12/IVNS1ABP/BCL2L1/NDUFA13/PLAGL2/CCAR2/BECN1/PRKRA/CAV
'NABP1/RAD9A/RPA2/NAE1/ZWILCH/BTG2/PIDD1/DCLRE1B/EME2/CHEK2/CLSPN/NBN/PLK3/ZFYVE

.1/SEH1L/HSPA13/ATR/RPTOR/STUB1/CXCL10/HSBP1L1/ATP2A2

2A2/PARL/TIMM23/TIMM8B/HUWE1/PITRM1/IMMP1L/TIMM9/TIMM22/USP36/NMT1/PAM16/HTRA2/A
IGA2/THOC1/FBXO6

3M8A/MAGOHB/SMG7/POLDIP3/THOC7/AAAS/THOC3/HNRNPA2B1/SUPT6H/U2AF1L4/SRSF3/NUP214/

ISFL1C/CHEK2/CCSAP/DCTN1/HNRNPU

2SF1/SRSF2/CASC3/FIP1L1/RBM8A/MAGOHB/SMG7/POLDIP3/THOC7/AAAS/THOC3/HNRNPA2B1/SUF

./PMM2/RPL32/RPL30/UBAC2/RPS9/RPS14/RPS27/RPL29/SRP68/TRAM1/RPL13/RPL4/RPL41/RPL
GOHB/SMG7/POLDIP3/THOC7/AAAS/THOC3/HNRNPA2B1/SUPT6H/U2AF1L4/SRSF3/NUP214/TSC1/IW
2L/TIMM23/TIMM8B/HUWE1/PITRM1/IMMP1L/TIMM9/TIMM22/USP36/NMT1/PAM16/HTRA2/ARIH2
.A/ANAPC15/TERF1/SUPT6H/KDM3A/CDK5RAP2
'PSMC5/AP2A1
.9/XCL1/CCL8/TMEM102
'PSMC5/AP2A1

2/CYTH3
ANP32B/SMARCA5/POLE3/IPO4/SMC2/HMGA2/PARP10/GRWD1

)/RPS9/RPS14/RPS27/RPL29/CASC3/RPL13/RBM8A/MAGOHB/RPL4/RPL41/SMG7/RPL9/RPS6/RPL2

ASCC3
IFSF13

'U2AF1L4/SRSF3/NUP214/IWS1/DDX19A/TPR/SRSF7/MCM3AP/NUP50

'U2AF1L4/SRSF3/NUP214/IWS1/DDX19A/TPR/SRSF7/MCM3AP/NUP50
SRP68/TRAM1/RPL13/RPL4/RPL41/RPL9/RPS6/RPL24/RPL11/RPL10/RPS24/RPS3A/RPL14/SRP19

)1/PAF1/HDAC8

'RAM1/RPL13/RPL4/RPL41/RPL9/RPS6/RPL24/RPL11/RPL10/RPS24/RPS3A/RPL14/SRP19/RPL10

/KIF22/CLTC/AP1S1/AP1S3/AP1S2/AP2B1/LGMN/AP2A1/ARF1

/CLTC/AP1S1/AP1S3/AP1S2/AP2B1/LGMN/AP2A1/ARF1
.9/RPS6/RPL24/RPL11/RPL10/RPS24/RPS3A/RPL14/SRP19/RPL10A/RPL12/SRP14/RPS18/RPS28

?1S3/AP1S2/AP2B1/LGMN/AP2A1/ARF1

/RPL26L1/EIF3C/ZC3H15/RPL31
B/FCGR1B
.24/RPL11/RPL10/RPS24/RPS3A/RPL14/SRP19/RPL10A/RPL12/SRP14/RPS18/RPS28/RPS17/RPL

P1/HAUS2/CDK5RAP2

/RAB7B/RRAS/ARHGEF19/GDI1/SH2B2/RAB35/ARFGAP1/RAB24/DYNLT1/GPSM2/CYTH2/FLOT1/ARH
MYD3/KMT5C/PADI2/UBE2E1/RUVBL2/POLE4/SAP30/PRKD2/UBE2A/USP22/JADE2/KAT2A/BEND3/M
1/EPHB3/ARFGAP1/TBC1D10B/DYNLT1/DVL3/NCKAP1L/ARHGAP1/CCL2/RTKN/RAP1GAP2/CXCL13/
KN2D/MAGED1/NCF1/DTNBP1/TNFAIP8L3/TRAF2/CCL5/MAPKAPK2/CXCR4/MIF/CD300A/IQGAP1/C
E41/LINGO1/HMGB2/TIMP2/TCF3/P3H1/CXCR4/SEMA4A/MAP2/BRSK1/IQGAP1/PTK7/EFNA5/SF3A
AC1/RELN/CSNK1E/MARK2/GDI1/NUMBL/FGFR1/DYNLT1/DVL3/VLDLR/CPNE1/HEYL/HDAC2/CSNK1
8/HLA-DRA/MUC1/HCST/EZR/IFNGR2/COL3A1/HLA-DQB1/SLAMF7/SCRIB/HMGB3/VTCN1/GRN/GBP
8/MAPK3/MAP2K2/CENPE/UBE2I/DKC1/PAK1/ACD/PEA15/FPR1/SOCS1/CKS1B/CCT2/RPS2/HSP90
3/MUC5B/DPAGT1/BGN/IL15/A4GALT/STT3A/GALNT6/FUT2/FUT6/AGO2/MUC20/TMEM258/CHPF/M
RB2/MTCP1/PRKCI/GRK2/APP/DMPK/MAPK7/BCAR3/VRK1/RACK1/CSNK1G2/STK38/NTMT1/SFRP2/
CR1/CD209/LYN/CARD11/MUC3A/PSMB4/CTSS/PSMB8/PSMB10/PSMA5/CD300LF/NRAS/SFPQ/PQBP
/LDOC1/PIM2/HIP1R/BCL3/FLOT1/SRI/GTPBP4/EIF3D/CPNE1/HDAC2/KDM1A/ITGB1BP1/ITGA2/
GAL/SLC27A4/MYO5A/KRT20/HPSE/S100A7/OVOL2/KDF1/TRADD/KRT10/PSAP/ACVR1B/NOTCH1/U
/SFPQ/PQBP1/PSMD13/IFI16/PSMC4/MUC5B/UBE2D1/PSMD10/PSMD11/PSMD1/PSMA7/TRAF3/PSM
/MAPK11/PQBP1/XPR1/IFI16/BANF1/MAP3K14/PML/IL12RB1/MST1R/IL15/ILF3/ISG20/BATF3/
3/NSF/GGA1/RAB3D/STAC/PALM/STX7/CSK/VAMP8/SYS1/VPS35/ARFRP1/OPTN/RAB3A/GGA3/TME
/MAVS/OAS3/TRIM21/TARBP2/PSMC3/VPS37B/PPIE/POLR2I/PPIB/POLR2J/POLR2D/DHX9/IFITM
/PLK3/SH3GLB1/MAVS/EDEM2/ZPR1/RAB29/KIF20B/CACNB3/CDK5R1/UBR5/CACNA1D/VPS11/ATG
/VHL/PSMA1/PSMB5/BAD/PSMA4/PSMD9/CHCHD2/NOTCH1/RPS27A/PLK3/VEGFA/BNIP3L/EGLN2/U
L2/ATP6V0D1/SUV39H2/PSMD8/VHL/PSMA1/PSMB5/BAD/PSMA4/PSMD9/CHCHD2/NOTCH1/RPS27A/
/STX4/SEMA5B/ANGPT2/SEMA3C/GPR183/SEMA3E/LBP/CCL3/S100A7/CXCL17/AKIRIN1/CYP19A1/

PHA2/CCL19/KLF5/HTT/CAV1/ATP7A/DZIP1/PLD1/EVI5L/NCKAP1/PPP1R9A/PLEKHM1/CCDC88A/N

SME3/SLC9A3R1/DVL3/FERMT1/PLEKHA4/FRAT2/DAPK3/CSNK1D/FZD2/AXIN1/NLE1/MITF/WNT7
P/PSMB4/GBA/KCTD10/WFS1/UBE2L3/PSMB8/RNF187/PSMB10/PSMA5/DNAJB2/TAF9/PSMD13/PS
0AB1/NPLOC4/CSNK1E/MAN1B1/PSMB2/PSME3/HSPBP1/BCAP31/UBE2J2/FBXO5/VLDLR/LAMP3/C
4/YWHAH/BBC3/PMAIP1/HCFC1/MTFR2/VDAC1/GBA/CDK5/UBE2L3/CTTN/PHB/ZNF205/GSK3A/WD
/NCKAP1L/MLST8/CAPZA1/DAPK3/MKKS/ARHGDIB/FHOD3/HCLS1/TESK1/ITGB1BP1/ARPC5L/RAC1
SIRPA/SOCS1/ARPC2/CTLA4/FSTL3/CD83/TFE3/PDCD1/TGM2/FERMT1/SIRPG/FLOT1/NCKAP1L/
MAPK7/BCAR3/VRK1/RACK1/CSNK1G2/STK38/SFRP2/PDCD10/CHEK2/CLSPN/MAPKAPK3/HSP90AA1
2D1/PSMD10/PSMD11/PSMD1/PSMA7/TRAF3/PSMB3/ARRB2/MUC20/TLR5/PSMD4/GPATCH3/PSMD3/
A/IL4R/PNP/LILRB4/AP3D1/AXL/FADD/BTK/ZNF675/BTN2A2/CCL3/ABL1/SART1/HLA-DOA/FCG
D1/FADD/GPR183/WNT4/BTN2A2/LFNG/ABL1/SART1/HLA-DOA/BAD/PTGER4/PTPN2/ADAM17/TME
S21/MRPS15/CCSAP/MRPL18/MAP1A/JMJD4/ADD3/LIMA1/ETF1/MRPL37/EIF5A/DSTN/PLEKHH2/
/PIK3R6/HAX1/INPP5D/PTPRC/ZNF16/ACIN1/NFKBID/ANKRD54/CD4/IFNG/ANXA1/PPP2R3C/GN
PJ/CCAR2/EGF/CAV1/ZBED2/PSMB1/PIN1/CSNK1G1/PSMB6/PSMA3/LYPD6/PSMB7/FZD9/WNK1/S

S/TRIM56/XRCC5/LILRB1/RNF135/OTUD5/ZBP1/TICAM1

S/TRIM56/XRCC5/LILRB1/RNF135/OTUD5/ZBP1/TICAM1

7/TRNAU1AP/NAALADL1/PPP1CA/MRPL9/CPD/PA2G4/ZC3H12A/PAM/NCBP2/EEF1D/ERAP2/BCL3/
/SVBP/PSME3/HSPBP1/UBE2J2/NPEPPS/OTUB1/DTX2/FBXO5/GTPBP4/PRR7/TRIM31/ANAPC7/SPS
P/PSMB4/GBA/KCTD10/WFS1/UBE2L3/PSMB8/RNF187/PSMB10/PSMA5/DNAJB2/TAF9/PSMD13/PS
E2J2/FBXO5/LAMP3/CSNK1D/ANAPC7/CDK2/KIF14/SPSB1/SNF8/AXIN1/PMAIP1/ADRM1/CCDC22/
SME3/SLC9A3R1/DVL3/FERMT1/PLEKHA4/FRAT2/DAPK3/CSNK1D/FZD2/AXIN1/NLE1/MITF/WNT7
UCY2C/PGK1/CHST4/PIGS/ALG3/MGAT5/ATIC/JAK3/DDOST/SIRT6/NUP210/CANT1/GPAA1/PUDP/
1/ZNF385A/MAGED1/FZD7/RHOG/HSF1/GLIPR2/NCF1/PRMT1/CARD9/MGAT5/AKR1C3/DDIT3/IGF
/SPOCK2/B4GALT2/XYLT2/UGDH/POMT2/FUT3/ADAMTS9/MUC5B/DPAGT1/BGN/IL15/A4GALT/STT
/AKAP8L/EHD2/LAMTOR1/RNF26/FA2H/KIFC3/RTN3/TGFB2/CHMP3/BANF1/RAB10/MYOF/CIT/VPS
GA2/LAMTOR5/APOBEC3G/XPR1/CHMP3/NUP85/IFI16/BANF1/NUP43/PML/VPS28/SMARCB1/RAE1/
2/NFAM1/CDH17/LCK/PTPN6/CD27/IL12RB1/CYP26B1/CD79A/IL15/MSH2/DROSHA/KAT2A/IL4R/
14/TNFRSF11A/NPLOC4/PSMB2/IRF7/TGM2/PSME3/TNIP2/LAG3/FLOT1/SMPDL3B/ADAM8/NONO/
/CROCC/FHDC1/OCRL/CETN2/LRRC61/WRAP73/TMEM107/CEP250/HAUS6/ATP6V0D1/NDE1/TTC26/
/C5AR1/MSH2/ARRB2/PRKCI/TGFB3/APP/PPP5C/IKBKG/AXL/FADD/PIGT/CBL/AKT1S1/RACK1/NA
H1/CORO1B/HIP1R/PAM/SRI/NCKAP1L/MLST8/CAPZA1/RANGRF/DAPK3/MKKS/ARHGDIB/FHOD3/H
/CROCC/FHDC1/OCRL/CETN2/LRRC61/WRAP73/TMEM107/CEP250/HAUS6/ATP6V0D1/NDE1/TTC26/

PG5/WDR1/GPR183/EIF2B5/MAPT/RHEB/CSPG4/MED12/NF2/CCL3/PRPF19/ABL1/CSK/SRGAP2/H

SF13B/PSMB10/PSMA5/CCL4/COMMD7/PSMD13/CD27/PSMC4/CASP1/MAP3K14/SMPD4/PSMD10/PS
/LTBR/FAIM/NKIRAS2/PPP5C/CARD16/IKBKG/FADD/ECM1/BTK/PELI1/PRDX4/ZNF675/TNIP1/T
2L1/EGLN2/ZPR1/MECP2/CDK5R1/RHOA/STXBP1/SIX1/LGMN

ELI3/GRINA/ACVR1/SLC25A5/SFRP2/TNFAIP3/TRADD/CTNNA1/THBS1/CSNK2A1/CASP8/PTPN1/

K2/IFITM2/DDB1/P4HB/MX1/TRIM32/VAPA/TBC1D20/ADAR/TRIM27/SETDB1/TRIM62/CD4/APOBE
ASC3/FIP1L1/RBM8A/CSTF3/MAGOHB/ZNF473/RPRD1A/POLDIP3/THOC7/THOC3/U2AF1L4/SRSF3/
/IRF4/IFNG/ANXA1/CCR2/CD80/SOX13/RASGRP1/FGL2/LILRB2/NLRP3/IL12A/ZBTB7B/PRDM1/S

4/GBA/KCTD10/WFS1/UBE2L3/PSMB8/RNF187/PSMB10/PSMA5/DNAJB2/TAF9/PSMD13/PSMC4/GSK
KIF18A/WIPF3/LIMK2/CCT3/HMGB2/YBX3/EIF5A2/BAX/MFGE8/NR0B1/SNRPA1/FANCG/ZP3/RUVE
/SLC26A6/TNFAIP6/HSF1/KIF18A/WIPF3/LIMK2/HMGB2/YBX3/EIF5A2/BAX/NR0B1/SNRPA1/CAS
/SLC26A6/TNFAIP6/HSF1/KIF18A/WIPF3/LIMK2/HMGB2/YBX3/EIF5A2/BAX/NR0B1/SNRPA1/CAS
/NPEPPS/EHD1/ARHGAP1/HCLS1/LYN/ITGB1BP1/REEP2/AKAP8L/IL1B/EHD2/CDK5/TXN/UBE2L3/

N6/MAP3K14/TNIK/DVL2/MST1R/C5AR1/RPS3/TGFB3/MUC20/ADRA2A/SPRY4/MAP3K6/IKBKG/ZNF6

C4/GSK3A/FGFR2/DVL2/RNF220/PSMD10/PSMD11/PSMD1/PSMA7/RUVBL2/PSMB3/SDHAF2/CTDNEP1

3/F2RL1/DYNLL1/PPARD/GPI/PPIA/ZC3H12A/BID/CCT4/BCAP31/UBE2J2/FERMT1/NPEPPS/SRI/A
/IKBKG/AXL/CBL/AKT1S1/RACK1/NAE1/BTBD10/TFAP2A/MAPT/CCL3/BTG2/ABL1/ZNF746/FCGR2B
.1/PSMD1/PSMA7/RUVBL2/PSMB3/SDHAF2/CTDNEP1/PSMD4/TRPM4/PSMD3/RBX1/PSMD7/TBL1XR1/

RD16/IKBKG/FADD/ECM1/PELI1/TNIP1/TRIM25/PIDD1/ABL1/TNFAIP3/TRADD/BCL10/CASP8/MAV
SFRP2/DBNL/HSP90AA1/TBX2/DNM2/NOTCH1/LLPH/VEGFA/RAB11A/MESP1/IFRD1/CDK5R1
IN

/USC2/CASP8/TRIM21/BCL2L1/THOC2/VPS37B/TNFRSF14/IFITM3/SLC22A5/EPHA2/IST1/KPNA6/
OR1/DRG1/MAPT/ARPC1A/WNT4/NF2/BRK1/ARPC5/ABL1/STMN2/ACTR3/SPIRE1/DCTN1/MECP2/CDK
BAG2/TBC1D7/BCL10/HSP90AA1/RPL5/PIAS4/MASTL/HSPA5/TRIM21/IVNS1ABP/UBR5/CHFR/NMI/
/CCL19/KLF5/HTT/CAV1/ATP7A/DZIP1/PLD1/EVI5L/NCKAP1/PPP1R9A/PLEKHM1/CCDC88A/NEURL
/HDAC8/ADD1/PPARG/PIN1/STK4/RPL11/EPB41/PARK7/IRF4/MBD2/IFNG/EIF4G1/DERL1/ERCC2/
/CD4/HYAL2/ANXA1/VPS33B/VAMP1/NKD2/RAB7A/VAV3
I/DYRK3/ANXA1/CCR2/ERCC2/RPS17/BAP1

AB2IP/FKBP14/STUB1/ADD1/SSR1

[illegible]GPRC5B/PDGFA/FGR/LCK/PTPN6/SAMSN1/SOCS3/SLA/FGFR2/DVL2/MST1R/IL15/CADM4/MATK/L[illegible]
[illegible]GPRC5B/PDGFA/FGR/LCK/PTPN6/SAMSN1/SOCS3/SLA/FGFR2/DVL2/MST1R/IL15/CADM4/MATK/L[illegible]
[illegible]/PSMB8/TNFSF13B/PSMB10/PSMA5/CCL4/COMMD7/PSMD13/CD27/PSMC4/CASP1/MAP3K14/SMPD4/
[illegible]R2B/TNFAIP3

[illegible]ARS2/TSEN15/DPH3/RPP30/DALRD3/TRMT2B/EXOSC2/CDK5RAP1/CARS2/QRSL1/SARS2/RTCB/ME[illegible]
[illegible]ETTL3/UCN/CASP3/POLDIP3/HNRNPD/PLA2G6/PYM1/FXR1/IFNG

[illegible]HIP1/SOCS5/SNX6/FASLG/PIGR

'UT1/AHCYL1/SRSF4/RPRD2/CLP1/NUDT21

3IP2/DLG3/CSF1R/MAGED1/PGK1/FZD7/HSF1/GLIPR2/NCF1/PRMT1/CARD9/IGFBP3/TNFRSF18/TN
;AP2/CXCL13/SIPA1L2/ARHGDIB/TBC1D30/CCL4/TBC1D13/RASA3/DVL2/ARHGAP30/AGFG1/ABR/S
2/RBBP7/TRIM71/MYBBP1A/ZNF335/SEC13/DOT1L/TDG/HAT1/MBD3/HNRNPU/TSN/EXOSC10/TAF1D
'POMGNT1/MGAT4A/NAGPA/MFNG/FUT1/LFNG/EXT1/VCP/DHDDS/B4GALNT2/DAD1/POFUT2/FUT8/GA
'POMGNT1/MGAT4A/NAGPA/MFNG/FUT1/LFNG/EXT1/VCP/DHDDS/B4GALNT2/DAD1/POFUT2/FUT8/GA
A5/IVNS1ABP/UBR5/CHFR/NMI/RASSF5/DAXX/GLMN/PRMT3/UBE2N/PEF1/CAV1/PARP10/STUB1/RA

'ZNF683/PTPRC/CLEC4E/LOXL3/CD180/RPS6/NFKBID/IRF4/UNG/IFNG/ANXA1/SUPT6H/DOCK11/C

A/TLR1/NOD2/ARG2/LILRB1/RASGRP1/TICAM1/UBE2J1/WNT5A/C5AR2/CACTIN/SLAMF1

2/SMCHD1/BRCC3/UBE2V2/PARG/NUDT16L1/CEBPG/FIGN/WRNIP1/RNF168/RECQL5/EYA2

;/WASF2/MAPKAPK2/VAV1/NDN/SOGA1/ITGA5/DOK1/ATP6V1C1/IQGAP1/ARPC4/PDGFRL/EFNA5/RG
'BL1/PYGO2/MAST2/SLC4A2/PPP2R1A/PHC2/TSPAN8/TTLL1/EIF2S2/DYNLL1/DPCD/FSTL3/NDC1/
;15/NPM1/SPRED1/BRCA1/THY1/CORO1C/UBASH3B/SIRPA/SOCS1/CTDSPL/COPS9/F2RL1/PPP1R9B
'CBFB/PSMB4/PSMB8/PSMB10/PSMA5/PSMD13/PSMC4/GSK3A/FGFR2/DVL2/IFT80/RNF220/ATP6VC
IP4K2C/RALB/PACS2/ATP6V0D1/RUBCN/RHEB/VMP1/SUPT5H/PAFAH1B2/VCP/NPRL3/ATG4B/NSFL1

CBL/RACK1/CSPG4/NF2/ACVR1/ABL1/SFRP2/PTPN2/ADAM17/TNK2/PTPN1/VEGFA/PAK2/DGKQ/PPF

IG/ABL1/SYMPK/NFATC4/TDG/TMC8/BAG2/SPPL3/NVL/TRAF4/HSPA5/MITD1/STK3/SNAPIN/LRPAF

A/ARFRP1/RGP1/GGA3/UBA52/LMTK2/RAB11A/RAB29/VPS37B/TMEM87A/AP3B1/CLTC/AP1S1/SNX1
2/CDC42EP2/STX4/VIM/TRIM25/CCL3/CIITA/ACTR3/VCAM1/PTPN2/IRF5/KYNU/OAS3/TLR2/TRIM
'PN1/OPTN/HSPA4L/COPS5/HSPA5/CANX/EDEM2/HSPA14/PPP2R5B/GFPT1/TLN1/DAXX/HSPB7/ANK
R6/INPP5D/ZNF683/PTPRC/IKZF3/METTL3/LOXL3/NFKBID/CLPTM1/SH3RF1/IRF4/IFNG/ANXA1/C
;/CD80/RASGRP1/LILRB2/NLRP3/IL12A/ZBTB7B/ROR2/SOCS5/MYB/ZAP70/CTNNBIP1/MALT1/CD1

;/RAB7A/CHMP6/EPN1/RASSF2/AFAP1L2/TSG101/HIP1

3PL1/CHMP4A

/MAGED1/FZD7/HSF1/GLIPR2/NCF1/PRMT1/CARD9/IGFBP3/TNFRSF18/TNFAIP8L3/TRAF2/AREG

ARK2/NCAPG2/FGFR1/MT3/DVL3/NCKAP1L/MLST8/DBF4/ADAM8/PAK4/VLDLR/SHC1/KIF14/AXIN1

D8/TAF10/DESI2/VCP/PSMA1/TNFAIP3/USP5/COPS6/CLSPN/PSMB5/SENP3/PSMA4/PSMD9/RPS2

2/VPS11/YWHAG/ATG13/TIGAR/TFDP1/ATG7/HTT/HAX1/TMEM102/CSNK2A2/PARL/HUWE1/BIK/D

1/HTT/DZIP1/KIAA1324/EVI5L/FEZ2/RAB1B/STAM/CCDC88A/PIP4K2B/UBQLN2/ULK1/TCHP/DY

/PSME2/MYB/PDGFRA/PSMD6/GATA3/PSMC2/PSMA6/NUDT21/YAP1/TP73/HSPA9/PSMD5/CDK12

:D1/ATP6AP1/PAK1/CRABP2/ANKRD27/NRCAM/XRCC2/BIN1/ARPC2/HDAC1/RELN/CSNK1E/MARK2/(
:FNB1/MAP1S/PAK1/CRABP2/ANKRD27/NRCAM/THY1/CORO1C/PALLD/HSP90AB1/EPHB6/ISL2/GAS7
'N4RL2/BIN1/ISL2/RELN/CSNK1E/ZSWIM5/MARK2/GDI1/EPHB3/FGFR1/MT3/DYNLT1/DVL3/VLDLF
./MAP1S/PAK1/CRABP2/ANKRD27/NRCAM/THY1/CORO1C/PALLD/HSP90AB1/EPHB6/ISL2/GAS7/REI
'A1/HLA-DMB/VSIG4/CTSK/WIPF3/TRAF2/RBCK1/PSMD14/HMGB2/TRIM6/WASF2/CCL5/BAX/CD86/
;6KB2/RACK1/VIM/TRIM71/DPH7/ZNF598/PLEKHN1/EIF2B5/RPL38/KHDRBS1/PIWIL4/BTG2/LARF
;ERPINE1/RNF34/SLPI/PERP/BCL2L12/NOL3/BIN1/HDAC1/CST2/PRELID1/LEF1/SERPINB1/HIP1
:/DDIT4/AGBL5/IRF7/ZC3H12A/STAB1/BCL3/SHC1/CXCL9/CXCL13/NLRC5/PLSCR1/DEFB1/SLC11
.R1/CDK5/LAMTOR1/CTTN/MEG3/SPOCK1/RNF157/PHB/DNAJB2/TGFB2/TAF9/GSK3A/PML/CXCL16/

.0/DESI2/VCP/PSMA1/TNFAIP3/USP5/CLSPN/PSMB5/PSMA4/PSMD9/RPS27A/USP21/CFTR/UIMC1/
:35C1/ST3GAL5/POMGNT1/MGAT4A/NAGPA/MFNG/FUT1/LFNG/EXT1/VCP/DHDDS/B4GALNT2/DAD1/F

'NK2/NBN/AAK1/VEGFA/PAK2/LMTK2/MAP3K10/PPP2R5B
.3

;RA2/CD84/THOC1/INPP5D/DUSP22/PTPRC/CNR1/LOXL3/NFKBID/CASP3/BCR

A9/NME1/ADORA1/PRNP/HRAS/RAB31/APLP1/SYK/MTMR2/HCK/UNC13D/LRP10/CD151/NCF4/EZR/T
/CCL13/RELN/S100B/ZSWIM5/MARK2/GDI1/EPHB3/CORO1B/SLC9A3R1/MT3/DVL3/SH3KBP1/CCL2/
NFRSF11A/RELN/MARK2/NCAPG2/FGFR1/MT3/DVL3/NCKAP1L/MLST8/DBF4/FGFR3/ADAM8/PAK4/V

31/EVPL/VDR/GORAB/MAFF/APCDD1/LAMB3/GAL/MYO5A/KRT20/HPSE/S100A7/OVOL2/KDF1/TRADD

3A/LRPPRC/KIF5B/DYNC2LI1/ARL3/TRIM46/TUB/TTC21A/ARL8A/CNIH2/BBS12/TRAK2/IFT140/B
3A/LRPPRC/KIF5B/DYNC2LI1/ARL3/TRIM46/TUB/TTC21A/ARL8A/CNIH2/BBS12/TRAK2/IFT140/B

1/SOCS5/STAT3

PL42/MRPL22/MRPL58/MRPL38/MRPS30/GSPT1/MRPS27/MRPS18A/MRPL43/MRPS9/MRPL50/GLE1/M

B/DNAJC6/RAB34/RHOQ/TFRC/MRC2/WIPF3/CARMIL1/WASF2/RABEP2/MAPKAPK2/VAV1/COLEC12/
SPH1/SCMH1/MKKS/ZFP41/DEFB1/OAZ3/FANCA/TESK1/RPL10L/ADGRG2/DAZAP1/POC1A/DNMT3A/
3/ILF3/GRB7/ZNF706/RPS3/SAMD4B/NCL/EIF3H/AGO2/CALR/APP/PPP1R15A/UQCC2/RPS6KB2/
1/GRINA/VCP/FCGR2B/SRPRB/WIPI1/ERP29/SGTA/RCN3/TATDN2/PTPN2/THBS1/DCTN1/PTPN1/
R1/GPATCH3/CARD16/AXL/BTK/UBE2L6/TRIM25/LBP/CARD18/BTN2A2/FCGR2B/TNFAIP3/CSK/R

A/C5AR2/ARRB1/SLAMF1/SOCS5/STAT3

DDR1/ZYX/CDK4/GIT1/CSF1R/NCF4/COL3A1/SH2D2A/GUCY2C/ESRP1/CREB3L1/COMP/NCF1/RHO

1S/PAK1/CRABP2/ANKRD27/NRCAM/THY1/CORO1C/PALLD/HSP90AB1/EPHB6/ISL2/GAS7/RELN/S1

MH1/MKKS/ZFP41/DEFB1/OAZ3/TESK1/RPL10L/ADGRG2/DAZAP1/POC1A/DNMT3A/CYP26B1/AGFG1
L12/NOL3/BIN1/HDAC1/CST2/PRELID1/LEF1/SERPINB1/HIP1R/PSME3/BID/MT3/BCAP31/PRR7/

CD38/GAK/ADCY6/DPYSL3/SNAPIN/EPHB2/CIB1/EVI5L/RYK/FSTL4/LPAR1/SEMA3F/TCHP/CEP97
CL3/CIITA/ACTR3/VCAM1/PTPN2/IRF5/OAS3/TLR2/TRIM21/HLA-F/CDC37/NMI/RPL13A/STXBP1
/SYT11/ADGRE2/CCR2/VAMP1/PRAM1/PRKCB/CHRNA5/SYP

/CARMIL1/CXCR4/SEMA4A/NDN/MAP2/DOK1/BRSK1/B4GALT5/EFNA5/RAB25/MAPK3/LAMC2/ST14/
CB1/SMYD3/RAE1/KMT5C/ZMIZ2/LOX/RUVBL2/POLE4/NUP155/USP22/JADE2/KAT2A/BEND3/MTF2

PTGER4/NOTCH1/SRGN/MAVS/TLR2/ALOX15B/TWIST1/GLMN/IL17RA/TNFRSF14/DHX9/RBPJ/CCL1
IP3/RNF216/MAVS/IRF5/DMBT1/BNIP3L/OAS3/IFNAR2/TARBP2/IFI6/DHX9/IFITM3/BECN1/TL
M13/MFF/BAD/HSP90AA1/PRKAA2/SLC8B1/VPS35/CASP8/MRPL18/SH3GLB1/TOMM22/BNIP3L/SR
SMA3/ARL11/HYAL2/PSMB7/ERCC2/ACE/ZBTB24/XRCC5/SLC7A6OS/SETD1A/TP53/PLD4/PSMD12/

B/SEMA4C/MAP4K4/PTPRS/TRAK2/TSC1

RAK2/DAB2IP/PARP10/TAF3/PKD1

POBEC3B/IFNGR2/RNASE6/ANKRD1/SCRIB/VTCN1/GRN/GBP5/HSF1/CFL1/CARD9/APOBEC3C/CHIT
D27/NRCAM/THY1/PALLD/HSP90AB1/EPHB6/ISL2/GAS7/RELN/S100B/CDH11/ZSWIM5/MARK2/GDI
POBEC3B/IFNGR2/RNASE6/ANKRD1/SCRIB/VTCN1/GRN/GBP5/HSF1/CFL1/CARD9/APOBEC3C/CHIT
/NPEPPS/CD68/RPLP2/LAMP3/HSPA4/TRAM1L1/SIX2/RPSA/YWHAH/KPNB1/SNF8/HCLS1/BBC3/P

PPARD/FERMT1/MYO1G/DAPK3/KIF14/JAG1/ITGB1BP1/CCL28/RAC1/ITGA2/CDK5/SPOCK2/CTTN/

1/ITGB1BP1/CCL28/TNC/CBFB/IL2RA/SPOCK1/MYADM/PLXND1/PTPN6/TACSTD2/IL4R/SEMA4D/

./TMEM67/ADD1/PPP1R9A

;T8/TRIM65/CAPN1/PIP4K2A/LAMP3/ATP6V1H/DAPK3/SNF8/CLN3/VDAC1/ATP6V1E1/GBA/LAMTOR
.POBEC3B/ADAM15/IFNGR2/EME1/RNASE6/ANKRD1/SCRIB/VTCN1/GRN/GBP5/HSF1/CFL1/CARD9/D
'PABPC1/TICRR/GGCT/MRPL47/TRNAU1AP/ORMDL2/B4GALT3/PPP1CA/MRPL9/PA2G4/ZC3H12A/NCE
;T8/TRIM65/CAPN1/PIP4K2A/LAMP3/ATP6V1H/DAPK3/SNF8/CLN3/VDAC1/ATP6V1E1/GBA/LAMTOR

.RG1/HLA-A/RNF19B/SASH3/IRF1/CTLA4/TNFRSF11A/TFE3/LEF1/PDCD1/IRF7/ZC3H12A/ERAP2/

./PSME2/PSMD6/MALT1/NFATC2/PSMC2/PSMA6/FBXW11/MAPK1/PSMD5

/CDH11/ZSWIM5/MARK2/GDI1/EPHB3/NUMBL/MT3/DYNLT1/FLOT1/SH3KBP1/VLDLR/LYPLA2/SHC1/

`1/SRI/ADAM8/HEYL/TRIM31/HDAC2/FZD2/NLRC5/HCLS1/KDM1A/FANCA/CARD11/CCDC22/BUD31/

`8/PTTG1IP/NF2/IPO13/PKIA/WRAP53/RPF2/TMEM98/HNRNPU/NVL/MAVS/CD2AP/HIKESHI/ZPR1/

IAPKAPK3/DBNL/ERP29/THBS1/PTPN1/RPS27A/VEGFA/PLA2G1B/UBA52/DUSP7/MAP3K10/TPD52L1

iR1/SRF/NIN/IST1/SLC9A6/CTDP1/ADNP/RYK/PIN1/FSTL4/MEX3C/SEMA3F/MEGF8/ULK1/ARIH2/

RAF2/CCT3/SAE1/TRIM6/P3H1/TRIM28/CCL5/NUTF2/PHPT1/MIF/MTCL1/PKDCC/EFNA5/CD2/HIF
V0C/ATP6V0E1/BAG3/DNM1L/IKBKG/PIP4K2C/RALB/DAP/ATP6V0D1/MAPT/RUBCN/RHEB/SUPT5H/

P1/PHAX/SNAPC4/GTF2F2/ELL/EXOSC6/MEPCE/SNAPC3/CCNT1/INTS10/INTS6L/INTS2/RPAP2/I

T20/PRKAG1/TIGAR/BECN1/HTT/RPTOR/ATP6V1B2/LARP1/KIAA1324/SNX5/FEZ2/RAB1B/MAP3K7
STL4/SEMA3F/MEGF8/TRIM46/XK/SEMA6B/SEMA4C/PTPRS/TRAK2/PLXNB3/SHTN1/TTL/DPYSL2/N

15/HSP90B1/HSPA13/DAB2IP/FKBP14/STUB1/BAG6/ADD1/SSR1/ATF6B/SDF2/FICD/RNF121/RNF

1/CARD9/PSMB9/IGFBP3/DTNBP1/TNFAIP8L3/TRAF2/PSMD14/AREG/TIMP2/JAK3/CCL5/MAPKAP
C12/PHPT1/CD300A/HLA-DPB1/ARPC4/UNC93B1/CFD/PRKDC/PSMD2/MAPK3/CD276/HLA-DQB2/C
8/FCHSD1/RAB3B/BIN1/HSP90AB1/F2RL1/ARPC2/ARPC3/GDI1/E2F4/PLOD3/PPP1CA/PPARD/CO
RX5/FAM53B/MARK2/PSMB2/ZNF703/TMEM237/PPP1CA/LEF1/SFRP5/PSME3/SLC9A3R1/DVL3/FE
ASAL1/DBNL/PLXNC1/STYXL1/FIG4/MOB2/LLPH/VEGFA/HSPA5/DDX56/RAB11A/PACSIN1/PPP2R

MRPS9/MRPL50/MTRF1L/MRPS35/MRPS33/MRPS10/MRPL45/MRPL41/MRPL49/MRRF/MRPS6

AS/AGO1/POLR2B/FXR1/EIF4G1/CNOT2/HNRNPA2B1/SMAD2/ELAVL1/ZMPSTE24/NUP214/SMAD3/

TRT2/DUS3L/TARBP1/TRMT10C/ANKRD16/CDKAL1/TRIT1/ADAT2/ELP6/NSUN2/CLP1/METTL8/TS

9B/PRMT2/OSGIN2/GDI1/PPARD/LEF1/MT3/PAK4/EXTL3/CPNE1/KIF14/BCAR1/CDK5/LAMTOR1/

M17/CSNK2A1/NOTCH1/VEGFA/EXOSC9/GLI1/RAB11A/ZPR1/PPM1F/PPIB/SRF/RHOA/CD38/HBEG

MRPS9/MRPL50/MRPS35/MRPS33/MRPS10/MRPL45/MRPL41/MRPL49/MRPS6/TUFM/OXA1L/MRPS28/

TLR8/MAP3K7/CNPY3/MFHAS1/IRF4/CTSL/CTSB/BPIFB1/PTPRS/APPL2/RPS6KA3/TLR1/S100A1
1C/PTPRC/CXCL10/CASP3/MYC/HUS1/FIGNL1/EYA3/ANXA1/TLK2/CLK2

/PELI1/ZNF675/IGBP1/CCL3/PELI3/PSMD8/PSMA1/PSMB5/PSMA4/PSMD9/RPS27A/MTHFR/UBA52/

NSMF/CTTNBP2NL/SBF1/PTPN23/PTP4A2/YWHAB/DUSP12/PPP4R1/SDHAF2/PTPRE/PTPA/CABIN1/C

F2/CYP4A11/PCK1

DUFA13/APBB1/CCAR2/CYP27B1/CTDP1/IP6K2/NAIF1/EAF2/PPARG/RYK/FSTL4/SEMA3F/TCHP/HY

/RPL23/AKT1S1/RACK1/ZNF675/RGS3/IGF1R/MAPT/STK38/RUBCN/NF2/PKIA/ABL1/SFRP2/TNFAI

`1/CARD9/PSMB9/IGFBP3/DTNBP1/TNFAIP8L3/TRAF2/PSMD14/AREG/TIMP2/JAK3/CCL5/MAPKAPK

RPS3/MTCP1/TGFB3/MUC20/ADRA2A/TELO2/MAP3K6/IKBKG/RALB/CSPG4/DBF4B/CEMIP/TRAF7/CX

:B/DVL2/LRP8/PRKCI/ZMYND8/SEMA4D/SYT1/DNM1L/SYT3/GDPD5/KATNB1/STK25/ARHGDIA/SCN1

;FRP2/TNFAIP3/PRKRIP1/PTPN2/PTPN1/DUS2/PAK2/DUSP7/TARBP2/PPM1F/PRKAR1B/GSKIP/YWH

IIN8/HEATR3/HYAL2/IFNG/GEMIN6/SMN2/IPO7/PIK3R2/APPL2/NOLC1/NUP214/HDAC3/SMAD3/GE

IAD3/MYCN/MAEL/TP53

IIZ1/RBPJ/ARRDC1/PSEN2/NOTCH2/EGF/LLGL2/BMP2/YTHDF2/CHAC1/METTL3/NCSTN/EGFL7/NEU

\4C/PTPRS/MUL1/SHTN1/UNC13A/TTL/BCL11A/DPYSL2/DVL1/SYT17/WNT5A/PPP3CB/OLFM1/WNT3

;ETDB1/PRMT6/BRD7/BRD4/ASF1A/NSD1/TLK2/AKAP8/KMT2B/SUPT6H/KDM3A/WBP2/ZMPSTE24/SE

[illegible]SC3/PIWIL4/REXO2/DNA2/KRI1/DCLRE1B/EME2/CPSF3/RAD1/CHD1L/TATDN2/RPS27A/POP5/ER[illegible]

GFB3/MUC20/ADRA2A/MAP3K6/IKBKG/STK25/CSPG4/DBF4B/TRAF7/CXCL17/ABL1/STK24/CLSPN/

IT3/FLOT1/SH3KBP1/VLDLR/LYPLA2/SHC1/PIK3CD/RAC1/ACTB/CDK5/CTTN/PLXND1/RAB10/C12

AJB2/F3/LCK/IFI16/PSMC4/CASP1/GSK3A/OSBPL7/PML/PSMD10/RPS3/TFAP4/APP/ADRA2A/RB

TRPM4/CCRL2/GPR157/MYO5A/LETM1/CCL3/CEMIP/ABL1/CALM2/PLCB2/PTGER4/SPPL3/SLC8B1/

AN/EDEM2/ACAN/NUDT18

)1/TRAPPC3/STX4/TRAPPC2L/PDCD6/NSF/SNX11/SEC13/HPS1/STX7/HOOK2/DNM2/SRGN/GNAI3/F

3/OR7E24/OR10J1/OR1D2/OR1D4/OR10G8/OR51G1/OR5F1/OR7A10/OR5P2/OR10T2/OR4C46/OR10H

./PAF1/ZNF16/ACIN1/MOV10/DPY30/ANKRD54/AGO1/MYC/BGLAP/PRMT6/CD4/IFNG/KMT2B/PRKCB

TF3C4/ICE1/GTF3A/GTF2B/INTS3/RPRD2/INTS5/NCBP1/BRF1/PHAX

DUS1L/APOBEC3F/THUMPD2/NSUN5P2/METTL2B/AARS2/METTL6/PARN/THUMPD3/QTRT2/DUS3L/TAR
/TRIM46/RNF126/VPS29/DNAJC13/VPS54/RAB7A/ACTR2/RAB6A/SRSF10/STX16/SNX6/SNX2/EHD3

./EREG

./MRPS2/MRPL21/NDUFA7/AARS2/MRPS26/C1QBP/MRPS11/MRPL42/TRMT10C/MRPL22/NOA1/MRPL5

/UBQLN2/ULK1/PIK3C2B/WDR45/ATG3/RAB7A/GAA/FEZ1/CHMP4A/TFEB/SMCR8/PHF23/CCDC115/U

24/NUP214/SMAD3/MYCN/MAEL/TP53

3A/CHD7

7/RBM22/USP36/KIF5B/HYAL2/HTRA2/IFNG/ARIH2/PIK3R2/UBL5/HDAC3/BAP1/SMAD3/TP53/TAR

RIP1/LTBR/PIEZO1/RUVBL2/BAG3/GRB2/CNN2/TLR5/XRCC6/CARD16/FADD/RAD9A/CUL4B/LETM1/
RIP1/LTBR/PIEZO1/RUVBL2/BAG3/GRB2/CNN2/TLR5/XRCC6/CARD16/FADD/RAD9A/CUL4B/LETM1/

RAPGEF1/PSMC5/NPHP4/TLE6/KLHL12/CAV1/DAB2IP/DKKL1/BMP2/PSMB1/WWTR1/STK4/KREMEN2/

'RPF19/PELI3/PSMD8/KLHL42/WSB1/PSMA1/TNFAIP3/FOXF2/RNF216/PSMB5/PSMA4/PSMD9/RPS2
'3K6/IKBKG/STK25/ZNF675/IGBP1/IGF1R/STK24/FCGR2B/SFRP2/PDCD10/DBNL/PTGER4/PTPN1/
BL1/TBCD/VCL/TRIP6/CDH24/MPP7/CTNNA1/PTK2/THBS1/VEGFA/ACTN1/PPM1F/PARD3/TLN1/SF

'XN/GNB1/DEAF1/SWI5/B4GALT2/DNMT3A/IFI16/PPP1CC/NDRG4/PML/PPP1R1B/PARP1/CRIP1/TI
'YBX3/CHMP4B/HMOX1/BAX/MAPKAPK2/FAM83D/SIRT6/SOGA1/NUP210/DHX34/PCBP4/MTCL1/MSN/

1/ACADL/ACOX2/CYP4F3/DCXR/AGXT2/SORD/PCK2/SLC27A2/PON3/ABAT/EHHADH/HAO2/AKR1D1/

5/PIH1D1/TRMT12/MECP2/ETF1/BAZ2A/NSUN4/MLLT6/GATAD2A/TGS1/PRMT3/DNMT3B/COPRS/RN

/TNFRSF14/DHX9/CCL19/OSM/IL17RB/PANX2/CLEC4E/CRLF2/TNFRSF1B/TLR8/FFAR2/TRIM27/S

4/NUP214/SMAD3/MYCN/MAEL/TP53
4F/HEY1/SEMA5B/ARHGDIA/VIM/SEMA3C/PMP22/SEMA3E/CBFA2T2/STMN2/NFATC4/GDF11/DENND

[illegible]4/PCBP4/MTCL1/MSN/ATP6V1C1/RRAGD/FOXK1/PSMD2/HIF1A/MAPK3/NUPR1/HSPA8/RRAGC/RHB[illegible]
[illegible]/AGXT/HPD/TAT

K7/COX4I1/ATP6V0A1
ARD6B/CDC42/LAMB3/PRKCI/TGFB3/WDR1/EPB41L3/PARD6G/WNT4/VMP1/NF2/LIMS1/ABL1/TBC[illegible]

RBBP8/BRCA1/XRCC2/ACTL6A/IFT52/EIF2S2/PELO/E2F8/HOPX/NCAPG2/PLOD3/LEF1/RRP7A/SA[illegible]
IF14/LYN/RAC1/KCTD10/STMN3/TGFB2/RASA3/ARHGAP30/ABR/CADM4/SIPA1L3/CDC42SE1/FGD[illegible]

;M/SEH1L/EIF6

AP2/ZP3/SH3PXD2B/PRKDC/SLC9A1/EFNA5/RUVBL1/FLVCR1/MAD2L2/HIF1A/MRGBP/PPP2R1A/PAK
RL1/STAT1/HDAC1/DDIAS/NPLOC4/DYRK2/CARD19/HYOU1/DDIT4/SFRP5/ZC3H12A/SLC9A3R1/BI

/TIGAR/BPGM/SEH1L/EIF6

3P2/ZMPSTE24/SUPT3H/BAZ1A/IWS1/PHF20/KAT5/MSL3/ARRB1/MCM3AP/OGT/KANSL2/BRD8/MSL1

/UBE2J2/FERMT1/NPEPPS/SRI/ADAM8/GIPR/MCU/FRMD8/HCLS1/ITGB1BP1/RAC1/IL1B/CDK5/UBE
2B/NFKB2/ITGAX/LAMC2/LCP1/MMP10/SERPINE1/FGFR4/LAMA5/MMP2/ITGB5/FMOD/PLOD2/PLOD3

LILRB1/FUT7/CD80/NLRP3/IL12A/ZBTB7B/PRDM1/SOCS5/CD274/STAT3

NF19A/RAD23A/SVIP/DVL1/CLEC16A/OGT/MAPK9/SOCS5

GNA12/RBBP8/BRCA1/XRCC2/ACTL6A/IFT52/EIF2S2/PELO/E2F8/HOPX/NCAPG2/PLOD3/LEF1/R

H1/SFRP4/TMEM64/GLI1/MESP1/RNF213/PSMC3/STK3/RAPGEF1/PSMC5/NPHP4/TLE6/BARX1/KL
HBDD3/GLMN/INHA/TNFRSF14/IDO1/RUNX3/XCL1/IL13RA2/CD84/THOC1/INPP5D/DUSP22/PTPR

M/SEH1L/EIF6

:NTPD6/CANT1/HLA-DPB1/CLTA/MOSPD1/UNC93B1/ZDHHC7/B4GALT5/CREB3/GBP2/TMEM165/GALN
IS1/SIRT6/GINS4/PRKDC/RUVBL1/HIF1A/CBX1/UBE2I/CHD3/ACD/BRCA1/PCNA/MCRS1/CHAF1A/S
)QB1/TRPM2/IFI30/TUBB4B/GRN/NCF1/CYB561/HLA-DPA1/HLA-DMB/TPCN1/CTSK/CHIT1/ATP6VC
)X11/E2F4/PPARD/INCENP/CBX3/DVL3/HDAC2/KDM1A/NASP/HMGN4/CHRAC1/EHMT2/BUD31/AKAP8
:APH/GINS2/MCM10/FANCD2/SMARCD3/OIP5/CBX6/POLD1/MCM7/CCDC137/PLCB1/PIF1/MCM3/RAD
'56/RAN/SNHG7/RRP12/HMGB2/S100A3/HMOX1/LIN28B/MAP2/NAA10/FANCG/SNHG12/MRTO4/PRKD
)QB1/TRPM2/IFI30/TUBB4B/GRN/NCF1/CYB561/HLA-DPA1/HLA-DMB/TPCN1/CTSK/CHIT1/ATP6VC
;/FLOT1/CD68/VLDLR/HLA-DQA2/LAMP3/CPNE1/ATP6V1H/CLN3/SLC36A1/HLA-DRB5/SLC26A11/A
'4/RNASE6/HLA-DQB1/TRPM2/IFI30/TUBB4B/GRN/NCF1/CYB561/ATP6V0B/HLA-DPA1/HLA-DMB/T
RRAGD/SSNA1/RGS14/SLC1A4/CHD3/NPM1/PCNA/XRCC2/SGO1/SFI1/DYNLRB1/IFT52/DYNLL1/DYN
IFT57/CHEK1/RASSF7/RAN/HSF1/RAB11FIP5/SAC3D1/KIF18A/CRMP1/ESPL1/RABEP2/MAPKAPK2/
'AM83D/MTCL1/CLTA/MAD2L2/RGS14/MAP1S/CBX1/CENPE/NPM1/SGO1/DYNLL1/SPDL1/CAPN6/KNT
IP/ITGA5/FLOT2/MSN/IQGAP1/PTK7/SLC9A1/EFNA5/MAPK3/HSPA8/FZD1/PLEC/LCP1/MAP2K2/S
IRG1/MICALL1/HLA-A/ATP6AP1/CD63/ABCB6/CHMP4C/RAP2B/CORO1C/SCAMP4/HGS/NPC1/RAB7B/
-DQB1/TRPM2/RHOG/SLC26A6/RAB11FIP5/CD53/RAB34/SNX7/PLAU/GIPC1/TFRC/ATP6V0B/HLA-
'CORO1C/BMF/BIN1/PALLD/NPFFR2/ARPC2/MYO19/KNTC1/GAS7/PPP1R9B/TIGD5/ARPC3/WIPF1/I
ICAPG2/RNPS1/REXO4/INCENP/UNC45A/NELFE/FBL/BLM/DBF4/NHP2/NONO/DAPK3/CDK2/LSM10/S
10/RAB35/VMA21/PAM/MT3/SYTL1/FGFR3/HLA-DQA2/SV2A/SYNGR3/KDELR1/CLN3/HLA-DRB5/MC
1/QSOX1/ITGAV/VAT1/MVP/RAB31/UNC13D/CD109/TUBB/CSTB/TMSB4X/S100A8/LYZ/NME2/F13A
C26A6/RAB11FIP5/CD53/RAB34/SNX7/PLAU/TFRC/ATP6V0B/HLA-DPA1/DTNBP1/ABCC4/ATAD3B/
M7/PLCB1/PIF1/MCM3/RAD51AP1/RCC1/SPAG5/CENPO/EZH2/EXOSC4/CDK4/MAD2L1/DNMT1/ALYF
/MUC1/IFNGR2/RNF24/BSG/HLA-DQB1/SLC50A1/CHST4/GRN/GBP5/RAB11FIP5/GLIPR2/RAB34/M
IAJC5/TCN2/DDOST/EPDR1/ATP6V1C1/HLA-DPB1/CLTA/RRAGD/TMEM165/HSPA8/RRAGC/NEU1/GM2
RRAGC/HLA-DQB2/TCIRG1/MICALL1/HLA-A/AP4M1/ATP6AP1/CD63/ABCB6/CHMP4C/ANKRD27/RAP2
IIF1A/CBX1/UBE2I/CHD3/ACD/BRCA1/PCNA/MCRS1/CHAF1A/SGO1/SMARCA4/ACTL6A/IRF1/COPS9
P1/TFRC/HMGB2/YBX3/HMOX1/DOK1/FLOT2/MSN/PDE4A/RAB15/MOSPD1/SLC9A1/GBP2/NUPR1/PL
'APBB1IP/ITGA5/FLOT2/MSN/IQGAP1/PTK7/SLC9A1/EFNA5/MAPK3/HSPA8/FZD1/PLEC/LCP1/MAF
/RAB34/MGAT5/HLA-DPA1/AREG/TRAPPC4/RAB6B/RAB32/ENTPD6/CANT1/HLA-DPB1/CLTA/MOSPD
;/FLOT1/CD68/VLDLR/HLA-DQA2/LAMP3/CPNE1/ATP6V1H/CLN3/SLC36A1/HLA-DRB5/SLC26A11/A
:OTL1/ARHGEF2/KLC2/KRT80/HSPB1/COL4A5/REEP4/NME1/TRIM54/TPGS2/KIF11/GTSE1/HCK/CC
:OTL1/ARHGEF2/KLC2/KRT80/HSPB1/COL4A5/REEP4/NME1/TRIM54/TPGS2/KIF11/GTSE1/HCK/CC
IT3/PRKCD/TOR4A/SERPINI1/QSOX1/ITGAV/SCAMP5/SLC6A9/VAT1/LAMP5/MVP/RAB31/MTMR2/UN
:OTL1/ARHGEF2/KLC2/KRT80/HSPB1/REEP4/NME1/TRIM54/TPGS2/KIF11/GTSE1/HCK/CCT5/SPAG
/SPAG5/MAPRE1/TUBB/RUSC1/NME2/TWF2/ASPM/EZR/KRT7/DCDC2/MID1IP1/TUBB4B/KIF12/TBC
/RRS1/RRP9/DYRK2/MRPL47/RRP1/MRPL9/PITPNM1/RRP7A/SNRPE/FBL/ZC3H12A/TXNL4A/MT3/S
C/TIPIN/CHD4/RUVBL2/CENPB/RBBP4/TBX19/DFFA/UBE2A/SMARCC1/BRD9/KAT2A/BEND3/CBX5/
KAP5L/MAP2/IQGAP1/RGS14/MAP1S/MAP2K2/CENPE/NUDC/KLC3/TUBB6/TUBA1A/TUBB2A/TTLL1/
'SLC4A2/PAK1/GNA12/NPM1/THY1/ITGB5/CORO1C/PALLD/RPS2/ARPC2/PABPC1/RRAS/ARPC3/HYC
2/PAK1/GNA12/NPM1/THY1/ITGB5/CORO1C/PALLD/RPS2/ARPC2/PABPC1/RRAS/ARPC3/HYOU1/CC
'SLC4A2/PAK1/GNA12/NPM1/THY1/ITGB5/CORO1C/PALLD/RPS2/ARPC2/PABPC1/RRAS/ARPC3/HYC
PNE1/SLC11A1/RAC1/LAMTOR1/CD47/NFAM1/NRAS/SERPINB6/AGPAT2/RAB10/DYNC1LI1/SLC44A
/ARPC2/GABRA3/PPP1R9B/ARPC3/WIPF1/S100B/SH2B2/CORO1B/PIP5K1C/HIP1R/SLC9A3R1/DYN
PP2R1A/ACD/NSMCE2/PCNA/SGO1/DYNLL1/SPDL1/XRCC3/KNTC1/ERCC6L/NUP37/PPP1CA/INCENF
3AR1/HSP90AB1/RAB7B/CLCN7/MFSD12/SORT1/GPR137B/FLOT1/CD68/VLDLR/HLA-DQA2/LAMP3/
6/MRPS15/RPL5/RPS27A/MRPL18/RPS12/RPS4X/RPL6/RPL37/RPL27A/RPL36/UBA52/CANX/HSPA
RPL23/RACK1/LAS1L/CCT8/RPL38/RPL23A/MCTS1/HPS1/UBQLN4/RPS7/RPL7A/RPS23/RPS10/RPS
DK2/SMC4/KNSTRN/DCTN5/NCAPH2/NUP85/BANF1/PPP1CC/CENPK/NUP43/DYNC1LI1/DSN1/KMT5C

./ARPC5/STMN2/ACTR3/DBNL/CTNNA1/SRGAP2/DNM2/PTK2/INPPL1/CD177/PDLIM4/TUBB3/APBB1
?1CC/CENPK/NUP43/DYNC1LI1/DSN1/KMT5C/CENPB/ZNF207/CBX5/NUP107/HELLS/RAD21/MTBP/C
I8/MAGOH/IVNS1ABP/RBMX/ZRSR2/HNRNPA1/PPIE/SF3B3/HNRNPR/DHX15/GPKOW/RBM28/USP39/S
RPL37/RPL27A/RPL36/UBA52/RPL19/MRPL37/RPS11/RPL22L1/NSUN4/RPL13A/MRPL27/MRPL28/F
SMD13/COMMD9/DYNC1LI1/IMPDH2/ATP6V0C/PSMD11/GSDMD/ALDOC/PNP/XRCC6/PSMD3/CTSD/PSM
GSH/S100A7/GCA/VCP/PSAP/HSP90AA1/MAN2B1/CYB5R3/HEXB/BCAN/ACAN/AGA/LGMN/TPP1/MAN
'CALM2/CEP19/BOD1/CSPP1/TCTEX1D2/DCTN1/KATNA1/PLK3/HNRNPU/RAB11A/PLEKHG6/NEDD1/K
N1/MYO6/PACSIN2/LIMA1/CD2AP/PACSIN1/TLN1/RHOA/PDPN/FAP/EPS8L1/EPHA2/KSR1/ATP6V1
KATNA1/SH3GLB1/HNRNPU/ZFYVE19/GNAI3/MARK4/HSPA5/KIF20B/MITD1/ARL8B/VPS37B/EXOC3/
CYSTM1/CTSD/CLEC5A/TBC1D10C/KCNAB2/STX7/VAMP8/DBNL/CD33/CD177/OLFM4/AP2A2/ATP6AF
2B1/CYB5R3/GLIPR1/HEXB

2B1/CYB5R3/GLIPR1/HEXB
'DDB1/TFIP11/THOC7/LRWD1/THOC3/POLR2B/TERF1/HNRNPA2B1/LRIF1/PPP1CB/ORC4/XRCC5/TE
PDIA3/SNX3/ATP7A/MYO1C/RAB8B/SYT11/HLA-C/VAMP3/KIF5B/RAB22A/ATP6V0A1/HLA-E/RAB7A
'CENPQ/BOD1/DCTN1/HNRNPU

)/MRPL33/RPL13/MRPL36/RPL4/RPL41/RPL9/MRPL14/RPL24/RPL11/RPL10/MRPL3/MRPL20/RPL1

'SB/ATP6V0A1/CTNS/RPN1/RAB2A/RAB7A
'SB/ATP6V0A1/CTNS/RPN1/RAB2A/RAB7A
'RPL9/RPS6/RPL24/RPL11/RPL10/RPS24/RPS3A/RPL14/RPL10A/ZNF622/RPL12/RPS18/RPS28/F

3/PRPF40B/BCAS2/SMU1/PRPF40A/XAB2/SYF2/CWC15/CRNKL1/ZMAT2/SF3A1

2NPH1/CWC15/WDR83/CRNKL1/SF3A1/GPATCH1

T6/NCF4/EZR/HLA-DQB1/FZD7/CD320/GRN/RAN/RAB11FIP5/TBC1D16/MICALL2/TFRC/ATP6V0B/
UBAP2L/DYRK2/ZSWIM5/WDR77/ORMDL2/POLR3K/SNRPE/POLR2H/CCNO/UBE2J2/SNRPF/HDAC2/PI
3/SLC2A3/TSPAN14/MLEC/ADGRG3/DOCK2/SPTAN1/ITGAL/ATP11A/TCN1/DNASE1L1/PLD1/SLCO

1/GMCL1/SPARC/HNRNPA2B1/AKAP8/LRIF1/MBD1/SMC1A/TP53/TEP1/DAG1/MEN1/TPR

A9/PLD1/KIAA1324/VPS16/SNX16/MVB12B/OSBPL9/AP5S1/VPS33B/ARL8A/RAB7A/CHMP6/CYB5

/NFYC/POU2AF1/TAF12/TAF10/RARG/NFATC4/FOXF2/TBX2/LMO4/USF1/GTF3C5/HNRNPU/THRA/
9A/MXRA8/GNB1/FGR/PLXND1/EVC2/CLTB/NDRG4/PIEZO1/ARF4/RPS3/MUC20/DPEP1/SPRY4/GP

P93/NXT1/SORT1/P2RX5/NUP205/NUP37/NDC1/NUP188/CBX3/CSE1L/SLC22A18/TXNL4A/DUSP2/

A2/VPS11/AP3B1/CLTC/AP1S1/RAB27B/AP2B1/HBEGF/BTC/AP2A1/EREG/VPS33A/STEAP2/SNX3/

A-F/AP2A2/COPB2/AP3B1/CLTC/AP1S1/GOSR2/AP2B1/HBEGF/COPB1/BTC/AP2A1/EREG/COPA/K

BF1/SLC17A5/BCL2L1/HLA-F/CLTC/AP1S1/GOSR2/RAB27B/SNAPIN/TMEM184A/AP2A1/KLHL12/

1D/UBTF/NUAK1/SMARCA5/RNMT/ZNF415/UBE2N/RPP30/POLR2E/SPATA2/SURF6/IMP4/TAF1B/A

R2/PFN1/GAD1/NSMF/RIMS3/NCL/PARD6B/NRBP1/LLGL1/CDC42/PPFIA3/MYO9B/ACTL8/GMFG/A
RF1/STEAP2/COPA/KLHL12/PEF1/LMAN2/ATP7A/PKD1/ACTR1A/LMAN2L/ERGIC2/RAB8B/CD59/I

B/POLR3G/SETD5/LEO1/RPRD2/CRCP/POLR1D/POLR2J3/TAF4B/RECQL5

3G/SETD5/LEO1/RPRD2/CRCP/POLR1D/POLR2J3/TAF4B/RECQL5

/WNT7B/RAB11FIP4/ANXA11/WAS/CACNG4/CLTB/AP2M1/RAB10/DVL2/AP2S1/ATP6V0C/HLA-B/A
5/MYO1C/ANTXR1/DOCK8/NCKAP1/ABCA7/KCNC4/THEM4/ATP2B1/VEZT/MYO1D/ADGRE2

3G/SETD5/LEO1/RPRD2/CRCP/POLR1D/POLR2J3/TAF4B/RECQL5

CAMP2/ARFRP1/BICD1/RGP1/OPTN/GGA3/RAB11A/RAB29/YIPF2/GOLPH3L/CHST3/CLTC/AP1S1/

A2/RAPGEF1/CLTC/TAPBP/TAP2/AP2B1/HBEGF/AP2A1/RAB9A/CAV1/ATP7A/RAB8B/GPR161/HLA-

/COPG1/TMEM199/PDCD6/RASSF9/CEMIP/HSPD1/SEC13/VAMP8/WIPI1/DBNL/COPG2/DNM2/TNK2/C
DTNBP1/SYNGR1/DNAJC5/TRAPPC4/RAB6B/SLC1A7/MAP2/FLOT2/BRSK1/RAB15/SRPX2/CLTA/DGKZ
2/GYG1/ARHGAP9/HK3/CCT2/HSP90AB1/DYNC1H1/ILF2/AMPD3/NPC2/GPI/PA2G4/SERPINB1/PPIA

1/EZH1/KMT2A/JARID2/RBM15/WDR82/CHD8/KANSL1/AEBP2/SETD1B/KDM6A/RBBP5/C17orf49/MG

EIF4A3/PRPF3/PRPF6/BARD1/USP22/ARHGAP18/SF3A3/ITPKC/TIMM50/CXXC1/OGG1/AAGAB/HAUS
LYN/CARD11/VDAC1/EHD2/LAMTOR5/LAMTOR1/LCP2/MYADM/LCK/MYOF/CD79A/LRP8/APP/HK1/CD4

LYN/CARD11/VDAC1/EHD2/LAMTOR5/LAMTOR1/LCP2/MYADM/LCK/MYOF/CD79A/LRP8/APP/HK1/CD4

BP5/RAB27B/ATG9A/DPYSL3/PSEN2/SNAPIN
FAM111A/KDM4A/NOLC1/ESRRA/MTDH/RPP38/PRMT7/STOX1/DDX46/DUSP11/IPO11/PSPC1/HOXB5/

NAPIN
/CLN3/LYN/CARD11/VDAC1/EHD2/LAMTOR5/LAMTOR1/LCP2/MYADM/LCK/MYOF/CD79A/LRP8/APP/H
TAN1/CTBP1/GMFB/MAEA/ACTR1A/LLGL2/PPP1R9A/UTRN/EPB41/CAPZA2/C2CD2L/TCHP/DMTN/MED
2B1/THADA/C2CD2L/HTRA2/GNG2/RPS28/CARMIL2/ESYT1/GEM/IKBKB/GNAS/CHMP4A/HIP1/GNAI2

A/DCTN2/FKBP4/CNTNAP1/ITGA2/ACTB/CDK5/CTTN/SPOCK1/ADRA2C/STMN3/AP3M2/INPP5J/GAD1

F1/TAF3/ERCC3/TAF1B/TADA3/TRRAP/PPARG/JUNB/NFYA/ATF6B/TCEA1/GTF2A2/ING2/POU2F1/H
L3/MRPS9/MRPL50/MRPS35/MRPS33/MRPS10/MRPL45/NDUFAB1/MRPL41/MRPL49/MRPS6
L3/MRPS9/MRPL50/MRPS35/MRPS33/MRPS10/MRPL45/NDUFAB1/MRPL41/MRPL49/MRPS6

/GES2/GYG1/ARHGAP9/HK3/CCT2/HSP90AB1/DYNC1H1/ILF2/AMPD3/NPC2/HYOU1/GPI/PA2G4/SER
/BLOC1S4/LASP1/GPSM2/FLOT1/TUBB4A/CAP1/CAPZA1/RTKN/GYPC/PHLDB1/HCLS1/GLRX3/ACTB/
P6V0D1/MAPT/KCNAB2/ARPC5/STMN2/HSP90AA1/DNM2/AAK1/RAB3A/LMTK2/ZPR1/TUBB3/KIF20B

D18/MED27/FBXO45/TRIM21/TRPC4AP/KLHL21/UBE2D2/GLMN/RNF8/DCAF7/MED7/FBXL7/UBE2N/

11/TNP1

GES2/GYG1/ARHGAP9/HK3/CCT2/HSP90AB1/DYNC1H1/ILF2/AMPD3/NPC2/HYOU1/GPI/PA2G4/SE

2E2/VAC14/CDK5R1/POLR2I/POLR2J/ATG13/POLR2D/PRKAG1/PIK3R5/SOCS7/WDR41/PHKA2/GT

/NIN/DPYSL3/PI4K2A/PREX1/CALM3/CIB1/KLC1/CNR1/PPP1R9A/UTRN/ADGRL1/KIF5B/TIAM2/

L51/MRPL11/COA3/NDUFS8/MRPL15/C12orf65/MTX2/MRPL4/NDUFA1/NDUFA8/MFN1/UQCRB/MRP

P1A/WDR91/CCZ1/MYO5A/M6PR/RUBCN/STX7/VAMP8/PSAP/VPS35/LTV1/FIG4/MCOLN3/CCZ1B/V
AP1/KIF21B/GIPC1/DTNBP1/TIMP2/PLXDC1/TRAPPC4/NDN/MAP2/FLOT2/ABHD12/GNAZ/RGS14/

/PREX1/CALM3/CIB1/KLC1/CNR1/PPP1R9A/UTRN/ADGRL1/KIF5B/TIAM2/FKBP15/WHRN/DOCK7/

L17/SEMA4F/SH3GL1/RPS19/RPLP0/RPL23/EPB41L3/RPL38/NSF/HNRNPA3/RHEB/KCNAB2/TANC

)A/TMUB1/PDIA3/ATP11A/RAB13/SLC9A6/SLC9A9/VPS16/ATP9A/RAB8B/CD22/GPR161/SYT11/HL

PRIN1/HNRNPL/POLR2D/DHX9/EDC4/CNOT3/LSM1/KHSRP/HAX1/RPTOR/PABPC1L/LARP1/YTHDF2/

/PRKCI/SYT1/PPFIA3/SH3GL1/AP3D1/STX4/MYO5A/CSPG5/HTR3A/GABRD/TANC2/VCP/RNF216/D

S19/RPLP0/RPL23/EPB41L3/RPL38/NSF/HNRNPA3/RHEB/KCNAB2/TANC2/PALM/PRR12/DBNL/SRC

LC8B1/SYS1/TMEM11/RAB3A/LBR/SYNE4/SLC35B1/SLC35A4/ZDHHC9/ERGIC3/ABHD17A/COA1/RA

DAC2/PHF21A/KDM1A/MITF/HMGN4/HCFC1/EHMT2/SSRP1/ACTB/MTA2/WDHD1/NCAPH2/CBX4/NPM3

O4/PPARD/NOC2L/MED15/HEYL/HTATIP2/KDM1A/ATN1/MED8/KMT5A/HCFC1/CBFB/BUD31/MTA2/M

/PRKX/FGFR4/EPHB6/CAD/DYRK2/CSNK1E/MARK2/EPHB3/RPS6KA4/FGFR1/PIM2/CCNO/FGFR3/PA

T1/MYL6B/CARD10/RGS14/PLEC/LAMC2/CD3E/RPL36A/MRPL17/LAMA5/UPF3B/TUBB6/TUBA1A/TU

RHGEF19/TBC1D1/RIN1/MARK2/ARHGAP39/GDI1/PSME3/ARFGAP1/MT3/TBC1D10B/NCKAP1L/MLST

HGEF2/KLC2/NCK2/REEP4/NME1/VASP/PRNP/TRIM54/USH1C/KIF11/NUP62/CCT5/SPAG5/FHL3/M

PK2/STK17A/BRSK1/PKDCC/PDGFRL/PTK7/PRKDC/DGKZ/MAPK3/MAST2/MAP2K2/PAK1/PRKX/FGFR

OG/TUBB4B/KIF12/CD320/GBP5/GNA15/RAN/RAB34/KIF3C/KIF18A/MCM5/RHOQ/KIF21B/ATP6V0

OG/TUBB4B/KIF12/CD320/GBP5/GNA15/RAN/RAB34/KIF3C/KIF18A/MCM5/RHOQ/KIF21B/ATP6V0

OG/TUBB4B/KIF12/CD320/GBP5/GNA15/RAN/RAB34/KIF3C/KIF18A/MCM5/RHOQ/KIF21B/ATP6V0

4B/KIF12/CD320/GBP5/GNA15/RAN/RAB34/KIF3C/KIF18A/MCM5/RHOQ/KIF21B/ATP6V0B/RRAD/

PK8IP2/DLG3/HDAC7/EZR/CDKN2D/RHOG/HSF1/PRMT1/RHOQ/DOK2/TRAF2/TRIM6/TCF3/RHOC/SL

F1/PRMT1/RHOQ/DOK2/TRAF2/TRIM6/TCF3/RHOC/SLC12A2/MAPKAPK2/FAM83D/MSN/BRSK1/IQGA

P2/IQGAP1/FAM189B/CARD10/SH3PXD2B/PRKDC/SIGLEC10/HIF1A/LSM4/HSPA8/FZD1/TRIM11/C

/CHML/NCAPH/PRKCD/SERPINI1/HSPB1/ITIH5/B3GAT3/ADAP1/PLCB1/DEPDC1/PIF1/GMIP/CD10

AN/RANGAP1/GIPC1/FSCN1/ATIC/TIMP2/CHMP4B/WASF2/COL5A1/MFGE8/ITGA5/MSN/BZW2/PRDX

BX1/DENND2D/ARHGEF19/NXT1/TBC1D1/RIN1/GDI1/XPO6/CSE1L/TBC1D10B/EEF1D/DVL3/CYTH2

/PPP1R9B/TIGD5/ARPC3/WIPF1/LIMCH1/PDLIM3/CORO1B/ABLIM2/HIP1R/LASP1/FERMT1/TNNI2

UNK/MAP3K14/GSK3A/CDK18/NUAK2/TNIK/CIT/PLK4/STK32C/LMTK3/CDK7/PRKD2/STK10/PRKCI

1H1/XRCC3/MYO19/ATP13A1/ERCC6L/RFC2/DDX11/KIFC2/BLM/ATP6V1H/KIF14/ATP6V1E1/KIFC

/TBC1D10B/DVL3/EHD1/NCKAP1L/SYTL1/PAK4/ARHGAP1/RTKN/RANGRF/DAPK3/ARHGDIB/KPNB1/

/TBC1D10B/DVL3/EHD1/NCKAP1L/SYTL1/PAK4/ARHGAP1/RTKN/RANGRF/DAPK3/ARHGDIB/KPNB1/

A3/PFN1/ARHGAP30/AGFG1/ABR/SIPA1L3/CDC42SE1/DNAJC10/BAG3/SYDE1/RUNDC1/LLGL1/ARH
PN6/BEX4/AGBL5/KIFC2/TPPP3/GAS2L1/KIF14/CAMSAP2/HSPH1/LYN/B9D2/KIFC3/VBP1/KIF15
DH6/RPS2/HSP90AB1/STAT1/PUF60/PLCB3/CDH11/MARK2/KRT18/PPP1CA/CORO1B/UNC45A/EEF1
1/GNG5/GNB2/RAP2A/GNB1/NRAS/EIF2S3/DIRAS2/RAB10/TUBB2B/TUBG2/ARF4/RHEBL1/GNL2/R
RB2/TGFB3/CXCL11/XCL2/TLR5/CCRL2/FADD/ECM1/TIMM50/CCL3/PIDD1/TRIP6/TRADD/GDF11/
42SE1/SYDE1/RUNDC1/LLGL1/ARHGAP18/RANBP1/AGAP1/CDC42EP2/MYO9B/OCRL/DNM1L/PGAM5/
/MYO9B/OCRL/DNM1L/PGAM5/ARHGDIA/RIC8A/RGS3/TBC1D10C/RASAL3/TBC1D22A/CHN1/TBCD/NP

7/FHDC1/CETN2/DNM1L/PPP5C/KATNB1/OGG1/NDE1/CLIP3/DRG1/MAPT/CKAP5/HOOK2/DNM2/CCS
/TDG/EME2/RAD1/CHD1L/TATDN2/DQX1/NBN/RAD18/ERCC1/ACTR5/RFC5/DNMT3B/DHX9/POLE3/S
NL/CTNNA1/FLII/NEBL/ACTN1/ADD3/MYO6/LIMA1/CDK5R1/TNNC1/TLN1/WIPF2/DSTN/ACTN4
/SRGAP2/ARHGEF25/ARHGEF11/PAK2/PLEKHG6/DOCK2/EPS8L1/CAV1/ATP7A/ARHGEF17/PREX1/AR
3/GSKIP/YWHAG/ATG13/PRKAG1/RPLP1/FAF1/SH3BP5L/GMFB/EGF/RPTOR/CALM3/CIB1
4/SARNP/ERCC1/OPTN/RAB3A/CD2AP/EML2/HSPB7/SNX17/TAF13/AP2A1/KSR1/CTBP1/ERCC3/CI
C2/COPRS/IPO9/HPF1/CDYL/MSH6/TP53BP1/SRCAP/GRWD1/SFMBT2/PWP1/USP3/PHF1/LRWD1/IN
PL37/RPS11/RPL22L1/RPL13A/MRPL27/MRPL28/RPL37A/MRPL51/MRPL11/RPLP1/RPL32/RPL30/

APC4/PPARG/KIF3A/PPP1R9A/SMG7/PXN/CD22/MFHAS1
/DAB2IP/SYNJ2/INPP5D/ARHGAP27/ARHGAP17

K6/TRIM28/POLA2/CCL5/MAPKAPK2/STK17A/BRSK1/PKDCC/PDGFRL/PTK7/PRKDC/NT5C3A/DGKZ/
SN/EXOSC9/DXO/THOC2/MECP2/ETF1/RBMX/HNRNPA1/PPIE/RPL13A/HNRNPR/DHX9/RSL1D1/MEX3
1/PTK2/STYXL1/PTPN1/PDLIM4/MASTL/TRPC4AP/PARD3/AP3B1/ATP2B4/ANAPC5/CDC27/HSP90B
X8/DHX30/MOV10/CHD7/DDX31/SNRNP200/DDX20/SLFN11/DDX51
R12/UNG/SRP19/NOLC1/GEMIN5/ERI1/RNF135/EIF1/C1QBP/SRP72

1/PRKAR1B/DAXX/STK3/GSKIP/YWHAG/ATG13/PRKAG1/PIK3R5/SOCS7/RPLP1/FAF1/SH3BP5L/PI

GFRL/PTK7/PRKDC/NT5C3A/DGKZ/MAPK3/MAST2/MAP2K2/PAK1/PRKX/FGFR4/HK3/EPHB6/CAD/D

B2/CALR/DNM1L/RBX1/IKBKG/RPL23/ABI2/UBE2L6/ZNF675/RALB/RPA2/CUL4B/NAE1/UBXN1/H

K1/MAPT/PDCD6/KHDRBS1/CHN1/COL11A1/TRADD/WWC1/MPP7/BICD1/MAP1A/MAVS/OPTN/SH3BG

BKG/RPL23/ABI2/UBE2L6/ZNF675/RALB/RPA2/CUL4B/NAE1/UBXN1/HSPD1/SLC25A5/ZNF746/V

UBB4A/RTKN/RAB20/FKBP4/RAC1/EHD2/RAP2A/NRAS/EIF2S3/DIRAS2/RAB10/DYNC1LI1/TUBB2B/
UBB4A/RTKN/RAB20/FKBP4/RAC1/EHD2/RAP2A/NRAS/EIF2S3/DIRAS2/RAB10/DYNC1LI1/TUBB2B/

RAB20/FKBP4/RAC1/EHD2/RAP2A/NRAS/EIF2S3/DIRAS2/RAB10/DYNC1LI1/TUBB2B/TUBG2/ARL16
RAB20/FKBP4/RAC1/EHD2/RAP2A/NRAS/EIF2S3/DIRAS2/RAB10/DYNC1LI1/TUBB2B/TUBG2/ARL16

WIPI1/ZFYVE19/GOLPH3L/MITD1/TECPR1/PLD2/SESTD1/PARD3/TLN1/SNX17/HS1BP3/SNX22/SNX

/FKBP4/RAC1/EHD2/RAP2A/NRAS/EIF2S3/DIRAS2/RAB10/TUBB2B/TUBG2/ARL16/ARF4/RHEBL1/G
19/CDYL/CAV2/FRMD4A/CAV1/RPTOR/CNKSR1/ACTR1A/STUB1/DDB1/STAM/DDX20/ANXA1/ERCC2/

3BP/JAG2/CCL2/TXLNA/CXCL9/CXCL3/CXCL13/JAG1/WNT7B/CCL28/GAST/IL1B/CDK5/PDGFA/TNF

'2/ADPRH/IFT20/STXBP5/WDR41/SH3BP5L/SGSM1/YIPF1/LLGL2/EVI5L/KIF3A/TBC1D20/MADD/U

RAB20/FKBP4/RAC1/EHD2/RAP2A/NRAS/EIF2S3/DIRAS2/RAB10/DYNC1LI1/TUBB2B/TUBG2/ARL16
RAB20/FKBP4/RAC1/EHD2/RAP2A/NRAS/EIF2S3/DIRAS2/RAB10/DYNC1LI1/TUBB2B/TUBG2/ARL16

IT2/E2F4/NCAPG2/ZNF703/PPARD/LEF1/FBL/GAS2L1/BCL3/SRI/NOC2L/CPNE1/SIX2/HEYL/DAPK

./RNF4/TFAP2A/NFYC/MED12/RERE/POU2AF1/TAF12/RARG/ABL1/MTA3/NFATC4/CIITA/WWC1/SFR
)LR2K/DDX54/POLR3D/POLR3H/POLR3C/EXOSC3/REXO2/FARSA/CPSF3/DDX23/DQX1/DUS2/BMT2/P

2/ADRA2A/GAL/CCRL2/SH3GL1/WNT4/CCL3/PALM/ARAP1/PSAP/DNM2/VPS35/BICD1/ARHGEF11/GN
/CENPJ/TCERG1/DAXX/DNMT3B/DMAP1/CTBP1/CDYL/UXT/TLE6/CCAR1/LDB1/SFMBT2/SRSF2/SIN3

3/OR7E24/OR10J1/OR1D2/OR1D4/OR10G8/OR51G1/OR5F1/OR7A10/OR5P2/OR10T2/OR4C46/OR10H

/CXCL13/JAG1/WNT7B/CCL28/GAST/IL1B/PDGFA/TNFSF13B/CCL4/TGFB2/CALCA/CXCL16/IL15/T

P6V0E1/CDK7/MSH2/RBBP4/ABCA3/TTF2/DNAL4/POLQ/KATNB1/ABCC10/CCT8/ATP6V0D1/NSF/ABC

)3/NOSIP/RNF7/TRIM71/TRIM25/ZNF598/UBE2O/RNF122/MED12/PRPF19/PELI3/TRAF7/KLHL42/

C/NFKB2/CREB3/FOXK1/HIF1A/MND1/RBBP8/MNDA/PCNA/SMARCA4/ZNF93/ACTL6A/ETV7/IRF1/I

NA5/STIL/RGS14/MAP1S/CENPE/CHD3/PPP2R1A/PAK1/NPM1/CHMP4C/BRCA1/TUBB6/XRCC2/TUBA
2/AURKA/GPNMB/UNC119/PSRC1/HMMR/NCAPD2/TYMS/CDC25C/DTL/SKA3/FLNA/SPC25/CEP131/CD
ND2/RHOG/FMNL1/CRMP1/CFL1/MICALL2/RHOQ/S100A10/DOK2/DTNBP1/LINGO1/CARMIL1/WASF2/
5/JAK3/HMOX1/CD86/MAPKAPK2/NUTF2/MIF/ZP3/IQGAP1/HLA-DPB1/PRKDC/NFKB2/CD2/EBI3/HI
2/NUTF2/MIF/ZP3/IQGAP1/HLA-DPB1/PRKDC/NFKB2/CD2/EBI3/HIF1A/MAPK3/CD276/SERPINE1/
3/POLR3K/POLR2H/IRF7/BCL3/FERMT1/FLOT1/ADAM8/HDAC2/ATF4/FRMD8/SLC11A1/CARD11/GPR
PSME3/SLC9A3R1/DVL3/JAG2/PLEKHA4/SIX2/FZD2/MKKS/JAG1/WNT7B/TNC/RAC1/PSMB4/KDM5B/
/ATAD3B/CHIT1/PSMD14/TIMP2/JAK3/SYNGR1/HMOX1/CD86/APBB1IP/DNAJC5/HSPA6/DDOST/SEM
G4/ATAD3B/CHIT1/PSMD14/TIMP2/SYNGR1/HMOX1/CCL5/DNAJC5/HSPA6/DDOST/ALOX5/PLEKHO2/
300A/CANT1/IQGAP1/CFD/FTH1/STXBP2/PSMD2/ITGAX/HSPA8/NEU1/GM2A/TCIRG1/SLPI/FUCA2/
GAX/HSPA8/NEU1/GM2A/TCIRG1/SLPI/FUCA2/CD63/DOK3/STK11IP/RNASET2/FPR1/MNDA/PTGES2
/ATAD3B/CHIT1/PSMD14/TIMP2/JAK3/SYNGR1/HMOX1/CD86/APBB1IP/DNAJC5/HSPA6/DDOST/SEM
34B/GRN/TNFAIP6/CD53/PLAU/TFRC/ATAD3B/TRAF2/CHIT1/PSMD14/TIMP2/JAK3/SYNGR1/HMOX1
MD2/ITGAX/HSPA8/NEU1/GM2A/TCIRG1/SLPI/FUCA2/CD63/DOK3/STK11IP/RNASET2/FPR1/MNDA/
IF1A/JMJD6/MAPK3/IL27RA/CD3E/TCIRG1/ATP6AP1/SASH3/ISG15/TPD52/PRKX/UBASH3B/CD248
ZC3H12A/SNX10/BCL3/LAG3/NCKAP1L/JAG2/ADAM8/PRR7/EVI2B/PIK3CD/KDELR1/HCLS1/FANCA/
-DRA/MUC1/EZR/HLA-DQB1/HMGB3/VTCN1/CD320/GBP5/PRMT1/CARD9/PSMB9/TFRC/HLA-DPA1/HL
F3/C3AR1/HSP90AB1/F2RL1/ARPC2/RAB7B/LRRC14/CTLA4/ARPC3/WIPF1/NPLOC4/PSMB2/SH2B2/
LSCR1/CD209/LYN/HLA-DRB5/CARD11/FPR3/MUC3A/RAC1/CBFB/PSMB4/ACTB/BCAR1/CD47/PSMB8
/HMGB3/CHEK1/NPAS2/RAN/HSF1/RHNO1/NABP2/GINS3/CCT6A/MCM5/TFRC/PSMC3IP/AP5Z1/APOB
C2/DDX11/REXO4/POLR2H/PNKP/OTUB1/BLM/NONO/AUNIP/CDK2/KDM1A/FANCA/LIG1/SSRP1/WDHD
/CBX3/MT3/OTUB1/NOC2L/SCMH1/ITGB3BP/DAPK3/HDAC2/CDK2/PHF21A/KDM1A/NASP/CHRAC1/KM
3L/ZC3H12A/TXNL4A/NCBP2/USB1/DDX27/SNRPF/NOP2/GTPBP4/NHP2/PUS1/LSM7/POP7/NONO/LS
/RPL39/RPL35A/FARSB/MRPL13/GCN1/CDC123/EIF4A3/ILF3/GRB7/ZNF706/RPS3/SAMD4B/RPL27
100A8/LYZ/GIT1/NME2/F13A1/YKT6/CTSA/SYNGR2/TRPM2/SCRIB/QPCT/RHOG/ISLR/TUBB4B/GRN
CL26/MIF/DOK1/EFNA5/CREB3/MAPK3/EFNB1/SERPINE1/JAML/NRCAM/FPR1/ZNF580/PALLD/C3AR
MGB2/HMOX1/TRIM28/BAX/MAPKAPK2/SIRT6/FAAP24/MIF/PCBP4/CASP2/FANCG/BRSK1/WDR76/GI
D1/KIF3C/SAC3D1/KIF18A/CRMP1/KIF21B/DTNBP1/CHMP4B/ESPL1/GAS2L3/NCKAP5L/MAP2/FLOT
/PSMB9/PSMD14/TIMP2/CHMP4B/ESPL1/BAX/LCMT1/NAA10/PCBP4/CASP2/BRSK1/IQGAP1/MIIP/P
CHLH/ASPM/CDC7/LAMC1/FZD7/RHOG/PRAME/VTCN1/COMP/CD320/GRN/HSF1/PRMT1/AKR1C3/TFRC
CF2/PRPF6/POLR2L/DDX41/SF3A3/CPSF4/POLR2K/DNAJC8/FUS/ZBTB80S/CWC27/SNRPG/RBM17/H
10/PSMD11/PSMD1/GPX1/PSMA7/UBE2E1/FBXL6/DNAJC10/PSMB3/UBE2A/SMARCC1/ARRB2/KCTD2/
A/CDC7/TUBB4B/CHEK1/RHNO1/PRMT1/PSMB9/GIPC1/PSMD14/CHMP4B/ESPL1/BAX/FAM83D/MIF/I
/ACD/BAIAP2L1/BRCA1/NSMCE2/FCHSD1/BMF/BIN1/CCT2/LMNA/F2RL1/ARPC2/DYNLL1/DYNC1H1/
3/NUPR1/PPP2R1A/PEA15/CAPN10/MNDA/NSMAF/BMF/BIN1/CD248/ARHGEF19/E2F3/PSENEN/CTLA
I/SLPI/ISG15/NPM1/CHMP4C/TNFRSF4/RBL1/POLR2G/PCNA/ITGB5/SMARCA4/PPIH/TRIM59/PDCL
SME3/NCBP2/SNRPF/RPL17/RPLP2/PUS1/CNOT9/LSM7/NONO/RPSA/ATF4/LSM10/U2AF2/SF3B5/KD
2/KAT2A/BEND3/MTF2/ATXN7L3/CBX8/HELLS/PCGF2/PPP5C/TBL1XR1/DR1/PRDM4/VRK1/CXXC1/C
1/LTB/TNFRSF18/FSCN1/TRAF2/RBCK1/PSMD14/TRIM6/JAK3/HMOX1/CCL5/CD86/VAV1/CXCR4/AI
CA4/MRPS12/PELO/F2RL1/MRPL47/MARK2/DDIT4/PPP1CA/MRPL9/DVL3/FLOT1/ADAM8/MRPS23/CA
1/BYSL/RPL35A/LYAR/RRP36/CPSF6/EIF4A3/ISG20/GRB7/RUVBL2/PRPF3/EXOSC5/SNU13/PRPF6
VIPF3/LIMK2/CARMIL1/WASF2/RHOC/PLEKHG2/GAS2L3/CCL26/PHPT1/TPM2/ARPC4/MYL6B/PTK7/
0/UNC119B/EHD1/HAUS1/NCKAP1L/SPAG1/RSPH1/TUBB4A/CSNK1D/DNAAF5/MKKS/TBC1D30/DCTN2
/RHOC/PLEKHG2/GAS2L3/CCL26/PHPT1/TPM2/ARPC4/PTK7/SH3PXD2B/SLC9A1/EFNA5/MAD2L2/LC
15/TPD52/PRKX/UBASH3B/SOCS1/IRF1/F2RL1/STAT1/RAB7B/RRS1/RRAS/HDAC1/CTLA4/TNFRSF1
M1/PARP1/IL15/PTBP3/LTBR/LOX/MB/BATF3/DNASE2/SBNO2/CDC42/APP/HOXB7/PABPC4/LILRB4
LA-DPA1/HLA-DMB/TNFRSF18/VSIG4/CARMIL1/JAK3/CCL5/CD86/APBB1IP/VAV1/CXCR4/ITGA5/T
PIN/HM13/GNA12/ISG15/CHMP4C/PDCL3/HGS/DDA1/HSP90AB1/NPLOC4/CSNK1E/MAN1B1/ODC1/PS
J/IGFBP3/TNFRSF18/ARHGEF39/CARMIL1/HMOX1/RHOC/BDKRB1/CCL5/CXCR4/SEMA4A/CCL26/ITG
ND2/GRN/CRMP1/PRMT1/CFL1/MICALL2/DOK2/DTNBP1/LINGO1/AREG/P3H1/TRAPPC4/CXCR4/SEMA

ABP2/ANKRD27/NRCAM/THY1/CORO1C/RTN4RL2/F2RL1/ARPC2/DYNLL1/RELN/ZSWIM5/MARK2/GDI1
/BP/PSME3/HSPBP1/UBE2J2/NPEPPS/OTUB1/DTX2/FBXO5/GTPBP4/PRR7/TRIM31/ANAPC7/SPSB1/
/CSF1R/EZR/ANKRD1/SLC25A6/RHOG/VTCN1/RAN/GLIS2/RAB11FIP5/ANXA13/CCT6A/MICALL2/RA
L1/TOMM34/AP4M1/PES1/DKC1/SEC61G/ACD/GDAP1/TIMM17B/MCRS1/BMF/HGS/CCT2/YBX1/RPS2
/CARMIL1/SNRPD1/PLEKHG2/CCL26/MAP2/FKBP1A/GMNN/CLTA/ARPC4/MRTO4/EFNA5/SF3A2/RUV
R2G/HK3/YBX1/RPS2/PDE7A/PELO/TPI1/PABPC1/FOXK2/AMPD3/NUP93/NUP205/NUP37/RNPS1/E
SPH6/BYSL/RPL35A/LYAR/INTS1/USP14/FARSB/RNF113A/SMARCB1/RRP36/EIF4A3/ISG20/CDK7
/FTH1/STXBP2/PSMD2/ITGAX/HSPA8/NEU1/GM2A/TCIRG1/SLPI/FUCA2/CD63/DOK3/STK11IP/RN
COL3A1/GRN/TNFAIP6/GLIPR2/MGAT5/PLAU/IGFBP3/TNFRSF18/ARHGEF39/CARMIL1/CHMP4B/HMG
APK3/ITGAX/DUOXA2/LAMC2/P2RY6/SERPINE1/PAK1/CHMP4C/ZNF580/THY1/DUOX2/C3AR1/F2RL
1/IRF1/F2RL1/CTLA4/CD83/PLA2G2D/PRELID1/LEF1/PDCD1/ZC3H12A/BCL3/LAG3/SIRPG/NCKA
/FTH1/STXBP2/PSMD2/ITGAX/HSPA8/NEU1/GM2A/TCIRG1/SLPI/FUCA2/CD63/DOK3/STK11IP/RN
L26/MIF/DOK1/EFNA5/CREB3/MAPK3/EFNB1/SERPINE1/JAML/NRCAM/FPR1/ZNF580/PALLD/C3AR
/EIF2S3/DENR/RPL39/RPL35A/FARSB/MRPL13/GCN1/CDC123/EIF4A3/ILF3/GRB7/ZNF706/RPS3
PN10/MNDA/NSMAF/BMF/BIN1/CD248/ARHGEF19/E2F3/PSENEN/CTLA4/PRMT2/S100B/SORT1/RNF
F18/TRAF2/HNF1B/HMGB2/YBX3/JAK3/HMOX1/ATAD3A/CCL5/BAX/DNAJC5/ITGA5/MIF/BEX3/CAS
PN10/MNDA/NSMAF/BMF/BIN1/CD248/ARHGEF19/E2F3/PSENEN/CTLA4/PRMT2/S100B/SORT1/RNF
F18/TRAF2/RBCK1/HNF1B/HMGB2/YBX3/JAK3/HMOX1/ATAD3A/CCL5/BAX/DNAJC5/ITGA5/MIF/BE
E2A/SMARCC1/ARRB2/KCTD2/PSMD4/PSMD3/RBX1/PSMD7/TMUB2/TBL1XR1/PELI1/SGTB/RNF4/RA
LEKHG2/RABEP2/BAX/SEMA4A/SIRT6/CCL26/MAP2/LCMT1/MSN/IQGAP1/SRPX2/ARPC4/AMIGO2/S
1B/TPBG/HIP1R/BID/TPPP3/GPSM2/FLOT1/NCKAP1L/PIP4K2A/CXCL13/SNF8/PHLDB1/PMAIP1/T
/RPL36A/UBE2I/SLPI/FUCA2/ISG15/NPM1/CHMP4C/TNFRSF4/RBL1/POLR2G/PCNA/ITGB5/SMARCA
PA8/TRIM11/EIF3B/RPL36A/UBE2I/SLPI/FUCA2/ISG15/NPM1/CHMP4C/TNFRSF4/RBL1/POLR2G/
SG/COL16A1/ZNF296/SPAG4/SLC26A6/TNFAIP6/HSF1/KIF18A/WIPF3/LIMK2/CCT3/HMGB2/YBX3
/CHEK1/PRMT1/PSMB9/MCM5/PSMD14/CHMP4B/ESPL1/POLA2/BAX/FAM83D/BCAT1/LCMT1/PCBP4/C
13A1/CTSA/SYNGR2/TRPM2/SCRIB/QPCT/RHOG/ISLR/TUBB4B/GRN/TNFAIP6/RAB11FIP5/CD53/F
/TUBB4B/TNFAIP8L2/GRN/GBP5/NCF1/CARD9/PSMB9/HLA-DPA1/VSIG4/CTSK/APOBEC3C/PSMD14/
9/LYN/CARD11/MUC3A/PSMB4/CTSS/PSMB8/PSMB10/FGR/PSMA5/CD300LF/NRAS/SFPQ/PQBP1/PT
IPR2/PRMT1/S100A10/IGFBP3/HMGB2/CARMIL1/TIMP2/TCF3/P3H1/CD86/CXCR4/TNFSF9/PKDCC
C/TAF6/PEA15/NPM1/RBBP8/CHMP4C/BRCA1/RBL1/PCNA/BIN1/IRF1/PPM1G/CTDSPL/HSP90AB1/
/MAPK3/MAP2K2/CENPE/PPP2R1A/PAK1/PEA15/NPM1/SPRED1/FPR1/RAP2B/THY1/CORO1C/UBASH
F/FKBP1A/CD300A/FLOT2/MSN/PRDX1/ZP3/HLA-DPB1/PRKDC/CD2/EBI3/MAD2L2/JMJD6/CD276/
SPL1/LCMT1/NAA10/SGO2/MAD2L2/MND1/CENPE/PPP2R1A/GDAP1/CHMP4C/NSMCE2/SGO1/MSTO1/
E/TCIRG1/ATP6AP1/SASH3/ISG15/TPD52/PRKX/UBASH3B/CD248/SOCS1/IRF1/F2RL1/STAT1/RA
/ARHGEF19/LZTS2/PLOD3/PSMB2/PPP1CA/LEF1/FGFR1/TGM2/PSME3/SLC9A3R1/DVL3/JAG2/COL
B1/IL27RA/CD3E/PAK1/SASH3/TNFRSF4/SLC39A10/MNDA/THY1/UBASH3B/SIRPA/SOCS1/IRF1/F
APK3/ITGAX/SERPINE1/JAML/IL27RA/ZNF580/THY1/SIRPA/CD58/C3AR1/F2RL1/CCL13/TNFRSF
/SEMA4A/MAP2/LCMT1/NAA10/CD300A/EFNA5/MAD2L2/NUPR1/HSPA8/ACD/NPM1/SLC35F6/ANKRD2
MIM22/CD151/COL3A1/GRN/TNFAIP6/GLIPR2/MGAT5/PLAU/IGFBP3/TNFRSF18/ARHGEF39/CARMI
PK3/ITGAX/DUOXA2/LAMC2/P2RY6/SERPINE1/PAK1/ZNF580/THY1/DUOX2/C3AR1/F2RL1/RELN/Z
BP1/TNFAIP8L3/TRAF2/CCL5/MAPKAPK2/CXCR4/PHPT1/MIF/CD300A/IQGAP1/CARD10/EFNA5/DO
/BEX3/MTCL1/IQGAP1/RGS14/MAPK3/GM2A/P2RY6/PERP/SLC39A10/ANKRD27/PCNA/THY1/ARHGA
/STIL/MAPK3/MAP1S/PAK1/NPM1/BAIAP2L1/CHMP4C/BRCA1/FCHSD1/RHOF/BIN1/F2RL1/ARPC2/
AB15/STXBP2/SSNA1/HIF1A/SYT8/MAP1S/CENPE/ATP6AP1/PPP2R1A/NPM1/CHMP4C/STX1A/TUBA
GFA/RRS1/TMED9/PIP5K1C/SLC9A3R1/BLOC1S4/DYNLT1/GPSM2/PIK3CD/CSNK1D/MKKS/KIF14/K
/CXCR4/SEMA4A/MAP2/BRSK1/IQGAP1/SRPX2/PTK7/AMIGO2/EFNA5/SF3A2/IL34/RGS14/HIF1A/
HB3/NUMBL/TPBG/FGFR1/MYRF/DYNLT1/DVL3/VLDLR/CPNE1/HEYL/HDAC2/CSNK1D/KDM1A/LYN/C
HE41/LINGO1/HMGB2/CARMIL1/TIMP2/TCF3/P3H1/CXCR4/SEMA4A/MAP2/BRSK1/IQGAP1/PTK7/E
/CCT6A/MICALL2/RANGAP1/RHOQ/GIPC1/EPB41L2/DTNBP1/GPD1L/CCT3/SAE1/HMOX1/TRIM28/E
AME/VTCN1/CREB3L1/COMP/GRN/NPAS2/RPS6KA1/HSF1/CFL1/TNFRSF18/TRAF2/RBCK1/HNF1B/HM
SPA8/GLTP/MICALL1/PPP2R1A/ATP8B2/BAIAP2L1/CHMP4C/SLC35F6/ANKRD27/NRCAM/STX1A/GL

RPS2/HSP90AB1/FOXK2/TSPAN17/NUP93/NUP205/NUP37/PSMB2/NDC1/NUP188/ZC3H12A/SVBP/PS
BRCA1/TUBA1A/TTLL1/HGS/SFI1/DYNLRB1/IFT52/MSTO1/DYNLL1/DYNC1H1/RAB7B/RRS1/CSNK1E
3/HGS/SLC51B/NPC1/RPS2/CNPY4/RELN/GDI1/KRT18/SLC9A3R1/BID/RPL17/FLOT1/RPLP2/HSPA
AK1/NPM1/SPRED1/BRCA1/RBL1/PCNA/THY1/BCL2L12/SIRPA/NOL3/HSP90AB1/F2RL1/PARPBP/TN
PLEKHG2/COL5A1/NCKAP5L/CCL26/MAP2/FKBP1A/TPM2/ARPC4/SH3PXD2B/SLC9A1/MAD2L2/HSPA8
C/HLA-DPA1/HLA-DMB/VSIG4/JAK3/CCL5/CD86/VAV1/ITGA5/TNFSF9/CD300A/SELPLG/FLOT2/MS
/EHD1/HAUS1/SPAG1/RSPH1/TUBB4A/CSNK1D/DNAAF5/MKKS/TBC1D30/DCTN2/RAC1/MIEN1/B9D2/
ANKRD27/NRCAM/THY1/CORO1C/RTN4RL2/F2RL1/ARPC2/DYNLL1/RELN/ZSWIM5/MARK2/GDI1/EPHB
RCA1/RBL1/NSMCE2/PCNA/TUBA1A/SFI1/CTDSPL/DYNLL1/DYNC1H1/SPDL1/XRCC3/TICRR/KNTC1/
MMR/NCAPD2/TYMS/CDC25C/DTL/FLNA/SPC25/CEP131/CDKN2A/MCM6/TUBG1/RACGAP1/EIF4EBP1/
/PSMA5/PTPN6/PSMD13/CD27/PSMC4/CCR1/IL12RB1/CYP26B1/IL15/PSMD10/PSMD11/PSMD1/LOX
MCRS1/STX6/SLC51B/CCT2/LMNA/HSP90AB1/CNPY4/CSNK1E/LZTS2/GDI1/ZC3H12A/BID/CCT4/BC
1/GRN/TNFAIP6/GLIPR2/MGAT5/PLAU/IGFBP3/TNFRSF18/ARHGEF39/CARMIL1/HMOX1/RHOC/BDKR
DUOXA2/LAMC2/P2RY6/SERPINE1/PAK1/ZNF580/THY1/DUOX2/C3AR1/F2RL1/RELN/ZNF703/LEF1/
PRPF31/ZMAT5/SNRPB2/PRPF19/SART1/SYMPK/PHF5A/SRPK1/RBM42/CPSF3/DDX23/PTBP1/PRPF3
PRPF31/ZMAT5/SNRPB2/PRPF19/SART1/SYMPK/PHF5A/SRPK1/RBM42/CPSF3/DDX23/PTBP1/PRPF3
PRPF31/ZMAT5/SNRPB2/PRPF19/SART1/SYMPK/PHF5A/SRPK1/RBM42/CPSF3/DDX23/PTBP1/PRPF3
D13/PSMC4/ASCL2/PML/PSMD10/PSMD11/ERO1A/PSMD1/MB/PSMA7/PSMB3/TGFB3/PSMD4/DNM1L/P
NT1/IQGAP1/CFD/FTH1/STXBP2/PSMD2/ITGAX/HSPA8/NEU1/GM2A/TCIRG1/SLPI/FUCA2/CD63/DO
NFRSF4/SLC39A10/MNDA/THY1/SIRPA/SOCS1/IRF1/F2RL1/CTLA4/CD83/PLA2G2D/PRELID1/PDCD
F1/IRF3/C3AR1/HSP90AB1/F2RL1/ARPC2/RAB7B/LRRC14/CTLA4/ARPC3/WIPF1/NPLOC4/PSMB2/S
/SRRT/TTF2/PRPF6/BARD1/POLR2L/DDX41/SF3A3/CPSF4/APP/POLR2K/DNAJC8/FUS/CWC27/SNRP
1/NUP107/RPL23/SNRPG/PTTG1IP/SLBP/KHDRBS1/CHTOP/SEC13/IPO13/PKIA/EMD/SYMPK/CPSF3
KAP1L/ADAM8/CCL2/HSPH1/CD209/LYN/CCL28/CARD11/RAC1/CBFB/IL1B/IL2RA/CD47/TNFSF13B
/RHOF/RAB3B/F2RL1/RAB7B/RRAS/ARHGEF19/RELN/ARHGAP39/GDI1/SH2B2/RAB35/ARFGAP1/RAB
EG3/PSMB8/PSMB10/PSMA5/EIF2S3/PSMD13/NUP85/PSMC4/NUP43/SET/PSMD10/PSMD11/MRPL13/
N2B/CCL2/CDK2/EHMT2/PSMB4/PSMB8/PSMB10/PSMA5/PSMD13/PSMC4/PML/DYNC1LI1/PSMD10/PS
RNF157/PLXND1/GSK3A/NDRG4/TNIK/TUBB2B/DVL2/INPP5J/NSMF/LRP8/DGUOK/SEMA4B/PRKCI/P
C1/COL5A2/FLOT1/ADAM8/COL6A3/CAPN1/PHLDB1/TNC/ITGA2/CAPNS1/CTSS/PDGFA/CD47/SPOCK
SERPINE1/PAK1/ZNF580/THY1/C3AR1/F2RL1/RELN/ZNF703/LEF1/GPI/FGFR1/ZC3H12A/NCKAP1L
MRPL52/CKAP2/NSF/RUBCN/CKAP5/MCTS1/MRPL48/DAP3/MRPS7/MRPL53/GSPT2/VCP/STMN2/UBQL
/NAP1L2/ANAPC11/PARP1/SET/SMARCB1/PADI2/RUVBL2/PRKD2/KAT2A/ZNF207/MTF2/RAD21/RNF
AP2/HCLS1/PMAIP1/MITF/ARPC5L/ATXN2L/HCFC1/FKBP4/RAC1/GBA/JCHAIN/CTTN/MYADM/WAS/C
DA/CANT1/IQGAP1/CFD/FTH1/STXBP2/PSMD2/ITGAX/HSPA8/NEU1/GM2A/TCIRG1/SLPI/FUCA2/CD
GAX/HSPA8/NEU1/GM2A/TCIRG1/SLPI/FUCA2/CD63/DOK3/STK11IP/RNASET2/FPR1/MNDA/PTGES2
/PRMT1/PSMB9/MCM5/PSMD14/CHMP4B/ESPL1/POLA2/BAX/BCAT1/LCMT1/PCBP4/CASP2/BRSK1/GM
K2/LSM10/KIF14/HCFC1/RAB11FIP4/IL1B/TGFB2/SFPQ/POC1A/THOC5/PML/DYNC1LI1/FGFR2/CI
55/CALR/CPSF4/NUP107/RPL23/SNRPG/PTTG1IP/SLBP/KHDRBS1/CHTOP/SEC13/IPO13/PKIA/EMD
PRE1/TUBB/CDC25A/CDK4/GIT1/MAD2L1/ASPM/CDC7/FZD7/RAN/SAC3D1/CFL1/GIPC1/EPB41L2/C
NO/DVL3/DUSP2/BLM/MLST8/DBF4/GTPBP4/ADAM8/CDKN2B/SHC1/TESK1/LYN/WNT7B/BIRC7/CDKN
LMNA/GGCT/HDAC1/DDIAS/DYRK2/SORT1/HYOU1/PRELID1/DDIT4/KRT18/PPP1CA/PPARD/FGFR1/H
CN/MYADM/WAS/VIL1/CIT/PFN1/WASF1/INPP5J/GPX1/TACSTD2/RPS3/CAPZB/GRB2/FHOD1/CDC42
HDRBS1/EXOSC3/SUPT5H/PRPF19/BTG2/PSMD8/PSMA1/SRPK1/MYEF2/RBM42/PTBP1/PSMB5/PSMA4
P1/IL1B/CDK5/UBE2L3/TCP1/VIL1/GSK3A/STAC3/VPS28/PARP1/YWHAQ/YWHAB/ACSL3/CROCC/BA
3/NSF/GGA1/RAB3D/STAC/PALM/STX7/CSK/VAMP8/SYS1/VPS35/ARFRP1/OPTN/RAB3A/GGA3/TMBI
1/NAA10/SGO2/MAD2L2/MND1/CENPE/PPP2R1A/CHMP4C/NSMCE2/SGO1/MSTO1/SPDL1/XRCC3/TGFA
9/LYN/HLA-DRB5/CARD11/FPR3/MUC3A/RAC1/CBFB/PSMB4/ACTB/BCAR1/CD47/PSMB8/NFAM1/PSM
D11/EIF4A3/PSMD1/ISG20/PSMA7/EXOSC5/YWHAB/RPS3/SAMD4B/PSMB3/RPL27/RNH1/DROSHA/AG
RNH1/AGO2/RPL35/RPS20/PSMD4/RPL18A/PSMD3/PABPC4/PSMD7/RPS19/NT5C3B/RPLP0/RPL18/R
/NCKAP1L/SH3KBP1/CAP1/CAPZA1/MKKS/FHOD3/HCLS1/PSTPIP1/TESK1/ITGB1BP1/ARPC5L/RAC1
L1/ATAD2/DYNC1LI1/PFN1/INPP5J/MAP4/PARP1/SET/PSMD10/SMARCB1/GPX1/TACSTD2/CAPZB/

ZC3H12A/LAG3/SIRPG/FLOT1/NCKAP1L/GTPBP4/ADAM8/CCL2/CXCL13/HSPH1/JAG1/CD209/LYN/C
AT2A/PRKCI/TGFB3/ZMYND8/SEMA4D/SYT1/CDC42EP2/DNM1L/SYT3/KATNB1/WRAP73/STK25/ARHC
3/HCLS1/KDM1A/WNT7B/ARPC5L/RAC1/LAMTOR5/CDK5/CTTN/MYADM/WAS/VIL1/AP2M1/CIT/PFN1/
X54/ZBTB80S/RPS19/NOC4L/RRP7BP/TSR3/UTP11/LAS1L/UTP4/EXOSC3/NAT10/UTP6/HEATR1/SA
A/PSMD13/PSMC4/ASCL2/PML/OXTR/PSMD10/PSMD11/ERO1A/PSMD1/MB/PSMA7/PSMB3/TGFB3/PSM
RNF26/CCL4/PQBP1/CHMP3/IFI16/BANF1/PML/IL12RB1/OXTR/IL15/SMARCB1/ILF3/ISG20/TFAF
/TNFSF13B/FGR/LCK/PTPN6/CD27/IL12RB1/IL15/MSH2/RPS3/GRB2/CD6/CDC42/IL4R/PNP/LILF
S/PML/PARP1/DONSON/TIPIN/MSH2/RPS3/USP1/PRKD2/DFFA/ARRB2/PARM1/CBX8/POLQ/FUS/BAE
ID1/PDCD1/ZC3H12A/TNIP2/LAG3/SIRPG/NCKAP1L/ADAM8/CCL2/FANCA/HSPH1/CD209/LYN/CARD
DAM8/NHP2/VSIG1/MKKS/CCT7/PRIM1/RAC1/ACTB/PFKM/CDK5/JCHAIN/TCP1/DNAJB2/HOMER1/CA
31/PSMA5/LOXL2/TGFB2/DNMT3A/PSMD13/PSMC4/ASCL2/PML/OXTR/PSMD10/PSMD11/ERO1A/PSMD
GGTB/RPL23/RACK1/ROMO1/RPL38/NSF/RPL23A/CEMIP/ZDHHC12/GGA1/TIMM13/MFF/RPS7/CSK/F
CNA/TUBA1A/SFI1/CTDSPL/DYNLL1/DYNC1H1/SPDL1/XRCC3/TICRR/KNTC1/PPP1R9B/E2F8/CSNK1
3/YWHAH/BBC3/NLE1/KDM1A/PMAIP1/IL1B/WFS1/CTTN/TAF9/ZNF205/SFPQ/LCK/MAZ/GSK3A/PML
/LCK/PTPN6/CD27/IL12RB1/IL15/MSH2/RPS3/GRB2/CD6/CDC42/IL4R/PNP/LILRB4/AP3D1/AXL/
P4/POLA1/BARD1/MCM8/TOPBP1/PRIM2/POLQ/RRM1/RFWD3/RBBP7/RAD9A/RPA2/POLD3/TIMELESS
YNC1H1/SPDL1/XRCC3/RRS1/BEX4/DDX11/CEP85/NUP37/INCENP/NDC1/FBXO5/KIF14/KPNB1/SMC
/CDK2/NLE1/PSMB4/PSMB8/PSMB10/PSMA5/PSMD13/PSMC4/PML/DYNC1LI1/PRCC/PSMD10/PSMD11
A7/TNFSF13B/LCK/PTPN6/CD27/IL12RB1/CYP26B1/IL15/RPS3/SIT1/GRB2/CD6/DROSHA/CDC42/
/ARHGAP18/CDC42EP2/DNM1L/GMFG/ABI2/NDE1/CLIP3/DRG1/MAPT/ARPC1A/CKAP5/MZT1/BRK1/A
5/RPS3/CD6/PNP/GAL/HELLS/FADD/BTK/PELI1/RASAL3/GPR183/WNT4/BTN2A2/ZNF335/HSPD1/A
/MRPS7/TRAF7/NAT10/UTP6/HEATR1/MPV17L2/SART1/NOP10/KRI1/RPS7/RPL7A/WDR55/GAR1/RF
CALCA/CCR1/CXCL16/LOX/C5AR1/PADI2/PF4V1/PRKD2/ARRB2/CALR/CXCL11/CCR5/XCL2/CMKLR1
/PTPN6/IL12RB1/IMPDH2/CD79A/LST1/IL15/RPS3/CD6/PNP/GAL/HELLS/FADD/BTK/PELI1/RASA
NFSF13B/LCK/PTPN6/CD27/IL12RB1/IL15/RPS3/GRB2/CD6/CDC42/IL4R/PNP/LILRB4/AP3D1/FA
MAPT/CYCS/LONP1/ABL1/STK24/MSRB3/TNFAIP3/PDCD10/PSAP/PRKAA2/DNM2
2/PPP4C/INO80C/TIMELESS/EXOSC3/EID3/HSPD1/RAD51C/RAD51D/WRAP53/UBQLN4/EME2/SFR1/
TCTEX1D2/SPA17/CCSAP/BICD1/HNRNPU/MAP1A/DYNC1I2/KIF20B/KIF22/ARL8B/AP3B1/IFT20/F
RPS15/NOL6/MRPL18/HNRNPU/LTV1/SARNP/MAGOH/THOC2/XPO7/SLC35B1/SLC35A4/HNRNPA1/EIF
RT1/NOP10/KRI1/RPS7/RPL7A/WDR55/GAR1/SENP3/RPS15/RPS16/RPL5/GTF3C5/NOL6/RPF2/BMT
OT10/POLE3/CTBP1/SEH1L/EIF6/CNOT3/SUV39H1/POLR2E/PRKRA/MORF4L1/SND1/SPOUT1/SRCAF
/RPS15/DNM2/TCTEX1D2/VPS35/NOL6/HNRNPU/LTV1/SARNP/MAGOH/RAB11A/THOC2/KLHL21/EIF5
/ERCC1/ADGRG3/CD79B/CD38/INHA/RNF8/LYL1/RBPJ/NOTCH2/DOCK10/MSH6/TP53BP1/ATAD5/TH
C/PLK4/PSMD10/PSMD11/NES/PSMD1/DONSON/ODF2/CDK7/PSMA7/PSMB3/TOPBP1/APP/PSMD4/CET
C42/IL4R/PNP/LILRB4/AP3D1/AXL/FADD/BTK/PELI1/CLEC7A/RASAL3/GPR183/EXOSC3/BTN2A2/
6A/STK25/RACK1/MSX1/RAD9A/IL20RA/MYBBP1A/PTTG1IP/GRINA/ABL1/STK24/SFRP2/RPS7/NFA
L5/ABR/CDC42SE1/IL2RB/GRB2/CDC42/CALR/CDC42SE2/AXL/CLEC7A/RACK1/LBP/ARPC1A/RUBCN
RHEB/BTG2/PIDD1/ABL1/CHEK2/SGSM3/PRKAA2/VASH1/THBS1/NOTCH1/NBN/PLK3/TBRG4/CRLF3/
L3/PSMC4/AP2M1/RAB10/DYNC1LI1/AP2S1/PSMD10/PSMD11/PSMD1/KIF2A/HLA-B/PSMA7/PSMB3/
P90AA1/PRKAA2/RPS15/EIF3M/STYXL1/RPL5/RPF2/BICD1/RPL6/PIH1D1/TARBP2/RBMX/ZRSR2/E
/ACTR3/HSP90AA1/CHD1L/MPP7/SPIRE1/DCTN1/SH3GLB1/ERCC1/VEGFA/PIH1D1/MECP2/PPP2R5E
MCM8/PRIM2/MTBP/RFWD3/RPA2/CUL4B/E2F6/TRIM71/KHDRBS1/BTN2A2/BTG2/ACVR1/TAF10/PID
3/CD6/PNP/GAL/HELLS/FADD/BTK/PELI1/RASAL3/GPR183/WNT4/BTN2A2/ZNF335/HSPD1/ABL1/F
31/PKNOX1/RPS14/INPP5D/ZNF16/ADD1/PIK3CB/ACIN1/ASXL1/RPS6/CASP3/ADAR/SMAD5/BCR/N
3/PSMD7/LILRB4/IKBKG/BTK/EIF2B5/BTN2A2/PSMD8/ABL1/FCGR2B/PSMA1/KCNN4/CSK/PSMB5/E

/TOPBP1/RPL23/MSX1/RBBP7/RAD9A/RPA2/MYBBP1A/PTTG1IP/TAF12/BTG2/TAF10/PIDD1/DNA2/
APC11/ZBTB17/CCND2/CDC42/RANBP1/APP/MED25/RAD21/MTBP/RPL23/MSX1/CUL4B/MYBBP1A/WN
EP85/INCENP/FBXO5/DAPK3/KIF14/KPNB1/SMC4/KNSTRN/CDKN1C/AKAP8L/IL1B/NCAPH2/SIRT7/
L1/PSMA1/SFRP2/TNFAIP3/PSMB5/PSMA4/TBX2/PSMD9/NOTCH1/VEGFA/GLI1/AP2A2/MESP1/SULF
BP/RFWD3/RPA2/CUL4B/E2F6/TRIM71/KHDRBS1/BTN2A2/BTG2/ACVR1/TAF10/PIDD1/CHEK2/CCND

/PSMD1/DONSON/ODF2/CDK7/PSMA7/PSMB3/TOPBP1/APP/PSMD4/CETN2/PSMD3/RBX1/PSMD7/RAD2

AP28/DSTN/PLEKHH2/RHOA/MTPN/SPTAN1/ARF1/RHOBTB2/GMFB/HAX1/PREX1/ARFGEF1/ADD1/MY(
/RPL27A/TRIM21/RPL36/UBA52/TARBP2/RPL19/PSMC3/POLR2I/RPS11/POLR2J/RPL13A/POLR2D/
A2/IL4R/PNP/LILRB4/AP3D1/FADD/RASAL3/BTN2A2/HSPD1/SART1/CSK/BAD/BCL10/VCAM1
./EIF6/KHSRP/THOC1/DHX38/ENY2/PABPN1/SRSF1/SRSF2/CASC3/FIP1L1/RBM8A/MAGOHB/MYO1(
./EIF6/KHSRP/THOC1/DHX38/ENY2/PABPN1/SRSF1/SRSF2/CASC3/FIP1L1/RBM8A/MAGOHB/MYO1(

P1A/CABIN1/HELLS/CETN2/XRCC6/POLQ/RBX1/CENPN/RBBP7/CUL4B/DNA2/ABL1/SRPK1/CENPQ/(
/EIF3K/HSP90AA1/PRKAA2/RPS15/EIF3M/STYXL1/RPL5/RPF2/BICD1/RPL6/PIH1D1/TARBP2/RBM
CRN/AKAP8L/NCAPH2/SFPQ/CENPK/DYNC1LI1/DSN1/ANAPC11/TUBG2/KLHDC8B
3I2/WDR1/ARPC1A/WNT4/NF2/BRK1/ARPC5/ABL1/ACTR3/ARAP1/DBNL/PTGER4/SPIRE1/ADD3/LIM
2C/PSMD1/DONSON/PSMA7/PSMB3/ZNF207/TOPBP1/PSMD4/PSMD3/RBX1/PSMD7/RAD21/BABAM1/RF
/VCP/SFRP2/TRADD/BAD/BCL10/TNFSF15/THBS1/CSNK2A1/CASP8/PDCD2/VEGFA/PAK2/PIH1D1/F
PBB1/RNF8/DHX9/TIGAR/CCAR2/UBE2N/MMS19/ATR/TP53BP1/HMGA2/ATAD5/THOC1
3/NOP10/CPSF3/RPS15/NOL6/MRPL18/BICD1/HNRNPU/LTV1/SARNP/EXOSC10/MAGOH/PIH1D1/TH(
./KIF20B/SYNE4/PARD3/RHOA/DOCK2/PARVB/CCL19/HTT/FRMD4A/KIF26B/ARFGEF1/PKD1/ACTR1

/VCAM1
ONSON/PSMA7/PSMB3/ZNF207/TOPBP1/PSMD4/PSMD3/RBX1/PSMD7/RAD21/RFWD3/RPA2/NAE1/BTN
2A2/HSPD1/SART1/CSK/BAD/BCL10/VCAM1
ZWILCH/PRPF19/BTG2/PIDD1/DNA2/DOT1L/DCLRE1B/EME2/CHEK2/CLSPN/RAD1/NBN/PLK3/ZFYVE
/ETF1/RPL19/RPS11/EXOSC1/EIF3E/RPL13A/POLR2D/RPL37A/RPLP1/EDC4/RPL32/RPL30/CNOT1
C10/EXOSC7/DDX56/PIH1D1/UTP20/EXOSC1/NOB1/NSUN4/DDX10/WDR75/FAM207A/BMS1/NOL10/F
/SGTA/TOR2A/CSNK2A1/PFDN6/NPPB/GNAI3/HSPA4L/CSNK2B/HSPA5/TTC1/CANX/HSPA14/AIP/CD
RPL19/VPS37B/PARD3/RPS11/ZDHHC9/RPL13A/RPL37A/RPLP1/RPL32/RPL30/RPS9/RPS14/RPS27
/HSP90AA1/SPIRE1/DCTN1/VDAC2/ADD3/EML2/MECP2/CDK5R1/ARHGAP28/RHOA/MTPN/NIN/SPTAN
/CSNK2A1/CASP8/PDCD2/VEGFA/PAK2/PIH1D1/PRDX5/PPM1F/NDUFA13/RHOA/IFI6/ANP32B
OLR2I/PPIB/POLR2J/POLR2D/DHX9/IFITM3/KPNA6/NELFA/POLR2E/SNX3/CCL8/CAV2/CAV1/SUPT
ST1/PSMC5/TIGAR/RBPJ/VASN/SUV39H1/PDK3
3A/UBA52/PIH1D1/PSMC3/BAZ2A/DAXX/RNF8/SART3/ANP32B/SMARCA5/GTF2H1/DMAP1/PSMC5/P(
A1/PTGER4/THBS1/CD177/VEGFA/PLA2G1B
2/PSMD3/RBX1/PSMD7/RAD21/BABAM1/CEP250/HAUS6/NAE1/NDE1/CKAP5/DBF4B/RAD51C/PSMD8/
3G2
./NBN/MAGEF1/PIAS4/ERCC1/UIMC1/UBR5/TWIST1/APBB1/ACTR5/RNF8/RPA1/UBE2N/MMS19/MUS
RSF1/SRSF2/CASC3/FIP1L1/RBM8A/MAGOHB/SMG7/POLDIP3/THOC7/RBM22/AAAS/PARK7/EGR2/TH
7/RAD21/CEP250/HAUS6/NAE1/NDE1/CKAP5/DBF4B/RAD51C/PSMD8/HSPA2/PKIA/PSMA1/MTA3/CD

.9/GPR183/LBP/CCL3/S100A7/CXCL17/AKIRIN1/CYP19A1/SLC8B1/THBS1/ADAM17
.D2/CDK2AP2/CCSAP/DCTN1/FBXW5/BICD1/HNRNPU/MAP1A/MARK4/EML2/TRIM37/MECP2/CENPJ/(
.10/PSMA4/SPPL3/PSMD9/PTPN2/PAK2/RAB29/CACNB3/PSMC3/STOML2/PSMC5/UBE2N/CD247/INF
5/LSG1/RANBP3/THOC1/DHX38/PKD1/ENY2/PABPN1/SRSF1/SRSF2/CASC3/FIP1L1/RBM8A/MAGOHE
./EIF6/KHSRP/ATR/THOC1/DHX38/ENY2/PABPN1/SRSF1/SRSF2/CASC3/FIP1L1/RBM8A/MAGOHB/M
/WNT4/NF2/BRK1/ARPC5/ABL1/STMN2/NSFL1C/ACTR3/SPIRE1/DCTN1/MECP2/CENPJ/CDK5R1/PPM
ANP32A/DHX9/TNPO1/PSMC5/RBM10/MEX3D/RBM24/KHSRP/EXOSC2/ZC3HAV1/LARP1/YTHDF2/METT
/SIX1/AP2B1/PSMC5/AP2A1
F3E/RPL13A/POLR2D/RPL37A/RPLP1/RPL32/RPL30/EIF6/EIF4G2/EIF4G3/RPS9/EIF3G/RPS14/F
CSAP/DCTN1/HAUS7/HNRNPU/HAUS5/RAB11A/MECP2/RHOA/NEK6/CLTC

PSMD1/KIF2A/HLA-B/PSMA7/PSMB3/CAPZB/CALR/PSMD4/PSMD3/CTSD/PSMD7/IKBKG/AP1B1/SEC1
./MECP2/HNRNPA1/STOML2/APBB1/PPHLN1/RFC5/RNF8/DHX9/MAPKAPK5/TIGAR/FAF1/UBE2N/ERE

/5/BICD1/HNRNPU/MAP1A/MARK4/EML2/TRIM37/MECP2/CENPJ/CDK5R1/RBM14/RHOA/CLTC
35/FCGR2B/TNFAIP3/VCAM1/TWSG1
/PSMD4/PSMD3/CTSD/PSMD7/IKBKG/AP1B1/SEC13/PSMD8/HLA-DOA/FCGR2B/PSMA1/VAMP8/PSMB5
RS/VAV3/TSC1/SHTN1/TTL

/PSMD4/PSMD3/CTSD/PSMD7/AP3D1/IKBKG/AP1B1/SEC13/PSMD8/HLA-DOA/FCGR2B/PSMA1/VAMP8
/NEDD1/CENPJ/CC2D2A/VPS11/YWHAG/STXBP1/B9D1/SNPH/PSEN2/VPS33A/NPHP4/SNX3/CAV2/AC
C/SPIRE2/NCKAP1/PPP1R9A
3A/CCDC88A/LRPPRC/KIF5B/DYNC2LI1/ARL3/TRIM46/TUB/TTC21A/ARL8A/CNIH2/BBS12/TRAK2/
3/HAX1/PREX1/ARFGEF1/ADD1/MYO1C/SPIRE2/NCKAP1/PPP1R9A
P3K1/ELMO2/PSMC5/CD247/CD3G/PTPRC/CLEC4E/PIK3CB/MYO1C/PSMB1/NCKAP1/PLCG1/MAP3K7/

110/MEX3D/RBM24/KHSRP/EXOSC2/LARP1/YTHDF2/METTL3/HNRNPA0/PSMB1/TNFRSF1B/MOV10/PS
THDF2/METTL3/VRK2/PPARG/TRIM32/ADAR/AGPAT1/MADD
PRKAG1/MAPKAPK5/RPA1/BRPF1/TAF13/TBP/ATR/ATAD5/TAF3/CSNK2A2/BDKRB2/PIN1/RPL11/PF
RD7/BRD4/MAPK15/TERF1/HNRNPA2B1/AKAP8/WBP2/XRCC5/TP53/PARN/SLX4/ARRB1/TPR/RNF40/
31/THBS1/ADAM17
P1/DNAJC4/BHLHA15/HSP90B1/HSPA13/DAB2IP/FKBP14/FBXO6/STUB1/FAF2/CHAC1/ADD1/SSR1
X1/PREX1/ARFGEF1/ADD1/MYO1C/SPIRE2/NCKAP1/PPP1R9A
/RPL19/PSMC3/POLR2I/RPS11/POLR2J/RPL13A/POLR2D/RPL37A/RPLP1/DHX9/IFITM3/RPL32/RF
RBPJ/CCAR2/EGF/CAV1/ZBED2/BMP2/PSMB1/PIN1/CSNK1G1/PSMB6/PSMA3/MBD2/LYPD6/PSMB7/S
/VPS4A/CTDP1/SRPK2/ZCCHC3/DDB1/P4HB/CD180/VAPA/TBC1D20/ADAR/PRF1/IGF2R/POLR2B/CI
RCA5/GTF2H1/PSMC5/POLE3/RPA1/TBP/IPO4/GRWD1/ERCC3/TAF1B
C2/ATR/PARP10
/PS11/YWHAG/STXBP1/B9D1/SNPH/PSEN2/VPS33A/NPHP4/CAV2/ACTR1A/OFD1/TMEM67/CEP72/RA

BECN1/TFDP1/PRKRA/PDIA3/CAV1/DAB2IP/PTPRC/BIK/STK4/RPL11/PLA2G6/NOX1/PARK7/NMT1/
AP/HMGA2/MORC2/HDAC5/PABPC1L
./SMG7/HNRNPD/DCP2/HNRNPC/MYC/HUS1/MAPK15/TERF1/HNRNPA2B1
3/RTKN2/PSMA3/AGO1/RELA/PSMB7/SKP1/NOD1/NOD2/CARD14/RASSF2
/RBM24/KHSRP/EXOSC2/LARP1/YTHDF2/METTL3/HNRNPA0/PSMB1/MOV10/PSMB6/HNRNPD/PSMA3/I

OLE2/ATAD5/THOC1/MMS22L

FSF15/PTPN2/ADAM17/CASP8/TXNDC17/PIAS4/TNFRSF25/TNFRSF9/PSMC3/ACTN4/TNFRSF14/PSM
SHISA5/TRIM62/CD4/BRD4/RELA/TFG/NOD1/PRKCB/JMJD8/MUL1/SLC20A1/NOD2/PLEKHG5/IKBKE

THOC7/AAAS/THOC3/HNRNPA2B1/SUPT6H/U2AF1L4/SRSF3/NUP214/BICD2/IWS1/DDX19A/TPR/SRS

/HNRNPD/DCP2/HNRNPC/MYC/HUS1/MAPK15/TERF1/HNRNPA2B1

ARG/TRIM32/ADAR/AGPAT1/MADD

SMB6/PSMA3/PDE6G/AGO1/CELSR3/PSMB7/FZD9/GNG2/DVL1/WNT5A/PPP3CB/PSMD12/PARD6A/SMU

'1/ATAD5/BDKRB2/P4HB/TRIM32/RPL11/RTKN2/NOX1/PARK7/ING2/FIGNL1/HTRA2
:19

ARIH2/RNF186/UBL5/BAP1

/TSC1/IWS1/DDX19A/TPR/SRSF7/MCM3AP/NUP50

'T6H/U2AF1L4/SRSF3/NUP214/TSC1/IWS1/DDX19A/TPR/SRSF7/MCM3AP/NUP50

.9/RPS6/VAPA/RPL24/RPL11/RPL10/RPS24/RPS3A/RPL14/SRP19/RPL10A/RPL12/SRP14/RPS18/
/S1/DDX19A/TPR/SRSF7/MCM3AP/NUP50

24/RPL11/RPL10/RPS24/DCP2/PYM1/RPS3A/RPL14/RPL10A/EIF4G1/RPL12/RPS18/RPS28/RPS1

)/RPL10A/RPL12/SRP14/RPS18/RPS28/RPS17/RPL22/RPS15A/CHMP4A/SRP72/RPL31

)A/RPL12/SRP14/RPS18/RPS28/RPS17/RPL22/RPS15A/CHMP4A/SRP72/RPL31

3/RPS17/RPL22/RPS15A/SRP72/RPL31

.22/RPS15A/SRP72/RPL31

IGAP1/RTKN/SHC1/RANGRF/CDK2/ARHGDIB/KIF14/KPNB1/RAB20/LYN/RAC1/KCTD10/RAP2A/GNB1
MTF2/ATXN7L3/CBX8/PCGF2/PPP5C/TBL1XR1/DR1/PRDM4/VRK1/CXXC1/CUL4B/DPF2/NTMT1/SUV3
/SIPA1L2/MKKS/ARHGDIB/TBC1D30/ITGB1BP1/STMN3/CCL4/PLXND1/TBC1D13/RASA3/DVL2/ARHG
CARD10/EFNA5/DGKZ/RGS14/MAPK3/MAP2K2/CENPE/PPP2R1A/PAK1/PEA15/NPM1/SPRED1/RBL1/F
A2/IL34/RGS14/HIF1A/FZD1/TRIM11/PAK1/CRABP2/ADGRG1/ANKRD27/NRCAM/THY1/XRCC2/RTN4
LD/KDM1A/LYN/GPRC5B/IL1B/ADRA2C/RNF157/PLXND1/PPP1CC/NDRG4/NAP1L2/TUBB2B/DVL2/NS
P5/CARD9/PSMB9/TFRC/HLA-DPA1/HLA-DMB/VSIG4/CTSK/WIPF3/TRAF2/RBCK1/PSMD14/HMGB2/T
DAB1/TGFA/TNFRSF11A/RELN/RFC2/MARK2/NCAPG2/FGFR1/CCT4/MT3/PNKP/DVL3/NCKAP1L/MLST
MVD/SLC35C1/ST3GAL5/POMGNT1/MGAT4A/NAGPA/CSPG5/TNIP1/CHST12/CSPG4/MFNG/FUT1/LFNG
/PDCD10/CHEK2/CLSPN/MAPKAPK3/HSP90AA1/PRKAA2/CSNK2A1/VEGFA/PAK2/MASTL/LMTK2/MAP3
P1/PSMD13/IFI16/PSMC4/MUC5B/COCH/UBE2D1/PSMD10/PSMD11/PSMD1/PSMA7/TRAF3/PSMB3/AR
/ZNF593/ACTB/CDK5/TXN/DNAJB2/PLXND1/IFI16/PARP1/NES/EIF4A3/LOX/RUVBL2/TFAP4/ARRB
UGCG/GJB3
MB3/ARRB2/MUC20/TLR5/PSMD4/XRCC6/GPATCH3/PSMD3/PSMD7/IKBKG/FADD/BTK/PELI1/CLEC7A
/EXOSC5/TRAF3/APOBEC3D/GPATCH3/IKBKG/FADD/TRIM25/POLR3D/POLR3H/POLR3C/TNFAIP3/RN
BIM1/BCL2L1/RILPL1/RAB11A/ZDHHC23/TSPAN14/GOLPH3L/CACNB3/PACSIN1/GORASP2/IFT20/C
M3/KPNA6/NELFA/POLR2E/SNX3/CCL8/CAV2/SUPT4H1/HMGA2/PARP10/MORC2/NELFCD/GTF2F1/ZC
G13/STXBP1/ANP32B/DHX9/DMAP1/ADORA2B/ARF1/CIB1/CSNK2A2/HUWE1/TM9SF4/C2CD5/CNR1/M
UBA52/CACNB3/PSMC3/TWIST1/PSMC5/TIGAR/RBPJ/VASN/SUV39H1/PDK3
/PLK3/VEGFA/BNIP3L/EGLN2/UBA52/CACNB3/PSMC3/TWIST1/DNMT3B/PSMC5/TIGAR/RBPJ/VASN/
/SLC8B1/PTPN2/THBS1/ADAM17/NOTCH1

NEURL1/TCHP/DMTN/DYNC2LI1/CEP97/FAM98A/MAPK15/DOCK11/ATG3/EPS8L2/CARMIL2/PLXNB3/

3/GPRC5B/RAC1/CBFB/PSMB4/VANGL1/PSMB8/PSMB10/GNB1/PSMA5/PSMD13/PSMC4/GSK3A/AP2M1
IC4/GSK3A/PML/ANAPC11/VPS28/UBE2D1/USP14/PSMD10/PSMD11/PSMD1/MVB12A/PTPN23/PSMA7
SNK1D/ANAPC7/CDK2/KIF14/SPSB1/SNF8/AXIN1/PMAIP1/ADRM1/CCDC22/MYLIP/PSMB4/GBA/CPA
R45B/MAP1LC3B/PARP1/PSMD10/GPX1/YWHAQ/YWHAB/SDHAF2/BAG3/ARRB2/KAT2A/CHCHD3/INF2/
./CDK5/PDGFA/CD47/CTTN/MYADM/WAS/VIL1/CIT/PFN1/WASF1/NEB/TACSTD2/CAPZB/GRB2/FHOD
ADAM8/CCL2/CXCL13/HSPH1/CD209/LYN/ITGB1BP1/CCL28/CARD11/RAC1/CBFB/ITGA2/IL1B/IL2
./PRKAA2/CSNK2A1/VEGFA/PAK2/MASTL/LMTK2/MAP3K10/DGKQ/CDK5R1/PPM1F/NEK6/ATP2B4/MA
'PSMD7/IKBKG/FADD/BTK/PELI1/CLEC7A/TNIP1/LBP/HSPD1/PSMD8/PSMA1/TNFAIP3/MAPKAPK3/
R2B/TOX/BAD/PTPN2/CASP8/TMEM64/NKAP/RHOA/INHA/AP3B1/RUNX3/ZMIZ1/KITLG/CCL19/NOTC
I98/NKAP/SRF/RHOA/AP3B1/DOCK2/RUNX3/ZMIZ1/CCL19/PIK3R6/ATP7A/PKNOX1/PREX1/ZNF683
IRPL27/MRPL28/GAK/MRPL51/MRPL11/MTPN/SPTAN1/MRPL15/C12orf65/MRPL4/CAMSAP1/CIB1/M
AS/CD80/RASGRP1/LILRB2/NLRP3/IL12A/ZBTB7B/ROR2/SOCS5/STAT3/SCIN/MYB/ZAP70/HSPA1A
IAD3/ADGRA2/PSMD12/SMURF2/WNT3A/ROR2/GID8/DACT1/PSME2/JUP/PSMD6/ILK/PSMC2/PSMA6/

GTPBP2/RPL17/NPEPPS/RPLP2/EIF3D/MRPS23/CNOT9/RPSA/DAPK3/ATF4/RPL10L/ITGA2/RPS5/F

31/AXIN1/U2AF2/KDM1A/BIRC7/MED8/CCDC22/CBFB/MYLIP/PSMB4/FBXO32/KCTD10/CDK5/WFS1/

MC4/GSK3A/PML/ANAPC11/VPS28/UBE2D1/USP14/PSMD10/PSMD11/PSMD1/MVB12A/PTPN23/PSMA7

/MYLIP/PSMB4/GBA/KCTD10/CTSS/WFS1/UBE2L3/PSMB8/RNF187/PSMB10/PSMA5/DNAJB2/TAF9/F

3/GPRC5B/RAC1/CBFB/PSMB4/VANGL1/PSMB8/PSMB10/GNB1/PSMA5/PSMD13/PSMC4/GSK3A/AP2M1

/ABCC5/B4GALT5/FOXK1/PDP1/TMEM165/HIF1A/GALNT10/NUPR1/ACOT7/B3GNT5/GBGT1/CHST1/C

3P3/TNFRSF18/TNFAIP8L3/TRAF2/RBCK1/AREG/TIMP2/HMOX1/RHOC/CCL5/BAX/CD86/MAPKAPK2/

3A/GALNT6/FUT2/FUT6/AGO2/MUC20/TMEM258/CHPF/MVD/SLC35C1/ST3GAL5/POMGNT1/MGAT4A/N

28/TANGO2/AGFG1/A4GALT/MVB12A/TMEM43/STX18/NUP155/CDC42/VPS18/CHMP1A/PLEKHJ1/CTL

/ILF3/MVB12A/ISG20/NUP155/CDC42/VPS18/CHMP1A/GRK2/APOBEC3D/CCR5/NUP107/AXL/CLEC5

/PNP/CD8A/LILRB4/AP3D1/AXL/FADD/BTK/GPR183/WNT4/BTN2A2/MFNG/LFNG/ABL1/SART1/HLA-

ULBP2/NLRC5/PLSCR1/CD209/LYN/CARD11/GPRC5B/MUC3A/ITGA2/PLA2G7/PSMB4/IL1B/CTSS/CL

CKAP5/HSPB11/IQCB1/TMEM216/ACTR3/CEP19/TBC1D7/CEP41/IFT172/HSP90AA1/DNM2/TCTEX1L

C1/BTBD10/TFAP2A/MAPT/CCL3/BTG2/ABL1/ZNF746/FCGR2B/NFATC4/BAD/VPS35/CASP8/CAPN2/

CLS1/TESK1/ITGB1BP1/ARPC5L/RAC1/CDK5/PDGFA/CD47/CTTN/MYADM/WAS/VIL1/CIT/PFN1/WAS

CKAP5/HSPB11/IQCB1/TMEM216/ACTR3/CEP19/TBC1D7/CEP41/IFT172/HSP90AA1/DNM2/TCTEX1L

DAC10/NOTCH1/TMEM98/HEXB/TLR2/LGI4/PHGDH/CDK5R1/METRN/PARD3

MD11/LTBR/PSMD1/TNFSF12/PSMA7/RPS3/TRAF3/PSMB3/KAT2A/XCL2/PSMD4/PSMD3/CARD16/PSM

RIM25/PIDD1/ABL1/TNFAIP3/TRADD/BCL10/CASP8/RPS27A/MAVS/OPTN/TLR2/UBA52/GOLT1B/RF

/DAC2/GNAI3/TMBIM1/TXNDC12/IVNS1ABP/BCL2L1/PIH1D1/MEIS3

C3F/FAM111A/TRIM15
/PTCD1/PNPT1/ERI1/GRSF1/PARN/TRMT10C/RNF40/SRSF7/PAPOLA/LEO1/TUT1/AHCYL1/SRSF4/F
SOCS5/MYB/PRDX2/ZAP70/MALT1/HLA-G/GATA3/NFATC2

K3A/PML/ANAPC11/VPS28/UBE2D1/USP14/PSMD10/PSMD11/PSMD1/MVB12A/PTPN23/PSMA7/UBE2E
BL1/SYT8/PYGO2/MAST2/SLC4A2/PPP2R1A/PHC2/TSPAN8/TTLL1/CCT2/EIF2S2/DYNLL1/DPCD/UE
SP2/FANCG/ZP3/SH3PXD2B/RUVBL1/MAPK3/PYGO2/MAST2/SLC4A2/PPP2R1A/PHC2/TSPAN8/PCNA/
SP2/FANCG/ZP3/SH3PXD2B/RUVBL1/MAPK3/PYGO2/MAST2/SLC4A2/PPP2R1A/PHC2/TSPAN8/PCNA/
/FGR/CHMP3/PPP1CC/GSK3A/ABCA2/NDRG4/CPSF6/PTPN23/XPO5/RIMS3/BARD1/STX18/BAG3/IL4

575/RGS3/IGF1R/STK38/CSPG4/TRAF7/CXCL17/SFRP2/PDCD10/CSK/MAPKAPK3/DBNL/ERP29/THI

./PSMD4/TRPM4/PSMD3/RBX1/PSMD7/TBL1XR1/CSNK1G2/WNT4/MED12/JRK/PSMD8/RARG/EMD/VCI

ADAM8/YWHAH/GIPR/MCU/FRMD8/HCLS1/BBC3/PMAIP1/CCT7/ITGB1BP1/RAC1/IL1B/CDK5/UBE2L3
3/NFATC4/BAD/VPS35/CASP8/CAPN2/GABRB3/BCL2L1/EGLN2/RILPL1/HLA-F/ZPR1/RAB29/MECP2
/CSNK1G2/JRK/PSMD8/EMD/VCP/PSMA1/SFRP2/PSMB5/PSMA4/PSMD9/VPS35/NOTCH1/SFRP4/TMEM

/S/OPTN/GOLT1B/RHOA/NEK6/CXXC5/UBE2N/CCL19/DAB2IP/TMEM106A/LITAF/LPAR1/MAP3K7/TI

/ATG7/SNX3/SERPINB9/CCL8/CAV2/CAV1/THOC1/VPS4A/NECTIN2/PABPN1/IFITM2/DDB1/P4HB/N
K5R1/PPM1F/DSTN/RHOA
/RASSF5/DAXX/GLMN/SART3/PRMT3/UBE2N/PEF1/CAV1/PARP10/STUB1/RAB1A/HUWE1/HDAC8/SKI
L1/TCHP/DMTN/DYNC2LI1/CEP97/FAM98A/MAPK15/DOCK11/ATG3/EPS8L2/CARMIL2/PLXNB3/IFT1
/ACE/PPP2CA/USP33/MAU2/EIF3C/PDX1/TICAM1/MEN1/WNT5A/ARRB1

P8/ARRB2/SEMA4D/VPS25/APP/ADRA2A/AXL/BTK/DSTYK/CBL/ABI2/RACK1/IGF1R/CSPG4/NF2/

P8/ARRB2/TPST1/SEMA4D/VPS25/APP/ADRA2A/AXL/BTK/DSTYK/CBL/ABI2/RACK1/IGF1R/CSPG

CXCL16/PSMD10/PSMD11/LTBR/PSMD1/TNFSF12/PSMA7/RPS3/TRAF3/PSMB3/KAT2A/XCL2/PSMD

TL2A/THADA/PPA1/DUS1L/THUMPD2/PTCD1/GTF3C3/DDX1/METTL2B/AARS2/METTL6/GRSF1/RPP

NFAIP8L3/TRAF2/AREG/TRIM6/TIMP2/CCL5/MAPKAPK2/CXCR4/CCL26/ITGA5/MIF/CD300A/IQGAP
SIPA1L3/SYDE1/RUNDC1/LLGL1/ARHGAP18/RANBP1/SEMA4D/XCL2/AGAP1/CDC42EP2/MYO9B/OCRL
D/MORF4L2/MECP2/TARBP2/BAZ2A/POLR2I/POLR2J/PPHLN1/GLMN/POLR2D/DNMT3B/DHX9/SMARCA
ALNT7/ALG1/LARGE2/GXYLT2/MUC6/B4GALT4/MUC15/GFPT1/TMEM115/POMGNT2/SLC35C2/FUT11/
ALNT7/ALG1/LARGE2/GXYLT2/MUC6/B4GALT4/MUC15/GFPT1/TMEM115/POMGNT2/SLC35C2/FUT11/
AB1A/HUWE1/HDAC8/SKP2/PIN1

CD19/TSC1/TNFSF13/LILRB1/CD80/TP53/FGL2/NLRP3/CD40/TNFSF18/ZBTB7B/SOCS5/STAT3

;S14/HIF1A/MAPK3/EFNB1/RHBDF1/CD3E/TCIRG1/ATP6AP1/PAK1/CD63/IGFBP6/BAIAP2L1/FGFF
/BCAP31/FBXO5/JAG2/RSPH1/SCMH1/MKKS/ZFP41/DEFB1/OAZ3/FANCA/TESK1/RPL10L/ADGRG2/I
3/BEX4/PPP1R35/CEP85/DDIT4/ZC3H12A/SVBP/SLC9A3R1/PNKP/PPME1/OTUB1/DUSP2/NCKAP1L/
)C/PSMD10/PSMD11/PSMD1/PSMA7/RUVBL2/PSMB3/SDHAF2/APCDD1/CTDNEP1/APP/PSMD4/TRPM4/
.C/UBQLN4/VAMP8/WIPI1/PRKAA2/HDAC10/CSNK2A1/VPS35/SH3GLB1/TOMM22/OPTN/GNAI3/BNIF

'2R5B/ENPP2/TNFRSF14/HBEGF/SH3BP5L/KITLG/EREG/CRLF1/OSM/HAX1/EGF/CAV1/TMEM102

'1/CAV1/DAB2IP/GTF2F1/HDAC5/DZIP1/PKD1/STUB1/BMP2/ADNP/ADD1/PIN1/TFIP11/STK4/BTE

.7/GOSR2/BET1L/RAB9A/RAB13/ARL1/AP5B1/VPS4A/SNX5/VPS16/STAM/VPS26A/MVB12B/TRIM27
121/HLA-F/CDC37/NMI/RPL13A/STXBP1/NR1H2/IFITM3/STXBP4/CYP27B1/CCL19/XCL1/CCL3L3/
ZF1/GOSR2/TPP1/DNAJC4/SDF2L1/BHLHA15/HSP90B1/HSPA13/DAB2IP/FKBP14/FBXO6/STUB1/E
CR2/PPP2R3C/CD80/SOX13/RASGRP1/FGL2/LILRB2/NLRP3/RNF41/IL12A/ZBTB7B/PRDM1/SOCS5
.01/HLA-G/GATA3

/TRIM6/TIMP2/CCL5/MAPKAPK2/CXCR4/CCL26/ITGA5/MIF/CD300A/IQGAP1/CARD10/EFNA5/SH2D
./SLC11A1/WNT7B/ITGB1BP1/BIRC7/GPRC5B/IL1B/CDK5/PDGFA/TXN/MLKL/ADRA2C/LCP2/MAPK1

7A/USP21/CFTR/UIMC1/USP27X/COPS5/TRIM21/UBA52/PSMC3/USP11/RHOA/ACTR5/SART3/GPS1/
DHD1/PLA2G6/PARK7/USP36/NMT1/PAM16/HTRA2/ARIH2/FZD9/UBL5/LIG3/MUL1/BAP1
NC2LI1/CEP97/MAPK15/CNOT2/ATG3/CDK5RAP2/PTPRS/KIF9/SPICE1/SMC1A/IFT140/TSG101/CN

;DI1/NUMBL/FGFR1/DYNLT1/DVL3/VLDLR/CPNE1/HEYL/HDAC2/CSNK1D/KDM1A/LYN/GPRC5B/RAC1
'/RELN/S100B/CDH11/ZSWIM5/MARK2/GDI1/EPHB3/NUMBL/CORO1B/MT3/DYNLT1/DVL3/FLOT1/NC
R/CPNE1/HEYL/HDAC2/RAP1GAP2/CSNK1D/YWHAH/CAMSAP2/KDM1A/JAG1/LYN/GPRC5B/FKBP4/MYL
.N/S100B/CDH11/ZSWIM5/MARK2/GDI1/EPHB3/NUMBL/CORO1B/MT3/DYNLT1/DVL3/FLOT1/NCKAP1
'MAPKAPK2/VAV1/COLEC12/PHPT1/MIF/CD300A/ZP3/HLA-DPB1/ARPC4/UNC93B1/CFD/PRKDC/MAI
'4B/NAT10/MPV17L2/EIF3K/THBS1/PUS7/RPL5/JMJD4/SARNP/RPS4X/C8orf88/MAGOH/RCC1L/NA
.R/PSME3/BID/MT3/BCAP31/PRR7/COL6A3/LAMP3/RARRES1/BBC3/NLE1/PMAIP1/LYN/BIRC7/ADF
.A1/PMAIP1/RNASE2/IL1B/IL2RA/APOBEC3G/JCHAIN/RNF26/FGR/PQBP1/IFI16/MAP3K14/PML/F
'PSMD10/CDKN2C/SEMA4B/CDC42/SERTAD3/SEMA4D/SYT1/GAL/SYT3/SEMA4F/SEMA5B/NME6/RACK

'USP27X/COPS5/TRIM21/UBA52/PSMC3/USP11/RHOA/ACTR5/SART3/JOSD1/PSMC5/USP39/BECN1/
'OFUT2/FUT8/GALNT7/ALG1/LARGE2/GXYLT2/MUC6/B4GALT4/MUC15/GFPT1/TMEM115/POMGNT2/S

:RPM2/SCRIB/DNAJC6/RAB34/RHOQ/TFRC/MRC2/WIPF3/CARMIL1/WASF2/RABEP2/SLC12A2/MAPKA
/DAPK3/YWHAH/ITGB1BP1/RAC1/RAP2A/CDK5/S100A13/CTTN/RNF157/FGR/MYADM/PLXND1/VIL1/
/LDLR/SHC1/KIF14/AXIN1/SLC11A1/LYN/WNT7B/ITGB1BP1/BIRC7/GPRC5B/IL1B/CDK5/PDGFA/T

)/KRT10/IFT172/PSAP/ACVR1B/NOTCH1/UGCG/SFRP4/SLC4A7/MCOLN3/GLI1/TRPC4AP/ALOX15B/

3ICD2/RAB6A/NEFH/RASGRP1/FEZ1/NEFL
3ICD2/RAB6A/NEFH/RASGRP1/FEZ1/NEFL

ITRF1L/MRPS35/MRPS33/MRPS10/ABCE1/MRPL45/MRPL41/MRPL49/MRRF/MRPS6/OXA1L/MRPS28/M

/MFGE8/CD300A/CLTA/ARPC4/JMJD6/MAPK3/SYT8/P2RY6/SERPINE1/MICALL1/PAK1/CD63/STX1A
/CYP26B1/AGFG1/OSBP2/SBF1/ODF2/MYCBP/GOLGA3/MCM8/VCX/CALR/TDRKH/HSF2/MYBL1/CETN2
RACK1/VIM/TRIM71/DPH7/ZNF598/PLEKHN1/EIF2B5/RPL38/KHDRBS1/ABCG1/PIWIL4/BTG2/LARP
CFTR/COPS5/HSPA5/TXNDC12/BCL2L1/CANX/EDEM2/PPP2R5B/GORASP2/GFPT1/TLN1/ANKZF1/GOS
NF216/CD33/PTGER4/TWSG1/TUSC2/THBS1/SRGN/MAVS/IL20RB/CD2AP/HLA-F/TWIST1/NMI/INHA

Q/GIPC1/DOK2/ATP6V0B/IGFBP3/AREG/ATP6V0D2/JAK3/WASF2/MAPKAPK2/VAV1/NDN/SOGA1/ITG

.00B/CDH11/ZSWIM5/MARK2/GDI1/EPHB3/NUMBL/CORO1B/MT3/DYNLT1/DVL3/FLOT1/NCKAP1L/SH

./OSBP2/SBF1/ODF2/MYCBP/GOLGA3/VCX/CALR/TDRKH/HSF2/MYBL1/CETN2/TMEM119/TESMIN/AX
/COL6A3/LAMP3/RARRES1/BBC3/NLE1/PMAIP1/LYN/BIRC7/ADRM1/LAMTOR5/SPOCK2/PSMB8/SPOC

7/TRIM46/SEMA6B/SEMA4C/MAP4K4/PTPRS/TRAK2/PLXNB3/TSC1
./NR1H2/STXBP4/CCL19/XCL1/CCL3L3/CCL8/TRIM68

/EFNB1/MAP1S/PAK1/CRABP2/LAMA5/ANKRD27/NRCAM/THY1/CORO1C/PALLD/HSP90AB1/EPHB6/IS
2/NUP107/PCGF2/TRPM4/DR1/CXXC1/DPF2/GNL3/SUV39H2/ZNF335/TAF12/DOHH/TAF10/DOT1L/F

.9/OSM/TMEM106A/IL17RB/PANX2/CLEC4E/RAB1A/CRLF2/TNFRSF1B/TLR8/FFAR2/TRIM27/SYT11
R7/ATG7/ZC3HAV1/PTPRC/ZCCHC3/CXCL10/IFITM2/SKP2/TLR8/MX1/ABCF3/ADAR/PRF1/POLR3F/
EBF1/BCL2L1/TIMM10/AIP/NDUFA13/STOML2/SLC25A12/VPS11/YWHAG/ATG13/PSEN2/MTX2/TFDF
/ZFAT/KMT2A/PSME2/MYB/PDGFRA/PDGFRB/PSMD6/GATA3/PSMC2/PSMA6/NUDT21/YAP1/TP73/RBM

C1/HMGB2/TRIM6/BDKRB1/CCL5/CD86/MAPKAPK2/CXCR4/SLFN13/MIF/UNC93B1/BPIFB2/NFKB2/N
I1/EPHB3/NUMBL/MT3/DYNLT1/DVL3/FLOT1/NCKAP1L/SH3KBP1/VLDLR/LYPLA2/CPNE1/SHC1/PIK
C1/HMGB2/TRIM6/BDKRB1/CCL5/CD86/MAPKAPK2/CXCR4/SLFN13/MIF/UNC93B1/BPIFB2/NFKB2/N
MAIP1/CCT7/RPS5/TSPAN10/UBE2L3/RPS21/TCP1/RPL7/RPL28/CACNG4/NUP85/GSK3A/RAB10/PM

/SPOCK1/MYADM/PLEKHA2/ADAMTS9/CCDC80/SIGLEC1/PARVG/TACSTD2/LYPD3/AKIP1/CDC42/LAM

SWAP70/MAPK7/PELI1/ANGPT2/ARHGDIA/SEMA3E/NF2/BTN2A2/ABL1/TBCD/FCGR2B/AKNA/SIPA1/

5/CAPNS1/CDK5/LAMTOR1/CTTN/CHMP3/IFI16/GSK3A/WDR45B/OSBPL7/DRAM2/MAP1LC3B/VPS2
DIT3/APOBEC3C/CHIT1/HMGB2/TRIM6/BDKRB1/CCL5/CD86/MAPKAPK2/CXCR4/SLFN13/MIF/UNC
P2/EEF1D/ACLY/BCL3/GTPBP2/SCD5/RPL17/RPLP2/EIF3D/MRPS23/CNOT9/RPSA/DAPK3/ATF4/
5/CAPNS1/CDK5/LAMTOR1/CTTN/CHMP3/IFI16/GSK3A/WDR45B/OSBPL7/DRAM2/MAP1LC3B/VPS2

BCL3/LAG3/TSTA3/OTUB1/IL18BP/PRR7/HLA-DQA2/LAMP3/MYO1G/ULBP2/PIK3CD/CXCL13/KDE

/PIK3CD/CAMSAP2/TNC/RAC1/ACTB/CDK5/NREP/CTTN/PLXND1/RAB10/C12orf57/TUBB2B/FGFR2/
/IL1B/WFS1/NFAM1/MAPK11/COMMD7/DVL2/PSMD10/SMARCB1/LRP8/RHEBL1/RPS3/ANXA4/PRKD2/

/UBR5/RASSF5/SIX1/TNPO1/DMAP1/IPO9/NXT2/KPNA6/IPO4/TAF3/PAF1/PIN1/SUPT7L/ABCA7/T
1/DAXX/RAPGEF1/PIK3R5/MAP3K1/MAPKAPK5/ADORA2B/UBE2N/KITLG/CCL19/PIK3R6/IRAK2/EGF
/TRIM46/SEMA6B/LHX2/SEMA4C/RGS4/PTPRS/MUL1/SHTN1/UNC13A/TTL/BCL11A/DPYSL2/DVL1/S

[illegible]1A/MAPK3/HSPA8/OAZ1/LCP1/RHBDF1/IL27RA/TCIRG1/DKC1/PAK1/CAPN10/TNFRSF4/STX1A/S[illegible]
[illegible]/PAFAH1B2/EEF1A2/ABL1/IL10RA/NPRL3/UBQLN4/BAD/PSAP/PRKAA2/PTK2/VPS35/PLK3/SH3GL[illegible]

[illegible]NTS12/CT45A1/EXOSC8/ELL3/CDK9

[illegible]/CASP3/VPS26A/PIP4K2B/USP36/UBQLN2/ULK1/HTRA2/ATP6V0A1/TBC1D25/SESN2/NOD1/TSC1/[illegible]
[illegible]EFL/LRP4/WNT5A/OLFM1/WNT3A/KIF13B/NRP1/ZFYVE27/SHOX2/ILK/ZEB2/SLIT2/ZSWIM6/SEM[illegible]

[illegible]126/YIF1A/DERL1/CUL7/UGGT1/UGGT2

{2/CXCR4/FAM83D/CCL26/MIF/CD300A/PRDX1/IQGAP1/SH2D3A/LEFTY1/PSMD2/RGS14/MAPK3/P2
)3E/PAK1/SLC39A10/FPR1/MNDA/THY1/THEMIS2/IRF1/IRF3/C3AR1/HSP90AB1/F2RL1/ARPC2/RA
RO1B/HIP1R/SLC9A3R1/MT3/NCKAP1L/CAPZA1/RAP1GAP2/MKKS/FHOD3/SNF8/HCLS1/KDM1A/WNT7
RMT1/PLEKHA4/FRAT2/DAPK3/CSNK1D/FZD2/AXIN1/NLE1/MITF/WNT7B/GPRC5B/RAC1/CBFB/PSME
5B/METRN/APBB1/SRF/CAPRIN1/RHOA/RAPGEF1/GPC2/NIN/DPYSL3/AP2A1/IST1/ARF1/SNX3

MYCN/ERI1/MAEL/TP53

EN2/TRPT1/OSGEP/ELP5

CTTN/MEG3/SPOCK1/RNF157/PHB/DNAJB2/TGFB2/TAF9/GSK3A/PML/CXCL16/WASF1/PSMD10/CDKN

F/MTPN/RBPJ/IST1/EIF4G2

/MRPL30

/RBMX/PSMC3/CD38/PSMC5/UBE2N/CCL19/XCL1/CCL3L3/IRAK2/CCL8/DAB2IP

CTDNEP1/PPP1R16A/SWAP70/FAM220A/DMPK/PGAM5/PPP1R15A/PPP5C/IGBP1/TIMM50/PPP4C/PTF

YAL2/TRIM46/SEMA6B/SESN2/SEMA4C/RGS4/PTPRS/MUL1/PPP2CA/SMAD3/TP53/BCL11A

[illegible]P3/RPS7/CSK/BAG2/PRKRIP1/PTPN2/PTPN1/RPL5/DUS2/HNRNPU/PAK2/DUSP7/TARBP2/PPM1F/[illegible]

[illegible]2/CXCR4/FAM83D/CCL26/MIF/CD300A/PRDX1/IQGAP1/SH2D3A/LEFTY1/PSMD2/RGS14/MAPK3/P[illegible]

[illegible]CL17/CALM2/PDCD10/CSK/MAPKAPK3/CCND3/DBNL/ERP29/THBS1/ADAM17/PTPN1/RPS27A/VEGF[illegible]

.B/MAPT/TGIF2/PRPF19/CBFA2T2/STMN2/RASAL1/DBNL/PLXNC1/STYXL1/FIG4/MOB2/LLPH/VEGF

IAG/GPS1/HEG1/SH3BP5L/GMFB/DUSP18/CAV1/DAB2IP/RPTOR/EPHB2/CIB1/DUSP22/PTPRC/CDK5

MIN5/TP53/TARDBP/TNPO2/TPR/NUP50/STAT3/IPO11/SNUPN/SMN1/JUP/IPO5/NUP133

RL1/WDR12/MYC/ERH/WWP2/HIF1AN/NOD2/DLK2/EPN1/MAML1/APH1A/MIB2

3A
TD1A/TP53/IWS1/ARRB1/TPR/RNF40/OGT/ZBTB7B/WDR70/TRIP12/KMT2A/JARID2

C1/TSN/EXOSC9/EXOSC10/DXO/EXOSC7/UBA52/POLR2I/UTP20/EXOSC1/NOB1/RFC5/ENPP2/TOE1

/MAPKAPK3/DBNL/ERP29/TNFSF15/THBS1/PTPN1/RPS27A/VEGFA/PLA2G1B/PAK2/TRAF4/UBA52/I

orf57/TUBB2B/FGFR2/GRB7/EMB/SEMA4B/GRB2/PARD6B/LLGL1/SEMA4D/APP/MAPK7/SEMA4F/SEM
K1/CTSD/HSPE1/PDCD5/FADD/CLEC7A/RACK1/DAP/TNIP1/MAPT/PDCD6/CYCS/MTCH1/HSPD1/PIDI

/MCHR1/S1PR3/GRIN2D/MCOLN3/PLA2G1B/JPH1/CACNB3/CXCR6/TRPV6/CACNA1D/CD38/ATP2B4/A

RAB3A/CD2AP/RAB11A/GOLPH3L/VPS37B/VPS11/AP3B1/STXBP1/TAPBP/TAP2/GOSR2/FNBP1L/SNA

H2/OR5I1/OR5P3/OR2J2/OR4C15/OR8H1/OR2F1/OR2W1/OR1A1/OR4S1/OR7D4/OR10C1/OR51A7/OF

3/ESRRA/SETD1A/RASSF2/LILRB1/GNAS/RNF41/L3MBTL1/PURB/ROR2/STAT3/KMT2A/SCIN/RBM15

RBP1/TRMT10C/ANKRD16/METTL16/MRM1/CDKAL1/RBM15/TRIT1/PCIF1/ADAT2/ELP6
3/STX8/DENND2A/GBF1

58/MRPL38/MRPS30/MRPS27/MRPS18A/MTG2/MRPL43/GATC/MRPS9/MRPL50/FASTKD3/MTRF1L/MRF

JBXN2A/HPS4/GABARAP/ATG9B/ARSB/STX12/TMEM41B/NPRL2/AMBRA1/WIPI2/CHMP5/ACP2/ZKSCA

RDBP

/POLD3/SFRP2/NFATC4/HVCN1/SIPA1/PALM/CHEK2/BAD/RAD1/BCL10/PTGER4/DNM2/CASP8/FNTE
/POLD3/SFRP2/NFATC4/HVCN1/SIPA1/PALM/CHEK2/BAD/RAD1/BCL10/PTGER4/DNM2/CASP8/FNTE

/PSMB6/PFDN5/PSMA3

27A/FBXW5/TRIM21/UBA52/RNF213/UBR5/PSMC3/UBE2D2/CHFR/NMI/TRIM69/RNF8/UNKL/PSMC5/
/RPS27A/VEGFA/PAK2/TRAF4/COPS5/UBA52/MAP3K10/CCDC88C/TPD52L1/DAXX/RHOA/STK3/EMC1
RF/RHOA/RAPGEF1/ACTN4/HEG1/EPHA2/CORO2B/NPHP4/GPBAR1/CAV1/LDB1/RAB13

PIN/GPX1/MSH2/RUVBL2/USP1/BEST1/UBE2A/GRB2/TOPBP1/APP/XRCC6/HMGN1/CARD16/RRM1/
/ATP6V1C1/RRAGD/FOXK1/MAD2L2/PSMD2/HIF1A/MAPK3/NUPR1/HSPA8/RRAGC/OAZ1/RHBDF1/DKC

/HAO1/CYP4F2/SULT2A1/AGXT/CYP4A11/PCK1

MT/DMAP1/TYW3/RBM15B/DIMT1/MECOM/SUV39H1/TRMT2B/METTL21A/PAGR1/METTL4/PAF1/METT

YT11/HYAL2/IFNG/ANXA1/AIM2/TLR1/NOD2/ARG2/CPTP/LILRB1/RASGRP1/MAPKBP1/PPP1R11/N

5A/CTNNA1/SRGAP2/PTK2/NOTCH1/TMEM98/S1PR3/VEGFA/RAB29/IFRD1/NOTCH3/CDK5R1/RHOA/

)F1/DKC1/RNF19B/GNA12/NPM1/POLR2G/BMF/PDCL3/NPC1/DDA1/YBX1/GPSM1/HSP90AB1/PABPC1

)/VCL/TRIP6/CSK/CDH24/MPP7/CTNNA1/PTK2/THBS1/CD177/VEGFA/ACTN1/PPM1F/PARD3/TLN1/

ALL2/FGFR1/DVL3/JAG2/SIX2/FZD2/KRT8/NLE1/NASP/EMG1/WNT7B/HCFC1/CDKN1C/TULP3/STMN
5/GRB2/SYDE1/CDC42/ARHGAP18/CDC42SE2/MYO9B/OCRL/SPRY4/CBL/ARHGDIA/RASAL3/FGD1/OE

K1/CRABP2/SASH3/NRCAM/BASP1/SMARCA4/ACTL6A/SOCS1/PPP1R9B/OSGIN2/GDI1/PPARD/LEF1/
ID/BCL3/DUSP2/SRI/NOC2L/CPNE1/NONO/KDM1A/LYN/ITGB1BP1/CCDC22/DUSP4/IL1B/GBA/KCTD

1/KMT2A/CPA4/GTF3C4/BRCA2/GTF2B/SETD5/GATA3/KAT7/KANSL1

C2L3/FGR/MAPK11/TGFB2/CASP1/GSK3A/VPS28/OXCT1/PTPN23/GSDMD/ACSL3/BAG3/IL4R/TGFB3
3/SOAT1/BCL3/FERMT1/COL5A2/FLOT1/ADAM8/COL6A3/CAPN1/PHLDB1/TNC/ITGA2/PLA2G7/CAPN

RP7A/SALL2/FGFR1/DVL3/JAG2/SIX2/FZD2/KRT8/NLE1/NASP/EMG1/WNT7B/HCFC1/CDKN1C/TULP

HL12/CAV1/DAB2IP/DKKL1/CBY1/BMP2/PSMB1/WWTR1/STK4/KREMEN2/PSMB6/NXN/PFDN5/PSMA3
C/CNR1/LOXL3/NFKBID/CASP3/BCR

NT10/RHBDF1/HLA-DQB2/HLA-A/AP4M1/B3GNT5/GBGT1/CHST1/ABCB6/PTGES2/FUT4/STX6/B3GNT
SGO1/SMARCA4/ACTL6A/IRF1/COPS9/STAT1/XRCC3/RRS1/HDAC1/DDX11/E2F4/PPP1CA/PPARD/IN
0D2/SYNGR1/DNAJC5/TCN2/CXCR4/DDOST/EPDR1/ATP6V1C1/HLA-DPB1/CLTA/UNC93B1/FTH1/RRA
3L/SSRP1/ACTB/MTA2/TCP1/LOXL2/SWI5/SFPQ/DNMT3A/SIRT7/PML/UBA1/NSMF/SMARCB1/CPSF6
D51AP1/RCC1/SPAG5/CENPO/EZH2/EXOSC4/CDK4/C11orf80/MAD2L1/DNMT1/ALYREF/MUC1/CENPU
0C/OLA1/DHX37/SDCBP2/CD2BP2/MAD2L2/JMJD6/HSPA8/APEX2/MAP1S/CHD3/PES1/DKC1/CDCA7L
0D2/SYNGR1/DNAJC5/TCN2/CXCR4/DDOST/EPDR1/ATP6V1C1/HLA-DPB1/CLTA/UNC93B1/FTH1/RRA
ATP6V1E1/GBA/GNB2/LAMTOR5/LAMTOR1/TMEM9/NFAM1/GNB1/KXD1/AP2M1/ABCA2/DRAM2/SLC29A
TPCN1/CTSK/CHIT1/ATP6V0D2/SYNGR1/DNAJC5/TCN2/CXCR4/DDOST/EPDR1/ATP6V1C1/HLA-DPB1
NC1H1/LZTS2/DDX11/CEP85/KRT18/CCT4/SNX10/CEP89/GPSM2/FLOT1/HAUS1/RAP1GAP2/AUNIP/
/HSPA6/NR0B1/NCKAP5L/NDN/TRAF5/CCDC124/BRSK1/STIL/OLA1/RRAGD/RUVBL1/SSNA1/RGS14/
TC1/BEX4/DDX11/CEP85/INCENP/AGBL5/CBX3/CEP89/DYNLT1/GPSM2/HAUS1/FBXO5/RSPH1/AUNI
SLC4A2/PAK1/GNA12/NPM1/BAIAP2L1/THY1/ITGB5/CORO1C/CDH6/PALLD/RPS2/ARPC2/PABPC1/F
/PLEKHM2/PSENEN/SORT1/RAB35/PIP5K1C/IRF7/SNX10/EHD1/CD68/ARHGAP1/HLA-DQA2/LAMP3/
-DPA1/DTNBP1/ABCC4/ATAD3B/TRAF2/AREG/ATP6V0D2/SYNGR1/VOPP1/DNAJC5/RAB32/COLEC12/
LIMCH1/MARK2/PDLIM3/SH2B2/CORO1B/NDC1/ABLIM2/HIP1R/SLC9A3R1/LASP1/FLOT1/ACTG1/TN
SMC4/U2AF2/SRSF9/SNAPC2/SNRPC/GEMIN7/ITGB1BP1/NOP58/ATXN2L/EHMT2/SNRNP40/AKAP8L/
CTP1/TMEM163/VDAC1/WFS1/COPZ1/CLTB/RAB10/NCALD/KCNK9/BGN/APH1B/HLA-B/SYT12/ICA1/
A1/ADAM15/CTSA/BSG/TRPM2/QPCT/RHOG/ISLR/TUBB4B/GRN/TNFAIP6/RAB11FIP5/CD53/PLAU/A
/AREG/ATP6V0D2/SYNGR1/VOPP1/DNAJC5/RAB32/COLEC12/DDOST/CD300A/ARFGEF3/FLOT2/IQGA
REF/MUC1/CENPU/EME1/ZNF385A/MAGED1/HMGB3/CREB3L1/CHEK1/RAN/HSF1/NABP2/KIF18A/RAN
MGAT5/HLA-DPA1/LIMK2/AREG/TRAPPC4/RAB6B/RAB32/ENTPD6/CANT1/HLA-DPB1/CLTA/MOSPD1/
2A/HLA-DQB2/TCIRG1/FUCA2/CD63/ABCB6/STK11IP/SLC35F6/RNASET2/FPR1/MNDA/PTGES2/GYC
2B/CORO1C/SCAMP4/HGS/NPC1/RAB7B/PLEKHM2/PSENEN/SORT1/RAB35/PIP5K1C/IRF7/SNX10/EH
9/STAT1/XRCC3/HDAC1/DDX11/E2F4/PPP1CA/PPARD/INCENP/CBX3/DVL3/BLM/NHP2/HDAC2/KDM1
LEC/LAMC2/LCP1/MAP1S/MAP2K2/BSPRY/TPD52/CYHR1/PTGES2/STX6/RAB3B/RASEF/NPC1/HSP90
P2K2/SLC4A2/PAK1/PERP/GNA12/NPM1/BAIAP2L1/THY1/ITGB5/CORO1C/CDH6/PALLD/RPS2/ARPC
D1/UNC93B1/ZDHHC7/B4GALT5/CREB3/GBP2/TMEM165/GALNT10/RHBDF1/HLA-DQB2/HLA-A/AP4M1
ATP6V1E1/GBA/GNB2/LAMTOR5/LAMTOR1/TMEM9/NFAM1/GNB1/KXD1/AP2M1/ABCA2/DRAM2/SLC29A
CT5/SPAG5/FHL3/MAPRE1/TUBB/RUSC1/NME2/TWF2/ASPM/EZR/KRT7/COL3A1/ANKRD1/DCDC2/MID
CT5/SPAG5/FHL3/MAPRE1/TUBB/RUSC1/NME2/TWF2/ASPM/EZR/KRT7/COL3A1/ANKRD1/DCDC2/MID
NC13D/CD109/TUBB/CSTB/TMSB4X/S100A8/LYZ/NME2/F13A1/ADAM15/CTSA/BSG/SYNGR2/TRPM2/
G5/FHL3/MAPRE1/TUBB/RUSC1/NME2/TWF2/ASPM/EZR/KRT7/COL3A1/ANKRD1/DCDC2/MID1IP1/TU
CB/KIF3C/KIF18A/CCT6A/RHOQ/KIF21B/WIPF3/CCT3/CARMIL1/GAS2L3/NCKAP5L/MAP2/IFFO2/T
SNRPF/RPL17/RPLP2/NOC2L/EIF3D/MRPS23/NHP2/CNOT9/LSM7/POP7/RPSA/LSM10/KPNB1/U2AF2
/NCOR2/CBX8/PCGF2/TELO2/RBBP7/DPF2/INO80C/TIMELESS/POLR3D/ESCO2/RARG/MTA3/EME2/S
/DYNLRB1/CCT2/DYNLL1/DYNC1H1/CAPN6/KNTC1/BEX4/LZTS2/INCENP/KIFC2/CCT4/MT3/TPPP3/
DU1/CORO1B/PIP5K1C/PPIA/TGM2/LASP1/FERMT1/FLOT1/ACTG1/RPLP2/FGFR3/SH3KBP1/PAK4/F
DRO1B/PIP5K1C/PPIA/TGM2/LASP1/FERMT1/FLOT1/ACTG1/RPLP2/FGFR3/SH3KBP1/PAK4/FOCAD/
DU1/CORO1B/PIP5K1C/PPIA/TGM2/LASP1/FERMT1/FLOT1/ACTG1/RPLP2/FGFR3/SH3KBP1/PAK4/F
A2/ATP6V0C/C5AR1/TMEM63A/ABCA3/ICA1/STK10/GPR84/SYT1/LAIR1/CYSTM1/CPE/CLEC5A/TBC
NLT1/FERMT1/FLOT1/MYO1G/ATF4/PSTPIP1/CDC42BPG/ITGB1BP1/RAC1/PDE9A/BCAR1/CDK5/CTT
P/CBX3/BLM/NHP2/SCMH1/ITGB3BP/CDK2/KDM1A/DCTN2/KNSTRN/SSB/DCTN5/CENPI/DNMT3A/THO
/CPNE1/ATP6V1H/CLN3/SLC36A1/HLA-DRB5/RPN2/SLC26A11/ATP6V1E1/GBA/GNB2/LAMTOR5/LAM
A14/RPL19/MRPL37/RPS11/RPL22L1/NSUN4/RPL13A/MRPL27/MRPL28/RPL37A/MRPL51/MRPL11/F
S15/SGTA/RPS16/CASP8/RPL5/RPS27A/RPS12/CTBP2/CYB5R3/RPS4X/HBE1/RPL6/RPL37/RPL27A
C/CENPB/ZNF207/CHMP1A/TOPBP1/NUP107/RAD21/NIFK/CENPN/RAD9A/NDE1/ZWILCH/CKAP5/EID

./PLEKHH2/RHOA/PDPN/FAP/PARVB/DPYSL3/EPHA2/KITLG/HAX1/RAB13/CIB1/DUSP22/ACTG2/AN
CENPN/NDE1/ZWILCH/CKAP5/SUV39H2/ESCO2/SEC13
SNRNP25/LUC7L/SF3B2/MFAP1/PRPF4/DHX38/GEMIN2/SRSF1/SRSF2/CASC3/SNRNP70/DHX8/RBM8
RPL37A/MRPL51/MRPL11/RPLP1/RPL32/RPL30/MRPL15/C12orf65/MRPL4/SURF6/RPS9/RPS14/RP
MD7/GMFG/PRDX4/CCT8/TBC1D10C/PAFAH1B2/ARPC5/VCP/VCL/PDAP1/BIN2/DBNL/HSP90AA1/CSN
NBA/GPC2/HEXA/IST1
KIF20B/KLHL21/NEK6
.B2/CIB1/CYTH3/SNX5/C2CD5/ASAP3/MTMR14/MYO1C/NCKAP1/PLCG1/LAYN/ABCA7
/RHOA/RNF8
P2/SLC2A3/TSPAN14

EP1/APEX1/SLX4/MEN1
A/APPL2/TLR1/MPEG1

.4/MRPL24/RPL10A/MRPL55/ZNF622/RPL12/RPL26L1/RPL22/GEMIN5/MRPL21/RPL31/MRPL42/MR

RPS17/RPL26L1/RPL22/GEMIN5/RPS15A

/DDIT3/HLA-DPA1/HLA-DMB/DTNBP1/TPCN1/CTSK/ATP6V0D2/CARMIL1/JAK3/CHMP4B/SNX8/WASI
IK3CD/ANAPC7/CDK2/SPSB1/CHRAC1/PRIM1/HCFC1/TRMT61A/KRTCAP2/RPN2/ACTB/FBXO32/PFKM
AC1/TNFRSF1B/CD59

61A3/TSG101/STOML1/CHMP4A/MCOLN1

ERCC1/MED27/BCL9L/TAF1D/PTOV1/IVNS1ABP/GTF2E2/AIP/RBM14/TEAD3/SIX1/MED7/NR1H2/RU
R157/SLC27A4/SLC26A2/EPB41L3/TBC1D10C/MAPT/CSPG4/NF2/CDHR2/SGCE/PALM/WWC1/TTYH1/

/DTX2/GTPBP4/MRPS23/CPNE1/RAP1GAP2/HTATIP2/KPNB1/XPOT/ANXA11/DCTN5/UTP18/DNAJB2/

/EGF/EPN3/ATP7A

LHL12/PEF1/EGF

PEF1/CALM3/CUZD1/RAB1A/CD59/SYT11/HLA-C/MCTP2/VAMP3/RAB3C/VAMP1/HLA-E/CHGA/SEMA4

CACA/IP6K2/COIL/TRIM27/CC2D1A/WDR12/YY1AP1/MLLT1/CHCHD1/TERF1/FAM111A/POLR1C/WDR

RFIP2/WDR1/RASAL3/PARD6G/NF2/EMD/KDF1/ACTR3/BIN2/DBNL/PTK2/SPIRE1/DCTN1/CAPN2/CT
ER3IP1/YIPF5/CCDC88A/HLA-C/IGF2R/ARCN1/HLA-E/YIF1A/CNIH2/SEC23B/TEX261

TP6V0E1/RALA/ARRB2/CALR/CDC42EP2/OCRL/STX4/VIM/ATP6V0D1/WNT4/CEMIP/HVCN1/STX7/VA

AP1S3/AP1S2/TJAP1/COG4/ATG9A/BECN1/COG2/ARF1/RAB9A/ATP11A/YIPF1/PI4K2A/RAB13/ATP

-C/VAMP3/RAB22A/CD207/ATP6V0A1/HLA-E/RAB7A/APPL2/TLR1/CD163/FCGR1B/HIP1/RAB11FIP

:FTR/AAK1/RAB3A/SREBF1/MYO6/HLA-F/AP2A2/PACSIN1/ERGIC3/COPB2/VPS11/AP3B1/CLTC/AI
[illegible]/RGS14/SYT8/HSPA8/CD3E/SHARPIN/PDZD11/LAMA5/NRCAM/STX1A/STX6/RAB3B/BIN1/CAD/GAI
[illegible]/ACLY/DYNLT1/GTPBP2/C1orf35/CAP1/KPNB1/RNASE2/MAGED2/PDGFA/GNS/FTL/FGR/PSMA5/TC

;A/PAXIP1

;6/POLR3D/S100PBP/MAPT/PRPF31/SNRPB2/PRPF19/CHTOP/SART1/PHF5A/NFATC4/RUFY1/PRKAA
18/CTSD/CD8A/FADD/BTK/CBL/LDHB/CLIP3/MAPT/HPSE/SLC25A5/CSK/TRADD/BCL10/CTNNA1/AI

18/CTSD/CD8A/FADD/BTK/CBL/LDHB/CLIP3/MAPT/HPSE/SLC25A5/CSK/TRADD/BCL10/CTNNA1/AI

/MALT1/LEO1/SUPT20H/WDR43/YBX2/TAF4B/PLRG1/CDK12/MBD6/TOP1/CRIPT

IK1/CD48/CTSD/CD8A/FADD/BTK/CBL/LDHB/CLIP3/MAPT/HPSE/SLC25A5/CSK/TRADD/BCL10/CTN
)28/EXOC6/MYO1A/ACTR2/UNC13A/MYL12B/MYH9/HIP1/EXOC6B/MYZAP
2/DTNA/MYZAP/ACP1/NPTXR

./APP/GDPD5/AP3D1/KATNB1/CBL/MYO5A/SCN1B/CCDC120/EPB41L3/ATP6V0D1/MAPT/KCNAB2/AI

IYAL2/MAX/SMAD2/ERCC2/E2F7/HOXB9/BRF2/GTF3C3/MED17/SMAD3/GEMIN5/SUPT3H/TP53/TBPI

RPINB1/PPIA/ACLY/DYNLT1/GTPBP2/C1orf35/CAP1/KPNB1/HSPH1/RNASE2/MAGED2/PDGFA/GNS/
/TULP3/CTTN/MYADM/WAS/SFPQ/AP3M2/CLTB/RAB10/MAP1LC3B/FGFR2/PFN1/WASF1/MAP4/GAD1/
3/PACSIN1/CDK5R1/PARD3/PRSS12/EXOC3/APBB1/HNRNPR/AP1S1/ACAP3/NIN/DPYSL3/PREX1/CA

/ANAPC5/CDC27/UBE4B/AUP1/ZER1/MAEA/KLHL12/PEF1/PCGF3/FBXO6/STUB1/ANAPC4/DDB1/FBX

RPINB1/PPIA/ACLY/DYNLT1/GTPBP2/C1orf35/CAP1/KPNB1/HSPH1/RNASE2/MAGED2/PDGFA/GNS/

F2H1/PHKG2/POLE3/BECN1/TAF13/TBP/MMS19/PIK3R6/POLR2E/POLE2/GTF2F1/TAF3/ERCC3/ZNF

FKBP15/WHRN/DOCK7/TRAK2/TSC1/SHTN1/EXOC6/RAC3/MAP3K12/FEZ1/NEFL/DVL1/OLFM1/NPTXR

S24/MRPL33/TIMM23/NDUFB4/TIMM8B/MRPS5/MRPL36/NDUFA9/NDUFB11/MRPL14/NDUFA12/COX5A

AC14/RAB11A/YIPF2/MITD1/ARL8B/VPS37B/VPS11/GOSR2/RAB27B/LGMN/IFITM3/ATG9A/VPS33A

SLC1A4/HSPA8/CD3E/MAP1S/PPP2R1A/SHARPIN/PAK1/THY1/FCHSD1/MCRS1/RTN4RL2/RBM3/IFT5

TRAK2/TSC1/SHTN1/EXOC6/RAC3/MAP3K12/FEZ1/NEFL/DVL1/OLFM1/NPTXR/ABI1/TAOK2/MAPK8I

2/PALM/PRR12/DBNL/SRGAP2/DNM2/VPS35/GRIN2D/RPL6/PAK2/LZTS1/ADD3/FBXO45/CDK5R1/AB

.A-C/VAMP3/ULK1/VPS33B/DENND6A/HLA-E/SORCS2/ACKR3/GRIPAP1/RAB11FIP1/ARHGAP44/ACA

/CASC3/DHX30/MOV10/RPS6/USP3/OGFOD1/BTBD1/POLDIP3/FASTKD5/DCP2/AGO1/DYRK3/APOBEC

)NM2/VPS35/CTBP2/ACTN1/PAK2/PACSIN2/FBXO45/BCAN/CANX/RAB11A/ACAN/DGKQ/PORCN/PRKA

;AP2/DNM2/VPS35/GRIN2D/RPL6/PAK2/LZTS1/ADD3/FBXO45/CDK5R1/ABHD17A/MINK1/RPL30/BC

AB27B/ACER3/COA3/FAR1/STEAP2/MFN1/BET1L/TIMM23/ERGIC2/CNR1/SCO2/QSOX2/IER3IP1/TE

3/SFPQ/DNMT3A/ATAD2/SIRT7/NAP1L4/NAP1L2/SMARCB1/MSH2/NCOA5/CHD4/RUVBL2/CENPB/RBB

MED19/PIAS3/KDM5B/UBE2L3/CBX4/MIER2/TAF9/PQBP1/DNMT3A/PML/KCTD1/SMARCB1/ZMIZ2/VD

AK4/CCL2/DAPK3/CSNK1D/CDK2/TESK1/STK17B/LYN/CDC42BPG/PKN3/ALPK3/CDK5/MLKL/FGR/MA

UBB2A/MRPS12/RPS2/ARPC2/FMOD/COPE/ARPC3/CLDN10/NUP93/MRPL47/NUP205/PDLIM3/KRT18/

[illegible]8/DBF4/ARHGAP1/RAP1GAP2/SIPA1L2/ARHGDIB/TBC1D30/GPRC5B/ADRM1/DNAJB2/TBC1D13/RAS

MAPRE1/LARP6/TMSB4X/MX2/RUSC1/S100A8/TWF2/MAPK8IP2/FMNL2/EZR/ANKRD1/DCDC2/LSP1/F

R4/HK3/EPHB6/CAD/DGKG/DYRK2/CSNK1E/MARK2/EPHB3/RPS6KA4/PIP5K1C/FGFR1/PIM2/CCNO/F

0B/RRAD/ABCC4/ATP6V0D2/RHOC/RAB6B/RAB32/HSPA6/ENTPD6/DHX34/CANT1/ATP6V1C1/RAB15/

0B/RRAD/ABCC4/ATP6V0D2/RHOC/RAB6B/RAB32/HSPA6/ENTPD6/DHX34/CANT1/ATP6V1C1/RAB15/

0B/RRAD/ABCC4/ATP6V0D2/RHOC/RAB6B/RAB32/HSPA6/ENTPD6/DHX34/CANT1/ATP6V1C1/RAB15/

/ABCC4/ATP6V0D2/RHOC/RAB6B/RAB32/HSPA6/ENTPD6/DHX34/ATP6V1C1/RAB15/MYL6B/ABCC5/C

C12A2/MAPKAPK2/FAM83D/MSN/BRSK1/IQGAP1/CD2/MAD2L2/RGS14/HIF1A/ITGAX/CD3E/PAK1/S

AP1/CD2/MAD2L2/RGS14/HIF1A/ITGAX/CD3E/PAK1/STK11IP/NPM1/SPRED1/PCNA/THY1/RHOF/SI

CD3E/MAP2K2/AP4M1/MRPL17/UBE2I/STX1A/RAP2B/BASP1/CHAF1A/TUBA1A/SIRPA/NOL3/HGS/IF

09/SERPINH1/CSTB/CDK4/GIT1/DPM2/ASAP1/GUCA2A/CTSA/ADAP2/NCF4/CDKN2D/RPS6KA1/TBC1

K1/IQGAP1/EFHD2/PROM1/OLA1/RUVBL1/LRRC59/HSPA8/PLEC/LCP1/JAML/MICALL1/PPP1R13L/N

2/EHD1/NCKAP1L/SYTL1/PAK4/ARHGAP1/RABIF/RTKN/RANGRF/DAPK3/ARHGDIB/KPNB1/TBC1D30/

2/MYO1G/CAP1/CAPZA1/MYO1F/YWHAH/FHOD3/HCLS1/PSTPIP1/ARPC5L/CTTN/CORO2A/WAS/VASH2

1/GRK2/DMPK/MAPK7/MAP3K6/RPS6KB2/DSTYK/STK25/VRK1/CSNK1G2/STK38/OBSCN/CCL3/CDK19

C3/KIF15/PSMC4/ATAD2/ABCA2/ATP6V0C/KIF2A/ATP6V0E1/CDK7/MSH2/RUVBL2/RBBP4/ABCA3/T

/TBC1D30/XPOT/PKN3/RAB11FIP4/RAC1/KCTD10/WAS/LSM2/TBC1D13/DVL2/PFN1/WASF1/ABR/SE

/TBC1D30/XPOT/PKN3/RAB11FIP4/RAC1/KCTD10/WAS/LSM2/TBC1D13/DVL2/PFN1/WASF1/ABR/SE

IGAP18/RANBP1/AHSA1/AGAP1/DNAJB6/GRPEL2/CDC42EP2/MYO9B/OCRL/DNM1L/PGAM5/RALGDS/A
5/STMN3/NCALD/MAP1LC3B/MAP4/RAE1/KIF2A/RPS3/ZNF207/FHDC1/CETN2/DNM1L/PPP5C/PDCD5
.D/LASP1/PPME1/SLC3A2/EHD1/PAK4/ARHGAP1/LYPLA2/CAPZA1/CSNK1D/SH3GLB2/ATXN2L/HCFC
RALA/NKIRAS2/CDC42/AGAP1/DNM1L/RALB/RGS3/DRG1/RERG/RHEB/RAB4B/EEF1A2/RAB3D/DIRAS
/TNFSF15/ADAM17/CASP8/VEGFA/TRAF4/TMBIM1/CD2AP/TRIM37/INHA/KITLG/CCL19/XCL1/BMP8
/RALGDS/ARHGDIA/RIC8A/RGS3/TBC1D10C/RASAL3/TBC1D22A/CHN1/TBCD/NPRL3/RASAL1/SIPA1
PRL3/RASAL1/SIPA1/ARAP1/TBC1D7/SGSM3/ARHGAP11A/SRGAP2/TBC1D22B/ARHGEF11/SMAP1/PL

SAP/DCTN1/HAUS7/KATNA1/MAP1A/MARK4/MAP7D3/LZTS1/FNTA/GLI1/RAB11A/CCDC88C/EML2/KI
SMARCAL1/MUS81/SMUG1/POLE2/HMGA2/DCLRE1C/ERCC3/DNASE1L1/METTL4/DHX30/EXO5/POLG2/

RHGAP17/RALBP1/ARHGEF18/NCKAP1/BCR/NOX1/RHOH/TIAM2/SRGAP1/TRIO/DOCK11/EPS8L2/KCT

IB1/VPS4A/COIL/ATP1B1/PPARG/MYO1C/PRRC2C/PPP1R9A/EFHC1/HIC2
NG2/PRMT6/BRD7/CTSL/SPIN4/PHF13/BRD4/ASF1A/IPO7/SPIN1/SUPT6H/PRKCB/KDM4A/SUPT16H
/MRPL15/MRPL4/RPS9/RPS14/RPS27/MRPS24/RPL29/MRPL33/MRPS5/RPL13/MRPL36/RPL4/RPL41

/MAPK3/MAST2/MAP2K2/DKC1/PAK1/PRKX/FGFR4/POLR2G/CD3EAP/HK3/EPHB6/CAD/DGKG/DYRK2/
3D/LUC7L/RBM24/EIF4G2/EIF4G3/UTP23/LSM1/KHSRP/RPS14/PABPC1L/LARP1/SRSF1/SNRNP70/
31/SNX3/DAB2IP/GTF2F1/ENSA/ANAPC4/PPARG/KIF3A/PPP1R9A/SMG7/PXN/CD22/MFHAS1

IK3R6/GMFB/EGF/RPTOR/CALM3/CIB1

;KG/DYRK2/CSNK1E/MARK2/EPHB3/RPS6KA4/PIP5K1C/FGFR1/PIM2/CCNO/PNKP/FGFR3/PAK4/PIF
;PD1/SLC25A5/ZNF746/VCP/VCL/WRAP53/BAG2/CHEK2/BCL10/HSP90AA1/ACVR1B/TNK2/CASP8/F
RL/AP2A2/RBM14/VPS11/GATAD2A/AP2B1/ARRDC1/AP2A1/KSR1/MMS19/CDYL/CAV2/PEF1/FRMD4A

CP/VCL/WRAP53/BAG2/CHEK2/BCL10/HSP90AA1/ACVR1B/TNK2/CASP8/RPL5/RPS27A/PIAS4/RAD1

/TUBG2/ARL16/ARF4/RHEBL1/POLA1/GNL2/RALA/NKIRAS2/CDC42/AGAP1/PNP/DNM1L/RALB/ARF[illegible]
/TUBG2/ARL16/ARF4/RHEBL1/GNL2/RALA/NKIRAS2/CDC42/AGAP1/DNM1L/RALB/ARFIP2/GNL3/D[illegible]

/ARF4/RHEBL1/GNL2/RALA/NKIRAS2/CDC42/AGAP1/DNM1L/RALB/ARFIP2/GNL3/DRG1/RERG/RHE
/ARF4/RHEBL1/GNL2/RALA/NKIRAS2/CDC42/AGAP1/DNM1L/RALB/ARFIP2/GNL3/DRG1/RERG/RHE

3/DAB2IP/SNX29/RPS6KC1/CYTH3/PLD1/PLEKHA8/SNX5/ARHGAP33/SNX16/CCDC88A/EPB41/SN

NL2/RALA/NKIRAS2/CDC42/AGAP1/DNM1L/RALB/ARFIP2/GNL3/DRG1/RERG/RHEB/RAB4B/DAP3/
VAV3/SLA2/TEX261/BICD2/NEFH/NEFL/SH2B1/HIP1/MEN1/ARRB1/BFAR

'SF13B/CCL4/TGFB2/CALCA/CXCL16/IL15/TNFSF12/PF4V1/SEMA4B/TGFB3/CXCL11/SEMA4D/XCL

JLK1/RAB3IP/TMEM127/PDE6D/TBC1D25/DENND6A

;/ARF4/RHEBL1/GNL2/RALA/NKIRAS2/CDC42/AGAP1/DNM1L/RALB/ARFIP2/GNL3/DRG1/RERG/RHE
;/ARF4/RHEBL1/GNL2/RALA/NKIRAS2/CDC42/AGAP1/DNM1L/RALB/ARFIP2/GNL3/DRG1/RERG/RHE

K3/HDAC2/ATF4/YWHAH/MKKS/SNF8/HCLS1/KDM1A/ATN1/NOP58/HCFC1/FKBP4/BUD31/ACTB/MTA2

R1/BCL10/PSMD9/USP21/CTBP2/THRA/MED27/BCL9L/ACTN1/COPS5/AIP/RBM14/TCERG1/DAXX/AC
'OP5/TSN/EXOSC9/EXOSC10/EXOSC7/WARS2/DDX56/TRMT12/POLR2I/DHX57/POLR2J/NOB1/NSUN4

NAI3
3B/HSBP1L1/JUNB/WWTR1/MNT/MXD4/NAB2/PRMT5/ZHX2/PFDN5/BCOR/TFEC/BRD7/NSD1/GON4L/[illegible]

I2/OR5I1/OR5P3/OR2J2/OR4C15/OR8H1/OR2F1/OR2W1/OR1A1/OR4S1/OR7D4/OR10C1/OR51A7/O[illegible]

NFSF12/PF4V1/SEMA4B/TGFB3/CXCL11/SEMA4D/XCL2/GAL/CDC42EP2/SEMA4F/GMFG/SEMA5B/C[illegible]

G1/LONP1/ATP2A3/RAD51C/DNA2/HSPA2/RAD51D/ABCB9/HSP90AA1/CFTR/KATNA1/RAD18/HSPA[illegible]

/VHL/TNFAIP3/RNF216/RNF2/PIAS4/RAD18/MED27/TRIM21/UBE2Q1/TRIM37/RNF213/RNF25/UBR

RF3/YBX1/STAT1/FOXK2/E2F8/HDAC1/E2F3/BATF2/TFE3/DDX11/SUB1/E2F4/KLF16/PPARD/LE

A1A/TUBB2A/SGO1/TTLL1/MSTO1/LMNA/DYNC1H1/SPDL1/XRCC3/CAPN6/LZTS2/MARK2/CEP85/E2F
DKN2A/MCM6/TUBG1/RACGAP1/EIF4EBP1/CENPW/ARHGEF2/DSCC1/NCAPH/MCM10/REEP4/SMARCD3/
/RHOC/CXCR4/SEMA4A/NDN/MAP2/DOK1/MSN/BRSK1/IQGAP1/B4GALT5/EFNA5/RAB25/MAPK3/LAMC
IF1A/MAPK3/CD276/SERPINE1/IL27RA/CD3E/TCIRG1/MAST2/SASH3/ADGRG1/ISG15/TNFRSF4/BR
/IL27RA/CD3E/MAST2/SASH3/ISG15/TNFRSF4/BRCA1/MNDA/ZNF580/SIRPA/SPON2/SOCS1/CD58/
RC5B/IL1B/NFAM1/FGR/MAPK11/GPSM3/PQBP1/IFI16/AGPAT2/CASP1/IL12RB1/IL15/C5AR1/RPS
/PDGFA/VANGL1/TULP3/PSMB8/PSMB10/PSMA5/DEAF1/TGFB2/PLXND1/PSMD13/PSMC4/AP2M1/NDE
MA4A/ALOX5/PLEKHO2/MIF/CD300A/CANT1/IQGAP1/CFD/FTH1/STXBP2/MAD2L2/PSMD2/ITGAX/HS
/MIF/CD300A/CANT1/IQGAP1/CFD/CD2/FTH1/STXBP2/PSMD2/JMJD6/ITGAX/HSPA8/NEU1/GM2A/T
/CD63/DOK3/STK11IP/RNASET2/FPR1/MNDA/PTGES2/RAP2B/GYG1/ARHGAP9/RHOF/SIRPA/DEGS1/
2/RAP2B/GYG1/ARHGAP9/RHOF/SIRPA/DEGS1/HK3/CCT2/CD58/C3AR1/HSP90AB1/PRSS3/DYNLL1/
MA4A/ALOX5/PLEKHO2/MIF/CD300A/CANT1/IQGAP1/CFD/FTH1/STXBP2/MAD2L2/PSMD2/ITGAX/HS
1/DNAJC5/VAV1/HSPA6/DDOST/ALOX5/PLEKHO2/MIF/CD300A/CANT1/PRDX1/ZP3/IQGAP1/CFD/FT
/PTGES2/RAP2B/GYG1/ARHGAP9/RHOF/SIRPA/DEGS1/HK3/CCT2/CD58/C3AR1/HSP90AB1/F2RL1/F
3/SOCS1/IRF1/F2RL1/STAT1/RAB7B/RRS1/RRAS/HDAC1/CTLA4/TNFRSF11A/NBEAL2/PIR/FSTL3/
/MITF/LYN/CARD11/CBFB/PIAS3/IL2RA/NCAPH2/NFAM1/CDH17/LCK/PTPN6/THOC5/CD27/IFI16/
LA-DMB/TNFRSF18/VSIG4/CTSK/WIPF3/TRAF2/RBCK1/PSMD14/HMGB2/TRIM6/JAK3/HMOX1/WASF2
/IRF7/ZC3H12A/PSME3/TNIP2/FLOT1/ACTG1/NCKAP1L/SMPDL3B/HLA-DQA2/MYO1G/PIK3CD/PLSC
3/NFAM1/PSMB10/FGR/LCP2/PSMA5/NRAS/WAS/LCK/PTPN6/PSMD13/PSMC4/MUC5B/CD79A/PSMD10
BEC3C/PSMD14/CCT3/HMGB2/TCF3/TRIM28/POLA2/CACYBP/BAX/SIRT6/FAAP24/FANCG/GMNN/GIN
D1/WAS/SWI5/SFPQ/PML/COPS7B/ISY1/PARP1/RNF113A/CDK7/MSH2/RUVBL2/RPS3/POLA1/USP1/
MT5A/HCFC1/EHMT2/AKAP8L/IL1B/ACTB/MTA2/CDK5/KDM5B/CBX4/PHB/MIER2/NPM3/CENPI/LOXL
SM10/URM1/U2AF2/SF3B5/KDM1A/SRSF9/SNRPC/GEMIN7/EMG1/NOP58/TRMT1/SSB/TRMT61A/BUD3
7/NCL/EIF3H/AGO2/CALR/RPL35/RPS20/APP/RPL18A/PABPC4/PPP1R15A/UQCC2/RPS19/RPS6KB2
N/TNFAIP6/RAB11FIP5/CD53/PLAU/GIPC1/DTNBP1/ABCC4/ATAD3B/CHIT1/PSMD14/TIMP2/SYNGR
R1/F2RL1/EPHB6/ISL2/CCL13/TNFRSF11A/RELN/ZSWIM5/EPHB3/CORO1B/LEF1/PIP5K1C/FGFR1/
INS4/PRKDC/RUVBL1/MAD2L2/CNOT11/MAPK3/NUPR1/APEX2/CBX1/PAK1/ACD/PEA15/FANCE/ISG1
T2/MTCL1/BRSK1/EFNA5/STIL/SSNA1/RGS14/HIF1A/HSPA8/MAP1S/CENPE/CHD3/PPP2R1A/PAK1/
PRKDC/SSNA1/MAD2L2/CNOT11/PSMD2/CENPE/PPP2R1A/CHMP4C/BRCA1/RBL1/NSMCE2/PCNA/TUBA
C/HLA-DPA1/HLA-DMB/ABCC4/HMGB2/AREG/JAK3/HMOX1/EIF5A2/CCL5/CD86/SIRT6/CCL26/TNFS
HNRNPM/KHDRBS1/HNRNPA3/PRPF31/ZMAT5/SNRPB2/PRPF19/FASTK/SART1/SYMPK/PHF5A/FAM98B
/PSMD4/PSMD3/RBX1/PSMD7/TMUB2/TBL1XR1/PELI1/SGTB/RNF4/RACK1/RNF7/CUL4B/TRIM71/TR
LCMT1/NAA10/PCBP4/CASP2/BRSK1/MIIP/PRKDC/STIL/SSNA1/MAD2L2/CNOT11/PSMD2/CENPE/PP
/TGFA/TMED9/ARPC3/LIMCH1/DDX11/CORO1B/NELFE/RPS6KA4/HIP1R/BID/CCT4/PNKP/UBE2J2/C
A4/PRMT2/S100B/SORT1/RNPS1/PRELID1/DDIT4/PPP1CA/PDCD1/TGM2/HIP1R/ZC3H12A/AIFM2/S
L3/PILRA/BIN1/NPC1/IRF3/RPS2/HSP90AB1/DYNLL1/STAT1/PABPC1/HDAC1/CCDC86/NUP93/DDX
DM1A/SLC11A1/SRSF9/SNRPC/GEMIN7/SSB/TRMT61A/BUD31/RPS5/DAZAP1/SNRNP40/AKAP8L/PSM
CUL4B/DPF2/NTMT1/SUV39H2/ZNF335/TAF12/CHTOP/TAF10/DOT1L/MTA3/PRKAA2/HDAC10/HAT1/
LOX5/NDN/SNRPA1/CCL26/TNFSF9/MIF/DOK1/GPR35/MSN/HLA-DPB1/TALDO1/IL34/EBI3/GBP2/F
APN1/CAPZA1/FRAT2/FZD2/KPNB1/CAMSAP2/CLN3/AXIN1/VDAC1/AKAP8L/GBA/CAPNS1/CDK5/CTS
5/GNL2/RPL27/FTSJ3/EIF3H/DROSHA/VCX/AGO2/SF3A3/RPL35/PPAN/DDX54/RPS19/RPLP0/NOC4
/SH3PXD2B/SLC9A1/EFNA5/MAD2L2/LCP1/TPM3/KCNE5/PAK1/CAPN10/BAIAP2L1/FCHSD1/RHOF/I
2/RAC1/MIEN1/B9D2/RAP2A/PDGFA/CTTN/WAS/POC1A/NUP85/HAUS8/TNIK/PLK4/PFN1/MAP4/IFT
CP1/TPM3/PAK1/CAPN10/BAIAP2L1/FCHSD1/RHOF/ITGB5/CORO1C/BIN1/CORO7/PALLD/F2RL1/AF
11A/NBEAL2/PIR/FSTL3/CD83/BATF2/PLA2G2D/TFE3/NCAPG2/PRELID1/PSMB2/LEF1/IRF7/ZC3H
4/RPS19/FADD/ZNF675/WDR1/CLEC5A/GPR183/CCL3/MEIS2/RARG/ACVR1B/PTPN2/THBS1/CASP8/
TNFSF9/CD300A/FLOT2/ZP3/IQGAP1/HLA-DPB1/SLC9A1/EFNA5/EBI3/MAD2L2/ADAMDEC1/CD276/
SMB2/PSME3/HSPBP1/BCAP31/UBE2J2/PRSS16/FBXO5/ADAM8/VLDLR/LAMP3/LYPLA2/CTSE/CSNK1
GA5/MIF/CD300A/MSN/ZP3/IQGAP1/SRPX2/MIIP/CARD10/CREB3/HIF1A/RAB25/MAPK3/ITGAX/LA
A4A/NDN/MAP2/DOK1/BRSK1/IQGAP1/PTK7/B4GALT5/EFNA5/SF3A2/MAPK3/MANF/FZD1/EFNB1/CD

l/EPHB3/CORO1B/FGFR1/MT3/DYNLT1/DVL3/VLDLR/HDAC2/RAP1GAP2/YWHAH/CAMSAP2/TBC1D30/
'URM1/AXIN1/U2AF2/KDM1A/BIRC7/MED8/CCDC22/CBFB/MYLIP/PSMB4/FBXO32/PIAS3/KCTD10/(
ANGAP1/RHOQ/EPB41L2/GPD1L/TRAF2/CCT3/SAE1/TRIM6/P3H1/TRIM28/CCL5/NUTF2/PHPT1/MAI
2/LMNA/SPDL1/RRS1/TSPAN17/LZTS2/NUP93/NXT1/FAM53B/RAB35/NUP188/ZC3H12A/CSE1L/BII
'BL1/CD2BP2/SMIM20/LSM4/EIF3B/CD3E/CENPE/PAK1/TAF6/NPM1/BAIAP2L1/ANKRD27/STX1A/I
ERI3/PSMB2/REXO4/DDIT4/GPI/NDC1/NUP188/ZC3H12A/PSME3/NCBP2/RPL17/RPLP2/ITPA/CNOT
7/XPO5/EXOSC5/SNU13/SRRT/RPL27/NCL/FTSJ3/HENMT1/POLR2L/DROSHA/AGO2/CPSF4/RPL35/T
NASET2/FPR1/MNDA/PTGES2/RAP2B/GYG1/ARHGAP9/RHOF/SIRPA/DEGS1/HK3/CCT2/CD58/C3AR1/
DX1/TRIM28/RHOC/BDKRB1/CCL5/CXCR4/SEMA4A/CCL26/PHPT1/ITGA5/MIF/CD300A/MSN/ZP3/I(
L1/RELN/ZNF703/LEF1/GPI/FGFR1/ZC3H12A/NCKAP1L/FBXO5/ADAM8/DAPK3/PIK3CD/CXCL13/DI
AP1L/JAG2/ADAM8/PRR7/CCL2/PIK3CD/KDELR1/FANCA/SLC11A1/HSPH1/CD209/LYN/CARD11/RA(
NASET2/FPR1/MNDA/PTGES2/RAP2B/GYG1/ARHGAP9/RHOF/SIRPA/DEGS1/HK3/CCT2/CD58/C3AR1/
R1/F2RL1/EPHB6/ISL2/CCL13/TNFRSF11A/RELN/ZSWIM5/EPHB3/CORO1B/LEF1/PIP5K1C/FGFR1/
3/SAMD4B/RPL27/NCL/EIF3H/AGO2/CALR/RPL35/RPS20/APP/RPL18A/PABPC4/PPP1R15A/UQCC2/
PS1/PRELID1/PPP1CA/PDCD1/TGM2/HIP1R/ZC3H12A/AIFM2/SLC9A3R1/BID/BCAP31/TNFRSF10C/
SP2/UBE2Z/AMIGO2/PRKDC/SLC9A1/STIL/CREB3/TNFAIP8/HIF1A/NUPR1/SERPINE1/RNF34/PEA1
PS1/PRELID1/PPP1CA/PDCD1/TGM2/HIP1R/ZC3H12A/AIFM2/SLC9A3R1/BID/BCAP31/TNFRSF10C/
EX3/CASP2/UBE2Z/AMIGO2/PRKDC/SLC9A1/STIL/CREB3/TNFAIP8/HIF1A/NUPR1/SERPINE1/RNF3
ACK1/RNF7/CUL4B/TRIM71/TRIM25/RNF122/UBXN1/PSMD8/KLHL42/VCP/PSMA1/USP5/NSFL1C/UI
SH3PXD2B/SLC9A1/EFNA5/STIL/NUPR1/HSPA8/FZD1/LCP1/PAK1/SMPD2/NPM1/BAIAP2L1/CHMP4(
TESK1/MITF/ITGB1BP1/ARPC5L/RAC1/MIEN1/JCHAIN/CD47/CTTN/WAS/CDH17/TRMT112/VIL1/PS
A4/PPIH/TRIM59/PDCL3/PILRA/BIN1/NPC1/IRF3/RPS2/HSP90AB1/F2RL1/DYNLL1/STAT1/PABP(
'PCNA/ITGB5/SMARCA4/PPIH/TRIM59/PDCL3/PILRA/BIN1/NPC1/IRF3/RPS2/HSP90AB1/F2RL1/I
3/EIF5A2/TRIM28/BAX/MFGE8/NR0B1/SNRPA1/ITGA5/FANCG/ZP3/SH3PXD2B/RUVBL1/MAPK3/SYT
CASP2/BRSK1/GMNN/MIIP/PRKDC/SSNA1/MAD2L2/CNOT11/PSMD2/CENPE/PPP2R1A/NPM1/RBBP8/(
PLAU/GIPC1/DTNBP1/ABCC4/ATAD3B/CHIT1/PSMD14/TIMP2/SYNGR1/HMOX1/DNAJC5/HSPA6/DDOS
'HMGB2/TRIM6/JAK3/TRIM28/CCL5/MAPKAPK2/VAV1/COLEC12/CCL26/MIF/CD300A/PRDX1/HLA-I
TPN6/PSMD13/IFI16/PSMC4/MUC5B/SOCS3/COCH/UBE2D1/PSMD10/PSMD11/PSMD1/PSMA7/LRP8/T
C/IQGAP1/PTK7/SH3PXD2B/EFNA5/SF3A2/PROM1/IL34/RGS14/HIF1A/FZD1/SERPINE1/SUCO/ATI
'SPDL1/XRCC3/TICRR/KNTC1/GAS7/PPP1R9B/E2F8/DDIAS/CGREF1/PRMT2/E2F4/PSMB2/CBX3/PS
H3B/HGS/SOCS1/CKS1B/HSP90AB1/NPFFR2/TGFA/TNFRSF11A/RELN/MARK2/CEP85/NCAPG2/FGFR1
'LCP1/EFNB1/JAML/IL27RA/CD3E/TCIRG1/PAK1/SASH3/TPD52/TNFRSF4/SLC39A10/MNDA/THY1/
'SPDL1/XRCC3/MYO19/TGFA/RRS1/KNTC1/PPP1R9B/CEP85/INCENP/NDC1/FBXO5/DAPK3/KIF14/I
AB7B/RRS1/RRAS/HDAC1/CTLA4/TNFRSF11A/NBEAL2/PIR/FSTL3/CD83/BATF2/PLA2G2D/TFE3/N(
L6A3/PLEKHA4/SIX2/HEYL/FZD2/MKKS/JAG1/WNT7B/TNC/RAC1/ITGA2/PSMB4/KDM5B/PDGFA/VAI
F2RL1/CTLA4/CD83/PLA2G2D/C1QTNF1/PRELID1/PDCD1/ZC3H12A/TNIP2/LAG3/SIRPG/NCKAP1L/
F11A/OLR1/SLC7A8/PIP5K1C/PPIA/SLC3A2/SIRPG/NCKAP1L/ADAM8/MYO1G/SLC7A10/CCL2/GYP(
27/BRCA1/THY1/CORO1C/RTN4RL2/SIRPA/NOL3/LMNA/SPDL1/XRCC3/GDI1/PRELID1/CORO1B/HII
IL1/HMOX1/RHOC/BDKRB1/CCL5/CXCR4/SEMA4A/CCL26/PHPT1/MAP2/ITGA5/MIF/CD300A/MSN/ZI
ZNF703/LEF1/GPI/FGFR1/ZC3H12A/NCKAP1L/FBXO5/ADAM8/DAPK3/PIK3CD/CXCL13/DEFB1/LYN/
GKZ/MAD2L2/RGS14/CHTF18/MAPK3/MAP2K2/CENPE/UBE2I/DKC1/PPP2R1A/PAK1/ACD/PEA15/NPI
AP9/CORO1C/HSP90AB1/F2RL1/DYNLL1/ARHGEF19/CCL13/TBC1D1/RIN1/DDX11/ARHGAP39/GDI1/
'DYNC1H1/XRCC3/CAPN6/ARHGEF19/ARPC3/LIMCH1/MARK2/CEP85/CORO1B/HIP1R/PAM/DYNLT1/(
A1A/STX6/RAB3B/BIN1/SFI1/PPP6R1/MSTO1/DYNLL1/DYNC1H1/SPDL1/MYO19/TGFA/RRS1/PLEKI
KPNB1/SV2A/CLN3/DCTN2/LYN/ZNF593/CDK5/S100A13/FGR/AP3M2/WASF1/MAP4/SYT12/TRAPPC1
'ITGAX/FZD1/TRIM11/PAK1/CRABP2/ADGRG1/ANKRD27/NRCAM/THY1/XRCC2/RTN4RL2/BIN1/ISL2
GPRC5B/IL1B/ADRA2C/RNF157/PLXND1/PPP1CC/NDRG4/NAP1L2/TUBB2B/DVL2/OXTR/NSMF/GORAI
EFNA5/SF3A2/IL34/RGS14/HIF1A/FZD1/TRIM11/ATP6AP1/PPP2R1A/PAK1/CRABP2/ADGRG1/ANKI
BDKRB1/BAX/DNAJC5/NUTF2/PHPT1/MAP2/FKBP1A/CD300A/MTCL1/MSN/BRSK1/PKDCC/IQGAP1/CA
MGB2/YBX3/JAK3/CHMP4B/HMOX1/ATAD3A/CACYBP/CCL5/BAX/DNAJC5/ITGA5/MIF/BEX3/CASP2/U
ULP1/FCHSD1/CORO1C/STX6/BMF/NOL3/HGS/BIN1/NPC1/PPP6R1/LMNA/F2RL1/ARPC2/RAB7B/TGI

SME3/HSPBP1/TNIP2/UBE2J2/NPEPPS/OTUB1/DTX2/FBXO5/GTPBP4/PRR7/TRIM31/ANAPC7/SPSB1
:/LZTS2/E2F4/TMEM237/BRIX1/RRP7A/SNX10/CEP89/CCNO/UNC119B/GPSM2/EHD1/HAUS1/ACTG1
A4/TRAM1L1/RPSA/RANGRF/YWHAH/BBC3/PMAIP1/ITGB1BP1/REEP2/RPS5/EHD2/ACTB/RAP2A/CDK
NFRSF11A/DDIAS/NUP93/DDX11/HYOU1/NUP205/NUP37/NDC1/NUP188/ZC3H12A/BID/BCAP31/DVL
3/LCP1/MAP1S/TPM3/PAK1/BAIAP2L1/FCHSD1/RHOF/ITGB5/CORO1C/BIN1/CORO7/HSP90AB1/F2R
SN/ZP3/NINJ2/HLA-DPB1/SRPX2/AMIGO2/EFNA5/CD2/EBI3/MAD2L2/ITGAX/CD276/EFNB1/JAML/
/RAP2A/CTTN/WAS/POC1A/NUP85/HAUS8/TNIK/PLK4/PFN1/MAP4/IFT80/GORAB/ODF2/PTPN23/TA
33/CORO1B/FGFR1/MT3/DYNLT1/DVL3/VLDLR/HDAC2/RAP1GAP2/YWHAH/CAMSAP2/TBC1D30/KDM1A
/PPP1R9B/E2F8/CSNK1E/PRMT2/E2F4/PSMB2/PSME3/BID/HAUS1/BLM/FBXO5/DBF4/TUBB4A/CNOT
/ARHGEF2/DSCC1/NCAPH/MCM10/REEP4/SMARCD3/MCM7/HRAS/PLCB1/USH1C/KIF11/MCM3/NUP62/
X/PSMA7/PSMB3/DROSHA/KAT2A/IL4R/PNP/PSMD4/PSMD3/PSMD7/LILRB4/AP3D1/AXL/FADD/BTK/
CAP31/UBE2J2/DVL3/GPSM2/FERMT1/NPEPPS/LEPROTL1/RANGRF/YWHAH/HCLS1/BBC3/KDM1A/PMA
RB1/CCL5/CXCR4/SEMA4A/CCL26/PHPT1/ITGA5/MIF/CD300A/MSN/ZP3/IQGAP1/SRPX2/MIIP/CAR
/GPI/FGFR1/ZC3H12A/NCKAP1L/FBXO5/ADAM8/DAPK3/PIK3CD/CXCL13/DEFB1/LYN/ITGB1BP1/RA
38A/WTAP/HNRNPU/HTATSF1/LSM8/MAGOH/RBMX/ZRSR2/HNRNPA1/PPIE/POLR2I/POLR2J/HNRNPL/
38A/WTAP/HNRNPU/HTATSF1/LSM8/MAGOH/RBMX/ZRSR2/HNRNPA1/PPIE/POLR2I/POLR2J/HNRNPL/
38A/WTAP/HNRNPU/HTATSF1/LSM8/MAGOH/RBMX/ZRSR2/HNRNPA1/PPIE/POLR2I/POLR2J/HNRNPL/
PSMD3/RBX1/CARD16/PSMD7/ANGPT2/TBL2/PLEKHN1/LONP1/SUV39H2/PSMD8/VHL/PSMA1/PSMB5/
OK3/STK11IP/RNASET2/FPR1/MNDA/PTGES2/RAP2B/GYG1/ARHGAP9/RHOF/SIRPA/DEGS1/SPON2/H
O1/ZC3H12A/TNIP2/LAG3/SIRPG/NCKAP1L/ADAM8/CCL2/PLSCR1/FANCA/HSPH1/CD209/LYN/CARD
SH2B2/IRF7/ZC3H12A/PSME3/TNIP2/FLOT1/ACTG1/NCKAP1L/SMPDL3B/HLA-DQA2/MYO1G/SHC1/F
PG/RBM17/HNRNPM/SLBP/KHDRBS1/HNRNPA3/PRPF31/ZMAT5/SUPT5H/SNRPB2/PRPF19/CHTOP/SAR
3/RPS15/NOL6/LTV1/MAVS/SARNP/MAGOH/RITA1/HIKESHI/ZPR1/THOC2/XPO7/HNRNPA1/UBR5/EI
3/LCK/PTPN6/CD27/IL12RB1/IL15/RPS3/GRB2/CD6/STK10/CDC42/IL4R/SEMA4D/PNP/LILRB4/A
324/DYNLT1/GPSM2/CYTH2/FLOT1/ARHGAP1/RABIF/RTKN/SHC1/RANGRF/RAP1GAP2/CDK2/SIPA1L
/GCN1/RAE1/CDC123/EIF4A3/PSMD1/ILF3/PSMA7/GRB7/ZNF706/XPO5/EXOSC5/YWHAB/RPS3/SRR
SMD11/CDKN2C/PSMD1/DONSON/PSMA7/TFAP4/RBBP4/PSMB3/KAT2A/ZNF207/CBX5/CALR/TOPBP1/
PRRX1/ZMYND8/SEMA4D/SYT1/DNM1L/SYT3/SEMA4F/KATNB1/SEMA5B/STK25/ABI2/ARHGDIA/VIM/
X2/ADAM12/LOXL2/TGFB2/ADAM19/ADAMTS9/CCDC80/BGN/COL6A2/ERO1A/LOX/IBSP/VWA1/LUM/C
L/FBXO5/ADAM8/DAPK3/PIK3CD/CXCL13/LYN/ITGB1BP1/RAC1/ITGA2/PLA2G7/MIEN1/IL1B/BCAR
LN4/VAMP8/MRPS17/MRPS21/MRPS15/CCSAP/MRPL18/MAP1A/JMJD4/ADD3/LIMA1/ETF1/TECPR1/M
F4/CCT8/GNL3/MAPT/ZNF335/CHTOP/NAT10/NBN/MTHFR/HNRNPU/ERCC1/VEGFA/EXOSC10/SREBF1
CDH17/VIL1/PSMC4/PFN1/WASF1/INPP5J/PARP1/PIEZO1/RPS3/CAPZB/GRB2/CDC42/VPS18/ARHC
O63/DOK3/STK11IP/RNASET2/FPR1/MNDA/PTGES2/RAP2B/GYG1/ARHGAP9/RHOF/SIRPA/DEGS1/HK
2/RAP2B/GYG1/ARHGAP9/RHOF/SIRPA/DEGS1/HK3/CCT2/CD58/C3AR1/HSP90AB1/PRSS3/DYNLL1/
MNN/MIIP/PRKDC/SSNA1/MAD2L2/CNOT11/PSMD2/CENPE/PPP2R1A/RBBP8/CHMP4C/BRCA1/RBL1/N
IT/PLK4/ANAPC11/PSMD10/ZBTB17/CCND2/TFAP4/USP22/CDC42/CALR/RANBP1/APP/MED25/GDPD
O/SYMPK/CPSF3/RPS15/NOL6/LTV1/MAVS/SARNP/MAGOH/RITA1/HIKESHI/ZPR1/THOC2/XPO7/HNR
CHMP4B/RHOC/ESPL1/FAM83D/CCDC124/SGO2/CLTA/RUVBL1/MAD2L2/RGS14/CENPE/UBE2I/NUDC/
N1C/DUSP4/IL1B/GBA/ACTB/CHORDC1/PDGFA/MLKL/MAPK11/PTPN6/MAP3K14/TNIK/DVL2/MST1R/
HIP1R/PSME3/SLC9A3R1/BID/BCAP31/BCL3/TNFRSF10C/FGFR3/NOC2L/PRR7/NONO/DAPK3/ATF4/
2/ARHGAP18/TGFB3/APP/SWAP70/CDC42EP2/KATNB1/GMFG/ABI2/WDR1/CLIP3/DRG1/MAPT/CKAP2
4/PSMD9/WTAP/RPS27A/HNRNPU/EXOSC9/MAGOH/CDK11B/EXOSC7/TBRG4/UBA52/NANOS1/RBMX/HN
AG3/PRKCI/GZMB/PDCD5/STX4/RACK1/CEP250/CCT8/GNL3/CLIP3/MAPT/CEMIP/EMD/WRAP53/MFF
IM1/BCL2L1/RILPL1/RAB11A/ZDHHC23/TSPAN14/RAB29/GOLPH3L/CACNB3/PACSIN1/GORASP2/IF
A/RRS1/KNTC1/PPP1R9B/CEP85/INCENP/NDC1/FBXO5/DAPK3/KIF14/KPNB1/SMC4/FANCA/KNSTRN
MB10/FGR/LCP2/PSMA5/NRAS/WAS/LCK/PTPN6/PSMD13/PSMC4/MUC5B/CD79A/PSMD10/PSMD11/PS
GO2/RPL35/RPS20/PSMD4/RPL18A/PSMD3/PABPC4/PSMD7/RPS19/NT5C3B/RPLP0/RPL18/RPL23/V
RPL23/VIM/TRIM71/PLEKHN1/RPL38/HNRNPM/RPL23A/EXOSC3/BTG2/PSMD8/GSPT2/PSMA1/RPS7/
1/BCAR1/CD47/CTTN/MYADM/WAS/VIL1/CIT/PFN1/WASF1/NEB/KPTN/TACSTD2/CAPZB/GRB2/FHOD
/BAG3/ARRB2/KAT2A/ZNF207/MTF2/SWAP70/DNM1L/RAD21/KATNB1/GMFG/MTBP/NME6/CLIP3/MAP

CCL28/CARD11/RAC1/CBFB/IL1B/IL2RA/CD47/TNFSF13B/MYADM/ADAM19/LCK/PTPN6/CD27/IL12
GDIA/SCN1B/MAPT/BRK1/AKIRIN1/CBFA2T2/STMN2/RASAL1/PALM/ARAP1/DBNL/PLXNC1/DNM2/ST
/WASF1/NEB/CAPZB/SEMA4B/GRB2/ARHGAP18/SLC12A7/SEMA4D/SWAP70/CDC42EP2/PPP1R15A/SE
ART1/NOP10/KRI1/FAM98B/RPS7/RPL7A/WDR55/GAR1/SENP3/GTPBP3/RPS15/ADAT3/RPS16/PUS7
MD4/DNM1L/PSMD3/RBX1/CARD16/PSMD7/CBL/ANGPT2/TBL2/PLEKHN1/LONP1/SUV39H2/PSMD8/VE
P4/TRAF3/SYDE1/POLR2L/AGO2/CALR/APOBEC3D/POLR2K/GPATCH3/CARD16/PRDX4/CLEC7A/TNIF
RB4/AP3D1/AXL/FADD/BTK/PELI1/STX4/CLEC7A/RASAL3/GPR183/LBP/EXOSC3/BTN2A2/CCL3/ZN
BAM1/GZMA/OGG1/RPA2/CCT8/GNL3/PPP4C/TIMELESS/EXOSC3/DBF4B/ESCO2/PTGES3/NAT10/DNA
D11/RAC1/CBFB/IL1B/IL2RA/CD47/NFAM1/TNFSF13B/LCK/PTPN6/CD27/SAMSN1/IL12RB1/CYP26
ALCA/PML/DRAM2/PARP1/LOX/POLE4/POLA1/CROCC/BARD1/BAG3/PRIM2/XRCC6/TMEM119/TELO2/
D1/MB/PSMA7/PSMB3/TGFB3/PSMD4/DNM1L/PSMD3/RBX1/CARD16/PSMD7/CBL/ANGPT2/TMEM199/T
RPL7A/RPS23/RPS10/BAD/HSP90AA1/RPS15/SGTA/RPS16/SYS1/VPS35/CASP8/RPL5/RPS27A/ARF
LE/PRMT2/E2F4/PSMB2/PSME3/BID/HAUS1/BLM/FBXO5/TUBB4A/CNOT9/CDKN2B/CCL2/CSNK1D/AN
L/PARP1/PSMD10/LTBR/GPX1/TNFSF12/YWHAQ/YWHAB/RPS3/TFAP4/FAIM/ARRB2/PCGF2/GZMB/DN
/FADD/BTK/PELI1/STX4/CLEC7A/RASAL3/GPR183/LBP/EXOSC3/BTN2A2/CCL3/ZNF335/HSPD1/SA
S/SLBP/DBF4B/SSBP1/ACHE/ESCO2/DNA2/EME2/CHEK2/CLSPN/RAD1/CDK2AP1/NBN/CCNE2/GLI1/
C4/KNSTRN/AKAP8L/NCAPH2/CDCA2/SFPQ/BANF1/CENPK/NUP43/DYNC1LI1/DSN1/ANAPC11/TUBG2
1/CDKN2C/PSMD1/DONSON/TIPIN/MSH2/PSMA7/PSMB3/ZNF207/TOPBP1/PSMD4/PSMD3/RBX1/PSMI
/KAT2A/IL4R/PNP/LILRB4/AP3D1/FADD/PELI1/RASAL3/BTN2A2/HSPD1/ABL1/SART1/HLA-DOA/F
ARPC5/ABL1/TBCD/STMN2/ACTR3/DBNL/HSP90AA1/SPIRE1/DCTN1/VDAC2/MAP7D3/ADD3/EML2/MF
ABL1/FCGR2B/CCND3/VCAM1/TWSG1
PS10/SENP3/RPS15/RPS16/RPL5/NOL6/RPF2/BMT2/POP5/NVL/LTV1/RPL6/EXOSC9/EXOSC10/PAK
1/SWAP70/TRPM4/CCRL2/RPS19/GPR183/LBP/CCL3/S100A7/CXCL17/AKIRIN1/CYP19A1/BIN2/VC
AL3/GPR183/WNT4/BTN2A2/ZNF335/HSPD1/ABL1/FCGR2B/TNFAIP3/CCND3/VCAM1/TWSG1
ADD/PELI1/RASAL3/BTN2A2/HSPD1/SART1/FCGR2B/CSK/BAD/BCL10/VCAM1/TWSG1/PTPN2

/HDAC10/NBN/MAGEF1/ERCC1/TSN/RBM14/TEX11/ACTR5/RNF8/RPA1/UBE2N/MMS19/MUS81/MSH6/
RAB27B/TNPO1/IFT22/SNAPIN/SSX2IP/UXT/HTT/CCDC65/ACTR1A/OFD1/KLC1/RAB1A/CCDC40/KI
F5A/SLC35C2/POLR2D/DHX9/NXT2/RBM15B/SEH1L/EIF6/KHSRP/THOC1/DHX38/ENY2/PABPN1/SRS
T2/POP5/NVL/EXOSC9/EXOSC10/EXOSC7/DDX56/PIH1D1/UTP20/EXOSC1/NOB1/NSUN4/DDX10/WDF
P/MORC2/MIR155HG/HDAC5/METTL3/PPARG/MOV10/PPP1R8/ADAR/AAAS/SETDB1/AGO1/MBD2/POLR
5A/EXOC3/IFT20/POLR2D/AP2B1/TNPO1/IFT22/SNAPIN/RBM15B/SSX2IP/SEH1L/EIF6/ATR/THOC
HOC1/DCLRE1C/INPP5D/HDAC5/PTPRC/IKZF3/CD180/CASP3/CD22/UNG/FZD9/SUPT6H/DOCK11/CI
TN2/PSMD3/RBX1/PSMD7/RAD21/BABAM1/CEP250/HAUS6/NAE1/NDE1/KHDRBS1/CKAP5/DBF4B/RAI
/ZNF335/HSPD1/SART1/TOX/CSK/BAD/BCL10/VCAM1/PAK2/HLA-F/NKAP/RHOA/CD38/AP3B1/TNFF
ATC4/PDCD10/CHEK2/BAD/ERP29/PTPN2/STYXL1/PTPN1/NBN/PIAS4/VDAC2/PTPMT1/TMEM109/TX
N/BRK1/ARPC5/ABL1/FCGR2B/CSK/ACTR3/BIN2/HSP90AA1/DNM2/TUSC2/PTK2/THBS1
/KIF20B/PPP2R5B/CDK5R1/APBB1/INHA/PRKAG1/FAP/CNOT10/WDR6/EIF4G2/NOTCH2/CNOT3/TFI
/CAPZB/CALR/PSMD4/PSMD3/CTSD/PSMD7/CD8A/AP3D1/IKBKG/AP1B1/SEC13/PSMD8/HLA-DOA/FC
EIF3E/NSUN4/RPL13A/POLR2D/SART3/TGS1/MTERF3/MRPL11/DHX9/USP39/LUC7L/EIF6/STRAP/S
B/CDK5R1/PSMC3/TWIST1/SRF/RHOA/GTF2H1/NIN/PSMC5/FAF1/RPA1/ARF1/RHOBTB2/GMFB/ATR/
D1/SUSD2/CHEK2/CCND3/ACVR1B/ADAM17/CDK2AP2/PLK3/CCNE2/GLI1/ZPR1/GPR132/CRLF3/AT
FCGR2B/CCND3/VCAM1/TWSG1
NCSTN/MFHAS1/RPS24/HOXB6/ANKRD54/PDE4B/DMTN/DYRK3/ANXA1/CCR2/DOCK11/ERCC2/VPS54/
BCL10/PSMA4/SPPL3/PSMD9/PTPN2/PAK2/RAB29/CACNB3/PSMC3/STOML2/CD79B/CD38/PSMC5/UE

/RPS7/CHEK2/RAD1/PRKAA2/CSNK2A1/RPL5/MBD3/NBN/RPF2/PLK3/PAK1IP1/TMEM109/MYO6/CSN
NT4/DBF4B/RAD51C/BTG2/PIDD1/HSPA2/MTA3/NSFL1C/CHEK2/CCND3/CSPP1/ADAM17/PLK3/HNRN
/CENPK/DYNC1LI1/DSN1/ANAPC11/TUBG2/KLHDC8B/KIF2A/ZNF207/CHMP1A/RANBP1/RAD21/KATN
F2/PSMC3/TWIST1/RHOA/CLTC/SIX1/AP2B1/PSMC5/RBPJ
D3/ACVR1B/ADAM17/CDK2AP2/PLK3/CCNE2/ZPR1/GPR132/CRLF3/PPAT/HINFP/CNOT10/POLE3/RF

21/CEP250/HAUS6/NAE1/NDE1/KHDRBS1/CKAP5/DBF4B/RAD51C/PSMD8/HSPA2/PKIA/PSMA1/MTA3

01C/SPIRE2/NCKAP1/PPP1R9A
/RPL37A/RPLP1/DHX9/IFITM3/RPL32/RPL30/SEH1L/NELFA/POLR2E/RPS9/EIF3G/SUPT4H1/HMGA

C/SMG7/LRPPRC/POLDIP3/THOC7/AAAS/THOC3/HNRNPA2B1/SUPT6H/U2AF1L4/SRSF3/PNPT1/NUP2
C/SMG7/LRPPRC/POLDIP3/THOC7/AAAS/THOC3/HNRNPA2B1/SUPT6H/U2AF1L4/SRSF3/PNPT1/NUP2

CHD1L/HAT1/DQX1/RPS27A/NBN/RAD18/MIS18A/UBA52/NCAPD3/DAXX/ACTR5/RFC5/RNF8/SART3/
MX/ZRSR2/EIF3E/NSUN4/RPL13A/POLR2D/SART3/TGS1/MTERF3/MRPL11/DHX9/USP39/LUC7L/EII

MA1/ARHGAP28/PPM1F/DSTN/PLEKHH2/RHOA/MTPN/SPTAN1/CORO2B/ARF1/RHOBTB2/GMFB/HAX1/I
WD3/RPA2/NAE1/BTN2A2/BTG2/PSMD8/PIDD1/SUSD2/PSMA1/CHEK2/CLSPN/PSMB5/PSMA4/PSMD9
PRDX5/PPM1F/NDUFA13/RHOA/IFI6/ANP32B/LGMN

OC2/XPO7/HNRNPA1/EIF5A/POLR2D/DHX9/NXT2/MEX3D/RBM15B/SEH1L/EIF6/KHSRP/ATR/THOC1/
1A/CD3G/LLGL2/CYTH3

N2A2/BTG2/PSMD8/PIDD1/PSMA1/CHEK2/CLSPN/PSMB5/PSMA4/PSMD9/CDK2AP2/NBN/PLK3/ZFYVI

E19/UIMC1/BCL2L1/INTS7/CHFR/HINFP/TIPRL/CNOT10/CCAR2/CNOT3/MUS81/TFDP1/ATR/TP53I
10/CNOT3/LSM1/RPS9/RPS14/RPS27/EXOSC2/RPL29/CASC3/RPL13/RBM8A/MAGOHB/RPL4/RPL41/
RSL1D1/DIMT1/EBNA1BP2/NOL9/UTP23/RPP30/SUV39H1/PELP1/RPS9/WDR46/IMP4/RBFA/RPS14/
OC37/MLEC/PPIE/PPIB/GAK/PDCL/DNAJC2/DNAJC4/HSP90B1/PDIA3/HSPA13/DNAJC1/CSNK2A2/S
7/CIB1/RPL29/ZDHHC24/SRP68/TRAM1/C2CD5/RPL13/SSR1/MYO1C/RAB8B/RPL4/RPL41/RPL9/RI
N1/ARF1/RHOBTB2/GMFB/HAX1/CAMSAP1/PREX1/ARFGEF1/ADD1/MYO1C/SPIRE2/NCKAP1/PPP1R9A

T4H1/HMGA2/PARP10/MORC2/NELFCD/GTF2F1/ZC3HAV1/LARP1/VPS4A/NECTIN2/CTDP1/SRPK2/II

OLE3/RPA1/TBP/IPO4/SRCAP/GRWD1/ERCC3/TAF1B/PAF1/DDB1/GTF2A2/NAA60/CAND1/MYC/CENI

/HSPA2/PKIA/PSMA1/MTA3/CLSPN/CEP41/PSMB5/PSMA4/HSP90AA1/PSMD9/DCTN1/CEP164/HAUS7

S81/MORF4L1/TP53BP1/HMGA2/DCLRE1C/CIB1/MMS22L
HOC3/HNRNPA2B1/SUPT6H/U2AF1L4/SRSF3/PTPN14/NUP214/TSC1/TP53/STRADA/IWS1
LSPN/CEP41/PSMB5/PSMA4/HSP90AA1/PSMD9/DCTN1/CEP164/HAUS7/NBN/ZFYVE19/HAUS5/DYNC1

CDK5R1/RBM14/RHOA/CLTC
PP5D/DUSP22/CD3G/PTPRC/NECTIN2/BTN2A1/PIK3CB/PSMB1/PLCG1/EIF2B4/MAP3K7/NFKBID/PS
3/SMG7/POLDIP3/THOC7/RBM22/AAAS/PARK7/EGR2/THOC3/HNRNPA2B1/SUPT6H/U2AF1L4/SRSF3/
MYO1C/SMG7/LRPPRC/POLDIP3/THOC7/AAAS/THOC3/TERF1/HNRNPA2B1/SUPT6H/U2AF1L4/SRSF3/
M1F/DSTN/RHOA
TL3/HNRNPA0/PSMB1/MOV10/PSMB6/GTPBP1/HNRNPD/PSMA3/DCP2/HNRNPC/FXR1/SYNCRIP/EIF4C

RPS27/RPL29/EIF2D/LARP1/YTHDF2/YTHDF1/RPL13/METTL3/RPL4/RPL41/RPL9/RPS6/EIF2B4/I

13/PSMD8/HLA-DOA/FCGR2B/PSMA1/VAMP8/PSMB5/PSMA4/PSMD9/DNM2/DCTN1/CANX/HLA-F/AP2A
EG/MMS19/ATR/TP53BP1/ATAD5/MORC2

5/PSMA4/PSMD9/DNM2/DCTN1/CANX/HLA-F/AP2A2/DYNC1I2/KIF22/PSMC3/CLTC/TAPBP/AP1S1/A

3/PSMB5/PSMA4/PSMD9/DNM2/DCTN1/CANX/HLA-F/AP2A2/DYNC1I2/KIF22/PSMC3/AP3B1/CLTC/T
CTR1A/OFD1/TMEM67/CEP72/RAB8B/C2CD3/CNTRL/TSNARE1/VAMP3/RAB3C/CEP97/RAB3IP/NPHP1

/IFT140/BICD2/RAB6A/NEFH/RASGRP1/FEZ1/NEFL

/PSMB6/PLA2G6/PSMA3/RELA/PSMB7/SKP1/PIK3R2/VAV3/APPL2/ACTR2/IKBKB/FCGR1B/NCKIPSD

SMB6/HNRNPD/PSMA3/DCP2/HNRNPC/FXR1/SYNCRIP/EIF4G1/PSMB7/ELAVL1/FASTKD1/SERBP1/NU

RMT5/ING2/HUS1/PRMT6/BRD7/DYRK3
/OGT/ZBTB7B

PL30/SEH1L/NELFA/POLR2E/RPS9/SUPT4H1/HMGA2/NELFCD/RPS14/GTF2F1/RPS27/RPL29/CTDP1
SPIN1/FZD9/WNK1/SMAD3/ADGRA2/WNT5A/PSMD12/SMURF2/WNT3A/ROR2/GID8/DACT1/PSME2/JUP
D4/IFNG/NOD1/TRIM15/RAB7A/PDE5A/TSG101/TAF11/C1QBP/RAD23A/SP1/POMZP3/SMURF2

AB8B/C2CD3/CNTRL/TSNARE1/VAMP3/RAB3C/CEP97/RAB3IP/NPHP1/VPS33B/HAUS2

/HYAL2/HTRA2

DCP2/HNRNPC/FXR1/SYNCRIP/EIF4G1/PSMB7/ELAVL1/FASTKD1/SERBP1/NUP214/TNFSF13/PAIP1

MC5
3/DDX1/MIB2/MTDH/CASP10/TICAM1/CD40

SF7/MCM3AP/NUP50

URF2/NFATC1/ROR2

/RPS28/RPS17/RPL22/RPS15A/CHMP4A/RER1/SRP72/RPL31

7/PPP2CA/RPL22/RPS15A/SMG8/PARN/RPL31/PNRC2/GSPT1/SKIV2L/DCP1A

l/STMN3/MAPK11/NRAS/WAS/TGFB2/RASSF1/RASA3/RAB10/WASF1/ABR/CADM4/CDC42SE1/FGD6/F
39H2/ZNF335/TAF12/CHTOP/TAF10/DOT1L/MTA3/PRKAA2/HDAC10/HAT1/MBD3/USP21/MTHFR/RNF
GAP30/AGFG1/ABR/SBF1/SIPA1L3/CDC42SE1/FGD6/ARRB2/SYDE1/RUNDC1/LLGL1/ARHGAP18/RAN
FPR1/RAP2B/THY1/CORO1C/UBASH3B/HGS/SOCS1/CKS1B/HSP90AB1/NPFFR2/TGFA/TNFRSF11A/RE
ARL2/BIN1/ISL2/HDAC1/RELN/CSNK1E/ZSWIM5/MARK2/GDI1/EPHB3/NUMBL/FGFR1/MT3/DYNLT1/
SMF/LRP8/SRRT/FAIM/PRKCI/ZMYND8/SEMA4D/SYT1/DNM1L/SYT3/GDPD5/KATNB1/STK25/ARHGDI
TRIM6/JAK3/HMOX1/WASF2/CCL5/BAX/CD86/MAPKAPK2/VAV1/COLEC12/PHPT1/MIF/FKBP1A/CD3C
T8/DBF4/FGFR3/ADAM8/PAK4/VLDLR/SHC1/KIF14/AXIN1/SLC11A1/LYN/WNT7B/ITGB1BP1/BIRC7
G/EXT1/VCP/DHDDS/ACOT8/B4GALNT2/NECAB3/DAD1/POFUT2/FUT8/GALNT7/ALG1/LARGE2/GXYLT
3K10/DGKQ/CDK5R1/PPM1F/NEK6/ATP2B4/MAPKAPK5/HPF1/SBK1/PDK3/OSM/HAX1/MAP4K1/CAV1/
RRB2/MUC20/TLR5/PSMD4/XRCC6/GPATCH3/PSMD3/PSMD7/RPS19/IKBKG/FADD/BTK/PELI1/CLEC7
32/APP/MED25/ANKRD33/CARD16/HEY1/GZMA/ZNF675/RALB/MSX1/STPG1/PTPRF/CARD18/MFNG/T

A/TNIP1/LBP/HSPD1/PSMD8/PSMA1/TNFAIP3/MAPKAPK3/PSMB5/BCL10/PSMA4/PSMD9/CASP8/RPS
NF216/BAD/MAVS/IRF5/DMBT1/BNIP3L/OAS3/IVNS1ABP/BCL2L1/IFNAR2/TARBP2/AP1S1/IFI6/I
CLTC/STXBP1/GAK
C3HAV1/LARP1/VPS4A/NECTIN2/CTDP1/SRPK2/IFITM2/DDB1/P4HB/MX1/TRIM32/VAPA/TBC1D20/
MYO1C/TBC1D20/EFCAB7/RBM22/USP36/KIF5B/HYAL2/HTRA2/IFNG/ARIH2/TRIM46/PIK3R2/UBL5

/SUV39H1/PDK3/ATG7/CAV1/ATP7A

/IFT140/CNTROB/CEP135

1/TNIK/FGFR2/DVL2/PFN1/IFT80/AP2S1/MST1R/RNF220/ATP6V0C/PSMD10/PSMD11/PSMD1/PSMA
7/UBE2E1/FBXL6/USP1/DNAJC10/PSMB3/UBE2A/SMARCC1/ARRB2/USP22/KCTD2/VPS25/PSMD4/PS
A2/KCTD10/CTSS/WFS1/UBE2L3/PSMB8/RNF187/PSMB10/PSMA5/DNAJB2/TAF9/POMT2/PSMD13/PS
/GRPEL2/GZMB/NDUFS6/DNM1L/UQCC2/TIMM44/PDCD5/TOMM40L/STPG1/TIMM50/ROMO1/LETM1/MA
01/CDC42/ARHGAP18/TGFB3/SWAP70/CDC42EP2/GMFG/ABI2/ARHGDIA/WDR1/SEMA3E/ARPC1A/WNT
2RA/CD47/SPOCK2/TNFSF13B/MYADM/PLEKHA2/TGFB2/ADAM19/LCK/PTPN6/CD27/IL12RB1/CCDC8
APKAPK5/SBK1/PDK3/OSM/HAX1/MAP4K1/CAV1/ATR/HMGA2/RPTOR/MKNK1/PKD1/CSNK2A2/VRK2/P
/PSMB5/BCL10/PSMA4/PSMD9/CASP8/RPS27A/DMBT1/PAK2/TLR2/MUC6/UBA52/MUC15/PSMC3/UBE
CH2/PIK3R6/HAX1/INPP5D/ZNF683/PTPRC/IKZF3/METTL3/LOXL3/ACIN1/NFKBID/CLPTM1/SH3RF
3/CD3G/PTPRC/CLEC4E/METTL3/LOXL3/RPS6/NFKBID/CHD7/CLPTM1/SH3RF1/IRF4/RHOH/CD4/IR
MRPS24/MRPL33/VPS4A/MRPS5/ADD1/MRPL36/MRPL14/OGFOD1/MRPL3/CAPZA2/ERAL1/AURKAIP1/
A/CTNNBIP1/MALT1/CD101/HLA-G/GATA3/MED1/NUDT21/FLT3LG/IL1RL2
/XIAP/YAP1/LRRK1/PRDM15/PSMD5

RPS21/MRPL12/RPL7/EIF3I/RPL28/MRPS34/TRMT112/EIF2S3/GSK3A/DENR/RPL39/RPL35A/FARS
/RNF26/UBE2L3/PSMB8/RNF187/RNF157/PSMB10/PSMA5/DNAJB2/ASB6/PSMD13/PSMC4/GSK3A/FI
7/UBE2E1/FBXL6/USP1/DNAJC10/PSMB3/UBE2A/SMARCC1/ARRB2/USP22/KCTD2/VPS25/PSMD4/PS
POMT2/PSMD13/PSMC4/GSK3A/OSBPL7/PML/ANAPC11/VPS28/UBE2D1/USP14/PSMD10/PSMD11/PSM
l/TNIK/FGFR2/DVL2/PFN1/IFT80/AP2S1/MST1R/RNF220/ATP6V0C/PSMD10/PSMD11/PSMD1/PTPN
CYC1/FUT4/B3GNT9/HK3/SLC51B/NPC1/GALNT18/B3GNT7/TPI1/FOXK2/GFPT2/CAD/AMPD3/FMOD/
/VAV1/CXCR4/CCL26/TRAF5/MIF/FKBP1A/CD300A/GPR35/CANT1/IQGAP1/CARD10/SLC9A1/RRAGI
NAGPA/CSPG5/TNIP1/CHST12/HPSE/CSPG4/SGSH/MFNG/COL11A1/FUT1/LFNG/EXT1/VCP/DHDDS/A
ONEP1/VPS25/FHDC1/DMPK/AP3D1/STK25/VRK1/EPB41L3/BNIP1/VMP1/ANKLE2/EMD/NSFL1C/PDC
5A/TNIP1/TRIM25/SEC13/VCP/SRPK1/VAMP8/NOTCH1/RPS27A/MAVS/CTBP2/HTATSF1/OAS3/TRIM
-DOA/FCGR2B/TOX/BAD/PTGER4/VCAM1/TUSC2/PTPN2/ADAM17/TMEM98/NKAP/ADGRG3/CD79B/SRI
D47/PSMB8/PSMB10/PSMA5/CD300LF/NRAS/GPSM3/SFPQ/PQBP1/PSMD13/IFI16/PSMC4/MUC5B/CC
D2/DCTN1/NOTCH1/CEP164/ODF2L/HAUS7/VDAC3/MARK4/HAUS5/RILPL1/DYNC1I2/RAB11A/NEDD1
/GABRB3/HSPA5/BCL2L1/EGLN2/RILPL1/HLA-F/ZPR1/RAB29/MECP2/CDK5R1/DAXX/RHOA/STXBP1
SF1/NEB/TACSTD2/CAPZB/GRB2/FHOD1/CNN2/CDC42/ARHGAP18/TGFB3/SWAP70/CDC42EP2/TRPM4
D2/DCTN1/NOTCH1/CEP164/ODF2L/HAUS7/VDAC3/MARK4/HAUS5/RILPL1/DYNC1I2/RAB11A/NEDD1

MD7/IKBKG/RACK1/ZNF675/CLIP3/CCL3/PELI3/PSMD8/LIMS1/PSMA1/TNFAIP3/TRADD/PSMB5/PS
HOA/NEK6/CXXC5/UBE2N/CCL19/TLR7/IRAK2/DAB2IP/TMEM106A/LITAF/NFKBIL1/TLR8/LPAR1/M

RPRD2/CLP1/NUDT21/INTS5/SAGE1/NCBP1/SUPV3L1

E1/FBXL6/USP1/DNAJC10/PSMB3/UBE2A/SMARCC1/ARRB2/USP22/KCTD2/VPS25/PSMD4/PSMD3/RI

3AP2L/FSTL3/NDC1/CCT4/BCAP31/FBXO5/JAG2/SPAG1/RSPH1/SCMH1/HDAC2/MKKS/ZFP41/DEFB1

/TTLL1/EIF2S2/DYNLL1/DPCD/FSTL3/PPARD/LDOC1/NDC1/PAM/BCAP31/KISS1/FBXO5/JAG2/RSI

/TTLL1/EIF2S2/DYNLL1/DPCD/FSTL3/PPARD/LDOC1/NDC1/PAM/BCAP31/KISS1/FBXO5/JAG2/RSI

4R/VPS18/SYT1/SNX12/DNM1L/PDCD5/CSPG5/KHDRBS1/CEMIP/PKIA/EMD/MFF/PDCD10/VAMP8/WV

3S1/PTPN1/RPS27A/VEGFA/PLA2G1B/UBA52/DUSP7/MAP3K10/TPD52L1/DAXX/RAPGEF1/PIK3R5/M

P/PSMA1/SFRP2/PSMB5/PSMA4/PSMD9/VPS35/NOTCH1/SFRP4/BCL9L/TMEM64/GLI1/MESP1/SULF2

3/TCP1/FGR/MAPK11/TGFB2/CASP1/GSK3A/VPS28/OXCT1/PTPN23/YWHAQ/YWHAB/GSDMD/ACSL3/P
2/CDK5R1/DAXX/RHOA/STXBP1/SIX1/AP2B1/LGMN/TIGAR/CDC34/GCLM/CRLF1/ATG7
M64/GLI1/MESP1/SULF2/UBR5/PSMC3/STK3/GSKIP/RAPGEF1/IFT20/PSMC5/RBPJ/CCAR2/NPHP4/

RIM32/NFKBID/VAPA/ATP2C1/CC2D1A/RHOH/SHISA5/TRIM62/CD4/BRD4/RELA/TFG/NOD1/PRKCB/

NCAM1/TRIM32/VAPA/THOC7/TRIM27/KPNA4/THOC3/TRIM62/CD4/WWP2/HYAL2/CTSB

P2/PIN1
140/CNTROB/CEP135

ACVR1/ABL1/SFRP2/CSK/PTK2/PTPN2/ADAM17/TNK2/PTPN1/VEGFA/PAK2/LMTK2/DGKQ/PPP2R5B/
4/NF2/ACVR1/ABL1/SFRP2/CSK/PTK2/PTPN2/ADAM17/TNK2/PTPN1/VEGFA/PAK2/LMTK2/DGKQ/PI
4/PSMD3/CARD16/PSMD7/IKBKG/RACK1/ZNF675/IGBP1/CLIP3/CCL3/PELI3/PSMD8/LIMS1/PSMA1

38/THUMPD3/QTRT2/DUS3L/TARBP1/TRMT10C/ANKRD16/AIMP1/CDKAL1/GTF3C4/TRIT1/ADAT2/EI

[illegible]1/CARD10/EFNA5/SH2D3A/IL34/DGKZ/LEFTY1/MAD2L2/HIF1A/MAPK3/NUPR1/FZD1/P2RY6/CD3[illegible]
[illegible]/DNM1L/PGAM5/RACK1/ARHGDIA/RIC8A/RGS3/TBC1D10C/RASAL3/WNT4/TBC1D22A/CCL3/CHN1/[illegible]
[illegible]5/ZMIZ1/POLE3/RBM15B/ELOF1/TBP/CTBP1/SEH1L/EIF6/CDYL/SUV39H1/POLR2E/PRKRA/MORF[illegible]
[illegible]XXYLT1/PMM2/SDF2L1/DOLK/EOGT/B3GALT6/ARFGEF1/ST8SIA4/GCNT1/B3GNT8
[illegible]XXYLT1/PMM2/SDF2L1/DOLK/EOGT/B3GALT6/ARFGEF1/ST8SIA4/GCNT1/B3GNT8

R4/SPRED1/POLR2G/MMP2/UBASH3B/CD3EAP/HGS/SOCS1/EPHB6/ARPC2/TGFA/PSENEN/ARPC3/SO[illegible]
DAZAP1/NCAPH2/ETV6/DEAF1/POC1A/PAQR8/DNMT3A/CYP26B1/AGFG1/OSBP2/SBF1/ODF2/MSH2/[illegible]
/FBXO5/NOC2L/GTPBP4/PRR7/CDKN2B/HDAC2/U2AF2/KDM1A/TESK1/LYN/ITGB1BP1/CDKN1C/DUS[illegible]
/PSMD3/RBX1/PSMD7/TBL1XR1/RACK1/CSNK1G2/JRK/PSMD8/ABL1/EMD/VCP/PSMA1/SFRP2/TNFA[illegible]
P3L/LZTS1/CSNK2B/CDC37/CDK5R1/TECPR1/VPS37B/ATG13/IFT20/PRKAG1/MTERF3/ATG9A/TIG[illegible]

3D1/RPL11/EPB41/PARK7

7/DPY30/TMCC1/VAMP3/AP5S1/ANXA8L1/RNF126/VPS29/VPS54/RAB7A/CHMP6/RAB6A/TSG101/G[illegible]
/CCL8/TRIM68/IFITM2/PPARG/MYO1C/NCAM1/IRF4/HLA-C/VAMP3/KIF5B/TRIM62/NUB1/IFNG/S[illegible]
BAG6/FAF2/CHAC1/ADD1/SSR1
5/MYB/PRDX2/ZAP70/MALT1/HLA-G/GATA3/NFATC2

[illegible]3A/IL34/LEFTY1/MAD2L2/MAPK3/FZD1/P2RY6/CD3E/MAP2K2/CENPE/ATP6AP1/PAK1/PEA15/FG[illegible]
[illegible]1/TGFB2/CALCA/MAP3K14/TNIK/DVL2/MST1R/PSMD10/C5AR1/LRP8/CCND2/RPS3/STK10/MTCP1/[illegible]

[illegible]/JOSD1/PSMC5/USP39/BECN1/USP48/SPATA2/SNX3/NEDD8/ENY2/TADA3/TRRAP/OTUD3/SKP2/TF[illegible]

[illegible]TROB/FEZ1/CEP135/ATXN2/CHMP4A/SMCR8

l/IL1B/ADRA2C/RNF157/MYADM/PLXND1/PPP1CC/NDRG4/NAP1L2/TUBB2B/DVL2/NSMF/LRP8/SRRT
CKAP1L/SH3KBP1/VLDLR/LYPLA2/CPNE1/SHC1/PIK3CD/YWHAH/WNT7B/RAC1/CNTNAP1/ACTB/RAP2
LIP/RAP2A/CDK5/NREP/CTTN/SPOCK1/ADRA2C/RNF157/PLXND1/GSK3A/NDRG4/NAP1L2/TNIK/TUE
lL/SH3KBP1/VLDLR/LYPLA2/CPNE1/SHC1/PIK3CD/YWHAH/WNT7B/RAC1/CNTNAP1/ACTB/RAP2A/CI
D2L2/PSMD2/MAPK3/CD276/IL27RA/HLA-DQB2/CD3E/HLA-A/PAK1/SASH3/SLC39A10/FPR1/MNDA/
ANOS1/TARBP2/ETF1/EIF5A/EIF3E/CAPRIN1/RPUSD3/NSUN4/RHOA/RPL13A/HNRNPR/POLR2D/RPI
RM1/LAMTOR5/SPOCK2/PSMB8/SPOCK1/SERPINB6/F3/LCK/VIL1/CD27/IFI16/CASP1/PML/USP14/
RPL39/IL12RB1/COCH/IL15/ILF3/C5AR1/ISG20/EXOSC5/GSDMD/TRAF3/DROSHA/IL4R/APOBEC3I
K1/MSX1/RBBP7/SEMA3C/EPB41L3/SEMA3E/MAPT/RERG/CDHR2/ABL1/GNG4/RASAL1/SFRP2/BRAT1

/USP48/SPATA2/SNX3/NEDD8/ENY2/TADA3/TRRAP/OTUD3/SKP2/TFPT/PSMB1/ASXL1/USP3/MAP3K
SLC35C2/FUT11/XXYLT1/PMM2/SDF2L1/DOLK/EOGT/B3GALT6/ARFGEF1/ST8SIA4/GCNT1/B3GNT8

APK2/VAV1/COLEC12/MFGE8/SLC1A7/CD300A/SLC12A9/CLTA/ARPC4/SLC9A1/JMJD6/MAPK3/SYT8
/TNIK/TUBB2B/COCH/DVL2/LST1/NSMF/LRP8/CDC42SE1/TACSTD2/CAPZB/FGD6/SEMA4B/CDC42/A
TXN/MLKL/ADRA2C/FGR/LCP2/MAPK11/TGFB2/CALCA/MAP3K14/TNIK/DVL2/MST1R/PSMD10/GCN1/

/PPHLN1/RBPJ/EPHA2/CYP27B1/EREG/TFDP1/LDB1/ATP7A/ERCC3/KRT15/FOSL2/ATP2A2/STK4/C

MRPL30/APEH/UPF1

A/GULP1/CORO1C/SIRPA/BIN1/SPON2/NPC1/GPR107/HSP90AB1/F2RL1/ARPC2/RAB7B/TIGD5/ARI
2/TMEM119/TESMIN/AXL/PRDX4/KHDRBS1/TTC26/SOHLH2/PIWIL4/HSPB11/RAD51C/PAFAH1B2/HS
P4B/NAT10/EEF1A2/MPV17L2/EIF3K/THBS1/PUS7/RPL5/PLK3/JMJD4/SARNP/RPS4X/C8orf88/MA
SR2/TPP1/TMUB1/SDF2L1/BHLHA15/HSP90B1/UBE4B/UBAC2/AUP1/PDIA3/HSPA13/CAV1/DAB2IP/
A/SIGIRR/IDO1/EPHA2/XCL1/CD84/INPP5D/PTPRC/NFKBIL1/PIN1/ADCY7/TLR8/TRIM27/SYT11/

GA5/FKBP1A/DOK1/ATP6V1C1/IQGAP1/ARPC4/PDGFRL/EFNA5/IGSF1/LEFTY1/RGS14/HIF1A/MAPI

H3KBP1/VLDLR/LYPLA2/CPNE1/SHC1/PIK3CD/YWHAH/WNT7B/RAC1/CNTNAP1/ACTB/RAP2A/CDK5/C

XL/PRDX4/KHDRBS1/TTC26/SOHLH2/PIWIL4/HSPB11/RAD51C/PAFAH1B2/HSPA2/ADAM28/SRPK1/
CK1/SERPINB6/F3/LCK/VIL1/CD27/IFI16/CASP1/PML/USP14/GPX1/RPS3/TFAP4/CST7/ARRB2/

SL2/ARPC2/SLC1A3/NBEAL2/RELN/S100B/CDH11/ZSWIM5/MARK2/GDI1/EPHB3/PLOD3/NUMBL/LE
EEF2KMT/SENP3/LOXL1/PRKAA2/HDAC10/HAT1/UBA2/MBD3/PIAS4/WDR5/PIH1D1/MORF4L2/MECP

1/HYAL2/IFNG/ANXA1/AIM2/NLRC4/TLR1/NOD2/BTN3A2/ARG2/CPTP/LILRB1/RASGRP1/MAPKBP1/
/IL10RB/SLFN11/APOBEC3F/HYAL2/IFNG/CD207/RELA/AIM2/FAM111A/POLR3A/TRIM15/ZMPSTE
P1/HAX1/TMEM102/CSNK2A2/PARL/TIMM23/ACACA/TIMM8B/HUWE1/PITRM1/NAIF1/CNP/THEM4/I
M47/HSPA9/ARMC6/PSMD5

NT5C3A/GBP2/MAPK3/TRIM11/SERPINE1/IL27RA/SLPI/FUCA2/ISG15/PDZD11/BAIAP2L1/LYPD8/
K3CD/YWHAH/WNT7B/RAC1/CNTNAP1/ACTB/RAP2A/CDK5/CTTN/RNF157/PLXND1/RAB10/C12orf57/
NT5C3A/GBP2/MAPK3/TRIM11/SERPINE1/IL27RA/SLPI/FUCA2/ISG15/PDZD11/BAIAP2L1/LYPD8/
ML/RPL39/VPS28/RPL35A/UBE2D1/YWHAQ/RUVBL2/YWHAB/RPS3/RPL27/BAG3/SSR3/NUP155/RPL3

MB3/CALR/RELL2/AXL/ANGPT2/CSPG5/SEMA3E/HPSE/WNT4/NF2/EMILIN1/LIMS1/ABL1/TBCD/VCI

/PLXNC1/B4GALNT2/TWSG1/PTK2/PTPN2/THBS1/NOTCH1

3/ATG4D/ATP6V0C/MVB12A/ATP6V0E1/BAG3/VPS18/VPS25/DNM1L/PGAM5/IKBKG/PIP4K2C/RALB/
93B1/BPIFB2/NFKB2/NT5C3A/GBP2/MAPK3/TRIM11/SERPINE1/IL27RA/SLPI/FUCA2/ISG15/PDZI
ASAH1/RPL10L/DLAT/ITGA2/RPS5/GBA/RPS21/FA2H/MRPL12/RPL7/EIF3I/RPL28/MRPS34/TRMT1
3/ATG4D/ATP6V0C/MVB12A/ATP6V0E1/BAG3/VPS18/VPS25/DNM1L/PGAM5/IKBKG/PIP4K2C/RALB/

LR1/SLC11A1/JAG1/CD209/LYN/HLA-DRB5/IL1B/JCHAIN/CTSS/TNFSF13B/WAS/PTPN6/CD27/SAM

/GRB7/EMB/SEMA4B/GRB2/PARD6B/LLGL1/SEMA4D/APP/MAPK7/SEMA4F/SEMA5B/STK25/ARHGDIA/
/TRAF3/ARRB2/PRKCI/CMKLR1/APP/CARD16/IKBKG/BTK/PELI1/ZNF675/DAP/TRIM25/TAF12/PII

[NPO3/EFCAB7/RPL11/MFHAS1/RBM22/KPNA4/PARK7/HEATR3/HYAL2/IFNG/IPO7/SKP1/SESN2/P]
[illegible]/MAP4K1/MAP4K2/DAB2IP
[illegible]YT17/WNT5A/PPP3CB/OLFM1/GATA4/WNT3A

)CS1/SLC51B/CCT2/CD58/IRF3/F2RL1/DYNLL1/NDUFAF2/GDI1/PPARD/GPI/SERPINB1/PPIA/ZC3
31/OPTN/GNAI3/BNIP3L/LZTS1/SREBF1/TRIM21/CDC37/CDK5R1/ATG13/IFT20/PRKAG1/WDR41/T

/TP53/FEZ1/SPTLC2/CHMP4A/SMCR8/ATP6V1D/CLEC16A/RNF41
A3A

2RY6/MAP2K2/ATP6AP1/PPP2R1A/PAK1/PEA15/FGFR4/SPRED1/RASGEF1A/FPR1/SIRPA/HGS/F2RI
AB7B/LRRC14/CTLA4/ARPC3/WIPF1/NPLOC4/PSMB2/SH2B2/IRF7/ZC3H12A/PSME3/TNIP2/FLOT1/
7B/ITGB1BP1/ARPC5L/RAC1/LAMTOR5/CDK5/CTTN/ADRA2C/MYADM/WAS/VIL1/AP2M1/CIT/PFN1/W
34/CDK5/VANGL1/PSMB8/PSMB10/GNB1/PSMA5/PSMD13/PSMC4/GSK3A/AP2M1/TNIK/FGFR2/DVL2/

N2C/SEMA4B/CDC42/SERTAD3/SEMA4D/SYT1/APP/GAL/SYT3/SEMA4F/SEMA5B/NME6/RACK1/MSX1/

RF/CYCS/ANKLE2/ILKAP/SYMPK/CALM2/PTBP1/PTPN12/CD33/BOD1/SPPL3/PTPN2/STYXL1/PTP

PRKAR1B/GSKIP/YWHAG/GPS1/HEG1/SH3BP5L/GMFB/DUSP18/CAV1/DAB2IP/RPTOR/EPHB2/CIB1/I

2RY6/MAP2K2/ATP6AP1/PPP2R1A/PAK1/PEA15/FGFR4/SPRED1/RASGEF1A/FPR1/SIRPA/HGS/F2RI

A/PLA2G1B/UBA52/DUSP7/MAP3K10/PIH1D1/CDK5R1/TPD52L1/DAXX/RHOA/STK3/RAPGEF1/ATP2I

A/HSPA5/DDX56/RAB11A/PACSIN1/PPP2R5B/METRN/APBB1/SRF/CAPRIN1/RHOA/RAPGEF1/DNMT[illegible]

[illegible]RAP1

l/TSEN34/EDC4/GTF2H1/RPA1/TSEN15/RNASEK/NOL9/UTP23/MUS81/RPP30/RNASEH1/SND1/ATAI

)USP7/MAP3K10/DGKQ/CDK5R1/PRKAR1B/DAXX/STK3/RAPGEF1/ATG13/MAP3K1/MINK1/RPLP1/MAI

MA5B/STK25/ARHGDIA/SCN1B/SEMA3C/SEMA3E/MAPT/CHN1/EXT1/ABL1/VCL/KEL/DBNL/PLXNC1/I
)1/VCP/SFRP2/USP5/TRADD/BAG2/BAD/BCL10/SGTA/RCN3/TNFSF15/PTK2/CASP8/PDCD2/PLK3/N

ADM/ADCY5/CCL19/XCL1/CLIC2/HTT/EDNRA/CAV1/CALM3/CCR10/PKD1/PTPRC/CXCL10/CNR1/GN/

APIN/IST1/ARF1/SNX3/KLHL12/SAMD9L/CAV2/PEF1/PI4K2A/CAV1/VPS4A/NECTIN2/RAB1A/RFX2

R5L2/OR5H1/OR8U1/OR10R2/OR11H12/OR6Y1/OR8K3/OR8J1/OR1A2/OR10A4/OR6N1/OR2T5/OR5AI

5/MEIS1/HSPA1A/CTNNBIP1/CLDN18/CD101/LEO1

PS35/MRPS33/MRPS10/MRPL45/NGRN/MRPL41/MRPL49/MRRF/MRPS6/TUFM/OXA1L/MRPS28/MRPL30

AN3/RAB23/VPS41/ATG16L1/RB1CC1/ULK3/HOOK3

3/ERCC1/C9orf116/TMEM109/FNTA/HSPA5/BCL2L1
3/ERCC1/C9orf116/TMEM109/FNTA/HSPA5/BCL2L1

/FBXL7/UBE2N/ANAPC5/CDC27/CDC34/UBE4B/UBE2E2/RNF114/PARP10/FBXO6/FBXO41/STUB1/AN
10/RAPGEF1/MAP3K1/GPS1/MINK1/ADORA2B/UBE2N/MECOM/CCL19/SH3RF3/IRAK2/MAP4K1/MAP4K

BABAM1/CBL/SEMA5B/ANGPT2/RFWD3/NABP1/RAD9A/XRCC4/CEP250/OGG1/RIC8A/CUL4B/POLD3/N
C1/RNF19B/GNA12/NPM1/POLR2G/BMF/PDCL3/HGS/NPC1/DDA1/YBX1/GPSM1/HSP90AB1/PABPC1/F

TL3/SNRPD3/PWP1/MRM2/PRMT5/METTL2A/PCMT1/PHF1/DPY30/TRMT2A/THADA/BCOR/SETDB1/MYC

NLRP3/WNT5A/C5AR2

/CD38/GAK/ACTN4

[illegible]/FOXK2/CSNK1E/NUP93/NUP205/NUP37/PSMB2/DDIT4/PPP1CA/NDC1/PIM2/NUP188/ZC3H12A/P[illegible]

/RASSF8/SRF/RHOA/RAPGEF1/ACTN4/HEG1/EPHA2/CORO2B/NPHP4/GPBAR1/CAV1/LDB1/RAB13

N3/DEAF1/TGFB2/VASH2/PSMC4/ASCL2/SOCS3/SMPD4/FGFR2/PLK4/DVL2/PFN1/BYSL/EIF4A3/G(
3SCN/CHN1/RHOT2/ABL1/RASAL1/SIPA1/ARAP1/SGSM3/ARHGAP11A/ARHGEF3/SRGAP2/DNM2/ARH(

/FGFR1/MT3/FGFR3/PAK4/EXTL3/SHC1/MKKS/KIF14/BCAR1/CDK5/WFS1/LAMTOR1/CTTN/MEG3/WW
)10/WFS1/WWC3/PHB/STMN3/MYADM/TGFB2/TAF9/PTPN6/RASA3/GSK3A/SOCS3/PSMD10/GPX1/KPT

3/HK1/DNM1L/TRPM4/PDCD5/STX4/RACK1/CLEC5A/HCAR2/ABCG1/CCL3/ACHE/CEMIP/ABL1/EMD/M
NS1/CTSS/PDGFA/CD47/SPOCK2/ADAM12/LOXL2/TGFB2/ADAM19/ADAMTS9/CCDC80/BGN/COL6A2/F

[illegible]3/STMN3/DEAF1/TGFB2/VASH2/PSMC4/ASCL2/SOCS3/SMPD4/FGFR2/PLK4/DVL2/PFN1/BYSL/EI[illegible]

T9/SCAMP4/ECE2/CORO7/GALNT18/PPP6R1/GPSM1/B3GNT7/TGFA/CHPF2/COPE/TMED9/PSENEN/SC
NCENP/CBX3/DVL3/BLM/RSPH1/NHP2/HDAC2/KDM1A/NASP/CHRAC1/PRIM1/SSB/EHMT2/BUD31/SSF
AGD/STXBP2/TMEM165/HSPA8/RRAGC/NEU1/GM2A/HLA-DQB2/TCIRG1/FUCA2/RNF19B/CD63/STK11
G/KMT5C/TIPIN/PADI2/CHD4/RUVBL2/CENPB/CCND2/EXOSC5/RBBP4/POLA1/TBX19/DFFA/UBE2A/
J/EME1/ZNF385A/MAGED1/HMGB3/CREB3L1/CHEK1/RAN/HSF1/RHNO1/NABP2/KIF18A/RANGAP1/MC
L/NPM1/NARF/UPF3B/MNDA/MCRS1/CD3EAP/B3GNT9/SMARCA4/NOL3/METTL1/RBM3/ZCCHC17/STAT
AGD/STXBP2/TMEM165/HSPA8/RRAGC/NEU1/GM2A/HLA-DQB2/TCIRG1/FUCA2/RNF19B/CD63/STK11
A3/SLC44A2/SLC15A3/AP2S1/OSTM1/UBA1/ATP6V0C/TMEM63A/KPTN/BORCS8/GLMP/STARD3/VPS1
1/CLTA/UNC93B1/FTH1/RRAGD/STXBP2/TMEM165/HSPA8/RRAGC/NEU1/GM2A/HLA-DQB2/TCIRG1/F
/CSNK1D/ATF4/CDK2/MKKS/NLRC5/CAMSAP2/DCTN2/RAB11FIP4/DCTN5/B9D2/TCP1/KIFC3/POC5/
/SLC1A4/CHD3/NPM1/UPF3B/BRCA1/PCNA/XRCC2/SGO1/SFI1/DYNLRB1/IFT52/DYNLL1/DYNC1H1/
IP/CSNK1D/KIF14/KNSTRN/RAB11FIP4/ANXA11/CTTN/KIF15/RASSF1/POC1A/NUP85/HAUS8/DYNC
RRAS/PPP1R9B/ARPC3/CDH11/HYOU1/PDLIM3/KRT18/PPP1CA/CORO1B/PIP5K1C/PPIA/TGM2/LASP
/SNF8/SLC11A1/HLA-DRB5/RAB11FIP4/RAC1/TMEM163/EHD2/LAMTOR5/RAP2A/LAMTOR1/TMEM9/C
/DDOST/CD300A/ARFGEF3/FLOT2/IQGAP1/HLA-DPB1/CLTA/STXBP2/ITGAX/SYT8/HSPA8/HLA-DQB
NNI2/ADAM8/MYO1G/CAP1/CAPZA1/RTKN/MYO1F/DAPK3/FHOD3/HCLS1/PSTPIP1/STK17B/DCTN2/A
/RBM19/MED19/PIAS3/CBX4/FIBP/CENPI/SFPQ/PQBP1/IFI16/SRSF12/PPP1CC/PML/C12orf57/I
/SYT1/APP/DNM1L/AP1G2/SYT3/CPE/STX4/RABAC1/M6PR/COPG1/ATP6V0D1/PDCD6/RASSF9/SEC1
ABCC4/ATAD3B/CHIT1/PSMD14/TIMP2/SYNGR1/DNAJC5/HSPA6/DDOST/ALOX5/PLEKHO2/MIF/CD3C
AP1/HLA-DPB1/CLTA/STXBP2/ITGAX/SYT8/HSPA8/HLA-DQB2/TCIRG1/HLA-A/CD63/DOK3/ANKRD2
NGAP1/MCM5/SMARCD1/CENPL/HMGB2/TCF3/TRIM28/POLA2/BRMS1/SIRT6/SGO2/ARPC4/WDR76/GI
/UNC93B1/ZDHHC7/B4GALT5/CREB3/GBP2/TMEM165/GALNT10/RHBDF1/HLA-DQB2/BCL9/HLA-A/AF
G1/NPC1/CCT2/C3AR1/HSP90AB1/DYNC1H1/RAB7B/FMOD/CLCN7/MFSD12/NPC2/SORT1/PLBD2/PA2
HD1/CD68/ARHGAP1/HLA-DQA2/LAMP3/SNF8/SLC11A1/HLA-DRB5/RAB11FIP4/RAC1/TMEM163/EHI
1A/NASP/CHRAC1/PRIM1/SSB/EHMT2/BUD31/SSRP1/ACTB/MTA2/WDHD1/TCP1/SWI5/DNMT3A/THOC
DAB1/STAT1/XRCC3/TGFA/CAPN6/CTLA4/GDPD3/S100B/SORT1/STX10/ODC1/KRT18/CORO1B/UNC4
C2/PABPC1/RRAS/PPP1R9B/ARPC3/CDH11/HYOU1/PDLIM3/KRT18/PPP1CA/CORO1B/PIP5K1C/PPIA
1/B3GNT5/GBGT1/CHST1/ABCB6/PTGES2/FUT4/STX6/B3GNT9/SCAMP4/ECE2/CORO7/GALNT18/PPF
A3/SLC44A2/SLC15A3/AP2S1/OSTM1/UBA1/ATP6V0C/TMEM63A/KPTN/BORCS8/GLMP/STARD3/VPS1
D1IP1/TUBB4B/KIF12/TBCB/KIF3C/KIF18A/CCT6A/RHOQ/KIF21B/WIPF3/CCT3/CARMIL1/COL5A1
D1IP1/TUBB4B/KIF12/TBCB/KIF3C/KIF18A/CCT6A/RHOQ/KIF21B/WIPF3/CCT3/CARMIL1/COL5A1
/QPCT/RHOG/ISLR/TUBB4B/GRN/TNFAIP6/RAB11FIP5/CD53/PLAU/GIPC1/DTNBP1/ABCC4/ATAD3E
UBB4B/KIF12/TBCB/KIF3C/KIF18A/CCT6A/RHOQ/KIF21B/WIPF3/CCT3/CARMIL1/COL5A1/GAS2L3
TPM2/IQGAP1/RGS14/SLC1A4/PLEC/LCP1/MAP1S/TPM3/MAP2K2/CENPE/PAK1/NUDC/NARF/KLC3/T
2/SF3B5/SNRPC/RPL10L/EMG1/NOP58/ATXN2L/SSB/RAC1/BUD31/RPS5/DAZAP1/SNRNP40/ZNF593
SFR1/HAT1/CSNK2A1/USF1/MBD3/RNF2/ANP32E/RBMX/NCAPD3/SRF/ACTR5/GATAD2A/DHX9/SMARC
/DYNLT1/HAUS1/TUBB4A/CSNK1D/KIF14/CAMSAP2/HSPH1/CCT7/DCTN2/KNSTRN/REEP2/FKBP4/CI
FOCAD/CAPN1/CAP1/FZD2/TSPAN4/ARPC5L/TNC/RAC1/ITGA2/RPS5/ACTB/GNB2/BCAR1/CTTN/RPI
/CAPN1/CAP1/FZD2/TSPAN4/ARPC5L/TNC/RAC1/ITGA2/RPS5/ACTB/GNB2/BCAR1/CTTN/RPL7/MCA
FOCAD/CAPN1/CAP1/FZD2/TSPAN4/ARPC5L/TNC/RAC1/ITGA2/RPS5/ACTB/GNB2/BCAR1/CTTN/RPI
C1D10C/FCGR3B/RAB4B/KCNAB2/RAB3D/HVCN1/VAMP8/RAB5C/MOXD1/PSAP/CD33/CD177/DMBT1/C
TN/FGR/MYADM/PLXND1/FAM89B/VIL1/SAMSN1/WASF1/INPP5J/PIEZO1/CADM4/KPTN/ARF4/ABI3/
OC5/NUP85/PPP1CC/SIRT7/CENPK/NUP43/PML/DYNC1LI1/DSN1/PARP1/THOC6/KMT5C/MSH2/CENF
MTOR1/TMEM9/NFAM1/GNB1/KXD1/AP2M1/ABCA2/DRAM2/SLC29A3/MAP1LC3B/SLC44A2/SLC15A3/A
RPLP1/DHX9/RPL32/RPL30/MRPL15/C12orf65/MRPL4/SURF6/RPS9/RPS14/RPS27/MRPS24/RPL29
A/FBXO45/RPL36/UBA52/BORCS6/RPL19/RPS11/RPL22L1/RPL13A/RPL37A/STXBP5/CTU1/RPLP1/
D3/HSPA2

NTXR1/DOCK8/NCKAP1/PLCG1/PPP1R9A/PXN/CCDC88A/SH3RF1/PTPN13

3A/SF3B6/MAGOHB/SNRPD3/PRPF38B/TFIP11/IK/PPP1R8/ADAR/SNRNP200/RBM22/HNRNPC/BUD13
PS27/MRPS24/RPL29/EIF2D/MRPL33/MRPS5/RPL13/MRPL36/RPL4/RPL41/RPL9/MRPL14/RPS6/R
NK2B/AP2A2/ATP6AP2/SLC2A3/PSMC3/PPIE/RHOA

RPL22/MRPL58/MRPL38/MRPS30

F2/RABEP2/VOPP1/TCN2/RAB32/CXCR4/FLOT2/VPS37C/HLA-DPB1/RAB15/CLTA/UNC93B1/EHBP1I
M/KCTD10/CDK5/KDM5B/UBE2L3/CBX4/TAF9/TRMT6/SOCS3/ANAPC11/KDM5C/UBE2D1/STT3A/DCAI

UNX3/GTF2H1/NAA15/RBPJ/TAF13/PMF1/TBP/MMS19/CTBP1/TFDP1/KLF5/TLE6/HAX1/LDB1/PKN(
/DNM2/MCHR1/ADAM17/ROM1/MYO6/PACSIN2/LIMA1/PACSIN1/TLN1/EVC/RHOA/PDPN/FAP/SLC22/

/NUP85/BANF1/NUP43/MYOF/INTS1/AGFG1/PARP1/NSMF/RAE1/CCND2/TMEM43/ANXA4/POLA1/NUI

4C/CNIH2/PTPRS/SEC23B/SYP

R3/KDM4A/NOLC1/ESRRA/NOL11/ZNRD1/MTDH/RPP38/PRMT7/STOX1/DDX46/DUSP11/TWISTNB/IP(

TBP2/ADD3/MYO6/PARD3/WIPF2/EXOC3/DSTN/CYTIP/PLEKHH2/RHOA/ACTN4/FNBP1L/SPTAN1/CTI

AMP8/RAB5C/GNLY/HSP90AA1/SRGAP2/DNM2/RPS27A/DMBT1/TLR2/MYO6/ANXA3/UBA52/CD2AP/HI

P7A/ARL1/CBY1/ARFGEF1/PLEKHA8/GCNT1/KIAA1324/CHAC1/ATP9A/AP4B1/ATP2C1/DPY30/IGF2

P1/MCOLN1/WNT5A/WNT3A/ROR2

?1S1/GOSR2/RAB27B/AP2B1/HBEGF/COPB1/BTC/AP2A1/EREG/VPS33A/ARF1/STEAP2/COPA/SNX3/
3RA3/PPP1R9B/TMED9/PLCB3/P2RX5/STX10/PPP1CA/RAB35/PIP5K1C/HIP1R/ARFGAP1/MT3/FLOT
GFB2/PTPN6/PSMD13/COMMD9/GALNS/DSN1/IMPDH2/PSMD11/PSMD1/PADI2/TRAPPC1/GSDMD/CNN2

A2/TATDN2/WTAP/INPPL1/FNDC1/HNRNPU/SARNP/BNIP3L/MAGOH/HSF4/PACSIN2/ARL6IP4/GTF2F
DAM17/CASP8/CAPN2/CD177/GNAI3/TLR2/ADD3/PACSIN2

DAM17/CASP8/CAPN2/CD177/GNAI3/TLR2/ADD3/PACSIN2

NNA1/ADAM17/CASP8/CAPN2/CD177/GNAI3/TLR2/ADD3/PACSIN2

RPC5/STMN2/HSP90AA1/DNM2/AAK1/MAP1A/RAB3A/LMTK2/ZPR1/TUBB3/KIF20B/PACSIN1/CDK5R1

L1/TAF11/GTF2E1/MED14/TAF4/SNAPC1/TAF2/GATA4/NFATC1/MED6

/FTL/FGR/PSMA5/TGFB2/PTPN6/PSMD13/COMMD9/GALNS/DSN1/IMPDH2/PSMD11/PSMD1/PADI2/TR
/NSMF/RIMS3/NCL/PARD6B/NRBP1/LLGL1/CDC42/PPFIA3/MYO9B/KLHL17/ACTL8/AP3D1/GMFG/AF
ALM3/CIB1/SLC9A6/KLC1/CNR1/PPP1R9A/UTRN/ADGRL1/UCN/KCNC4/SYT11/KIF5B/TIAM2/FKBP1

[illegible]O44/SKP2/FBXO10/ENC1/CDC23/KLHL25/RNF144A/BRAP/BCOR/CAND1/MED20/WWP2/ARIH2/ANA[illegible]

/FTL/FGR/PSMA5/TGFB2/PTPN6/PSMD13/COMMD9/GALNS/DSN1/IMPDH2/PSMD11/PSMD1/PADI2/T[illegible]

[illegible]768/TADA3/PAF1/TRRAP/PIK3CB/RPRD1A/MAP3K7/TCEA1/GTF2A2/CCNG2/POLR3F/ULK1/POLR2[illegible]

[illegible]/ABI1/TAOK2/MAPK8IP3/GAP43/PTBP2/NRP1/ZFYVE27

[illegible]/MRPL3/IMMP1L/TIMM9/TIMM22/MRPL20/MRPL24/PAM16/MRPS16/COX4I1/MRPL55/NDUFB2/CHC[illegible]

[illegible]/ARF1/RAB9A/YIPF1/HTT/ATP7A/SLC9A6/LITAF/VPS4A/SLC9A9/PLD1/KIAA1324/SDF4/VPS16/
[illegible]2/HSP90AB1/STAT1/SLC1A3/CAD/GABRA3/PPP1R9B/HDAC1/RELN/S100B/SORT1/RIN1/MARK2/G[illegible]

[illegible]P3/GAP43/PTBP2/NRP1/ZFYVE27

[illegible]HD17A/MINK1/RPL30/BCAS1/MPP2/ARF1/RPS14/RPS27/ADD1/LIN7B/PPP1R9A/ADGRL1/BCR/EP[illegible]

AP1/NISCH/LZTR1/CD274/ITGB1/EHD3/INPP5F/STX8/AVL9

C3F/FXR1/SMN2/CNOT2/ELAVL1/MAEL/DDX1/LSM3/TARDBP/ATXN2/GRSF1/TDRD7/DDX19A/PNRC2/

AR1B/RHOA/ABHD17A/STXBP1/ATP2B4/AP2B1/ADORA2B/CTBP1/ARF1/ARHGAP22/EPHB2/CNR1/PI

CAS1/MPP2/ARF1/RPS14/RPS27/ADD1/LIN7B/PPP1R9A/ADGRL1/BCR/EPB41/NEURL1/SYT11/PDE

3C1D20/SLC25A3/SYT11/ZDHHC20/VAMP3/RAB3C/FAR2/VAMP1/YIF1A/ENTPD4/PTPRS/TEX261/S

3P4/POLA1/SMARCC1/MCM8/VCX/KAT2A/CBX5/CABIN1/MTF2/NCOR2/PCGF2/HMGN1/POLQ/FUS/RA
OR/CDK7/DRAP1/BATF3/RUVBL2/MYCBP/SAP30/PRPF6/SMARCC1/USP22/KAT2A/MED24/PRRX1/ZM
APK11/LCK/HUNK/MAP3K14/GSK3A/CDK18/NUAK2/TNIK/SLA/FGFR2/CIT/PLK4/MST1R/STK32C/L
/MRPL9/NDC1/NUP188/RPL17/COL5A2/PLLP/ACTG1/RPLP2/MRPS23/COL6A3/TUBB4A/RPSA/JAG1/
SA3/CASP1/ARHGAP30/AGFG1/ABR/SIPA1L3/DNAJC10/SYDE1/PTPA/RUNDC1/LLGL1/MTCP1/ARHG
FMNL1/KIF12/RAB11FIP5/TMEM201/KIF3C/KIF18A/CRMP1/CFL1/MICALL2/KIF21B/GIPC1/EPB4
PNKP/FGFR3/PAK4/PIP4K2A/PAPSS1/CCL2/DAPK3/PIK3CD/CSNK1D/CDK2/TESK1/STK17B/LYN/C
/MYL6B/ABCC5/GINS4/GNAZ/OLA1/DHX37/DCTPP1/RRAGD/RUVBL1/GBP2/RGS14/RAB25/CHTF18/
/MYL6B/ABCC5/GINS4/GNAZ/OLA1/DHX37/DCTPP1/RRAGD/RUVBL1/GBP2/RGS14/RAB25/CHTF18/
/MYL6B/ABCC5/GINS4/GNAZ/OLA1/DHX37/DCTPP1/RRAGD/RUVBL1/GBP2/RGS14/RAB25/CHTF18/
GINS4/GNAZ/OLA1/DHX37/RRAGD/RUVBL1/GBP2/RGS14/RAB25/CHTF18/RIT1/HSPA8/RRAGC/TCI
STK11IP/NPM1/SPRED1/STX1A/PCNA/THY1/RHOF/SGO1/SIRPA/SOCS1/CKS1B/HSP90AB1/LRRC14/
IRPA/SOCS1/CKS1B/HSP90AB1/SH2B2/PITPNM1/PAM/TNIP2/CCNO/PPME1/DUSP2/FBXO5/AATF/G
RF3/TCEAL8/HSP90AB1/DYNLL1/HDAC1/WIPF1/CARD19/E2F4/LEF1/FGFR1/TGM2/HIP1R/CBX3/S
LD16/NCF1/RANGAP1/MGAT5/IGFBP3/PSMD14/SAE1/PPP1R37/TIMP2/RABEP2/CCL5/BEX3/IQGAP
NUDC/ISG15/BAIAP2L1/LAMA5/TSPAN8/THY1/ITGB5/CDH6/RPS2/HSP90AB1/STAT1/PUF60/PLCB
/XPOT/PKN3/RAB11FIP4/RAC1/GNB2/LAMTOR5/KCTD10/LAMTOR1/GNB1/WAS/LSM2/TBC1D13/DVL
2/VIL1/NCALD/PFN1/WASF1/NEB/KPTN/PARVG/CROCC/CAPZB/SMTN/FHOD1/CNN2/VPS18/INF2/C
9/ACVR1/FASTK/STK24/PIM3/SRPK1/CHEK2/MAPKAPK3/CCND3/PRKAA2/ACVR1B/STK40/CSNK2A1/
TTF2/PTPA/DNAL4/MYO9B/POLQ/KATNB1/ABCC10/CCT8/ATP6V0D1/NSF/ABCG1/LONP1/ATP2A3/H
3F1/CDC42SE1/XPO5/TRAPPC1/DENND6B/RIMS3/FGD6/PARD6B/RUNDC1/LLGL1/INF2/RANBP1/CD
3F1/CDC42SE1/XPO5/TRAPPC1/DENND6B/RIMS3/FGD6/PARD6B/RUNDC1/LLGL1/INF2/RANBP1/CD

ARHGDIA/RIC8A/RGS3/TBC1D10C/RASAL3/TBC1D22A/CHN1/TBCD/NPRL3/RASAL1/SIPA1/ARAP1/I
5/KATNB1/OGG1/NDE1/PIFO/CLIP3/DRG1/MAPT/CKAP5/EMD/TBCD/RAD51D/STMN2/HOOK2/DNM2/(
C1/LDHA/CTTN/CDH17/EIF2S3/RAB10/UBAP2/EVPL/PFN1/EPS15L1/GCN1/TACSTD2/YWHAB/GOLG/
S1/RHOT2/GSPT2/GNG4/GNGT2/RAB5C/GTPBP3/GPN2/DNM2/RASL12/ARFRP1/GNAI3/RAB3A/RAB1
3B/CCL3L3/CRLF1/OSM/CCL8/DAB2IP
1/ARAP1/TBC1D7/SGSM3/ARHGAP11A/SRGAP2/TNK2/TBC1D22B/ARHGEF11/SMAP1/PLEKHG6/ARHG/
LEKHG6/ARHGAP28/ADPRH/AGAP3/ACAP3/STXBP5/DOCK2/SGSM1/RANBP3/DAB2IP/PREX1/ARHGAP2

IF20B/KIF22
/CHD7/MGME1/UNG/DNASE1/TERF1/ERCC2/RAD54B/LIG3/HELB/XRCC5/DDX1/TDP2/TEP1/ASCC3/T

TD13/DOCK7/MAP3K11/VAV3/PLEKHG5

H/KAT5/SUZ12/MSL3/BAZ1B/L3MBTL1/EZH1/MCM3AP/PRMT7
1/RPL9/MRPL14/RPS6/RPL24/RPL11/RPL10/RPS24/MRPL3/RPS3A/MRPL20/RPL14/MRPL24/MRPS1

/CSNK1E/MARK2/POLR3K/CDS1/EPHB3/POLR2H/RPS6KA4/PIP5K1C/FGFR1/PIM2/CCNO/PNKP/FGF
/RBM8A/METTL3/CSTF3/SF3B6/HNRNPA0/CELF2/RPL41/PPP1R8/UNK/HNRNPD/PARK7/HNRNPC/RP

[illegible]4K2A/PAPSS1/CCL2/DAPK3/PIK3CD/CSNK1D/CDK2/TESK1/STK17B/LYN/CDC42BPG/PKN3/ALPK3/
[illegible]PL5/RPS27A/PIAS4/RAD18/TRAF4/HSPA5/UBA52/TRIM37/UBE2D2/APBB1/DAXX/NEK6/GLMN/RN[illegible]
[illegible]/CAV1/DAB2IP/RPTOR/CNKSR1/ACTR1A/STUB1

[illegible]8/TRAF4/HSPA5/UBA52/TRIM37/UBE2D2/APBB1/DAXX/NEK6/GLMN/RNF8/TNFRSF14/KCNH2/FAF[illegible]

[illegible]P2/GNL3/DRG1/RERG/RHEB/RAB4B/DAP3/EEF1A2/RAB3D/DIRAS1/RHOT2/GSPT2/CIITA/SRPRB/[illegible]
[illegible]G1/RERG/RHEB/RAB4B/DAP3/EEF1A2/RAB3D/DIRAS1/RHOT2/GSPT2/CIITA/SRPRB/RAB5C/GTPB[illegible]

:B/RAB4B/DAP3/EEF1A2/RAB3D/DIRAS1/RHOT2/GSPT2/CIITA/SRPRB/RAB5C/GTPBP3/GPN2/DNM2
:B/RAB4B/DAP3/EEF1A2/RAB3D/DIRAS1/RHOT2/GSPT2/CIITA/SRPRB/RAB5C/GTPBP3/GPN2/DNM2

X24/C2CD2L/ING2/PIK3C2B/WDR45/APPL2/ESYT1/HIP1/MAPKAP1/PITPNC1/PLCD1/DAPP1/FGD2/

:EF1A2/RAB3D/DIRAS1/RHOT2/GSPT2/CIITA/SRPRB/RAB5C/GTPBP3/GPN2/DNM2/RASL12/ARFRP1

L2/APP/GAL/CDC42EP2/SEMA4F/GMFG/SEMA5B/CSPG5/SEMA3C/SEMA3E/MAPT/WNT4/CCL3/SFRP2/

EB/RAB4B/DAP3/EEF1A2/RAB3D/DIRAS1/RHOT2/GSPT2/CIITA/SRPRB/RAB5C/GTPBP3/GPN2/DNM2
EB/RAB4B/DAP3/EEF1A2/RAB3D/DIRAS1/RHOT2/GSPT2/CIITA/SRPRB/RAB5C/GTPBP3/GPN2/DNM2

2/MED19/PIAS3/WFS1/MIER2/TAF9/COMMD7/FAM89B/DNMT3A/IFI16/ASCL2/E2F2/KCTD1/PARP1/

CTN4/NFKBIB/HINFP/DHX9/MED7/NR1H2/GMEB2/ZMIZ1/CCAR2/PMF1/MMS19/TFDP1/ARID5B/CCAR
4/DDX10/POLR2D/DHX15/TOE1/TGS1/DARS2/TSEN34/DHX9/RNMT/TYW3/YARS2/PARS2/DDX55/HAR

E2F7/PBXIP1/KCNIP3/HDAC3/AEBP1/TSG101/TDP2/C1QBP/APEX1/ZNF85/NACC2/EZH1/ZNF653/T

R5L2/OR5H1/OR8U1/OR10R2/OR11H12/OR6Y1/OR8K3/OR8J1/OR1A2/OR10A4/OR6N1/OR2T5/OR5A

SPG5/SEMA3C/SEMA3E/MAPT/WNT4/CCL3

5/DYNC1I2/HSPA14/KIF20B/RFC5/TAPBP/ATP2B4/TAP2/DHX9/SMARCA5/GTF2H1/SMARCAL1/MSH

R5/KLHL21/UBE2D2/CHFR/TRIM69/RNF8/UBE2F/MED7/ZMIZ1/FBXL7/UBE2N/ANAPC5/HECTD3/CD(

[illegible]1/SALL2/IRF7/PNKP/NONO/SIX2/HEYL/HDAC2/ATF4/NLRC5/SPIB/MITF/MED8/HCFC1/RFX5/SS[illegible]

F4/TPPP3/DYNLT1/GPSM2/HAUS1/FBXO5/SPAG1/RSPH1/TUBB4A/RANGRF/AUNIP/CSNK1D/DNAAF5/
/MCM7/HRAS/PLCB1/USH1C/KIF11/MCM3/NUP62/RCC1/GTSE1/SPAG5/MAPRE1/TUBB/EZH2/CDC25
C2/ST14/EFNB1/MAP1S/PAK1/CRABP2/GNA12/LAMA5/ANKRD27/NRCAM/THY1/RHOF/CORO1C/CDH6/
RCA1/MNDA/ZNF580/SIRPA/SPON2/SOCS1/CD58/IRF1/IRF3/C3AR1/HSP90AB1/F2RL1/STAT1/RAI
/IRF1/IRF3/C3AR1/HSP90AB1/F2RL1/STAT1/RAB7B/NPLOC4/CD83/POLR3K/LEF1/POLR2H/RPS6I
S3/GSDMD/PRKD2/LUM/CD6/POLR2L/DDX41/IL4R/APP/ADRA2A/TLR5/POLR2K/HK1/XRCC6/GPATCI
RG4/RAB10/PML/SOCS3/FGFR2/DVL2/PFN1/AP2S1/PSMD10/PSMD11/VDR/PSMD1/GORAB/PSMA7/T
SPA8/NEU1/LCP1/GM2A/IL27RA/TCIRG1/SLPI/FUCA2/CD63/DOK3/STK11IP/RNASET2/FPR1/MND
TCIRG1/SLPI/FUCA2/CD63/DOK3/STK11IP/RNASET2/FPR1/MNDA/PTGES2/RAP2B/GYG1/ARHGAP9/
/HK3/CCT2/CD58/C3AR1/HSP90AB1/F2RL1/PRSS3/DYNLL1/DYNC1H1/ILF2/AMPD3/NPC2/NBEAL2/
/DYNC1H1/ILF2/AMPD3/NPC2/NBEAL2/OLR1/SLC2A5/GPI/PA2G4/SERPINB1/PPIA/RAB24/ACLY/I
SPA8/NEU1/LCP1/GM2A/IL27RA/TCIRG1/SLPI/FUCA2/CD63/DOK3/STK11IP/RNASET2/FPR1/MND
TH1/STXBP2/MAD2L2/PSMD2/ITGAX/HSPA8/NEU1/GM2A/IL27RA/TCIRG1/HLA-A/SLPI/FUCA2/CD
PRSS3/DYNLL1/DYNC1H1/ILF2/AMPD3/NPC2/NBEAL2/OLR1/SLC2A5/GPI/PA2G4/SERPINB1/PPIA/
/CD83/BATF2/PLA2G2D/TFE3/NCAPG2/EPHB3/PRELID1/PSMB2/LEF1/PDCD1/IRF7/ZC3H12A/PSMI
/CCR1/IL12RB1/CYP26B1/CD79A/OSTM1/PARP1/IL15/LTBR/MSH2/BATF3/SBNO2/DROSHA/CDC42/
2/BDKRB1/CCL5/BAX/CD86/MAPKAPK2/VAV1/COLEC12/PHPT1/TNFSF9/MIF/CD300A/FLOT2/ZP3/I
CR1/CD209/LYN/HLA-DRB5/CARD11/FPR3/MUC3A/RAC1/CBFB/PSMB4/ACTB/BCAR1/CTSS/CD47/P
0/PSMD11/PSMD1/C5AR1/PSMA7/RPS3/PRKD2/PSMB3/GRB2/CDC42/MUC20/CMKLR1/PSMD4/PSMD3/
NS4/PRKDC/DCTPP1/RUVBL1/MAD2L2/CHTF18/MAPK3/APEX2/IL27RA/MAP1S/MND1/DKC1/PPP2R1
/BARD1/UBE2A/MCM8/POLR2L/TOPBP1/CBX8/POLR2K/CETN2/XRCC6/HMGN1/POLQ/RBX1/MUTYH/PI
L2/TAF9/SFPQ/DNMT3A/ATAD2/SIRT7/CENPK/NAP1L4/NAP1L2/KDM5C/SET/SMARCB1/SMYD3/KMT
31/DAZAP1/SNRNP40/AKAP8L/UTP18/RBM19/PPIL1/RPS21/UTP14A/RPL7/RBMX2/NPM3/POP1/SFI
2/RPLP0/RPL18/RPL23/RACK1/GADD45GIP1/VIM/MRPL10/TRIM71/TNIP1/DPH7/ZNF598/PLEKHN
R1/HMOX1/CCL5/DNAJC5/HSPA6/DDOST/ALOX5/PLEKHO2/MIF/CD300A/CANT1/ZP3/IQGAP1/RAB1
/NCKAP1L/SH3KBP1/ADAM8/VLDLR/LYPLA2/CCL2/SHC1/PIK3CD/CXCL9/CXCL3/CXCL13/DEFB1/RI
15/NPM1/RBBP8/SPRED1/BRCA1/NSMCE2/MNDA/POLR2G/PCNA/BCL2L12/MCRS1/XRCC2/CHAF1A/A
/NPM1/CHMP4C/BRCA1/KLC3/TUBB6/XRCC2/TUBA1A/TUBB2A/SGO1/TTLL1/DYNLRB1/IFT52/MSTO
A1A/CKS1B/SFI1/CTDSPL/DYNLL1/DYNC1H1/SPDL1/XRCC3/TICRR/TGFA/KNTC1/PPP1R9B/E2F8/I
SF9/MIF/ZP3/HLA-DPB1/PRKDC/IL34/EBI3/HIF1A/RAB25/ITGAX/CD276/LAMC2/P2RY6/EFNB1/
3/SCAF1/SRPK1/RBM42/CPSF3/DDX23/PTBP1/PRKRIP1/PRPF38A/WTAP/HNRNPU/HTATSF1/LSM8/
RIM25/RNF122/PRPF19/UBXN1/PSMD8/KLHL42/VCP/PSMA1/USP5/NSFL1C/UBQLN4/KCTD5/FOXF2/
PP2R1A/NPM1/RBBP8/CHMP4C/BRCA1/RBL1/NSMCE2/PCNA/TUBA1A/BIN1/SFI1/CTDSPL/HSP90AB
GPSM2/NPEPPS/NCKAP1L/PIP4K2A/YWHAH/HCLS1/BBC3/KDM1A/PMAIP1/CCT7/TESK1/ITGB1BP1/
SLC9A3R1/BID/MT3/BCAP31/TNFRSF10C/ADAM8/PRR7/ITGB3BP/CCL2/DAPK3/PIK3CD/ATF4/YWH
X11/NUP205/NUP37/PSMB2/KRT18/PPP1CA/LEF1/NDC1/NELFE/POLR2H/IRF7/PPIA/NUP188/ZC3I
MB4/RBM19/PPIL1/RPS21/PSMB8/RPL7/RPL28/PSMB10/RBMX2/PSMA5/SFPQ/CPSF1/LSM2/PQBP1/
/MBD3/USP21/MTHFR/RNF2/VEGFA/HSF4/UIMC1/SREBF1/WDR5/PIH1D1/MORF4L2/TRIM37/MECP2/
PSMD2/HIF1A/MAPK3/ITGAX/HSPA8/LCP1/IL27RA/HLA-DQB2/HLA-A/SHARPIN/ISG15/TNFRSF4/I
SS/CTTN/MRPL12/STMN3/MRPS34/TRMT112/VIL1/CHMP3/GSK3A/DENR/WDR45B/MAP1LC3B/DVL2/
4L/RRP7BP/TSR3/UTP11/LAS1L/SNRPG/MRPL10/CUL4B/UTP4/MYBBP1A/RPL38/MAPT/PRPF31/RPI
ITGB5/CORO1C/BIN1/CORO7/PALLD/F2RL1/ARPC2/MYO19/GAS7/ARHGEF19/PPP1R9B/TIGD5/ARP
T80/GORAB/ODF2/PTPN23/PARVG/TACSTD2/CROCC/CAPZB/FGD6/RALA/CDC42/TGFB3/ZMYND8/FHI
RPC2/GAS7/ARHGEF19/PPP1R9B/TIGD5/ARPC3/WIPF1/LIMCH1/PDLIM3/SH2B2/CORO1B/ABLIM2/I
H12A/PSME3/SNX10/BCL3/LAG3/NCKAP1L/JAG2/ADAM8/PRR7/PIP4K2A/EVI2B/CDKN2B/PIK3CD/I
/MYL9/HOXA5/THRA/VEGFA/ACTN1/LBR/SMAP1/TLR2/TMEM64/WDR5/NKAP/SRF/INHA/RBPJ/BPGM/
/EFNB1/SERPINE1/CD3E/PPP2R1A/PAK1/SASH3/ADGRG1/PRKX/LAMA5/THY1/CORO1C/UBASH3B/S
1D/ANAPC7/CDK2/KIF14/SPSB1/SNF8/CLN3/AXIN1/OAZ3/PMAIP1/ADRM1/CCDC22/MYLIP/PSMB4/
AMC2/P2RY6/SERPINE1/IL27RA/PAK1/TMIGD3/GNA12/ADGRG1/PRKX/LAMA5/SPRED1/RAP2B/ZNF
D3E/MAP1S/MICALL1/PAK1/CRABP2/ANKRD27/NRCAM/THY1/RTN4RL2/PALLD/HSP90AB1/EPHB6/I

/KDM1A/LYN/FKBP4/RAC1/ITGA2/MYLIP/MIEN1/RAP2A/CDK5/CTTN/SPOCK1/RNF157/HOMER1/WAS
CDK5/WFS1/RNF26/UBE2L3/CBX4/PSMB8/RNF187/RNF157/PSMB10/PSMA5/DNAJB2/ASB6/PSMD13/
P2/MIF/FKBP1A/MTCL1/MSN/PKDCC/IQGAP1/CARD10/EFNA5/CD2/HIF1A/MAPK3/HSPA8/OAZ1/LCI
)/CCT4/SNX10/BCAP31/TOMM5/UBE2J2/BCL3/RPL17/FERMT1/NPEPPS/EHD1/CD68/RPLP2/LAMP3/
FCHSD1/CHAF1A/SMARCA4/NOL3/BIN1/CORO7/CCT2/EIF2S2/HSP90AB1/ARPC2/DYNC1H1/TICRR/I
T9/LSM7/RPSA/KPNB1/SLC11A1/RNASE2/SSB/LDHA/PDE9A/RPS5/PSMB4/PFKM/APOBEC3G/RPS21/
TDRKH/MYBL1/GTF3C2/POLR2K/KTI12/DDX54/ZBTB8OS/RPS19/NOC4L/RRP7BP/GTF3C1/TSR3/UTI
/HSP90AB1/F2RL1/PRSS3/DYNLL1/DYNC1H1/ILF2/AMPD3/NPC2/NBEAL2/OLR1/SLC2A5/GPI/PA2(
QGAP1/SRPX2/MIIP/CARD10/CREB3/HIF1A/RAB25/MAPK3/ITGAX/DUOXA2/LAMC2/TRIM11/P2RY6/
EFB1/LYN/ITGB1BP1/RAC1/ITGA2/PLA2G7/MIEN1/IL1B/BCAR1/PDGFA/FAM83H/SPOCK2/MCAM/F(
C1/CBFB/IL1B/IL2RA/NCAPH2/CD47/TNFSF13B/PSMB10/WAS/LCK/PTPN6/CD27/IL12RB1/CYP26I
/HSP90AB1/F2RL1/PRSS3/DYNLL1/DYNC1H1/ILF2/AMPD3/NPC2/NBEAL2/OLR1/SLC2A5/GPI/PA2(
/NCKAP1L/SH3KBP1/ADAM8/VLDLR/LYPLA2/CCL2/SHC1/PIK3CD/CXCL9/CXCL3/CXCL13/DEFB1/RI
/RPS19/RPS6KB2/RPLP0/RPL18/RPL23/RACK1/GADD45GIP1/VIM/MRPL10/TRIM71/TNIP1/DPH7/Z
/ADAM8/PRR7/ITGB3BP/CCL2/DAPK3/PIK3CD/ATF4/YWHAH/BBC3/PMAIP1/STK17B/LYN/LDHA/CDI
15/NPM1/SLC39A10/SLC35F6/BRCA1/BCL2L12/XRCC2/NOL3/LMNA/HSP90AB1/HDAC1/DDIAS/HYOU
/ADAM8/PRR7/ITGB3BP/CCL2/DAPK3/PIK3CD/ATF4/YWHAH/BBC3/PMAIP1/STK17B/LYN/LDHA/CDI
34/PEA15/NPM1/SLC39A10/SLC35F6/BRCA1/BCL2L12/XRCC2/NOL3/LMNA/HSP90AB1/HDAC1/DDI/
3QLN4/KCTD5/FOXF2/RNF216/PSMB5/PSMA4/SGTA/PSMD9/RPS27A/FBXW5/PLK3/MAP1A/HSPA5/FI
C/ANKRD27/BRCA1/STX1A/THY1/NSMAF/FCHSD1/CORO1C/BMF/HGS/BIN1/F2RL1/ARPC2/DYNLL1/I
SMC4/PLK4/PFN1/WASF1/OXTR/PARP1/PIEZO1/RPS3/CROCC/RALA/STX18/GRB2/FHOD1/CDC42/T(
C1/HDAC1/CCDC86/NUP93/DDX11/NUP205/NUP37/PSMB2/KRT18/PPP1CA/LEF1/NDC1/NELFE/POLI
DYNLL1/STAT1/PABPC1/CCL13/HDAC1/CCDC86/NUP93/DDX11/NUP205/NUP37/PSMB2/KRT18/PPP1
T8/PYGO2/MAST2/SLC4A2/PPP2R1A/PHC2/TSPAN8/MMP2/TTLL1/CCT2/EIF2S2/DYNLL1/CAD/DPCI
CHMP4C/BRCA1/RBL1/NSMCE2/PCNA/TUBA1A/SFI1/CTDSPL/DYNLL1/DYNC1H1/SPDL1/XRCC3/TICI
ST/ALOX5/PLEKHO2/MIF/CD300A/CANT1/ZP3/IQGAP1/RAB15/CFD/OLA1/FTH1/STXBP2/PSMD2/I
DPB1/SEC61A1/UNC93B1/CFD/PRKDC/SIGLEC10/IL34/STXBP2/GBP2/PSMD2/TRIM11/HLA-DQB2/I
TRAF3/PSMB3/ARRB2/MUC20/TLR5/PSMD4/XRCC6/GPATCH3/PSMD3/PSMD7/RPS19/IKBKG/FADD/B
P6AP1/PPP1R13L/PAK1/CRABP2/SASH3/ISG15/ANKRD27/NRCAM/XRCC2/BIN1/SOCS1/HSP90AB1/
SME3/SLC9A3R1/GAS2L1/BLM/MLST8/FBXO5/CNOT9/EVI2B/CDKN2B/CCL2/CDK2/NLE1/EHMT2/CDI
1/SLC9A3R1/MT3/CCNO/DVL3/DUSP2/BLM/NCKAP1L/MLST8/DBF4/GTPBP4/ADAM8/PAK4/VLDLR/CI
/SIRPA/SOCS1/IRF1/F2RL1/CTLA4/CD83/PLA2G2D/PRELID1/LEF1/PDCD1/ZC3H12A/TNIP2/BCL
KPNB1/SMC4/FANCA/KNSTRN/MTFR2/CDKN1C/AKAP8L/IL1B/NCAPH2/PSMD13/SIRT7/CENPK/DYNC
CAPG2/EPHB3/PRELID1/PSMB2/LEF1/IRF7/ZC3H12A/PSME3/SNX10/BCL3/LAG3/NCKAP1L/JAG2/
NGL1/TULP3/PSMB8/PSMB10/PSMA5/DEAF1/TGFB2/PLXND1/PSMD13/PSMC4/AP2M1/NDRG4/RAB10/
/ADAM8/CCL2/PLSCR1/FANCA/HSPH1/CD209/LYN/CARD11/RAC1/CBFB/IL1B/IL2RA/PDGFA/CD47/
C/SHC1/SLC7A6/PIK3CD/CXCL9/CXCL3/CXCL13/LYN/CCL28/RAC1/ITGA2/PLA2G7/IL1B/JCHAIN/
P1R/MT3/OTUB1/FBXO5/NOC2L/TUBB4A/CAPZA1/HDAC2/RAP1GAP2/YWHAH/MKKS/KIF14/FHOD3/C/
P3/IQGAP1/SRPX2/MIIP/CARD10/CREB3/HIF1A/RAB25/MAPK3/ITGAX/DUOXA2/LAMC2/P2RY6/SEI
/ITGB1BP1/RAC1/ITGA2/PLA2G7/MIEN1/IL1B/BCAR1/PDGFA/FAM83H/SPOCK2/MCAM/FGR/CCL4/I
M1/SPRED1/RBL1/FPR1/RAP2B/THY1/CORO1C/UBASH3B/HGS/SOCS1/CKS1B/CCT2/RPS2/HSP90AB
/PRELID1/FGFR1/HIP1R/PSME3/BID/ARFGAP1/TBC1D10B/BCAP31/DVL3/NCKAP1L/FGFR3/PRR7/
GPSM2/NCKAP1L/MLST8/TUBB4A/CAPZA1/RANGRF/DAPK3/MKKS/ARHGDIB/FHOD3/PHLDB1/CAMSAP
HM2/TMED9/CSNK1E/MARK2/STX10/PIP5K1C/SLC9A3R1/CEP89/BLOC1S4/DYNLT1/GPSM2/HAUS1/
1/CROCC/RIMS3/LLGL1/FHOD1/CDC42/STARD3/IL4R/VPS18/CHMP1A/SYT1/PPFIA3/DNM1L/ACTL
2/HDAC1/RELN/CSNK1E/S100B/ZSWIM5/MARK2/GDI1/EPHB3/NUMBL/TPBG/FGFR1/MYRF/MT3/DYNI
3/LRP8/SRRT/FAIM/CST7/PRKCI/ZMYND8/SEMA4D/SYT1/DNM1L/SYT3/GDPD5/KATNB1/STK25/ARI
RD27/NRCAM/THY1/CORO1C/XRCC2/RTN4RL2/BIN1/ISL2/ARPC2/HDAC1/RELN/CSNK1E/ZSWIM5/M/
ARD10/EFNA5/STXBP2/HIF1A/MAPK3/ASIC1/OAZ1/LCP1/P2RY6/MAP2K2/DKC1/PAK1/CAPN10/ST
JBE2Z/AMIGO2/PRKDC/SLC9A1/STIL/CREB3/TNFAIP8/HIF1A/NUPR1/FZD1/SERPINE1/RNF34/PE/
FA/PLEKHM2/TMED9/RELN/ARPC3/NUP93/TBC1D1/STX10/PRELID1/PIP5K1C/PPIA/HIP1R/MYRF/I

l/URM1/AXIN1/U2AF2/KDM1A/RCE1/BIRC7/MED8/HCFC1/ADRM1/CCDC22/CBFB/MYLIP/PSMB4/AC
l/ATG101/NOP2/FBXO5/SPAG1/RSPH1/PIP4K2A/TUBB4A/RPSA/CSNK1D/DNAAF5/CDK2/KRT8/MKK
K5/ZDHHC18/RPS21/RPL7/RPL28/MYADM/VIL1/ZDHHC1/AP2M1/RAB10/RPL39/TNIK/STAC3/RPL3
L3/OTUB1/MLST8/FBXO5/NONO/AUNIP/ATF4/AXIN1/KDM1A/PMAIP1/HSPH1/LYN/WNT7B/BIRC7/K
RL1/ARPC2/DYNC1H1/GAS7/FMOD/PPP1R9B/ARPC3/WIPF1/PLOD2/LIMCH1/PDLIM3/PLOD3/CORO1
/CD3E/PAK1/PERP/SASH3/BAIAP2L1/LAMA5/NRCAM/RAP2B/THY1/ITGB5/UBASH3B/SIRPA/CDH6/
ACSTD2/CROCC/CAPZB/FGD6/RALA/CDC42/TGFB3/ZMYND8/FHDC1/CDC42EP2/OCRL/CETN2/LRRC6
A/LYN/FKBP4/RAC1/MYLIP/MIEN1/RAP2A/CDK5/CTTN/SPOCK1/RNF157/HOMER1/WAS/PLXND1/VI
T9/CDKN2B/CCL2/CSNK1D/ANAPC7/CDK2/LSM10/KIF14/DCTN2/CDKN1C/PSMB4/PSMB8/PSMB10/P
/RCC1/GTSE1/SPAG5/MAPRE1/TUBB/EZH2/CDC25A/CDK4/MAD2L1/MUC1/EME1/CDKN2D/ZNF385A/
/ZNF675/DPF2/BTN2A2/CCL3/MEIS2/PSMD8/RARG/ABL1/SART1/HLA-DOA/FCGR2B/PSMA1/TOX/P
AIP1/CCT7/ITGB1BP1/AKAP8L/IL1B/ACTB/CDK5/TXN/UBE2L3/TCP1/VIL1/GSK3A/AP2M1/STAC3/
RD10/CREB3/HIF1A/RAB25/MAPK3/ITGAX/DUOXA2/LAMC2/P2RY6/SERPINE1/IL27RA/PAK1/TMIG
AC1/ITGA2/PLA2G7/MIEN1/IL1B/BCAR1/PDGFA/FAM83H/SPOCK2/MCAM/FGR/CCL4/MYADM/TGFB2/
/SF3B3/HNRNPR/POLR2D/SART3/DHX15/TGS1/GPKOW/KHDRBS3/DHX9/RBM10/USP39/SNRNP25/RB
/SF3B3/HNRNPR/POLR2D/SART3/DHX15/TGS1/GPKOW/KHDRBS3/DHX9/RBM10/USP39/SNRNP25/RB
/SF3B3/HNRNPR/POLR2D/SART3/DHX15/TGS1/GPKOW/KHDRBS3/DHX9/RBM10/USP39/SNRNP25/RB
/BAD/PSMA4/VCAM1/PSMD9/THBS1/ADAM17/USF1/CHCHD2/NOTCH1/RPS27A/CAPN2/MTHFR/PLK3/
HK3/CCT2/CD58/C3AR1/HSP90AB1/F2RL1/PRSS3/DYNLL1/DYNC1H1/ILF2/AMPD3/NPC2/NBEAL2/
D11/RAC1/CBFB/IL1B/IL2RA/CD47/NFAM1/TNFSF13B/FGR/CD300LF/LCK/PTPN6/CD27/SAMSN1/
PIK3CD/PLSCR1/CD209/LYN/HLA-DRB5/CARD11/FPR3/MUC3A/RAC1/CBFB/PSMB4/ACTB/BCAR1/C
RT1/SYMPK/PHF5A/SCAF1/SRPK1/RBM42/CPSF3/DDX23/PTBP1/PRKRIP1/PRPF38A/WTAP/HNRNPU/
IF5A/POLR2D/PRKAG1/ANP32A/ANP32B/DHX9/TNPO1/DMAP1/IPO9/NXT2/CCHCR1/RBM15B/KPNA6/
AP3D1/FADD/PELI1/TNIP1/RASAL3/BTN2A2/HSPD1/SART1/FCGR2B/CSK/BAD/BCL10/VCAM1/TWS
L2/ARHGDIB/KIF14/KPNB1/RAB20/LYN/RAC1/KCTD10/RAP2A/GNB1/STMN3/MAPK11/NRAS/WAS/T
RT/SAMD4B/PSMB3/NCL/HENMT1/EIF3H/POLR2L/NUP155/DROSHA/AGO2/CALR/APP/NUP107/PSMD
/PSMD4/PCGF2/PSMD3/RBX1/PSMD7/RAD21/BABAM1/MTBP/RPL23/NME6/RFWD3/RBBP7/RPA2/E2F
/SCN1B/SEMA3C/PMP22/PTPRF/SEMA3E/MAPT/CHN1/CBFA2T2/ABL1/STK24/STMN2/RASAL1/SFRP
COL4A2/LAMB3/MATN3/APP/DNAJB6/COL8A2/PRDX4/MMP19/ITGB8/RIC8A/EMILIN1/COL11A1/AB
R1/PDGFA/FAM83H/MCAM/FGR/CCL4/MYADM/TGFB2/GPSM3/F3/FAM89B/VIL1/MAZ/CCR1/CXCL16/
MRPL37/EIF5A/DSTN/PLEKHH2/MRPL27/MRPL28/GAK/MRPL51/MRPL11/MTPN/SPTAN1/MRPL15/C1
l/PIH1D1/MECP2/HNRNPA1/UBR5/TWIST1/PPHLN1/NEK6/MLLT6/SART3/DNMT3B/MAPKAPK5/BECN
GAP18/NCLN/CDC42EP2/MED25/DNM1L/RBX1/GMFG/DR1/RNF4/ABI2/CLEC7A/RACK1/RALB/RPA2/
K3/CCT2/CD58/C3AR1/HSP90AB1/F2RL1/PRSS3/DYNLL1/DYNC1H1/ILF2/AMPD3/NPC2/NBEAL2/O
/DYNC1H1/ILF2/AMPD3/NPC2/NBEAL2/OLR1/SLC2A5/GPI/PA2G4/SERPINB1/PPIA/RAB24/ACLY/
NSMCE2/PCNA/TUBA1A/SFI1/CTDSPL/DYNLL1/DYNC1H1/SPDL1/XRCC3/TICRR/KNTC1/PPP1R9B/E
D5/RAD21/MTBP/RPL23/MSX1/CUL4B/MYBBP1A/WNT4/DBF4B/MEIS2/RAD51C/BTG2/PIDD1/LFNG/
RNPA1/UBR5/EIF5A/POLR2D/PRKAG1/ANP32A/ANP32B/DHX9/TNPO1/DMAP1/IPO9/NXT2/CCHCR1/
/RBBP8/CHMP4C/RBL1/NSMCE2/TUBA1A/SGO1/CKS1B/YBX1/PELO/DYNC1H1/SPDL1/MYO19/TGFA/
/PSMD10/CDKN2C/C5AR1/CDK7/CCND2/RPS3/TFAP4/MTCP1/TGFB3/MUC20/ADRA2A/SPRY4/TELO2/
/KRT8/YWHAH/BBC3/NLE1/KDM1A/PMAIP1/IL1B/WFS1/CTTN/TGFB2/TAF9/ZNF205/SFPQ/LCK/CD
2/ARPC1A/WNT4/NF2/EMILIN1/BRK1/ARPC5/ABL1/TBCD/STMN2/ACTR3/ARAP1/DBNL/PTGER4/SP
NRNPA1/PSMC3/EXOSC1/HNRNPL/HNRNPR/SART3/ANP32A/KHDRBS3/DHX9/TNPO1/PSMC5/RBM10/M
F/PDCD10/STAC/BAD/CENPQ/PRKAA2/CASP8/PLK3/BICD1/SH3GLB1/MAP1A/MAVS/MARK4/CD2AP/
FT20/CLTC/STXBP1/GAK
N/CDKN1C/AKAP8L/IL1B/NCAPH2/PSMD13/SIRT7/CENPK/DYNC1LI1/DSN1/ANAPC11/CYP26B1/TU
SMD1/C5AR1/PSMA7/RPS3/PRKD2/PSMB3/GRB2/CDC42/MUC20/CMKLR1/PSMD4/PSMD3/PSMD7/LIL
VIM/TRIM71/PLEKHN1/RPL38/HNRNPM/RPL23A/EXOSC3/BTG2/PSMD8/DNA2/GSPT2/PSMA1/RPS7/
/RPL7A/RPS23/MYEF2/RPS10/PSMB5/PSMA4/RPS15/PSMD9/RPS16/RPL5/RPS27A/HNRNPU/RPS12/
D1/CDC42/ARHGAP18/PRKCI/TGFB3/FHDC1/SWAP70/CDC42EP2/GMFG/ABI2/WDR1/ARPC1A/WNT4/
PT/CKAP2/NAT10/SLC25A5/TBCD/STMN2/ARAP1/TBC1D7/RAD1/DNM2/STYXL1/ODF2L/NBN/HNRNP

2RB1/IL15/PIEZO1/PTPN23/RPS3/GRB2/CD6/CDC42/IL4R/PNP/SWAP70/MAPK7/LILRB4/AP3D1/I
TYXL1/FIG4/MOB2/LLPH/VEGFA/MARK4/HSPA5/DDX56/DEF8/RAB11A/CENPJ/PACSIN1/PPP2R5B/M
EMA4F/GMFG/SCTR/SEMA5B/AKT1S1/ABI2/WDR1/SEMA3C/SEMA3E/MAPT/ARPC1A/BRK1/CDHR2/RAI
7/RPL5/DUS2/NOL6/RPF2/CTU2/BMT2/POP5/NVL/EXOSC9/EXOSC10/EXOSC7/DDX56/INTS9/PIH1I
HL/PSMA1/PSMB5/BAD/PSMA4/VCAM1/PSMD9/THBS1/ADAM17/USF1/CHCHD2/NOTCH1/RPS27A/CAPN
P1/TRIM25/HPGDS/SUPT5H/CCL3/TNFAIP3/SRPK1/RNF216/BAD/ACVR1B/NOTCH1/MAVS/OPTN/OAS
NF335/HSPD1/SART1/TOX/CSK/VAMP8/BAD/BCL10/VCAM1/THBS1/CD177/PAK2/HLA-F/NKAP/RHOA
A2/WRAP53/UBQLN4/CHEK2/HSP90AA1/HDAC10/SPIRE1/MBD3/NBN/MAGEF1/HNRNPU/NVL/ERCC1/I
6B1/LST1/IL15/MSH2/RPS3/SIT1/GRB2/CD6/DROSHA/CDC42/KAT2A/IL4R/PNP/GAL/LILRB4/AP3
/ZNF675/IL20RA/RPA2/CCT8/GNL3/POLD3/PIWIL4/RAD51C/IQCB1/PTGES3/NAT10/RAB3D/DNA2/
TBL2/ATP6V0D1/PLEKHN1/LONP1/SUV39H2/PSMD8/VHL/PSMA1/PSMB5/BAD/PSMA4/VCAM1/PSMD9/
FRP1/RPS12/TOMM22/RPS4X/RPL6/OPTN/RPL37/RPL27A/GGA3/HSPA5/RPL36/RILPL1/UBA52/RAI
NAPC7/CDK2/LSM10/KIF14/DCTN2/CDKN1C/PSMB4/PSMB8/PSMB10/PSMA5/PTPN6/PSMD13/PSMC4/
NM1L/MAPK7/PDCD5/FADD/STX4/RACK1/MSX1/RAD9A/IL20RA/PTTG1IP/WNT4/PELI3/GRINA/ACVI
ART1/TOX/CSK/VAMP8/BAD/BCL10/VCAM1/THBS1/CD177/PAK2/HLA-F/NKAP/RHOA/CD38/AP3B1/S
/ZPR1/STOML2/RBM14/RFC5/DHX9/DNAJC2/FAF1/POLE3/RPA1/EREG/CDC34/SMARCAL1/MSH6/ATC
2/KLHDC8B/RPS3/CDC42/ZNF207/CHMP1A/RAD21/KATNB1/CENPN/NDE1/NTMT1/ESCO2/RAD51C/SI
O7/RAD21/MTBP/NME6/RFWD3/GADD45GIP1/NABP1/RAD9A/RPA2/NAE1/ZWILCH/BTN2A2/BTG2/PSM
FCGR2B/CSK/BAD/BCL10/VCAM1/TWSG1/PTPN2
ECP2/CENPJ/CDK5R1/ARHGAP28/RHOA/MTPN/NIN/SPTAN1/ARF1/RHOBTB2/GMFB/HAX1/CAMSAP1/I

X1IP1/EXOSC7/DDX56/PIH1D1/UTP20/EXOSC1/NOB1/NSUN4/DDX10/MTERF3/WDR75/MRPL11/FAM2
CAM1/SLC8B1/THBS1/ADAM17/NOTCH1

/MORF4L1/TP53BP1/ATAD5/THOC1/DCLRE1C/PAGR1/MMS22L/PTPRC
IF3A/LRPPRC/KIF5B/DYNC2LI1/ARL3/TRIM46/TUB/TTC21A/ARL8A/CNIH2/KIF9/BBS12/TRAK2/A
SF1/SRSF2/CASC3/FIP1L1/RBM8A/MAGOHB/MYO1C/SMG7/LRPPRC/POLDIP3/THOC7/AAAS/SLC25A3
R75/FAM207A/BMS1/NOL10/RSL1D1/DIMT1/EBNA1BP2/NOL9/UTP23/RPP30/SUV39H1/PELP1/RPS9
R2B/FXR1/ASF1A/EIF4G1/CNOT2/HNRNPA2B1/KMT2B/MBD1/SMAD2/ELAVL1/ZMPSTE24/NUP214/SM
C1/DHX38/ENY2/PABPN1/SRSF1/SRSF2/CASC3/FIP1L1/RBM8A/CEP72/MAGOHB/KIF3A/SMG7/POLI
D19/POU2F2/GON4L/PRKCB/VAV3/PPP2R3C/SLA2/NOD2/TNFSF13
D51C/PSMD8/HSPA2/PKIA/PSMA1/MTA3/CHEK2/CLSPN/CEP41/PSMB5/PSMA4/HSP90AA1/PSMD9/DN
RSF14/RUNX3/ZMIZ1/CCL19/XCL1/PIK3R6/CD5/CAV1/TP53BP1/ATAD5/INPP5D
XNDC12/IVNS1ABP/BCL2L1/PPM1F/NDUFA13/PLAGL2/GSKIP/IFI6/CCAR2/BECN1/EPHA2/MSH6/PI

DP1/DAB2IP/HMGA2/RPTOR
CGR2B/PSMA1/VAMP8/PSMB5/PSMA4/PSMD9/DNM2/THBS1/DCTN1/CANX/HLA-F/AP2A2/DYNC1I2/KI
SFSWAP/EIF3G/ATR/RPS14/RPS27/EIF2D/GEMIN2/SRSF1/SRPK2/COIL/DHX30/SNRPD3/CELF2/NI
/ERCC3/STUB1/BIK/DDB1/MYO1C/SPIRE2/NCKAP1
TP2B4/PPAT/HINFP/CNOT10/STXBP4/POLE3/RPA1/CDC34/CNOT3/TFDP1/UBE2E2/CACUL1/POLE2/

/RPS17/PPP2R3C/BAP1/RAC3/RASSF2
BE2N/CD247/INPP5D/DUSP22/CD3G/PTPRC/NECTIN2/BTN2A1/PIK3CB/PSMB1/PLCG1/EIF2B4/MAI

NK2B/TP53RK/CDK5R1/NUAK1/TWIST1/RFC5/PRKAG1/MAPKAPK5/CNOT10/RPA1/BRPF1/TAF13/TBI
NPU/GLI1/RAB11A/CRLF3/KIF20B/MECP2/CENPJ/PPP2R5B/CDK5R1/RHOA/FAP/CNOT10/STXBP4/I
NB1/MTBP/WRAP73/NME6

PA1/CDC34/CNOT3/TFDP1/UBE2E2/CACUL1/POLE2

3/CHEK2/CLSPN/CEP41/PSMB5/PSMA4/HSP90AA1/PSMD9/DNM2/DCTN1/CEP164/HAUS7/NBN/PLK3/

A2/NELFCD/RPS14/GTF2F1/RPS27/RPL29/EIF2D/CTDP1/RPL13/RPL4/RPL41/RPL9/RPS6/TRIM3[illegible]

214/TSC1/BICD2/IWS1/ATXN2/RFTN1/DDX19A/TPR/SRSF7/MCM3AP/NUP50
214/TSC1/BICD2/IWS1/ATXN2/RFTN1/DDX19A/TPR/SRSF7/MCM3AP/NUP50

/ANP32B/DHX9/SMARCA5/GTF2H1/POLE3/RPA1/SMARCAL1/IPO4/SMC2/HMGA2/PARP10/GRWD1
F6/STRAP/SFSWAP/EIF3G/ATR/RPS14/RPS27/EIF2D/KLC1/GEMIN2/SRSF1/SRPK2/COIL/DHX8/D[illegible]

PREX1/ARFGEF1/ADD1/ASAP3/MYO1C/SPIRE2/NCKAP1/PPP1R9A/LPAR1/PXN
9/CDK2AP2/NBN/PLK3/ZFYVE19

/DHX38/EXOSC2/ENY2/PABPN1/SRSF1/SRSF2/CASC3/FIP1L1/RBM8A/MAGOHB/MYO1C/SMG7/LRPP[illegible]

E19

BP1/HMGA2/THOC1/FBXO6
/SMG7/RPL9/RPS6/RPL24/RPL11/RPL10/RPS24/DCP2/PYM1/RPS3A/AGO1/RPL14/RPL10A/EIF4G[illegible]
/RPS27/EXOSC2/DDX52/TEX10/RPS6/RPF1/MRM2/CHD7/RIOK1/RPL11/RPS24/WDR12/PDCD11/US[illegible]
SACS/LMAN2L/PPIC/SNRNP70/HSPB6/P4HB
PS6/RPL24/RPL11/RPL10/RPS24/RPS3A/ZDHHC20/RPL14/DMTN/RAB3IP/SRP19/RPL10A/RPL12/[illegible]
A

FITM2/DDB1/P4HB/MX1/TRIM32/VAPA/TBC1D20/ADAR/TRIM27/IGF2R/SETDB1/TRIM62/POLR2B/[illegible]

PT/ASF1A/TERF1/SUPT6H/ERCC2/NAP1L3/BRF2/GTF3C3/SUPT16H/TP53/TBPL1/TAF11/GTF2E1

7/NBN/ZFYVE19/HAUS5/UIMC1/DYNC1I2/RAB11A/GPR132/NEDD1/MECP2/CENPJ/PSMC3

1I2/RAB11A/GPR132/NEDD1/MECP2/CENPJ/PSMC3

SMB6/PSMA3/PDE4B/EIF2B1/CD4/RELA/PSMB7/PRAM1/SKP1/EIF2B2/PIK3R2/WNK1/BTN3A2/EIF[illegible]
/PTPN14/NUP214/TSC1/TP53/STRADA/IWS1/DDX19A/TPR/SRSF7/MCM3AP/NUP50/EIF4ENIF1
/PNPT1/NUP214/TSC1/BICD2/IWS1/ATXN2/RFTN1/DDX19A/TPR/SRSF7/MCM3AP/NUP50

G1/PSMB7/ELAVL1/FASTKD1/PNPT1/SERBP1/NUP214/TNFSF13/PAIP1

RPL24/RPL11/RPL10/KLHL25/RPS24/EIF4E2/EIF3F/RPS3A/EIF2B1/RPL14/EIF5B/RPL10A/EIF[illegible]

A2/DYNC1I2/KIF22/PSMC3/CLTC/TAPBP/AP1S1/AP1S3/TAP2/AP1S2/AP2B1/LGMN/PSMC5/AP2A1/

AP1S3/TAP2/AP1S2/AP2B1/LGMN/PSMC5/AP2A1/ARF1/PDIA3

TAPBP/AP1S1/AP1S3/TAP2/AP1S2/AP2B1/LGMN/PSMC5/AP2A1/ARF1/PDIA3/ACTR1A/KLC1/CD1A/
1/VPS33B/HAUS2/CDK5RAP2/RAB7A/ESYT1/EXOC6/UNC13A/CEP135/PEX16/EXOC6B/STX16/HAUS

)/PPP3CB/PSMD12/NFATC1/ABI1/MAPK9/TAB1/PIGR/RAP1A/PSME2

JP214/TNFSF13/PAIP1/TARDBP/APEX1/PARN

1/RPL13/RPL4/RPL41/RPL9/RPS6/TRIM32/RPL24/RPL11/RPL10/RPS24/TRIM27/AAAS/RPS3A/T
P/PSMD6/ILK/ZEB2/PSMC2/PSMA6/XIAP/ATP6V1C2/HHEX/YAP1/LRRK1/PRDM15/PSMD5

1/TARDBP/APEX1/PARN

RALA/GRB2/CDC42/CDC42SE2/ADRA2A/CDC42EP2/MYO9B/SPRY4/CBL/RALGDS/ABI2/RALB/ARHGD
F2/VEGFA/HSF4/UIMC1/SREBF1/WDR5/PIH1D1/MORF4L2/TRIM37/MECP2/UBR5/PPM1F/BAZ2A/TW
NBP1/SEMA4D/XCL2/AGAP1/CDC42EP2/MYO9B/OCRL/DNM1L/PGAM5/RACK1/ARHGDIA/RIC8A/RGS3/
ELN/MARK2/CEP85/NCAPG2/FGFR1/SLC9A3R1/MT3/CCNO/DVL3/DUSP2/BLM/NCKAP1L/MLST8/DBF
/DVL3/VLDLR/CPNE1/HEYL/HDAC2/RAP1GAP2/CSNK1D/YWHAH/CAMSAP2/KDM1A/JAG1/LYN/GPRC5
IA/SCN1B/MAPT/TGIF2/RHEB/CCL3/PRPF19/ZNF335/CBFA2T2/STMN2/RASAL1/DBNL/PLXNC1/ST
OOA/ZP3/HLA-DPB1/ARPC4/UNC93B1/CFD/PRKDC/STXBP2/MAD2L2/PSMD2/MAPK3/CD276/JAML/I
7/GPRC5B/IL1B/CDK5/PDGFA/TXN/MLKL/UBE2L3/TCP1/ADRA2C/FGR/LCP2/MAPK11/TGFB2/CALC
T2/MUC6/BCAN/B4GALT4/ACAN/MUC15/GFPT1/TMEM115/CHST3/POMGNT2/SLC35C2/FUT11/XXYLT
/ATR/HMGA2/RPTOR/MKNK1/PKD1/CSNK2A2/VRK2/PWP1/BDKRB2/CSNK1G1/STK4/UCN/PARK7/ULK
7A/TNIP1/POLR3D/LBP/POLR3C/HSPD1/PSMD8/PSMA1/TNFAIP3/MAPKAPK3/PSMB5/BCL10/PSMA4/
TAF10/LFNG/DOT1L/ABL1/SYMPK/NFATC4/TDG/TMC8/BAG2/SPPL3/NVL/CTBP2/TRAF4/CSNK2B/H

S27A/MAVS/DMBT1/PAK2/TLR2/MUC6/UBA52/MUC15/PSMC3/UBE2D2/RBM14/MAP3K1/LGMN/PSMC5/
DHX9/IFITM3/BECN1/CCL19/XCL1/TLR7/ARF1/PRKRA/CCDC130/ATG7/CCL8/HMGA2/GTF2F1/ZC3

/ADAR/TRIM27/IGF2R/SETDB1/TRIM62/POLR2B/CD4/APOBEC3F
5/HDAC3/BAP1/SMAD3/TP53/TARDBP/FEZ1

A7/RUVBL2/PSMB3/SDHAF2/ARRB2/CDC42/APCDD1/AGO2/CTDNEP1/APP/PSMD4/TRPM4/PSMD3/RB
SMD3/RBX1/PSMD7/TMUB2/TBL1XR1/PELI1/CBL/SGTB/RPL23/RNF4/UBE2L6/RACK1/RNF7/CUL4B/
SMC4/GSK3A/AP2M1/OSBPL7/PML/ANAPC11/VPS28/AP2S1/UBE2D1/USP14/PSMD10/PSMD11/PSMD
APT/CYCS/NDUFS5/LONP1/TAZ/NDUFB9/SSBP1/HSPD1/DNA2/RHOT2/SLC25A5/TIMM13/MFF/OPA3/
T4/NF2/BRK1/ARPC5/ABL1/ACTR3/ARAP1/DBNL/PTGER4/SPIRE1/ADD3/LIMA1/CD2AP/CDK5R1/A
30/IL15/PIEZO1/IBSP/PTPN23/RPS3/PRKD2/GRB2/CD6/CDC42/IL4R/CALR/PNP/RELL2/LILRB4/
PWP1/BDKRB2/CSNK1G1/STK4/UCN/PARK7/ULK1/DMTN/DYRK3/IFNG/NSD1/TLK2/EIF4G1/PRKCB/
E2D2/MAP3K1/LGMN/PSMC5/UBE2N/TLR7/HSP90B1/IRAK2/CAV1/DAB2IP/CLEC4E/ZCCHC3/NFKBI
F1/IRF4/MYC/BGLAP/CD4/IFNG/ANXA1/CCR2/PPP2R3C/ESRRA/RASSF2/LILRB1/GNAS/CD80/SOX
FNG/ANXA1/CCR2/TSC1/RPL22/FUT7/CD80/TP53/SOX13/RASGRP1/FGL2/LILRB2
/MRPL20/DMTN/MRPL24/MRPS16/MRPL55/CHCHD1/MRPS2/CARMIL2/SLN/MRPL21/MRPS26/MRPS11/

SB/MRPL13/GCN1/CDC123/EIF4A3/ILF3/GPX1/GRB7/ZNF706/RPS3/SAMD4B/RPL27/NCL/BAG3/E

BXW9/SOCS3/ANAPC11/VPS28/UBE2D1/RNF220/UBA1/PSMD10/RNF113A/PSMD11/PSMD1/DCAF16/

SMD3/RBX1/PSMD7/TMUB2/TBL1XR1/PELI1/CBL/SGTB/RPL23/RNF4/UBE2L6/RACK1/RNF7/CUL4B/

MD1/MVB12A/PTPN23/GPX1/PSMA7/UBE2E1/FBXL6/USP1/DNAJC10/PSMB3/UBE2A/SMARCC1/ARRB

N23/PSMA7/RUVBL2/PSMB3/SDHAF2/ARRB2/CDC42/APCDD1/AGO2/CTDNEP1/APP/PSMD4/TRPM4/P

/CHPF2/NUP93/MAN1B1/NUP205/NUP37/PLOD3/DDIT4/B4GALT3/GPI/B3GALNT1/NDC1/ST3GAL4/

)/MAPK3/NUPR1/RRAGC/P2RY6/CD3E/MAP2K2/UBE2I/ATP6AP1/SHARPIN/PAK1/ADGRG1/PEA15/F

ACOT8/B4GALNT2/NECAB3/DAD1/POFUT2/FUT8/GALNT7/MAN2B1/ALG1/LARGE2/GXYLT2/HEXB/GN

CD10/BIN2/PSAP/LEMD2/HOOK2/SGTA/DNM2/DCTN1/COG1/UGCG/SRGN/PLK3/SH3GLB1/OPTN/RAB

M21/UBA52/RAB29/TARBP2/VPS37B/CXCR6/PPIE/PPIB/TNFRSF14/IFITM3/EPHA2/IST1/KPNA6/

F/RHOA/INHA/AP3B1/DOCK2/RUNX3/ZMIZ1/LYL1/RBPJ/CCL19/NOTCH2/PIK3R6/DOCK10/ATP7A/

DCH/UBE2D1/IL15/PSMD10/PSMD11/PSMD1/PSMA7/TRAF3/PSMB3/ARRB2/CD6/MUC20/TLR5/PSMD

1/CENPJ/TMEM138/CC2D2A/YWHAG/IFT20/B9D1/FNBP1L/TNPO1/IFT22/TTLL3/SSX2IP/NPHP4/H

1/SIX1/AP2B1/LGMN

4/GMFG/ABI2/ARHGDIA/WDR1/SEMA3E/ARPC1A/WNT4/NF2/BRK1/ARPC5/ABL1/ACTR3/ARAP1/DBN

1/CENPJ/TMEM138/CC2D2A/YWHAG/IFT20/B9D1/FNBP1L/TNPO1/IFT22/TTLL3/SSX2IP/NPHP4/H

SMA4/VCAM1/PSMD9/TNFSF15/PTPN2/THBS1/ADAM17/CASP8/TXNDC17

MAP3K7/TRIM32/NFKBID/VAPA/ATP2C1/CC2D1A/RHOH/SHISA5/TRIM62/CD4/BRD4/RELA/TFG/NO

3X1/PSMD7/TMUB2/TBL1XR1/PELI1/CBL/SGTB/RPL23/RNF4/UBE2L6/RACK1/RNF7/CUL4B/TRIM7

1/OAZ3/FANCA/CCT7/TESK1/RPL10L/ADGRG2/DAZAP1/KDM5B/NCAPH2/TCP1/ETV6/DEAF1/POC1A/

PH1/SCMH1/HDAC2/MKKS/ZFP41/DEFB1/OAZ3/FANCA/TESK1/RPL10L/ADGRG2/DAZAP1/NCAPH2/E

PH1/SCMH1/HDAC2/MKKS/ZFP41/DEFB1/OAZ3/FANCA/TESK1/RPL10L/ADGRG2/DAZAP1/NCAPH2/E

WC1/C12orf4/TCTEX1D2/PTPN1/PLK3/SH3GLB1/MAVS/BNIP3L/RAB3A/SREBF1/EDEM2/ZPR1/RAB

MAP3K1/GPS1/MAPKAPK5/ADORA2B/UBE2N/KITLG/KSR1/CCL19/PIK3R6/IRAK2/DUSP18/EGF/MAP[illegible]

2/UBR5/PORCN/PSMC3/STK3/GSKIP/RAPGEF1/IFT20/PSMC5/RBPJ/CCAR2/NPHP4/TLE6/KLHL12/[illegible]

BAG3/IL4R/TGFB3/HK1/GZMB/DNM1L/TRPM4/PDCD5/STX4/RACK1/CLEC5A/HCAR2/CCT8/ABCG1/C[illegible]

/TLE6/KLHL12/EGF/CAV1/DAB2IP/DKKL1/ZBED2/BMP2/PSMB1/WWTR1/PIN1/CSNK1G1/STK4/KRE[illegible]

/JMJD8/MUL1/SLC20A1/NOD2/PLEKHG5/IKBKB/DDX1/MAPKBP1/MIB2/MTDH/CASP10/TICAM1/WNT[illegible]

/ENPP2/TNFRSF14/HBEGF/SH3BP5L/EPHA2/KITLG/EREG/CRLF1/OSM/HAX1/EGF/CAV1/TMEM102/I
PP2R5B/ENPP2/TNFRSF14/HBEGF/SH3BP5L/EPHA2/KITLG/EREG/CRLF1/OSM/HAX1/EGF/CAV1/TMI
1/TNFAIP3/TRADD/PSMB5/PSMA4/VCAM1/PSMD9/TNFSF15/PTPN2/THBS1/ADAM17/CASP8/TXNDC1'

LP6

E/MAP2K2/CENPE/ATP6AP1/PAK1/PEA15/FGFR4/FPR1/RAP2B/SOCS1/CKS1B/HSP90AB1/F2RL1/T[illegible]
LIMS1/TBCD/NPRL3/RASAL1/SIPA1/ARAP1/TBC1D7/SGSM3/ARHGAP11A/SRGAP2/RGP1/TBC1D22B/
4L1/SPOUT1/PCGF3/MORC2/MIR155HG/HDAC5/PABPC1L/TAF1B/METTL3/PPARG/MYO1C/MOV10/PP[illegible]

RT1/EPHB3/DDIT4/SH2B2/POLR2H/FGFR1/HIP1R/SLC9A3R1/NCBP2/MT3/ACTG1/NCKAP1L/FGFR3/
MYCBP/GOLGA3/MCM8/VCX/IL4R/CALR/CTDNEP1/TDRKH/HSF2/MYBL1/CETN2/TMEM119/TESMIN/AX
P4/DBNDD2/IL1B/GBA/PIAS3/CDK5/CHORDC1/PHB/MYADM/PTPN6/SAMSN1/SOCS3/VPS28/INPP5J/
IP3/NFATC4/PSMB5/PSMA4/PSMD9/CSNK2A1/VPS35/NOTCH1/SFRP4/TLR2/TMEM64/GLI1/MESP1/
AR/BECN1/SNAPIN/VPS33A/MFN1/ATG7/HTT/RPTOR/ATP6V1B2/CSNK2A2/LARP1/VPS4A/RAB1A/K

RIPAP1/CHMP4A/CLEC16A/VTA1/ARHGAP44/STX16/SNX6/SNX2/EHD3/INPP5F/DENND2A/GBF1/CHM
YNCRIP

FR4/FPR1/RAP2B/SOCS1/CKS1B/HSP90AB1/F2RL1/TGFA/CCL13/TNFRSF11A/RELN/LIMCH1/MARK
/TGFB3/MUC20/ADRA2A/XRCC6/TELO2/MAP3K6/IKBKG/STK25/RALB/CSPG4/DBF4B/CEMIP/TRAF7/

PT/PSMB1/ASXL1/USP3/MAP3K7/STAM/PSMB6/COPS8/PSMA3/PARK7/EIF3F/USP36/MYC/PSMB7/S

/FAIM/CDC42/CALR/PRKCI/ZMYND8/SEMA4D/SYT1/DNM1L/SYT3/GDPD5/KATNB1/STK25/CLEC7A/
2A/CDK5/CTTN/RNF157/PLXND1/VIL1/RAB10/C12orf57/TNIK/TUBB2B/FGFR2/DVL2/WASF1/NSM
B2B/DVL2/INPP5J/NSMF/LRP8/DGUOK/SEMA4B/CALR/PRKCI/PRRX1/ZMYND8/SEMA4D/SYT1/APP/
K5/CTTN/RNF157/PLXND1/VIL1/RAB10/C12orf57/TNIK/TUBB2B/FGFR2/DVL2/WASF1/NSMF/NE
/THY1/SPON2/THEMIS2/IRF1/IRF3/C3AR1/HSP90AB1/F2RL1/ARPC2/RAB7B/LRRC14/CTLA4/ARP
P1/DHX9/MAPKAPK5/COA3/MTPN/CNOT10/MEX3D/RBM24/DPH3/EIF6/EIF4G2/EIF4G3/CNOT3/RP
/GPX1/RPS3/TFAP4/CST7/ARRB2/SPINK4/APP/DNAJB6/DPEP1/CTSD/HSPE1/CARD16/PDCD5/FAD
/TLR5/GPATCH3/RPS19/FADD/CLEC7A/ROMO1/TRIM25/POLR3D/LBP/POLR3H/POLR3C/S100A7/T
/SIPA1/TMC8/DBNL/ACVR1B/DNM2/ADAM17/CSNK2A1/CCDC85B/NPPB/ARHGEF11/VEGFA/EXOSC9/

7/STAM/PSMB6/PSMA3/PARK7/EIF3F/USP36/MYC/PSMB7/STAMBPL1/SMAD2/BAP1/SMAD3/USP33/

3/P2RY6/SERPINE1/MICALL1/PAK1/CD63/SLC39A10/STX1A/SLC12A8/GULP1/CORO1C/SIRPA/RAI
ARHGAP18/CALR/CDC42SE2/SEMA4D/SYT1/CDC42EP2/DNM1L/SYT3/SEMA4F/SEMA5B/STK25/ABI2/
/C5AR1/LRP8/CCND2/RPS3/DUSP12/STK10/MTCP1/TGFB3/MUC20/ADRA2A/XRCC6/TELO2/MAP3K6/

CASP3/BCR/DSC2/ATP2C1/NAB2/CTSL/KRT16/RBP2/ANXA1/RELA/NSDHL/TMEM79/HOXB13/LHX2/V

PC3/WIPF1/CSNK1E/SORT1/RIN1/HYOU1/PIP5K1C/TGM2/HIP1R/ARFGAP1/SNX10/STAB1/SIRPG/(
SPA2/ADAM28/SRPK1/BAD/DNM2/SPA17/NOTCH1/CFTR/GJB3/ERCC1/PRSS21/BCL2L1/GLI1/MEA1/
AGOH/RCC1L/GGA3/NANOS1/TARBP2/ETF1/EIF5A/EIF3E/CAPRIN1/RPUSD3/NSUN4/RHOA/RPL13A/
/FKBP14/GET4/FBXO6/STUB1/BAG6/FAF2/CHAC1/TMEM67/ADD1/FBXO44/SSR1/P4HB/ATP2A2/PD
/ATP2B1/IFNG/ANXA1/AKAP8/CHID1/PTPRS/CLEC4A/APPL2/MUL1/ARG2/CPTP/LILRB1/RNF135/

K3/FZD1/EFNB1/RHBDF1/CD3E/TCIRG1/ATP6AP1/PAK1/CD63/IGFBP6/BAIAP2L1/FGFR4/SPRED1/

CTTN/RNF157/PLXND1/VIL1/RAB10/C12orf57/TNIK/TUBB2B/FGFR2/DVL2/WASF1/NSMF/GRB7/L

3AD/DNM2/SPA17/NOTCH1/CFTR/GJB3/ERCC1/PRSS21/BCL2L1/GLI1/MEA1/SSTR2/TARBP2/GORAS
SPINK4/APP/DNAJB6/DPEP1/CTSD/HSPE1/CARD16/PDCD5/FADD/CLEC7A/RACK1/IGBP1/DAP/MAP

F1/SLC9A3R1/MT3/FLOT1/SH3KBP1/VLDLR/COL6A3/LYPLA2/VSIG1/SHC1/PIK3CD/YWHAH/KIF14/
2/TWIST1/APBB1/MLLT6/DNMT3B/ZMIZ1/DMAP1/CRTC2/POLE3/BRPF1/MECOM/CTBP1/SEH1L/SUV

/PPP1R11/NLRP3/WNT5A/C5AR2/CLECL1
24/MUL1/TRIM56/LILRB1/DDX1/FGL2/C1QBP/NLRP3/TICAM1/CD40/LSM14A/TRIM44/AIMP1/PCB
MMP1L/TIMM9/SLC25A36/TIMM22/USP36/NMT1/PAM16/HTRA2/ARIH2/FZD9/PNPT1/UBL5/MUL1/B

/SIRPA/SPON2/IRF1/DUOX2/IRF3/F2RL1/PRSS3/STAT1/CCL13/NPC2/TNFRSF11A/WIPF1/NPLOC4
/TNIK/TUBB2B/FGFR2/DVL2/WASF1/NSMF/GRB7/LRP8/EMB/BTBD3/SEMA4B/GRB2/PARD6B/LLGL1/
/SIRPA/SPON2/IRF1/DUOX2/IRF3/F2RL1/PRSS3/STAT1/CCL13/NPC2/TNFRSF11A/WIPF1/NPLOC4
35/RPS20/VPS25/NUP107/GRPEL2/HK1/RPL18A/GZMB/TIMM44/PDCD5/TOMM40L/AP3D1/RPS19/RI

L/SGCE/TRIP6/TTYH1/EMID1/VCAM1/SRGAP2/DNM2/PTK2/THBS1/NOTCH1/OLFM4/VEGFA/ACTN1

/DAP/PACS2/ATP6V0D1/MAPT/RUBCN/RHEB/VMP1/SUPT5H/PAFAH1B2/EEF1A2/ABL1/VCP/IL10RA/
)11/BAIAP2L1/LYPD8/SIRPA/SPON2/IRF1/DUOX2/IRF3/F2RL1/PRSS3/STAT1/CCL13/NPC2/TNF[illegible]
112/EIF2S3/DENR/RPL39/SMPD4/RPL35A/FARSB/MRPL13/GCN1/CDC123/EIF4A3/ILF3/GRB7/ZN[illegible]
/DAP/PACS2/ATP6V0D1/MAPT/RUBCN/RHEB/VMP1/SUPT5H/PAFAH1B2/EEF1A2/ABL1/VCP/IL10RA/

MSN1/IL12RB1/CD79A/HLA-B/MSH2/PRKD2/SIT1/CD6/IL4R/GZMM/LAIR1/SWAP70/CD48/TRPM4/[illegible]

/SCN1B/SEMA3C/PTPRF/SEMA3E/MAPT/CHN1/EXT1/ABL1/STK24/VCL/KEL/DBNL/PLXNC1/HSP90A
D1/TNFAIP3/TRADD/BCL10/LPAR5/RPS27A/SFRP4/RNF2/PIAS4/MAVS/VEGFA/PLA2G1B/TLR2/C

IK3R2/CNEP1R1/APPL2/NOLC1/NUP214/HDAC3/SMAD3

3H12A/PAM/BID/CCT4/BCAP31/UBE2J2/FERMT1/NPEPPS/SRI/ADAM8/YWHAH/GIPR/MCU/FRMD8/H[illegible]
TIGAR/BECN1/WDR6/EIF4G2/ATG7/HTT/HAX1/RPTOR/ATP6V1B2/CSNK2A2/LARP1/KIAA1324/SNX[illegible]

1/NPFFR2/TGFA/RRAS/CCL13/TNFRSF11A/C1QTNF1/PSMB2/NELFE/FGFR1/ZC3H12A/PSME3/SLC
/ACTG1/NCKAP1L/SMPDL3B/HLA-DQA2/MYO1G/NONO/PIK3CD/PLSCR1/CD209/LYN/HLA-DRB5/CAR
WASF1/OXTR/NEB/GPX1/CAPZB/SEMA4B/GRB2/ARHGAP18/SLC12A7/SEMA4D/ADRA2A/SWAP70/CDC
/PFN1/IFT80/AP2S1/MST1R/RNF220/ATP6V0C/PSMD10/PSMD11/EIF4A3/PSMD1/PSMA7/RUVBL2/

/RBBP7/SEMA3C/EPB41L3/SEMA3E/MAPT/RERG/CDHR2/RARG/ABL1/VCL/GNG4/RASAL1/SFRP2/BR

N1/PPP2R3A/SSH2/PTPMT1/MASTL/DUSP7/LMTK2/SSH3/PPP2R5B/NUAK1/PPM1F/MTMR7/TIPRL/P

DUSP22/PTPRC/CDK5RAP1/PPARG/WWTR1/CASP3/ADAR/RPL11/TRIM27/PARK7/RHOH/MLLT1/HYAL2

L1/NPFFR2/TGFA/RRAS/CCL13/TNFRSF11A/C1QTNF1/PSMB2/NELFE/FGFR1/ZC3H12A/PSME3/SLC9

34/PIK3R5/MAP3K1/MAPKAPK5/ADORA2B/UBE2N/KITLG/CCL19/PIK3R6/IRAK2/EGF/MAP4K1/MAP4

3B/GPC2/NIN/DPYSL3/AP2A1/IST1/ZC4H2/ARF1

)5/DCLRE1C/ERCC3/DNASE1L1/EXOSC2/DBR1/FIP1L1/CSTF3/DDB1/EXO5/MOV10/PPP1R8/CASP3/

PKAPK5/ADCY6/ADORA2B/UBE2N/ADCY5/CCL19/IRAK2/EGF/MAP4K1/MAP4K2/DAB2IP/RPTOR/BOR[illegible]

HSP90AA1/DNM2/PTK2/NOTCH1/MAP1A/VEGFA/RAB3A/FBXO45/RAB11A/TUBB3/IFRD1/CDK5R1/ME[illegible]
MAGEF1

A13/BDKRB2/CCR3/PLCG1/LPAR1/TMEM38A/CHD7

2/VPS16/C2CD5/RAB8B/CD59/RAB1B/VPS8/STAM/VAPA/TBC1D20/TMCC1/TSNARE1/VAMP3/KIF5B/

P2/OR2T8/OR4D2/OR2M2/OR2A25/OR10A5/OR1L4/OR5A1/OR10A2/OR8G1/OR56B1/OR4F4/OR8D2/(

)/SHMT2

NAPC4/HUWE1/FBXO44/SKP2/PSMB1/FBXO10/TRIM32/PSMB6/CDC23/TRIM27/PSMA3/RNF144A/UB
K2/DAB2IP/DUSP22/BMP2/STK4/MAP3K7/FGF19/MFHAS1/SH3RF1/NOX1/MYC/ZNF622/SKP1/MAP3

MEIS2/SFRP2/BRAT1/GNGT2/NFATC4/CHEK2/RAD1/VCAM1/DNM2/USF1/PLK3/RAD18/FNTB/ERCC1/
OXK2/CSNK1E/NUP93/NUP205/ODC1/NUP37/PSMB2/DDIT4/PPP1CA/NDC1/PIM2/NUP188/ZC3H12

/MBD2/PRMT6/BRD4/NSD1/FAM98A/DYDC2/THUMPD2/KMT2B/SUPT6H/KDM3A/MBD1/ZMPSTE24/SE

SME3/HSPBP1/MT3/BCAP31/ATG101/MLST8/ADAM8/TRIM65/CAPN1/PIP4K2A/LAMP3/ATP6V1H/DAI

[illegible]RAB/MSH2/TBX19/RALA/UBE2A/GRB2/RUNDC1/MAFF/KAT2A/TGFB3/PRRX1/DNAJB6/OCRL/PCGF2/[illegible]
[illegible]GEF25/NOTCH1/ARHGEF11/CD2AP/PLEKHG6/DENND4B/ARHGAP28/RHOA/RAPGEF1/HEG1/EPS8L1/K[illegible]

WC3/SPOCK1/RNF157/PHB/MAPK11/DNAJB2/TGFB2/TAF9/VIL1/GSK3A/PML/RAI1/SOCS3/FGFR2/[illegible]
TN/ARRB2/SPRY4/GPATCH3/MAPK7/AKT1S1/RACK1/ZNF675/IGBP1/RGS3/TNIP1/RASAL3/PTTG1I[illegible]

MFF/PDCD10/KCNN4/BAD/CD33/PTGER4/PSMD9/VPS35/CFTR/PLK3/SH3GLB1/MAVS/PFKFB2/PLA2[illegible]
ERO1A/LOX/IBSP/ACSL3/VWA1/LUM/COL4A2/LAMB3/MATN3/APP/DNAJB6/COL8A2/PRDX4/MMP19/[illegible]

F4A3/GORAB/MSH2/TBX19/RALA/UBE2A/GRB2/USP22/RUNDC1/MAFF/KAT2A/TGFB3/PRRX1/DNAJB[illegible]

ORT1/STX10/B4GALT3/B3GALNT1/RAB35/PITPNM1/ST3GAL4/ARF3/ARFGAP1/BCAP31/CHST10/CY
RP1/ACTB/MTA2/WDHD1/TCP1/SWI5/DNMT3A/THOC5/PPP1CC/SIRT7/CENPK/PML/DSN1/PARP1/TH
1IP/SLC35F6/ANKRD27/RNASET2/FPR1/MNDA/PTGES2/GYG1/HGS/NPC1/CCT2/C3AR1/HSP90AB1/
/SMARCC1/BRD9/KAT2A/BEND3/CBX5/NCOR2/CBX8/HELLS/PCGF2/HMGN1/TELO2/RAD21/MTBP/RB
CM5/SMARCD1/CENPL/HMGB2/TCF3/TRIM28/POLA2/BRMS1/SIRT6/SGO2/ARPC4/WDR76/GINS4/PR
T1/ILF2/RRS1/E2F8/CCDC86/RRP9/PRMT2/DDX11/CEP85/SUB1/POLR3K/REXO4/RRP1/KRT18/PP
1IP/SLC35F6/ANKRD27/RNASET2/FPR1/MNDA/PTGES2/GYG1/HGS/NPC1/CCT2/C3AR1/HSP90AB1/
18/CTSD/AP3D1/AP1B1/CCZ1/ABCC10/M6PR/GNB4/ATP6V0D1/NSF/HPSE/SLC48A1/RHEB/SEC13/
FUCA2/RNF19B/CD63/ABCB6/STK11IP/SLC35F6/ANKRD27/RNASET2/FPR1/MNDA/PTGES2/GYG1/G
/KIF15/POC1A/LCK/C7orf31/HAUS8/DYNC1LI1/PLK4/IFT80/TUBG2/TBC1D31/MVB12A/KIF2A/O
/SPDL1/LZTS2/WDR13/DDX11/CEP85/KRT18/PITPNM1/CCT4/SNX10/CEP89/GPSM2/FLOT1/HAUS1/
C1LI1/DSN1/MAP4/TUBG2/RAE1/KIF2A/ODF2/RPS3/CDC42/KAT2A/ZNF207/TOPBP1/MZT2B/APP/
P1/FERMT1/CYTH2/FLOT1/ACTG1/RPLP2/FGFR3/SH3KBP1/PAK4/FOCAD/CAPN1/CAP1/FZD2/JAG1/
CHMP3/AP2M1/ABCA2/RAB10/PML/SLC29A3/VPS28/AP2S1/UBA1/SNX27/ATP6V0C/APH1B/MVB12A/
B2/TCIRG1/HLA-A/CD63/DOK3/ANKRD27/FPR1/STX1A/RAP2B/RHOF/STX6/SIRPA/RAB3B/DEGS1/
ARPC5L/RAC1/ACTB/BCAR1/CTTN/FGR/NPM3/MYADM/WAS/VIL1/SPECC1/CIT/WASF1/MST1R/CTTN
DSN1/PRCC/DVL2/SURF2/IL15/THOC6/RNF113A/CPSF6/EIF4A3/ISG20/SBF1/KIF2A/PTPN23/CE
13/RAB3D/MFF/SIPA1/STX7/COPG2/ERP29/SGTA/VPS45/SCAMP2/VDAC2/RAB3A/SREBF1/COPS5/
0OA/FLOT2/CANT1/IQGAP1/CFD/OLA1/FTH1/STXBP2/PSMD2/TFF3/ITGAX/SYT8/HSPA8/NEU1/GM
27/FPR1/STX1A/RAP2B/RHOF/STX6/SIRPA/RAB3B/DEGS1/ECE2/CD58/C3AR1/DYNLL1/RAB7B/TG
INS4/PRKDC/RUVBL1/MAD2L2/HIF1A/CHTF18/CBX1/CENPE/UBE2I/CHD3/PPP2R1A/ACD/RBBP8/P
P4M1/B3GNT5/GBGT1/CHST1/ABCB6/PTGES2/FUT4/STX6/B3GNT9/SCAMP4/ECE2/CORO7/GALNT18/
2G4/GPR137B/ACLY/FLOT1/CD68/VLDLR/HLA-DQA2/LAMP3/CPNE1/ATP6V1H/CAP1/ASAH1/CLN3/
D2/LAMTOR5/RAP2A/CTSS/LAMTOR1/TMEM9/CHMP3/AP2M1/ABCA2/RAB10/PML/SLC29A3/VPS28/A
C5/PPP1CC/SIRT7/CENPK/PML/DSN1/PARP1/THOC6/NSMF/SMARCB1/CPSF6/KMT5C/ZMIZ2/TIPIN/
45A/HIP1R/ARF3/SLC9A3R1/PAM/MT3/TPPP3/BCAP31/BCL3/GTPBP4/PRR7/ARHGAP1/SLC39A13/
A/TGM2/LASP1/FERMT1/CYTH2/FLOT1/ACTG1/RPLP2/FGFR3/SH3KBP1/PAK4/FOCAD/CAPN1/CAP1/
P6R1/GPSM1/B3GNT7/RAB7B/TGFA/CHPF2/COPE/TMED9/PSENEN/SORT1/STX10/PLOD3/B4GALT3/
18/CTSD/AP3D1/AP1B1/CCZ1/ABCC10/M6PR/GNB4/ATP6V0D1/NSF/HPSE/SLC48A1/RHEB/SEC13/
1/GAS2L3/NCKAP5L/MAP2/IFFO2/FKBP1A/TPM2/IQGAP1/MYL6B/RGS14/SLC1A4/PLEC/LCP1/MAP
1/GAS2L3/NCKAP5L/MAP2/IFFO2/FKBP1A/TPM2/IQGAP1/MYL6B/RGS14/SLC1A4/PLEC/LCP1/MAP
3/CHIT1/PSMD14/TIMP2/SYNGR1/DNAJC5/TRAPPC4/HSPA6/DDOST/ALOX5/PLEKHO2/MIF/CD300A/
3/NCKAP5L/MAP2/IFFO2/FKBP1A/TPM2/IQGAP1/MYL6B/RGS14/SLC1A4/PLEC/LCP1/MAP1S/TPM3/
TUBB6/TUBA1A/TUBB2A/TTLL1/PALLD/DYNLRB1/CCT2/LMNA/DYNLL1/DYNC1H1/CAPN6/KNTC1/GA
3/AKAP8L/UTP18/ACTB/APOBEC3G/PPIL1/RPS21/UTP14A/MEG3/MRPL12/RPL7/EIF3I/VBP1/RPL
CA5/RUNX3/DMAP1/CCAR2/TBP/IPO4/MSH6/SAP130/MORF4L1/EP400/LDB1/SRCAP/SIN3B/TRRAP/
DK5/TCP1/KIFC3/KIF15/RASSF1/HAUS8/DYNC1LI1/MAP1LC3B/TUBB2B/MAP4/TUBG2/KIF2A/ODF
L7/MCAM/PPP1CC/RAB10/PFN1/WASF1/ATP6V0C/YWHAQ/GRB7/PARVG/YWHAB/RPS3/RPL27/RALA/
AM/PPP1CC/RAB10/PFN1/WASF1/ATP6V0C/YWHAQ/GRB7/PARVG/YWHAB/RPS3/RPL27/RALA/CNN2/
L7/MCAM/PPP1CC/RAB10/PFN1/WASF1/ATP6V0C/YWHAQ/GRB7/PARVG/YWHAB/RPS3/RPL27/RALA/
GLIPR1/RAB3A/CYB5R1/TLR2/TMBIM1/SLC15A4/AP2A2/ATP6AP2/SLC2A3/TSPAN14/MLEC/PLA1A/
/RPS3/FGD6/NRBP1/PRKCI/APP/SWAP70/SPRY4/STX4/ABI2/MYO5A/VIM/ARFIP2/EPB41L3/MAPT/
PB/ZNF207/CBX5/NUP107/HELLS/XRCC6/TELO2/RAD21/MTBP/CENPN/NABP1/RPA2/NDE1/ZWILCH/
AP2S1/OSTM1/UBA1/ATP6V0C/TMEM63A/KPTN/YWHAB/SLC22A17/BORCS8/GLMP/STARD3/VPS18/C
9/EIF2D/MRPL33/LARP1/MRPS5/SRP68/RPL13/MRPL36/RPL4/RPL41/RPL9/MRPL14/RPS6/RPL24/
/RPL32/RPL30/PSMC5/SNAPIN/MMS19/HPS3/SURF6/RPS9/HTT/PI4K2A/RPS14/GET4/RPS27/RPL

3/SYNCRIP/CCDC12/HNRNPA2B1/U2AF1L4/LUC7L3/TRA2B/SUGP1/LSM3/PRPF40B/CACTIN/BCAS2/
PL24/RPL11/RPL10/RPS24/MRPL3/RPS3A/MRPL20/RPL14/MRPL24/MRPS16/RPL10A/MRPL55/ZNF

1/RRAGD/TMEM165/RAB25/MAPK3/RRAGC/HLA-DQB2/TCIRG1/MICALL1/HLA-A/MAP2K2/AP4M1/A
16/CDK7/PDSS1/UBE2E1/RUVBL2/CCND2/FBXL6/POLE4/PIGC/RBBP4/POLA1/BARD1/UBE2A/ASB

X1/GTF2F1/TAF3/ERCC3/SIX5/TAF1B/TADA3/TRRAP/SNAI3/PPARG/JUNB/WWTR1/SRA1/NFYA/S
5/EPS8L1/EPHA2/KSR1/MPP2/ATP7A/CIB1/PKD1/BBS7/PROM2/TMEM67/C2CD5/GNA13/MYO1C/A

155/TMEM120B/CBX5/CALR/CTDNEP1/RANBP1/APP/NUP107/DMPK/CETN2/RRM1/NOC4L/BNIP1/C

)11/PSPC1/HOXB5/MALT1/LEO1/SUPT20H/WDR43/YBX2/POLR1D/TAF4B/PLRG1/CDK12/MBD6/TOP1

3P1/GMFB/MAEA/HAX1/CAV1/RIC8B/CNKSR1/ACTR1A/LLGL2/C2CD5/SPIRE2/PPP1R9A/UTRN/PXN/

LA-F/AP2A2/RAB11A/VPS11/RAPGEF1/CLTC/STXBP1/TAPBP/TAP2/AP2B1/HBEGF/STXBP4/BECN1/

2R/VAMP3/TMEM79/CHID1/FAM91A1/VPS54/RAB6A/PLD4/GGA2/ATXN2/TEPSIN/MMP24/ARFGAP3/S

/KLHL12/PEF1/EGF/EPN3/LMAN2/ATP7A/ACTR1A/CD3G/LMAN2L/VPS16/ERGIC2/RAB8B/CD59/IEI
T1/ACTG1/SRI/PRR7/GABRR3/SV2A/SYNGR3/CLN3/LRFN1/LYN/MCTP1/RAC1/TMEM163/CNTNAP1/
2/ALDOC/TGFB3/APP/PNP/XRCC6/PSMD3/CTSD/PSMD7/ECM1/GMFG/PRDX4/CCT8/HPSE/S100A7/G(

E2/THOC2/KIF22/DGKQ/BAZ2A/PPIE/RBM14/APBB1/TRIM69/NEK6/POLR2D/GATAD2A/SART3/DHX

1/ARL8B/PARD3/PRSS12/EXOC3/APBB1/AP3B1/HNRNPR/AP1S1/ACAP3/RAB27B/NIN/DPYSL3/SNAI

RAPPC1/GSDMD/CNN2/CALR/ALDOC/TGFB3/APP/PNP/XRCC6/PSMD3/CTSD/PSMD7/ECM1/GMFG/PRD
RFIP2/WDR1/RASAL3/PARD6G/MAPT/NF2/EMD/KDF1/ACTR3/BIN2/IFT172/DBNL/TCTEX1D2/PTK2/
15/WHRN/PRKCB/DOCK7/KCNIP3/TRAK2/SYP/TSC1/SHTN1/EXOC6/RAC3/UNC13A/MAP3K12/ELK1/I

PC15/SKP1/ANKIB1/MED30/ATG3/DERL1/KCTD13/CUL7/RNF19A/ABTB1/USP33/FBXL18/FBXL2/CI

RAPPC1/GSDMD/CNN2/CALR/ALDOC/TGFB3/APP/PNP/XRCC6/PSMD3/CTSD/PSMD7/ECM1/GMFG/PRD

3/PIK3C2B/CCNJ/POLR1C/POLR3A/SESN2/PRPS1/ERCC2/PIK3R2/SLA2/IKBKB/SUPT3H/ZNRD1/TI

HD1/MRPS2/PNPT1/MRPL21/NDUFA7/MRPS26/DNAJC11/MRPS11/NDUFA6/TIMM21/UQCR10/MRPL42/

/SNX16/MVB12B/OSBPL9/IGF2R/RAB22A/AP5S1/CTSL/VPS33B/VPS29/CTNS/CHID1/ARL8A/DERL

DI1/EPHB3/PPP1CA/HIP1R/PAM/MT3/DYNLT1/KISS1/SRI/PRR7/TUBB4A/ATF4/SV2A/GLRX3/HCF

341/NEURL1/SYT11/PDE4B/RPL14/DMTN/RPL10A/FXR1/NETO2/RPL12/SEMA4C/CNIH2/RPS18/PTI

/NUFIP2/LSM14A/EIF4ENIF1/ZC3H12D/DCP1A/TDRD5/CARHSP1/TRIM5/SMN1/CNOT1/MEX3B/NSUN

N1/PLCG1/PPP1R9A/BCR/SEMA3F/ATP2B1/SPARC/FXR1/RELA/VAMP1/FZD9/CNIH2/PTPRS/ELAVL

4B/RPL14/DMTN/RPL10A/FXR1/NETO2/RPL12/SEMA4C/CNIH2/RPS18/PTPRS/TSC1/SORCS2/ACTR

YP/MUL1/PPOX/RER1/DNAJC11/PEX16/ECE1/UBIAD1/PISD/STX16/IMMT/CSGALNACT1

D21/RNF4/PRDM4/VRK1/POLR3D/TFAP2A/MED12/RERE/SUPT5H/SSBP1/OVOL2/MTA3/NFATC4/DNT

YND8/HSF2/RBPMS/HSBP1/ATXN7L3/NCOR2/GMEB1/DDX54/FUS/TBL1XR1/DR1/RNF4/E2F6/DPF2/

MTK3/CDK7/MATK/CCND2/PRKD2/STK10/PRKCI/GRK2/DMPK/MAPK7/MAP3K6/AXL/RPS6KB2/BTK/D

/RPL10L/TNC/MUC3A/RPS5/MGP/ACTB/RPS21/MRPL12/RPL7/RPL28/MRPS34/PSMD13/NUP85/CLT

AP18/RANBP1/AHSA1/AGAP1/APP/DNAJB6/CDC42EP2/MYO9B/OCRL/DNM1L/PGAM5/PPP1R15A/PDC

1L2/FSCN1/WIPF3/WASF2/CACYBP/RAB6B/CXCR4/FAM83D/GAS2L3/NDN/MAP2/TPM2/MTCL1/MSN/

DC42BPG/PKN3/CARD11/NAGK/ALPK3/PFKM/CDK5/MLKL/FGR/MAPK11/PI4KB/LCK/HUNK/MAP3K14/

RIT1/HSPA8/RRAGC/TCIRG1/CENPE/CHD3/ATP6AP1/GNA12/ABCB6/RAP2B/GNGT1/KLC3/TUBB6/R

RIT1/HSPA8/RRAGC/TCIRG1/CENPE/CHD3/ATP6AP1/GNA12/ABCB6/RAP2B/GNGT1/KLC3/TUBB6/R

RIT1/HSPA8/RRAGC/TCIRG1/CENPE/CHD3/ATP6AP1/GNA12/ABCB6/RAP2B/GNGT1/KLC3/TUBB6/R

RG1/CENPE/CHD3/ATP6AP1/GNA12/ABCB6/RAP2B/GNGT1/KLC3/TUBB6/RHOF/EFTUD2/ABCF2/XRC

/SH2B2/PITPNM1/PAM/TNIP2/CCNO/PPME1/DUSP2/FBXO5/AATF/GYS1/PRR7/CNOT9/CDKN2B/SHC

YS1/PRR7/CDKN2B/SHC1/KCNQ1/ATF4/KIF14/SV2A/HCLS1/AXIN1/GLRX3/TESK1/DCTN2/ITGB1B

LC9A3R1/GPSM2/AATF/SH3KBP1/TCEAL3/ARHGAP1/CNOT9/HEYL/DAPK3/GABRR3/FZD2/ATF4/CDK

1/UBE2Z/SH3PXD2B/EFNA5/TNFAIP8/PSMD2/RGS14/NUPR1/PYGO2/OAZ1/GM2A/SERPINE1/MAP2K

3/CDH11/MARK2/KRT18/PPP1CA/CORO1B/UNC45A/EEF1D/LASP1/PPME1/SLC3A2/EHD1/ADAM8/PA

2/PFN1/WASF1/ABR/SBF1/ARF4/CDC42SE1/XPO5/TRAPPC1/DENND6B/RIMS3/FGD6/PARD6B/RUND

CR5/FHDC1/MYO9B/KLHL17/AFAP1/EPB41L1/GMFG/MYO5A/WDR1/EPB41L3/FMNL3/MAPT/ARPC1A/

/TNK2/AAK1/PLK3/MARK4/PAK2/MASTL/CDK11B/CSNK2B/LMTK2/MAP3K10/TP53RK/NUAK1/CDK20/

SPD1/RAD51C/DNA2/HSPA2/RAD51D/VCP/ABCB9/HSP90AA1/CHD1L/TOR2A/CFTR/KATNA1/NVL/RA

C42SE2/CDC42EP2/MYO9B/OCRL/DNM1L/TRAPPC3/ABI2/MYO5A/ARHGDIA/ARFIP2/TBC1D10C/FMN

C42SE2/CDC42EP2/MYO9B/OCRL/DNM1L/TRAPPC3/ABI2/MYO5A/ARHGDIA/ARFIP2/TBC1D10C/FMN

3AG2/TBC1D7/SGSM3/ARHGAP11A/SRGAP2/TNK2/TBC1D22B/ARHGEF11/RAB3A/SMAP1/PLEKHG6/AI
CCSAP/DCTN1/HAUS7/KATNA1/MAP1A/MARK4/RITA1/MAP7D3/LZTS1/FNTA/GLI1/RAB11A/CCDC88(
A3/CAPZB/BAG3/CNN2/ARHGAP18/RANBP1/AHSA1/SWAP70/SH3GL1/CBL/RACK1/ARFIP2/CCT8/TR
1A/TUBB3/RAB29/ARL8B/AGAP3/RHOA/ARF5/MRAS/RAB27B/BMS1/IFT22/ARF1/LSG1/MFN1/RHOB

AP28/ADPRH/AGAP3/RUNDC3A/GPS1/ACAP3/STXBP5/DOCK2/SGSM1/RANBP3/DAB2IP/PREX1/ARHG
22/ARHGAP27/RIC8B/ARHGAP17/LLGL2/RALBP1/ARHGAP33/EVI5L/ASAP3/RALGAPB

TREX2/APEX1/SETMAR/SLX4/POLD2/ACTR8

16/RPL10A/MRPL55/RPL12/MRPS2/RPS18/RPS28/RPS17/RPL26L1/RPL22/MRPL21/RPS15A/NDUF

R3/PAK4/GYS1/PIP4K2A/PAPSS1/CCL2/DAPK3/PIK3CD/CSNK1D/CDK2/TESK1/STK17B/LYN/CDC4
53A/MBD2/FXR1/SYNCRIP/EIF4G1/HNRNPA2B1/RBMS2/ELAVL1/LUC7L3/SRSF3/SERBP1/TRA2B/H

/PFKM/CDK5/MLKL/FGR/MAPK11/PI4KB/LCK/HUNK/MAP3K14/GSK3A/CDK18/NUAK2/TNIK/SLA/FG[illegible]
[illegible]8/TNFRSF14/KCNH2/FAF1/ARRDC1/BECN1/UBE2N/CACUL1/NEDD8/STUB1/BAG6/CASC3/FAF2/SU[illegible]

[illegible]1/ARRDC1/BECN1/UBE2N/CACUL1/NEDD8/STUB1/BAG6/CASC3/FAF2/SUMO2/TNFRSF1B/PXN/SMAD[illegible]

RAB5C/GTPBP3/GPN2/DNM2/RASL12/ARFRP1/GNAI3/RAB3A/RCC1L/RAB11A/SEPHS1/TUBB3/RAB2[illegible]
[illegible]3/GPN2/DNM2/RASL12/ARFRP1/GNAI3/RAB3A/RCC1L/RAB11A/SEPHS1/TUBB3/RAB29/ARL8B/AG[illegible]

2/RASL12/ARFRP1/GNAI3/RAB3A/RCC1L/RAB11A/SEPHS1/TUBB3/RAB29/ARL8B/AGAP3/RHOA/ARI
2/RASL12/ARFRP1/GNAI3/RAB3A/RCC1L/RAB11A/SEPHS1/TUBB3/RAB29/ARL8B/AGAP3/RHOA/ARI

/PASK/MARK1/NISCH/OGT/OSBPL5/SNX6/SNX21/ZFYVE26/SNX2/GSDMA/SCIN/GAP43/PLEKHF2/OS

1/GNAI3/RAB3A/RCC1L/RAB11A/SEPHS1/TUBB3/RAB29/ARL8B/AGAP3/RHOA/ARF5/GMPPB/MRAS/I

/GDF11/FAM3B/TNFSF15/NPPB/VEGFA/FNTA/DGKQ/METRN/INHA/GFER/HBEGF/NRTN/ADM/BTC/LRI

2/RASL12/ARFRP1/GNAI3/RAB3A/RCC1L/RAB11A/SEPHS1/TUBB3/RAB29/ARL8B/AGAP3/RHOA/ARI
2/RASL12/ARFRP1/GNAI3/RAB3A/RCC1L/RAB11A/SEPHS1/TUBB3/RAB29/ARL8B/AGAP3/RHOA/ARI

/PSMD10/SMARCB1/ZMIZ2/MAMSTR/VDR/CDK7/ZBTB17/PADI2/CHD4/RUVBL2/RPS3/ANXA4/TBX19/

R1/SRCAP/GTF2F1/EDF1/ENY2/TADA3/PPARG/JUNB/WWTR1/SUPT7L/MNT/TRIP4/ASXL1/TRIM32/S
RS2/DIMT1/TSEN15/RNASEK/RPP30/POLR2E/RNASEH1/SND1/DALRD3/TRMT2B/DHX38/EXOSC2/DBI

TGIF1/HDGF/HOXC6/HR/HSPA1A/E4F1/SF1/DNAJB1/GPS2/CIR1/MED1/RLIM/ID4/AEBP2/YAP1

P2/OR2T8/OR4D2/OR2M2/OR2A25/OR10A5/OR1L4/OR5A1/OR10A2/OR8G1/OR56B1/OR4F4/OR8D2/(

6/HSPA13/ATP7A/ATP6V1B2/ATAD5/ERCC3/VPS4A/RALBP1/ATP1B1/MYO1C/ATP2A2/KIF3A/ABCA

C34/UBE4B/UBE2E2/SH3RF3/MAEA/TRIM68/FBXO6/STUB1/ANAPC4/HUWE1/FBXO44/SKP2/WDSUB1/

3P4/ACTB/MTA2/KDM5B/ETV6/DEAF1/ZNF205/SFPQ/PQBP1/DNMT3A/IFI16/ASCL2/MAZ/ZNF692/I

/CDK2/KPNB1/PHLDB1/CAMSAP2/DCTN2/KNSTRN/FKBP4/RAC1/CDK5/CHORDC1/STMN3/RASSF1/PO
A/CDK4/MAD2L1/MUC1/EME1/CDKN2D/ZNF385A/ARID3A/CDC7/SCRIB/TUBB4B/CHEK1/RAN/HSF1/
/PALLD/HSP90AB1/EPHB6/ISL2/ARPC2/SLC1A3/GAS7/CCL13/NBEAL2/RELN/S100B/CDH11/ZSWI
37B/NPLOC4/CD83/POLR3K/LEF1/POLR2H/RPS6KA4/SERPINB1/IRF7/FGFR1/ZC3H12A/BCL3/LAG
KA4/SERPINB1/IRF7/FGFR1/ZC3H12A/BCL3/LAG3/FERMT1/FLOT1/NCKAP1L/ADAM8/HDAC2/ATF4,
H3/FADD/PELI1/CLEC7A/CLEC5A/POLR3D/LBP/POLR3H/HPSE/POLR3C/CCL3/HSPD1/CXCL17/ABL
ACSTD2/PRKD2/PSMB3/RALA/PAX8/GRB2/ARRB2/CDC42/KAT2A/PSMD4/HOXB7/PSMD3/PSMD7/WDR
A/PTGES2/RAP2B/GYG1/ARHGAP9/RHOF/SIRPA/DEGS1/HK3/CCT2/CD58/C3AR1/HSP90AB1/F2RL1,
/RHOF/SIRPA/DEGS1/HK3/CCT2/CD58/C3AR1/HSP90AB1/F2RL1/PRSS3/DYNLL1/DYNC1H1/ILF2/
/OLR1/SLC2A5/GPI/PA2G4/SERPINB1/PPIA/RAB24/ACLY/DYNLT1/CD68/NCKAP1L/ADAM8/CAPN1,
DYNLT1/CD68/NCKAP1L/ADAM8/CAPN1/CPNE1/C1orf35/CAP1/ASAH1/KPNB1/SLC11A1/RNASE2/R
A/PTGES2/RAP2B/GYG1/ARHGAP9/RHOF/SIRPA/DEGS1/HK3/CCT2/CD58/C3AR1/HSP90AB1/F2RL1,
63/DOK3/SASH3/STK11IP/RNASET2/FPR1/MNDA/PTGES2/RAP2B/GYG1/ARHGAP9/RHOF/SIRPA/DE
/RAB24/ACLY/DYNLT1/CD68/NCKAP1L/ADAM8/CAPN1/CPNE1/C1orf35/CAP1/ASAH1/KPNB1/SLC1
E3/SNX10/BCL3/LAG3/NCKAP1L/JAG2/ADAM8/PRR7/PIP4K2A/EVI2B/CDKN2B/PIK3CD/KDELR1/H
/KAT2A/IL4R/APP/PNP/CD8A/LILRB4/AP3D1/AXL/FADD/BTK/ZNF675/GPR183/WNT4/BTN2A2/CC
HLA-DPB1/ARPC4/UNC93B1/CFD/PRKDC/CD2/IL34/CREB3/STXBP2/EBI3/MAD2L2/PSMD2/HIF1A/
SMB8/NFAM1/PSMB10/FGR/LCP2/PSMA5/CD300LF/NRAS/WAS/LCK/PTPN6/PSMD13/PSMC4/MUC5B/
/PSMD7/LILRB4/IKBKG/BTK/EIF2B5/ARPC1A/BTN2A2/BRK1/PSMD8/ARPC5/ABL1/FCGR2B/PSMA1,
A/ACD/FANCE/ISG15/NPM1/RBBP8/FGFR4/BRCA1/NSMCE2/POLR2G/PCNA/MCRS1/XRCC2/CHAF1A/
PP5C/FUS/RAD21/BABAM1/UBE2L6/RFWD3/NABP1/RAD9A/XRCC4/OGG1/RPA2/CUL4B/PPP4C/RBM1
5C/ZMIZ2/PADI2/CHD4/UBE2E1/RUVBL2/POLE4/BCORL1/RBBP4/SAP30/PRKD2/UBE2A/SMARCC1/
PQ/CPSF1/LSM2/PQBP1/TRMT112/THOC5/SRSF12/NSUN5/TRMT6/MPHOSPH6/PRCC/ISY1/BYSL/RP
1/EIF2B5/RPL38/DRG1/MRPL52/KHDRBS1/RPL23A/MCTS1/PIWIL4/MRPL48/DAP3/BTG2/MRPS7/L
5/CFD/OLA1/FTH1/STXBP2/PSMD2/RAB25/ITGAX/MANF/SYT8/HSPA8/NEU1/GM2A/SERPINE1/TCI
NASE2/LYN/CCL28/FPR3/RAC1/ITGA2/PLA2G7/IL1B/BCAR1/CDK5/PDGFA/CXCR3/CCL4/TGFB2/G
CTL6A/IRF3/XRCC3/TICRR/E2F8/PARPBP/CTLA4/DDIAS/NPLOC4/DYRK2/CSNK1E/RFC2/DDX11/E
1/LMNA/DYNLL1/DYNC1H1/SPDL1/XRCC3/CAPN6/DPCD/LZTS2/MARK2/CEP85/E2F4/KIFC2/TPPP3,
DDIAS/CSNK1E/PRMT2/CEP85/E2F4/PSMB2/INCENP/FGFR1/PSME3/SLC9A3R1/BID/GPSM2/HAUS1,
JAML/CD3E/TCIRG1/CDCA7L/PAK1/SASH3/ADGRG1/NPM1/TNFRSF4/FGFR4/SLC39A10/SLC35F6/Z
MAGOH/ARL6IP4/IVNS1ABP/ZPR1/THOC2/RBMX/ZRSR2/HNRNPA1/PPIE/POLR2I/POLR2J/HNRNPL/
/BAG2/RNF216/PSMB5/PSMA4/SGTA/PSMD9/RCN3/RPS27A/FBXW5/PLK3/MAP1A/HSPA5/FBXO45/U
1/DYNLL1/DYNC1H1/SPDL1/XRCC3/MYO19/TICRR/TGFA/KNTC1/PPP1R9B/E2F8/CSNK1E/PRMT2/D
ARPC5L/RAC1/AKAP8L/IL1B/GBA/CDK5/CD47/UBE2L3/PFDN2/CTTN/TCP1/WAS/ZNF205/SFPQ/PO
AH/BBC3/PMAIP1/STK17B/LYN/LDHA/CDK5/PHB/TGFB2/ZNF205/SFPQ/F3/DNMT3A/LCK/PTRH2/C
H12A/BCAP31/DYNLT1/RPL17/RPLP2/EIF3D/LAMP3/ATP6V1H/ULBP2/CCL2/RPSA/SHC1/TRIM31/
/THOC5/PSMD13/PSMC4/SRSF12/TRMT6/RPL39/PRCC/EDC3/ISY1/RPL35A/SET/THOC6/PSMD10/R
/UBR5/PPM1F/BAZ2A/TWIST1/RBM14/APBB1/PPHLN1/MLLT6/RNF8/SART3/PRMT3/DNMT3B/COPRS,
LAMA5/FPR1/STX1A/EDA/MMP2/SMARCA4/NOL3/SOCS1/IRF1/DUOX2/IRF3/HSP90AB1/F2RL1/STA
SET/MRPL13/SMARCB1/NES/KIF2A/PADI2/DFFA/CAPZB/DNASE2/SMARCC1/CHMP1A/TGFB3/CTDNE
L23A/EXOSC3/MCTS1/PRPF19/MRPS7/PTGES3/TRAF7/NAT10/UTP6/HEATR1/MPV17L2/SART1/NOP
C3/WIPF1/LIMCH1/PDLIM3/SH2B2/CORO1B/ABLIM2/PIP5K1C/HIP1R/SLC9A3R1/PAM/CYTH2/ACT
OC1/CDC42EP2/OCRL/CETN2/LRRC61/WRAP73/TMEM107/ARFIP2/CEP250/HAUS6/PMP22/ATP6V0D
PIP5K1C/HIP1R/SLC9A3R1/PAM/CYTH2/ACTG1/NCKAP1L/MLST8/SH3KBP1/CAP1/CAPZA1/RTKN/S
KDELR1/HCLS1/FANCA/JAG1/MITF/LYN/CARD11/CBFB/CDKN1C/PSMB4/PIAS3/IL2RA/NCAPH2/PS
/EPHA2/KITLG/CCL19/VPS33A/EIF6/NOTCH2/MAEA/HAX1/LDB1/PKNOX1/RPS14/INPP5D/CIB1/P
IRPA/SOCS1/IRF1/ARPC2/CTLA4/FSTL3/CD83/LIMCH1/PLA2G2D/TFE3/C1QTNF1/EPHB3/ZNF703,
/IL1B/GBA/CPA2/KCTD10/CTSS/WFS1/UBE2L3/CLN6/PSMB8/RNF187/PSMB10/PHB/PSMA5/DNAJB
580/THY1/RHOF/CORO1C/C3AR1/LMNA/F2RL1/RRAS/RELN/BEX4/LIMCH1/ZNF703/PPARD/CORO1B,
SL2/GAS7/PPP1R9B/RELN/S100B/CDH11/ZSWIM5/MARK2/GDI1/EPHB3/NUMBL/RAB35/FGFR1/SLC

S/PLXND1/VIL1/GSK3A/NDRG4/TNIK/TUBB2B/DVL2/PFN1/INPP5J/MAP4/NSMF/LRP8/TACSTD2/C
/NUP85/PSMC4/GSK3A/NUP43/FBXW9/SOCS3/ANAPC11/VPS28/UBE2D1/RNF220/UBA1/PSMD10/RN
P1/RHBDF1/IL27RA/TCIRG1/DKC1/PAK1/CAPN10/TNFRSF4/STX1A/MCRS1/STX6/SOCS1/SLC51B/
/HSPA4/TRAM1L1/SIX2/RTKN/RPSA/CSNK1D/YWHAH/KPNB1/SNF8/KDELR1/HCLS1/BBC3/PMAIP1/
RRS1/GAS7/FMOD/ARPC3/NDUFAF2/NUP93/WDR77/HOPX/NUP205/BRIX1/CORO1B/RRP7A/NDC1/SN
/PSMB8/RPL7/RPL28/PSMB10/PSMA5/POP1/LSM2/PSMD13/NUP85/PSMC4/GSK3A/NUP43/RPL39/E
P11/NIFK/NABP1/LAS1L/TRIM71/UTP4/MAPT/EXOSC3/PIWIL4/NAT10/UTP6/HEATR1/SART1/NOP
G4/SERPINB1/PPIA/RAB24/ACLY/DYNLT1/CD68/NCKAP1L/ADAM8/CAPN1/CPNE1/C1orf35/CAP1/
/SERPINE1/IL27RA/PAK1/TMIGD3/GNA12/ADGRG1/PRKX/CHMP4C/LAMA5/SPRED1/RAP2B/ZNF580
GR/CCL4/MYADM/TGFB2/GPSM3/F3/FAM89B/VIL1/CHMP3/MAZ/CCR1/TUBB2B/CXCL16/PFN1/NSMF
B1/IL15/RPS3/SIT1/GRB2/CD6/DROSHA/CDC42/KAT2A/IL4R/PNP/CD8A/LILRB4/AP3D1/FADD/P
G4/SERPINB1/PPIA/RAB24/ACLY/DYNLT1/CD68/NCKAP1L/ADAM8/CAPN1/CPNE1/C1orf35/CAP1/
NASE2/LYN/CCL28/FPR3/RAC1/ITGA2/PLA2G7/IL1B/BCAR1/CDK5/PDGFA/CXCR3/CCL4/TGFB2/G
ZNF598/PLEKHN1/EIF2B5/RPL38/DRG1/MRPL52/KHDRBS1/RPL23A/MCTS1/PIWIL4/MRPL48/DAP3
K5/TGFB2/ZNF205/SFPQ/F3/LCK/PTRH2/CASP1/GSK3A/PML/ABR/APH1B/LTBR/VDR/TNFSF12/YW
J1/PRELID1/KRT18/PPARD/LEF1/PDCD1/PA2G4/IRF7/PIM2/HIP1R/ZC3H12A/PSME3/BID/MT3/T
K5/TGFB2/ZNF205/SFPQ/F3/LCK/PTRH2/CASP1/GSK3A/PML/ABR/APH1B/LTBR/VDR/TNFSF12/YW
AS/HYOU1/PRELID1/KRT18/PPARD/LEF1/PDCD1/PA2G4/IRF7/PIM2/HIP1R/ZC3H12A/PSME3/BID
BXO45/UBA52/CD2AP/EDEM2/PSMC3/CHFR/AGAP3/GLMN/ANKZF1/TMUB1/PSMC5/FAF1/CCAR2/FBX
DYNC1H1/ARPC3/LIMCH1/SUB1/ORMDL2/EPHB3/CORO1B/TPBG/HIP1R/BID/TPPP3/GPSM2/FLOT1/
GFB3/ZMYND8/SEMA4D/SWAP70/CDC42EP2/MED25/RBX1/PIP4K2C/GMFG/WRAP73/ABI2/CLEC7A/R
R2H/IRF7/PPIA/NUP188/ZC3H12A/BCAP31/DYNLT1/RPL17/RPLP2/EIF3D/LAMP3/ATP6V1H/ULBP
1CA/LEF1/NDC1/NELFE/POLR2H/IRF7/PPIA/NUP188/ZC3H12A/BCAP31/DYNLT1/RPL17/RPLP2/E
D/UBAP2L/FSTL3/PPARD/LDOC1/NDC1/PAM/CCT4/BCAP31/FBXO5/JAG2/SPAG1/RSPH1/SCMH1/HD
RR/KNTC1/PPP1R9B/E2F8/CSNK1E/PRMT2/E2F4/PSMB2/PIM2/PSME3/BID/CCNO/PPME1/HAUS1/B
TGAX/MANF/SYT8/HSPA8/NEU1/GM2A/SERPINE1/TCIRG1/SLPI/FUCA2/CD63/DOK3/STK11IP/RNA
HLA-A/SLPI/PAK1/ISG15/MNDA/SIRPA/TRIM59/SPON2/SOCS1/CD58/IRF1/IRF3/HSP90AB1/F2R
TK/PELI1/CLEC7A/TNIP1/POLR3D/LBP/POLR3C/HSPD1/PSMD8/FCGR2B/PSMA1/TNFAIP3/MAPKAP
ARPC2/STAT1/RAB7B/HDAC1/RELN/CSNK1E/CD83/MARK2/GDI1/ZNF703/NUMBL/PPARD/LEF1/PA2
KN1C/PSMB4/LAMTOR5/CDK5/LAMTOR1/PSMB8/PSMB10/PSMA5/TGFB2/RASSF1/THOC5/PSMD13/PS
DKN2B/SHC1/KIF14/AXIN1/SLC11A1/TESK1/LYN/WNT7B/ITGB1BP1/BIRC7/GPRC5B/CDKN1C/DUS
B/LAG3/SIRPG/NCKAP1L/JAG2/ADAM8/PRR7/ULBP2/CCL2/TXLNA/PIK3CD/KDELR1/FANCA/SLC11
1LI1/DSN1/ANAPC11/CYP26B1/TUBG2/KLHDC8B/KIF2A/ZNF207/CHMP1A/CALR/INF2/TDRKH/RAN
ADAM8/PRR7/PIP4K2A/EVI2B/CDKN2B/PIK3CD/KDELR1/HCLS1/FANCA/JAG1/MITF/LYN/CARD11/
/PML/SOCS3/FGFR2/COCH/DVL2/PFN1/AP2S1/COL6A2/PSMD10/PSMD11/VDR/PSMD1/GORAB/PSMA
/NFAM1/TNFSF13B/FGR/CD300LF/LCK/PTPN6/CD27/SAMSN1/IL12RB1/CYP26B1/LST1/IL15/ABR
/CD47/CXCR3/CCL4/TGFB2/GPSM3/LCK/PTPN6/NUP85/CALCA/CCR1/CXCL16/ABR/C5AR1/PADI2/
AMSAP2/TBC1D30/KDM1A/ITGB1BP1/FKBP4/MCTP1/RAC1/MYLIP/GBA/CDK5/CD47/ANKRD13B/SPO
RPINE1/IL27RA/PAK1/TMIGD3/GNA12/ADGRG1/PRKX/LAMA5/SPRED1/RAP2B/ZNF580/THY1/RHOF
MYADM/TGFB2/GPSM3/F3/FAM89B/VIL1/MAZ/CCR1/TUBB2B/CXCL16/PFN1/NSMF/C5AR1/GRB7/PR
1/NPFFR2/TGFA/TNFRSF11A/RELN/RFC2/MARK2/CEP85/NCAPG2/FGFR1/SLC9A3R1/CCT4/MT3/CC
ARHGAP1/CCL2/RAP1GAP2/CXCL13/SIPA1L2/ARHGDIB/PLSCR1/TBC1D30/BBC3/PMAIP1/LYN/ADR
2/HCLS1/TESK1/ITGB1BP1/ARPC5L/FKBP4/RAC1/CDK5/CHORDC1/PDGFA/CD47/PFDN2/CTTN/STM
TUBB4A/PIK3CD/CSNK1D/MKKS/KIF14/KPNB1/SV2A/CLN3/DCTN2/LYN/ZNF593/B9D2/CDK5/LAMT
B/AP3D1/TRAPPC3/KATNB1/IKBKG/BTK/STX4/CBL/STK25/MYO5A/CSPG5/COPG1/TRAPPC2L/NDE1
LT1/DVL3/VLDLR/CPNE1/HEYL/HDAC2/RAP1GAP2/CSNK1D/FZD2/YWHAH/KIF14/CAMSAP2/KDM1A/
HGDIA/SCN1B/MAPT/TGIF2/RHEB/CCL3/PRPF19/ZNF335/CBFA2T2/STMN2/RASAL1/THBS2/DBNL/
ARK2/GDI1/EPHB3/NUMBL/FGFR1/MT3/DYNLT1/DVL3/VLDLR/CPNE1/HEYL/HDAC2/RAP1GAP2/CSN
X1A/THY1/MCRS1/UBASH3B/STX6/RAB3B/SLC51B/CCT2/LMNA/HSP90AB1/DYNLL1/DYNC1H1/CNPY
A15/NPM1/SLC39A10/SLC35F6/BRCA1/BCL2L12/XRCC2/NOL3/NPC1/LMNA/HSP90AB1/HDAC1/DDI
BID/MT3/DYNLT1/FLOT1/ACTG1/UQCC3/NCKAP1L/SH3KBP1/HSPA4/RABIF/CSNK1D/YWHAH/MSR1/

TB/FBXO32/PIAS3/KCTD10/CDK5/WFS1/RNF26/UBE2L3/CBX4/PSMB8/RNF187/RNF157/PSMB10/P[illegible]
S/KPNB1/FHOD3/SNF8/TBC1D30/NLE1/LRFN1/DCTN2/RPL10L/RAB20/ATXN2L/RPS5/B9D2/POC1A/
5A/YWHAQ/YWHAB/RPS3/ACSL3/RPL27/SSR3/NUP155/PRKCI/RPL35/RPS20/ZMYND8/RPL18A/GZM[illegible]
MT5A/FKBP4/UNC5CL/IL1B/RAP2A/CHORDC1/WFS1/TXN/MLKL/WAS/TGFB2/TAF9/SFPQ/THOC5/CD[illegible]
B/HIP1R/TPPP3/COL5A2/ACTG1/NCKAP1L/FBXO5/SH3KBP1/TUBB4A/CAP1/CAPZA1/RANGRF/CSNK[illegible]
SOCS1/PALLD/CD58/IRF1/CTLA4/CLDN10/FSTL3/CD83/CDH11/PLA2G2D/C1QTNF1/EPHB3/ZNF70[illegible]
1/WRAP73/TMEM107/ARFIP2/CEP250/HAUS6/PMP22/ATP6V0D1/NDE1/FGD1/TTC26/CKAP5/HSPB1[illegible]
L1/GSK3A/NDRG4/TNIK/TUBB2B/DVL2/PFN1/INPP5J/MAP4/NSMF/LRP8/TACSTD2/CROCC/CAPZB/[illegible]
SMA5/PTPN6/PSMD13/PSMC4/HAUS8/PML/DYNC1LI1/PLK4/ANAPC11/UBE2D1/PSMD10/PSMD11/CD[illegible]
ARID3A/CDC7/TUBB4B/CHEK1/RAN/NABP2/KIF18A/PRMT1/CFL1/PSMB9/MCM5/PSMD14/CHMP4B/R[illegible]
SMB5/BAD/PSMA4/ACVR1B/PSMD9/PTPN2/THBS1/PUS7/CASP8/NOTCH1/PDCD2/MYL9/HOXA5/SMAP[illegible]
/VPS28/BYSL/PARP1/YWHAQ/XPO5/YWHAB/ACSL3/CROCC/BARD1/BAG3/PRKCI/ZMYND8/GZMB/LIL[illegible]
D3/GNA12/ADGRG1/PRKX/LAMA5/SPRED1/RAP2B/ZNF580/THY1/RHOF/CORO1C/DUOX2/C3AR1/LMN[illegible]
/GPSM3/F3/FAM89B/VIL1/MAZ/CCR1/CXCL16/PFN1/NSMF/C5AR1/GRB7/PRKD2/SEMA4B/SYDE1/A[illegible]
M15B/LUC7L/RBM24/SF3B2/STRAP/LSM1/SFSWAP/POLR2E/CCAR1/KHSRP/MFAP1/PRPF4/GTF2F1/[illegible]
M15B/LUC7L/RBM24/SF3B2/STRAP/SFSWAP/POLR2E/CCAR1/MFAP1/PRPF4/GTF2F1/DHX38/DBR1/[illegible]
M15B/LUC7L/RBM24/SF3B2/STRAP/SFSWAP/POLR2E/CCAR1/MFAP1/PRPF4/GTF2F1/DHX38/DBR1/[illegible]
VEGFA/BNIP3L/TLR2/EGLN2/UBA52/MECP2/PSMC3/TWIST1/SRF/RHOA/CD38/ACTN4/DNMT3B/PSM[illegible]
OLR1/SLC2A5/GPI/PA2G4/SERPINB1/PPIA/RAB24/ACLY/DYNLT1/CD68/NCKAP1L/ADAM8/CAPN1/[illegible]
IL12RB1/CYP26B1/LST1/IL15/ABR/MSH2/RPS3/SIT1/CST7/GRB2/CD6/DROSHA/CDC42/KAT2A/I[illegible]
TSS/CD47/PSMB8/NFAM1/PSMB10/FGR/LCP2/PSMA5/CD300LF/NRAS/WAS/LCK/PTPN6/PSMD13/PS[illegible]
/SARNP/HTATSF1/LSM8/MAGOH/CDK11B/ARL6IP4/TBRG4/ZPR1/THOC2/RBMX/ZRSR2/HNRNPA1/PP[illegible]
/SEH1L/EIF6/IPO4/LSG1/RANBP3/THOC1/DHX38/PKD1/ENY2/GEMIN2/PABPN1/SRSF1/SRSF2/CA[illegible]
G1/PTPN2
GFB2/RASSF1/RASA3/RAB10/WASF1/ARHGAP30/ABR/CADM4/SIPA1L3/YWHAQ/CDC42SE1/RHEBL1/[illegible]
4/NCOR2/POLR2K/PSMD3/PABPC4/PPP1R15A/UQCC2/PSMD7/RPS6KB2/RACK1/VIM/TRIM71/DPH7/[illegible]
6/NAE1/BTN2A2/BTG2/PSMD8/PIDD1/SUSD2/PSMA1/CHEK2/CLSPN/PSMB5/RAD1/PSMA4/PSMD9/C[illegible]
2/NFATC4/KEL/DENND5A/DBNL/PLXNC1/DNM2/PTK2/STYXL1/FIG4/MOB2/LLPH/VEGFA/LZTS1/HS[illegible]
L1/SFRP2/FOXF2/LAMA4/LOXL1/COL9A3/VCAM1/EFEMP2/PTK2/THBS1/NOTCH1/CAPN2/ADAMTS5
PFN1/NSMF/C5AR1/GRB7/PRKD2/SEMA4B/SYDE1/AGO2/CALR/SEMA4D/XCL2/CMKLR1/APP/ADRA2A/
2orf65/SNAPIN/MRPL4/IST1/VPS33A/CAV1/GRWD1/CAMSAP1/CIB1/MRPS24/EIF2D/KLC1/MRPL3[illegible]
1/UBE2N/ANAPC5/CDC27/CTBP1/ATR/PARP10/MORC2/ANAPC4/TADA3/PAF1/HDAC8/IK/SMG7/UCN/
CUL4B/CLIP3/DRG1/MAPT/ARPC1A/BRK1/ARPC5/ABL1/TBCD/VCP/STMN2/TMC8/ACTR3/DBNL/PTG[illegible]
LR1/SLC2A5/GPI/PA2G4/SERPINB1/PPIA/RAB24/ACLY/DYNLT1/CD68/NCKAP1L/ADAM8/CAPN1/C[illegible]
DYNLT1/CD68/NCKAP1L/ADAM8/CAPN1/CPNE1/C1orf35/CAP1/ASAH1/KPNB1/SLC11A1/RNASE2/R[illegible]
2F8/CSNK1E/PRMT2/E2F4/PSMB2/PIM2/PSME3/BID/CCNO/PPME1/HAUS1/BLM/FBXO5/DBF4/TUBB[illegible]
HSPA2/ABL1/MTA3/NSFL1C/CHEK2/CCND3/CSPP1/ADAM17/PLK3/HNRNPU/TRIM21/GLI1/RAB11A/[illegible]
RBM15B/KPNA6/SEH1L/EIF6/IPO4/LSG1/RANBP3/THOC1/DHX38/PKD1/ENY2/GEMIN2/PABPN1/SR[illegible]
KNTC1/E2F8/LZTS2/ERCC6L/NCAPG2/NUP37/NUMBL/PPP1CA/INCENP/LEF1/RAB35/FGFR1/TXNL4[illegible]
/MAP3K6/IKBKG/ZNF675/RALB/RGS3/IGF1R/STK38/CSPG4/DBF4B/CEMIP/TRAF7/CXCL17/PKIA/[illegible]
27/IFI16/CASP1/MAZ/GSK3A/PML/E2F2/PARP1/PSMD10/ERO1A/CRIP1/LTBR/GPX1/MSH2/TNFSF[illegible]
IRE1/DCTN1/MAP1A/ADD3/LIMA1/EML2/MECP2/CDK5R1/ARHGAP28/PPM1F/DSTN/PLEKHH2/RHOA/[illegible]
EX3D/RBM15B/RBM24/CDK11A/SFSWAP/SAFB2/KHSRP/EXOSC2/ZC3HAV1/LARP1/PABPN1/YTHDF2/[illegible]
EDEM2/RAB11A/ZPR1/RAB29/KIF20B/CACNB3/CDK5R1/UBR5/VPS11/YWHAG/ATG13/ABHD17A/ANP[illegible]

BG2/KLHDC8B/KIF2A/ZNF207/CHMP1A/CALR/TDRKH/RANBP1/MYBL1/RAD21/KATNB1/MTBP/WRAP7[illegible]
RB4/IKBKG/BTK/EIF2B5/ARPC1A/BTN2A2/BRK1/PSMD8/ARPC5/ABL1/FCGR2B/PSMA1/KCNN4/CSK/
RPL7A/RPS23/MYEF2/RPS10/PSMB5/PSMA4/RPS15/PSMD9/RPS16/RPL5/RPS27A/HNRNPU/RPS12/[illegible]
/RPS4X/RPL6/EXOSC9/EXOSC10/MAGOH/RPL37/RPL27A/DXO/EXOSC7/TBRG4/RPL36/UBA52/NANO[illegible]
NF2/BRK1/ARPC5/ABL1/ACTR3/ARAP1/DBNL/PTGER4/SPIRE1/INPPL1/NEBL/ACTN1/PLA2G1B/AD[illegible]
J/ERCC1/EXOSC10/ADD3/LIMA1/BCL2L1/PIH1D1/EML2/TRIM37/ARHGAP28/HNRNPA1/UBR5/TWIS[illegible]

FADD/PELI1/RASAL3/WNT4/NF2/BTN2A2/HSPD1/ABL1/SART1/FCGR2B/AKNA/CSK/BAD/BCL10/B4[illegible]
METRN/APBB1/SRF/CAPRIN1/RHOA/RAPGEF1/ENPP2/FNBP1L/GPC2/NIN/DPYSL3/EPS8L1/AP2A1/[illegible]
RG/ARPC5/ABL1/KCNN4/ACTR3/KEL/DBNL/DNM2/SPIRE1/VEGFA/ADD3/LIMA1/RAB11A/IFRD1/CD[illegible]
D1/SEPHS1/TRMT12/TARBP2/TP53RK/INTS7/UTP20/EXOSC1/NOB1/NSUN4/INTS4/DDX10/TOE1/W[illegible]
N2/MTHFR/PLK3/VEGFA/BNIP3L/TLR2/EGLN2/UBA52/MECP2/CACNB3/PSMC3/TWIST1/SRF/RHOA/[illegible]
S3/TRIM21/TARBP2/PSMC3/VPS37B/PPIE/POLR2I/PPIB/POLR2J/STXBP1/POLR2D/SIGIRR/DHX9/
A/CD38/AP3B1/STXBP1/PDPN/TNFRSF14/RUNX3/ZMIZ1/ADORA2B/CCL19/XCL1/PIK3R6/CD5/CAV[illegible]
EXOSC10/UIMC1/MIS18A/ANXA3/GLI1/MECP2/HNRNPA1/UBR5/TWIST1/STOML2/APBB1/PPHLN1/R[illegible]
3D1/AXL/FADD/BTK/PELI1/CLEC7A/RASAL3/GPR183/EXOSC3/BTN2A2/ZNF335/HSPD1/ABL1/SAR[illegible]
/RAD51D/WRAP53/TNFAIP3/NOP10/DCLRE1B/CSK/GAR1/HSP90AA1/RCN3/DCTN1/NOTCH1/NBN/HN[illegible]
/THBS1/ADAM17/USF1/CHCHD2/NOTCH1/RPS27A/CAPN2/MTHFR/PLK3/VEGFA/BNIP3L/TLR2/EGLN[illegible]
B11A/ZDHHC23/GOLPH3L/CACNB3/TIMM10/GORASP2/CDK5R1/RPL19/VPS37B/PARD3/NDUFA13/RP[illegible]
/HAUS8/PML/DYNC1LI1/PLK4/ANAPC11/UBE2D1/PSMD10/PSMD11/CDKN2C/PSMD1/DONSON/ODF2/[illegible]
R1/TRAF7/SLC25A5/SFRP2/TNFAIP3/MFF/RPS7/NFATC4/TMC8/TRADD/BAD/BCL10/ERP29/CTNNA[illegible]
STXBP1/TNFRSF14/RUNX3/ZMIZ1/ADORA2B/CCL19/XCL1/PIK3R6/CD5/CAV1/TP53BP1/ATAD5/IN[illegible]
G7/RNASEH1/POLE2/ATR/ATAD5/GRWD1/THOC1
LC25A5/EME2/SRPK1/ACTR3/CENPQ/BOD1/CSNK2A1/HNRNPU/RAD18/SYCE1L/CCNE2/MIS18A/RAB[illegible]
MD8/PIDD1/ABL1/PSMA1/DCLRE1B/EME2/CHEK2/CLSPN/PSMB5/PSMA4/PSMD9/CDK2AP2/NBN/PLK[illegible]

PREX1/ARFGEF1/ADD1/MYO1C/SPIRE2/NCKAP1/PPP1R9A/MX1

207A/BMS1/NOL10/RSL1D1/DIMT1/EBNA1BP2/NOL9/EIF6/UTP23/RPP30/SUV39H1/SURF6/PELP1/

ACTR2/IFT140/BICD2/RAB6A/NEFH/RASGRP1/FEZ1/NEFL
B6/THOC3/HNRNPA2B1/SUPT6H/U2AF1L4/SRSF3/PNPT1/NUP214/TSC1/BICD2/IWS1/ATXN2/RFTN[illegible]
9/WDR46/IMP4/RBFA/RPS14/RPS27/EXOSC2/TAF1B/DDX52/TEX10/PWP1/RPS6/RPF1/MRM2/CHD7/
MAD3/MYCN/ERI1/MAEL/TP53
DIP3/THOC7/AAAS/THOC3/DYNC2LI1/ARL3/TUB/TERF1/HNRNPA2B1/SUPT6H/TTC21A/CNIH2/U2A[illegible]

NM2/DCTN1/CEP164/HAUS7/NBN/PLK3/ZFYVE19/OPTN/HAUS5/MASTL/UIMC1/DYNC1I2/RAB11A/G[illegible]

RKRA/CAV1/DAB2IP/ATAD5/BAG6/CHAC1/BDKRB2/P4HB/TNFRSF1B/TRIM32/CASP3/RPL11/RTKN2/

IF22/PSMC3/AP3B1/CLTC/TAPBP/AP1S1/AP1S3/TAP2/AP1S2/AP2B1/LGMN/PSMC5/AP2A1/CCL19/
IP7/RPL24/OGFOD1/ADAR/RPL11/RPL10/PRMT5/SNRNP200/DDX20/ERAL1/GEMIN8/EIF3F/MRPL2[illegible]

/RPTOR/PAGR1/PKD1

P3K7/NFKBID/PSMB6/CD22/PSMA3/PDE4B/EIF2B1/CD4/RELA/PSMB7/KLHL6/PRAM1/SKP1/EIF2B[illegible]

P/CNOT3/TFDP1
BTC/BECN1/ANAPC5/EREG/CNOT3/TFDP1/UBE2E2/EGF/DAB2IP/HMGA2/RPTOR/ATAD5/PAGR1/ANA[illegible]

/ZFYVE19/OPTN/HAUS5/MASTL/DYNC1I2/RAB11A/GPR132/NEDD1/MECP2/CENPJ/PSMC3

2/RPL24/RPL11/RPL10/RPS24/TRIM27/AAAS/EIF3F/RPS3A/TRIM62/RPL14/POLR2B/RPL10A/RP[illegible]

HX30/SNRPD3/TFIP11/CELF2/NIP7/RPL24/OGFOD1/ADAR/RPL11/RPL10/PRMT5/SNRNP200/DDX2[illegible]

RC/POLDIP3/THOC7/AAAS/DCP2/THOC3/ZNHIT3/TERF1/HNRNPA2B1/SUPT6H/U2AF1L4/SRSF3/PN[illegible]

1/CNOT2/RPL12/RPS18/RPS28/RPS17/PPP2CA/RPL22/LSM3/RPS15A/SMG8/PARN/RPL31/PNRC2/[illegible]
P36/RPL14/DDX51/RPL10A/ZNHIT3/RRP15/WDR3/RPS28/RPS17/NOLC1/NOL11/ERI1/NSUN5P2/Z[illegible]

SRP14/ATG3/RPS18/RPS28/RPS17/RPL22/RPS15A/CHMP4A/SRP72/PEX16/RPL31

CD4/APOBEC3F

2B3/IKBKB

2AK1/EIF4G1/RPL12/EIF2B2/RPS18/RPS28/RPS17/PAIP1/EIF2B3/RPL22/EIF3C/DDX1/EIF1/R[illegible]

/ARF1/PDIA3

/PSMB1/KIF3A/PSMB6/CAPZA2/PSMA3/HLA-C/HLA-DOB/VAMP3/CTSL/CD207/PSMB7/HLA-E/RAB7A
3/KIF24

RIM62/RPL14/POLR2B/RPL10A/RPL12/RPS18/RPS28/RPS17/NUP214/RPL22/TARDBP/RPS15A/TA

IA/RASAL3/FGD1/RERG/RAB4B/OBSCN/BRK1/RAB3D/RHOT2/ABL1/RASAL1/RAB5C/SGSM3/DBNL/A[illegible]
IST1/RBM14/APBB1/MLLT6/RNF8/SART3/PRMT3/DNMT3B/COPRS/DMAP1/CRTC2/POLE3/BRPF1/UB[illegible]
/TBC1D10C/RASAL3/FGD1/WNT4/TBC1D22A/CCL3/CHN1/LIMS1/TBCD/NPRL3/RASAL1/SIPA1/ARA[illegible]
4/FGFR3/GTPBP4/ADAM8/PAK4/VLDLR/CDKN2B/SHC1/KIF14/NLRC5/AXIN1/SLC11A1/TESK1/LYN/
3/FKBP4/MYLIP/IL1B/RAP2A/CDK5/NREP/CTTN/SPOCK1/ADRA2C/RNF157/PLXND1/ASCL2/PPP1C[illegible]
YXL1/NOTCH1/FIG4/MOB2/LLPH/VEGFA/TLR2/HSPA5/DDX56/RAB11A/KIF20B/PACSIN1/PPP2R5B/
L27RA/HLA-DQB2/CD3E/HLA-A/PAK1/SASH3/SLC39A10/FPR1/MNDA/THY1/PILRA/SPON2/SOCS1/[illegible]
A/MAP3K14/GSK3A/TNIK/DVL2/MST1R/PSMD10/GCN1/C5AR1/LRP8/CCND2/RPS3/DUSP12/PARM1/[illegible]
1/HBEGF/PMM2/SDF2L1/CCL19/DOLK/ATP7A/EOGT/B3GALT6/HS6ST2/ARFGEF1/TMEM106A/HS2ST[illegible]
1/DMTN/DYRK3/IFNG/NSD1/TLK2/EIF4G1/PRKCB/DOCK7/RPS6KA3/WNK1/RASSF2/IKBKB/MAP3K1[illegible]
/PSMD9/CASP8/RPS27A/MAVS/DMBT1/PAK2/TLR2/MUC6/UBA52/HLA-F/MUC15/PSMC3/UBE2D2/RB[illegible]
SPA5/MITD1/ARHGAP28/TWIST1/EIF3E/STK3/DHX9/SNAPIN/LRPAP1/DPH3/CLIC2/EGF/CAV1/DA[illegible]

/UBE2N/TLR7/HSP90B1/IRAK2/CAV1/DAB2IP/CLEC4E/ZCCHC3/NFKBIL1/PSMB1/TLR8/MAP3K7/P[illegible]
HAV1/PTPRC/ZCCHC3/HNRNPUL1/CXCL10/IFITM2/SKP2/TLR8/MX1/ABCF3/ADAR/PRF1/POLR3F/I[illegible]

X1/PSMD7/CPE/AXL/TBL1XR1/RACK1/CSNK1G2/WNT4/MED12/JRK/PSMD8/RARG/ABL1/EMD/VCP/PS
/TRIM71/TRIM25/RNF122/LONP1/EXOSC3/UBXN1/PSMD8/KLHL42/VCP/PSMA1/TNFAIP3/USP5/NS
1/MVB12A/PTPN23/GPX1/PSMA7/UBE2E1/FBXL6/USP1/DNAJC10/PSMB3/UBE2A/SMARCC1/ARRB2/
/APOO/BAD/COX19/WIPI1/HSP90AA1/VPS35/CASP8/CHCHD2/TMEM11/SH3GLB1/TOMM22/OPTN/BN
RHGAP28/PPM1F/DSTN/PLEKHH2/RHOA/MTPN/SPTAN1/CORO2B/NOTCH2/ARF1/RHOBTB2/GMFB/HAX
/AP3D1/FADD/STX4/CSPG5/RASAL3/WNT4/BTN2A2/EMILIN1/HSPD1/LIMS1/ABL1/SART1/SFRP2/
OCK7/RPS6KA3/WNK1/RASSF2/IKBKB/MAP3K12
L1/PSMB1/TLR8/MAP3K7/PSMB6/FFAR2/CNPY3/MFHAS1/ICAM2/PSMA3/IRF4/CTSL/CTSB/RELA/PS
13/RASGRP1/FGL2/LILRB2

/TAOK1/MRPL42/KIF24/MRPL22/MRPL58/MRPL38/MRPS30/GSPT1/MRPS27/SPEF1/SCIN/NAPB/MR

IF3H/AGO2/CALR/RPL35/RPS20/APP/DPEP1/RPL18A/PABPC4/PPP1R15A/UQCC2/CPE/RPS19/RPS[illegible]
PSMA7/UBE2E1/WSB2/FBXL6/RPS3/TRAF3/PSMB3/BARD1/UBE2A/ASB1/ARRB2/USP22/JADE2/CDC[illegible]
/TRIM71/TRIM25/RNF122/LONP1/UBXN1/PSMD8/KLHL42/VCP/PSMA1/TNFAIP3/USP5/NSFL1C/UB[illegible]
2/USP22/KCTD2/VPS25/PSMD4/PSMD3/RBX1/PSMD7/TMUB2/TBL1XR1/PELI1/CBL/SGTB/GZMA/RP[illegible]
SMD3/RBX1/PSMD7/CPE/AXL/TBL1XR1/RACK1/CSNK1G2/WNT4/MED12/JRK/PSMD8/RARG/ABL1/EM[illegible]
SOAT1/NUP188/MT3/ACLY/CHST10/SCD5/TSTA3/UQCC3/AATF/EXTL3/PAPSS1/ADSL/AMD1/SLC35[illegible]
GFR4/SPRED1/RASGEF1A/FPR1/EDA/BMF/SOCS1/SECTM1/IRF3/HSP90AB1/F2RL1/TGFA/CCL13/T[illegible]
PTAB/MUC6/BCAN/EDEM2/B4GALT4/ACAN/MUC15/GFPT1/SULF2/PORCN/TMEM115/CHST3/AGA/POM[illegible]
3A/PACSIN2/RAB11A/HIKESHI/RAB29/GOLPH3L/PACSIN1/GORASP2/VPS37B/NEK6/VPS11/XKR8/[illegible]
CTBP1/SEH1L/SNX3/CCL8/CAV2/CAV1/HMGA2/PARP10/MORC2/ZC3HAV1/LARP1/VPS4A/NECTIN2/[illegible]
PKNOX1/PREX1/DCLRE1C/INPP5D/HDAC5/ZNF683/CD3G/PTPRC/CLEC4E/IKZF3/METTL3/LOXL3/R[illegible]
4/XRCC6/GPATCH3/PSMD3/PSMD7/RPS19/IKBKG/FADD/BTK/PELI1/CLEC7A/TNIP1/POLR3D/LBP/[illegible]
TT/CBY1/DZIP1/CCDC65/ACTR1A/OFD1/BBS7/RFX2/CCDC40/EVI5L/TMEM67/CEP72/WWTR1/KIF3[illegible]

L/PTGER4/SPIRE1/ADD3/LIMA1/CD2AP/CDK5R1/ARHGAP28/PPM1F/TNNC1/DSTN/PLEKHH2/RHOA/[illegible]
TT/CBY1/DZIP1/CCDC65/ACTR1A/OFD1/NECTIN2/BBS7/RFX2/CCDC40/EVI5L/TMEM67/CEP72/WW[illegible]

D1/PRKCB/JMJD8/MUL1/SLC20A1/NOD2/PLEKHG5/IKBKB/DDX1/MAPKBP1/MIB2/MTDH/CASP10/TI[illegible]

1/TRIM25/RNF122/UBXN1/PSMD8/KLHL42/VCP/PSMA1/TNFAIP3/USP5/NSFL1C/UBQLN4/RPS7/KC
/PAQR8/DNMT3A/CYP26B1/FOLR2/AGFG1/OSBP2/MST1R/PPP1R1B/OXTR/SPESP1/SBF1/ODF2/MSH
TV6/DEAF1/TGFB2/POC1A/PAQR8/DNMT3A/CYP26B1/FOLR2/AGFG1/OSBP2/PPP1R1B/OXTR/SPESP
TV6/DEAF1/TGFB2/POC1A/PAQR8/DNMT3A/CYP26B1/AGFG1/OSBP2/PPP1R1B/OXTR/VDR/SBF1/OD
29/KIF20B/CACNB3/CDK5R1/UBR5/TNNC1/CACNA1D/VPS11/ATG13/STXBP1/ANP32B/DHX9/DMAP1/

4K1/CAV1/MAP4K2/DAB2IP/EPHB2/PTPRC/BMP2

EGF/CAV1/DAB2IP/DKKL1/ZBED2/BMP2/RYK/PSMB1/WWTR1/PIN1/CSNK1G1/STK4/KREMEN2/PSMB[illegible]

CL3/ACHE/CEMIP/ABL1/EMD/WRAP53/MFF/PDCD10/KCNN4/BAD/CD33/PTGER4/PSMD9/VPS35/CAS[illegible]

MEN2/PSMB6

5A/CD40

EPHB2/DUSP22/EPHA10/PTPRC
EM102/EPHB2/DUSP22/EPHA10/PTPRC
7

GFA/CCL13/TNFRSF11A/RELN/LIMCH1/MARK2/NCAPG2/C1QTNF1/NELFE/RPS6KA4/FGFR1/ZC3H12

/ARHGEF11/SMAP1/PLEKHG6/ARHGAP28/ADPRH/AGAP3/RAPGEF1/ACAP3/STXBP5/DOCK2/EPHA2/S

P1R8/ADAR/TRIM27/PHF1/AAAS/SETDB1/AGO1/MBD2/POLR2B/FXR1/ASF1A/EIF4G1/LHX2/HNRNP

/SH3KBP1/CNOT9/ATP6V1H/SHC1/LYN/RAC1/ATP6V1E1/IL1B/ACTB/BCAR1/PDGFA/FIBP/FGR/LC[illegible]
[illegible]XL/PRDX4/ANGPT2/MMP19/KHDRBS1/HPGDS/TTC26/WNT4/SOHLH2/PIWIL4/HSPB11/RAD51C/PAFA[illegible]
/PPP1R1B/SET/PSMD10/SMARCB1/SMYD3/CDKN2C/CADM4/YWHAB/RPS3/TFAP4/DNAJC10/ARRB2/P[illegible]
[illegible]ATP6AP2/RNF213/SULF2/UBR5/PSMC3/STK3/GSKIP/RAPGEF1/IFT20/PSMC5/RBPJ/CCAR2/NPHP4/
[illegible]IAA1324/SNX5/VPS16/FEZ2/MTMR14/RAB1B/MAP3K7/STAM/CASP3/VPS26A/PIP4K2B/USP36/UBQ[illegible]

[illegible]MP2B/TOM1/CHMP2A/STX5/CHMP7/VPS37A/ACAP2/BLOC1S1/ALMS1/CHMP5/PICALM/DENND1C/UBC

2/NCAPG2/C1QTNF1/NELFE/RPS6KA4/FGFR1/ZC3H12A/MT3/DVL3/FLOT1/KISS1/NCKAP1L/MLST8/
/CXCL17/ABL1/STK24/CALM2/PDCD10/CSK/CLSPN/MAPKAPK3/CCND3/DBNL/ERP29/HSP90AA1/TN[illegible]

TAMBPL1/SMAD2/SENP5/BAP1/SMAD3/USP33/USP24/SUPT3H/TP53/INO80E/COPS7A/RAD23A/OTU[illegible]

/ARHGDIA/SCN1B/CSPG5/SEMA3C/MAPT/TGIF2/RHEB/CCL3/PRPF19/ZNF335/LIMS1/CBFA2T2/ABI
F/NES/GRB7/LRP8/EMB/BTBD3/SEMA4B/GRB2/PARD6B/LLGL1/CDC42/SEMA4D/SYT1/APP/NDUFS6/
/DNM1L/SYT3/SEMA4F/GDPD5/HEY1/KATNB1/SEMA5B/STK25/ABI2/ARHGDIA/VIM/SCN1B/SEMA3C/
S/GRB7/LRP8/EMB/BTBD3/SEMA4B/GRB2/PARD6B/LLGL1/CDC42/SEMA4D/SYT1/APP/DNM1L/MAPK
C3/WIPF1/NPLOC4/PSMB2/SH2B2/IRF7/ZC3H12A/PSME3/TNIP2/LAG3/FLOT1/ACTG1/NCKAP1L/SI
S9/KHSRP/DNAJC1/RPS14/MKNK1/LARP1/YTHDF2/CDK5RAP1/CASC3/RBM4B/YTHDF1/RBM8A/METTI
D/ECM1/CLEC7A/RACK1/IGBP1/DAP/MAPT/PDCD6/CYCS/CARD18/UBXN1/MTCH1/HSPD1/PIDD1/VCI
NFAIP3/RNF216/GNLY/TUSC2/ADAM17/ADAMTS5/MAVS/IRF5/DMBT1/OPTN/BNIP3L/PLA2G1B/OAS
/CDK11B/EGLN2/RAB11A/NANOS1/CRLF3/IFRD1/CDK5R1/NDUFA13/APBB1/SRF/RHOA/CD38/HBEGI

/USP24/SUPT3H/TP53/INO80E/RAD23A/OTUD5/NLRP3/ACTR8/NFRKB/PSMD12/SMURF2/OGT/TAB1/

33B/BIN1/SPON2/NPC1/SLC39A6/GPR107/HSP90AB1/F2RL1/ARPC2/RAB7B/SLC1A3/TIGD5/ARPC3
/ARHGDIA/CSPG5/WDR1/SEMA3C/EPB41L3/FMNL3/SEMA3E/MAPT/FGD1/CCL3/CHN1/LIMS1/ABL1/I
/IKBKG/DSTYK/STK25/RALB/PIFO/MAPT/CSPG4/DBF4B/CEMIP/TRAF7/EEF1A2/CXCL17/ABL1/STI

WHRN/ERCC2/ZMPSTE24/CSTA

CYTH2/FLOT1/EHD1/ACTG1/NCKAP1L/SH3KBP1/VLDLR/MYO1G/ATP6V1H/CAP1/CCL2/MSR1/CLN3/I
/SSTR2/TARBP2/GORASP2/TEX11/IFT20/RNF8/ATP2B4/PRKAG1/SPATA24/DEDD/TBP/MORN2/CDYL
/HNRNPR/POLR2D/RPLP1/DHX9/MAPKAPK5/COA3/MTPN/TIGAR/CNOT10/MEX3D/RBM24/DPH3/EIF6/
IA4/ATF6B/PLA2G6/PARK7/SDF2/FICD/UBQLN2/RNF121/SESN2/YIF1A/RNF186/DERL1/PIK3R2/C
MAPKBP1/KAT5/C1QBP/PPP1R11/OTUD5/NLRP3/C5AR2/ARRB1/IL12A/CACTIN/SLAMF1/PDCD1LG2/

/POLR2G/MMP2/ZC3H3/ITGB5/VEPH1/PEG10/UBASH3B/CD3EAP/HGS/SOCS1/PELO/HSP90AB1/EPHI

RP8/EMB/BTBD3/SEMA4B/GRB2/PARD6B/LLGL1/CDC42/SEMA4D/SYT1/APP/DNM1L/MAPK7/SYT3/SI

SP2/IFT20/RNF8/ATP2B4/PRKAG1/SPATA24/DEDD/TBP/MORN2/CDYL/SPATA2/RNF114/DZIP1/CI
T/PDCD6/CYCS/CARD18/MTCH1/HSPD1/PIDD1/VCP/SFRP2/TRADD/BAD/BCL10/TNFSF15/THBS1/C

/ITGB1BP1/RAC1/ACTB/RAP2A/CDK5/CTTN/MYADM/PLXND1/PTPN6/RAB10/C12orf57/TNIK/TUBB
39H1/MORF4L1/EP400/LDB1/ATP7A/SRCAP/METTL21A/PAGR1/TADA3/BAG6/TRRAP/PWP1/LOXL3/

P2/TRIM5/BST2/MICB/TRAF3IP1/POLR3G/GBP3/APOBEC3H/EXOC1
AP1

4/BATF2/POLR3K/ODC1/DDIT4/LY86/PPARD/AGBL5/LDOC1/IRF7/PIM2/ZC3H12A/STAB1/TNIP2/I
/CDC42/SEMA4D/SYT1/APP/DNM1L/MAPK7/SYT3/SEMA4F/SEMA5B/STK25/ABI2/ARHGDIA/SCN1B/S
4/BATF2/POLR3K/ODC1/DDIT4/LY86/PPARD/AGBL5/LDOC1/IRF7/PIM2/ZC3H12A/STAB1/TNIP2/I
PLP0/RPL18/SGTB/RPL23/NAGPA/M6PR/TIMM50/CCT8/ROMO1/RPL38/PTTG1IP/MAPT/RPL23A/HSI

/NPRL3/ATG4B/NSFL1C/UBQLN4/VAMP8/BAD/WIPI1/PSAP/HSP90AA1/PRKAA2/HDAC10/PTK2/CSN
RSF11A/WIPF1/NPLOC4/BATF2/POLR3K/ODC1/DDIT4/LY86/PPARD/AGBL5/LDOC1/IRF7/PIM2/ZC
F706/RPS3/ACSL3/SAMD4B/RPL27/NCL/EIF3H/AGO2/CALR/RPL35/RPS20/APP/RPL18A/PABPC4/
/NPRL3/ATG4B/NSFL1C/UBQLN4/VAMP8/BAD/WIPI1/PSAP/HSP90AA1/PRKAA2/HDAC10/PTK2/CSN

CD8A/LILRB4/FADD/BTK/GPR183/EXOSC3/HSPD1/HLA-DOA/FCGR2B/TNFAIP3/STX7/CSK/DBNL/B

A1/CTNNA1/DNM2/PTK2/NOTCH1/MAP1A/VEGFA/RAB3A/FBXO45/RAB11A/ZPR1/TUBB3/IFRD1/CDK[illegible]
[illegible]PS5/TRIM21/ANXA3/UBA52/MAP3K10/TRIM37/RNF25/PPP2R5B/TWIST1/ADGRG3/SRF/STK3/SIG[illegible]

CLS1/BBC3/PMAIP1/CCT7/LYN/ITGB1BP1/RAC1/AKAP8L/IL1B/CDK5/TXN/UBE2L3/TCP1/ADRA2C/
5/SNRNP70/FEZ2/PIK3CB/RAB1B/MAP3K7/CASP3/VPS26A/PIP4K2B/PARK7/USP36/UBQLN2/ULK1/

9A3R1/MT3/TNIP2/DVL3/LBH/DUSP2/KISS1/FGFR3/ADAM8/PAK4/CCL2/SHC1/AXIN1/LYN/WNT7B/
D11/FPR3/MUC3A/RAC1/CBFB/PSMB4/IL1B/ACTB/BCAR1/CTSS/CD47/PSMB8/NFAM1/PSMB10/PHB/
42EP2/TRPM4/PPP1R15A/SEMA4F/GMFG/SCTR/SEMA5B/AKT1S1/ABI2/WDR1/SEMA3C/SEMA3E/MAP
PSMB3/SDHAF2/ARRB2/CDC42/APCDD1/AGO2/CTDNEP1/ZMYND8/APP/PSMD4/TRPM4/PSMD3/RBX1/

AT1/SIPA1/TMC8/DBNL/HSP90AA1/ACVR1B/DNM2/ADAM17/CSNK2A1/CCDC85B/NPPB/LLPH/ARHGE

ALD1/PPP1R7/SSU72/HSP90B1/PPP1R14C/DUSP18/HTT/CALM3/ENSA/DUSP22/PTPRC/CTDP1/MTMI

2/IFNG/TERF1/IPO7/DUSP14/RGS4/CHMP6/WNK1/PPP2CA/TSG101/TP53/PRKAR2A/SMCR8/DUSP2[illegible]

[illegible]A3R1/MT3/TNIP2/DVL3/LBH/DUSP2/KISS1/FGFR3/ADAM8/PAK4/CCL2/SHC1/AXIN1/LYN/WNT7B[illegible]

[illegible]K2/DAB2IP/HMGA2/RPTOR/CALM3/CIB1/PKD1/PTPRC/BMP2

/MGME1/DCP2/AGO1/PAM16/DNASE1/CNOT2/ERCC2/PNPT1/ERI1/ZNRD1/DDX1/TDP2/HARBI1/TRE[illegible]

A/PTPRC/BMP2/ADCY3/ADNP/PIK3CB/ADCY7/LPAR1/STK4/MAP3K7/UCN/CCDC88A/COPS8/MADD/PA

TRN/PARD3/APBB1/SRF/RHOA/STXBP1/ROBO3/NIN/NRTN/SPTAN1/EPHA2/ARTN/IST1/NEO1/EPHB2

/RAB22A/ANXA8L1/ANXA1/VPS33B/MAPK15/VAMP1/NKD2/TMEM127/TFG/DNAJC13/CNIH2/RAB7A/'

OR4X2/OR1E1/OR5AK2/OR4C3/OR10J5/OR2C1/OR1N2/OR7D2

E2E3/WWP2/ARIH2/RNF126/PSMB7/RNF115/SKP1/ANKIB1/MKRN1/RNF186/RNF19A/TRIM56/NOD2/
K9/NOD1/SEMA4C/MAP4K4/DUSP15/MAP3K11/ZMPSTE24/MUL1/NOD2/HDAC3/RASSF2/IKBKB/MAP3K

/C9orf116/TMEM109/UIMC1/FNTA/HSPA5/BCL2L1/MECP2/INTS7/LZIC/ACTR5/IFT20/RNF8/DNMT
A/PSME3/HSPBP1/MT3/BCAP31/ATG101/MLST8/ADAM8/TRIM65/CAPN1/PIP4K2A/LAMP3/ATP6V1H/

TD1A/MAEL/RAB6A/GNAS/IWS1/NSUN5P2/METTL2B/METTL6/SUZ12/SETMAR/THUMPD3/MEN1/TARBP

PK3/CSNK1D/AXIN1/SLC11A1/CCDC22/VDAC1/MYLIP/ATP6V1E1/PSMB4/IL1B/GBA/LAMTOR5/CAP

/HOXB7/HOXD9/HEY1/TBL1XR1/TMEM107/MSX1/SEMA3C/RIC8A/TRIM71/TFAP2A/PDCD6/MED12/P[illegible]
ITLG/SSX2IP/NOTCH2/RHOBTB2/DAB2IP/ARHGEF17/PREX1/ARHGAP22/EPHB2/ARFGEF1/ARHGAP1[illegible]

CXCL16/PSMD10/CDKN2C/RUVBL2/SEMA4B/CDC42/SERTAD3/SEMA4D/SYT1/APP/GAL/SYT3/SEMA4[illegible]
[illegible]/IGF1R/PDCD6/STK38/NF2/BTN2A2/EMILIN1/GRINA/ABL1/VHL/NPRL3/RASAL1/SFRP2/TNFAIP[illegible]

[illegible]1B/TLR2/CD2AP/EDEM2/ZPR1/RAB29/KIF20B/GOLPH3L/CACNB3/ALOX15B/CDK5R1/UBR5/TWIST[illegible]
ITGB8/RIC8A/ABCG1/EMILIN1/COL11A1/ABL1/SFRP2/FOXF2/LAMA4/LOXL1/COL9A3/VCAM1/EFE[illegible]

6/OCRL/PCGF2/HOXB7/HOXD9/HEY1/TBL1XR1/TMEM107/MSX1/SEMA3C/RIC8A/TRIM71/TFAP2A/PI

TH2/SLC39A13/HLA-DQA2/CSNK1D/SLC35B2/KDELR1/CLN3/SLC35A2/UXS1/HLA-DRB5/B4GALT7/I
OC6/NSMF/SMARCB1/CPSF6/KMT5C/ZMIZ2/TIPIN/MSH2/CHD4/RUVBL2/CENPB/POLE4/RBBP4/POLA
DYNC1H1/RAB7B/FMOD/CLCN7/MFSD12/NPC2/SORT1/PLBD2/PA2G4/GPR137B/VMA21/ACLY/FLOT1/
BP7/RPA2/DPF2/INO80C/TIMELESS/POLR3D/EXOSC3/SUV39H2/ESCO2/RARG/MTA3/EME2/SFR1/LI
KDC/RUVBL1/MAD2L2/HIF1A/CHTF18/CBX1/CENPE/UBE2I/CHD3/PES1/PPP2R1A/PAK1/ACD/RBBP8
P1CA/BRIX1/LDOC1/RRP7A/PA2G4/XPO6/POLR2H/FGFR1/FBL/DDX27/EEF1D/PNKP/BLM/NOP2/AA
DYNC1H1/RAB7B/FMOD/CLCN7/MFSD12/NPC2/SORT1/PLBD2/PA2G4/GPR137B/VMA21/ACLY/FLOT1/
EEF1A2/RAB3D/HLA-DOA/NPRL3/ABCB9/STX7/VAMP8/RAB5C/PSAP/VPS35/CFTR/MCOLN3/CCZ1B/
LB1L/HGS/NPC1/CCT2/C3AR1/HSP90AB1/DYNC1H1/RAB7B/FMOD/CLCN7/MFSD12/NPC2/SORT1/PLI
DF2/CHD4/RUVBL2/CCDC77/CROCC/CDC42/KAT2A/MZT2B/RANBP1/CETN2/ACTL8/KATNB1/WRAP73/
/RAP1GAP2/AUNIP/CSNK1D/ATF4/CDK2/MKKS/NLRC5/CAMSAP2/TBC1D30/DCTN2/CDC42BPG/ITGB
RAD21/KATNB1/IKBKG/TBL1XR1/VRK1/HAUS6/NDE1/CKAP2/CKAP5/PRPF19/MZT1/IQCB1/HSPA2/
/LYN/TSPAN4/ARPC5L/TNC/RAC1/ITGA2/RPS5/ACTB/GNB2/BCAR1/CTTN/KIFC3/RPL7/MCAM/CDH
/HLA-B/ATP6V0E1/PARM1/LLGL1/STARD3/VPS18/CHMP1A/VPS25/SLC9A7/OCRL/CTSD/AP1G2/SH
ECE2/CD58/C3AR1/DYNLL1/RAB7B/TGFA/COPE/NBEAL2/SORT1/OLR1/STX10/SLC2A5/RAB35/VMA
BP2NL/NEB/SIPA1L3/KPTN/NCOA5/PARVG/CROCC/CAPZB/BAG3/SMTN/LLGL1/FHOD1/CNN2/VPS18/
NPB/PRPF3/PRPF6/BARD1/USP22/CBX5/ARHGAP18/TOPBP1/SF3A3/NCOR2/PCGF2/HOXB7/TELO2/
SLC17A5/BCL2L1/HLA-F/RAB11A/YIPF2/COPB2/CLTC/AP1S1/STXBP5/GOSR2/RAB27B/ATG9A/CO
2A/SERPINE1/TCIRG1/SLPI/FUCA2/CD63/DOK3/STK11IP/RNASET2/FPR1/STX1A/MNDA/PTGES2/
FA/COPE/NBEAL2/SORT1/OLR1/STX10/SLC2A5/RAB35/VMA21/HIP1R/PAM/RAB24/STAB1/SRI/CD
HC2/BRCA1/RBL1/NSMCE2/PCNA/MCRS1/XRCC2/CHAF1A/SGO1/SMARCA4/ACTL6A/IRF1/COPS9/AR
/PPP6R1/GPSM1/B3GNT7/RAB7B/TGFA/FMOD/CHPF2/COPE/TMED9/PSENEN/SORT1/STX10/PLOD3/
SLC36A1/RNASE2/HLA-DRB5/RPN2/SLC26A11/ATP6V1E1/GBA/GNB2/LAMTOR5/CTSS/LAMTOR1/TM
P2S1/UBA1/SNX27/ATP6V0C/APH1B/MVB12A/HLA-B/ATP6V0E1/PARM1/LLGL1/STARD3/VPS18/CH
/MSH2/CHD4/RUVBL2/CENPB/POLE4/RBBP4/POLA1/TBX19/DFFA/UBE2A/SMARCC1/BRD9/KAT2A/B
LAMP3/RANGRF/RAP1GAP2/CSNK1D/SNF8/PLSCR1/AXIN1/PSTPIP1/LYN/ITGB1BP1/ATN1/PKN3/F
/FZD2/JAG1/LYN/TSPAN4/ARPC5L/TNC/RAC1/ITGA2/RPS5/ACTB/GNB2/BCAR1/CTTN/KIFC3/RPL
B3GALNT1/RAB35/PITPNM1/ST3GAL4/ARF3/PAM/ARFGAP1/BCAP31/CHST10/CYTH2/SLC39A13/HL
EEF1A2/RAB3D/HLA-DOA/NPRL3/ABCB9/STX7/VAMP8/RAB5C/PSAP/VPS35/CFTR/MCOLN3/CCZ1B/
1S/TPM3/MAP2K2/CENPE/PAK1/NUDC/NARF/KLC3/MMP2/TUBB6/CORO1C/TUBA1A/TUBB2A/TTLL1/
1S/TPM3/MAP2K2/CENPE/PAK1/NUDC/NARF/KLC3/MMP2/TUBB6/CORO1C/TUBA1A/TUBB2A/TTLL1/
/FLOT2/CANT1/BRSK1/IQGAP1/RAB15/CLTA/CFD/OLA1/FTH1/STXBP2/PSMD2/TFF3/ITGAX/SYT8/
/MAP2K2/CENPE/PAK1/NUDC/NARF/KLC3/MMP2/TUBB6/CORO1C/TUBA1A/TUBB2A/TTLL1/BIN1/PA
S7/TIGD5/ARPC3/BEX4/WIPF1/LZTS2/MARK2/PDLIM3/SH2B2/KRT18/INCENP/CORO1B/KIFC2/CC
28/RBMX2/POP1/TAF9/LSM2/MRPS34/FBXW9/RPL39/EDC3/ISY1/BYSL/RPL35A/RNF113A/MRPL13/
/JUNB/TFPT/TRPS1/ASXL1/USP3/TTI1/IRF4/HNRNPC/ING2/MYC/YY1AP1/MBD2/ASF1A/MAX/REL
2/ZNF207/DNAL4/FHDC1/DNM1L/KATNB1/SCTR/MYO5A/CCT8/HAUS6/NDE1/MAPT/CKAP2/CKAP5/K
CNN2/CDC42/CALR/MTF2/SPRY4/AFAP1/RPS19/RPLP0/RPL18/CBL/RPL23/VIM/ITGB8/RPL38/CS
CDC42/CALR/MTF2/SPRY4/AFAP1/RPS19/RPLP0/RPL18/CBL/RPL23/VIM/ITGB8/RPL38/CSPG4/S
CNN2/CDC42/CALR/MTF2/SPRY4/AFAP1/RPS19/RPLP0/RPL18/CBL/RPL23/VIM/ITGB8/RPL38/EP
/ADGRG3/EXOC3/SIGLEC9/RHOA/CD38/RAB27B/MANBA/COPB1/BRI3/TMEM184A/ITGAL/ATP11A/C
/FGD1/CSPG4/NF2/BRK1/ARPC5/ABL1/STMN2/KDF1/SGCE/PALM/ACTR3/WWC1/DBNL/CTNNA1/SRG
/CKAP5/SUV39H2/EID3/ESCO2/SEC13/PTGES3/NAT10/DNA2/DOT1L/RAD51D/WRAP53/DCLRE1B/G
TSD/AP3D1/AP1B1/CCZ1/ABCC10/M6PR/TMEM199/GNB4/ATP6V0D1/NSF/HPSE/SLC48A1/RHEB/VM
/RPL11/RPL10/RPS24/MRPL3/RPS3A/AURKAIP1/MRPL20/ABCF1/RPL14/MRPL24/MRPS16/RPL10A/
29/EIF2D/BAG6/RPL13/PIN1/RPL4/RPL41/RPL9/RPS6/MAP3K7/RPL24/RPL11/RPL10/RPS24/RP

/SNRNP35/ZCRB1/SMU1/AAR2/PRPF40A/AKAP17A/XAB2/SYF2/PTBP2/HNRNPH1/CWC15/WDR83/CR[illegible]
[illegible]22/RPL12/MRPS2/RPS18/RPS28/RPS17/RPL26L1/RPL22/GEMIN5/MRPL21/RPS15A/MRPS26/MRP[illegible]

TP6AP1/CD63/ABCB6/CHMP4C/FGFR4/ANKRD27/RAP2B/FCHSD1/CORO1C/TUBA1A/STX6/SCAMP4/RA
1/POLR2L/USP22/JADE2/KCTD2/KAT2A/CBX5/MTF2/TMEM258/ATXN7L3/PRIM2/CBX8/POLR2K/DCU

MAD5/ATF6B/NCOA6/TCEA1/GTF2A2/ING2/POU2F1/EYA3/NR6A1/HYAL2/MAX/RELA/HOXB13/SMAD
NTXR1/DOCK8/NCKAP1/CASK/UTRN/ABCA7/KCNC4/EFCAB7/GPR161/THEM4/NOX1/ATP2B1/VEZT/PI

ERS6/ATP2A3/PLCD4/NEMP1/SEC13/AKIRIN1/ABL1/EMD/GHRHR/TMC8/LEMD2/TOR2A/PRPF38A/DC

1/PWP2/TAF1A/CRIPT

/EPB41/CAPZA2/RHOH/C2CD2L/MYO1D/TCHP/DMTN/MED28/KCNC3/RHOD/TSC1/EXOC6/MYO1A/ACT[illegible]

/AP2A1/TLR7/HSP90B1/RAB9A/PDIA3/SNX3/CAV1/RAB13/ATP7A/DAB2IP/PLD1/MYO1C/RAB8B/L[illegible]

SYT17/AFTPH/COG5/STX16/TRAPPC9

R3IP1/YIPF5/CCDC88A/HLA-C/IGF2R/VAMP3/CD4/CD207/VPS33B/ARCN1/HLA-E/YIF1A/CNIH2/S
ITGA2/VDAC1/ACTB/CDK5/WFS1/STRN4/CTTN/SPOCK1/RPL7/ADRA2C/HOMER1/CACNG4/CLTB/PPP
CA/PAFAH1B2/ARPC5/VCP/VCL/APRT/BIN2/DBNL/HSP90AA1/THBS1/SRGN/MAN2B1/OLFM4/CYB5R3

15/TAP2/TOE1/ZMIZ1/RBM10/RBM15B/MECOM/CDC34/CDYL/SF3B2/POMP/SAP130/MORF4L1/EP400

PIN/PREX1/CALM3/CIB1/SLC9A6/KLC1/CNR1/TNFRSF1B/KIF3A/PPP1R9A/UTRN/ADGRL1/UCN/KCN

X4/CCT8/HPSE/S100A7/GCA/PAFAH1B2/ARPC5/VCP/VCL/APRT/BIN2/GNLY/DBNL/HSP90AA1/THBS
/SPIRE1/CCSAP/DCTN1/CAPN2/HNRNPU/MAP1A/CTBP2/ADD3/MYO6/CANX/GLI1/ARL8B/PARD3/WIP
FEZ1/NEFL

DC16/MIB2/DERL3/ARMC8/SMURF2/RNF40/ASB2/LZTR1/CDC26/GID8/KLHL22/ANAPC1/WDR26/PC[illegible]

X4/CCT8/HPSE/S100A7/GCA/PAFAH1B2/ARPC5/VCP/VCL/APRT/BIN2/GNLY/DBNL/HSP90AA1/THB[illegible]

P53/TBPL1/TAF11/GTF2E1/PRKAR2A/SMCR8

/TRMT10C/IMMT/MRPL22/MRPL58/MRPL38/MRPS30/CHCHD6/MRPS27/NDUFB7/COX6A1/NDUFA11/M[illegible]

1/RAB7A/CHMP6/CYB561A3/SLA2/ANKRD13A/TSG101/STOML1/CHMP4A/MCOLN1/CLEC16A
C1/FKBP4/RAC1/PDE9A/PIAS3/CDK5/WFS1/STRN4/DNER/CTTN/ADRA2C/RPL28/GNB1/HOMER1/TG[illegible]

PRS/TSC1/SORCS2/ACTR2/NEFH/BCL11A/DVL1/NLGN4X/LRP4/ARRB1/ARHGAP44/ABI1/CRTC1/LR[illegible]

N2/APOBEC3H/PSMC2/DIS3L2/CTSH/PSMA6/RBM4/HOXD10/EIF2S1

1/RNF19A/CADPS2/RPL22/UNC13A/GRIPAP1/HIP1/DAG1/C1QBP/DVL1/NLGN4X/WNT5A/PPP3CB/C

2/NEFH/BCL11A/DVL1/NLGN4X/LRP4/ARRB1/ARHGAP44/ABI1/CRTC1/LRFN3/STAT3/PCBP2/GAP4

TIP1/PRKAA2/NOTCH1/ZNF711/MBD3/HNRNPU/RNF2/CTBP2/THRA/UBTF/SREBF1/CSNK2B/GLI1/T

MYBBP1A/TFAP2A/NFYC/WNT4/MED12/RERE/POU2AF1/MEIS2/TAF12/ARID5A/RARG/CBFA2T2/ABL

STYK/STK25/VRK1/CSNK1G2/IGF1R/STK38/OBSCN/CCL3/CDK19/ACVR1/FASTK/ABL1/STK24/PIM

3/RPL39/TUBB2B/EVPL/MAP4/RPL35A/BGN/TUBG2/COL6A2/PSMD11/MRPL13/NEB/ODF2/RPS3/CR

D5/GMFG/STX4/RACK1/ARHGDIA/RIC8A/RGS3/TBC1D10C/RASAL3/TBC1D22A/DBF4B/CCL3/CHN1/

3RSK1/GPAA1/PPP1R18/ARPC4/PROM1/RGS14/RAB25/PLEC/LCP1/MAP1S/TPM3/MAST2/CENPE/FA

/GSK3A/CDK18/NUAK2/TNIK/SLA/FGFR2/CIT/ALDH18A1/PLK4/MST1R/STK32C/LMTK3/CDK7/COA

HOF/EFTUD2/ABCF2/XRCC2/TUBA1A/TUBB2A/SMARCA4/RAB3B/RASEF/DYNLRB1/HSP90AB1/DYNLL

HOF/EFTUD2/ABCF2/XRCC2/TUBA1A/TUBB2A/SMARCA4/RAB3B/RASEF/DYNLRB1/HSP90AB1/DYNLL

HOF/EFTUD2/ABCF2/XRCC2/TUBA1A/TUBB2A/SMARCA4/RAB3B/RASEF/DYNLRB1/HSP90AB1/DYNLL

C2/TUBA1A/TUBB2A/SMARCA4/RAB3B/RASEF/DYNLRB1/HSP90AB1/DYNLL1/DYNC1H1/RAB7B/XRCC

1/KCNQ1/ATF4/KIF14/SV2A/HCLS1/AXIN1/GLRX3/TESK1/DCTN2/ITGB1BP1/GPRC5B/LDHA/RAC1/

P1/GPRC5B/RAC1/VDAC1/ACTB/BCAR1/CDK5/MLKL/FAM83H/FGR/WAS/LCK/PTPN6/PPP1CC/GSK3A/

2/YWHAH/KIF14/KPNB1/SYNGR3/PLSCR1/HCLS1/AXIN1/U2AF2/SRSF9/LYN/ATN1/CARD11/EHMT2/

2/SLPI/PPP2R1A/NPM1/SPRED1/ANKRD27/PCNA/THY1/NSMAF/ARHGAP9/PPP2R2C/SIRPA/NOL3/S

K4/ARHGAP1/LYPLA2/CAPZA1/CSNK1D/SH3GLB2/LYN/TSPAN4/ITGB1BP1/ATXN2L/HCFC1/LDHA/I

C1/LLGL1/INF2/RANBP1/CDC42SE2/CDC42EP2/MYO9B/OCRL/DNM1L/SH3GL1/TRAPPC3/RALGDS/A

NF2/ARPC5/TNNI3/ABL1/EMD/VCL/ZNF185/ACTR3/DBNL/VASH1/CTNNA1/PTK2/SPIRE1/SSH2/IN

/STK3/NEK6/GAK/PRKAG1/MAP3K1/MINK1/MAPKAPK5/PHKA2/GTF2H1/PHKG2/ADCK5/SBK1/CDK11

D18/HSPA5/DYNC1I2/HSPA14/KIF20B/RNF213/KIF22/PSMC3/RFC5/TAPBP/ATP2B4/TAP2/DHX9/

L3/PARD6G/SERGEF/PIFO/BICDL2/FGD1/NSF/TBC1D22A/OBSCN/BRK1/IPO13/DENND3/TBC1D7/D

L3/PARD6G/SERGEF/PIFO/BICDL2/FGD1/NSF/TBC1D22A/OBSCN/BRK1/GGA1/IPO13/DENND3/TBC

RHGAP28/ADPRH/AGAP3/RUNDC3A/GPS1/ACAP3/STXBP5/DOCK2/SGSM1/RANBP3/DNAJC1/DAB2IP/I
C/PHF6/EML2/KIF20B/CENPJ/KIF22/CDK5R1/ARL8B
IM25/STK38/RPL23A/CKAP5/ACVR1/STK24/EMD/VCL/RPL7A/CDH24/TXNDC9/DBNL/MPP7/CTNNA1/
TB2/RAB9A/RAB40C/RAB13/ARL1/RAB1A/RAB36/GNA13/RAB8B/RAB1B/MX1/GPN1/GTPBP1/RHOH/I

AP22/ARHGAP27/RIC8B/ARHGAP17/LLGL2/RALBP1/ARHGAP33/EVI5L/ASAP3/RALGAPB/TBC1D20/I

A7/MRPS11/RPL31/MRPL42/MRPL22/MRPS30

2BPG/CHRAC1/PKN3/PRIM1/CARD11/NAGK/ALPK3/PFKM/CDK5/MLKL/FGR/MAPK11/PI4KB/LCK/FL
NRNPLL/GEMIN5/TP53/TARDBP/GRSF1/C1QBP/PARN/TDRD7/MRPS11/NOVA1/TPR/RNF40/PURB/EI

FR2/CIT/PLK4/MST1R/STK32C/LMTK3/CDK7/COASY/MATK/CCND2/PRKD2/STK10/CHKA/PRKCI/GR
MO2/TNFRSF1B/TRIP4/PXN/STAM/SMAD5/LRPPRC/RPL11/MFHAS1/CKB/SYT11/EIF4E2/PSMA3/EG

5/LRPPRC/RPL11/MFHAS1/CKB/SYT11/EIF4E2/PSMA3/EGR2/RELA/NKD2/TRAF1/BLZF1/SMAD2/D

9/ARL8B/AGAP3/RHOA/ARF5/GMPPB/MRAS/ACTN4/RAB27B/BMS1/NIN/IFT22/ARF1/LSG1/MFN1/R[illegible]
AP3/RHOA/ARF5/GMPPB/MRAS/RAB27B/BMS1/NIN/IFT22/ARF1/LSG1/MFN1/RHOBTB2/RAB9A/RAB[illegible]

F5/GMPPB/MRAS/RAB27B/BMS1/NIN/IFT22/ARF1/LSG1/MFN1/RHOBTB2/RAB9A/RAB40C/RAB13/G
F5/GMPPB/MRAS/RAB27B/BMS1/NIN/IFT22/ARF1/LSG1/MFN1/RHOBTB2/RAB9A/RAB40C/RAB13/G

SBPL2/TWF1/GBF1/SH3PXD2A

RAB27B/BMS1/NIN/IFT22/ARF1/LSG1/MFN1/RHOBTB2/RAB9A/RAB40C/RAB13/GTPBP6/ARL1/RAB

PAP1/KITLG/ARTN/EREG/CCL19/XCL1/MRAP2/BMP8B/COPA/CCL3L3/GMFB/CRLF1/OSM/IL17D/CC

F5/GMPPB/MRAS/RAB27B/BMS1/NIN/IFT22/ARF1/LSG1/MFN1/RHOBTB2/RAB9A/RAB40C/RAB13/G
F5/GMPPB/MRAS/RAB27B/BMS1/NIN/IFT22/ARF1/LSG1/MFN1/RHOBTB2/RAB9A/RAB40C/RAB13/G

/PRPF6/KAT2A/MED24/CBX5/CALR/ZMYND8/NCOR2/MED25/RBX1/DDX54/FUS/HEY1/DR1/RPL23/R

SRA1/NCOA6/GTF2A2/PPRC1/PARK7/TFEC/SCAND1/MED20/BRD7/HYAL2/MAX/MED30/PRKCB/MED1
R1/RPAP1/DDX52/PTRHD1/DHX8/DHX30/METTL3/CARS2/MOV10/QRSL1/SARS2/MRM2/PPP1R8/RTC

OR4X2/OR1E1/OR5AK2/OR4C3/OR10J5/OR2C1/OR1N2/OR7D2

7

/FBXO10/KCMF1/TRIM32/CDC23/TRIM27/SH3RF1/NEURL1/RNF149/EGR2/RNF144A/BRAP/BCOR/U

E2F2/PARP1/OTX1/SMARCB1/SMYD3/CRIP1/ZNF239/VDR/MYPOP/MSH2/ZBTB17/BATF3/CHD4/RUV

C1A/CHMP3/GSK3A/HAUS8/DYNC1LI1/TUBB2B/PLK4/INPP5J/MAP4/TUBG2/RAE1/KIF2A/ODF2/RP
NABP2/KIF18A/PRMT1/CFL1/PSMB9/MCM5/PSMD14/TIMP2/CHMP4B/RHOC/ESPL1/POLA2/BAX/BCA
M5/MARK2/GDI1/EPHB3/PLOD3/NUMBL/CORO1B/LEF1/SLC9A3R1/MT3/DYNLT1/DVL3/FLOT1/NCKA
3/FERMT1/FLOT1/NCKAP1L/ADAM8/HDAC2/PIK3CD/ATF4/NLRC5/FRMD8/KDELR1/SLC11A1/LYN/C
/NLRC5/FRMD8/SLC11A1/LYN/CARD11/GPRC5B/RAC1/IL1B/GBA/CD47/RNF26/NFAM1/FGR/MAPK1
1/NFATC4/BCL10/PTGER4/TUSC2/THBS1/ADAM17/CASP8/MAVS/IRF5/IL20RB/TLR2/HLA-F/ATP6
1/SEMA3C/TRIM71/TIMELESS/SEMA3E/TFAP2A/WNT4/MED12/OVOL2/PSMD8/ACVR1/LUZP1/RARG/
/PRSS3/DYNLL1/DYNC1H1/ILF2/AMPD3/NPC2/NBEAL2/OLR1/SLC2A5/LEF1/GPI/PA2G4/SERPINB
AMPD3/NPC2/NBEAL2/BATF2/OLR1/SLC2A5/GPI/PA2G4/SERPINB1/PPIA/ZC3H12A/RAB24/ACLY/
/CPNE1/C1orf35/CAP1/PIK3CD/ASAH1/KPNB1/SLC11A1/RNASE2/LYN/RAC1/CTSS/LAMTOR1/GNS
AC1/CTSS/LAMTOR1/GNS/CD47/FTL/NFAM1/FGR/PSMA5/NRAS/SERPINB6/PTPN6/PSMD13/AGPAT2
/PRSS3/DYNLL1/DYNC1H1/ILF2/AMPD3/NPC2/NBEAL2/OLR1/SLC2A5/LEF1/GPI/PA2G4/SERPINB
GS1/SPON2/HK3/CCT2/CD58/C3AR1/HSP90AB1/F2RL1/PRSS3/DYNLL1/DYNC1H1/ILF2/AMPD3/NP
1A1/RNASE2/RAC1/CTSS/LAMTOR1/GNS/CD47/FTL/NFAM1/FGR/PSMA5/NRAS/SERPINB6/PTPN6/P
CLS1/FANCA/JAG1/MITF/LYN/CARD11/CBFB/CDKN1C/PSMB4/PIAS3/IL2RA/NCAPH2/PSMB8/NFAM
L3/MFNG/LFNG/ABL1/SART1/HLA-DOA/FCGR2B/TOX/BAD/PTGER4/VCAM1/TUSC2/PTPN2/ADAM17/
MAPK3/CD276/EFNB1/SERPINE1/IL27RA/HLA-DQB2/CD3E/HLA-A/ATP6AP1/PAK1/SASH3/ISG15/
CD79A/UBE2D1/PSMD10/PSMD11/PSMD1/C5AR1/PSMA7/RPS3/PRKD2/TRAF3/PSMB3/GRB2/ARRB2/
/KCNN4/CSK/ACTR3/PSMB5/BCL10/PSMA4/SPPL3/HSP90AA1/PSMD9/PTK2/PTPN2/PAK2/MUC6/RA
ZNF93/TOP1MT/ACTL6A/CCT2/HSP90AB1/XRCC3/TICRR/E2F8/HDAC1/PARPBP/NPLOC4/CSNK1E/R
7/POLD3/INO80C/TRIM25/TIMELESS/EID3/PRPF19/ESCO2/RAD51C/BTG2/DNA2/ABL1/RAD51D/V
BRD9/USP22/JADE2/VCX/KAT2A/BEND3/CABIN1/MTF2/ATXN7L3/CBX8/HELLS/PCGF2/HMGN1/PPP
L35A/RTCA/LYAR/INTS1/USP14/THOC6/RNF113A/RRP36/PTBP3/CPSF6/EIF4A3/ISG20/CDK7/PR
ARP4B/NAT10/EEF1A2/MRPL53/MPV17L2/GSPT2/RPS7/FARSA/RPL7A/RPS23/RPS10/MRPS17/MRP
RG1/SLPI/FUCA2/ATP6AP1/PAK1/CD63/DOK3/STK11IP/RNASET2/FPR1/STX1A/MNDA/PTGES2/RA
PSM3/PLXND1/F3/NUP85/CALCA/CCR1/TUBB2B/CXCL16/USP14/LOX/C5AR1/PADI2/GRB7/PF4V1/
2F4/REXO4/DDIT4/POLR2H/IRF7/CBX3/ZC3H12A/BID/PNKP/BCL3/OTUB1/BLM/FBXO5/AATF/CNO
/BLOC1S4/DYNLT1/GPSM2/HAUS1/FBXO5/SPAG1/RSPH1/TUBB4A/RANGRF/AUNIP/CSNK1D/DNAAF5
/BLM/FBXO5/AATF/TUBB4A/CNOT9/CDKN2B/CCL2/DAPK3/CSNK1D/ANAPC7/CDK2/LSM10/KIF14/N
NF580/MMP2/PDCL3/CD248/COPS9/STAT1/TGFA/HDAC1/E2F3/NACC1/TNFRSF11A/S100B/WDR77/
SF3B3/SUGP2/HNRNPR/POLR2D/SART3/DHX15/TGS1/GPKOW/KHDRBS3/TSEN34/DHX9/RBM28/CCAR
BA52/CD2AP/EDEM2/PSMC3/CHFR/AGAP3/GLMN/ANKZF1/TMUB1/PSMC5/FAF1/CCAR2/SDF2L1/FBX
DX11/CEP85/E2F4/PSMB2/INCENP/FGFR1/CBX3/PSME3/BID/GPSM2/HAUS1/BLM/FBXO5/DBF4/TU
C1A/VIL1/GSK3A/PML/NAP1L2/PLK4/ANAPC11/PFN1/WASF1/SMARCB1/NES/YWHAQ/RUVBL2/YWHA
ASP1/GSK3A/PML/PARP1/ABR/APH1B/LTBR/VDR/TNFSF12/YWHAQ/YWHAB/RPS3/TFAP4/DFFA/BAR
KRT8/HTATIP2/KPNB1/SNF8/PLSCR1/CD209/LYN/HCFC1/SSB/RAB11FIP4/RAC1/ITGA2/VDAC1/R
NF113A/PSMD11/PTBP3/CPSF6/EIF4A3/PSMD1/C5AR1/CDK7/PSMA7/PRPF3/EXOSC5/YWHAB/SNU1
/DMAP1/CRTC2/POLE3/BRPF1/UBE2N/MECOM/CTBP1/SUV39H1/MORF4L1/EP400/LDB1/PCGF3/SRC
T1/CCL13/TNFRSF11A/IFI35/PSMB2/SH2B2/KRT18/NUMBL/RPS6KA4/IRF7/PPIA/PSME3/MT3/TN
P1/SWAP70/DNM1L/SH3GL1/KATNB1/RPL23/VRK1/MMP19/GADD45GIP1/WDR1/MRPL10/IGF1R/MRP
10/KRI1/RPS7/SRPK1/RPL7A/RPS23/WDR55/GAR1/RPS10/DDX23/SENP3/EIF3K/HSP90AA1/PRKA
G1/SRI/NCKAP1L/MLST8/TNNI2/SH3KBP1/CAP1/CAPZA1/RTKN/SHC1/KCNQ1/DAPK3/KRT8/MKKS/
1/NDE1/FGD1/TTC26/CKAP5/HSPB11/BRK1/IQCB1/AKIRIN1/TMEM216/ABL1/VCL/PALM/ACTR3/A
HC1/DAPK3/KRT8/MKKS/ARHGDIB/FHOD3/HCLS1/PSTPIP1/TESK1/CDC42BPG/ITGB1BP1/ARPC5L/
MB8/NFAM1/PSMB10/ETV6/PSMA5/TGFB2/CDH17/LCK/PTPN6/THOC5/PSMD13/CD27/IFI16/PSMC4
AF1/ZNF16/ADD1/PPARG/JUNB/ACIN1/MOV10/RPS6/CASP3/ADAR/SMAD5/MFHAS1/NCOA6/DPY30/
/LEF1/PDCD1/TGM2/ZC3H12A/LAG3/FERMT1/SIRPG/FLOT1/NCKAP1L/JAG2/GTPBP4/ADAM8/CCL2
2/TAF9/POMT2/PSMD13/ADAMTS9/PSMC4/GSK3A/AP2M1/OSBPL7/PML/ANAPC11/VPS28/AP2S1/UB
/LEF1/GPI/FGFR1/ZC3H12A/SVBP/SLC9A3R1/NCKAP1L/FBXO5/GTPBP4/ADAM8/CCL2/DAPK3/PIK
9A3R1/MT3/BLOC1S4/DYNLT1/DVL3/FLOT1/EHD1/NCKAP1L/SH3KBP1/PAK4/VLDLR/LYPLA2/CPNE

ROCC/CAPZB/RALA/DGUOK/SEMA4B/CDC42/KAT2A/PRKCI/TGFB3/PRRX1/ZMYND8/SEMA4D/SYT1/C

F113A/PSMD11/RAE1/ZMIZ2/PSMD1/DCAF16/PSMA7/UBE2E1/WSB2/FBXL6/RPS3/TRAF3/PSMB3/B

CCT2/CD58/IRF3/LMNA/HSP90AB1/F2RL1/DYNLL1/PLEKHM2/CNPY4/NDUFAF2/CSNK1E/LZTS2/TB

CCT7/DCTN2/SSB/RPS5/TSPAN10/LAMTOR5/CDK5/TXN/UBE2L3/TULP3/FAM83H/RPS21/TCP1/RPL

RPE/VMA21/HIP1R/TXNL4A/TPPP3/SNRPF/UQCC3/NOP2/NCKAP1L/FBXO5/SPAG1/EIF3D/TUBB4A/

DC3/RPL35A/SET/PSMD10/PSMD11/RAE1/EIF4A3/PSMD1/ISG20/GPX1/PSMA7/EXOSC5/YWHAB/RP

10/KRI1/FAM98B/RPS7/FARSA/RPL7A/WDR55/GAR1/SENP3/GTPBP3/RPS15/ADAT3/RPS16/PUS7/

ASAH1/KPNB1/SLC11A1/RNASE2/RAC1/CTSS/LAMTOR1/GNS/CD47/FTL/NFAM1/FGR/PSMA5/NRAS/

/THY1/RHOF/CORO1C/DUOX2/C3AR1/LMNA/F2RL1/RRAS/RELN/BEX4/LIMCH1/ZSWIM5/ZNF703/PP

/C5AR1/GRB7/PRKD2/SEMA4B/SYDE1/AGO2/CALR/SEMA4D/XCL2/CMKLR1/APP/ADRA2A/SWAP70/S

ELI1/CLEC7A/RASAL3/GPR183/WNT4/BTN2A2/HSPD1/LFNG/ABL1/SART1/HLA-DOA/FCGR2B/CSK/

ASAH1/KPNB1/SLC11A1/RNASE2/RAC1/CTSS/LAMTOR1/GNS/CD47/FTL/NFAM1/FGR/PSMA5/NRAS/

PSM3/PLXND1/F3/NUP85/CALCA/CCR1/TUBB2B/CXCL16/USP14/LOX/C5AR1/PADI2/GRB7/PF4V1/

/BTG2/MRPS7/LARP4B/NAT10/EEF1A2/MRPL53/MPV17L2/GSPT2/RPS7/FARSA/RPL7A/RPS23/RPS

HAQ/YWHAB/RPS3/TFAP4/DFFA/BARD1/ARRB2/PTPA/PRKCI/TGFB3/GAL/GZMB/DNM1L/CTSD/HSPE

NIP2/BCL3/NCKAP1L/AATF/NOC2L/ADAM8/PAK4/LAMP3/EVI2B/NONO/CCL2/SHC1/HDAC2/HTATIP

HAQ/YWHAB/RPS3/TFAP4/DFFA/BARD1/ARRB2/PTPA/PRKCI/TGFB3/GAL/GZMB/DNM1L/CTSD/HSPE

/MT3/TNIP2/BCL3/NCKAP1L/AATF/NOC2L/ADAM8/PAK4/LAMP3/EVI2B/NONO/CCL2/SHC1/HDAC2/

L7/ANAPC5/HECTD3/CDC27/CDC34/HSP90B1/UBE4B/AUP1/MAEA/HSPA13/CAV1/FBXO6/STUB1/AN

NCKAP1L/PIP4K2A/TUBB4A/CAPZA1/RANGRF/DAPK3/CXCL13/MKKS/KIF14/FHOD3/SNF8/PHLDB1/

ACK1/RALB/RPA2/CUL4B/DRG1/MAPT/ARPC1A/WNT4/NF2/EMILIN1/BRK1/LIMS1/AKIRIN1/ARPC5

2/CCL2/RPSA/SHC1/TRIM31/KRT8/HTATIP2/KPNB1/SNF8/PLSCR1/CD209/LYN/HCFC1/SSB/RAB1

IF3D/LAMP3/ATP6V1H/ULBP2/CCL2/RPSA/SHC1/TRIM31/KRT8/HTATIP2/KPNB1/SNF8/PLSCR1/C

AC2/MKKS/ARHGDIB/ZFP41/DEFB1/OAZ3/FANCA/CCT7/TESK1/RPL10L/FKBP4/ITGA2/ADGRG2/DA

LM/FBXO5/DBF4/TUBB4A/CNOT9/CDKN2B/CCL2/CSNK1D/ANAPC7/CDK2/LSM10/KIF14/NASP/DCTN

SET2/FPR1/STX1A/MNDA/PTGES2/RAP2B/GYG1/ARHGAP9/RHOF/SIRPA/DEGS1/HK3/CCT2/CD58/C

L1/STAT1/RAB7B/CCL13/LRRC14/NPLOC4/IFI35/S100B/TRAFD1/POLR3K/PSMB2/LY86/IRF7/PS

K3/PSMB5/BCL10/PSMA4/PSMD9/PTPN2/CASP8/PTPN1/RPS27A/MAVS/DMBT1/PAK2/TLR2/MUC6/U

G4/FGFR1/ZC3H12A/DYNLT1/DVL3/FLOT1/EHD1/NCKAP1L/FBXO5/ADAM8/VLDLR/CPNE1/EVI2B/H

MC4/PML/DYNC1LI1/MLF1/PRCC/PSMD10/PSMD11/CDC123/CDKN2C/PSMD1/DONSON/TIPIN/CDK7/

P4/DBNDD2/IL1B/GBA/ACTB/CDK5/CHORDC1/PDGFA/TXN/MLKL/ADRA2C/FGR/LCP2/MAPK11/TGFB

A1/HSPH1/CD209/LYN/CARD11/RAC1/CBFB/IL1B/IL2RA/NCAPH2/CD47/NFAM1/TNFSF13B/PSMB1

BP1/MYBL1/DNM1L/RAD21/KATNB1/MTBP/WRAP73/NME6/MSX1/NDE1/DRG1/MAPT/WNT4/MZT1/RAD

CBFB/CDKN1C/PSMB4/PIAS3/IL2RA/NCAPH2/PSMB8/NFAM1/PSMB10/ETV6/PSMA5/TGFB2/CDH17/

7/TACSTD2/TBX19/PRKD2/PSMB3/VWA1/RALA/PAX8/GRB2/ARRB2/CDC42/KAT2A/MATN3/PSMD4/H

/MSH2/RPS3/SIT1/CST7/GRB2/CD6/DROSHA/CDC42/KAT2A/IL4R/PNP/GAL/LILRB4/AP3D1/AXL/

GRB7/PF4V1/GRB2/STK10/CALR/CXCL11/CCR5/XCL2/CMKLR1/APP/SWAP70/CD48/TRPM4/RPS19/

CK1/CD300LF/MYADM/DNAJB2/WAS/VIL1/ATAD2/GSK3A/DNAJA4/DYNC1LI1/PFN1/INPP5J/MAP4/

/CORO1C/BIN1/DUOX2/C3AR1/LMNA/F2RL1/RRAS/RELN/BEX4/LIMCH1/ZSWIM5/ZNF703/PPARD/C

KD2/SEMA4B/SYDE1/AGO2/CALR/SEMA4D/XCL2/CMKLR1/APP/ADRA2A/SWAP70/TRPM4/SEMA4F/RP

NO/PNKP/DVL3/DUSP2/BLM/NCKAP1L/MLST8/FBXO5/DBF4/FGFR3/GTPBP4/ADAM8/PAK4/VLDLR/C

M1/ITGA2/CCL4/DNAJB2/F3/LCK/TBC1D13/RASA3/IFI16/CASP1/PML/FGFR2/DVL2/PFN1/ARHGA

N3/MYADM/WAS/RASSF1/POC1A/VIL1/CHMP3/GSK3A/CIT/PLK4/PFN1/WASF1/INPP5J/NES/RAE1/

OR1/S100A13/FGR/KXD1/AP3M2/HAUS8/NDRG4/RAB10/PLK4/WASF1/MAP4/ODF2/SYT12/TRAPPC1

/MAPT/PDCD6/BICDL2/NSF/SEC13/RHOT2/NSFL1C/PDCD10/ACTR3/CEP19/VAMP8/MILR1/WIPI1/

LRFN1/JAG1/LYN/GPRC5B/FKBP4/MYLIP/IL1B/RAP2A/CDK5/TULP3/NREP/CTTN/SPOCK1/ADRA2C

PLXNC1/STYXL1/NOTCH1/FIG4/MOB2/LLPH/VEGFA/TLR2/HSPA5/DDX56/GLI1/RAB11A/KIF20B/P

K1D/YWHAH/KIF14/CAMSAP2/KDM1A/JAG1/LYN/ITGB1BP1/GPRC5B/FKBP4/RAC1/MYLIP/IL1B/RA

4/NDUFAF2/CSNK1E/LZTS2/P2RX5/GDI1/ZC3H12A/BID/NCBP2/CCT4/BCAP31/UBE2J2/DVL3/GPS

AS/HYOU1/PRELID1/KRT18/PPARD/LEF1/PDCD1/PA2G4/IRF7/PIM2/HIP1R/ZC3H12A/PSME3/BID

PLSCR1/CLN3/BBC3/PMAIP1/RAB20/REEP2/ITGA2/AKAP8L/EHD2/ACTB/GLDN/CTTN/FA2H/MYADM

SMA5/DNAJB2/ASB6/PSMD13/NUP85/PSMC4/GSK3A/NUP43/FBXW9/SOCS3/ANAPC11/COPS7B/VPS2
/VIL1/CHMP3/LRFN4/WDR45B/CENPK/HAUS8/MAP1LC3B/PLK4/EDC3/VPS28/MAP4/IFT80/AGFG1/
B/CPE/PDCD5/AP3D1/RPS19/RPLP0/STX4/RPL18/ICMT/SGTB/RPL23/RACK1/MYO5A/EPB41L3/RO
27/NUP85/NUP43/PML/TNIK/DVL2/PARP1/USP14/PSMD10/RAE1/LTBR/GPX1/RPS3/USP1/BAG3/N
1D/KRT8/MKKS/KPNB1/FHOD3/CAMSAP2/HCLS1/PSTPIP1/TESK1/ITGB1BP1/ARPC5L/FKBP4/B4GA
3/KRT18/PPP1CA/LEF1/PDCD1/PIP5K1C/ZC3H12A/BLOC1S4/LAG3/SIRPG/TSTA3/FLOT1/ACTG1/
1/BRK1/IQCB1/AKIRIN1/TMEM216/ABL1/VCL/PALM/ACTR3/ARAP1/CEP19/TBC1D7/CEP41/TTYH1
RALA/DGUOK/SEMA4B/CDC42/PRKCI/TGFB3/PRRX1/ZMYND8/SEMA4D/SYT1/CDC42EP2/DNM1L/SYT
KN2C/PSMD1/DONSON/ODF2/PSMA7/UBE2E1/CCND2/TFAP4/PSMB3/ZNF207/TOPBP1/APP/PSMD4/C
HOC/ESPL1/POLA2/BAX/BCAT1/LCMT1/NAA10/PCBP4/CASP2/BRSK1/GMNN/MIIP/PRKDC/STIL/SS
1/TMEM64/WDR5/NKAP/PSMC3/RHOA/INHA/AP3B1/RUNX3/ZMIZ1/PSMC5/KITLG/CCL19/EIF6/NOT
RB4/PDCD5/STX4/RACK1/RALB/CEP250/CCT8/GNL3/CLIP3/MAPT/NF2/KCNAB2/CEMIP/PKIA/EMD
A/F2RL1/RRAS/RELN/BEX4/LIMCH1/ZNF703/PPARD/CORO1B/LEF1/GPI/FGFR1/ZC3H12A/SVBP/S
GO2/CALR/SEMA4D/XCL2/CMKLR1/APP/ADRA2A/SWAP70/SEMA4F/FADD/STX4/SEMA5B/CLEC7A/RA
DHX38/DBR1/GEMIN2/PABPN1/SRSF1/SRSF2/SRPK2/HNRNPUL1/CASC3/SNRNP70/COIL/FIP1L1/R
GEMIN2/PABPN1/SRSF1/SRSF2/SRPK2/HNRNPUL1/CASC3/SNRNP70/COIL/FIP1L1/RBM4B/DHX8/R
GEMIN2/PABPN1/SRSF1/SRSF2/SRPK2/HNRNPUL1/CASC3/SNRNP70/COIL/FIP1L1/RBM4B/DHX8/R
C5/TIGAR/RBPJ/ADM/BECN1/PSEN2/VASN/HSP90B1/SUV39H1/PDK3/EDNRA/CAV1/ATP7A
CPNE1/C1orf35/CAP1/PIK3CD/ASAH1/KPNB1/SLC11A1/RNASE2/LYN/RAC1/CTSS/LAMTOR1/GNS/
L4R/PNP/GAL/LILRB4/AP3D1/AXL/FADD/BTK/PELI1/STX4/CLEC7A/RASAL3/GPR183/LBP/EXOSC
MC4/MUC5B/CD79A/UBE2D1/PSMD10/PSMD11/PSMD1/C5AR1/PSMA7/RPS3/PRKD2/TRAF3/PSMB3/G
IE/POLR2I/POLR2J/RPUSD3/HNRNPL/SF3B3/SUGP2/HNRNPR/POLR2D/SART3/DHX15/TGS1/GPKOW
SC3/FIP1L1/RBM8A/MAGOHB/SNRPD3/SMG7/TNPO3/EFCAB7/POLDIP3/THOC7/RBM22/DDX20/KPNA

FGD6/RALA/GRB2/SYDE1/CDC42/ARHGAP18/CDC42SE2/ADRA2A/CDC42EP2/MYO9B/OCRL/SPRY4/C
ZNF598/PLEKHN1/EIF2B5/RPL38/HNRNPM/KHDRBS1/EXOSC3/PIWIL4/BTG2/SEC13/PSMD8/LARP4
DK2AP2/NBN/PLK3/RNF2/ZFYVE19
PA5/DDX56/RAB11A/RAB29/IFRD1/PACSIN1/PPP2R5B/CDK5R1/METRN/APBB1/SRF/CAPRIN1/RHO

/SWAP70/SEMA4F/FADD/STX4/SEMA5B/CLEC7A/RACK1/SEMA3C/SEMA3E/IGF1R/LBP/PDCD6/CCL3
3/VPS4A/MRPS5/VPS16/DHX8/ADD1/MRPL36/TFIP11/MRPL14/OGFOD1/MRPL3/CAPZA2/ERAL1/PY
/CDC23/HNRNPD/PHF1/DCP2/BUB3/HNRNPC/ING2/BCOR/SETDB1/MYC/PRMT6/BRD7/BRD4/ASF1A/
ER4/HSP90AA1/PRKAA2/CHD1L/MPP7/LMO4/SPIRE1/STYXL1/DCTN1/VPS35/SH3GLB1/THRA/VDAC
PNE1/C1orf35/CAP1/PIK3CD/ASAH1/KPNB1/SLC11A1/RNASE2/LYN/RAC1/CTSS/LAMTOR1/GNS/C
AC1/CTSS/LAMTOR1/GNS/CD47/FTL/NFAM1/FGR/PSMA5/NRAS/SERPINB6/PTPN6/PSMD13/AGPAT2
4A/CNOT9/CDKN2B/CCL2/CSNK1D/ANAPC7/CDK2/LSM10/KIF14/NASP/DCTN2/PRIM1/CDKN1C/PSM
CRLF3/KIF20B/MECP2/CENPJ/PPP2R5B/CDK5R1/RHOA/FAP/LGMN/CNOT10/STXBP4/BTC/BECN1/A
SF1/SRSF2/CASC3/FIP1L1/RBM8A/MAGOHB/SNRPD3/SMG7/TNPO3/EFCAB7/POLDIP3/THOC7/RBM2
A/CCNO/DYNLT1/LBH/GPSM2/HAUS1/BLM/FBXO5/AATF/EVI2B/ITGB3BP/RTKN/ANAPC7/CDK2/KIF
ABL1/SFRP2/TNFAIP3/CALM2/PDCD10/CSK/MAPKAPK3/CCND3/DBNL/ERP29/THBS1/ADAM17/PTPN
12/YWHAQ/YWHAB/RPS3/TFAP4/DNAJC10/FAIM/BAG3/ARRB2/PCGF2/GZMB/DNM1L/MAPK7/PPP1R1
MTPN/NIN/SPTAN1/CORO2B/ARF1/RHOBTB2/GMFB/HAX1/CAMSAP1/PREX1/ARFGEF1/CIB1/ADD1/A
SRSF1/SRSF2/SRPK2/PAF1/SNRNP70/RBM4B/RBM8A/METTL3/HNRNPA0/PSMB1/MOV10/CELF2/PSM
32B/AP2B1

3/NME6/MSX1/NDE1/DRG1/WNT4/MZT1/RAD51C/HSPA2/RAD51D/NSFL1C/EME2/ACTR3/CHEK2/RAD
/ACTR3/PSMB5/BCL10/PSMA4/SPPL3/HSP90AA1/PSMD9/PTK2/PTPN2/PAK2/MUC6/RAB29/MUC15/
RPS4X/RPL6/EXOSC9/EXOSC10/MAGOH/RPL37/RPL27A/DXO/EXOSC7/TBRG4/RPL36/UBA52/ZPR1/
S1/ETF1/RPL19/PSMC3/RPS11/EXOSC1/EIF3E/RPL13A/HNRNPR/POLR2D/ANP32A/RPL37A/RPLP1
D3/LIMA1/CD2AP/PACSIN1/ARHGAP28/PPM1F/FAM171A1/SRF/DSTN/PLEKHH2/RHOA
T1/RBM14/PLEKHH2/MLLT6/DNMT3B/MTPN/SPTAN1/TIGAR/BECN1/CORO2B/CTBP1/GMFB/ATG7/CA

GALNT2/VCAM1/TWSG1/PTK2/PTPN2
CCL19/IST1/ARF1/SNX3/HTT/ATP7A/DAB2IP/DZIP1/PROM2/ADNP/CNR1/PLXNB2/RAB8B/NCKAP1
K5R1/ARHGAP28/SRF/DSTN/PLEKHH2/RHOA/MTPN/SPTAN1/IST1/ARF1/RHOBTB2/GMFB/HAX1/ATP
DR75/CTU1/TSEN34/FAM207A/BMS1/TPRKB/NOL10/RSL1D1/TYW3/DIMT1/EBNA1BP2/TSEN15/NOL
CD38/ACTN4/DNMT3B/PSMC5/TIGAR/RBPJ/ADM/BECN1/PSEN2/VASN/HSP90B1/SUV39H1/PDK3/ED
/IFITM3/FAM3A/KPNA6/NELFA/POLR2E/SNX3/CCL8/CAV2/CAV1/SUPT4H1/HMGA2/PARP10/MORC2
1/TP53BP1/ATAD5/INPP5D/PTPRC/NECTIN2/CRLF2
FC5/RNF8/DHX9/MAPKAPK5/DNAJC2/TIGAR/FAF1/UBE2N/EREG/MMS19/SMARCAL1/TFDP1/MSH6/A
T1/HLA-DOA/FCGR2B/TNFAIP3/TOX/CSK/BAD/BCL10/VCAM1/TWSG1/PTPN2/IL20RB/PAK2/HLA-F
RNPU/MAP1A/ERCC1/LARGE2/VEGFA/EXOSC10/CCNE2/IL20RB/TMEM64/MUC6/DEF8/NANOS1/HNRN
2/UBA52/MECP2/CACNB3/PSMC3/TWIST1/SRF/RHOA/CD38/ACTN4/DNMT3B/PSMC5/TIGAR/RBPJ/A
S11/ZDHHC9/RPL13A/YWHAG/RPL37A/RPLP1/RPL32/RPL30/TFDP1/RPS9/RPS14/CALM3/GET4/RP
PSMA7/UBE2E1/CCND2/TFAP4/PSMB3/ZNF207/TOPBP1/APP/PSMD4/CETN2/PSMD3/RBX1/PSMD7/R
1/PTPN2/THBS1/CSNK2A1/STYXL1/CASP8/PTPN1/PIAS4/VDAC2/GNAI3/PTPMT1/PAK2/TMBIM1/T
PP5D/PTPRC/NECTIN2/CRLF2

11A/KIF22/ARL8B/NCAPD3/TEX11/NEK6/STAG3L4/BECN1/ANAPC5/PMF1/MMS19/CDC27/SEH1L/M
3/ZFYVE19

/RPS9/WDR46/IMP4/RBFA/RPS14/RPS27/EXOSC2/DDX52/DHX30/AAMP/TEX10/PWP1/MRPL36/NIP

1/DDX19A/TPR/SRSF7/MCM3AP/NUP50
/RIOK1/RPL11/RPS24/WDR12/PDCD11/USP36/RPL14/DDX51/RPL10A/ZNHIT3/SPIN1/RRP15/WDR

F1L4/SRSF3/BBS12/NUP214/TSC1/IFT140/IWS1/GRIPAP1/HIP1/DAG1/NACC2/ARHGAP44/DDX19

PR132/NEDD1/MECP2/CENPJ/PSMC3

/NOX1/PARK7/ING2/SHISA5

/ARF1/PDIA3/ACTR1A/KLC1/YTHDF1/CD1A/PSMB1/RAB8B/KIF3A/PSMB6/CAPZA2/PSMA3/HLA-C/
0/AGO1/ZNHIT3/GEMIN6/SMN2/CNOT2/RPL12/MRPS2/RPS28/LUC7L3/GEMIN5/EIF3C/DDX1/LSM3

2/CD19/PRKCB/PIK3R2/VAV3/SLA2/WNK1/BTN3A2/EIF2B3/IKBKB

PC4/PAF1/ZNF16

L12/RPS18/RPS28/RPS17/NUP214/RPL22/TARDBP/RPS15A/TAF11

0/ERAL1/GEMIN8/EIF3F/MRPL20/AGO1/KIF5B/DYRK3/ZNHIT3/GEMIN6/SMN2/CNOT2/RPL12/MRP[illegible]

PT1/NUP214/TSC1/BICD2/IWS1/ZNHIT6/ATXN2/RFTN1/PARN/DDX19A/TPR/SRSF7/MCM3AP/NUP5[illegible]

TNKS1BP1/GSPT1/SKIV2L/DCP1A/CSDE1
NHIT6/TSR1/RPP38/MRPS11

PS15A

A/CLEC4A

F11

RHGEF3/DNM2/ARHGEF25/NOTCH1/ARHGEF11/RAB3A/RAB11A/PLEKHG6/DENND4B/RAB29/RHOA/RA
E2N/MECOM/CTBP1/SUV39H1/MORF4L1/EP400/LDB1/PCGF3/SRCAP/HMGA2/HDAC5/PAGR1/ENY2/T
P1/TBC1D7/SGSM3/ARHGAP11A/PLXNC1/SRGAP2/PTK2/TNK2/RGP1/TBC1D22B/ARHGEF11/SMAP1/
/WNT7B/ITGB1BP1/BIRC7/GPRC5B/CDKN1C/DUSP4/DBNDD2/IL1B/GBA/ACTB/CDK5/CHORDC1/PDG
C/GSK3A/NDRG4/NAP1L2/TNIK/TUBB2B/DVL2/INPP5J/NSMF/LRP8/SRRT/FAIM/DGUOK/SEMA4B/C
/METRN/APBB1/SRF/CAPRIN1/RHOA/RAPGEF1/DNMT3B/GPC2/NIN/DPYSL3/AP2A1/IST1/ZC4H2/A
THEMIS2/IRF1/IRF3/C3AR1/HSP90AB1/F2RL1/ARPC2/STAT1/RAB7B/LRRC14/CTLA4/ARPC3/WIP
STK10/MTCP1/TGFB3/MUC20/ADRA2A/DCUN1D5/XRCC6/HMGN1/TELO2/MAP3K6/IKBKG/DSTYK/STK
1/ST8SIA4/GCNT1/B3GNT8
2/GALNT3
M14/MAP3K1/LGMN/DHX9/PSMC5/UBE2N/EREG/TLR7/HSP90B1/IRAK2/CAV1/DAB2IP/NECTIN2/CL
B2IP/HMGA2/GTF2F1/HDAC5/CALM3/DZIP1/PKD1/EDF1/STUB1/BMP2/HDAC8/ADNP/ADD1/PPARG/

SMB6/FFAR2/CNPY3/MFHAS1/ICAM2/PSMA3/IRF4/CTSL/CTSB/RELA/AIM2/PSMB7/SKP1/NOD1/TR
L10RB/SLFN11/APOBEC3F/HYAL2/IFNG/CD207/RELA/AIM2/FAM111A/POLR3A/TRIM15/ZMPSTE24

SMA1/SFRP2/TNFAIP3/NFATC4/PLCB2/PSMB5/PSMA4/PRKAA2/PSMD9/CSNK2A1/VPS35/NOTCH1/R
FL1C/UBQLN4/RPS7/KCTD5/FOXF2/RNF216/PSMB5/PSMA4/SGTA/PSMD9/PTK2/UBL7/CSNK2A1/RP
USP22/KCTD2/VPS25/PSMD4/PSMD3/RBX1/UQCC2/PSMD7/TMUB2/TBL1XR1/PELI1/CBL/SGTB/GZM
IP3L/RAB3A/RCC1L/SREBF1/NDUFB3/BCL2L1/RAB29/TIMM10/AIP/CDC37/TIMMDC1/NDUFA13/ST
1/PREX1/ARFGEF1/ARHGAP17/ADD1/ASAP3/MYO1C/SPIRE2/NCKAP1/PPP1R9A/LPAR1/PXN/CCDC8
CSK/BAD/EMID1/BCL10/VCAM1/DNM2

SMB7/SKP1/NOD1/TRIM15/BPIFB1/PTPRS/CLEC4A/APPL2/RPS6KA3/TLR1/S100A1/NOD2

PS18A/TWF1/MRPL43/VILL/MRPS9/MRPL50/EPS8/GLE1/MTRF1L/MRPS35/MRPS33/MID1/MRPS10/

6KB2/RPLP0/RPL18/RPL23/RACK1/GADD45GIP1/VIM/MRPL10/TRIM71/TNIP1/DPH7/ZNF598/PLE[illegible]
42/ATXN7L3/PSMD4/CBX8/DCUN1D5/KLHL17/PCGF2/DNM1L/PSMD3/RBX1/PSMD7/PELI1/MTBP/CB[illegible]
QLN4/RPS7/KCTD5/FOXF2/RNF216/PSMB5/PSMA4/SGTA/PSMD9/PTK2/UBL7/CSNK2A1/RPL5/RPS2[illegible]
L23/RNF4/UBE2L6/RACK1/RNF7/CUL4B/TRIM71/TRIM25/RNF122/LONP1/PRPF19/UBXN1/PSMD8/[illegible]
D/VCP/PSMA1/SFRP2/TNFAIP3/NFATC4/PLCB2/PSMB5/PSMA4/PRKAA2/PSMD9/CSNK2A1/VPS35/N[illegible]
B2/UXS1/B4GALT7/MUC3A/LDHA/DLAT/NAGK/KRTCAP2/RPN2/IL1B/PFKM/FA2H/B4GALT2/GNPDA2[illegible]
NFRSF11A/RELN/S100B/P2RX5/C1QTNF1/PPARD/NELFE/FGFR1/PIM2/TGM2/ZC3H12A/SLC9A3R1/[illegible]
GNT2/SLC35C2/FUT11/XXYLT1/HBEGF/MANBA/PMM2/SDF2L1/HEXA/CCL19/DOLK/ATP7A/EOGT/B3[illegible]
GAK/TJAP1/FNBP1L/COG4/COG2/IST1/BHLHA15/SNX3/HTT/CAV2/LMAN2/PI4K2A/CAV1/ARL1/GE[illegible]
RAB1A/SRPK2/IFITM2/DDB1/P4HB/RAB1B/MX1/NCAM1/TRIM32/VAPA/TBC1D20/ADAR/MVB12B/TR[illegible]
PS6/NFKBID/CHD7/CLPTM1/SH3RF1/IRF4/RHOH/CD4/IFNG/ANXA1/CCR2/FZD9/DOCK11/CD19/PO[illegible]
POLR3C/CCL3/CDK19/HSPD1/PSMD8/PSMA1/TNFAIP3/TRADD/VAMP8/MAPKAPK3/PSMB5/BCL10/PS[illegible]
A/ARMC9/CCDC88A/NEURL1/C2CD3/CNTRL/TCHP/DYNC2LI1/CEP97/RAB3IP/NPHP1/ARL3/CELSR3[illegible]

PDPN/MTPN/SPTAN1/CORO2B/NOTCH2/ARF1/RHOBTB2/GMFB/HAX1/CAV1/PREX1/ARFGEF1/ARHGAP
TR1/KIF3A/ARMC9/CCDC88A/NEURL1/C2CD3/CNTRL/TCHP/DYNC2LI1/CEP97/RAB3IP/NPHP1/ARL[illegible]

CAM1/WNT5A/CD40

TD5/FOXF2/RNF216/PSMB5/PSMA4/SGTA/PSMD9/PTK2/UBL7/CSNK2A1/RPL5/RPS27A/FBXW5/USP

2/MYCBP/SNU13/GOLGA3/MCM8/VCX/IL4R/CALR/CTDNEP1/TDRKH/HSF2/APP/MYBL1/CETN2/TMEM

1/VDR/SBF1/ODF2/MSH2/MYCBP/GOLGA3/UBE2A/ARRB2/MCM8/VCX/IL4R/CALR/TGFB3/CTDNEP1/

F2/MSH2/MYCBP/GOLGA3/UBE2A/ARRB2/MCM8/VCX/IL4R/CALR/TGFB3/CTDNEP1/TDRKH/HSF2/AP

/ADORA2B/SNAPIN/ARF1/UBAC2/SNX3/HAX1/IL13RA2/CD84/CALM3/CIB1/CSNK2A2/PARL/HUWE1

6

P8/CFTR/PLK3/SH3GLB1/MAVS/PFKFB2/PLA2G1B/TLR2/CD2AP/EDEM2/ZPR1/RAB29/KIF20B/GOL

A/MT3/DVL3/FLOT1/KISS1/NCKAP1L/MLST8/DBF4/FGFR3/ADAM8/PAK4/VLDLR/CNOT9/CCL2/SHC
GSM1/CCL19/XCL1/RANBP3/CCL3L3/DOCK10/CCL8/CAV2/DAB2IP/PREX1/ARHGAP22/ARHGAP27/A
A2B1/POLR1C/KMT2B/LRIF1/MBD1/SMAD2/ELAVL1/WBP2/ZMPSTE24/NUP214/SMAD3/MYCN/TFAP2

P2/MAPK11/F3/LCK/VIL1/GSK3A/AP2M1/NDRG4/SOCS3/MYOF/FGFR2/WASF1/AP2S1/MST1R/ATP6

H1B2/ACVR1/HSPA2/ADAM28/SRPK1/BAD/DNM2/SPIRE1/SPA17/NOTCH1/CFTR/GJB3/PAQR7/RNF2

TPA/CABIN1/MTF2/SEMA4D/VPS25/SWAP70/SPRY4/PPP1R15A/PPP5C/GMFG/CBL/RPL23/AKT1S1/

/SNX3/TLE6/BARX1/KLHL12/EGF/CAV1/DAB2IP/DKKL1/CBY1/ZBED2

LN2/ULK1/LIX1L/PIK3C2B/HTRA2/WDR45/ATP6V0A1/TBC1D25/SESN2/NOD1/ATG3/RAB7A/CHMP6

/DBF4/FGFR3/ADAM8/PAK4/VLDLR/CNOT9/CCL2/SHC1/HDAC2/CSNK1D/KIF14/HCLS1/AXIN1/SLC
FSF15/PTK2/THBS1/ADAM17/PTPN1/RPS27A/VEGFA/PLA2G1B/PAK2/TRAF4/UBA52/DUSP7/MAP3K

D5/NLRP3/ACTR8/NFRKB/PSMD12/SMURF2/OGT/TAB1/USP46/USP13/PSME2/BRCC3/OTUD6B/COPS

L1/STMN2/RASAL1/BAD/DBNL/PLXNC1/DNM2/STYXL1/NOTCH1/FIG4/MOB2/OLFM4/LLPH/VEGFA/T
/DNM1L/MAPK7/SYT3/SEMA4F/SEMA5B/STK25/ABI2/ARHGDIA/SCN1B/CSPG5/SEMA3C/EPB41L3/S
/PMP22/PTPRF/SEMA3E/MAPT/TGIF2/CHN1/PRPF19/CBFA2T2/ABL1/STK24/STMN2/RASAL1/SFRP
7/SYT3/SEMA4F/SEMA5B/STK25/ABI2/ARHGDIA/SCN1B/CSPG5/SEMA3C/EPB41L3/SEMA3E/MAPT/
MPDL3B/ADAM8/HLA-DQA2/MYO1G/NONO/ULBP2/PIK3CD/NLRC5/PLSCR1/SLC11A1/CD209/LYN/HL
L3/MOV10/EIF2B4/UCN/OGFOD1/UNK/LRPPRC/POLDIP3/ENC1/RPL10/KLHL25/HNRNPD/LSM14B/N
P/SFRP2/TRADD/BAD/BCL10/RCN3/TNFSF15/THBS1/CSNK2A1/CASP8/PDCD2
3/TLR2/ANXA3/IFNAR2/TARBP2/IFI6/IL17RA/TNFRSF14/DHX9/IFITM3/RPL30/RBPJ/BECN1/EP
F/MTPN/CCAR2/CYP27B1/IST1/EIF4G2/CDK11A/OGFR/RPTOR/CIB1/IL17RB/EXOSC2/CTDP1/IP6

/USP46/USP13/PSME2/BRCC3/OTUD6B/SUDS3/YOD1/USP28/PSMD6/GATA3/USP4/TGFBR1/PSMC2/

3/WIPF1/CSNK1E/SORT1/RIN1/HYOU1/SLC2A5/PIP5K1C/TGM2/HIP1R/ARFGAP1/SNX10/STAB1/S[illegible]
RASAL1/NFATC4/PALM/ARAP1/KEL/DBNL/PLXNC1/DNM2/PTK2/ZMYM3/OLFM4/VEGFA/BCL9L/HEXB[illegible]
K24/CALM2/PDCD10/CSK/CLSPN/MAPKAPK3/CCND3/BAD/DBNL/BCL10/ERP29/HSP90AA1/TNFSF15[illegible]

PSTPIP1/SLC11A1/HSPH1/CD209/RAB20/LYN/APOBR/MCTP1/RAC1/ITGA2/ANXA11/IL1B/EHD2/A
L/SPATA2/RNF114/DZIP1/CIB1/SIX5/CSNK2A2/ACTR1A/NECTIN2/BAG6/RFX2/SLCO4C1/CNR1/Z
/EIF4G2/EIF4G3/CNOT3/PDK3/RPS9/KHSRP/DNAJC1/RPS14/MKNK1/PGAM1/LARP1/YTHDF2/CDK5
CUL7/UGGT1/UGGT2/TP53/TARDBP/SVIP/PDX1/DERL3
/SOCS5/CD274/PCBP2

B6/ARPC2/TGFA/FMOD/PSENEN/ARPC3/NUP93/FSTL3/SORT1/SUB1/EPHB3/ZNF703/DDIT4/SH2B2

EMA4F/SEMA5B/STK25/ABI2/ARHGDIA/SCN1B/SEMA3C/EPB41L3/SEMA3E/MAPT/RERE/CHN1/ZNF3

B1/SIX5/CSNK2A2/ACTR1A/NECTIN2/BAG6/RFX2/SLCO4C1/CNR1/ZSCAN2/METTL3
SNK2A1/CASP8/PDCD2

2B/FGFR2/COCH/COL6A2/NSMF/SIPA1L3/GRB7/LRP8/PARVG/TACSTD2/EMB/VWA1/BTBD3/SEMA4B/
SUMO2/SUPT7L/MAP3K7/PHF1/DPY30/NAA60/AAAS/PARK7/IRF4/EGR2/BCOR/SETDB1/PRMT6/BRD

BCL3/CD68/CCL2/SHC1/HDAC2/CXCL9/CXCL3/KRT8/CXCL13/NLRC5/PLSCR1/DEFB1/SLC11A1/PM[illegible]
SEMA3C/EPB41L3/SEMA3E/MAPT/RERE/CHN1/ZNF335/EXT1/ABL1/STK24/VCL/RASAL1/NFATC4/K[illegible]
BCL3/CD68/CCL2/SHC1/HDAC2/CXCL9/CXCL3/KRT8/CXCL13/NLRC5/PLSCR1/DEFB1/SLC11A1/PM[illegible]
PD1/IPO13/PKIA/IL10RA/TIMM13/WRAP53/TNFAIP3/MFF/RPS7/RPL7A/RPS23/RPS10/BAD/ACOT[illegible]

K2A1/VPS35/PLK3/SH3GLB1/TOMM22/OPTN/GNAI3/BNIP3L/LZTS1/SREBF1/CSNK2B/TRIM21/CDC

3H12A/STAB1/TNIP2/BCL3/CD68/CCL2/SHC1/HDAC2/CXCL9/CXCL3/KRT8/CXCL13/NLRC5/PLSCR

PPP1R15A/UQCC2/RPS19/RPS6KB2/ST3GAL5/RPLP0/RPL18/RPL23/RACK1/GADD45GIP1/VIM/MRP

K2A1/VPS35/PLK3/SH3GLB1/TOMM22/OPTN/GNAI3/BNIP3L/LZTS1/SREBF1/CSNK2B/TRIM21/CDC

CL10/ADAM17/NBN/TMEM98/ERCC1/IL20RB/HLA-F/CD79B/RNF8/TAP2/TNFRSF14/CCL19/XCL1/C

5R1/METRN/PARD3/APBB1/SRF/RHOA/STXBP1/ROBO3/NIN/NRTN/SPTAN1/EPHA2/ARTN/IST1/NEO
IRR/DHX9/MTPN/FAF1/CRTC2/UBE2N/XCL1/ARID5B/IRAK2/CAV1/TP53BP1/DAB2IP/PARP10/CRT

/FGR/MAPK11/TGFB2/CASP1/GSK3A/MIDN/PML/VPS28/OXCT1/PTPN23/IL1R2/YWHAQ/XPO5/YWHA[illegible]
/HTRA2/IFNG/TLK2/MAPK15/ATP6V0A1/EIF4G1/TBC1D25/SESN2/NOD1/PIK3R2/KDM4A/ZMPSTE2[illegible]

/ITGB1BP1/BIRC7/UNC5CL/DUSP4/PSMB4/IL1B/GBA/RAP2A/IL2RA/PDGFA/LAMTOR1/MLKL/PSMB
/FGR/LCP2/PSMA5/CD300LF/NRAS/WAS/SFPQ/PQBP1/LCK/PTPN6/PSMD13/IFI16/PSMC4/MUC5B/
T/ARPC1A/BRK1/CDHR2/RARG/ARPC5/ABL1/KCNN4/ACTR3/KEL/DBNL/DNM2/SPIRE1/NPPB/VEGFA
PSMD7/SH3GL1/CPE/AXL/TBL1XR1/RACK1/CSNK1G2/WNT4/MED12/JRK/PSMD8/RARG/ABL1/EMD/V

F11/VEGFA/EXOSC9/CDK11B/EGLN2/RAB11A/NANOS1/CRLF3/IFRD1/CDK5R1/NDUFA13/APBB1/SR

R14/PIN1/RPRD1A/PPP1R8/MFHAS1/PTPN13/DUSP13/UBLCP1/EYA3/PPP2R3B/RCAN2/PPP1R12A/[illegible]

6/DVL1

/ITGB1BP1/BIRC7/UNC5CL/DUSP4/PSMB4/IL1B/GBA/RAP2A/IL2RA/PDGFA/LAMTOR1/MLKL/PSMB[illegible]

X2/RPP38/APEX1/PARN/SETMAR/POLD2/PNRC2

ARK7/PDE6G

2/SLC9A6/EPHA10/YTHDF1/ADNP/FEZ2/RYK/PIK3CB/PLXNB2/KIF3A/FSTL4/PLCG1/CNP/NCAM1/[illegible]

VAV3/CHMP6/SEC23B/SYP/UNC13A/TSG101/TBPL1/CHMP4A/ARFGAP3/VTA1/STX16/ALS2CL/TRAP[illegible]

/FBXL18/CDC16/MIB2/UBE2R2/RNF41/PSMD12/BFAR/SMURF2/RNF40/CDC26/TRIP12/TRIM44/AN
K12/STRADA/RASGRP1/MARVELD3/TAOK1/MEN1/WNT5A/FGD2/ROR2/MAPK9/SLAMF1/TAOK2/TAB1/

T3B/TIGAR/CCAR2/POLE3/UBE4B/MSH6/NPHP4/ATR/TP53BP1/HMGA2/DCLRE1C/ERCC3/PTPRC/HR
/DAPK3/CSNK1D/SNF8/AXIN1/OAZ3/SLC11A1/CCDC22/VDAC1/MYLIP/ATP6V1E1/PSMB4/IL1B/GB

P1/TRMT10C/EZH1/OGT/PRMT7/METTL16/SETD4/GSPT1/MRM1/KMT2A/JARID2/TDRD5/RBM15/PCI

NS1/CDK5/LAMTOR1/CTTN/PSMB8/PSMB10/PSMA5/DNAJB2/TAF9/PSMD13/NUP85/IFI16/PSMC4/G[illegible]

RPF19/ZNF335/BRK1/MFNG/COL11A1/CHTOP/OVOL2/ACVR1/LUZP1/LFNG/RARG/TANC2/EXT1/ABL
7/CYTH3/RALBP1/ARHGAP33/RALGAPB/GNA13/ARHGEF18/LPAR1/BCR/MADD/RHOH/F2RL2/TIAM2/.

F/SEMA5B/NME6/RACK1/MSX1/CSPG5/RBBP7/SEMA3C/EPB41L3/SEMA3E/MAPT/EPPK1/RERG/MCTS
3/CALM2/CSK/TBC1D7/WWC1/LEMD2/PRKAA2/PTPN2/THBS1/CASP8/PTPN1/BMT2/VDAC2/OPTN/TX

1/APBB1/GLMN/VPS11/CD38/ATG13/IL17RA/ANP32B/TNFRSF14/DHX9
MP2/PTK2/THBS1/NOTCH1/CAPN2/ADAMTS5

DCD6/MED12/PRPF19/ZNF335/BRK1/MFNG/COL11A1/CHTOP/OVOL2/ACVR1/LUZP1/LFNG/RARG/TA

RAC1/PDGFA/RTN3/NRAS/B4GALT2/XYLT2/PI4KB/COPZ1/FUT3/RAB10/SMPD4/LST1/A4GALT/CHS
A1/TBX19/DFFA/UBE2A/SMARCC1/BRD9/KAT2A/BEND3/CBX5/CHMP1A/TOPBP1/PRIM2/NCOR2/CBX
/PRSS16/CD68/ADAM8/VLDLR/CAPN1/HLA-DQA2/LAMP3/CPNE1/ATP6V1H/CAP1/KCNQ1/PIK3CD/A
EMD2/HAT1/CSNK2A1/USF1/MBD3/CAPN2/RNF2/ANP32E/EXOSC10/CSNK2B/COPS5/MIS18A/MECP2
8/PHC2/BRCA1/RBL1/NSMCE2/PCNA/MLLT3/MCRS1/XRCC2/CHAF1A/CD3EAP/SGO1/SMARCA4/TOP1
TF/NOC2L/GTPBP4/NHP2/POP7/RPS19BP1/NONO/IDH3G/PLSCR1/DTD1/NLE1/SRSF9/EMG1/NOP58
/PRSS16/CD68/ADAM8/VLDLR/CAPN1/HLA-DQA2/LAMP3/CPNE1/ATP6V1H/CAP1/KCNQ1/PIK3CD/A
GNAI3/GLIPR1/MYO6/TMBIM1/SLC17A5/SLC15A4/UBA52/HLA-F/AP2A2/BORCS6/TECPR1/ARL8B/
BD2/PA2G4/GPR137B/VMA21/RAB24/ACLY/FLOT1/PRSS16/CD68/ADAM8/VLDLR/CAPN1/PIP4K2A/
/CEP250/CCT8/HAUS6/DPF2/ATP6V0D1/NDE1/TFAP2A/POLR3H/CKAP2/TTC26/CKAP5/HSPB11/MZ
1BP1/BIRC7/RAB11FIP4/DCTN5/B9D2/TCP1/KIFC3/POC5/KIF15/RASSF1/POC1A/LCK/SPECC1/C
SLC25A5/EMD/KLHL42/NSFL1C/KDF1/CALM2/CEP19/BOD1/CSPP1/TCTEX1D2/CCSAP/DCTN1/HAUS
17/PPP1CC/RAB10/PFN1/WASF1/OXTR/ATP6V0C/YWHAQ/GRB7/PARVG/YWHAB/RPS3/RPL27/RALA/
3GL1/AP3D1/WDR91/ATP6V0D1/MMGT1/CLIP3/SLC48A1/GGA1/HLA-DOA/STX7/SNX20/RUFY1/VAM
21/HIP1R/PAM/RAB24/STAB1/SRI/CD68/NCKAP1L/SH3KBP1/ADAM8/HLA-DQA2/LAMP3/CPNE1/KC
/TOPBP1/MYO9B/KLHL17/CCDC102A/ACTL8/AFAP1/SH3GL1/GMFG/MYO5A/WDR1/ARPC1A/NF2/ARP
MAPK7/UQCC2/BABAM1/RNF4/RFWD3/ITPKC/TIMM50/CXXC1/OGG1/RPA2/AAGAB/HAUS6/GNL3/TRI
PB1/SSPN/DPYSL3/PSEN2/SNAPIN/TMEM184A/AP2A1/STEAP2/COPA/RAB9A/KLHL12/YIPF1/CAV2
RAP2B/GYG1/ARHGAP9/RHOF/BMF/SIRPA/RAB3B/DEGS1/HK3/CCT2/CD58/C3AR1/HSP90AB1/PRSS
68/NCKAP1L/SH3KBP1/ADAM8/HLA-DQA2/LAMP3/CPNE1/KCNQ1/FZD2/SV2A/MSR1/SYNGR3/KDELR
PC2/DYNLL1/STAT1/SPDL1/XRCC3/KNTC1/HDAC1/PARPBP/ARPC3/ERCC6L/RFC2/DDX11/E2F4/NC
B4GALT3/B3GALNT1/RAB35/PITPNM1/ST3GAL4/ARF3/PAM/ARFGAP1/BCAP31/CHST10/CYTH2/SLC
EM9/GNS/FTL/NFAM1/GNB1/KXD1/AP2M1/ABCA2/DRAM2/SLC29A3/GALNS/DSN1/MAP1LC3B/SLC44
MP1A/VPS25/APP/SLC9A7/OCRL/CTSD/AP1G2/SH3GL1/AP3D1/WDR91/CCZ1/ATP6V0D1/MMGT1/CL
END3/CBX5/PRIM2/NCOR2/CBX8/PCGF2/XRCC6/TELO2/NABP1/RBBP7/RAD9A/RPA2/DPF2/POLD3/
KBP4/RAB11FIP4/PDE9A/ITGA2/EHD2/GNB2/S100A13/ANKRD13B/TNFSF13B/PI4KB/TRMT112/OS
7/MCAM/CDH17/PPP1CC/RAB10/EVPL/PFN1/WASF1/OXTR/UBA1/ATP6V0C/YWHAQ/GRB7/PARVG/YW
A-DQA2/LYPLA2/CSNK1D/SLC35B2/KDELR1/CLN3/SLC35A2/UXS1/HLA-DRB5/B4GALT7/RAC1/GBA
GNAI3/GLIPR1/MYO6/TMBIM1/SLC17A5/SLC15A4/UBA52/HLA-F/AP2A2/BORCS6/TECPR1/ARL8B/
BIN1/PALLD/DYNLRB1/CCT2/LMNA/ADAMTSL5/DYNLL1/DYNC1H1/CAPN6/KNTC1/GAS7/TIGD5/ARP
BIN1/PALLD/DYNLRB1/CCT2/LMNA/ADAMTSL5/DYNLL1/DYNC1H1/CAPN6/KNTC1/GAS7/TIGD5/ARP
/HSPA8/NEU1/GM2A/SERPINE1/TCIRG1/SLPI/FUCA2/CD63/DOK3/STK11IP/RNASET2/FPR1/STX1
LLD/DYNLRB1/CCT2/LMNA/ADAMTSL5/DYNLL1/DYNC1H1/CAPN6/KNTC1/GAS7/TIGD5/ARPC3/BEX4
T4/MT3/TPPP3/DYNLT1/HAUS1/ACTG1/RSPH1/TUBB4A/DAPK3/CSNK1D/KRT8/KIF14/CAMSAP2/HC
/GCN1/RRP36/CPSF6/MSI2/EIF4A3/ILF3/GRB7/RUVBL2/PRPF3/XPO5/SNU13/RPS3/SAMD4B/MSI
A/MBD1/SMAD2/WBP2/SMAD3/SETD1A/BAZ1A/TP53/INO80E/TARDBP/KAT5/ETV3/SUZ12/HMGXB4/
CNAB2/RAB3D/EMD/TBCD/CALM2/AKNA/BAG2/SRPRB/BCL10/HOOK2/BOD1/CSPP1/DNM2/CDK2AP2/
PG4/S100A7/REXO2/LIMS1/ARPC5/VCL/ZNF185/RPS7/TRIP6/RPL7A/ACTR3/RPS10/PTPN12/RPS
100A7/REXO2/LIMS1/ARPC5/VCL/ZNF185/RPS7/TRIP6/RPL7A/ACTR3/RPS10/PTPN12/RPS15/CT
PK1/CSPG4/S100A7/REXO2/LIMS1/ARPC5/VCL/ZNF185/RPS7/TRIP6/RPL7A/ACTR3/RPS10/PTPN
AV2/CAV1/CUZD1/PTPRC/PLD1/SLCO4C1/TNFRSF1B/CD59/VAPA/ATP8B3/NCSTN/SLC30A5/SURF4
AP2/DNM2/LMO4/PTK2/ADAM17/INPPL1/AAK1/CD177/PDLIM4/ACTN1/MYO6/PACSIN2/LIMA1/CD2
AR1/CHEK2/CENPQ/BOD1/HAT1/DCTN1/NBN/HNRNPU/ERCC1/MIS18A/PHF6/THOC2/KIF22
P1/SEC13/EEF1A2/RAB3D/HLA-DOA/NPRL3/ABCB9/STX7/VAMP8/RAB5C/WIPI1/PSAP/VPS35/CFT
/MRPL55/CHCHD1/ZNF622/RPL12/MRPS2/RPS18/RPS28/RPS17/PNPT1/RPL26L1/RRBP1/RPL22/G
S3A/RPL14/RPL10A/AIM2/ZNF622/RPL12/PRKCB/NLRC4/RPS18/RPS28/RPS17/RPL26L1/TSC1/R

NKL1/SF1/HNRNPH3/ZMAT2/CWF19L1/SF3A1/GPATCH1/PDCD7/WAC
S11/RPL31/MRPL42/MRPL22/MRPL58/MRPL38/MRPS30/MRPS27

AB3B/HGS/BIN1/NPC1/GPR107/F2RL1/ARPC2/RAB7B/PLEKHM2/PSENEN/SORT1/RAB35/PIP5K1C/
UN1D5/PCGF2/RBX1/IKBKG/DR1/PRDM4/UBE2L6/RBBP7/CXXC1/LAS1L/SNRPG/RNF7/CUL4B/E2F6/

2/ERCC2/E2F7/HOXB9/BRF2/PBX3/GTF3C3/MED17/SMAD3/GEMIN5/SUPT3H/TP53/TBPL1/TAF11/
DE6G/MYO1D/ADGRE2

CTN1/WTAP/NUCB2/SP140/BNIP3L/LBR/TMEM109/SREBF1/MYO6/BCL2L1/SEPHS1/SYNE4/XPO7/E

R2/UNC13A/FNBP1/MYL12B/MYH9/HIP1/EXOC6B/BFSP1/MYZAP/CTTNBP2/PARD6A

PAR1/GPR161/SYT11/HLA-C/IGF2R/VAMP3/KIF5B/RAB22A/SPARC/PIK3C2B/HYAL2/CD207/ATP6[illegible]

SEC23B/TEX261/GGA2/FCGR1B/HIP1/TEPSIN/DVL1/ECE1/ARFGAP3/AFTPH/WNT5A/LRP2/SCYL1/
1CC/GSK3A/LRFN4/RAB10/TNIK/ADAM23/WASF1/KCNK9/MAP4/GAD1/PPP1R1B/ABR/NSMF/EIF4A3
3/VEGFA/HEXB/ACTN1

0/PRPF4/THOC1/SFMBT2/HDAC5/CBY1/PABPN1/SRSF1/SRSF2/CASC3/SNRNP70/RBM4B/RBM8A/ME

NC4/SYT11/MYO1D/KIF5B/TIAM2/TRIM46/FKBP15/ARL8A/WHRN/PRKCB/DOCK7/KCNIP3/TRAK2/S

S1/SRGN/MAN2B1/OLFM4/CYB5R3/VEGFA/HEXB/ACTN1
PF2/EXOC3/DSTN/CYTIP/PLEKHH2/RHOA/AP3B1/ACTN4/RAB27B/FNBP1L/SPTAN1/SNAPIN/TTLL3

GF1/BRCC3/PHC1/MED31

S1/SRGN/MAN2B1/OLFM4/CYB5R3/VEGFA/HEXB/ACTN1

RPS18A/NDUFS3/MTG2/MRPL43/MRPS9/MRPL50/NDUFA3/TIMM10B/SUPV3L1/TOMM20/UQCRFS1/MR

FB2/SFPQ/CACNG4/PPP1CC/GSK3A/CIT/WASF1/INPP5J/AGFG1/OSBP2/PPP1R1B/NSMF/ERO1A/EI

FN3/STAT3/PCBP2/CHRNA1/GAP43/CAMK2N1/TANC1/SYN1/SH2D5/DLG5/HNRNPH2/PICK1/DNAJB1

TTNBP2/ARHGAP44/NPTXR/NPTX1/WNT3A/CRTC1/LRFN3/USP46/STAT3/ITGB1

3/CAMK2N1/TANC1/SYN1/SH2D5/DLG5/HNRNPH2/PICK1/DNAJB1/RPS25/ADAM10/CHMP2B

RIM37/MEIS3/RBMX/NKAP/NCAPD3/APBB1/SRF/HMGN2/MLLT6/RNF8/SIX1/ACTN4/HINFP/DNMT3B/
1/LMCD1/MTA3/NFATC4/TDG/CIITA/WWC1/SFR1/BCL10/PSMD9/USP21/HNRNPU/PIAS4/CTBP2/TH
3/SRPK1/CSK/CHEK2/MAPKAPK3/CCND3/PRKAA2/ACVR1B/STK40/PTK2/CSNK2A1/TNK2/CCNJL/AA
OCC/VWA1/RPL27/LUM/COL4A2/NUP155/SMTN/LLGL1/LAMB3/RPL35/MATN3/RPS20/VPS25/NUP10
TBCD/VCP/NPRL3/RASAL1/SFRP2/CALM2/SIPA1/ARAP1/TBC1D7/BAD/SGSM3/DBNL/PSAP/ARHGAP
BP3/CAPN10/BAIAP2L1/NRCAM/BRCA1/STX1A/KLC3/CORO1C/RAB3B/CDH6/BIN1/CORO7/PALLD/H
SY/MATK/CCND2/PRKD2/DGUOK/STK10/CHKA/PRKCI/GRK2/HK1/DMPK/IP6K1/MAPK7/MAP3K6/AXL/
1/DYNC1H1/RAB7B/XRCC3/MYO19/RRAS/DDX49/ATP13A1/ERCC6L/RFC2/DDX11/RAB35/ARF3/KIF
1/DYNC1H1/RAB7B/XRCC3/MYO19/RRAS/DDX49/ATP13A1/ERCC6L/RFC2/DDX11/RAB35/ARF3/KIF
1/DYNC1H1/RAB7B/XRCC3/MYO19/RRAS/DDX49/ATP13A1/ERCC6L/RFC2/DDX11/RAB35/ARF3/KIF
3/MYO19/RRAS/DDX49/ATP13A1/ERCC6L/RFC2/DDX11/RAB35/ARF3/KIFC2/DDX27/RAB24/DYNLT
/VDAC1/ACTB/BCAR1/PFKM/CDK5/MLKL/FAM83H/WWC3/FGR/WAS/LCK/PTPN6/CASP1/PPP1CC/GSK
/DVL2/PARP1/GCN1/CDKN2C/PTPN23/CADM4/MSH2/GRB7/CCND2/RPS3/POLA1/ACSL3/PRKD2/TRA
/CNTNAP1/GNG5/IL1B/EHD2/BCAR1/PPIL1/STRN4/ETV6/STMN3/ADAM12/PLEKHA2/WAS/TAF9/PL
OCS1/CKS1B/GPSM1/HSP90AB1/DYNLL1/RCAN3/ARHGEF19/PPP1R9B/PPP1R35/TBC1D1/CST2/RIN
TGA2/IL1B/CTTN/CDH17/EIF2S3/PTPN6/RAB10/UBAP2/ADAM23/EVPL/PFN1/EPS15L1/GCN1/IBS
BI2/BCAR3/CCZ1/MYO5A/ARHGDIA/ARFIP2/RIC8A/TBC1D10C/FMNL3/PARD6G/SERGEF/EIF2B5/P
PPL1/PDLIM4/HNRNPU/FLII/MAP1A/NEBL/ACTN1/ADD3/MYO6/LIMA1/SSH3/CDK5R1/TNNC1/TLN1/
A/PDK3/IRAK2/MAP4K1/ATR/MAP4K2/MKNK1/ERCC3/CSNK2A2/RPS6KC1/SRPK2/STK35/VRK2/ADC
SMARCA5/GTF2H1/PSMC5/TOR3A/SMARCAL1/MSH6/HSPA13/ATP7A/SRCAP/ATP6V1B2/ATAD5/MORC
ENND5A/SGSM3/ARHGEF3/SRGAP2/ARHGEF25/BICD1/RGP1/TBC1D22B/OPTN/ARHGEF11/RABGGTB/
1D7/DENND5A/SGSM3/ARHGEF3/SRGAP2/ARHGEF25/BICD1/RGP1/TBC1D22B/OPTN/ARHGEF11/RAB

PREX1/ARHGAP22/ARHGAP27/RIC8B/ARHGAP17/LLGL2/RALBP1/ARHGAP33/EVI5L/ASAP3/RALGAP

/PTPN1/SH3GLB1/OLFM4/RPL6/PAK2/PACSIN2/HSPA5/LIMA1/CD2AP/NOTCH3/CDK5R1/TLN1/EIF

RAB22A/RAB3C/ARL3/EIF5B/RAB33A/ARL8A/GNG2/RGS4/RAB2A/RHOD/RAB7A/TUBD1/RAC3/RRAS

BCR/RHOH/TIAM2/ARHGAP19/ARHGAP25/SRGAP1/IPO7/PDE6D/TBC1D25/SESN2/RGS4/VAV3/SEC2

AD1/HUNK/MAP3K14/GSK3A/DPAGT1/CDK18/NUAK2/TNIK/SLA/FGFR2/CIT/ALDH18A1/PLK4/MST1

F4ENIF1/SNRNP35/RBMS1

K2/HK1/DMPK/MAPK7/MAP3K6/AXL/PIP4K2C/RPS6KB2/BTK/DSTYK/STK25/VRK1/ITPKC/CSNK1G2/
R2/RELA/NKD2/TRAF1/BLZF1/SUMO3/SMAD2/DERL1/KDM4A/CUL7/MUL1/XRCC5/SMAD3

ERL1/KDM4A/CUL7/MUL1/XRCC5/SMAD3/TSG101/TP53/PRKAR2A/CASP10/UBE2J1/CD40/ARRB1/R

HOBTB2/RAB9A/RAB40C/RAB13/GTPBP6/ARL1/RAB1A/RAB36/GNA13/RAB8B/RAB1B/MX1/GPN1/GT
40C/RAB13/GTPBP6/ARL1/RAB1A/RAB36/GNA13/RAB8B/RAB1B/MX1/GPN1/GTPBP1/MFHAS1/ERAL

TPBP6/ARL1/RAB1A/RAB36/GNA13/RAB8B/RAB1B/MX1/GPN1/GTPBP1/MFHAS1/ERAL1/RHOH/RAB2
TPBP6/ARL1/RAB1A/RAB36/GNA13/RAB8B/RAB1B/MX1/GPN1/GTPBP1/MFHAS1/ERAL1/RHOH/RAB2

1A/RAB36/GNA13/RAB8B/RAB1B/MX1/GPN1/GTPBP1/MFHAS1/ERAL1/RHOH/RAB22A/RAB3C/ARL11

L8/EGF/PYY2/DKKL1

TPBP6/ARL1/RAB1A/RAB36/GNA13/RAB8B/RAB1B/MX1/GPN1/GTPBP1/MFHAS1/ERAL1/RHOH/PDE6
TPBP6/ARL1/RAB1A/RAB36/GNA13/RAB8B/RAB1B/MX1/GPN1/GTPBP1/MFHAS1/ERAL1/RHOH/PDE6

NF4/E2F6/MYBBP1A/DRG1/NFYC/RERG/MED12/RERE/MEIS2/TAF12/ARID5A/TAF10/RARG/VGLL4/

6/WBP2/MED17/NCOA7/MAML1/SUPT3H/TSG101/TBPL1/TAF11/KAT5/MTDH/APEX1/MED14/TAF4/M
B/METTL2A/DDX31/TRMT2A/SNRNP200/DDX20/DCP2/POLR3F/DDX51/MED20/POLR2B/DUS1L/CNOT

BE2E3/TRIM62/MED20/RNF121/WWP2/ARIH2/RNF126/RNF115/SKP1/ANKIB1/MED30/MKRN1/ATG3/

BL2/CENPB/RPS3/TFAP4/RBBP4/POLA1/TBX19/HNRNPAB/PAX8/SMARCC1/MCM8/MAFF/BEND3/MTF

S3/CROCC/PARD6B/PTPA/LLGL1/KAT2A/ZNF207/CHMP1A/RANBP1/CETN2/LRRC61/ACTL8/KATNB1
T1/LCMT1/NAA10/PCBP4/CASP2/BRSK1/IQGAP1/GMNN/MIIP/PRKDC/STIL/SSNA1/MAD2L2/CNOT1
P1L/SH3KBP1/ADAM8/VLDLR/COL6A3/LYPLA2/CPNE1/VSIG1/CAP1/CCL2/SHC1/DAPK3/PIK3CD/Y
ARD11/GPRC5B/RAC1/IL1B/GBA/CD47/RNF26/S100A13/NFAM1/FGR/LCP2/MAPK11/TGFB2/GPSM3
1/TGFB2/GPSM3/PQBP1/PTPN6/IFI16/AGPAT2/CASP1/PML/IL12RB1/IL15/C5AR1/IL1R2/RPS3/
AP2/ALOX15B/SULF2/TWIST1/GLMN/IL17RA/TNFRSF14/DHX9/IDO1/HEG1/ADORA2B/EREG/CCL19
ABL1/VCL/PSMA1/SFRP2/KDF1/RPS7/NFATC4/PDCD10/FOXF2/PSMB5/IFT172/BCL10/PSMA4/TBX
1/PPIA/ZC3H12A/RAB24/ACLY/BCL3/DYNLT1/CD68/NCKAP1L/ADAM8/CAPN1/CPNE1/C1orf35/CA
TNIP2/DYNLT1/CD68/NCKAP1L/ADAM8/CAPN1/CPNE1/C1orf35/CAP1/PIK3CD/ASAH1/KPNB1/PLS
/CD47/S100A13/FTL/NFAM1/FGR/PSMA5/NRAS/SERPINB6/PTPN6/PSMD13/AGPAT2/COMMD9/RAB1
/COMMD9/RAB10/DYNC1LI1/GALNS/DSN1/SLC44A2/IMPDH2/ABR/ATP6V0C/PSMD11/PSMD1/C5AR1
1/PPIA/ZC3H12A/RAB24/ACLY/BCL3/DYNLT1/CD68/NCKAP1L/ADAM8/CAPN1/CPNE1/C1orf35/CA
C2/NBEAL2/OLR1/SLC2A5/GPI/PA2G4/SERPINB1/IRF7/PPIA/RAB24/ACLY/BCL3/DYNLT1/LAG3/
SMD13/AGPAT2/COMMD9/RAB10/DYNC1LI1/GALNS/DSN1/SLC44A2/IMPDH2/ABR/ATP6V0C/PSMD11
1/PSMB10/ETV6/PSMA5/TGFB2/CDH17/LCK/PTPN6/THOC5/PSMD13/CD27/IFI16/PSMC4/PML/CCR
CASP8/TMEM98/VEGFA/LBR/TLR2/TMEM64/NKAP/ADGRG3/CD79B/SRF/RHOA/INHA/AP3B1/DOCK2/
TNFRSF4/SLC39A10/FPR1/MNDA/ZNF580/THY1/SIRPA/SPON2/SOCS1/THEMIS2/IRF1/IRF3/C3AR
CDC42/MUC20/CMKLR1/TLR5/PSMD4/GPATCH3/PSMD3/PSMD7/LILRB4/IKBKG/FADD/BTK/PELI1/C
B29/MUC15/CACNB3/PLD2/PSMC3/STOML2/WIPF2/CD79B/CD38/RAPGEF1/MAP3K1/ELMO2/PSMC5/
FC2/DDX11/ERI3/REXO4/LEF1/POLR2H/FGFR1/PPIA/CCT4/PNKP/OTUB1/BLM/FBXO5/DBF4/GTPB
CP/WRAP53/UBQLN4/DCLRE1B/FAAP20/TDG/COPS6/EME2/CHEK2/CLSPN/RAD1/SFR1/CHD1L/HDAC
5C/TBL1XR1/BABAM1/DR1/PRDM4/VRK1/CENPN/RBBP7/CXXC1/CUL4B/DPF2/INO80C/NTMT1/RERE
PF3/EXOSC5/SNU13/SRRT/TTF2/PRPF6/RPL27/BARD1/FTSJ3/HENMT1/POLR2L/DDX41/DROSHA/A
S21/EIF3K/RPS15/RPS16/MRPS15/THBS1/EIF3M/PUS7/RPL5/RPS27A/MRPL18/RPS12/JMJD4/SA
P2B/GYG1/ARHGAP9/RHOF/TNFAIP2/SIRPA/RAB3B/HGS/DEGS1/HK3/CCT2/CD58/C3AR1/HSP90AB
EMB/PRKD2/RALA/SEMA4B/GRB2/ARRB2/CALR/CXCL11/CCR5/SEMA4D/XCL2/CMKLR1/APP/SWAP70
T9/NONO/AUNIP/CDK2/KDM1A/FANCA/PMAIP1/LYN/LIG1/KMT5A/SSRP1/WDHD1/WAS/TAF9/SWI5/
/CDK2/MKKS/KIF14/KPNB1/PHLDB1/CAMSAP2/CLN3/DCTN2/KNSTRN/FKBP4/RAC1/CDK5/CHORDC1
LE1/DCTN2/CDKN1C/PSMB4/IL1B/PSMB8/PSMB10/PSMA5/PTPN6/PSMD13/PSMC4/HAUS8/PML/DYN
ODC1/ZNF703/PPARD/LEF1/CLEC11A/FGFR1/TGM2/LBH/SIRPG/NOP2/NCKAP1L/FBXO5/FGFR3/CA
2/RBM10/USP39/SNRNP25/RBM15B/AHNAK2/LUC7L/TSEN15/RBM24/SF3B2/STRAP/LSM1/SFSWAP/
L7/ANAPC5/HECTD3/CDC27/CDC34/HSP90B1/UBE4B/UBAC2/AUP1/MAEA/HSPA13/CAV1/DAB2IP/G
BB4A/CNOT9/EVI2B/CDKN2B/CCL2/DAPK3/CSNK1D/ANAPC7/CDK2/LSM10/KIF14/DCTN2/KNSTRN/
B/RPS3/PRKD2/CROCC/STX18/GRB2/FHOD1/CDC42/KAT2A/TGFB3/MTF2/RANBP1/SYT1/SWAP70/C
D1/ARRB2/PTPA/PRKCI/TGFB3/GAL/GZMB/DNM1L/CTSD/HSPE1/PDCD5/KATNB1/FADD/GZMA/UTP1
PS5/NELFB/MXRA8/PSMB4/LAMTOR5/APOBEC3G/RPS21/PSMB8/RPL7/RPL28/RTN3/PSMB10/CCL4/
3/RPS3/SRRT/SAMD4B/PSMB3/TTF2/PRPF6/RPL27/BARD1/POLR2L/RNH1/DDX41/AGO2/SF3A3/CP
AP/HMGA2/MORC2/HDAC5/PAGR1/ENY2/TADA3/SIN3B/HUWE1/PAF1/TRRAP/HDAC8/PWP1/DDB1/SU
IP2/IL18BP/HLA-DQA2/CNOT9/CPNE1/CCL2/CAPZA1/SHC1/TRIM31/TXLNA/PIK3CD/CXCL9/CXCL
L52/CKAP2/NSF/RUBCN/CKAP5/MCTS1/MRPL48/DAP3/MRPS7/HSPA2/MRPL53/GSPT2/VCP/STMN2/
A2/RPS15/RPS16/EIF3M/STYXL1/RPL5/NOL6/RPF2/BICD1/BMT2/POP5/NVL/LTV1/RPL6/EXOSC9
ARHGDIB/FHOD3/HCLS1/PSTPIP1/TESK1/CDC42BPG/ITGB1BP1/ARPC5L/RAC1/EHD2/ACTB/BCAR1
RAP1/CEP19/TBC1D7/CEP41/TTYH1/IFT172/DBNL/HSP90AA1/SRGAP2/DNM2/TCTEX1D2/DCTN1/N
RAC1/EHD2/ACTB/BCAR1/RAP2A/CDK5/PDGFA/CD47/CTTN/MYADM/WAS/VIL1/SPECC1/NUAK2/TNI
/PML/CCR1/MLF1/IL12RB1/FGFR2/CYP26B1/CD79A/LYAR/OSTM1/PARP1/IL15/PSMD10/PSMD11/
ANKRD54/IRF4/AGO1/MYC/BGLAP/DMTN/PRMT6/DYRK3/CD4/IFNG/EIF2AK1
/DAPK3/CXCL13/ARHGDIB/KIF14/HSPH1/JAG1/CD209/LYN/ITGB1BP1/CCL28/CARD11/TNC/RAC1
E2D1/USP14/PSMD10/PSMD11/PSMD1/MVB12A/PTPN23/GPX1/TNFSF12/PSMA7/UBE2E1/FBXL6/US
3CD/CXCL13/ARHGDIB/KIF14/JAG1/MITF/LYN/ITGB1BP1/CCL28/MCTP1/RAC1/ITGA2/PLA2G7/M
1/SHC1/HDAC2/PIK3CD/RAP1GAP2/YWHAH/CAMSAP2/KDM1A/LYN/WNT7B/FKBP4/TNC/RAC1/CNTNA

DC42EP2/DNM1L/SYT3/SEMA4F/KATNB1/WRAP73/SEMA5B/STK25/ABI2/ARHGDIA/VIM/SCN1B/SEM
ARD1/UBE2A/ASB1/ARRB2/NUP155/USP22/JADE2/CDC42/ATXN7L3/NUP107/PSMD4/CBX8/DCUN1D
C1D1/GDI1/STX10/PPARD/GPI/SERPINB1/PPIA/ZC3H12A/PAM/BID/CCT4/BCAP31/UBE2J2/DVL3
7/RPL28/CACNG4/NUP85/GSK3A/RAB10/PML/RPL39/VPS28/BYSL/RPL35A/UBE2D1/PARP1/MSH2/
HSPA4/ITGB3BP/CAPZA1/RPSA/RANGRF/CSNK1D/DNAAF5/MKKS/SV2A/FHOD3/CAMSAP2/HCLS1/AX
S3/SAMD4B/PSMB3/DFFA/RPL27/DNASE2/RNH1/NUP155/DROSHA/AGO2/APOBEC3D/ALDOC/RPL35/
RPL5/DUS2/GTF3C5/NOL6/RPF2/CTU2/BMT2/POP5/NVL/EXOSC9/EXOSC10/EXOSC7/WARS2/DDX56
SERPINB6/PTPN6/PSMD13/AGPAT2/COMMD9/RAB10/DYNC1LI1/GALNS/DSN1/SLC44A2/IMPDH2/IL
ARD/CORO1B/LEF1/GPI/FGFR1/ZC3H12A/SVBP/SLC9A3R1/NCKAP1L/FBXO5/GTPBP4/ADAM8/CCL2
EMA4F/FADD/STX4/SEMA5B/CLEC7A/RACK1/SEMA3C/SEMA3E/IGF1R/LBP/PDCD6/CCL3/S100A7/C
CCND3/BAD/BCL10/PTGER4/VCAM1/TWSG1/PTPN2/ADAM17/CASP8/TMEM98/IL20RB/PAK2/RAB29/
SERPINB6/PTPN6/PSMD13/AGPAT2/COMMD9/RAB10/DYNC1LI1/GALNS/DSN1/SLC44A2/IMPDH2/IL
EMB/PRKD2/RALA/SEMA4B/GRB2/ARRB2/CALR/CXCL11/CCR5/SEMA4D/XCL2/CMKLR1/APP/SWAP70
10/MRPS17/MRPS21/EIF3K/RPS15/RPS16/MRPS15/THBS1/EIF3M/PUS7/RPL5/RPS27A/MRPL18/R
1/PDCD5/FADD/GZMA/UTP11/CLEC7A/RACK1/MSX1/STPG1/DAP/RAD9A/IL20RA/HCAR2/PLEKHN1/
2/KIF14/HCLS1/CLN3/NLE1/KDM1A/MITF/BIRC7/MIEN1/IL1B/LAMTOR5/WFS1/CBX4/CTTN/RNF1
1/PDCD5/FADD/GZMA/UTP11/CLEC7A/RACK1/MSX1/STPG1/DAP/RAD9A/IL20RA/HCAR2/PLEKHN1/
HTATIP2/KIF14/HCLS1/CLN3/NLE1/KDM1A/MITF/BIRC7/MIEN1/IL1B/LAMTOR5/WFS1/CBX4/CTT
APC4/BAG6/BBS7/FAF2/TMEM67/DDB1/FBXO44/SKP2/PSMB1/WWTR1/SUMO2/PSMB6/RPL11/CDC23
CAMSAP2/HCLS1/TBC1D30/PMAIP1/LRFN1/TESK1/MITF/ITGB1BP1/ARPC5L/ATXN2L/HCFC1/FKBP
/ABL1/HEATR1/VCP/THBS2/PALM/ACTR3/ARAP1/HSP90AA1/CHD1L/MPP7/DNM2/SPIRE1/DCTN1/S
1FIP4/RAC1/ITGA2/VDAC1/RPS5/NELFB/MXRA8/PSMB4/LAMTOR5/APOBEC3G/RPS21/PSMB8/TCP1
D209/LYN/HCFC1/SSB/RAB11FIP4/RAC1/ITGA2/VDAC1/RPS5/NELFB/MXRA8/PSMB4/LAMTOR5/AP
ZAP1/IL1B/MAGED2/KDM5B/NCAPH2/TCP1/ADRA2C/ETV6/DEAF1/POC1A/PAQR8/DNMT3A/CALCA/P
2/PRIM1/CDKN1C/AKAP8L/PSMB4/UBE2L3/PSMB8/PSMB10/PSMA5/PTPN6/PSMD13/PSMC4/SIRT7/
3AR1/HSP90AB1/F2RL1/PRSS3/DYNLL1/DYNC1H1/ILF2/AMPD3/NPC2/NBEAL2/OLR1/SLC2A5/GPI
ME3/TNIP2/LAG3/FLOT1/ACTG1/SMPDL3B/ADAM8/HLA-DQA2/NONO/ULBP2/CCL2/CAPZA1/TRIM31
BA52/HLA-F/IFNAR2/MUC15/CDC37/PSMC3/UBE2D2/RBM14/NMI/MAP3K1/LGMN/DHX9/NR1H2/PSM
EYL/HDAC2/CXCL9/CSNK1D/SULT2B1/MSR1/HCLS1/KDM1A/JAG1/LYN/WNT7B/GPRC5B/RAC1/CBFB
MSH2/ZBTB17/PSMA7/TFAP4/RBBP4/PSMB3/BARD1/KAT2A/ZNF207/CBX5/CHMP1A/CALR/TOPBP1/
2/PTPN6/CALCA/MAP3K14/SOCS3/TNIK/DVL2/MST1R/PPP1R1B/PSMD10/GCN1/SMYD3/CDKN2C/C5
0/WAS/CDH17/LCK/PTPN6/CD27/SAMSN1/IL12RB1/CYP26B1/IMPDH2/CD79A/LST1/IL15/SNX27/
51C/HSPA2/RAD51D/MFF/NSFL1C/EME2/ACTR3/CHEK2/RAD1/ACOT8/BOD1/SPIRE1/CCSAP/VPS35
LCK/PTPN6/THOC5/PSMD13/CD27/IFI16/PSMC4/PML/CCR1/MLF1/IL12RB1/FGFR2/CYP26B1/CD7
OXB7/PSMD3/PSMD7/HEY1/MSX1/WDR1/SEMA3C/TRIM71/TIMELESS/SEMA3E/TFAP2A/WNT4/MED12
FADD/BTK/PELI1/STX4/CLEC7A/RASAL3/GPR183/LBP/EXOSC3/BTN2A2/CCL3/EMILIN1/ZNF335/
FADD/ECM1/TREM1/ANGPT2/WDR1/GPR183/LBP/CCL3/S100A7/GLG1/EMILIN1/CXCL17/AKIRIN1
PARP1/SET/PSMD10/SMARCB1/GPX1/TACSTD2/DFFA/CAPZB/BAG3/DGUOK/SEMA4B/ARRB2/CDC42/
ORO1B/LEF1/GPI/FGFR1/ZC3H12A/SVBP/SLC9A3R1/SRI/NCKAP1L/FBXO5/GTPBP4/ADAM8/CCL2/
S19/FADD/STX4/SEMA5B/CLEC7A/RACK1/SEMA3C/SEMA3E/IGF1R/LBP/PDCD6/CCL3/S100A7/CEM
DKN2B/SHC1/KIF14/NLRC5/AXIN1/SLC11A1/TESK1/LYN/WNT7B/ITGB1BP1/BIRC7/GPRC5B/CDKN
P30/AGFG1/ABR/C5AR1/SIPA1L3/MSH2/ARF4/RPS3/TFAP4/DNAJC10/PRKD2/SYDE1/PTPA/RUNDC
NEB/TACSTD2/RPS3/CAPZB/GRB2/SYDE1/FHOD1/CDC42/KAT2A/CHMP1A/ARHGAP18/TGFB3/RANBP
/CROCC/RIMS3/LLGL1/FHOD1/CDC42/KAT2A/STARD3/IL4R/VPS18/CHMP1A/SYT1/PPFIA3/CETN2
CENPQ/BOD1/COPG2/RPS15/DNM2/C12orf4/PTK2/SPIRE1/DCTN1/BICD1/HNRNPU/LTV1/CTBP2/R
/RNF157/PLXND1/ASCL2/PPP1CC/GSK3A/LRFN4/NDRG4/NAP1L2/TNIK/TUBB2B/DVL2/INPP5J/OX
ACSIN1/PPP2R5B/METRN/PARD3/APBB1/SRF/CAPRIN1/RHOA/SLC25A12/RAPGEF1
P2A/CDK5/NREP/CTTN/SPOCK1/ADRA2C/RNF157/MYADM/PLXND1/ASCL2/PPP1CC/GSK3A/NDRG4/N
M2/FERMT1/NPEPPS/EHD1/SRI/ARHGAP1/LEPROTL1/RANGRF/CXCL9/YWHAH/HCLS1/BBC3/KDM1A/
/MT3/TNIP2/BCL3/DYNLT1/NCKAP1L/AATF/NOC2L/ADAM8/PAK4/LAMP3/EVI2B/NONO/CCL2/SHC1
/TGFB2/ZNF205/CACNG4/CHMP3/BANF1/CLTB/GSK3A/AP2M1/MYOF/DVL2/FOLR2/AP2S1/AGFG1/A

8/UBE2D1/RNF220/UBA1/USP14/PSMD10/RNF113A/PSMD11/RAE1/ZMIZ2/PSMD1/DCAF16/PSMA7/
NEB/GORAB/MVB12A/KIF2A/ODF2/PTPN23/GRB7/RPS3/CROCC/STX18/BAG3/VCX/KAT2A/ZNF207/
MO1/PACS2/RPL38/CLIP3/NSF/RPL23A/CEMIP/ZDHHC12/GGA1/RAB3D/TIMM13/MFF/RPS7/STAC/
UP155/PRRX1/HSBP1/APP/DNAJB6/NUP107/CBX8/IER5/POLQ/MAPK7/RELL2/PPP1R15A/FUS/MAP
LT7/RAC1/BCAR1/CD47/CTTN/STMN3/MYADM/LOXL2/WAS/TGFB2/VIL1/CIT/PFN1/WASF1/INPP5J
NCKAP1L/GTPBP4/ADAM8/PAK4/CCL2/SHC1/CXCL13/HSPH1/JAG1/CD209/LYN/WNT7B/CCL28/CAR
/IFT172/DBNL/HSP90AA1/SRGAP2/DNM2/TCTEX1D2/DCTN1/NOTCH1/CEP164/ODF2L/HAUS7/INPP
3/SEMA4F/KATNB1/WRAP73/SEMA5B/STK25/ABI2/ARHGDIA/VIM/SCN1B/SEMA3C/PMP22/PTPRF/S
ETN2/PSMD3/RBX1/PSMD7/RAD21/BABAM1/MTBP/RFWD3/CEP250/RPA2/CUL4B/HAUS6/NAE1/NDE1
NA1/MAD2L2/CNOT11/PSMD2/CENPE/PPP2R1A/RBBP8/CHMP4C/BRCA1/RBL1/NSMCE2/PCNA/TUBA1
CH2/PIK3R6/OSM/IL17D/HAX1/LDB1/INPP5D/CIB1/ZNF683/PTPRC/YTHDF2/PAF1/IKZF3/ZNF16
/WRAP53/MFF/NSFL1C/PDCD10/STAC/STX7/CSK/VAMP8/BAD/CENPQ/HSP90AA1/PRKAA2/VPS35/C
LC9A3R1/NCKAP1L/FBXO5/GTPBP4/ADAM8/CCL2/DAPK3/PIK3CD/CXCL13/MKKS/ARHGDIB/KIF14/
CK1/SEMA3C/SEMA3E/IGF1R/LBP/PDCD6/CCL3/S100A7/CEMIP/ACVR1/CXCL17/AKIRIN1/ABL1/P
BM4B/DHX8/RBM8A/METTL3/CSTF3/SF3B6/MAGOHB/SNRPD3/HNRNPA0/TFIP11/CELF2/IK/HNRNPD
BM8A/METTL3/CSTF3/SF3B6/MAGOHB/SNRPD3/HNRNPA0/TFIP11/CELF2/IK/HNRNPD/PRMT5/SNRN
BM8A/METTL3/CSTF3/SF3B6/MAGOHB/SNRPD3/HNRNPA0/TFIP11/CELF2/IK/HNRNPD/PRMT5/SNRN

CD47/S100A13/FTL/NFAM1/FGR/PSMA5/NRAS/SERPINB6/PTPN6/PSMD13/AGPAT2/COMMD9/RAB10
3/BTN2A2/CCL3/ZNF335/HSPD1/ABL1/SART1/HLA-DOA/FCGR2B/TNFAIP3/TOX/CSK/VAMP8/MILR
RB2/ARRB2/CDC42/MUC20/CMKLR1/TLR5/PSMD4/GPATCH3/PSMD3/PSMD7/LILRB4/IKBKG/FADD/B
/KHDRBS3/TSEN34/DHX9/RBM28/GTF2H1/RNMT/CCAR2/RBM10/USP39/SNRNP25/RBM15B/LUC7L/T
4/AAAS/ANKRD54/PARK7/GEMIN8/HEATR3/EGR2/THOC3/HYAL2/IFNG/GEMIN6/SMN2/IPO7/PPP1R

BL/RALGDS/ABI2/BCAR3/RALB/ARHGDIA/ARFIP2/RASAL3/FGD1/RERG/RHEB/RAB4B/OBSCN/CHN1
B/NAT10/MPV17L2/PSMA1/MYEF2/PSMB5/PSMA4/EIF3K/PSMD9/THBS1/PUS7/RPL5/RPS27A/HNRN

A/CD38/RAPGEF1/GAK/ACAP3/GPC2/NIN/ADCY6/DPYSL3/SNAPIN/AP2A1/IST1/ARF1/SNX3/DAB2

/S100A7/CEMIP/ACVR1/CXCL17/AKIRIN1/ABL1/PDCD10/TRIP6/TRADD/ACVR1B/PTK2/THBS1/AD
M1/AURKAIP1/MRPL20/KIF5B/MYC/DMTN/LIX1L/DYRK3/MRPL24/MRPS16/MRPL55/CHCHD1/TBC1D
NSD1/TLK2/MAPK15/ANAPC15/TERF1/HNRNPA2B1/AKAP8/KMT2B/SUPT6H/KDM3A/CDK5RAP2/WBP2
2/ERCC1/VEGFA/RAB3A/ADD3/PIH1D1/EML2/MECP2/PPP2R5B/CDK5R1/ARHGAP28/PSMC3/TWIST1
D47/S100A13/FTL/NFAM1/FGR/PSMA5/NRAS/SERPINB6/PTPN6/PSMD13/AGPAT2/COMMD9/RAB10/
/COMMD9/RAB10/DYNC1LI1/GALNS/DSN1/SLC44A2/IMPDH2/ABR/ATP6V0C/PSMD11/PSMD1/C5AR1
B4/PSMB8/PSMB10/PSMA5/PTPN6/PSMD13/PSMC4/SIRT7/HAUS8/PML/DYNC1LI1/CIT/PLK4/ANAP
NAPC5/EREG/CNOT3/TFDP1/UBE2E2/EGF/DAB2IP/HMGA2/RPTOR/ATAD5/THOC1/PAGR1/PKD1/ANA
2/DDX20/KPNA4/AAAS/ANKRD54/PARK7/GEMIN8/HEATR3/EGR2/THOC3/HYAL2/IFNG/GEMIN6/SMN
14/SMC4/ITGB1BP1/KNSTRN/LIG1/KMT5A/RAB11FIP4/ANXA11/IL1B/BCAR1/CDK5/PDGFA/CDCA2
1/CCNJL/RPS27A/VEGFA/CCNE2/PLA2G1B/UBA52/DUSP7/MAP3K10/PIH1D1/CDC37/CDK5R1/PRKA
5A/PDCD5/FADD/BTK/STX4/STK25/RACK1/MSX1/DAP/RAD9A/TIMM50/IL20RA/DPF2/MYBBP1A/PT
SAP3/MYO1C/SPIRE2/NCKAP1/PPP1R9A/LPAR1/PXN
B6/GTPBP1/HNRNPD/PSMA3/DCP2/HNRNPC/FXR1/SYNCRIP/EIF4G1/PSMB7/HNRNPA2B1/SUPT6H/E

1/BOD1/SPIRE1/CCSAP/FBXW5/HNRNPU/SYCE1L/CCNE2/MASTL/RAB11A/KIF20B/KIF22/NCAPD3/
CACNB3/PLD2/PSMC3/STOML2/WIPF2/CD79B/CD38/RAPGEF1/ELMO2/PSMC5/UBE2N/CD247/INPP5
NANOS1/ETF1/RPL19/PSMC3/RPS11/EXOSC1/EIF3E/RPL13A/HNRNPR/RNASEH2C/POLR2D/ANP32A
/DHX9/EDC4/RPL32/TNPO1/RPL30/PSMC5/CNOT10/RBM10/MEX3D/RBM24/CNOT3/LSM1/RPS9/KHS

MSAP1/ARFGEF1/CIB1/EVI5L/TMEM67/ADD1/FEZ2/PPP1R9A/IK/UCN/PHF1/CAPZA2/DCP2/BUB3/

/PPP1R9A/LPAR1/PLEKHM1/CCDC88A
7A/RPTOR/PREX1/ARFGEF1/ADNP/ADD1/RYK/MYO1C/SPIRE2/FSTL4/NCKAP1/PPP1R9A
9/DPH3/UTP23/RPP30/SUV39H1/PRKRA/PELP1/RPS9/WDR46/IMP4/TRMT2B/RBFA/RPS14/RPS27/
NRA/CAV1/ATP7A
/NELFCD/GTF2F1/CIB1/ZC3HAV1/LARP1/VPS4A/NECTIN2/YTHDF2/CTDP1/SRPK2/ZCCHC3/CNR1/

TG7/ATR/TP53BP1/HMGA2/ATAD5/MORC2/THOC1/PTPRC/HDAC8/PPARG/TTF1/TFIP11/SPIRE2/SM
/RHBDD3/NKAP/RHOA/GLMN/CD38/INHA/AP3B1/TNFRSF14/IDO1/RUNX3/ZMIZ1/CCL19/XCL1/PIK
PA1/SRF/CACNA1D/CD38/RFC5/MAPKAPK5/TPP1/SLC22A5/POLE3/RPA1/ANKRD11/SMARCAL1/STR
DM/BECN1/PSEN2/VASN/HSP90B1/SUV39H1/PDK3/ATG7/EDNRA/CAV1/ATP7A/ERCC3/STUB1/BMP2
S27/CIB1/RPL29/ZDHHC24/BAG6/SRP68/TRAM1/C2CD5/RPL13/ATP1B1/SSR1/MYO1C/RAB8B/RPL
AD21/MTBP/RFWD3/CEP250/RPA2/CUL4B/HAUS6/NAE1/NDE1/CKAP5/DBF4B/BTN2A2/RAD51C/BTG
XNDC12/IVNS1ABP/BCL2L1/PIH1D1/MEIS3/NDUFA13/TPD52L1/PLAGL2/STK3/YWHAG/IFI6/FAF1

US81

7/RPS6/RPF1/MRM2/RPL24/CHD7/RIOK1/RPL11/RPL10/RPS24/DDX31/ERAL1/WDR12/PDCD11/HE

3/RPS28/RPS17/NOLC1/GTF3C3/NOL11/ERI1/TP53/NSUN5P2/ZNHIT6/TSR1/RPP38/MRPS11

A/TPR/SRSF7/MCM3AP/NUP50/IFT81/USP46/IQSEC2

HLA-DOB/VAMP3/RAB3C/CTSL/CD207/RAB33A/PSMB7/HLA-E/NOD1/RAB7A/CLEC4A/ACE/NOD2/IK
/ZNHIT6/ATXN2/C1QBP/MRPS11/SRSF10/LSM14A/MRPL22/PRMT7/PSIP1/AAR2/XAB2/PTBP2/SNU

S2/RPS28/LUC7L3/GEMIN5/EIF3C/DDX1/LSM3/ZNHIT6/ATXN2/C1QBP/MRPS11/SRSF10/LSM14A/

0

PGEF1/MRAS/PDPN/RAB27B/MAPKAPK5/HEG1/IFT22/EPS8L1/KITLG/KSR1/SSX2IP/NOTCH2/RHOB
ADA3/SIN3B/HUWE1/PAF1/TRRAP/HDAC8/PWP1/DDB1/SUPT7L/ASXL1/USP3/MAP3K7/UCN/PRMT5/
PLEKHG6/ARHGAP28/ADPRH/AGAP3/RAPGEF1/GPS1/ACAP3/STXBP5/DOCK2/EPHA2/SGSM1/CCL19/
FA/TXN/MLKL/ADRA2C/FGR/LCP2/MAPK11/TGFB2/PTPN6/CALCA/MAP3K14/MIDN/SOCS3/TNIK/DV
ALR/PRKCI/PRRX1/ZMYND8/SEMA4D/SYT1/APP/DNM1L/SYT3/SEMA4F/GDPD5/HEY1/KATNB1/SEMA
RF1
F1/NPLOC4/TRAFD1/PSMB2/SH2B2/PDCD1/IRF7/ZC3H12A/PSME3/TNIP2/LAG3/FLOT1/ACTG1/NC
25/RALB/PIFO/MAPT/CSPG4/DBF4B/CEMIP/PTGES3/TRAF7/EEF1A2/CXCL17/ABL1/STK24/WRAP5

EC4E/ZCCHC3/NFKBIL1/PSMB1/TLR8/MAP3K7/PSMB6/FFAR2/CNPY3/MFHAS1/ICAM2/PSMA3/IRF4
PIN1/TFIP11/STK4/BTBD1/RPL11/EPB41/PARK7/IRF4/MBD2/IFNG/NSD1/EIF4G1/LHX2/SUMO3/

IM15/NLRC4/BPIFB1/PTPRS/CLEC4A/APPL2/RPS6KA3/TLR1/S100A1/NOD2/XRCC5
/MUL1/TRIM56/IKBKB/LILRB1/DDX1/FGL2/RPS15A/C1QBP/NLRP3/TICAM1/CD40/CCL11/IL12A/

PS27A/SFRP4/PIAS4/BCL9L/TLR2/CSNK2B/TMEM64/UBA52/GLI1/AP2A2/CCDC88C/MESP1/ATP6AL5/RPS27A/FBXW5/USP21/PLK3/MAGEF1/MAP1A/EXOSC9/EXOSC10/EXOSC7/USP27X/HSPA5/FBXOA/RPL23/RNF4/WDR91/UBE2L6/RACK1/TMEM199/RNF7/CUL4B/TRIM71/TRIM25/PTTG1IP/RNF122OML2/VPS11/YWHAG/ATG13/COA1/IFI6/COA3/ATG9A/NRF1/NDUFS8/TIGAR/CCAR2/BECN1/NDUFA8A

ABCE1/MRPL45

KHN1/EIF2B5/RPL38/XPNPEP1/DRG1/MRPL52/KHDRBS1/HPGDS/ABCG1/RPL23A/MCTS1/PIWIL4/M
L/RPL23/RNF4/UBE2L6/RACK1/RFWD3/NOSIP/RNF7/CUL4B/TRIM71/TRIM25/ZNF598/UBE2O/PTT
7A/FBXW5/USP21/PLK3/MAGEF1/MAP1A/USP27X/HSPA5/FBXO45/UBA52/CD2AP/EDEM2/TRPC4AP/
KLHL42/VCP/PSMA1/TNFAIP3/USP5/NSFL1C/UBQLN4/RPS7/KCTD5/FOXF2/BAG2/RNF216/PSMB5/
OTCH1/RPS27A/SFRP4/PIAS4/BCL9L/TLR2/CSNK2B/TMEM64/UBA52/GLI1/AP2A2/CCDC88C/MESP
/XYLT2/UGDH/POMT2/FUT3/NUP85/MUC5B/NUP43/DPAGT1/GMPS/IMPDH2/BGN/PARP1/IL15/A4GA
BID/MT3/BCAP31/EEF1D/TNIP2/DYNLT1/DVL3/KISS1/MLST8/FGFR3/ADAM8/CPNE1/CCL2/SHC1/
GALT6/HS6ST2/ARFGEF1/FBXO6/TMEM106A/HS2ST1/ST8SIA4/BMP2/RAB1A/GCNT1/CST3/B3GNT8
T4/ARFGEF1/PTPRC/LMAN2L/VPS4A/NECTIN2/RAB1A/BAG6/RFX2/ATP2A2/ABCA7/STAM/TMEM38A
IM27/AAAS/SETDB1/TRIM62/CD4/APOBEC3F/WWP2/HYAL2/CTSB
U2F2/GON4L/PPP2R3C/TSC1/RPL22/FUT7/CD80/TP53/SOX13/RASGRP1/FGL2/LILRB2
MA4/PTGER4/PSMD9/CASP8/RPS27A/MAVS/DMBT1/OPTN/PAK2/TLR2/MUC6/UBA52/HLA-F/MUC15/
/MAPK15/HAUS2/IQCG/ATG3/CDK5RAP2/PTPDC1/ACTR2/IFT140/CNTROB/CEP135/TMEM80/RILPL

17/FRMD6/ADD1/ASAP3/MYO1C/ATP2A2/SPIRE2/ABRACL/NCKAP1/PPP1R9A/LPAR1/PXN/CCDC88A
3/CELSR3/TUB/MAPK15/HAUS2/IQCG/TTC21A/ATG3/CDK5RAP2/BBS12/PTPDC1/ACTR2/IFT140/C

21/PLK3/MAGEF1/MAP1A/USP27X/HSPA5/FBXO45/UBA52/CD2AP/EDEM2/TRPC4AP/RNF213/PSMC3
119/TESMIN/HOXD9/AXL/PRDX4/ANGPT2/MMP19/CCT8/KHDRBS1/HPGDS/TTC26/WNT4/SOHLH2/PI
TDRKH/HSF2/APP/MYBL1/NCOR2/CETN2/TMEM119/TESMIN/AXL/PRDX4/ANGPT2/MMP19/APOL2/KH
P/MYBL1/NCOR2/CETN2/TMEM119/TESMIN/AXL/PRDX4/ANGPT2/MMP19/APOL2/KHDRBS1/HPGDS/T
/TM9SF4/C2CD5/CNR1/MYO1C/ATP2A2/CASK/YIPF5/TBC1D20/EFCAB7/RBM22/AAAS/PARK7/USP3

PH3L/CENPJ/CACNB3/ALOX15B/CDK5R1/UBR5/TWIST1/APBB1/GLMN/VPS11/CD38/YWHAG/ATG13/

1/HDAC2/CSNK1D/KIF14/HCLS1/AXIN1/SLC11A1/LYN/WNT7B/ITGB1BP1/BIRC7/GPRC5B/RAC1/U
RFGEF1/RIC8B/ARHGAP17/LLGL2/RALBP1/ARHGAP33/EVI5L/ASAP3/RALGAPB/RSU1/DOCK8/PIN1
C/MAEL/ZNRD1/TP53/EIF1/SUZ12/MSL3/BAZ1B/TPR/EZH1/PRMT7/NUP50/STAT3/TWISTNB/JARI

VOC/APH1B/EPS15L1/LOX/MVB12A/CADM4/ATP6V0E1/ARF4/MATK/GRB7/NCOA5/PRKD2/SMARCC1/
/ERCC1/PRSS21/HEXB/MASTL/BCL2L1/GLI1/MEA1/SSTR2/TARBP2/GORASP2/TEX11/IFT20/RNF8
RACK1/ZNF675/IGBP1/RGS3/TNIP1/IGF1R/MAPT/STK38/NF2/BTN2A2/EMILIN1/UBXN1/PKIA/AB

/TSC1/TSG101/TP53/FEZ1/SPTLC2/CHMP4A/SMCR8/MCOLN1/ATP6V1D/CLEC16A/VTA1/RNF41

11A1/LYN/WNT7B/ITGB1BP1/BIRC7/GPRC5B/RAC1/UNC5CL/IL1B/RAP2A/CDK5/PDGFA/LAMTOR1/
10/PIH1D1/DGKQ/CDK5R1/PRKAR1B/TPD52L1/DAXX/RHOA/STK3/RAPGEF1/ATG13/ATP2B4/PRKAG

3/SUDS3/YOD1/USP28/PSMD6/GATA3/USP4/TGFBR1/PSMC2/USP32/PSMA6

LR2/HSPA5/DDX56/RAB11A/KIF20B/PACSIN1/PPP2R5B/METRN/APBB1/SRF/CAPRIN1/RHOA/RAPG
EMA3E/MAPT/RERE/SSBP1/CHN1/ZNF335/EXT1/ABL1/STK24/VCL/RASAL1/MFF/NFATC4/OPA3/KE
2/NFATC4/GDF11/KEL/DENND5A/DBNL/PLXNC1/DNM2/PTK2/STYXL1/NOTCH1/FIG4/MOB2/LLPH/V
RERE/CHN1/ZNF335/EXT1/ABL1/STK24/VCL/RASAL1/NFATC4/KEL/DBNL/PLXNC1/HSP90AA1/SRG
A-DRB5/CARD11/GPRC5B/FPR3/MUC3A/RAC1/CBFB/PSMB4/IL1B/ACTB/BCAR1/CTSS/CD47/PSMB8
EURL1/EIF4E2/PYM1/EIF2B1/AGO1/EIF5B/FXR1/EIF2AK1/SYNCRIP/EIF4G1/CNOT2/EIF2B2/SE

HA2/FAM3A/SEH1L/TLR7/ATG7/ZC3HAV1/PTPRC/CLEC4E/RAB1A/ZCCHC3/CXCL10/IFITM2/SKP2/
K2/ADNP/NAIF1/EAF2/PPARG/RYK/PIN1/FSTL4/UCN/TRIM32/SEMA3F/MEGF8/TCHP/KAZALD1/HY

USP32/PSMA6

LC3A2/SIRPG/CYTH2/FLOT1/EHD1/ACTG1/NCKAP1L/SH3KBP1/VLDLR/MYO1G/ATP6V1H/CAP1/CCL
/LZTS1/ZMYM4/RAB11A/IFRD1/CDK5R1/METRN/FAM171A1/SRF/CAPRIN1/RHOA/ENPP2/ACTN4/PD
/LMO4/PTK2/THBS1/ADAM17/PTPN1/RPS27A/NBN/VEGFA/PFKFB2/PLA2G1B/PAK2/TRAF4/UBA52/

CTB/CDK5/JCHAIN/DNER/CD47/ANKRD13B/CTTN/FGR/CD300LF/LOXL2/WAS/CACNG4/PI4KB/NECA
SCAN2/METTL3
RAP1/CASC3/SNRNP70/RBM4B/YTHDF1/RBM8A/METTL3/PIN1/MOV10/ABCA7/EIF2B4/UCN/OGFOD1

/LEF1/POLR2H/SFRP5/FGFR1/HIP1R/SLC9A3R1/NCBP2/MT3/FERMT1/ACTG1/NCKAP1L/FGFR3/SH

35/EXT1/ABL1/STK24/VCL/RASAL1/NFATC4/KEL/DBNL/PLXNC1/HSP90AA1/SRGAP2/DNM2/PTK2/

/GRB2/PARD6B/AKIP1/LLGL1/CDC42/LAMB3/CALR/MATN3/SEMA4D/APP/DNM1L/MAPK7/SEMA4F/A
7/BRD4/RELA/NSD1/VPS33B/DYDC2/KMT2B/NAA50/SUPT6H/KDM3A/SUMO3/WBP2/SENP5/ZMPSTE2

AIP1/RNASE2/LYN/IL1B/LAMTOR5/IL2RA/APOBEC3G/JCHAIN/CD47/RNF26/TMEM229B/FGR/CCL4
EL/DBNL/PLXNC1/HSP90AA1/SRGAP2/DNM2/PTK2/NOTCH1/MAP1A/LLPH/VEGFA/RAB3A/LZTS1/FB
AIP1/RNASE2/LYN/IL1B/LAMTOR5/IL2RA/APOBEC3G/JCHAIN/CD47/RNF26/TMEM229B/FGR/CCL4
8/HSP90AA1/RPS15/SGTA/RPS16/CASP8/RPL5/RPS27A/SH3GLB1/RPS12/TOMM22/MAVS/RPS4X/R

37/CDK5R1/TECPR1/VPS37B/VPS11/ATG13/IFT20/CLTC/PRKAG1/MTERF3/FNBP1L/WDR41/ATG9A
1/DEFB1/SLC11A1/PMAIP1/RNASE2/LYN/IL1B/LAMTOR5/IL2RA/APOBEC3G/JCHAIN/WFS1/CD47/
L10/TRIM71/TNIP1/DPH7/ZNF598/PLEKHN1/EIF2B5/RPL38/DRG1/MRPL52/KHDRBS1/RPL23A/CE
37/CDK5R1/TECPR1/VPS37B/VPS11/ATG13/IFT20/CLTC/PRKAG1/MTERF3/FNBP1L/WDR41/ATG9A

D247/MSH6/IL13RA2/TP53BP1/CD84/ATAD5/THOC1/DCLRE1C/INPP5D/DUSP22/ZNF683/CD3G/PT

1/EPHB2/SLC9A6/EPHA10/YTHDF1/ADNP/FEZ2/CNR1/RYK/PIK3CB/PLXNB2/KIF3A/FSTL4/PLCG1
C3/HDAC5/TAF3/CIB1/PKD1

B/ANXA4/GSDMD/ACSL3/BARD1/BAG3/ICA1/IL4R/TGFB3/ADRA2A/SNX12/TLR5/HK1/GZMB/DNM1L
4/MUL1/TSC1/USP33/CPTP/TP53/FEZ1/SPTLC2/DAPL1/CHMP4A/FBXL2/TFEB/KAT5/SMCR8/MTDH

8/ADRA2C/PSMB10/PHB/PSMA5/CCL4/MAPK11/NRAS/TGFB2/PTPN6/PSMD13/RASA3/CD27/PSMC4/
CD79A/UBE2D1/PSMD10/PSMD11/PSMD1/C5AR1/PSMA7/RPS3/PRKD2/TRAF3/PSMB3/GRB2/ARRB2/
/ADD3/LIMA1/RAB11A/IFRD1/CDK5R1/ARHGAP28/SRF/DSTN/PLEKHH2/RHOA/HTR1D/CD38/MTPN/
CP/PSMA1/SFRP2/TNFAIP3/NFATC4/PLCB2/PSMB5/PSMA4/PRKAA2/PSMD9/CSNK2A1/VPS35/NOTC

F/RHOA/CD38/HBEGF/MTPN/NIN/CCAR2/CYP27B1/IST1/EIF4G2/CDK11A/OGFR/RPTOR/CIB1/IL1

MTMR1/DUSP14/PTPRA/PPP1CB/DUSP15/PTPRS/PPP2R5D/CNEP1R1/PTPN14/TSC1/PPP2CA/PTPDC

8/ADRA2C/PSMB10/PHB/PSMA5/CCL4/MAPK11/NRAS/TGFB2/PTPN6/PSMD13/RASA3/CD27/PSMC4/

RPL24/ETV1/SEMA3F/MEGF8/EGR2/ULK1/CELSR3/TRIM46/XK/SEMA6B/TRIO/LHX2/PTPRA/SEMA4

PC9/FASLG/HPS4/TGFBRAP1/SAMD9/STX8/MFSD14A/RAB38/GBF1/SNAP23/STX12/ZEB2/CTSZ/TR

APC1/UBE2K/PSME2/TRIM5/TRIM3/TRIM36/HACE1/UBE2V2/PSMD6/GPS2/FBXO30/PSMC2/PSMA6/
MAP4K3/DACT1

H1/SDF4/CXCL10/RBM4B/METTL3/DDB1/JUNB/SCARA3/TRIM32/CASP3/PDE6G/NMT1/MYC/HUS1/F
A/LAMTOR5/CAPNS1/CDK5/LAMTOR1/CTTN/PSMB8/PSMB10/PHB/PSMA5/DNAJB2/TAF9/PSMD13/NU

F1/MYB/WDR82/SETD5/PICK1/METTL22/GATA3/NSUN2

SK3A/NUP43/OSBPL7/PML/DRAM2/SET/ATP6V0C/USP14/PSMD10/PSMD11/RAE1/PSMD1/ATP6V0E1

1/SFRP2/RPS7/IFT172/BCL10/ACVR1B/VASH1/OSR2/LMO4/DAD1/CASP8/NOTCH1/FUT8/MBD3/FK
ARHGAP19/ARHGAP25/SRGAP1/TRIO/MAP4K4/RHOD/PIK3R2/EPS8L2/KCTD13/VAV3

1/CDHR2/ABL1/GNG4/RASAL1/SFRP2/GHRHR/BRAT1/OPA3/SIPA1/TMC8/WWC1/DBNL/TBX2/ACVR1
NDC12/IVNS1ABP/BCL2L1/CD2AP/DUSP7/PPP2R5B/NDUFA13/TWIST1/ADGRG3/RHOA/RAPGEF1/AT

NC2/EXT1/ABL1/SFRP2/RPS7/IFT172/BCL10/ACVR1B/VASH1/OSR2/LMO4/DAD1/CASP8/NOTCH1/

T2/KIAA0319L/LMTK3/HLA-B/QPCTL/GALNT6/GOLGA3/TRAPPC1/STX18/FUT2/FUT6/ICA1/PARM1
8/PCGF2/XRCC6/TELO2/RAD21/NIFK/NABP1/RBBP7/RAD9A/RPA2/DPF2/POLD3/INO80C/TIMELES
SAH1/CLN3/SLC36A1/BBC3/SLC11A1/RNASE2/LYN/HLA-DRB5/SLC26A11/ANXA11/ATP6V1E1/IL1
/RBMX/KIF22/BAZ2A/NCAPD3/EIF3E/SRF/HMGN2/ACTR5/GATAD2A/DHX9/SMARCA5/STAG3L4/RUN
MT/ACTL6A/IRF1/COPS9/ARPC2/DYNLL1/STAT1/SPDL1/XRCC3/RRS1/KNTC1/HDAC1/PARPBP/ARP
/GPRC5B/ZNF593/SSRP1/UTP18/RBM19/MAGED2/TULP3/S100A13/UTP14A/RPL7/ETV6/NPM3/RCN
SAH1/CLN3/SLC36A1/BBC3/SLC11A1/RNASE2/LYN/HLA-DRB5/SLC26A11/ANXA11/ATP6V1E1/IL1
VPS11/AP3B1/CLTC/AP1S1/AP1S3/AP1S2/AP2B1/WDR41/IFITM3/MANBA/SPNS1/BRI3/SNAPIN/A
HLA-DQA2/LAMP3/CPNE1/ATP6V1H/CAP1/KCNQ1/PIK3CD/ASAH1/CLN3/SLC36A1/BBC3/SLC11A1/
T1/IQCB1/EMD/TBCD/RAD51D/PSMA1/NSFL1C/DCLRE1B/RPS7/CALM2/AKNA/NME7/CEP19/CEP41/
7orf31/ABCA2/HAUS8/DYNC1LI1/MLF1/PLK4/IFT80/TUBG2/TBC1D31/MVB12A/KIF2A/ODF2/PTP
7/KATNA1/PLK3/HNRNPU/HAUS5/MAP7D3/RAB11A/PLEKHG6/NEDD1/EML2/KIF20B/KIF22/ARL8B/
CNN2/CDC42/CALR/MTF2/SPRY4/AFAP1/RPS19/RPLP0/RPL18/CBL/RPL23/ABI2/VIM/ITGB8/RPL
P8/RAB5C/WIPI1/VPS35/VPS45/RPS27A/SCAMP2/CFTR/PDLIM4/LTV1/FIG4/MCOLN3/OPTN/PACS
NQ1/FZD2/SV2A/MSR1/SYNGR3/KDELR1/SLC11A1/RAB20/HLA-DRB5/WNT7B/GPRC5B/MCTP1/RAC1
C5/TNNI3/ABL1/VCL/ZNF185/TRIP6/ACTR3/BIN2/CENPQ/DBNL/PTPN12/VCAM1/CTNNA1/PTK2/A
M25/UBE2O/POLR3D/HNRNPM/S100PBP/DRG1/MAPT/PRPF31/OBSCN/FAM118B/SNRPB2/PRPF19/CH
/PEF1/ATP7A/CALM3/CUZD1/RAB1A/RAB8B/CD59/RAB1B/CCDC88A/NCSTN/SYT11/HLA-C/MCTP2/
3/DYNLL1/DYNC1H1/ILF2/AMPD3/NPC2/NBEAL2/FSTL3/OLR1/SLC2A5/GPI/PA2G4/SERPINB1/PP
1/SLC11A1/RAB20/HLA-DRB5/WNT7B/GPRC5B/MCTP1/RAC1/TMEM163/WFS1/LAMTOR1/CD47/SMAG
APG2/NUP37/PPP1CA/PPARD/INCENP/CBX3/DVL3/BLM/NHP2/SCMH1/ITGB3BP/HDAC2/AUNIP/CDK
39A13/HLA-DQA2/LYPLA2/CSNK1D/SLC35B2/KDELR1/CLN3/SLC35A2/DEFB1/UXS1/HLA-DRB5/WN
A2/SLC15A3/AP2S1/OSTM1/BGN/UBA1/ATP6V0C/PSMD1/TMEM63A/KPTN/PADI2/YWHAB/SLC22A17
IP3/SLC48A1/GGA1/HLA-DOA/STX7/SNX20/RUFY1/VAMP8/RAB5C/WIPI1/VPS35/VPS45/RPS27A/
INO80C/TIMELESS/POLR3D/PRPF19/ESCO2/NAT10/RARG/DNA2/HSPA2/RAD51D/MTA3/DCLRE1B/E
BPL7/RAB10/GDPD1/OSBP2/SET/SBF1/CDK7/TNFSF12/NDOR1/YWHAB/GALNT6/ANXA4/ACSL3/CAL
HAB/RPS3/RPL27/RALA/CNN2/CDC42/CALR/MTF2/SPRY4/AFAP1/RPS19/RPLP0/RPL18/CBL/RPL2
/PDGFA/RTN3/NRAS/B4GALT2/XYLT2/PI4KB/COPZ1/FUT3/CLTB/RAB10/SMPD4/CIT/LST1/A4GAL
VPS11/AP3B1/CLTC/AP1S1/AP1S3/AP1S2/AP2B1/WDR41/IFITM3/MANBA/SPNS1/BRI3/SNAPIN/A
C3/BEX4/WIPF1/LZTS2/MARK2/PDLIM3/SH2B2/KRT18/INCENP/CORO1B/KIFC2/CCT4/MT3/TPPP3
C3/BEX4/WIPF1/LZTS2/MARK2/PDLIM3/SH2B2/KRT18/INCENP/CORO1B/KIFC2/CCT4/MT3/TPPP3
A/MNDA/PTGES2/RAP2B/GYG1/ARHGAP9/RHOF/STX6/BMF/SIRPA/RAB3B/DEGS1/BIN1/HK3/CCT2/
/WIPF1/LZTS2/MARK2/PDLIM3/SH2B2/KRT18/INCENP/CORO1B/KIFC2/CCT4/MT3/TPPP3/DYNLT1
LS1/PSTPIP1/HSPH1/CCT7/DCTN2/KNSTRN/REEP2/FKBP4/RAC1/CDK5/FAM83H/CTTN/TCP1/KIFC
1/TTF2/PRPF6/RPL27/BARD1/HNRNPAB/NCL/FTSJ3/HENMT1/EIF3H/DDX41/CDC42/AGO2/CBX5/C
TAF4/SLX4/ACTR8/NACC2/MEN1/NFRKB/SP1/TNKS1BP1/ACTR6/NFATC1
CCSAP/DCTN1/HAUS7/KATNA1/BICD1/HNRNPU/MAP1A/HAUS5/DYNC1I2/RAB11A/TUBB3/EML2/KIF
15/CTNNA1/DNM2/PTK2/RPS16/ADAM17/RPL5/CAPN2/FLII/RPS4X/RPL6/ACTN1/PACSIN2/HSPA5
NNA1/DNM2/PTK2/RPS16/ADAM17/RPL5/CAPN2/FLII/RPS4X/RPL6/ACTN1/PACSIN2/HSPA5/LIMA
12/RPS15/CTNNA1/DNM2/PTK2/RPS16/ADAM17/RPL5/CAPN2/FLII/RPS4X/RPL6/ACTN1/PACSIN2
/IGF2R/MOSPD2/SPARC/DGAT1/ATP6V0A1/VAMP1/ARL8A/DNAJC13/MMP25/RAB7A/UBR4/MCEMP1
AP/TUBB3/PACSIN1/DUOX1/TLN1/APBB1/PLEKHH2/RHOA/PDPN/FAP/PARVB/DPYSL3/EPS8L1/EPH

R/SH3GLB1/MAN2B1/LTV1/MCOLN3/CCZ1B/GNAI3/GLIPR1/MYO6/TMBIM1/SLC17A5/SLC15A4/UBA
EMIN5/MRPL21/RPS15A/NDUFA7/MRPS26/APEX1/MRPS11/RPL31/NUFIP2/MRPL42/BTF3/MRPL22/
PL22/GEMIN5/IKBKB/RPS15A/DAG1/CASP10/RPL31/NLRP3/TICAM1

IRF7/SNX10/RAB24/FLOT1/PRSS16/EHD1/CD68/ARHGAP1/LEPROTL1/HLA-DQA2/LAMP3/CTSE/KC
/DPF2/POLD3/INO80C/POLR3D/IGF1R/DCAF15/PDCD6/POLR3H/PRPF31/POLR3C/MED12/EID3/ZN

GTF2E1/KAT5/APEX1/MED14/TAF4/SNAPC1/TAF2/GATA4/NFATC1

IF5A/DNAJC2/IPO9/NXT2/FAF1/PSEN2/RBM15B/TNRC18/TOR3A/IST1/KPNA6/SEH1L/RANBP3/HA

V0A1/HLA-E/RAB7A/APPL2/TLR1/MPEG1/CD163/PLEKHG5

DDHD2
/KPTN/ARF4/PSMA7/LRP8/SYT12/RPS3/RIMS3/ICA1/ARRB2/DROSHA/CDC42/VPS18/ZMYND8/SYT

TTL3/EAF2/ACIN1/PIN1/TFIP11/IK/PPP1R8/PSKH1/POLDIP3/THOC7/USP36/ZC3H18/THOC3/DY

YP/TSC1/SHTN1/EXOC6/RAC3/UNC13A/MAP3K12/ELK1/FEZ1/NEFL

/CTBP1/GMFB/MAEA/ATG7/HAX1/CAV1/CCDC65/RIC8B/CNKSR1/ACTR1A/LLGL2/BBS7/CCDC40/C2

PS35/MRPS33/MRPS10/NDUFA2/MRPL45/NDUFB10/BCS1L/NDUFB5/AGK/NDUFAB1/MRPL41/MRPL49

F4A3/LMTK3/DMWD/ARF4/LRP8/RPS3/ARRB2/CDC42/AGO2/TMEM50A/TGFB3/ZMYND8/APP/GAL/KL

/NLGN2/RPS25/ADAM10/CHMP2B

/DHX9/SMARCA5/DNAJC2/STAG3L4/GTF2H1/CRTC2/RBPJ/TNRC18/CDYL/UXT/SUV39H1/MSH6/PEL
RA/MED27/BCL9L/ACTN1/HSF4/COPS5/MAP3K10/MECP2/CENPJ/AIP/RBM14/TCERG1/NMI/DAXX/A
K1/PLK3/CCNE2/MARK4/PAK2/MASTL/CDK11B/CSNK2B/TBRG4/LMTK2/MAP3K10/TP53RK/CDK5R1/
7/RPL18A/COL8A2/EPB41L1/RPS19/ECM1/RPLP0/RPL18/RPL23/VIM/KRT20/COPG1/EPB41L3/MR
11A/BCL10/SRGAP2/UBA2/TBC1D22B/ARHGEF11/RAB3A/SMAP1/PAK2/PLEKHG6/DGKQ/PPP2R5B/C
SP90AB1/ARPC2/MYO19/CAPN6/PLEKHM2/GAS7/RCAN3/PPP1R9B/TIGD5/ARPC3/BEX4/WIPF1/S10
/PIP4K2C/RPS6KB2/BTK/DSTYK/STK25/NME6/VRK1/ITPKC/CSNK1G2/IGF1R/STK38/OBSCN/CCL3
C2/DDX27/RAB24/DYNLT1/GTPBP2/BLM/GTPBP4/ITPA/TUBB4A/MYO1G/ATP6V1H/MYO1F/ARHGDIB
C2/DDX27/RAB24/DYNLT1/GTPBP2/BLM/GTPBP4/ITPA/TUBB4A/MYO1G/ATP6V1H/MYO1F/ARHGDIB
C2/DDX27/RAB24/DYNLT1/GTPBP2/BLM/GTPBP4/ITPA/TUBB4A/MYO1G/ATP6V1H/MYO1F/ARHGDIB
1/GTPBP2/BLM/GTPBP4/TUBB4A/MYO1G/ATP6V1H/MYO1F/ARHGDIB/KIF14/DCTN2/RAB20/RAC1/A
3A/MIDN/DVL2/PARP1/GCN1/CDKN2C/PTPN23/CADM4/MSH2/GRB7/CCND2/RPS3/POLA1/ACSL3/DU
F3/GRB2/ARRB2/PARD6B/LLGL1/CDC42/MTCP1/SLC12A7/ADRA2A/TELO2/MAPK7/PPP1R15A/CD8A
XND1/ADAM19/LCK/PTPN6/BANF1/SRSF12/CASP1/PPP1CC/MLF1/CIT/DVL2/INPP5J/MVB12A/GPX
1/MARK2/ARHGAP39/GDI1/LEF1/SERPINB1/PSME3/ARFGAP1/HSPBP1/MT3/TBC1D10B/CCNO/PPME
P/TACSTD2/YWHAB/GOLGA3/CAPZB/BAG3/CNN2/ARHGAP18/CALR/RANBP1/AHSA1/SWAP70/SH3GL1
IFO/BICDL2/FGD1/NSF/TBC1D22A/OBSCN/BRK1/GGA1/IPO13/HPS1/DENND3/TBC1D7/DENND5A/S
/WIPF2/DSTN/PLEKHH2
K2/CSNK1G1/CASK/STK4/MAP3K7/PSKH1/RIOK1/BCR/ULK1/DYRK3/BRD4/EIF2AK1/TLK2/MAPK15
2/ERCC3/VPS4A/RALBP1/ATP1B1/MYO1C/AK6/ATP2A2/ACIN1/KIF3A/ABCA7/ABCF3/ATP2C1/ATP
PAK2/RUSC2/PLEKHG6/DENND4B/RAB29/YIPF2/XPO7/ADPRH/RASSF5/RAPGEF1/IFT20/STXBP5/D
GGTB/PAK2/RUSC2/GGA3/RILPL1/PLEKHG6/DENND4B/RAB29/YIPF2/XPO7/ADPRH/RASSF5/RAPGE

B/ATP1B1

3E/EXOC3/FNBP1L/RSL1D1/SPTAN1/EPS8L1/EPHA2/IST1/VASN/EIF4G2
2/GEM/RAB6A/GNAS/TBCC/RAB28/TSR1

3B

R/STK32C/USP14/LMTK3/CDK7/COASY/MATK/CCND2/POLE4/POLA1/PRKD2/DGUOK/NANS/PGS1/ST

/IGF1R/STK38/OBSCN/CCL3/CDK19/ACVR1/FASTK/ABL1/STK24/PIM3/SRPK1/CSK/CHEK2/MAPKA

NF40/CCDC50/HLTF/PCBP2/GABARAP/USP13/UBE2K/STX8

PBP1/MFHAS1/ERAL1/RHOH/RAB22A/RAB3C/ARL11/POLR2B/ARL3/EIF5B/NUDT2/RAB33A/EIF2B2
1/RHOH/RAB22A/RAB3C/ARL11/ARL3/EIF5B/NUDT2/RAB33A/EIF2B2/ARL8A/RAB2A/RHOD/RAB7A

2A/RAB3C/ARL11/POLR2B/ARL3/EIF5B/NUDT2/RAB33A/EIF2B2/ARL8A/RAB2A/RHOD/RAB7A/TUB
2A/RAB3C/ARL11/ARL3/EIF5B/NUDT2/RAB33A/EIF2B2/ARL8A/RAB2A/RHOD/RAB7A/TUBD1/NOLC

/ARL3/EIF5B/NUDT2/RAB33A/EIF2B2/ARL8A/RAB2A/RHOD/RAB7A/TUBD1/NOLC1/RAC3/RRAS2/G

G/RAB22A/RAB3C/ARL11/ARL3/EIF5B/NUDT2/RAB33A/EIF2B2/ARL8A/RAB2A/RHOD/RAB7A/TUBD
G/RAB22A/RAB3C/ARL11/ARL3/EIF5B/NUDT2/RAB33A/EIF2B2/ARL8A/RAB2A/RHOD/RAB7A/TUBD

VHL/MTA3/NFATC4/TDG/FOXF2/TRIP6/CIITA/WIPI1/BCL10/TBX2/PSMD9/LMO4/USF1/UBA2/CHC

AML2/GATA4/WNT3A/MED6
2/THUMPD2/POLR1C/POLR3A/PNPT1/RAD54B/ERI1/ZNRD1/DDX1/TDP2/METTL2B/AARS2/METTL6/

/RNF186/KCTD13/UBR4/RNF19A/MUL1/TRIM56/RNF10/RNF135/FBXL18/FBXL2/MIB2/UBE2R2/PP

2/PRRX1/HSF2/ZNF589/MYBL1/CC2D1B/GMEB1/XRCC6/HOXB7/TESMIN/MUTYH/HOXD9/HEY1/GTF3

/WRAP73/RNF4/CCDC120/CEP250/HAUS6/PARD6G/NDE1/CLIP3/DRG1/MAPT/CKAP2/TTC26/NTMT1
1/PSMD2/RGS14/CENPE/PPP2R1A/RBBP8/CHMP4C/BRCA1/RBL1/NSMCE2/PCNA/TUBB6/PPP2R2C/X
WHAH/KIF14/WNT7B/ITGB1BP1/RAC1/CNTNAP1/ACTB/RAP2A/CDK5/S100A13/CTTN/RNF157/PSMB
/PQBP1/PTPN6/IFI16/AGPAT2/CASP1/PML/IL12RB1/IL15/C5AR1/IL1R2/RPS3/ANXA4/GSDMD/P
ANXA4/GSDMD/PRKD2/TRAF3/ASB1/LUM/ARRB2/CD6/POLR2L/DDX41/IL4R/TGFB3/CMKLR1/APP/A
/KPNA6/XCL1/TLR7/POLR2E/OSM/IL17D/IL17RB/PANX2/PTPRC/CLEC4E/CRLF2/ZCCHC3/TLR8/M
2/PSMD9/LMO4/ADAM17/NOTCH1/MTHFR/HOXA5/VEGFA/CSNK2B/AP2A2/MESP1/KIF20B/SYNE4/PS
P1/PIK3CD/ASAH1/KPNB1/SLC11A1/RNASE2/LYN/RAC1/CTSS/LAMTOR1/GNS/CD47/S100A13/FTL
CR1/SLC11A1/RNASE2/LYN/RAC1/CTSS/LAMTOR1/GNS/CD47/S100A13/FTL/NFAM1/FGR/LCP2/PS
O/DYNC1LI1/GALNS/DSN1/SLC44A2/IMPDH2/ABR/ATP6V0C/PSMD11/PSMD1/C5AR1/TMEM63A/PAD
/TMEM63A/PADI2/TRAPPC1/GSDMD/STK10/GPR84/CNN2/ALDOC/LAIR1/PNP/CYSTM1/XRCC6/PSMD
P1/PIK3CD/ASAH1/KPNB1/SLC11A1/RNASE2/LYN/RAC1/CTSS/LAMTOR1/GNS/CD47/S100A13/FTL
TSTA3/CD68/NCKAP1L/ADAM8/CAPN1/MYO1G/CPNE1/C1orf35/CAP1/ULBP2/PIK3CD/ASAH1/KPNB
/PSMD1/C5AR1/TMEM63A/PADI2/TRAPPC1/GSDMD/STK10/GPR84/CNN2/ALDOC/LAIR1/PNP/CYSTM
1/MLF1/IL12RB1/FGFR2/CYP26B1/CD79A/LYAR/OSTM1/PARP1/IL15/PSMD10/PSMD11/PTBP3/LT
RUNX3/ZMIZ1/LYL1/RBPJ/EPHA2/KITLG/CCL19/NOTCH2/PIK3R6/DOCK10/HAX1/ATP7A/PKNOX1/
1/HSP90AB1/F2RL1/ARPC2/STAT1/RAB7B/LRRC14/CTLA4/ARPC3/WIPF1/NPLOC4/CD83/PSMB2/S
LEC7A/TNIP1/EIF2B5/LBP/ARPC1A/BTN2A2/BRK1/HSPD1/PSMD8/ARPC5/ABL1/FCGR2B/PSMA1/T
UBE2N/CD247/INPP5D/DUSP22/CD3G/PTPRC/NECTIN2/CLEC4E/BAG6/BTN2A1/PIK3CB/MYO1C/PS
P4/NHP2/NONO/AUNIP/CDK2/KPNB1/DTD1/KDM1A/FANCA/CCT7/NASP/CHRAC1/LIG1/PRIM1/EHMT
10/SPIRE1/CEP164/RPS27A/NBN/MAGEF1/PIAS4/RAD18/ERCC1/UIMC1/COPS5/UBA52/MORF4L2/
/SUV39H2/ZNF335/TAF12/CHTOP/TAF10/DOT1L/MTA3/TDG/SRPK1/CENPQ/PRKAA2/CHD1L/HDAC1
GO2/SF3A3/CPSF4/RPL35/RBPMS/APP/NCOR2/POLR2K/DNAJC8/KTI12/DDX54/PABPC4/FUS/ZBTB
RNP/RPS4X/RPL6/C8orf88/MAGOH/RPL37/RPL27A/RCC1L/COPS5/WARS2/EIF1AD/RPL36/UBA52/
1/F2RL1/PRSS3/DYNLL1/DYNC1H1/ILF2/AMPD3/NPC2/NBEAL2/OLR1/SLC2A5/GPI/PA2G4/SERPI
/TRPM4/CCRL2/MAPK7/SEMA4F/RPS19/STX4/SEMA5B/ANGPT2/SCN1B/SEMA3C/GPR183/SEMA3E/L
SFPQ/THOC5/IFI16/PML/COPS7B/ISY1/PARP1/UBA1/PSMD10/RNF113A/CRIP1/DONSON/TIPIN/C
/KIFC3/VBP1/KIF15/STMN3/RASSF1/SFPQ/POC1A/AP3M2/CHMP3/GSK3A/HAUS8/DYNC1LI1/TUBB
C1LI1/PRCC/PLK4/ANAPC11/UBE2D1/PSMD10/PSMD11/RAE1/CDKN2C/PSMD1/DONSON/TIPIN/ODF
PN1/SIX2/SHC1/HDAC2/CDK2/KIF14/HCLS1/KDM1A/CD209/LYN/ITGB1BP1/CARD11/TNC/ITGA2/
POLR2E/CCAR1/KHSRP/MFAP1/PRPF4/THOC1/GTF2F1/DHX38/DBR1/GEMIN2/PABPN1/SRSF1/SRSF
ET4/FBXO6/PKD1/STUB1/ANAPC4/BAG6/BBS7/FAF2/TMEM67/DDB1/FBXO44/SKP2/PSMB1/WWTR1/
EHMT2/RAB11FIP4/CDKN1C/PSMB4/IL1B/CDK5/CHORDC1/PSMB8/PSMB10/PSMA5/SFPQ/POC1A/PT
DC42EP2/GZMB/DNM1L/RAD21/PDCD5/KATNB1/PIP4K2C/GMFG/WRAP73/ABI2/RALB/MSX1/WDR1/C
1/CLEC7A/RACK1/MSX1/STPG1/DAP/RAD9A/IL20RA/HCAR2/PLEKHN1/EIF2B5/MYBBP1A/RNF122/
LCK/XPR1/CHMP3/NUP85/IFI16/BANF1/DENR/AP2M1/NUP43/PML/RPL39/DYNC1LI1/COPS7B/VPS
SF4/RPL35/RPS20/APP/PSMD4/POLR2K/DNAJC8/RPL18A/PSMD3/PABPC4/FUS/PSMD7/CWC27/RPS
PT7L/ASXL1/USP3/MAP3K7/UCN/PRMT5/PHF1/DPY30/NAA60/IRF4/ING2/USP36/BCOR/SETDB1/M
3/KRT8/CXCL13/MKKS/NLRC5/HLA-DRB5/CBFB/PSMB4/IL1B/PIAS3/IL2RA/PSMB8/TCP1/TNFSF1
UBQLN4/VAMP8/WIPI1/MRPS17/MRPS21/MRPS15/CCSAP/DCTN1/CAPN2/MRPL18/PLK3/ADAMTS5/M
/EXOSC10/PAK1IP1/EXOSC7/DDX56/PIH1D1/TARBP2/RBMX/ZRSR2/UTP20/EXOSC1/EIF3E/NOB1/
/RAP2A/CDK5/PDGFA/CD47/CTTN/MYADM/WAS/VIL1/SPECC1/NUAK2/TNIK/CIT/PFN1/WASF1/ABR
OTCH1/CEP164/ODF2L/HAUS7/INPPL1/VDAC3/MARK4/HAUS5/DEF8/RILPL1/CD2AP/DYNC1I2/RAB
K/CIT/PFN1/WASF1/ABR/NEB/KPTN/PARVG/TACSTD2/CAPZB/FGD6/RALA/GRB2/SMTN/LLGL1/FHO
PTBP3/LTBR/PSMD1/LOX/MB/SIPA1L3/MSH2/PSMA7/BATF3/PSMB3/CIAPIN1/DNASE2/SBNO2/DRO

/CBFB/ITGA2/IL1B/IL2RA/CD47/SPOCK2/SPOCK1/TNFSF13B/CXCR3/MYADM/PLEKHA2/TGFB2/PL
P1/DNAJC10/PSMB3/BARD1/UBE2A/BAG3/SMARCC1/ARRB2/USP22/KCTD2/VPS25/ADRA2A/SNX12/
IEN1/IL1B/MTA2/BCAR1/RAP2A/CDK5/PDGFA/FAM83H/MCAM/FGR/CXCR3/CCL4/MYADM/TGFB2/GP
P1/MYLIP/ACTB/RAP2A/CDK5/NREP/CTTN/SPOCK1/RNF157/STMN3/PLXND1/PQBP1/GSK3A/NDRG4

A3C/PMP22/PTPRF/SEMA3E/PIFO/MAPT/CHN1/BRK1/CDHR2/AKIRIN1/CBFA2T2/ABL1/STK24/FCG
5/KLHL17/PCGF2/DNM1L/TRPM4/PSMD3/RBX1/PSMD7/UFC1/PELI1/MTBP/CBL/RPL23/RNF4/UBE2
/GPSM2/FERMT1/NPEPPS/SRI/ADAM8/LEPROTL1/RANGRF/YWHAH/GIPR/MCU/FRMD8/HCLS1/BBC3/
KPTN/YWHAQ/ARF4/RUVBL2/YWHAB/RPS3/CROCC/RPL27/BARD1/BAG3/SSR3/MCM8/NUP155/CALR/
IN1/PSTPIP1/NLE1/SRSF9/NASP/SNRPC/GEMIN7/RPL10L/ARPC5L/ATXN2L/FKBP4/ADRM1/RAC1/
RPS20/APP/NUP107/PNP/PSMD4/HK1/RPL18A/PSMD3/PABPC4/PSMD7/RPS19/NT5C3B/RPLP0/RPL
/INTS9/PIH1D1/GTF2E2/SEPHS1/TRMT12/TARBP2/TP53RK/INTS7/POLR2I/UTP20/POLR2J/EXOS
15/ABR/ATP6V0C/PSMD11/PSMD1/C5AR1/TMEM63A/PADI2/TRAPPC1/GSDMD/STK10/GPR84/CNN2/
/TRIM31/DAPK3/PIK3CD/CXCL13/MKKS/ARHGDIB/KIF14/DEFB1/JAG1/MITF/LYN/ITGB1BP1/CCL
EMIP/ACVR1/CXCL17/AKIRIN1/ABL1/PDCD10/TRIP6/TRADD/ACVR1B/PTK2/THBS1/ADAM17/VPS3
NKAP/STOML2/SRF/RHOA/GLMN/AP3B1/DOCK2/TNFRSF14/IDO1/RUNX3/ZMIZ1/CCL19/XCL1/PIK3
15/ABR/ATP6V0C/PSMD11/PSMD1/C5AR1/TMEM63A/PADI2/TRAPPC1/GSDMD/STK10/GPR84/CNN2/
/TRPM4/CCRL2/MAPK7/SEMA4F/RPS19/STX4/SEMA5B/ANGPT2/SCN1B/SEMA3C/GPR183/SEMA3E/L
PS12/JMJD4/SARNP/RPS4X/RPL6/C8orf88/MAGOH/RPL37/RPL27A/RCC1L/COPS5/WARS2/EIF1AD
EIF2B5/MYBBP1A/RNF122/CLIP3/TFAP2A/MAPT/PDCD6/CYCS/FGD1/OBSCN/CCL3/CDK19/MTCH1/
57/TAF9/VIL1/CD27/PTRH2/MAZ/PTGFR/NUAK2/SOCS3/FGFR2/SET/THOC6/PSMD10/NES/C5AR1/
EIF2B5/MYBBP1A/RNF122/CLIP3/TFAP2A/MAPT/PDCD6/CYCS/FGD1/OBSCN/CCL3/CDK19/MTCH1/
N/RNF157/TAF9/VIL1/CD27/PTRH2/MAZ/PTGFR/NUAK2/SOCS3/FGFR2/SET/THOC6/PSMD10/NES/
/PSMA3/PARK7/BUB3/RNF144A/NUB1/UBQLN2/RNF121/WWP2/ARIH2/RNF126/TLK2/ANAPC15/PSM
4/RAC1/CBFB/MIEN1/GBA/JCHAIN/CD47/CTTN/MYADM/DNAJB2/WAS/CDH17/TRMT112/VIL1/PSMC
H3GLB1/ERCC1/VEGFA/MARK4/DEF8/PIH1D1/MECP2/CENPJ/PPP2R5B/CDK5R1/PSMC3/PPM1F/TWI
/RPL7/RPL28/RTN3/PSMB10/CCL4/LCK/THOC5/XPR1/CHMP3/NUP85/IFI16/BANF1/DENR/AP2M1/
OBEC3G/RPS21/PSMB8/TCP1/RPL7/RPL28/RTN3/PSMB10/CCL4/LCK/THOC5/XPR1/CHMP3/NUP85/
TGFR/CYP26B1/BYSL/FOLR2/AGFG1/OSBP2/MST1R/PPP1R1B/OXTR/SPESP1/VDR/SBF1/ODF2/MSH
HAUS8/PML/DYNC1LI1/CIT/PLK4/ANAPC11/UBE2D1/PSMD10/PSMD11/NES/CDKN2C/PSMD1/DONSO
/PA2G4/SERPINB1/PIP5K1C/PPIA/RAB24/ACLY/DYNLT1/GTPBP2/CD68/NCKAP1L/ADAM8/CAPN1/
/DAPK3/PIK3CD/NLRC5/PLSCR1/PSTPIP1/DEFB1/SLC11A1/CD209/RAB20/LYN/HLA-DRB5/CARD1
C5/UBE2N/EREG/FAM3A/TLR7/HSP90B1/PIK3R6/IRAK2/SERPINB9/CAV1/DAB2IP/NECTIN2/YTHD
/IL1B/EHD2/IL2RA/ADRA2C/RNF157/MAPK11/MYADM/LOXL2/TGFB2/PLXND1/CD27/ADAMTS9/PPP
PSMD4/PCGF2/MED25/PSMD3/RBX1/PPP1R15A/PSMD7/RAD21/BABAM1/MTBP/RPL23/NME6/RFWD3/
AR1/CDK7/LRP8/CCND2/RPS3/TFAP4/STK10/MTCP1/TGFB3/MUC20/VPS25/APP/ADRA2A/XRCC6/S
MSH2/RPS3/SIT1/GRB2/CD6/DROSHA/CDC42/KAT2A/IL4R/PNP/GAL/SWAP70/HELLS/SKAP2/CD8A
/FBXW5/HNRNPU/SYCE1L/CCNE2/MASTL
9A/LYAR/OSTM1/PARP1/IL15/PSMD10/PSMD11/PTBP3/LTBR/PSMD1/LOX/MB/SIPA1L3/MSH2/PSM
/NF2/COL11A1/OVOL2/PSMD8/ACVR1/LUZP1/RARG/TNNI3/ABL1/VCL/PSMA1/SFRP2/KDF1/RPS7/
HSPD1/ABL1/SART1/HLA-DOA/FCGR2B/TNFAIP3/TOX/CSK/VAMP8/MILR1/BAD/BCL10/VCAM1/TWS

KAT2A/ZNF207/MTF2/SEMA4D/DNAJB6/SWAP70/DNM1L/SEMA4F/RAD21/KATNB1/GMFG/MTBP/SEMA
RANGRF/DAPK3/PIK3CD/CXCL13/MKKS/ARHGDIB/KIF14/DEFB1/JAG1/MITF/LYN/ITGB1BP1/CCL2
IP/ACVR1/CXCL17/AKIRIN1/ABL1/PDCD10/TRIP6/TRADD/ACVR1B/PTK2/THBS1/ADAM17/NOTCH1
1C/DUSP4/DBNDD2/IL1B/GBA/ACTB/CDK5/CHORDC1/PDGFA/TXN/MLKL/UBE2L3/TCP1/ADRA2C/FG
1/LLGL1/ARHGAP18/DNAL4/RANBP1/AHSA1/SEMA4D/XCL2/AGAP1/APP/DNAJB6/CDC42EP2/MYO9B
1/SWAP70/CDC42EP2/AFAP1/KATNB1/GMFG/RNF4/ABI2/ARHGDIA/WDR1/CEP250/SEMA3E/CLIP3/
/DNM1L/ACTL8/AP3D1/TRAPPC3/KATNB1/IKBKG/BTK/STX4/CBL/STK25/MYO5A/RALB/CSPG5/COP
AB3A/RAB11A/KIF22/PARD3/CACNA1D/AP3B1/STXBP1/ACTN4/GOSR2/RAB27B/FNBP1L/SNPH/ADO
TR/NSMF/GORAB/LRP8/SRRT/FAIM/DGUOK/CST7/SEMA4B/CALR/PRKCI/PRRX1/ZMYND8/SEMA4D/S

AP1L2/TNIK/TUBB2B/DVL2/INPP5J/NSMF/LRP8/TACSTD2/SRRT/FAIM/DGUOK/SEMA4B/CDC42/CA
PMAIP1/CCT7/LYN/ITGB1BP1/REEP2/MCTP1/RAC1/AKAP8L/IL1B/EHD2/ACTB/CDK5/TXN/UBE2L3
/HDAC2/ATF4/HTATIP2/KIF14/HCLS1/CLN3/NLE1/KDM1A/MITF/BIRC7/MIEN1/IL1B/GBA/LAMTO
4GALT/SPESP1/EPS15L1/YWHAQ/TMEM43/YWHAB/TRAPPC1/RALA/STX18/GRB2/ARRB2/NUP155/CD

UBE2E1/WSB2/FBXL6/RPS3/USP1/TRAF3/PSMB3/BARD1/UBE2A/ASB1/ARRB2/NUP155/USP22/JAD
CHMP1A/VPS25/FHDC1/PPAN/OCRL/CETN2/LRRC61/RPS19/PIP4K2C/RPLP0/RRP7BP/WRAP73/TME
PALM/STX7/CSK/RPL7A/RPS23/RPS10/VAMP8/BAD/HSP90AA1/RPS15/SGTA/RPS16/SYS1/VPS35/
3K6/BABAM1/STK25/AKT1S1/DNAJC7/RACK1/RFWD3/ZNF675/IGBP1/MSX1/RAD9A/OGG1/RPA2/LE
/AGFG1/TUBG2/LOX/NEB/KIF2A/GPX1/KPTN/TACSTD2/RPS3/CAPZB/LUM/GRB2/FHOD1/CDC42/ZN
D11/RAC1/CBFB/IL1B/ACTB/IL2RA/CD47/GLDN/KIFC3/TNFSF13B/FIBP/MYADM/TGFB2/CDH17/A
L1/VDAC3/MARK4/HAUS5/DEF8/RILPL1/CD2AP/DYNC1I2/RAB11A/NEDD1/CENPJ/TMEM138/SRF/C
EMA3E/MAPT/CHN1/BRK1/CDHR2/AKIRIN1/CBFA2T2/ABL1/STK24/FCGR2B/STMN2/RASAL1/SFRP2
/CKAP5/DBF4B/BTN2A2/RAD51C/BTG2/PSMD8/PIDD1/HSPA2/SUSD2/PKIA/PSMA1/MTA3/CHEK2/C
A/SGO1/SFI1/CTDSPL/MSTO1/LMNA/DYNLL1/DYNC1H1/SPDL1/XRCC3/TICRR/TGFA/RRS1/KNTC1/
/METTL3/LOXL3/PSMB1/ACIN1/MOV10/NFKBID/PSMB6/CLPTM1/SH3RF1/DPY30/PSMA3/ANKRD54/
ASP8/PTPN1/TMEM98/PLK3/BICD1/SH3GLB1/NVL/MAP1A/MAVS/BNIP3L/CCNE2/MARK4/SREBF1/T
DEFB1/JAG1/MITF/LYN/ITGB1BP1/CCL28/MCTP1/RAC1/ITGA2/PLA2G7/MIEN1/IL1B/MTA2/BCAR
DCD10/TRIP6/TRADD/ACVR1B/PTK2/THBS1/ADAM17/NOTCH1/STAT5A/VEGFA/HSPA5/ANXA3/RAB1
/PRMT5/SNRNP200/RBM22/DDX20/GEMIN8/U2SURP/HNRNPC/POLR2B/BUD13/GEMIN6/SYNCRIP/SM
P200/RBM22/DDX20/GEMIN8/U2SURP/HNRNPC/POLR2B/BUD13/GEMIN6/SYNCRIP/SMN2/HNRNPA2B
P200/RBM22/DDX20/GEMIN8/U2SURP/HNRNPC/POLR2B/BUD13/GEMIN6/SYNCRIP/SMN2/HNRNPA2B

/DYNC1LI1/GALNS/DSN1/SLC44A2/IMPDH2/ABR/ATP6V0C/PSMD11/PSMD1/C5AR1/TMEM63A/PADI
1/BAD/BCL10/VCAM1/TWSG1/C12orf4/PTPN2/THBS1/CD177/IL20RB/PAK2/HLA-F/RHBDD3/NKAP
TK/PELI1/CLEC7A/TNIP1/EIF2B5/LBP/ARPC1A/BTN2A2/BRK1/HSPD1/PSMD8/ARPC5/ABL1/FCGR
SEN15/RBM24/CDK11A/SSU72/SF3B2/STRAP/LSM1/SFSWAP/POLR2E/SAFB2/CCAR1/KHSRP/MFAP1
12A/HNRNPA2B1/SUPT6H/U2AF1L4/PIK3R2/SRSF3/APPL2/NOLC1/PTPN14/NUP214/TSC1/HDAC3/

/BRK1/RAB3D/RHOT2/ABL1/RASAL1/SIPA1/ARAP1/RUFY1/RAB5C/SGSM3/DBNL/ARHGAP11A/ARHG
PU/JMJD4/SARNP/RPS4X/C8orf88/TSN/EXOSC9/MAGOH/DXO/RCC1L/EXOSC7/TBRG4/UBA52/NANO

IP/PREX1/EPHB2/CIB1/ARHGAP33/YTHDF1/ADNP/CNR1/RYK/PLXNB2/FSTL4/MOV10/PPP1R9A/LP

AM17
25/MRPS2/ZMPSTE24/CARMIL2/SLN/SUPT16H/MRPL21/TSG101/SMCR8/MRPS26/DVL1/MRPS11/MC
/ZMPSTE24/XRCC5/SETD1A/TP53/IWS1/PSMG2/PARN/SETMAR/SLX4/ARRB1/TPR/RNF40/OGT/ZBT
/SRF/RHOA/RPL13A/VPS11/STXBP1/GTF2H1/MTPN/NIN/SPTAN1/PSMC5/FAF1/RPA1/ARF1/RHOBT
DYNC1LI1/GALNS/DSN1/SLC44A2/IMPDH2/ABR/ATP6V0C/PSMD11/PSMD1/C5AR1/TMEM63A/PADI2
/TMEM63A/PADI2/TRAPPC1/GSDMD/STK10/GPR84/CNN2/ALDOC/LAIR1/PNP/CYSTM1/XRCC6/PSMD
C11/UBE2D1/PSMD10/PSMD11/NES/CDKN2C/PSMD1/DONSON/ODF2/CDK7/PSMA7/UBE2E1/CCND2/P
PC4/SRPK2/PAF1/ZNF16
2/IPO7/PPP1R12A/HNRNPA2B1/SUPT6H/U2AF1L4/PIK3R2/SRSF3/APPL2/NOLC1/PTPN14/NUP214
/TGFB2/CHMP3/PPP1CC/NUP43/HAUS8/RAB10/DYNC1LI1/NAP1L2/DSN1/FGFR2/CIT/ANAPC11/MA
R1B/TPD52L1/DAXX/RHOA/STK3/RAPGEF1/YWHAG/CNPPD1/ATP2B4/PRKAG1/PIK3R5/MAP3K1/GPS
TG1IP/PDCD6/WNT4/DAP3/PELI3/GRINA/ACVR1/TRAF7/PIDD1/FASTK/RHOT2/SLC25A5/ABL1/ST

LAVL1/FASTKD1/SRSF3/PNPT1/SERBP1/NUP214/TRA2B/TNFSF13/PAIP1/SAFB/IWS1/TARDBP/C1

TEX11/RHOA/NEK6
D/DUSP22/CD3G/PTPRC/NECTIN2/CLEC4E/BAG6/BTN2A1/PIK3CB/MYO1C/PSMB1/NCKAP1/PLCG1/
/RPL37A/RPLP1/DHX9/EDC4/RPL32/TNPO1/RPL30/PSMC5/CNOT10/RBM10/MEX3D/RBM24/CNOT3/
RP/RPS14/RPS27/EXOSC2/ZC3HAV1/RPL29/LARP1/YTHDF2/CASC3/RPL13/RBM8A/METTL3/MAGOH

HNRNPC/BCOR/AURKAIP1/TCHP/DMTN/PRMT6/CEP97/PAM16/HTRA2/ASF1A/ANAPC15/TERF1/FZD9

EXOSC2/CDK5RAP1/DDX52/METTL3/TEX10/RPS6/RPF1/MRM2/CHD7/RIOK1/ADAR/RTCB/RPL11/RP

IFITM2/NFKBIL1/DDB1/P4HB/CD180/MX1/TRIM32/VAPA/TBC1D20/ADAR/TRIM27/SYT11/PRF1/I

G7/UCN/CCDC88A/HNRNPD/PRMT5/DCP2/HNRNPC/SETDB1/MYC/SLFN11/MBD2/HUS1/FIGNL1/EYA3
3R6/CD5/CAV1/TP53BP1/ATAD5/THOC1/INPP5D/DUSP22/ZNF683/PTPRC/IKZF3/METTL3/LOXL3/
AP/NPHP4/POLE2/LDB1/ATR/DCLRE1C/INPP5D
/TM9SF4/ATP1B1/PPARG/P4HB/PSMB1/PIN1
4/RPL41/RPL9/RPS6/RPL24/RPL11/RPL10/RPS24/TIMM9/RPS3A/TIMM22/ZDHHC20/NMT1/VAMP3
2/PSMD8/PIDD1/HSPA2/PKIA/PSMA1/MTA3/CHEK2/CLSPN/CEP41/CCND3/PSMB5/PSMA4/HSP90AA
/CCAR2/BECN1/GCLM/TFDP1/PRKRA/PDIA3/HTT/CAV1/TMEM102/DAB2IP/ATAD5/CSNK2A2/PTPRC

ATR3/USP36/MRPL20/URB2/RPL14/DDX51/RPL10A/ZNHIT3/ZNF622/RPL12/MRPS2/RRP15/WDR3/

BKB/RAB6A/FGL2/LILRB2/FCGR1B
PN/SHQ1/SMN1/ABT1/CNOT1/EIF3J/CRNKL1/SF1/USP4/SF3A1/CLP1/NUDT21/EIF2A/EIF2S1/GE

MRPL22/PRMT7/PSIP1/AAR2/XAB2/TDRD5/PTBP2/SNUPN/SHQ1/SMN1/ABT1/CNOT1/EIF3J/CRNKL

TB2/RAB9A/RAB40C/RAB13/DAB2IP/ARHGEF17/PREX1/EPHB2/ARFGEF1/CNKSR1/CYTH3/RAB1A/P
PHF1/DPY30/NAA60/IRF4/ING2/USP36/BCOR/SETDB1/PRMT6/BRD7/EYA3/BRD4/NSD1/DYDC2/SK
XCL1/RANBP3/CCL3L3/DOCK10/CCL8/CAV2/DAB2IP/PREX1/ARHGAP22/ARHGAP27/ARFGEF1/RIC8
L2/MST1R/PPP1R1B/PSMD10/GCN1/SMYD3/CDKN2C/C5AR1/CDK7/LRP8/CCND2/RPS3/TFAP4/DUSP
5B/STK25/ABI2/ARHGDIA/VIM/SCN1B/WDR1/SEMA3C/GPR183/PMP22/PTPRF/SEMA3E/MAPT/TGIF

KAP1L/SMPDL3B/ADAM8/HLA-DQA2/MYO1G/NONO/ULBP2/SHC1/PIK3CD/CXCL13/NLRC5/PLSCR1/S
3/CALM2/PDCD10/CSK/CLSPN/MAPKAPK3/CCND3/BAD/DBNL/BCL10/ERP29/HSP90AA1/TNFSF15/L

/POLR3F/CTSL/CTSB/RELA/AIM2/PSMB7/SKP1/HLA-E/NOD1/TRIM15/NLRC4/BPIFB1/PTPRS/CLE
SMAD2/DERL1/ERCC2/ZMPSTE24/ACE/PPP2CA/SLN/SMAD3/USP33/MAU2

LSM14A/TRIM44/AIMP1/PCBP2

P2/RNF213/SULF2/UBR5/PORCN/PSMC3/RHOA/STK3/GSKIP/RAPGEF1/IFT20/CLTC/AP2B1/PSMC5
45/UBA52/CD2AP/EDEM2/TRPC4AP/PCYOX1L/RNF213/PSMC3/VPS37B/USP11/UBE2D2/CHFR/AGAP
/LONP1/PRPF19/UBXN1/PSMD8/KLHL42/VCP/PSMA1/TNFAIP3/USP5/NSFL1C/UBQLN4/RPS7/DENN
1/NDUFA8/BHLHA15/GCLM/TFDP1/MFN1/ATG7/HTT/CAV2/HAX1/UQCRB/ATP7A/TMEM102/NDUFAF6

RPL48/DAP3/BTG2/MRPS7/LARP4B/NAT10/EEF1A2/MRPL53/MPV17L2/GSPT2/RPS7/FARSA/RPL7A
G1IP/RNF122/DCAF15/PDCD6/MED12/LRRC41/PRPF19/UBXN1/PELI3/PSMD8/TRAF7/ABL1/KLHL4
PCYOX1L/RNF213/PSMC3/VPS37B/USP11/UBE2D2/CHFR/AGAP3/GLMN/RNF8/ANKZF1/TMUB1/PSMC
PSMA4/SGTA/PSMD9/RCN3/PTK2/UBL7/CSNK2A1/CASP8/RPL5/RPS27A/CAPN2/FBXW5/USP21/PLK
1/ATP6AP2/RNF213/SULF2/UBR5/PORCN/PSMC3/RHOA/STK3/GSKIP/RAPGEF1/IFT20/CLTC/AP2B
LT/CHST2/RAE1/PRTFDC1/STT3A/COASY/GALNT6/PIGC/ACSL3/LUM/FUT2/NANS/FUT6/NUP155/A
HDAC2/PIK3CD/SLC35B2/GIPR/HCLS1/AXIN1/BBC3/PMAIP1/LYN/WNT7B/ITGB1BP1/BIRC7/CARD

/TBC1D20/ATP8B3/DYM/PRMT5/NOX1/SYT11/TMCC1/SURF4/RAB22A/ANXA8L1/VPS33B/MAPK15/T

PSMC3/UBE2D2/RBM14/MAP3K1/IL17RA/LGMN/DHX9/IDO1/PSMC5/ADORA2B/UBE2N/EREG/KPNA6/
2/HYLS1/ATP6V1D/HAUS3/KIF24/GAS8/DZIP1L/IFT81/PCNT/CEP78/EHD3/SPATA6/ATXN10/CFA

/DSC2/TRIM27/CAPZA2/RHOH/PDE4B/KCNE3
NTROB/CEP135/TMEM80/RILPL2/HYLS1/ATP6V1D/HAUS3/KIF24/GAS8/DZIP1L/IFT81/PCNT/CEP

/VPS37B/USP11/UBE2D2/CHFR/AGAP3/GLMN/RNF8/ANKZF1/TMUB1/PSMC5/FAF1/ARRDC1/CCAR2/
WIL4/HSPB11/PLCD4/RAD51C/PAFAH1B2/ACVR1/HSPA2/ADAM28/HVCN1/SRPK1/BAD/DNM2/SPIRE
DRBS1/HPGDS/TTC26/WNT4/SOHLH2/PIWIL4/HSPB11/RAD51C/PAFAH1B2/ACVR1/HSPA2/GHRHR/A
TC26/WNT4/SOHLH2/PIWIL4/HSPB11/RAD51C/PAFAH1B2/ACVR1/HSPA2/GHRHR/ADAM28/SRPK1/B
6/KIF5B/ADGRE2/DMTN/HYAL2/HTRA2/IFNG/ARIH2/TRIM46/MAPK15/VAMP1/PPP1R12A/SUPT6H/

IL17RA/ANP32B/TNFRSF14/DHX9

NC5CL/IL1B/RAP2A/CDK5/PDGFA/LAMTOR1/TXN/MLKL/ADRA2C/PHB/FGR/LCP2/CCL4/MAPK11/TG

D2/PCGF1/RBM15/SMCHD1

PTPRE/GRB2/COL4A2/POLR2L/CDC42/MUC20/VPS25/APP/ADRA2A/POLR2K/SPRY4/CD8A/AXL/DST
/ATP2B4/PRKAG1/ZMIZ1/SPATA24/DEDD/TBP/EREG/MORN2/CDYL/SPATA2/RNF114/DZIP1/CIB1/
L1/SFRP2/TNFAIP3/RPS7/CALM2/CSK/BAG2/LEMD2/BOD1/PRKAA2/PRKRIP1/PTPN2/STYXL1/PTP

TXN/MLKL/ADRA2C/PHB/LCP2/CCL4/MAPK11/TGFB2/CD27/CALCA/MAP3K14/NDRG4/CCR1/SOCS3/
1/PIK3R5/MAP3K1/MINK1/RPLP1/MAPKAPK5/ADCY6/ADORA2B/UBE2N/ADCY5/KITLG/EREG/CCL19

EF1
L/DBNL/PLXNC1/HSP90AA1/SRGAP2/DNM2/PTK2/NOTCH1/MAP1A/LLPH/VEGFA/RAB3A/LZTS1/PAC
EGFA/LZTS1/HSPA5/DDX56/RAB11A/RAB29/IFRD1/PACSIN1/NOTCH3/PPP2R5B/CDK5R1/METRN/A
AP2/DNM2/PTK2/NOTCH1/MAP1A/LLPH/VEGFA/RAB3A/LZTS1/PACSIN2/FBXO45/RAB11A/TUBB3/K
/NFAM1/TNFSF13B/PSMB10/PHB/FGR/LCP2/PSMA5/CD300LF/NRAS/WAS/TGFB2/SFPQ/PQBP1/LCK
SN2/ELAVL1/RPS6KA3/NOLC1/TSC1/PAIP1/RPL22/GEMIN5/EIF3C/DDX1/EIF1/ATXN2/C1QBP/TR

TLR8/MX1/ABCF3/ADAR/PLA2G6/PLA2G2A/SYT11/PRF1/IRF4/POLR3F/IL10RB/SLFN11/CD4/APO
AL2/TRIM46/SEMA6B/EIF4G1/SESN2/BLZF1/SEMA4C/RGS4/ADNP2/PTPRS/PRSS2/RPS6KA3/MUL1

2/MSR1/SYNGR3/CLN3/PSTPIP1/SLC11A1/HSPH1/CD209/RAB20/LYN/APOBR/MCTP1/RAC1/ITGA2
PN
DUSP7/MAP3K10/PIH1D1/DGKQ/CDK5R1/PRKAR1B/TPD52L1/DAXX/RHOA/STK3/RAPGEF1/ATG13/A

P2/CLTB/AP2M1/CXCL16/WASF1/VPS28/FOLR2/LYAR/AP2S1/IL15/SIGLEC1/ABR/EPS15L1/LRP8

/CASP3/UNK/LRPPRC/POLDIP3/ENC1/RPL10/KLHL25/HNRNPD/PLA2G6/LSM14B/NEURL1/EIF4E2/

3KBP1/CNOT9/ATP6V1H/CDKN2B/SHC1/LYN/RAC1/CDKN1C/ATP6V1E1/IL1B/ACTB/BCAR1/PDGFA/

NOTCH1/MAP1A/LLPH/VEGFA/RAB3A/LZTS1/FBXO45/RAB11A/TUBB3/KIF20B/IFRD1/PACSIN1/CD

XL/SEMA5B/STK25/ABI2/ARHGDIA/SCN1B/CSPG5/WDR1/SEMA3C/SEMA3E/MAPT/RERE/CHN1/LIMS
4/NUP214/MUL1/SETD1A/SUPT3H/BAZ1A/IWS1/BCL11A/PHF20/KAT5/SUZ12/MSL3/SETMAR/MEN1

/MAPK11/PQBP1/VIL1/XPR1/ADAMTS9/IFI16/BANF1/CASP1/MAP3K14/PTGFR/PML/RPL39/IL12R
XO45/RAB11A/TUBB3/KIF20B/IFRD1/PACSIN1/CDK5R1/METRN/PARD3/APBB1/SRF/CAPRIN1/RHO
/MAPK11/PQBP1/VIL1/XPR1/ADAMTS9/IFI16/BANF1/CASP1/MAP3K14/PTGFR/PML/RPL39/IL12R
PL6/BNIP3L/RPL37/RPL27A/GNPTAB/SREBF1/HSPA5/RPL36/UBA52/PIH1D1/RAB11A/HIKESHI/Z

/TIGAR/BECN1/SNAPIN/WDR6/VPS33A/EIF4G2/MFN1/ATG7/HTT/HAX1/CD84/RPTOR/ATP6V1B2/C
RNF26/TMEM229B/FGR/CCL4/MAPK11/PQBP1/VIL1/XPR1/ADAMTS9/IFI16/BANF1/CASP1/MAP3K1
RS6/HACD1/MCTS1/PIWIL4/MRPL48/DAP3/BTG2/MRPS7/LARP4B/NAT10/EEF1A2/MRPL53/MPV17L
/TIGAR/BECN1/SNAPIN/WDR6/VPS33A/EIF4G2/MFN1/ATG7/HTT/HAX1/CD84/RPTOR/ATP6V1B2/C

PRC/NECTIN2/CD1A/LOXL3/TNFRSF1B/ADCY7/TLR8/MAP3K7/NFKBID/TRIM27/PRF1/IRF4/HLA-C

/CNP/NCAM1/TRIM32/RPL24/CASP3/ETV1/SEMA3F/MEGF8/EGR2/ULK1/CELSR3/TRIM46/XK/SEMA

/TRPM4/CTSD/CARD16/UQCC2/PDCD5/STX4/RACK1/KRT20/CLEC5A/HCAR2/CCT8/SERGEF/MAPT/A
/SVIP/MCL1/ABL2/ATP6V1D/CLEC16A/RNF41/TICAM1

MAP3K14/NDRG4/CCR1/TNIK/FGFR2/DVL2/MST1R/OXTR/PSMD10/PSMD11/LTBR/PSMD1/C5AR1/PS
CDC42/MUC20/CMKLR1/TLR5/PSMD4/XRCC6/GPATCH3/PSMD3/PSMD7/LILRB4/IKBKG/FADD/BTK/P
SPTAN1/ADCY6/ADM/ADORA2B/IST1/GCLM/ARF1/RHOBTB2/GMFB/EDNRA/HAX1/CAV1/ATP7A/RPTO
H1/RPS27A/SFRP4/GRIN2D/PIAS4/BCL9L/GABRB3/TLR2/CSNK2B/TMEM64/UBA52/GLI1/AP2A2/C

7RB/SLC9A6/EXOSC2/CTDP1/IP6K2/ADNP/NAIF1/EAF2/PPARG/RYK/PIN1/FSTL4/MEX3C/UCN/TR

1/IKBKB/LILRB2/PPM1N/DUSP26/PPP1R11/PPP1R3D/GNAI2/ACP1/DAPP1/PPP3CB/PPP1R36

MAP3K14/NDRG4/CCR1/TNIK/FGFR2/DVL2/MST1R/OXTR/PSMD10/PSMD11/LTBR/PSMD1/C5AR1/PS

C/PTPRS/DOCK7/TRAK2/PLXNB3/SHTN1/LAMA1/USP33/TTL/NEFH/FEZ1/DPYSL2/NEFL/DAG1/DVL

APPC2/CHMP2B/AP1M1/CHMP2A/STX5/VAPB/GORASP1/CHMP7/VPS37A/STX2/BLOC1S1/STX11/CHM

RLIM/XIAP/FBXW11/UBE3C/FBXO27/PLAA/RNF168/PSMD5/UBC/FBXL15/UBE2W

IGNL1/NPHP1/EYA3/HYAL2/ANXA1/RELA/TLK2/FKBPL/CLK2/CTNS
P85/IFI16/PSMC4/GSK3A/NUP43/OSBPL7/PML/DRAM2/VPS28/SET/ATP6V0C/USP14/PSMD10/PSM

/GPX1/PSMA7/EXOSC5/YWHAB/SAMD4B/PSMB3/DFFA/BAG3/SMARCC1/NUP155/AGO2/APP/ADRA2A/

BP8/NBN/CAPN2/MTHFR/GJB3/HOXA5/VEGFA/BCL2L1/PHGDH/ZPR1/MESP1/ATP6AP2/PHF6/SLC39

B/DNM2/PTK2/ADAM17/CSNK2A1/NOTCH1/CCDC85B/STAT5A/NPPB/ARHGEF11/VEGFA/EXOSC9/PAK
P2B4/GPS1/MAPKAPK5/HEG1/CCAR2/EPHA2/MECOM/CDC34/MRAP2/CLIC2/DUSP18/CAV1/DAB2IP/

FUT8/MBD3/FKBP8/NBN/CAPN2/MTHFR/GJB3/HOXA5/VEGFA/BCL2L1/PHGDH/ZPR1/MESP1/ATP6AP

/LLGL1/CDC42/PRKCI/TPST1/CHPF/APP/SLC9A7/DNM1L/AP1G2/AP3D1/TRAPPC3/SLC35C1/AP1B
S/POLR3D/PRPF19/ESCO2/NAT10/RARG/DNA2/HSPA2/RAD51D/MTA3/DCLRE1B/EME2/GAR1/RAD1/
B/GBA/GNB2/LAMTOR5/CTSS/LAMTOR1/TMEM9/GNS/S100A13/FTL/NFAM1/GNB1/KXD1/AP2M1/WDR
X3/DMAP1/CCAR2/TBP/IST1/UXT/IPO4/SUV39H1/MSH6/SAP130/PELP1/MORF4L1/EP400/LDB1/S
C3/ERCC6L/RFC2/DDX11/E2F4/NCAPG2/NUP37/PPP1CA/PPARD/INCENP/FBL/CBX3/DDX27/DVL3/
2/DEAF1/POP1/PTPN6/KNOP1/PUM3/IFI16/PPP1CC/MAP3K14/NSUN5/SIRT7/MIDN/PML/FBXW9/M
B/GBA/GNB2/LAMTOR5/CTSS/LAMTOR1/TMEM9/GNS/S100A13/FTL/NFAM1/GNB1/KXD1/AP2M1/WDR
P2A1/VPS33A/SEH1L/VASN/TLR7/NAPG/ATP11A/EGF/PI4K2A/DAGLB/RPTOR/ATP6V1B2/AP5B1/L
RNASE2/LYN/HLA-DRB5/RPN2/SLC26A11/ANXA11/ATP6V1E1/IL1B/GBA/GNB2/CPA2/LAMTOR5/CT
TXNDC9/PSMB5/HOOK2/BOD1/CSPP1/DNM2/TCTEX1D2/CCSAP/DCTN1/CEP164/ODF2L/HAUS7/KATN
N23/TMEM63A/CHD4/RUVBL2/CCDC77/CROCC/SMTN/CDC42/KAT2A/CHMP1A/TOPBP1/MZT2B/RANBP
KLHL21
38/CSPG4/NF2/S100A7/REXO2/LIMS1/ARPC5/TBCD/VCL/ZNF185/RPS7/TRIP6/RPL7A/ACTR3/RP
IN2/GGA3/TMBIM1/UBA52/HLA-F/AP2A2/VAC14/RAB11A/YIPF2/MITD1/ARL8B/VPS37B/VPS11/A
/TMEM163/WFS1/LAMTOR1/CD47/SMAGP/KIFC3/NFAM1/NRAS/WAS/SERPINB6/CACNG4/COPZ1/AP3
DAM17/CAPN2/PDLIM4/MYL9/NEBL/ACTN1/MYO6/LIMA1/CD2AP/NOTCH3/TNNC1/STOML2/WIPF2/D
TOP/ABL1/SART1/PHF5A/WRAP53/NOP10/DCLRE1B/FAAP20/NFATC4/TDG/CIITA/GAR1/FLYWCH1/
PDE4B/SURF4/IGF2R/VAMP3/RAB3C/VAMP1/NKD2/ARCN1/HLA-E/CHGA/SEMA4C/CNIH2/PTPRS
IA/PAM/SNX10/RAB24/ACLY/DYNLT1/GTPBP2/SRI/CD68/NCKAP1L/ADAM8/CAPN1/LAMP3/CPNE1/
P/KIFC3/NFAM1/NRAS/SERPINB6/CACNG4/COPZ1/AP3M2/NECAP2/AGPAT2/CLTB/AP2M1/RAB10/D
2/SMC4/KDM1A/NASP/DCTN2/HMGN4/KNSTRN/CHRAC1/PRIM1/SSB/EHMT2/BUD31/DCTN5/AKAP8L/
T7B/B4GALT7/MUC3A/RAC1/GBA/PDGFA/RTN3/NRAS/B4GALT2/XYLT2/PI4KB/COPZ1/FUT3/MUC5B
/TRAPPC1/BORCS8/GLMP/LUM/STARD3/VPS18/CTSD/AP3D1/AP1B1/CCZ1/ABCC10/M6PR/CSPG5/T
SCAMP2/CFTR/PDLIM4/LTV1/FIG4/MCOLN3/OPTN/CCZ1B/PACSIN2/GGA3/TMBIM1/UBA52/HLA-F/
ME2/GAR1/RAD1/SFR1/HAT1/CSNK2A1/USF1/MBD3/NBN/RNF2/ANP32E/SYCE1L/ERCC1/THOC2/RB
R/INF2/APP/DNAJB6/MYO9B/DNM1L/FUS/GDPD5/BTK/STX4/CBL/RACK1/ZNF675/M6PR/CEP250/T
3/ABI2/VIM/ITGB8/RPL38/CSPG4/NF2/S100A7/REXO2/LIMS1/ARPC5/TBCD/VCL/ZNF185/RPS7/
T/CHST2/KIAA0319L/LMTK3/HLA-B/QPCTL/GALNT6/GOLGA3/TRAPPC1/STX18/FUT2/FUT6/ICA1/
P2A1/VPS33A/SEH1L/VASN/TLR7/NAPG/ATP11A/EGF/PI4K2A/DAGLB/RPTOR/ATP6V1B2/AP5B1/L
/DYNLT1/COL5A2/HAUS1/ACTG1/SRI/TNNI2/RSPH1/COL6A3/TUBB4A/DAPK3/CSNK1D/KRT8/KIF1
/DYNLT1/COL5A2/HAUS1/ACTG1/SRI/TNNI2/RSPH1/COL6A3/TUBB4A/DAPK3/CSNK1D/KRT8/KIF1
CD58/C3AR1/HSP90AB1/PRSS3/DYNLL1/DYNC1H1/ILF2/AMPD3/TMED9/NPC2/NBEAL2/FSTL3/OLR
/COL5A2/HAUS1/ACTG1/SRI/TNNI2/RSPH1/COL6A3/TUBB4A/DAPK3/CSNK1D/KRT8/KIF14/FHOD3
3/KIF15/WAS/RASSF1/SPECC1/HAUS8/DYNC1LI1/MAP1LC3B/TUBB2B/EVPL/MAP4/TUBG2/NES/KI
ALR/APOBEC3D/SF3A3/RPL35/TDRKH/RPS20/RBPMS/PPAN/RPL18A/PABPC4/FUS/CWC27/RPS19/R

20B/CENPJ/KIF22/KLHL21/RASSF5/NEK6/CLTC/ENKD1
/LIMA1/RPL19/PPIB/TLN1/RPS11/PROCR/RHOA/RPL13A/YWHAG/CLTC/GAK/ACTN4/RPL37A/FAP/
1/RPL19/PPIB/TLN1/RPS11/PROCR/RHOA/RPL13A/YWHAG/CLTC/GAK/ACTN4/RPL37A/FAP/RPLP1
/HSPA5/LIMA1/RPL19/PPIB/TLN1/RPS11/PROCR/RHOA/RPL13A/YWHAG/CLTC/GAK/ACTN4/RPL37

A2/KITLG/KSR1/SSX2IP/MPP2/ARF1/HAX1/LDB1/RAB13/ATP7A/ATP6V1B2/CIB1/DUSP22/CYTH3

52/HLA-F/AP2A2/VAC14/BORCS6/TMEM138/TECPR1/ARL8B/VPS11/AP3B1/CLTC/AP1S1/AP1S3/A
MRPL58/MRPL38/MRPS30/MRPS27

NQ1/CDK2/SNF8/CLN3/SLC11A1/RAB20/HLA-DRB5/RAB11FIP4/MCTP1/CCDC22/RAC1/TMEM163/A
F335/TAF12/ACVR1/TRAF7/TAF10/DNA2/KLHL42/VHL/DHDDS/KCTD5/CCND3/SENP3/PRKAA2/ACV

X1/DNAJC1/TAF3/ENY2/NDUFB4/ATRAID/PCYT1A/CASC3/TFPT/MYO1C/QSOX2/MX1/VAPA/TMEM38

1/APP/PPFIA3/KLHL17/DNM1L/FUS/SEMA4F/SH3GL1/CPE/AP3D1/RPS19/RPLP0/STX4/RPL23/RA

RK3/ATP6V0A1/CLK2/MBD1/U2AF1L4/E2F7/LUC7L3/SRSF3/SETD1A/MAML1/CLK3/TARDBP/PIP5K

CD5/SPIRE2/KIF3A/PPP1R9A/EFHC1/UTRN

/PMPCA/MRPS6

HL17/TRPM4/CNNM4/PPP5C/FUS/SEMA4F/GDPD5/CPE/KATNB1/RRM1/STX4/ABI2/RACK1/MYO5A/S

P1/MORF4L1/EP400/LDB1/HMGA2/MORC2/GRWD1/GTF2F1/HDAC5/ZNF683/ENY2/SIN3B/PAF1/ADN
CTN4/NFKBIB/HINFP/DNMT3B/DHX9/MED7/NR1H2/GMEB2/ZMIZ1/DMAP1/CCAR2/TAF13/PMF1/MMS
NUAK1/CDK20/STK3/NEK6/GAK/PRKAG1/MAP3K1/MINK1/MAPKAPK5/PHKA2/GTF2H1/PHKG2/ADCK5
PL10/RPL38/MRPL52/RPL23A/EPPK1/OBSCN/EMILIN1/DAP3/COL11A1/SEC13/MRPS7/ARPC5/CCD
DK5R1/ARHGAP28/ADPRH/AGAP3/DAXX/STK3/ACAP3/STXBP5/RPLP1/DOCK2/SGSM1/ARF1/RANBP3
OB/CDH11/LIMCH1/MARK2/PDLIM3/CORO1B/AGBL5/ABLIM2/HIP1R/KIFC2/SLC9A3R1/TPPP3/LAS
/CDK19/ACVR1/FASTK/ABL1/STK24/PIM3/NME7/SRPK1/CSK/CIITA/CHEK2/MAPKAPK3/CCND3/PR
/KIF14/DCTN2/RAB20/RAC1/ATP6V1E1/GNG5/GNB2/RAP2A/KIFC3/KIF15/GNB1/NRAS/EIF2S3/D
/KIF14/DCTN2/RAB20/RAC1/ATP6V1E1/GNG5/GNB2/RAP2A/KIFC3/KIF15/GNB1/NRAS/EIF2S3/D
/KIF14/DCTN2/RAB20/RAC1/ATP6V1E1/GNG5/GNB2/RAP2A/KIFC3/KIF15/GNB1/NRAS/EIF2S3/D
TP6V1E1/GNG5/GNB2/RAP2A/KIFC3/KIF15/GNB1/NRAS/EIF2S3/DIRAS2/PSMC4/ATAD2/ABCA2/R
SP12/PRKD2/TRAF3/BARD1/SIT1/GRB2/ARRB2/PARD6B/LLGL1/CDC42/MTCP1/SLC12A7/ADRA2A/
/RPS19/AP1B1/CBL/ANGPT2/VRK1/RACK1/RAD9A/CEP250/TBL2/TNIP1/PARD6G/PIFO/MAPT/NSF
1/YWHAQ/ABI3/YWHAB/ACSL3/DFFA/STX18/SIT1/GRB2/ICA1/ARRB2/FHOD1/CDC42/CHMP1A/PRR
1/GPSM2/NCKAP1L/MLST8/FBXO5/DBF4/COL6A3/ARHGAP1/ATP6V1H/CDKN2B/RTKN/RAP1GAP2/SI
/CPE/CBL/RACK1/ARFIP2/ITGB8/CCT8/TRIM25/PTPRF/STK38/RPL23A/CKAP5/EMILIN1/ACVR1/
GSM3/HSP90AA1/ARHGEF3/SRGAP2/ARHGEF25/BICD1/RGP1/TBC1D22B/OPTN/ARHGEF11/RABGGTB

/TRIO/CLK2/MAP3K9/PRKCB/MAP4K4/ERCC2/MAP3K11/RPS6KA3
2B1/MYO1D/KIF5B/SLFN11/ABCF1/DYNC2LI1/FIGNL1/TNNT2/MYL6/ATP6V0A1
OCK2/WDR41/TNPO1/IPO9/SH3BP5L/EPS8L1/SGSM1/IPO4/RANBP3/YIPF1/EGF/CAV1/ATP7A/ARH
F1/IFT20/STXBP5/DOCK2/WDR41/TNPO1/IPO9/SH3BP5L/EPS8L1/SGSM1/IPO4/RANBP3/YIPF1/E

K10/POLR2L/CHKA/PRKCI/GRK2/PRIM2/POLR2K/HK1/DMPK/IP6K1/POLQ/MAPK7/MAP3K6/AXL/PI

PK3/CCND3/PRKAA2/ACVR1B/STK40/DGKA/PTK2/CSNK2A1/TNK2/CCNJL/AAK1/PLK3/PFKFB2/CCN

/ARL8A/RAB2A/RHOD/RAB7A/TUBD1/NOLC1/RAC3/RRAS2/GEM/RAB6A/GNAS
/TUBD1/NOLC1/RAC3/RRAS2/GEM/RAB6A/GNAS

D1/NOLC1/RAC3/RRAS2/GEM/RAB6A/GNAS
1/RAC3/RRAS2/GEM/RAB6A/GNAS

EM/RAB6A/GNAS

1/NOLC1/RAC3/RRAS2/GEM/PDE5A/RAB6A/GNAS
1/NOLC1/RAC3/RRAS2/GEM/PDE5A/RAB6A/GNAS

HD2/GTF3C5/NBN/HNRNPU/PIAS4/CTBP2/THRA/ERCC1/EXOSC9/CSNK2B/MAP3K10/GTF2E2/MECP2

RPP38/APEX1/PARN/THUMPD3/QTRT2/DDX19A/DUS3L/TARBP1/TRMT10C/MRPL58/METTL16/DDX46

P1R11/RNF214/RNF41/UBE2J1/BFAR/SMURF2/RNF40/ASB2/TRIP12/HLTF/ANAPC1/UBE2K/FANCL

C1/PRDM4/MSX1/CXXC1/EHF/E2F6/DPF2/MYBBP1A/TFAP2A/MAPT/NFYC/MED12/SUV39H2/POU2AF

/CKAP5/MZT1/ABL1/TBCD/VCP/STMN2/NSFL1C/CEP19/CHEK2/IFT172/HOOK2/PRKAA2/SRGAP2/P
RCC2/TUBA1A/TUBB2A/SGO1/CKS1B/SFI1/CTDSPL/MST01/LMNA/DYNLL1/DYNC1H1/SPDL1/XRCC3
10/FGR/MYADM/TGFB2/CDH17/PLXND1/PTPN6/VIL1/RAB10/C12orf57/TNIK/TUBB2B/FGFR2/COC
RKD2/TRAF3/ASB1/LUM/ARRB2/CD6/POLR2L/DDX41/KAT2A/IL4R/TGFB3/CMKLR1/APP/ADRA2A/P
DRA2A/TLR5/POLR2K/HK1/XRCC6/GPATCH3/TRPM4/CARD16/AXL/FADD/BTK/PELI1/CLEC7A/UBE2
AP3K7/UCN/TRIM32/FFAR2/AGPAT1/NOX1/PARK7/IRF4/PDE4B/POLR3F/CD4/HYAL2/IFNG/ANXA1
MC3/TWIST1/SRF/CC2D2A/RHOA/STK3/GLMN/IFT20/CLTC/SIX1/PDPN/AP2B1/HBEGF/PSMC5/RBP
/NFAM1/FGR/PSMA5/NRAS/CDH17/SERPINB6/PTPN6/PSMD13/AGPAT2/COMMD9/RAB10/DYNC1LI1/
MA5/CD300LF/NRAS/SERPINB6/PTPN6/PSMD13/AGPAT2/COMMD9/RAB10/DYNC1LI1/GALNS/DSN1/
I2/TRAPPC1/GSDMD/SBNO2/STK10/GPR84/CNN2/IL4R/ALDOC/LAIR1/PNP/CYSTM1/XRCC6/PSMD3
3/CTSD/PSMD7/GMFG/PRDX4/CLEC5A/CCT8/TBC1D10C/FCGR3B/HPSE/RAB4B/S100A7/KCNAB2/GC
/NFAM1/FGR/PSMA5/NRAS/CDH17/SERPINB6/PTPN6/PSMD13/AGPAT2/COMMD9/RAB10/DYNC1LI1/
1/KDELR1/SLC11A1/RNASE2/JAG1/LYN/RAC1/IL1B/CTSS/LAMTOR1/GNS/CD47/S100A13/FTL/NF
1/XRCC6/PSMD3/CTSD/PSMD7/GMFG/PRDX4/WDR1/CLEC5A/CCT8/TBC1D10C/FCGR3B/HPSE/RAB4B
BR/PSMD1/LOX/MB/SIPA1L3/MSH2/PSMA7/BATF3/PSMB3/CIAPIN1/DNASE2/SBNO2/DROSHA/CDC4
PREX1/DCLRE1C/INPP5D/HDAC5/ZNF683/CD3G/PTPRC/CLEC4E/IKZF3/METTL3/PPARG/JUNB/LOX
H2B2/LEF1/GPI/PDCD1/IRF7/ZC3H12A/PSME3/TNIP2/LAG3/SIRPG/FLOT1/ACTG1/NCKAP1L/SMP
NFAIP3/KCNN4/CSK/ACTR3/MAPKAPK3/PSMB5/BCL10/PSMA4/SPPL3/HSP90AA1/PSMD9/PTK2/PTP
MB1/NCKAP1/PLCG1/EIF2B4/MAP3K7/NFKBID/PSMB6/FFAR2/CD22/PLA2G6/ICAM2/PSMA3/PDE4B
2/RAC1/SSRP1/MTA2/WDHD1/APOBEC3G/TCP1/PHB/WAS/SWI5/SFPQ/DNMT3A/BANF1/PML/COPS7B
KIF22/FANCF/UBR5/PPIE/POLR2I/TWIST1/POLR2J/RBM14/APBB1/ACTR5/RFC5/RNF8/POLR2D/G
0/HAT1/MBD3/USP21/MTHFR/HNRNPU/RNF2/ANP32E/VEGFA/HSF4/UIMC1/SREBF1/MIS18A/WDR5/
80S/CWC27/RPS19/NOC4L/RRP7BP/TSR3/UTP11/LAS1L/SNRPG/TRIM71/UTP4/RBM17/HNRNPM/SL
HSPA14/NANOS1/TARBP2/ETF1/RPL19/MRPL37/EIF5A/RPS11/EIF3E/CAPRIN1/RPL22L1/RPUSD3
NB1/PIP5K1C/PPIA/RAB24/ACLY/DYNLT1/GTPBP2/CD68/NCKAP1L/SYTL1/ADAM8/CAPN1/MYO1G/
BP/CCL3/S100A7/CHN1/CXCL17/AKIRIN1/EXT1/CYP19A1/BIN2/PLXNC1/VCAM1/SLC8B1/PTK2/P
DK7/MSH2/RUVBL2/RPS3/TFAP4/POLA1/USP1/BARD1/UBE2A/GRB2/MCM8/POLR2L/CBX5/TOPBP1/
2B/PLK4/WASF1/INPP5J/MAP4/IFT80/TUBG2/RAE1/KIF2A/ODF2/RPS3/CROCC/BAG3/PARD6B/PT
2/MSH2/PSMA7/UBE2E1/CCND2/TFAP4/PSMB3/USP22/ZNF207/CHMP1A/TOPBP1/RANBP1/APP/PSM
IL1B/CAPNS1/IL2RA/KDM5B/PDGFA/CD47/S100A13/RNF187/TNFSF13B/NRAS/TGFB2/VASH2/F3/
2/SRPK2/HNRNPUL1/CASC3/SNRNP70/COIL/FIP1L1/RBM4B/DHX8/RBM8A/METTL3/CSTF3/SF3B6/
SUMO2/PSMB6/RPL11/ENC1/CDC23/PSMA3/PARK7/BUB3/RNF144A/SDF2/NUB1/UBQLN2/RNF121/W
PN6/PSMD13/CHMP3/PSMC4/HAUS8/PML/DYNC1LI1/CIT/PLK4/ANAPC11/UBE2D1/PSMD10/PSMD11
CT8/GNL3/DRG1/MAPT/ARPC1A/WNT4/NF2/BRK1/CHTOP/ARPC5/ABL1/STMN2/MFF/NSFL1C/ACTR3
CLIP3/TFAP2A/MAPT/PDCD6/CYCS/FGD1/OBSCN/CCL3/CDK19/MTCH1/HSPD1/TRAF7/PIDD1/EEF1
28/RPL35A/AP2S1/SET/ATP6V0C/SMARCB1/RAE1/LTBR/ILF3/MVB12A/ISG20/PSMA7/YWHAB/RPS
19/NT5C3B/RPLP0/RPL18/RPL23/VIM/SNRPG/TRIM71/RBM17/PLEKHN1/RPL38/HNRNPM/SLBP/KH
BD2/PRMT6/BRD7/EYA3/BRD4/NSD1/DYDC2/SKP1/AKAP8/KMT2B/NAA50/SUPT6H/KDM3A/PRKCB/M
3B/PSMB10/CXCR3/PSMA5/CD300LF/CCL4/COMMD7/F3/PTPN6/PSMD13/CD27/PSMC4/AGPAT2/CAS
AP1A/JMJD4/OPTN/BNIP3L/ADD3/SREBF1/LIMA1/ETF1/CDC37/TECPR1/MRPL37/EIF5A/DSTN/PL
NSUN4/RPL13A/DDX10/POLR2D/SART3/TGS1/MTERF3/WDR75/MRPL11/DHX9/FAM207A/BMS1/NOL1
/NEB/KPTN/PARVG/TACSTD2/CAPZB/FGD6/RALA/GRB2/NUP155/SMTN/LLGL1/FHOD1/CNN2/CDC42
11A/NEDD1/CENPJ/TMEM138/SRF/CC2D2A/YWHAG/IFT20/B9D1/FNBP1L/TNPO1/IFT22/PARVB/DP
D1/CNN2/CDC42/ARHGAP18/CALR/PRKCI/TGFB3/INF2/ADRA2A/DNAJB6/FHDC1/SWAP70/CDC42EP
SHA/CDC42/KAT2A/IL4R/APP/PNP/PSMD4/HOXB7/PSMD3/PABPC4/PSMD7/CD8A/LILRB4/AP3D1/R

XND1/ADAM19/LCK/PTPN6/CD27/PML/IL12RB1/CCDC80/IL15/PIEZO1/IBSP/PTPN23/TACSTD2/R
PSMD4/PSMD3/RBX1/CTSD/UQCC2/PSMD7/TMUB2/TBL1XR1/PELI1/CBL/SGTB/GZMA/RPL23/RNF4/
SM3/PLXND1/F3/FAM89B/VIL1/ADAMTS9/MAZ/DNAJA4/NDRG4/CCR1/CXCL16/PFN1/ABR/NSMF/C5
/RAB10/C12orf57/TNIK/TUBB2B/FGFR2/DVL2/WASF1/INPP5J/MAP4/LST1/NSMF/ARF4/GRB7/LR

R2B/STMN2/RASAL1/SFRP2/NFATC4/PALM/ARAP1/TBC1D7/KEL/DENND5A/DBNL/PLXNC1/DNM2/TC
L6/RACK1/RFWD3/NOSIP/RNF7/CUL4B/TRIM71/GNL3/NAE1/TRIM25/ZNF598/UBE2O/PTTG1IP/RN
KDM1A/PMAIP1/CCT7/TESK1/LYN/ITGB1BP1/RAC1/AKAP8L/IL1B/ACTB/CDK5/TXN/UBE2L3/TCP1
CTDNEP1/RPL35/RPS20/VPS25/DNAJB6/NUP107/GRPEL2/HK1/RPL18A/GZMB/DNM1L/PPP1R15A/R
RPS5/CTTN/TCP1/EIF3I/VBP1/MYADM/CENPI/WAS/TAF9/SWI5/EIF2S3/PSMD13/VIL1/CHMP3/PS
18/RPL23/VIM/OGG1/TRIM71/PLEKHN1/RPL38/HNRNPM/RPL23A/EXOSC3/BTG2/SEC13/PSMD8/DN
C1/NOB1/GPAT2/NSUN4/INTS4/DDX10/POLR2D/TOE1/DARS2/WDR75/CTU1/TSEN34/FAM207A/BMS
ALDOC/LAIR1/PNP/CYSTM1/XRCC6/PSMD3/CTSD/PSMD7/GMFG/STX4/PRDX4/CLEC5A/CCT8/TBC1D
28/MCTP1/RAC1/ITGA2/PLA2G7/MIEN1/IL1B/MTA2/BCAR1/RAP2A/CDK5/PDGFA/FAM83H/SPOCK2
5/NOTCH1/STAT5A/VEGFA/HSPA5/ANXA3/RAB11A/KIF20B/DUOX1/VPS37B/PPM1F/TWIST1/RHOA/
R6/ITGAL/CD5/CAV1/ATP7A/PKNOX1/PREX1/DUSP22/ZNF683/CD3G/PTPRC/CLEC4E/METTL3/LOX
ALDOC/LAIR1/PNP/CYSTM1/XRCC6/PSMD3/CTSD/PSMD7/GMFG/PRDX4/CLEC5A/CCT8/TBC1D10C/F
BP/CCL3/S100A7/CHN1/CXCL17/AKIRIN1/EXT1/CYP19A1/BIN2/PLXNC1/VCAM1/SLC8B1/PTK2/P
/RPL36/UBA52/HSPA14/NANOS1/TARBP2/ETF1/RPL19/MRPL37/EIF5A/RPS11/EIF3E/CAPRIN1/R
HSPD1/TRAF7/PIDD1/EEF1A2/RARG/ABL1/VCP/SFRP2/MFF/RPS7/NFATC4/TRADD/CHEK2/BAD/BC
GPX1/MSH2/ARF4/CCND2/ANXA4/FAIM/DFFA/IL2RB/BARD1/CIAPIN1/PAX8/BAG3/ARRB2/PRKCI/
HSPD1/TRAF7/PIDD1/EEF1A2/RARG/ABL1/VCP/SFRP2/MFF/RPS7/NFATC4/TRADD/CHEK2/BAD/BC
C5AR1/GPX1/MSH2/ARF4/CCND2/ANXA4/FAIM/DFFA/IL2RB/BARD1/CIAPIN1/PAX8/BAG3/ARRB2/
B7/NKD2/SKP1/ANKIB1/RNF186/DERL1/KCTD13/RNF19A/FBXL18/FBXL2/CDC16/KAT5/UBE2R2/R
4/LRFN4/DNAJA4/PLK4/PFN1/WASF1/INPP5J/MAP4/OXTR/PARP1/PIEZO1/TACSTD2/RPS3/CROCC
ST1/SRF/RHOA/WDR75/FNBP1L/GTF2H1/NIN/PSMC5/FAF1/RPA1/BECN1/DPYSL3/EPS8L1/DIMT1/
NUP43/PML/RPL39/DYNC1LI1/COPS7B/VPS28/RPL35A/AP2S1/SET/ATP6V0C/THOC6/SMARCB1/RA
IFI16/BANF1/DENR/AP2M1/NUP43/PML/RPL39/DYNC1LI1/COPS7B/VPS28/RPL35A/AP2S1/SET/A
2/MYCBP/SNU13/GOLGA3/UBE2A/MCM8/SYDE1/MAFF/VCX/AGO2/IL4R/CALR/TGFB3/CTDNEP1/TDR
N/TIPIN/ODF2/CDK7/PSMA7/UBE2E1/CCND2/POLE4/TFAP4/POLA1/PSMB3/MCM8/ZNF207/TOPBP1
CPNE1/C1orf35/CAP1/PIK3CD/ASAH1/KPNB1/SV2A/SYNGR3/SLC11A1/RNASE2/LYN/RAC1/MAGED
1/MUC3A/PSMB4/APOBEC3G/JCHAIN/CTSS/CD47/PSMB8/PSMB10/FGR/PSMA5/CD300LF/CCL4/NRA
F2/CLEC4E/ZCCHC3/METTL3/PPARG/NFKBIL1/PSMB1/TLR8/MAP3K7/PSMB6/ADAR/FFAR2/CNPY3/
1CC/NDRG4/CCR1/SOCS3/NAP1L2/TUBB2B/IL12RB1/CYP26B1/DVL2/PARP1/IL15/NSMF/MAMSTR/
GADD45GIP1/NABP1/RBBP7/RAD9A/RPA2/E2F6/NAE1/TIMELESS/MYBBP1A/ZWILCH/RHEB/BTN2A2
PRY4/TELO2/MAP3K6/IKBKG/GMFG/CBL/STK25/AKT1S1/RACK1/ZNF675/RALB/RGS3/IGF1R/STK3
/LILRB4/AP3D1/AXL/FADD/BTK/PELI1/CLEC7A/IGBP1/RASAL3/GPR183/WNT4/EXOSC3/BTN2A2/

A7/BATF3/PSMB3/CIAPIN1/DNASE2/SBNO2/DROSHA/CDC42/KAT2A/IL4R/APP/PNP/PSMD4/HOXB7
NFATC4/PDCD10/FOXF2/PSMB5/IFT172/BCL10/PSMA4/TBX2/TWSG1/PSMD9/LMO4/ADAM17/NOTCH
G1/LMO4/C12orf4/PTPN2/THBS1/CD177/IL20RB/PAK2/HLA-F/RHBDD3/NKAP/RHOA/GLMN/CD38/

5B/NME6/RACK1/ARHGDIA/VIM/SEMA3C/PMP22/SEMA3E/CLIP3/MAPT/CKAP2/RUBCN/EMILIN1/NA
8/MCTP1/RAC1/ITGA2/PLA2G7/MIEN1/IL1B/MTA2/BCAR1/RAP2A/CDK5/PDGFA/FAM83H/SPOCK2/
/STAT5A/VEGFA/HSPA5/ANXA3/RAB11A/KIF20B/DUOX1/PPM1F/TWIST1/RHOA/EMC10/ENPP2/ACT
R/LCP2/MAPK11/TGFB2/PTPN6/CALCA/MAP3K14/GSK3A/MIDN/SOCS3/TNIK/DVL2/MST1R/PPP1R1
/OCRL/DNM1L/DNAJB11/CTSD/HSPE1/PGAM5/PPP1R15A/PDCD5/FADD/DNAJC9/CLEC7A/RACK1/AR
DRG1/MAPT/CKAP2/ARPC1A/WNT4/NF2/BRK1/ARPC5/ABL1/TBCD/STMN2/NSFL1C/ACTR3/ARAP1/D
G1/CEP250/HAUS6/TRAPPC2L/NDE1/MAPT/PDCD6/BICDL2/NSF/CKAP5/IQCB1/SEC13/RAB3D/RHO
RA2B/BECN1/SNAPIN/SEH1L/EIF6/UXT/ARF1/KLHL12/HTT/PEF1/IL13RA2/CD84/MAP4K2/CALM3
YT1/APP/DNM1L/SYT3/SEMA4F/GDPD5/HEY1/KATNB1/SEMA5B/STK25/ABI2/ARHGDIA/VIM/SCN1B

LR/PRKCI/PRRX1/ZMYND8/SEMA4D/SYT1/APP/GAL/DNM1L/SYT3/SEMA4F/GDPD5/HEY1/RPS19/KA
/TCP1/FGR/PTPN6/VIL1/CHMP3/PPP1CC/GSK3A/AP2M1/ABCA2/NDRG4/PML/STAC3/VPS28/BYSL/
R5/CDK5/WFS1/TXN/CBX4/CTTN/RNF157/TAF9/VIL1/CD27/PTRH2/MAZ/PTGFR/NUAK2/SOCS3/FG
C42/VPS18/CHCHD3/CHMP1A/CALR/PRKCI/CTDNEP1/ZMYND8/CCR5/SYT1/OCRL/DMPK/GZMB/DNM1

E2/CDC42/KAT2A/ATXN7L3/NUP107/PSMD4/CBX8/DCUN1D5/KLHL17/PCGF2/DNM1L/TRPM4/PSMD3
M107/RNF4/RALB/CENPN/WDR1/CEP250/HAUS6/PACS2/ATP6V0D1/NDE1/RPL38/DRG1/MAPT/TTC2
CASP8/RPL5/RPS27A/ARFRP1/SH3GLB1/RPS12/TOMM22/RPS4X/RPL6/OPTN/RAB3A/RPL37/RPL27
TM1/PPP4C/PTPRF/TIMELESS/PTTG1IP/IGF1R/MAPT/EID3/UBXN1/SEC13/GRINA/PTGES3/ABL1/
F207/ARHGAP18/PRKCI/TGFB3/APP/DNAJB6/FHDC1/SWAP70/CDC42EP2/KATNB1/GMFG/ABI2/VIM
DAM19/LCK/PTPN6/CD27/LRFN4/RAB10/IL12RB1/IL15/SIGLEC1/PIEZO1/PTPN23/TACSTD2/RPS
C2D2A/YWHAG/IFT20/B9D1/FNBP1L/TNPO1/IFT22/PARVB/DPYSL3/EPS8L1/EPHA2/TTLL3/CCL19
/NFATC4/PALM/ARAP1/TBC1D7/KEL/DENND5A/DBNL/PLXNC1/DNM2/TCTEX1D2/PTK2/STYXL1/VPS
LSPN/CEP41/CCND3/PSMB5/PSMA4/HSP90AA1/PSMD9/ADAM17/CDK2AP2/DCTN1/CEP164/HAUS7/N
PPP1R9B/E2F8/CSNK1E/PRMT2/LZTS2/CEP85/E2F4/PSMB2/INCENP/RAB35/FGFR1/PIM2/PSME3/
IRF4/AGO1/MYC/BGLAP/PRMT6/CD4/IFNG/ANXA1/PSMB7/CCR2/KMT2B/PRKCB/PPP2R3C/ACE/ESR
MBIM1/BCL2L1/CD2AP/EDEM2/RAB11A/ZPR1/RAB29/KIF20B/CACNB3/CDK5R1/UBR5/PPM1F/RASS
1/RAP2A/CDK5/PDGFA/FAM83H/SPOCK2/MCAM/FGR/CXCR3/CCL4/MYADM/WAS/TGFB2/GPSM3/PLXN
1A/KIF20B/DUOX1/PPM1F/TWIST1/RHOA/EMC10/ENPP2/ACTN4/PDPN/LGMN/TNFRSF14/HBEGF
N2/HNRNPA2B1/U2AF1L4/ELAVL1/LUC7L3/SRSF3/UBL5/TRA2B/SUGP1/GEMIN5/DDX1/LSM3/C1QB
1/U2AF1L4/ELAVL1/LUC7L3/SRSF3/UBL5/TRA2B/SUGP1/GEMIN5/DDX1/LSM3/C1QBP/NOVA1/SRS
1/U2AF1L4/ELAVL1/LUC7L3/SRSF3/UBL5/TRA2B/SUGP1/GEMIN5/DDX1/LSM3/C1QBP/NOVA1/SRS

2/TRAPPC1/GSDMD/STK10/GPR84/CNN2/IL4R/ALDOC/LAIR1/PNP/CYSTM1/XRCC6/PSMD3/CTSD/P
/RHOA/GLMN/CD38/INHA/AP3B1/STXBP1/TNFRSF14/IDO1/RUNX3/ZMIZ1/ADORA2B/CCL19/XCL1/
2B/PSMA1/TNFAIP3/KCNN4/CSK/ACTR3/MAPKAPK3/PSMB5/CD33/BCL10/PSMA4/SPPL3/HSP90AA1
/PRPF4/THOC1/GTF2F1/DHX38/ERCC3/PABPC1L/DBR1/GEMIN2/PABPN1/SRSF1/SRSF2/SRPK2/PA
SMAD3/GEMIN5/TP53/STRADA/IWS1/TARDBP

EF3/SRGAP2/DNM2/ARHGEF25/TNK2/NOTCH1/ARHGEF11/RAB3A/CD2AP/RAB11A/PLEKHG6/DENND4
S1/TARBP2/ETF1/CDC37/PSMC3/POLR2I/EIF5A/POLR2J/EXOSC1/EIF3E/CAPRIN1/RPUSD3/NSUN

AR1

OLN1/CLEC16A/VTA1/TAOK1/MRPL42/KIF24/PHF23/WNT3A/MRPL22/MRPL58/MRPL38/MRPS30/GS
B7B/CDC26/WDR70/TRIP12/KLHL22/ANAPC1
B2/GMFB/HAX1/ATR/DAB2IP/CAMSAP1/PREX1/ARFGEF1/ERCC3/STUB1/VPS16/BIK/ADD1/DDB1/M
/TRAPPC1/GSDMD/STK10/GPR84/CNN2/IL4R/ALDOC/LAIR1/PNP/CYSTM1/XRCC6/PSMD3/CTSD/PS
3/CTSD/PSMD7/GMFG/PRDX4/CLEC5A/CCT8/TBC1D10C/FCGR3B/HPSE/RAB4B/S100A7/KCNAB2/GC
OLE4/TFAP4/POLA1/PSMB3/MCM8/ZNF207/TOPBP1/PRIM2/APP/PSMD4/CETN2/PSMD3/RBX1/PSMD

/TSC1/HDAC3/SMAD3/GEMIN5/TP53/STRADA/IWS1/TARDBP
P4/KLHDC8B/RAE1/CDC123/TIPIN/KIF2A/CDK7/CCND2/RPS3/RALA/PARD6B/CDC42/ZNF207/CHM
1/MAPKAPK5/HEG1/GTF2H1/ADORA2B/UBE2N/KITLG/KSR1/CCL19/PIK3R6/IRAK2/DUSP18/HTT/E
K24/SFRP2/TNFAIP3/MFF/RPS7/NFATC4/PDCD10/TMC8/TRADD/CHEK2/BAD/BCL10/ERP29/TM2D1

QBP/APEX1/PARN/SRSF10/PSMD12/RNF40/SRSF7/TRA2A/METTL16/ZC3H12D/DCP1A/SMU1/PSME2

EIF2B4/MAP3K7/NFKBID/PSMB6/FFAR2/CD22/PLA2G6/ICAM2/PSMA3/PDE4B/EIF2B1/CD4/RELA/
LSM1/RPS9/RNASEH1/SND1/KHSRP/RPS14/RPS27/EXOSC2/ZC3HAV1/RPL29/LARP1/YTHDF2/CASC
B/HNRNPA0/PSMB1/MOV10/RPL4/RPL41/SMG7/RPL9/RPS6/RPL24/PSMB6/GTPBP1/RPL11/RPL10/

/SUPT6H/KDM3A/CDK5RAP2/RAB7A/CARMIL2/LIG3/MUL1/XRCC5/TP53/LILRB2/FEZ1/ARPIN/CHM

'S24/METTL2A/WDR12/THADA/PDCD11/USP36/AGO1/RPL14/DDX51/DUS1L/RPL10A/ZNHIT3/HNRNP

GF2R/SETDB1/TRIM62/POLR2B/CD4/APOBEC3F/IFNG/AIM2/FAM111A/NOD1/TRIM15/RAB7A/ZMPS

/PAM16/MAPK15/TERF1/HNRNPA2B1/SUPT6H/MBD1/ERCC2/E2F7/KCTD13/ZMPSTE24/TMEM161A/L
DOCK8/TNFRSF1B/NFKBID/CASP3/CLPTM1/CD22/ICOS/SH3RF1/IRF4/CD4/IFNG/ANXA1/CCR2/HL

/RPL14/DMTN/RAB3IP/SRP19/RPL10A/RPL12/SRP14/BLZF1/ATG3/RPS18/RPS28/RPS17/RPL22/
.1/PSMD9/ADAM17/CDK2AP2/DCTN1/CEP164/HAUS7/NBN/PLK3/ZFYVE19/HAUS5/DYNC1I2/RAB11A
:/BIK/BDKRB2/P4HB/MNT/STK4/TRIM32/RPL11/SH3RF1/RTKN2/PLA2G6/MADD/NOX1/PARK7/ING2

RPS28/RPS17/NOLC1/RPL26L1/TSC1/NOL11/ERI1/NSUN5P2/ZNHIT6/TSR1/RPP38/C1QBP/MRPS1

MIN4/NCBP1

.1/SF1/USP4/SF3A1/CLP1/NUDT21/EIF2A/EIF2S1/GEMIN4/NCBP1

LD1/RAB36/GNA13/RAB8B/RAB1B/ARHGEF18/NCKAP1/LPAR1/BCR/MADD/PARK7/RHOH/F2RL2/BRA
P1/AKAP8/KMT2B/NAA50/SUPT6H/KDM3A/PRKCB/KDM4A/WBP2/ZMPSTE24/HDAC3/BAP1/SETD1A/S
B/ARHGAP17/LLGL2/RALBP1/ARHGAP33/EVI5L/PROM2/ASAP3/RALGAPB/RSU1/DOCK8/PLXNB2/PI
12/STK10/MTCP1/TGFB3/MUC20/VPS25/APP/ADRA2A/XRCC6/SPRY4/TELO2/MAP3K6/IKBKG/DSTY
2/RHEB/NF2/CCL3/CHN1/PRPF19/ZNF335/CBFA2T2/ABL1/STK24/STMN2/RASAL1/SFRP2/NFATC4

LC11A1/CD209/LYN/HLA-DRB5/CARD11/GPRC5B/FPR3/MUC3A/RAC1/CBFB/PSMB4/IL1B/ACTB/BC
MO4/PTK2/THBS1/ADAM17/PTPN1/RPS27A/NBN/NVL/VEGFA/PFKFB2/PLA2G1B/PAK2/MASTL/TRAF

C4A/APPL2/RPS6KA3/TLR1/S100A1/NOD2/XRCC5

/RBPJ/CCAR2/AP2A1/NPHP4/SNX3/TLE6/BARX1/KLHL12/EGF/CAV1/LDB1/DAB2IP/DKKL1/CBY1/
3/GLMN/RNF8/ANKZF1/TMUB1/PSMC5/FAF1/ARRDC1/CCAR2/FBXL7/UBE2N/ANAPC5/HECTD3/CDC2
D3/KCTD5/FOXF2/BAG2/CHEK2/RNF216/PSMB5/PSMA4/HSP90AA1/SGTA/PSMD9/RCN3/PTK2/UBL7
/COX14/CALM3/CSNK2A2/PARL/TIMM23/NECTIN2/NDUFB4/TIMM8B/HUWE1/PITRM1/BIK/NAIF1/S

/RPS23/RPS10/MRPS17/MRPS21/SPPL3/EIF3K/HSP90AA1/RPS15/RPS16/MRPS15/THBS1/EIF3M/
2/VCP/VHL/WSB1/PSMA1/TNFAIP3/USP5/RPS7/FOXF2/BAG2/TBC1D7/RNF216/PSMB5/BCL10/PSM
5/FAF1/ARRDC1/CCAR2/FBXL7/UBE2N/ANAPC5/HECTD3/CDC27/CDC34/HSP90B1/USP48/UBE4B/A
3/MAGEF1/MAP1A/USP27X/HSPA5/FBXO45/UBA52/CD2AP/EDEM2/TRPC4AP/PCYOX1L/RNF213/PSM
1/PSMC5/RBPJ/CCAR2/AP2A1/NPHP4/SNX3/TLE6/BARX1/KLHL12/EGF/CAV1/LDB1/DAB2IP/DKKL
GO2/ALDOC/MUC20/TMEM258/CHPF/APP/NUP107/MVD/HK1/SLC35C1/RRM1/ST3GAL5/PIGT/POMGN
11/GPRC5B/CCDC22/RAC1/UNC5CL/IL1B/LAMTOR5/PDGFA/LAMTOR1/TXN/MLKL/S100A13/ADRA2C

MEM127/BLZF1/DNAJC13/PRKCB/RAB2A/RAB7A/CNEP1R1/ZMPSTE24/CHMP6/CUL7/SYP/PPP2CA/S

TLR7/HSP90B1/CCL3L3/IRAK2/OSM/IL17D/CAV1/DAB2IP/IL17RB/NECTIN2/CLEC4E/ZCCHC3/CN
P157/TRAF3IP1/ATAT1/CEP126/CLUAP1/CFAP53/CEP162/KIFAP3/DCTN3/NEK8/ALMS1/SCLT1/M

78/EHD3/SPATA6/ATXN10/CFAP157/PKD2/TRAF3IP1/ATAT1/CEP126/CLUAP1/CFAP53/CEP162/K

FBXL7/UBE2N/ANAPC5/HECTD3/CDC27/CDC34/HSP90B1/USP48/UBE4B/AUP1/MAEA/CACUL1/EGF/

1/SPA17/NOTCH1/CFTR/GJB3/PAQR7/RNF2/THRA/VDAC2/ERCC1/RAB3A/PRSS21/HEXB/MASTL/BC

DAM28/SRPK1/BAD/DNM2/SPIRE1/SPA17/NOTCH1/CFTR/GJB3/PAQR7/RNF2/THRA/ERCC1/PRSS21

AD/DNM2/SPIRE1/SPA17/NOTCH1/CFTR/GJB3/PAQR7/RNF2/THRA/ERCC1/PRSS21/HEXB/MASTL/H

DNAJC13/PRKCB/CNIH2/PIK3R2/CHRNA5/NOLC1/PTPN14/UBL5/SYP/NUP214/HDAC3/BAP1/SMAD3

FB2/CD27/CALCA/MAP3K14/NDRG4/CCR1/SOCS3/TNIK/FGFR2/DVL2/MST1R/IL15/PSMD10/GCN1/

YK/CBL/AKT1S1/ANGPT2/TRIM71/ATP6V0D1/IGF1R/HNRNPM/PDCD6/ARPC1A/WNT4/CSPG4/CHN1/
SIX5/CSNK2A2/PABPC1L/ACTR1A/NECTIN2/YTHDF2/BAG6/RFX2/SLCO4C1/CNR1/ZSCAN2/METTL3
N1/RPL5/DUS2/FKBP8/PAK2/MASTL/TRIM21/IVNS1ABP/DUSP7/LMTK2/PIH1D1/TARBP2/DGKQ/UB

TNIK/FGFR2/DVL2/MST1R/IL15/PSMD10/SMYD3/LTBR/C5AR1/LRP8/CCND2/RPS3/PRKD2/ARRB2/
/PIK3R6/IRAK2/CACUL1/EGF/MAP4K1/MAP4K2/DAB2IP/HMGA2/RPTOR/CALM3/CIB1/PKD1/BORA/

SIN2/FBXO45/BCL2L1/RAB11A/TUBB3/KIF20B/IFRD1/PACSIN1/CDK5R1/METRN/PARD3/APBB1/S
PBB1/SRF/CAPRIN1/RHOA/CD38/RAPGEF1/YWHAG/GAK/SIX1/ACAP3/DNMT3B/GPC2/NIN/ADCY6/D
IF20B/IFRD1/PACSIN1/CDK5R1/METRN/PARD3/APBB1/SRF/CAPRIN1/RHOA/STXBP1/ENPP2/ROBO
/PTPN6/PSMD13/IFI16/PSMC4/MUC5B/IL12RB1/COCH/CD79A/UBE2D1/IL15/PSMD10/PSMD11/PS
MT10C/TPR/PASK/LSM14A/METTL16/EIF4ENIF1/ZC3H12D/STAT3/MRPS27

BEC3F/HYAL2/IFNG/CD207/RELA/AIM2/FAM111A/POLR3A/HLA-E/CHGA/NOD1/TRIM15/NLRC4/ZM
/PPP2CA/SHTN1/BAP1/SMAD3/UNC13A/TTL/TSG101/TP53/BCL11A/DPYSL2

/ANXA11/IL1B/EHD2/ACTB/CDK5/JCHAIN/DNER/CD47/ANKRD13B/CTTN/FGR/CD300LF/LOXL2/WA

TP2B4/PRKAG1/PIK3R5/MAP3K1/MINK1/RPLP1/MAPKAPK5/TIGAR/ADCY6/ADORA2B/UBE2N/ADCY5

/CDC42SE1/LDLRAD3/IL2RB/GRB2/ARRB2/CD6/CDC42/CALR/GRK2/CDC42SE2/SYT1/APP/SNX12/

PYM1/EIF2B1/AGO1/EIF5B/FXR1/IFNG/EIF2AK1/SYNCRIP/EIF4G1/CNOT2/EIF2B2/SESN2/ELAV

NREP/FIBP/FGR/LCP2/MAPK11/TGFB2/F3/FAM89B/LCK/VIL1/GSK3A/AP2M1/NDRG4/PML/SOCS3/

K5R1/METRN/PARD3/APBB1/SRF/CAPRIN1/RHOA/STXBP1/ENPP2/ROBO3/PDPN/MINK1/NIN/NRTN/

1/EXT1/ABL1/TBCD/VCL/NFATC4/KEL/DBNL/PLXNC1/HSP90AA1/SRGAP2/DNM2/PTK2/NOTCH1/MA
/ARRB1/TPR/EZH1/MCM3AP/OGT/KANSL2/NUP50/BRD8/SETD4/MSL1/KMT2A/HMG20B/CPA4/JARID

B1/FGFR2/COCH/CCDC80/MST1R/IL15/ABR/ILF3/C5AR1/ISG20/GPX1/BATF3/PF4V1/EXOSC5/GS
A/STXBP1/ROBO3/MINK1/NIN/NRTN/SPTAN1/EPHA2/ARTN/IST1/DOCK10/NEO1/ATP7A/DAB2IP/E
B1/FGFR2/COCH/CCDC80/MST1R/IL15/ABR/ILF3/C5AR1/ISG20/GPX1/BATF3/PF4V1/EXOSC5/GS
PR1/TIMM10/AIP/RPL19/UBR5/VPS37B/UBE2D2/NDUFA13/RPS11/RPL13A/VPS11/AP3B1/YWHAG/

:SNK2A2/STUB1/LARP1/VPS4A/RAB1A/KIAA1324/SNX5/VPS16/SNRNP70/FEZ2/MTMR14/PIK3CB/R
4/PTGFR/PML/RPL39/IL12RB1/FGFR2/COCH/CCDC80/MST1R/IL15/ABR/ILF3/C5AR1/ISG20/GPX
2/GSPT2/RPS7/FARSA/RPL7A/RPS23/RPS10/MRPS17/MRPS21/EIF3K/RPS15/RPS16/MRPS15/THB
:SNK2A2/STUB1/LARP1/VPS4A/RAB1A/KIAA1324/SNX5/VPS16/SNRNP70/FEZ2/MTMR14/PIK3CB/R

:/UNG/HLA-DOB/CTSL/CD4/IFNG/ANXA1/CCR2/KLHL6/HLA-E/SUPT6H/CD19/POU2F2/PRKCB/CLEC

6B/TRIO/LHX2/PTPRA/SEMA4C/PTPRS/DOCK7/TRAK2/PLXNB3/SHTN1/LAMA1/USP33/TTL/NEFH/F

BCG1/CARD18/BTN2A2/CCL3/ACHE/CEMIP/SLC25A5/PKIA/ABL1/EMD/FCGR2B/PIM3/WRAP53/TNF

MA7/YWHAB/RPS3/PRKD2/PSMB3/IL2RB/GRB2/ARRB2/TGFB3/MUC20/CCR5/XCL2/APP/ADRA2A/CS
ELI1/CLEC7A/TNIP1/EIF2B5/LBP/ARPC1A/BTN2A2/BRK1/HSPD1/PSMD8/ARPC5/ABL1/FCGR2B/P
R/PREX1/ARFGEF1/HRH1/ADNP/ADD1/RYK/MYO1C/BDKRB2/SPIRE2/FSTL4/NCKAP1/PPP1R9A/KCN
CDC88C/MESP1/ATP6AP2/RNF213/MECP2/CACNB3/SULF2/UBR5/PORCN/PSMC3/RHOA/STK3/GSKIP

IM32/SEMA3F/MEGF8/ULK1/TCHP/KAZALD1/HYAL2/ARIH2/TRIM46/SEMA6B/EIF4G1/LHX2/SESN2

MA7/YWHAB/RPS3/PRKD2/PSMB3/IL2RB/GRB2/ARRB2/STK10/TGFB3/MUC20/CCR5/XCL2/APP/ADR

[illegible]1/ANOS1/LRP4/WNT5A/PPP3CB/OLFM1

P5/PICALM/TMED2/VPS41/GOLGA2/SDCBP/TRIM9/SNAP47/HOOK3/CREB1

D11/RAE1/PSMD1/ATP6V0E1/GPX1/TNFSF12/PSMA7/EXOSC5/YWHAB/SAMD4B/PSMB3/DFFA/BARD1

NUP107/PSMD4/DNM1L/PSMD3/RBX1/PABPC4/UQCC2/PSMD7/IKBKG/PIP4K2C/RPL23/WDR91/RACK

A3/KIF20B/RIPPLY2/SULF2/PSMC3/TWIST1/SRF/CC2D2A/STK3/GLMN/B9D1/SIX1

2/CDK11B/ENOX2/BCL2L1/EGLN2/GLI1/RAB11A/ZPR1/NANOS1/CRLF3/MORF4L2/IFRD1/ALOX15B
ATAD5/CRTC3/CALM3/EPHB2/CIB1/PTPRC/LITAF/OTUD3/BDKRB2/PIN1/LPAR1/TRIM32/NFKBID/

2/PHF6/SLC39A3/KIF20B/RIPPLY2/CDK5R1/SULF2/PSMC3/TWIST1/SRF/CC2D2A/STK3/GLMN/B9

1/ST3GAL5/POMGNT1/MGAT4A/NAGPA/STK25/ARFIP2/NOSIP/M6PR/CSPG5/COPG1/B4GALNT3/TRA
SFR1/HAT1/CSNK2A1/USF1/MBD3/NBN/NOL6/HNRNPU/RNF2/ANP32E/SYCE1L/ERCC1/EXOSC9/ADD
45B/ABCA2/PLA2G15/DRAM2/SLC29A3/GALNS/DSN1/SLC44A2/SLC15A3/AP2S1/OSTM1/BGN/UBA1
RCAP/HMGA2/MORC2/CSNK2A2/SIN3B/PAF1/TRRAP/JUNB/TFPT/TRPS1/ASXL1/USP3/TTI1/PARK7
BLM/RSPH1/NHP2/SCMH1/ITGB3BP/HDAC2/AUNIP/CDK2/SMC4/KDM1A/NASP/DCTN2/HMGN4/KNSTR
PHOSPH6/CDKN2AIPNL/DSN1/PLK4/ANAPC11/BYSL/ZNF692/SURF2/LYAR/PARP1/SMARCB1/RAE1/
45B/ABCA2/PLA2G15/DRAM2/SLC29A3/GALNS/DSN1/SLC44A2/SLC15A3/AP2S1/OSTM1/BGN/UBA1
ITAF/ATRAID/PLD1/KIAA1324/SLCO4C1/VPS16
SS/LAMTOR1/TMEM9/GNS/S100A13/FTL/NFAM1/GNB1/KXD1/AP2M1/WDR45B/OSBPL7/ABCA2/PLA2
A1/PLK3/BICD1/CCDC85B/HNRNPU/ZFYVE19/RAD18/GNAI3/CCNE2/MARK4/RITA1/HAUS5/MASTL/
1/CETN2/ACTL8/KATNB1/WRAP73/CCDC120/CEP250/CCT8/HAUS6/DPF2/PPP4C/ATP6V0D1/NDE1/

S10/CDH24/PTPN12/RPS15/MPP7/CTNNA1/DNM2/PTK2/RPS16/ADAM17/TNK2/RPL5/NOTCH1/CAPN
BHD17A/CLTC/SNX17/GOSR2/RAB27B/AP2B1/IFITM3/ATG9A/BECN1/TMEM184A/AP2A1/VPS33A/T
M2/NECAP2/AGPAT2/CLTB/AP2M1/RAB10/DYNC1LI1/ZPLD1/NCALD/MYOF/SLC44A2/GAD1/AP2S1/
STN/PLEKHH2
RUFY1/CHEK2/PRKAA2/TATDN2/WTAP/NBN/INPPL1/FNDC1/HNRNPU/RNF2/SP140/PIAS4/RAD18/S

C1orf35/CAP1/ASAH1/KPNB1/SLC11A1/RNASE2/RAC1/ANXA11/IL1B/MAGED2/CTSS/PDGFA/LAMT
YNC1LI1/ZPLD1/NCALD/MYOF/SLC44A2/GAD1/AP2S1/ATP6V0C/C5AR1/TMEM63A/HLA-B/ATP6V0E
SSRP1/ACTB/MTA2/WDHD1/NCAPH2/TCP1/CENPI/LOXL2/WAS/SWI5/SFPQ/DNMT3A/THOC5/NUP85/
/CLTB/RAB10/NCALD/SMPD4/CIT/BGN/LST1/A4GALT/CHST2/KIAA0319L/LMTK3/HLA-B/QPCTL/G
MEM199/CCT8/GNB4/ATP6V0D1/NSF/HPSE/SLC48A1/RHEB/VMP1/CSPG4/SGSH/S100A7/GCA/SEC1
AP2A2/VAC14/RAB11A/YIPF2/MITD1/ARL8B/VPS37B/VPS11/ABHD17A/CLTC/SNX17/GOSR2/RAB2
MX/NCAPD3/SRF/TEX11/ACTR5/GATAD2A/DHX9/SMARCA5/RUNX3/DMAP1/CCAR2/POLE3/RPA1/PMF
RAPPC2L/RAB4B/NF2/MEIS2/ACHE/RAD51C/LIMS1/TAF10/ABL1/VCP/STMN2/UBQLN4/SIPA1/STX
TRIP6/RPL7A/ACTR3/RPS10/CDH24/PTPN12/RPS15/MPP7/CTNNA1/DNM2/PTK2/RPS16/ADAM17/T
PARM1/LLGL1/CDC42/PRKCI/PLEKHJ1/TPST1/CHPF/APP/SLC9A7/OCRL/DNM1L/AP1G2/AP3D1/TR
ITAF/ATRAID/PLD1/KIAA1324/SLCO4C1/VPS16
4/FHOD3/CAMSAP2/HCLS1/PSTPIP1/HSPH1/CCT7/GLRX3/DCTN2/KNSTRN/REEP2/FKBP4/RAC1/FB
4/FHOD3/CAMSAP2/HCLS1/PSTPIP1/HSPH1/CCT7/GLRX3/DCTN2/KNSTRN/REEP2/FKBP4/RAC1/FB
1/STX10/SLC2A5/GPI/RAB35/PA2G4/SERPINB1/PPIA/PAM/SNX10/MT3/RAB24/ACLY/DYNLT1/GT
/CAMSAP2/HCLS1/PSTPIP1/HSPH1/CCT7/GLRX3/DCTN2/KNSTRN/REEP2/FKBP4/RAC1/FBXO32/CD
F2A/ODF2/KPTN/SMTN/ZNF207/VPS18/DNAL4/FHDC1/MYO9B/DNM1L/KATNB1/SCTR/MYO5A/VIM/K
PLP0/NOC4L/RPL18/RRP7BP/GTF3C1/RPL23/UTP11/RACK1/GADD45GIP1/VIM/LAS1L/SNRPG/MRP

RPLP1/RPL30/PARVB/EPHA2/CORO2B/HSP90B1/ARF1/PDIA3/RPS9/CAV2/CAV1/RPS14/ARHGAP22
/RPL30/PARVB/EPHA2/CORO2B/HSP90B1/ARF1/PDIA3/RPS9/CAV2/CAV1/RPS14/ARHGAP22/PTPR
A/FAP/RPLP1/RPL30/PARVB/EPHA2/CORO2B/HSP90B1/ARF1/PDIA3/RPS9/CAV2/CAV1/RPS14/AR

/ACTG2/SNX5/C2CD5/ASAP3/MTMR14/MYO1C/ANTXR1/DOCK8/NCKAP1/PLCG1/PPP1R9A/LAYN/ABC

P1S2/AP2B1/WDR41/IFITM3/MANBA/SPNS1/BRI3/SNAPIN/AP2A1/VPS33A/SEH1L/VASN/TLR7/NA

TP6V1E1/EHD2/LAMTOR5/RAP2A/CTSS/LAMTOR1/TMEM9/DNER/ANKRD13B/ADRA2C/PHB/PI4KB/CH
R1B/DAD1/WTAP/CCNJL/FBXW5/RNF2/PIAS4/RAD18/FNTB/ERCC1/MED27/CCNE2/RABGGTB/FNTA/

A/TBC1D20/RTCB/LRPPRC/TRIM27/KPNA4/AAAS/BRAP/IGF2R/SHISA5/ABCF1/PLPP7

BAC1/ABI2/MYO5A/CSPG5/EPB41L3/HTR3A/ATP6V0D1/RPL38/MAPT/NSF/HNRNPA3/RHEB/GABRD/

1A/ASCC3/TEPSIN/APEX1/PDX1/TMEM179B/SRSF10/MAML2/SMURF2/SRSF7/PRPF40B/CACTIN/RS

CN1B/HTR3A/PTPRF/MAPT/NSF/TMEM266/TMEM185A/UBXN1/EEF1A2/ABL1/FCGR2B/STMN2/SGCE/

P/DHX30/PPARG/FOSL2/TTF1/MNT/ASXL1/CIC/USP3/CHD7/HNRNPD/NCOA6/PHF1/HNRNPC/LRWD1
19/CTBP1/CDYL/UXT/TFDP1/ARID5B/TLE6/CCAR1/SND1/LDB1/TP53BP1/SRCAP/HMGA2/GTF2F1/
/EPHA2/SBK1/KSR1/CDK11A/PDK3/IRAK2/CCL8/MAP4K1/ATR/MAP4K2/MKNK1/EPHB2/ERCC3/CSN
C6/VCL/THBS2/RPS7/RPL7A/ACTR3/RPS23/RPS10/LAMA4/KRT10/MRPS17/COPG2/COL9A3/MRPS2
/PRKRA/GMFB/CCL8/EGF/CAV1/ATP7A/DNAJC1/DAB2IP/RPTOR/ARL1/PREX1/GTF2F1/ARHGAP22/
P1/GAS2L1/FERMT1/AATF/TNNI2/MYO1G/CAP1/CAPZA1/MYO1F/YWHAH/KIF14/FHOD3/CAMSAP2/H
KAA2/ACVR1B/STK40/DGKA/PTK2/CSNK2A1/TNK2/CCNJL/GUK1/NME3/AAK1/PLK3/PFKFB2/CCNE2
IRAS2/PSMC4/ATAD2/ABCA2/RAB10/DYNC1LI1/TUBB2B/GMPS/TUBG2/ATP6V0C/EIF4A3/KIF2A/A
IRAS2/PSMC4/ATAD2/ABCA2/RAB10/DYNC1LI1/TUBB2B/GMPS/TUBG2/ATP6V0C/EIF4A3/KIF2A/A
IRAS2/PSMC4/ATAD2/ABCA2/RAB10/DYNC1LI1/TUBB2B/GMPS/TUBG2/ATP6V0C/EIF4A3/KIF2A/A
AB10/DYNC1LI1/TUBB2B/TUBG2/ATP6V0C/EIF4A3/KIF2A/ATP6V0E1/CDK7/MSH2/ARF4/CHD4/RU
TELO2/MAPK7/PPP1R15A/CARD16/CD8A/RPS19/AP1B1/CBL/ABI2/ANGPT2/VRK1/RACK1/RAD9A/C
/STK38/RHEB/CSPG4/DBF4B/LIMS1/ACVR1/EEF1A2/PKIA/TNNI3/ABL1/RPS7/CALM2/PDCD10/TD
X1/ZMYND8/APP/KLHL17/DNM1L/CARD16/AFAP1/SH3GL1/IKBKG/FADD/RRM1/CBL/ABI2/RACK1/M
PA1L2/ARHGDIB/TBC1D30/RARRES1/OAZ3/HSPH1/ITGB1BP1/BIRC7/GPRC5B/ADRM1/CDKN1C/UBE
CDHR2/STK24/EMD/VCL/SFRP2/RPL7A/CDH24/TXNDC9/DBNL/VCAM1/MPP7/CTNNA1/PTPN2/THBS1
/PAK2/RUSC2/RCC1L/GGA3/RILPL1/PLEKHG6/DENND4B/RAB29/YIPF2/XPO7/ADPRH/STOML2/RAS

GEF17/PREX1/CIB1/ARHGAP17/LLGL2/RALBP1/EVI5L/MYO1C/KIF3A/ARHGEF18/NCKAP1/TBC1D2
GF/CAV1/ATP7A/ARHGEF17/PREX1/CIB1/ARHGAP17/LLGL2/RALBP1/EVI5L/MYO1C/KIF3A/ARHGE

P4K2C/RPS6KB2/BTK/DSTYK/STK25/NME6/VRK1/ITPKC/GMPPA/CSNK1G2/POLD3/POLR3D/IGF1R/

E2/MARK4/PAK2/MASTL/CDK11B/CSNK2B/TBRG4/LMTK2/MAP3K10/TP53RK/DGKQ/CDK5R1/NUAK1/

/RNF25/AIP/DGKQ/BAZ2A/TWIST1/APBB1/TCERG1/SRF/DAXX/TEAD3/NEK6/HES2/ENPP2/SIX1/A

/TWISTNB/SKIV2L/MRM1/CDKAL1/XRN2/TRIT1/PCIF1/CNOT1/POLR3G/DHX32/TUT1/LRRC47/NSU

/TRIM5/TRIM3/TRIM36/MED31/HACE1/UBE2V2/G2E3/RMND5B/NSMCE1/MALT1/FBXO30/RNF139/N

1/MEIS2/ZNF335/HSPD1/RARG/ABL1/ZNF746/NFATC4/TDG/AKNA/FOXF2/ZNF605/MYEF2/TBX2/[illegible]

'TK2/SPIRE1/CDK2AP2/CCSAP/DCTN1/HAUS7/FBXW5/KATNA1/PLK3/BICD1/HNRNPU/MAP1A/MARK4
;/TICRR/TGFA/RRS1/KNTC1/PPP1R9B/E2F8/DDIAS/CSNK1E/PRMT2/LZTS2/CEP85/E2F4/PSMB2/I
H/DVL2/WASF1/LST1/COL6A2/NSMF/NES/VDR/SIPA1L3/GRB7/LRP8/PARVG/CDC42SE1/TACSTD2/
'NP/TLR5/POLR2K/HK1/XRCC6/GPATCH3/TRPM4/CARD16/AXL/FADD/BTK/PELI1/CLEC7A/UBE2L6/
L6/ITGB8/CLEC5A/TRIM25/POLR3D/LBP/POLR3H/HPSE/POLR3C/CARD18/BTN2A2/CCL3/HSPD1/C
/RELA/AIM2/CCR2/POLR1C/POLR3A/HLA-E/NOD1/TRIM15/TLR1/NOD2/XRCC5/SMAD3/LILRB1/RN
'J/CRYGS/ADM/EPHA2/AP2A1
'GALNS/DSN1/IL12RB1/SLC44A2/IMPDH2/ABR/ATP6V0C/PSMD11/PSMD1/C5AR1/TMEM63A/MSH2/F
'SLC44A2/IMPDH2/IL15/ABR/ATP6V0C/PSMD11/LTBR/PSMD1/C5AR1/TMEM63A/PADI2/BATF3/TRA
;/CTSD/PSMD7/BTK/GMFG/STX4/CBL/PRDX4/CLEC5A/CCT8/TBC1D10C/LBP/FCGR3B/HPSE/RAB4B/
A/PAFAH1B2/RAB3D/ARPC5/VCP/VCL/APRT/PDAP1/HVCN1/VAMP8/BIN2/RAB5C/DBNL/PSAP/CD33
'GALNS/DSN1/IL12RB1/SLC44A2/IMPDH2/ABR/ATP6V0C/PSMD11/PSMD1/C5AR1/TMEM63A/MSH2/F
'AM1/FGR/PSMA5/NRAS/WAS/SERPINB6/PTPN6/PSMD13/CD27/AGPAT2/COMMD9/RAB10/DYNC1LI1/
;/S100A7/KCNAB2/GCA/PAFAH1B2/RAB3D/ARPC5/VCP/VCL/APRT/PDAP1/HVCN1/VAMP8/BIN2/RAB
2/KAT2A/IL4R/APP/PNP/SWAP70/PSMD4/HOXB7/POLQ/PSMD3/PABPC4/PSMD7/CD8A/LILRB4/AP3
L3/ACIN1/RPS6/NFKBID/CHD7/CLPTM1/SH3RF1/IRF4/RHOH/MYC/BGLAP/CD4/IFNG/ANXA1/EIF2
'DL3B/ADAM8/HLA-DQA2/MYO1G/EVI2B/NONO/ULBP2/CCL2/PIK3CD/CXCL13/NLRC5/PLSCR1/HCLS
'N2/CASP8/RPS27A/DMBT1/PAK2/TLR2/MUC6/UBA52/RAB29/MUC15/CACNB3/PLD2/PSMC3/UBE2D2
;/EIF2B1/CD4/RELA/PSMB7/KLHL6/PRAM1/SKP1/EIF2B2/CD19/PRKCB/PIK3R2/CLEC4A/VAV3/AP
;/ISY1/PARP1/SET/RNF113A/SMARCB1/LOX/DONSON/TIPIN/ISG20/CDK7/MSH2/RUVBL2/EXOSC5/
PS1/HINFP/DHX9/GTF2H1/DMAP1/TIGAR/RPA1/UBE2N/MMS19/SMARCAL1/MUS81/POLR2E/SMUG1/
'PIH1D1/MORF4L2/TRIM37/MECP2/UBR5/PPM1F/BAZ2A/TWIST1/RBM14/APBB1/DAXX/PPHLN1/ACT
BP/KHDRBS1/HNRNPA3/PRPF31/ZMAT5/EXOSC3/SUPT5H/SNRPB2/PRPF19/CHTOP/NAT10/FASTK/U
;/NSUN4/RHOA/RPL13A/MRPL27/MRPL28/HNRNPR/POLR2D/RPL37A/MTERF3/DARS2/MRPL51/MRPL1
'CPNE1/C1orf35/CAP1/TXLNA/PIK3CD/ASAH1/KPNB1/SV2A/SNF8/SYNGR3/SLC11A1/RNASE2/LYN
'TPN2/THBS1/ADAM17
'CBX8/POLR2K/CETN2/XRCC6/HMGN1/POLQ/RBX1/MUTYH/PPP1R15A/PPP5C/FUS/RAD21/IKBKG/BA
'PA/LLGL1/CDC42/KAT2A/ZNF207/CHMP1A/DNAL4/RANBP1/APP/CETN2/LRRC61/ACTL8/AP3D1/KA
D4/CETN2/PSMD3/RBX1/PSMD7/RAD21/MTBP/NME6/RFWD3/GADD45GIP1/NABP1/RAD9A/CEP250/R
'PTPN6/PPP1CC/MAZ/PTGFR/PML/IL12RB1/FGFR2/FOLR2/MST1R/IL15/NES/CDC123/MAB21L2/C5
'MAGOHB/SNRPD3/PRPF38B/HNRNPA0/ACIN1/TFIP11/CELF2/RP9/IK/PPP1R8/RTCB/THOC7/HNRNP
'WP2/ARIH2/RNF126/TLK2/ANAPC15/PSMB7/NKD2/SKP1/ANKIB1/RNF186/DERL1/KCTD13/RNF19A
/RAE1/CDKN2C/PSMD1/DONSON/TIPIN/ODF2/ZBTB17/PSMA7/UBE2E1/CCND2/TFAP4/RBBP4/PSME
;/BAD/SPIRE1/DCTN1/VPS35/CASP8/NBN/SH3GLB1/HNRNPU/ERCC1/OPTN/VEGFA/MARK4/SREBF1/
A2/RARG/ABL1/ZNF746/VCP/FCGR2B/SFRP2/MFF/RPS7/NFATC4/TRADD/CHEK2/BAD/BCL10/ARHG
3/TFAP4/POLA1/PSMB3/IL2RB/RPL27/GRB2/POLR2L/NUP155/CDC42/KAT2A/VPS18/CBX5/CHMP1
DRBS1/HNRNPA3/PRPF31/ZMAT5/RPL23A/EXOSC3/SUPT5H/SNRPB2/PRPF19/CHTOP/BTG2/PSMD8/
BD1/KDM4A/WBP2/ZMPSTE24/HDAC3/BAP1/SETD1A/SUPT3H/MAP3K12/BAZ1A/TP53/IWS1/PHF20/
P1/MAP3K14/PML/CCR1/SOCS3/IL12RB1/MST1R/IL15/PSMD10/RNF113A/PSMD11/LTBR/PSMD1/I
EKHH2/NEK6/MRPL27/MRPL28/ATG13/GAK/MRPL51/PDPN/MRPL11/FAP/ATG9A/MTPN/SPTAN1/TIG
0/RSL1D1/USP39/DIMT1/EBNA1BP2/LUC7L/NOL9/EIF6/UTP23/STRAP/RPP30/SUV39H1/SFSWAP/
/ARHGAP18/CALR/PRKCI/TGFB3/INF2/ADRA2A/DNAJB6/FHDC1/SWAP70/CDC42EP2/MYO9B/KLHL1
'YSL3/EPS8L1/EPHA2/TTLL3/CCL19/SSX2IP/KLF5/NPHP4/HTT/CAV1/ATP7A/CBY1/DZIP1/CCDC6
'2/KLHL17/EPB41L1/GMFG/ABI2/ARHGDIA/ARFIP2/WDR1/EPB41L3/FMNL3/SEMA3E/FGD1/ARPC1A
PS19/AXL/FADD/BTK/BABAM1/ZNF675/WDR1/CLEC5A/DPF2/GPR183/WNT4/TAZ/BTN2A2/CCL3/KC

PS3/PRKD2/GRB2/CD6/CDC42/IL4R/CALR/SEMA4D/PNP/SWAP70/MAPK7/RELL2/LILRB4/AP3D1/F
'WDR91/UBE2L6/RACK1/TMEM199/RNF7/CUL4B/TRIM71/TRIM25/PTTG1IP/RNF122/NSF/HPSE/LON
AR1/KIF2A/PTPN23/PADI2/ABI3/GRB7/TACSTD2/PRKD2/SEMA4B/PARD6B/SYDE1/STK10/AGO2/C
P8/EMB/BTBD3/DGUOK/SEMA4B/GRB2/PARD6B/LLGL1/CDC42/PRKCI/PRRX1/ZMYND8/SEMA4D/SYT

'TEX1D2/PTK2/STYXL1/VPS35/ODF2L/VDAC3/FIG4/MOB2/LLPH/VEGFA/MARK4/LZTS1/HSPA5/DDX
IF122/DCAF15/PDCD6/MED12/LRRC41/PRPF19/UBXN1/PELI3/PSMD8/TRAF7/ABL1/KLHL42/VCP/V
/ADRA2C/FGR/MAPK11/TGFB2/VIL1/CASP1/GSK3A/AP2M1/MIDN/PML/STAC3/VPS28/BYSL/PARP1
AD21/CPE/LILRB4/TIMM44/PDCD5/TOMM40L/AP3D1/RPS19/RPLP0/RPL18/MTBP/SGTB/RPL23/NA
MC4/SRSF12/CLTB/DENR/AP2M1/CENPK/NAP1L4/NAP1L2/COPS7B/PFN1/EDC3/WASF1/ISY1/INPF
IA2/GSPT2/VCP/PSMA1/RPS7/TDG/RPL7A/RPS23/MYEF2/RPS10/PSMB5/PSMA4/PRKAA2/RPS15/PS
1/TPRKB/NOL10/RSL1D1/TYW3/YARS2/PARS2/TAF13/HARS2/DIMT1/EBNA1BP2/TBP/TSEN15/NOL
10C/FCGR3B/HPSE/RAB4B/CCL3/S100A7/KCNAB2/GCA/PAFAH1B2/RAB3D/ARPC5/VCP/VCL/FCGR2
/MCAM/FGR/CXCR3/CCL4/MYADM/WAS/TGFB2/GPSM3/PLXND1/F3/FAM89B/VIL1/CHMP3/ADAMTS9/
EMC10/ENPP2/ACTN4/PDPN/LGMN/TNFRSF14/HBEGF/KITLG/ARTN/CCL19/XCL1/CCAR1/CCL8/CAV
L3/DOCK8/TNFRSF1B/RPS6/NFKBID/CASP3/CHD7/CLPTM1/ICOS/NCSTN
CGR3B/HPSE/RAB4B/S100A7/KCNAB2/GCA/PAFAH1B2/RAB3D/ARPC5/VCP/VCL/FCGR2B/APRT/PDA
TPN2/THBS1/ADAM17
PL22L1/RPUSD3/NSUN4/RHOA/RPL13A/MRPL27/MRPL28/HNRNPR/POLR2D/RPL37A/MTERF3/DARS2
L10/ARHGEF3/CTNNA1/DNM2/TNFSF15/PTPN2/THBS1/STYXL1/CASP8/NOTCH1/PDCD2/RPS27A/CA
TGFB3/CCR5/SEMA4D/DNAJB6/DPEP1/PCGF2/MAPK7/CARD16/AXL/FADD/DSTYK/CBL/IGBP1/ARHG
L10/ARHGEF3/CTNNA1/DNM2/TNFSF15/PTPN2/THBS1/STYXL1/CASP8/NOTCH1/PDCD2/RPS27A/CA
PRKCI/TGFB3/CCR5/SEMA4D/DNAJB6/DPEP1/PCGF2/MAPK7/CARD16/AXL/FADD/PELI1/DSTYK/CE
AD23A/SVIP/DVL1/DERL3/CLEC16A/UBE2J1/ARRB1/PSMD12/BFAR/ARMC8/SMURF2/OGT/MAPK9/C
/CAPZB/RALA/STX18/GRB2/FHOD1/CDC42/KAT2A/VPS18/CHMP1A/ARHGAP18/TGFB3/NCLN/ZMYND
EPHA2/CCL19/CDC34/ARF1/RHOBTB2/GMFB/NPHP4/HTT/CAV1/ATP7A/ATR/DZIP1/EPHB2/ERCC3/
E1/LTBR/ILF3/MVB12A/ISG20/GPX1/PSMA7/YWHAB/RPS3/TFAP4/POLA1/PSMB3/IL2RB/RPL27/G
TP6V0C/THOC6/SMARCB1/RAE1/LTBR/ILF3/MVB12A/ISG20/GPX1/PSMA7/YWHAB/RPS3/TFAP4/PC
KH/HSF2/APP/MYBL1/CETN2/TMEM119/TESMIN/HOXD9/AXL/PRDX4/ANGPT2/MMP19/APOL2/CCT8/
/PRIM2/APP/PSMD4/CETN2/PSMD3/RBX1/PSMD7/RAD21/BABAM1/MTBP/RFWD3/CEP250/RPA2/CUL
2/CDK5/CTSS/PDGFA/LAMTOR1/GNS/CD47/S100A13/FTL/NFAM1/FGR/PSMA5/NRAS/TGFB2/SERPI
S/WAS/SFPQ/PQBP1/PTPN6/PSMD13/IFI16/PSMC4/MUC5B/CASP1/PML/RPL39/SOCS3/IL12RB1/C
MFHAS1/ICAM2/PSMA3/IRF4/POLR3F/CTSL/IFNG/CTSB/RELA/AIM2/PSMB7/SKP1/HLA-E/NOD1/T
VDR/LRP8/TACSTD2/SRRT/FAIM/PAX8/ARRB2/CDC42/KAT2A/IL4R/CALR/PRKCI/TGFB3/ZMYND8/
/PRPF19/BTG2/PSMD8/PIDD1/DNA2/SUSD2/DOT1L/ABL1/PSMA1/TNFAIP3/DCLRE1B/SIPA1/EME2
8/CSPG4/DBF4B/NF2/CEMIP/TRAF7/CXCL17/PKIA/ABL1/STK24/SFRP2/TNFAIP3/CALM2/PDCD1C
ZNF335/MFNG/HSPD1/LFNG/ABL1/SART1/HLA-DOA/FCGR2B/TNFAIP3/TOX/CSK/CCND3/BAD/BCL1

/PSMD3/PABPC4/PSMD7/CD8A/LILRB4/AP3D1/RPS19/AXL/FADD/BTK/BABAM1/ZNF675/WDR1/CLE
I1/POFUT2/MTHFR/HOXA5/VEGFA/CSNK2B/AP2A2/MESP1/KIF20B/SYNE4/PSMC3/TNNC1/TWIST1/S
INHA/AP3B1/STXBP1/PDPN/TNFRSF14/IDO1/RUNX3/ZMIZ1/ADORA2B/CCL19/XCL1/PIK3R6/CD5/

T10/HSPA2/SLC25A5/CBFA2T2/TBCD/FCGR2B/STMN2/UBQLN4/NFATC4/TMC8/CSK/ARAP1/TBC1D7
MCAM/FGR/CXCR3/CCL4/MYADM/WAS/TGFB2/GPSM3/PLXND1/F3/FAM89B/VIL1/ADAMTS9/MAZ/DNA
N4/PDPN/LGMN/TNFRSF14/HBEGF
B/PSMD10/GCN1/SMYD3/CDKN2C/C5AR1/CDK7/LRP8/CCND2/RPS3/TFAP4/DUSP12/PARM1/STK10/
HGDIA/DAP/RIC8A/RGS3/TBC1D10C/RASAL3/MAPT/PDCD6/CYCS/WNT4/TBC1D22A/SSBP1/CCL3/C
BNL/PTGER4/PRKAA2/PTK2/SPIRE1/CDK2AP2/CCSAP/DCTN1/CAPN2/FBXW5/BICD1/HNRNPU/MAP1
T2/TMEM216/NSFL1C/BRAT1/PDCD10/STX7/ACTR3/CEP19/VAMP8/CEP41/MILR1/WIPI1/CENPQ/E
/ACTR1A/LLGL2/VPS4A/NECTIN2/RAB1A/BBS7/CNR1/MYO1C/ATP2A2/SPIRE2/KIF3A/CD59/RAB1
/WDR1/SEMA3C/GPR183/PMP22/PTPRF/SEMA3E/MAPT/TGIF2/RHEB/NF2/CCL3/CHN1/PRPF19/ZNF

TNB1/SEMA5B/STK25/ABI2/CLEC7A/ARHGDIA/VIM/SCN1B/CSPG5/WDR1/SEMA3C/GPR183/PMP22/
PARP1/OXCT1/CPSF6/PTPN23/YWHAQ/XPO5/YWHAB/SYT12/ACSL3/CROCC/RIMS3/BARD1/STX18/E
FR2/SET/THOC6/PSMD10/NES/C5AR1/GPX1/MSH2/ARF4/CCND2/ANXA4/FAIM/DFFA/IL2RB/BARD1
L/PDCD5/AP3D1/TRAPPC3/STX4/CBL/VRK1/M6PR/STPG1/TIMM50/EPB41L3/BNIP1/ROMO1/LETM1

/RBX1/PSMD7/IKBKG/UFC1/PELI1/BABAM1/MTBP/CBL/RPL23/RNF4/UBE2L6/RACK1/RFWD3/NOSI
6/RPL23A/VMP1/CKAP5/OBSCN/HSPB11/MZT1/IQCB1/MRPS7/TRAF7/TMEM216/HPS1/MPV17L2/N
A/FNTA/GGA3/HSPA5/TMBIM1/BCL2L1/RPL36/RILPL1/UBA52/RAB11A/ZDHHC23/TSPAN14/RAB2
STK24/FCGR2B/WRAP53/SFRP2/UBQLN4/PDCD10/BAG2/CHEK2/BAD/DBNL/PSAP/ERP29/HSP90AA1
/KRT20/WDR1/NDE1/CLIP3/DRG1/MAPT/CKAP2/ARPC1A/WNT4/EPPK1/CKAP5/NF2/OBSCN/EMILI
3/EMB/GRB2/CD6/STK10/CDC42/IL4R/SEMA4D/DNAJB6/PNP/SWAP70/COL8A2/MAPK7/LILRB4/A
/SSX2IP/KLF5/NPHP4/HTT/CAV1/ATP7A/CBY1/DZIP1/CCDC65/ACTR1A/OFD1/PLD1/BBS7/RFX2/
35/ODF2L/VDAC3/FIG4/MOB2/LLPH/VEGFA/MARK4/LZTS1/HSPA5/DDX56/DEF8/RAB11A/RAB29/I
BN/PLK3/ZFYVE19/HAUS5/UIMC1/GLI1/DYNC1I2/RAB11A/GPR132/NEDD1/MECP2/CENPJ
BID/CCNO/PPME1/DYNLT1/GPSM2/HAUS1/BLM/FBXO5/DBF4/TUBB4A/CNOT9/CDKN2B/CCL2/RTKN/
RA/SETD1A/RASSF2/LILRB1/GNAS/CD80/SOX13/RASGRP1/FGL2/LILRB2/NLRP3/RNF41/L3MBTL1
F5/VPS11/YWHAG/ATG13/ABHD17A/CLTC
D1/F3/FAM89B/VIL1/ADAMTS9/MAZ/DNAJA4/NDRG4/CCR1/CXCL16/PFN1/ABR/NSMF/C5AR1/KIF2

P/NOVA1/SRSF10/SRSF7/PRPF40B/TRA2A/PRMT7/CACTIN/RSRC1/METTL16/BCAS2/SNRNP35/DD
F10/SRSF7/PRPF40B/TRA2A/PRMT7/CACTIN/RSRC1/METTL16/BCAS2/SNRNP35/DDX46/ZCRB1/P
F10/SRSF7/PRPF40B/TRA2A/PRMT7/CACTIN/RSRC1/METTL16/BCAS2/SNRNP35/DDX46/ZCRB1/P

SMD7/BTK/GMFG/STX4/CBL/PRDX4/WDR1/CLEC5A/CCT8/TBC1D10C/FCGR3B/HPSE/RAB4B/CCL3/S
PIK3R6/CD5/IL13RA2/CAV1/TP53BP1/CD84/ATAD5/THOC1/INPP5D/DUSP22/ZNF683/PTPRC/NE
/PSMD9/PTK2/PTPN2/CASP8/RPS27A/DMBT1/IL20RB/PAK2/TLR2/MUC6/UBA52/RAB29/MUC15/CA
F1/HNRNPUL1/CASC3/SNRNP70/COIL/FIP1L1/RBM4B/DHX8/RBM8A/METTL3/CSTF3/SF3B6/MAGOH

B/RAB29/ARHGAP28/PLD2/RUNDC3A/RHOA/RAPGEF1/MRAS/PDPN/RAB27B/DOCK2/MAPKAPK5/HEG1
4/RHOA/RPL13A/HNRNPR/POLR2D/ANP32A/RPLP1/DHX9/MAPKAPK5/COA3/MTPN/TNPO1/PSMC5/C

PT1/MRPS27/SPEF1/GABARAP/SCIN/NAPB/MRPS18A/TWF1/MRPL43/CHMP2B/VILL/SMARCE1/CHM

YO1C/TFIP11/SPIRE2/NCKAP1/PPP1R9A
MD7/BTK/GMFG/STX4/CBL/PRDX4/CLEC5A/CCT8/TBC1D10C/FCGR3B/HPSE/RAB4B/CCL3/S100A7/
A/PAFAH1B2/RAB3D/ARPC5/VCP/VCL/APRT/PDAP1/HVCN1/VAMP8/BIN2/RAB5C/DBNL/PSAP/CD3
7/RAD21/MTBP/RFWD3/CEP250/RPA2/CUL4B/E2F6/TRIM71/HAUS6/NAE1/NDE1/KHDRBS1/CKAP5/

P1A/TGFB3/HELLS/CETN2/RAD21/KATNB1/BABAM1/VRK1/RACK1/RALB/HAUS6/GNL3/PARD6G/TI
GF/MAP4K1/CAV1/MAP4K2/DAB2IP/HMGA2/RPTOR/CALM3/EPHB2/CIB1/PKD1/PTPRC/BMP2/CDK5
/ACVR1B/CTNNA1/PTPN2/THBS1/CSNK2A1/STYXL1/CASP8/PTPN1/NBN/SRGN/PIAS4/VDAC2/GNAI

/RBM15/CARHSP1/PAPOLA/HSPA1A/CNOT1/PSMD6/LEO1/AHCYL1/NBAS/PSMC2/SRSF4/PSMA6/NU

PSMB7/KLHL6/PRAM1/SKP1/EIF2B2/CD19/PRKCB/PIK3R2/CLEC4A/VAV3/APPL2/SLA2/WNK1/BT
3/RPL13/RBM8A/METTL3/MAGOHB/HNRNPA0/PSMB1/TNFRSF1B/MOV10/RPL4/RPL41/SMG7/RPL9/
HNRNPD/RPS24/ZHX2/PSMA3/DCP2/PYM1/HNRNPC/RPS3A/AGO1/RPL14/RPL10A/FXR1/SYNCRIP/

P4A/PSMG2/SMCR8/CLEC16A/SETMAR/RNF41/TAOK1/SLX4/TMSB15A/TPR/PHF23

'A2B1/THUMPD2/RRP15/SMAD2/WDR3/RPS28/RPS17/PTCD1/NOLC1/SMAD3/NOL11/ERI1/DDX1/NSU

;TE24/PPP2R3C/MUL1/ARG2/LILRB1/PDE5A/TSG101/TARDBP/FGL2/MAPKBP1/TAF11/FBXL2/C1QB

.IG3/PPP2CA/HELB/TNFSF13/XRCC5/SMC1A/ACTR2
.A-E/SUPT6H/CD19/VAV3/PPP2R3C/SLA2/NOD2/ARG2/TNFSF13/LILRB1/PDE5A/CD80/SOX13/RAS

'TP53/GRIPAP1/GGA2/RPS15A/CHMP4A/RILPL2/RFTN1/SRP72/PEX16
./GPR132/NEDD1/MECP2/CENPJ
:/NMT1/FIGNL1/EYA3/PAM16/HYAL2/HTRA2/RELA

1

.P/SETDB1/RAB22A/TIAM2/RAB3C
;UPT3H/MAP3K12/BAZ1A/TP53/IWS1/PHF20/KAT5/KDM3B/NEK11/SUZ12/MSL3/BAZ1B/SETMAR/NA
N1
'K/GMFG/CBL/STK25/AKT1S1/WDR91/RACK1/ZNF675/RALB/RGS3/PIF0/IGF1R/MAPT/STK38/RUBC
:/GDF11/KEL/DENND5A/DBNL/PLXNC1/CTNNA1/SRGAP2/DNM2/PTK2/STYXL1/NOTCH1/TMEM98/FIG

'AR1/IL2RA/CTSS/CD47/PSMB8/NFAM1/TNFSF13B/PSMB10/PHB/FGR/LCP2/PSMA5/CD300LF/NRAS
'4/UBA52/DUSP7/MAP3K10/PIH1D1/DGKQ/CDK5R1/PRKAR1B/TPD52L1/DAXX/RHOA/STK3/RFC5/RA

/ZBED2/PKD1/CSNK2A2/BMP2/PAF1/DDB1/RYK/PSMB1/WWTR1/PIN1/CSNK1G1/MOV10/STK4/MAP3K
27/CDC34/HSP90B1/USP48/UBE4B/AUP1/MAEA/CACUL1/EGF/HSPA13/CAV1/NEDD8/FBXO6/EXOSC2
/CSNK2A1/VPS35/CASP8/RPL5/RPS27A/CAPN2/FBXW5/USP21/PLK3/MAGEF1/RGP1/MAP1A/BNIP3
SCO2/COX7A2/MX1/NDUFA9/NDUFB11/CNP/POLG2/NDUFA12/DDHD1/NDUFAF4/PLA2G6/MGME1/THEM

/PUS7/RPL5/RPS27A/MRPL18/PLK3/RPS12/JMJD4/SARNP/RPS4X/RPL6/C8orf88/NLN/MAGOH/RPL
IA4/HSP90AA1/PSMD9/RPL5/RPS27A/FBXW5/MAGEF1/RNF2/RAD18/MED27/MASTL/HSPA5/FBXO45/
.UP1/MAEA/CACUL1/EGF/HSPA13/CAV1/NEDD8/FBXO6/CSNK2A2/STUB1/ANAPC4/VPS4A/BAG6/HUW
IC3/VPS37B/USP11/UBE2D2/CHFR/AGAP3/GLMN/RNF8/ANKZF1/FAP/LGMN/TMUB1/PSMC5/FAF1/AR
.1/CBY1/ZBED2/PKD1/CSNK2A2/BMP2/PAF1/DDB1/RYK/PSMB1/WWTR1/PIN1/CSNK1G1/MOV10/STK
IT1/GMDS/MGAT4A/NAGPA/NME6/SLC26A2/CSPG5/TNIP1/PPCDC/CHST12/TAZ/HACD1/CSPG4/CEMI
:/PHB/FGR/CCL4/MAPK11/TGFB2/SFPQ/F3/TMEM101/LCK/PTPN6/CD27/CASP1/MAP3K14/MAZ/GSK

SHTN1/TSG101/TARDBP/TBPL1/MYH9/CHMP4A

IR1/NFKBIL1/PSMB1/TLR8/MAP3K7/UCN/PSMB6/FFAR2/CNPY3/MFHAS1/PLA2G2A/ICAM2/PSMA3/F
IKS1/DNAAF3/LRRC6/CCDC66/ZMYND10/DNAL1/MNS1/IFT27/YWHAE/TPGS1/RAB23/CCDC13/SPAG1

.IFAP3/DCTN3/NEK8/ALMS1/SCLT1/MKS1/DNAAF3/LRRC6/CCDC66/ZMYND10/DNAL1/MNS1/IFT27/

/HSPA13/CAV1/NEDD8/FBXO6/CSNK2A2/STUB1/ANAPC4/VPS4A/BAG6/HUWE1/BBS7/FAF2/TMEM67/
L2L1/GLI1/MEA1/SSTR2/UBE2Q1/TARBP2/GORASP2/TEX11/IFT20/STXBP1/RNF8/ATP2B4/PRKAG
/HEXB/MASTL/HSPA5/BCL2L1/GLI1/MEA1/SSTR2/UBE2Q1/TARBP2/GORASP2/NPAS1/TEX11/IFT2
SPA5/BCL2L1/GLI1/MEA1/SSTR2/UBE2Q1/TARBP2/GORASP2/NPAS1/TEX11/IFT20/RNF8/ATP2B4
/TP53/IWS1/GRIPAP1/NEFH/TARDBP/FEZ1

/SMYD3/LTBR/C5AR1/LRP8/CCND2/RPS3/DUSP12/PRKD2/ARRB2/STK10/MTCP1/TGFB3/MUC20/SEM

/EMILIN1/BRK1/ARPC5/ABL1/GHRHR/NFATC4/ACTR3/ARAP1/TRADD/MAPKAPK3/CCND3/PTBP1/PTP
/SPIRE2/ADAMTS2/RPS6/TBC1D20/SMAD5/INSL3/STRBP/TRIM27/NEURL1/DDX20/ING2/IGF2R/G
R5/PPM1F/PARD3/PRKAR1B/TWIST1/GLMN/GSKIP/RAPGEF1/YWHAG/MLLT6/ATP2B4/GPS1/PRMT3/

/STK10/MTCP1/TGFB3/MUC20/SEMA4D/XCL2/RBPMS/APP/ADRA2A/XRCC6/TMEM119/TELO2/RELL2/
/PTPRC/BMP2/ADCY3/ADNP/PIK3CB/ADCY7/PPP1R9A/LPAR1/STK4/MAP3K7/UCN/CCDC88A/COPS8/

RF/CAPRIN1/RHOA/STXBP1/ENPP2/ROBO3/PDPN/MINK1/NIN/NRTN/SPTAN1/EPHA2/ARTN/IST1/D
PYSL3/SNAPIN/AP2A1/IST1/ZC4H2/ARF1/LSM1/SNX3/DAB2IP/PREX1/EPHB2/CIB1/BMP2/CDK5R
3/PDPN/MINK1/NIN/NRTN/SPTAN1/EPHA2/ARTN/IST1/DOCK10/NEO1/ATP7A/DAB2IP/EPHB2/SLC
MD1/C5AR1/HLA-B/MSH2/PSMA7/RPS3/PRKD2/TRAF3/PSMB3/GRB2/ARRB2/CDC42/IL4R/MUC20/C

PSTE24/MPEG1/MUL1/TRIM56/NOD2/ARG2

[illegible]S/CACNG4/PI4KB/NECAP2/CLTB/AP2M1/CXCL16/WASF1/VPS28/KCNK9/FOLR2/LYAR/AP2S1/IL15

[illegible]/KITLG/EREG/CCL19/PIK3R6/IRAK2/CACUL1/EGF/MAP4K1/MAP4K2/DAB2IP/HMGA2/RPTOR/CAL[illegible]

/DNM1L/SH3GL1/AXL/CBL/CLEC7A/RACK1/M6PR/CSNK1G2/LBP/CLIP3/ARPC1A/RUBCN/ILDR1/RAB

L1/RPS6KA3/NOLC1/TSC1/PAIP1/RPL22/PDPR/GEMIN5/EIF3C/DDX1/EIF1/ATXN2

/MYOF/FGFR2/WASF1/AP2S1/UBE2D1/MST1R/PARP1/ATP6V0C/APH1B/EPS15L1/LOX/MVB12A/CADM

/SPTAN1/EPHA2/ARTN/IST1/DOCK10/NEO1/ATP7A/DAB2IP/EPHB2/SLC9A6/EPHA10/ARHGAP33/YT

P1A/OLFM4/VEGFA/RAB3A/ACTN1/LZTS1/FBXO45/RILPL1/RAB11A/TUBB3/IFRD1/CDK5R1/METRN
2/GTF3C4/MYB/BRCA2/WDR82/GTF2B/NUP133/SETD5/ATAT1/METTL22/GATA3/KAT7/KANSL1/RAS

DMD/TRAF3/REG1B/SBNO2/CD6/DROSHA/IL4R/APOBEC3D/CXCL11/CCR5/TLR5/GPATCH3/CARD16/
PHB2/SLC9A6/EPHA10/ARHGAP33/YTHDF1/ADNP/FEZ2/RYK/PIK3CB/PLXNB2/DPYSL4/KIF3A/FST
DMD/TRAF3/REG1B/SBNO2/CD6/DROSHA/IL4R/APOBEC3D/CXCL11/CCR5/TLR5/GPATCH3/CARD16/
ATG13/RPL37A/GOSR2/RPLP1/PMM2/RPL32/TNPO1/DMAP1/IPO9/RPL30/NXT2/KPNA6/TFDP1/IPC

AB1B/MAP3K7/STAM/PLEKHM1/CASP3/VPS26A/PIP4K2B/PARK7/USP36/UBQLN2/ULK1/LIX1L/PI
1/BATF3/PF4V1/EXOSC5/GSDMD/TRAF3/REG1B/SBNO2/CD6/DROSHA/IL4R/APOBEC3D/CXCL11/C
S1/EIF3M/PUS7/RPL5/RPS27A/UGCG/MRPL18/RPS12/JMJD4/SARNP/RPS4X/RPL6/C8orf88/MAG
AB1B/MAP3K7/STAM/PLEKHM1/CASP3/VPS26A/PIP4K2B/PARK7/USP36/UBQLN2/ULK1/LIX1L/PI

4A/SLA2/TSC1/BTN3A2/ARG2/TNFSF13/LILRB1/CD80/FGL2/LILRB2/FCGR1B/TFEB/C1QBP/CD8

`EZ1/DPYSL2/NEFL

`AIP3/MFF/PDCD10/KCNN4/CSK/BAD/CD33/ERP29/PTGER4/SLC8B1/PSMD9/VPS35/CASP8/PTPN1/

F2RA/PSMD4/SPRY4/PSMD3/RBX1/MAPK7/RELL2/PPP5C/PSMD7/MAP3K6/IKBKG/DSTYK/STK25/ZN
SMA1/TNFAIP3/KCNN4/CSK/ACTR3/MAPKAPK3/PSMB5/BCL10/PSMA4/SPPL3/HSP90AA1/PSMD9/PT
MB4
/RAPGEF1/IFT20/CLTC/AP2B1/PSMC5/RBPJ/CCAR2/AP2A1/MPP2/NPHP4/SNX3/TLE6/BARX1/KLH

/BLZF1/SEMA4C/RGS4/ADNP2/PTPRS/PRSS2/RPS6KA3/MUL1/PPP2CA/SHTN1/BAP1/SMAD3/UNC13

A2A/CSF2RA/PSMD4/SPRY4/PSMD3/RBX1/MAPK7/RELL2/PPP5C/PSMD7/MAP3K6/IKBKG/DSTYK/ST

/BAG3/SMARCC1/NUP155/AGO2/APP/ADRA2A/NUP107/SNX12/PSMD4/DNM1L/PSMD3/RBX1/PABPC4

1/RALB/VIM/DAP/TRIM71/ATP6V0D1/PLEKHN1/PTTG1IP/HNRNPM/MAPT/RUBCN/EXOSC3/RHEB/SU

[illegible]/CDK5R1/PPM1F/PPIB/NDUFA13/APBB1/SRF/RHOA/STK3/CD38/SIX1/SOCS7/LGMN/HBEGF/MTPN/
[illegible]CD22/RTKN2/THEM4/RNF149/PARK7/RHOH/ING2/MYC/FIGNL1/DYRK3/ARHGAP25/HYAL2/HTRA2/[illegible]

D1/SIX1

PPC2L/MMGT1/CLIP3/CHST12/PDCD6/NSF/B4GALNT4/ABCG1/TMEM87B/RHEB/GLG1/MFNG/FUT1/S
3/THOC2/RBMX/NCAPD3/SRF/TEX11/ACTR5/GATAD2A/DHX9/SMARCA5/RUNX3/DMAP1/CCAR2/POLE
/ATP6V0C/PSMD1/TMEM63A/KPTN/PADI2/TRAPPC1/BORCS8/GLMP/LUM/DNASE2/CST7/STARD3/V
/IRF4/HNRNPC/LRWD1/ING2/MYC/YY1AP1/MBD2/HTRA2/ASF1A/MAX/RELA/NSD1/FAM111A/SUPT
N/CHRAC1/KMT5A/PRIM1/SSB/EHMT2/BUD31/DCTN5/AKAP8L/SSRP1/ACTB/RBM19/MTA2/WDHD1/
RRP36/EIF4A3/C19orf53/ILF3/GORAB/ISG20/KIF2A/S100A16/CCND2/EXOSC5/SNU13/RPS3/G
/ATP6V0C/PSMD1/TMEM63A/KPTN/PADI2/TRAPPC1/BORCS8/GLMP/LUM/DNASE2/CST7/STARD3/V

G15/DRAM2/SLC29A3/GALNS/DSN1/MAP1LC3B/SLC44A2/SLC15A3/AP2S1/OSTM1/BGN/MST1R/UBA
BCL2L1/RILPL1/DYNC1I2/RAB11A/PLEKHG6/CCDC88C/NEDD1/KIF20B/MECP2/CENPJ/SASS6/PR
PIFO/TFAP2A/POLR3H/CKAP2/TTC26/CKAP5/HSPB11/MZT1/IQCB1/EMD/TBCD/RAD51D/PSMA1/NS

2/PDLIM4/FLII/RPS4X/RPL6/VEGFA/ACTN1/PACSIN2/HSPA5/LIMA1/RPL19/PARD3/PPIB/TLN1/
LR7/STEAP2/IRAK2/PDIA3/SNX3/YIPF1/CAV1/RAB13/SLC9A6/LITAF/VPS4A/SLC9A9/PLD1/KIA
ATP6V0C/C5AR1/TMEM63A/HLA-B/ATP6V0E1/SYT12/ANXA4/ABCA3/RALA/GRB2/ICA1/STK10/GP

ARNP/BNIP3L/MAGOH/HSF4/UIMC1/PACSIN2/ARL6IP4/GTF2E2/ZPR1/THOC2/TARBP2/KIF22/DGK

OR1/GNS/CD47/FTL/TCP1/NFAM1/FGR/PSMA5/NRAS/TGFB2/SERPINB6/PTPN6/PSMD13/AGPAT2/
1/SYT12/ABCA3/RALA/ICA1/STK10/GPR84/CALR/SYT1/LAIR1/CYSTM1/OCRL/DNM1L/AP1G2/CY
PPP1CC/SIRT7/CENPK/NUP43/PML/DYNC1LI1/DSN1/PARP1/UBA1/THOC6/NSMF/SMARCB1/CPSF6/
ALNT6/GOLGA3/TRAPPC1/LUM/STX18/FUT2/FUT6/ICA1/PARM1/LLGL1/CDC42/PRKCI/PLEKHJ1/
3/EEF1A2/RAB3D/VCP/HLA-DOA/NPRL3/ABCB9/STX7/VAMP8/RAB5C/WIPI1/PSAP/HSP90AA1/VPS
7B/AP2B1/LGMN/IFITM3/ATG9A/BECN1/TMEM184A/AP2A1/LRPAP1/VPS33A/TLR7/STEAP2/IRAK2
1/TBP/SMARCAL1/IPO4/MSH6/SAP130/MORF4L1/POLE2/EP400/LDB1/ATR/TP53BP1/SRCAP/THOC
7/VAMP8/WWC1/BCL10/HSP90AA1/CDK2AP1/DNM2/SPIRE1/TNK2/VPS35/SERHL2/CAPN2/BICD1/
NK2/RPL5/NOTCH1/CAPN2/PDLIM4/FLII/RPS4X/RPL6/VEGFA/ACTN1/PACSIN2/HSPA5/LIMA1/R
APPC3/SLC35C1/AP1B1/ST3GAL5/POMGNT1/STX4/MGAT4A/NAGPA/STK25/VRK1/ARFIP2/NOSIP/

X032/CDK5/FAM83H/CTTN/TCP1/KIFC3/KIF15/HOMER1/WAS/RASSF1/SPECC1/HAUS8/DYNC1LI1/
X032/CDK5/FAM83H/CTTN/TCP1/KIFC3/KIF15/HOMER1/WAS/RASSF1/SPECC1/HAUS8/DYNC1LI1/
PBP2/SRI/CD68/NCKAP1L/SYTL1/ADAM8/CAPN1/LAMP3/CPNE1/C1orf35/CAP1/ASAH1/KPNB1/SV
K5/FAM83H/CTTN/TCP1/KIFC3/KIF15/HOMER1/WAS/RASSF1/SPECC1/HAUS8/DYNC1LI1/MAP1LC
RT20/WDR1/CCT8/HAUS6/NDE1/MAPT/CKAP2/EPPK1/CKAP5/KCNAB2/RAB3D/EMD/TBCD/CALM2/AK
L10/TRIM71/UTP4/RBM17/RPL38/HNRNPM/DRG1/MAPT/MRPL52/SLBP/HNRNPA3/PRPF31/ZMAT5/

/PTPRC/NECTIN2/SRP68/ADD1/ASAP3/GNA13/RSU1/P4HB/CD59/NCKAP1/CASK/LAYN/RPL4/RPL
C/NECTIN2/SRP68/ADD1/ASAP3/GNA13/RSU1/P4HB/CD59/NCKAP1/CASK/LAYN/RPL4/RPL9/PXN/
HGAP22/PTPRC/NECTIN2/SRP68/ADD1/ASAP3/GNA13/RSU1/P4HB/CD59/NCKAP1/CASK/LAYN/RPL

A7/PXN/KCNC4/CCDC88A/SRA1/SH3RF1/PTPN13/THEM4/ATP2B1/VEZT/MYO1D/ADGRE2/RAB22A/

PG/ATP11A/EGF/PI4K2A/DAGLB/RPTOR/ATP6V1B2/AP5B1/LITAF/VPS4A/ATRAID/PLD1/KIAA132

MP3/AP2M1/ABCA2/RAB10/PML/SLC29A3/TNIK/SLA/VPS28/CD79A/AP2S1/IL15/UBA1/SNX27/AT
MYO6/FBXO45/TRIM21/WDR5/TRPC4AP/GTF2E2/VAC14/MORF4L2/TRIM37/CDK5R1/KLHL21/POLR2

/KCNAB2/ACHE/RAB3D/TANC2/ABL1/FCGR2B/MFF/PALM/STX7/RNF216/PRR12/DBNL/SGTA/SRGAP2

RC1/EIF4ENIF1/BCAS2/TAB1/DDX46/TRIP12/DUSP11/THAP7/SMU1/PRPF40A/FNBP4/AKAP17A/S

/PALM/KCNN4/BAG2/DBNL/HSP90AA1/PRKAA2/SRGAP2/DNM2/PTK2/DCTN1/VPS35/CAPN2/PLK3/PD

/EGR2/ING2/SETDB1/MBD2/POLR2B/PRMT6/PHF13/BRD4/ASF1A/RELA/NSD1/MAPK15/LHX2/POL

SFMBT2/EDF1/ENY2/TADA3/SRSF2/SIN3B/TRRAP/HSBP1L1/PPARG/JUNB/WWTR1/SUPT7L/MNT/T

K2A2/EPHA10/RPS6KC1/SRPK2/STK35/VRK2/ADCK2/CSNK1G1/CASK/STK4/MAP3K7/PSKH1/RIOK1

1/EFEMP2/RPS15/CTNNA1/RPS16/MRPS15/THBS1/RPL5/RPS27A/MRPL18/BICD1/MYL9/RPS12/MA

CALM3/ARHGAP27/RIC8B/ARHGAP17/MMP15/LLGL2/BMP2/RALBP1/PITRM1/ARHGAP33/EVI5L/ASA

CLS1/PSTPIP1/HSPH1/DCTN2/LYN/ARPC5L/FKBP4/MYLIP/ACTB/B9D2/CDK5/CTTN/KIFC3/CORO2

/MARK4/PAK2/MASTL/CDK11B/CSNK2B/TBRG4/DCK/LMTK2/MAP3K10/SEPHS1/TP53RK/DGKQ/CDK5

TP6V0E1/CDK7/MSH2/ARF4/CHD4/RUVBL2/RHEBL1/RBBP4/ABCA3/TTF2/GNL2/RALA/MCM8/PTPA/

TP6V0E1/CDK7/MSH2/ARF4/CHD4/RUVBL2/RHEBL1/RBBP4/ABCA3/TTF2/GNL2/RALA/MCM8/PTPA/

TP6V0E1/CDK7/MSH2/ARF4/CHD4/RUVBL2/RHEBL1/RBBP4/ABCA3/TTF2/GNL2/RALA/MCM8/PTPA/

VBL2/RHEBL1/RBBP4/ABCA3/TTF2/GNL2/RALA/MCM8/PTPA/DDX41/NKIRAS2/CDC42/DNAL4/AGA

EP250/LDHB/TBL2/TNIP1/PARD6G/PIFO/MAPT/NSF/STK38/RHEB/CSPG4/DBF4B/LIMS1/ACVR1/

G/CSK/TRADD/CHEK2/MAPKAPK3/CCND3/BAD/BCL10/HSP90AA1/DNM2/PRKRIP1/PTK2/PTPN2/DC

YO5A/VIM/ARFIP2/M6PR/DAP/RAD9A/CEP250/NDE1/HNRNPM/MAPT/NSF/KHDRBS1/CARD18/MED12

2L3/SPOCK2/SPOCK1/DNAJB2/WAS/GPSM3/SERPINB6/TBC1D13/VIL1/RASA3/CD27/CASP1/MAP3K

/ADAM17/PTPN1/S1PR3/SH3GLB1/CD177/ADAMTS5/OLFM4/RPL6/ACTN1/PAK2/PACSIN2/HSPA5/

SF5/RAPGEF1/IFT20/STXBP5/FNBP1L/DOCK2/WDR41/TNPO1/IPO9/SH3BP5L/BECN1/EPS8L1/SG

0/BCR/MADD/NOX1/RHOH/TIAM2/ULK1/KNDC1/RAB3IP/SRGAP1/TRIO/IPO7/TMEM127/PDE6D/TB

F18/NCKAP1/TBC1D20/BCR/MADD/NOX1/RHOH/TIAM2/ULK1/KNDC1/RAB3IP/SRGAP1/TRIO/IPO7/

/POLR3H/STK38/POLR3C/OBSCN/CCL3/CDK19/ACVR1/PTGES3/FASTK/ABL1/STK24/PIM3/NME7/SR

/CDK20/STK3/NEK6/GAK/PRKAG1/MAP3K1/MINK1/MAPKAPK5/PHKA2/GTF2H1/PHKG2/ADCK5/EPHA2

[illegible]CTN4/CXXC5/DHX9/NR1H2/ZMIZ1/DMAP1/PSMC5/FAF1/CRTC2/RBPJ/METTL23/TBP/MMS19/CTBP1

[illegible]N2/GATC/DIS3L2/DDX50/CRCP/METTL8/DDX18/RNASEH2B/POLR1D/TSEN2/POLR2J3/EXOG/TRPT1

[illegible]EURL1B/MED1/RLIM/XIAP/UHRF2/UBA3/RNF215/FBXW11

SR2/LMO4/USF1/ZIC5/NOTCH1/ZNF711/MBD3/GTF3C5/HNRNPU/STAT5A/HOXA5/SARNP/THRA/HSF

/HAUS5/MAP7D3/RAB11A/ZPR1/CCDC88C/TUBB3/EML2/TRIM37/MECP2/CENPJ/CDK5R1/PARD3/S
NCENP/RAB35/FGFR1/PIM2/PSME3/SLC9A3R1/BID/CCNO/PPME1/DYNLT1/GPSM2/HAUS1/BLM/FB
EMB/CAPZB/VWA1/FGD6/BTBD3/SEMA4B/GRB2/PARD6B/AKIP1/LLGL1/CDC42/LAMB3/ARHGAP18/
ITGB8/CLEC5A/TRIM25/POLR3D/LBP/POLR3H/HPSE/POLR3C/CARD18/BTN2A2/CCL3/HSPD1/CXC
XCL17/ABL1/FCGR2B/TNFAIP3/NFATC4/CSK/RNF216/CD33/BCL10/PTGER4/TWSG1/TUSC2/THBS1
F135/AFAP1L2/CD80/DDX1/RASGRP1/LILRB2

ADI2/TRAPPC1/GSDMD/SBNO2/STK10/GPR84/CNN2/IL4R/ALDOC/APP/LAIR1/PNP/SWAP70/CYST
PPC1/GSDMD/SBNO2/CST7/STK10/GPR84/CNN2/IL4R/ALDOC/APP/LAIR1/PNP/CYSTM1/XRCC6/P
CCL3/S100A7/KCNAB2/GCA/PAFAH1B2/RAB3D/ARPC5/VCP/VCL/APRT/PDAP1/HVCN1/VAMP8/MIL
/HSP90AA1/CD177/MAN2B1/OLFM4/CYB5R3/GLIPR1/RAB3A/HEXB/TLR2/CSNK2B/TMBIM1/SLC15
ADI2/TRAPPC1/GSDMD/SBNO2/STK10/GPR84/CNN2/IL4R/ALDOC/LAIR1/PNP/SWAP70/CYSTM1/X
GALNS/DSN1/IL12RB1/SLC44A2/IMPDH2/ABR/ATP6V0C/PSMD11/PSMD1/C5AR1/TMEM63A/HLA-B/
5C/DBNL/PSAP/CD33/HSP90AA1/TUSC2/ADAM17/CD177/MAN2B1/OLFM4/CYB5R3/GLIPR1/RAB3A/
D1/RPS19/AXL/FADD/BTK/BABAM1/ZNF675/WDR1/XRCC4/CLEC5A/DPF2/GPR183/WNT4/TAZ/EXO
AK1/CCR2/FZD9/DOCK11/CD19/POU2F2/GON4L/PPP2R3C/ESRRA/TSC1/RPL22/RASSF2/LILRB1/
1/SLC11A1/HSPH1/JAG1/CD209/LYN/HLA-DRB5/CARD11/GPRC5B/FPR3/MUC3A/RAC1/CBFB/ITG
/STOML2/WIPF2/CD79B/CD38/RAPGEF1/MAP3K1/LGMN/ELMO2/PSMC5/UBE2N/CD247/TLR7/HSP9
PL2/SLA2/WNK1/BTN3A2/EIF2B3/ACTR2/IKBKB/LILRB1/LILRB2/FCGR1B/RFTN1/NCKIPSD/BTN
POLE4/RPS3/RBBP4/POLA1/USP1/PRKD2/DFFA/BARD1/UBE2A/DNASE2/ARRB2/MCM8/PARM1/POL
MSH6/MORF4L1/POLE2/ATR/TP53BP1/HMGA2/PARP10/DCLRE1C/PAGR1/FBXO6/ERCC3/CIB1/MMS
R5/MLLT6/RNF8/SART3/PRMT3/ANP32B/DNMT3B/SMARCA5/DNAJC2/COPRS/ZMIZ1/DMAP1/CRTC2/
TP6/HEATR1/SART1/SYMPK/PHF5A/NOP10/KRI1/FAM98B/SCAF1/RPS7/SRPK1/RPL7A/WDR55/GA
1/RPLP1/DHX9/MAPKAPK5/COA3/RPL32/MTPN/RPL30/CNOT10/YARS2/MEX3D/MRPL15/C12orf65/
/RAC1/MAGED2/CDK5/CTSS/PDGFA/LAMTOR1/GNS/CD47/S100A13/FTL/NFAM1/FGR/PSMA5/NRAS/

BAM1/CBL/ZBTB40/UBE2L6/RFWD3/MSX1/NABP1/RAD9A/XRCC4/OGG1/RPA2/CUL4B/PPP4C/RBM1
TNB1/WRAP73/RNF4/IGBP1/COPG1/CCDC120/CEP250/HAUS6/PARD6G/NDE1/CLIP3/DRG1/MAPT/
PA2/CUL4B/HAUS6/NAE1/NDE1/ZWILCH/DRG1/CKAP5/DBF4B/BTN2A2/MEIS2/RAD51C/BTG2/PSM
AR1/TIPIN/TNFSF12/MATK/CCND2/RPS3/PRKD2/REG1B/UBE2A/CD6/CALR/PRKCI/TGFB3/PRRX1/
D/PRMT5/SNRNP200/RBM22/DDX20/GEMIN8/U2SURP/HNRNPC/THOC3/SREK1IP1/POLR2B/BUD13/
/UGGT1/UGGT2/FBXL18/FBXL2/CDC16/KAT5/UBE2R2/RAD23A/SVIP/DVL1/DERL3/CLEC16A/RNF
3/CDC42/KAT2A/ZNF207/CBX5/CHMP1A/CALR/TOPBP1/RANBP1/APP/PSMD4/CETN2/PCGF2/MED2
PIH1D1/MECP2/CENPJ/CDC37/CDK5R1/HNRNPA1/PPM1F/STOML2/DSTN/RHOA/PPHLN1/VPS11/YW
EF3/CTNNA1/DNM2/TNFSF15/PTPN2/THBS1/STYXL1/CASP8/NOTCH1/PDCD2/RPS27A/CAPN2/SFR
A/GRK2/APOBEC3D/CPSF4/RPL35/RANBP1/RPS20/CCR5/NUP107/POLR2K/XRCC6/RPL18A/RBX1/
GSPT2/SART1/SYMPK/PHF5A/PSMA1/SCAF1/RPS7/SRPK1/RPL7A/RPS23/MYEF2/RPS10/RBM42/C
KAT5/KDM3B/NEK11/SUZ12/MSL3/BAZ1B/SETMAR/NACC2/MEN1/ARRB1/RNF40/EZH1/MCM3AP/OG
SG20/HLA-B/IL1R2/TNFSF12/PADI2/PSMA7/LRP8/PF4V1/TRAF3/PSMB3/IL2RB/RALA/GRB2/CN
AR/MRPL15/BECN1/C12orf65/SNAPIN/MRPL4/IST1/VPS33A/ATG7/HTT/HAX1/CAV1/GRWD1/CAM
SURF6/PELP1/RPS9/WDR46/IMP4/EIF3G/ATR/RBFA/RPS14/RPS27/EXOSC2/EIF2D/GEMIN2/SRS
7/TRPM4/EPB41L1/GMFG/ABI2/MYO5A/ARHGDIA/VIM/ARFIP2/SCN1B/WDR1/EPB41L3/FMNL3/SE
5/ACTR1A/OFD1/PLD1/BBS7/RFX2/CCDC40/EVI5L/TMEM67/CEP72/WWTR1/KIF3A/NCKAP1/PPP1
/WNT4/NF2/OBSCN/BRK1/ARPC5/ABL1/ACTR3/ARAP1/DBNL/PTGER4/SPIRE1/PTPN1/SSH2/INPP
NAB2/MEIS2/MFNG/ESCO2/PSMD8/LFNG/RARG/ABL1/SART1/HLA-DOA/FCGR2B/PSMA1/SFRP2/TO

ADD/PELI1/STX4/ANGPT2/ARHGDIA/CSPG5/RASAL3/SEMA3E/WNT4/NF2/BTN2A2/EMILIN1/HSPD1
P1/SGSH/PRPF19/UBXN1/GGA1/PSMD8/EEF1A2/KLHL42/VCP/PSMA1/ATG4B/TNFAIP3/USP5/NSF
ALR/ZMYND8/SEMA4D/XCL2/CMKLR1/APP/ADRA2A/SWAP70/DPEP1/SEMA4F/FADD/ECM1/STX4/SE
1/APP/DNM1L/MAPK7/SYT3/SEMA4F/GDPD5/KATNB1/SEMA5B/STK25/ABI2/ARHGDIA/VIM/SCN1B/

56/DEF8/RAB11A/RAB29/IFRD1/CENPJ/PACSIN1/PPP2R5B/CDK5R1/METRN/APBB1/SRF/CAPRIN1
HL/WSB1/PSMA1/TNFAIP3/USP5/RPS7/FOXF2/BAG2/TBC1D7/RNF216/PSMB5/BCL10/PSMA4/HSP9
/OXCT1/PTPN23/IL1R2/YWHAQ/XPO5/YWHAB/ANXA4/GSDMD/ACSL3/CROCC/BARD1/BAG3/ICA1/IL
GPA/TMEM107/MSX1/M6PR/TIMM50/CEP250/RPA2/CCT8/ROMO1/GNL3/RPL38/PTTG1IP/MAPT/TTC
5J/AP2S1/TUBG2/PARP1/SET/PSMD10/PSMD11/SMARCB1/SMYD3/CPSF6/CDK7/GRB7/RUVBL2/PRF
MD9/RPS16/RPL5/RPS27A/HNRNPU/RPS12/RPS4X/RPL6/PFKFB2/EXOSC9/EXOSC10/MAGOH/RPL37
9/DPH3/UTP23/RPP30/SUV39H1/POLR2E/PRKRA/PELP1/RPS9/WDR46/SND1/IMP4/KHSRP/DALRD3
B/APRT/PDAP1/HVCN1/VAMP8/BIN2/RAB5C/DBNL/PSAP/CD33/HSP90AA1/CD177/MAN2B1/OLFM4/
MAZ/DNAJA4/NDRG4/PML/CCR1/TUBB2B/CXCL16/PFN1/ABR/USP14/NSMF/C5AR1/KIF2A/PTPN23/
2/EGF/CAV1/ATP7A/TMEM102/DAB2IP/PREX1/CIB1/PTPRC/VPS4A/BMP2

P1/HVCN1/VAMP8/BIN2/RAB5C/DBNL/PSAP/CD33/HSP90AA1/CD177/MAN2B1/OLFM4/CYB5R3/GL

/MRPL51/MRPL11/RPLP1/DHX9/MAPKAPK5/COA3/RPL32/MTPN/RPL30/CNOT10/YARS2/MEX3D/MRF
PN2/SFRP4/TOMM22/PIAS4/HOXA5/IRF5/ARHGEF11/BNIP3L/PAK2/USP27X/BCL2L1/UBA52/MAP3
DIA/MSX1/OGG1/CLEC5A/BNIP1/PTTG1IP/IGF1R/TFAP2A/WNT4/PELI3/HSPD1/ANKLE2/BTG2/GF
PN2/SFRP4/TOMM22/PIAS4/HOXA5/IRF5/ARHGEF11/BNIP3L/MARK4/PAK2/USP27X/BCL2L1/UBA5
L/IGBP1/ARHGDIA/MSX1/OGG1/CLEC5A/BNIP1/PTTG1IP/IGF1R/TFAP2A/WNT4/PELI3/HSPD1/AN
DC26/SOCS5/KLHL22/ANAPC1/PCBP2/DNAJC18/UBXN2A/ZFAND2B/UBE2K/PSME2
8/SEMA4D/APP/DNAJB6/SWAP70/CDC42EP2/MED25/DNM1L/RBX1/PIP4K2C/GMFG/DR1/WRAP73/RN
STUB1/VPS4A/KIAA1324/BIK/ADNP/DDB1/MYO1C/SPIRE2/NCKAP1/LPAR1/ADGRL1/PXN/STAM/PL
RB2/POLR2L/NUP155/CDC42/KAT2A/VPS18/CBX5/CHMP1A/GRK2/APOBEC3D/CPSF4/RPL35/RANBF
LA1/GSDMD/PSMB3/IL2RB/REG1B/RPL27/GRB2/POLR2L/NUP155/CDC42/KAT2A/VPS18/CBX5/CHM
KHDRBS1/HPGDS/TTC26/WNT4/VMP1/SOHLH2/PIWIL4/HSPB11/PLCD4/RAD51C/PAFAH1B2/ACVR1/
4B/E2F6/TRIM71/HAUS6/NAE1/TIMELESS/NDE1/SLBP/KHDRBS1/CKAP5/DBF4B/BTN2A2/RAD51C/
NB6/PTPN6/PSMD13/AGPAT2/COMMD9/RAB10/DYNC1LI1/GALNS/DSN1/SLC44A2/IMPDH2/ABR/ATF
OCH/CXCL16/UBE2D1/MST1R/USP14/PSMD10/PSMD11/PSMD1/ISG20/HLA-B/PSMA7/LRP8/GSDMD/
RIM15/NLRC4/BPIFB1/PTPRS/CLEC4A/APPL2/RPS6KA3/TLR1/S100A1/MUL1/NOD2/XRCC5/IKBKE
SEMA4D/SYT1/CMKLR1/PNP/TMEM119/DNM1L/TRPM4/SYT3/GDPD5/LILRB4/AP3D1/KATNB1/AXL/F
/CHEK2/CLSPN/PSMB5/RAD1/SGSM3/PSMA4/PRKAA2/VASH1/PSMD9/THBS1/CDK2AP2/NOTCH1/NBN
/CSK/CLSPN/MAPKAPK3/CCND3/DBNL/ERP29/HSP90AA1/TNFSF15/PRKRIP1/PTK2/PTPN2/THBS1/
0/PTGER4/VCAM1/TWSG1/TUSC2/PTPN2/ADAM17/CASP8/NBN/TMEM98/ERCC1/IL20RB/PAK2/HLA-

C5A/DPF2/GPR183/WNT4/TAZ/BTN2A2/CCL3/KCNAB2/MEIS2/MFNG/ESCO2/PSMD8/LFNG/RARG/AE
RF/CC2D2A/RHOA/STK3/GLMN/IFT20/CLTC/SIX1/PDPN/AP2B1/HBEGF/HEG1/PSMC5/RBPJ/CRYGS
IL13RA2/CAV1/TP53BP1/CD84/ATAD5/THOC1/INPP5D/DUSP22/ZNF683/PTPRC/NECTIN2/CRLF2/

/RAD1/DENND5A/PTGER4/DNM2/LMO4/PTK2/THBS1/STYXL1/VPS35/NOTCH1/ODF2L/NBN/HNRNPU/
JA4/NDRG4/CCR1/TUBB2B/CXCL16/PFN1/ABR/NSMF/C5AR1/KIF2A/PTPN23/CADM4/PADI2/ABI3/

MTCP1/TGFB3/MUC20/VPS25/APP/ADRA2A/DCUN1D5/XRCC6/HMGN1/SPRY4/DNM1L/TELO2/MAP3K6
HN1/MTCH1/HSPD1/CHTOP/LIMS1/PIDD1/HSPA2/TBCD/VCP/NPRL3/RASAL1/SFRP2/CALM2/SIPA1
A/MARK4/ADD3/LIMA1/CD2AP/EML2/TRIM37/MECP2/CENPJ/CDK5R1/ARHGAP28/PPM1F/RBM14/DS
OD1/COPG2/HSP90AA1/RPS15/DNM2/C12orf4/PTK2/SPIRE1/DCTN1/VPS45/CEP164/HAUS7/CFTF
B/CASK/TBC1D20/LRPPRC/CDC23/SYT11/KIF5B/ADGRE2/TRIM46/MAPK15/TERF1/VAMP1/TFG/CH
335/RARG/CBFA2T2/ABL1/STK24/STMN2/RASAL1/SFRP2/THBS2/NFATC4/GDF11/KEL/DENND5A/D

PTPRF/SEMA3E/MAPT/TGIF2/RHEB/NF2/CCL3/CHN1/PRPF19/ZNF335/LIMS1/CBFA2T2/ABL1/STK
AG3/PARD6B/IL4R/VPS18/PRKCI/CXCL11/ZMYND8/SYT1/ADRA2A/SNX12/GZMB/DNM1L/TRPM4/SF
/CIAPIN1/PAX8/BAG3/ARRB2/PRKCI/TGFB3/CCR5/SEMA4D/DNAJB6/DPEP1/PCGF2/MAPK7/PPP5C
/TRAPPC2L/PDCD6/ARPC1A/NSF/TAZ/GCA/MTCH1/ANKLE2/SEC13/ARPC5/RHOT2/SLC25A5/EMD/F

IP/RNF7/CUL4B/TRIM71/GNL3/TNIP1/NAE1/INO80C/TRIM25/ZNF598/UBE2O/PTTG1IP/RNF122/I
PRL3/ATG4B/NSFL1C/STX7/ACTR3/RPS23/CEP19/RPS10/CHEK2/TBC1D7/CEP41/BIN2/IFT172/WI
)/GOLPH3L/CACNB3/PACSIN1/TIMM10/GORASP2/CDK5R1/RPL19/VPS37B/PARD3/NDUFA13/RPS11/
./VASH1/SGTA/HDAC10/PTPN2/SPIRE1/PTPN1/CHCHD2/FUT8/MAGEF1/PIAS4/ERCC1/VEGFA/UIMC
N1/MZT1/BRK1/COL11A1/ARPC5/ABL1/TBCD/STMN2/SFRP2/ACTR3/ARAP1/IFT172/DBNL/HOOK2/F
P3D1/FADD/PELI1/RIC8A/TNIP1/RASAL3/PTPRF/WNT4/VMP1/NF2/BTN2A2/HSPD1/LIMS1/ACVR1/
'CCDC40/EVI5L/TMEM67/CEP72/WWTR1/KIF3A/NCKAP1/PPP1R9A/LPAR1/PLEKHM1/ARMC9/CCDC88
IFRD1/CENPJ/PACSIN1/PPP2R5B/CDK5R1/METRN/APBB1/SRF/CAPRIN1/RHOA/CD38/RAPGEF1/IFT

'DAPK3/CSNK1D/ANAPC7/CDK2/LSM10/KIF14/KPNB1/SMC4/NASP/DCTN2/KNSTRN/LIG1/PRIM1/CI
./PSMD12/PURB/IL12A/ZBTB7B/ROR2/PRDM1/SOCS5/STAT3/KMT2A/SCIN/PSME2/RBM15/MYB/PRI

2A/PTPN23/CADM4/PADI2/ABI3/GRB7/TACSTD2/PRKD2/SEMA4B/PARD6B/SYDE1/STK10/AGO2/ARH

K46/ZCRB1/PCBP2/PSIP1/SMU1/AAR2/PRPF40A/XAB2/SYF2/RBM15/PTBP2/SNUPN/HNRNPH1/CWC1
CBP2/PSIP1/SMU1/AAR2/PRPF40A/XAB2/SYF2/RBM15/PTBP2/SNUPN/HNRNPH1/CWC15/WDR83/PAF
CBP2/PSIP1/SMU1/AAR2/PRPF40A/XAB2/SYF2/RBM15/PTBP2/SNUPN/HNRNPH1/CWC15/WDR83/PAF

S100A7/KCNAB2/GCA/PAFAH1B2/RAB3D/ARPC5/VCP/VCL/FCGR2B/APRT/PDAP1/HVCN1/VAMP8/MII
CTIN2/CRLF2/IKZF3/CNR1/METTL3/LOXL3/DOCK8/TNFRSF1B/NFKBID/CASP3/CLPTM1/CD22/BCR/
ACNB3/PLD2/PSMC3/UBE2D2/STOML2/WIPF2/CD79B/CD38/RAPGEF1/MAP3K1/LGMN/ELMO2/PSMC5/
IB/SNRPD3/PRPF38B/HNRNPA0/ZNF473/ACIN1/TFIP11/CELF2/IK/RPRD1A/PPP1R8/ADAR/POLDIF

./IFT22/EPS8L1/KITLG/KSR1/SSX2IP/NOTCH2/RHOBTB2/RAB9A/DOCK10/RAB40C/RAB13/DAB2IF
NOT10/RBM10/MEX3D/SEH1L/RBM24/DPH3/EIF6/EIF4G2/EIF4G3/CNOT3/POLR2E/PRKRA/RPS9/KH

P2A/MRPS9/SMARCC2/MRPL50/CHMP7/EPS8/SNX14/CHMP5/GLE1/MTRF1L/MRPS35/MRPS33/MID1/M

'KCNAB2/GCA/PAFAH1B2/RAB3D/ARPC5/VCP/VCL/FCGR2B/APRT/PDAP1/HVCN1/VAMP8/MILR1/BIN
3/HSP90AA1/CD177/MAN2B1/OLFM4/CYB5R3/GLIPR1/RAB3A/HEXB/TLR2/CSNK2B/TMBIM1/SLC15A
'DBF4B/BTN2A2/RAD51C/BTG2/PSMD8/ACVR1/TAF10/PIDD1/HSPA2/PKIA/PSMA1/MTA3/CHEK2/CI

MELESS/NDE1/ZWILCH/CKAP2/CKAP5/ANKLE2/SUSD2/KLHL42/SFRP2/KDF1/CALM2/AKNA/ACTR3/C
RAP1
[3/PTPMT1/BNIP3L/PAK2/TMEM109/TNFRSF25/TMBIM1/TXNDC12/IVNS1ABP/BCL2L1/PIH1D1/MEI

OT21/RBM4

N3A2/EIF2B3/ACTR2/IKBKB
RPS6/PPP1R8/RPL24/PSMB6/GTPBP1/LRPPRC/RPL11/RPL10/HNRNPD/RPS24/ZHX2/PSMA3/DCP2/F
EIF4G1/PSMB7/CNOT2/RPL12/RPS18/RPS28/ELAVL1/FASTKD1/RPS17/PNPT1/SERBP1/NUP214/PF

N5P2/METTL2B/ZNHIT6/AARS2/TSR1/METTL6/GRSF1/RPP38/PARN/MRPS11/THUMPD3/QTRT2/WD[illegible]

[illegible]P/RAD23A

[illegible]GRP1/FGL2/LILRB2/NLRP3/RNF41/TICAM1/CD40/TNFSF18/CLECL1/IL12A/WNT3A/ZBTB7B/PRD[illegible]

ACC2/MEN1/ARRB1/RNF40/EZH1/MCM3AP/OGT/KANSL2/PRMT7/ZBTB7B/BRD8/WDR70/TRIP12/MSL1

CN/CSPG4/DBF4B/NF2/CEMIP/TRAF7/EEF1A2/CXCL17/PKIA/ABL1/STK24/SFRP2/TNFAIP3/CALM2
G4/MOB2/LLPH/VEGFA/TLR2/LZTS1/HSPA5/DDX56/RAB11A/RAB29/KIF20B/IFRD1/PACSIN1/NOTC

S/WAS/TGFB2/SFPQ/PQBP1/LCK/PTPN6/PSMD13/IFI16/PSMC4/MUC5B/SAMSN1/SOCS3/IL12RB1/C
APGEF1/ATG13/ATP2B4/PRKAG1/PIK3R5/MAP3K1/MINK1/RPLP1/MAPKAPK5/TIGAR/ADCY6/ADORA2

K7/KREMEN2/PSMB6/VPS26A/NXN/PFDN5/PSMA3/PDE6G/AGO1/MBD2/BRD7/CELSR3/LYPD6/BCL7B/
2/CSNK2A2/STUB1/ANAPC4/VPS4A/BAG6/HUWE1/BBS7/FAF2/TMEM67/DDB1/FBXO44/SKP2/PSMB1/
3L/USP27X/HSPA5/FBXO45/UBA52/CD2AP/AP2A2/EDEM2/TRPC4AP/PCYOX1L/RNF213/PSMC3/VPS3
M4/COA4/PPRC1/PARK7/IMMP1L/TIMM9/SLC25A36/TIMM22/USP36/NMT1/PAM16/HTRA2/WDR45/A[illegible]

L37/RPL27A/RCC1L/COPS5/GGA3/WARS2/EIF1AD/RPL36/UBA52/HSPA14/NANOS1/ATP6AP2/TARBP
/TRIM21/IVNS1ABP/LRR1/UBA52/TRPC4AP/UBE2Q1/TRIM37/RNF213/RNF25/UBR5/PSMC3/KLHL21
WE1/BBS7/FAF2/TMEM67/DDB1/FBXO44/SKP2/PSMB1/WWTR1/SUMO2/USP3/FBXO10/TRIM32/PSMB6
RDC1/CCAR2/CTSW/SDF2L1/FBXL7/UBE2N/ANAPC5/HECTD3/CDC27/CDC34/HSP90B1/USP48/UBE4
K4/MAP3K7/KREMEN2/PSMB6/VPS26A/NXN/PFDN5/PSMA3/PDE6G/AGO1/MBD2/BRD7/CELSR3/LYPD6
IP/MFNG/FUT1/SEC13/LFNG/EXT1/VCP/APRT/DHDDS/NME7/AMDHD2/ACOT8/PRKAA2/B4GALNT2/NE
K3A/NDRG4/CCR1/SOCS3/TNIK/FGFR2/DVL2/SLC44A2/MST1R/IL15/LTBR/C5AR1/GPX1/RPS3/PRK

PARK7/IRF4/POLR3F/CTSL/HYAL2/CTSB/RELA/AIM2/PSMB7/CCR2/SKP1/HLA-E/NOD1/TRIM15/NL
16/BBIP1/PARVA/WDR35/TCTN3/WDR90/SDCCAG8/POC1B/STK36/NUDCD3/KIF3B/ARL13B/AHI1/AT

/YWHAE/TPGS1/RAB23/CCDC13/SPAG16/BBIP1/PARVA/WDR35/TCTN3/WDR90/SDCCAG8/POC1B/STK

/DDB1/FBXO44/SKP2/PSMB1/WWTR1/SUMO2/USP3/FBXO10/TRIM32/PSMB6/RPL11/CDC23/KLHL25/
G1/ZMIZ1/SPATA24/DEDD/TBP/EREG/MORN2/CDYL/SPATA2/RNF114/DKKL1/DZIP1/CIB1/SIX5/CS
20/RNF8/ATP2B4/PPAT/PRKAG1/ZMIZ1/SPATA24/CYP27B1/DEDD/TBP/EREG/MORN2/KPNA6/CDYL/
4/PPAT/PRKAG1/ZMIZ1/SPATA24/CYP27B1/DEDD/TBP/EREG/MORN2/KPNA6/CDYL/SPATA2/RNF114

IA4D/XCL2/RBPMS/APP/ADRA2A/XRCC6/TMEM119/TELO2/RELL2/MAP3K6/IKBKG/DSTYK/STK25/BC

PN12/HSP90AA1/PTK2/PTPN2/THBS1/ADAM17/PTPN1/STAT5A/VEGFA/PAK2/SREBF1/LMTK2/AP2A2
GMCL1/DUSP13/FIGNL1/NPHP1/SPIN4/NR6A1
/DNMT3B/HEG1/TIPRL/SH3BP5L/MECOM/CTBP1/CDYL/STRAP/PPP1R14C/GMFB/DUSP18/CAV1/DAB2

/MAP3K6/IKBKG/DSTYK/STK25/BCAR3/RACK1/RALB/GPR183/IGF1R/CLIP3/CSPG4/DBF4B/CCL3/S
/NAB2/MADD/NEURL1/PARK7/PDE6G/SLC27A1/CD4/JTB/IFNG/MAP3K9/NOD1/MAP4K4/MAP3K11/PI

OCK10/NEO1/ATP7A/DAB2IP/EPHB2/SLC9A6/EPHA10/NECTIN2/ARHGAP33/YTHDF1/ADNP/FEZ2/
AP1/ARHGAP33/YTHDF1/ADNP/CNR1/RYK/PLXNB2/FSTL4/MOV10/PPP1R9A/LPAR1/TRIM32/CCDC
9A6/EPHA10/ARHGAP33/YTHDF1/ADNP/FEZ2/RYK/PIK3CB/PLXNB2/DPYSL4/KIF3A/FSTL4/NCKA
MKLR1/TLR5/PSMD4/HK1/XRCC6/GPATCH3/PSMD3/PSMD7/LILRB4/RPS19/IKBKG/FADD/BTK/PEL

[illegible]/SIGLEC1/ABR/EPS15L1/LRP8/CDC42SE1/LDLRAD3/IL2RB/GRB2/ARRB2/CD6/CDC42/SLC12A7/[illegible]

[illegible]3/CIB1/PKD1/BORA/PTPRC/BMP2/ADCY3/ZNF16/ADNP/PIK3CB/ADCY7/PPP1R9A/LPAR1/STK4/[illegible]

4B/ACHE/BRK1/ARPC5/SUSD2/ABL1/FCGR2B/IL10RA/CSK/ACTR3/RUFY1/MAPKAPK3/BIN2/RAB5

4/ATP6V0E1/ARF4/MATK/GRB7/NCOA5/PRKD2/SMARCC1/PTPRE/GRB2/ARRB2/COL4A2/POLR2L/C

HDF1/ADNP/FEZ2/RYK/PIK3CB/PLXNB2/DPYSL4/KIF3A/FSTL4/NCKAP1/PLCG1/MOV10/PPP1R9A/

/PARD3/APBB1/SRF/CAPRIN1/RHOA/STXBP1/ROBO3/ACTN4/PDPN/MINK1/HEG1/NIN/NRTN/SPTA[illegible]
D2/GNL3L/BLOC1S1/SETD1B/KDM6A/PIAS2/NAT8B/SIRT2/NCOA3/NUP35/RBBP5/FLCN/RWDD3/R[illegible]

/RPS19/IKBKG/AXL/FADD/BTK/PELI1/GZMA/CLEC7A/VIM/CSF2RB/ROMO1/TRIM25/POLR3D/LBP/F
L4/NCKAP1/PLCG1/MOV10/PPP1R9A/CNP/NCAM1/RPL24/ETV1/SEMA3F/MEGF8/EGR2/ULK1/MNX1/
/RPS19/IKBKG/AXL/FADD/BTK/PELI1/GZMA/CLEC7A/VIM/CSF2RB/ROMO1/TRIM25/POLR3D/LBP/F
)4/PEX10/RPS9/HAX1/RPS14/CALM3/RPS27/CSNK2A2/NUDT19/PARL/RPL29/TIMM23/VPS4A/TIMM

[illegible]3C2B/HTRA2/IFNG/WDR45/TLK2/MAPK15/ATP6V0A1/EIF4G1/TMEM208/TBC1D25/SESN2/NOD1/A[illegible]
[illegible]R5/TLR5/GPATCH3/CARD16/RPS19/IKBKG/AXL/FADD/BTK/PELI1/GZMA/CLEC7A/VIM/CSF2RB/R[illegible]
[illegible]H/RPL37/RPL27A/RCC1L/COPS5/WARS2/EIF1AD/RPL36/UBA52/HSPA14/NANOS1/TARBP2/ETF1/[illegible]
[illegible]3C2B/HTRA2/IFNG/WDR45/TLK2/MAPK15/ATP6V0A1/EIF4G1/TMEM208/TBC1D25/SESN2/NOD1/A[illegible]

[illegible]/RFTN1/BTNL8/MCOLN1/TNFRSF17/NLRP3/CD40/PPP3CB/TNFSF18/GNL1/IL12A/ZBTB7B/SLAMF[illegible]

'SRGN/CFTR/PLK3/SH3GLB1/MAVS/PFKFB2/BNIP3L/PLA2G1B/TLR2/SREBF1/CD2AP/EDEM2/RAB11

NF675/IGBP1/RGS3/CSF2RB/TNIP1/RASAL3/GPR183/IGF1R/STK38/CSPG4/NF2/BTN2A2/CCL3/S1
K2/PTPN2/CASP8/RPS27A/MAVS/DMBT1/PAK2/TLR2/MUC6/UBA52/RAB29/MUC15/CACNB3/PLD2/F

IL12/EGF/CAV1/LDB1/DAB2IP/DKKL1/CBY1/ZBED2/PKD1/CSNK2A2/BMP2/PAF1/DDB1/RYK/PSMB1

3A/TTL/TSG101/TP53/BCL11A/DPYSL2

K25/ZNF675/IGBP1/RGS3/CSF2RB/TNIP1/RASAL3/GPR183/IGF1R/STK38/CSPG4/NF2/BTN2A2/C

4/UQCC2/PSMD7/IKBKG/PIP4K2C/RPL23/WDR91/RACK1/RALB/VIM/DAP/HCAR2/CUL4B/TRIM71/AT

JPT5H/UBXN1/BTG2/PAFAH1B2/SEC13/PSMD8/EEF1A2/ABL1/VCP/IL10RA/PSMA1/NPRL3/TNFAIP3

/DMAP1/RBPJ/CCAR2/CYP27B1/IST1/EIF4G2/NOTCH2/CDK11A/OSM/MORF4L1/OGFR/DAB2IP/HMG[illegible]
[illegible]ELA/TMEM127/SESN2/DUSP14/RGS4/PIK3R2/KCTD13/TMEM161A/SLA2/C3orf33/MUL1/TSC1/PP[illegible]

SEC13/LFNG/EXT1/LPCAT2/PDCD10/APOO/ARAP1/DENND5A/WIPI1/DBNL/COPG2/B4GALNT2/DNM2/
3/RPA1/PMF1/TBP/SMARCAL1/IPO4/SUV39H1/MSH6/SAP130/SMC2/MORF4L1/POLE2/EP400/LDB1
S18/CTSD/AP3D1/AP1B1/BTK/CBL/CCZ1/MYO5A/ABCC10/M6PR/CSPG5/TMEM199/CCT8/GNB4/ATP
H/KDM3A/MBD1/SMAD2/WBP2/SMAD3/MYCN/SETD1A/MAU2/MAEL/BAZ1A/TP53/INO80E/TARDBP/KA
CAPH2/TCP1/CDCA2/CENPI/LOXL2/WAS/SWI5/SFPQ/DNMT3A/THOC5/PUM3/NUP85/BANF1/PPP1CC
LGA3/POLA1/GNL2/CIAPIN1/NCL/SDHAF2/FTSJ3/GRB2/SPATS2L/POLR2L/ZNF354A/DROSHA/VCX
S18/CTSD/AP3D1/AP1B1/BTK/CBL/CCZ1/MYO5A/ABCC10/M6PR/CSPG5/TMEM199/CCT8/GNB4/ATP

1/ATP6V0C/PSMD1/TMEM63A/KPTN/PADI2/YWHAB/SLC22A17/TRAPPC1/BORCS8/GLMP/LUM/DNASE
CR/NEK6/IFT20/B9D1/DHX9/NIN/IFT22/PSEN2/FBXL7/IST1/CDC27/SSX2IP/UXT/NPHP4/SPOUT
FL1C/DCLRE1B/RPS7/CALM2/AKNA/NME7/CEP19/TBC1D7/CEP41/TXNDC9/PSMB5/IFT172/HOOK2/

RPS11/PROCR/RHOA/RPL13A/YWHAG/CLTC/GAK/ACTN4/RPL37A/FAP/RPLP1/RPL30/PARVB/EPHA2
A1324/SNX5/VPS16/CD1A/SNX16/ANTXR1/ATP9A/RAB8B/TLR8/ABCA7/MAP3K7/KREMEN2/STAM/
84/CALR/SYT1/LAIR1/CYSTM1/OCRL/DNM1L/AP1G2/CYB561D2/SYT3/CPE/AP1B1/STX4/M6PR/CO

Q/INTS7/BAZ2A/PPIE/RBM14/EIF3E/APBB1/CHFR/DAXX/TRIM69/HSPB7/PLEKHH2/SUGP2/NEK6/

OMMD9/RAB10/DYNC1LI1/GALNS/DSN1/SLC44A2/IMPDH2/ATP6V0C/PSMD11/SPESP1/PSMD1/C5AR
561D2/SYT3/CPE/AP1B1/STX4/M6PR/COPG1/CLEC5A/TMEM199/TBC1D10C/ATP6V0D1/PDCD6/RAS
KMT5C/ZMIZ2/DONSON/TIPIN/MSH2/PADI2/CHD4/RUVBL2/CENPB/CCND2/EXOSC5/POLE4/RBBP4/
UC20/TPST1/CHPF/APP/SLC9A7/OCRL/DNM1L/AP1G2/AP3D1/TRAPPC3/SLC35C1/AP1B1/ST3GAL5
35/CFTR/SH3GLB1/MAN2B1/LTV1/MCOLN3/CYB5R3/CCZ1B/GNAI3/GLIPR1/HEXB/MYO6/TMBIM1/S
/PDIA3/SNX3/YIPF1/CAV1/RAB13/SLC9A6/LITAF/VPS4A/SLC9A9/PLD1/KIAA1324/SNX5/VPS16
1/DCLRE1C/MMS22L/SIN3B/TRRAP/DDB1/JUNB/TFPT/TRPS1/TFIP11/ASXL1/USP3/THOC7/TTI1/
DLIM4/MOB2/OLFM4/OPTN/RAB3A/PAK2/MYO6/TRAF4/COPS5/PTOV1/CD2AP/LMTK2/RAB11A/ZPR1
L19/PARD3/PPIB/TLN1/RPS11/PROCR/RHOA/RPL13A/YWHAG/CLTC/GAK/ACTN4/RPL37A/FAP/RPL
6PR/CSPG5/COPG1/B4GALNT3/TRAPPC2L/PIFO/MMGT1/CLIP3/CHST12/PDCD6/NSF/B4GALNT4/AB

MAP1LC3B/TUBB2B/EVPL/MAP4/TUBG2/NES/NEB/KIF2A/ODF2/KPTN/CAPZB/LUM/BAG3/COL4A2/S
MAP1LC3B/TUBB2B/EVPL/MAP4/TUBG2/NES/NEB/KIF2A/ODF2/KPTN/CAPZB/LUM/BAG3/COL4A2/S
2A/SYNGR3/CLN3/SLC11A1/RNASE2/MCTP1/RAC1/TMEM163/VDAC1/ANXA11/IL1B/MAGED2/CTSS/
B/TUBB2B/EVPL/MAP4/TUBG2/NES/NEB/KIF2A/ODF2/KPTN/CAPZB/LUM/BAG3/SMTN/ZNF207/VPS
NA/BAG2/KRT10/SRPRB/DBNL/BCL10/HOOK2/BOD1/CSPP1/DNM2/CDK2AP2/CCSAP/DCTN1/HAUS7/
PL23A/RHEB/MCTS1/JRK/PIWIL4/MRPL48/SNRPB2/PRPF19/DAP3/CHTOP/MRPS7/LARP4B/PTGES3

/PXN/CSRP2
CSRP2
4/RPL9/PXN/CSRP2

IAM2

4/SLCO4C1/VPS16

[illegible]P6V0C/APH1B/MVB12A/PTPN23/HLA-B/ATP6V0E1/PTP4A2/DENND6B/TRAF3/IL2RB/CST7/GRB2/[illegible]
[illegible]I/UBE2D2/POLR2J/ZDHHC9/GLMN/ATG13/RNF8/DCAF7/POLR2D/PRKAG1/PIK3R5/SOCS7/WDR41/[illegible]

2/DNM2/PTK2/VPS35/VPS45/AAK1/PDLIM4/GRIN2D/CTBP2/VDAC2/RPL6/RAB3A/GABRB3/PAK2/L[illegible]

[illegible]YF2/RBM15/DAZAP2/CWC15/PSPC1/LPXN/HSPA1A/CRNKL1/GPATCH2/TUT1/CIR1/SF3A1/SRSF4/[illegible]

)LIM4/HNRNPU/MAP1A/MARK4/LZTS1/ANXA3/CANX/LMTK2/ZPR1/TUBB3/SLC2A3/CDK5R1/PARD3/F

R3A/SUPT6H/KDM3A/PRKCB/MBD1/SMAD2/WBP2/ZNF431/HDAC3/BAP1/SMC1A/SMAD3/SUPT16H/SAF
RIP4/ASXL1/PXN/TRIM32/MXD4/SRA1/NAB2/PRMT5/NCOA6/GTF2A2/ZHX2/PFDN5/PPRC1/PARK7/E
/BCR
AP1A/CTBP2/NEBL/RPS4X/RPL6/ACTN1/JPH1/RPL37/RPL27A/ADD3/MUC6/RPL36/UBA52/CD2AP/T
AP3/RALGAPB/ATP1B1
2A/VBP1/KIF15/GNB1/STMN3/WAS/CDH17/VASH2/VIL1/GSK3A/RAB10/NCALD/MAP1LC3B/PFN1/WA
5R1/NUAK1/CDK20/STK3/NEK6/GAK/PRKAG1/MAP3K1/MINK1/MAPKAPK5/PHKA2/GTF2H1/NME4/PHK
/DDX41/NKIRAS2/CDC42/DNAL4/AGAP1/MYO9B/HELLS/XRCC6/POLQ/DNM1L/CCDC102A/DDX54/KAT
/DDX41/NKIRAS2/CDC42/DNAL4/AGAP1/MYO9B/HELLS/XRCC6/POLQ/DNM1L/CCDC102A/DDX54/KAT
/DDX41/NKIRAS2/CDC42/DNAL4/AGAP1/MYO9B/HELLS/XRCC6/POLQ/DNM1L/CCDC102A/DDX54/KAT
P1/MYO9B/HELLS/XRCC6/POLQ/DNM1L/CCDC102A/DDX54/KATNB1/MYO5A/RALB/ABCC10/RGS3/CCT
EEF1A2/PKIA/TNNI3/ABL1/TNFAIP3/RPS7/CALM2/PDCD10/TDG/TRIP6/CSK/TRADD/CHEK2/MAPKA
N1/USF1/PTPN1/CCNJL/FBXW5/BICD1/PPP1R12C/MAVS/CTBP2/PFKFB2/CCNE2/PAK2/FNTA/SREE
2/HSPA2/CCDC6/TNNI3/ABL1/VCP/SCAF1/CALM2/TDG/TRADD/RUFY1/DBNL/PTPN12/BCL10/HSP90
K14/SOCS3/PFN1/ARHGAP30/AGFG1/PPP1R1B/ABR/SET/USP14/GCN1/CDKN2C/PSMD1/SBF1/SIPA1
LIMA1/CD2AP/NOTCH3/CDK5R1/TLN1/EIF3E/EXOC3/ACTN4/EGFL6/FNBP1L/FAP/RSL1D1/SPTAN1/
SM1/IPO4/RANBP3/DOCK10/YIPF1/EGF/CAV1/ATP7A/ARHGEF17/PREX1/ARFGEF1/RIC8B/CIB1/AF

C1D25/DENND6A/DOCK11/EPS8L2/KCTD13/DOCK7/MAP3K11/VAV3/PLEKHG5/USP33/BICD2/RASGRF
/TMEM127/PDE6D/TBC1D25/DENND6A/DOCK11/EPS8L2/KCTD13/DOCK7/MAP3K11/VAV3/PLEKHG5/U

RPK1/CSK/CIITA/CHEK2/MAPKAPK3/CCND3/PRKAA2/ACVR1B/STK40/DGKA/PTK2/CSNK2A1/TNK2/C

2/SBK1/KSR1/NOL9/CDK11A/DOLK/PIK3R6/PDK3/IRAK2/CCL8/PGM2L1/MAP4K1/PI4K2A/ATR/CHK

./TFDP1/KLF5/PELP1/ATG7/TLE6/LDB1/TP53BP1/HMGA2/RPTOR/TRIM68/GTF2F1/CRTC3/HDAC5/

./SUPV3L1/RNASE7/MED21/DGCR8/TDRD9/DDX21

[illegible]4/UBTF/SREBF1/GLI1/PIH1D1/MESP1/MECP2/RBMX/VEZF1/ARID3B/BAZ2A/TWIST1/SRF/HES2/[illegible]

ASS6/RBM14/CC2D2A/RHOA/NEK6/CLTC
XO5/DBF4/AATF/TUBB4A/CNOT9/CDKN2B/CCL2/RTKN/DAPK3/CSNK1D/ANAPC7/CDK2/LSM10/KIF14
CALR/MATN3/CDC42SE2/SEMA4D/SYT1/APP/CDC42EP2/DNM1L/MAPK7/SYT3/SEMA4F/AXL/SEMA5B/
17/ABL1/FCGR2B/TNFAIP3/NFATC4/CSK/RNF216/CD33/BCL10/PTGER4/TWSG1/TUSC2/THBS1/AI
/ADAM17/CASP8/CAPN2/SRGN/S1PR3/MAVS/IRF5/IL20RB/TLR2/TRIM21/CD2AP/HLA-F/ATP6AP2

I1/XRCC6/PSMD3/CTSD/PSMD7/BTK/GMFG/STX4/CBL/PRDX4/CLEC5A/CCT8/TBC1D10C/GPR183/LI
SMD3/CTSD/PSMD7/BTK/GMFG/STX4/CBL/PRDX4/CLEC5A/CCT8/TBC1D10C/LBP/MAPT/FCGR3B/HPS
R1/BIN2/RAB5C/DBNL/PSAP/CD33/HSP90AA1/C12orf4/CD177/MAN2B1/OLFM4/CYB5R3/GLIPR1/I
A4/ANXA3/AP2A2/ATP6AP2/SLC2A3/TSPAN14/MLEC/PSMC3/PPIE/AGA/ADGRG3/SIGLEC9/RHOA
RCC6/PSMD3/CTSD/PSMD7/BTK/GMFG/STX4/CBL/PRDX4/CLEC5A/CCT8/TBC1D10C/GPR183/LBP/FC
/MSH2/PADI2/TRAPPC1/GSDMD/ARRB2/STK10/GPR84/CNN2/IL4R/ALDOC/GZMM/LAIR1/PNP/SWAP7
/HEXB/PLA2G1B/TLR2/CSNK2B/TMBIM1/SLC15A4/ANXA3/AP2A2/ATP6AP2/SLC2A3/TSPAN14/MLEC
SC3/BTN2A2/CCL3/KCNAB2/MEIS2/MFNG/HSPD1/ESCO2/PSMD8/LFNG/RARG/ABL1/SART1/HLA-DOA
FUT7/GNAS/CD80/TP53/SOX13/RASGRP1/FGL2/LILRB2/MYH9/NRROS
A2/PLA2G7/PSMB4/IL1B/ACTB/BCAR1/IL2RA/CTSS/CD47/PSMB8/NFAM1/TNFSF13B/PSMB10/PHB/
OB1/IRAK2/CAV1/DAB2IP/INPP5D/DUSP22/CD3G/PTPRC/NECTIN2/CLEC4E/BAG6/ZCCHC3/BTN2A1
18/C5AR2/CD40/PPP3CB/PSMD12
R2L/BEND3/TOPBP1/APOBEC3D/TDRKH/PRIM2/SWAP70/CBX8/POLR2K/HELLS/CETN2/XRCC6/HMGN1
22L/STUB1/HUWE1/TRRAP/DDB1/TFPT/EXO5/TFIP11/SPIRE2/USP3/POLG2/COPS8/NCOA6/TCEA1/
/POLE3/BRPF1/UBE2N/ELOF1/MECOM/CTBP1/IPO4/SUV39H1/MORF4L1/EP400/LDB1/SUPT4H1/PCC
R1/RBM42/CPSF3/DDX23/PTBP1/SENP3/GTPBP3/RPS15/ADAT3/PRKRIP1/RPS16/PUS7/PRPF38A/W
/PARS2/HARS2/MRPL4/RBM24/DPH3/EIF6/EIF4G2/EIF4G3/CNOT3/RPS9/KHSRP/DALRD3/EIF3G/I
/TGFB2/SERPINB6/PTPN6/PSMD13/AGPAT2/COMMD9/RAB10/DYNC1LI1/CCR1/GALNS/DSN1/SLC44A

7/POLD3/INO80C/TRIM25/TIMELESS/PTTG1IP/MAPT/MCTS1/EID3/PRPF19/ESCO2/RAD51C/BTG2/
BICDL2/CKAP2/TTC26/NTMT1/CKAP5/HSPB11/MZT1/RHOT2/ABL1/TBCD/KLHL42/VCP/STMN2/NSFI
D8/PIDD1/HSPA2/PKIA/ABL1/PSMA1/PIM3/MTA3/NSFL1C/DCLRE1B/EME2/CHEK2/CLSPN/CEP41/C
/ADRA2A/MVD/PNP/CBX8/TMEM119/TRPM4/FADD/ECM1/PELI1/STX4/RPL23/CLEC7A/ROMO1/RASAI
GEMIN6/SYNCRIP/SMN2/CLK2/HNRNPA2B1/SUPT6H/U2AF1L4/ELAVL1/LUC7L3/SRSF3/UBL5/TRA2I
A1/UBE2J1/ARRB1/PSMD12/BFAR/ARMC8/SMURF2/RNF40/OGT/MAPK9/CDC26/SOCS5/KLHL22/ANAI
5/PSMD3/RBX1/PSMD7/RAD21/BABAM1/MTBP/RPL23/RNF4/NME6/RFWD3/MSX1/RBBP7/CEP250/RPA
HAG/ATG13/SART3/DNMT3B/MAPKAPK5/NIN/BTC/BECN1/UBE2N/ANAPC5/EREG/CTBP1/ARF1/TFDP1
P4/SAP30BP/TOMM22/PIAS4/HOXA5/IRF5/ARHGEF11/BNIP3L/MARK4/PAK2/USP27X/BCL2L1/UBA5
AP1G2/RPS19/IKBKG/AXL/AP1B1/FADD/RPLP0/RPL18/TBKBP1/RPL23/ABI2/RACK1/GADD45GIP1/
PSF3/DDX23/PTBP1/PSMB5/PSMA4/RPS15/PSMD9/PRKRIP1/RPS16/PUS7/PRPF38A/WTAP/RPL5/RI
C/KANSL2/PRMT7/ZBTB7B/BRD8/WDR70/TRIP12/MSL1/KMT2A/CPA4/JARID2/PCGF1/BRCC3/GTF3C
N2/CDC42/IL4R/CXCL11/CCR5/XCL2/CMKLR1/CSF2RA/PSMD4/PSMD3/CCRL2/RBX1/CARD16/PSMD7
SAP1/CIB1/MRPS24/CSNK2A2/MMP15/EIF2D/KLC1/MRPL33/VPS4A/CST3/MRPS5/VPS16/DHX8/ADI
F1/SRPK2/DDX52/COIL/DHX30/AAMP/TEX10/SNRPD3/PWP1/MRPL36/CELF2/NIP7/RPS6/RPF1/MRM
MA3E/FGD1/ARPC1A/WNT4/NF2/OBSCN/BRK1/ARPC5/TNNI3/ABL1/ACTR3/ARAP1/DBNL/PTGER4/SI
R9A/LPAR1/PLEKHM1/ARMC9/CCDC88A/NEURL1/C2CD3/CNTRL/TCHP/DMTN/DYNC2LI1/CEP97/RAB3
L1/PDLIM4/FLII/MOB2/NEBL/ARHGEF11/ACTN1/PLA2G1B/ADD3/PACSIN2/LIMA1/CD2AP/PACSIN1
X/PSMB5/BAD/PSMA4/PTGER4/ACVR1B/VCAM1/TWSG1/PSMD9/TUSC2/PTPN2/THBS1/ADAM17/PUS7/

1/LIMS1/ABL1/TBCD/SART1/FCGR2B/SFRP2/AKNA/SIPA1/CSK/LAMA4/BAD/EMID1/PLXNC1/BCL10
L1C/UBQLN4/RPS7/DENND3/KCTD5/FOXF2/BAG2/CHEK2/RNF216/PSMB5/SGSM3/PSMA4/HSP90AA1/
MA5B/ANGPT2/CLEC7A/RACK1/SEMA3C/GPR183/SEMA3E/IGF1R/LBP/PDCD6/WNT4/EPPK1/NF2/CCI
/SEMA3C/EPB41L3/CUL4B/PMP22/PTPRF/SEMA3E/MAPT/RERE/CHN1/ZNF335/BTG2/EXT1/CBFA2T2

./RHOA/CD38/RAPGEF1/IFT20/ENPP2/GAK/ACAP3/PDPN/FNBP1L/GPC2/NIN/ADCY6/DPYSL3/SNAI
)OAA1/PSMD9/UBA2/RPL5/RPS27A/FBXW5/CTU2/MAGEF1/RNF2/PIAS4/RAD18/MED27/MASTL/HSPA
.4R/PRKCI/TGFB3/ZMYND8/ADRA2A/DNAJB6/SWAP70/SNX12/TLR5/HK1/GZMB/DNM1L/TRPM4/CTSI
C26/RPL23A/NF2/OBSCN/HSPD1/ESCO2/GGA1/IPO13/PKIA/IL10RA/TIMM13/WRAP53/TNFAIP3/MI
PF3/SNU13/RPS3/RBBP4/CAPZB/PRPF6/SDHAF2/GRB2/EIF3H/VCX/ZNF207/AGO2/VPS18/ARHGAP1
7/RPL27A/DXO/EXOSC7/TBRG4/RPL36/UBA52/ZPR1/NANOS1/NUDT18/ETF1/RPL19/PSMC3/RPS11/
3/TRMT2B/RBFA/RPS14/GTF2F1/RPS27/EXOSC2/TAF1B/CDK5RAP1/DDX52/METTL3/TEX10/PWP1/(
/CYB5R3/GLIPR1/RAB3A/HEXB/TLR2/CSNK2B/TMBIM1/SLC15A4/ANXA3/AP2A2/ATP6AP2/SLC2A3/
/CADM4/PADI2/ABI3/GRB7/TACSTD2/PRKD2/SEMA4B/PARD6B/SYDE1/STK10/AGO2/ARHGAP18/CAI

IPR1/RAB3A/HEXB/TLR2/CSNK2B/TMBIM1/SLC15A4/ANXA3/AP2A2/ATP6AP2/SLC2A3/TSPAN14/MI

PL15/C12orf65/PARS2/HARS2/MRPL4/RBM24/DPH3/EIF6/EIF4G2/EIF4G3/GCLM/CNOT3/RPS9/KI
3K10/CDK5R1/PPM1F/NDUFA13/APBB1/TPD52L1/PLAGL2/RHOA/STK3/YWHAG/PNMA3/ANP32B/FAP/
RINA/ACVR1/PIDD1/SLC25A5/ABL1/VHL/PIM3/SFRP2/TNFAIP3/NFATC4/PDCD10/TRADD/BCL10/I
52/MAP3K10/CDK5R1/PPM1F/NDUFA13/APBB1/TPD52L1/PLAGL2/RHOA/STK3/YWHAG/PNMA3/ANP32
NKLE2/BTG2/GRINA/ACVR1/PIDD1/SLC25A5/ABL1/VHL/PIM3/SFRP2/TNFAIP3/NFATC4/PDCD10/T

NF4/ABI2/CLEC7A/RACK1/RALB/RPA2/CUL4B/CLIP3/DRG1/MAPT/ARPC1A/WNT4/NF2/EMILIN1/BI
LEKHM1/CCDC88A/RIOK1/TRIM27/PIP4K2B/NEURL1/CAND1/ULK1/NPHP1/IFNG/FAM98A/AIM2/MAI
P1/RPS20/CCR5/NUP107/POLR2K/XRCC6/RPL18A/RBX1/AP1G2/RPS19/IKBKG/AXL/AP1B1/FADD/I
MP1A/GRK2/APOBEC3D/CPSF4/RPL35/RANBP1/RPS20/CCR5/NUP107/POLR2K/XRCC6/RPL18A/RBX1
/HSPA2/ADAM28/HVCN1/SRPK1/BAD/ACVR1B/DNM2/SPIRE1/SPA17/NOTCH1/CAPN2/CFTR/GJB3/PA
/BTG2/PSMD8/ACVR1/TAF10/PIDD1/HSPA2/SUSD2/PKIA/PSMA1/MTA3/CHEK2/CLSPN/CEP41/CCNI
P6V0C/PSMD11/PSMD1/C5AR1/TMEM63A/PADI2/SYT12/TRAPPC1/GSDMD/RIMS3/STK10/GPR84/CNN
/TRAF3/PSMB3/ARRB2/CD6/APOBEC3D/GZMM/MUC20/XCL2/APP/CDC42EP2/TLR5/PSMD4/XRCC6/GZ
3/LILRB1/RNF135
FADD/BTK/STK25/CLEC7A/ARHGDIA/SCN1B/CSPG5/SEMA3C/MAPT/WNT4/TGIF2/RHEB/NF2/BTN2A2
N/PLK3/RNF2/ZFYVE19/UIMC1/TBRG4/BCL2L1/GPR132/CRLF3/TRIM37/KIF20B/ALOX15B/BTG3/I
/ADAM17/PTPN1/CCNJL/DUS2/RPS27A/VEGFA/CCNE2/PLA2G1B/PAK2/TRAF4/CSNK2B/UBA52/DUSI
-F/RAB29/RHBDD3/NKAP/IL21R/STOML2/ADGRG3/CD79B/SRF/RHOA/GLMN/CD38/INHA/AP3B1/RNI

3L1/SART1/HLA-DOA/FCGR2B/PSMA1/SFRP2/TOX/PSMB5/BAD/PSMA4/PTGER4/ACVR1B/VCAM1/TWS
S/ADM/EPHA2/AP2A1/ARTN
/IKZF3/CNR1/METTL3/LOXL3/DOCK8/TNFRSF1B/NFKBID/CASP3/CLPTM1/CD22/BCR/ICOS/MFHAS1

/THRA/VDAC2/ERCC1/VEGFA/EXOSC10/TLR2/ADD3/PACSIN2/LIMA1/BCL2L1/PIH1D1/EML2/RAB29
/GRB7/TACSTD2/PRKD2/SEMA4B/PARD6B/SYDE1/STK10/AGO2/ARHGAP18/CALR/ZMYND8/SEMA4D/X

S/IKBKG/DSTYK/GMFG/CBL/RPL23/STK25/AKT1S1/WDR91/RACK1/ZNF675/RALB/RGS3/PIFO/IGF1
./PLCB2/ARAP1/TRADD/TBC1D7/BAD/SGSM3/PSAP/ARHGAP11A/CD33/BCL10/SRGAP2/RCN3/DNM2/
STN/PLEKHH2/RHOA
R/BICD1/HNRNPU/LTV1/CTBP2/RAB3A/MARK4/HAUS5/DEF8/DYNC1I2/RAB11A/NEDD1/BORCS6/CEN
HGA/PRKCB/CNIH2/CDK5RAP2/FAM91A1/DOCK7/CHRNA5/CHMP6/SPICE1/TRAK2/SYP/CADPS2/ACTI
DBNL/PLXNC1/CTNNA1/SRGAP2/DNM2/PTK2/STYXL1/VPS35/NOTCH1/TMEM98/FIG4/MOB2/LLPH/VI

K24/STMN2/RASAL1/SFRP2/NFATC4/GDF11/KEL/BAD/DENND5A/DBNL/PLXNC1/CTNNA1/SRGAP2/DN
H3GL1/LILRB4/PDCD5/STX4/RACK1/MYO5A/RALB/CSPG5/CEP250/CCT8/GNL3/PARD6G/CLIP3/MAI
C/CARD16/IKBKG/AXL/FADD/PELI1/DSTYK/CBL/RACK1/IGBP1/ARHGDIA/MSX1/OGG1/CLEC5A/BNI
FCGR2B/TIMM13/NSFL1C/LPCAT2/APOO/STX7/KCNN4/ACTR3/VAMP8/BIN2/BAD/PSAP/LEMD2/HSP9

)CAF15/PDCD6/MED12/LRRC41/PRPF19/UBXN1/PELI3/PSMD8/TRAF7/TAF10/DESI2/ABL1/KLHL42
IPI1/DBNL/HSP90AA1/PRKAA2/RPS15/DNM2/TCTEX1D2/CCSAP/STYXL1/DCTN1/RPL5/NOTCH1/CEI
/ZDHHC9/RPL13A/YWHAG/ABHD17A/IFT20/CLTC/STXBP1/GAK/RPL37A/AP2B1/RPLP1/GPC2/RPL32
C1/TRAF4/COPS5/HSPA5/TXNDC12/BCL2L1/EDEM2/MAP3K10/HIKESHI/NUAK1/UBR5/TWIST1/APBI
PTGER4/EFEMP2/SPIRE1/CCSAP/DCTN1/INPPL1/KATNA1/PLK3/MAP1A/NEBL/ACTN1/PLA2G1B/MAI
/CDHR2/ABL1/SART1/VCL/FCGR2B/AKNA/CSK/CDH24/TTYH1/BAD/CD33/BCL10/B4GALNT2/VCAM1/
3A/NEURL1/C2CD3/CNTRL/TCHP/DMTN/DYNC2LI1/CEP97/RAB3IP/NPHP1/ARL3/CELSR3/FAM98A/M
C20/ENPP2/GAK/ACAP3/PDPN/FNBP1L/GPC2/NIN/ADCY6/DPYSL3/SNAPIN/EPS8L1/EPHA2/AP2A1/

)KN1C/AKAP8L/PSMB4/IL1B/NCAPH2/PSMB8/PSMB10/PSMA5/POC1A/PTPN6/PSMD13/CHMP3/PSMC4
)X2/MEIS1/ZAP70/HSPA1A/CTNNBIP1/PSMD6/MALT1/CLDN18/CD101/LEO1/HLA-G/GATA3/NFATC2

IGAP18/CALR/ZMYND8/SEMA4D/XCL2/CMKLR1/APP/ADRA2A/SWAP70/DPEP1/SEMA4F/FADD/ECM1/S

.5/WDR83/PAPOLA/SMN1/HNRNPH2/CRNKL1/SF1/HNRNPH3/ZMAT2/USP4/CWF19L1/SF3A1/SRSF4/C
POLA/SMN1/HNRNPH2/CRNKL1/SF1/HNRNPH3/ZMAT2/USP4/CWF19L1/SF3A1/SRSF4/GPATCH1/PDCI
POLA/SMN1/HNRNPH2/CRNKL1/SF1/HNRNPH3/ZMAT2/USP4/CWF19L1/SF3A1/SRSF4/GPATCH1/PDCI

.R1/BIN2/RAB5C/DBNL/PSAP/CD33/HSP90AA1/C12orf4/TUSC2/ADAM17/CD177/MAN2B1/MAVS/OI
/ICOS/MFHAS1
/UBE2N/CD247/TLR7/HSP90B1/IRAK2/CAV1/DAB2IP/INPP5D/DUSP22/CD3G/PTPRC/NECTIN2/CLI
P3/THOC7/HNRNPD/PRMT5/FASTKD5/SNRNP200/RBM22/DDX20/GEMIN8/U2SURP/PDCD11/HNRNPC/T

P/ARHGEF17/PREX1/ARHGAP22/EPHB2/ARFGEF1/ARHGAP17/CNKSR1/CYTH3/RAB1A/PLD1/RALBP1/
HSRP/CAV1/SPOUT1/DNAJC1/RPS14/MIR155HG/MKNK1/EXOSC2/LARP1/YTHDF2/CDK5RAP1/CASC3/

MRPS10/ABCE1/MRPL45

N2/RAB5C/DBNL/PSAP/CD33/HSP90AA1/C12orf4/CD177/MAN2B1/OLFM4/CYB5R3/GLIPR1/RAB3A/
A4/ANXA3/AP2A2/ATP6AP2/SLC2A3/TSPAN14/MLEC/PSMC3/PPIE/AGA/ADGRG3/SIGLEC9/RHOA
.SPN/CEP41/CCND3/PSMB5/PSMA4/HSP90AA1/ACVR1B/PSMD9/DNM2/ADAM17/CDK2AP2/DCTN1/CCN

CHEK2/CCND3/BOD1/CSPP1/SPIRE1/CDK2AP2/CCSAP/DCTN1/NOTCH1/CEP164/HAUS7/KATNA1/PLK

IS3/PPM1F/NDUFA13/TPD52L1/PLAGL2/DAXX/STK3/GSKIP/CD38/YWHAG/IFI6/FAF1/CCAR2/BECN

PYM1/HNRNPC/RPS3A/AGO1/RPL14/RPL10A/FXR1/SYNCRIP/EIF4G1/PSMB7/CNOT2/RPL12/RPS18/
P2CA/TNFSF13/PAIP1/RPL22/LSM3/TARDBP/RPS15A/SMG8

R74/DUS3L/WDR18/TARBP1/TRMT10C/ANKRD16/METTL16/MRM1/CDKAL1/XRN2/TRIT1/ADAT2/ELP6

M1/PDCD1LG2/CD70/SOCS5/CD274

[illegible]/KMT2A/CPA4/JARID2/PCGF1/BRCC3/GTF3C4/PHC1/MYB/BRCA2/HR/SUDS3/WDR82/GTF2B/SETD[illegible]

[illegible]/PDCD10/CSK/CLSPN/MAPKAPK3/CCND3/BAD/DBNL/BCL10/ERP29/HSP90AA1/TNFSF15/LMO4/PR[illegible]
[illegible]CH3/PPP2R5B/CDK5R1/METRN/APBB1/SRF/CAPRIN1/RHOA/CD38/HES2/RAPGEF1/YWHAG/GAK/SIX[illegible]

[illegible]OCH/CD79A/UBE2D1/IL15/ABR/PSMD10/PSMD11/PSMD1/C5AR1/HLA-B/GPX1/MSH2/PSMA7/LRP8/[illegible]
[illegible]B/UBE2N/ADCY5/KITLG/EREG/CCL19/PIK3R6/IRAK2/CACUL1/EGF/MAP4K1/MAP4K2/DAB2IP/HM[illegible]

/VPS29/FOXL1/PSMB7/NKD2/SPIN1/SKP1/FZD9/GNG2
/WWTR1/SUMO2/USP3/FBXO10/TRIM32/PSMB6/RPL11/CDC23/KLHL25/MVB12B/RNF149/PSMA3/PAI
37B/USP11/UBE2D2/CHFR/AGAP3/GLMN/VPS11/CLTC/RNF8/ANKZF1/AP2B1/FAP/LGMN/TMUB1/PSM
RIH2/NDUFB2/EIF4G1/NDUFAF3/FZD9/SESN2/ATG3/PNPT1/TRAK2/UBL5/LIG3/ESRRA/MUL1/BAP1

2/ETF1/RPL19/MRPL37/EIF5A/CPM/RPS11/EIF3E/CAPRIN1/RPL22L1/RPUSD3/NSUN4/RHOA/RP

/UBE2D2/CHFR/NMI/RASSF5/DAXX/TRIM69/GLMN/RNF8/DCAF7/PRMT3/SOCS7/MED7/UNKL/PSMC

/RPL11/CDC23/KLHL25/MVB12B/RNF149/PSMA3/PARK7/BUB3/RNF144A/USP36/NUB1/UBQLN2/R

B/UBAC2/AUP1/MAEA/CACUL1/EGF/HSPA13/CAV1/NEDD8/DAB2IP/GET4/FBXO6/PKD1/CSNK2A2/

/BCL7B/VPS29/FOXL1/PSMB7/NKD2/SPIN1/SKP1/FZD9/GNG2

CAB3/PIGX/DAD1/GUK1/POFUT2/UGCG/FUT8/NME3/GALNT7/ALG1/NPPB/LARGE2/PFKFB2/GXYLT

D2/GRB2/ARRB2/CALR/TGFB3/MUC20/SEMA4D/XCL2/APP/ADRA2A/DNM1L/TELO2/RELL2/PPP5C/

RC4/BPIFB1/PTPRS/CLEC4A/APPL2/RPS6KA3/TLR1/S100A1/NOD2/XRCC5

G5/KIF17/RFX3/INTU/RPGR/RPGRIP1L/HAP1/PRKAR2B/CEP152/DRC1/BBS5/CFAP44/LRRC49/C

36/NUDCD3/KIF3B/ARL13B/AHI1/ATG5/KIF17/RFX3/INTU/RPGR/RPGRIP1L/HAP1/PRKAR2B/CE

/MVB12B/RNF149/PSMA3/PARK7/BUB3/RNF144A/USP36/NUB1/UBQLN2/RNF121/WWP2/ARIH2/RNF1
SNK2A2/PABPC1L/ACTR1A/NECTIN2/YTHDF2/ADCY3/BAG6/RFX2/SLCO4C1/CNR1/ZSCAN2/METTL3/
/SPATA2/RNF114/TLE6/DKKL1/DZIP1/CIB1/SIX5/CSNK2A2/PABPC1L/ACTR1A/NECTIN2/YTHDF2/
4/TLE6/DKKL1/DZIP1/CIB1/SIX5/CSNK2A2/PABPC1L/ACTR1A/NECTIN2/YTHDF2/BAG6/RFX2/SL[illegible]

[illegible]AR3/RACK1/RALB/GPR183/BTBD10/PIFO/IGF1R/CLIP3/MAPT/CSPG4/DBF4B/CCL3/S100A7/CEM[illegible]

2/DGKQ/PPP2R5B/CDC37/CDK5R1/SULF2/HNRNPA1/POLR2I/POLR2J/RHOA/RAPGEF1/IFT20/POLR2

2IP/RPTOR/PARP10/CALM3/ENSA/EPHB2/CIB1/DUSP22/PTPRC/CDK5RAP1/HDAC8/PWP1/BDKRB2/W

S100A7/CEMIP/ACVR1/TRAF7/CXCL17/HSPA2/ABL1/STK24/FCGR2B/SFRP2/BRAT1/CALM2/PDCD1[illegible]
[illegible]P2R3C/ACE/MUL1/WNK1/NOD2/PPP2CA/XRCC5/CARD14/RASSF2/MAP3K12/PDE5A/AFAP1L2/STRA[illegible]

RYK/PIK3CB/PLXNB2/DPYSL4/KIF3A/FSTL4/NCKAP1/PLCG1/MOV10/PPP1R9A/CNP/NCAM1/POLG2/
38A
P1/PLCG1/MOV10/PPP1R9A/CNP/NCAM1/RPL24/ETV1/SEMA3F/MEGF8/EGR2/ULK1/MNX1/KNDC1/CE
I1/STX4/CLEC7A/TNIP1/EIF2B5/POLR3D/LBP/ARPC1A/POLR3C/EXOSC3/BTN2A2/BRK1/HSPD1/PS

:ALR/GRK2/FAM155B/CDC42SE2/SYT1/APP/SNX12/SLC9A7/DNM1L/TRPM4/SH3GL1/AXL/CBL/CLE(

\P3K7/UCN/CCDC88A/COPS8/NAB2/MADD/NEURL1/PARK7/PDE6G/SLC27A1/CD4/JTB/IFNG/ZNF62[illegible]

:/DBNL/HOOK2/HSP90AA1/DNM2/TUSC2/PTK2/THBS1/TNK2/PTPN1/INPPL1/AAK1/SFRP4/BICD1/(

)C42/TGFB3/MUC20/RBPMS/VPS25/APP/ADRA2A/POLR2K/SPRY4/CD8A/AXL/DSTYK/CBL/AKT1S1/

/CNP/NCAM1/RPL24/ETV1/SEMA3F/MEGF8/EGR2/ULK1/MNX1/KNDC1/CELSR3/TRIM46/XK/SEMA6B/

N1/PARVB/EPHA2/ARTN/IST1/VPS33A/DOCK10/NEO1/PREX1/EPHB2/CIB1/SLC9A6/FRMD6/EPHA1(
IF1/MSL3P1/SIN3A/PAXIP1/NUP98/NDUFAB1/PRDM2/HDAC4/JADE3/DHPS/ING4/ATRX/LIPT2/GF]

POLR3H/POLR3C/CCL3/S100A7/CDK19/ABL1/FCGR2B/IL10RA/TNFAIP3/RNF216/MAPKAPK3/BAD/[illegible]
/KNDC1/CELSR3/TRIM46/XK/SEMA6B/TRIO/LHX2/PTPRA/SEMA4C/MAP4K4/PTPRS/DOCK7/CUL7/T[illegible]
POLR3H/POLR3C/CCL3/S100A7/CDK19/ABL1/FCGR2B/IL10RA/TNFAIP3/RNF216/MAPKAPK3/BAD/[illegible]
M8B/HUWE1/PITRM1/SRP68/TRAM1/RPL13/SNX16/RAB8B/RPL4/RPL41/RPL9/RPS6/TNPO3/RPL24/

G3/PIK3R2/RAB7A/KDM4A/ZMPSTE24/CHMP6/MUL1/TSC1/USP33/CPTP/TSG101/TP53/FEZ1/SPTI
MO1/TRIM25/POLR3D/LBP/POLR3H/POLR3C/CCL3/S100A7/CDK19/HSPD1/ABL1/FCGR2B/IL10RA/
PL19/MRPL37/EIF5A/RPS11/EIF3E/CAPRIN1/RPL22L1/RPUSD3/NSUN4/RHOA/RPL13A/MRPL27/
G3/PIK3R2/RAB7A/KDM4A/ZMPSTE24/CHMP6/MUL1/TSC1/USP33/CPTP/TSG101/TP53/FEZ1/SPTI

/PRDM1/PDCD1LG2/CD70/SOCS5/CD274/STAT3

.A/ZPR1/RAB29/KIF20B/GOLPH3L/RHBDD3/CENPJ/CACNB3/ALOX15B/CDK5R1/UBR5/PPM1F/TWIST

.00A7/EMILIN1/PSMD8/TRAF7/CXCL17/ABL1/STK24/FCGR2B/PSMA1/RASAL1/SFRP2/PDCD10/CSK
PSMC3/UBE2D2/STOML2/WIPF2/RBM14/CD79B/CD38/RAPGEF1/MAP3K1/LGMN/ELMO2/PSMC5/UBE2N

./WWTR1/PIN1/GLRB/CSNK1G1/MOV10/PPP1R9A/STK4/MAP3K7/KREMEN2/PSMB6/VPS26A/NXN/PFI

CL3/S100A7/EMILIN1/PSMD8/TRAF7/CXCL17/ABL1/STK24/FCGR2B/PSMA1/RASAL1/SFRP2/PDCI

P6V0D1/PLEKHN1/PTTG1IP/HNRNPM/MAPT/NSF/RUBCN/EXOSC3/RHEB/SUPT5H/UBXN1/BTG2/GGA1

3/USP5/UBQLN4/RPS7/FOXF2/MYEF2/BAG2/PSMB5/BAD/PSAP/PSMA4/HSP90AA1/PRKAA2/SGTA/PS

A2/RPTOR/GPAT4/CIB1/IL17RB/EXOSC2
P2CA/HDAC3/RASSF2/ACKR3/MAPKBP1/MAPKAP1/DUSP26/DAG1/C1QBP/MCL1/PDX1/MARVELD3/ABI

/SYS1/COG1/NOTCH1/VPS45/UGCG/FUT8/SCAMP2/ARFRP1/GALNT7/SH3GLB1/RGP1/FIG4/OPTN/LA
./ATR/TP53BP1/SRCAP/HMGA2/THOC1/DCLRE1C/MMS22L/SIN3B/TRRAP/HDAC8/DDB1/JUNB/TFPT/
P6V0D1/NSF/RUBCN/HPSE/SLC48A1/RHEB/CSPG4/SGSH/S100A7/GCA/SEC13/EEF1A2/RAB3D/HPS1
AT5/KDM3B/ETV3/SMCR8/HMGN3/SUZ12/BAZ1B/HMGXB4/L3MBTL1/TAF4/SLX4/ACTR8/NACC2/MEN1
C/SIRT7/CENPK/NUP43/PML/DYNC1LI1/DSN1/PLK4/PARP1/UBA1/THOC6/NSMF/SMARCB1/CPSF6/K
X/ZNF207/BEND3/CBX5/RPL35/SERTAD3/PPAN/POLR2K/XRCC6/IP6K1/DDX54/HOXD9/RPS19/NOC4
P6V0D1/NSF/RUBCN/HPSE/SLC48A1/RHEB/CSPG4/SGSH/S100A7/GCA/SEC13/EEF1A2/RAB3D/HPS1

E2/CST7/STARD3/VPS18/CTSD/AP3D1/AP1B1/PIP4K2C/BTK/STX4/CBL/CCZ1/MYO5A/ABCC10/M6P
C1/CALM3/RIC8B/CIB1/ACTR1A/OFD1/VPS4A/CTDP1/BBS7/TMEM67/CEP72/KIF3A/TRIP4/EFHC1/
/BOD1/CSPP1/DNM2/TCTEX1D2/PTK2/CCSAP/DCTN1/CEP164/ODF2L/HAUS7/KATNA1/PLK3/BICD1/

2/CORO2B/SSX2IP/EIF4G2/HSP90B1/ARF1/PDIA3/RPS9/CAV2/FRMD4A/CAV1/RPS14/ARHGAP22/P
PLEKHM1/VPS26A/NCSTN/MVB12B/HLA-C/RNF144A/OSBPL9/HLA-DOB/VAMP3/RAB22A/AP5S1/ANXA
OPG1/CLEC5A/TMEM199/TBC1D10C/ATP6V0D1/PDCD6/RASSF9/FCGR3B/WNT4/RAB4B/KCNAB2/CEMI

/DCAF7/POLR2D/GATAD2A/SART3/DHX15/ACTN4/TAP2/TOE1/TGS1/HINFP/DHX9/MED7/ZMIZ1/RBM

R1/TMEM63A/PADI2/TRAPPC1/GSDMD/ABCA3/ICA1/STK10/GPR84/CNN2/CALR/ALDOC/TGFB3/SYT1
SSF9/FCGR3B/WNT4/RAB4B/KCNAB2/CEMIP/SEC13/RAB3D/HVCN1/VAMP8/RAB5C/MOXD1/DBNL/PSA
/POLA1/TBX19/DFFA/UBE2A/SMARCC1/BRD9/KAT2A/ZNF207/BEND3/CBX5/PRIM2/NUP107/NCOR2/
5/POMGNT1/STX4/MGAT4A/NAGPA/STK25/VRK1/ARFIP2/NOSIP/M6PR/CSPG5/COPG1/B4GALNT3/TF
SLC17A5/SLC15A4/BCAN/UBA52/HLA-F/AP2A2/VAC14/ACAN/BORCS6/TMEM138/TECPR1/ARL8B/AC
5/CD1A/SNX16/ANTXR1/ATP9A/RAB8B/VPS8/TLR8/AP4B1/ABCA7/MAP3K7/KREMEN2/STAM/PLEKHM
/IRF4/HNRNPC/LRWD1/ING2/THOC3/MYC/YY1AP1/MBD2/HUS1/POLR2B/ASF1A/MAX/RELA/TERF1/S
./NANOS1/PRDX5/RAB29/TRIM37/KIF20B/TARBP2/PACSIN1/CDK5R1/UBR5/PPM1F/PPIB/PROCR/P
LP1/RPL30/PARVB/EPHA2/CORO2B/SSX2IP/EIF4G2/HSP90B1/ARF1/PDIA3/RPS9/CAV2/FRMD4A/C
3CG1/TMEM87B/RHEB/GLG1/MFNG/FUT1/SEC13/LFNG/EXT1/NSFL1C/LPCAT2/PDCD10/APOO/ARAP1

SMTN/ZNF207/VPS18/DNAL4/DNAJB6/FHDC1/MYO9B/DNM1L/KATNB1/SCTR/MYO5A/VIM/KRT20/WDR
SMTN/ZNF207/VPS18/DNAL4/DNAJB6/FHDC1/MYO9B/DNM1L/KATNB1/SCTR/MYO5A/VIM/KRT20/WDR
/PDGFA/WFS1/LAMTOR1/GNS/CD47/FTL/TCP1/NFAM1/FGR/PSMA5/NRAS/TGFB2/SERPINB6/PTPN6/
518/DNAL4/DNAJB6/FHDC1/MYO9B/DNM1L/KATNB1/SCTR/MYO5A/VIM/KRT20/WDR1/CCT8/HAUS6/N
/KATNA1/BICD1/PDLIM4/HNRNPU/MAP1A/ACTN1/HAUS5/MYO6/CD2AP/DYNC1I2/RAB11A/TUBB3/EM
3/NAT10/UTP6/MRPL53/HEATR1/MPV17L2/SART1/SYMPK/VCP/PHF5A/PSMA1/WRAP53/NOP10/KRI1

ARRB2/PARM1/LLGL1/STARD3/VPS18/CHMP1A/PRKCI/PLEKHJ1/CCR5/VPS25/APP/SNX12/SLC9A7/
MED7/PHKA2/GTF2H1/NAA15/RNMT/DMAP1/PHKG2/POLE3/BRPF1/BECN1/TAF13/RBM15B/FBXL7/U[illegible]

ZTS1/ADD3/COPS5/FBXO45/SLC17A5/BCL2L1/CANX/RAB11A/MECP2/PACSIN1/DGKQ/CDK5R1/PLD2

WAC/RBM4/HP1BP3/ZNF217/SPRTN/SETD1B/PLRG1/PIAS2/CDK12/IL16/OSGEP/NSRP1/CWC22

PRSS12/APBB1/CAPRIN1/RHOA/HTR1D/INHA/HNRNPR/ABHD17A/IFT20/MINK1/SNPH/PMM2/NIN/N4

'B/TP53/DDX1/ELK1/KDM3B/HMGN3/SUZ12/APEX1/PDX1/L3MBTL1/MEN1/ACTR6/TAF2/TPR/PHF23
3COR/TFEC/SCAND1/MED20/BRD7/HYAL2/MAX/NSD1/CNOT2/MED30/GON4L/PRKCB/MED16/E2F7/PI

'UBB3/ACAN/RPL19/MRPL37/TLN1/RPS11/RPL22L1/COPB2/RPL13A/MRPL27/MRPL28/CLTC/RPL37

ASF1/MAP4/RAE1/NEB/KIF2A/KPTN/LRP8/PARVG/RPS3/CROCC/CAPZB/RALA/SMTN/LLGL1/FHOD1/
KG2/ADCK5/EPHA2/SBK1/KSR1/NOL9/CDK11A/DOLK/PIK3R6/PDK3/IRAK2/CCL8/PGM2L1/MAP4K1/
'NB1/MYO5A/RALB/ABCC10/RGS3/CCT8/NUDT22/ATP6V0D1/DRG1/NSF/ABCG1/LONP1/RERG/ATP2A
'NB1/MYO5A/RALB/ABCC10/RGS3/CCT8/NUDT22/ATP6V0D1/DRG1/NSF/ABCG1/LONP1/RERG/ATP2A
'NB1/MYO5A/RALB/ABCC10/RGS3/CCT8/NUDT22/ATP6V0D1/DRG1/NSF/ABCG1/LONP1/RERG/ATP2A
'8/ATP6V0D1/DRG1/NSF/ABCG1/LONP1/RERG/ATP2A3/RHEB/RAB4B/HSPD1/RAD51C/EEF1A2/RAB3
APK3/WWC1/CCND3/BAD/BCL10/HSP90AA1/DNM2/PRKRIP1/PTK2/PTPN2/DCTN1/USF1/PTPN1/CCNJ
3F1/TRAF4/BCL2L1/AP2A2/PIH1D1/ZPR1/IFNAR2/CENPJ/CACNB3/CDC37/CDK5R1/PRKAR1B/DAXX
)AA1/MPP7/DNM2/PTK2/ADAM17/TNK2/CASP8/FUT8/FKBP8/INPPL1/CFTR/RNF2/MAVS/SARNP/THI
L3/CDC42SE1/CCND2/ANXA4/DNAJC10/DFFA/PPP4R1/BAG3/CST7/SYDE1/PTPA/RNH1/RUNDC1/LI
'EPS8L1/EPHA2/IST1/EDIL3/VASN/EIF4G2
RHGAP17/LLGL2/CYTH3/RALBP1/EVI5L/MYO1C/DOCK8/KIF3A/ARHGEF18/NCKAP1/PPP1R9A/EIF2I

P1/MAPKAP1/RAB11FIP1/SMCR8/DVL1/CLEC16A/DIAPH3/TNPO2/RNF41/AP3M1/RGL1/ARHGAP44/I
JSP33/BICD2/RASGRP1/GGA2/MAPKAP1/RAB11FIP1/SMCR8/RILPL2/DVL1/CLEC16A/DIAPH3/TNPO

CCNJL/GUK1/NME3/CTU2/AAK1/PLK3/PFKFB2/CCNE2/MARK4/PAK2/MASTL/OAS3/CDK11B/GNPTAB/

KB/MAP4K2/MKNK1/EPHB2/ERCC3/CSNK2A2/EPHA10/RPS6KC1

/PAGR1/ERCC3/EDF1/TAF1B/TADA3/CTDP1/NIF3L1/BBS7/HDAC8/ADD1/PPARG/JUNB/SUMO2/TRI[illegible]

[illegible]IX1/ACTN4/MTERF3/HINFP/DHX9/MLXIP/NR1H2/GMEB2/RUNX3/FOXJ3/NRF1/LYL1/TOX2/RBPJ/[illegible]

1/KPNB1/SMC4/NLE1/NASP/DCTN2/KNSTRN/LIG1/PRIM1/CDKN1C/AKAP8L/PSMB4/IL1B/WDHD1/N[illegible]
/STK25/ABI2/ARHGDIA/MSX1/SCN1B/CSPG5/WDR1/SEMA3C/EPB41L3/FMNL3/SEMA3E/MAPT/FGD1/
DAM17/CASP8/NOTCH1/CAPN2/SRGN/S1PR3/MAVS/IRF5/PLA2G1B/IL20RB/TLR2/TRIM21/CD2AP/[illegible]
2/ALOX15B/SULF2/TWIST1/NMI/GLMN/INHA/IL17RA/SIGIRR/TNFRSF14/DHX9/IDO1/HEG1/ADOR[illegible]

3P/FCGR3B/HPSE/EXOSC3/RAB4B/CCL3/S100A7/KCNAB2/GCA/MFNG/HSPD1/PAFAH1B2/LFNG/RAB[illegible]
SE/RAB4B/CCL3/S100A7/KCNAB2/GCA/HSPD1/PAFAH1B2/RAB3D/ARPC5/VCP/VCL/FCGR2B/APRT/[illegible]
RAB3A/HEXB/TLR2/CSNK2B/TMBIM1/SLC15A4/ANXA3/AP2A2/ATP6AP2/SLC2A3/TSPAN14/MLEC/P[illegible]

CGR3B/HPSE/EXOSC3/RAB4B/CCL3/S100A7/KCNAB2/GCA/MFNG/HSPD1/PAFAH1B2/LFNG/RAB3D/A[illegible]
70/CYSTM1/XRCC6/GZMB/TRPM4/PSMD3/CTSD/PSMD7/CD8A/FADD/BTK/GMFG/STX4/CBL/PRDX4/W[illegible]
C/PSMC3/PPIE/AGA/ADGRG3/SIGLEC9/RHOA
A/FCGR2B/PSMA1/SFRP2/TNFAIP3/TOX/PSMB5/BAD/PSMA4/PTGER4/ACVR1B/VCAM1/TWSG1/PSMD[illegible]

/FGR/LCP2/PSMA5/CD300LF/CCL4/NRAS/WAS/TGFB2/GPSM3/SFPQ/PQBP1/LCK/PTPN6/PSMD13/C[illegible]
1/NFKBIL1/PIK3CB/MYO1C/PSMB1/NCKAP1/PLCG1/TLR8/EIF2B4/MAP3K7/NFKBID/PSMB6/FFAR2/

1/POLQ/RBX1/MUTYH/TELO2/PPP5C/FUS/RAD21/RRM1/TATDN1/BABAM1/GZMA/UBE2L6/RFWD3/NA[illegible]
/MGME1/PARK7/UNG/AP5S1/HUS1/POLR2B/PRMT6/FIGNL1/EYA3/ASF1A/ERCC2/ZMPSTE24/TMEM1[illegible]
GF3/SRCAP/HMGA2/PARP10/MORC2/GRWD1/HDAC5/PAGR1/PABPC1L/ENY2/TADA3/BAG6/SIN3B/HU[illegible]
VTAP/RPL5/DUS2/NOL6/RPF2/CTU2/BICD1/BMT2/POP5/HNRNPU/NVL/SARNP/HTATSF1/TSN/EXOS[illegible]
DNAJC1/RPS14/MKNK1/RPS27/MRPS24/RPL29/EIF2D/MRPL33/LARP1/YTHDF2/CDK5RAP1/MRPS5/[illegible]
A2/IMPDH2/ABR/ATP6V0C/PSMD11/PSMD1/C5AR1/TMEM63A/PADI2/SYT12/TRAPPC1/GSDMD/RIMS[illegible]

/PIDD1/DNA2/DOT1L/ABL1/RAD51D/VCP/WRAP53/SFRP2/UBQLN4/BRAT1/DCLRE1B/FAAP20/NFAT[illegible]
L1C/ACTR3/CEP19/CHEK2/IFT172/HOOK2/COPG2/PRKAA2/SRGAP2/TCTEX1D2/PTK2/SPIRE1/CDK[illegible]
CCND3/PSMB5/PSMA4/HSP90AA1/PSMD9/ADAM17/CDK2AP2/CCSAP/DCTN1/CEP164/HAUS7/NBN/FB[illegible]
L3/GPR183/IGF1R/PDCD6/HPSE/DBF4B/MCTS1/MEIS2/ZNF335/BRK1/CHTOP/RARG/DOT1L/SLC25[illegible]
3/PPP2CA/HNRNPLL/SUGP1/ZRANB2/GEMIN5/CLK3/IWS1/DDX1/LSM3/TARDBP/C1QBP/NOVA1/SRS[illegible]
PC1/PCBP2/DNAJC18/UBXN2A/ZFAND2B/USP13/UBE2K/PSME2
A2/CUL4B/E2F6/HAUS6/NAE1/MYBBP1A/NDE1/DRG1/WNT4/CKAP5/DBF4B/BTN2A2/RAD51C/BTG2/[illegible]
1/RHOBTB2/GMFB/HTT/HAX1/EGF/ATR/MORC2/DZIP1/CSNK2A2/ANAPC4/HUWE1/PAF1/KIAA1324/[illegible]
52/MAP3K10/CDK5R1/PPM1F/NDUFA13/APBB1/TPD52L1/PLAGL2/DAXX/RHOA/STK3/YWHAG/PNMA3/
/VIM/XRCC4/CLEC5A/TNIP1/PACS2/TRIM25/RPL38/RPL23A/SUPT5H/MCTS1/CCL3/UBXN1/HSPD1/
PS27A/HNRNPU/RPS12/SARNP/RPS4X/RPL6/HTATSF1/EXOSC9/EXOSC10/LSM8/MAGOH/RPL37/RPL[illegible]
C4/PHC1/MYB/BRCA2/HR/SUDS3/WDR82/GTF2B/SETD5/LEO1
7/IKBKG/AXL/FADD/ECM1/RPLP0/PELI1/STX4/CBL/RACK1/ZNF675/VIM/IL20RA/CSF2RB/TRIM2[illegible]
D1/MRPL36/ACIN1/TFIP11/MRPL14/OGFOD1/CASP3/MRPL3/CAPZA2/ERAL1/PARK7/PYM1/USP36/[illegible]
M2/RPL24/OGFOD1/CHD7/RIOK1/ADAR/RPL11/RPL10/RPS24/PRMT5/DDX31/SNRNP200/DDX20/ER[illegible]
RGAP2/SPIRE1/PTPN1/SSH2/INPPL1/PDLIM4/KCNE4/FLII/MOB2/NEBL/ARHGEF11/ACTN1/PLA2G[illegible]
3IP/NPHP1/ARL3/CELSR3/FAM98A/MAPK15/HAUS2/IQCG/DOCK11/ATG3/CDK5RAP2/RHOD/EPS8L2/
1/CDK5R1/ARHGAP28/PPM1F/FAM171A1/TLN1/WIPF2/SRF/DSTN/PLEKHH2/RHOA/MRAS/ACTN4/MI[illegible]
/CASP8/NOTCH1/PDCD2/TMEM98/MYL9/HOXA5/THRA/ERCC1/VEGFA/ACTN1/LBR/SMAP1/TLR2/TME[illegible]

D/B4GALNT2/VCAM1/TWSG1/DNM2/PTK2/PTPN2/THBS1
/SGTA/PSMD9/RCN3/PTK2/UBL7/CSNK2A1/VPS35/CASP8/RPL5/RPS27A/CAPN2/FBXW5/USP21/PL[illegible]
L3/S100A7/EMILIN1/CEMIP/ACVR1/CXCL17/AKIRIN1/ABL1/STK24/VCL/SFRP2/CYP19A1/PDCD1[illegible]
2/ABL1/STK24/VCL/STMN2/RASAL1/SFRP2/NFATC4/KEL/DENND5A/DBNL/PLXNC1/HSP90AA1/CTN[illegible]

PIN/EPS8L1/EPHA2/AP2A1/CCL19/IST1/ARF1/KLF5/SNX3/HTT/CAV1/ATP7A/DAB2IP/PREX1/DZI
A5/FBXO45/TRIM21/IVNS1ABP/LRR1/UBA52/TRPC4AP/UBE2Q1/TRIM37/RNF213/RNF25/UBR5/PSM
D/CARD16/UQCC2/LILRB4/PDCD5/STX4/RACK1/RALB/KRT20/CEP250/CLEC5A/HCAR2/CCT8/GNL3/
F/NSFL1C/RPS7/RPL7A/RPS23/RPS10/BAD/CENPQ/ACOT8/LEMD2/BOD1/HSP90AA1/RPS15/SGTA/
18/CALR/CABIN1/SF3A3/NUP107/PPAN/CDC42EP2/PSMD4/HELLS/CETN2/LRRC61/MED25/NDUFS6/
/EXOSC1/EIF3E/SLC25A12/RPL13A/HNRNPR/RNASEH2C/POLR2D/PRKAG1/ANP32A/RPL37A/RPLP1/
CARS2/QRSL1/SARS2/RPS6/RPF1/RPRD1A/MRM2/CHD7/RIOK1/ADAR/RTCB/RPL11/RPS24/METTL2A
/TSPAN14/MLEC/PSMC3/PPIE/AGA/ADGRG3/SIGLEC9/RHOA
LR/ZMYND8/SEMA4D/XCL2/CMKLR1/APP/ADRA2A/SWAP70/DPEP1/SEMA4F/FADD/ECM1/STX4/SEMA5

LEC/PSMC3/PPIE/AGA/ADGRG3/SIGLEC9/RHOA

HSRP/DALRD3/GSS/EIF3G/DNAJC1/RPS14/MKNK1/RPS27/MRPS24/RPL29/EIF2D/MRPL33/LARP1/Y
/IDO1/TIGAR/FAF1/CCAR2/RBM10/ADM/BECN1/PSEN2/CDC34/TFDP1/PRKRA/PDIA3/ATG7/HTT/CA
PRKAA2/CTNNA1/STK40/PTK2/THBS1/HIGD2A/CSNK2A1/DAD1/CASP8/PTPN1/NOTCH1/RPS27A/FKI
2B/FAP/IDO1/TIGAR/FAF1/CCAR2/RBM10/ADM/BECN1/PSEN2/CDC34/TFDP1/PRKRA/PDIA3/ATG7/
TRADD/BAD/BCL10/PRKAA2/CTNNA1/STK40/PTK2/THBS1/HIGD2A/CSNK2A1/DAD1/CASP8/PTPN1/N

RK1/LIMS1/AKIRIN1/HSPA2/ARPC5/ABL1/HEATR1/TBCD/VCP/NPRL3/STMN2/THBS2/TMC8/PALM/A
PK15/EIF4G1/TERF1/CNOT2/CHGA/DOCK11/CDK5RAP2/MAP4K4/ERCC2/RAB7A/EPS8L2
RPLP0/RPL18/TBKBP1/CBL/RPL23/ABI2/RACK1/GADD45GIP1/VIM/XRCC4/CLEC5A/TNIP1/PACS2/
1/AP1G2/RPS19/IKBKG/AXL/AP1B1/FADD/RPLP0/RPL18/TBKBP1/CBL/RPL23/ABI2/CLEC7A/RACK
AQR7/RNF2/THRA/VDAC2/ERCC1/RAB3A/PRSS21/HEXB/MASTL/BCL2L1/GLI1/MEA1/SSTR2/UBE2Q1
D3/PSMB5/PSMA4/HSP90AA1/ACVR1B/PSMD9/DNM2/ADAM17/CDK2AP2/DCTN1/CCNJL/CEP164/HAUS
N2/IL4R/VPS18/ALDOC/TGFB3/SYT1/APP/LAIR1/ADRA2A/PNP/CYSTM1/PPFIA3/XRCC6/DNM1L/PS
ZMB/GPATCH3/PSMD3/PSMD7/RPS19/IKBKG/AXL/FADD/BTK/PELI1/STX4/TBKBP1/TREM1/CLEC7A/

2/CCL3/PRPF19/ZNF335/OVOL2/LIMS1/ACVR1/AKIRIN1/CBFA2T2/ABL1/SART1/VHL/STMN2/RASA
PPP2R5B/INTS7/CDK5R1/PSMC3/RBM14/APBB1/CHFR/INHA/PRKAG1/HINFP/FAP/RUNX3/PSMC5/TI
P7/MAP3K10/PIH1D1/TARBP2/DGKQ/CDC37/CDK5R1/PPM1F/PRKAR1B/TPD52L1/DAXX/RHOA/STK3/
F8/DOCK2/TNFRSF14/IDO1/RUNX3/ZMIZ1/LYL1/RBPJ/CCL19/XCL1/NOTCH2/PIK3R6/DOCK10/MSI

SG1/PSMD9/LMO4/TUSC2/PTPN2/THBS1/ADAM17/PUS7/CASP8/NOTCH1/PDCD2/TMEM98/MYL9/HOXA

1

9/TRIM37/IFRD1/PACSIN1/CDK5R1/ARHGAP28/HNRNPA1/UBR5/TWIST1/RBM14/PLEKHH2/RHOA/RI
XCL2/CMKLR1/APP/ADRA2A/SWAP70/DPEP1/TRPM4/SEMA4F/RPS19/FADD/ECM1/STX4/SEMA5B/ANG

1R/MAPT/STK38/RUBCN/CSPG4/DBF4B/NF2/CEMIP/PTGES3/TRAF7/EEF1A2/CXCL17/PKIA/ABL1/S
/TNFSF15/CASP8/PDCD2/RGP1/HNRNPU/TBC1D22B/ARHGEF11/VEGFA/RAB3A/SMAP1/PLA2G1B/FNT

NPJ/KIF22/ARL8B/PARD3/CACNA1D/CC2D2A/VPS11/AP3B1/YWHAG/IFT20/STXBP1/B9D1/ACTN4/C
R2/GEM/UNC13A/TTL/BICD2/RAB6A/GRIPAP1/NEFH/RASGRP1/FEZ1/NEFL/MYH9/CHMP4A
EGFA/TLR2/LZTS1/LGI4/HSPA5/DDX56/GLI1/RAB11A/ZPR1/RAB29/KIF20B/IFRD1/PACSIN1/NOT

NM2/PTK2/STYXL1/NOTCH1/TMEM98/S1PR3/FIG4/MOB2/OLFM4/LLPH/VEGFA/TLR2/LZTS1/HSPA5/
PT/KHDRBS1/NF2/KCNAB2/CEMIP/PKIA/ABL1/EMD/PIM3/WRAP53/MFF/NSFL1C/CALM2/PDCD10/ST
IP1/BTBD10/PTTG1IP/IGF1R/TFAP2A/WNT4/PELI3/HSPD1/ANKLE2/BTG2/GRINA/ACVR1/PIDD1/S
90AA1/ATP10D/SGTA/DNM2/SPIRE1/THBS1/DCTN1/VPS35/CASP8/RPS27A/UGCG/TMEM11/CFTR/AA

2/VCP/VHL/WSB1/PSMA1/TNFAIP3/USP5/RPS7/COPS6/FOXF2/BAG2/CLSPN/TBC1D7/RNF216/PSMI
P164/ODF2L/HAUS7/RPF2/VDAC3/BICD1/SH3GLB1/HNRNPU/NEBL/RPL6/MARK4/HAUS5/RILPL1/DY
2/RPL30/EPHA2/MRAP2/TFDP1/ITGAL/RPS9/CAV1/RAB13/RPS14/CALM3/EPHB2/GET4/RPS27/CII
31/TPD52L1/DAXX/STK3/NEK6/EMC10/RAPGEF1/RNF8/MINK1/DHX9/DNAJC2/RSL1D1/TIGAR/CCAI
P7D3/ADD3/LIMA1/CD2AP/RAB11A/CCDC88C/ACAN/EML2/MECP2/CENPJ/PACSIN1/CDK5R1/ARHGAI
/TWSG1/CTNNA1/PTK2/PTPN2
MAPK15/HAUS2/IQCG/DOCK11/ATG3/CDK5RAP2/RHOD/EPS8L2/VAV3/CARMIL2/PLXNB3/SHTN1/PTI
/CCL19/IST1/ARF1/KLF5/SNX3/HTT/CAV1/ATP7A/DAB2IP/PREX1/DZIP1/EPHB2/CIB1/PLD1/ARI

4/BANF1/SIRT7/CENPK/HAUS8/PML/DYNC1LI1/DSN1/CIT/PRCC/PLK4/ANAPC11/MAP4/UBE2D1/TU
2/PSMC2/MED1/PSMA6/NUDT21

STX4/SEMA5B/ANGPT2/CLEC7A/RACK1/SEMA3C/GPR183/SEMA3E/IGF1R/LBP/PDCD6/WNT4/EPPK1/

GPATCH1/PDCD7/CLP1/NUDT21/RBM4
D7/CLP1/NUDT21/RBM4
D7/CLP1/NUDT21/RBM4

LFM4/CYB5R3/GLIPR1/RAB3A/HEXB/PLA2G1B/TLR2/CSNK2B/TMBIM1/SLC15A4/ANXA3/AP2A2/ATI

EC4E/BAG6/ZCCHC3/BTN2A1/NFKBIL1/PIK3CB/MYO1C/PSMB1/NCKAP1/PLCG1/TLR8/EIF2B4/MAP3
THOC3/AURKAIP1/SREK1IP1/POLR2B/BUD13/GEMIN6/SYNCRIP/SMN2/HNRNPA2B1/SUPT6H/ERCC2/

/RAB36/ARHGAP33/RALGAPB/GNA13/DOCK8/RAB8B/RAB1B/ARHGEF18/NCKAP1/LPAR1/BCR/MADD/I
/RBM4B/YTHDF1/RBM8A/METTL3/PPARG/HNRNPA0/PSMB1/TNFRSF1B/MOV10/EIF2B4/UCN/PPP1R8/

/HEXB/TLR2/CSNK2B/TMBIM1/SLC15A4/ANXA3/HLA-F/AP2A2/ATP6AP2/SLC2A3/TSPAN14/MLEC/I

NJL/CEP164/HAUS7/NBN/PLK3/ZFYVE19/OPTN/CCNE2/HAUS5/MASTL/LZTS1/DYNC1I2/RAB11A/ZI

K3/SH3GLB1/HNRNPU/ZFYVE19/VEGFA/GNAI3/CCNE2/MARK4/HAUS5/MASTL/MIS18A/BCL2L1/CD2/

N1/EPHA2/DEDD/KITLG/GCLM/UBE4B/TFDP1/MSH6/PRKRA/PDIA3/HTT/CD5/CAV1/TMEM102/DAB2I

/RPS28/ELAVL1/FASTKD1/RPS17/PNPT1/SERBP1/NUP214/PPP2CA/TNFSF13/PAIP1/RPL22/LSM3/

[illegible]/SHQ1/NOP14/ABT1/TUT1/INTS3/NSUN2/WDR43/NSA2/CLP1/MRPS9/TSR2/INTS5/METTL8/DDX1[illegible]

5/LEO1/GATA3/KAT7/WAC/KANSL1

KRIP1/PTK2/PTPN2/THBS1/ADAM17/PTPN1/CCNJL/DUS2/RPS27A/NBN/HNRNPU/VEGFA/PFKFB2/C[illegible]
1/ACAP3/DNMT3B

/RPS3/PRKD2/TRAF3/PSMB3/DNASE2/GRB2/ARRB2/CDC42/IL4R/TGFB3/MUC20/CMKLR1/LAIR1/T[illegible]
GA2/RPTOR/CALM3/CIB1/PKD1/BORA/PTPRC/STUB1/BMP2/PABPN1/ADCY3/RAB1A/ZNF16/ADNP/P[illegible]

RK7/BUB3/RNF144A/USP36/NUB1/UBQLN2/RNF121/WWP2/ARIH2/RNF126/TLK2/ANAPC15/PSMB7/N
MC5/FAF1/ARRDC1/CCAR2/CTSW/SDF2L1/FBXL7/UBE2N/ANAPC5/HECTD3/AP2A1/CDC27/CDC34/HS
1/TP53/FEZ1/NDUFA7/AARS2/DNAJC11/CLEC16A/RNF41/NDUFA6/TIMM21/UQCR10/DDHD2/IMMT/S

L13A/MRPL27/MRPL28/HNRNPR/TAPBP/POLR2D/RPL37A/MTERF3/DARS2/MRPL51/MRPL11/RPLP1/I
5/ARRDC1/FBXL7/UBE2N/ANAPC5/HECTD3/CDC27/CDC34/UBE4B/UBE2E2/SH3RF3/PEX10/RAB40C/
NF121/WWP2/ARIH2/RNF126/TLK2/ANAPC15/PSMB7/NKD2/RNF115/SKP1/ANKIB1/RNF186/DERL1/
STUB1/ANAPC4/VPS4A/BAG6/HUWE1/BBS7/FAF2/TMEM67/DDB1/FBXO44/SKP2/PSMB1/WWTR1/SUM

2/DCK/MUC6/BCAN/B4GALT4/ACAN/TRMT12/MUC15/GFPT1/TMEM115/CHST3/STOML2/POMGNT2/SL
CARD16/MAP3K6/IKBKG/AXL/FADD/ECM1/PELI1/DSTYK/CBL/SCTR/RPL23/STK25/RACK1/MSX1/R

DC14A/SNAP29/CCDC113/TMEM17/DYNC2H1/PKHD1/IFT74/CCDC114/CEP290/CCDC151/PAFAH1B1/

P152/DRC1/BBS5/CFAP44/LRRC49/CDC14A/SNAP29/CCDC113/TMEM17/DYNC2H1/PKHD1/IFT74/C

126/TLK2/ANAPC15/PSMB7/NKD2/RNF115/SKP1/ANKIB1/RNF186/DERL1/KCTD13/UBR4/CUL7/RN[illegible]
/SPACA6/SPIRE2/GLRB/ADAMTS2/RPS6/TBC1D20/ATP8B3/SMAD5/INSL3/STRBP/TRIM27/NEURL1/
/BAG6/RFX2/SLCO4C1/ADNP/CNR1/ZSCAN2/METTL3/SPACA6/JUNB/SPIRE2/ADCY7/ADAMTS2/RPS[illegible]
CO4C1/ADNP/CNR1/ZSCAN2/METTL3/JUNB/SPIRE2/ADCY7/ADAMTS2/RPS6/CASP3/TBC1D20/SMAD[illegible]

[P/ACVR1/PTGES3/TRAF7/EEF1A2/CXCL17/HSPA2/ABL1/STK24/VCP/FCGR2B/SFRP2/BRAT1/CALM

2D/IFI6/AP2B1/LGMN/HBEGF/ELMO2/NRTN/RBPJ/STXBP4/BTC/PSEN2/EPHA2/AP2A1/EREG/SERP[illegible]

WWTR1/PIN1/UCN/PPP1R8/CASP3/ADAR/RPL11/NXN/MFHAS1/TRIM27/PHF1/PTPN13/RNF149/PAR[illegible]

)/CSK/CHEK2/CLSPN/MAPKAPK3/WWC1/GDF11/CCND3/DBNL/PSAP/ERP29/HSP90AA1/ACVR1B/CDK2
)A/RASGRP1/TNFRSF10A/PRKAR2A/MAPKAP1/DAG1

/RPL24/ETV1/SEMA3F/MEGF8/EGR2/ULK1/MNX1/KNDC1/CELSR3/TRIM46/XK/SEMA6B/TRIO/LHX2/

ELSR3/TRIM46/XK/SEMA6B/TRIO/LHX2/PTPRA/SEMA4C/MAP4K4/PTPRS/DOCK7/CUL7/TRAK2/PLXN
SMD8/ARPC5/ABL1/FCGR2B/PSMA1/TNFAIP3/STX7/KCNN4/CSK/ACTR3/VAMP8/MAPKAPK3/PSMB5/P

C7A/RACK1/M6PR/CSNK1G2/SLC36A4/LBP/CLIP3/ARPC1A/RUBCN/ILDR1/RAB4B/ACHE/BRK1/ARPC

2/MAP3K9/NOD1/CD19/MAP4K4/MAP3K11/VAV3/PPP2R3C/ACE/MUL1/WNK1/NOD2/PPP2CA/XRCC5/C

CD177/DMBT1/VEGFA/SMAP1/TLR2/MYO6/PACSIN2/ANXA3/BCL2L1/CANX/CD2AP/AP2A2/PACSIN1/

ANGPT2/VIM/MSX1/TRIM71/PTPRF/ATP6V0D1/UBE2O/IGF1R/HNRNPM/PDCD6/ARPC1A/WNT4/TGIF2

/TRIO/LHX2/PTPRA/SEMA4C/MAP4K4/PTPRS/DOCK7/CUL7/TRAK2/PLXNB3/MUL1/SHTN1/LAMA1/U[illegible]

)/YTHDF2/RAB1A/ARHGAP33/YTHDF1/ADNP/FEZ2/METTL3/RYK/PIK3CB/ANTXR1/PLXNB2/SCARA3/
[1/LIPT1/ESCO1/SNW1/RLF/SMYD2/ATG5/ING5/NUP160

GNLY/BCL10/LOXL1/PTGER4/VCAM1/TUSC2/ADAM17/CASP8/NOTCH1/CAPN2/ADAMTS5/MAVS/IRF5/
RAK2/PLXNB3/MUL1/SHTN1/LAMA1/USP33/ACTR2/UNC13A/TTL/STRADA/NEFH/BCL11A/FEZ1/DPYS
GNLY/BCL10/LOXL1/PTGER4/VCAM1/TUSC2/ADAM17/CASP8/NOTCH1/CAPN2/ADAMTS5/MAVS/IRF5/
/EFCAB7/RPL11/RPL10/RPS24/RBM22/KPNA4/IMMP1L/TIMM9/LRWD1/HEATR3/RPS3A/TIMM22/USP

LC2/DAPL1/CHMP4A/FBXL2/TFEB/KAT5/SMCR8/MTDH/SVIP/MCL1/MCOLN1/ABL2/ATP6V1D/CLEC16
/TNFAIP3/RNF216/MAPKAPK3/BAD/GNLY/BCL10/LOXL1/PTGER4/VCAM1/TUSC2/ADAM17/CASP8/NC
MRPL28/HNRNPR/POLR2D/RPL37A/MTERF3/DARS2/MRPL51/MRPL11/RPLP1/DHX9/MAPKAPK5/COA3/
LC2/DAPL1/CHMP4A/FBXL2/TFEB/KAT5/SMCR8/MTDH/SVIP/MCL1/MCOLN1/ABL2/ATP6V1D/CLEC16

[illegible]1/APBB1/CACNA1D/GLMN/VPS11/CD38/YWHAG/ATG13/IL17RA/ANP32B/TNFRSF14/DHX9/DMAP1/[illegible]

[illegible]/MAPKAPK3/WWC1/GDF11/PSMB5/DBNL/PSAP/PSMA4/LEMD2/ERP29/PTGER4/PSMD9/STK40/PTK2/
[illegible]/CD247/TLR7/HSP90B1/IRAK2/CAV1/DAB2IP/INPP5D/DUSP22/CD3G/PTPRC/NECTIN2/CLEC4E/[illegible]

[illegible]N5/PSMA3/PDE6G/AGO1/MBD2/BRD7/CELSR3/LYPD6/BCL7B/VPS29/FOXL1/PSMB7/NKD2/SPIN1/[illegible]

)10/CSK/MAPKAPK3/WWC1/GDF11/PSMB5/DBNL/PSAP/PSMA4/LEMD2/ERP29/PTGER4/PSMD9/STK4(

[illegible]/PAFAH1B2/SEC13/PSMD8/EEF1A2/ABL1/VCP/IL10RA/PSMA1/NPRL3/ATG4B/TNFAIP3/USP5/UB[illegible]

SMD9/PTK2/CSNK2A1/VPS35/PTPN1/RPL5/RPS27A/PLK3/SH3GLB1/MAGEF1/RGP1/HNRNPU/MAP1A/

[illegible]2/GNAI2/NLRP3/MEN1/ARRB1/ARHGAP44

ARGE2/GNAI3/GNPTAB/SREBF1/HLA-F/RTN1/B4GALT4/VAC14/GOLPH3L/NOTCH3/GORASP2/SLC35I
/TRPS1/TFIP11/ASXL1/IK/USP3/LRPPRC/THOC7/TTI1/IRF4/HNRNPC/LRWD1/ING2/THOC3/MYC/Y
l/VCP/HLA-DOA/NPRL3/TNFAIP3/USP5/PON2/ABCB9/STX7/VAMP8/MILR1/RAB5C/GNLY/PSAP/BCI
l/NFRKB/ARRB1/SP1/TNKS1BP1/ACTR6/RNF40/NFATC1
KMT5C/ZMIZ2/DONSON/TIPIN/MSH2/PADI2/CHD4/RUVBL2/CENPB/CCND2/EXOSC5/POLE4/RBBP4/I
4L/RPL18/RRP7BP/GTF3C1/RPL23/UTP11/NIFK/VRK1/VCX3A/LAS1L/NOL12/UTP4/GNL3/INO80C/
l/VCP/HLA-DOA/NPRL3/TNFAIP3/USP5/PON2/ABCB9/STX7/VAMP8/MILR1/RAB5C/GNLY/PSAP/BCI

PR/CSPG5/TMEM199/CCT8/GNB4/ATP6V0D1/NSF/RUBCN/HPSE/SLC48A1/RHEB/VMP1/CSPG4/SGSH/
/CCDC88A/NUBP2/LRRC45/PHF1/AAAS/C2CD3/PRICKLE3/PDE4B/CNTRL/TCHP/DYNC2LI1/CEP97/I
/CCDC85B/HNRNPU/FLII/ZFYVE19/RAD18/GNAI3/CCNE2/MARK4/RITA1/HAUS5/MASTL/TAF1D/DD/

PTPRC/CYTH3/NECTIN2/SRP68/ADD1/ASAP3/LIN7B/GNA13/RSU1/P4HB/ATP2A2/CD59/NCKAP1/C/
A1/CD207/VPS33B/VPS29/ATP6V0A1/HLA-E/ARL8A/DNAJC13/RHOD/RAB7A/CHMP6/APPL2/CYB561
IP/SEC13/RAB3D/HVCN1/VAMP8/RAB5C/MOXD1/DBNL/PSAP/CD33/COPG2/SGTA/DNM2/SPIRE1/TNI

M10/YARS2/RPA1/RBM15B/AFF3/MECOM/CDC34/CDYL/SF3B2/NELFA/LYRM4/LSG1/POMP/SAFB2/S/

l/APP/LAIR1/PNP/GAL/CYSTM1/PPFIA3/XRCC6/PSMD3/CTSD/PSMD7/CPE/ECM1/GMFG/STX4/PRD)
AP/CD33/COPG2/SGTA/DNM2/SPIRE1/TNK2/RPS27A/CFTR/CD177/DMBT1/GLIPR1/RAB3A/GABRB3/
/CBX8/HELLS/PCGF2/XRCC6/HMGN1/TELO2/RAD21/MTBP/RFWD3/CENPN/NABP1/RBBP7/RAD9A/RP/
RAPPC2L/PIFO/MMGT1/CLIP3/CHST12/PDCD6/NSF/B4GALNT4/ABCG1/WNT4/TMEM87B/RHEB/CSPG4
GA/VPS11/AP3B1/CLTC/AP1S1/AP1S3/AP1S2/AP2B1/LGMN/WDR41/IFITM3/TPP1/MANBA/GPC2/SI
M1/VPS26A/NCSTN/MVB12B/PRF1/HLA-C/RNF144A/OSBPL9/HLA-DOB/VAMP3/RAB22A/AP5S1/CTSI
SMC1B/LRIF1/MBD1/PPP1CB/SMAD2/WBP2/REPIN1/ORC4/HELB/XRCC5/SMAD3/SETD1A/BAZ1A/TP5
EXOC3/APBB1/TPD52L1/PHEX/ARF5/RAPGEF1/STXBP1/GAK/ACTN4/ANP32A/LGMN/MTPN/KCNH2/N)
CAV1/RPS14/ARHGAP22/PTPRC/CYTH3/NECTIN2/SRP68/ADD1/ASAP3/LIN7B/GNA13/RSU1/P4HB/A
l/DENND5A/WIPI1/DBNL/COPG2/B4GALNT2/DNM2/NECAB3/SYS1/COG1/NOTCH1/VPS45/UGCG/FUT8

R1/CCT8/HAUS6/NDE1/MAPT/CKAP2/EPPK1/CKAP5/OBSCN/KCNAB2/COL11A1/RAB3D/TNNI3/EMD/T
R1/CCT8/HAUS6/NDE1/MAPT/CKAP2/EPPK1/CKAP5/OBSCN/KCNAB2/COL11A1/RAB3D/TNNI3/EMD/T
/PSMD13/AGPAT2/CLTB/COMMD9/RAB10/DYNC1LI1/GALNS/DSN1/SLC44A2/KCNK9/IMPDH2/ATP6V(
NDE1/MAPT/CKAP2/EPPK1/CKAP5/OBSCN/KCNAB2/COL11A1/RAB3D/TNNI3/EMD/TBCD/VCL/CALM2/
ML2/KIF20B/CENPJ/KIF22
l/RPS7/RPL7A/RPS23/MYEF2/GAR1/RPS10/RBM42/DDX23/MRPS17/PSMA4/MRPS21/EIF3K/PRKAA2

/CCDC93/OCRL/CTSD/AP1G2/SYT3/SH3GL1/AP3D1/STX4/WDR91/CCZ1/MYO5A/M6PR/CCDC120/ATI
3E2N/ANAPC5/TBP/MMS19/CDC27/UBE4B/PIK3R6/POLR2E/AUP1/ZER1/PELP1/MAEA/MORF4L1/KLI

2/PRSS12/EXOC3/APBB1/CACNA1D/RHOA/YWHAG/HNRNPR/ABHD17A/STXBP1/AP1S1/ATP2B4/GAK/[illegible]

4BP3/BECN1/PSEN2/MPP2/LSM1/DOCK10/GLRX2/HTT/MAGEE1/PI4K2A/ATP7A/DAB2IP/RPTOR/PRI

3/EZH1/NFATC1/MCM3AP/PLAC8
3XIP1/WBP2/KCNIP3/PTPN14/HDAC3/MED17/SMAD3/NCOA7/MAML1/SUPT3H/AEBP1/TSG101/DDX1/

7A/MRPL51/MRPL11/RPLP1/RPL32/COPB1/RPL30/SPTAN1/CRYBB1/CRYGS/MRPL15/MRPL4/KSR1/

/CNN2/ZNF207/VPS18/ALDOC/INF2/CCR5/FHDC1/MYO9B/KLHL17/CETN2/DNM1L/ACTL8/PPP5C/A
/PI4K2A/ATR/CHKB/AK1/MAP4K2/MKNK1/EPHB2/ERCC3/CSNK2A2/EPHA10/RPS6KC1/SRPK2/IP6K
A3/RHEB/RAB4B/HSPD1/RAD51C/EEF1A2/RAB3D/DIRAS1/DNA2/HSPA2/RHOT2/GSPT2/RAD51D/VC
A3/RHEB/RAB4B/HSPD1/RAD51C/EEF1A2/RAB3D/DIRAS1/DNA2/HSPA2/RHOT2/GSPT2/RAD51D/VC
A3/RHEB/RAB4B/HSPD1/RAD51C/EEF1A2/RAB3D/DIRAS1/DNA2/HSPA2/RHOT2/GSPT2/RAD51D/VC
3D/DIRAS1/DNA2/HSPA2/RHOT2/GSPT2/RAD51D/VCP/GNG4/GNGT2/ABCB9/DDX23/RAB5C/GTPBP3/
JL/FBXW5/BICD1/PPP1R12C/MAVS/CTBP2/PFKFB2/CCNE2/PAK2/FNTA/SREBF1/TRAF4/BCL2L1/A
X/RHOA/NEK6/GSKIP/YWHAG/ATG13/CLTC/CNPPD1/STXBP1/ATP2B4/PRKAG1/MAP3K1/MAPKAPK5/
RA/ERCC1/GNAI3/SH3BGRL/LBR/TRAF4/CSNK2B/HSPA5/BCL2L1/CD2AP/AP2A2/CCDC88C/SSTR2/
LGL1/MTCP1/ARHGAP18/CABIN1/RANBP1/AHSA1/SPINK4/AGAP1/PPP1R16A/APP/DNAJB6/DPEP1/

34/TBC1D20/BCR/MADD/NOX1/RHOH/EIF2B1/TIAM2/ULK1/KNDC1/RAB3IP/SRGAP1/TRIO/IPO7/T

PARD6A/FGD2/GAS8/NUP50/LZTR1/EXOC2
D2/RNF41/AP3M1/RGL1/ARHGAP44/PARD6A/FGD2/GAS8/NUP50/LZTR1/EXOC2

/CSNK2B/TBRG4/DCK/LMTK2/MAP3K10/SEPHS1/TP53RK/DGKQ/CDK5R1/NUAK1/POLR2I/CDK20/POI

P4/ASXL1/STK4/TRIM32/NFKBID/NAB2/HNRNPD/NCOA6/GTF2A2/DDX20/PPRC1/PARK7/IRF4/PDCI

ZNF668/AFF3/TBP/BHLHA15/TFDP1/SUV39H1/ARID5B/KLF5/MSH6/CCAR1/BARX1/LDB1/PCGF3/H[illegible]

CAPH2/PSMB8/KIF15/PSMB10/PSMA5/POC1A/DNMT3A/PTPN6/PSMD13/CHMP3/PSMC4/BANF1/SIRT
/RERE/CCL3/CHN1/ZNF335/LIMS1/EXT1/ABL1/STK24/TBCD/VHL/VCL/RASAL1/NFATC4/PALM/AR
HLA-F/ATP6AP2/ALOX15B/SULF2/TWIST1/STOML2/NMI/GLMN/INHA/IL17RA/SIGIRR/TNFRSF14/
A2B/EPHA2/EREG/CCL19/KPNA6/XCL1/TLR7/POLR2E/OSM/IL17D/CD84/INPP5D/IL17RB/PANX2/

3D/ARPC5/ABL1/VCP/VCL/FCGR2B/APRT/PDAP1/HVCN1/VAMP8/MILR1/BIN2/RAB5C/DBNL/PSAP/
PDAP1/HVCN1/VAMP8/MILR1/BIN2/RAB5C/DBNL/PSAP/CD33/HSP90AA1/C12orf4/THBS1/CD177/
SMC3/PPIE/AGA/ADGRG3/SIGLEC9/RHOA

RPC5/ABL1/VCP/VCL/FCGR2B/APRT/PDAP1/HVCN1/VAMP8/MILR1/BIN2/RAB5C/DBNL/PSAP/CD33/
OR1/CLEC5A/CCT8/TBC1D10C/FCGR3B/HPSE/EXOSC3/RAB4B/CCL3/S100A7/KCNAB2/GCA/HSPD1/

9/LMO4/TUSC2/PTPN2/THBS1/ADAM17/PUS7/CASP8/NOTCH1/PDCD2/NBN/TMEM98/MYL9/HOXA5/T

D27/IFI16/PSMC4/MUC5B/CCR1/IL12RB1/COCH/CD79A/UBE2D1/IL15/PSMD10/PSMD11/PSMD1/C
/CD22/CNPY3/MFHAS1/PLA2G6/ICAM2/PSMA3/IRF4/PDE4B/EIF2B1/CTSL/CD4/CTSB/RELA/PSMB

3P1/RBBP7/RAD9A/XRCC4/OGG1/RPA2/CUL4B/CCT8/GNL3/PPP4C/RBM17/POLD3/INO80C/TRIM25/
61A/RAD54B/LIG3/HELB/XRCC5/SMC1A/FAAP100/ACTR2/SUPT16H/TP53/INO80E/DDX1/TDP2/CO
WE1/PAF1/TRRAP/HDAC8/PWP1/DDB1/SUPT7L/ASXL1/USP3/MAP3K7/UCN/CHD7/PRMT5/PHF1/DPY
C9/EXOSC10/LSM8/MAGOH/CDK11B/EXOSC7/ARL6IP4/TBRG4/DDX56/IVNS1ABP/INTS9/PIH1D1/Z
CASC3/RBM4B/YTHDF1/RPL13/RBM8A/METTL3/CARS2/MRPL36/MOV10/QRSL1/RPL4/SARS2/RPL41/
3/RALA/STK10/LLGL1/GPR84/CNN2/IL4R/VPS18/ALDOC/TGFB3/SYT1/APP/LAIR1/ADRA2A/PNP/

C4/TDG/COPS6/EME2/CHEK2/CLSPN/BAD/RAD1/SFR1/CHD1L/HDAC10/SPIRE1/CEP164/RPS27A/N
2AP2/SPA17/CCSAP/DCTN1/HAUS7/FBXW5/KATNA1/PLK3/BICD1/HNRNPU/MAP1A/MARK4/HAUS5/M
XW5/PLK3/HNRNPU/ZFYVE19/HAUS5/BCL2L1/DYNC1I2/RAB11A/GPR132/NEDD1/KIF20B/MECP2/C
A5/ABL1/SFRP2/TNFAIP3/GHRHR/MTA3/FAM98B/PDCD10/BAD/TBX2/VCAM1/OSR2/PTK2/THBS1/A
F10/SRSF7/PRPF40B/TRA2A/PRMT7/CACTIN/RSRC1/METTL16/BCAS2/SNRNP35/DDX46/ZCRB1/BR

PSMD8/PIDD1/HSPA2/SUSD2/PKIA/PSMA1/MTA3/NSFL1C/CALM2/CHEK2/CLSPN/CEP41/CCND3/PS
BIK/C2CD5/MYO1C/SPIRE2/NCKAP1/LPAR1/PXN/CCDC88A/HNRNPD/DDHD1/TRIM27/PHF1/PIP4K2
/ANP32B/FAP/IDO1/TIGAR/FAF1/CCAR2/RBM10/ADM/BECN1/PSEN2/CDC34/TFDP1/PRKRA/PDIA3/
/SEC13/SLC25A5/VCP/FCGR2B/RPS7/KCTD5/COPS6/SRPK1/RPL7A/RPS23/RPS10/VAMP8/RNF216/
27A/DXO/CDK11B/EXOSC7/SREBF1/ARL6IP4/TBRG4/RPL36/UBA52/ZPR1/NANOS1/THOC2/ETF1/R

5/LBP/CLIP3/CCL3/PELI3/PSMD8/LIMS1/IL10RA/PSMA1/TNFAIP3/CIITA/TRADD/PSMB5/BAD/P
AURKAIP1/MRPL20/KIF5B/MYC/DMTN/CTSL/LIX1L/DYRK3/MRPL24/PRSS1/PAM16/MRPS16/HTRA2/
AL1/WDR12/GEMIN8/PDCD11/EIF3F/HEATR3/USP36/MRPL20/AGO1/URB2/RPL14/DDX51/RPL10A/
1B/ADD3/MYO6/PACSIN2/LIMA1/CD2AP/PACSIN1/CDK5R1/ARHGAP28/PPM1F/TNNC1/FAM171A1/T
/VAV3/CARMIL2/PLXNB3/SHTN1/PTPDC1/RAC3/MYO1A/ACTR2/IFT140/CNTROB/NEFH/CEP135/TM
NK1/DOCK2/MTPN/SPTAN1/PARVB/DPYSL3/CORO2B/NOTCH2/HSP90B1/ARF1/RHOBTB2/GMFB/NPHP
M64/WDR5/NKAP/PSMC3/ADGRG3/CD79B/SRF/RHOA/STK3/INHA/AP3B1/ZNF160/DOCK2/RUNX3/ZM

K3/MAGEF1/RGP1/MAP1A/BNIP3L/USP27X/GGA3/HSPA5/FBXO45/EGLN2/UBA52/CD2AP/AP2A2/ED
D/TRIP6/TRADD/LAMA4/PLXNC1/PTGER4/ACVR1B/VASH1/SLC8B1/SRGAP2/LMO4/PTK2/THBS1/AD
NA1/SRGAP2/DNM2/PTK2/STYXL1/NOTCH1/USP21/FIG4/MAP1A/MOB2/LLPH/VEGFA/RAB3A/PAK2/

IP1/EPHB2/CIB1/PLD1/ARHGAP33/EVI5L/PROM2/YTHDF1/ADNP/CNR1/RYK/PLXNB2/RAB8B/FSTL
MC3/KLHL21/UBE2D2/CHFR/NMI/RASSF5/DAXX/TRIM69/GLMN/RNF8/DCAF7/PRMT3/SOCS7/CTU1/
/SERGEF/CLIP3/MAPT/ABCG1/CARD18/NF2/BTN2A2/CCL3/KCNAB2/ACHE/CEMIP/SLC25A5/PKIA/
/RPS16/SYS1/VPS35/CASP8/RPL5/RPS27A/RPF2/SRGN/TMEM98/ARFRP1/BICD1/SH3GLB1/HNRNP
/DNM1L/RBX1/UQCC2/RPS19/RPLP0/GMFG/STX4/COA6/DR1/RRP7BP/RNF4/ABI2/RALB/CENPN/RB
/DHX9/EDC4/RPL32/TNPO1/RPL30/PSMC5/TIGAR/CNOT10/RBM10/MEX3D/BPGM/SEH1L/RBM24/EI
A/GTF2A2/ZNF143/WDR12/THADA/PDCD11/USP36/AGO1/RPL14/DDX51/POU2F1/PPA1/POLR2B/DU

5B/ANGPT2/CLEC7A/RACK1/SEMA3C/GPR183/TRIM25/SEMA3E/IGF1R/LBP/PDCD6/WNT4/EPPK1/N

YTHDF2/CDK5RAP1/MRPS5/CASC3/CHAC1/RBM4B/YTHDF1/RPL13/RBM8A/METTL3/CARS2/MRPL36/
AV1/DAB2IP/HMGA2/ARHGEF17/DKKL1/PREX1/INPP5D/ERCC3/PTPRC/BMP2/SRPK2/LGALS14/ZNF
3P8/PLK3/VDAC2/VEGFA/GNAI3/BNIP3L/GABRB3/PAK2/DDAH2/COPS5/HSPA5/TMBIM1/TXNDC12/
/HTT/CAV1/DAB2IP/HMGA2/ARHGEF17/DKKL1/PREX1/INPP5D/ERCC3/PTPRC/BMP2/SRPK2/LGALS
NOTCH1/RPS27A/FKBP8/PLK3/VDAC2/VEGFA/GNAI3/BNIP3L/GABRB3/PAK2/DDAH2/COPS5/HSPA5/

ACTR3/ARAP1/TBC1D7/DBNL/PTGER4/HSP90AA1/PRKAA2/CHD1L/MPP7/DNM2/LMO4/TCTEX1D2/PT

/TRIM25/RPL38/RPL23A/SUPT5H/MCTS1/CCL3/UBXN1/HSPD1/SEC13/SLC25A5/VCP/FCGR2B/RPS
K1/GADD45GIP1/VIM/XRCC4/CLEC5A/ROMO1/TNIP1/PACS2/TRIM25/RPL38/RPL23A/SUPT5H/MCT
1/TARBP2/GORASP2/TEAD3/TEX11/CD38/IFT20/STXBP1/RNF8/ATP2B4/PPAT/PRKAG1
S7/NBN/PLK3/ZFYVE19/OPTN/CCNE2/HAUS5/MASTL/UIMC1/LZTS1/GLI1/DYNC1I2/RAB11A/ZPR1/
SMD3/CTSD/SYT3/PSMD7/ECM1/BTK/GMFG/STX4/CBL/PRDX4/CSPG5/WDR1/CLEC5A/CCT8/TBC1D1
/VIM/CLEC5A/TNIP1/TRIM25/POLR3D/LBP/POLR3H/POLR3C/CCL3/S100A7/CD300LB/HSPD1/ARI

AL1/SFRP2/KDF1/TOX/BAD/DBNL/PLXNC1/ACVR1B/CTNNA1/DNM2/STYXL1/CASP8/NOTCH1/CAPN2/
IPRL/CNOT10/CCAR2/FBXL7/WDR6/EIF4G2/NOTCH2/CNOT3/UXT/MUS81/TFDP1/MSH6/ATR/TP53B
/GSKIP/RAPGEF1/YWHAG/ATG13/CNPPD1/ATP2B4/PRKAG1/PIK3R5/MAP3K1/GPS1/MINK1/RPLP1/
H6/ITGAL/CD5/CAV1/ATP7A/TP53BP1/PKNOX1/ATAD5/THOC1/PREX1/DCLRE1C/INPP5D/HDAC5/D

A5/THRA/ERCC1/VEGFA/ACTN1/LBR/SMAP1/TLR2/TMEM64/WDR5/NKAP/PSMC3/ADGRG3/CD79B/SR

PL13A/CD38/STXBP1/MLLT6/GAK/ACTN4/DNMT3B/FAP/NR1H2/MTPN/SPTAN1/TIGAR/ADCY6/BECN
GPT2/CLEC7A/RACK1/IGBP1/SEMA3C/GPR183/SEMA3E/IGF1R/LBP/PDCD6/WNT4/EPPK1/NF2/CCL

STK24/WRAP53/SFRP2/TNFAIP3/RPS7/CALM2/PDCD10/CSK/BAG2/CLSPN/MAPKAPK3/CCND3/BAD/
TA/PLEKHG6/ARHGAP28/PPM1F/ADPRH/NDUFA13/AGAP3/RHOA/RAPGEF1/ACAP3/STXBP5/ANP32B/

GOSR2/RAB27B/FNBP1L/SNPH/NIN/ADORA2B/BECN1/PSEN2/SNAPIN/VPS33A/SEH1L/BHLHA15/EI

TCH3/PPP2R5B/CDK5R1/METRN/PARD3/APBB1/SRF/CAPRIN1/RHOA/SLC25A12/CD38/HES2/RAPGE

/DDX56/RAB11A/RAB29/KIF20B/IFRD1/PACSIN1/NOTCH3/PPP2R5B/CDK5R1/METRN/APBB1/SRF/
TAC/STX7/CSK/VAMP8/WWC1/BAD/CENPQ/HSP90AA1/PRKAA2/C12orf4/TCTEX1D2/VPS35/CASP8/
SLC25A5/ABL1/VHL/PIM3/SFRP2/TNFAIP3/NFATC4/PDCD10/TRADD/BAD/PSAP/BCL10/PRKAA2/C
AK1/SH3GLB1/TOMM22/GNAI3/BNIP3L/RAB3A/RCC1L/FNTA/PACSIN2/BCL2L1/RILPL1/UBA52/AP

35/SENP3/BCL10/PSMA4/HSP90AA1/PSMD9/UBA2/RPL5/RPS27A/FBXW5/USP21/CFTR/CTU2/MAGE[illegible]
YNC1I2/RAB11A/NEDD1/TRIM37/CENPJ/GORASP2/TMEM138/VPS37B/SASS6/RBM14/SQLE/SRF/CC[illegible]
31/RPL29/ZDHHC24/VPS4A/BAG6/TM9SF4/SRP68/TRAM1/C2CD5/RPL13/LIN7B/ATP1B1/SSR1/MY[illegible]
R2/RPA1/UBE2N/CORO2B/MMS19/MECOM/CCL19/SEH1L/UBAC2/SH3RF3/ATG7/MAP4K1/CAV1/ATR/[illegible]
P28/PPM1F/FAM171A1/SRF/DSTN/PLEKHH2/RHOA

PDC1/MYO1A/ACTR2/IFT140/CNTROB/CEP135/TMEM80/PIP5K1A/SH2B1
HGAP33/EVI5L/YTHDF1/ADNP/CNR1/RYK/PLXNB2/FSTL4/NCKAP1/MOV10/PPP1R9A/LPAR1/PLEKH[illegible]

JBG2/KLHDC8B/PSMD10/PSMD11/NES/RAE1/CDKN2C/PSMD1/DONSON/TIPIN/KIF2A/ODF2/CDK7/M[illegible]

/NF2/CCL3/S100A7/EMILIN1/CEMIP/ACVR1/CXCL17/AKIRIN1/ABL1/STK24/VCL/SFRP2/CYP19A[illegible]

P6AP2/SLC2A3/TSPAN14/MLEC/PSMC3/PPIE/AGA/ADGRG3/SIGLEC9/RHOA

3K7/NFKBID/PSMB6/FFAR2/CD22/CNPY3/MFHAS1/PLA2G6/ICAM2/PSMA3/IRF4/PDE4B/EIF2B1/C[illegible]
/U2AF1L4/ELAVL1/LUC7L3/SRSF3/PNPT1/UBL5/TRA2B/HNRNPLL/SUGP1/ZRANB2/GEMIN5/SAFB/[illegible]

PARK7/RHOH/F2RL2/BRAP/SETDB1/RAB22A/TIAM2/RAB3C/KNDC1/ARHGAP19/ARL3/ARHGAP25/RA[illegible]
/OGFOD1/PSMB6/ADAR/UNK/LRPPRC/POLDIP3/ENC1/RPL10/KLHL25/HNRNPD/LSM14B/NEURL1/EI[illegible]

PSMC3/PPIE/AGA/ADGRG3/SIGLEC9/RHOA

PR1/GPR132/CRLF3/NEDD1/MECP2/CENPJ/PSMC3/TPD52L1/NEK6/YWHAG/PPAT/HINFP/PSMC5/CN[illegible]

AP/RAB11A/NEDD1/KIF20B/CENPJ/MITD1/ARL8B/KLHL21/PARD3/CDK20/NCAPD3/CHFR/TEAD3/R[illegible]

IP/ATAD5/CIB1/CSNK2A2/PTPRC/BAG6/CHAC1/BIK/TFPT/BDKRB2/P4HB/TNFRSF1B/MNT/STK4/T[illegible]

/TARDBP/RPS15A/SMG8

3/SAGE1/GEMIN4/TSEN2/TRPT1

CNE2/PLA2G1B/PAK2/TRAF4/CSNK2B/UBA52/DUSP7/MAP3K10/PIH1D1/TARBP2/DGKQ/CDC37/CDK[illegible]

LR5/PSMD4/HK1/CD48/XRCC6/GPATCH3/TRPM4/PSMD3/PSMD7/CD8A/LILRB4/RPS19/IKBKG/FADD/

IK3CB/PIN1/ADCY7/PPP1R9A/LPAR1/STK4/MAP3K7/UCN/CCDC88A/COPS8/NAB2/HNRNPD/MADD/N[illegible]

NKD2/RNF115/SKP1/ANKIB1/RNF186/DERL1/KCTD13/ZMPSTE24/UBR4/PNPT1/CUL7/RNF19A/BAP[illegible]
SP90B1/USP48/UBE4B/UBAC2/AUP1/MAEA/CACUL1/EGF/HSPA13/CAV1/NEDD8/DAB2IP/GET4/FBX[illegible]
SDHAF1/COX7A2L/ADGRG6/COX10/STOX1/STAT3/PANK2/CHCHD6/CHCHD4/HPS4/NDUFB7/GABARAP/

HX9/MAPKAPK5/TPP1/COA3/RPL32/MTPN/RPL30/CNOT10/YARS2/MEX3D/MRPL15/BECN1/C12orf
/MAEA/ATG7/RNF114/KLHL12/PEF1/CAV1/UBAC1/NEDD8/PCGF3/TRIM68/PARP10/FBXO6/FBXO41
/KCTD13/ZMPSTE24/UBR4/CUL7/RNF19A/BAP1/USP33/USP24/FBXL18/TSG101/FBXL2/CDC16/KA
2/USP3/FBXO10/TRIM32/PSMB6/RPL11/ENC1/CDC23/KLHL25/MVB12B/RNF149/PSMA3/PARK7/B

25A12/SLC35C2/FUT11/CLTC/XXYLT1/PPAT/GMPPB/PRKAG1/HBEGF/GPC2/NME4/PMM2/TYW3/TI
AD9A/IL20RA/GPR183/TRIM25/IGF1R/HPSE/RHEB/CSPG4/CCL3/S100A7/SEC13/LIMS1/TRAF7/C

/ARL6/PCM1/RAB8A

DC114/CEP290/CCDC151/PAFAH1B1/ARL6/PCM1/RAB8A

F19A/BAP1/USP33/USP24/FBXL18/TSG101/FBXL2/CDC16/KAT5/UBE2R2/RAD23A/SVIP/PPP1R11/
/DDX20/AAAS/PARK7/ING2/IGF2R/GMCL1/DUSP13/FIGNL1/NPHP1/SPIN4/NR6A1
6/CASP3/TBC1D20/SMAD5/INSL3/STRBP/TRIM27/PLA2G6/NEURL1/DDX20/ING2/IGF2R/GMCL1/DU
5/INSL3/STRBP/TRIM27/PLA2G6/NEURL1/DDX20/ING2/IGF2R/GMCL1/DUSP13/MBD2/FIGNL1/NPI

M2/PDCD10/CSK/CHEK2/CLSPN/MAPKAPK3/WWC1/GDF11/CCND3/BAD/DBNL/PSAP/BCL10/ERP29/H

INA12/POLR2E/ARID5B/CAV2/EGF/DAB2IP/ATP6V1B2/GTF2F1/EPHB2/SLC9A6/CNKSR1/EPHA10/[illegible]

[illegible]7/BCOR/MYC/MLLT1/DMTN/PRMT6/HYAL2/IFNG/RELA/EIF4G1/IPO7/SUPT6H/KDM3A/DUSP14/RG[illegible]

2AP1/TWSG1/TNFSF15/PTK2/THBS1/ADAM17/TNK2/PTPN1/NOTCH1/RPS27A/NBN/MAVS/MOB2/VEGI

/PTPRA/SEMA4C/MAP4K4/PTPRS/DOCK7/PNPT1/CUL7/TRAK2/PLXNB3/MUL1/SHTN1/LAMA1/USP33/

NB3/MUL1/SHTN1/LAMA1/USP33/ACTR2/UNC13A/TTL/STRADA/NEFH/BCL11A/FEZ1/DPYSL2/NEFL/
BCL10/PSMA4/SPPL3/HSP90AA1/PSMD9/PTK2/PTPN2/CASP8/RPS27A/CD177/MAVS/DMBT1/PLA2G

C5/SUSD2/ABL1/FCGR2B/IL10RA/CSK/ACTR3/RUFY1/MAPKAPK3/BIN2/RAB5C/DBNL/HOOK2/HSP9[illegible]

CARD14/RASSF2/MAP3K12/PDE5A/AFAP1L2/STRADA/RASGRP1/TNFRSF10A/PRKAR2A/MAPKAP1/DA[illegible]

/DGKQ/PLD2/PRSS12/WIPF2/RAPGEF1/CLTC/ENPP2/AP1S1/XKR8/GAK/ACTN4/SNX17/RAB27B/AP2

2/CSPG4/GLG1/CHN1/EMILIN1/BRK1/OVOL2/ACVR1/ARPC5/ABL1/SFRP2/GHRHR/NFATC4/ACTR3/

SP33/ACTR2/UNC13A/TTL/STRADA/NEFH/BCL11A/FEZ1/DPYSL2/NEFL/ARPIN/DAG1/DVL1/ANOS1/

/KIF3A/FSTL4/PLCG1/PPP1R9A/CNP/NCAM1/PXN/RPL24/CASP3/TBC1D20

/DMBT1/OPTN/BNIP3L/PLA2G1B/OAS3/TLR2/SLC17A5/IVNS1ABP/ANXA3/BCL2L1/IFNAR2/RAB29/
SL2/NEFL/DAG1/DVL1/ANOS1/LRP4/SYT17/WNT5A/LRP2/PPP3CB/ARHGAP44/OLFM1
/DMBT1/OPTN/BNIP3L/PLA2G1B/OAS3/TLR2/SLC17A5/IVNS1ABP/ANXA3/BCL2L1/IFNAR2/RAB29/
P36/NMT1/RPL14/AGPS/SRP19/PAM16/HYAL2/RPL10A/HTRA2/IFNG/ARIH2/TERF1/IPO7/RPL12/S

5A/VTA1/RNF41/TICAM1
OTCH1/CAPN2/ADAMTS5/MAVS/IRF5/DMBT1/OPTN/BNIP3L/PLA2G1B/OAS3/TLR2/HSPA5/SLC17A5/
/RPL32/MTPN/RPL30/CNOT10/YARS2/MEX3D/MRPL15/C12orf65/PARS2/HARS2/MRPL4/RBM24/DP
5A/VTA1/RNF41/TICAM1

STXBP4/ADCY5/CORO2B/CCL19/DPH3/ARF1/TFDP1/UBAC2/OSM/SNX3/HAX1/FRMD4A/DNAJC1/ENS

/PTPN2/THBS1/PTPN1/NOTCH1/RPS27A/VEGFA/PLA2G1B/PAK2/TRAF4/COPS5/UBA52/DUSP7/MAP[illegible]
BAG6/ZCCHC3/BTN2A1/NFKBIL1/PIK3CB/MYO1C/PSMB1/SCARA3/CD59/NCKAP1/PLCG1/TLR8/EIF[illegible]

SKP1/FZD9/GNG2/RGS4

)/PTK2/PTPN2/THBS1/PTPN1/NOTCH1/RPS27A/VEGFA/PLA2G1B/PAK2/TRAF4/COPS5/UBA52/DUSI

QLN4/RPS7/FOXF2/MYEF2/BAG2/CHEK2/PSMB5/BAD/SGSM3/PSAP/PSMA4/HSP90AA1/PRKAA2/SGTA

/OPTN/PFKFB2/GNAI3/EXOSC9/BNIP3L/DXO/LZTS1/EXOSC7/SREBF1/TBRG4/TRIM21/UBA52/EDEM

31/SLC35A4/TMEM115/CHST3/TMEM87A/GOLT1B/ZDHHC9/ERGIC3/TMEM130/COPB2/FUT11/IFT20/
YY1AP1/MBD2/HUS1/POLR2B/FIGNL1/BRD4/ASF1A/MAX/RELA/TERF1/SMC1B/LRIF1/MBD1/PPP1CI
L10/HSP90AA1/VPS35/CAPN2/SRGN/CFTR/MAN2B1/MCOLN3/CYB5R3/CCZ1B/GNAI3/GLIPR1/RAB3/

POLA1/TBX19/DFFA/UBE2A/SMARCC1/MCM8/BRD9/KAT2A/ZNF207/BEND3/CBX5/CHMP1A/TOPBP1/I
/BTBD10/MYBBP1A/HNRNPM/SLBP/RPL23A/EXOSC3/VMP1/CKAP5/NF2/EID3/DNTTIP2/SNRPB2/CH
L10/HSP90AA1/VPS35/CAPN2/SRGN/CFTR/MAN2B1/MCOLN3/CYB5R3/CCZ1B/GNAI3/GLIPR1/RAB3/

/S100A7/GCA/SEC13/EEF1A2/RAB3D/HPS1/VCP/HLA-DOA/NPRL3/TNFAIP3/USP5/UBQLN4/PON2/
DYRK3/EYA3/ARL3/TTLL5/MAMLD1/JTB/HAUS2/PPP1R12A/SKP1/CDK5RAP2/CCDC8/MAP3K11/UBR
AH2/BCL2L1/RILPL1/DYNC1I2/RAB11A/PLEKHG6/CCDC88C/NEDD1/KIF20B/MECP2/CENPJ/SASS6/

ASK/LAYN/RPL4/RPL9/PXN/CSRP2
1A3/SLA2/SORCS2/CPTP/TSG101/GRIPAP1/APH1A/GGA2/STOML1/FCGR1B/CHMP4A/ECE1/MCOLN1/
X2/RPS27A/CFTR/CD177/DMBT1/GLIPR1/RAB3A/GABRB3/CYB5R1/TLR2/SREBF1/MYO6/PACSIN2/

AP130/MORF4L1/POLE2/EP400/ATR/TP53BP1/SRCAP/PRPF4/THOC1/SFMBT2/HDAC5/CBY1/EIF2D/

X4/MYO5A/CLEC5A/CCT8/TBC1D10C/FCGR3B/HPSE/RAB4B/S100A7/KCNAB2/GCA/HSPD1/PAFAH1B
/CYB5R1/TLR2/SREBF1/MYO6/PACSIN2/TMBIM1/SLC17A5/SLC15A4/ANXA3/BCL2L1/UBA52/HLA-I
A2/DPF2/POLD3/INO80C/TIMELESS/POLR3D/NDE1/ZWILCH/ARPC1A/EXOSC3/CKAP5/SUV39H2/EII
4/GLG1/MFNG/FUT1/SEC13/LFNG/EXT1/NSFL1C/LPCAT2/PDCD10/APOO/ARAP1/DENND5A/WIPI1/I
PNS1/BRI3/HEXA/SNAPIN/AP2A1/IST1/VPS33A/SEH1L/VASN/TLR7/NAPG/ATP11A/EGF/PI4K2A/I
L/CTSB/ANXA1/CD207/VPS33B/VPS29/ATP6V0A1/HLA-E/ARL8A/DNAJC13/RHOD/RAB7A/CHMP6/AI
53/INO80E/TARDBP/KAT5/ETV3/SUZ12/APEX1/BAZ1B/HMGXB4/TAF4/SLX4/POLD2/ACTR8/NACC2/
XT2/FAF1/MEX3D/PSEN2/SNAPIN/TMEM184A/HECTD3/VPS33A/HSP90B1/ARF1/PRKRA/RAB40C/CC/
ATP2A2/CD59/NCKAP1/CASK/LAYN/RPL4/RPL9/PXN/CSRP2
3/SCAMP2/ARFRP1/PLK3/GALNT7/BICD1/SH3GLB1/RGP1/FIG4/OPTN/LARGE2/GNAI3/GNPTAB/SRI

TBCD/VCL/CALM2/AKNA/BAG2/KRT10/SRPRB/DBNL/BCL10/HOOK2/BOD1/CSPP1/DNM2/ARHGEF25/
TBCD/VCL/CALM2/AKNA/BAG2/KRT10/SRPRB/DBNL/BCL10/HOOK2/BOD1/CSPP1/DNM2/ARHGEF25/
OC/PSMD11/SPESP1/PSMD1/C5AR1/TMEM63A/PADI2/SYT12/TRAPPC1/GSDMD/ABCA3/ICA1/STK10/
/AKNA/BAG2/KRT10/SRPRB/DBNL/BCL10/HOOK2/BOD1/CSPP1/DNM2/ARHGEF25/CDK2AP2/CCSAP/I

2/RPS15/PRKRIP1/RPS16/RBM45/MRPS15/EIF3M/PRPF38A/BTBD2/RPL5/RPS27A/NOL6/MRPL18/I

P6V0D1/MMGT1/CLIP3/PDCD6/RASSF9/RUBCN/ABCG1/SLC48A1/RAB4B/NF2/SNX11/HSPD1/GGA1/[illegible]
HL12/POLE2/EP400/PEF1/PCGF3/GTF2F1/PAGR1/FBXO6/TAF3/ERCC3/ENY2/STUB1/ANAPC4/ZNF[illegible]

STXBP5/RAB27B/AP2B1/MINK1/SNPH/ATG9A/TMUB1/RPL30/SSPN/PSMC5/BCAS1/PSEN2/SNAPIN/

EX1/EPHB2/CIB1/SLC9A6/SACS/HRH1/ARHGAP33/CASC3/VPS16/ADNP/RBM8A/MAGOHB/TNFRSF1B/

/TBPL1/TDP2/TAF11/KAT5/MTDH/C1QBP/APEX1/MED14/ZNF85/TAF4/NACC2/MAML2/GATA4/EZH1/

MMS19/EDIL3/SEH1L/COPA/NPNT/NPHP4/RPS9/CAV2/CAV1/RPS14/RPS27/KRT15/MRPS24/CD3G/

FAP1/FUS/PDCD5/EPB41L1/KATNB1/AXL/GMFG/ABI2/MYO5A/WDR1/OGG1/EPB41L3/FMNL3/NDE1/

2/STK35/VRK2/PIK3CB/AK6/ADCK2/CSNK1G1/CASK/STK4/MAP3K7/PSKH1/RIOK1/PPIP5K1/BCR/

P/GNG4/GNGT2/ABCB9/DDX23/RAB5C/GTPBP3/HSP90AA1/CHD1L/GPN2/DNM2/RASL12/TOR2A/DQX

P/GNG4/GNGT2/ABCB9/DDX23/RAB5C/GTPBP3/HSP90AA1/CHD1L/GPN2/DNM2/RASL12/TOR2A/DQX

P/GNG4/GNGT2/ABCB9/DDX23/RAB5C/GTPBP3/HSP90AA1/CHD1L/GPN2/DNM2/RASL12/TOR2A/DQX

/HSP90AA1/CHD1L/GPN2/DNM2/RASL12/TOR2A/DQX1/DCTN1/NBN/CFTR/ARFRP1/KATNA1/NVL/RA

P2A2/PIH1D1/ZPR1/IFNAR2/CENPJ/CACNB3/DGKQ/CDC37/CDK5R1/PRKAR1B/DAXX/RHOA/NEK6/G

TPRKB/ELMO2/FAF1/ADCY6/BECN1/AP2A1/KSR1/FIZ1/CD247/RHOBTB2/CACUL1/CAV2/CAV1/MAP

KIF20B/MECP2/RBMX/CENPJ/MITD1/AIP/HNRNPA1/TLN1/TWIST1/POLR2J/RHOA/VPS11/RAPGEF1/

GRPEL2/CDC42EP2/CBX8/MYO9B/OCRL/DMPK/DNM1L/PSMD3/PGAM5/PPP1R15A/CARD16/PDCD5/GM

MEM127/PDE6D/TBC1D25/DENND6A/EIF2B2/DOCK11/EPS8L2/KCTD13/DOCK7/MAP3K11/VAV3/DOC

LR2J/STK3/NEK6/POLR2D/GAK/GMPPB/PRKAG1/MAP3K1/CTU1/MINK1/MAPKAPK5/PHKA2/GTF2H1/I

[illegible]11/EGR2/BCOR/CAND1/MYC/BRD7/WWP2/BRD4/ZNHIT3/RELA/NSD1/LHX2/CNOT2/AKAP8/MED30/[illegible]

MGA2/RPTOR/GRWD1/HDAC5/SIX5/THAP11/TAF1B/LITAF/ZCCHC3/IKZF3/RFX2/SNAI3/EAF2/PPA[illegible]

7/CENPK/HAUS8/PML/DYNC1LI1/DSN1/TUBB2B/CIT/PRCC/PLK4/ANAPC11/MAP4/UBE2D1/TUBG2/[illegible]
AP1/CDH24/KEL/DBNL/PLXNC1/HSP90AA1/SRGAP2/DNM2/PTK2/NOTCH1/SSH2/ZMYM3/MAP1A/OLF[illegible]
DHX9/IDO1/HEG1/RBPJ/ADORA2B/EPHA2/EREG/CCL19/KPNA6/SEH1L/XCL1/CD247/TLR7/POLR2E[illegible]
PTPRC/LITAF/CLEC4E/CRLF2/ZCCHC3/NFKBIL1/TNFRSF1B/PIN1/ADCY7/TLR8/MAP3K7/UCN/TRI[illegible]

CD33/PTGER4/HSP90AA1/C12orf4/NBN/TMEM98/CD177/MAN2B1/OLFM4/CYB5R3/ERCC1/GLIPR1/[illegible]
MAN2B1/OLFM4/CYB5R3/GLIPR1/RAB3A/HEXB/TLR2/CSNK2B/TMBIM1/SLC15A4/ANXA3/AP2A2/AT[illegible]

/PTGER4/HSP90AA1/C12orf4/NBN/TMEM98/CD177/MAN2B1/OLFM4/CYB5R3/ERCC1/GLIPR1/RAB3[illegible]
PAFAH1B2/RAB3D/ARPC5/VCP/VCL/FCGR2B/APRT/PDAP1/HVCN1/STX7/VAMP8/MILR1/BIN2/RAB5[illegible]

HRA/ERCC1/VEGFA/ACTN1/LBR/SMAP1/TLR2/TMEM64/WDR5/NKAP/PSMC3/ADGRG3/CD79B/SRF/RH[illegible]

5AR1/HLA-B/MSH2/PSMA7/RPS3/PRKD2/TRAF3/PSMB3/GRB2/ARRB2/CD6/CDC42/IL4R/CALR/MUC[illegible]
7/KLHL6/PRAM1/SKP1/EIF2B2/NOD1/CD19/TRIM15/PRKCB/BPIFB1/PTPRS/PIK3R2/CLEC4A/VAV[illegible]

/TIMELESS/SLBP/LONP1/EXOSC3/DBF4B/SSBP1/EID3/PIWIL4/ACHE/PRPF19/HSPD1/ESCO2/RAD[illegible]
PS7A/ASCC3/KAT5/TREX2/RAD23A/APEX1/FAM168A/SETMAR/TAOK1/SLX4/POLD2/ACTR8/MEN1/N[illegible]
30/NAA60/IRF4/HNRNPC/LRWD1/ING2/USP36/BCOR/SETDB1/MYC/CENPT/MBD2/PRMT6/BRD7/EYA[illegible]
PR1/SEPHS1/TRMT12/THOC2/TARBP2/RBMX/TP53RK/INTS7/ZRSR2/HNRNPA1/PPIE/POLR2I/UTP2[illegible]
/RPL9/MRPL14/RPS6/EIF2B4/UCN/RPL24/OGFOD1/GTPBP1/UNK/LRPPRC/POLDIP3/RPL11/ENC1/[illegible]
CYSTM1/PPFIA3/XRCC6/DNM1L/PSMD3/CTSD/SYT3/PSMD7/ECM1/BTK/GMFG/STX4/CBL/PRDX4/MY[illegible]

BN/PLK3/MAGEF1/PIAS4/RAD18/ERCC1/C9orf116/MASTL/TMEM109/UIMC1/MYO6/COPS5/FBXO45[illegible]
AP7D3/DYNC1I2/RAB11A/ZPR1/CCDC88C/TUBB3/EML2/TRIM37/KIF20B/MECP2/CENPJ/KIF22/CD[illegible]
ENPJ/BTG3/PSMC3/CHFR/NEK6/YWHAG/CLTC/FAP/LGMN/PSMC5/CNOT10/BTC/BECN1/FBXL7/ANAP[illegible]
DAM17/CSNK2A1/NOTCH1/PDCD2/S1PR3/HNRNPU/STAT5A/RPS4X/VEGFA/GNAI3/PLA2G1B/HSF4/T[illegible]
DT/PCBP2/PSIP1/SMU1/AAR2/PRPF40A/AKAP17A/XAB2/ECD/SYF2/RBM15/PTBP2/SNUPN/HNRNPH

MB5/RAD1/PSMA4/HSP90AA1/PSMD9/CSPP1/ADAM17/CDK2AP2/CCSAP/CSNK2A1/DCTN1/CEP164/H[illegible]
B/PLA2G6/PARK7/ING2/SURF4/USP36/NMT1/SETDB1/KIF5B/ULK1/BRD7/BRD4/HTRA2/ANXA1/AR[illegible]
/ATG7/HTT/CAV1/DAB2IP/HMGA2/ARHGEF17/DKKL1/PREX1/INPP5D/ERCC3/PTPRC/BMP2/SRPK2/[illegible]
/PTBP1/PSMB5/BAD/ACOT8/PSMA4/RPS15/SGTA/RPS16/VPS35/CASP8/USF1/RPL5/NOTCH1/RPS2[illegible]
BMX/ZRSR2/RPL19/HNRNPA1/PSMC3/PPIE/POLR2I/RPS11/POLR2J/EXOSC1/EIF3E/SRF/RPUSD3/[illegible]

SMA4/HSP90AA1/VCAM1/PSMD9/TNFSF15/PTPN2/ADAM17/CASP8/TXNDC17/PTPN1/RPS27A/UGCG/[illegible]
/WDR45/MRPL55/CHCHD1/TBC1D25/MRPS2/PRKCB/ATG3/KIF9/CNEP1R1/PRSS2/ZMPSTE24/CARMI[illegible]
ZNHIT3/GEMIN6/SMN2/CNOT2/ZNF622/RPL12/MRPS2/RRP15/WDR3/RPS28/LUC7L3/RPS17/NOLC1[illegible]
LN1/WIPF2/SRF/CACNA1D/DSTN/PLEKHH2/RHOA/MRAS/ACTN4/PDPN/MINK1/FNBP1L/DOCK2/MTPN[illegible]
EM80/PIP5K1A/SH2B1
4/HAX1/RAB13/ARHGEF17/PREX1/ARFGEF1/ARHGAP17/FRMD6/LLGL2/VPS4A/ADD1/ASAP3/MYO1C[illegible]
IZ1/LYL1/PSMC5/RBPJ/BPGM/EPHA2/KITLG/MECOM/CCL19/VPS33A/EIF6/NOTCH2/PIK3R6/DOCK

EM2/TRPC4AP/PCYOX1L/RNF213/RHBDD3/PSMC3/VPS37B/USP11/UBE2D2/NDUFA13/NRDC/CHFR/A[illegible]
AM17
LZTS1/HSPA5/FBXO45/DDX56/WDR5/PHGDH/RAB11A/ZPR1/TUBB3/RAB29/KIF20B/IFRD1/MECP2/[illegible]

4/NCKAP1/MOV10/PPP1R9A/LPAR1/PLEKHM1/CCDC88A/SEMA3F/NEURL1/MEGF8/TCHP/KNDC1/DMT
JBE2F/MED7/UNKL/ZMIZ1/PSMC5/ARRDC1/FBXL7/UBE2N/ANAPC5/HECTD3/CDC27/SEH1L/CDC34/
ABL1/EMD/FCGR2B/PIM3/WRAP53/TNFAIP3/MFF/NSFL1C/PDCD10/STAC/STX7/KCNN4/CSK/VAMP8/
J/NVL/RPS12/TOMM22/MAP1A/MAVS/RPS4X/RPL6/OPTN/BNIP3L/MARK4/RPL37/RPL27A/GNPTAB/
BP7/SNRPG/TMEM199/RPA2/CUL4B/NDE1/RPL38/CLIP3/DRG1/MAPT/ARPC1A/PRPF31/RPL23A/ND
F6/CNOT3/MUS81/LSM1/SMUG1/ENTPD1/RPS9/RNASEH1/SND1/PGM2L1/KHSRP/RPS14/PGAM1/RPS
S1L/RPL10A/ZNHIT3/RELA/SPIN1/HNRNPA2B1/THUMPD2/RRP15/POU2F2/SMAD2/SNAPC5/WDR3/R

F2/CCL3/S100A7/EMILIN1/CEMIP/ACVR1/CXCL17/AKIRIN1/ABL1/STK24/VCL/SFRP2/CYP19A1/

MOV10/QRSL1/RPL4/SARS2/RPL41/RPL9/MRPL14/RPS6/EIF2B4/UCN/RPL24/OGFOD1/GTPBP1/UN
346/IP6K2/BIK/CNR1/GNA13/PPARG/PIK3CB/TFPT/TNFRSF1B/PIN1/ARHGEF18/PLCG1/LPAR1/S
IVNS1ABP/BCL2L1/UBA52/PIH1D1/ZPR1/PRDX5/MEIS3/MECP2
14/ZNF346/IP6K2/BIK/CNR1/GNA13/PPARG/PIK3CB/TFPT/TNFRSF1B/PIN1/ARHGEF18/PLCG1/L
/TMBIM1/TXNDC12/IVNS1ABP/BCL2L1/UBA52/PIH1D1/ZPR1/PRDX5/MEIS3/MECP2/NDUFA13/TWI

K2/SPIRE1/THBS1/CCSAP/STYXL1/DCTN1/VPS35/NOTCH1/ODF2L/VDAC3/SH3GLB1/HNRNPU/THRA

7/CALM2/KCTD5/COPS6/SRPK1/RPL7A/RPS23/RPS10/VAMP8/RNF216/PTBP1/PSMB5/BAD/ACOT8/
S1/CCL3/UBXN1/HSPD1/SEC13/SLC25A5/VCP/FCGR2B/RPS7/CALM2/KCTD5/COPS6/SRPK1/RPL7A

/GPR132/CRLF3/NEDD1/MECP2/CENPJ/PSMC3/TPD52L1/NEK6/YWHAG/ATP2B4/PPAT/HINFP/PSMC
OC/ENDOD1/FCGR3B/HPSE/RAB4B/CCL3/S100A7/KCNAB2/GCA/PAFAH1B2/RAB3D/ARPC5/VCP/VCL
D5A/PSMD8/FCGR2B/PSMA1/TNFAIP3/SRPK1/CIITA/ACTR3/MAPKAPK3/PSMB5/BCL10/PSMA4/VCA

/SFRP4/HNRNPU/FIG4/HOXA5/MOB2/OLFM4/LLPH/VEGFA/BCL9L/SMAP1/HSF4/TLR2/HSPA5/DDX5
P1/DAB2IP/HMGA2/RPTOR/THOC1
MAPKAPK5/HEG1/GTF2H1/FAF1/SH3BP5L/ADCY6/ADORA2B/UBE2N/ADCY5/KITLG/KSR1/EREG/CCL
USP22/ZNF683/CD3G/PTPRC/CLEC4E/BAG6/IKZF3/METTL3/LOXL3/DOCK8/TNFRSF1B/CD180/RPS

F/RHOA/STK3/INHA/AP3B1/SIX1/ZNF160/PDPN/DOCK2/RUNX3/ZMIZ1/LYL1/PSMC5/RBPJ/BPGM/

1/DPYSL3/SNAPIN/CORO2B/LRPAP1/CTBP1/GMFB/ATG7/SNX3/CAV1/CAMSAP1/EPHB2/ARFGEF1/C
3/S100A7/EMILIN1/CEMIP/ACVR1/CXCL17/AKIRIN1/ABL1/STK24/VCL/SFRP2/CYP19A1/PDCD10

DBNL/BCL10/ERP29/HSP90AA1/TNFSF15/LMO4/PRKRIP1/PTK2/PTPN2/THBS1/ADAM17/PTPN1/CC
LGMN/DOCK2/DHX9/NR1H2/EPHA2/SGSM1/CCL19/XCL1/RANBP3/CCL3L3/DOCK10/NPNT/MSH6/CCL

F6/UXT/ARF1/MFN1/NPHP4/KLHL12/HTT/CAV2/PEF1/IL13RA2/SPOUT1/CD84/MAP4K2/CALM3/PA

F1/YWHAG/GAK/SIX1

CAPRIN1/RHOA/CD38/HES2/RAPGEF1/YWHAG/GAK/SIX1/ACTN4/ACAP3/PDPN/DNMT3B
PTPN1/TMEM98/CFTR/PLK3/BICD1/SH3GLB1/HNRNPU/NVL/MAP1A/MAVS/BNIP3L/RAB3A/CCNE2/M
TNNA1/STK40/PTK2/THBS1/HIGD2A/CSNK2A1/DAD1/VPS35/CASP8/PTPN1/DUS2/NOTCH1/RPS27A
2A2/RAB11A/GOLPH3L/PACSIN1/TIMM10/MITD1/NDUFA13/RHOA/NEK6/VPS11/AP3B1/YWHAG/CLT

F1/RNF2/PIAS4/RAD18/MED27/MASTL/UIMC1/USP27X/COPS5/HSPA5/FBXO45/TRIM21/IVNS1ABP/
2D2A/NSUN4/RHOA/NEK6/VPS11/YWHAG/ATG13/IFT20/B9D1/MTERF3/MRPL11/FNBP1L/ATG9A/TN
01C/RAB8B/GLRB/RPL4/RPL41/RPL9/RPS6/RPL24/EFCAB7/RPL11/RPL10/RPS24/TIMM9/RPS3A/
TP53BP1/MAP4K2/DAB2IP/HMGA2/RPTOR/ATAD5/THOC1

M1/CCDC88A/SEMA3F/NEURL1/MEGF8/TCHP/KNDC1/DMTN/DYNC2LI1/CEP97/TRIM46/XK/SEMA6B/

SH2/PSMA7/UBE2E1/CCND2/POLE4/TFAP4/POLA1/PSMB3/MCM8/PTPA/ZNF207/CHMP1A/TOPBP1/C

1/PDCD10/TRIP6/TRADD/LAMA4/PLXNC1/PTGER4/ACVR1B/VASH1/SLC8B1/CTNNA1/SRGAP2/LMO4/

TSL/CD4/CTSB/RELA/PSMB7/KLHL6/PRAM1/SKP1/EIF2B2/NOD1/CD19/TRIM15/PRKCB/BPIFB1/P
IWS1/DDX1/LSM3/TARDBP/GRSF1/C1QBP/NOVA1/SRSF10/RNF40/SRSF7/PRPF40B/TRA2A/PRMT7/

B33A/SRGAP1/TRIO/DOCK11/RAB2A/MAP4K4/RHOD/PIK3R2/RAB7A/EPS8L2/KCTD13/DOCK7/VAV3/
F4E2/PSMA3/AAAS/DCP2/PYM1/HNRNPC/EIF2B1/AGO1/POLR2B/EIF5B/FXR1/EIF2AK1/SYNCRIP/

OT10/POLE3/RPA1/FBXL7/ANAPC5/CDC27/CDC34/CNOT3/TFDP1/UBE2E2/CACUL1/POLE2/HMGA2/

HOA/NEK6/CLTC/RNF8/CABLES2/BTC/USP39/BECN1/FBXL7/ANAPC5/PMF1/EREG/IST1/SEH1L/AR

RIM32/CASP3/RPL11/SH3RF1/RTKN2/PLA2G6/MADD/NOX1/PARK7/ING2/SHISA5/NMT1/FIGNL1/E

5R1/PPM1F/PRKAR1B/TPD52L1/DAXX/RHOA/STK3/GSKIP/RAPGEF1/YWHAG/ATG13/CNPPD1/ATP2B

/ECM1/BTK/PELI1/STX4/TREM1/CLEC7A/TNIP1/EIF2B5/POLR3D/LBP/ARPC1A/POLR3C/EXOSC3/
EURL1/PARK7/PDE6G/MYC/SLC27A1/CD4/JTB/IFNG/MAPK15/ZNF622/HNRNPA2B1/MAP3K9/NOD1/

1/USP33/USP24/FBXL18/TSG101/FBXL2/CDC16/KAT5/UBE2R2/RAD23A/SVIP/PPP1R11/DVL1/DE[illegible]
[illegible]6/PKD1/CSNK2A2/STUB1/ANAPC4/VPS4A/ATRAID/BAG6/HUWE1/BBS7/SNX5/FAF2/TMEM67/DDB1[illegible]
/MFSD14A/ATG9B/NDUFA11/SREBF2/NDUFS3/RAB38/SLIRP

55/PARS2/PSEN2/HARS2/MRPL4/RBM24/DPH3/EIF6/EIF4G2/EIF4G3/GCLM/CNOT3/GLRX2/ATG7/
/STUB1/ANAPC4/RAB1A/HUWE1/PAF1/HDAC8/DDB1/FBXO44/SKP2/PSMB1/WWTR1/PIN1/WDSUB1/F
T5/UBE2R2/RAD23A/SVIP/PPP1R11/DVL1/DERL3/CLEC16A/UBE2J1/ARRB1/PSMD12/BFAR/ARMC8
JB3/RNF144A/SDF2/USP36/NUB1/UBQLN2/RNF121/CTSL/WWP2/CTSB/ARIH2/RNF126/TLK2/ANAP

GAR/ADCY6/SDF2L1/HEXA/BPGM/ADCY5/AP2A1/CCL19/SEH1L/EIF6/DOLK/PDK3/PGM2L1/EGF/AT
XCL17/ABL1/LMCD1/FCGR2B/IL10RA/RPS7/CALM2/PDCD10/TRIP6/CSK/TRADD/MAPKAPK3/WWC1/

/DVL1/DERL3/CLEC16A/UBE2J1/ARRB1/PSMD12/BFAR/ARMC8/SMURF2/RNF40/OGT/MAPK9/CDC26/

JSP13/MBD2/FIGNL1/NPHP1/SPIN4/NR6A1
HP1/SPIN4/NR6A1

SP90AA1/PRKAA2/ACVR1B/CDK2AP1/TWSG1/TNFSF15/LMO4/PTK2/THBS1/ADAM17/TNK2/PTPN1/N

STUB1/BMP2/SNX5/C2CD5/RYK/PIK3CB/MYO1C/BDKRB2/FSTL4/NCKAP1/PLCG1

S4/CRTAP/PPP2R5D/PIK3R2/CHMP6/C3orf33/WNK1/PPP2CA/HDAC3/RASSF2/NCOA7/IKBKB/TSG1

FA/PLA2G1B/PAK2/TRAF4/CSNK2B/UBA52/DUSP7/MAP3K10/PIH1D1/DGKQ/CDK5R1/PRKAR1B/TPD5

/ACTR2/UNC13A/TTL/STRADA/NEFH/BCL11A/FEZ1/DPYSL2/NEFL/ARPIN/DAG1/DVL1/ANOS1/LRP[illegible]

/ARPIN/DAG1/DVL1/ANOS1/LRP4/SYT17/WNT5A/LRP2/PPP3CB/ARHGAP44/OLFM1
1B/PAK2/TLR2/MUC6/UBA52/HLA-F/RAB29/MUC15/CACNB3/PLD2/PSMC3/UBE2D2/STOML2/WIPF2[illegible]

OAA1/DNM2/TUSC2/PTK2/THBS1/KCNK5/TNK2/PTPN1/INPPL1/AAK1/SFRP4/BICD1/CD177/DMBT1/

G1

2B1/FNBP1L/DOCK2/NR1H2/ELMO2/ADM/BECN1/AP2A1/LRPAP1/CCL19/CD247/HSP90B1/ARF1/ST[illegible]

ARAP1/TRADD/MAPKAPK3/GDF11/CCND3/PTBP1/PTPN12/LEMD2/HSP90AA1/ACVR1B/TWSG1/DNM2/[illegible]

/LRP4/SYT17/WNT5A/LRP2/PPP3CB/ARHGAP44/OLFM1

/TARBP2/CD79B/RHOA/HMGN2/AP1S1/IFI6/IL17RA/NFKBIB/SIGIRR/TNFRSF14/DHX9/NR1H2/IF

/TARBP2/CD79B/RHOA/HMGN2/AP1S1/IFI6/IL17RA/NFKBIB/SIGIRR/TNFRSF14/DHX9/NR1H2/IF
SRP14/RPS18/RPS28/PIK3R2/RAB7A/TYSND1/RPS17/APPL2/NOLC1/UBL5/NUP214/HDAC3/BAP1/

/IVNS1ABP/ANXA3/BCL2L1/IFNAR2/RAB29/TARBP2/CD79B/RHOA/HMGN2/AP1S1/IFI6/IL17RA/N[illegible]

[illegible]H3/EIF6/EIF4G2/EIF4G3/GCLM/CNOT3/PDK3/RPS9/KHSRP/DALRD3/GSS/EIF3G/DNAJC1/RPS14/[illegible]

A/CIB1/IL17RB/PANX2/CSNK2A2/ENY2/PARL/CLEC4E/CRLF2/HUWE1/TM9SF4/C2CD5/CNR1/MYO1

3K10/ATP6AP2/RHBDD3/PSMC3/TPD52L1/DAXX/STK3/INHA/EMC10/RAPGEF1/PIK3R5/MAP3K1/GP[illegible]
2B4/MAP3K7/NFKBID/PSMB6/FFAR2/CD22/CNPY3/MFHAS1/PLA2G6/ICAM2/PSMA3/IRF4/PDE4B/E[illegible]

P7/MAP3K10/ATP6AP2/RHBDD3/PSMC3/TPD52L1/DAXX/STK3/INHA/EMC10/RAPGEF1/PIK3R5/MAP[illegible]

A/PSMD9/PTK2/CSNK2A1/VPS35/PTPN1/RPL5/RPS27A/PLK3/SH3GLB1/MAGEF1/RGP1/HNRNPU/MA[illegible]

M2/NANOS1/CDC37/CDK5R1/PSMC3/TWIST1/EXOSC1/NRDC/CHFR/SLC25A12/GLMN/VPS11/HNRNPR/

/CLTC/TAPBP/AP1S1/AP1S3/AP1S2/GOSR2/ACER3/COG4/COPB1/PSEN2/SNAPIN/COG2/TMEM167A/
3/SMAD2/WBP2/REPIN1/ORC4/HELB/XRCC5/SMC1A/SMAD3/SETD1A/BAZ1A/TP53/INO80E/TARDBP/
A/HEXB/MYO6/TMBIM1/SLC17A5/SLC15A4/BCAN/UBA52/HLA-F/AP2A2/ACAN/BORCS6/TECPR1/ARI

PRIM2/NUP107/NCOR2/CBX8/HELLS/PCGF2/XRCC6/HMGN1/POLQ/TELO2/RAD21/MTBP/NIFK/RFWD
TOP/ARID5A/PAFAH1B2/REXO2/LARP4B/TRAF7/NAT10/UTP6/ABL1/STK24/HEATR1/TIMM13/NOP1
A/HEXB/MYO6/TMBIM1/SLC17A5/SLC15A4/BCAN/UBA52/HLA-F/AP2A2/ACAN/BORCS6/TECPR1/ARI

ABCB9/STX7/VAMP8/MILR1/RAB5C/GNLY/WIPI1/PSAP/BCL10/HSP90AA1/VPS35/CAPN2/SRGN/CF
4/CUL7/PPP2R3C/RNF19A/SPICE1/USP33/IFT140/BICD2/CEP95/CNTROB/FEZ1/CEP135/CDC16/
/PROCR/EVC/NEK6/IFT20/B9D1/PIK3R5/DHX9/TMUB1/NIN/IFT22/CCHCR1/PSEN2/FBXL7/IST1/

/CLEC16A/VTA1/TICAM1/ACAP1/FGD2/WNT3A/SPPL2B/LZTR1/SNX6/TAB1/CD274/SNX21/SNX2/E
TMBIM1/SLC17A5/SLC15A4/ANXA3/BCL2L1/UBA52/HLA-F/AP2A2/RAB11A/ATP6AP2/SLC2A3/TSP

/GEMIN2/PABPN1/SRSF1/SRSF2/CASC3/SNRNP70/COIL/STK35/RBM4B/DHX8/ADD1/RBM8A/METTL

2/RAB3D/ARPC5/VCP/VCL/APRT/PDAP1/GHRHR/THBS2/HVCN1/STX7/VAMP8/BIN2/RAB5C/MOXD1/
F/AP2A2/RAB11A/ATP6AP2/SLC2A3/TSPAN14/PACSIN1/MLEC/PLA1A/ADGRG3/EXOC3/SIGLEC9/C
D3/PRPF19/ESCO2/RAD51C/SEC13/PTGES3/NAT10/RARG/DNA2/HSPA2/ARPC5/DOT1L/RAD51D/VC
DBNL/COPG2/B4GALNT2/DNM2/NECAB3/SYS1/COG1/NOTCH1/VPS45/UGCG/FUT8/SCAMP2/ARFRP1/
DPP7/DAGLB/RPTOR/ATP6V1B2/AP5B1/LITAF/VPS4A/ATRAID/PLD1/KIAA1324/SLCO4C1/FAF2/V
PPL2/CYB561A3/SLA2/SORCS2/CPTP/TSG101/GRIPAP1/APH1A/GGA2/STOML1/FCGR1B/CHMP4A/E
/MEN1/NFRKB/SP1/TNKS1BP1/ACTR6/PURB/NFATC1
AR1/HTT/MAGEE1/CAV2/EPN3/CAV1/EIF3G/ATP7A/SRCAP/TRIM68/SYNJ2/PREX1/ARFGEF1/CIB1/

EBF1/GGA3/HLA-F/RTN1/B4GALT4/VAC14/RAB11A/RAB29/YIPF2/GOLPH3L/NOTCH3/GORASP2/SL

CDK2AP2/CCSAP/DCTN1/HAUS7/KATNA1/BICD1/PDLIM4/MYL9/HNRNPU/MAP1A/NEBL/ACTN1/JPH1/
CDK2AP2/CCSAP/DCTN1/HAUS7/KATNA1/BICD1/PDLIM4/MYL9/HNRNPU/MAP1A/NEBL/ACTN1/JPH1/
/GPR84/CNN2/CALR/ALDOC/TGFB3/SYT1/APP/LAIR1/PNP/GAL/CYSTM1/PPFIA3/XRCC6/DNM1L/P
DCTN1/HAUS7/KATNA1/BICD1/PDLIM4/MYL9/HNRNPU/MAP1A/NEBL/ACTN1/JPH1/HAUS5/MYO6/CD

POP5/HNRNPU/NVL/LTV1/RPS12/SARNP/RPS4X/RPL6/HTATSF1/LSM8/MAGOH/RPL37/RPL27A/TRI

RAB3D/ARPC5/HLA-DOA/STMN2/STX7/SNX20/RUFY1/VAMP8/RAB5C/WIPI1/DBNL/PSAP/VCAM1/DN[illegible]
768/TADA3/PAF1/TRRAP/METTL3/TEX10/SNRPD3/DDB1/FBXO44/SKP2/PIK3CB/SUPT7L/RPRD1A/[illegible]

KCTD12/CTBP1/MPP2/ZC4H2/ARF1/DOCK10/HTT/MAGEE1/PI4K2A/DAGLB/SYNJ2/RPS14/CALM3/E[illegible]

/GLRB/PPP1R9A/EFHC1/LPAR1/RPS6/UCN/KCNC4/CASP3/TTLL7/CD22

/WNT3A/MED6/ZNF653/TGIF1/HDGF/BRDT/ECD/SS18L2/HOXC6/MED31/HR/LPXN/HSPA1A/ABT1/JU

RPL29/MRPL33/MRPS5/HSPB6/ADD1/RPL13/MRPL36/RPL4/RPL41/RPL9/MRPL14/RPS6/CSRP2/RPI

PIFO/CLIP3/DRG1/MAPT/ARPC1A/CKAP5/NF2/OBSCN/RAB3D/HSPA2/ARPC5/TNNI3/ABL1/EMD/TB(
FASTKD5/PIP4K2B/CKB/IGF2R/CCNG2/ULK1/UCKL1/DYRK3/DGKD/PIK3C2B/BRD4/EIF2AK1/TLK2/
1/DCTN1/NBN/CFTR/ARFRP1/KATNA1/NVL/RAD18/GNAI3/RAB3A/DXO/MYO6/HSPA5/DDX56/DYNC1I
1/DCTN1/NBN/CFTR/ARFRP1/KATNA1/NVL/RAD18/GNAI3/RAB3A/DXO/MYO6/HSPA5/DDX56/DYNC1I
1/DCTN1/NBN/CFTR/ARFRP1/KATNA1/NVL/RAD18/GNAI3/RAB3A/DXO/MYO6/HSPA5/DDX56/DYNC1I
D18/GNAI3/RAB3A/MYO6/HSPA5/DDX56/DYNC1I2/HSPA14/RAB11A/TUBB3/RAB29/KIF20B/RNF21
SKIP/YWHAG/ATG13/CLTC/CNPPD1/STXBP1/ATP2B4/PRKAG1/MAP3K1/MAPKAPK5/TPRKB/ELMO2/N
4K2/DAB2IP/RPTOR/HDAC5/CALM3/PGAM1/CIB1/PKD1/DUSP22/BORA/PTPRC/CDK5RAP1/SRSF2/AI
/YWHAG/CLTC/STXBP1/ATP2B4/RAB27B/SOCS7/KHDRBS3/ELMO2/KCNH2/SLC22A5/FAF1/DPYSL3/I
FG/STX4/RALGDS/RPL23/WDR91/RACK1/IGBP1/ARHGDIA/RIC8A/RGS3/TBC1D10C/RASAL3/PPP4C/

K6/PLEKHG5/USP33/EIF2B3/BICD2/RASGRP1/GGA2/TAGAP/MAPKAP1/RAB11FIP1/SMCR8/RILPL2/

NME4/PHKG2/ADCK5/POLE3/EPHA2/SBK1/KSR1/NOL9/CDK11A/DOLK/PIK3R6/POLR2E/PDK3/IRAK[illegible]

LRIF1/KDM3A/HIF1AN/PRKCB/MED16/SMAD2/E2F7/WBP2/NOLC1/BRF2/RNF19A/GTF3C3/HDAC3/M

RG/FOSL2/JUNB/TRPS1/MNT/USP3/HIC2/CHD7/NFYA/SMAD5/SSBP2/ATF6B/DLX4/HNRNPD/PRMT5/

KLHDC8B/PSMD10/PSMD11/NES/RAE1/CDKN2C/PSMD1/DONSON/TIPIN/KIF2A/ODF2/CDK7/MSH2/P
M4/LLPH/VEGFA/RAB3A/BCL9L/HEXB/ACTN1/LZTS1/PACSIN2/FBXO45/RILPL1/ZMYM4/RAB11A/T
/OSM/IL17D/CD84/INPP5D/TMEM106A/IL17RB/PANX2/PTPRC/LITAF/CLEC4E/RAB1A/CRLF2/ZCC
M32/FFAR2/AGPAT1/TRIM27/NOX1/SYT11/ATP2B1/PARK7/IRF4/PDE4B/POLR3F/CD4/HYAL2/IFN

RAB3A/HEXB/TLR2/CSNK2B/TMBIM1/SLC15A4/ANXA3/HLA-F/AP2A2/ATP6AP2/SLC2A3/TSPAN14/
P6AP2/SLC2A3/TSPAN14/MLEC/PSMC3/PPIE/AGA/ADGRG3/SIGLEC9/RHOA

A/HEXB/TLR2/CSNK2B/TMBIM1/SLC15A4/ANXA3/HLA-F/AP2A2/ATP6AP2/SLC2A3/TSPAN14/MLEC
C/DBNL/PSAP/CD33/BCL10/HSP90AA1/C12orf4/TUSC2/ADAM17/NBN/CD177/MAN2B1/MAVS/OLFM

OA/STK3/INHA/AP3B1/RNF8/SIX1/ZNF160/PDPN/DOCK2/IDO1/RUNX3/ZMIZ1/LYL1/PSMC5/RBPJ

20/XCL2/CMKLR1/APP/PNP/SWAP70/TLR5/PSMD4/HK1/XRCC6/GPATCH3/PSMD3/PSMD7/LILRB4/A
3/APPL2/RPS6KA3/SLA2/TLR1/S100A1/WNK1/NOD2/BTN3A2/EIF2B3/ACTR2/IKBKB

51C/BTG2/PTGES3/NAT10/DNA2/ABL1/RAD51D/VCP/WRAP53/NOP10/UBQLN4/DCLRE1B/FAAP20/T
FRKB
3/PHF13/BRD4/ASF1A/RELA/NSD1/TLK2/DYDC2/SPIN1/SKP1/AKAP8/KMT2B/NAA50/SUPT6H/KDM
0/POLR2J/EXOSC1/NOB1/RPUSD3/HNRNPL/SF3B3/NSUN4/SUGP2/HNRNPR/INTS4/DDX10/POLR2D/
RPL10/KLHL25/HNRNPD/RPS24/MRPL3/LSM14B/NEURL1/EIF4E2/ERAL1/PYM1/EIF3F/RPS3A/EIF
05A/RALB/CSPG5/WDR1/CLEC5A/CCT8/TBC1D10C/ENDOD1/FCGR3B/NSF/HPSE/RAB4B/CCL3/S100

/BCL2L1/UBA52/MORF4L2/KIF22/INTS7/NUAK1/FANCF/UBR5/PPIE/POLR2I/TWIST1/POLR2J/RB
K5R1/ARL8B/PARD3/SASS6/RBM14/CC2D2A/RHOA/NEK6/AP3B1/IFT20/CLTC
C5/EREG/CDC27/CNOT3/MUS81/TFDP1/UBE2E2/MAEA/CAV2/EGF/HMGA2/RPTOR/ATAD5/ERCC3/PK
BRG4/BCL2L1/GLI1
1/CWC15/WDR83/PAPOLA/SMN1/HSPA1A/HNRNPH2/CRNKL1/SF1/HNRNPH3/CIR1/ZMAT2/USP4/CWF

AUS7/NBN/FBXW5/PLK3/SH3GLB1/HNRNPU/RNF2/ZFYVE19/MARK4/HAUS5/UIMC1/BCL2L1/GLI1/D
IH2/MAPK15/TERF1/CNOT2/HNRNPA2B1/AKAP8
LGALS14/ZNF346/IP6K2/BIK/CNR1/GNA13/PPARG/PIK3CB/TFPT/TNFRSF1B/PIN1/ARHGEF18/PL
7A/FKBP8/BICD1/RPS12/MAVS/CTBP2/DMBT1/RPS4X/RPL6/HTATSF1/BNIP3L/RPL37/PAK2/RPL2
HNRNPL/SF3B3/SUGP2/RPL13A/HNRNPR/POLR2D/SART3/DHX15/TGS1/ANP32A/RPL37A/GPKOW/KH

INPPL1/STAT5A/PIAS4/MAVS/IRF5/VEGFA/IL20RB/PAK2/OAS3/TNFRSF25/CSNK2B/TRIM21/BCL
L2
/RPL26L1/TSC1/NOL11/GEMIN5/ERI1/EIF3C/DDX1/NSUN5P2/LSM3/ZNHIT6/ATXN2/TSR1/RPP38
/KCNH2/SPTAN1/PARVB/DPYSL3/CORO2B/NOTCH2/HSP90B1/ARF1/RHOBTB2/GMFB/NPHP4/HAX1/C

/ANTXR1/SPIRE2/ARHGEF18/NCKAP1/PPP1R9A/LPAR1/PXN/TRIM32/CSRP2/CCDC88A/BCR/ATP2C
10/MAEA/OSM/IL17D/HAX1/LDB1/ATP7A/PKNOX1/PREX1/DCLRE1C/RPS14/INPP5D/HDAC5/CIB1/

GAP3/SF3B3/GLMN/VPS11/ABHD17A/CLTC/RNF8/ANKZF1/AP2B1/FAP/LGMN/TPP1/MANBA/TMUB1/

PACSIN1/PPP2R5B/CDK5R1/METRN/PARD3/APBB1/SRF/CAPRIN1/RHOA/CD38/RAPGEF1/IFT20/ST

N/DYNC2LI1/CEP97/TRIM46/XK/SEMA6B/FAM98A/MAPK15/DOCK11/SEMA4C/ATG3/MAP4K4/PTPRS
UBE4B/UBE2E2/SH3RF3/PEX10/RAB40C/MAEA/ATG7/RNF114/KLHL12/PEF1/CAV1/UBAC1/NEDD8/
/BAD/CENPQ/CD33/ERP29/PTGER4/HSP90AA1/PRKAA2/SLC8B1/PSMD9/VPS35/CASP8/PTPN1/SRG
SREBF1/PACSIN2/GGA3/HSPA5/RPL36/UBA52/CD2AP/PIH1D1/RAB11A/HIKESHI/ZPR1/NEDD1/RA
UFS5/LONP1/TAZ/CKAP5/MCTS1/NDUFB9/PRPF19/TAF12/MZT1/BRK1/HSPD1/SEC13/MRPS7/PTGE
27/DNASE1L1/EXOSC2/ZC3HAV1/RPL29/LARP1/YTHDF2/CASC3/RPL13/RBM8A/METTL3/MAGOHB/H
PS28/RPS17/PTCD1/PNPT1/NOLC1/GTF3C3/SMAD3/NOL11/ERI1/MAEL/TP53/DDX1/NSUN5P2/MET

PDCD10/TRIP6/TRADD/LAMA4/PLXNC1/PTGER4/ACVR1B/VASH1/SLC8B1/CTNNA1/SRGAP2/LMO4/P

K/LRPPRC/POLDIP3/RPL11/ENC1/RPL10/KLHL25/HNRNPD/RPS24/MRPL3/LSM14B/NEURL1/EIF4E
TK4/RPS6/NFKBID/CASP3/CALHM2/RPL11/NCSTN/PLA2G6/NOX1/NEURL1/DDX20/PARK7/PNMA5

PAR1/STK4/RPS6/NFKBID/CASP3/CALHM2/RPL11/NCSTN/PLA2G6/NOX1/NEURL1/DDX20/PARK7/P
ST1/TEX11/RHOA/CD38/STXBP1/SIX1/IFI6/PDPN/LGMN/IDO1/NAA15/PSMC5/TIGAR/CCAR2/BTC

/VDAC2/ERCC1/VEGFA/RAB3A/MARK4/TLR2/ADD3/DEF8/PIH1D1/EML2/TRIM37/MECP2/CENPJ/PP

PSMA4/RPS15/SGTA/TUSC2/RPS16/VPS35/CASP8/USF1/RPL5/NOTCH1/RPS27A/FKBP8/BICD1/RP
/RPS23/RPS10/VAMP8/RNF216/PTBP1/PSMB5/BAD/GNLY/ACOT8/PSMA4/RPS15/SGTA/TUSC2/RPS

5/CNOT10/STXBP4/POLE3/RPA1/FBXL7/ANAPC5/CDC27/CDC34/CNOT3/TFDP1/UBE2E2/CACUL1/P
/FCGR2B/APRT/PDAP1/HVCN1/VAMP8/MILR1/BIN2/RAB5C/DBNL/PSAP/CD33/HSP90AA1/C12orf4
M1/PSMD9/PTPN2/CASP8/PTPN1/RPS27A/CD177/MAVS/IRF5/KYNU/DMBT1/OPTN/PLA2G1B/PAK2/

6/TMEM64/RAB11A/MESP1/MORF4L2/KIF20B/TARBP2/PACSIN1/ALOX15B/VEZF1/PPP2R5B/NKAP/

19/PIK3R6/IRAK2/GMFB/DUSP18/CACUL1/HTT/EGF/MAP4K1/CAV1/MAP4K2/DAB2IP/HMGA2/RPTO
6/NFKBID/CASP3/CHD7/CLPTM1/CD22/ICOS/NCSTN/SH3RF1/PRF1/IRF4/RHOH/UNG/CD4/IFNG/A

EPHA2/KITLG/ARTN/MECOM/CCL19/VPS33A/EIF6/NOTCH2/PIK3R6/DOCK10/MAEA/OSM/IL17D/BA

IB1/DUSP22/SACS/CST3/EVI5L/PROM2/CXCL10/TMEM67/ADD1/FEZ2/RYK/ANTXR1/TNFRSF1B/TF
/TRIP6/ARAP1/TRADD/LAMA4/PLXNC1/PTGER4/ACVR1B/VASH1/SLC8B1/CTNNA1/SRGAP2/LMO4/T

NJL/RPL5/DUS2/RPS27A/NBN/HNRNPU/NVL/VEGFA/PFKFB2/CCNE2/PLA2G1B/PAK2/MASTL/TRAF4
8/EDNRA/CAV2/CAV1/DNAJC1/DAB2IP/ARL1/PREX1/ARHGAP22/CALM3/ARHGAP27/ARFGEF1/RIC8

BPC1L/ACTR1A/OFD1/LLGL2/VPS4A/NECTIN2/RAB1A/BBS7/TMEM67/CNR1/CEP72/MYO1C/ATP2A2

ARK4/PLA2G1B/JPH1/SREBF1/TMBIM1/BCL2L1/CD2AP/EDEM2/RAB11A/ZPR1/PNKD/RAB29/KIF20
/FKBP8/PLK3/RAD18/VDAC2/VEGFA/GNAI3/BNIP3L/GABRB3/PAK2/TMEM109/DDAH2/COPS5/HSPA
C/STXBP1/TAPBP/XKR8/GAK/TAP2/GOSR2/AP2B1/FNBP1L/DOCK2/HBEGF/BTC/BECN1/SNAPIN/AP

/LRR1/UBA52/TRPC4AP/UBE2Q1/TRIM37/RNF213/RNF25/UBR5/PSMC3/KLHL21/USP11/UBE2D2/C
PO1/IFT22/BECN1/TTLL3/IST1/SSX2IP/EIF6/NPHP4/ATG7/HTT/RPS14/CBY1/DZIP1/CCDC65/R
TIMM22/ZDHHC20/NMT1/VAMP3/KIF5B/RAB3C/RPL14/DMTN/RAB3IP/ARL3/SRP19/RPL10A/IFNG/

FAM98A/MAPK15/DOCK11/SEMA4C/ATG3/MAP4K4/PTPRS/EPS8L2/CARMIL2/CUL7/TRAK2/PLXNB3/

TDNEP1/RANBP1/PRIM2/APP/PSMD4/CETN2/PSMD3/RBX1/ACTL8/PSMD7/RAD21/KATNB1/MTBP/WR

/PTK2/THBS1/ADAM17/CCSAP

TPRS/PIK3R2/CLEC4A/VAV3/APPL2/RPS6KA3/SLA2/TLR1/S100A1/WNK1/NOD2/BTN3A2/EIF2B3/
CACTIN/RSRC1/METTL16/BCAS2/SNRNP35/PHRF1/DDX46/ZCRB1/BRDT/PCBP2/PSIP1/SMU1/AAR2

/DOCK6/PLEKHG5/SHTN1/RAC3/RRAS2/ARHGAP15/RAB6A/TP53/RASGRP1/RAB28/TAGAP/MAPKAP1
EIF4G1/PSMB7/CNOT2/HNRNPA2B1/EIF2B2/SESN2/SMAD2/ELAVL1/FASTKD1/ZMPSTE24/RPS6KA3

RPTOR/ATAD5/CALM3/ENSA/ERCC3/PKD1/BORA/ACTR1A/OFD1/ANAPC4/VPS4A

F1/MAEA/SMC2/OSM/SPOUT1/HMGA2/CALM3/ENSA/CIB1/BORA/CUZD1/LLGL2/ANAPC4/VPS4A

YA3/PAM16/HYAL2/HTRA2/IFNG

4/PRKAG1/PIK3R5/MAP3K1/GPS1/SOCS7/MINK1/RPLP1/MAPKAPK5/HEG1/GTF2H1/TIGAR/FAF1/S

BTN2A2/CD300LB/BRK1/HSPD1/PSMD8/ARPC5/ABL1/FCGR2B/PSMA1/TNFAIP3/CD300C/STX7/KCN
CD19/MAP4K4/MAP3K11/VAV3/PPP2R3C/ACE/MUL1/WNK1/NOD2/PPP2CA/XRCC5/CARD14/RASSF2/

RL3/CLEC16A/UBE2J1/ARRB1/PSMD12/BFAR/ARMC8/SMURF2/RNF40/OGT/MAPK9/CDC26/SOCS5/U
/FBXO44/SKP2/PSMB1/WWTR1/SUMO2/USP3/FBXO10/TRIM32/PSMB6/RPL11/ENC1/CDC23/KLHL25

RPS9/KHSRP/DALRD3/GSS/EIF3G/DNAJC1/RPS14/MKNK1/RPS27/MRPS24/RPL29/STUB1/EIF2D/M
BXO10/KCMF1/TRIM32/PSMB6/BTBD1/RPL11/ENC1/NXN/CDC23/KLHL25/TRIM27/SH3RF1/NEURL1
/SMURF2/RNF40/OGT/MAPK9/CDC26/SOCS5/USP46/TRIP12/KLHL22/ANAPC1/PCBP2/DNAJC18/UB
C15/PSMB7/NKD2/RNF115/SKP1/ANKIB1/ADAMTS12/RNF186/DERL1/KCTD13/ZMPSTE24/UBR4/CU

P7A/AK1/EOGT/B3GALT6/HS6ST2/PGAM1/ARFGEF1/TMEM106A/HS2ST1/ST8SIA4/ACACA/ADCY3
PTBP1/BAD/DBNL/PSAP/BCL10/ERP29/SPPL3/HSP90AA1/TNFSF15/PTK2/PTPN2/THBS1/STYXL1/

/SOCS5/USP46/TRIP12/KLHL22/ANAPC1/PCBP2/DNAJC18/UBXN2A/ZFAND2B/USP13/UBE2K/PSME

OTCH1/RPS27A/NBN/MAVS/MOB2/VEGFA/PFKFB2/PLA2G1B/PAK2/TRAF4/CSNK2B/UBA52/DUSP7/M

01/TARDBP/PRKAR2A/SNTA1/SMCR8/DUSP26/DAG1/PPP1R11/DVL1/MARVELD3

52L1/DAXX/RHOA/STK3/INHA/EMC10/RAPGEF1/ATG13/ENPP2/ATP2B4/PRKAG1/PIK3R5/MAP3K1/[illegible]

4/SYT17/WNT5A/LRP2/PPP3CB/ARHGAP44/OLFM1

/RBM14/CD79B/CD38/RAPGEF1/STXBP1/MAP3K1/LGMN/TNFRSF14/DHX9/IDO1/ELMO2/PSMC5/ADO

/VEGFA/SMAP1/TLR2/MYO6/PACSIN2/ANXA3/BCL2L1/CANX/CD2AP/AP2A2/PACSIN1/DGKQ/PLD2/

EAP2/RHOBTB2/ITGAL/SNX3/CD5/CAV2/EGF/LMAN2/CAV1/CALM3/ARHGAP27/CD3G/PTPRC/RAB1A

PTK2/PTPN2/THBS1/ADAM17/PTPN1/NOTCH1/RPS27A/FUT8/FKBP8/SFRP4/STAT5A/NPPB/VEGFA/[illegible]

ITM3/IDO1/RPL30/RBPJ/ADM/BECN1/EPHA2/CYP27B1/CCL19/FAM3A/KPNA6/SEH1L/XCL1/TLR7/[illegible]

ITM3/IDO1/RPL30/RBPJ/ADM/BECN1/EPHA2/CYP27B1/CCL19/FAM3A/KPNA6/SEH1L/XCL1/TLR7/[illegible]
SMAD3/RPL22/GNPAT/TSG101/TP53/TARDBP/RPS15A

FKBIB/SIGIRR/TNFRSF14/DHX9/NR1H2/IFITM3/IDO1/RPL30/TIGAR/RBPJ/ADM/BECN1/EPHA2/C
MKNK1/RPS27/MRPS24/RPL29/EIF2D/MRPL33/LARP1/ST8SIA4/ACACA/YTHDF2/CDK5RAP1/MRPS5

C/TNFRSF1B/TLR8/EFCAB7/FFAR2/CD22/TRIM27/PLA2G6/RBM22/SYT11/PARK7/C2CD2L/USP36/[illegible]

S1/MINK1/MAPKAPK5/HBEGF/NRTN/SPTAN1/PSMC5/ADORA2B/BTC/UBE2N/EPHA2/KITLG/ARTN/KS[illegible]
IF2B1/CTSL/CD4/CTSB/RELA/AIM2/PSMB7/KLHL6/PRAM1/SKP1/EIF2B2/NOD1/CD19/TRIM15/PR[illegible]

3K1/GPS1/MINK1/MAPKAPK5/HBEGF/NRTN/SPTAN1/PSMC5/ADORA2B/BTC/UBE2N/EPHA2/KITLG/A

P1A/THRA/OPTN/PFKFB2/GNAI3/EXOSC9/BNIP3L/DXO/LZTS1/EXOSC7/SREBF1/GGA3/TBRG4/TRI

/ATG13/IFT20/ATP2B4/PRKAG1/ANP32A/WDR41/DHX9/TNPO1/PSMC5/TIGAR/FAF1/PHKG2/CCAR2

/NOTCH2/TLR7/TMEM167B/ARF1/STEAP2/BET1L/COPA/RAB9A/RAB40C/KLHL12/CAV2/PEF1/LMAN
/KAT5/ETV3/SUZ12/APEX1/BAZ1B/HMGXB4/TAF4/SLX4/POLD2/ACTR8/NACC2/MEN1/NFRKB/SP1/
L8B/AGA/VPS11/AP3B1/CLTC/AP1S1/AP1S3/AP1S2/AP2B1/LGMN/WDR41/IFITM3/TPP1/MANBA/G

3/CENPN/NABP1/RBBP7/RAD9A/RPA2/UTP4/DPF2/POLD3/INO80C/TIMELESS/POLR3D/NDE1/ZWIL
0/KRI1/RPS7/AKNA/DNTTIP1/RPL7A/WDR55/GAR1/RPS10/DDX23/PTBP1/SENP3/PRKRIP1/MRPS1
L8B/AGA/VPS11/AP3B1/CLTC/AP1S1/AP1S3/AP1S2/AP2B1/LGMN/WDR41/IFITM3/TPP1/MANBA/G

TR/SH3GLB1/MAN2B1/LTV1/MCOLN3/CYB5R3/OPTN/CCZ1B/GNAI3/GLIPR1/RAB3A/HEXB/MYO6/TM
PRKAR2A/RILPL2/MACROD2/HYLS1/APEX1/PATJ/ATP6V1D/GNAI2/ACTR8/PARD6A/HAUS3/LRRCC1
CDC27/SSX2IP/UXT/NPHP4/KLHL12/HTT/SPOUT1/CALM3/CBY1/DZIP1/RIC8B/CIB1/ACTR1A/OFD

HD3/SLC30A3/INPP5F/STX8/PLEKHF2
AN14/PACSIN1/DGKQ/MLEC/PLA1A/ADGRG3/EXOC3/SIGLEC9/COPB2/RHOA/CD38/AP3B1/RAPGEF1

3/SNRPD3/EAF2/MYO1C/AK6/MRPL36/ZNF473/WWTR1/ACIN1/SUMO2/RAB8B/PIN1/TFIP11/TRIP4

DBNL/PSAP/CD33/LOXL1/HSP90AA1/CTNNA1/THBS1/NOTCH1/SRGN/CD177/MAN2B1/DMBT1/OLFM4
OPB2/RHOA/CD38/AP3B1/RAPGEF1/CLTC/TAPBP/AP1S1/AP1S3/TAP2/AP1S2/SNX17/STXBP5/GOS
P/WRAP53/MTA3/UBQLN4/DCLRE1B/EME2/ACTR3/GAR1/CHEK2/RAD1/SFR1/CENPQ/LEMD2/BOD1/H
PLK3/GALNT7/BICD1/SH3GLB1/RGP1/FIG4/OPTN/LARGE2/PHTF1/GNAI3/GNPTAB/SREBF1/GGA3/
PS16
CE1/MCOLN1/CLEC16A/VTA1/TICAM1/LRP2/ACAP1/FGD2/IL12A/WNT3A/SPPL2B/LZTR1/SNX6/TA

/FAM168B/VPS4A/ATRAID/PLD1/CASC3/NMRAL1/MTMR14/RHBDD2/PPARG/ATP2A2/ATP9A/TNFRSF

C35B1/SULF2/SLC35A4/TMEM115/CHST3/TMEM87A/GOLT1B/ZDHHC9/ERGIC3/TMEM130/COPB2/FU

/HAUS5/MYO6/CD2AP/DYNC1I2/RAB11A/TUBB3/EML2/KIF20B/CENPJ/KIF22/CDK5R1/KLHL21/TN
/HAUS5/MYO6/CD2AP/DYNC1I2/RAB11A/TUBB3/EML2/KIF20B/CENPJ/KIF22/CDK5R1/KLHL21/TN
SMD3/CTSD/SYT3/PSMD7/CPE/ECM1/GMFG/STX4/PRDX4/RABAC1/MYO5A/CLEC5A/CCT8/TBC1D10C
2AP/DYNC1I2/RAB11A/TUBB3/EML2/KIF20B/CENPJ/KIF22/CDK5R1/KLHL21/TNNC1/WIPF2/CACN

M21/IVNS1ABP/RPL36/UBA52/CANX/HSPA14/PIH1D1/TARBP2/RBMX/ZRSR2/RPL19/HNRNPA1/PSM

M2/TNK2/VPS35/PTPN1/VPS45/RPS27A/SCAMP2/CFTR/PDLIM4/LTV1/FIG4/MCOLN3/OPTN/CCZ1B
FBXO10/MAP3K7/RIOK1/ENC1/NAA25/CDC23/KLHL25/OST4/PRMT5/NCOA6/TCEA1/PHF1/GTF2A2/

PHB2/RPS27/SLC9A6/ARHGAP33/ADD1/CNR1/LIN7B/RAB8B/PIN1/GLRB/PPP1R9A/CASK/UTRN/LP

UP/MTF1/E4F1/SF1/DNAJB1/GPS2/CIR1/SUPT20H/GATA3

L24/RPL11/RPL10/RPS24/EPB41/MRPL3/SH3RF1/RPS3A/MRPL20/BGLAP/RPL14/SPARC/NPHP1/K

CD/RAD51D/VCL/STMN2/ZNF185/CALM2/ACTR3/BAG2/CDH24/DBNL/HOOK2/HSP90AA1/VASH1/CTN
/MAPK15/FN3KRP/CCNJ/TRIO/CLK2/ADPGK/MAP3K9/PRKCB/MAP4K4/PRPS1/ERCC2/FASTKD1/MAP
I2/HSPA14/RAB11A/TUBB3/NUDT18/RAB29/KIF20B/RNF213/KIF22/ARL8B/PSMC3/DHX57/AGAP3
I2/HSPA14/RAB11A/TUBB3/NUDT18/RAB29/KIF20B/RNF213/KIF22/ARL8B/PSMC3/DHX57/AGAP3
I2/HSPA14/RAB11A/TUBB3/NUDT18/RAB29/KIF20B/RNF213/KIF22/ARL8B/PSMC3/DHX57/AGAP3
3/KIF22/ARL8B/PSMC3/DHX57/AGAP3/RHOA/ARF5/ACTR5/RFC5/DDX10/TAPBP/ATP2B4/DHX15/M
IN/FAF1/ADCY6/BECN1/WDCP/AP2A1/KSR1/FIZ1/CD247/RHOBTB2/CACUL1/HTT/CAV2/CAV1/MAP
RHGAP33/ATP1B1/VRK2/HNRNPA0/PIN1/PLCG1/PPP1R9A/TRIP4/UTRN/RPS6/MAP3K7/PXN/CCDC8
LUC7L/PMF1/IST1/SSX2IP/CTBP1/ARF1/TFDP1/EPN3/LDB1/DAB2IP/HMGA2/ARL1/SYNJ2/GTF2F
/CARD18/TBC1D22A/DBF4B/CCL3/CHN1/ANKLE2/PKIA/TBCD/VCP/NPRL3/RASAL1/SFRP2/RPS7/C

/DVL1/CLEC16A/DIAPH3/TNPO2/RNF41/AP3M1/RGL1/ARHGAP44/PARD6A/FGD2/GAS8

2/CCL8/PGM2L1/POLE2/MAP4K1/PI4K2A/YRDC/ATR/CHKB/AK1/MAP4K2/MKNK1/EPHB2/ERCC3/CS[illegible]

ED17/SMAD3/SETD1A/NCOA7/TP53/ELK1/TBPL1/TNFRSF10A/TAF11/ZNHIT6/GTF2E1/KAT5/MTDH

/HOXB6/CC2D1A/ETV1/ZNF143/IRF4/HNRNPC/EGR2/BCOR/TFEC/POLR3F/AGO1/TAL2/MYC/POU2F

SMA7/UBE2E1/CCND2/POLE4/TFAP4/POLA1/PSMB3/MCM8/PTPA/USP22/ZNF207/CHMP1A/TOPBP1/
UBB3/SLC39A3/KIF20B/SSH3/IFRD1/PACSIN1/CDK5R1/METRN/PARD3/FAM171A1/APBB1/SRF/CA
HC3/NFKBIL1/TNFRSF1B/PIN1/ADCY7/TLR8/MAP3K7/UCN/TRIM32/FFAR2/AGPAT1/TRIM27/NOX1
G/ANXA1/RELA/AIM2/CCR2/AKAP8/POLR1C/CHID1/POLR3A/HLA-E/NOD1/TRIM15/PTPRS/CLEC4A

MLEC/PSMC3/PPIE/AGA/ADGRG3/SIGLEC9/RHOA/STXBP1/RNF8/DOCK2/MANBA/COPB1/SPTAN1/AD

/PSMC3/PPIE/AGA/ADGRG3/SIGLEC9/RHOA/STXBP1/RNF8/DOCK2/MANBA/COPB1/SPTAN1/ADORA2
4/CYB5R3/ERCC1/GLIPR1/RAB3A/HEXB/PLA2G1B/IL20RB/TLR2/CSNK2B/TMBIM1/SLC15A4/ANXA

/BPGM/EPHA2/KITLG/ARTN/MECOM/CCL19/VPS33A/EIF6/NOTCH2/PIK3R6/DOCK10/MSH6/MAEA/O

P3D1/RPS19/IKBKG/AXL/FADD/BTK/PELI1/STX4/CLEC7A/HCAR2/TNIP1/RASAL3/GPR183/EIF2B

DG/COPS6/EME2/GAR1/CHEK2/CLSPN/RAD1/SFR1/HSP90AA1/CHD1L/CDK2AP1/TATDN2/HDAC10/S

3A/PRKCB/MBD1/KDM4A/WBP2/ZMPSTE24/NAP1L3/HDAC3/BAP1/SUPT16H/SETD1A/SUPT3H/SAFB/
SART3/DHX15/TOE1/TGS1/WDR75/CTU1/GPKOW/KHDRBS3/TSEN34/DHX9/FAM207A/RBM28/BMS1/T
2B1/AURKAIP1/MRPL20/AGO1/ABCF1/RPL14/TMA7/PPA1/MRPL24/MRPS16/EIF5B/RPL10A/FXR1/
A7/KCNAB2/GCA/PAFAH1B2/RAB3D/ARPC5/VCP/VCL/FCGR2B/APRT/PDAP1/HVCN1/VAMP8/MILR1/

M14/APBB1/ACTR5/RFC5/RNF8/POLR2D/GPS1/HINFP/DHX9/GTF2H1/DMAP1/TIGAR/TIPRL/CNOT1

D1/BORA/ACTR1A/OFD1/ANAPC4/VPS4A

19L1/SF3A1/SRSF4/GPATCH1/PDCD7/CLP1/NUDT21/CLASRP/RBM4/RBMXL1/GEMIN4/NCBP1/TSEN

YNC1I2/RAB11A/GPR132/CRLF3/NEDD1/TRIM37/KIF20B/MECP2/CENPJ/PPP2R5B/CDK5R1/PSMC3

CG1/LPAR1/STK4/RPS6/NFKBID/CASP3/CALHM2/RPL11/NCSTN/PLA2G6/NOX1/NEURL1/DDX20/PA
7A/OAS3/TRIM21/IVNS1ABP/BCL2L1/RPL36/UBA52/AP2A2/DYNC1I2/VAC14/RAB29/TARBP2/RPL
DRBS3/RPLP1/TSEN34/DHX9/EDC4/RBM28/GTF2H1/RNMT/RPL32/TNPO1/RPL30/PSMC5/CNOT10/C

2L1/UBA52/CANX/HLA-F/IFNAR2/TNFRSF9/AIP/CDC37/DUOX1/PSMC3/CXCR6/IL21R/TWIST1/NM

/C1QBP/MRPS11/SRSF10/WDR74/WDR18/LSM14A/MRPL22/PRMT7/METTL16/MRM1/PSIP1/AAR2/XA
AV1/RAB13/ARHGEF17/PREX1/ARFGEF1/ARHGAP17/FRMD6/LLGL2/VPS4A/ADD1/ASAP3/MYO1C/AN

1/EPB41/TRIM27/CAPZA2
ZNF683/CD3G/PTPRC/YTHDF2/CLEC4E/PAF1/IKZF3/ZNF16/ADD1/METTL3/PPARG/JUNB/LOXL3/P

PSMC5/FAF1/ARRDC1/CCAR2/CTSW/SDF2L1/FBXL7/UBE2N/ANAPC5/HECTD3/AP2A1/CDC27/CDC34

XBP1/GAK/ROBO3/ACAP3/MINK1/GPC2/NIN/NRTN/SPTAN1/ADCY6/ADM/DPYSL3/SNAPIN/EPHA2/A

/EPS8L2/CARMIL2/CUL7/TRAK2/PLXNB3/MUL1/TSC1/SHTN1/ACTR2/UNC13A/TTL/IFT140/CNTRO
PCGF3/TRIM68/PARP10/FBXO6/FBXO41/STUB1/ANAPC4/RAB1A/HUWE1/PAF1/HDAC8/DDB1/FBXO4
N/TMEM98/CFTR/AAK1/PLK3/BICD1/SH3GLB1/NVL/MAP1A/MAVS/PFKFB2/BNIP3L/CCNE2/MARK4/
B29/KIF20B/TIMM10/AIP/RPL19/UBR5/VPS37B/KLHL21/UBE2D2/NDUFA13/RPS11/CC2D2A/RASS
S3/TRAF7/TAF10/ARPC5/ABL1/MPV17L2/TBCD/SART1/STMN2/SRPK1/ACTR3/RPS23/RPS10/DDX2
NRNPA0/PSMB1/TNFRSF1B/MOV10/RPL4/RPL41/SMG7/RPL9/RPS6/CNP/PPP1R8/RPL24/PSMB6/CA
TL2B/TAF11/ZNHIT6/GTF2E1/AARS2/TSR1/METTL6/GRSF1/RPP38/PARN/MRPS11/THUMPD3/QTRT

TK2/PTPN2/THBS1/ADAM17/CCSAP/VPS35/NOTCH1

2/ERAL1/PYM1/EIF3F/RPS3A/EIF2B1/AURKAIP1/MRPL20/AGO1/ABCF1/RPL14/TMA7/PPA1/MRPL

NMA5
/BECN1/KITLG/MECOM/CCL19/NOTCH2/HSP90B1/GCLM/CRLF1/MAEA/SERPINB9/HTT/HAX1/CAV1/

P2R5B/CDK5R1/ARHGAP28/PSMC3/PPM1F/TWIST1/RBM14/SRF/RHOA/RPL13A/VPS11/RAPGEF1/IF

S12/MAVS/CTBP2/DMBT1/RPS4X/RPL6/HTATSF1/BNIP3L/RPL37/PAK2/RPL27A/OAS3/TRIM21/IV
16/VPS35/CASP8/USF1/RPL5/NOTCH1/RPS27A/FKBP8/BICD1/RPS12/MAVS/CTBP2/DMBT1/RPS4X

OLE2/HMGA2/RPTOR/ATAD5/CALM3/ENSA/PAGR1/ERCC3/PKD1/BORA/ACTR1A/PTPRC/OFD1/ANAPC
/THBS1/NOTCH1/SRGN/CD177/MAN2B1/CTBP2/OLFM4/CYB5R3/VEGFA/GLIPR1/RAB3A/HEXB/ACTN
OAS3/TLR2/TRIM21/MUC6/UBA52/HLA-F/IFNAR2/MUC15/CDC37/PSMC3/UBE2D2/RBM14/NMI/RPL

METRN/TWIST1/APBB1/SRF/CAPRIN1/RHOA/STK3/AP3B1/RAPGEF1/PDPN/DNMT3B/GPC2/RUNX3/Z

R/CALM3/EPHB2/CIB1/PKD1/DUSP22/BORA/PTPRC/BMP2/ADCY3/CDK5RAP1/CXCL10/ADNP/PIK3C
NXA1/CCR2/HLA-E/FZD9/SUPT6H/DOCK11/CD19/POU2F2/GON4L/PRKCB/CLEC4A/VAV3/PPP2R3C/

RX1/HAX1/LDB1/ATP7A/PKNOX1/PREX1/DCLRE1C/RPS14/INPP5D/HDAC5/CIB1/ZNF683/CD3G/PT

IP11/FSTL4/PPP1R9A/LPAR1/ABCA7/IK/UCN/PHF1/CAPZA2/SEMA3F/SYT11/PARK7/DCP2/BUB3/
CTEX1D2/PTK2/THBS1/ADAM17/CCSAP

/CSNK2B/UBA52/DUSP7/MAP3K10/PIH1D1/TARBP2/DGKQ/CDC37/CDK5R1/PPM1F/PRKAR1B/TPD52
B/ARHGAP17/PTPRC/LLGL2/BMP2/RALBP1/ARHGAP33/EVI5L/ASAP3/RALGAPB/ATP1B1/GNA13/PP

/RAB8B/SPIRE2/KIF3A/CD59/RAB1B/CASK/PLEKHM1/TBC1D20/LRPPRC/CDC23/SYT11/C2CD3/CN

B/CACNB3/DGKQ/CDK5R1/UBR5/PPM1F/PARD3/TNNC1/PRKAR1B/CACNA1D/RASSF5/VPS11/YWHAG/
5/TMBIM1/TXNDC12/IVNS1ABP/BCL2L1/UBA52/HLA-F/PIH1D1/ZPR1/PRDX5/MEIS3/MECP2
2A1/EREG/ARF1/TFDP1/MFN1/RHOBTB2/BET1L/NAPG/ATP11A/ATG7/SNX3/KLHL12/SAMD9L/CAV2

HFR/NMI/RASSF5/DAXX/TRIM69/RHOA/GLMN/ACTR5/RNF8/DCAF7/SART3/GPS1/PRMT3/SOCS7/CT
PS27/ACTR1A/OFD1/VPS4A/NECTIN2/RAB1A/KIAA1324/BBS7/RFX2/CCDC40/EVI5L/TMEM67/DHX
TUB/NKD2/PRAM1/RPL12/SRP14/FZD9/BLZF1/ATG3/RPS18/RPS28/RPS17/KCNIP3/RPL22/IKBKB

MUL1/TSC1/SHTN1/ACTR2/UNC13A/TTL/IFT140/CNTROB/BCL11A/FEZ1/CEP135/DPYSL2/NEFL/A

AP73/NME6/VRK1/RFWD3/NABP1/RAD9A/CEP250/RPA2/CUL4B/E2F6/TRIM71/HAUS6/NAE1/NDE1/

ACTR2
/PRPF40A/AKAP17A/XAB2/ECD/SYF2/RBM15/XRN2/PTBP2/SNUPN/HNRNPH1/CWC15/WDR83/PAPOL

/NOLC1/SERBP1/ACE/NUP214/TSC1/TNFSF13/SMAD3/MYCN/PAIP1/RPL22/GEMIN5/MAEL/EIF3C/

H3BP5L/ADCY6/ADORA2B/UBE2N/ADCY5/KITLG/KSR1/EREG/CCL19/PIK3R6/IRAK2/GMFB/DUSP18

N4/CSK/ACTR3/VAMP8/MAPKAPK3/PSMB5/CD33/BCL10/PSMA4/SPPL3/HSP90AA1/VCAM1/PSMD9/C
MAP3K12/PDE5A/AFAP1L2/STRADA/RASGRP1/TNFRSF10A

SP46/TRIP12/KLHL22/ANAPC1/PCBP2/DNAJC18/UBXN2A/ZFAND2B/USP13/UBE2K/PSME2/TRIM3/
/MVB12B/RNF149/PSMA3/PARK7/BUB3/RNF144A/SDF2/USP36/NUB1/UBQLN2/RNF121/CTSL/WWP2

RPL33/LARP1/YTHDF2/CDK5RAP1/MRPS5/CASC3/CHAC1/SNRNP70/RBM4B/YTHDF1/RPL13/RBM8A/
/RNF149/PSMA3/PARK7/RNF144A/BRAP/BCOR/UBE2E3/CAND1/TRIM62/NUB1/MED20/RNF121/WWP
XN2A/ZFAND2B/USP13/UBE2K/PSME2
L7/RNF19A/BAP1/USP33/UGGT1/UGGT2/USP24/FBXL18/TSG101/FBXL2/CDC16/KAT5/UBE2R2/RA

CASP8/PTPN1/NOTCH1/RPS27A/PIAS4/MAVS/VEGFA/PLA2G1B/TLR2/TRAF4/BCL2L1/UBA52/DUSP

AP3K10/PIH1D1/DGKQ/CDK5R1/PRKAR1B/TPD52L1/DAXX/RHOA/STK3/GLMN/INHA/EMC10/RAPGEF

MINK1/RPLP1/TNFRSF14/MAPKAPK5/HBEGF/ADCY6/ADORA2B/UBE2N/ADCY5/KITLG/KSR1/EREG/C

RA2B/UBE2N/EREG/CCL19/XCL1/CD247/TLR7/HSP90B1/IRAK2/CAV1/TP53BP1/DAB2IP/ATAD5/I

TRPV6/PRSS12/WIPF2/KCNJ16/RAPGEF1/CLTC/ENPP2/AP1S1/ATP2B4/XKR8/GAK/ACTN4/SNX17/

/RALBP1/TM9SF4/SNX5/PROM2/PPARG/PIK3CB/MYO1C/LOXL3/ATP9A/SCARA3/SSC4D/NCKAP1/PL

BCL9L/PAK2/FNTA/SREBF1/CSNK2B/HSPA5/UBA52/LMTK2/AP2A2/DGKQ/PPP2R5B/CDC37/CDK5R1

ARF1/PRKRA/CCDC130/IRAK2/ATG7/SNX3/SERPINB9/CCL8/CAV1/ATP7A/DAB2IP/HMGA2/GTF2F1

ARF1/PRKRA/CCDC130/IRAK2/ATG7/SNX3/SERPINB9/CCL8/CAV1/ATP7A/DAB2IP/HMGA2/GTF2F1

YP27B1/CCL19/FAM3A/KPNA6/SEH1L/XCL1/TLR7/MUS81/ARF1/PRKRA/CCDC130/IRAK2/ATG7/SN
/CASC3/CHAC1/RBM4B/YTHDF1/NMRAL1/RPL13/RBM8A/METTL3/CARS2/MRPL36/MOV10/QRSL1/RP

NMT1/KIF5B/DMTN/WWP2/HYAL2/HTRA2/IFNG/ANXA1/ARIH2/AIM2/TERF1/HLA-E/CHGA/SUPT6H/

R1/EREG/MECOM/CCL19/XCL1/NOTCH2/BMP8B/PIK3R6/CCL3L3/SH3RF3/NPNT/IRAK2/DUSP18/OS
KCB/NLRC4/BPIFB1/PTPRS/PIK3R2/CLEC4A/VAV3/APPL2/RPS6KA3/SLA2/TLR1/S100A1/WNK1/N

RTN/KSR1/EREG/MECOM/CCL19/XCL1/NOTCH2/BMP8B/PIK3R6/CCL3L3/SH3RF3/NPNT/IRAK2/DUS

M21/EGLN2/UBA52/EDEM2/NANOS1/RHBDD3/CDC37/CDK5R1/PSMC3/NDUFA13/TWIST1/EXOSC1/NR

/RBM10/MEX3D/BECN1/WDR6/SEH1L/RBM24/EIF6/EIF4G2/UBAC2/ATG7/HTT/KHSRP/HAX1/EGF/C

2/CAV1/MAP4K2/ARL1/B3GALT6/HS6ST2/ARFGEF1/PKD1/LITAF/LMAN2L/CYTH3/HS2ST1/ST8SIA
TNKS1BP1/ACTR6/PURB/NFATC1
PC2/SPNS1/CTSW/BRI3/HEXA/SNAPIN/AP2A1/IST1/VPS33A/SEH1L/VASN/TLR7/RAB9A/NAPG/AT

CH/ARPC1A/EXOSC3/CKAP5/SUV39H2/EID3/PRPF19/ESCO2/RAD51C/SEC13/PTGES3/NAT10/RARG
5/RPL5/RPS27A/NBN/NOL6/RPF2/PLK3/BMT2/POP5/NVL/SP140/MOB2/LLPH/MED27/GNAI3/EXOS
PC2/SPNS1/CTSW/BRI3/HEXA/SNAPIN/AP2A1/IST1/VPS33A/SEH1L/VASN/TLR7/RAB9A/NAPG/AT

BIM1/SLC17A5/SLC15A4/TRIM21/BCAN/UBA52/HLA-F/AP2A2/VAC14/ACAN/RAB29/BORCS6/TMEM
/SPPL2B/BCAS2/IFT81/STOX1/PCNT/KLHL22/TUBGCP2/ZFYVE26/CEP78
1/VPS4A/YTHDF2/CTDP1/BBS7/TMEM67/C2CD5/SPACA9/CEP72/KIF3A/TRIP4/EFHC1/PSKH1/ARM

/CLTC/TAPBP/AP1S1/AP1S3/TAP2/AP1S2/SNX17/STXBP5/GOSR2/RAB27B/AP2B1/HBEGF/MANBA/

/STK4/IK/PPP1R8/PSKH1/POLDIP3/THOC7/TRIM27/DDX20/PARK7/GEMIN8/USP36/ZC3H18/THOC

/CYB5R3/VDAC2/VEGFA/GNAI3/GLIPR1/RAB3A/HEXB/ACTN1/CYB5R1/PLA2G1B/TLR2/CSNK2B/TM
R2/RAB27B/AP2B1/HBEGF/MANBA/COPB1/BTC/BRI3/SNX22/SNAPIN/TMEM184A/AHNAK2/SGSM1/A
AT1/CSNK2A1/DCTN1/USF1/MBD3/NBN/CAPN2/HNRNPU/RNF2/ANP32E/RAD18/SYCE1L/ERCC1/EXO
MUC6/BCAN/HLA-F/RTN1/B4GALT4/VAC14/RAB11A/ACAN/RAB29/YIPF2/MUC15/GOLPH3L/NOTCH3

B1/CD274/SNX21/SNX2/EHD3/TGFBRAP1/SLC30A3/INPP5F/STX8/PLEKHF2

1B/RAB8B/RAB1B/MX1/RPS6/CNP/VAPA/LRPPRC/COPS8/RHPN2/GLB1/SH3RF1/PLA2G2A/SLC30A7

T11/IFT20/CLTC/TAPBP/AP1S1/AP1S3/AP1S2/TJAP1/GOSR2/RAB27B/ACER3/COG4/ATG9A/COPB

NC1/WIPF2/CACNA1D/RASSF5/NEK6/CLTC/ATP2B4/ENKD1/ACTN4
NC1/WIPF2/CACNA1D/RASSF5/NEK6/CLTC/ATP2B4/ENKD1/ACTN4
/ATP6V0D1/FCGR3B/HPSE/RAB4B/S100A7/KCNAB2/GCA/HSPD1/PAFAH1B2/RAB3D/ARPC5/VCP/VC
A1D/RASSF5/NEK6/CLTC/ATP2B4/ENKD1/ACTN4

C3/MRPL37/PPIE/UTP20/RPS11/RBM14/EIF3E/CAPRIN1/RPL22L1/NOB1/HNRNPL/SF3B3/NSUN4/

/RAB3A/PACSIN2/GGA3/TMBIM1/UBA52/HLA-F/LMTK2/AP2A2/VAC14/RAB11A/RAB29/YIPF2/MIT
DPY30/RNF144A/BRAP/BCOR/CCNG2/POLR3F/CAND1/ERH/ULK1/MED20/PIGA/POLR2B/WWP2/PIK3

AR1/ADGRL1/UCN/KCNC4/BCR/NCSTN/EPB41/NEURL1/IGSF21/SYT11/ATP2B1/PARK7/MCTP2/PDE

RT16/CD4/PXDN/MRPL24/MRPS16/RPL10A/ANXA1/MRPL55/MYL6/RPL12/MRPS2/RPS18/RPS28/CO

NA1/DNM2/PTK2/SPIRE1/CCSAP/DCTN1/SSH2/HAUS7/INPPL1/CAPN2/KATNA1/BICD1/PDLIM4/MY
3K11
/RHOA/ARF5/ACTR5/RFC5/DDX10/ENPP2/TAPBP/ATP2B4/DHX15/MRAS/TAP2/RAB27B/DHX9/SMAR
/RHOA/ARF5/ACTR5/RFC5/DDX10/ENPP2/TAPBP/ATP2B4/DHX15/MRAS/TAP2/RAB27B/DHX9/SMAR
/RHOA/ARF5/ACTR5/RFC5/DDX10/ENPP2/TAPBP/ATP2B4/DHX15/MRAS/TAP2/RAB27B/DHX9/SMAR
RAS/TAP2/RAB27B/DHX9/SMARCA5/BMS1/GTF2H1/PSMC5/IFT22/DDX55/TOR3A/SMARCAL1/NTPCR
4K2/DAB2IP/RPTOR/HDAC5/CALM3/PGAM1/CIB1/PKD1/DUSP22/BORA/PTPRC/STUB1/CDK5RAP1/S
8A/PLA2G6/RHOH/CCNG2/KIF5B/CD4/HYAL2/JTB/PRKCSH/WDR45/RELA/CCNJ/PRAM1/PPP1R12A/
1/INPP5D/CALM3/ARHGAP27/PKD1/ARHGAP17/STUB1/LITAF/LLGL2/TADA3/VPS4A
ALM2/SIPA1/ARAP1/BAG2/TBC1D7/CCND3/BAD/SGSM3/DBNL/PSAP/ARHGAP11A/BCL10/BOD1/HSP

NK2A2/EPHA10/RPS6KC1/RPAP1/SRPK2/PCYT1A/IP6K2/STK35/VRK2/PIK3CB/AK6/ADCK2/CSNK1

/DUSP26/HMGN3/C1QBP/APEX1/PDX1/MED14/NLRP3/TAF4/ARRB1/SP1/PARD6A/ZNF516/TAF2/PU

1/MBD2/NKRF/NR6A1/MAX/RELA/NSD1/AIM2/TERF1/LHX2/AKAP8/ZNF219/KDM3A/POU2F2/MBD1/

CTDNEP1/RANBP1/PRIM2/APP/MYBL1/PSMD4/CETN2/PSMD3/RBX1/ACTL8/PPP5C/PSMD7/RAD21/K
PRIN1/RHOA/STXBP1/ENPP2/ROBO3/ACTN4/PDPN/MINK1/HEG1/NIN/NRTN/SPTAN1/PARVB/EPHA2
/SYT11/ATP2B1/PARK7/IRF4/PDE4B/POLR3F/CD4/HYAL2/NFATC2IP/IFNG/ANXA1/RELA/AIM2/C
/APPL2/TLR1/MUL1/TRIM56/NOD2/BAP1/ARG2/XRCC5/SMAD3/CPTP/LILRB1/RNF135/AFAP1L2/C

ORA2B/BRI3/CCL19/IST1/NOTCH2/DOCK10/MSH6/ITGAL/ATP11A/ATG7/DPP7/IL13RA2/ATP7A/T

B/BRI3/CCL19/IST1/NOTCH2/DOCK10/MSH6/ITGAL/ATP11A/ATG7/DPP7/IL13RA2/ATP7A/TP53B
3/HLA-F/AP2A2/ATP6AP2/SLC2A3/TSPAN14

SM/IL17D/BARX1/HAX1/LDB1/ATP7A/TP53BP1/PKNOX1/ATAD5/THOC1/PREX1/DCLRE1C/RPS14/I

5/POLR3D/LBP/ARPC1A/POLR3C/EXOSC3/BTN2A2/CCL3/S100A7/ZNF335/BRK1/HSPD1/PSMD8/CX

PIRE1/CEP164/RPS27A/MBD3/NBN/MAGEF1/HNRNPU/NVL/PIAS4/RAD18/ERCC1/TSN/EXOSC10/CC

MAP3K12/BAZ1A/TP53/IWS1/INO80E/PHF20/KAT5/KDM3B/NEK11/HMGN3/SUZ12/MSL3/BAZ1B/SE
PRKB/NOL10/RSL1D1/GTF2H1/RNMT/ZFC3H1/TYW3/CCAR2/RBM10/USP39/SNRNP25/RBM15B/AHNA
MRPL55/EIF2AK1/SYNCRIP/CHCHD1/EIF4G1/CNOT2/RPL12/EIF2B2/MRPS2/SESN2/RPS18/RPS28
BIN2/RAB5C/DBNL/PSAP/CD33/HSP90AA1/C12orf4/THBS1/NOTCH1/VPS45/SRGN/CFTR/CD177/M

0/STXBP4/HPF1/CCAR2/POLE3/RPA1/UBE2N/EPHA2/MMS19/RBM24/SMARCAL1/CNOT3/MUS81/TFD

2/TRPT1/RBM41/PLRG1

/KLHL21/RBM14/RHOA/NEK6/YWHAG/CLTC/ATP2B4/FAP/PSMC5/CNOT10/STXBP4/BTC/BECN1/FBX

RK7/PNMA5/IGF2R/NMT1/MYC/SLFN11/TIAM2
19/HNRNPA1/UBR5/PSMC3/VPS37B/CXCR6/PPIE/POLR2I/PPIB/TLN1/RPS11/POLR2J/DAXX/RHOA
CAR2/RBM10/MEX3D/USP39/SNRNP25/RBM15B/LUC7L/EREG/TSEN15/RBM24/CDK11A/SSU72/SF3B

I/RAPGEF1/IFI6/ACTN4/IL17RA/SIGIRR/TNFRSF14/NR1H2/IFITM3/PSMC5/UBE2N/EREG/CCL19

B2/XRN2/PTBP2/SNUPN/SHQ1/SMN1/NOP14/ABT1/CNOT1/GTF3A/EIF3J/CRNKL1/MTG2/SF1/USP4
TXR1/SPIRE2/ABRACL/ARHGEF18/NCKAP1/PPP1R9A/LPAR1/PXN/TRIM32/CSRP2/CCDC88A/BCR/D

SMB1/ACIN1/PRRC2C/MOV10/ASXL1/STK4/RPS6/NFKBID/PSMB6/CASP3/CHD7/ADAR/CLPTM1/SMA

/HSP90B1/USP48/UBE4B/UBAC2/AUP1/MAEA/ATG7/SNX3/CACUL1/EGF/HSPA13/CAV1/NEDD8/DAB

P2A1/ARTN/IST1/UBE4B/ARF1/DOCK10/NEO1/SNX3/RAB13/ATP7A/DAB2IP/CAMSAP1/PREX1/EPH

B/BCL11A/FEZ1/CEP135/DPYSL2/NEFL/ARPIN/RTN4IP1
4/SKP2/PSMB1/WWTR1/SUMO2/PIN1/WDSUB1/FBXO10/KCMF1/TRIM32/PSMB6/BTBD1/RPL11/ENC1
PLA2G1B/TLR2/SREBF1/TMBIM1/BCL2L1/CD2AP/EDEM2/RAB11A/ZPR1/RAB29/KIF20B/GOLPH3L/
F5/PPHLN1/RPL13A/VPS11/AP3B1/YWHAG/ATG13/ABHD17A/IFT20/GAK/SIX1/RPL37A/GOSR2/RP
3/COX19/CENPQ/DBNL/EIF3K/HSP90AA1/PRKAA2/RPS15/CHD1L/PSMD9/PTK2/SPIRE1/EIF3M/HA
SP3/GTPBP1/LRPPRC/RPL11/RPL10/HNRNPD/RPS24/ZHX2/PSMA3/AAAS/DCP2/PYM1/HNRNPC/UNG
2/SNAPC1/WDR74/SP1/DUS3L/WDR18/TARBP1/TRMT10C/ANKRD16/METTL16/AIMP1/MRM1/CDKAL1

24/MRPS16/EIF5B/RPL10A/FXR1/MRPL55/EIF2AK1/SYNCRIP/CHCHD1/EIF4G1/CNOT2/RPL12/EI

HMGA2/ATAD5

T20/STXBP1/SIX1/WDR75/FNBP1L/GTF2H1/MTPN/NIN/SPTAN1/PSMC5/FAF1/RPA1/BECN1/DPYSL

NS1ABP/BCL2L1/RPL36/UBA52/AP2A2/DYNC1I2/VAC14/THOC2/RAB29/TARBP2/RPL19/HNRNPA1/
/RPL6/HTATSF1/BNIP3L/RPL37/PAK2/RPL27A/OAS3/TRIM21/IVNS1ABP/BCL2L1/RPL36/UBA52/

4/VPS4A/CTDP1/PAF1/ZNF16/CEP72/DDB1/SKP2/PSMB1/IK/RPS6/RPL24/PSMB6/CDC23/PSMA3/
1/CYB5R1/TLR2/CSNK2B/TMBIM1/SLC15A4/ANXA3/HLA-F/AP2A2/PCYOX1L/ATP6AP2/SLC2A3/TS
13A/STXBP1/IFI6/MAP3K1/LGMN/TNFRSF14/DHX9/NR1H2/IFITM3/PSMC5/STXBP4/UBE2N/CYP27

MIZ1/NIN/RBPJ/ADM/BTC/DPYSL3/CYP27B1/AP2A1/KITLG/CCL19/IST1/RBM24/ZC4H2/ARF1/PI

B/WWTR1/ADCY7/PPP1R9A/LPAR1/STK4/MAP3K7/UCN/CASP3/CCDC88A/ADAR/COPS8/NAB2/TRIM2
SLA2/TSC1/NOD2/ARG2/TNFSF13/SMAD3/RPL22/LILRB1/PDE5A/FUT7/CD80/TP53/SOX13/RASGR

PRC/YTHDF2/CLEC4E/PAF1/IKZF3/ZNF16/ADD1/METTL3/PPARG/JUNB/LOXL3/PSMB1/ACIN1/PRR

HNRNPC/BCOR/AURKAIP1/UBQLN2/ULK1/TCHP/DMTN/PRMT6/CEP97/PAM16/HTRA2/ASF1A/TRIM46

L1/DAXX/RHOA/STK3/GSKIP/RFC5/RAPGEF1/YWHAG/ATG13/CNPPD1/ATP2B4/PRKAG1/PIK3R5/MA
ARG/RSU1/ANTXR1/DOCK8/PIN1/PLCG1

TRL/TSNARE1/VAMP3/KIF5B/ADGRE2/RAB3C/CEP97/RAB3IP/NPHP1/TRIM46/VPS33B/FAM98A/MA

ATG13/ABHD17A/CLTC/STXBP1/ANP32B/AP2B1/DHX9/GPC2/DMAP1/STXBP4/ADORA2B/BECN1/SNA

/PEF1/EGF/CAV1/TMEM102/SYNJ2/CALM3/EPHB2/GET4/CD3G/PTPRC/VPS4A/NECTIN2/RAB1A/BA

U1/JOSD1/UBE2F/MED7/UNKL/ZMIZ1/PSMC5/ARRDC1/USP39/BECN1/FBXL7/UBE2N/ANAPC5/HECT
30/FEZ2/CEP72/WWTR1/KIF3A/RAB1B/NIP7/STAM/CSRP2/RPL24/OGFOD1/ARMC9/CCDC88A/TBC1
/TP53/GRIPAP1/GGA2/RPS15A/PIP5K1A/CHMP4A/RER1/RILPL2/DAG1/RFTN1/DVL1/SRP72/PEX1

RPIN/RTN4IP1

ZWILCH/DRG1/CKAP2/KHDRBS1/CKAP5/DBF4B/BTN2A2/MZT1/ANKLE2/RAD51C/BTG2/PSMD8/ACVR

A/SMN1/HNRNPH2/CRNKL1/SF1/HNRNPH3/LEO1/TUT1/AHCYL1/CIR1/ZMAT2/USP4/CWF19L1/SF3A

TP53/DDX1/TARDBP/EIF1/ATXN2/C1QBP/APEX1/PARN/TDRD7

/CACUL1/HTT/EGF/MAP4K1/CAV1/LDB1/MAP4K2/DAB2IP/HMGA2/RPTOR/CALM3/EPHB2/CIB1/PKD

12orf4/PTK2/PTPN2/CASP8/PTPN1/RPS27A/CD177/MAVS/DMBT1/PLA2G1B/IL20RB/PAK2/TLR2/

HACE1/COPS3/YOD1/HSPA1A/RMND5B/USP28/PSMD6/PCNP/USP4/RNF139/PSMC2/DIS3L2/USP32/
/HTRA2/CTSB/ARIH2/RNF126/TLK2/ANAPC15/PSMB7/NKD2/RNF115/SKP1/ANKIB1/ADAMTS12/RN

METTL3/CARS2/MRPL36/PIN1/MOV10/QRSL1/RPL4/SARS2/RPL41/ABCA7/RPL9/MRPL14/RPS6/EI
2/ARIH2/RNF126/PSMB7/RNF115/SKP1/ANKIB1/MED30/MKRN1/ATG3/RNF186/DERL1/KCTD13/UB

D23A/SVIP/PPP1R11/DVL1/DERL3/CLEC16A/RNF41/UBE2J1/ARRB1/PSMD12/BFAR/ARMC8/SMURF

7/MAP3K10/PIH1D1/CRLF3/MEIS3/GOLT1B/TPD52L1/PLAGL2/RUNDC3A/DAXX/RHOA/STK3/NEK6/

1/ATG13/ENPP2/ATP2B4/PRKAG1/PIK3R5/MAP3K1/MINK1/RPLP1/TNFRSF14/MAPKAPK5/HBEGF/T

CL19/XCL1/FIZ1/NOTCH2/BMP8B/PIK3R6/CCL3L3/SH3RF3/NPNT/IRAK2/CRLF1/OSM/CACUL1/CC

NPP5D/DUSP22/CD3G/PTPRC/NECTIN2/CLEC4E/BAG6/ZCCHC3/BTN2A1/CNR1/CD1A/NFKBIL1/PIK

RAB27B/AP2B1/FNBP1L/DOCK2/NR1H2/ELMO2/KCNH2/ADM/BECN1/AP2A1/LRPAP1/CCL19/CD247/

CG1/CSNK1G1

/SULF2/HNRNPA1/PARD3/POLR2I/POLR2J/RBM14/RHOA/INHA/RAPGEF1/IFT20/POLR2D/IFI6/AP

/HDAC5/ZC3HAV1/PTPRC/LITAF/BMP2/PABPN1/CLEC4E/RAB1A/ZCCHC3/PAF1/IKZF3/HNRNPUL1/

/HDAC5/ZC3HAV1/PTPRC/LITAF/BMP2/PABPN1/CLEC4E/RAB1A/ZCCHC3/PAF1/IKZF3/HNRNPUL1/

X3/SERPINB9/CCL8/CAV1/ATP7A/DAB2IP/HMGA2/GTF2F1/HDAC5/ZC3HAV1/PTPRC/LITAF/BMP2/
L4/SARS2/RPL41/RPL9/MRPL14/RPS6/EIF2B4/UCN/RPL24/OGFOD1/GTPBP1/UNK/LRPPRC/POLDI

MAP4K4/PIK3R2/NOLC1/PTPN14/KCNS3/UBL5/TLR1/NOD2/HDAC3/BAP1/ARG2/SMAD3/CPTP/LILR

M/CCL8/CAV2/EGF/MAP4K1/CAV1/MAP4K2/DAB2IP
OD2/BTN3A2/XRCC5/EIF2B3/ACTR2

P18/OSM/CCL8/CAV2/EGF/MAP4K1/CAV1/MAP4K2/DAB2IP

DC/CHFR/SF3B3/SLC25A12/GLMN/VPS11/HNRNPR/ATG13/IFT20/ATP2B4/PRKAG1/ANP32A/FAP/W

AV1/DAB2IP/RPTOR/ATP6V1B2/PGAM1/PKD1/EXOSC2/CSNK2A2/ZC3HAV1/STUB1/LARP1/YTHDF2/

4/RAB1A/PLD1/PLEKHA8/RAB36/GCNT1/B3GNT8/ERGIC2/RHBDD2/QSOX2/ACBD3/SCARA3/CD59/R

P11A/EGF/PI4K2A/DPP7/DAGLB/RPTOR/ATP6V1B2

/DNA2/HSPA2/ARPC5/DOT1L/RAD51D/VCP/WRAP53/MTA3/NSFL1C/UBQLN4/DCLRE1B/FAAP20/EME
C9/EXOSC10/BCL9L/MOB1A/TMA16/PAK1IP1/NOL7/TAF1D/EXOSC7/UBTF/ARL6IP4/DDX56/RPL36
P11A/EGF/PI4K2A/DPP7/DAGLB/RPTOR/ATP6V1B2

138/TECPR1/ARL8B/AGA/VPS11/AP3B1/CLTC/AP1S1/AP1S3/AP1S2/AP2B1/LGMN/WDR41/IFITM3

C9/CCDC88A/NUBP2/LRRC45/CC2D1A/PHF1/PLA2G6/AAAS/C2CD3/PRICKLE3/LRWD1/PDE4B/CNTR

COPB1/BTC/BRI3/SNX22/SNAPIN/TMEM184A/AHNAK2/SGSM1/AP2A1/EREG/COPA/RAB9A/ITGAL/A

3/CENPT/DYRK3/MAMLD1/RDM1/MAX/GEMIN6/SMN2/ATP6V0A1/TERF1/CLK2/HNRNPA2B1/GON4L/M

BIM1/SLC15A4/ANXA3/AP2A2/PCYOX1L/ATP6AP2/SLC2A3/TSPAN14/MLEC/PSMC3/PPIE/PLA1A/A
P2A1/EREG/COPA/RAB9A/ITGAL/ATP11A/KLHL12/HTT/CAV2/PEF1/EGF/CAV1/RAB13/ATP7A/CAL
SC10/CSNK2B/COPS5/MIS18A/PHF6/THOC2/MECP2/RBMX/KIF22/BAZ2A/NCAPD3/EIF3E/SRF/DAX
/GORASP2/SLC35B1/SULF2/SLC35A4/TMEM115/CHST3/TMEM87A/GOLT1B/ZDHHC9/ERGIC3/TMEM1

/NEURL1/SYT11/PARK7/IGF2R/ZDHHC20/VAMP3/KIF5B/RAB3C/DMTN/FIGNL1/HYAL2/FXR1/CTSB

1/BECN1/PSEN2/SNAPIN/COG2/TMEM167A/NOTCH2/TLR7/TMEM167B/ARF1/STEAP2/BET1L/COPA/

L/APRT/PDAP1/GHRHR/MFF/THBS2/HVCN1/STX7/VAMP8/BIN2/RAB5C/MOXD1/DBNL/PSAP/CD33/L

RPL13A/MRPL27/MRPL28/HNRNPR/POLR2D/SART3/DHX15/ACTN4/TGS1/RPL37A/MRPL51/MRPL11/

D1/DGKQ/ARL8B/VPS37B/CYTIP/RHOA/VPS11/RAPGEF1/ABHD17A/CLTC/SNX17/TJAP1/GOSR2/RAC2B/MAX/ARIH2/DYDC2/ANAPC15/CCNJ/RPN1/SKP1/POLR1C/ANKIB1/MED30/POLR3A/KMT2B/SES

4B/VAMP3/RAB3C/RPL14/DMTN/RPL10A/FXR1/ANXA1/VAMP1/NETO2/RPL12/FZD9/PTPRA/SEMA4C

L24A1/TUBD1/RPS17/MXRA5/NUP214/RPL26L1/LAMA1/ACTR2/RPL22/AEBP1/MRPL21/COL4A1/CO

L9/HNRNPU/FLII/MAP1A/NEBL/RAB3A/ACTN1/MARK4/RITA1/MAP7D3/LZTS1/ADD3/FNTA/MYO6/P

CA5/BMS1/GTF2H1/PSMC5/IFT22/DDX55/TOR3A/SMARCAL1/NTPCR/ARF1/LSG1/MFN1/RHOBTB2/R
CA5/BMS1/GTF2H1/PSMC5/IFT22/DDX55/TOR3A/SMARCAL1/NTPCR/ARF1/LSG1/MFN1/RHOBTB2/R
CA5/BMS1/GTF2H1/PSMC5/IFT22/DDX55/TOR3A/SMARCAL1/NTPCR/ARF1/LSG1/MFN1/RHOBTB2/R
/ARF1/LSG1/MFN1/RHOBTB2/RAB9A/MSH6/RAB40C/EP400/HSPA13/RAB13/ATP7A/SRCAP/ATP6V1
RSF2/ARHGAP33/ATP1B1/VRK2/HNRNPA0/PIN1/PLCG1/PPP1R9A/TRIP4/UTRN/RPS6/MAP3K7/PXN
NELL2/PRKCB/CDK5RAP2/PPP1CB/RHOD/ELAVL1/PIK3R2/MAP3K11/RPS6KA3/PTPN14/ACE/WNK1/

90AA1/SRGAP2/PRKRIP1/STYXL1/TNK2/CCNJL/UBA2/PPP2R3A/RPL5/DUS2/NOTCH1/PPP1R12C/A

G1/CASK/STK4/POLG2/MAP3K7/PSKH1/RIOK1/PPIP5K1/BCR/FASTKD5/PIP4K2B/CKB/IGF2R/CCN

RB/GATA4/NFATC1/MAFK/MED6/MAPK9/ZNF653/CRTC1/HDGF/TRIP12/STAT3

ZNF202/SMAD2/E2F7/WBP2/ZMPSTE24/HOXB9/PLAGL1/KCNIP3/BRF2/ZNF431/PBX3/ORC4/ESRRA

ATNB1/RRM1/MTBP/WRAP73/NME6/VRK1/RFWD3/GADD45GIP1/NABP1/RAD9A/CEP250/RPA2/CUL4B
/ARTN/IST1/VPS33A/RHOBTB2/DOCK10/NEO1/ATP7A/DAB2IP/CAMSAP1/PREX1/EPHB2/CIB1/SLC
CR2/AKAP8/POLR1C/CHID1/POLR3A/HLA-E/CHGA/NOD1/LTBP3/TRIM15/NLRC4/PTPRS/CLEC4A/A
D80/DDX1/RASGRP1/LILRB2/MAPKBP1/KAT5/C1QBP/PPP1R11/OTUD5/ZBP1/NLRP3/TICAM1/UBE2

TP53BP1/CD84/ATAD5/THOC1/TCN1/PGAM1/DNASE1L1/ZNF683/PTPRC/CLEC4E/PLD1/HUWE1/CST3

P1/CD84/ATAD5/THOC1/TCN1/PGAM1/DNASE1L1/ZNF683/PTPRC/CLEC4E/PLD1/HUWE1/CST3/SLC

NPP5D/HDAC5/CIB1/ZNF683/CD3G/PTPRC/YTHDF2/CLEC4E/PAF1/IKZF3/ZNF16/ADD1/METTL3/F

CL17/AKIRIN1/ARPC5/ABL1/SART1/FCGR2B/PSMA1/TNFAIP3/TOX/STX7/KCNN4/CSK/ACTR3/VAM

NE2/UIMC1/COPS5/MIS18A/ANXA3/UBA52/GLI1/ZPR1/MORF4L2/MECP2/KIF22/FANCF/HNRNPA1/

TMAR/L3MBTL1/ACTR8/NACC2/MEN1/ARRB1/ACTR6/TPR/RNF40/EZH1/MCM3AP/OGT/RCCD1/KANSL
K2/DIMT1/EBNA1BP2/LUC7L/TSEN15/NOL9/RBM24/DPH3/CDK11A/SSU72/UTP23/SF3B2/STRAP/R
/ELAVL1/RPS17/RPS6KA3/NOLC1/ABTB1/RPL26L1/TSC1/PAIP1/RRBP1/EIF2B3/RPL22/GEMIN5/
AN2B1/CTBP2/OLFM4/CYB5R3/VEGFA/GLIPR1/RAB3A/HEXB/ACTN1/CYB5R1/TLR2/CSNK2B/COPS5

P1/SUV39H1/POLR2E/UBE2E2/SMUG1/MSH6/MORF4L1/POLE2/ATR/TP53BP1/HMGA2/ATAD5/PARP1

L7/ANAPC5/EREG/CDC27/CNOT3/UXT/TFDP1/UBE2E2/MSH6/SND1/CAV2/EGF/DAB2IP/HMGA2/RPT

/RPL13A/AP1S1/AP1S3/POLR2D/TAP2/AP1S2/RPL37A/AP2B1/RPLP1/DOCK2/TNFRSF14/DHX9/IF
2/CNOT3/STRAP/LSM1/SFSWAP/POLR2E/SAFB2/RPS9/CCAR1/KHSRP/MFAP1/PRPF4/THOC1/RPS14

/XCL1/HSP90B1/ARF1/CCL3L3/SPATA2/IRAK2/CRLF1/OSM/CCL8/IL13RA2/CAV1/TRIM68/INPP5

/WDR43/SF3A1/NSA2/CLP1/MRPS9/NUDT21/EIF2A/TSR2/DDX18/EIF2S1/GEMIN4/GNL3L/NCBP1
SC2/ATP2C1/EPB41/TRIM27/CAPZA2

D5/BCR/MFHAS1/NCOA6/SH3RF1/RTKN2/DPY30/PSMA3/ANKRD54/IRF4/RHOH/AGO1/MYC/BGLAP/A

2IP/GET4/FBXO6/PKD1/CSNK2A2/STUB1/ANAPC4/VPS4A/ATRAID/BAG6/HUWE1/BBS7/CST3/SNX5

B2/ARFGEF1/CIB1/SLC9A6/EPHA10/ARHGAP33/YTHDF1/ADNP/FEZ2/CNR1/RYK/PIK3CB/PLXNB2/

/NXN/CDC23/KLHL25/TRIM27/SH3RF1/NEURL1/RNF149/PSMA3/AAAS/PARK7/EGR2/RNF144A/BRA
RHBDD3/CENPJ/CACNB3/ALOX15B/CDK5R1/UBR5/PPM1F/TWIST1/APBB1/CACNA1D/RASSF5/GLMN/
LP1/DHX9/PMM2/RPL32/TNPO1/DMAP1/IPO9/RPL30/NXT2/RPA1/BECN1/KPNA6/TFDP1/IPO4/UBA
T1/STYXL1/DCTN1/RPL5/RPS27A/GTF3C5/RPF2/PFDN6/BICD1/THRA/VDAC2/RPL6/RAB3A/MAP7D
/PDE4B/RPS3A/AGO1/RPL14/APOBEC3F/PAM16/RPL10A/FXR1/IFNG/SYNCRIP/EIF4G1/PSMB7/DN
/XRN2/GTF3C4/TRIT1/ADAT2/ELP6/SHQ1/NOP14/ABT1/ICE1/GTF3A/GTF2B/DEDD2/TUT1/INTS3

F2B2/MRPS2/SESN2/RPS18/RPS28/ELAVL1/GGT6/RPS17/RPS6KA3/NOLC1/ABTB1/RPL26L1/TSC1

3/EPS8L1/DIMT1/EPHA2/CORO2B/CCL19/CDC34/ARF1/RHOBTB2/KLF5/GMFB/NPHP4/GPBAR1/HTT

UBR5/PSMC3/VPS37B/CXCR6/PPIE/POLR2I/PPIB/TLN1/RPS11/POLR2J/DAXX/RHOA/RPL13A/AP1
AP2A2/DYNC1I2/VAC14/THOC2/RAB29/MECP2/TARBP2/RPL19/HNRNPA1/UBR5/PSMC3/VPS37B/CX

BUB3/CNTRL/CCNG2/MYC/SLFN11/HUS1/BRD7/DYRK3/BRD4/MAX/ANXA1/MAPK15/EIF4G1/HAUS2/
PAN14/MLEC/PSMC3/PPIE/TRPV6/TLN1/AGA/ADGRG3/CACNA1D/SIGLEC9
B1/EREG/CCL19/FAM3A/XCL1/TLR7/HSP90B1/PIK3R6/CCL3L3/IRAK2/SERPINB9/CCL8/CAV1/CD

K3R6/KLF5/NPNT/SNX3/HAX1/DAB2IP/DKKL1/PREX1/INPP5D/EPHB2/CIB1/PTPRC/BMP2/ATRAID

7/MADD/NEURL1/ANKRD54/PARK7/PDE6G/CCNG2/SLC27A1/MLLT1/CD4/HYAL2/JTB/IFNG/CCNJ/I
P1/FGL2/LILRB2

C2C/MOV10/ASXL1/STK4/RPS6/NFKBID/PSMB6/CASP3/CHD7/ADAR/CLPTM1/SMAD5/BCR/MFHAS1/

/SEMA6B/ANAPC15/TERF1/FZD9/SUPT6H/KDM3A/SEMA4C/ATG3/CDK5RAP2/MAP4K4/PTPRS/RAB7A

P3K1/GPS1/SOCS7/MINK1/RPLP1/MAPKAPK5/HEG1/GTF2H1/TIGAR/FAF1/SH3BP5L/ADCY6/ADORA

PK15/HAUS2/TERF1/VAMP1/ARCN1/TFG/CHGA/PRKCB/CNIH2/CDK5RAP2/FAM91A1/DOCK7/CHRNA5

PIN/EPHA2/ADCY5/XCL1/MRAP2/CLIC2/CD247/ARF1/TFDP1/UBAC2/SNX3/HTT/HAX1/EGF/IL13R

G6/PLEKHA8/VPS16/C2CD5/NAIF1/ATP1B1/SPACA6/PPARG/ATP2A2/ATP9A/RAB8B/SPIRE2/CD59

'D3/CDC27/SEH1L/CDC34/USP48/UBE4B/UBE2E2/SH3RF3/PEX10/SPATA2/RAB40C/MAEA/ATG7/RN
D20/RPL11/RPL10/PIP4K2B/NEURL1/AAAS/ERAL1/C2CD3/CNTRL/MRPL20/CENPT/UBQLN2/ULK1/
6

:1/TAF10/PIDD1/DNA2/HSPA2/PKIA/EMD/VCP/PSMA1/MTA3/NSFL1C/DCLRE1B/EME2/CHEK2/CLSF

.1/SRSF4/GPATCH1/RPRD2/PDCD7/CLP1/NUDT21/CLASRP/RBM4

[illegible]1/DUSP22/BORA/PTPRC/BMP2/ADCY3/CDK5RAP1/ZNF16/CXCL10/ADNP/PIK3CB/WWTR1/ADCY7/P[illegible]

[illegible]MUC6/UBA52/HLA-F/IFNAR2/RAB29/MUC15/CACNB3/CDC37/PLD2/PSMC3/UBE2D2/STOML2/WIPF2

WAC/PSMA6/RLIM/RNF215/CUL2/FBXW11/VPS37A/ERLEC1
F186/DERL1/RAB7A/KCTD13/ZMPSTE24/UBR4/CUL7/RNF19A/BAP1/USP33/UGGT1/UGGT2/USP24/

F2B4/UCN/RPL24/OGFOD1/CASP3/GTPBP1/UNK/LRPPRC/POLDIP3/RPL11/ENC1/RPL10/NCSTN/KL
R4/CUL7/RNF19A/MUL1/TRIM56/NOD2/HDAC3/TULP4/RNF10/RNF135/FBXL18/TSG101/FBXL2/CD

2/RNF40/OGT/MAPK9/CDC26/SOCS5/USP46/TRIP12/KLHL22/ANAPC1/PCBP2/DNAJC18/UBXN2A/A

EMC10/RAPGEF1/ACTN4/PIK3R5/MAP3K1/MINK1/CXXC5/MAPKAPK5/HBEGF/ADORA2B/BTC/BECN1/

'IGAR/ADCY6/ADORA2B/UBE2N/ADCY5/KITLG/KSR1/EREG/CCL19/XCL1/FIZ1/NOTCH2/BMP8B/PIK

L8/CAV2/HAX1/EGF/MAP4K1/CAV1/MAP4K2/DAB2IP/HMGA2/RPTOR/CALM3/CIB1/TMEM106A/PKD1

[illegible]3CB/MYO1C/PSMB1/SCARA3/CD59/NCKAP1/PLCG1/TLR8/EIF2B4/MAP3K7/NFKBID/PSMB6/FFAR2/

'HSP90B1/ARF1/STEAP2/RHOBTB2/ITGAL/SNX3/CD5/CAV2/EGF/LMAN2/CAV1/CALM3/ARHGAP27/S

'2B1/LGMN/HBEGF/ELMO2/NRTN/RBPJ/STXBP4/BTC/PSEN2/EPHA2/AP2A1/EREG/VASN/SERPINA12

CXCL10/CNR1/IFITM2/NFKBIL1/SKP2/JUNB/HNRNPA0/ATP2A2/TNFRSF1B/CD180/TLR8/MX1/CN[illegible]

CXCL10/CNR1/IFITM2/NFKBIL1/SKP2/JUNB/HNRNPA0/ATP2A2/TNFRSF1B/CD180/TLR8/MX1/CN[illegible]

NECTIN2/PABPN1/CLEC4E/RAB1A/ZCCHC3/PAF1/IKZF3/HNRNPUL1/CXCL10/CNR1/IFITM2/NFKBIP3/RPL11/ENC1/RPL10/KLHL25/HNRNPD/RPS24/MRPL3/PLA2G6/LSM14B/NEURL1/EIF4E2/ERAL1

B1/TSG101/TP53/IWS1/GRIPAP1/TARDBP/RASGRP1/CEP135/MAPKBP1/RAB11FIP1/HMGN3/SVIP/

'DR41/DHX9/TNPO1/PSMC5/TIGAR/FAF1/PHKG2/CCAR2/RBM10/MEX3D/BECN1/WDR6/SEH1L/RBM24

/ATRAID/BAG6/KIAA1324/BBS7/CST3/SNX5/SNRNP70/FEZ2/CNR1/METTL3/PIK3CB/HNRNPA0/PSM

AB1B/IER3IP1/TLR8/AP4B1/ABCA7/NCAM1/VAPA/TBC1D20/ST3GAL2

2/ACTR3/GAR1/CHEK2/RAD1/SFR1/CENPQ/LEMD2/BOD1/HAT1/CSNK2A1/DCTN1/USF1/MBD3/NBN/
/PIH1D1/PNO1/ZPR1/PHF6/MORF4L2/KIF20B/RNF213/EWSR1/NUAK1/BAZ2A/POLR2I/UTP20/RPS

/TPP1/MANBA/GPC2/ATG9A/SPNS1/CTSW/BECN1/BRI3/HEXA/SNAPIN/AP2A1/IST1/VPS33A/SEH1

L/KIF5B/TCHP/DYNC2LI1/CEP97/DYRK3/EYA3/ARL3/TTLL5/MAMLD1/JTB/MAPK15/HAUS2/PPP1R

TP11A/KLHL12/HTT/CAV2/PEF1/EGF/CAV1/RAB13/ATP7A/CALM3/CUZD1/CD3G/PTPRC/RAB1A/PL

BD1/SUMO3/SNAPC5/U2AF1L4/E2F7/KCTD13/LUC7L3/SRSF3/PLAGL1/NOLC1/SETD1A/MAU2/GEMI

GA/ADGRG3/EXOC3/SIGLEC9/RHOA/CD38/STXBP1/ACTN4/RAB27B/DOCK2/MANBA/COPB1/SPTAN1/
M3/CUZD1/CD3G/PTPRC/RAB1A/PLD1/SNX5/SLCO4C1/TMEM67/C2CD5/MYO1C/TNFRSF1B/RAB8B/S
X/TEX11/HMGN2/ACTR5/RFC5/RNF8/GATAD2A/DHX9/SMARCA5/STAG3L4/RUNX3/DMAP1/CCAR2/PC
30/COPB2/SLC35C2/FUT11/IFT20/CLTC/TAPBP/AP1S1/AP1S3/AP1S2/TJAP1/GOSR2/RAB27B/AC

/VPS33B/TLK2/ATP6V0A1/CCR2/FZD9/CHGA/CCDC78/HIF1AN/CDK5RAP2/VPS54/SERBP1/CUL7/S

RAB9A/RAB40C/ATP11A/KLHL12/YIPF1/CAV2/PEF1/LMAN2/PI4K2A/CAV1/RAB13/ATP7A/MAP4K2

OXL1/HSP90AA1/SGTA/CTNNA1/THBS1/NOTCH1/VPS45/SRGN/CD177/MAN2B1/DMBT1/OLFM4/CYB5

GPKOW/RPLP1/DHX9/FAM207A/EDC4/RBM28/BMS1/NOL10/RSL1D1/RPL32/RPL30/MRPL15/USP39/

B27B/AP2B1/LGMN/IFITM3/ATG9A/TMUB1/NIPA1/BECN1/PSEN2/TMEM184A/AP2A1/LRPAP1/VPS3
N2/NAA50/ATG3/NAA20/PRPS1/DERL1/ERCC2/PIK3R2/KDM4A/KCTD13/CUL7/RNF19A/SLA2/ABTB

[illegible]/PRKCB/CNIH2/RPS18/KCNC3/PTPRS/ELAVL1/CHRNA5/KCNIP3/RNF19A/TMEM240/SYP/TSC1/PLE

L13A1/NEFH/FGL2/RPS15A/NEFL/NDUFA7/SNTA1/HIP1/DAG1/MRPS11/ANOS1/BFSP1/COL10A1/

ACSIN2/LIMA1/GLI1/LMTK2/RAB11A/CCDC88C/PHF6/EML2/RAB29/KIF20B/SSH3/CENPJ/PACSI

AB9A/MSH6/RAB40C/EP400/HSPA13/RAB13/ATP7A/SRCAP/ATP6V1B2/ATAD5/MORC2/ARL1/KIF26
AB9A/MSH6/RAB40C/ENTPD1/EP400/HSPA13/RAB13/ATP7A/SRCAP/ATP6V1B2/ATAD5/MORC2/ARL
AB9A/MSH6/RAB40C/ENTPD1/EP400/HSPA13/RAB13/ATP7A/SRCAP/ATP6V1B2/ATAD5/MORC2/ARL
B2/ATAD5/MORC2/ARL1/KIF26B/DHX38/ERCC3/KLC1/VPS4A/RAB1A/RALBP1/RAB36/DDX52/DHX
/CCDC88A/SH3RF1/PLA2G6/PARK7/RHOH/CCNG2/KIF5B/CD4/HYAL2/JTB/PRKCSH/WDR45/RELA/
NOD2/SMAD3/RAC3/IKBKB/MAML1/MAP3K12/TP53/NEFH/BCL11A/FEZ1/SH2B1/PRKAR2A/MAPKAP1

NP32E/TBC1D22B/ARHGEF11/RAB3A/CCNE2/SMAP1/PAK2/CSNK2B/ANXA3/LMTK2/PLEKHG6/PRDX5

G2/POLR3F/FICD/ULK1/MED20/POLR2B/UCKL1/DYRK3/DGKD/PIK3C2B/BRD4/PIGF/EIF2AK1/TLK

./XRCC5/SMAD3/MYCN/MAU2/TFAP2C/SAFB/MACC1/AEBP1/TP53/SOX13/ELK1/BCL11A/TARDBP/TB

/E2F6/TRIM71/HAUS6/NAE1/NDE1/ZWILCH/DRG1/CKAP2/KHDRBS1/CKAP5/DBF4B/BTN2A2/MEIS2
9A6/FRMD6/STRIP2/EPHA10/YTHDF2/RAB1A/ARHGAP33/YTHDF1/ADNP/ADD1/FEZ2/METTL3/GNA1
PPL2/TLR1/MUL1/TRIM56/NOD2/BTN3A2/BAP1/ARG2/XRCC5/SMAD3/CPTP/LILRB1/RNF135/AFAP
J1/WNT5A/C5AR2/CD40/ARRB1/IL12A/GATA4/WNT3A/LSM14A/CACTIN/SLAMF1/PDCD1LG2/SOCS5

/SLCO4C1/FAF2

O4C1/FAF2

PARG/JUNB/LOXL3/PSMB1/ACIN1/PRRC2C/MOV10/ASXL1/STK4/RPS6/NFKBID/PSMB6/CASP3/CHD

P8/MAPKAPK3/PSMB5/BAD/BCL10/PSMA4/SPPL3/HSP90AA1/ACVR1B/VCAM1/PSMD9/C12orf4/PTK

UBR5/BAZ2A/PPIE/POLR2I/TWIST1/STOML2/POLR2J/RBM14/APBB1/TEX11/PPHLN1/ACTR5/RFC5

2/PRMT7/ZBTB7B/BRD8/WDR70/TRIP12/HLTF/BRDT/MSL1/KMT2A/HMG20B/CPA4/JARID2/PCGF1/
PP30/SUV39H1/LSM1/SFSWAP/POLR2E/SAFB2/PRKRA/PELP1/RPS9/CCAR1/WDR46/IMP4/KHSRP/S
MRPL21/EIF3C/DDX1/EIF1/RPS15A/ATXN2/NDUFA7/AARS2/MRPS26/C1QBP/MRPS11/ZC3H15/RPL
/TMBIM1/SLC15A4/ANXA3/HLA-F/AP2A2/PCYOX1L/RAB11A/ATP6AP2/SLC2A3/TSPAN14/MLEC/PS

0/MORC2/THOC1/DCLRE1C/PAGR1/FBXO6/ERCC3/CIB1/MMS22L/STUB1/BAG6/HUWE1/TRRAP/METT

OR/ATAD5/CALM3/PAGR1/ERCC3/PKD1/CSNK2A2/BORA/ACTR1A/OFD1/ANAPC4/VPS4A/YTHDF2

ITM3/RPL32/TNPO1/COPB1/RPL30/CRTC2/BECN1/SNAPIN/RBM15B/EPHA2/AP2A1/TBP/IST1/KPN
/GTF2F1/DHX38/RPS27/ERCC3/EXOSC2/PABPC1L/ZC3HAV1/DBR1/RPL29/GEMIN2/LARP1/PABPN1

D/CCR10/CIB1/IL17RB/PTPRC/YTHDF2/CRLF2/IP6K2/CXCL10/METTL3/IFITM2/VRK2/PPARG/PI

RL11/DMTN/PRMT6/DYRK3/CD4/HYAL2/IFNG/ANXA1/EIF2AK1/ARIH2/PSMB7/CCR2/KMT2B/FZD9/

/FAF2/SNRNP70/TMEM67/DDB1/FBXO44/SKP2/PSMB1/WWTR1/SUMO2/TNFRSF1B/PIN1/USP3/FBXO

DPYSL4/KIF3A/FSTL4/NCKAP1/PLCG1/MOV10/PPP1R9A/LPAR1/CNP/NCAM1/UCN/TRIM32/VAPA/

AP/BCOR/UBE2E3/CAND1/TRIM62/NUB1/MED20/RNF121/WWP2/ARIH2/RELA/RNF126/PSMB7/RNF11
/VPS11/CD38/YWHAG/ATG13/ABHD17A/CLTC/B9D1/SIX1/IL17RA/ANP32B/AP2B1/TNFRSF14/DHX9
AC2/PEX10/NPHP4/RPS9/HAX1/EGF/ATR/ARL1/RPS14/CALM3/DZIP1/GET4/TAF3/RPS27/CSNK2A2
D3/ADD3/UBTF/NDUFB3/CSNK2B/MIS18A/PSMG1/UBA52/PIH1D1/EML2/MECP2/TARBP2/RBMX/CENP
IASE1/CNOT2/ADPGK/RPL12/RPS18/ENTPD4/RPS28/ELAVL1/FASTKD1/RPS17/NUDT3/PNPT1/SERB
3/NSUN2/WDR43/GATC/DIS3L2/NSA2/RPRD2/CLP1/MRPS9/TSR2/INTS5/METTL8/DDX18/SAGE1/GE

1/PAIP1/RRBP1/EIF2B3/RPL22/GEMIN5/MRPL21/EIF3C/DDX1/EIF1/RPS15A/ATXN2/NDUFA7/AAR

7/HAX1/CAV1/LDB1/ATP7A/ATR/DAB2IP/PARP10/CAMSAP1/PREX1/DZIP1/EPHB2/ARFGEF1/ERCC3

S1/AP1S3/POLR2D/TAP2/AP1S2/RPL37A/AP2B1/RPLP1/DOCK2/TNFRSF14/DHX9/IFITM3/RPL32/
CR6/PPIE/POLR2I/PPIB/TLN1/RPS11/POLR2J/DAXX/RHOA/RPL13A/HMGN2/AP1S1/AP1S3/POLR2

/ANAPC15/PSMB7/CCNJ/PPP1R12A/CNOT2/SKP1/AKAP8

D84/MAP4K2/DAB2IP/TRIM68/TMEM106A/ZNF683/ZC3HAV1/NECTIN2/YTHDF2/CLEC4E/SRPK2/ZCC

D/ZNF16/ADNP/ADD1/CNR1/PWP1/PPARG/JUNB/WWTR1/ACIN1/PLXNB2/TNFRSF1B/PIN1

PO7/SESN2/MAP3K9/NOD1/DUSP14/RGS4/MAP4K4/MAP3K11/CHMP6/PPP2R3C/ACE/MUL1/WNK1/TS

/NCOA6/SH3RF1/RTKN2/DPY30/PSMA3/ANKRD54/IRF4/RHOH/AGO1/MYC/BGLAP/ARL11/DMTN/PRMT

A/CARMIL2/ACE/TRAK2/PLXNB3/LIG3/MUL1/TSC1/XRCC5/ANKRD13A/SLN/IKBKB/CPTP/LILRB1/T

A2B/UBE2N/ADCY5/KITLG/KSR1/EREG/CCL19/PIK3R6/IRAK2/GMFB/DUSP18/CACUL1/HTT/EGF/MA

6/CHMP6/SPICE1/TRAK2/ESYT1/SYP/MUL1/EXOC6/CADPS2/MYO1A/ACTR2/GEM/UNC13A/TTL/BICD

2A2/CAV1/CD84/CALM3/EPHB2/CIB1/CSNK2A2/ENY2/PARL/VPS4A/PLD1/HUWE1/TM9SF4/HDAC8/C

D/RAB1B/GLRB/VPS8/MX1/ABCA7/CNP/STAM/VAPA/CCDC88A/TBC1D20/ATP8B3/THEM4/NOX1/SYT1

NF114/SNX3/KLHL12/PEF1/CAV1/UBAC1/NEDD8/PCGF3/TRIM68/PARP10/FBXO6/ENY2/FBXO41/ST
/TCHP/DYNC2LI1/CEP97/RAB3IP/NPHP1/ARL3/CELSR3/TNNT2/WDR45/MAPK15/HAUS2/CNOT2/RPL

PN/CEP41/CCND3/PSMB5/PSMA4/BOD1/HSP90AA1/ACVR1B/PSMD9/DNM2/ADAM17/CDK2AP2/CCSAP/

P1R9A/LPAR1/STK4/MAP3K7/UCN/CASP3/CCDC88A/ADAR/COPS8/NAB2/TRIM27/MADD/NEURL1/AN

/RBM14/CD79B/SIGLEC9/NMI/CD38/RAPGEF1/STXBP1/MAP3K1/LGMN/TNFRSF14/DHX9/NR1H2/ID

/FBXL18/TSG101/FBXL2/CDC16/KAT5/UBE2R2/RAD23A/SVIP/PPP1R11/DVL1/DERL3/CLEC16A/RN

.HL25/HNRNPD/RPS24/MRPL3/LSM14B/NEURL1/EIF4E2/ERAL1/PARK7/PYM1/EIF3F/RPS3A/EIF2B
)C16/MIB2/UBE2R2/SUZ12/PPP1R11/RNF214/KCTD9/RNF41/TICAM1/UBE2J1/ARRB1/PSMD12/BFA

.DAMTS7/ZFAND2B/USP13/UBE2K/PSME2

'UBE2N/KITLG/KSR1/EREG/CCL19/SEH1L/XCL1/MRAP2/NOTCH2/TLR7/SERPINA12/PIK3R6/CCL3L

[illegible]3R6/CCL3L3/SH3RF3/NPNT/IRAK2/CRLF1/OSM/CACUL1/CCL8/CAV2/HAX1/EGF/MAP4K1/CAV1/MA[illegible]

/DUSP22/BORA/PTPRC/BMP2/ADCY3/PROM2/GDF10/ADNP/RYK/PIK3CB/NENF/PIN1/ADCY7/PPP1R

'CD22/CNPY3/MFHAS1/PLA2G6/ICAM2/PSMA3/PARK7/IRF4/HLA-C/PDE4B/POLR3F/EIF2B1/CTSL/

SLC9A6/CD3G/PTPRC/SLC9A9/RAB1A/RALBP1/TM9SF4/SNX5/PROM2/ATP1B1/PPARG/PIK3CB/MYO1

[illegible]/BMP8B/STRAP/POLR2E/ARID5B/NPNT/NEO1/CAV2/EGF/CAV1/DAB2IP/ATP6V1B2/GTF2F1/EPHB[illegible]

[illegible]/CASP3/CHD7/ABCF3/ADAR/FGF19/BCR

[illegible]/CASP3/CHD7/ABCF3/ADAR/FGF19/BCR

L1/SKP2/JUNB/HNRNPA0/ATP2A2/TNFRSF1B/CD180/TLR8/MX1/CNP/CASP3/CHD7/ABCF3/ADAR/[illegible]
/PYM1/GART/EIF3F/RPS3A/EIF2B1/AURKAIP1/MRPL20/AGO1/ABCF1/RPL14/TMA7/PPA1/MRPL24

/PPP1R11/PDX1/DERL3/NLRP3/UBE2J1/JAGN1/WNT5A/C5AR2/CD40/ARRB1/PPP3CB/ARHGAP44/PA

/EIF6/EIF4G2/UBAC2/ATG7/SNX3/HTT/KHSRP/HAX1/EGF/CAV1/DAB2IP/RPTOR/ATP6V1B2/CRTC

IB1/SUMO2/TNFRSF1B/RAB1B/MOV10/MAP3K7/PSMB6/CASP3/VPS26A/GTPBP1/LRPPRC/RPL11/HNR

/NOL6/CAPN2/HNRNPU/RNF2/ANP32E/RAD18/LLPH/SYCE1L/ERCC1/EXOSC9/EXOSC10/ADD3/CSNK2
11/EXOSC1/RBM14/DAXX/SF3B3/RPL13A/SIX1/DHX15/ZNF300/TOE1/TGS1/PNMA3/WDR75/ANP32

L/VASN/TLR7/RAB9A/NAPG/ATP11A/HTT/EGF/PI4K2A/DPP7/DAGLB/RPTOR/ATP6V1B2

12A/SKP1/LRIF1/CCDC78/WHRN/CDK5RAP2/CCDC8/TUBD1/CCDC96/MAP3K11/UBR4/CUL7/PPP2R3

D1/SNX5/SLCO4C1/TMEM67/C2CD5/MYO1C/ATP2A2/TNFRSF1B/RAB8B/SPIRE2/CD59/VAPA/ATP8B

N5/MAML1/CLK3/TP53/DDX1/TARDBP/TDP2/PIP5K1A/ASCC3/MTDH/TEPSIN/SUZ12/APEX1/PDX1/

/LY6K/CTSW/BRI3/HEXA/SNAPIN/TMEM184A
PIRE2/CD59/VAPA/ATP8B3/GPR161/NCSTN/SLC30A5/SYT11/HLA-C/SNX24/MCTP2/RNF144A/SUR
LE3/RPA1/PSEN2/PMF1/TBP/IST1/SEH1L/SMARCAL1/UXT/IPO4/SUV39H1/MSH6/SAP130/PELP1/
ER3/COG4/GPC2/ATG9A/COPB1/BECN1/PSEN2/SNAPIN/AP2A1/LRPAP1/COG2/TMEM167A/NOTCH2/

EC23B/DOCK6/SYP/TSC1/PLEKHG5/SHTN1/EXOC6/ANKRD13A/RAC3/USP33

/ARL1/B3GALT6/HS6ST2/CBY1/ARFGEF1/PKD1/LITAF/LMAN2L/CYTH3/HS2ST1/ST8SIA4/RAB1A/

R3/VDAC2/VEGFA/GNAI3/GLIPR1/RAB3A/HEXB/ACTN1/CYB5R1/PLA2G1B/TLR2/CSNK2B/COPS5/T

/SNRNP25/C12orf65/MRPL4/EBNA1BP2/LUC7L/RBM24/EIF6/UTP23/SF3B2/CNOT3/RPP30/LSM1/S

3A/TLR7/ARF1/STEAP2/BET1L/RAB9A/IRAK2/PDIA3/ATP11A/SNX3/YIPF1/HTT/SAMD9L/PI4K2A
1/USP33/SETD1A/IKBKB/SUPT3H/FBXL18/ZNRD1/TP53/TBPL1/PHF20/SPTLC2/TAF11/FBXL2/CD

KHG5/CADPS2/SORCS2/ACTR2/RPL22/UNC13A/COL13A1/GRIPAP1/ELK1/NEFH/BCL11A/APH1A/CD

:PL31

I1/KIF22/CDK5R1/ARL8B/TNNC1/TLN1/WIPF2/APBB1/CACNA1D/DSTN/PLEKHH2/RHOA/NEK6

5B/DHX38/ERCC3/KLC1/VPS4A/RAB1A/RALBP1/RAB36/DDX52/DHX8/DHX30/ATP1B1/GNA13/MYO1C
.1/KIF26B/DHX38/ERCC3/KLC1/VPS4A/RAB1A/RALBP1/RAB36/DDX52/DHX8/DHX30/ATP1B1/GNA1
.1/KIF26B/DHX38/ERCC3/KLC1/VPS4A/RAB1A/RALBP1/RAB36/DDX52/DHX8/DHX30/ATP1B1/GNA1
3/DHX30/ATP1B1/GNA13/MYO1C/AK6/ATP2A2/ACIN1/RAB8B/PIN1/KIF3A/RAB1B/MOV10/MX1/ABC
CNJ/PRAM1/PPP1R12A/NELL2/PRKCB/CDK5RAP2/PPP1CB/RHOD/ELAVL1/PIK3R2/MAP3K11/RPS6K
./SMCR8/C1QBP/DVL1/PARN/MARVELD3/LRP4/RNF41/TICAM1/ARRB1/PARD6A/TPR/GATA4/NFATC1

5/DGKQ/PPP2R5B/CDC37/CDK5R1/ARHGAP28/ADPRH/PRKAR1B/AGAP3/RUNDC3A/DAXX/STK3/GLMN/

2/MAPK15/FN3KRP/TERF1/CCNJ/TRIO/CLK2/POLR1C/ADPGK/POLR3A/MAP3K9/PRKCB/MAP4K4/PR

[illegible]PL1/STAT4/KDM3B/ETV3/SUZ12/APEX1/PDX1/SETMAR/ZBP1/NACC2/MEN1/SP1/ZNF516/ZNF704/

2/MZT1/ANKLE2/RAD51C/BTG2/PSMD8/ACVR1/TAF10/PIDD1/DNA2/HSPA2/PKIA/ABL1/EMD/TBCD/
.3/RYK/PIK3CB/ANTXR1/PLXNB2/DPYSL4/SCARA3/KIF3A/FSTL4/ARHGEF18/NCKAP1/PLCG1/MOV1
P1L2/CD80/DDX1/RASGRP1/LILRB2/MAPKBP1/NRROS/KAT5/C1QBP/RFTN1/PPP1R11/OTUD5/ZBP1/
5/CD274/STAT3/PCBP2

)7/ADAR/CLPTM1/SMAD5/BCR/ICOS/MFHAS1/NCOA6/SH3RF1/RTKN2/DPY30/PSMA3/ANKRD54/IRF4

K2/PTPN2/THBS1/ADAM17/CASP8/PDCD2/RPS27A/CD177/HOXA5/MAVS/DMBT1/VEGFA/SMAP1/PLA2

5/RNF8/POLR2D/GATAD2A/GPS1/HINFP/DNMT3B/DHX9/MAPKAPK5/DNAJC2/GTF2H1/DMAP1/TIGAR/

/SMCHD1/BRCC3/GTF3C4/PHC1
SPOUT1/MFAP1/TRMT2B/RBFA/PRPF4/THOC1/RPS14/GTF2F1/DHX38/RPS27/ERCC3/EXOSC2/PABPC
L31/MRPL42/TRMT10C/TPR/PASK/LSM14A/MRPL22/NOA1/MRPL58/METTL16/EIF4ENIF1/ZC3H12D/
SMC3/PPIE/TRPV6/TLN1/AGA/PRSS12/ADGRG3/EXOC3/CACNA1D/SIGLEC9

CL3/DDB1/TFPT/EXO5/TNFRSF1B/TFIP11/SPIRE2/USP3/POLG2/TRIM32/CASP3/COPS8/NCOA6/TC

NA6/CTBP1/SEH1L/CD247/SF3B2/NELFA/ARF1/SUV39H1/POLR2E/MSH6/PRKRA/ATG7/RPS9/SNX3/
./YTHDF2/SRSF1/SRSF2/SRPK2/PAF1/HNRNPUL1/CASC3/SNRNP70/COIL/FIP1L1/RBM4B/DHX8/RP

IK3CB/JUNB/CCR3/P4HB/PSMB1/TNFRSF1B/MX1/IL32/NCAM1/MAP3K7/TRIM32/PSMB6/CASP3/ADA

/DOCK11/CD19/POU2F2/GON4L/PRKCB/ERCC2/PPP2R3C/ACE/ZBTB24/ESRRA/TSC1/XRCC5/SLC7A6

)10/TRIM32/PSMB6/RPL11/ENC1/CDC23/KLHL25/MVB12B/RNF149/PSMA3/PARK7/BUB3/RNF144A/

RPL24/CASP3/CCDC88A

.5/SKP1/ANKIB1/MED30/MKRN1/ATG3/SUMO3/RNF186/DERL1/KCTD13/SENP5/UBR4/CUL7/RNF19A
)/GPC2/RSL1D1/DMAP1/STXBP4/EPHA2/ADCY5/CORO2B/CCL19/DPH3/MRAP2/CD247/ARF1/TFDP1/
2/NUDT19/PARL/RPL29/TIMM23/VPS4A/BAG6/TIMM8B/HUWE1/PAF1/PITRM1/SRP68/HDAC8/TRAM1
PJ/DGKQ/CDK5R1/ZRSR2/ARHGAP28/PSMC3/BAZ2A/TIMMDC1/NDUFA13/EIF3E/DAXX/NSUN4/RHOA/
3P1/JMJD8/NUP214/PPP2CA/TNFSF13/PAIP1/RPL22/ERI1/PDE5A/TP53/LSM3/TARDBP/RPS15A/S
EMIN4/NCBP1/TSEN2/TRPT1

RS2/MRPS26/C1QBP/MRPS11/ZC3H15/RPL31/MRPL42/TRMT10C/TPR/PASK/LSM14A/MRPL22/NOA1/

3/DUSP22/STUB1/SACS/VPS4A/PLD1/KIAA1324/VPS16/EVI5L/BIK/ADNP/ADD1/FEZ2/ASAP3/DDB

/TNPO1/COPB1/RPL30/SLC22A5/CRTC2/BECN1/SNAPIN/RBM15B/EPHA2/AP2A1/TBP/IST1/KPNA6/
2D/TAP2/AP1S2/RPL37A/AP2B1/RPLP1/DOCK2/TNFRSF14/DHX9/IFITM3/RPL32/TNPO1/COPB1/RP

CHC3/IP6K2/METTL3/IFITM2/PPARG/NFKBIL1/SKP2/MYO1C/PSMB1/SCARA3/CD180/TLR8/MX1/NO

SC1/NOD2/PPP2CA/XRCC5/CARD14/RASSF2/MAP3K12/PDE5A/AFAP1L2/TSG101/STRADA/RASGRP1/

C6/DYRK3/CD4/HYAL2/IFNG/ANXA1/EIF2AK1/ARIH2/FOXL1/PSMB7/CCR2/KMT2B/FZD9/DOCK11/C

TP53/BCL11A/LILRB2/FEZ1/ATXN2/ARPIN/CHMP4A/PSMG2/SMCR8/SVIP/CLEC16A/LRP4/SETMAR/

AP4K1/CAV1/LDB1/MAP4K2/DAB2IP/HMGA2/RPTOR/CALM3/EPHB2/CIB1/PKD1/DUSP22/BORA/PTPR

D2/RAB6A/GRIPAP1/NEFH/RASGRP1/FEZ1/CEP135/NEFL/MYH9/CHMP4A/TFEB

CXCL10/C2CD5/CNR1/CEP72/MYO1C/ATP2A2/PIN1/PLCG1/PPP1R9A/CASK/KCNMB4/YIPF5/ABCA7/

.1/TMCC1/TIMM9/TIMM22/IGF2R/TSNARE1/RFT1/NMT1/VAMP3/KIF5B/CD4/ARHGAP25/HYAL2/ANX

UB1/ANAPC4/TADA3/RAB1A/HUWE1/PAF1/TRRAP/HDAC8/OTUD3/DDB1/FBXO44/SKP2/TFPT/PSMB1
12/MRPS2/IQCG/CCDC78/ATG3/CDK5RAP2/RPS28/PTPRS/KIF9/RAB7A/CHMP6/SPICE1/HDAC3/P

DCTN1/CCNJL/CEP164/HAUS7/NBN/FBXW5/PLK3/HNRNPU/ZFYVE19/OPTN/CCNE2/HAUS5/MASTL/

[illegible]KRD54/PARK7/RHOH/PDE6G/CCNG2/SLC27A1/MLLT1/CD4/HYAL2/JTB/IFNG/CCNJ/IPO7/ZNF622/[illegible]

[illegible]O1/ELMO2/PSMC5/ADORA2B/UBE2N/EREG/CCL19/FAM3A/XCL1/CD247/TLR7/HSP90B1/PIK3R6/I[illegible]

NF41/UBE2J1/LRP2/ARRB1/PSMD12/BFAR/ARMC8/SMURF2/RNF40/OGT/MAPK9/CDC26/SOCS5/USP4

31/AURKAIP1/MRPL20/AGO1/ABCF1/RPL14/TMA7/PPA1/MRPL24/MRPS16/EIF5B/RPL10A/FXR1/II

AR/SMURF2/RNF40/PHF23/OGT/ASB2/MAPK9/LZTR1/CDC26/SOCS5/WDR70/TRIP12/TRIM44/HLTF/

L3/SH3RF3/NPNT/PRKRA/IRAK2/CRLF1/OSM/HTT/CCL8/CAV2/HAX1/EGF/MAP4K1/CAV1/ATR/MAP4

AP4K2/DAB2IP/HMGA2/RPTOR/CALM3/CIB1/TMEM106A/PKD1/DUSP22/BORA/PTPRC/BMP2/ADCY3/Z

R9A/LPAR1/ABCA7/STK4/MAP3K7/UCN/CCDC88A/FGF19/MFHAS1/COPS8/NAB2/SH3RF1/PLA2G6/PI

/CD4/IFNG/CTSB/ANXA1/RELA/AIM2/PSMB7/CCR2/KLHL6/PRAM1/SKP1/HLA-E/EIF2B2/NOD1/CD1

.C/LOXL3/ATP9A/SCARA3/SSC4D/NCKAP1/PLCG1/CSNK1G1

2/SLC9A6/DUSP22/CNKSR1/EPHA10/STUB1/BMP2/SNX5/GDF10/C2CD5/RYK/PIK3CB/MYO1C/BDKRE

ˈGF19/BCR
ˈ/MRPS16/EIF5B/RPL10A/FXR1/MRPL55/EIF2AK1/SYNCRIP/CHCHD1/EIF4G1/CNOT2/RPL12/EIF2

ARD6A

C3/PGAM1/PKD1/EXOSC2/CSNK2A2/ZC3HAV1/STUB1/LARP1/YTHDF2/ATRAID/BAG6/KIAA1324/BBS

RNPD/PIP4K2B/PSMA3/AAAS/PARK7/DCP2/HNRNPC/RNF144A/USP36/NUB1/UBQLN2/ULK1/PAM16/[illegible]

2B/COPS5/MIS18A/PHF6/THOC2/MECP2/RBMX/KIF22/INTS7/BAZ2A/USP11/NCAPD3/EIF3E/SRF/
2B/TSEN34/DHX9/FAM207A/SMARCA5/RBM28/BMS1/NOL10/RUNX3/RSL1D1/RNMT/ZNF415/TMUB1/

3C/RNF19A/SPICE1/USP33/IFT140/BICD2/CEP95/TSG101/CNTROB/FEZ1/CEP135/CDC16/RAB28/

33/GPR161/NCSTN/SLC30A5/SYT11/HLA-C/SNX24/MCTP2/RNF144A/SURF4/IGF2R/VAMP3/RAB22

/BAZ1B/TMEM179B/NACC2/SRSF10/ARRB1/MAML2/NUFIP2/SMURF2/SRSF7/GATA4/NFATC1/PRPF4

RF4/IGF2R/VAMP3/RAB22A/RAB3C/MOSPD2/SPARC/DGAT1/CD4/ANXA1/CD207/ATP6V0A1/VAMP1/
/SMC2/MORF4L1/POLE2/EP400/LDB1/ATR/TP53BP1/SPOUT1/SRCAP/HMGA2/ATAD5/MORC2/THOC1/
/TLR7/TMEM167B/ARF1/STEAP2/BET1L/COPA/RAB9A/RAB40C/ATP11A/KLHL12/YIPF1/CAV2/PEF

/PLD1/PLEKHA8/RAB36/GCNT1/KIAA1324/B3GNT8/CHAC1/ERGIC2/RHBDD2/ATP9A/QSOX2/ACBD3/

MBIM1/SLC17A5/SLC15A4/ANXA3/BCL2L1/AP2A2/PCYOX1L/ATP6AP2/SLC2A3/TSPAN14/MLEC/P

SURF6/RPS9/WDR46/SND1/IMP4/KHSRP/HAX1/EIF3G/MFAP1/RPTOR/PRPF4/RPS14/MIR155HG/AR

A/CAV1/RAB13/ATP7A/SLC9A6/LITAF/RPS6KC1/VPS4A/SLC9A9/RAB1A/PLD1/TM9SF4/KIAA1324/
DC16/GTF2E1/PIGQ/PRKAR2A/MIB2/KAT5/SMCR8/SUZ12/MSL3/DERL3/TAF4/POLD2/MEN1/ARMC8/

)H9/NEFL

C/AK6/ATP2A2/ACIN1/RAB8B/PIN1/KIF3A/RAB1B/MOV10/MX1/ABCA7/GPN1/CHD7/ABCF3/GTPBP1

13/MYO1C/AK6/ATP2A2/ACIN1/RAB8B/PIN1/KIF3A/RAB1B/MOV10/MX1/ABCA7/GPN1/CHD7/ABCF3

13/MYO1C/AK6/ATP2A2/ACIN1/RAB8B/PIN1/KIF3A/RAB1B/MOV10/MX1/ABCA7/GPN1/CHD7/ABCF3

CA7/GPN1/CHD7/ABCF3/GTPBP1/ATP2C1/DDX31/SNRNP200/DDX20/ATP2B1/RHOH/MYO1D/KIF5B/M

KA3/PTPN14/ACE/WNK1/NOD2/SMAD3/MYCN/RAC3/IKBKB/MAML1/MAP3K12/TP53/STRADA/NEFH/BC

1/NISCH/MAPRE2/ROR2/EXOC2/TAOK2/SOCS5/TAB1

/GSKIP/YWHAG/ATG13/ATP2B4/PRKAG1/PIK3R5/GPS1/ACAP3/STXBP5/SOCS7/RPLP1/DOCK2/FAF1

RPS1/ERCC2/FASTKD1/MAP3K11/RPS6KA3/PNPT1

/PURB/GATA4/NFATC1/MAFK/LSM14A/ZBTB7B/NFXL1/PRDM1/HOXC10/RBMS1/STOX1/TGIF1/HLTF/

/VCP/PSMA1/PIM3/MTA3/NSFL1C/DCLRE1B/EME2/CHEK2/CLSPN/CEP41/CCND3/PSMB5/TTYH1/PSM
10/PPP1R9A/ASXL1/LPAR1/STK4/CNP/NCAM1/PXN/RPL24/CASP3/TBC1D20
/NLRP3/TICAM1/UBE2J1/WNT5A/C5AR2/CD40/ARRB1/CLECL1/IL12A/GATA4/NFATC1/WNT3A/LSM1

1/RHOH/UNG/AGO1/MYC/BGLAP/ARL11/DMTN/PRMT6/DYRK3/CD4/HYAL2/IFNG/ANXA1/EIF2AK1/AI

2G1B/PAK2/TLR2/TMEM64/MUC6/UBA52/HLA-F/RAB29/MUC15/CACNB3/NKAP/PLD2/PSMC3/UBE2D2

/FAF1/POLE3/RPA1/UBE2N/EREG/MMS19/CDC34/SMARCAL1/MUS81/TFDP1/POLR2E/SMUG1/MSH6/C

C1L/DBR1/GEMIN2/PABPN1/SRSF1/CDK5RAP1/SRSF2/SRPK2/PAF1/DDX52/HNRNPUL1/CASC3/SNRN
/MRPL38/MRPS30/GSPT1/AIMP1/STAT3/MRPS27

CEA1/PHF1/MGME1/PARK7/UNG/ING2/SHISA5/MYC/SLFN11/AP5S1/HUS1/POLR2B/PRMT6/FIGNL1/

/SND1/CCL8/CAV2/CAV1/EIF3G/SUPT4H1/SRCAP/HMGA2/RPTOR/PARP10/MORC2/NELFCD/RPS14/C
PL13/RBM8A/METTL3/CSTF3/SF3B6/MAGOHB/SNRPD3/PRPF38B/HNRNPA0/PSMB1/ZNF473/ACIN1/T

AR

5OS/RPL22/SETD1A/RASSF2/LILRB1/FUT7/GNAS/CD80/TP53/SOX13/PLD4/RASGRP1/FGL2/LILRI

/SDF2/USP36/NUB1/UBQLN2/RNF121/CTSL/WWP2/HTRA2/IFNG/CTSB/ARIH2/RELA/RNF126/TLK2/

A/NUP214/MUL1/TRIM56/NOD2/HDAC3/TULP4/RNF10/RNF135/FBXL18/TSG101/BCL11A/FBXL2/CI
/UBAC2/RAB9A/OSM/SNX3/HAX1/EGF/FRMD4A/DNAJC1/ENSA/EPHB2/CIB1/IL17RB/PANX2/CSNK2A
1/RPL13/SNX16/CEP72/RAB8B/PIN1/SUPT7L/RPL4/RPL41/ABCA7/IK/RPL9/RPS6/TNPO3/VAPA/I
/RPL13A/VPS11/CLTC/STXBP1/TAPBP/COA1/POLR2D/SART3/TGS1/MTERF3/MRPL11/ANP32B/AP2I
SMG8

/MRPL58/METTL16/EIF4ENIF1/ZC3H12D/MRPL38/MRPS30/GSPT1/AIMP1/STAT3/MRPS27

31/MYO1C/ANTXR1/TFIP11/SPIRE2/RAB1B/NCKAP1/PPP1R9A/LPAR1/ADGRL1/PXN/STAM/PLEKHM1

/CTBP1/SEH1L/CD247/SF3B2/NELFA/ARF1/SUV39H1/POLR2E/RAB9A/MSH6/PRKRA/ATG7/RPS9/SN
PL30/SLC22A5/CRTC2/BECN1/SNAPIN/RBM15B/EPHA2/AP2A1/TBP/IST1/KPNA6/CTBP1/SEH1L/CI

CAM1/MAP3K7/TRIM32/PSMB6/ADAR/FFAR2/CNPY3/MFHAS1/TRIM27/CAPZA2/ICAM2/PSMA3/IRF4/

/TNFRSF10A/SMG8/PRKAR2A/MAPKAP1/SMCR8/DUSP26/DAG1

CD19/POU2F2/GON4L/PRKCB/ERCC2/ZMPSTE24/PPP2R3C/ACE/ZBTB24/ESRRA/TSC1/XRCC5/SLC7A

/RNF41/TAOK1/SLX4

RC/STUB1/BMP2/PABPN1/ADCY3/RAB1A/CDK5RAP1/ZNF16/CXCL10/ADNP/PPARG/PIK3CB/WWTR1/I

/KCNC4/TMEM38A/CCDC88A/CHD7/TBC1D20/EFCAB7/MFHAS1/EPB41/PLA2G6/RBM22/SYT11/AAAS/

KA1/VPS33B/MAPK15/VAMP1/NKD2/TFG/FZD9/PRKCB/CNIH2/RAB7A/CNEP1R1/ZMPSTE24/VAV3/CI

1/WWTR1/SUMO2/PIN1/ASXL1/WDSUB1/USP3/FBXO10/KCMF1/MAP3K7/TRIM32/STAM/PSMB6/BTBD1
TPDC1/SMC1A/ACTR2/IFT140/TSG101/CNTROB/LSM3/TBPL1/FEZ1/CEP135/TMEM80/ATXN2/CHMP4

LZTS1/BCL2L1/DYNC1I2/RAB11A/ZPR1/GPR132/CRLF3/NEDD1/KIF20B/MECP2/CENPJ/MITD1/KIF

/SESN2/MAP3K9/NOD1/CD19/DUSP14/RGS4/MAP4K4/PIK3R2/MAP3K11/VAV3/CHMP6/PPP2R3C/AC[illegible]

[illegible]AK2/ITGAL/SERPINB9/IL13RA2/CAV1/TP53BP1/CLEC2B/CD84/DAB2IP/ATAD5/THOC1/INPP5D/[illegible]

46/TRIP12/KLHL22/ANAPC1/PCBP2/DNAJC18/CCDC115/UBXN2A/DACT1/ADAMTS7/ZFAND2B/USP13

FNG/MRPL55/EIF2AK1/SYNCRIP/CHCHD1/EIF4G1/CNOT2/CTNS/RPL12/EIF2B2/MRPS2/SESN2/RPS
/KLHL22/ANAPC1/UBXN2A/ASB16/PCGF1/UBE2K/PSME2/PHC1/FANCL/TRIM5/TRIM3/TRIM36/ZC3H

4K2/DAB2IP/RPTOR/CALM3/ARFGEF1/CIB1/TMEM106A/DUSP22

[illegible]NF16/PROM2/GDF10/ADNP/RYK/PIK3CB/NENF/PIN1/ADCY7/PPP1R9A/LPAR1/ABCA7/STK4/MAP3[illegible]

LA2G2A/MADD/NOX1/NEURL1

[illegible]9/TRIM15/PRKCB/NLRC4/BPIFB1/PTPRS/PIK3R2/CLEC4A/VAV3/APPL2/RPS6KA3/SLA2/TLR1/S[illegible]

32/WWTR1/PIN1/FSTL4/ARHGEF18/NCKAP1/PLCG1

2B2/MRPS2/SESN2/RPS18/RPS28/ELAVL1/GGT6/RPS17/RPS6KA3/NOLC1/ABTB1/RPL26L1/TSC1/[illegible]

37/CST3/SNX5/SNRNP70/FEZ2/CNR1/METTL3/DDB1/PIK3CB/HNRNPA0/PSMB1/SUMO2/TNFRSF1B/F

FXR1/HTRA2/IFNG/ARIH2/SYNCRIP/TLK2/MAPK15/ATP6V0A1/EIF4G1/PSMB7/NKD2/TBC1D25/AN[illegible]

DAXX/TEX11/PPHLN1/HMGN2/ACTR5/RFC5/RNF8/GATAD2A/DHX9/SMARCA5/STAG3L4/RUNX3/DMAP1
NIN/RBPJ/SPATA24/DDX55/MTX2/TAF13/UBE2N/DIMT1/EBNA1BP2/DEDD/TSEN15/NOL9/EIF6/UTP

/PRKAR2A/HARBI1/RILPL2/MACROD2/RAD23A/HYLS1/APEX1/PATJ/ATP6V1D/GNAI2/ACTR8/SCYL1

A/RAB3C/MOSPD2/SPARC/DGAT1/CD4/ANXA1/CD207/ATP6V0A1/VAMP1/PDE6D/ARCN1/HLA-E/CHGA

DB/CACTIN/RSRC1/EIF4ENIF1/BCAS2/HOXC10/CRTC1/TAB1/DDX46/TRIP12/DUSP11/FOXC2/THAP

PDE6D/ARCN1/HLA-E/CHGA/SAYSD1/ARL8A/DNAJC13/SEMA4C/CNIH2/MMP25/PTPRS/RAB7A/UBR4/
/DCLRE1C/CSNK2A2/MMS22L/SIN3B/LIN37/PAF1/TRRAP/DDB1/JUNB/TFPT/TRPS1/TFIP11/ASXL1
1/LMAN2/PI4K2A/CAV1/RAB13/ATP7A/MAP4K2/ARL1/B3GALT6/HS6ST2/CBY1/ARFGEF1/PKD1/LIT

/SCARA3/CD59/RAB1B/IER3IP1/TLR8/AP4B1/ABCA7/NCAM1/VAPA/TBC1D20/ST3GAL2

SMC3/PPIE/PLA1A/AGA/ADGRG3/EXOC3/SIGLEC9/RHOA/CD38/STXBP1/ACTN4/STXBP5/RAB27B/DC

FGEF1/DHX38/RPS27/MRPS24/PABPC1L/RPL29/EIF2D/GEMIN2/MRPL33/LARP1/PABPN1/YTHDF2/S

/SNX5/SDF4/VPS16/CD1A/SNX16/BDKRB2/ANTXR1/ATP9A/RAB8B/NIPA2/VPS8/TLR8/AP4B1/LPA[illegible]
/SMURF2/TAF2/RNF40/NAA11/EZH1/OGT/KANSL2/SPTSSA/DPM1/ASB2/LZTR1/CDC26/BRD8/SOCS[illegible]

l/ATP2C1/DDX31/SNRNP200/DDX20/ATP2B1/DCP2/RHOH/MYO1D/KIF5B/MYO18B/RAB22A/SLFN11/

3/GTPBP1/ATP2C1/DDX31/SNRNP200/DDX20/ATP2B1/DCP2/RHOH/MYO1D/KIF5B/MYO18B/RAB22A/

3/GTPBP1/ATP2C1/DDX31/SNRNP200/DDX20/ATP2B1/DCP2/RHOH/MYO1D/KIF5B/MYO18B/RAB22A/

MYO18B/RAB22A/SLFN11/ABCF1/RAB3C/DDX51/DYNC2LI1/FIGNL1/ARL3/TNNT2/EIF5B

CL11A/FEZ1/PIP5K1A/SH2B1/PRKAR2A/MAPKAP1/SMCR8/C1QBP/RAD23A/DVL1/PARN/MARVELD3

l/CCAR2/SH3BP5L/PPP1R7/SERPINB8/SGSM1/GCLM/SERPINA12/ARF1/PIK3R6/RANBP3/AUP1/PRI

/STAT3/DLX6/PSIP1/FOXC2/KMT2A/JARID2/XRN2/ZW10/TFCP2/HOXC6/MYB/MZF1/SREBF2/ZBTB4

MA4/BOD1/HSP90AA1/ACVR1B/PSMD9/DNM2/ADAM17/CDK2AP2/CCSAP/DCTN1/CCNJL/CEP164/HAU

14A/ZBTB7B/CACTIN/SLAMF1/PDCD1LG2/SOCS5/CD274

RIH2/FOXL1/PSMB7/CCR2/HLA-E/KMT2B/FZD9/SUPT6H/DOCK11/CD19/POU2F2/GON4L/PRKCB/ER

2/STOML2/WIPF2/RBM14/CD79B/RHOA/CD38/AP3B1/RAPGEF1/STXBP1/MAP3K1/LGMN/TNFRSF14/

GLRX2/ATG7/MORF4L1/RNASEH1/POLE2/ATR/TP53BP1/HMGA2/ATAD5/PARP10/MORC2/GRWD1/THO

NP70/COIL/FIP1L1/RBM4B/DHX8/RBM8A/METTL3/CSTF3/TEX10/SF3B6/MAGOHB/SNRPD3/PRPF38

/DYRK3/EYA3/BRD4/HTRA2/ASF1A/TLK2/MAPK15/CNOT2/SESN2/ERCC2/E2F7/ZMPSTE24/VAV3/P

GTF2F1/CRTC3/RPS27/ERCC3/ZC3HAV1/RPL29/EIF2D/KLC1/LARP1/VPS4A/NECTIN2/PABPN1/RA
TFIP11/MOV10/RPL4/CELF2/RPL41/IK/SMG7/RPL9/RPS6/RPRD1A/PPP1R8/RPL24/PSMB6/ADAR/

32

/ANAPC15/PSMB7/NKD2/RNF115/SKP1/ANKIB1/ADAMTS12/RNF186/DERL1/RAB7A/KCTD13/ZMPST

)C16/MIB2/UBE2R2/SUZ12/PPP1R11/RNF214/KCTD9/RNF41/TICAM1/UBE2J1/ARRB1/PSMD12/BF/
A2/BORA/ENY2/PARL/VPS4A/CLEC4E/CRLF2/HUWE1/TM9SF4
RPL24/CCDC88A/EFCAB7/RPL11/RPL10/MFHAS1/RPS24/RBM22/KPNA4/SYT11/C2CD3/PARK7/BUB:
31/DHX9/COG4/SMARCA5/COA3/GTF2H1/MTPN/NDUFS8/NIN/SPTAN1/HCCS/PSMC5/POLE3/RPA1/U:

1/CCDC88A/RIOK1/TRIM27/PIP4K2B/PLA2G6/CAPZA2/NEURL1/SYT11/PARK7/USP36/CAND1/UBQI

NX3/SND1/SERPINB9/CCL8/CAV2/CAV1/EIF3G/SUPT4H1/SRCAP/HMGA2/RPTOR/PARP10/MORC2/TI
)247/SF3B2/NELFA/ARF1/SUV39H1/POLR2E/RAB9A/MSH6/PRKRA/ATG7/RPS9/SNX3/SND1/SERPII

/HLA-C/POLR3F/VAMP3/KIF5B/TRIM62/NUB1/CTSL/KRT16/APOBEC3F/IFNG/CTSB/ANXA1/SYNCR

A60S/RPL22/SETD1A/RASSF2/LILRB1/FUT7/GNAS/CD80/TP53/SOX13/PLD4/RASGRP1/FGL2/LILI

PIN1/ADCY7/PPP1R9A/LPAR1/STK4/MAP3K7/UCN/CASP3/CCDC88A/ADAR/RPL11/COPS8/NAB2/HNI

/PARK7/MCTP2/C2CD2L/USP36/NMT1/KIF5B/ADGRE2/DMTN/CD4/HYAL2/HTRA2/IFNG/ARIH2/TRI

HMP6/APPL2/SEC23B/SYP/MUL1/PPP2CA/EPN1/RAC3/ACTR2/CPTP/GBA2/GNPAT/FNBP1/TP53/GR

1/RPL11/ENC1/NXN/CDC23/KLHL25/COPS8/TRIM27/SH3RF1/NEURL1/RNF149/PSMA3/AAAS/PARK[illegible]
4A/SMCR8/RILPL2/C1QBP/HYLS1/MRPS11/ATP6V1D/VTA1/HAUS3/KIF24/GAS8/TPR/PHF23/LSM1[illegible]

F22/PSMC3/NCAPD3/CHFR/TPD52L1/RHOA/NEK6/YWHAG/CLTC/PPAT/HINFP/PSMC5/CNOT10/POLE[illegible]

E/SLA2/MUL1/WNK1/TSC1/NOD2/PPP2CA/XRCC5/CARD14/RASSF2/MAP3K12/PDE5A/AFAP1L2/TSG[illegible]

[illegible]USP22/ZNF683/CD3G/PTPRC/NECTIN2/YTHDF2/CLEC4E/BAG6/ZCCHC3/SIGLEC7/BTN2A1/CNR1/[illegible]

3/UBE2K/PSME2

[illegible]18/RPS28/ELAVL1/GGT6/RPS17/RPS6KA3/NOLC1/ACE/ABTB1/RPL26L1/TSC1/PAIP1/RRBP1/EI[illegible]
[illegible]C1/MED31/HACE1/RAB40B/UBE2V2/G2E3/HSPA1A/RMND5B/NSMCE1/KLHL36/PSMD6/MALT1/PCNP/[illegible]

[illegible]7/UCN/CCDC88A/FGF19/MFHAS1/COPS8/NAB2/SH3RF1/PLA2G6/PLA2G2A/MADD/NOX1/NEURL1

100A1/WNK1/NOD2/BTN3A2/TNFSF13/XRCC5

ARG2/PAIP1/RRBP1/EIF2B3/RPL22/PDPR/GEMIN5/MRPL21/EIF3C/DDX1/EIF1/RPS15A/SPTLC2/

[illegible]IN1/RAB1B/MOV10/MAP3K7/TRIM32/PSMB6/CASP3/VPS26A/GTPBP1/LRPPRC/RPL11/HNRNPD/PI[illegible]

(IB1/SESN2/NOD1/PPP1CB/ELAVL1/PIK3R2/TYSND1/KDM4A/FASTKD1/ZMPSTE24/PNPT1/SERBP1/

1/CCAR2/POLE3/RPA1/PSEN2/PPP1R7/PMF1/TBP/IST1/SEH1L/SMARCAL1/CDYL/UXT/IPO4/SUV3
P23/MUS81/RPP30/POLR2E/SMUG1/PDK3/SPATA2/SURF6/PELP1/SMC2/RPS9/WDR46/IMP4/TRIM6

1/PARD6A/HAUS3/DDHD2/KIF24/GAS8/LRRCC1/MAPRE2/SPPL2B/DZIP1L/BCAS2/IFT81/STOX1/P

A/SAYSD1/ARL8A/DNAJC13/SEMA4C/CNIH2/MMP25/PTPRS/RAB7A/UBR4/APPL2/MCEMP1/SEC23B/

P7/HMG20B/SMU1/MDC1/PRPF40A/FNBP4/AKAP17A/ANKRD2/SYF2/RBM15/FANCL/DAZAP2/HACE1/

/APPL2/MCEMP1/SEC23B/TLR1/SYP/MPEG1/CD163
1/USP3/THOC7/TTI1/PHF1/PARK7/IRF4/BUB3/HNRNPC/LRWD1/ING2/THOC3/MYC/SLFN11/YY1AP
TAF/LMAN2L/CYTH3/HS2ST1/ST8SIA4/RAB1A/PLD1/PLEKHA8/RAB36/GCNT1/KIAA1324/B3GNT8/

CK2/MANBA/ATG9A/COPB1/SPTAN1/LY6K/CTSW/BRI3/HEXA/DPYSL3/PSEN2/SNAPIN/TMEM184A

SRSF1/SRSF2/MRPS5/SRP68/CASC3/SNRNP70/DHX8/DHX30/RPL13/RBM8A/SF3B6/MAGOHB/SNRPD

[illegible]1/ABCA7/MAP3K7/KREMEN2/STAM/PLEKHM1/VPS26A/CD22/GPR161/NCSTN/MVB12B/TRIM27/NOX[illegible]
[illegible]5/NAA38/GID8/KLHL22/ANAPC1/TWISTNB/WDR26/MSL1/KMT2A/JARID2/PCGF1/BCCIP/RBM15/BR[illegible]

/ABCF1/RAB3C/DDX51/PPA1/DYNC2LI1/FIGNL1/ARL3/TNNT2/EIF5B/NUDT2/RAB33A/MYL6/ATP6[illegible]
/SLFN11/ABCF1/RAB3C/DDX51/PPA1/DYNC2LI1/FIGNL1/ARL3/TNNT2/EIF5B/NUDT2/RAB33A/MY[illegible]
/SLFN11/ABCF1/RAB3C/DDX51/PPA1/DYNC2LI1/FIGNL1/ARL3/TNNT2/EIF5B/NUDT2/RAB33A/MY[illegible]

[illegible]RA/PPP1R14C/GMFB/SERPINB9/CCL8/EGF/CAV1/ATP7A/DNAJC1/DAB2IP/RPTOR/ARL1/PREX1/G[illegible]

4/HR/RUNX2/MEIS1/PSPC1/HOXB5

S7/NBN/FBXW5/PLK3/HNRNPU/RNF2/ZFYVE19/OPTN/CCNE2/HAUS5/MASTL/CDK11B/LZTS1/BCL2L

CC2/ZMPSTE24/PPP2R3C/ACE/ZBTB24/ESRRA/TSC1/TNFSF13/XRCC5/SMAD3/SLC7A6OS/RPL22/S

DHX9/IDO1/RUNX3/ELMO2/ZMIZ1/PSMC5/ADORA2B/UBE2N/KITLG/EREG/CCL19/XCL1/CD247/TLR

C1/DCLRE1C/PAGR1/FBXO6/ERCC3/CIB1/DNASE1L1/MMS22L/PTPRC/STUB1/METTL4/HUWE1/TRRA

3/HNRNPA0/ZNF473/ACIN1/TFIP11/CELF2/RP9/IK/RPS6/RPF1/RPRD1A/MRM2/PPP1R8/CHD7/RI

LAGL1/TMEM161A/RAD54B/LIG3/HELB/XRCC5/SMC1A/FAAP100/ACTR2/SUPT16H/ACKR3/MAEL/TP

31A/CTDP1/SRPK2/TRAM1/RPL13/IFITM2/VRK2/DDB1/SKP2/CCR3/P4HB/PSMB1/RAB1B/NCKAP1/
GTPBP1/POLDIP3/RPL11/THOC7/RPL10/HNRNPD/RPS24/PRMT5/FASTKD5/SNRNP200/RBM22/DDX2

E24/UBR4/CHMP6/CUL7/RNF19A/PLEKHG5/BAP1/SMAD3/USP33/UGGT1/UGGT2/USP24/FBXL18/TS

AR/SMURF2/TPR/RNF40/PHF23/OGT/NUP50/ASB2/MAPK9/LZTR1/CDC26/SOCS5/WDR70/TRIP12/T[illegible]

3/IMMP1L/TIMM9/LRWD1/HEATR3/RPS3A/TIMM22/USP36/NMT1/RPL14/AGPS/ARL3/SRP19/PAM16/
SP39/NDUFA1/LUC7L/NDUFA8/TBP/EIF6/STRAP/ARF1/IPO4/RHOBTB2/SFSWAP/POMP/GMFB/HAX1/

LN2/ULK1/TCHP/DMTN/DYNC2LI1/CEP97/NPHP1/IFNG/FAM98A/AIM2/MAPK15/EIF4G1/TERF1/CN[illegible]

HOC1/NELFCD/RPS14/GTF2F1/CRTC3/CALM3/RPS27/ERCC3/ZC3HAV1/RPL29/EIF2D/KLC1/LARP1/
NB9/CCL8/CAV2/CAV1/EIF3G/SUPT4H1/SRCAP/HMGA2/RPTOR/PARP10/MORC2/THOC1/NELFCD/RP[illegible]

IP/RELA/AIM2/PSMB7/IPO7/SKP1/AKAP8/CHID1/POLR3A/HLA-E/CHGA/NOD1/TRIM15/NLRC4/BP[illegible]

RB2

RNPD/TRIM27/MADD/NEURL1/ANKRD54/PARK7/RHOH/PDE6G/CCNG2/MYC/SLC27A1/MLLT1/CD4/HY[illegible]

M46/MAPK15/TERF1/VAMP1/NKD2/PPP1R12A/FZD9/SESN2/SUPT6H/CD19/DNAJC13/PRKCB/CNIH2/

IPAP1/TARDBP/MYH9/CHMP4A/RER1/HIP1/RILPL2/ALKBH4/ANO7/RFTN1/DVL1/DNAJC11/NLGN4X/

7/EIF3F/EGR2/RNF144A/BRAP/USP36/BCOR/UBE2E3/CAND1/TRIM62/MYC/NUB1/MED20/RNF121/[illegible]
4A/MRPL22/MAPRE2/MAPK9/DZIP1L/IFT81/PCNT/TUBGCP2/ITGB1/CEP78/UBXN2A/EHD3/HPS4/S[illegible]

3/BTC/RPA1/BECN1/FBXL7/ANAPC5/EREG/CDC27/SEH1L/CDC34/CNOT3/MUS81/ARF1/TFDP1/UBE[illegible]

101/STRADA/RASGRP1/TNFRSF10A/SMG8/PRKAR2A/MAPKAP1/SMCR8/DUSP26/DAG1

METTL3/CD1A/PPARG/NFKBIL1/PIK3CB/MYO1C/LOXL3/PSMB1/TNFRSF1B/SCARA3/CD59/NCKAP1/[illegible]

[illegible]F2B3/RPL22/UNC13A/GEMIN5/AEBP1/MRPL21/EIF3C/LHB/DDX1/EIF1/APH1A/LTA4H/RPS15A/AT[illegible]
/E4F1/LEO1/GPS2/FBXO30/USP4/TGFBR1/RNF139/PSMC2/NEURL1B/WAC/MED1/GORASP1/PSMA6/[illegible]

ATXN2/NDUFA7/AARS2/MRPS26/C1QBP/MRPS11/ZC3H15/RPL31/B3GALT2/MRPL42/TRMT10C/TPR/

P4K2B/PSMA3/AAAS/PARK7/DCP2/HNRNPC/RNF144A/USP36/NUB1/UBQLN2/ULK1/WWP2/PAM16/FX[illegible]

/JMJD8/RNF19A/NUP214/MUL1/TSC1/TNFSF13/PAIP1/USP33/CPTP/TP53/TARDBP/FEZ1/SPTLC2/

9H1/MSH6/SAP130/PELP1/SMC2/MORF4L1/POLE2/EP400/LDB1/ATR/TP53BP1/SPOUT1/PCGF3/SR[illegible]
3/PARP10/GRWD1/RPS14/GET4/ARFGEF1/EXOSC2/EDF1/TAF1B/GEMIN2/ACACA/SRPK2/ZNF346/S[illegible]

CNT/KLHL22/TUBGCP2/ZFYVE26/CEP78

TLR1/SYP/MPEG1/CD163

NDUFS3/CWC15/ZBTB4/HR/PSPC1/LPXN/SUDS3/SMN1/HSPA1A/ICE1/USP28/RP2/CRNKL1/GTF2B/[illegible]

1/CENPT/MBD2/HUS1/POLR2B/HTRA2/ASF1A/MAX/RELA/NSD1/TERF1/PPP1R12A/HNRNPA2B1/FAM[illegible]
CHAC1/SDF4/ERGIC2/RHBDD2/ATP9A/QSOX2/ACBD3/SCARA3/CD59/RAB1B/IER3IP1/TLR8/AP4B1/

3/PRPF38B/HNRNPA0/MRPL36/TFIP11/MOV10/RPL4/CELF2/RPL41/IK/NIP7/SMG7/RPL9/MRPL14/

1/SYT11/PRF1/HLA-C/RNF144A/OSBPL9/HLA-DOB/IGF2R/MYO1D/VAMP3/RAB22A/RAB3C/AP5S1/[illegible]
[illegible]C3/PHC1/MED31/SPTSSB/UBE2V2/ZNFX1/NSMCE1/WDR82/GTF2B/POLR3G/SETD5/LEO1/MAT2A/O[illegible]

V0A1/ARL8A/GNG2/RGS4/RAB2A/ENTPD4/RHOD/ERCC2/KIF9/RAB7A/TUBD1
L6/ATP6V0A1/ARL8A/GNG2/RGS4/RAB2A/ENTPD4/RHOD/ERCC2/KIF9/RAB7A/TUBD1
L6/ATP6V0A1/ARL8A/GNG2/RGS4/RAB2A/ENTPD4/RHOD/ERCC2/KIF9/RAB7A/TUBD1

TF2F1/ARHGAP22/CALM3/ENSA/ARHGAP27/RIC8B/CIB1/ARHGAP17/MMP15/LLGL2/BMP2/RALBP1/(

1/DYNC1I2/RAB11A/ZPR1/TUBB3/GPR132/CRLF3/NEDD1/KIF20B/MECP2/CENPJ/MITD1/KIF22/B

ETD1A/RASSF2/LILRB1/FUT7/GNAS/CD80/TP53/SOX13/PLD4/RASGRP1/FGL2/LILRB2

7/HSP90B1/PIK3R6/IRAK2/CCL8/CD5/HAX1/IL13RA2/CAV1/TMEM102/TP53BP1/CD84/DAB2IP/A

P/HDAC8/PPARG/DDB1/TFPT/EXO5/TTF1/TFIP11/SPIRE2/SMG7/USP3/POLG2/UCN/CASP3/CCDC8

OK1/ADAR/RTCB/POLDIP3/RPL11/THOC7/HNRNPD/RPS24/PRMT5/FASTKD5/METTL2A/TRMT2A/SNR

53/INO80E/DDX1/TDP2/COPS7A/ASCC3/KAT5/TREX2/NEK11/MRPS26/MACROD2/RAD23A/APEX1/Z

PLCG1/MX1/RPL4/RPL41/IK/RPL9/RPS6/NCAM1/MAP3K7/TRIM32/VAPA/RPL24/PSMB6/TBC1D20/
0/ZHX2/PSMA3/DCP2/PYM1/GEMIN8/U2SURP/PDCD11/HNRNPC/RPS3A/THOC3/AURKAIP1/SREK1IP

G101/FBXL2/CDC16/KAT5/UBE2R2/RAD23A/SVIP/PPP1R11/DVL1/DERL3/CLEC16A/RNF41/UBE2J

RIM44/HLTF/KLHL22/ANAPC1/UBXN2A/ASB16/HMG20B/PCGF1/UBE2K/PSME2/PHC1/FANCL/TRIM5[illegible]

/HYAL2/RPL10A/HTRA2/IFNG/ARIH2/TUB/MAPK15/TERF1/IPO7/SKP1/RPL12/SRP14/CHGA/SESN[illegible]
/EGF/EIF3G/UQCRB/ATR/NDUFAF6/GRWD1/CAMSAP1/PREX1/COX14/RPS14/CCDC65/ARFGEF1/RPS[illegible]

OT2/CHGA/DOCK11/ATG3/CDK5RAP2/MAP4K4/RHOD/ERCC2/PTPRS/KIF9/RAB7A/EPS8L2/CARMIL2[illegible]

/VPS4A/NECTIN2/PABPN1/RAB1A/CTDP1/SRPK2/TRAM1/RPL13/IFITM2/VRK2/DDB1/SKP2/CCR3/[illegible]
S14/GTF2F1/CRTC3/CALM3/RPS27/ERCC3/ZC3HAV1/RPL29/EIF2D/KLC1/LARP1/VPS4A/NECTIN2[illegible]

IFB1/PTPRS/CLEC4A/APPL2/RPS6KA3/TLR1/S100A1/MPEG1/MUL1/TRIM56/NOD2/ARG2/XRCC5

AL2/JTB/IFNG/MAPK15/TERF1/CCNJ/IPO7/ZNF622/HNRNPA2B1/SESN2/MAP3K9/NOD1/CD19/DUS[illegible]

/MAP4K4/PIK3R2/CHRNA5/NOLC1/PTPN14/UBL5/SYP/NUP214/HDAC3/BAP1/ANKRD13A/SMAD3/UN[illegible]

/ARFGAP3/LRP4

WWP2/ARIH2/RELA/RNF126/PSMB7/STAMBPL1/RNF115/SKP1/ANKIB1/MED30/MKRN1/ATG3/SUMO3/
PATA6/GABARAP/BCCIP/GAP43/MFSD14A/ATG9B/ATXN10/PDGFRA/RAB38/CFAP157/PDGFRB/HSPA

2E2/SMC2/CACUL1/HTT/POLE2/CAV2/EGF/HMGA2/RPTOR/ATAD5/CALM3/ENSA/ERCC3/PKD1/BORA/

PLCG1/ADCY7/TLR8/EIF2B4/MAP3K7/NFKBID/PSMB6/CLEC2D/ADAR/FFAR2/CD22/BCR/CNPY3/MF[illegible]

XN2/NDUFA7/AARS2/DNPEP/MRPS26/C1QBP/ECE1/MRPS11/ZC3H15/RPL31/MRPL42/TRMT10C/TPR
RLIM/XIAP/UHRF2/RNF215/CUL2

PASK/LSM14A/MRPL22/SPTSSA/NOA1/MRPL58/METTL16/EIF4ENIF1/ZC3H12D/MRPL38/MRPS30/G[illegible]

R1/HTRA2/IFNG/ARIH2/SYNCRIP/RELA/TLK2/MAPK15/ATP6V0A1/EIF4G1/PSMB7/NKD2/TBC1D25/

/DAPL1/CHMP4A/FBXL2/TFEB/KAT5/SMCR8/MTDH/RAD23A/SVIP/APEX1/MCL1/DVL1/PARN/DERL3/

CAP/HMGA2/ATAD5/MORC2/GRWD1/THOC1/DCLRE1C/CSNK2A2/MMS22L/SIN3B/LIN37/PAF1/TRRAP
RP68/ZNF16/DDX52/IP6K2/COIL/STK35/RBM4B/RPL13/ERGIC2/TEX10/PWP1/SKP2/PIK3CB/MYO

PCNP/GPATCH2/SF1/TUT1/MAGEA11/CIR1

111A/SMC1B/LRIF1/SUPT6H/KDM3A/MBD1/PPP1CB/SUMO3/SMAD2/WBP2/REPIN1/ORC4/PPP2CA/H
/ABCA7/NCAM1/VAPA/TBC1D20/ST3GAL2

/RPS6/RPF1/USP3/PPP1R8/RPL24/OGFOD1/SRA1/RIOK1/ADAR/BTBD1/UNK/LRPPRC/POLDIP3/RP

ULK1/CTSL/CD4/CTSB/ANXA1/CD207/VPS33B/VPS29/ATP6V0A1/STAMBPL1/FKBP15/TMEM127/CT
RMDL1/SUPT20H/TGFBR1/CHD8/ZSWIM6/KAT7/RPRD2/MED1/KANSL1/POLD4/PFKL/CUL2/CRCP/FB

CST3/PITRM1/LXN/ARHGAP33/FAF2/EVI5L/CXCL10/ASAP3/RALGAPB/ATP1B1

TG3/PSMC3/NCAPD3/CHFR/TPD52L1/RHOA/NEK6/YWHAG/CLTC/PPAT/HINFP/FAP/LGMN/PSMC5/CN

TAD5/INPP5D/DUSP22/CD3G/PTPRC/NECTIN2/CLEC4E/BAG6/CRLF2/ZCCHC3/BTN2A1/ZNF16/CXC

8A/COPS8/HNRNPD/PRMT5/NCOA6/TCEA1/MGME1/PARK7/DCP2/HNRNPC/LRWD1/UNG/SETDB1/LIN9

NP200/RBM22/DDX20/WDR12/THADA/GEMIN8/U2SURP/PDCD11/HNRNPC/USP36/THOC3/AURKAIP1/

BTB47/MCL1/FAM168A/BAZ1B/MRPS11/SETMAR/TAOK1/SLX4/POLD2/ACTR8/NACC2/MEN1/NFRKB

ADAR/RPL11/RPL10/RPS24/FDPS/MVB12B/TRIM27/GTF2A2/KPNA4/PSMA3/AAAS/EIF3F/UNG/RPS
1/AGO1/RPL14/POLR2B/BUD13/RPL10A/FXR1/GEMIN6/SYNCRIP/SMN2/EIF4G1/PSMB7/CNOT2/HN

1/WNT5A/LRP2/ARRB1/PSMD12/BFAR/ARMC8/SMURF2/RNF40/OGT/MAPK9/CDC26/SOCS5/USP46/T

/TRIM3/TRIM36/ZC3HC1/MED31/HACE1/RAB40B/UBE2V2/G2E3/HSPA1A/RMND5B/NSMCE1/NUP133

2/TTC21A/RPS18/RNF186/RPS28/PIK3R2/RAB7A/TYSND1/CNEP1R1/RPS17/APPL2/NOLC1/UBL5/
27/ERCC3/TAF1B/STUB1/EIF2D/GEMIN2/NDUFB4/SRSF1/RAB1A/SRPK2/TM9SF4/CCDC40/VPS16/

/ACE/SPICE1/PLXNB3/TSC1/XRCC5/SMC1A/SMAD3/RAC3/ACTR2/IKBKB/IFT140/CPTP/GBA2/TSG

P4HB/PSMB1/RAB1B/NCKAP1/PLCG1/MX1/RPL4/RPL41/IK/RPL9/RPS6/NCAM1/MAP3K7/TRIM32/V
/PABPN1/RAB1A/CTDP1/SRPK2/TRAM1/RPL13/IFITM2/VRK2/DDB1/SKP2/CCR3/P4HB/PSMB1/RAB

P14/RGS4/MAP4K4/PIK3R2/MAP3K11/VAV3/CHMP6/PPP2R3C/JMJD8/ACE/SLA2/MUL1/WNK1/TSC1

C13A/TTL/TSG101/TP53/IWS1/GRIPAP1/NEFH/TARDBP/FEZ1

/SMAD2/RNF186/DERL1/KCTD13/SENP5/UBR4/CUL7/RNF19A/NUP214/MUL1/TRIM56/NOD2/HDAC3
1A/TRAF3IP1/ABT1/CNOT1/FARP2/STX12/TMEM41B/ATAT1/NLGN2/CEP126/CLUAP1/CHMP2B/MAP

/ACTR1A/OFD1/ANAPC4/VPS4A

HAS1/TRIM27/PLA2G6/ICAM2/PSMA3/PARK7/IRF4/HLA-C/PDE4B/POLR3F/EIF2B1/ADGRE2/CTSL

/PASK/LSM14A/MRPL22/NOA1/MRPL58/METTL16/EIF4ENIF1/ZC3H12D/MRPL38/MRPS30/GSPT1/A

SPT1/AIMP1/STAT3

/ANKIB1/SESN2/NOD1/PPP1CB/ELAVL1/PIK3R2/RAB7A/TYSND1/KDM4A/FASTKD1/ZMPSTE24/CHM

/ABL2/ATP6V1D/CLEC16A/PPP1R3D/RNF41/TICAM1/UBE2J1

/HDAC8/PWP1/DDB1/JUNB/TFPT/TRPS1/TFIP11/PRR14/ASXL1/IK/USP3/POLG2/LRPPRC/THOC7/
1C/ACIN1/TTF1/TFIP11/CASK/RPL4/NIP7/RPS6/RPF1/MRM2/PLEKHM1/CHD7/ADAR/RPL11/ATF6

ELB/XRCC5/SMC1A/SMAD3/MYCN/ACTR2/SETD1A/MAU2/RASSF2/MAEL/BAZ1A/TP53/INO80E/TARD

L11/RPL10/HNRNPD/RPS24/FASTKD5/MRPL3/SNRNP200/RBM22/DCP2/WDR12/PDCD11/HNRNPC/EI

NS/DENND6A/CHID1/HLA-E/ARL8A/DNAJC13/DERL1/RHOD/RAB7A/CHMP6/APPL2/CARMIL2/CYB56
XW11/PRPS2/POLR1D/AEBP2/FBXO27/POLR2J3/SETD1B/RNF168/TAF4B/PLRG1/RECQL5/KEAP1/K

OT10/POLE3/BTC/RPA1/BECN1/FBXL7/ANAPC5/EREG/CDC27/SEH1L/CDC34/CDK11A/CNOT3/MUS8

L10/CNR1/CD1A/NFKBIL1/PIK3CB/MYO1C/PSMB1/DOCK8/ACIN1/SCARA3/CD59/NCKAP1/PLCG1

/MYC/SLFN11/AP5S1/MBD2/HUS1/POLR2B/PRMT6/FIGNL1/APOBEC3F/EYA3/PAM16/ASF1A/MAPK1

SREK1IP1/AGO1/RPL14/DDX51/POLR2B/DUS1L/BUD13/RPL10A/ZNHIT3/GEMIN6/SYNCRIP/SMN2/

3A/IGF2R/SETDB1/TRIM62/RPL14/POLR2B/PRMT6/UCKL1/CD4/APOBEC3F/WWP2/HYAL2/BRD4/RP
RNPA2B1/RPL12/SUPT6H/RPS18/ERCC2/RPS28/U2AF1L4/ELAVL1/FASTKD1/LUC7L3/SRSF3/RPS1

RIP12/KLHL22/ANAPC1/PCBP2/DNAJC18/CCDC115/UBXN2A/DACT1/ADAMTS7/ZFAND2B/USP13/AZ

/KLHL36/PSMD6/MALT1/PCNP/E4F1/LEO1/GPS2/FBXO30/USP4/TGFBR1/RNF139/PSMC2/NEURL1B

NUP214/HDAC3/BAP1/XRCC5/ANKRD13A/SMAD3/RPL22/MAU2/IFT140/BICD2/GNPAT/RAB6A/TSG1
COIL/DHX30/ADD1/SCO2/SNRPD3/DDB1/MYO1C/SPIRE2/NCKAP1/COX7A2/PPP1R9A/MX1/CELF2/N

101/TP53/CNTROB/FEZ1/CEP135/ATXN2/ARPIN/CHMP4A

APA/RPL24/PSMB6/TBC1D20/ADAR/RPL11/THOC7/RPL10/RPS24/FDPS/MVB12B/TRIM27/GTF2A2/
1B/NCKAP1/PLCG1/MX1/RPL4/RPL41/IK/RPL9/RPS6/NCAM1/MAP3K7/TRIM32/VAPA/RPL24/PSMB

/NOD2/PPP2CA/XRCC5/CARD14/RASSF2/MAP3K12/PDE5A/AFAP1L2/TSG101/TP53/STRADA/RASGR

/BAP1/SMAD3/USP33/TULP4/RNF10/USP24/SUPT3H/RNF135/FBXL18/TSG101/TP53/INO80E/BCLRE3/CHMP2A/CFAP53/NPRL2/CEP162/ACTA1/KIFAP3/EIF2A/NTNG2/DCTN3/NEK8/CHMP7/VPS37A

/CD4/IFNG/CTSB/ANXA1/RELA/AIM2/PSMB7/CCR2/KLHL6/PRAM1/SKP1/HLA-E/EIF2B2/SUPT6H/

IMP1/STAT3

P6/PNPT1/SERBP1/JMJD8/RNF19A/NUP214/MUL1/TSC1/PLEKHG5/TNFSF13/SMAD3/PAIP1/USP33

TTI1/PHF1/PARK7/IRF4/BUB3/HNRNPC/LRWD1/ING2/THOC3/SETDB1/MYC/SLFN11/YY1AP1/CENP
B/TRIM27/TCEA1/CC2D1A/DDX31/SLC30A5/PYM1/WDR12/PDCD11/LRWD1/RPS3A/USP36/ZNF506/

BP/TEP1/KAT5/KDM3B/ETV3/SMCR8/HMGN3/SUZ12/SKA2/APEX1/BAZ1B/HMGXB4/SETMAR/L3MBTL

F3F/RPS3A/AURKAIP1/MRPL20/AGO1/ABCF1/RPL14/DYRK3/BUD13/APOBEC3F/SRP19/MRPL24/MR

1A3/ACE/SLA2/TRAK2
DM6A/CDK12/MED21

1/ARF1/TFDP1/UBE2E2/MAEA/SMC2/CACUL1/HTT/POLE2/CAV2/EGF/HMGA2/RPTOR/ATAD5/CALM3

5/DNASE1/TERF1/HNRNPA2B1/FAM111A/SESN2/SUPT6H/MBD1/ERCC2/E2F7/KCTD13/ZMPSTE24/T

CLK2/HNRNPA2B1/THUMPD2/RBMS2/SUPT6H/RRP15/SMAD2/WDR3/ERCC2/RPS28/U2AF1L4/ELAVL1

L10A/CTSB/SYNCRIP/RELA/VPS29/EIF4G1/PSMB7/CCR2/IPO7/FAM111A/SKP1/RPL12/SUPT6H/T
7/PNPT1/SERBP1/UBL5/NUP214/TRA2B/PPP2CA/HNRNPLL/TNFSF13/PAIP1/SUGP1/RPL22/ZRANB

IN1/UBE2K/PSME2

/WAC/MED1/GORASP1/PSMA6/RLIM/XIAP/UHRF2/UBA3/RNF215/CUL2/FBXW11/RASD2/UBE3C/GNL

01/TP53/DDX1/TARDBP
DUFA9/NDUFB11/NIP7/PXN/RPL24/OGFOD1/TBC1D20/ADAR/NDUFA12/RPL11/RPL10/COPS8/PRMT

KPNA4/PSMA3/AAAS/EIF3F/UNG/RPS3A/IGF2R/THOC3/SETDB1/TRIM62/RPL14/ULK1/POLR2B/PR
6/TBC1D20/ADAR/RPL11/THOC7/RPL10/RPS24/FDPS/MVB12B/TRIM27/GTF2A2/KPNA4/PRF1/PSM

P1/TNFRSF10A/SMG8

11A/FBXL2/CDC16/COPS7A/MIB2/UBE2R2/SUZ12/RAD23A/PPP1R11/RNF214/KCTD9/OTUD5/NLRP
/AMBRA1/EIF2S1/WIPI2/ALMS1/SCLT1/MKS1/CEP295/DNAAF3/LRRC6/CHMP5

NOD1/CD19/TRIM15/PRKCB/NLRC4/BPIFB1/PTPRS/PIK3R2/CLEC4A/VAV3/APPL2/RPS6KA3/PPP2

/CPTP/TP53/TARDBP/FEZ1/SPTLC2/DAPL1/CHMP4A/FBXL2/TFEB/KAT5/SMCR8/MTDH/RAD23A/SV

T/MBD2/HUS1/POLR2B/FIGNL1/BRD4/HTRA2/PBX4/ASF1A/MAX/RELA/NSD1/TERF1/C1orf131/PP
MYC/URB2/YY1AP1/DDX51/MNX1/MLLT1/AGPS/HUS1/PRMT6/UBLCP1/SRP19/NKRF/FXR1/RDM1/CT

1/TAF4/SLX4/POLD2/ACTR8/NACC2/MEN1/NFRKB/ARRB1/SP1/TNKS1BP1/ACTR6/TPR/RNF40/PUR

PS16/RPL10A/ZNHIT3/FXR1/MRPL55/SYNCRIP/SMN2/CHCHD1/EIF4G1/CNOT2/ZNF622/CCDC12/H

/ENSA/ERCC3/CIB1/PKD1/BORA/ACTR1A/OFD1/ANAPC4/TADA3/VPS4A

MEM161A/REPIN1/RAD54B/LIG3/ORC4/PPP2CA/HELB/TNFSF13/XRCC5/SMC1A/FAAP100/ACTR2/

/LUC7L3/SRSF3/ZMPSTE24/RPS17/PTCD1/PNPT1/NOLC1/UBL5/TRA2B/PPP2CA/HNRNPLL/SMAD3/

RIM15/RPS18/DERL1/ERCC2/RPS28/RAB7A/KDM4A/UBR4/CHMP6/RPS17/CUL7/NUP214/XRCC5/SM
2/GEMIN5/SAFB/ZNRD1/TP53/IWS1/DDX1/LSM3/TARDBP/RPS15A/SMG8/GRSF1/C1QBP/APEX1/PA

3L/FBXO27/SPRTN/PLAA/RNF168/PSMD5/KEAP1/PIAS2/MED21/UBC/SOCS4/DNAJA1/NUP35/FBXL

5/TRIM27/GTF2A2/NDUFAF4/CAPZA2/COA4/SNRNP200/DDX20/NAA60/ERAL1/GEMIN8/EIF3F/MRP

MT6/UCKL1/CD4/APOBEC3F/WWP2/HYAL2/BRD4/RPL10A/CTSB/SYNCRIP/RELA/VPS29/EIF4G1/PS
A3/AAAS/EIF3F/UNG/RPS3A/IGF2R/THOC3/SETDB1/TRIM62/RPL14/ULK1/POLR2B/PRMT6/UCKL1

[illegible]3/RNF41/TICAM1/UBE2J1/ACTR8/NFRKB/ARRB1/PSMD12/BFAR/SMURF2/TPR/RNF40/PHF23/OGT/

R3C/SLA2/TLR1/S100A1/MUL1/WNK1/NOD2/BTN3A2/ARG2/TNFSF13/XRCC5/SMAD3/EIF2B3/ACTR

'IP/APEX1/MCL1/DVL1/PARN/DERL3/ABL2/ATP6V1D/CLEC16A/PPP1R3D/RNF41/TICAM1/UBE2J1/

'P1R12A/HNRNPA2B1/FAM111A/AKAP8/SMC1B/LRIF1/SUPT6H/KDM3A/MBD1/PPP1CB/SUMO3/SMAD2
'SB/CHCHD1/TERF1/ZNF622/SPIN1/FAM111A/AKAP8/POLR1C/RPL12/PPP1CB/SNAPC5/WDR3/KDM4

!B/EZH1/NFATC1

INRNPA2B1/RPL12/SRP14/MRPS2/RBMS2/RRP15/RPS18/WDR3/DERL1/RPS28/U2AF1L4/ELAVL1/LU

SUPT16H/ERI1/MAEL/BAZ1A/GNAS/TP53/INO80E/DDX1/TDP2/WIZ/TEP1/SH2B1/COPS7A/ASCC3/K

/MYCN/SUGP1/ZRANB2/NOL11/GEMIN5/SAFB/ERI1/CLK3/TP53/IWS1/DDX1/NSUN5P2/LSM3/TARD

AD3/RPL22/IKBKB/ACKR3/RAB6A/TSG101/CD80/TP53/TARDBP/RPS15A/TAF11/CHMP4A/FBXL2/

RN/NOVA1/RPL31/SRSF10/PSMD12/PNRC2/TNKS1BP1/TRMT10C/RNF40/SRSF7/PRPF40B/TRA2A/

.15/UBE2W/RWDD3/UCHL3/GOLGA2/CDK5RAP3/TRIM9/ANGPT1/UBOX5/RNF181/PTPN22/MKRN2/RN[illegible]

PL20/VAMP3/AGO1/CAND1/CENPT/MBD2/DMTN/SRP19/ZNHIT3/ASF1A/GEMIN6/SMN2/NDUFB2/EIF4

SMB7/CCR2/IPO7/FAM111A/SKP1/RPL12/SUPT6H/TRIM15/RPS18/DERL1/ERCC2/RPS28/RAB7A/K[illegible]
./CD4/APOBEC3F/WWP2/HYAL2/BRD4/RPL10A/IFNG/CTSB/SYNCRIP/RELA/VPS29/EIF4G1/PSMB7/

/NUP50/ASB2/MAPK9/LZTR1/CDC26/SOCS5/TAB1/WDR70/USP46/TRIP12/TRIM44/HLTF/KLHL22/[illegible]

R2/IKBKB/LILRB1/RNF135/CD80/DDX1/RASGRP1/FGL2/LILRB2

'WNT5A/LRP2/PSMD12/SMURF2/TPR/RNF40/PHF23/GATA4

2/WBP2/REPIN1/ORC4/PPP2CA/HELB/XRCC5/SMC1A/SMAD3/MYCN/ACTR2/SUPT16H/SETD1A/MAU2/
4A/SENP5/YPEL4/PNMA2/NOLC1/RBM34/ORC4/ESRRA/ABTB1/GTF3C3/XRCC5/MYCN/STAU2/NOL11/

JC7L3/RPS17/PNPT1/NOLC1/TRA2B/RPL26L1/XRCC5/SUGP1/RRBP1/RPL22/GEMIN5/ERI1/MAEL/M

KAT5/TREX2/RAD23A/APEX1/PARN/FAM168A/TEFM/SETMAR/TAOK1/SLX4/POLD2/ACTR8/MEN1/NF[illegible]

BP/METTL2B/ZNHIT6/AARS2/TSR1/METTL6/GRSF1/RPP38/C1QBP/PARN/MRPS11/NOVA1/THUMPD3/

GTF2E1/COPS7A/KAT5/DAG1/C1QBP/RAD23A
PRMT7/CACTIN/RSRC1/METTL16/BCAS2/ZC3H12D/SNRNP35/PHRF1/DDX46/ZCRB1/GSPT1/STAT3/[illegible]

[illegible]208/FBXO22/PAXIP1/NUP98/KLHL7/ANAPC13/DCAF12/RABGEF1/KCTD6/HDAC4/DDRGK1/MTOR/C[illegible]

[illegible]G1/NDUFAF3/TERF1/VAMP1/CNOT2/RPL12/MRPS2/TBCE/CDK5RAP2/SMAD2/ERCC2/RPS28/LUC7L[illegible]

[illegible]M4A/UBR4/CHMP6/RPS17/CUL7/NUP214/XRCC5/SMAD3/RPL22/IKBKB/ACKR3/RAB6A/TSG101/CD[illegible]
[illegible]CCR2/IPO7/FAM111A/SKP1/RPL12/CHGA/SUPT6H/TRIM15/RPS18/DERL1/ERCC2/RPS28/RAB7A/[illegible]

ANAPC1/UBXN2A/ASB16/HMG20B/USP13/PCGF1/UBE2K/PSME2/BRCC3/OTUD6B/PHC1/FANCL/TRIM5

/RASSF2/MAEL/BAZ1A/TP53/INO80E/TARDBP/TEP1/KAT5/KDM3B/ETV3/SMCR8/HMGN3/SUZ12/SK[illegible]
/ERI1/ZNRD1/TSG101/TP53/INO80E/NSUN5P2/TDP2/ABCB8/KAT5/TSR1/MTDH/NEK11/MACROD2/[illegible]

MRPL21/EIF3C/DDX1/LSM3/TARDBP/EIF1/RPS15A/TEP1/ZNHIT6/ATXN2/NDUFA7/TSR1/GRSF1/M[illegible]

RKB/CD40

/QTRT2/SRSF10/WDR74/DUS3L/WDR18/TARBP1/TRMT10C/RNF40/SRSF7/PRPF40B/ANKRD16/TRA2A

BRDT/PCBP2/SKIV2L/PSIP1/DCP1A/CSDE1/SMU1/AAR2/PRPF40A/AKAP17A/XAB2/ECD/PSME2/SYF

)C73/PSME4/RNF38/KBTBD8/NEDD4L/TRIM33/CAMLG/KCTD11/ANAPC2/CDC20B/UBE2V1/PSME1/AS

3/NAP1L3/CARMIL2/BRF2/BBS12/SEC23B/GTF3C3/CADPS2/SMAD3/RAC3/ACTR2/UNC13A/GEMIN5/

30/TP53/TARDBP/RPS15A/TAF11/CHMP4A/FBXL2/GTF2E1/COPS7A/KAT5/DAG1/C1QBP/RAD23A
KDM4A/UBR4/CHMP6/RPS17/CUL7/MPEG1/NUP214/XRCC5/SMAD3/RPL22/IKBKB/ACKR3/RAB6A/TS(

5/TRIM3/TRIM36/ZC3HC1/MED31/HACE1/RAB40B/COPS3/UBE2V2/G2E3/SUDS3/YOD1/HSPA1A/RMN

A2/APEX1/BAZ1B/HMGXB4/SETMAR/L3MBTL1/TAF4/SLX4/POLD2/ACTR8/NACC2/MEN1/NFRKB/ARR[illegible]
RPP38/SUZ12/C1QBP/APEX1/PARN/MAK16/ZC3H15/TMEM179B/SETMAR/NOVA1/THUMPD3/SNAPC1/[illegible]

RPS26/RPP38/APEX1/TDRD7/SRP72/MAK16/MRPS11/DERL3/TEFM/RPL31/WDR74/DDX19A/PNRC2/[illegible]

A/PRMT7/CACTIN/RSRC1/METTL16/BCAS2/SNRNP35/RBMS1/PHRF1/DDX46/ZCRB1/BRDT/PCBP2/DU

F2/RBM15/XRN2/TFCP2/PCIF1/PTBP2/CARHSP1/SNUPN/HNRNPH1/CWC15/WDR83/PAPOLA/SMN1/HS

GB3/CUL1/PEX2/ATG5/RNF31/RNF20/RNF175/NUP160/CBLB/DCAF4/UBE3D/PPIL2/RRAGA/TRIM1

/CPTP/GBA2/EIF3C/TP53/DDX1/LSM3/TBPL1/NEFL/TAF11/ZNHIT6/ATXN2/ARPIN/NDUFA7/CHMP

G101/CD80/TP53/TARDBP/RPS15A/TAF11/CHMP4A/FBXL2/GTF2E1/COPS7A/KAT5/DAG1/C1QBP/R

ND5B/NSMCE1/USP28/NUP133/KLHL36/PSMD6/MALT1/PCNP/E4F1/LEO1/GPS2/GATA3/FBXO30/USI

31/SP1/TNKS1BP1/ACTR6/TPR/RNF40/PURB/EZH1/NFATC1/MCM3AP/RCCD1
NACC2/PIN4/WDR74

NUFIP2/MRPL42/BTF3/TRMT10C/PRPF40B/LSM14A/TRA2A/MRPL22/CACTIN/MRPL58/EIF4ENIF1/I

USP11/MRM1/PSIP1/CDKAL1/SMU1/AAR2/PRPF40A/AKAP17A/XAB2/ECD/SYF2/RBM15/XRN2/TRIT

SPA1A/HNRNPH2/CNOT1/CRNKL1/PSMD6/SF1/HNRNPH3/LEO1/TUT1/AHCYL1/CIR1/RPS25/ZMAT2/

7

4A/TBCC/COPS7A/AARS2/HIP1/PSMG2

AD23A

P4/TGFBR1/RNF139/PSMC2/NEURL1B/USP32/WAC/MED1/GORASP1/PSMA6/RLIM/XIAP/UHRF2/UBA[illegible]

BCAS2/ZC3H12D/SNRNP35/MRPL38/MRPS30/RBMS1/NAA38/ZCRB1/MRPS27/DCP1A/SMU1/AAR2/PR[illegible]

1/PTBP2/SNUPN/ADAT2/HNRNPH1/ELP6/SHQ1/CWC15/WDR83/PAPOLA/SMN1/NOP14/HSPA1A/ABT1

USP4/CWF19L1/NBAS/SF3A1/PSMC2/DIS3L2/SRSF4/GPATCH1/RPRD2/PDCD7/MED1/PSMA6/CLP1/

3/RNF215/CUL2

PF40A/AKAP17A/XAB2/SYF2/TDRD5/PTBP2/CARHSP1/TRIM5/HNRNPH1/MRPS18A/CWC15/TOP2B/S

/HNRNPH2/CRNKL1/DEDD2/SF1/HNRNPH3/LEO1/TUT1/INTS3/AHCYL1/CIR1/ZMAT2/USP4/CWF19L

NUDT21

LIRP/WDR83/SMN1/NOP14/HSPA1A/HNRNPH2/CNOT1/EIF3J/CRNKL1/MTG2/MEX3B/RWDD1/SF1/HN

1/NSUN2/WDR43/SF3A1/NSA2/SRSF4/GPATCH1/RPRD2/PDCD7/CLP1/MRPS9/NUDT21/CLASRP/TSR

RNPH3/MRPL43/RPS25/ZMAT2/NFATC2/CWF19L1/NSUN2/SF3A1/APOBEC3H/PSMC2/DIS3L2/CTSH/

2/RBM4

NSA2/GPATCH1/PDCD7/WAC/PSMA6/MRPS9/EIF2A/RBM4/MRPL50
