# Supplementary material for: The Landscape of Using Glycosyltransferase Gene Signatures for Overall Survival Prediction in Hepatocellular Carcinoma
Source: J Oncol. 2022 Jun 21;2022:5989419. doi: 10.1155/2022/5989419 (PMC9239767; doi:10.1155/2022/5989419)
Supplement: Supplementary Materials — Figure S1: Venn diagram shows the intersections of co-differentially expressed glycosyltransferase (GT) genes between the 210 GTs obtained from the literature and those obtained from TCGA database. Figure S2: identification of a four-gene signature in the training set of TCGA database constructed using the LASSO method. Figure S3: survival analyses grouped by the optimal cutoff expression value of each gene in the training dataset of TCGA database. (A) ALG3. (B) B3GAT3. (C) GLA. (D) ST6GALNAC4. Figure S4: mRNA expression levels of the four genes in matched HCC and adjacent noncancerous samples in TCGA database were compared. (A) ALG3. (B) B3GAT3. (C) GLA. (D) ST6GALNAC4. Figure S5: the interactions among signature genes displayed in a PPI network. Figure S6: stratified survival analysis between high- and low-risk groups. (A)NAFLD. (B) HBV infection. (C) HCV infection. (D) Both HBV and HCV infections. (E) No treatment and therapy. (F) Pharmaceutical therapy. (G) Radiation therapy, NOS. Figure S7: the nomogram to predict prognostic probabilities in TCGA database. (A) The nomogram for predicting OS in the training set. The calibration plots for predicting (B) 3-year and (C) 5-year survival probabilities. Figure S8: survival analyses grouped by the optimal cutoff expression value of each signature gene in the validation dataset of TCGA database. (A) ALG3. (B) B3GAT3. (C) GLA. (D) ST6GALNAC4. Table S1: the clinical information of 371 primary HCC samples and 50 normal samples. Table S2: combining the clinical information and expression profiles of 210 human glycosyltransferase (GT) genes. Table S3: DEGs of 371 samples in TCGA database. Table S4: all significantly enriched GO terms. Table S5: all significantly enriched KEGG pathways. [file 5989419.f1.zip › 5989419.f1/Supplementary Table.5 risk H_L_AllenrichKEGG.sig.pdf]

ID	Descriptic	setSize	enrichment	NES	pvalue	p.adjust	qvalues
hsa05165	Human papi	321	0.4535176	1.5168867	0.001001	0.0102477	0.0054098
hsa05016	Huntingtor	269	0.4404965	1.4626777	0.001006	0.0102477	0.0054098
hsa05131	Shigellosi	212	0.4757418	1.5658992	0.001007	0.0102477	0.0054098
hsa05132	Salmonella	212	0.479945	1.579734	0.001007	0.0102477	0.0054098
hsa05020	Prion dise	240	0.4396212	1.4531522	0.0010081	0.0102477	0.0054098
hsa05166	Human T-ce	215	0.5028772	1.6542871	0.0010081	0.0102477	0.0054098
hsa04144	Endocytosi	233	0.4833499	1.5943948	0.0010091	0.0102477	0.0054098
hsa04810	Regulatior	209	0.4567972	1.5006145	0.0010091	0.0102477	0.0054098
hsa05170	Human immu	198	0.4541426	1.4872467	0.0010111	0.0102477	0.0054098
hsa05169	Epstein-Ba	192	0.5056904	1.6547689	0.0010121	0.0102477	0.0054098
hsa05130	Pathogenic	182	0.4960999	1.616796	0.0010132	0.0102477	0.0054098
hsa04621	NOD-like r	167	0.4721422	1.5300177	0.0010194	0.0102477	0.0054098
hsa05152	Tuberculos	168	0.5362255	1.7376987	0.0010194	0.0102477	0.0054098
hsa05203	Viral carc	161	0.4844461	1.5674392	0.0010235	0.0102477	0.0054098
hsa04218	Cellular s	153	0.489643	1.5808431	0.0010246	0.0102477	0.0054098
hsa05164	Influenza	158	0.4596661	1.487073	0.0010246	0.0102477	0.0054098
hsa05206	MicroRNAs	158	0.5086495	1.6455401	0.0010246	0.0102477	0.0054098
hsa03013	RNA transp	152	0.4779992	1.5435713	0.0010256	0.0102477	0.0054098
hsa04145	Phagosome	145	0.6062024	1.9532161	0.0010256	0.0102477	0.0054098
hsa03040	Spliceosom	132	0.5179121	1.6556848	0.0010406	0.0102477	0.0054098
hsa04110	Cell cycle	123	0.6030942	1.9167031	0.0010406	0.0102477	0.0054098
hsa03010	Ribosome	127	0.5559621	1.7708309	0.0010417	0.0102477	0.0054098
hsa04380	Osteoclast	122	0.5291945	1.6810332	0.0010417	0.0102477	0.0054098
hsa04142	Lysosome	128	0.5245409	1.6687912	0.0010449	0.0102477	0.0054098
hsa04512	ECM-recept	86	0.5554744	1.706101	0.0010684	0.0102477	0.0054098
hsa05323	Rheumatoic	88	0.6431027	1.9764459	0.0010695	0.0102477	0.0054098
hsa04666	Fc gamma F	87	0.5544505	1.7004796	0.001073	0.0102477	0.0054098
hsa03008	Ribosome b	71	0.5534781	1.6709042	0.0010834	0.0102477	0.0054098
hsa05140	Leishmania	71	0.6154584	1.8580177	0.0010834	0.0102477	0.0054098
hsa04612	Antigen pr	65	0.5894278	1.762247	0.0010893	0.0102477	0.0054098
hsa05100	Bacterial	65	0.5467699	1.6347101	0.0010893	0.0102477	0.0054098
hsa03460	Fanconi ar	48	0.5505983	1.585471	0.0011236	0.0102477	0.0054098
hsa04672	Intestinal	45	0.5937936	1.6978776	0.0011261	0.0102477	0.0054098
hsa05320	Autoimmune	45	0.5664258	1.6196231	0.0011261	0.0102477	0.0054098
hsa04940	Type I dia	41	0.5848803	1.6441119	0.0011494	0.0102477	0.0054098
hsa05219	Bladder ca	41	0.6465432	1.8174476	0.0011494	0.0102477	0.0054098
hsa03030	DNA replic	36	0.655346	1.7971769	0.0011587	0.0102477	0.0054098
hsa05310	Asthma	28	0.693903	1.8476145	0.0011765	0.0102477	0.0054098
hsa05200	Pathways i	516	0.3894626	1.3218613	0.001998	0.0147347	0.0077785
hsa05010	Alzheimer	332	0.4097425	1.3726759	0.002002	0.0147347	0.0077785
hsa05014	Amyotrophi	327	0.4074267	1.3630543	0.002004	0.0147347	0.0077785
hsa04060	Cytokine-c	282	0.4042104	1.3434671	0.0020101	0.0147347	0.0077785
hsa05012	Parkinson	214	0.4258017	1.4006152	0.0020182	0.0147347	0.0077785
hsa04668	TNF signal	111	0.4872861	1.5355801	0.0020921	0.0147347	0.0077785
hsa05145	Toxoplasmc	108	0.4948755	1.5555628	0.0020964	0.0147347	0.0077785
hsa04658	Th1 and Th	90	0.5134139	1.5834196	0.0021299	0.0147347	0.0077785
hsa04657	IL-17 sigr	93	0.5235706	1.6220295	0.0021322	0.0147347	0.0077785
hsa04640	Hematopoie	92	0.5200986	1.6056066	0.0021368	0.0147347	0.0077785
hsa03050	Proteasome	44	0.5568883	1.5869139	0.0022624	0.015283	0.008068
hsa03440	Homologous	37	0.5646549	1.5551169	0.0023202	0.0153596	0.0081084

hsa05163	Human cytc	212	0.433618	1.427249	0.0030211	0.0189634	0.0100109
hsa04015	Rap1 signa	207	0.4326736	1.4197072	0.0030303	0.0189634	0.0100109
hsa04062	Chemokine	189	0.4439228	1.4521324	0.0030364	0.0189634	0.0100109
hsa04933	AGE-RAGE s	99	0.4951643	1.5477114	0.0031546	0.0193364	0.0102078
hsa00232	Caffeine m	5	-0.952029	-1.882854	0.0037594	0.0226247	0.0119437
hsa04510	Focal adhe	197	0.4361227	1.4274144	0.0040445	0.0233386	0.0123206
hsa04141	Protein pr	163	0.4516493	1.4620669	0.0040858	0.0233386	0.0123206
hsa00072	Synthesis	10	-0.811109	-2.051631	0.0041322	0.0233386	0.0123206
hsa05135	Yersinia i	125	0.4638739	1.4738078	0.0041797	0.0233386	0.0123206
hsa05120	Epithelial	69	0.5148075	1.5571464	0.0043011	0.0233386	0.0123206
hsa05230	Central ca	69	0.5144033	1.5559238	0.0043011	0.0233386	0.0123206
hsa05416	Viral myoc	57	0.5256095	1.5498304	0.0044248	0.0236226	0.0124705
hsa05332	Graft-vers	36	0.573052	1.5714994	0.004635	0.0243521	0.0128556
hsa05162	Measles	132	0.4554392	1.4559685	0.0052029	0.0267047	0.0140976
hsa00120	Primary bi	17	-0.904342	-2.607291	0.0053191	0.0267047	0.0140976
hsa04620	Toll-like	95	0.4829092	1.4992889	0.0053248	0.0267047	0.0140976
hsa00220	Arginine b	21	-0.810452	-2.507095	0.0054645	0.0267453	0.014119
hsa00053	Ascorbate	19	-0.707744	-2.120635	0.0054945	0.0267453	0.014119
hsa00340	Histidine	22	-0.821071	-2.594334	0.005618	0.02695	0.0142271
hsa00040	Pentose ar	24	-0.517127	-1.672129	0.005848	0.0276525	0.0145979
hsa00591	Linoleic a	25	-0.773121	-2.487519	0.0059524	0.0277498	0.0146493
hsa00650	Butanoate	26	-0.805173	-2.627552	0.006135	0.0278175	0.014685
hsa04950	Maturity c	26	-0.516514	-1.685561	0.006135	0.0278175	0.014685
hsa00410	beta-Alani	29	-0.720841	-2.388366	0.0067114	0.0282809	0.0149297
hsa00630	Glyoxylate	29	-0.692604	-2.29481	0.0067114	0.0282809	0.0149297
hsa00250	Alanine, a	34	-0.544471	-1.879822	0.0068493	0.0282809	0.0149297
hsa00020	Citrate cy	30	-0.470949	-1.57461	0.0068966	0.0282809	0.0149297
hsa05340	Primary in	34	0.5583156	1.5179755	0.0070093	0.0282809	0.0149297
hsa00640	Propanoate	33	-0.697913	-2.365583	0.0070423	0.0282809	0.0149297
hsa00760	Nicotinate	33	-0.440816	-1.49415	0.0070423	0.0282809	0.0149297
hsa00860	Porphyrin	33	-0.441138	-1.495241	0.0070423	0.0282809	0.0149297
hsa00350	Tyrosine m	36	-0.764205	-2.665297	0.0071942	0.0282809	0.0149297
hsa04625	C-type lec	102	0.4637085	1.4536796	0.0073222	0.0282809	0.0149297
hsa00260	Glycine, s	38	-0.812756	-2.8379	0.0074074	0.0282809	0.0149297
hsa00620	Pyruvate m	38	-0.547086	-1.910261	0.0074074	0.0282809	0.0149297
hsa00380	Tryptophar	42	-0.789632	-2.851053	0.0074627	0.0282809	0.0149297
hsa04975	Fat digest	42	-0.545201	-1.968511	0.0074627	0.0282809	0.0149297
hsa00071	Fatty acic	43	-0.860306	-3.151222	0.0075188	0.0282809	0.0149297
hsa04914	Progesterc	91	0.4628383	1.4273774	0.0085562	0.0310216	0.0163765
hsa00270	Cysteine a	49	-0.507322	-1.896175	0.0086957	0.0310216	0.0163765
hsa00140	Steroid hc	51	-0.737302	-2.763698	0.0088496	0.0310216	0.0163765
hsa00830	Retinol me	52	-0.836335	-3.151115	0.0088496	0.0310216	0.0163765
hsa04979	Cholesterc	50	-0.556904	-2.086402	0.0088496	0.0310216	0.0163765
hsa00330	Arginine a	48	-0.502682	-1.853957	0.0089286	0.0310216	0.0163765
hsa04623	Cytosolic	53	0.524409	1.5277511	0.0089586	0.0310216	0.0163765
hsa05110	Vibrio chc	49	0.52018	1.4935103	0.0090192	0.0310216	0.0163765
hsa00280	Valine, le	47	-0.75276	-2.758385	0.0090909	0.0310216	0.0163765
hsa04064	NF-kappa E	99	0.4607527	1.4401526	0.0094637	0.0317415	0.0167565
hsa04061	Viral prot	97	0.4650014	1.4485724	0.0094937	0.0317415	0.0167565
hsa05150	Staphylocc	84	0.4758035	1.4592894	0.009636	0.0318951	0.0168376
hsa05205	Proteoglyc	198	0.4209615	1.3785838	0.0101112	0.0327918	0.017311

hsa00590	Arachidoni	56	-0.48698	-1.834197	0.0102041	0.0327918	0.017311
hsa01212	Fatty acic	56	-0.538917	-2.029814	0.0102041	0.0327918	0.017311
hsa00982	Drug metak	58	-0.806647	-3.093366	0.0103093	0.0328031	0.017317
hsa04650	Natural ki	114	0.450367	1.424513	0.0104058	0.0328031	0.017317
hsa00360	Phenylalar	17	-0.66264	-1.910445	0.0106383	0.0332196	0.0175368
hsa05321	Inflammatc	63	0.4983318	1.4835685	0.0109529	0.0336793	0.0177795
hsa00310	Lysine deg	60	-0.425712	-1.643743	0.010989	0.0336793	0.0177795
hsa00980	Metabolism	64	-0.760185	-2.972792	0.0114943	0.0349046	0.0184263
hsa00010	Glycolysis	65	-0.477323	-1.878179	0.0119048	0.0357318	0.018863
hsa05211	Renal cell	66	0.4888732	1.4653312	0.0119826	0.0357318	0.018863
hsa05204	Chemical c	67	-0.794663	-3.129247	0.0121951	0.0360409	0.0190262
hsa04114	Oocyte mei	118	0.4516066	1.4317428	0.012487	0.0365769	0.0193092
hsa00983	Drug metak	68	-0.522893	-2.056139	0.0126582	0.0367533	0.0194023
hsa04664	Fc epsilor	65	0.4921575	1.4714322	0.0130719	0.0375454	0.0198204
hsa03320	PPAR signa	75	-0.685438	-2.73843	0.0131579	0.0375454	0.0198204
hsa01230	Biosynthes	69	-0.397162	-1.543291	0.0138889	0.0386321	0.0203941
hsa03015	mRNA surve	92	0.4575021	1.4123635	0.0138889	0.0386321	0.0203941
hsa04920	Adipocytok	69	-0.436383	-1.695697	0.0138889	0.0386321	0.0203941
hsa04659	Th17 cell	104	0.4479328	1.4060178	0.0146597	0.0404363	0.0213466
hsa04976	Bile secre	82	-0.61826	-2.489994	0.0149254	0.0404448	0.021351
hsa04540	Gap juncti	84	0.4671779	1.4328346	0.0149893	0.0404448	0.021351
hsa02010	ABC transp	43	-0.433901	-1.589341	0.0150376	0.0404448	0.021351
hsa04146	Peroxisome	81	-0.653444	-2.622469	0.0151515	0.0404448	0.021351
hsa05202	Transcript	167	0.423414	1.3721098	0.0152905	0.0404893	0.0213745
hsa04514	Cell adhes	138	0.4333077	1.3911269	0.0155119	0.0407495	0.0215119
hsa04670	Leukocyte	109	0.4449478	1.4001649	0.0157068	0.0409366	0.0216107
hsa04610	Complement	83	-0.690815	-2.769248	0.015873	0.0410466	0.0216688
hsa04260	Cardiac mu	80	0.4625944	1.4134818	0.01606	0.0412081	0.021754
hsa05330	Allograft	35	0.5370848	1.469554	0.0162791	0.041449	0.0218812
hsa04151	PI3K-Akt s	344	0.3751756	1.2584184	0.0180361	0.0453411	0.0239358
hsa05222	Small cell	92	0.4509947	1.3922744	0.0181624	0.0453411	0.0239358
hsa05167	Kaposi sar	185	0.4085907	1.3343302	0.0182186	0.0453411	0.0239358
hsa05161	Hepatitis	156	0.4176635	1.350271	0.0184615	0.0456028	0.024074
hsa00520	Amino suga	46	0.5129374	1.4715624	0.0191226	0.0468858	0.0247513
hsa04217	Necroptosi	124	0.428601	1.3626916	0.0198123	0.0482197	0.0254554
hsa04931	Insulin re	105	-0.400815	-1.736655	0.0212766	0.0510902	0.0269708
hsa00600	Sphingolip	47	0.5046469	1.4527467	0.0213004	0.0510902	0.0269708
hsa04390	Hippo sigr	153	0.4156388	1.3419161	0.0215164	0.0512369	0.0270482
hsa04922	Glucagon s	102	-0.401346	-1.707105	0.0217391	0.0513975	0.0271331
hsa04926	Relaxin si	128	0.4229212	1.3454949	0.0219436	0.0515129	0.027194
hsa04961	Endocrine	53	0.4940943	1.4394356	0.0223964	0.0522057	0.0275597
hsa04066	HIF-1 sigr	105	0.4368019	1.3718093	0.0230366	0.0533226	0.0281493
hsa03430	Mismatch r	22	0.5815784	1.4663912	0.0242718	0.055677	0.0293922
hsa01200	Carbon met	114	-0.405064	-1.756154	0.0243902	0.055677	0.0293922
hsa04120	Ubiquitin	132	0.4190255	1.3395596	0.024974	0.0566191	0.0298896
hsa04152	AMPK signa	119	-0.316338	-1.343334	0.0263158	0.0592553	0.0312812
hsa00770	Pantothena	20	-0.542418	-1.660635	0.0265957	0.059481	0.0314004
hsa04210	Apoptosis	134	0.4168494	1.3338149	0.026999	0.0599776	0.0316625
hsa00500	Starch anc	30	-0.443917	-1.484229	0.0275862	0.0608736	0.0321355
hsa04923	Regulatior	54	-0.397144	-1.503375	0.0277778	0.0608904	0.0321444
hsa00561	Glycerolip	58	-0.363224	-1.392908	0.0309278	0.0673494	0.0355542

hsa04330	Notch sigr	52	0.4814323	1.3951404	0.0326209	0.0705721	0.0372554
hsa04974	Protein di	99	0.4298129	1.3434458	0.0336488	0.0723231	0.0381798
hsa04115	p53 signal	72	0.4525861	1.3664272	0.0346696	0.0740363	0.0390842
hsa04915	Estrogen s	133	0.4063425	1.3002431	0.0353063	0.0749128	0.0395469
hsa01210	2-Oxocarbo	18	-0.54227	-1.595333	0.0378378	0.0797728	0.0421125
hsa05133	Pertussis	73	0.4446337	1.3450983	0.0389189	0.0815327	0.0430416
hsa00601	Glycosphir	26	0.5378422	1.4071277	0.0417163	0.0865679	0.0456997
hsa05235	PD-L1 expr	87	0.4347953	1.3335014	0.0418455	0.0865679	0.0456997
hsa00061	Fatty acic	17	-0.54679	-1.576441	0.0425532	0.0869451	0.0458988
hsa00430	Taurine ar	9	-0.679078	-1.660291	0.0425532	0.0869451	0.0458988
hsa05168	Herpes sin	452	0.3466833	1.1736636	0.045045	0.0914718	0.0482885
hsa04137	Mitophagy	64	0.4483534	1.3367962	0.0469945	0.0948487	0.0500712
hsa04071	Sphingolip	114	0.4088892	1.2933187	0.0489074	0.0981112	0.0517935

rank	leading_ec	core_enric	geneSymbol
4207	tags=36%,	6696/5315/	SPP1/PKM/E2F1/COL1A1/IKBKE/BAK1/ITGA3/CCNA2/CCNE1
5288	tags=45%,	2877/84790	GPX2/TUBA1C/NDUFA4L2/TUBA4A/COX7B2/TUBA1B/TUBA3C/
4847	tags=45%,	80201/2910	HKDC1/PYCARD/CXCL8/PFN2/ARPC1B/ITPR3/MAPK12/MAPK1
5388	tags=48%,	29108/2364	PYCARD/LY96/CXCL8/ANXA2/PFN2/ARPC1B/BAK1/MAPK12/M
5344	tags=48%,	1535/84790	CYBA/TUBA1C/NCF2/NDUFA4L2/TUBA4A/COX7B2/ITPR3/MAP
5351	tags=52%,	991/4316/9	CDC20/MMP7/PTTG1/CCNB2/SLC2A1/E2F1/IL2RG/ICAM1/BU
6921	tags=64%,	10095/3561	ARPC1B/IL2RG/DAB2/SRC/DNM1/FOLR1/CYTH4/RAB31/HRAS
3797	tags=35%,	3984/5217/	LIMK1/PFN2/ARPC1B/ITGA3/ITGB4/RAC2/LPAR2/ENAH/SRC
5851	tags=47%,	10053/891/	AP1M2/CCNB1/CCNB2/LIMK1/CDK1/BAK1/ITPR3/MAPK12/RA
5143	tags=52%,	1869/9641/	E2F1/IKBKE/BAK1/ICAM1/CCNA2/CCNE1/MAPK12/CD3D/MAP
4182	tags=43%,	1364/29108	CLDN4/PYCARD/TUBA1C/CXCL8/TUBA4A/ARPC1B/BAK1/MAPK
4189	tags=37%,	2919/29108	CXCL1/PYCARD/CYBA/CXCL8/IKBKE/ITPR3/MAPK12/MAPK13
4185	tags=44%,	8877/2207/	SPHK1/FCER1G/MAPK12/MAPK13/FCGR3A/SRC/RIPK2/ITGAM
5764	tags=57%,	5315/991/9	PKM/CDC20/CDK1/BAK1/CCNA2/CCNE1/SRC/YWHAZ/CDKN2A/
3826	tags=40%,	4605/891/9	MYBL2/CCNB1/CCNB2/FOXM1/E2F1/CXCL8/CDK1/ITPR3/CCN
5796	tags=47%,	29108/3576	PYCARD/CXCL8/KPNA2/IKBKE/BAK1/ICAM1/HLA-DMA/HLA-D
2724	tags=34%,	4318/6659/	MMP9/SOX4/CDCA5/E2F1/STMN1/BAK1/KIF23/CCNE1/CDC25
6495	tags=61%,	10460/5491	TACC3/RPP25/EIF4EBP1/NUP62/ALYREF/RAN/NUP210/RANG
3917	tags=57%,	1535/84790	CYBA/TUBA1C/NCF2/TUBA4A/TUBA1B/TUBA3C/FCGR3A/ITGA
6588	tags=70%,	6628/10262	SNRPB/SF3B4/ALYREF/HSPA6/SNRPD2/SNRPD1/SNRPA1/HSP
2874	tags=50%,	991/2810/5	CDC20/SFN/PLK1/CCNB1/PTTG1/CCNB2/CDC6/E2F1/CDK1/P
6453	tags=78%,	6132/6173/	RPL8/RPL36A/MRPL17/MRPS12/RPS2/MRPL9/RPL17/RPSA/R
3126	tags=36%,	54209/1535	TREM2/CYBA/NCF2/MAPK12/MAPK13/TYROBP/FCGR3A/CSF1/
4487	tags=51%,	10053/5535	AP1M2/LAPTM4B/CTSC/GLA/LAPTM5/PPT1/CTSV/ACP5/ATP6
3494	tags=38%,	6696/1277/	SPP1/COL1A1/ITGA3/ITGB4/COL1A2/HMMR/COL9A2/CD44/A
2888	tags=52%,	6364/2919/	CCL20/CXCL1/CXCL6/CXCL8/CXCL5/ICAM1/CSF1/HLA-DMA/
3764	tags=46%,	8612/8877/	PLPP2/SPHK1/LIMK1/MARCKSL1/ARPC1B/RAC2/FCGR3A/MAR
4903	tags=62%,	54913/6949	RPP25/TCOF1/NOP56/RAN/DKC1/NXT1/GTPBP4/NHP2/POP7/
2881	tags=46%,	1535/65108	CYBA/MARCKSL1/NCF2/MAPK12/MAPK13/FCGR3A/ITGAM/CYB
5731	tags=68%,	3108/972/3	HLA-DMA/CD74/HLA-DRB1/HLA-DQA1/HLA-DRA/TAP1/HLA-D
5486	tags=60%,	10095/6714	ARPC1B/SRC/DNM1/RHOG/WASF2/ITGA5/ARPC4/CLTA/MAD2L
4647	tags=46%,	29089/1160	UBE2T/RMI2/RAD51/FANCI/FANCD2/EME1/FANCG/FAAP24/F
2867	tags=42%,	3108/7040/	HLA-DMA/TGFB1/HLA-DRB1/HLA-DQA1/HLA-DRA/IL15RA/HL
3537	tags=38%,	3108/3123/	HLA-DMA/HLA-DRB1/HLA-DQA1/HLA-DRA/HLA-DPA1/HLA-DQ
3537	tags=51%,	3108/3123/	HLA-DMA/HLA-DRB1/HLA-DQA1/HLA-DRA/HLA-DPA1/HLA-DQ
2542	tags=46%,	4318/1869/	MMP9/E2F1/CXCL8/SRC/CDKN2A/HRAS/CDK4/TYMP/MMP1/UP
4703	tags=78%,	4171/4173/	MCM2/MCM4/RFC4/RNASEH2A/MCM6/FEN1/POLD1/MCM7/MCM3
2162	tags=39%,	2207/3108/	FCER1G/HLA-DMA/HLA-DRB1/HLA-DQA1/HLA-DRA/HLA-DPA1
4212	tags=32%,	4318/11239	MMP9/EGLN3/BIRC5/NQO1/SLC2A1/E2F1/CXCL8/EPO/IL2RG
5455	tags=41%,	25825/8479	BACE2/TUBA1C/NDUFA4L2/TUBA4A/COX7B2/ITPR3/TUBA1B/
7364	tags=55%,	2877/84790	GPX2/TUBA1C/NDUFA4L2/TUBA4A/PFN2/COX7B2/ITPR3/MAP
5019	tags=33%,	6364/2919/	CCL20/CXCL1/CXCL6/TNFRSF21/CXCL8/EPO/CXCL5/IL2RG/
5664	tags=47%,	7345/84790	UCHL1/TUBA1C/NDUFA4L2/TUBA4A/COX7B2/ITPR3/TUBA1B/
3096	tags=33%,	4318/6364/	MMP9/CCL20/CXCL1/CXCL6/CXCL5/ICAM1/MAPK12/MAPK13/
4098	tags=42%,	23643/6300	LY96/MAPK12/MAPK13/HLA-DMA/TGFB1/IRAK1/BIRC3/HLA-
6866	tags=60%,	3561/6300/	IL2RG/MAPK12/CD3D/MAPK13/NFKBIE/HLA-DMA/HLA-DRB1/
5764	tags=48%,	4318/6280/	MMP9/S100A9/CCL20/CXCL1/LCN2/CXCL6/CXCL8/CXCL5/IK
3500	tags=37%,	100133941/	CD24/EPO/ITGA3/CD3D/CSF1/ITGAM/HLA-DMA/CD7/CD44/H
5664	tags=73%,	10213/5698	PSMD14/PSMB9/PSMD2/PSMB2/PSME3/PSMB4/ADRM1/PSMB8/
4568	tags=57%,	5888/8438/	RAD51/RAD54L/POLD1/EME1/RBBP8/BRCA1/XRCC2/XRCC3/B

6148	tags=47%,	1869/3576/	E2F1/CXCL8/BAK1/ITPR3/MAPK12/RAC2/MAPK13/SRC/CDKN
4809	tags=36%,	5909/5217/	RAP1GAP/PFN2/VEGFB/MAPK12/RAC2/MAPK13/LPAR2/ENAH/
6104	tags=49%,	6364/2919/	CCL20/CXCL1/CXCL6/CXCL8/CXCL5/RAC2/SRC/HCK/PRKCD/
3551	tags=37%,	3576/1277/	CXCL8/COL1A1/ICAM1/VEGFB/MAPK12/MAPK13/COL1A2/CYB
201	tags=100%,	10/7498/15	NAT2/XDH/CYP1A2/CYP2A6
4373	tags=35%,	6696/1277/	SPP1/COL1A1/VEGFB/ITGA3/ITGB4/RAC2/SRC/COL1A2/FLN
5965	tags=50%,	1410/578/1	CRYAB/BAK1/CKAP4/TUSC3/HSPA6/TRAF2/DDIT3/SSR2/DDO
1005	tags=60%,	56898/3157	BDH2/HMGCS1/HMGCL/ACAT1/BDH1/HMGCS2
2911	tags=30%,	29108/3984	PYCARD/LIMK1/CXCL8/MAPK12/RAC2/MAPK13/SRC/IRAK1/P
2911	tags=36%,	2919/3576/	CXCL1/CXCL8/MAPK12/MAPK13/SRC/ATP6V1F/GIT1/ATP6V0
2385	tags=32%,	2539/5315/	G6PD/PKM/SLC16A3/SLC1A5/HKDC1/PFKP/SLC2A1/HK2/GLS
6179	tags=67%,	3383/5880/	ICAM1/RAC2/HLA-DMA/ITGB2/HLA-DRB1/HLA-DQA1/HLA-DR
3537	tags=47%,	3108/3123/	HLA-DMA/HLA-DRB1/HLA-DQA1/HLA-DRA/HLA-DPA1/HLA-DQ
5143	tags=43%,	9641/3561/	IKBKE/IL2RG/BAK1/CCNE1/CD3D/IRAK1/MX2/CDK4/HSPA6/
483	tags=71%,	3295/1109/	HSD17B4/AKR1C4/AMACR/SCP2/CYP39A1/CYP27A1/BAAT/AC
5150	tags=40%,	6696/23643	SPP1/LY96/CXCL8/IKBKE/MAPK12/MAPK13/IRAK1/CTSK/CC
893	tags=67%,	435/2805/9	ASL/GOT1/ACY1/GLUD1/ASS1/GLUL/GOT2/GPT2/ARG1/NAGS
974	tags=58%,	7367/219/2	UGT2B17/ALDH1B1/ALDH3A2/ALDH9A1/UGT2B4/ALDH7A1/UG
1168	tags=68%,	3067/3034/	HDC/HAL/MAOA/ALDH1B1/ALDH3A2/ASPA/ALDH9A1/HNMT/MA
974	tags=42%,	7367/9942/	UGT2B17/XYLB/UGP2/UGT2B4/CRYL1/UGT2B7/DCXR/UGT2B1
358	tags=28%,	1573/84647	CYP2J2/PLA2G12B/CYP1A2/CYP2E1/CYP2C9/CYP2C8/CYP3A
1005	tags=69%,	79944/5689	L2HGDH/BDH2/HMGCS1/HMGCL/ACSM1/HADH/ACADS/ECHS1/A
734	tags=31%,	3171/3375/	FOXA3/IAPP/FOXA2/NR5A2/HNF4A/GCK/PKLR/SLC2A2
940	tags=55%,	23417/2627	MLYCD/HIBCH/ALDH1B1/ALDH3A2/ALDH9A1/ACADS/ECHS1/A
1191	tags=55%,	55902/5095	ACSS2/PCCA/AMT/PCCB/ACO1/MCEE/GLUL/ACAT1/HOGA1/GL
1053	tags=47%,	8659/435/2	ALDH4A1/ASL/GOT1/ASPA/GLUD1/ASS1/GLUL/GOT2/GPT2/G
1520	tags=27%,	2271/6392/	FH/SDHD/ACO1/SUCLG2/PC/OGDHL/PCK2/PCK1
6180	tags=65%,	3561/915/1	IL2RG/CD3D/ADA/TAP1/JAK3/CD3E/RFXANK/RFX5/CD79A/L
1191	tags=58%,	593/55902/	BCKDHA/ACSS2/PCCA/PCCB/DBT/MLYCD/HIBCH/ACACB/SUCL
1500	tags=30%,	23410/5498	SIRT3/NMRK1/SIRT1/NT5E/SIRT5/NNT/ENPP1/NADK2/AOX1
974	tags=24%,	7367/1356/	UGT2B17/CP/UGT2B4/ALAS1/ALAD/UGT2B7/UGT2B15/UGT2B
875	tags=50%,	128/1644/4	ADH5/DDC/MAOA/FAH/GOT1/MAOB/GSTZ1/GOT2/DBH/HGD/AD
4182	tags=37%,	29108/2207	PYCARD/FCER1G/IKBKE/ITPR3/MAPK12/MAPK13/SRC/RELB/
1128	tags=58%,	23464/275/	GCAT/AMT/MAOA/CBS/MAOB/GAMT/GLYCTK/ALDH7A1/ALAS1/
1191	tags=45%,	55902/98/2	ACSS2/ACYP2/ALDH1B1/ALDH3A2/ACACB/ALDH9A1/PC/ACAT
952	tags=60%,	169355/111	IDO2/INMT/MAOA/ALDH1B1/ALDH3A2/ALDH9A1/HADH/ECHS1
834	tags=31%,	948/8613/2	CD36/PLPP3/GOT2/ACAT1/DGAT2/PLA2G12B/MTTP/APOB/FA
1242	tags=74%,	128/37/303	ADH5/ACADVL/HADHB/CPT2/ALDH1B1/ALDH3A2/ALDH9A1/HA
5072	tags=40%,	5347/891/9	PLK1/CCNB1/CCNB2/CDK1/PKMYT1/BUB1/CCNA2/MAPK12/MA
824	tags=29%,	2729/2805/	GCLC/GOT1/CBS/GOT2/TST/ADI1/BHMT2/SDS/CTH/AGXT2/M
771	tags=45%,	7367/1583/	UGT2B17/CYP11A1/CYP17A1/SULT1E1/HSD17B2/HSD17B8/C
974	tags=56%,	7367/54884	UGT2B17/RETSAT/DHRS4/CYP2C18/ALDH1A1/CYP26A1/CYP3
1075	tags=46%,	1071/55908	CETP/ANGPTL8/LDLR/CD36/APOE/APOC2/LIPG/ANGPTL4/LI
1229	tags=44%,	6303/11248	SAT1/SAT2/ALDH4A1/AGMAT/MAOA/ALDH1B1/GOT1/ALDH3A2
6248	tags=55%,	29108/9641	PYCARD/IKBKE/IL18/CCL5/POLR3K/IRF3/POLR2H/IRF7/CC
5661	tags=55%,	11015/9296	KDELR3/ATP6V1F/ATP6V0B/SLC12A2/SEC61A1/SEC61G/ATP
1166	tags=66%,	64087/3032	MCCC2/HADHB/PCCA/PCCB/DBT/HMGCS1/HMGCL/HIBCH/ALDH
4112	tags=42%,	2919/23643	CXCL1/LY96/CXCL8/ICAM1/IRAK1/BIRC3/BCL2A1/RELB/SY
2913	tags=32%,	6364/2919/	CCL20/CXCL1/CXCL6/CXCL8/CXCL5/IL2RG/CSF1/IL18/CSF
2162	tags=35%,	3880/3383/	KRT19/ICAM1/FCGR3A/ITGAM/KRT23/C1QB/HLA-DMA/C1QC/
3339	tags=31%,	4318/1277/	MMP9/COL1A1/ITPR3/MAPK12/PLAUR/MAPK13/SRC/GPC3/CO

358	tags=18%,	1573/84647	CYP2J2/PLA2G12B/EPHX2/CYP4F3/CYP4F2/CYP2B6/CYP2E1
1313	tags=43%,	79071/37/3	ELOVL6/ACADVL/HADHB/CPT2/CBR4/HADH/CPT1A/ACADS/EC
1242	tags=59%,	128/4257/2	ADH5/MGST1/GSTM5/FMO2/GSTO1/MGST2/UGT2B17/MAOA/MA
6866	tags=46%,	2207/3383/	FCER1G/ICAM1/RAC2/TYROBP/FCGR3A/ITGB2/SYK/HRAS/IF
875	tags=53%,	1644/4128/	DDC/MAOA/GOT1/MAOB/GOT2/PAH/HPD/GLYAT/TAT
3615	tags=37%,	3561/3108/	IL2RG/HLA-DMA/TGFB1/HLA-DRB1/HLA-DQA1/IL18/HLA-DR
843	tags=25%,	219/224/22	ALDH1B1/ALDH3A2/ALDH9A1/HADH/ECHS1/BBOX1/AADAT/AC
610	tags=52%,	128/4257/2	ADH5/MGST1/GSTM5/GSTO1/MGST2/UGT2B17/AKR7L/UGT2B4
922	tags=29%,	5236/219/2	PGM1/ALDH1B1/ALDH3A2/ALDH9A1/GCK/ALDH7A1/ENO3/PKL
4490	tags=41%,	112399/651	EGLN3/SLC2A1/TGFB1/HRAS/ARNT2/HIF1A/MAP2K2/MAPK3/
1242	tags=55%,	128/4257/9	ADH5/MGST1/NAT1/GSTM5/SULT1A1/GSTO1/MGST2/UGT2B17
2951	tags=28%,	991/5347/8	CDC20/PLK1/CCNB1/PTTG1/CCNB2/CDK1/PKMYT1/BUB1/ITP
1211	tags=35%,	4257/9/294	MGST1/NAT1/GSTM5/GSTO1/MGST2/UGT2B17/TPMT/UGT2B4/
2881	tags=29%,	2207/6300/	FCER1G/MAPK12/ALOX5AP/RAC2/MAPK13/SYK/HRAS/VAV1/A
948	tags=45%,	3157/1376/	HMGCS1/CPT2/CD36/AQP7/PPARA/CPT1A/ACAA1/ACADM/ACO
999	tags=33%,	48/435/280	ACO1/ASL/GOT1/CBS/ACY1/ASS1/PC/GLUL/GOT2/GPT2/ARG
6224	tags=53%,	23381/1018	SMG5/ALYREF/CSTF2/UPF3B/PPP2R1A/PELO/PABPC1/PPP2R
834	tags=22%,	948/32/514	CD36/ACACB/PRKAG2/PPARA/CPT1A/IRS1/IRS2/ACSL6/PPA
3551	tags=34%,	3561/6300/	IL2RG/MAPK12/CD3D/MAPK13/NFKBIE/HLA-DMA/TGFB1/HLA
974	tags=38%,	7367/8671/	UGT2B17/SLC4A4/NR0B2/ABCC2/CA2/SLCO1A2/AQP8/LDLR/
2322	tags=26%,	84790/983/	TUBA1C/CDK1/TUBA4A/ITPR3/TUBA1B/TUBA3C/SRC/HRAS/T
445	tags=28%,	23461/1034	ABCA5/ABCA10/ABCC2/ABCA9/ABCC9/ABCG2/ABCA8/ABCB11
1830	tags=53%,	83594/5825	NUDT12/ABCD3/MPV17L/PAOX/PEX13/PEX1/NOS2/PEX12/PE
2986	tags=25%,	4318/6692/	MMP9/SPINT1/ETV4/SSX1/CXCL8/BAK1/CCNA2/ETV5/ITGAM
3537	tags=30%,	1364/3383/	CLDN4/ICAM1/MPZL1/ITGAM/HLA-DMA/MPZ/ITGB2/SDC3/VC
3779	tags=32%,	1364/4318/	CLDN4/MMP9/CYBA/NCF2/ICAM1/MAPK12/RAC2/MAPK13/ITG
1103	tags=60%,	1191/2157/	CLU/F8/C8G/C1R/SERPINA1/C2/PROS1/VWF/CFI/C5/C1S/S
5288	tags=38%,	486/483/17	FXYD2/ATP1B3/COX7B2/ATP1A1/TPM4/ASPH/UQCRH/TPM2/T
6818	tags=71%,	3108/3123/	HLA-DMA/HLA-DRB1/HLA-DQA1/HLA-DRA/HLA-DPA1/HLA-DQ
4212	tags=32%,	6696/2056/	SPP1/EPO/COL1A1/IL2RG/VEGFB/ITGA3/CCNE1/ITGB4/LPA
3826	tags=37%,	1869/578/3	E2F1/BAK1/ITGA3/CCNE1/CKS2/BIRC3/ITGAV/COL4A5/LAM
4462	tags=34%,	2919/1869/	CXCL1/E2F1/CXCL8/IKBKE/BAK1/ITPR3/ICAM1/MAPK12/MA
4093	tags=32%,	4318/332/1	MMP9/BIRC5/E2F1/CXCL8/IKBKE/CCNA2/CCNE1/MAPK12/MA
4370	tags=57%,	80201/5973	HKDC1/RENBP/UAP1L1/GNPDA1/HK2/NPL/CHIT1/HK3/GPI/G
4882	tags=38%,	29108/1536	PYCARD/CYBB/PYGB/SQSTM1/BIRC3/IFNGR2/SLC25A6/TRAF
917	tags=20%,	84699/5836	CREB3L3/PYGL/CD36/ACACB/PRKAG2/PPARA/CPT1A/FOXO1/
4259	tags=34%,	8612/8877/	PLPP2/SPHK1/GLA/GAL3ST1/SGPP2/NEU1/CERS5/DEGS1/SM
3461	tags=29%,	332/174/84	BIRC5/AFP/TEAD2/TGFB1/YWHAZ/ITGB2/TEAD4/BIRC3/FZD
917	tags=20%,	84699/5836	CREB3L3/PYGL/PDE3B/ACACB/PRKAG2/CAMK2B/PPARA/CPT1
3396	tags=27%,	4318/1277/	MMP9/COL1A1/VEGFB/MAPK12/MAPK13/SRC/COL1A2/TGFB1/
5086	tags=43%,	486/483/17	FXYD2/ATP1B3/DNM1/ATP1A1/PLCB1/CLTA/PLCB3/CLTB/AP
3129	tags=30%,	112399/226	EGLN3/ALDOA/HKDC1/ENO2/PFKP/SLC2A1/ENO1/EPO/TIMP1
4451	tags=59%,	5984/9156/	RFC4/EXO1/POLD1/PCNA/RFC2/LIG1/MSH2/POLD3/RPA2/SS
1024	tags=33%,	128/55902/	ADH5/ACSS2/PCCA/AMT/PCCB/ACO1/HIBCH/GOT1/SUCLG2/M
8241	tags=65%,	991/27338/	CDC20/UBE2S/BIRC3/SAE1/UBE2M/UBE2Z/UBE2I/UBE2Q2/B
940	tags=17%,	23417/8469	MLYCD/CREB3L3/CD36/ACACB/PRKAG2/ADRA1A/HNF4A/CPT1
843	tags=45%,	1806/79646	DPYD/PANK3/ALDH1B1/ALDH3A2/ENPP1/PANK1/ALDH2/DPYS
3099	tags=31%,	332/84790/	BIRC5/TUBA1C/CTSC/TUBA4A/BAK1/ITPR3/TUBA1B/LMNB2/
926	tags=33%,	178/5236/5	AGL/PGM1/PYGL/ENPP1/TREH/UGP2/GCK/GBA3/GYS2/G6PC
1865	tags=28%,	196883/364	ADCY4/INSR/NPY1R/ADCY8/ADRB1/NPR1/ADCY1/PDE3B/AQP
843	tags=26%,	219/224/22	ALDH1B1/ALDH3A2/ALDH9A1/LPIN2/PNPLA3/PLPP3/LIPG/T

6359	tags=58%,	196403/30	DTX3/HDAC1/PSENEN/NUMBL/HDAC2/DVL3/HEYL/DTX2/JAG2
3544	tags=28%,	6510/486/	SLC1A5/FXYD2/ATP1B3/COL1A1/SLC7A7/COL1A2/ATP1A1/C
3512	tags=36%,	2810/891/	SFN/CCNB1/CCNB2/RRM2/CDK1/CCNE1/CDKN2A/CDK4/GTSE1
5072	tags=33%,	4318/3880/	MMP9/KRT19/ITPR3/SRC/KRT23/KRT17/PRKCD/TFF1/HRAS/
999	tags=44%,	48/2805/9	ACO1/GOT1/ACY1/AADAT/GOT2/GPT2/NAGS/GPT
2196	tags=27%,	29108/236	PYCARD/LY96/CXCL6/CXCL8/CXCL5/MAPK12/MAPK13/ITGAM
3485	tags=46%,	10331/252	B3GNT3/FUT4/B3GNT5/B4GALT3/B3GALNT1/ST3GAL4/FUT3/
6866	tags=53%,	6300/915/	MAPK12/CD3D/MAPK13/NFKBIE/BATF/HRAS/IFNGR2/CD3E/H
794	tags=35%,	32/84869/	ACACB/CBR4/HSD17B8/ACSL6/ACSL5/ACSL1
360	tags=33%,	51380/570/	CSAD/BAAT/CDO1
8379	tags=51%,	9641/578/	IKBKE/BAK1/SRC/HLA-DMA/IRAK1/CD74/BIRC3/HLA-DRB1/
6747	tags=58%,	1869/6714/	E2F1/SRC/SQSTM1/HRAS/FUNDC1/HIF1A/RAB7B/RRAS/TFE3
3975	tags=32%,	8877/2207/	SPHK1/FCER1G/MAPK12/RAC2/MAPK13/S1PR2/SGPP2/HRAS/

/ITGB4/COL1A2/COL9A2/TUBG1/EIF4EBP1/ITGAV/COL4A5/LAMB1/HES6/MX2/HRAS/ATP6V1F/CD
KLC2/TUBB/PLCB1/SLC25A6/CREB3L1/IFT57/PSMD14/TUBB4B/TRAF2/UQCRH/BAX/CREB3/PSMD2
3/SRC/RIPK2/CD44/HK2/SQSTM1/ARHGEF2/CYTH4/IL18/PRKCD/CASP4/PLCD3/PLCB1/TRAF2/CC
APK13/RIPK2/GAPDH/FLNC/IRAK1/FLNA/BIRC3/KLC2/PKN1/CYTH4/IL18/HRAS/CASP4/S100A10
K12/TUBA1B/RAC2/MAPK13/TUBA3C/CYBB/C1QB/STIP1/C1QC/C1QA/KLC2/PRNP/PRKCD/TUBB/NC
B1B/CCNA2/CCNE1/CD3D/TSPO/SPI1/HLA-DMA/TGFB1/ITGB2/CDKN2A/HLA-DRB1/RELB/HLA-DQA
/GIT1/ASAP1/HSPA6/DNAJC6/TFRC/CXCR4/GRK6/CHMP4B/RAB11FIP5/WIPF3/HSPA8/VPS37C/AR
/ITGAM/IQGAP3/ITGB2/ITGAV/HRAS/GIT1/TMSB4X/EZR/FGD3/CFL1/WASF2/CXCR4/VAV1/BDKRB
C2/CD3D/MAPK13/CDC25C/IRAK1/APOBEC3B/TAP1/HRAS/CHEK1/CFL1/TRAF2/APOBEC3C/CXCR4/
K13/NFKBIE/HLA-DMA/IRAK1/CD44/HLA-DRB1/RELB/HLA-DQA1/HLA-DRA/SYK/TAP1/CDK4/HLA-
12/TUBA1B/MAPK13/TUBA3C/LPAR2/SRC/GAPDH/IRAK1/ARHGEF2/CYTH4/IL18/NCK2/CASP4/TUB
/RIPK2/CYBB/BIRC3/PKN1/IL18/PRKCD/CASP4/PLCB1/TRPV2/TRPM2/GBP5/TRAF2/CCL5/GBP2/
/HLA-DMA/TGFB1/IRAK1/CORO1A/FCGR2A/ITGB2/CD74/HLA-DRB1/HLA-DQA1/IL18/HLA-DRA/SY
HDAC11/SYK/HRAS/CDK4/HDAC7/CHEK1/CREB3L1/SCRIB/TRAF2/JAK3/MAPKAPK2/BAX/NFKB2/CR
A2/CCNE1/MAPK12/MAPK13/TGFB1/E2F5/SQSTM1/CDKN2A/EIF4EBP1/CDC25A/HRAS/CDK4/CHEK1
RB1/HLA-DQA1/IL18/HLA-DRA/MX2/CDK4/HLA-DPA1/IFNGR2/HLA-DQB1/SLC25A6/HLA-DMB/CCL
B/MARCKS/EFNA3/CDC25C/CD44/SLC7A1/CDKN2A/GLS/IGF2BP1/EFNA4/CDC25A/HRAS/DNMT1/EZ
AP1/EIF3B/UBE2I/UPF3B/EIF2S2/PABPC1/NUP93/NUP37/RNPS1/NDC1/NXT1/NUP205/NUP188/N
M/CYBB/HLA-DMA/CORO1A/FCGR2A/ITGB2/HLA-DRB1/ITGAV/HLA-DQA1/HLA-DRA/TAP1/ATP6V1F
A8/SNRPA/SF3A2/LSM4/PPIH/EFTUD2/PUF60/SNRPE/SNRPF/NCBP2/LSM7/TXNL4A/SF3B5/SNRPC
KMYT1/CDC45/MCM2/BUB1/BUB1B/CCNA2/ORC6/MCM4/CCNE1/CDC25B/ORC1/TTK/TGFB1/YWHAZ/C
PLP2/RPL10L/RPL39/RPL28/RPS5/MRPL12/RPL7/MRPL13/RPS21/RPL35A/RPS20/RPS3/RPL27/R
SPI1/TGFB1/FCGR2A/SQSTM1/RELB/ACP5/SYK/CSF1R/IFNGR2/NCF4/CTSK/NCF1/TNFRSF11B/TR
VOB/CTSK/CTSA/CLTA/GM2A/TCIRG1/ATP6V0D2/NEU1/ATP6AP1/FUCA2/AP4M1/SORT1/CD63/NPC
GRN/ITGAV/COL4A5/LAMB1/COMP/LAMC1/ITGA5/RELN/LAMC2/LAMA5/FRAS1/COL6A3/TNC/ITGB5
TGFB1/ITGB2/HLA-DRB1/HLA-DQA1/ACP5/IL18/HLA-DRA/ATP6V1F/HLA-DPA1/HLA-DQB1/ATP6V
CKS/HCK/SYK/VASP/PRKCD/ASAP1/NCF1/CFL1/WASF2/VAV1/LIMK2/ARPC4/MAPK3/ARPC3/ARPC2
FBL/NOP58/RRP7A/UTP14A/UTP18/EMG1/POP1/GNL2/SNU13/DROSHA/UTP4/GNL3/UTP6/POP5/NO
B/HLA-DMA/TGFB1/IRAK1/FCGR2A/ITGB2/HLA-DRB1/HLA-DQA1/HLA-DRA/HLA-DPA1/IFNGR2/NC
PA1/HLA-DQB1/IFI30/HSPA6/HLA-DMB/HLA-DPB1/HSPA8/HLA-A/HSP90AB1/RFXANK/CTSS/PSME
2/ARPC3/ARPC2/HCLS1/PIK3CD/ACTG1/ARPC5L/SHC1/RAC1/BCAR1/ACTB/WASF1/CLTB/CTTN/CD
ANCE/BRCA1/BLM/FANCA/USP1/RAD51C/TELO2/EME2/RPA2/ERCC1/FANCF/RPA1/MUS81/ATR
A-DPA1/HLA-DQB1/HLA-DMB/CD86/CXCR4/HLA-DPB1/HLA-DQA2/CCL28/HLA-DRB5/IL15/TNFSF1
B1/HLA-DMB/CD86/HLA-DPB1/HLA-A/CTLA4/HLA-DQA2/HLA-B/HLA-DRB5/GZMB/HLA-DOA/HLA-F
B1/HLA-DMB/CD86/HLA-DPB1/HLA-A/HLA-DQA2/HLA-B/HLA-DRB5/IL1B/ICA1/GAD1/CPE/GZMB/
K3A/MMP2/MAP2K2/MAPK3/E2F3/DAPK3/NRAS/FGFR3/RASSF1/E2F2
/MCM5/POLA2/PCNA/RFC2/LIG1/PRIM1/POLA1/POLE4/PRIM2/POLD3/DNA2/RPA2/SSBP1/RFC5/R
/HLA-DQB1/HLA-DMB/HLA-DPB1/HLA-DQA2/HLA-DRB5
/BAK1/VEGFB/ITGA3/CCNA2/CCNE1/RAC2/CKS2/LPAR2/RAD51/SPI1/TXNRD1/GSTP1/TGFB1/CDK
TUBA3C/CSF1/GAPDH/CYBB/KLC2/HRAS/DKK1/TUBB/FZD6/PLCB1/SLC25A6/PSMD14/FZD7/TUBB4
K12/TUBA1B/MAPK13/TUBA3C/SQSTM1/KLC2/NUP62/TUBB/ALYREF/TOMM40/PSMD14/TUBB4B/TRA
CSF1/LIF/TNFRSF12A/OSMR/TGFB1/GDF15/CLCF1/IL18/CSF1R/CSF3R/IL15RA/RELT/IFNGR2/L
TUBA3C/KLC2/TUBB/SLC25A6/PSMD14/TUBB4B/DDIT3/UQCRH/BAX/PSMD2/TUBA1A/CYC1/TUBB6/
MMP14/CSF1/LIF/BIRC3/CREB3L1/TRAF2/CCL5/CREB3/TRAF5/MAPK3/IRF1/SOCS3/RPS6KA4/PI
DRB1/LAMB1/HLA-DQA1/HLA-DRA/HLA-DPA1/IFNGR2/HLA-DQB1/HSPA6/HLA-DMB/LAMC1/HLA-DP
HLA-DQA1/HLA-DRA/HLA-DPA1/IFNGR2/HLA-DQB1/HLA-DMB/JAK3/HLA-DPB1/CD3E/MAPK3/STAT
BKE/MAPK12/MAPK13/S100A8/MMP1/TRAF2/TRAF5/MAPK3/HSP90AB1/CCL2/CXCL3/IL1B/MAPK11
LA-DRB1/HLA-DQA1/HLA-DRA/CSF1R/CSF3R/HLA-DPA1/HLA-DQB1/HLA-DMB/TFRC/CD37/ITGA5/
PSMA5/PSMC4/PSMB10/PSMD13/PSMD11/PSMA7/PSMD1/PSMD7/PSMB3/PSMD4/PSMD3/PSMD9/PSMD
LM/TOPBP1/BARD1/POLD3/RAD51C/BABAM1/RPA2/SSBP1/NBN/RAD51D/UIMC1/RPA1/MUS81

2A/EIF4EBP1/ITGAV/TAP1/HRAS/CDK4/PLCB1/CREB3L1/TRAF2/CCL5/CXCR4/BAX/CREB3/HLA-A
SRC/CSF1/ITGAM/EFNA3/ITGB2/EFNA4/VASP/HRAS/CSF1R/PGF/PLCB1/VAV1/APBB1IP/EFNA5/R
HRAS/PLCB1/NCF1/CCL5/JAK3/CXCR4/VAV1/GRK6/CCL26/MAPK3/GNGT1/CXCL13/STAT1/PAK1/C
B/TGFB1/COL4A5/PRKCD/HRAS/PLCD3/CDK4/COL3A1/PLCB1/BAX/SERPINE1/MMP2/MAPK3/STAT1

C/FLNA/COL9A2/BIRC3/ITGAV/COL4A5/LAMB1/VASP/HRAS/PGF/ZYX/COMP/LAMC1/VAV1/ITGA5/
ST/BAX/DNAJC5/HSPA8/SEC61A1/SEC61G/HSP90AB1/HYOU1/HSPH1/NPLOC4/MAN1B1/HSPBP1/UB

KN1/IL18/RHOG/RPS6KA1/TRAF2/WASF2/VAV1/ITGA5/WIPF3/MAP2K2/MAPK3/WIPF1/PIP5K1C/I
B/CCL5/ATP6V1C1/TCIRG1/ATP6V0D2/ATP6AP1/PAK1/ATP6V1H/LYN/CXCL3/RAC1/ATP6V1E1/AT
/SLC7A5/HRAS/SIRT6/HIF1A/MAP2K2/MAPK3/HK3/FGFR1/PIK3CD/LDHA/NRAS/PFKM/FGFR3
A/HLA-DPA1/HLA-DQB1/HLA-DMB/CD86/HLA-DPB1/HLA-A/HLA-DQA2/ACTG1/HLA-B/RAC1/HLA-D
B1/HLA-DMB/CD86/HLA-DPB1/HLA-A/HLA-DQA2/HLA-B/HLA-DRB5/IL1B/GZMB/HLA-DOA/HLA-F
JAK3/MSN/BAX/HSPA8/CD3E/STAT1/IRF3/IRF7/PIK3CD/CDK2/BBC3/CD209/BID/IL1B/IL2RA/C
OX2/CYP7A1/AKR1D1/SLC27A5/CYP8B1
L5/CD86/MAP2K2/MAPK3/STAT1/IRF3/IRF7/CCL4/PIK3CD/RAC1/IL1B/CXCL9/CCL3/MAPK11/CX
/GLS2/GPT/CPS1/OTC
T2B7/ALDH2/UGT2B15/RGN/UGT2B10
OB/ALDH7A1/CNDP1/ALDH2/AMDHD1/FTCD/UROC1
5/SORD/UGT2B10
4
CAT1/ACSM3/ALDH5A1/BDH1/ABAT/ACSM5/EHHADH/HMGCS2/ACSM2B/ACSM2A

COX1/ALDH7A1/CNDP1/ALDH2/ALDH6A1/ABAT/DPYS/EHHADH/UPB1
YCTK/GRHPR/SHMT1/CAT/HAO2/HAO1/AGXT
LS2/GPT/ALDH5A1/AGXT2/ABAT/CPS1/AGXT

CK/CD8A/BTK/IKBKG/CIITA/TAP2/PTPRC/CD4/DCLRE1C/ICOS/UNG/CD19/CD8B
G2/BCKDHB/MCEE/ACADS/ECHS1/ACSS3/ACAT1/ACOX1/ALDH6A1/ABAT/EHHADH
/ASPDH
10
H6/AOX1/HPD/ADH1A/ADH1C/TAT/ADH4/ADH1B
SYK/PRKCD/HRAS/LSP1/MAPKAPK2/NFKB2/CARD9/MAPK3/RRAS/STAT1/PAK1/IRF1/PIK3CD/BCL3
GRHPR/SDS/SHMT1/SARDH/CTH/GATM/AGXT2/PIPOX/DMGDH/DAO/AGXT/GNMT/BHMT
1/HAGH/ALDH7A1/GRHPR/LDHD/PKLR/ACOT12/ALDH2/PCK2/PCK1
/MAOB/AADAT/KMO/ACMSD/ACAT1/GCDH/ALDH7A1/TDO2/DHTKD1/CAT/HAAO/ALDH2/CYP1A1/ALDH
BP1/ABCG8/ABCG5/APOA1/MOGAT2
DH/CPT1A/ACADS/ECHS1/ECI2/ACAA1/ACADM/ACAT1/ACOX1/GCDH/ACAA2/ACSL6/ALDH7A1/ACSL
PK13/CDC25B/AURKA/CDC25C/MAD2L1/CDC25A/RPS6KA1/MAPK3/HSP90AB1/MAD2L2/ANAPC7/PIK
AT1A/CDO1/BHMT/TAT
YP3A7/SRD5A1/UGT2B4/AKR1C4/UGT2B7/SRD5A2/CYP3A5/CYP1A1/UGT2B15/CYP7A1/UGT2B10/A
A7/UGT2B4/RDH5/UGT2B7/CYP3A5/CYP1A1/UGT2B15/ADH6/AOX1/UGT2B10/RDH16/CYP1A2/HSD1
PC/CYP27A1/APOB/APOC1/ABCB11/ABCG8/LPA/APOH/ABCG5/APOA2/LCAT/ANGPTL3/CYP7A1/APO
/ALDH9A1/PRODH/MAOB/GAMT/GOT2/HOGA1/ALDH7A1/ARG1/CNDP1/PRODH2/ALDH2/GATM/DAO
L4/CASP1/IL1B/POLR2K/IKBKG/POLR2L/POLR3C/POLR3D/POLR3H/MAVS/NFKBIB/CXCL10/POLR2
6V1C1/TCIRG1/ATP6V0D2/ATP6AP1/ATP6V1H/ACTG1/KCNQ1/KDELR1/ACTB/ATP6V1E1/ATP6V0C/
1B1/ALDH3A2/ALDH9A1/BCKDHB/HADH/MCEE/ACADS/HIBADH/ECHS1/ACAA1/ACADM/ACAT1/IVD/A
K/LTB/TRAF2/PLAU/NFKB2/UBE2I/TRAF5/EDA/CARD10/TNFRSF11A/CCL13/LYN/CCL4/CARD11/C
1R/CCL5/CXCR4/CCL26/IL34/CXCL13/CCL13/CCL4/CCL2/CCL28/CXCL3/TNFRSF10C/IL2RA/CXC
FCGR2A/ITGB2/KRT17/C1QA/HLA-DRB1/PTAFR/HLA-DQA1/HLA-DRA/HLA-DPA1/HLA-DQB1/HLA-D
L1A2/FLNC/TGFB1/FLNA/CD44/ITGAV/HRAS/GPC1/FZD6/EZR/FZD7/PLAU/VAV1/MSN/ITGA5/IQG

/CYP4A11/CYP2C9/CYP2C8
HS1/ELOVL2/HSD17B8/ACAA1/ACADM/HSD17B4/ACAT1/ACOX1/ACAA2/ACSL6/SCP2/ACSL5/ACADL
OB/UGT2B4/GSTA2/FMO4/UGT2B7/FMO5/CYP3A5/GSTM1/UGT2B15/CYP2D6/ADH6/GSTA1/AOX1/UG
NGR2/HCST/VAV1/HLA-A/MAP2K2/MAPK3/PAK1/ULBP2/PIK3CD/SHC1/LCP2/HLA-B/RAC1/BID/NR

A/HLA-DPA1/IFNGR2/HLA-DQB1/HLA-DMB/HLA-DPB1/STAT1/HLA-DQA2/TGFB2/HLA-DRB5/IL1B/
AT1/GCDH/ALDH7A1/DHTKD1/AASS/ALDH2/PIPOX/EHHADH
/CYP2A13/EPHX1/GSTA2/UGT2B7/CYP3A5/CYP1A1/GSTM1/UGT2B15/AKR7A3/CYP2D6/ADH6/GSTA
R/ALDH2/PCK2/ADH6/FBP1/G6PC/ADH1A/ALDOB/ADH1C/PCK1/ADH4/ADH1B
PAK1/TFE3/PAK4/PIK3CD/TGFA/TGFB2/RAC1/NRAS/PRCC/GRB2/TGFB3/CDC42/RBX1/VHL/BAD/P
/SULT1A2/CYP2C18/CYP3A7/UGT2B4/CYP2A13/EPHX1/GSTA2/CYP3A43/UGT2B7/NAT2/CYP3A5/C
R3/CCNE1/MAPK12/MAPK13/AURKA/YWHAZ/CDC25C/MAD2L1/RPS6KA1/ESPL1/PPP2R1A/MAPK3/SG
UPP2/CES1/GSTA2/UGT2B7/NAT2/CES2/XDH/GSTM1/UGT2B15/GSTA1/DPYS/UGT2B10/UPB1/CYP2
LOX5/MAP2K2/MAPK3/LYN/PIK3CD/LCP2/RAC1/NRAS/GRB2/BTK/MAPK11
X1/ANGPTL4/ACSL6/PLIN5/SCP2/ACSL5/PLIN1/CYP27A1/ACADL/ACSL1/FABP1/ACOX2/APOA2/S
1/NAGS/SDS/SHMT1/GPT/ENO3/PKLR/CTH/MAT1A/PAH/CPS1/OTC/ALDOB
2C/RNPS1/NXT1/NCBP2/PPP1CA/PPP1CC/DAZAP1/CPSF1/EIF4A3/CPSF6/PABPC4/MSI2/FUS/CPS
RGC1A/ACSL5/LEPR/ACSL1/PCK2/G6PC/PCK1
-DRB1/HLA-DQA1/HLA-DRA/HLA-DPA1/IFNGR2/HLA-DQB1/HLA-DMB/JAK3/HLA-DPB1/CD3E/HIF1
ADCY1/UGT2B4/ABCG2/EPHX1/BAAT/UGT2B7/ABCB11/ABCG8/SLC51A/ABCG5/UGT2B15/SLCO1B3/
UBB/PLCB1/TUBB4B/TUBA1A/MAP2K2/MAPK3/TUBB6/TUBB2A/PLCB3/CSNK1D/TUBB4A/TUBB2B/NR
/ABCG8/ABCG5/ABCA6/ABCB4
X19/DECR2/CRAT/ECH1/SOD1/MLYCD/HMGCL/DHRS4/NUDT7/PEX11A/ECI2/PEX11G/ACAA1/HSD17
/SPI1/BIRC3/BCL2A1/CSF1R/ARNT2/TCF3/DDIT3/IGFBP3/PLAU/CD86/BAX/PROM1/MLLT3/HDAC
AN/HLA-DRB1/ITGAV/HLA-DQA1/HLA-DRA/HLA-DPA1/NECTIN1/HLA-DQB1/VTCN1/HLA-DMB/CD86
AM/CYBB/ITGB2/VASP/EZR/NCF4/NCF1/CXCR4/VAV1/MSN/MMP2/THY1/PIK3CD/ACTG1/RAC1/BCA
ERPING1/CFH/CFB/FGB/F10/C3/FGG/VTN/F5/C4BPB/CFHR1/FGA/PROC/SERPINA5/CFHR2/C7/MB
PM3/CYC1/SLC9A1/SLC9A7/ATP2A3/COX6B1/COX8A/ATP1B1/CACNG4/COX7B/TNNI3/UQCRB/CACN
B1/HLA-DMB/CD86/HLA-DPB1/HLA-A/HLA-DQA2/HLA-B/HLA-DRB5/GZMB/HLA-DOA/HLA-F/HLA-C
R2/CSF1/COL1A2/OSMR/EFNA3/YWHAZ/COL9A2/EIF4EBP1/PKN1/ITGAV/COL4A5/LAMB1/SYK/EFN
B1/CDK4/LAMC1/TRAF2/BAX/LAMC2/TRAF5/LAMA5/CKS1B/E2F3/CDKN2B/PIK3CD/CDK2/BIRC7/I
PK13/SRC/HCK/SYK/HRAS/CDK4/TRAF2/CD86/MAPKAPK2/BAX/HLA-A/HIF1A/MAP2K2/MAPK3/GNG
PK13/SRC/TGFB1/YWHAZ/IRAK1/HRAS/CREB3L1/JAK3/BAX/CREB3/MAP2K2/ATP6AP1/MAPK3/PCN
FPT2/TSTA3/UGDH/GNPDA2/UXS1/NAGK/NANS/HK1/GMDS/GMPPA/AMDHD2/HEXB/GMPPB/CYB5R1/G
2/JAK3/RBCK1/CHMP4B/BAX/TRAF5/CHMP4C/HSP90AB1/FTH1/SHARPIN/STAT1/PPIA/CASP1/FTL
IRS1/PIK3R1/IRS2/PPARGC1A/MLXIPL/PPP1R3B/SLC27A2/PCK2/GYS2/SLC2A2/G6PC/SLC27A5/
PD2/ASAH1/GBA/SMPD4/CERS6/PSAP/ACER3/UGCG
6/DLG3/AREG/SCRIB/FZD7/SERPINE1/FZD1/PPP2R1A/PPP2R2C/CSNK1E/DVL3/YWHAH/CSNK1D/A
A/SIK2/FOXO1/GCK/PPARGC1A/ITPR2/PFKFB1/PCK2/FBP1/GCGR/GYS2/SLC2A2/G6PC/PCK1
COL4A5/HRAS/COL3A1/PLCB1/GNA15/CREB3L1/MMP1/CREB3/MMP2/MAP2K2/MAPK3/GNGT1/PLCB3
2M1/AP2S1/PLCB2/ATP1B1/VDR/DNM2/CLTC/RAB11A/ATP2B4/ADCY6/AP2A2/AP2B1/AP2A1/BDKR
/GAPDH/CYBB/HK2/EIF4EBP1/PFKFB3/PGK1/IFNGR2/TFRC/HMOX1/SERPINE1/HIF1A/MAP2K2/MA
BP1/RFC5/MSH6/RPA1
CEE/ACADS/ECHS1/IDNK/GLUD1/PC/GOT2/ACAT1/ACOX1/GCK/TKFC/GLYCTK/GPT2/SDS/SHMT1/G
RCA1/SOCS1/UBE2J2/ANAPC7/SOCS3/BIRC7/UBE2D1/UBE2L3/UBE2E1/UBE2A/PIAS3/ANAPC11/U
A/FOXO1/IRS1/PIK3R1/IRS2/PPARGC1A/LEPR/PFKFB1/PCK2/FBP1/GYS2/G6PC/PCK1
/UPB1
TUBA3C/LMNB1/CTSV/BIRC3/BCL2A1/HRAS/CTSK/TRAF2/DDIT3/BAX/CASP2/TUBA1A/MAP2K2/MA

7/IRS1/PIK3R1/IRS2/ADRB2/PLIN1/FABP4
KFC/GLYCTK/DGAT2/ALDH7A1/LIPC/GPAM/ALDH2/MOGAT2

/JAG1/DVL2/KAT2A/APH1B/HEY1/NCOR2/MFNG/CTBP2/ADAM17/NOTCH3/NOTCH1/LFNG/PSEN2/RB
OL9A2/COL4A5/COL3A1/COL16A1/COL5A1/COL5A2/SLC7A8/MEP1A/COL6A3/SLC3A2/KCNQ1/SLC3
/CHEK1/IGFBP3/BAX/SERPINE1/PERP/AIFM2/PMAIP1/CDK2/BBC3/BID/CCND2/CYCS/GORAB/THB
PLCB1/HSPA6/CREB3L1/HSPA8/CREB3/MMP2/MAP2K2/MAPK3/HSP90AB1/PLCB3/KRT18/FKBP4/PI

/C1QB/C1QC/IRAK1/ITGB2/C1QA/CFL1/ITGA5/MAPK3/IRF1/IRF3/CASP1/IL1B
B4GALT2/A4GALT/FUT2/FUT1/FUT6
IF1A/MAP2K2/MAPK3/STAT1/PIK3CD/BATF2/PDCD1/NRAS/LCK/PTPN6/BATF3/MAPK11/IKBKG/RP

EIF4EBP1/HLA-DQA1/HLA-DRA/SYK/TAP1/ALYREF/HLA-DPA1/NECTIN1/IFNGR2/HLA-DQB1/ZNF8
/MITF/ATF4/NRAS/PGAM5/MRAS/RHOT2/RPS27A/CSNK2B/BNIP3L/CSNK2A1/OPTN/UBA52/BECN1/
PLCB1/ADORA1/TRAF2/BAX/PPP2R1A/MAP2K2/MAPK3/CERS5/NSMAF/GNA12/PPP2R2C/DEGS1/SMP

K4/FZD6/HES4/DLG3/ATP6V0B/CREB3L1/SCRIB/FZD7/COMP/LAMC1/ITGA5/RELN/BAX/CREB3/LA
/CLTA/TUBA1A/CYC1/STX1A/TUBB6/HDAC1/KLC3/GPX8/TUBB2A/PSMB2/POLR2G/GPX7/HDAC2/PL
L5/WASF2/RBCK1/ITGA5/BAX/RRAGD/ARPC4/RRAGC/TRAF5/MAPK3/SHARPIN/HK3/ARPC3/ARPC2/
/RHOG/TRAF2/BAX/ARPC4/MAP2K2/MAPK3/HSP90AB1/RAB7B/KLC3/DYNLL1/RRAS/DYNC1H1/ARPC
F4/HSPA6/SLC25A6/CREB3L1/PSMD14/NCF1/LAMC1/TUBB4B/DDIT3/CCL5/UQCRH/BAX/HSPA8/CR
1/MAD2L1/HLA-DRA/HRAS/CDK4/IL15RA/HLA-DPA1/HLA-DQB1/CHEK1/RAN/SLC25A6/CREB3L1/H
PC4/CLTA/HLA-A/CHMP4C/WIPF1/HGS/FGFR4/ARPC3/ARPC2/RAB35/PIP5K1C/ARF3/CYTH2/SH3K
1/MSN/ITGA5/IQGAP1/ITGAX/LIMK2/ARPC4/SLC9A1/MAP2K2/MAPK3/GNA12/FGFR4/RRAS/ARPC3
BAX/LIMK2/CD3E/HLA-A/TRAF5/MAP2K2/MAPK3/GNGT1/PAK1/IRF3/PAK4/PIK3CD/GNG5/GNB2/H
DPA1/HLA-DQB1/PSMD14/HLA-DMB/TRAF2/JAK3/HLA-DPB1/BAX/NFKB2/PSMD2/CD3E/ISG15/HLA
B/EZR/TUBB4B/TRAF2/WASF2/BAX/WIPF3/ARPC4/TUBA1A/MAPK3/BAIAP2L1/TUBB6/GNA12/WIPF
RBCK1/CARD9/TRAF5/MAPK3/HSP90AB1/SHARPIN/STAT1/IRF3/PLCB3/IRF7/MCU/PSTPIP1/CCL2
K/HLA-DPA1/LSP1/IFNGR2/HLA-DQB1/ATP6V0B/HLA-DMB/MRC2/HLA-DPB1/BAX/ITGAX/CARD9/T
EB3/HLA-A/TRAF5/ATP6V0D2/MAPK3/HDAC1/RBL1/IRF3/HDAC2/LYN/IRF7/CDKN2B/YWHAH/PMAI
/SLC25A6/IGFBP3/MAPKAPK2/SERPINE1/HLA-A/MAP2K2/MAPK3/E2F3/RRAS/RBL1/E2F4/CDKN2B
5/HLA-DPB1/BAX/MAP2K2/MAPK3/STAT1/NXT1/IRF3/HLA-DQA2/IRF7/SOCS3/PIK3CD/ACTG1/CC
H2/EZR/FSCN1/PLAU/ITGA5/HMOX1/EFNA5/UBE2I/MAP2K2/MAPK3/BRCA1/HDAC1/E2F3/ST14/BM
CBP2/POP7/EIF3I/KPNB1/XPOT/EIF3D/EEF1A2/GEMIN7/NUP85/THOC6/POP1/EIF4A3/NUP43/TH
/TUBB/HLA-DPA1/NCF4/HLA-DQB1/ATP6V0B/COMP/NCF1/HLA-DMB/TUBB4B/TFRC/ITGA5/MRC2/H
/PPIL1/SRSF9/SNRNP40/U2AF2/BUD31/LSM2/PQBP1/EIF4A3/PRPF3/ISY1/SF3A3/SNU13/PRPF6
DC25C/E2F5/MCM6/CDKN2A/MCM7/MAD2L1/MCM3/CDC25A/CDK4/CDKN2D/CDC7/CHEK1/MCM5/ESPL
PLP0/RPL18A/RPL35/RPS19/RPL38/RPL23/RPL18/RPL23A/MRPL10/RPS10/MRPS15/MRPS7/RPL5
AF2/NFKB2/MAPK3/TNFRSF11A/STAT1/SOCS1/MITF/SIRPG/SOCS3/PIK3CD/SIRPA/LCP2/TGFB2/
1/CTSS/NPC2/ATP6V1H/ASAH1/CTSE/SLC11A1/CD68/LAMP3/CLN3/GBA/AP3M2/CLTB/GNS/GALNS
/SV2A/ITGA2/COL6A2/COL4A2/LAMB3/CD47/IBSP/ITGB8/THBS1/THBS2/LAMA4
0B/CTSK/MMP1/HLA-DMB/LTB/CCL5/CD86/HLA-DPB1/ATP6V1C1/TCIRG1/ATP6V0D2/ATP6AP1/TN
/PAK1/PIP5K1C/LYN/BIN1/PIK3CD/ARPC5L/RAC1/WAS/WASF1/CDC42/FCGR3B/RPS6KB2/ARPC1A
P10/HEATR1/GAR1/REXO2/NAT10/CSNK2B/NVL/NOL6/CSNK2A1/BMS1/NOB1/WDR75/LSG1/RBM28/
F4/HLA-DQB1/NCF1/HLA-DMB/HLA-DPB1/MAPK3/STAT1/HLA-DQA2/EEF1A2/TGFB2/HLA-DRB5/IL
3/HLA-DQA2/HSPA4/HLA-B/HLA-DRB5/RFX5/CALR/CD8A/NFYC/HLA-DOA/CIITA/HSPA5/HSP90AA
C42/VCL/CBL/ARPC1A/ARPC5/CTNNA1/DNM2/PTK2/CD2AP/CLTC/RHOA/ELMO2/CAV1/PIK3CB/CAV

3B/MAP3K14/LTBR

HSPD1/HLA-DOA/HLA-F

NASEH2C/POLE3/RPA1/RNASEH1/POLE2

N2A/BIRC3/ITGAV/COL4A5/LAMB1/HRAS/CSF1R/PGF/CDK4/CSF3R/FZD6/IL15RA/PLCB1/IFNGR2
B/TRAF2/DDIT3/UQCRH/PSMD2/FZD1/TUBA1A/CYC1/MAP2K2/MAPK3/TUBB6/KLC3/TUBB2A/PSMB2
F2/DDIT3/UQCRH/BAX/NUP210/PSMD2/TUBA1A/CYC1/TUBB6/KLC3/GPX8/TUBB2A/NUP93/PSMB2/
TB/TNFRSF11B/CCL5/TNFRSF18/CXCR4/TNFSF9/CCL26/IL34/IL27RA/EDA/TNFRSF4/TNFRSF11A
KLC3/TUBB2A/PSMB2/UBE2J2/MCU/TUBB4A/ATF4/PSMB4/ADRM1/VDAC1/PSMA5/PSMC4/TUBB2B/P
K3CD/BCL3/CCL2/ATF4/CXCL3/IL1B/MLKL/JAG1/IL15/MAP3K14/DNM1L/PGAM5/MAPK11/IKBKG/
B1/HSPA8/LAMC2/ALOX5/LAMA5/MAPK3/STAT1/SOCS1/HLA-DQA2/BIRC7/TGFB2/HLA-DRB5/LAMB
1/HLA-DQA2/JAG2/HLA-DRB5/IL2RA/LCK/JAG1/IL12RB1/IL2RB/MAPK11/IKBKG/IL4R/HLA-DOA
/IKBKG/TRAF3/FADD/MAPK7/TRAF4/S100A7/HSP90AA1/TRADD/HSP90B1/MUC5B/CASP8/TNFAIP3
HLA-DPB1/CD2/CD3E/HLA-DQA2/HLA-DRB5/IL1B/IL2RA/ITGA2/IL1R2/CD8A/IL4R/CSF2RA/CD3
8/PSMA1/PSMA4/PSMB5/PSMC5/PSMC3/POMP/PSMB1/PSMB6/PSMA3/IFNG/PSMB7

/TRAF5/MAP2K2/MAPK3/GNGT1/GNA12/E2F3/IRF3/PLCB3/CCL4/PIK3CD/CCL2/ATF4/GNG5/GNB2
GS14/MAP2K2/MAPK3/FPR1/FGFR4/RRAS/PLCB3/FGFR1/PIK3CD/ACTG1/LCP2/RAC1/BCAR1/NRAS
CL13/PLCB3/LYN/CCL4/PIK3CD/SHC1/CCL2/GNG5/CXCL16/CCL28/GNB2/CXCL3/RAC1/BCAR1/GN
/PLCB3/PIK3CD/CCL2/F3/TGFB2/RAC1/IL1B/NRAS/COL4A2/TGFB3/CDC42/MAPK11/VCAM1/PLCB

RELN/LAMC2/LAMA5/MAPK3/PAK1/PIP5K1C/COL6A3/PAK4/PIK3CD/ACTG1/SHC1/TNC/ITGB5/PPP
E2J2/BCAP31/TRAM1L1/ATF4/RPN2/CAPN1/UBE2D1/STT3A/DNAJC10/DNAJB2/WFS1/BAG2/SSR3/

RF3/PIK3CD/ACTG1/CCL2/LCP2/CASP1/RAC1/BCAR1/IL1B/ACTB/WAS/LCK/CD8A/CDC42/MAPK11
P6V0C/MAP3K14/CDC42/MAPK11/ATP6V0E1/IKBKG

RB5/BID/ACTB/CYCS/HLA-DOA/HLA-F/ABL1/CASP8/EIF4G3/CAV1/EIF4G2/HLA-C/CASP3/ITGAL

CND2/CYCS/IL2RB/EIF3H/IKBKG/TRAF3/FADD/RACK1/FCGR2B/CCND3/STAT5A/TLR2/TRADD/CSN

CL11/IKBKG/LBP/TRAF3/FADD/TLR5/TLR2/CASP8/IFNAR2/CCL3L3/IRF5/CXCL10/TLR7/PIK3CB

/CASP1/CD209/IL1B/NRAS/MAP3K14/MRAS/CLEC7A/MAPK11/IKBKG/CALM2/PLK3/NFATC4/BCL10

8A1/AOX1/EHHADH/CYP1A2

5/ACADL/ACSL1/ACADSB/ALDH2/ADH6/EHHADH/ADH1A/ADH1C/CYP4A11/ADH4/ADH1B
3CD/CDK2/ANAPC11/STK10/MAPK11/IGF1R/HSP90AA1/GNAI3/ADCY6/ANAPC5/KIF22/ADCY5/CDC

KR1D1/CYP1A2/HSD11B1/HSD17B6/CYP2E1/CYP3A4
7B6/ADH1A/CYP2B6/ADH1C/CYP4A11/CYP2C9/CYP2C8/ADH4/ADH1B/CYP2A6/CYP3A4
A1/APOC3

E/ADAR/POLR3F/AIM2/POLR1C/RELA/POLR3A/IKBKB/ZBP1
ATP6V0E1/ERO1A/ATP6V0D1/CFTR/ARF1/ADCY3/ATP6V1B2/PDIA4/PLCG1/GNAS
CAA2/ALDH7A1/ACADSB/ALDH2/AGXT2/ALDH6A1/ABAT/AOX1/EHHADH/HMGCS2
XCL3/IL1B/LCK/TNFSF13B/PARP1/MAP3K14/BTK/LTBR/IKBKG/LBP/TRAF3/TRIM25/VCAM1/PIDD
L9/CCR1/CXCR3/XCL2/IL2RB/CCL3/LTBR/CXCL11/CCR5/PF4V1
MB/SELPLG/HLA-DPB1/CFD/FPR1/C3AR1/KRT18/HLA-DQA2/FPR3/C5AR1/HLA-DRB5
AP1/FZD1/HIF1A/MMP2/SLC9A1/MAP2K2/MAPK3/CD63/RRAS/PAK1/FGFR1/HCLS1/PIK3CD/ACTG1

/ACSL1/ACADSB/EHHADH
T2B10/CYP1A2/FMO3/ADH1A/CYP2B6/CYP2E1/ADH1C/CYP2C9/CYP2C8/ADH4/ADH1B/CYP2A6/CYP
AS/LCK/PTPN6/GRB2/CD48/GZMB/FCGR3B/IFNAR2/CD247/HLA-C/ICAM2/PIK3CB/CASP3/ITGAL/

IL12RB1/TGFB3/IL4R/TLR5/HLA-DOA/TLR2

1/UGT2B10/CYP1A2/HSD11B1/SULT2A1/ADH1A/CYP2B6/CYP2E1/ADH1C/CYP2C9/ADH4/ADH1B/CY

AK2/VEGFA/RAPGEF1/EGLN2
YP1A1/GSTM1/UGT2B15/ADH6/GSTA1/UGT2B10/CYP1A2/HSD11B1/SULT2A1/ADH1A/CYP2E1/ADH1
O1/MAD2L2/FBXO5/ANAPC7/YWHAH/CDK2/PPP1CA/PPP1CC/YWHAQ/ANAPC11/YWHAB/MAPK11/IGF1
E1/CYP2A6/CYP3A4

LC27A2/PCK2/EHHADH/FABP4/CYP7A1/APOA1/APOA5/HMGCS2/APOC3/CYP4A11/SLC27A5/PCK1/C

F4/GSPT2/CPSF3/MSI1/PPP2R3A/SYMPK/ETF1/MAGOH/PPP2R5B/RNMT/FIP1L1/SSU72/MAGOHB/N

A/IL27RA/MAPK3/HSP90AB1/STAT1/HLA-DQA2/HLA-DRB5/IL1B/IL2RA/LCK/IL12RB1/IL2RB/MA
SLCO1B1/ABCB4/CYP7A1/SLC22A7/UGT2B10/SULT2A1/SLC22A1/SLC27A5/AQP9/SLC10A1/CYP3A
AS/PDGFA

B4/ACOX1/PXMP2/ACSL6/AMACR/PECR/PHYH/SCP2/ACSL5/ACSL1/CAT/BAAT/EPHX2/ACOX2/XDH/
1/NUPR1/ETV7/TFE3/HDAC2/MITF/PDGFA/MLF1/CDKN2C/CCND2/ETV6/PRCC/IL1R2/IL2RB/PAX8
/SELPLG/HLA-DPB1/CD2/HLA-A/CD276/NRCAM/CTLA4/CD58/HLA-DQA2/CNTNAP1/HLA-B/HLA-DR
R1/ACTB/CDC42/MAPK11/VCL/VCAM1/CLDN10/MYL9/SIPA1/CTNNA1/PTK2/GNAI3
L2/MASP1/C8B/F11/F2/F7/SERPINF2/F13B/MASP2/C4BPA/CPB2/KLKB1/C8A/CFHR3/SERPIND1/
B1/CACNB3/SLC9A6/COX7A2/CACNA1D/COX6C/ATP2A2/COX5A/TNNC1/COX4I1
/PRF1/HLA-DOB/IFNG/HLA-E/CD80/IL12A/FASLG/HLA-G
A4/HRAS/CSF1R/PGF/CDK4/CSF3R/G6PC3/AREG/CREB3L1/COMP/LAMC1/JAK3/ITGA5/RELN/EFNA
TGA2/COL4A2/LAMB3/E2F2/CYCS/ZBTB17/IKBKG/TRAF3/TRAF4/LAMA4/PTK2/CCNE2
T1/E2F3/STAT1/IRF3/LYN/IRF7/PIK3CD/LEF1/GNG5/GNB2/HLA-B/CXCL3/RAC1/GNB1/BID/NRA
A/E2F3/STAT1/IRF3/IRF7/PIK3CD/CDK2/ATF4/TGFB2/BID/NRAS/YWHAQ/E2F2/CYCS/GRB2/TGF
FPT1/CYB5R3/PMM2
/VDAC1/BID/IL1B/CAPN1/MLKL/CHMP3/PARP1/DNM1L/CHMP1A/PGAM5/SLC25A5/FADD/VDAC2/HS
PCK1

CTG1/AXIN1/BBC3/LEF1/FZD2/WNT7B/PPP1CA/TGFB2/PPP1CC/ACTB/YWHAQ/RASSF1/CCND2/DVL

/PIK3CD/SHC1/ATF4/GNG5/GNB2/GNB1/NRAS/COL4A2/GRB2/MAPK11/ARRB2/GNB4/GNGT2/PLCB2
B2/ATP2B1
PK3/HK3/PIK3CD/LDHA/PFKM/HK1/LTBR/IGF1R/LDHB/ANGPT2/RBX1

PT/ENO3/PKLR/CAT/OGDHL/RGN/ALDH6A1/FBP1/EHHADH/HAO2/HAO1/CPS1/AGXT/ALDOB
BA1/PML/CUL4B/RBX1/CBL/RNF7/UBE2O/UBE2L6/VHL/TRIM37/UBA2/PRPF19/RPS27A/UBE2D2/P

PK3/LMNA/CTSS/PMAIP1/PIK3CD/ACTG1/BBC3/ATF4/BID/CAPN1/NRAS/ACTB/PARP1/CYCS/MAP3

PJ/CTBP1/NOTCH2/NCSTN/MAML1/APH1A/DVL1/MAML2
6A1/CPA2/COL6A2/COL4A2/COL8A2/SLC36A4/COL11A1/PRSS3/KCNN4/ATP1B1
S1/CHEK2/CCND3/PIDD1
K3CD/TGFA/SHC1/ATF4/NRAS/GRB2/KRT20/CTSD/PLCB2/KRT10/CALM2/HSP90AA1/GNAI3/HSP90

S6KB2/TLR2/CSNK2B/CSNK2A1/CD247/NFKBIB/CD4/EGF/CSNK2A2/CD3G/PIK3CB/IFNG/PLCG1/R

3/HLA-DMB/TRAF2/CCL5/ITGA5/HLA-DPB1/BAX/ZNF320/CARD9/HLA-A/TRAF5/PILRA/ZNF93/ST
BCL2L1/CSNK2A2/MFN1/ATG9A/ULK1/TP53/RELA/TFEB/RAB7A/MAPK9/KRAS/SP1/GABARAP/RRAS
D2/PLCB3/ASAH1/PIK3CD/RAC1/BID/NRAS/MAPK11/CTSD/CERS6/PLCB2/PPP2R3A/TRADD/PLD2/

MC2/ATP6V1C1/ISG15/FZD1/HLA-A/TCIRG1/ATP6V0D2/PPP2R1A/LAMA5/MAP2K2/ATP6AP1/MAPK
CB3/POLR2H/SLC1A3/DCTN2/TUBB4A/BBC3/ATG101/PSMB4/ADRM1/VDAC1/TGM2/PSMA5/PSMC4/D
IRF3/PLCB3/HCLS1/CYTH2/CBX3/PIK3CD/ACTG1/ARPC5L/CASP1/CAPNS1/VDAC1/RAC1/BCAR1/I
3/ARPC2/DYNLRB1/PAK1/PLEKHM2/NCKAP1L/DYNLT1/CYTH2/CSE1L/STX10/PIK3CD/ACTG1/ARPC
EB3/PSMD2/TUBA1A/CYC1/MAPK3/TUBB6/KLC3/TUBB2A/PSMB2/MCU/PIK3CD/TUBB4A/ATF4/PSMB
LA-DMB/TCF3/JAK3/HLA-DPB1/BAX/ESPL1/NFKB2/CREB3/CD3E/HLA-A/MAP2K2/MAPK3/E2F3/HL
BP1/BIN1/ARFGAP1/CAPZA1/ARPC5L/SNF8/RAB11FIP4/EHD1/HLA-B/IL2RA/FOLR2/SH3GLB2/RA
/ARPC2/PAK1/PIP5K1C/NCKAP1L/FGFR1/PAK4/PIK3CD/ACTG1/ARPC5L/ITGB5/PPP1CA/RAC1/BC
LA-B/RAC1/GNB1/BID/APOBEC3G/NRAS/CYCS/CALR/MAPK11/CCR5/IKBKG/AP1G2/GNB4/CUL4B/F
-A/TRAF5/HDAC1/E2F3/STAT1/CD58/IRF3/HDAC2/LYN/HLA-DQA2/IRF7/PIK3CD/CDK2/SAP30/A
1/ABCF2/TUBB2A/ARPC3/ARPC2/PAK1/NCKAP1L/HCLS1/CYTH2/MYO1F/MYO1G/SLC9A3R1/ACTG1/
/CASP1/IFI16/VDAC1/CXCL3/IL1B/TXN/CARD16/DNM1L/GSDMD/MAPK11/IKBKG/TRAF3/CARD18/
CIRG1/ATP6V0D2/ATP6AP1/MAPK3/RFXANK/STAT1/CTSS/ATP6V1H/HLA-DQA2/CD209/TGFB2/HLA
P1/PIK3CD/CDK2/ATF4/HLA-B/RAC1/NRAS/YWHAQ/CCND2/CHD4/GRB2/CDC42/YWHAB/KAT2A/LTB
/MCU/PIK3CD/CDK2/PPP1CA/TGFB2/HLA-B/VDAC1/PPP1CC/CAPN1/NRAS/CCND2/RBBP4/E2F2/TG
L2/CASP1/VDAC1/HLA-DRB5/BID/IL1B/ACTB/CYCS/RAE1/PML/IKBKG/SLC25A5/TRAF3/CPSF4/T
IF/SOCS1/HDAC2/DDIT4/PAK4/PIK3CD/SHC1/TNC/TGFB2/NRAS/DNMT3A/PDGFA/FGFR3/RASSF1/C
OC5/EIF2S3/XPO5/RAE1/PABPC4/EIF3H/NUP107/NUP155/FUS/POP5/EIF2B5/SEC13/MAGOH/THC
LA-DPB1/COLEC12/SEC61A1/SEC61G/ATP6V1C1/HLA-A/TUBA1A/TCIRG1/ATP6V0D2/ATP6AP1/RA
/FUS/HNRNPM/HNRNPA3/RBM17/SNRPG/PRPF31/DDX23/SART1/SNRPB2/PRPF19/PRPF38A/HNRNPU
1/PRKDC/PCNA/MAD2L2/HDAC1/E2F3/RBL1/E2F4/HDAC2/DBF4/CDKN2B/ANAPC7/YWHAH/CDK2/TG
/MRPS21/RPS23/RPS15/MRPL18/RPS12/RPS16/RPL7A/RPL36/RPS27A/RPL6/RPS7/RPL22L1/RPL
RAC1/IL1B/LCK/GRB2/MAP3K14/BTK/MAPK11/IKBKG/LILRB4/FCGR3B
/ATP6V0C/PLA2G15/AP1G2/CTSD/AP3D1/M6PR/AP1B1/NAGPA/SGSH/DNASE2/ATP6V0D1/AGA/MAN

FRSF11A/CTLA4/ATP6V1H/HLA-DQA2/CCL2/TGFB2/CXCL3/HLA-DRB5/IL1B/ATP6V1E1/IL15/TNF
/ARPC5/INPPL1/FCGR2B/DNM2/PLD2
RPP30/EIF6/CSNK2A2/IMP4/RIOK1/AK6/NXT2
1B/PTPN6/TGFB3/MAPK11
1/HLA-F/CANX/HSPA2/TAPBP/LGMN/TAP2/PDIA3/CD4/HLA-C/NFYA/HLA-DOB/IFNG/CTSL/HLA-E
2/ELMO3/PXN

/ARNT2/MMP1/FZD7/LAMC1/TRAF2/GSTM3/JAK3/CXCR4/BDKRB1/HMOX1/BAX/NFKB2/LAMC2/TPM3
/CSNK1E/FRAT2/PSENEN/PLCB3/DVL3/MCU/PIK3CD/AXIN1/TUBB4A/ATF4/FZD2/ATG101/WNT7B/
NUP37/NDC1/GPX7/NXT1/NUP205/NUP188/DCTN2/MCU/ACTG1/TUBB4A/ATF4/CASP1/ATG101/PSM
/CXCL13/CCL13/CCL4/CCL2/CXCL16/CCL28/TGFB2/CXCL3/IL1B/TNFRSF10C/IL2RA/CXCL9/CCR
SMD13/TXN/PSMD11/UBE2L3/CYCS/PSMA7/PSMD1/UBA1/PSMD7/PSMB3/PSMD4/NDUFS6/PSMD3/SL
TRAF3/FADD
3/CYCS/TGFB3/MAPK11/CCR5/IKBKG/IL10RA/LAMA4/HLA-DOA/CIITA/TLR2/GNAI3/HSPA2/CASP
/STAT5A/NOTCH3/NOTCH1/RUNX3/CD247/NFKBIB/CD4/RBPJ/CD3G/NOTCH2/HLA-DOB/IFNG/PLCG
/ANAPC5/IL17D/IL17RA/CXCL10/SRSF1/CASP3/MAP3K7/IL17RB/IFNG/ELAVL1/MAPK15/RELA
3/HLA-DOA

/HLA-B/RAC1/BCAR1/GNB1/BID/IL1B/NRAS/CCR1/E2F2/CYCS/CALR/GRB2/CCL3/MAPK11/CCR5/
/ACTB/PDGFA/FGFR3/PFN1/RALA/SIPA1L2/CDC42/PRKCI/SIPA1L3/MRAS/PRKD2/MAPK11/IGF1
B1/CXCL9/NRAS/FGR/CCR1/WAS/CXCR3/GSK3A/XCL2/GRB2/CDC42/CCL3/CXCL11/CCR5/IKBKG/
2/PLCD4/STAT5A

1CA/RAC1/BCAR1/PPP1CC/ACTB/ITGA2/PDGFA/COL6A2/COL4A2/CCND2/LAMB3/PARVG/GRB2/CDC
CALR/PPP1R15A/DNAJB11/ERO1A/PDIA6/RBX1/HSPA4L/SSR4/VCP/SEC13/UBXN1/HSPA5/HSP90A

/IKBKG

/PRF1/HLA-DOB/EIF4G1/HLA-E/RAC3/CD80/LAMA1

K2B/CCNE2/HSPA2/MAVS/CSNK2A1/OAS3/CASP8/BAD/TNFAIP3/IFNAR2/NFKBIB/BCL2L1/TLR7/

/MAP3K7/TLR8

/KSR1/CASP8/RHOA

27/ADCY3/ANAPC4/ADCY7/PIK3CB

1/TRADD/BCL10/CSNK2B/CSNK2A1/PIAS4/TNFAIP3

/ITGB5/FZD2/WNT7B/PPP1CA/TGFB2/RAC1/PPP1CC/NRAS/ACTB/ITGA2/PTPN6/CTTN/TFAP4/GR

'3A4
'PRF1/IFNG/PLCG1/HLA-E/RAC3/PRKCB/NFATC1/VAV3/TNFRSF10A/PIK3R2/MICB/KRAS/FASLG/F

P2A6/CYP3A4

C/CYP2C9/CYP2C8/ADH4/ADH1B/CYP2A6/CYP3A4
R

'YP8B1

IXT2/RBM8A/PABPC1L/SMG7/PABPN1/CASC3/CSTF3/PYM1/ACIN1/PPP1CB/PPP2R3B/PPP2R5D/PPF

.PK11/IKBKG/IL4R/HLA-DOA/HSP90AA1/STAT5A
.4

'SLC27A2/PIPOX/EHHADH/HAO2/DAO/HAO1/AGXT
;/PML/ZBTB17/IGF1R/FUS/GZMB
B5/PDCD1/SIGLEC1/CD6/CD8A/ITGB8/GLG1/VCAM1/CLDN10/PTPRF/HLA-DOA/HLA-F

'F12/F9/CFHR5/KNG1/C6/PLG/CFHR4/SERPINC1

.5/CREB3/LAMC2/PPP2R1A/LAMA5/MAP2K2/MAPK3/HSP90AB1/GNGT1/BRCA1/PPP2R2C/FGFR4/FGF

.S/CCR1/E2F2/CYCS/MAPK11/CCR5/IKBKG/TRAF3/GNB4/FADD/ANGPT2/GNGT2/CALM2/HLA-F/NFA
B3/YWHAB/MAPK11/IKBKG/TRAF3/FADD/STAT5A/TLR2/NFATC4/VDAC3/CCNE2/MAVS/CASP8/BAD

;P90AA1/STAT5A/TRADD/VDAC3/USP21/CASP8/TNFAIP3/IFNAR2/FAF1/PLA2G4A/SPATA2L/CAPN2

.2/TGFB3/YWHAB/PRKCI/PARD6B/LLGL1/PARD6G/WWC1/WNT4/NF2/CCND3

'IAS4/UBE2Q1/ANAPC5/UBA52/UBE2N/UBR5/UBE2E2/UBE2F/CDC27/STUB1/MAP3K1/UBE4B/ANAPC

;K14/IKBKG/GZMB/CTSD/DFFA/FADD/CSF2RB

B1/HSPA2/ADCY6/ADCY5/CALM3/HBEGF/ADCY3/KRT15/ADCY7/PIK3CB

ELA/NFATC1/IKBKB/RASGRP1/PIK3R2/STAT3/CD274/TICAM1/KRAS/PPP3CB/MAP3K3/ZAP70

AT1/IRF3/HLA-DQA2/IRF7/SOCS3/PIK3CD/CCL2/SRSF9/PPP1CA/HCFC1/HLA-B/HLA-DRB5/PPP1
2/ATG9B
GNAI3/S1PR3/PPP2R5B

3/HDAC1/PPP2R2C/RBL1/STAT1/IRF1/ATP6V1H/IRF3/HDAC2/COL6A3/DVL3/HEYL/BCAP31/SLC9
DCTN5/TUBB2B/PSMD13/PSMD11/CLTB/AP2M1/CYCS/PSMA7/AP2S1/PSMD1/PSMD7/POLR2K/PSMB3/
L1B/CAPN1/ACTB/UBE2D1/WASF1/GSK3A/PFN1/CYCS/CTTN/HK1/CDC42/MAPK11/IKBKG/VCL/AKT
5L/LEF1/CASP1/RAC1/IL1B/MLKL/ACTB/TXN/DYNC1LI1/PFN1/CYCS/FHOD1/RALA/RPS3/GSDMD/
4/ADRM1/VDAC1/RAC1/PSMA5/IL1B/PSMC4/TUBB2B/PSMD13/PSMD11/CYCS/PSMA7/PSMD1/PSMD7
A-DQA2/CDKN2B/ANAPC7/PIK3CD/CDK2/ATF4/TGFB2/HLA-B/VDAC1/HLA-DRB5/IL2RA/NRAS/LCK
B10/EHD2/FGFR3/ARF4/CHMP3/CLTB/AP2M1/EPS15L1/IL2RB/AP2S1/VPS28/CHMP1A/CDC42/PML
AR1/PPP1CC/PIP4K2A/NRAS/ACTB/ITGA2/PDGFA/FGFR3/WASF1/PFN1/PIP4K2C/CDC42/MRAS/IT
ADD/RBX1/AP1B1/APOBEC3D/RNF7/GNGT2/RPS6KB2/CALM2/HLA-F/TLR2/NFATC4/TRADD/PTK2/C
DRM1/HLA-B/RAC1/HLA-DRB5/BID/PSMC4/PSMD13/CCND2/PSMD11/E2F2/CYCS/PSMD1/CALR/MAP
ARPC5L/TUBB4A/CASP1/RAC1/IL1B/TUBB2B/ACTB/WASF1/PTPN6/CYCS/CTTN/RPS3/NCL/CDC42/
FADD/CARD6/TRIP6/VDAC2/PLCB2/HSP90AA1/VDAC3/MAVS/OAS3/CASP8/TNFAIP3/RHOA/IFNAR2
-DRB5/BID/IL1B/RFX5/CYCS/ATP6V0C/TGFB3/CLEC7A/MAPK11/LBP/CTSD/NFYC/FADD/FCGR3B/
R/RANBP1/CCR5/IKBKG/TRAF3/ATP6V0D1/CCND3/HLA-F/STAT5A/TRADD/VDAC3/CCNE2/GTF2E2/
FB3/MRAS/MAPK11/SLC25A5/CHEK2/VDAC2/RHEB/CCND3/RAD9A/CALM2/HLA-F/NBN/RAD1/NFATC
RIM25/FADD/PRSS3/CCND3/HLA-DOA/CIITA/TRADD/MAVS/OAS3/RAB11A/CASP8/IFNAR2/KPNA6/
CND2/E2F2/GRB2/VIM
C2/TGS1/EIF3E/STRAP/EIF3G/RPP30/SEH1L/MAGOHB/THOC1/SUMO2/EIF4G3/EIF4G2/NXT2/RBM
B7B/TUBB6/HGS/TUBB2A/DYNC1H1/CTSS/MSR1/OLR1/ATP6V1H/HLA-DQA2/ACTG1/TUBB4A/ITGB5
/LSM8/RBMX/HSPA2/MAGOH/PHF5A/THOC2/TCERG1/DHX15/HNRNPA1/SF3B3/PPIE/USP39/SF3B2/
FB2/CDKN2C/YWHAQ/CCND2/ANAPC11/E2F2/CDK7/RAD21/TGFB3/YWHAB/ZBTB17
37/MRPS17/MRPL15/MRPL28/RPL27A/RPS11/RPS4X/RPL19/RPLP1/MRPL27/UBA52/RPL13A/RPL3

2B1/ABCA2/PSAP/GNPTAB/HEXB/CTSW/AP3B1/SLC17A5/CLTC/GGA1/GGA3/ABCB9/AP1S3/AP1S2/

SF13B/ATP6V0C/TGFB3/CCL3/ATP6V0E1

[illegible]

/FZD1/TRAF5/HIF1A/MMP2/LAMA5/MAP2K2/MAPK3/HSP90AB1/GNGT1/CKS1B/HDAC1/GNA12/E2F3
PSMB4/ADRM1/VDAC1/BID/PSMA5/IL1B/CAPN1/PSMC4/TUBB2B/NRAS/RTN3/CDK5/PSMD13/APP/F
B4/ANXA11/ATXN2L/ADRM1/VDAC1/RAC1/BID/PSMA5/PSMC4/DCTN5/TUBB2B/ACTB/NUP85/PSMD1
1/TNFSF12/IL15/CD27/TNFSF13B/CXCR3/IL12RB1/XCL2/IL1R2/IL2RB/TGFB3/CCL3/LTBR/CXC
C25A5/NDUFS5/NDUFB9/COX6B1/MAPT/PSMD9/PSMD8/VDAC2/UBE2L6/PSMA1/COX8A/CALM2/HSPA

8/BAD/PIK3R5
1/RELA/STAT4/NFATC1/IKBKB/MAML1/MAML2/MAPK9/IL12A/PPP3CB/GATA3/ZAP70

/IKBKG/GNB4/FADD/IL10RA/GNGT2/RPS6KB2/RHEB/PLCB2/CALM2/HLA-F/NFATC4/TRADD/PTK2/[illegible]
/PARD6B/PARD6G/ANGPT2/RALGDS/THBS1/RALB/PLCB2/LPAR5/CALM2/SIPA1/GNAI3/FGFR2/TL[illegible]
F4V1/ARRB2/GNB4/GRK2/GNGT2/PLCB2/PTK2/GNAI3/GNG4/CXCR6/BAD/PIK3R5/ADCY6/RHOA/D[illegible]

42/IBSP/IGF1R/ITGB8/VCL/THBS1/THBS2/CCND3/LAMA4/MYL9/PTK2/TLN1/PPP1R12C/BAD/PA[illegible]
A1/ERP29/UBQLN4/NSFL1C/CANX/HSP90B1/HSPA2/EDEM2/UBE2D2/DAD1/SSR1/PDIA3/TRAM1/S[illegible]

AB9A/CSNK2A2/MX1/CD3G/PIK3CB/CASP3/MAP3K7

2/CDC42/LUM/MRAS/MAPK11/DROSHA/IGF1R/HPSE/CBL/THBS1/WNT4/RPS6KB2

PPP3CB/SH2D1A/HLA-G/ZAP70

P2CA/GSPT1/DDX19A/TARDBP

R1/COL6A3/MLST8/GYS1/DDIT4/YWHAH/PIK3CD/CDK2/TGFA/TNC/ITGB5/ATF4/GNG5/GNB2/RAC1

TC4/TRADD/RPS27A/GNG4/CASP8/PIK3R5/IFNAR2/VEGFA/UBA52/BECN1/CLEC2B/PIK3R6

2/VPS4A

4/RHOBTB2/HUWE1/CDC34/SKP2/DDB1/TRIM32/CDC23/UBE2E3/CUL7/WWP2/SKP1/UBE2J1/UBE2

CC/BID/IL1B/ZNF14/ZNF205/CYCS/CALR/PML/ZNF611/IKBKG/ZNF486/TRAF3/SRPK1/ZNF600/[illegible]

)A3R1/PIK3CD/AXIN1/CDK2/TNC/ITGB5/FZD2/WNT7B/HLA-B/NRAS/ATP6V1E1/ITGA2/COL6A2/JA
'GPX1/PSMD4/NDUFS6/PSMD3/SLC25A5/POLR2L/NDUFS5/NDUFB9/DNAL4/WIPI1/COX6B1/PSMD9/F
'1S1/RBX1/WIPI1/TLR5/RPS6KB2/ARPC1A/ARPC5/PLCB2/TNIP1/PLCD4/MYL9/TRADD/PTK2/BCL1
'CDC42/MAPK11/IKBKG/FADD/M6PR/VPS18/TLR5/ARPC1A/ARPC5/HSP90AA1/MYL9/TLR2/DNM2/TF
'/PSMB3/PSMD4/NDUFS6/MAPK11/PSMD3/SLC25A5/NDUFS5/NDUFB9/COX6B1/PSMD9/PSMD8/VDAC2
(/CDKN2C/IL15/CCND2/ANAPC11/E2F2/IL1R2/IL2RB/CALR/MAP3K14/TGFB3/KAT2A/LTBR/RANBF
./CAPZB/PRKCI/AGAP1/SH3GL1/CCR5/VPS25/ARRB2/IGF1R/PARD6B/PARD6G/CBL/MVB12A/SNX12
'GB8/VCL/FGD1/ABI2/ARPC1A/ARPC5/LPAR5/MYL9/PTK2/BRK1/FGFR2/SSH2
;NAI3/GNG4/CASP8/BAD/AP1S3/AP1S2/PAK2/TAPBP/CD247/AP1S1/TAP2/PDIA3/CD4/BCL2L1/AT
'3K14/PSMD7/VIM/BTK/PSMD4/MAPK11/IKBKG/PSMD3/TRAF3/FADD/NCOR2/PSMD8/CCND3/HLA-DO
'MAPK11/IKBKG/MYO5A/FADD/TLR5/CLDN10/ARPC1A/ARPC5/LPAR5/TRADD/BRK1/ABL1/MYO6/TUE
2
'HSPD1/IL10RA/ATP6V0D1/FCGR2B/HLA-DOA/CIITA/CALM2/VDR/TLR2/PLK3/TRADD/BCL10/RAB5
'YWHAG/HDAC10/SRF/CASP8/BAD/VAC14/RHOA/ACTN1/TBP/ACTN4/GTF2H1/SND1/RBPJ/HDAC8/HI
C4/VDAC3/CCNE2
'NFKBIB/CXCL10/TLR7/MX1/NXT2/PIK3CB/CASP3/PABPN1/HNRNPUL1/FDPS/HLA-DOB/ADAR/IFNG

18A/PABPC1L/EIF2B1/GEMIN2/CASC3/THOC7/EIF2B4/EIF3F/DDX20/AAAS/PYM1/THOC3/ACIN1/F
5/CD209/HLA-B/RAC1/HLA-DRB5/TUBB2B/ACTB/ATP6V1E1/ITGA2/DYNC1LI1/ATP6V0C/CALR/CLE
'SRSF2/MAGOHB/THOC1/SRSF1/PRPF4/DHX38/PRPF38B/SF3B6/RBM8A/SNRNP70/DHX8/RP9/SNRNF

37A/MRPL4/RPL30/RPL32/MRPL33/MRPS5/RPS27/MRPL36/RPL13/RPS9/RPL41/RPS14/MRPL11/RF

'LGMN/AP1S1/MANBA

3/FGFR4/PIM2/STAT1/FRAT2/HDAC2/PLCB3/FGFR1/PPARD/MITF/DVL3/DAPK3/CDKN2B/HEYL/PMA
'SMD11/CYCS/PSMA7/DVL2/PSMD1/PSMD7/PSMB3/PSMD4/NDUFS6/PSMD3/SLC25A5/APH1B/NDUFS5
.3/NUP43/PSMD11/PFN1/CYCS/PSMA7/RAE1/PSMD1/PSMD7/PSMB3/GPX1/PSMD4/TOMM40L/NDUFS6
'L11/CCR5/PF4V1/CXCL17/IL4R/CSF2RB/CSF2RA/IL10RA/INHA/GDF11/TNFRSF25/IL20RB/CXCF
A5/PSMA4/VDAC3/RPS27A/GNAI3/PSMB5/NDUFB3/TUBB3/COX7B/DAXX/PSMC5/NDUFA8/UBA52/UQC

GNAI3/PTGER4/GNG4/CASP8/ARHGEF11/ADCY6/RHOA/TAPBP/VEGFA/CCL3L3/TAP2/ADCY5/PDIA3/
N1/ADCY6/RHOA/VEGFA/KITLG/RAPGEF1/ADORA2B/ADCY5/PARD3/EPHA2/EGF/RASSF5/CALM3/ADC
OCK2/CCL3L3/PIK3R6/XCL1/ADCY5/PARD3/NFKBIB/CXCL10/CCL8/PREX1/ADCY3/CCR10/ADCY7/P

K2/RHOA/VEGFA/RAPGEF1/ACTN1/ACTN4
UB1/UBE4B/CAPN2/P4HB/DNAJC1/LMAN2/PDIA4/ATF6B/SEC23B/FBXO6/RPN1/UGGT1/SVIP/UBQL

[illegible]/GNB1/IL2RA/NRAS/ITGA2/PDGFA/COL6A2/FGFR3/YWHAQ/COL4A2/CCND2/LAMB3/IL2RB/GRB2/[illegible]

[illegible]2/UBE2K/CDC16/RHOBTB1/ANAPC1/TRIP12/SMURF2/MID1/CDC26/FANCL/UBA3/FBXW11/CUL2/[illegible]

[illegible]ADD/ZNF589/ZNF544/ZNF195/ZNF354A/ZNF675/ZNF229/EIF2B5/RHEB/ZNF783/HLA-DOA/HLA-[illegible]

AG1/COL4A2/CCND2/LAMB3/ATP6V0C/DVL2/CHD4/GRB2/TUBG2/CDC42/PRKCI/ATP6V0E1/IKBKG/
PSMD8/VDAC2/PLCB2/PSMA1/COX8A/PSMA4/VDAC3/PSMB5/NDUFB3/TUBB3/DCTN1/COX7B/CLTC/C
10/RPS27A/TLN1/UBE2D2/GLMN/RHOA/UBA52/UBE2N/TECPR1/ACTN1/BECN1/ACTN4/ARF1/NFKBI
RADD/BRK1/RAB5C/MYO6/HSP90B1/DYNC1I2/CASP8/RHOA/AHNAK2/VPS11/ARF1/PTPRC/VPS33A/
2/PSMA1/COX8A/HSPA5/PSMA4/VDAC3/CSNK2B/PSMB5/HSPA2/NDUFB3/TUBB3/NOTCH1/CSNK2A1/
P1/IKBKG/SLC25A5/MSX1/CHEK2/VDAC2/CCND3/HLA-DOA/HLA-F/STAT5A/NFATC4/VDAC3/CANX/
2/GRK2/ARPC1A/ARPC5/SH3GLB1/HLA-F/ARAP1/RUFY1/VPS45/VPS35/SMAP1/DNM2/PLD2/RAB5C/

TR/CALM3/CD3G/HLA-C/TNFRSF1B/PIK3CB/CASP3/MAP3K7/DDB1/PXN/GNG2/PLCG1/HLA-E/RELA/
OA/HLA-F/TLR2/TRADD/CCNE2/MAVS/OAS3/CASP8/TNFAIP3/PSMC5/IFNAR2/TAPBP/RUNX3/PSMC
3B3/CASP8/ARHGEF11/PAK2/RHOA

5C/KSR1/CASP8/BAD/RHOA/CYP27B1
LA-C/PIK3CB/CASP3/SKP2/GTF2A2/EGR2/ATF6B/DDB1/GTF2E1/CCR3/PXN/TP53/HDAC5/HLA-E/

G/PRSS1/RELA/PRKCB

PRMT5/SUMO3/EIF2B3/EIF4E2/EIF4G1/FXR1/EIF2B2/GEMIN8/PAIP1/EIF3C/EIF5B/GEMIN6/EI
EC7A/STX18/ATP6V0E1/FCGR3B/M6PR/THBS1/STX7/ATP6V0D1/THBS2/FCGR2B/HLA-DOA/HLA-F/
P200/U2SURP/THOC3/ACIN1/HNRNPC/RBM22/SRSF3/SNRPD3/U2AF1L4/CCDC12/LSM3/TRA2B/HSP

PL29/RPS3A/RPS6/MRPL14/RPS24/RPL10/RPL9/RPL4/MRPL3/RPL14/MRPS16/RPL24/RPL11/MRP

AIP1/PIK3CD/AXIN1/CDK2/TGFA/BBC3/LEF1/BIRC7/FZD2/GNG5/WNT7B/JAG2/GNB2/TGFB2/RAC
5/FADD/NDUFB9/NAE1/WIPI1/ATP2A3/COX6B1/MAPT/PSMD9/WNT4/PSMD8/VDAC2/PLCB2/PSMA1/
6/NUP107/NUP155/MAPK11/PSMD3/FUS/NDUFS5/NDUFB9/DNAL4/HNRNPA3/WIPI1/COX6B1/PSMD9/
R6/ACVR1/IL21R/IFNAR2/GDF10/CCL3L3/IL17D/IL20RA/ACVR1B/XCL1/IL32/OSM/CD4/IL17RA/
CRB/NDUFA1/PSMC3/NDUFA13/NDUFS8/ADCY5/COX7A2/BCL2L1/COX6C/PSMB1/CALM3/MFN1/NDUF

/CALM3/ADCY3/GNA13/HLA-C/ADCY7/PIK3CB/CASP3/ATF6B/CCR3/PXN/TP53/GNG2/GNAS/HLA-E/
CY3
PIK3CB/CCL19/CCR3/PXN/GNG2/PLCG1/RELA/RAC3/PRKCB/ARRB1/CCR2/VAV3/IKBKB

LN2/PRKCSH/MOGS/DERL1/SKP1/DERL3/UBE2J1/EIF2AK1/RAD23A/UGGT2

ITCP1/PKN3/YWHAB/IKBKG/IBSP/IGF1R/IL4R/GNB4/ITGB8/ANGPT2/THBS1/GNGT2/RPS6KB2/THI

3C/XIAP/KEAP1/UBE2W/SMURF1/UBE3C/PIAS2/ANAPC13/ANAPC2/NEDD4L/UBOX5/CBLB/ERCC8/UI

[illegible]/TLR2/ZNF605/ZNF426/TRADD/MAVS/ZNF607/ZNF746/OAS3/ZNF7/ZNF623/CASP8/DAXX/BAD/[illegible]

IBSP/HEY1/TRAF3/PARD6B/ITGB8/LLGL1/PARD6G/FADD/THBS1/WNT4/RPS6KB2/MFNG/ATP6V0D1/
ASP8/MAP3K10/PSMC5/NDUFA8/POLR2I/UQCRB/NDUFA1/PSMC3/POLR2D/NDUFA13/BECN1/NRF1/TI
3/BCL2L1/ELMO2/RPTOR/CYTH3/CAPN2/FNBP1L
ELMO2/CYTH3/RAB9A/ACBD3/KLC1/PIK3CB/CASP3/MAP3K7/VPS16/NCKAP1/CASP7/KPNA4/RHOH/I
COX7B/BAD/PSMC5/NDUFA8/GRIN2D/UQCRB/NDUFA1/PSMC3/NDUFA13/NDUFS8/COX7A2/CACNA1D/C
CCNE2/TLN1/SRF/VAC14/ADCY6/ANAPC5/TBP/ADCY5/CRTC2/CD4/CDC27/BCL2L1/ATR/RANBP3/MA
/FGFR2/HSPA2/ACAP3/CLTC/RAB11A/RHOA/ARF1/AP2A2/WIPF2/SNX3/PARD3/ARF5/IST1/AP2B1/

/RAC3/PRKCB/SKP1
3/CD247/ENTPD1/TAP2/PDIA3/NFKBIB/CXCL10/RBPJ/CD3G/HLA-C/PIK3CB/CASP3/SKP2/MAP3K7

RELA

F1/NUP214/DDX19A/TARDBP/GEMIN5/RPP38/EIF3J/SMN2/TPR
TLR2/CANX/RAB5C/DYNC1I2/TUBB3
A1A/BCAS2/TRA2A/SRSF10/PRPF40A/DDX46/SRSF7/PRPF40B/SRSF4/CWC15

L20/RPL10A/RPS18/MRPL21/RPL12/RPS28/MRPL24/RPL26L1/MRPS2/RPL22/RPS17/RPS15A/MRPI

1/GNB1/BID/IL2RA/NRAS/ITGA2/PDGFA/JAG1/IL15/FGFR3/RASSF1/COL4A2/CCND2/LAMB3/IL1
COX8A/CALM2/ADAM17/PSMA4/VDAC3/CSNK2B/PSMB5/NDUFB3/TUBB3/CSNK2A1/COX7B/CASP8/BAI
/PSMD8/VCP/PSMA1/COX8A/SEC13/HSPA5/UBQLN4/PSMA4/PSMB5/FIG4/NDUFB3/TUBB3/DCTN1/CC
/CXCL10/IL13RA2/CCL8/BMP8B/TNFSF15/CRLF2/TNFRSF14/CCR10/BMP2/TNFRSF1B
A12/COX5A/PSMB6/NDUFB4/KLC1/NDUFA9/CASP3/NDUFB11/PSMA3/COX4I1/KIF5B/HTRA2/TP53/I

/RELA/MYC/RAC3/PRKCB/NFATC1/IL10RB/IKBKB/TSC1/ELK1

3S2/RHEB/CCND3/LPAR5/LAMA4/HSP90AA1/TLR2/PPP2R3A/PTK2/FGFR2/HSP90B1/CCNE2/YWHAG/

3A6

FNAR2/TAPBP/ZNF415/ZNF404/ZNF813/TAP2/ZNF432/PDIA3/BCL2L1/SRSF2/ZNF251/SRSF1/ZN

/THBS2/RHEB/CCND3/LAMA4/HLA-F/PPP2R3A/TRADD/PTK2/CCNE2/PTGER4/NOTCH3/NOTCH1/PPP[illegible]
3P/NDUFS8/POLR2J/ATG13/AP2A2/HTT/ACTR1A/COX7A2/AP2B1/COX6C/PSMB1/NDUFA12/COX5A/[illegible]

DYNC2LI1
COX6C/CSNK2A2/PSMB1/CAV1/NDUFA12/COX5A/PSMB6/NDUFB4/KLC1/NDUFA9/PIK3CB/CASP3/ND[illegible]
AP3K1/ADCY3/ANAPC4/CD3G/HLA-C/ADCY7/TRRAP/PIK3CB/BUB3/ITGAL/FDPS/EGR2/ATF6B/HLA[illegible]
/EPN3/VPS37B/CYTH3/CAV1/VPS4A/SNX5/AGAP3/ARFGEF1/AP2A1/HLA-C/VPS26A/VPS29/STAM/[illegible]

7

L22/RPL31/MRPS11

2RB1/E2F2/CYCS/DVL2/MSH2/IL2RB/RALA/GRB2/PAX8/TGFB3/CDC42/PML/ZBTB17/IKBKG/IGF1[illegible]
D/PSMC5/NDUFA8/GRIN2D/UQCRB/NDUFA1/PSMC3/NDUFA13/BECN1/NDUFS8/ATG13/CDK5R1/COX7[illegible]
OX7B/OPTN/DAXX/BAD/PSMC5/HNRNPA1/NDUFA8/GRIN2D/UQCRB/NDUFA1/PSMC3/NDUFA13/BECN1[illegible]

PLCG1/PARK7/GNAS/PSMB7

/PPP2R5B/CDC37/GNG4/BAD/PIK3R5/PRKAA2/IFNAR2/VEGFA/KITLG

F282/ZNF43/ZNF74/ZNF480/ZNF256/ZNF300/ZNF124/TNFRSF14/HLA-C/ZNF765/ZNF160/ZNF22[illegible]

2R5B/CASP8/BAD/IFNAR2/VEGFA/LFNG

PSMB6/NDUFB4/KLC1/AP2A1/POLR2E/NDUFA9/POLR2B/CASP3/NDUFB11/PSMA3/COX4I1

UFB11/PSMA3/CAV2/COX4I1/ATF6B

-DOB

CAV2/IGF2R/CAPZA2/ASAP3/KIF5B/HLA-E/ARRB1/MVB12B/VTA1/RAB22A/RAB11FIP1/RAB7A/TS[illegible]

R/HEY1/TRAF3/IL4R/GNB4/FADD/CSF2RB/RBX1/CBL/RALGDS/CSF2RA/CCDC6/GNGT2/WNT4/RPS6[illegible]

A2/PSEN2/CACNA1D/COX6C/APBB1/ATP2A2/CSNK2A2/PSMB1/CAPN2/CALM3/NDUFA12/COX5A/PSM[illegible]

/NDUFS8/WDR41/ATG13/ACTR1A/COX7A2/BCL2L1/SEH1L/COX6C/PSMB1/RAB1A/NDUFA12/COX5A/[illegible]

2/PIK3CB/ZNF267/CASP3/MAP3K7/EIF2B1/ZNF549/ZNF792/ZNF530/ZNF101/ZNF528/ZNF768/E

G101/SMAD2/HSPA1A/GIT2/TGFBR1/PLD1/PIP5K1A/RNF41/CHMP6/ACAP1/CHMP2B/SNX2/EPN1/A

KB2/RALB/TRAF4/PLCB2/CCND3/LPAR5/VHL/LAMA4/CALM2/HSP90AA1/STAT5A/CTBP2/CTNNA1/P
B6/NDUFB4/KLC1/NDUFA9/PIK3CB/CASP3/NDUFB11/PSMA3/COX4I1/NCSTN/CASP7/NOX1/KIF5B
PSMB6/NXT2/NDUFB4/KLC1/NDUFA9/TNFRSF1B/CASP3/NDUFB11/PSMA3/COX4I1/KIF5B/ULK1/TP

IF2B4/HLA-DOB/ZNF430/ZNF473/ZNF669/TP53/IFNG/ZNF587/ZNF682/EIF2B3/NECTIN2/PPP1C

RFGAP3/IQSEC2/SMURF2/ZFYVE27/GBF1/HLA-G/SMAD3/CHMP4A/SNX6/PARD6A

TK2/PLD2/ABL1/GNAI3/FGFR2/HSP90B1/CCNE2/PTGER4/NOTCH3/NOTCH1/GLI1/MSH6/GNG4/CAS

53/HNRNPA2B1/PSMB7/UBQLN2/SRSF3/DERL1/NDUFA7/NDUFB2/ATXN2/NUP214/NEFL/PSMD12/NE

B/SRSF3/ZNF785/HLA-E/EIF2B2/RELA/ZNF808/ZNF701/ZNF107/ZNF419/ZNF135/POU2F1/POU2

P8/ARHGEF11/BAD/ADCY6/RHOA/IFNAR2/VEGFA/KITLG

FH/TARDBP/SMCR8/DNAH14/CHMP2B/SRSF7/NDUFA6/TPR/COX6A1/COX7A2L/PSMD6/GABARAP/PPP

F2/EIF2AK1/ZFP69/ZNF257/ZFP82/ZNF845/ZNF841/ZNF347/IKBKB/ZNF766/TSC1/ZNF431/ZNF

[illegible]3CB/NUP133/UQCR10/NDUFA11/UQCRFS1/NUP50/NDUFS3/PSMC2/NDUFB10/NDUFB7/NCBP1/EIF2[illegible]

[illegible]211/PIK3R2/ZNF506/ZNF8/ZNF714/ZNF331/SRSF7/ZNF439/ZNF84/ZNF57/ZNF550/ZNF736/TI[illegible]

[illegible]1/NDUFA3/NRG2/DCTN3/PSMA6/NDUFB5/RB1CC1/WIPI2/VAPB/NDUFAB1

CAM1/ZNF614/SRSF4/IL12A/FASLG/ZNF85/HLA-G/ZNF777/ZFP69B/TAB1/ZNF226/ZNF250/ZNF12

2/EIF2S1/ZNF562/ZNF764/ZFP30/ZNF416/ZNF90/TYK2/ZNF559/ZNF26/CASP9/ZNF773/ZIK1/ZN

NF133/IRAK4/ZNF547/ZNF234/ZNF200/TNFRSF1A/TNFSF14/AKT3/ZNF786/TBK1/ZNF286A/ZNF2[illegible]

[illegible]4/ZNF551/ZNF182/ZNF778/ZNF613/JAK2/ZNF552/ZNF514/MTOR/ZNF517/ZNF285/ZNF667/ZNF[illegible]

665/ZNF227/ZNF850/ZNF230/APAF1/ZNF713/ZNF630/ZNF77/ZNF708/BST2/EIF2AK2/ZNF670/Z[illegible]

NF233/ZNF248/ZNF585B/NXF1/BCL2/ZNF350
