# Supplementary figures and images for: The Landscape of Using Glycosyltransferase Gene Signatures for Overall Survival Prediction in Hepatocellular Carcinoma
Source: J Oncol. 2022 Jun 21;2022:5989419. doi: 10.1155/2022/5989419 (PMC9239767; doi:10.1155/2022/5989419)

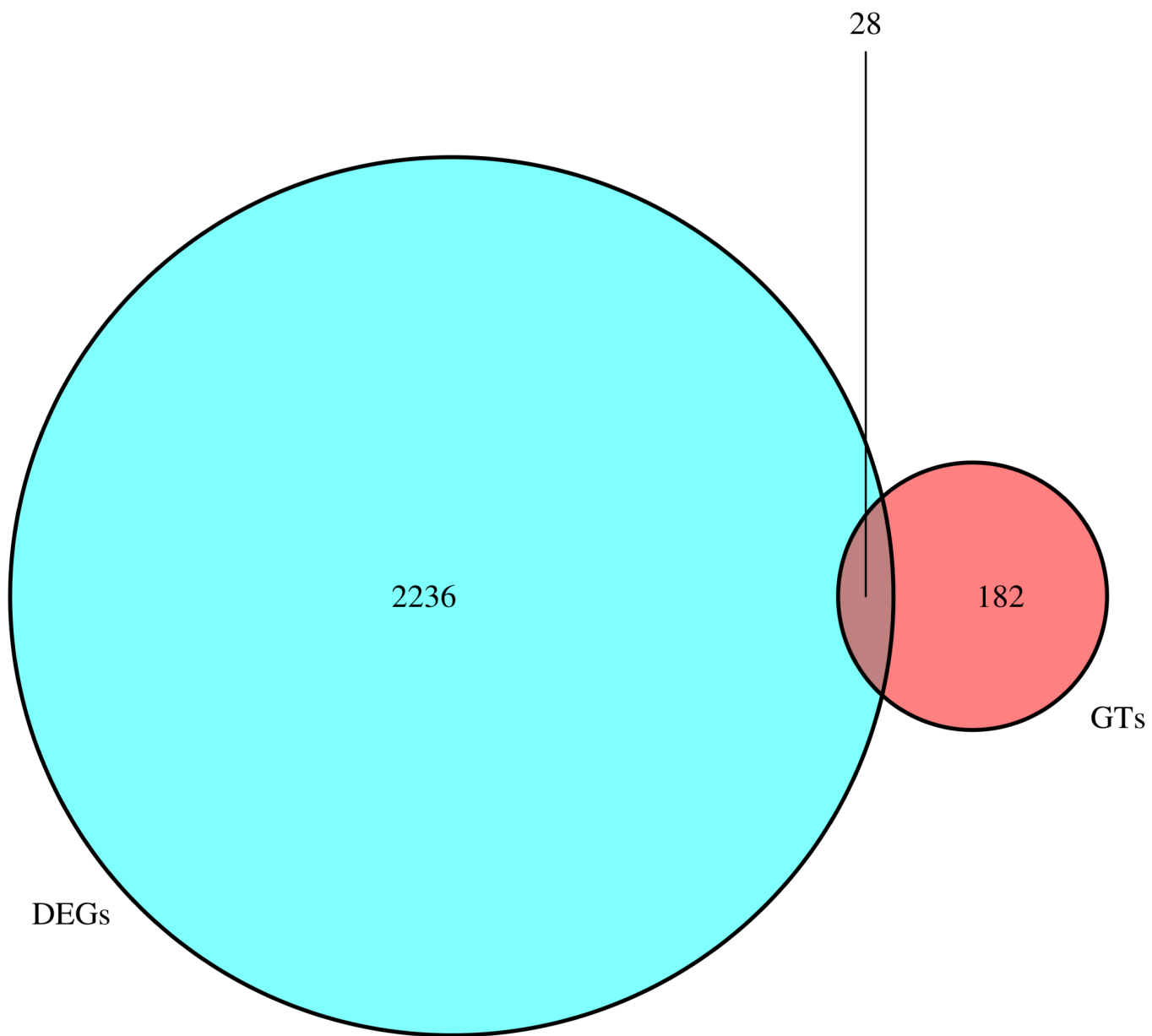

Supplement: Supplementary Materials — Figure S1: Venn diagram shows the intersections of co-differentially expressed glycosyltransferase (GT) genes between the 210 GTs obtained from the literature and those obtained from TCGA database. Figure S2: identification of a four-gene signature in the training set of TCGA database constructed using the LASSO method. Figure S3: survival analyses grouped by the optimal cutoff expression value of each gene in the training dataset of TCGA database. (A) ALG3. (B) B3GAT3. (C) GLA. (D) ST6GALNAC4. Figure S4: mRNA expression levels of the four genes in matched HCC and adjacent noncancerous samples in TCGA database were compared. (A) ALG3. (B) B3GAT3. (C) GLA. (D) ST6GALNAC4. Figure S5: the interactions among signature genes displayed in a PPI network. Figure S6: stratified survival analysis between high- and low-risk groups. (A)NAFLD. (B) HBV infection. (C) HCV infection. (D) Both HBV and HCV infections. (E) No treatment and therapy. (F) Pharmaceutical therapy. (G) Radiation therapy, NOS. Figure S7: the nomogram to predict prognostic probabilities in TCGA database. (A) The nomogram for predicting OS in the training set. The calibration plots for predicting (B) 3-year and (C) 5-year survival probabilities. Figure S8: survival analyses grouped by the optimal cutoff expression value of each signature gene in the validation dataset of TCGA database. (A) ALG3. (B) B3GAT3. (C) GLA. (D) ST6GALNAC4. Table S1: the clinical information of 371 primary HCC samples and 50 normal samples. Table S2: combining the clinical information and expression profiles of 210 human glycosyltransferase (GT) genes. Table S3: DEGs of 371 samples in TCGA database. Table S4: all significantly enriched GO terms. Table S5: all significantly enriched KEGG pathways. [file 5989419.f1.zip › 5989419.f1/Supplementary Figure 1.pdf]

A

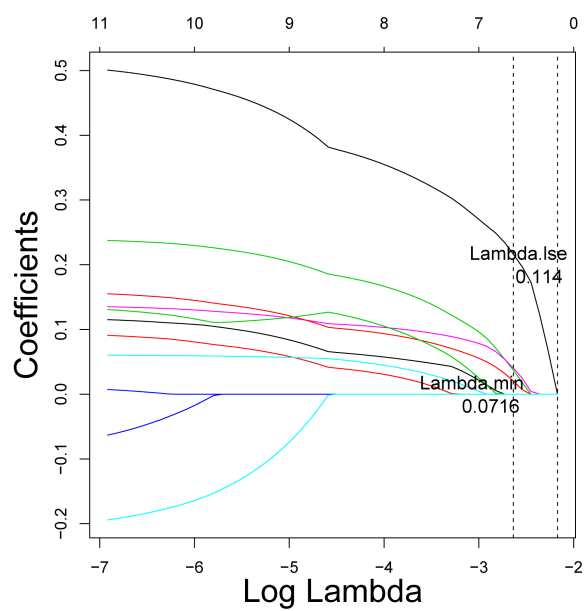

B

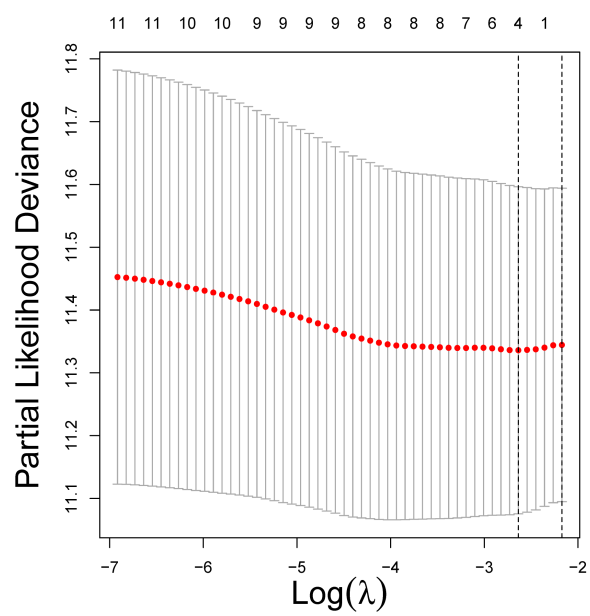

Supplement: Supplementary Materials — Figure S1: Venn diagram shows the intersections of co-differentially expressed glycosyltransferase (GT) genes between the 210 GTs obtained from the literature and those obtained from TCGA database. Figure S2: identification of a four-gene signature in the training set of TCGA database constructed using the LASSO method. Figure S3: survival analyses grouped by the optimal cutoff expression value of each gene in the training dataset of TCGA database. (A) ALG3. (B) B3GAT3. (C) GLA. (D) ST6GALNAC4. Figure S4: mRNA expression levels of the four genes in matched HCC and adjacent noncancerous samples in TCGA database were compared. (A) ALG3. (B) B3GAT3. (C) GLA. (D) ST6GALNAC4. Figure S5: the interactions among signature genes displayed in a PPI network. Figure S6: stratified survival analysis between high- and low-risk groups. (A)NAFLD. (B) HBV infection. (C) HCV infection. (D) Both HBV and HCV infections. (E) No treatment and therapy. (F) Pharmaceutical therapy. (G) Radiation therapy, NOS. Figure S7: the nomogram to predict prognostic probabilities in TCGA database. (A) The nomogram for predicting OS in the training set. The calibration plots for predicting (B) 3-year and (C) 5-year survival probabilities. Figure S8: survival analyses grouped by the optimal cutoff expression value of each signature gene in the validation dataset of TCGA database. (A) ALG3. (B) B3GAT3. (C) GLA. (D) ST6GALNAC4. Table S1: the clinical information of 371 primary HCC samples and 50 normal samples. Table S2: combining the clinical information and expression profiles of 210 human glycosyltransferase (GT) genes. Table S3: DEGs of 371 samples in TCGA database. Table S4: all significantly enriched GO terms. Table S5: all significantly enriched KEGG pathways. [file 5989419.f1.zip › 5989419.f1/Supplementary Figure 2.pdf]

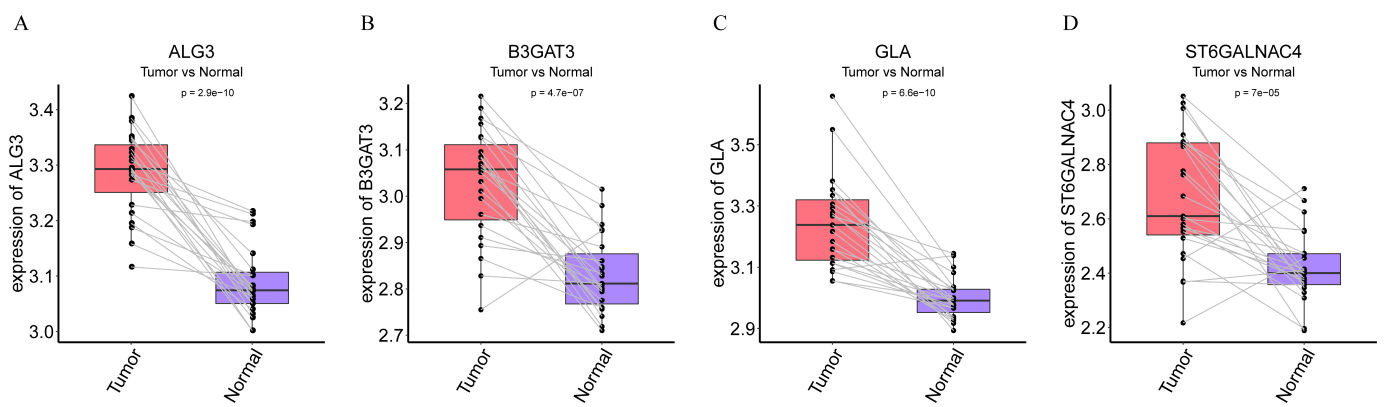

Supplement: Supplementary Materials — Figure S1: Venn diagram shows the intersections of co-differentially expressed glycosyltransferase (GT) genes between the 210 GTs obtained from the literature and those obtained from TCGA database. Figure S2: identification of a four-gene signature in the training set of TCGA database constructed using the LASSO method. Figure S3: survival analyses grouped by the optimal cutoff expression value of each gene in the training dataset of TCGA database. (A) ALG3. (B) B3GAT3. (C) GLA. (D) ST6GALNAC4. Figure S4: mRNA expression levels of the four genes in matched HCC and adjacent noncancerous samples in TCGA database were compared. (A) ALG3. (B) B3GAT3. (C) GLA. (D) ST6GALNAC4. Figure S5: the interactions among signature genes displayed in a PPI network. Figure S6: stratified survival analysis between high- and low-risk groups. (A)NAFLD. (B) HBV infection. (C) HCV infection. (D) Both HBV and HCV infections. (E) No treatment and therapy. (F) Pharmaceutical therapy. (G) Radiation therapy, NOS. Figure S7: the nomogram to predict prognostic probabilities in TCGA database. (A) The nomogram for predicting OS in the training set. The calibration plots for predicting (B) 3-year and (C) 5-year survival probabilities. Figure S8: survival analyses grouped by the optimal cutoff expression value of each signature gene in the validation dataset of TCGA database. (A) ALG3. (B) B3GAT3. (C) GLA. (D) ST6GALNAC4. Table S1: the clinical information of 371 primary HCC samples and 50 normal samples. Table S2: combining the clinical information and expression profiles of 210 human glycosyltransferase (GT) genes. Table S3: DEGs of 371 samples in TCGA database. Table S4: all significantly enriched GO terms. Table S5: all significantly enriched KEGG pathways. [file 5989419.f1.zip › 5989419.f1/Supplementary Figure 4.pdf]

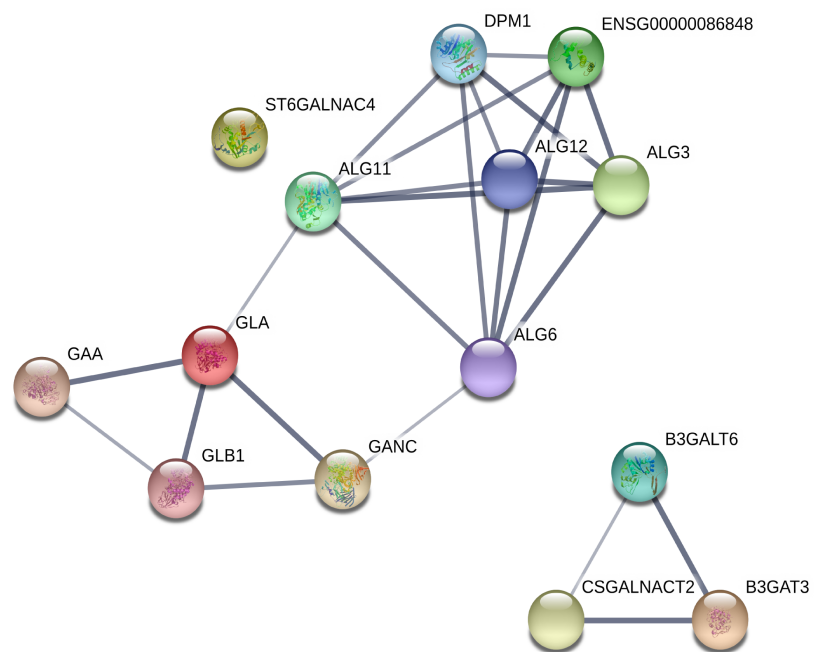

Supplement: Supplementary Materials — Figure S1: Venn diagram shows the intersections of co-differentially expressed glycosyltransferase (GT) genes between the 210 GTs obtained from the literature and those obtained from TCGA database. Figure S2: identification of a four-gene signature in the training set of TCGA database constructed using the LASSO method. Figure S3: survival analyses grouped by the optimal cutoff expression value of each gene in the training dataset of TCGA database. (A) ALG3. (B) B3GAT3. (C) GLA. (D) ST6GALNAC4. Figure S4: mRNA expression levels of the four genes in matched HCC and adjacent noncancerous samples in TCGA database were compared. (A) ALG3. (B) B3GAT3. (C) GLA. (D) ST6GALNAC4. Figure S5: the interactions among signature genes displayed in a PPI network. Figure S6: stratified survival analysis between high- and low-risk groups. (A)NAFLD. (B) HBV infection. (C) HCV infection. (D) Both HBV and HCV infections. (E) No treatment and therapy. (F) Pharmaceutical therapy. (G) Radiation therapy, NOS. Figure S7: the nomogram to predict prognostic probabilities in TCGA database. (A) The nomogram for predicting OS in the training set. The calibration plots for predicting (B) 3-year and (C) 5-year survival probabilities. Figure S8: survival analyses grouped by the optimal cutoff expression value of each signature gene in the validation dataset of TCGA database. (A) ALG3. (B) B3GAT3. (C) GLA. (D) ST6GALNAC4. Table S1: the clinical information of 371 primary HCC samples and 50 normal samples. Table S2: combining the clinical information and expression profiles of 210 human glycosyltransferase (GT) genes. Table S3: DEGs of 371 samples in TCGA database. Table S4: all significantly enriched GO terms. Table S5: all significantly enriched KEGG pathways. [file 5989419.f1.zip › 5989419.f1/Supplementary Figure 5.pdf]

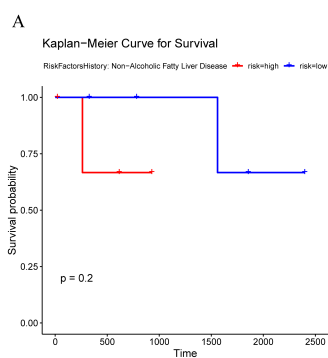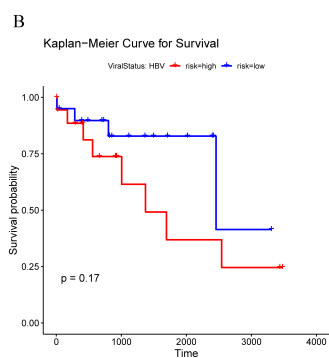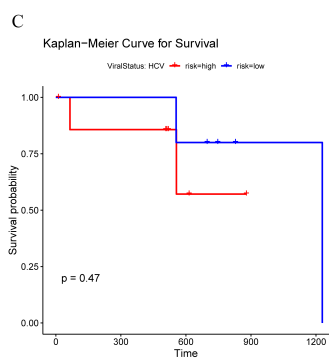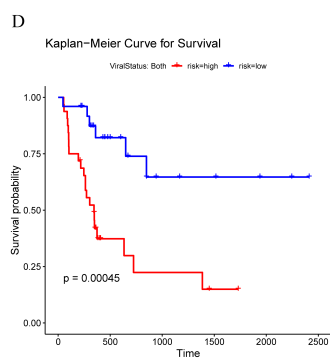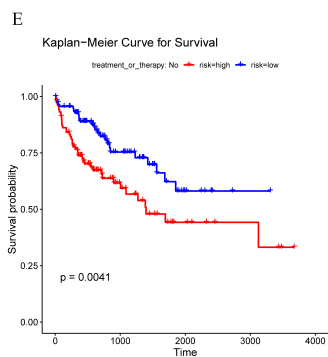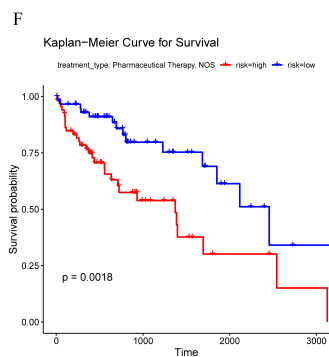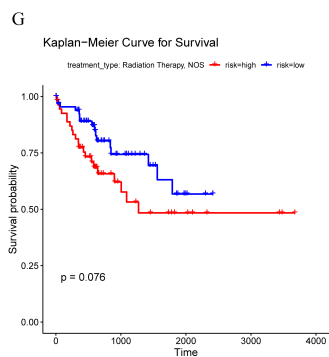

Supplement: Supplementary Materials — Figure S1: Venn diagram shows the intersections of co-differentially expressed glycosyltransferase (GT) genes between the 210 GTs obtained from the literature and those obtained from TCGA database. Figure S2: identification of a four-gene signature in the training set of TCGA database constructed using the LASSO method. Figure S3: survival analyses grouped by the optimal cutoff expression value of each gene in the training dataset of TCGA database. (A) ALG3. (B) B3GAT3. (C) GLA. (D) ST6GALNAC4. Figure S4: mRNA expression levels of the four genes in matched HCC and adjacent noncancerous samples in TCGA database were compared. (A) ALG3. (B) B3GAT3. (C) GLA. (D) ST6GALNAC4. Figure S5: the interactions among signature genes displayed in a PPI network. Figure S6: stratified survival analysis between high- and low-risk groups. (A)NAFLD. (B) HBV infection. (C) HCV infection. (D) Both HBV and HCV infections. (E) No treatment and therapy. (F) Pharmaceutical therapy. (G) Radiation therapy, NOS. Figure S7: the nomogram to predict prognostic probabilities in TCGA database. (A) The nomogram for predicting OS in the training set. The calibration plots for predicting (B) 3-year and (C) 5-year survival probabilities. Figure S8: survival analyses grouped by the optimal cutoff expression value of each signature gene in the validation dataset of TCGA database. (A) ALG3. (B) B3GAT3. (C) GLA. (D) ST6GALNAC4. Table S1: the clinical information of 371 primary HCC samples and 50 normal samples. Table S2: combining the clinical information and expression profiles of 210 human glycosyltransferase (GT) genes. Table S3: DEGs of 371 samples in TCGA database. Table S4: all significantly enriched GO terms. Table S5: all significantly enriched KEGG pathways. [file 5989419.f1.zip › 5989419.f1/Supplementary Figure 6.pdf]

A

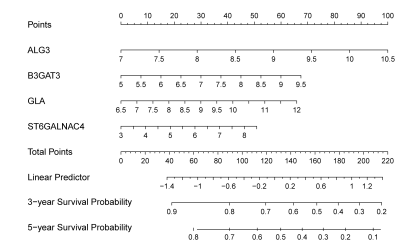

B

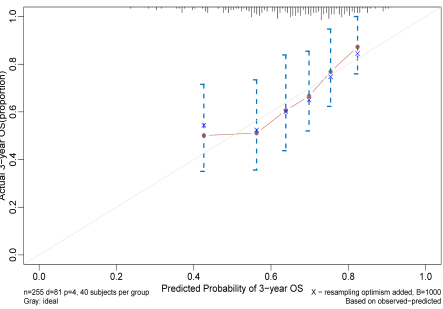

C

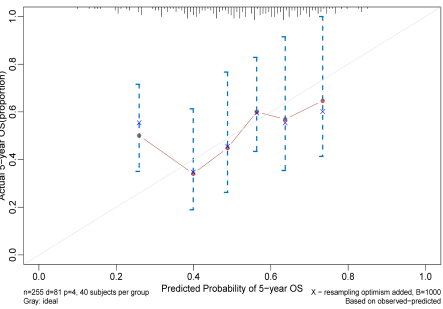

Supplement: Supplementary Materials — Figure S1: Venn diagram shows the intersections of co-differentially expressed glycosyltransferase (GT) genes between the 210 GTs obtained from the literature and those obtained from TCGA database. Figure S2: identification of a four-gene signature in the training set of TCGA database constructed using the LASSO method. Figure S3: survival analyses grouped by the optimal cutoff expression value of each gene in the training dataset of TCGA database. (A) ALG3. (B) B3GAT3. (C) GLA. (D) ST6GALNAC4. Figure S4: mRNA expression levels of the four genes in matched HCC and adjacent noncancerous samples in TCGA database were compared. (A) ALG3. (B) B3GAT3. (C) GLA. (D) ST6GALNAC4. Figure S5: the interactions among signature genes displayed in a PPI network. Figure S6: stratified survival analysis between high- and low-risk groups. (A)NAFLD. (B) HBV infection. (C) HCV infection. (D) Both HBV and HCV infections. (E) No treatment and therapy. (F) Pharmaceutical therapy. (G) Radiation therapy, NOS. Figure S7: the nomogram to predict prognostic probabilities in TCGA database. (A) The nomogram for predicting OS in the training set. The calibration plots for predicting (B) 3-year and (C) 5-year survival probabilities. Figure S8: survival analyses grouped by the optimal cutoff expression value of each signature gene in the validation dataset of TCGA database. (A) ALG3. (B) B3GAT3. (C) GLA. (D) ST6GALNAC4. Table S1: the clinical information of 371 primary HCC samples and 50 normal samples. Table S2: combining the clinical information and expression profiles of 210 human glycosyltransferase (GT) genes. Table S3: DEGs of 371 samples in TCGA database. Table S4: all significantly enriched GO terms. Table S5: all significantly enriched KEGG pathways. [file 5989419.f1.zip › 5989419.f1/Supplementary Figure 7.pdf]
